# Supplementary material for: Exhaustive identification of conserved upstream open reading frames with potential translational regulatory functions from animal genomes
Source: Sci Rep. 2020 Oct 1;10:16289. doi: 10.1038/s41598-020-73307-6 (PMC7530721; doi:10.1038/s41598-020-73307-6)
Supplement: Supplementary file 1 — Supplementary Information. [file 41598_2020_73307_MOESM1_ESM.pdf]

Title: Exhaustive identification of conserved upstream open reading frames with potential translational regulatory functions from animal genomes

Authors: Hiro Takahashi[1,2,3#]*, Shido Miyaki[2#], Hitoshi Onouchi[4#], Taichiro Motomura[1], Nobuo Idesako[2], Anna Takahashi[5,6], Masataka Murase[1], Shuichi Fukuyoshi[7], Toshinori Endo[8], Kenji Satou[9] , Satoshi Naito[4,10], and Motoyuki Itoh[11]*

Affiliations:

[1]Graduate School of Medical Sciences, Kanazawa University, Kanazawa 920-1192, Japan

[2]Graduate School of Horticulture, Chiba University, Matsudo 271-8510, Japan

[3]Fundamental Innovative Oncology Core Center, National Cancer Center, 5-1-1 Tsukiji, Chuo-ku, Tokyo 104-0045, Japan

[4]Graduate School of Agriculture, Hokkaido University, Sapporo 060-8589, Japan

[5]Faculty of Information Technologies and Control, Belarusian State University of Informatics and Radio Electronics, Minsk 220013, Belarus

[6]College of Bioscience and Biotechnology, Chubu University, 1200 Matsumoto-cho, Kasugai, Aichi 487-8501, Japan

[7]Institute of Medical, Pharmaceutical and Health Sciences, Kanazawa University, Kakuma-machi, Kanazawa, Ishikawa 920-1192, Japan

[8]Graduate School of Information Science and Technology, Hokkaido University, Sapporo 060-0814, Japan

[9]Faculty of Biological Science and Technology, Institute of Science and Engineering, Kanazawa University, Kanazawa 920-1192, Japan

[10]Graduate School of Life Science, Hokkaido University, Sapporo 060-0810, Japan

[11]Graduate School of Pharmaceutical Science, Chiba University, Chuo-ku, Chiba 260-8675, Japan

*Correspondence. Tel: +81-76-234-4484; Fax: +81-76-234-4484; Email: takahasi@p.kanazawa-u.ac.jp

Correspondence may also be addressed to Motoyuki Itoh. Email: mito@chiba-u.jp

[#]Joint first authors.

Supplementary Figures: S1 to S6

Supplementary Tables: S1 to S5

Supplementary Figure S1

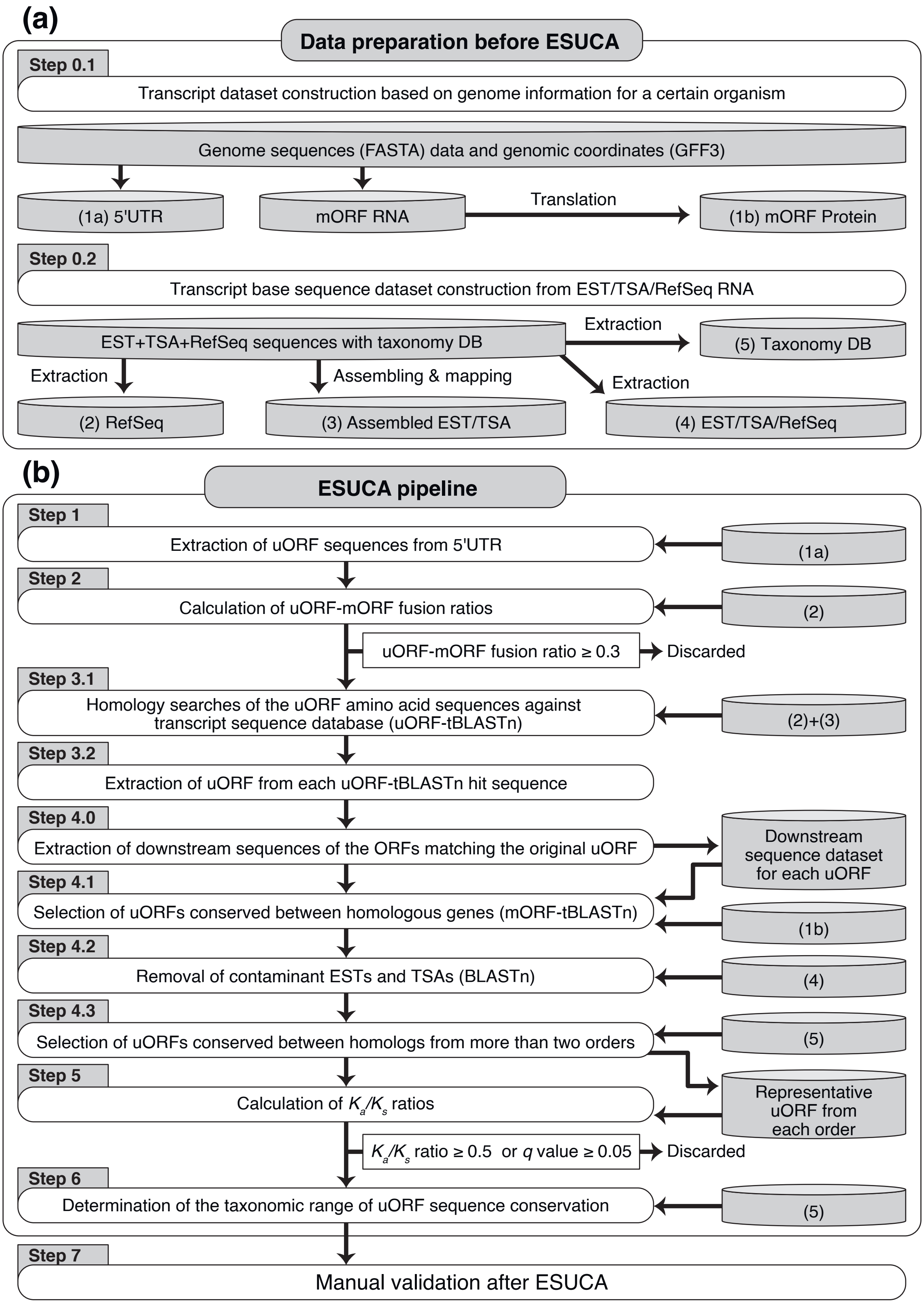

Supplementary Figure S1. Identification of animal CPuORFs using ESUCA. (**a**) Data preparation. (**b**) Outline of the ESUCA pipeline. Numbers with parenthesis indicate datasets labeled with the same numbers in (**a**).

Supplementary Figure S2

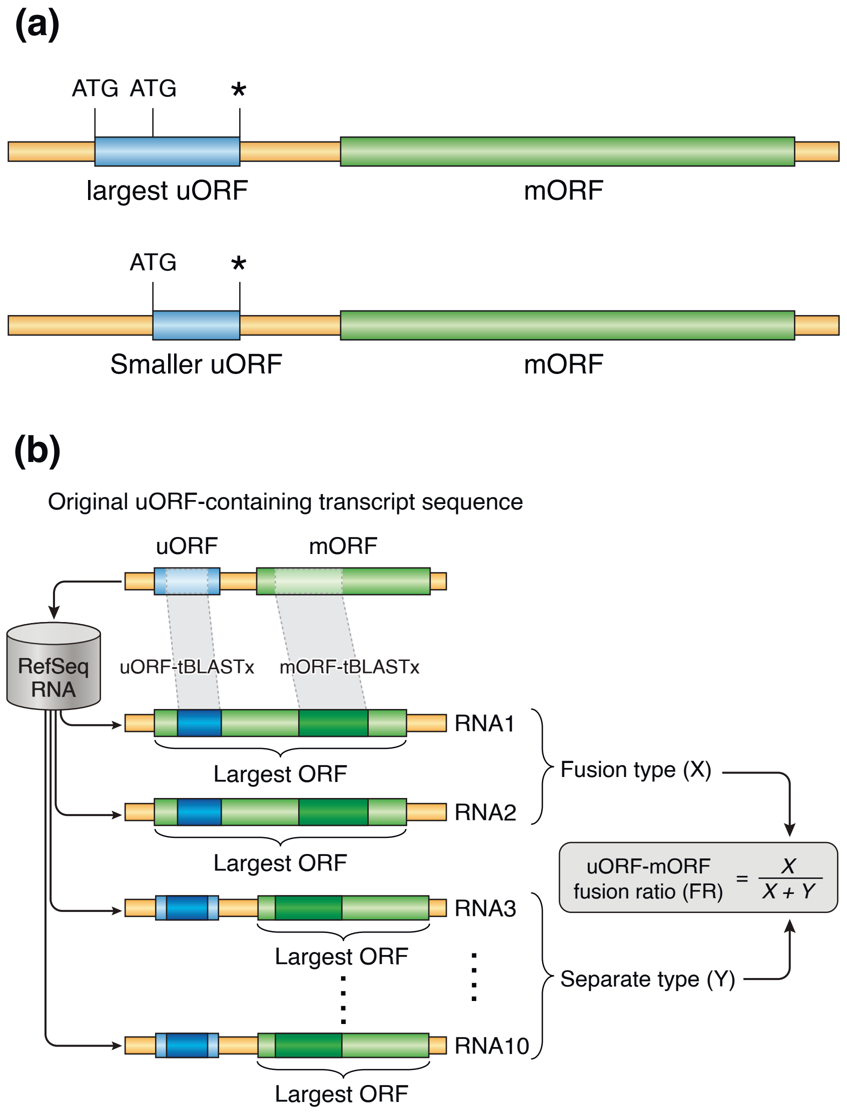

Supplementary Figure S2. Extraction of the largest uORF sequences and calculations of uORF-mORF fusion ratio. **(a)** Extraction of the largest uORF sequences from the 5′-UTR. Extraction of the largest uORF sequences from the 5′-UTR. After data preparation for ESUCA (Supplementary Fig. S1b), we conducted the extraction of uORF sequences by searching the 5′-UTR sequences for an ATG codon and its nearest downstream in-frame stop codon at step 1 of ESUCA (Supplementary Fig. S1b). Sequences starting with an ATG codon and ending with the nearest in-frame stop codon were extracted as uORF sequences. When multiple uORFs shared the same stop codon in a transcript, only the longest uORF sequence was used for further analyses. **(b)** Outline for uORF-mORF fusion ratio calculations. For each original uORF-containing transcript sequence, RefSeq RNAs containing both sequences similar to the uORF and the mORF of each uORF-containing transcript were selected using uORF-tBLASTx and mORF-tBLASTx from the RefSeq RNA database (database (2) in Supplementary Fig. S1a). For example, the selected RNA sequences are RNA1, 2, 3...10, as illustrated. Based on whether the uORF-tBLASTx-hit region was included in the largest RefSeq RNA ORF, the selected RefSeq RNAs were classified into two types, namely fusion (*X*) (RNA1 and 2) and separate types (*Y*) (RNA3-10). For each original uORF-containing transcript, the uORF-mORF fusion ratio was calculated as $X/(X+Y)$.

Supplementary Figure S3

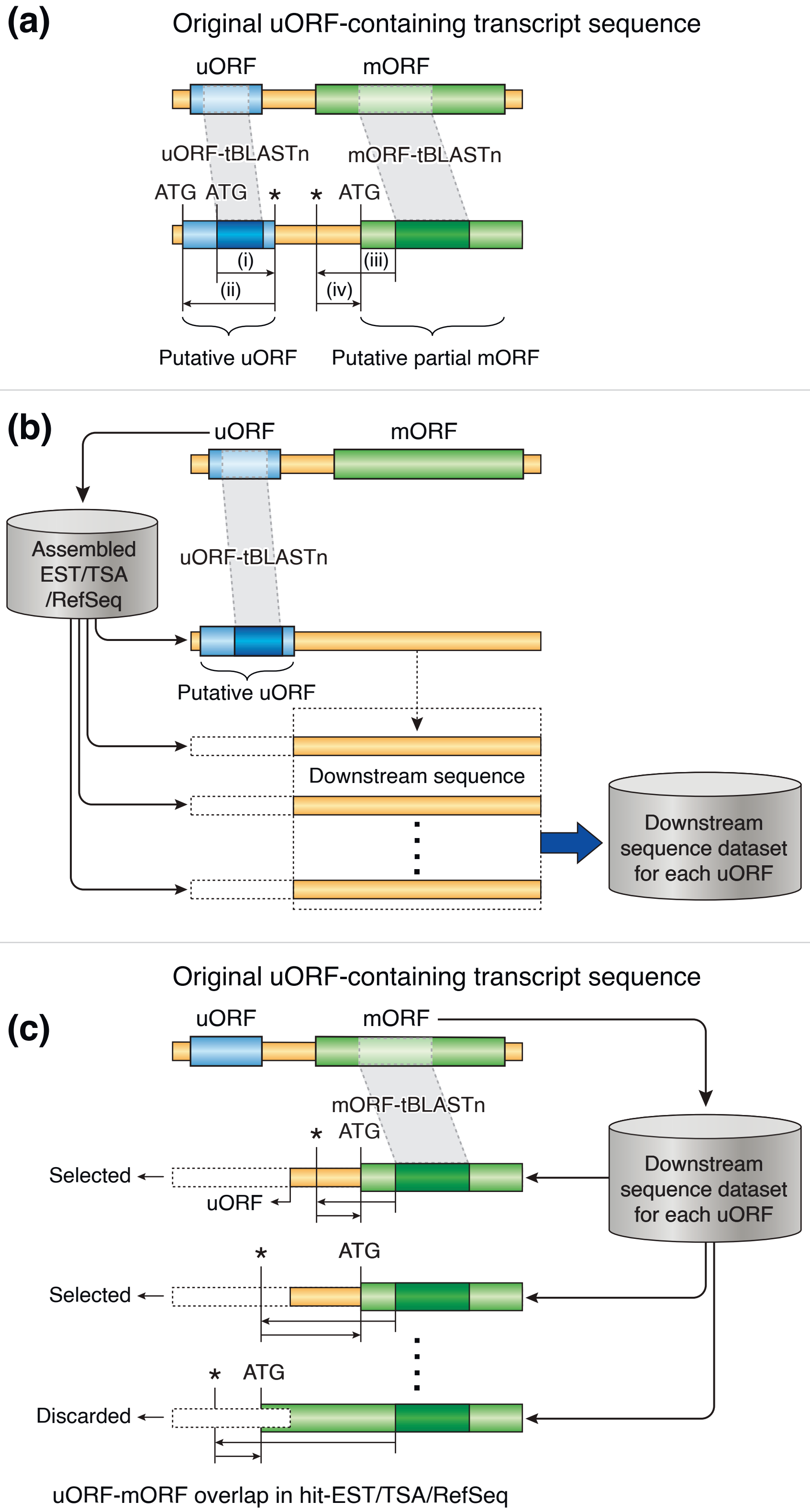

Supplementary Figure S3. Outline of homology searches for uORFs with amino acid sequences conserved between homologous genes. **(a)** For each original uORF-containing transcript, sequences containing both similar regions to the uORF and the mORF of uORF-containing transcripts were selected using uORF-tBLASTn (step 3.1 of ESUCA) and mORF-tBLASTn (step 4.1 of ESUCA). A transcript sequence database consisting of RefSeq RNAs (database (2) in Supplementary Fig. S1a) served as data source, while an assembled EST/TSA (database (3) in Supplementary Fig. S1a) was generated at step 0.2 of data preparation for ESUCA. Asterisks represent stop codons. At step 3.2 of ESUCA, the largest tBLASTn-hit region-overlapping uORF was extracted. **(b)** Detailed illustration of step 4.0 of ESUCA. Putative uORF extraction and downstream sequence dataset construction were conducted systematically for each uORF-tBLASTn hit sequence. **(c)** Detailed illustration of step 4.1 of ESUCA. After mORF-tBLASTn, the 5'-most in-frame ATG codon located downstream of the selected stop codon was identified as the initiation codon of the putative partial or intact mORF. uORF-mORF overlaps were discarded as fusion types, according to the positional relationship between them, when found in the hit-assembled EST/TSA+RefSeq sequences.

Supplementary Figure S4

HG0001

ENSDARG00000006242 (ptp4a1)

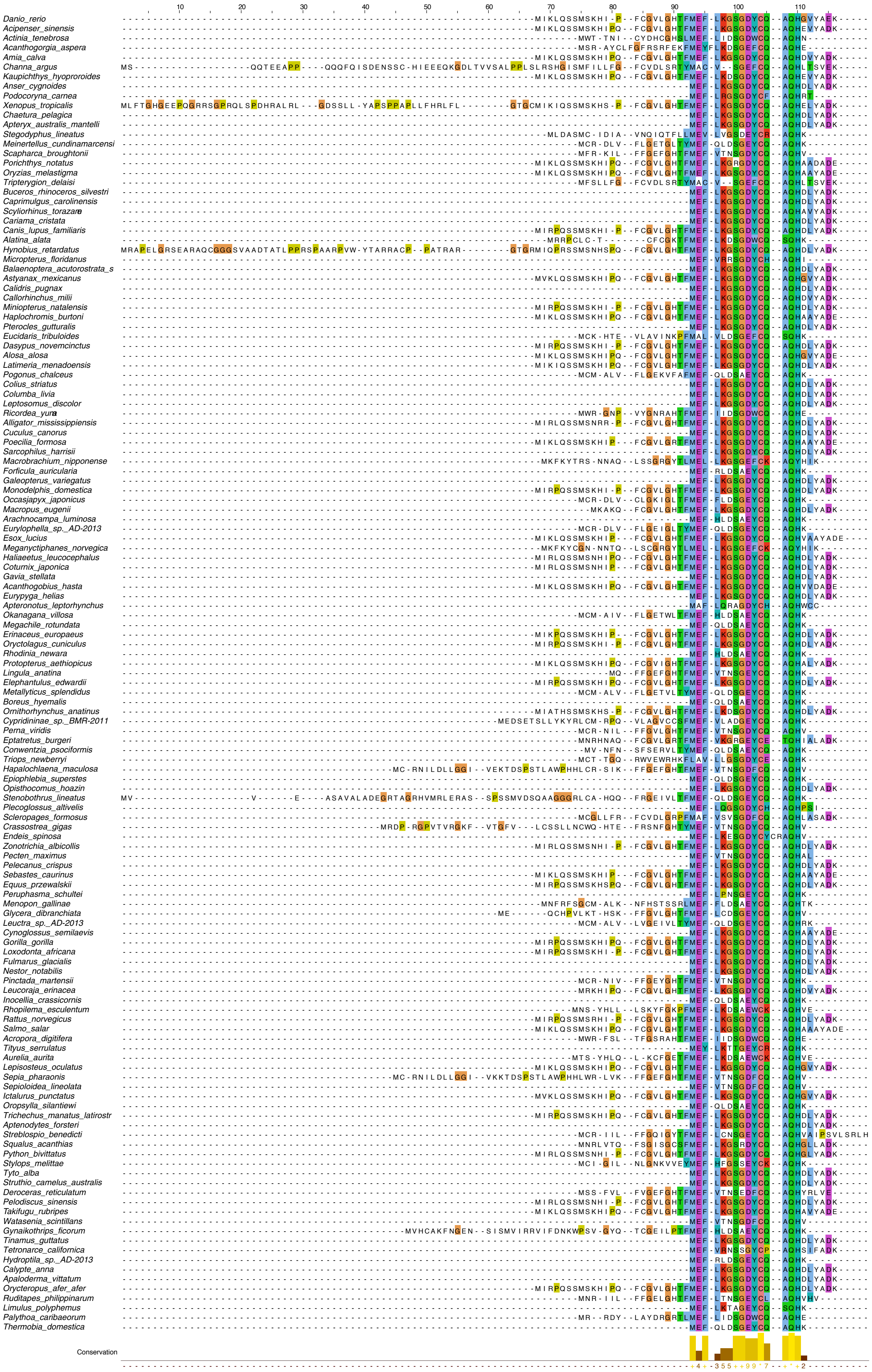

HG0001

ENSDARG00000035676 (ptp4a2b)

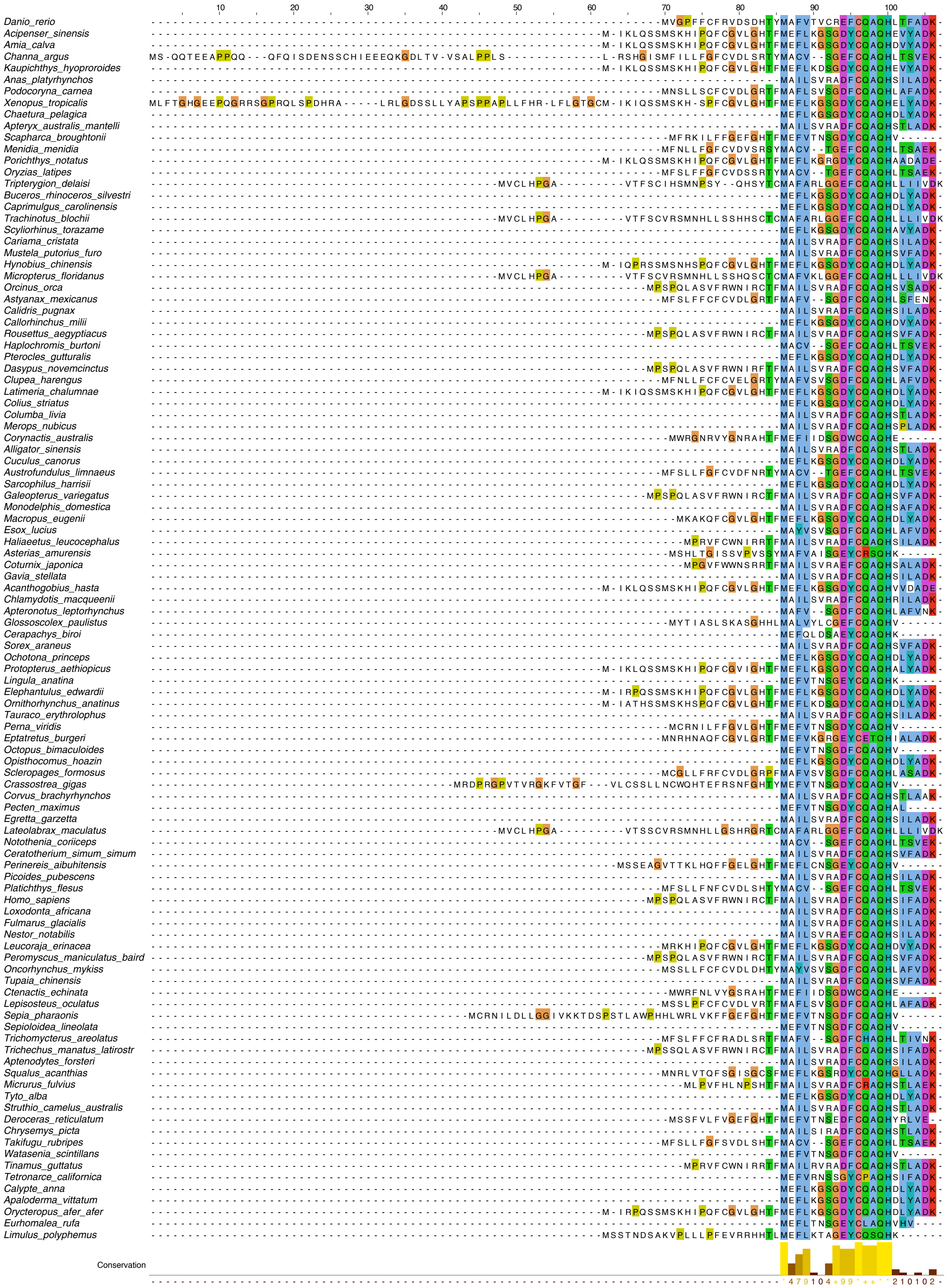

HG0001

ENSDARG00000039997 (ptp4a3)

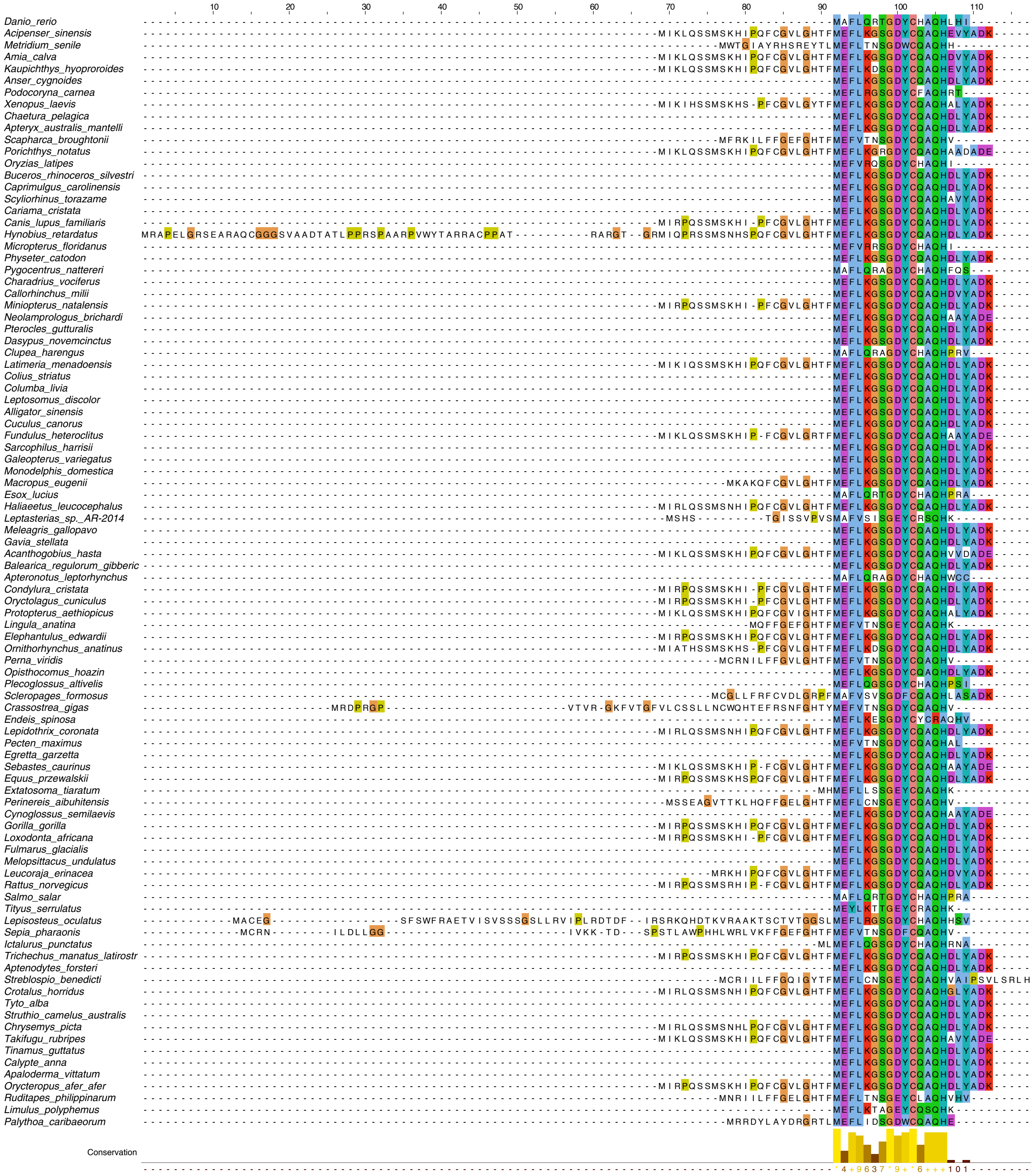

HG0001

ENSDARG00000054814 (PTP4A3)

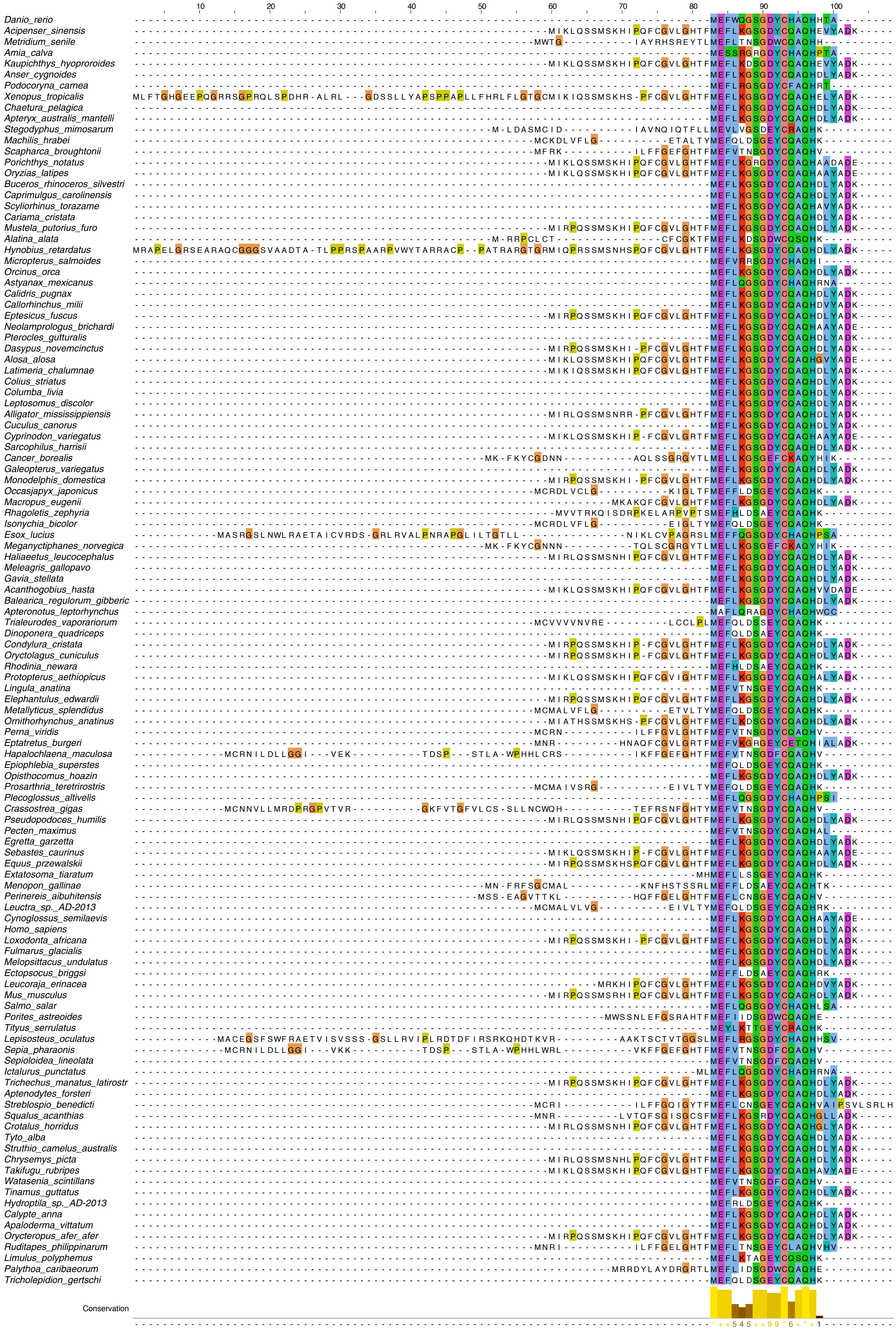

HG0001

ENSDARG00000087443 (ptp4a2a)

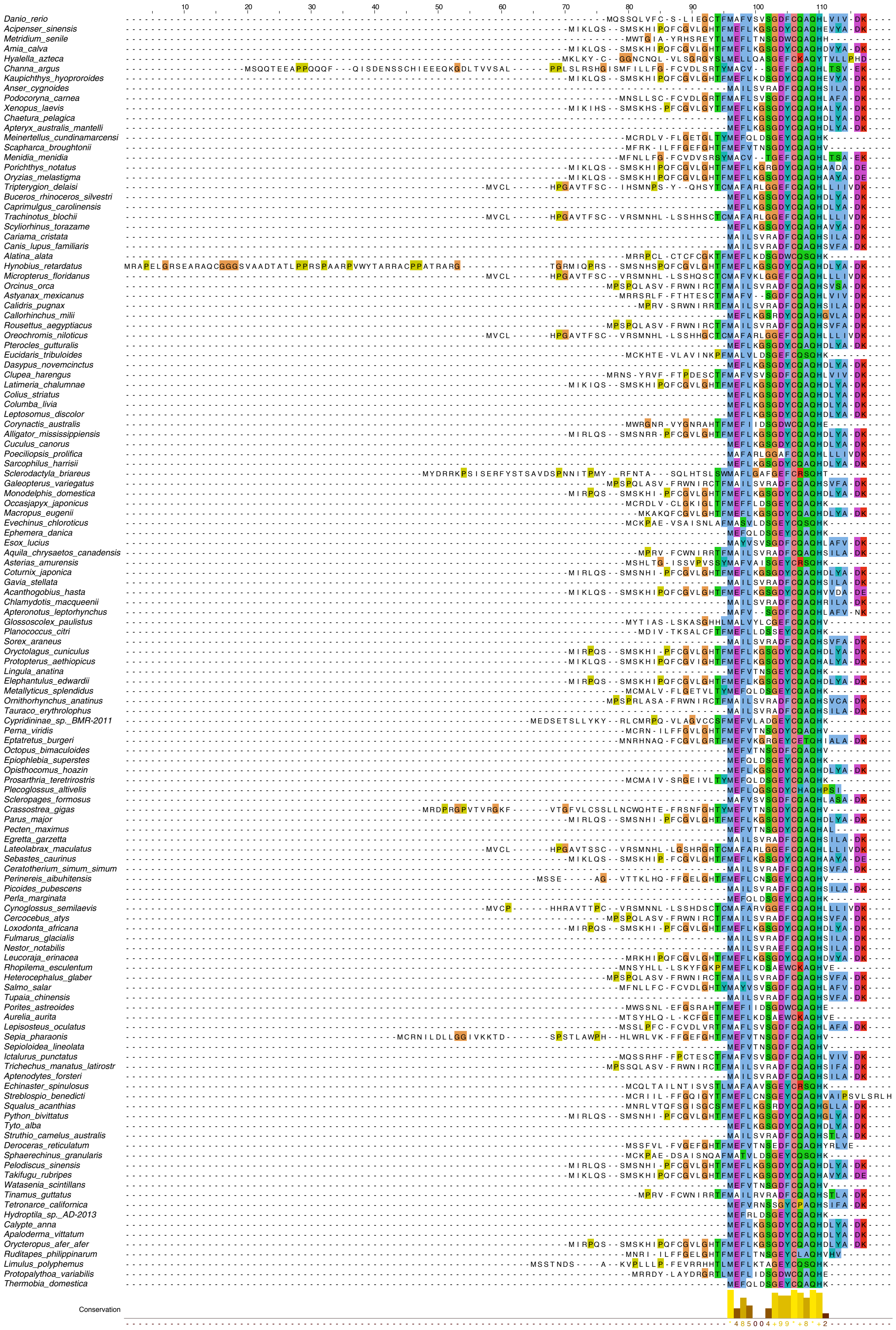

HG0001

ENSG00000112245 (PTP4A1)

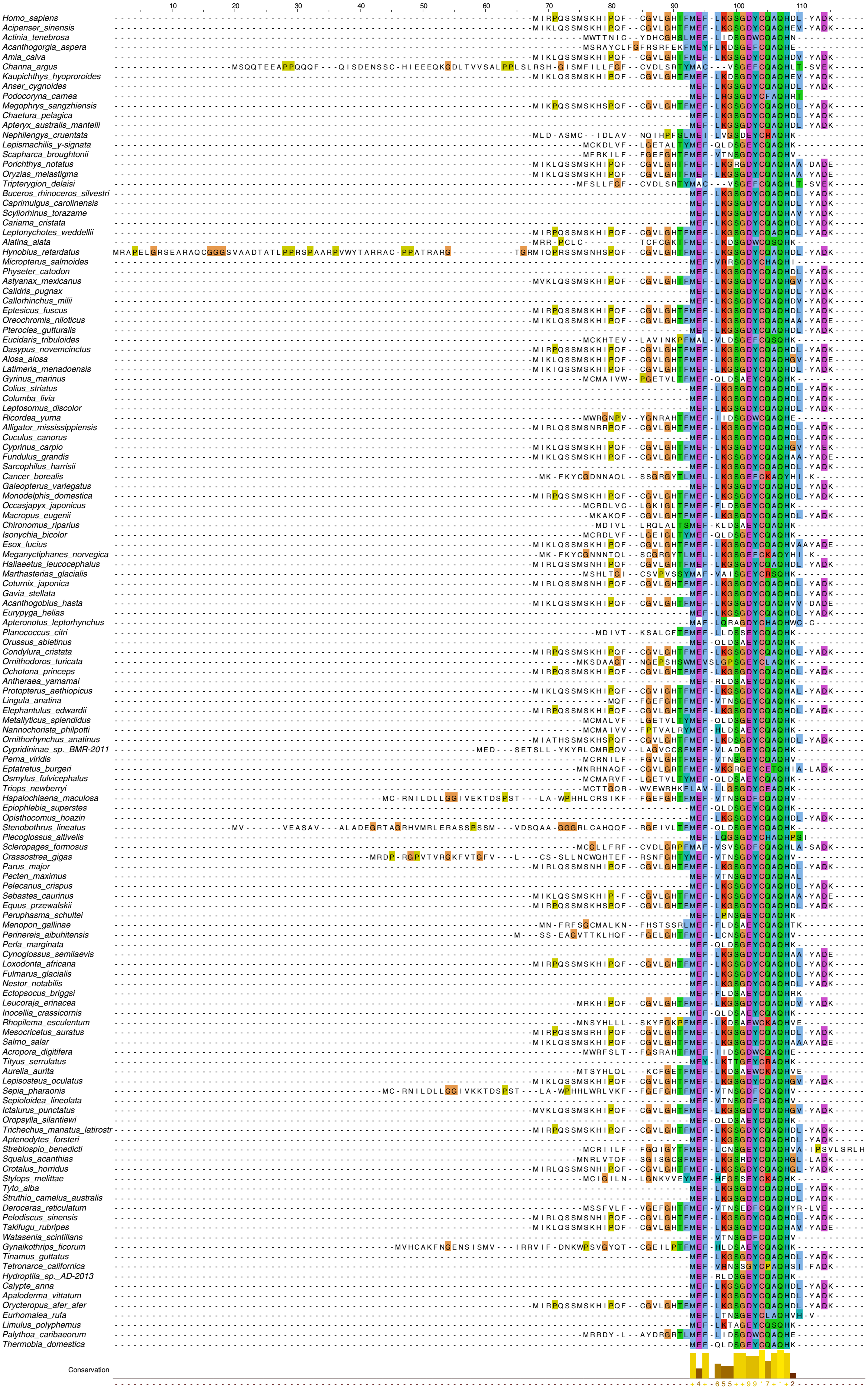

HG0001

ENSG00000184007 (PTP4A2)

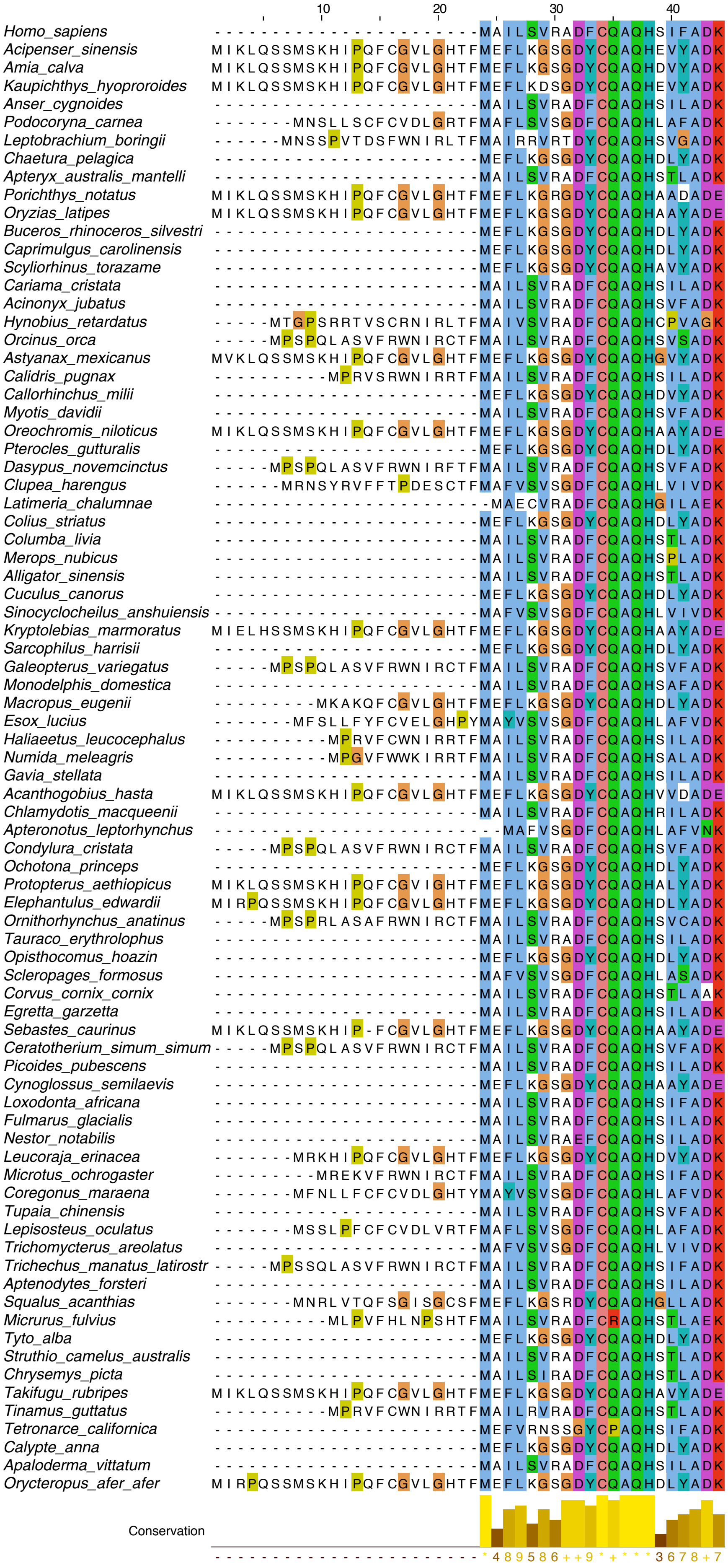

HG0001

ENSGALG00000003265 (PTP4A2)

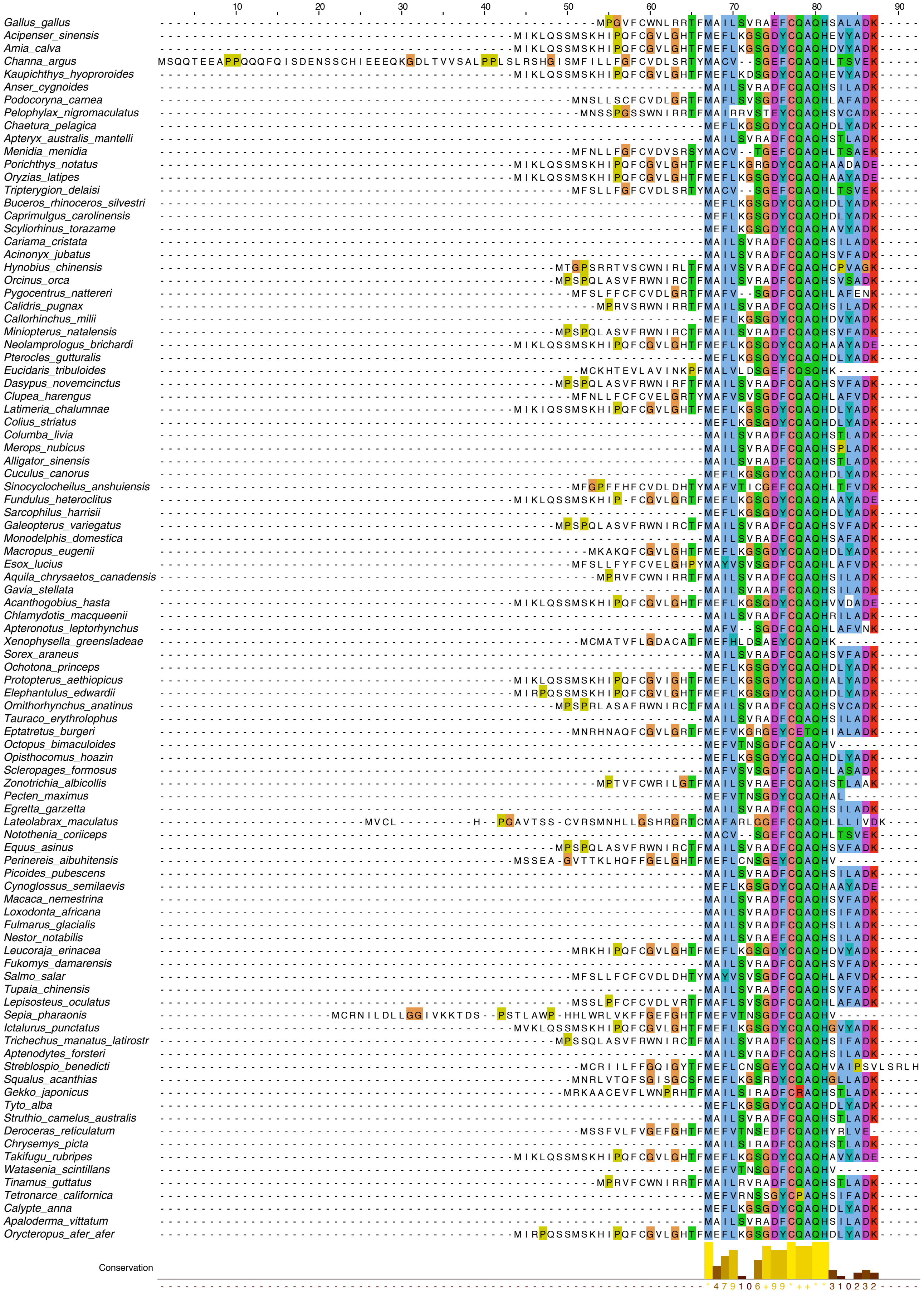

HG0001

ENSGALG00000016271 (PTP4A1)

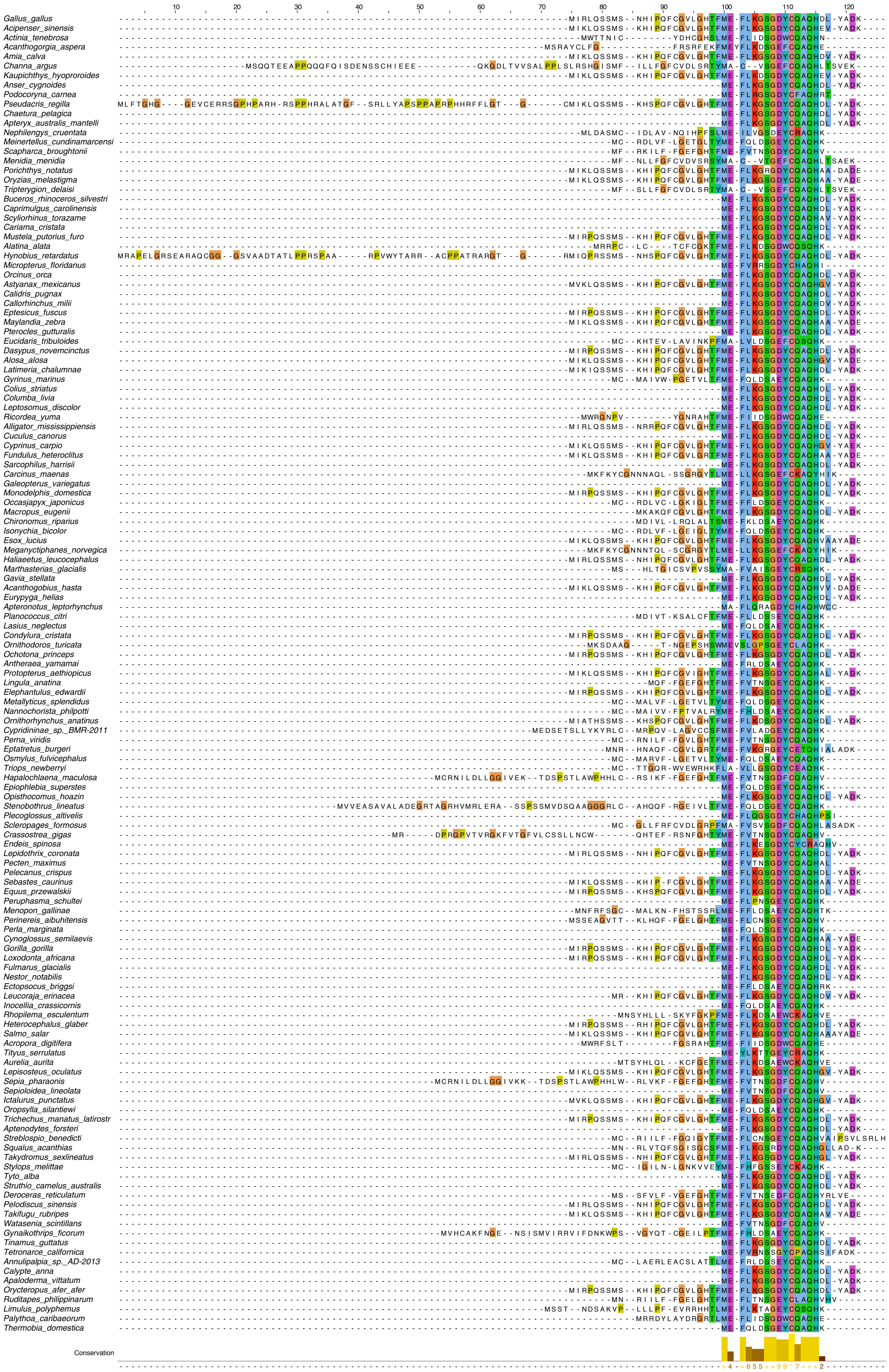

HG0001

FBgn0024734 (PRL-1)

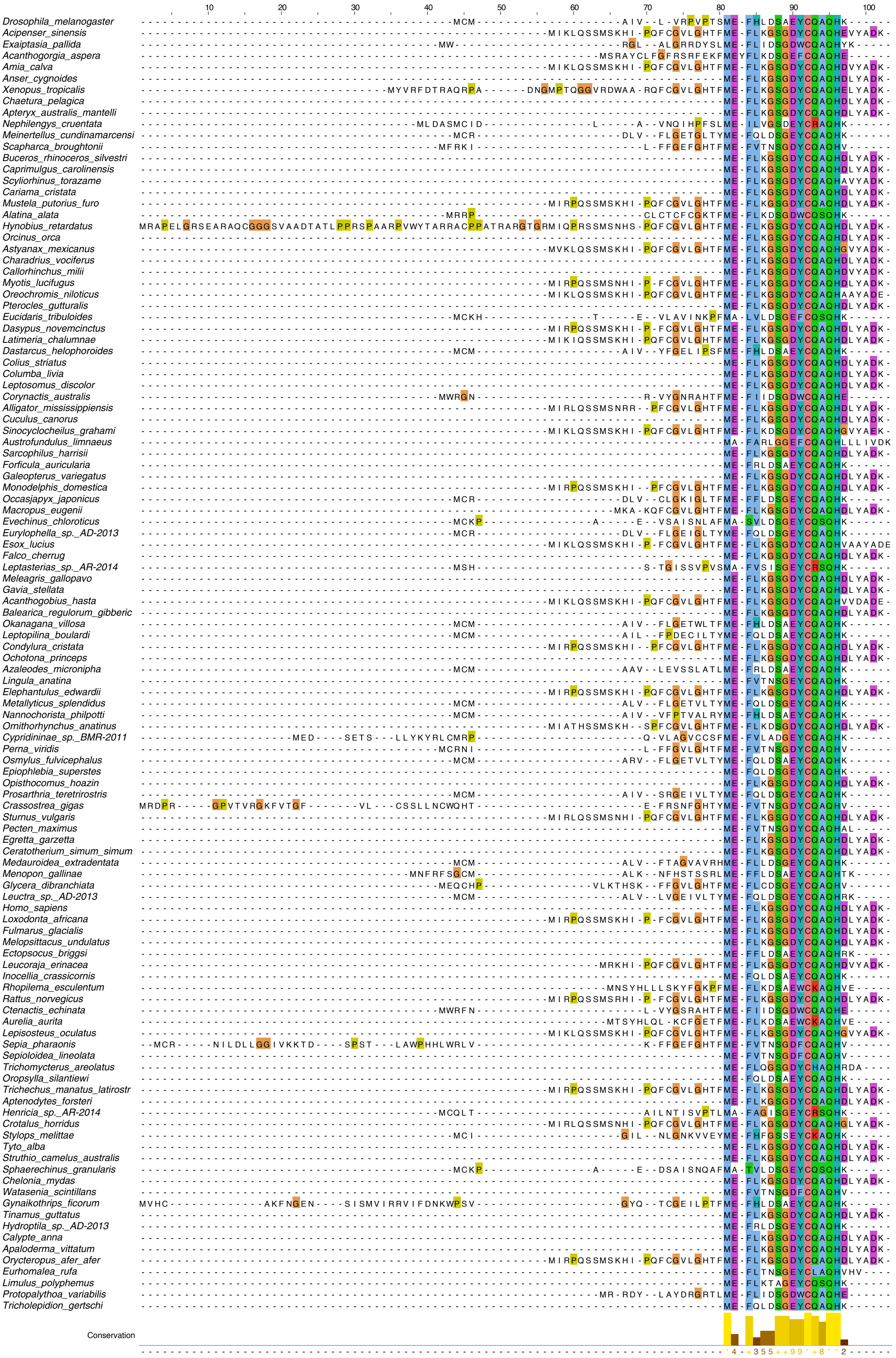

HG0002

ENSDARG00000003077

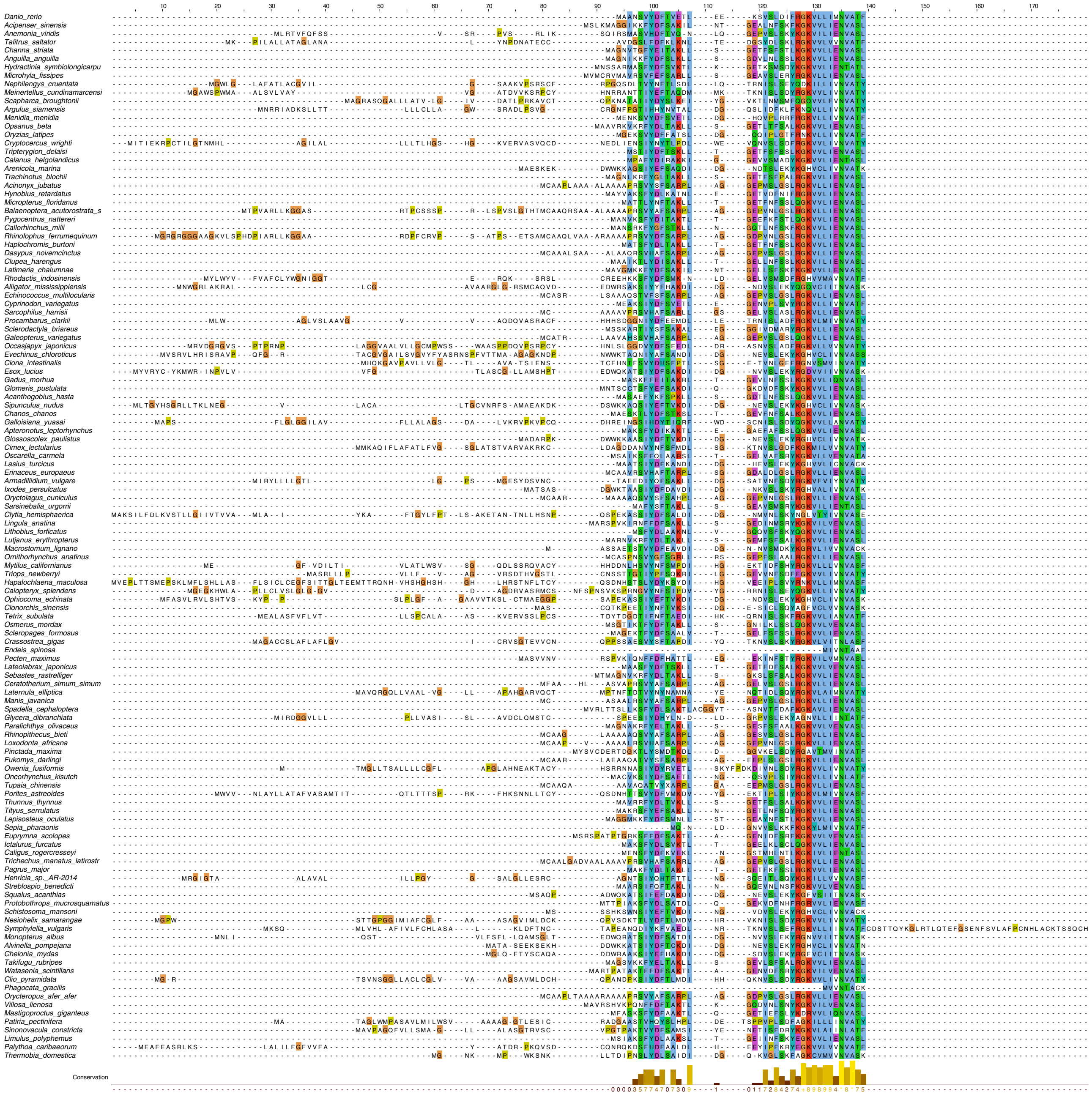

HG0003

ENSGALG00000014906 (MOCS2)

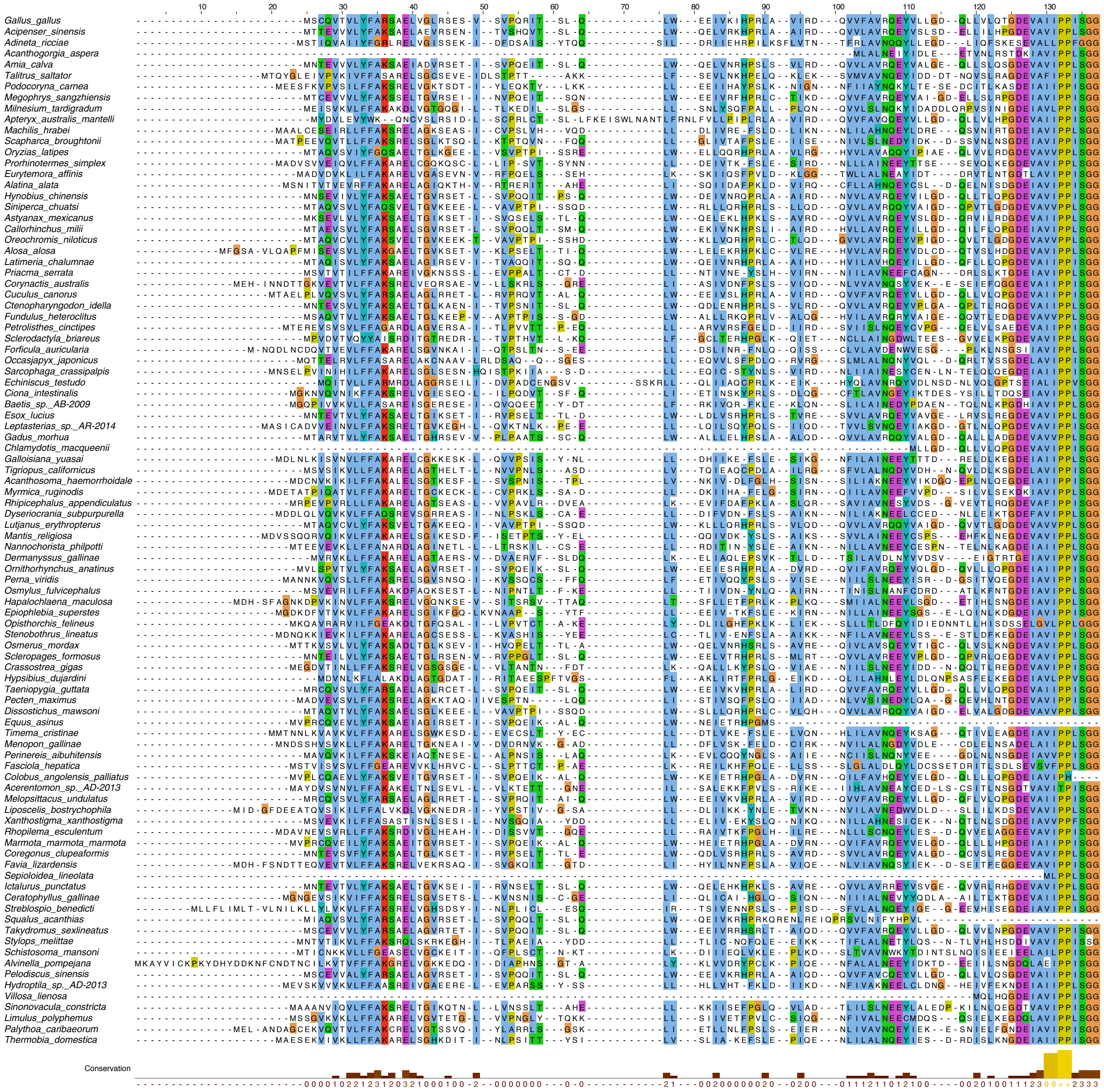

HG0003

FBgn0039280 (Mocs2)

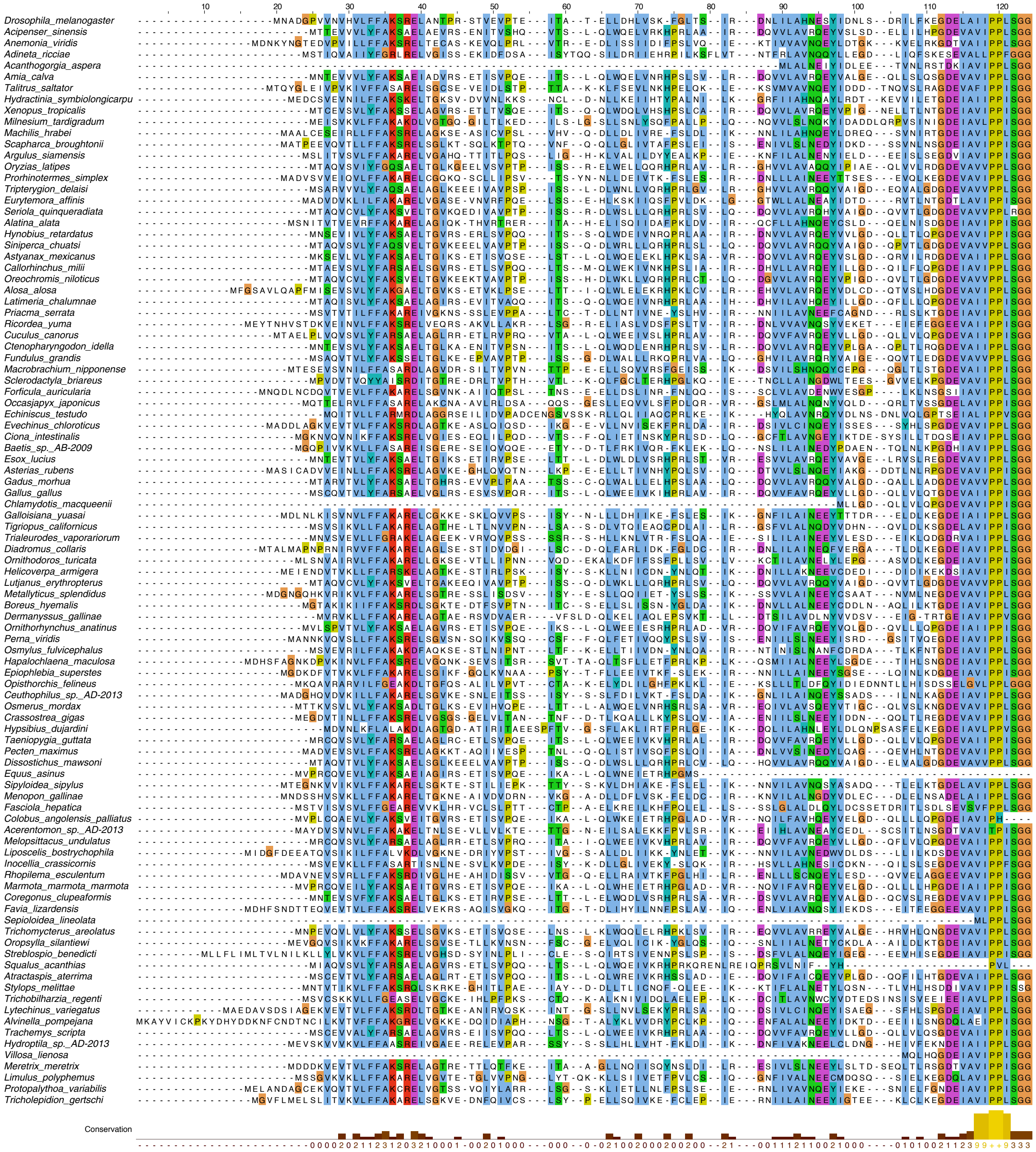

HG0004.1

ENSDARG00000076779 (fam13b)

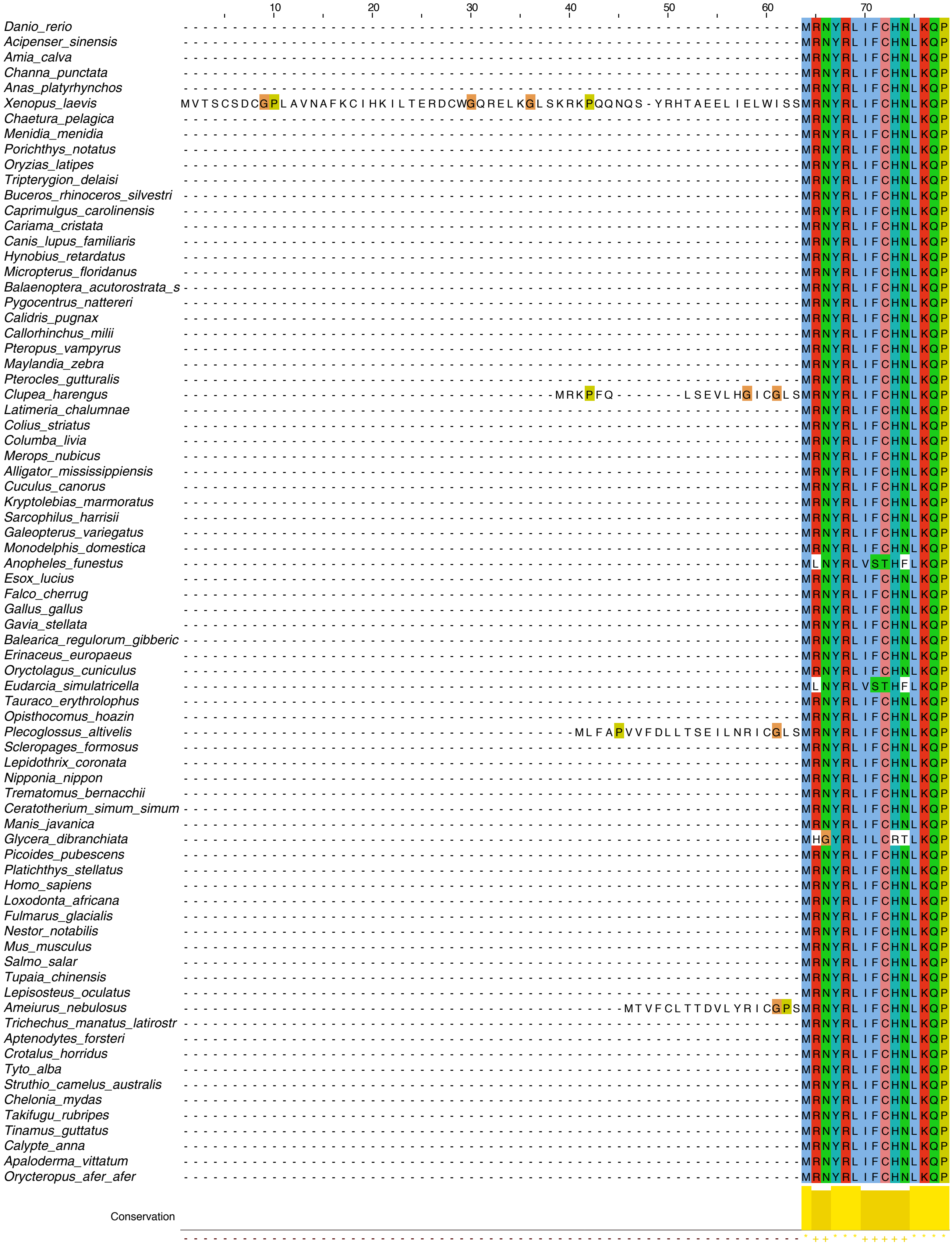

HG0004.1

ENSG00000031003 (FAM13B)

```
                              10        20        30        40        50        60        70
Homo_sapiens                  ---------------------------------------------------------------MRNYRLIFCHNLKQP
Acipenser_sinensis            ---------------------------------------------------------------MRNYRLIFCHNLKQP
Amia_calva                    ---------------------------------------------------------------MRNYRLIFCHNLKQP
Channa_punctata               ---------------------------------------------------------------MRNYRLIFCHNLKQP
Anas_platyrhynchos            ---------------------------------------------------------------MRNYRLIFCHNLKQP
Xenopus_laevis                MVTSCSDCGPLAVNAFKCIHKILTERDCWGQRELKGLSKRKPQQNQS-YRHTAEELIELWISSMRNYRLIFCHNLKQP
Chaetura_pelagica             ---------------------------------------------------------------MRNYRLIFCHNLKQP
Menidia_menidia               ---------------------------------------------------------------MRNYRLIFCHNLKQP
Porichthys_notatus            ---------------------------------------------------------------MRNYRLIFCHNLKQP
Oryzias_latipes               ---------------------------------------------------------------MRNYRLIFCHNLKQP
Tripterygion_delaisi          ---------------------------------------------------------------MRNYRLIFCHNLKQP
Buceros_rhinoceros_silvestri  ---------------------------------------------------------------MRNYRLIFCHNLKQP
Caprimulgus_carolinensis      ---------------------------------------------------------------MRNYRLIFCHNLKQP
Cariama_cristata              ---------------------------------------------------------------MRNYRLIFCHNLKQP
Canis_lupus_familiaris        ---------------------------------------------------------------MRNYRLIFCHNLKQP
Hynobius_retardatus           ---------------------------------------------------------------MRNYRLIFCHNLKQP
Micropterus_floridanus        ---------------------------------------------------------------MRNYRLIFCHNLKQP
Balaenoptera_acutorostrata_s  ---------------------------------------------------------------MRNYRLIFCHNLKQP
Pygocentrus_nattereri         ---------------------------------------------------------------MRNYRLIFCHNLKQP
Calidris_pugnax               ---------------------------------------------------------------MRNYRLIFCHNLKQP
Callorhinchus_milii           ---------------------------------------------------------------MRNYRLIFCHNLKQP
Pteropus_vampyrus             ---------------------------------------------------------------MRNYRLIFCHNLKQP
Maylandia_zebra               ---------------------------------------------------------------MRNYRLIFCHNLKQP
Pterocles_gutturalis          ---------------------------------------------------------------MRNYRLIFCHNLKQP
Clupea_harengus               --------------------------------------MRKPFQ-------LSEVLHGICGLSMRNYRLIFCHNLKQP
Latimeria_chalumnae           ---------------------------------------------------------------MRNYRLIFCHNLKQP
Colius_striatus               ---------------------------------------------------------------MRNYRLIFCHNLKQP
Columba_livia                 ---------------------------------------------------------------MRNYRLIFCHNLKQP
Merops_nubicus                ---------------------------------------------------------------MRNYRLIFCHNLKQP
Alligator_mississippiensis    ---------------------------------------------------------------MRNYRLIFCHNLKQP
Cuculus_canorus               ---------------------------------------------------------------MRNYRLIFCHNLKQP
Danio_rerio                   ---------------------------------------------------------------MRNYRLIFCHNLKQP
Kryptolebias_marmoratus       ---------------------------------------------------------------MRNYRLIFCHNLKQP
Sarcophilus_harrisii          ---------------------------------------------------------------MRNYRLIFCHNLKQP
Galeopterus_variegatus        ---------------------------------------------------------------MRNYRLIFCHNLKQP
Monodelphis_domestica         ---------------------------------------------------------------MRNYRLIFCHNLKQP
Esox_lucius                   ---------------------------------------------------------------MRNYRLIFCHNLKQP
Falco_cherrug                 ---------------------------------------------------------------MRNYRLIFCHNLKQP
Gallus_gallus                 ---------------------------------------------------------------MRNYRLIFCHNLKQP
Gavia_stellata                ---------------------------------------------------------------MRNYRLIFCHNLKQP
Balearica_regulorum_gibberic  ---------------------------------------------------------------MRNYRLIFCHNLKQP
Erinaceus_europaeus           ---------------------------------------------------------------MRNYRLIFCHNLKQP
Oryctolagus_cuniculus         ---------------------------------------------------------------MRNYRLIFCHNLKQP
Tauraco_erythrolophus         ---------------------------------------------------------------MRNYRLIFCHNLKQP
Opisthocomus_hoazin           ---------------------------------------------------------------MRNYRLIFCHNLKQP
Plecoglossus_altivelis        ----------------------------------------MLFAPVVFDLLTSEILNRICGLSMRNYRLIFCHNLKQP
Scleropages_formosus          ---------------------------------------------------------------MRNYRLIFCHNLKQP
Lepidothrix_coronata          ---------------------------------------------------------------MRNYRLIFCHNLKQP
Nipponia_nippon               ---------------------------------------------------------------MRNYRLIFCHNLKQP
Trematomus_bernacchii         ---------------------------------------------------------------MRNYRLIFCHNLKQP
Ceratotherium_simum_simum     ---------------------------------------------------------------MRNYRLIFCHNLKQP
Manis_javanica                ---------------------------------------------------------------MRNYRLIFCHNLKQP
Picoides_pubescens            ---------------------------------------------------------------MRNYRLIFCHNLKQP
Platichthys_stellatus         ---------------------------------------------------------------MRNYRLIFCHNLKQP
Loxodonta_africana            ---------------------------------------------------------------MRNYRLIFCHNLKQP
Fulmarus_glacialis            ---------------------------------------------------------------MRNYRLIFCHNLKQP
Nestor_notabilis              ---------------------------------------------------------------MRNYRLIFCHNLKQP
Mus_musculus                  ---------------------------------------------------------------MRNYRLIFCHNLKQP
Salmo_salar                   ---------------------------------------------------------------MRNYRLIFCHNLKQP
Tupaia_chinensis              ---------------------------------------------------------------MRNYRLIFCHNLKQP
Lepisosteus_oculatus          ---------------------------------------------------------------MRNYRLIFCHNLKQP
Ictalurus_punctatus           ---------------------------------------------------------------MRNYRLIFCHNLKQP
Trichechus_manatus_latirostr  ---------------------------------------------------------------MRNYRLIFCHNLKQP
Aptenodytes_forsteri          ---------------------------------------------------------------MRNYRLIFCHNLKQP
Crotalus_horridus             ---------------------------------------------------------------MRNYRLIFCHNLKQP
Tyto_alba                     ---------------------------------------------------------------MRNYRLIFCHNLKQP
Struthio_camelus_australis    ---------------------------------------------------------------MRNYRLIFCHNLKQP
Chelonia_mydas                ---------------------------------------------------------------MRNYRLIFCHNLKQP
Takifugu_rubripes             ---------------------------------------------------------------MRNYRLIFCHNLKQP
Tinamus_guttatus              ---------------------------------------------------------------MRNYRLIFCHNLKQP
Calypte_anna                  ---------------------------------------------------------------MRNYRLIFCHNLKQP
Apaloderma_vittatum           ---------------------------------------------------------------MRNYRLIFCHNLKQP
Orycteropus_afer_afer         ---------------------------------------------------------------MRNYRLIFCHNLKQP

Conservation
```

HG0004.1

ENSGALG00000041706

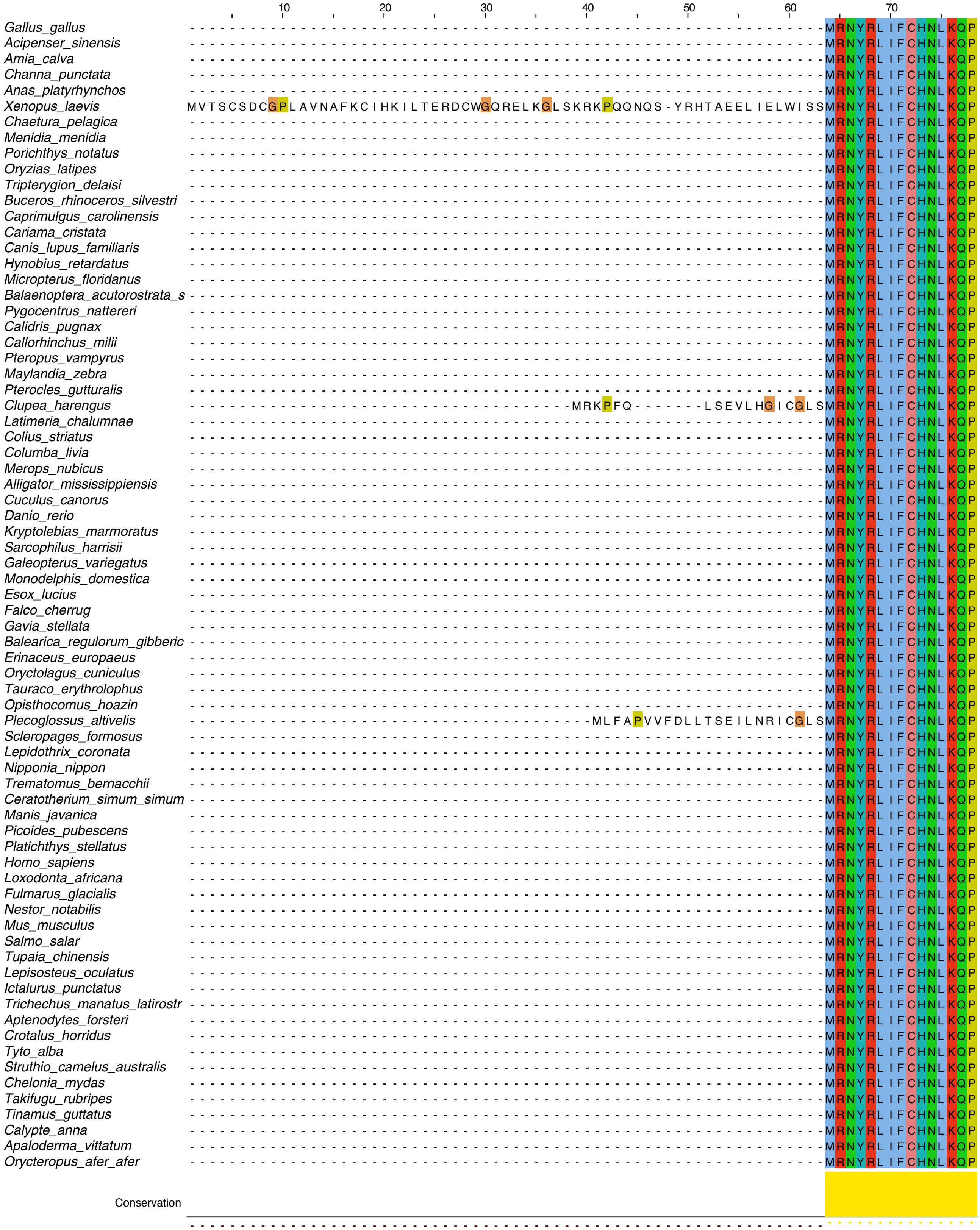

HG0004.1

FBgn0028494

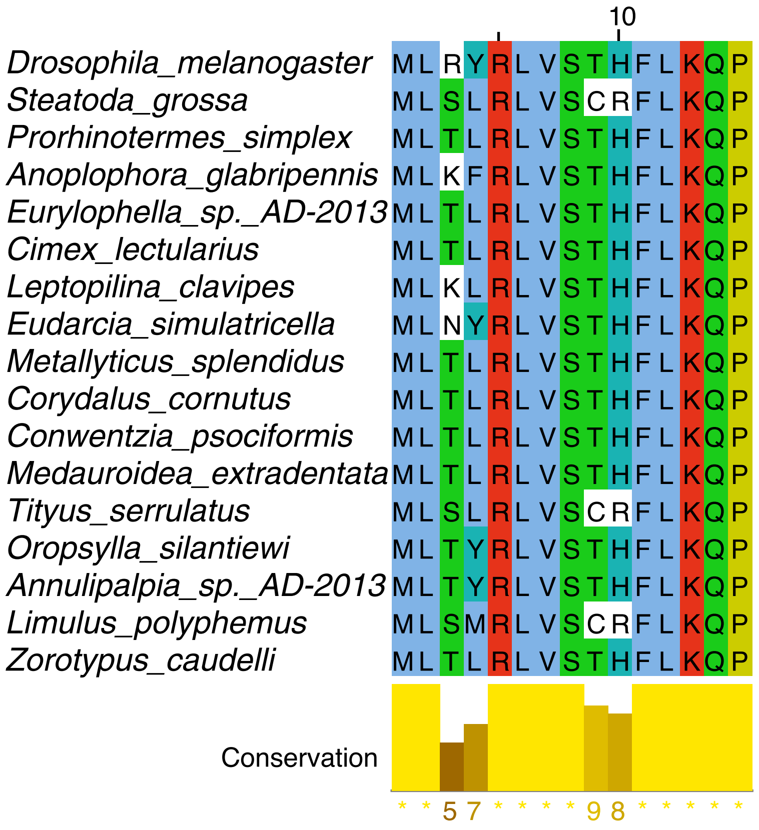

HG0004.2

ENSG00000138640 (FAM13A)

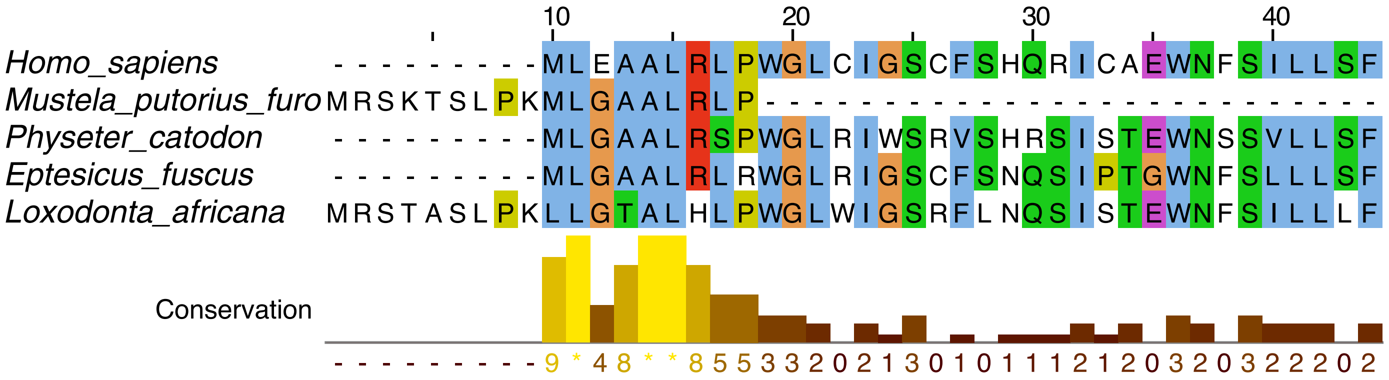

HG0005

ENSDARG00000011055 (fbxo9)

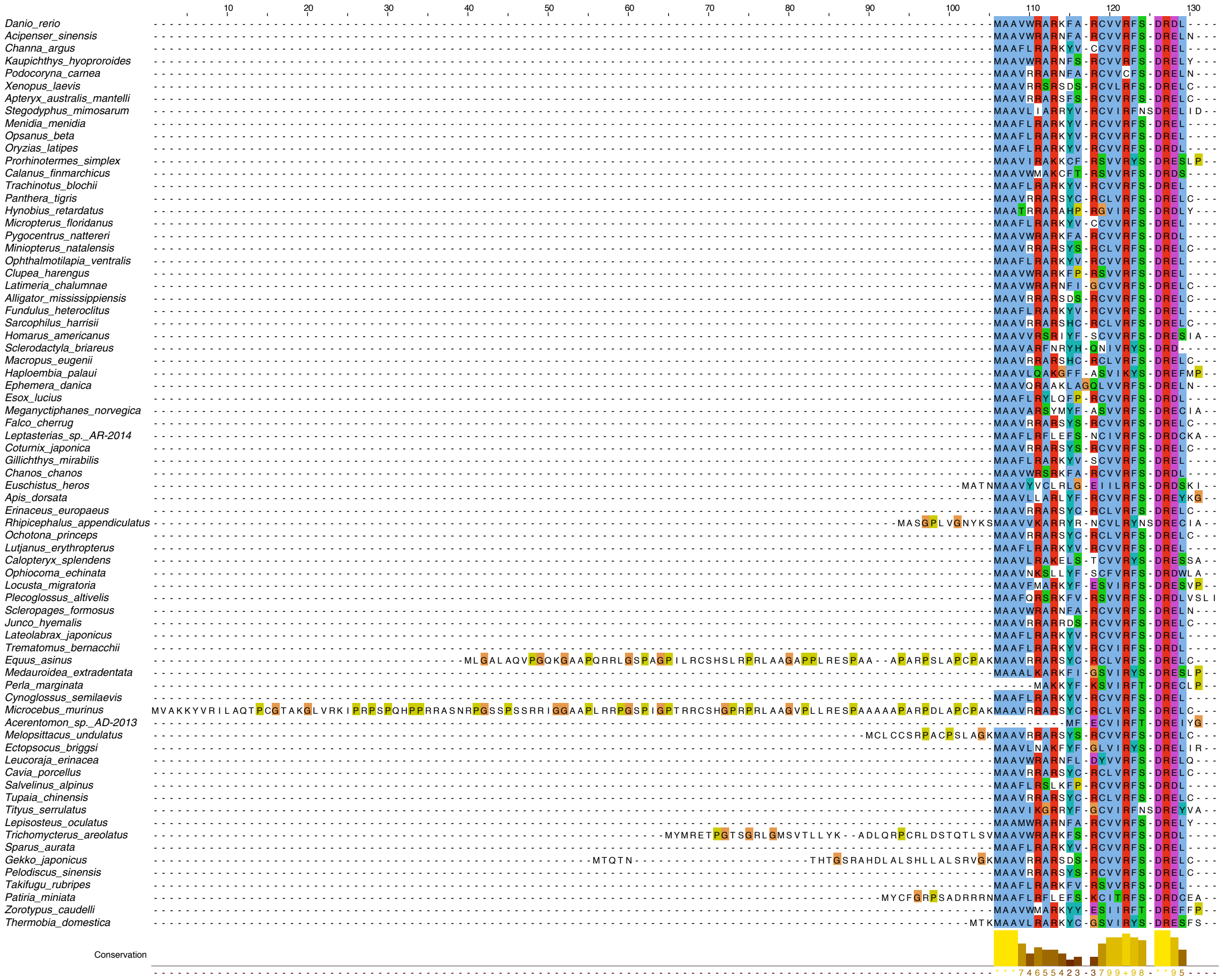

HG0005

ENSGALG00000016320

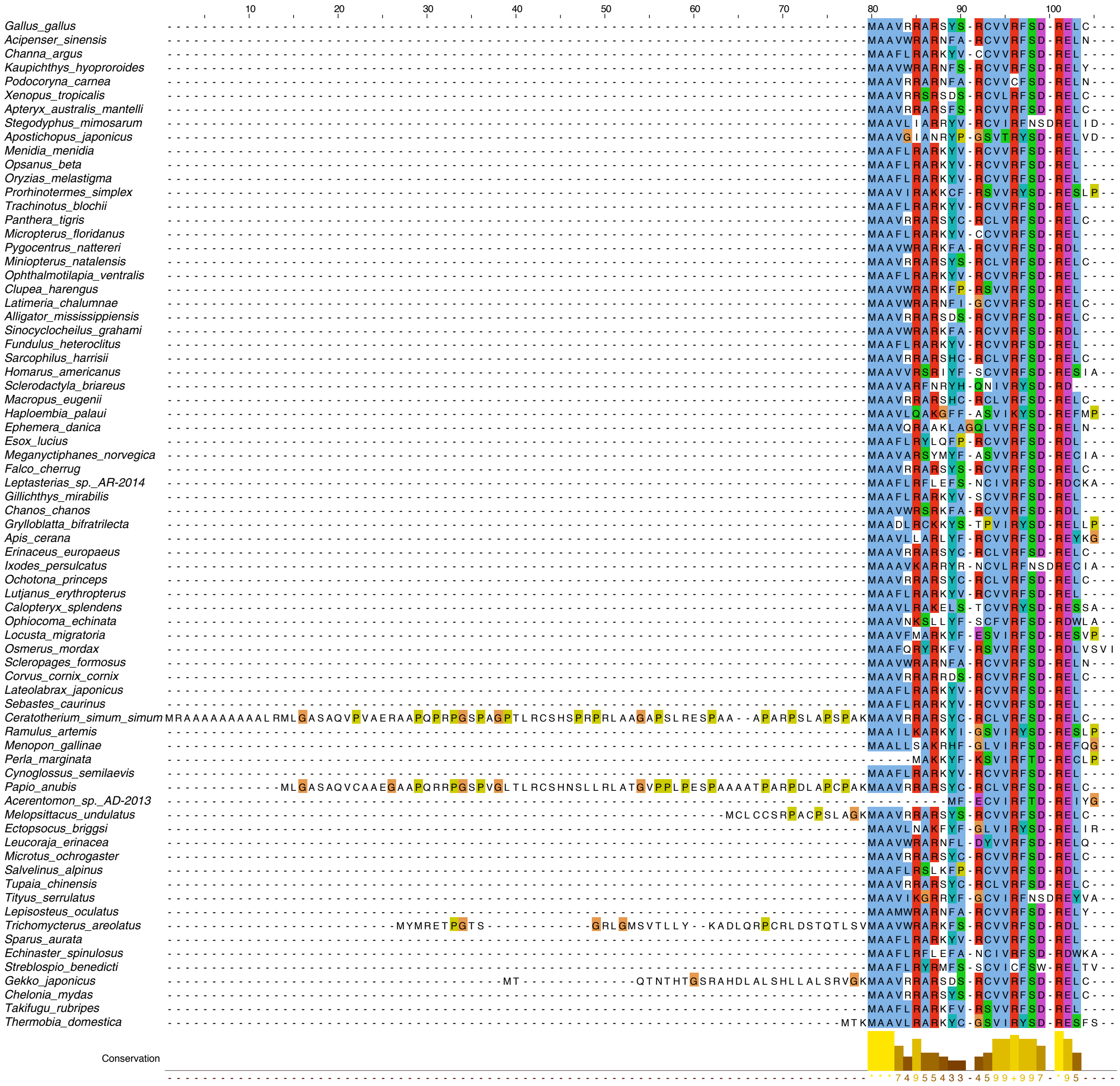

HG0006

FBgn0029971 (CG18624)

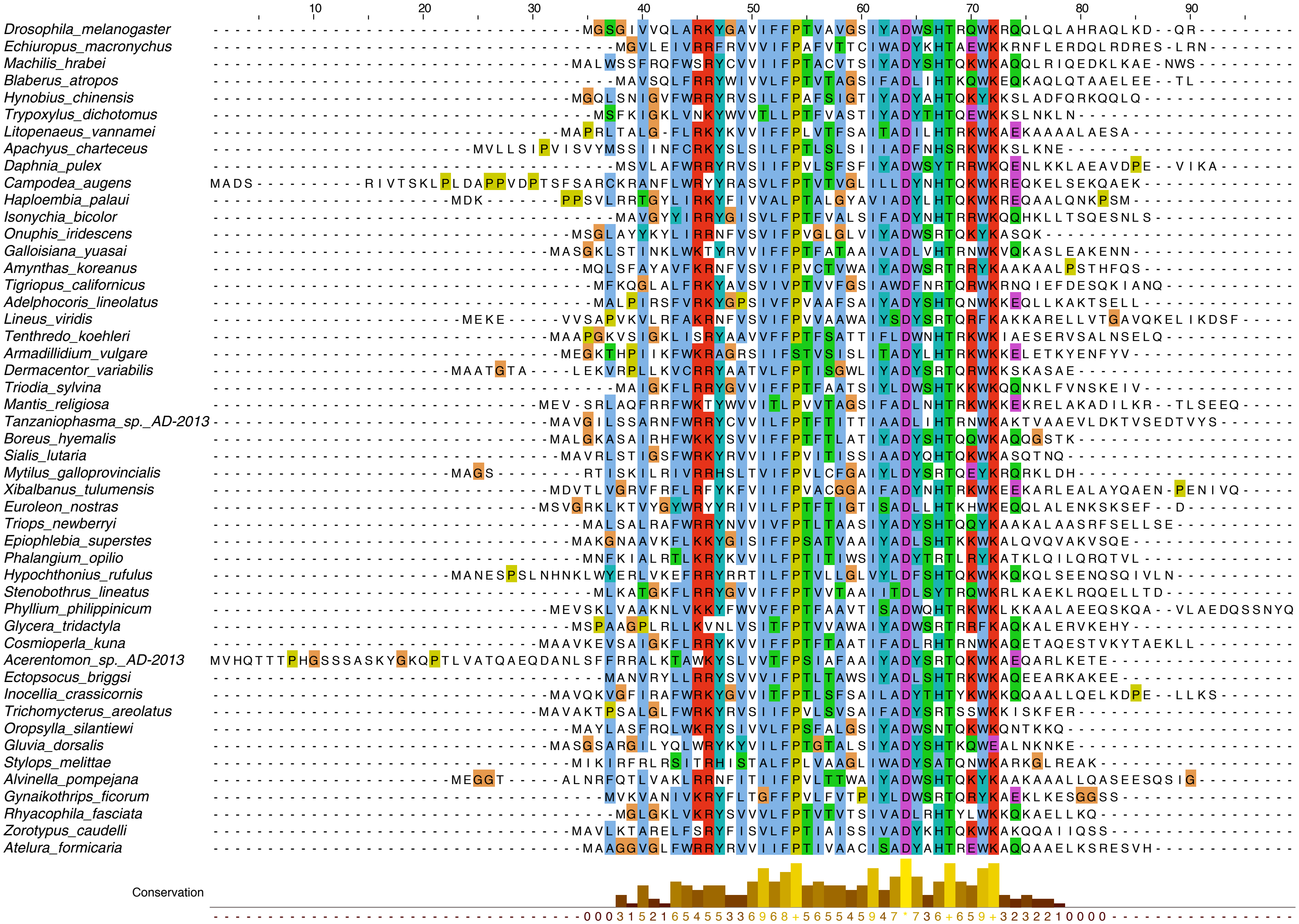

HG0007

FBgn0034372 (Gint3)

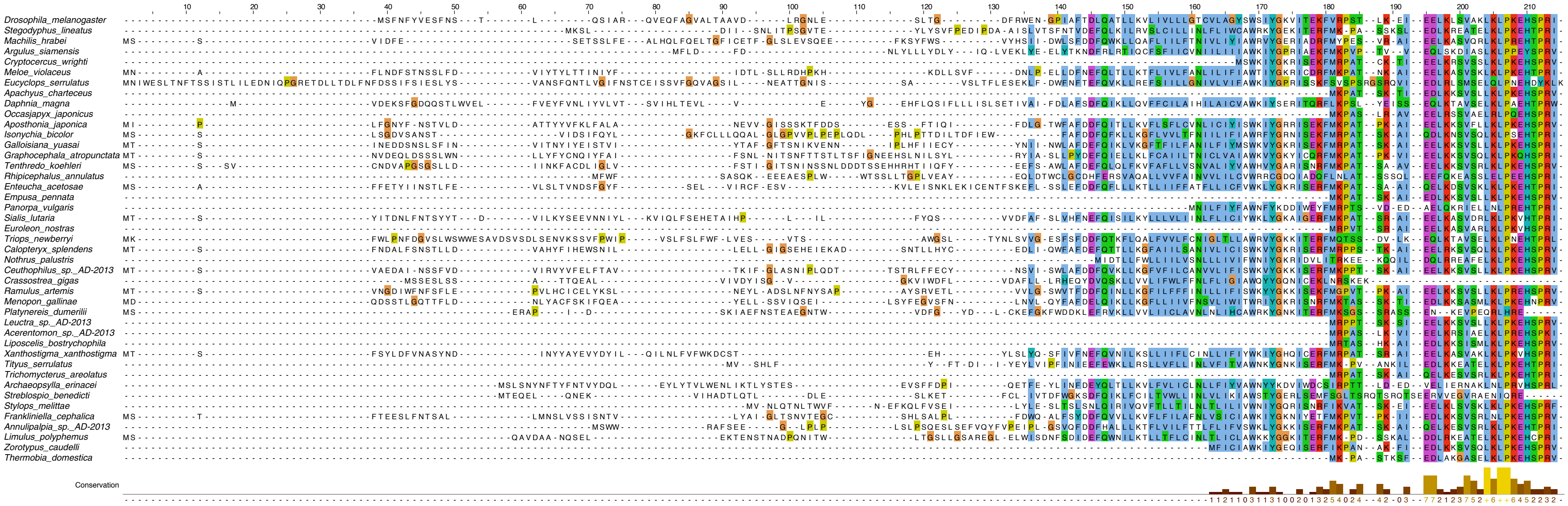

HG0008

ENSG00000175567 (UCP2)

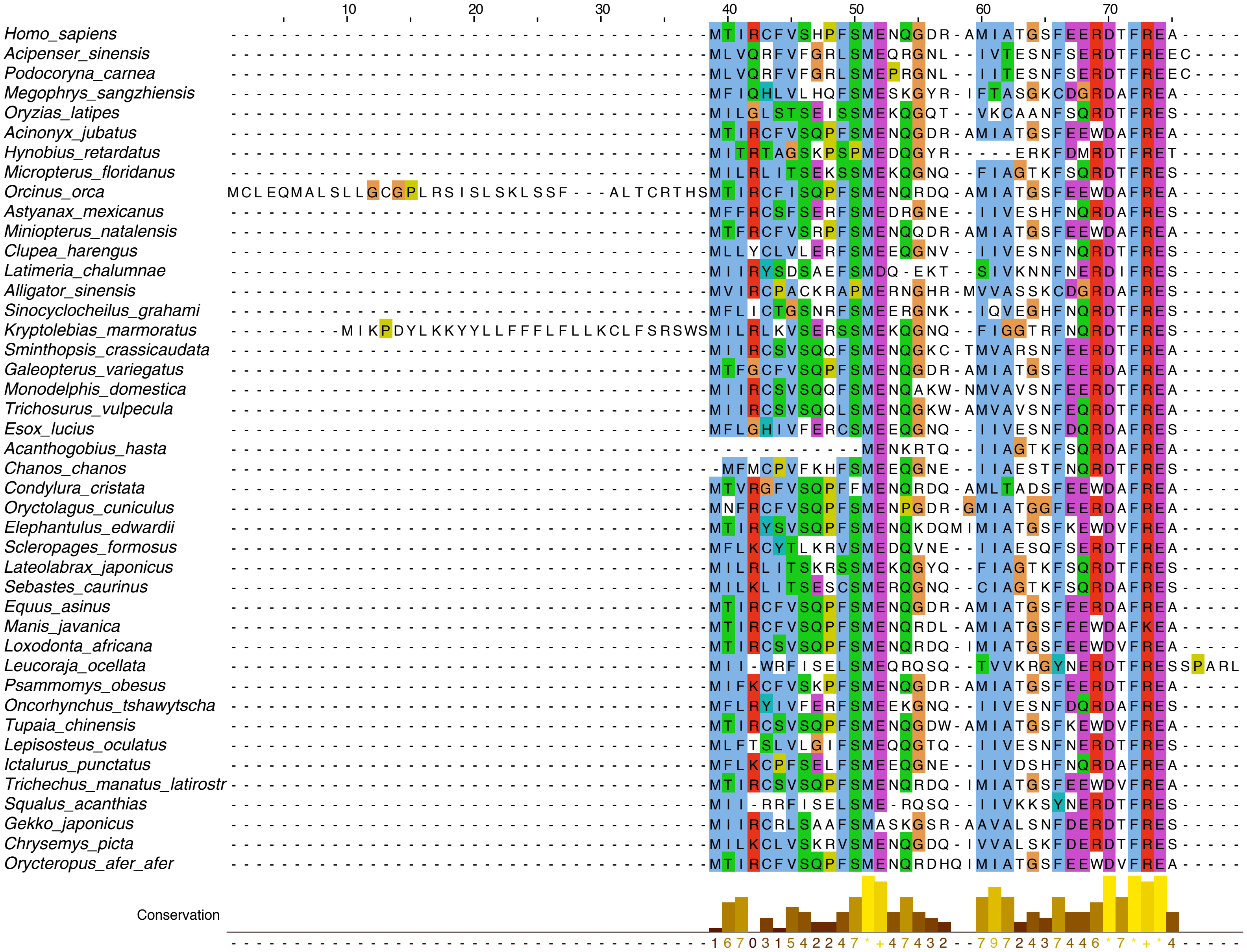

HG0009

FBgn0050100 (CG30100)

```
                                          10        20        30        40        50        60        70
Drosophila_melanogaster        -----MKMVQPSRQQILRLYKHLIRYGNHLKLTDKNYFLGRVRHEFRDNRSLTNPVEVEFSFKRGETLLKKGRIL
Talitrus_saltator              -----------------------------MELSDRRFFEKRIRREFTKQRNS--PEELDTAVQRAEAFLEKGRLL
Latrodectus_geometricus        --------MTVSRQKVLSLYKELLKYSKNLKYTDKSYYLNRLKNEFLENRNLQNASEINFQIQRGKNFLEKKRLL
Meinertellus_cundinamarcensi   -------MQIPNKQQVLCLYKSLLRYGETLRLTDKQYFRTRVRSEFQKNKSSDDSKEIVFQYEKGLKVLNSKRIL
Blattella_germanica            -------MSNPTAREVLKLYRDILKYGRQLQFTDKNYFGQRIRNEFKAGKDLKNQEDINFHFQRGEALLKNKRVV
Calanus_finmarchicus           ------------MSQATLLYKSLLRYGRTLQMTDKDYFYRRVRFEFEKNSVLLEKQEVQFQLDKARQFLKNKRLV
Photinus_pyralis               --------MHPNAKDILKLYKNLLRYGQELQYTDKNYFARRIKNEFRRNRDITDEADIQFNYKRGISLLEKRAVV
Apachyus_charteceus            -----MASTLPNTRQILSLYKNLMRYGQKLKFTNQSYFYERVRREFENNKTIPED-KREFYYKKGLSLLEKKRVV
Daphnia_pulex                  MNTIFIIMNVPNKTQIFELFRSCIRYSGQLKLSDKRLVSKRIKSEFHKNKTLSNPEEICKAYEKGQSFLSKRRLL
Occasjapyx_japonicus           ---MAAYMGVPSPVQVLRLYRSLVRYGKELKYTDRDFYQRRIRNEFERHRLLTKDEDIRFQYEKGLAFISRKRII
Haploembia_palaui              -------MSSPSASEVLRLYRELLRFGRQLQFTDQKYFLRRIKTQFQENKHLEDKEQIEFQFNKGQSLLKRKRVV
Baetis_sp._AD-2013             --------MSVARSEVLNLYRSILKYGKDIKLTDKDYFRKRIRQEFRSNQKVTNAEEIQFHVERAKAFLANKRVV
Glomeris_pustulata             --------MTIQRQSVLKLYRELLRYGQTLQYTDKKFYFRRIRKEFEQNRSLTAPADIQYYYEKGLTFLTNKRLI
Galloisiana_yuasai             -------MTNIGGRQIIKLYRDLLRYGKRLEFTDINYFNRRIKTEFLKNKNLASLDEIQFQFERGISLLKNKRVV
Tigriopus_californicus         ---------MSQRSEALRLYKSLLRYGQSLRLTDSAYLHRRCTAEFRTPRST--PAQIERALAQARALLSQRRLL
Cercopis_vulnerata             -------MNKPTVKQIVQLYKDLLRYGNNLKLTDKEYYIDRIHLEFRKNQSLDNPKDIQFCFDRGLALLKNKRVV
Argochrysis_armilla            -----------MRSAILRLYKEILRYGETLKYTDKTYFRERIRTTFRNNKDLTNEKDIDFQFKKGQVFLEKRRVV
Rhipicephalus_appendiculatus   -----MSKPPSKQAVLSLYRSLVRYGHRLELSDKNYYFKRLREEFEARKTLTEPKELQFYFNKGKAFLERKRLI
Papilio_machaon                --------MSITRSEILNLYKKLMQYSQSLNLTDVQYFRTRVSNEFRRNKTLTKPEDISFAYKKGEALLRKGSVL
Mantis_religiosa               -------MSIPSPRQVLQLYKSILRYGQELKFTDKEYFYYRINQEFKNNKDIKRTEDIIFNFQRGQALLKNKRVV
Panorpa_vulgaris               -------MSVPNSSHILRLYKDLLRYGQSLQLTDKLYFRSKIVNEFKKNKTIENHKDISFNYEKGKSLLKKGRVV
Dermanyssus_gallinae           ---MGPVSGAPSVQAMRSLYKQLARYGQQLVLTDRRWFERQLRNEFAKPRVT--AEEQNFAYRKGQRFLELKRLV
Osmylus_fulvicephalus          -------MLIPNNGQVRNLYRHILRYGQTLRYTDKIYFYNRVRNEFKQNKSLTDPADIVFNFKKGEELLRKKTVC
Triops_newberryi               -------MSGPSPYQVLKLYRSLLRYSSTLQYTDKSFYIKRIRSEFKKKKDLTNEEEIRKAFEKGLSFLSKKRIV
Calopteryx_splendens           -------MCQPSPSQVLSLYRSLLRYGNQLKYTDKKYFIKRIRKEFESNRNLIP-KERGFQYERGLVLLHNKRVI
Hypochthonius_rufulus          --------MSVSREHILRLYKNILRYSNSLVYTDKSYYLQRVKREFRENKQLTDDSDIRFYYEKGITFLNKKRLI
Prosarthria_teretrirostris     -------MNAPTAHEVLRLYRKLLQYGKQLKLTDKKYFFDRIRKEFKTNKTLTNQEDVTFQFKRGLALLENKRVV
Ramulus_artemis                -------MNTPARVQVLRLYRDLLRYGQNLQLTDKDYFCKRVREEFRNNRVLENKNDLLFHFNKGESLLKTKRVV
Menopon_gallinae               -------MCVPTTREVLTLYKRLLRYGRQLKYTDQDYYSKRIKQEFQSNKDVKGN-EAIFHYEKGLQLLKLKRVV
Perla_marginata                -------MSGPTAKQVIQLYRDLLRYSKHLKFTDKNFFTKRVRKEFKTNKRLSNEEDIQFHYQKGLILLKNKSVI
Polydesmus_angustus            -----MSASVVGKSQILHLYRQLLRYGRTLTYTDKNYYYGRIRAEFEKHREAEL-KTQLFQYKKGKALLENGRLI
Acerentomon_sp._AD-2013        --MCWQQMGIPTNGQILKLYRSLLRYGRTLQYTDKDFFKRKVQQEFHRNGAITAPNEIKFQYEKGISFLSRKRLV
Liposcelis_bostrychophila      -------MSAPSRTQVLRLYKDILRYGEELTYTNKTYFKNRVKNEFLSAKSAKDNKEIDNLYQKGVALLMRRRVV
Xanthostigma_xanthostigma      -------MEVPSSTQVLRLYRQLIRYGQKLQYTDKNYYYKRVIAEFKRNRNLEDPKEIKFQFEKGQSLLKRKSVI
Trichomycterus_areolatus       ---------MPSRSEILKLYKQLLRYGQQLRLTDKSYYQERINQEFKQHKNLASNKDITFNFEKGKKLLEKGLVM
Ctenocephalides_felis          -------MNTPSAAQVLRLYKNLLRYGQNLQYTDKDYFCNRIRKEFRNNKTCSRTEDLIFNFEVCI---------
Stylops_melittae               -----------MRLKVLNLYKSLMRYGKSLRLTDNEYFLERIRSEFRRNKDLYSNQEIDYHFKKGETLLKKKLLV
Symphylella_vulgaris           --------MNPTRVQILSLYKTLLRYADNLKYSDTNYLKQRVRVEFSQHRSLTAKKDIEFYFKRGKSFLERKRLV
Frankliniella_cephalica        ------MSTLINSNQVLQLYRSLIRYGQSLKLTDQKYYLKRVRKEFRANKDLQDPEKIEFMYKKGVSLLDRKRLI
Philopotamus_ludificatus       ---------MPSKREILKLYKQLLRYGQQLQLTDKTYFQQRIVNEFKKNKDLSKSDDITFNFKKGQQLLNKGLVL
Zorotypus_caudelli             --------MNPGKPGILILYKSLIRYGKNLKYTDKKYFIERIRQEFKNRKELENEEDIKFAFQRGVSVLNNKRII
Atelura_formicaria             -------MVAPSSKQVLQLYRNLMRYGRQLKYTGKDYYSKRVREEFKRNKSLENDEKKIFHFEKGQALLSQKRIV
```

Conservation

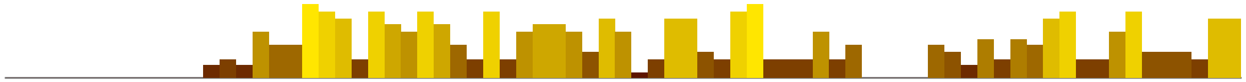

```
--------0000232755*+93+87+853+37887497139943+*333735000054263659+337+444399
```

HG0010.1

ENSG00000125863 (MKKS)

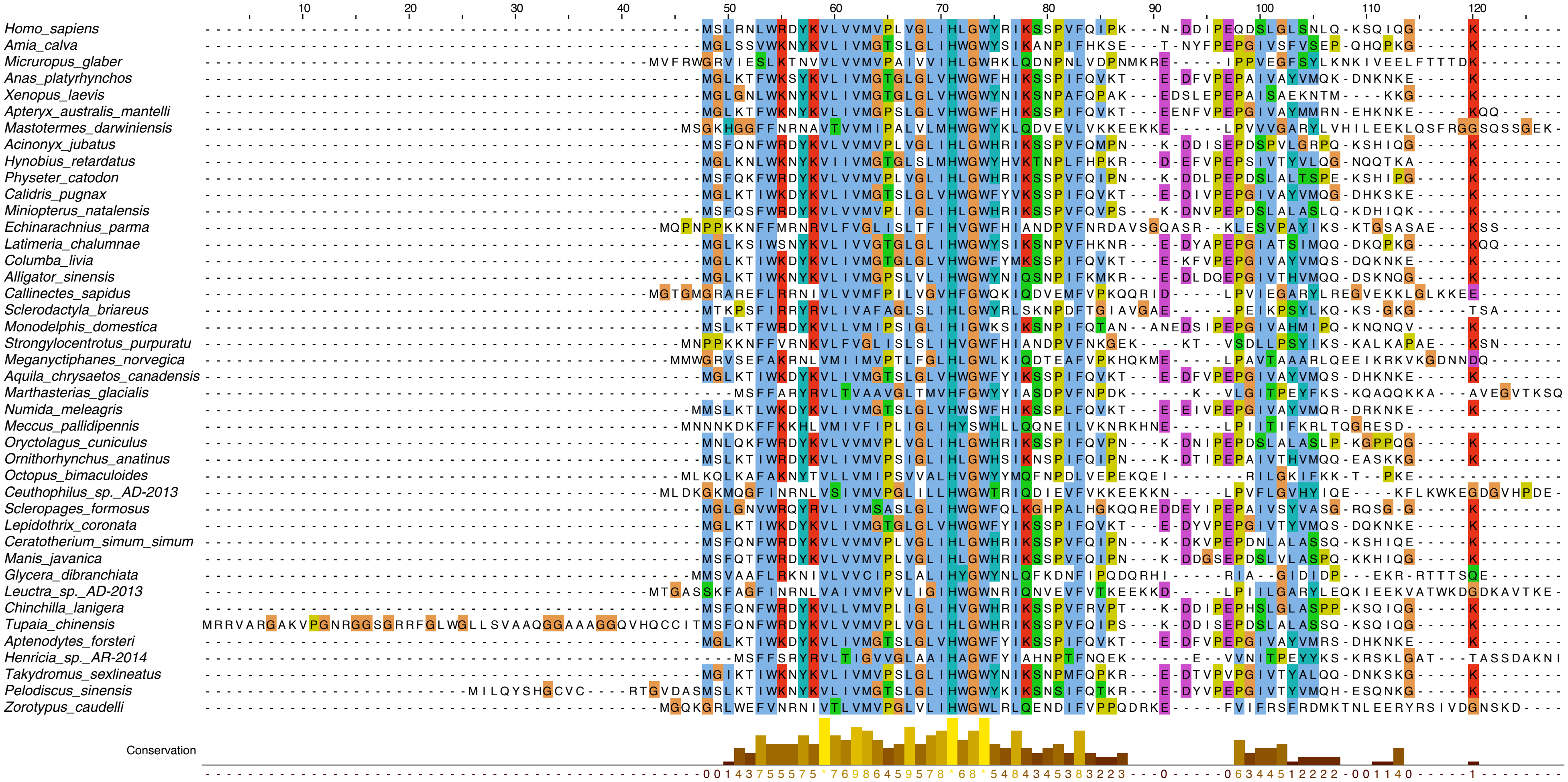

HG0010.1

ENSGALG00000009013 (MKKS)

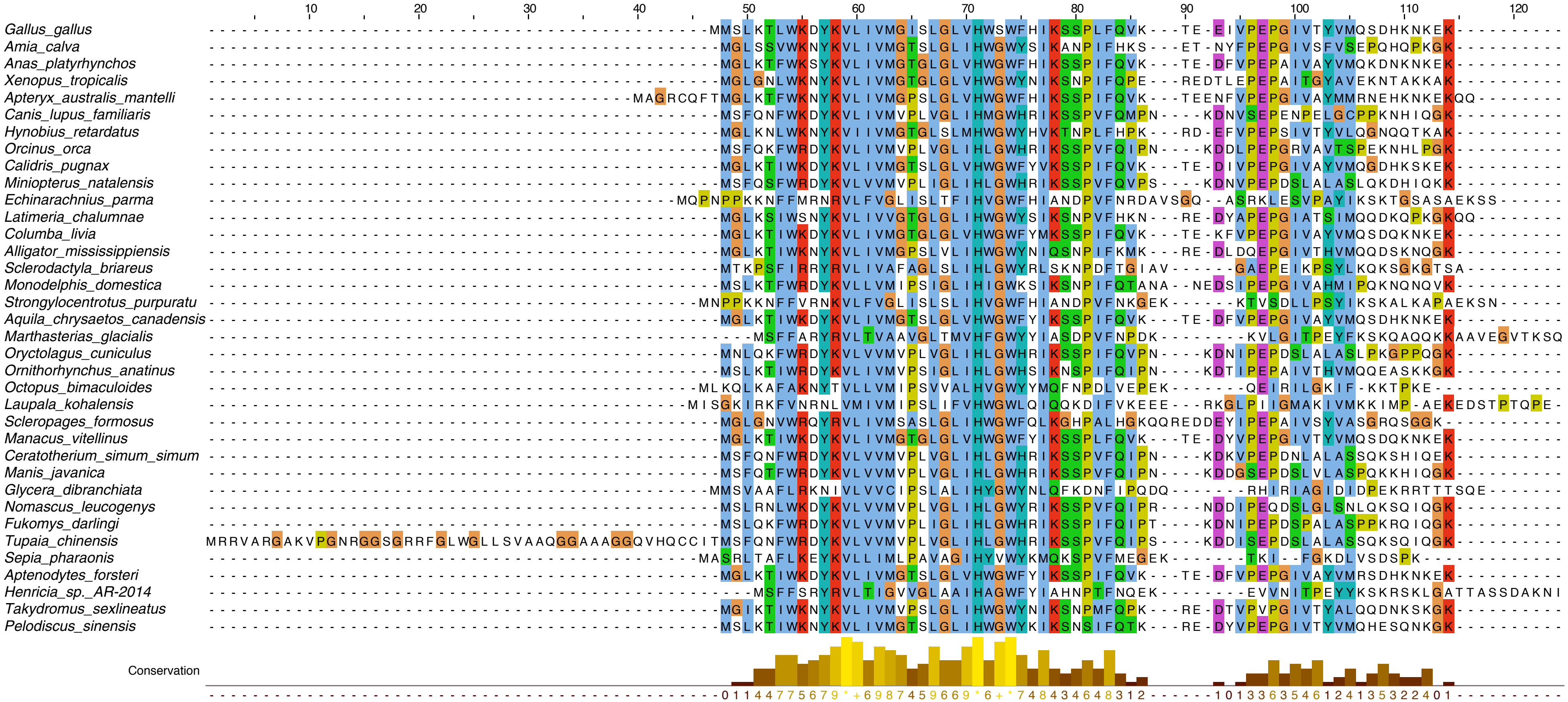

HG0010.2

ENSG00000125863 (MKKS)

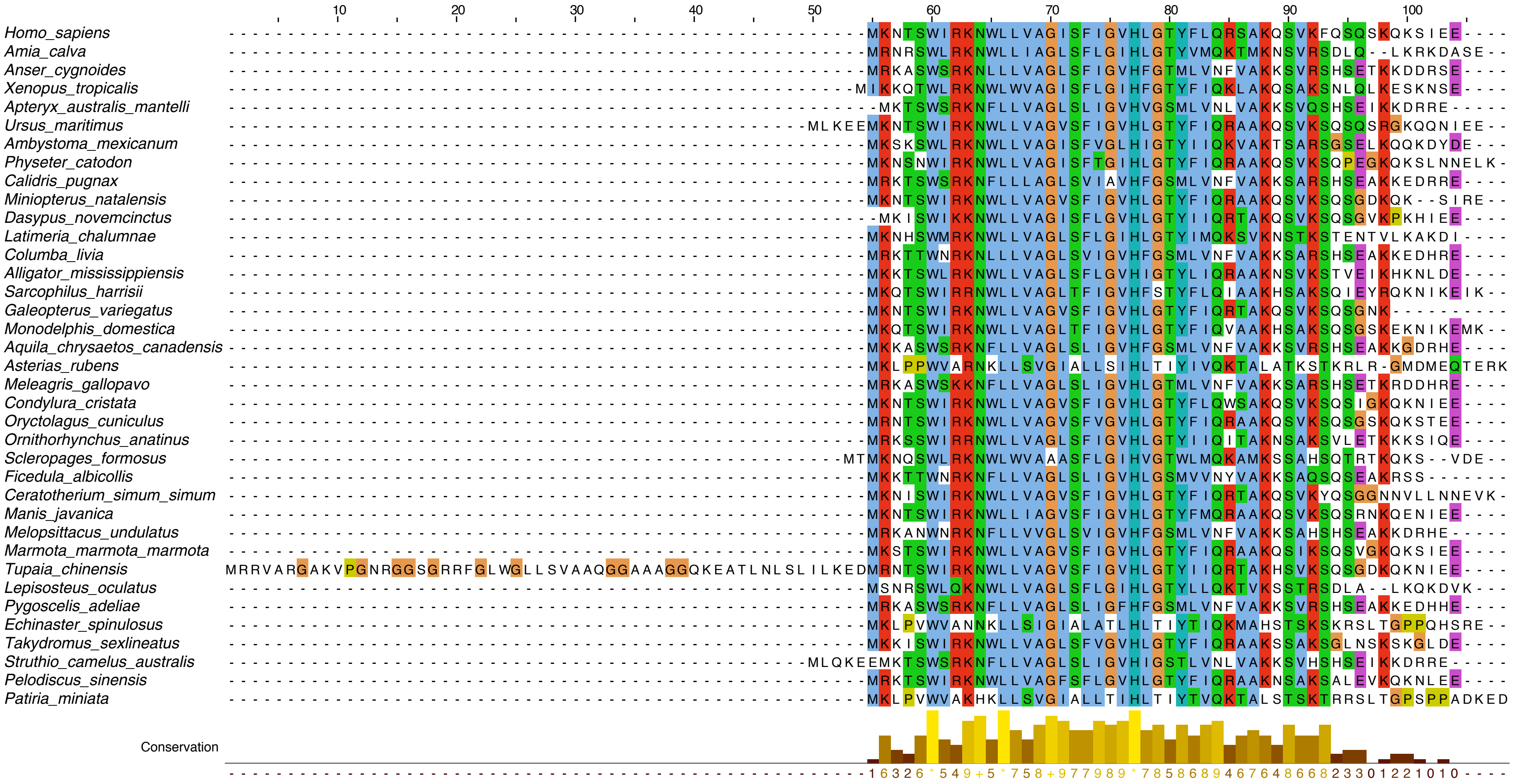

HG0010.2

ENSGALG00000009013 (MKKS)

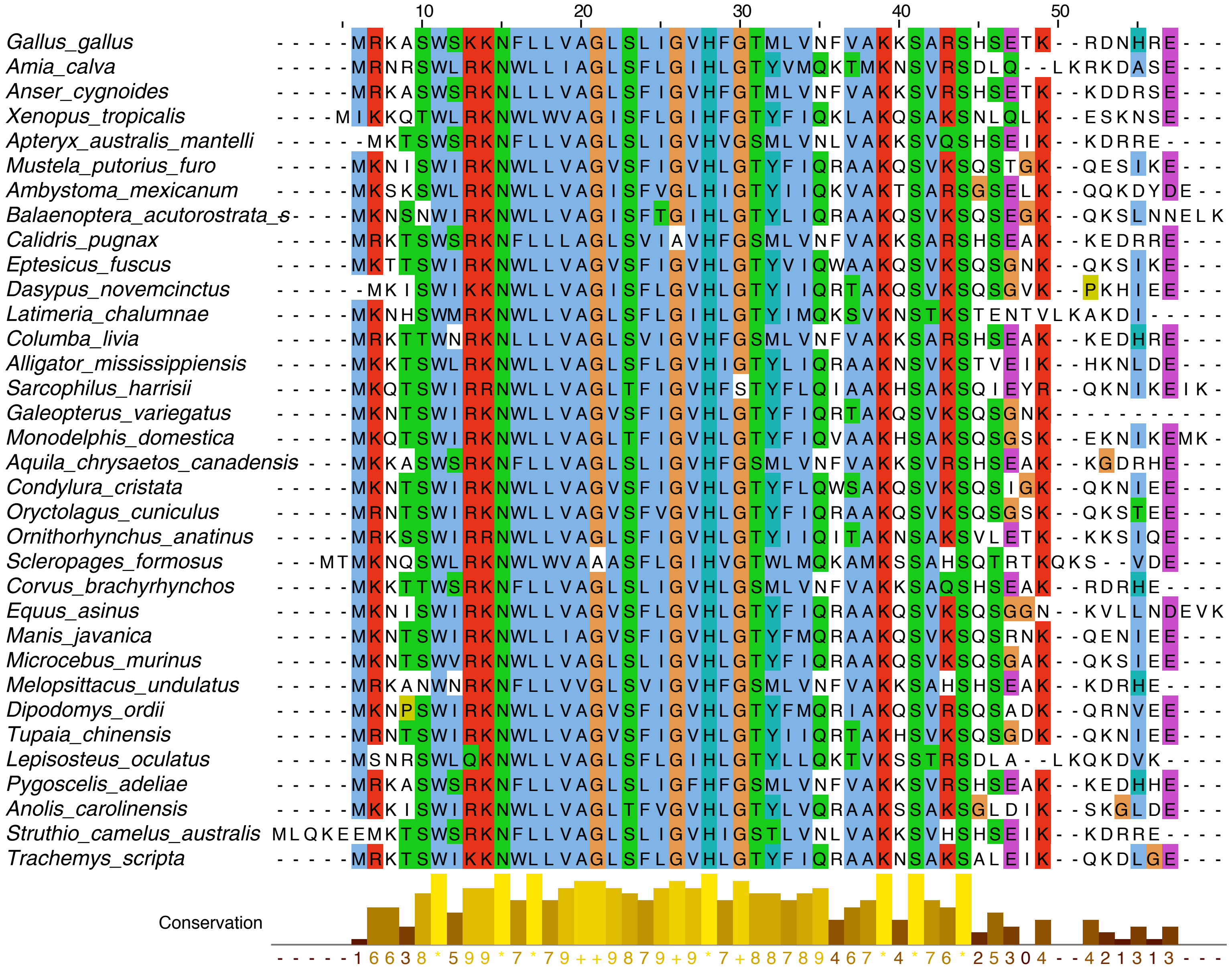

HG0010.3

ENSG00000125863 (MKKS)

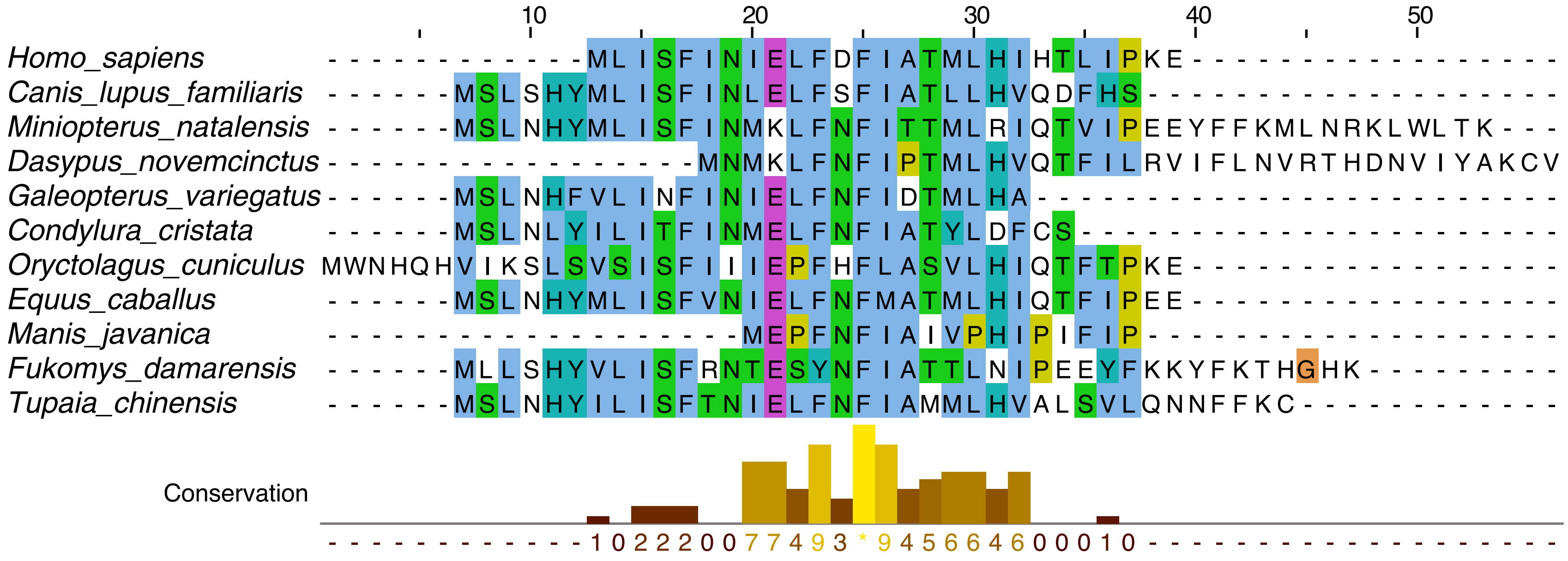

HG0011

FBgn0027360 (Tim10)

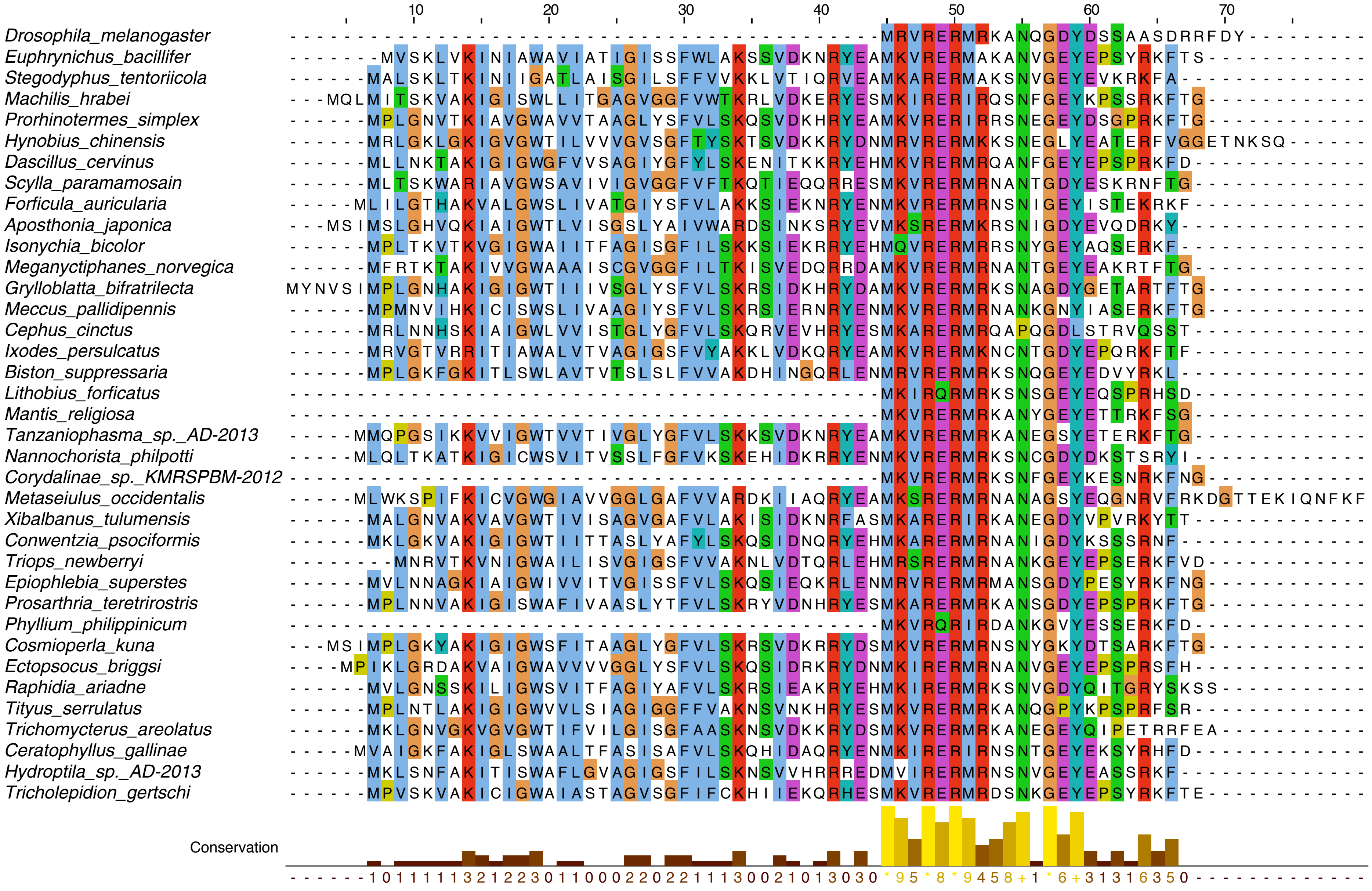

HG0012

FBgn0064116 (CG33713)

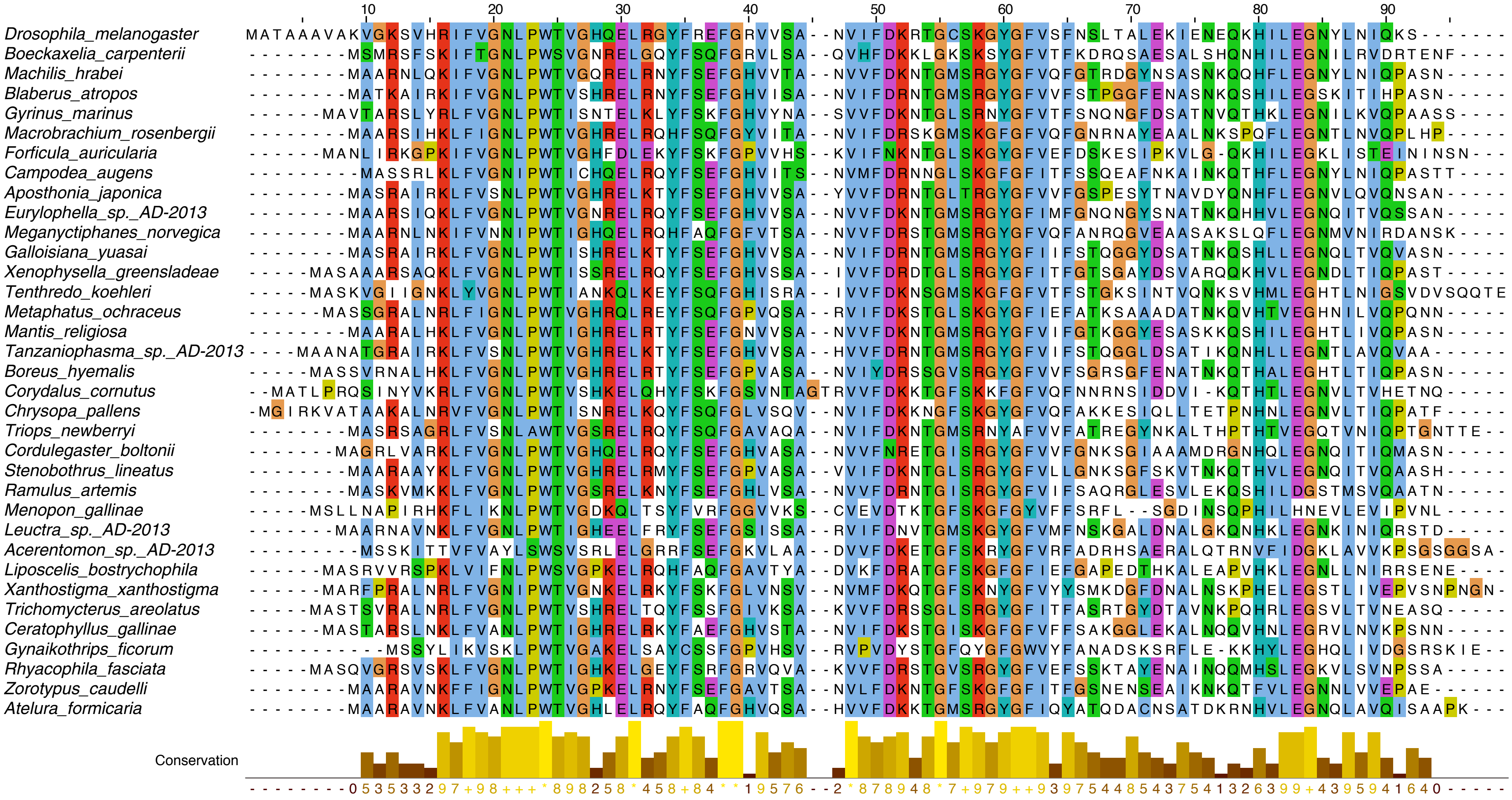

HG0013

FBgn0261381 (mtTFB1)

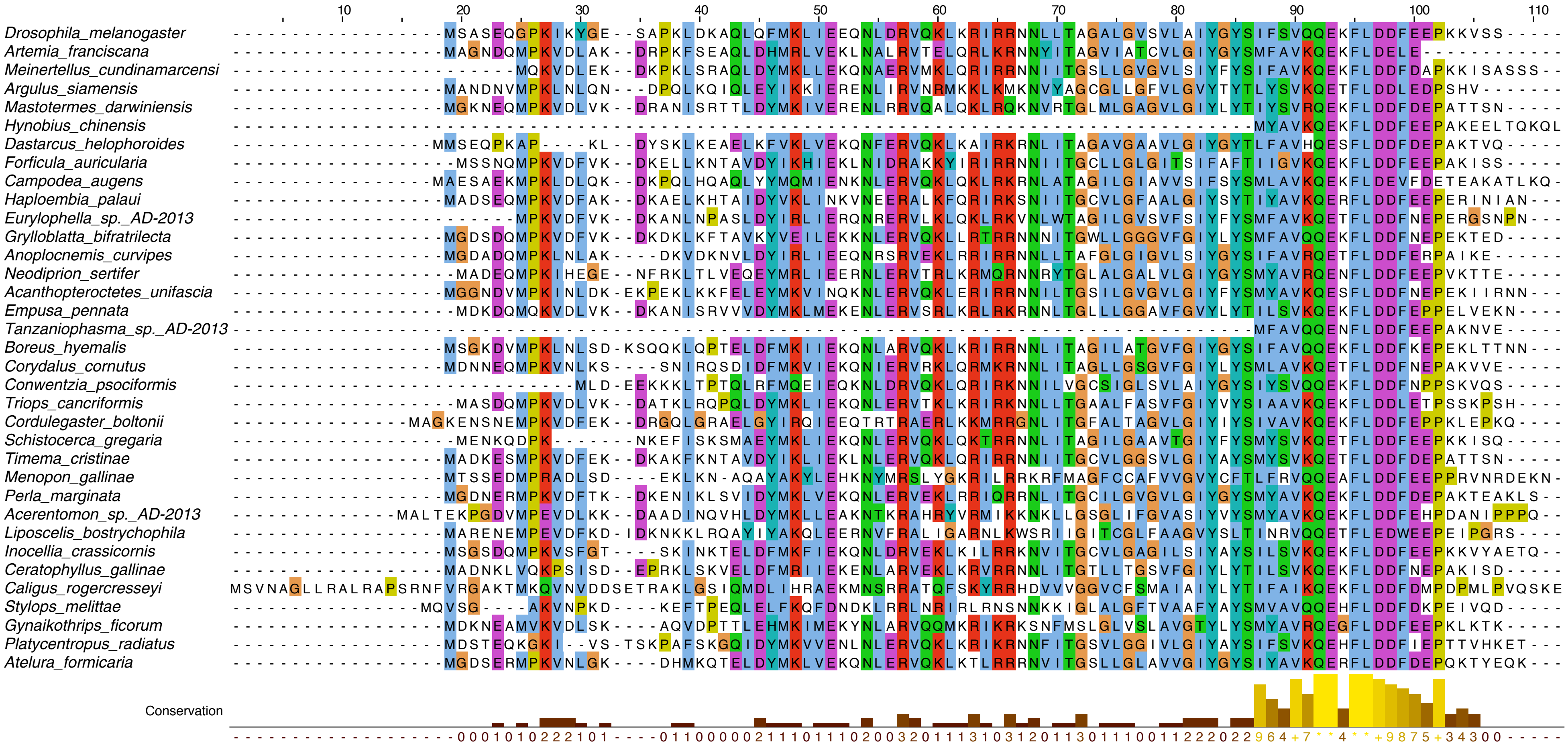

HG0014

FBgn0260392 (CG42518)

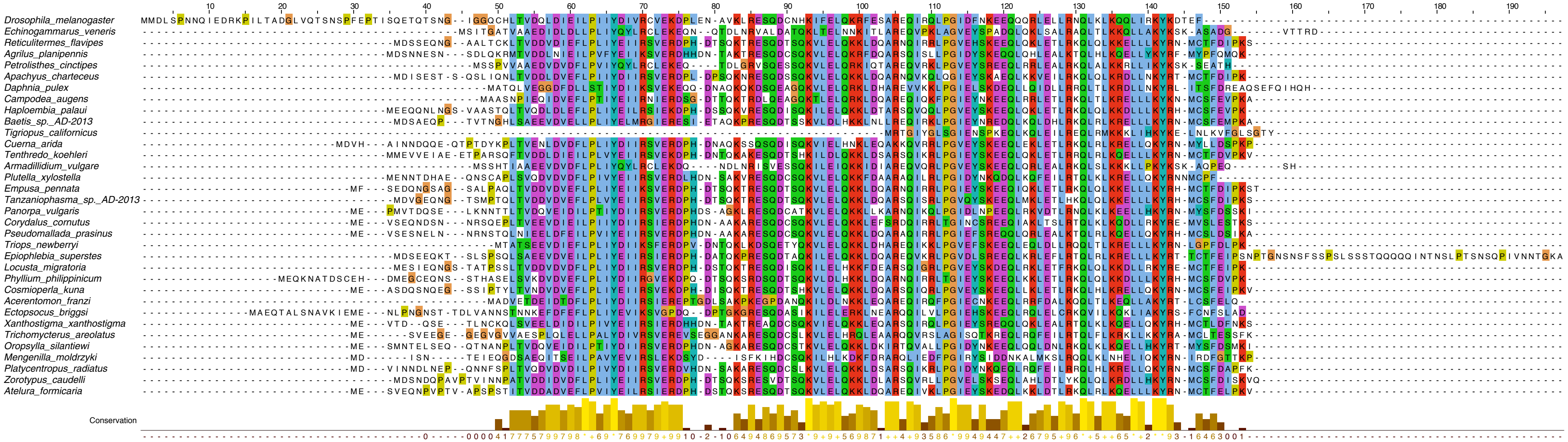

HG0015

ENSDARG00000007377 (odc1)

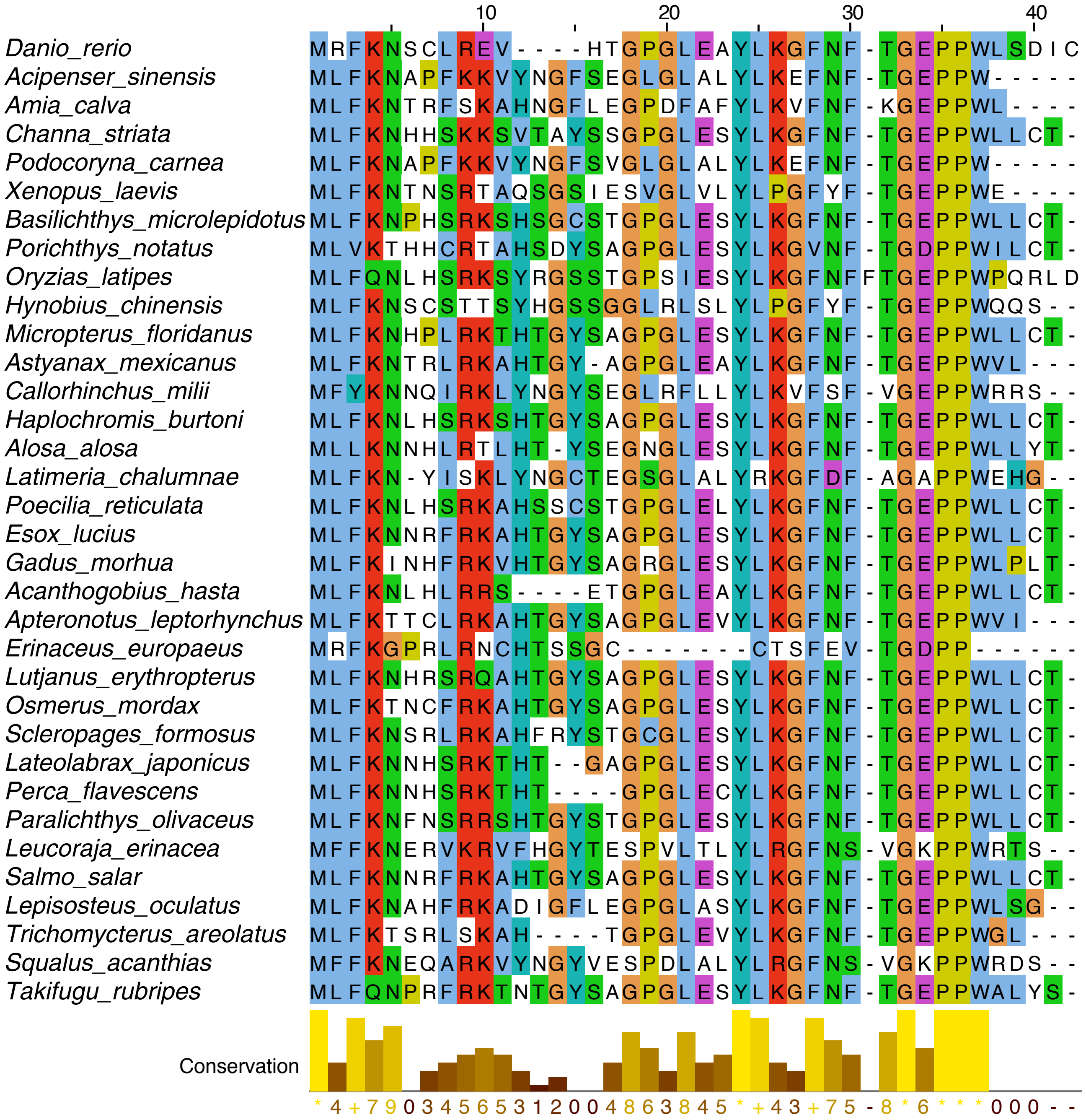

HG0016

ENSGALG00000010399 (SELENOT)

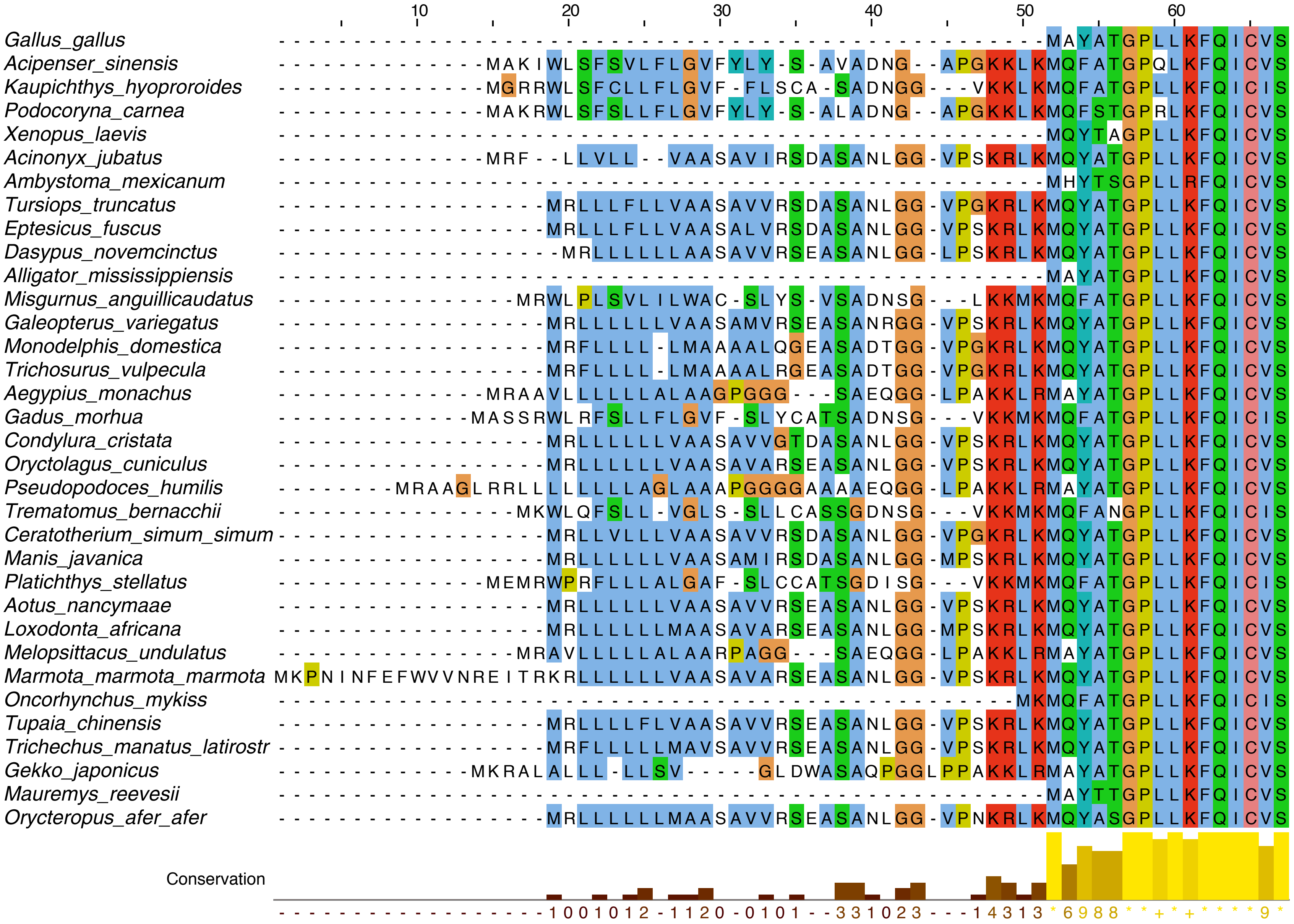

HG0017

ENSDARG00000043154 (ucp2)

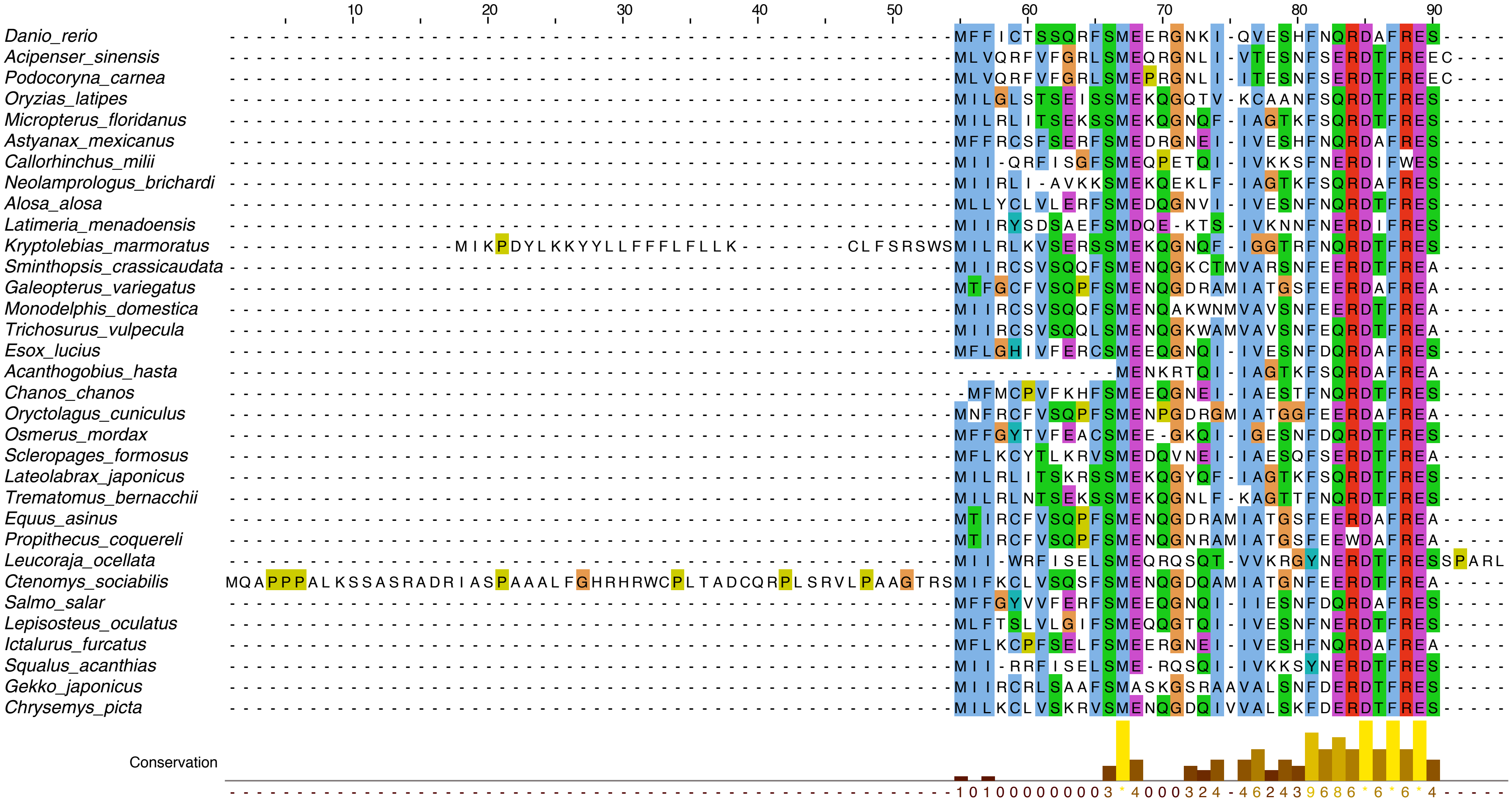

HG0018

ENSDARG00000053291 (pnrc2)

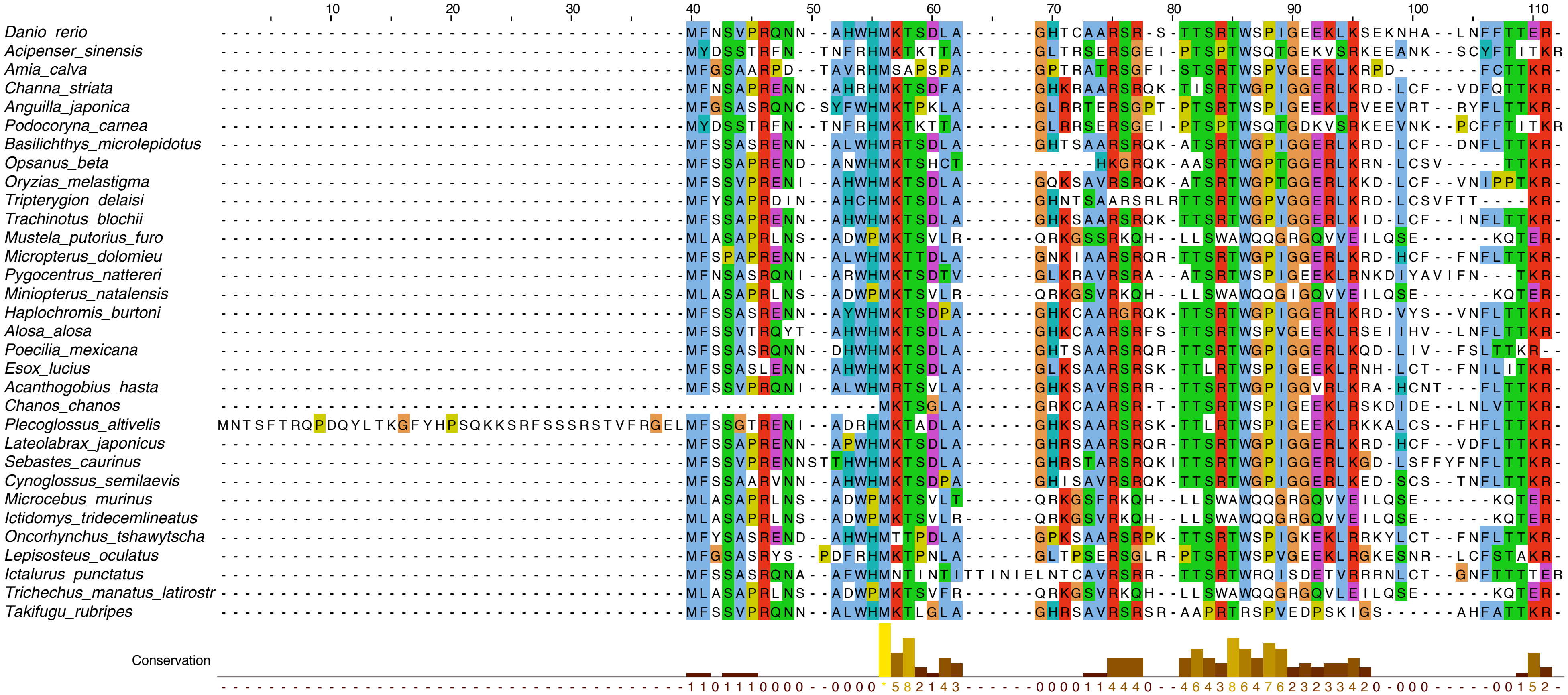

HG0018

ENSG00000189266 (PNRC2)

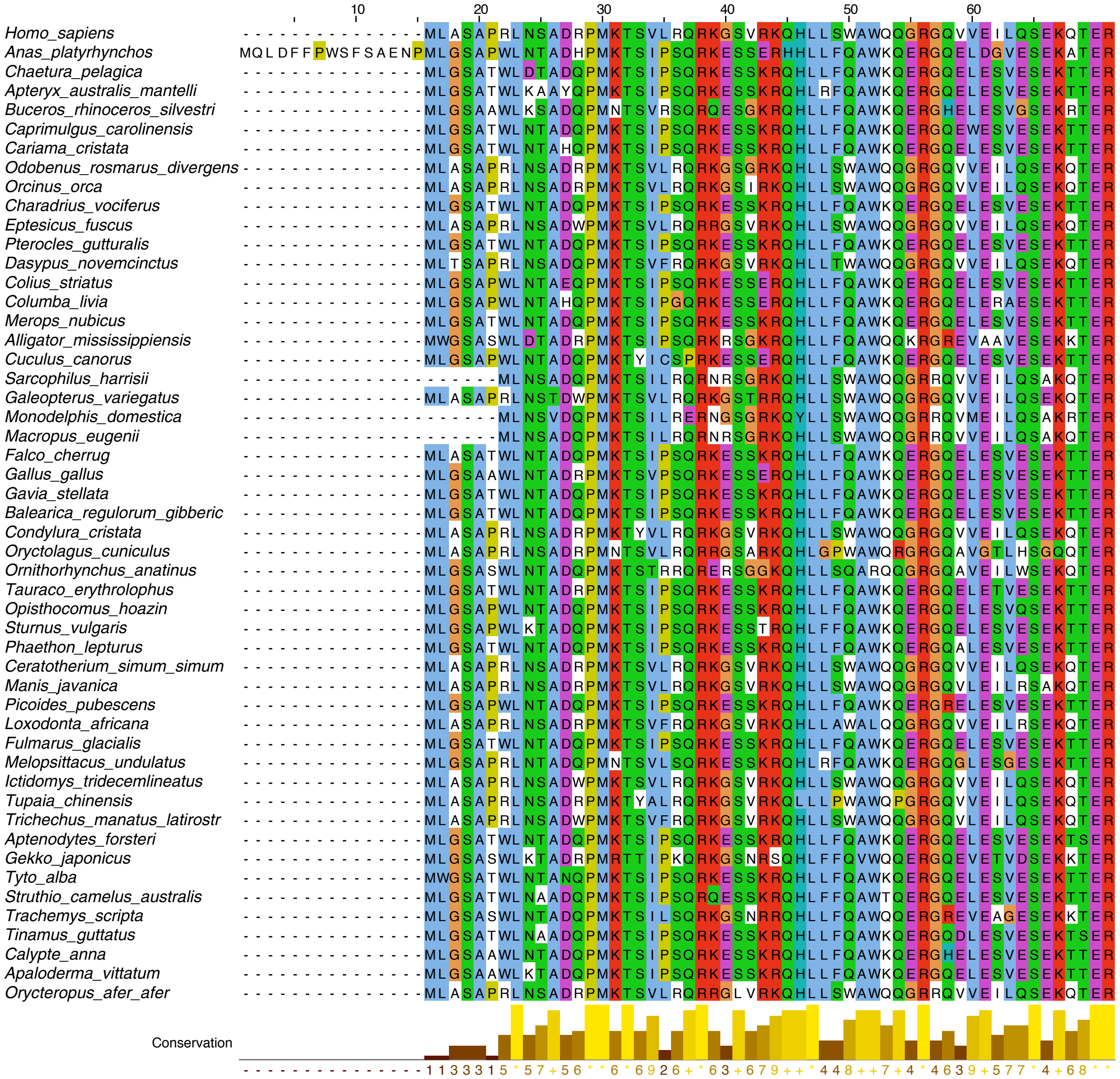

HG0018

ENSGALG00000004122 (PNRC2)

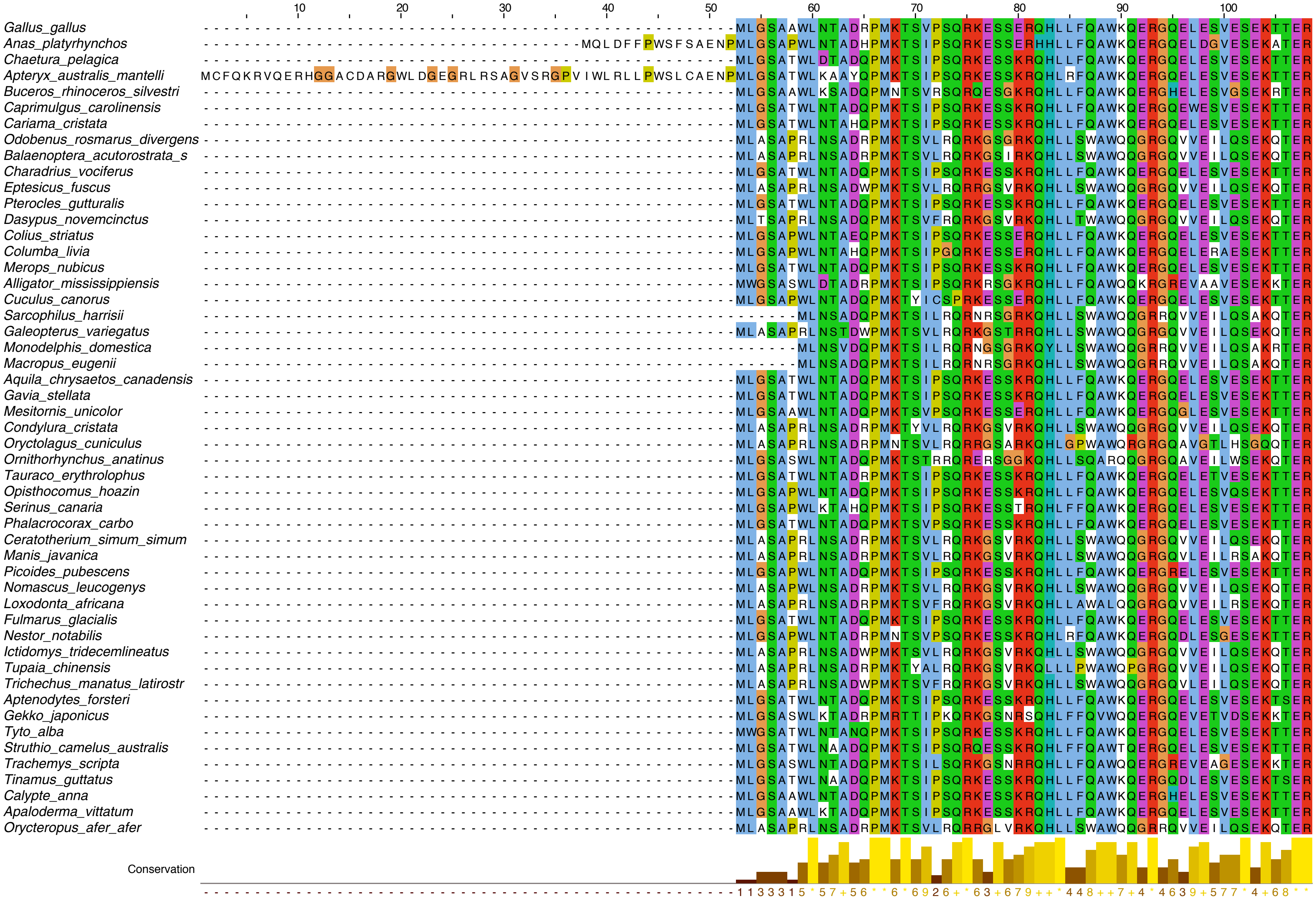

HG0019

ENSG00000178397 (FAM220A)

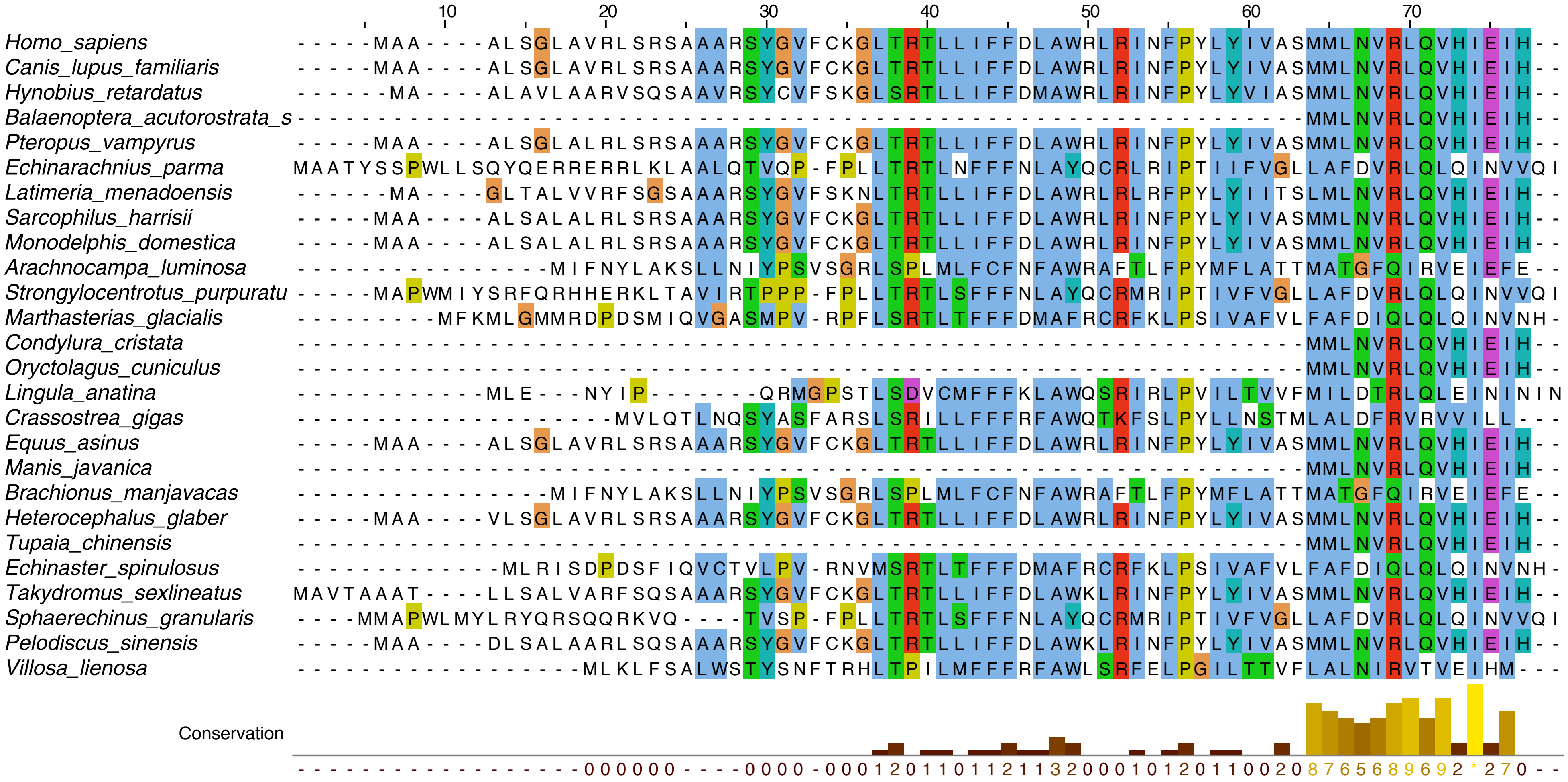

HG0020

FBgn0035436 (CG12016)

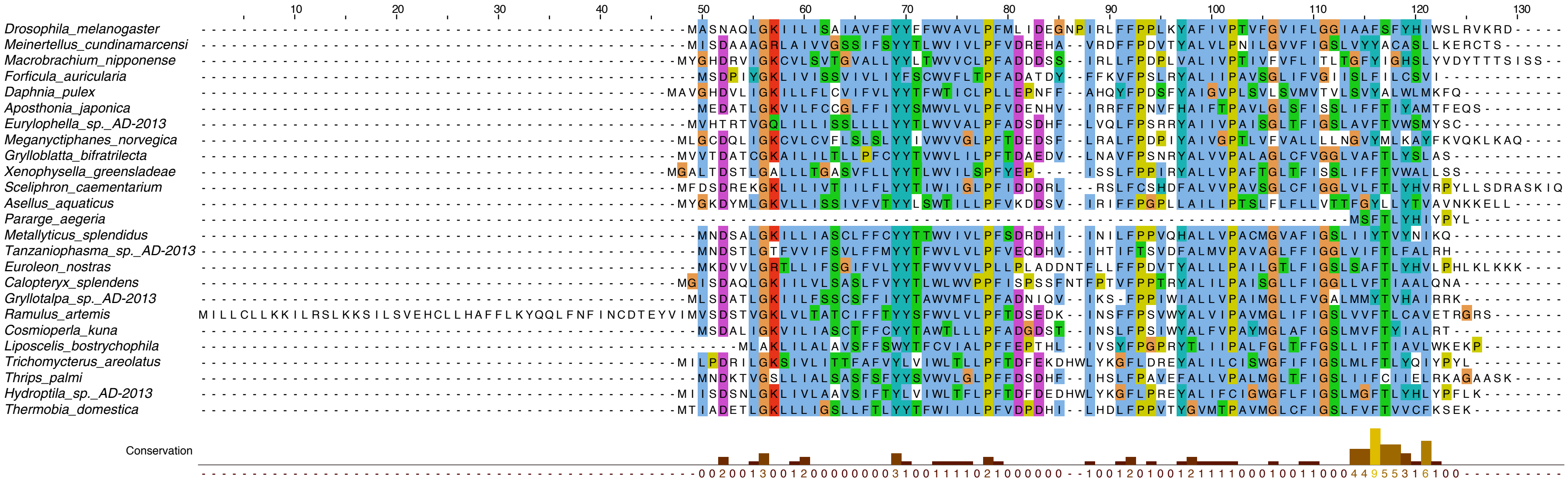

HG0021.1

ENSDARG00000068708 (ifrd1)

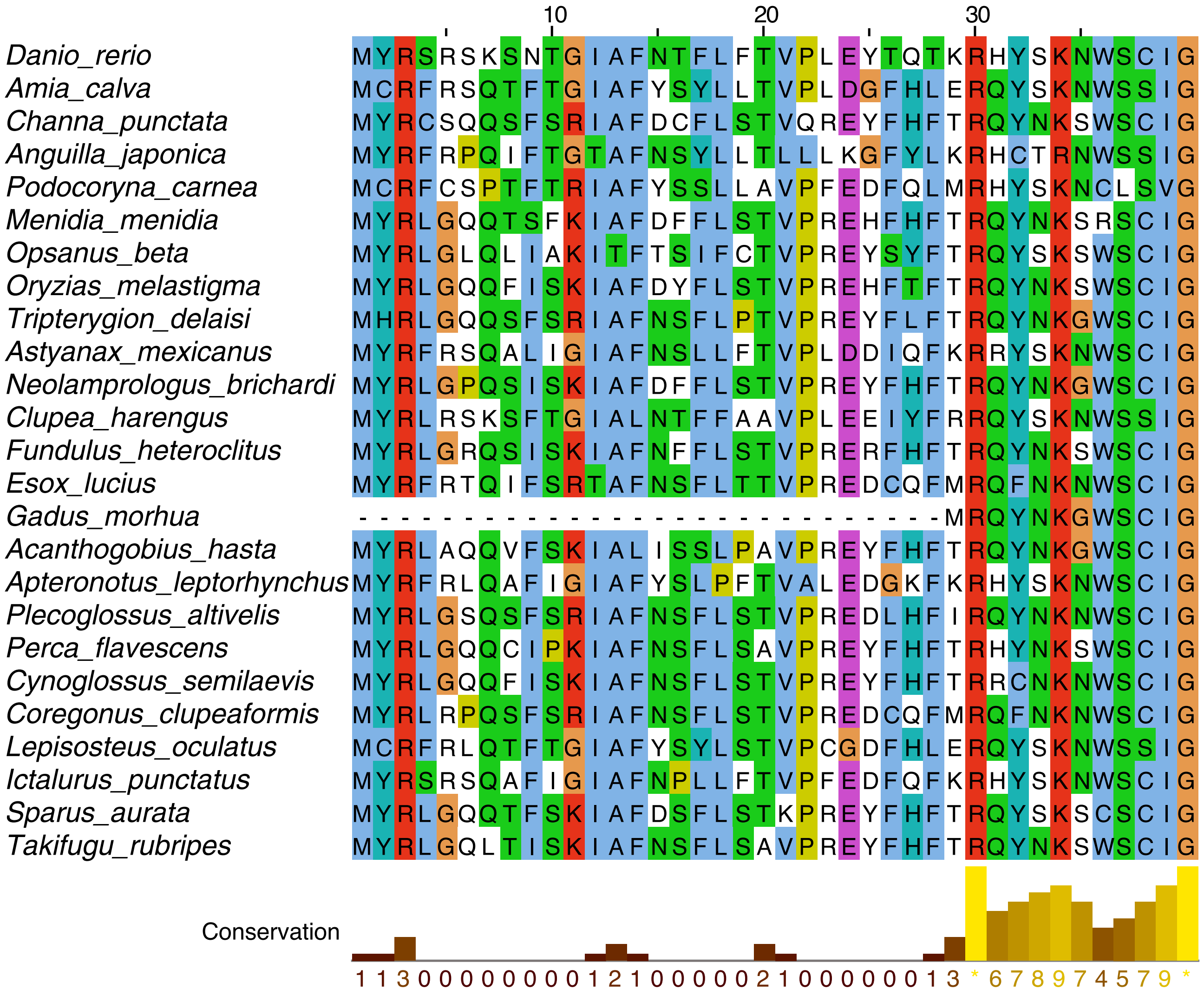

HG0021.1

ENSG00000006652 (IFRD1)

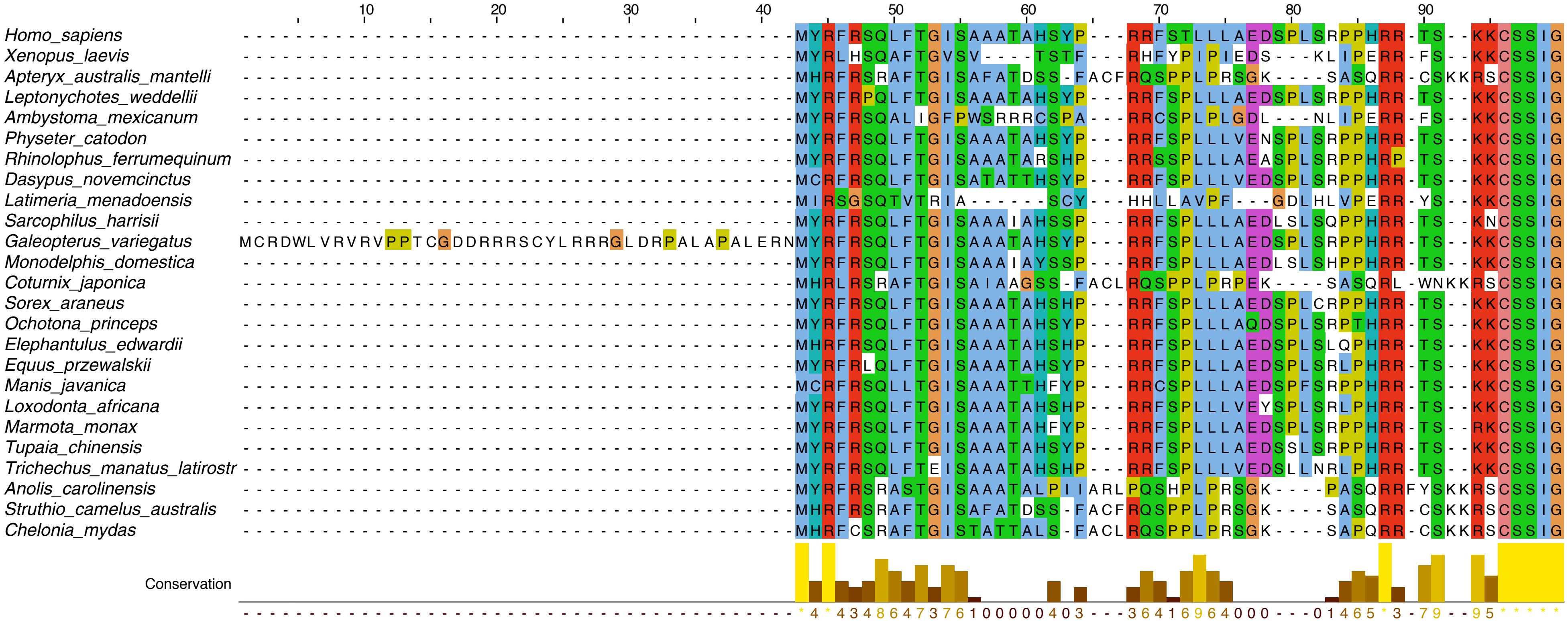

HG0021.1

ENSGALG00000009448 (IFRD1)

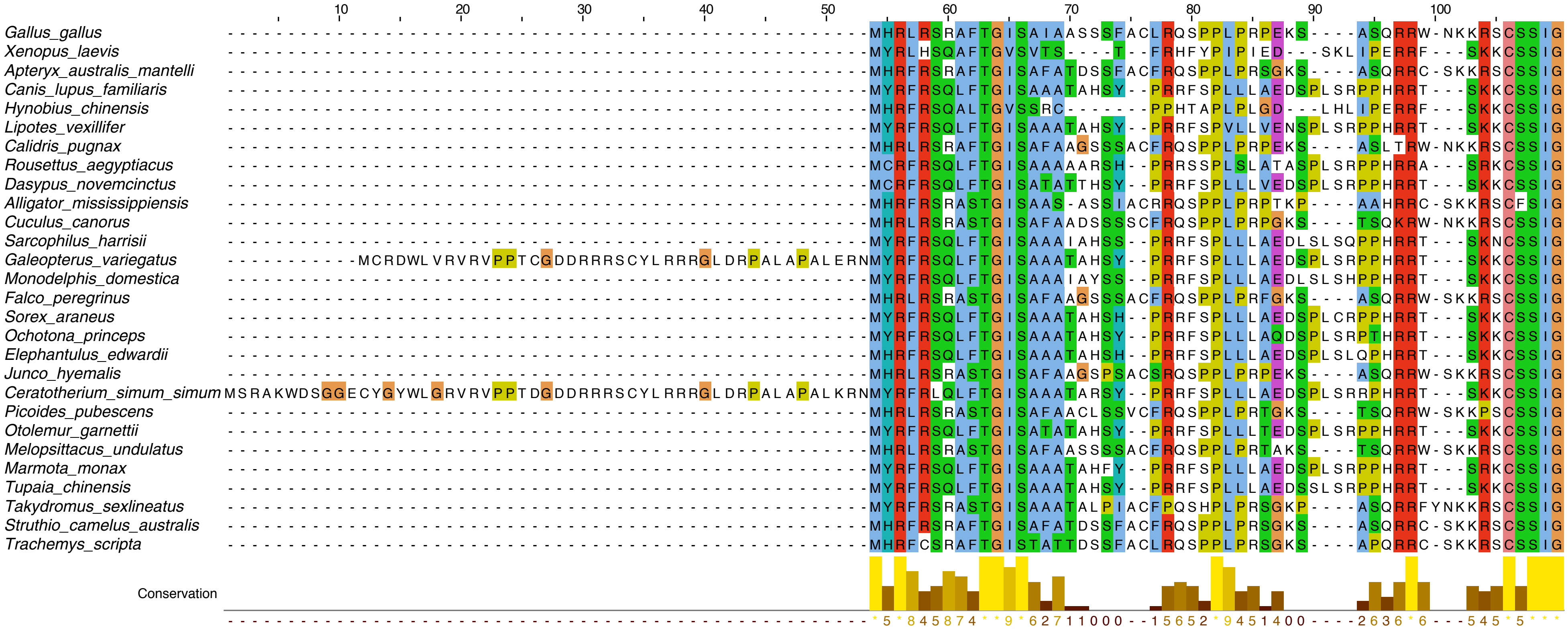

HG0021.2

ENSDARG00000036811 (ifrd2)

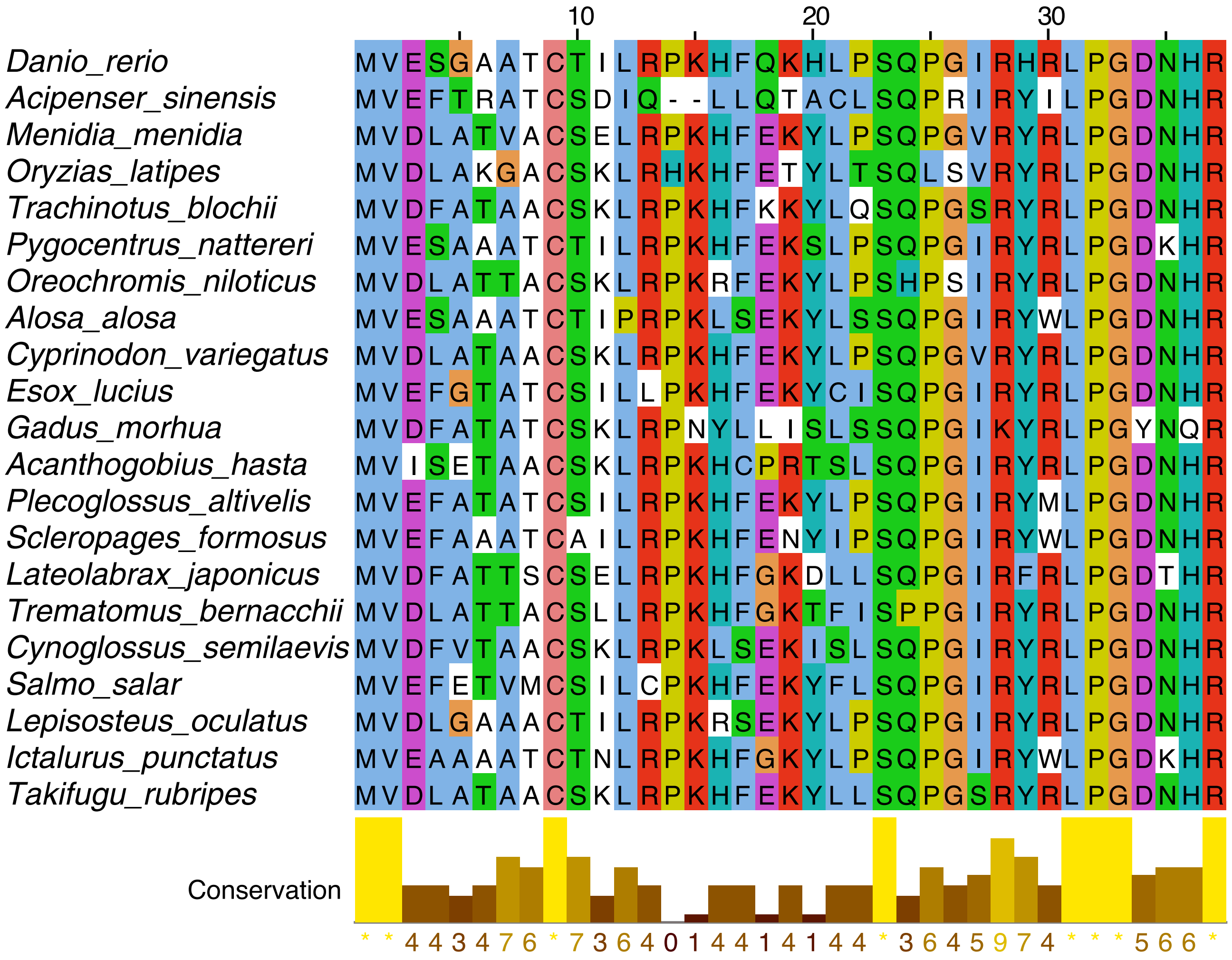

HG0022

FBgn0061359 (CG33671)

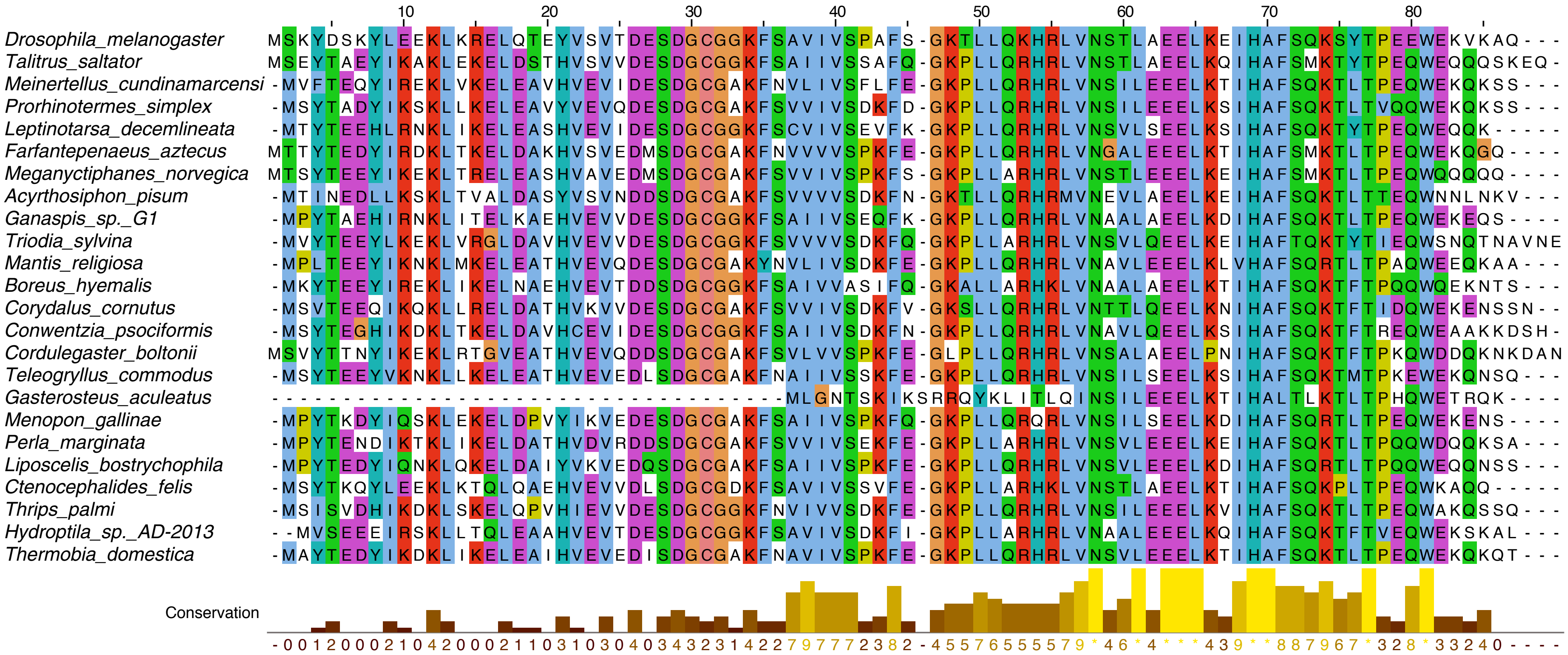

HG0023.1

ENSG00000175197 (DDIT3)

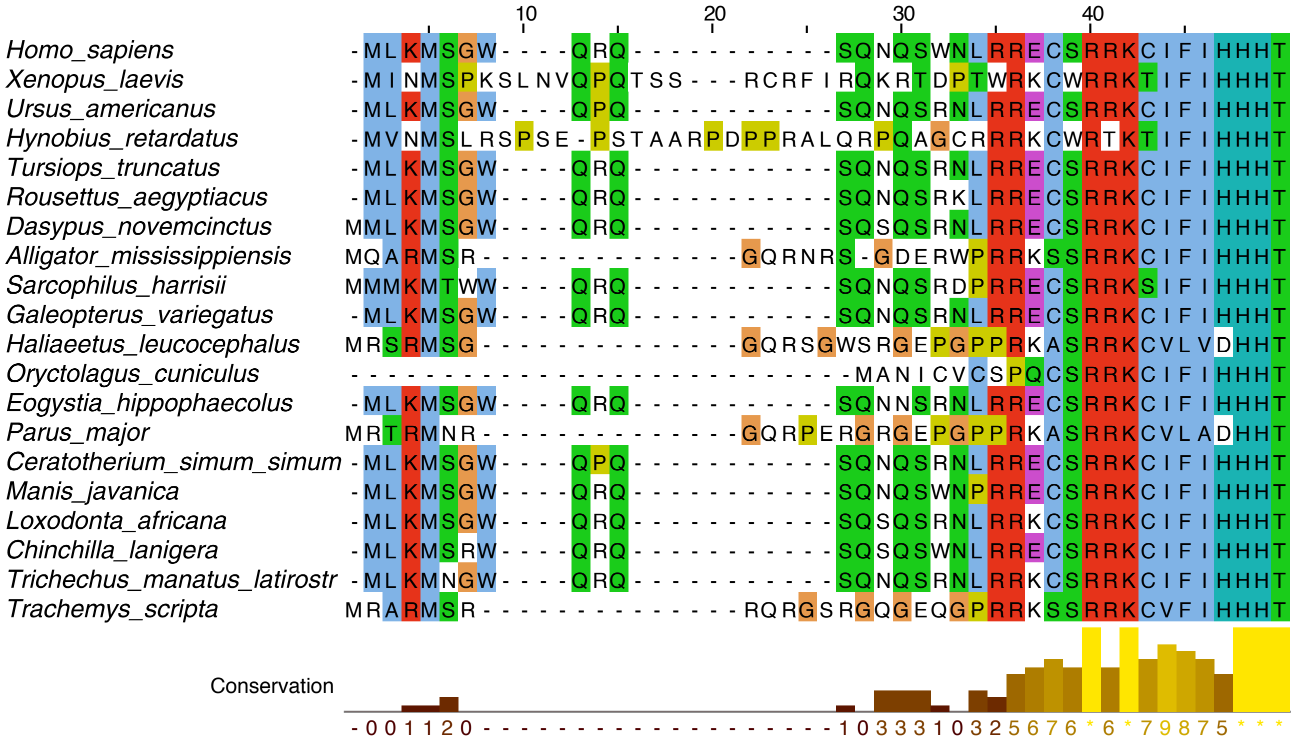

HG0023.2

ENSDARG00000059836 (ddit3)

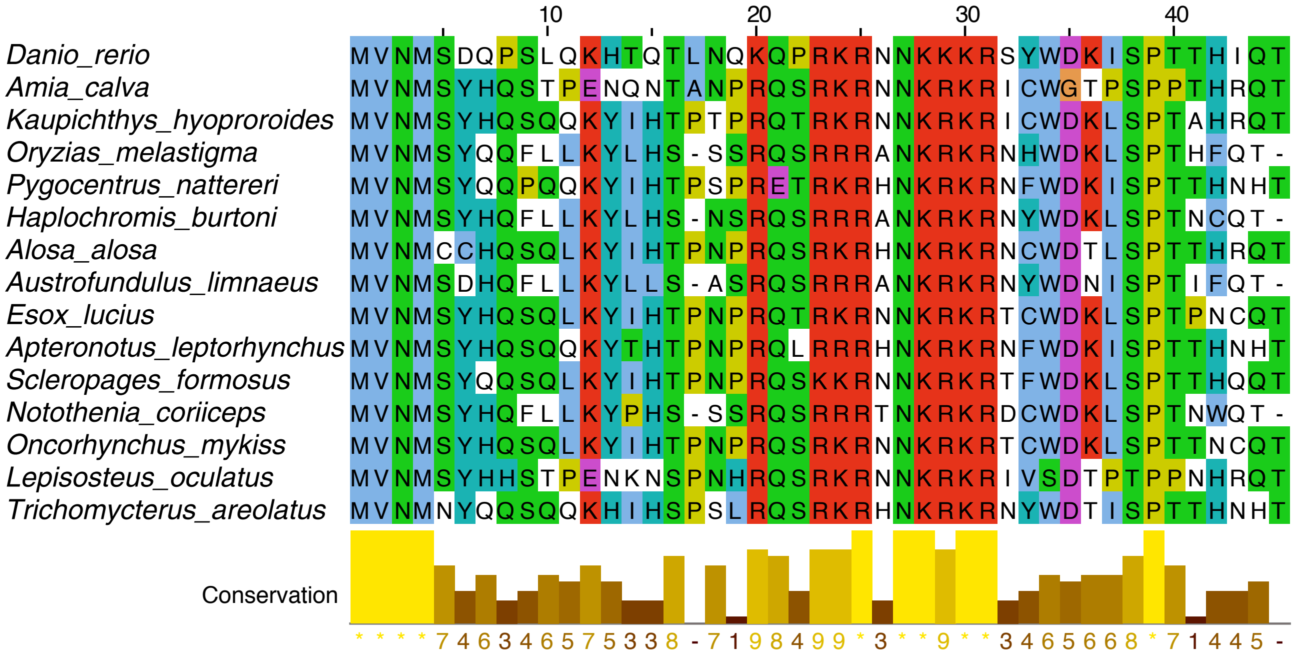

HG0024.1

ENSDARG00000093406

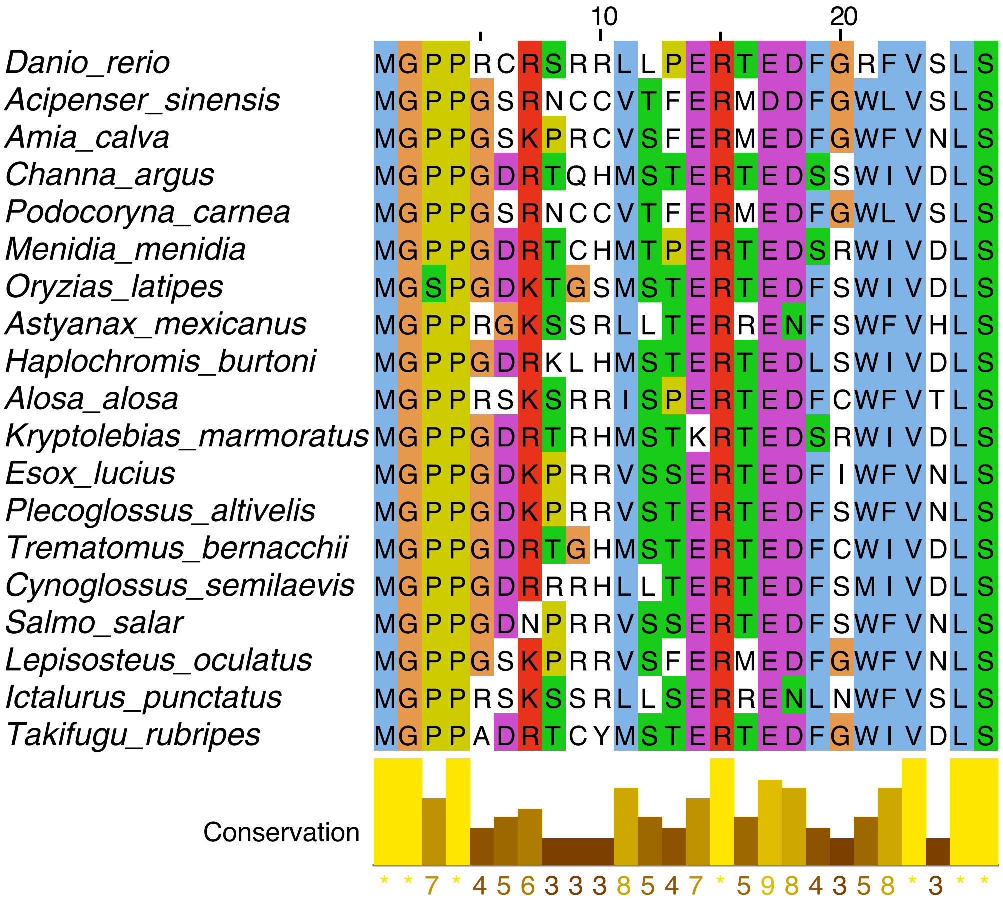

HG0024.2

ENSG00000112308 (C6orf62)

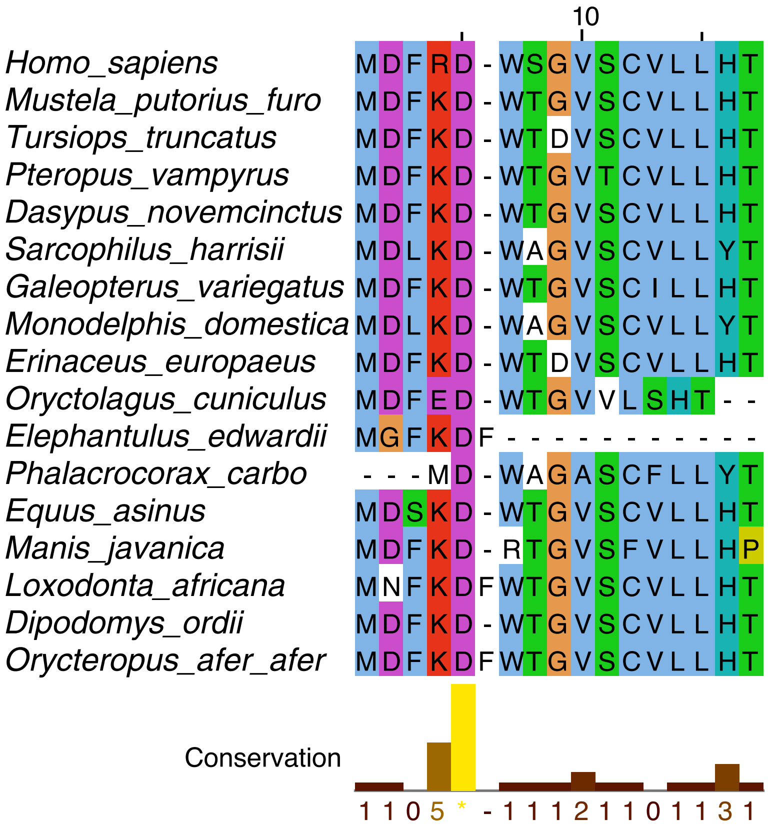

HG0024.2

ENSGALG00000013628 (C6orf62)

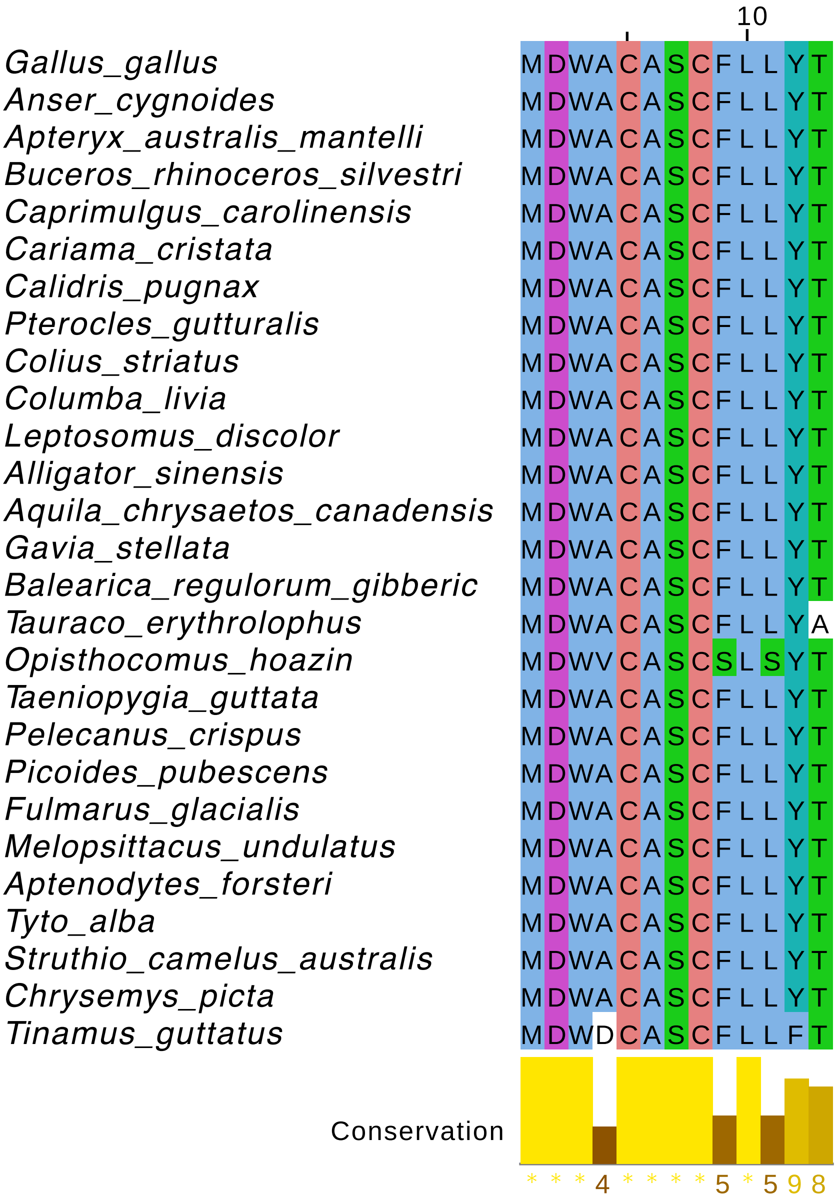

HG0024.3

ENSGALG00000013628 (C6orf62)

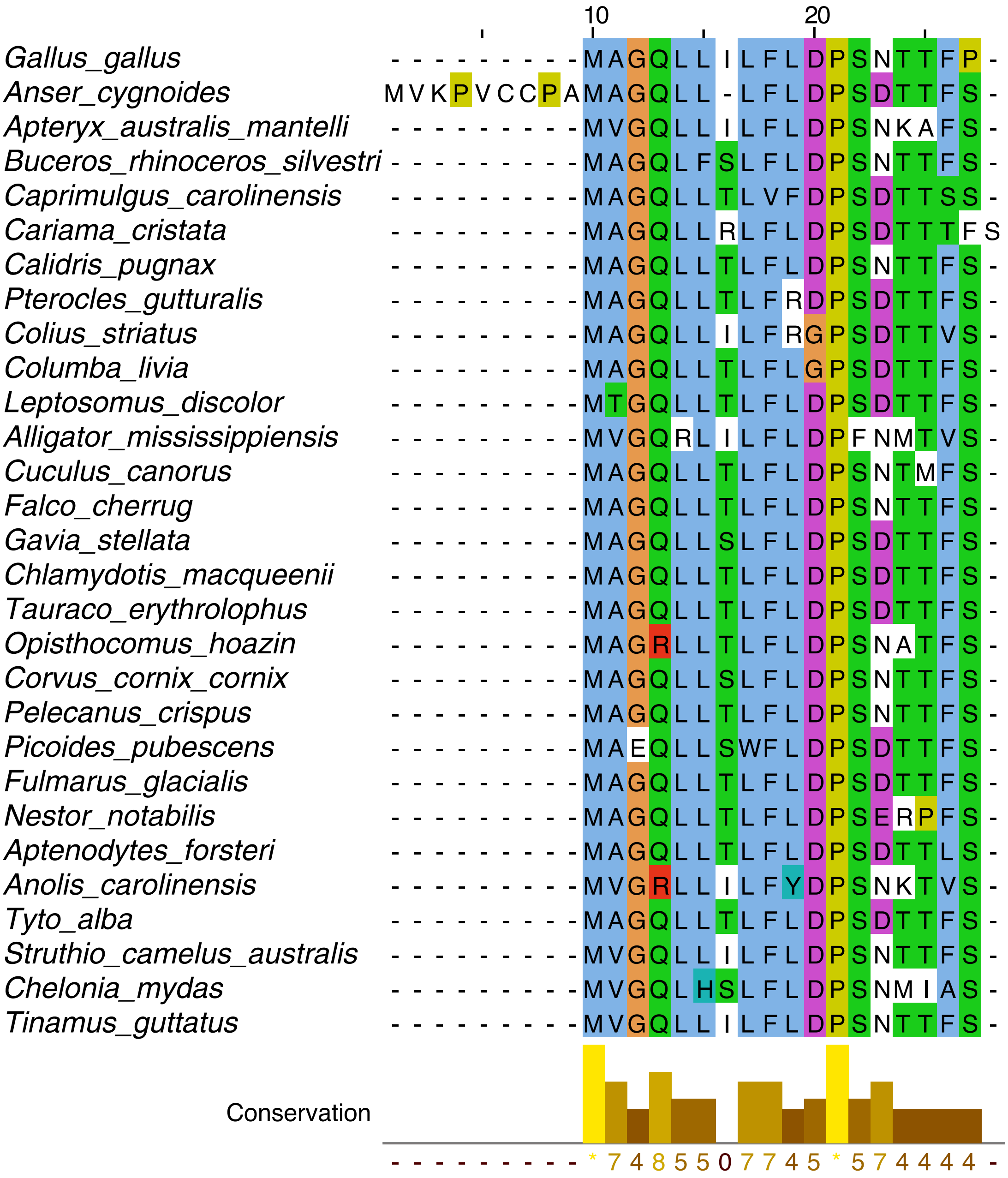

HG0025

ENSDARG00000060054 (epc1b)

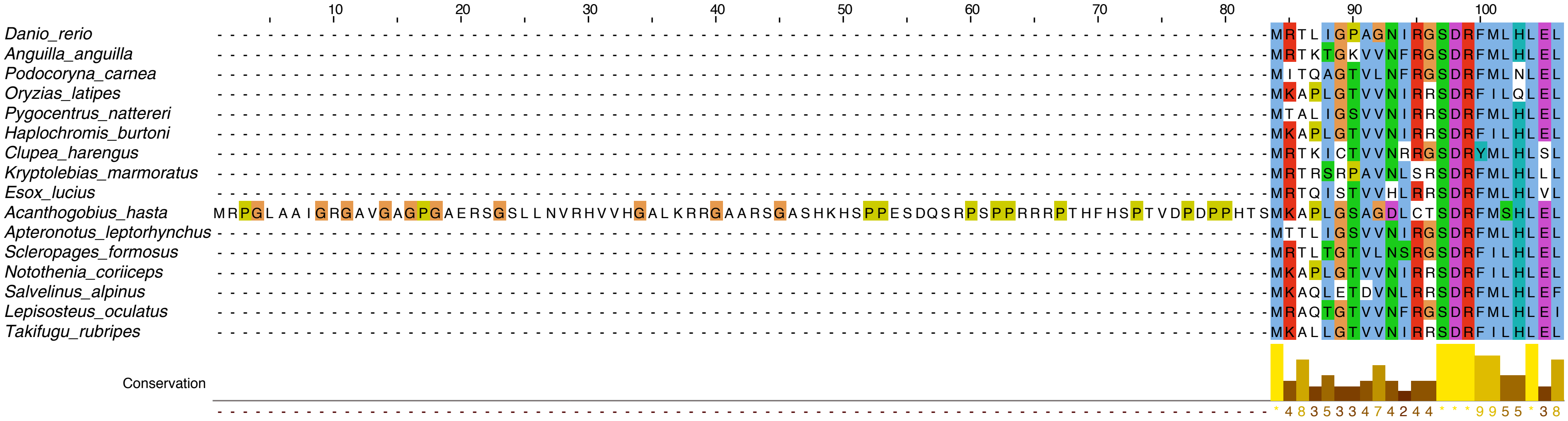

HG0026

ENSDARG00000087059 (fam213ab)

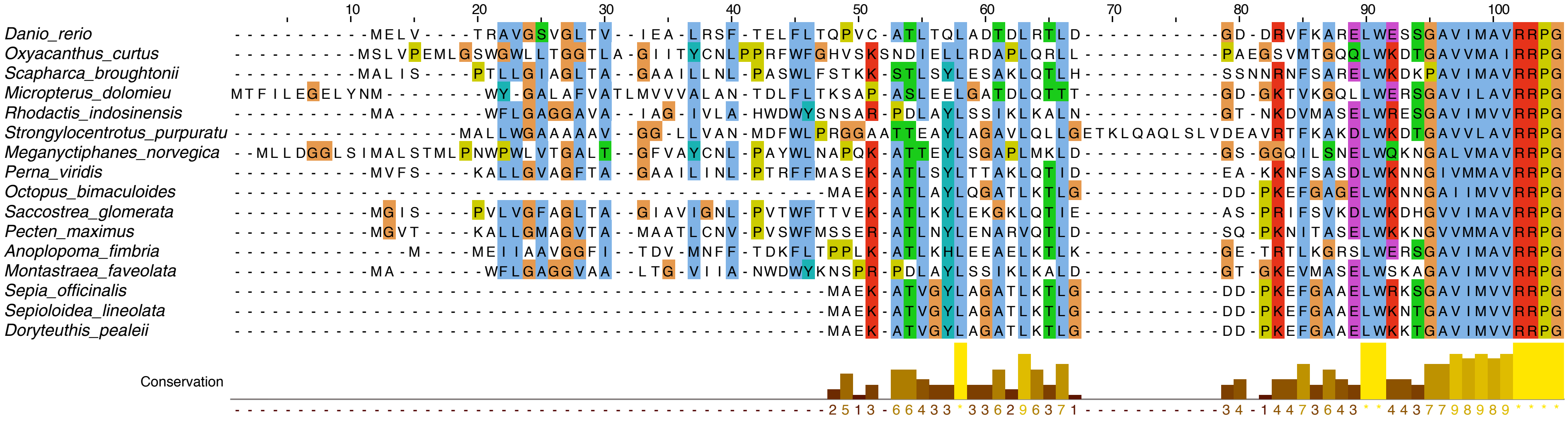

HG0027

ENSG00000077254 (USP33)

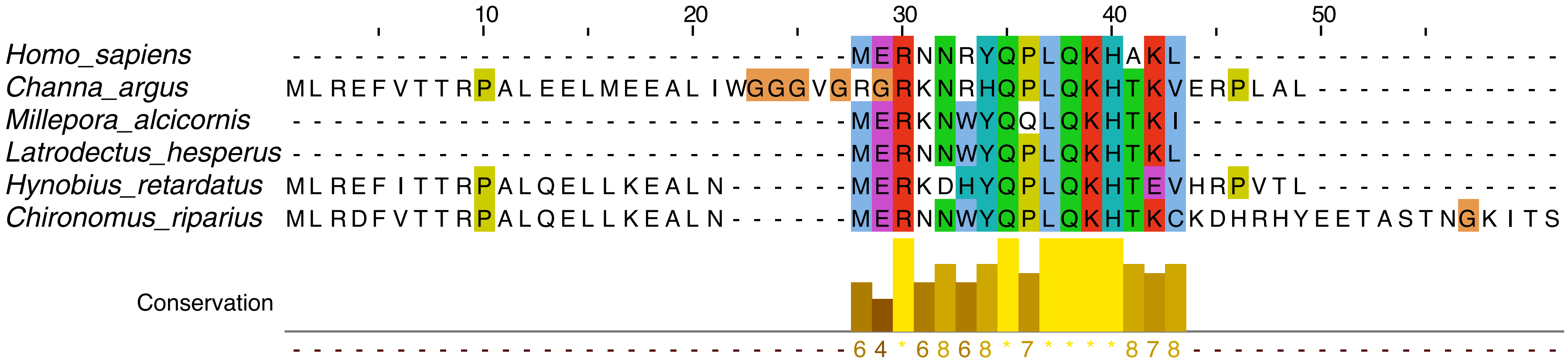

HG0028.1

ENSDARG00000032103 (mapk6)

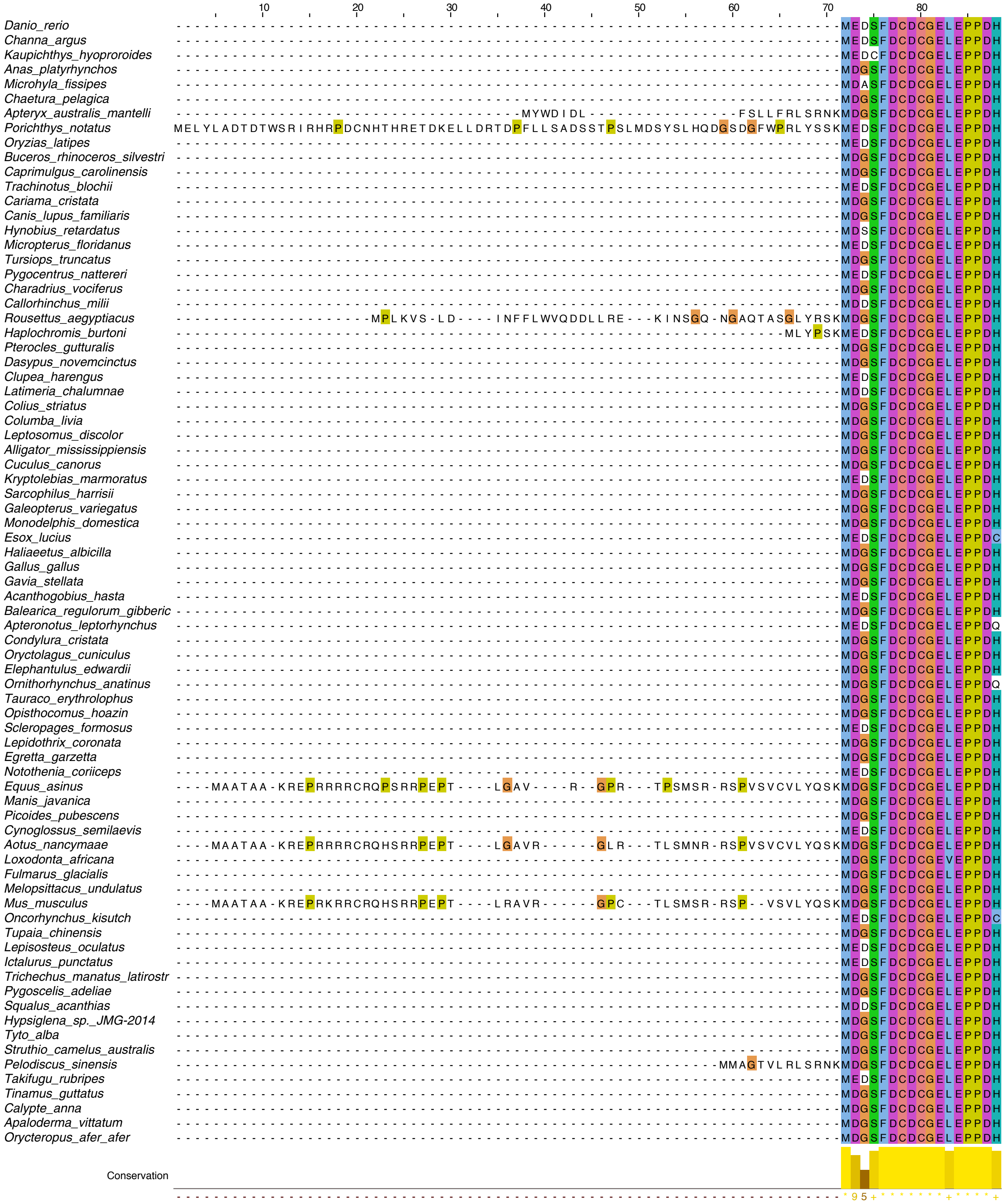

HG0028.1

ENSG00000069956 (MAPK6)

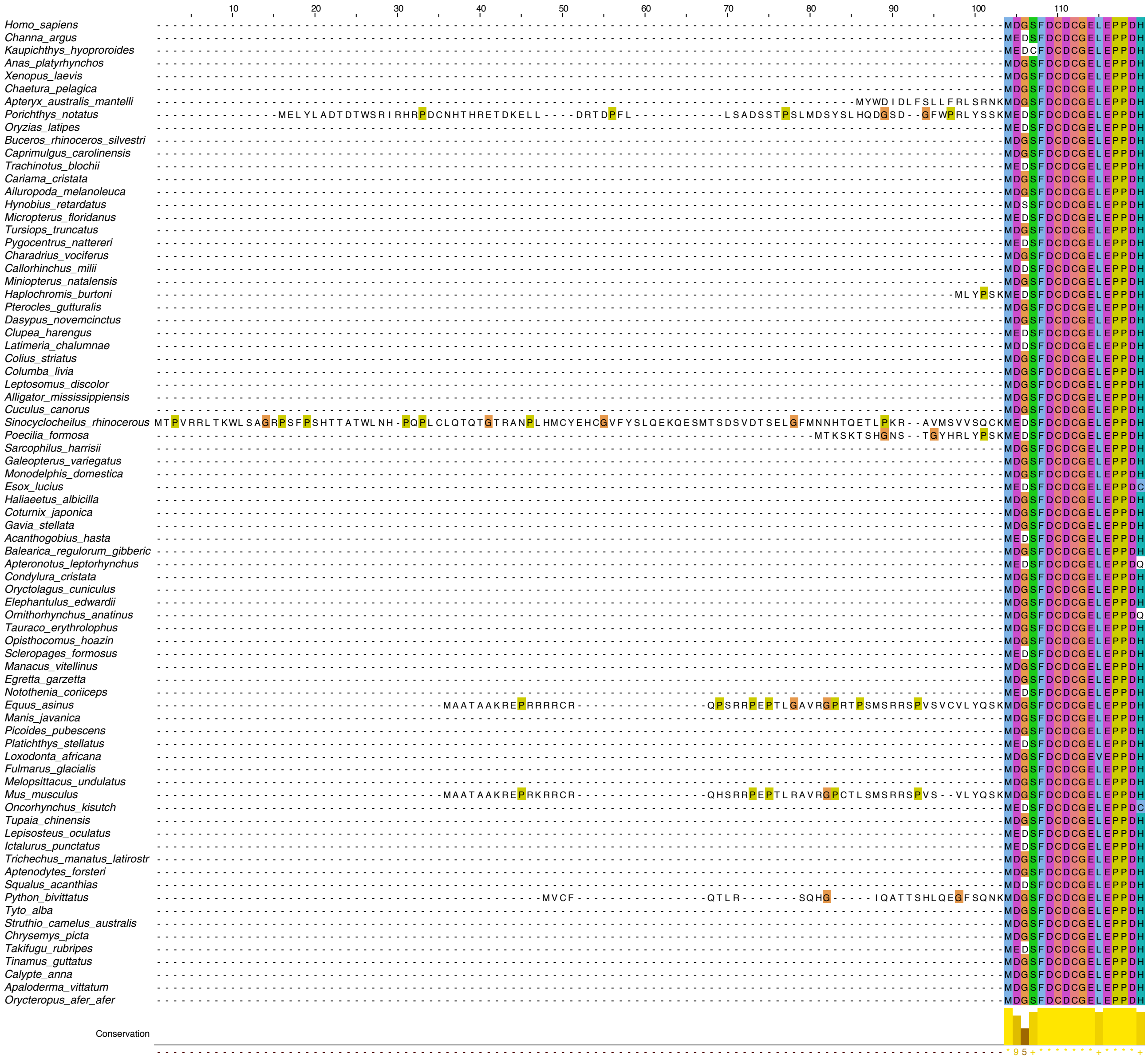

HG0028.1

ENSGALG00000031448 (MAPK6)

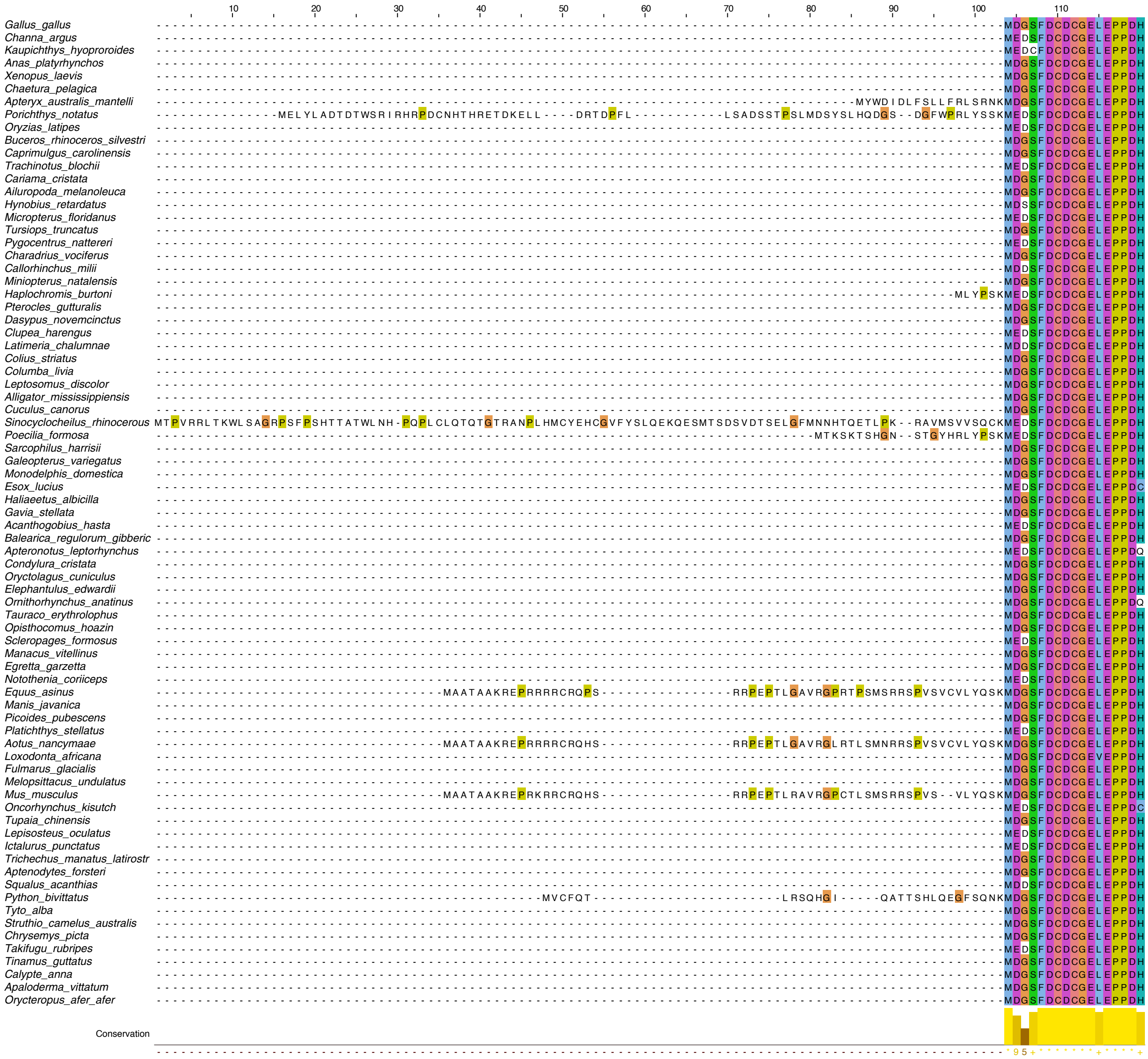

HG0028.2

ENSG00000069956 (MAPK6)

Species	Alignment (positions 1–23; ticks at 10, 20)
Homo_sapiens	MFIEACCVHIYSHFC--------
Anser_cygnoides	MLIEAYYVHIYSHFC--------
Rhacophorus_omeimontis	MMIEAYILHIYSHFCSMSTCFAH
Chaetura_pelagica	MLIEAYYVHIYSHFC--------
Apteryx_australis_mantelli	MLIEAYYVHIYSHFC--------
Buceros_rhinoceros_silvestri	MLIEAYYVHIYSHFC--------
Caprimulgus_carolinensis	MLIEAYYVHIYSHFC--------
Cariama_cristata	MLIEAYYVHIYSHFC--------
Acinonyx_jubatus	MFIEACCVHIYSHFC--------
Balaenoptera_acutorostrata_s	MFIEACCVHIYSHFC--------
Calidris_pugnax	MLIEAYYVHIYSHFC--------
Eptesicus_fuscus	MFIEACCVHIYSHFC--------
Pterocles_gutturalis	MLIEAYYVHIYSHFC--------
Dasypus_novemcinctus	MFIEACCVHIYSHFC--------
Colius_striatus	MLIEAYYVHIYSHFC--------
Columba_livia	MLIEAYYVHIYSHFC--------
Leptosomus_discolor	MLIEAYYVHIYSHFC--------
Alligator_sinensis	MLIEAYYVHIYSYFC--------
Cuculus_canorus	MLIEAYYVHIYSHFC--------
Sarcophilus_harrisii	MFREAYCVHIYSLFC--------
Galeopterus_variegatus	MFIEACCVHIYSHFC--------
Aquila_chrysaetos_canadensis	MLIEAYYVHIYSHFC--------
Coturnix_japonica	MLIEAYYVHIYSHFC--------
Gavia_stellata	MLIEAYYVHIYSHFC--------
Chlamydotis_macqueenii	MFIEAYYVHIYSHFC--------
Condylura_cristata	MFIEACCVHIYSHFC--------
Oryctolagus_cuniculus	MFIEACCVHIYSHFC--------
Elephantulus_edwardii	MSIEACCVHIYSHFC--------
Ornithorhynchus_anatinus	MLIEACFVHIYSHFC--------
Tauraco_erythrolophus	MLKEAYYVHIYSHFC--------
Opisthocomus_hoazin	MLIETYYVHIYSHFC--------
Acanthisitta_chloris	MLIEAYYVHIYSHFC--------
Egretta_garzetta	MLIEAYYVHIYSHFC--------
Ceratotherium_simum_simum	MFIEACCVHIYSHFC--------
Picoides_pubescens	MLIEAYYVHIYSHFC--------
Loxodonta_africana	MFIEACCVHIYSHFC--------
Fulmarus_glacialis	MLIEAYYVHIYSHFC--------
Melopsittacus_undulatus	MLIEAYYVHIYSHFC--------
Marmota_marmota_marmota	MFIEACCVHIYSHFC--------
Tupaia_chinensis	MFLEACCVHIYSHFC--------
Trichechus_manatus_latirostr	MFIEARCVHIYSRFC--------
Pygoscelis_adeliae	MLIEAYYVHIYSHFC--------
Takydromus_sexlineatus	MLIEAYYVHIYSHFC--------
Tyto_alba	MLIEAYYVHIYSHFC--------
Struthio_camelus_australis	MLIEAYYVHIYSHFC--------
Pelodiscus_sinensis	MLLEAHYVHIYSHFC--------
Tinamus_guttatus	MLIEAYYVHIYSHFC--------
Calypte_anna	MLIEAYYVHIYSHFC--------
Apaloderma_vittatum	MLIEAYYVHIYSHFC--------
Orycteropus_afer_afer	MFIEACCVHIYSHFC--------

Conservation

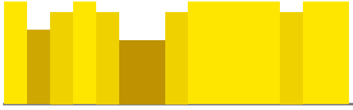

* 8 + * + 7 7 + * * * * + * * - - - - - - - -

HG0029

ENSDARG00000007523 (kmt2e)

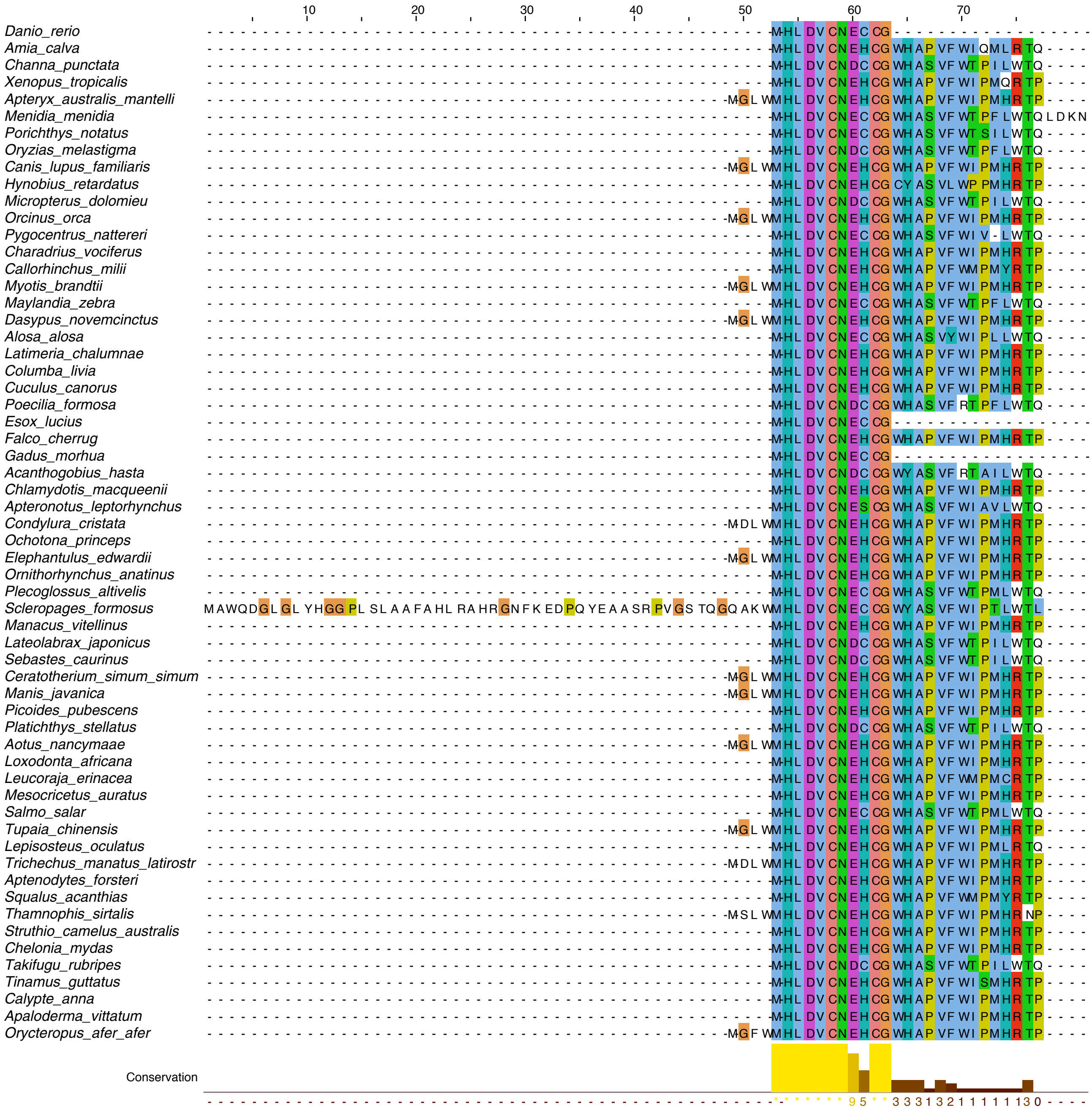

HG0029

ENSG00000005483 (KMT2E)

```
                             10        20        30        40        50
Homo_sapiens                 ---------------------------MGLWMHLDVCNEHCGWHAPVFWIPMHRTP----
Acipenser_sinensis           -------------------------------MHLDVCNEHFGWHASVFWIPMLRTP----
Amia_calva                   -------------------------------MHLDVCNEHCGWHAPVFWIQMLRTQ----
Channa_punctata              -------------------------------MHLDVCNDCCGWHASVFWTPILWTQ----
Anser_cygnoides              -------------------------------MHLDVCNEHCGWHAPVFWIPMHRTL----
Xenopus_tropicalis           -------------------------------MHLDVCNEHCGWHAPVFWIPMQRTP----
Chaetura_pelagica            -------------------------------MHLDVCNEHCGWHAPVFWIPMHRTP----
Apteryx_australis_mantelli   ---------------------------MGLWMHLDVCNEHCGWHAPVFWIPMHRTP----
Menidia_menidia              -------------------------------MHLDVCNECCGWHASVFWTPFLWTQLDKN
Porichthys_notatus           -------------------------------MHLDVCNECCGWHASVFWTSILWTQ----
Oryzias_latipes              -------------------------------MHLDVCNDCCGWHASVFWTPFLWTQ----
Buceros_rhinoceros_silvestri -------------------------------MHLDVCNEHCGWHAPVFWIPMHRTP----
Caprimulgus_carolinensis     -------------------------------MHLDVCNEHCGWHAPVFWIPMHRTP----
Cariama_cristata             -------------------------------MHLDVCNEHCGWHAPVFWIPMHRTP----
Mustela_putorius_furo        MLYRSILDSRFCEDIYSFLDLRFARHLMGLWMHLDVCNEHCGWHAPVFWIPMHRTP----
Hynobius_retardatus          -------------------------------MHLDVCNEHCGCYASVLWPPMHRTP----
Micropterus_dolomieu         -------------------------------MHLDVCNDCCGWHASVFWTPILWTQ----
Orcinus_orca                 ---------------------------MGLWMHLDVCNEHCGWHAPVFWIPMHRTP----
Pygocentrus_nattereri        -------------------------------MHLDVCNECCGWHASVFWIV-LWTQ----
Calidris_pugnax              -------------------------------MHLDVCNEHCGWHAPVFWIPMHRTP----
Callorhinchus_milii          -------------------------------MHLDVCNEHCGWHAPVFWMPMYRTP----
Myotis_brandtii              ---------------------------MGLWMHLDVCNEHCGWHAPVFWIPMHRTP----
Haplochromis_burtoni         -------------------------------MHLDVCNECCGWHASVFWTPFLWTQ----
Pterocles_gutturalis         -------------------------------MHLDVCNEHCGWHAPVFWIPMHRTP----
Dasypus_novemcinctus         ---------------------------MGLWMHLDVCNEHCGWHAPVFWIPMHRTP----
Clupea_harengus              -------------------------------MHLDVCNECCGWHASVFWIPLLWTQ----
Latimeria_chalumnae          -------------------------------MHLDVCNEHCGWHAPVFWIPMHRTP----
Columba_livia                -------------------------------MHLDVCNEHCGWHAPVFWIPMHRTP----
Merops_nubicus               -------------------------------MHLDVCNEHCGWHAPVFWIPMHRTP----
Alligator_mississippiensis   -------------------------------MHLDVCNEHCGWHAPVFWIPMHRTP----
Cuculus_canorus              -------------------------------MHLDVCNEHCGWHAPVFWIPMHRTP----
Sinocyclocheilus_angustiporu -------------------------------MHLDVCNECCG------------------
Austrofundulus_limnaeus      -------------------------------MHLDVCNDCCGWHASVFWTPFLWTQ----
Sarcophilus_harrisii         -------------------------------MHLDVCNEHCGWHAPVFWIPMHRTP----
Galeopterus_variegatus       ---------------------------MGLWMHLDVCNEHCGWHAPVFWIPMHRTP----
Monodelphis_domestica        -------------------------------MHLDVCNEHCGWHAPVFWIPMHRTP----
Esox_lucius                  -------------------------------MHLDVCNECCG------------------
Falco_cherrug                -------------------------------MHLDVCNEHCGWHAPVFWIPMHRTP----
Gadus_morhua                 -------------------------------MHLDVCNECCG------------------
Coturnix_japonica            -------------------------------MHLDVCNEHCGWHAPVFWIPMHRTL----
Acanthogobius_hasta          -------------------------------MHLDVCNDCCGWYASVFRTAILWTQ----
Chlamydotis_macqueenii       -------------------------------MHLDVCNEHCGWHAPVFWIPMHRTP----
Apteronotus_leptorhynchus    -------------------------------MHLDVCNESCGWHASVFWIAVLWTQ----
Erinaceus_europaeus          ---------------------------MGLWMHLDVCNEHCGWHAPVFWIPMHRTP----
Ochotona_princeps            -------------------------------MHLDVCNEHCGWHAPVFWIPMHRTP----
Elephantulus_edwardii        ---------------------------MGLWMHLDVCNEHCGWHAPVFWIPMHRTP----
Ornithorhynchus_anatinus     -------------------------------MHLDVCNEHCGWHAPVFWIPMHRTP----
Plecoglossus_altivelis       -------------------------------MHLDVCNECCGWHASVFWTPMLWTQ----
Scleropages_formosus         -------------------------------MHLDVCNECCGWYASVFWIPTLWTL----
Parus_major                  -----------------------MHLFMGLWMHLDVCNEHCGWHAPVFWIPMHRTP----
Nipponia_nippon              -------------------------------MHLDVCNEHCGWHAPVFWIPMHRTP----
Lateolabrax_japonicus        -------------------------------MHLDVCNDCCGWHASVFWTPILWTQ----
Sebastes_caurinus            -------------------------------MHLDVCNDCCGWHASVFWTPILWTQ----
Ceratotherium_simum_simum    ---------------------------MGLWMHLDVCNEHCGWHAPVFWIPMHRTP----
Manis_javanica               ---------------------------MGLWMHLDVCNEHCGWHAPVFWIPMHRTP----
Picoides_pubescens           -------------------------------MHLDVCNEHCGWHAPVFWIPMHRTP----
Platichthys_stellatus        -------------------------------MHLDVCNDCCGWHASVFWTPILWTQ----
Loxodonta_africana           ---------------------------MGLWMHLDVCNEHCGWHAPVFWIPMHRTP----
Leucoraja_erinacea           -------------------------------MHLDVCNEHCGWHAPVFWMPMCRTP----
Mesocricetus_auratus         ---------------------------MGLWMHLDVCNEHCGWHAPVFWIPMHRTP----
Salmo_salar                  -------------------------------MHLDVCNECCGWHASVFWTPMLWTQ----
Tupaia_chinensis             ---------------------------MGLWMHLDVCNEHCGWHAPVFWIPMHRTP----
Lepisosteus_oculatus         -------------------------------MHLDVCNEHCGWHAPVFWIPMLRTQ----
Trichomycterus_areolatus     -------------------------------MHLDVCHACSGWYASVFGIAQLWTH----
Trichechus_manatus_latirostr ---------------------------MDLWMHLDVCNEHCGWHAPVFWIPMHRTP----
Aptenodytes_forsteri         -------------------------------MHLDVCNEHCGWHAPVFWIPMHRTP----
Squalus_acanthias            -------------------------------MHLDVCNEHCGWHAPVFWMPMYRTP----
Thamnophis_sirtalis          ---------------------------MSLWMHLDVCNEHCGWHAPVFWIPMHRNP----
Tyto_alba                    -------------------------------MHLDVCNEHCGWHAPVFWIPMHRTP----
Struthio_camelus_australis   -------------------------------MHLDVCNEHCGWHAPVFWIPMHRTP----
Chelonia_mydas               -------------------------------MHLDVCNEHCGWHAPVFWIPMHRTP----
Takifugu_rubripes            -------------------------------MHLDVCNDCCGWHASVFWTPILWTQ----
Tinamus_guttatus             -------------------------------MHLDVCNEHCGWHAPVFWISMHRTP----
Calypte_anna                 -------------------------------MHLDVCNEHCGWHAPVFWIPMHRTP----
Apaloderma_vittatum          -------------------------------MHLDVCNEHCGWHAPVFWIPMHRTP----
Orycteropus_afer_afer        ---------------------------MGFWMHLDVCNEHCGWHAPVFWIPMHRTP----
```

Conservation

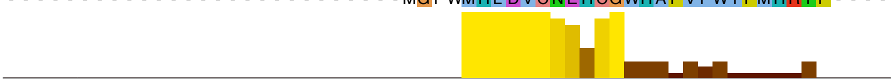

```
-------------------------------******+95+*33313231111130----
```

HG0029

ENSG00000168137

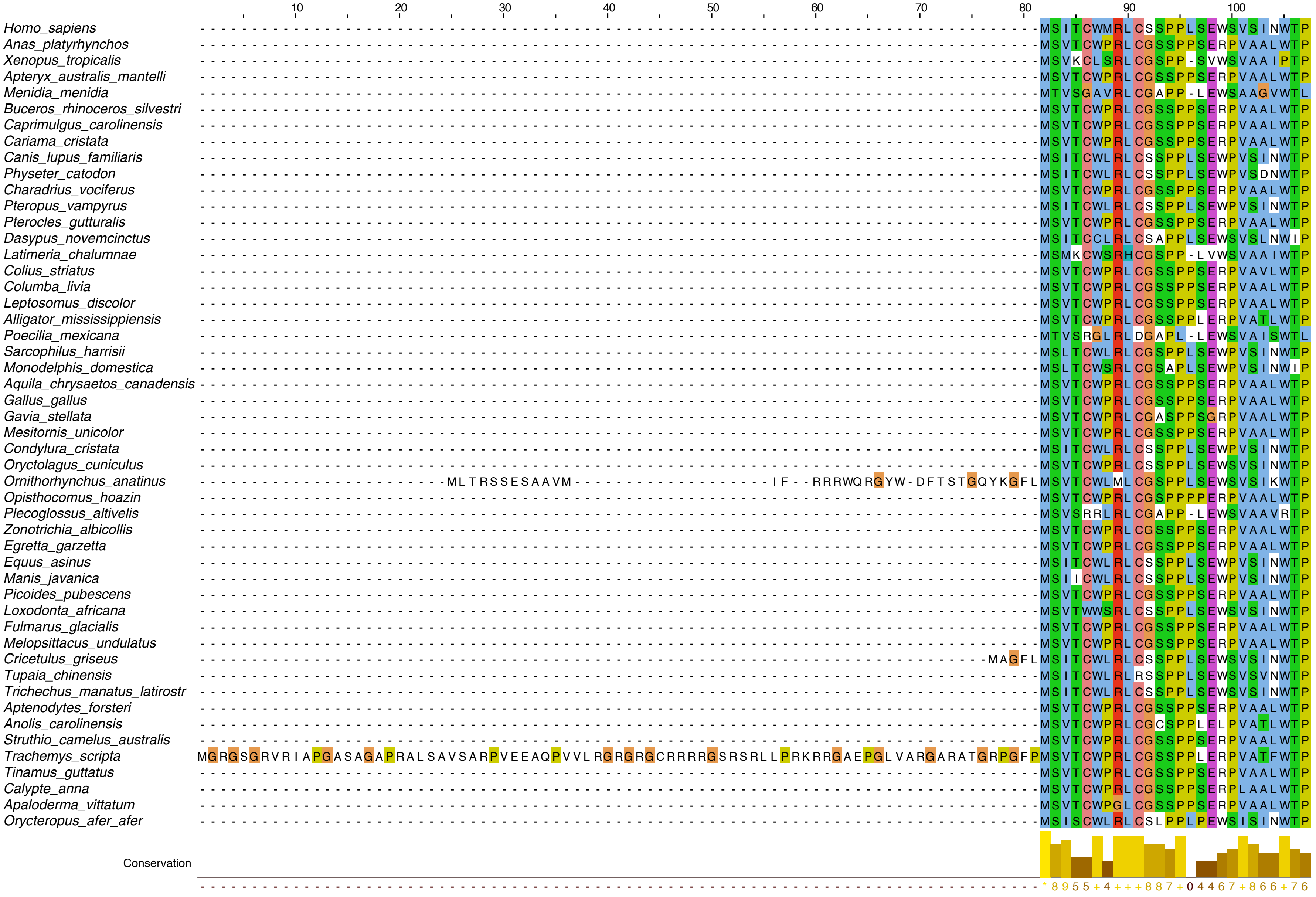

HG0029

ENSGALG00000008167

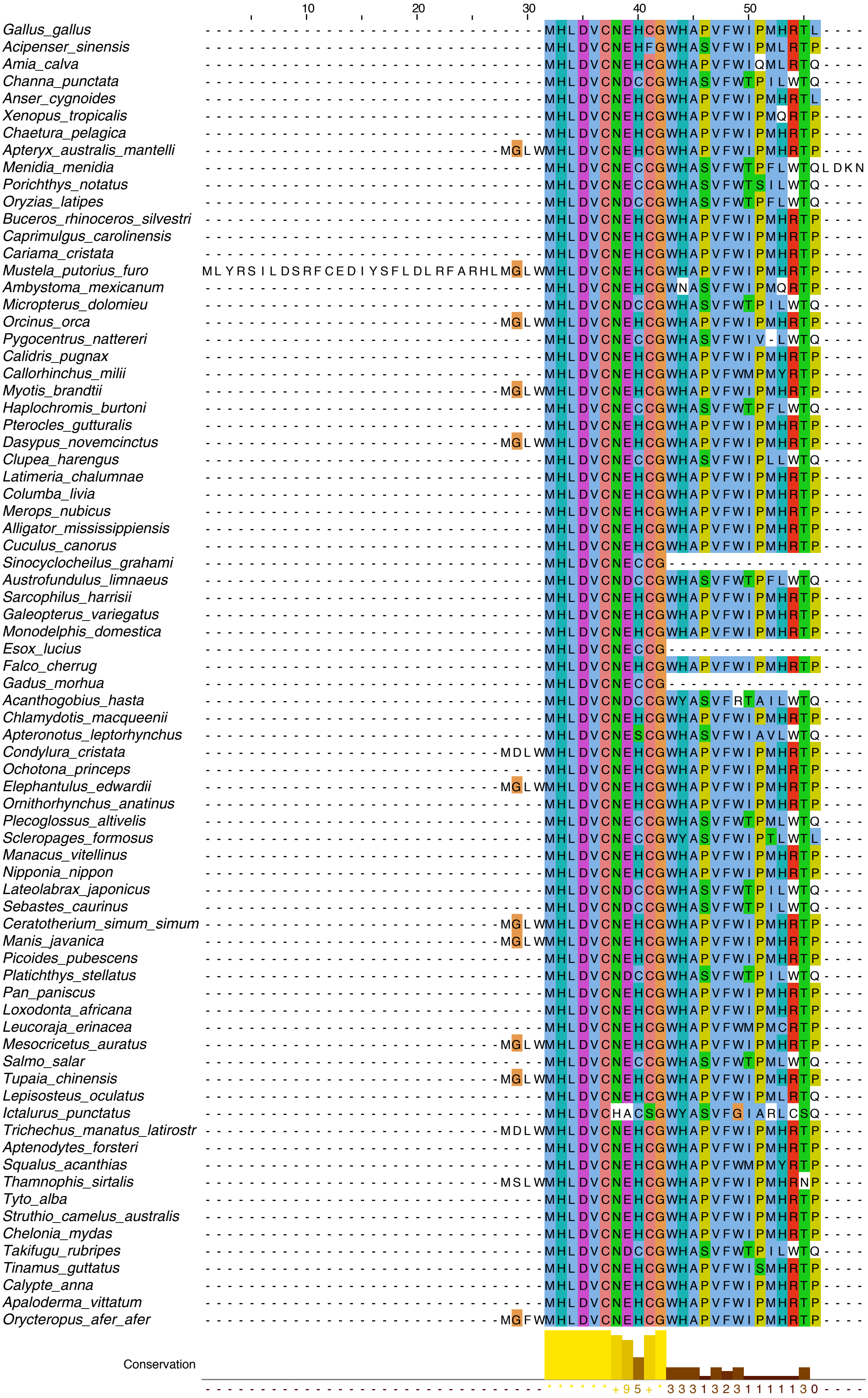

HG0030.1

ENSG00000100664 (EIF5)

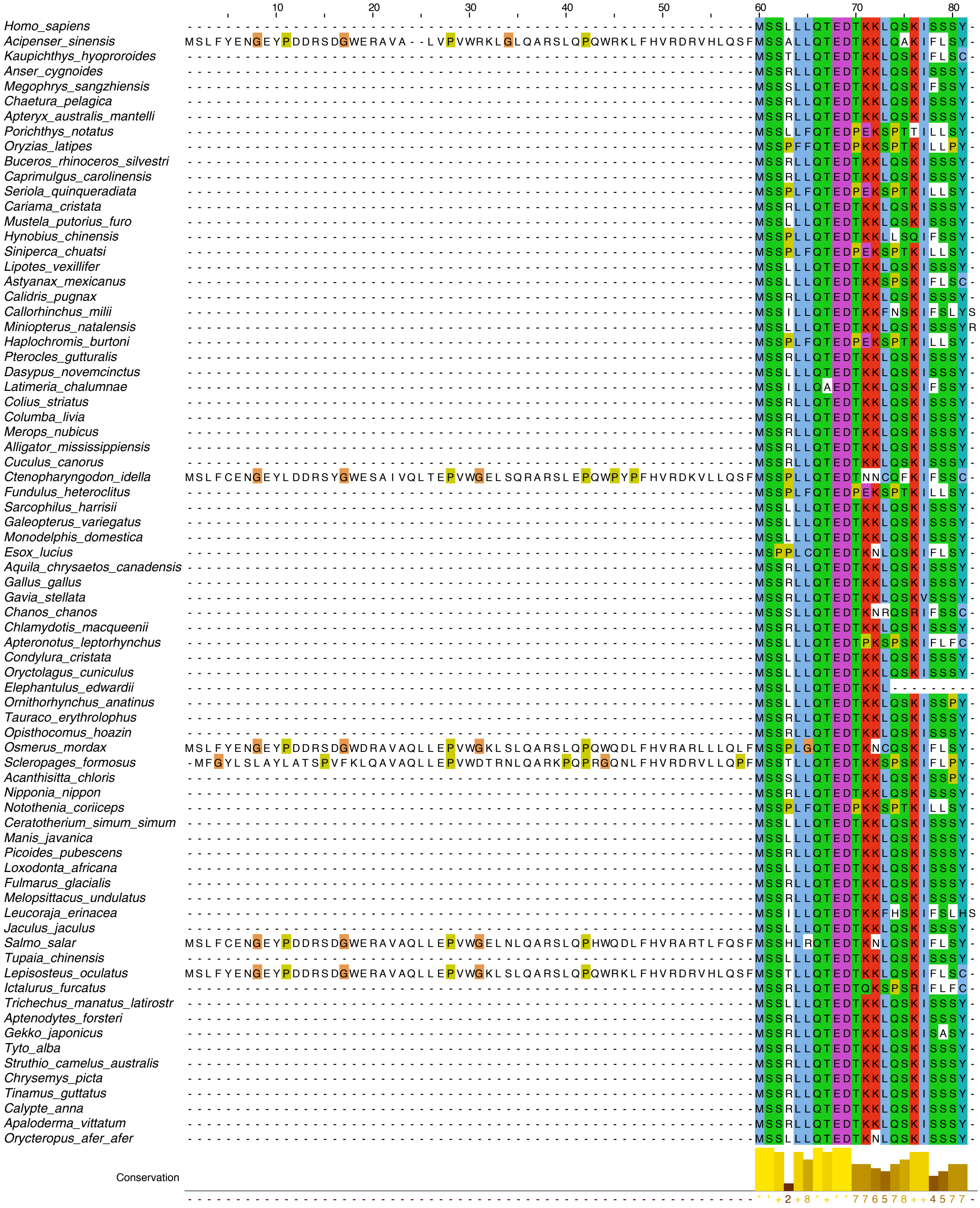

HG0030.1

ENSGALG00000011464 (EIF5)

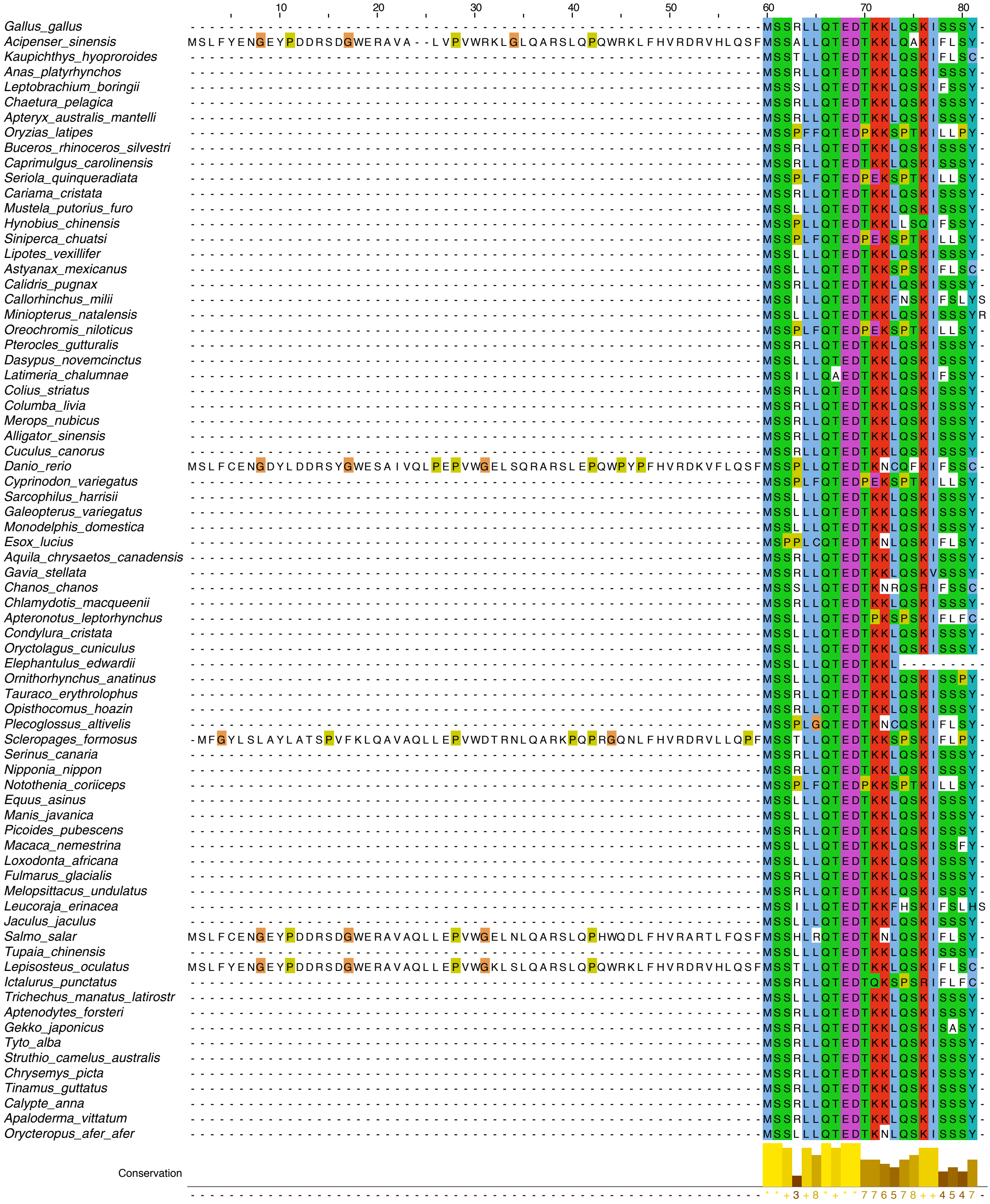

HG0030.2

FBgn0030719 (eIF5)

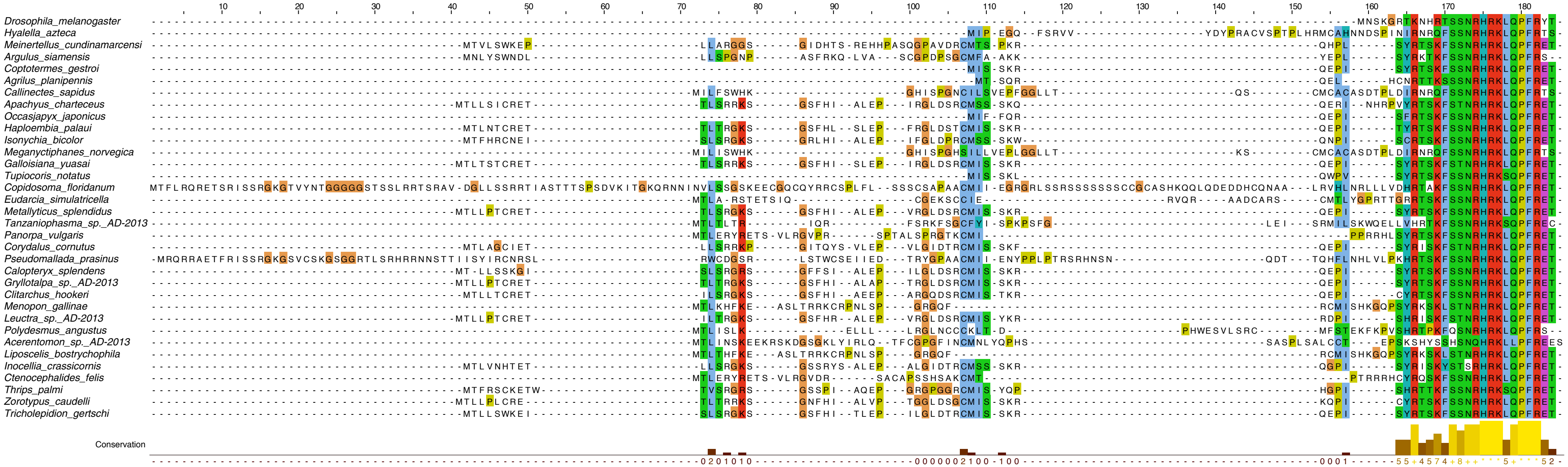

HG0031

ENSDARG00000000540 (asnsd1)

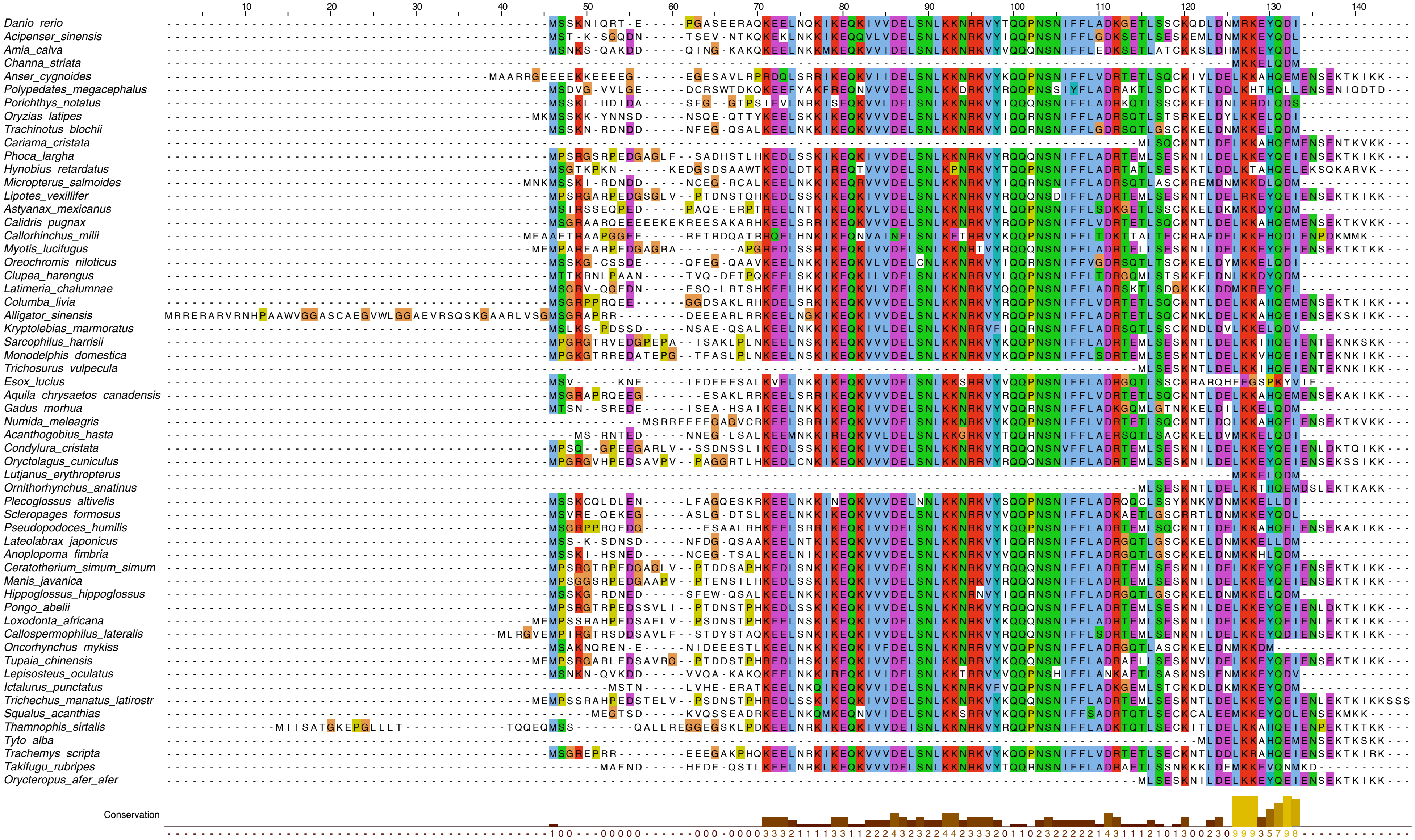

HG0031

ENSG00000138381 (ASNSD1)

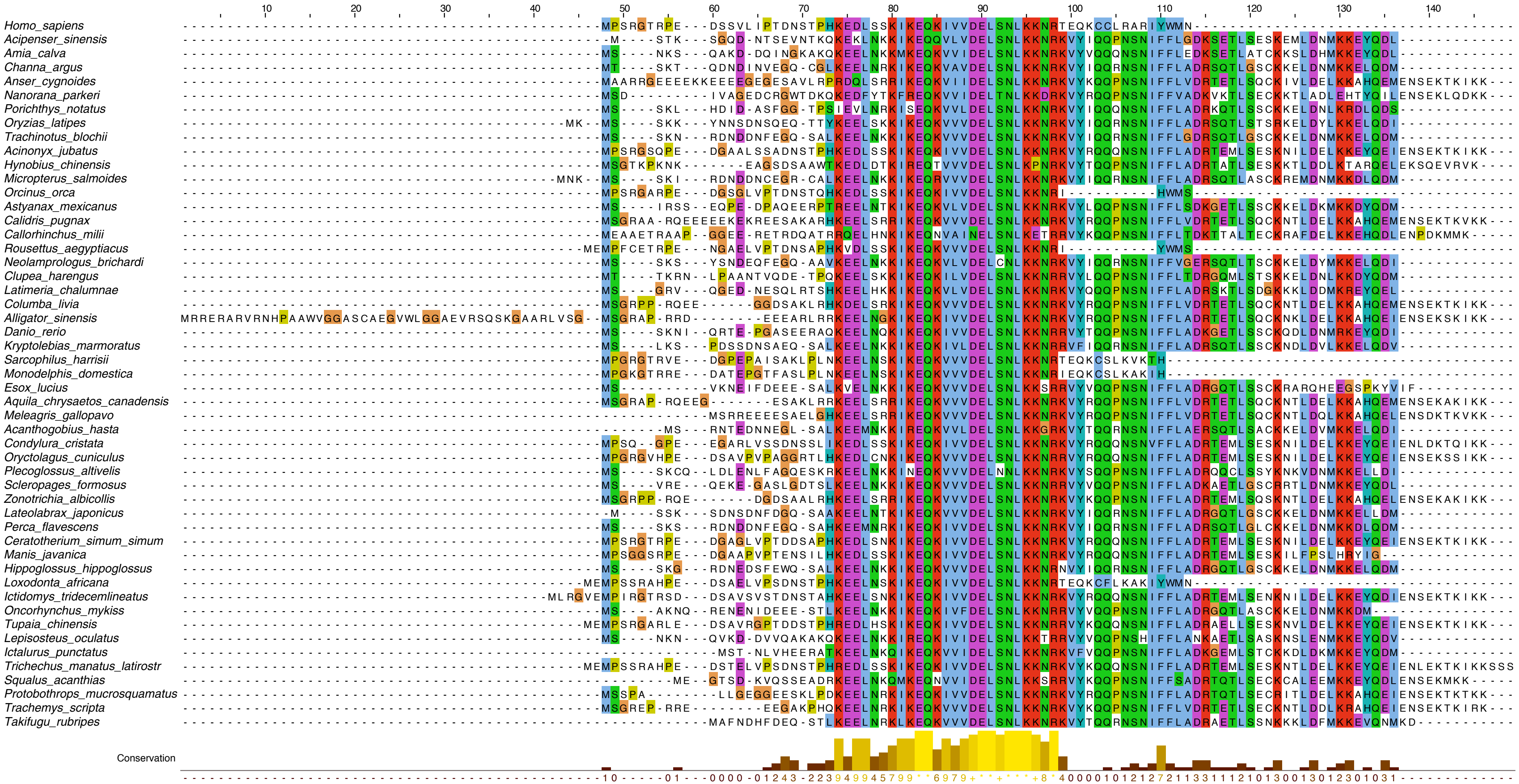

HG0031

ENSGALG00000041666

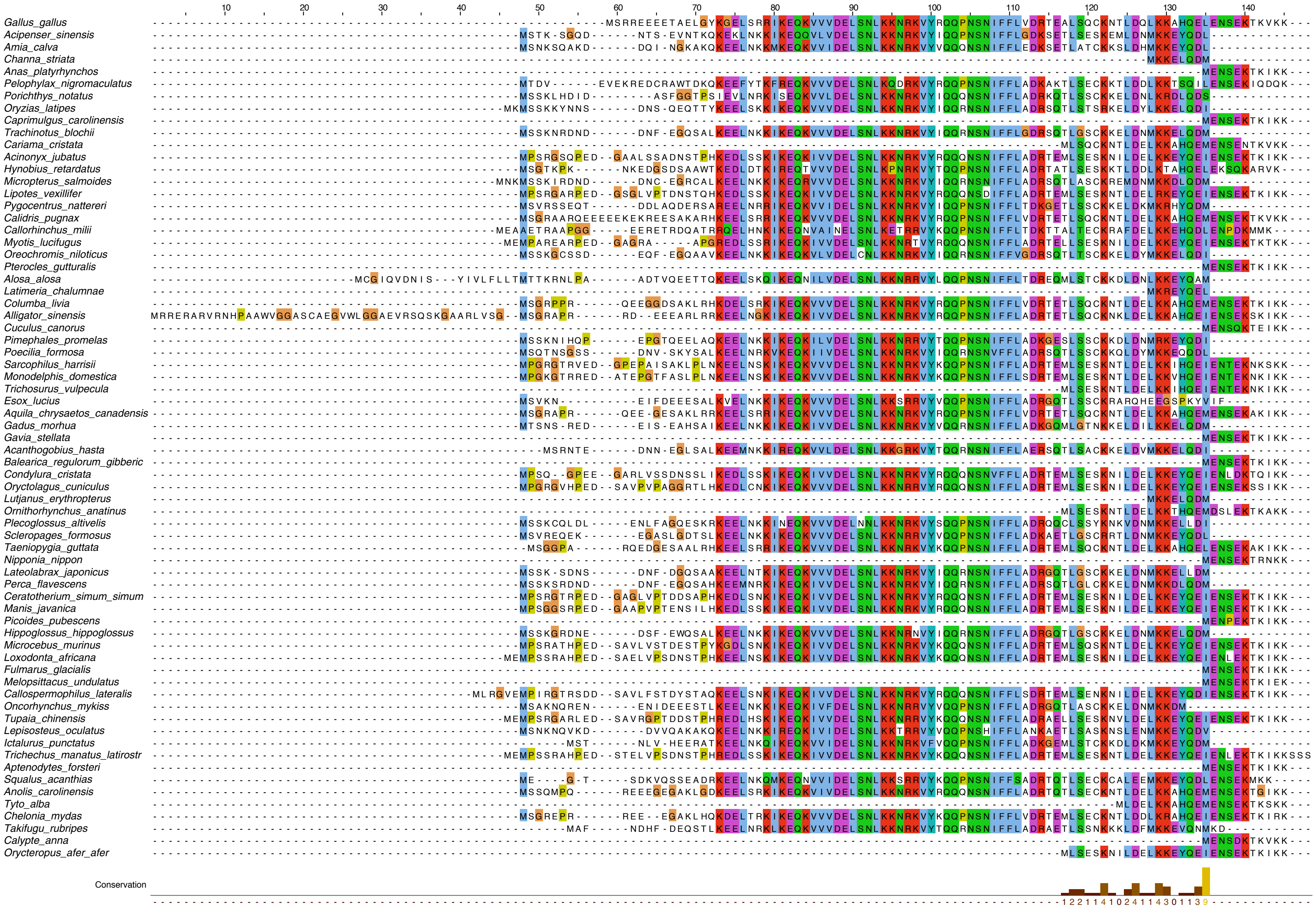

HG0032

ENSDARG00000104981 (SLC45A4)

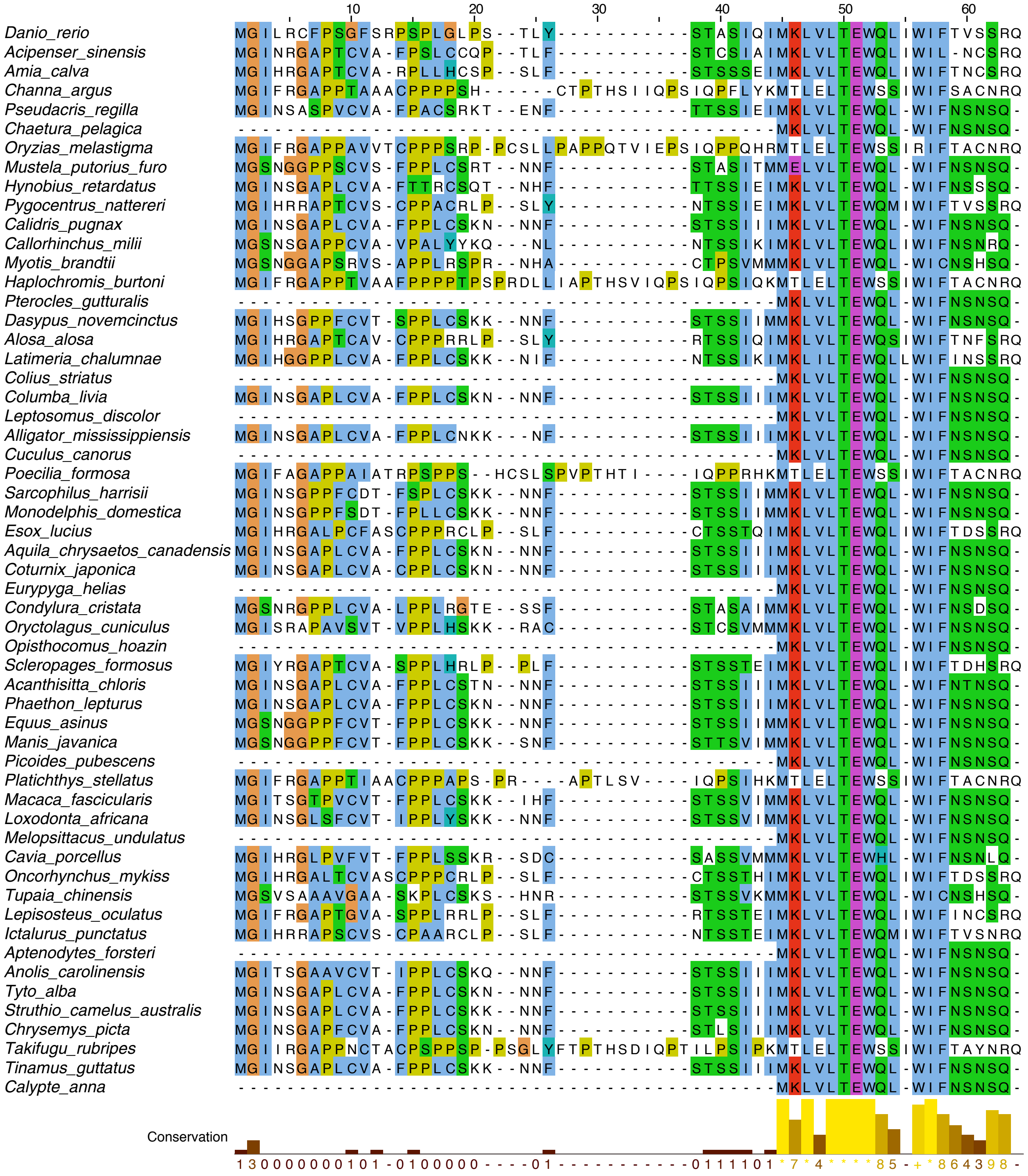

HG0033

ENSG00000112144 (ICK)

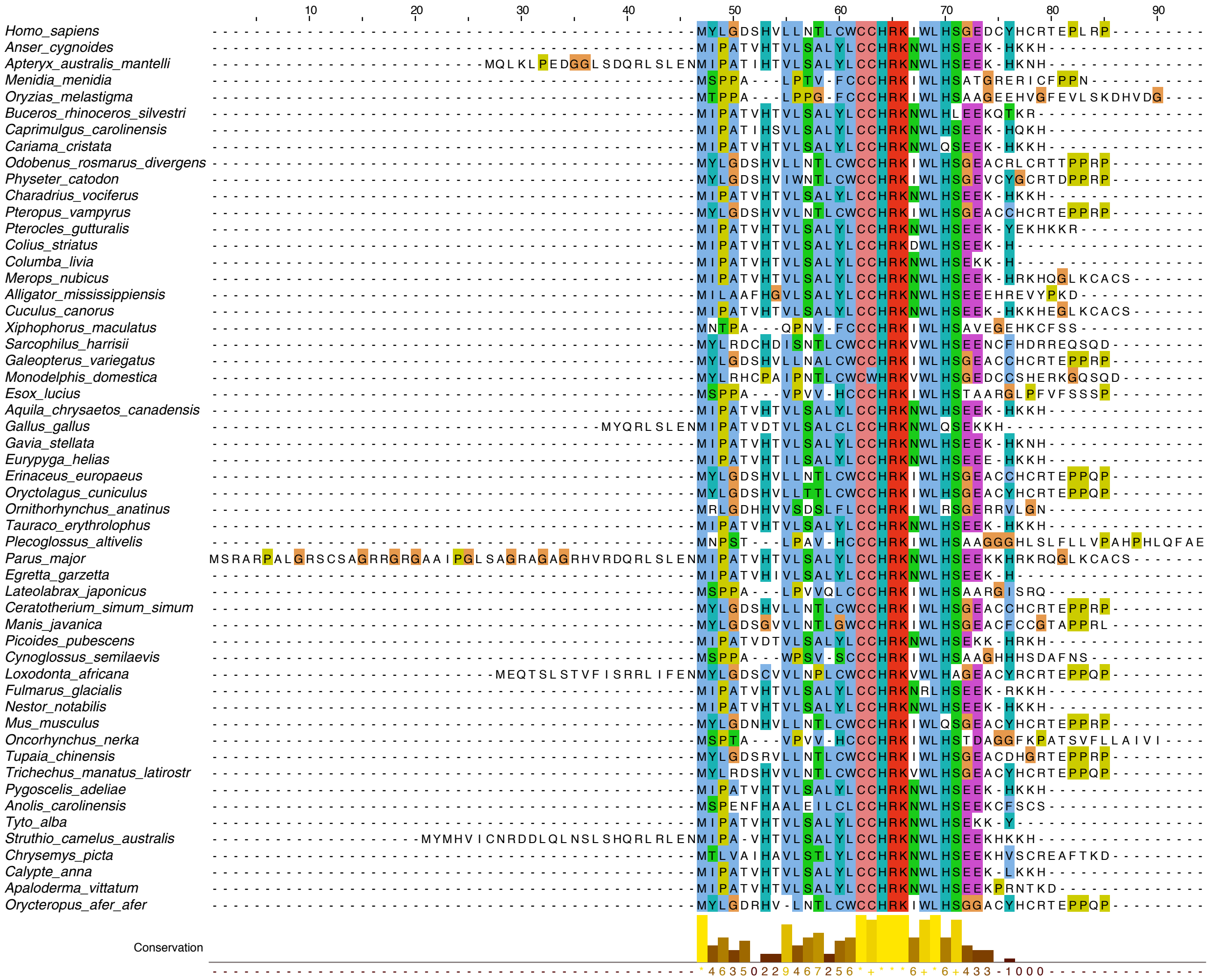

HG0033

ENSGALG00000016321 (ICK)

```
                                      10        20        30        40        50        60
Gallus_gallus                 --------------------------MIPATVDTVLSALCLCCHRKNWLQSEKKH-----------
Anas_platyrhynchos            ----------MRVWSGQADQRLSLENMIPATVDTVLSALYLCCHRKNWLQSEEQHKNSGH------
Apteryx_australis_mantelli    --MQL--KLPEDGGLSGNDQRLSLENMIPATIHTVLSALYLCCHRKNWLHSEEKHKNH--------
Buceros_rhinoceros_silvestri  --------------------------MIPATVHTVLSALYLCCHRKNWLHLEEKQTKR--------
Caprimulgus_carolinensis      --------------------------MIPATIHSVLSALYLCCHRKNWLHSEEKHQKH--------
Cariama_cristata              --------------------------MIPATVHTVLSALYLCCHRKNWLQSEEKHKKH--------
Ailuropoda_melanoleuca        --------------------------MYLGDSHVLLNTLCWCCHRKIWLHSGEACRLCRTAPPRP-
Balaenoptera_acutorostrata_s  --------------------------MYLGDSHVLLSTLCWCCHRKIWLHSGEVCCGCRTDPPRP-
Charadrius_vociferus          --------------------------MIPATVHTVLSALYLCCHRKNWLHSEEKHKKH--------
Pteropus_alecto               --------------------------MYLGDSHVVLNTLCWCCHRKIWLHSGEACCHCRTEPPRP-
Pterocles_gutturalis          --------------------------MIPATVHTVLSALYLCCHRKNWLHSEEKYEKHKKR-----
Latimeria_menadoensis         --------------------------MSTVDLPVLLCYMYCCCHRKGWLHSKK-------------
Colius_striatus               --------------------------MIPATVHTVLSALYLCCHRKDWLHSEEKH-----------
Columba_livia                 --------------------------MIPATVHTVLSALYLCCHRKNWLHSEKKH-----------
Leptosomus_discolor           --------------------------MIPATVHTVLSALYLCCHRKNWLYSEKKH-----------
Alligator_mississippiensis    --------------------------MILAAFHGVLSALYLCCHRKNWLHSEEEHREVYPKD----
Cuculus_canorus               --------------------------MIPATVHTVLSALYLCCHRKNWLHSEEKHKKHEGLKCACS
Sarcophilus_harrisii          --------------------------MYLRDCHDISNTLCWCCHRKVWLHSEENCFHDRREQSQD-
Galeopterus_variegatus        --------------------------MYLGDSHVLLNALCWCCHRKIWLHSGEACCHCRTEPPRP-
Haliaeetus_leucocephalus      --------------------------MIPATVHTVLSALYLCCHRKNWLHSEEKH-----------
Gavia_stellata                --------------------------MIPATVHTVLSALYLCCHRKNWLHSEEKHKNH--------
Balearica_regulorum_gibberic  --------------------------MIPATVHTVLSALYLCCHRKNWLHSEEKHKKH--------
Erinaceus_europaeus           --------------------------MYLGDSHVLLNTLCWCCHRKIWLHSGEACCHCRTEPPQP-
Oryctolagus_cuniculus         --------------------------MYLGDSHVLLTTLCWCCHRKIWLHSGEACYHCRTEPPQP-
Tauraco_erythrolophus         --------------------------MIPATVHTVLSALYLCCHRKNWLHSEEKHKKH--------
Opisthocomus_hoazin           --------------------------MIHATVLTVLSALYLCCHRKNWLHLEEKHRKHYGLKCACS
Lepidothrix_coronata          --------------------------MIPATVHTVLSALYLCCHRKNWLQSKEKHKKH--------
Nipponia_nippon               --------------------------MIPATVHTVLSALYLCCHRKNWLHSEEKHKKH--------
Equus_asinus                  --------------------------MYLGDSHVLLNTLCWCCHRKIWLHSGEACCRCRTGPPRP-
Manis_javanica                --------------------------MYLGDSGVVLNTLGWCCHRKIWLHSGEACFCCGTAPPRL-
Picoides_pubescens            --------------------------MIPATVDTVLSALYLCCHRKNWLHSEKKHRKH--------
Homo_sapiens                  --MG-YSFFPRSCGSLLPGRRLIFGNMYLGDSHVLLNTLCWCCHRKIWLHSGEDCYHCRTEPLRP-
Loxodonta_africana            -------MEQTSLSTVFISRRLIFENMYLGDSCVVLNPLCWCCHRKVWLHAGEACYRCRTEPPQP-
Fulmarus_glacialis            --------------------------MIPATVHTVLSALYLCCHRKNRLHSEEKRKKH--------
Nestor_notabilis              --------------------------MIPATVHTVLSALYLCCHRKNWLHSEEKHKKH--------
Cricetulus_griseus            --------------------------MYLGDNHVLLNTLCWCCHRKIWLQSGEACYCCRTDPPRP-
Tupaia_chinensis              --------------------------MYLGDSRVLLNTLCWCCHRKIWLHSGEACDHGRTEPPRP-
Trichechus_manatus_latirostr  --------------------------MYLRDSHVVLNTLCWCCHRKVWLHSGEACYHCRTEPPQP-
Aptenodytes_forsteri          --------------------------MIPATVHTVLSALYLCCHRKNWLHSEEKHKKH--------
Anolis_carolinensis           --------------------------MSPENFHAALEILCLCCHRKNWLHSEEKCFSCS-------
Tyto_alba                     --------------------------MIPATVHTVLSALYLCCHRKNWLHSEKKY-----------
Struthio_camelus_australis    MYMHVICNRDDLQLNSLSHQRLRLENMIPA-VHTVLSALYLCCHRKNWLHSEEKHKKH--------
Chrysemys_picta               --------------------------MTLVAIHAVLSTLYLCCHRKNWLHSEEKHVSCREAFTKD-
Calypte_anna                  --------------------------MIPATVHTVLSALYLCCHRKNWLHSEEKLKKH--------
Apaloderma_vittatum           --------------------------MIPATVHTVLSALYLCCHRKNWLHSEEKPRNTKD------
Orycteropus_afer_afer         --------------------------MYLGDRHV-LNTLCWCCHRKIWLHSGGACYHCRTEPPQP-
```

Conservation

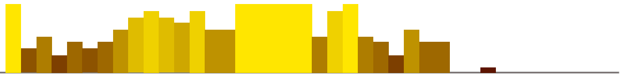

```
--------------------------*4635457 9+98+77*****6+*653755001--------
```

HG0034

ENSDARG00000060991 (mief1)

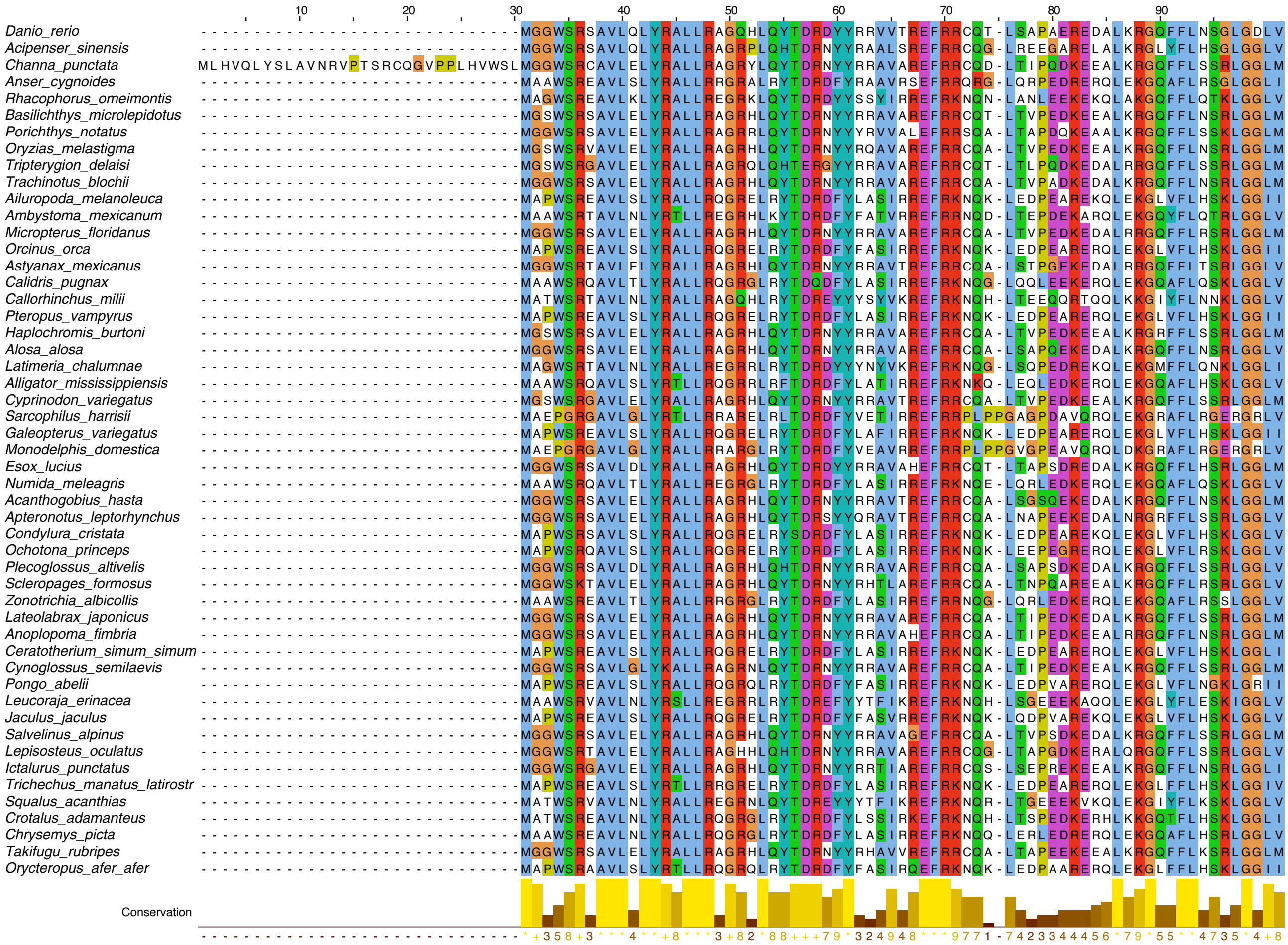

HG0034

ENSG00000100335 (MIEF1)

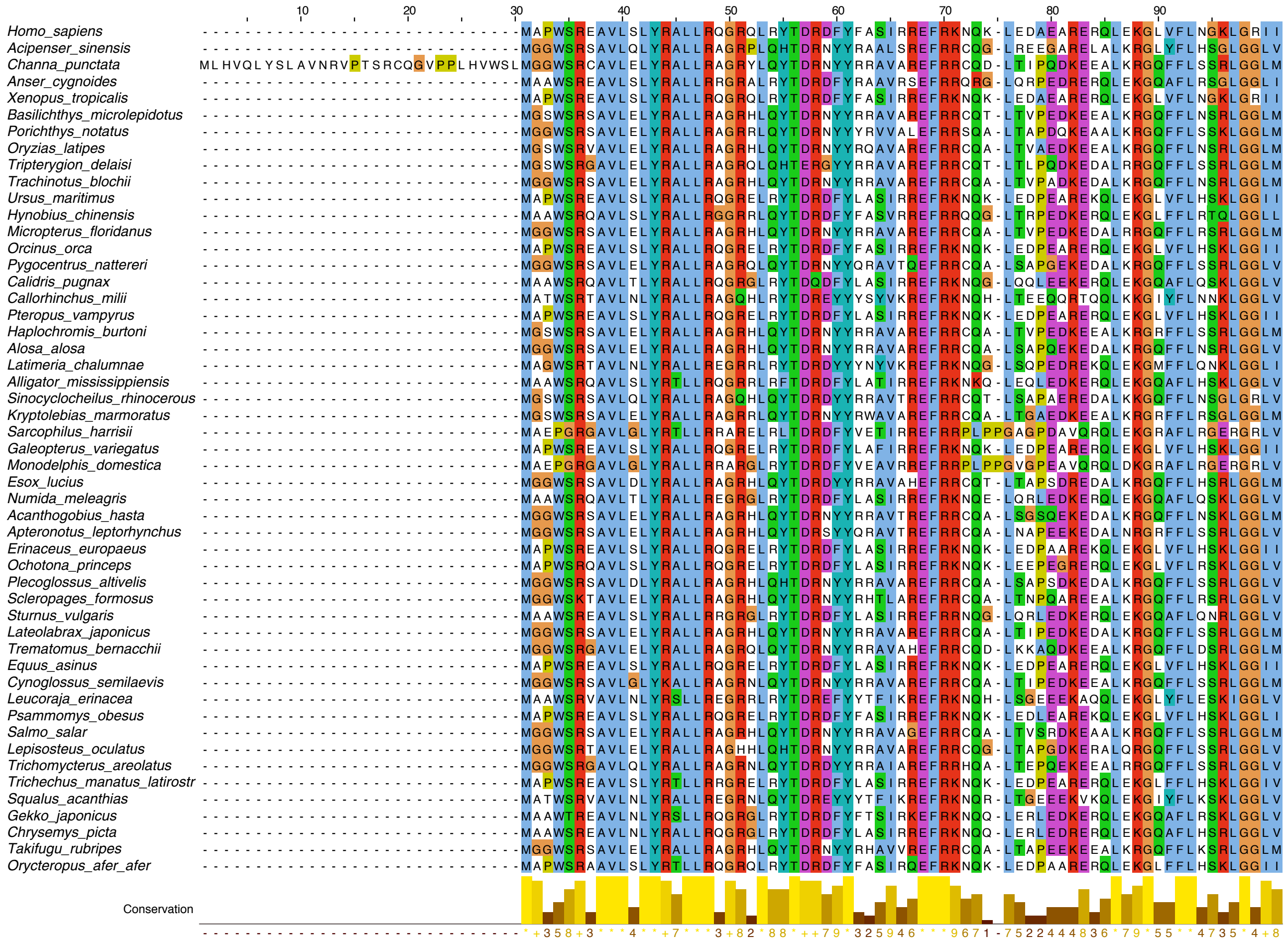

HG0035

ENSG00000197147 (LRRC8B)

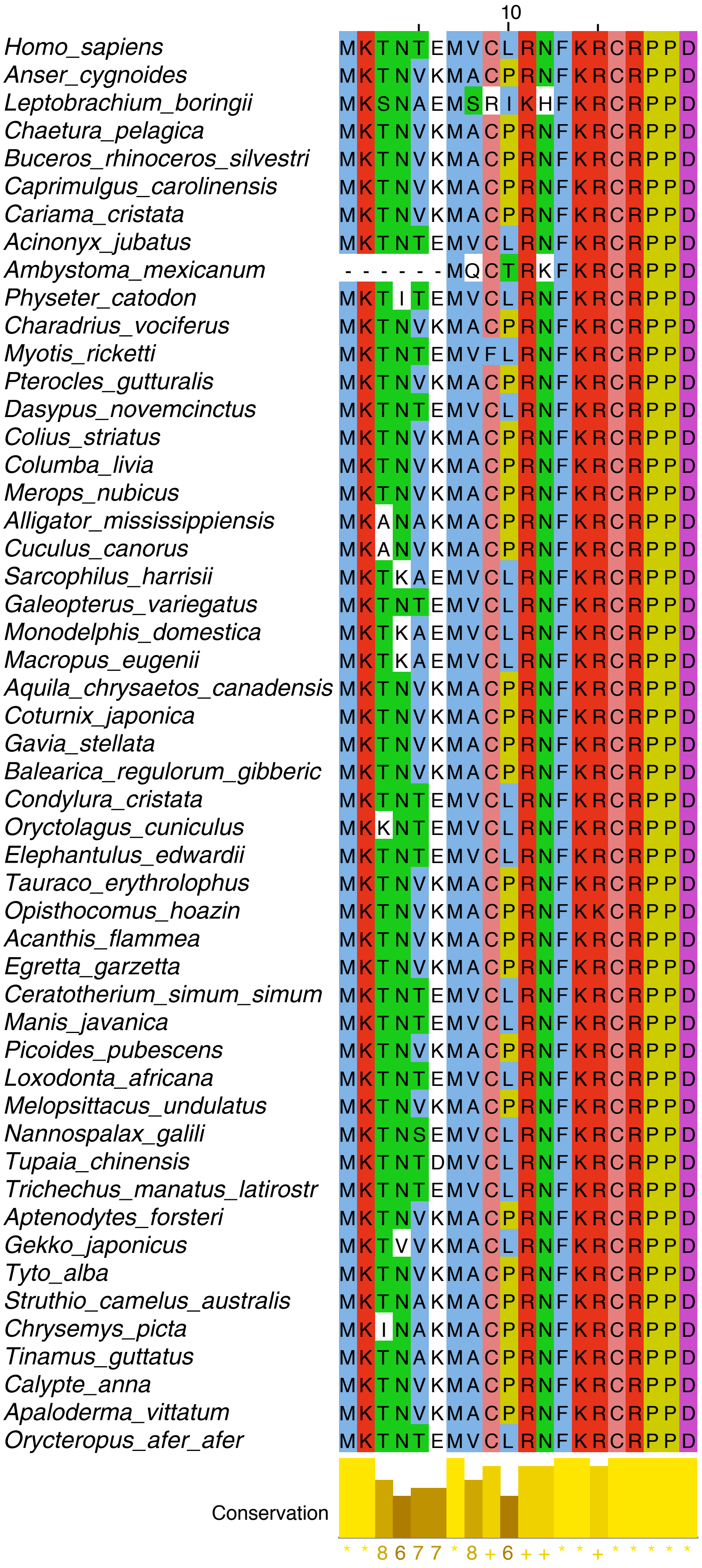

HG0035

ENSGALG00000006167 (LRRC8B)

```
                              10        20        30        40
Gallus_gallus                 ----------------------MKTNVKMACPRNFKRCRPPD
Anser_cygnoides               ----------------------MKTNVKMACPRNFKRCRPPD
Leptobrachium_boringii        ----------------------MKSNAEMSRIKHFKRCRPPD
Chaetura_pelagica             ----------------------MKTNVKMACPRNFKRCRPPD
Buceros_rhinoceros_silvestri  ----------------------MKTNVKMACPRNFKRCRPPD
Caprimulgus_carolinensis      ----------------------MKTNVKMACPRNFKRCRPPD
Cariama_cristata              ----------------------MKTNVKMACPRNFKRCRPPD
Felis_catus                   --------------MLAAHKCRMKTNTEMVCLRNFKRCRPPD
Ambystoma_mexicanum           ----------------------------MQCTRKFKRCRPPD
Physeter_catodon              ----------------------MKTITEMVCLRNFKRCRPPD
Charadrius_vociferus          ----------------------MKTNVKMACPRNFKRCRPPD
Myotis_ricketti               ----------------------MKTNTEMVFLRNFKRCRPPD
Pterocles_gutturalis          ----------------------MKTNVKMACPRNFKRCRPPD
Dasypus_novemcinctus          ----------------------MKTNTEMVCLRNFKRCRPPD
Colius_striatus               ----------------------MKTNVKMACPRNFKRCRPPD
Columba_livia                 ----------------------MKTNVKMACPRNFKRCRPPD
Merops_nubicus                ----------------------MKTNVKMACPRNFKRCRPPD
Alligator_mississippiensis    ----------------------MKANAKMACPRNFKRCRPPD
Cuculus_canorus               ----------------------MKANVKMACPRNFKRCRPPD
Sarcophilus_harrisii          ----------------------MKTKAEMVCLRNFKRCRPPD
Galeopterus_variegatus        ----------------------MKTNTEMVCLRNFKRCRPPD
Monodelphis_domestica         ----------------------MKTKAEMVCLRNFKRCRPPD
Macropus_eugenii              ----------------------MKTKAEMVCLRNFKRCRPPD
Aquila_chrysaetos_canadensis  ----------------------MKTNVKMACPRNFKRCRPPD
Gavia_stellata                ----------------------MKTNVKMACPRNFKRCRPPD
Balearica_regulorum_gibberic  ----------------------MKTNVKMACPRNFKRCRPPD
Condylura_cristata            ----------------------MKTNTEMVCLRNFKRCRPPD
Oryctolagus_cuniculus         ----------------------MKKNTEMVCLRNFKRCRPPD
Elephantulus_edwardii         ----------------------MKTNTEMVCLRNFKRCRPPD
Tauraco_erythrolophus         ----------------------MKTNVKMACPRNFKRCRPPD
Opisthocomus_hoazin           ----------------------MKTNVKMACPRNFKKCRPPD
Acanthis_flammea              ----------------------MKTNVKMACPRNFKRCRPPD
Egretta_garzetta              ----------------------MKTNVKMACPRNFKRCRPPD
Ceratotherium_simum_simum     ----------------------MKTNTEMVCLRNFKRCRPPD
Manis_javanica                ----------------------MKTNTEMVCLRNFKRCRPPD
Picoides_pubescens            ----------------------MKTNVKMACPRNFKRCRPPD
Homo_sapiens                  ----------------------MKTNTEMVCLRNFKRCRPPD
Loxodonta_africana            ----------------------MKTNTEMVCLRNFKRCRPPD
Melopsittacus_undulatus       ----------------------MKTNVKMACPRNFKRCRPPD
Nannospalax_galili            ----------------------MKTNSEMVCLRNFKRCRPPD
Tupaia_chinensis              ----------------------MKTNTDMVCLRNFKRCRPPD
Trichechus_manatus_latirostr  ----------------------MKTNTEMVCLRNFKRCRPPD
Aptenodytes_forsteri          ----------------------MKTNVKMACPRNFKRCRPPD
Anolis_carolinensis           ----------------------MKTLVRMACPRNFKRCRPPD
Tyto_alba                     ----------------------MKTNVKMACPRNFKRCRPPD
Struthio_camelus_australis    ----------------------MKTNAKMACPRNFKRCRPPD
Chelonia_mydas                MRVLFWKREPTELESHLKCLSRMKINAKMACPRNFKRCRPPD
Tinamus_guttatus              ----------------------MKTNAKMACPRNFKRCRPPD
Calypte_anna                  ----------------------MKTNVKMACPRNFKRCRPPD
Apaloderma_vittatum           ----------------------MKTNVKMACPRNFKRCRPPD
Orycteropus_afer_afer         ----------------------MKTNTEMVCLRNFKRCRPPD

Conservation                  ----------------------**8677*8+6++**+*****
```

HG0036

ENSG00000155096 (AZIN1)

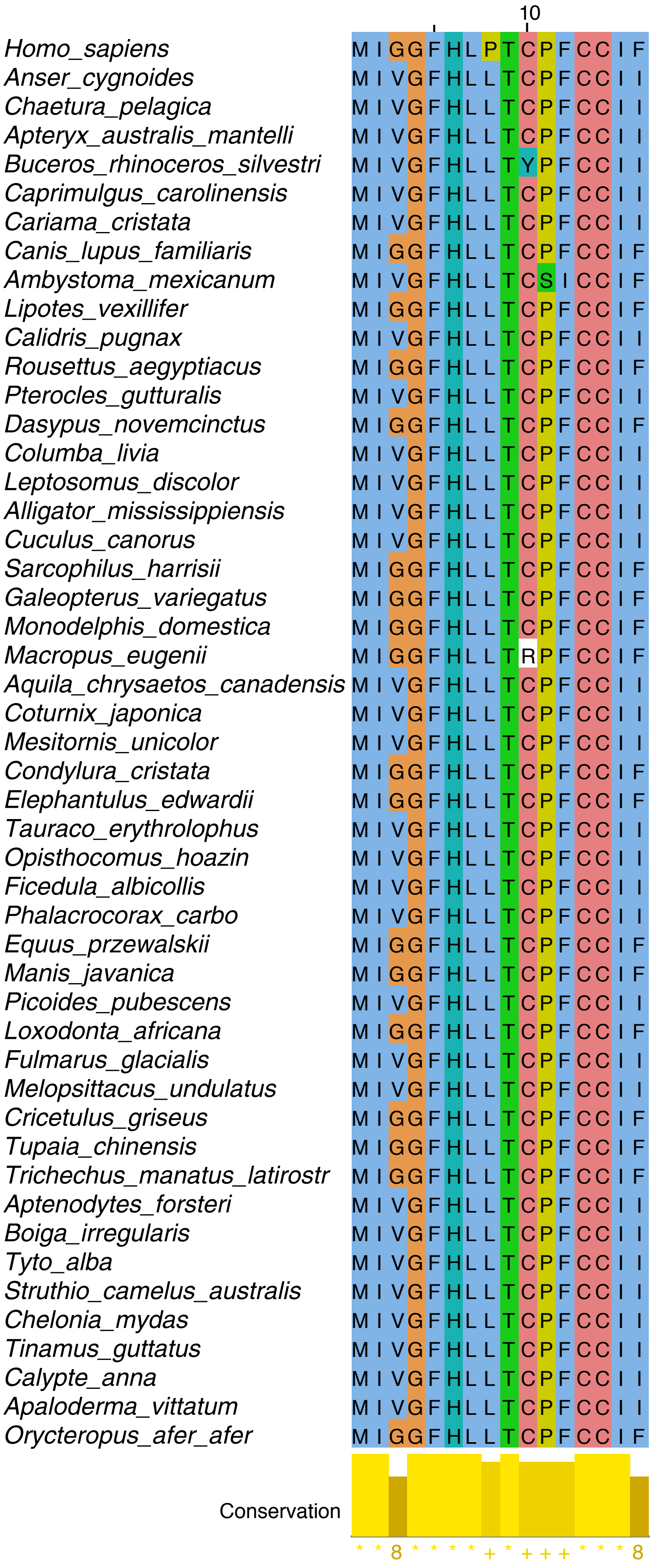

HG0036

ENSGALG00000029295

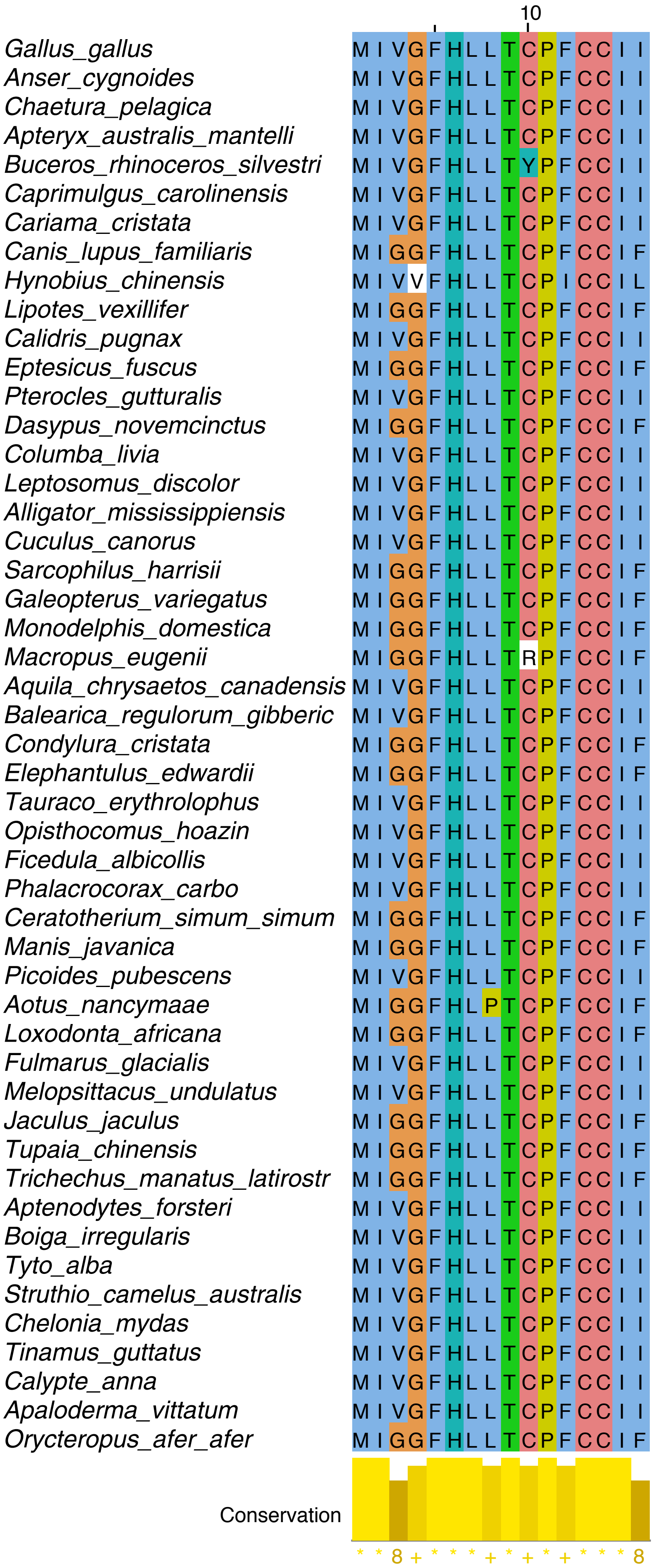

HG0037

ENSG00000140092 (FBLN5)

Species	Sequence (positions 1–13; tick at 10)
Homo_sapiens	MKCSFLTFLLQLV
Anser_cygnoides	MKCSFLTFLLQLV
Xenopus_laevis	MKCSFLTFLQQLV
Chaetura_pelagica	MKCSFLTFLLQLV
Apteryx_australis_mantelli	MKCSFLTFLLQLV
Buceros_rhinoceros_silvestri	MKCSFLTFLLQLV
Caprimulgus_carolinensis	MKCSFLTFLLQLV
Cariama_cristata	MKCSFLTFLLQLV
Felis_catus	MKCSFLKFLLQLV
Hynobius_retardatus	MKCSFLTFLLQLV
Balaenoptera_acutorostrata_s	MKCSFLTFLLQLV
Calidris_pugnax	MKCSFLTFLLQLV
Myotis_brandtii	MKCSFLTFLLQLV
Pterocles_gutturalis	MKCSFLTFLLQLV
Dasypus_novemcinctus	MKCSFLAFLLQLV
Colius_striatus	MKCSFLTFLLQLV
Columba_livia	MKCSFLTFLLQLV
Leptosomus_discolor	MKCSFLTFLLQLV
Cuculus_canorus	MKCSFLTFLLQLV
Monodelphis_domestica	MKCSFLTFLLQLV
Trichosurus_vulpecula	MKCSFLTFLLQLV
Aquila_chrysaetos_canadensis	MKCSFLTFLLQLV
Coturnix_japonica	MKCSFLTFLLQLV
Gavia_stellata	MKCSFLTFLLQLV
Chlamydotis_macqueenii	MKCSFLTFLLQLV
Condylura_cristata	MKCSFLTFLLQLV
Oryctolagus_cuniculus	MKCSFLTFLLQLV
Ornithorhynchus_anatinus	MKCSFLTFLLQLV
Tauraco_erythrolophus	MKCSFLTFLLQLV
Opisthocomus_hoazin	MKCSFLTFLLQLV
Corvus_brachyrhynchos	MKCSFLTFLLQLV
Egretta_garzetta	MKCSFLTFLLQLV
Ceratotherium_simum_simum	MKCSFLTFLLQLV
Picoides_pubescens	MKCSFLTFLLQLV
Loxodonta_africana	MKCSFLTFLLQLV
Fulmarus_glacialis	MKCSFLTFLLQLV
Nestor_notabilis	MKCSFLTFLLQLV
Chinchilla_lanigera	MKCSFLTFLLQLV
Tupaia_chinensis	MKCSFLTFLLQLV
Trichechus_manatus_latirostr	MKCSFLTFLLQLV
Aptenodytes_forsteri	MKCSFLTFLLQLV
Tyto_alba	MKCSFLTFLLQLV
Struthio_camelus_australis	MKCSFLTFLLQLV
Pelodiscus_sinensis	MKCSFLTFLLQLV
Tinamus_guttatus	MKCSFLTFLLQLV
Calypte_anna	MKCSFLTFLLQLV
Apaloderma_vittatum	MKCSFLTFLLQLV

Conservation

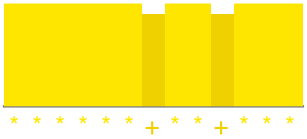

HG0037

ENSGALG00000010747 (FBLN5)

	10
Gallus_gallus	MKCSFLTFLLQLV
Anser_cygnoides	MKCSFLTFLLQLV
Xenopus_laevis	MKCSFLTFLQQLV
Chaetura_pelagica	MKCSFLTFLLQLV
Apteryx_australis_mantelli	MKCSFLTFLLQLV
Buceros_rhinoceros_silvestri	MKCSFLTFLLQLV
Caprimulgus_carolinensis	MKCSFLTFLLQLV
Cariama_cristata	MKCSFLTFLLQLV
Felis_catus	MKCSFLKFLLQLV
Hynobius_retardatus	MKCSFLTFLLQLV
Balaenoptera_acutorostrata_s	MKCSFLTFLLQLV
Calidris_pugnax	MKCSFLTFLLQLV
Myotis_brandtii	MKCSFLTFLLQLV
Pterocles_gutturalis	MKCSFLTFLLQLV
Dasypus_novemcinctus	MKCSFLAFLLQLV
Colius_striatus	MKCSFLTFLLQLV
Columba_livia	MKCSFLTFLLQLV
Leptosomus_discolor	MKCSFLTFLLQLV
Cuculus_canorus	MKCSFLTFLLQLV
Monodelphis_domestica	MKCSFLTFLLQLV
Trichosurus_vulpecula	MKCSFLTFLLQLV
Aquila_chrysaetos_canadensis	MKCSFLTFLLQLV
Gavia_stellata	MKCSFLTFLLQLV
Chlamydotis_macqueenii	MKCSFLTFLLQLV
Condylura_cristata	MKCSFLTFLLQLV
Oryctolagus_cuniculus	MKCSFLTFLLQLV
Ornithorhynchus_anatinus	MKCSFLTFLLQLV
Tauraco_erythrolophus	MKCSFLTFLLQLV
Opisthocomus_hoazin	MKCSFLTFLLQLV
Corvus_brachyrhynchos	MKCSFLTFLLQLV
Egretta_garzetta	MKCSFLTFLLQLV
Ceratotherium_simum_simum	MKCSFLTFLLQLV
Picoides_pubescens	MKCSFLTFLLQLV
Aotus_nancymaae	MKCSFLTFLLQLV
Loxodonta_africana	MKCSFLTFLLQLV
Fulmarus_glacialis	MKCSFLTFLLQLV
Nestor_notabilis	MKCSFLTFLLQLV
Chinchilla_lanigera	MKCSFLTFLLQLV
Tupaia_chinensis	MKCSFLTFLLQLV
Trichechus_manatus_latirostr	MKCSFLTFLLQLV
Aptenodytes_forsteri	MKCSFLTFLLQLV
Tyto_alba	MKCSFLTFLLQLV
Struthio_camelus_australis	MKCSFLTFLLQLV
Pelodiscus_sinensis	MKCSFLTFLLQLV
Tinamus_guttatus	MKCSFLTFLLQLV
Calypte_anna	MKCSFLTFLLQLV
Apaloderma_vittatum	MKCSFLTFLLQLV

Conservation

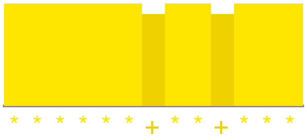

HG0038

ENSGALG00000006571

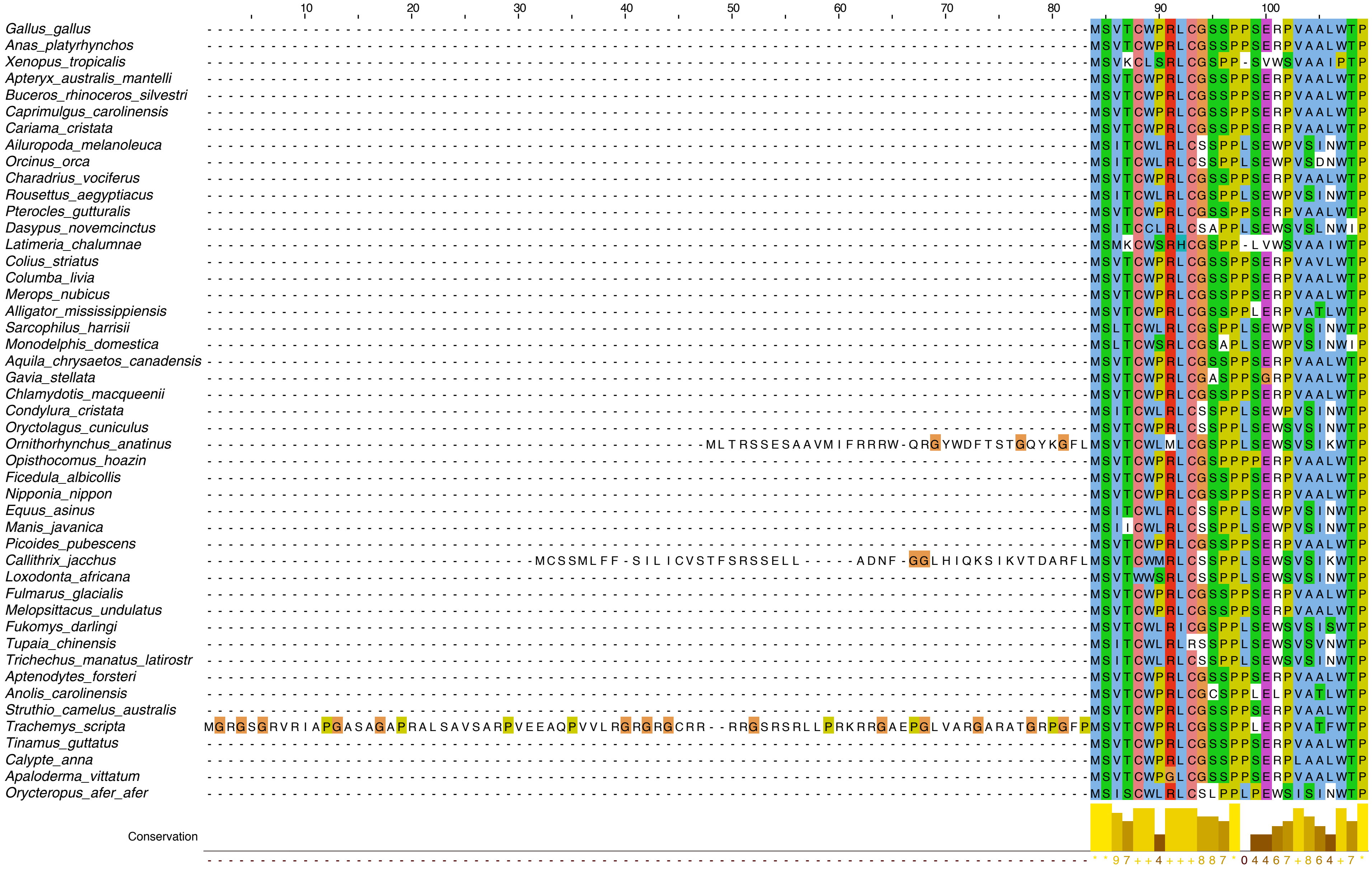

HG0039.1

ENSG00000077092 (RARB)

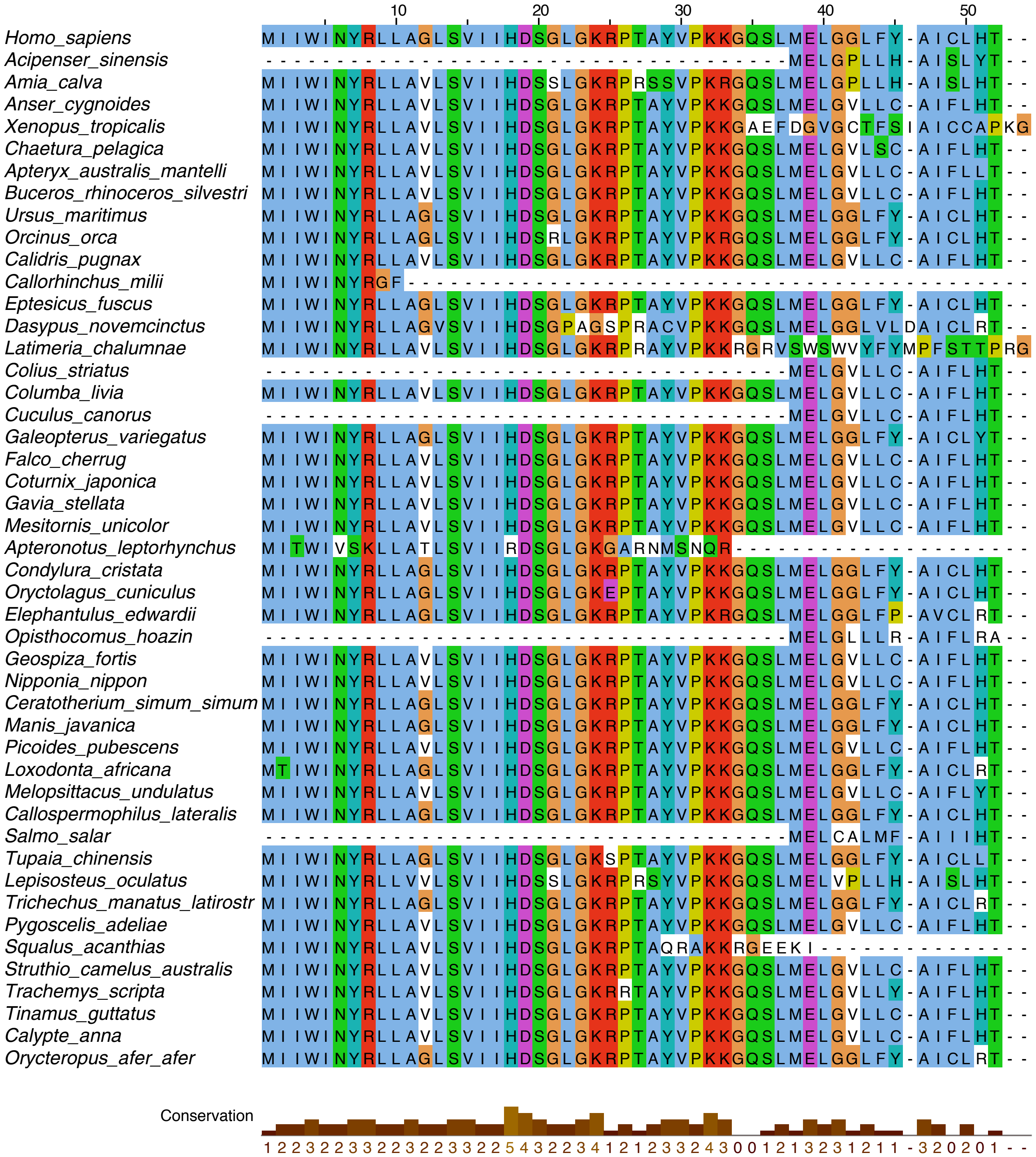

HG0039.2

ENSG00000077092 (RARB)

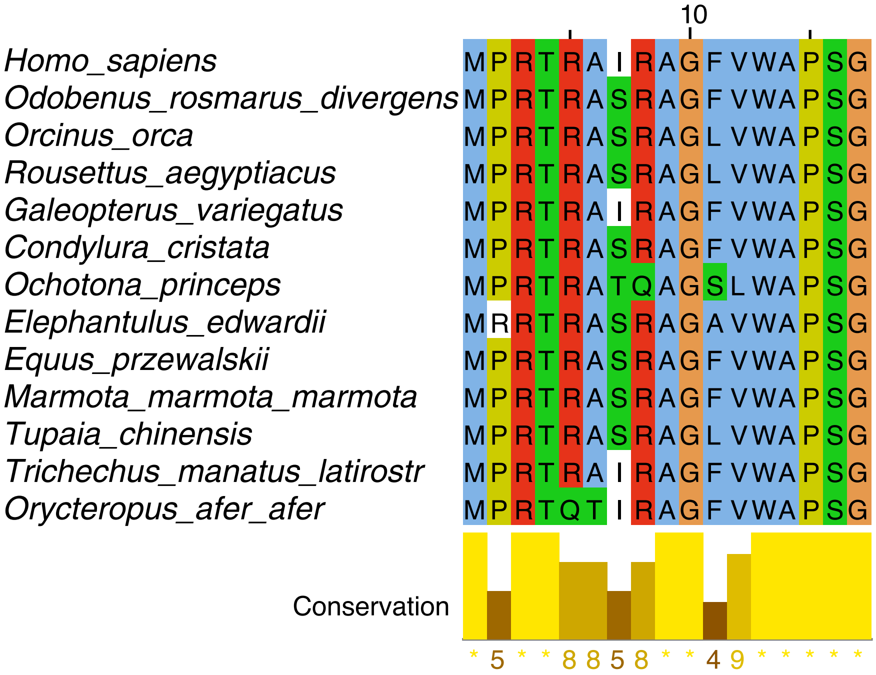

HG0040.1

ENSG00000164190 (NIPBL)

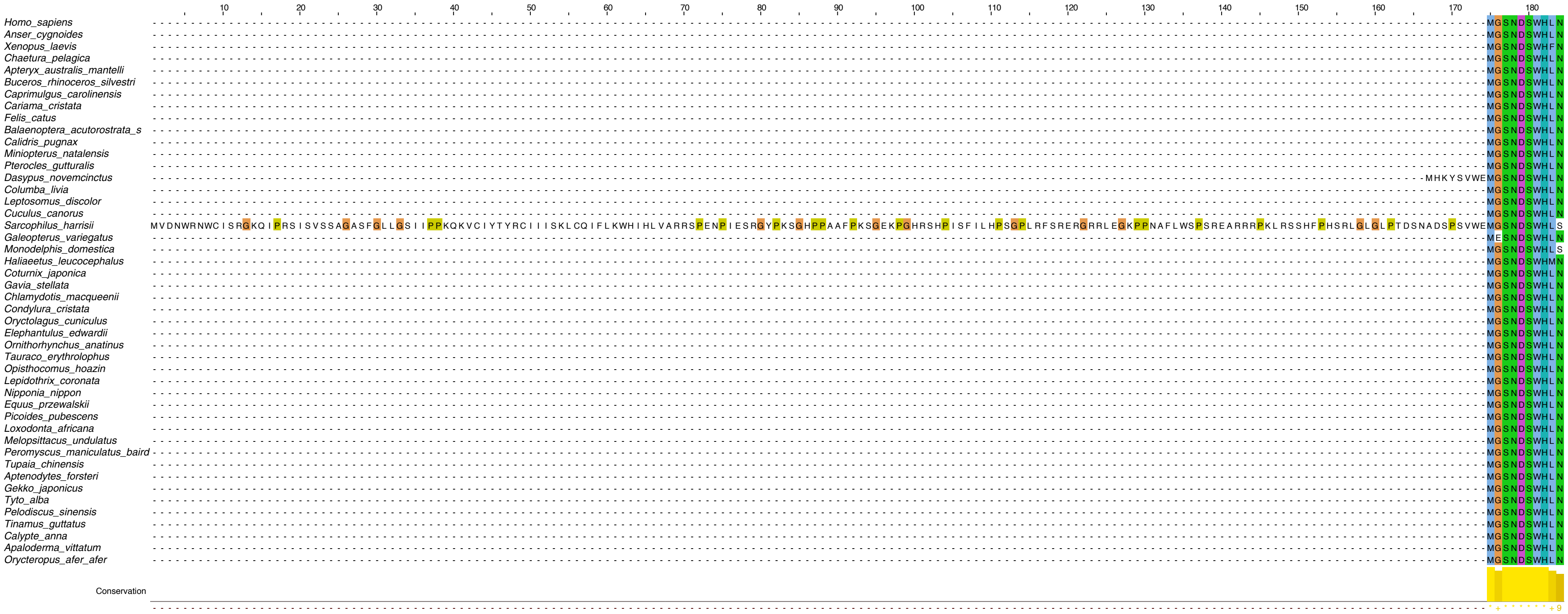

HG0040.2

ENSG00000164190 (NIPBL)

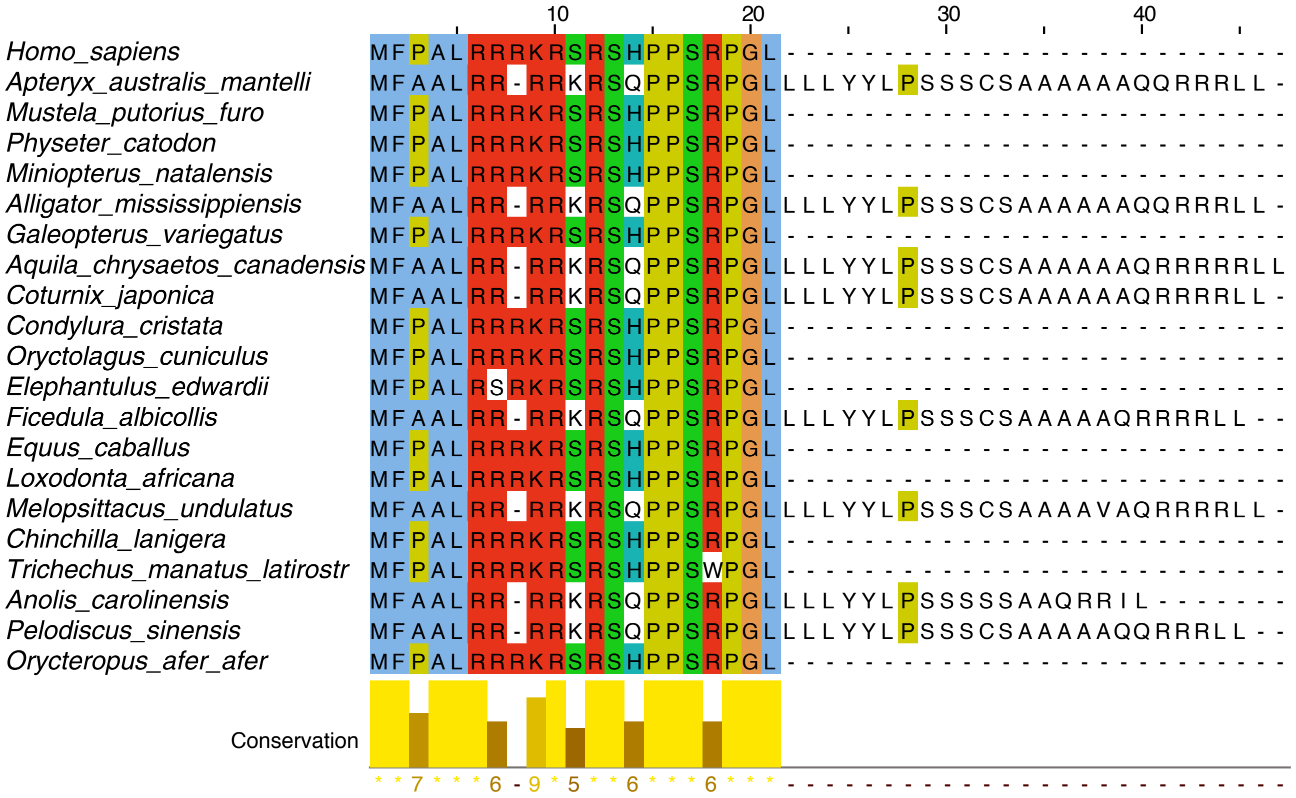

HG0041

ENSDARG00000061352 (bcl11aa)

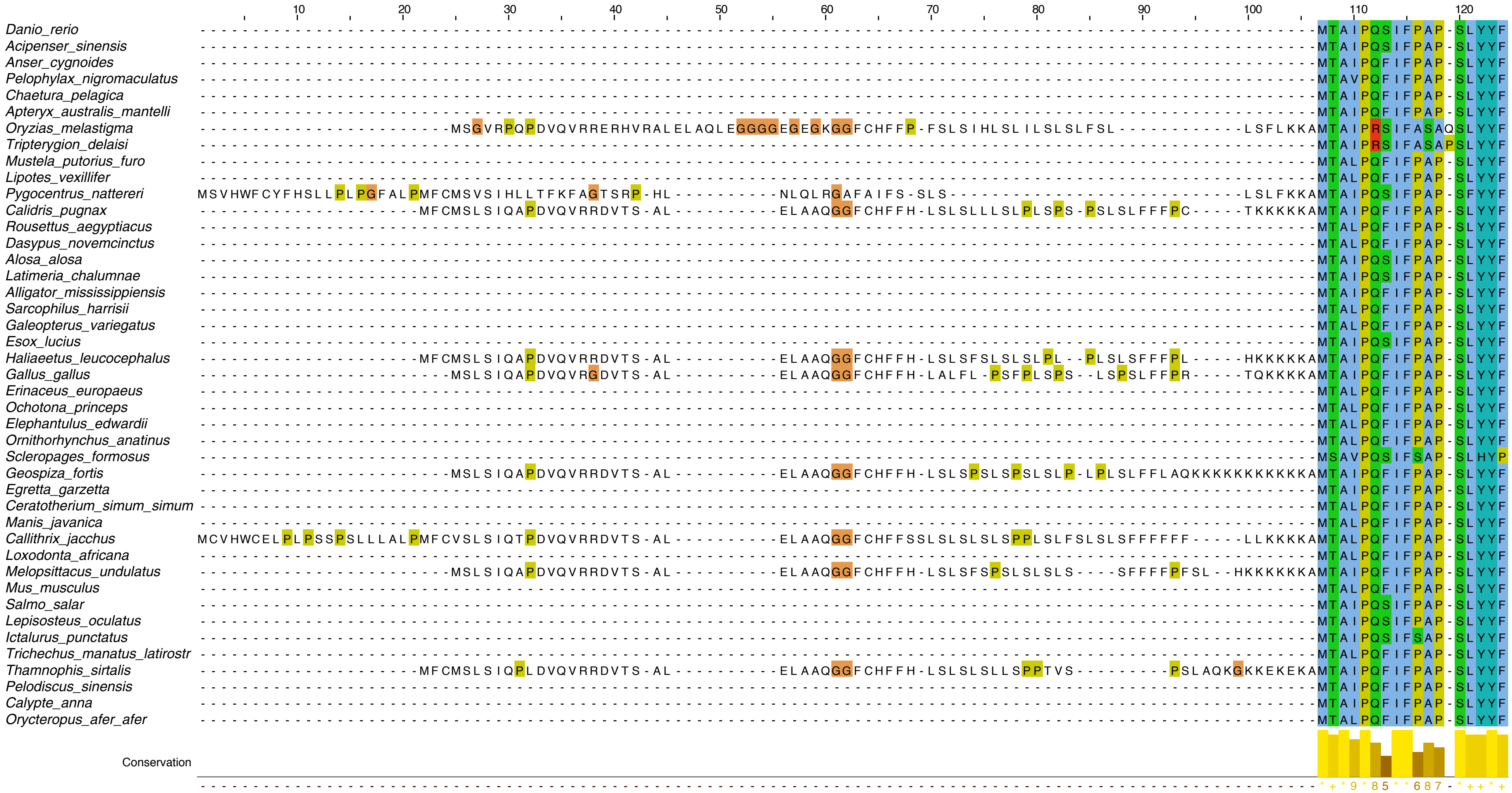

HG0041

ENSG00000119866 (BCL11A)

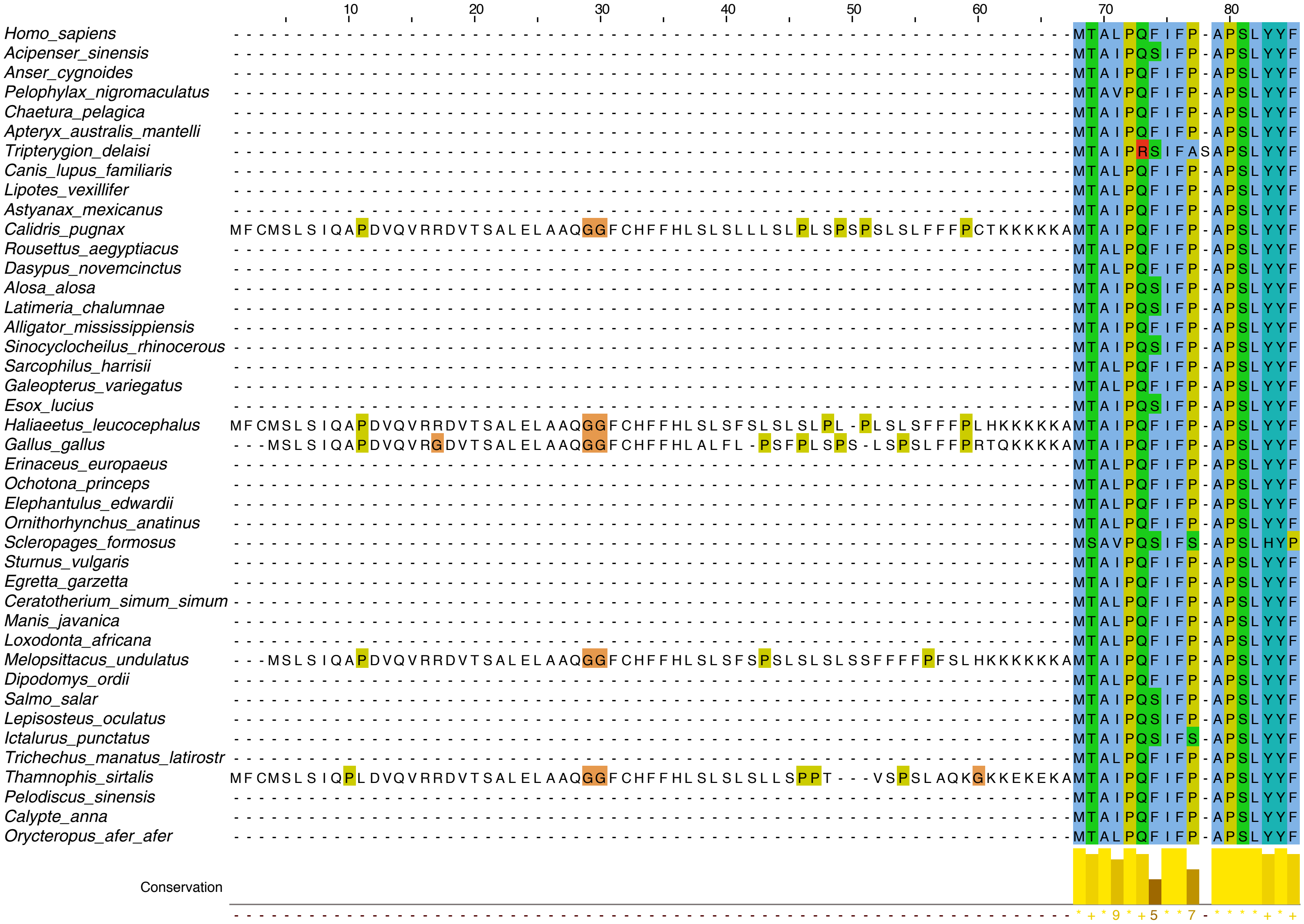

HG0041

ENSGALG00000034048

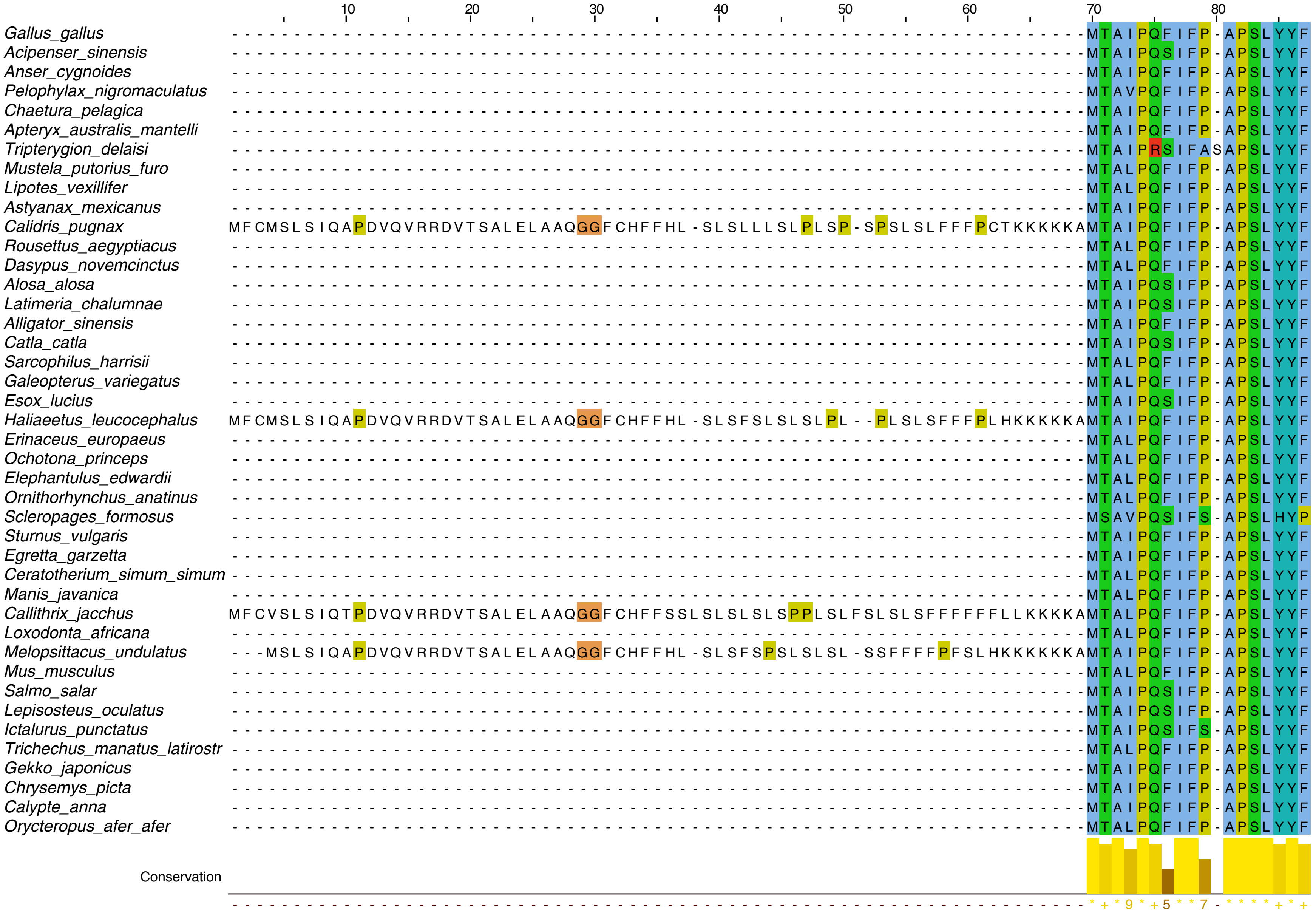

HG0042.1

ENSG00000140937 (CDH11)

Species	Alignment (position 10 marked)
Homo_sapiens	- - - - M G G - V C Y R N W Q Q V S N G
Anas_platyrhynchos	- - - - M C G - I C Y V N W Q Q V S S G
Chaetura_pelagica	- - - - M C G - I C Y E N W Q R V S S G
Apteryx_australis_mantelli	- - - - M C G - I C Y V S W Q Q V S S G
Buceros_rhinoceros_silvestri	- - - - M C G - I C Y I N W Q Q V S S G
Caprimulgus_carolinensis	- - - - M C G - I C Y I N W Q Q V S S G
Acinonyx_jubatus	- - - - M G G - V C Y R N W Q Q V S N G
Balaenoptera_acutorostrata_s	M T N Q M G G V L R Y R N W Q Q V S N G
Charadrius_vociferus	- - - - M C G - I C Y I N W Q Q V S S G
Eptesicus_fuscus	- - - - M G G - V C Y R N W Q Q V S N G
Pterocles_gutturalis	- - - - M C G - I C Y I N W Q Q V S S G
Dasypus_novemcinctus	- - - - M G E - L S Y R N W Q Q V S N G
Columba_livia	- - - - M C G - I C Y I N W Q Q V S S G
Leptosomus_discolor	- - - - M C G - I C Y I N W Q Q V S S G
Alligator_mississippiensis	- - - - M G G - I C Y I N W Q Q V T N G
Galeopterus_variegatus	- - - - M G R - V C Y R N W Q Q V S N G
Monodelphis_domestica	- - - - M G G - V C Y R N W Q Q V S N G
Falco_cherrug	- - - - M C G - I C Y I N W Q Q V S S G
Coturnix_japonica	- - - - M C G - I C Y I N W Q R V S S G
Gavia_stellata	- - - - M C G - I C Y I N W Q Q V S S G
Chlamydotis_macqueenii	- - - - M C G - I C Y I N W Q Q V S S G
Sorex_araneus	- - - - M G G - V C Y R N W Q Q V S N G
Oryctolagus_cuniculus	- - - - M G G - V C Y R N W Q Q V S N G
Tauraco_erythrolophus	- - - - M C G - I C Y I N W Q Q V S S G
Opisthocomus_hoazin	- - - - M C G - I C Y I N W Q Q V S S G
Pseudopodoces_humilis	- - - - M C G - I C Y I N W Q Q V S S G
Phaethon_lepturus	- - - - M C G - I C Y I N W Q Q V S N G
Ceratotherium_simum_simum	- - - - M G G - V C Y R N W Q Q V S N G
Manis_javanica	- - - - M G G - V C Y R N W Q Q V S N G
Picoides_pubescens	- - - - M C G - I C Y I N W Q Q V P S G
Loxodonta_africana	- - - - M G G - V Y Y R N W Q Q V S N G
Fulmarus_glacialis	- - - - M C G - I C Y I N W Q Q V S S G
Melopsittacus_undulatus	- - - - M C G - I C Y I N W Q Q V S S G
Heterocephalus_glaber	- - - - M G G - V C Y R N W Q Q V S N G
Tupaia_chinensis	- - - - M G G - L C Y R N W Q Q V S N G
Aptenodytes_forsteri	- - - - M S G - I C Y I T W Q Q V S S G
Takydromus_sexlineatus	- - - - M G G - I C Y F N W Q W V S S G
Struthio_camelus_australis	- - - - M C G - I C Y R S W Q Q V S S G
Chelonia_mydas	- - - - M G G - I C Y I N W Q Q V S S G
Tinamus_guttatus	- - - - M C G - I C Y I S W Q Q V S S G
Calypte_anna	- - - - M C G - I C Y I N W Q Q V S S G
Apaloderma_vittatum	- - - - M C G - T C Y I N W Q Q V S S G
Orycteropus_afer_afer	M T N Q M G R - V C Y R N W Q Q V S N G

Conservation

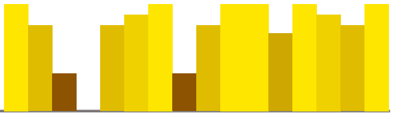

- - - - * 9 4 - 9 + * 4 9 * * 8 * + 9 *

HG0042.2

ENSGALG00000039985

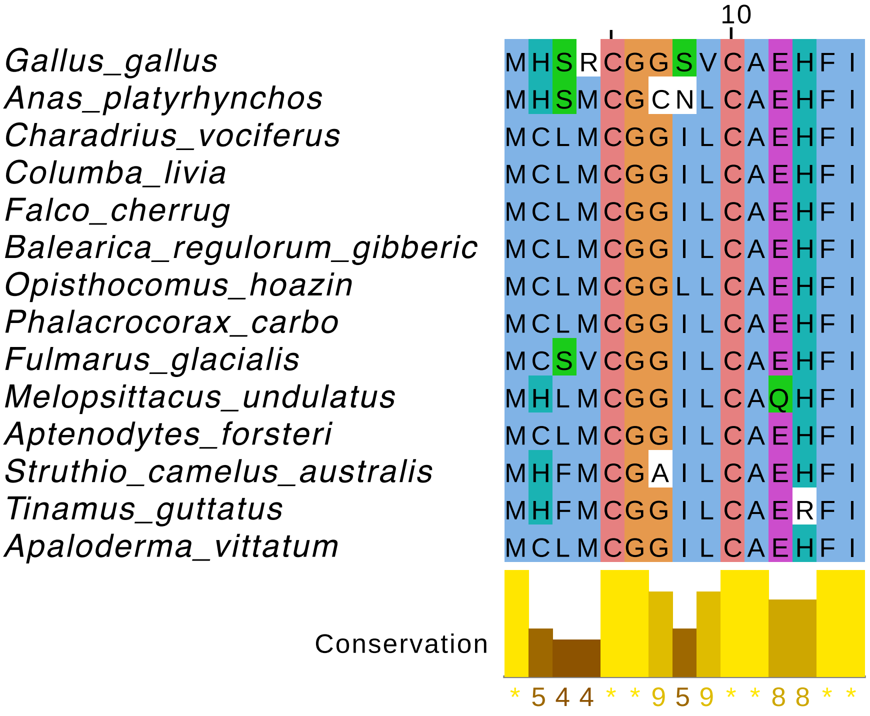

HG0042.3

ENSG00000140937 (CDH11)

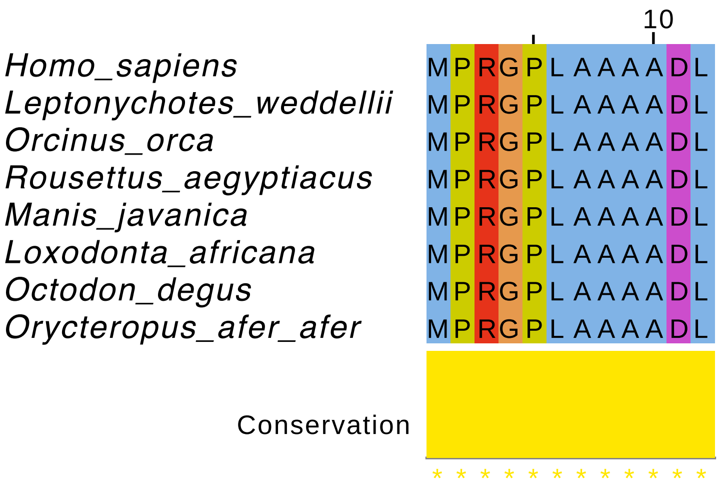

HG0042.4

ENSG00000140937 (CDH11)

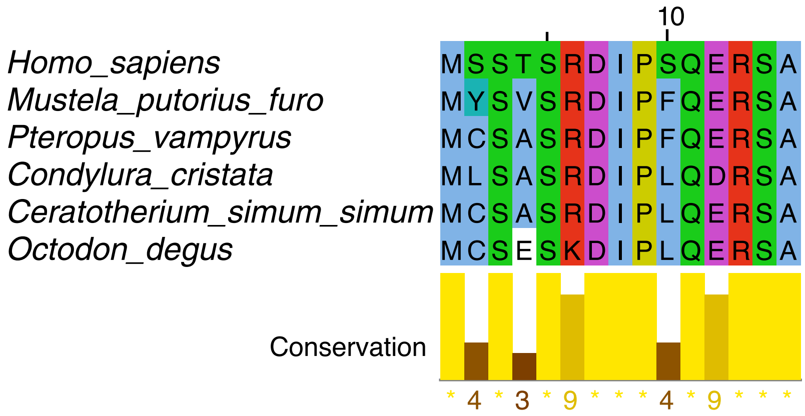

HG0042.5

ENSG00000140937 (CDH11)

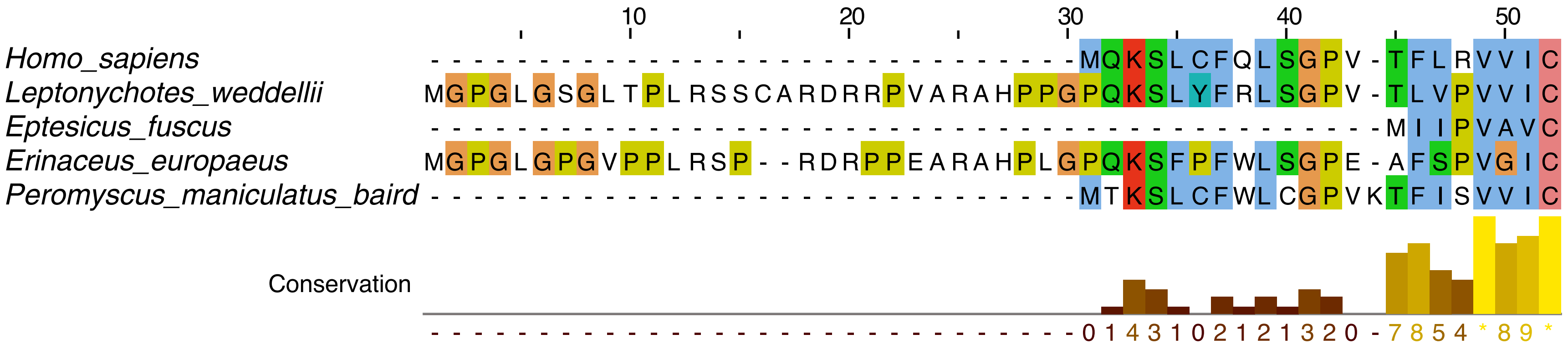

HG0043

ENSG00000172995 (ARPP21)

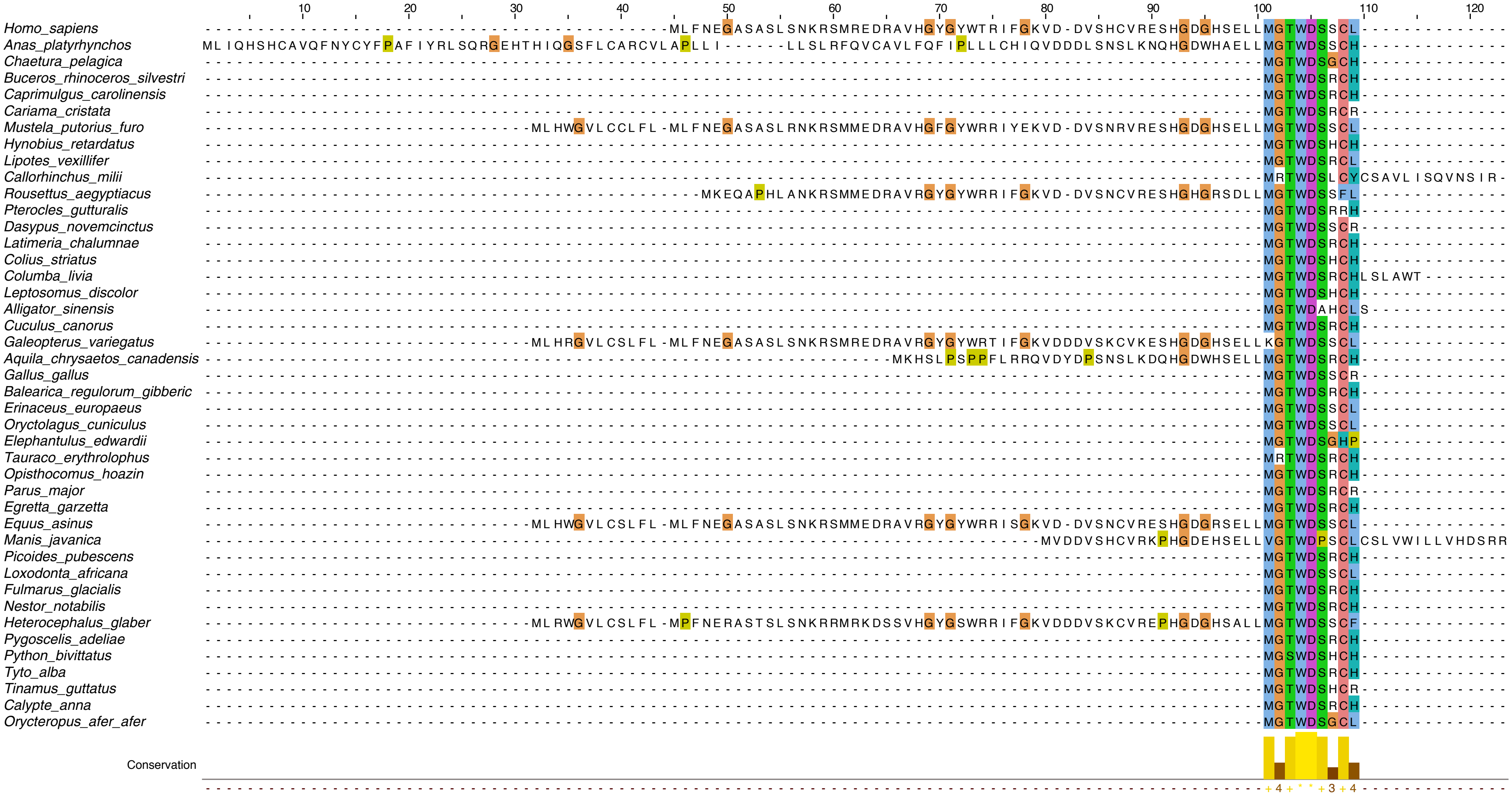

HG0044

ENSDARG00000061687 (atxn1a)

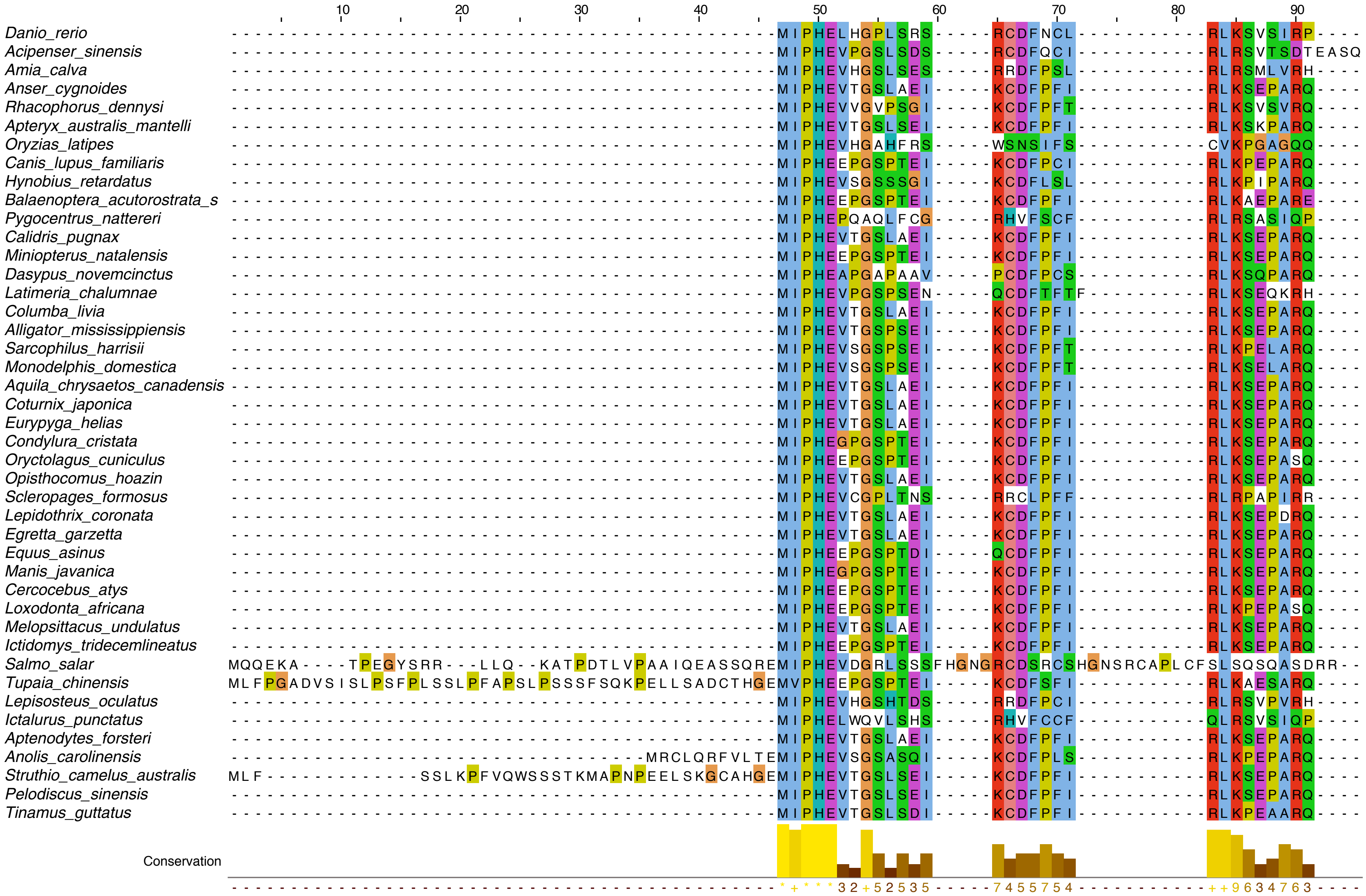

HG0044

ENSG00000124788 (ATXN1)

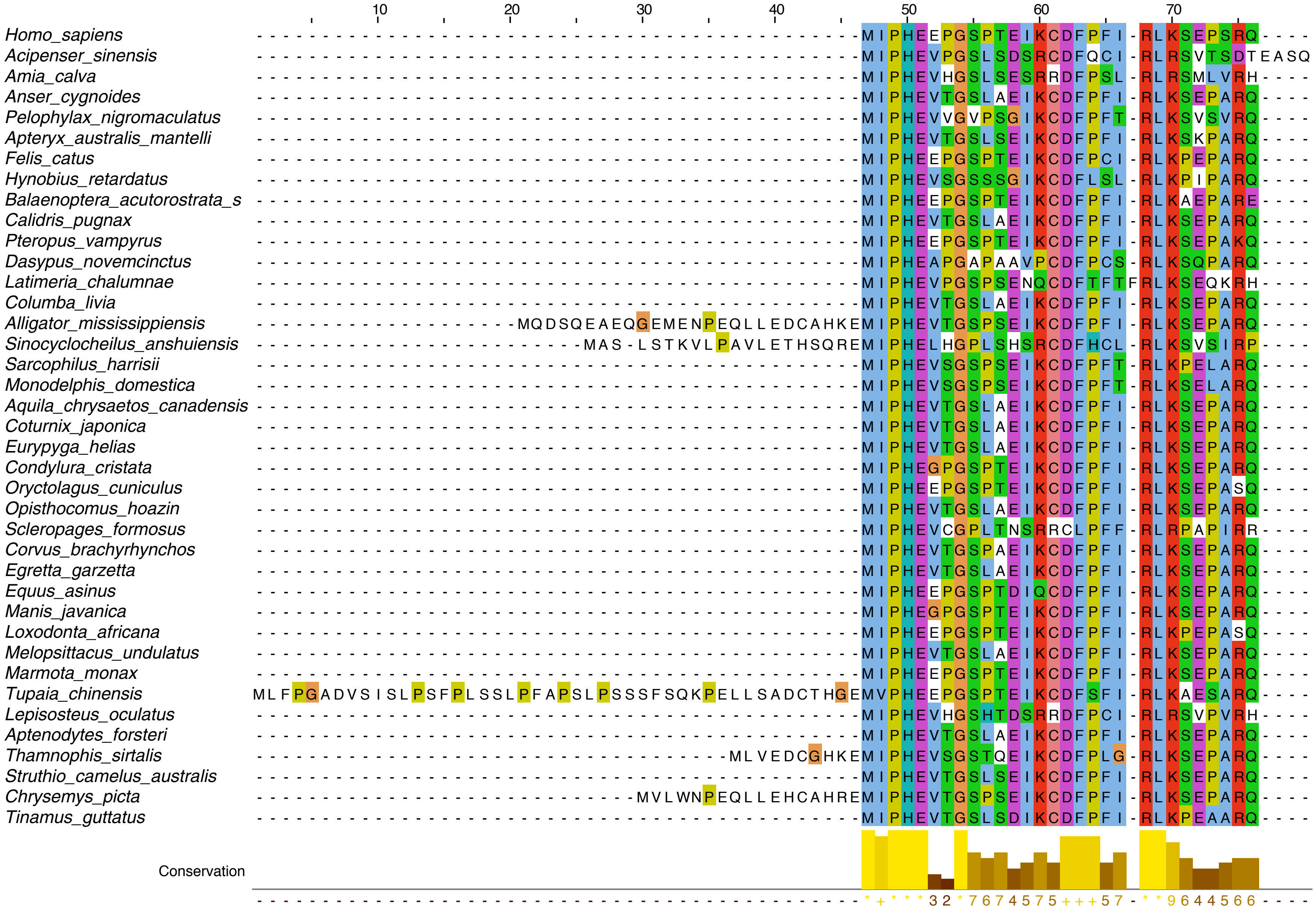

HG0045.1

ENSG00000083168 (KAT6A)

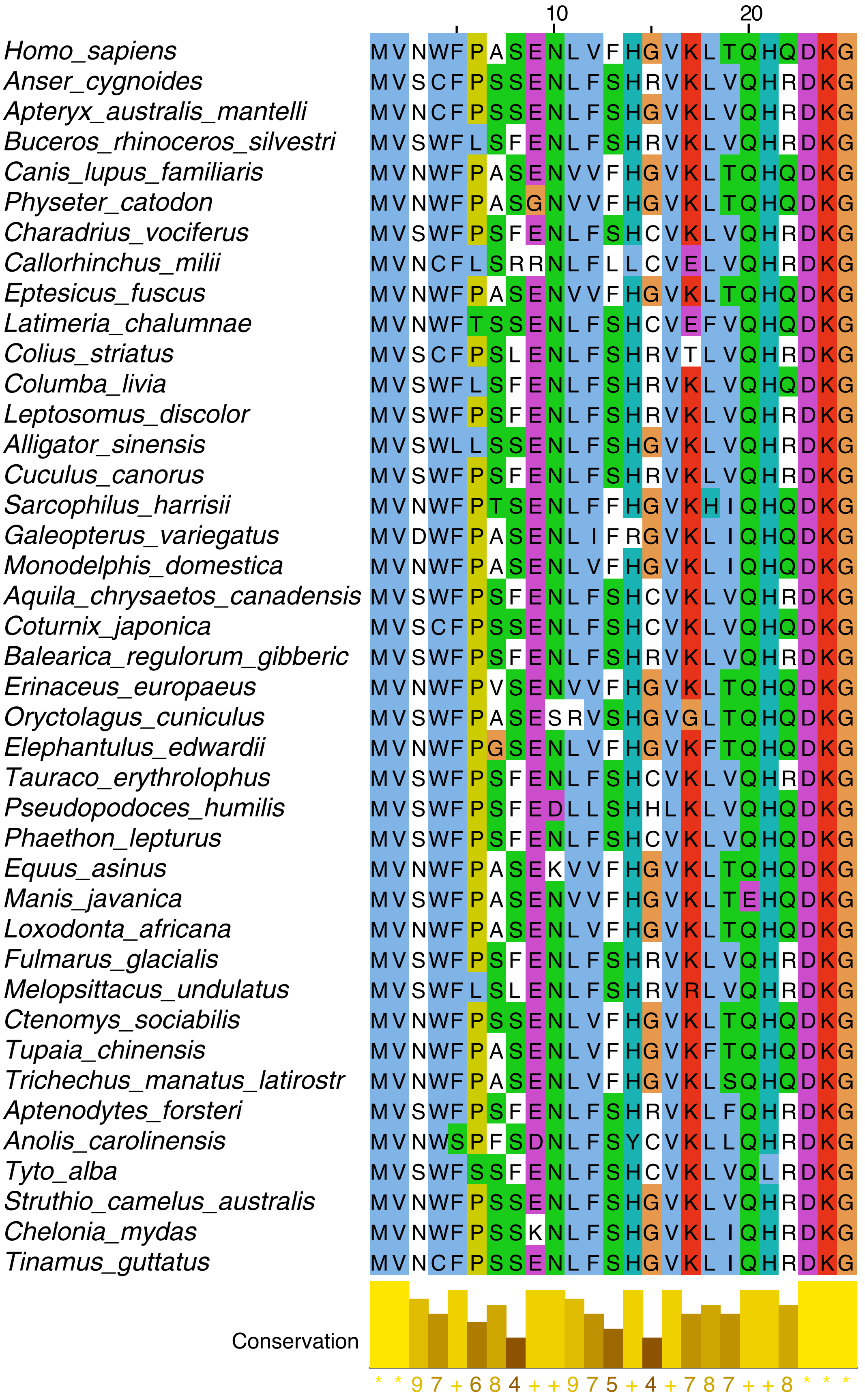

HG0045.2

ENSG00000083168 (KAT6A)

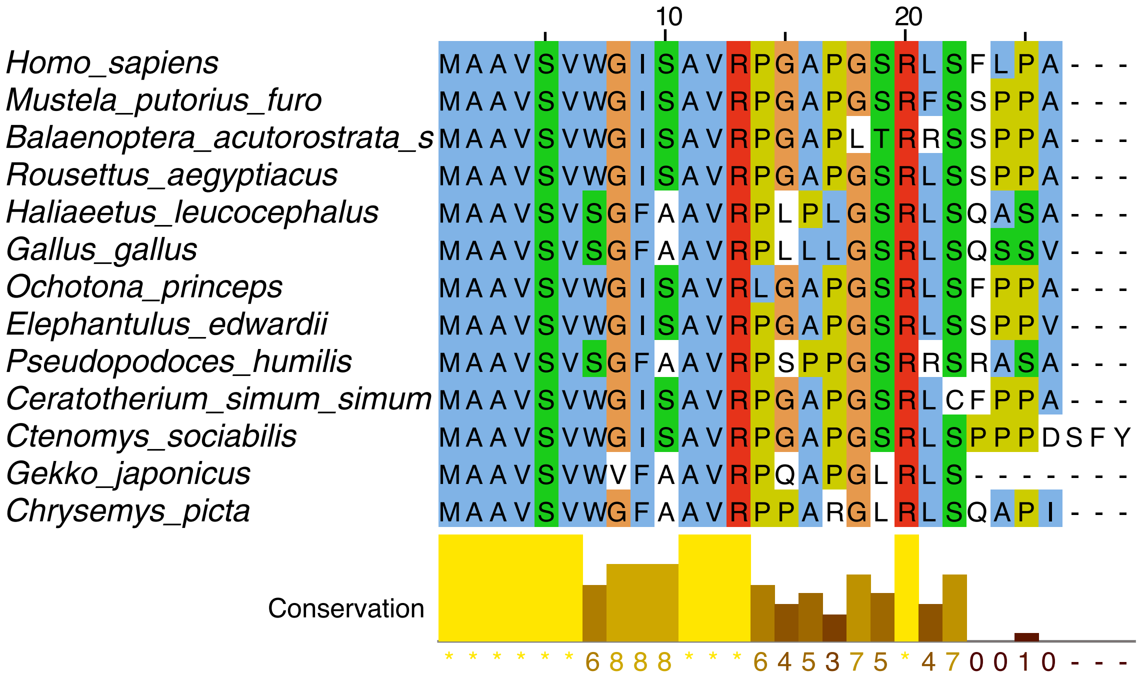

HG0046

ENSDARG00000018817 (bdnf)

Species	Alignment (position 10 marked at column 10)
Danio_rerio	MMTSNKTHYLPASVGVCGY
Anser_cygnoides	MMTSNKTHYLPASVGETGF
Nanorana_parkeri	MMTSNKRHYLPASVGETGF
Chaetura_pelagica	MMTSNKTHYLPASVGETGF
Canis_lupus_familiaris	MMTSNKTHYLPASVGETGF
Lipotes_vexillifer	MMTSNKTHYLPASVGETGF
Calidris_pugnax	MMTSNKTHYLPASVGETGF
Miniopterus_natalensis	MMTSNKTHYLPASVGETGF
Haplochromis_burtoni	MMTSNKTHYLPASVGVSGY
Columba_livia	MMTSNKTHYLPASVGETGF
Cuculus_canorus	MMTSNKTHYLPASVGETGF
Austrofundulus_limnaeus	MMTSNKTHYLPASVGVSGY
Galeopterus_variegatus	MMTSNKTHYLPASVGETRF
Monodelphis_domestica	MMTSNKTHYLPASVGETGF
Aquila_chrysaetos_canadensis	MMTSNKTHYLPASVGETGF
Coturnix_japonica	MMTSNKTHYLPASVGETRF
Condylura_cristata	MMTSNKTHYLPASVGETGF
Ochotona_princeps	MMTSNKTHYLPASVGETRF
Elephantulus_edwardii	MMTSNKTHYLPASVGETGF
Opisthocomus_hoazin	MMTSNKTHYLPASVRETGF
Corvus_brachyrhynchos	MMTSNKTHYLPASVGETGF
Egretta_garzetta	MMTSNKTHYLPASVGETGF
Notothenia_coriiceps	MMTSNKTHYLPASVGVSGY
Ceratotherium_simum_simum	MMTSNKTHYLPASVGETGF
Manis_javanica	MMTSNKTHYLPASVGETGF
Cynoglossus_semilaevis	MMTSNKTHYLPASVGVSGY
Aotus_nancymaae	MMTSNKTHYLPASVGETRF
Loxodonta_africana	MMTSNKTHYLPASVGETGF
Melopsittacus_undulatus	MMTSNKTHYLPASVGETGF
Dipodomys_ordii	MMTSNKTHYLPASVGETGF
Salmo_salar	MMTSNKTHYLPASVGVSGY
Lepisosteus_oculatus	MMTSNKTHYLPASVGESGF
Trichechus_manatus_latirostr	MMTSNKTHYLPASVGETGF
Aptenodytes_forsteri	MMTSNKTHYLPASVGETGF
Anolis_carolinensis	MMTSNETHSLPASGGETGL
Struthio_camelus_australis	MMTSNKTHYLPASVGETGF
Chelonia_mydas	MMTSNKTHYLPASVGETGF
Tinamus_guttatus	MMTSNKTHYLPASVGETGF
Calypte_anna	MMTSNKTHYLPASVGETGF
Orycteropus_afer_afer	MMTSNKTHYLPASVGETRF

Conservation 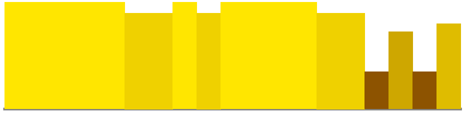

* * * * * + + * + * * * * + + 4 8 4 9

HG0046

ENSG00000176697 (BDNF)

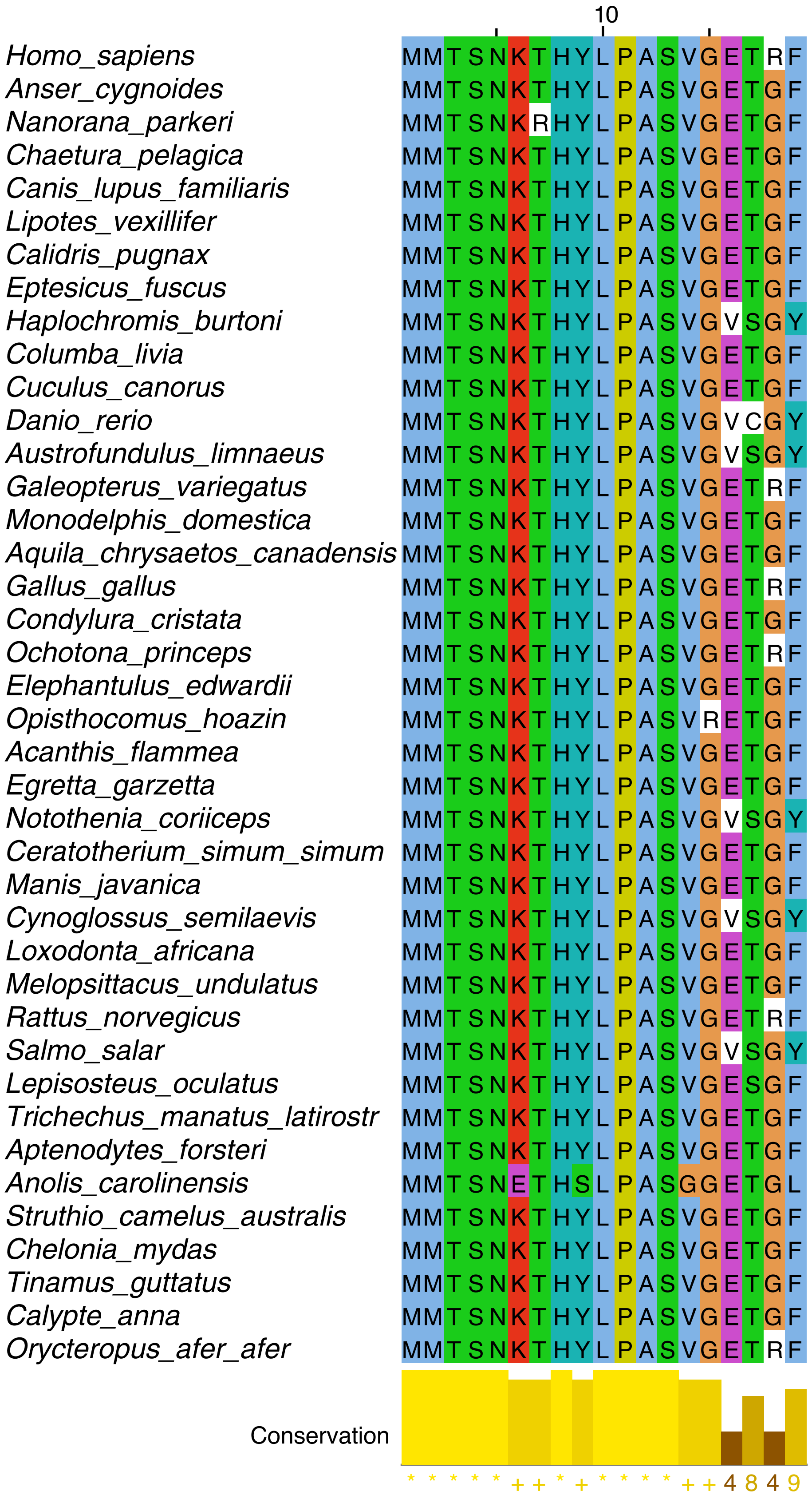

HG0047

ENSGALG00000015422 (NRG1)

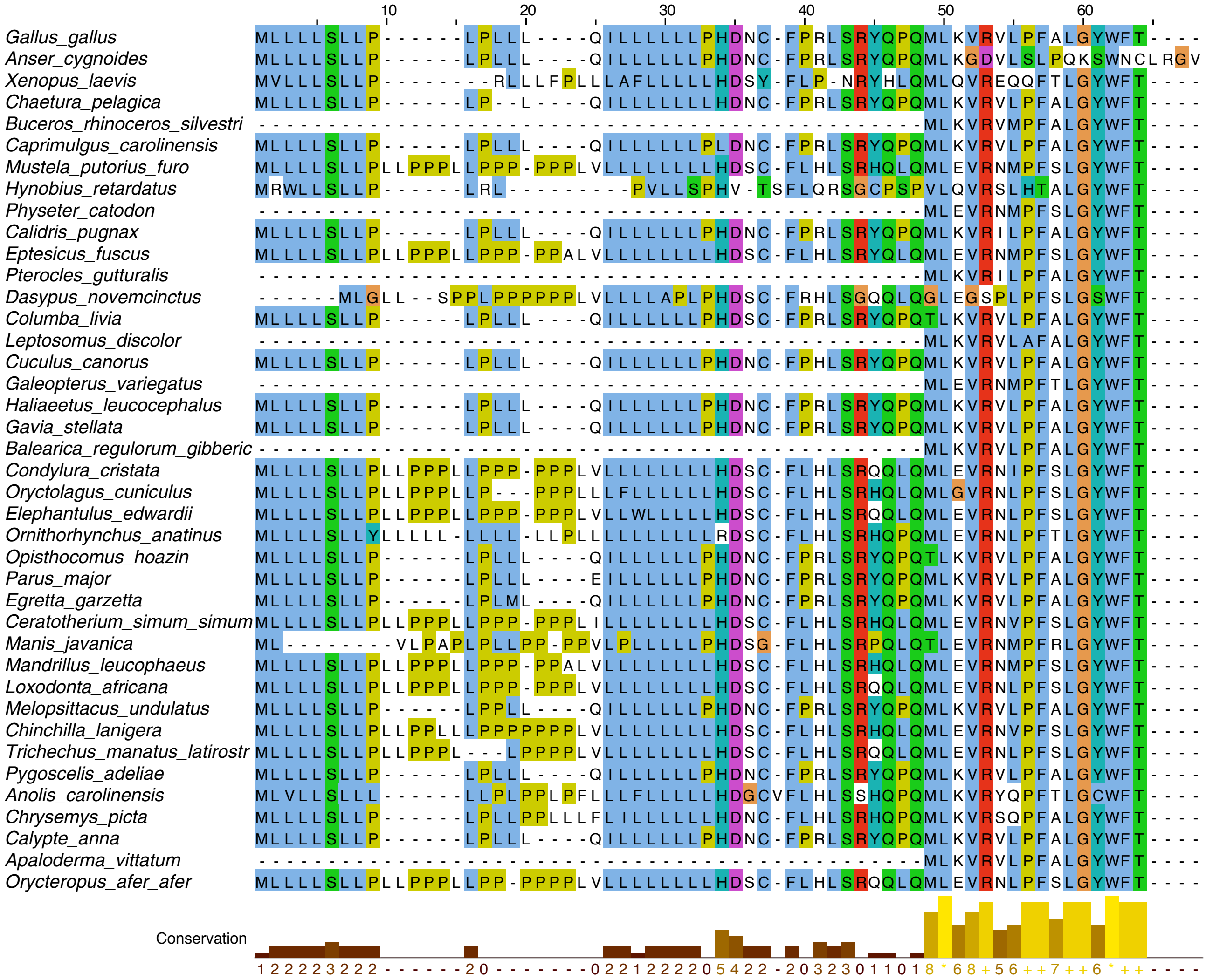

HG0048

ENSG00000177565 (TBL1XR1)

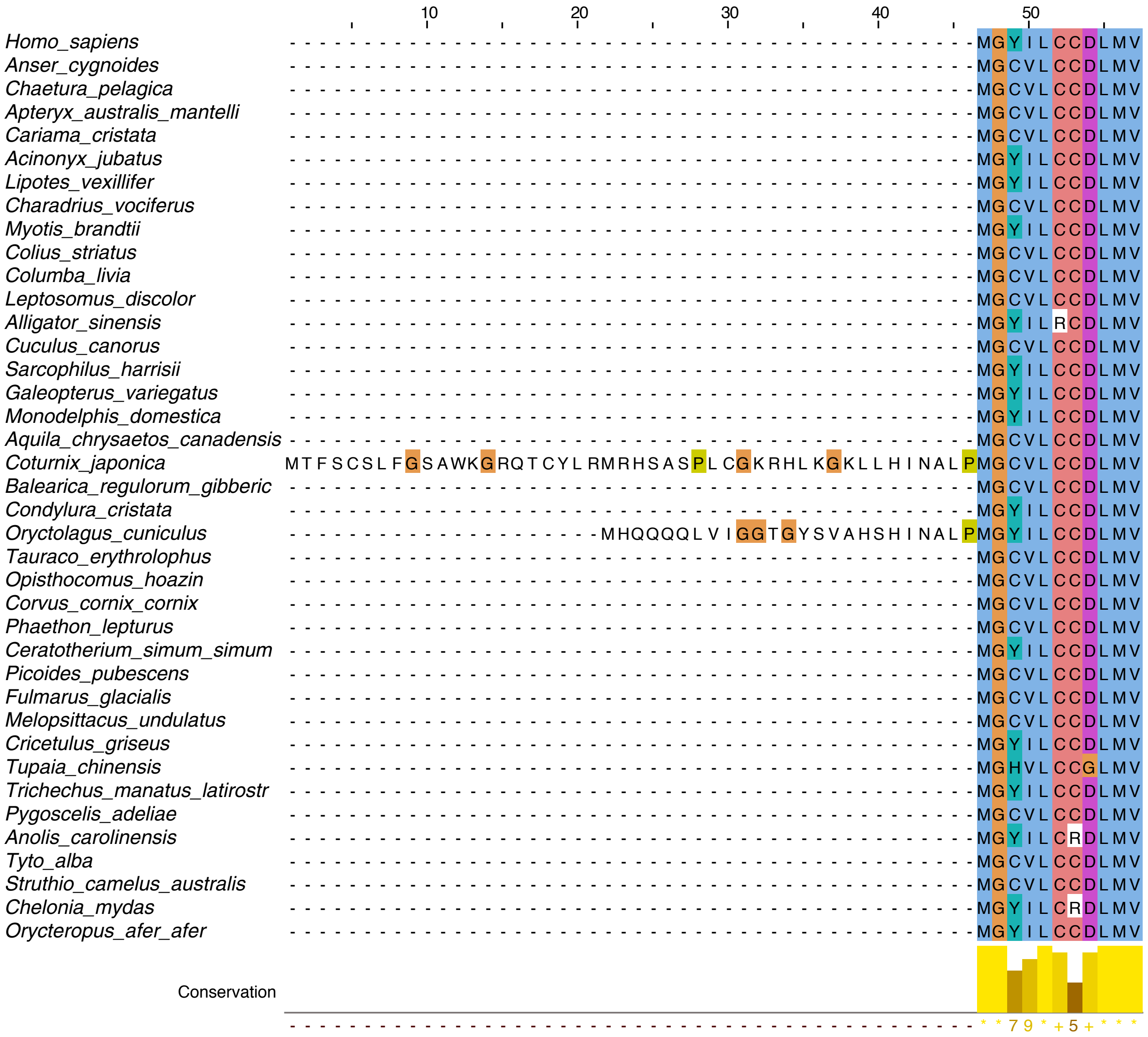

HG0048

ENSGALG00000039182

Species	Alignment (positions 1–36; ticks at 10, 20, 30)
Gallus_gallus	-------------------------MGCVLCCDLMV
Anser_cygnoides	-------------------------MGCVLCCDLMV
Chaetura_pelagica	-------------------------MGCVLCCDLMV
Apteryx_australis_mantelli	-------------------------MGCVLCCDLMV
Cariama_cristata	-------------------------MGCVLCCDLMV
Ailuropoda_melanoleuca	-------------------------MGYILCCDLMV
Lipotes_vexillifer	-------------------------MGYILCCDLMV
Calidris_pugnax	-------------------------MGCVLCCDLMV
Rousettus_aegyptiacus	-------------------------MGYILCCDLMV
Colius_striatus	-------------------------MGCVLCCDLMV
Columba_livia	-------------------------MGCVLCCDLMV
Leptosomus_discolor	-------------------------MGCVLCCDLMV
Cuculus_canorus	-------------------------MGCVLCCDLMV
Sarcophilus_harrisii	-------------------------MGYILCCDLMV
Galeopterus_variegatus	-------------------------MGYILCCDLMV
Monodelphis_domestica	-------------------------MGYILCCDLMV
Aquila_chrysaetos_canadensis	-------------------------MGCVLCCDLMV
Balearica_regulorum_gibberic	-------------------------MGCVLCCDLMV
Condylura_cristata	-------------------------MGYILCCDLMV
Oryctolagus_cuniculus	MHQQQQLVIGGTGYSVAHSHINALPMGYILCCDLMV
Tauraco_erythrolophus	-------------------------MGCVLCCDLMV
Opisthocomus_hoazin	-------------------------MGCVLCCDLMV
Acanthisitta_chloris	-------------------------MGCVLCCDLMV
Egretta_garzetta	-------------------------MGCVLCCDLMV
Equus_asinus	-------------------------MGFILCCDLMV
Picoides_pubescens	-------------------------MGCVLCCDLMV
Cercocebus_atys	-------------------------MGYILCCDLMV
Fulmarus_glacialis	-------------------------MGCVLCCDLMV
Melopsittacus_undulatus	-------------------------MGCVLCCDLMV
Chinchilla_lanigera	-------------------------MGYVLCCDLMV
Tupaia_chinensis	-------------------------MGHVLCCGLMV
Trichechus_manatus_latirostr	-------------------------MGYILCCDLMV
Aptenodytes_forsteri	-------------------------MGCVLCCDLMV
Gekko_japonicus	-------------------------MGCILRCDLMV
Tyto_alba	-------------------------MGCVLCCDLMV
Struthio_camelus_australis	-------------------------MGCVLCCDLMV
Apaloderma_vittatum	-------------------------MDCVLCCDLMV
Orycteropus_afer_afer	-------------------------MGYILCCDLMV
Conservation	-------------------------*+79*+*+***

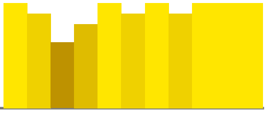

HG0049

ENSDARG00000044485 (sall4)

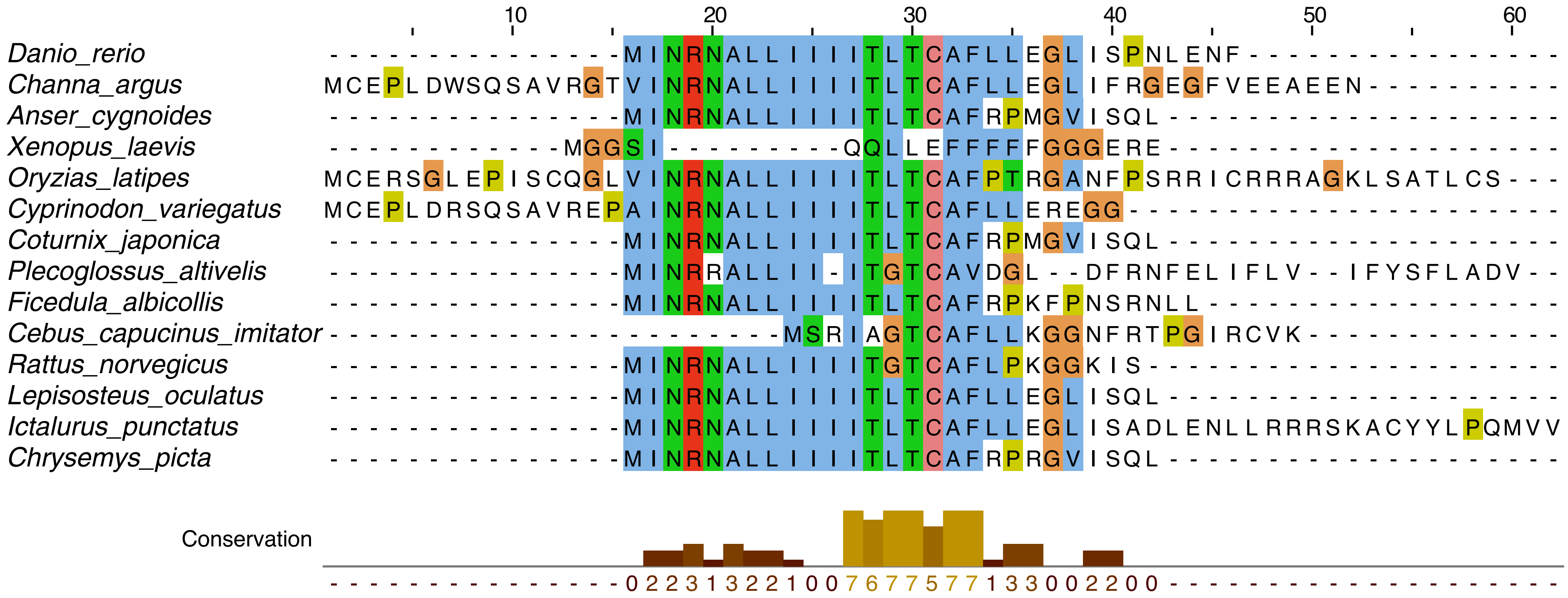

HG0049

ENSGALG00000039238

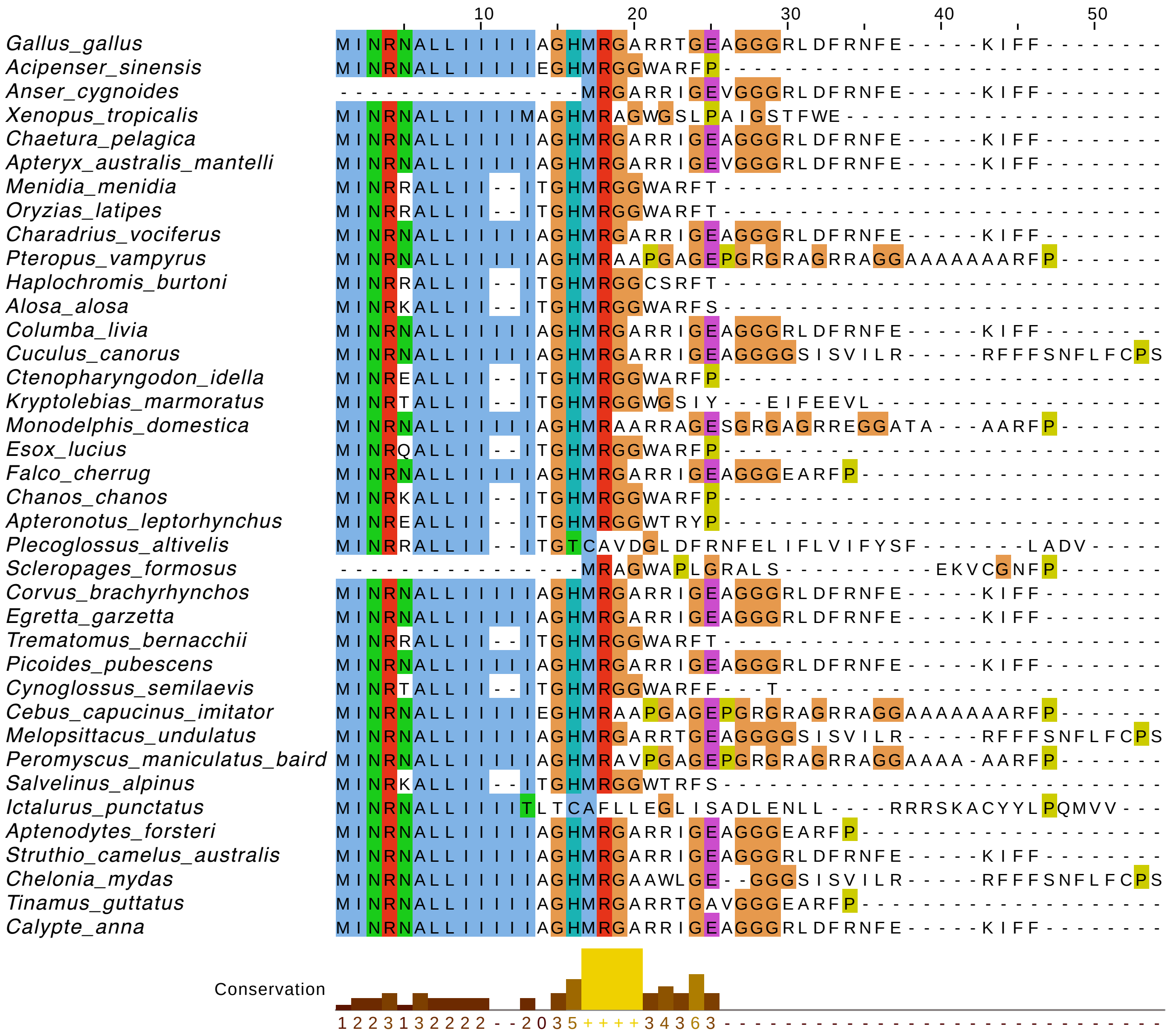

HG0050.1

ENSG00000074054 (CLASP1)

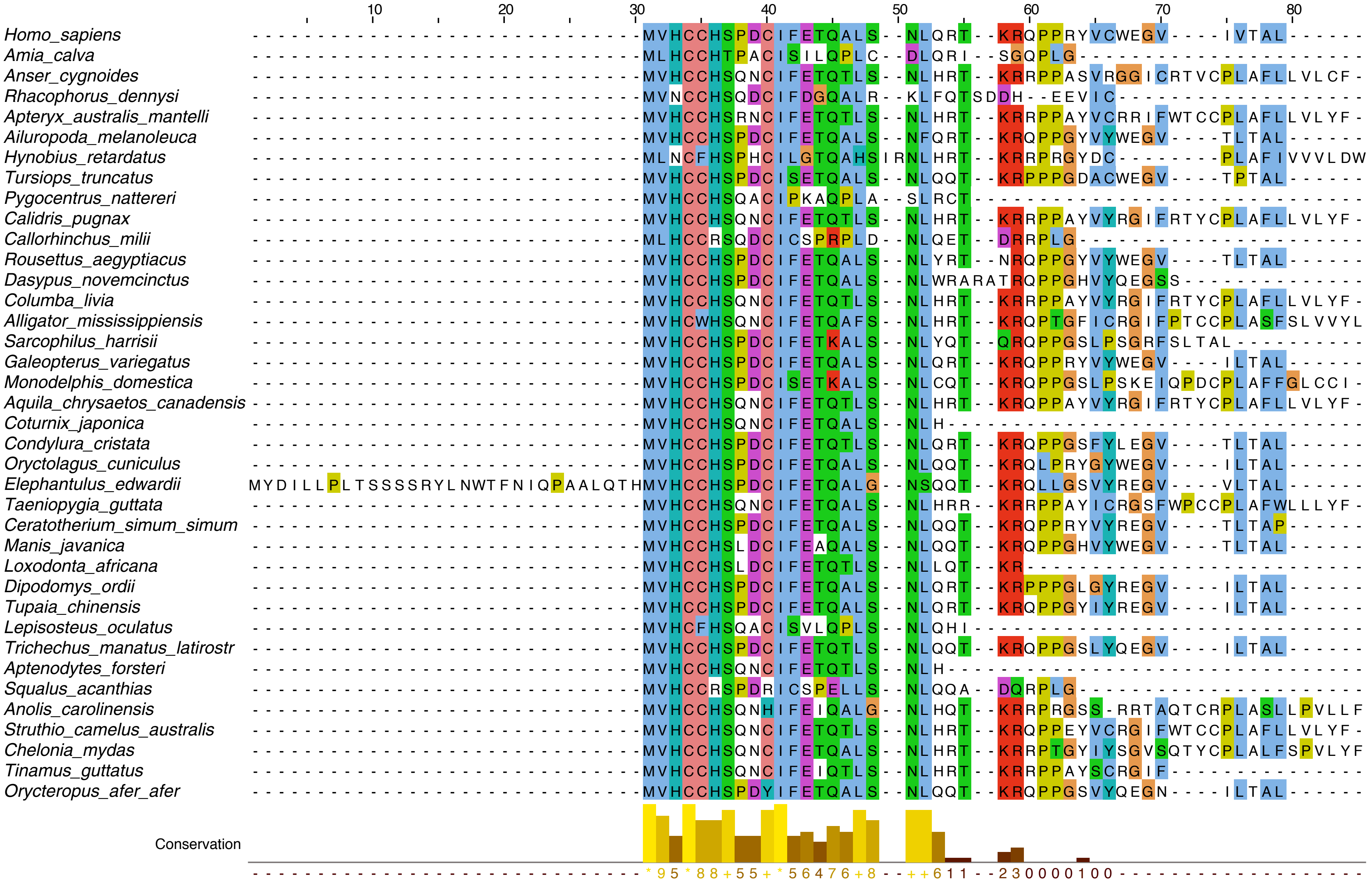

HG0050.2

ENSG00000074054 (CLASP1)

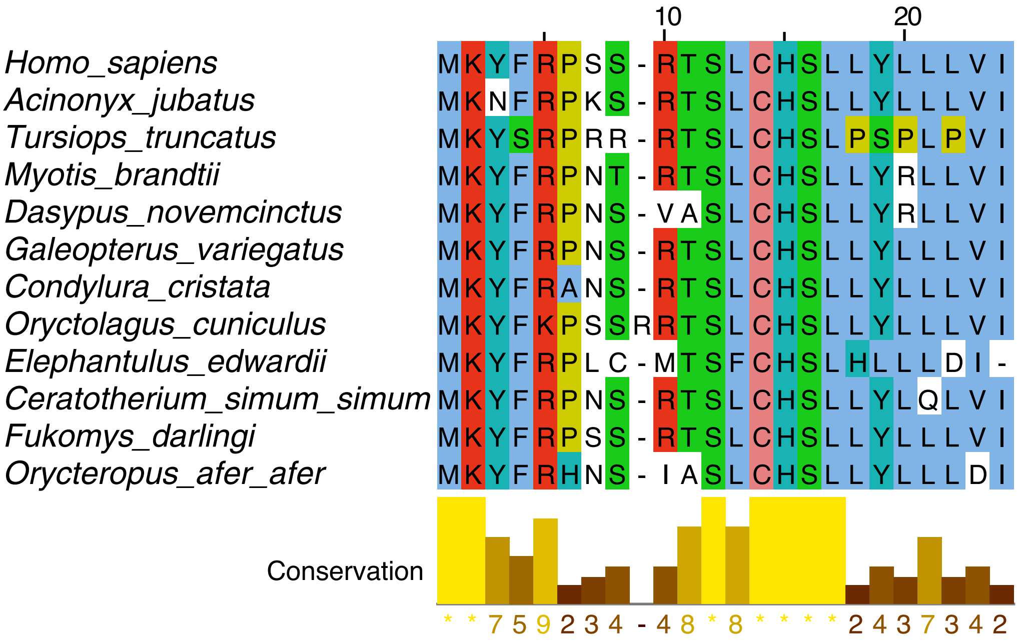

HG0051

ENSG00000116679 (IVNS1ABP)

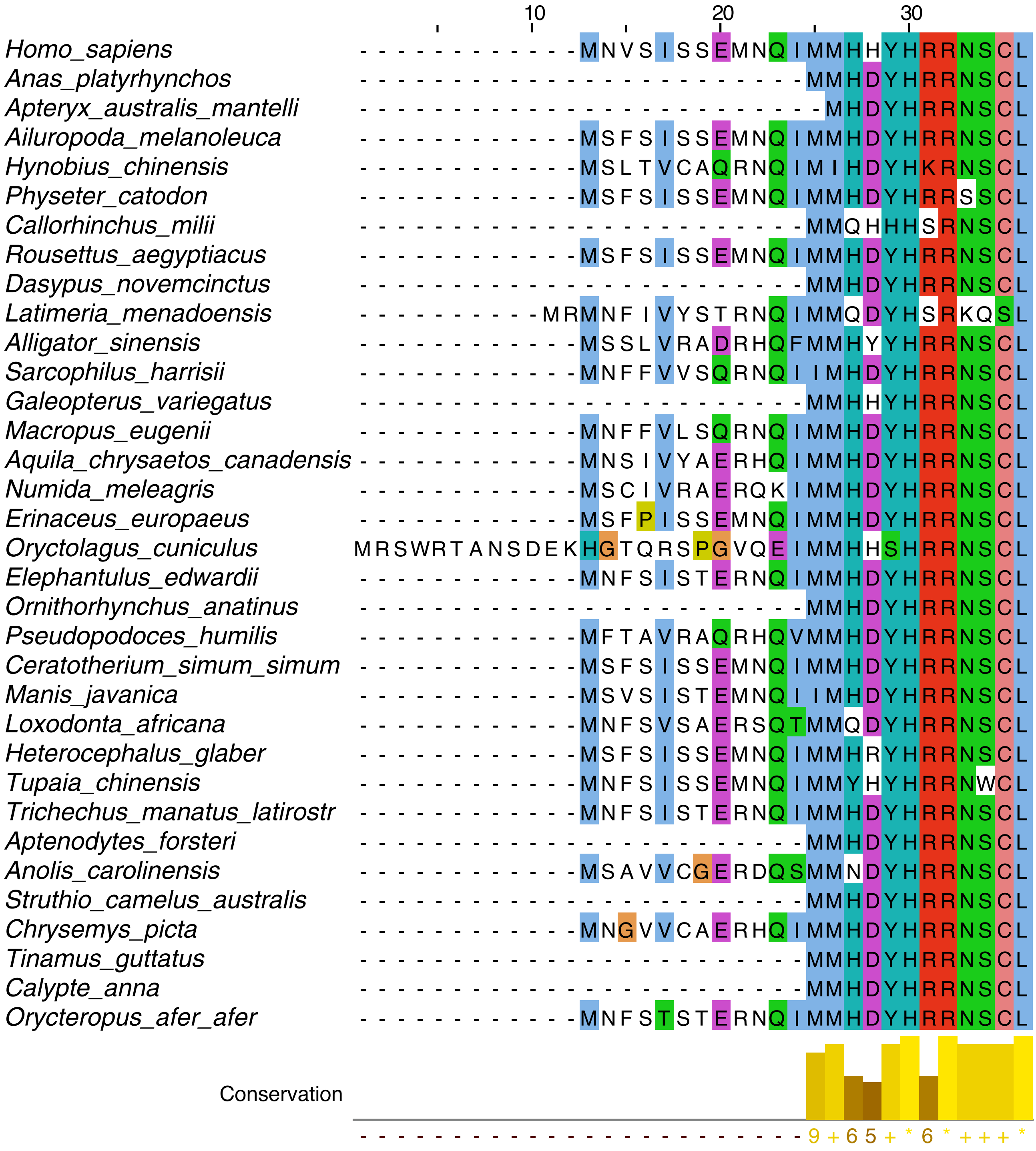

HG0051

ENSGALG00000037162

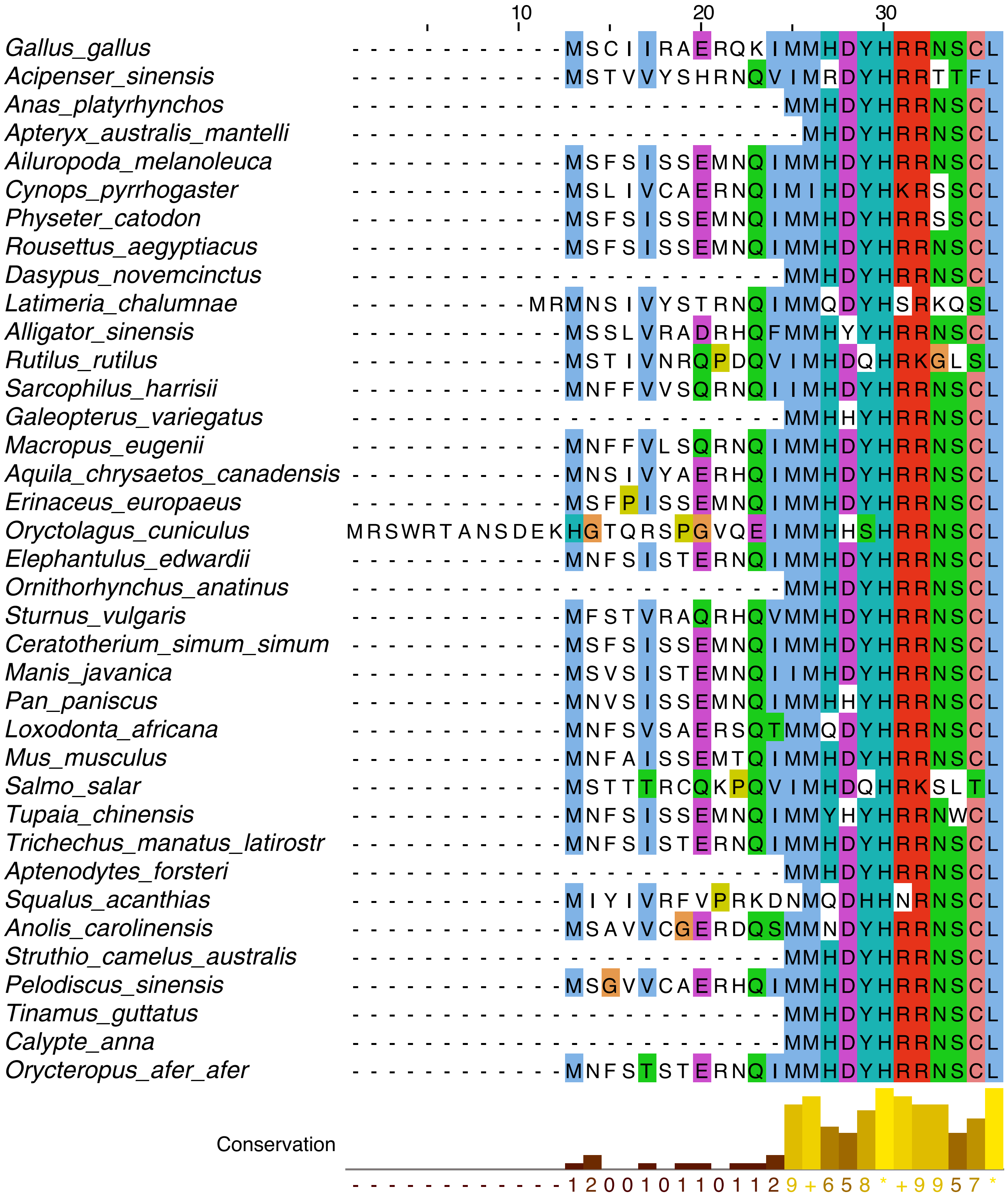

HG0052

ENSG00000178235 (SLITRK1)

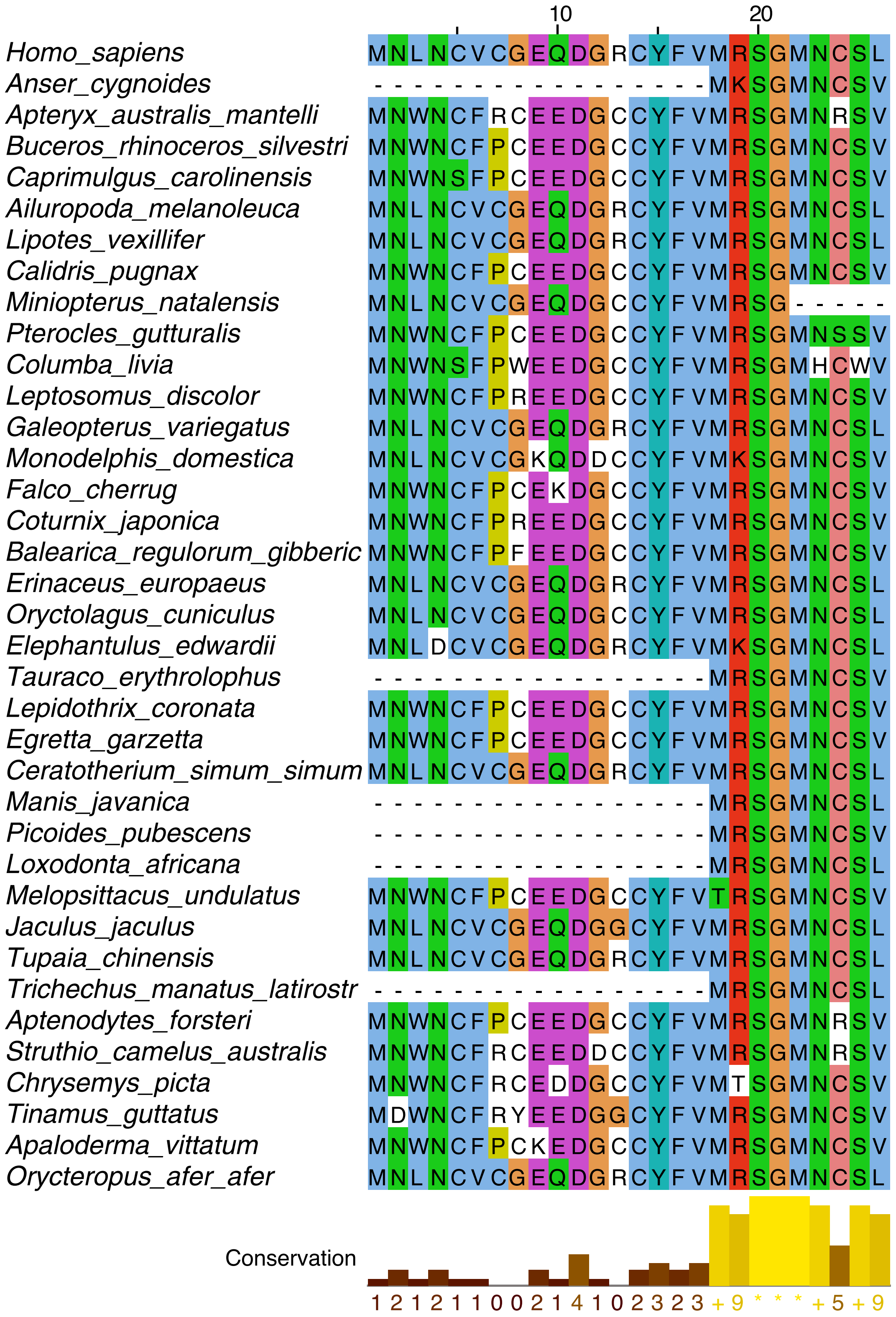

HG0053.1

ENSG00000184564 (SLITRK6)

	10
Homo_sapiens	---MVNYMQKSQHSCEVCREF
Anser_cygnoides	-------MQKSQHSCEVCCEF
Chaetura_pelagica	-------MQKSQHSSEVCCEF
Buceros_rhinoceros_silvestri	MLQTVNYMQKSQHSCEVCCEF
Caprimulgus_carolinensis	-------MQKSQHSCEVCCEF
Cariama_cristata	-------MQKSQHSCEVCCEF
Acinonyx_jubatus	---MVNYMQKSQHSCEVCREF
Charadrius_vociferus	-------MQKKQRSCEVCCEF
Eptesicus_fuscus	---MVNYMQKSQHSCEVCPEF
Pterocles_gutturalis	-------MQKSQHSCEVCCEF
Colius_striatus	-------MQKSQHSCEVCSEF
Columba_livia	-------MQKTQHSCEVCCEF
Merops_nubicus	-------MQKSQHSCEVCCEF
Cuculus_canorus	-------MQKSQHCCEVCCEF
Falco_cherrug	-------MQKSQHSCEVCCEF
Gallus_gallus	-------MRKSQHSCEVCCEF
Gavia_stellata	-------MQKSQHSCEVCCEF
Mesitornis_unicolor	-------MQKSQRSCEVCREF
Condylura_cristata	---MVNYMQKTQHSCEVCCEF
Ornithorhynchus_anatinus	---MVNYMQKSQHSCEVCCEF
Tauraco_erythrolophus	-------MQKSQHSYEVCCEC
Opisthocomus_hoazin	-------MQKGQHSCEVCFEY
Acanthisitta_chloris	-------MQKSQPSCEVCCEF
Pelecanus_crispus	-------MQKSQHSCEVCCEF
Equus_caballus	---MVNYMQKSQHSCEVCCEF
Manis_javanica	---MVNYMQKSQHSCEVCCEF
Picoides_pubescens	-------MQKSQHSCEVCCEF
Loxodonta_africana	---MVNYMQKSQRSCEVCREF
Fulmarus_glacialis	-------MQKSQHSCEVCCEF
Nestor_notabilis	-------MQISQHSCEVCCEF
Marmota_marmota_marmota	---MVNYMQKSQHSCEVCCEF
Trichechus_manatus_latirostr	---MVNYMQKSQRSCEVCREF
Aptenodytes_forsteri	-------MQKSQHSCEVCCEF
Chelonia_mydas	---MVNYMQKSQHRWEVCCEF
Calypte_anna	-------MQKSQHSCEVCCEF
Apaloderma_vittatum	-------MQTTQHSCEVCCEF
Orycteropus_afer_afer	---MVNYMQKSQHSCEVCREF
Conservation	-------*++8*8++***5*+

HG0053.2

ENSG00000184564 (SLITRK6)

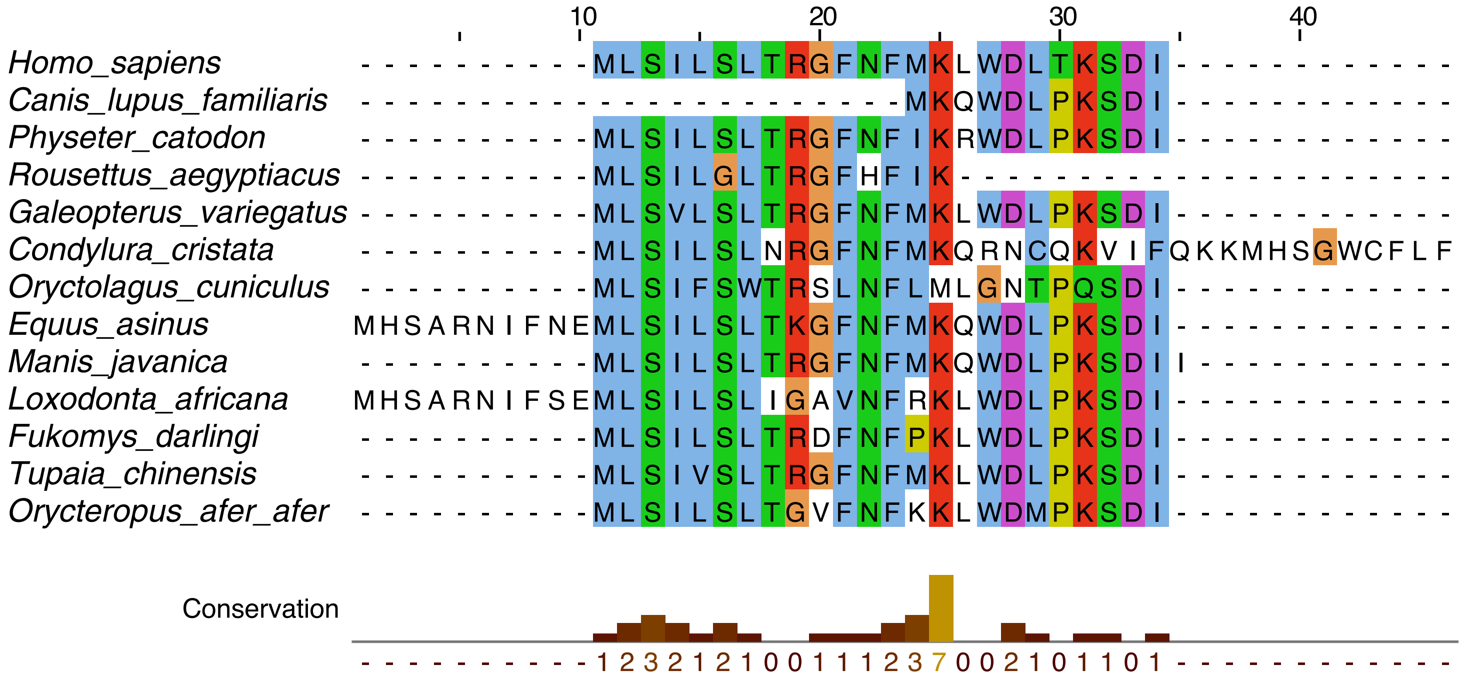

HG0054

ENSG00000124479 (NDP)

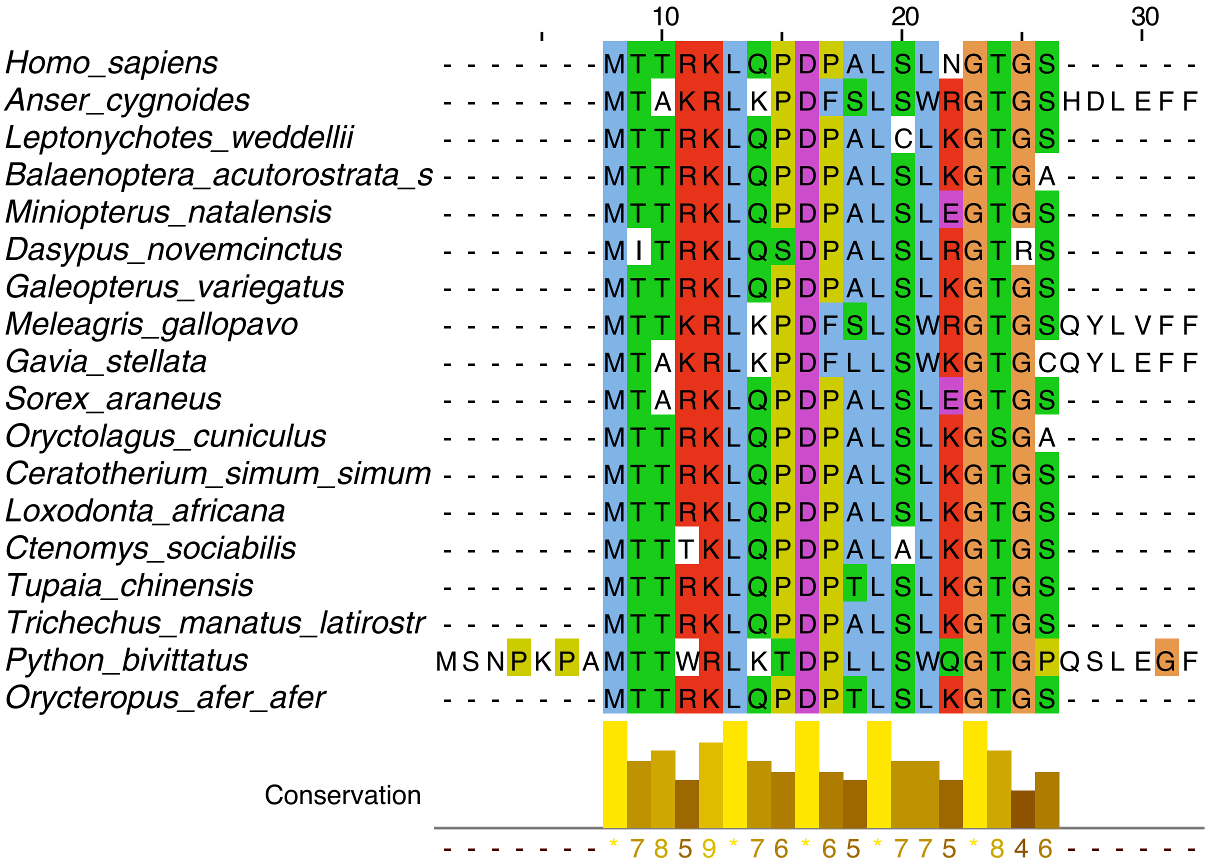

HG0054

ENSGALG00000016222 (NDP)

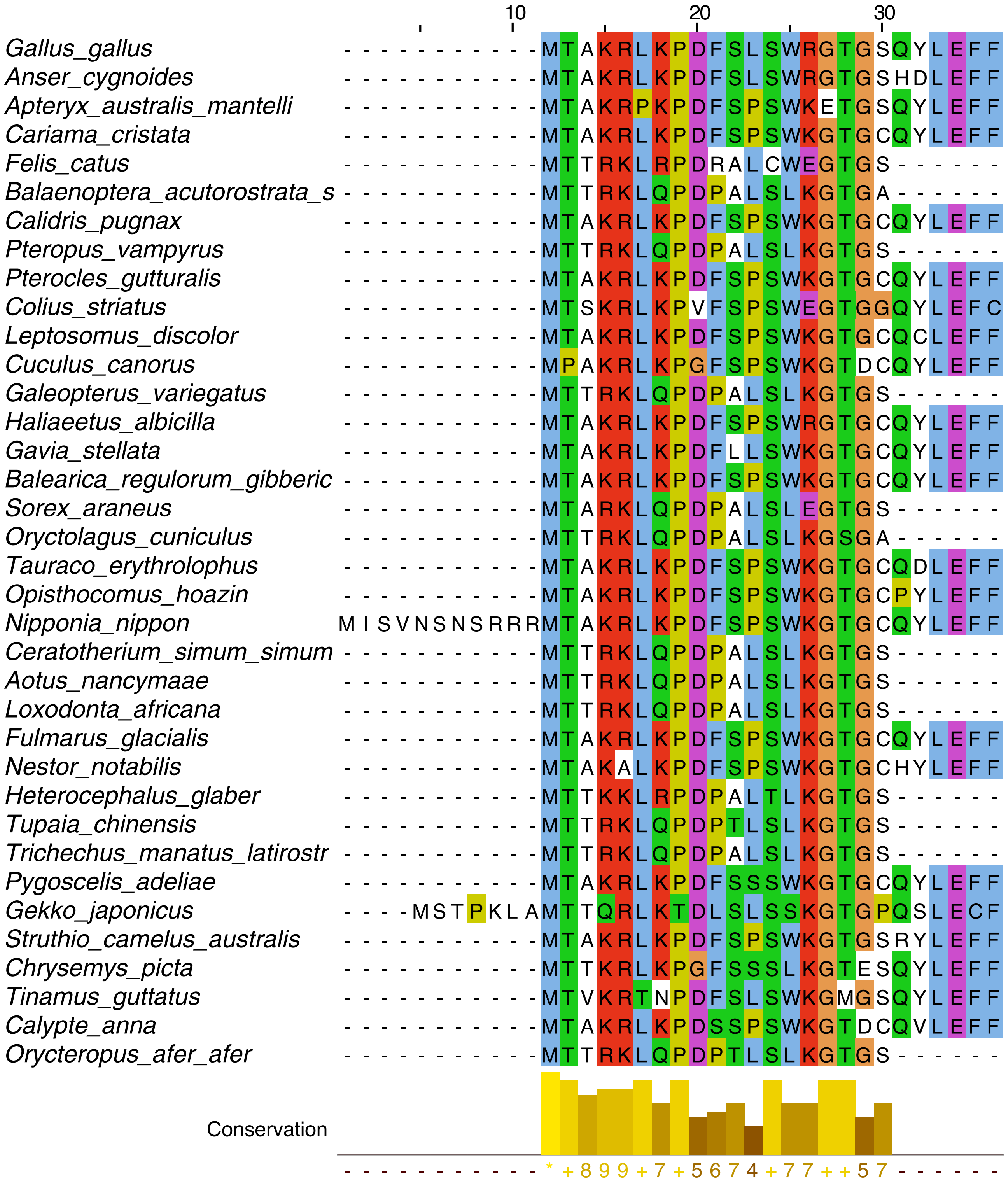

HG0055.1

ENSGALG00000010533 (ELAVL4)

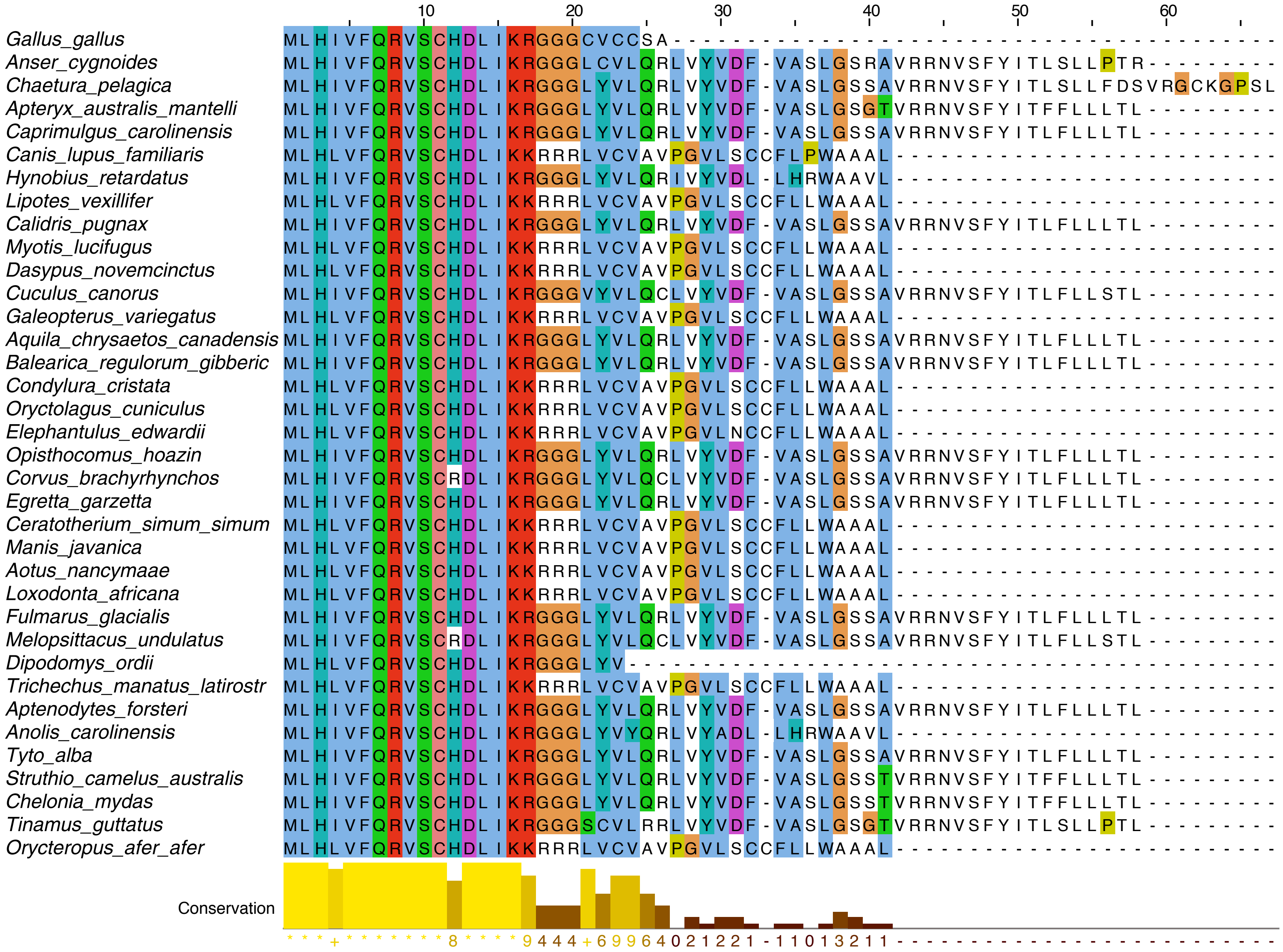

HG0055.2

ENSGALG00000010533 (ELAVL4)

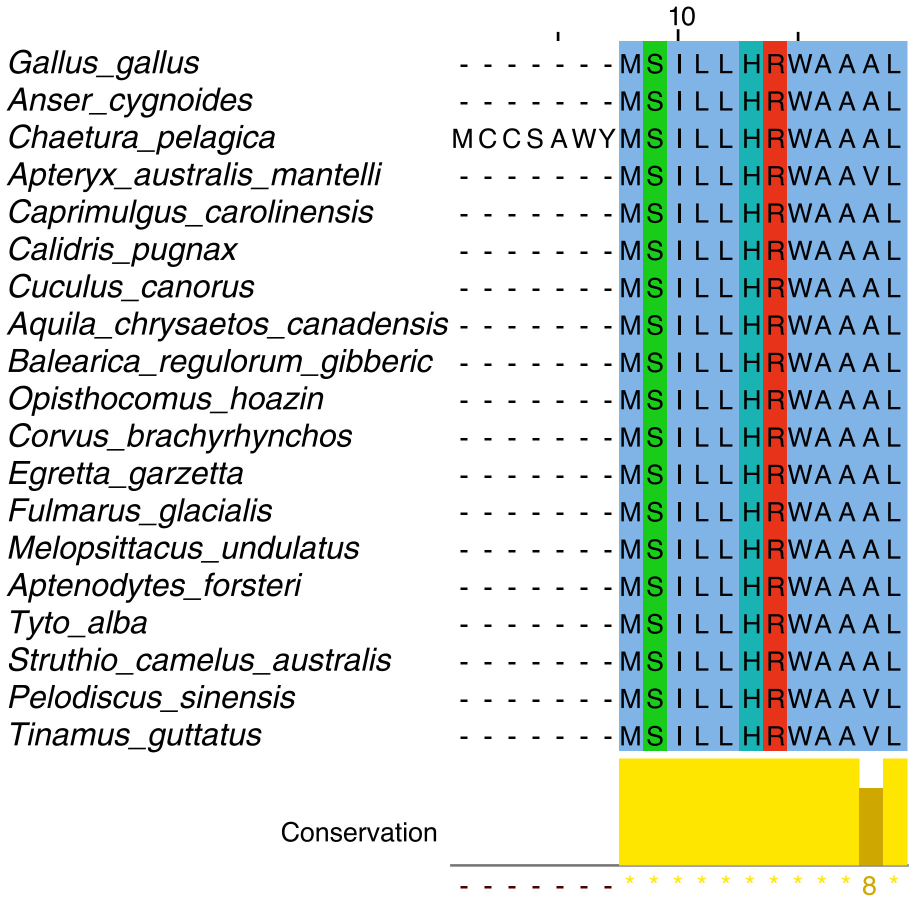

HG0056

ENSG00000139329 (LUM)

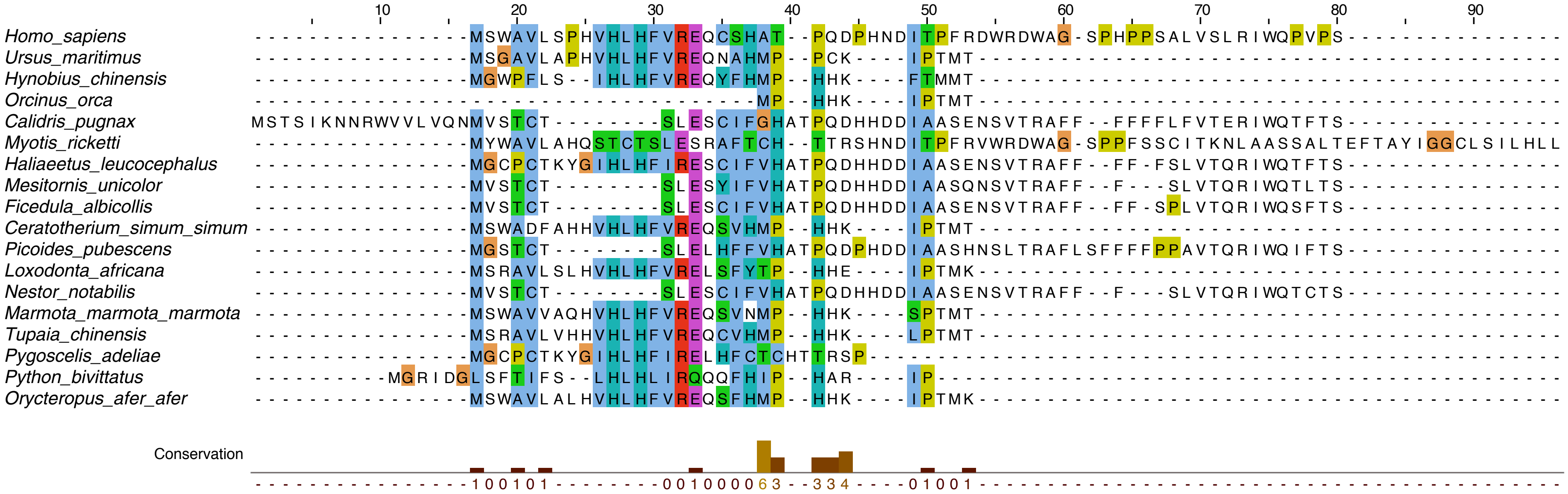

HG0056

ENSGALG00000011271 (LUM)

```
                                     10        20        30
Gallus_gallus                MGCPCTECGIHLHFIKRAAFSYMPHHKITMMT
Anser_cygnoides              ----------------------MPHHKITMMT
Apteryx_australis_mantelli   ----------------------MPHHKITMMT
Caprimulgus_carolinensis     ----------------------MPHHKITMMT
Cariama_cristata             ----------------------MPHHKITMMT
Hynobius_chinensis           MGWPF--LSIHLHFVRE-QYFHMPHHKFTMMT
Charadrius_vociferus         ----------------------MPHHKITMMT
Myotis_ricketti              ----------------------MPHHKIPQ--
Pterocles_gutturalis         ----------------------MPHHKITMMT
Latimeria_chalumnae          ---------MHLHLVKE-LCFFIPHHKLIMMT
Colius_striatus              MDCPYTKYGIHLHFIRE-LHFCSCHTTISMMT
Columba_livia                ----------------------MPHHKITMMT
Merops_nubicus               ----------------------MPHHKITMMT
Alligator_sinensis           ----------------------MPHHKITMMT
Haliaeetus_leucocephalus     MVS-------TCTSLERAAFLYMPHHRITMMT
Gavia_stellata               ----------------------MPHHKITMMT
Chlamydotis_macqueenii       MGCPCTKCGIHLHFIRE-LHFRTCYTTNSP--
Condylura_cristata           ----------------------MPHHKIPTMT
Ficedula_albicollis          MGCPCTKYGIHLHFIRE-LHFCTCHTTRSP--
Nipponia_nippon              MGCPCTKYGIHLHFIRD-LHFSTCHTTRSP--
Equus_przewalskii            ----------------------MPHHKIPTMT
Picoides_pubescens           MGCPYTKYGIHLHFIRA-AFFCPCHTTRSP--
Propithecus_coquereli        MNWAVLAPHVHLHFVRE-QSVHMPHHKIPTMT
Loxodonta_africana           MSRAVLSLHVHLHFVRE-LSFYTPHHEIPTMK
Fulmarus_glacialis           ----------------------MPHHKITMMT
Nestor_notabilis             ----------------------MPHHKITMMT
Tupaia_chinensis             MSRAVLVHHVHLHFVRE-QCVHMPHHKLPTMT
Pygoscelis_adeliae           ----------------------MPHHKITMMT
Gekko_japonicus              ----------------------MPHHQNPMMT
Tyto_alba                    ----------------------MPHHKITMMT
Struthio_camelus_australis   ----------------------MPHHKITMMT
Pelodiscus_sinensis          MGCPCTVYTVHLHFIRE-QHLYIPHHKIIMMT
Tinamus_guttatus             MGCPCTKYGIHLHFITE-LHFCTYHTTRSL--
Apaloderma_vittatum          ----------------------MPHHKITMMT
Orycteropus_afer_afer        MSWAVLALHVHLHFVRE-QSFHMPHHKIPTMK
```

Conservation

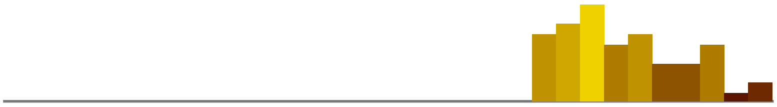

```
----------------------78+6744612
```

HG0057

ENSG00000280987 (MATR3)

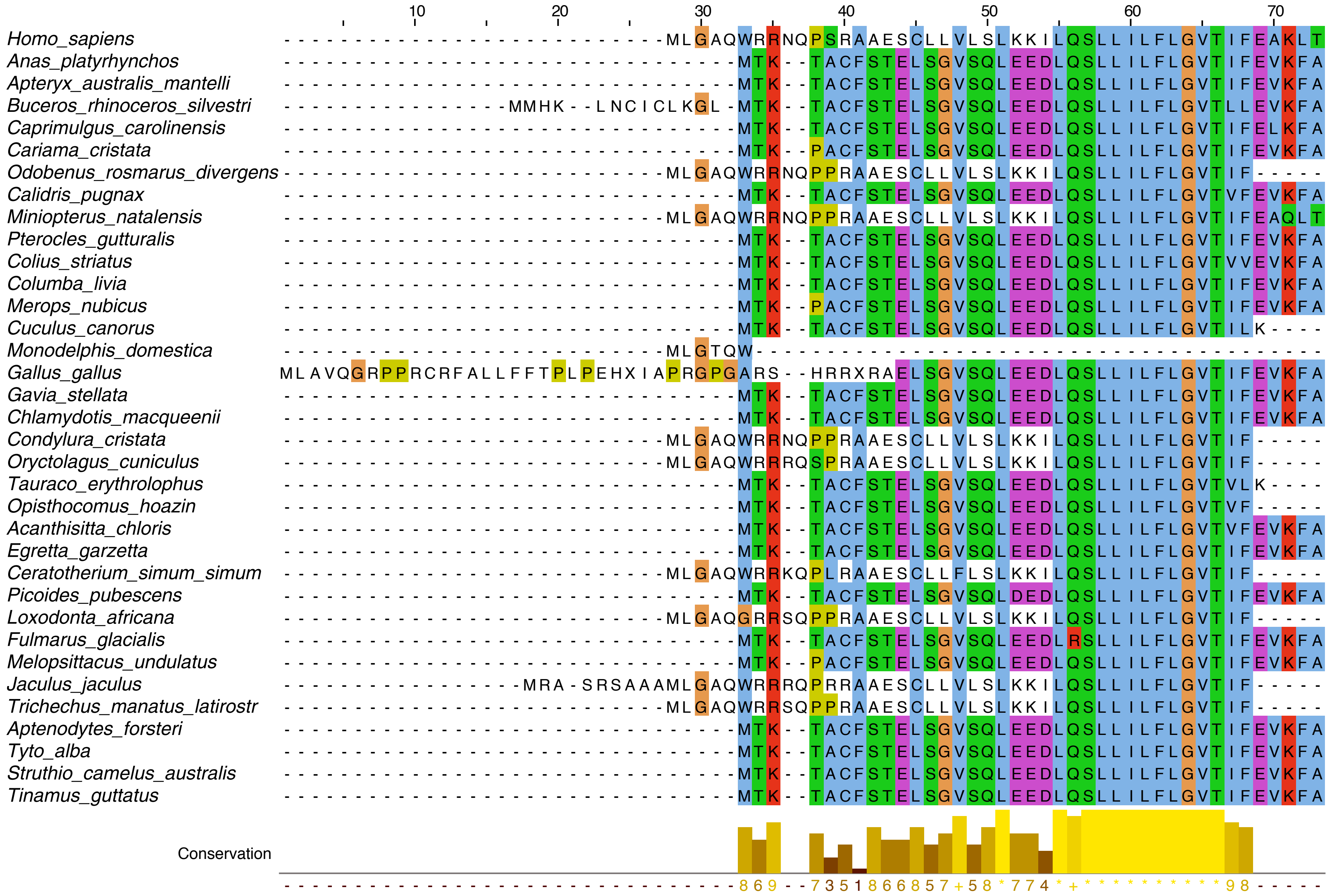

HG0058

ENSG00000180332 (KCTD4)

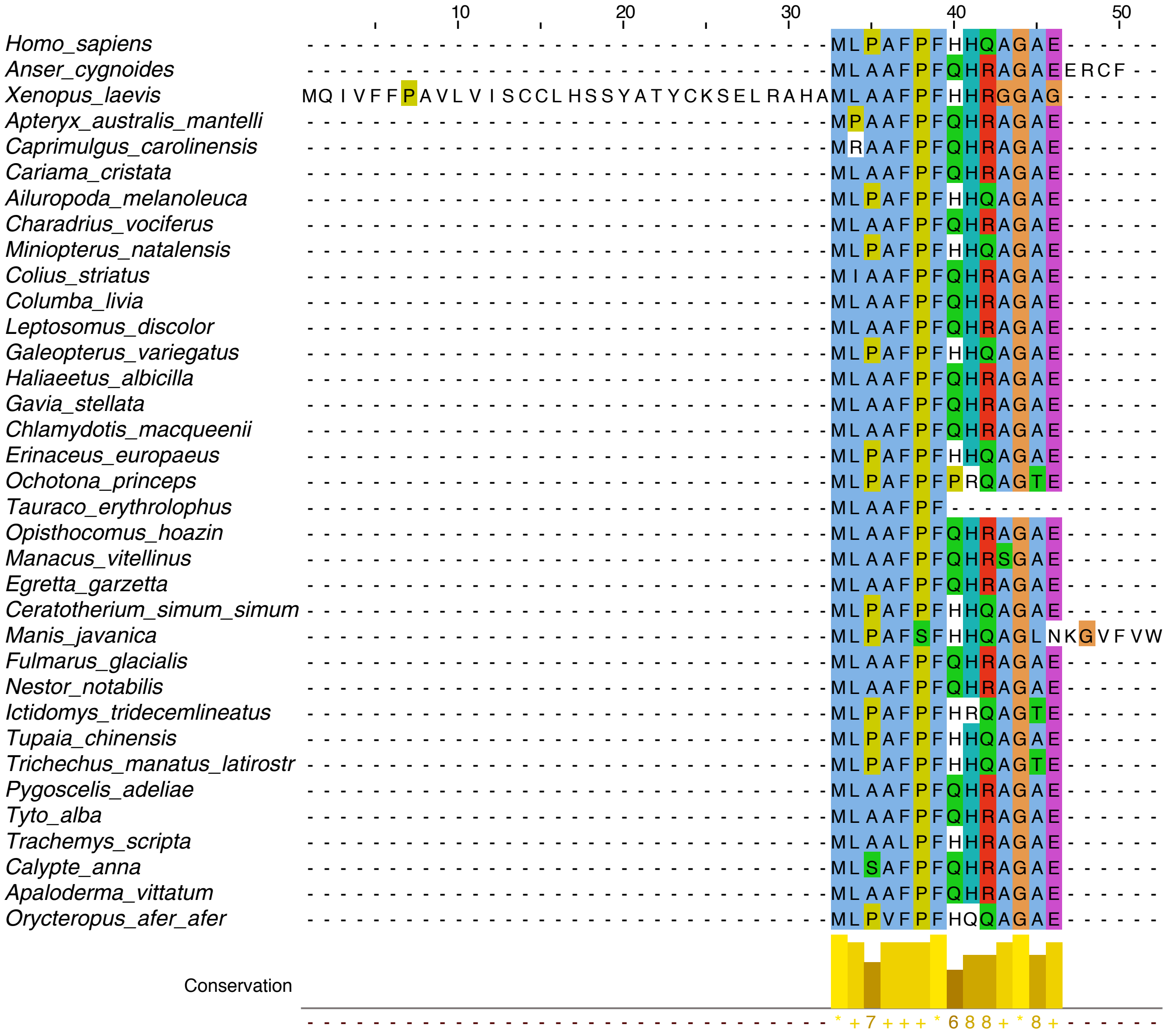

HG0059.1

ENSDARG00000018060 (pik3r2)

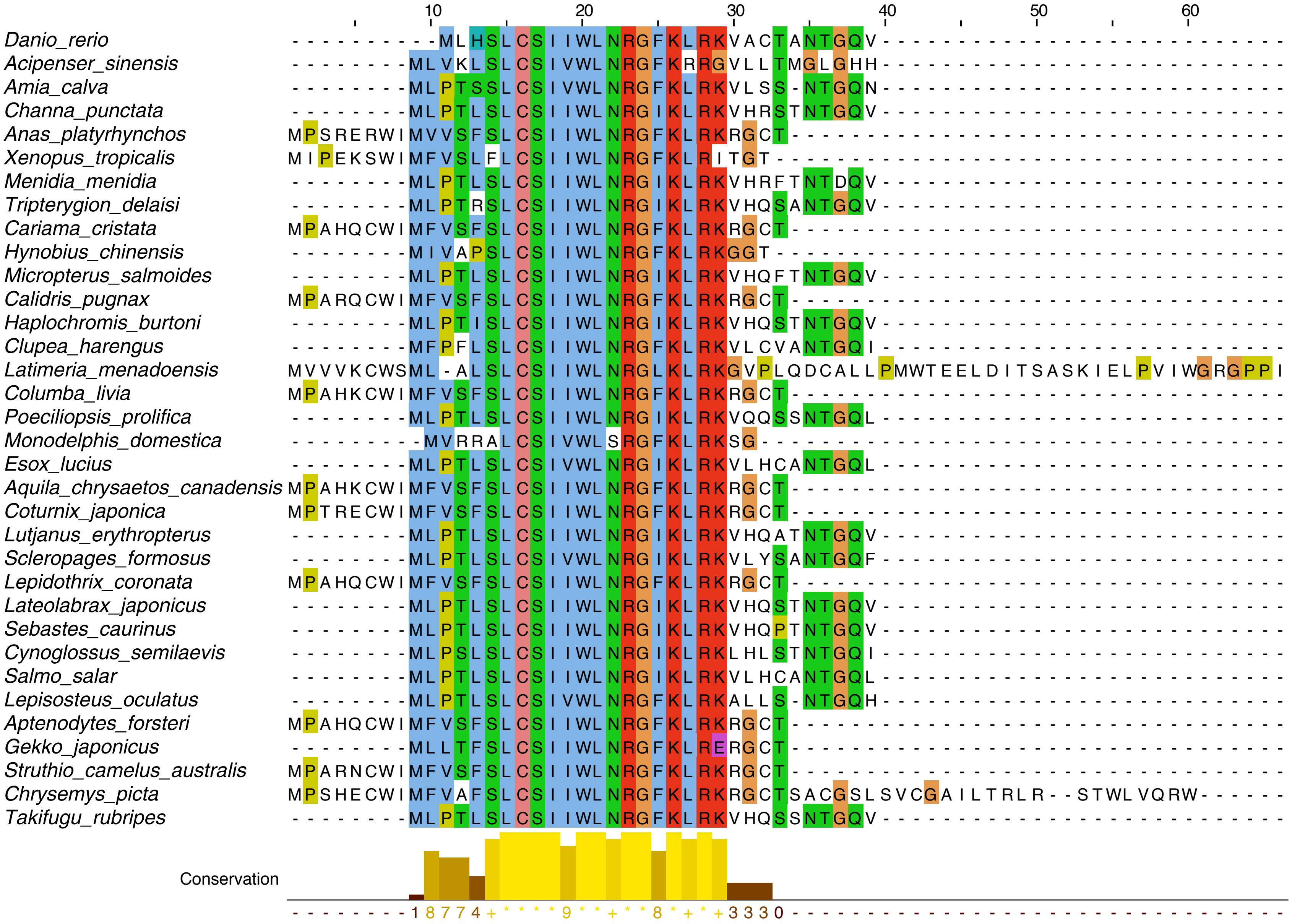

HG0059.1

ENSGALG00000003428

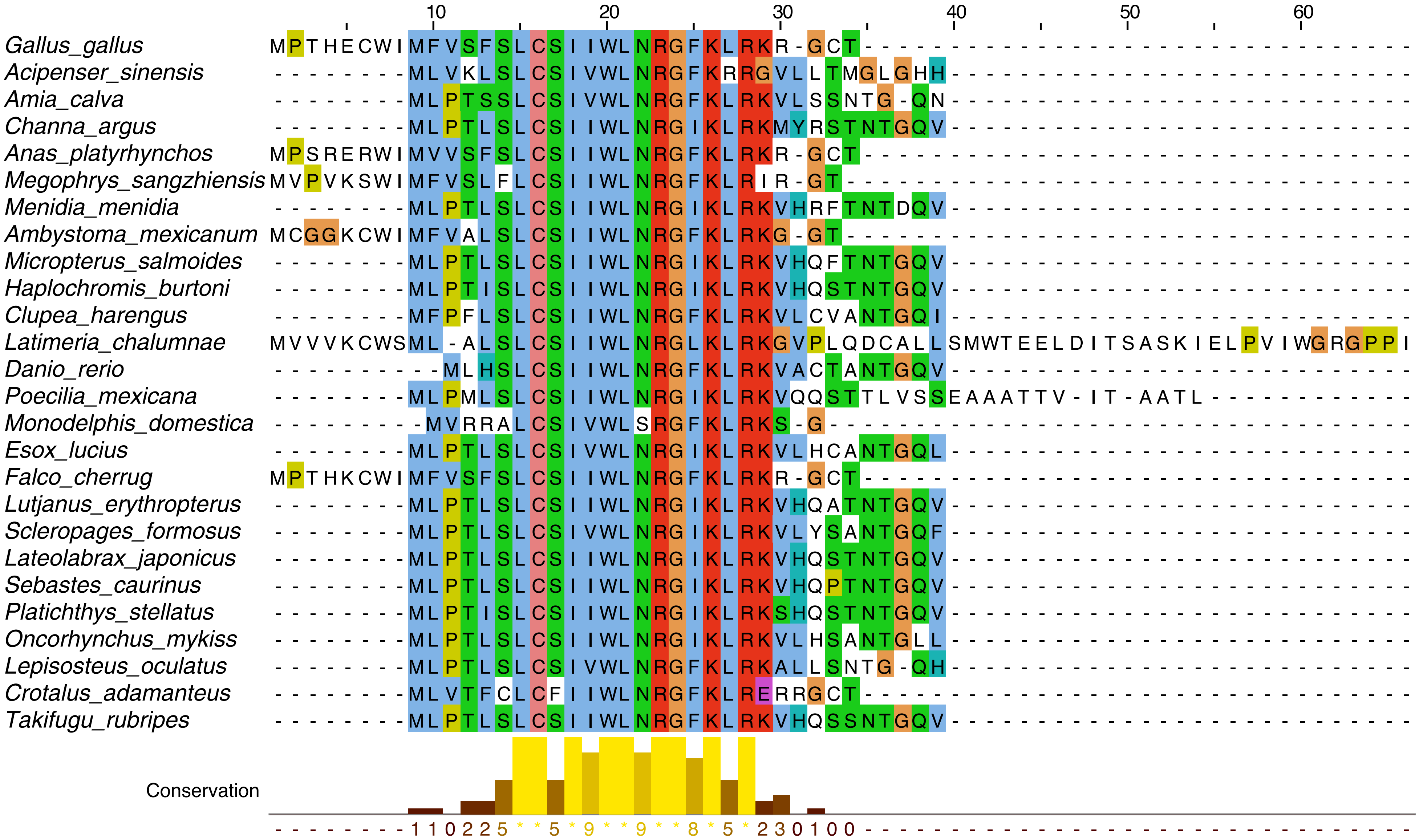

HG0059.2

ENSG00000145675 (PIK3R1)

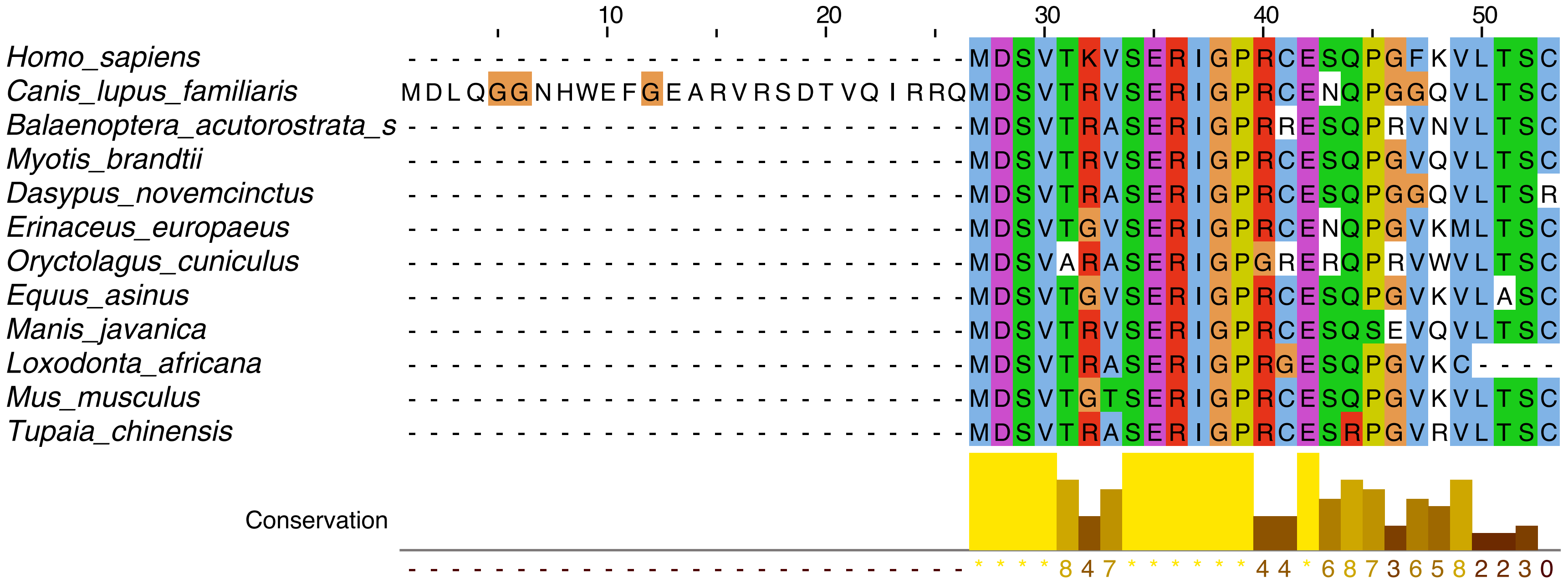

HG0059.3

ENSG00000105647 (PIK3R2)

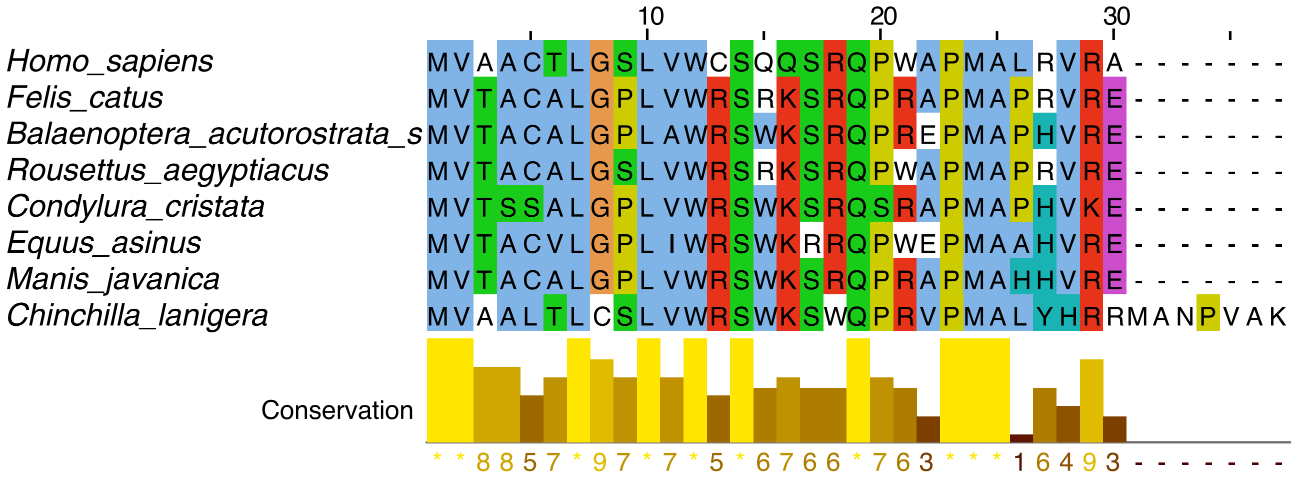

HG0059.4

ENSG00000145675 (PIK3R1)

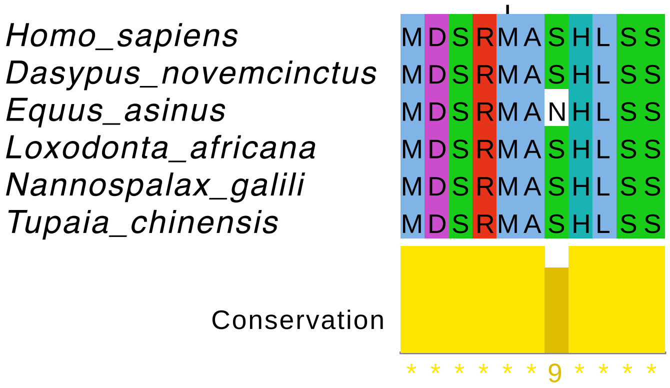

HG0059.5

ENSDARG00000038524 (pik3r1)

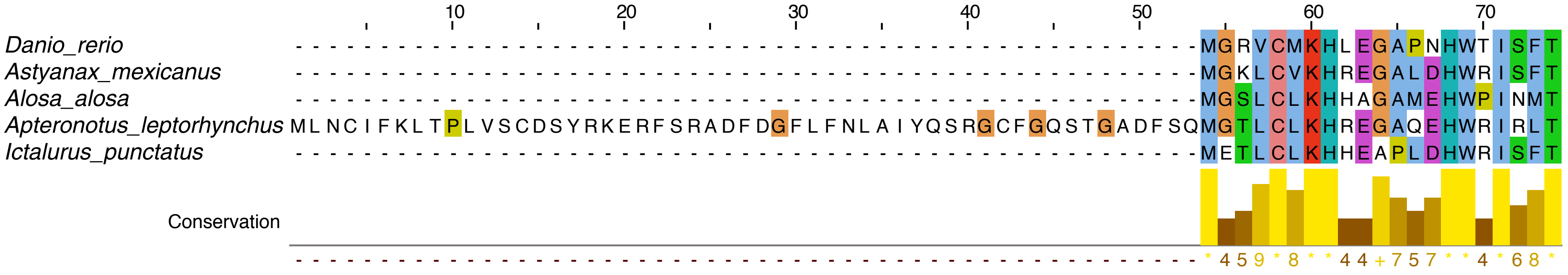

HG0060.1

ENSG00000049192 (ADAMTS6)

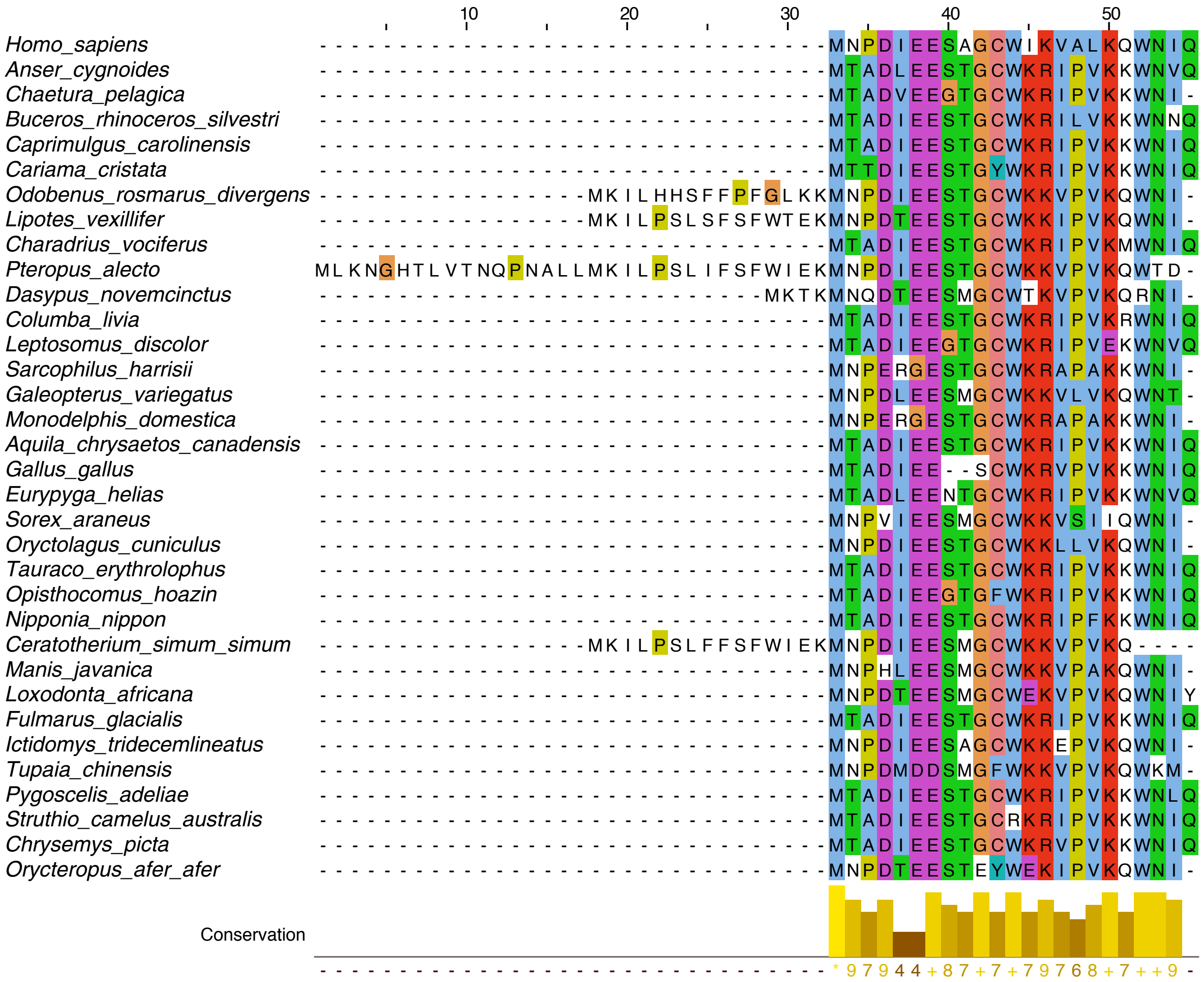

HG0060.1

ENSGALG00000014751

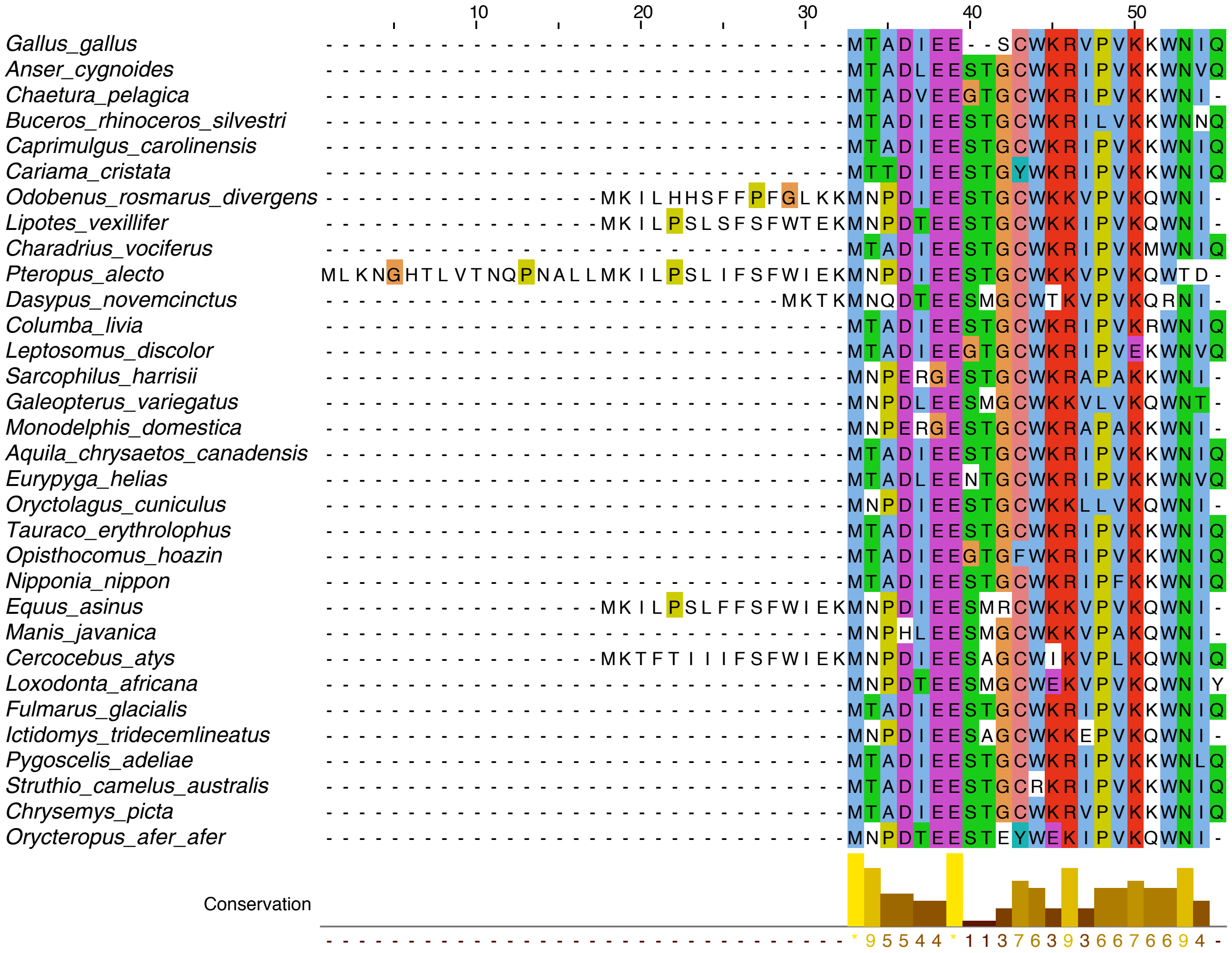

HG0060.2

ENSG00000049192 (ADAMTS6)

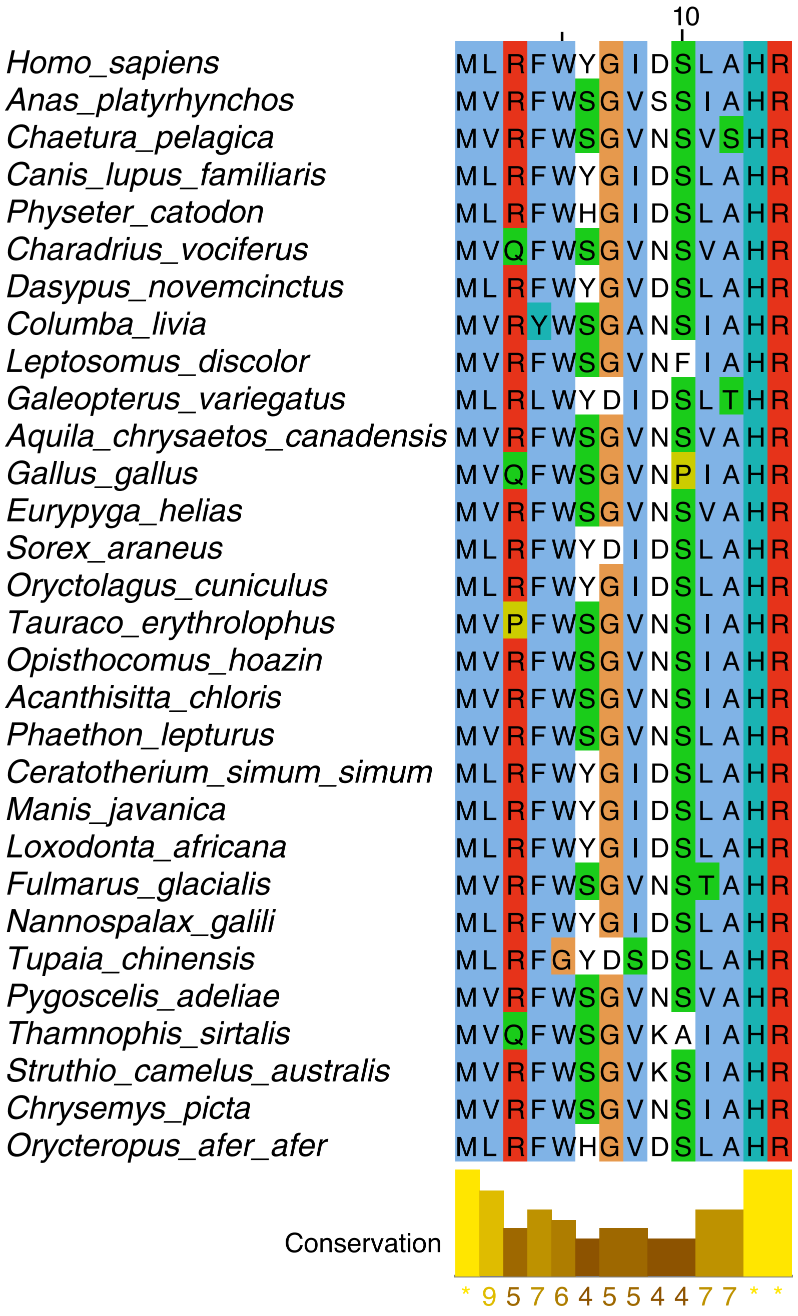

HG0060.2

ENSGALG00000014751

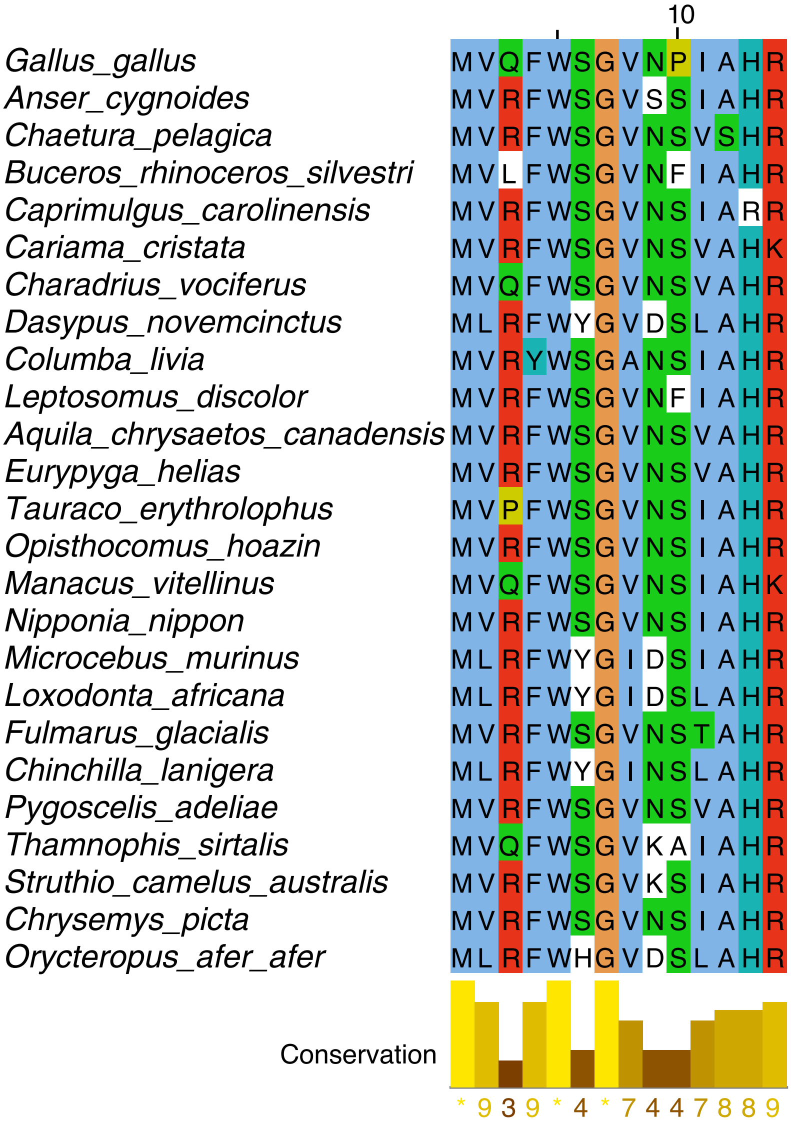

HG0061.1

ENSG00000166398

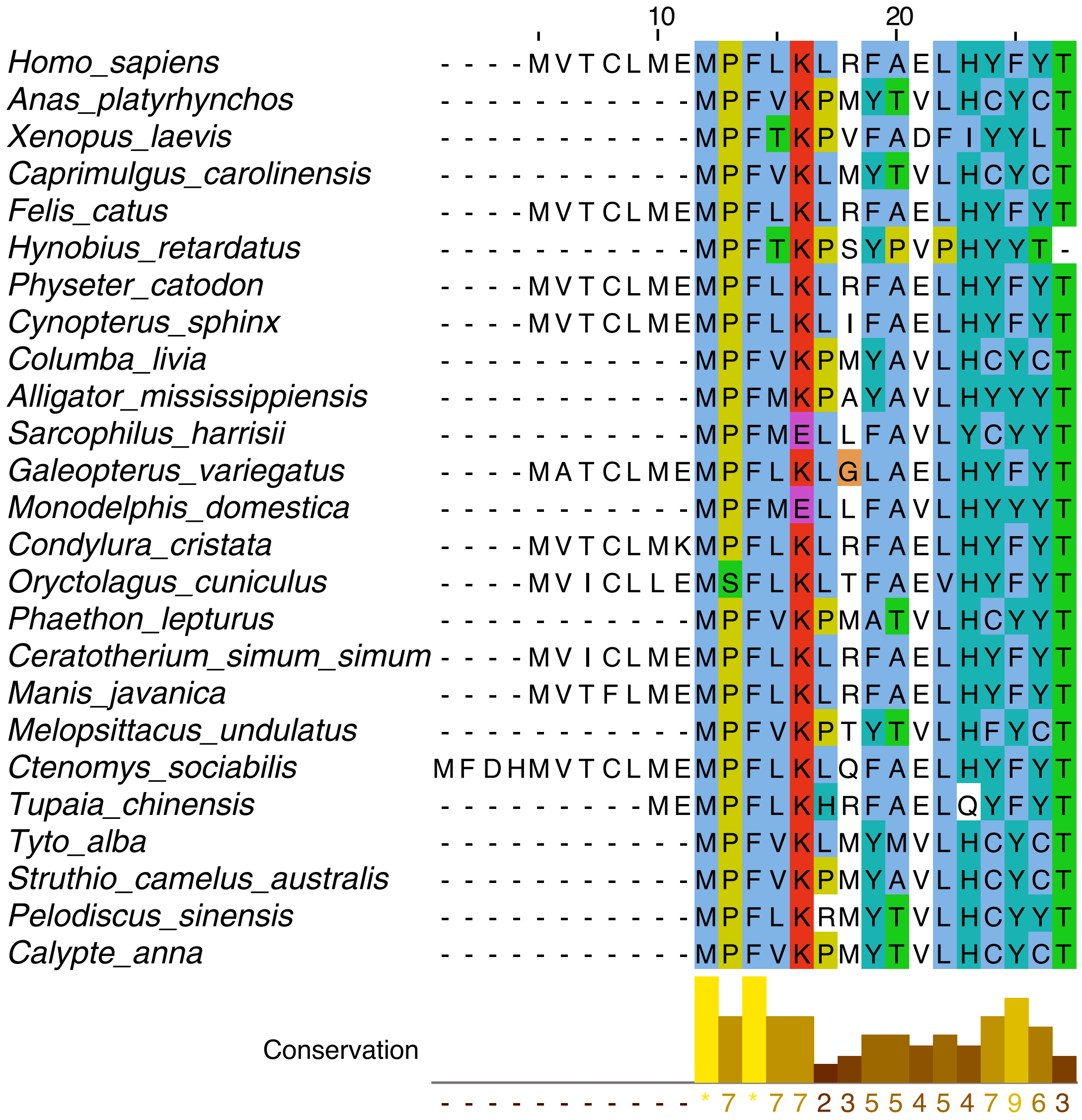

HG0061.1

ENSGALG00000004949

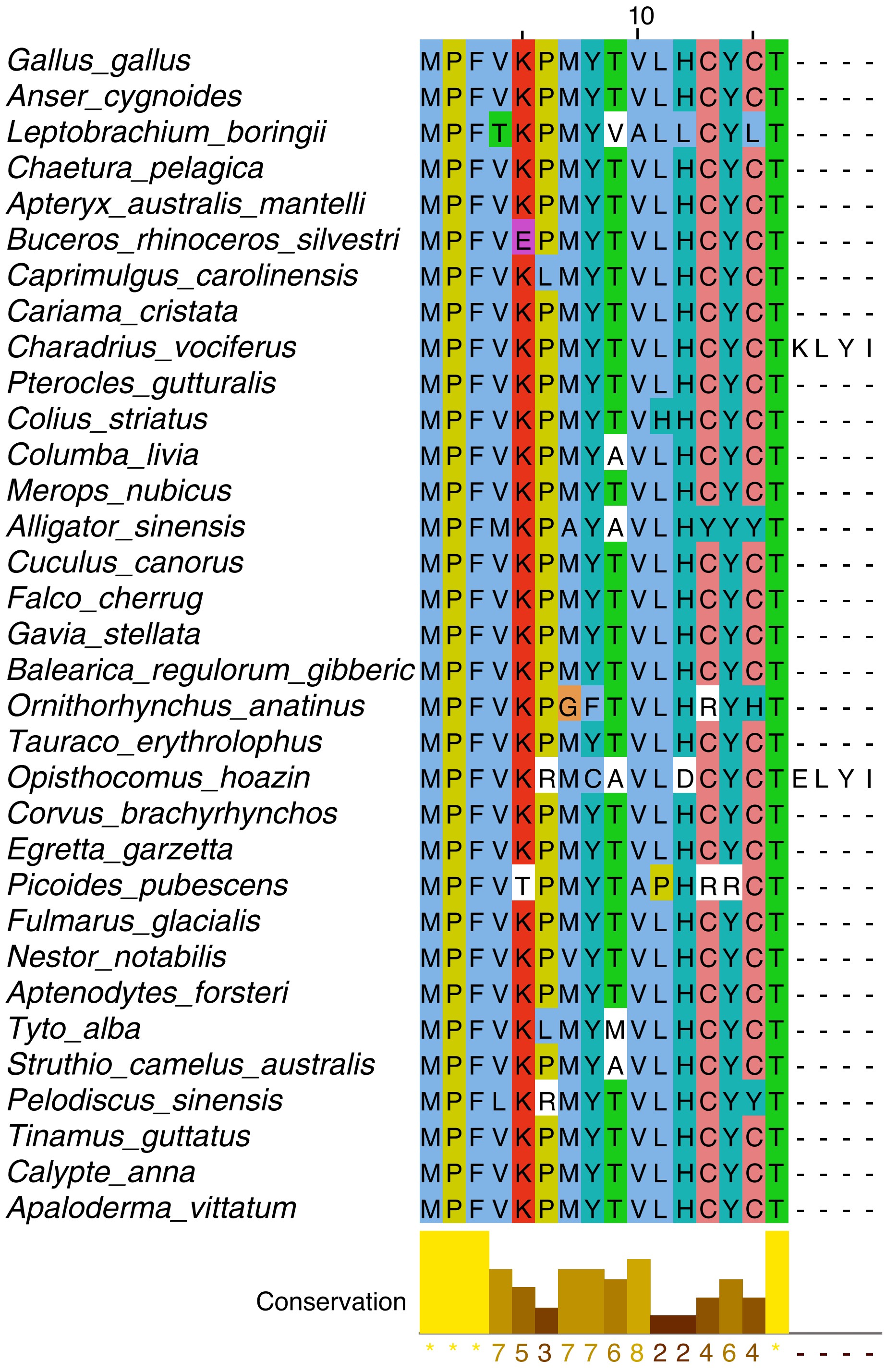

HG0061.2

ENSGALG00000004949

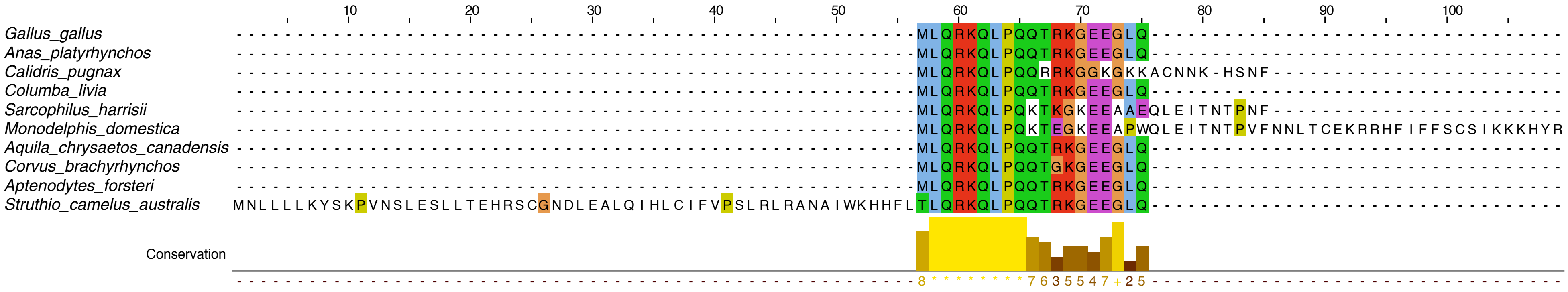

HG0061.3

ENSG00000166398

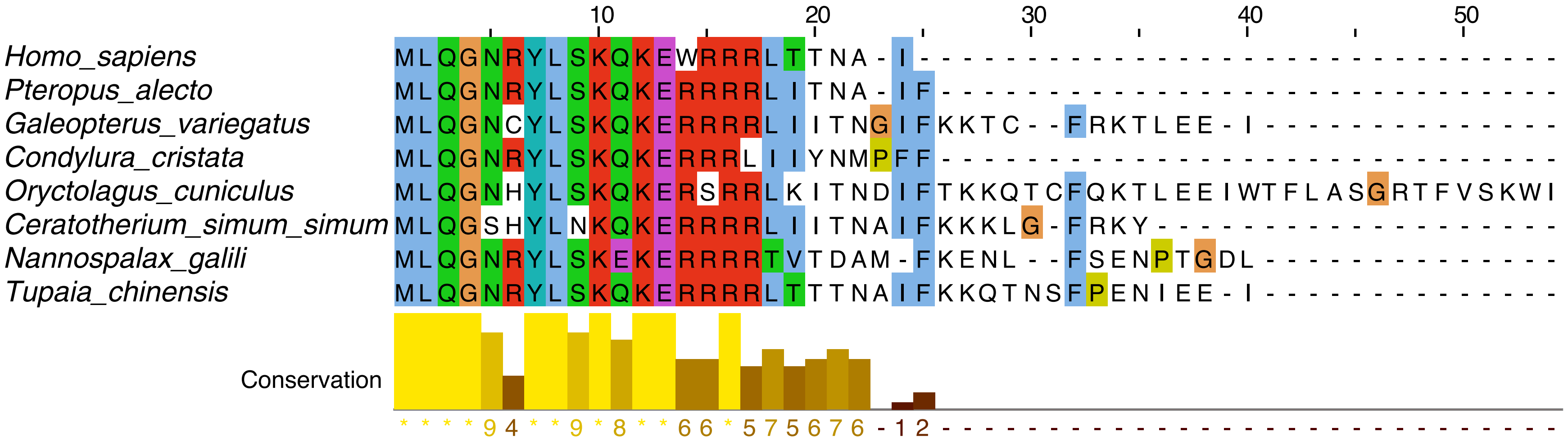

HG0062.1

ENSDARG00000075569 (gpbp1l1)

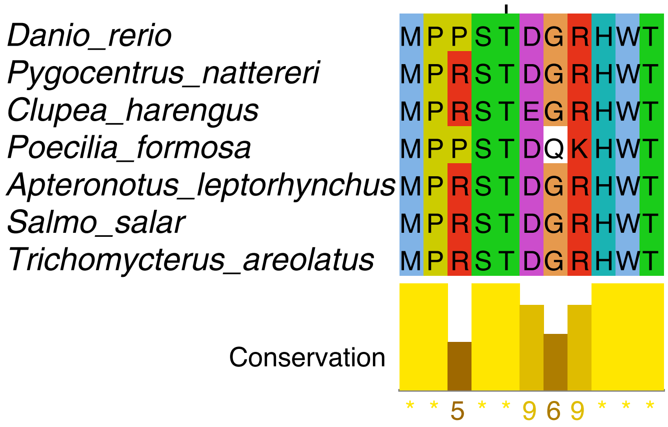

HG0062.1

ENSGALG00000010276 (GPBP1L1)

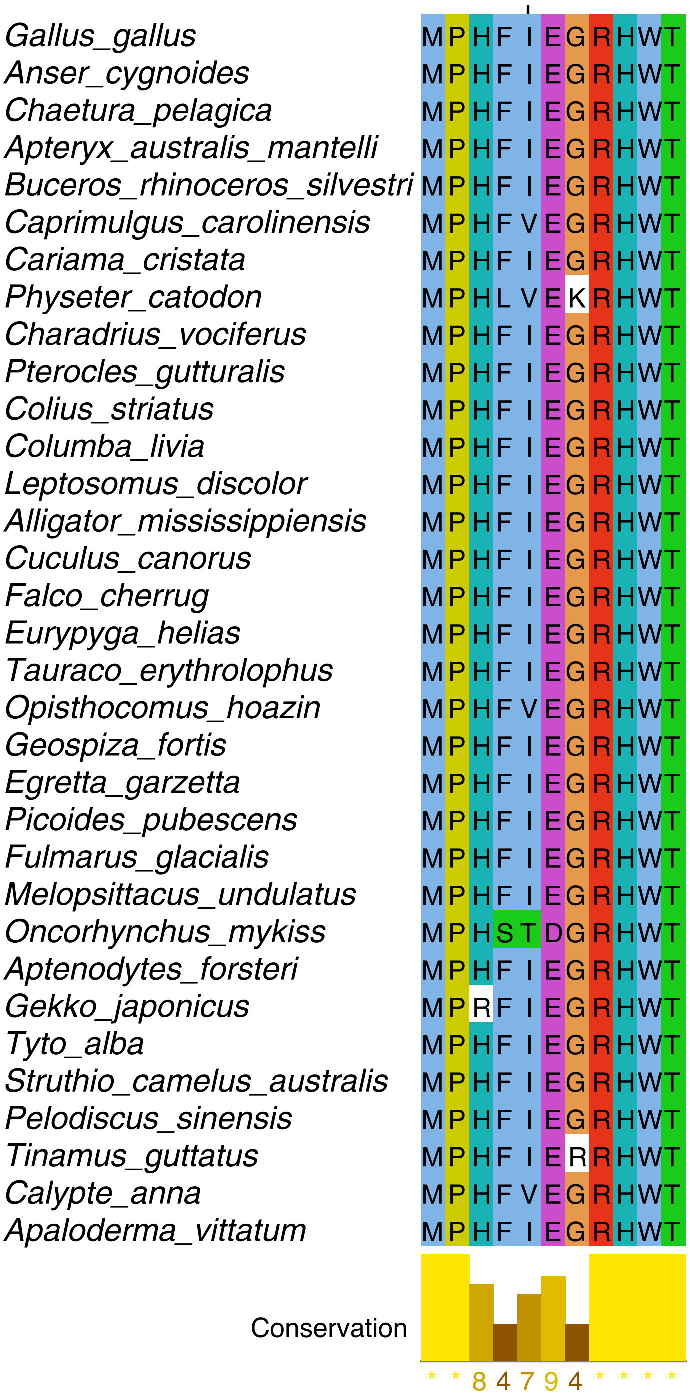

HG0062.2

ENSGALG00000010276 (GPBP1L1)

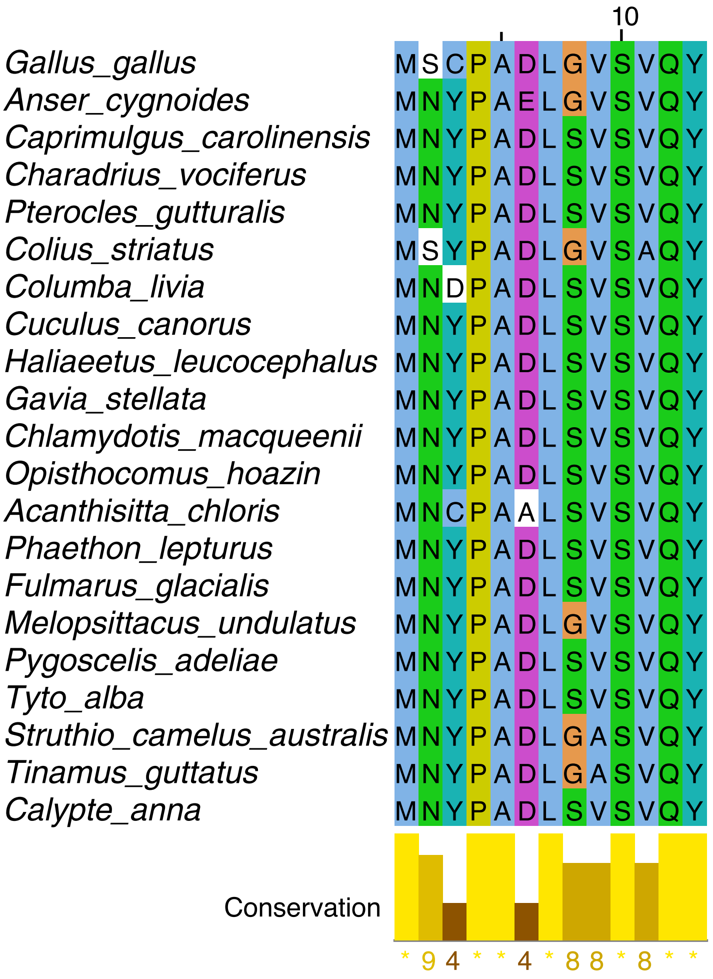

HG0062.3

ENSG00000159592 (GPBP1L1)

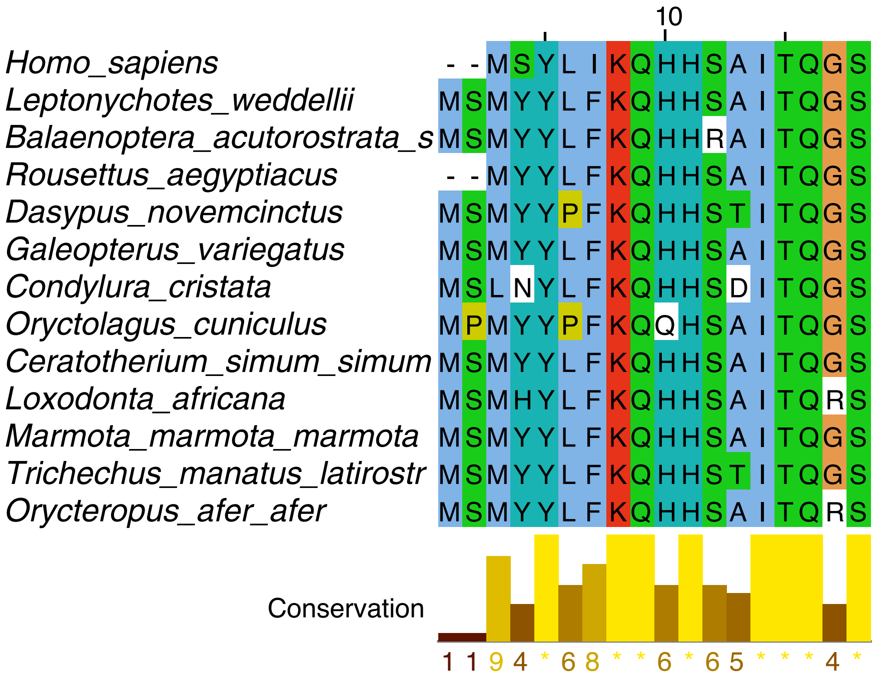

HG0063.1

ENSG00000162630 (B3GALT2)

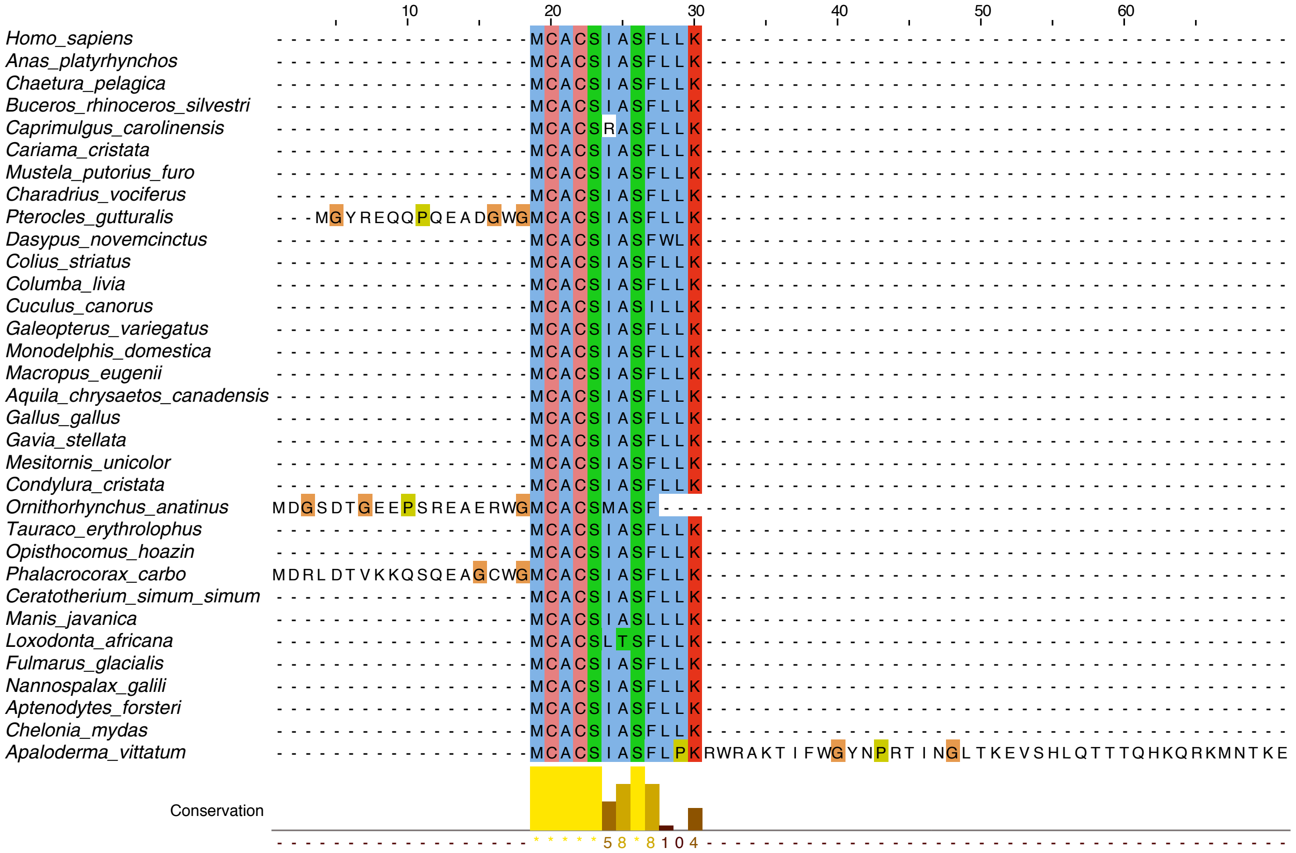

HG0063.1

ENSGALG00000002477

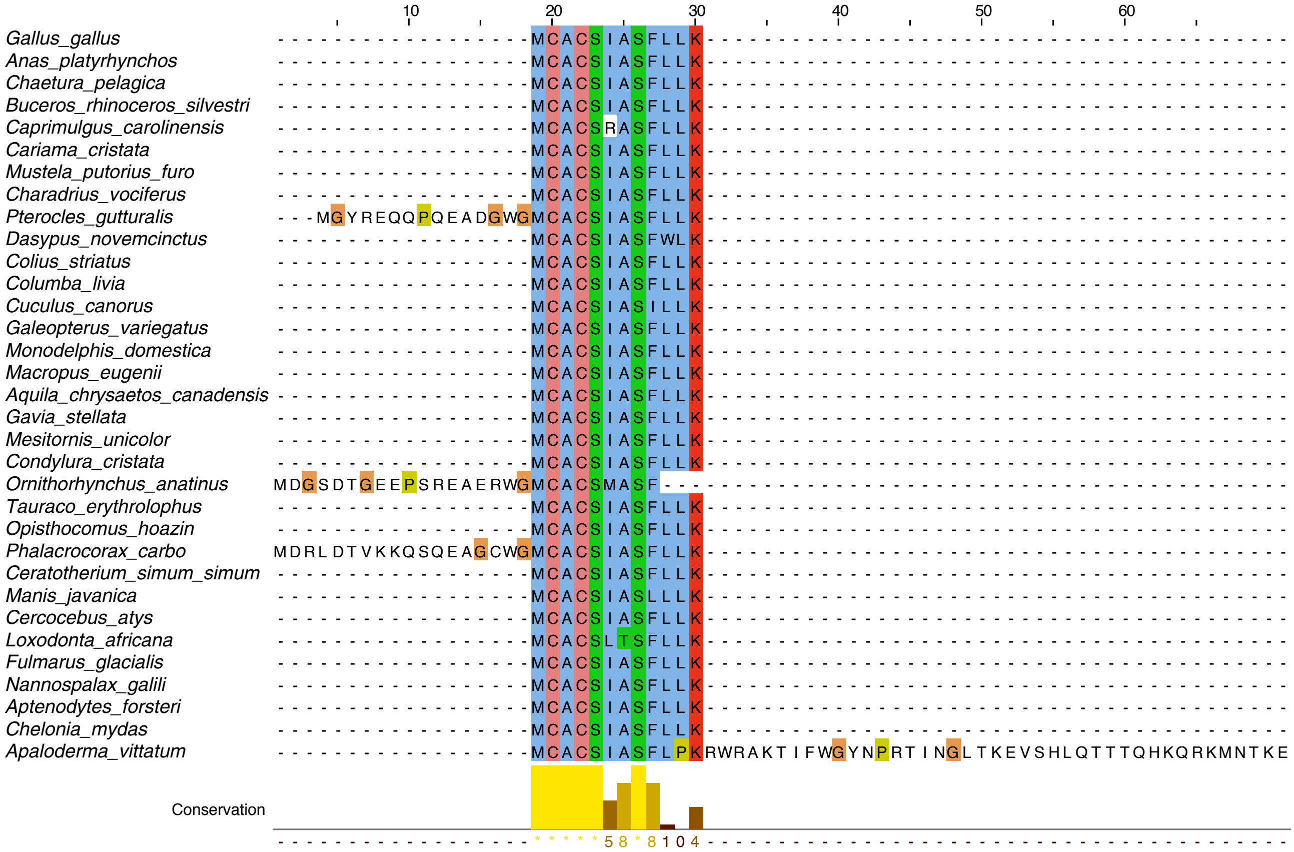

HG0063.2

ENSG00000162630 (B3GALT2)

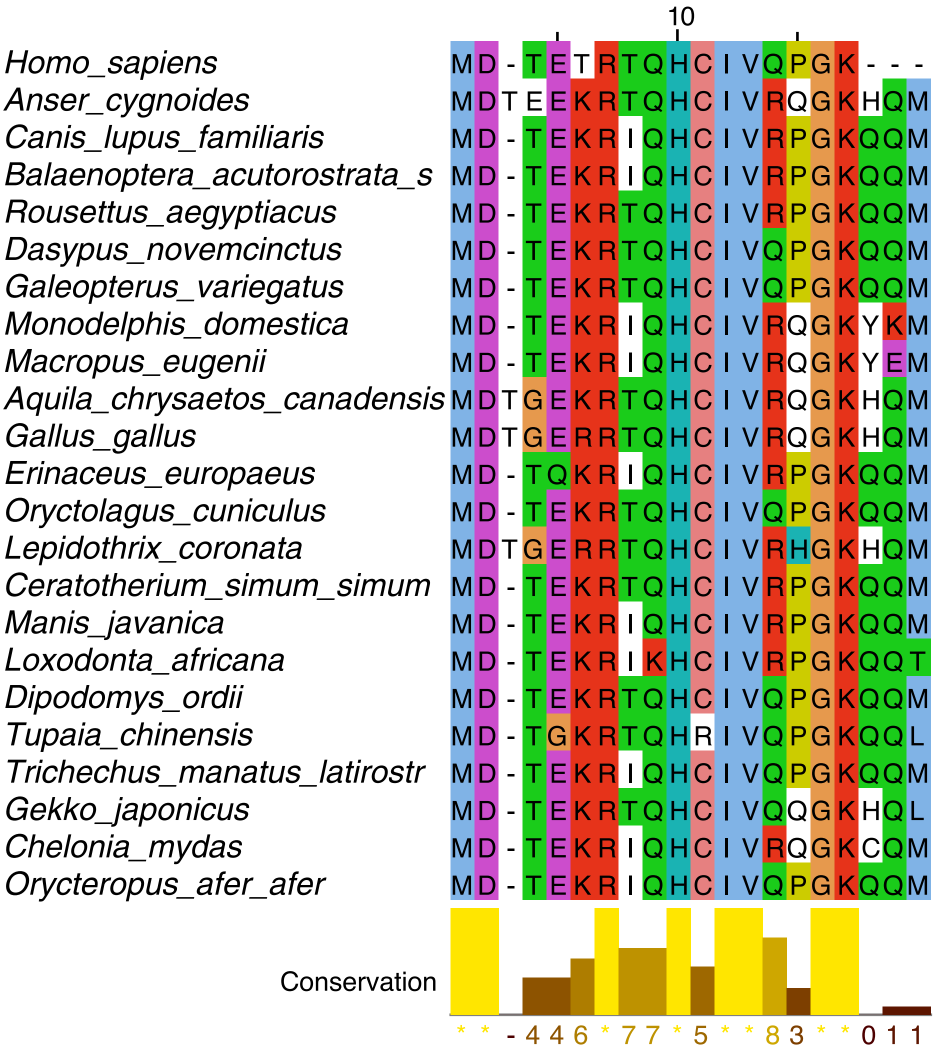

HG0064.1

ENSG00000102678 (FGF9)

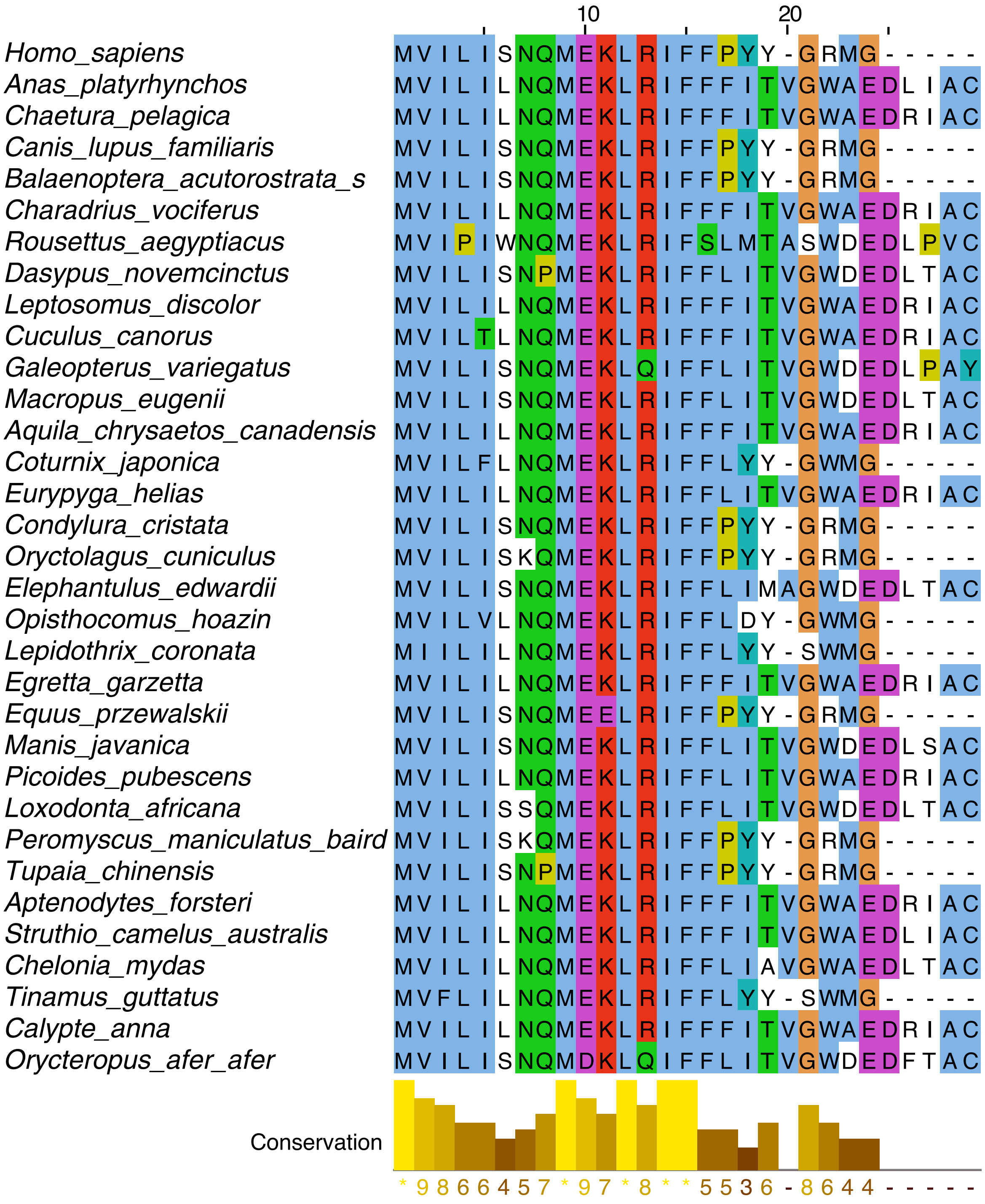

HG0064.2

ENSG00000113578 (FGF1)

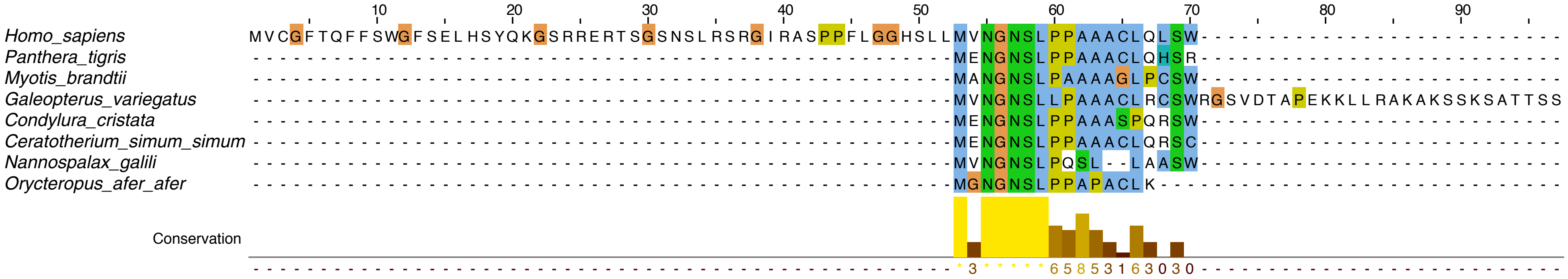

HG0065

ENSGALG00000029927 (STRBP)

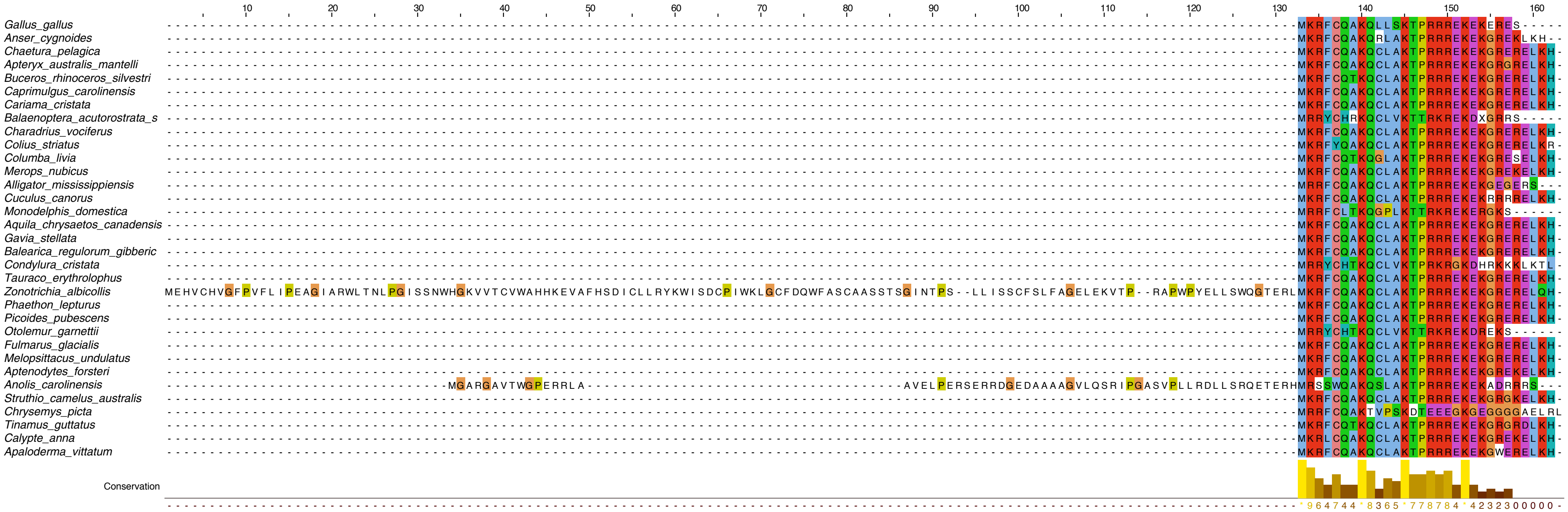

HG0066

ENSG00000112182 (BACH2)

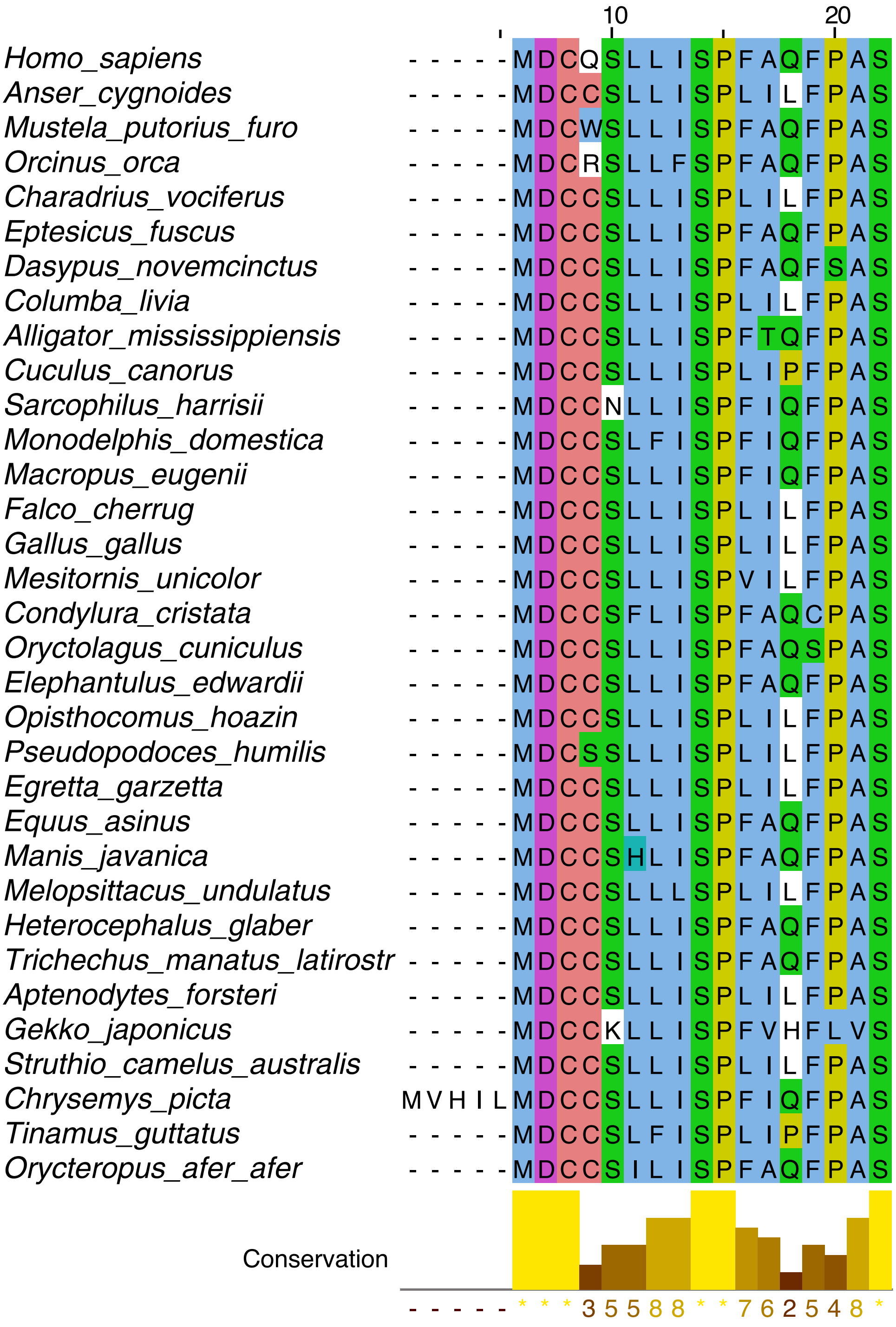

HG0067

ENSG00000166862 (CACNG2)

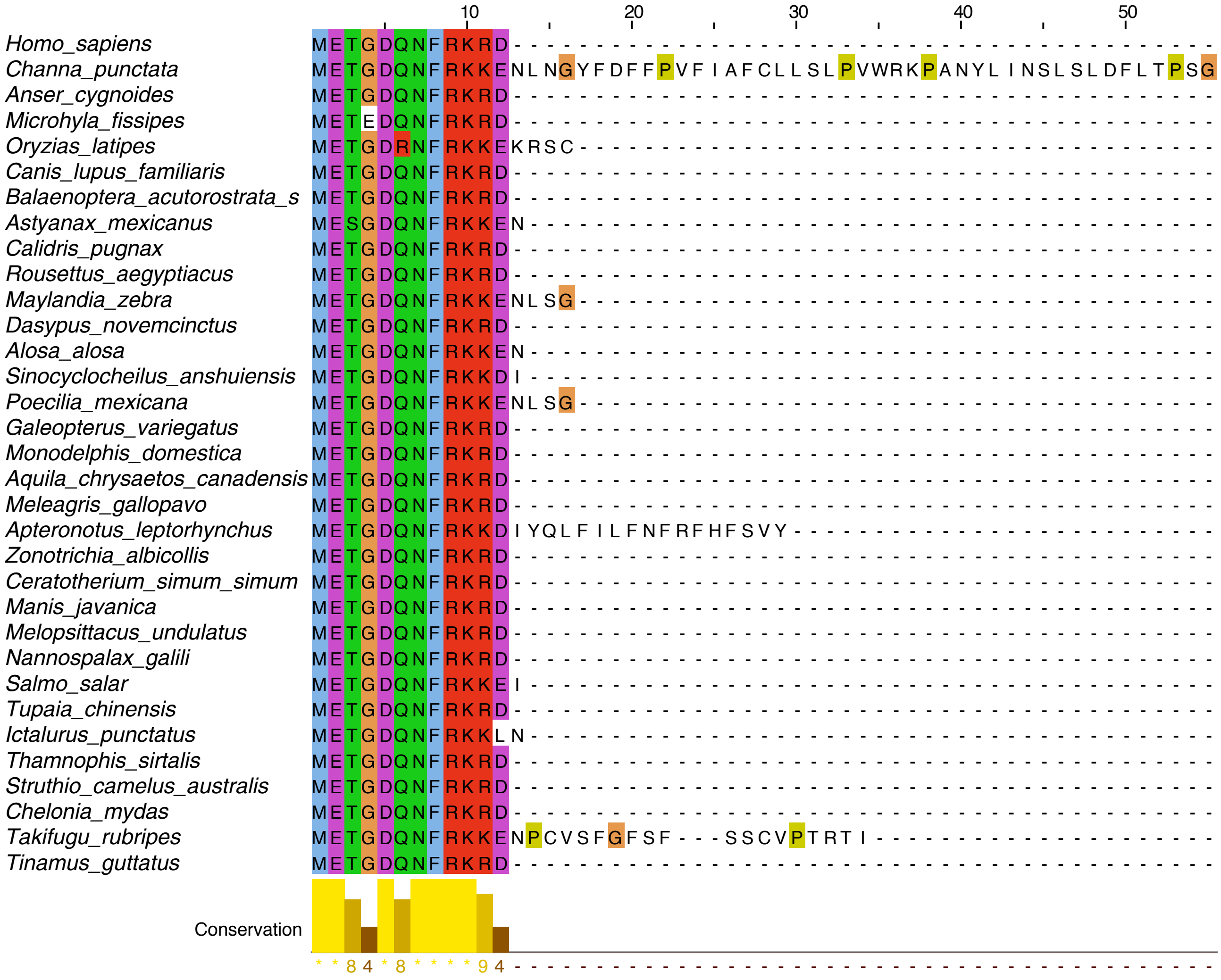

HG0068

ENSDARG00000071235

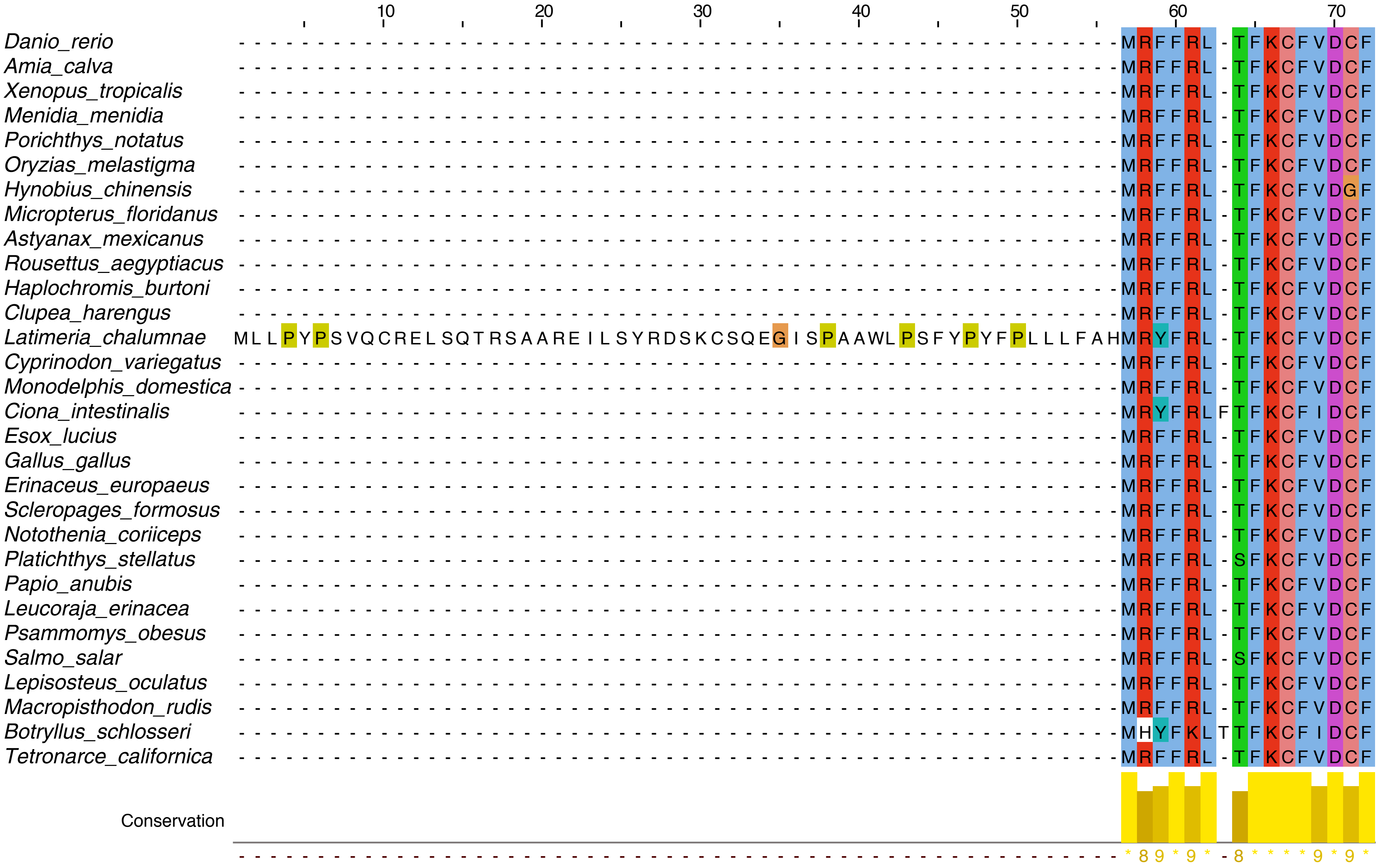

HG0068

ENSG00000130821 (SLC6A8)

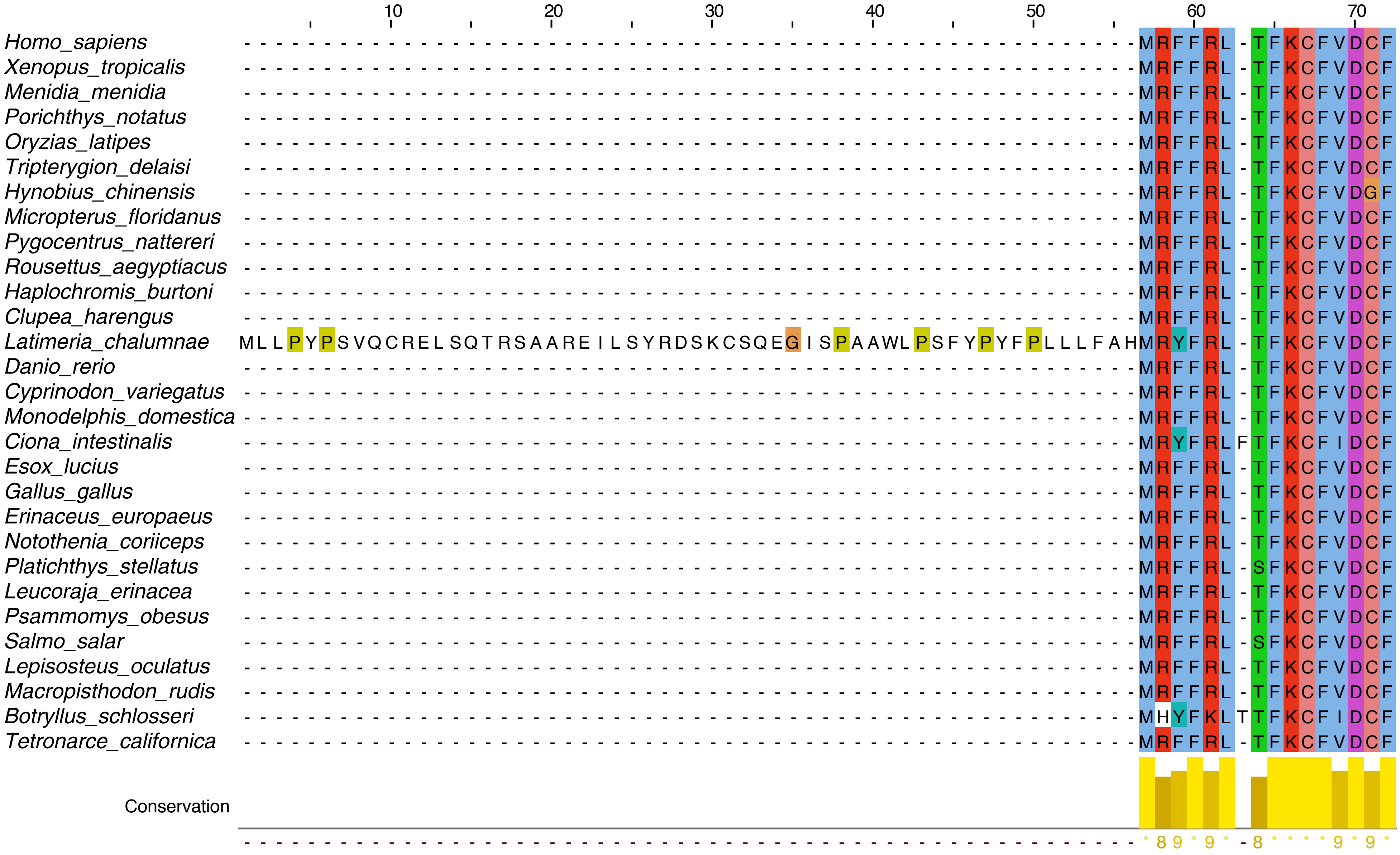

HG0068

ENSGALG00000041419

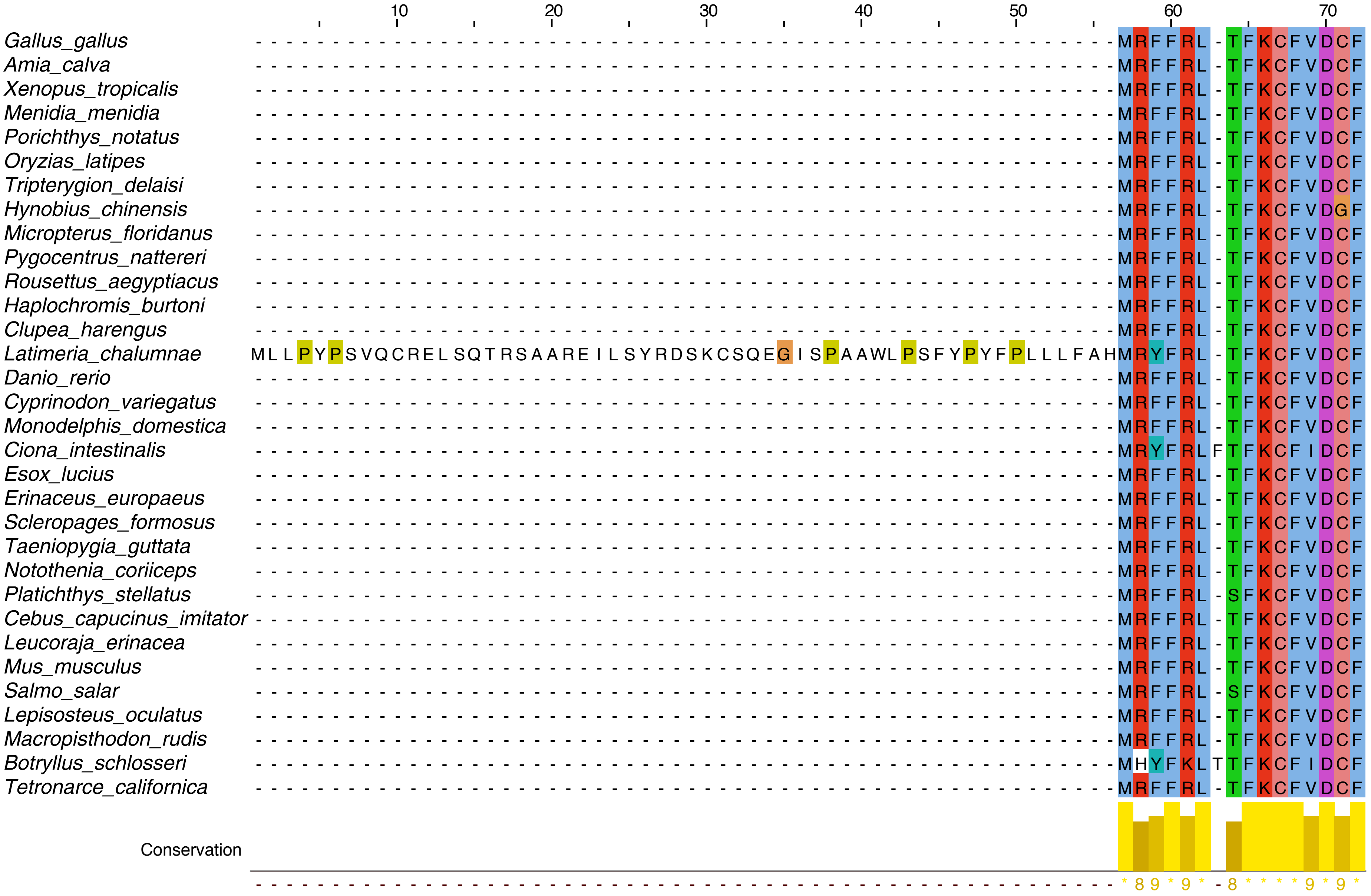

HG0069

ENSG00000100697 (DICER1)

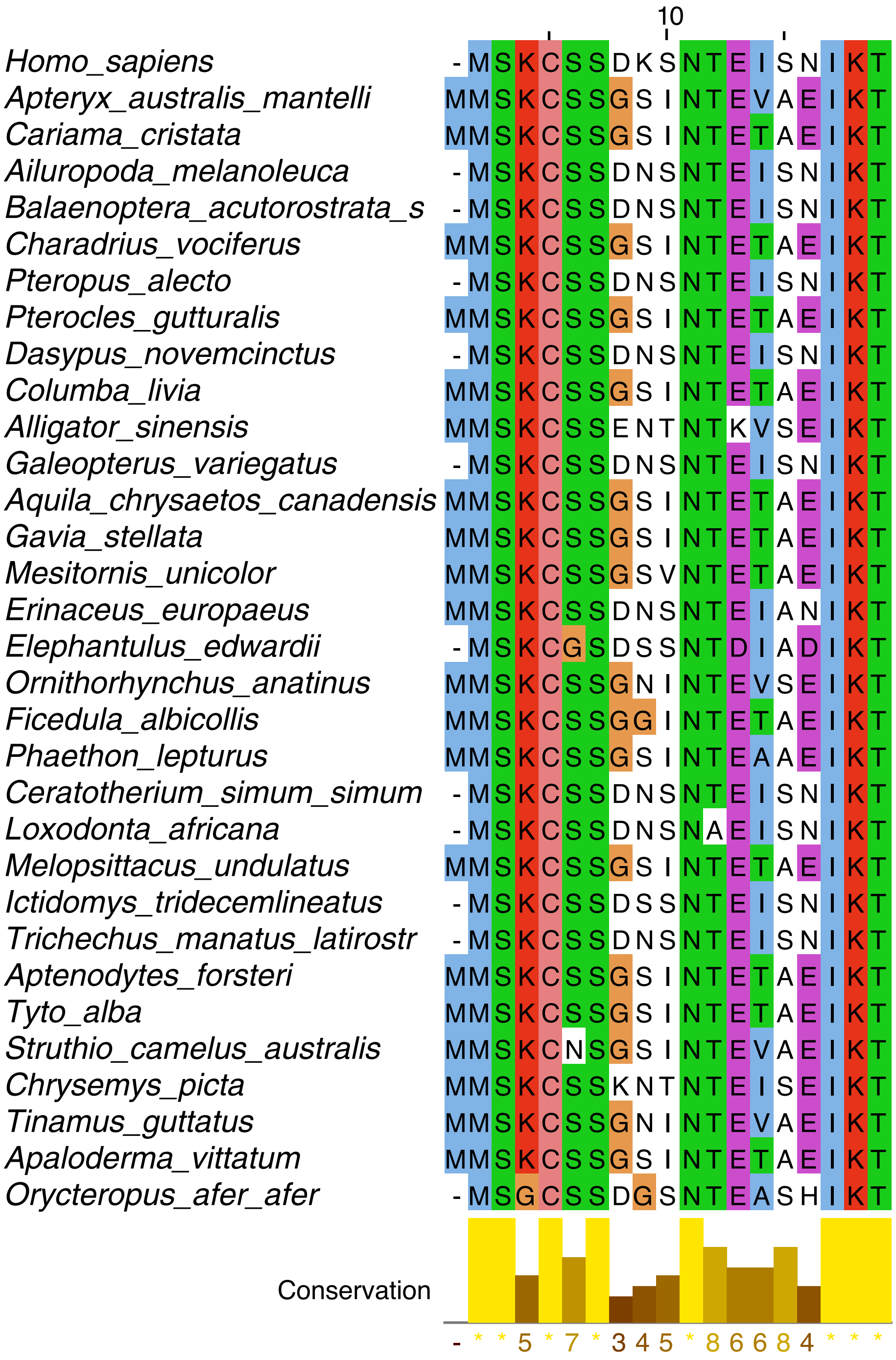

HG0069

ENSGALG00000010999 (DICER1)

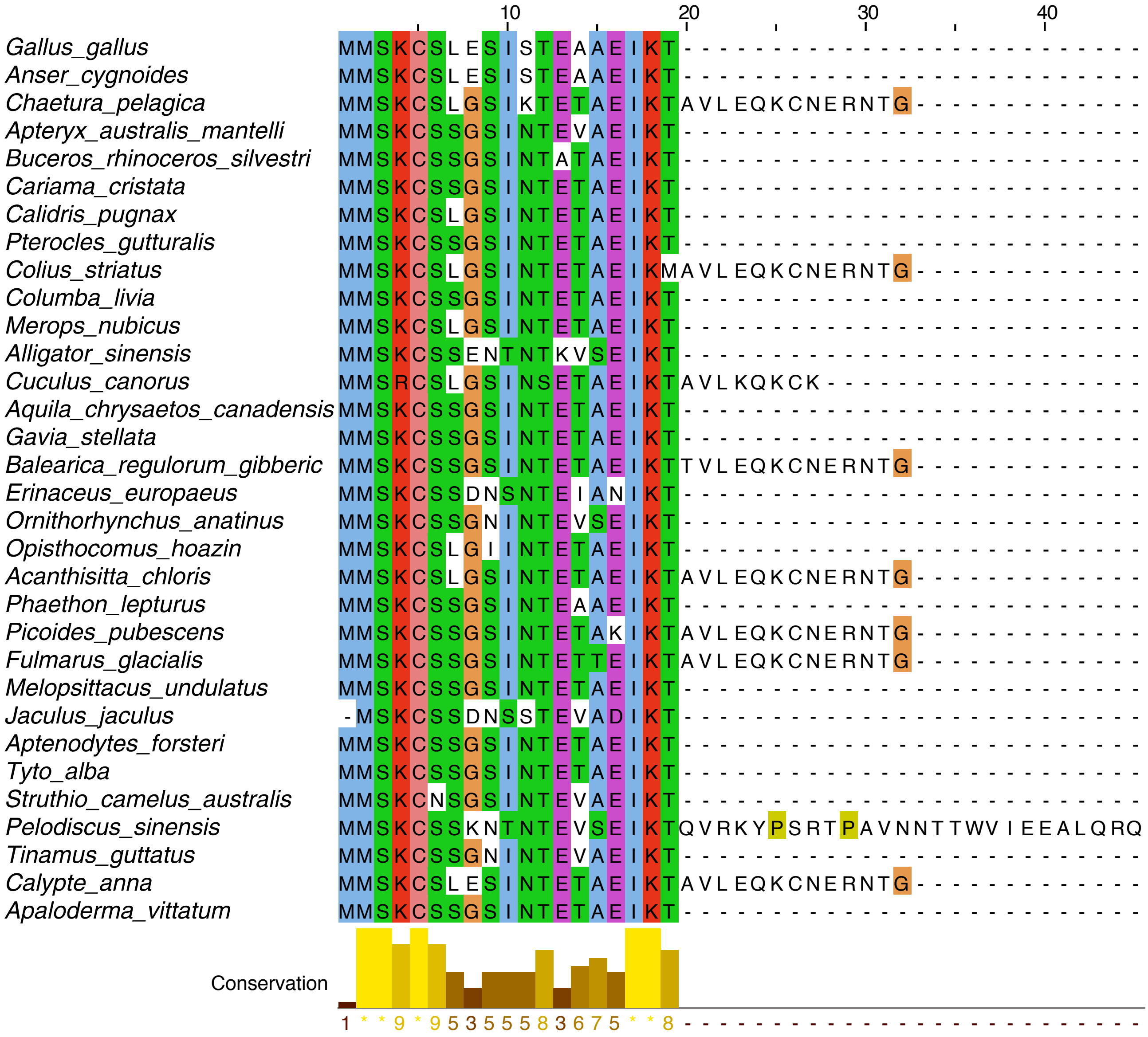

HG0070

ENSDARG00000040926 (nr2f2)

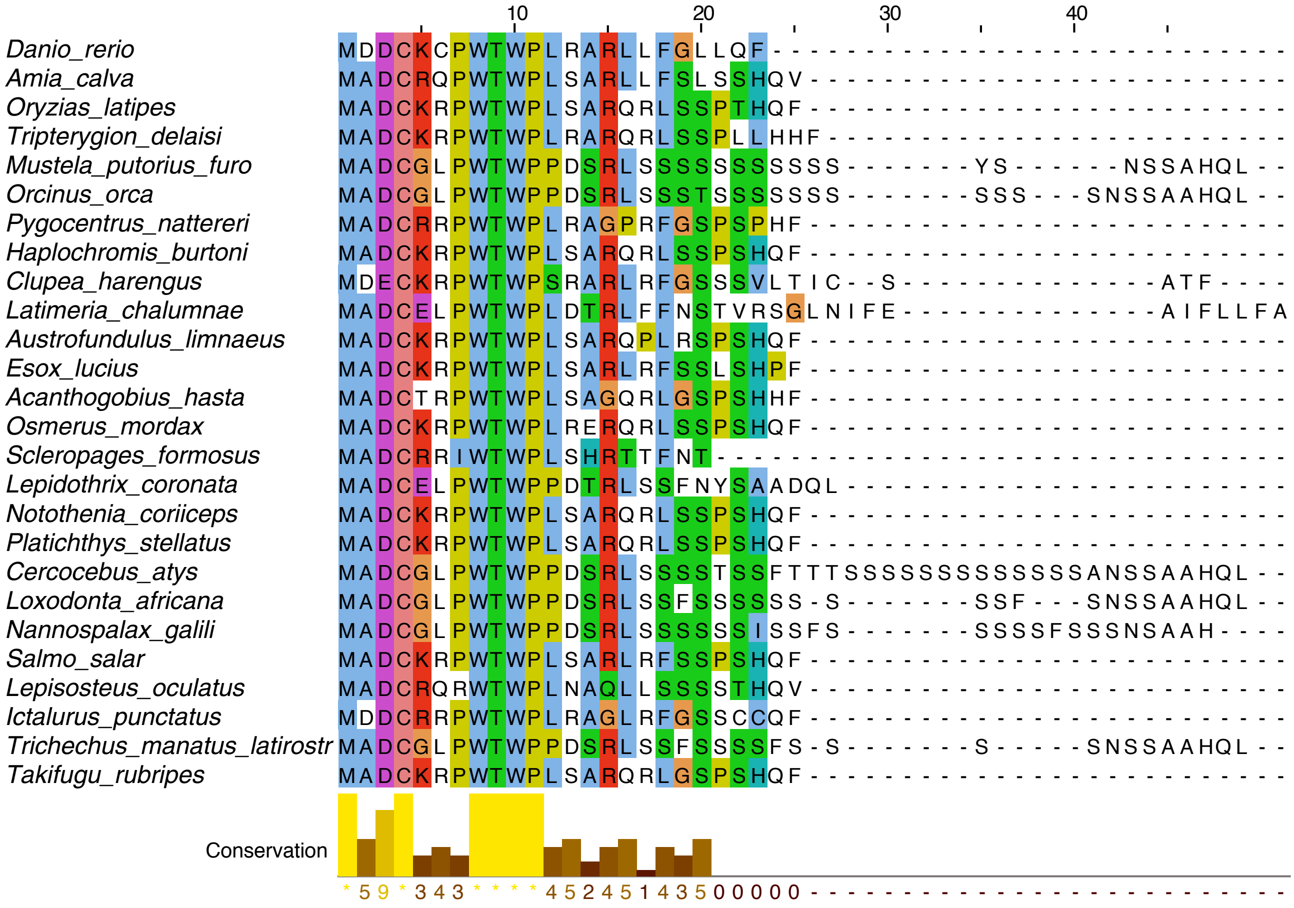

HG0070

ENSDARG00000052695 (nr2f1a)

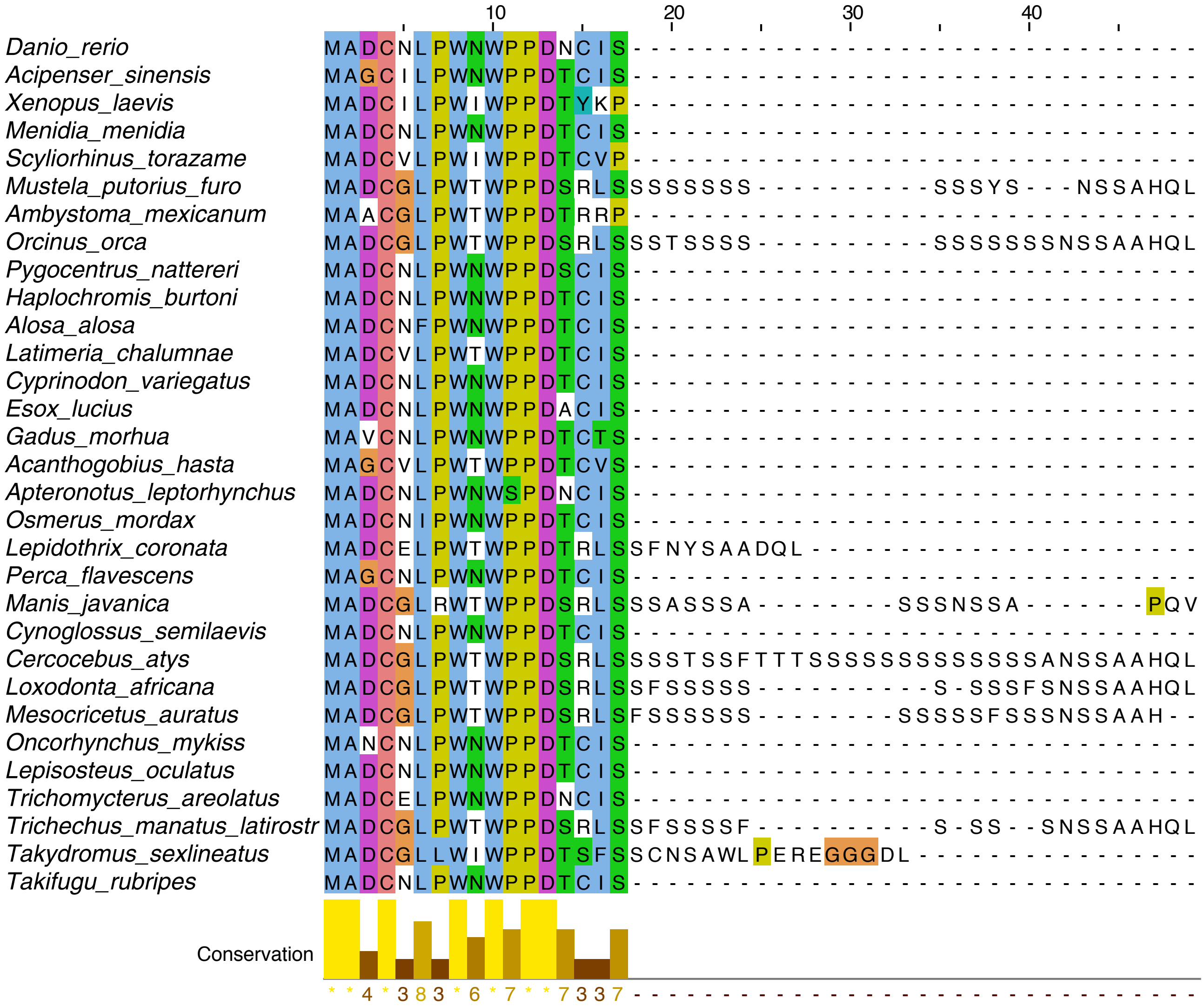

HG0070

ENSG00000175745 (NR2F1)

```
                                      10        20        30        40        50
Homo_sapiens                 MAGCVRPWTWPPDTRAP-------------------------------------
Acipenser_sinensis           MAGCILPWNWPPDTCIS-------------------------------------
Xenopus_laevis               MADCILPWIWPPDTYKP-------------------------------------
Menidia_menidia              MADCNLPWNWPPDTCIS-------------------------------------
Oryzias_latipes              MADCKRPWTWPLSARQRLSSPTHQF-----------------------------
Scyliorhinus_torazame        MADCVLPWIWPPDTCVP-------------------------------------
Canis_lupus_familiaris       MADCGLPWTWPPDSRLSSSSSSSSSSSSSSSSSSSSSSSSSSSSSYSNSSAHQL
Hynobius_retardatus          MAGCALPWTWPPDTSEP-------------------------------------
Orcinus_orca                 MADCGLPWTWPPDSRLSSSTSSSSSSSSSSSNS---------------SAAHQL
Pygocentrus_nattereri        MADCNLPWNWPPDSCIS-------------------------------------
Haplochromis_burtoni         MADCNLPWNWPPDTCIS-------------------------------------
Clupea_harengus              MAGCNIPWNWPPDTCIS-------------------------------------
Latimeria_chalumnae          MADCVLPWTWPPDTCIS-------------------------------------
Catostomus_commersonii       MAGCNLPWNWPPDNCIS-------------------------------------
Austrofundulus_limnaeus      MADCKRPWTWPLSARQPLRSPSHQF-----------------------------
Esox_lucius                  MADCKRPWTWPLSARLRFSSLSHPF-----------------------------
Gadus_morhua                 MAVCNLPWNWPPDTCTS-------------------------------------
Acanthogobius_hasta          MAGCVLPWTWPPDTCVS-------------------------------------
Osmerus_mordax               MADCNIPWNWPPDTCIS-------------------------------------
Lepidothrix_coronata         MADCELPWTWPPDTRLSSFNYSAADQL---------------------------
Perca_flavescens             MAGCNLPWNWPPDTCIS-------------------------------------
Manis_javanica               MADCGLRWTWPPDSRLSSSASSSASSSNSSAPQV--------------------
Platichthys_stellatus        MAGCNIPWNWPPDTCIS-------------------------------------
Loxodonta_africana           MADCGLPWTWPPDSRLSSFSSSSSS-SSSFSNS---------------SAAHQL
Nannospalax_galili           MADCGLPWTWPPDSRLSSSSSSISSFSSSSSFSSSN------------SAAH--
Salmo_salar                  MANCNLPWNWPPDTCIS-------------------------------------
Lepisosteus_oculatus         MADCNLPWNWPPDTCIS-------------------------------------
Trichomycterus_areolatus     MADCELPWNWPPDNCIS-------------------------------------
Trichechus_manatus_latirostr MADCGLPWTWPPDSRLSSFSSSSFS-SS--SNS---------------SAAHQL
Takifugu_rubripes            MADCNLPWNWPPDTCIS-------------------------------------
```

Conservation

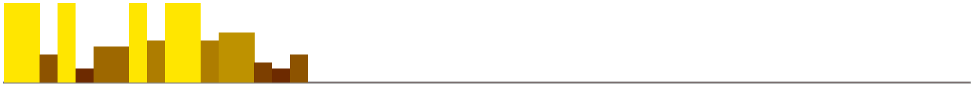

```
                             **4*255*6**67732 4-------------------------------------
```

HG0070

ENSG00000185551 (NR2F2)

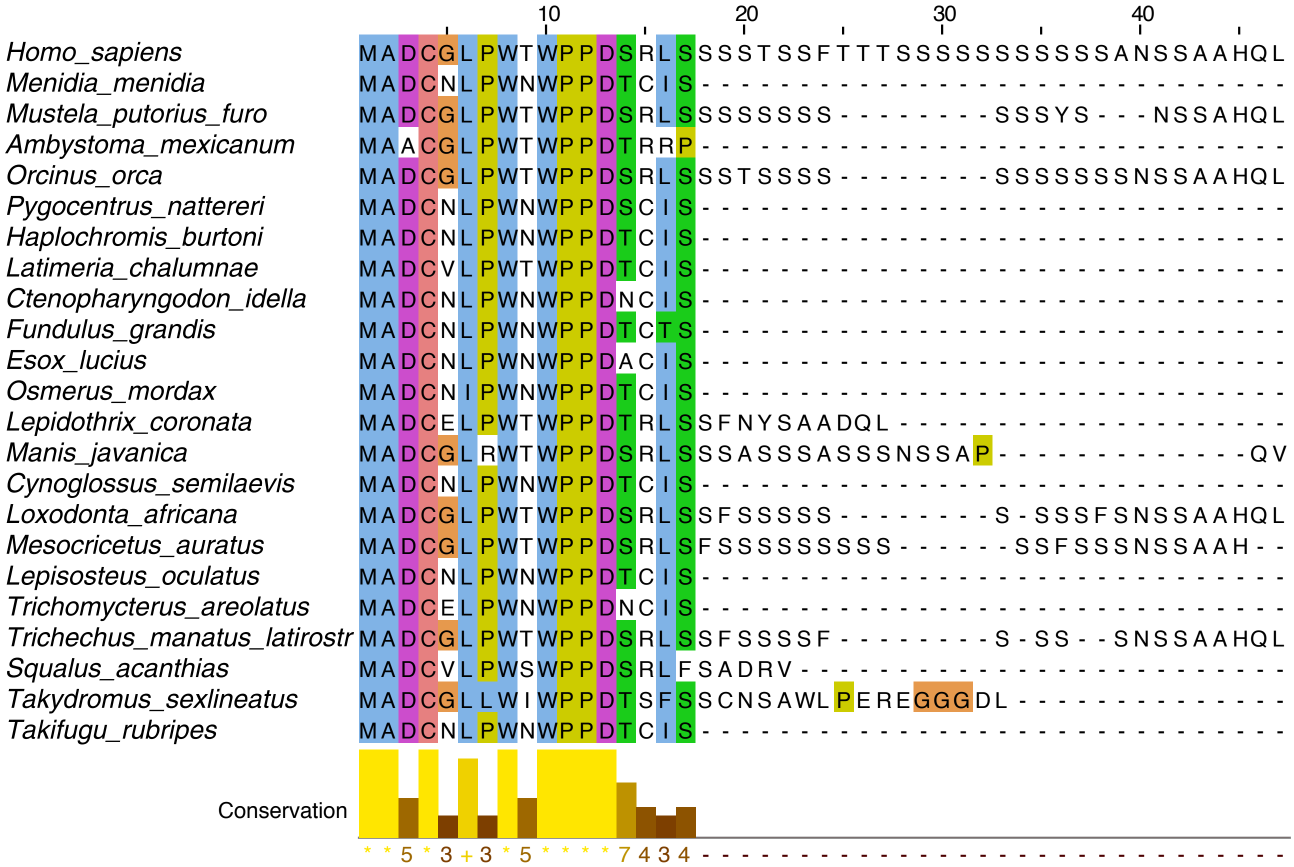

HG0071.1

ENSDARG00000098240 (meis2a)

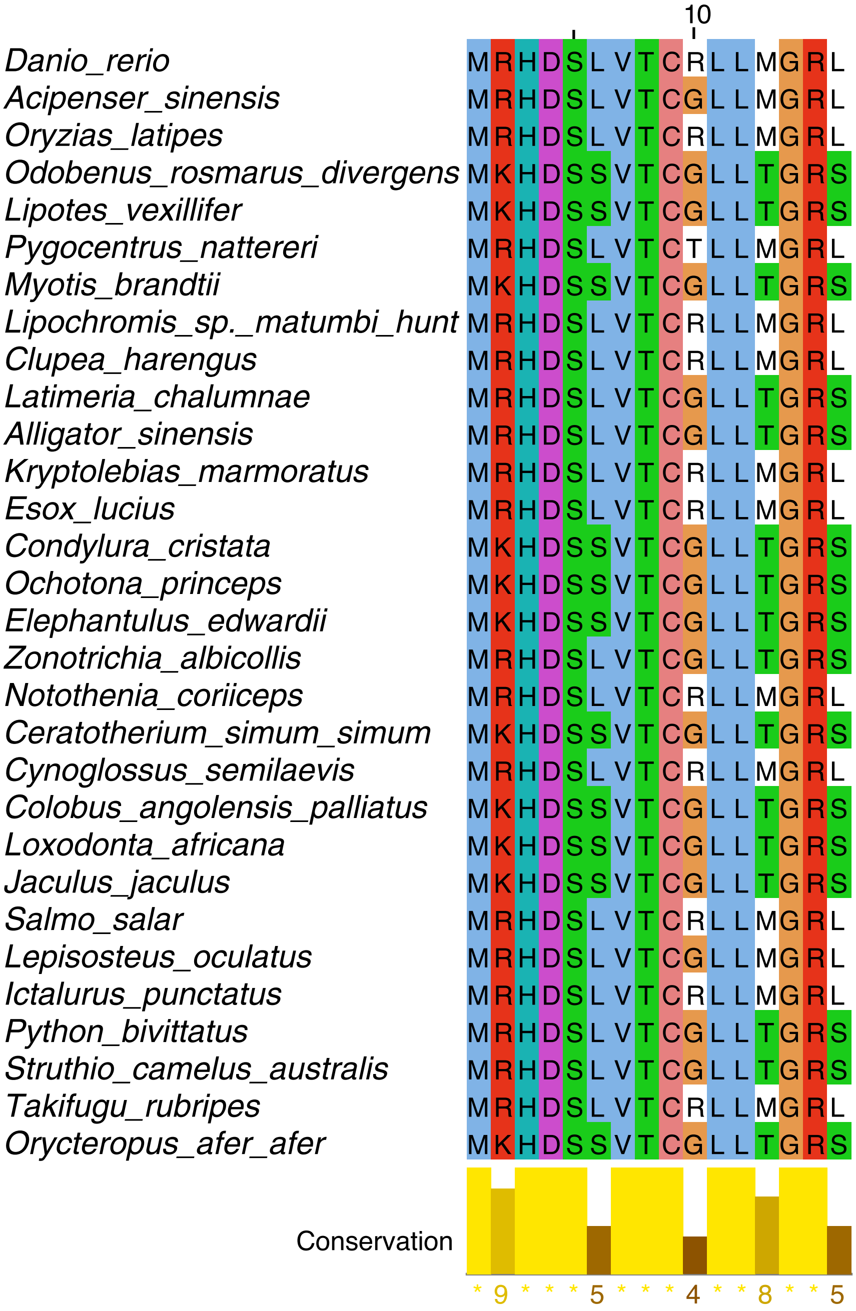

HG0071.1

ENSG00000134138 (MEIS2)

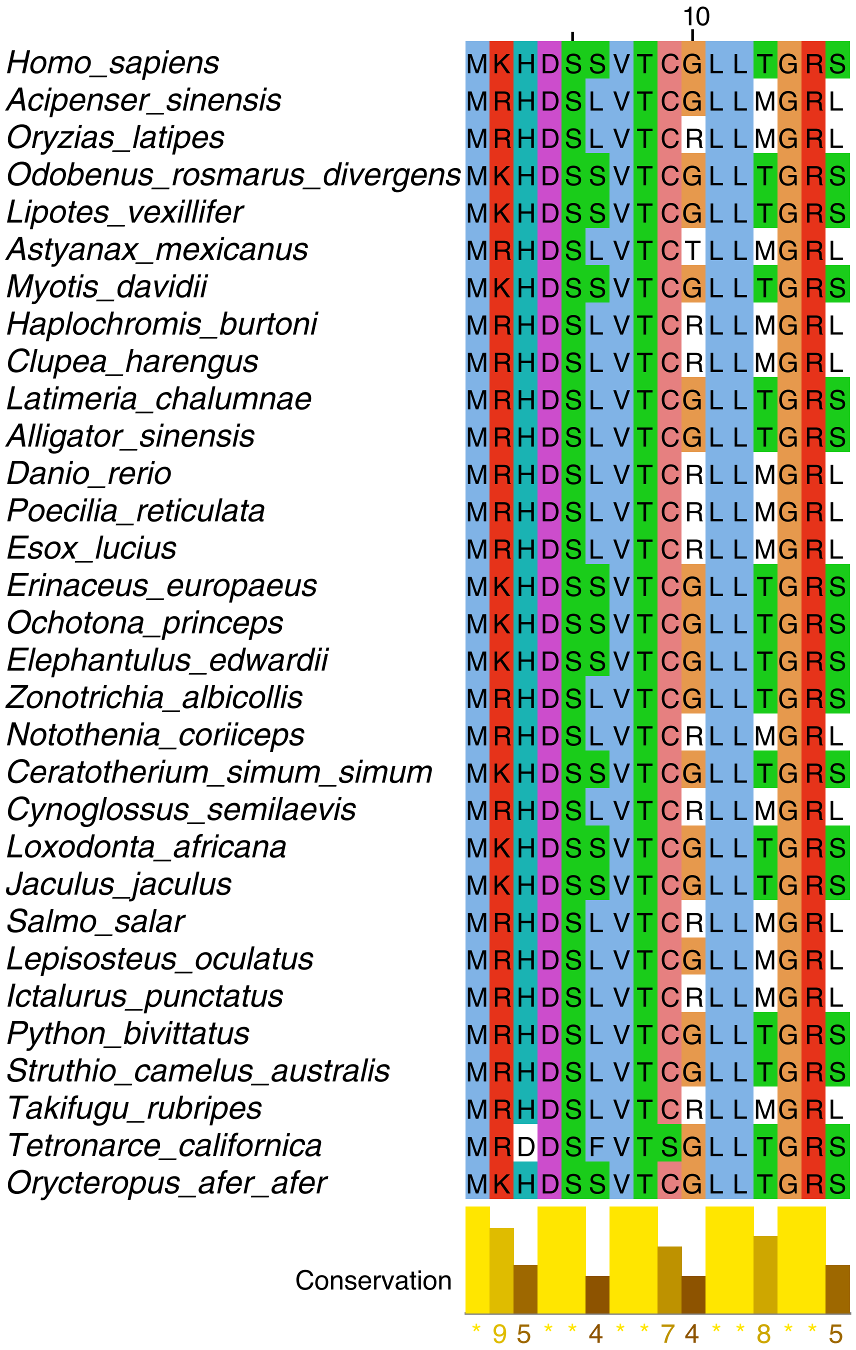

HG0071.2

ENSG00000143995 (MEIS1)

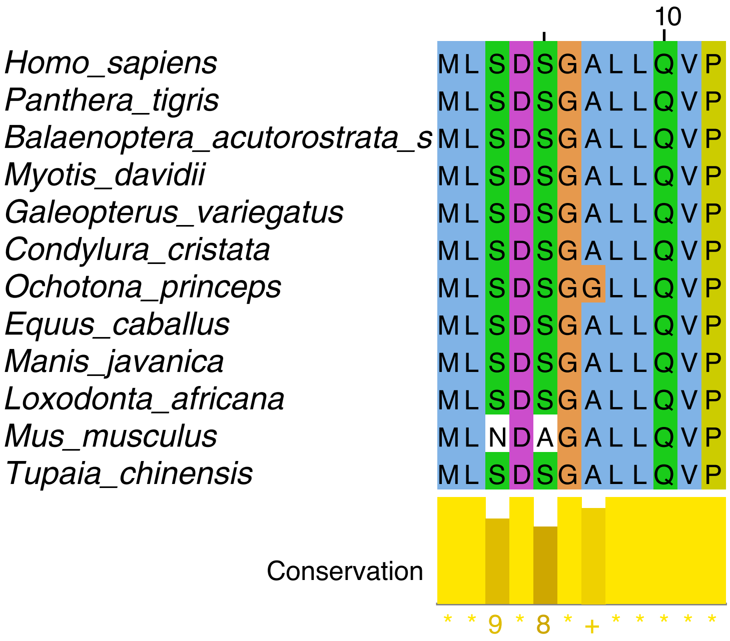

HG0072

ENSG00000104435 (STMN2)

```
                                     10        20        30        40        50
Homo_sapiens                ---MGGADREGLISISNCILVLCTPAPLADQYLMLGDSDSAGVMSGDLGSQSA----
Chaetura_pelagica           ---------------------------------MPRDSGSAGVMSGDL---------
Odobenus_rosmarus_divergens ---------------------------------MLGDSDSAGVMSGDLGSQSA----
Hynobius_retardatus         -------------------------------------------MSGDLWSQSAGAEP
Tursiops_truncatus          ---------------------------------MLGDSDSAGVMSGDLGSQSA----
Charadrius_vociferus        ---------------------------------MPGDSGSAGVMSGDL---------
Myotis_davidii              ---------------------------------MLGGSDSARVMSGDLGSQSARASG
Dasypus_novemcinctus        -------------------------------------------MSGDLGSQSA----
Columba_livia               ---------------------------------MPRDSGSAGVMSGDL---------
Cuculus_canorus             ---------------------------------MPRDSGSAGVMSGDL---------
Galeopterus_variegatus      ---MGGAAREGLISISNCILVLCTPAPLADQYLMLGDSDSAGVMSGDLGSQSA----
Falco_cherrug               ---------------------------------MPGDSGSAGVMSGDL---------
Gallus_gallus               ---------------------------------MPGDSGSAGVMSGDL---------
Condylura_cristata          ---------------------------------MLGDSDSAGVVSGDLGSQSA----
Ochotona_princeps           ---MGGAAREGLISISNCIVVLCTPAPLADQYLMLRDSDSAGVMSGDLGSQSA----
Opisthocomus_hoazin         ---------------------------------MPGGSGSAGVMSGDL---------
Sturnus_vulgaris            ---------------------------------MPGDSGSAGVMSGDLSSQSAWVEE
Egretta_garzetta            ---------------------------------MPGDSGSAGVMSGDL---------
Ceratotherium_simum_simum   ---------------------------------MLGDSDSAGVMSGDLGSQSA----
Picoides_pubescens          ---------------------------------MPGDSGSAGVMSGDL---------
Loxodonta_africana          ---MGGAAREGLISISNCILVLCTLAPLADQYLMLGDSDSAGVMSGDLGSQSA----
Melopsittacus_undulatus     ---------------------------------MPGDSGSAGVMSGDL---------
Marmota_marmota_marmota     ---MGGAAREGLISISNCILVLCTPAPLADQYSMLGDSDSAGVMSGDLGSQSA----
Tupaia_chinensis            ---------------------------------MLGDSDSAGVMSGDLGSQSA----
Aptenodytes_forsteri        ---------------------------------MPGDSGSAGVMSGDL---------
Squalus_acanthias           MFHWKKRGGPILICISNCILALCTLASLVDQYLMPGDPENAPVMSRDLFSQSALLGR
Anolis_carolinensis         ---------------------------------MLADSGSAGVMSGELWSQSAWLEE
Struthio_camelus_australis  ---------------------------------MPGDSGSAGVMSGDL---------
Chelonia_mydas              ---------------------------------MLGDSSSAGVMSGDL---------
Tinamus_guttatus            ---------------------------------MPGDSGSAGVMSGDL---------
Calypte_anna                ---------------------------------MPGDSGSAGVMSGDL---------

Conservation                ---------------------------------100110230383*49*---------
```

HG0072

ENSGALG00000039690 (STMN2)

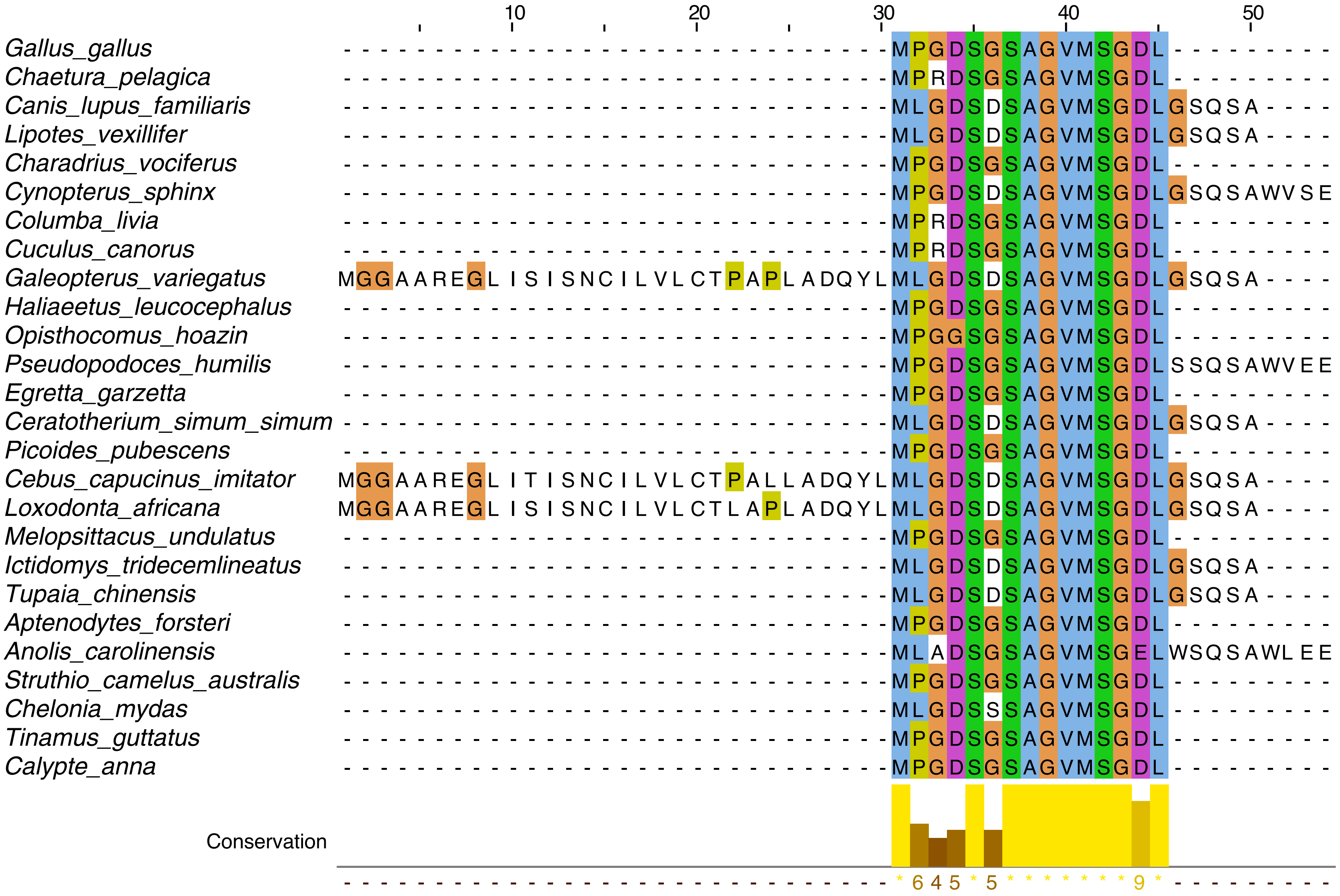

HG0073

ENSDARG00000041708 (csrp2bp)

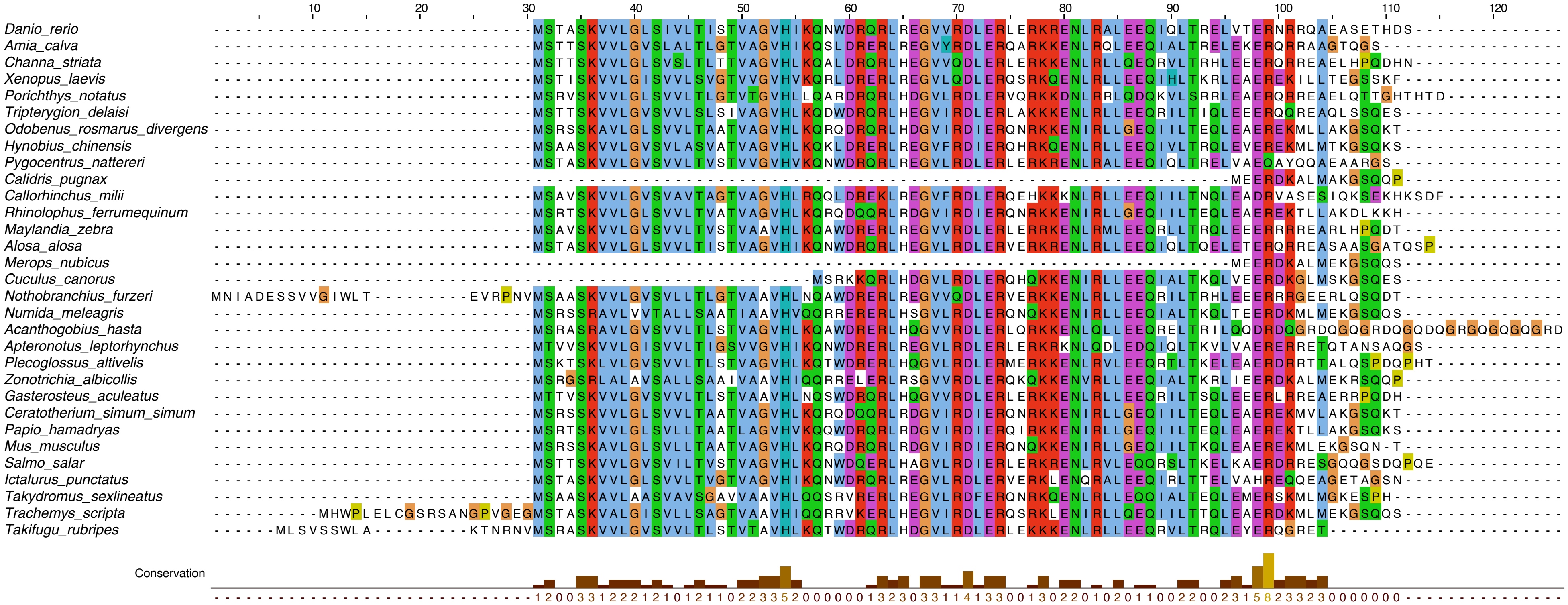

HG0074

ENSDARG00000078624 (arhgef9b)

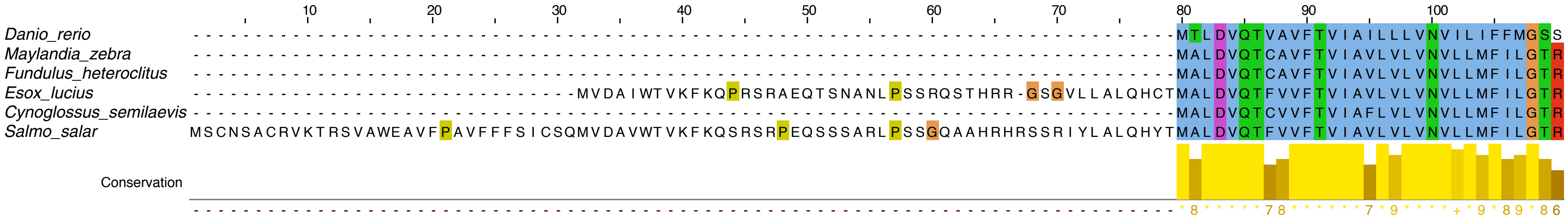

HG0074

ENSG00000131089 (ARHGEF9)

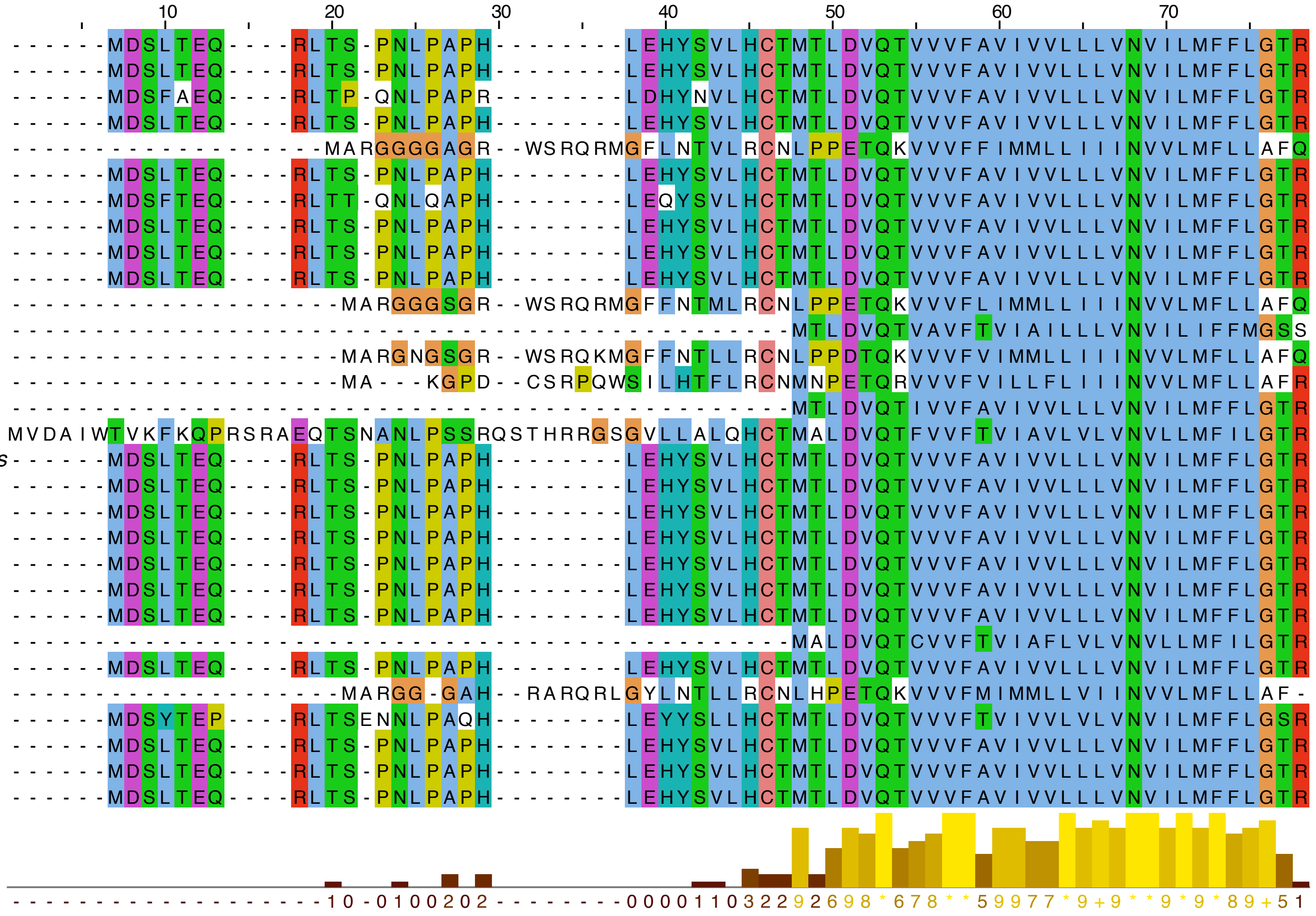

HG0075

ENSG00000165699 (TSC1)

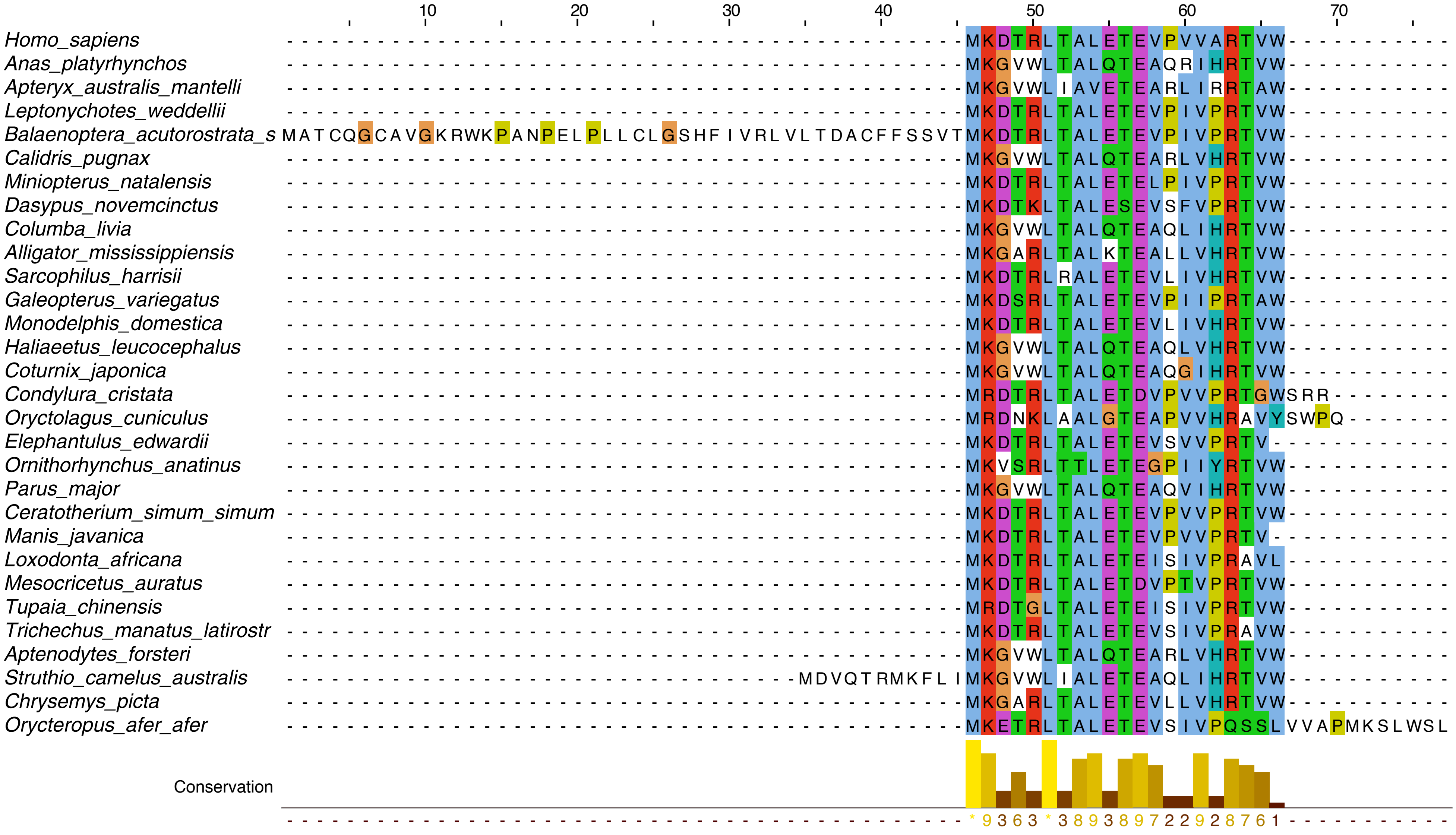

HG0076

ENSDARG00000040008 (neurod6a)

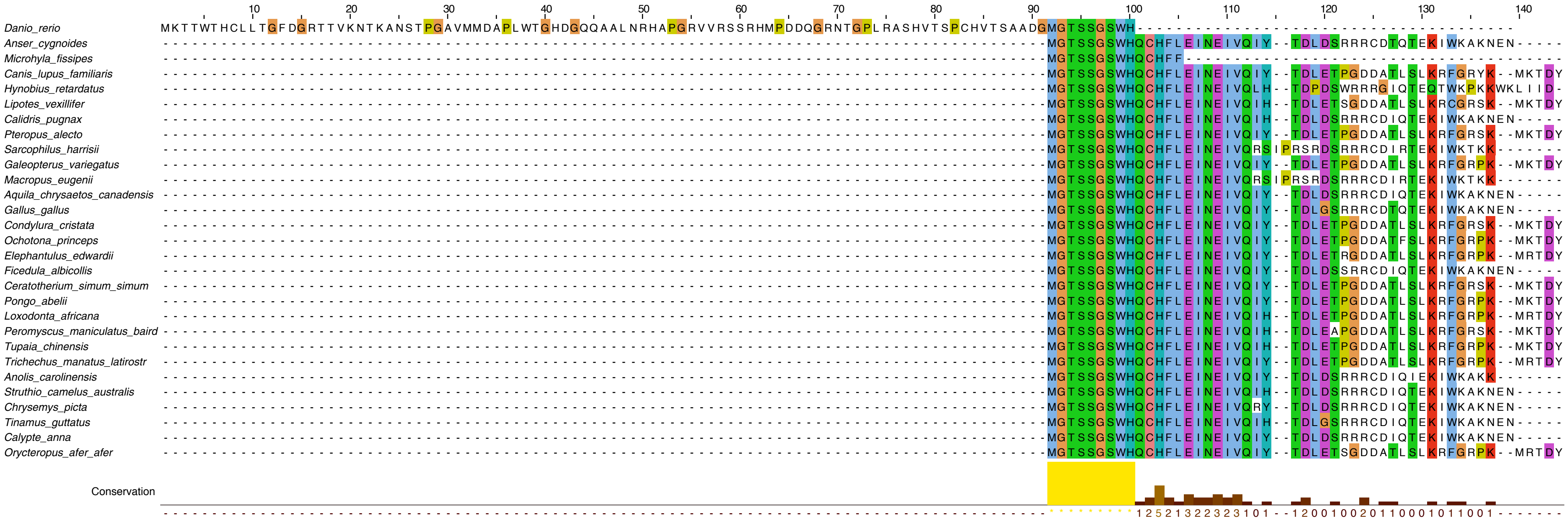

HG0076

ENSG00000164600 (NEUROD6)

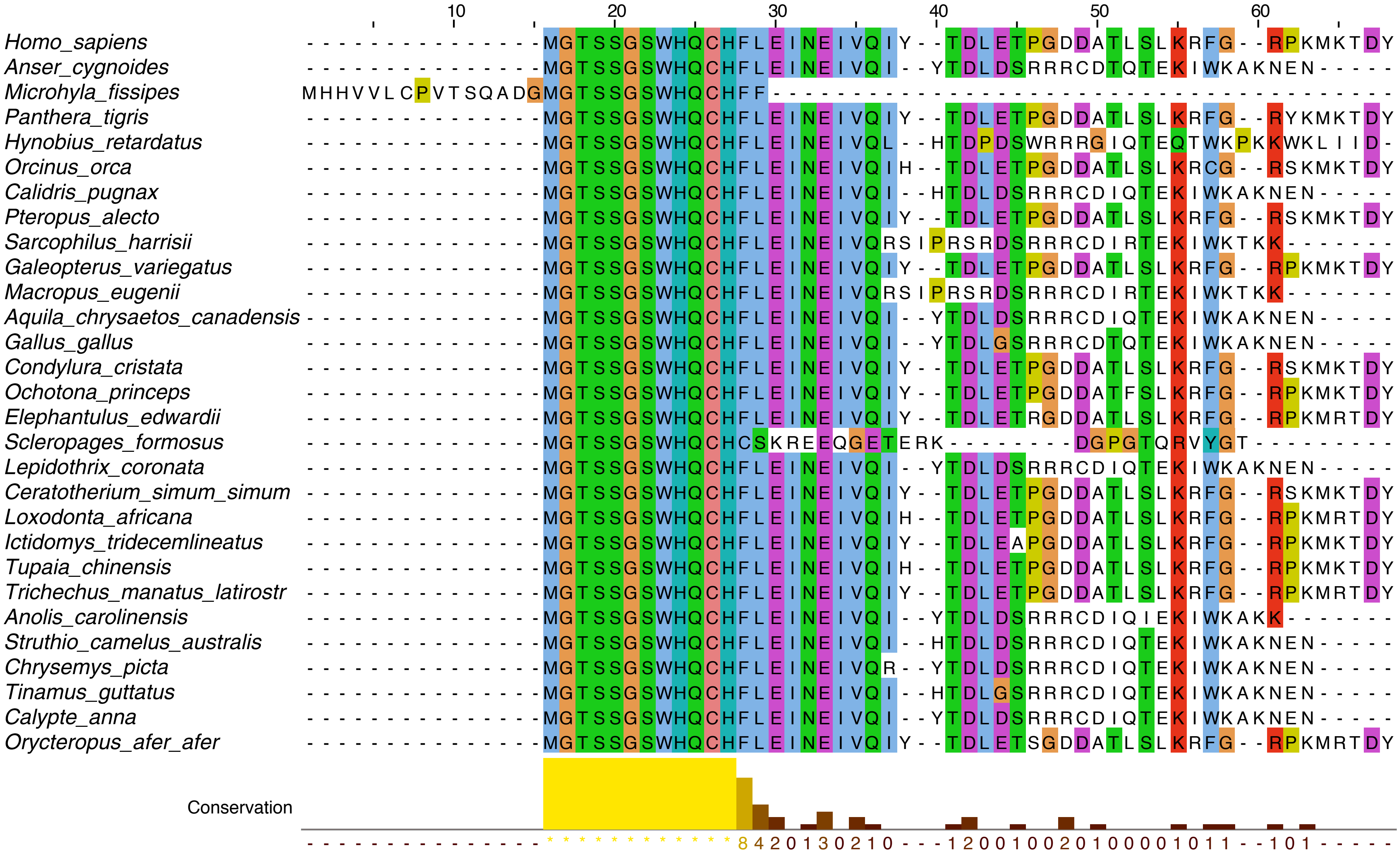

HG0077.1

ENSGALG00000005074

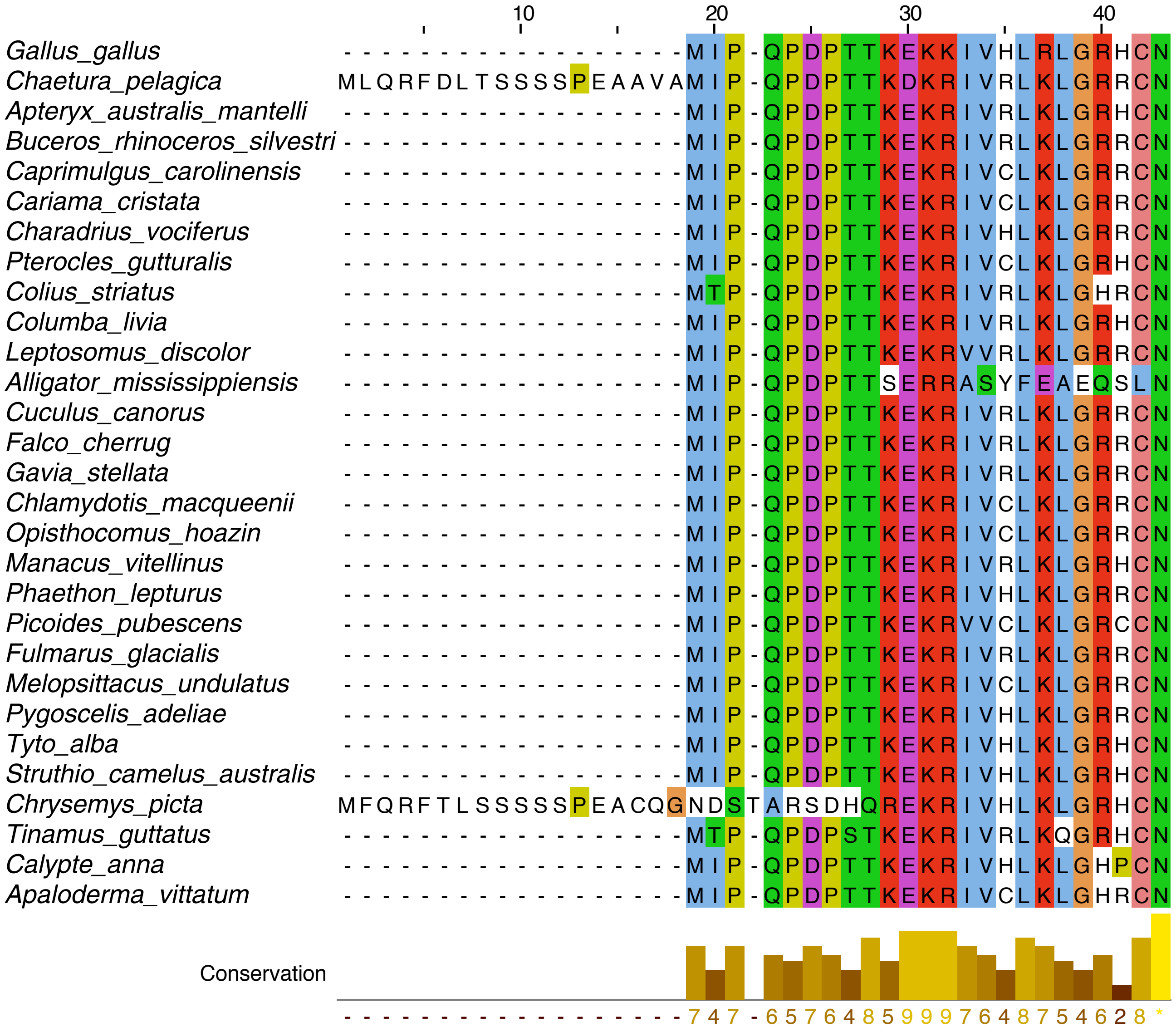

HG0077.2

ENSGALG00000005074

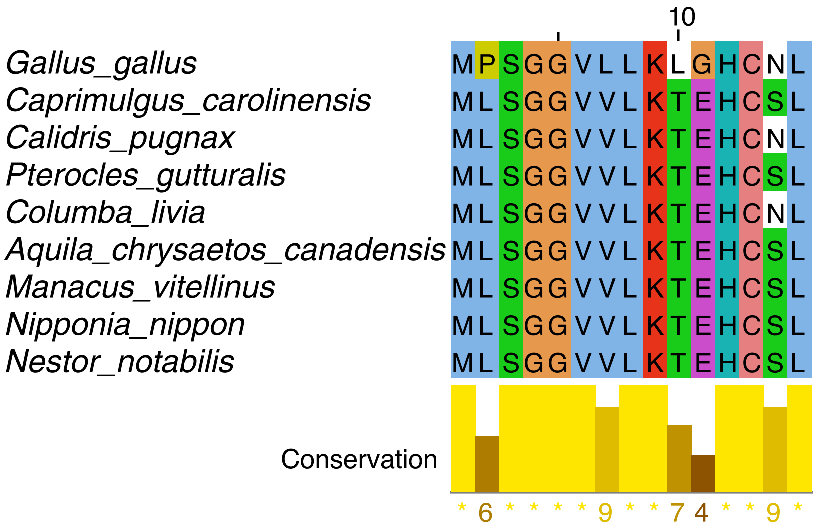

HG0078.1

ENSGALG00000016633 (HMBOX1)

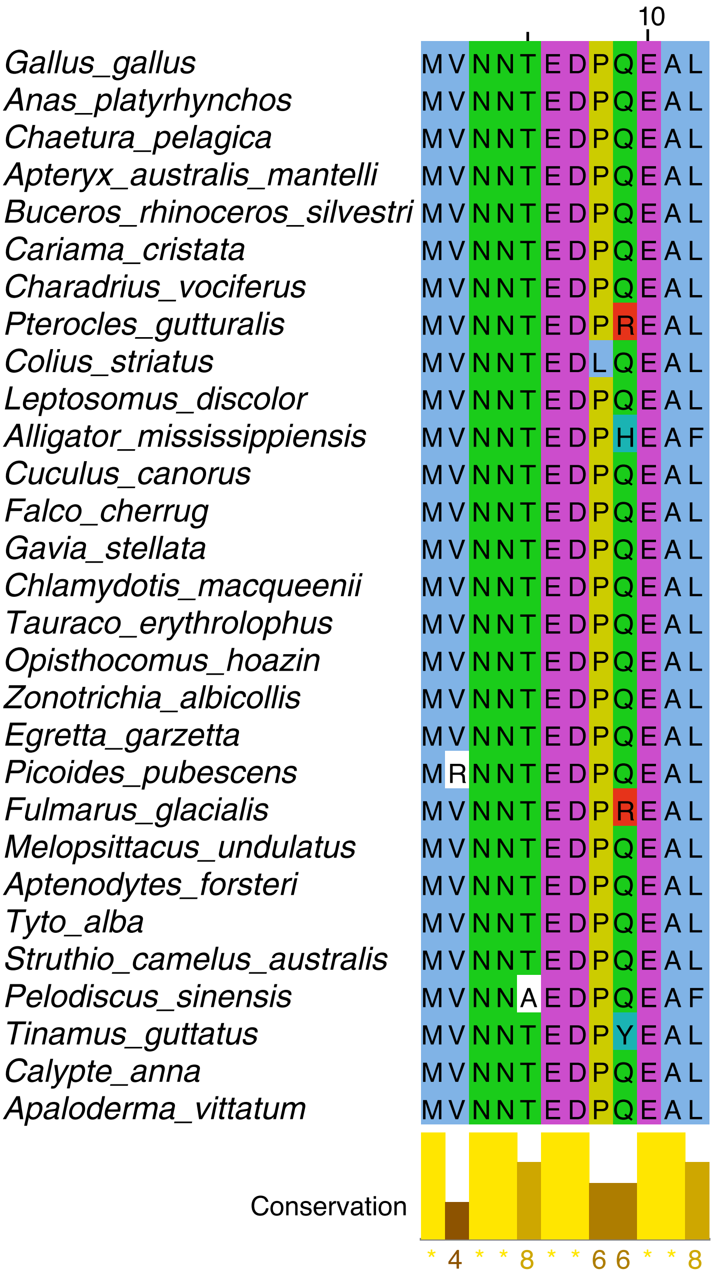

HG0078.2

ENSG00000147421 (HMBOX1)

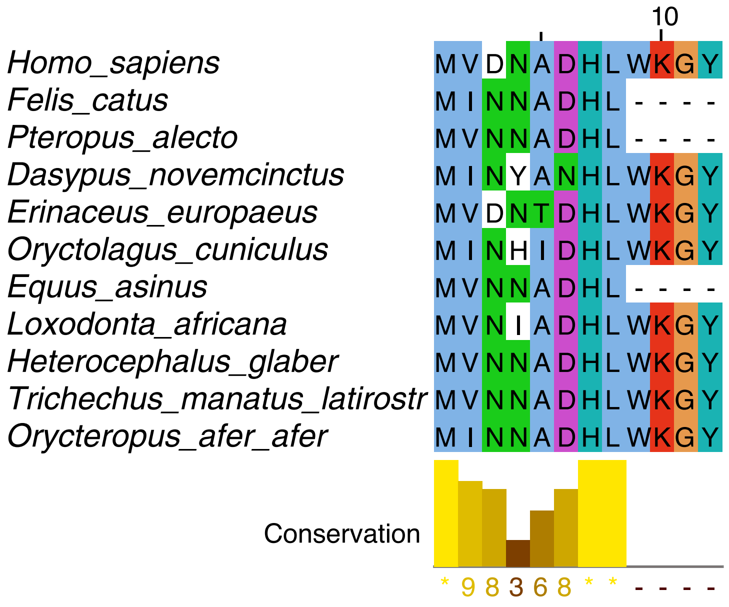

HG0079

ENSG00000204406 (MBD5)

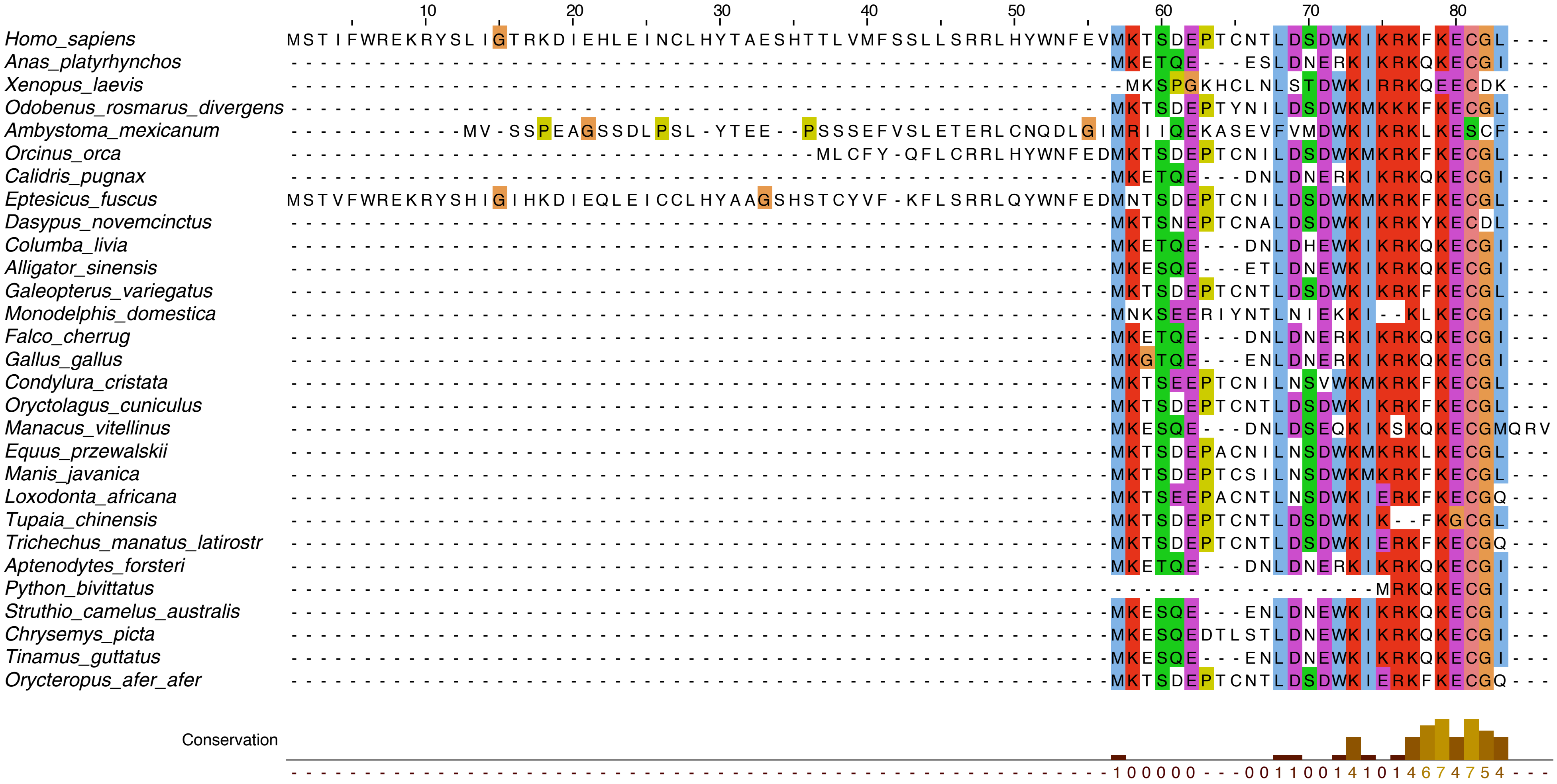

HG0080.1

ENSG00000179603 (GRM8)

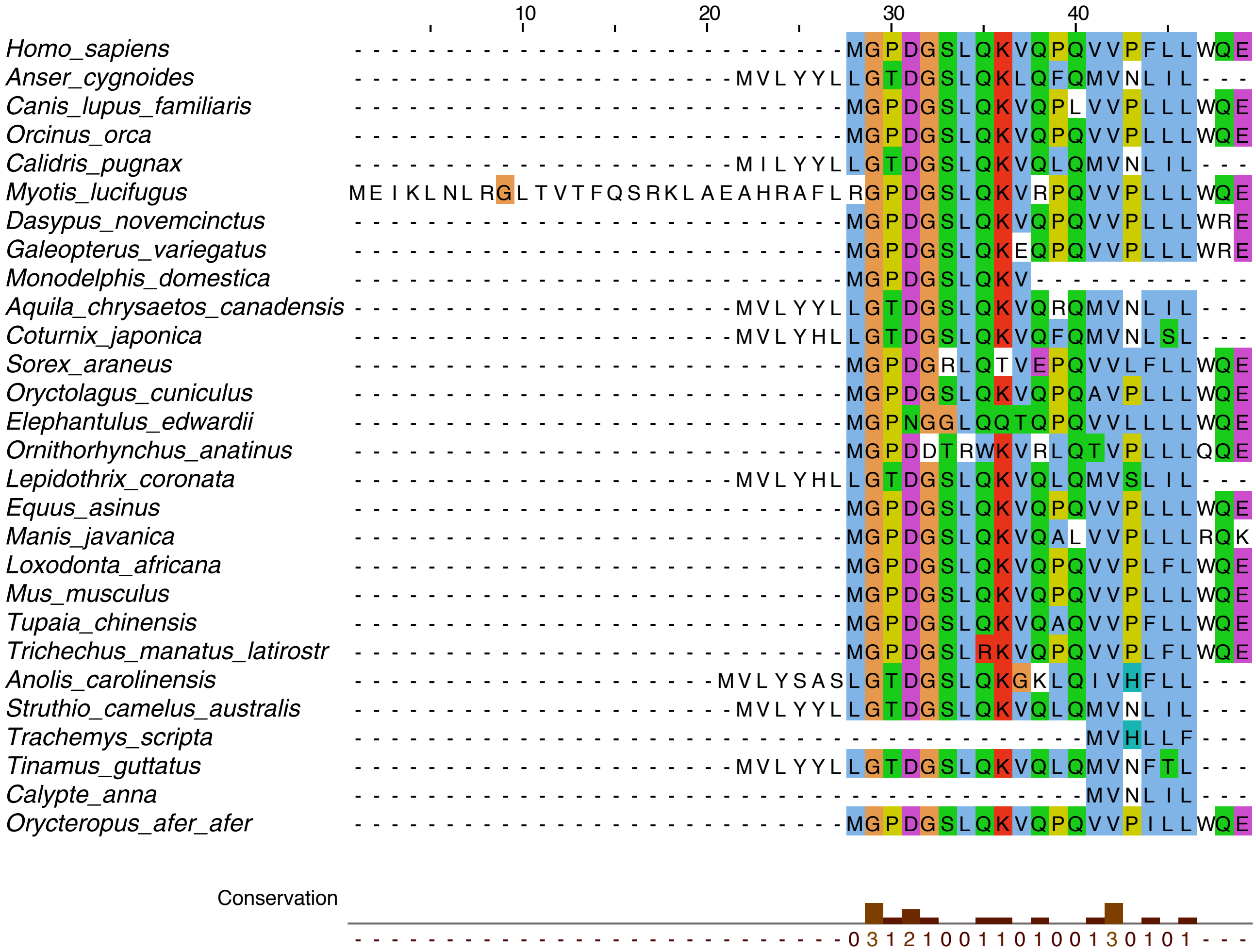

HG0080.1

ENSGALG00000039403

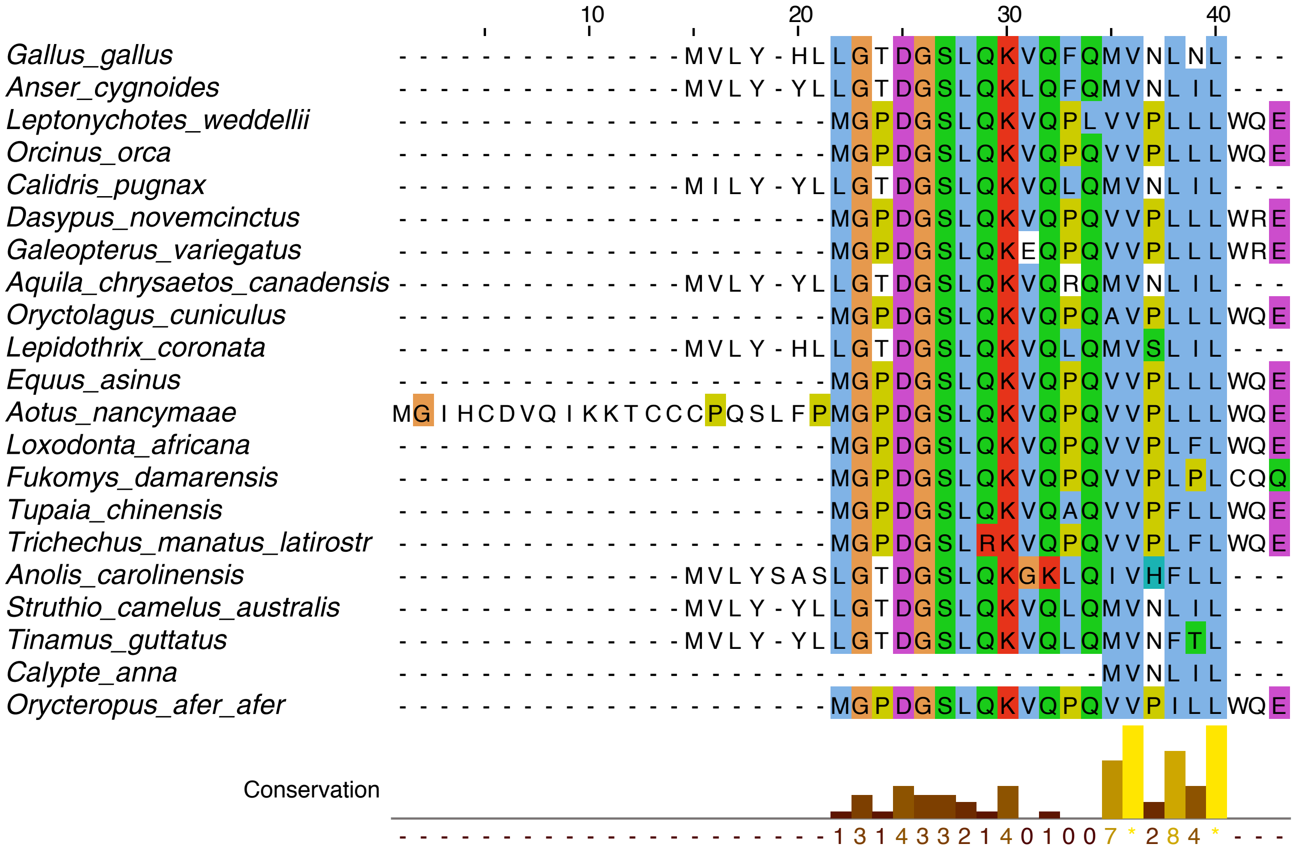

HG0080.2

ENSG00000179603 (GRM8)

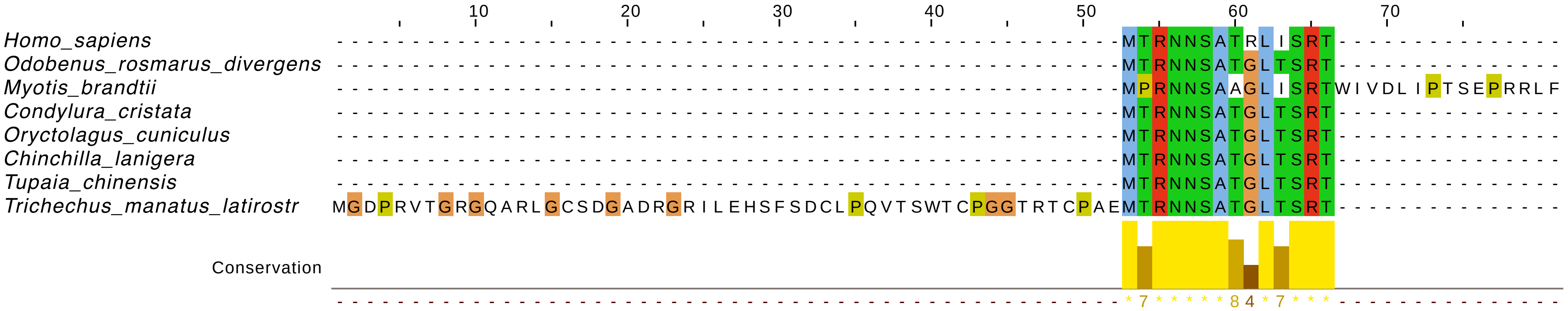

HG0080.3

ENSG00000179603 (GRM8)

```
                                  10        20        30        40        50        60        70        80        90        100       110
Homo_sapiens              -------------------------------------------------MEETWRLLGQLSPLSPEISHKCGRCARLTRRRRRRRRR--------------------------------
Mustela_putorius_furo     -------------------------------------------------MEDTWRRPGQLSPLSPETSHKCGRGACLARRRRRR-----------------------------------
Erinaceus_europaeus       -------------------------------------------------MEATWGWPAQLCPLGPQISHKCGRGAGQARRRRRHGSAGFL--TAPSAGSRE------------------
Oryctolagus_cuniculus     -------------------------------------------------MEETWRRLGQLSPLNPESSHKCGRGARLARGGGGGGGGADMELRAPQRASSPRPLRGAGNNSATGLTSRT
Mus_musculus              MVGAKRLVIPGAIPAAAPRAAWGEWPRAEGREAPGVDQSRPPVGVFVIHMEETRRWLGRLSPLSPETSHKCGRGARLSRWPRRRQR---------------------------------
Conservation              -------------------------------------------------**4*6446+8*7**7*85******9*477*3344300---------------------------------
```

HG0081.1

ENSDARG00000028228 (zbtb18)

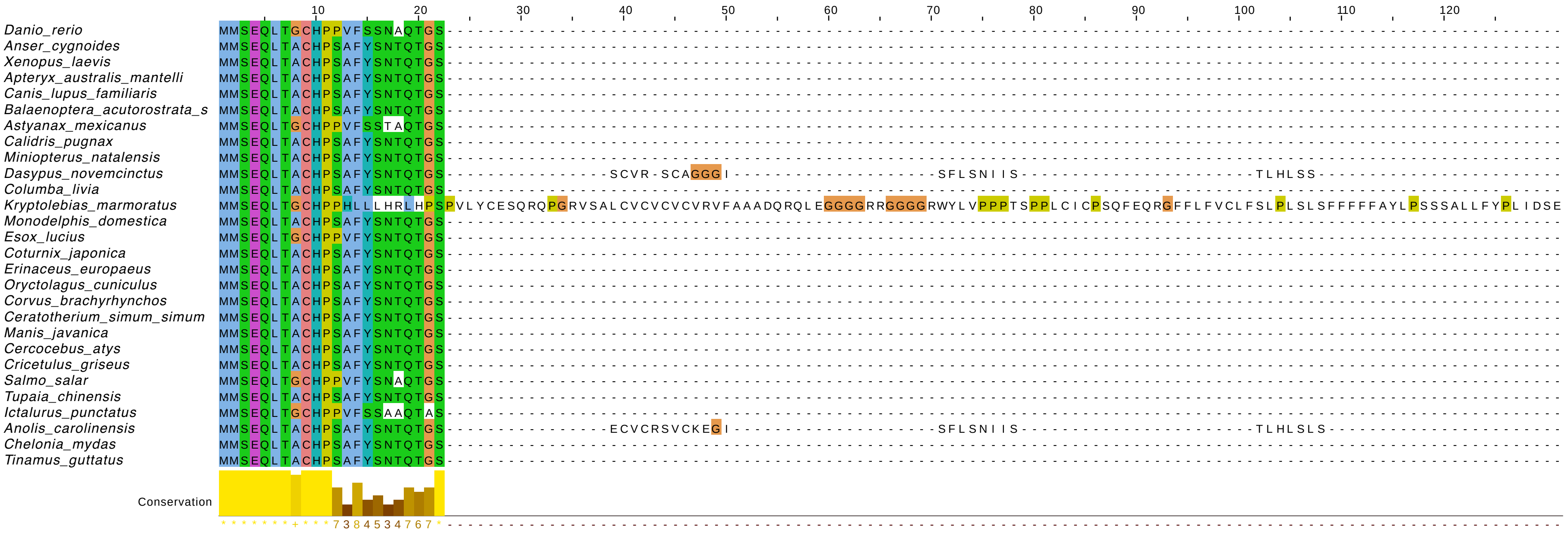

HG0081.2

ENSDARG00000028228 (zbtb18)

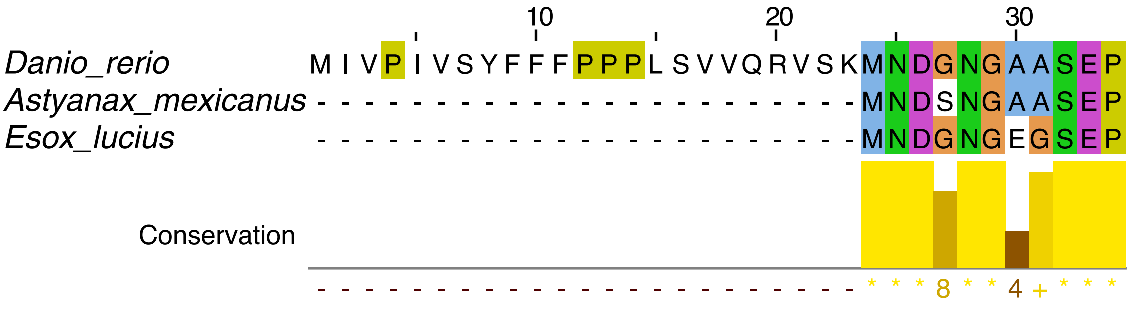

HG0082

ENSDARG00000062379 (slc35a4)

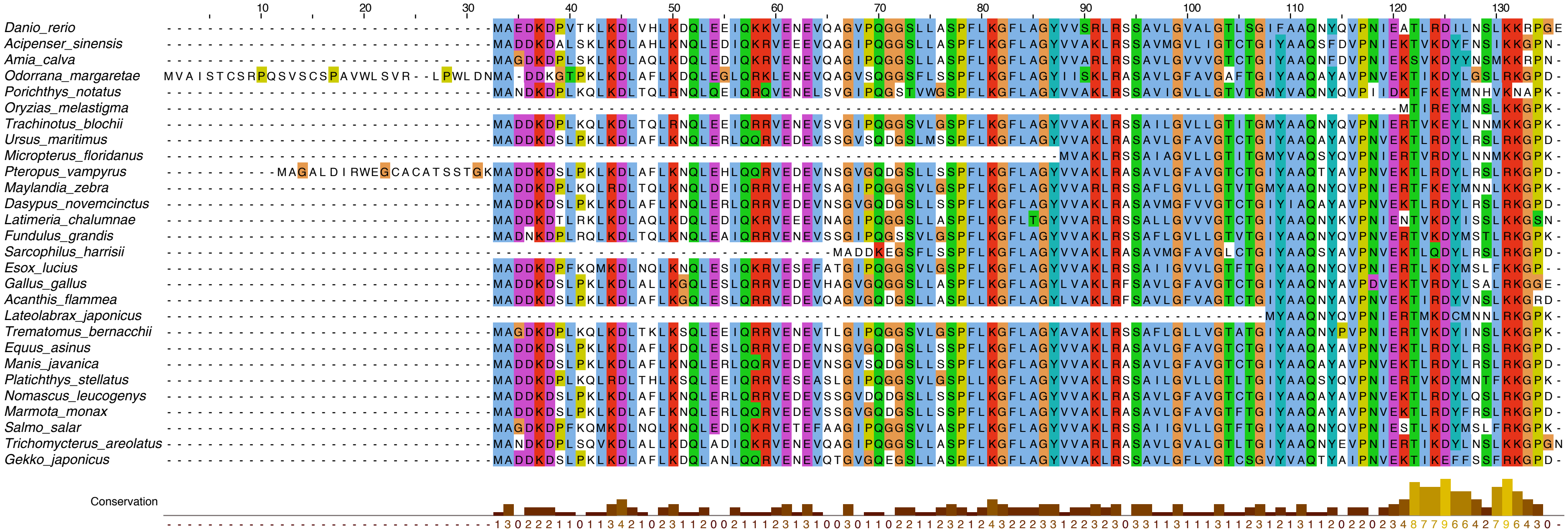

HG0082

ENSG00000176087 (SLC35A4)

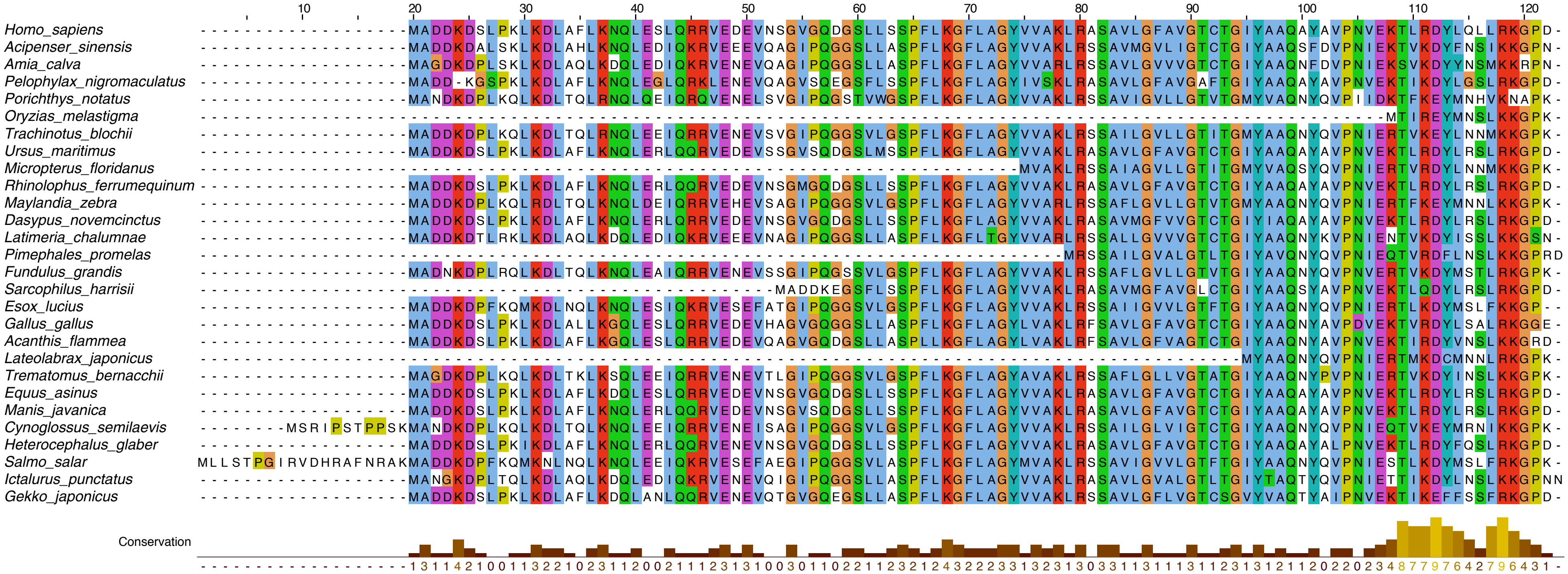

HG0083.1

ENSGALG00000008039 (MFSD13A)

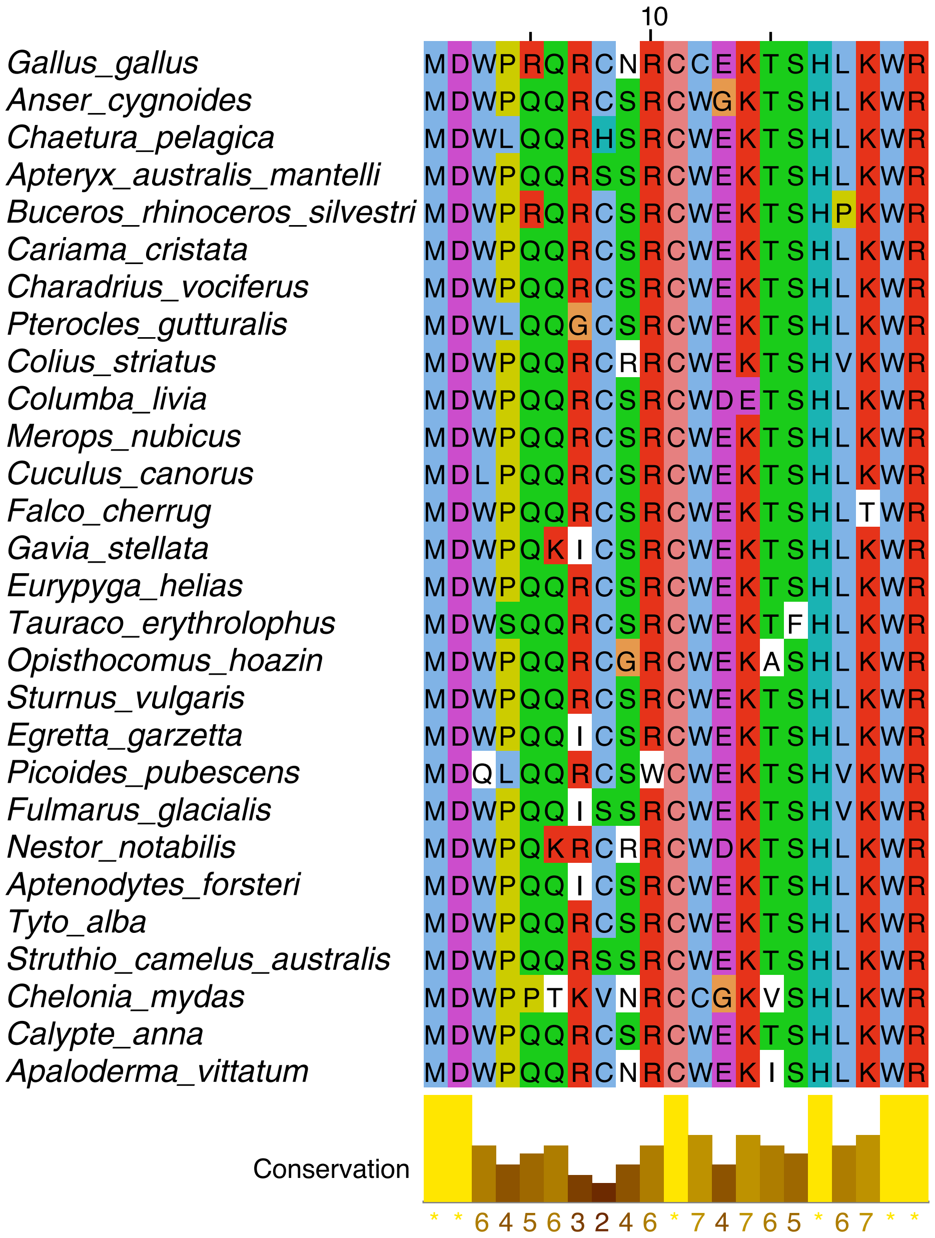

HG0083.2

ENSG00000138111 (MFSD13A)

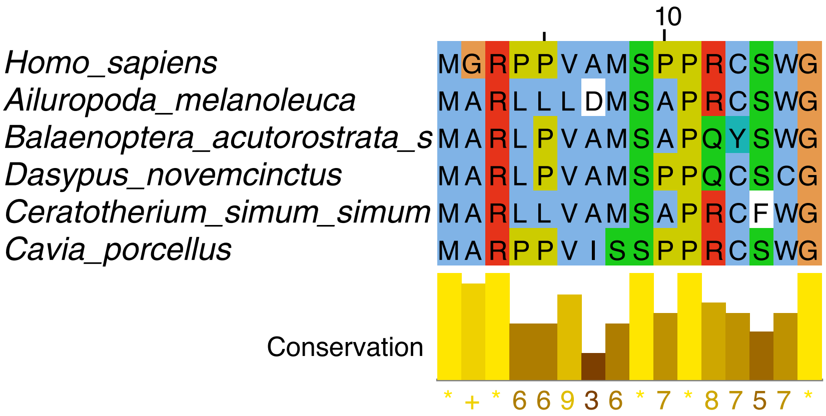

HG0084.1

ENSGALG00000009207 (TRIM2)

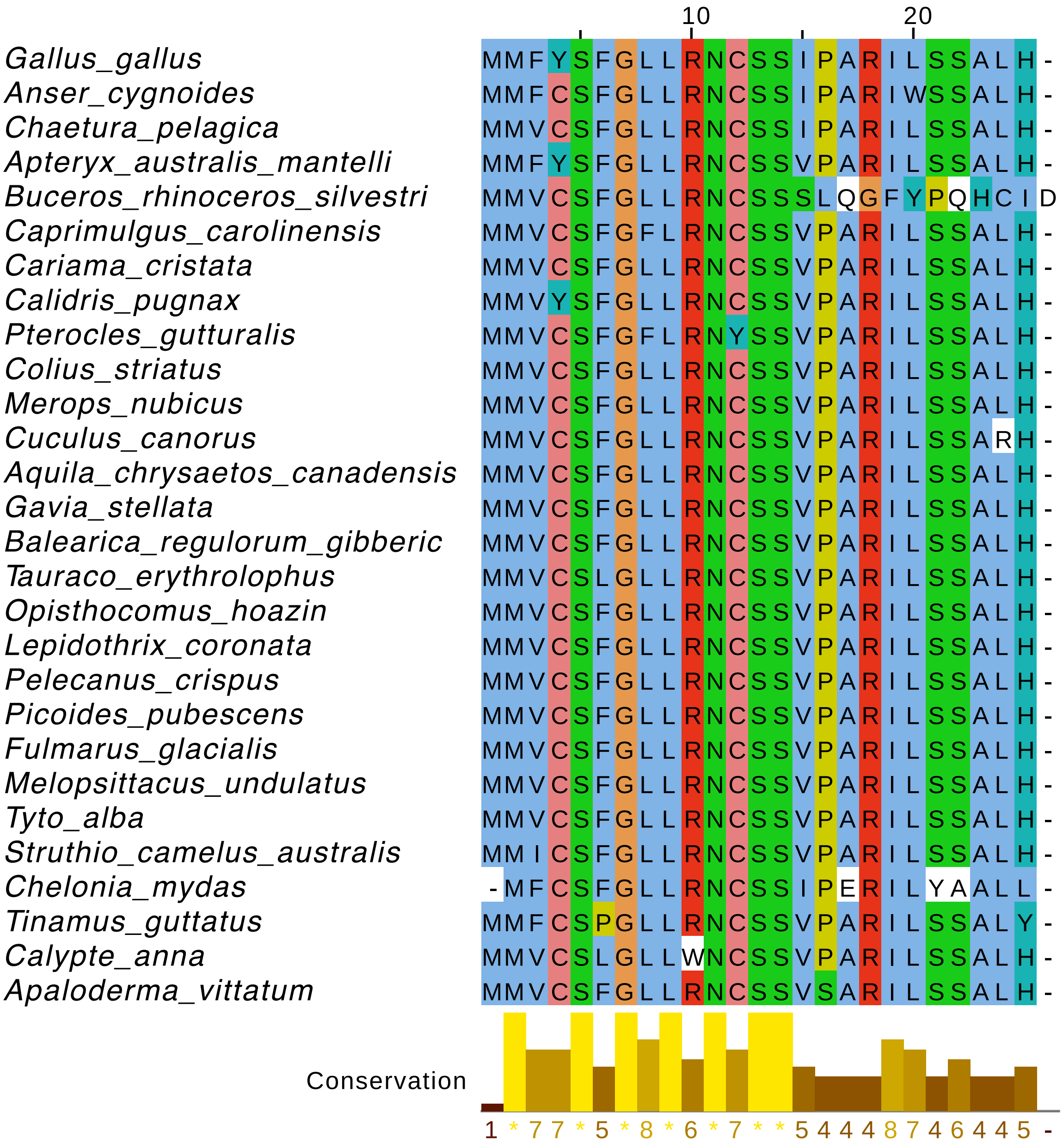

HG0084.2

ENSG00000154447 (SH3RF1)

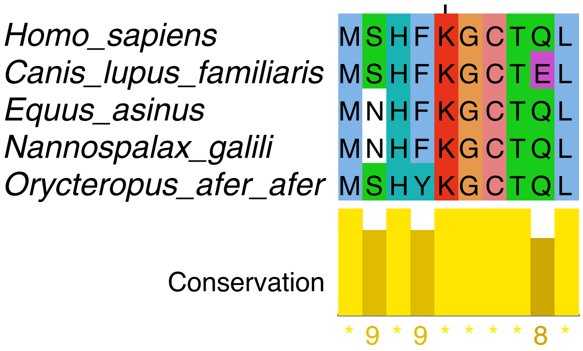

HG0085

ENSG00000107779 (BMPR1A)

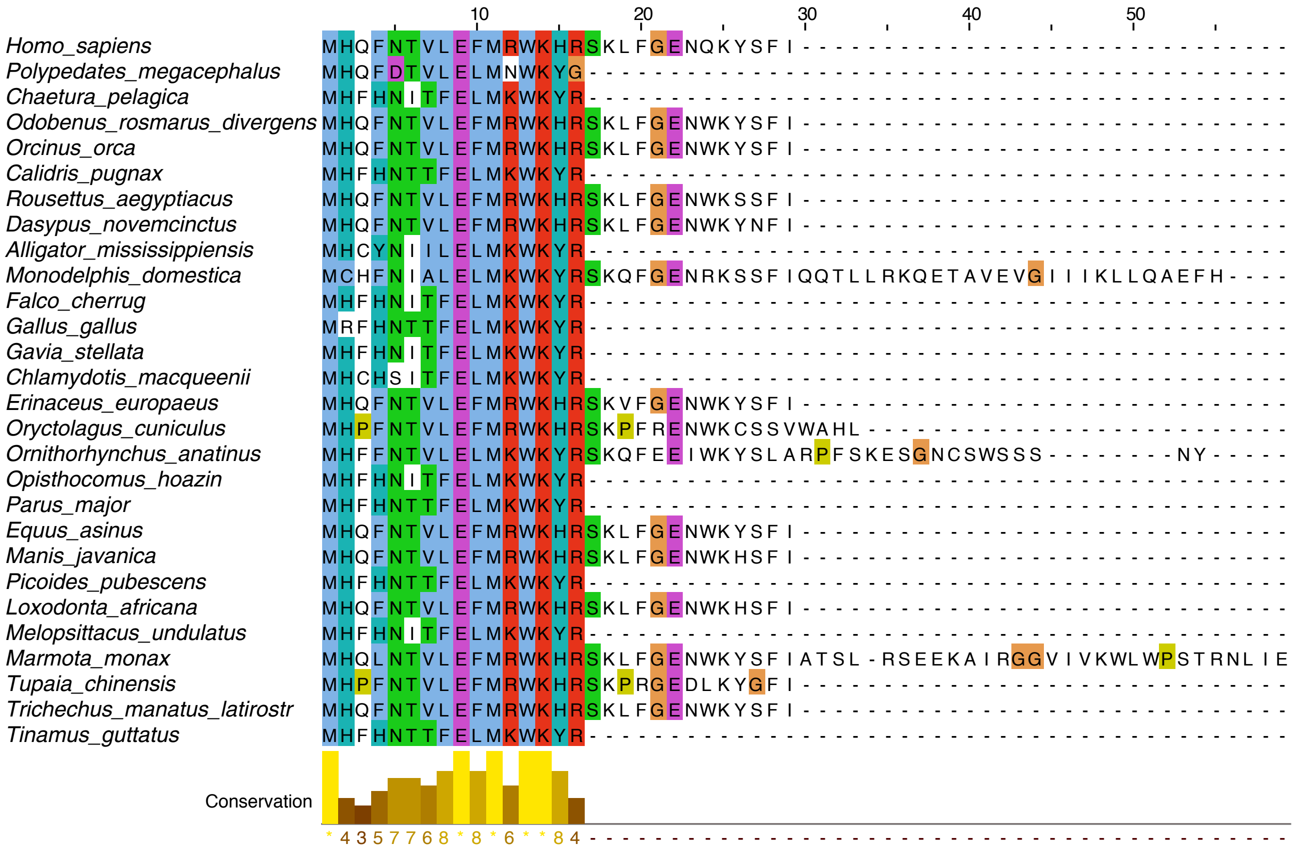

HG0085

ENSGALG00000002003 (BMPR1A)

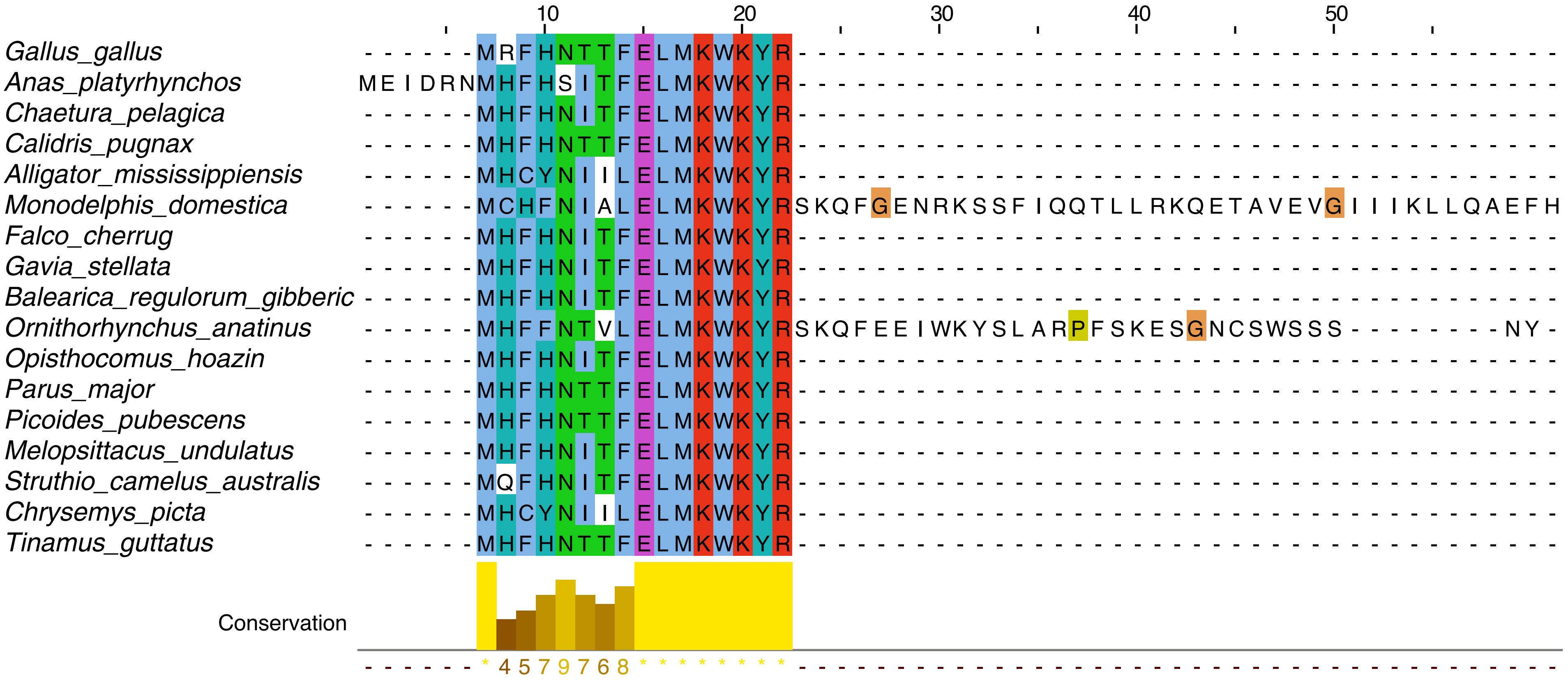

HG0086

ENSGALG00000009835 (XPOT)

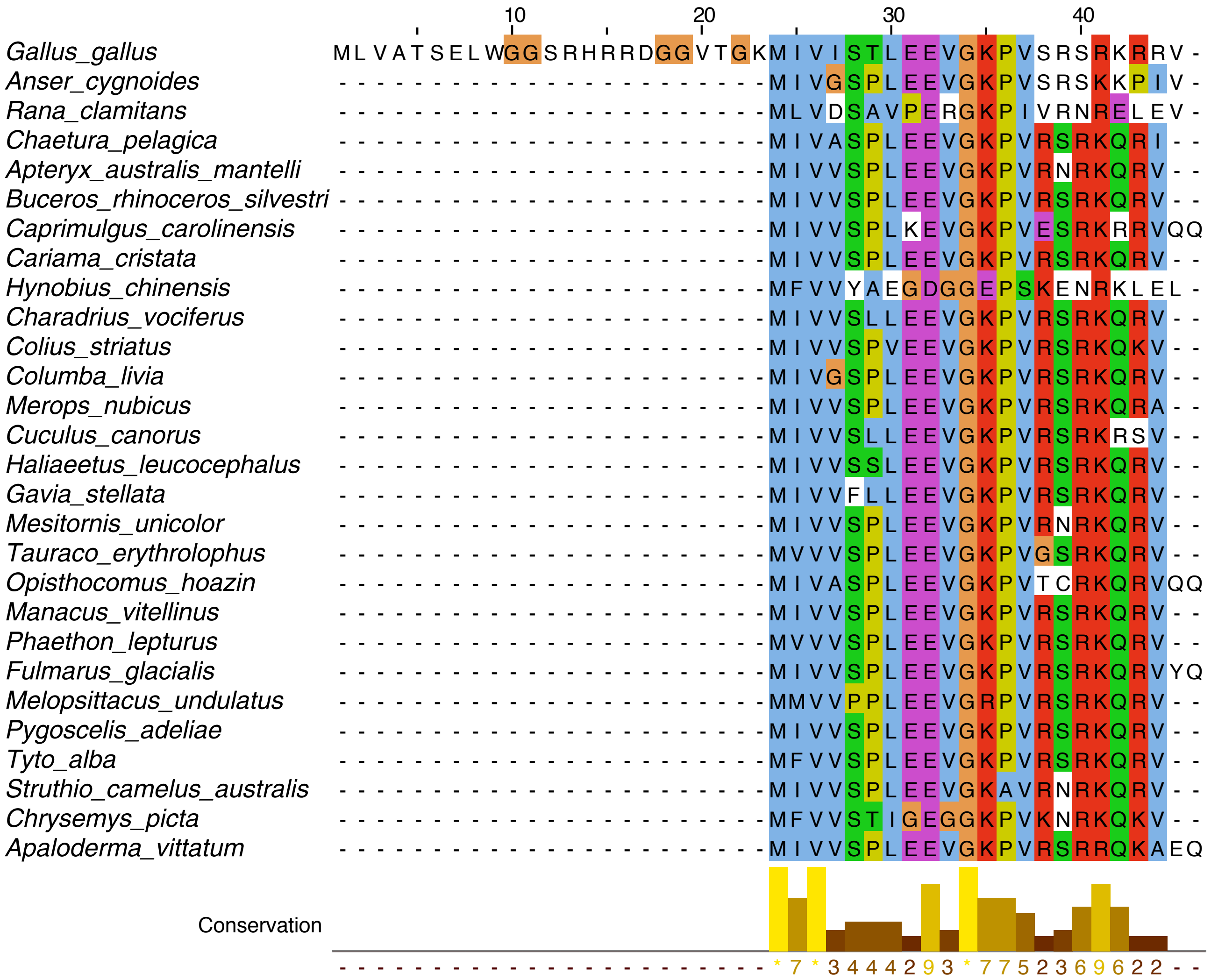

HG0087

ENSG00000164548 (TRA2A)

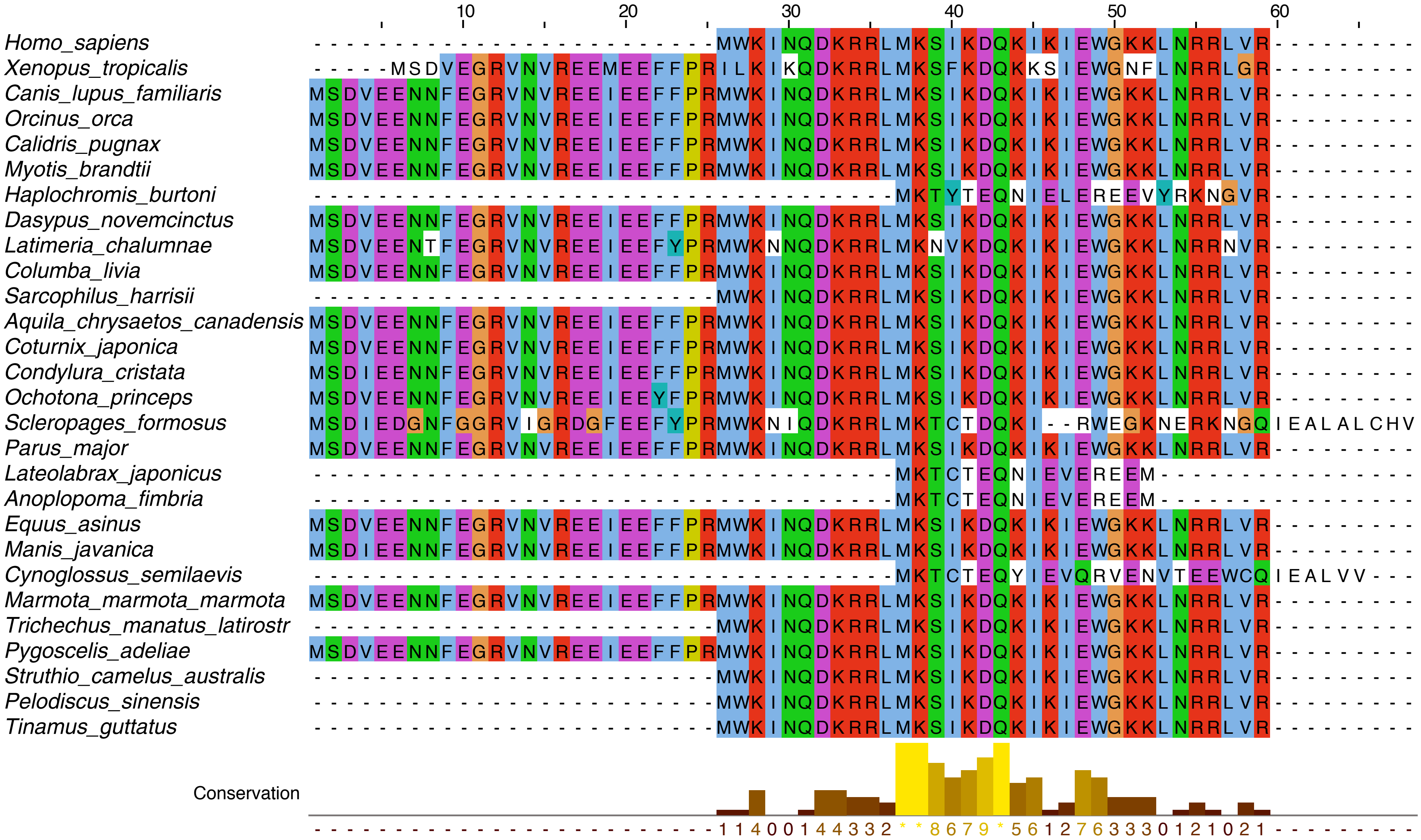

HG0088

ENSG00000273079 (GRIN2B)

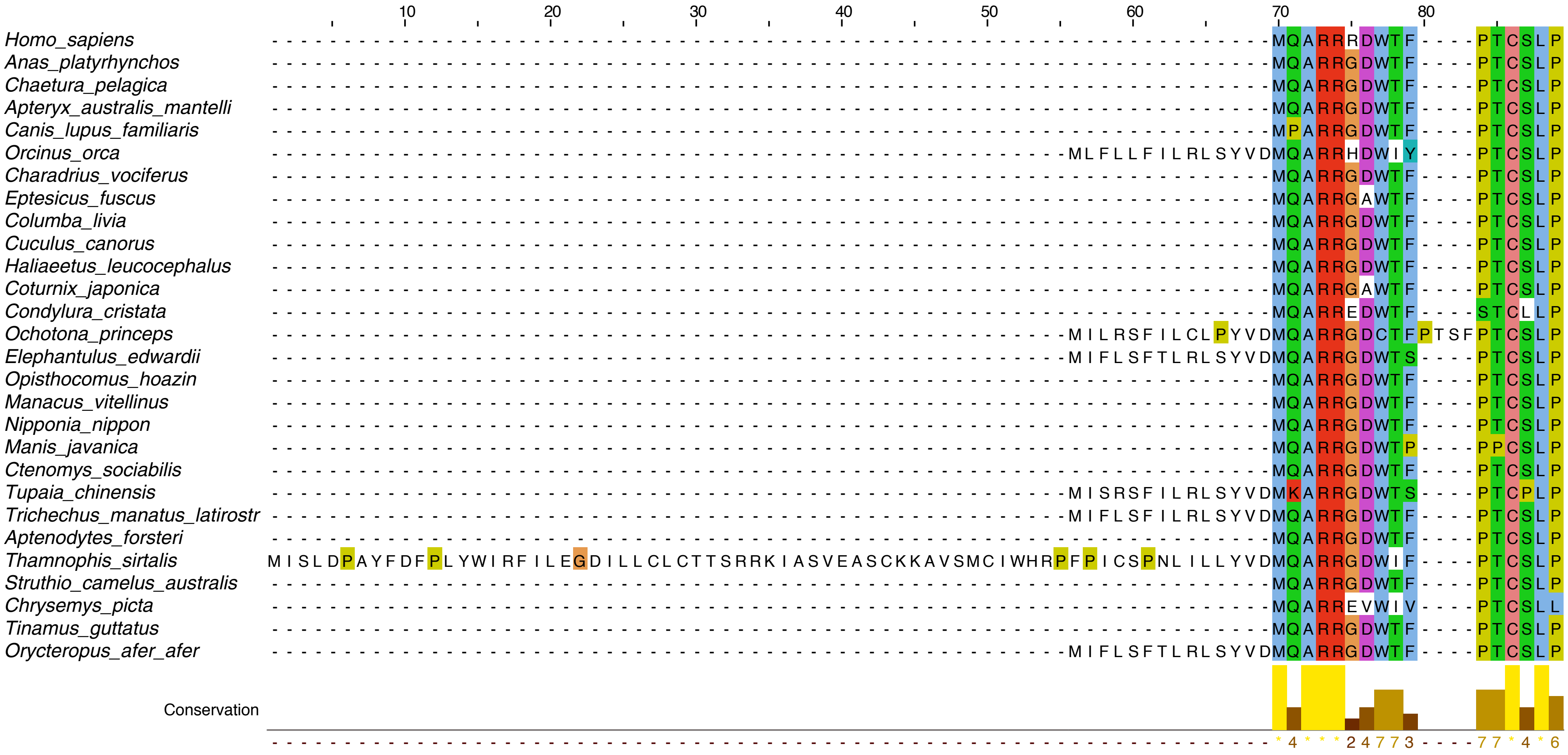

HG0089.1

ENSG00000092421 (SEMA6A)

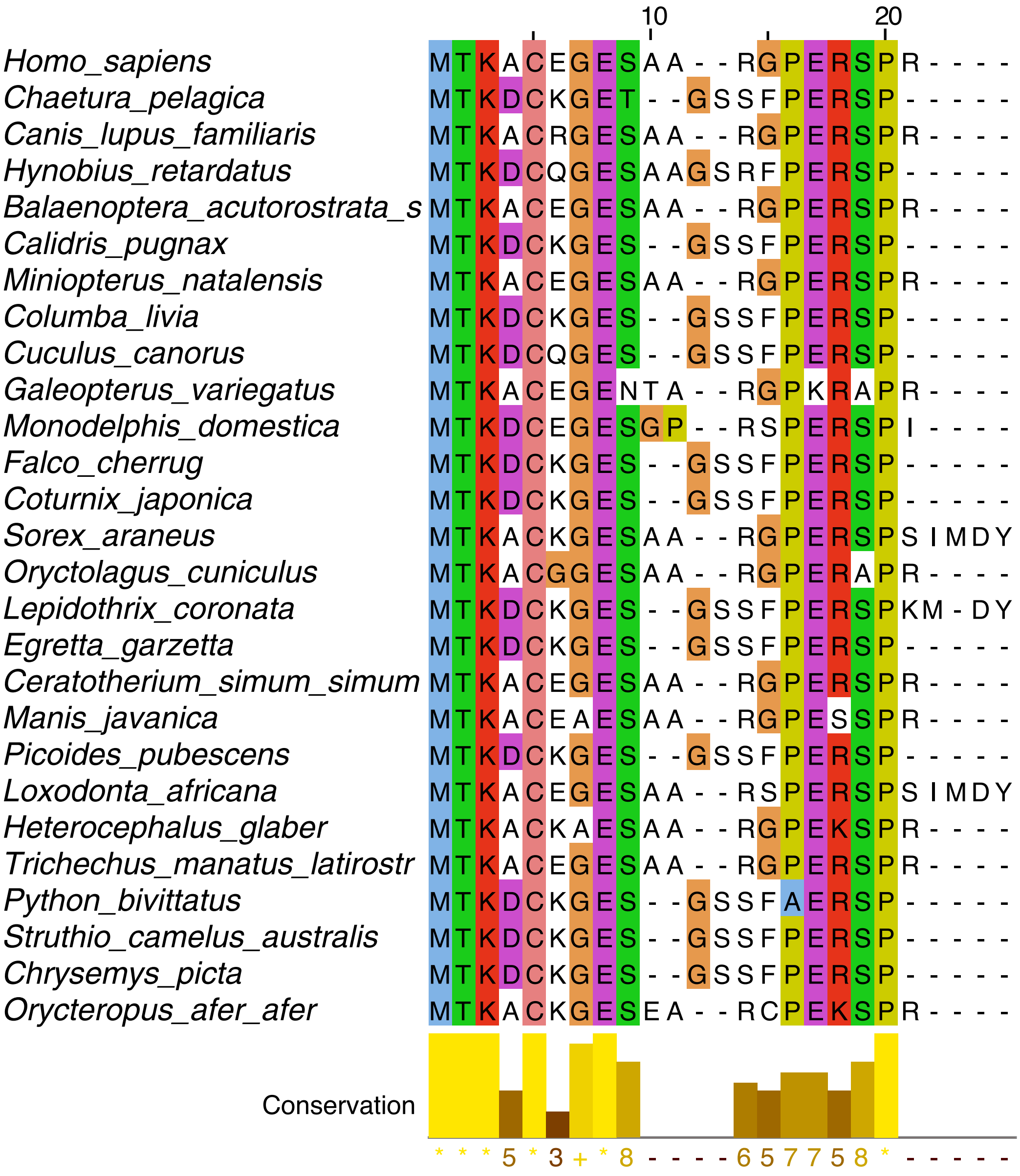

HG0089.1

ENSGALG00000002294 (SEMA6A)

```
                                      10        20        30
Gallus_gallus                 MTKDCKGES--GSSFPERSP-------------------
Nanorana_parkeri              MTKDCQRVP-TGSAFPERSLSV---DY------------
Chaetura_pelagica             MTKDCKGET--GSSFPERSP-------------------
Odobenus_rosmarus_divergens   MTKACKGESAA--CGPERSPR------------------
Hynobius_retardatus           MTKDCQGESAAGSRFPERSP-------------------
Lipotes_vexillifer            MTKACEGESAA--RGPERSPR------------------
Calidris_pugnax               MTKDCKGES--GSSFPERSP-------------------
Miniopterus_natalensis        MTKACEGESAA--RGPERSPR------------------
Columba_livia                 MTKDCKGES--GSSFPERSP-------------------
Alligator_mississippiensis    MTKDCTGERGCGV--PRAVPGHGLLNGTLPQLGLSSGCR
Cuculus_canorus               MTKDCQGES--GSSFPERSP-------------------
Monodelphis_domestica         MTKDCEGESGP--RSPERSPI------------------
Falco_cherrug                 MTKDCKGES--GSSFPERSP-------------------
Sorex_araneus                 MTKACKGESAA--RGPERSPSI--MDY------------
Oryctolagus_cuniculus         MTKACGGESAA--RGPERAPR------------------
Ficedula_albicollis           MTKDCKGES--GSSFPERSPKM---DY------------
Egretta_garzetta              MTKDCKGES--GSSFPERSP-------------------
Equus_caballus                MTKACKGESTA--RGPERSPR------------------
Picoides_pubescens            MTKDCKGES--GSSFPERSP-------------------
Cebus_capucinus_imitator      MTKACEGESAA--RGPERSPR------------------
Loxodonta_africana            MTKACEGESAA--RSPERSPSI--MDY------------
Heterocephalus_glaber         MTKACKAESAA--RGPEKSPR------------------
Trichechus_manatus_latirostr  MTKACEGESAA--RGPERSPR------------------
Takydromus_sexlineatus        MTKDCKGES--GSSFAERSP-------------------
Struthio_camelus_australis    MTKDCKGES--GSSFPERSP-------------------
Chrysemys_picta               MTKDCKGES--GSSFPERSP-------------------
Orycteropus_afer_afer         MTKACKGESEA--RCPEKSPR------------------

Conservation                  ***5*3443----0078466-------------------
```

HG0089.2

ENSG00000092421 (SEMA6A)

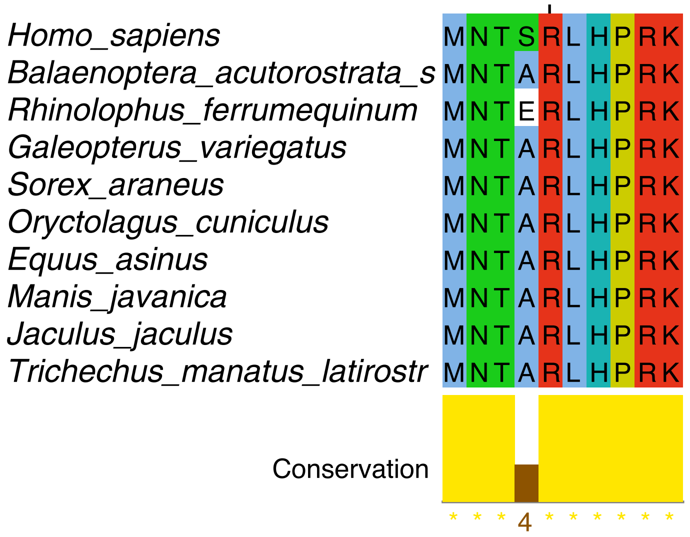

HG0089.3

ENSGALG00000002294 (SEMA6A)

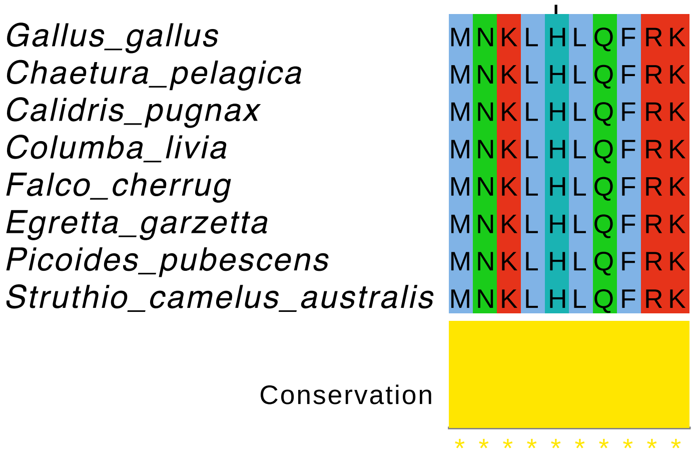

HG0090.1

ENSG00000172765 (TMCC1)

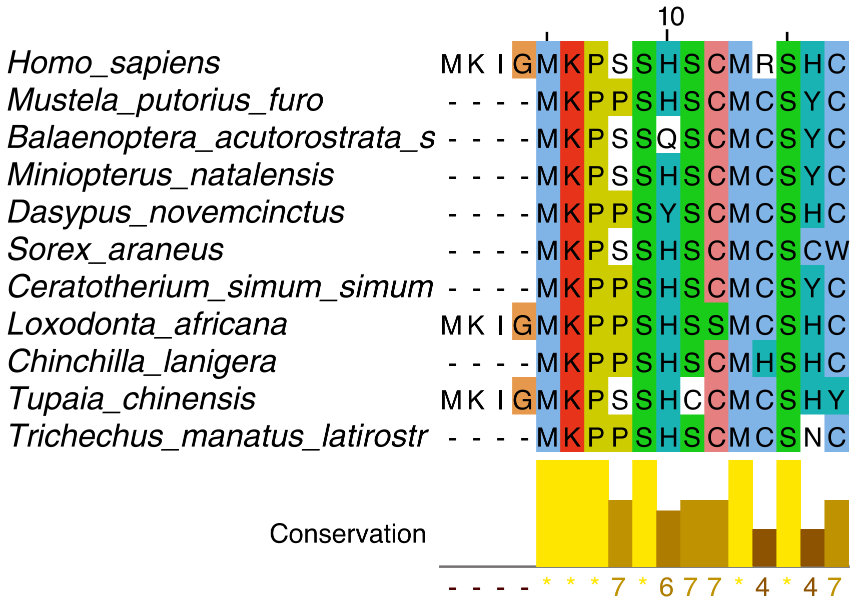

HG0090.1

ENSGALG00000008487 (TMCC1)

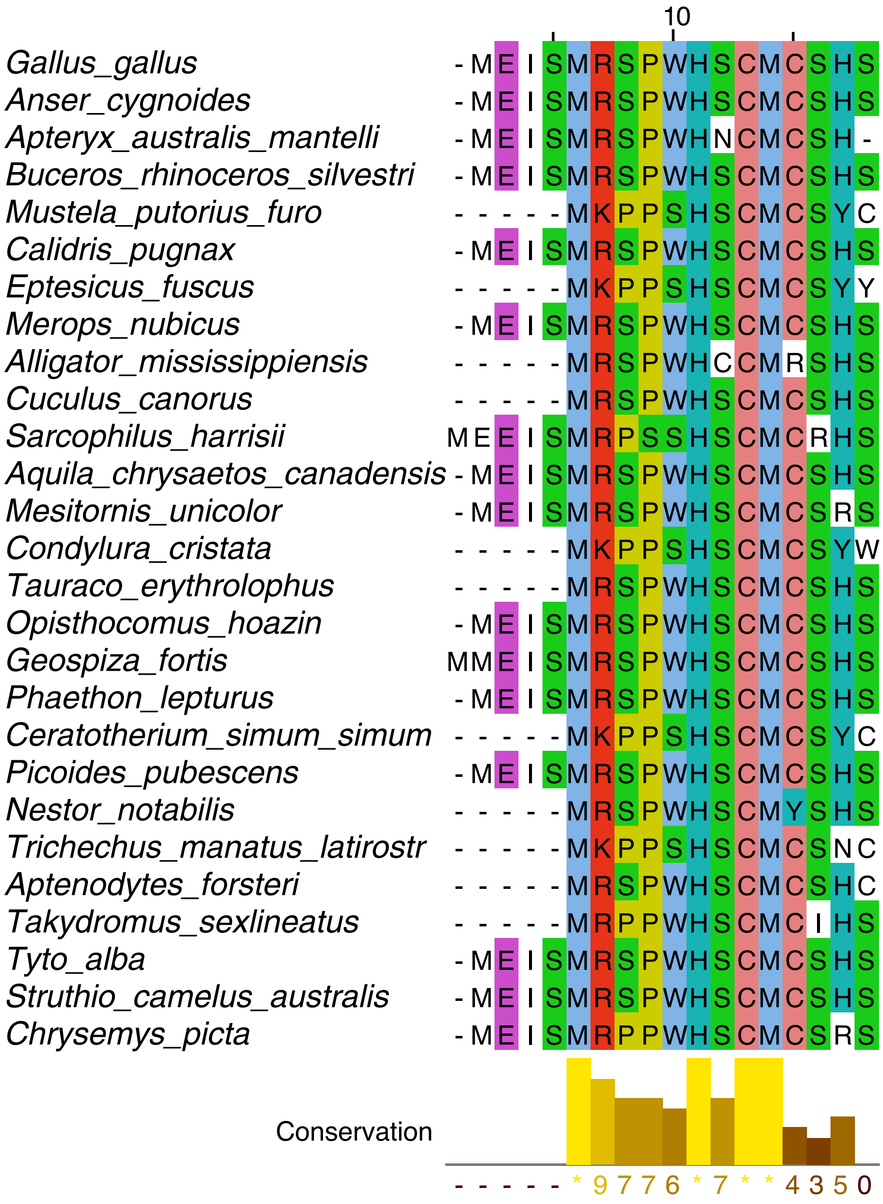

HG0090.2

ENSDARG00000060954 (tmcc1b)

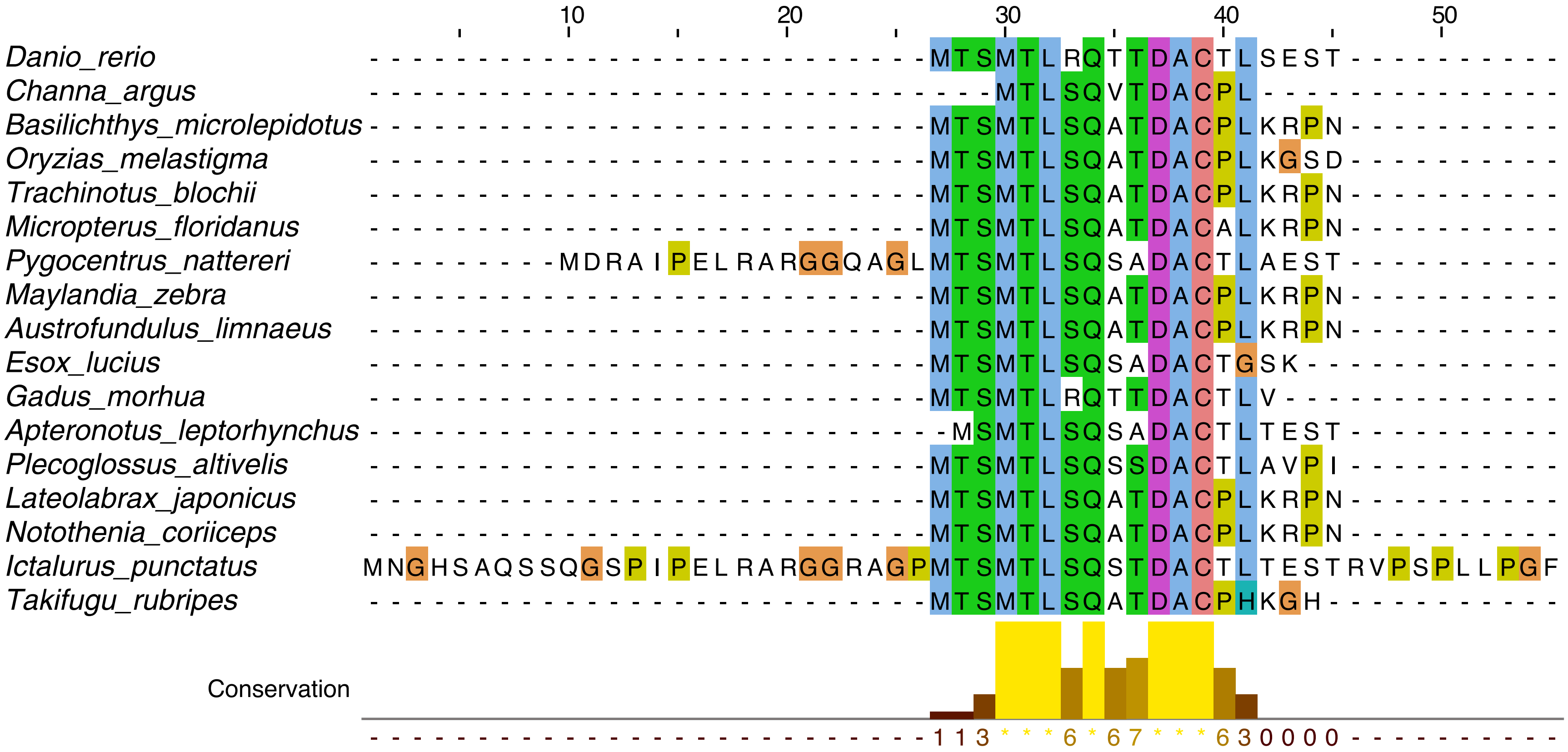

HG0090.3

ENSGALG00000008487 (TMCC1)

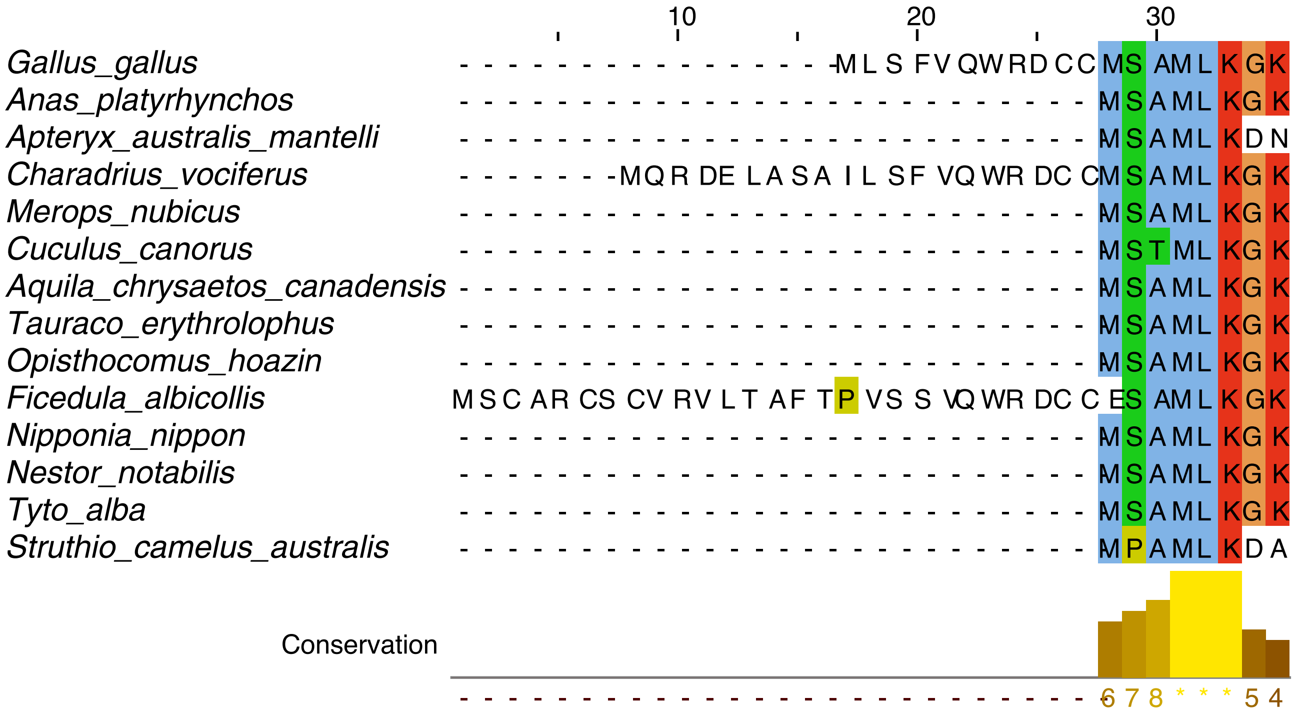

HG0091.1

ENSG00000172209 (GPR22)

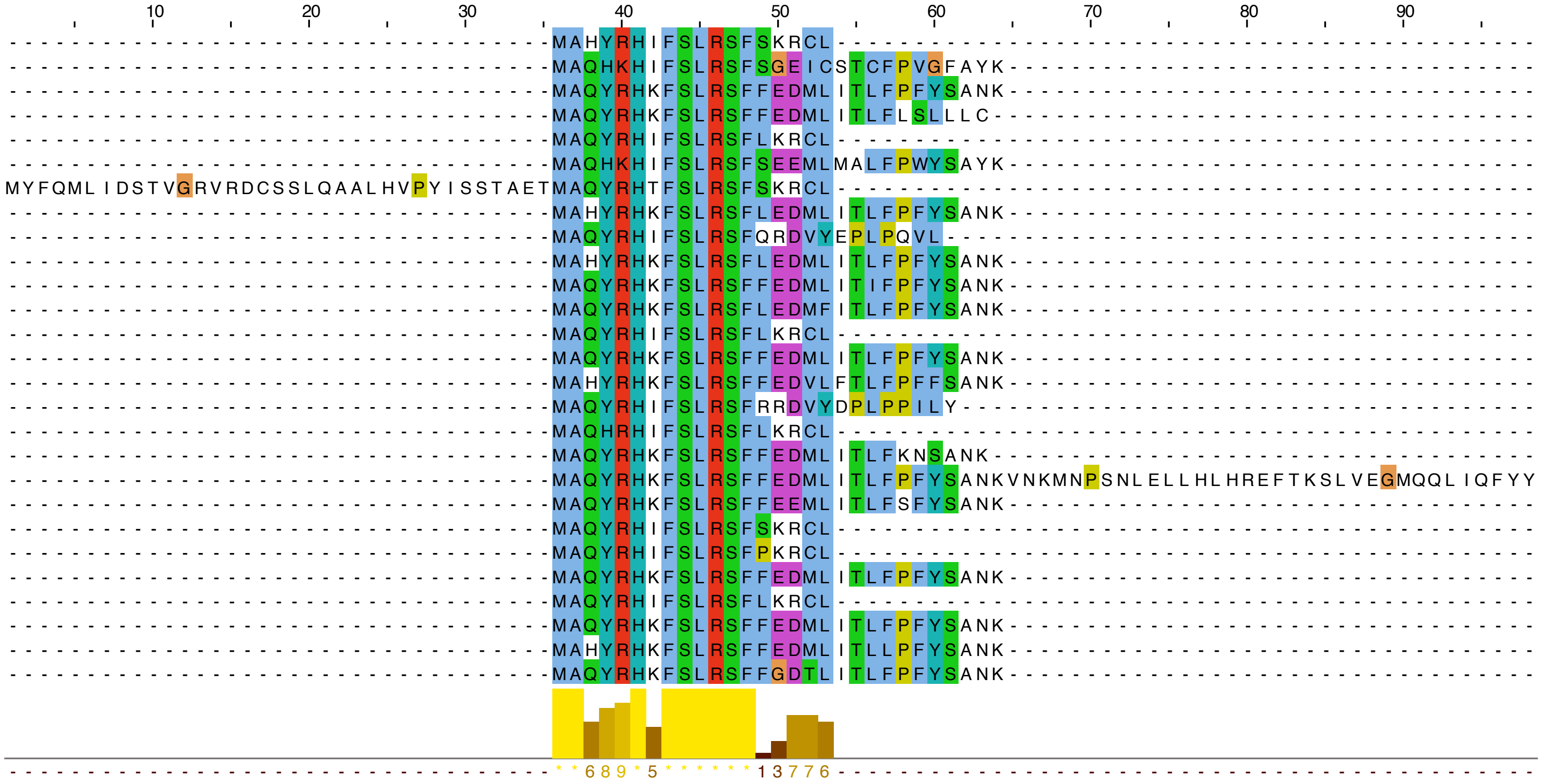

HG0091.2

ENSG00000172209 (GPR22)

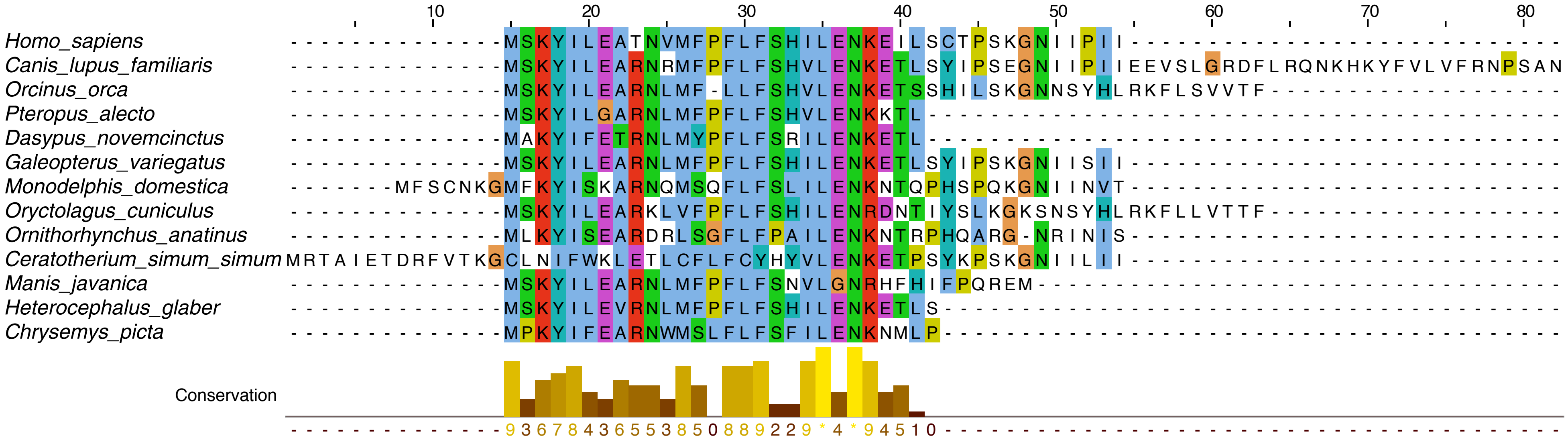

HG0091.3

ENSG00000172209 (GPR22)

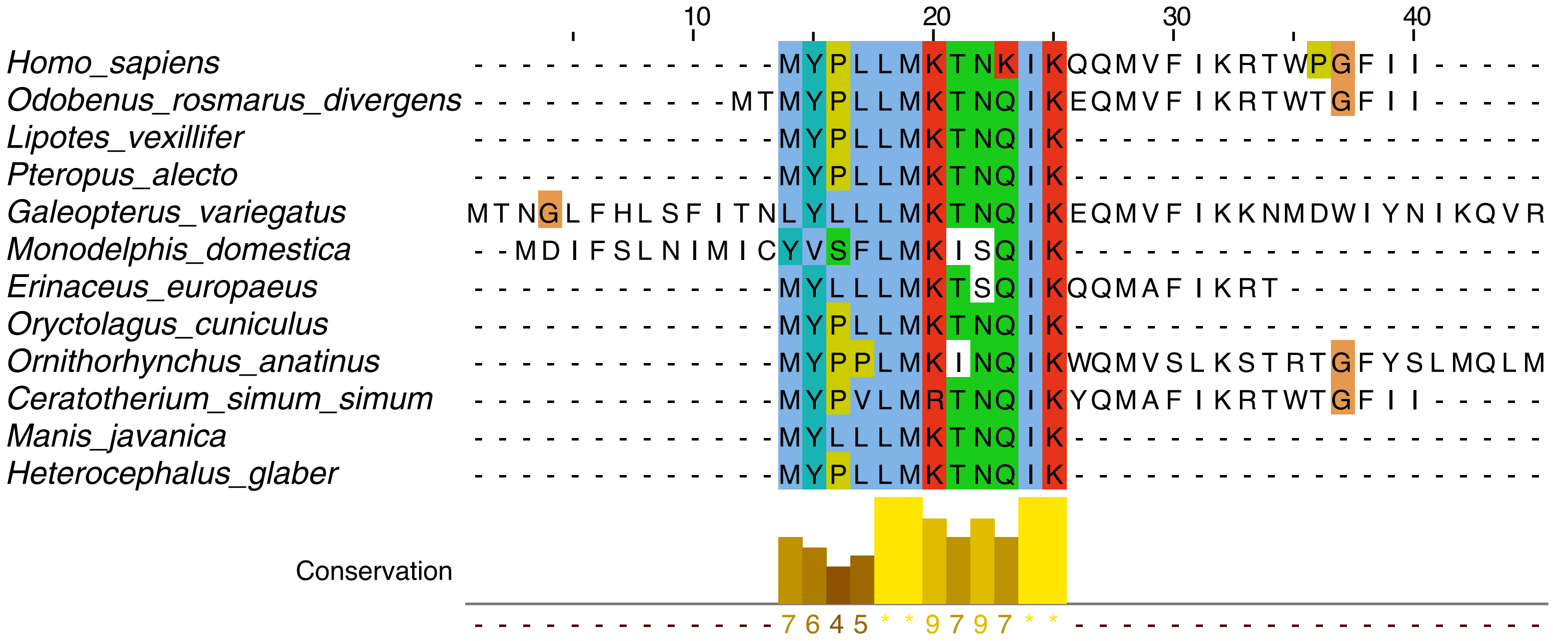

HG0092.1

ENSGALG00000009966 (FRS2)

```
                                     10        20        30        40        50        60
Gallus_gallus                        --------------------------------------MISSVTRQHDTLKYDRSPDAATEAGNINGQI
Anser_cygnoides                      --------------MVIKQLLSCSAAVC----LHVLGQMISSVTRQHDTLKYDRTPDAATEAGNINGQI
Xenopus_laevis                       --------------------------------------------MRHVILV------------------
Apteryx_australis_mantelli           ------------MLHGAKCLMLMTLGWK----VTFLDQMITSVTRQHDTLKYDRSPDAAAEAGNINGQI
Buceros_rhinoceros_silvestri         MI-SSVTRQHDTLSMTEALMLQQRLGILMAESRDHMVF-------------------------------
Charadrius_vociferus                 MI-SSVTRQHDALSMTEALMLQQRLGILMAKSRDHMVF-------------------------------
Pteropus_alecto                      MRGIWFKLQYREFRLDIKNFQKIKYQILKR-------FVMSSNKKKHGILKYD----------------
Dasypus_novemcinctus                 --------------MQLKLFKQIGTKLHLFFSEESSTRLSQQT--KHVILKYD----------------
Latimeria_menadoensis                -------------------------------------------------MKYERGPDVARGIRNLNGQI
Colius_striatus                      MI-SSMTRQHDTLSMTEALMLQQRLGILMAKSRDLMVF-------------------------------
Columba_livia                        --------------------------------------MISSVTRHHDTLKYDRSPDAAAEPGNINGQI
Leptosomus_discolor                  MI-SSVTRQHDTLSMTEALMLQQRLGILMAKSRDHMVF-------------------------------
Alligator_mississippiensis           ------------MSPFDDC---SDMGACI---ECILDPVVAHVKRQHDILKYDRSPDAATEARNINGQI
Cuculus_canorus                      MI-SSVTRQHDTSSMTEALMLQQRLGILMAKSRDHMVF-------------------------------
Sarcophilus_harrisii                 --------------MQLLLFSHIRRKLHLIFSKEKFARLCKQA--KYGILKYD----------------
Aquila_chrysaetos_canadensis         MI-SSVTRQHDTLSMTEALMLQQRLGILMAKSRDHMVF-------------------------------
Gavia_stellata                       MI-SSVTRQHDPLSMTEALMLQQRLGILMAKSRDHMVF-------------------------------
Balearica_regulorum_gibberic         --------------------------------------MISSVTRQHDTLKYDRSPDAAAEAGNINGQI
Condylura_cristata                   --------------MQLWLFNQIRAKLYLFFSEDNSTRISHHTEKSHGILKYD----------------
Tauraco_erythrolophus                MI-SSVTRQHDTLSMTEALMLQQRLGILMAKCRDHMVF-------------------------------
Opisthocomus_hoazin                  --------------------------------------MISSVTRQHDTLKYDRSPDAAAEAGNINGQI
Zonotrichia_albicollis               MI-SSVTRQHDTLSMTEALMLQQRLGILMAKSRDQMVF-------------------------------
Phaethon_lepturus                    --------------------------------------MISSVTRQHDTLKYDRSPDAAAEAGNINGQI
Fulmarus_glacialis                   MI-SSVTRQYDTLSMTEALMLQQRLGILMARSRDHMVF-------------------------------
Aptenodytes_forsteri                 MI-SSVTRQHDTLSMTEALMLQQRLGILMAKSRDHMVF-------------------------------
Pelodiscus_sinensis                  --------------------------------------MFVYVRRQHAILKYDRSPDAATEARNINGQI
Calypte_anna                         MI-SSVARQHDTLSMTEALMLQQRLGILMAKSRDPMVF-------------------------------

Conservation
                                     ---------------------------------------------------------------------
```

HG0092.2

ENSG00000166225 (FRS2)

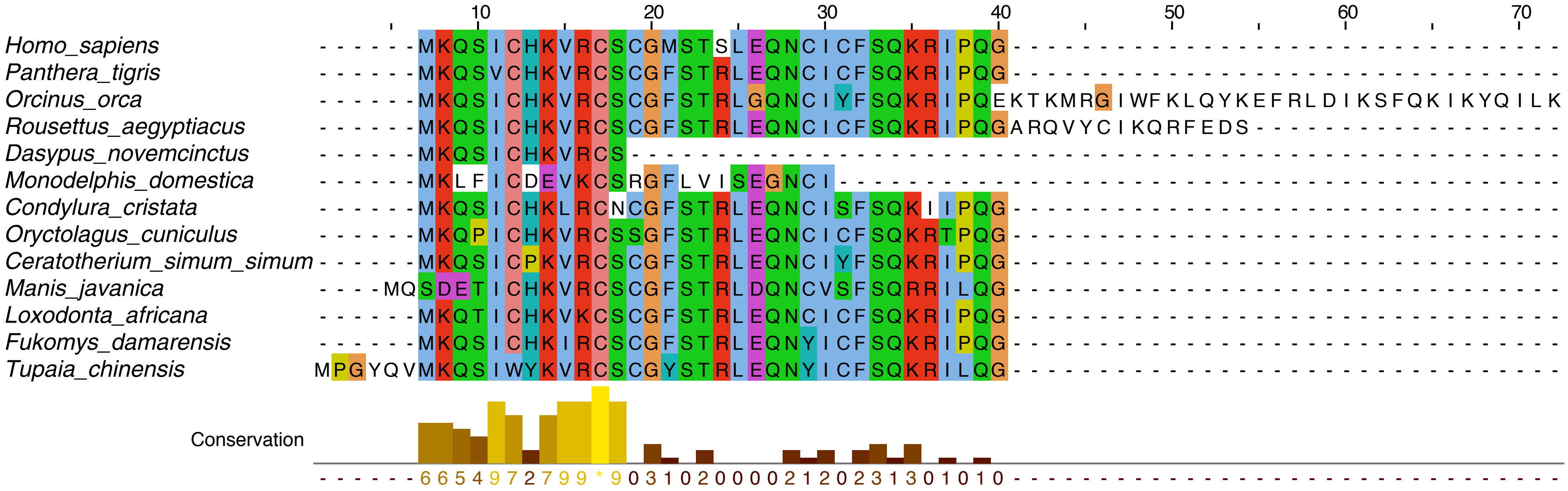

HG0093

ENSG00000169554 (ZEB2)

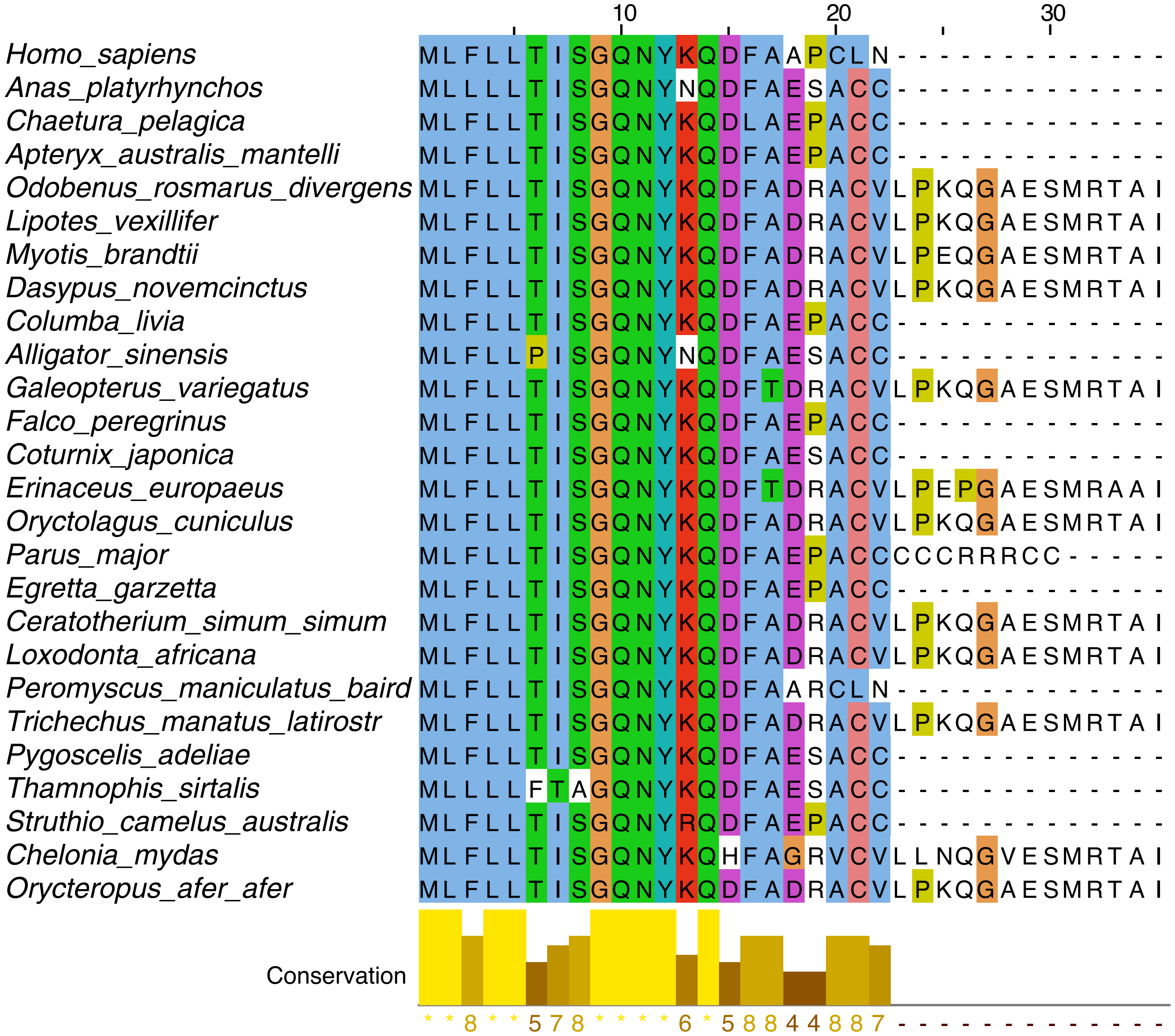

HG0093

ENSGALG00000040465 (Sip1)

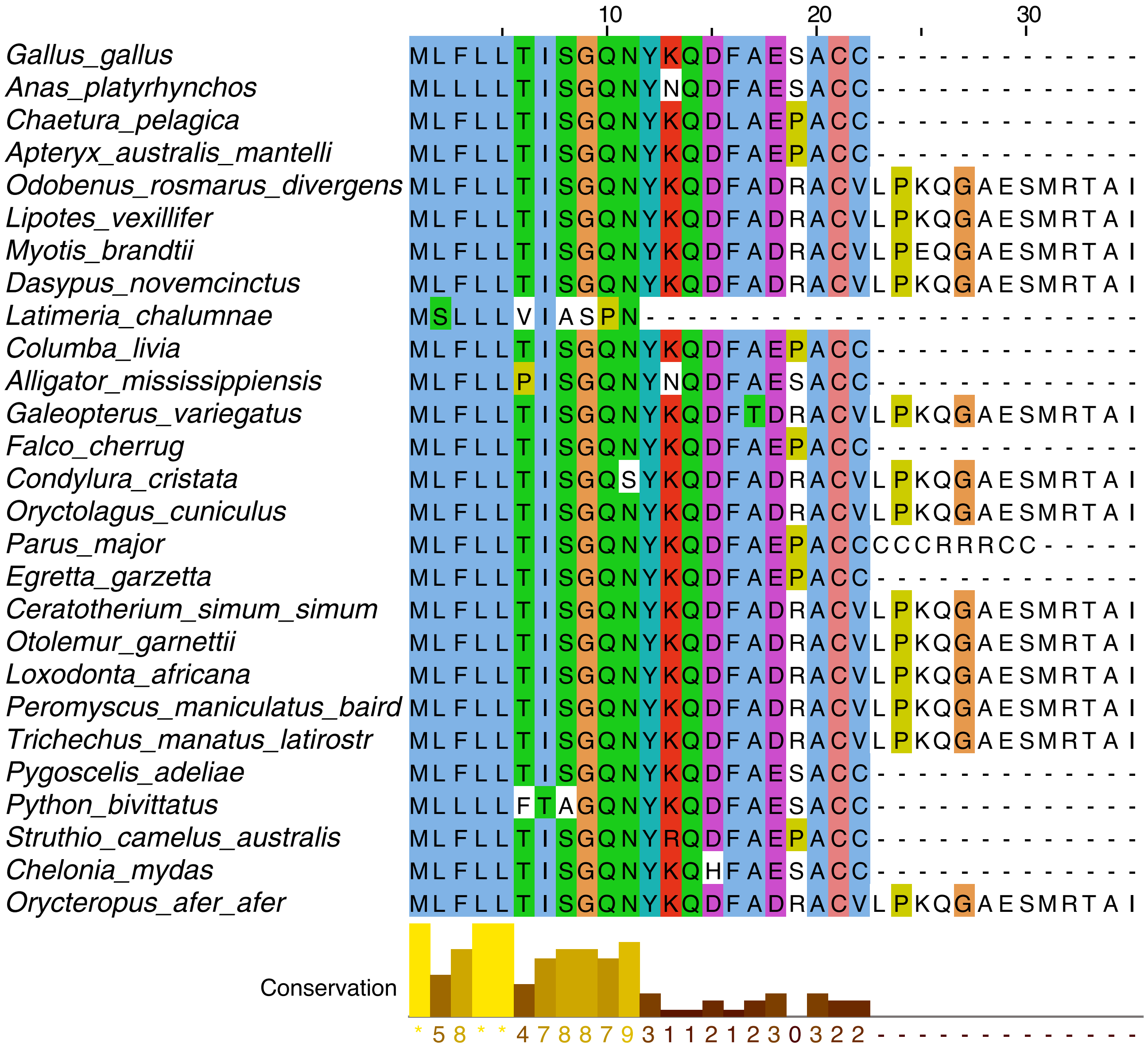

HG0094.1

ENSG00000112175 (BMP5)

```
                                       10        20        30
Homo_sapiens                   ---------------------MQQKLLTTVDNWKSEFQLS
Anser_cygnoides                -----MPRSADK--ILVFCSINAEKTITTVENWKSEFQLS
Apteryx_australis_mantelli     -----MSRSADK--ILVFCSISAEKTITTVENWKSEFQLS
Mustela_putorius_furo          ---------------------MQQKLLTTVDNWKSKFQLS
Balaenoptera_acutorostrata_s   ---------------------MQQKLLTTVDNWKSEFQLS
Calidris_pugnax                -----MPRSADK--ILVFCSINAEKTITTVENWKSEFQLS
Pteropus_alecto                ---------------------MQQKLLTTVDNWKSEFQPS
Columba_livia                  -----MPRSADK--ILVFCSINAEKTITTVENWKSEFQLS
Alligator_mississippiensis     -----MPRSADK--VLFFCSINAEKTITTVENWKSEFQLS
Monodelphis_domestica          -----MHRSFDQIILAVFCYINAVQTITTVDNWKSEFQLS
Aquila_chrysaetos_canadensis   -----MPRSADK--ILVFCSINAEKTITTVENWKSEFQLS
Coturnix_japonica              -----MPRSADK--ILVFCSINAEKTITTVENWKSEFQLS
Sorex_araneus                  ---------------------MQQKLLTTVDNWKSEFQLS
Ochotona_princeps              ---------------------MKQKLLTTVDNWRSEFQLS
Elephantulus_edwardii          ---------------------MQQKLLTTVDNWKFEFQLS
Corvus_brachyrhynchos          -----MPRSADK--ILVFCSINAEKTITTVENWKSEFQLS
Ceratotherium_simum_simum      ---------------------MQQKLLTTVDNWKSEFQLS
Manis_javanica                 ---------------------MQQKLLTTVDNWKSEFQLS
Loxodonta_africana             ---------------------MQQKLLTTVDNWKSKFQLS
Marmota_marmota_marmota        ---------------------MQQKLLATVDNWKSEFQLS
Trichechus_manatus_latirostr   ---------------------MQQKLLTTVDNWKSKFQLS
Aptenodytes_forsteri           -----MPRSADK--ILVFCSINAEKTITTVENWKSEFQLS
Anolis_carolinensis            MLCTGMPASTNQ--VLVFCSIHVEKTVMTVENWKSEFQLS
Struthio_camelus_australis     -----MPRSADK--ILVFCSINAEKTITTVENWKSEFQLS
Pelodiscus_sinensis            -----MPRSADK--ILVFCSINAEKAITTVENWKSEFQLS
Tinamus_guttatus               -----MSRSADK--ILVFCSINAEKTITTVENWKSEFQLS
Orycteropus_afer_afer          ---------------------MQQKLLTTVDNWKYEFQLS

Conservation                   ---------------------3347697**9**957**6*
```

HG0094.2

ENSG00000112175 (BMP5)

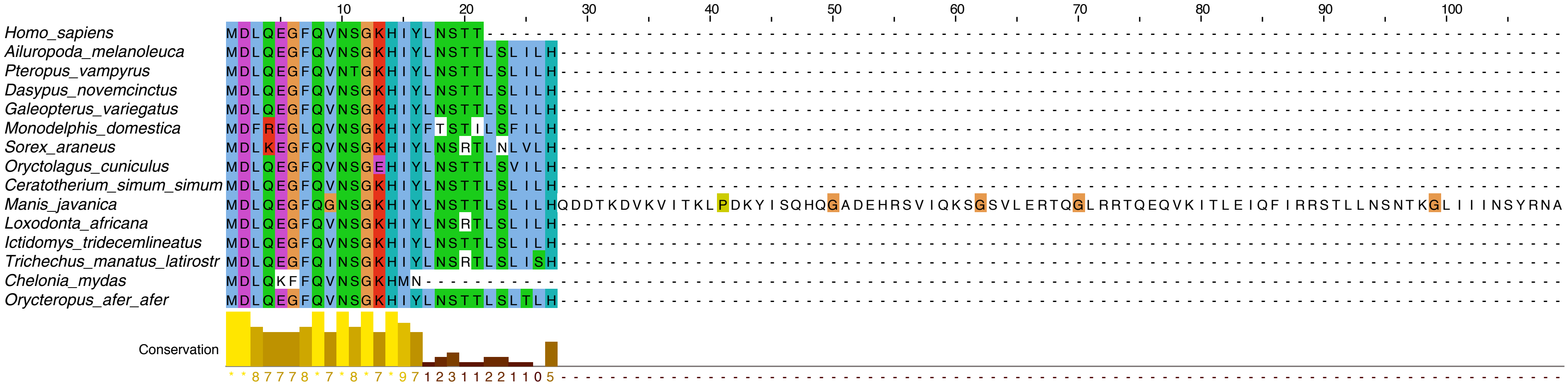

HG0095

ENSDARG00000013708 (usp9)

```
                                     10        20        30        40        50
Danio_rerio                 MEERSSLILGYAMVSATYPVPQVVLSLSEVCRFSVA-----PEE----KLISSGVPAT
Amia_calva                  ------------MVSAKHPVLQVVLSYTDVCWFSVI-----VEE----AIISSLTTTT
Channa_argus                ------------MVSAAHPDLQVLVSLSCVFG--FSSRCSTAEVA----VSSLLACAP
Microhyla_fissipes          ------------MVSAKRSVFEVELCKADICRLAF------LEG----TIISSLVTAT
Menidia_menidia             ------------MVSAAHPDLQVLVSLSRAFG--LSSLCSTAEVA----VISLLVSAP
Porichthys_notatus          ------------MVSAAHPDLQVLVSLSRVLG--LDSLCSTAELA----VISLLASAP
Oryzias_melastigma          ------------MVSATHPDLQVLVSLSCVFG--LSSLCSTAEVA----VISLLASAP
Ambystoma_mexicanum         ------------MVSAKCGVHEVVLCQPHTCWYYVY-----IKG----SPISSLTLAT
Pygocentrus_nattereri       ------------MVSAAHPVLQVVLSLSDVCRFSIS-----PEE----AVIFSCTPAT
Callorhinchus_milii         ------------MVSARQPELEVVLLCTDLNWFSIF-----TEE----AIHSSLATTT
Haplochromis_burtoni        ------------MVSAAHPDLQVLVSLSRVFR--LSSLCSTAEVA----VISLLASAP
Dasypus_novemcinctus        ------------MVSARWFILDLELFKTDICWYFVF-----YKW----TIISFFKTTT
Alosa_alosa                 ------------MVSAIPPVLQVVLSLSRACRLSVS-----LEE----AVISPRAPAT
Latimeria_chalumnae         ------------MLSAKQSVLEVVLLYTDTCWFAIF-----TEG----VIISSLKTTT
Poeciliopsis_prolifica      ------------MVSAVHPDLQVLATLSRVFR--LSFLRCTAEVA----VISLLASAP
Sarcophilus_harrisii        ------------MVSARWFVLNLELFKTDICWYFIF-----YKR----TLISPLTTTT
Esox_lucius                 ------------MVSAARPDLQVVLSLSLAPRPQCSSLRSVSEVAEAPPVISLLASAP
Gadus_morhua                ------------MVSAAHPDLQVLVSLSCVFG--LVYLCSTAEAA----VIPLVASAP
Apteronotus_leptorhynchus   ------------MVTATQPKPQVLLSCLLALWIRPA-----ALQ----AVISRLAPTT
Ornithorhynchus_anatinus    ------------MVAATWLALELELFKTDTCRYCIV-----SKR----TIIPSLPTTT
Scleropages_formosus        ------------MVAASRPALQLVLSHTDVCWFSVT-----TDE----ALISSPAIAT
Trematomus_bernacchii       ------------MVSAAHPDLQVLVSLSRAFG--LSSLCSTAEVA----VISLLASAP
Platichthys_stellatus       ------------MVSAAHPDLQVLVSLSRVFG--LSSLCSTAEVA----VISLLASAP
Macaca_nemestrina           ------------MVSARWFILELDLFKTDKCWYFIF-----YKW----TIISFLKTTT
Oncorhynchus_mykiss         ------------MVSAARPDLQVVLSLSLVPRPLLSSLCSSSEVAEAPPVISLVVSAA
Lepisosteus_oculatus        ------------MVSAKHPVLQVVLSSTDACWFSII-----VEE----AIISSLTTTT
Ictalurus_punctatus         ------------MVS------ASALALSAVCYFSIS-----PEE----AVISSRAPVT

Conservation                ------------*9730000045374241443--50-----331-----624112476
```

HG0096

ENSGALG00000014186 (MPPED1)

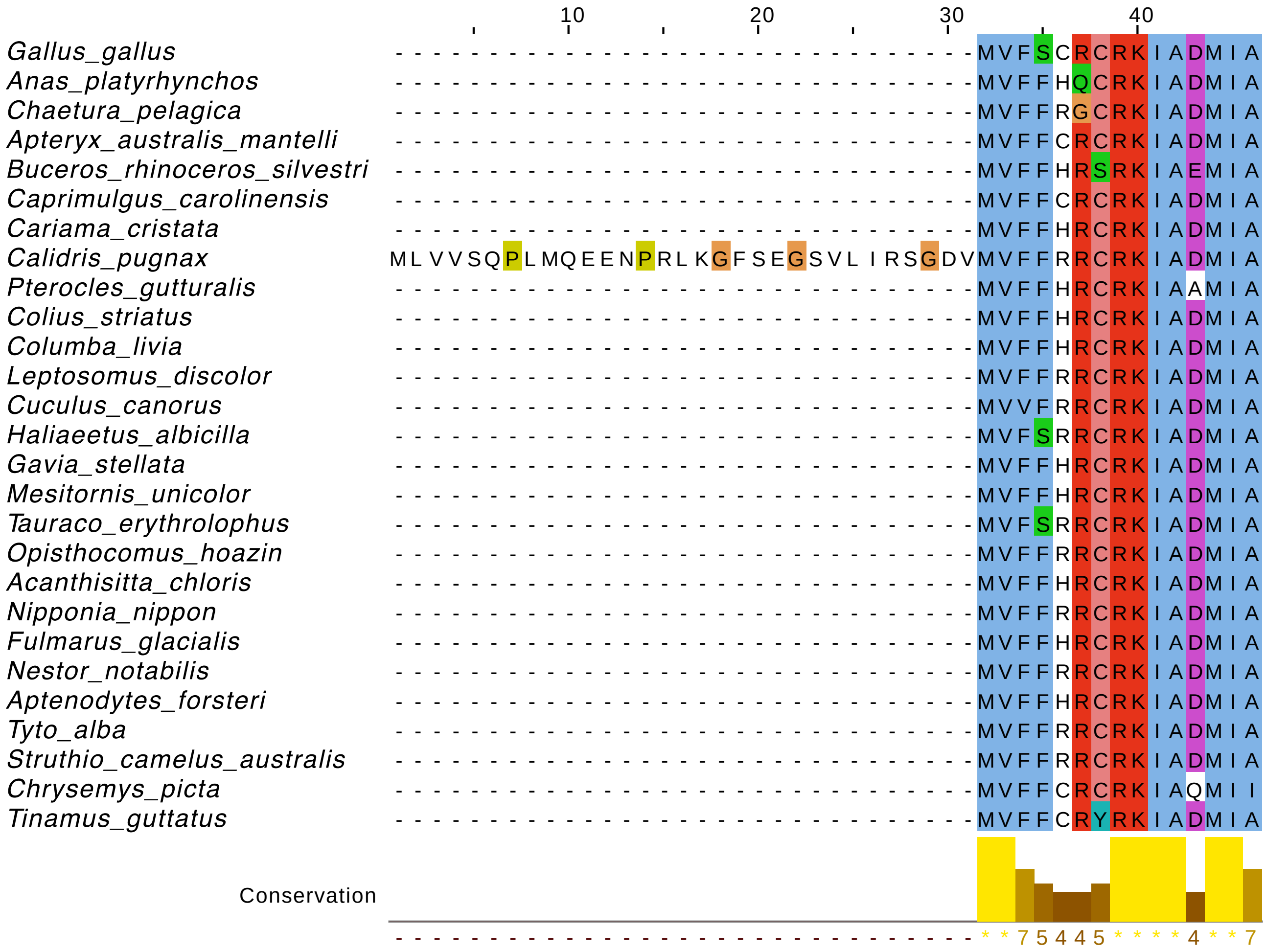

HG0097

ENSGALG00000037496

Species	Alignment (positions 1–40)
Gallus_gallus	MELQPRAGAADTPSAFLRSSHHERGRFHLVKMAVWDRCNL
Ailuropoda_melanoleuca	-------------------------------MAVRDRCNL
Orcinus_orca	-------------------------------MAVRDRCNL
Calidris_pugnax	-------------------------------MAVWDRCNL
Rousettus_aegyptiacus	-------------------------------MAVRDRCNL
Dasypus_novemcinctus	-------------------------------MAVRGRCHL
Columba_livia	-------------------------------MAVWDRCNL
Alligator_mississippiensis	-------------------------------MAVGDRCNL
Sarcophilus_harrisii	-------------------------------MAVRDHCNL
Galeopterus_variegatus	-------------------------------MAVRDRCNL
Monodelphis_domestica	-------------------------------MAVRDHCNL
Falco_peregrinus	-------------------------------MAVWDRCNL
Erinaceus_europaeus	-------------------------------MAVRDRCNL
Elephantulus_edwardii	-------------------------------MAVRDRCNL
Lepidothrix_coronata	-------------------------------MAVWDRCNL
Nipponia_nippon	-------------------------------MAVWDRCNL
Ceratotherium_simum_simum	-------------------------------MAVRDRCNL
Manis_javanica	-------------------------------MAVRDRCNL
Pongo_abelii	-------------------------------MAVRDRCNL
Loxodonta_africana	-------------------------------MAVRDRCNL
Melopsittacus_undulatus	-------------------------------MAVRDRCNL
Microtus_ochrogaster	-------------------------------MAVCDRCNL
Trichechus_manatus_latirostr	-------------------------------MAVRDRCNL
Aptenodytes_forsteri	-------------------------------MAVWDRCNL
Gekko_japonicus	-------------------------------MAVWDRCNL
Pelodiscus_sinensis	-------------------------------MAVWDHCNL
Calypte_anna	-------------------------------MAVWDRYNL
Conservation	-------------------------------***358 75*

Position markers: 10, 20, 30

HG0098

ENSG00000198739 (LRRTM3)

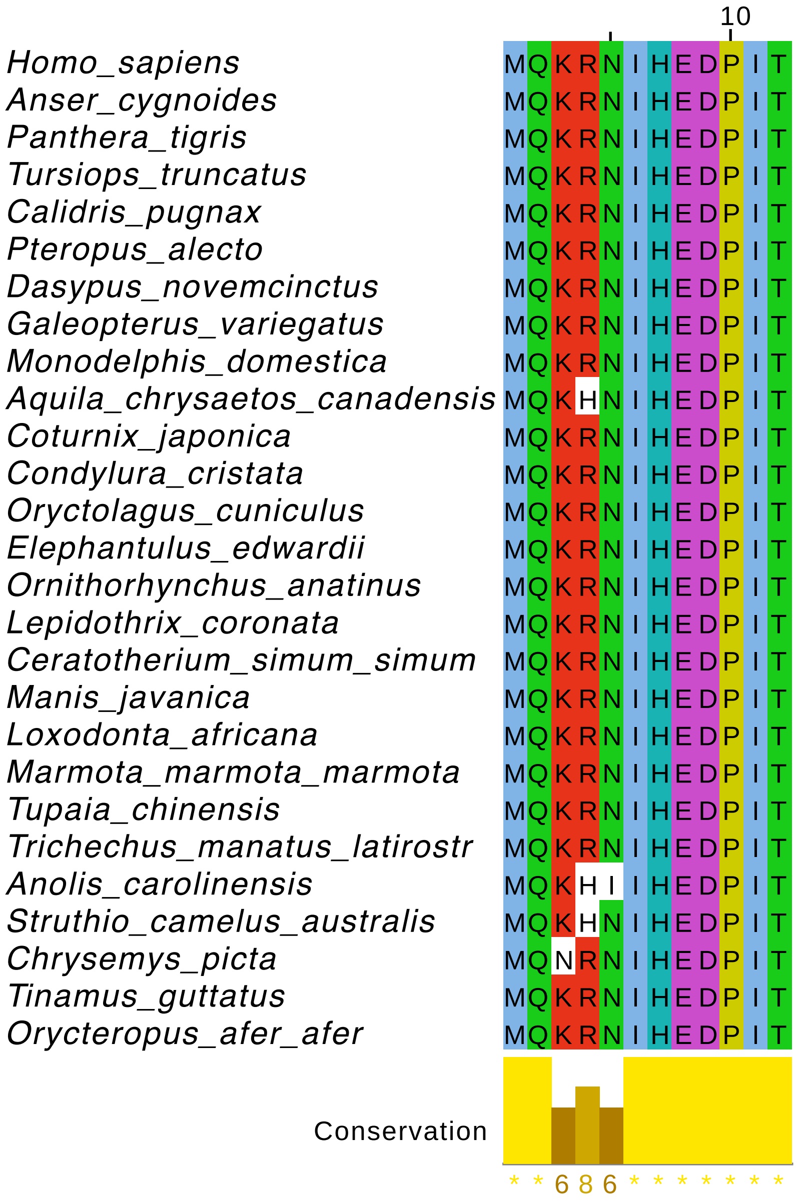

HG0099.1

ENSG00000168575 (SLC20A2)

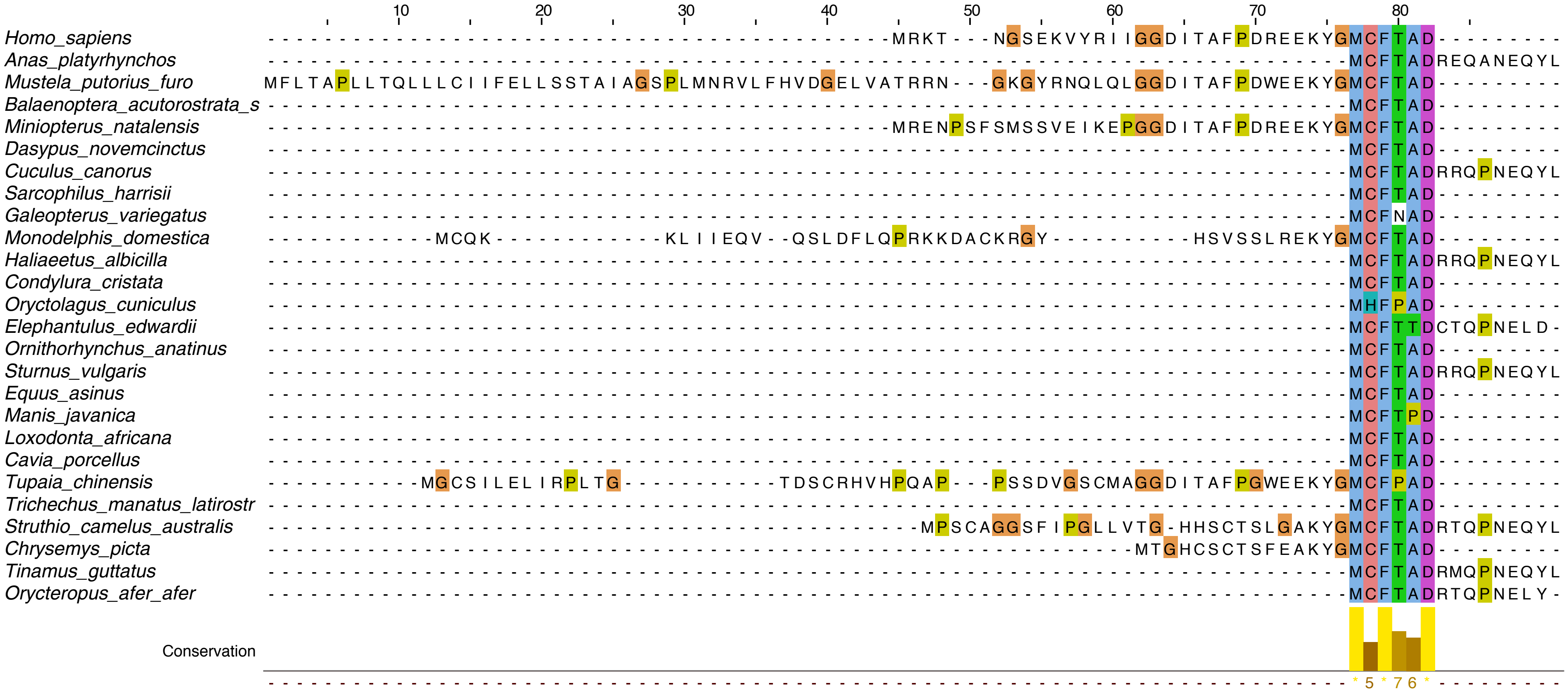

HG0099.2

ENSG00000144136 (SLC20A1)

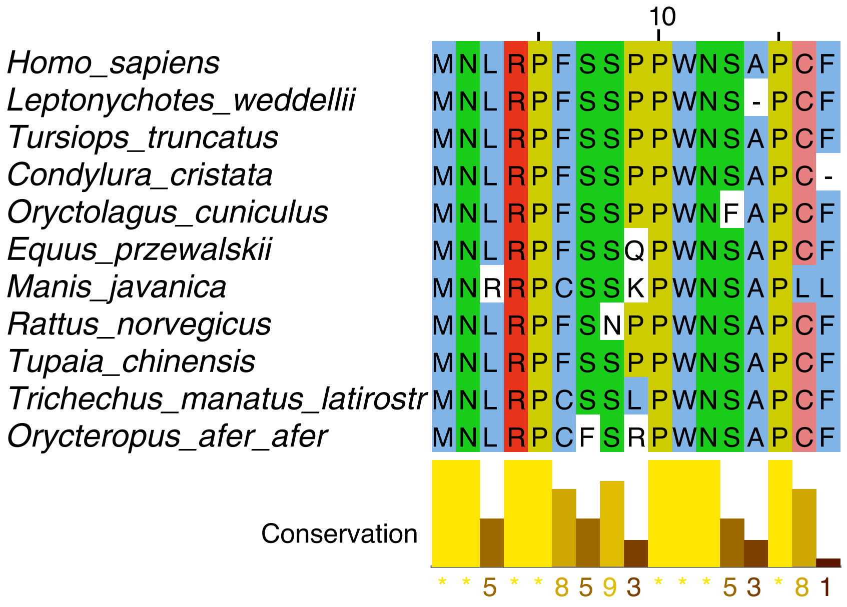

HG0099.3

ENSG00000168575 (SLC20A2)

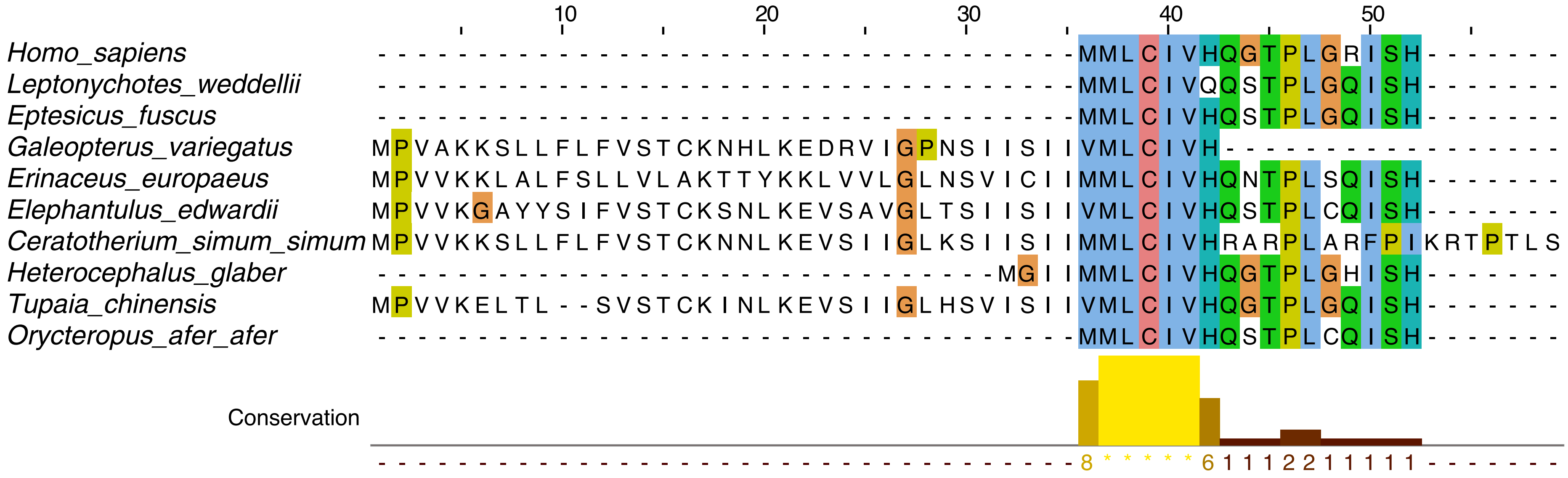

HG0099.4

ENSDARG00000010641 (slc20a1b)

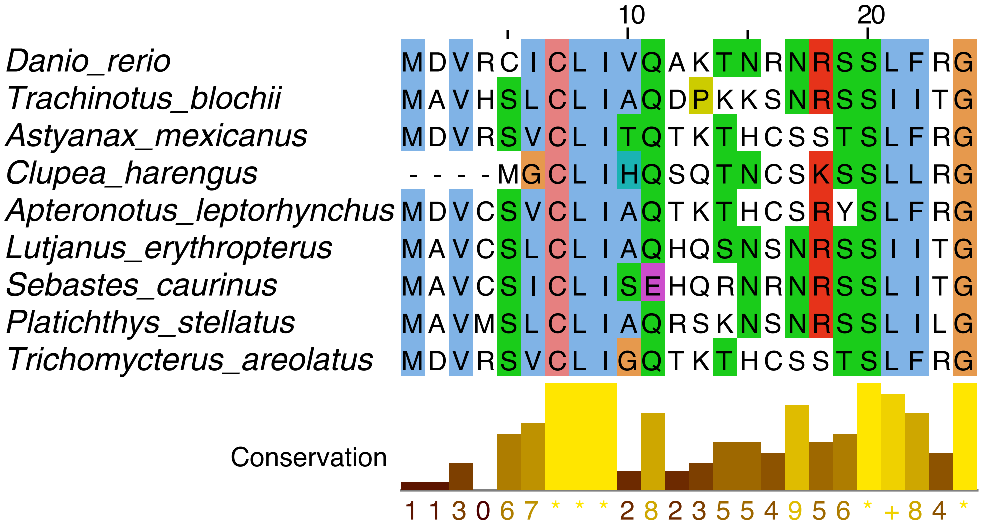

HG0100.1

ENSDARG00000104148 (crebbpb)

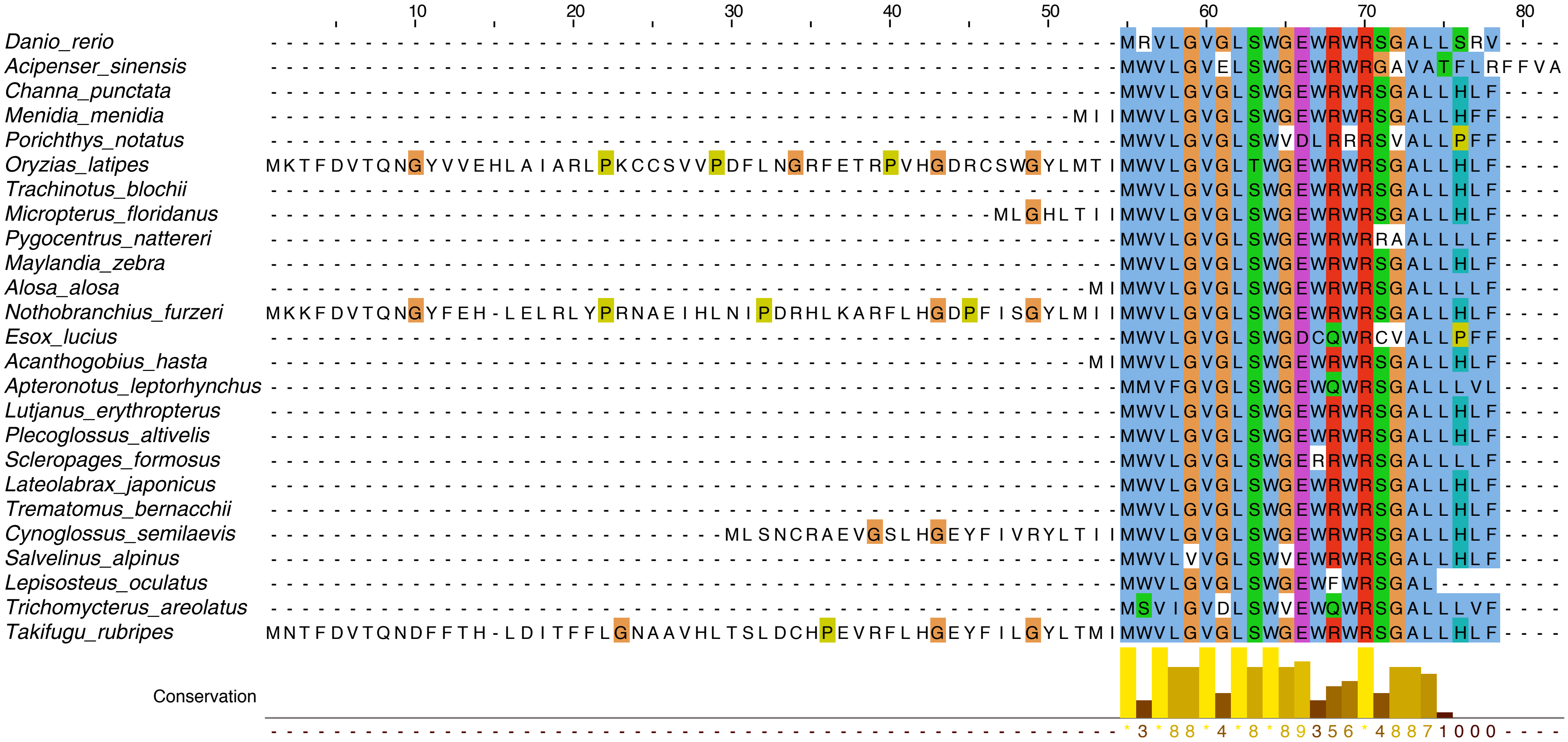

HG0100.1

ENSDARG00000104609 (crebbpa)

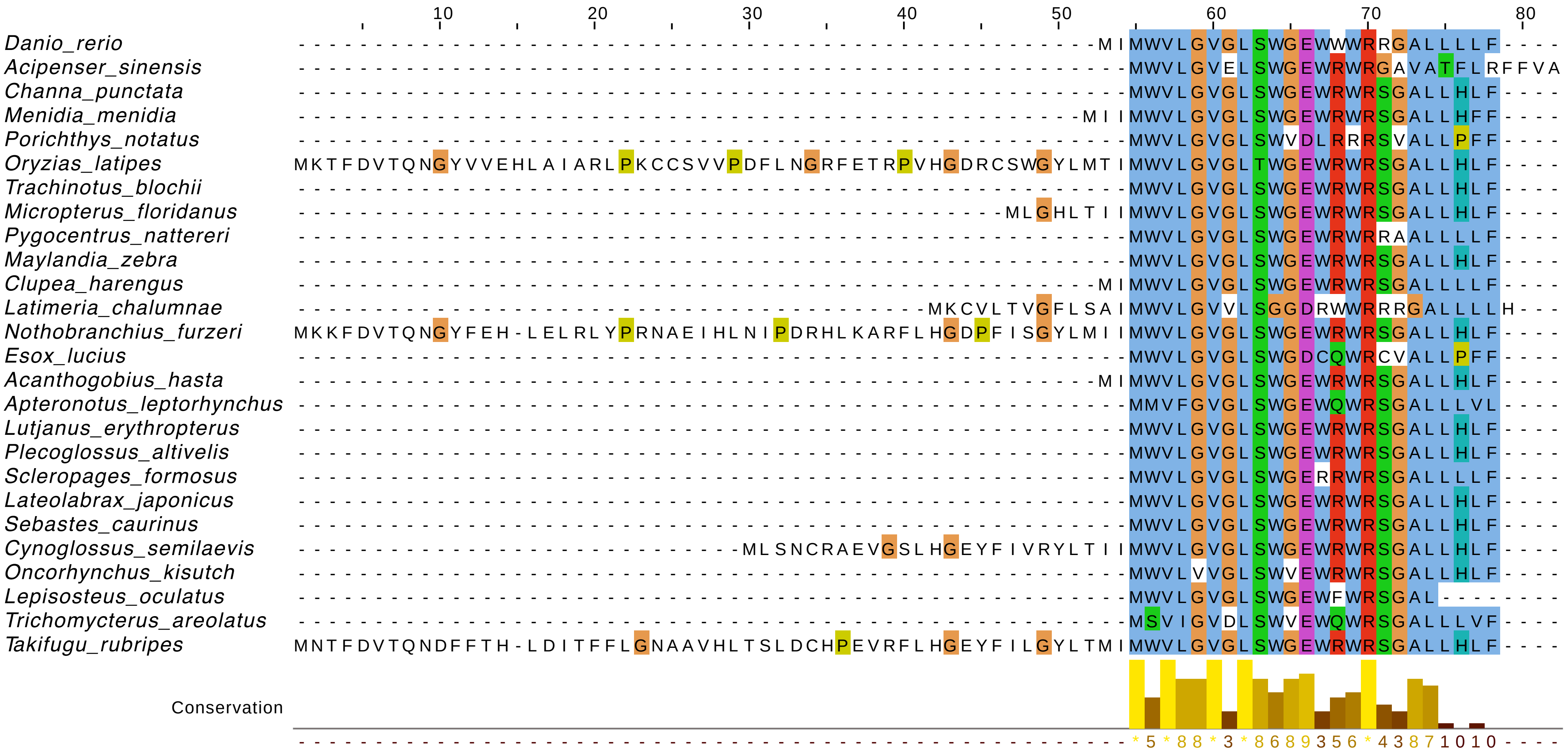

HG0100.2

ENSDARG00000061108 (ep300b)

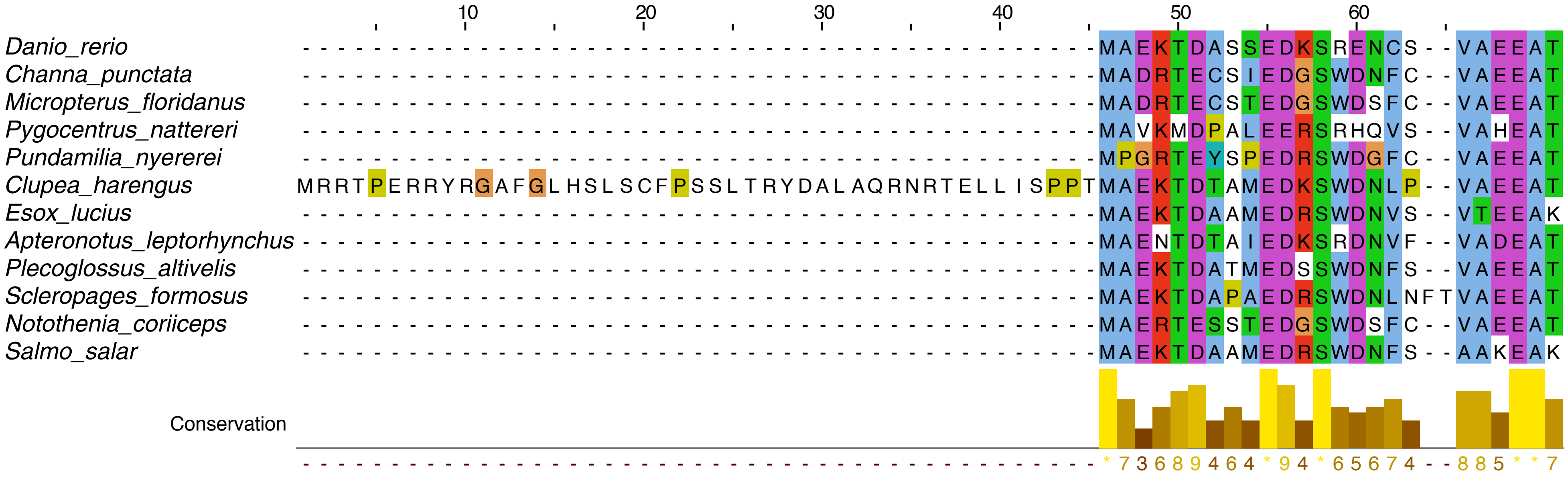

HG0101

ENSG00000099250 (NRP1)

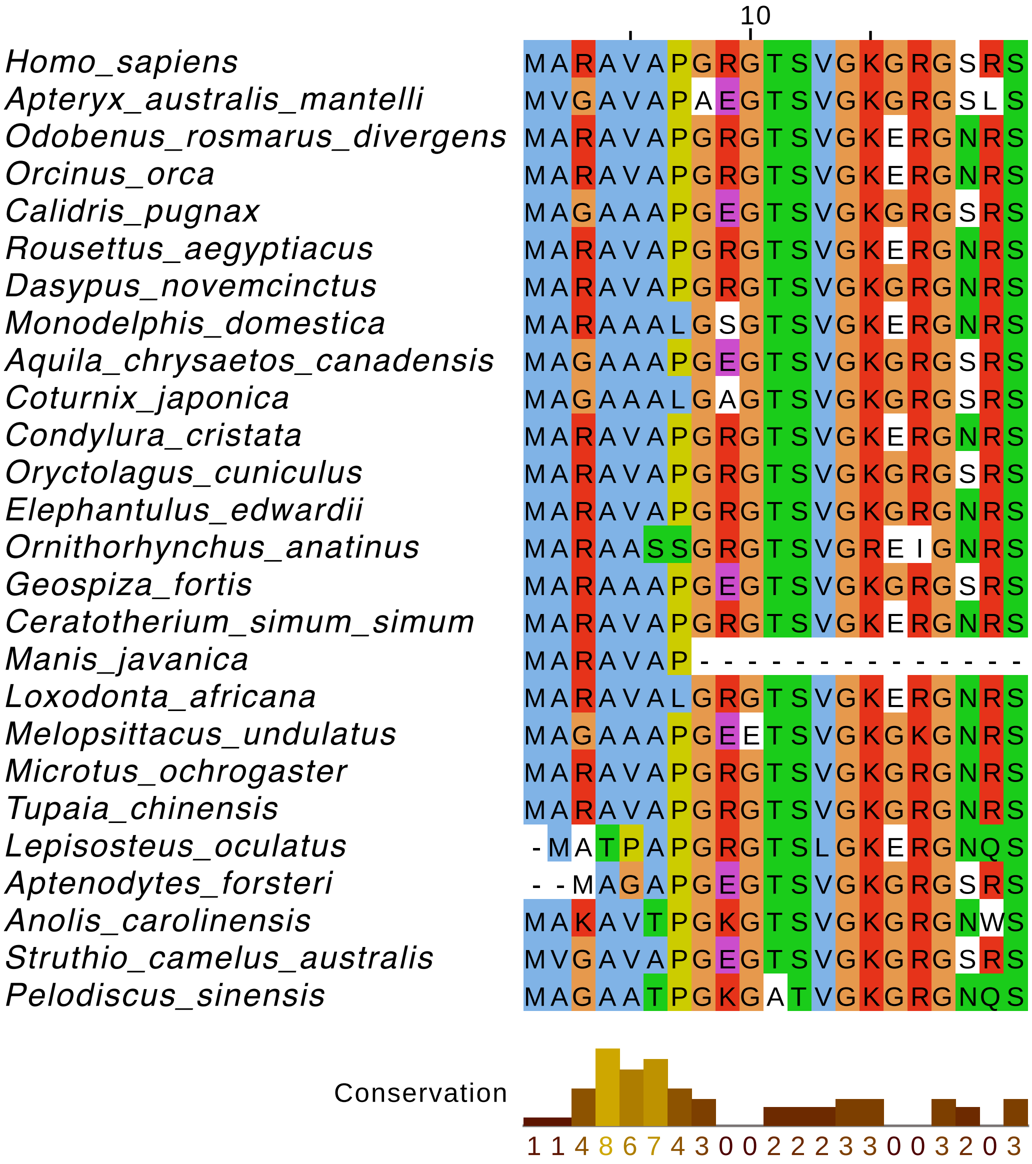

HG0102

ENSG00000182263 (FIGN)

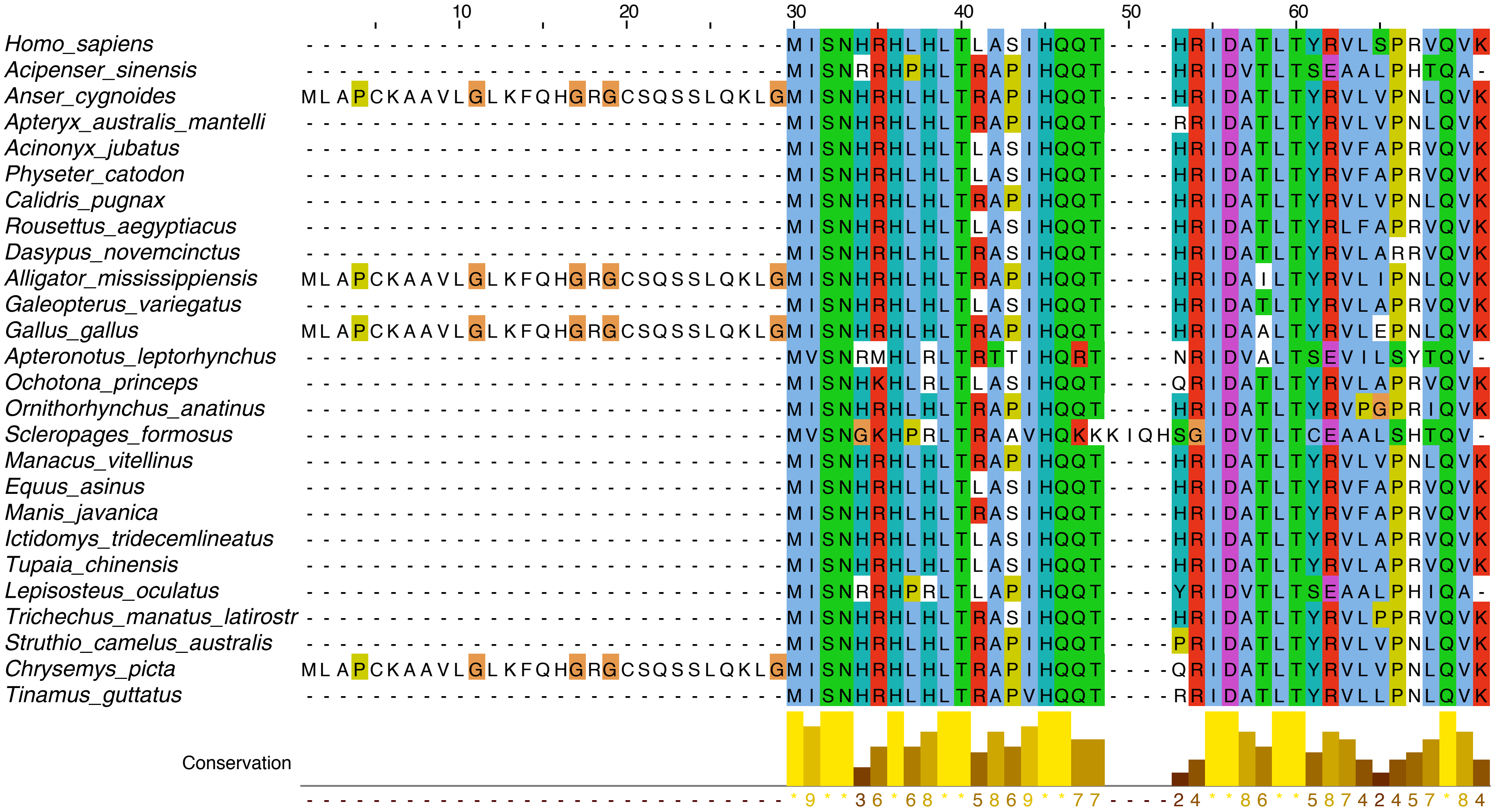

HG0103

ENSG00000165197 (VEGFD)

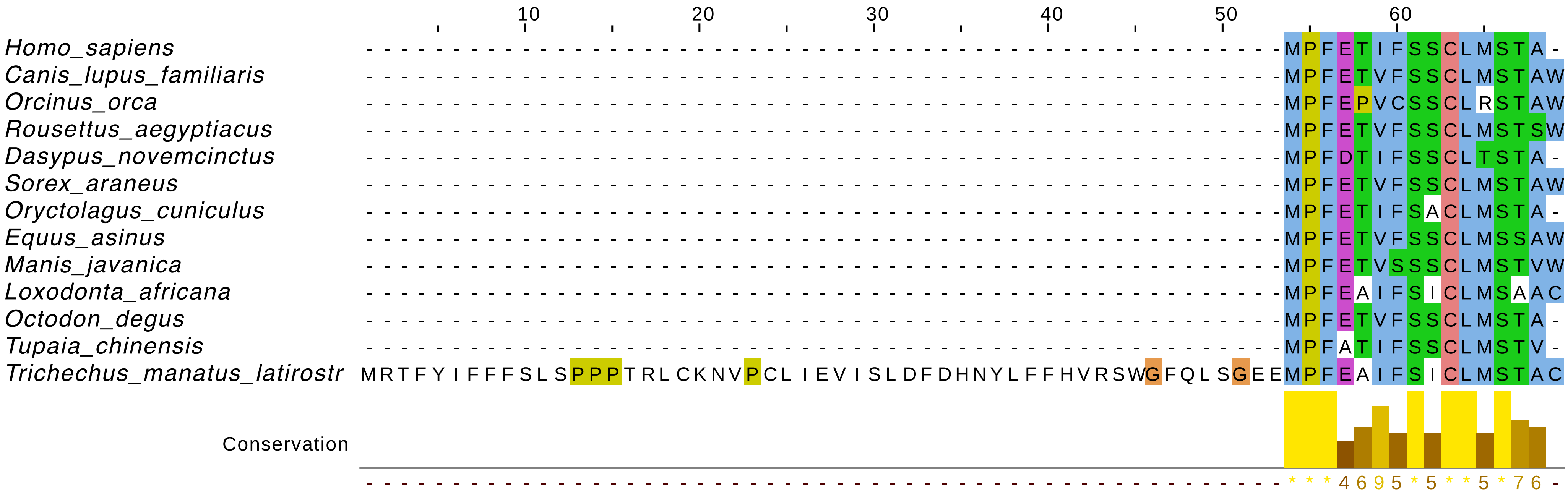

HG0103

ENSGALG00000016558 (VEGFD)

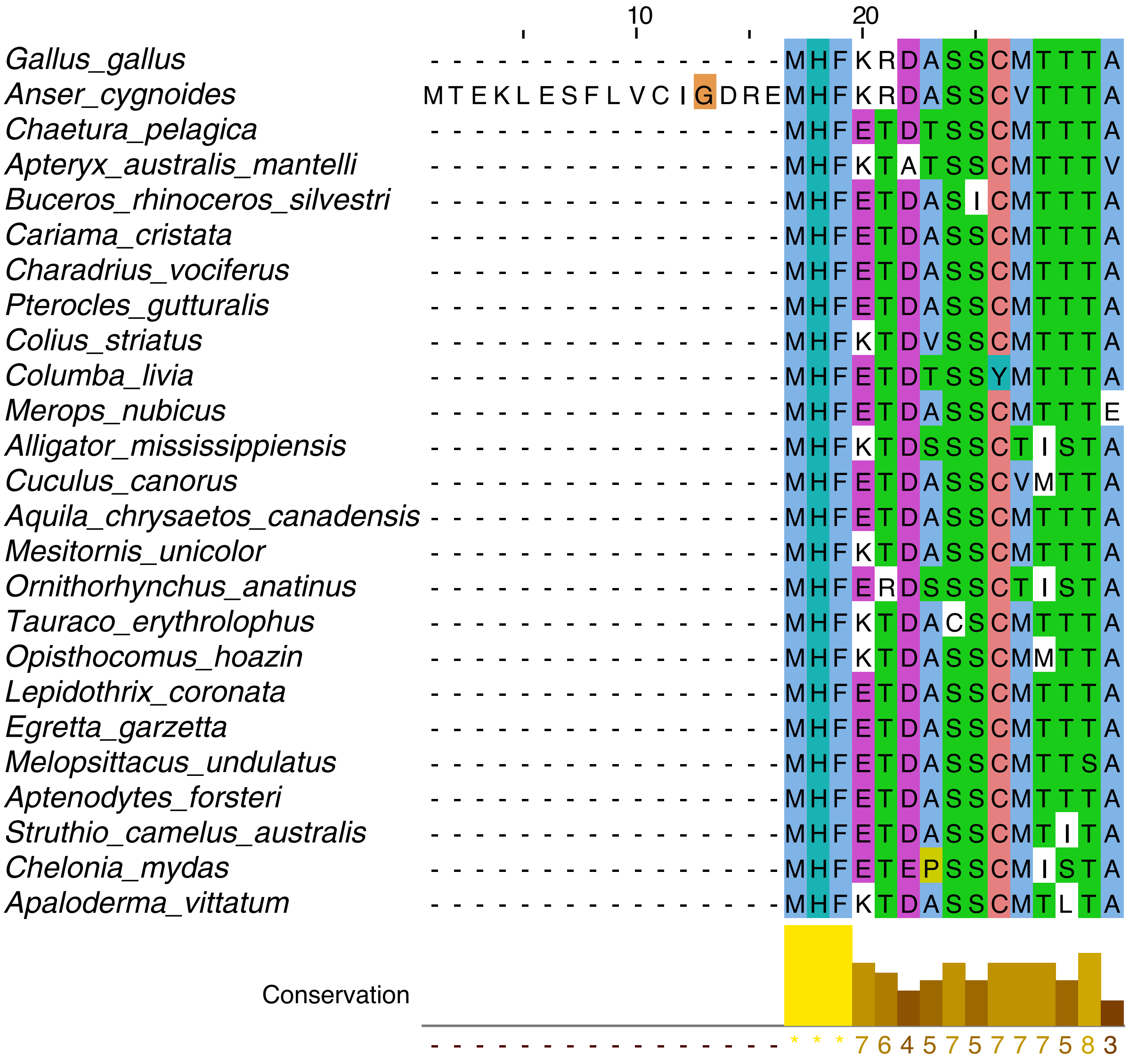

HG0104.1

ENSGALG00000038097 (EI24)

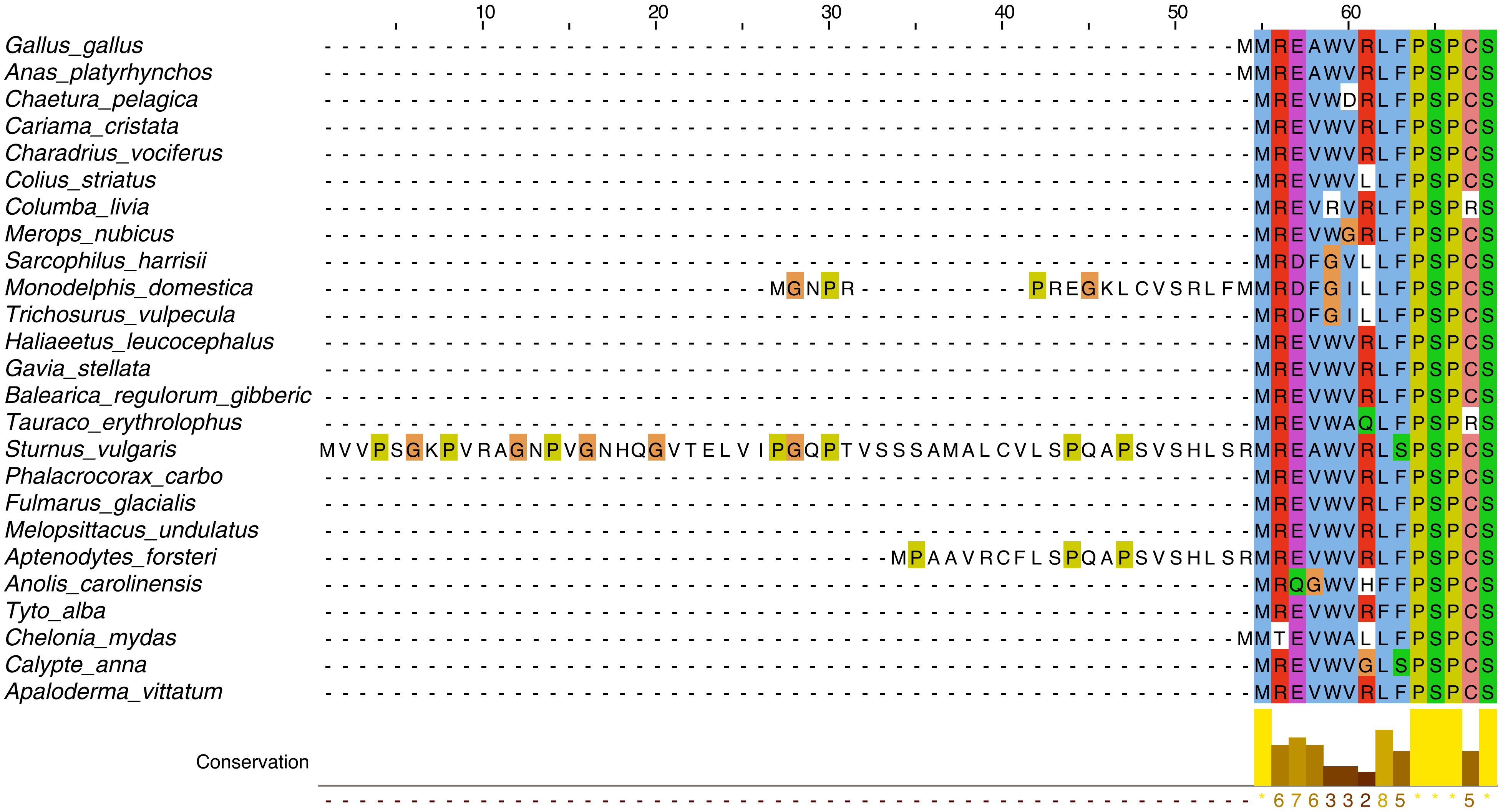

HG0104.2

ENSG00000149547 (EI24)

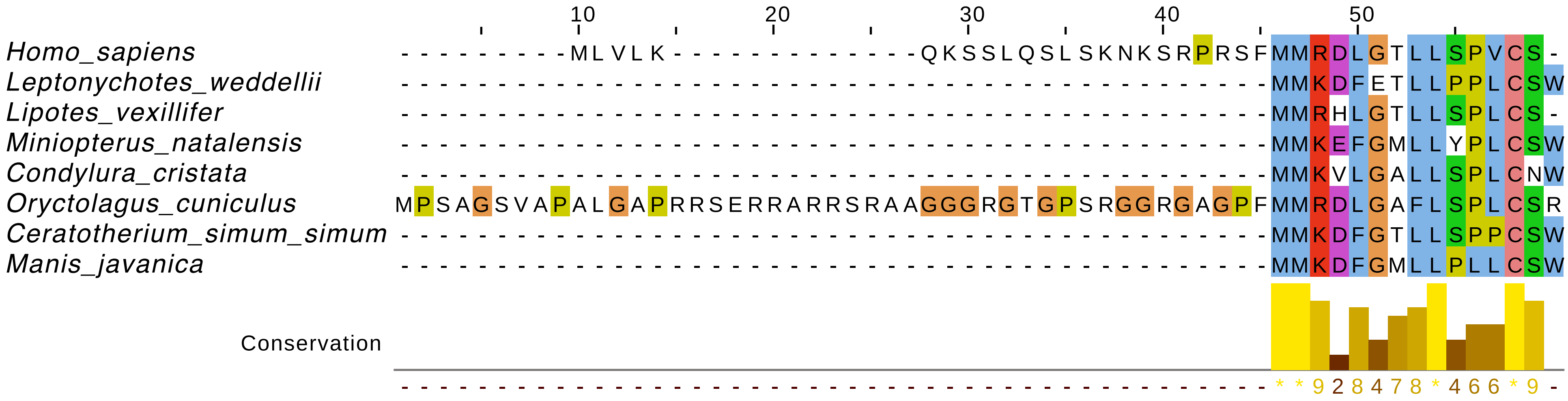

HG0105

ENSG00000135298 (ADGRB3)

```
                                           10        20        30        40        50        60        70
Homo_sapiens                  ------------------------------------------------MGNGDWDRRGVSPSLLLGRVPVLVS--
Anser_cygnoides               MWLDLVDPEPKLLFSLGGVCVCRAGVFSPRFLSSAAFFNLGFEPEPGKMGNGASDRRGISPSLLLGRVPVVLVF-
Chaetura_pelagica             ------------------------------------------------MGNGASDRRGISPSLLLGRVPVVLVF-
Odobenus_rosmarus_divergens   ------------------------------------------------MGNGDWDRRGVSPSLLLGRVPVLVS--
Lipotes_vexillifer            ------------------------------------------------MGNGTWDRRGVSPSLLLGRVPVLVS--
Pteropus_alecto               ------------------------------------------------MGNGDWDRRGVSPSLLLGRVPVLVS--
Dasypus_novemcinctus          ------------------------------------------------MGNGDWDRRGVSPSLLLGRVPVLVS--
Galeopterus_variegatus        ------------------------------------------------MGNGDWDRRGVSPSLLLGRVPVLVS--
Monodelphis_domestica         ------------------------------------------------MGNGDSARRGVSPSLLLGRVPVLGS--
Aquila_chrysaetos_canadensis  ------------------------------------------------MGNGASDRRGISPSLLLGRVPVVLVF-
Coturnix_japonica             ------------------------------------------------MGNGASDRRGISPSLVSGRVPVVLEV-
Condylura_cristata            ------------------------------------------------MGNGDWDRRGVSPSLLLGRVPVLVS--
Oryctolagus_cuniculus         ------------------------------------------------MGNGDWERRGVSPSLLLGRVPVLVS--
Parus_major                   ------------------------------------------------MGNGASVRRGISPSLLLGRVPVVLVF-
Nipponia_nippon               ------------------------------------------------MGNGASDRRGISPSLLLGRVPVVLVF-
Equus_caballus                ------------------------------------------------MGNGDWDRRGVSPSLLLGRVPVLVS--
Manis_javanica                ------------------------------------------------MGNGAWDRRGVSPSLLPGRVPALVS--
Loxodonta_africana            ------------------------------------------------MGNGDGARRGVSPSLLLGRVAVLVS--
Ictidomys_tridecemlineatus    ------------------------------------------------MGNGDWDRRGVSPSLLLGRVPVLVS--
Trichechus_manatus_latirostr  ------------------------------------------------MGNGDWDRRGVSPSLLLGRVPVLVS--
Pygoscelis_adeliae            ------------------------------------------------MGNGASDRRGISPSLLLGRVPVVLVF-
Thamnophis_sirtalis           ------------------------------------------------MGNDASARRGMSPCLFLGHVLVVLGSS
Struthio_camelus_australis    MWLDLVDPEPKLLFFLGGVYVWRAGVFSLRFSSSAAFFNLGFEPEPGKMGNGASDRRGISPSLLSGRVPVVLVF-
Chrysemys_picta               ------------------------------------------------MGNGASDRRGISPSLLLGRVPVVLVF-
Calypte_anna                  MWLDLVDPESKLLFFLCGVCVWRAGVFSLRFLSSTAFLNLGFEPEPGKMGNGASDRRGISPSLLLGRVPVVLVF-
```

Conservation

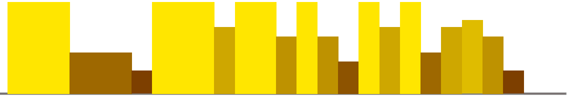

```
------------------------------------------------***5553***8**7*74*8*58973--
```

HG0105

ENSGALG00000016176

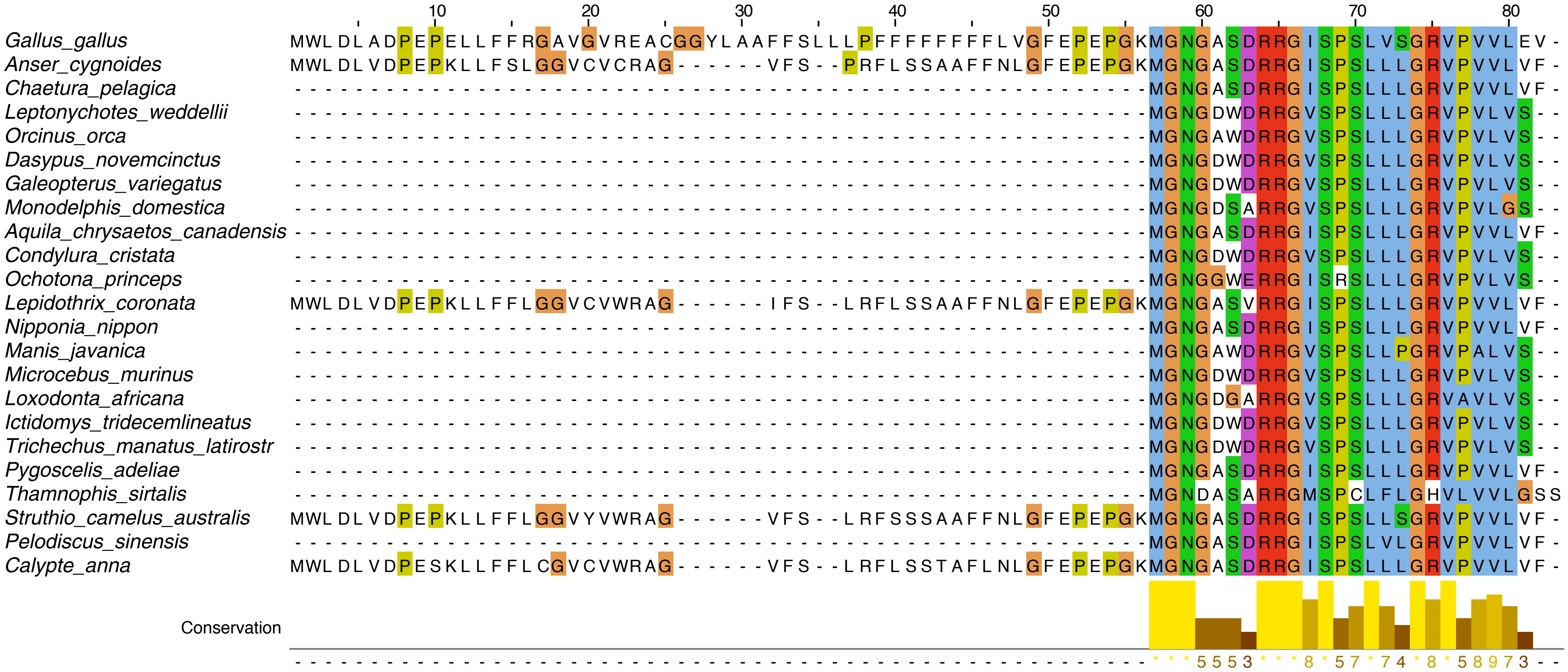

HG0106

ENSG00000147548 (NSD3)

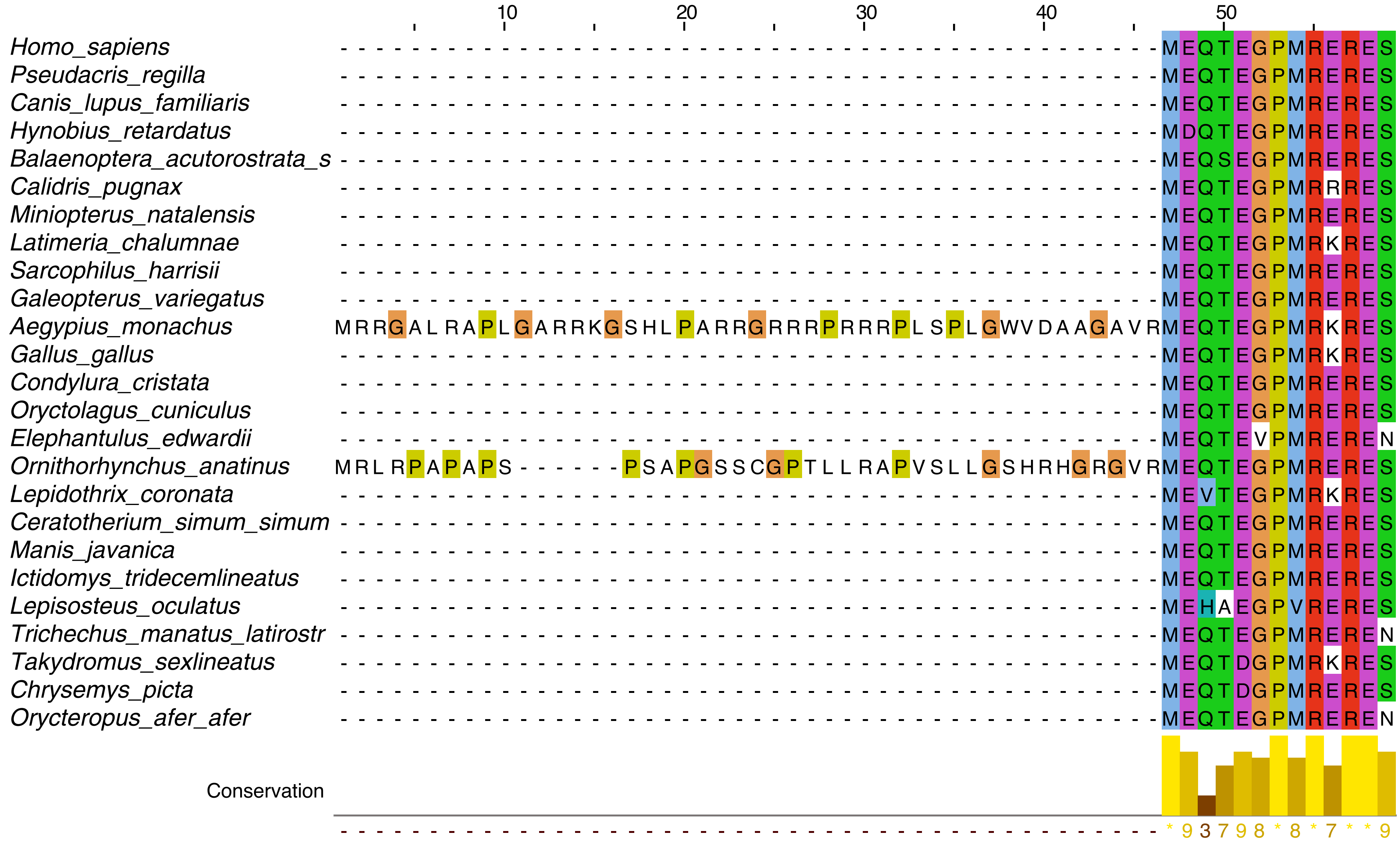

HG0107.1

ENSG00000058668 (ATP2B4)

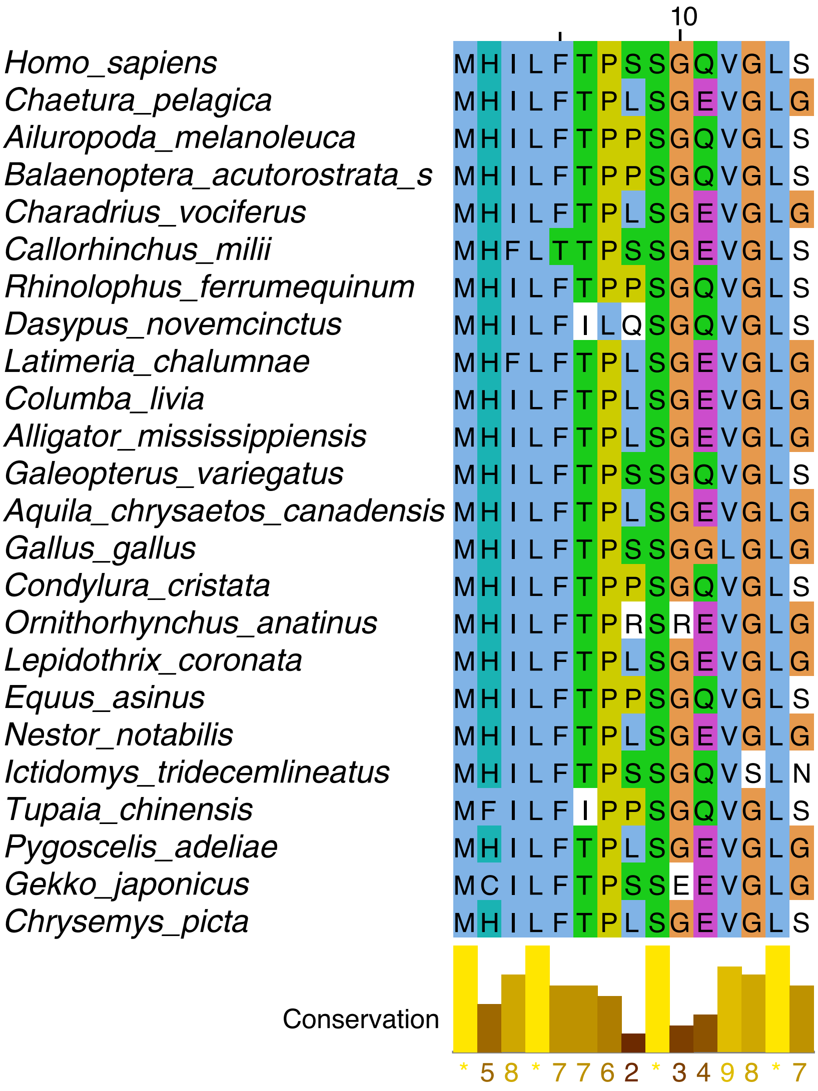

HG0107.1

ENSGALG00000003601 (ATP2B4)

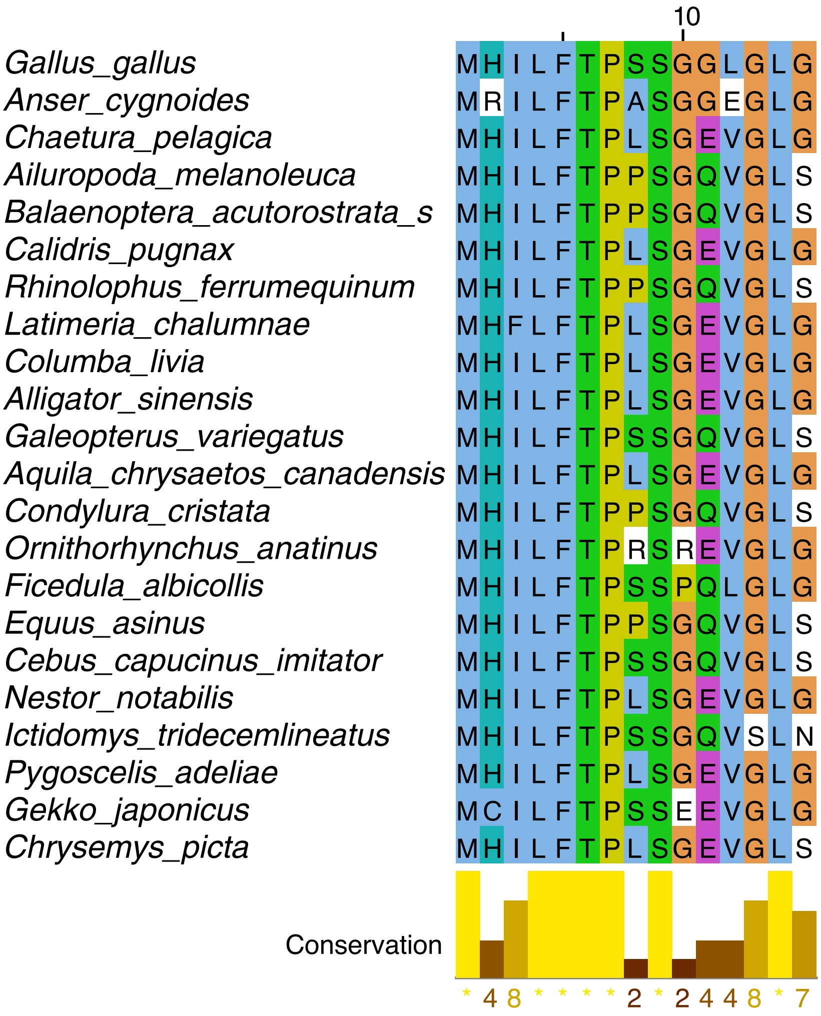

HG0107.2

ENSG00000070961 (ATP2B1)

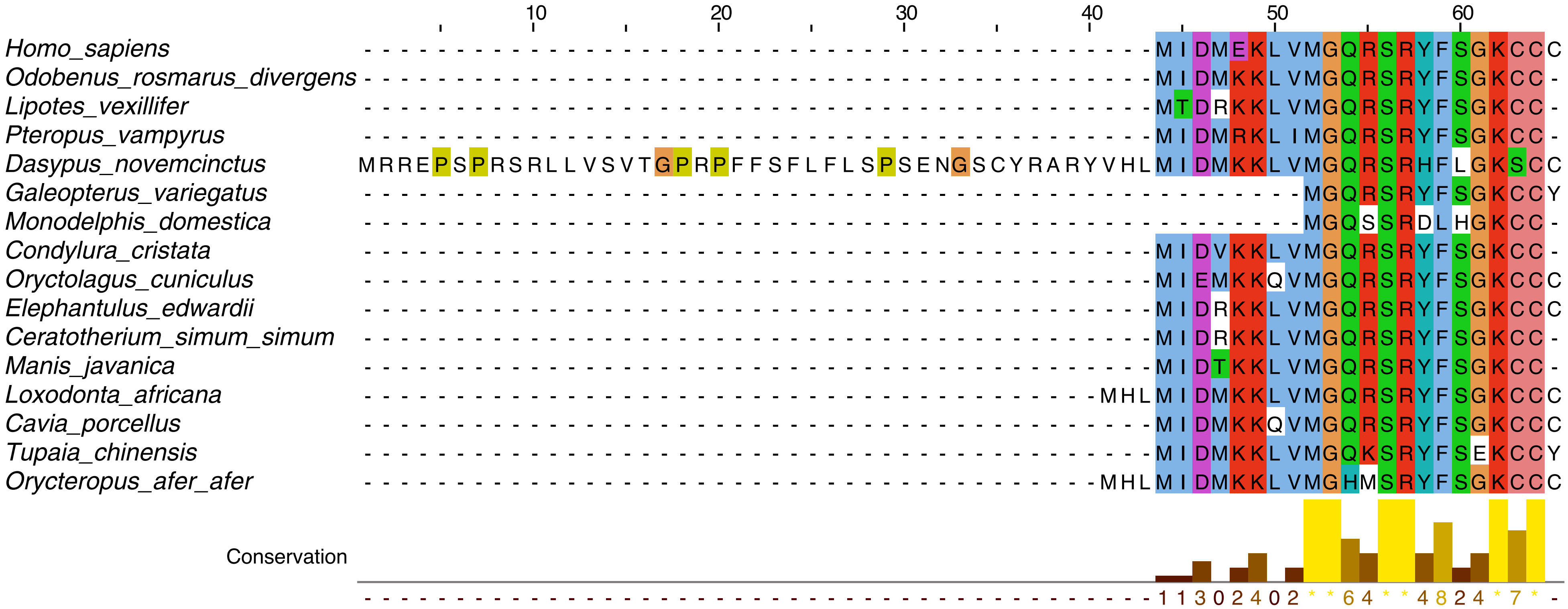

HG0107.2

ENSGALG00000030550

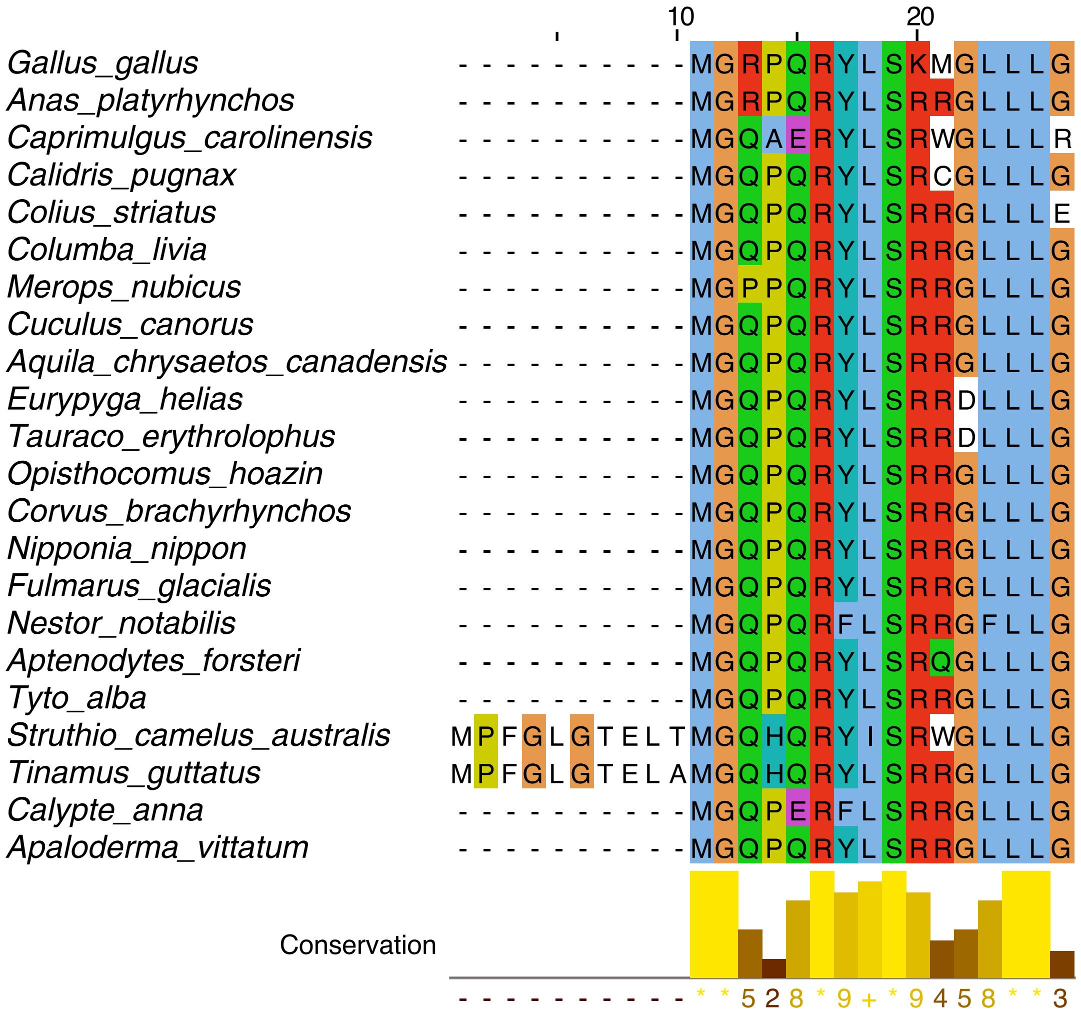

HG0107.3

ENSDARG00000063433 (atp2b2)

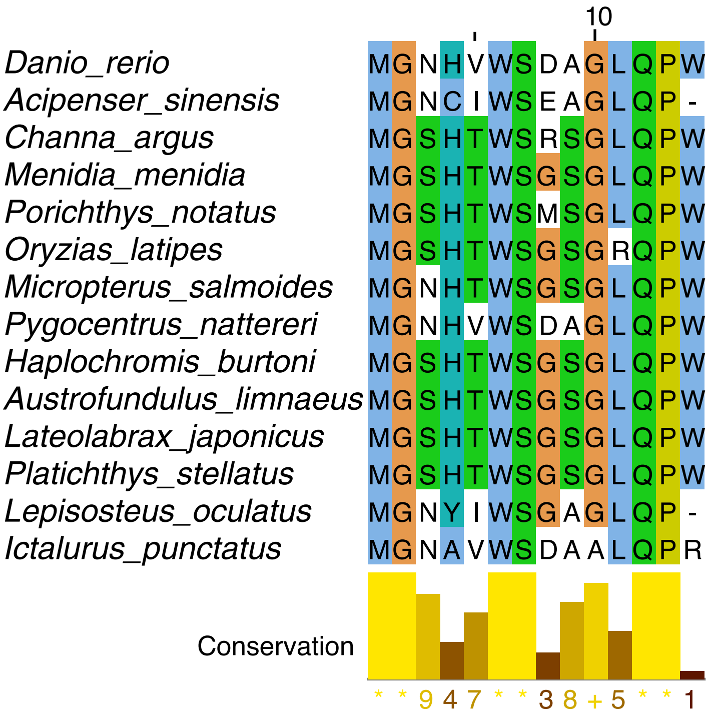

HG0107.3

ENSG00000157087 (ATP2B2)

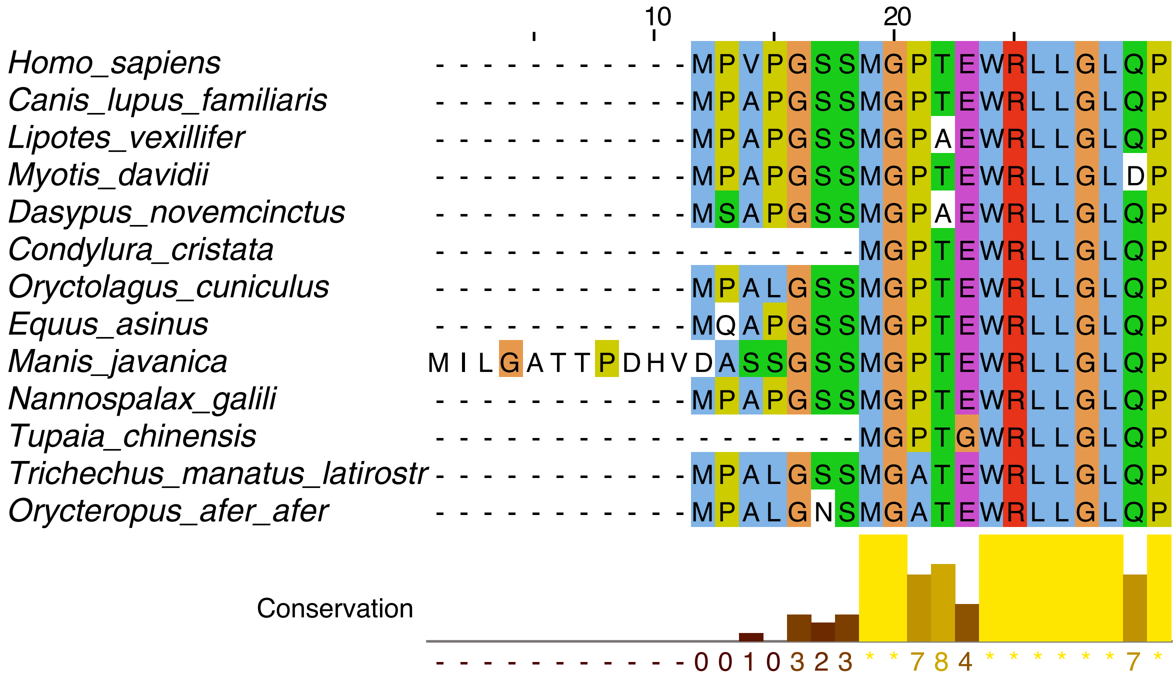

HG0107.4

ENSG00000070961 (ATP2B1)

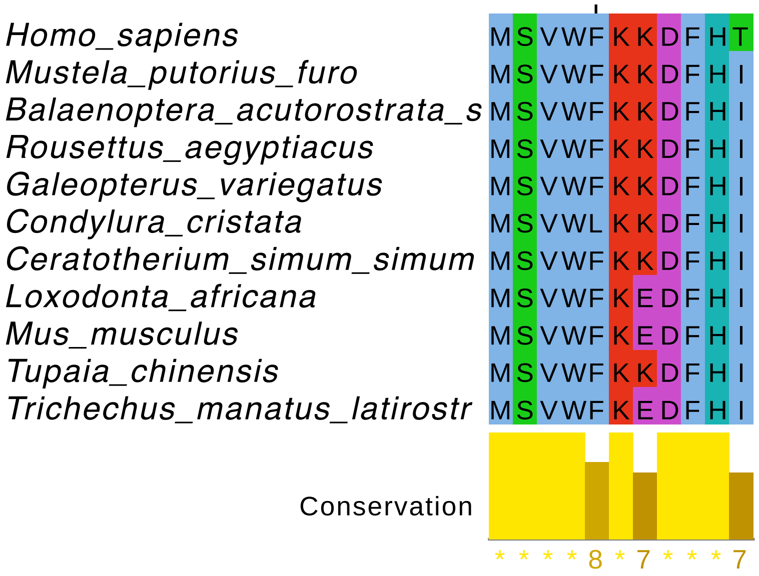

HG0108.1

ENSGALG00000007396 (TAOK3)

```
                                          10        20        30
                                 ....|....|....|....|....|....|....
Gallus_gallus                    -------------------MKEETDWDLKAPTSS
Anas_platyrhynchos               -------------------MKEEIDWELKARASS
Apteryx_australis_mantelli       -------------------MKEEIDWESKARTSS
Buceros_rhinoceros_silvestri     -------------------MKEETDWKSKAQTGS
Cariama_cristata                 -------------------MKEEIVWKLKARTSS
Calidris_pugnax                  -------------------MKEEIDWELKARTSS
Colius_striatus                  -------------------MKEEIDWKLKAQTSS
Columba_livia                    -------------------MKEETDWKLKARTGS
Merops_nubicus                   -------------------MKEEIDWELKAQTS-
Alligator_mississippiensis       -------------------MKAETDWEFKARTRS
Cuculus_canorus                  -------------------MKEESDWELKARTSS
Falco_cherrug                    -------------------MKEEIDWELKARTSS
Gavia_stellata                   -------------------MKEEIDWELKVRTSS
Eurypyga_helias                  -------------------MKEETDWELKARASS
Tauraco_erythrolophus            -------------------MKEEIDWKLKARTSS
Opisthocomus_hoazin              -------------------MKEEVDWELKGRTSS
Corvus_cornix_cornix             -------------------MKEEIGWDLRAQTSS
Phaethon_lepturus                -------------------MKEEIDWELKARTSS
Nestor_notabilis                 -------------------MKEETDWELKAQTSL
Aptenodytes_forsteri             -------------------MKEEIDWELKARTSS
Takydromus_sexlineatus           MLIAIVTQILNCYLQTPLSMREETDWELEAGTSS
Tyto_alba                        -------------------MKEEIGWKLKARTSS
Pelodiscus_sinensis              -------------------MREETDWEFKARTRS
Tinamus_guttatus                 -------------------MKEEVDWESKARTSS

Conservation                     -------------------*94*54*647838 40
```

HG0108.2

ENSG00000135090 (TAOK3)

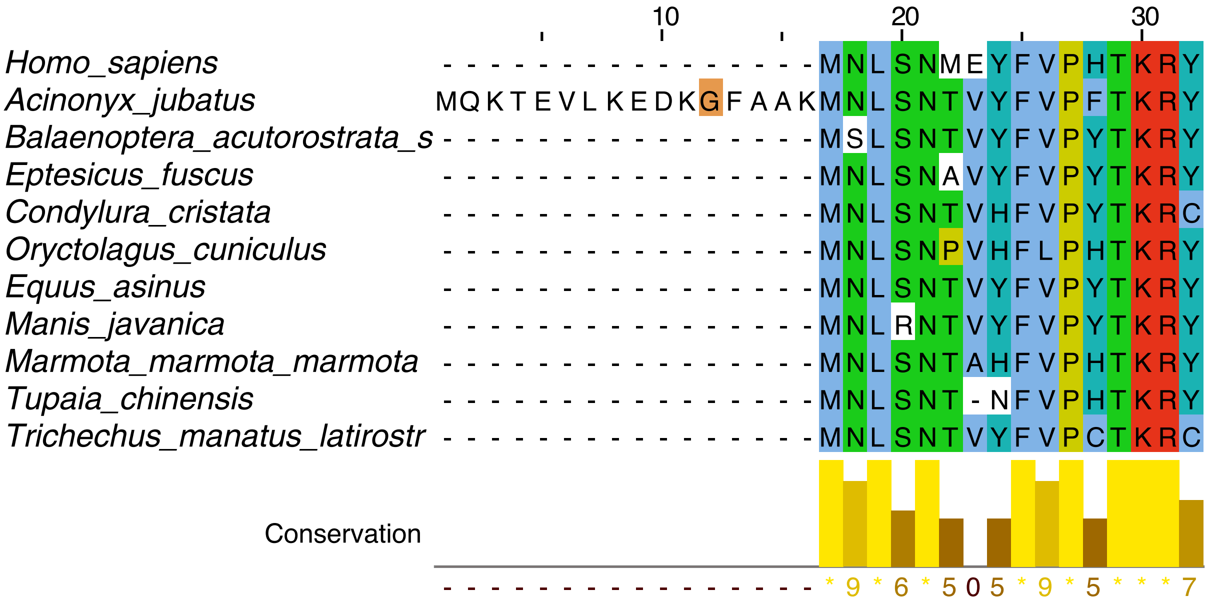

HG0108.3

ENSDARG00000098304 (taok1b)

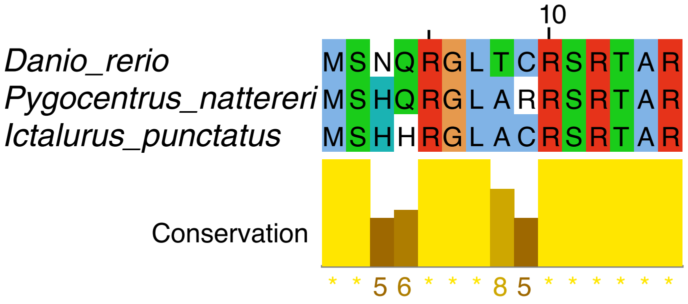

HG0109.1

ENSG00000048540 (LMO3)

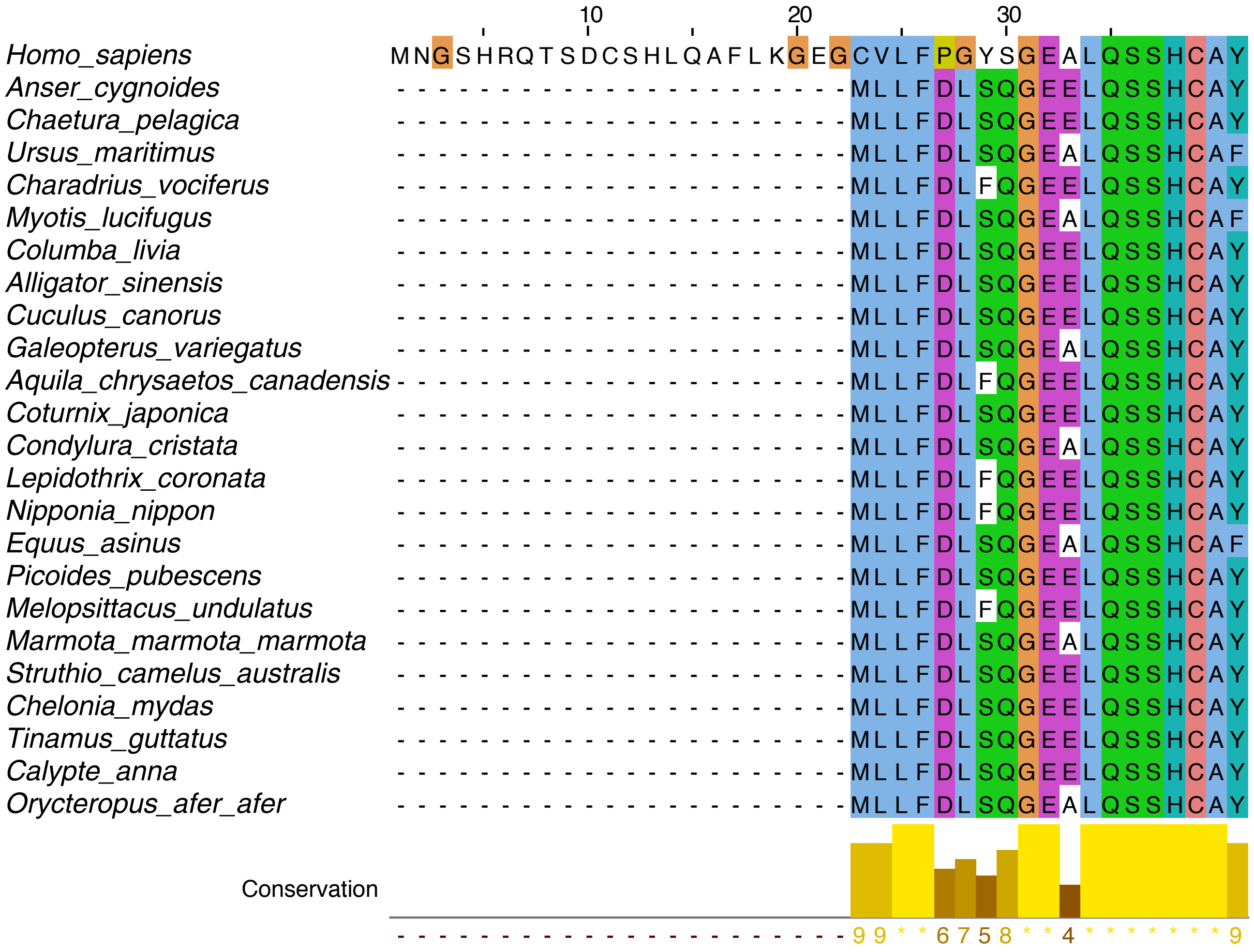

HG0109.2

ENSG00000048540 (LMO3)

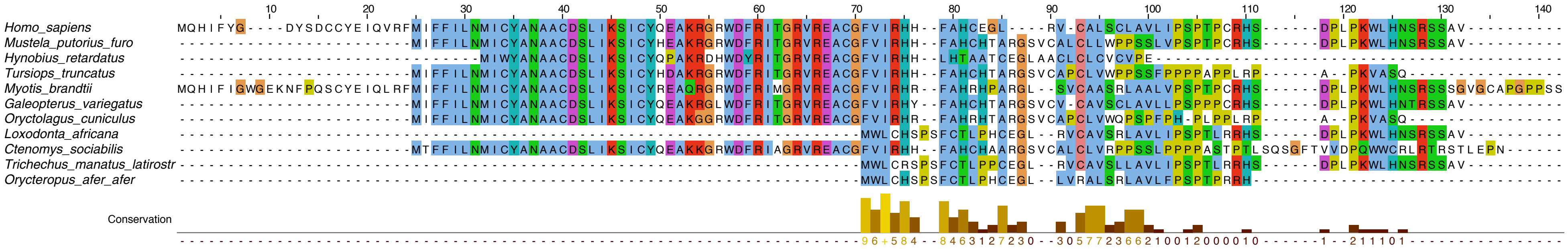

HG0109.3

ENSG00000048540 (LMO3)

```
                                       10        20        30        40        50        60        70
Homo_sapiens                   -----------MYACLDGPEKPNLRNLPMYRTSQLRGSH---------------------------------
Canis_lupus_familiaris         -----------MCACLDGPEKPNLRNLPMYRTNQLKGFLLIRRL----------------------------
Orcinus_orca                   -----------MCACLDRPEKPSLRELPMYRTNPLKGFLLICRL----------------------------
Rousettus_aegyptiacus          -----------MCACLDGPEKPNLRNLPMYRTNQLKGFLLICRL----------------------------
Dasypus_novemcinctus           -----------MCACSDGPAKPNLRKLPMYRTNQLKGVLLICRL----------------------------
Galeopterus_variegatus         ------MG-EVRVRLLRGPEQPNLRNLPMHRPNQLKGFPLICRLSVNFVIFQEKRYKAATVLTERPPPISLN
Monodelphis_domestica          MSKDLFIQMRCMCACISIRERQKLRNLLIYRSNQLKGVVLTC------------------------------
Oryctolagus_cuniculus          -----------MCACIDGPGKPNLRNLPMHRTSQRKGFSLICRL----------------------------
Elephantulus_edwardii          ----------------------------MYRTNQLKGVSLICRL----------------------------
Equus_asinus                   -----------MCSCLDGPEKPNLRNLPMYRTNQLKGSY---------------------------------
Manis_javanica                 ------MGEVHMCLLR-PARKAKHRKLPMYRTNQLKGFLLICRL----------------------------
Cavia_porcellus                ----------------------------MFRTNQLKGFSLICRL----------------------------
Tupaia_chinensis               --------MRCMCACLDGPEKPNLRISAYVQNKSAE-GVLINLPSVNFVLFQEKRYKAASVLTEQPPPVSLN
Trichechus_manatus_latirostr   -----------MCACLNRPAKPNLRNLPMYRTNQLKGVLLFCRL----------------------------
Orycteropus_afer_afer          ----------------------------MYRTNQLKGVLLICRL----------------------------

Conservation                   -----------01010-000101130007486563734121000----------------------------
```

HG0110.1

ENSG00000091656 (ZFHX4)

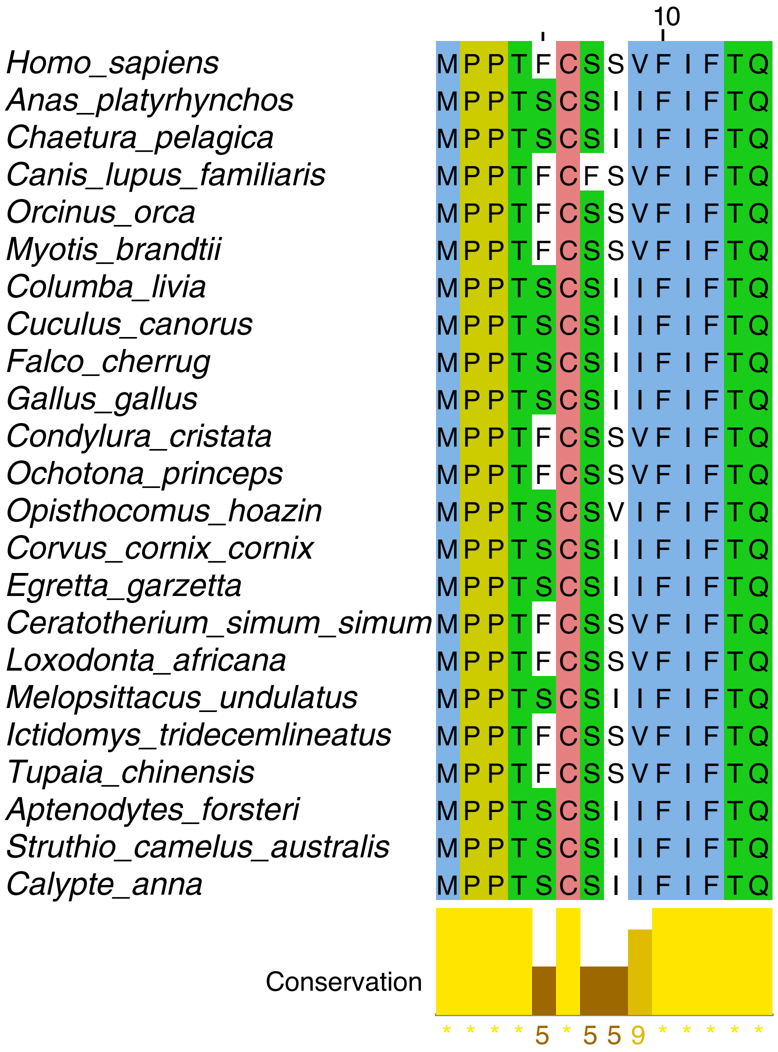

HG0110.2

ENSDARG00000073944

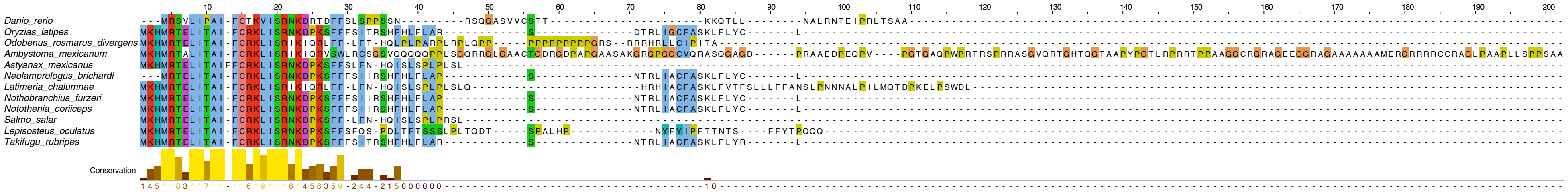

HG0111

ENSGALG00000034944

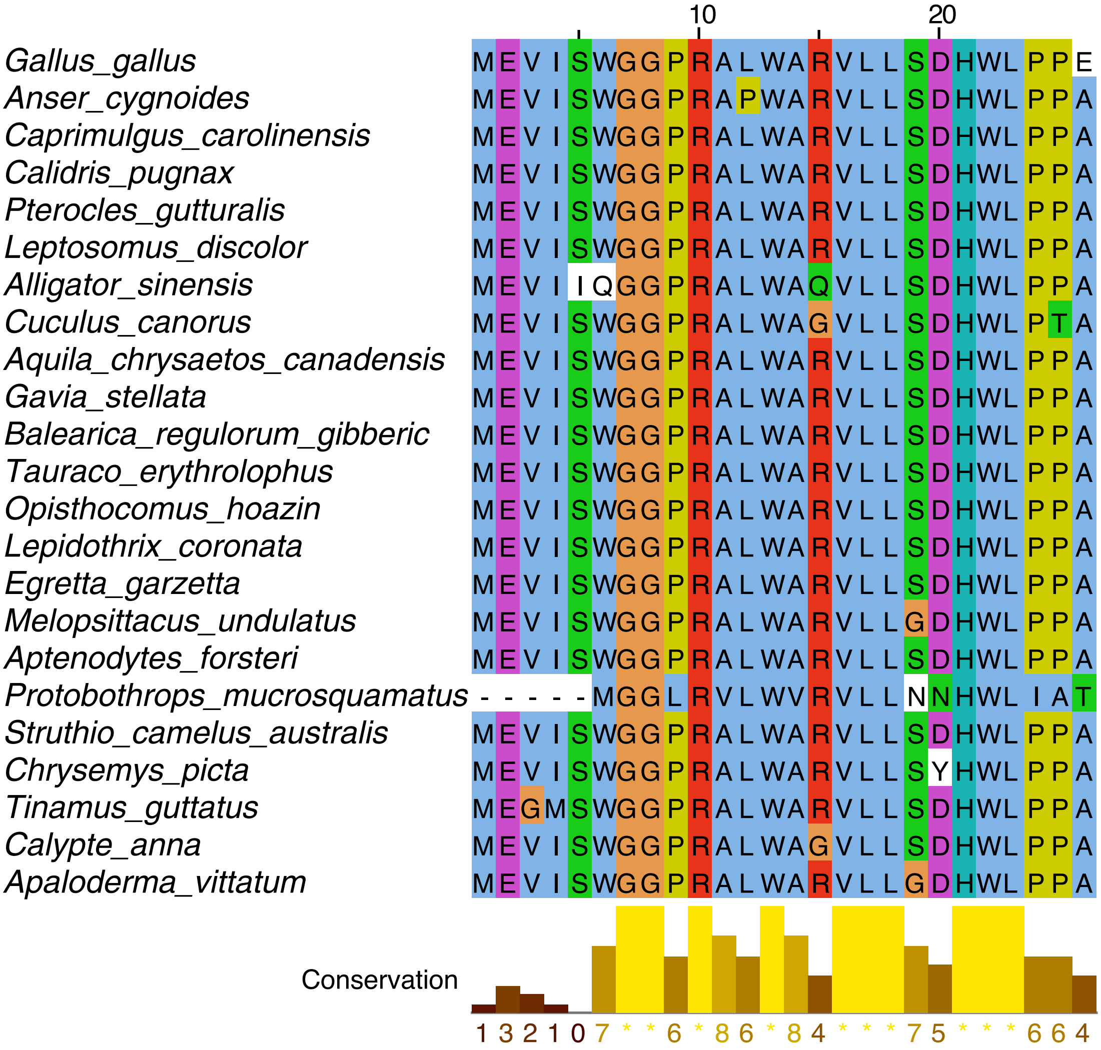

HG0112

ENSG00000105997 (HOXA3)

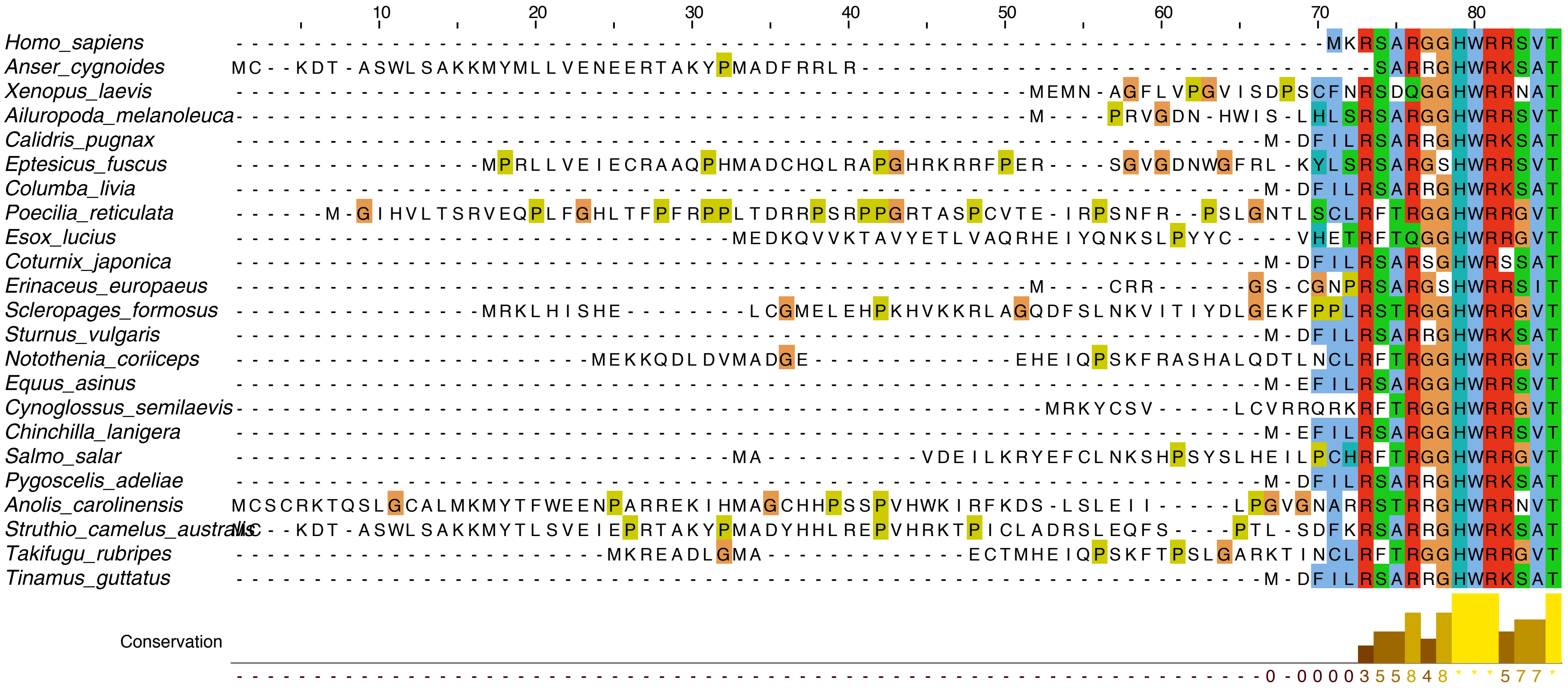

HG0113

ENSDARG00000099727 (ssbp2)

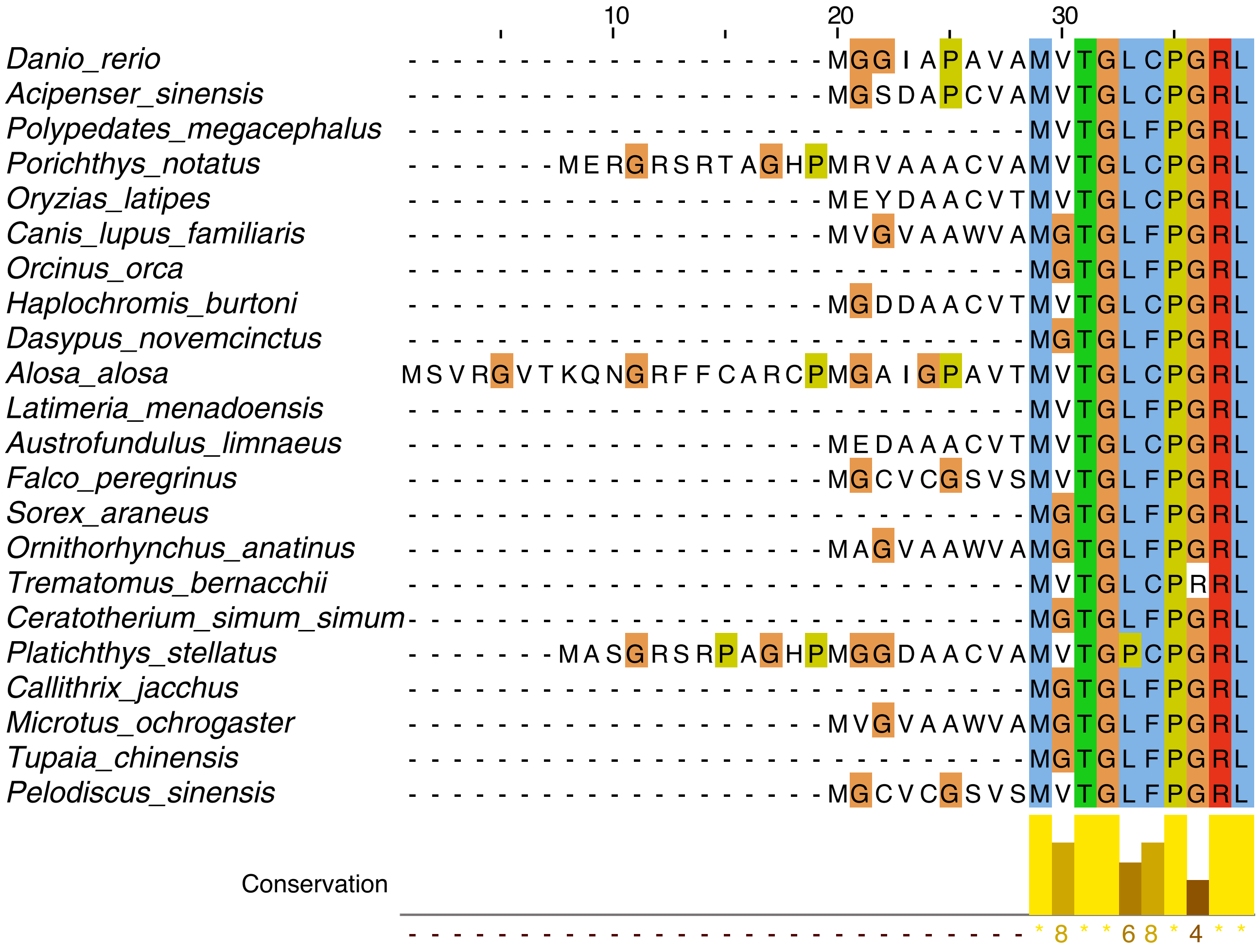

HG0113

ENSGALG00000023089 (SSBP2)

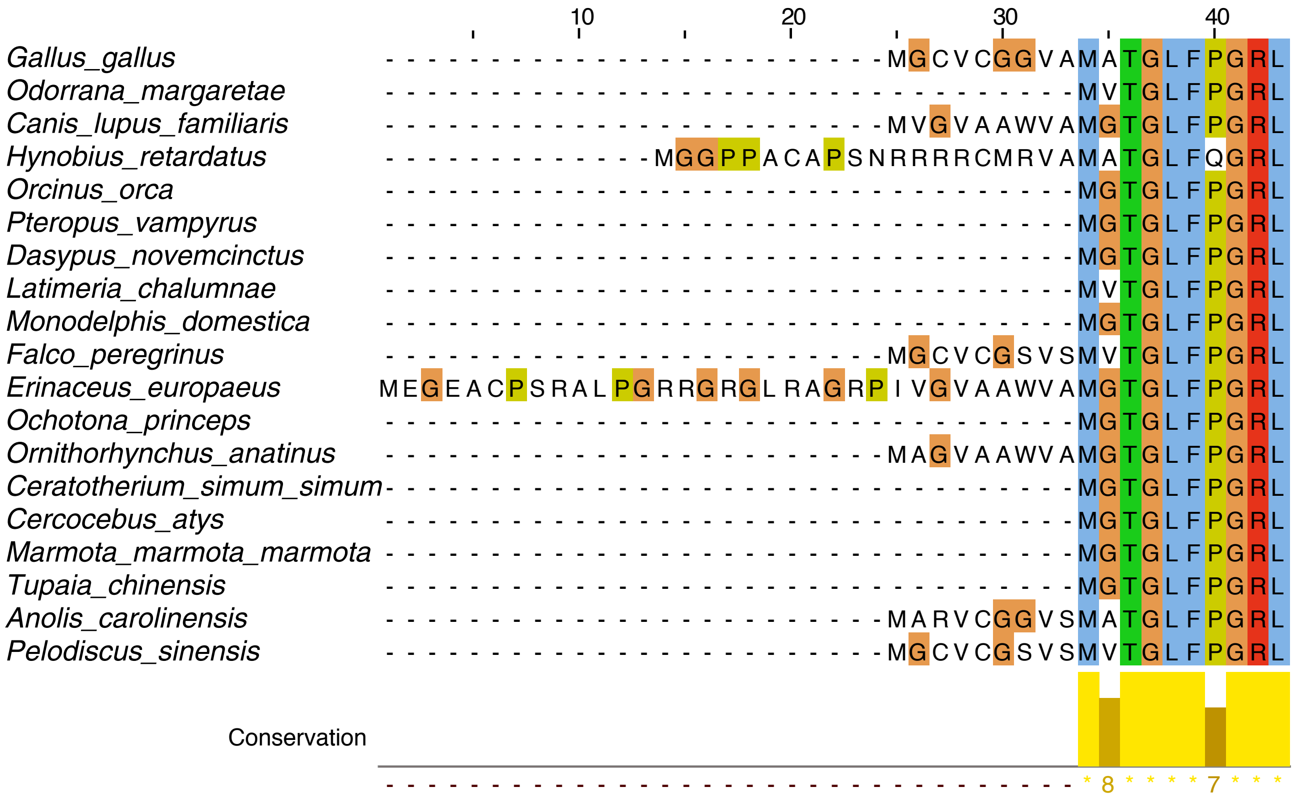

HG0114.1

ENSGALG00000003701 (SGMS1)

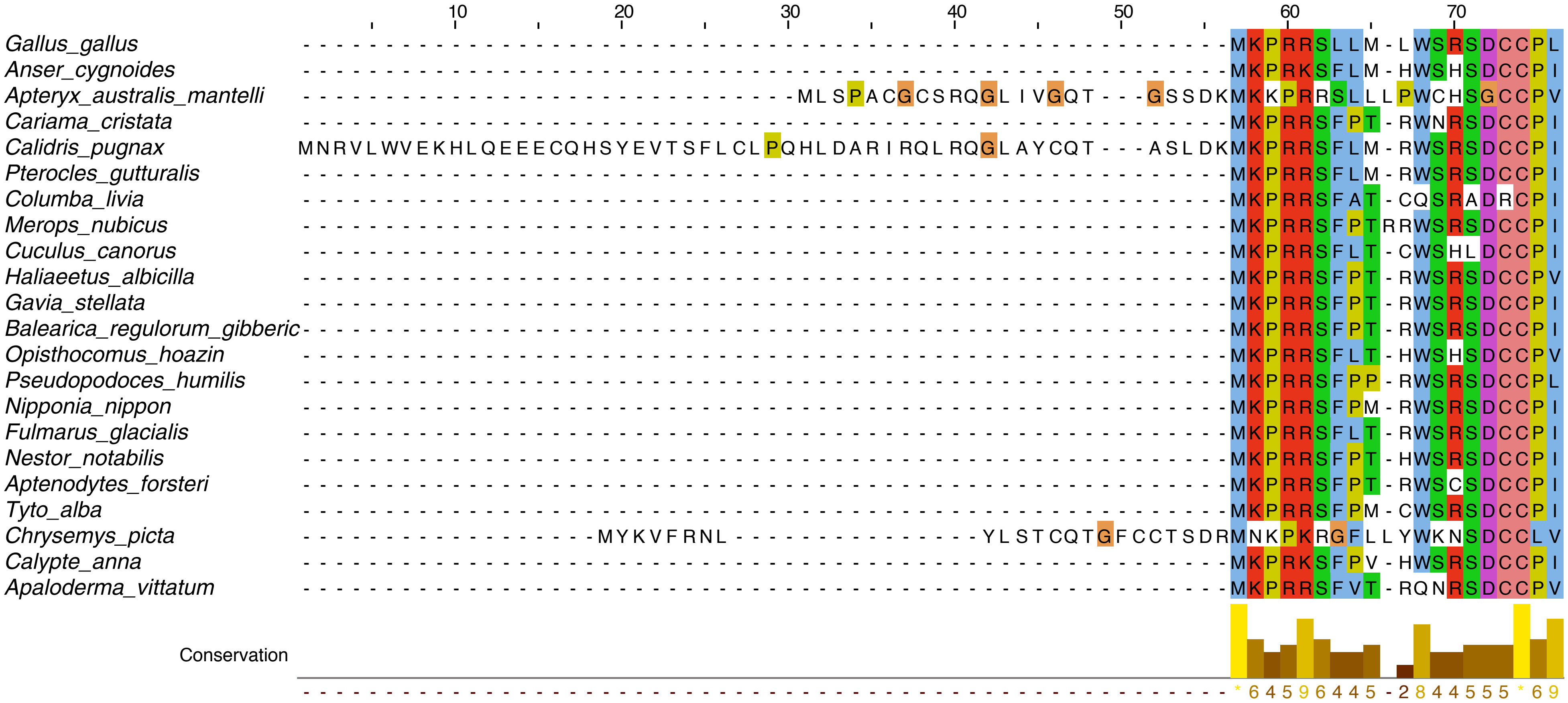

HG0114.2

ENSG00000198964 (SGMS1)

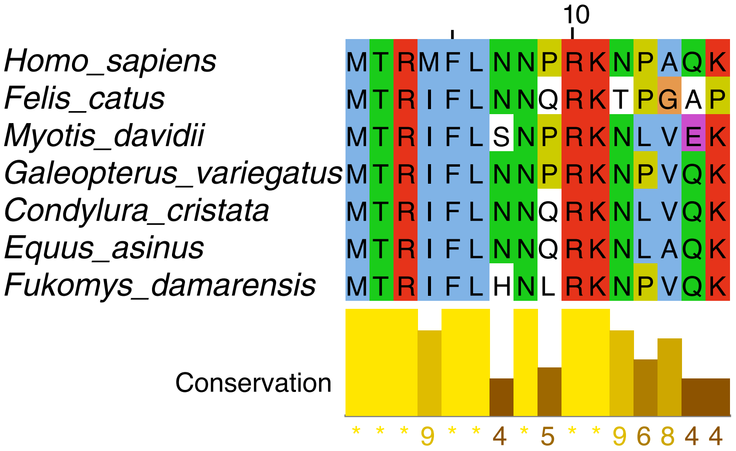

HG0115.1

ENSGALG00000010976 (CCDC126)

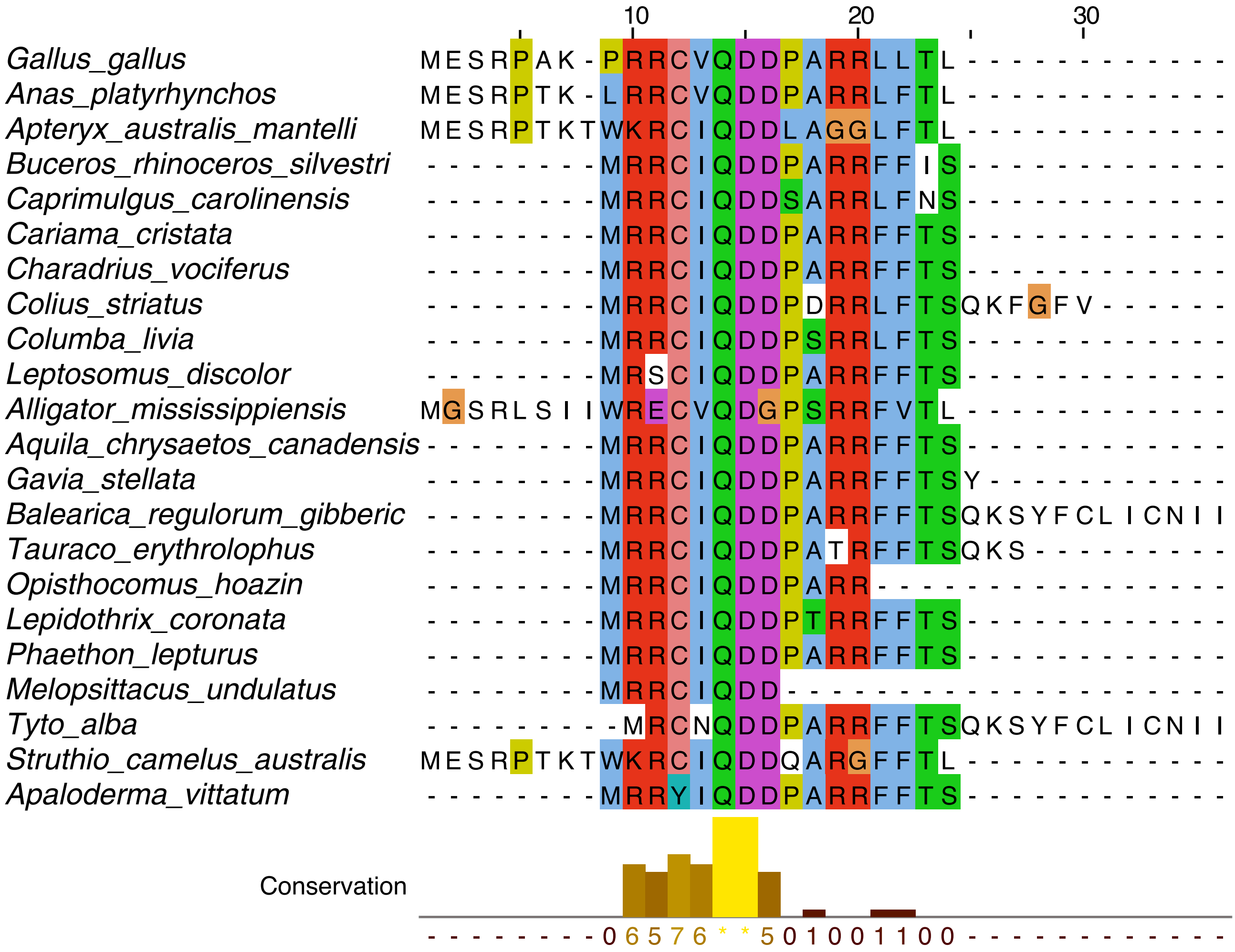

HG0115.2

ENSG00000169193 (CCDC126)

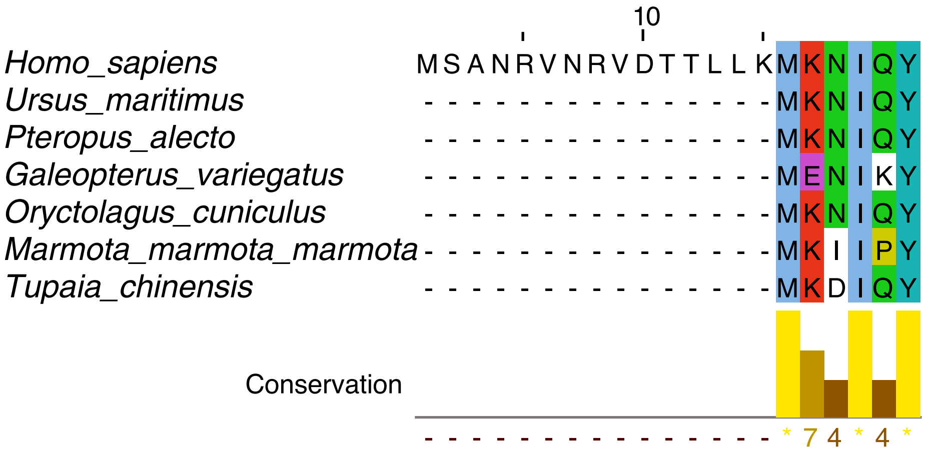

HG0116

ENSG00000010270 (STARD3NL)

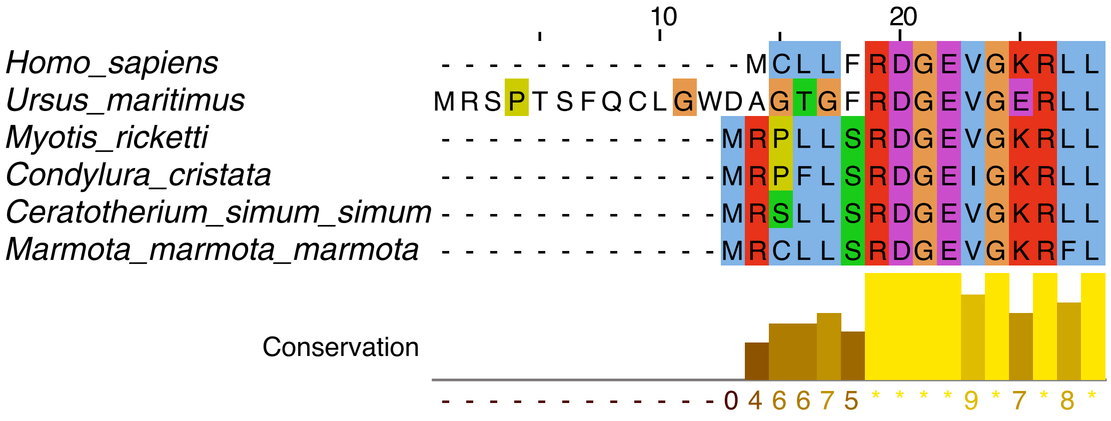

HG0116

ENSGALG00000033038

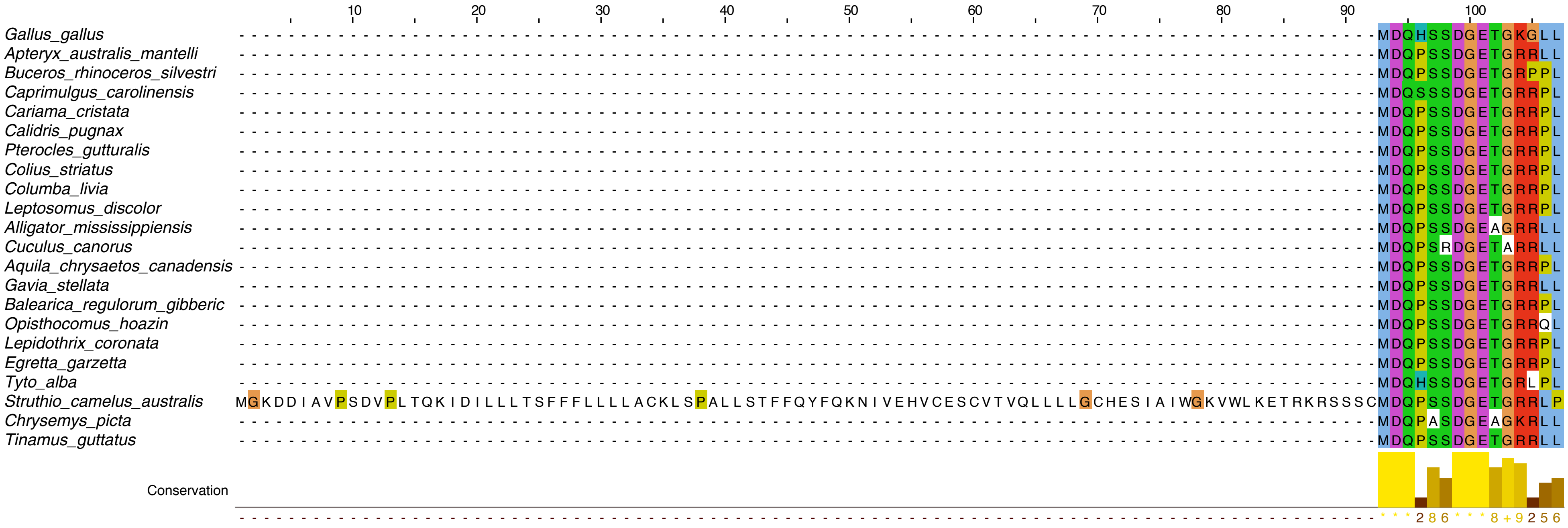

HG0117.1

ENSG00000140945 (CDH13)

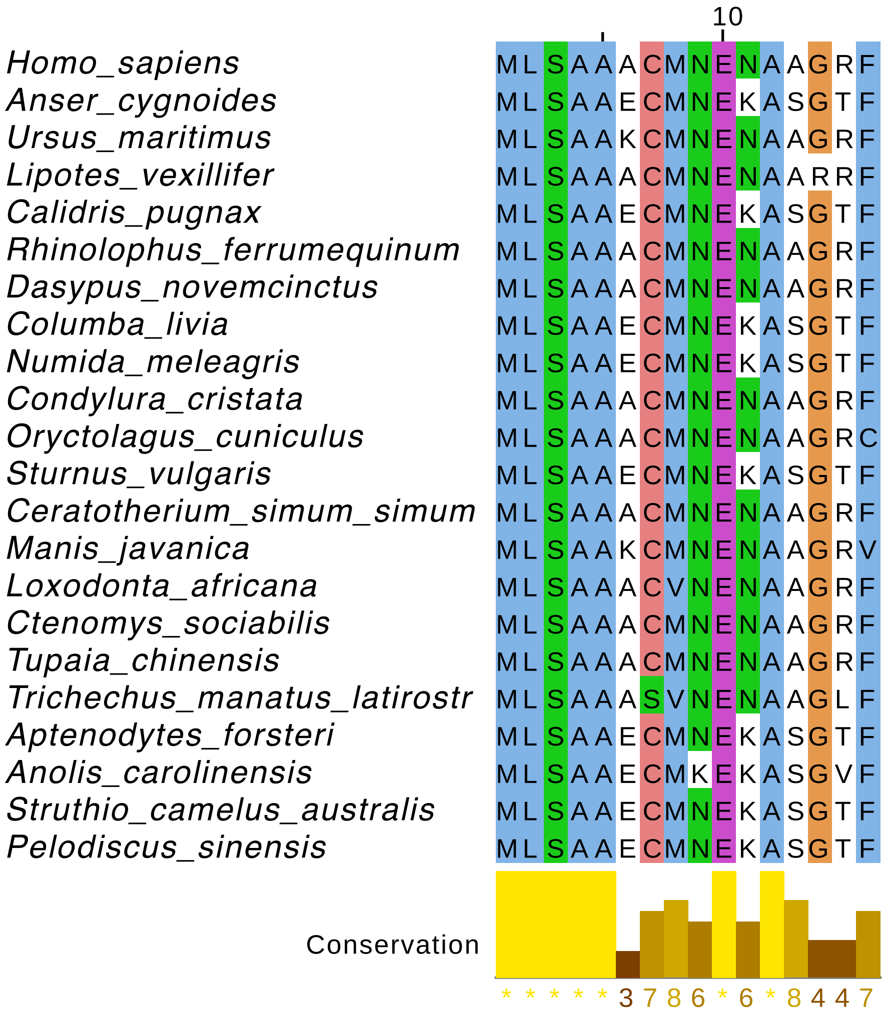

HG0117.2

ENSG00000140945 (CDH13)

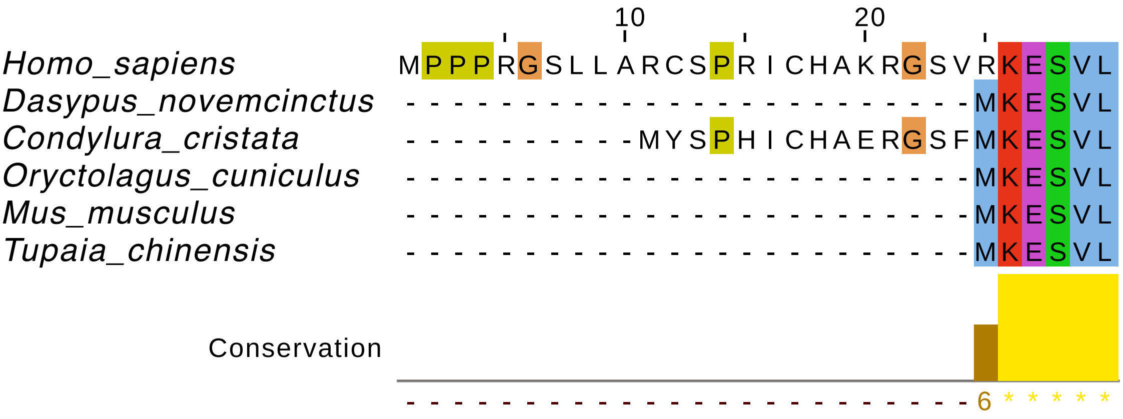

HG0118.1

ENSG00000183662 (FAM19A1)

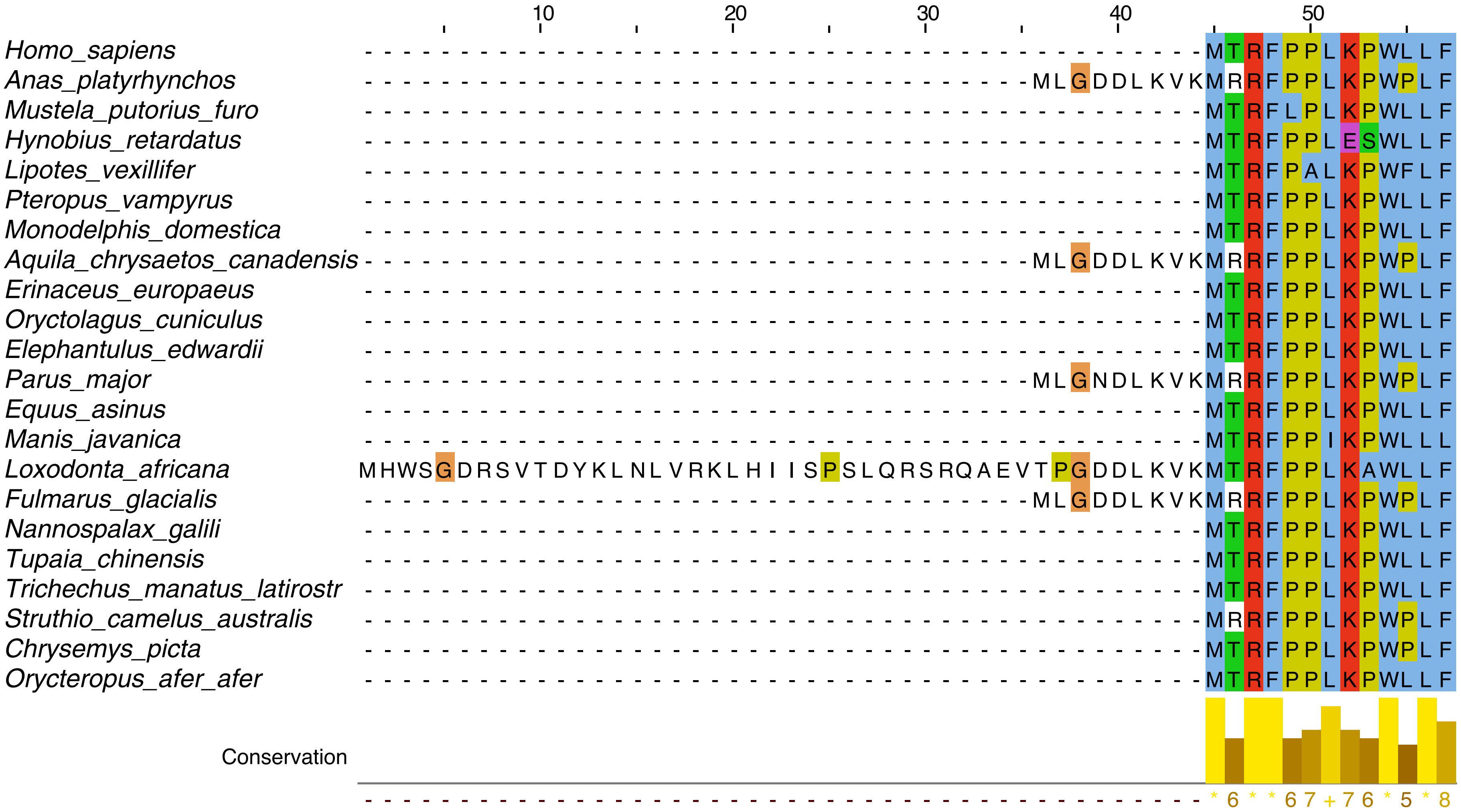

HG0118.2

ENSG00000183662 (FAM19A1)

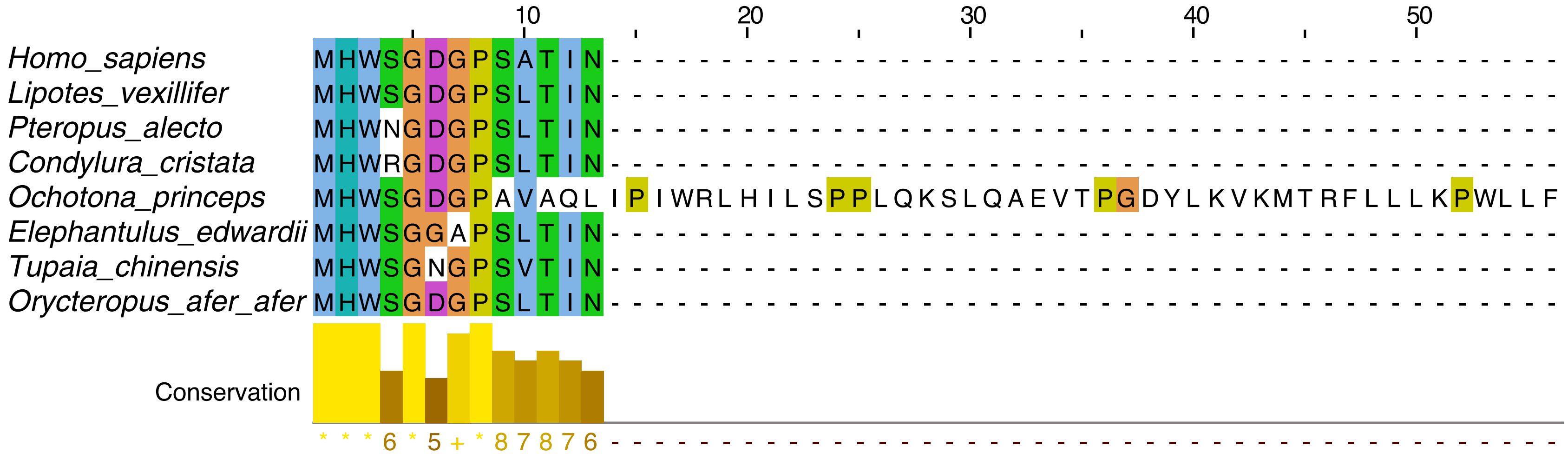

HG0119

ENSGALG00000043570 (SGK3)

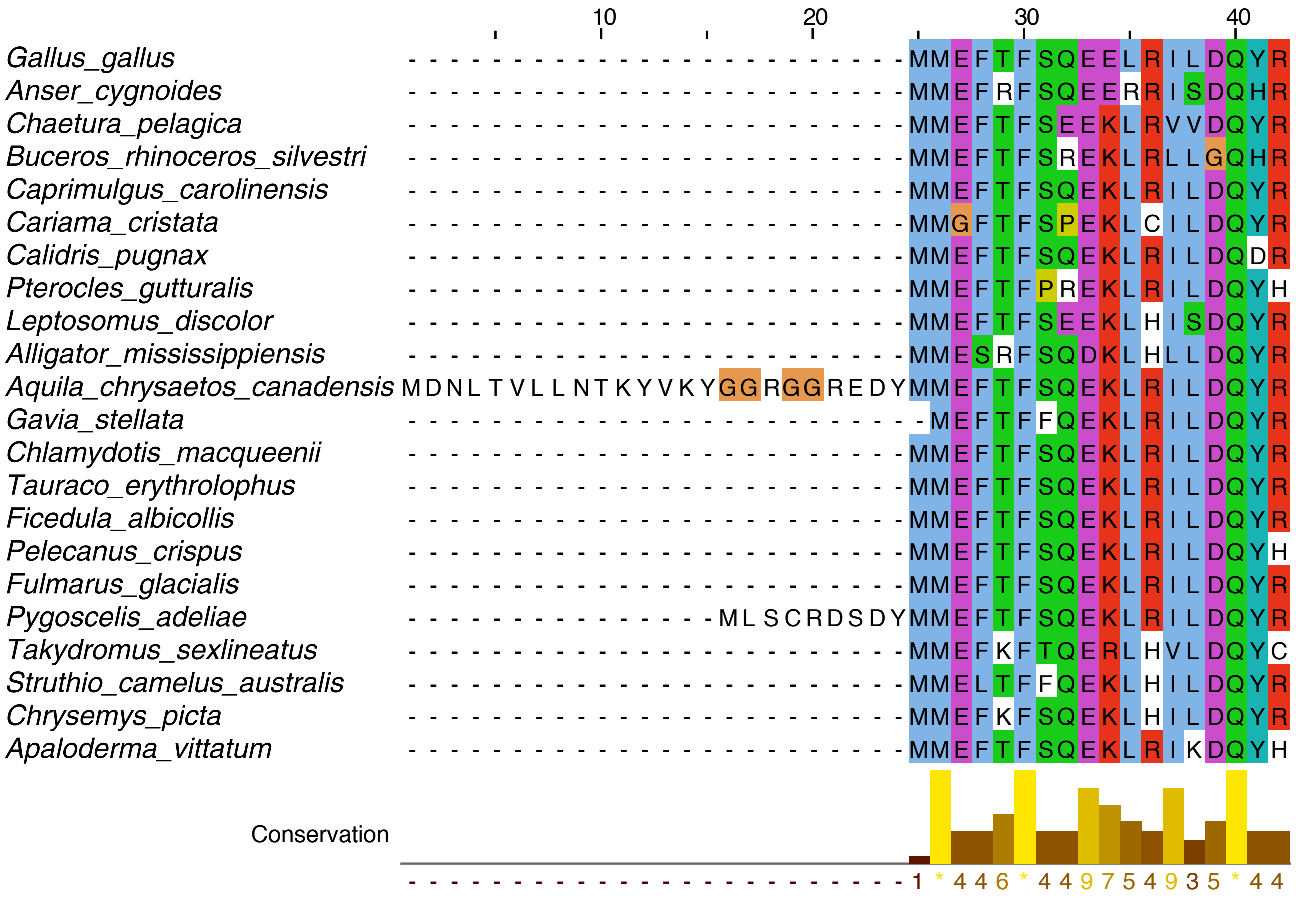

HG0120

ENSG00000022355 (GABRA1)

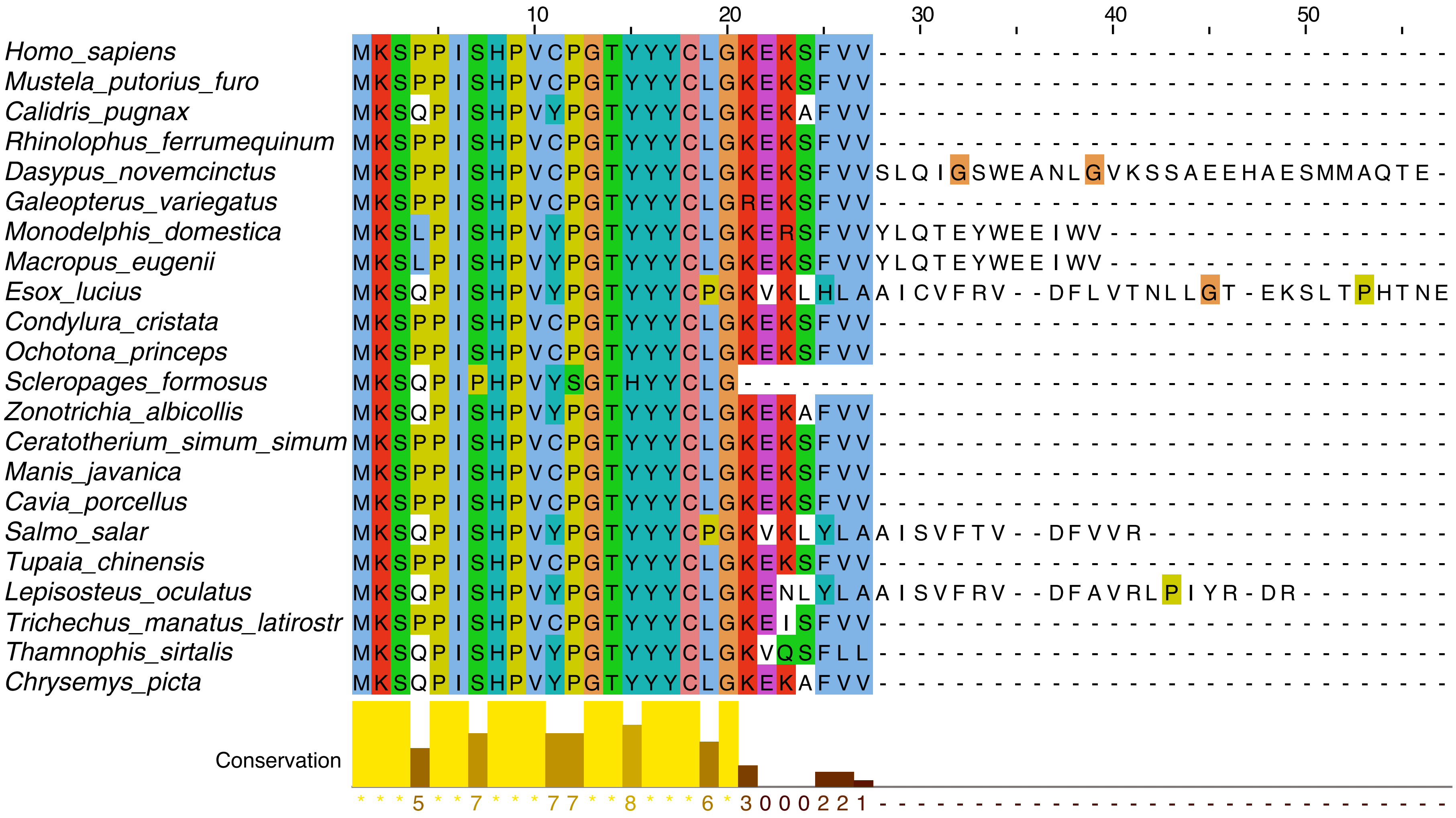

HG0121

ENSG00000122786 (CALD1)

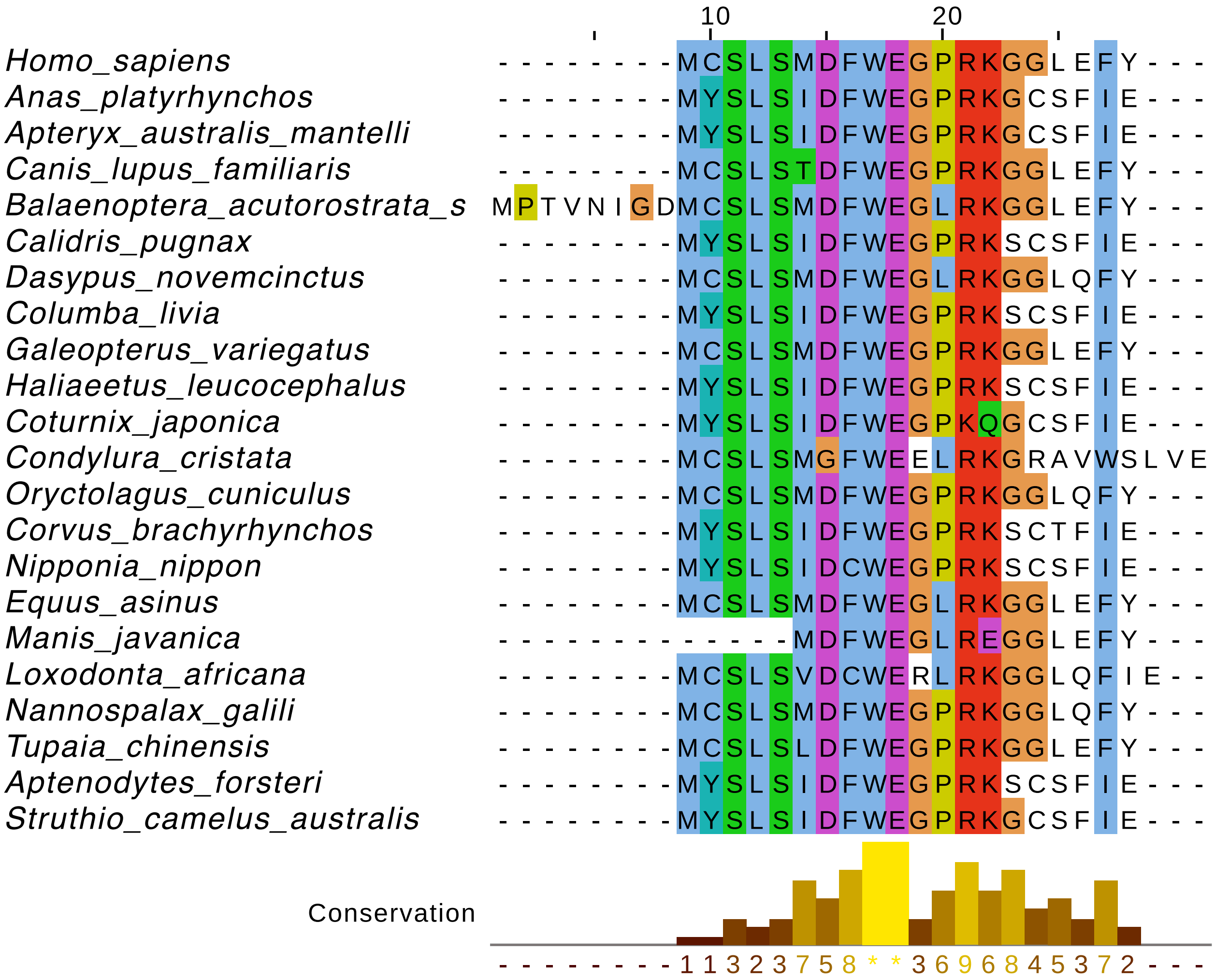

HG0122

ENSG00000169925 (BRD3)

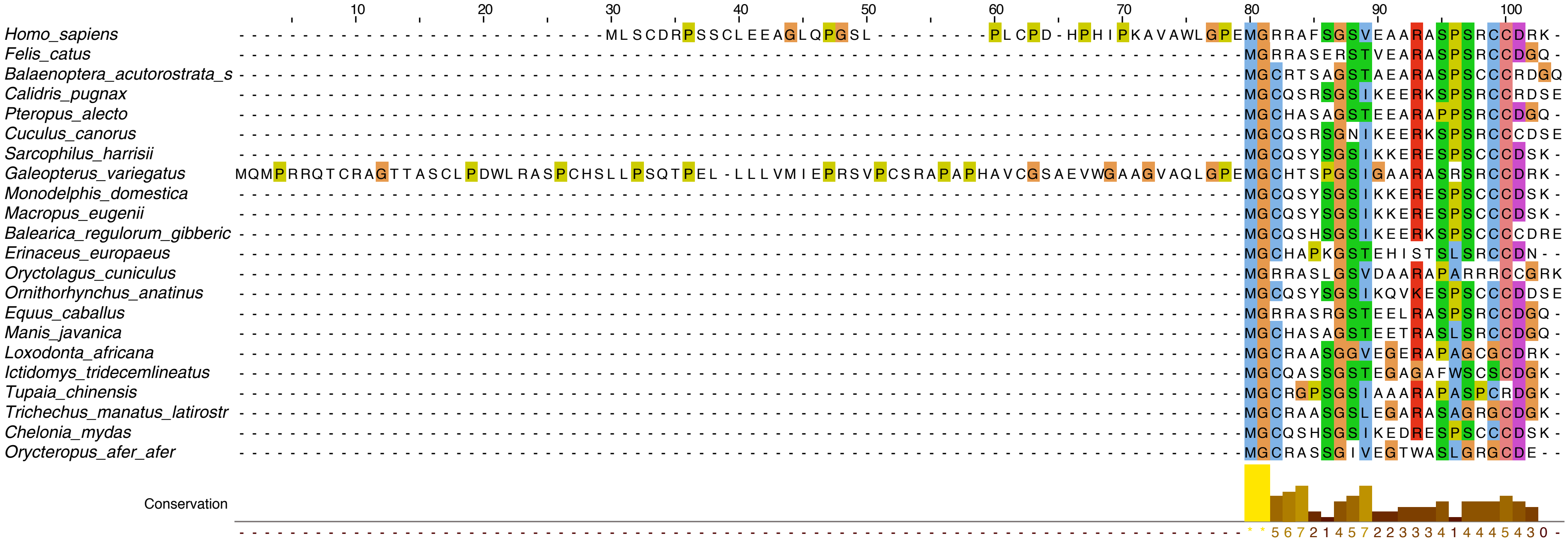

HG0123

ENSG00000173276 (ZBTB21)

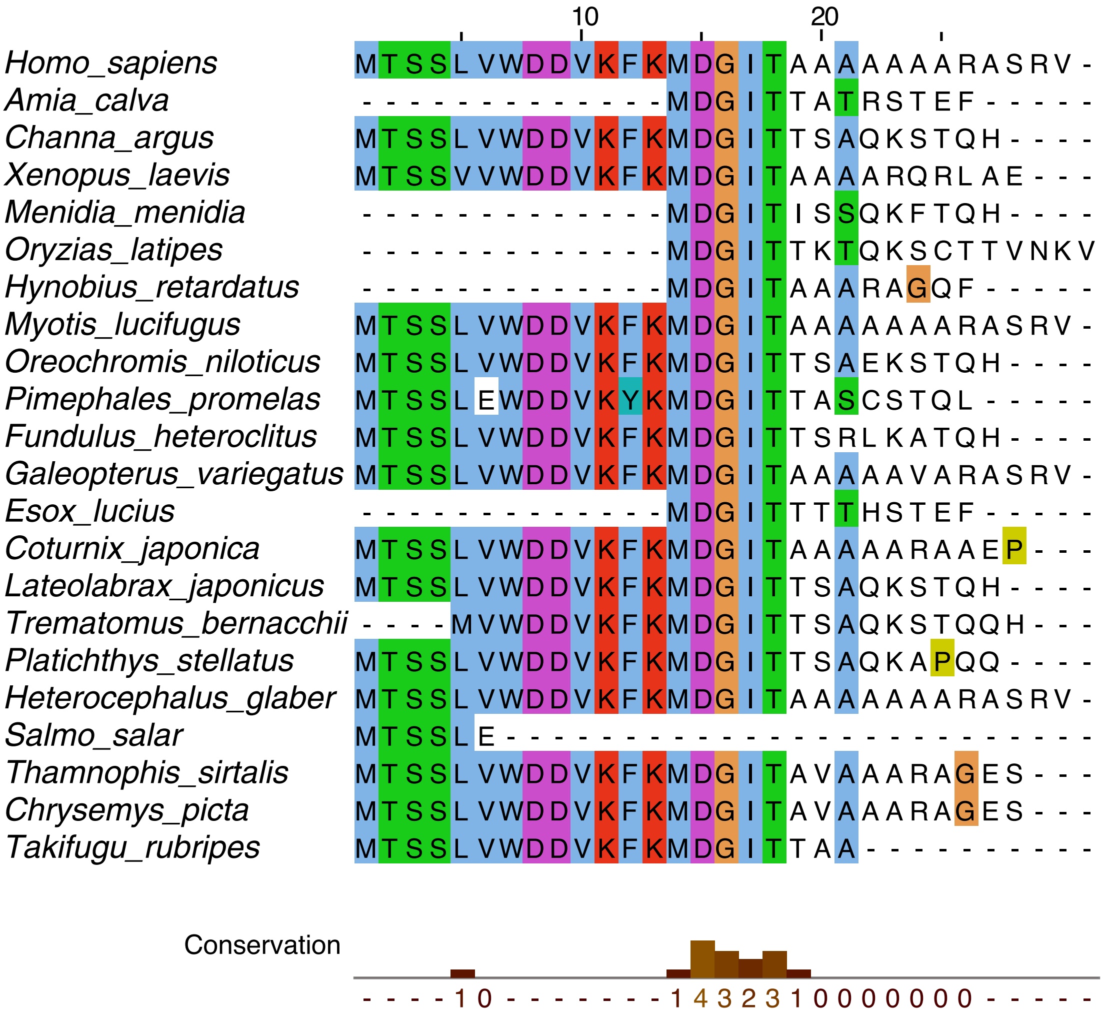

HG0124.1

ENSDARG00000031763 (smad6b)

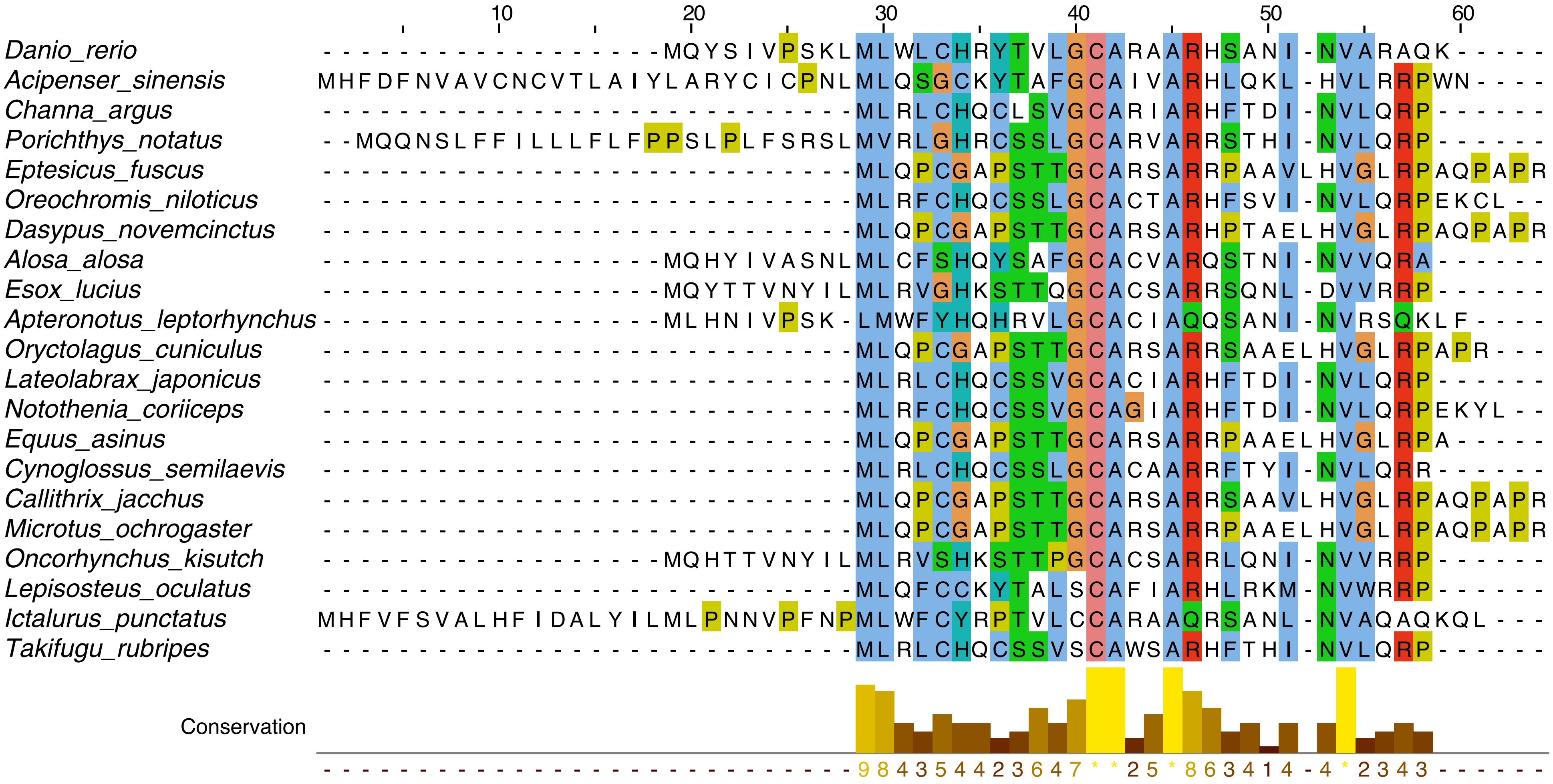

HG0124.2

ENSDARG00000053209 (smad6a)

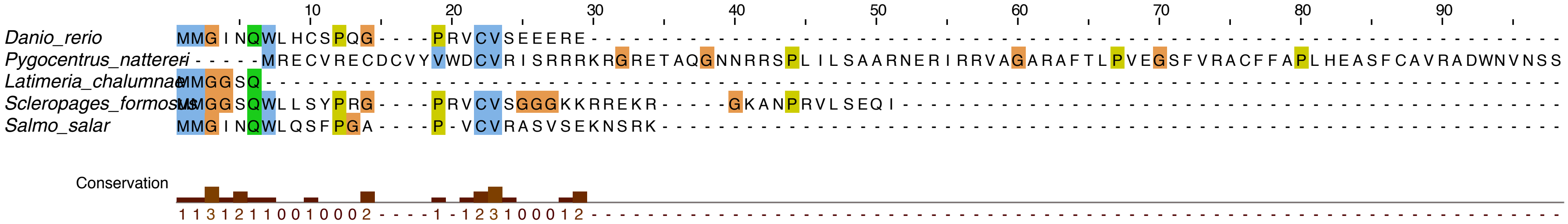

HG0125.1

ENSDARG00000051926 (plag1)

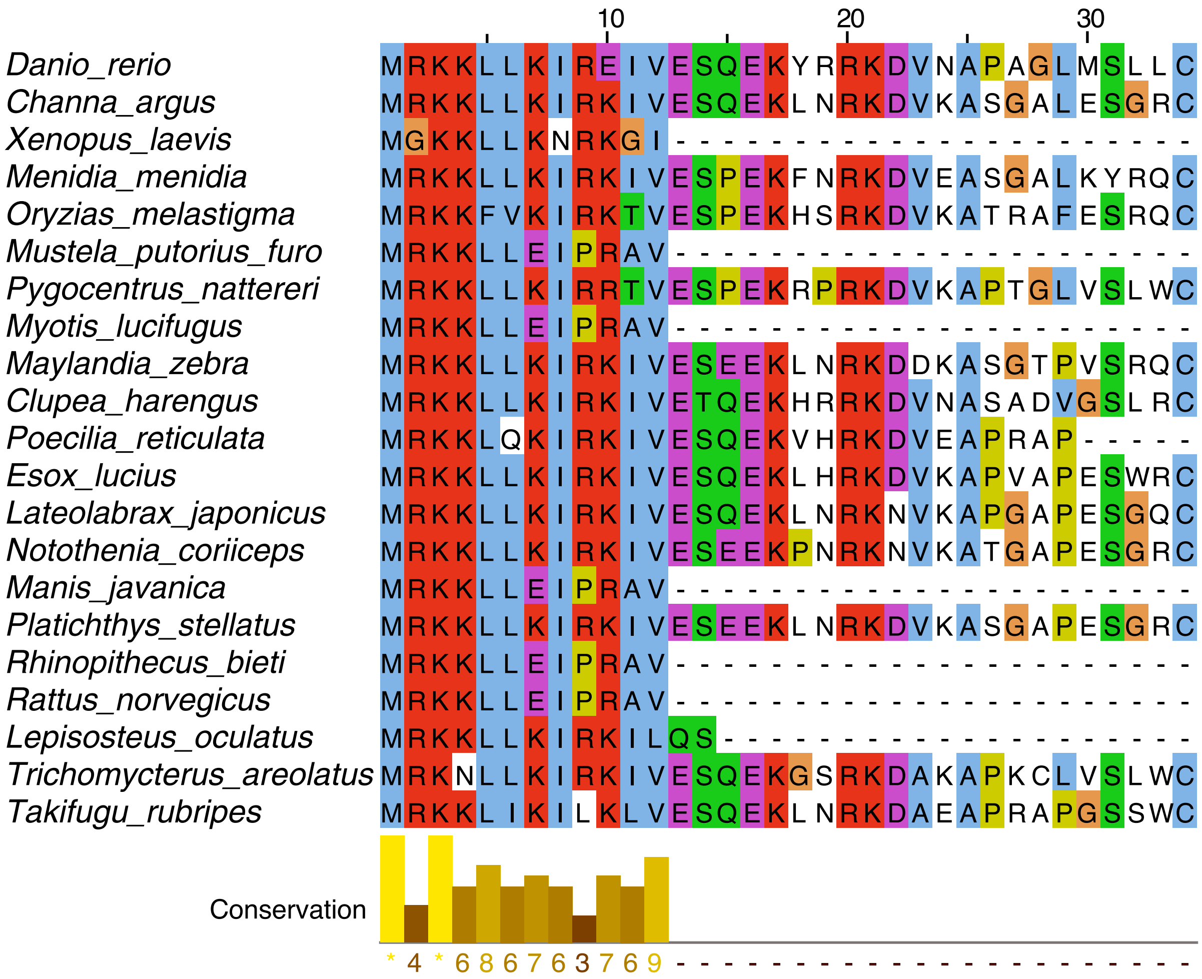

HG0125.2

ENSG00000181690 (PLAG1)

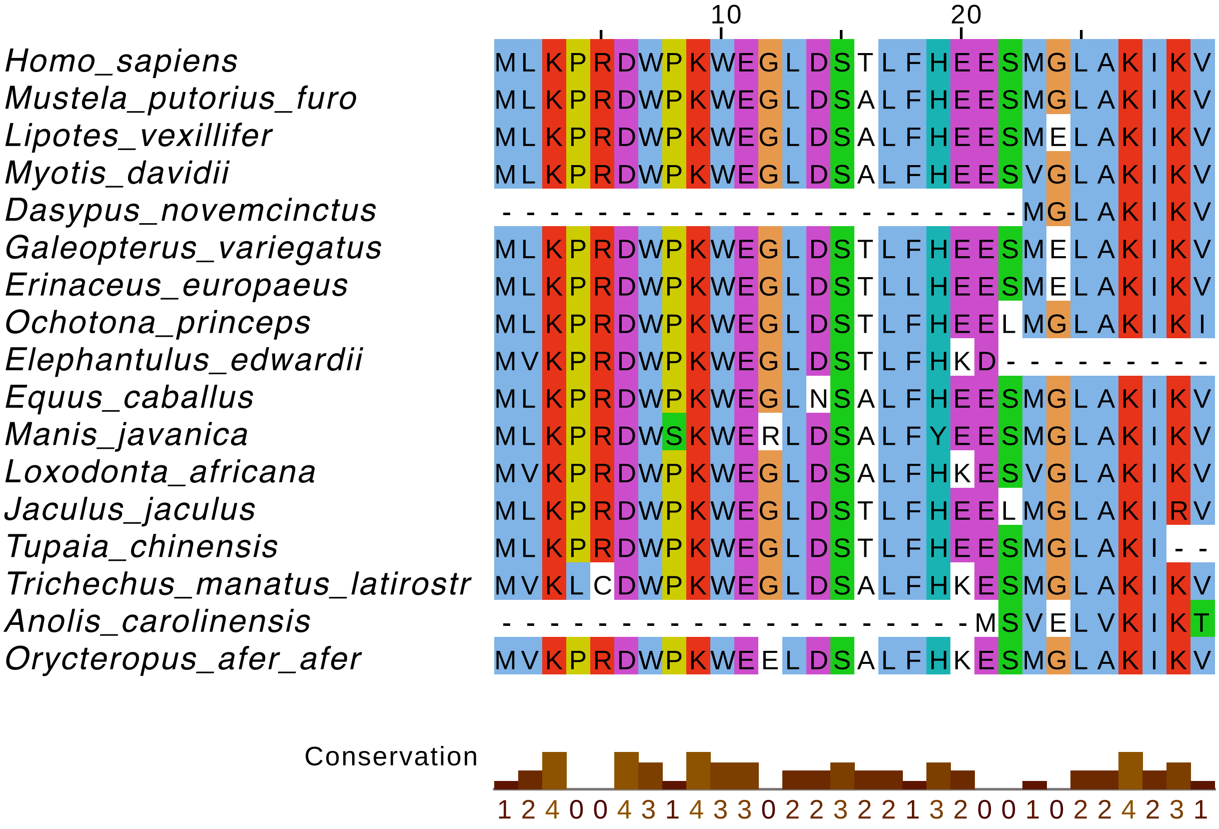

HG0126.1

ENSGALG00000010899 (G6PC2)

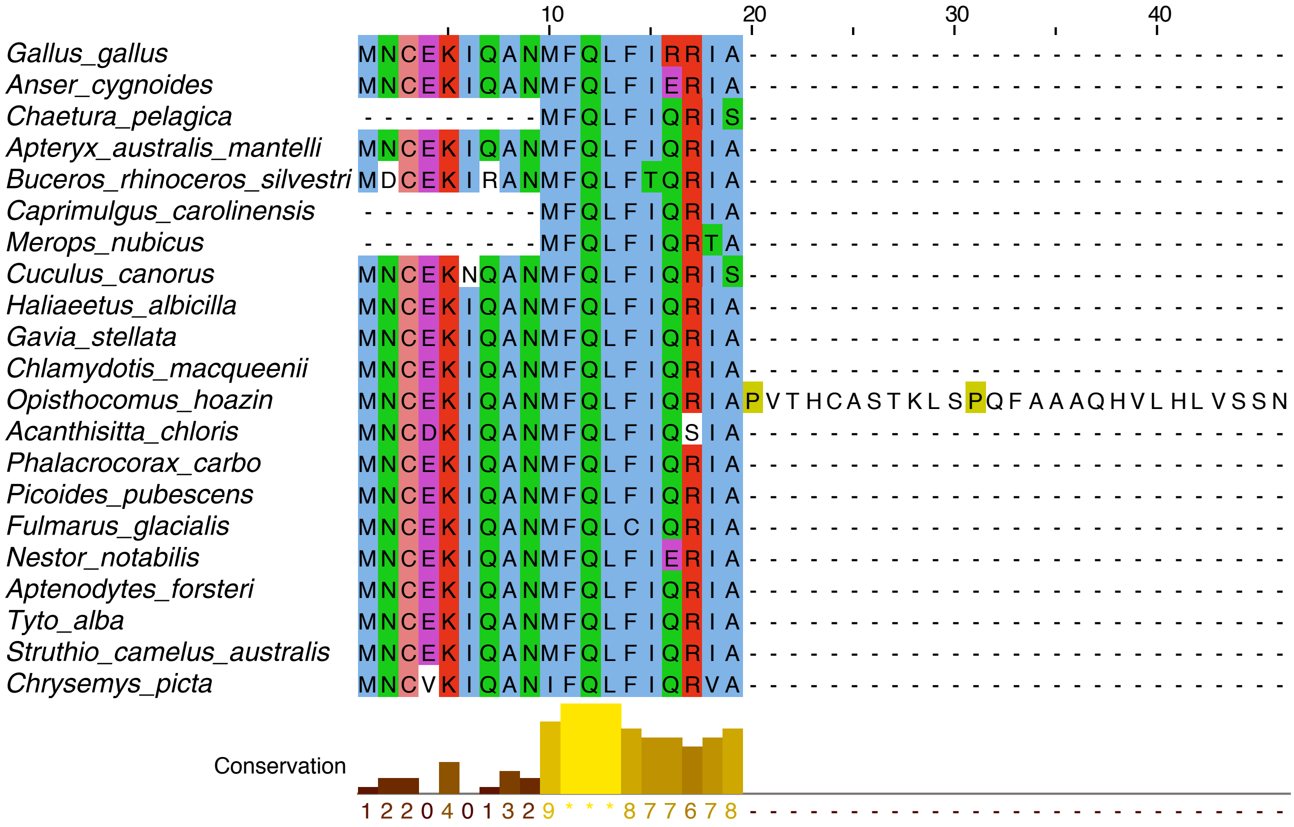

HG0126.2

ENSGALG00000010899 (G6PC2)

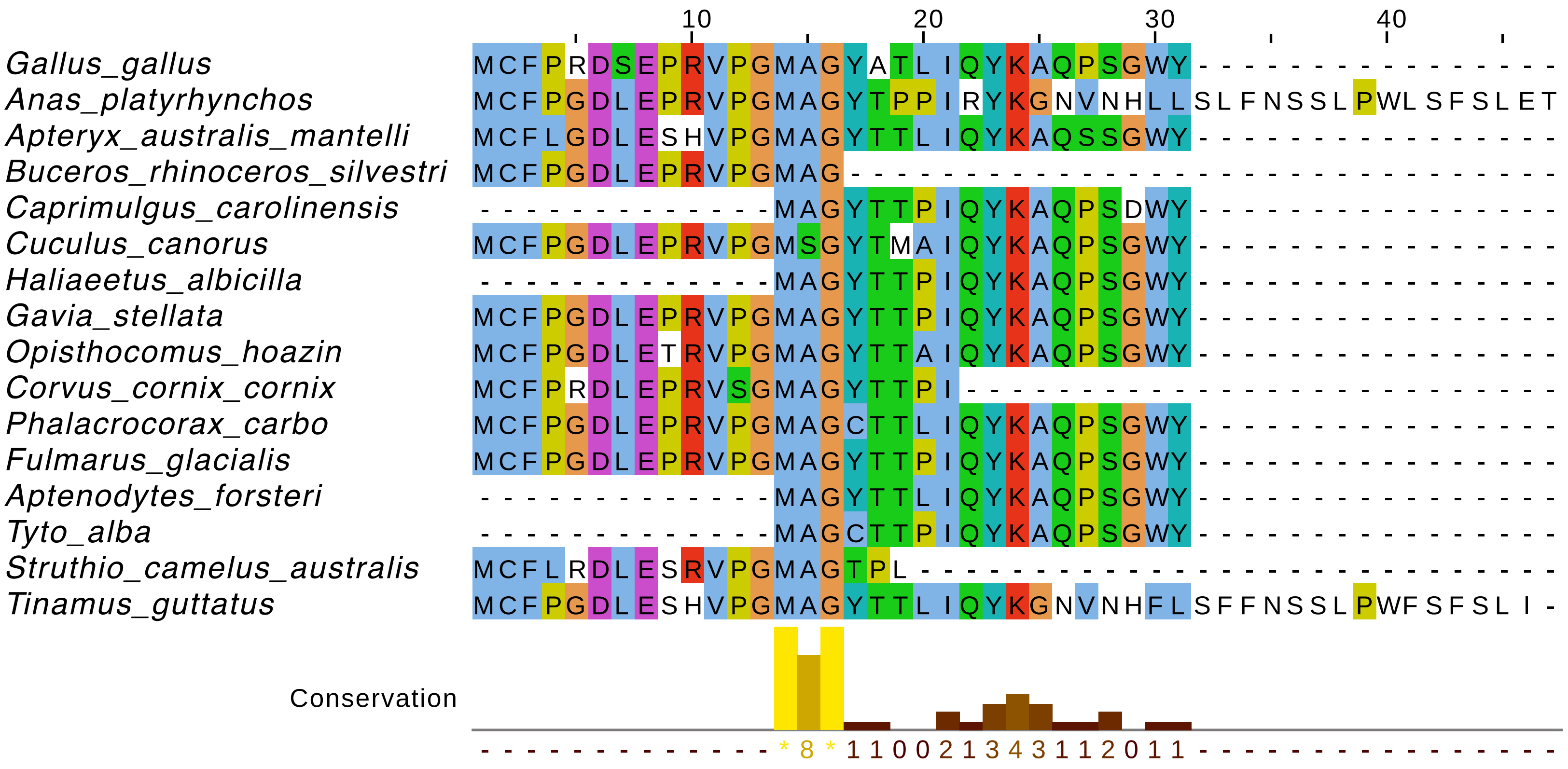

HG0127.1

ENSGALG00000029709

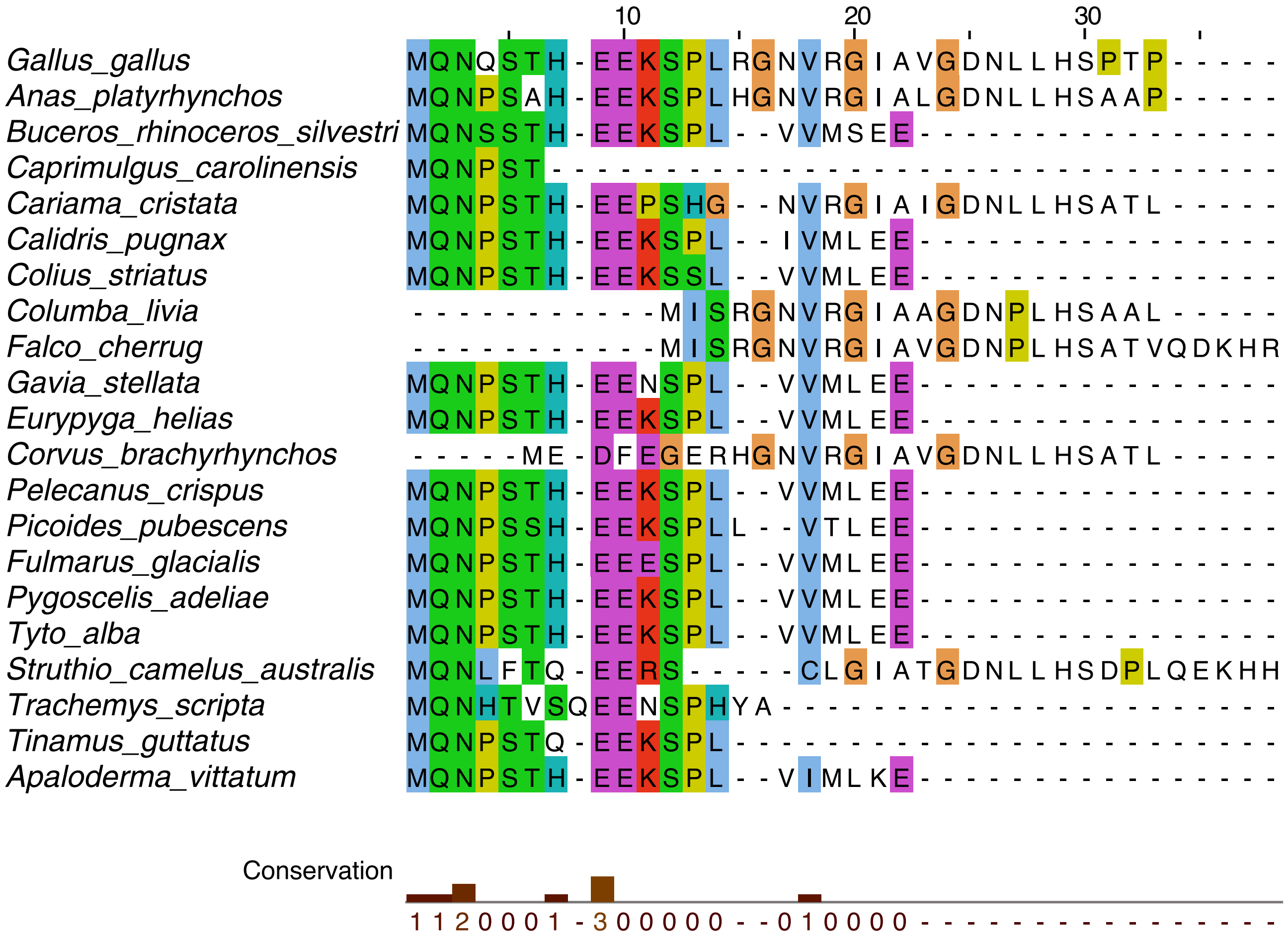

HG0127.2

ENSG00000109452 (INPP4B)

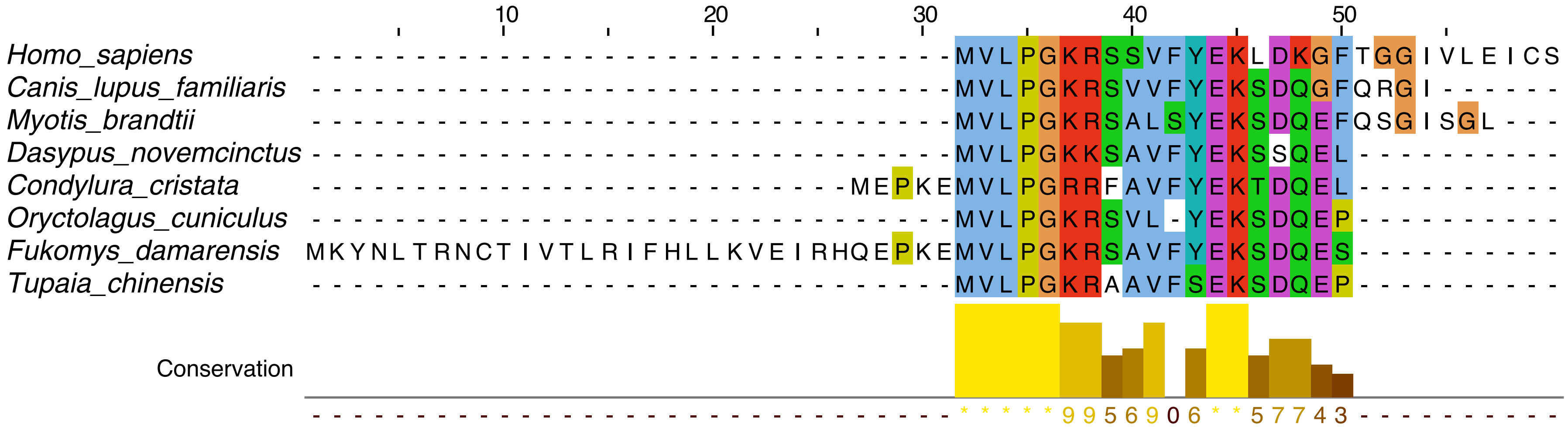

HG0128

ENSDARG00000091029 (phox2bb)

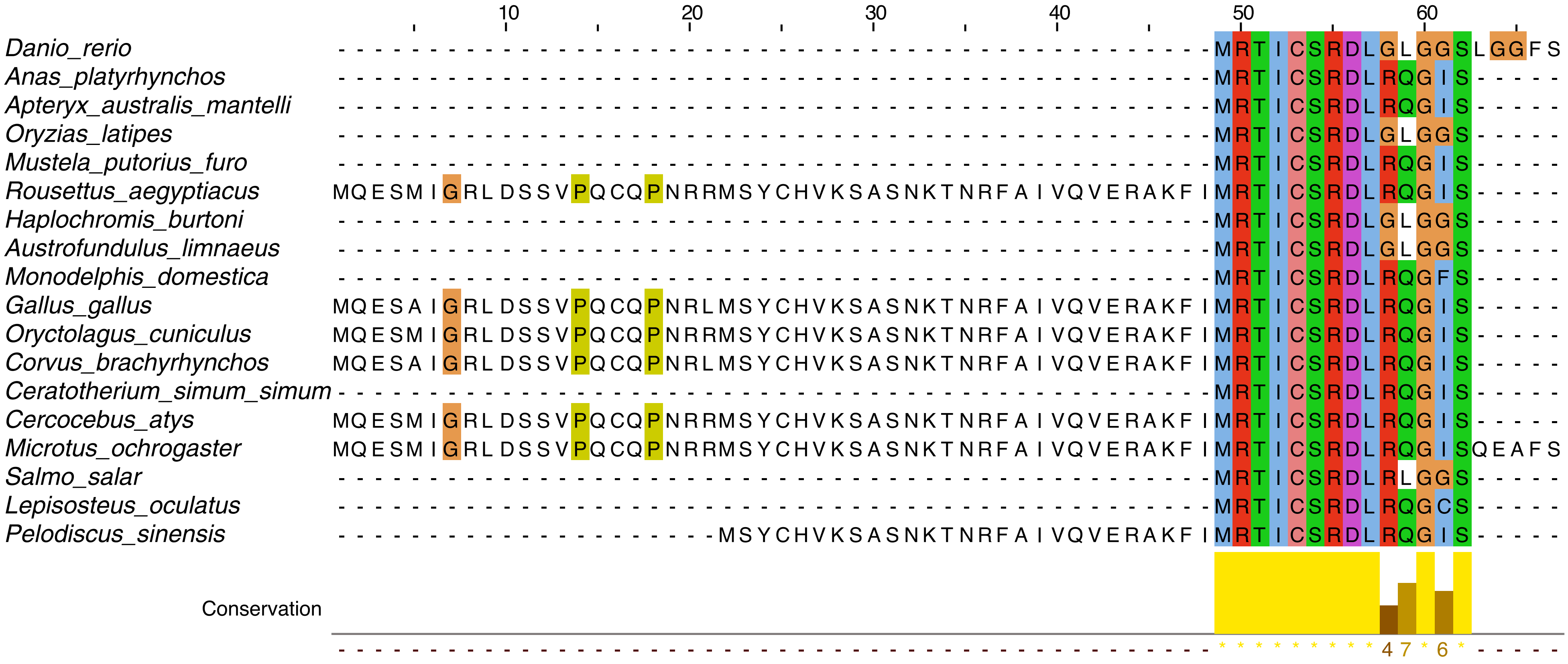

HG0128

ENSG00000109132 (PHOX2B)

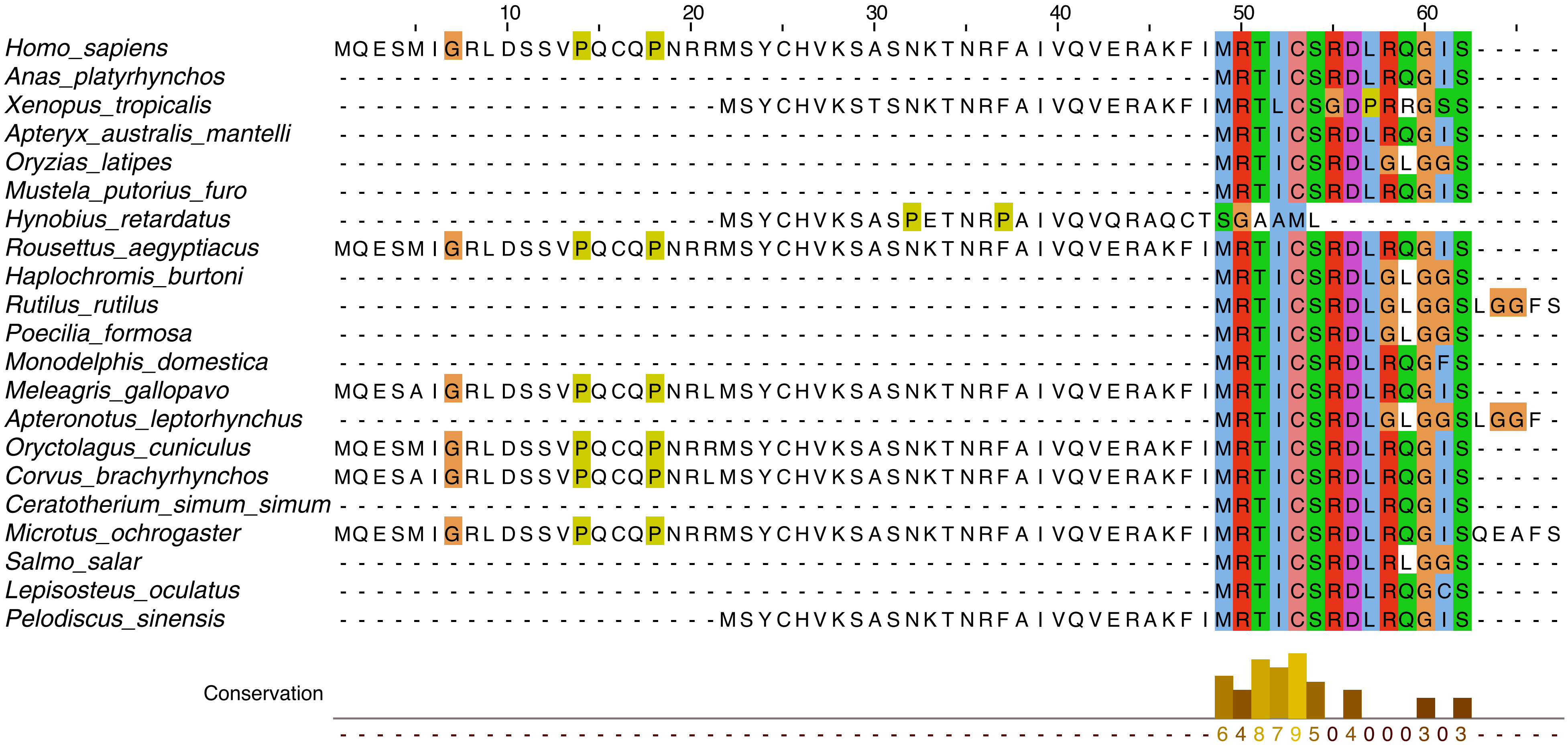

HG0129

ENSDARG00000099437

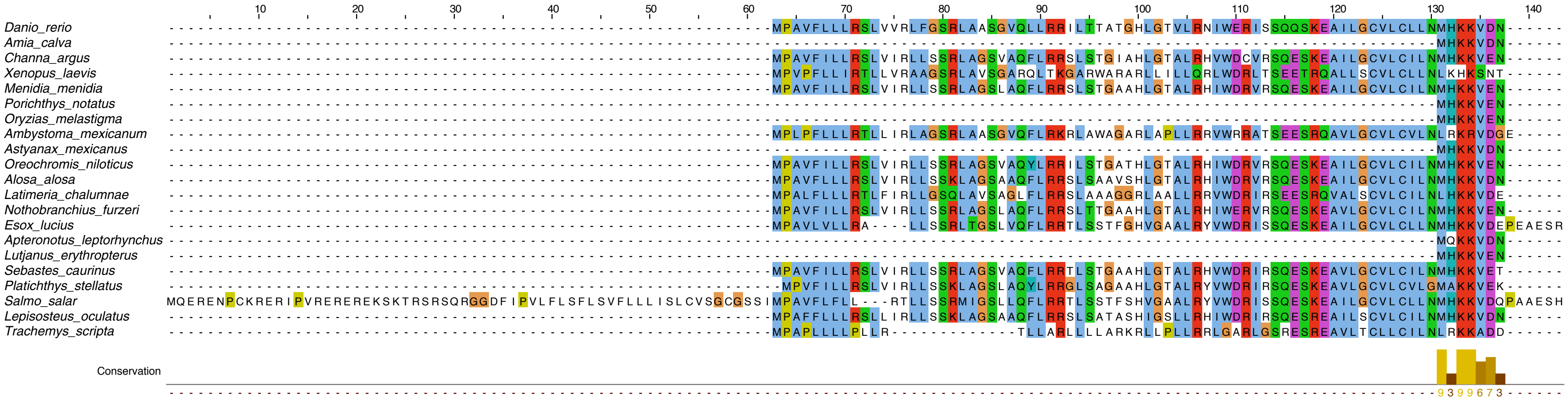

HG0130

ENSGALG00000002993

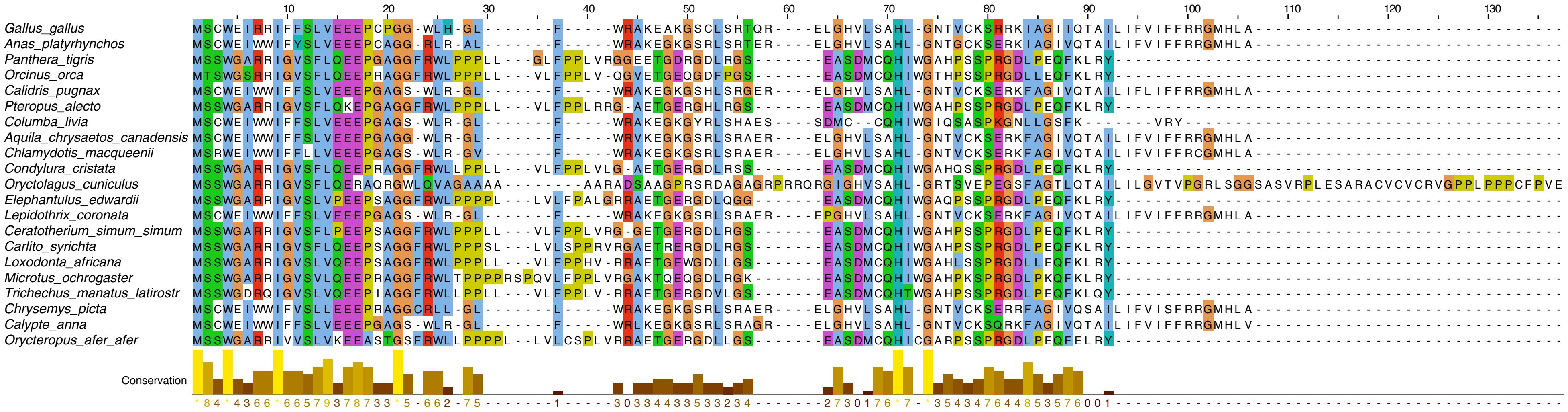

HG0131

ENSGALG00000013135 (GALNT1)

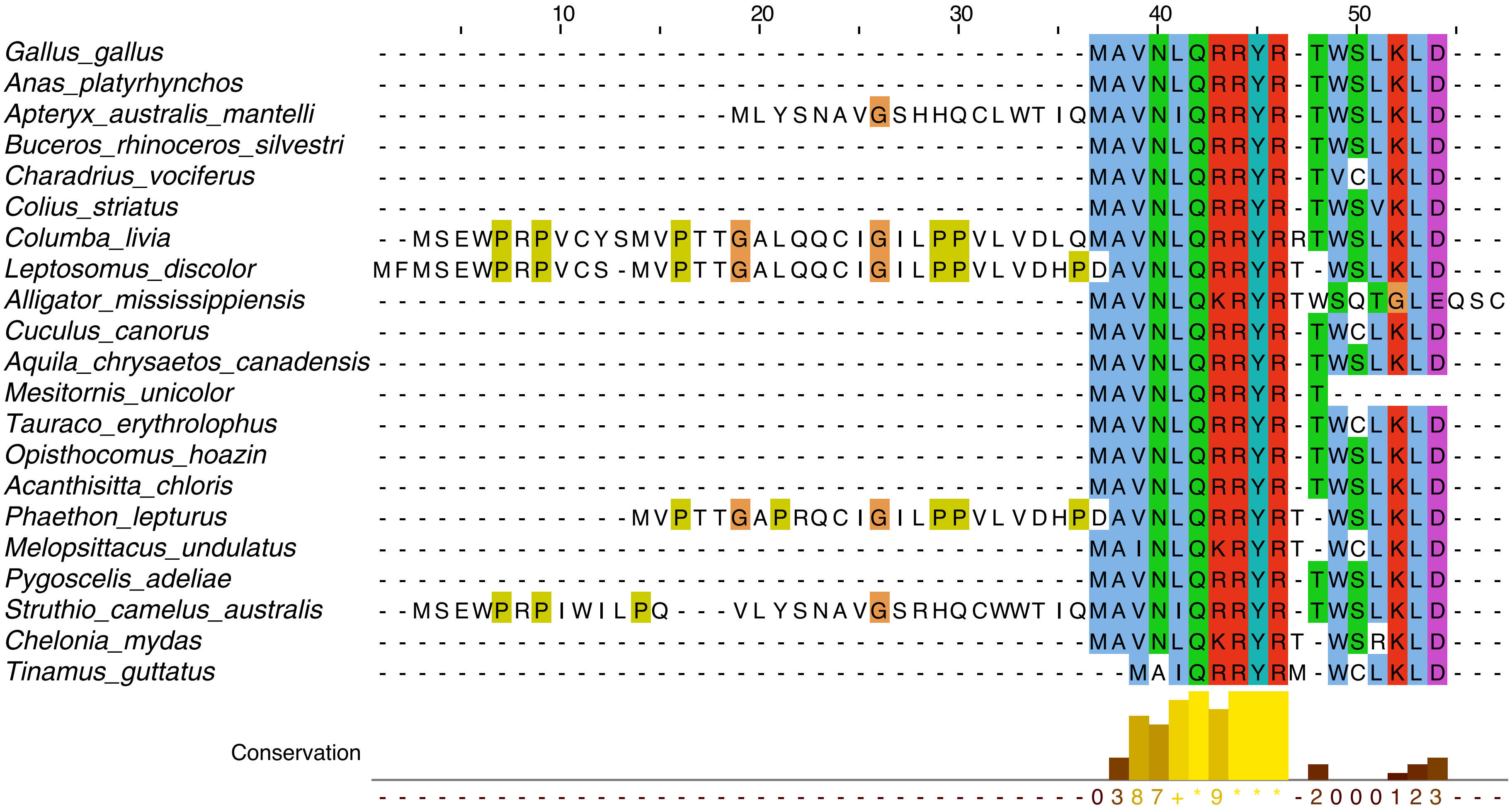

HG0132

ENSG00000151067 (CACNA1C)

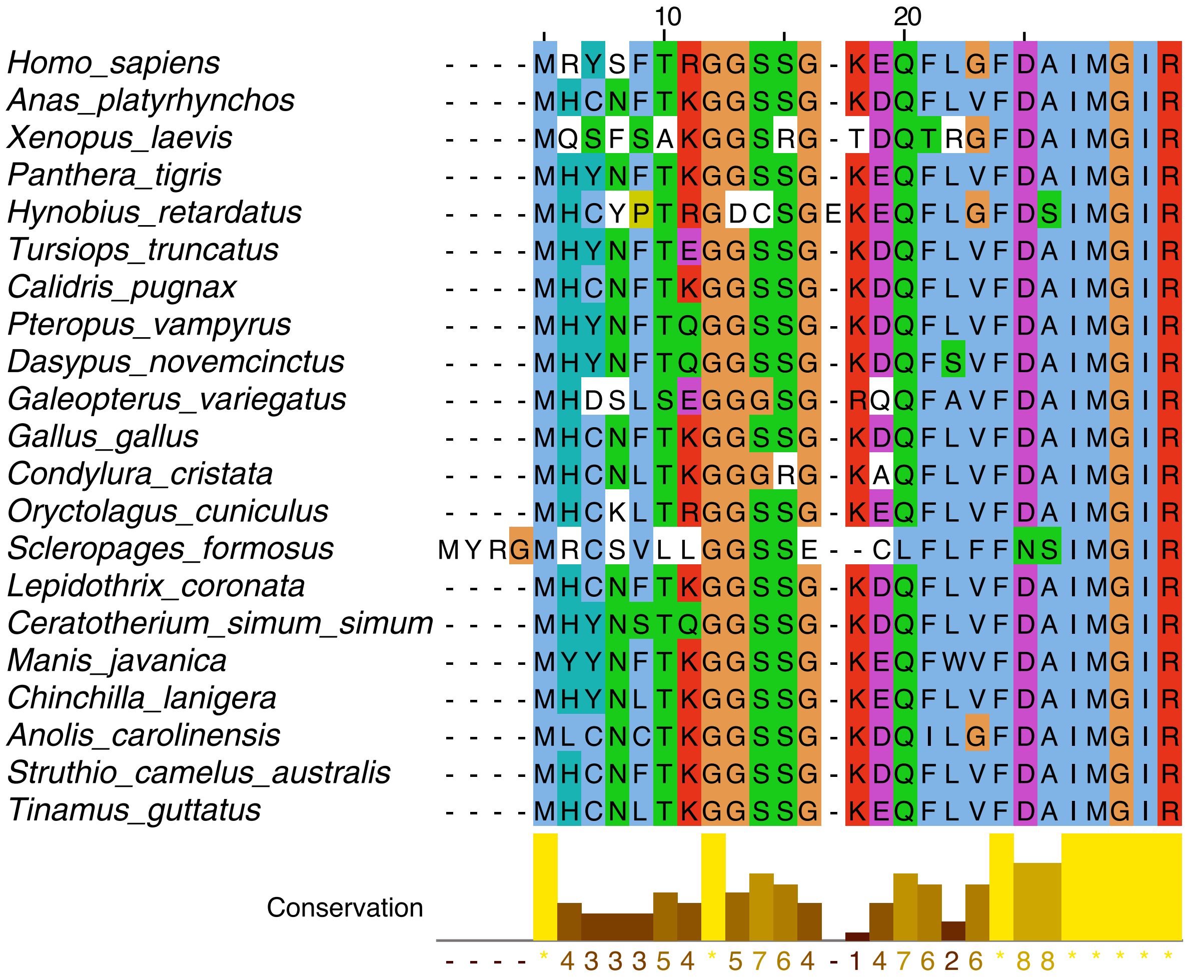

HG0133.1

ENSDARG00000051886 (ankrd11)

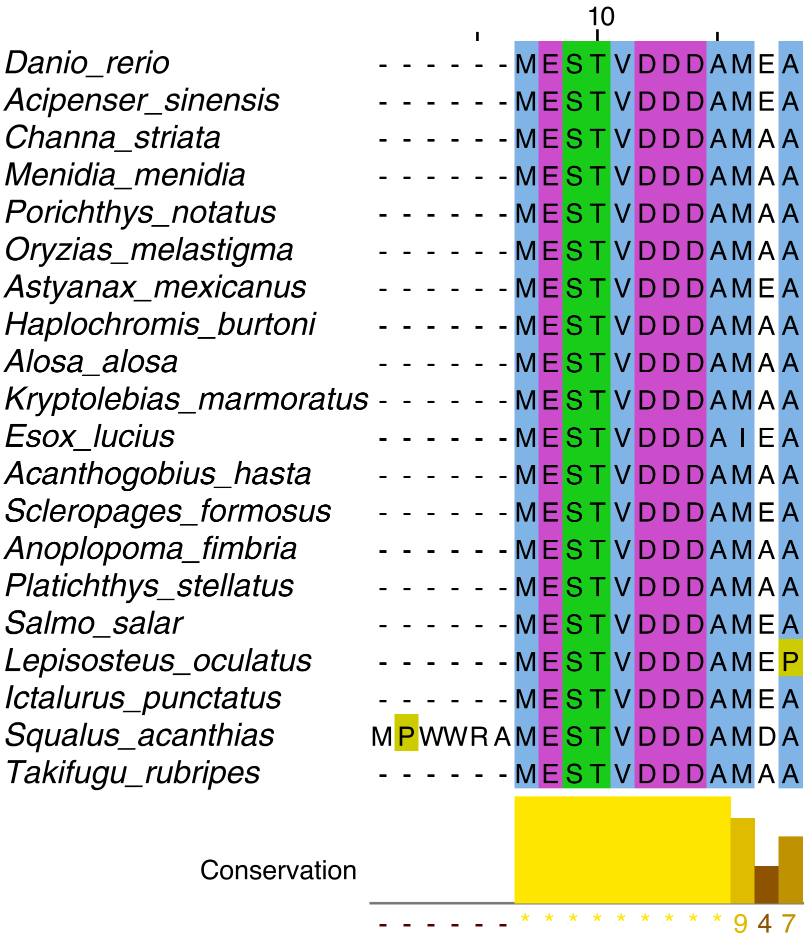

HG0133.1

ENSG00000167522 (ANKRD11)

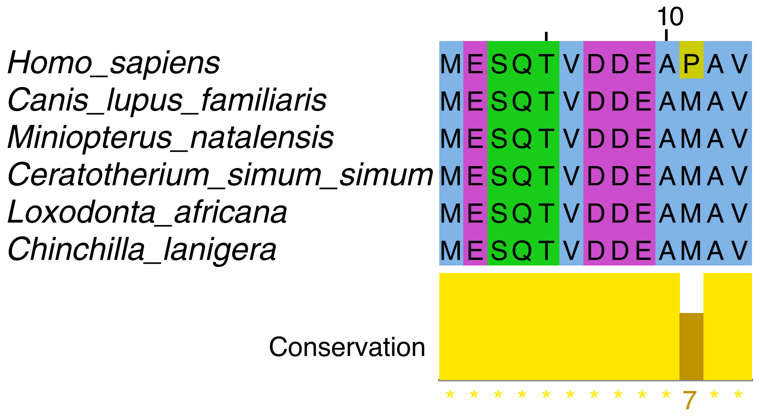

HG0133.2

ENSDARG00000051886 (ankrd11)

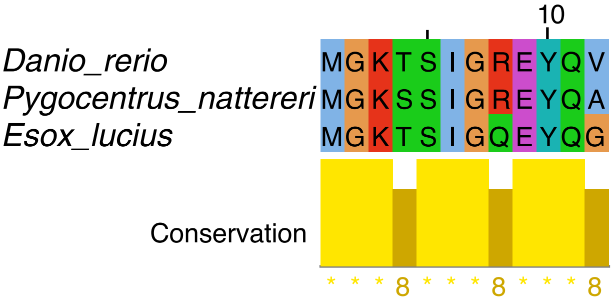

HG0133.3

ENSG00000167522 (ANKRD11)

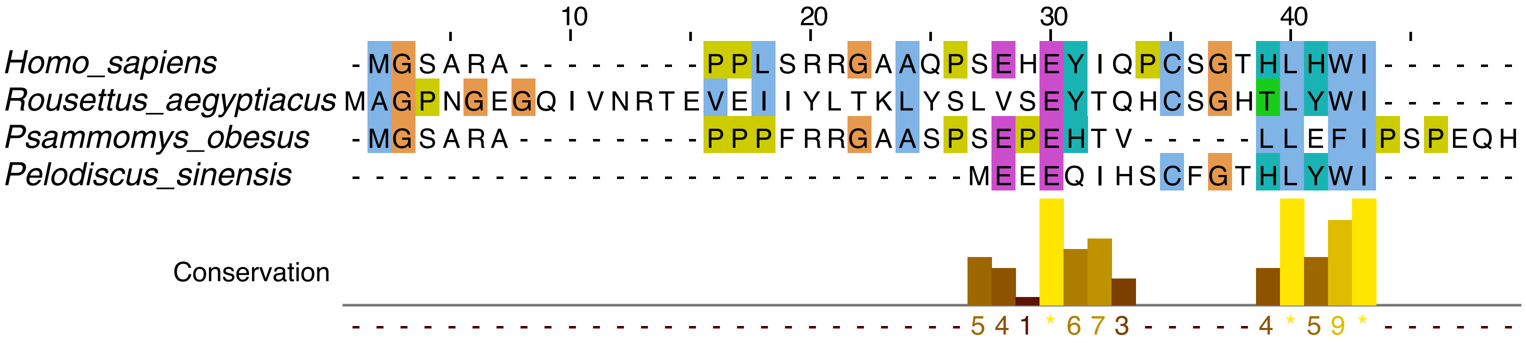

HG0134.1

ENSGALG00000041267 (BMI1)

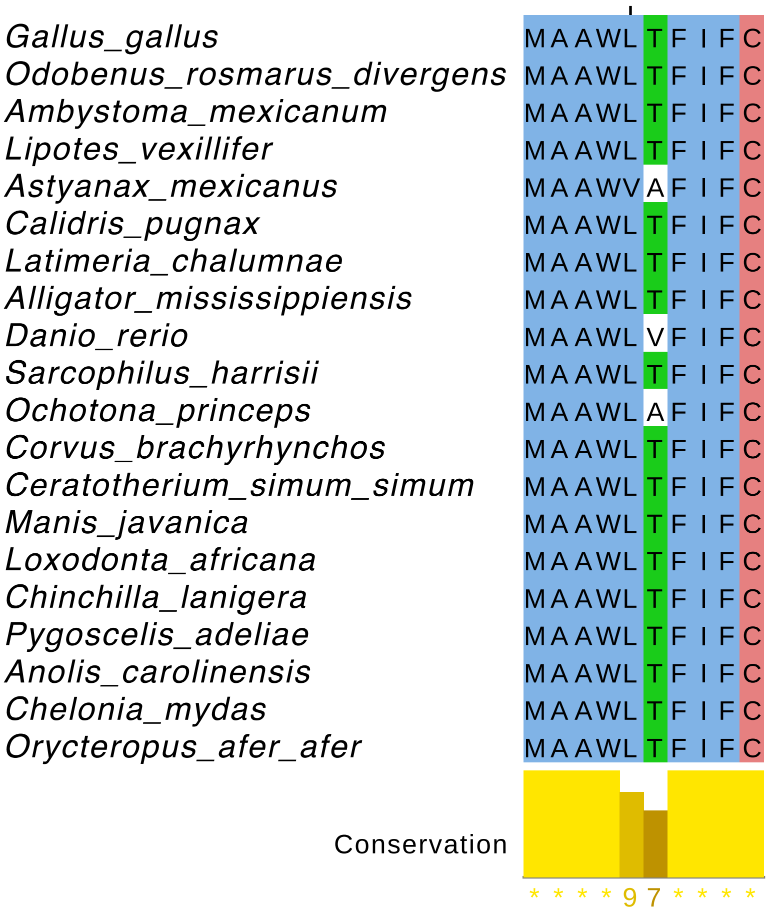

HG0134.2

ENSG00000168283 (BMI1)

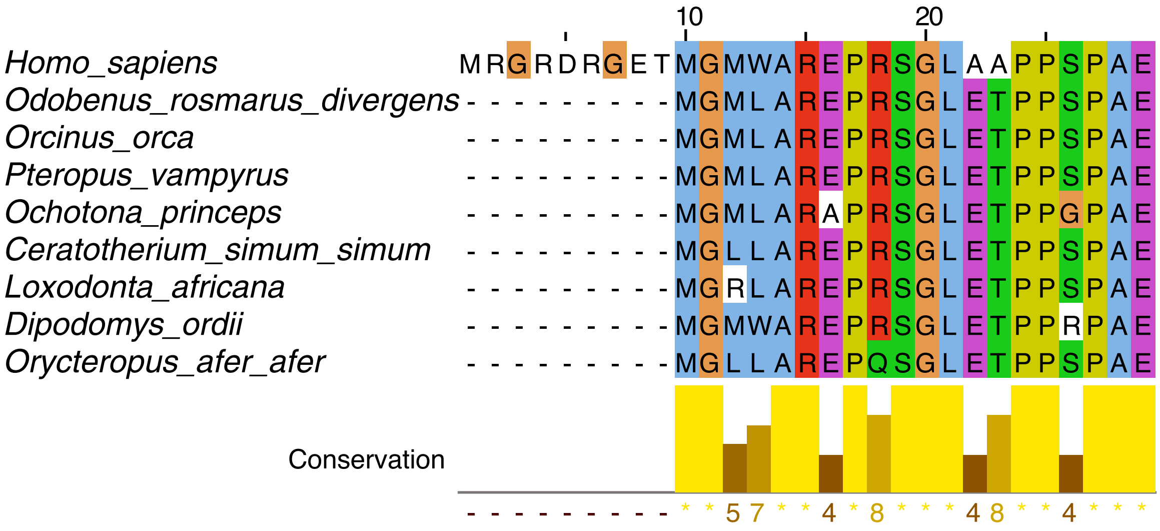

HG0135

ENSDARG00000094132 (igf1)

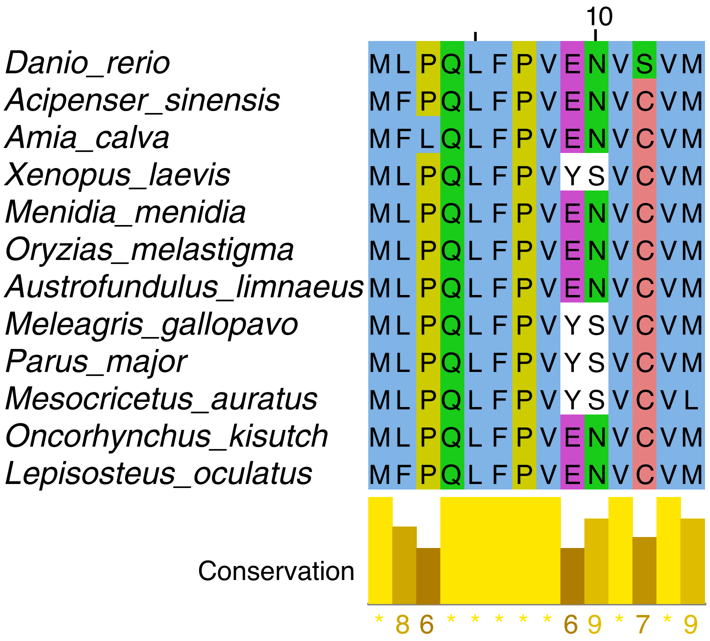

HG0135

ENSG00000017427 (IGF1)

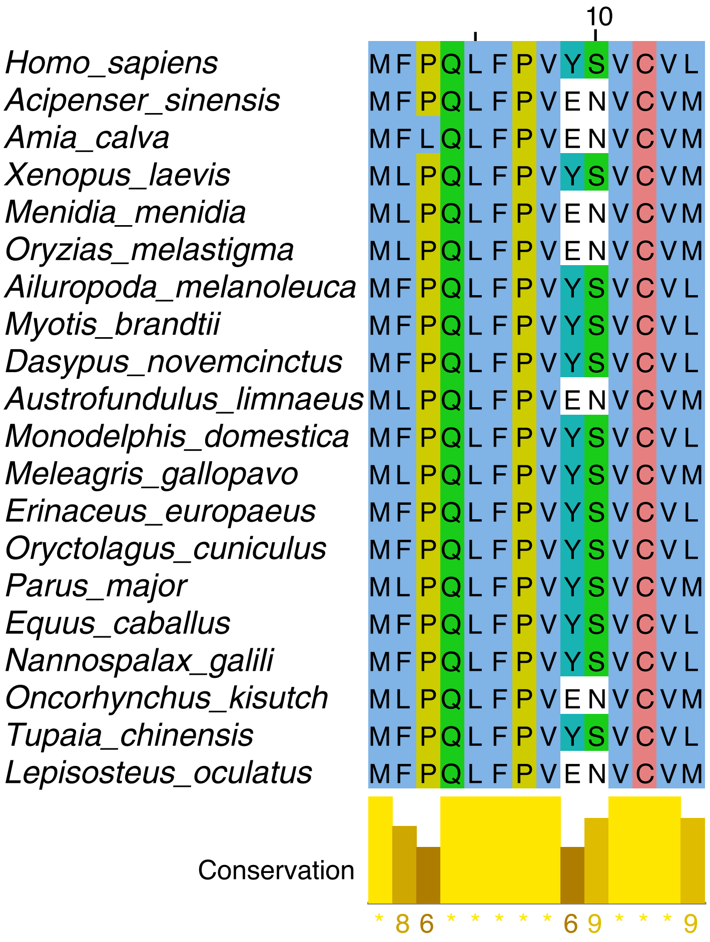

HG0136.1

ENSG00000125845 (BMP2)

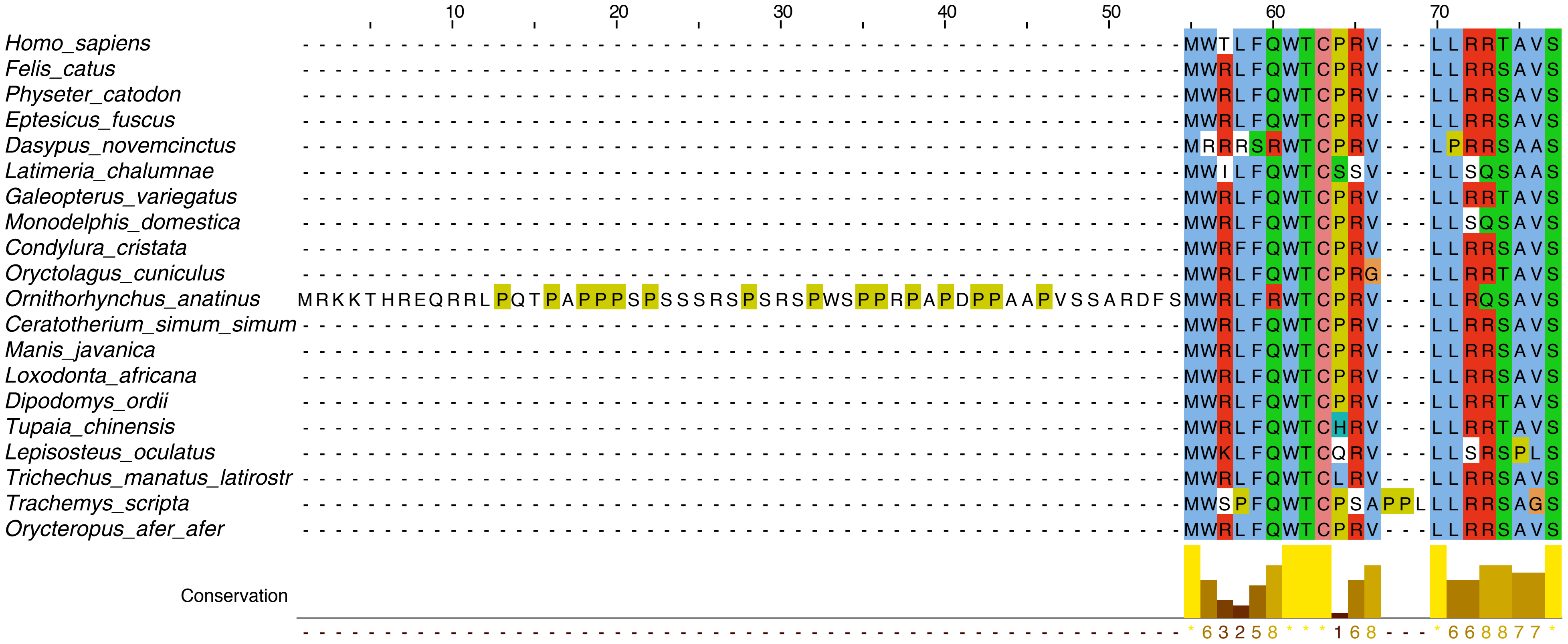

HG0136.2

ENSG00000125845 (BMP2)

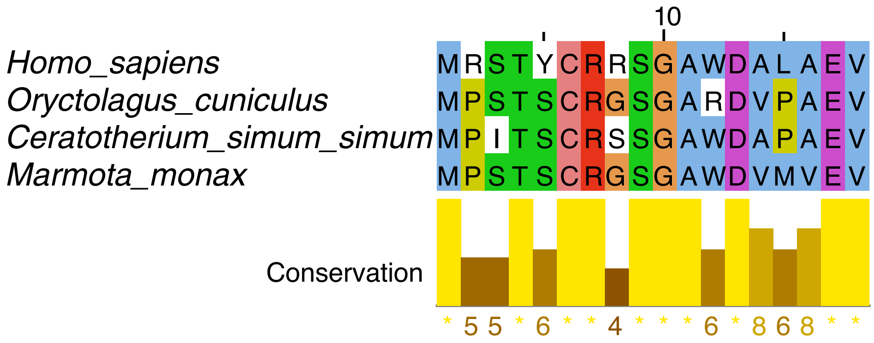

HG0137.1

ENSG00000181722 (ZBTB20)

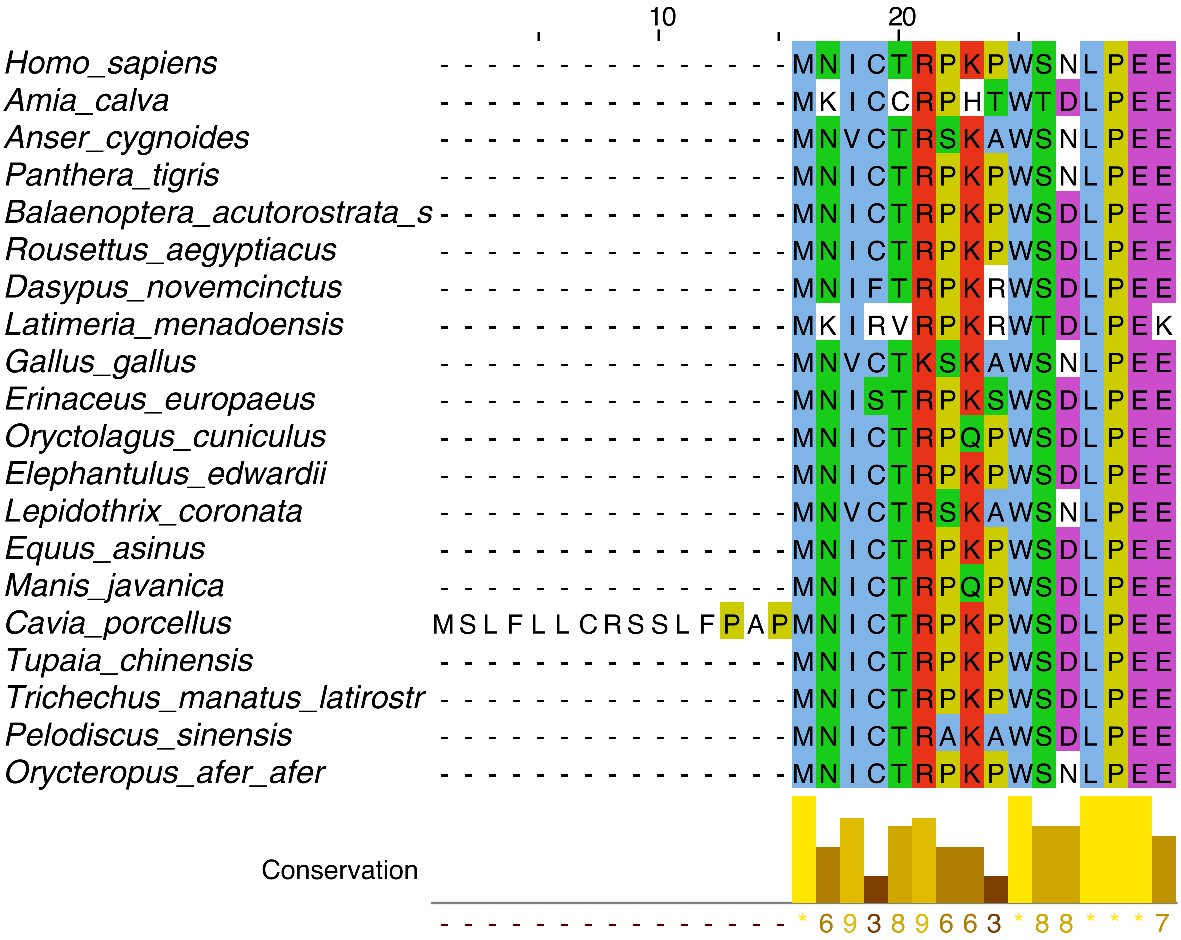

HG0137.2

ENSG00000181722 (ZBTB20)

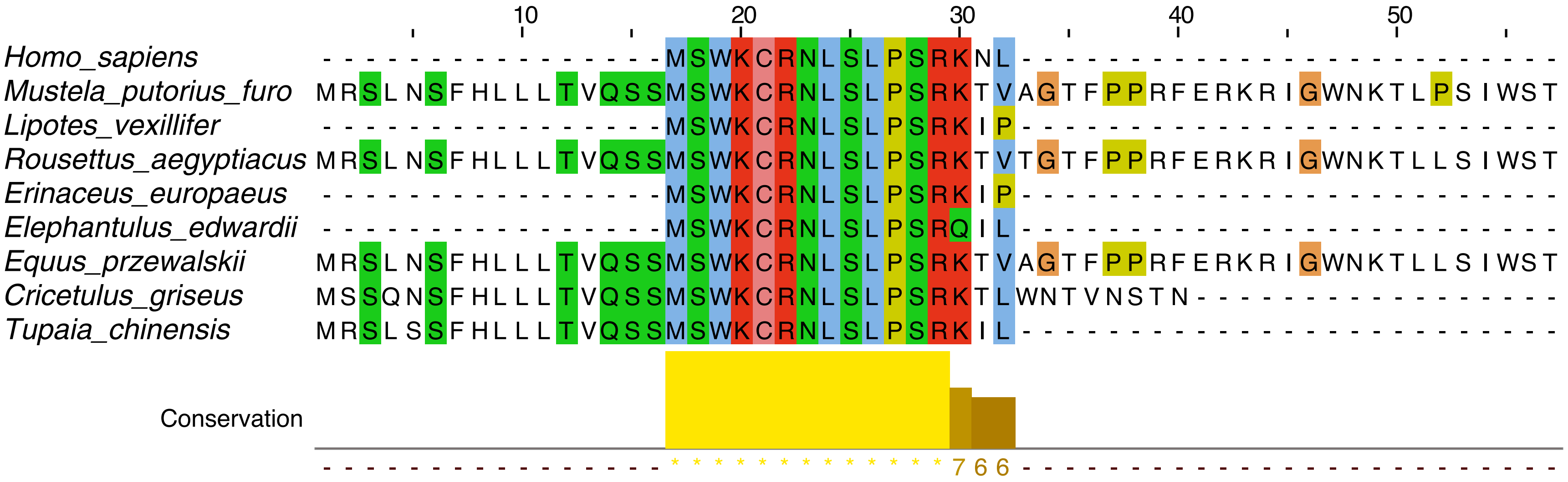

HG0138.1

ENSG00000185070 (FLRT2)

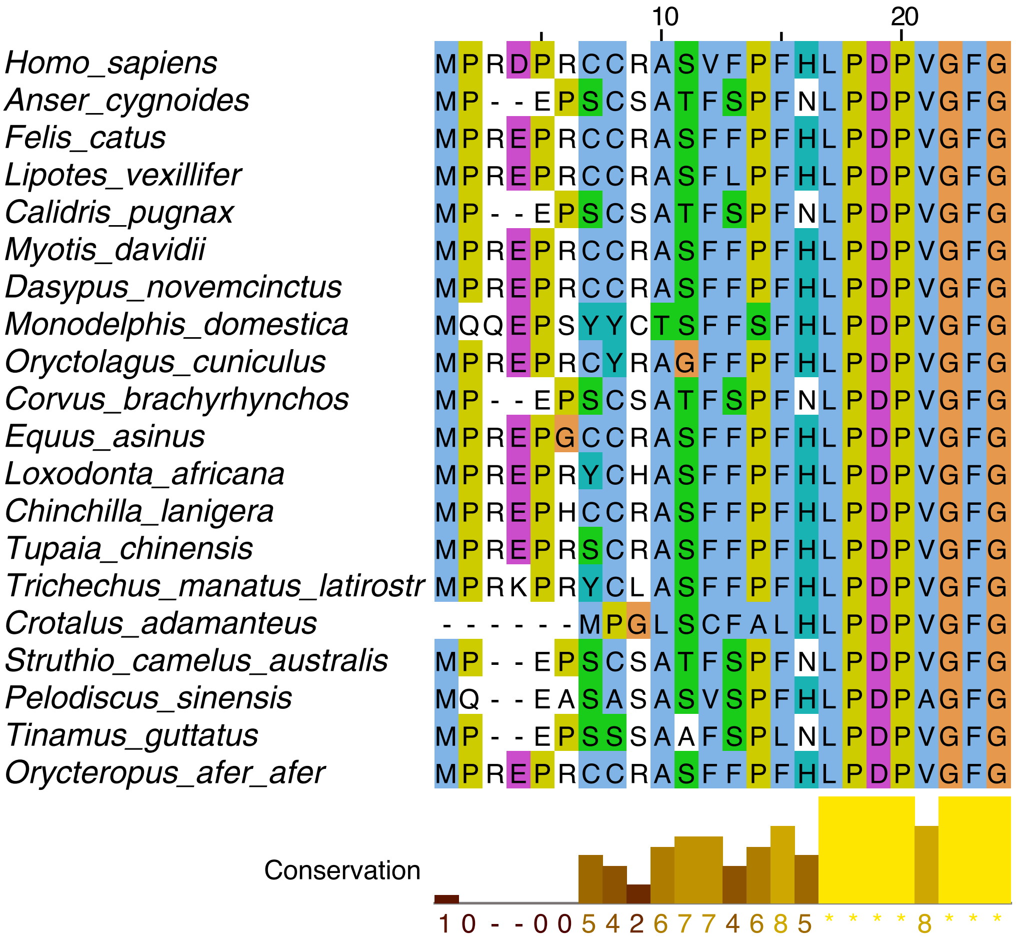

HG0138.2

ENSG00000185070 (FLRT2)

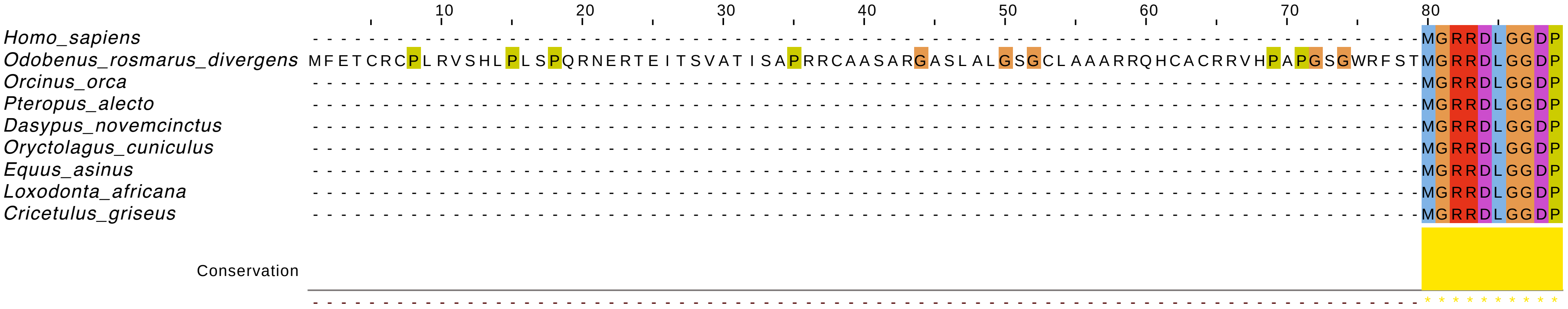

HG0139

ENSGALG00000006379 (SHH)

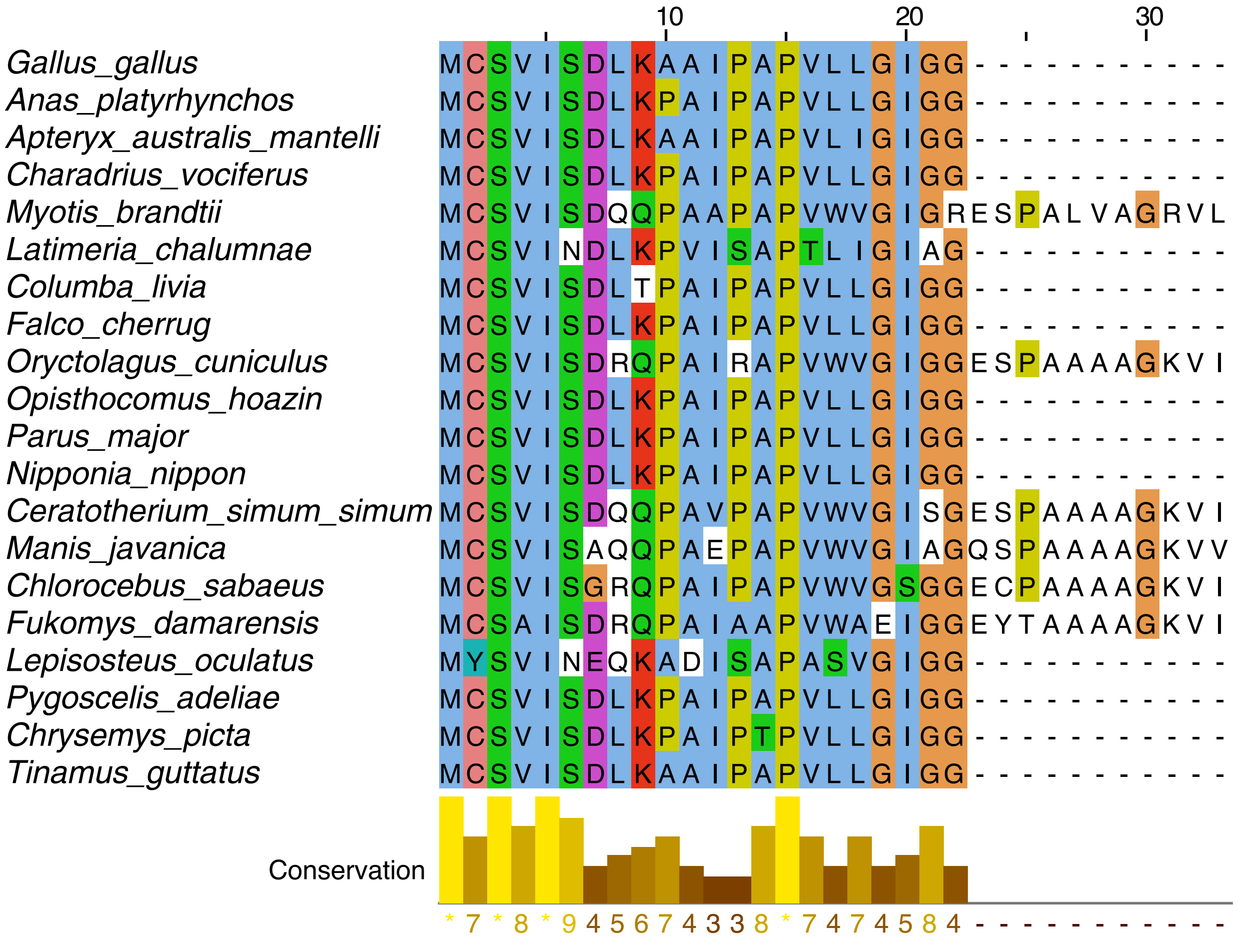

HG0140

ENSG00000009307 (CSDE1)

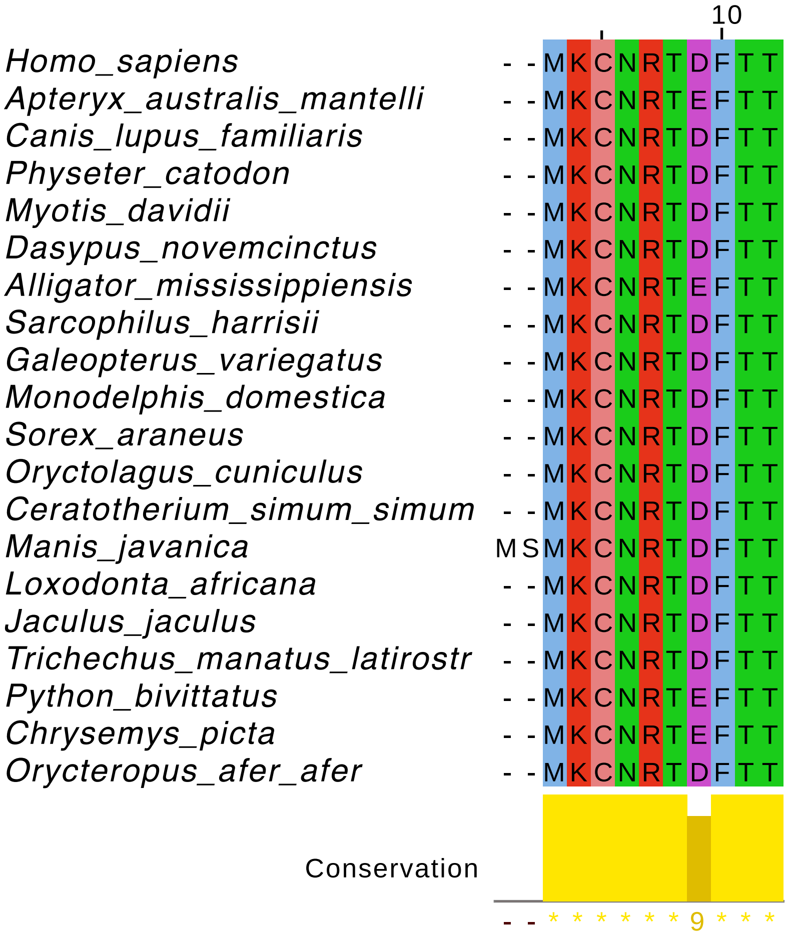

HG0141.1

ENSGALG00000010435 (RASGRP3)

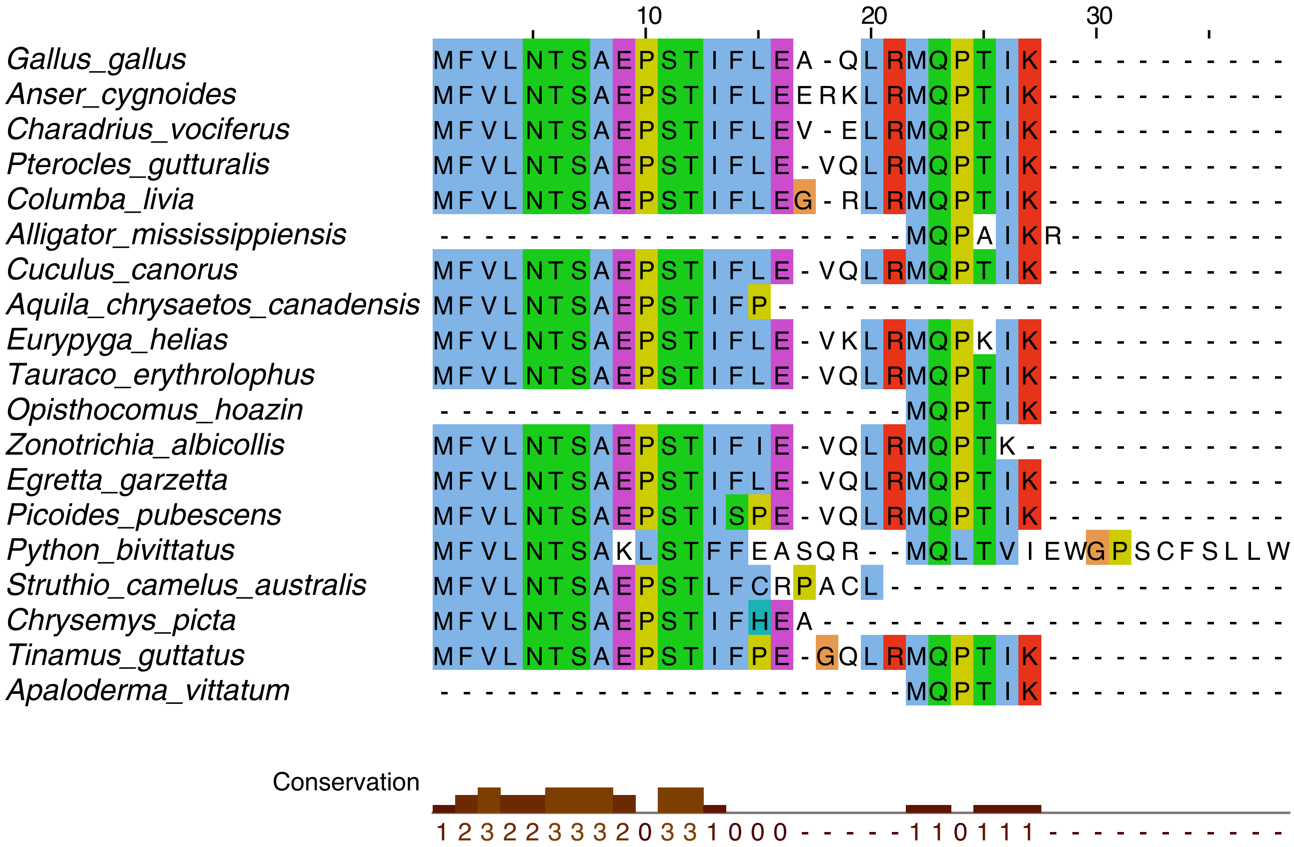

HG0141.2

ENSG00000152689 (RASGRP3)

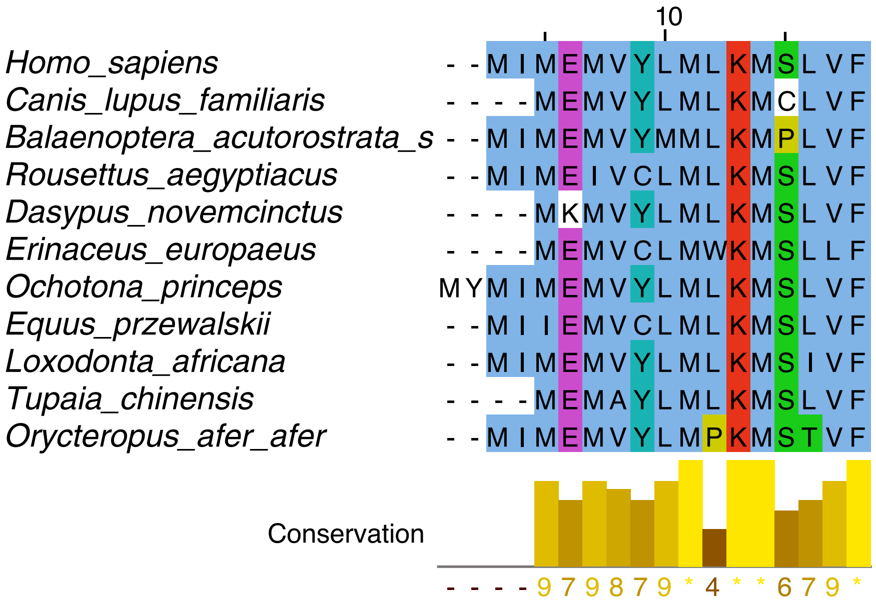

HG0141.3

ENSG00000152689 (RASGRP3)

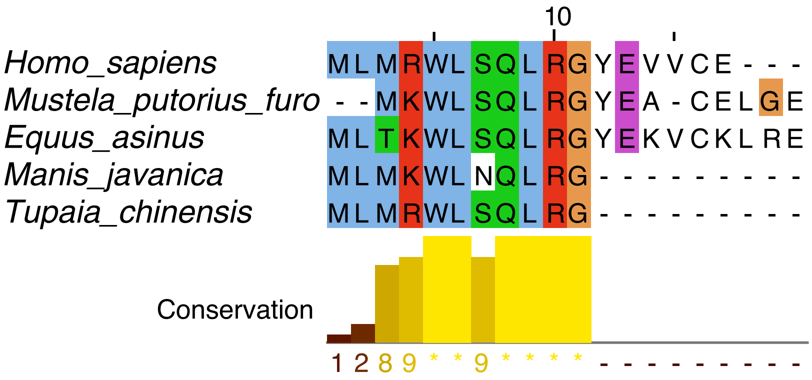

HG0142.1

ENSG00000204217 (BMPR2)

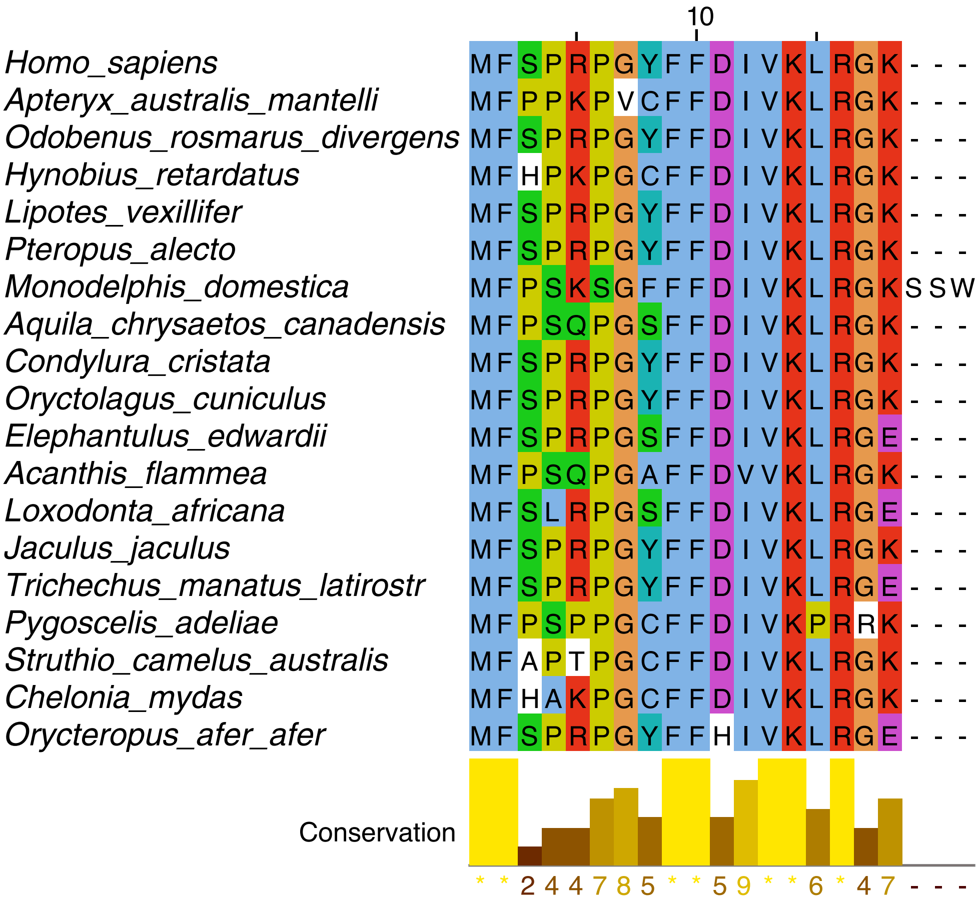

HG0142.2

ENSG00000204217 (BMPR2)

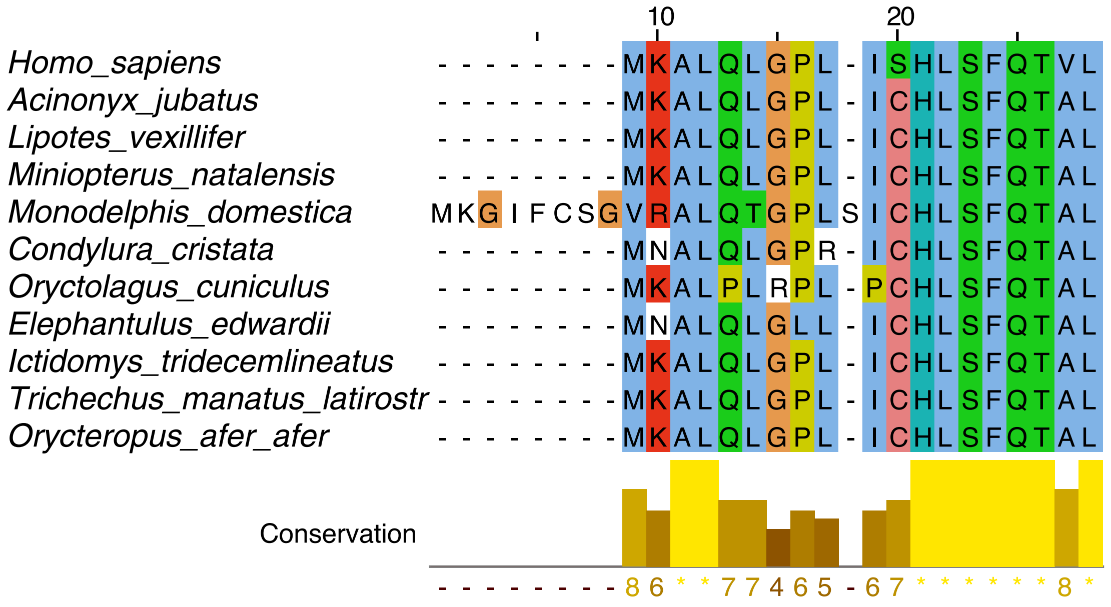

HG0143

ENSG00000101958 (GLRA2)

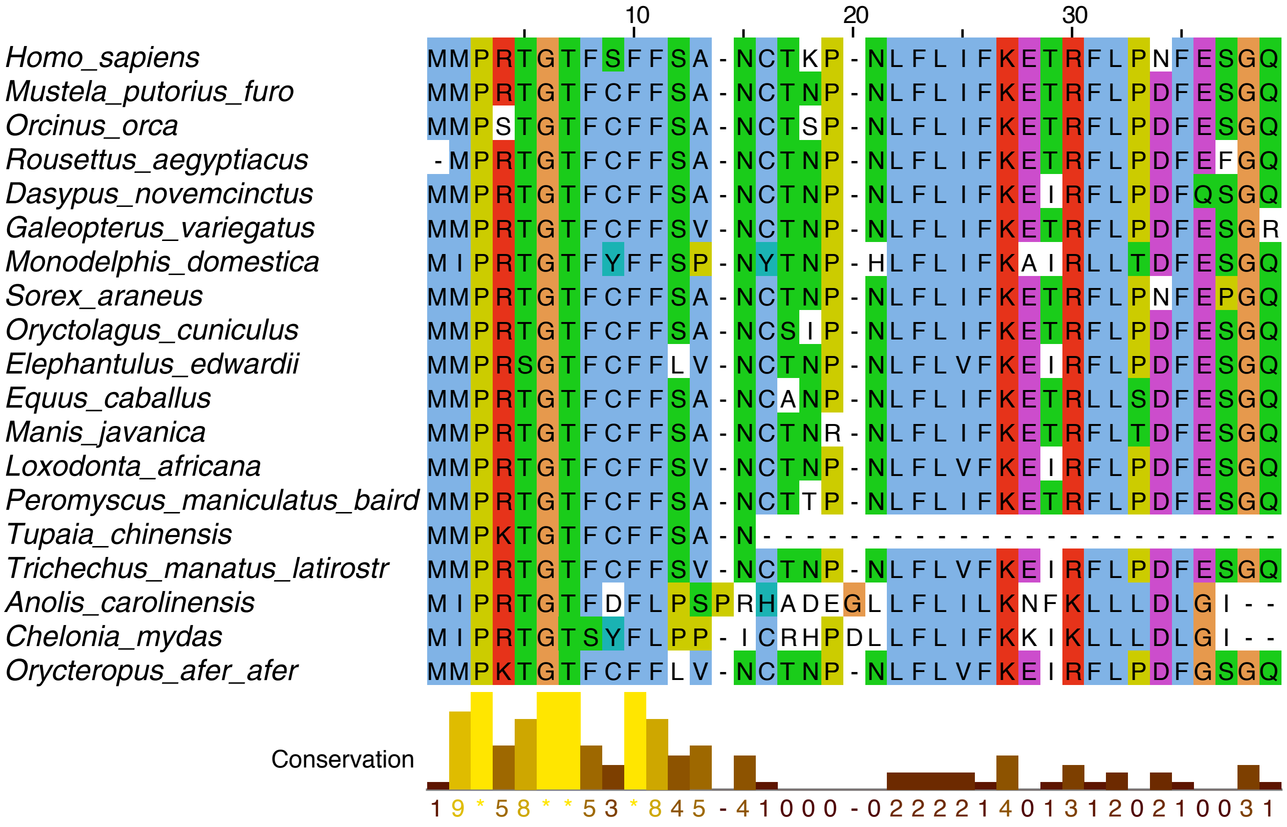

HG0144

ENSG00000117114 (ADGRL2)

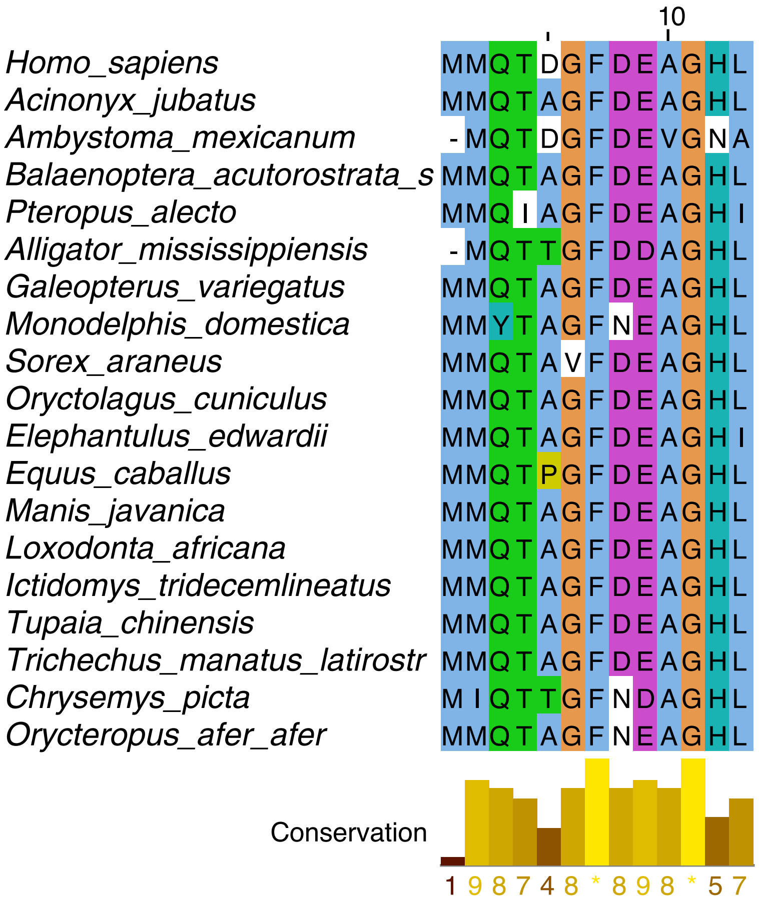

HG0145

ENSG00000162599 (NFIA)

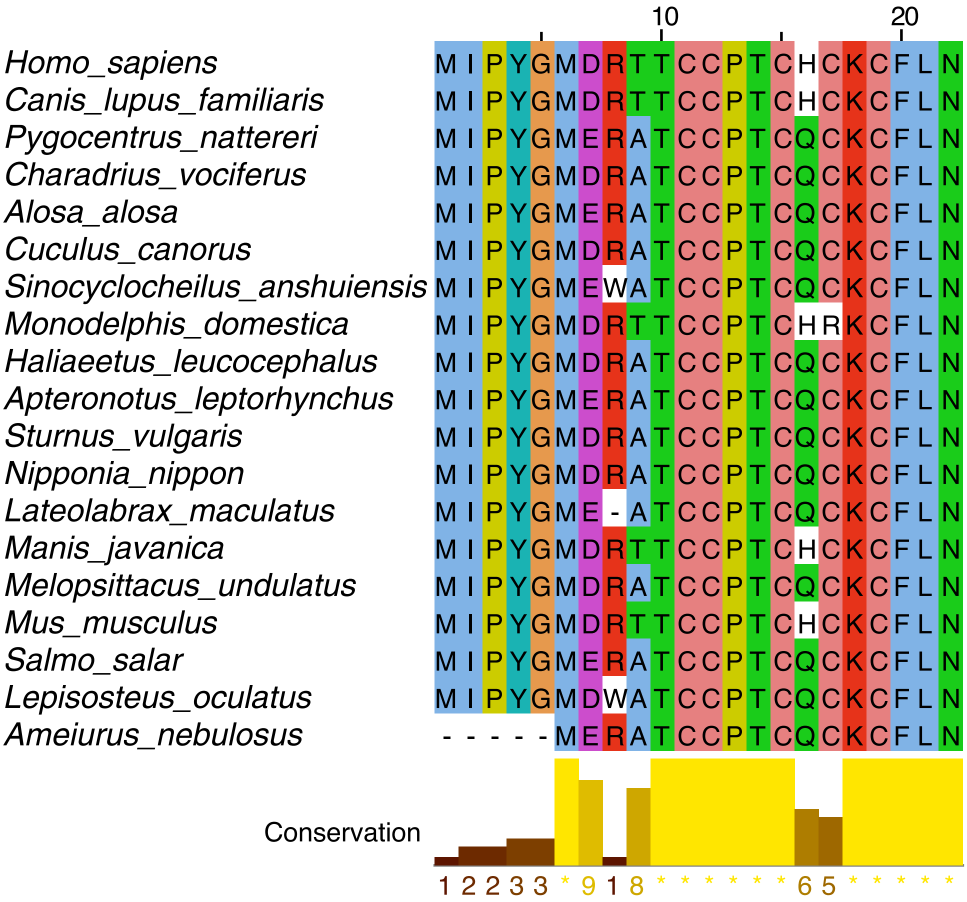

HG0146.1

ENSG00000163681 (SLMAP)

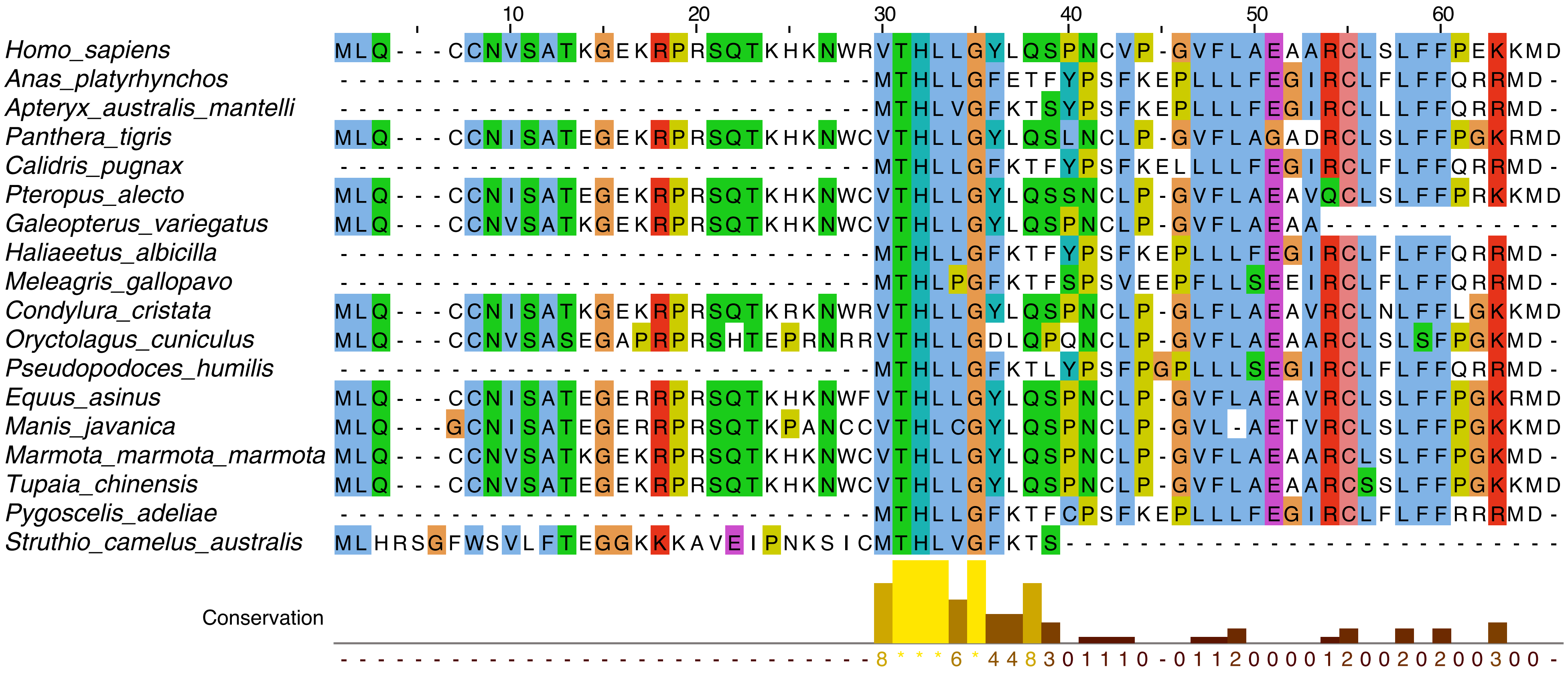

HG0146.2

ENSG00000163681 (SLMAP)

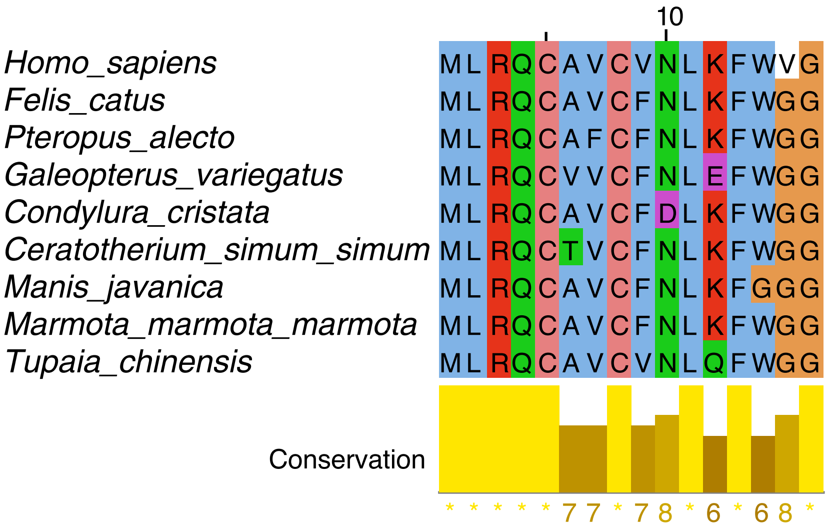

HG0146.3

ENSG00000163681 (SLMAP)

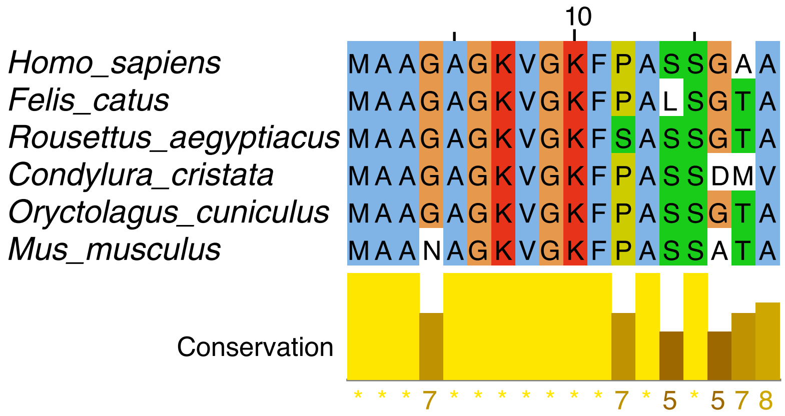

HG0147

ENSG00000108984 (MAP2K6)

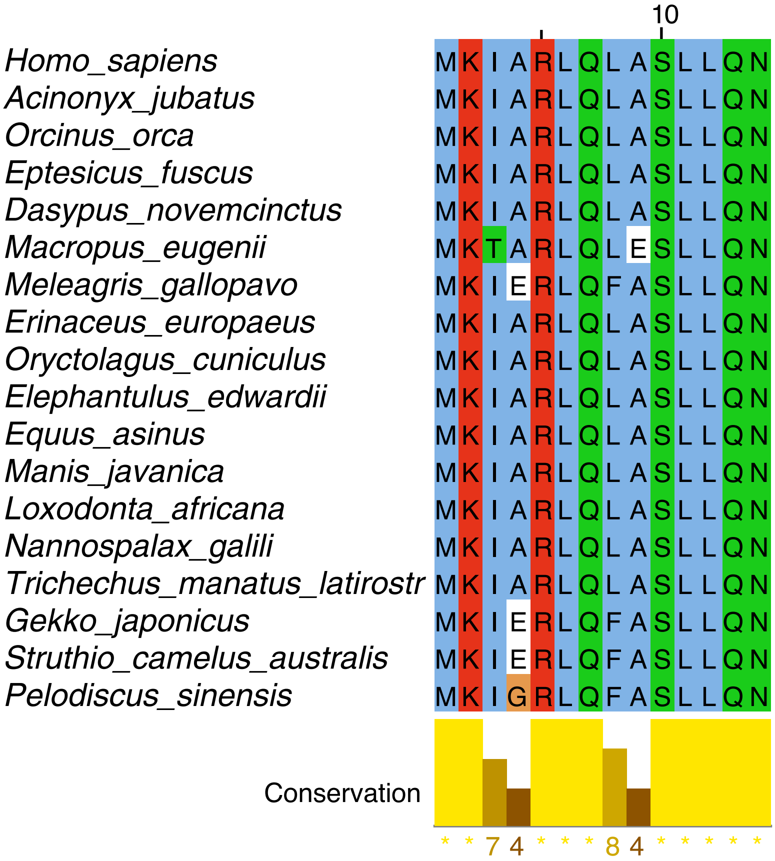

HG0147

ENSGALG00000004370 (MAP2K6)

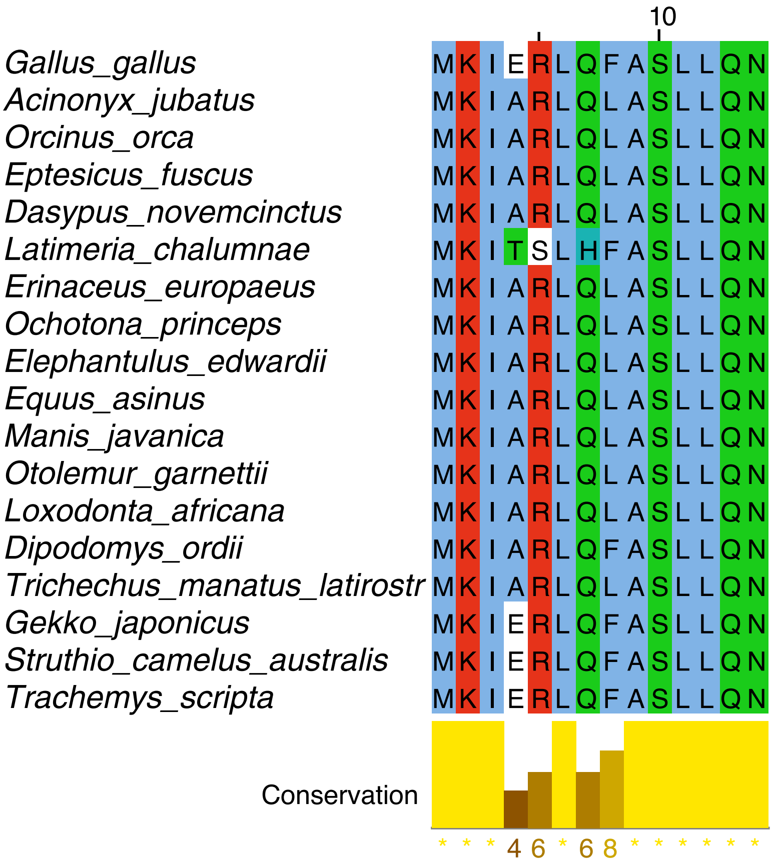

HG0148.1

ENSGALG00000011855 (RHOJ)

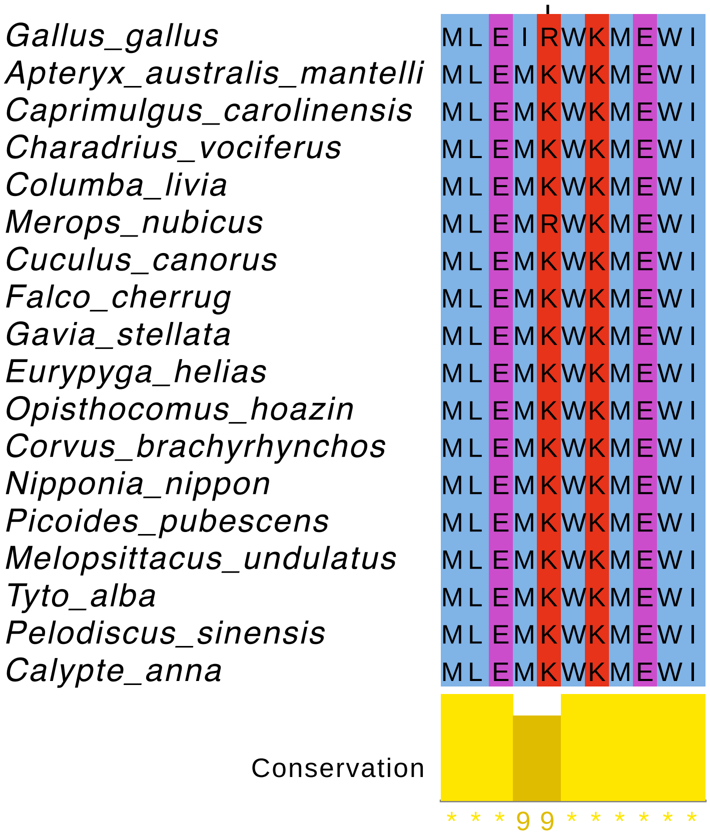

HG0148.2

ENSGALG00000011855 (RHOJ)

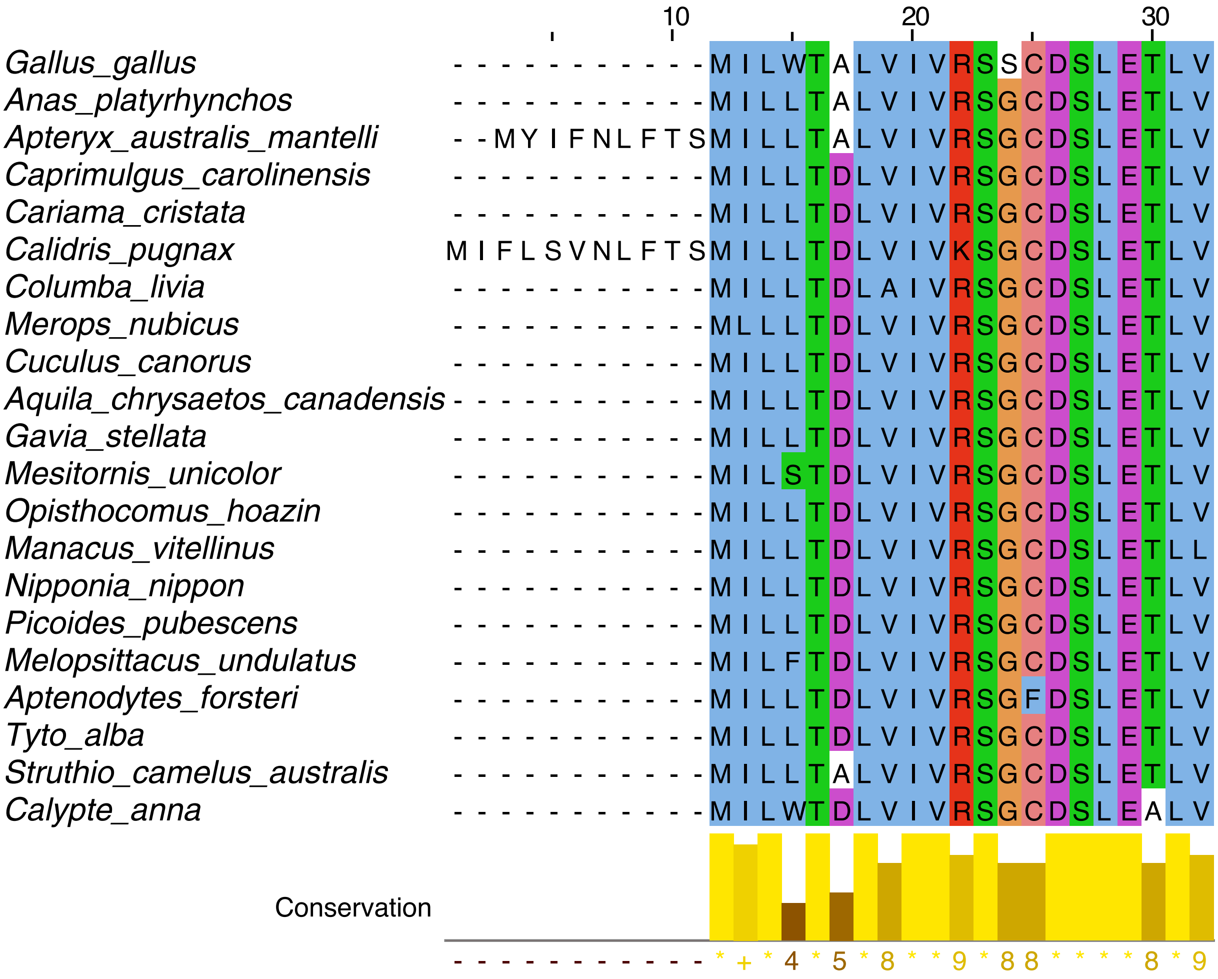

HG0149.1

ENSGALG00000014645 (MEF2C)

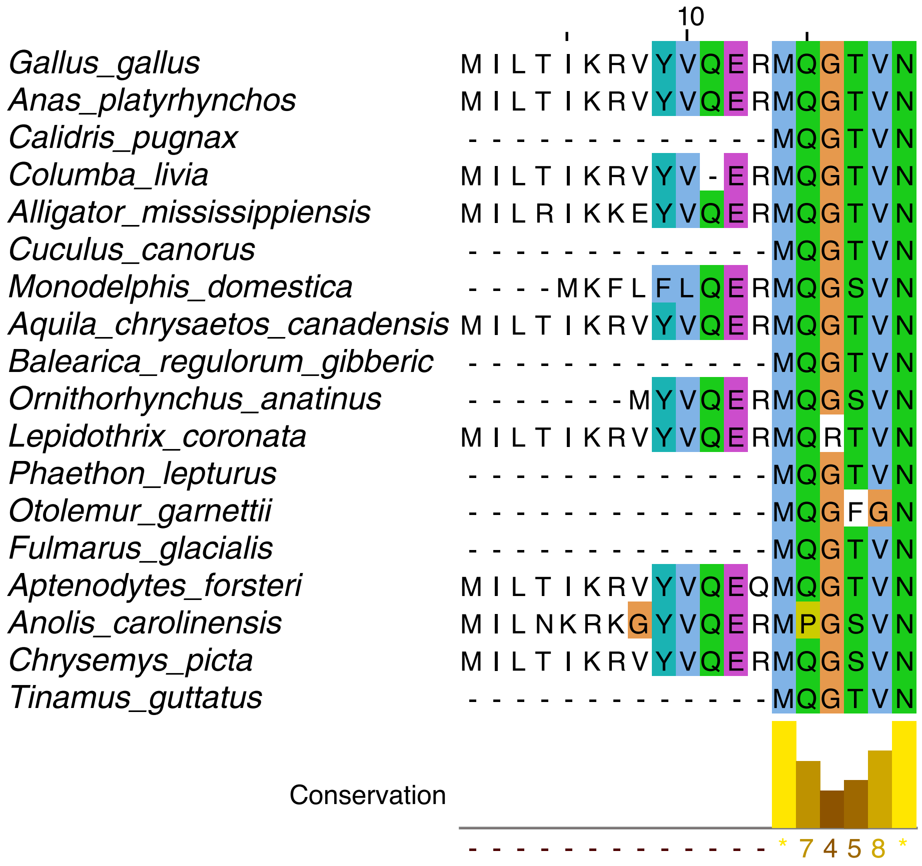

HG0149.2

ENSG00000081189 (MEF2C)

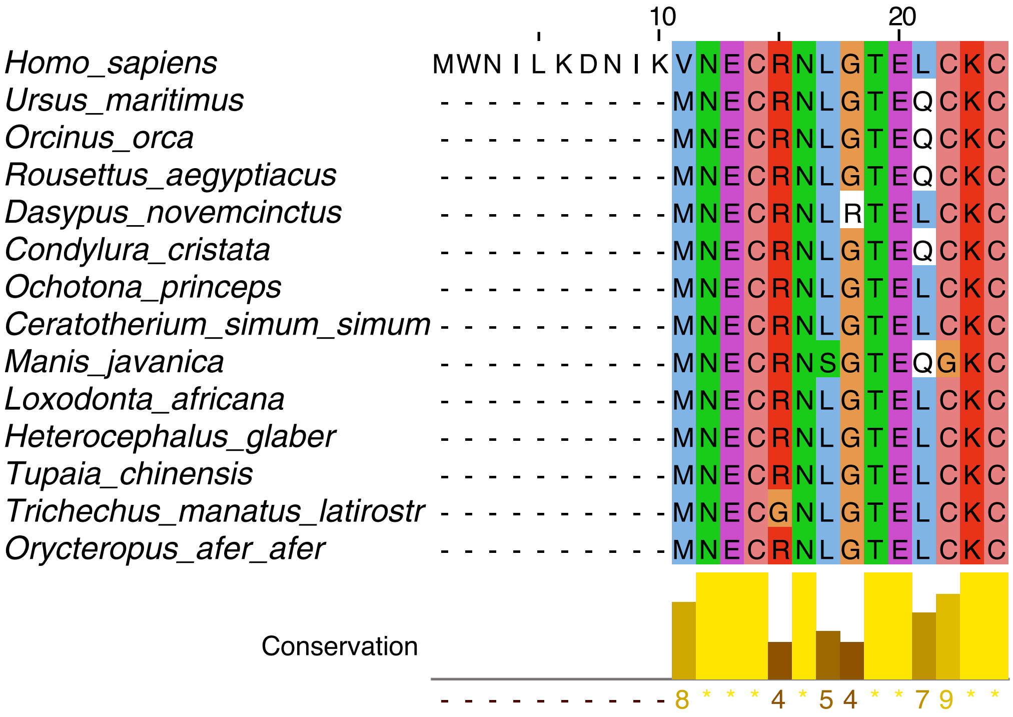

HG0150.1

ENSGALG00000032289 (IKZF5)

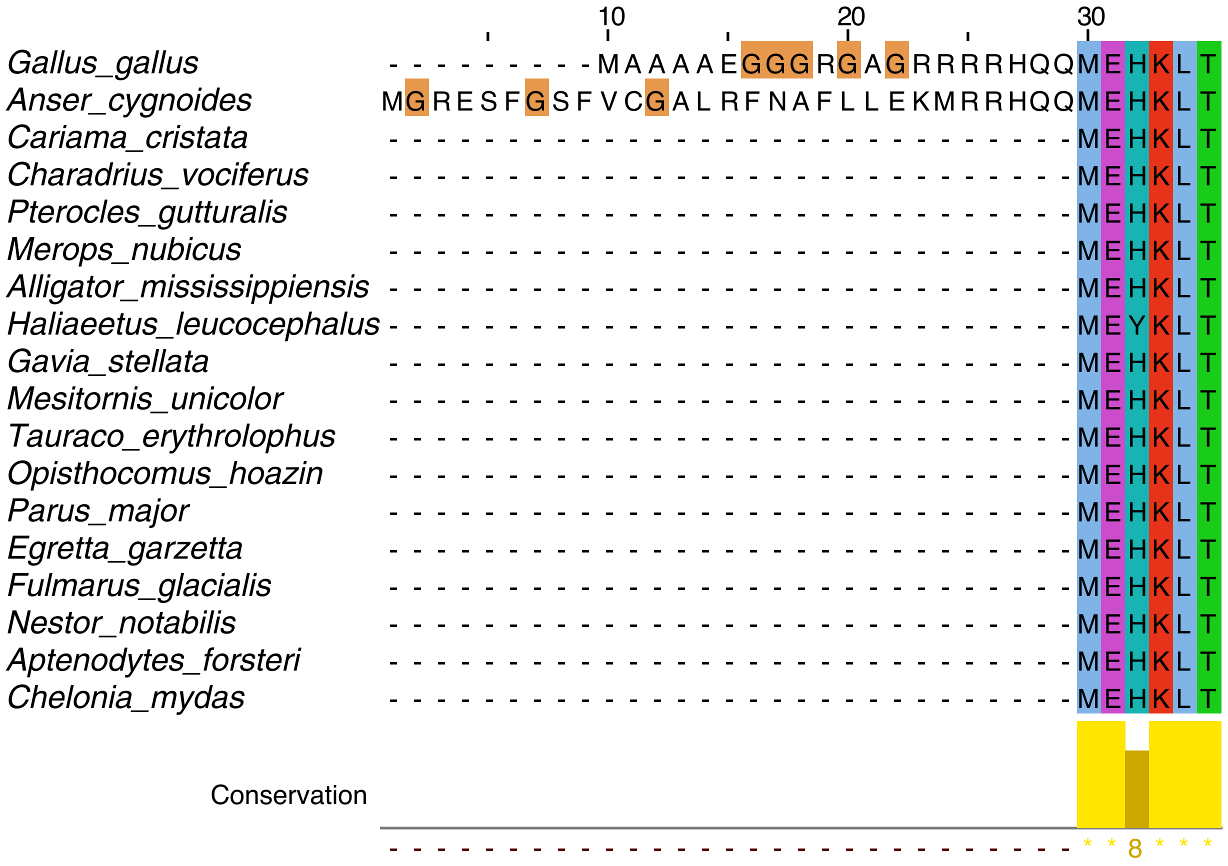

HG0150.2

ENSG00000095574 (IKZF5)

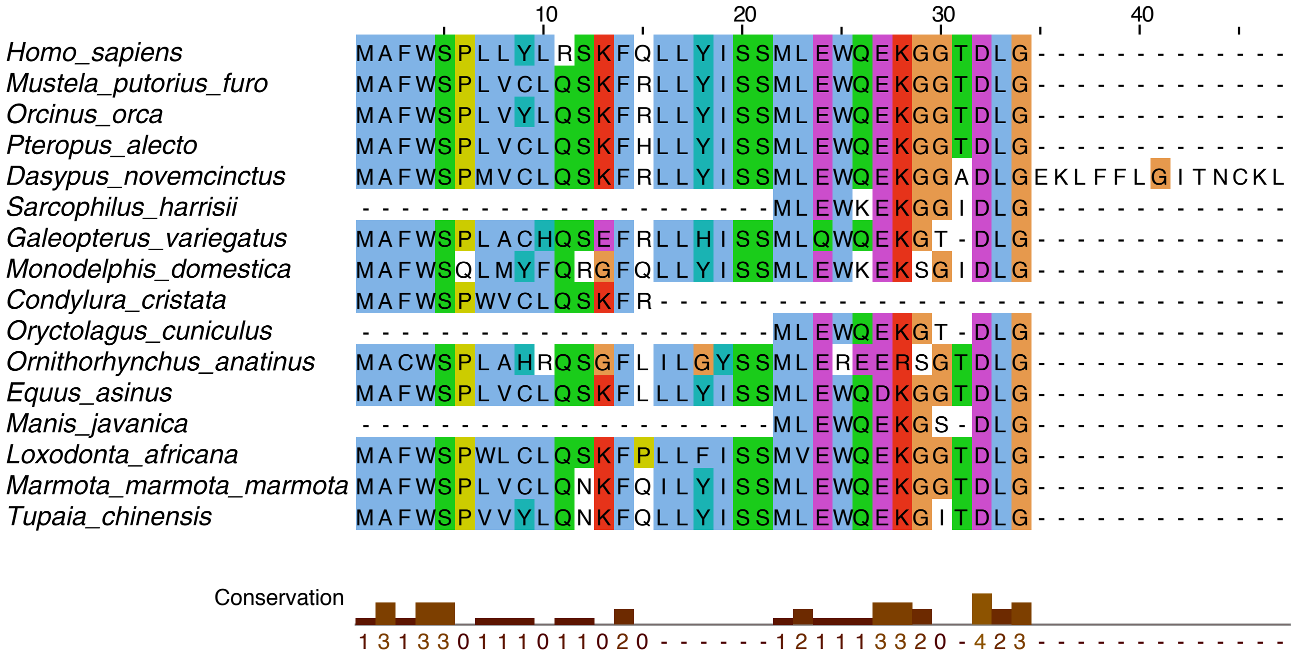

HG0151

ENSG00000006468 (ETV1)

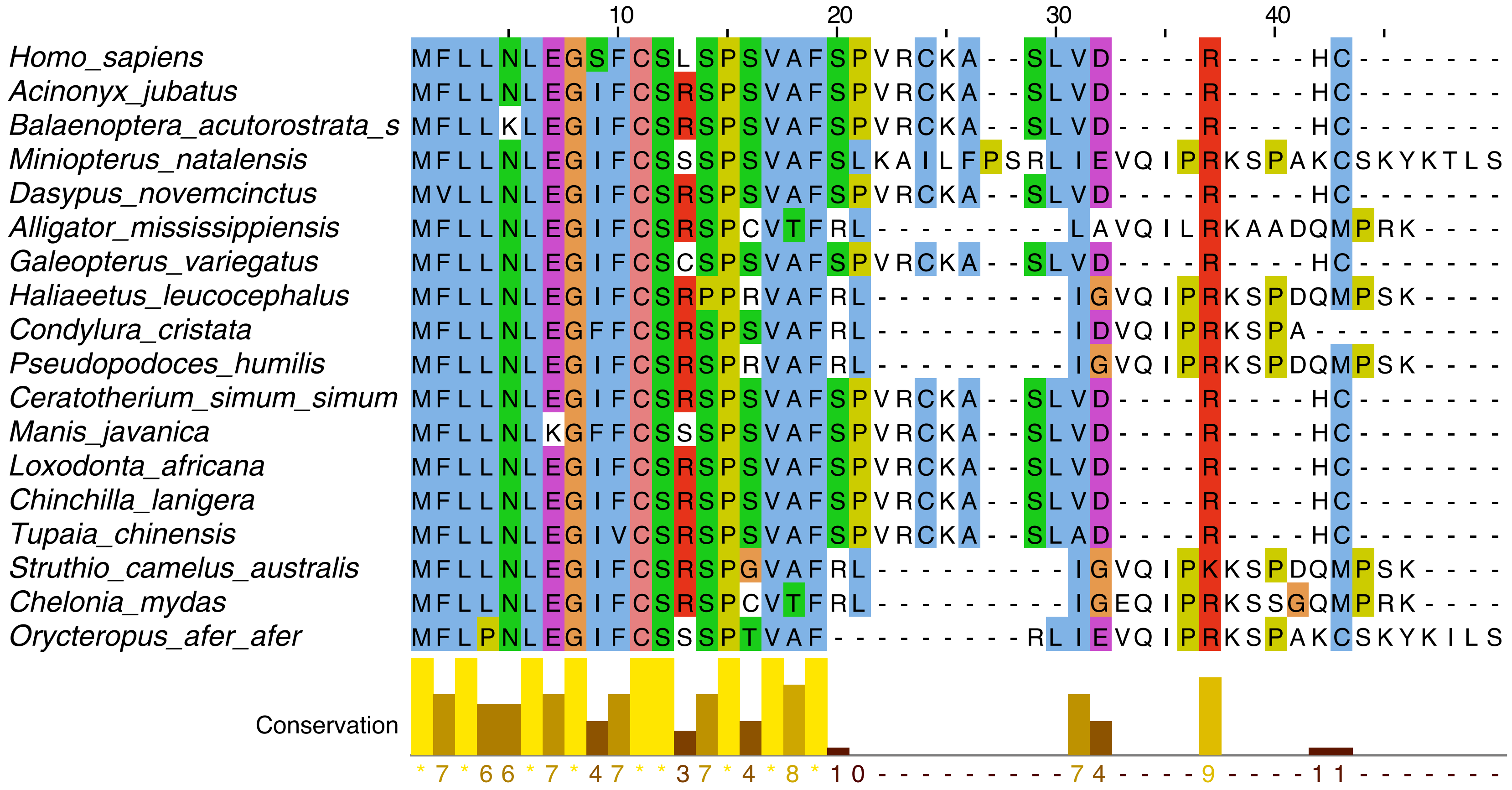

HG0151

ENSGALG00000035504 (ER81)

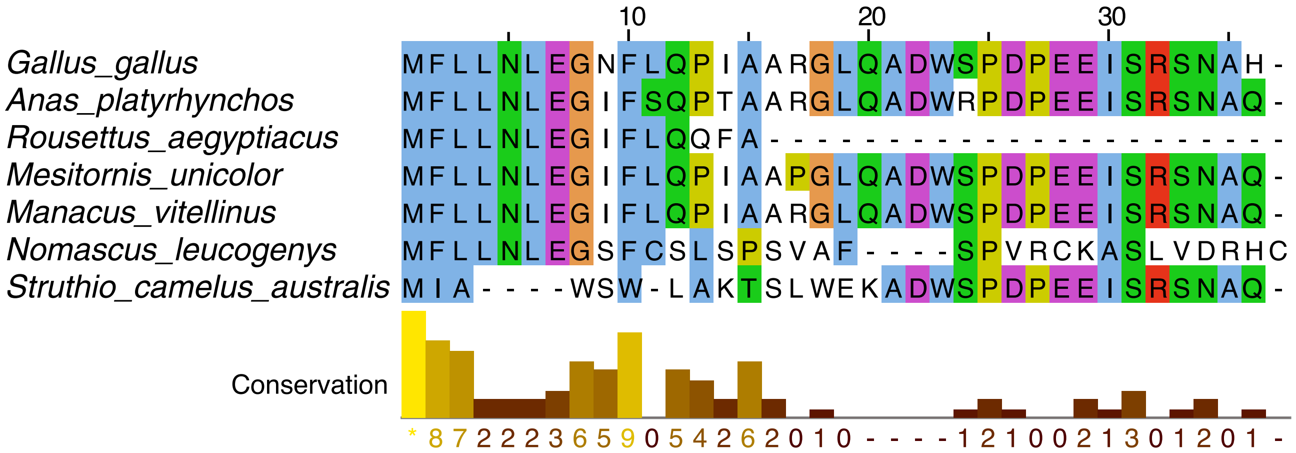

HG0152

ENSDARG00000015536 (sox6)

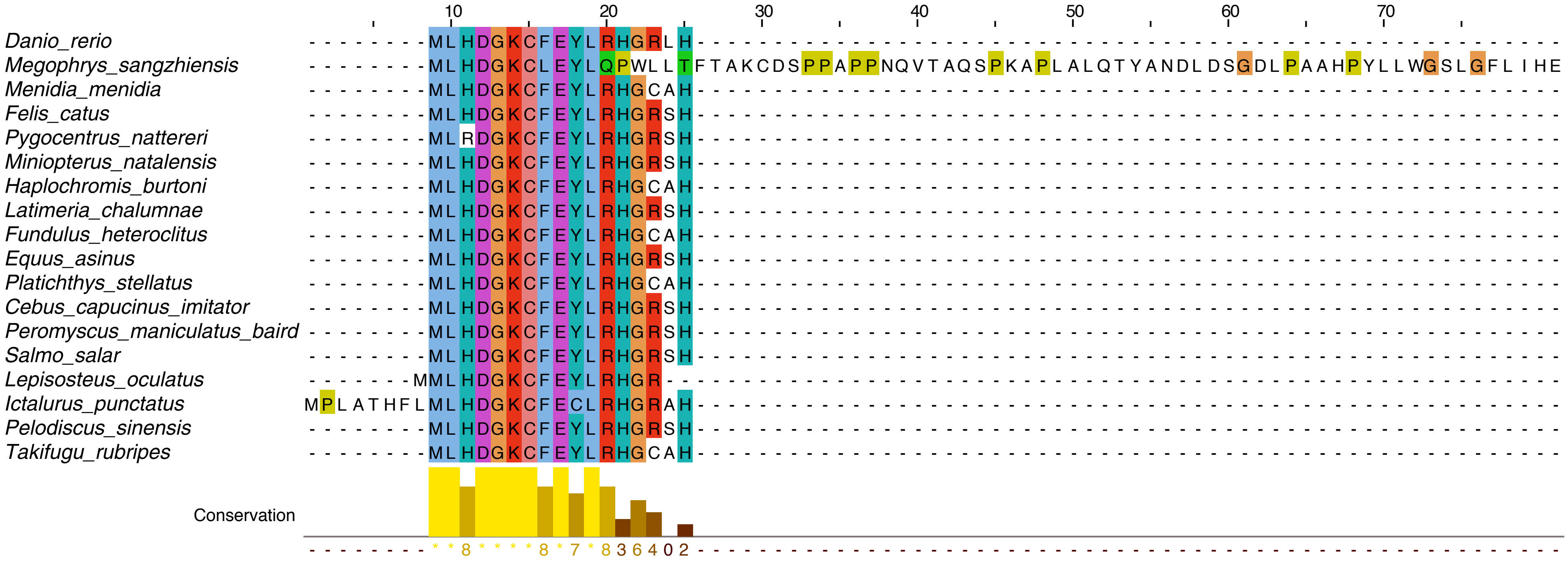

HG0153

ENSGALG00000003136 (IKZF2)

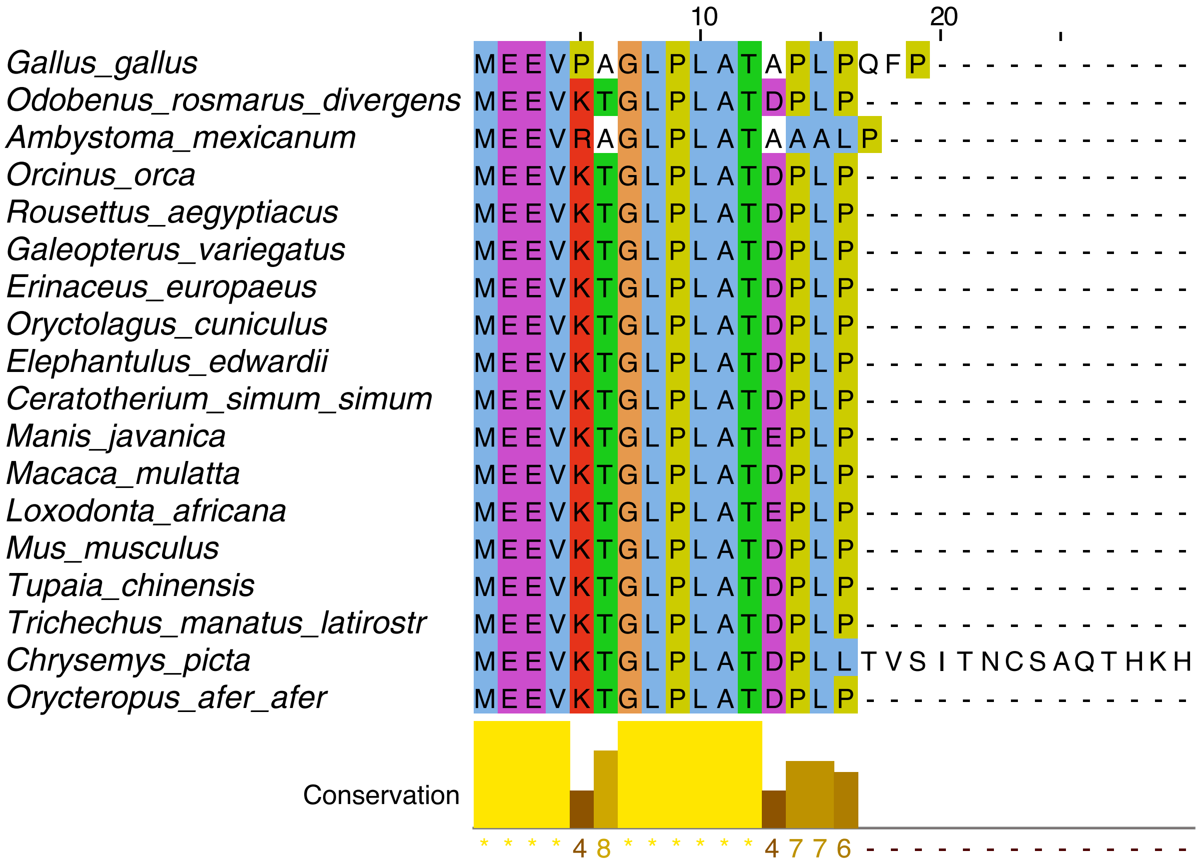

HG0154

ENSG00000128573 (FOXP2)

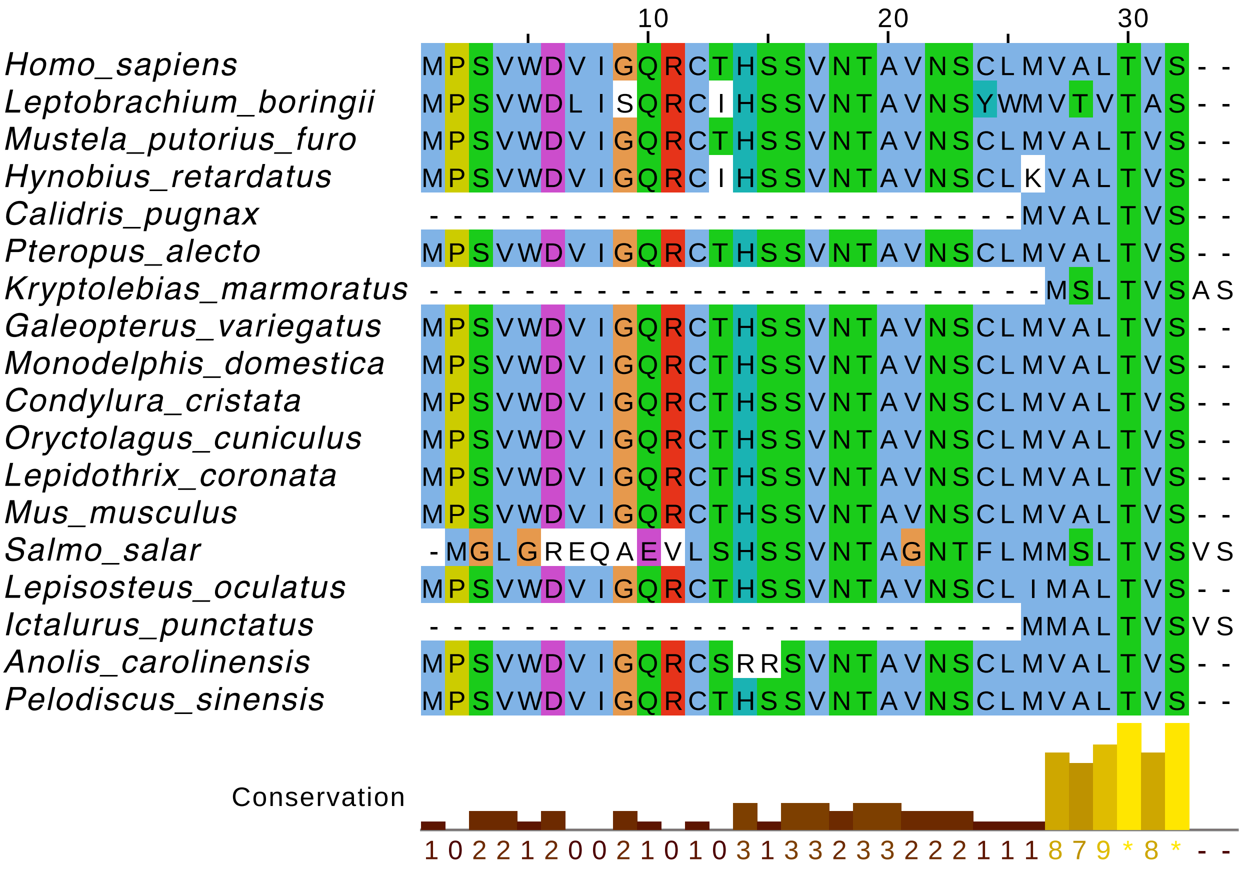

HG0155

ENSG00000136535 (TBR1)

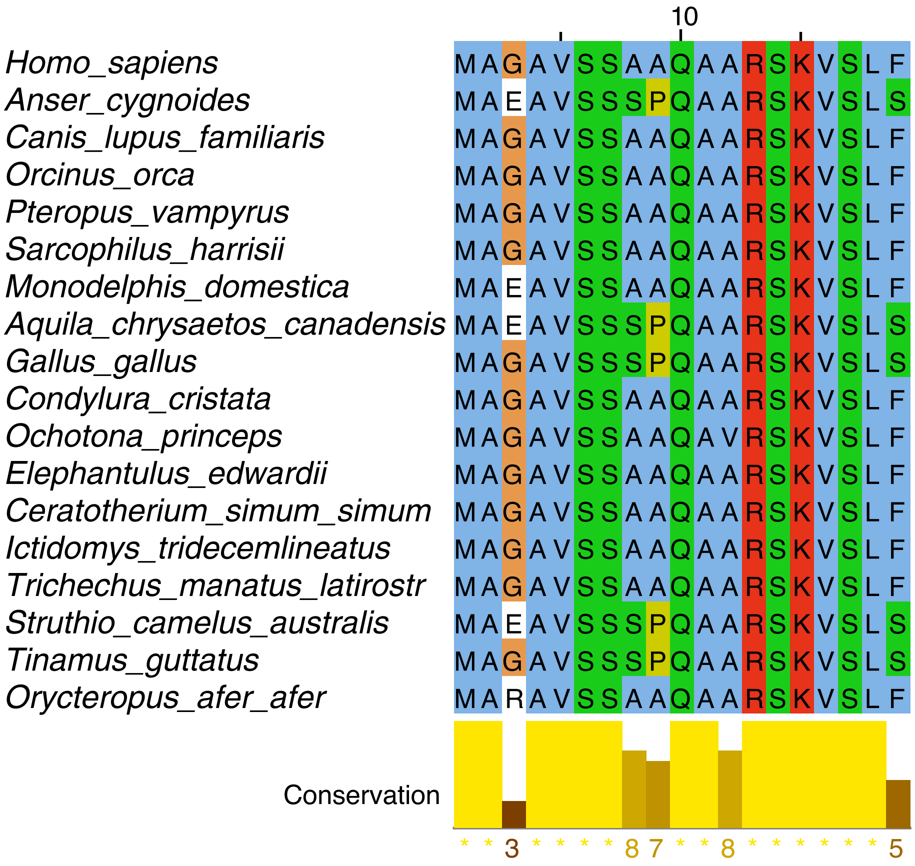

HG0156

ENSG00000156113 (KCNMA1)

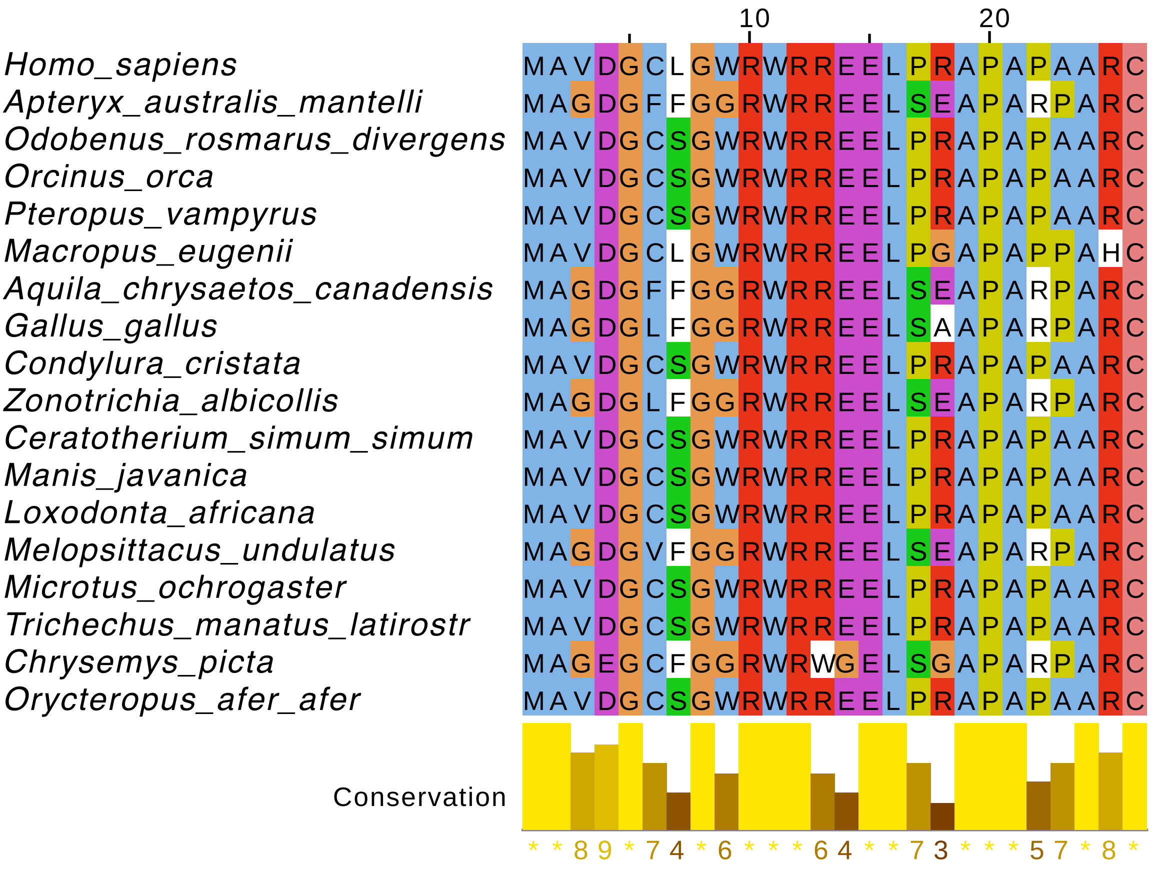

HG0157

ENSG00000173926 (3-Mar)

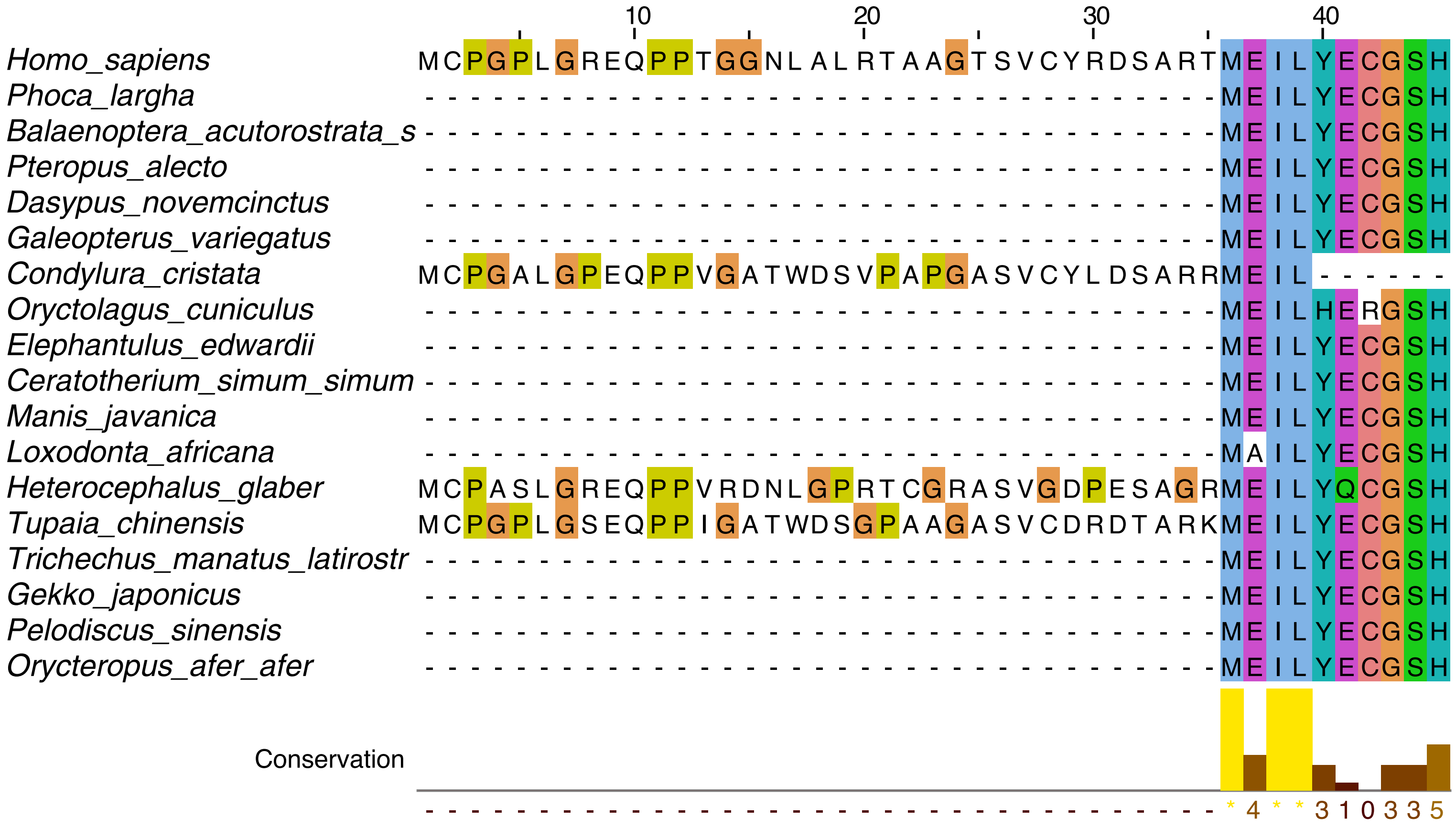

HG0158

ENSG00000198939 (ZFP2)

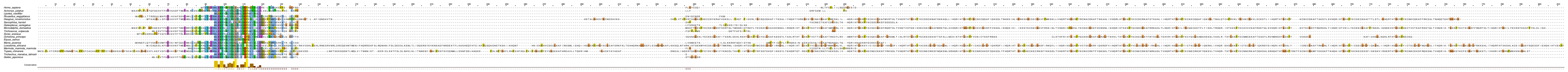

HG0159.1

ENSG00000182197 (EXT1)

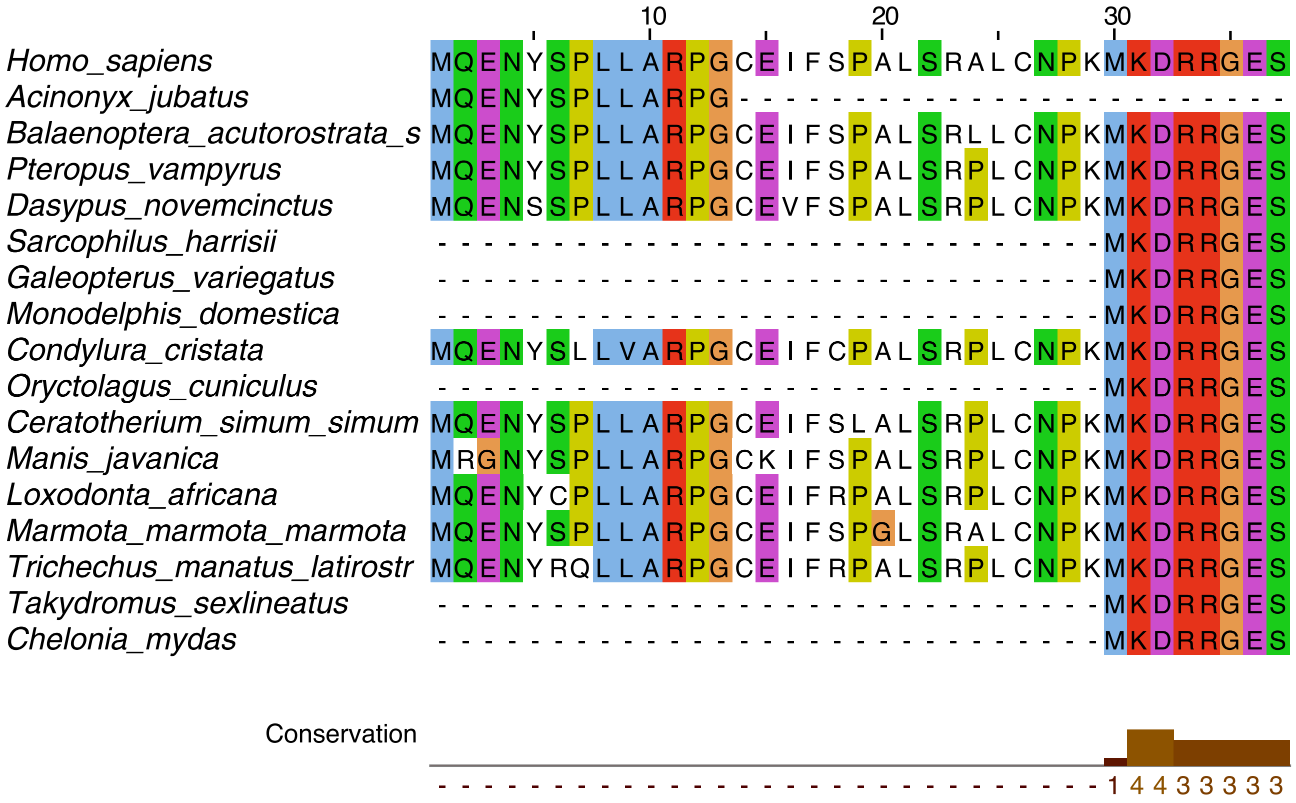

HG0159.2

ENSG00000182197 (EXT1)

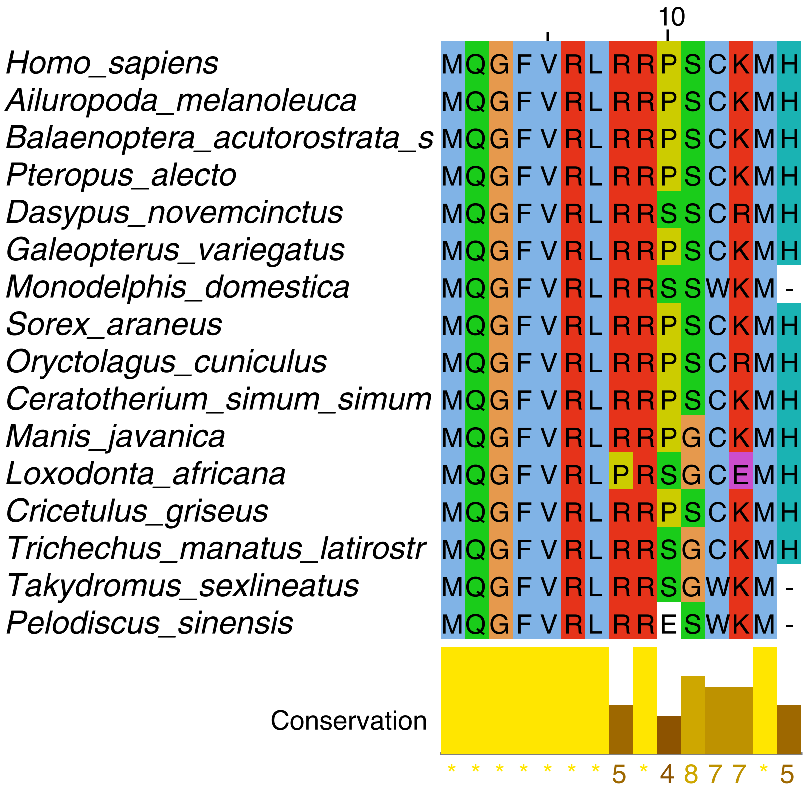

HG0160

ENSDARG00000060089 (btaf1)

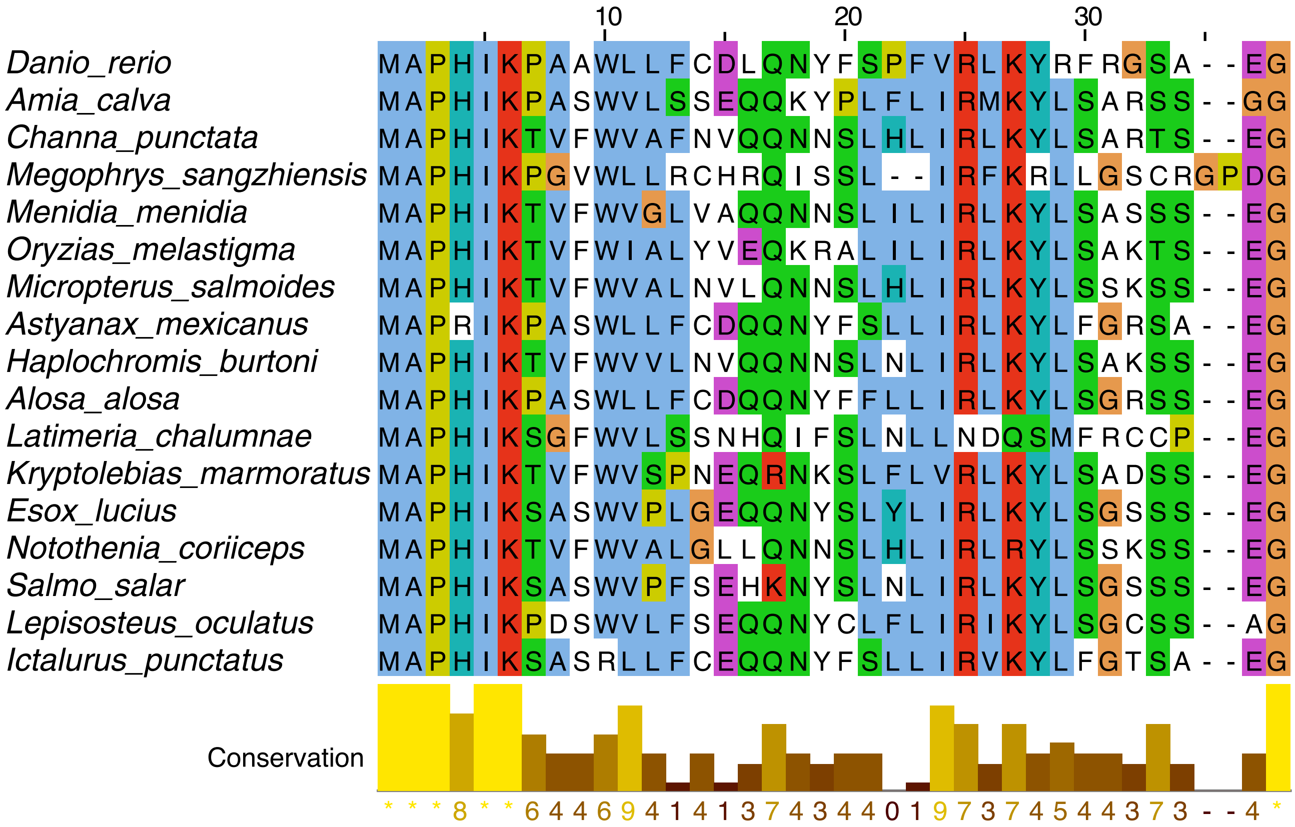

HG0161

ENSGALG00000017191

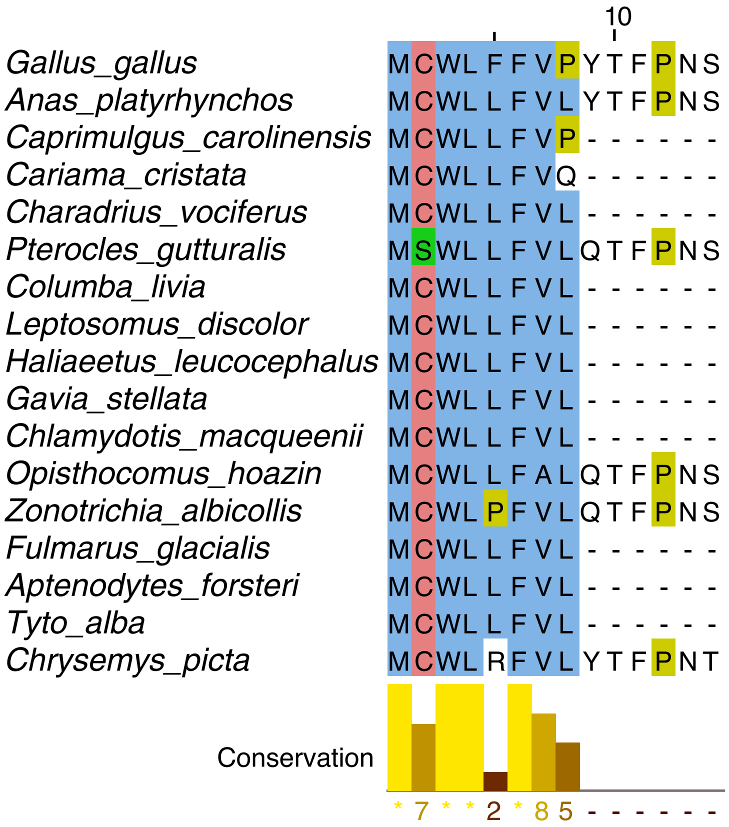

HG0162

ENSG00000138347 (MYPN)

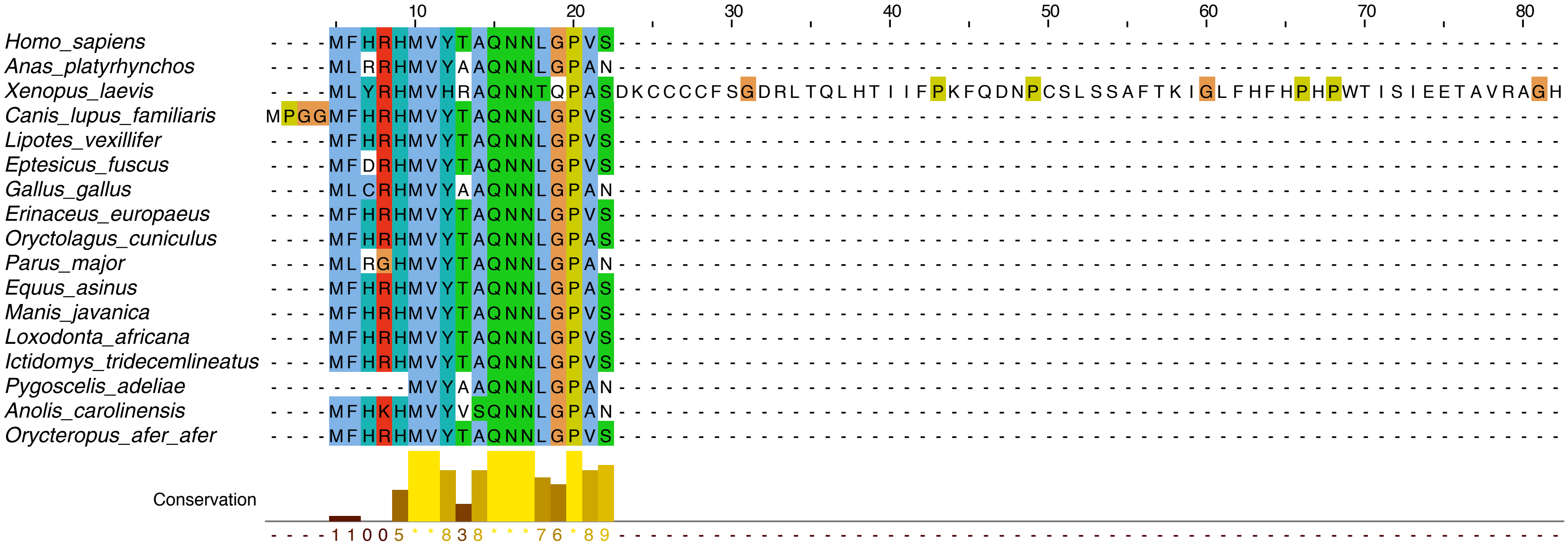

HG0163

ENSG00000146285 (SCML4)

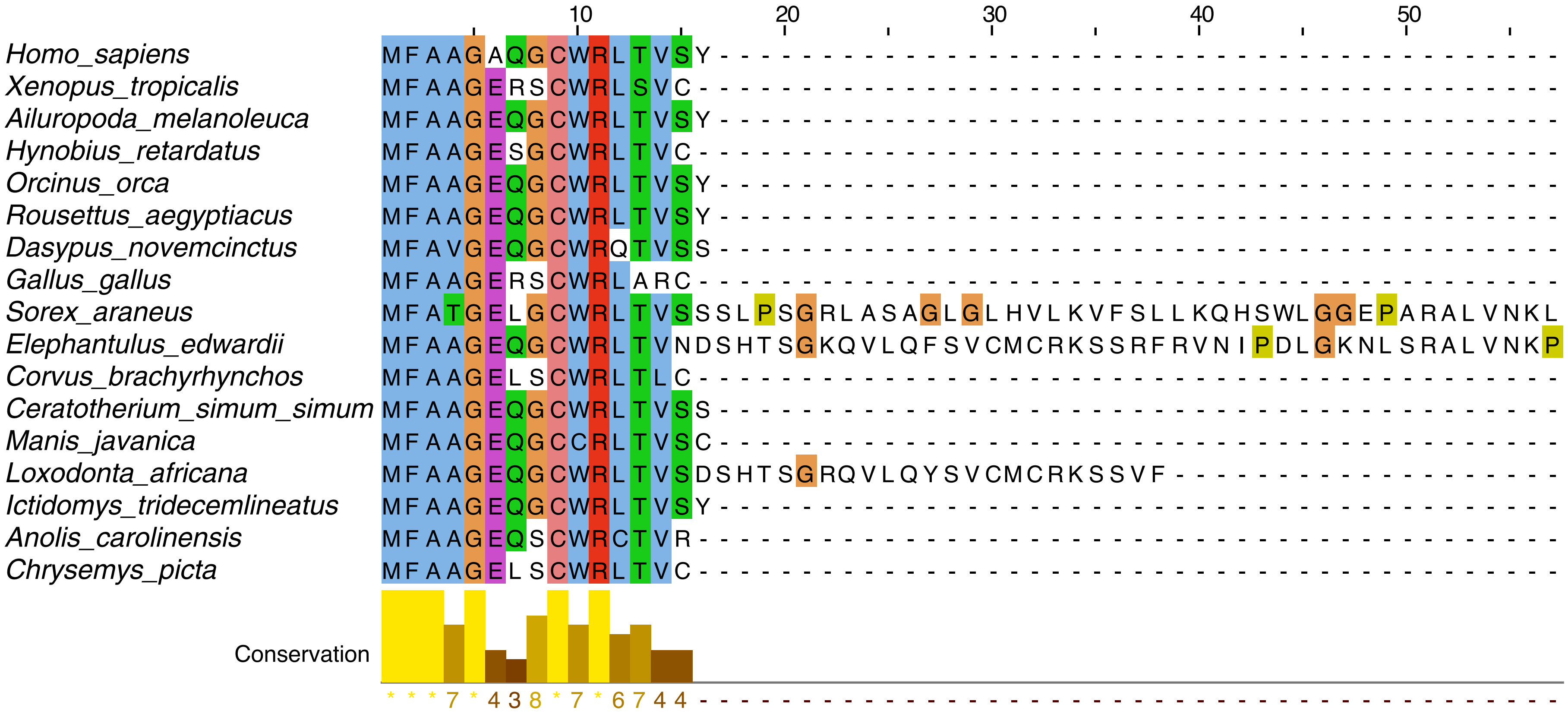

HG0164

ENSG00000151292 (CSNK1G3)

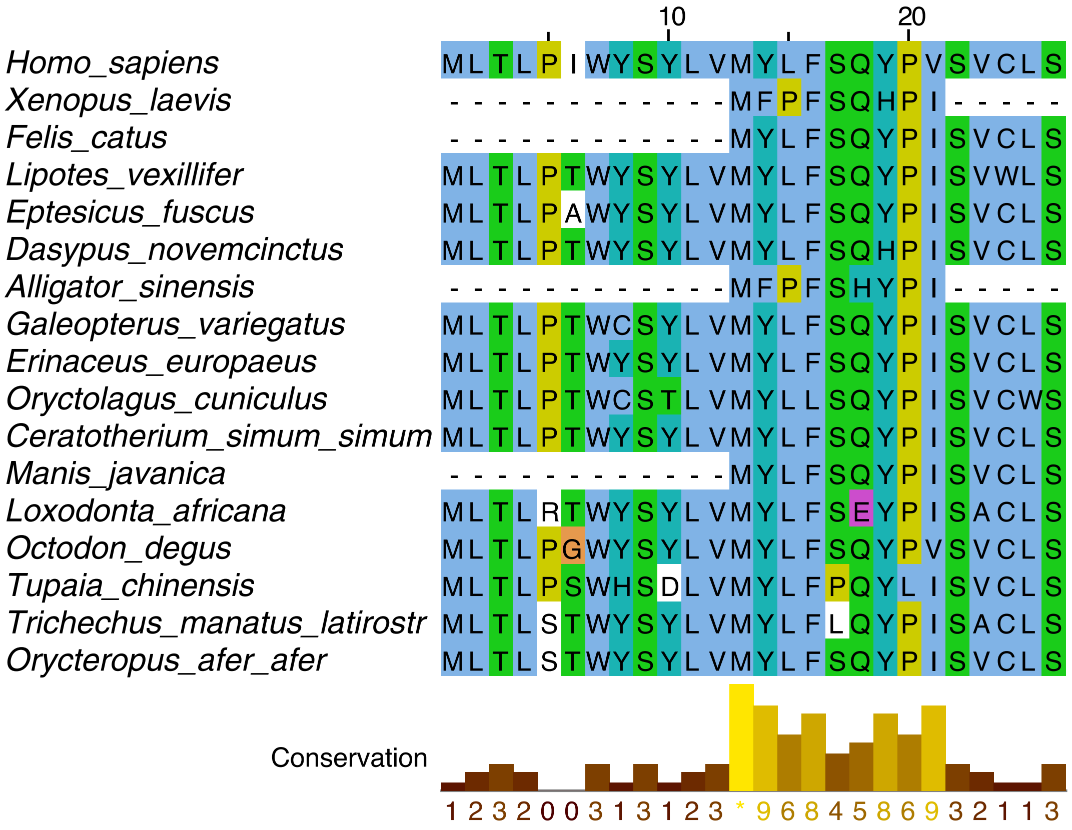

HG0165

ENSG00000177508 (IRX3)

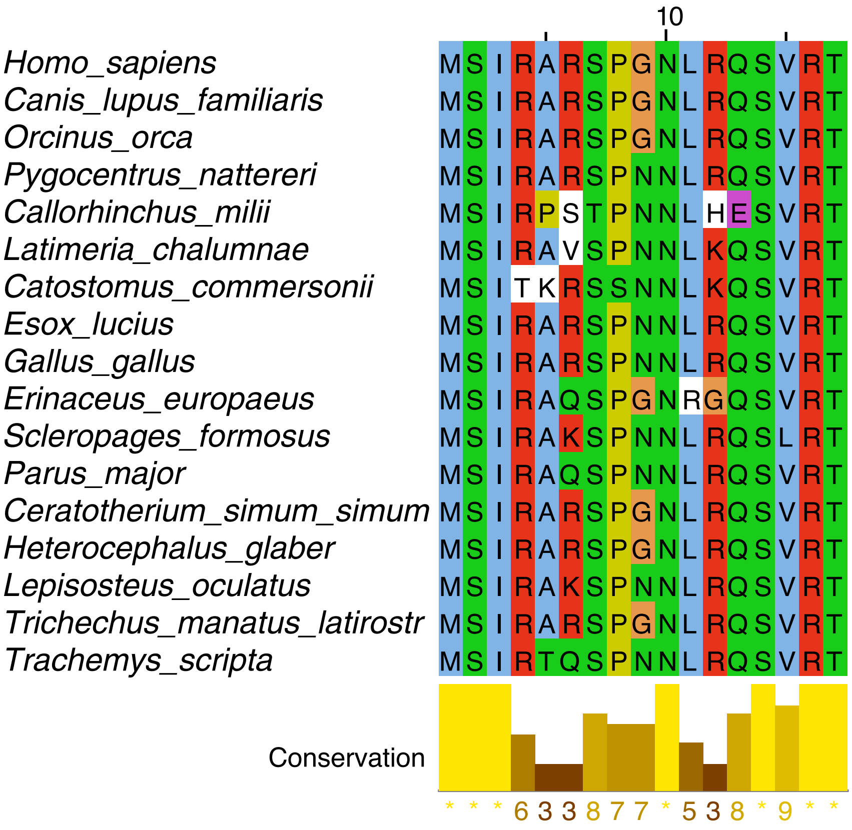

HG0166.1

ENSG00000148948 (LRRC4C)

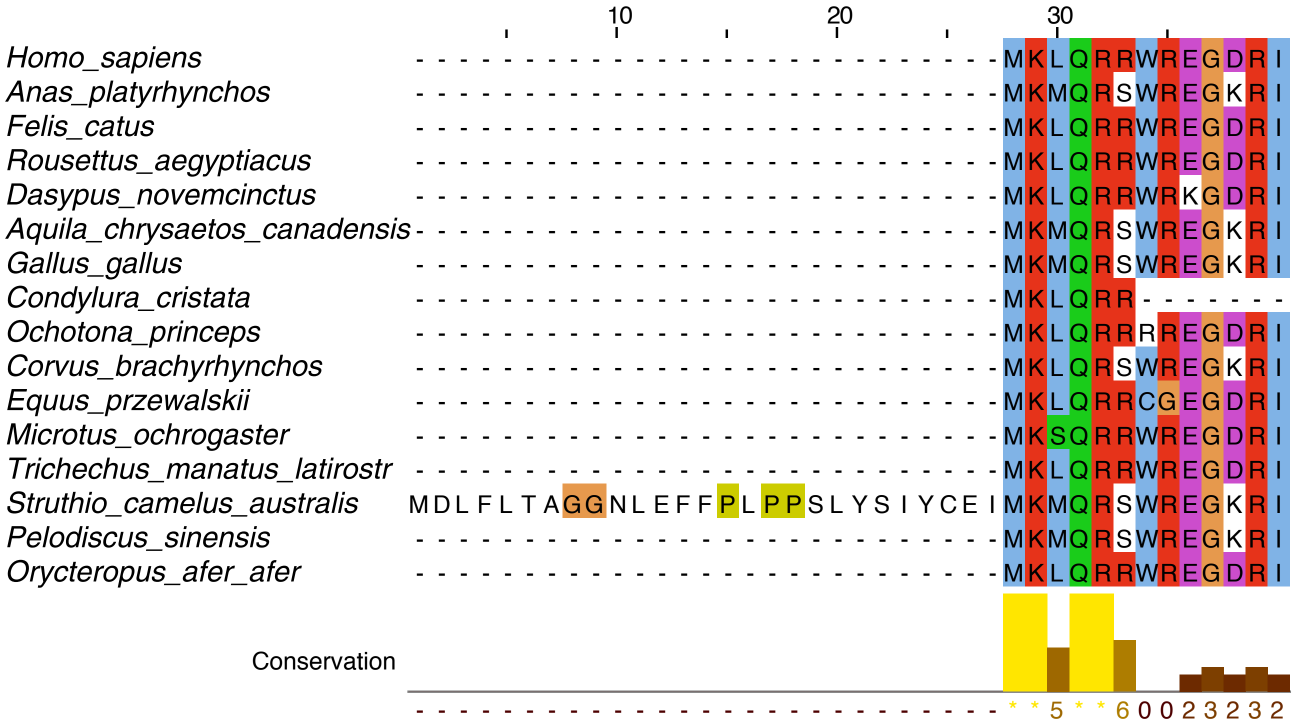

HG0166.2

ENSG00000148948 (LRRC4C)

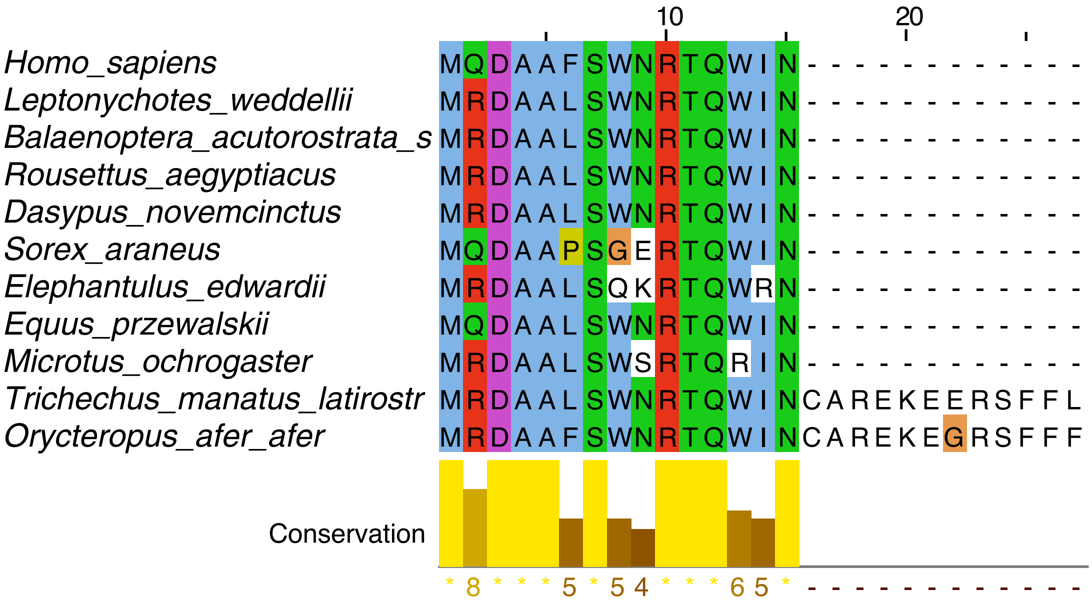

HG0166.3

ENSG00000148948 (LRRC4C)

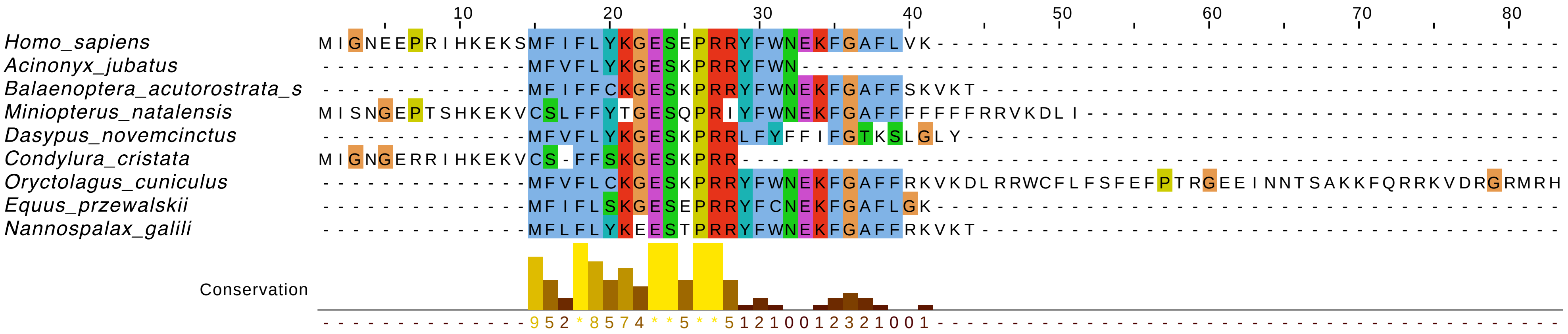

HG0166.4

ENSG00000148948 (LRRC4C)

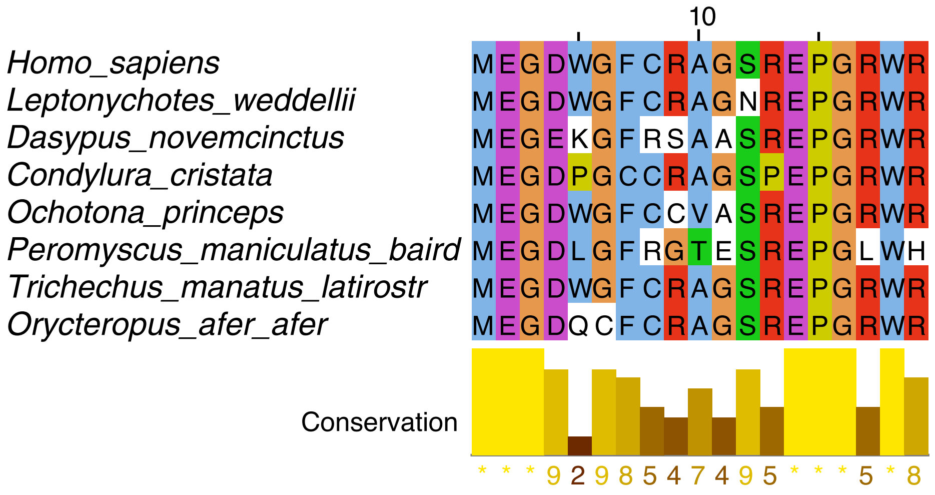

HG0167

ENSDARG00000104005 (slc4a3)

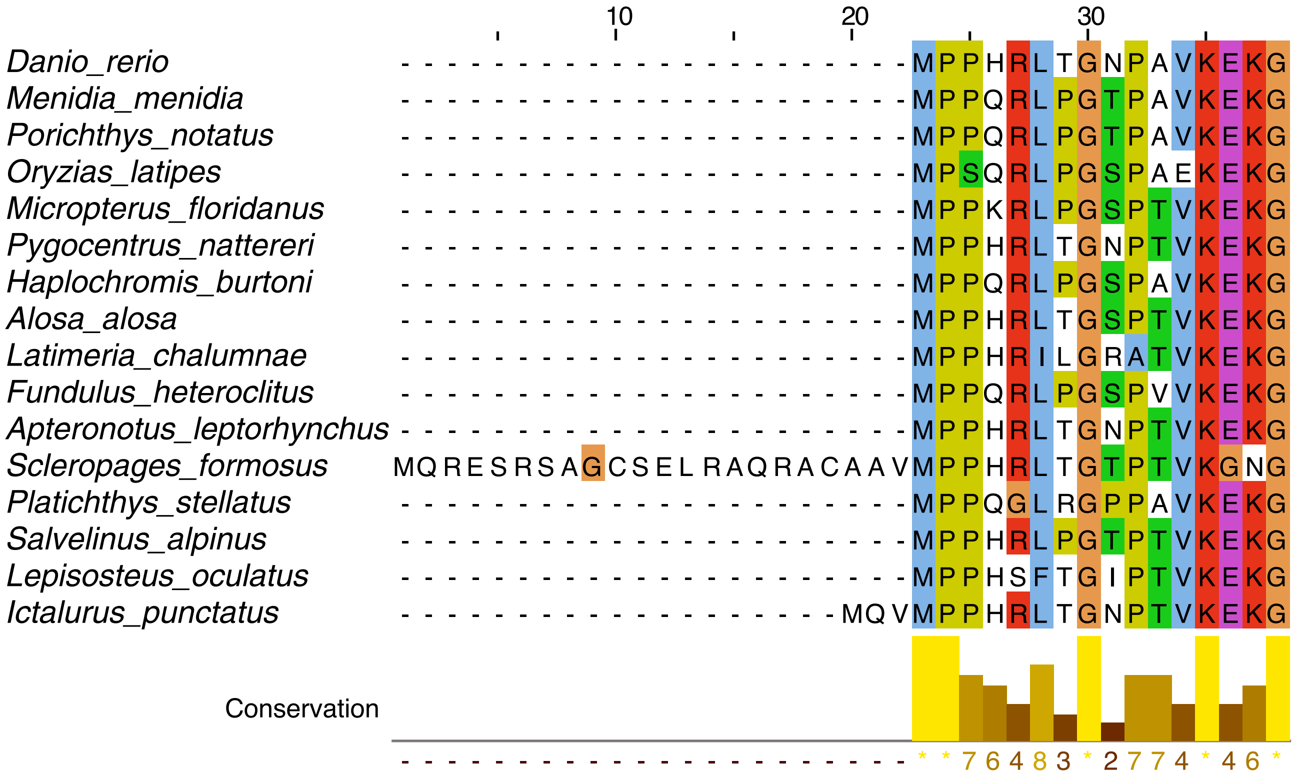

HG0168

ENSGALG00000006997 (BRINP1)

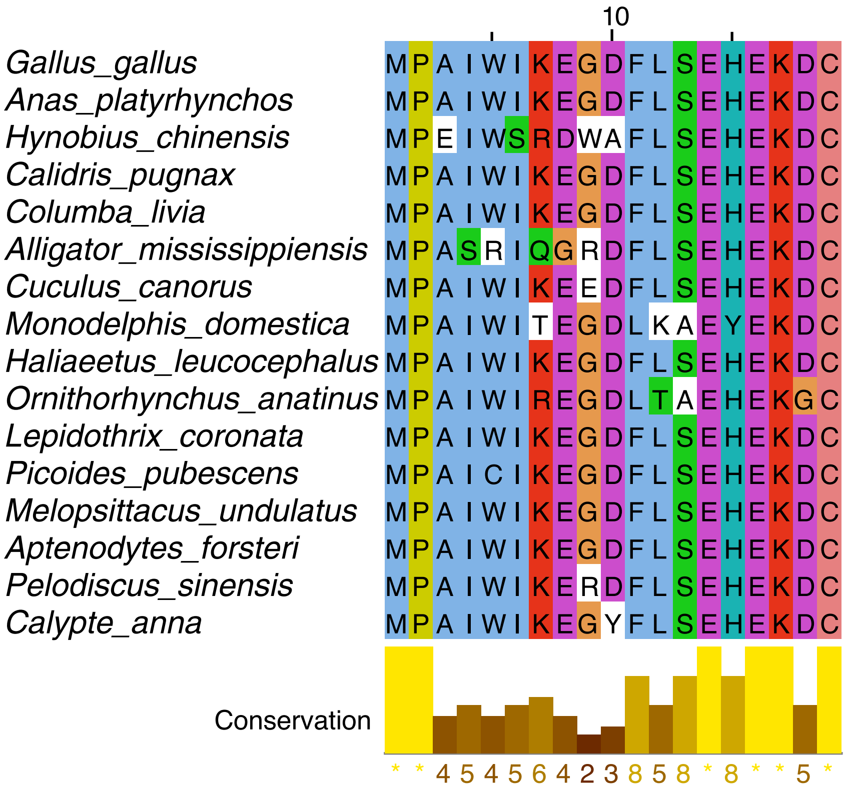

HG0169

ENSG00000114933 (INO80D)

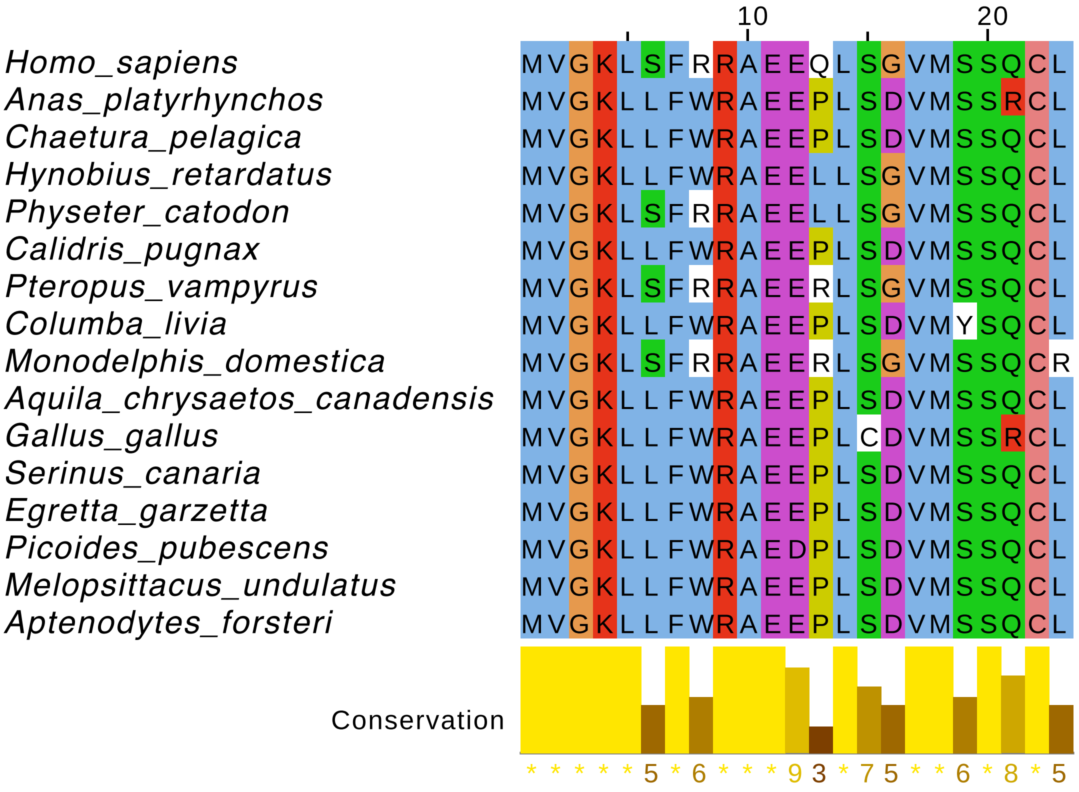

HG0170

ENSG00000180530 (NRIP1)

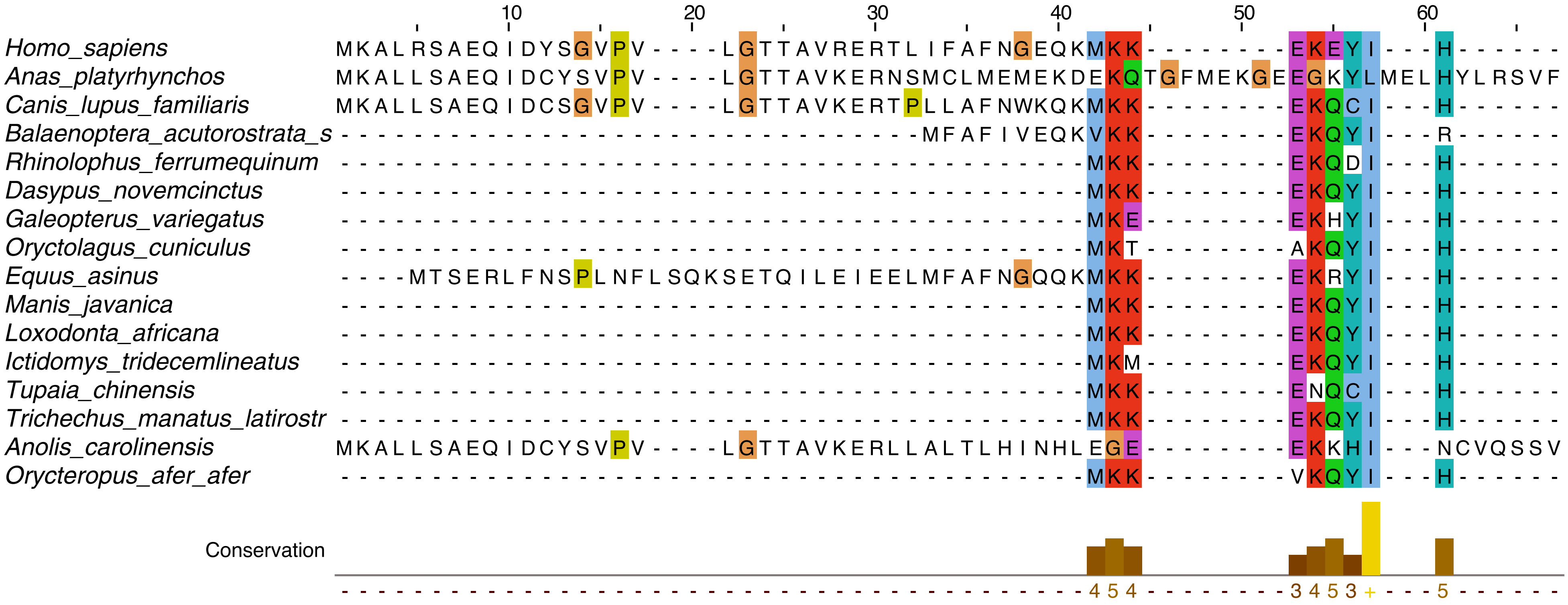

HG0171.1

ENSG00000177311 (ZBTB38)

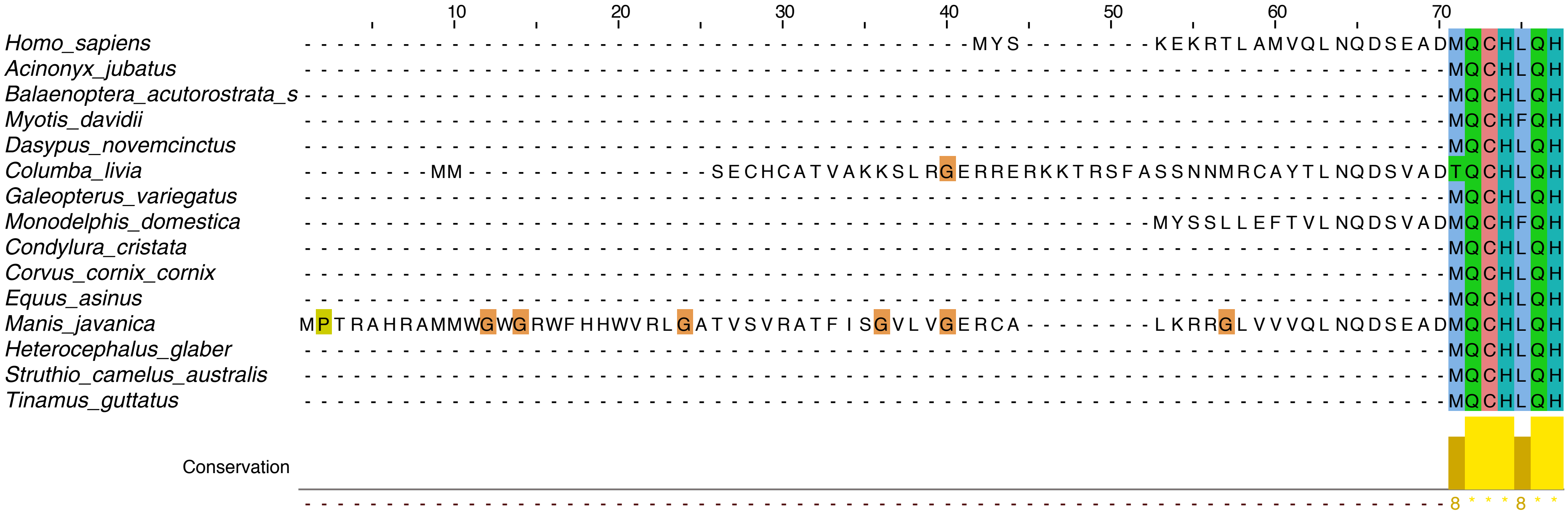

HG0171.2

ENSG00000177311 (ZBTB38)

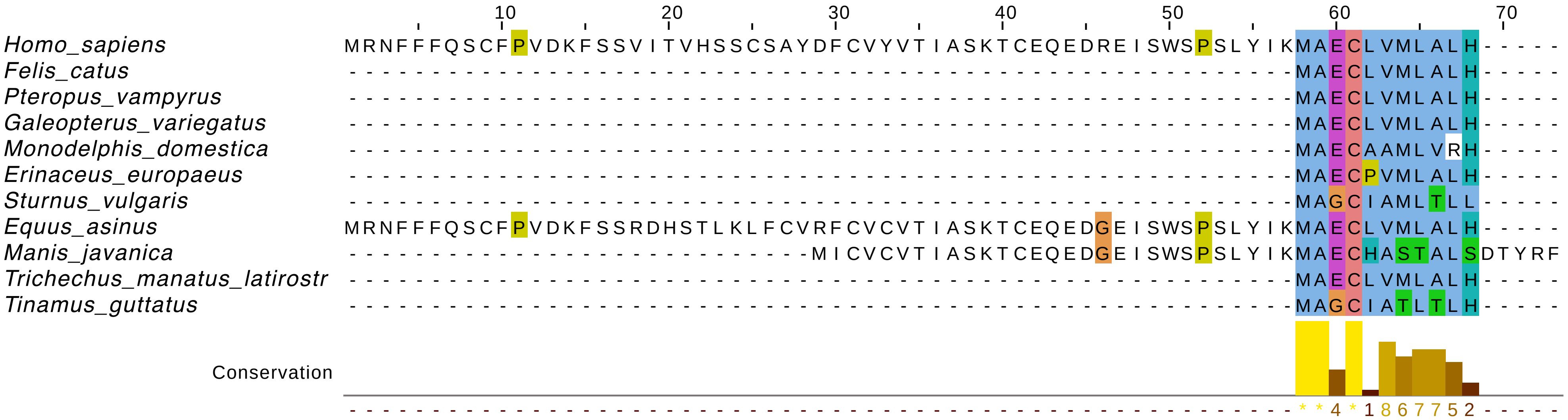

HG0171.3

ENSG00000177311 (ZBTB38)

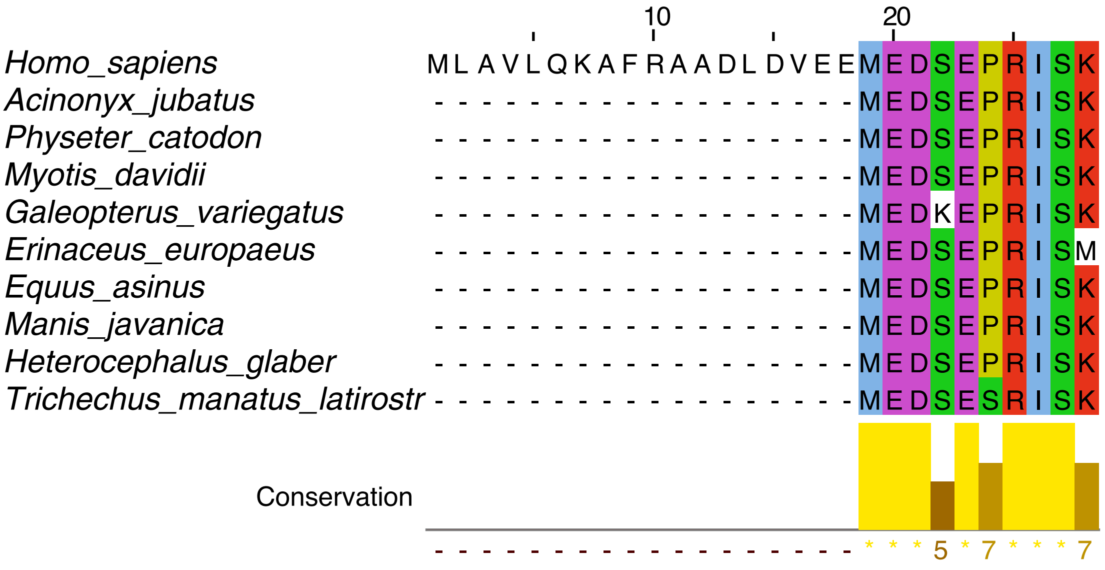

HG0171.4

ENSG00000177311 (ZBTB38)

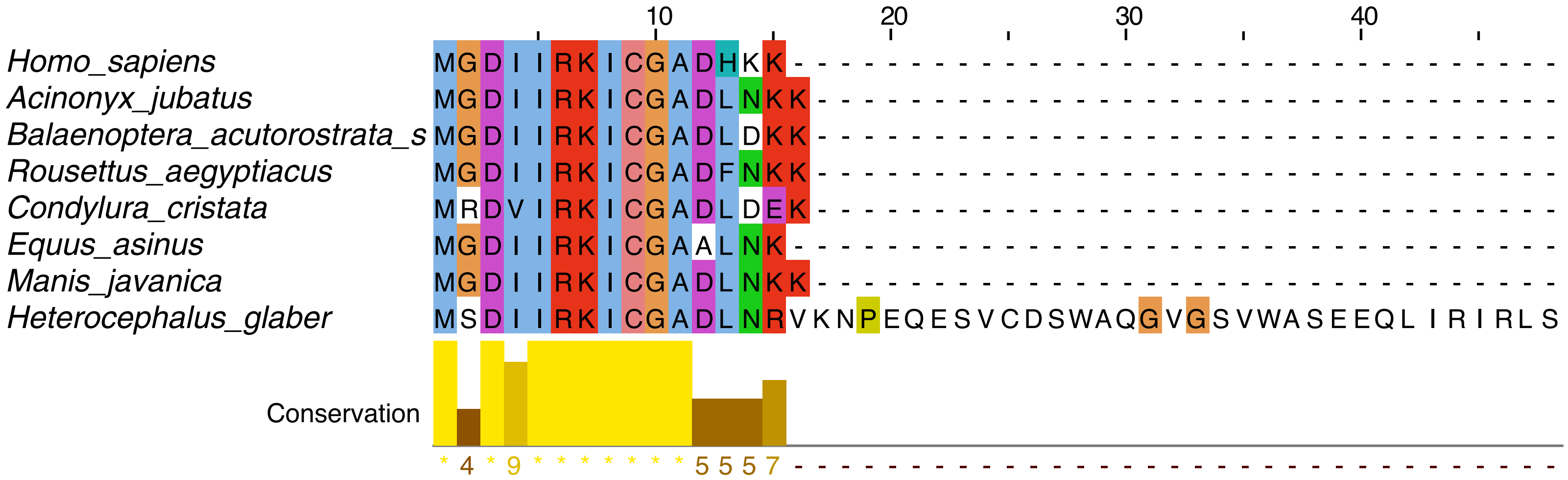

HG0172.1

ENSG00000196782 (MAML3)

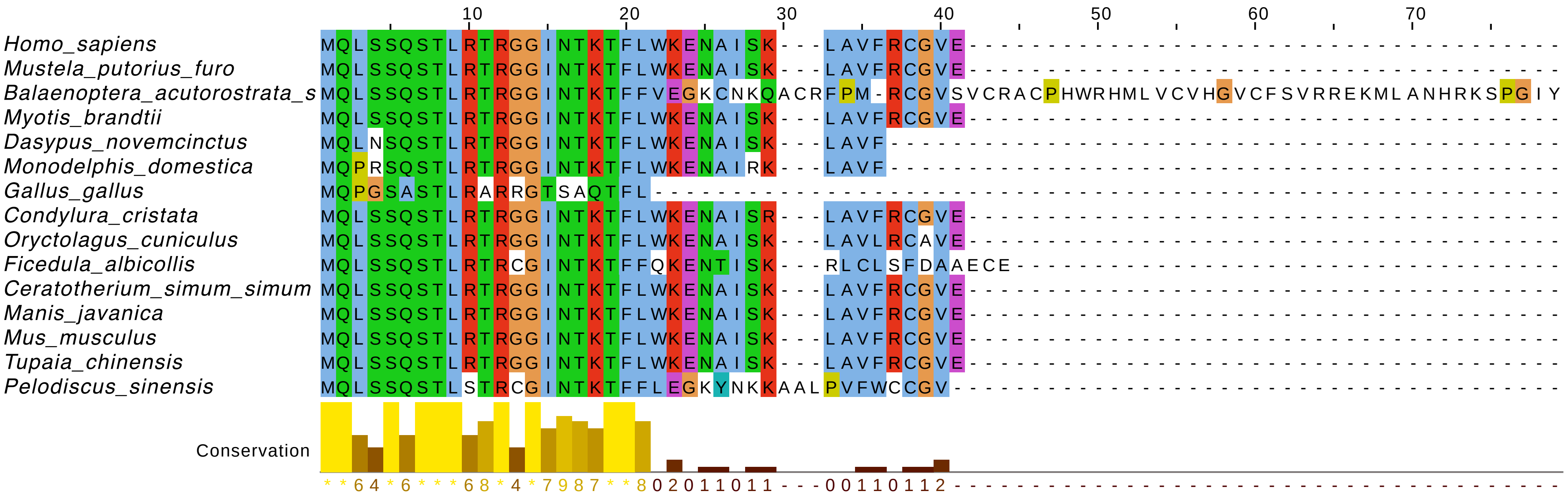

HG0172.2

ENSG00000196782 (MAML3)

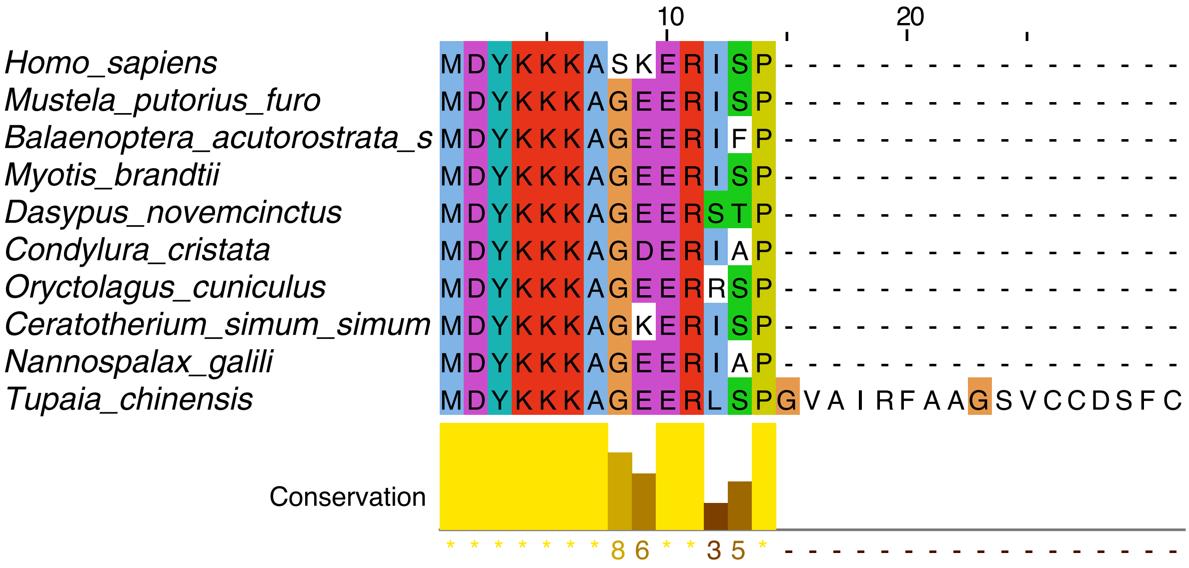

HG0173

ENSG00000101746 (NOL4)

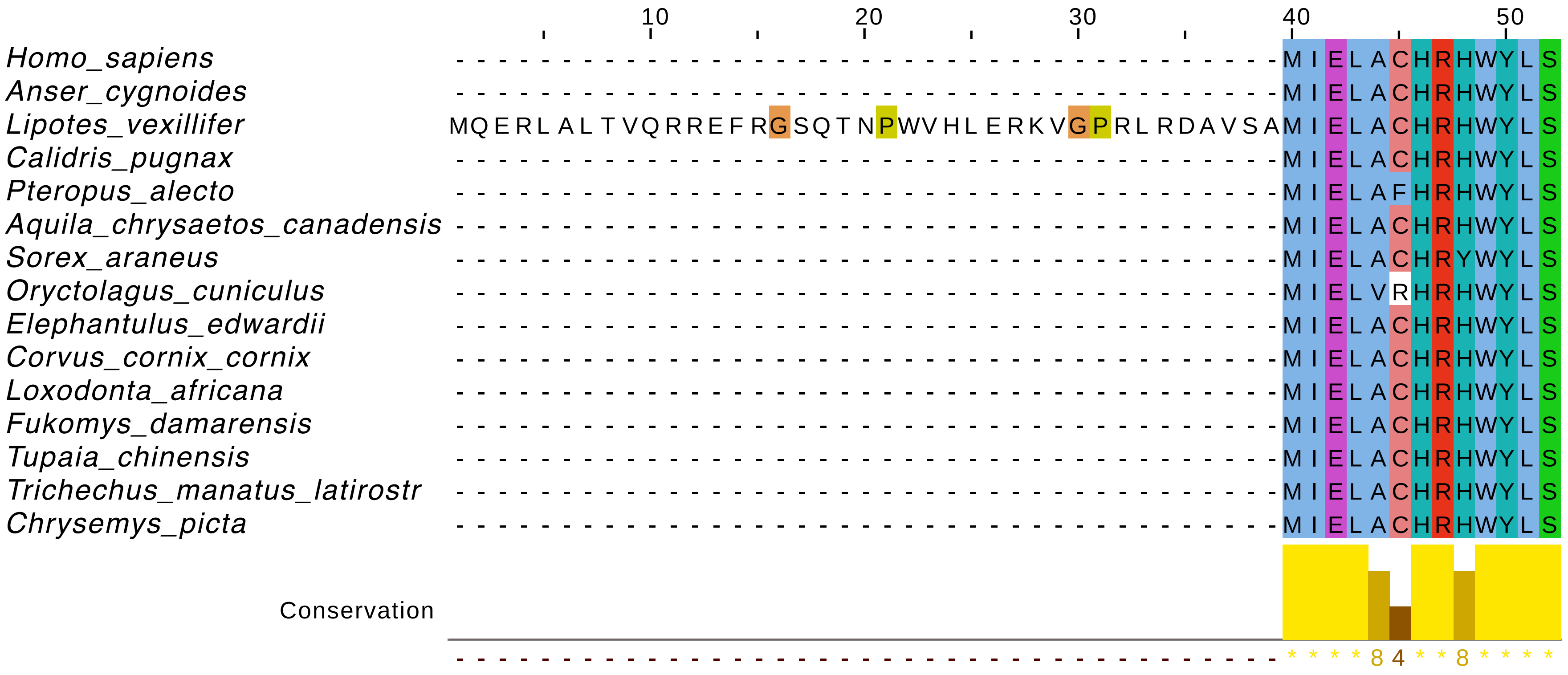

HG0174

ENSG00000144355 (DLX1)

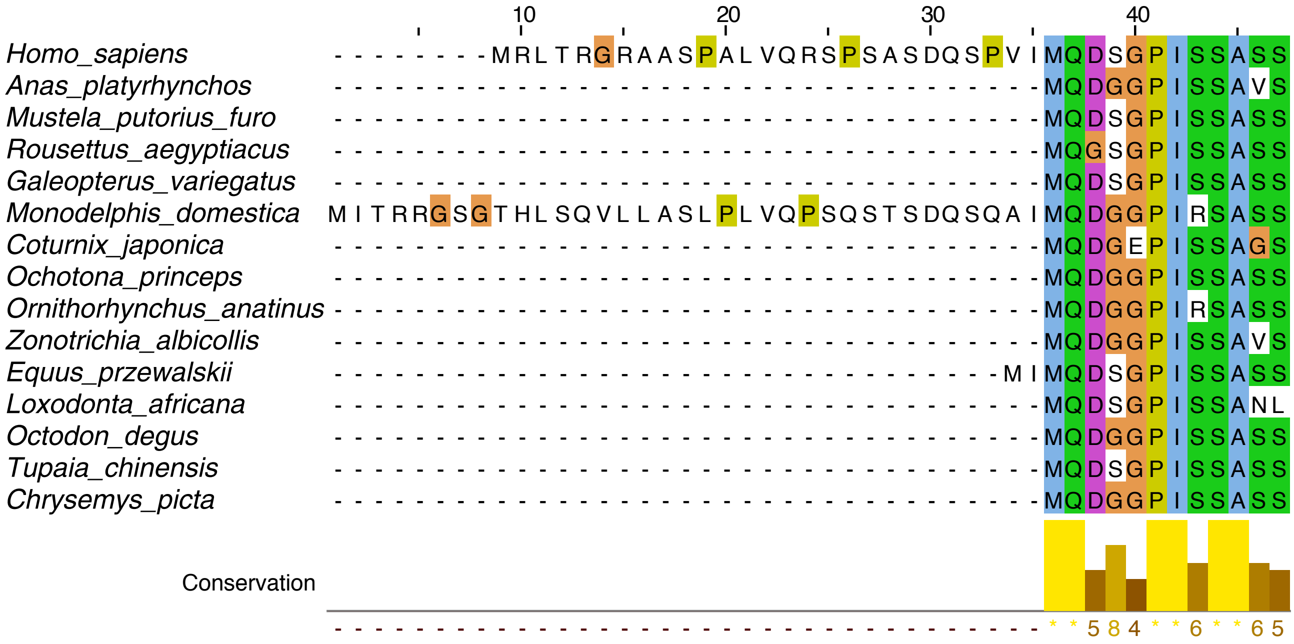

HG0175

ENSG00000151320 (AKAP6)

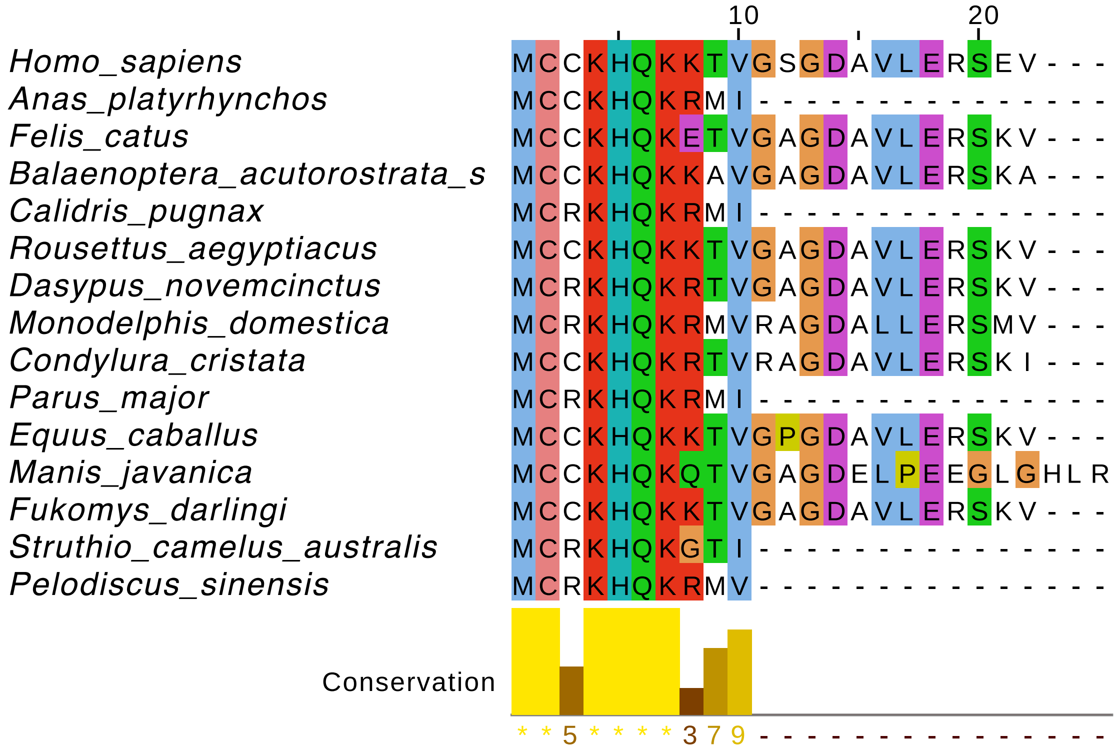

HG0176

ENSG00000170962 (PDGFD)

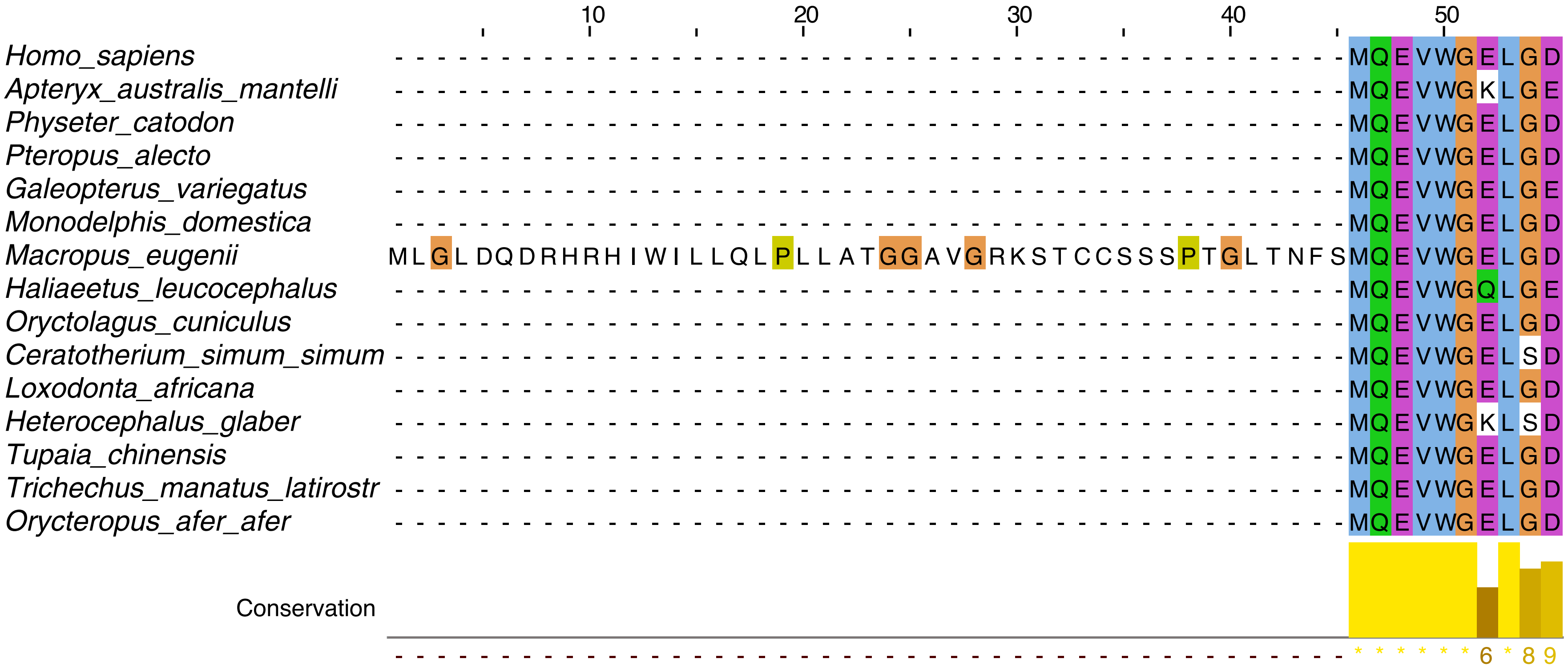

HG0177

ENSG00000171843 (MLLT3)

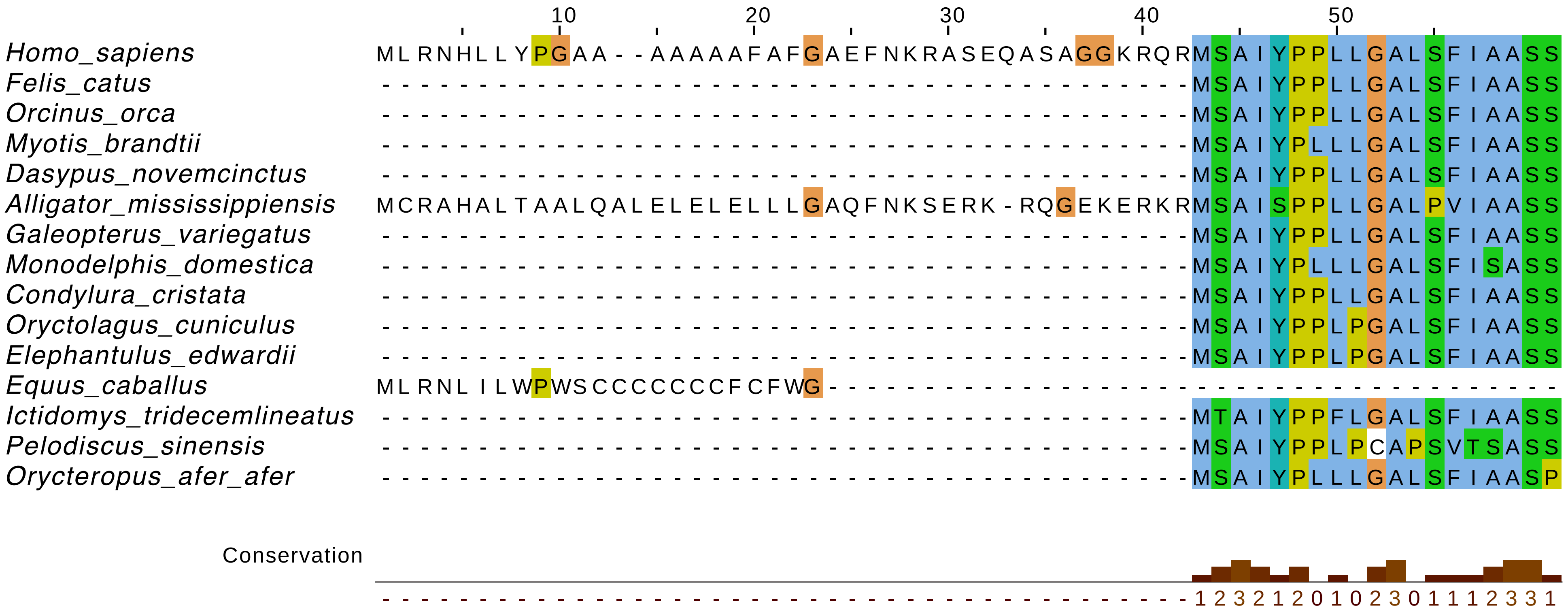

HG0178.1

ENSG00000131634 (TMEM204)

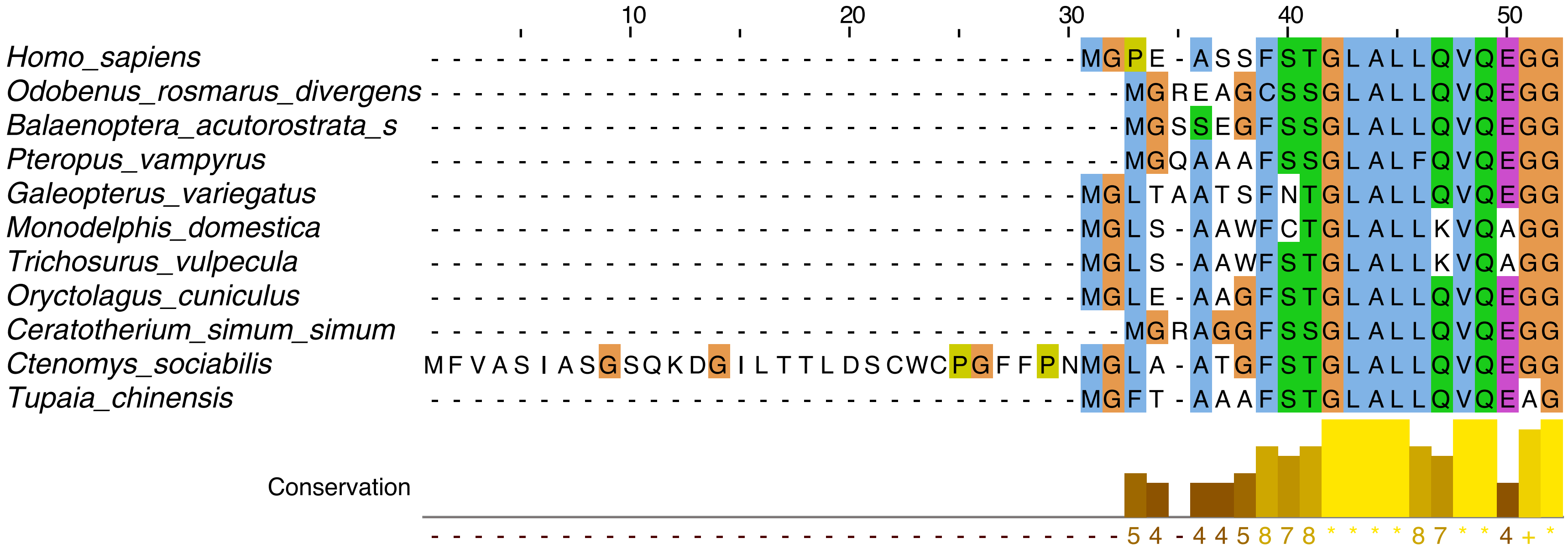

HG0178.1

ENSGALG00000029047 (TMEM204)

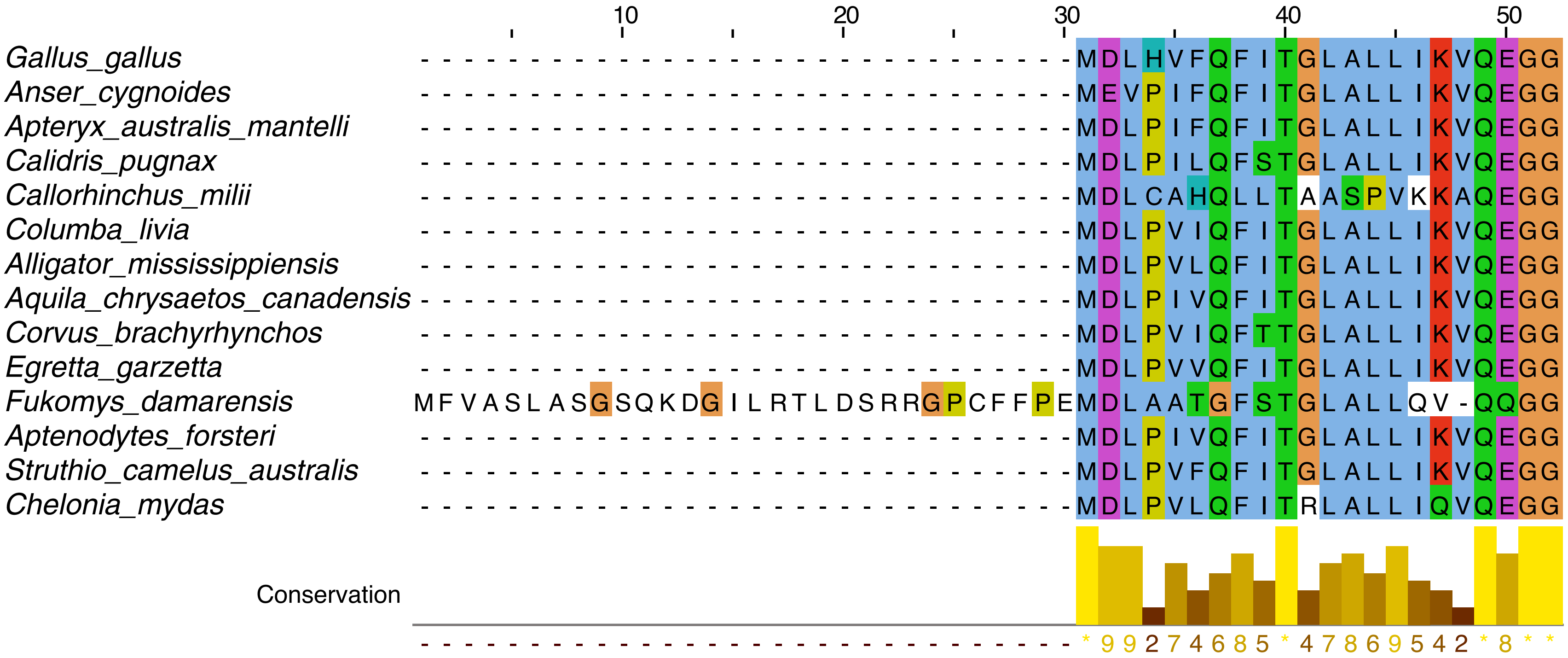

HG0178.2

ENSGALG00000029047 (TMEM204)

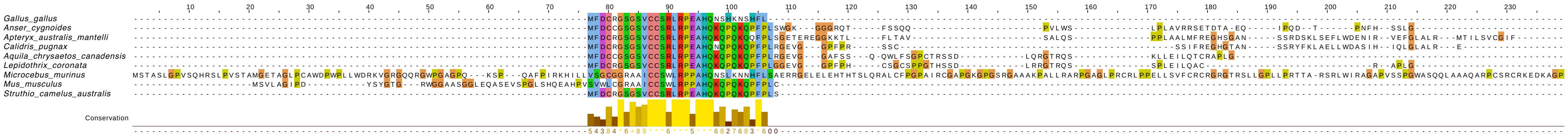

HG0179

ENSDARG00000036549 (agpat3)

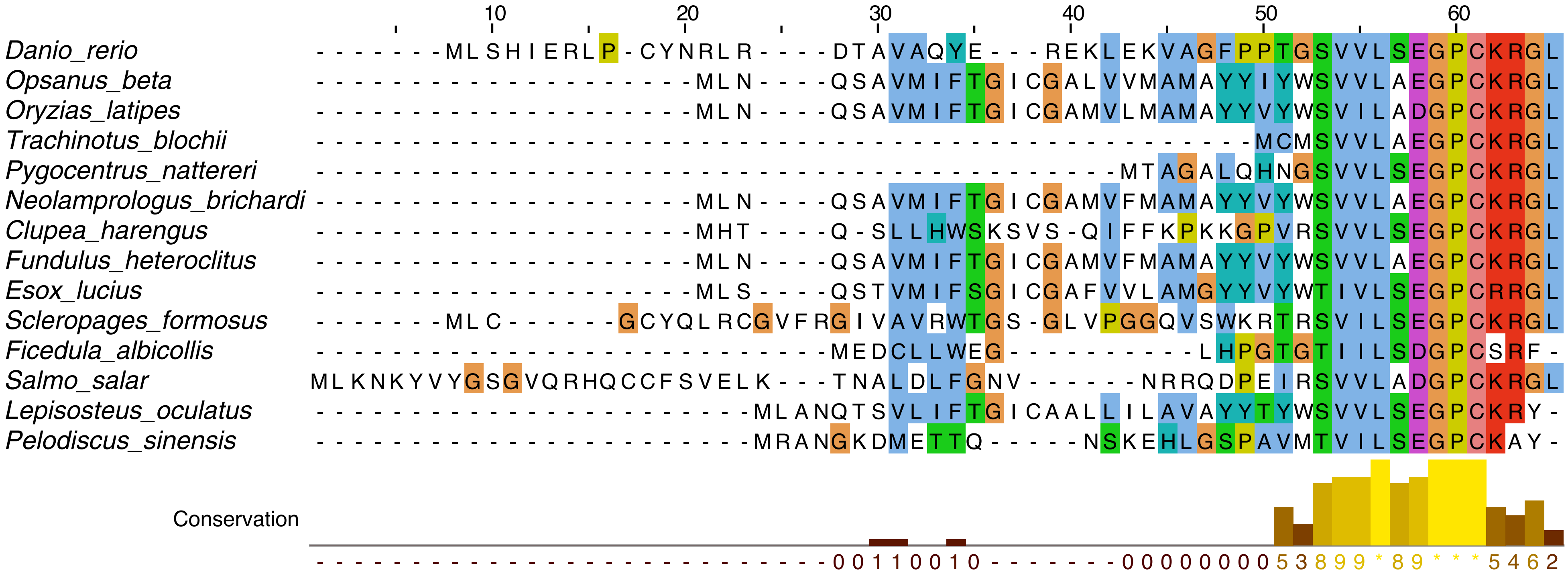

HG0179

ENSG00000160216 (AGPAT3)

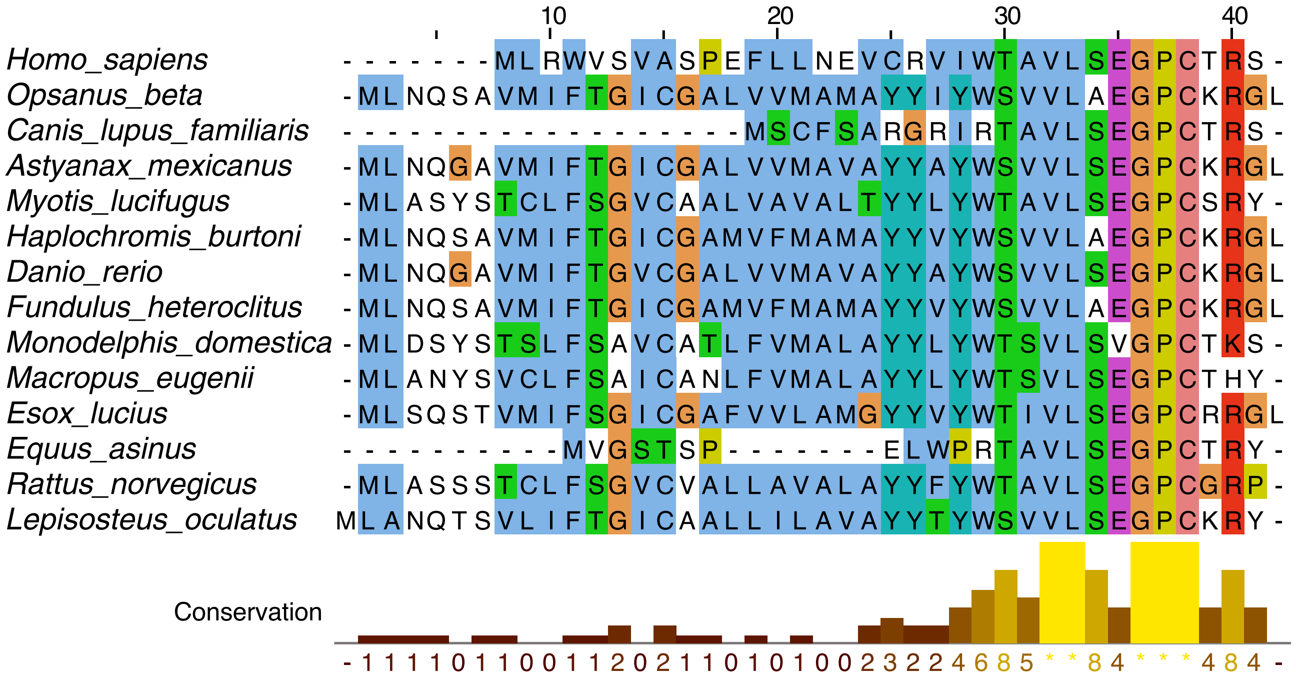

HG0180.1

ENSG00000112246 (SIM1)

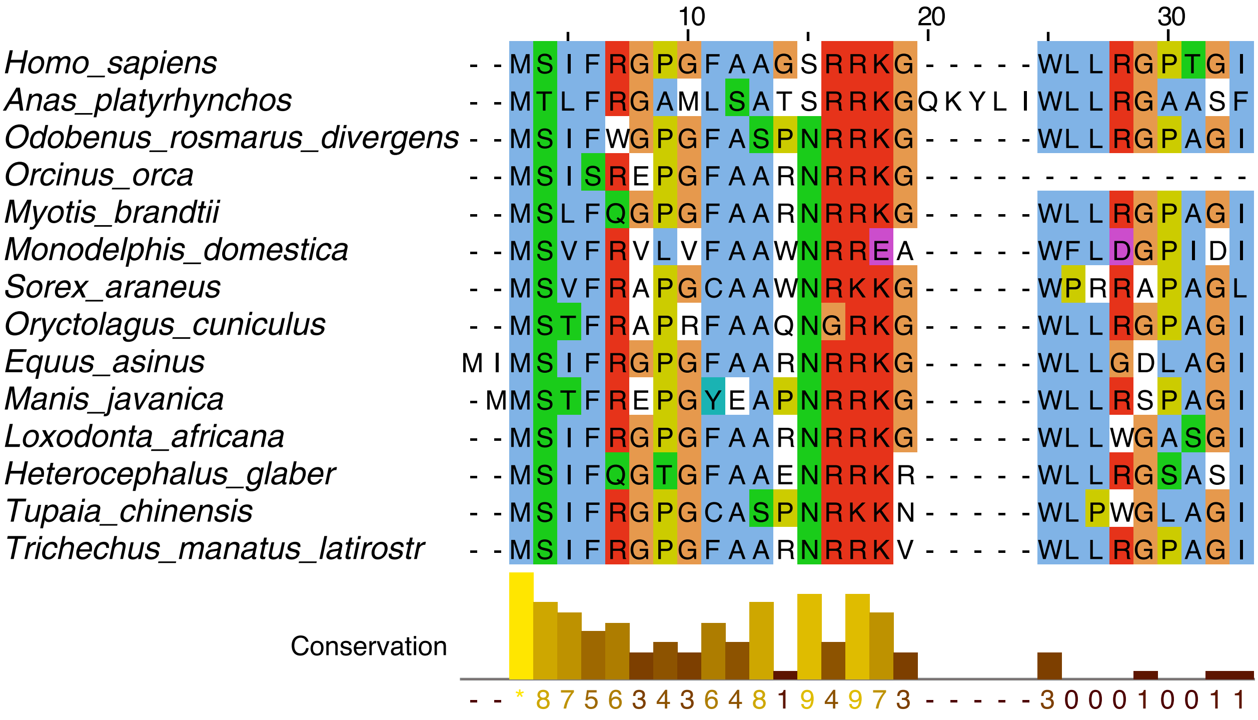

HG0180.2

ENSG00000112246 (SIM1)

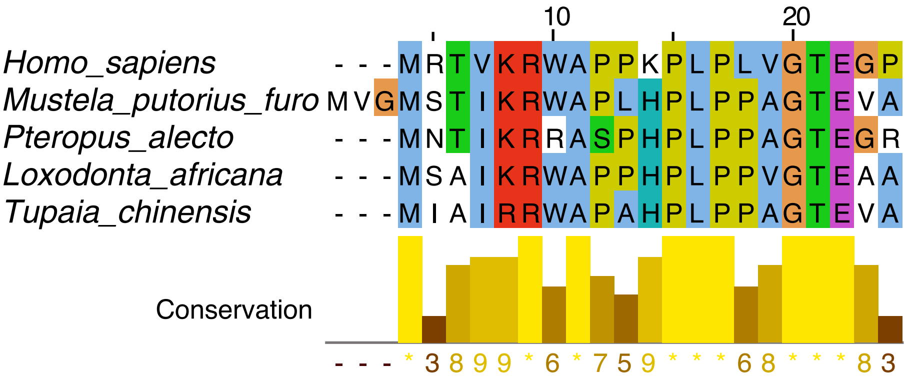

HG0181.1

ENSG00000143776 (CDC42BPA)

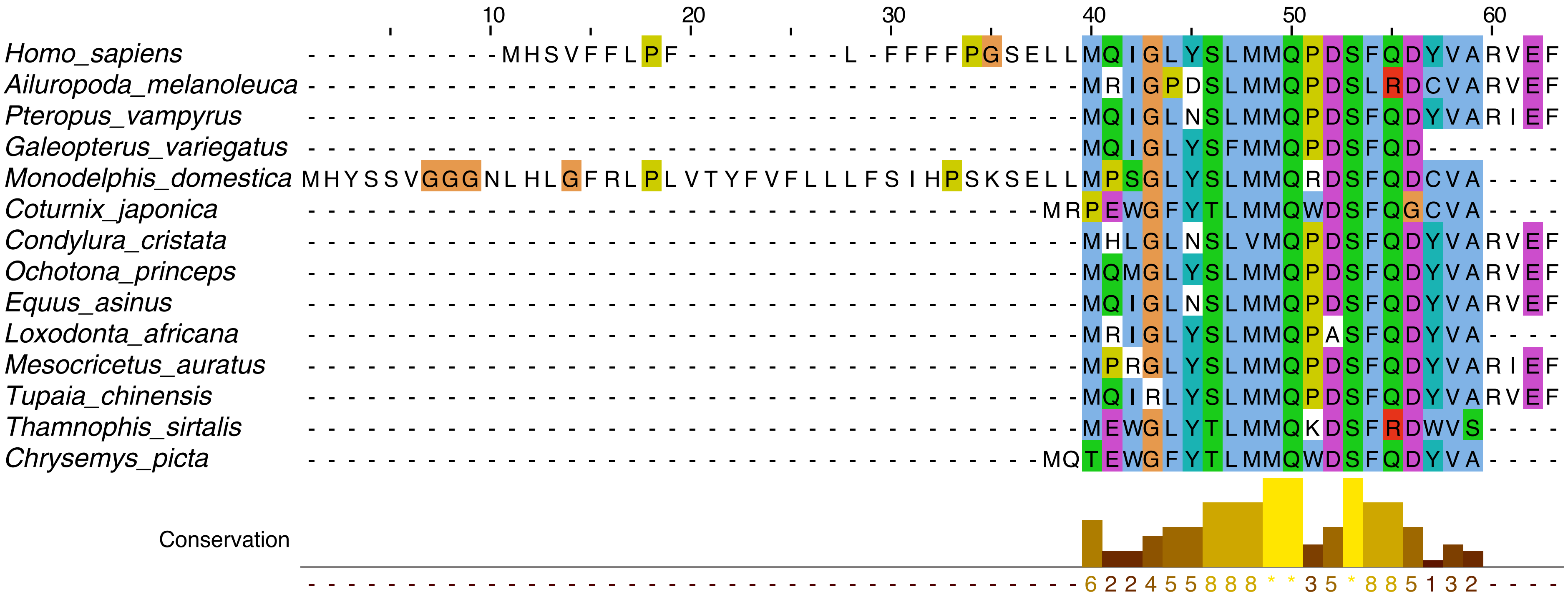

HG0181.2

ENSG00000143776 (CDC42BPA)

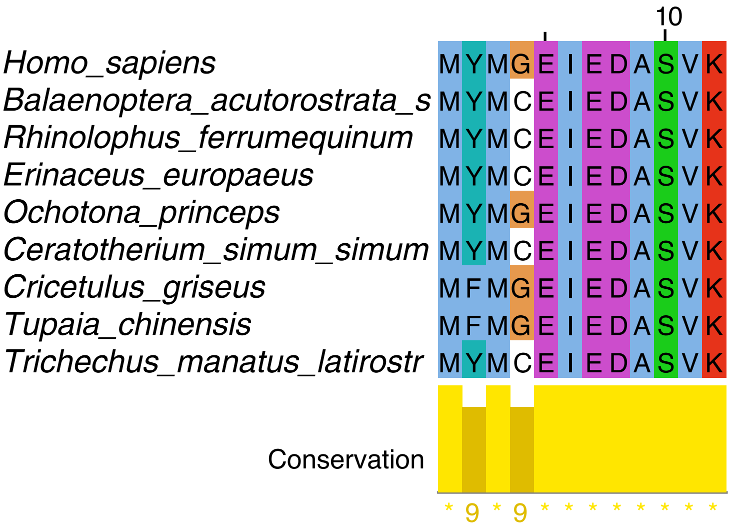

HG0182

ENSGALG00000031991

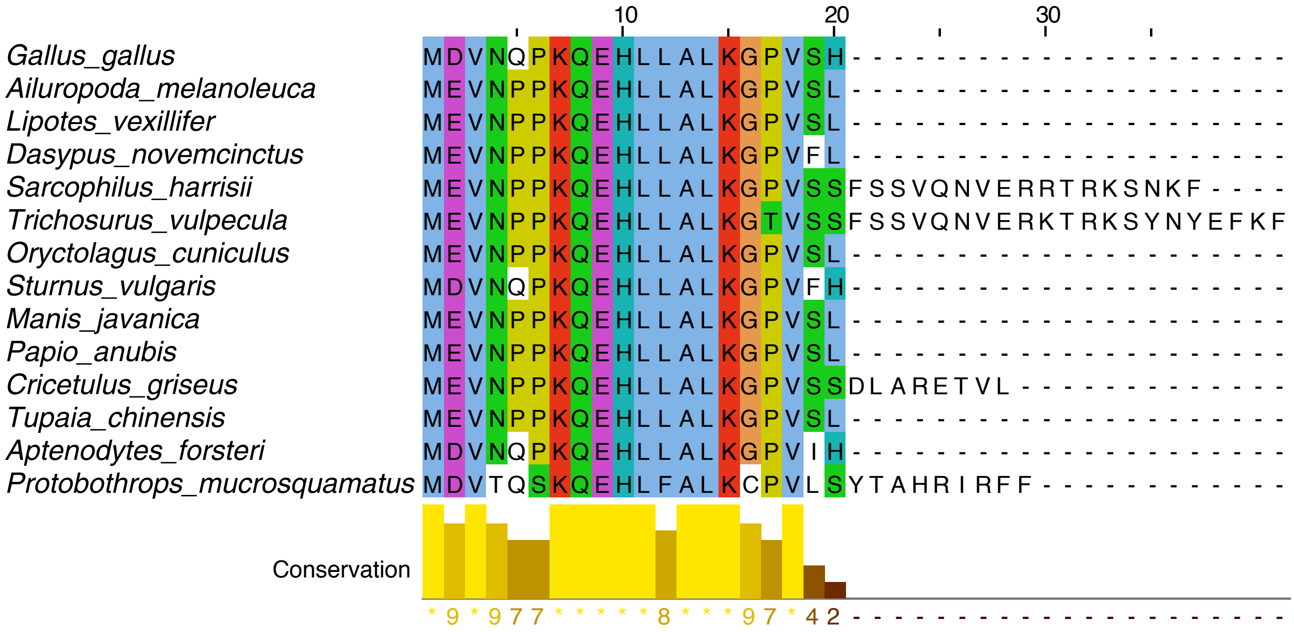

HG0183

ENSG00000011465 (DCN)

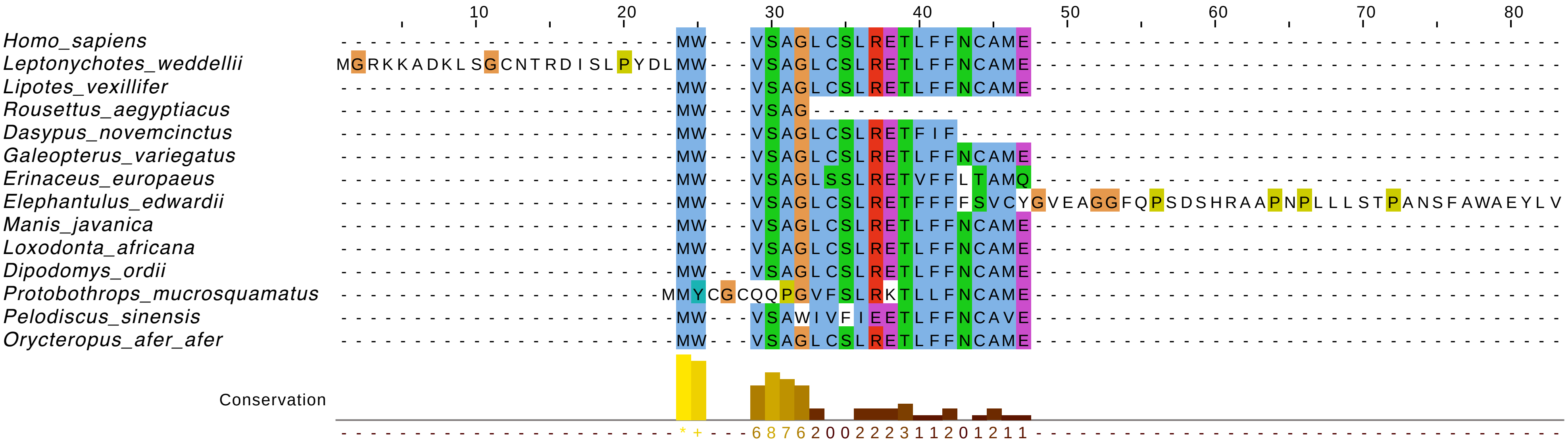

HG0184

ENSG00000136542 (GALNT5)

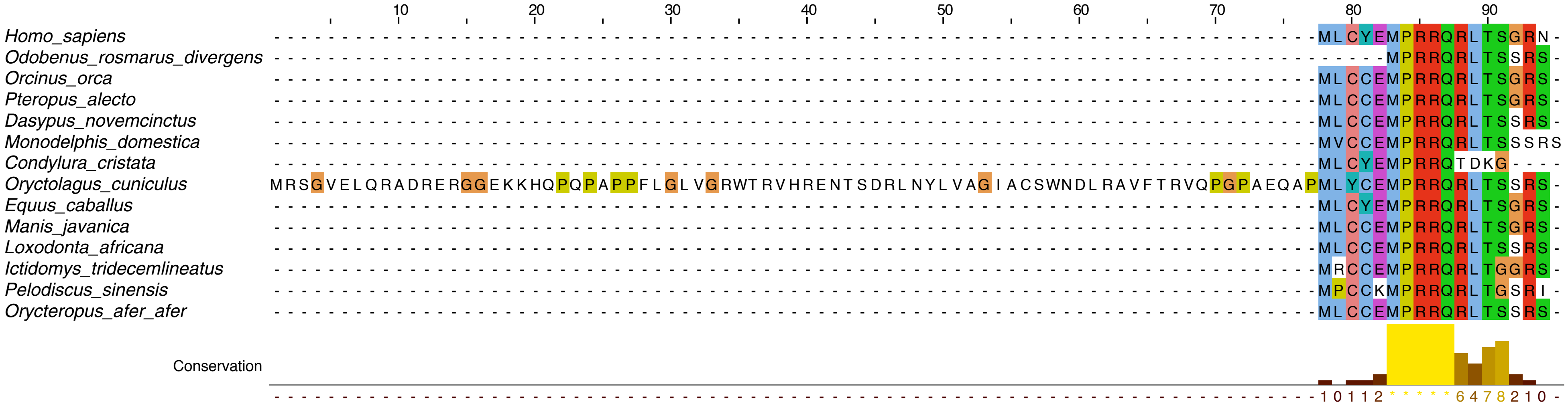

HG0185

ENSG00000143033 (MTF2)

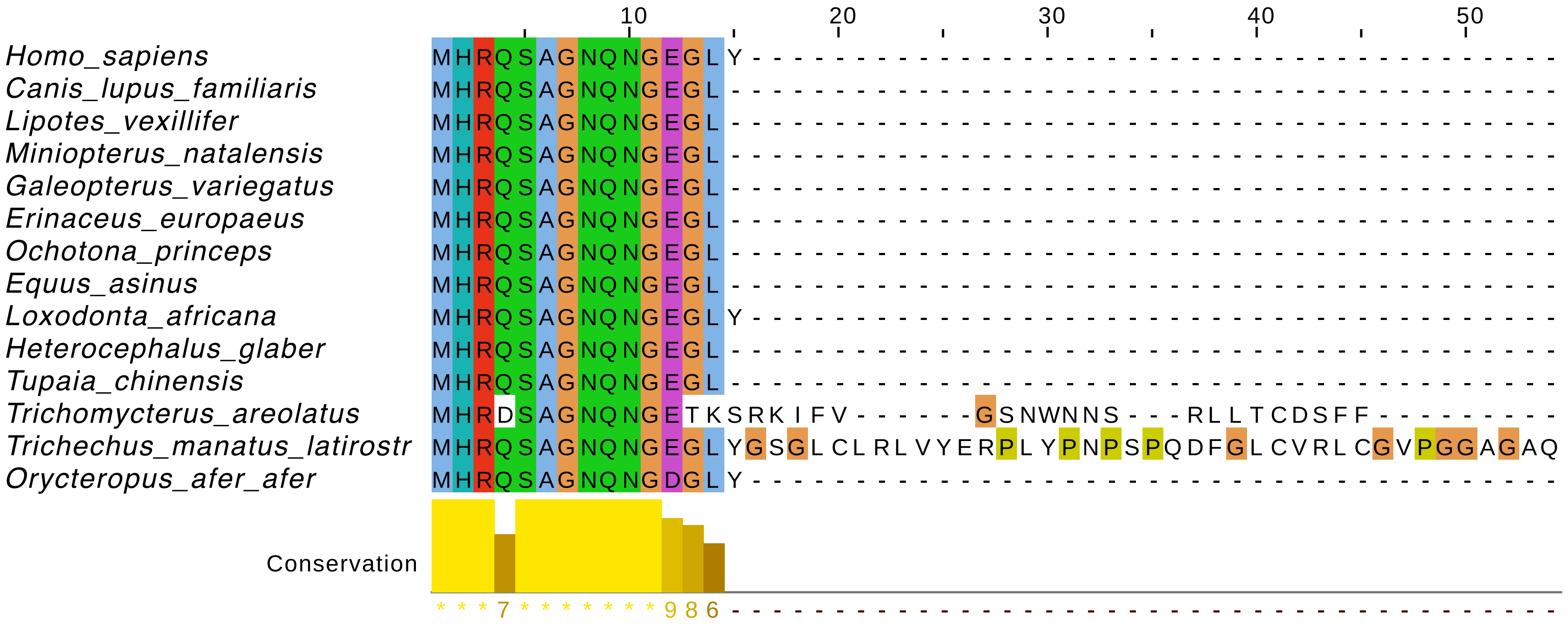

HG0186

ENSG00000167552 (TUBA1A)

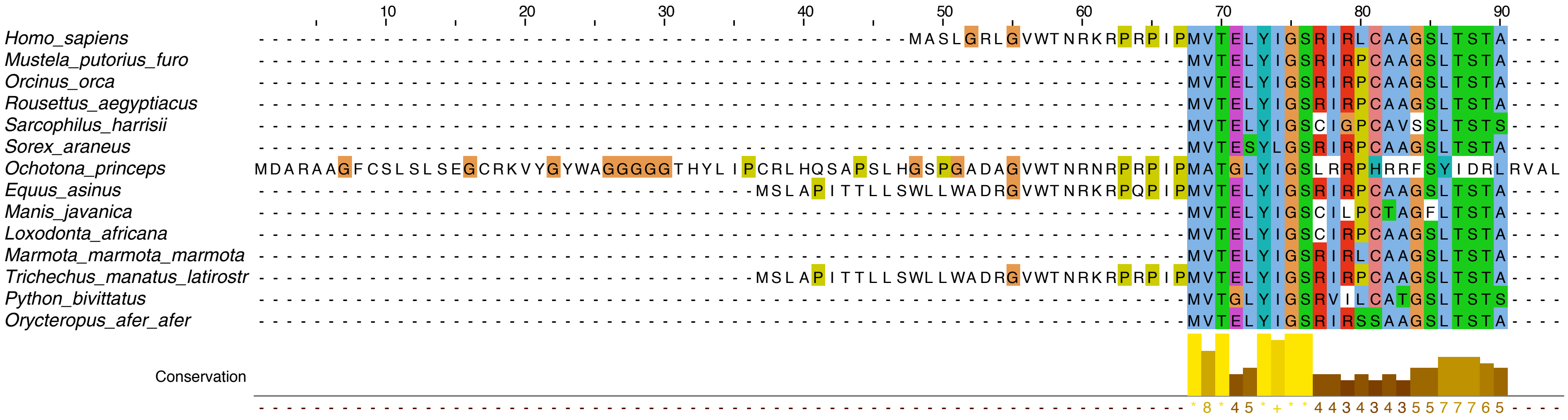

HG0187.1

ENSGALG00000004058 (GPR146)

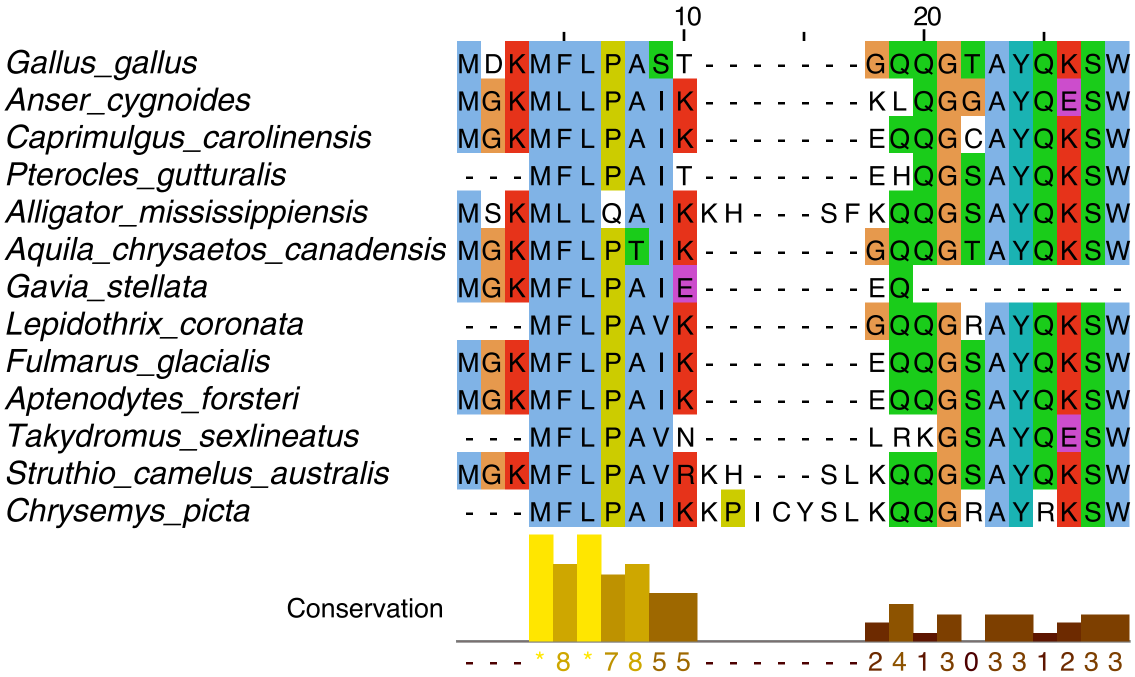

HG0187.2

ENSDARG00000059610 (gpr146)

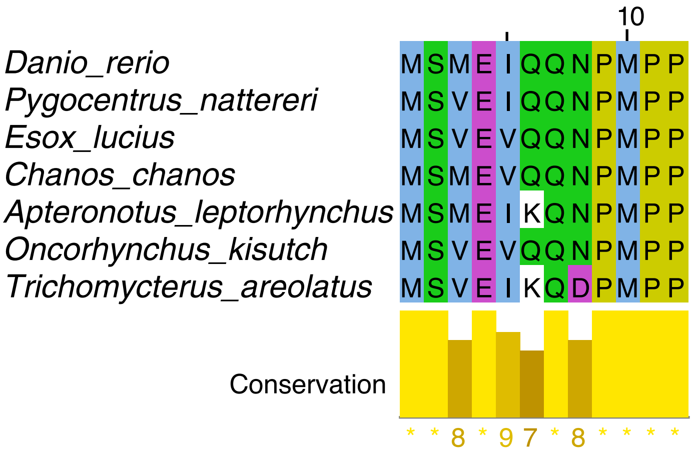

HG0187.3

ENSGALG00000004058 (GPR146)

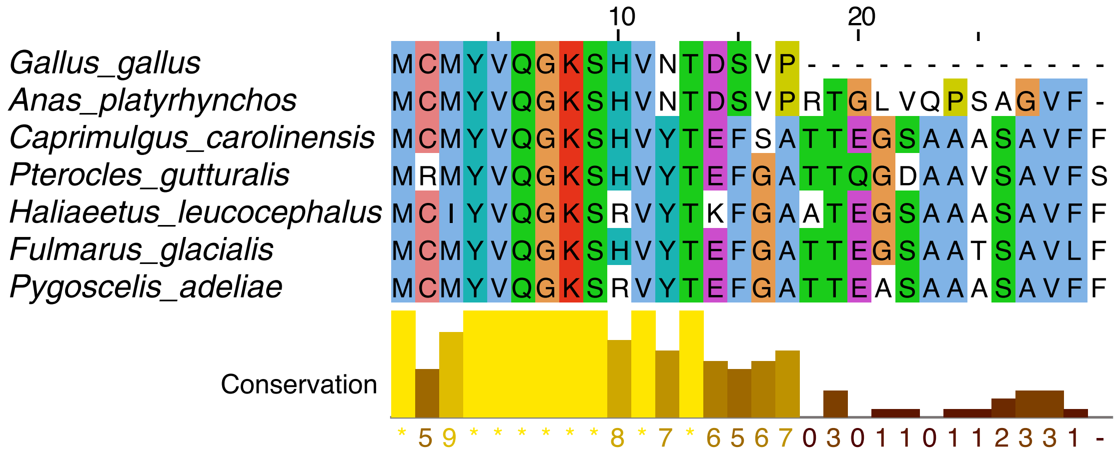

HG0188

ENSG00000157625 (TAB3)

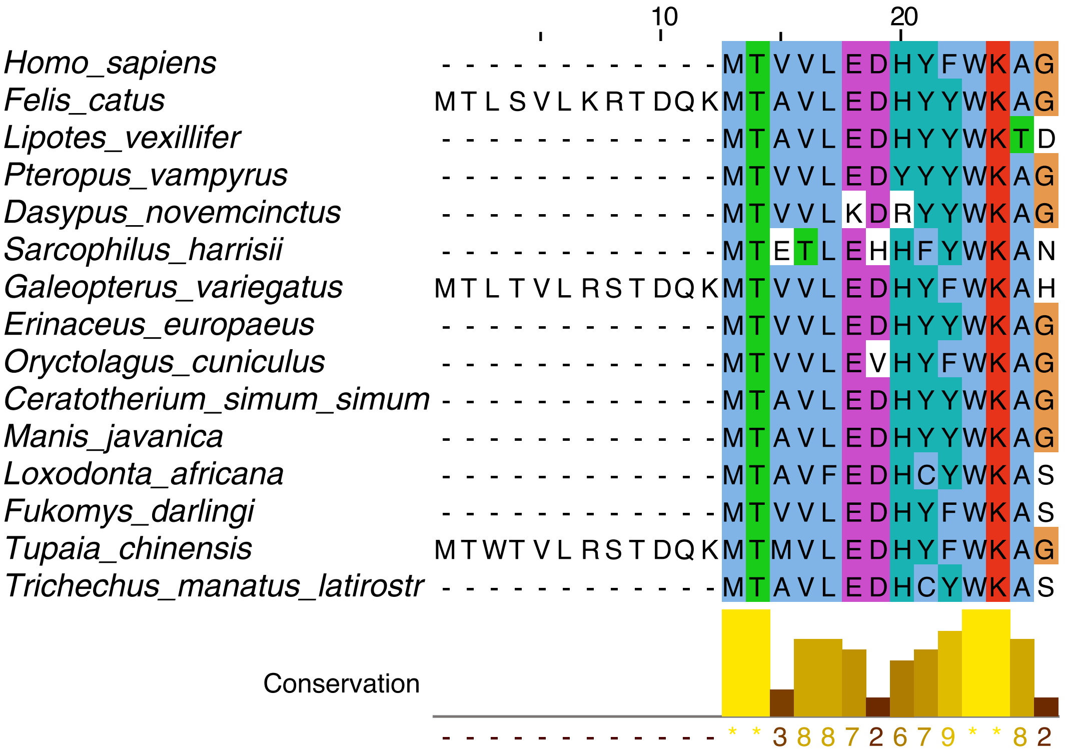

HG0188

ENSGALG00000016284 (TAB3)

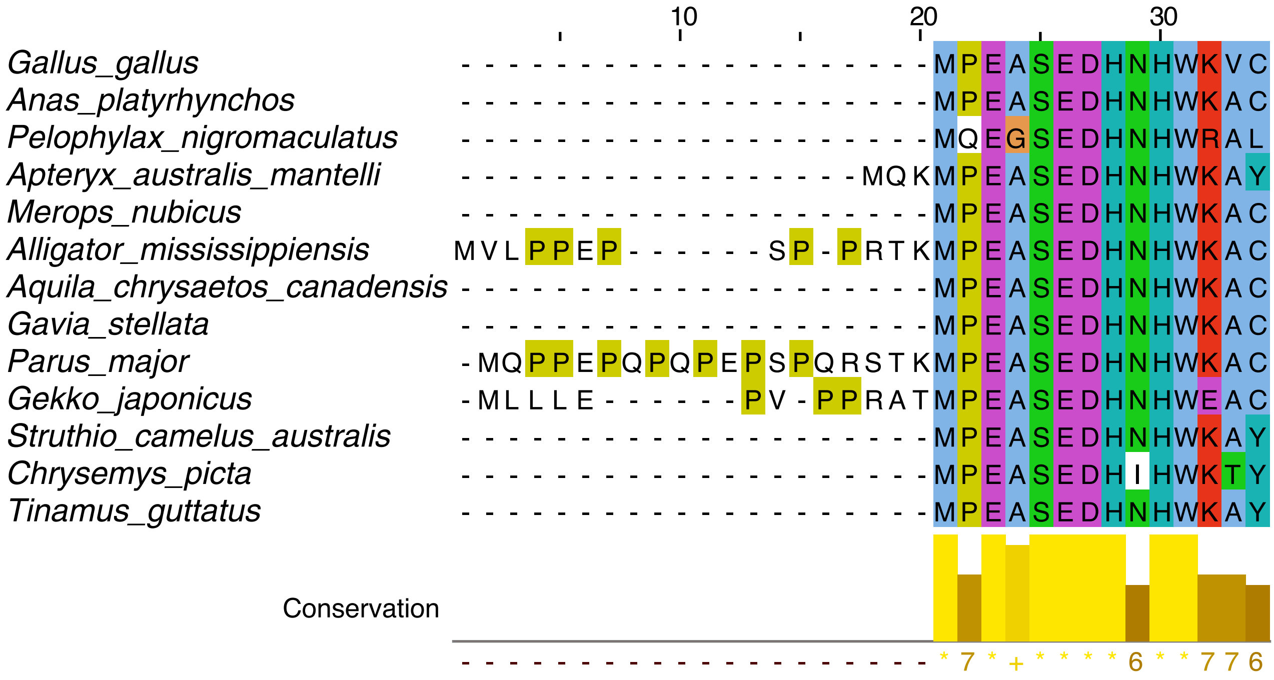

HG0189

ENSDARG00000001807 (tnfrsf21)

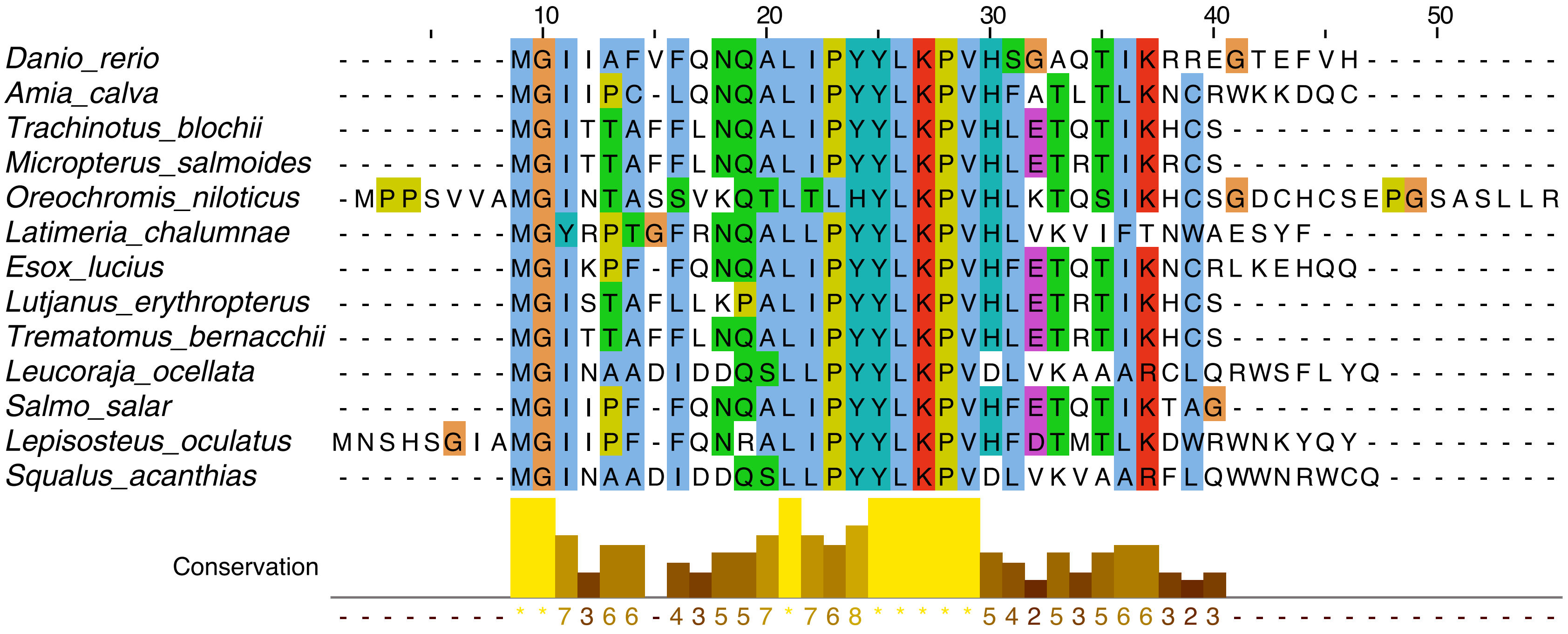

HG0190

ENSGALG00000006949 (CHD2)

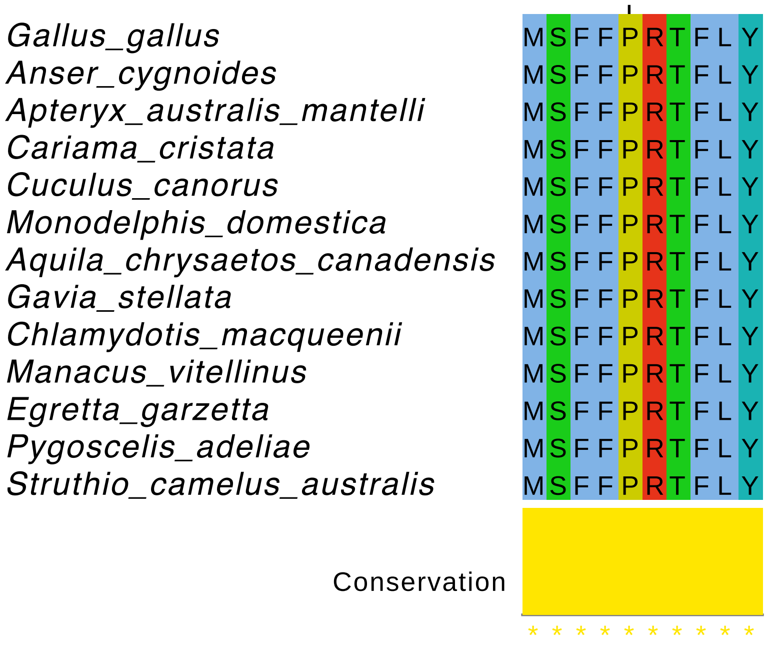

HG0191

ENSGALG00000036097

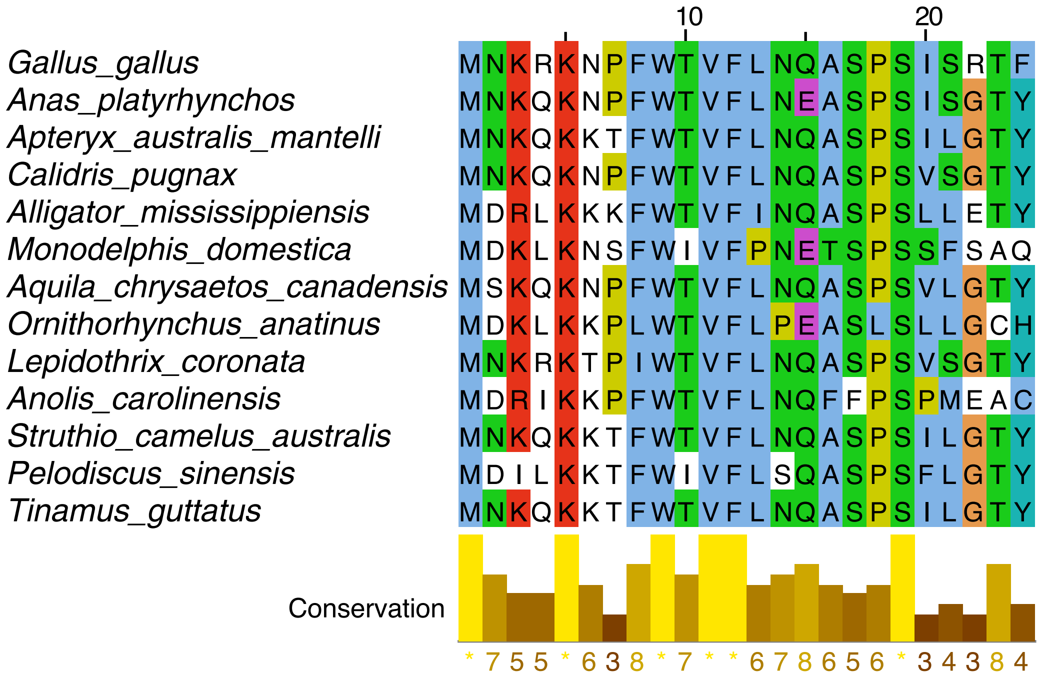

HG0192

ENSG00000125107 (CNOT1)

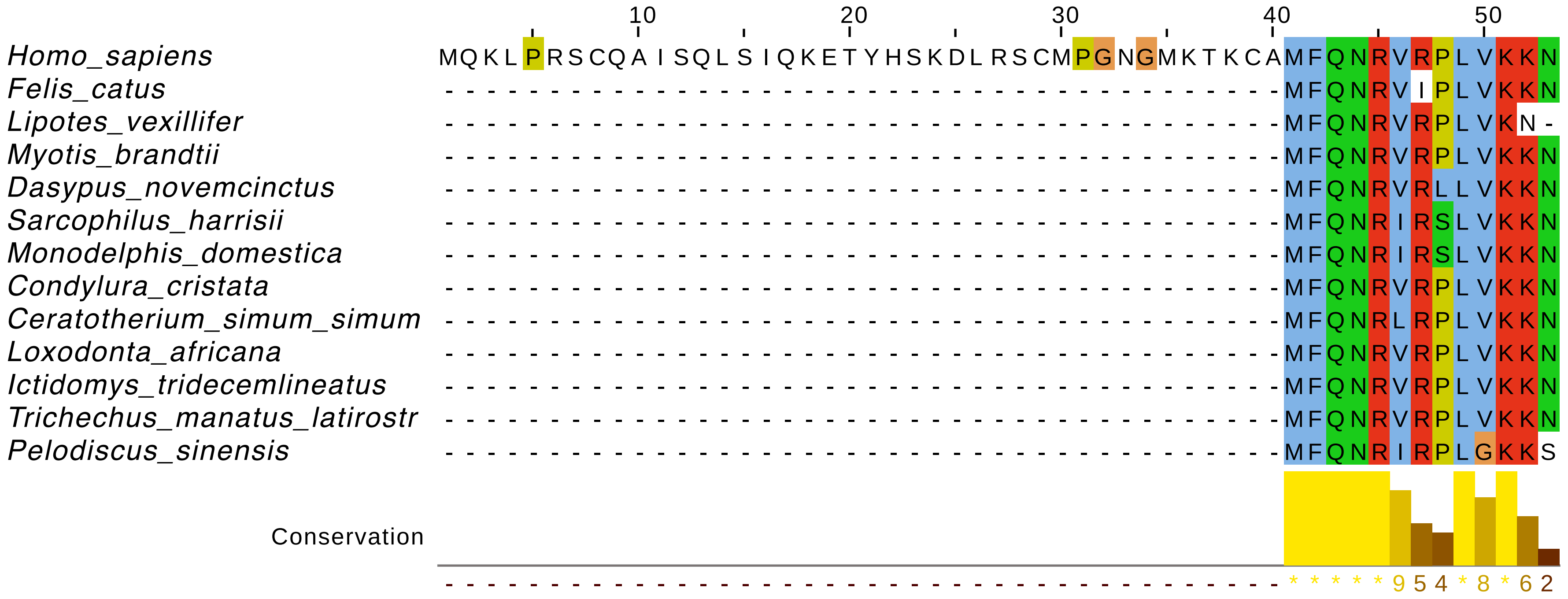

HG0193

ENSG00000134853 (PDGFRA)

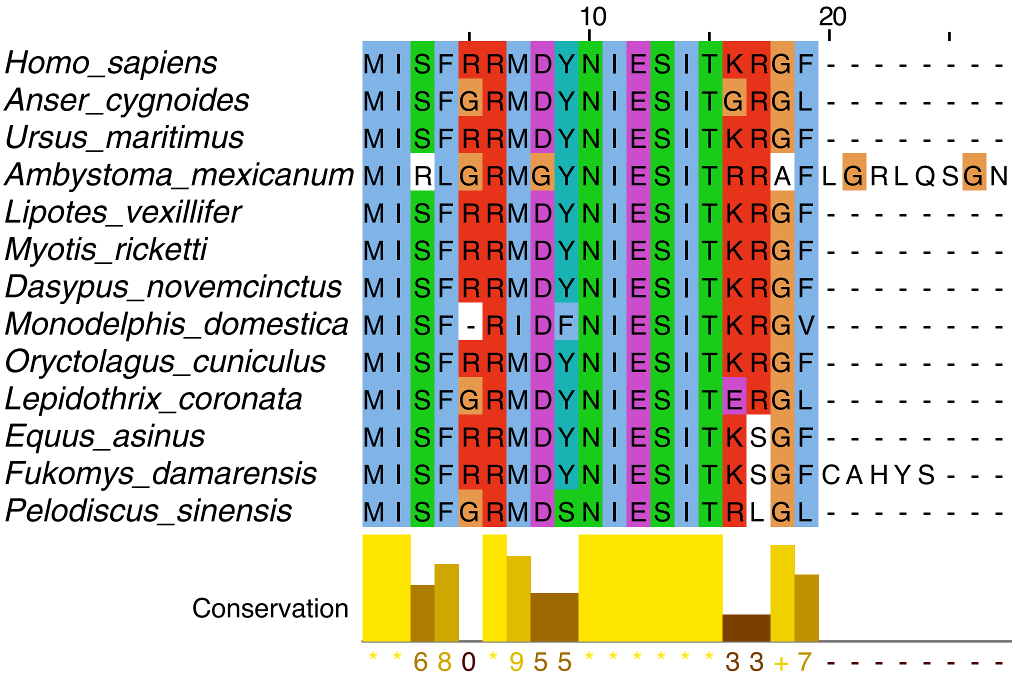

HG0194

ENSG00000143507 (DUSP10)

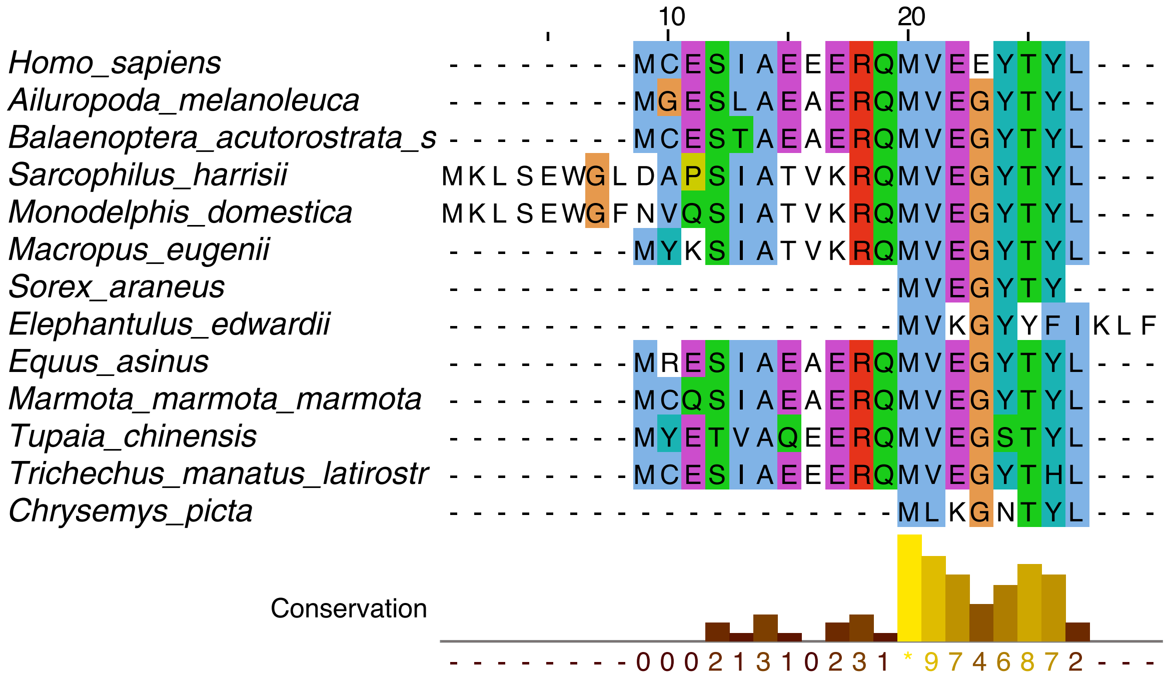

HG0195

ENSG00000165966 (PDZRN4)

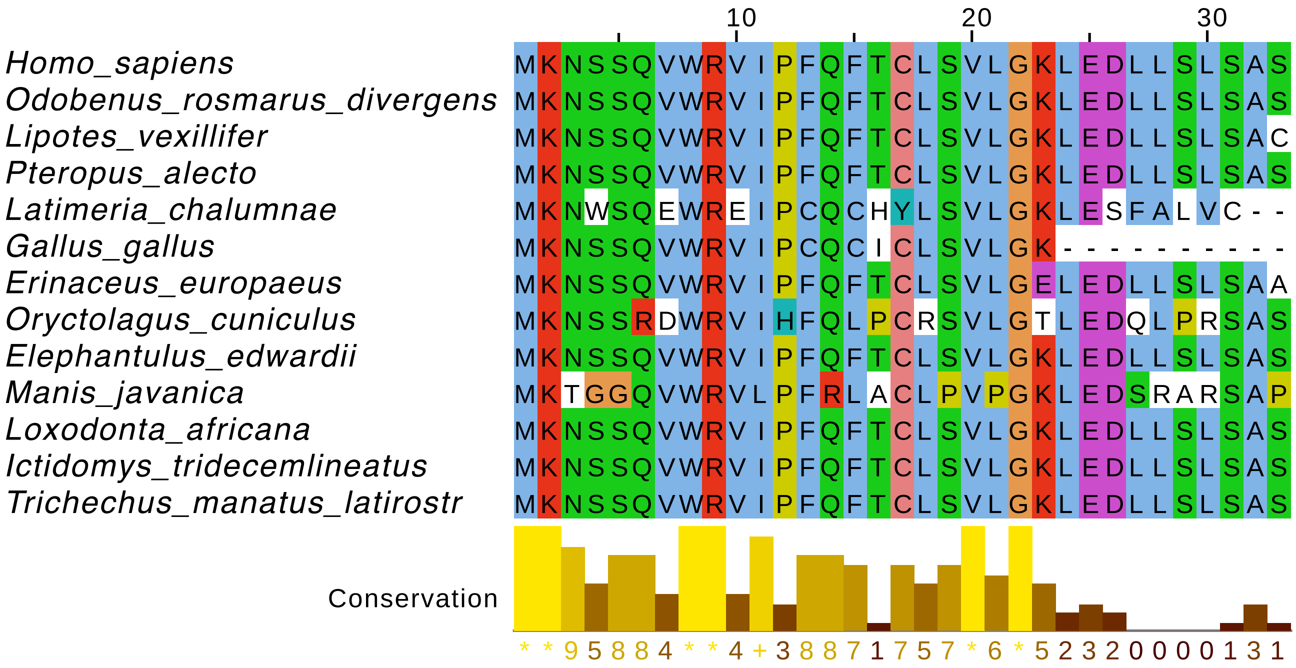

HG0196

ENSG00000189403 (HMGB1)

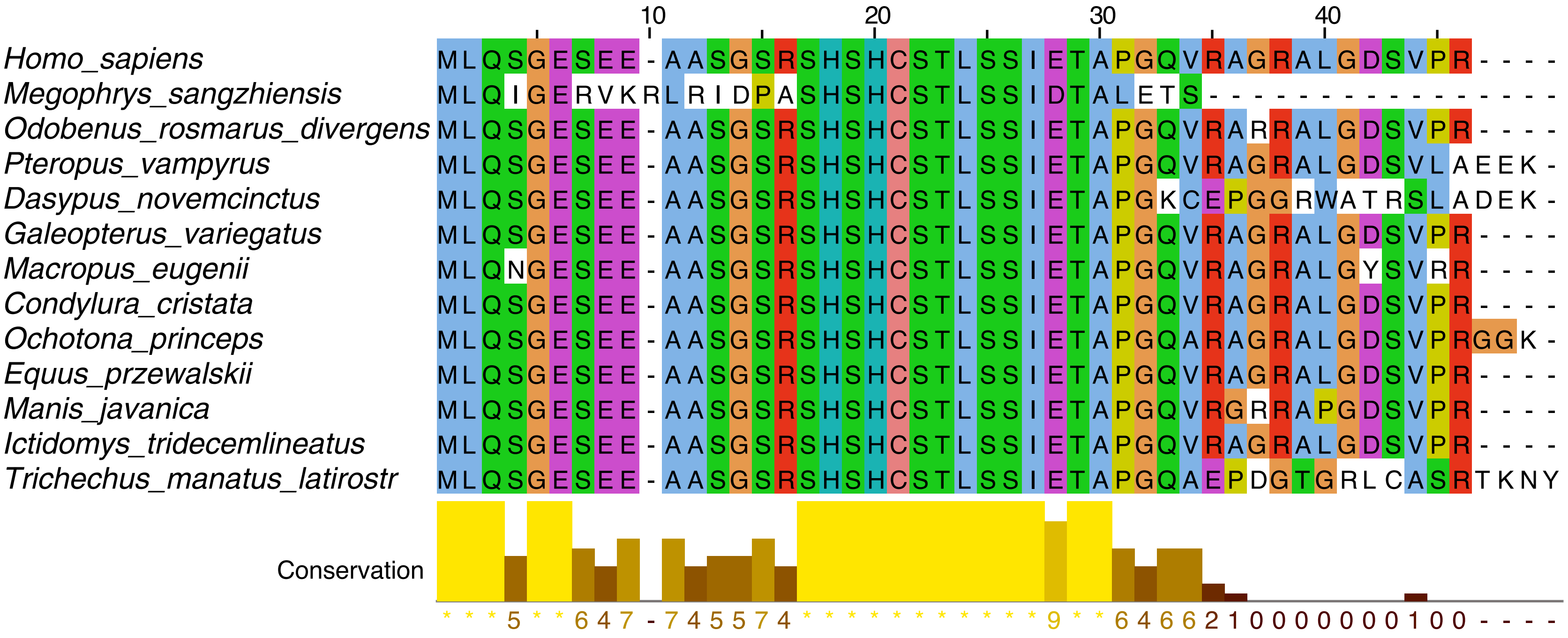

HG0197

ENSG00000196132 (MYT1)

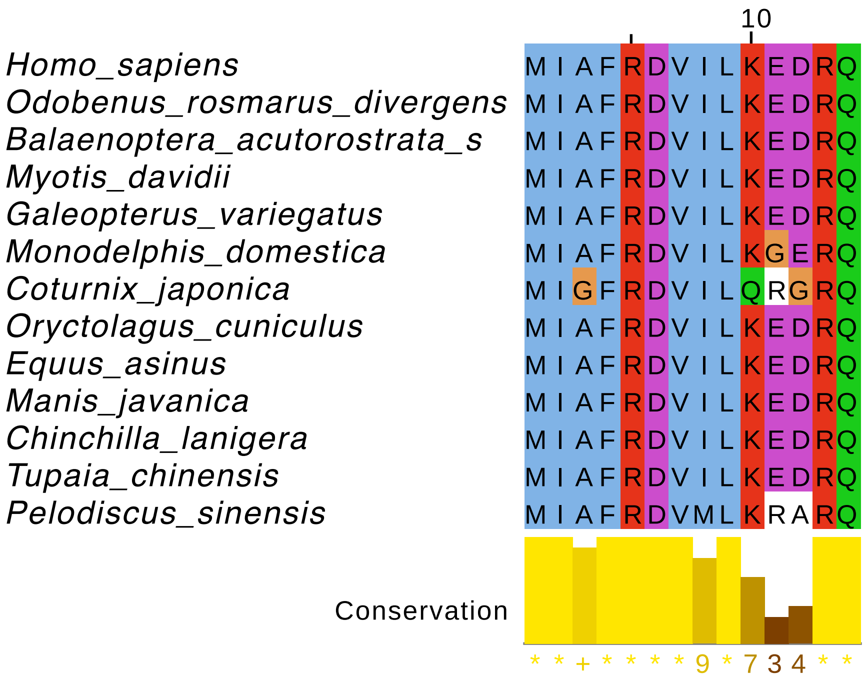

HG0198.1

ENSDARG00000018688 (elk3)

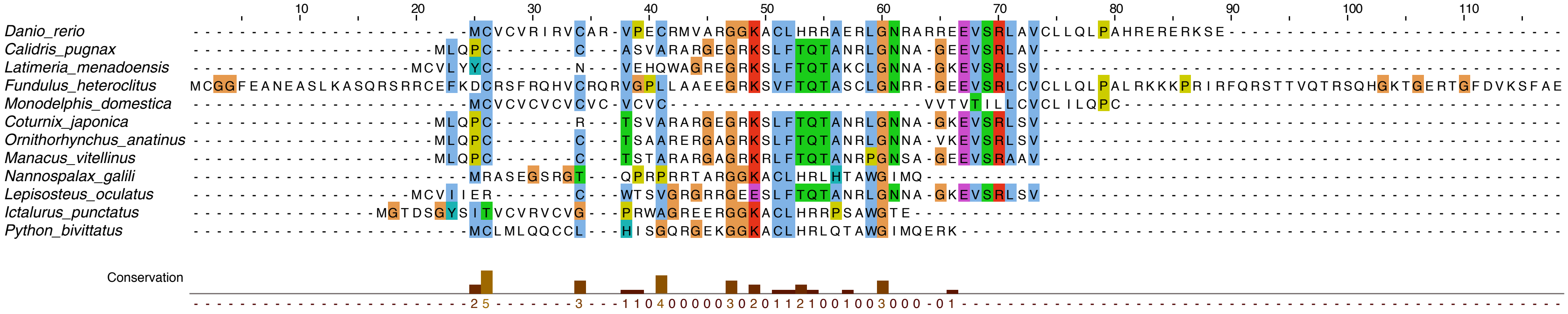

HG0198.2

ENSG00000111145 (ELK3)

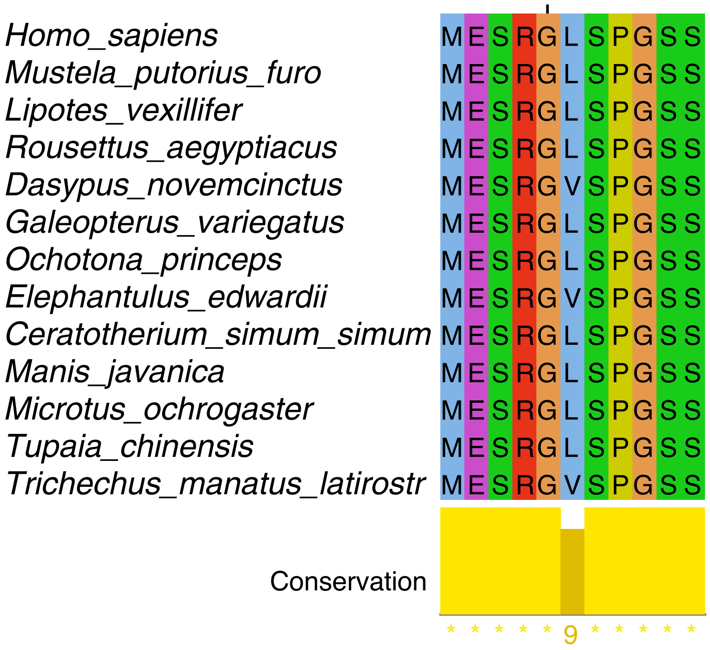

HG0199

ENSGALG00000007989 (ADNP)

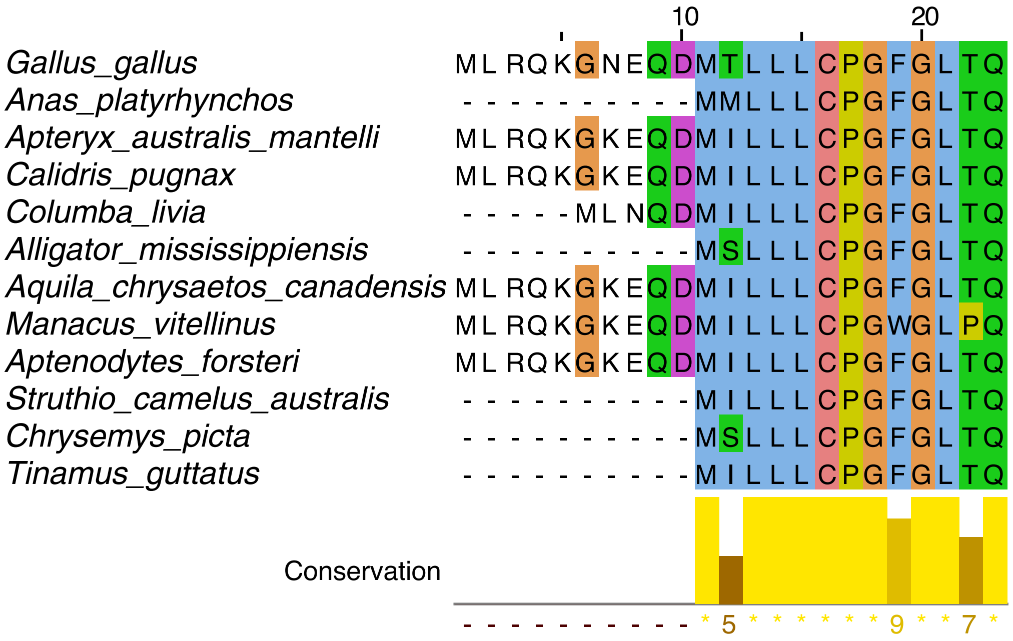

HG0200

ENSGALG00000012015 (NDST4)

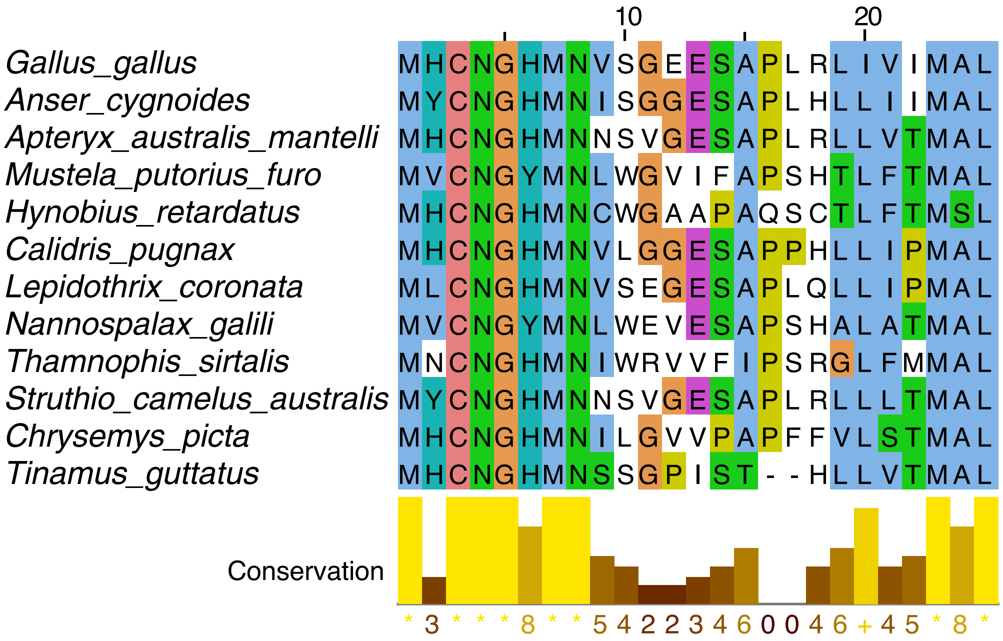

HG0201

ENSGALG00000015256 (PAM)

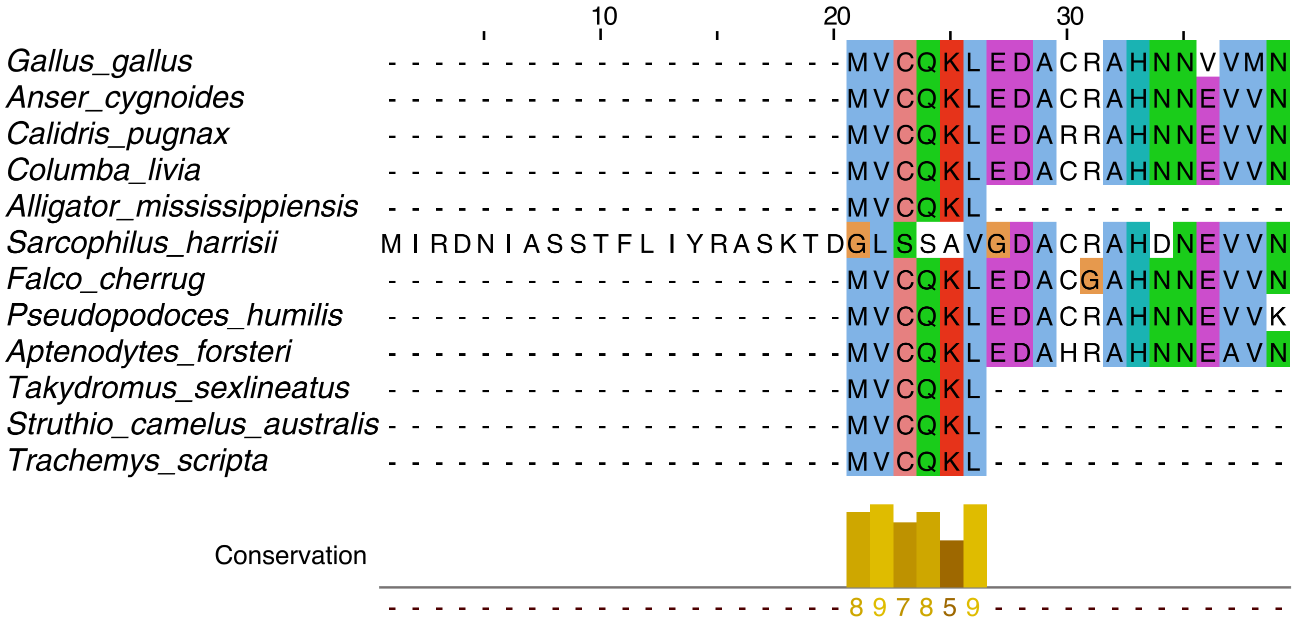

HG0202

ENSG00000124766 (SOX4)

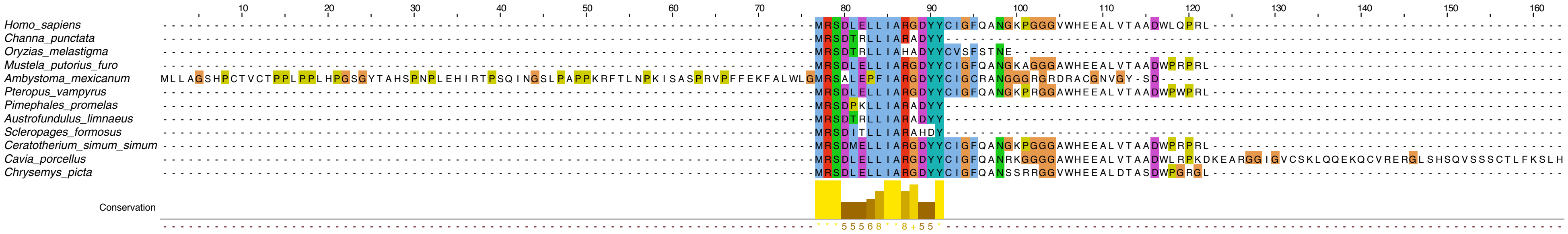

HG0203

ENSG00000128606 (LRRC17)

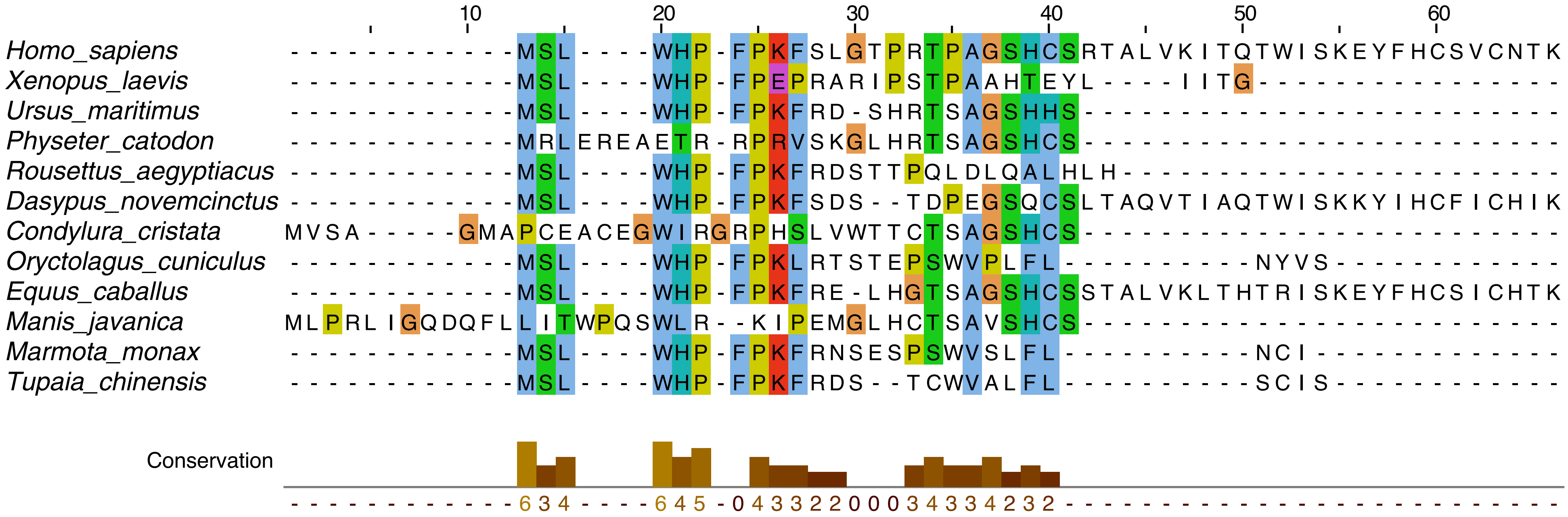

HG0204

ENSG00000162599 (NFIA)

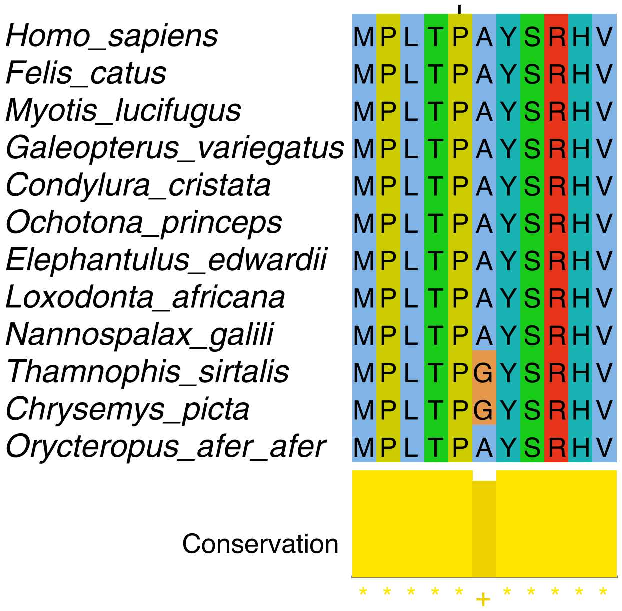

HG0205

ENSG00000184611 (KCNH7)

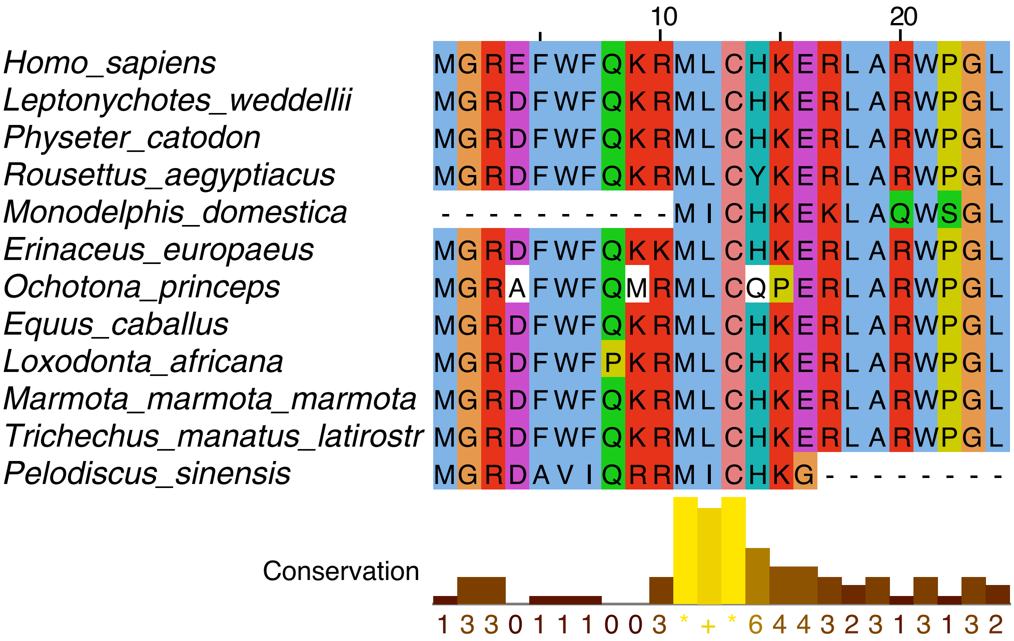

HG0206.1

ENSGALG00000002069

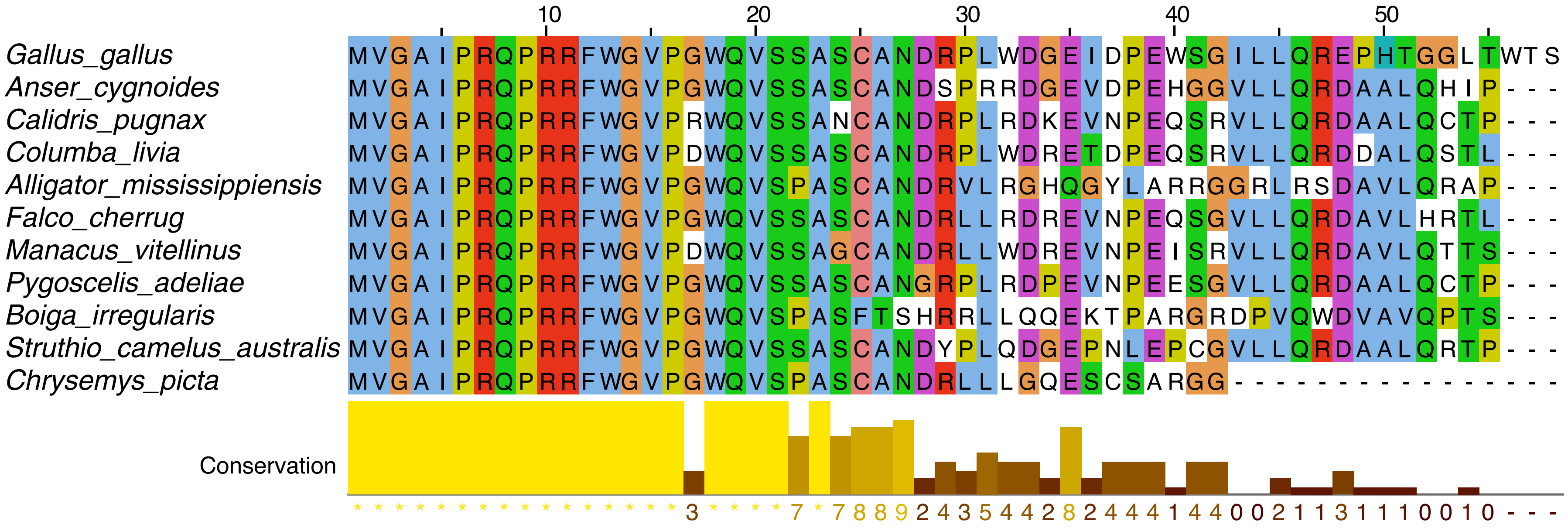

HG0206.2

ENSG00000005238 (FAM214B)

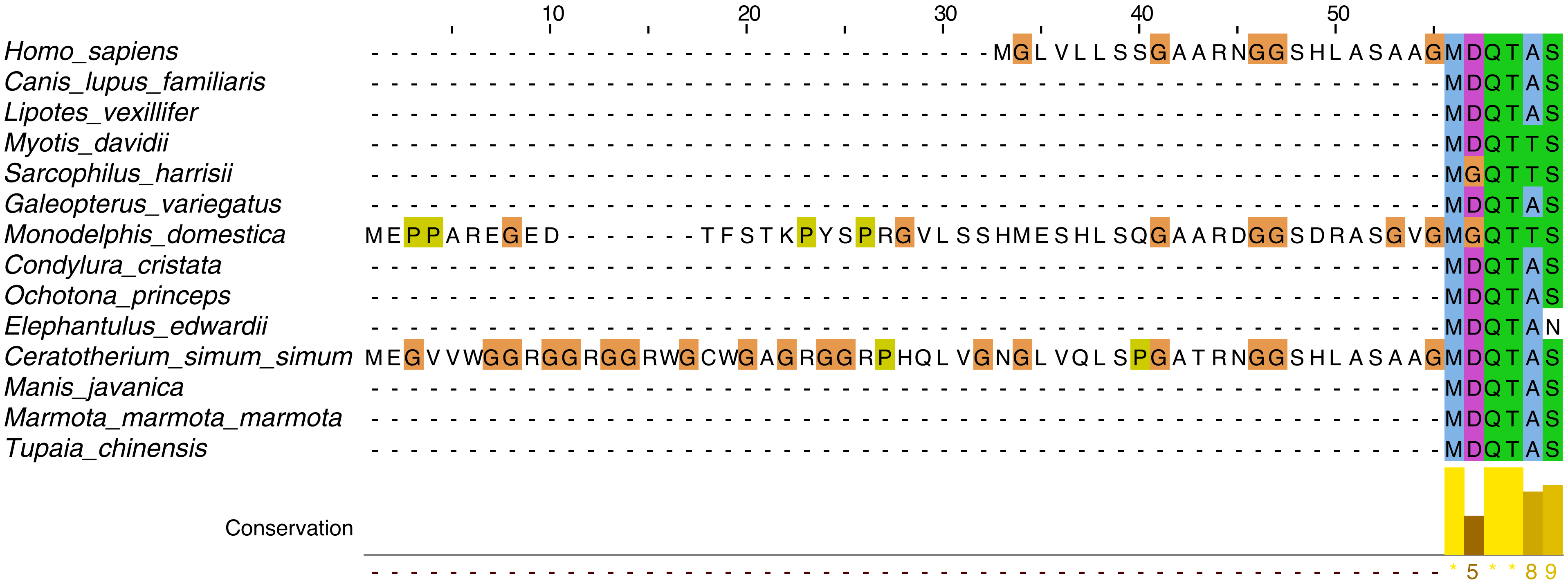

HG0207.1

ENSGALG00000007121 (TNFSF8)

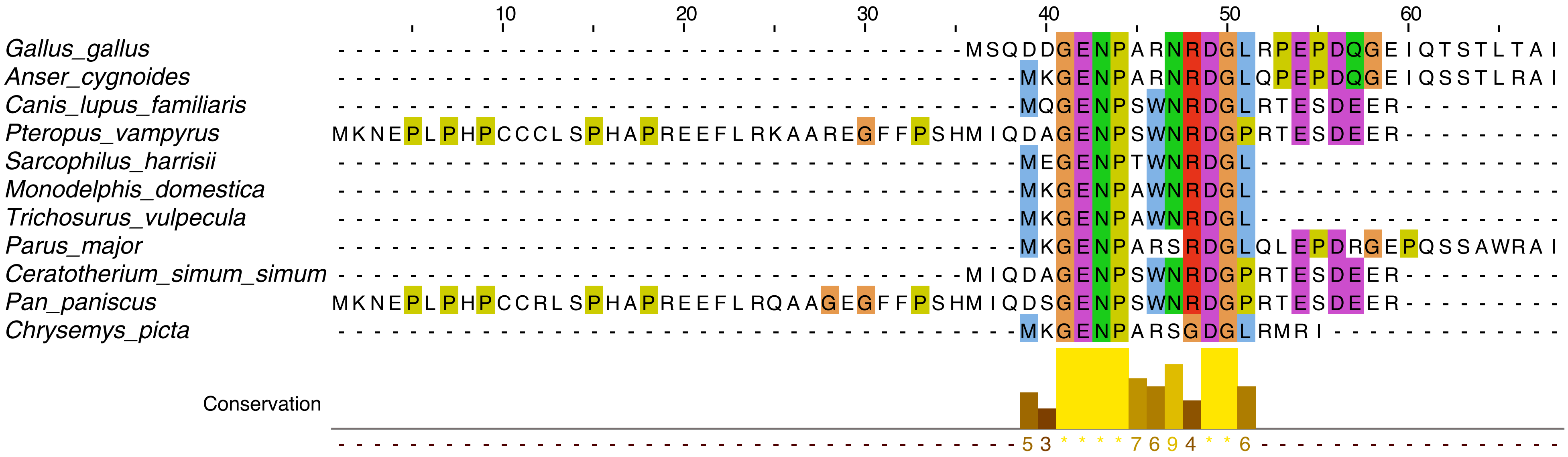

HG0207.2

ENSG00000106952 (TNFSF8)

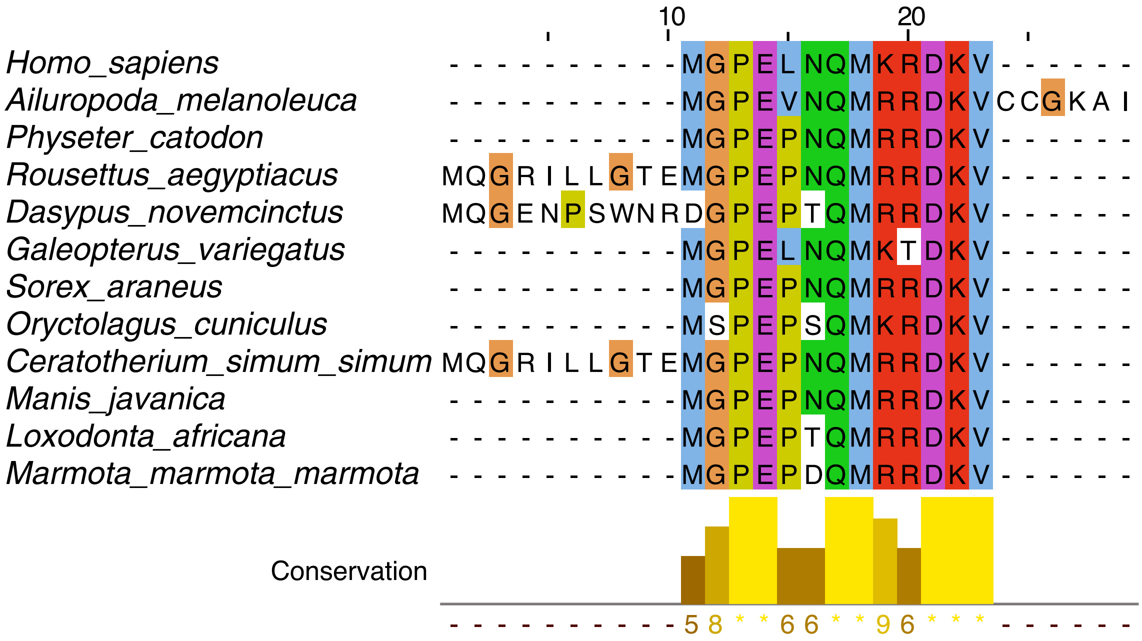

HG0208

ENSGALG00000013272 (PRMT8)

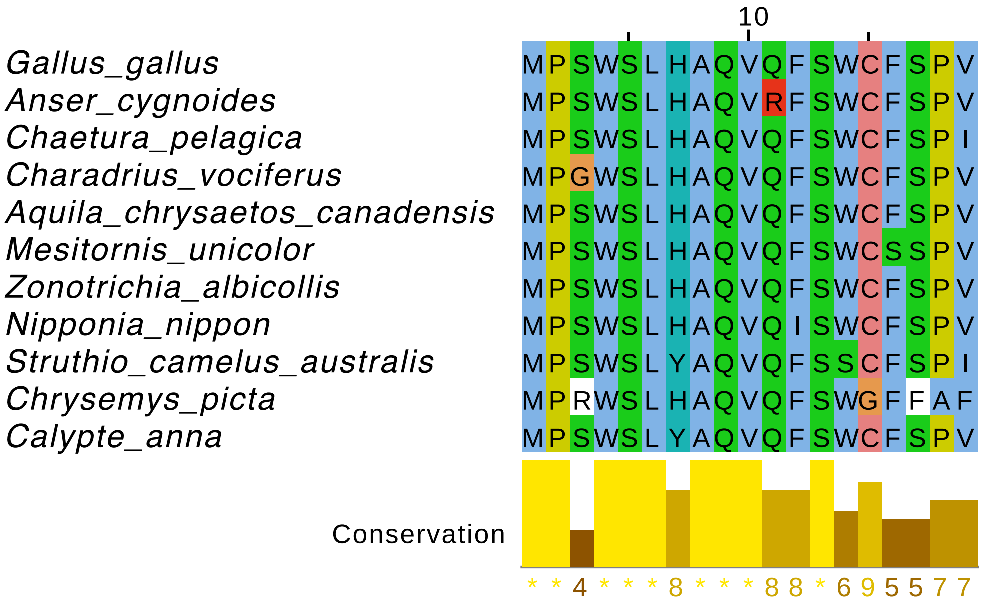

HG0209

ENSG00000101040 (ZMYND8)

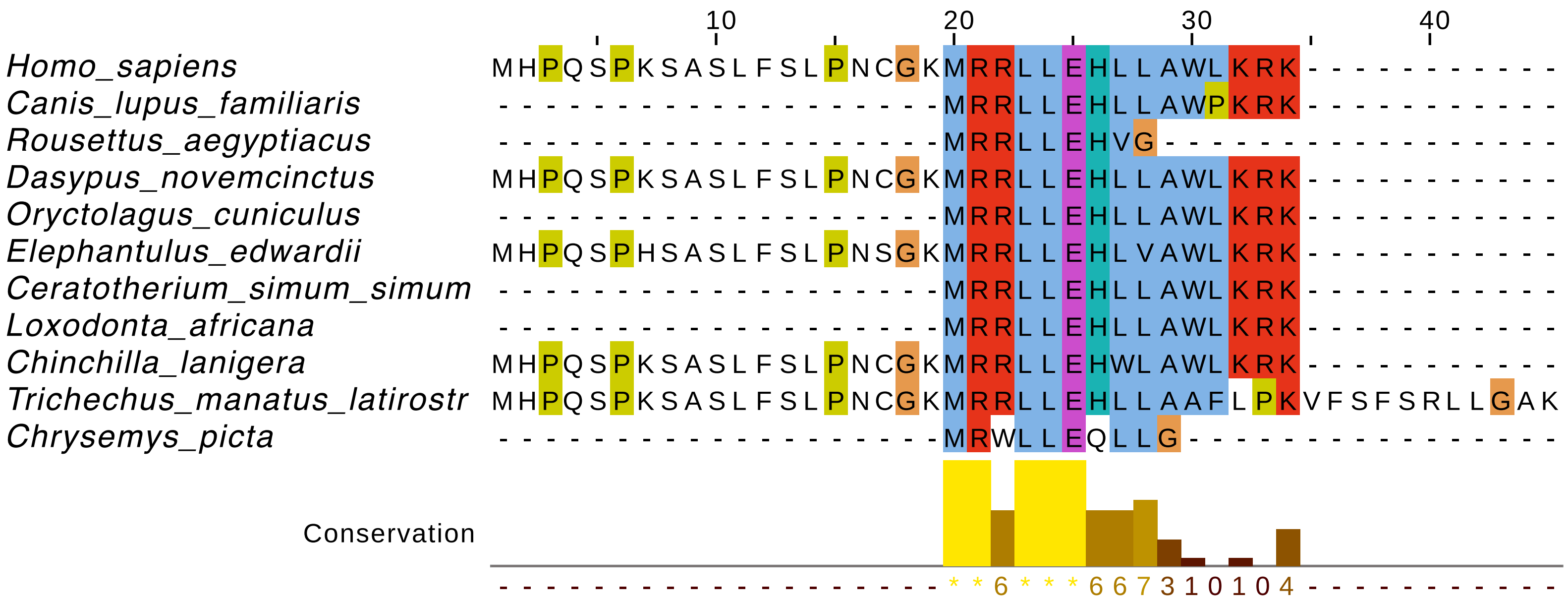

HG0210

ENSG00000155849 (ELMO1)

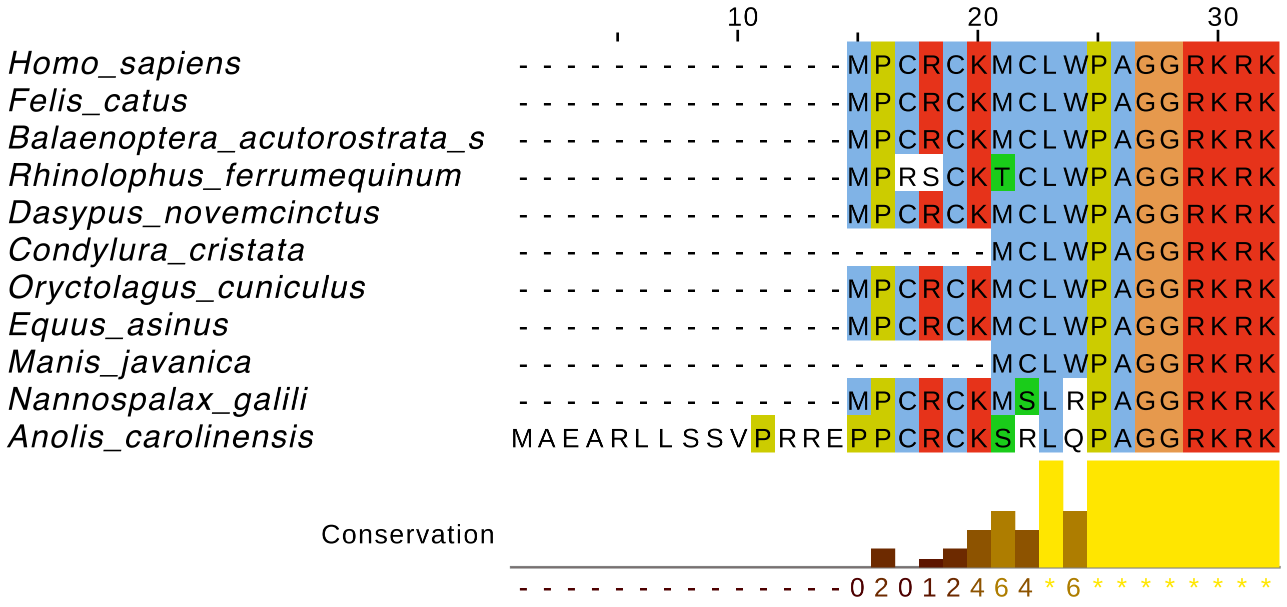

HG0211

ENSG00000176204 (LRRTM4)

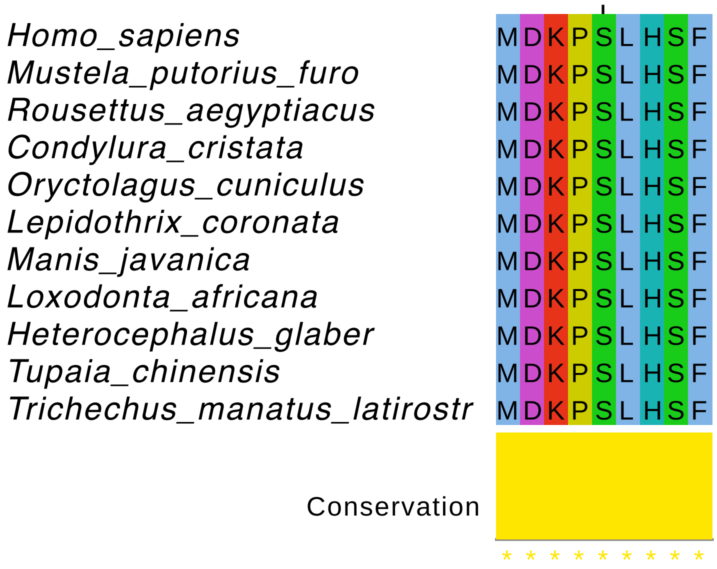

HG0212

ENSDARG00000100244 (ebf3a)

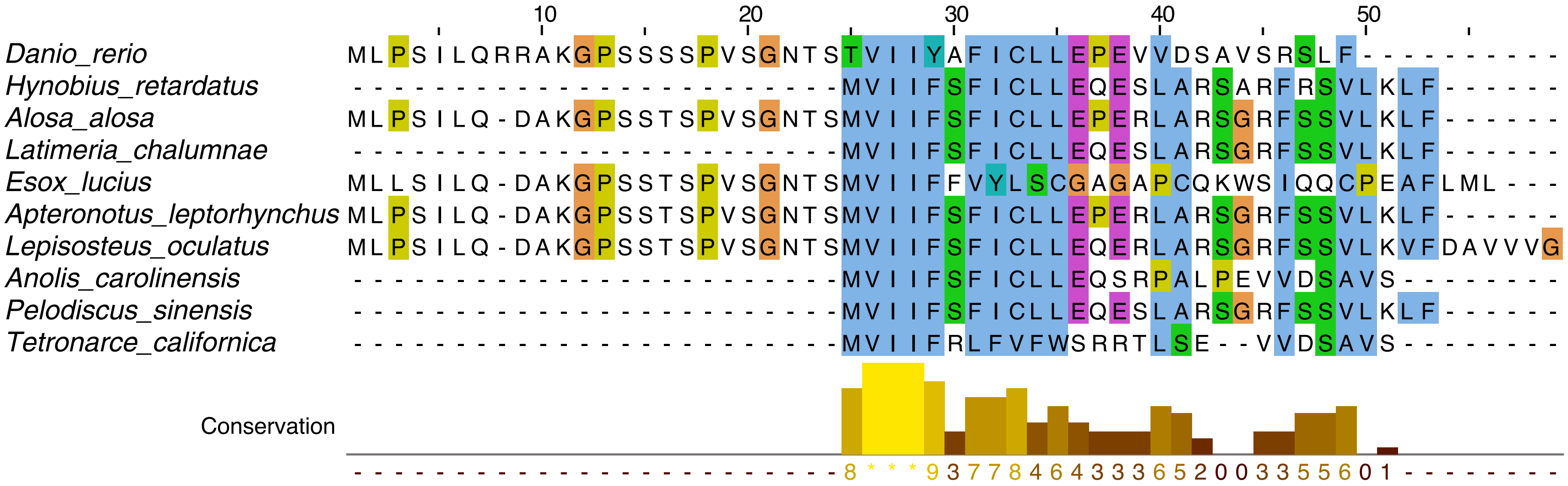

HG0213.1

ENSGALG00000001946 (RSPO1)

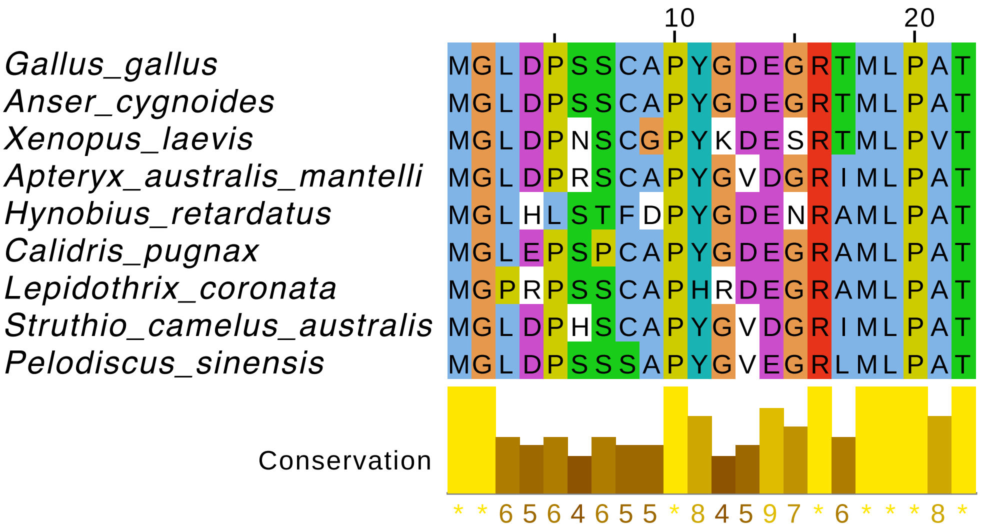

HG0213.2

ENSGALG00000001946 (RSPO1)

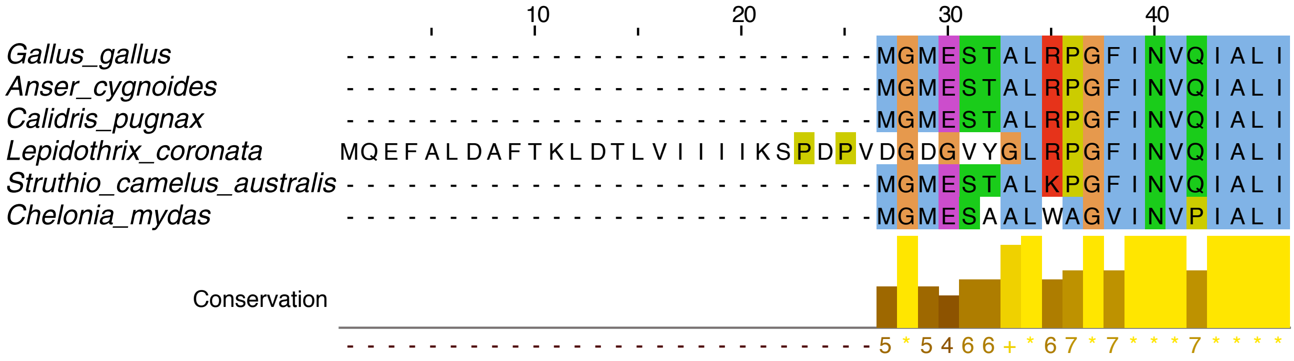

HG0214.1

ENSGALG00000012917 (CDH6)

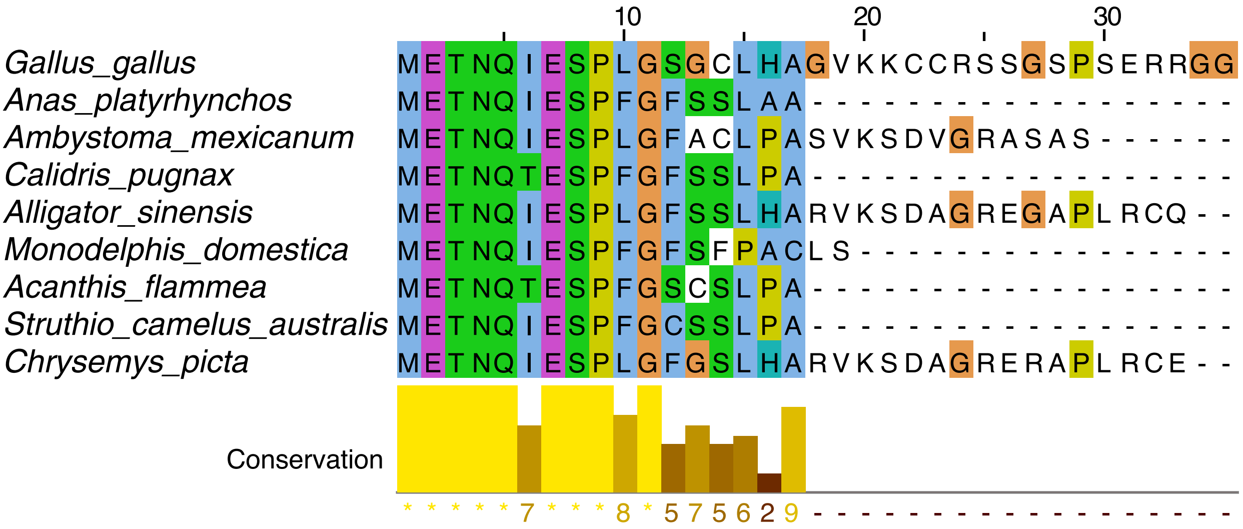

HG0214.2

ENSG00000040731 (CDH10)

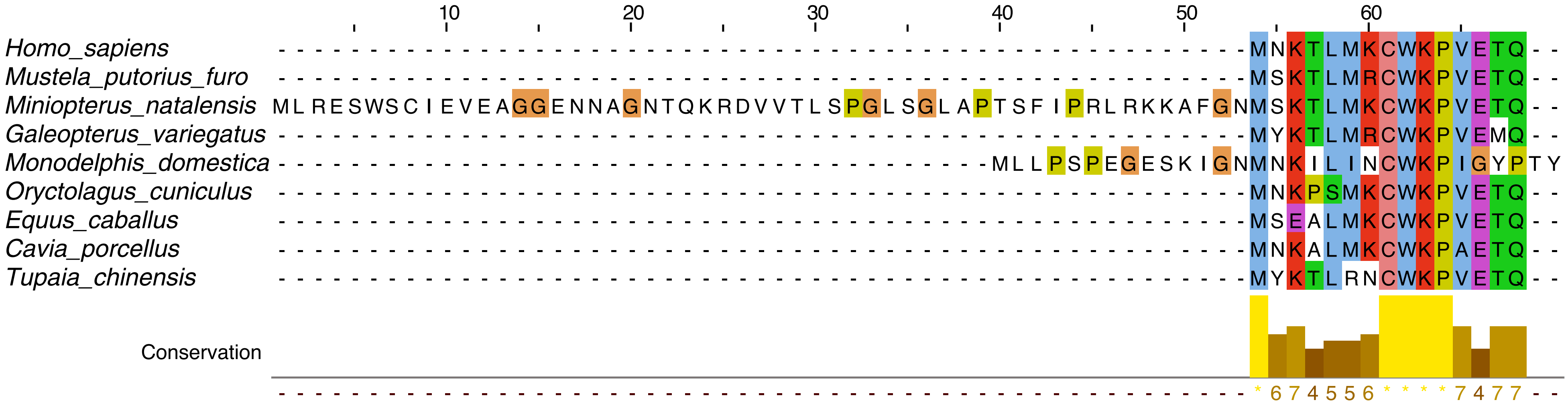

HG0215

ENSDARG00000079280 (dhrsx)

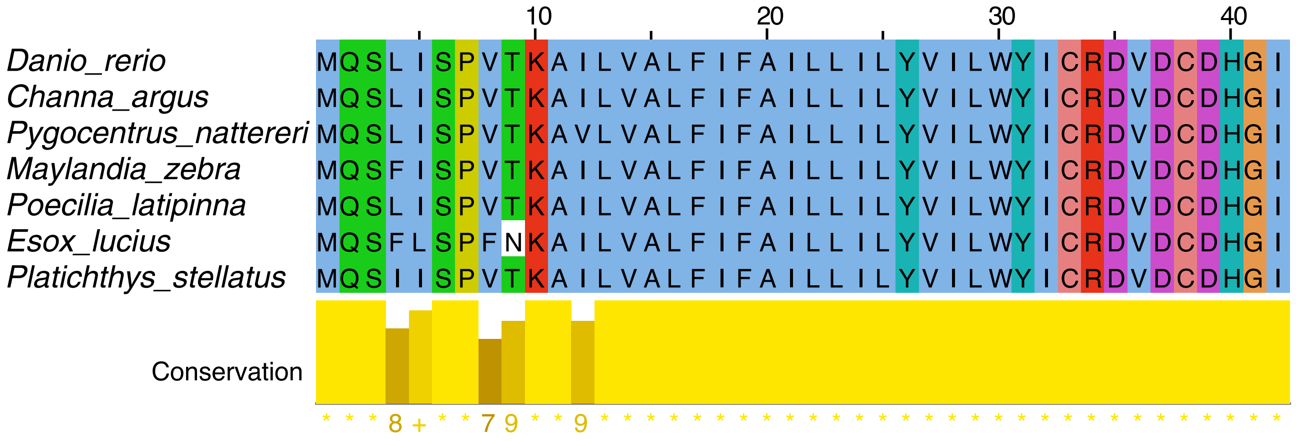

HG0215

ENSGALG00000016681 (DHRSX)

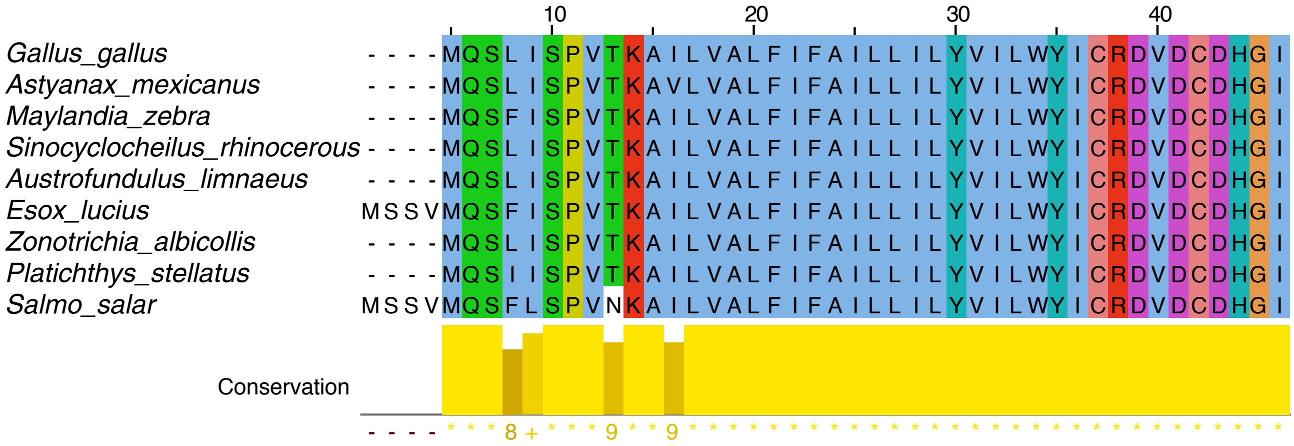

HG0216.1

ENSGALG00000020922

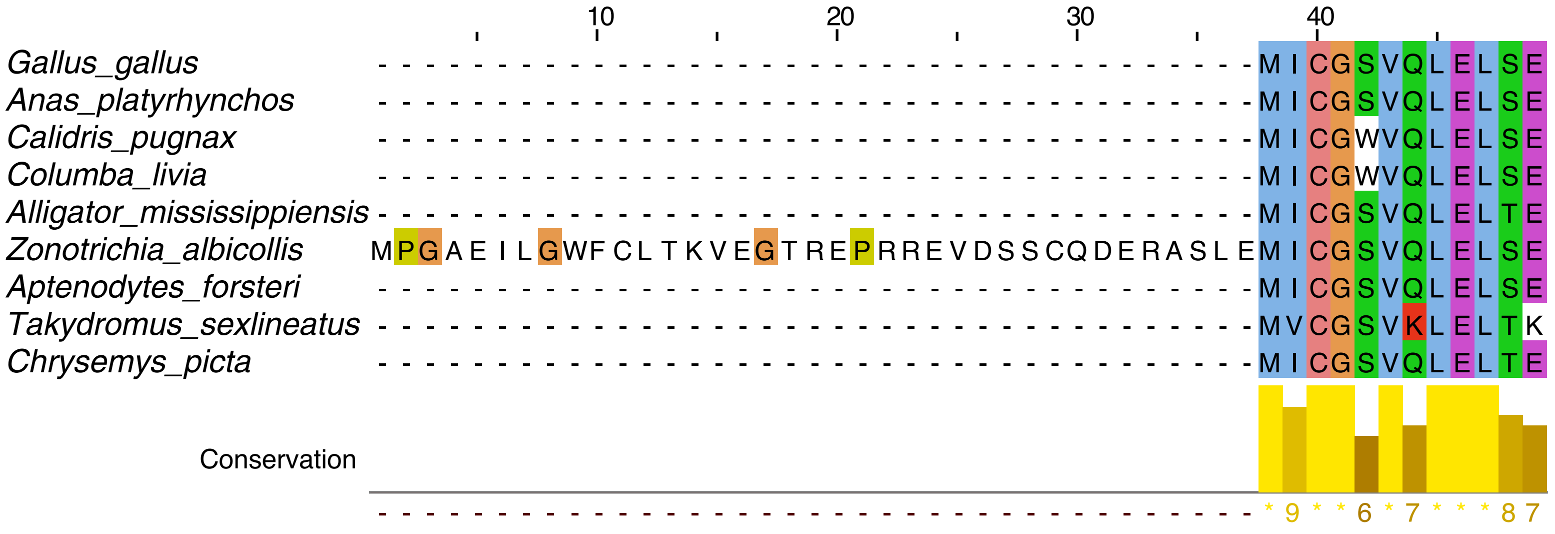

HG0216.2

ENSG00000116667 (C1orf21)

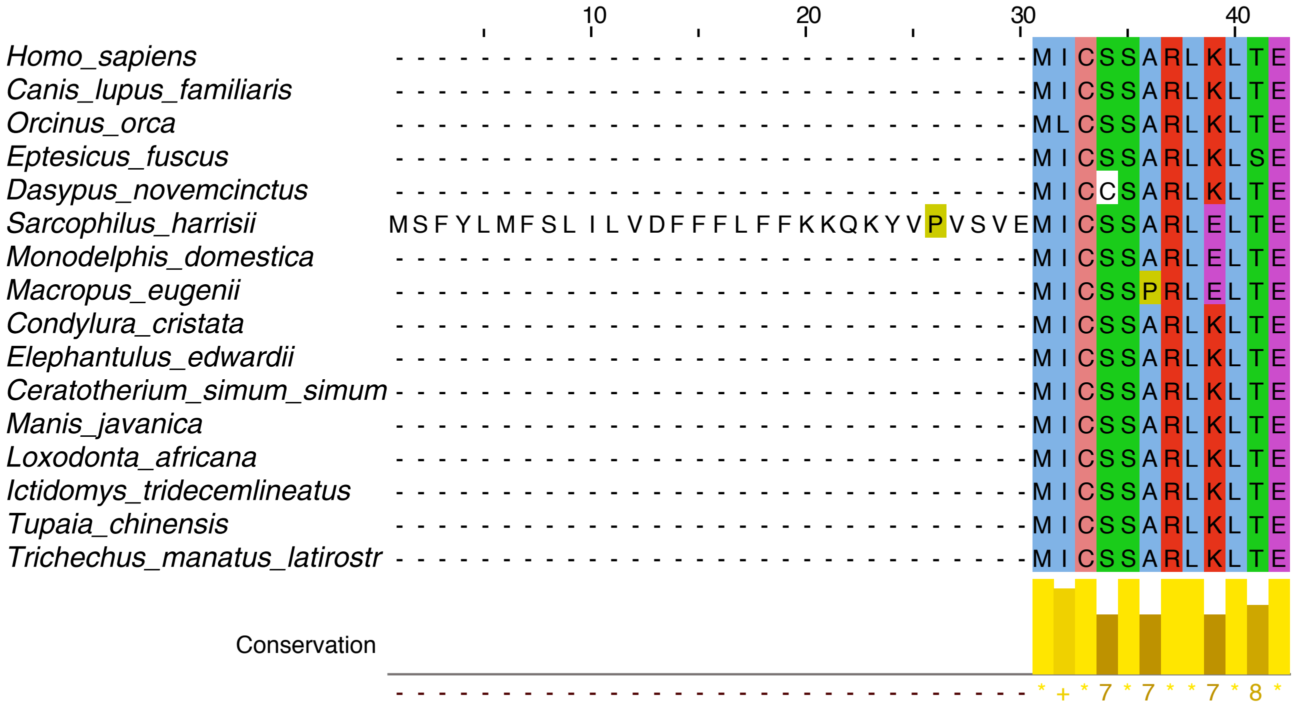

HG0217.1

ENSG00000150394 (CDH8)

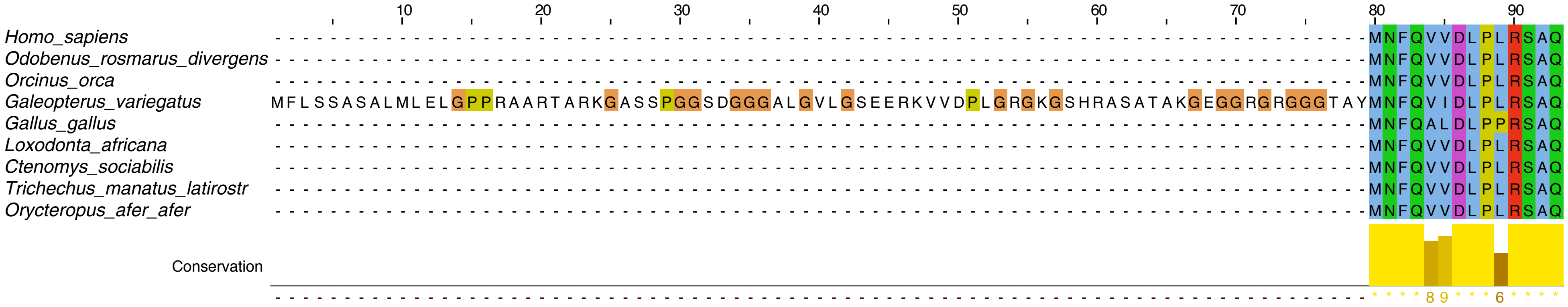

HG0217.2

ENSGALG00000005319 (CDH8)

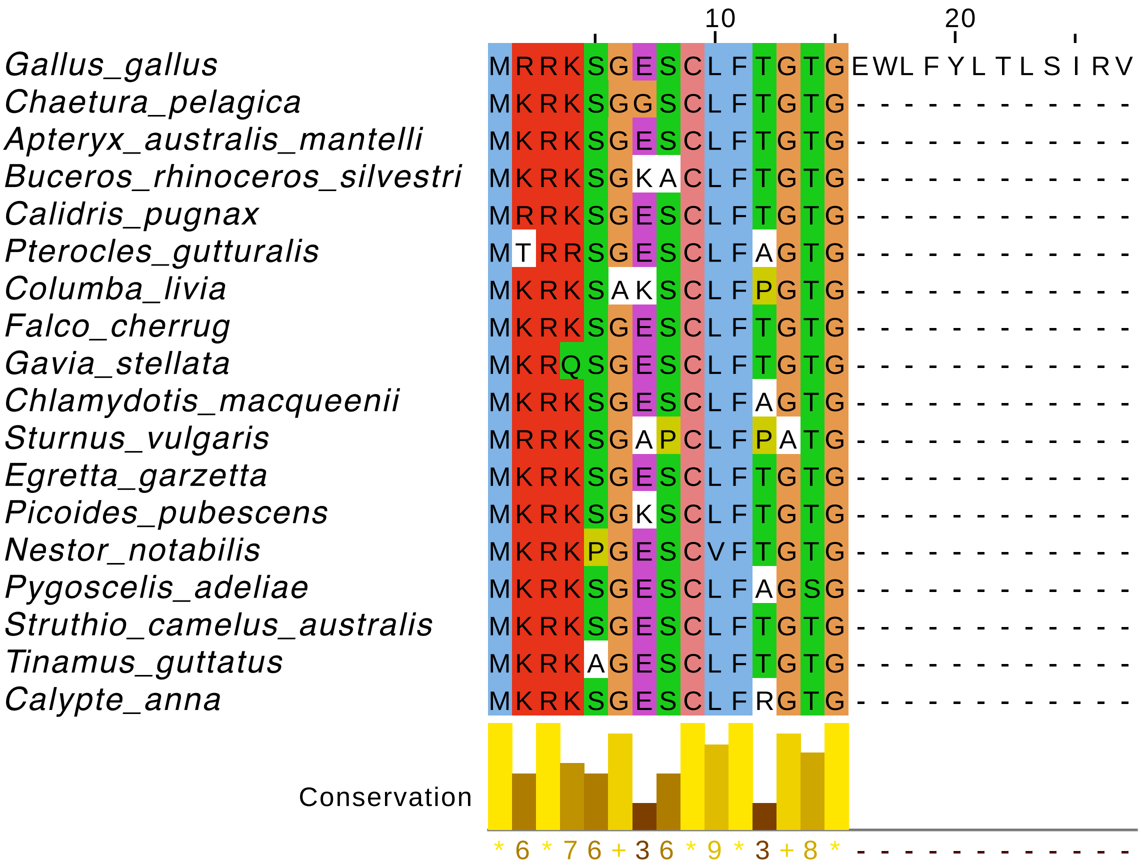

HG0218

ENSDARG00000100149 (axin2)

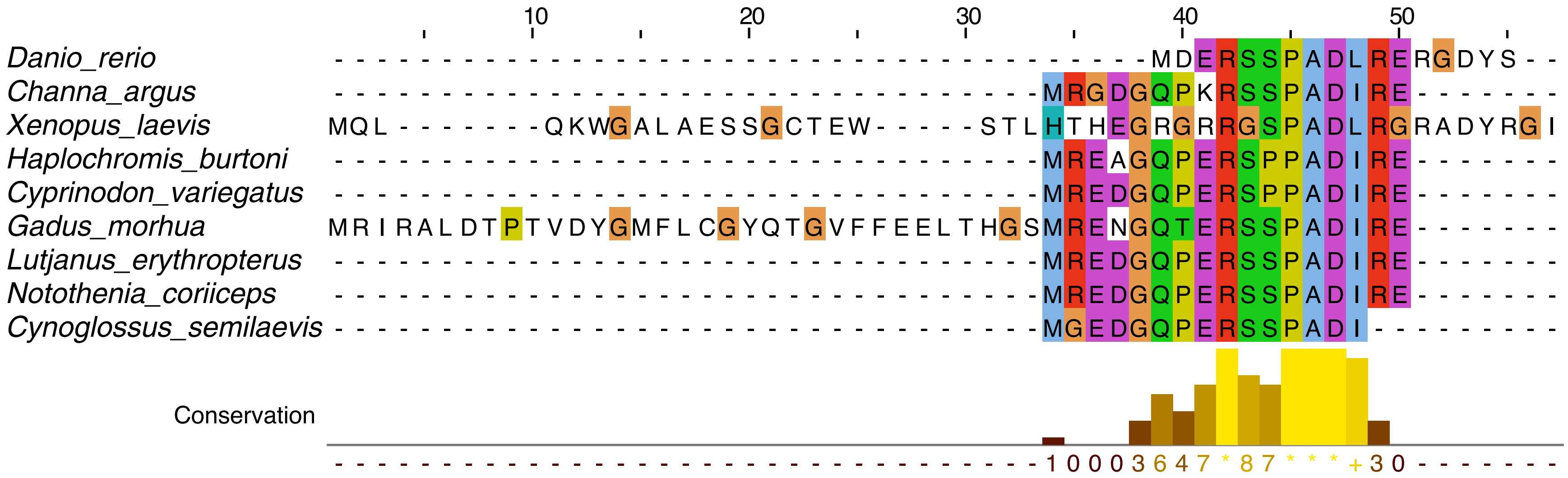

HG0219

ENSDARG00000104082 (trps1)

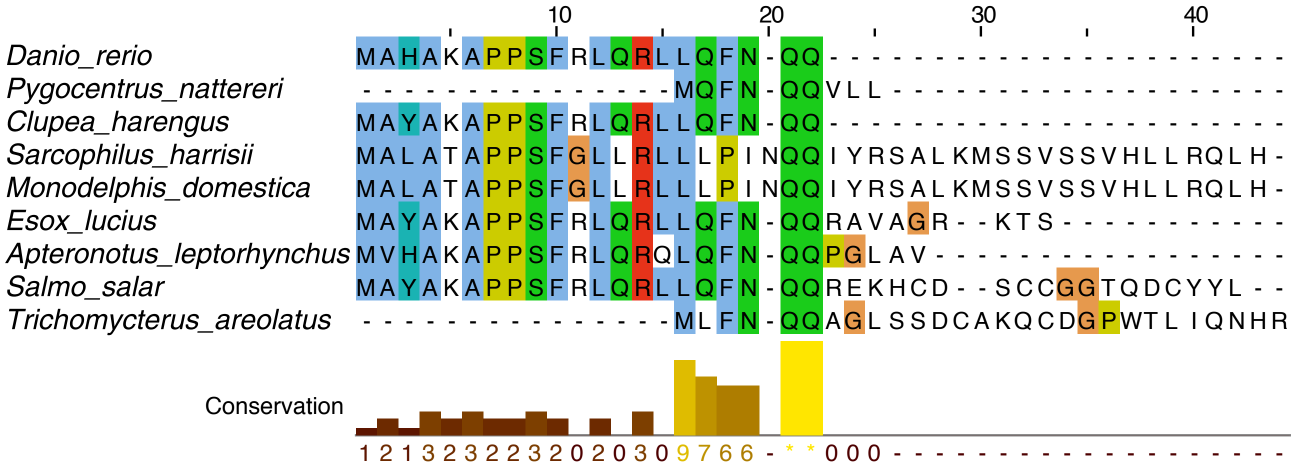

HG0220

ENSGALG00000008418 (LARGE2)

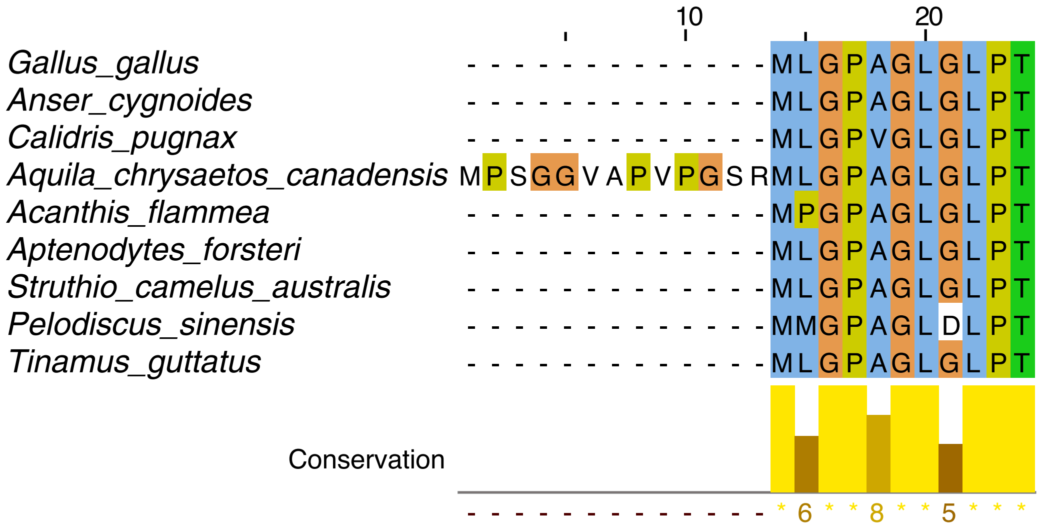

HG0221

ENSGALG00000010859 (EPGN)

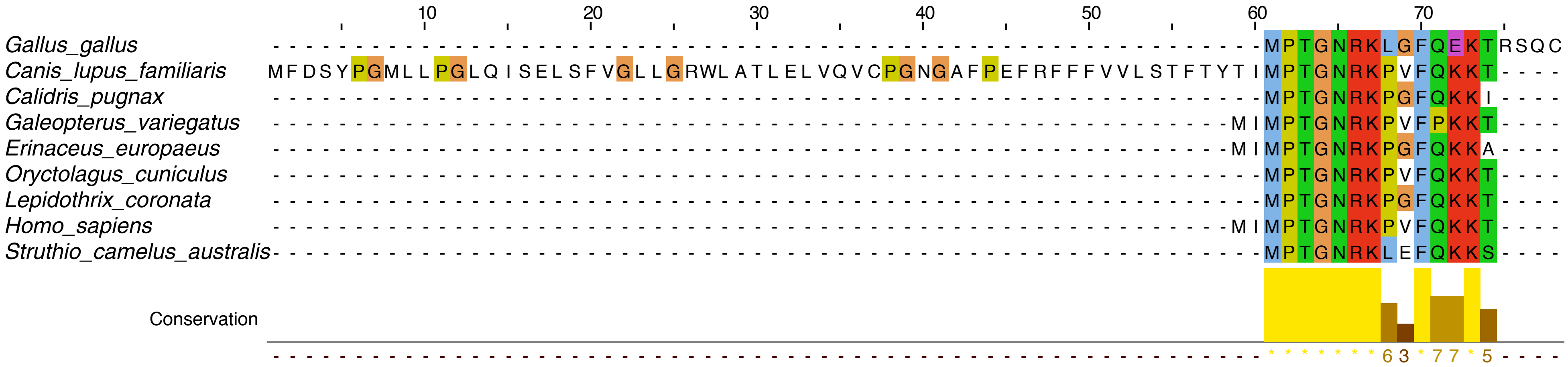

HG0222

ENSGALG00000011564 (RUFY3)

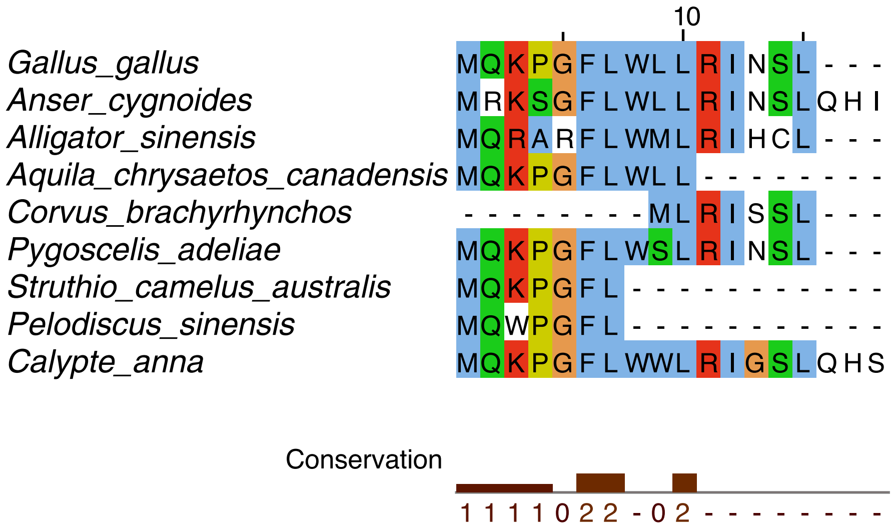

HG0223

ENSG00000091656 (ZFHX4)

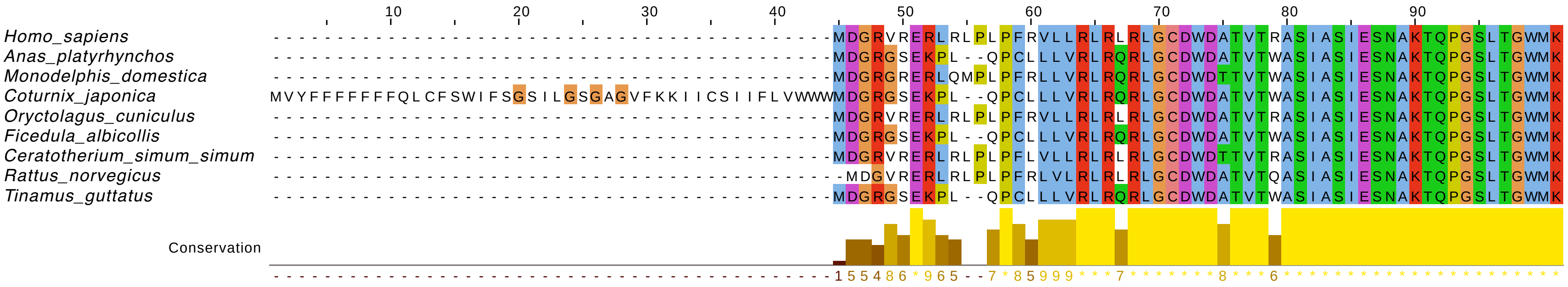

HG0224

ENSG00000118007 (STAG1)

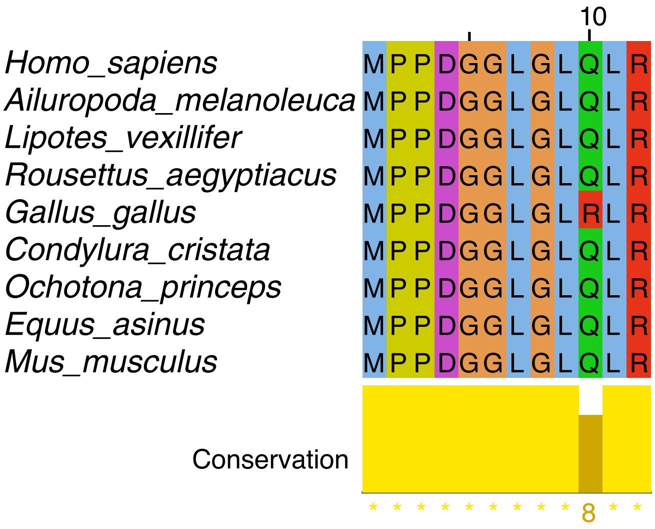

HG0225

ENSG00000150051 (MKX)

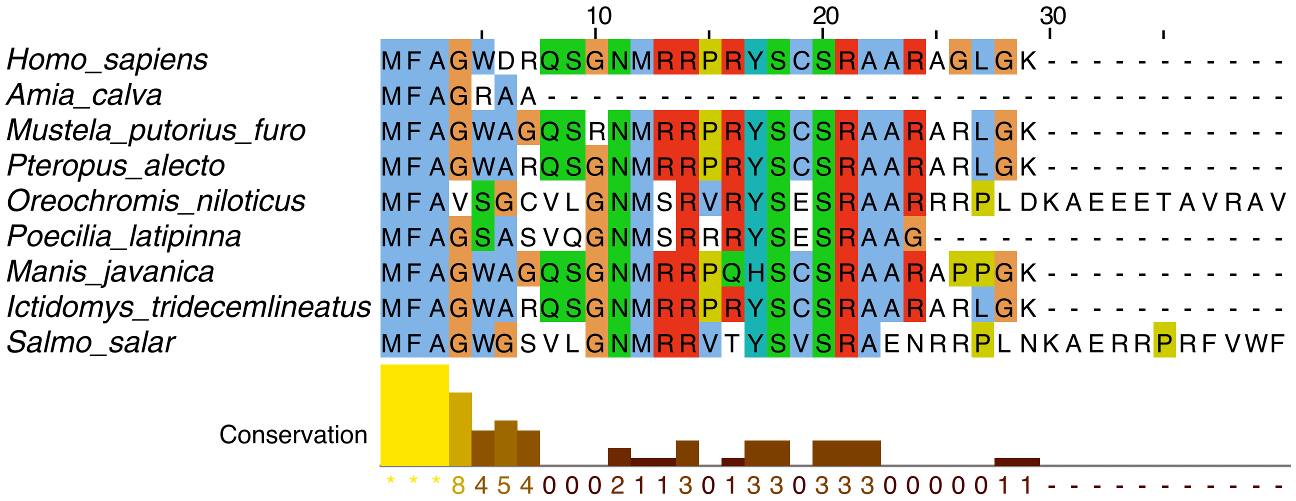

HG0226.1

ENSG00000180592

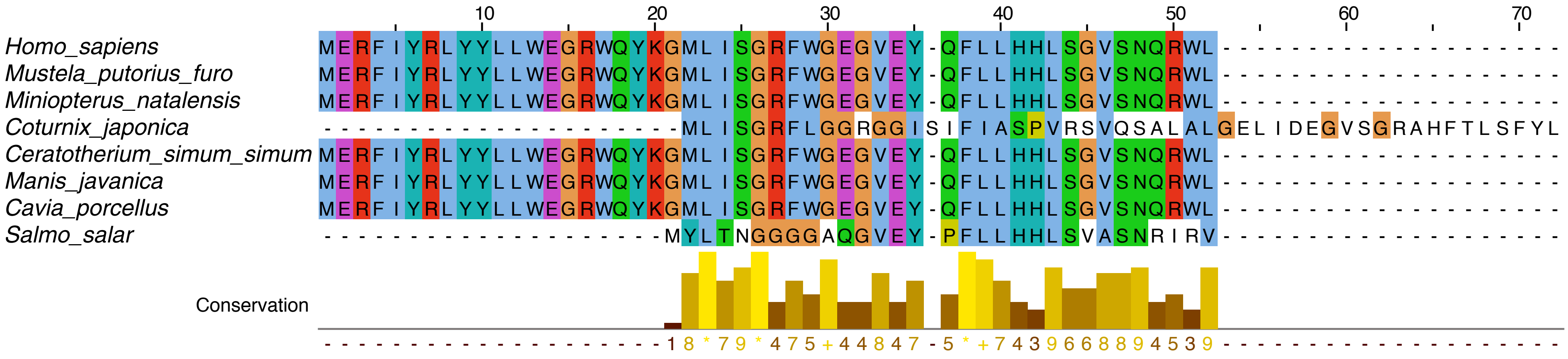

HG0226.2

ENSG00000180592

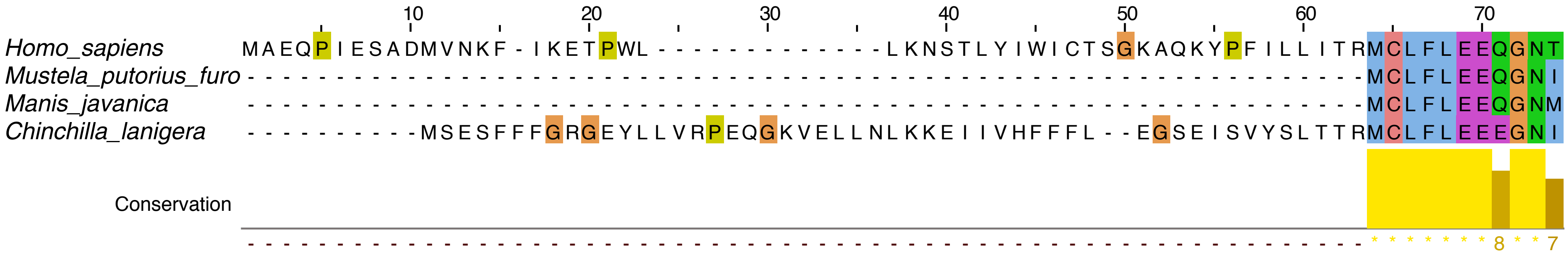

HG0226.3

ENSG00000180592

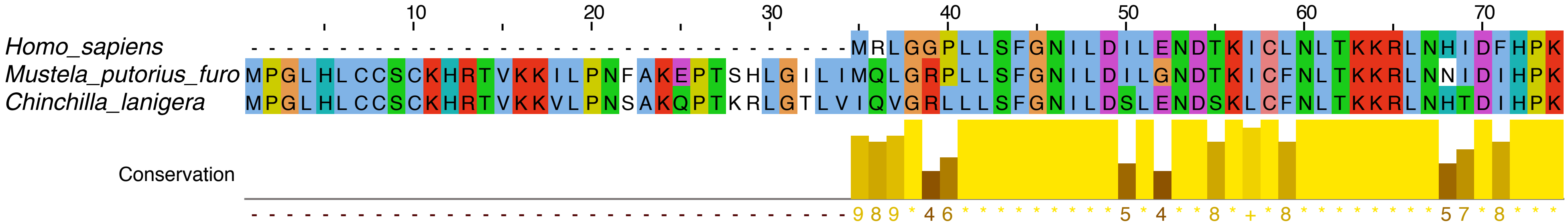

HG0227.1

ENSGALG00000031929

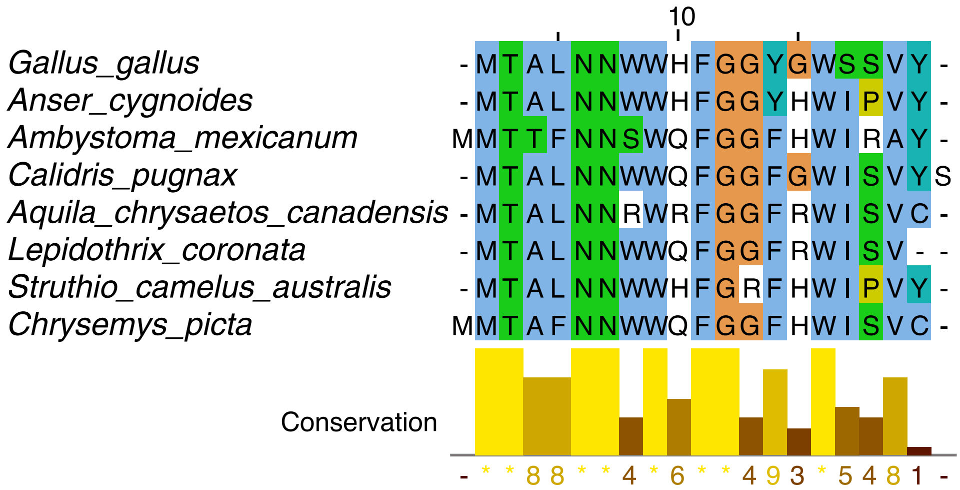

HG0227.2

ENSG00000275163

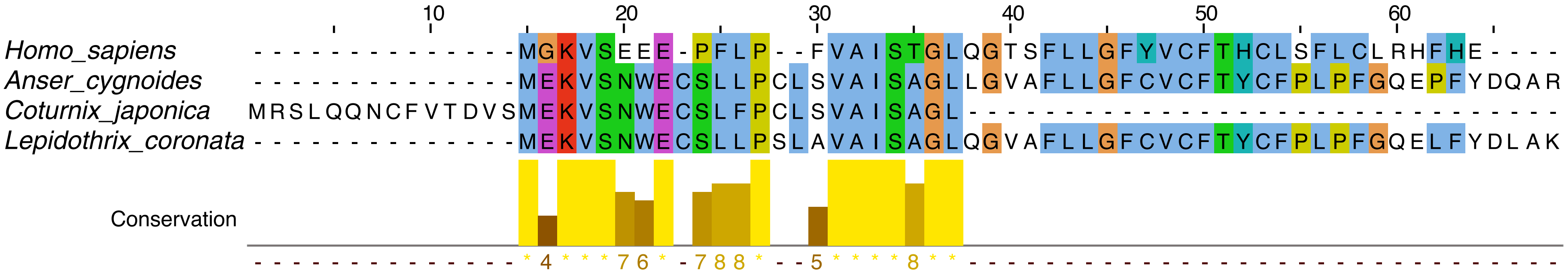

HG0228

ENSDARG00000087887 (phf21b)

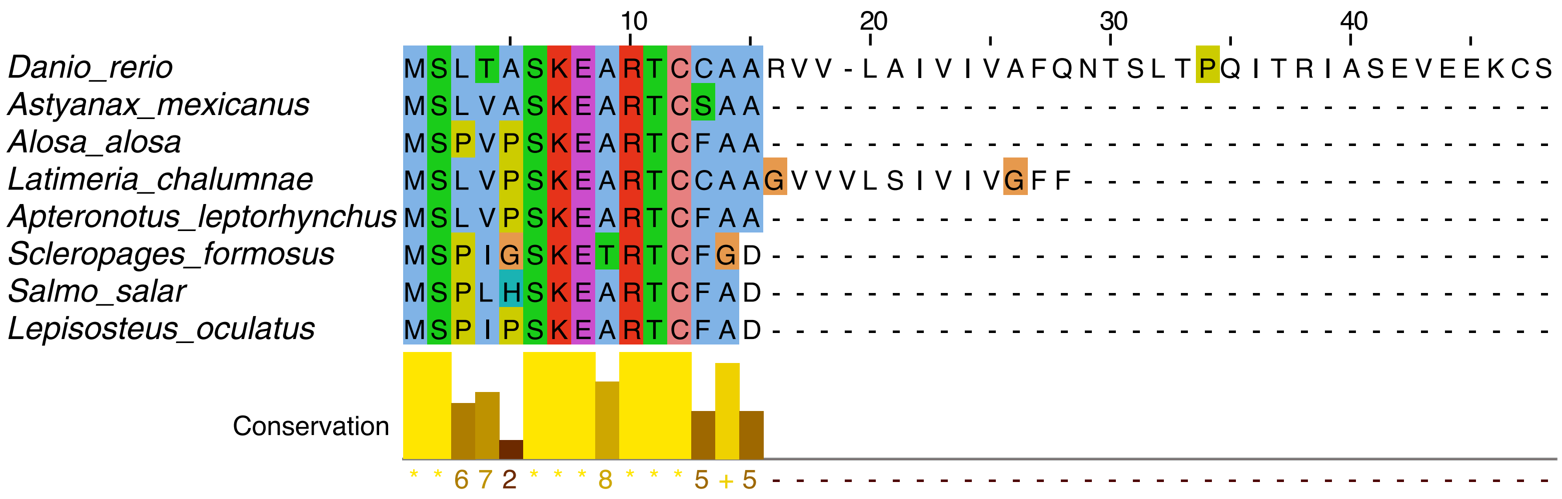

HG0229

ENSGALG00000005474

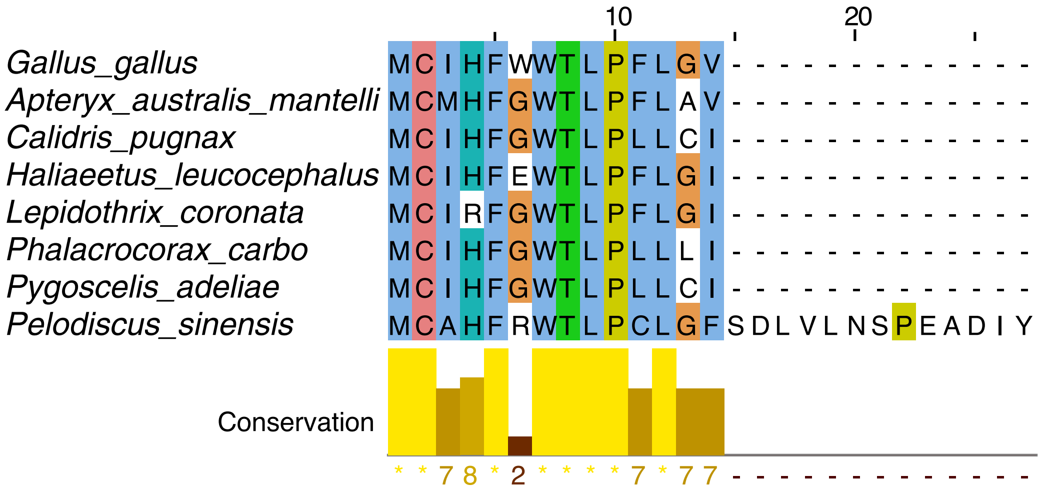

HG0230

ENSGALG00000011007 (OSBPL3)

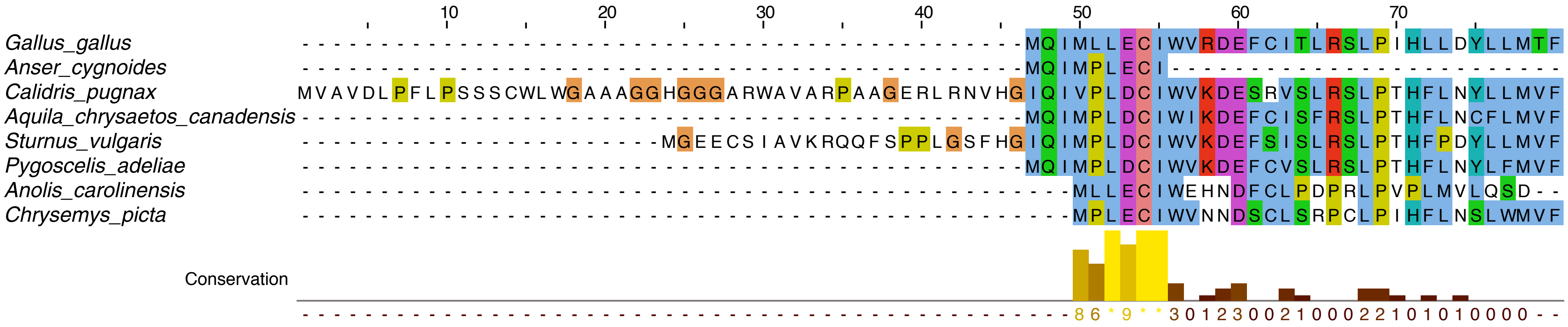

HG0231

ENSG00000150636

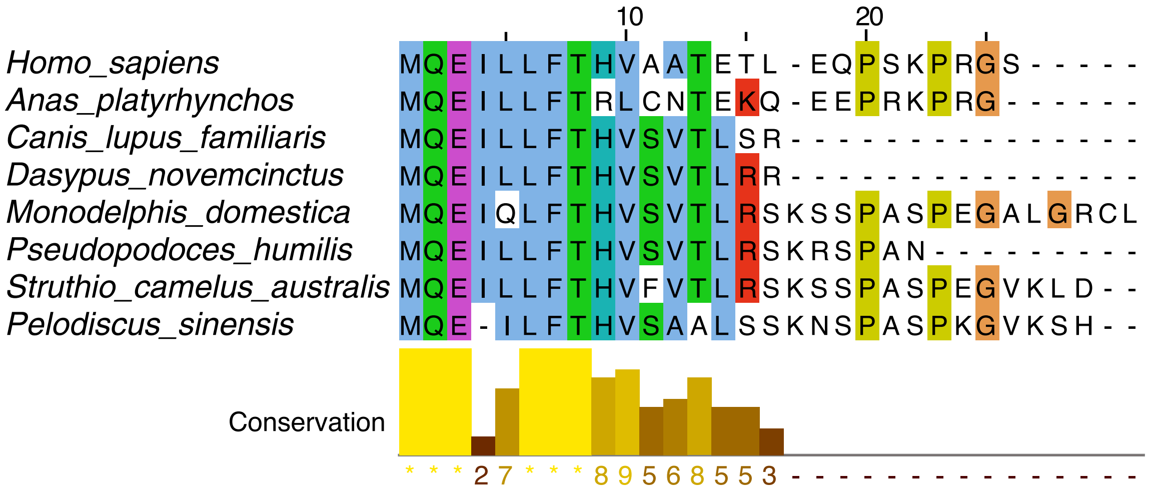

HG0232

ENSG00000180875 (GREM2)

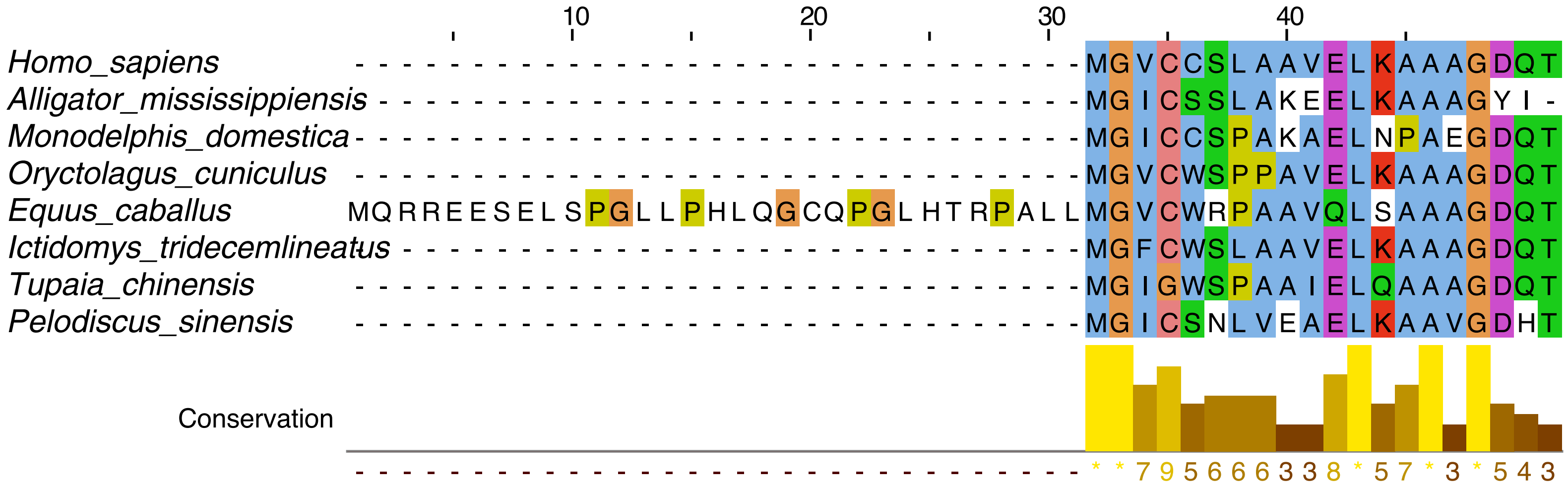

HG0233

ENSG00000206432 (TMEM200C)

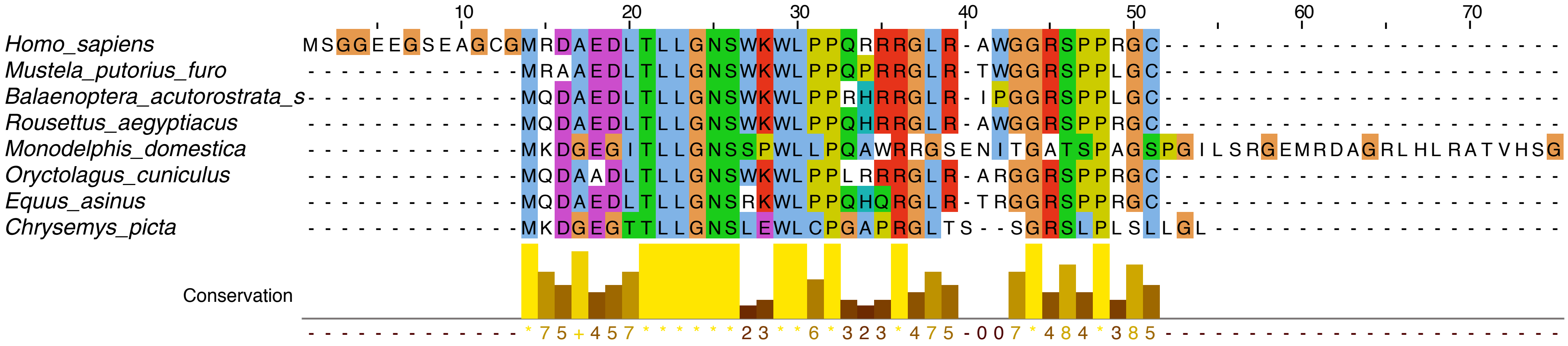

HG0234.1

ENSGALG00000011250 (ASIC4)

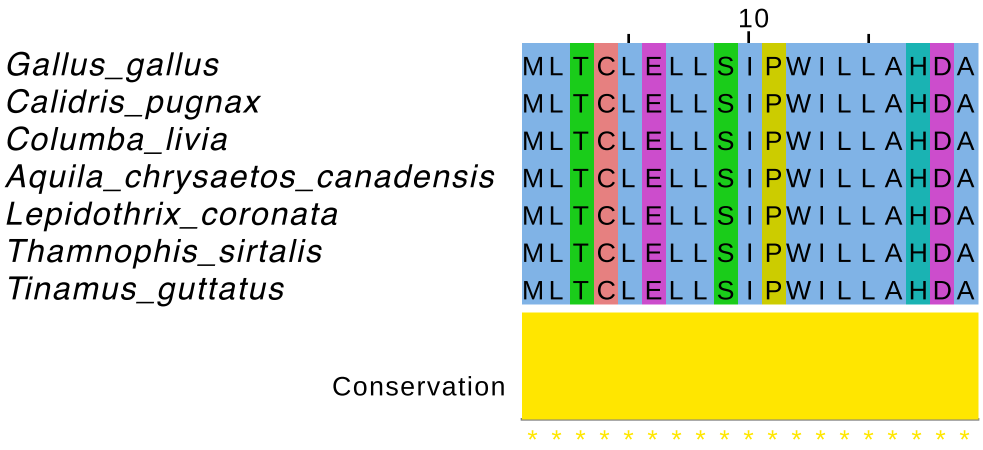

HG0234.2

ENSG00000108684 (ASIC2)

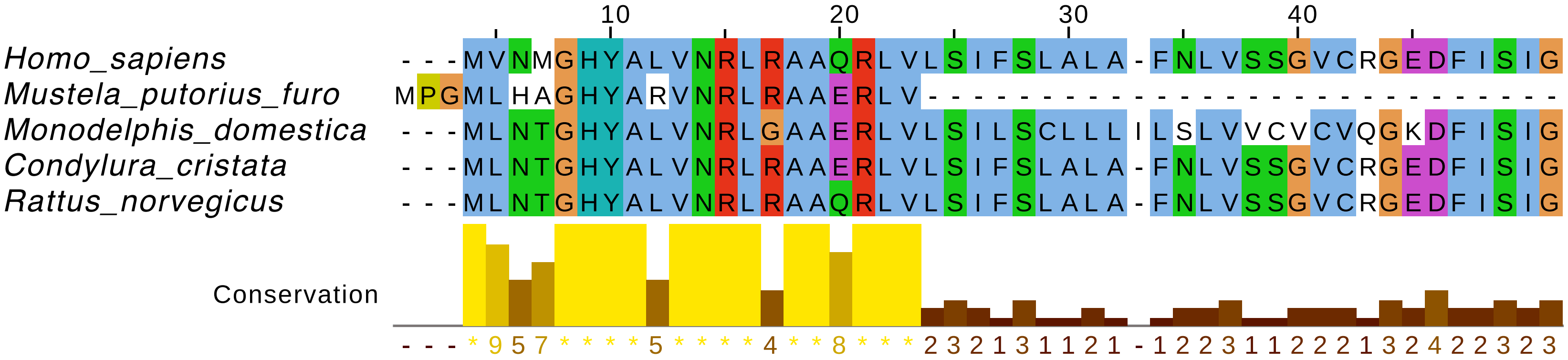

HG0235.1

ENSGALG00000035927

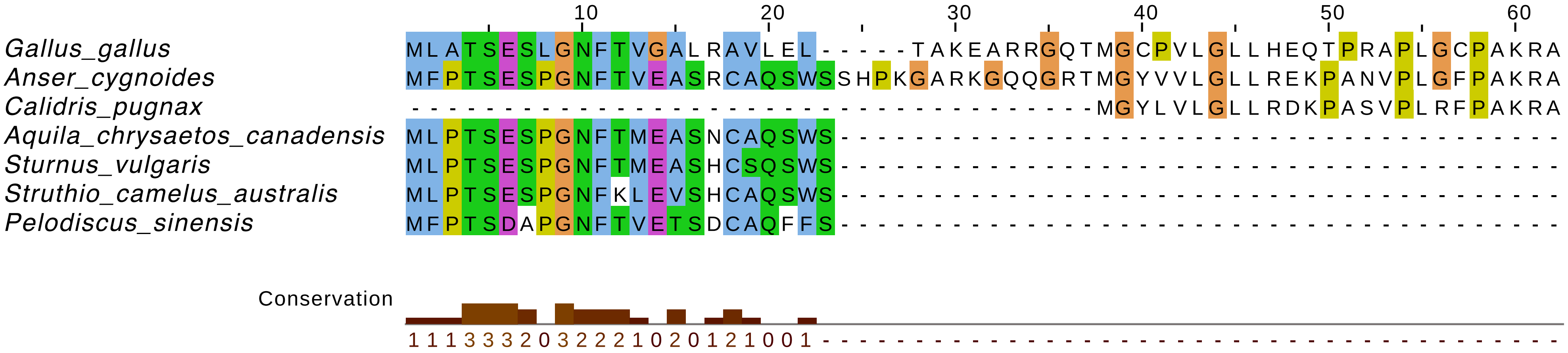

HG0235.2

ENSG00000101638 (ST8SIA5)

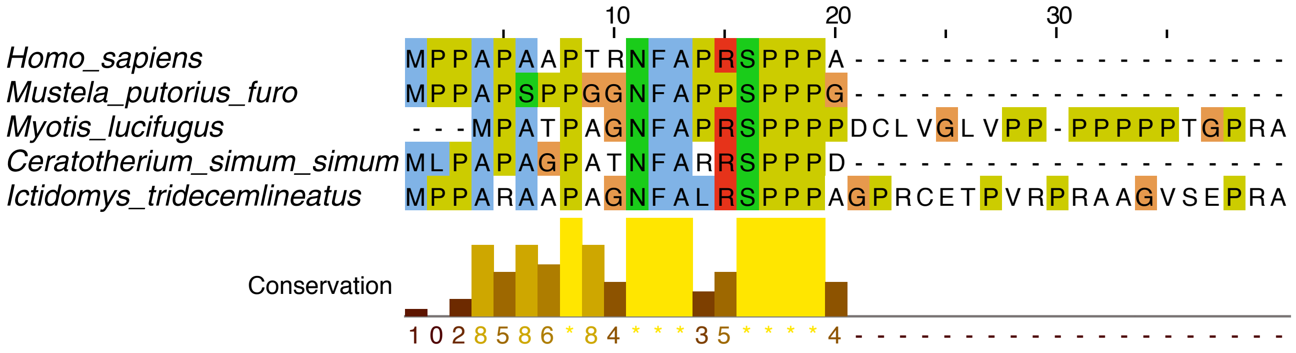

HG0236

ENSGALG00000004437 (LHFPL2)

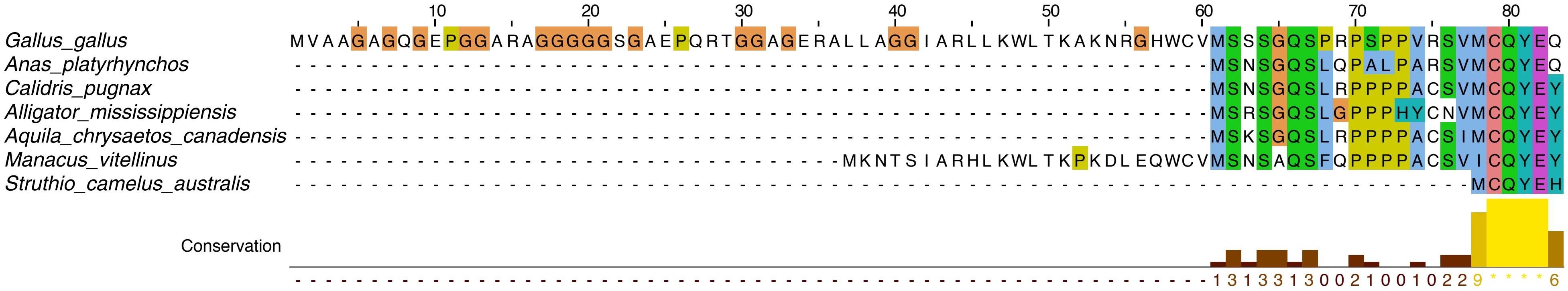

HG0237

ENSGALG00000012123 (PAX6)

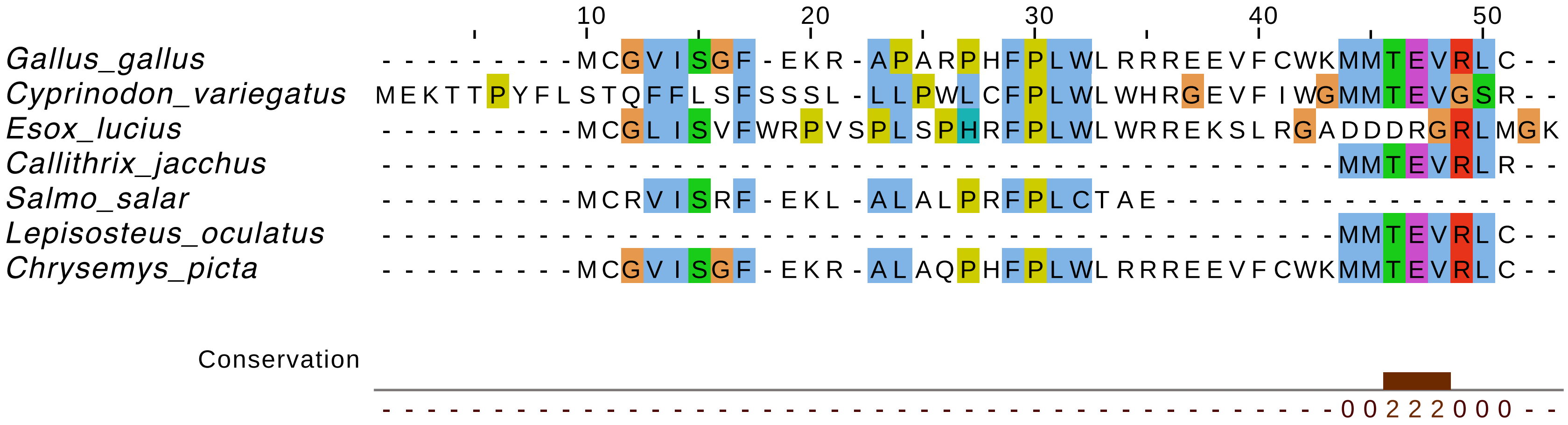

HG0238

ENSG00000049618 (ARID1B)

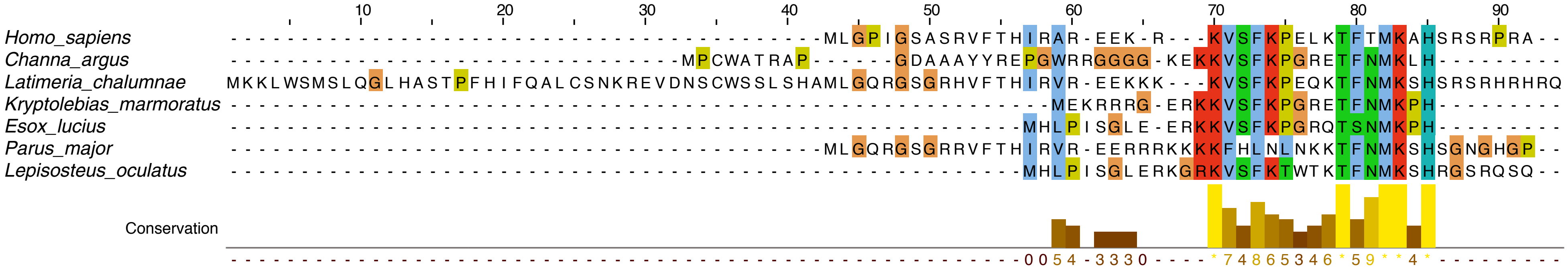

HG0239

ENSG00000149256 (TENM4)

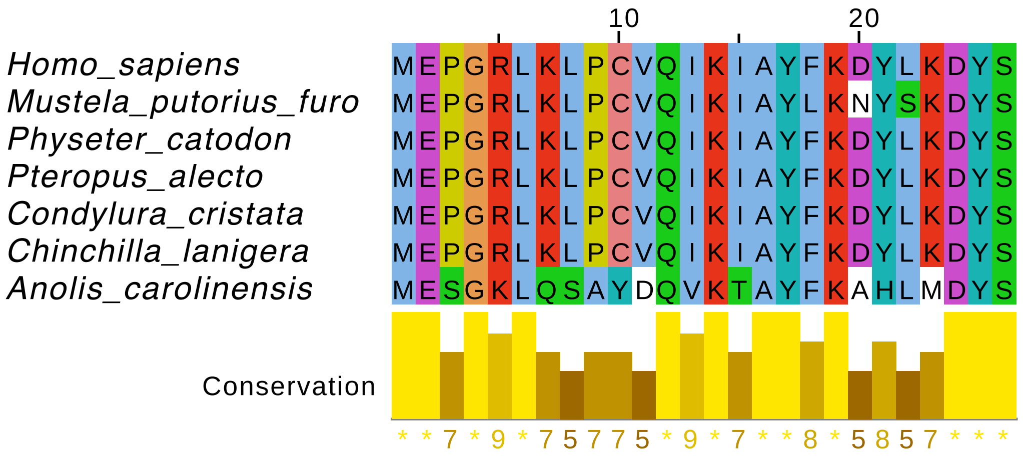

HG0240

ENSG00000166342 (NETO1)

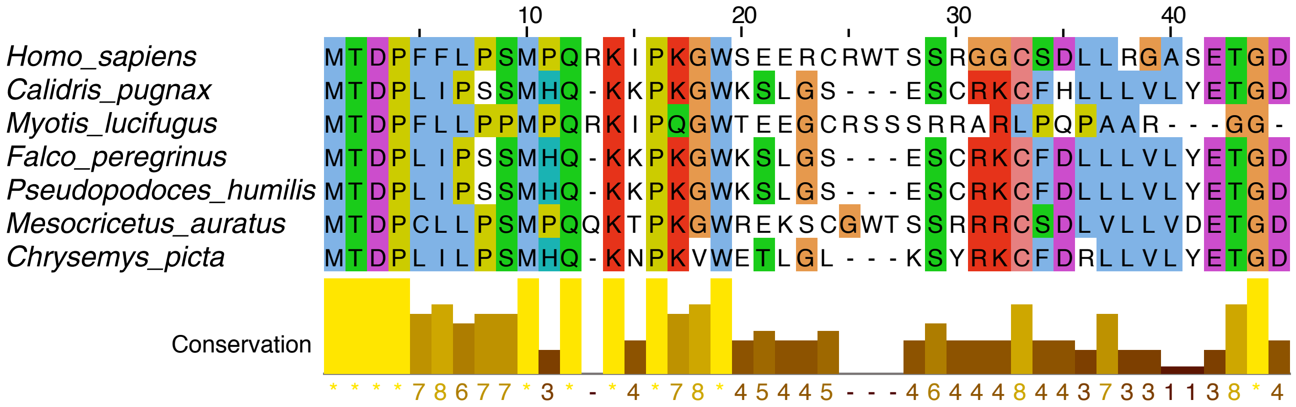

HG0241

ENSG00000198561 (CTNND1)

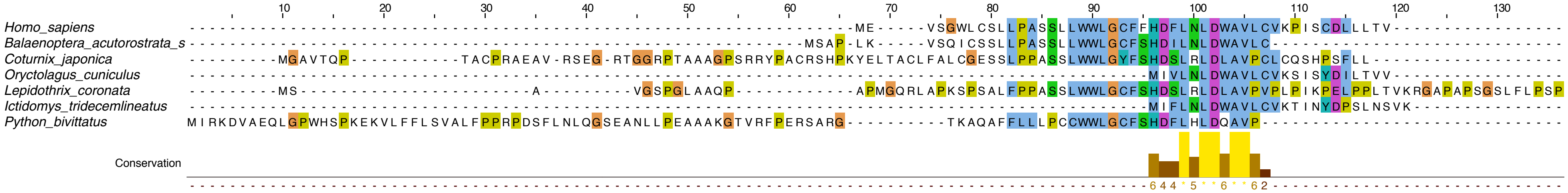

HG0242.1

ENSGALG00000007112 (LINS1)

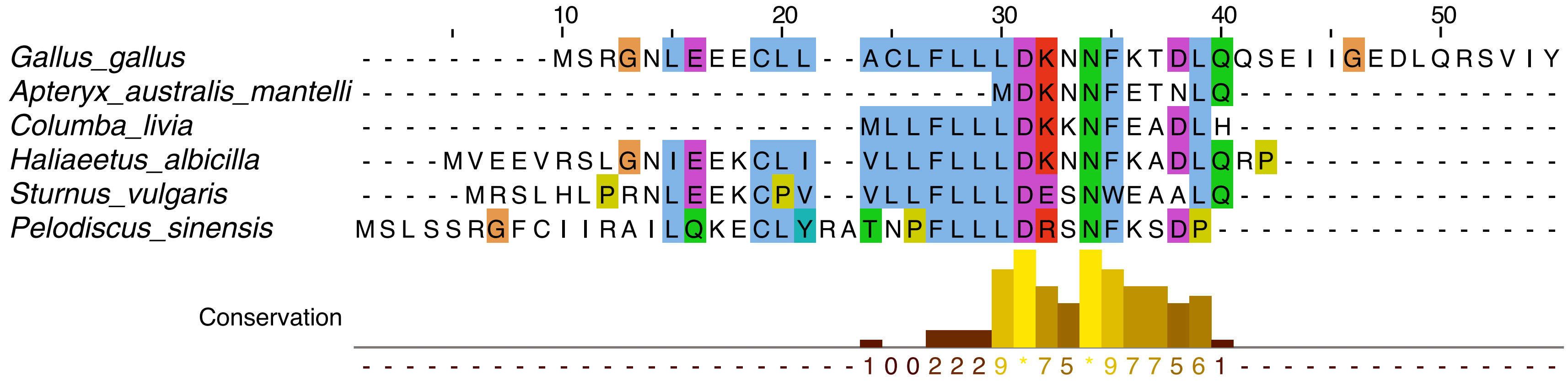

HG0242.2

ENSG00000140471 (LINS1)

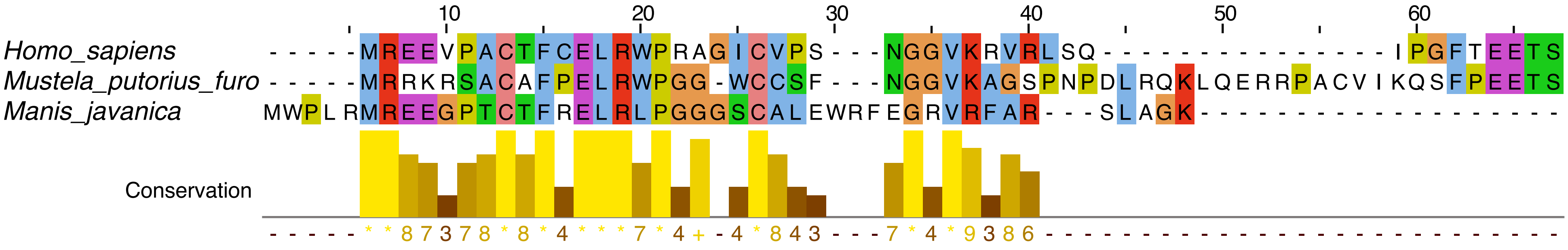

HG0243.1

ENSG00000163697 (APBB2)

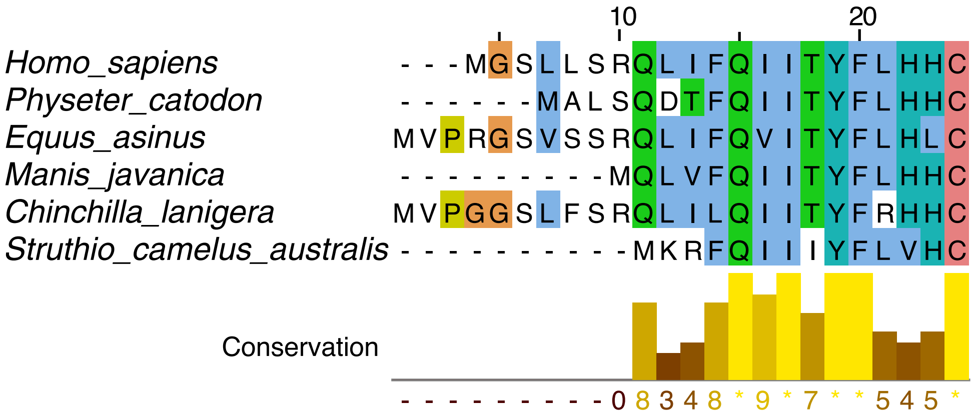

HG0243.2

ENSG00000163697 (APBB2)

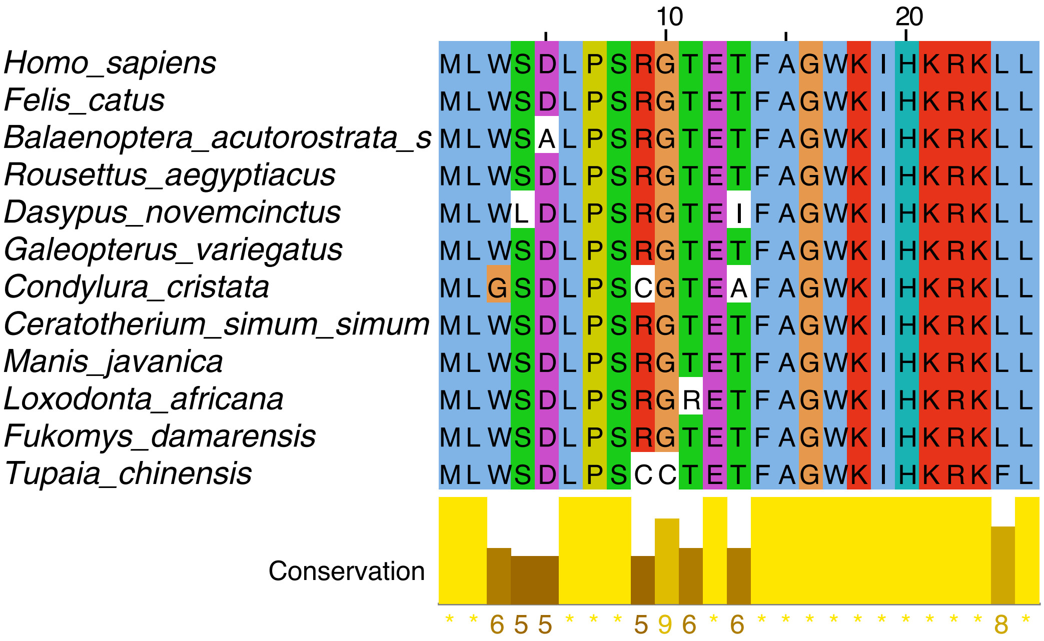

HG0244

ENSGALG00000010837 (ASB5)

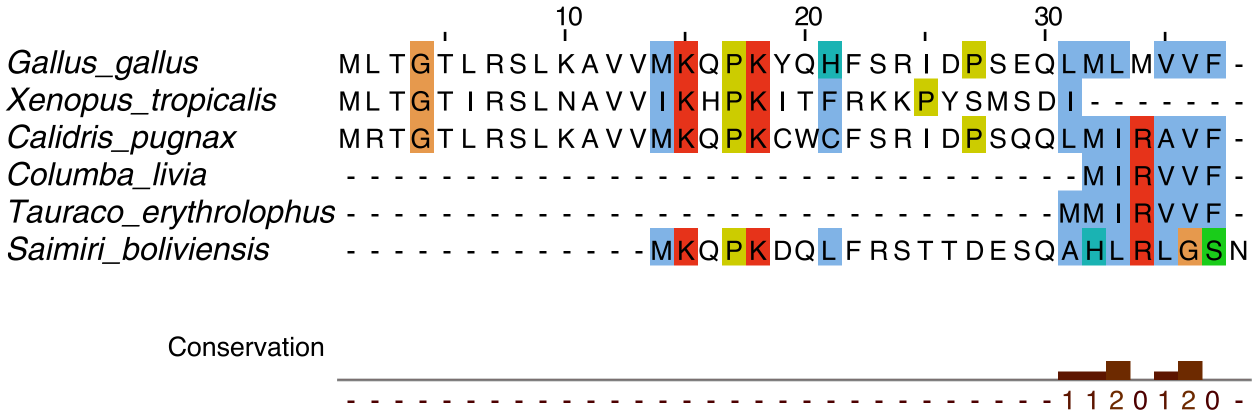

HG0245

ENSG00000091831 (ESR1)

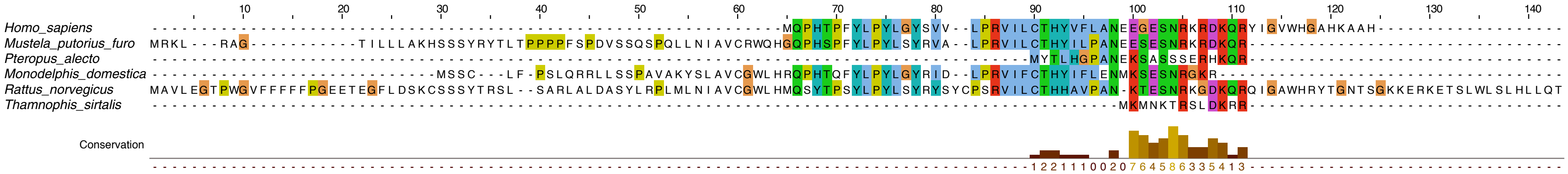

HG0246

ENSG00000162676 (GFI1)

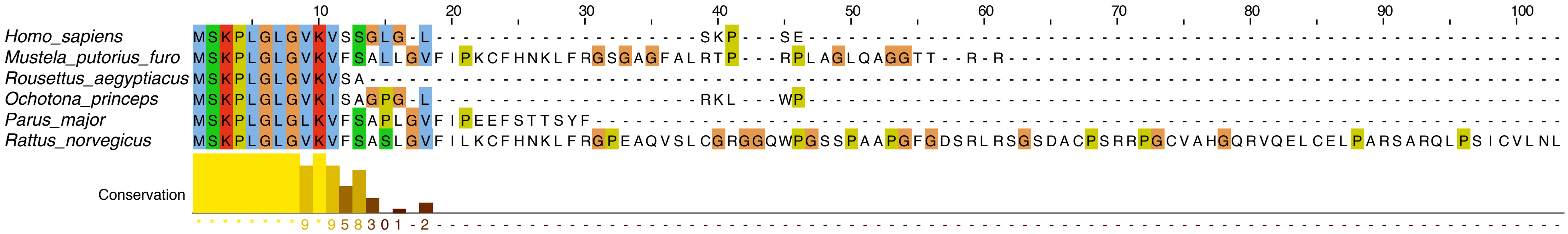

HG0247.1

ENSDARG00000014746 (rbfox1)

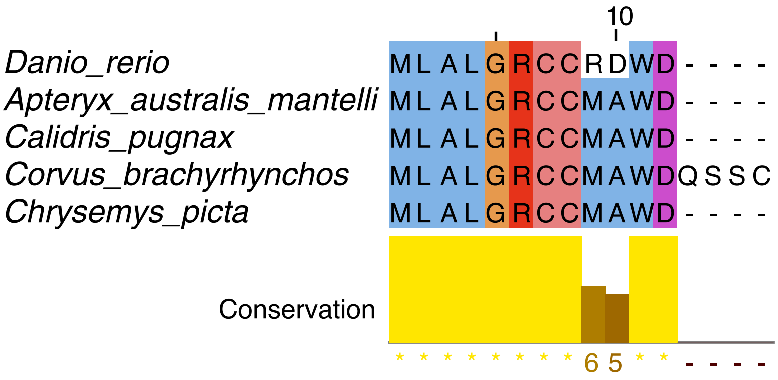

HG0247.2

ENSG00000100320 (RBFOX2)

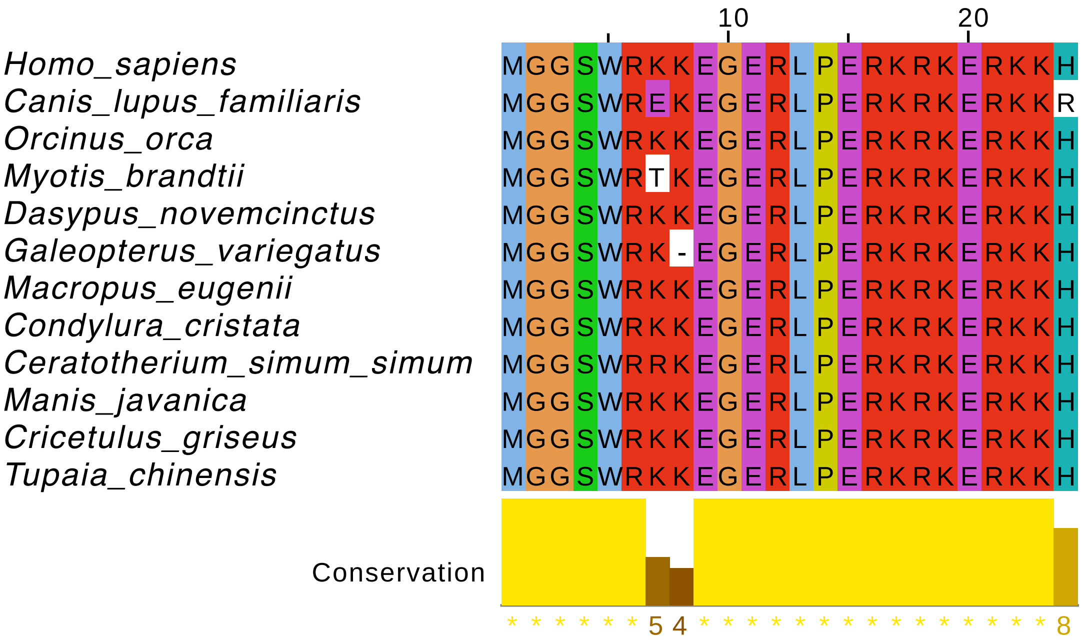

HG0248.1

ENSGALG00000004570 (NCOA3)

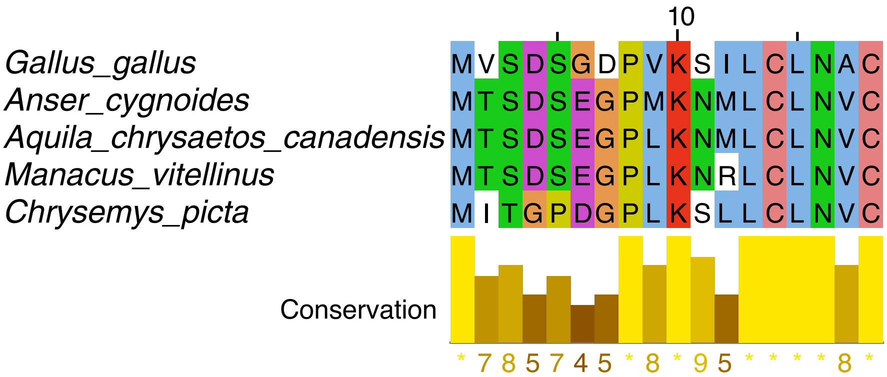

HG0248.2

ENSG00000124151 (NCOA3)

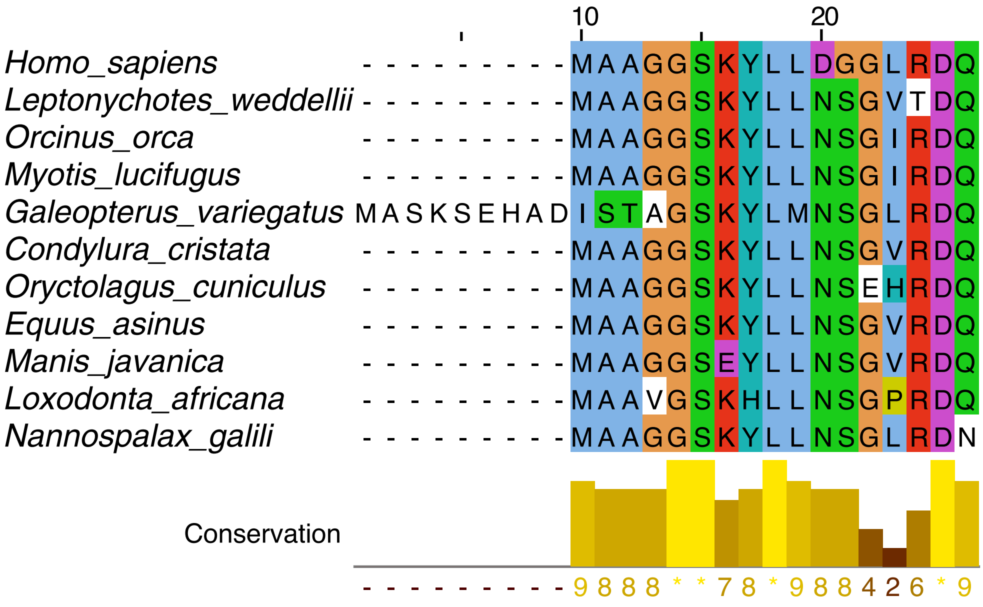

HG0249

ENSDARG00000062550 (rc3h1a)

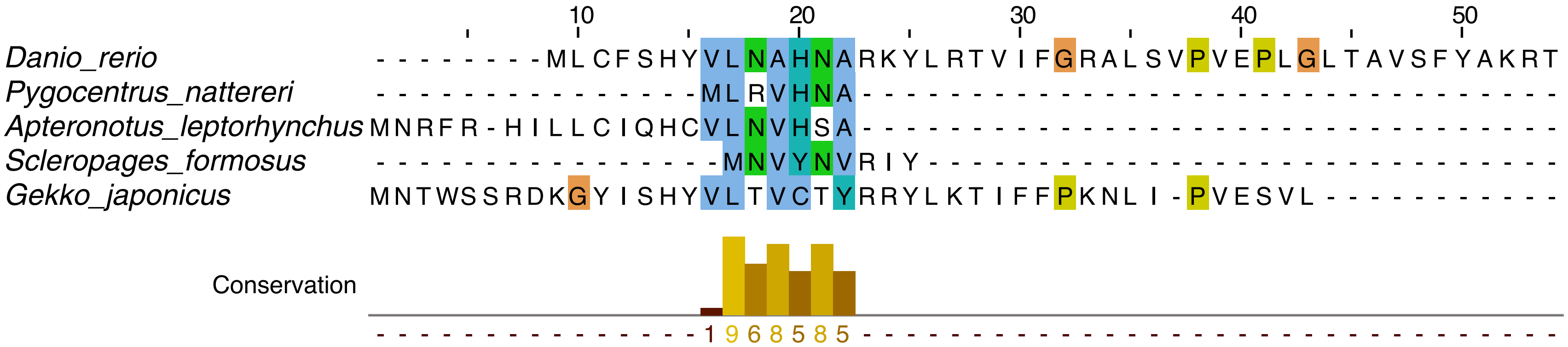

HG0250

ENSGALG00000014861

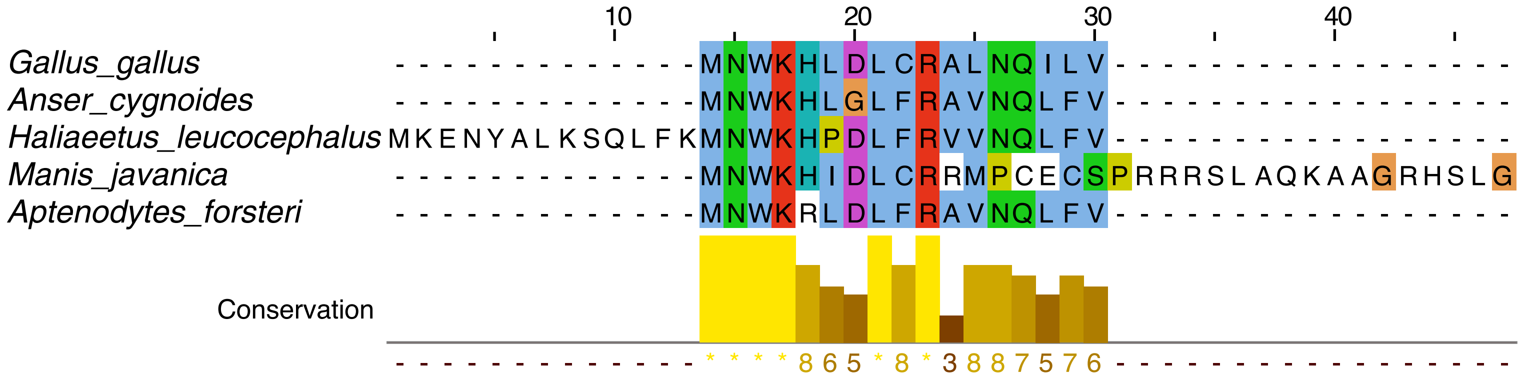

HG0251

ENSG00000185053 (SGCZ)

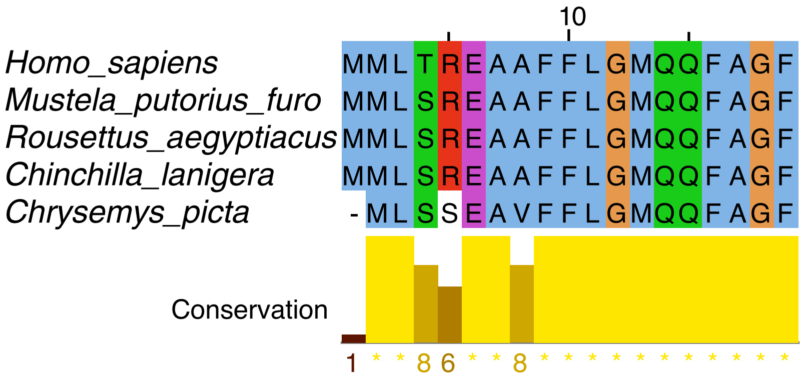

HG0252.1

ENSGALG00000009645 (ESRRG)

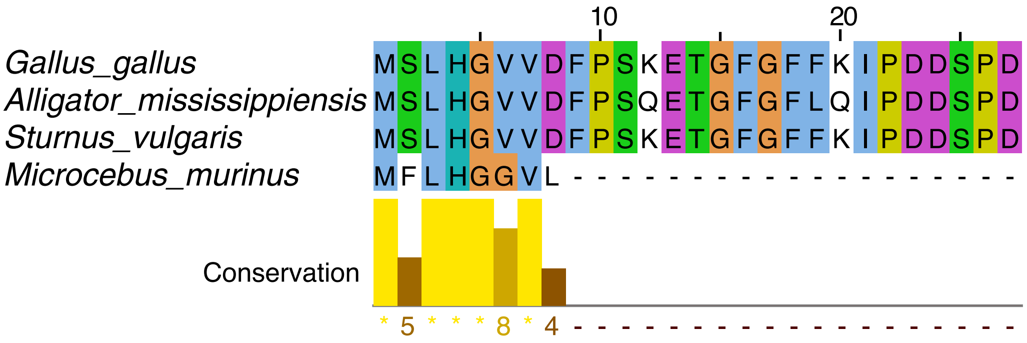

HG0252.2

ENSG00000119715 (ESRRB)

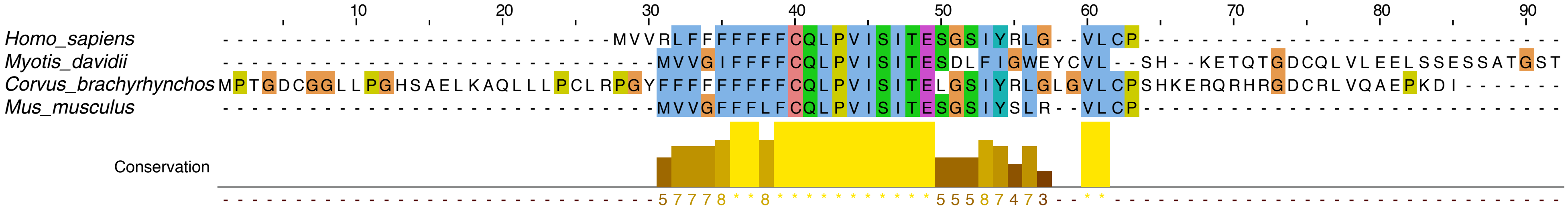

HG0252.3

ENSG00000196482 (ESRRG)

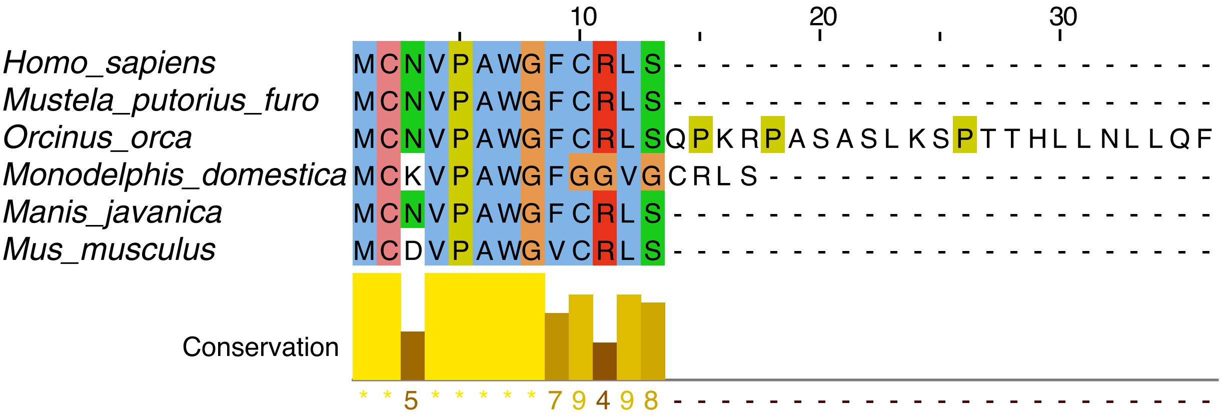

HG0253.1

ENSGALG00000015184

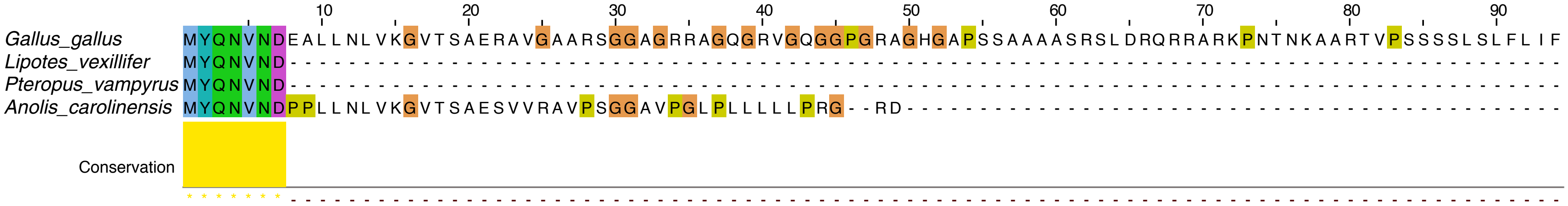

HG0253.2

ENSG00000140332 (TLE3)

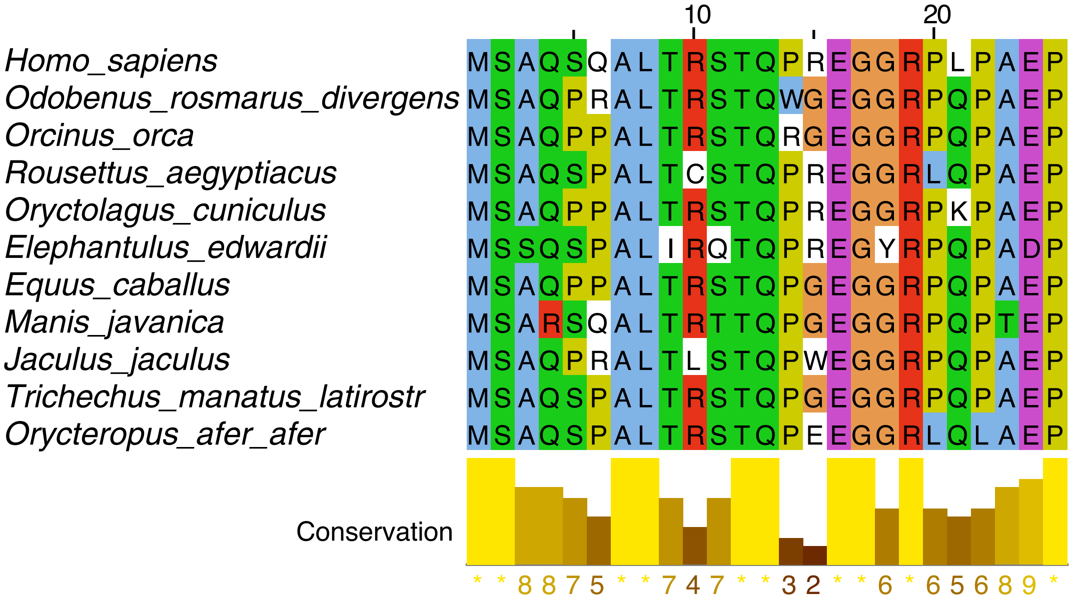

HG0253.3

ENSG00000140332 (TLE3)

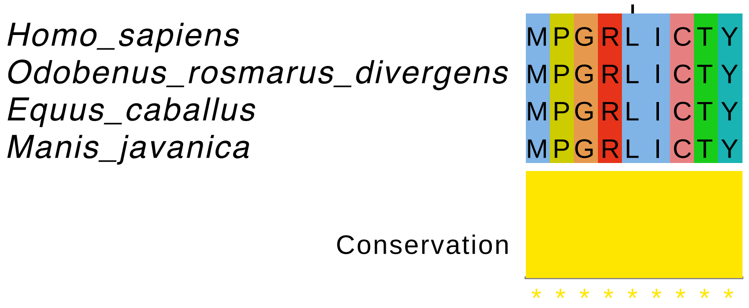

HG0254.1

ENSGALG00000035919

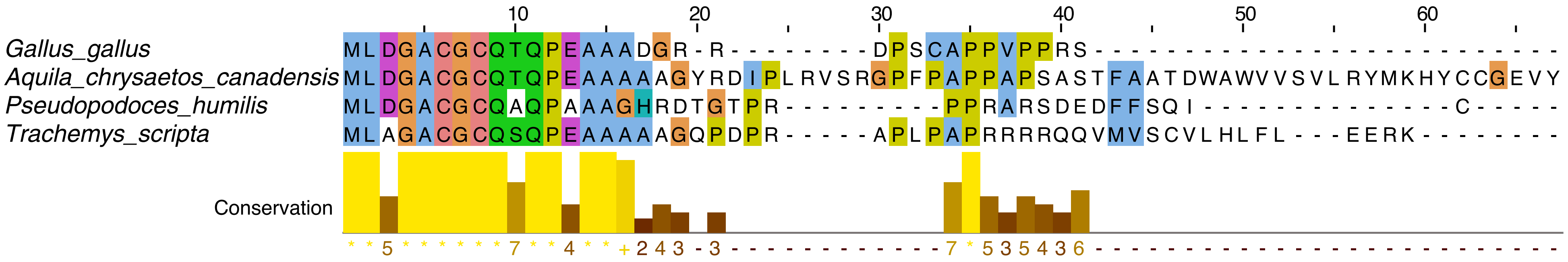

HG0254.2

ENSG00000072134 (EPN2)

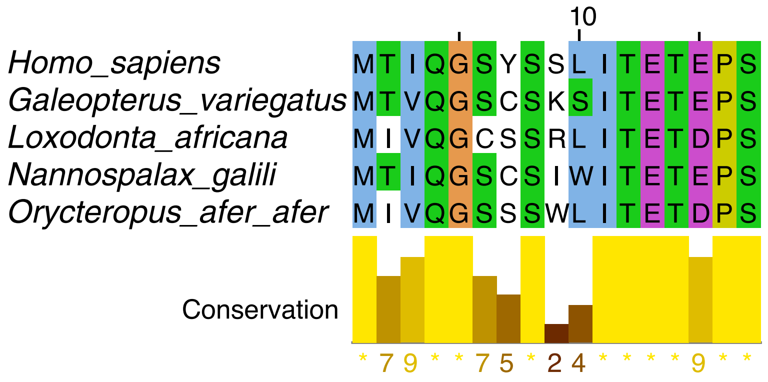

HG0255

ENSGALG00000012139 (ARL14EP)

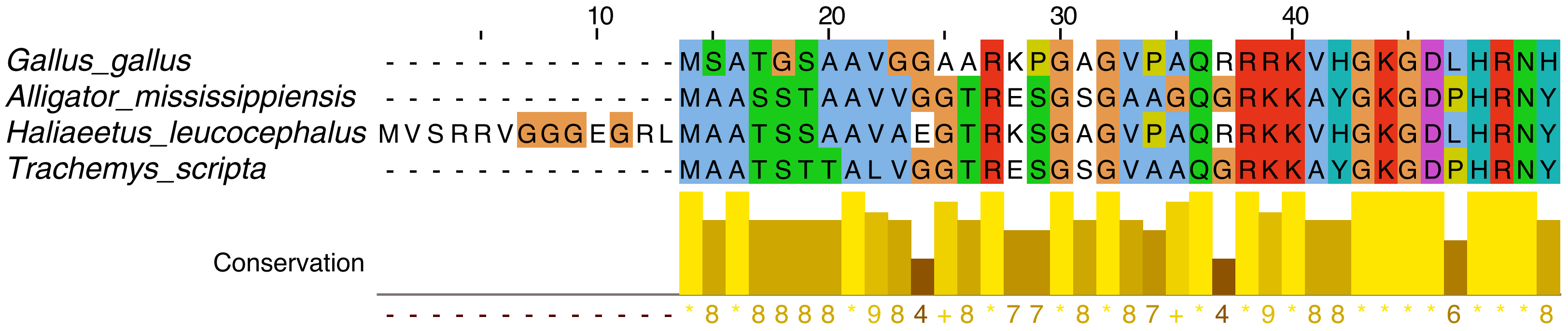

HG0256

ENSGALG00000039901

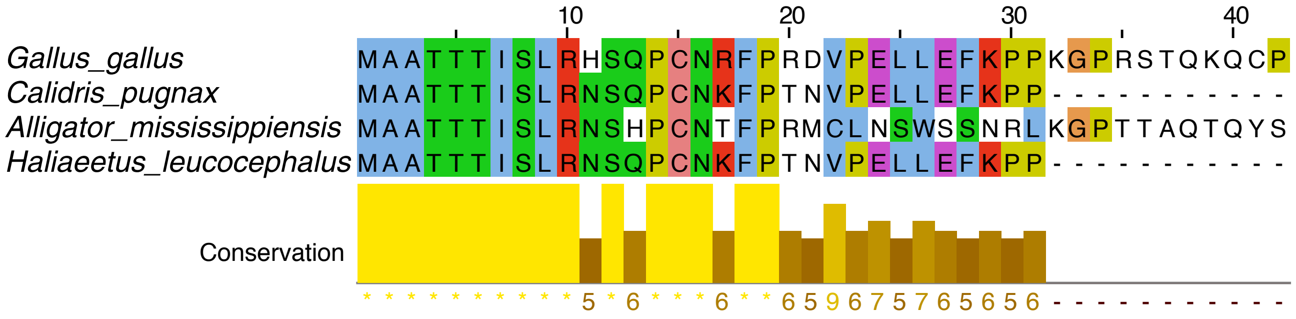

HG0257

ENSG00000198791 (CNOT7)

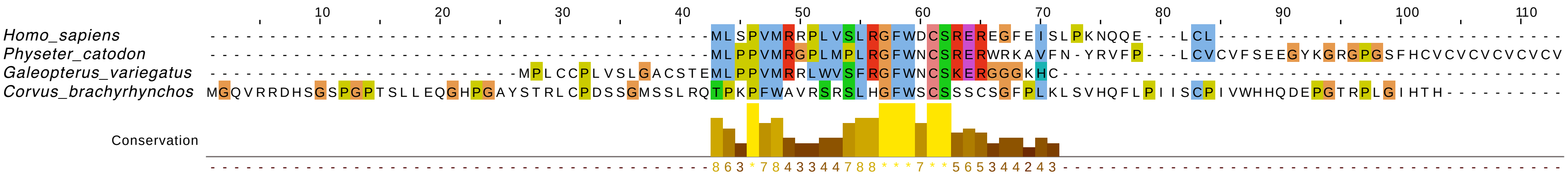

HG0258.1

ENSGALG00000016797 (FHL2)

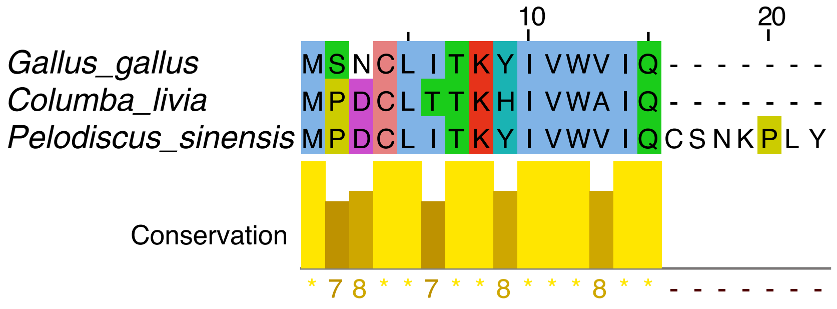

HG0258.2

ENSGALG00000016797 (FHL2)

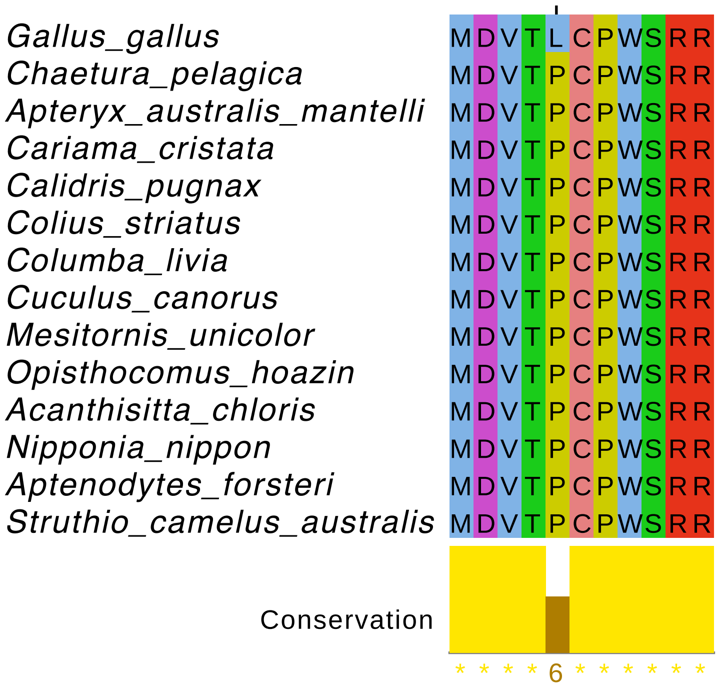

HG0259

ENSGALG00000009936 (AP4S1)

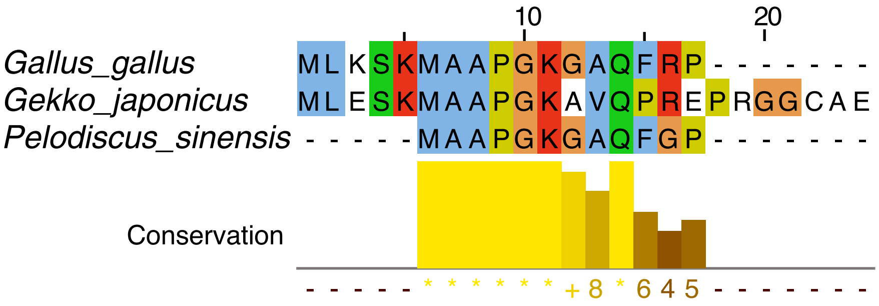

HG0260.1

ENSGALG00000016890 (MBNL2)

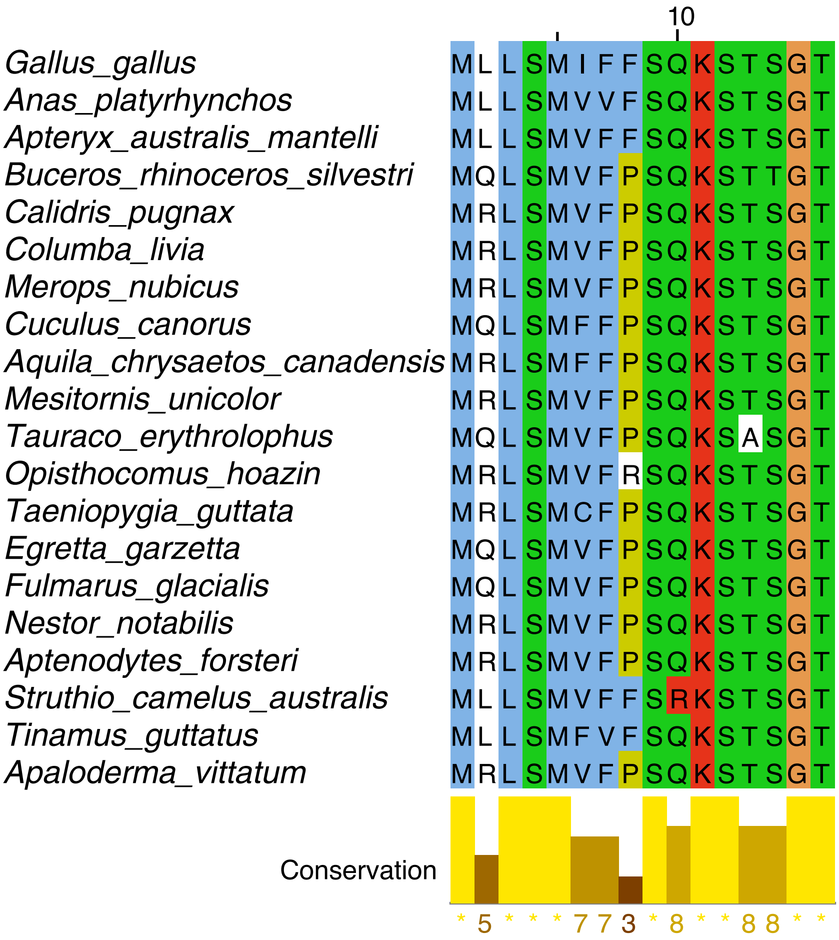

HG0260.2

ENSG00000139793 (MBNL2)

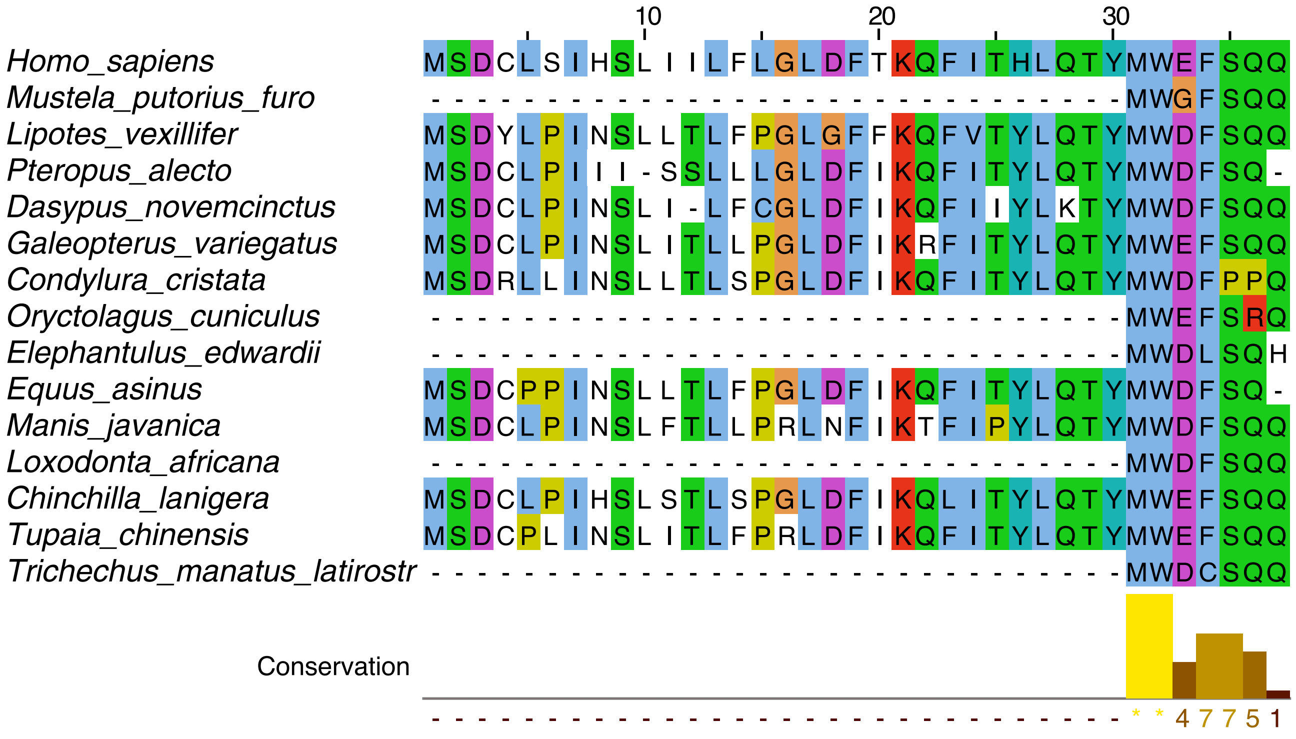

HG0260.3

ENSG00000152601 (MBNL1)

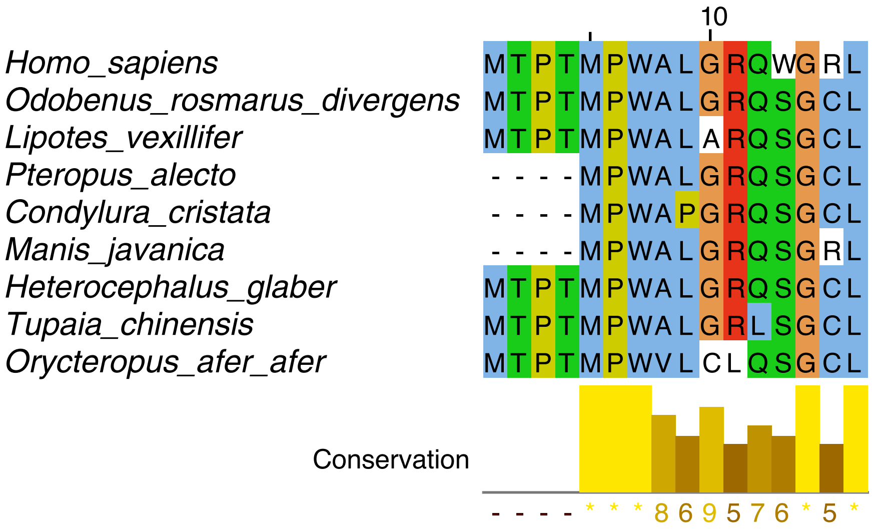

HG0260.4

ENSG00000152601 (MBNL1)

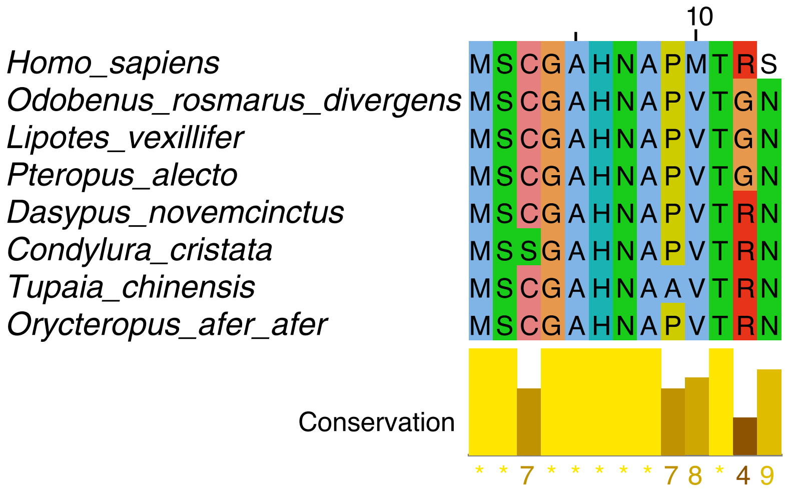

HG0260.5

ENSG00000152601 (MBNL1)

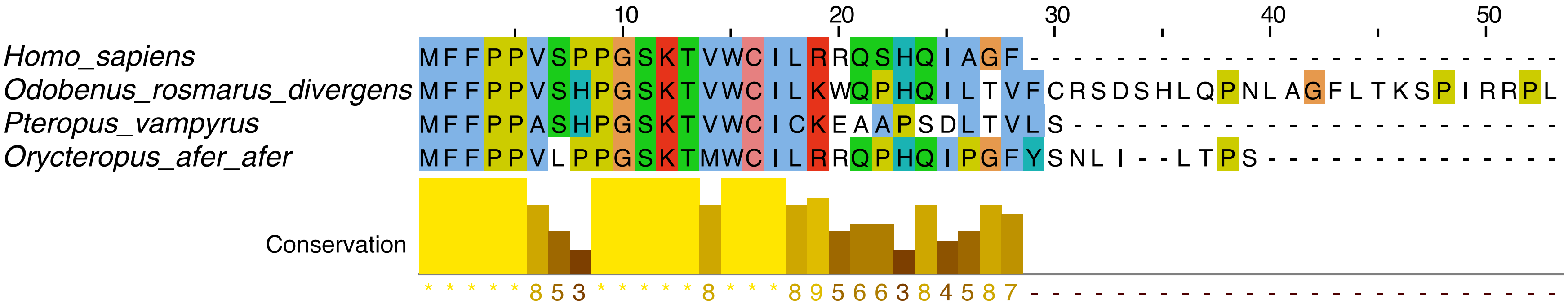

HG0260.6

ENSG00000152601 (MBNL1)

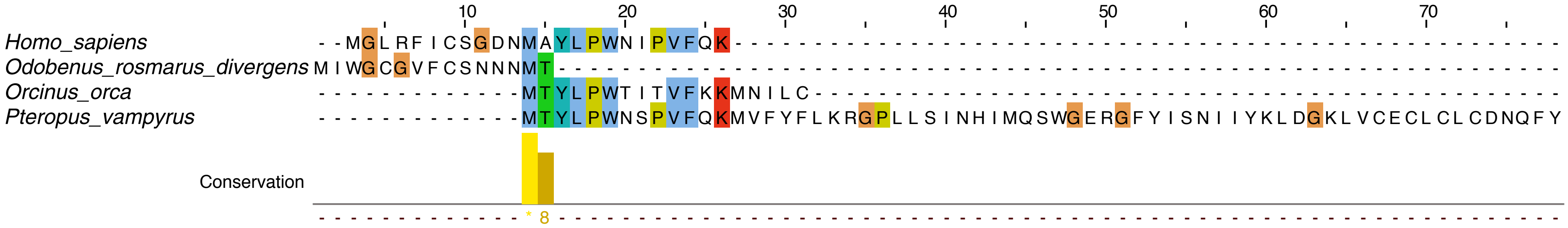

HG0261.1

ENSGALG00000005867 (8-Mar)

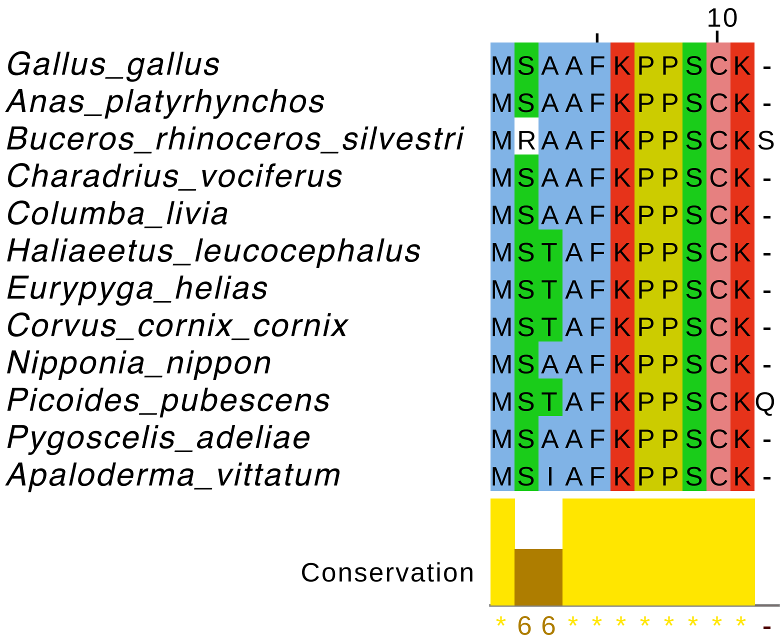

HG0261.2

ENSGALG00000009520 (1-Mar)

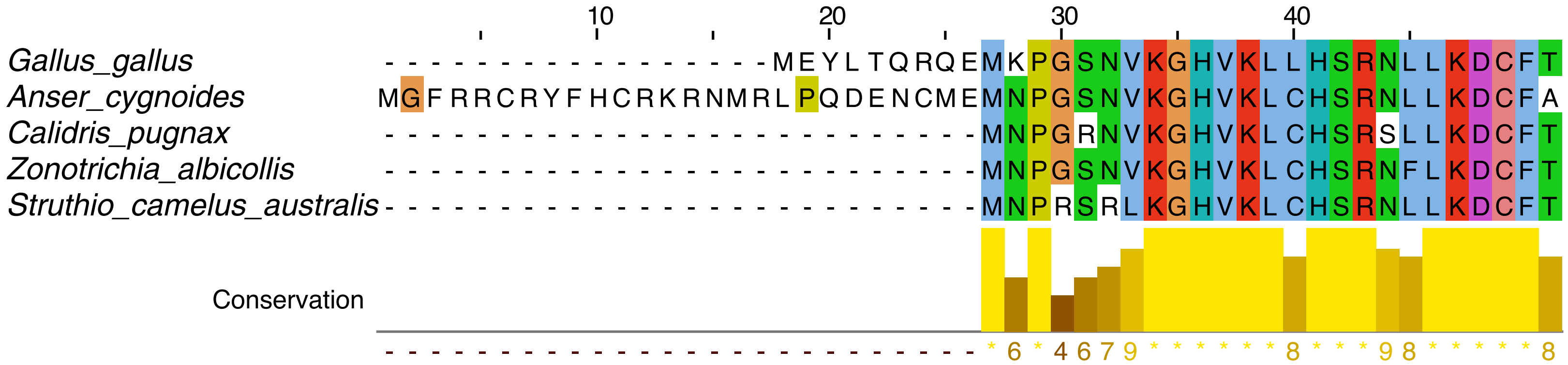

HG0261.3

ENSG00000145416 (1-Mar)

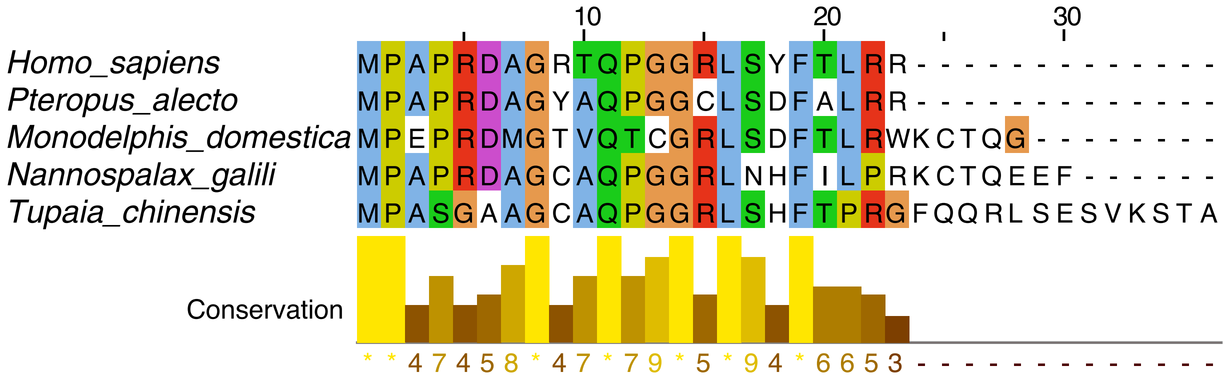

HG0261.4

ENSG00000165406 (8-Mar)

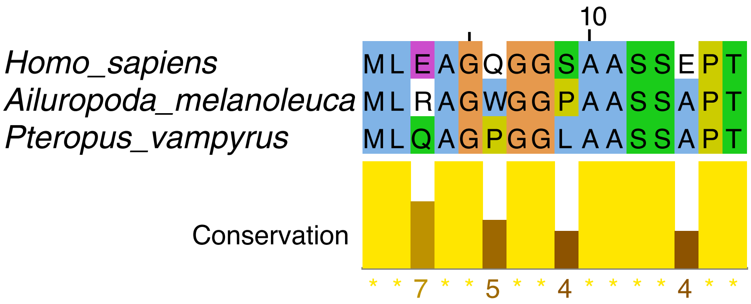

HG0262.1

ENSG00000023445 (BIRC3)

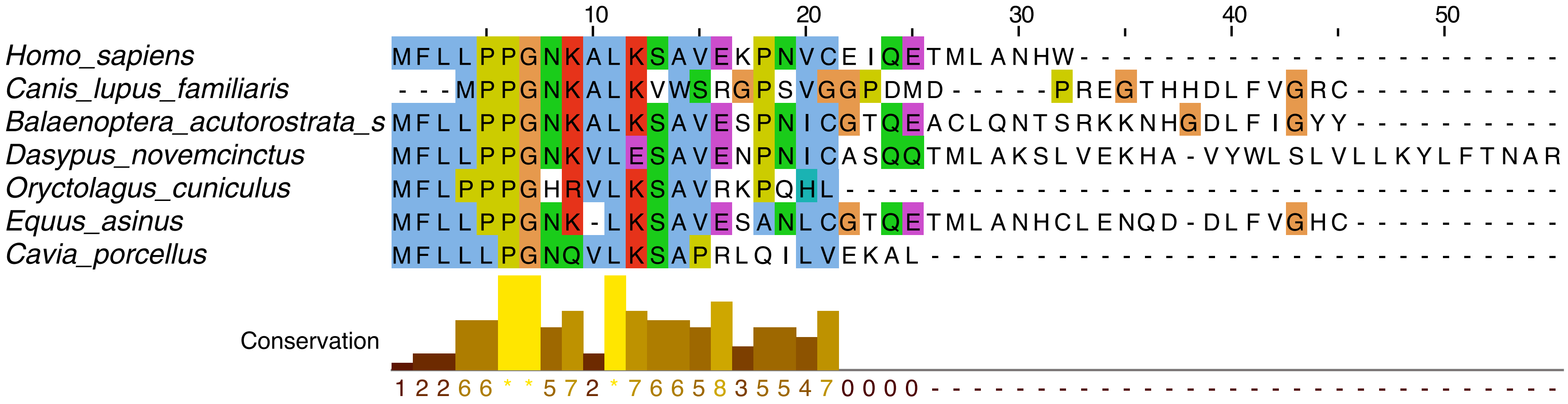

HG0262.2

ENSG00000023445 (BIRC3)

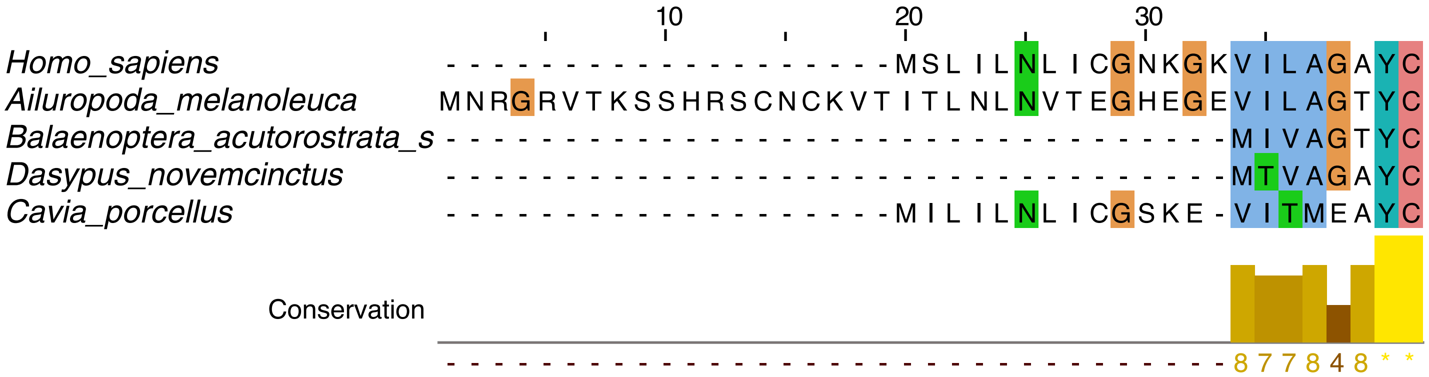

HG0262.3

ENSGALG00000017186

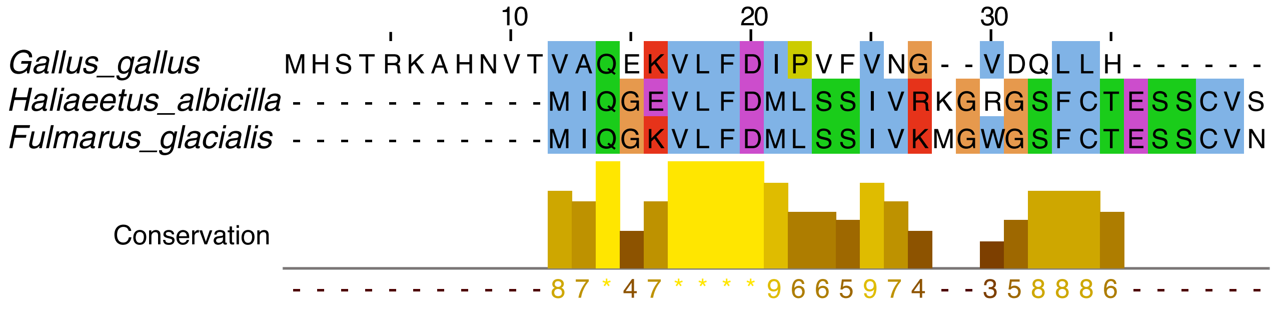

HG0262.4

ENSG00000023445 (BIRC3)

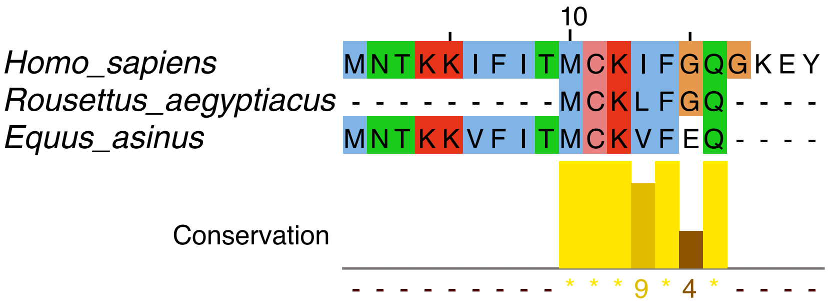

HG0263.1

ENSG00000169826 (CSGALNACT2)

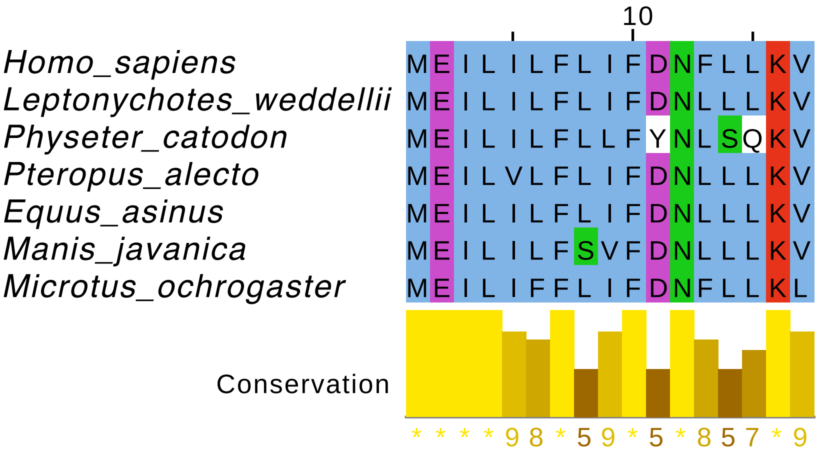

HG0263.2

ENSGALG00000002543 (CSGALNACT2)

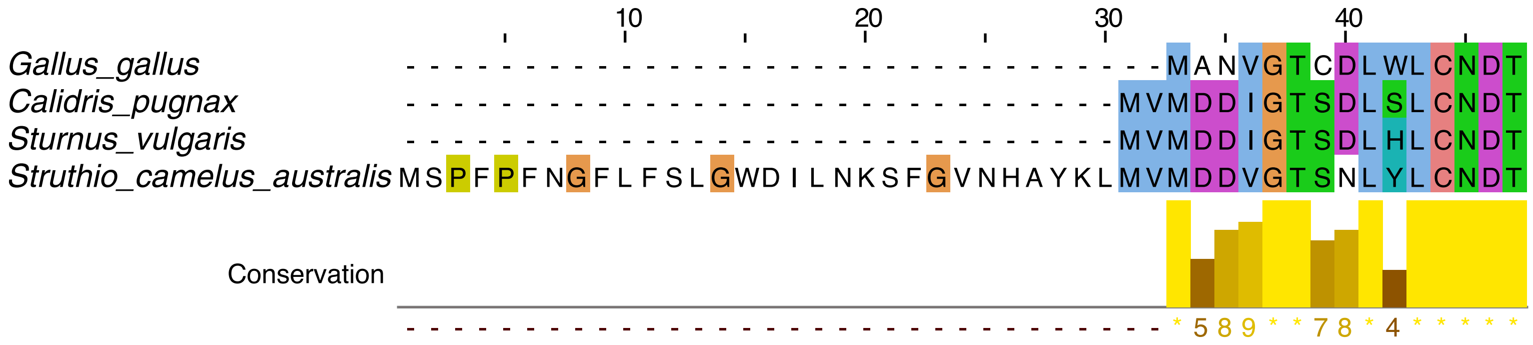

HG0263.3

ENSGALG00000002543 (CSGALNACT2)

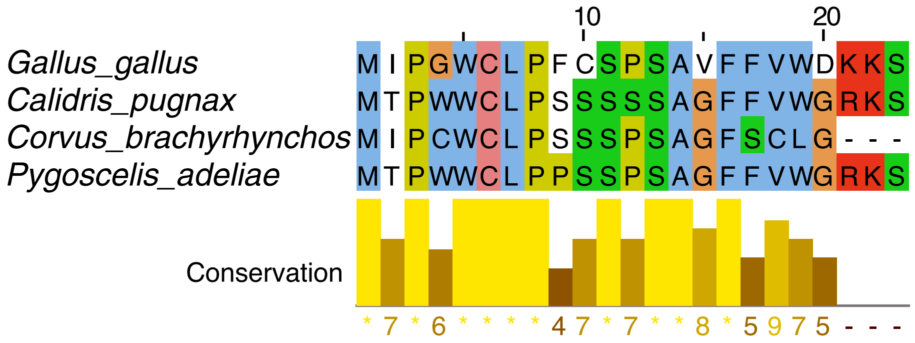

HG0263.4

ENSG00000147408 (CSGALNACT1)

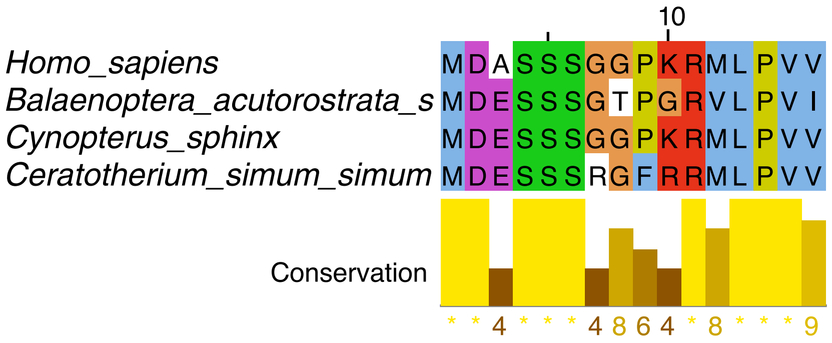

HG0264.1

ENSG00000151458 (ANKRD50)

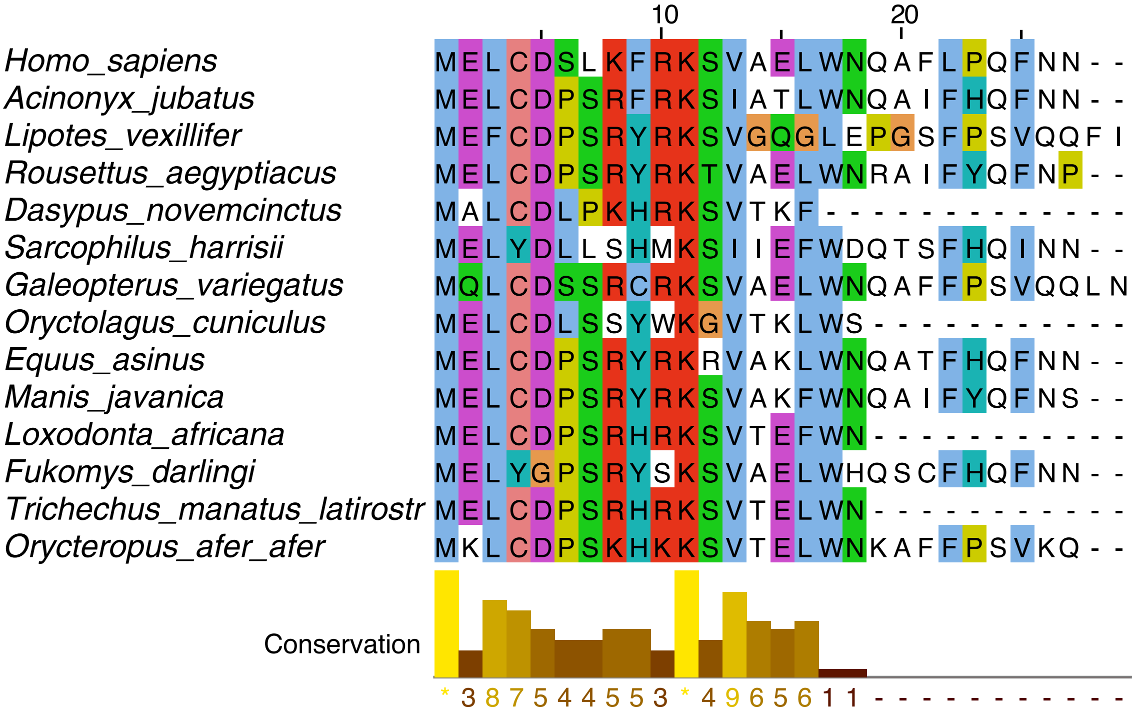

HG0264.2

ENSG00000151458 (ANKRD50)

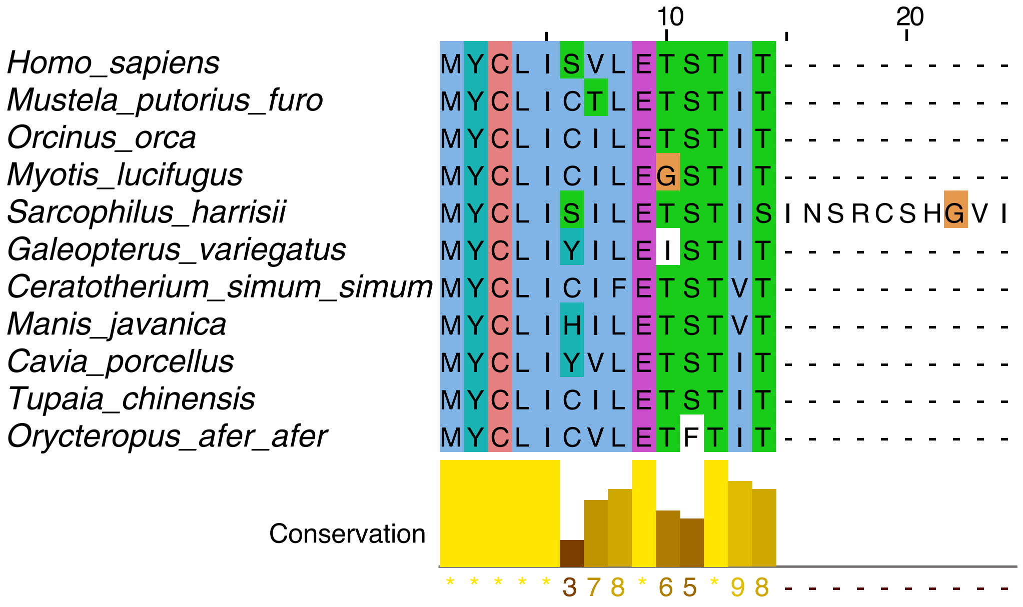

HG0264.3

ENSGALG00000011831 (ANKRD50)

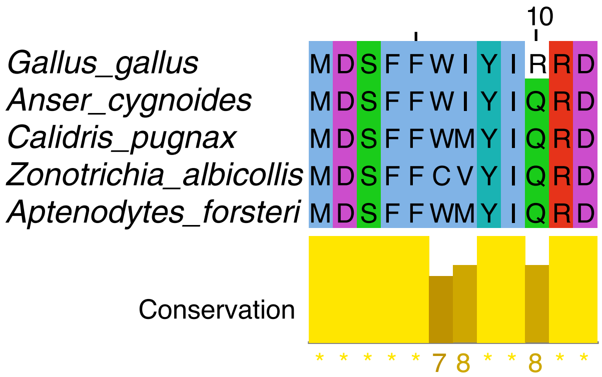

HG0264.4

ENSG00000151458 (ANKRD50)

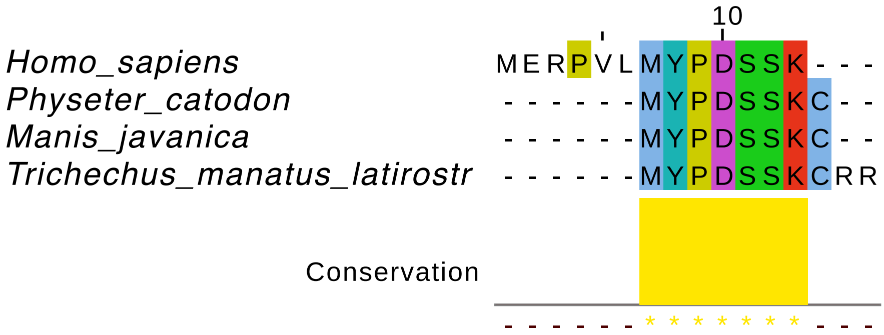

HG0265.1

ENSG00000144285 (SCN1A)

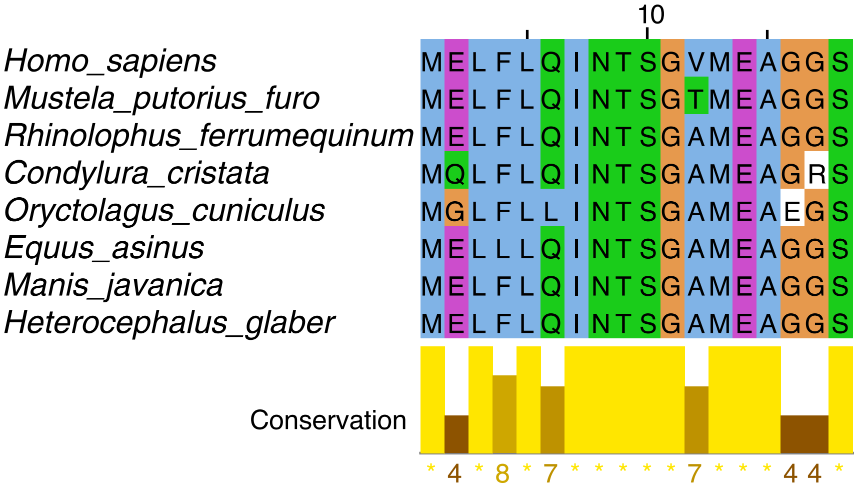

HG0265.2

ENSG00000196876 (SCN8A)

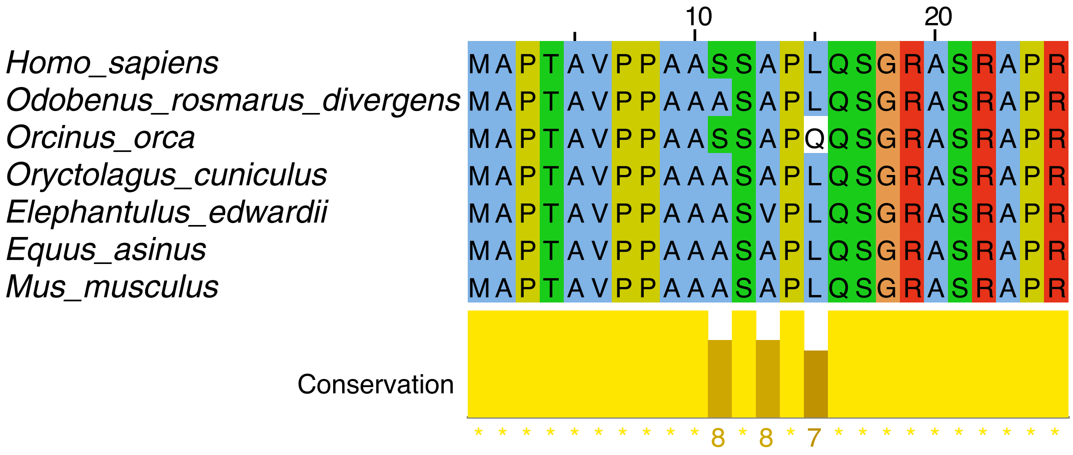

HG0265.3

ENSDARG00000018032 (scn8ab)

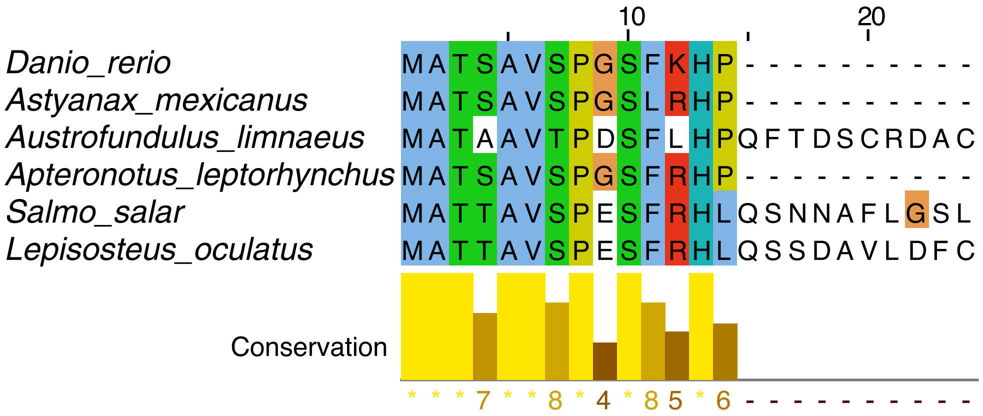

HG0266.1

ENSG00000184185 (KCNJ12)

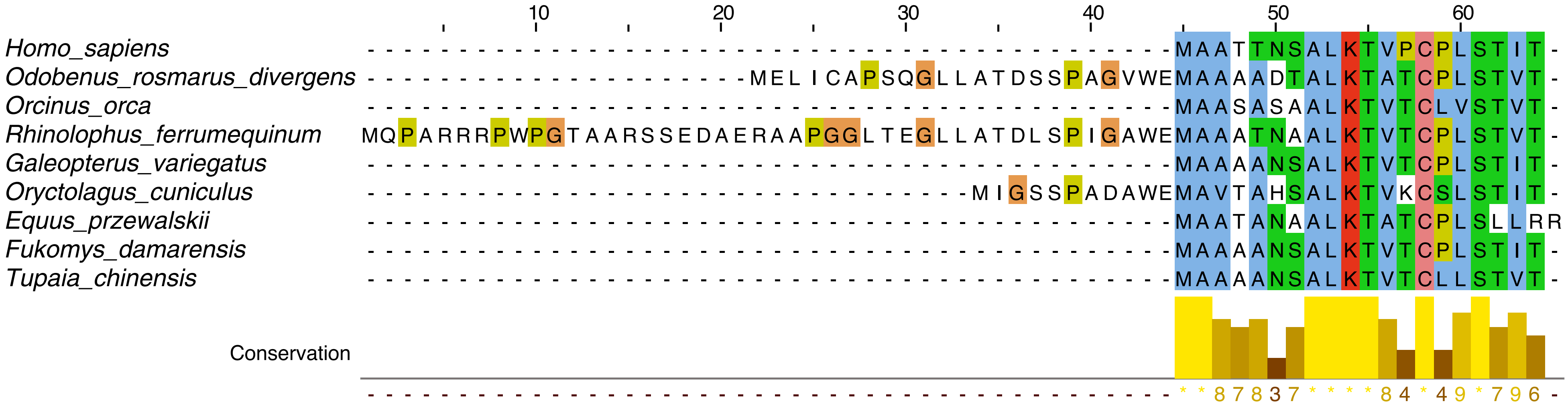

HG0266.1

ENSG00000260458 (KCNJ18)

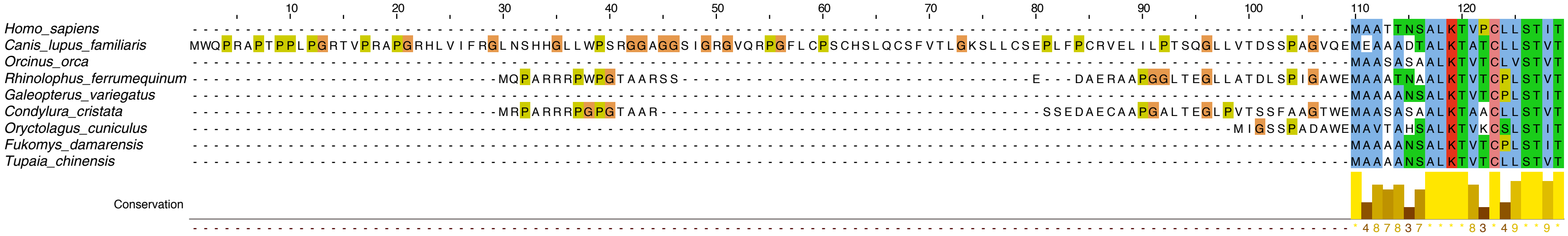

HG0266.2

ENSGALG00000001181 (KCNJ5)

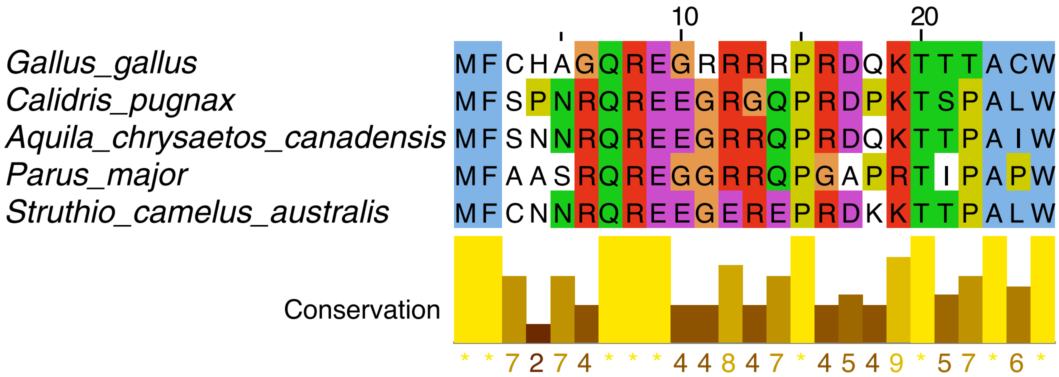

HG0267

ENSDARG00000028173 (slc4a2a)

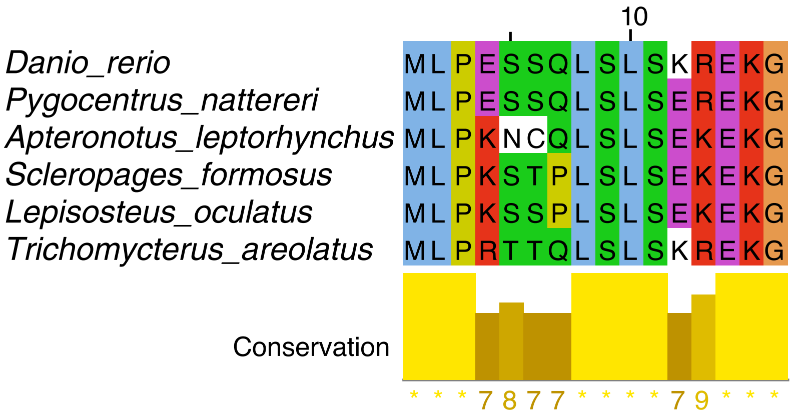

HG0267

ENSDARG00000052330 (slc4a2b)

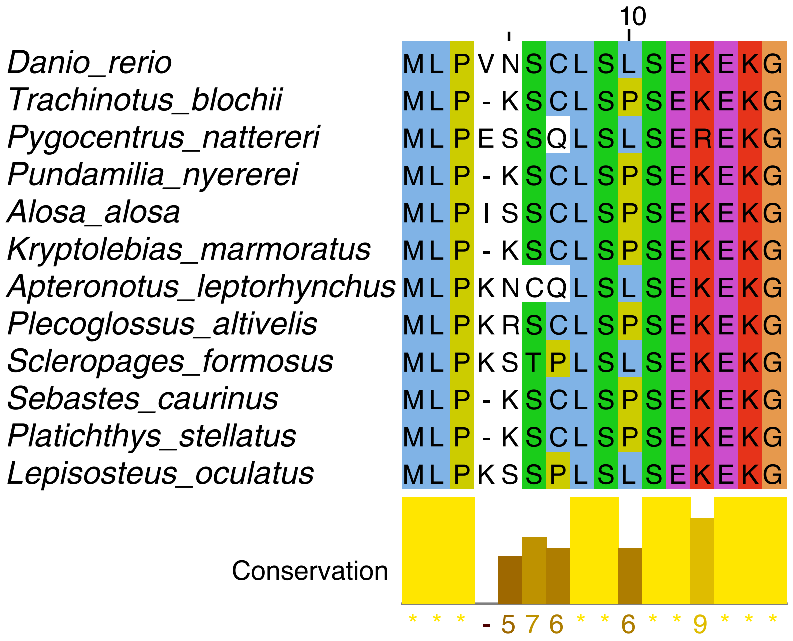

HG0267

ENSG00000164889 (SLC4A2)

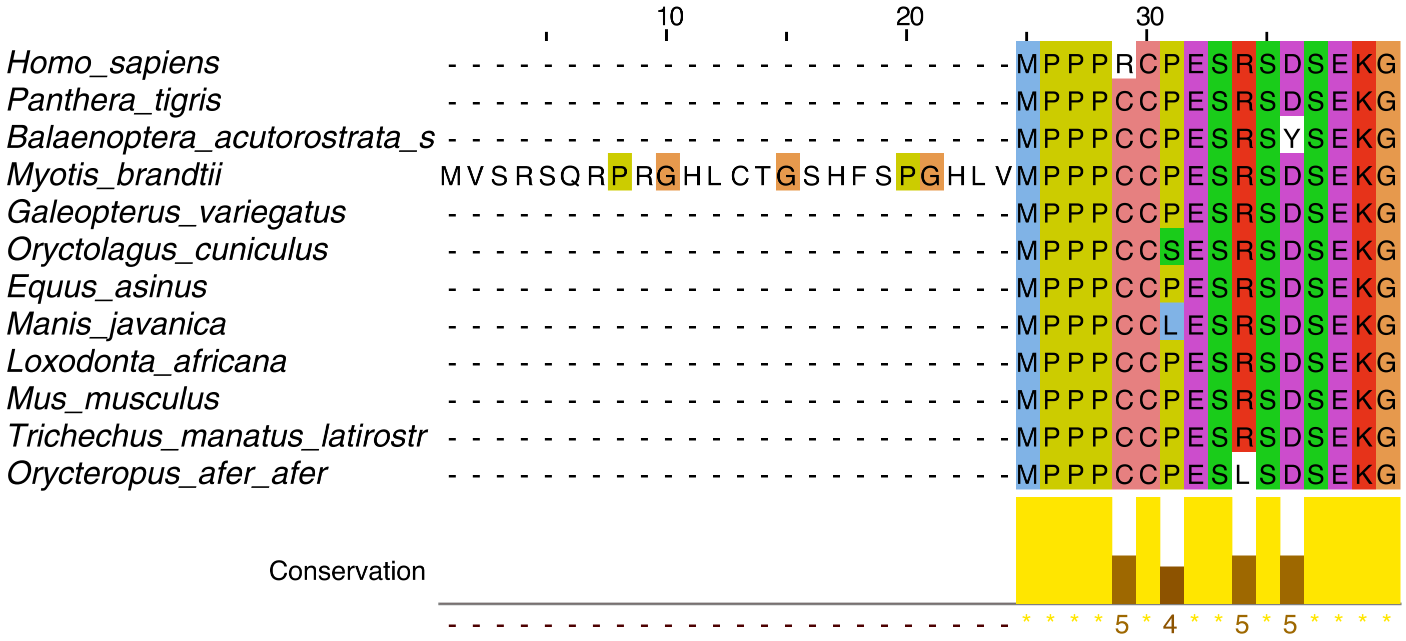

HG0268.1

ENSDARG00000011703 (clocka)

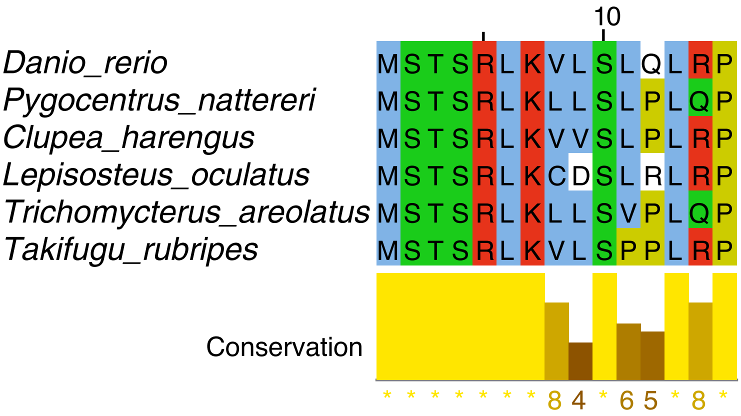

HG0268.2

ENSG00000134852 (CLOCK)

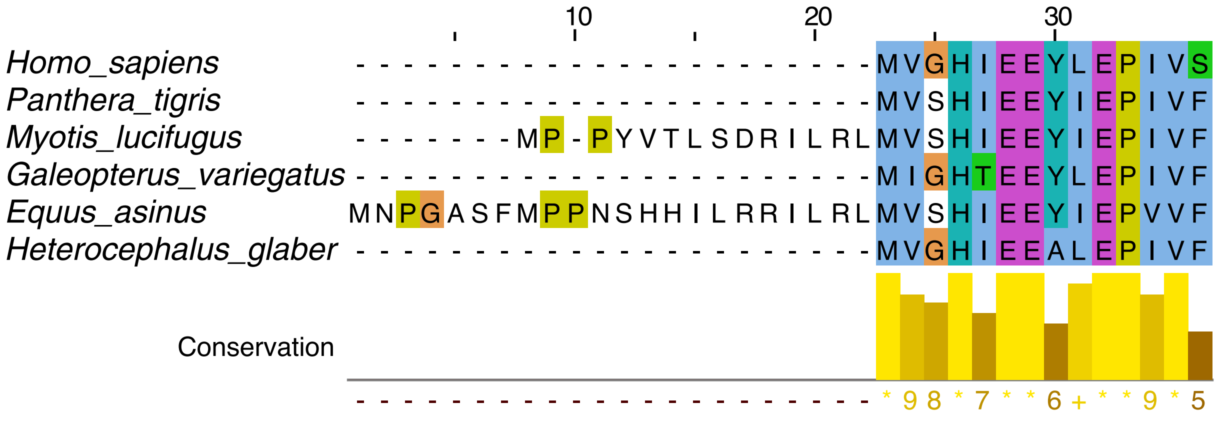

HG0268.3

ENSG00000134852 (CLOCK)

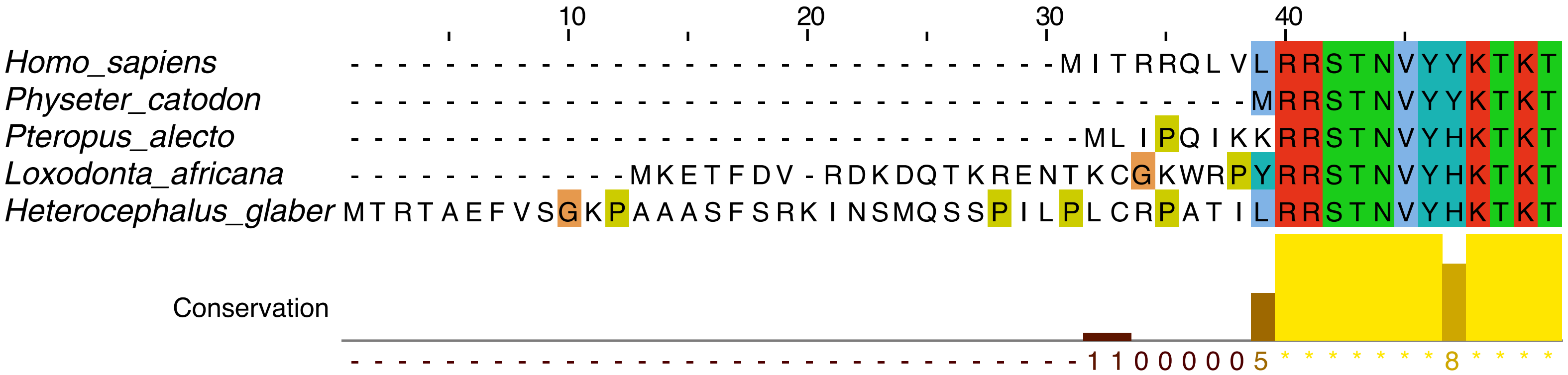

HG0269.1

ENSGALG00000028685 (SEMA5A)

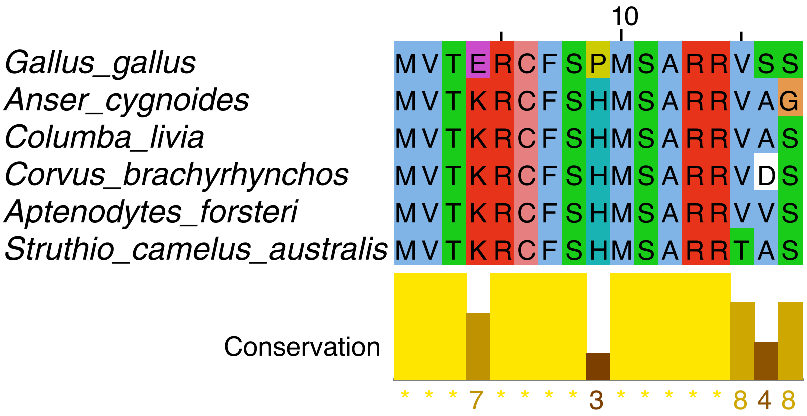

HG0269.2

ENSDARG00000061471 (sema5ba)

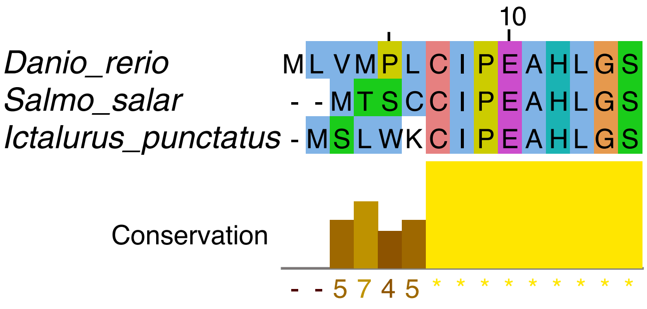

HG0269.3

ENSGALG00000011686 (SEMA5B)

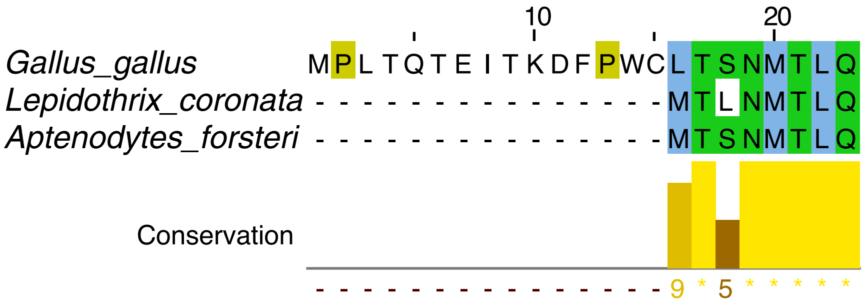

HG0270.1

ENSGALG00000015147 (ALDH1A1)

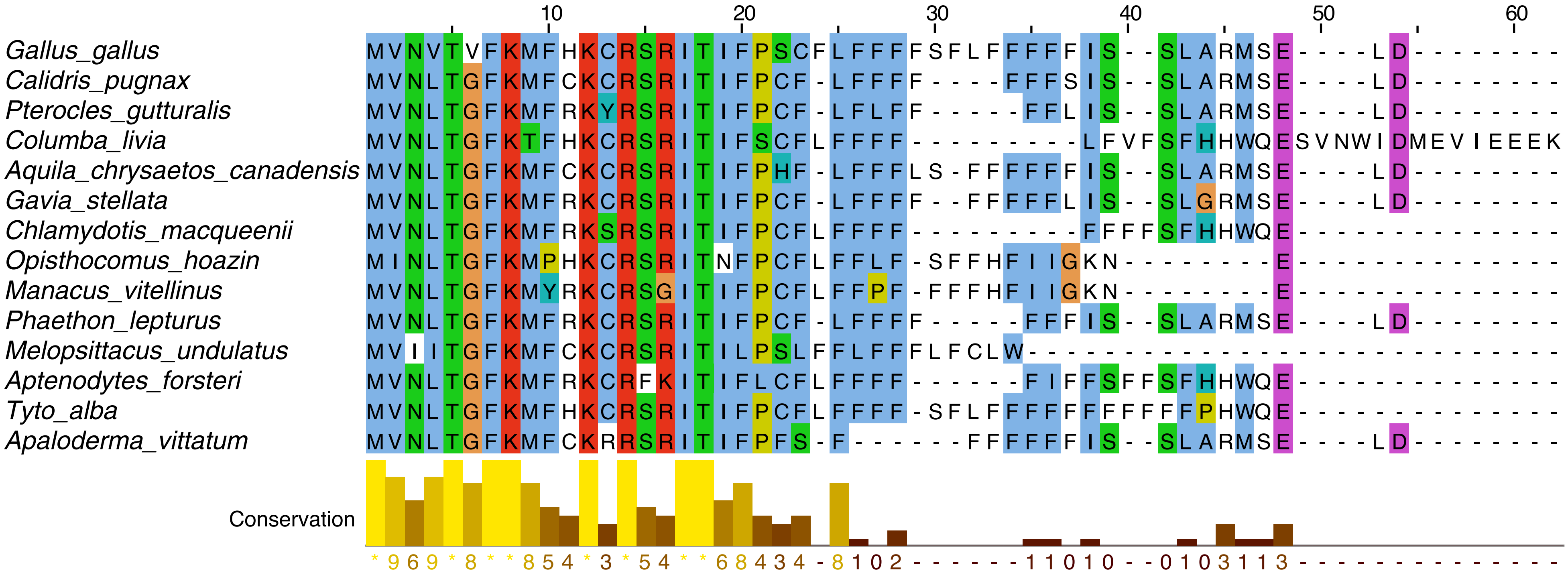

HG0270.2

ENSGALG00000015147 (ALDH1A1)

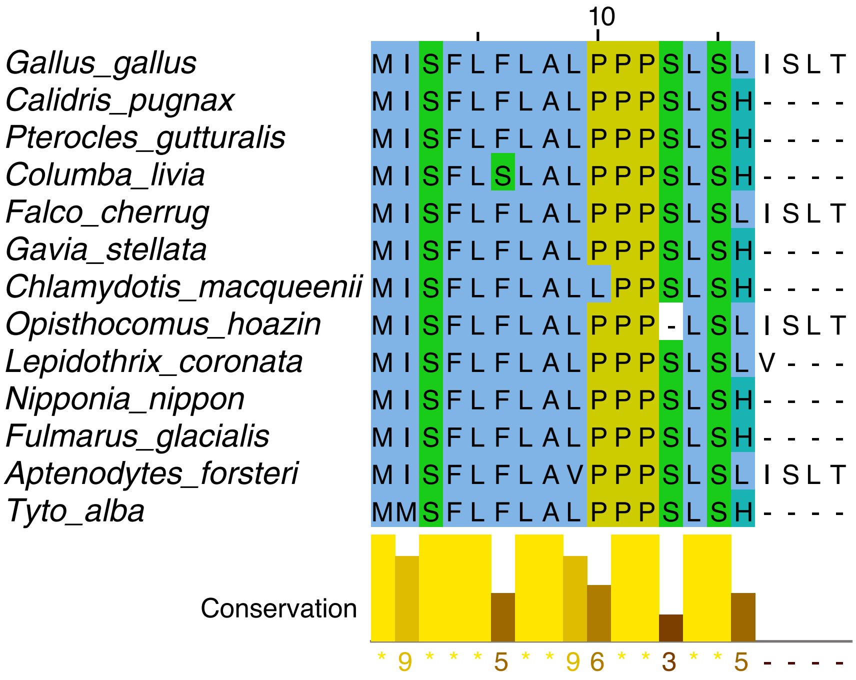

HG0270.3

ENSG00000128918 (ALDH1A2)

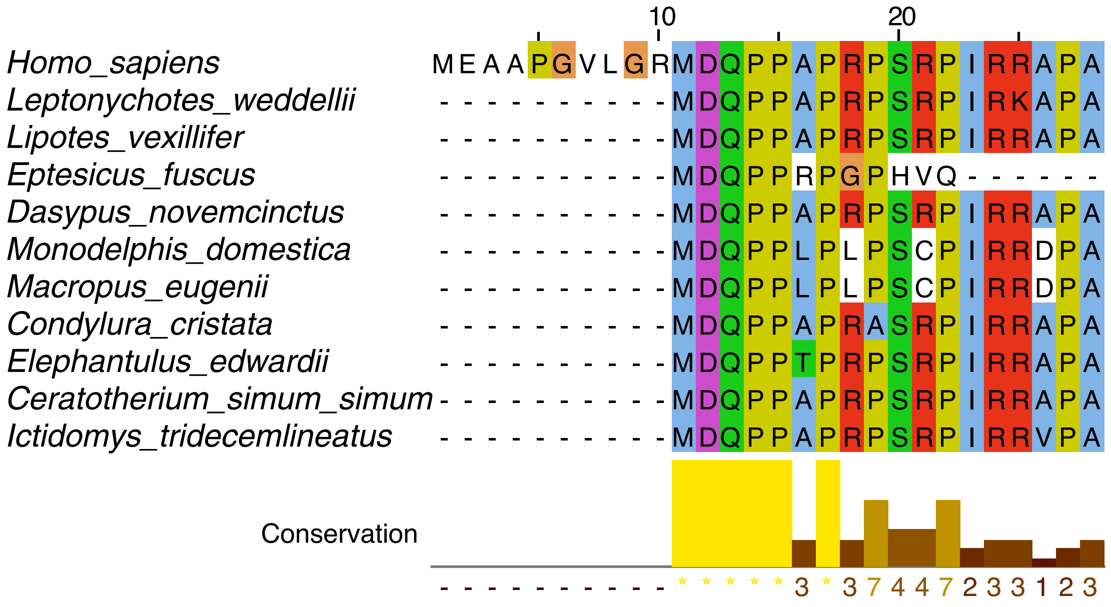

HG0271.1

ENSGALG00000004064 (GPER1)

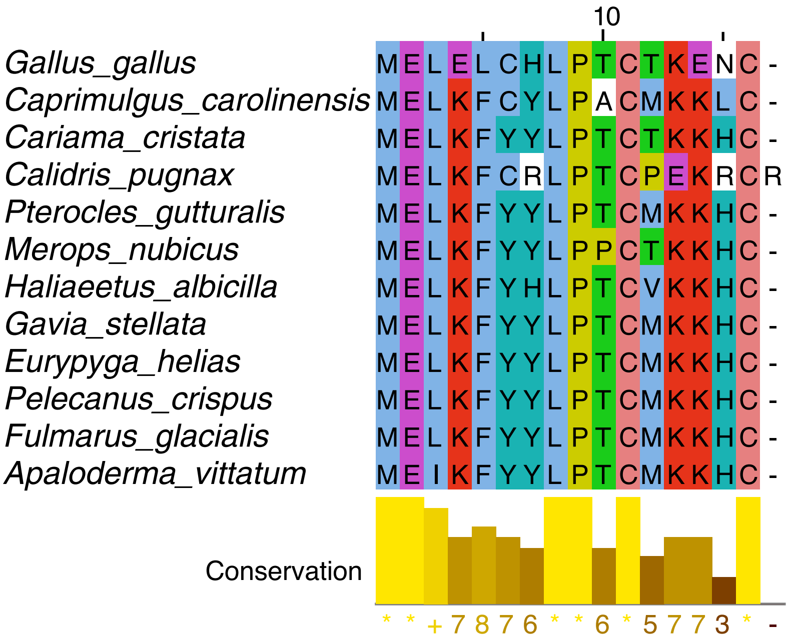

HG0271.2

ENSG00000164850 (GPER1)

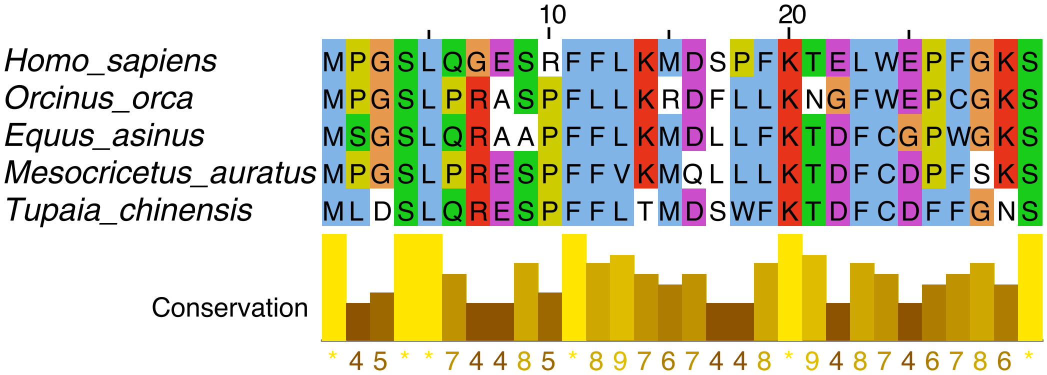

HG0271.3

ENSDARG00000074661 (gper1)

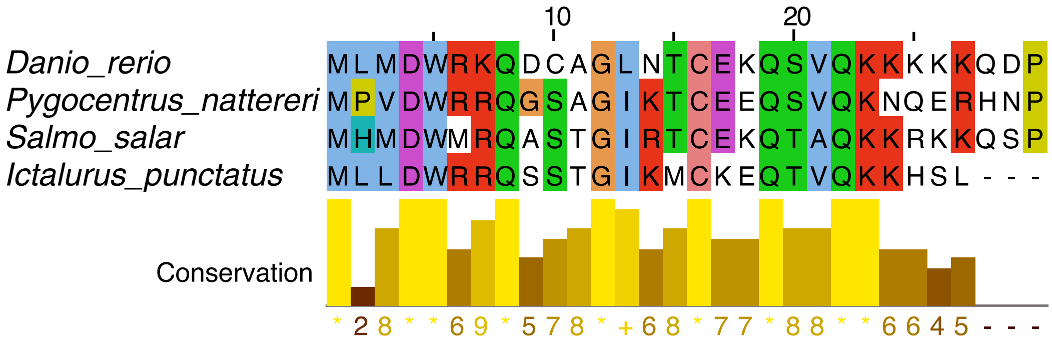

HG0272.1

FBgn0034649 (CG9865)

```
                                  10        20        30        40        50        60        70        80        90        100
Drosophila_melanogaster           ------MSDTRKDPCKPNACRIQACLTKNHYQEDKCLDVLEAMRQCCLKWHKES--LCCSGIDLEKTYLPDASKEKNATGKSNK--------------------------
Boeckaxelia_potanini              MDREYNSVLKPRDPCKPKACAIQRCLERTHYNVVLCRPFYEQLTECCRQFAARS--DACGSEEQPPASALDREPVA----------------------------------
Parasteatoda_tepidariorum         --------MQKKEPCQKYACELQKCLKENNFQQEHCLSVIKSLLQCCEKWNFKS--KCCEGFIESSDMVPDL--------------------------------------
Meinertellus_cundinamarcensi      ---------MKKDPCKIFACEIQRCLQENKFQESACQAAINALKKCCKKWREDS--LVCSGIKIDDELNKDIPQK-----------------------------------
Mastotermes_darwiniensis          ---------MKKDPCKPHACSIQKCLKENNYQEASCQEFIELLKECCRKWSSAS--ISCSGIDTQEAPSQTPEDKKQ---------------------------------
Carabus_granulatus                -------MPVTKDPCKATACKIQKCLKENNYQESACADVLEEMKECCRKWAKVS--LCCSGIKIEDKAGSSSFNQSVK--------------------------------
Carcinus_maenas                   ---------MKKDPCQRQACDIQDCLQRNKYQEAACEIQINALQECCRKWHEES--GCCAGVKLKGTVTSDAALKTKPSSQKSNVK------------------------
Forficula_auricularia             -----MPRIDPKNPCMHYSCDYNKCLTETNYKEDKCKYYLDQLRNCCKKFAHLNS-LSCEGIKFDPPKENDHTETVTK--------------------------------
Occasjapyx_japonicus              ------MPAKPNDPCKPLACEIQRCLVANDFLESKCEEEVKRMLACCDLWGETSGGRSCSGFWINRPGEAPRKRQEAKK-------------------------------
Aposthonia_japonica               --------MPKKDPCEPHACKIQKCLTENNFQEQKCKAVIDDMINCCKKWGGES--LCCSGFLSKGNKTDSSKEGSSAVTNEGKPSSET---------------------
Eurylophella_sp._AD-2013          ---------MKGDPCQLQACAIQKCLQVNNYKEAACPKEFEEMRKCCEKWGQLS--GCCSGFKDPELISSENRQGDKVASK-----------------------------
Galloisiana_yuasai                ----------MKDPCKPYACKIQKCLKEHNYQENACKDVIEEMVICCRKWGDIS--LCCSGIDTGKTNGSSGSKTVSGKT------------------------------
Pachypsylla_venusta               -------MSKKKDPCKVHACAIQKCLKENDFQESKCLQYFEYMRQCCVKFHADS--ICCSGIDINKSYLKDNEKSSPSKTDHIERSTLVAKISSYYSSRSNNNLFERFFQ
Brachycistis_timberlakei          --------MTTTDPCKPYACKLQKCLKDNVYQPSRCEEVIEEIRQCCIKHSAKS--IVCEGIDTSKQYEHNTVDYKKATK------------------------------
Ixodes_persulcatus                --------MKAVDPCQKEACAIQKCLQEHNYQEDACVKFIKEMLKCCEKWREMS--GCCSGFLKDEASKKAKGELKSKT-------------------------------
Antheraea_yamamai                 ---------MSKDPCKRFACDIQRCLVENNYQEVRCASVFEAMRRCCLKFKPSS--LVCEGYDLEPRKFAPVTDRAKK--------------------------------
Mantis_religiosa                  ---------MKKDPCKPYACEIQKCLKEHNYQIEPCQKFVEYLRDCCRKWGDVS--ISCSGIDTRTSPNKASDKE-----------------------------------
Panorpa_vulgaris                  ------MSNVPKDPCKPSACKIQQCLQEHNFQEQSCIDVIEQMRLCCVKWEGKS--FVCNGINTAEPADLAISPKHTKHS----RY------------------------
Dermanyssus_gallinae              --------MPKGDPCQKQACDIQKCLQANNYQEALCVEAFRAMDLCCEKWGLQS--GCCTGFIKEQQLKKKIANESTKTPISFN--------------------------
Pseudomallada_prasinus            ------MPKPYIDPCKEFACRIQKCLRENNFQEHRCKQVIEELRQCCVKWKSES--ISCSGIDINSPKEEQ---------------------------------------
Triops_newberryi                  ------------------------------------------MKECCSKWQERS--QSCAGFLVPEIEKKKNEPLS----------------------------------
Calopteryx_splendens              ------------------------------------------MQKCCDKWFDES--LCCSGFKKPSES------------------------------------------
Stenobothrus_lineatus             ---------MRKDPCKPIACKIQKCLTENRFQEPACQRVLEEMKECCRKWAHIS--LCCSGIDTSDKPKD----------------------------------------
Extatosoma_tiaratum               ----------MKDPCKPQACKIQKCLTENNYQEAACKDVIKEMVECCRKWADVS--LCCSGINTKETTANSPEEI-----------------------------------
Menopon_gallinae                  --------MTKKDPCKSLACALQKCLKENDFQEERCKKVLDDIRTCCKKFKEIS--VVCSGIDVSEKIK-----------------------------------------
Cosmioperla_kuna                  --------MKKRDPCKAHACRIQKCLAENNFQESACQAVLEDMRSCCRKFKNVS--LCCSGIDIGDTPTLETASEPKK--------------------------------
Ectopsocus_briggsi                -------MPKKVEPCKPLACALQKCLTENNYQEAACKEAISALKDCCAKWGETSP-VVCSGFNK----------------------------------------------
Xanthostigma_xanthostigma         ----------MKDPCKLTACKIQKCLQENHFQEDACADVLEEMRQCCLKWYKES--LCCSGIRLDEPKIKEKKSINQSKTN-----------------------------
Ctenocephalides_felis             --------MRSKDPCKANACMIQKCLSENNFQESACTDVIELMRLCCVKWGTKS--FVCNGIKTEKETSSTSSTVENN----KIPYKLDHKS------------------
Thrips_palm    i                  ------MSKRSKDPCKPFACAIQDCLNAKNFQESACVDVIENLNECCRHWKHKS--LVCSGMLIGPAEVEKSDVKSDGTSGVEVRKA-----------------------
Hydroptila_sp._AD-2013            --------MKQKDPCKATACLIQKCLRERGFQEERCADVLEEMRQCCLKWHKVS--LCCSGIKLDPPKIEKETK------------------------------------
Zorotypus_caudelli                -------MKKKKDPCELYACMLQKCLTENNFRESCCIAVLEQLRECCRIWGNAS--ICCSGFDNSPKAEKEPENKEPESSAKK---------------------------
Thermobia_domestica               --------MKGGDPCQKQACDIQKCLKIHNYQVSACKDFFEAMKKCCEKWGDLS--GCCSGFKKERELEEAQKTK-----------------------------------
```

Conservation

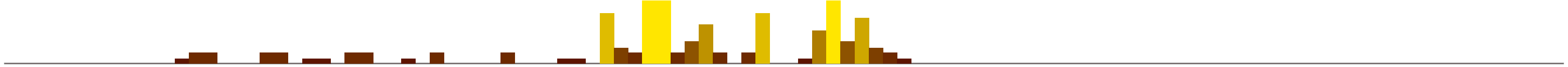

```
---------000122000220110220010200002000110932**2472029--16*48321000000000000----------------------------------
```

HG0272.2

FBgn0034649 (CG9865)

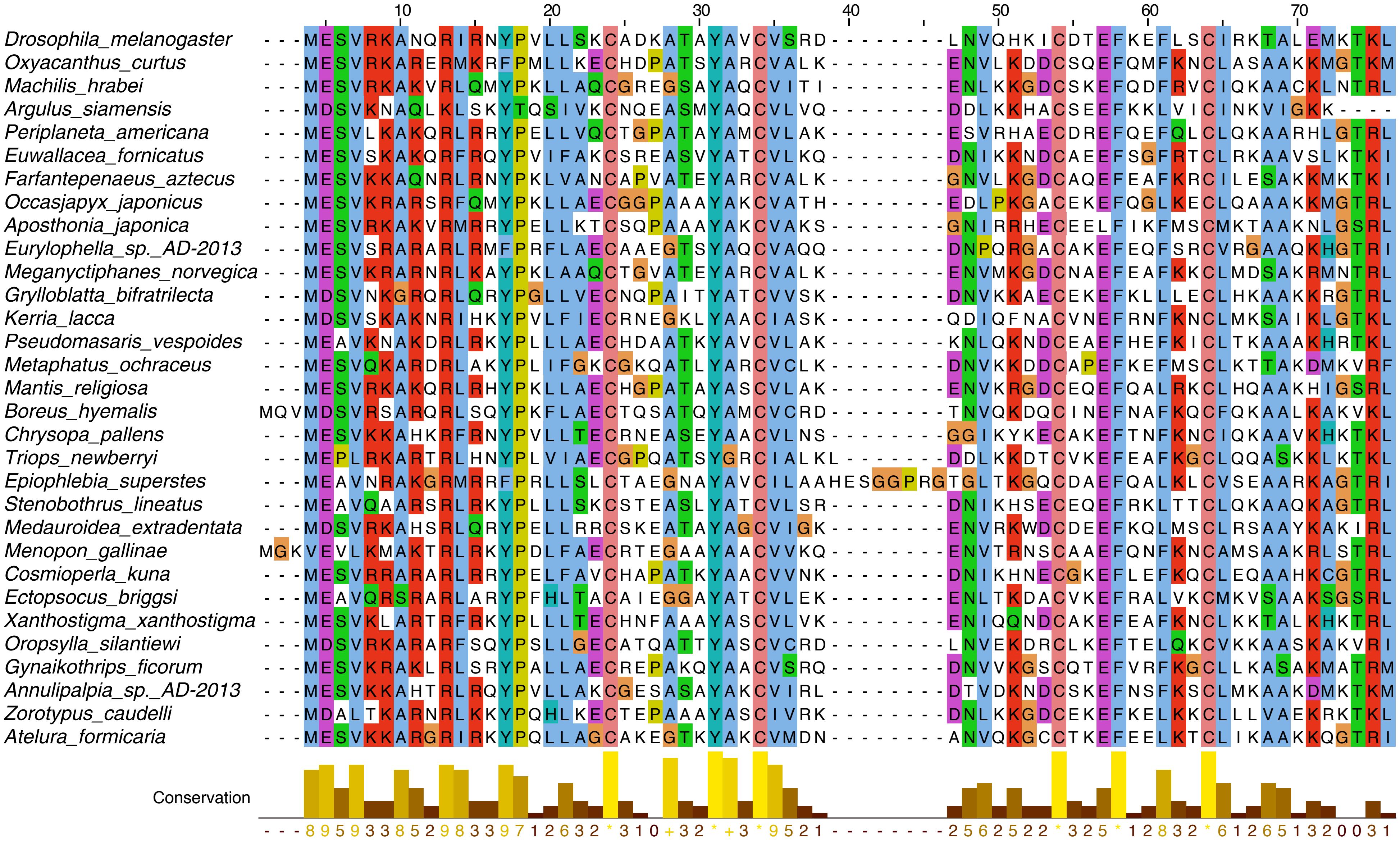

HG0272.3

ENSG00000143315 (PIGM)

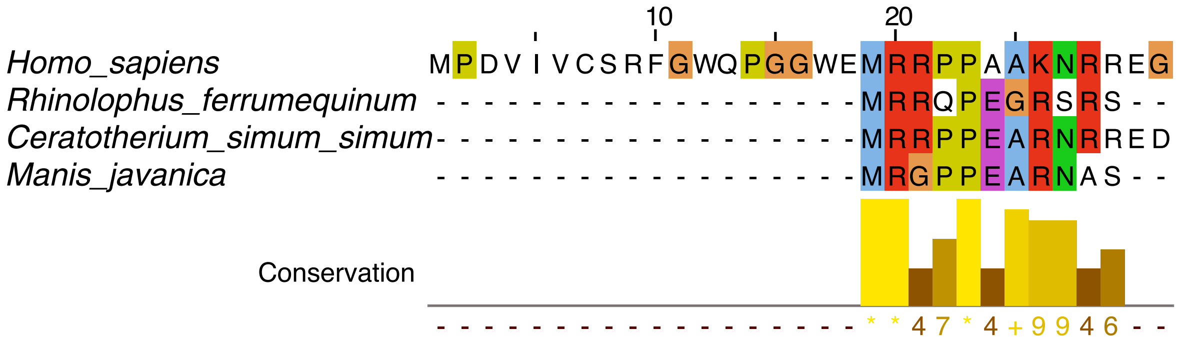

HG0273.1

ENSGALG00000003293 (MBTPS1)

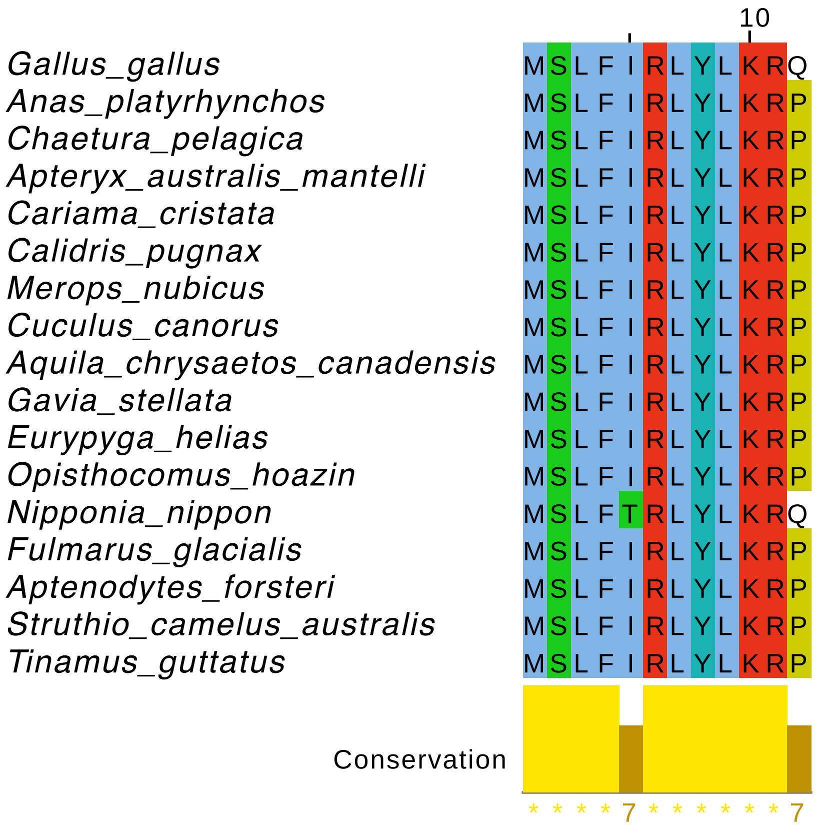

HG0273.2

ENSG00000140943 (MBTPS1)

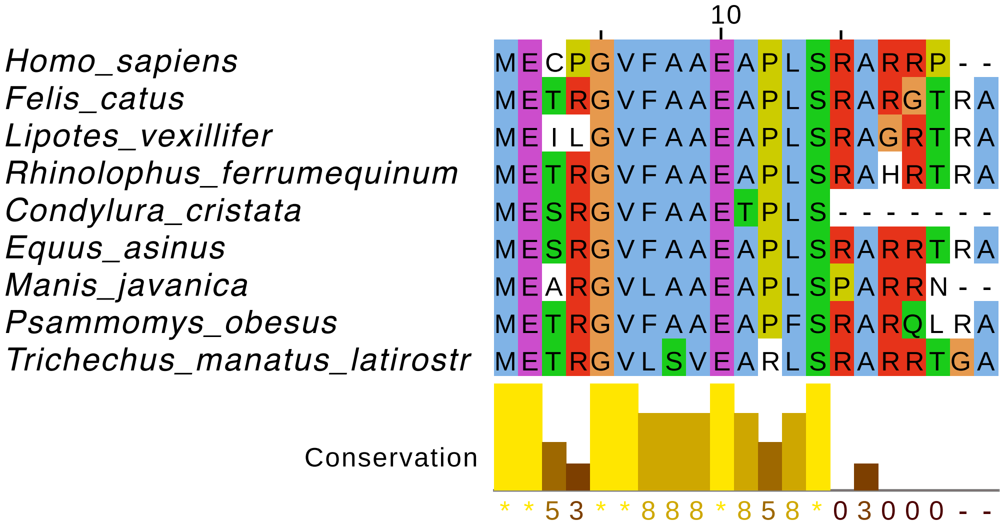

HG0273.3

ENSG00000140943 (MBTPS1)

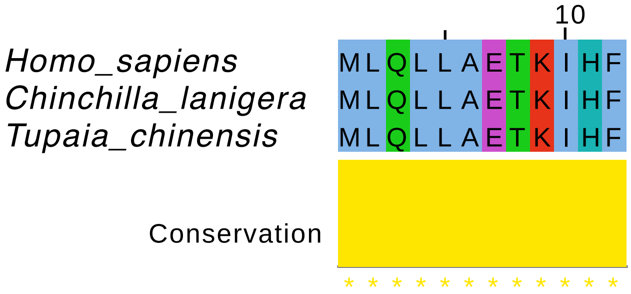

HG0274.1

ENSG00000204256 (BRD2)

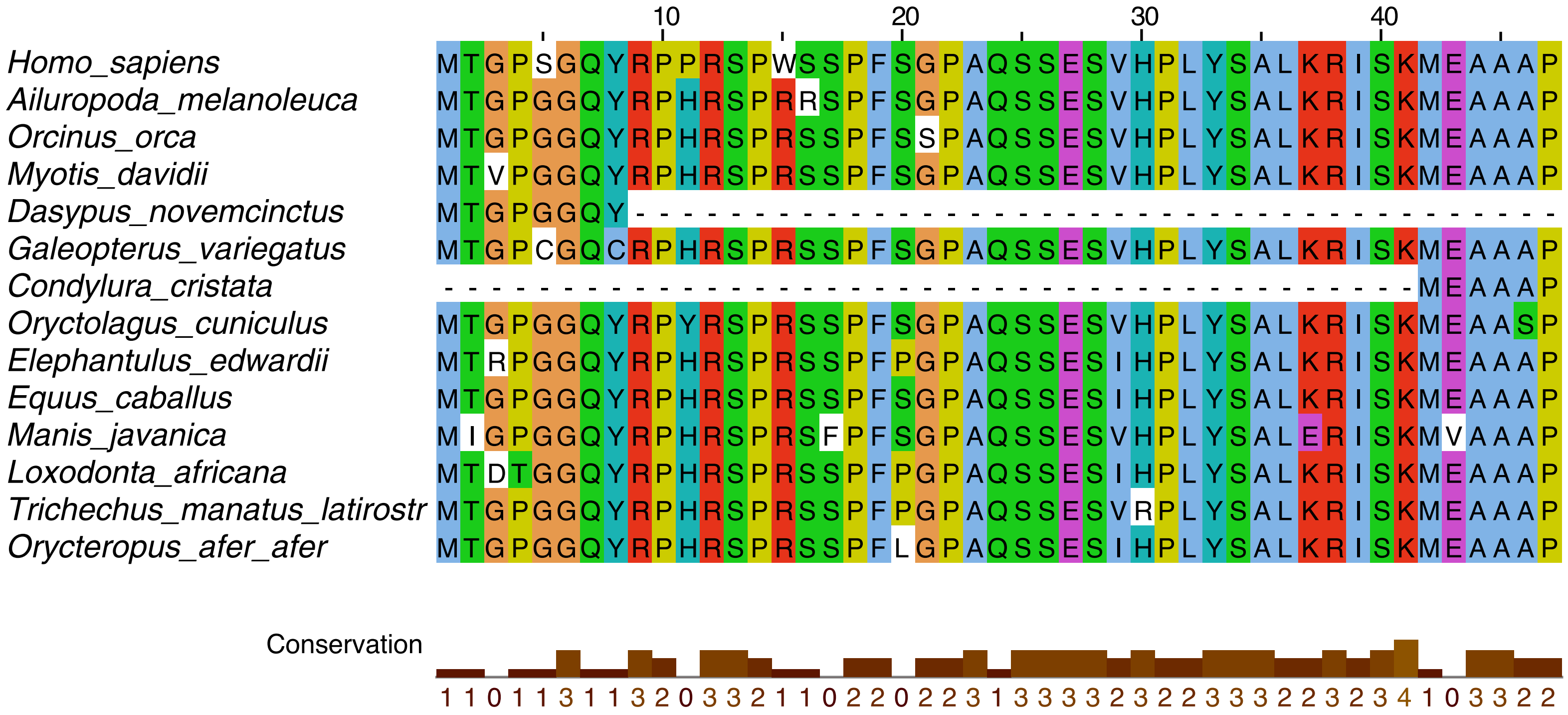

HG0274.2

ENSDARG00000100129

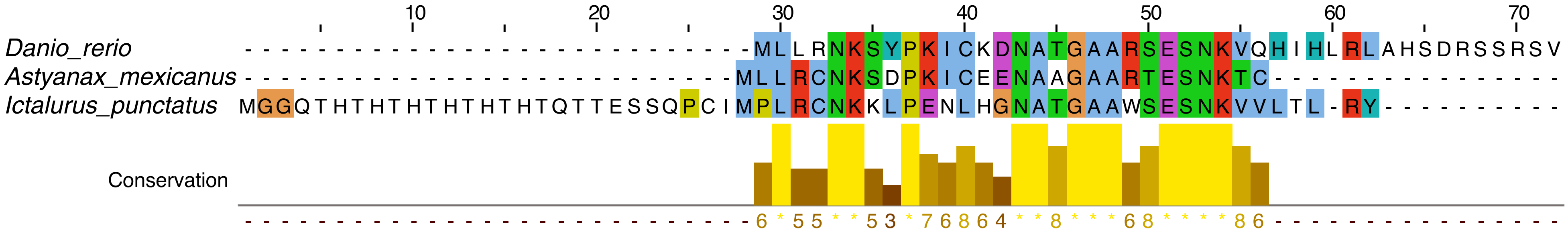

HG0275.1

ENSG00000100239 (PPP6R2)

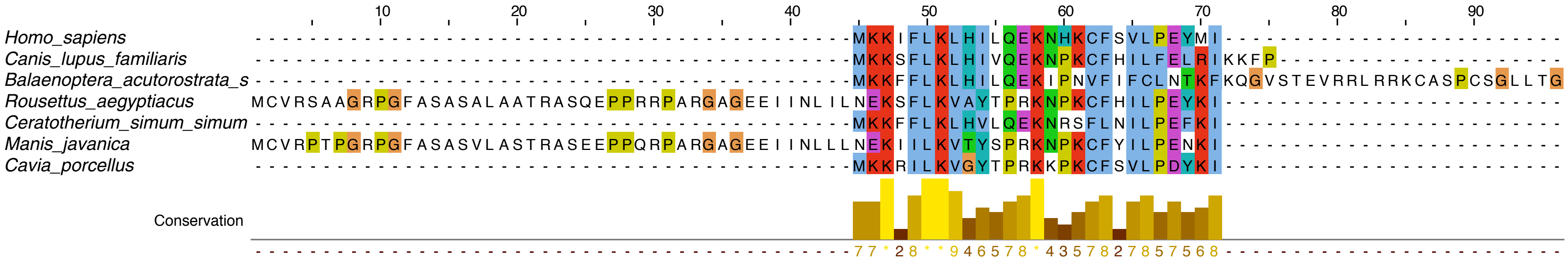

HG0275.2

ENSDARG00000045540 (ppp6r2a)

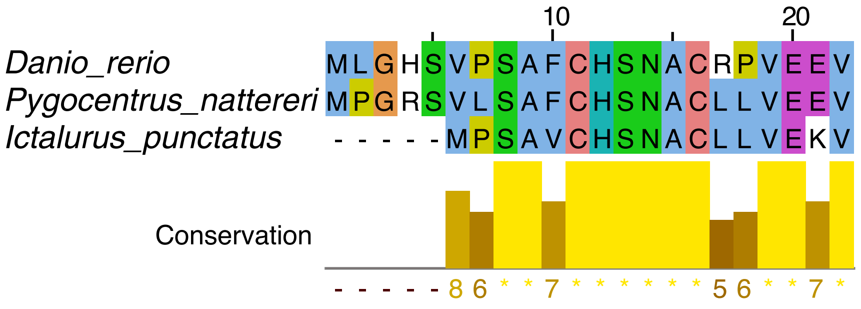

HG0276.1

ENSGALG00000008912

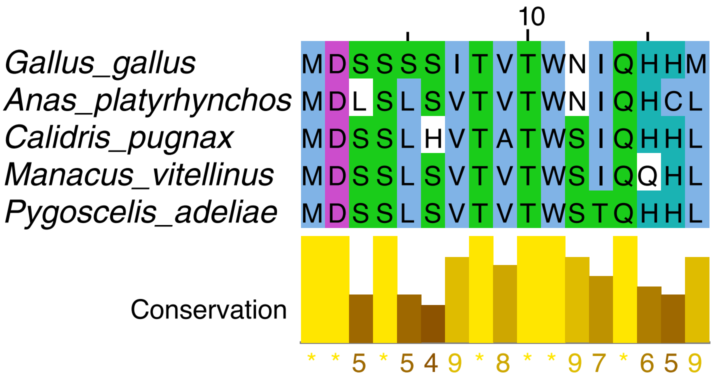

HG0276.2

ENSG00000073734 (ABCB11)

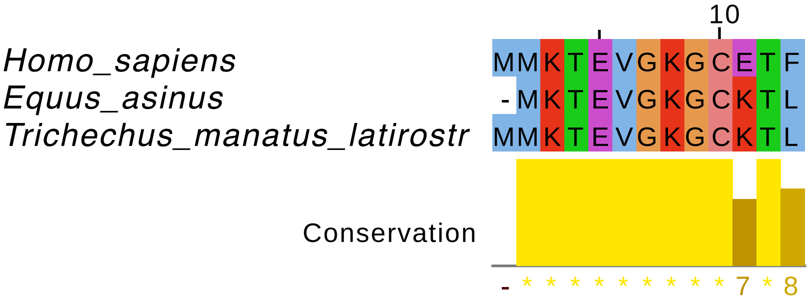

HG0277.1

ENSG00000102531 (FNDC3A)

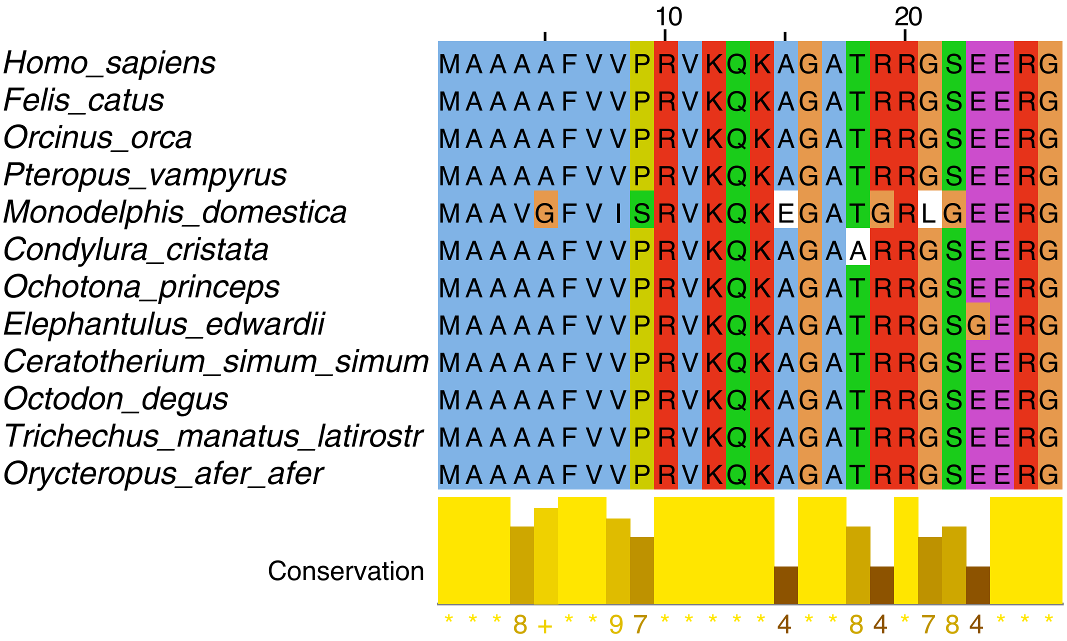

HG0277.2

ENSGALG00000004169

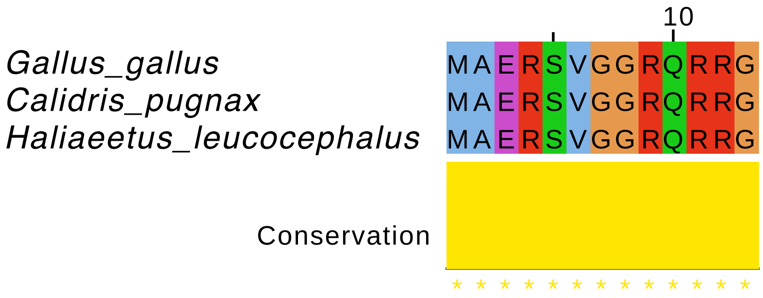

HG0278.1

ENSG00000119946 (CNNM1)

```
                               10        20        30        40        50        60        70        80        90
Homo_sapiens          -----------------------------------------------------------MPGHLQPPAGTSCPGGV----------------
Myotis_lucifugus      MGNFPGQWGRRGAGRELLPGRSSLRCCLLLKQEALLQAKLSLGCSSDIPECVRVMHRAVMRGHFQPPAGTSCPDRVRTPTASSPRAQGACTW
Dasypus_novemcinctus  ------------------------------------------------------MLCAVMRGHLQPSSGTSCPG------------------
Condylura_cristata    -----------------------------------------------------------MRGHLWPPAGTTCPGRV----------------
Oryctolagus_cuniculus -----------------------------------------------------------MRGHLQPPAPTPCPGRVCAPTASSPRAQGARTW
Equus_caballus        ----------------------------------MLQGKLSPDCNSDTTKCVWVMQSAVMRGHLQPPAKTSCPGRV----------------
```

Conservation

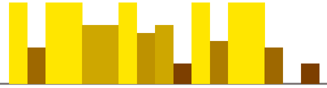

```
                      -----------------------------------------------------------*5**88*783*6**503----------------
```

HG0278.2

ENSDARG00000078733 (cnnm2b)

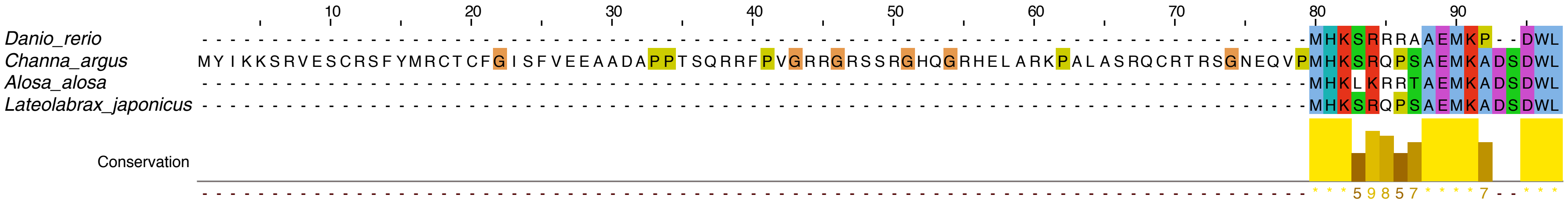

HG0279.1

ENSG00000175928 (LRRN1)

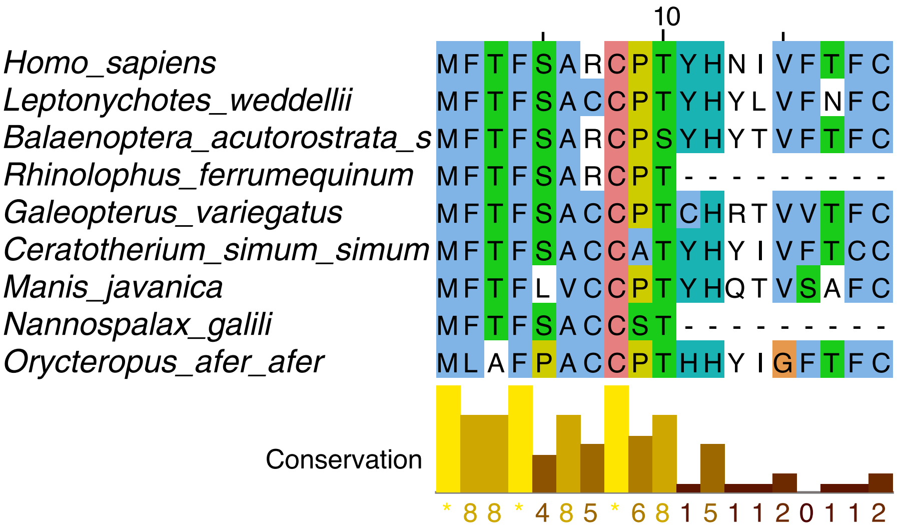

HG0279.2

ENSGALG00000035099 (Lrrn1)

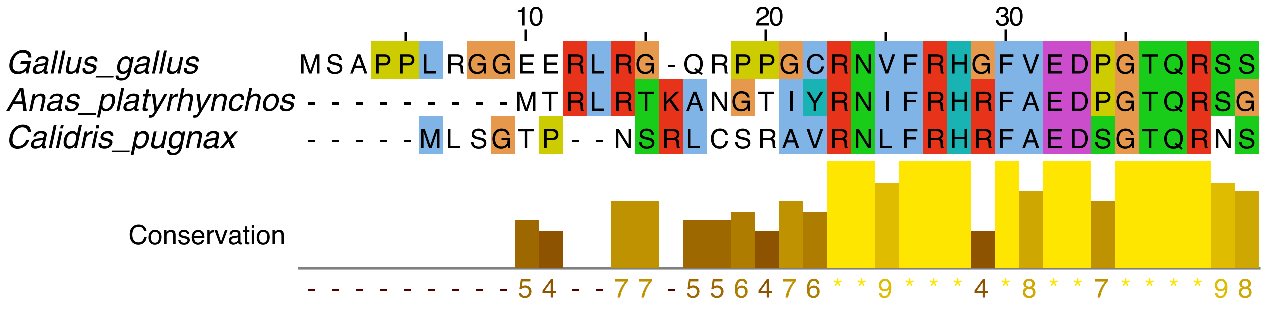

HG0280.1

ENSG00000156299 (TIAM1)

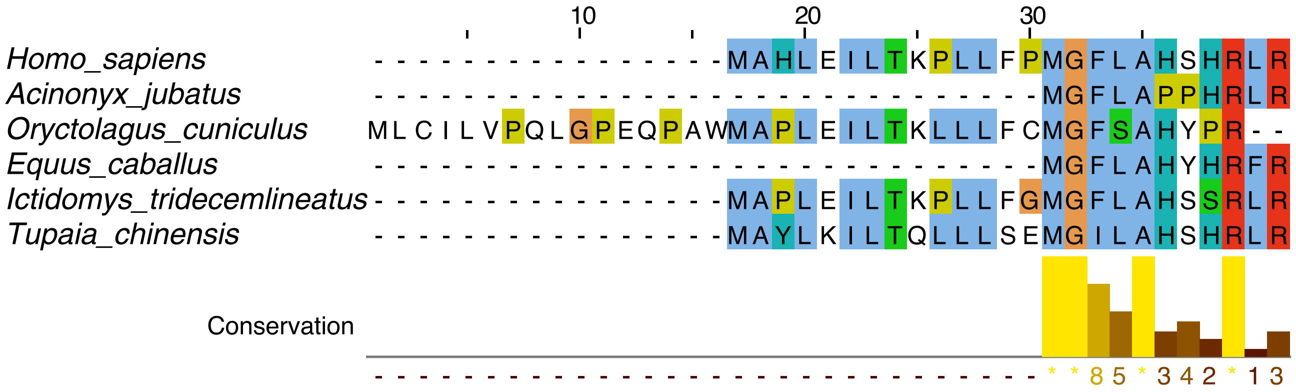

HG0280.2

FBgn0085447 (sif)

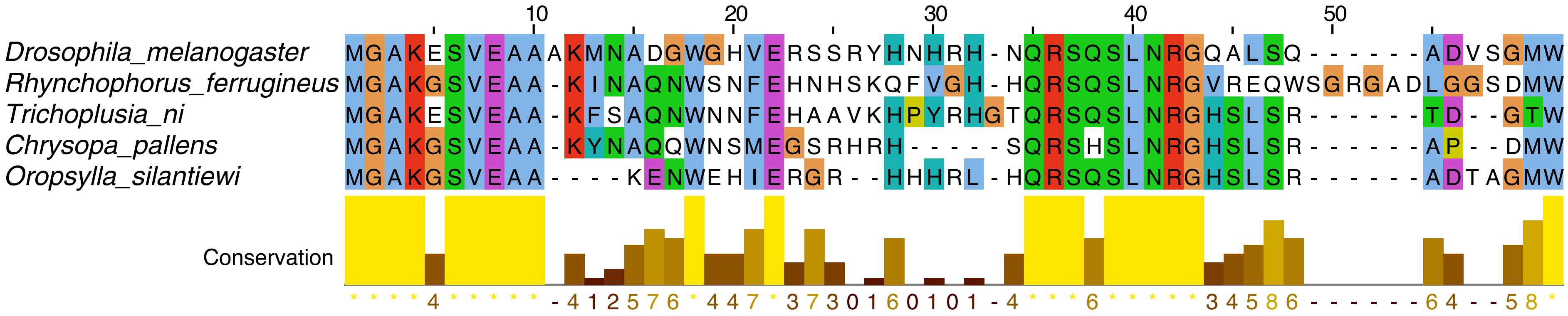

HG0281.1

ENSGALG00000006766 (DEPDC5)

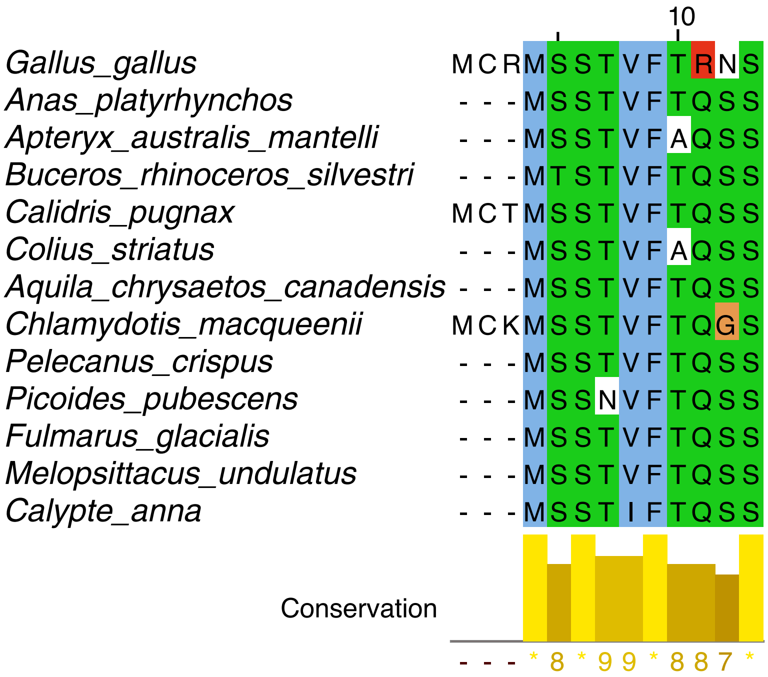

HG0281.2

ENSG00000100150 (DEPDC5)

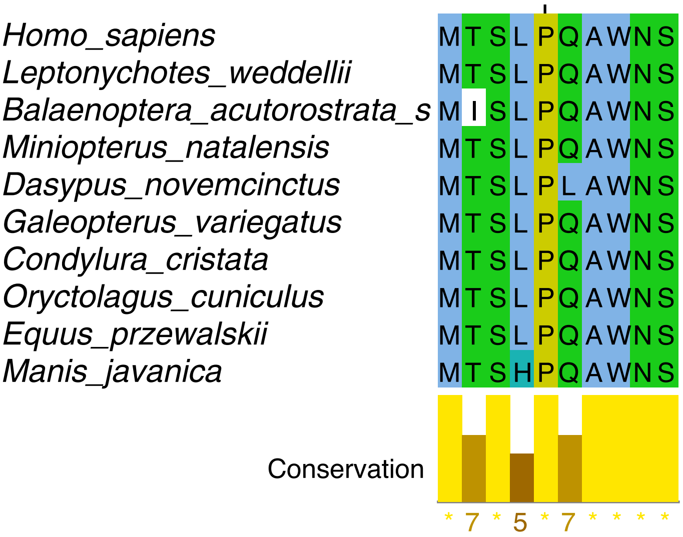

HG0282.1

ENSDARG00000061328 (cdon)

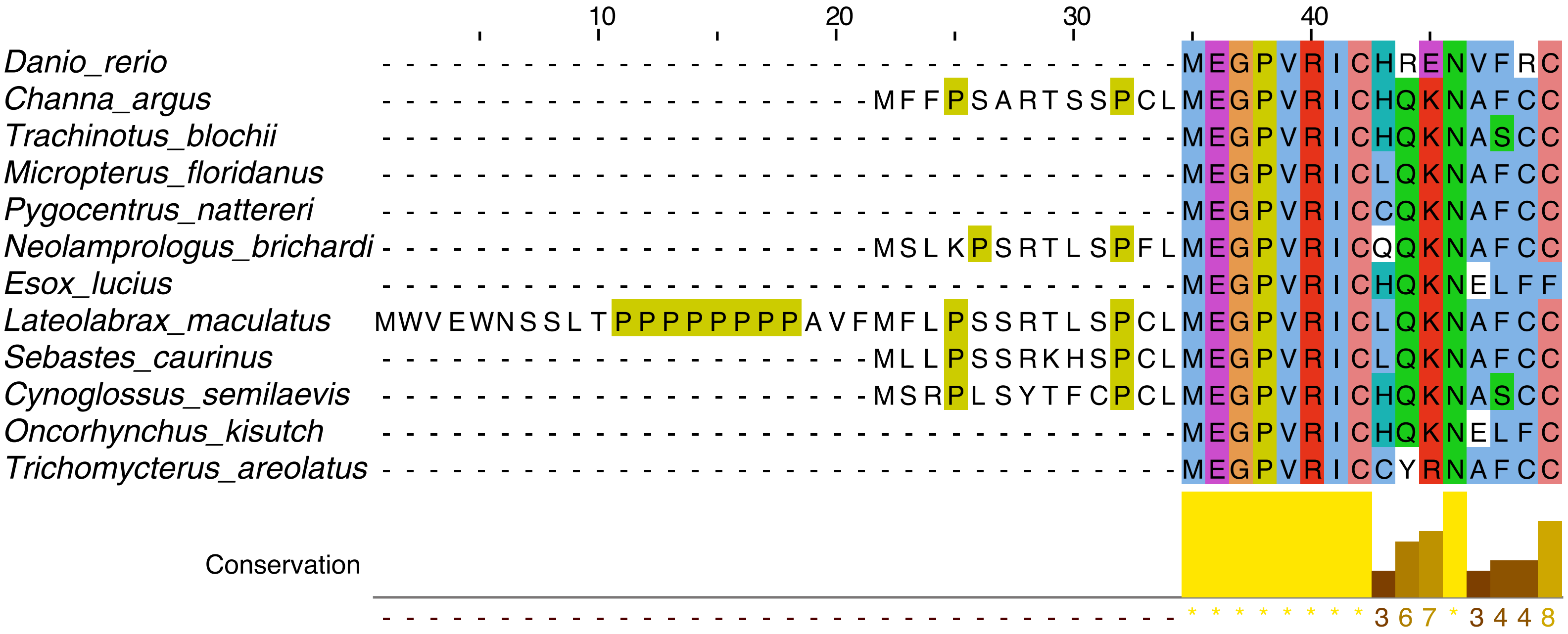

HG0282.2

ENSG00000144857 (BOC)

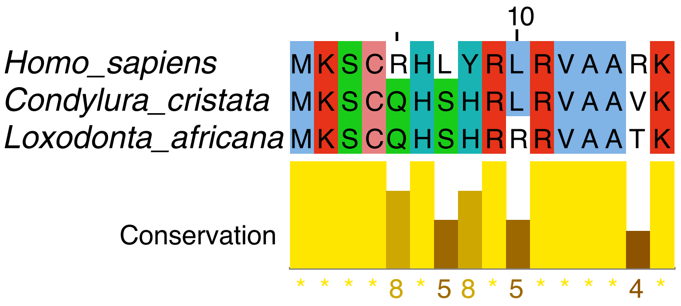

HG0283.1

ENSGALG00000010270 (AREL1)

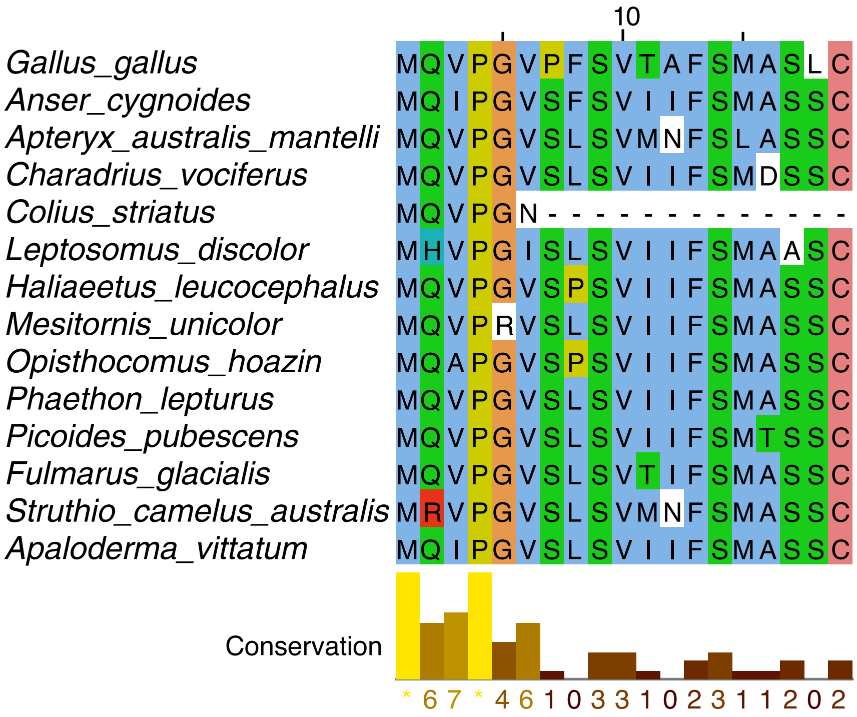

HG0283.2

ENSG00000119682 (AREL1)

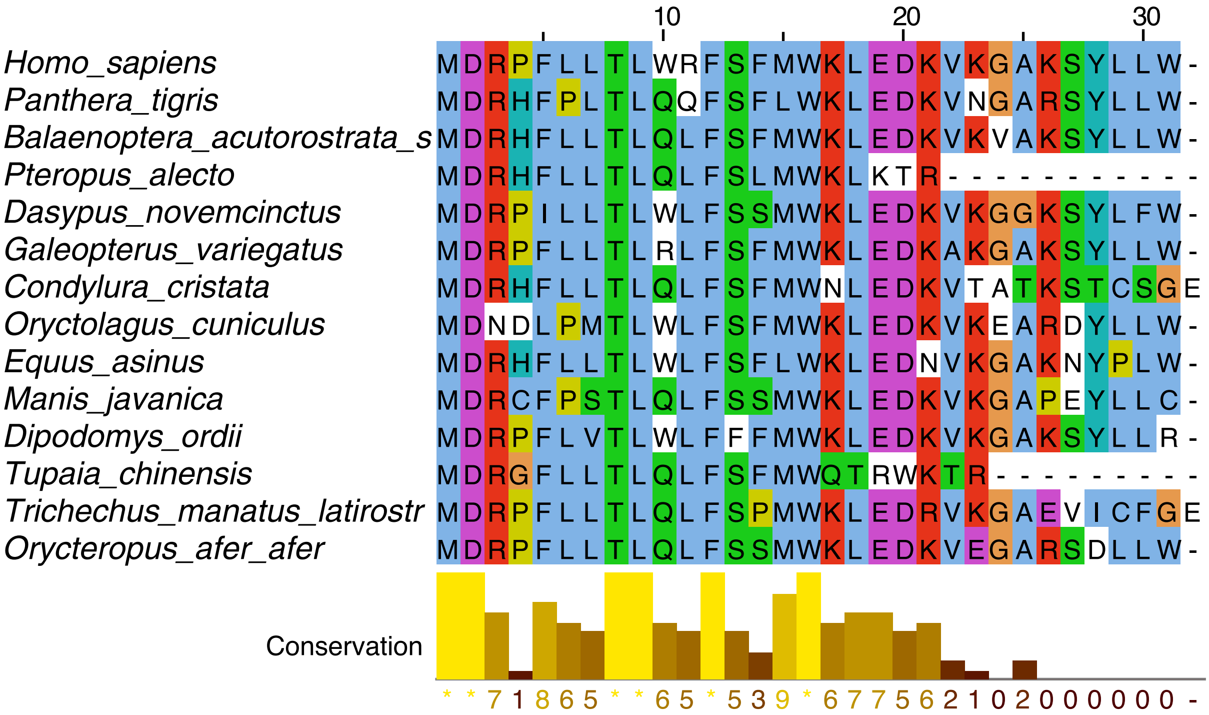

HG0284.1

ENSG00000007168 (PAFAH1B1)

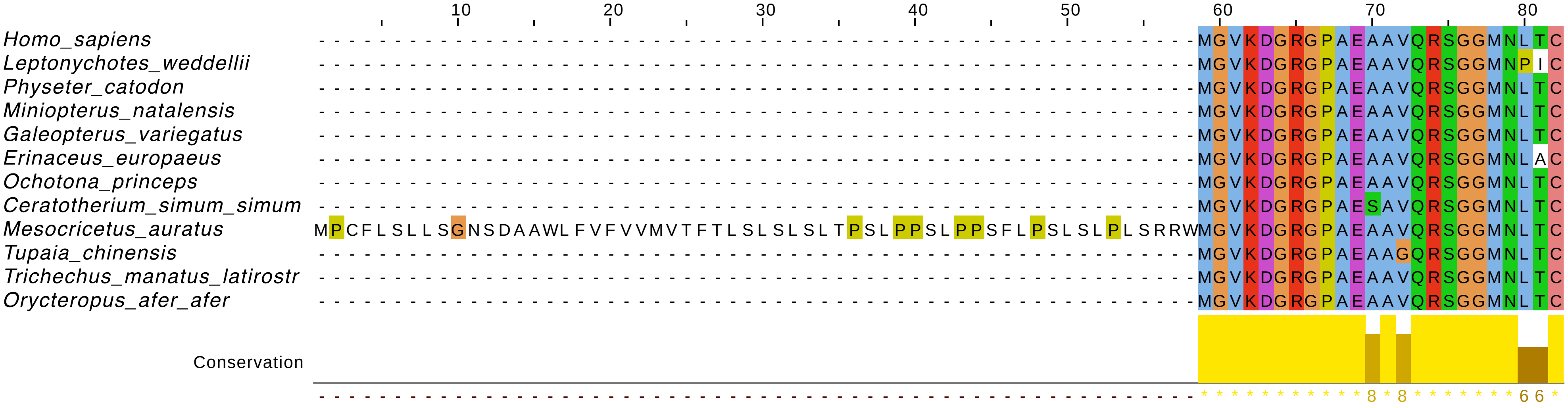

HG0284.2

ENSGALG00000005834 (PAFAH1B1)

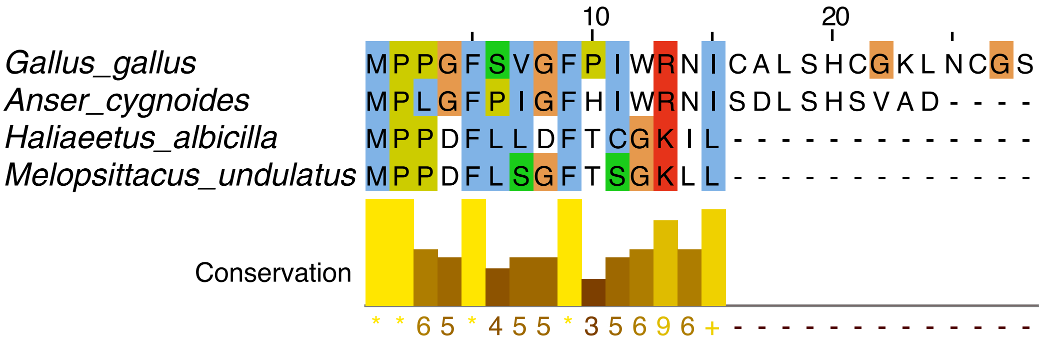

HG0285.1

ENSGALG00000012156 (DPP10)

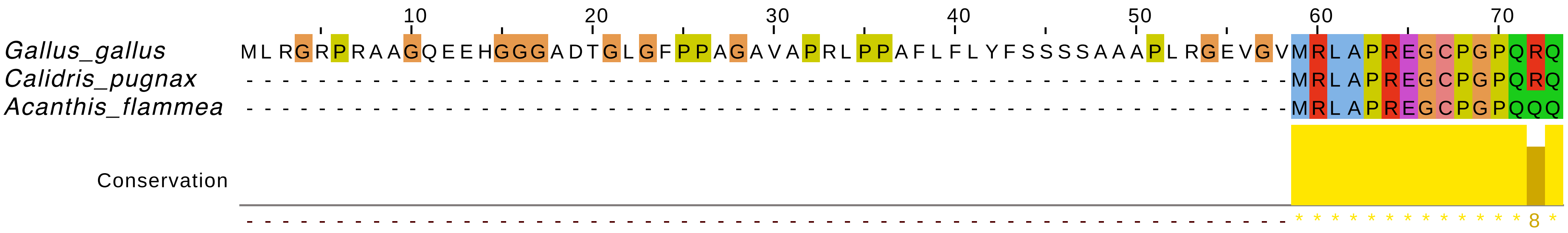

HG0285.2

ENSG00000175497 (DPP10)

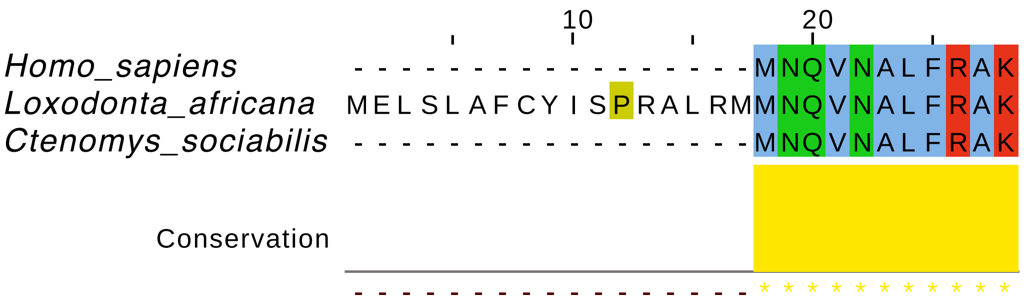

HG0286.1

ENSG00000116698 (SMG7)

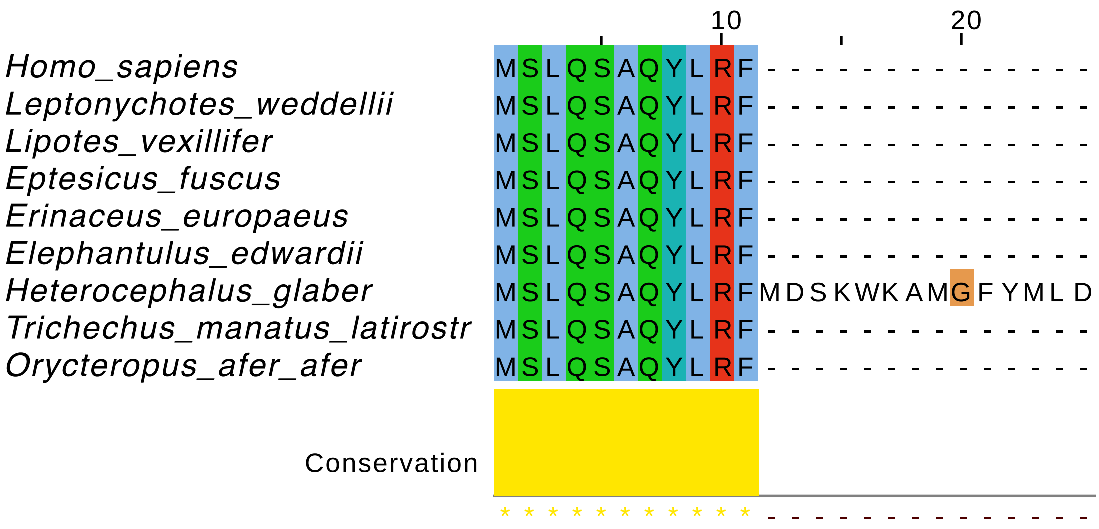

HG0286.2

ENSDARG00000060767 (smg7)

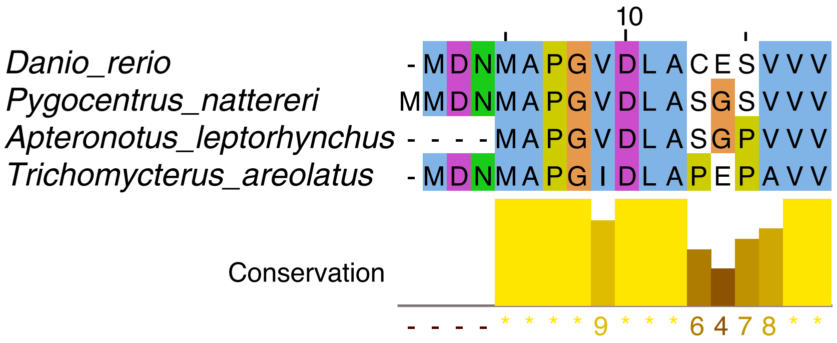

HG0287.1

ENSG00000170325 (PRDM10)

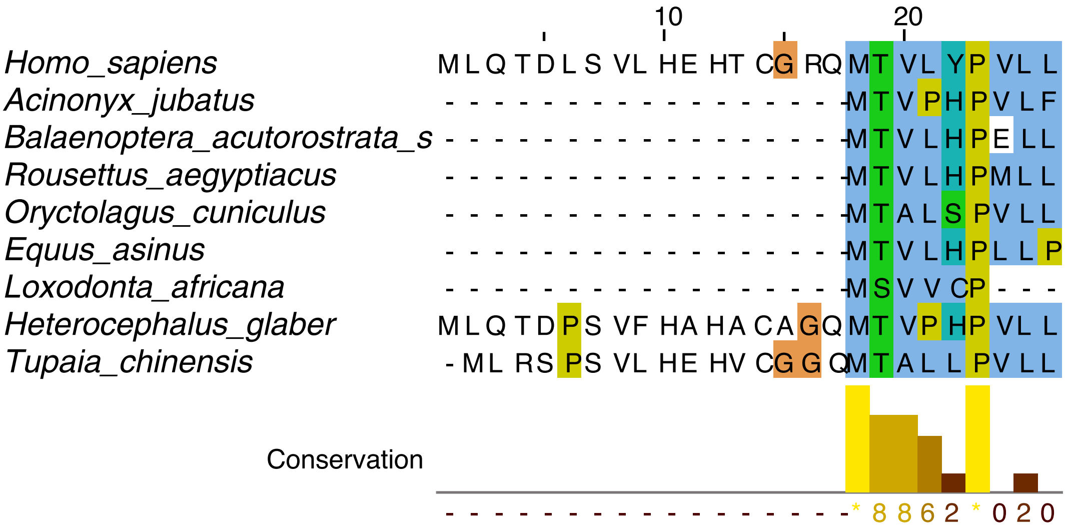

HG0287.2

ENSDARG00000104251 (prdm10)

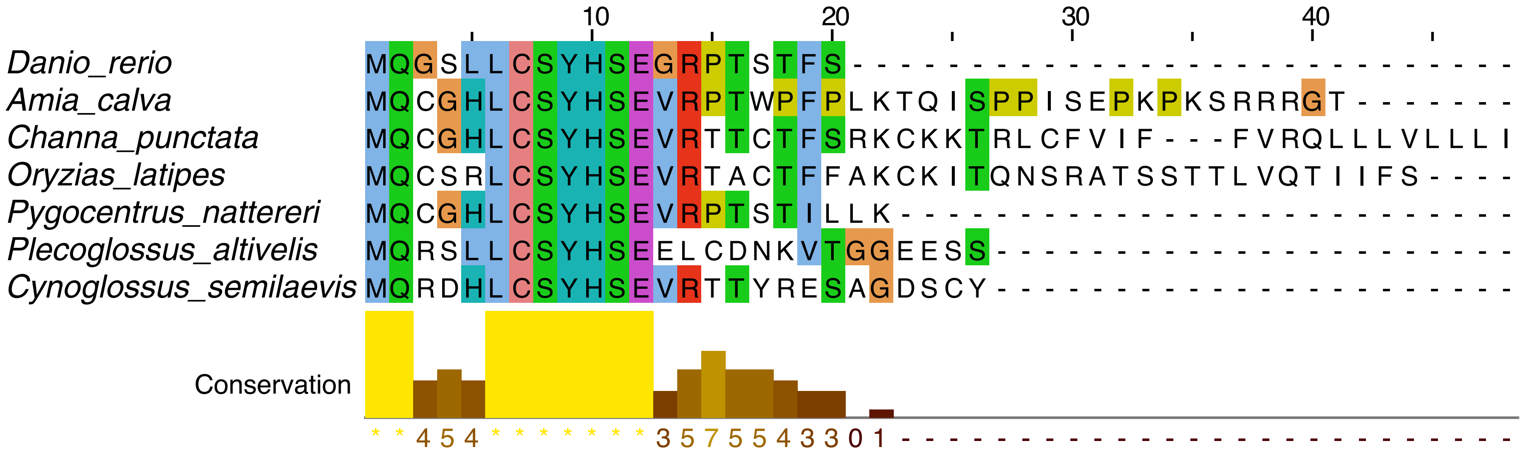

HG0288.1

ENSGALG00000006655 (MAP3K13)

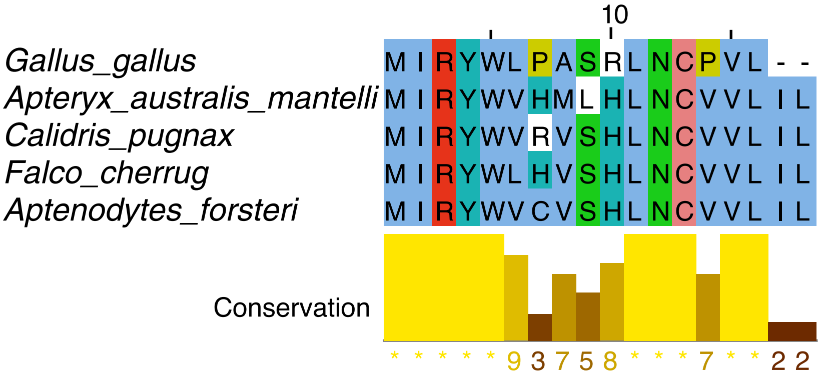

HG0288.2

ENSG00000073803 (MAP3K13)

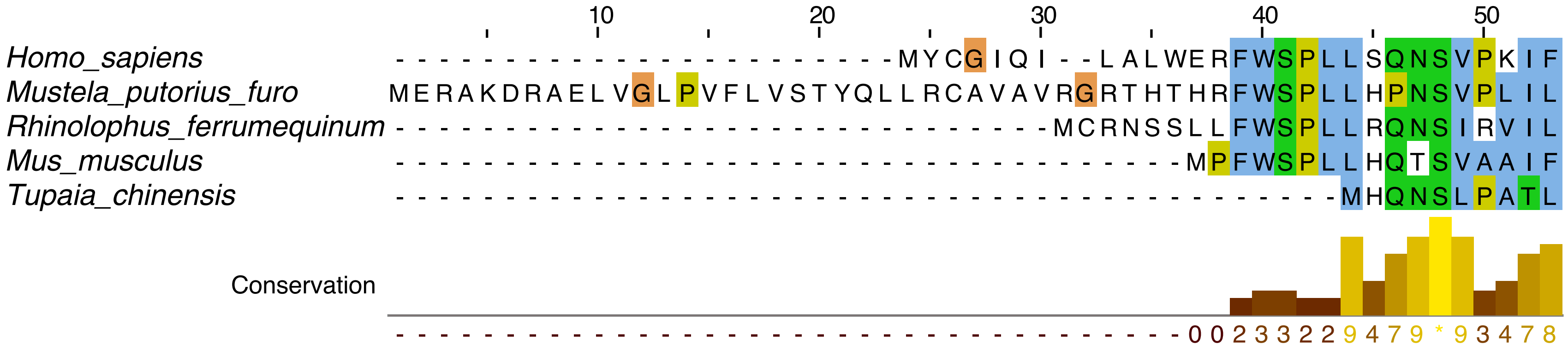

HG0289

ENSG00000072415 (MPP5)

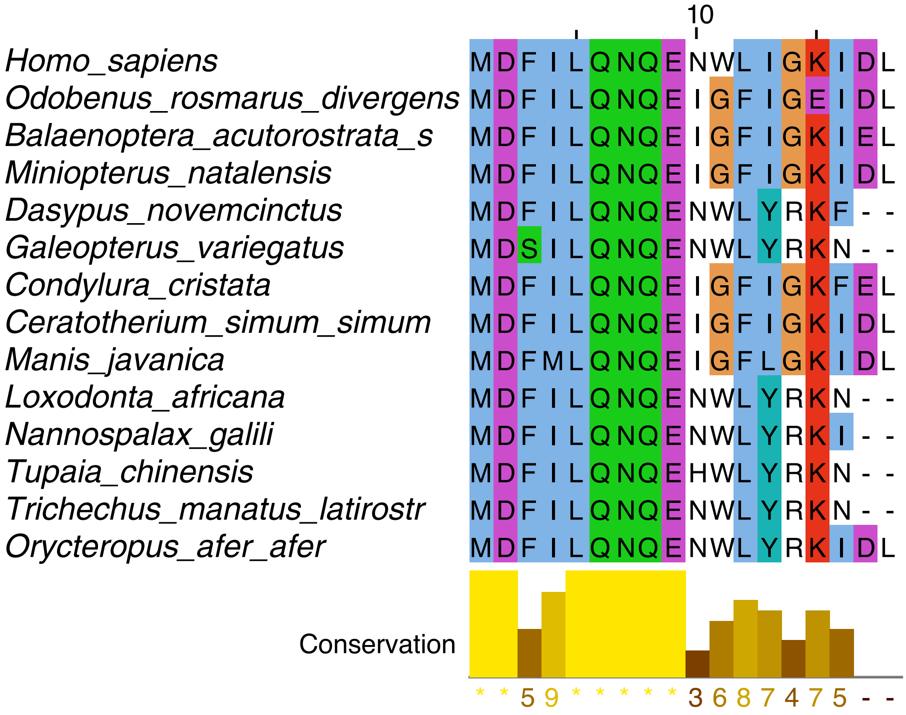

HG0289

ENSGALG00000009581 (MPP5)

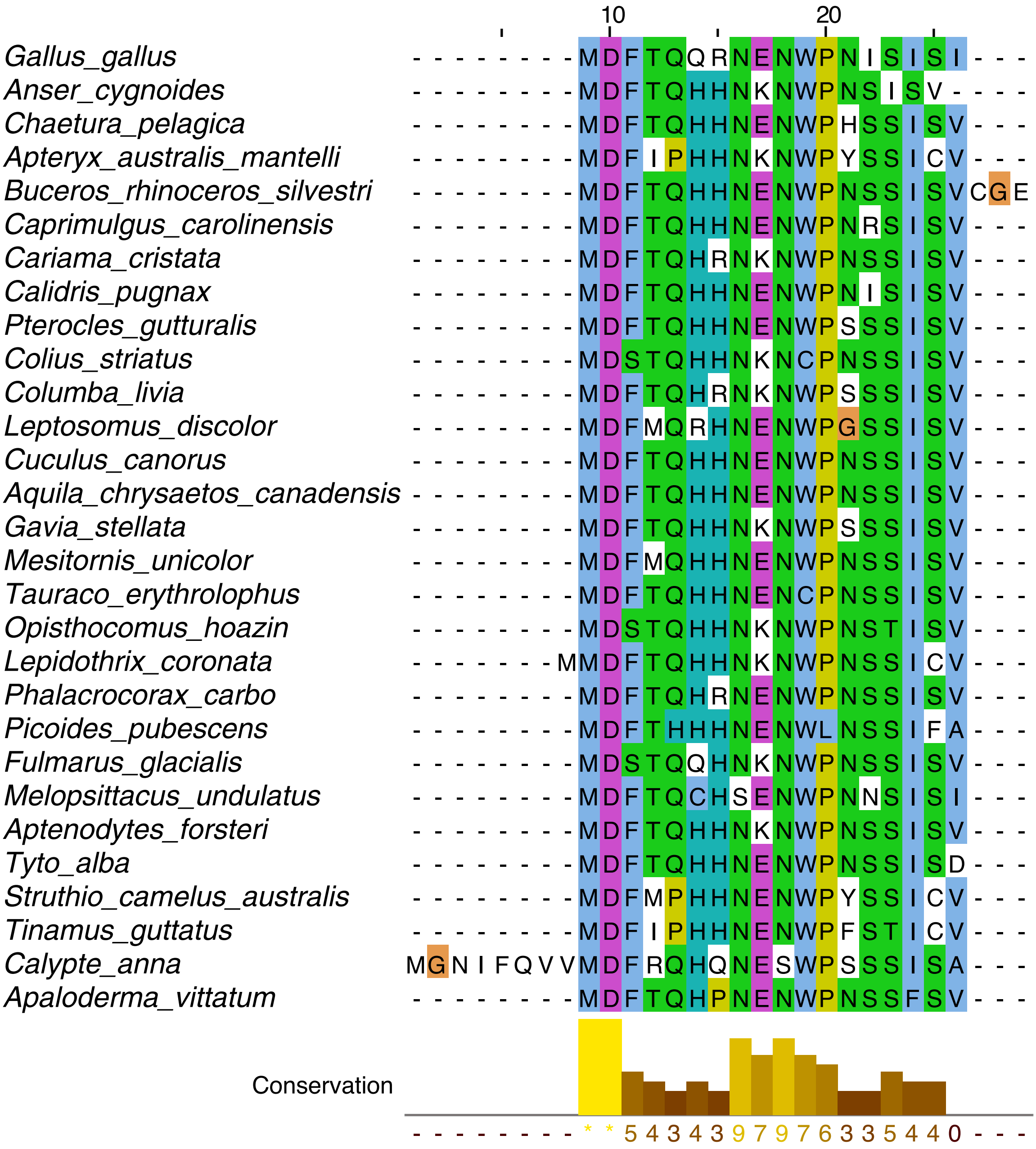

HG0290.1

ENSG00000116539 (ASH1L)

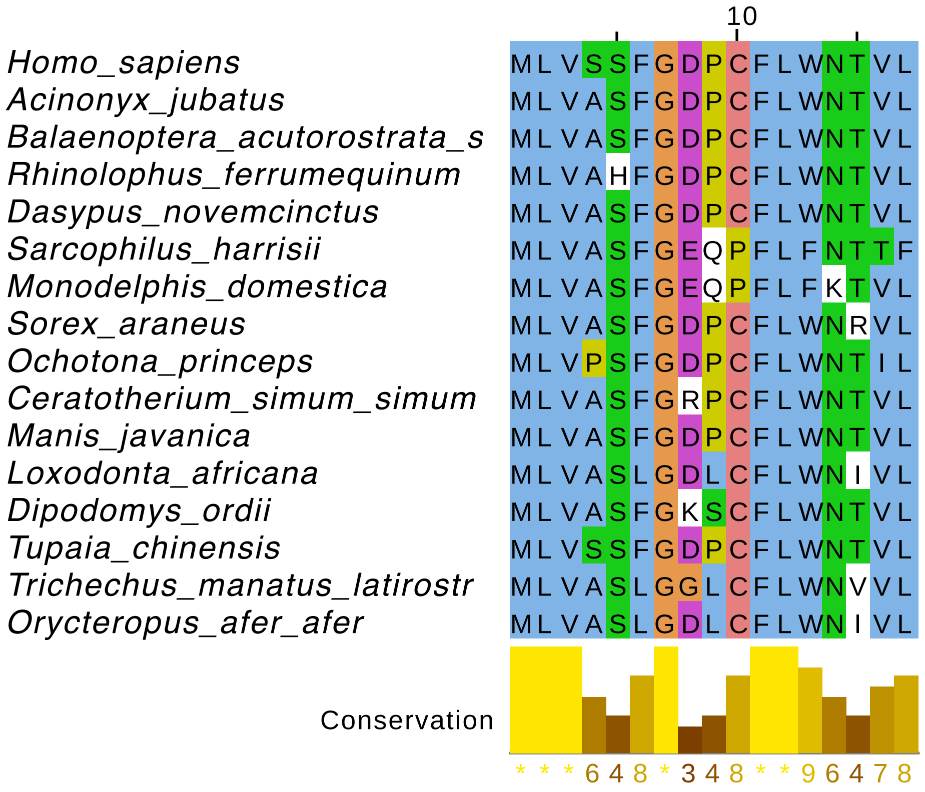

HG0290.2

ENSDARG00000070981 (ash1l)

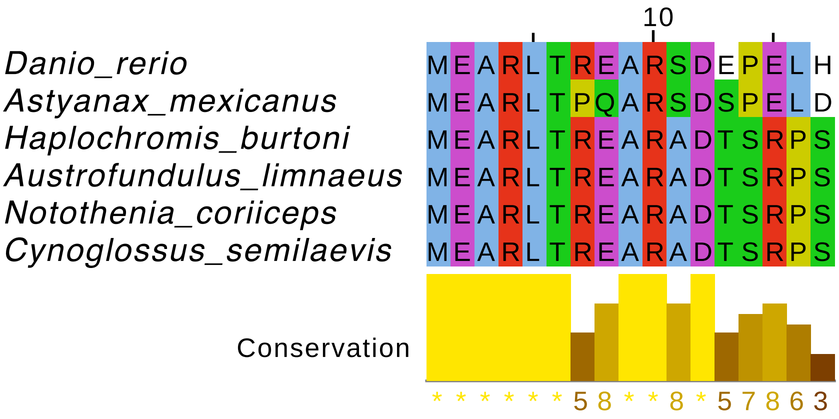

HG0291.1

ENSGALG00000009173 (GFRA1)

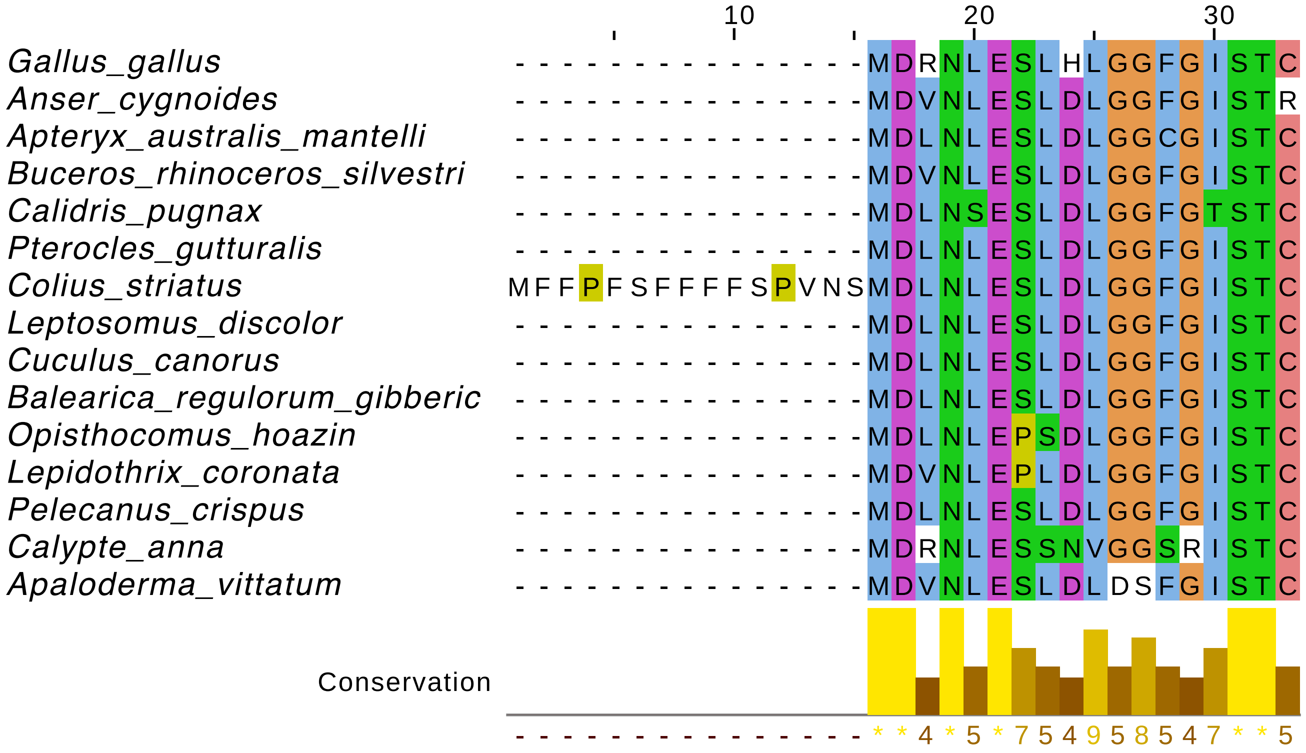

HG0291.2

ENSG00000151892 (GFRA1)

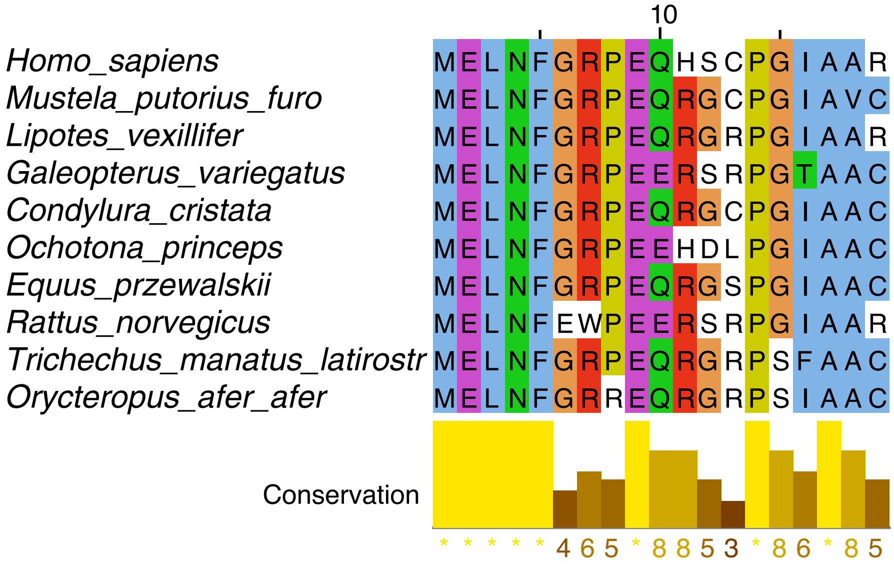

HG0292.1

ENSGALG00000006502 (PCGF5)

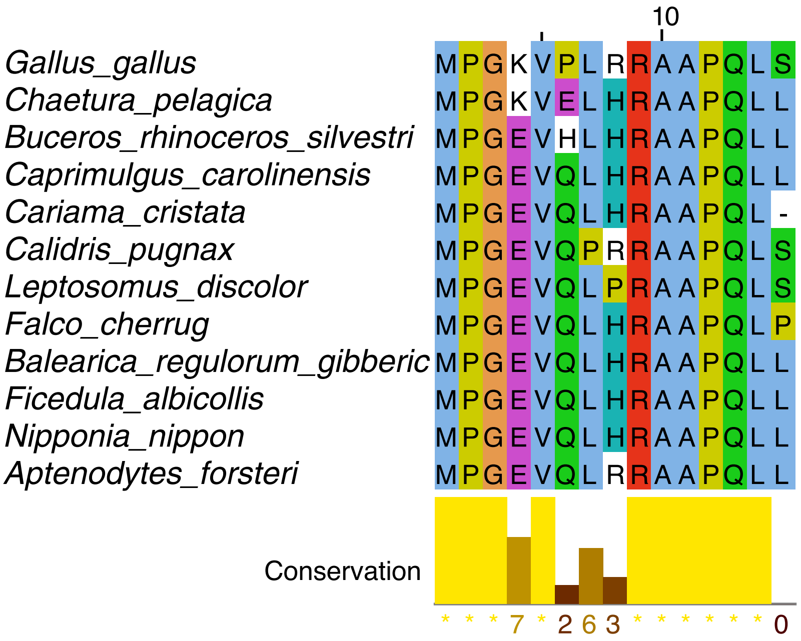

HG0292.2

ENSG00000180628 (PCGF5)

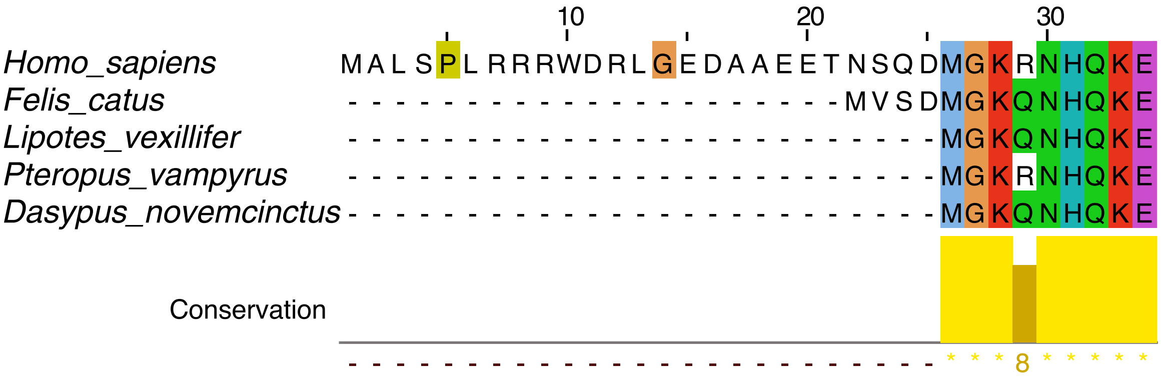

HG0293

ENSG00000152127 (MGAT5)

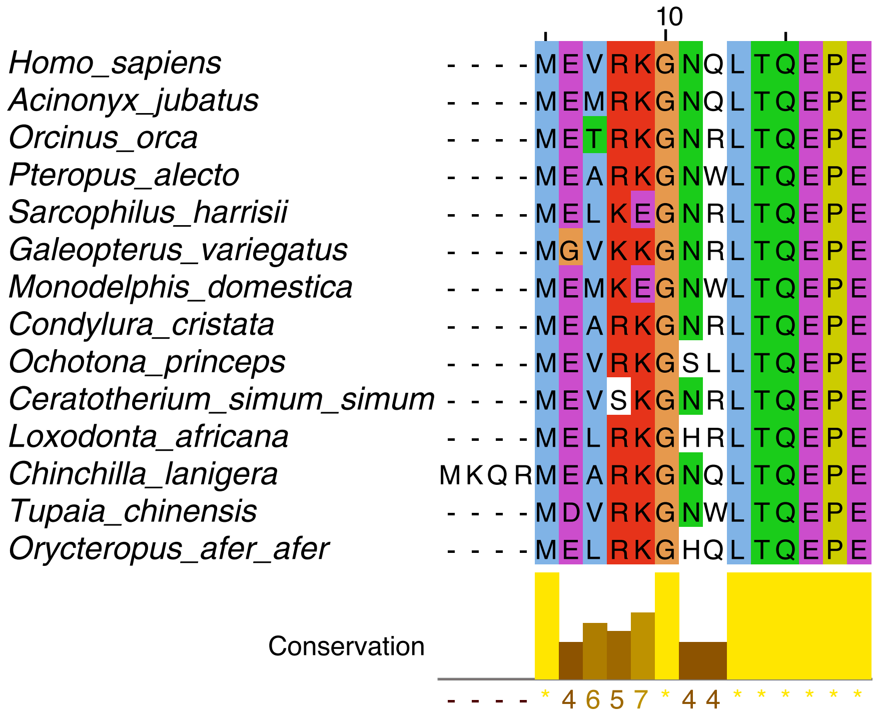

HG0293

ENSGALG00000012187 (MGAT5)

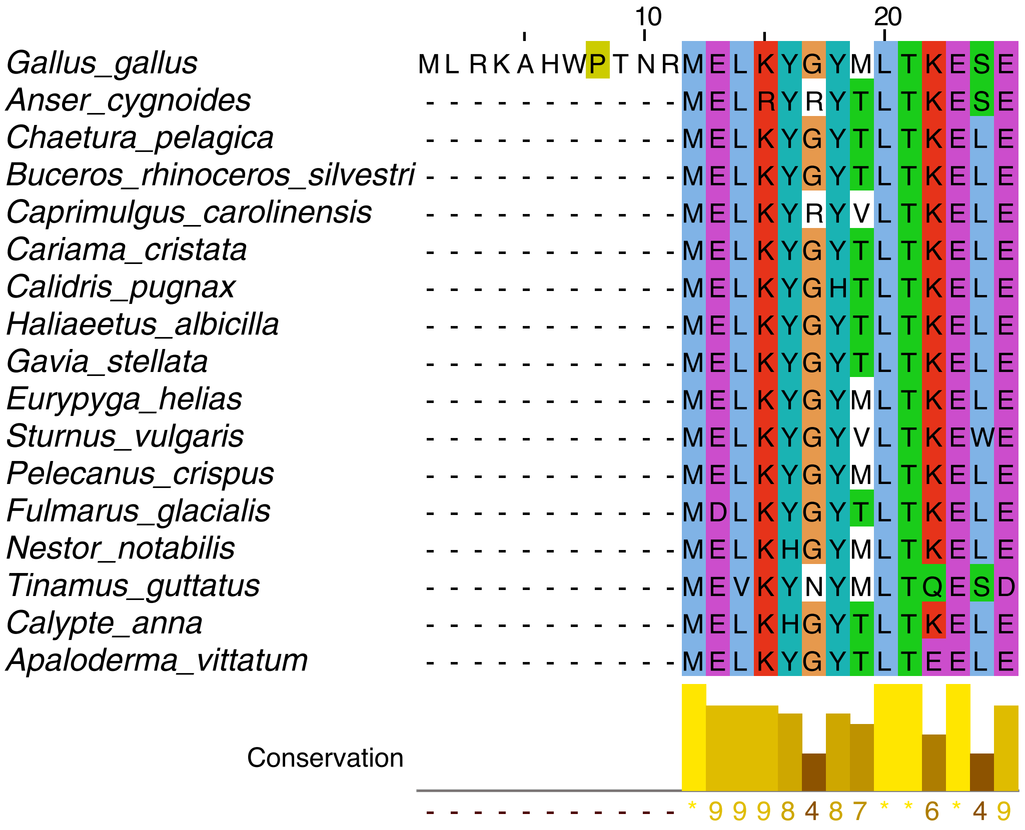

HG0294.1

ENSGALG00000017036 (LHFP)

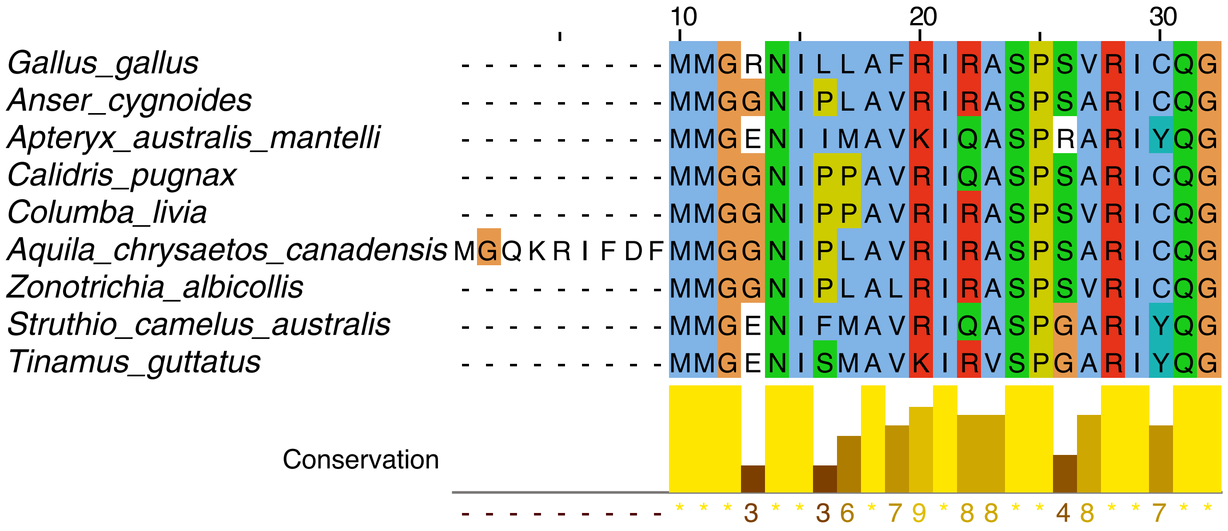

HG0294.2

ENSG00000183722 (LHFP)

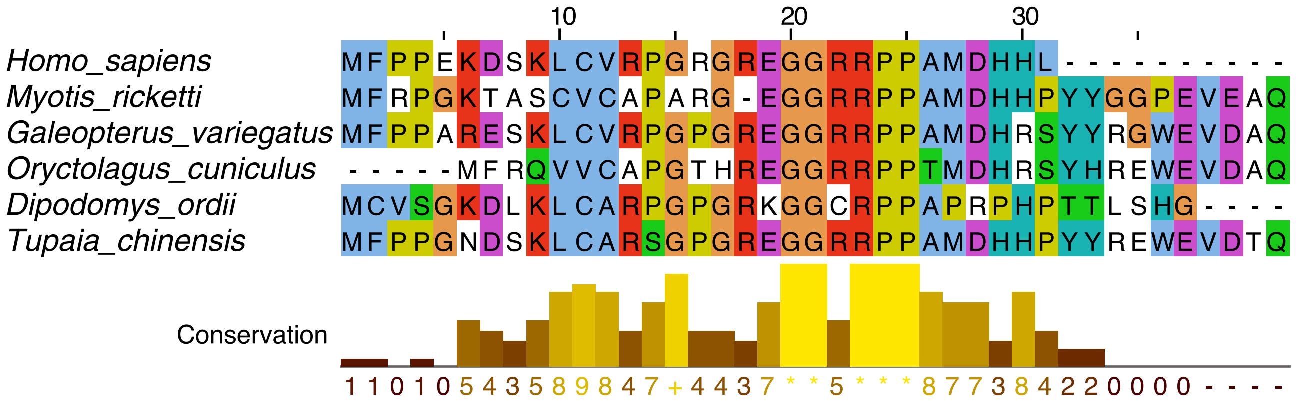

HG0295.1

ENSG00000166888 (STAT6)

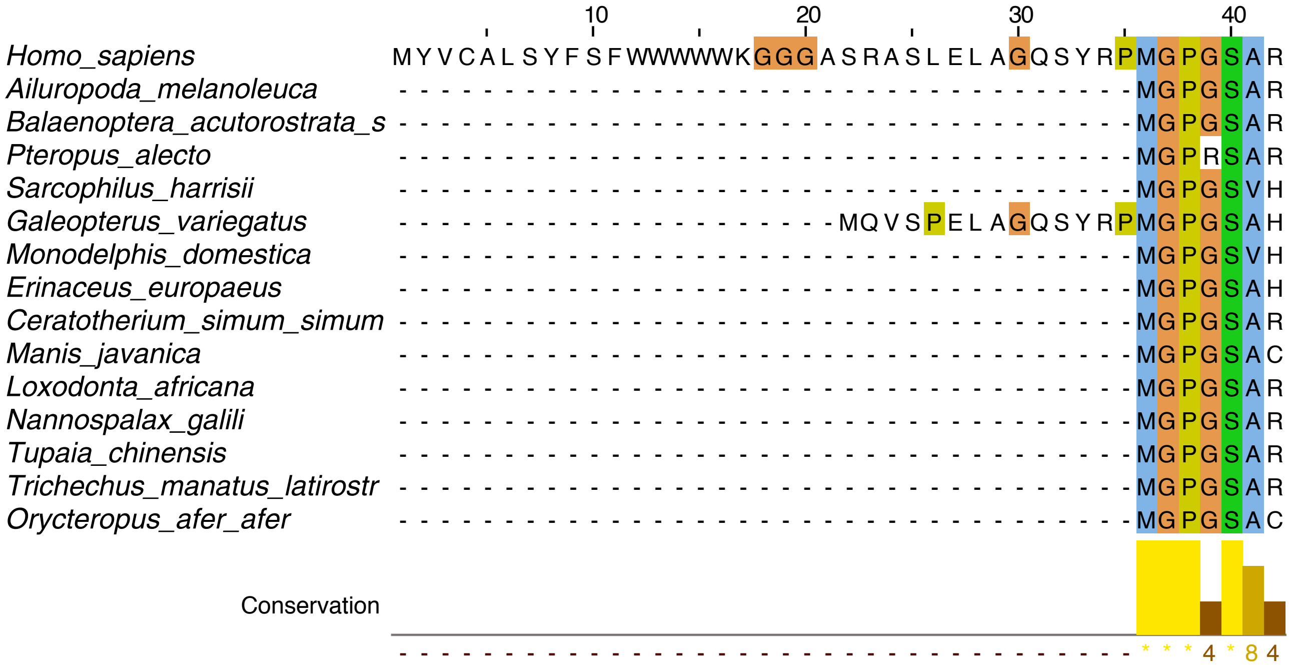

HG0295.2

ENSGALG00000003282 (STAT5B)

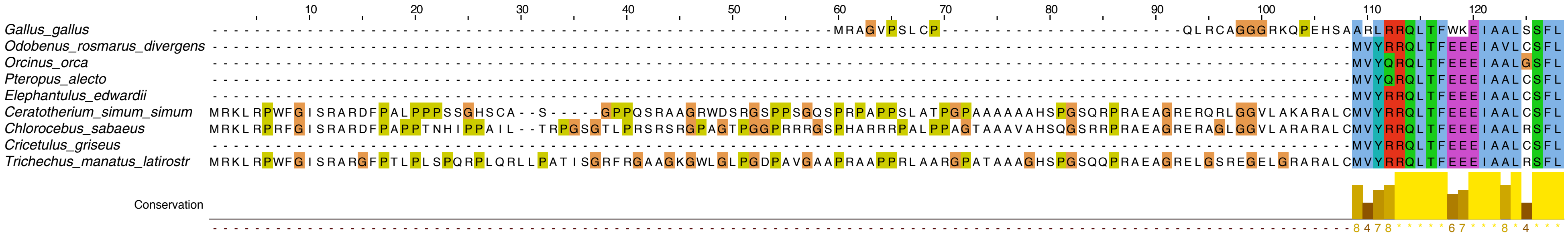

HG0296

ENSGALG00000006912 (CPEB3)

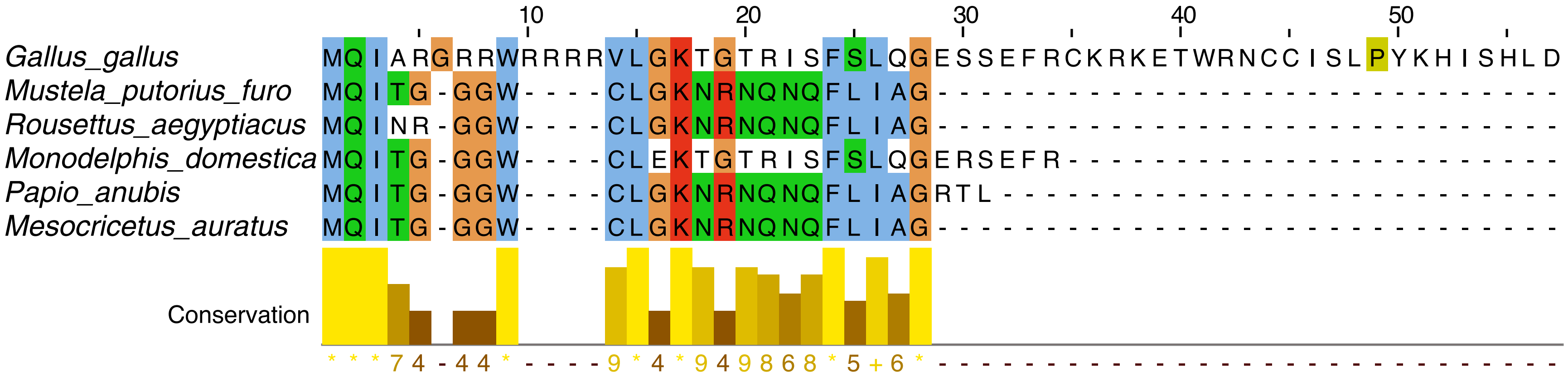

HG0297

ENSGALG00000031684

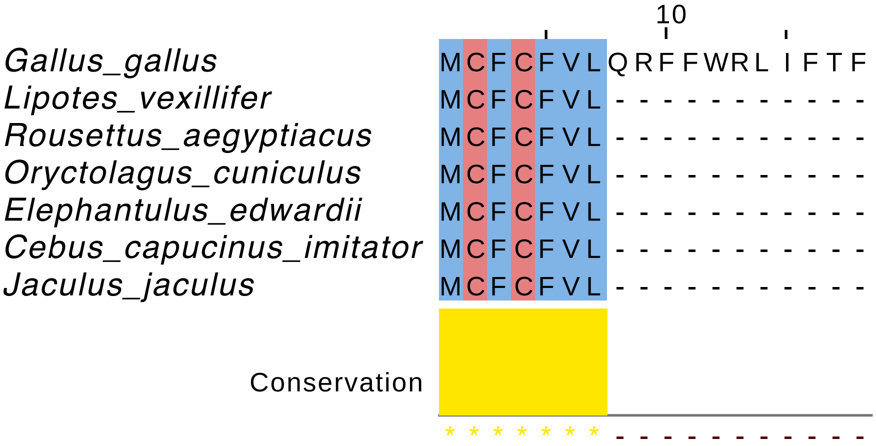

HG0298

ENSGALG00000042308

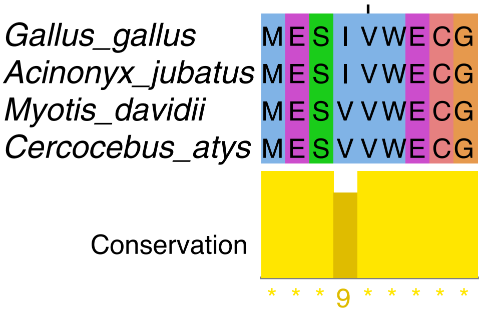

HG0299

ENSGALG00000040465 (Sip1)

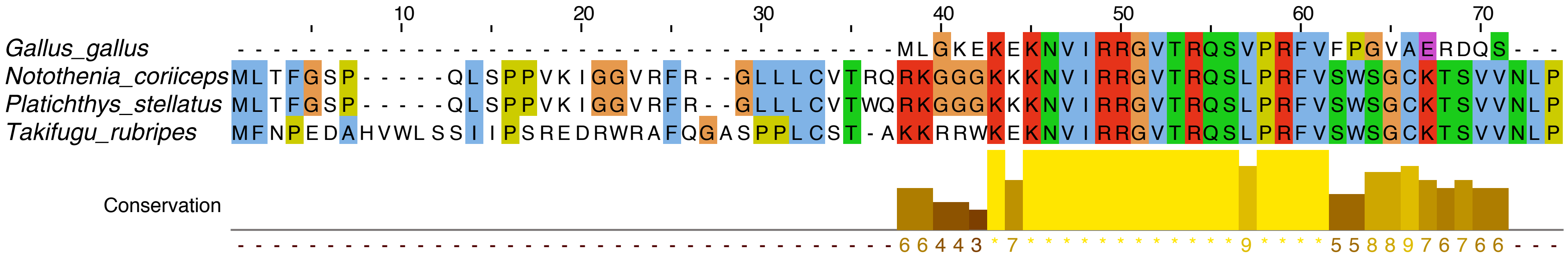

HG0300

FBgn0031668 (CG31917)

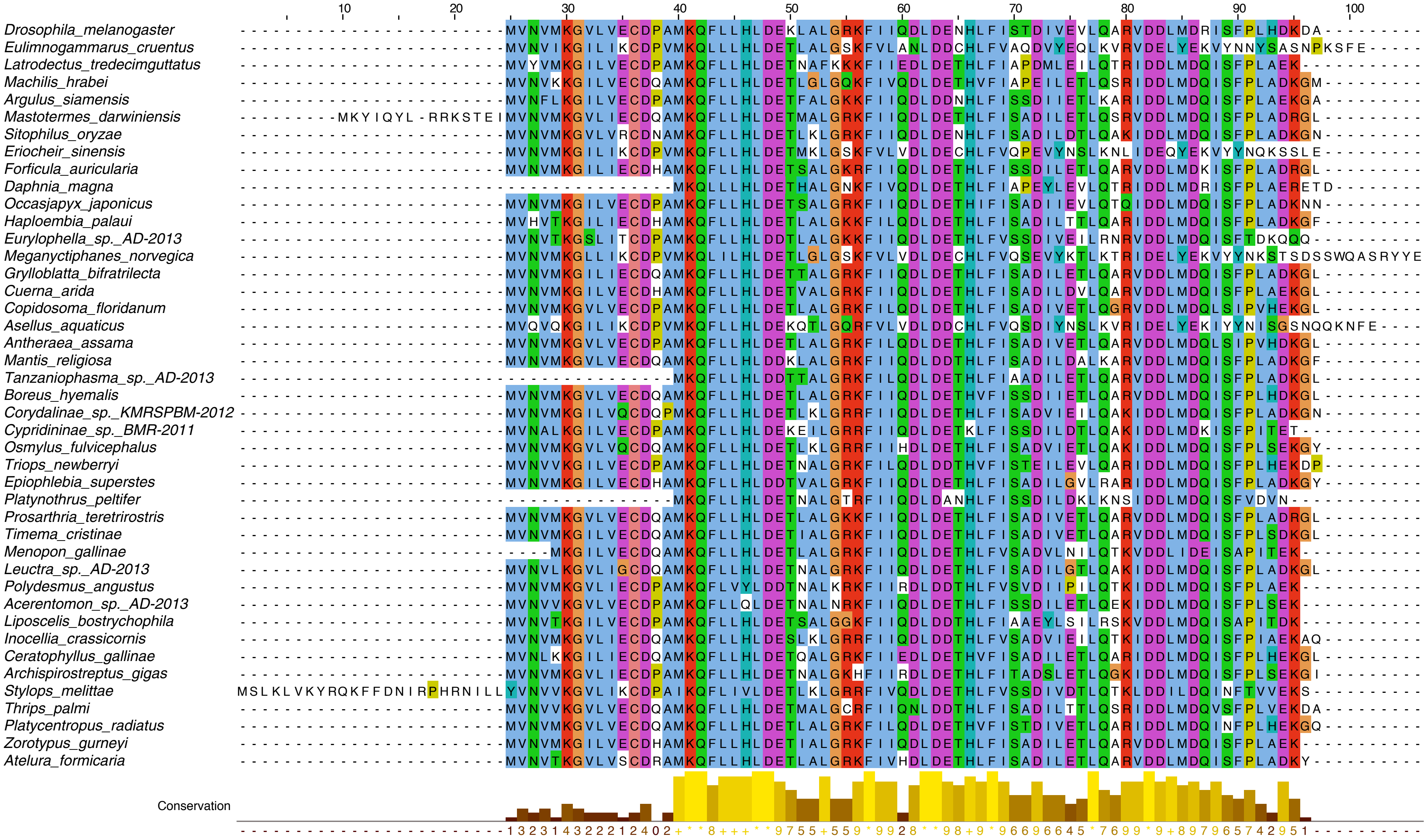

HG0301

FBgn0036856 (CG9666)

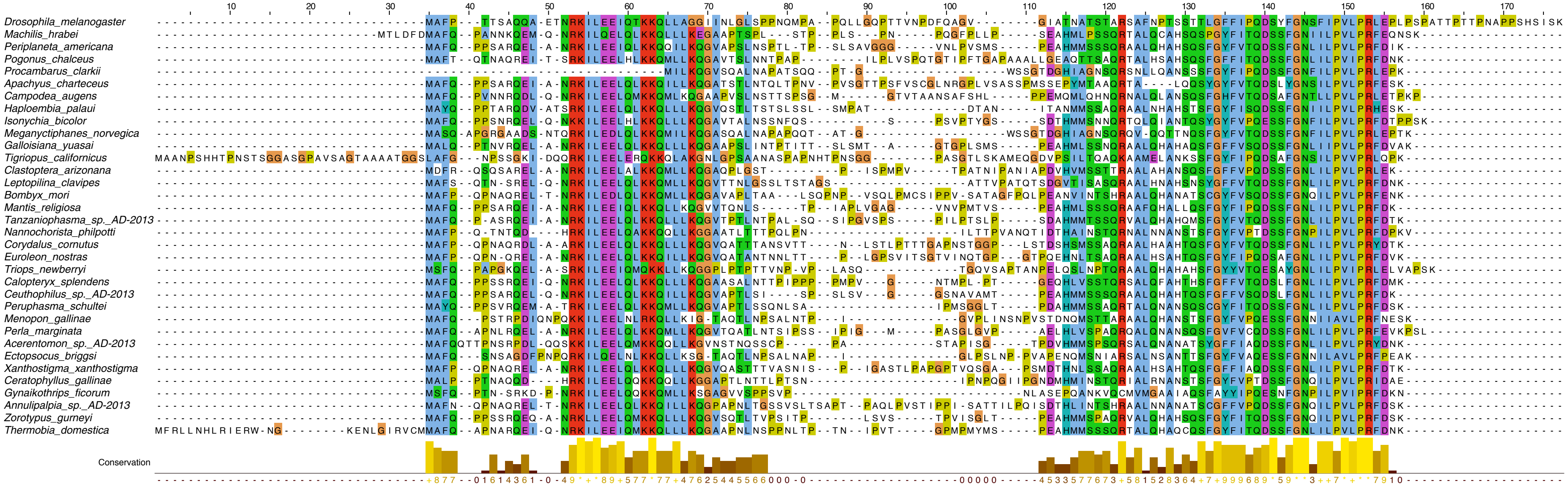

HG0302

FBgn0031454 (CG9960)

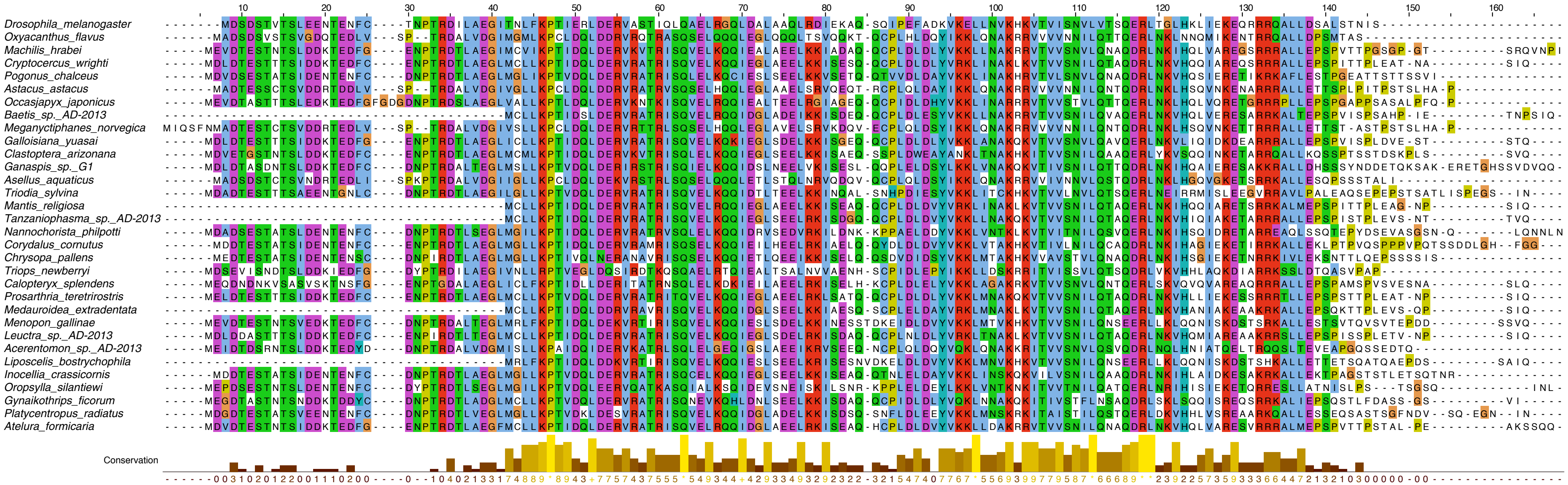

HG0303

FBgn0260464

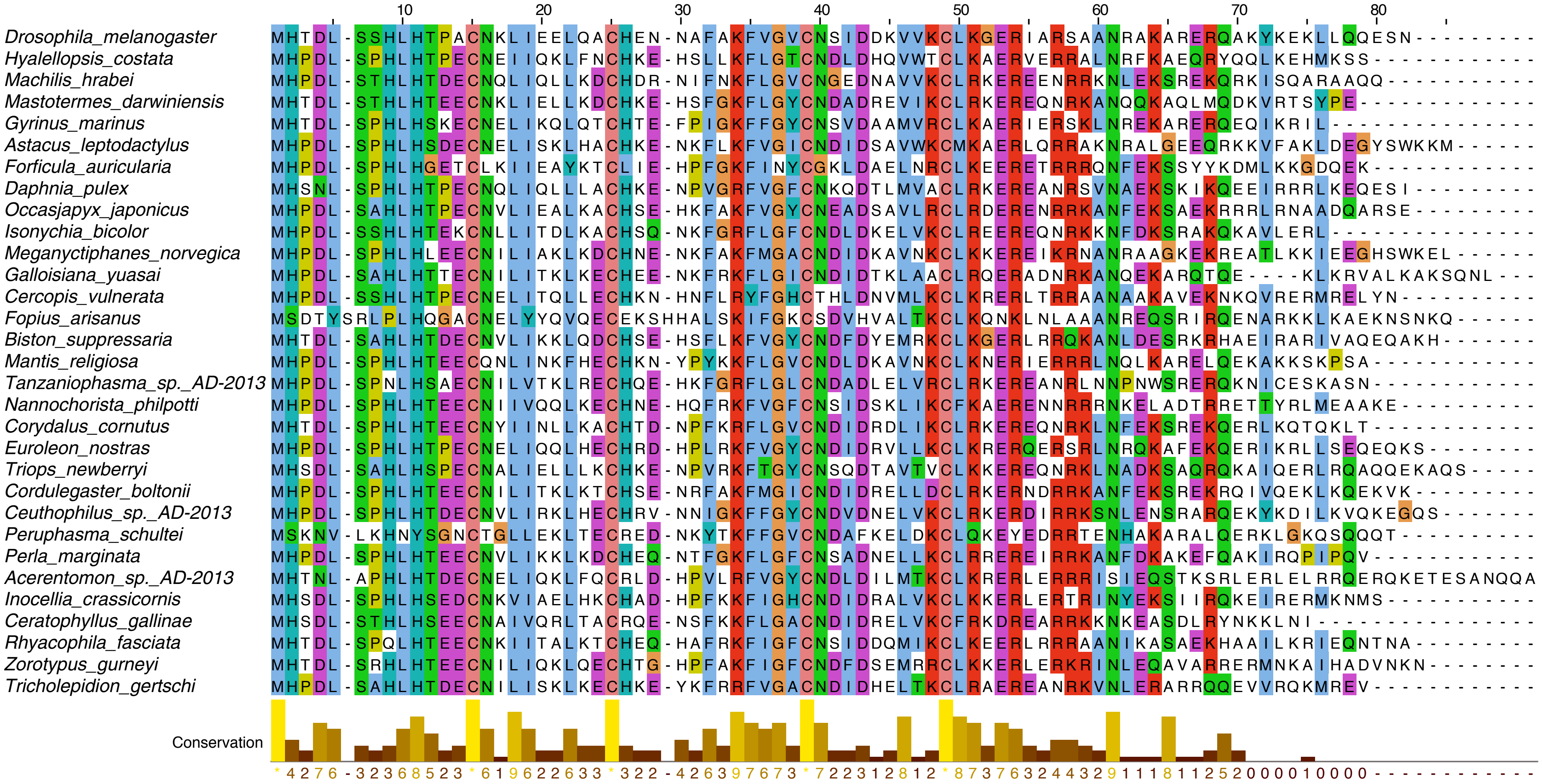

HG0304

FBgn0260467 (CG7071)

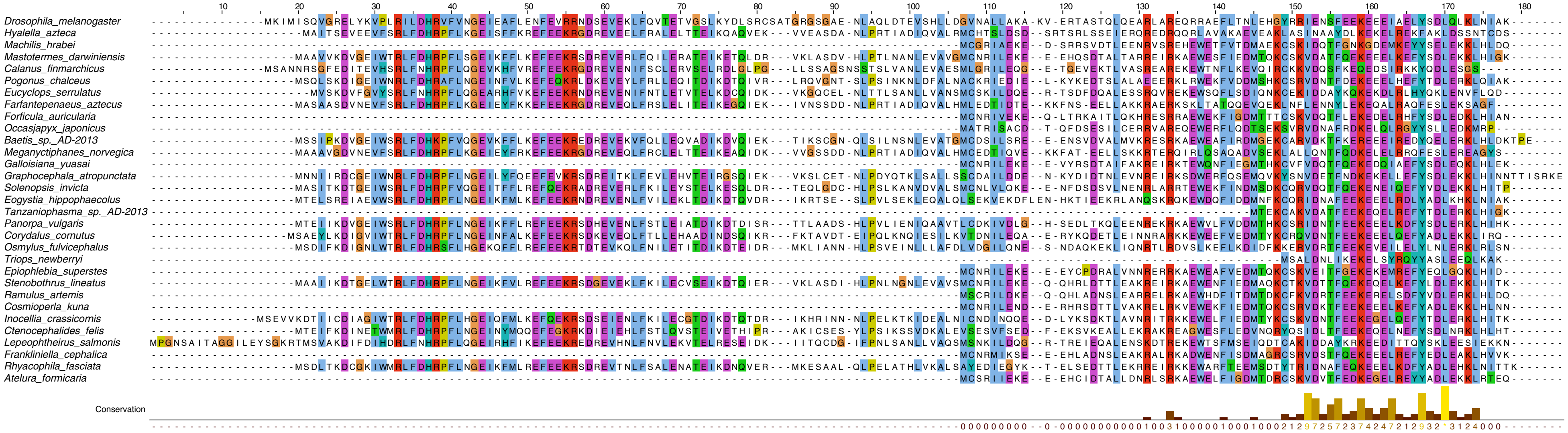

HG0305

FBgn0050290 (Ppcdc)

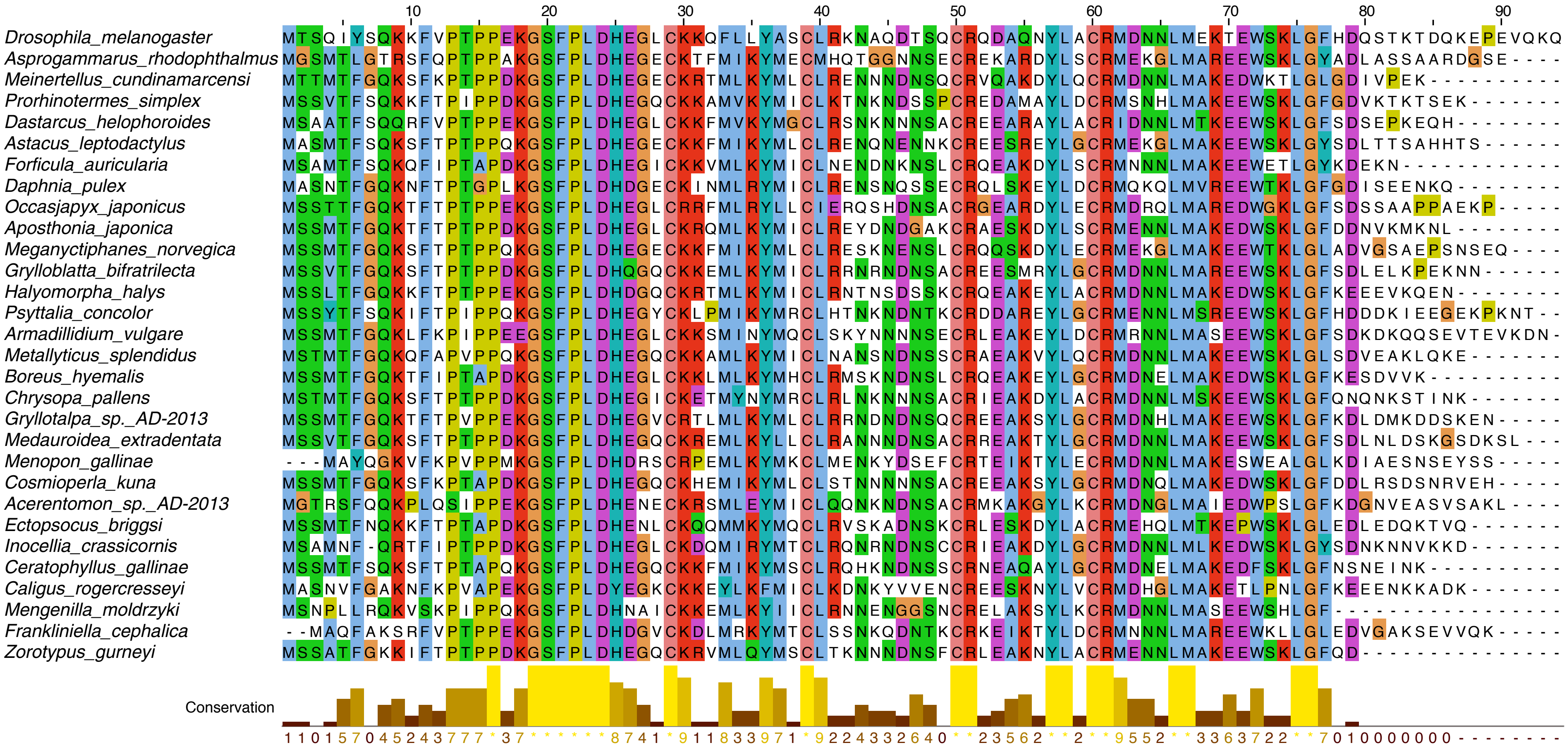

HG0306

FBgn0259726

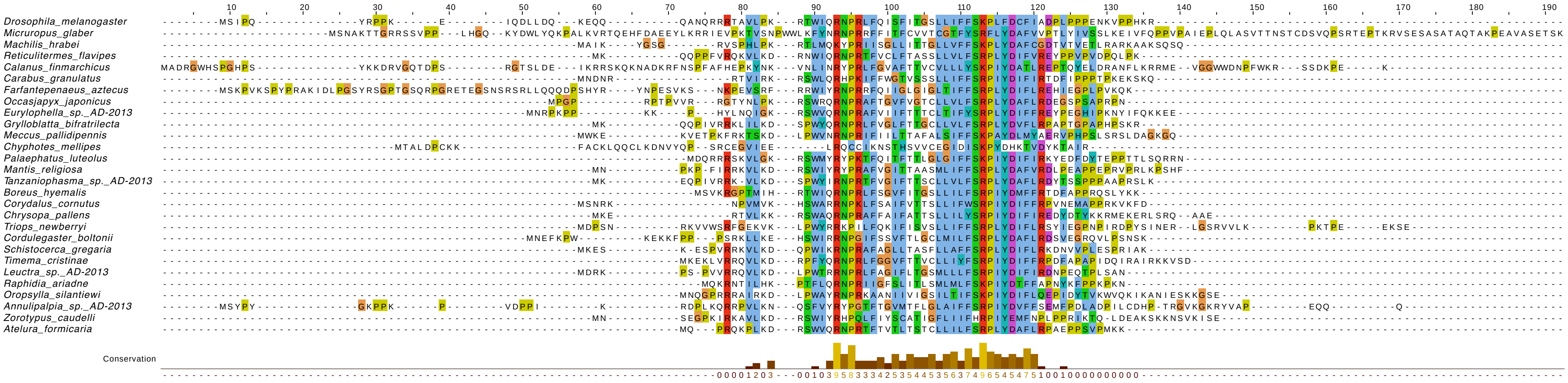

HG0307

FBgn0037822 (CG14683)

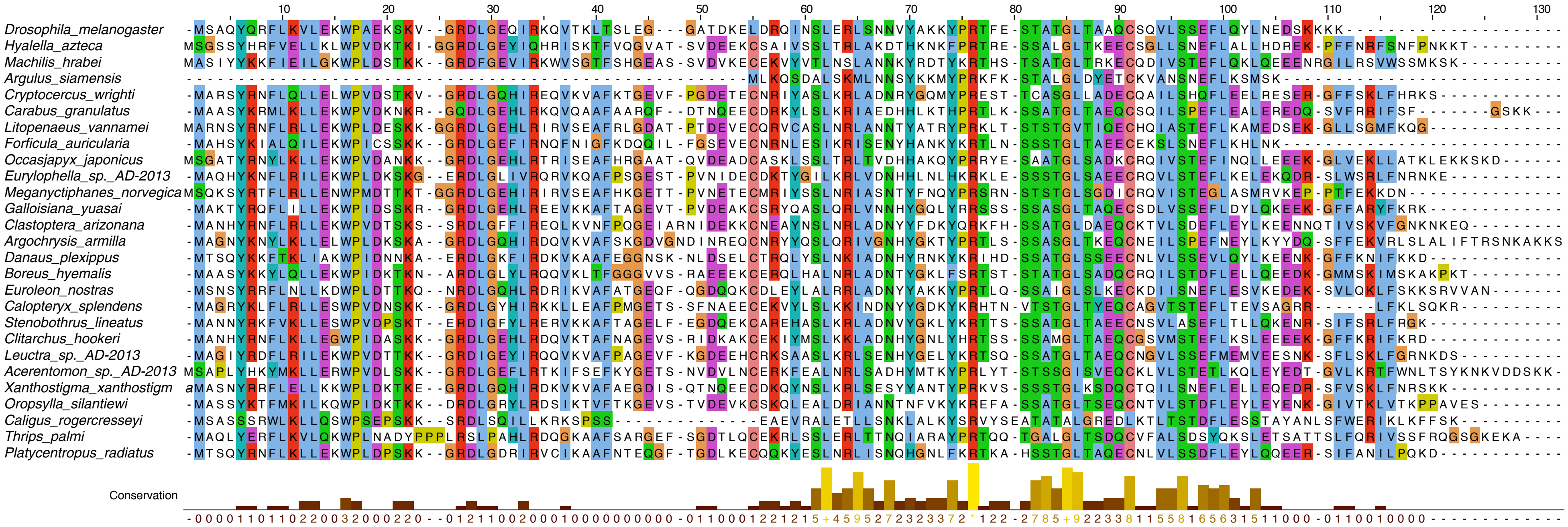

HG0308

FBgn0264743 (CG44001)

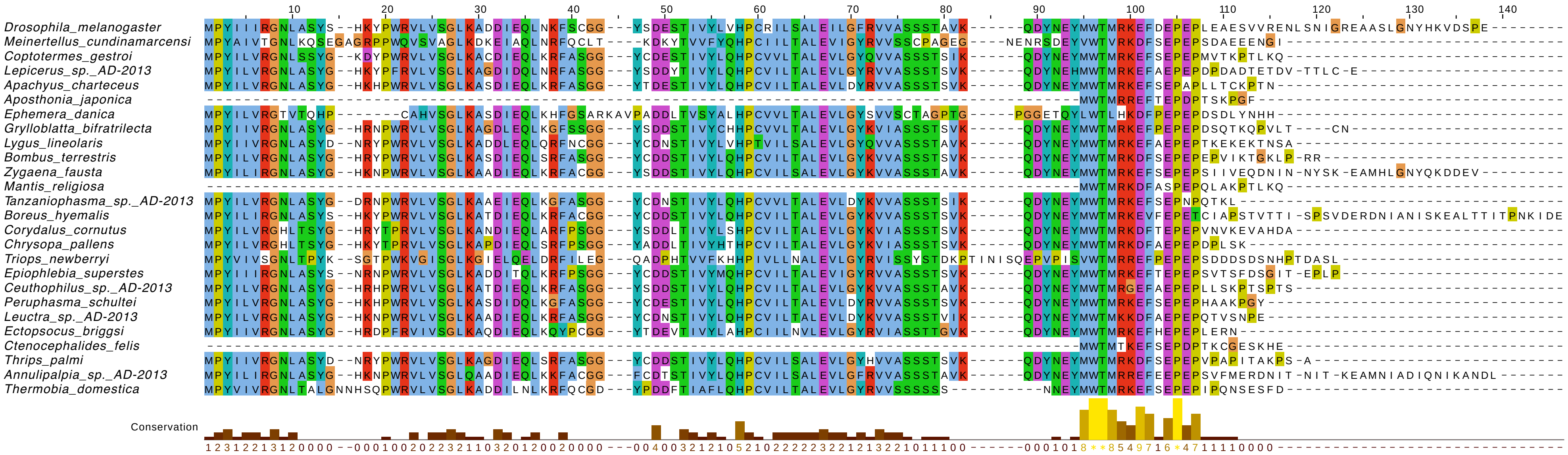

HG0309

FBgn0037689 (CG8135)

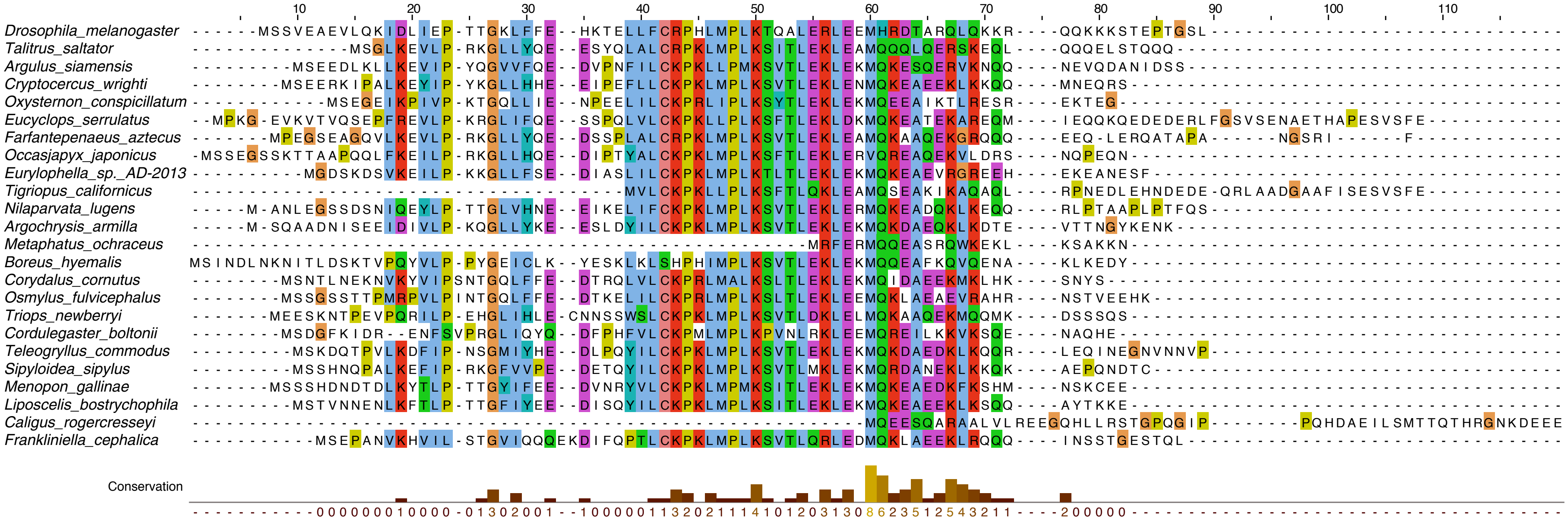

HG0310

FBgn0039339 (CG5116)

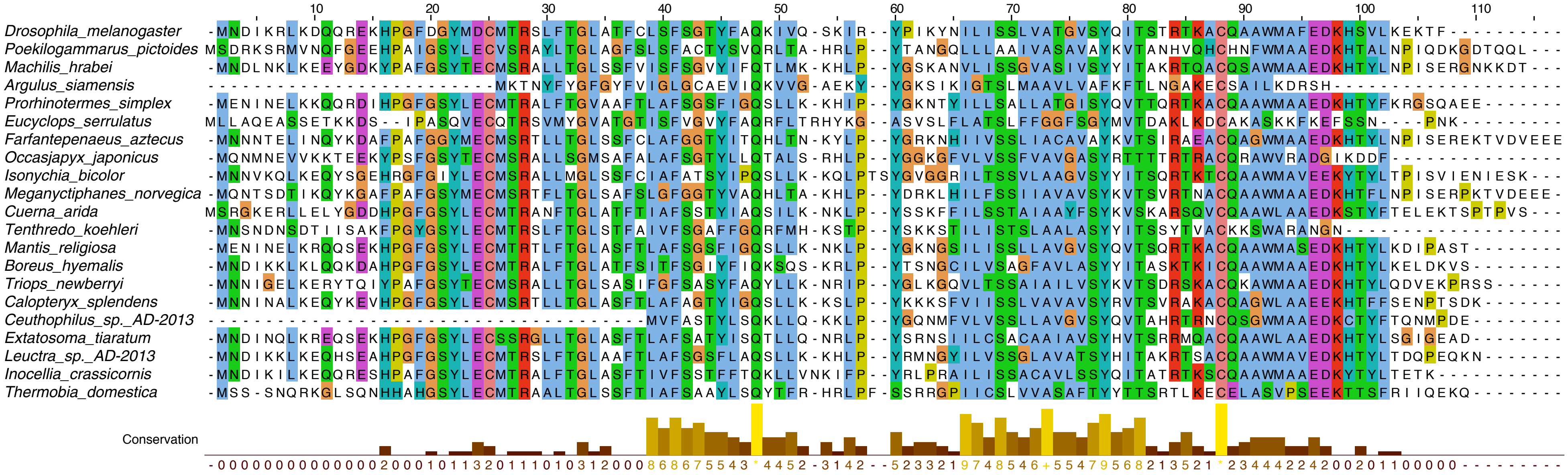

HG0311

FBgn0263251 (vnc)

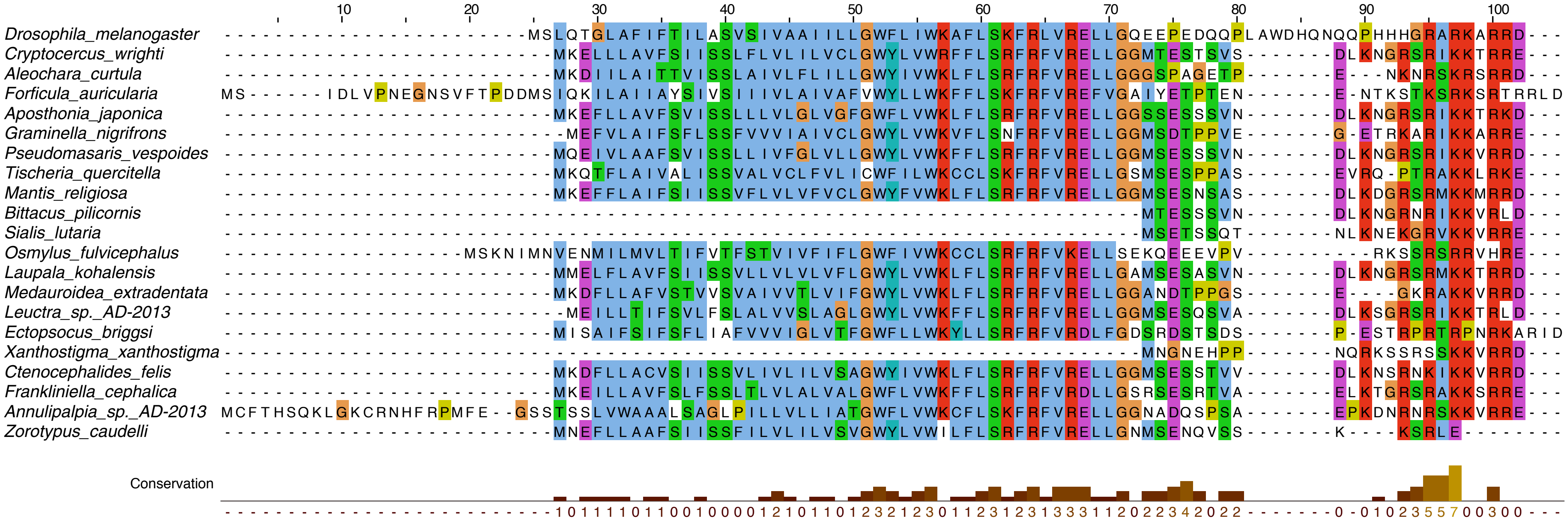

HG0312

FBgn0038641 (CG7708)

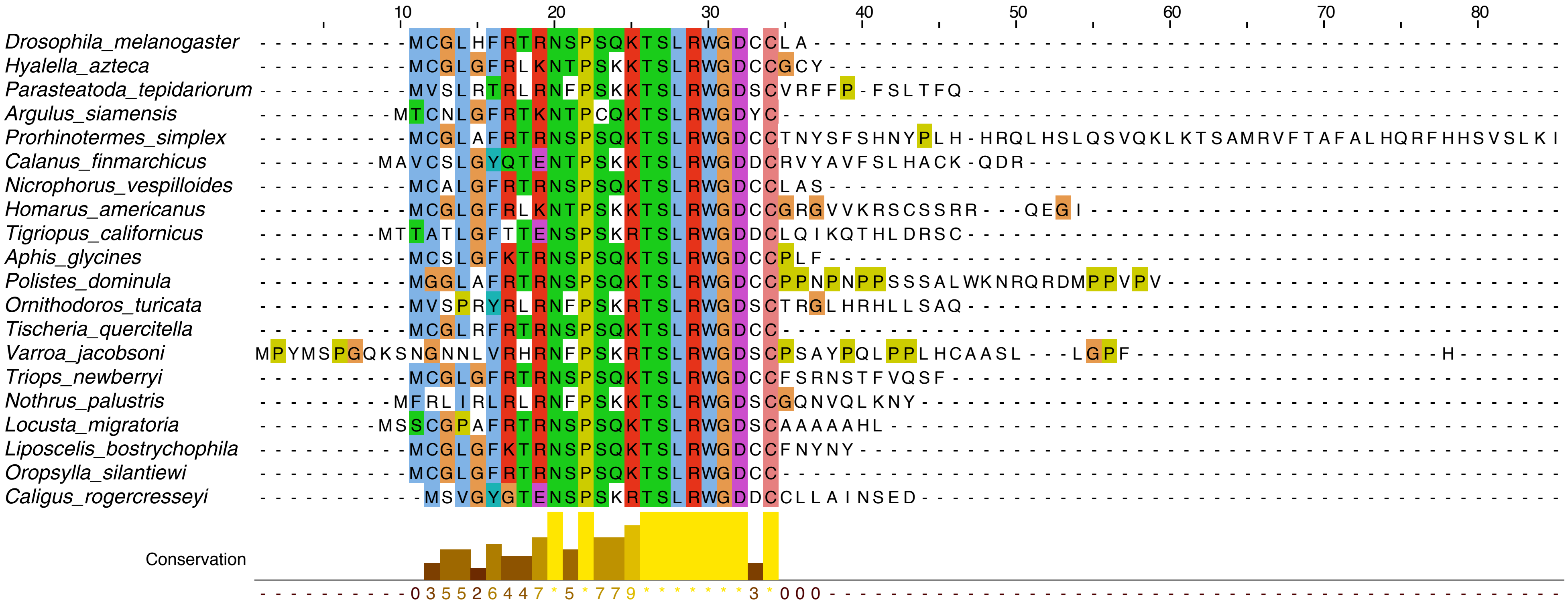

HG0313

FBgn0259725

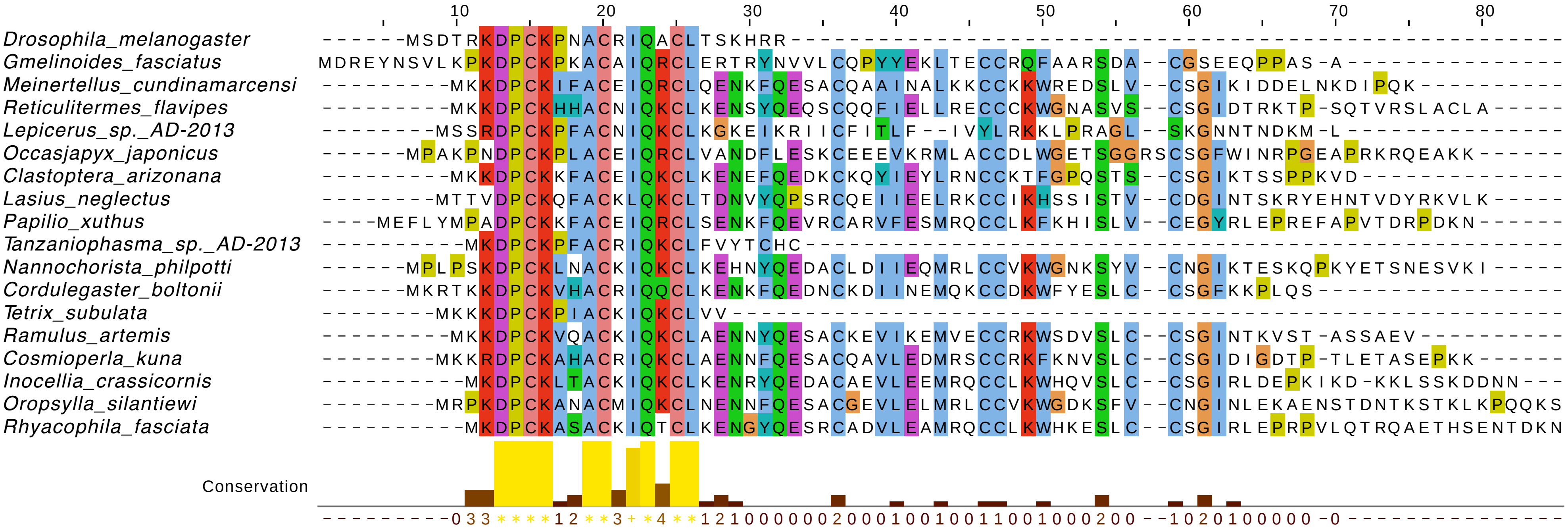

HG0314

FBgn0260468 (CG7950)

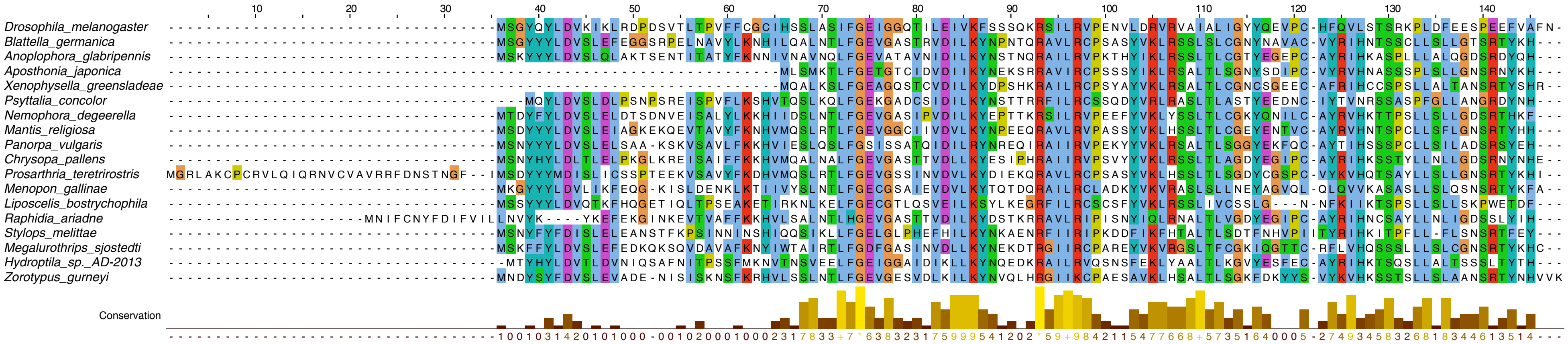

HG0315

FBgn0260234 (CG42508)

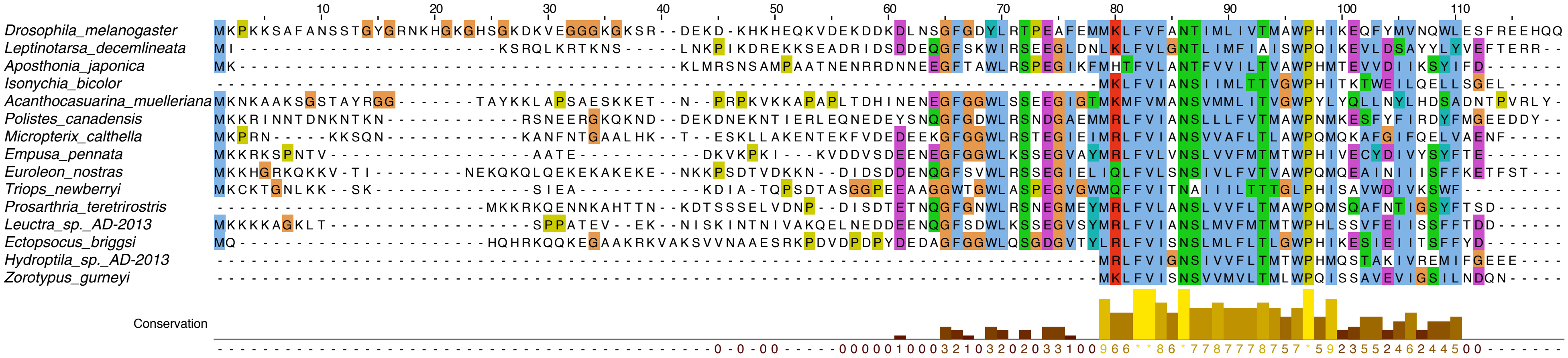

HG0316

FBgn0041164 (armi)

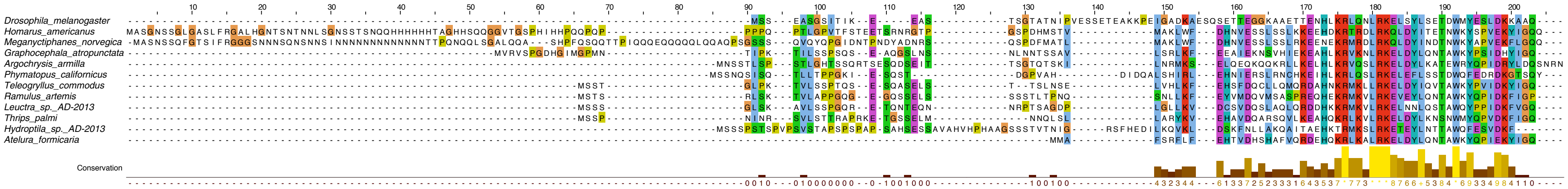

HG0317

FBgn0050011 (gem)

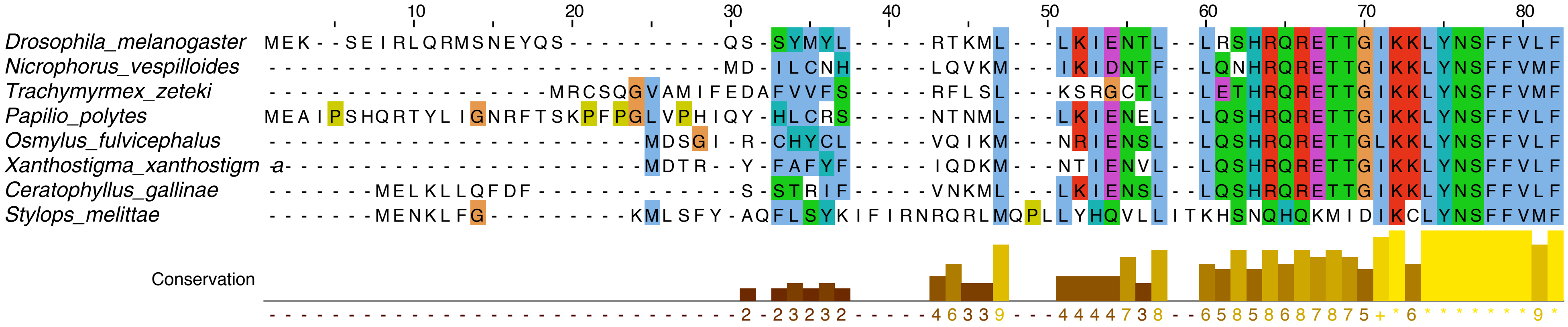

HG0318

FBgn0035293 (CG5687)

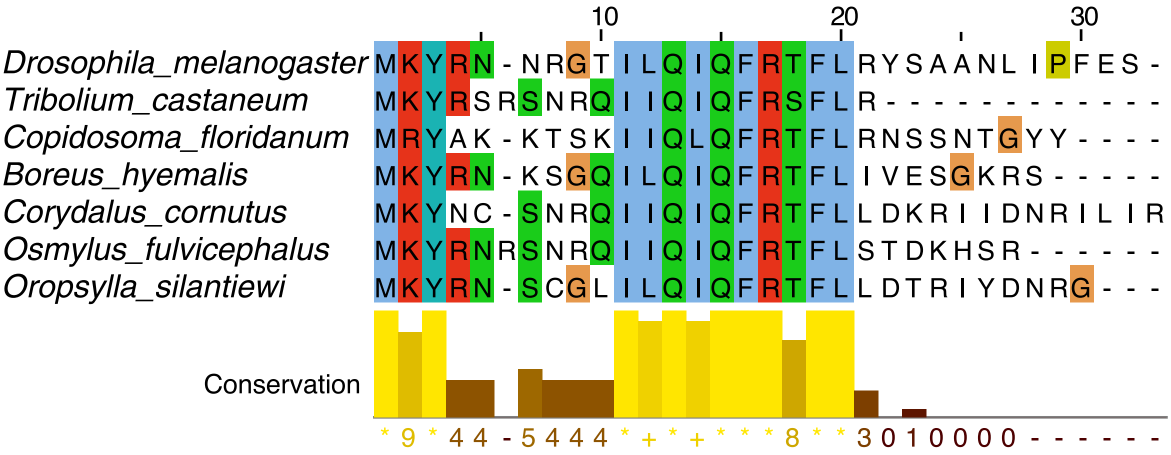

HG0319

FBgn0026778 (Rad1)

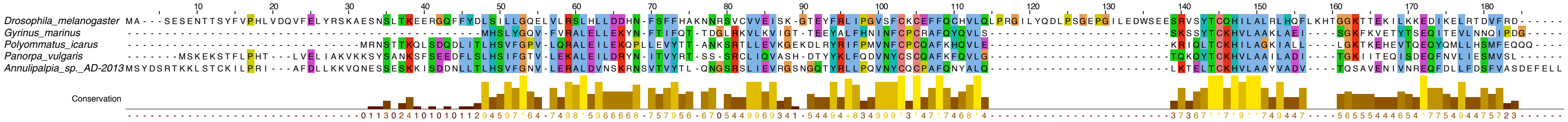

HG0320

FBgn0040993

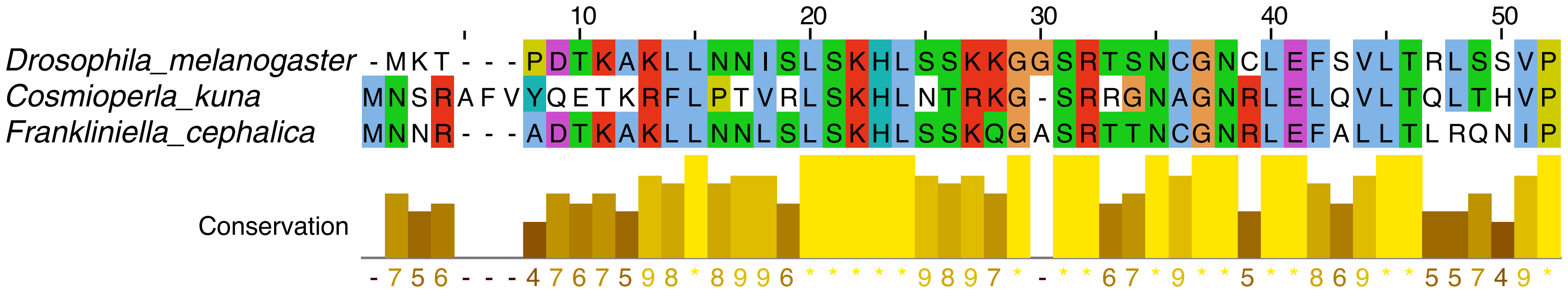

HG0321

ENSDARG00000070426 (chac1)

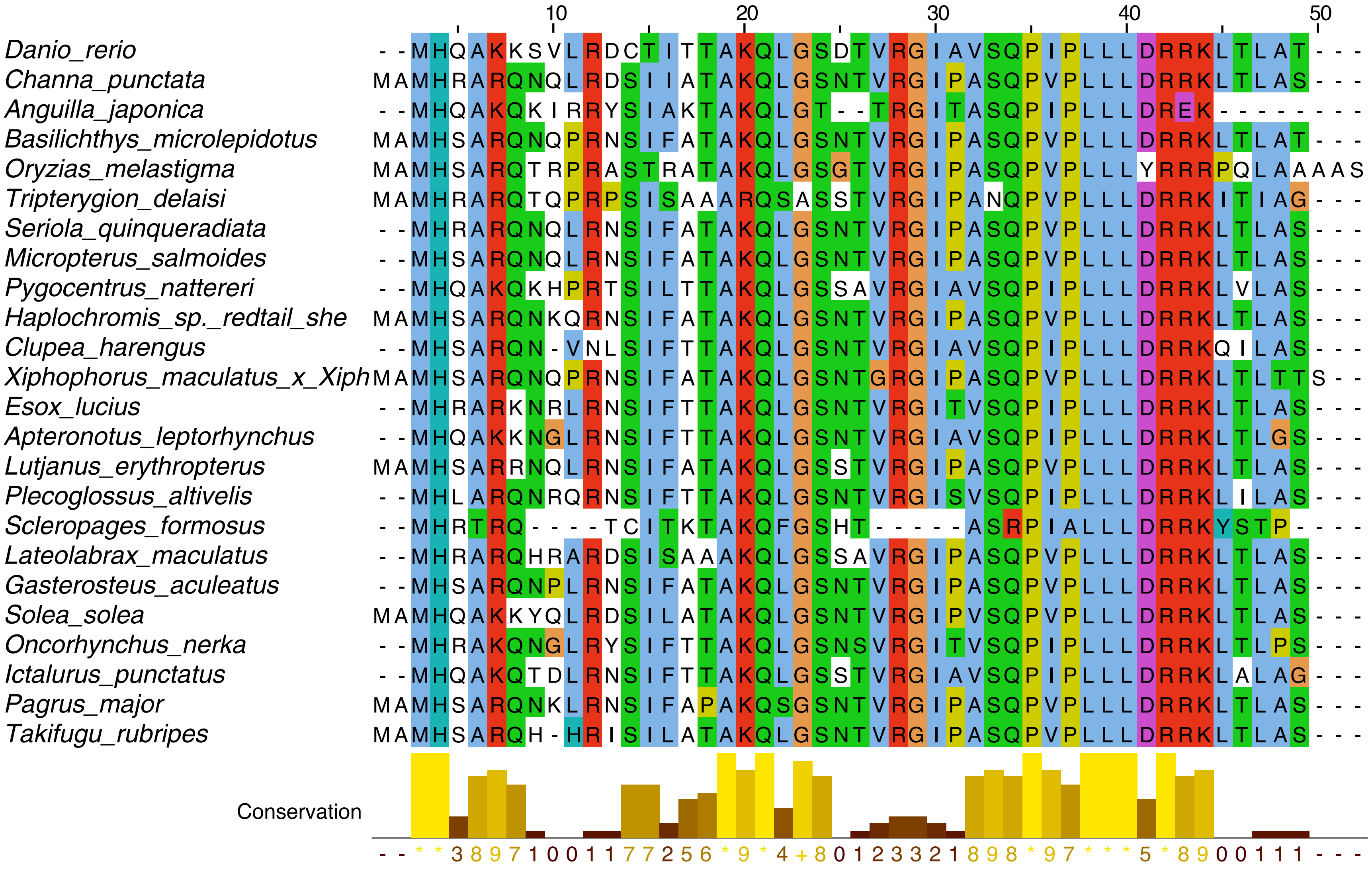

HG0322

ENSDARG00000070917 (kitlga)

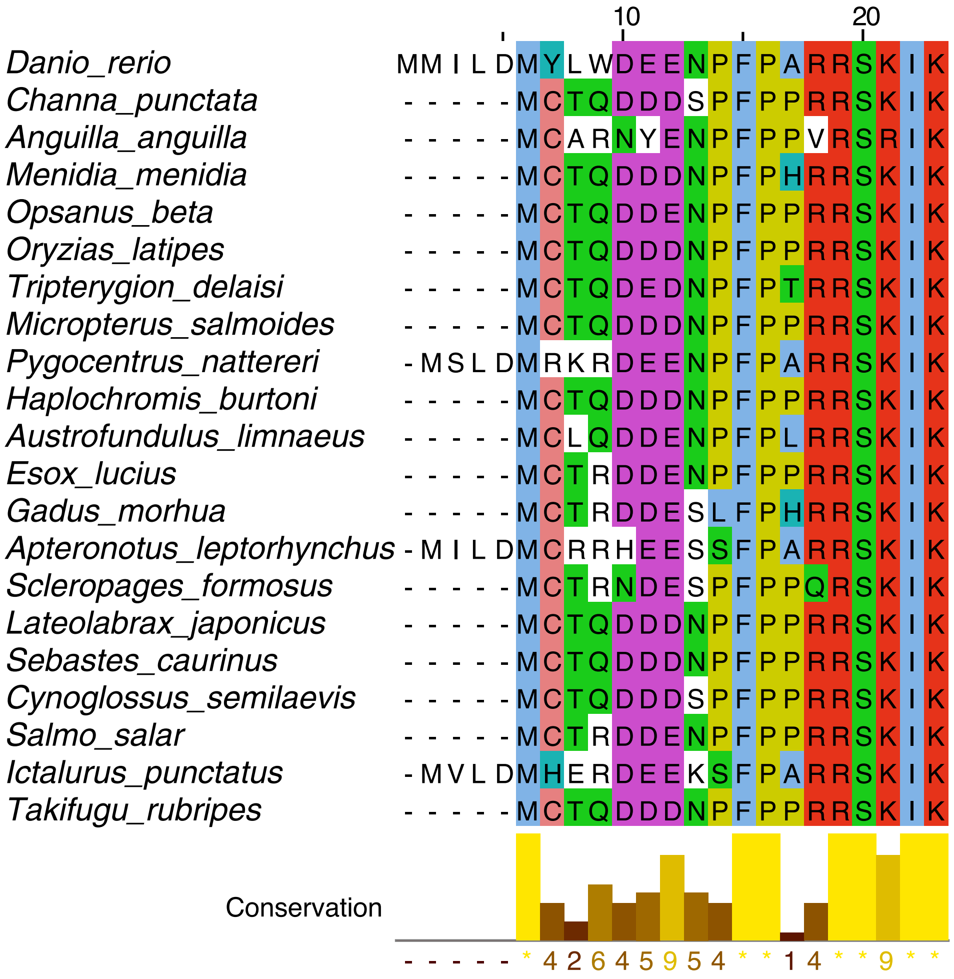

HG0323

ENSDARG00000071197 (usp40)

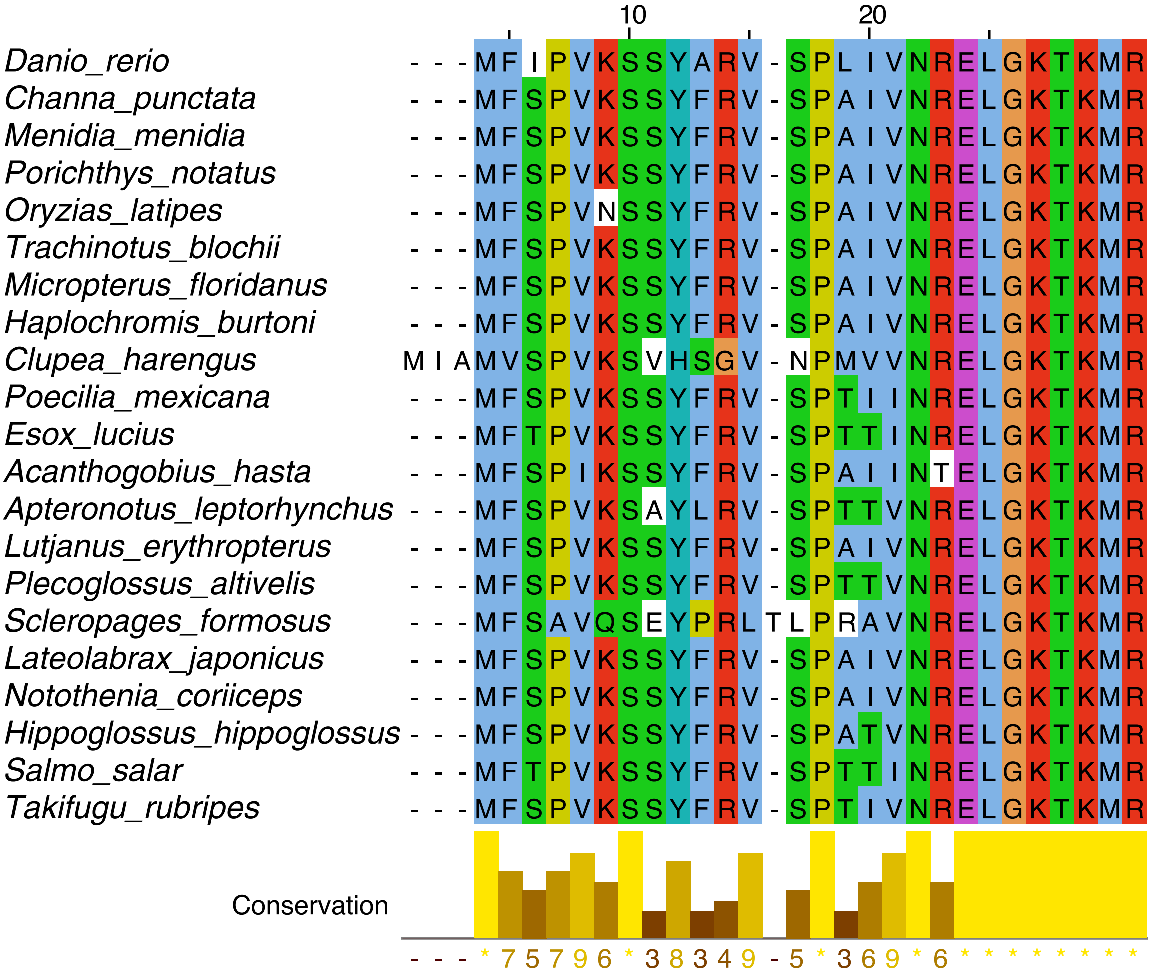

HG0324

ENSDARG00000094965 (nfil3-5)

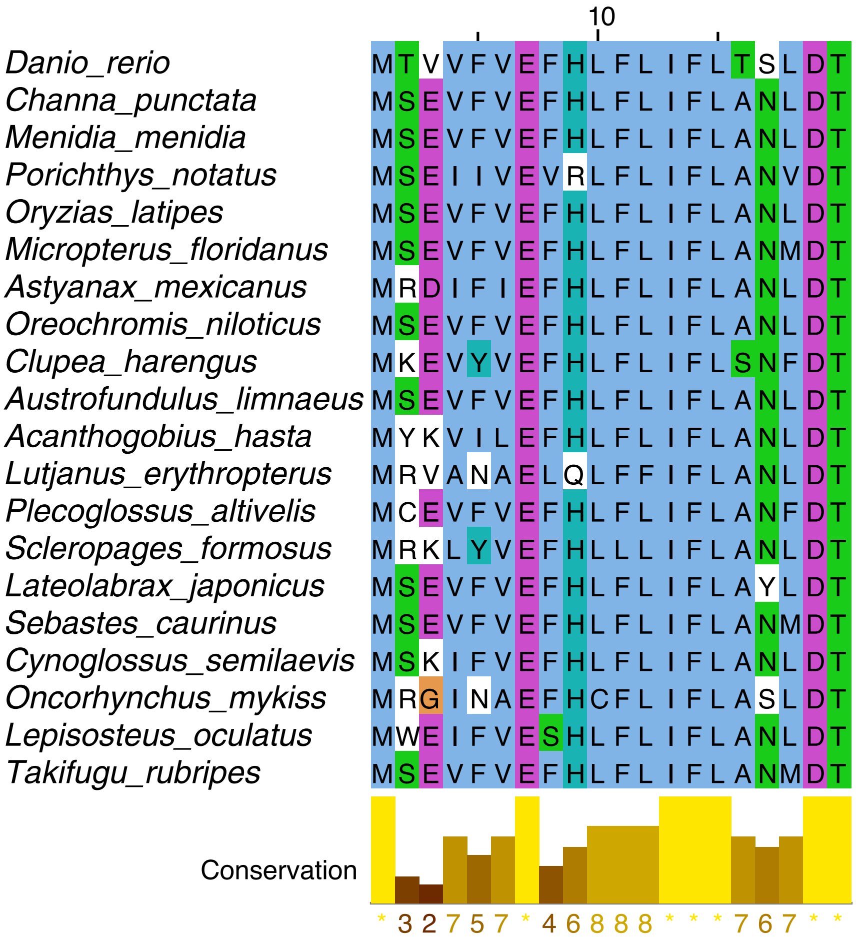

HG0325

ENSDARG00000079738 (znf219)

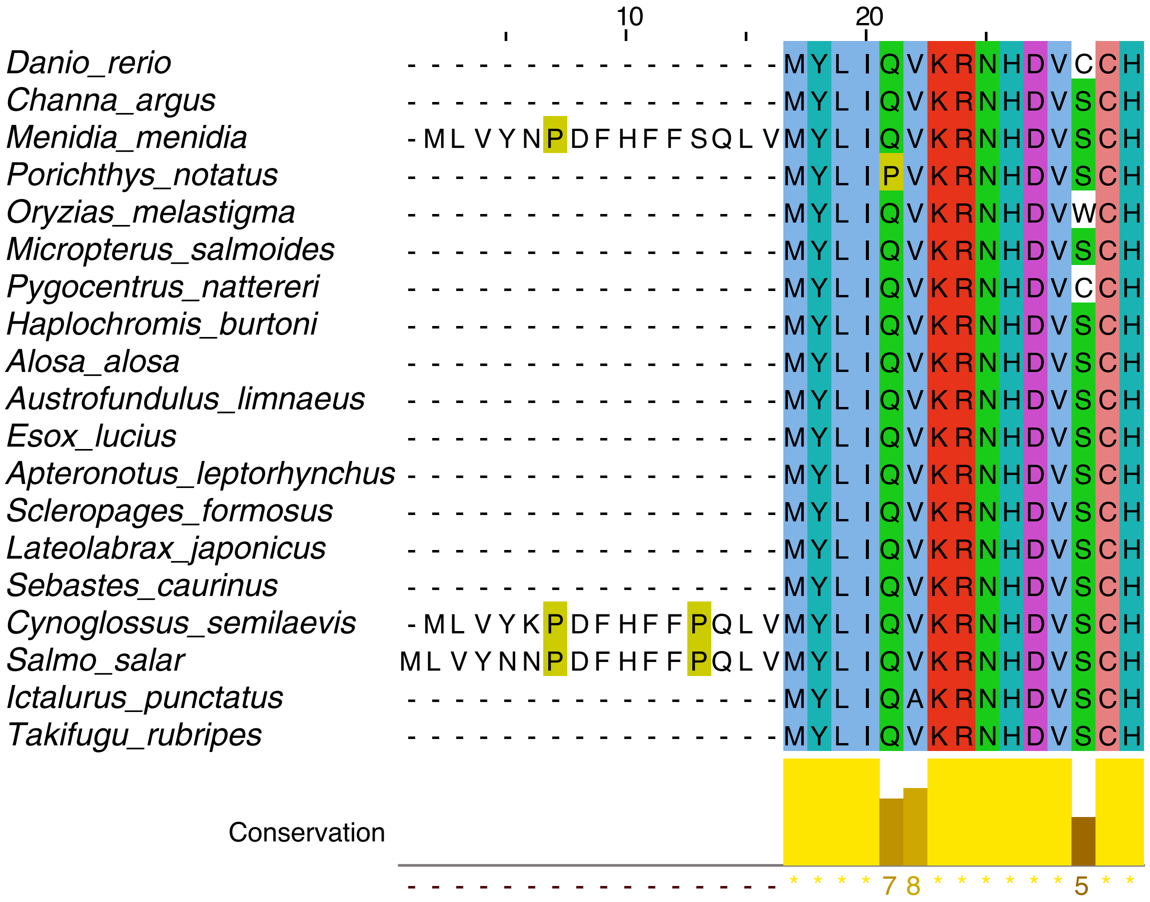

HG0326

ENSDARG00000012848 (arih2)

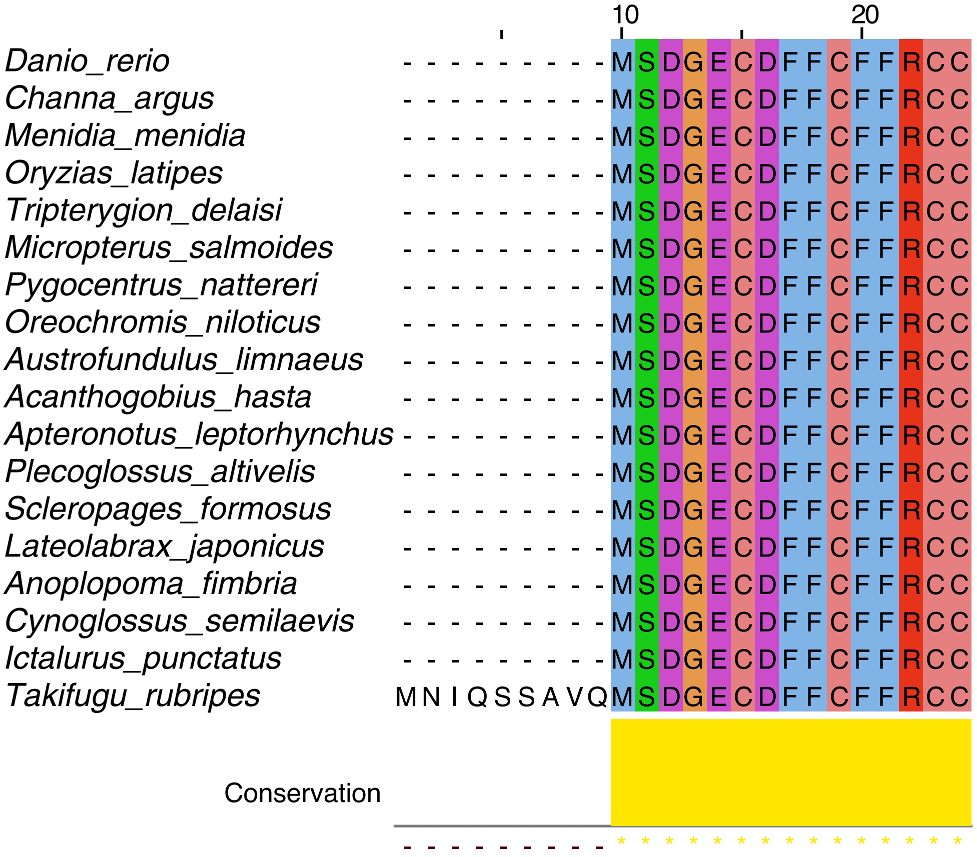

HG0327

ENSDARG00000022203 (gtf2ird1)

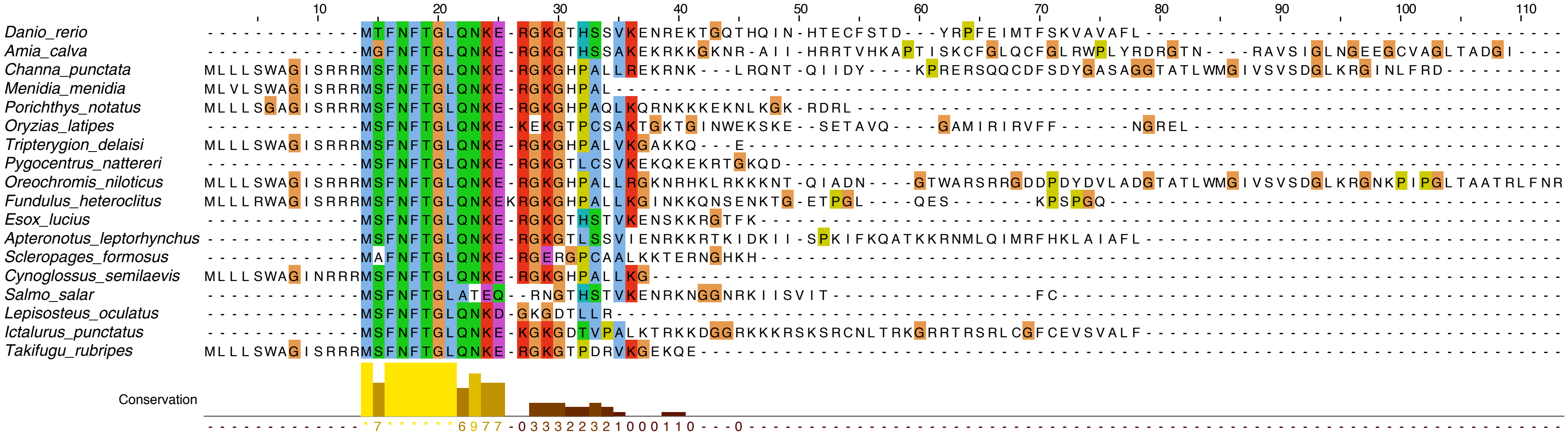

HG0328

ENSDARG00000099999 (tcf3b)

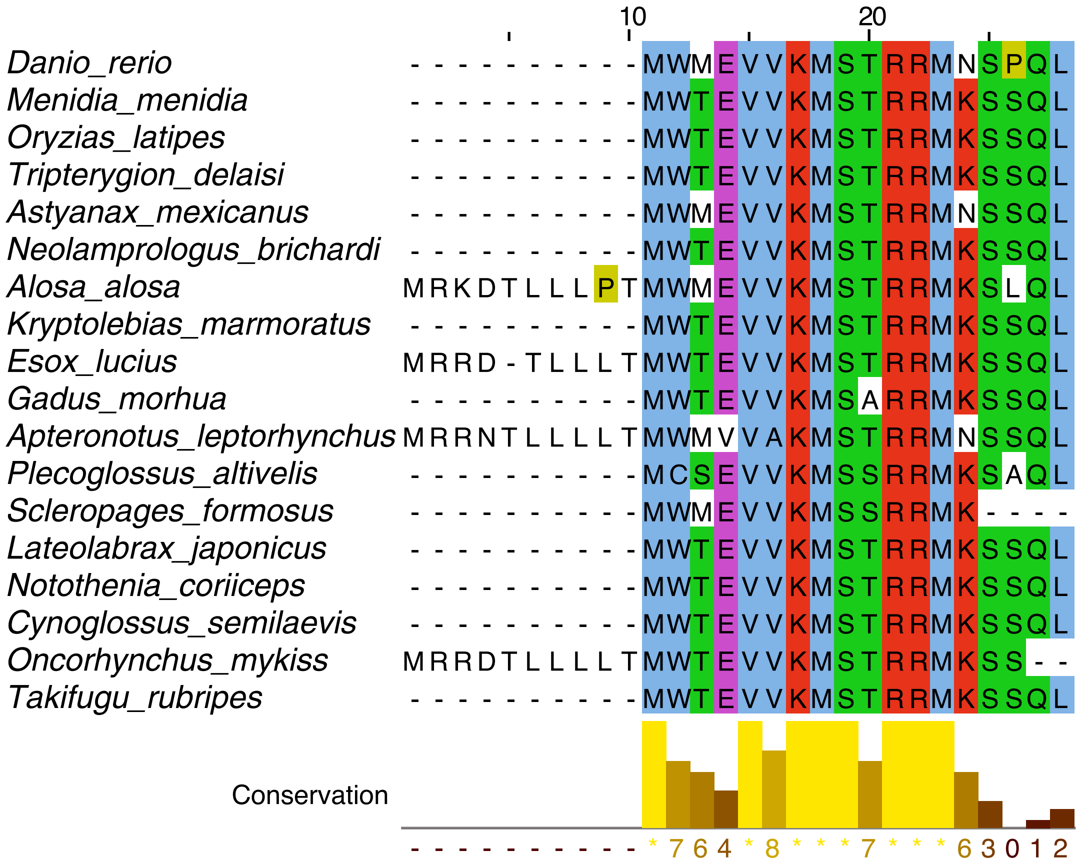

HG0329

ENSDARG00000036442 (atp11c)

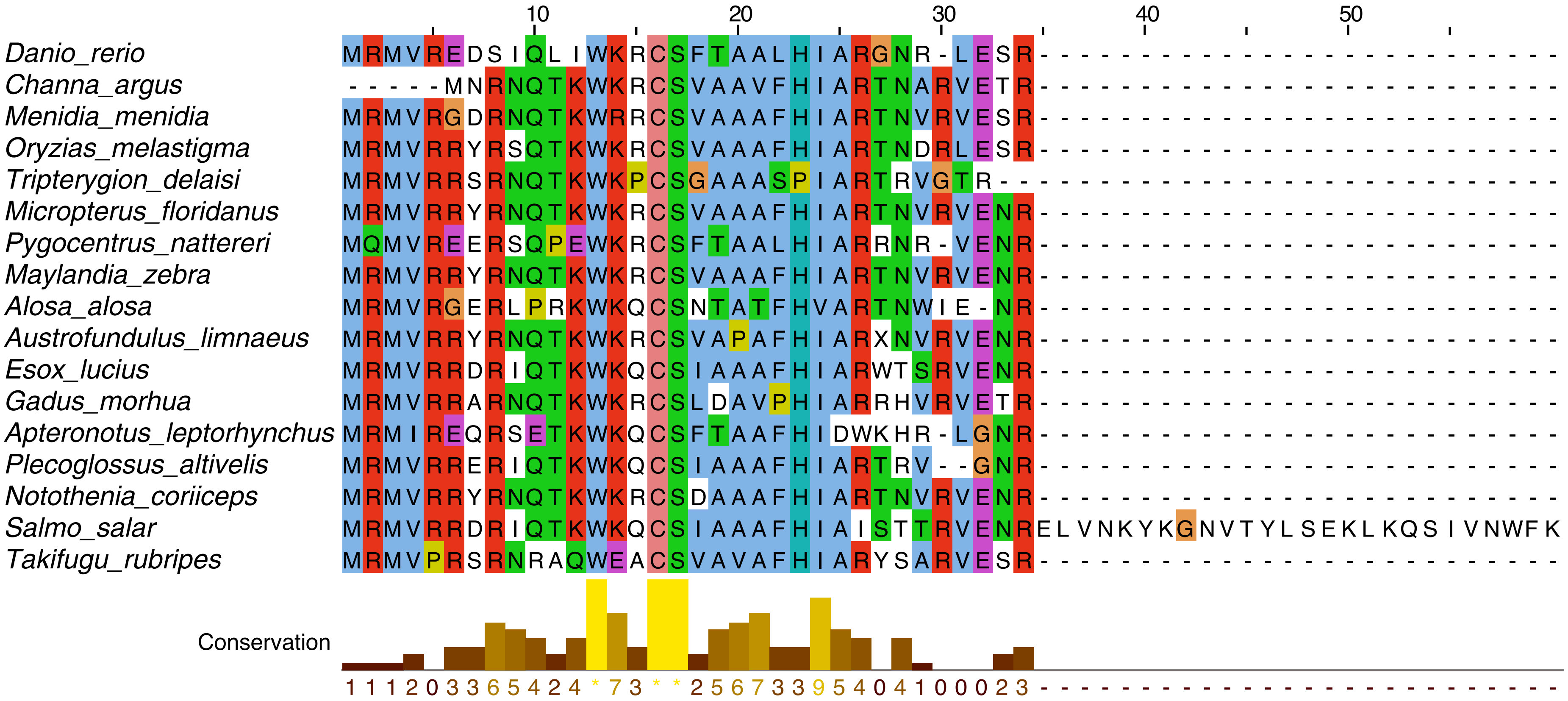

HG0330

ENSDARG00000039392 (wnk1b)

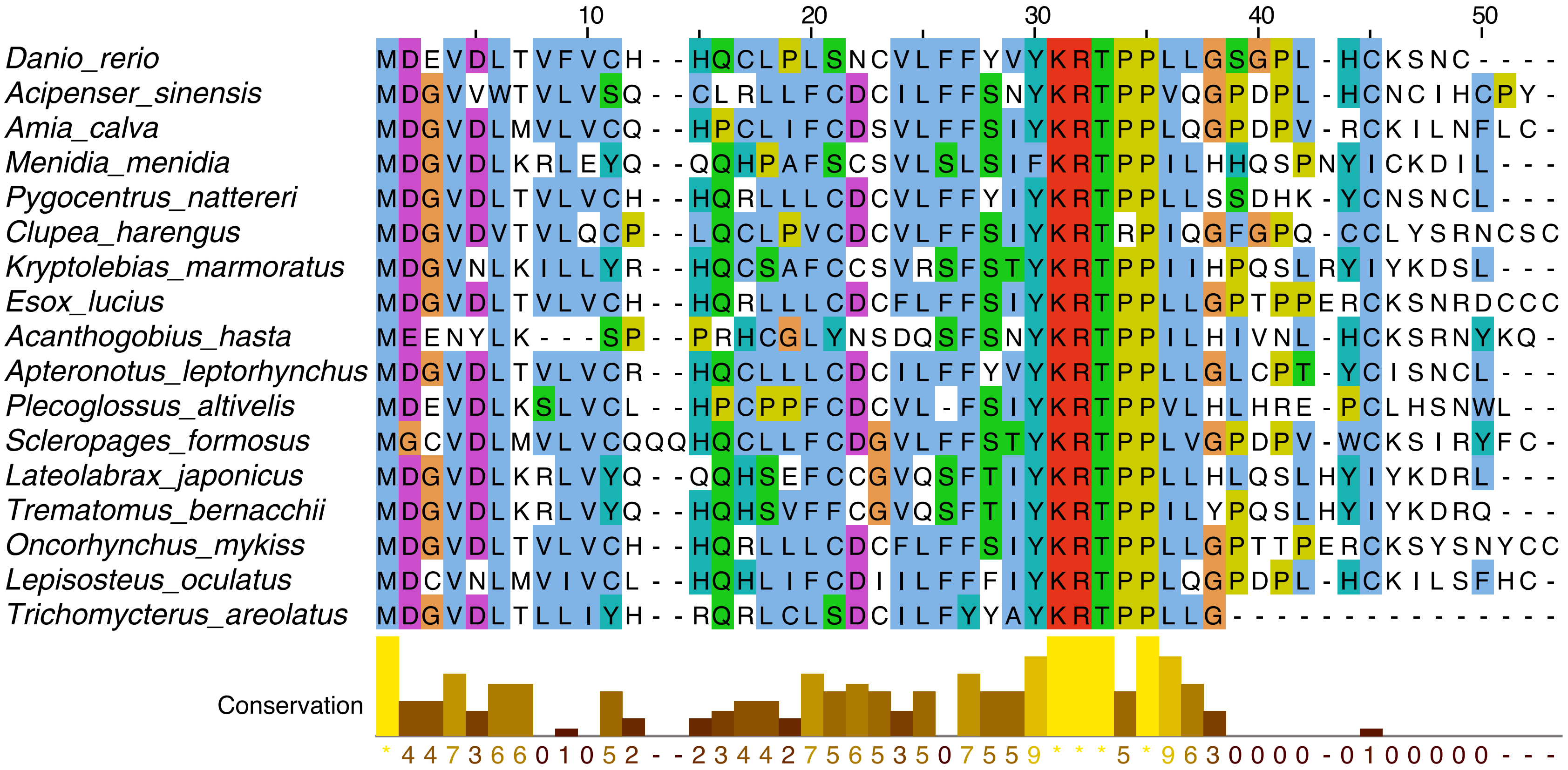

HG0331

ENSDARG00000044899 (tmem183a)

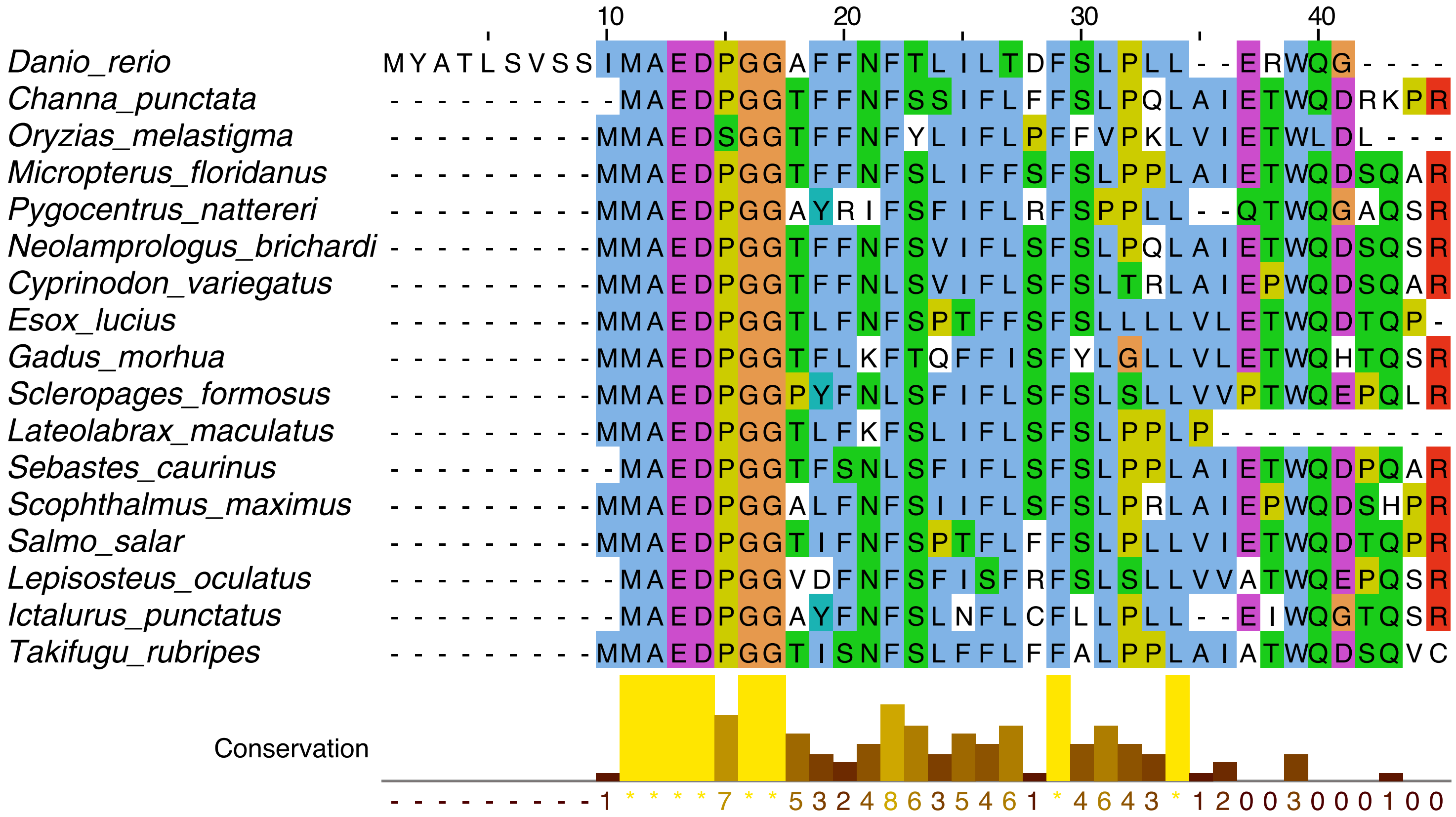

HG0332

ENSDARG00000102893 (atg7)

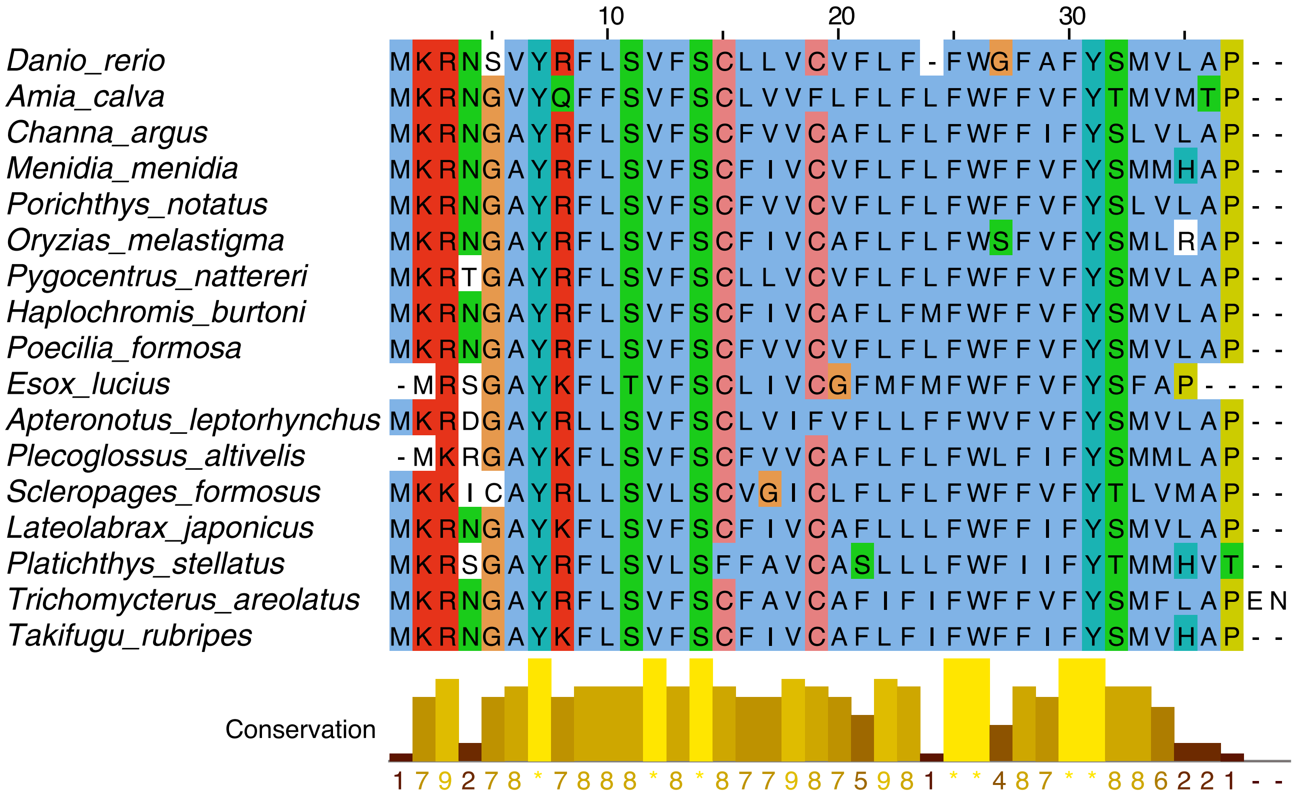

HG0333

ENSDARG00000042518 (hipk2)

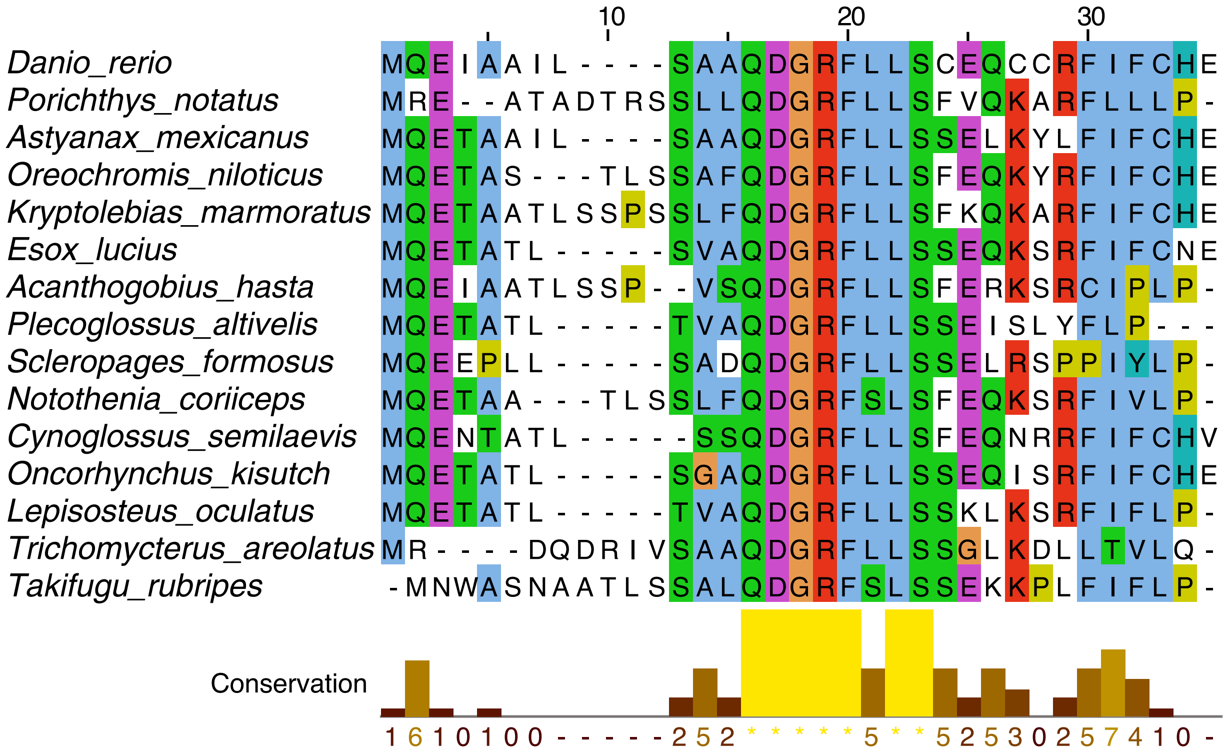

HG0334

ENSDARG00000075349

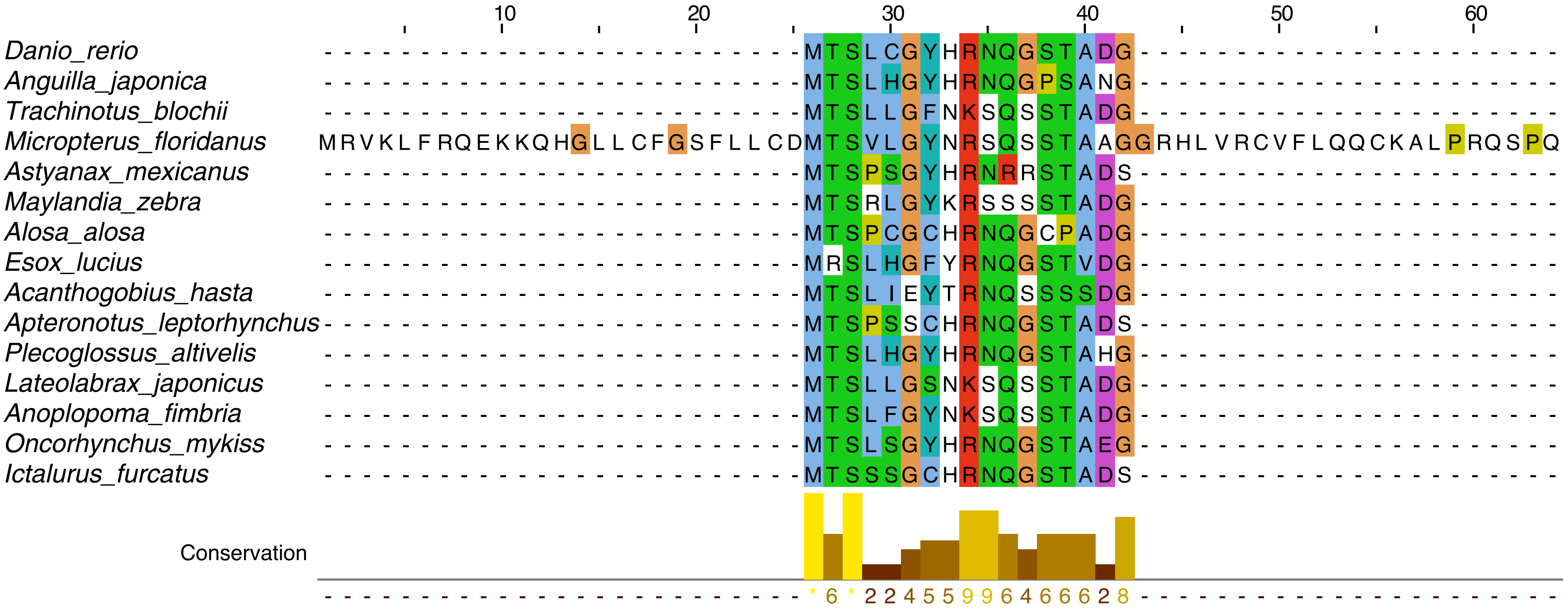

HG0335

ENSDARG00000034700 (vegfab)

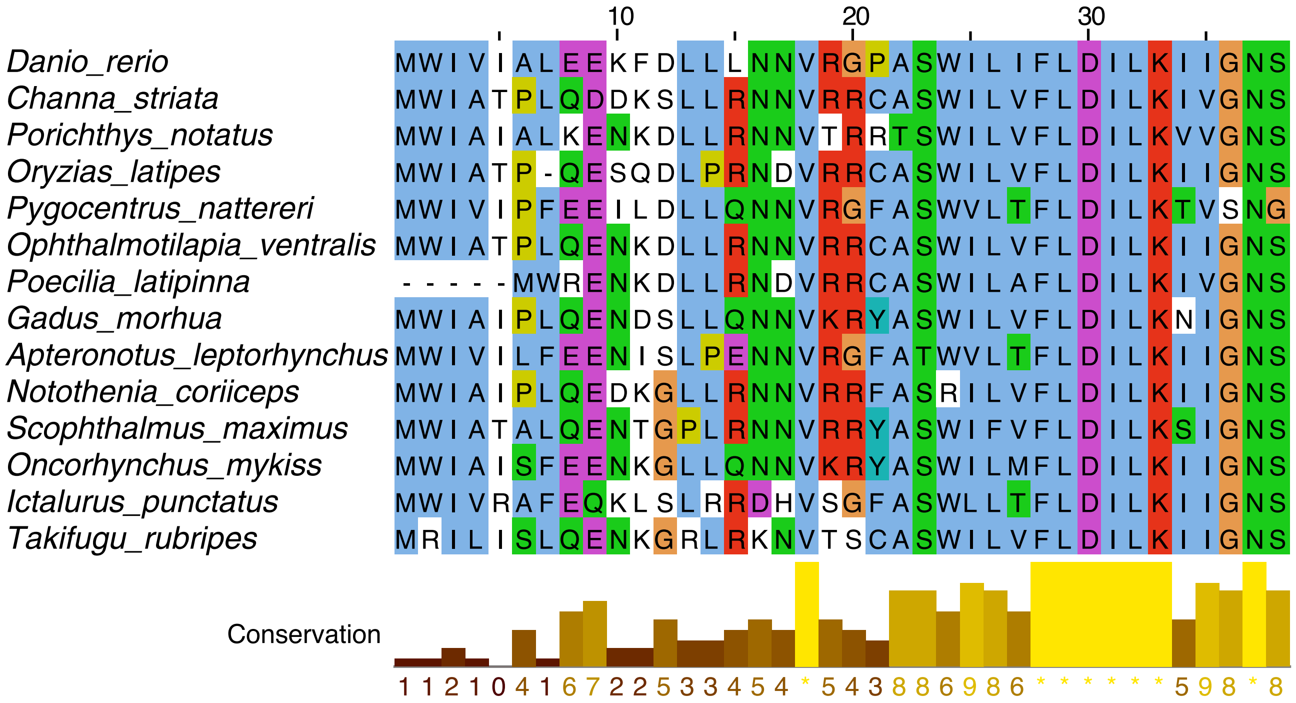

HG0336

ENSDARG00000028676 (nuak1b)

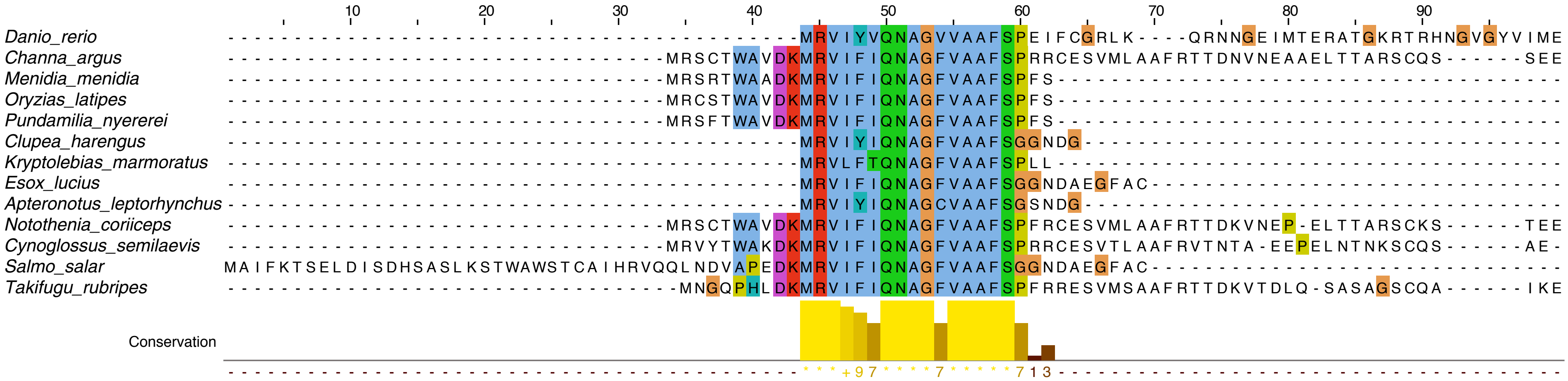

HG0337

ENSDARG00000054906

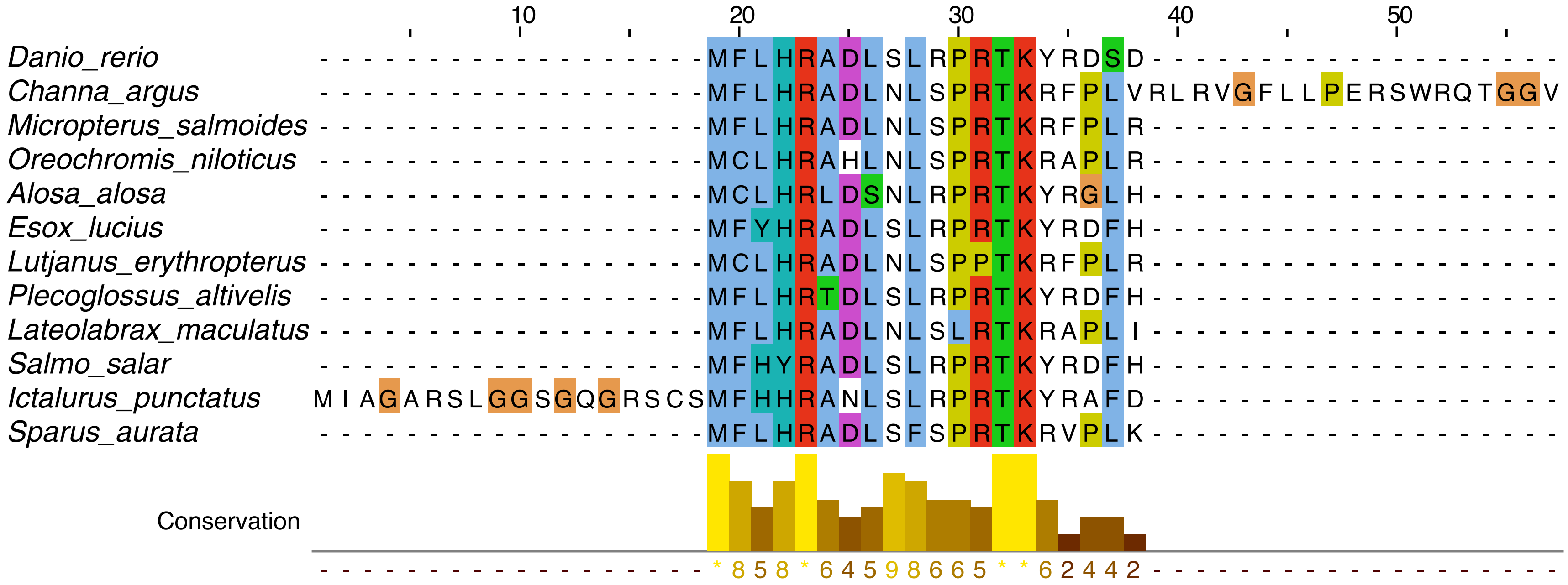

HG0338

ENSDARG00000000151 (thraa)

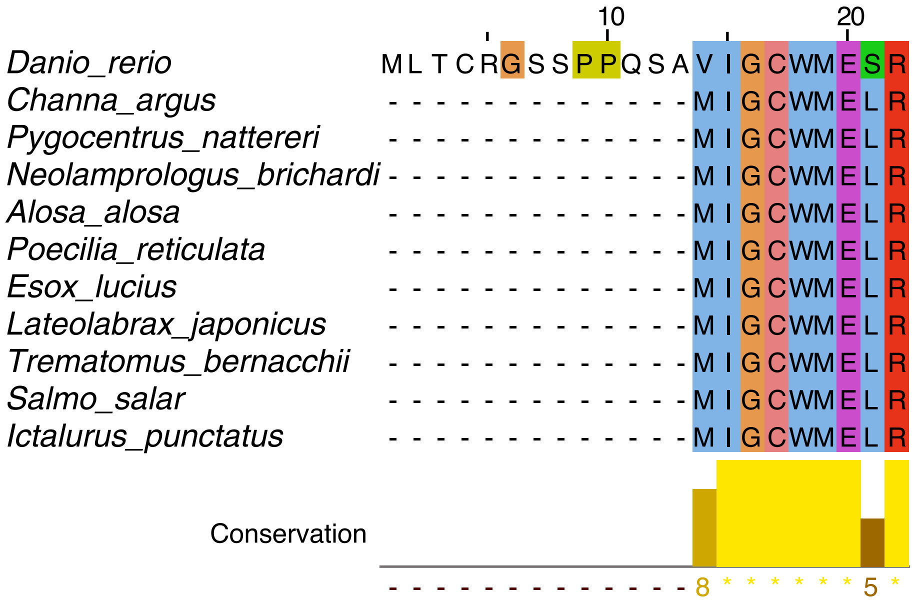

HG0339

ENSDARG00000063144 (shisa7b)

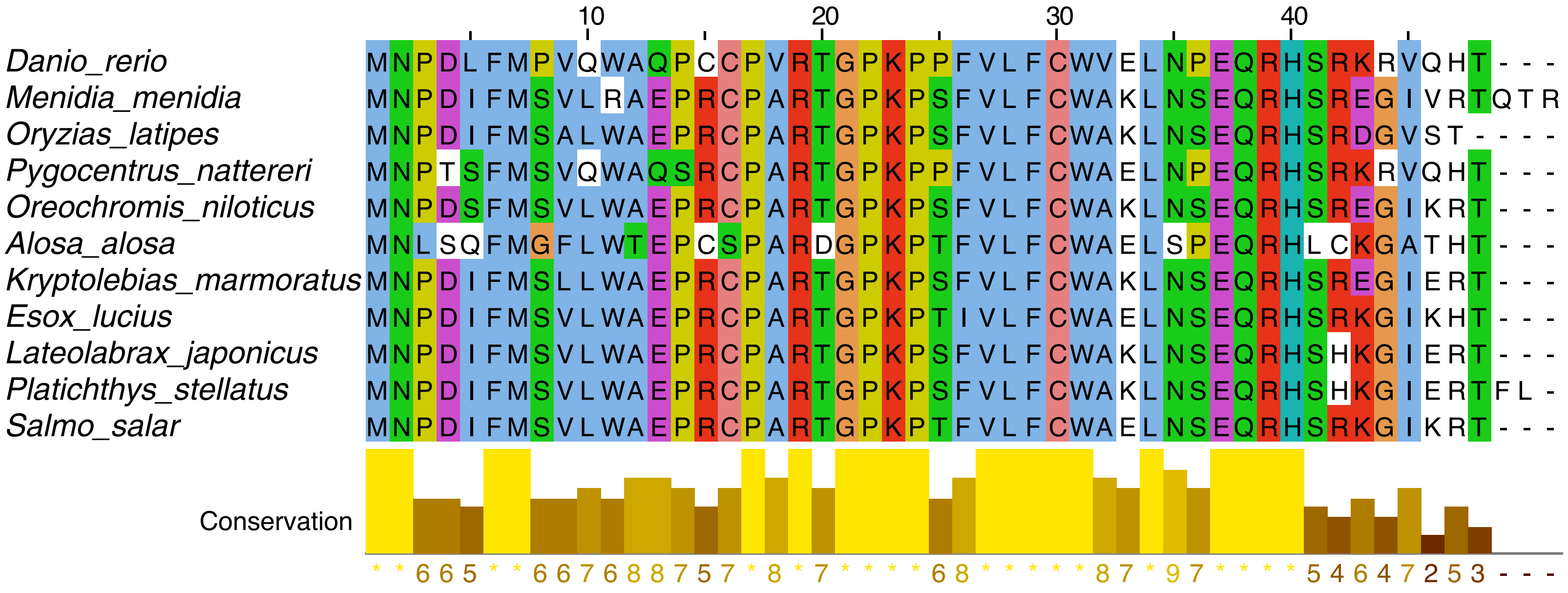

HG0340

ENSDARG00000017591 (fat1a)

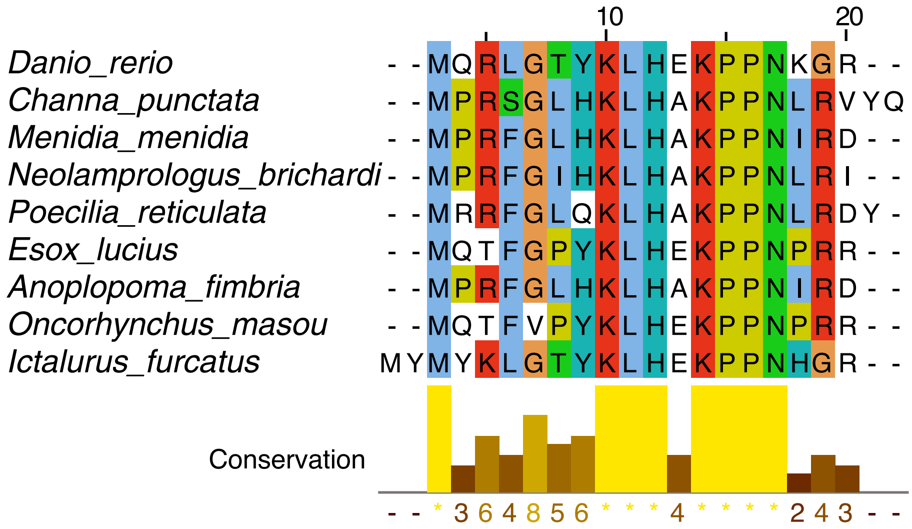

HG0341

ENSDARG00000039430 (msl2b)

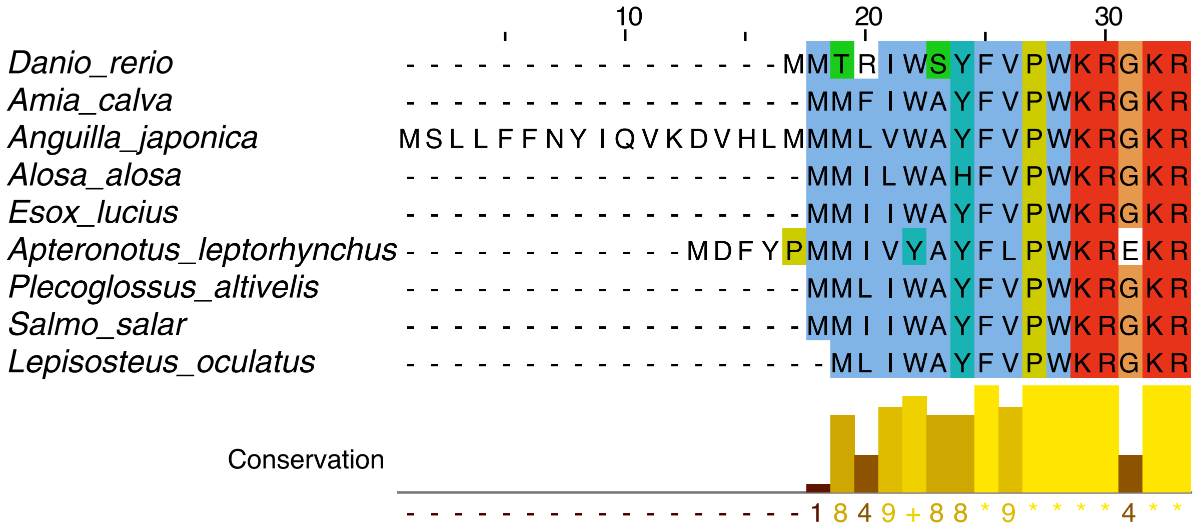

HG0342

ENSDARG00000045801 (fbxl14b)

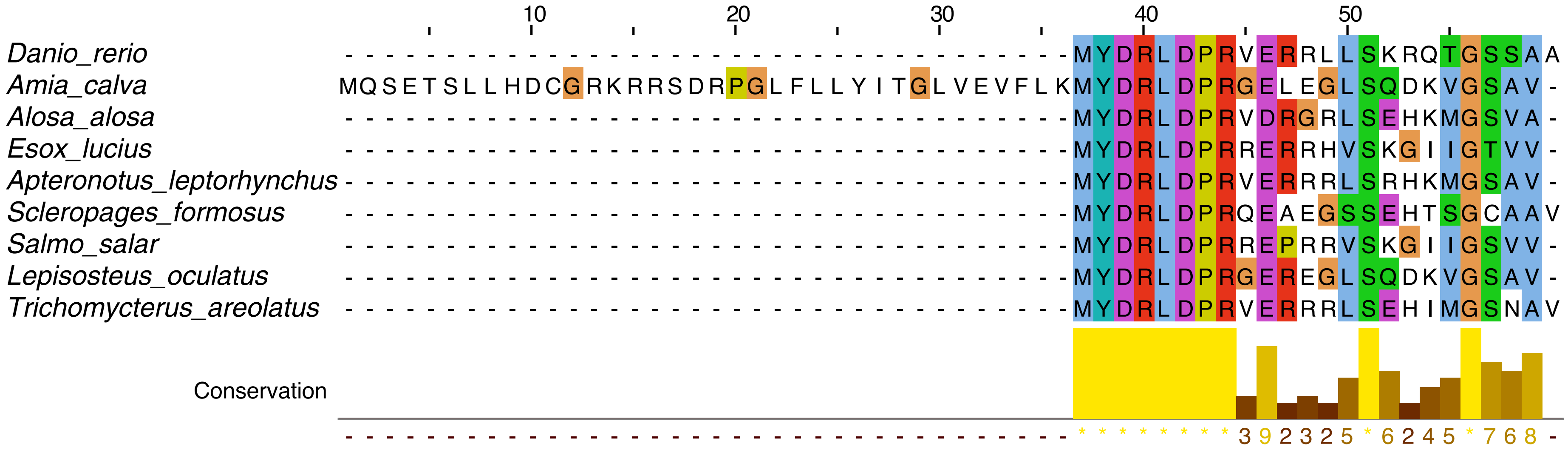

HG0343

ENSDARG00000057062

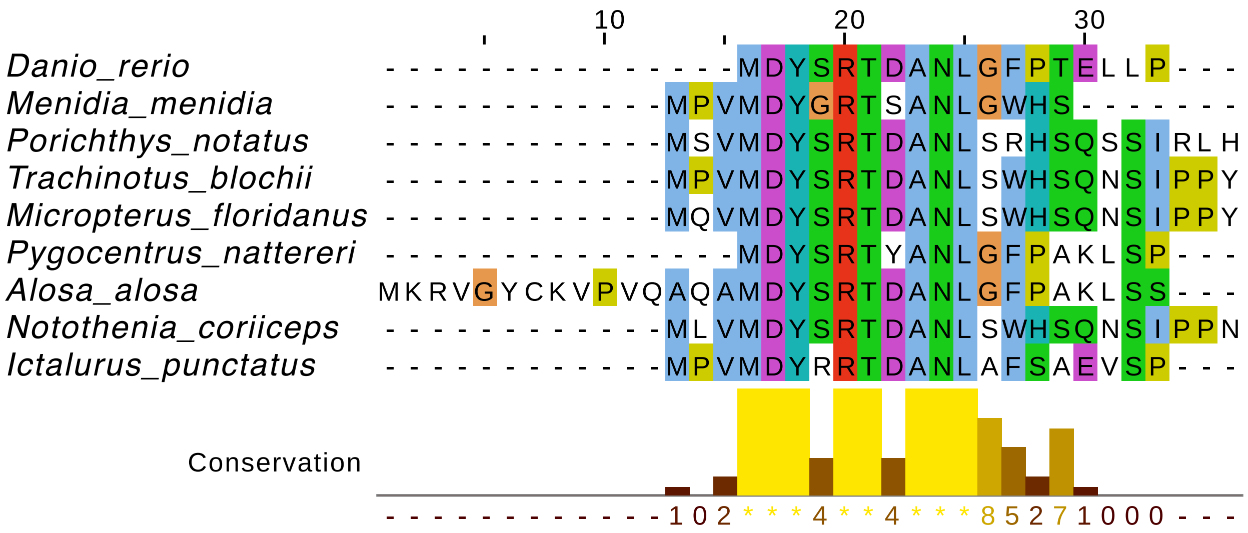

HG0344

ENSDARG00000032072 (cdk16)

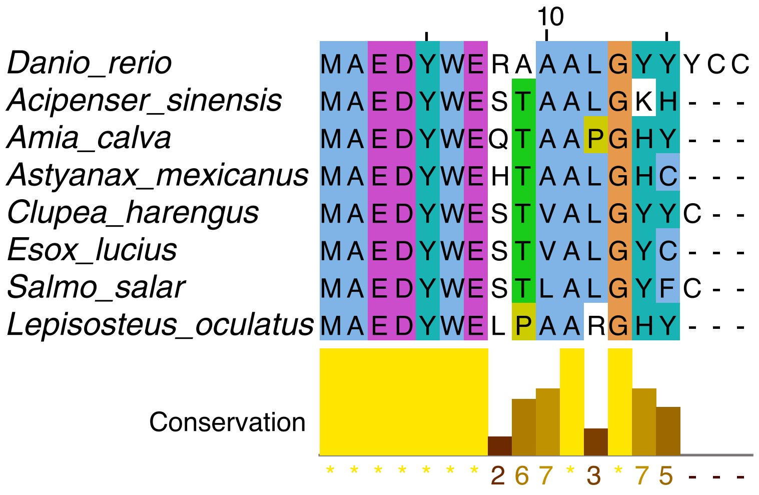

HG0345

ENSDARG00000035910 (znf281b)

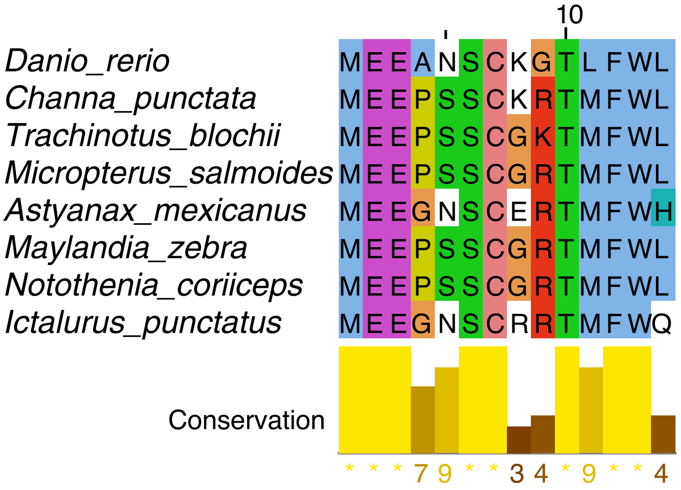

HG0346

ENSDARG00000041982 (adamts6)

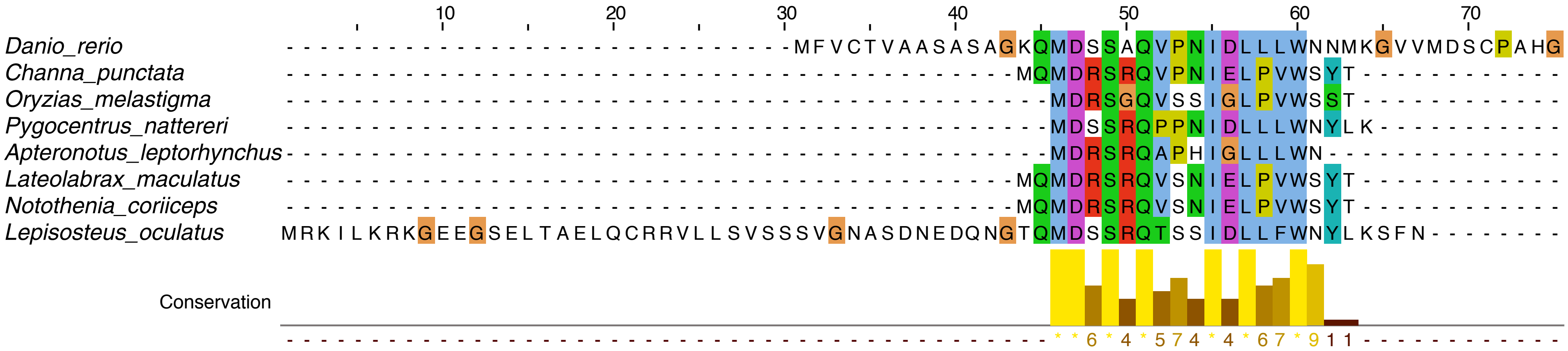

HG0347

ENSDARG00000063194

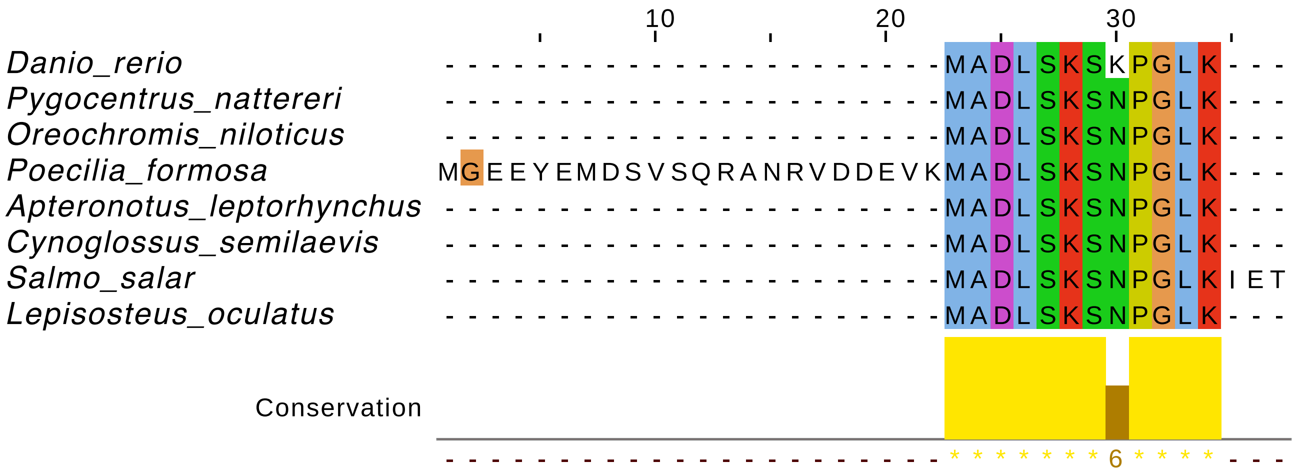

HG0348

ENSDARG00000036139 (lctla)

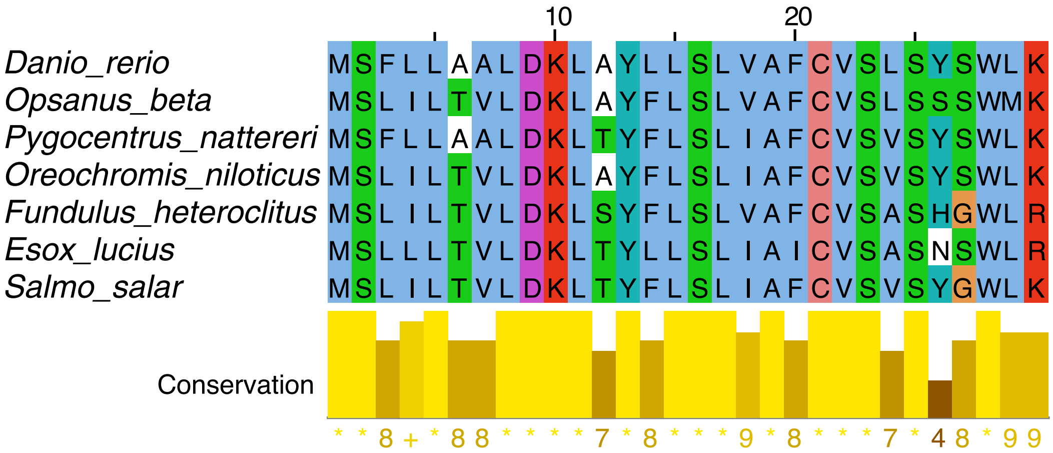

HG0349

ENSDARG00000028699 (crkl)

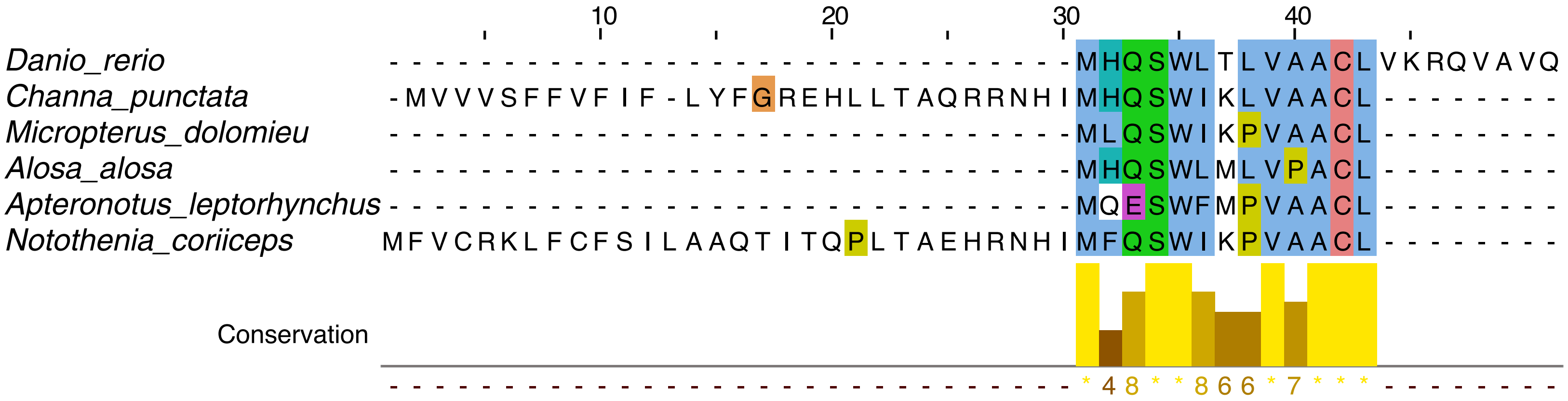

HG0350

ENSDARG00000036194 (pitx2)

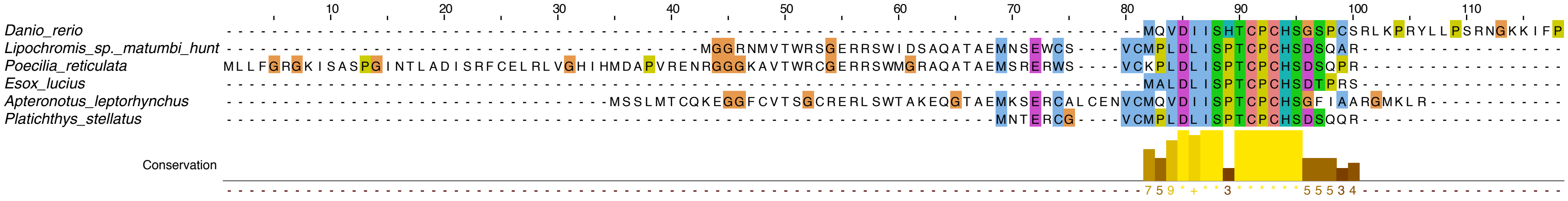

HG0351

ENSDARG00000068096 (atf5a)

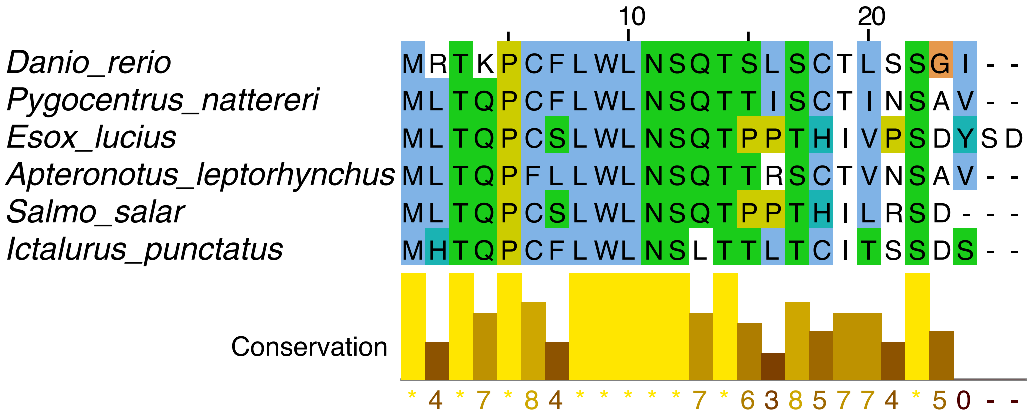

HG0352

ENSDARG00000070214 (slc41a1)

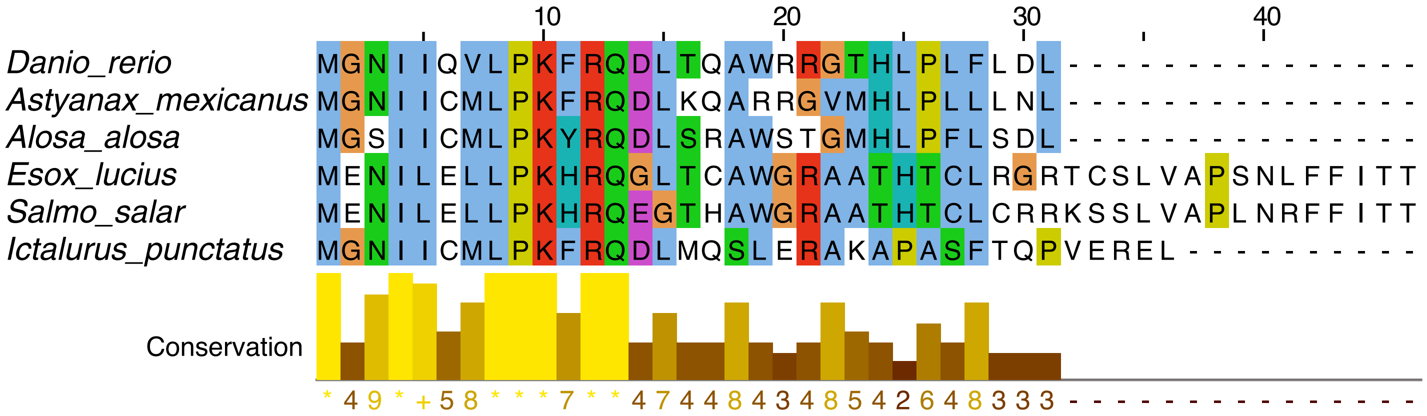

HG0353

ENSDARG00000078864 (lats2)

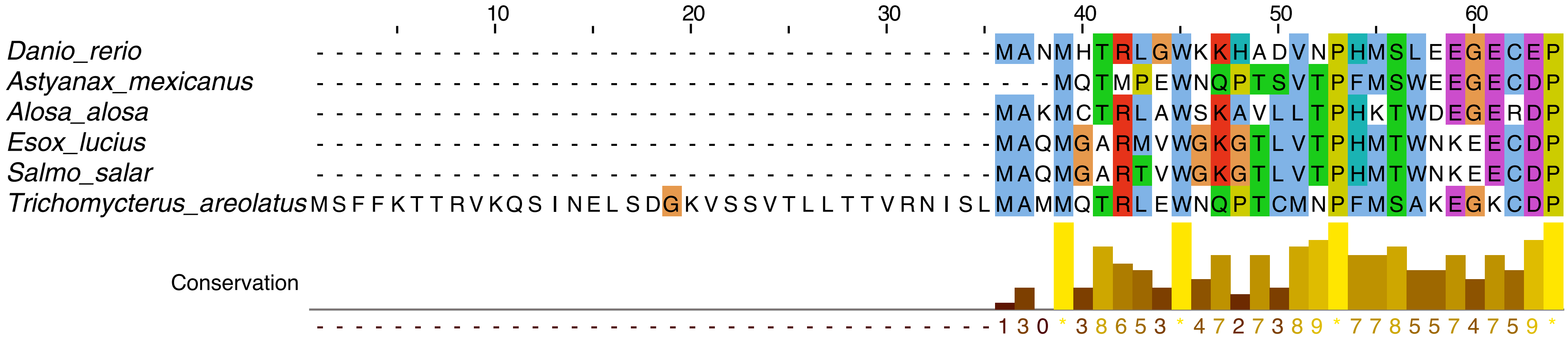

HG0354

ENSDARG00000019897 (gpat4)

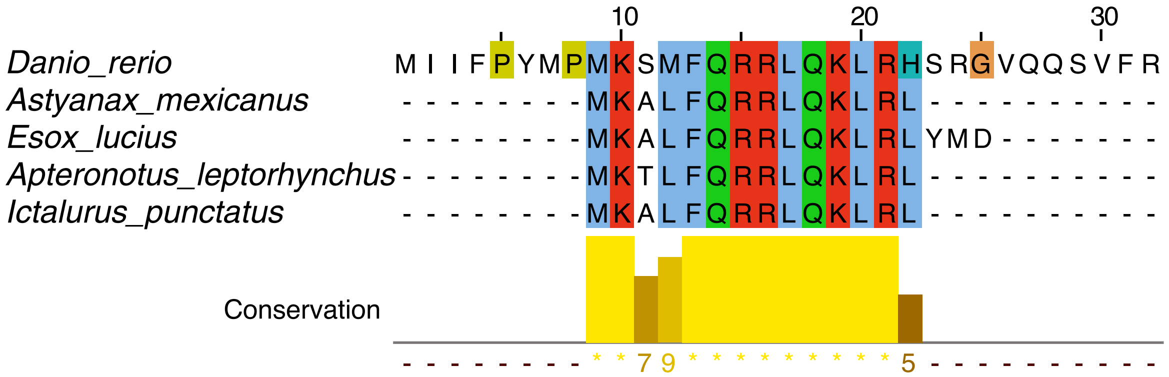

HG0355

ENSDARG00000019930 (tal1)

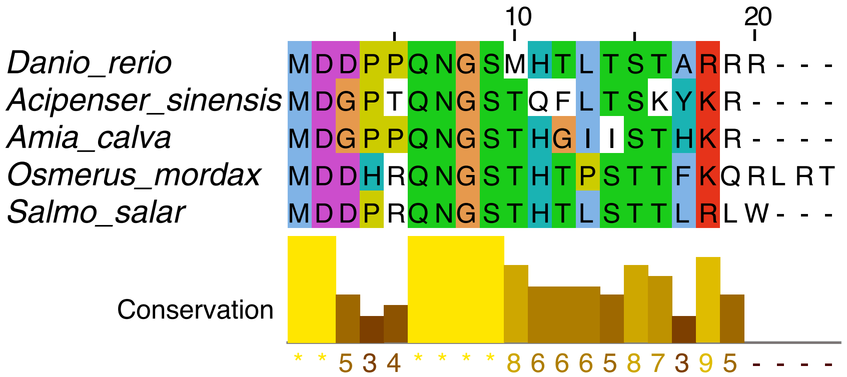

HG0356

ENSDARG00000020475 (znrf1)

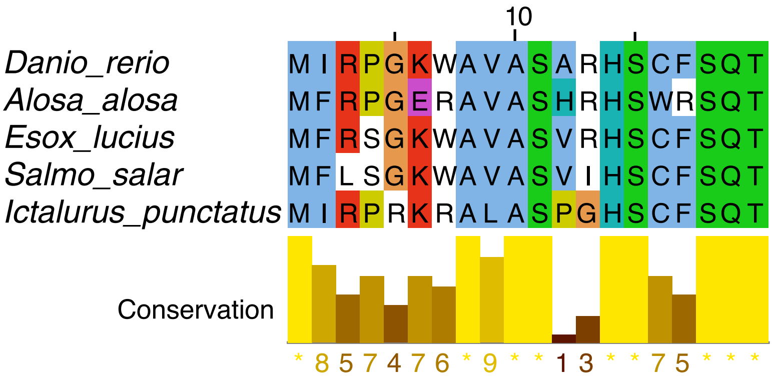

HG0357

ENSDARG00000037285 (mipa)

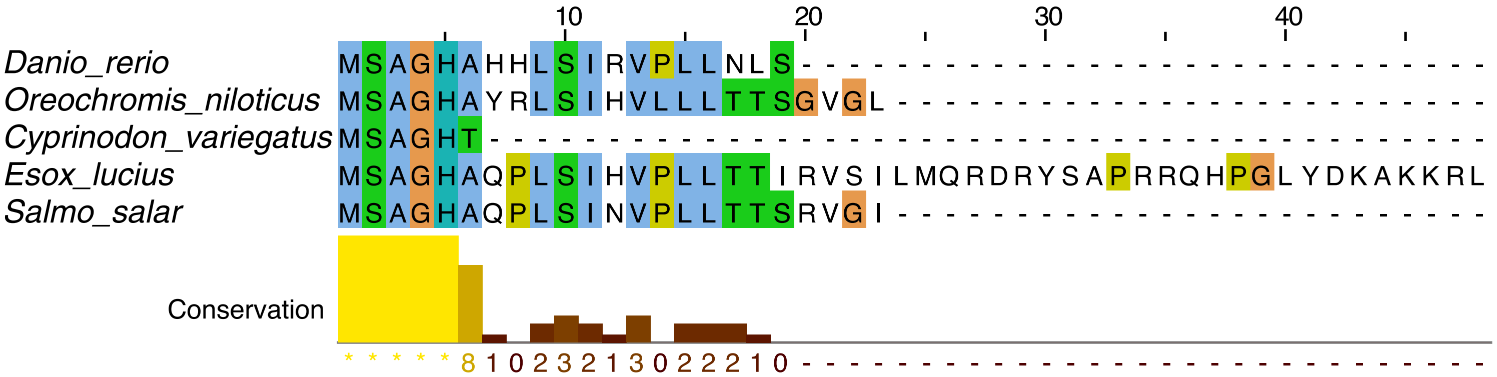

HG0358

ENSDARG00000062506 (zc3h18)

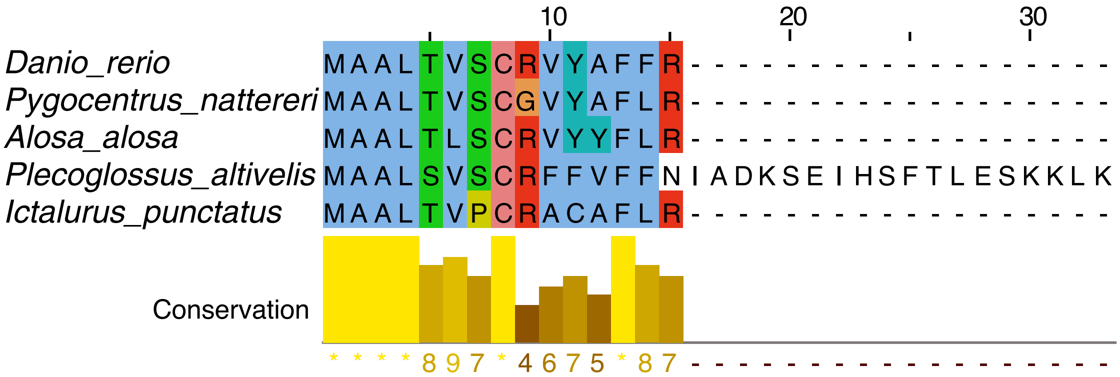

HG0359

ENSDARG00000070348 (hoxc10a)

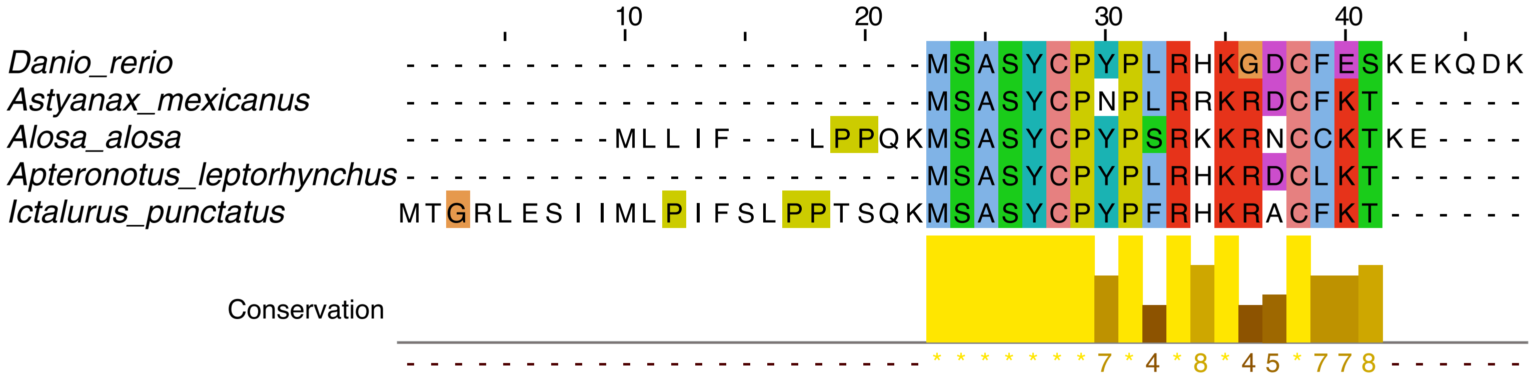

HG0360

ENSDARG00000070801 (opa1)

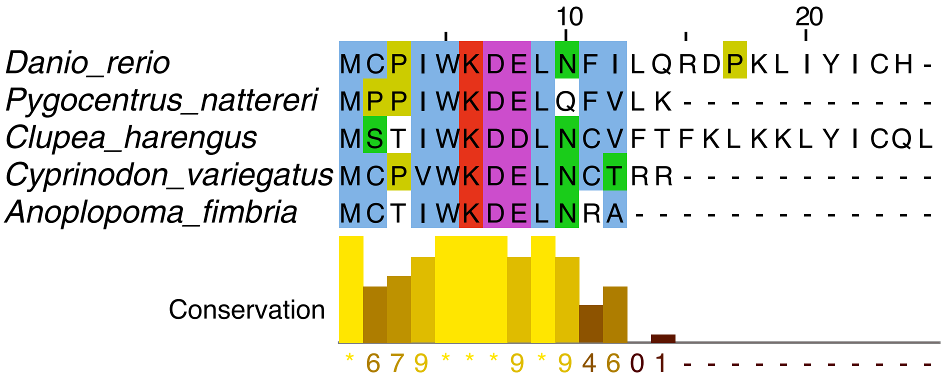

HG0361

ENSDARG00000077581 (zzz3)

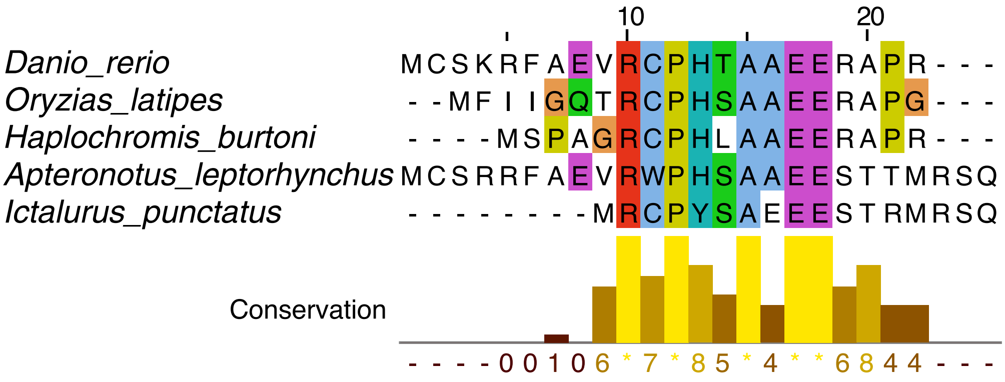

HG0362

ENSDARG00000078355 (zc3h4)

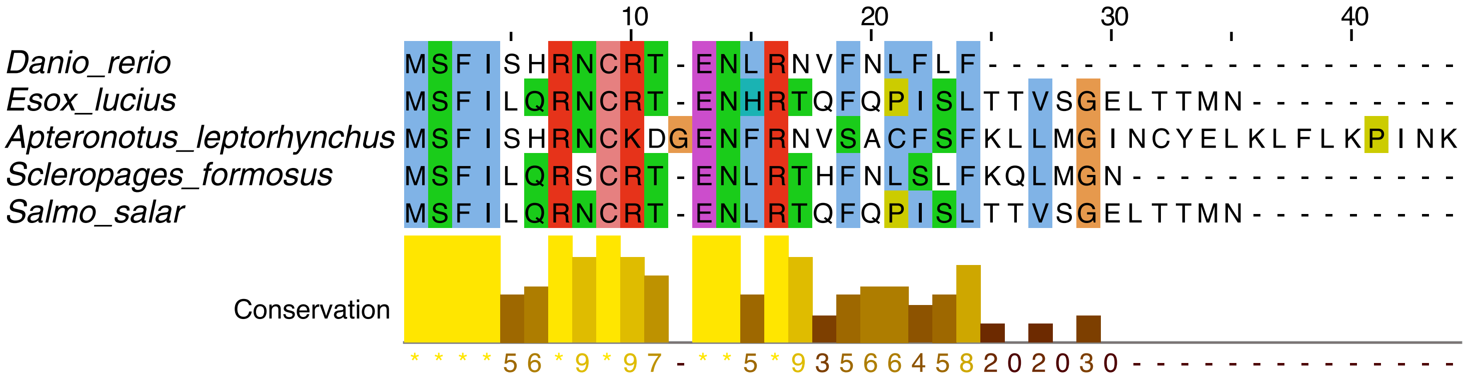

HG0363

ENSDARG00000095896 (pou3f3b)

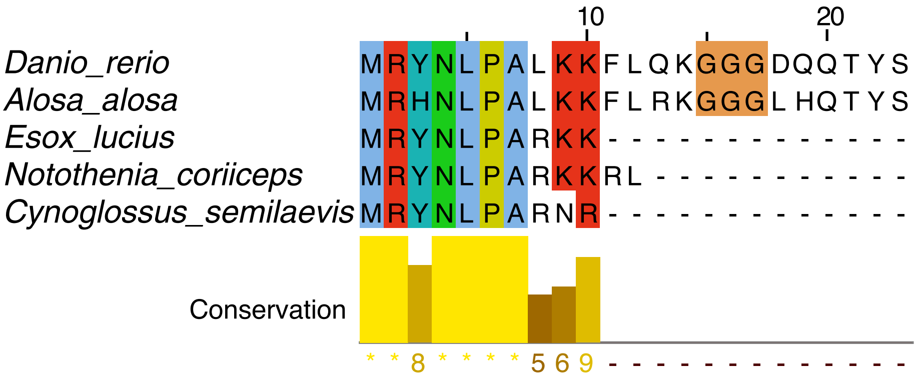

HG0364

ENSDARG00000004988 (ppp3ca)

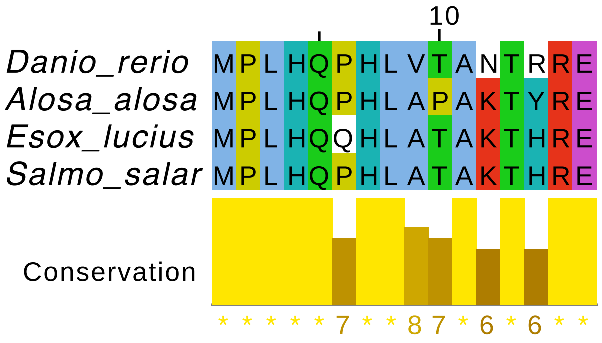

HG0365

ENSDARG00000016132 (keap1a)

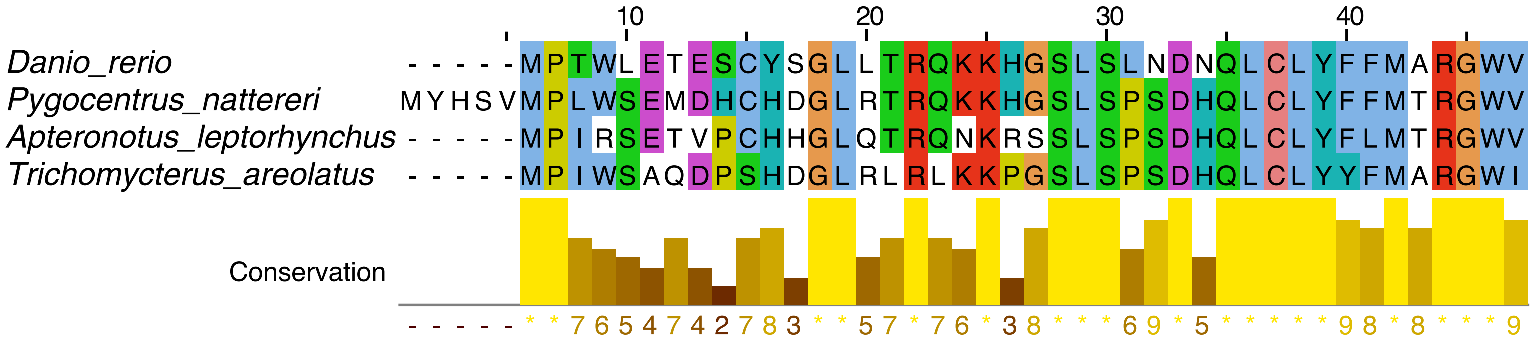

HG0366

ENSDARG00000017803 (gsk3b)

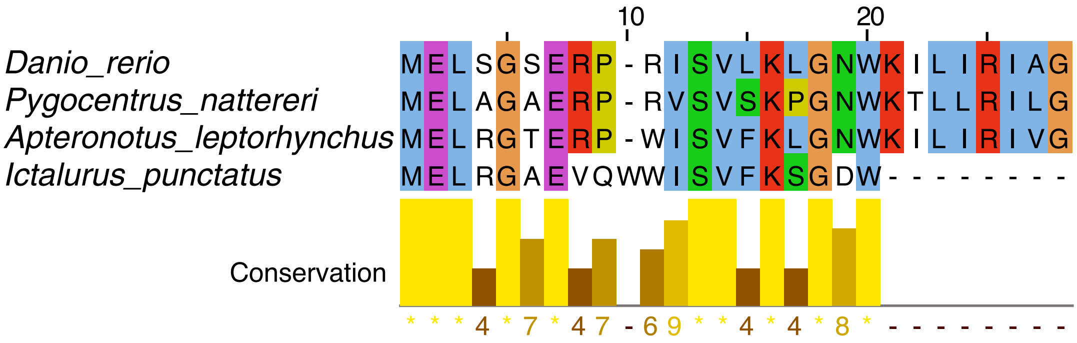

HG0367

ENSDARG00000034056 (csnk1g2b)

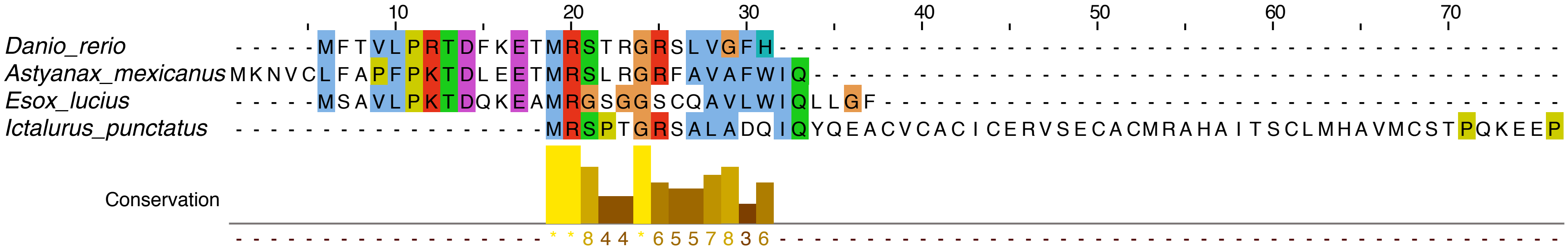

HG0368

ENSDARG00000058606 (sik1)

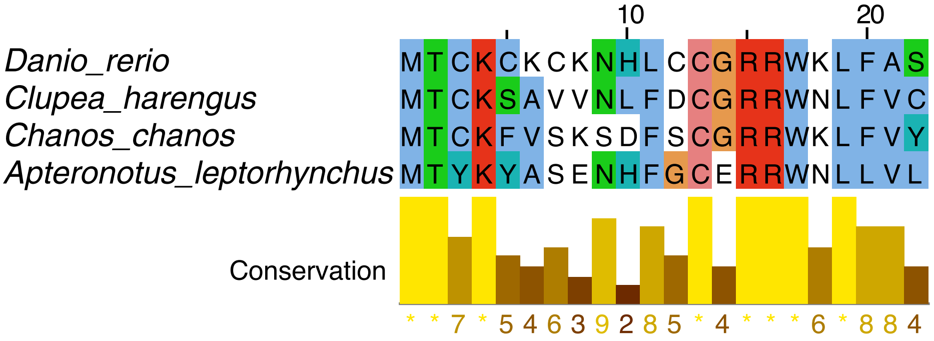

HG0369

ENSDARG00000059276 (hoxd4a)

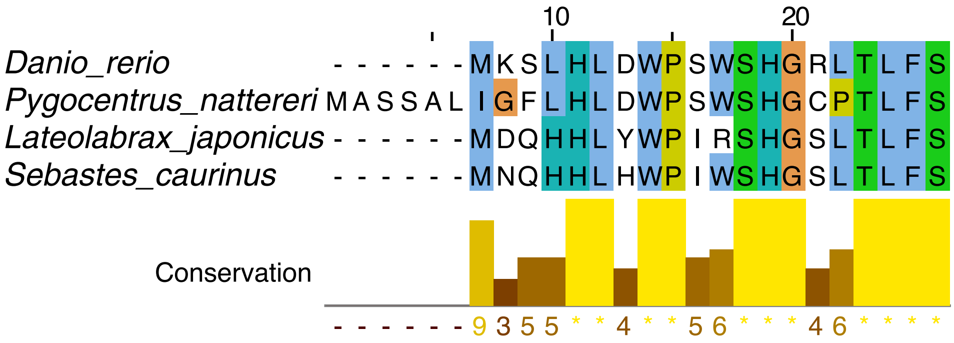

HG0370

ENSDARG00000062693 (nrxn3b)

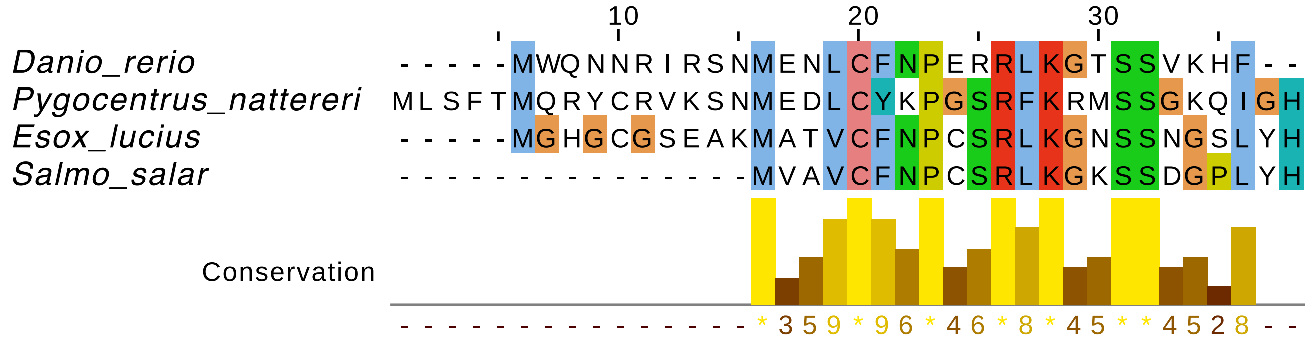

HG0371

ENSDARG00000062765 (whsc1l1)

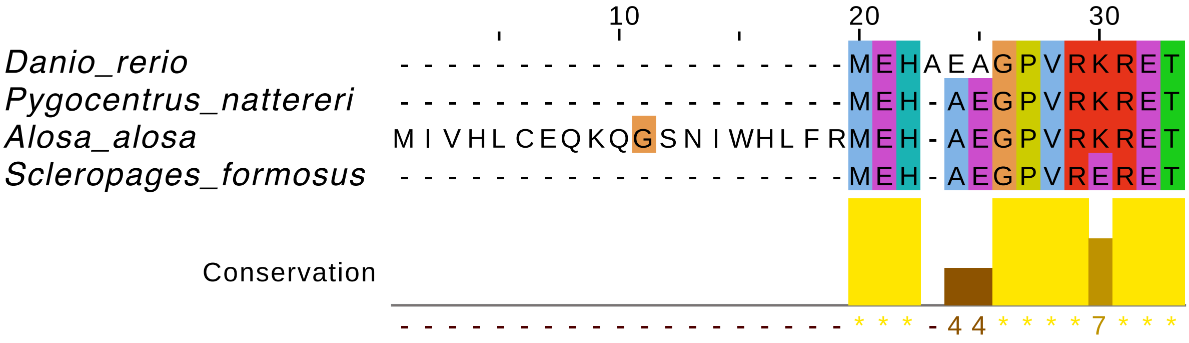

HG0372

ENSDARG00000069467 (igsf9bb)

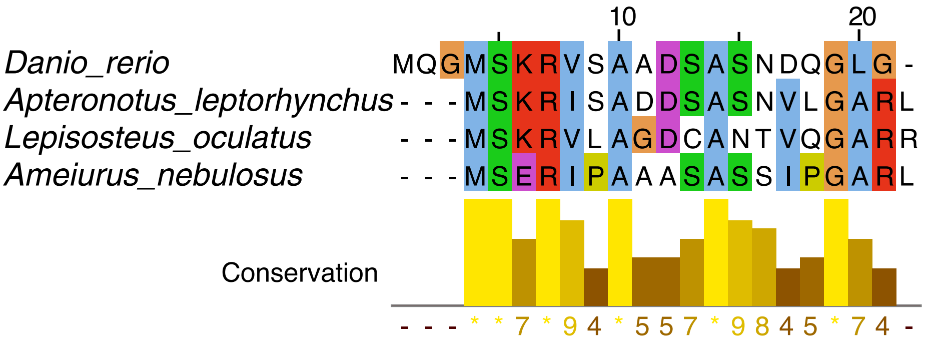

HG0373

ENSDARG00000074611 (wdr37)

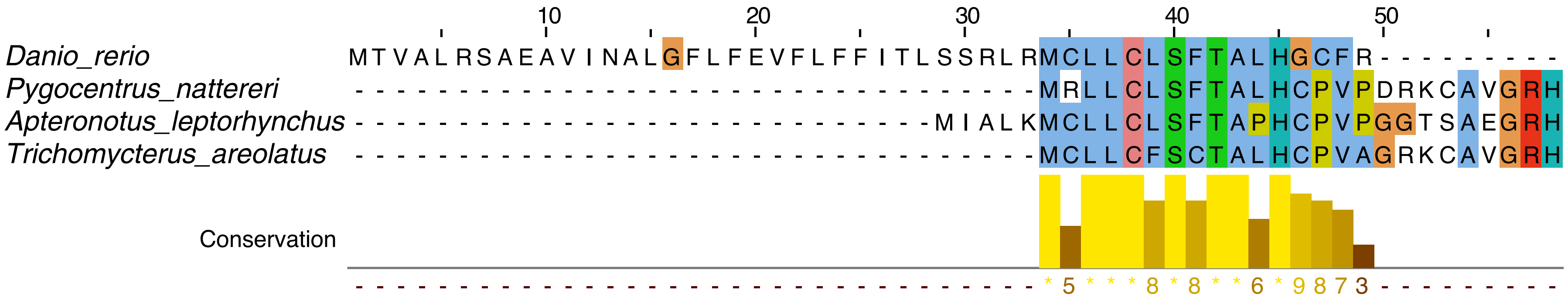

HG0374

ENSDARG00000075147 (lrrc38a)

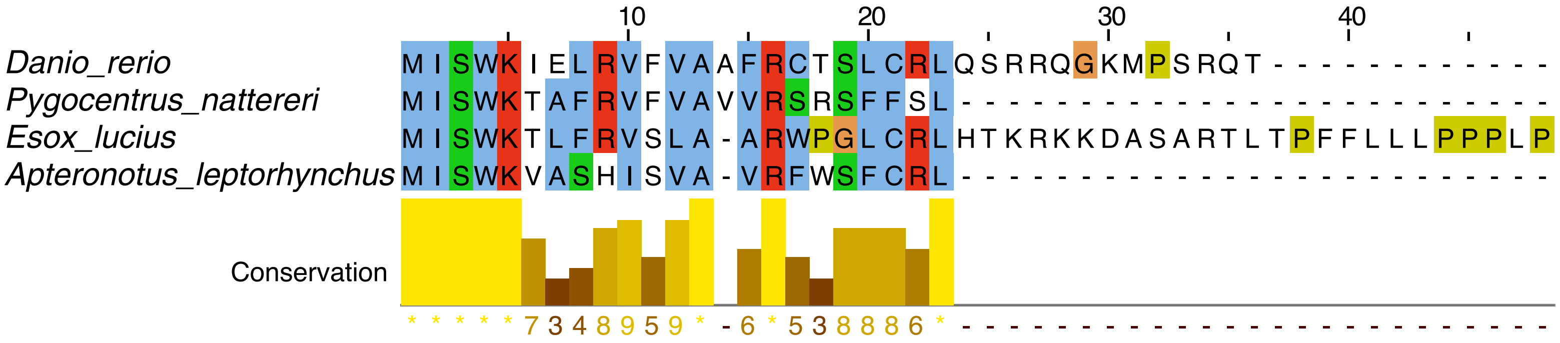

HG0375

ENSDARG00000076171 (znf827)

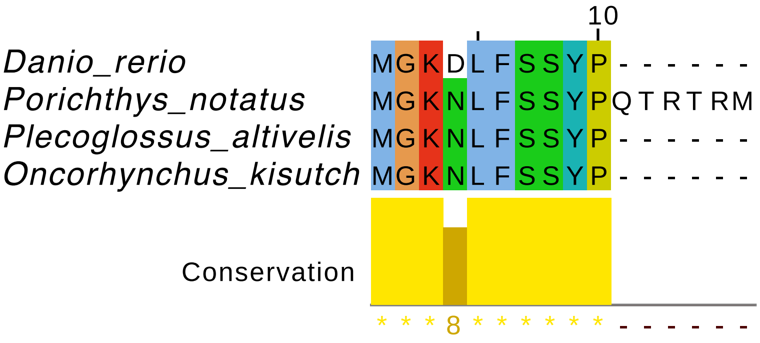

HG0376

ENSDARG00000077229 (ano8b)

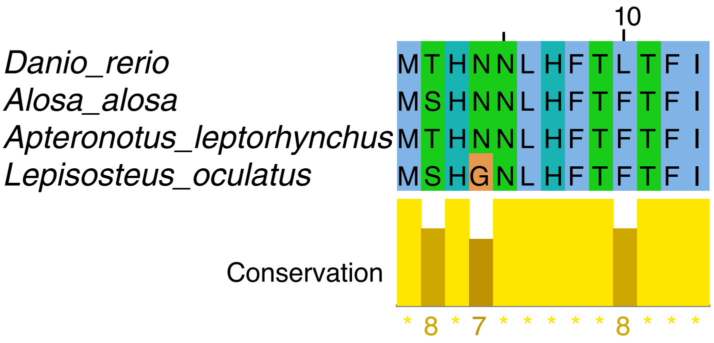

HG0377

ENSDARG00000079549 (cdc42ep1a)

```
                              10        20        30        40        50        60
Danio_rerio          MQPRFSGIGVMHIFSSHLSSPNLSGYEQRPMAKTGHS----------------------------------
Astyanax_mexicanus   ----------MHIFNSHPSQLEHFGHGQEPATKELCVFLMEKAFLRNISLSCSFFSFALRSPEPGIVLHEW
Alosa_alosa          ----------MHIFNSHFS----------------------------------------------------
Ictalurus_punctatus  ----------MSIFISHLSQWDHFGYGQRPVAKNLCVHTYGKFSLRNISLSCTFFSLTFLEKP--------

Conservation         ----------*4**5**5*----------------------------------------------------
```

HG0378

ENSDARG00000080009 (bahcc1b)

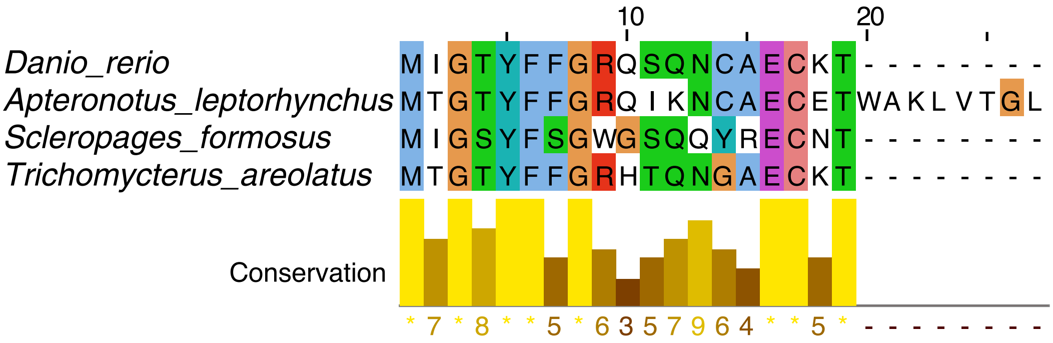

HG0379

ENSDARG00000104372 (gnb1b)

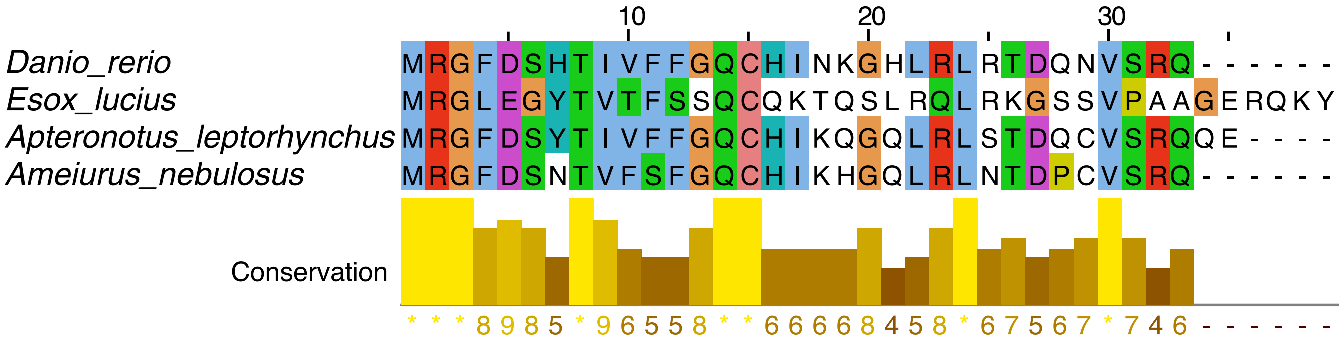

HG0380

ENSDARG00000005350 (dip2ba)

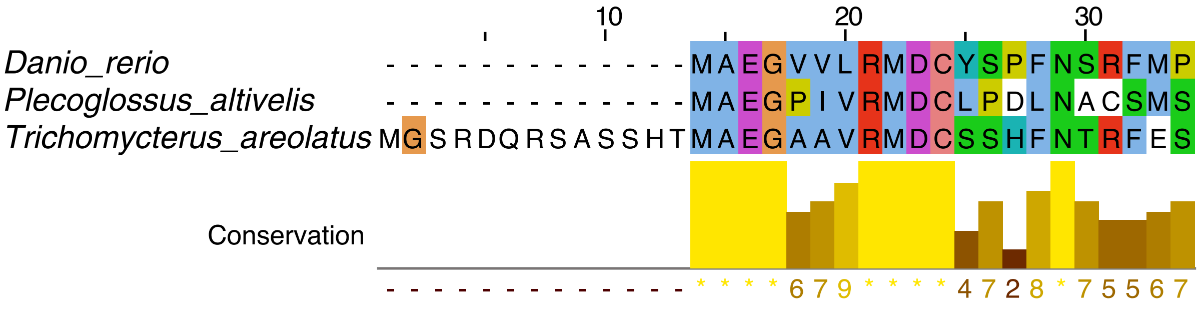

HG0381

ENSDARG00000006124 (kdm5c)

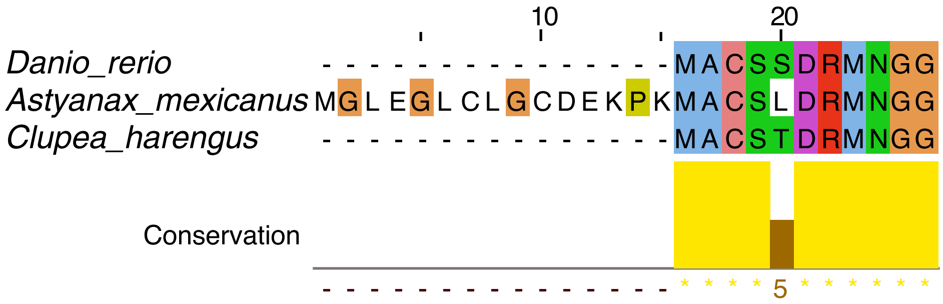

HG0382

ENSDARG00000017242

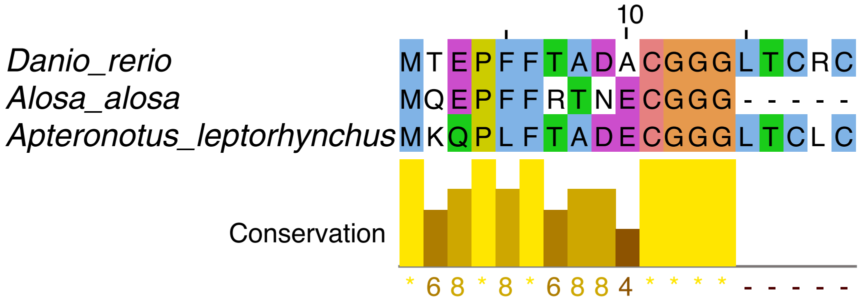

HG0383

ENSDARG00000036501 (rab39bb)

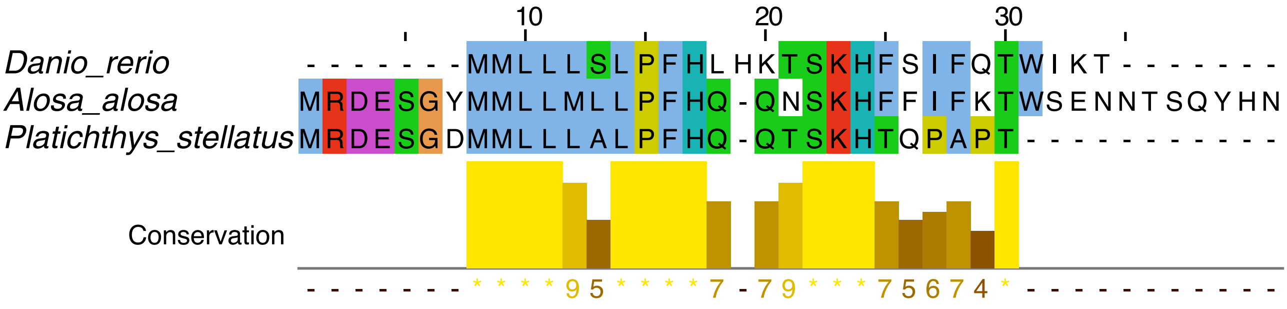

HG0384

ENSDARG00000054748 (cuedc1b)

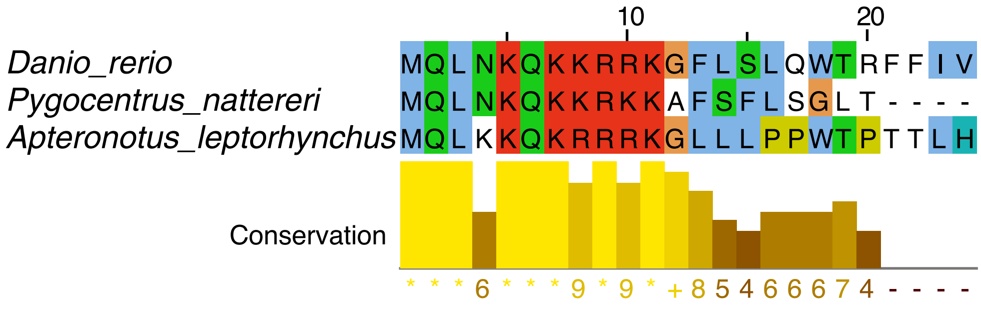

HG0385

ENSDARG00000059549 (akap10)

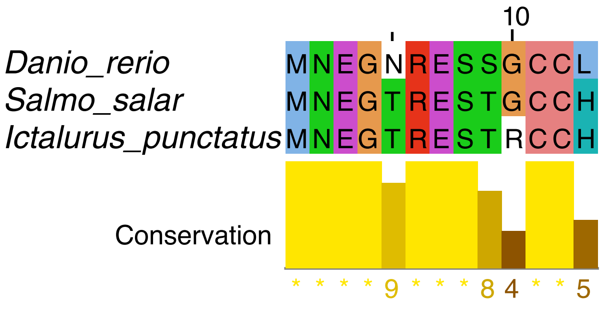

HG0386

ENSDARG00000062084 (clcn1a)

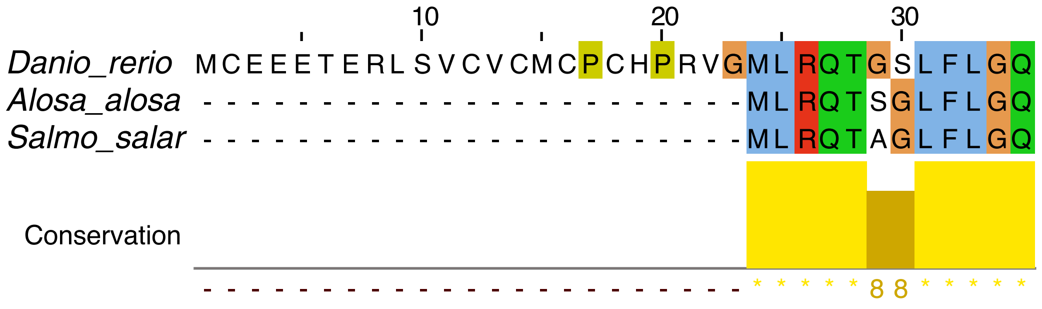

HG0387

ENSDARG00000071150 (cica)

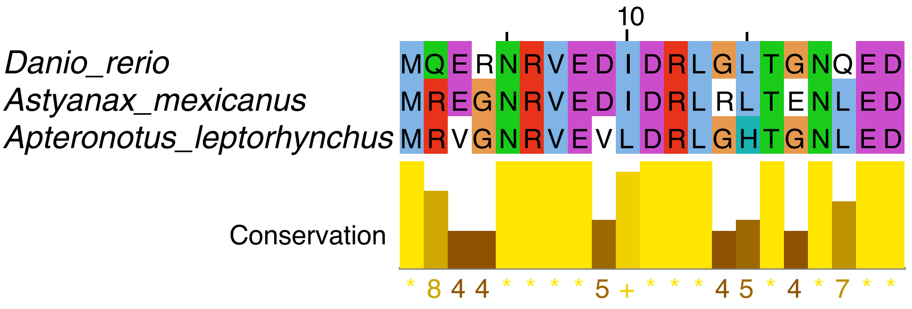

HG0388

ENSDARG00000077228 (ntrk3a)

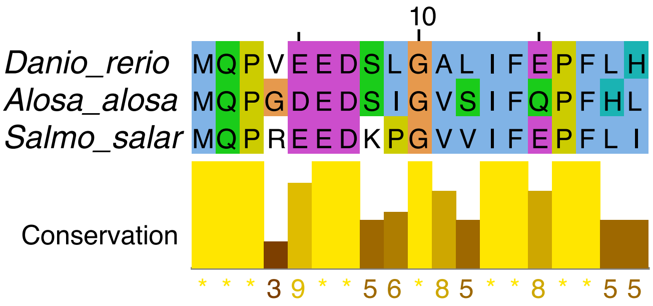

HG0389

ENSDARG00000077361 (bptf)

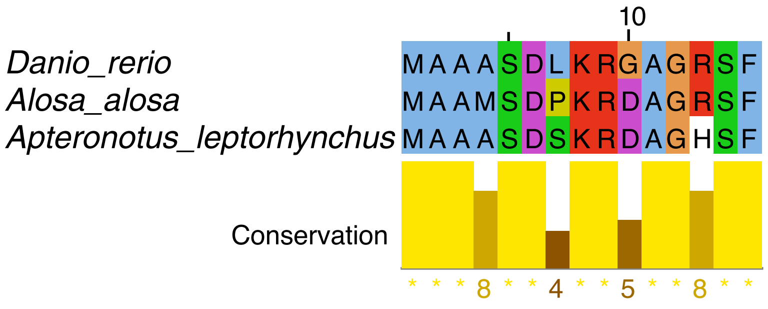

HG0390

ENSDARG00000090035 (rtn4r)

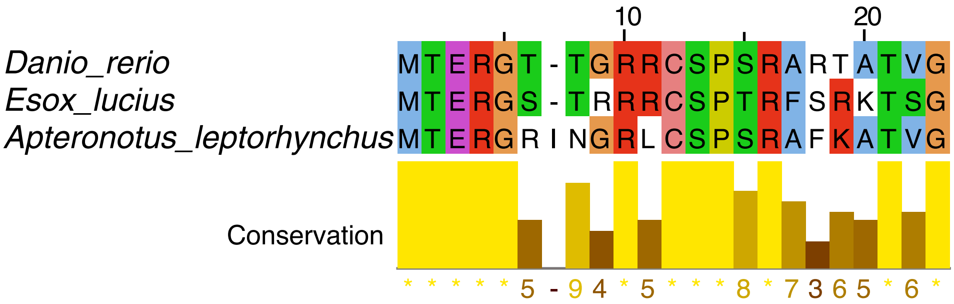

HG0391

ENSDARG00000099634 (elna)

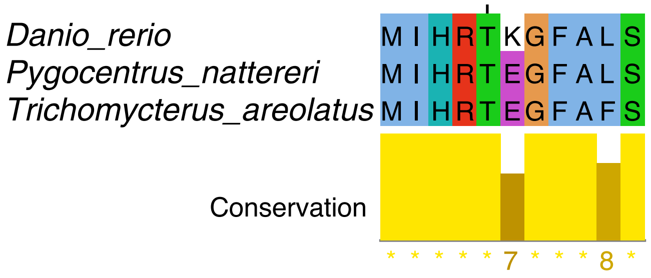

HG0392

ENSDARG00000100003 (glulb)

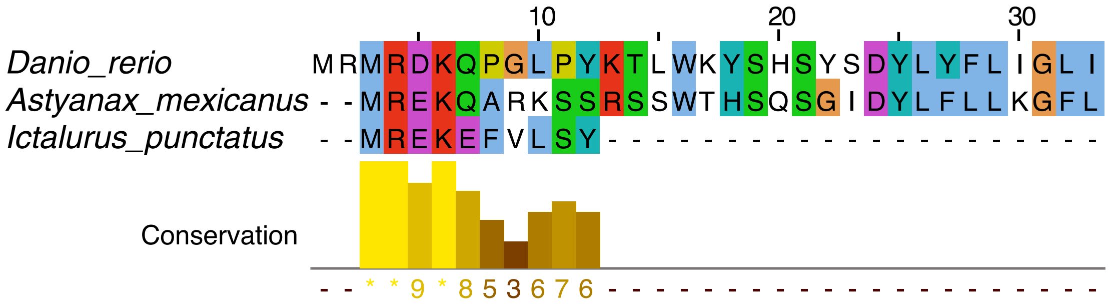

HG0393

ENSGALG00000002090 (GPSM2)

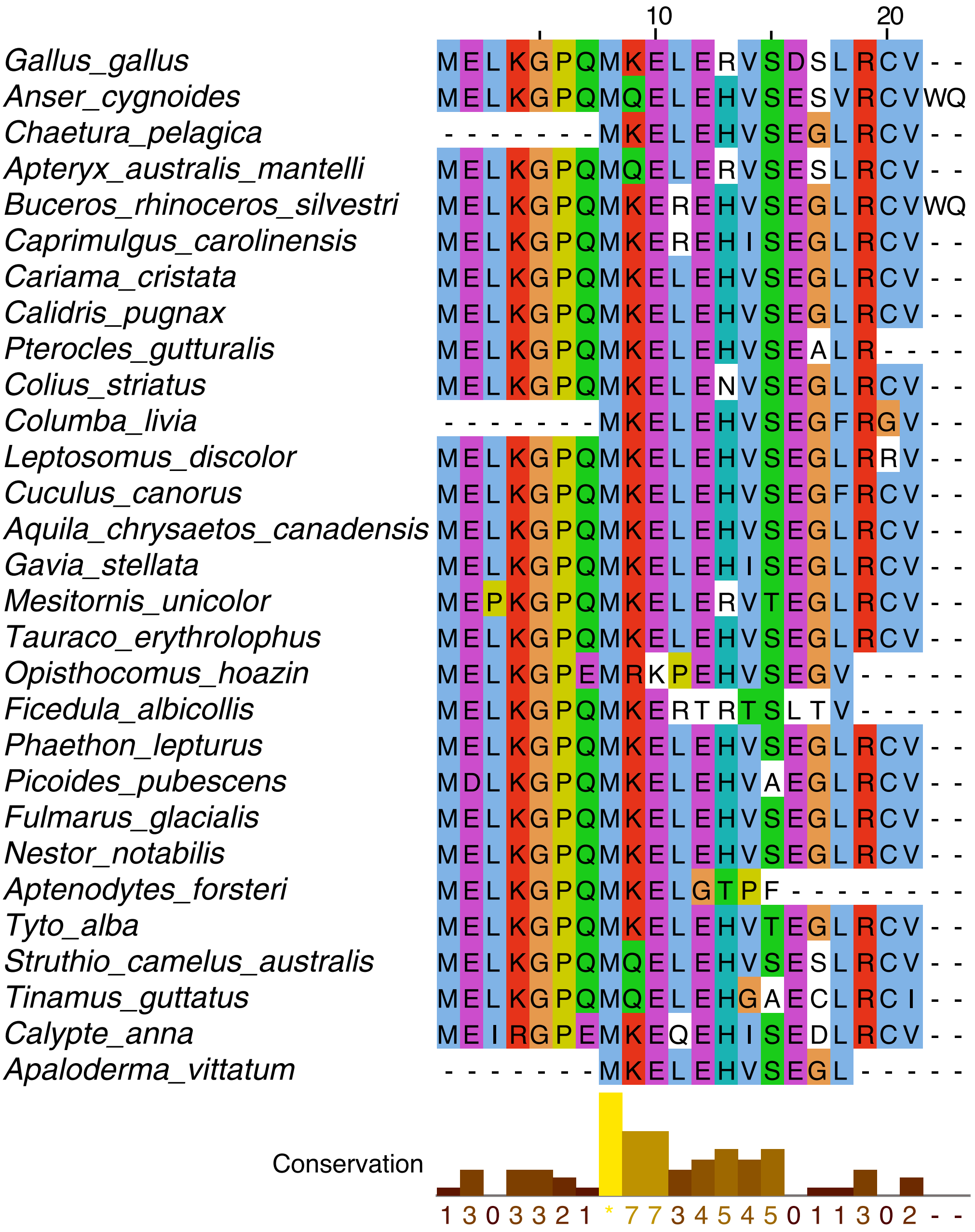

HG0394

ENSGALG00000002479 (MAT1A)

Species	Alignment (positions 10, 20 marked)
Gallus_gallus	MRSPCMHTQIQAHADFTD----
Anser_cygnoides	MHTPCVHAQIQAHAEFTR----
Chaetura_pelagica	MHSPCMCTQMQAHADFTH----
Apteryx_australis_mantelli	MHTPCTHTQIQAHADFTH----
Caprimulgus_carolinensis	MHTPCMRTQMQAHADFTH----
Cariama_cristata	MHAPCMRTQMQAHAEFTH----
Charadrius_vociferus	MHTPCMRTQMQARADFTY----
Pterocles_gutturalis	MHTPCMRTQMQAHADFTH----
Colius_striatus	MHTPCMHAQMQAHAGFTH----
Columba_livia	MHTPCMCTQMQAHADFTH----
Merops_nubicus	---------MQAHAEFTHSNLS
Aquila_chrysaetos_canadensis	MHAPCMHTQMQAHADFTH----
Gavia_stellata	MHTACMRTQMQAHADFTH----
Chlamydotis_macqueenii	MHAPCMRTQMQAHADFTR----
Tauraco_erythrolophus	MHTPCMCTRMQAHADFTH----
Manacus_vitellinus	MRSPCVHAQMQAYAAFTH----
Egretta_garzetta	MHTPCMCTQMQAHADFTH----
Fulmarus_glacialis	MHTPCMRTQMQAHADFTH----
Nestor_notabilis	MHTPCMCTQMQAHAVFTH----
Pygoscelis_adeliae	MHTPCMRTQMQAHADFTH----
Tyto_alba	MHTPCMRTQMQAHADFTH----
Struthio_camelus_australis	MHAPCTHT--------------
Tinamus_guttatus	MHTACTHSQLQARADFTH----
Calypte_anna	MHAPCMCTQMKAHADFIH----
Apaloderma_vittatum	MHTPCVCTQMQTHADFTH----

Conservation

1 3 1 1 2 1 0 1 1 1 1 2 1 3 0 2 1 1 - - - -

HG0395

ENSGALG00000014936 (ESCO1)

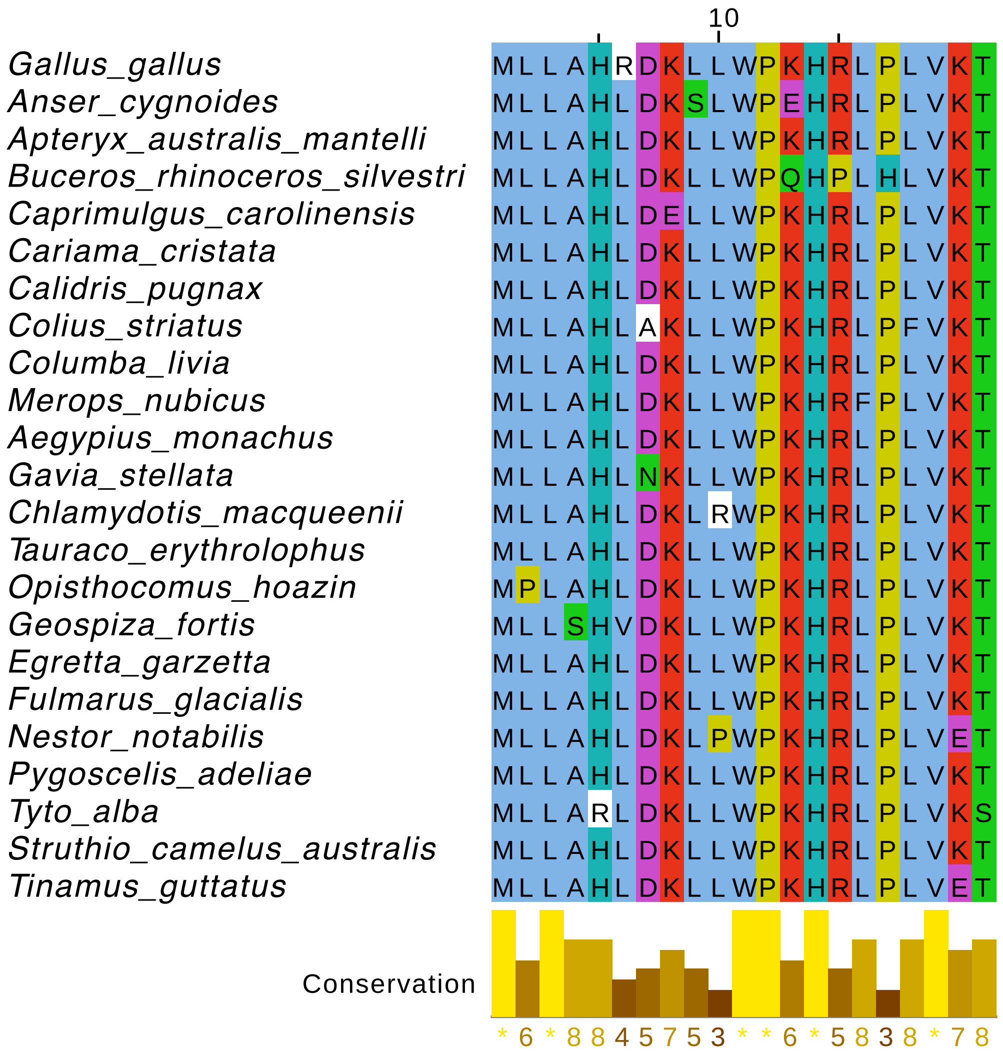

HG0396.1

ENSGALG00000034253

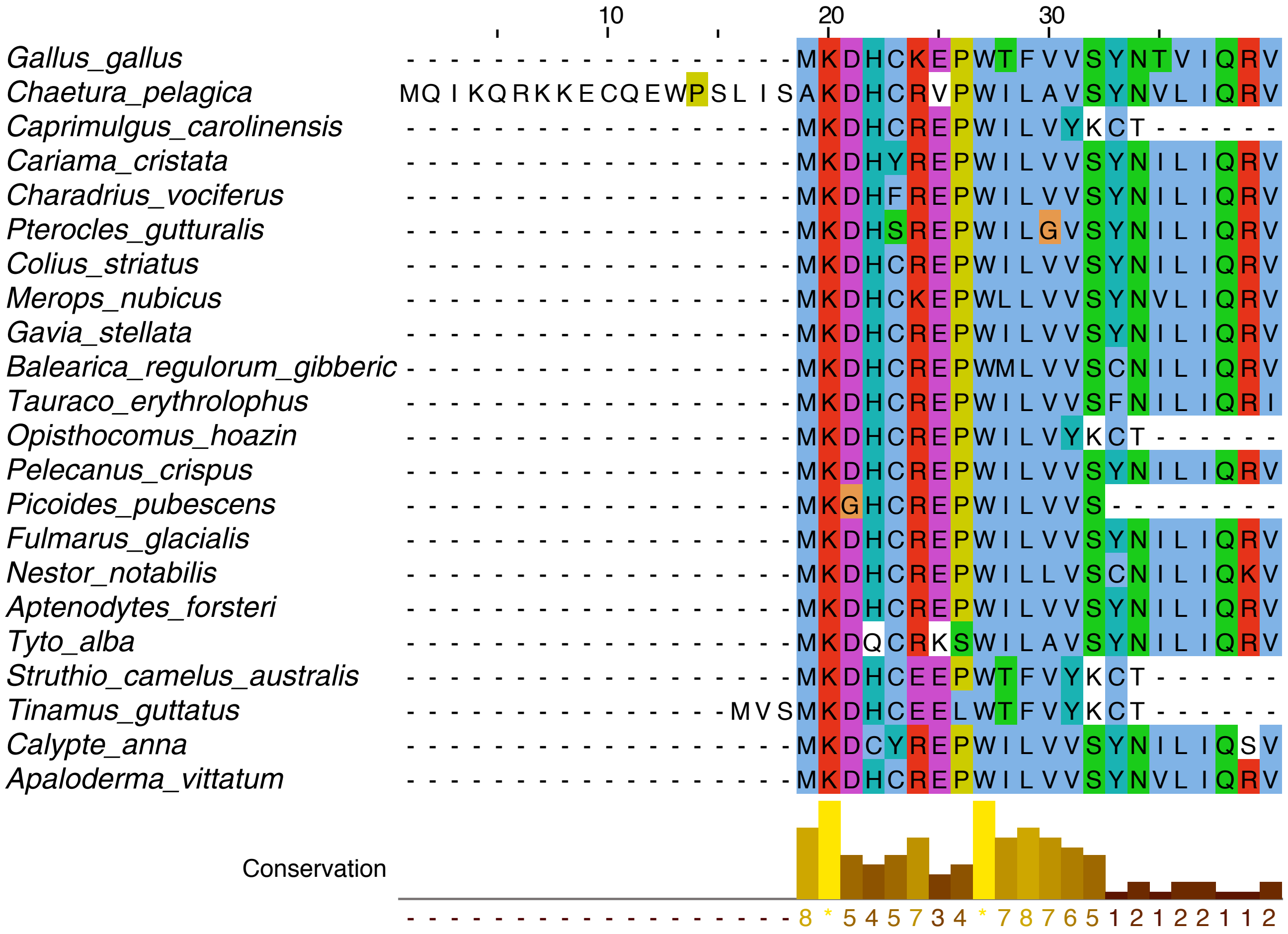

HG0396.2

ENSGALG00000034253

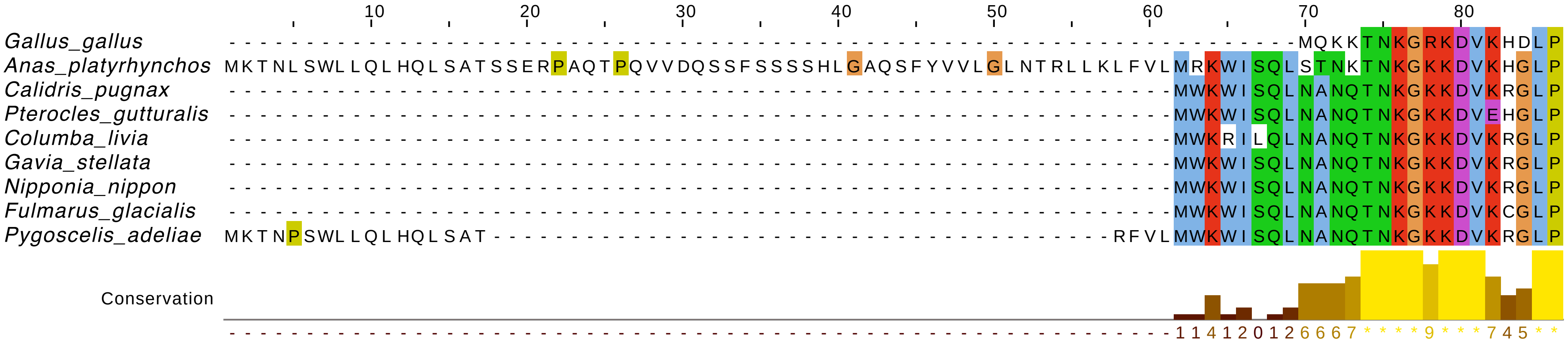

HG0397

ENSGALG00000009438 (SLC39A9)

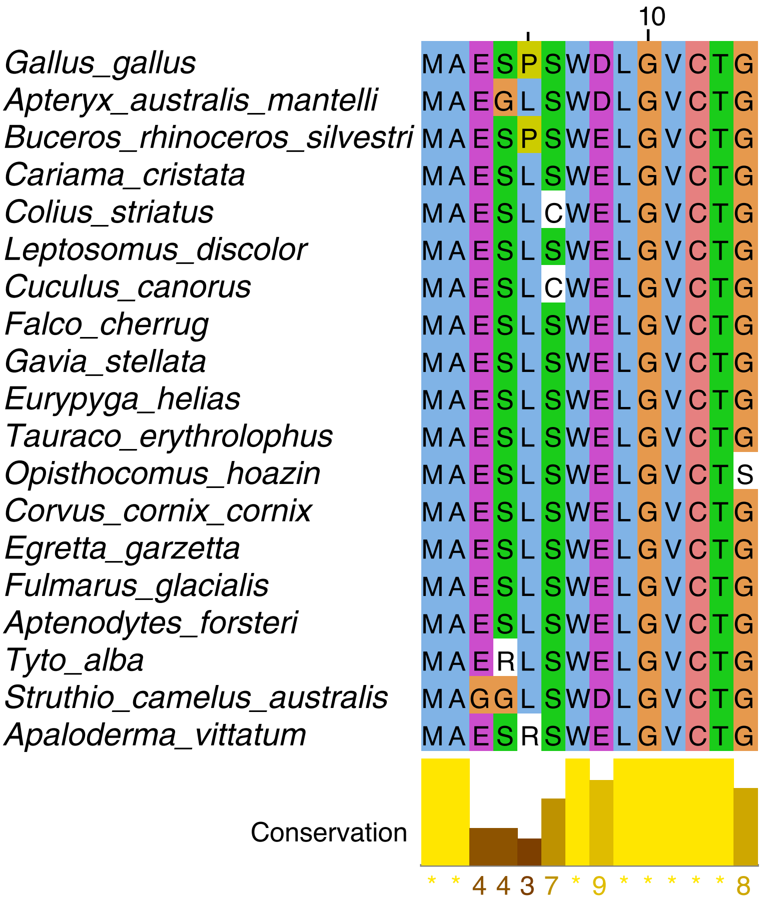

HG0398

ENSGALG00000005369 (BTBD10)

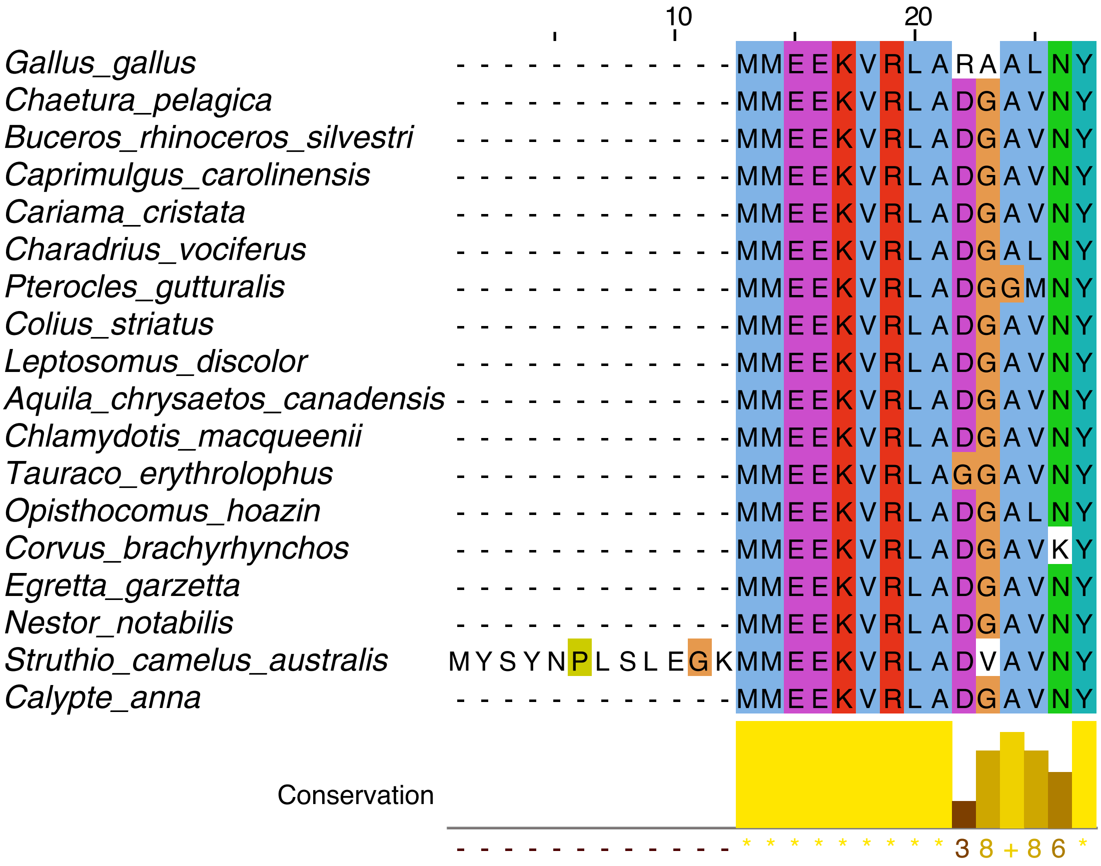

HG0399

ENSGALG00000032659

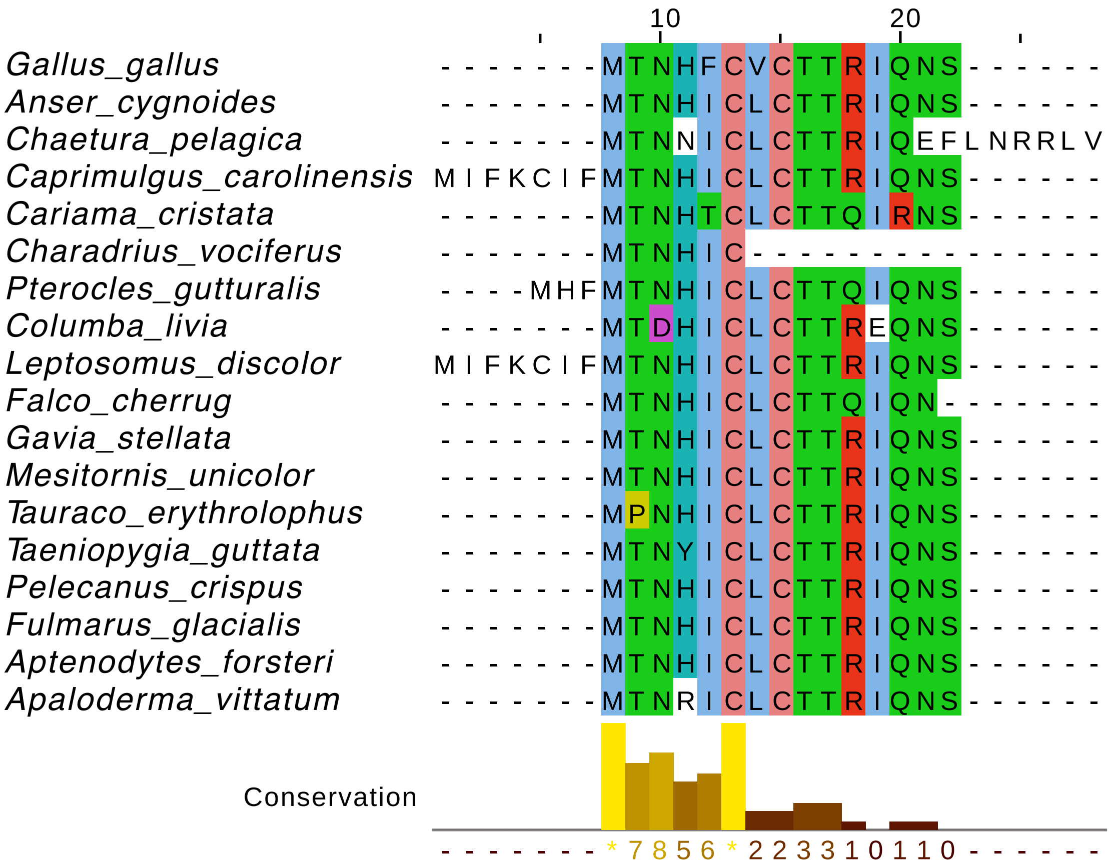

HG0400

ENSGALG00000004261 (AQP9)

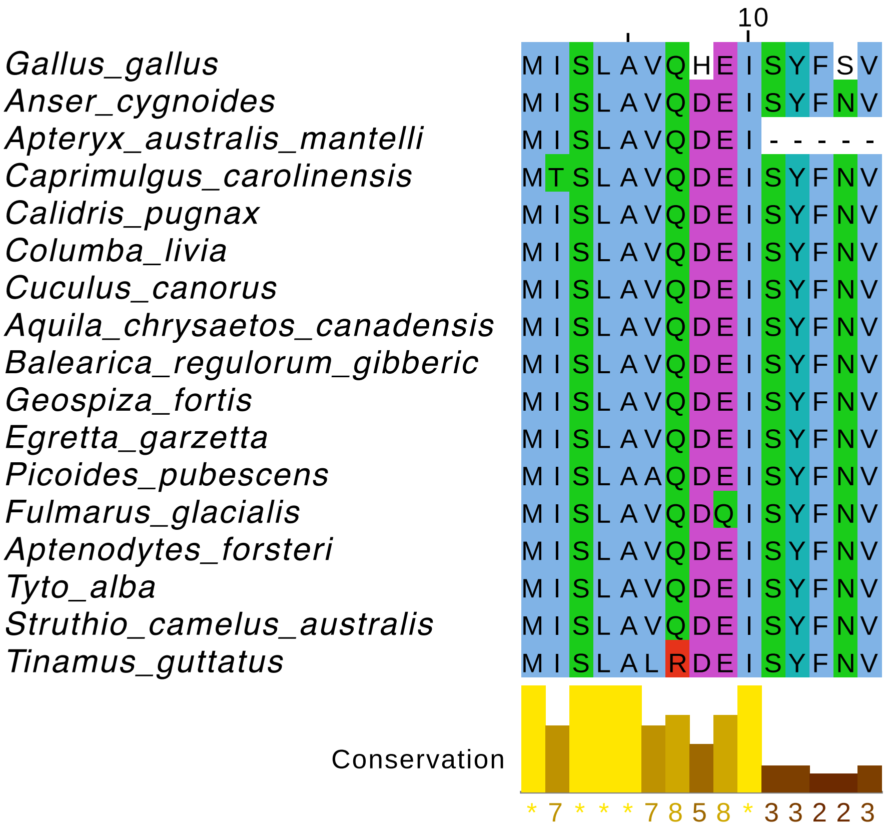

HG0401

ENSGALG00000009057 (TASP1)

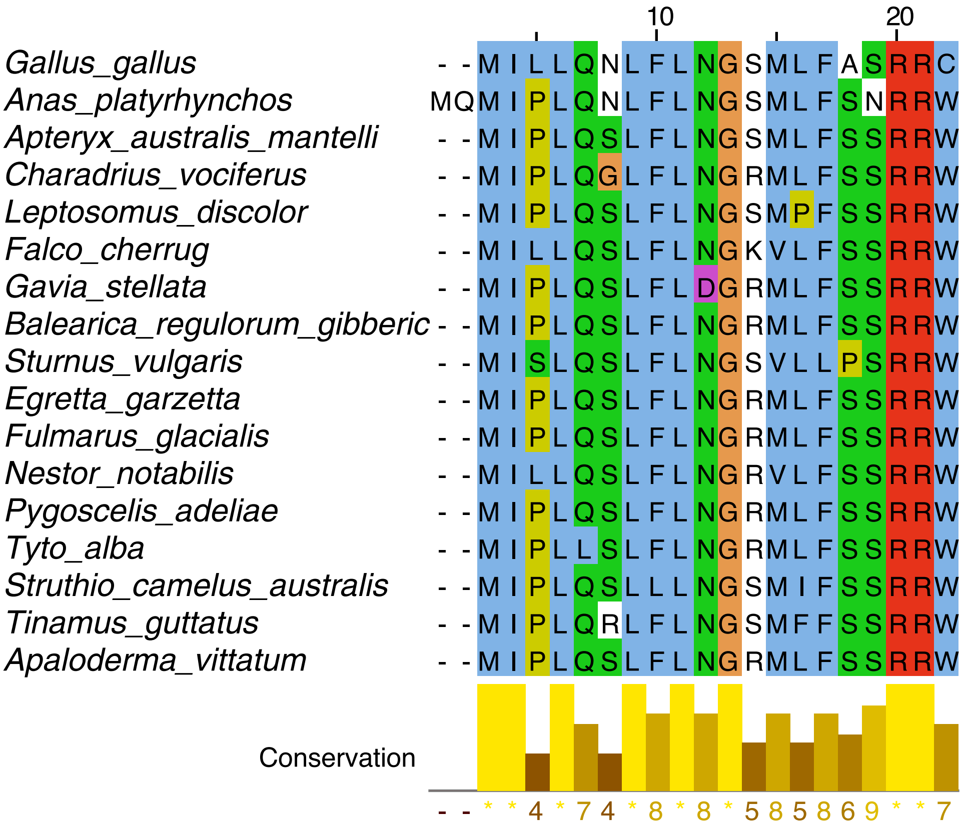

HG0402

ENSGALG00000035584

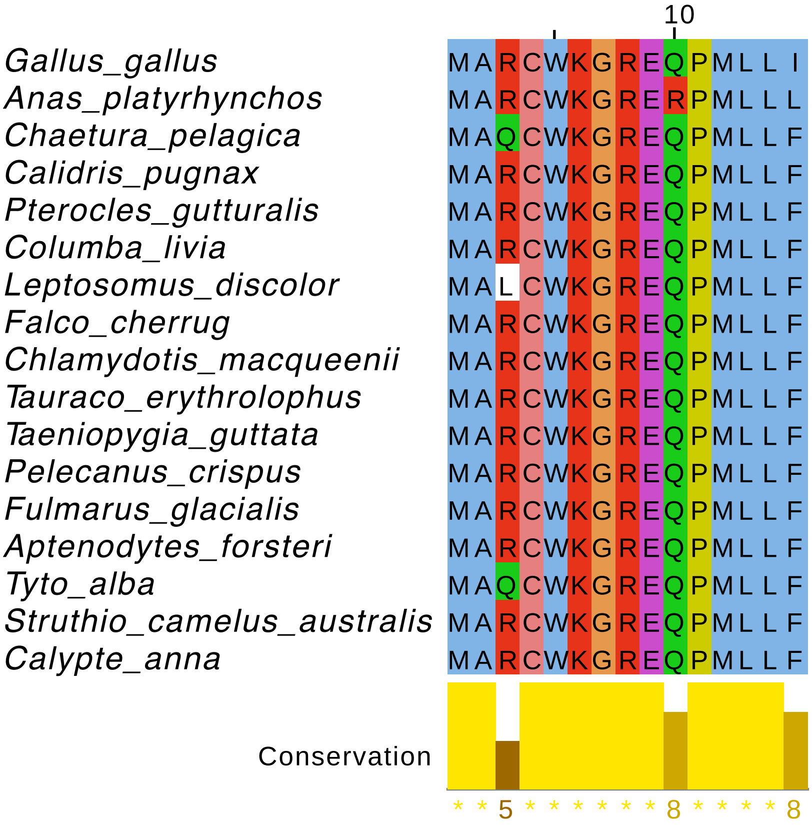

HG0403.1

ENSGALG00000007717 (GCNT7)

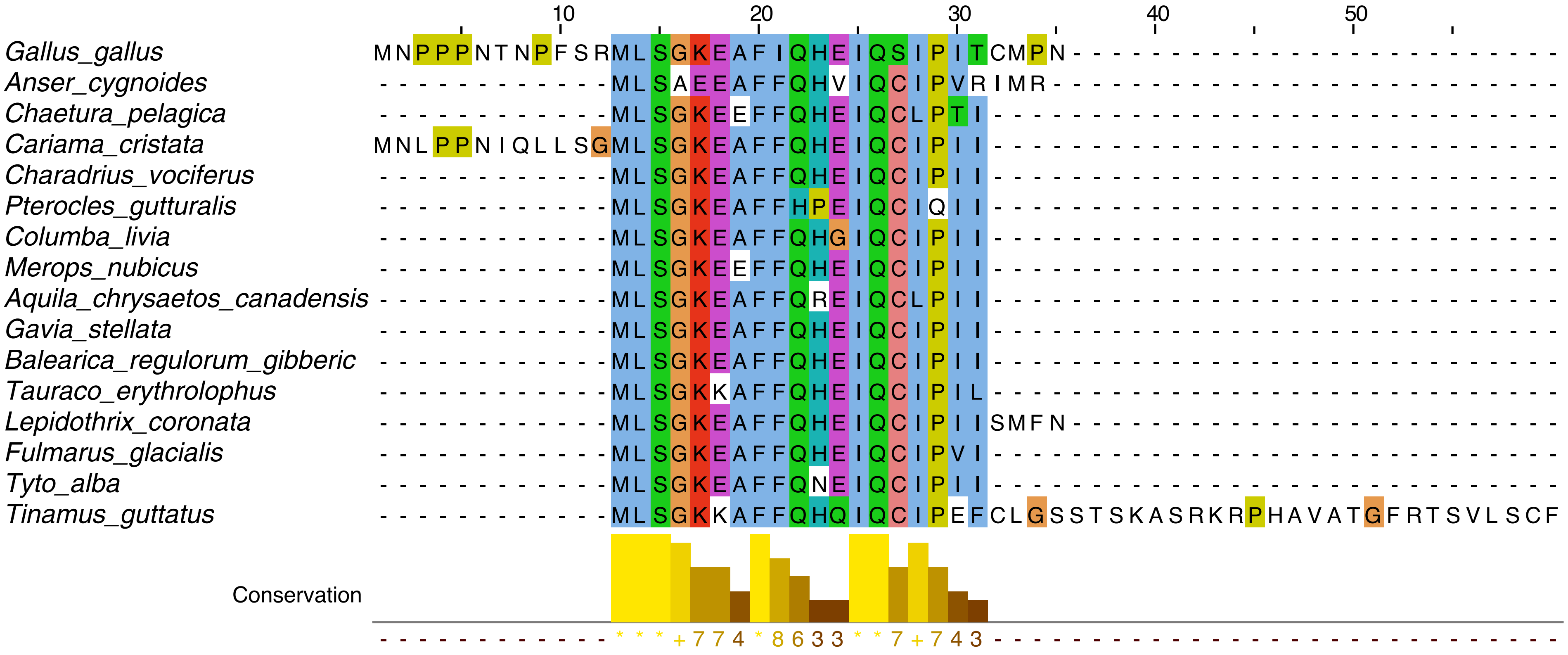

HG0403.2

ENSGALG00000007717 (GCNT7)

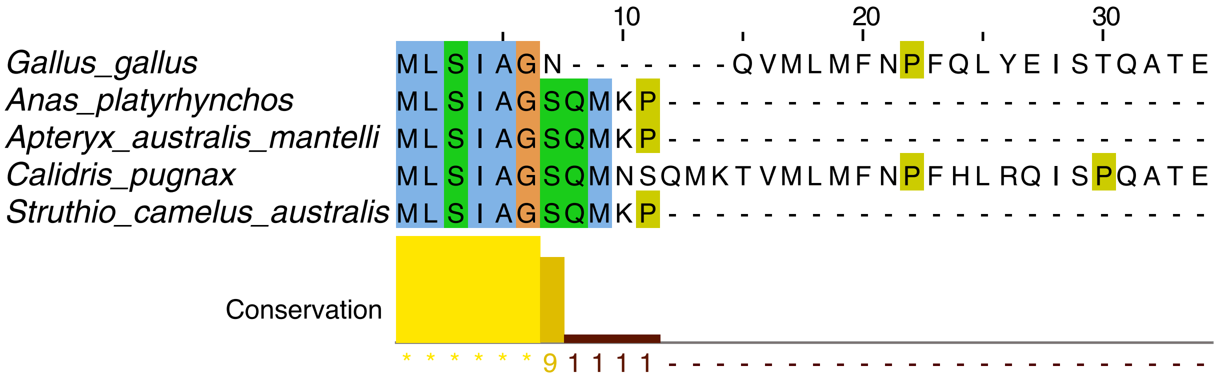

HG0404

ENSGALG00000008700 (PDCD4)

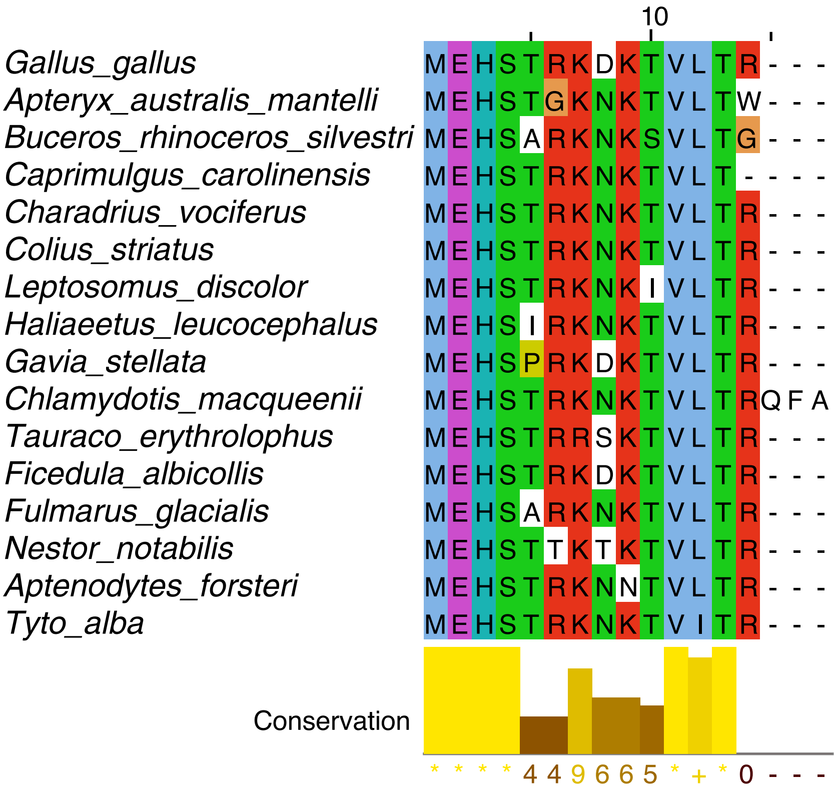

HG0405.1

ENSGALG00000009185

```
                                      10        20        30        40        50        60        70        80
Gallus_gallus                 ------------------------------------------------------------------------MLLSSLWAFLDLRFFS
Anser_cygnoides               ------------MW------NTLL-AYLRVRE--AVQEVLCRLYKYSVRQ-----AVISLSLLQISAACLGHMLLSRLRAFLDLRFFS
Cariama_cristata              ------------------------------------------------------------------------MLLISLRAFLDLRFFS
Charadrius_vociferus          MKERLARVGDLKSFSPSPLTHSQTEELHRHQEITLHQDARSR-YFLPCTQQVTSFEILFSPLKQISAACLGYMLLISLRAFLDLRFFS
Pterocles_gutturalis          ------------------------------------------------------------------------MLLSSLQAFLDLRCFS
Colius_striatus               ------------------------------------------------------------------------MLLIGLRAFLDLRFFS
Leptosomus_discolor           ------------------------------------------------------------------------MLLISLRAFLDLRFFS
Aquila_chrysaetos_canadensis  ----------------------------------------------------------MDNHGKISTACLGYMLLISLRAFLDLRFFS
Gavia_stellata                ----------------------------------------------------------------MSTACLGYMLLISLRAFLDLRFFS
Chlamydotis_macqueenii        ------------------------------------------------------------------------MLLISLQAFLDLGFFS
Opisthocomus_hoazin           ----------------------------------------------------------------MSTACLGCMLLISLRAFLDLRFSS
Acanthisitta_chloris          ------------------------------------------------------------------------MLLISLLASLDLRFFS
Phalacrocorax_carbo           ----------------------------------------------------------------MSTACLGYMLLISLQAFLDLRFSS
Struthio_camelus_australis    ------------------------------------------------------------------------MLLSSLRAFLDLRFFS

Conservation                  ------------------------------------------------------------------------***54*4*5***485*
```

HG0405.2

ENSGALG00000009185

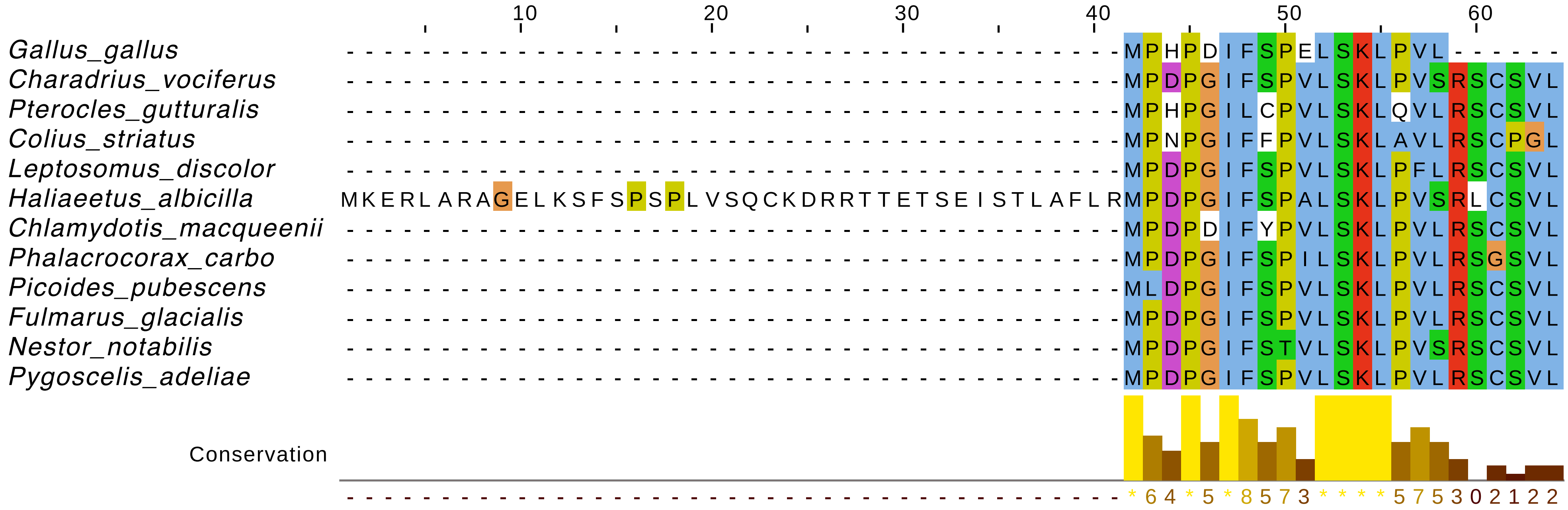

HG0406

ENSGALG00000004935 (LSM14A)

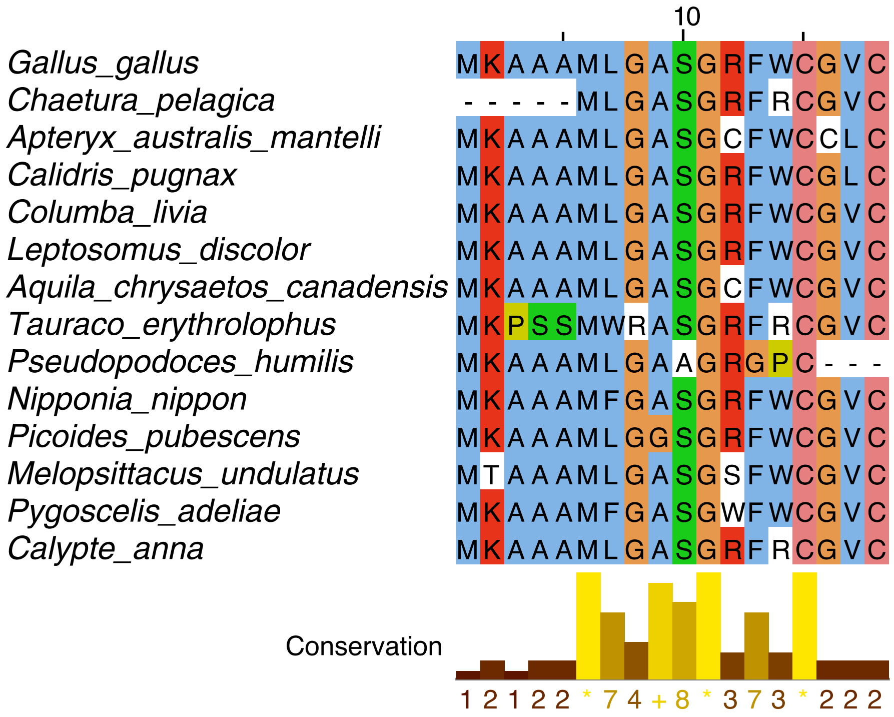

HG0407

ENSGALG00000015027 (JAK2)

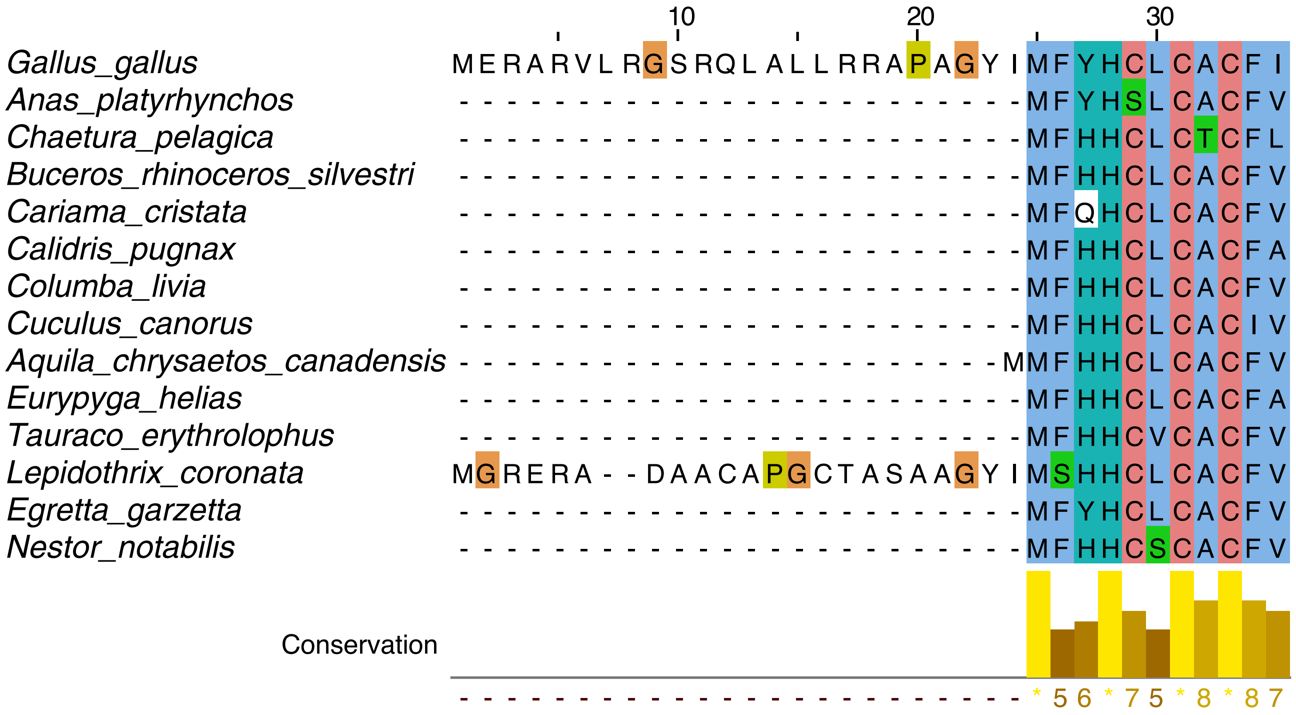

HG0408

ENSGALG00000036255 (WNT7B)

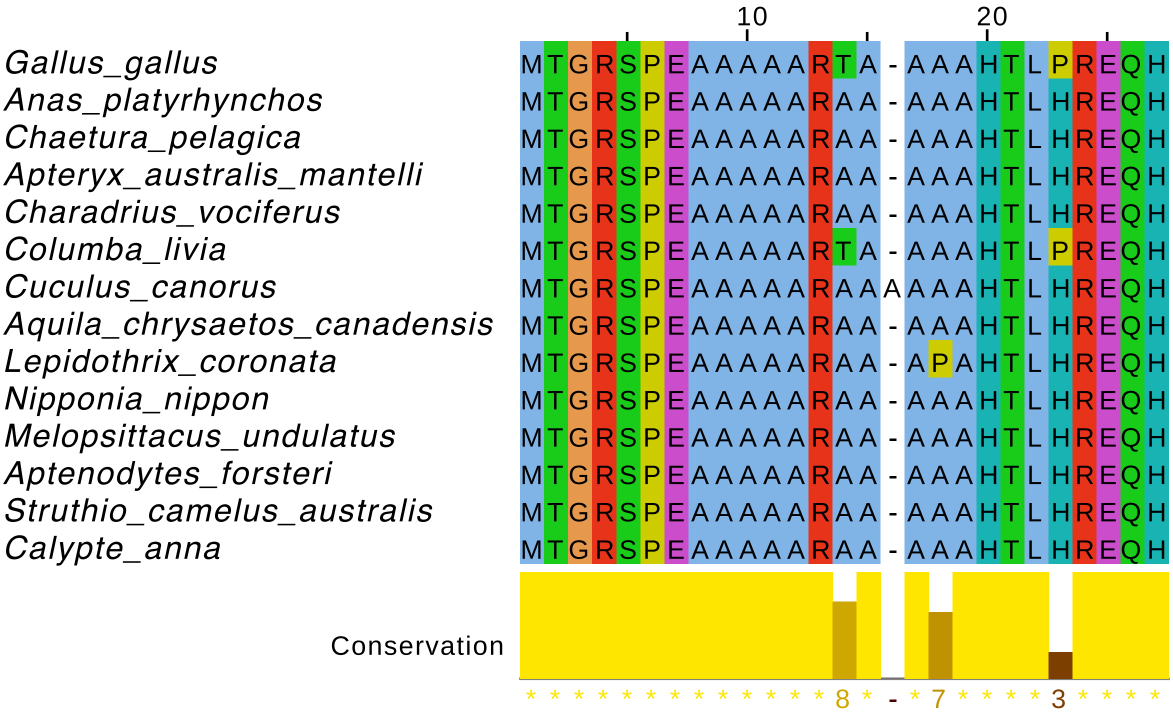

HG0409

ENSGALG00000002315

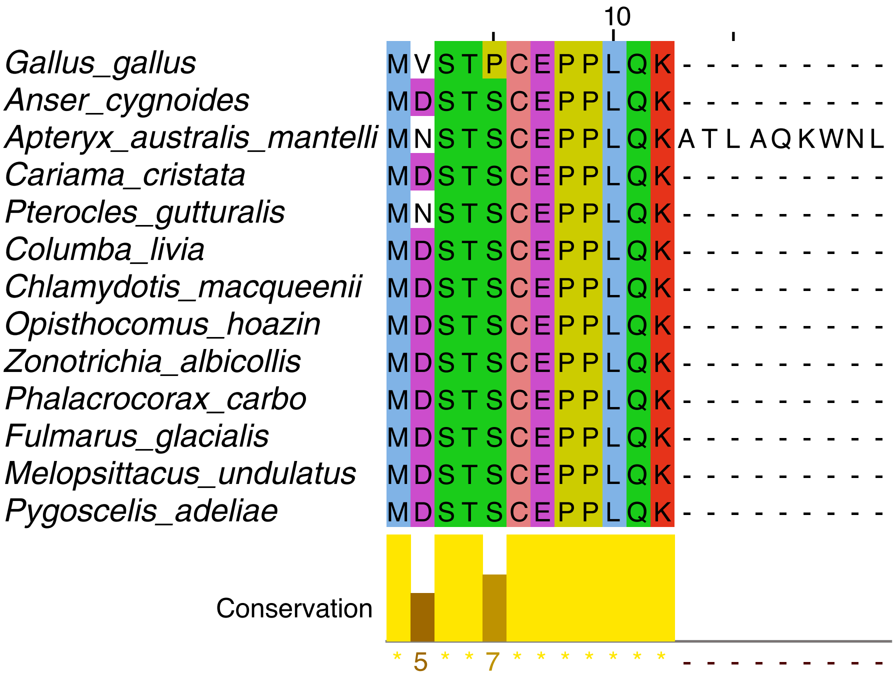

HG0410

ENSGALG00000008148 (MYBPC3)

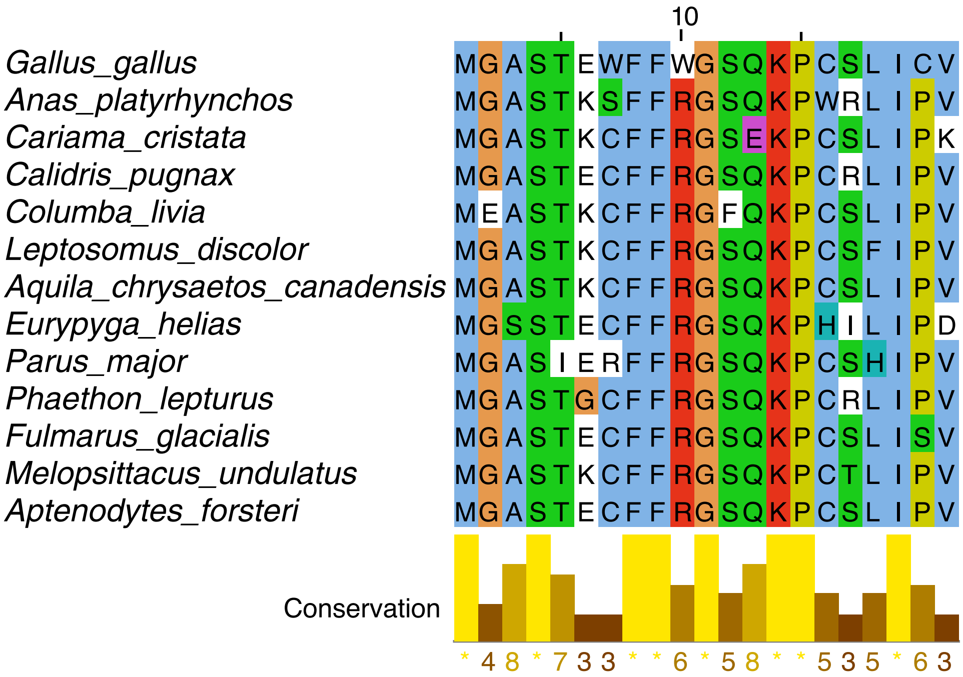

HG0411

ENSGALG00000012166 (SLC35F5)

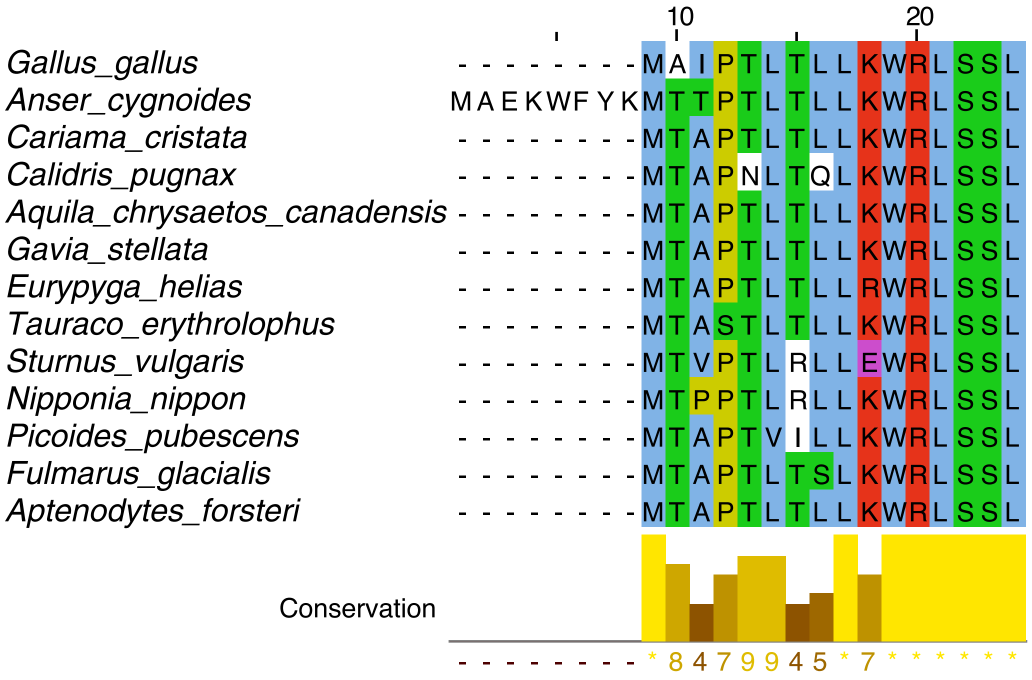

HG0412

ENSGALG00000003446 (PRLR)

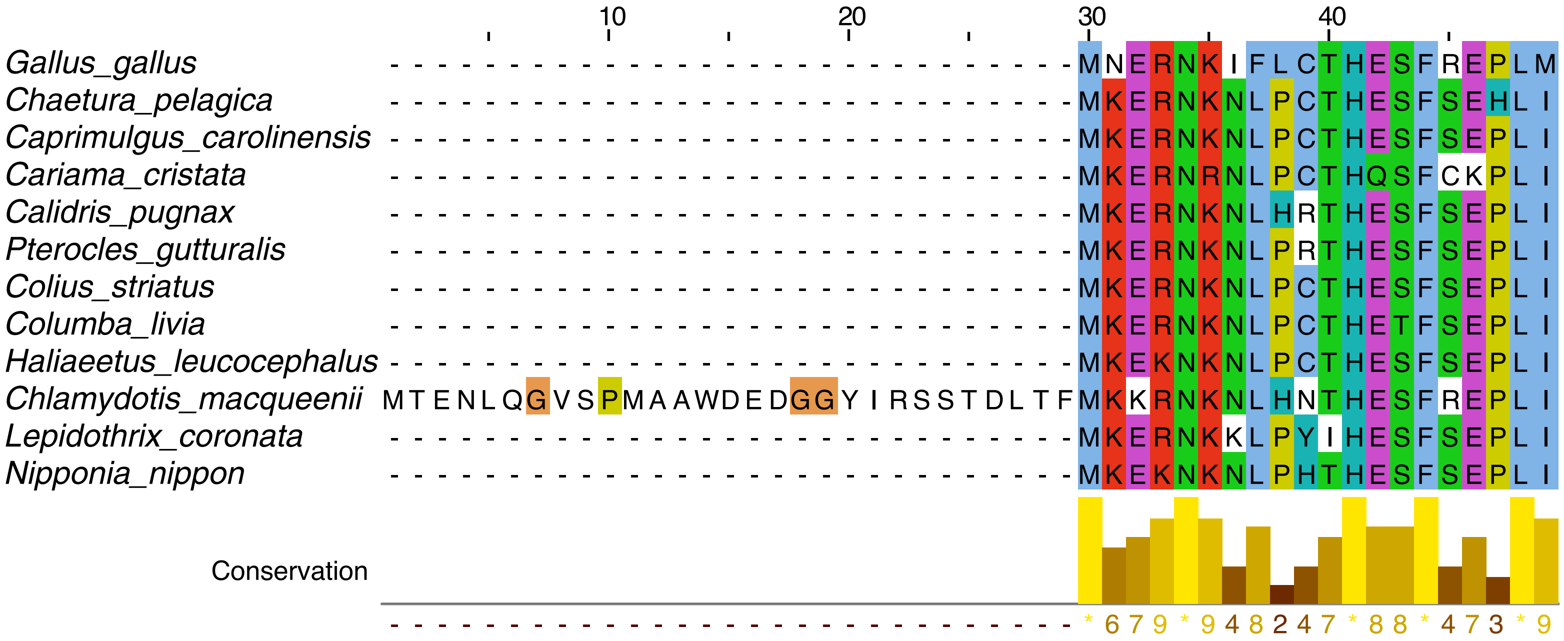

HG0413

ENSGALG00000005475

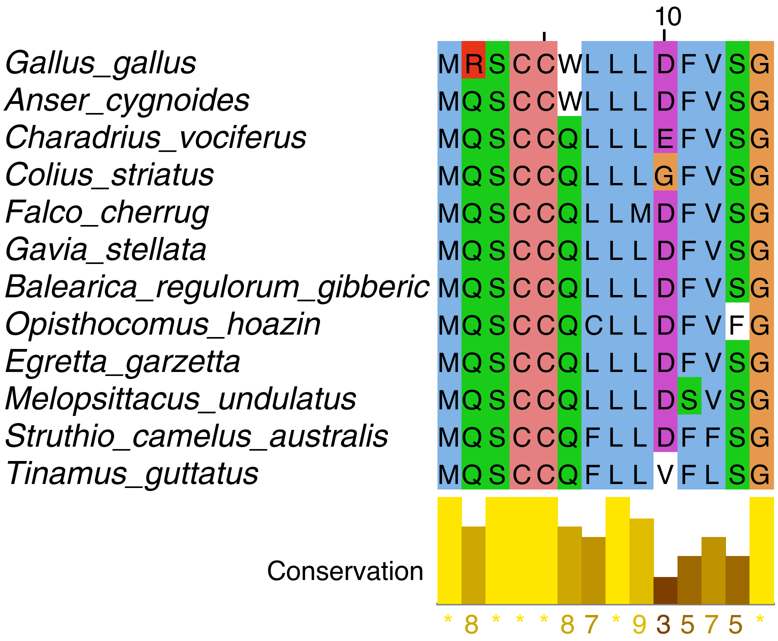

HG0414

ENSGALG00000032599

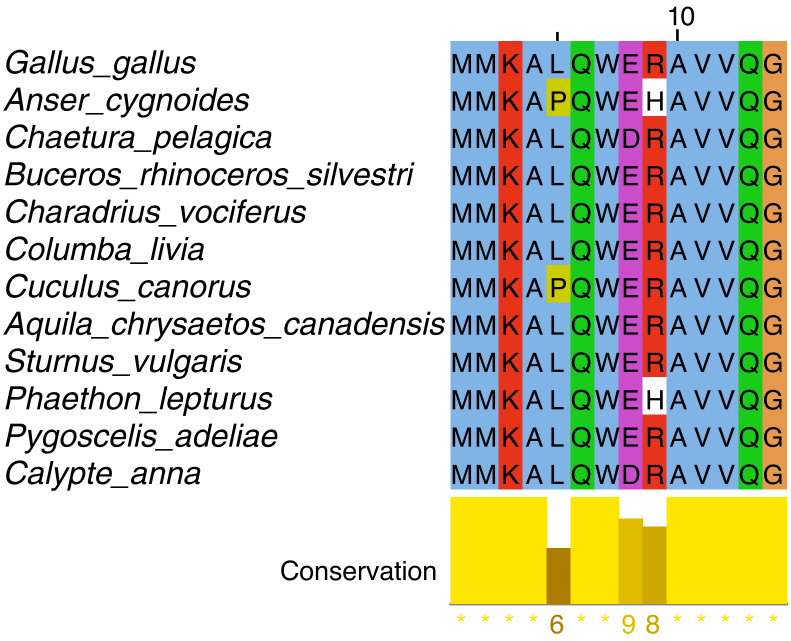

HG0415

ENSGALG00000034970

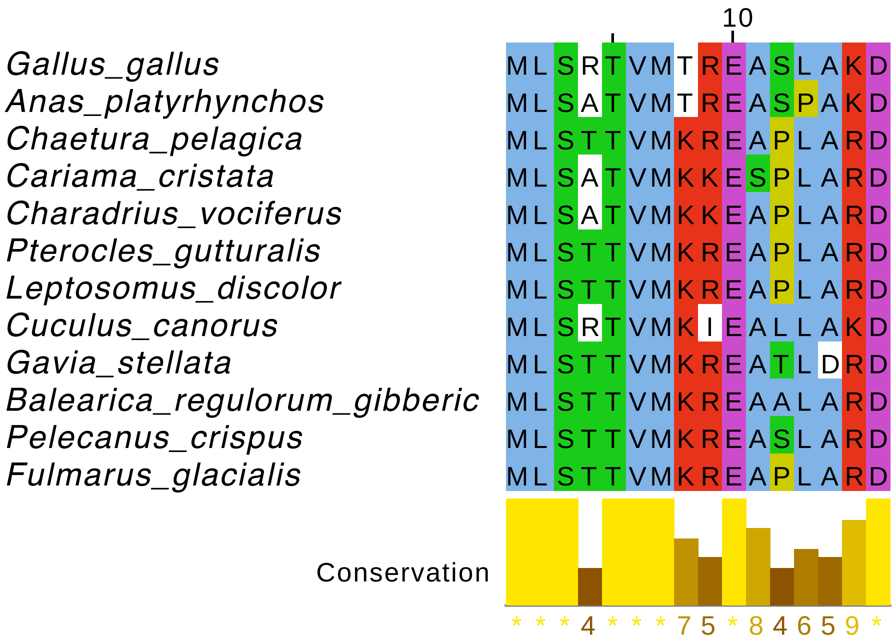

HG0416

ENSGALG00000015874 (TTK)

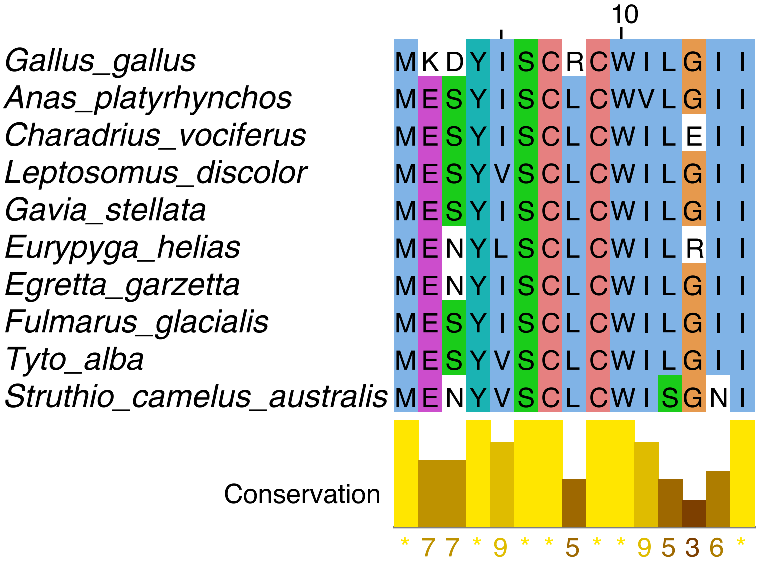

HG0417

ENSGALG00000001522 (PHF20)

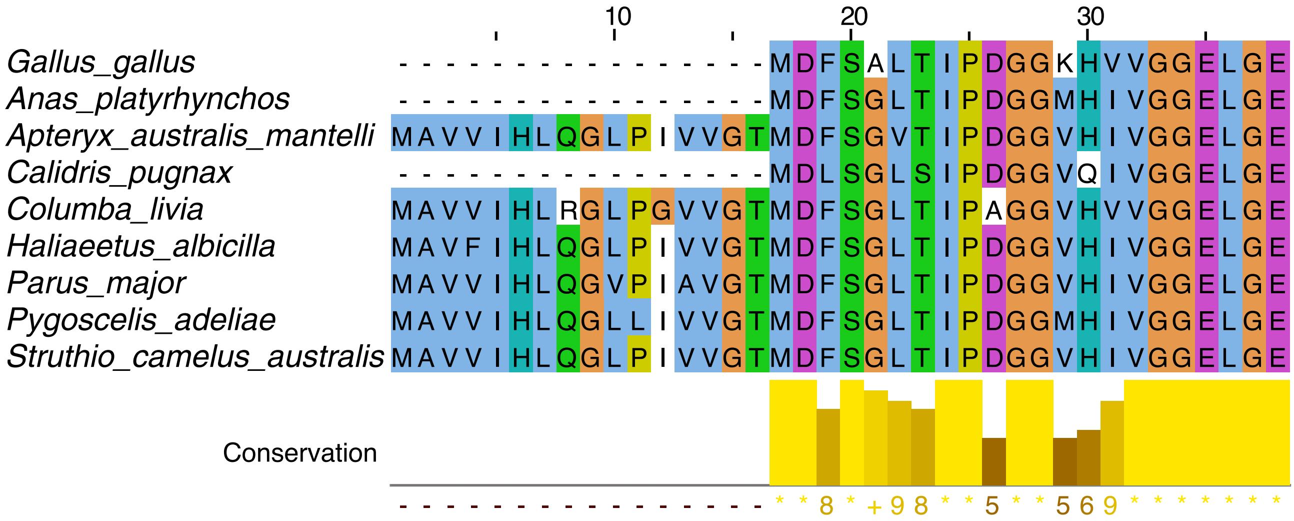

HG0418

ENSGALG00000010337 (GPR149)

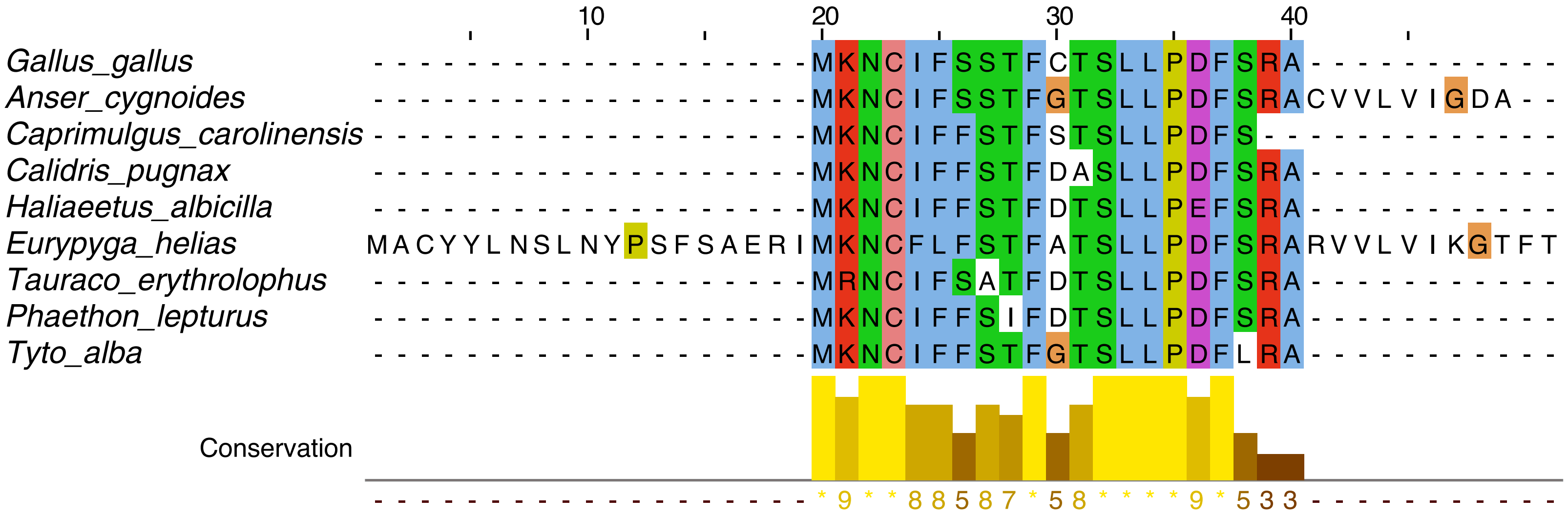

HG0419

ENSGALG00000012542 (RASD2)

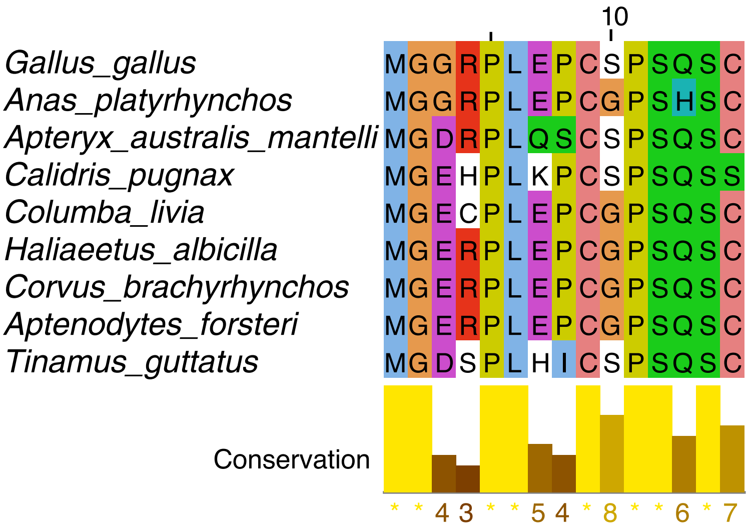

HG0420

ENSGALG00000000336 (PPP1R12B)

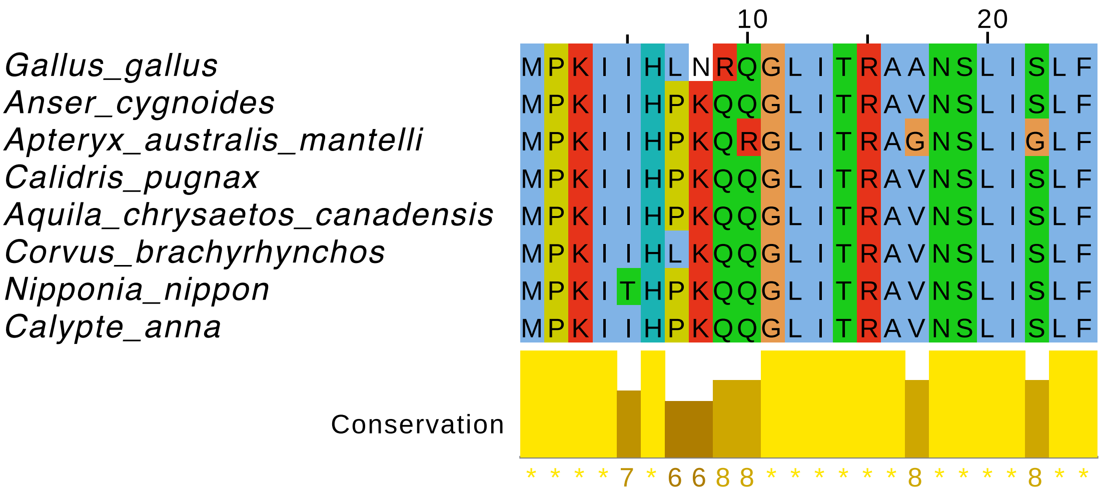

HG0421

ENSGALG00000003492

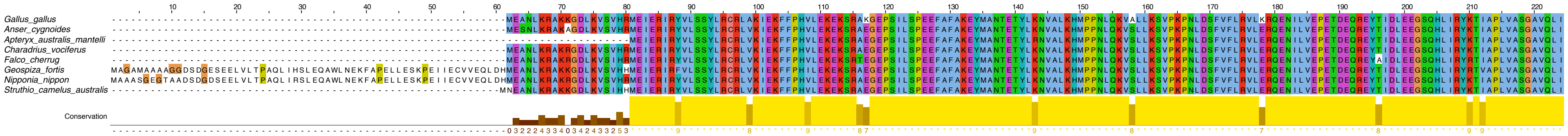

HG0422

ENSGALG00000005772 (BOK)

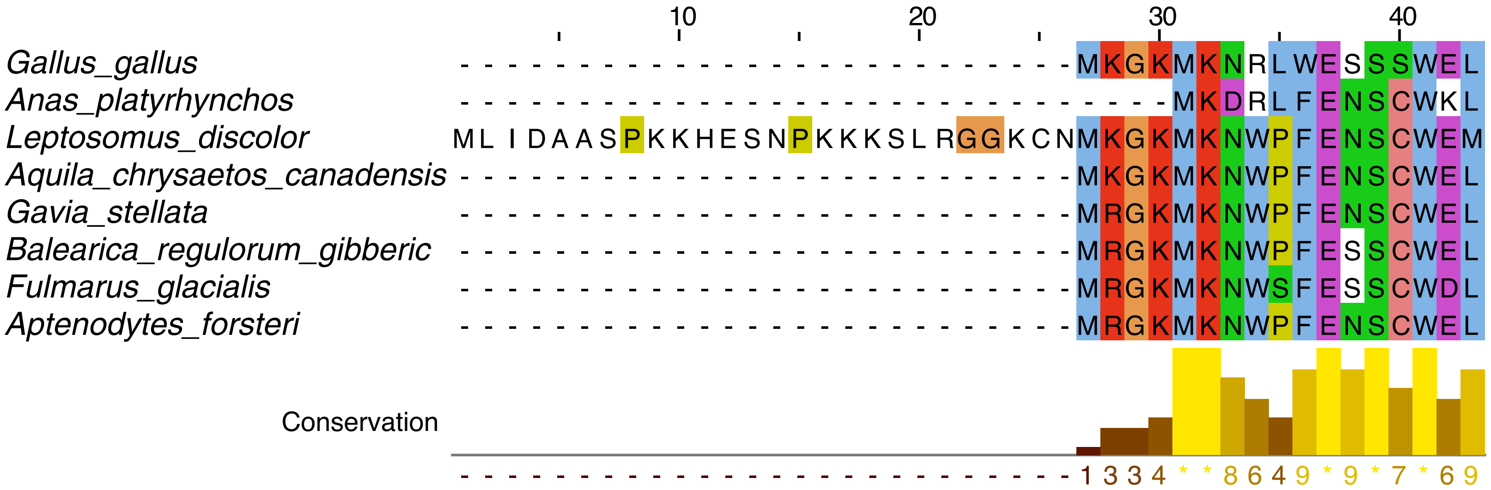

HG0423

ENSGALG00000006329

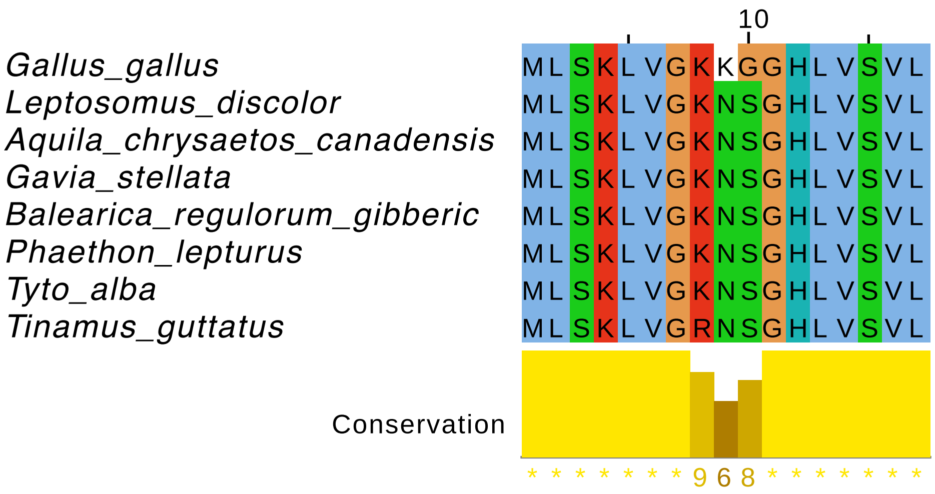

HG0424

ENSGALG00000039553

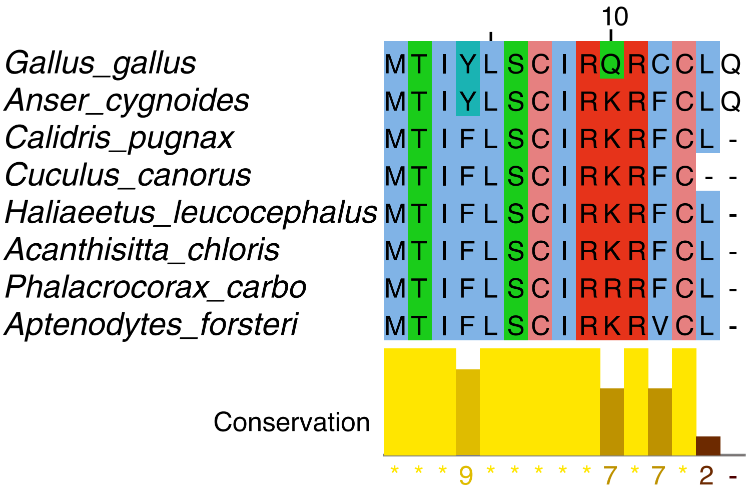

HG0425

ENSGALG00000043044

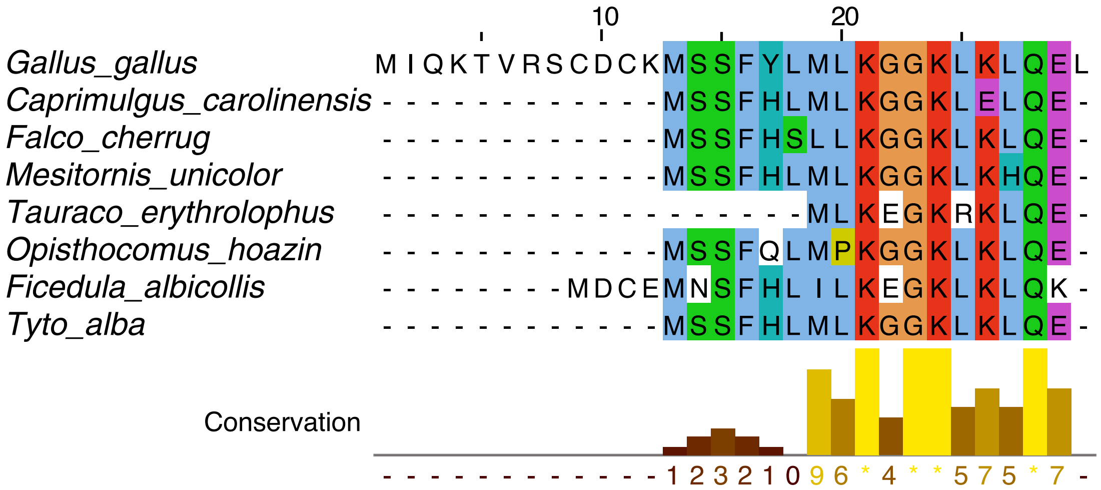

HG0426

ENSGALG00000002192 (PTPRC)

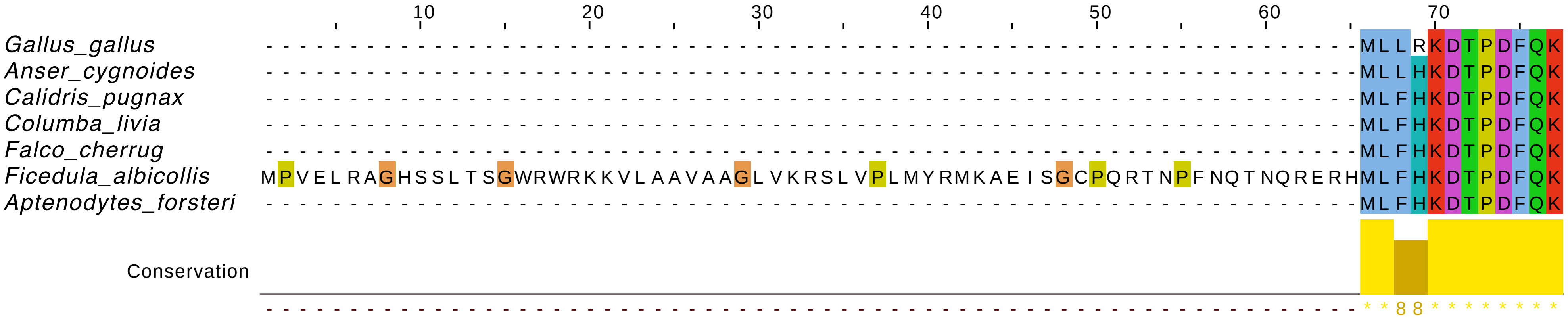

HG0427

ENSGALG00000005550 (ST6GAL1)

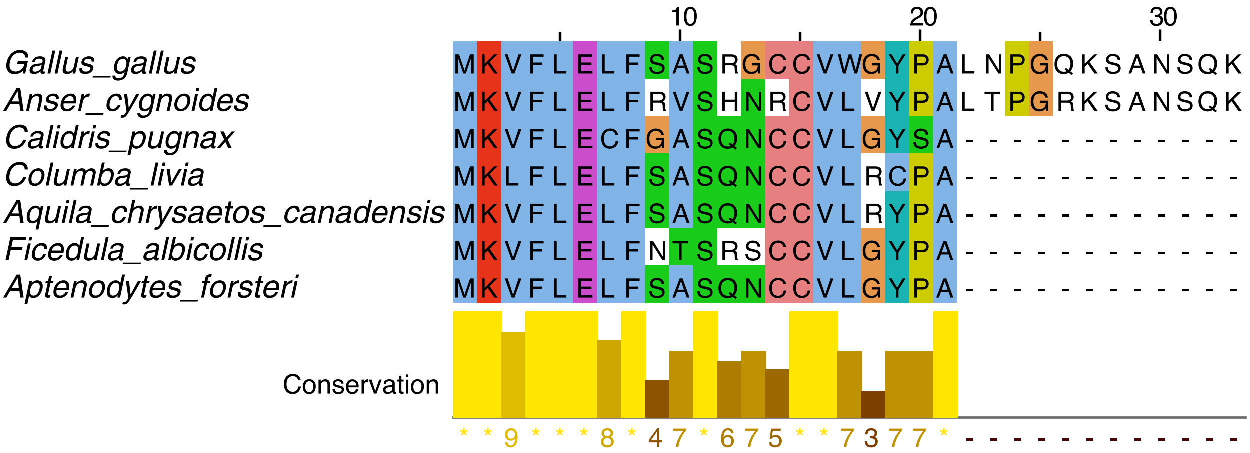

HG0428

ENSGALG00000005976 (TCEA2)

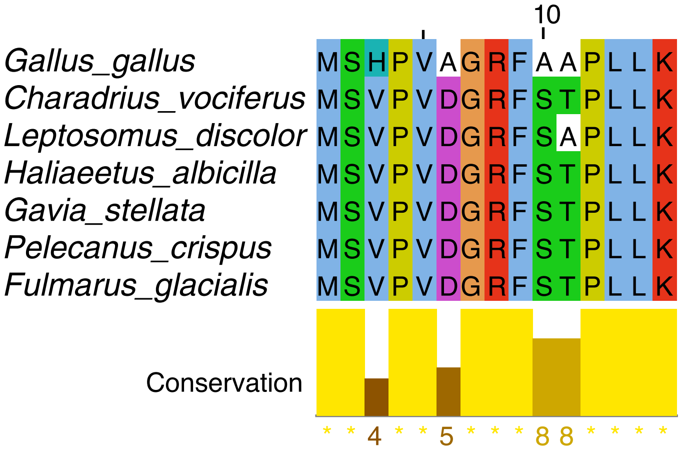

HG0429

ENSGALG00000006626 (PDE6C)

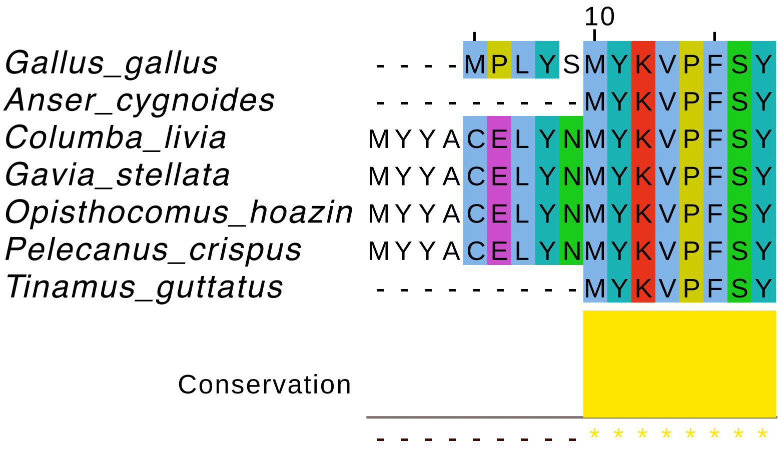

HG0430

ENSGALG00000015490

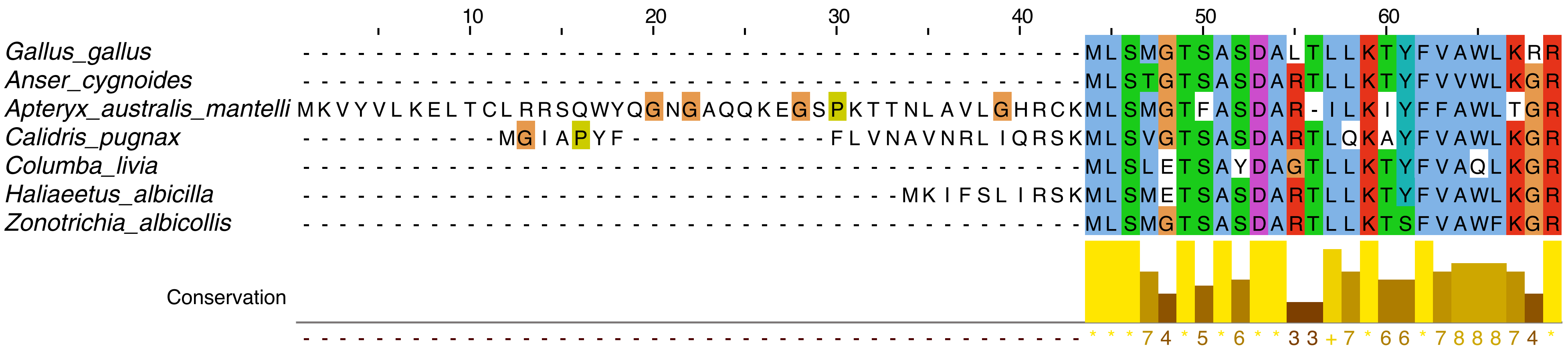

HG0431

ENSGALG00000026542 (FAM217B)

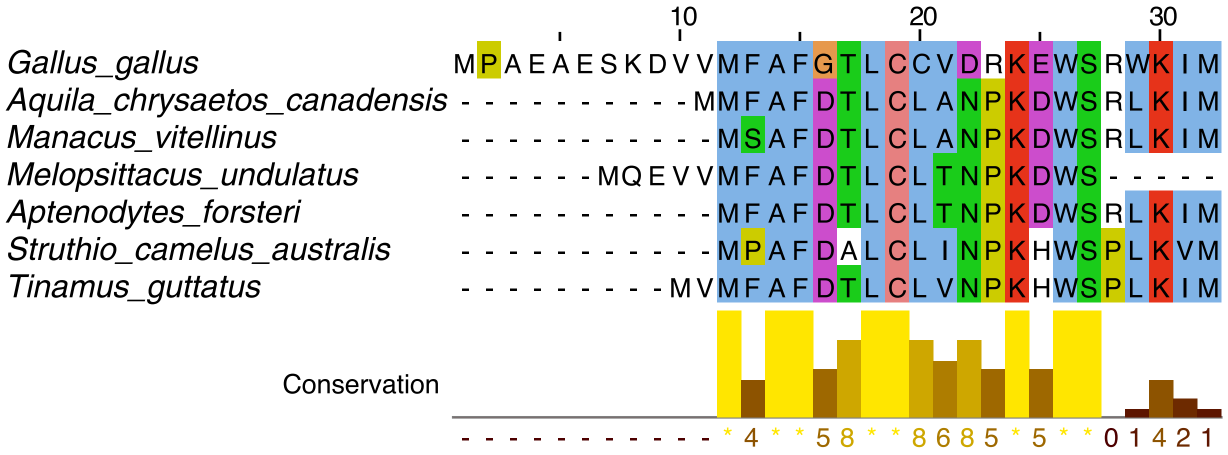

HG0432

ENSGALG00000040546

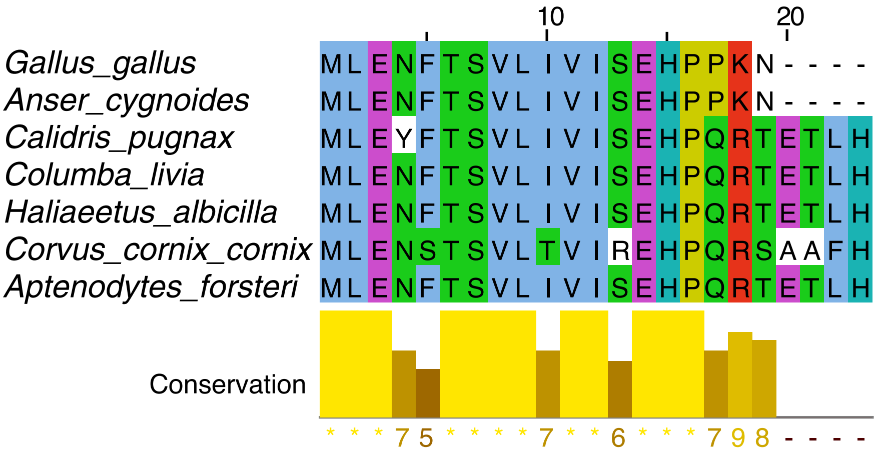

HG0433

ENSGALG00000002952 (NRG4)

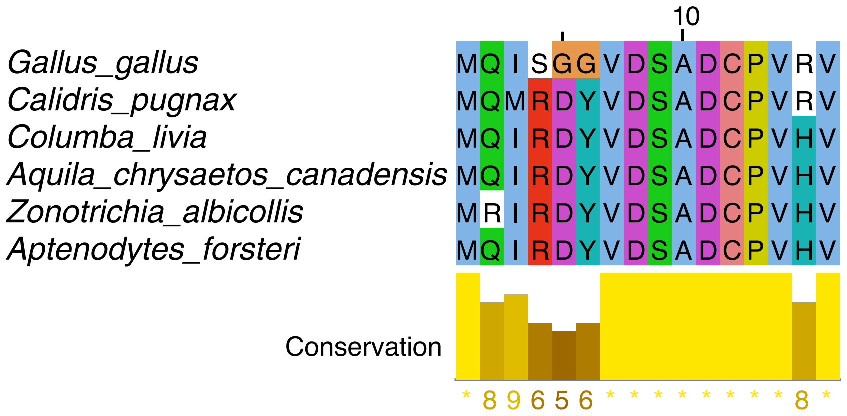

HG0434

ENSGALG00000008357 (TPCN1)

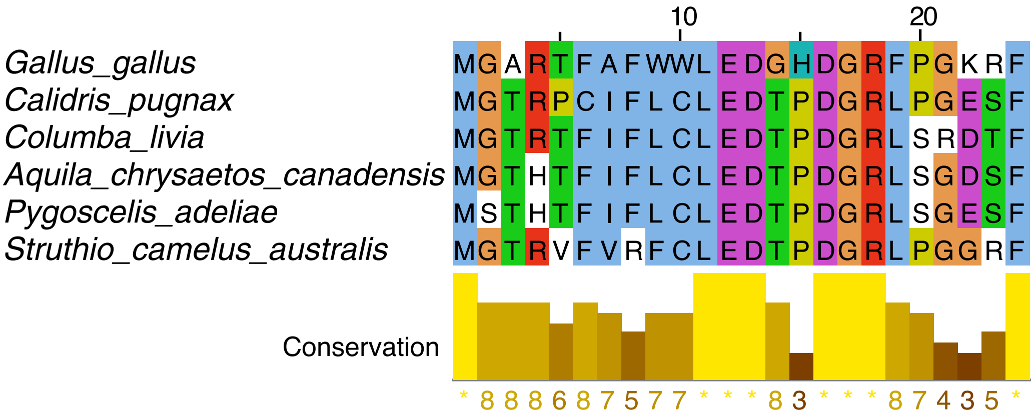

HG0435

ENSGALG00000010778 (CHRM3)

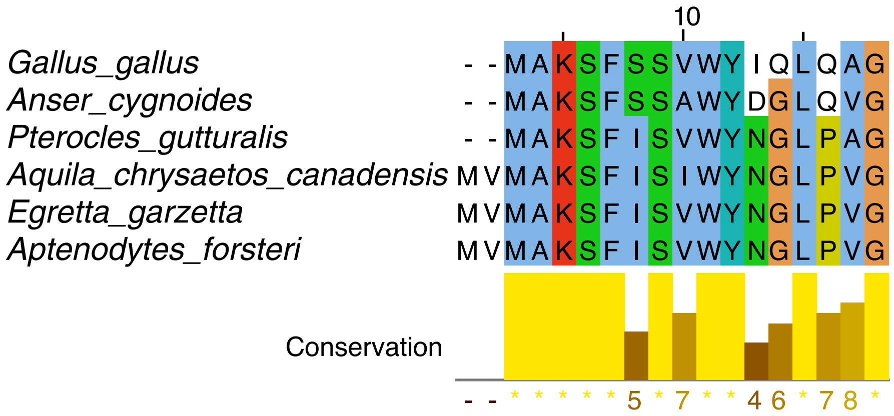

HG0436

ENSGALG00000026736 (OGN)

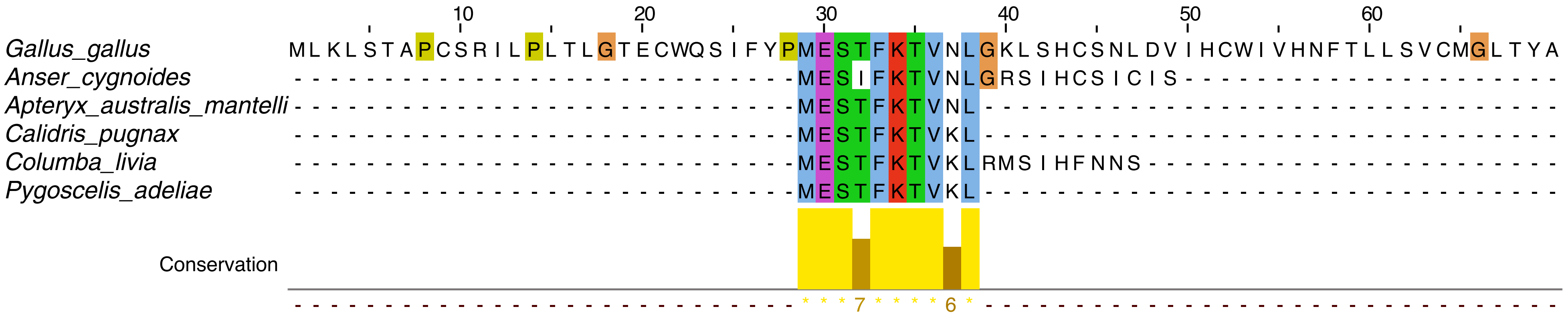

HG0437

ENSGALG00000001728 (NACC2)

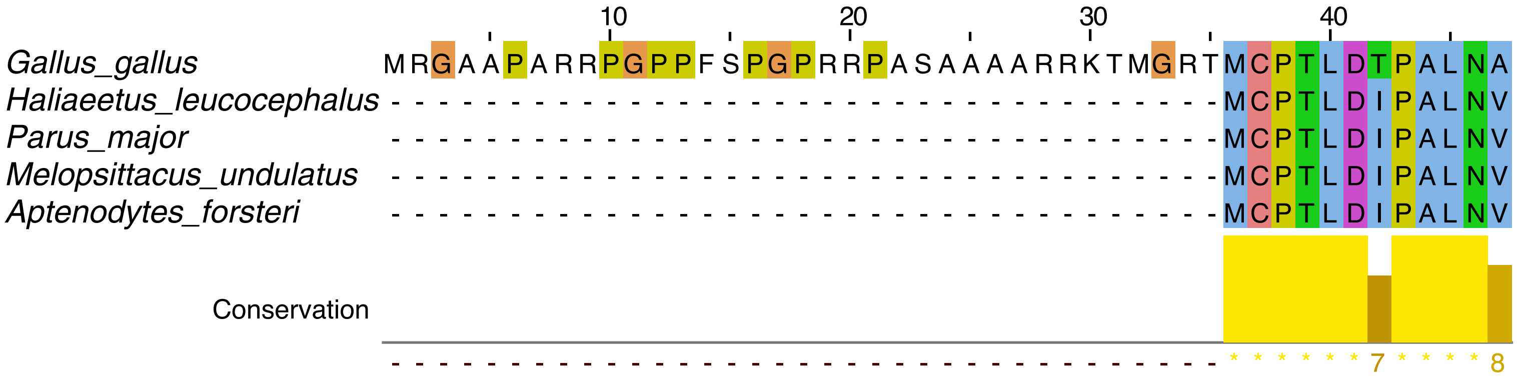

HG0438

ENSGALG00000008545 (SENP5)

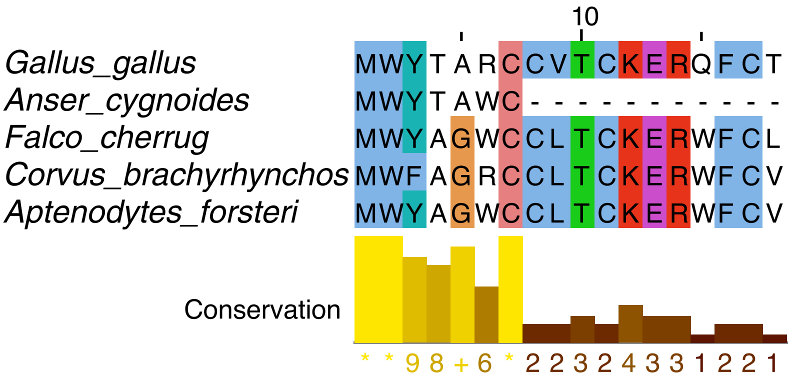

HG0439

ENSGALG00000009172 (OSBPL6)

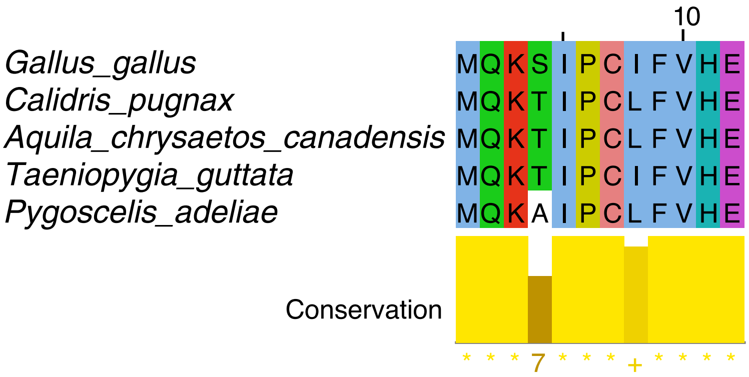

HG0440

ENSGALG00000010090

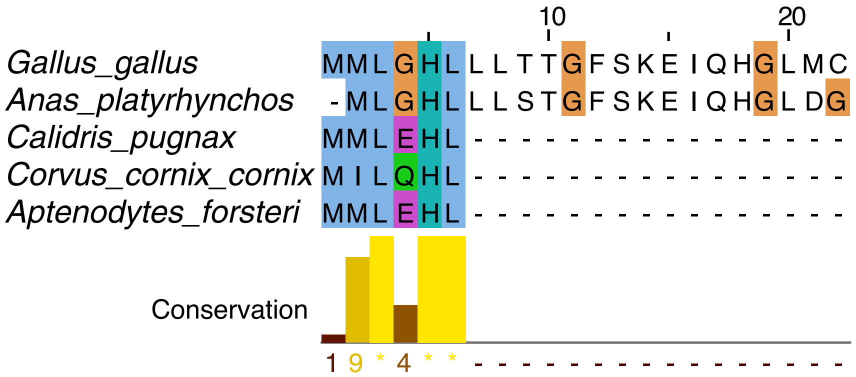

HG0441

ENSGALG00000015069 (B4GALT4)

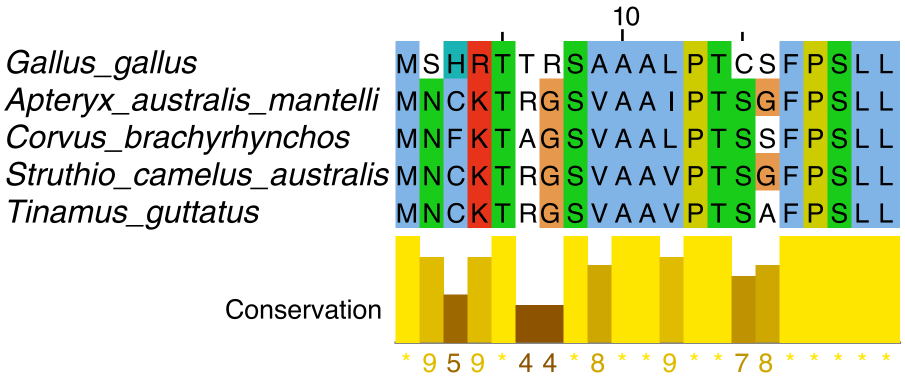

HG0442

ENSGALG00000030250

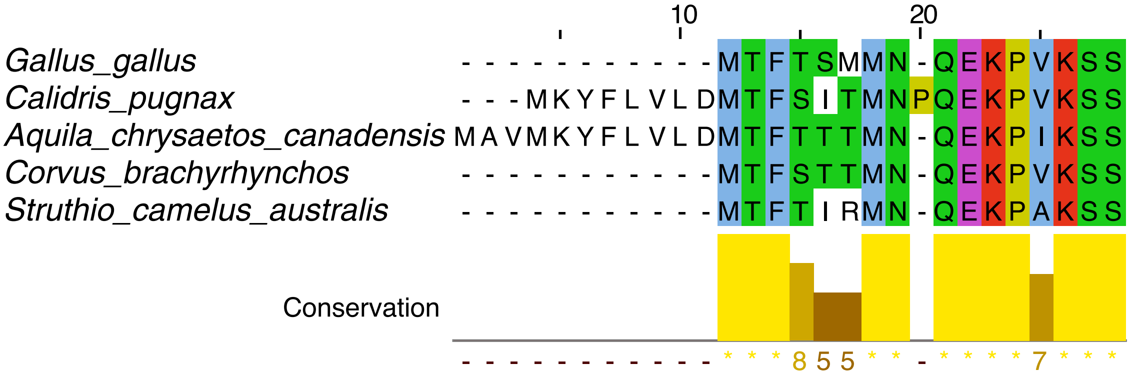

HG0443

ENSGALG00000039454

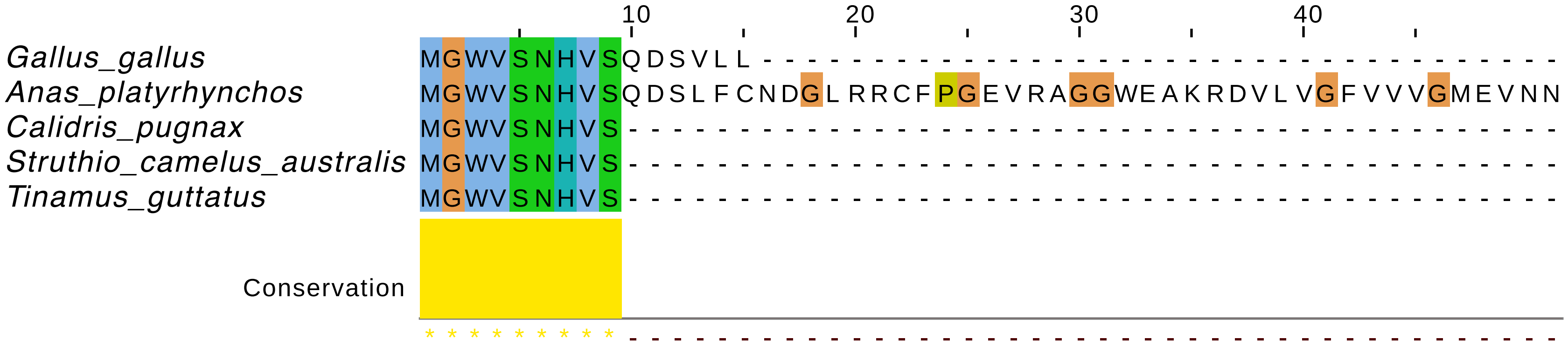

HG0444

ENSGALG00000000558 (SLC1A6)

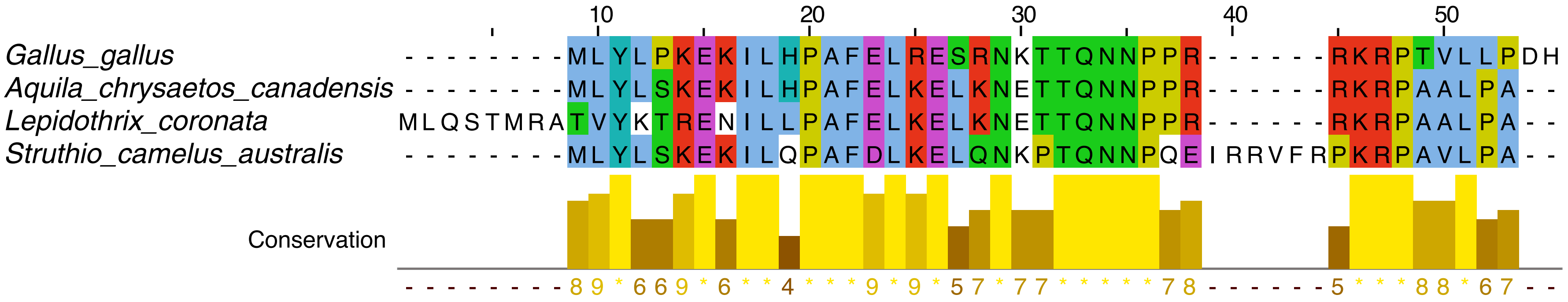

HG0445

ENSGALG00000004017

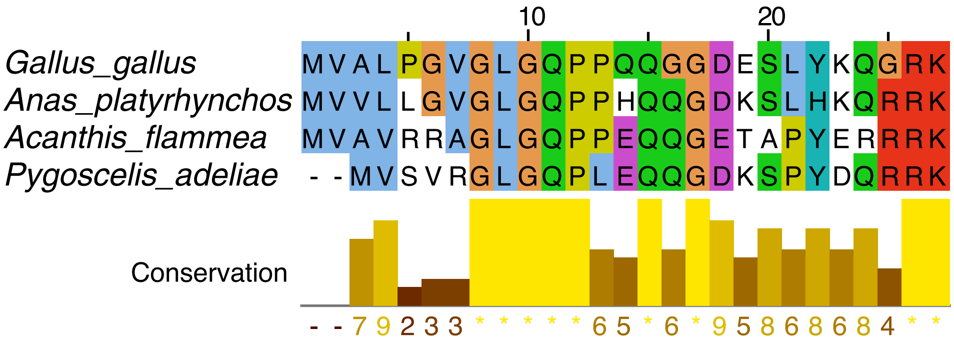

HG0446

ENSGALG00000006807 (PLSCR5)

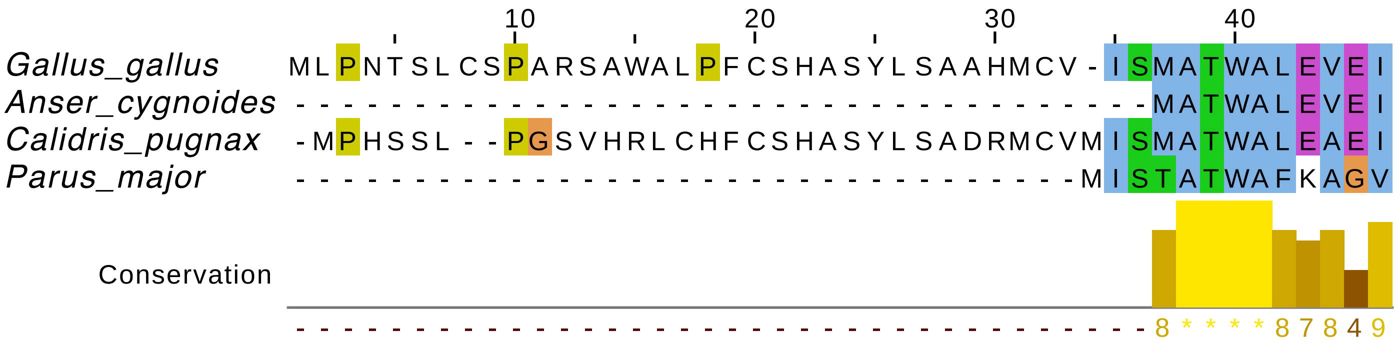

HG0447

ENSGALG00000008784

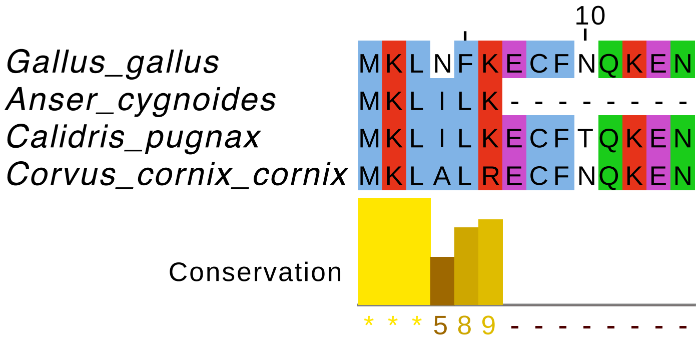

HG0448

ENSGALG00000011762 (UBA6)

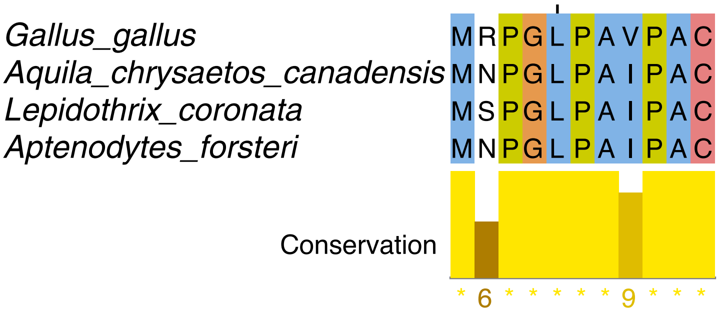

HG0449

ENSGALG00000014106 (PPFIBP1)

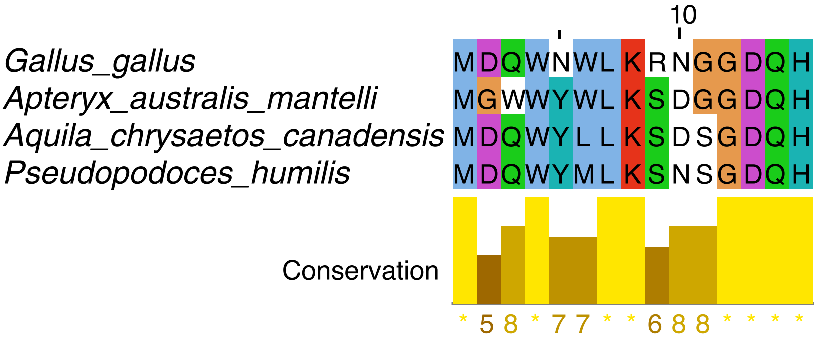

HG0450

ENSGALG00000015528 (JAKMIP1)

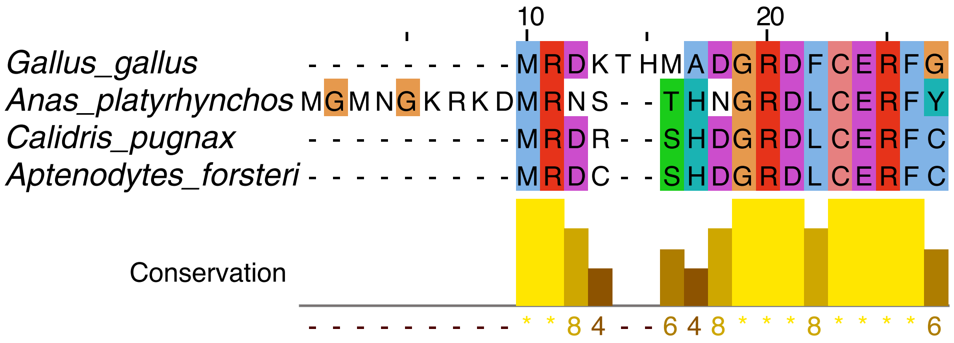

HG0451

ENSGALG00000019489 (CHST9)

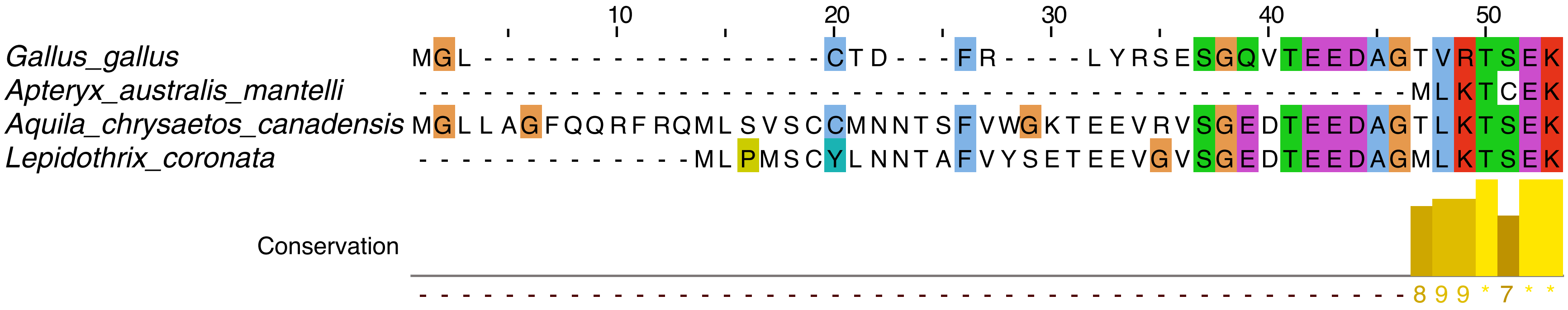

HG0452

ENSGALG00000004227 (FAM20B)

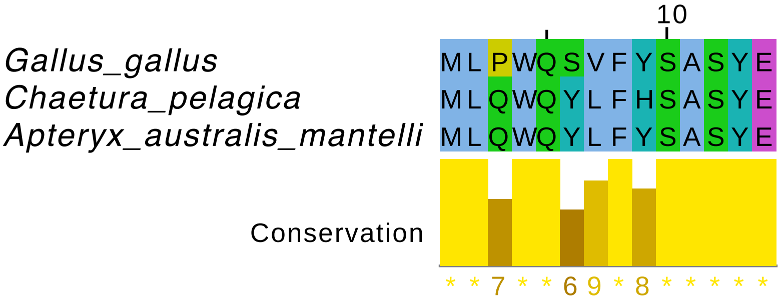

HG0453

ENSGALG00000005995 (SLC13A5)

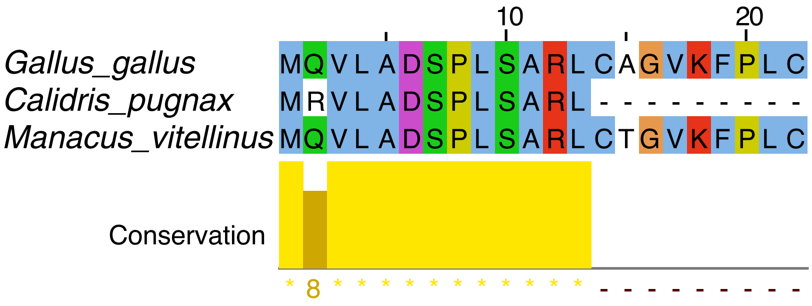

HG0454

ENSGALG00000007186 (TMEM268)

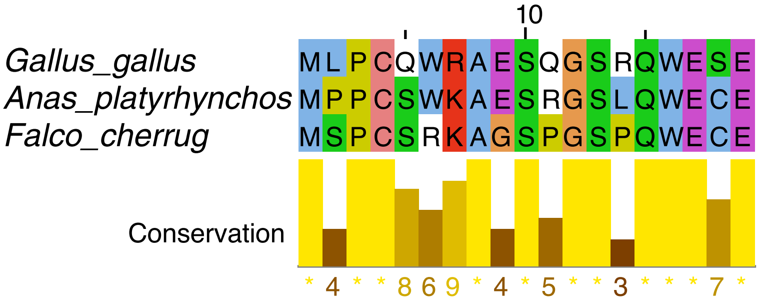

HG0455

ENSGALG00000010718 (THSD7A)

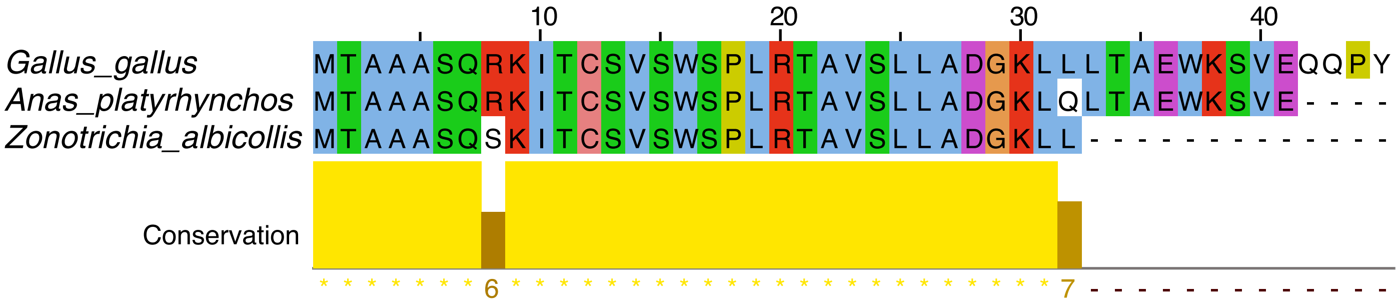

HG0456

ENSGALG00000033256

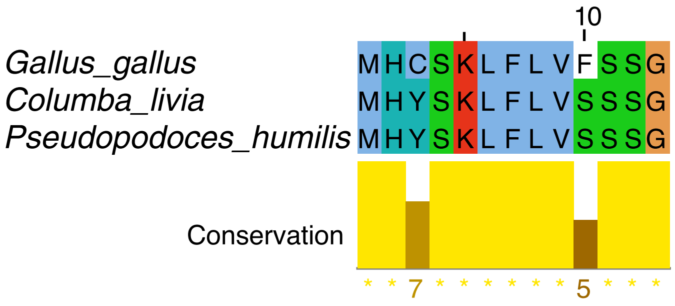

HG0457

ENSGALG00000039538 (CLDND1)

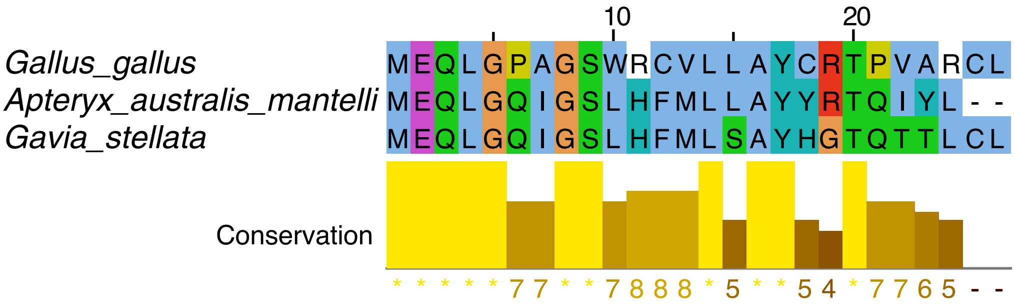

HG0458

ENSG00000060339 (CCAR1)

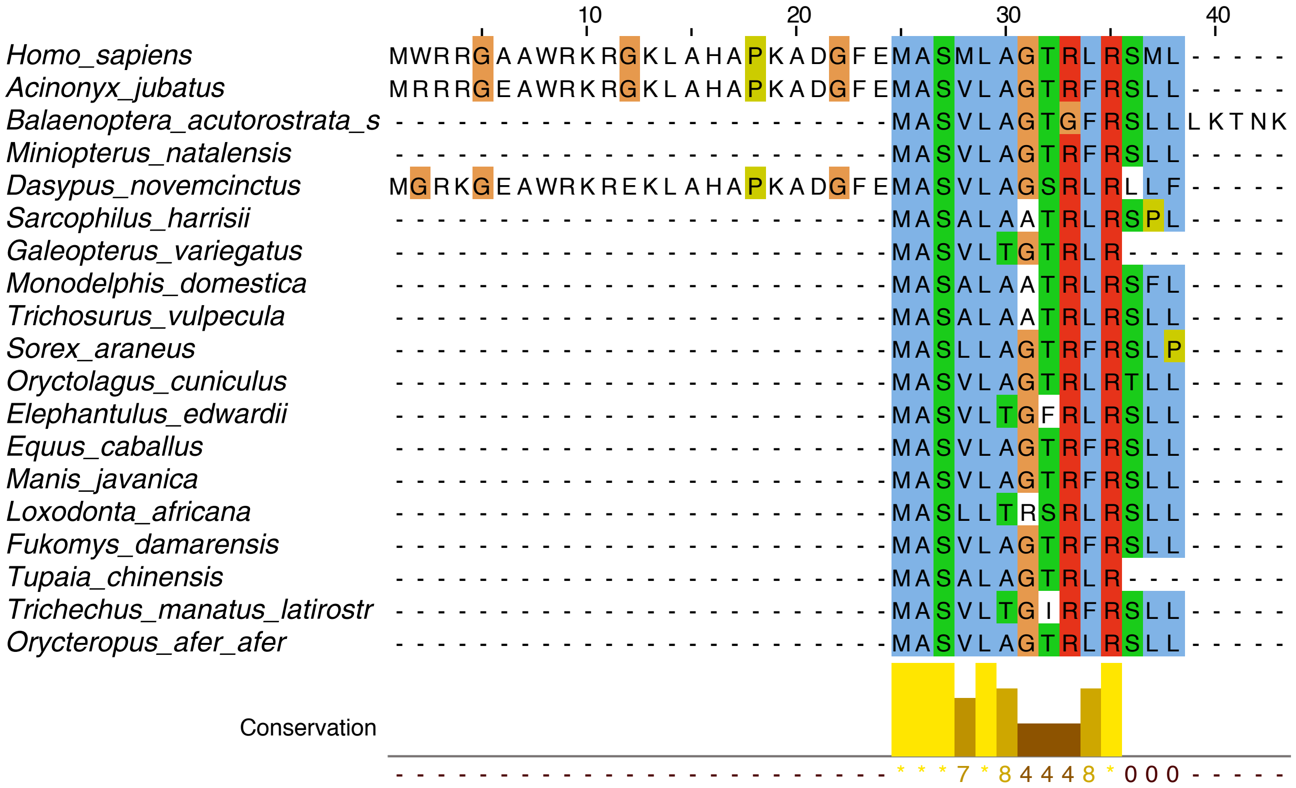

HG0459.1

ENSG00000163848 (ZNF148)

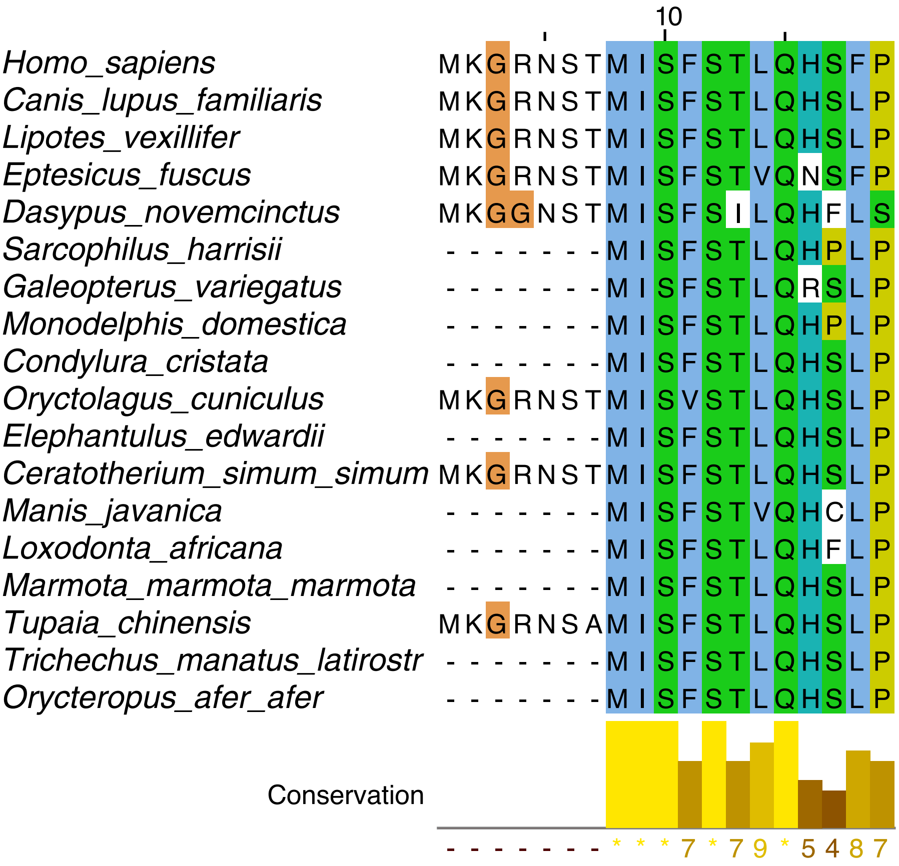

HG0459.2

ENSG00000163848 (ZNF148)

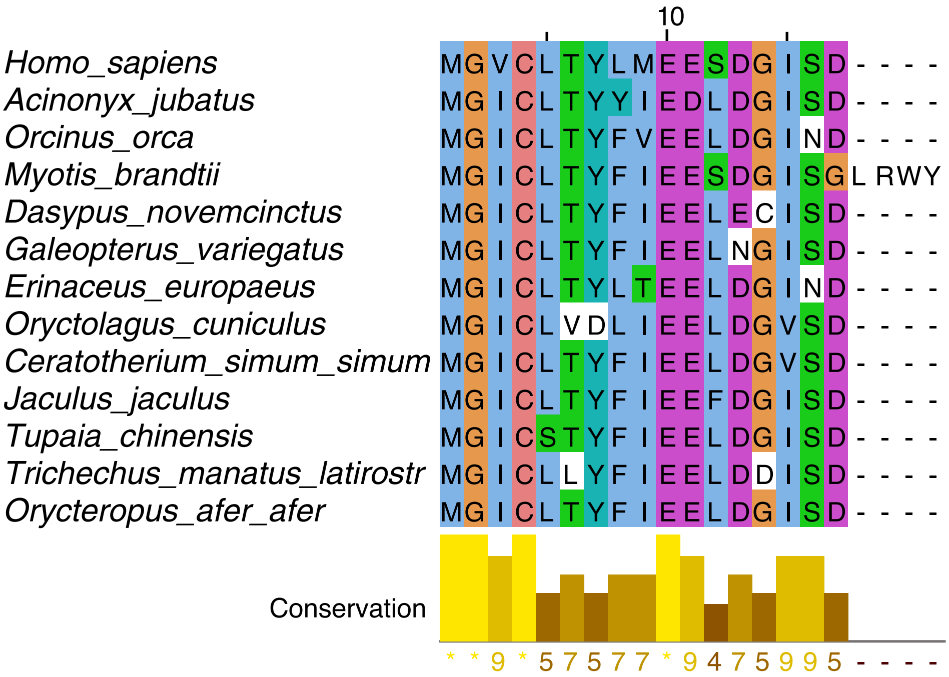

HG0459.3

ENSG00000163848 (ZNF148)

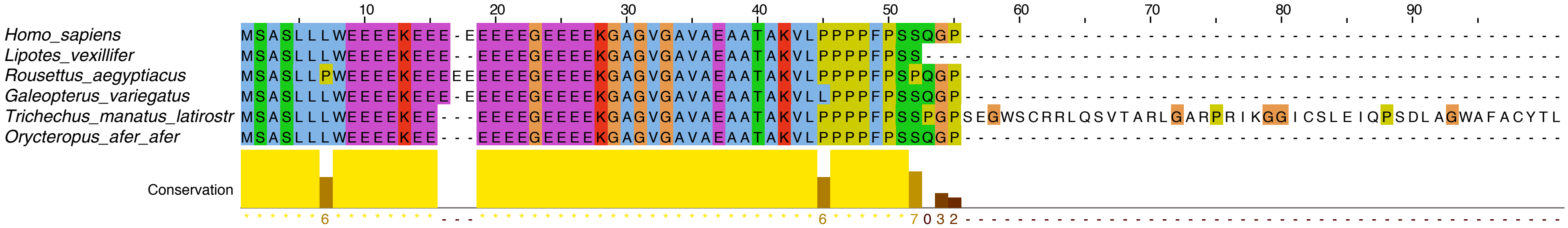

HG0460

ENSG00000180008 (SOCS4)

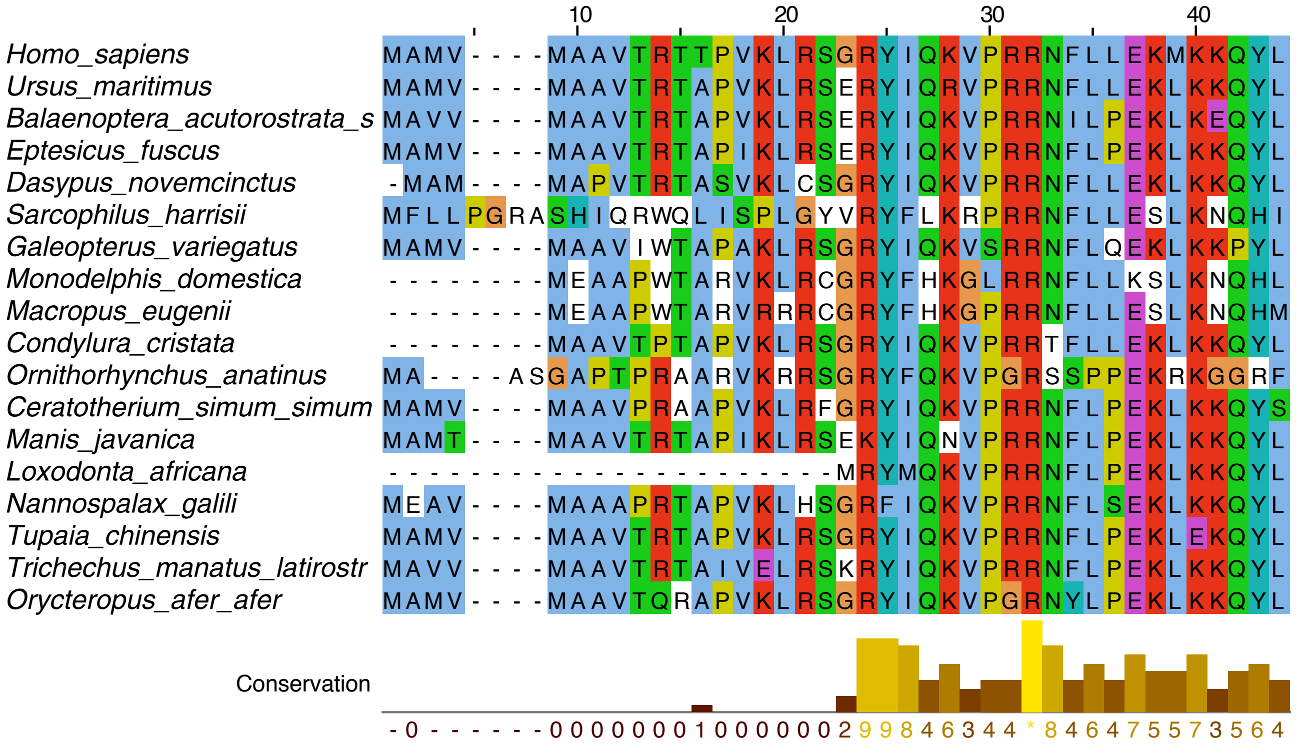

HG0461

ENSG00000064419 (TNPO3)

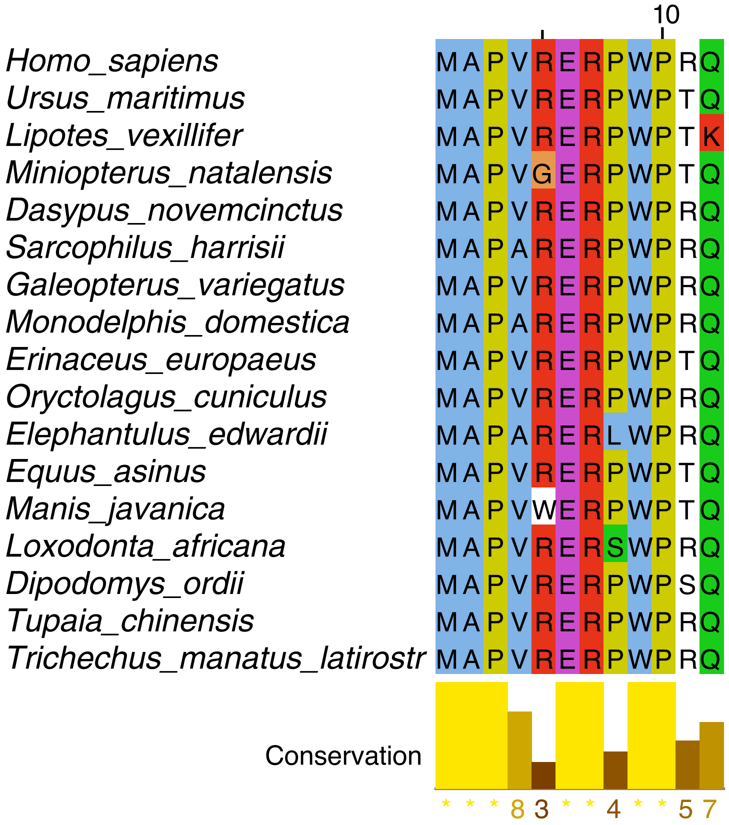

HG0462

ENSG00000125686 (MED1)

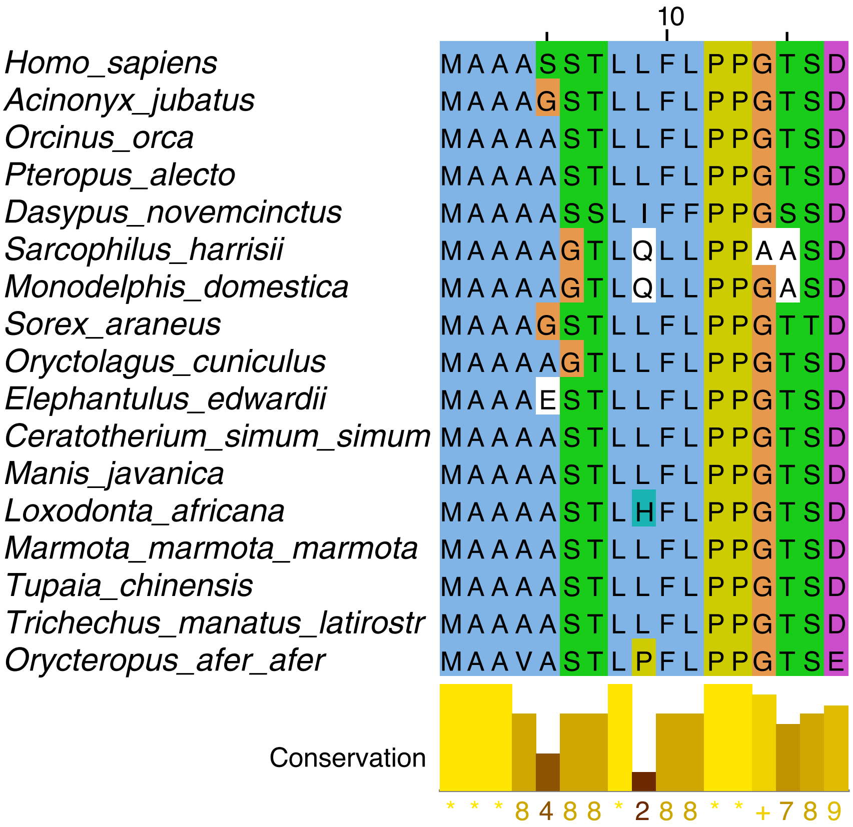

HG0463

ENSG00000129473 (BCL2L2)

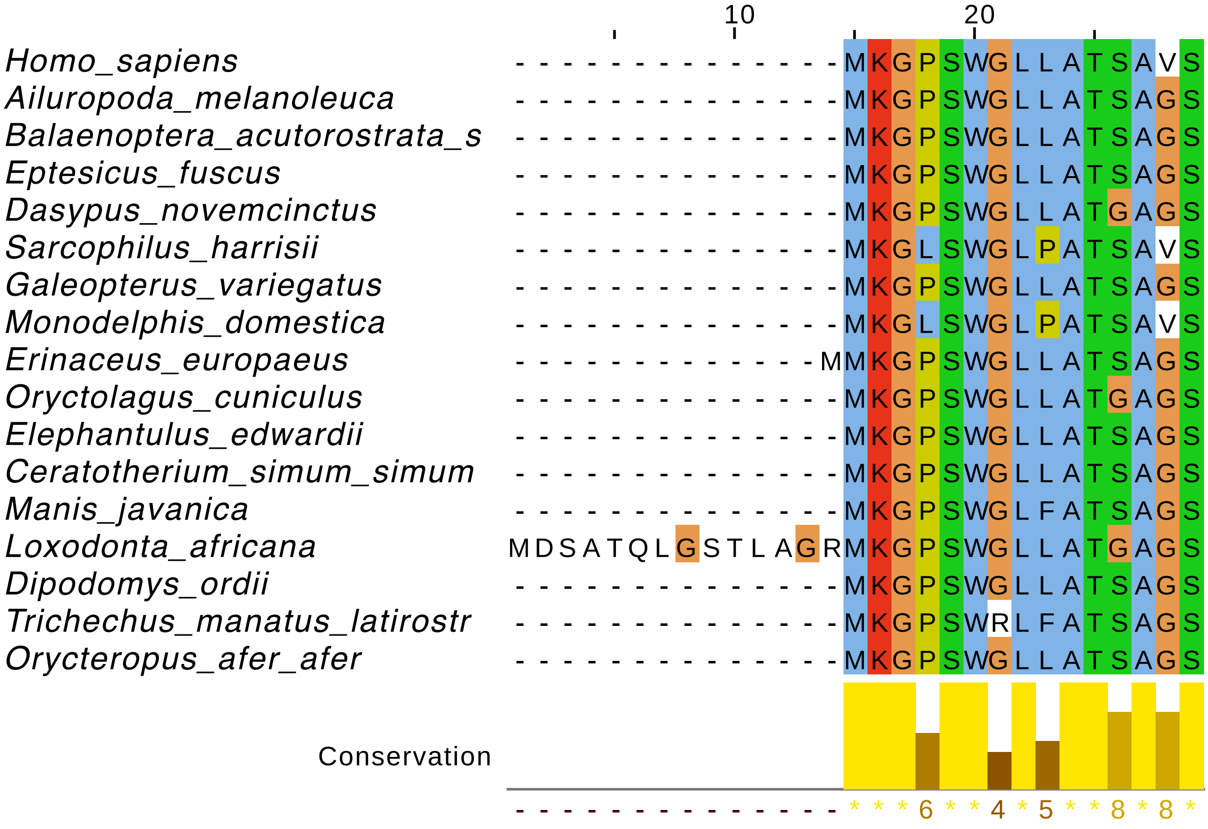

HG0464

ENSG00000188215 (DCUN1D3)

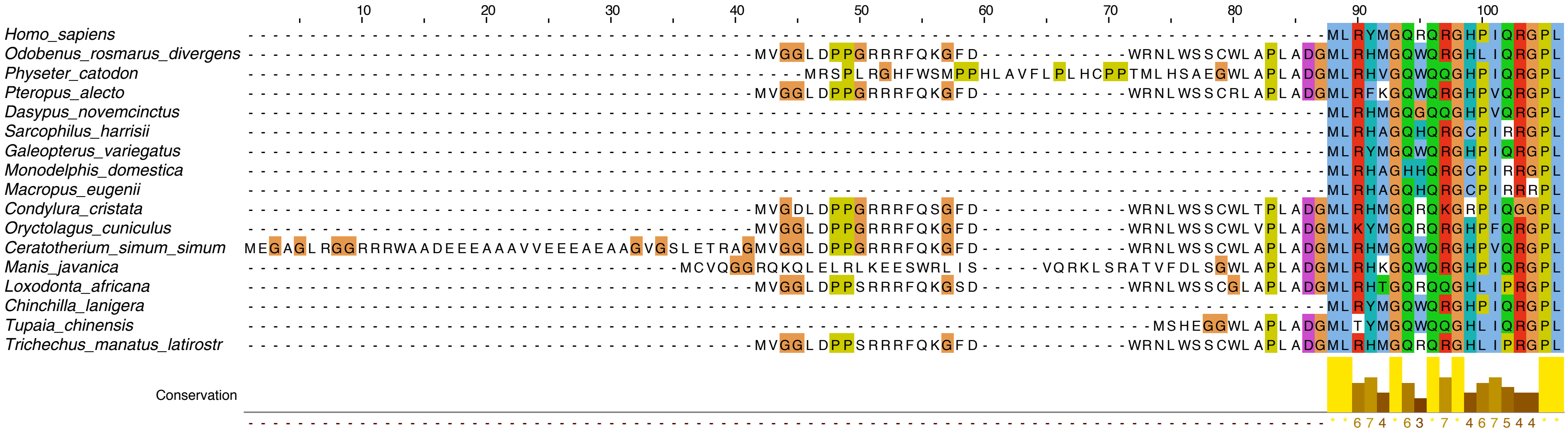

HG0465

ENSG00000132153 (DHX30)

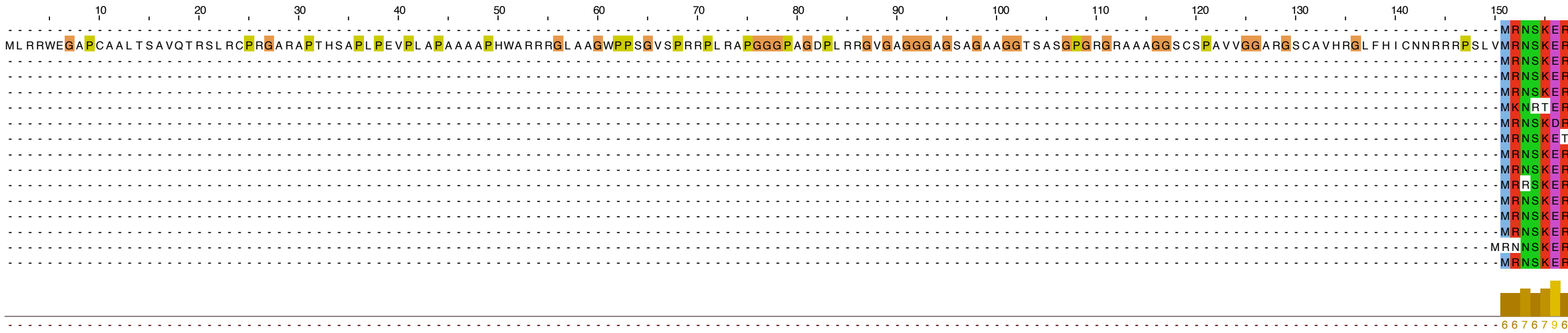

HG0466

ENSG00000139679 (LPAR6)

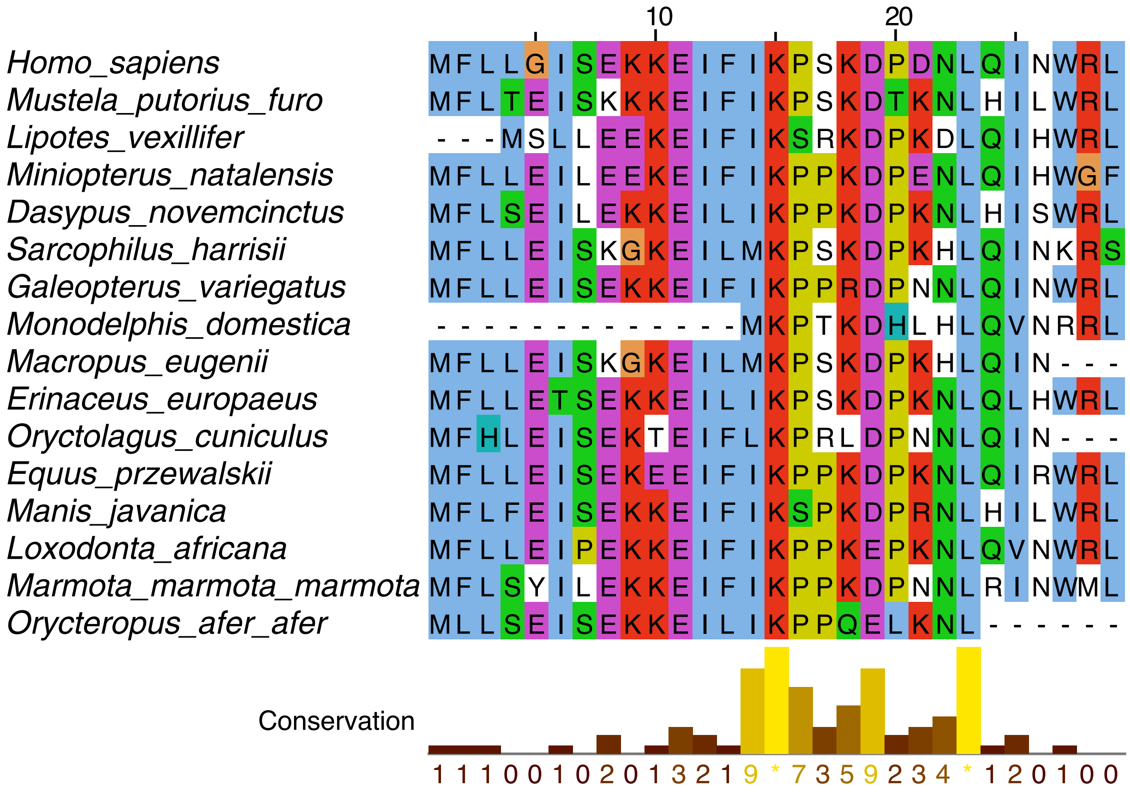

HG0467.1

ENSG00000163320 (CGGBP1)

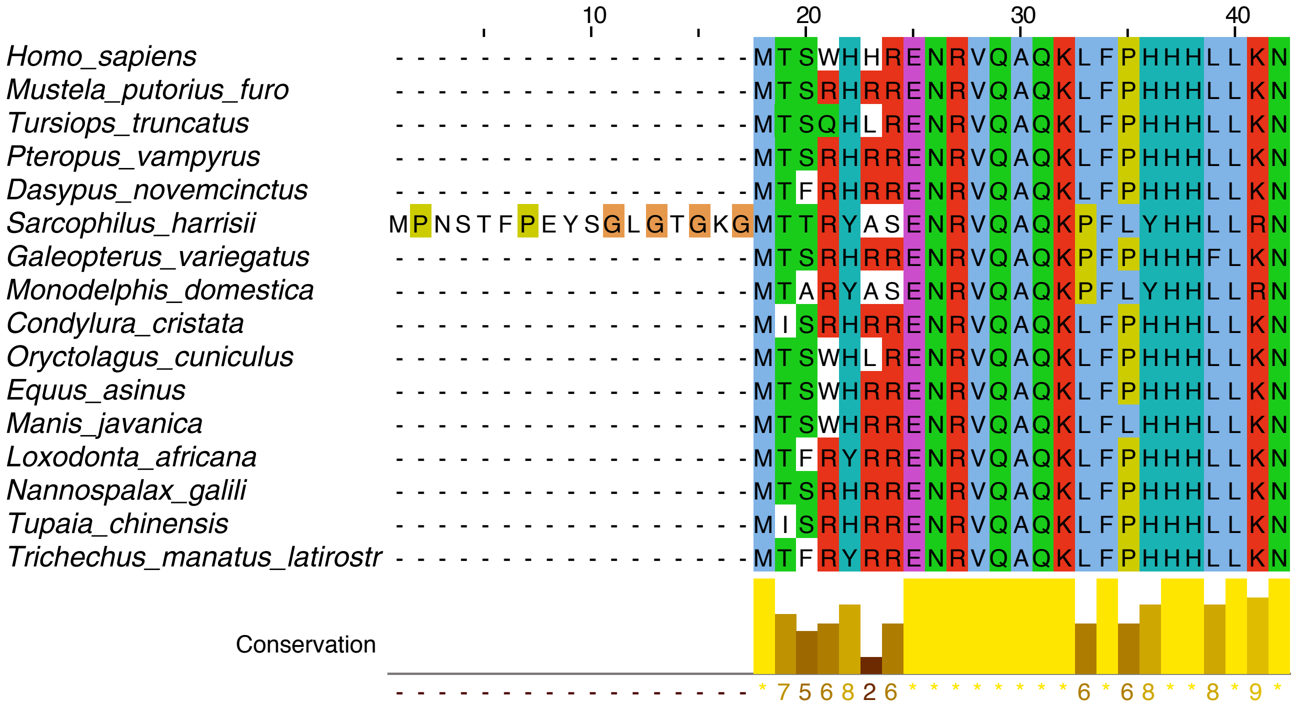

HG0467.2

ENSG00000163320 (CGGBP1)

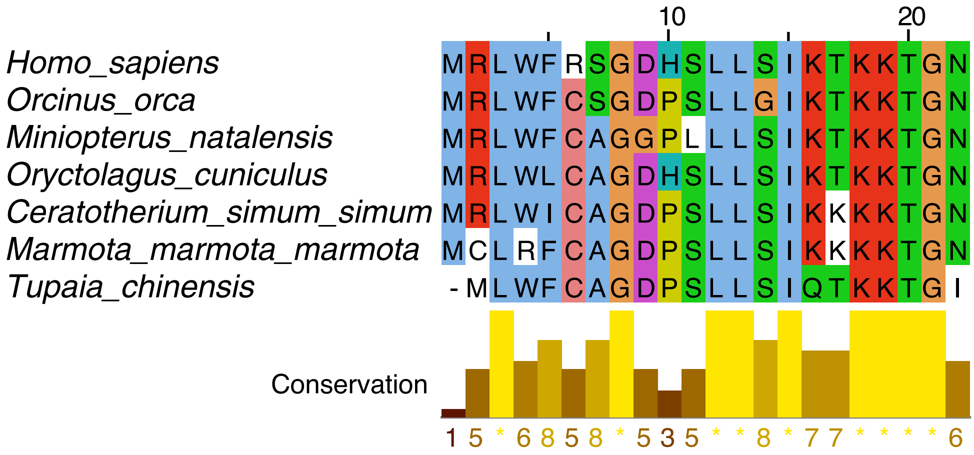

HG0468

ENSG00000187778 (MCRS1)

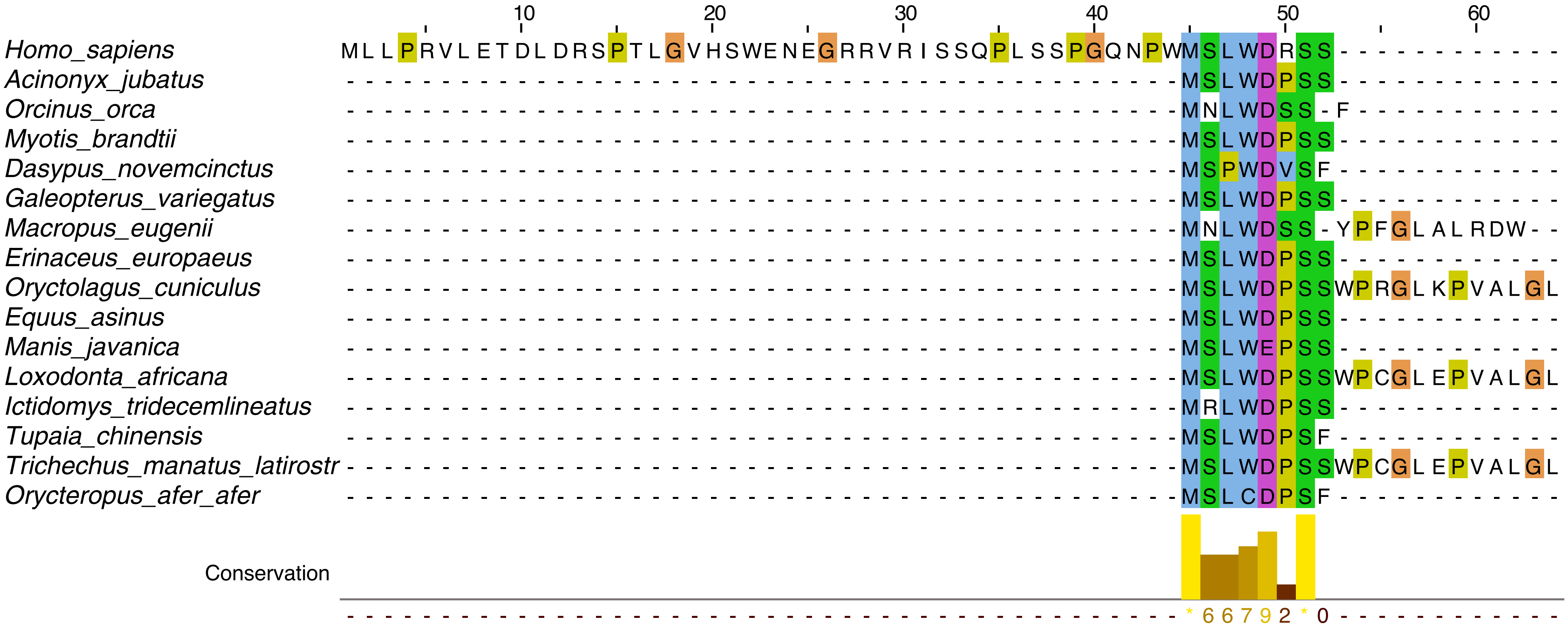

HG0469

ENSG00000198369 (SPRED2)

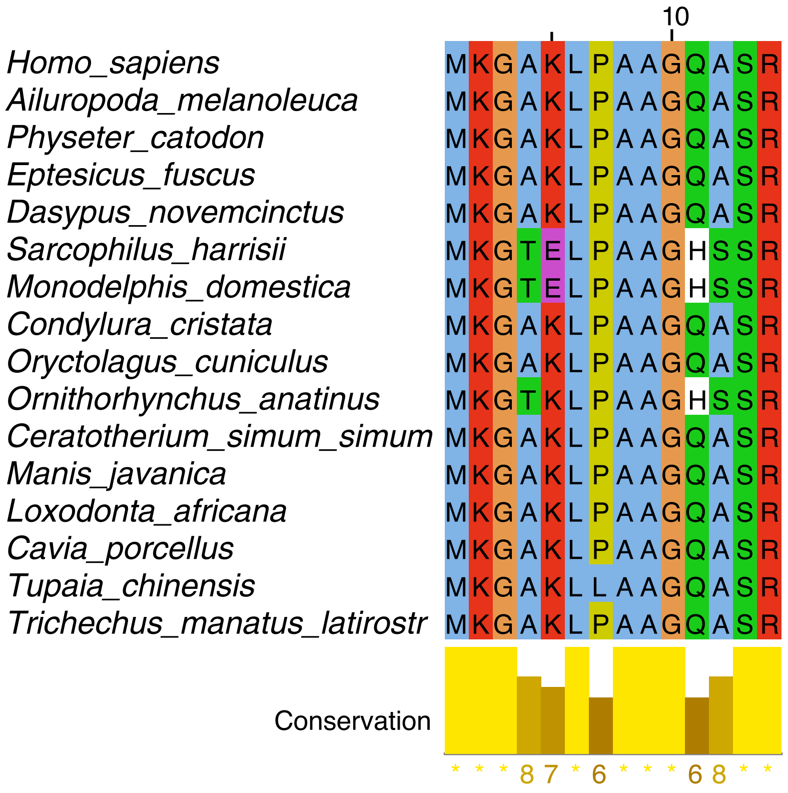

HG0470

ENSG00000224470 (ATXN1L)

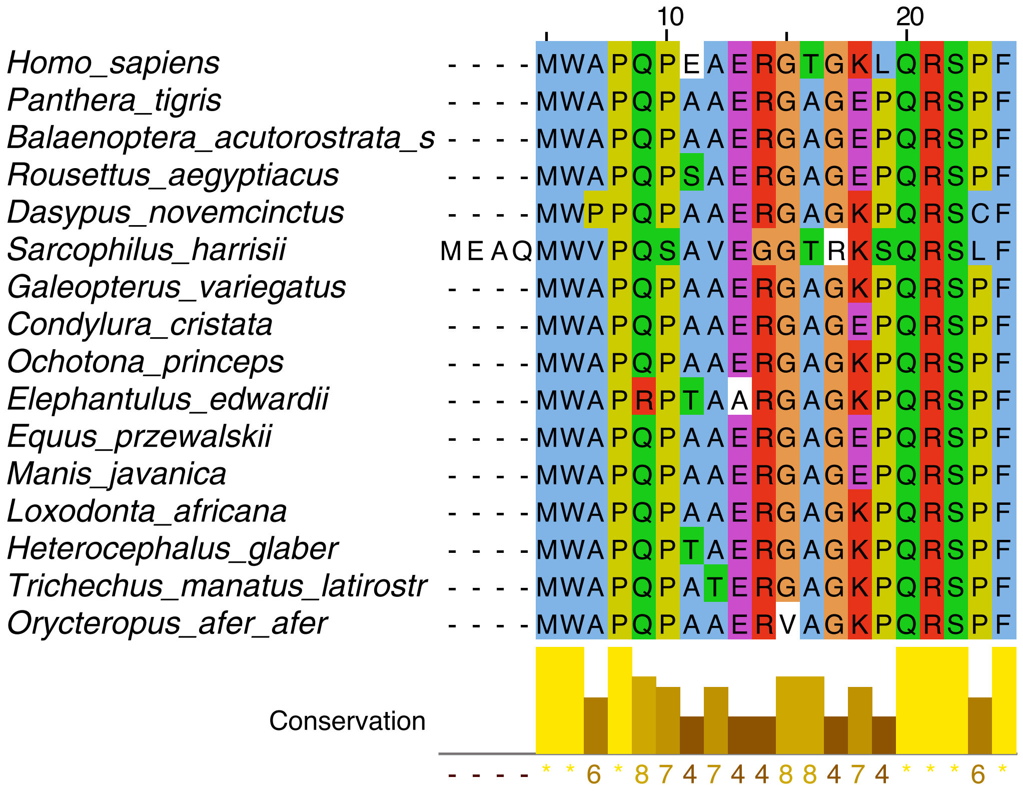

HG0471

ENSG00000023041 (ZDHHC6)

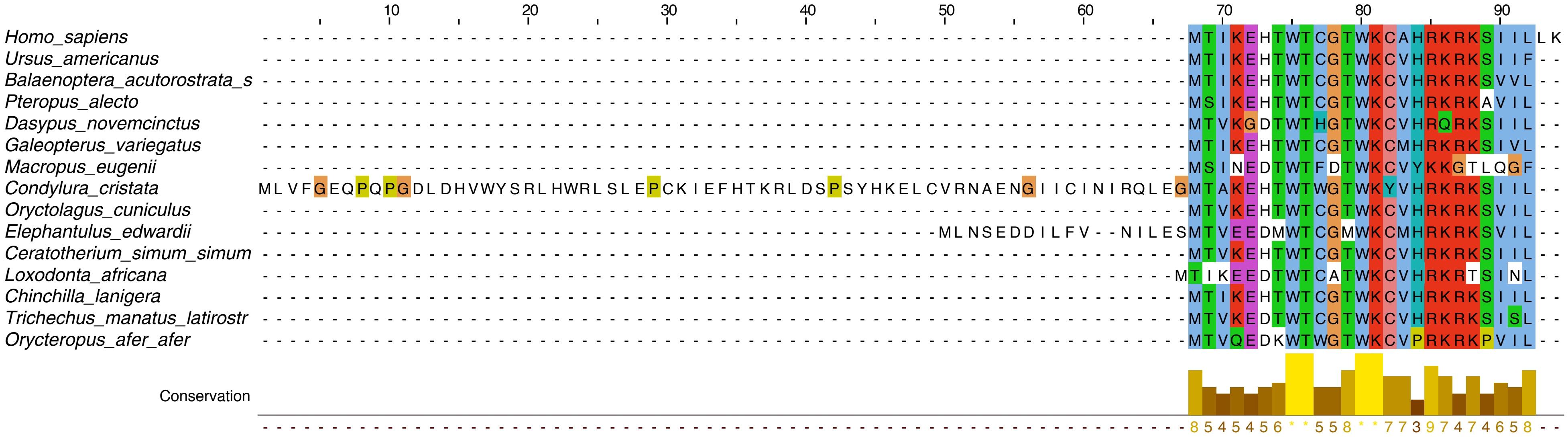

HG0472

ENSG00000105991 (HOXA1)

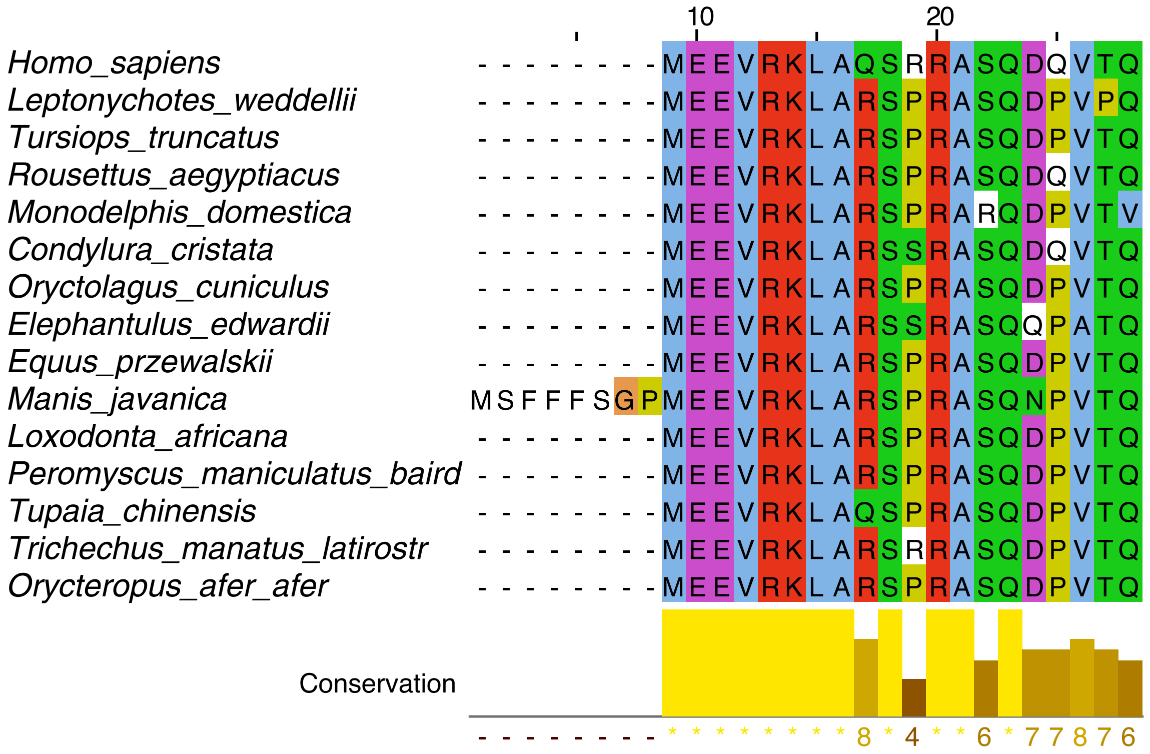

HG0473.1

ENSG00000138650 (PCDH10)

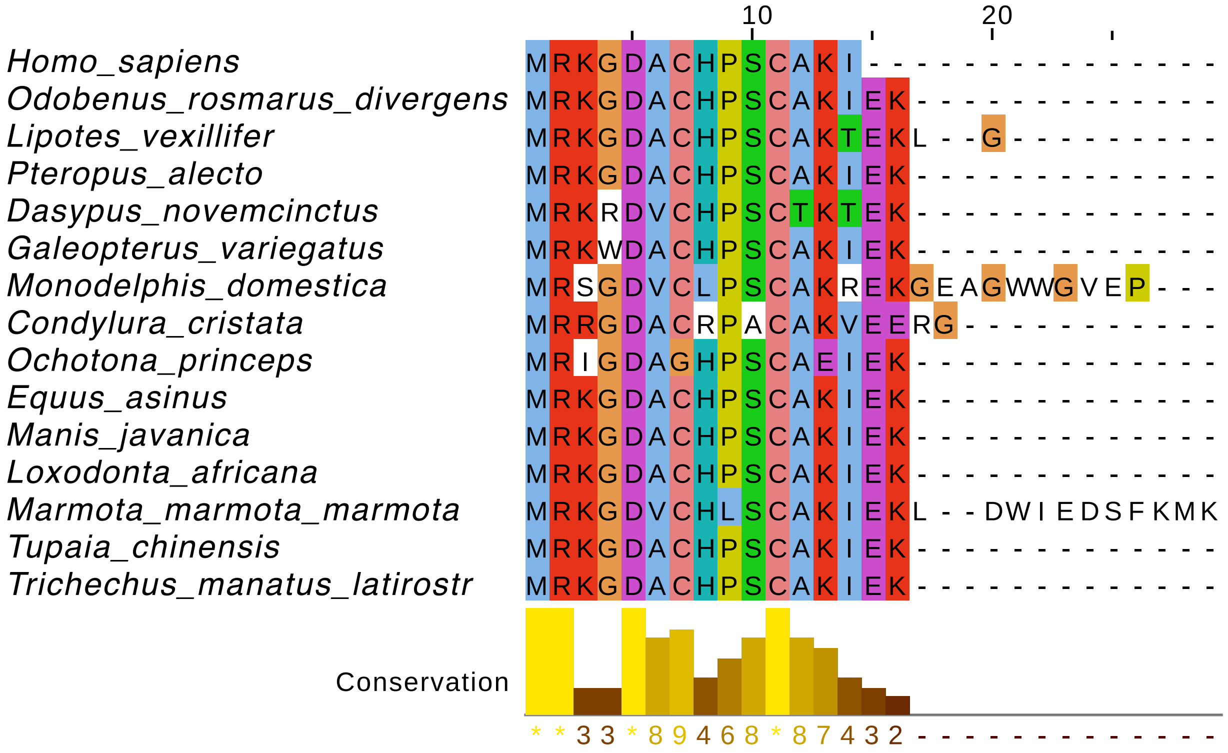

HG0473.2

ENSG00000240184 (PCDHGC3)

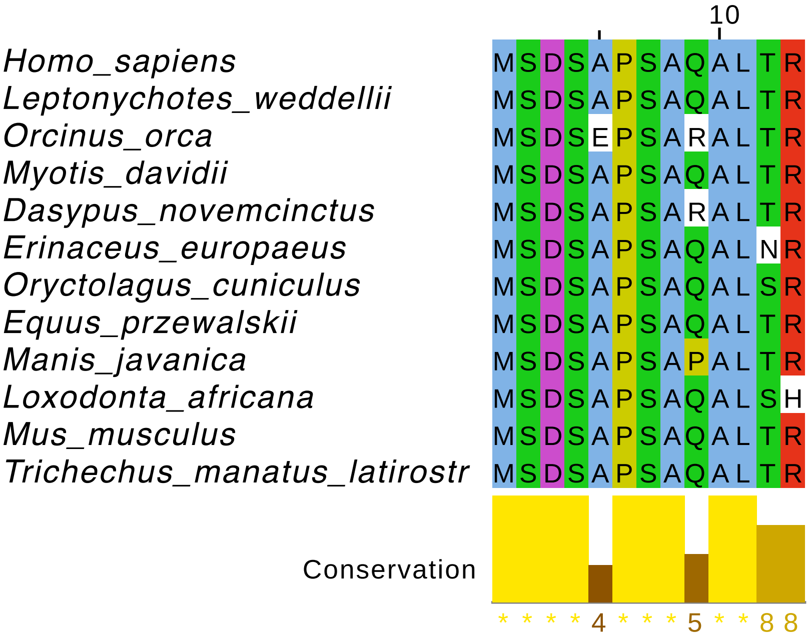

HG0474

ENSG00000139083 (ETV6)

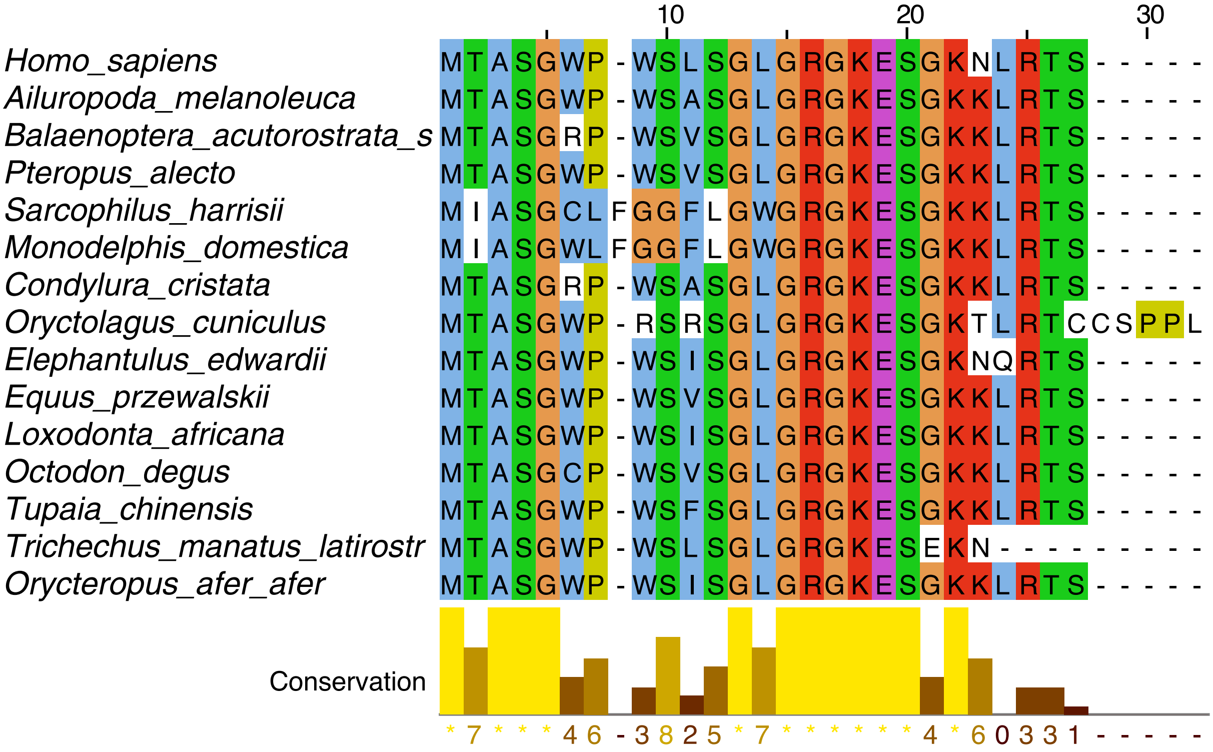

HG0475

ENSG00000109118 (PHF12)

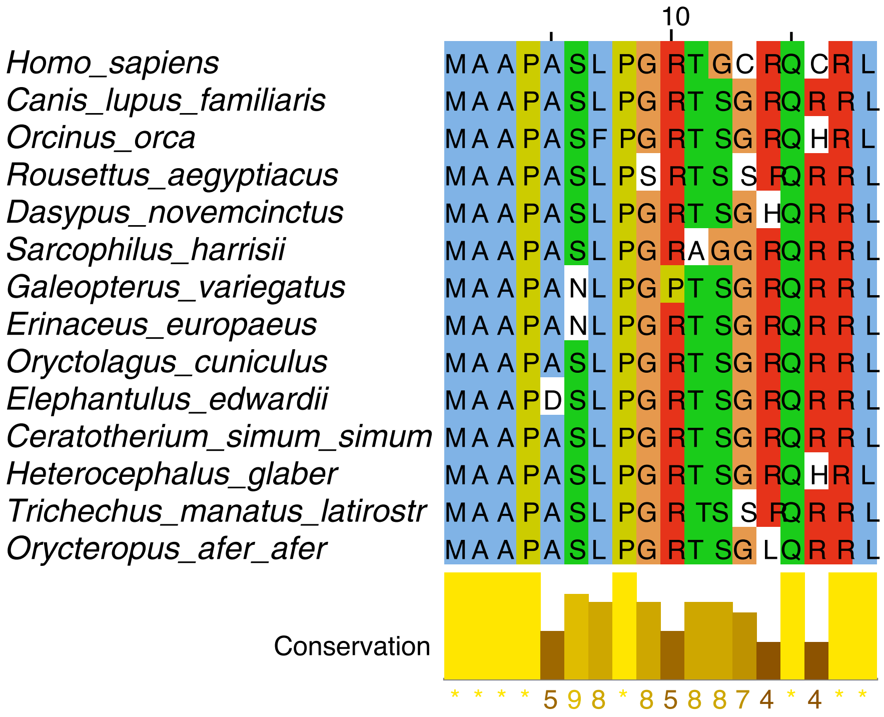

HG0476

ENSG00000130939 (UBE4B)

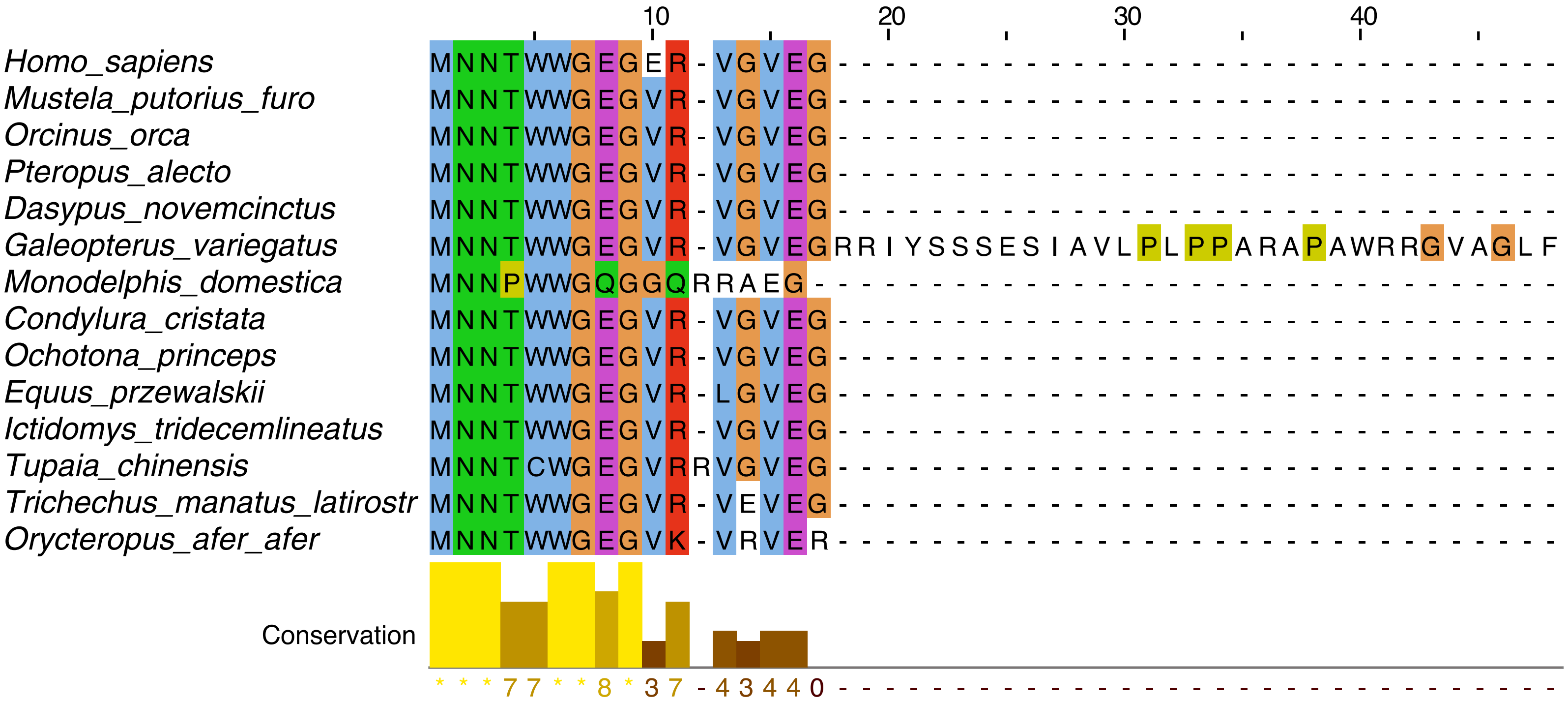

HG0477

ENSG00000166925 (TSC22D4)

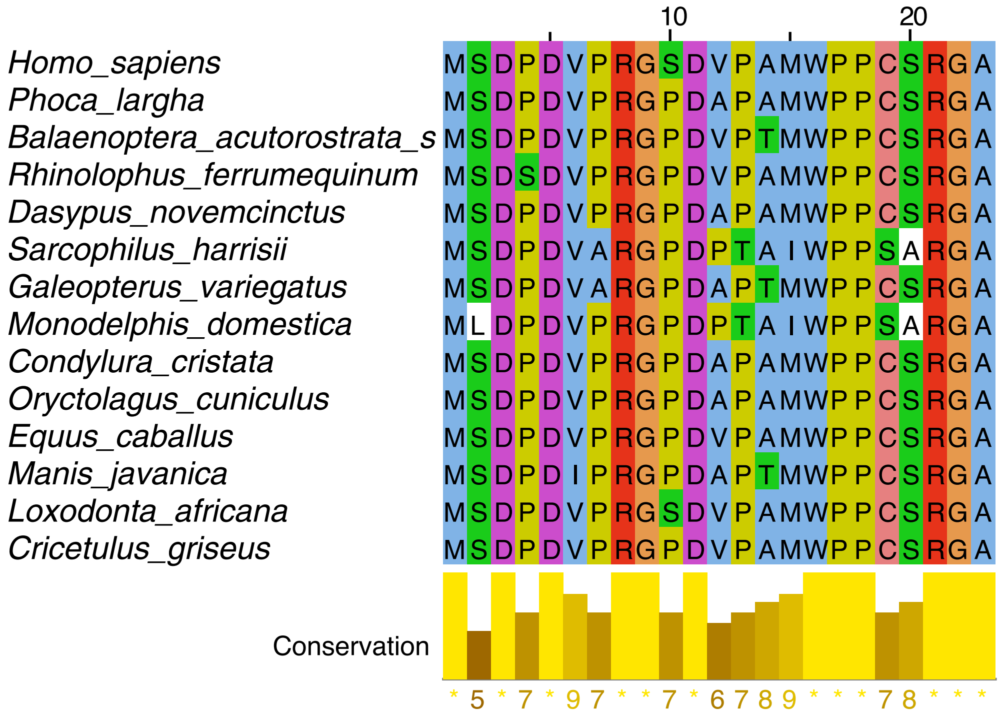

HG0478

ENSG00000168214 (RBPJ)

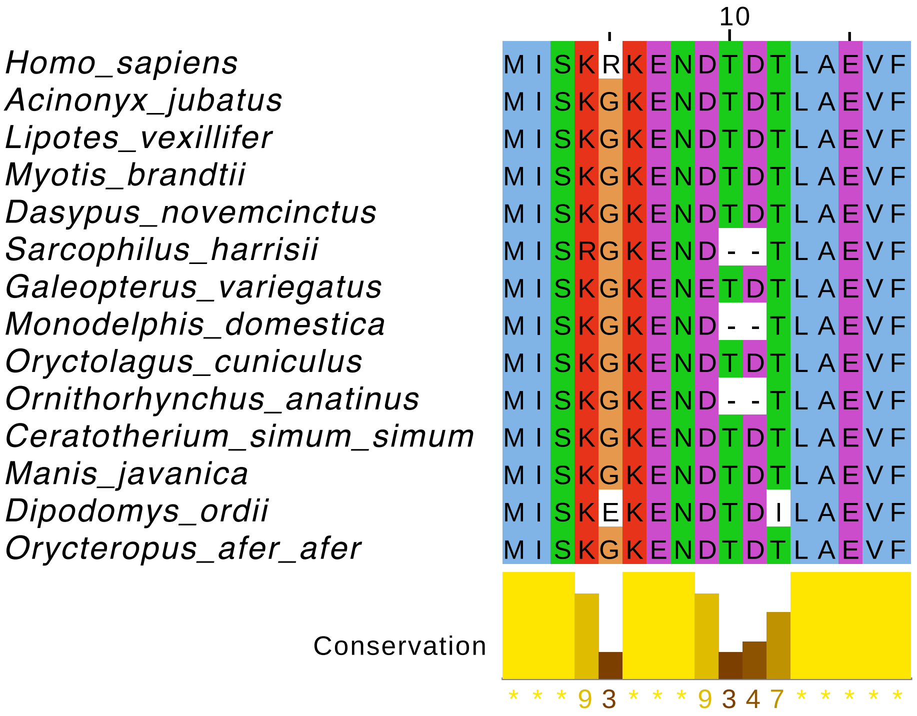

HG0479.1

ENSG00000168453 (HR)

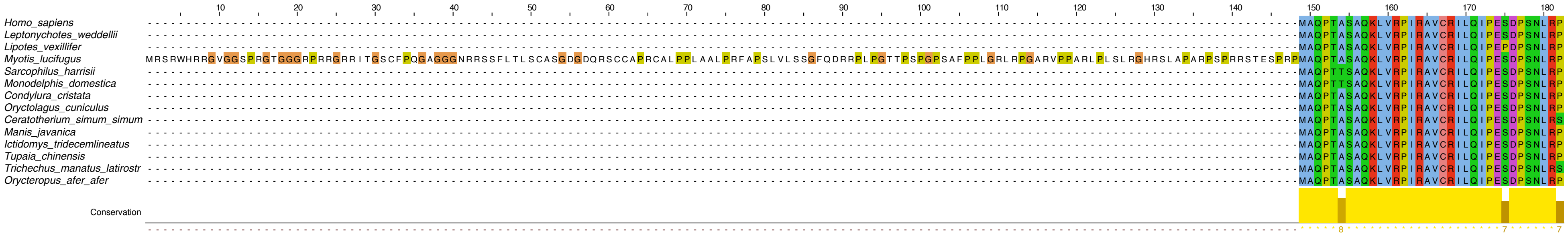

HG0479.2

ENSG00000168453 (HR)

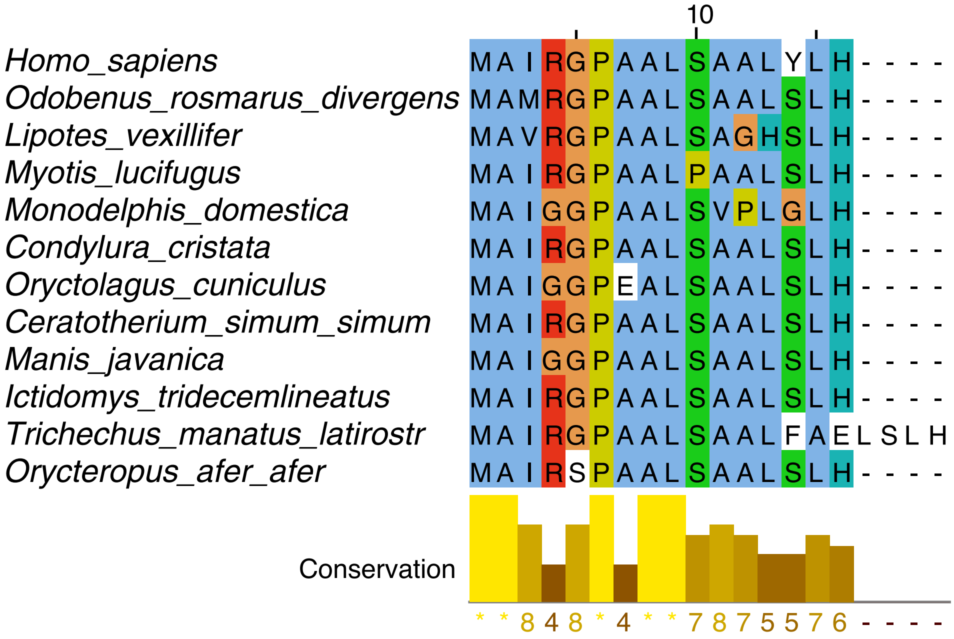

HG0480

ENSG00000198018 (ENTPD7)

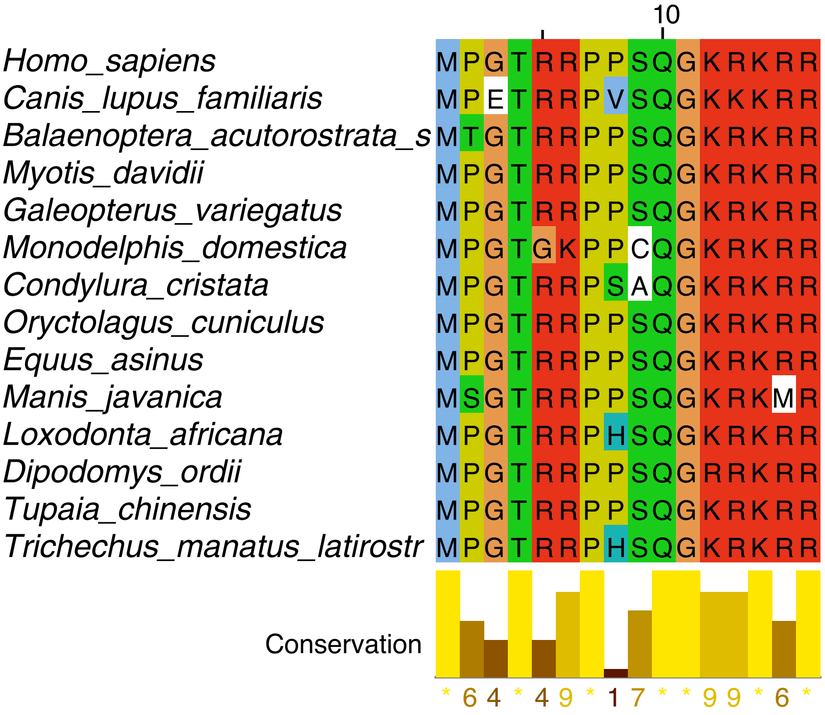

HG0481

ENSG00000198963 (RORB)

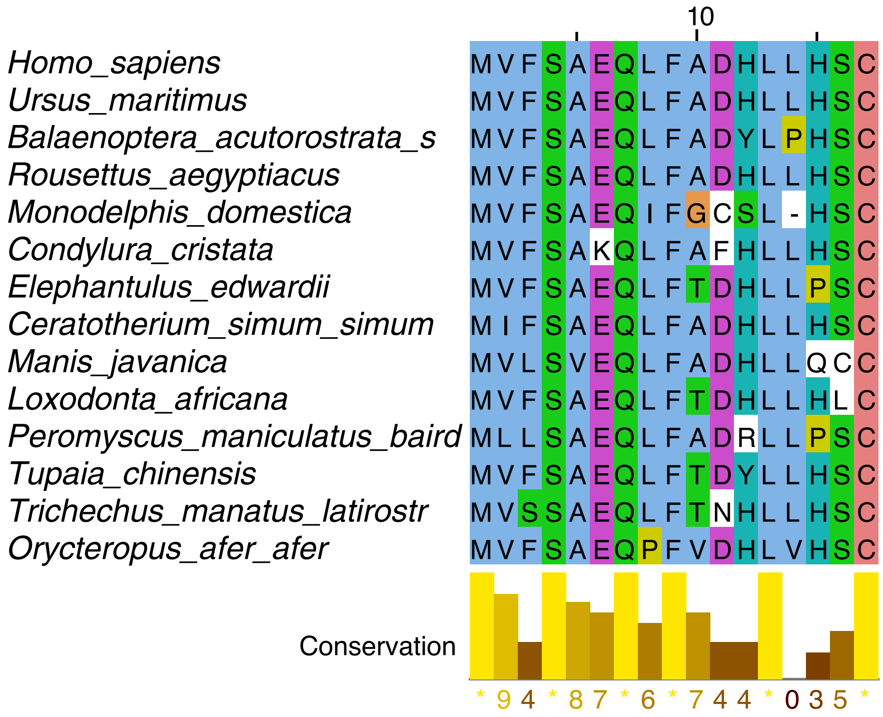

HG0482

ENSG00000067167 (TRAM1)

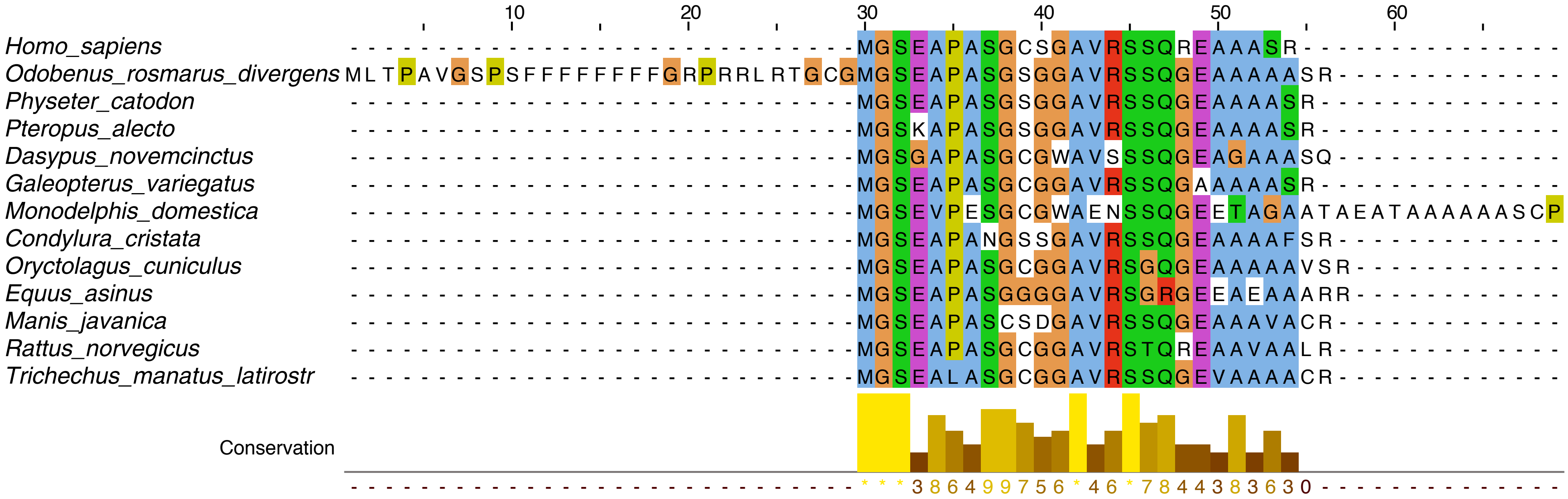

HG0483

ENSG00000164463 (CREBRF)

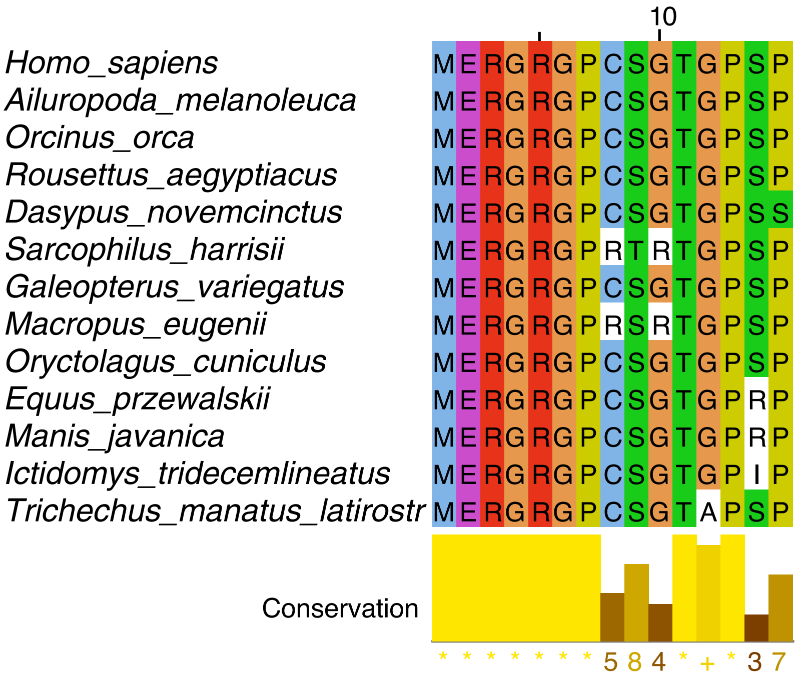

HG0484

ENSG00000166444 (ST5)

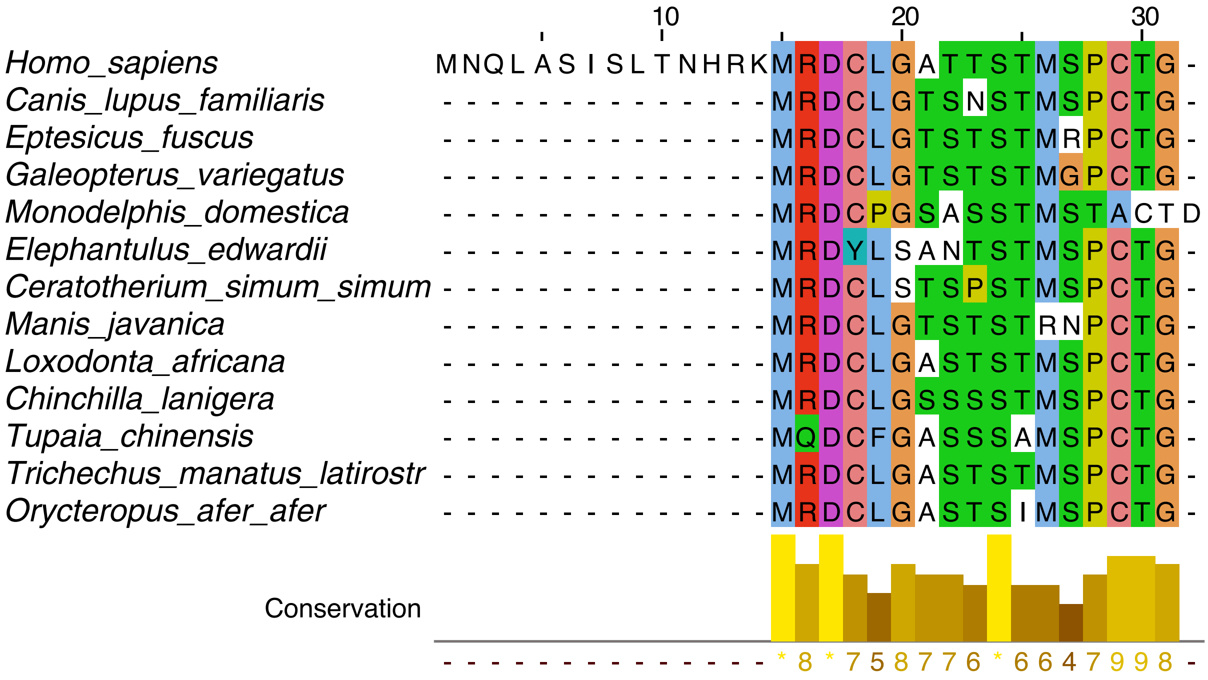

HG0485.1

ENSG00000182667 (NTM)

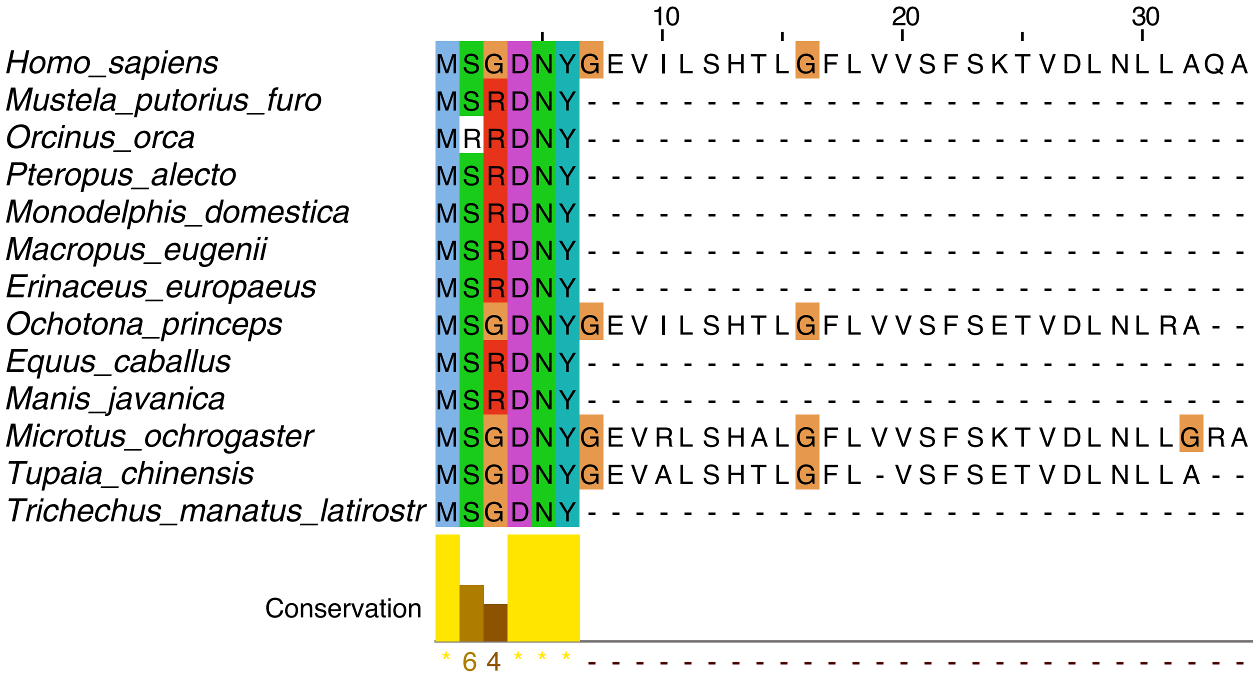

HG0485.2

ENSG00000183715 (OPCML)

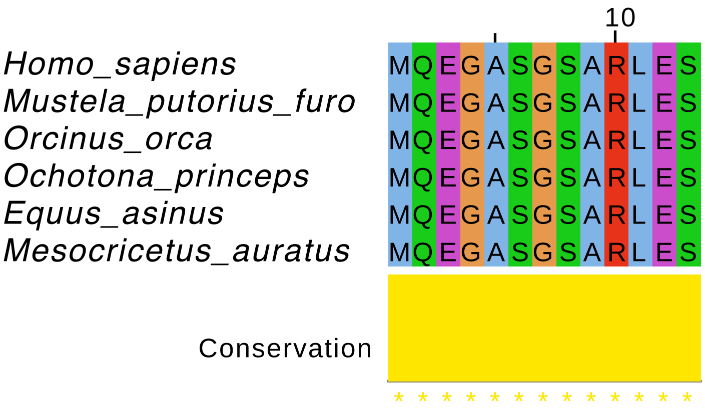

HG0486

ENSG00000067900 (ROCK1)

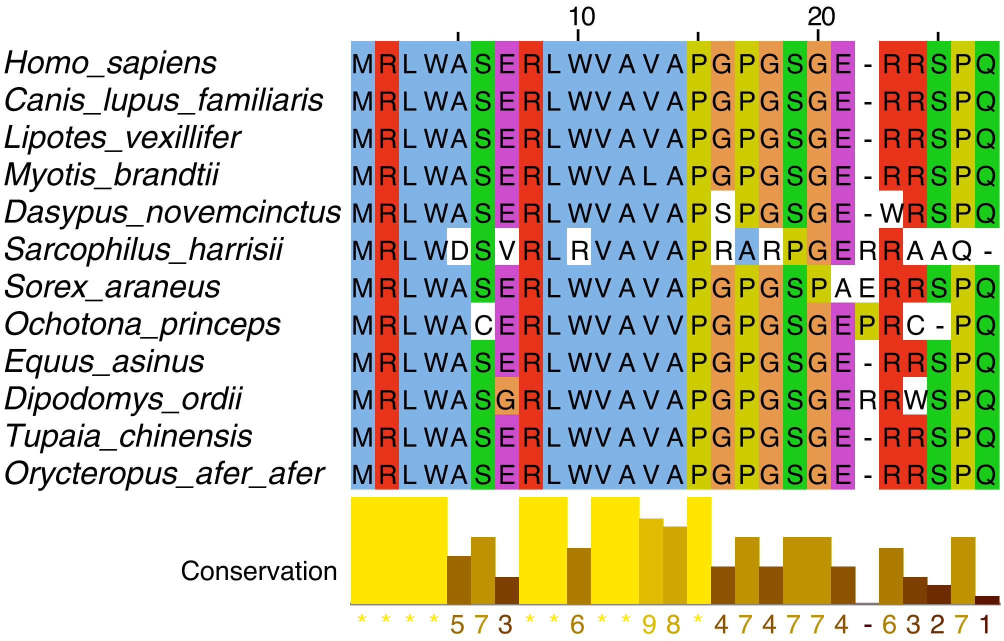

HG0487

ENSG00000091831 (ESR1)

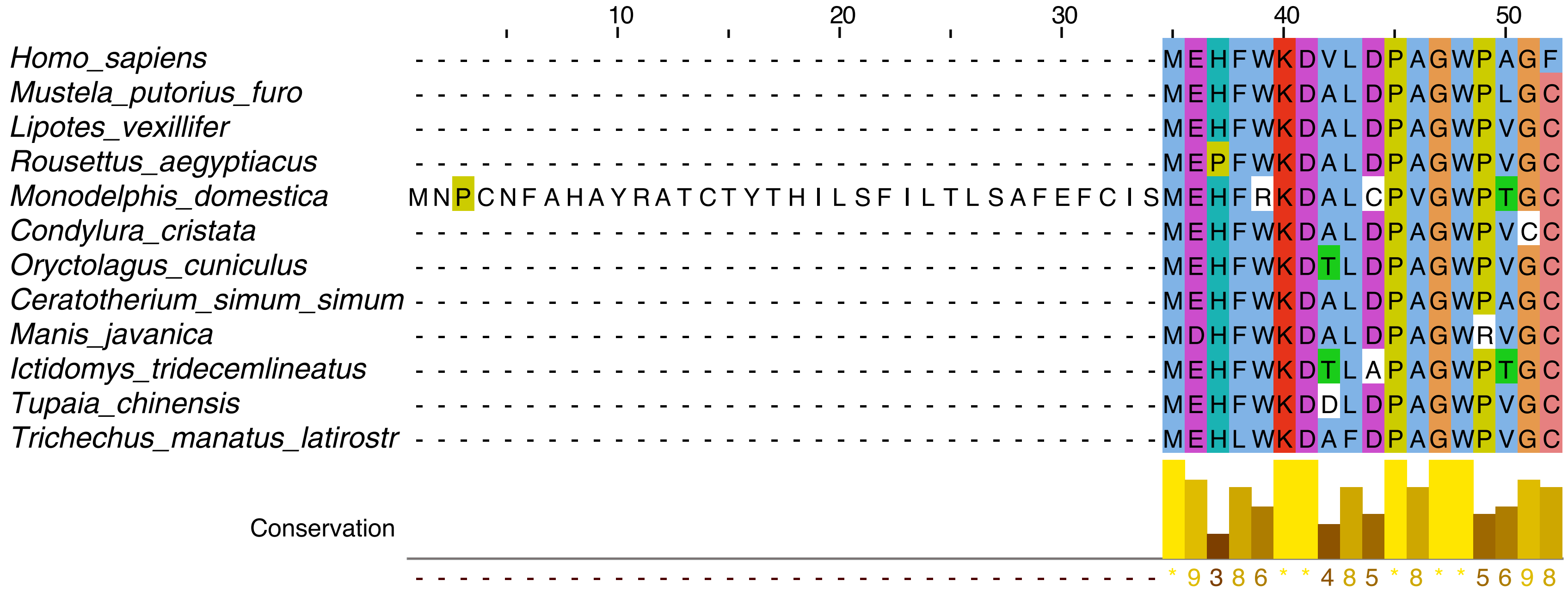

HG0488.1

ENSG00000116194 (ANGPTL1)

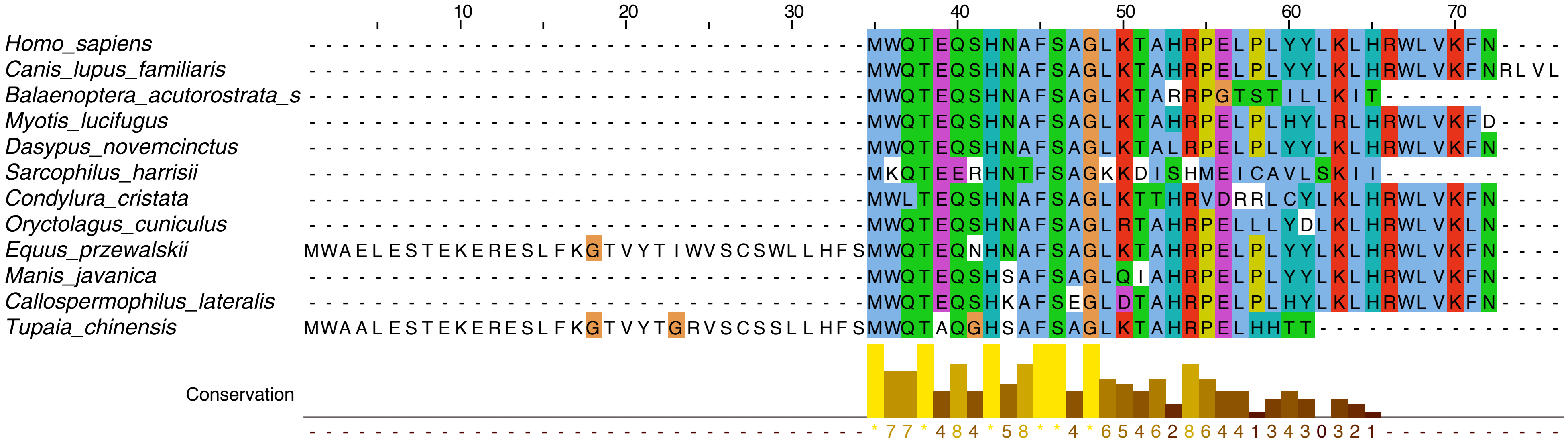

HG0488.2

ENSG00000116194 (ANGPTL1)

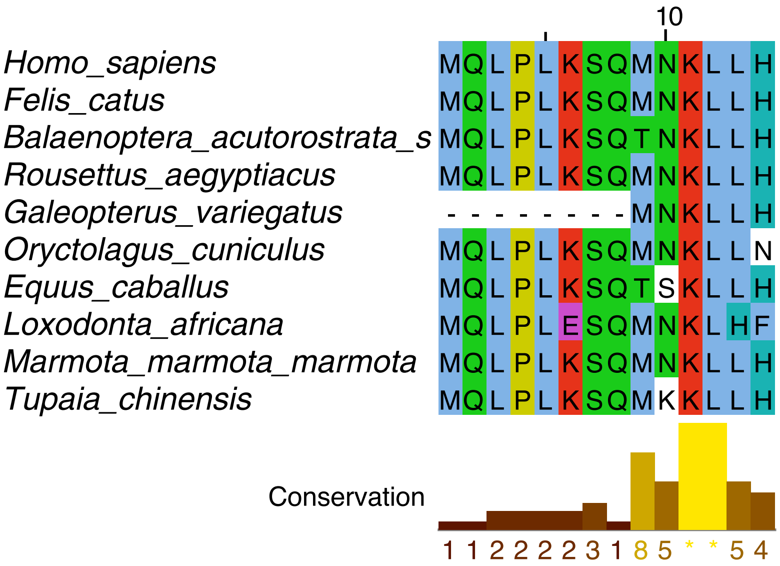

HG0489

ENSG00000143847 (PPFIA4)

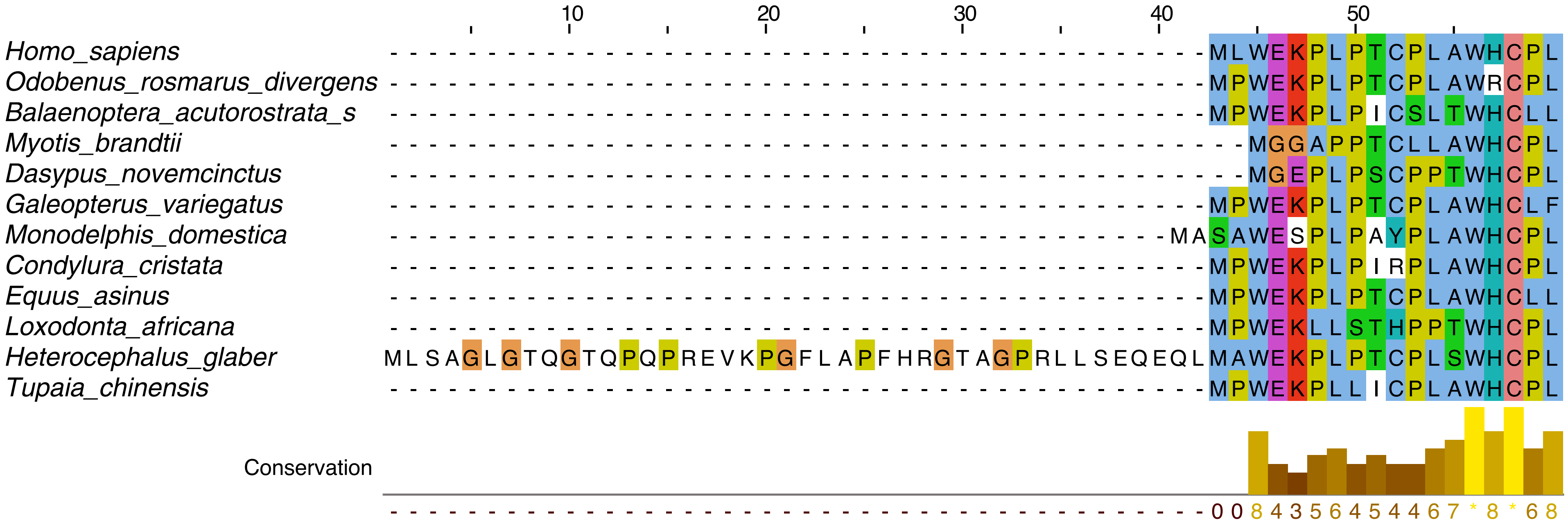

HG0490.1

ENSG00000152413 (HOMER1)

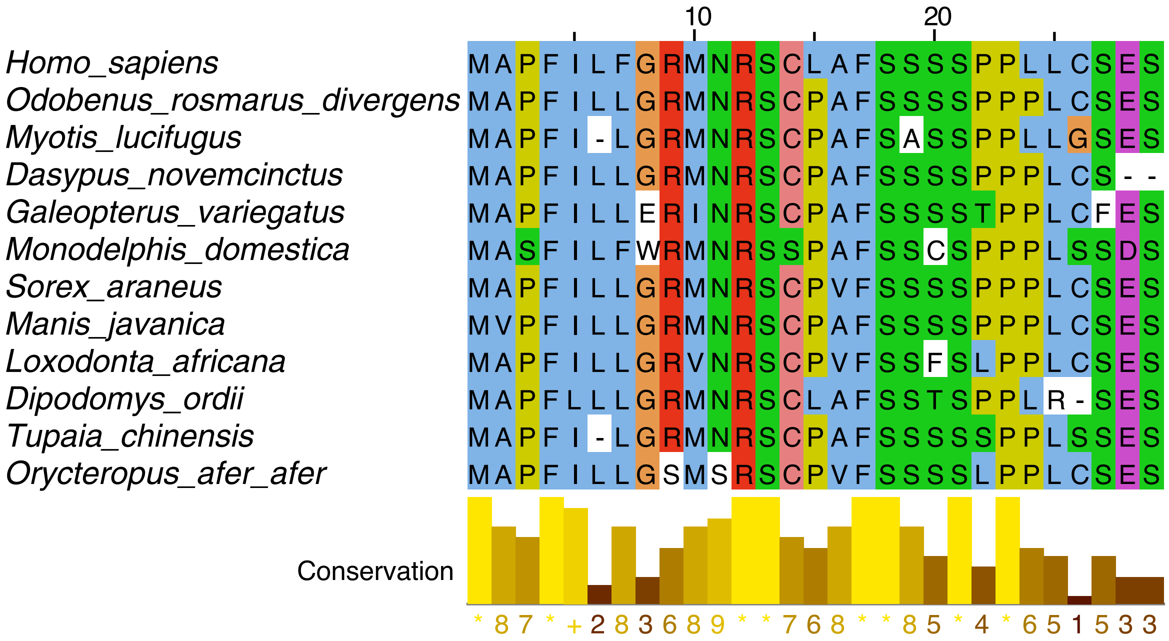

HG0490.2

ENSG00000152413 (HOMER1)

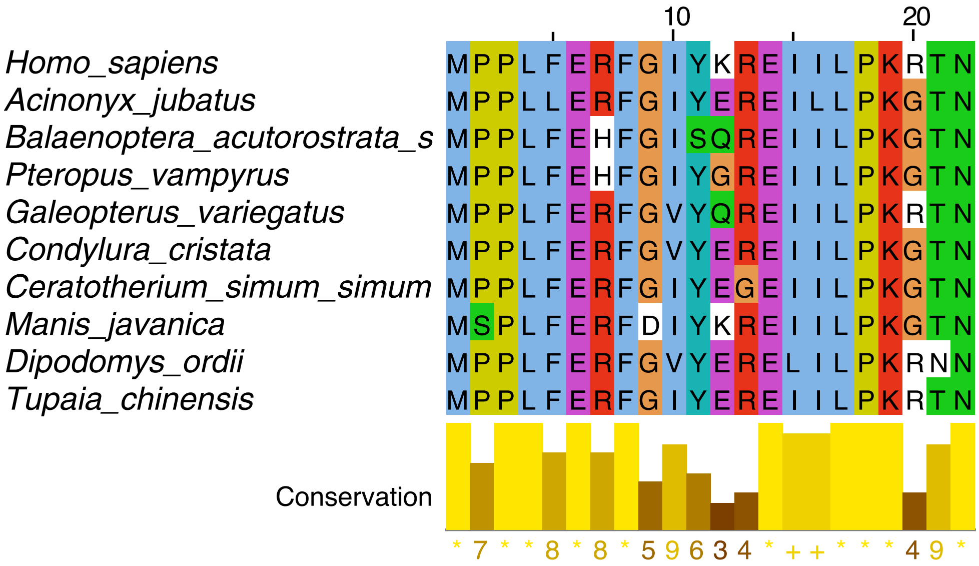

HG0491

ENSG00000154114 (TBCEL)

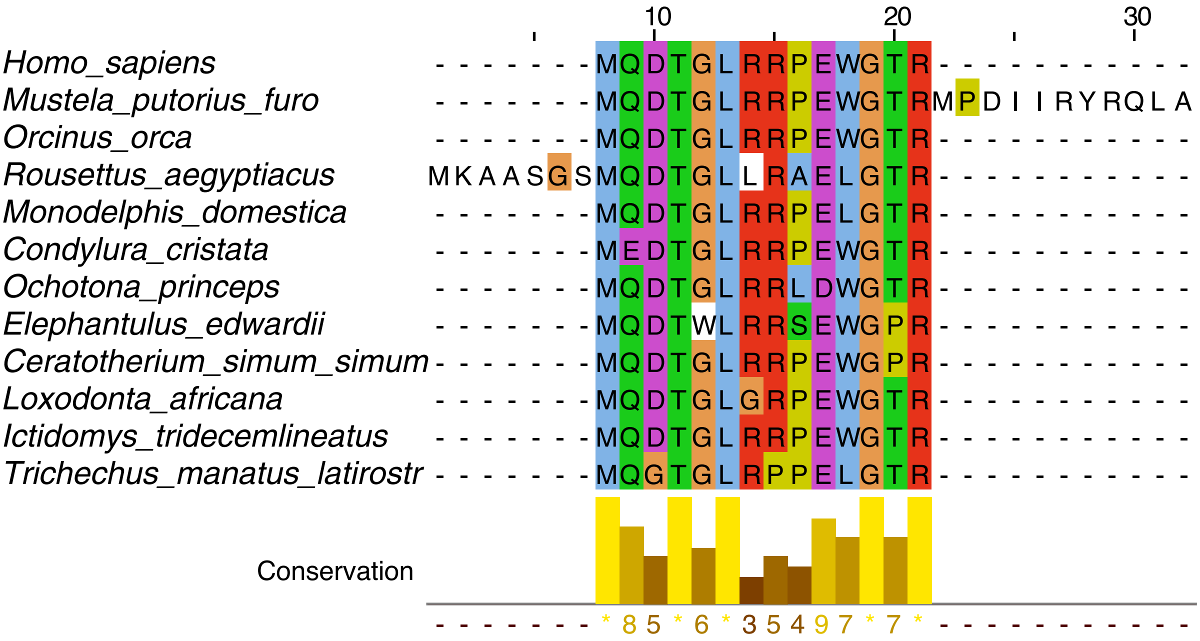

HG0492

ENSG00000162670 (BRINP3)

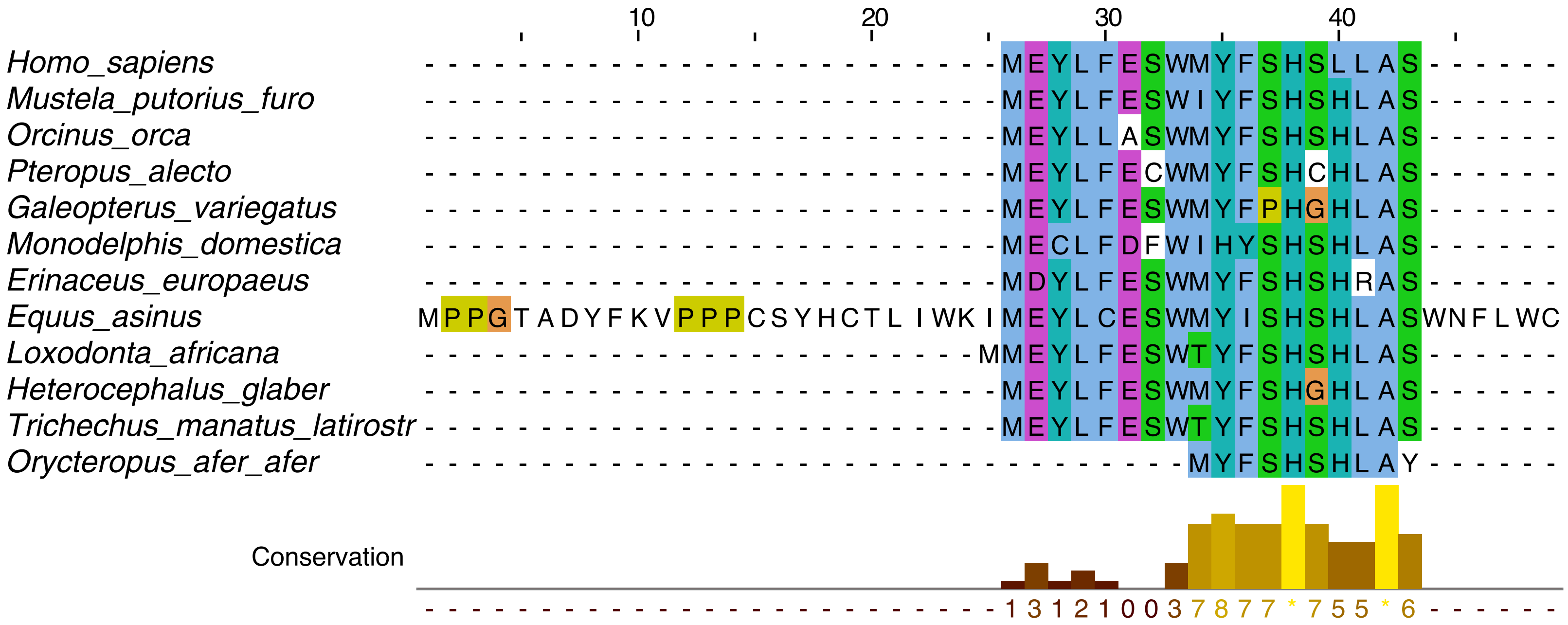

HG0493

ENSG00000164707 (SLC13A4)

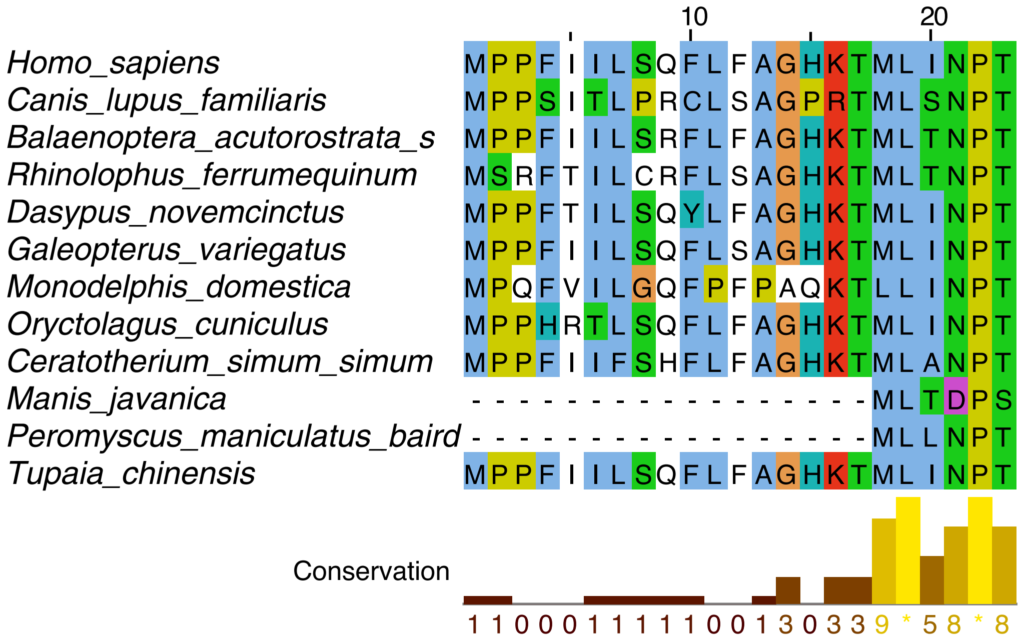

HG0494

ENSG00000184226 (PCDH9)

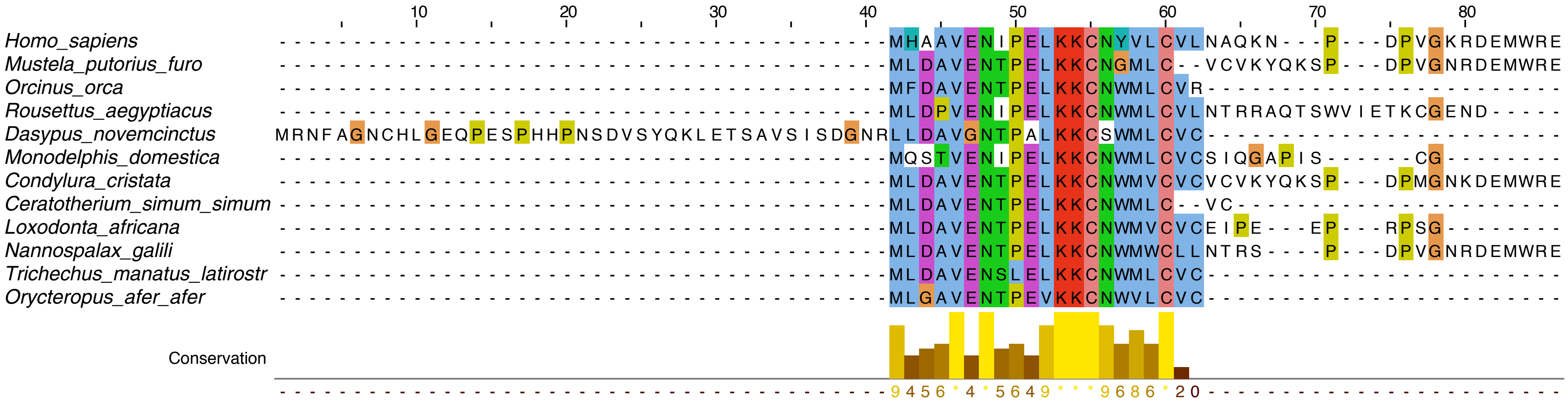

HG0495.1

ENSG00000196730 (DAPK1)

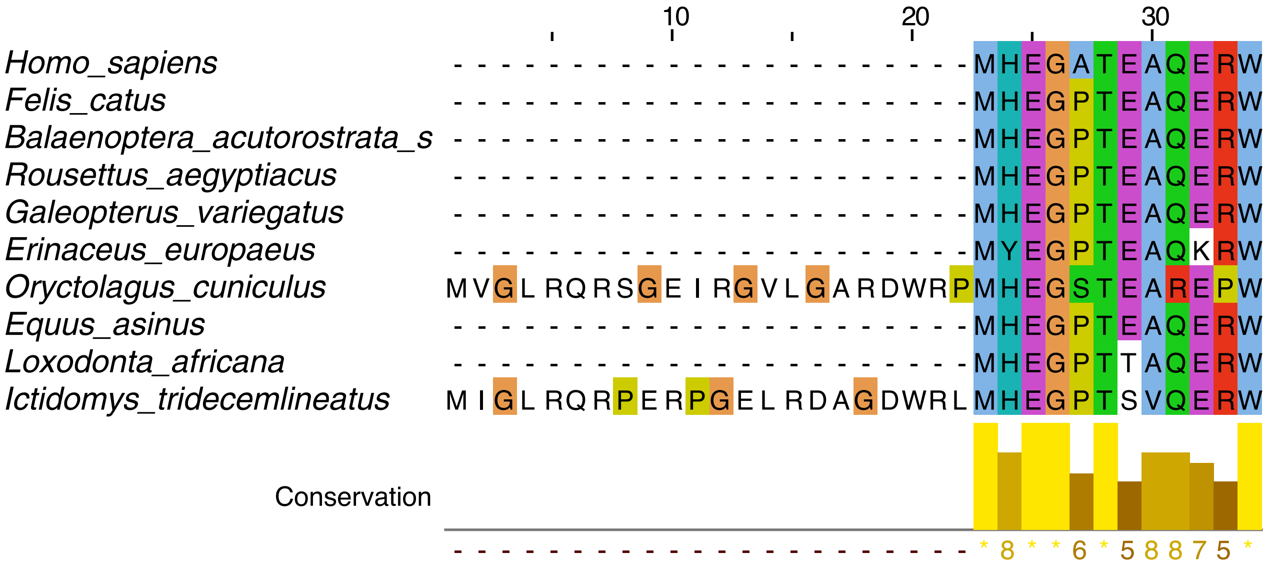

HG0495.2

ENSG00000196730 (DAPK1)

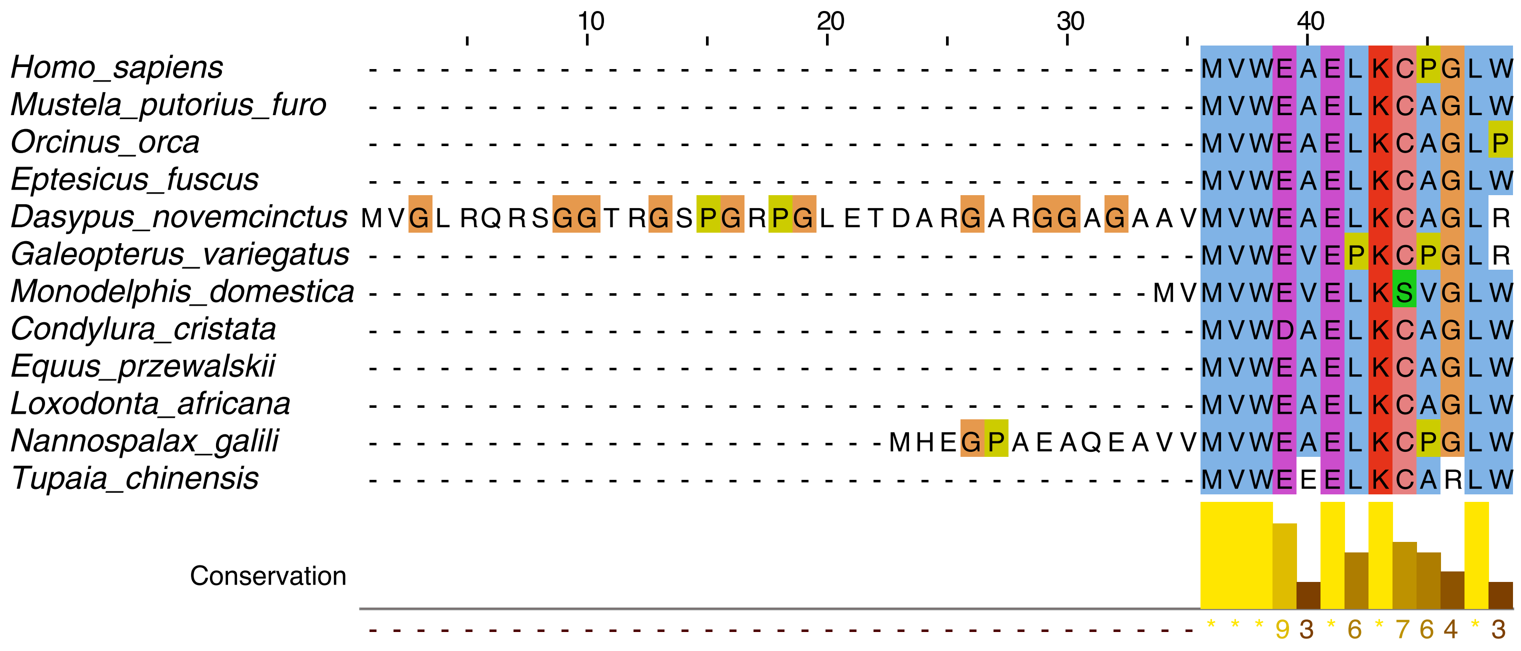

HG0496

ENSG00000135913 (USP37)

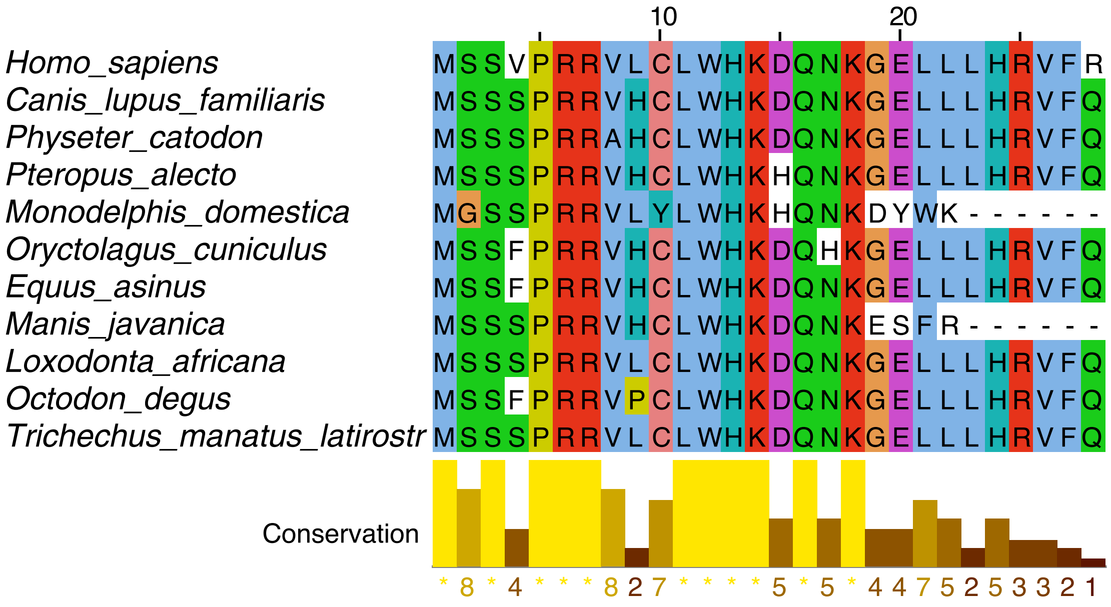

HG0497

ENSG00000147571 (CRH)

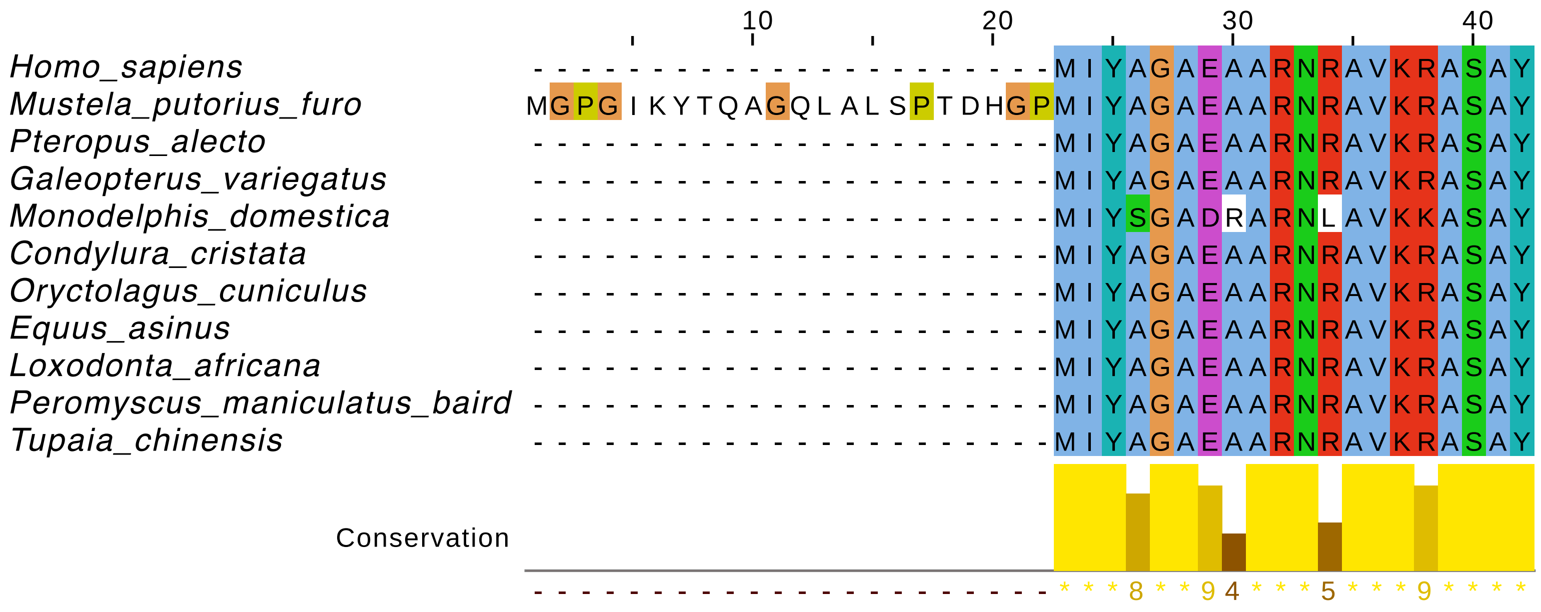

HG0498

ENSG00000005379 (TSPOAP1)

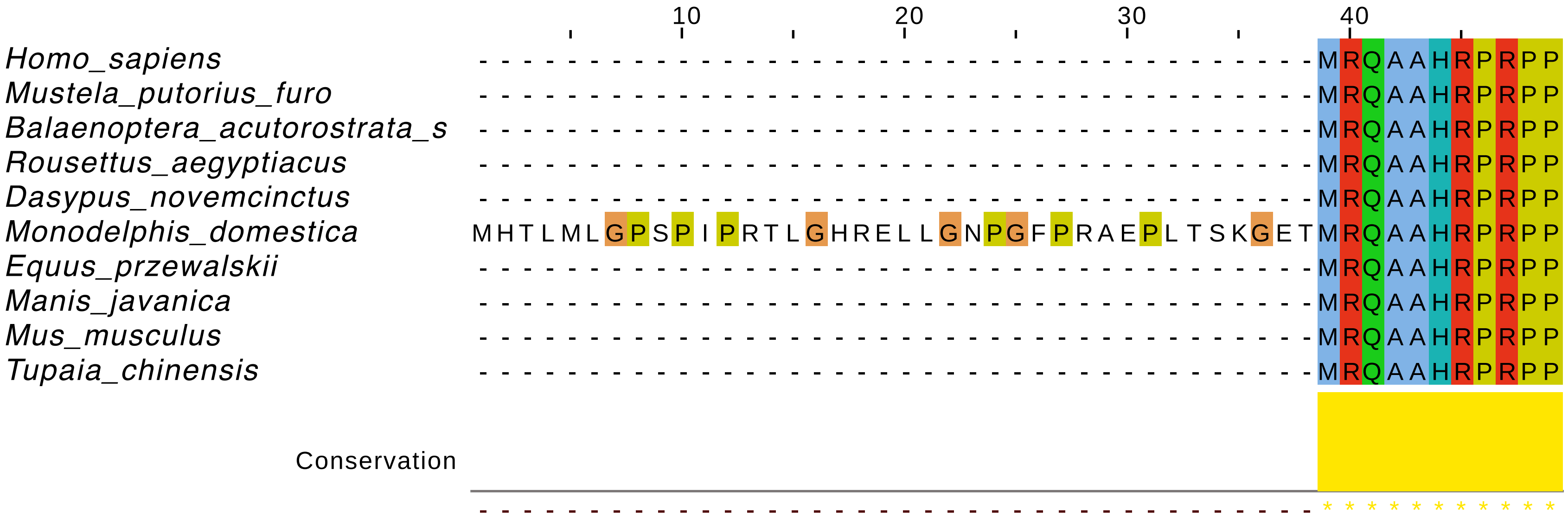

HG0499

ENSG00000011258 (MBTD1)

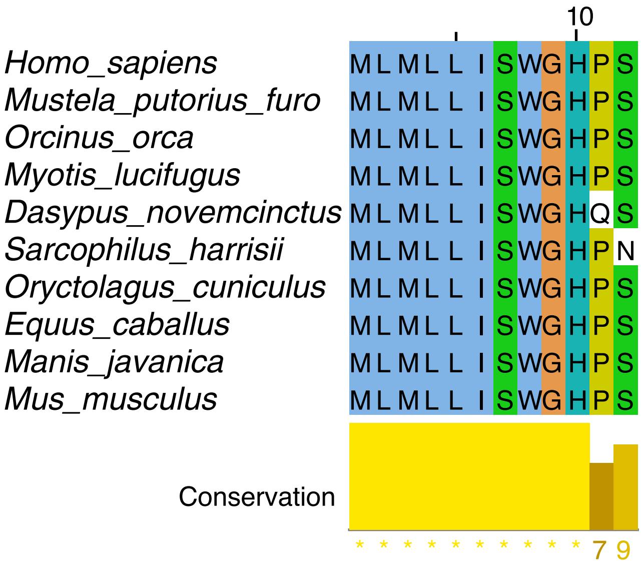

HG0500

ENSG00000070778 (PTPN21)

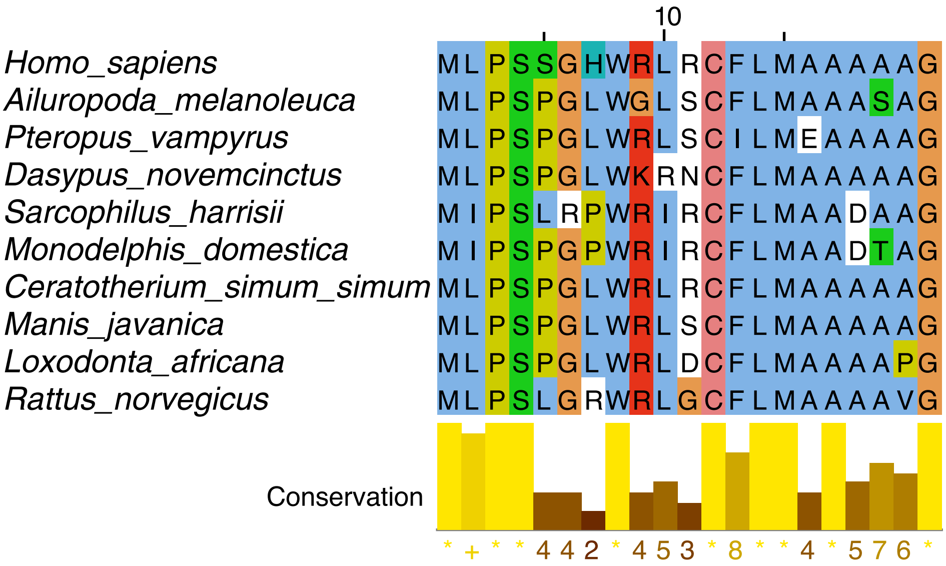

HG0501

ENSG00000110400 (NECTIN1)

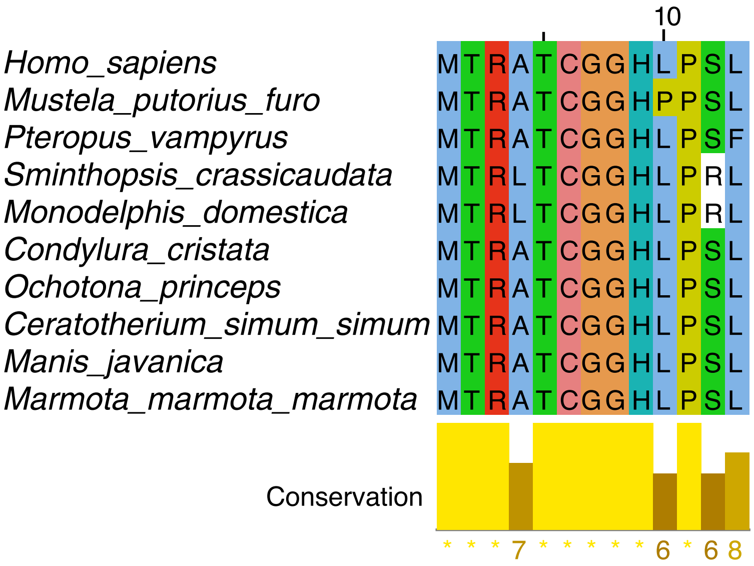

HG0502

ENSG00000126882

```
                                    10        20        30        40
Homo_sapiens                MSEPHNYSLNYKLFLTQRISVIFVHCEEKKREPRAHEMQQNPYL
Canis_lupus_familiaris      MSEQHDYPLTSRLSFF----------------------------
Rhinolophus_ferrumequinum   -------------------------------------MQQNPYL
Galeopterus_variegatus      -------------------------------------MQQSPRL
Monodelphis_domestica       -------------------------------------MQQNPYL
Erinaceus_europaeus         MSEQYDYLLTSKLFFF----------------------------
Oryctolagus_cuniculus       -------------------------------------MQQNPYL
Equus_caballus              -------------------------------------MQQNPYL
Microtus_ochrogaster        MSEQHAYPLNYKLFFF----------------------------
Orycteropus_afer_afer       -------------------------------------MQQNPYL

Conservation
                            --------------------------------------------
```

HG0503

ENSG00000141564 (RPTOR)

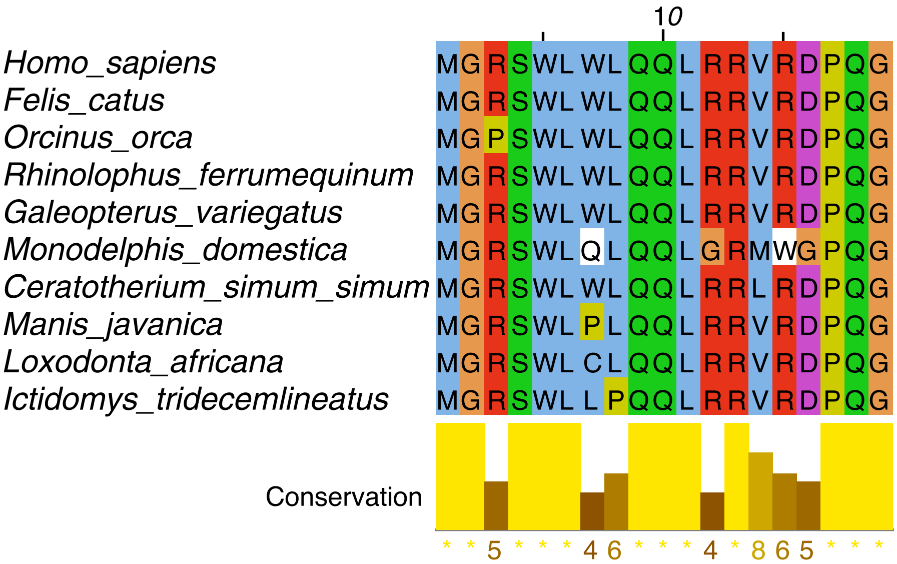

HG0504

ENSG00000144460 (NYAP2)

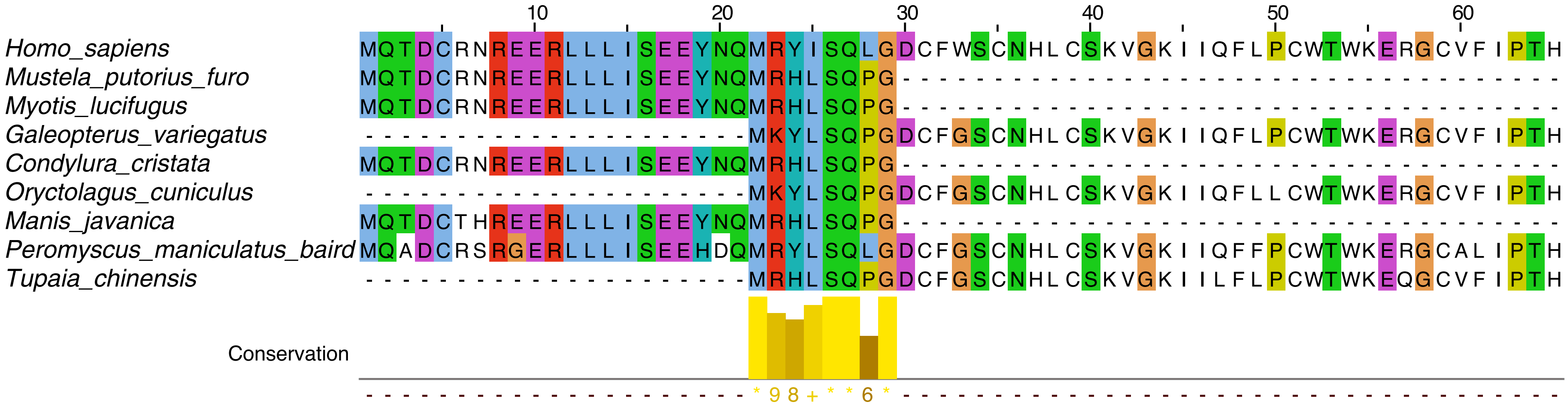

HG0505

ENSG00000157470

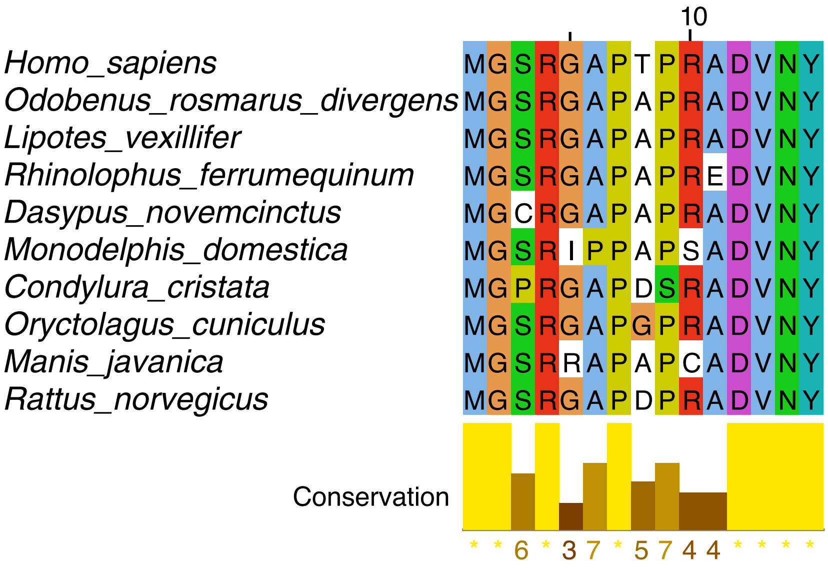

HG0506.1

ENSG00000177853 (ZNF518A)

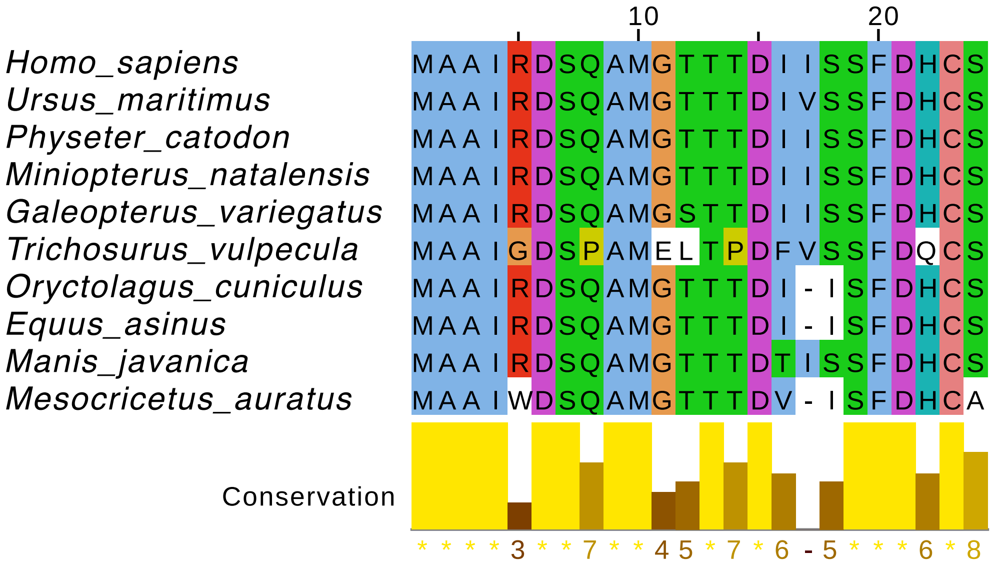

HG0506.2

ENSG00000177853 (ZNF518A)

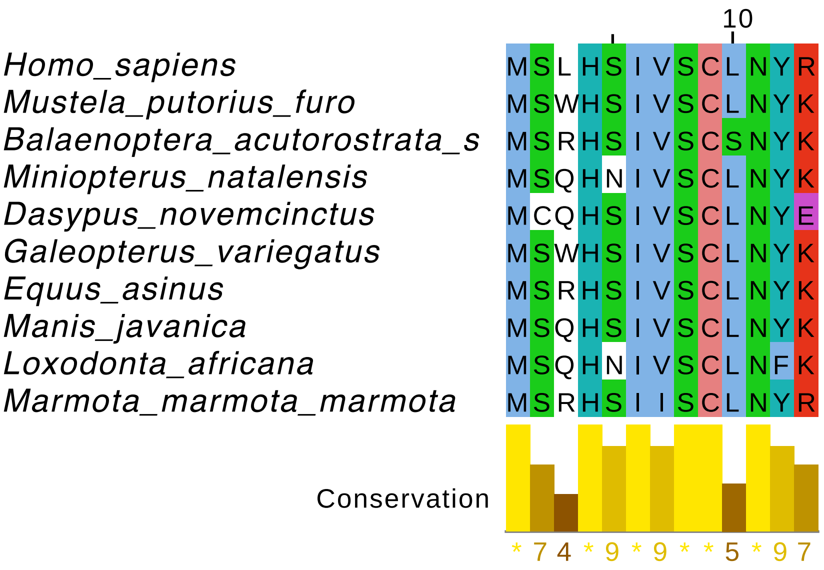

HG0507

ENSG00000060237 (WNK1)

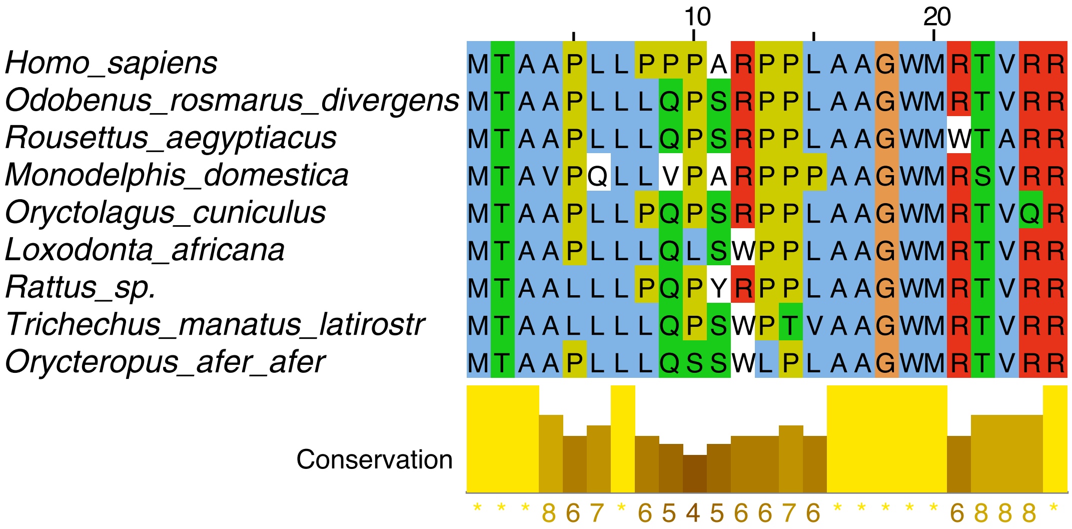

HG0508

ENSG00000061936 (SFSWAP)

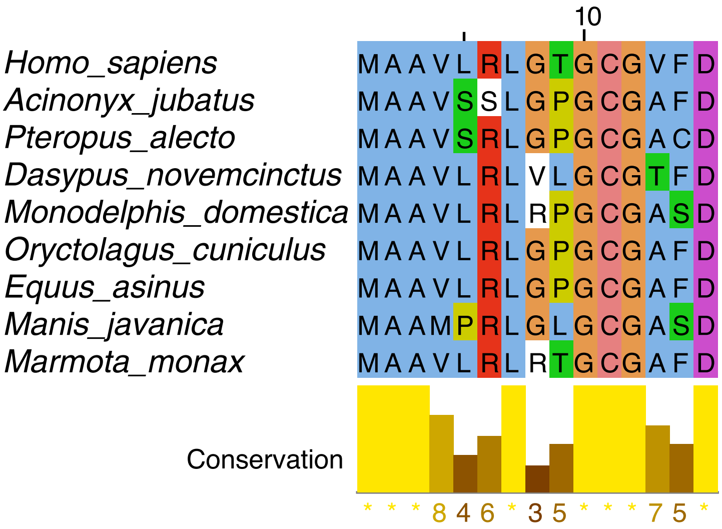

HG0509

ENSG00000068024 (HDAC4)

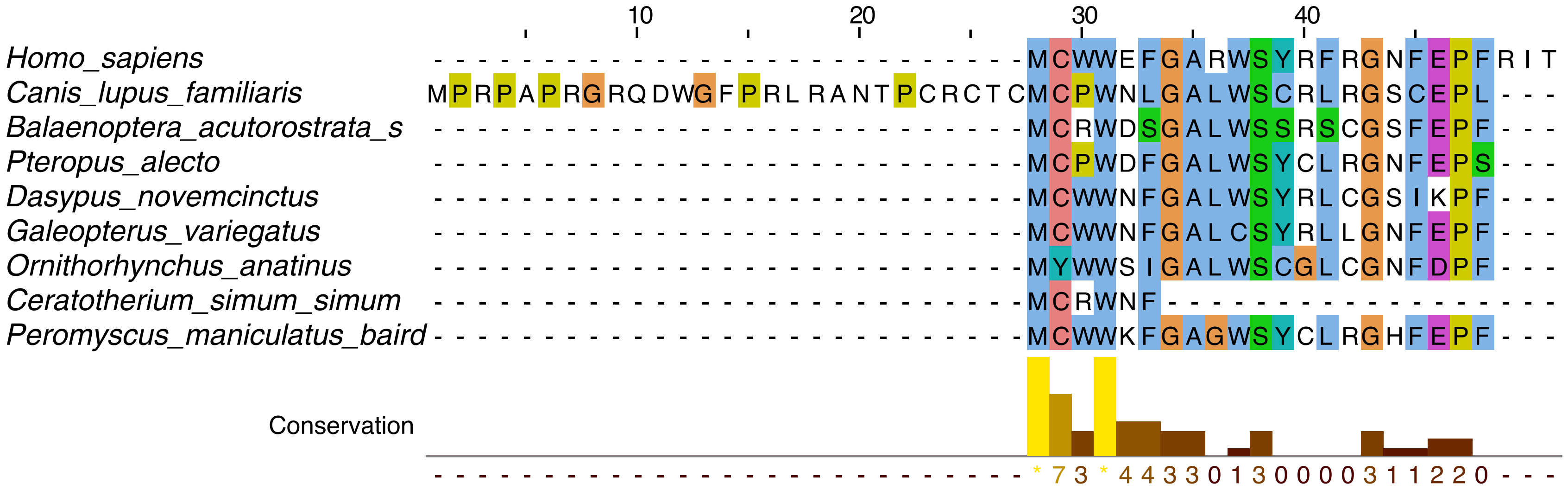

HG0510

ENSG00000134769 (DTNA)

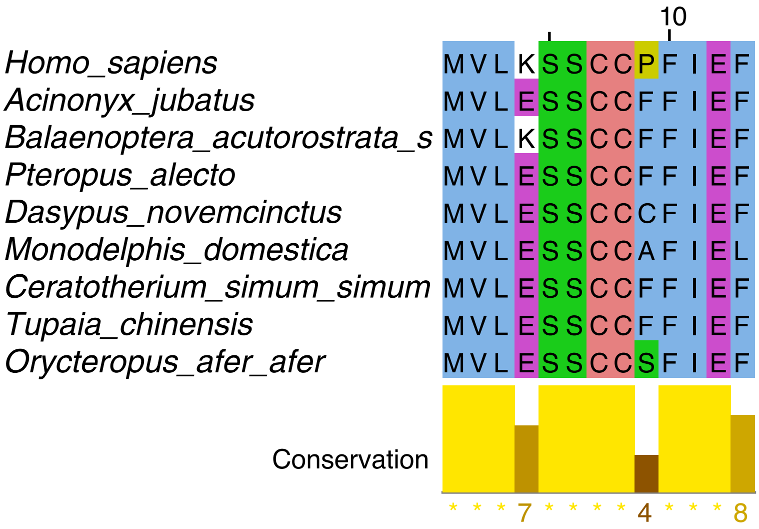

HG0511

ENSG00000143337 (TOR1AIP1)

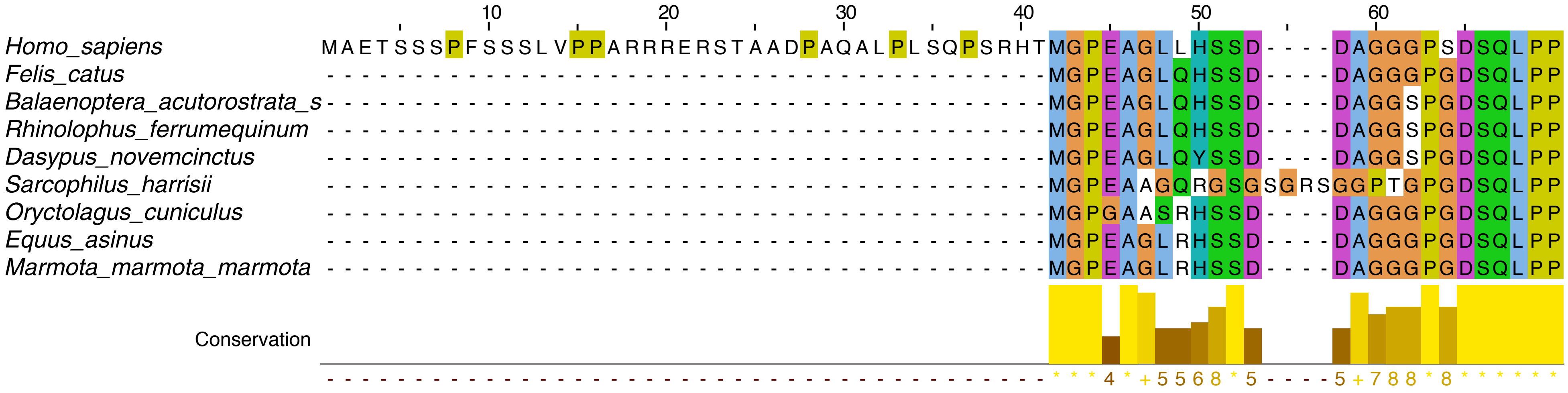

HG0512.1

ENSG00000112319 (EYA4)

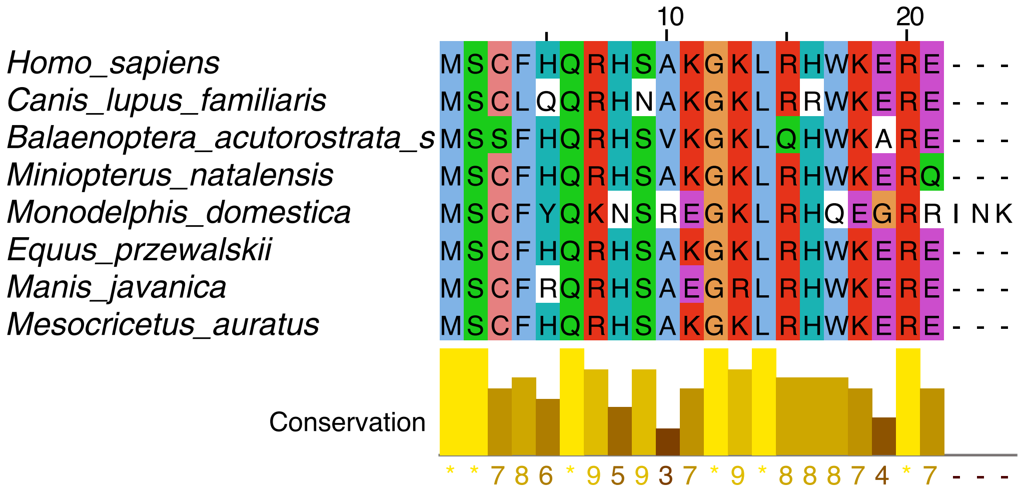

HG0512.2

ENSG00000104313 (EYA1)

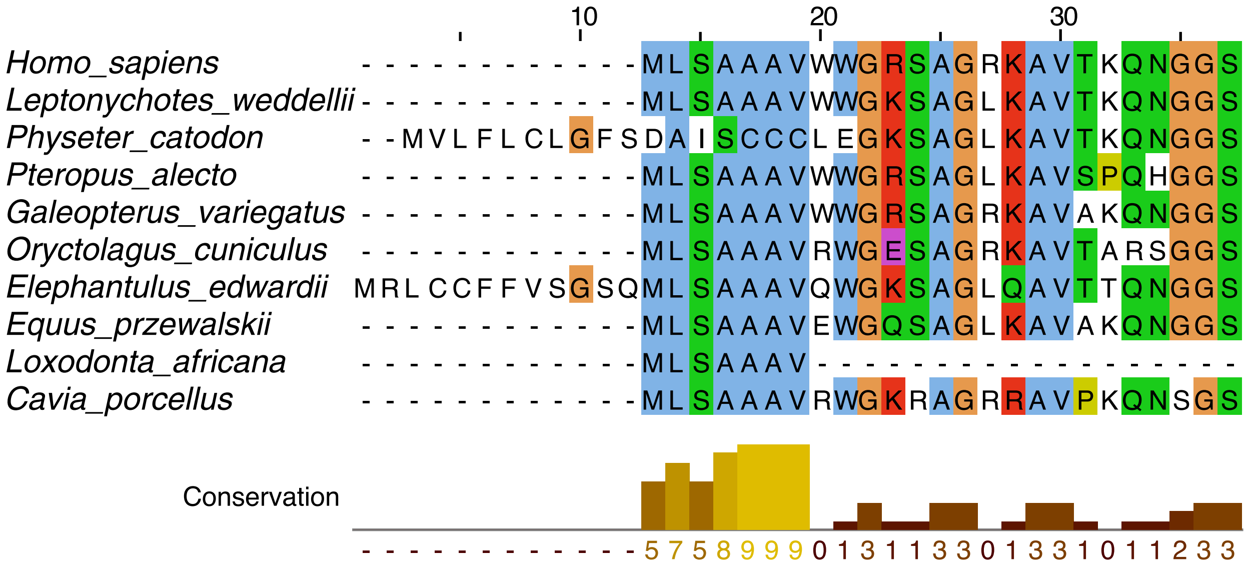

HG0512.3

ENSG00000104313 (EYA1)

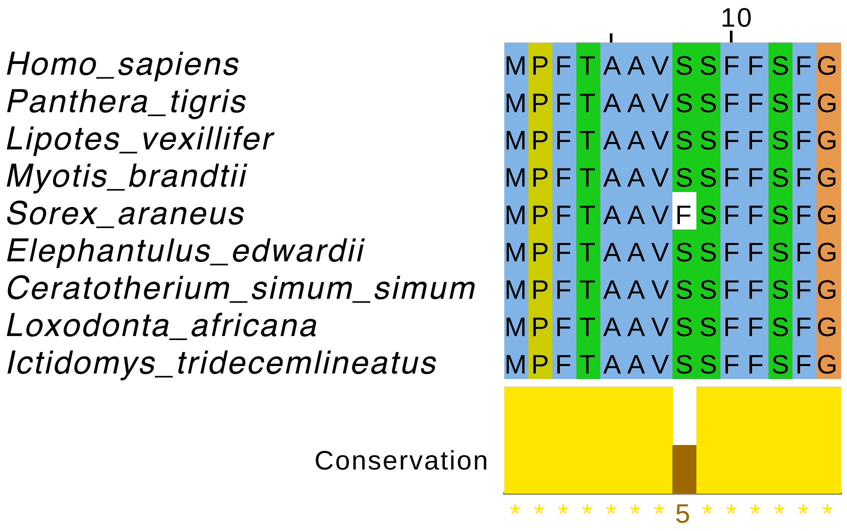

HG0513

ENSG00000126860 (EVI2A)

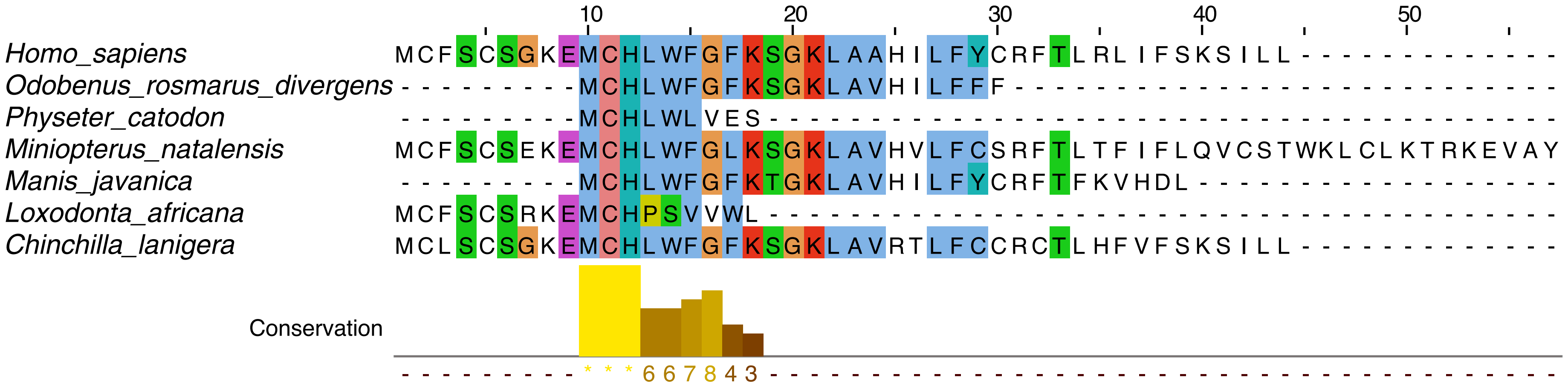

HG0514

ENSG00000147570 (DNAJC5B)

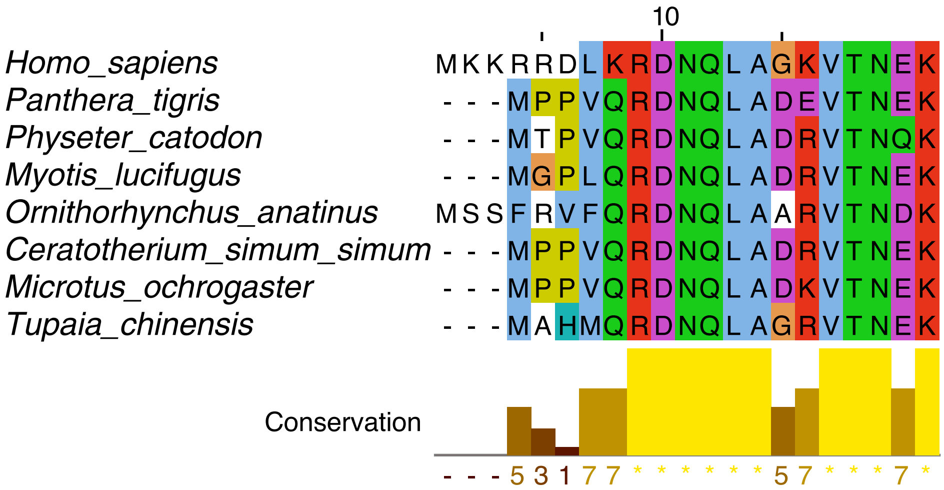

HG0515

ENSG00000170471 (RALGAPB)

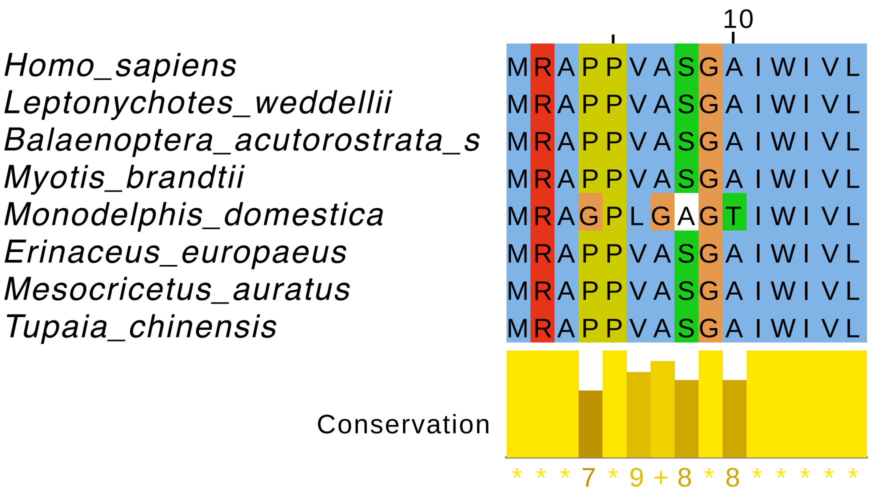

HG0516

ENSG00000041515 (MYO16)

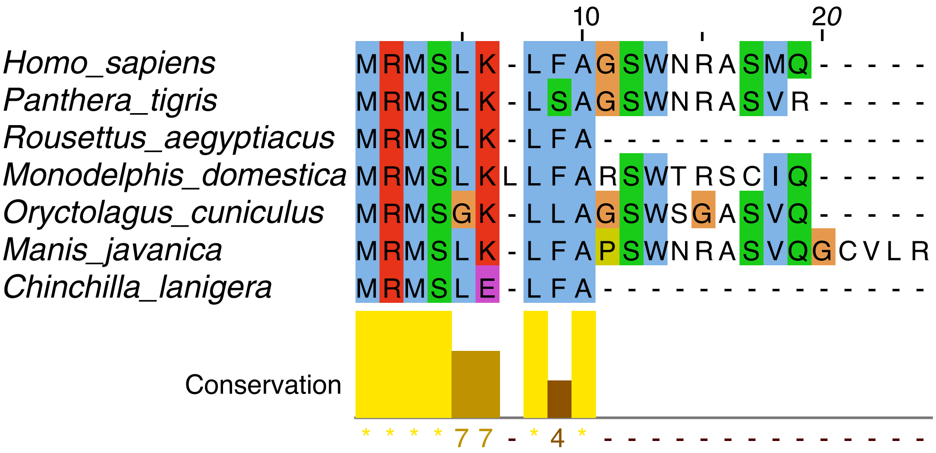

HG0517

ENSG00000086717 (PPEF1)

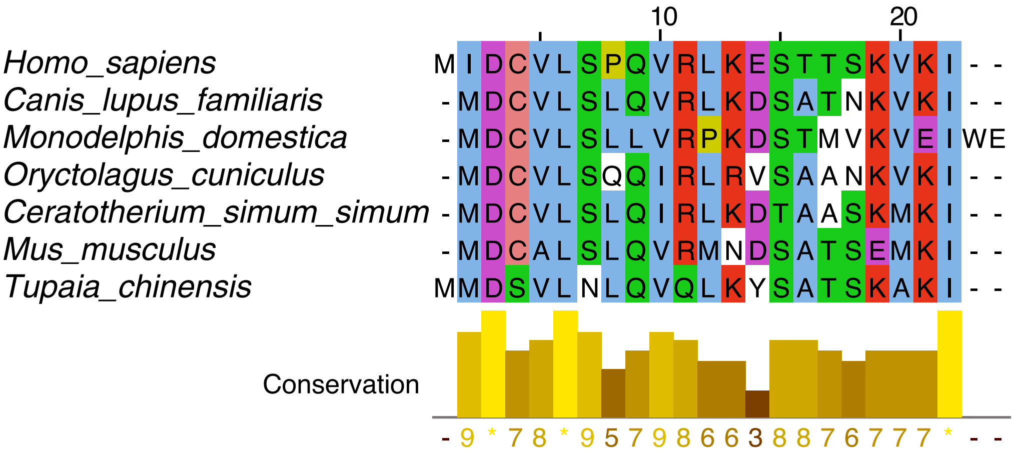

HG0518

ENSG00000111254 (AKAP3)

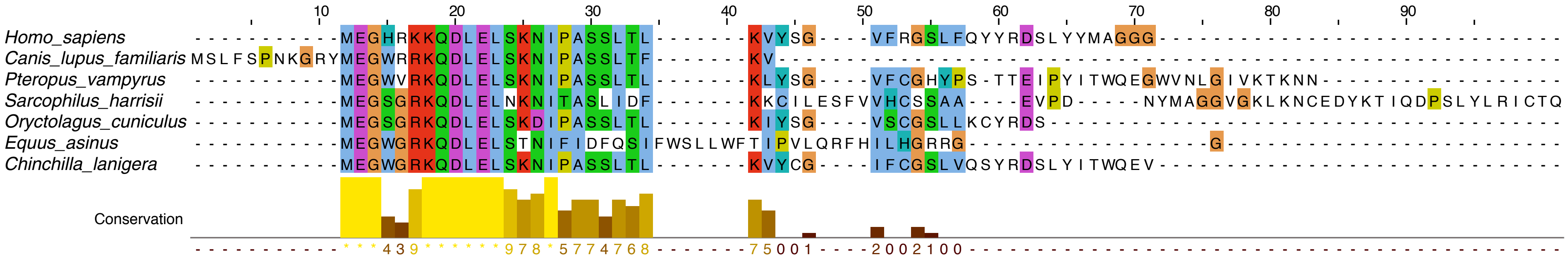

HG0519

ENSG00000127074 (RGS13)

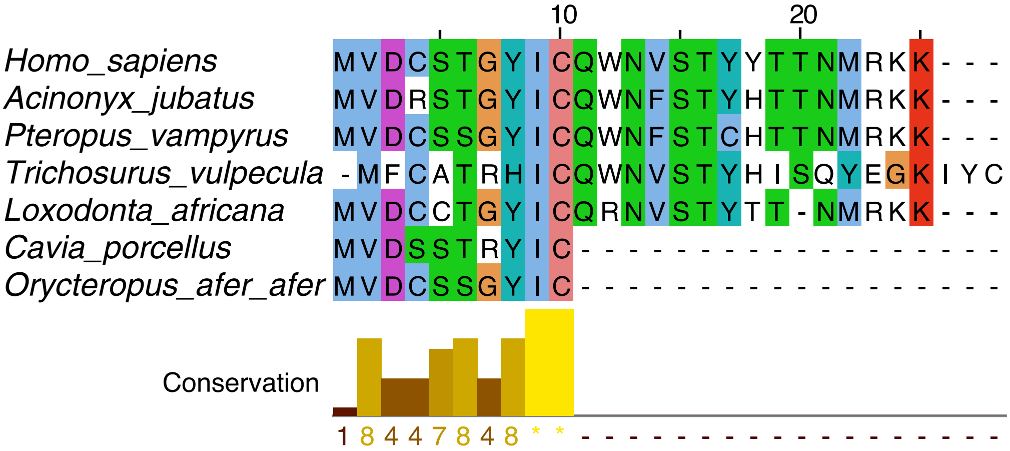

HG0520

ENSG00000197415 (VEPH1)

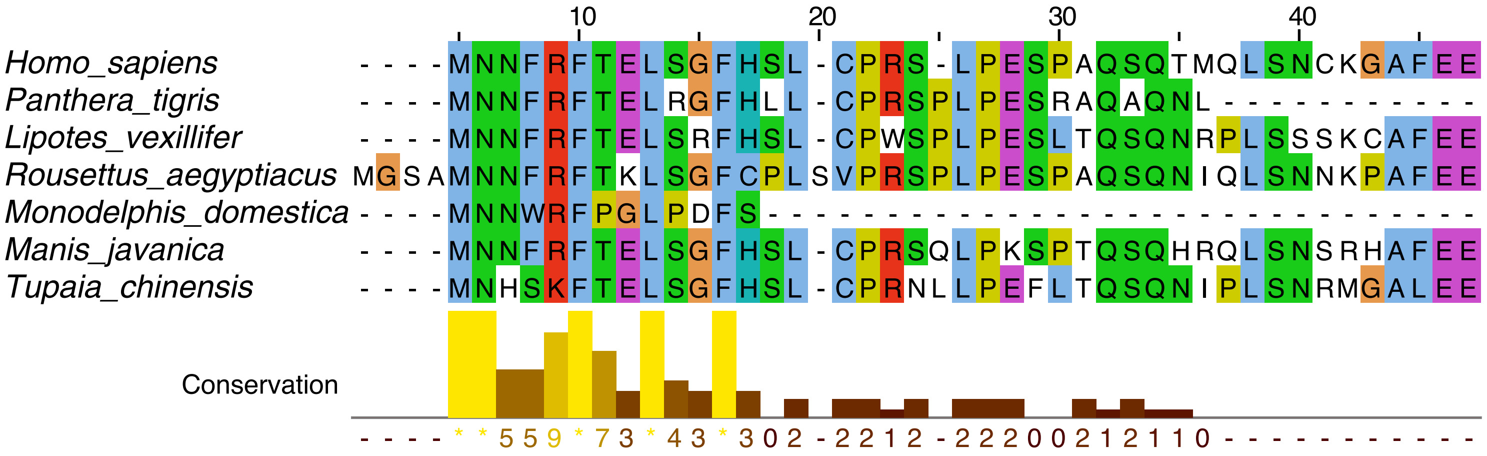

HG0521

ENSG00000038274 (MAT2B)

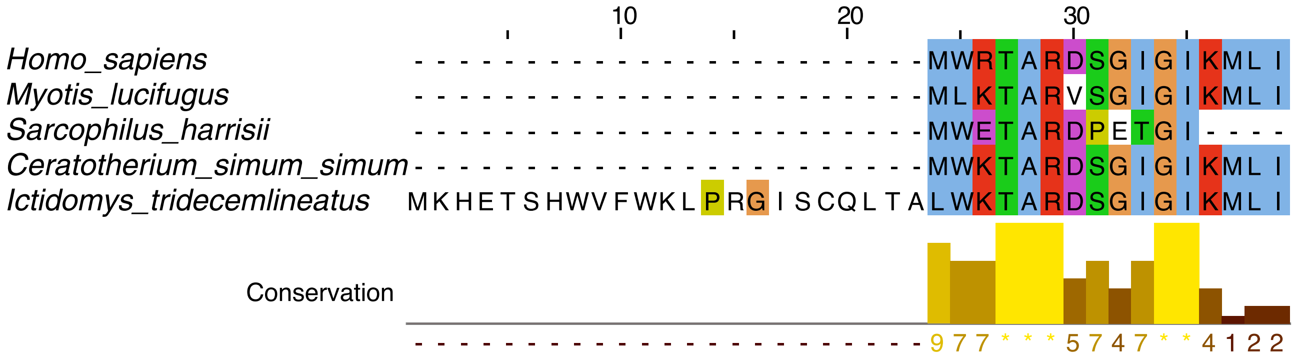

HG0522

ENSG00000140396 (NCOA2)

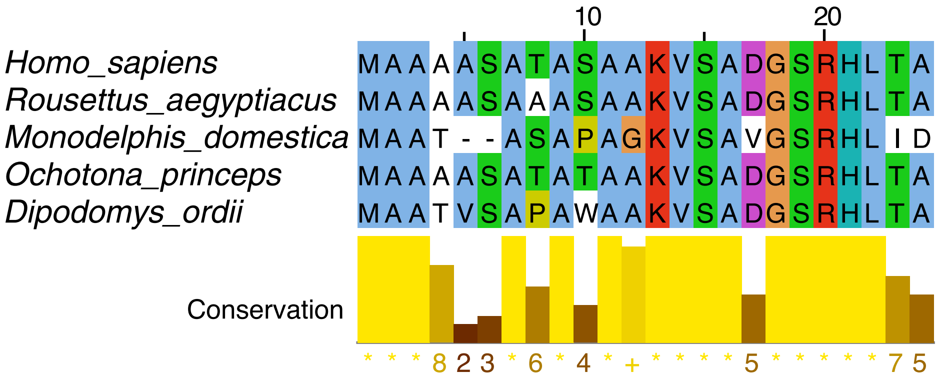

HG0523

ENSG00000075213 (SEMA3A)

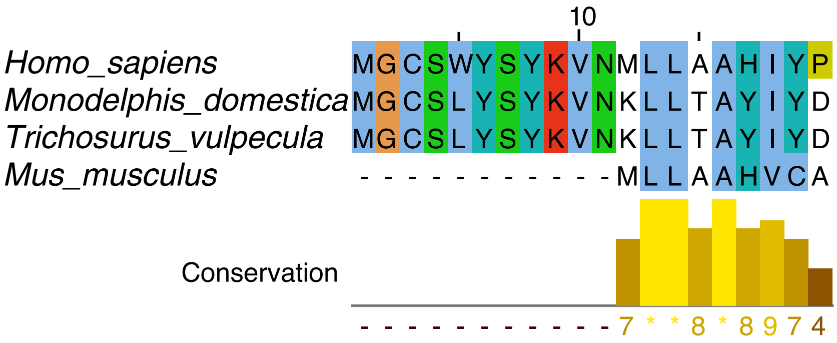

HG0524

ENSG00000107929 (LARP4B)

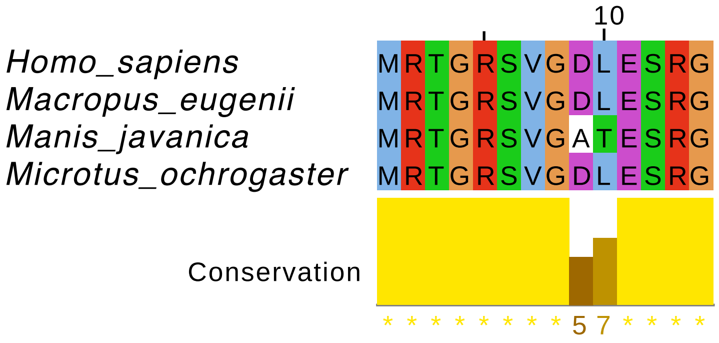

HG0525

ENSG00000136167 (LCP1)

```
                           10        20        30        40        50        60        70        80        90        100       110
Homo_sapiens               MTSPEIAVVQRKQSRGIELLQNARVSNIVSAAASYLTKVEEILYFQLRKLKIPPASDDQAPLDTEHCAFLLQVTSCLVTTHPGLTKLFCRSERRGSWSYTSTTKDSFFPARSVT
Canis_lupus_familiaris     -----------------------------------------------------------------MICHSSHFLPC--DRTAGVDSCSADPRGGGFWSHASTTEDSFSTRSVA-
Ornithorhynchus_anatinus   ------------------------------------------------------------------------MTSCLVTT----------------------------------
Mus_musculus               ------------------------------------------MLLERRKWK----------VASRFHCDLLSVTSCLVTTQPCLTRLLCRSEGLGFLTTQQCC-----------
```

Conservation

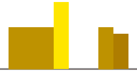

```
------------------------------------------------------------------------777*--76----------------------------------
```

HG0526

ENSG00000143569 (UBAP2L)

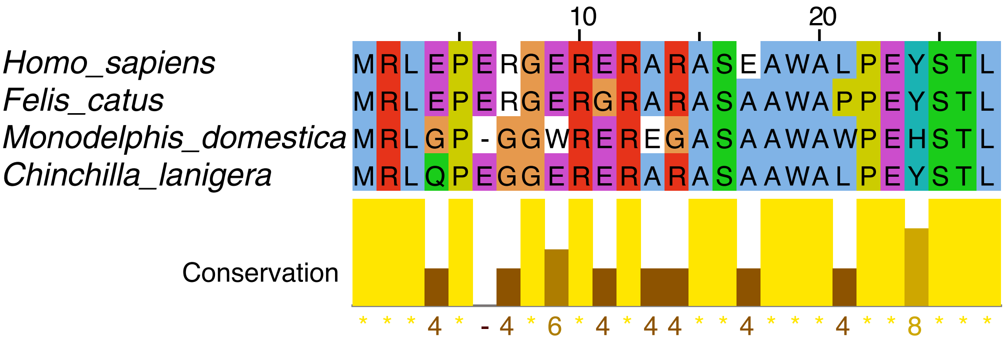

HG0527

ENSG00000198382 (UVRAG)

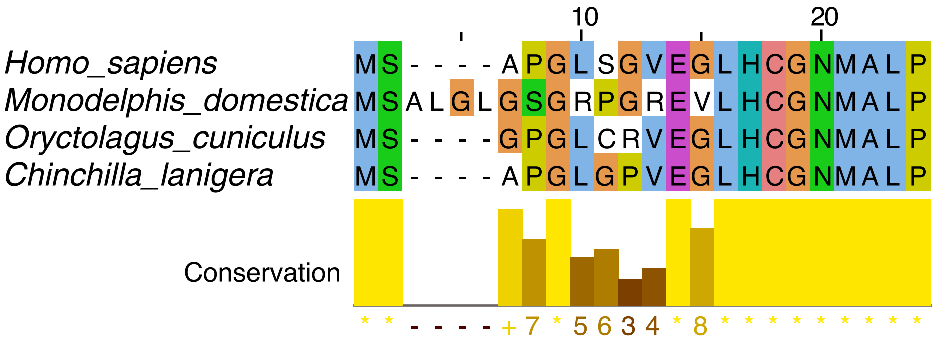

HG0528

ENSG00000204767

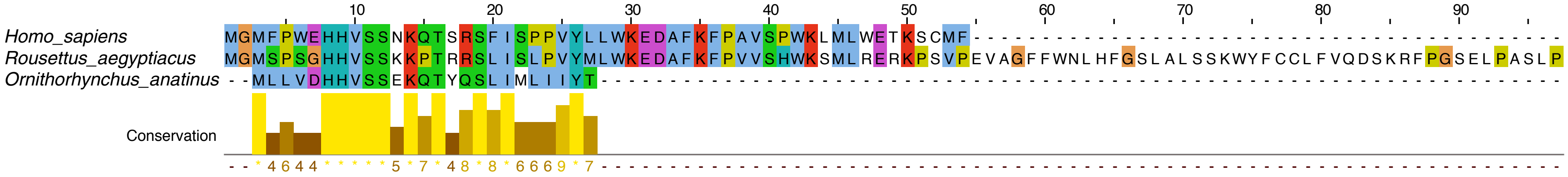

HG0529

ENSG00000112851 (ERBIN)

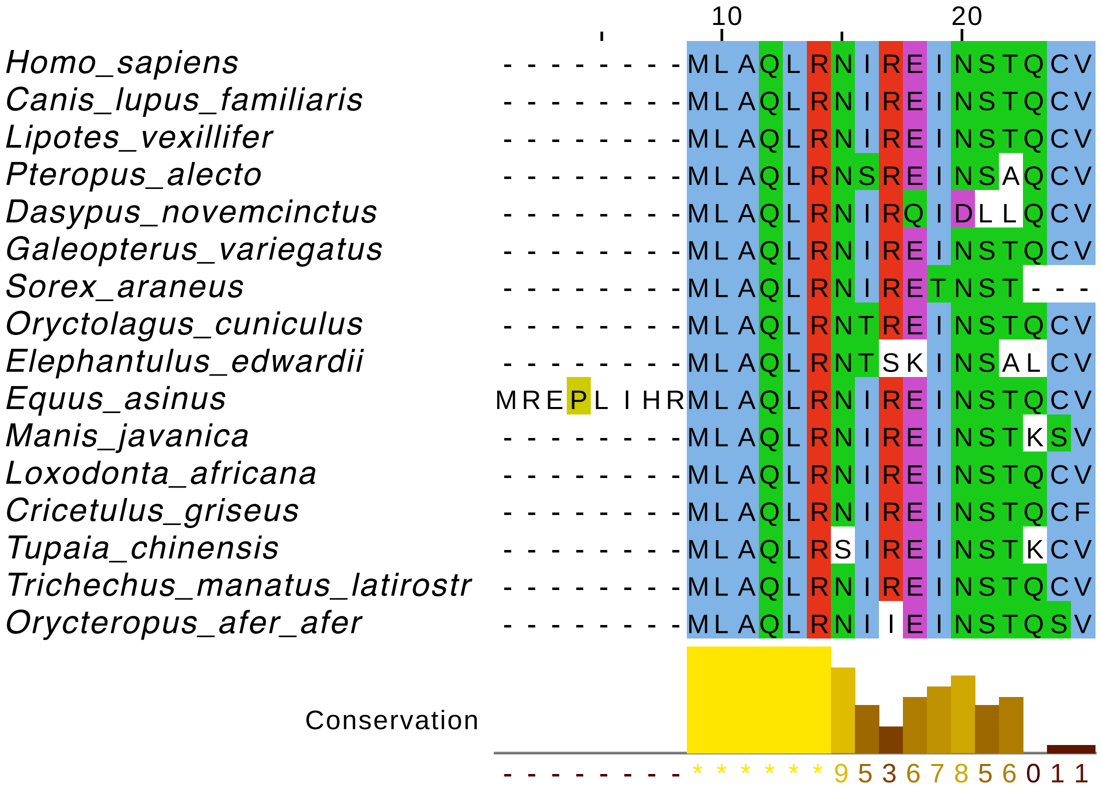

HG0530

ENSG00000128594 (LRRC4)

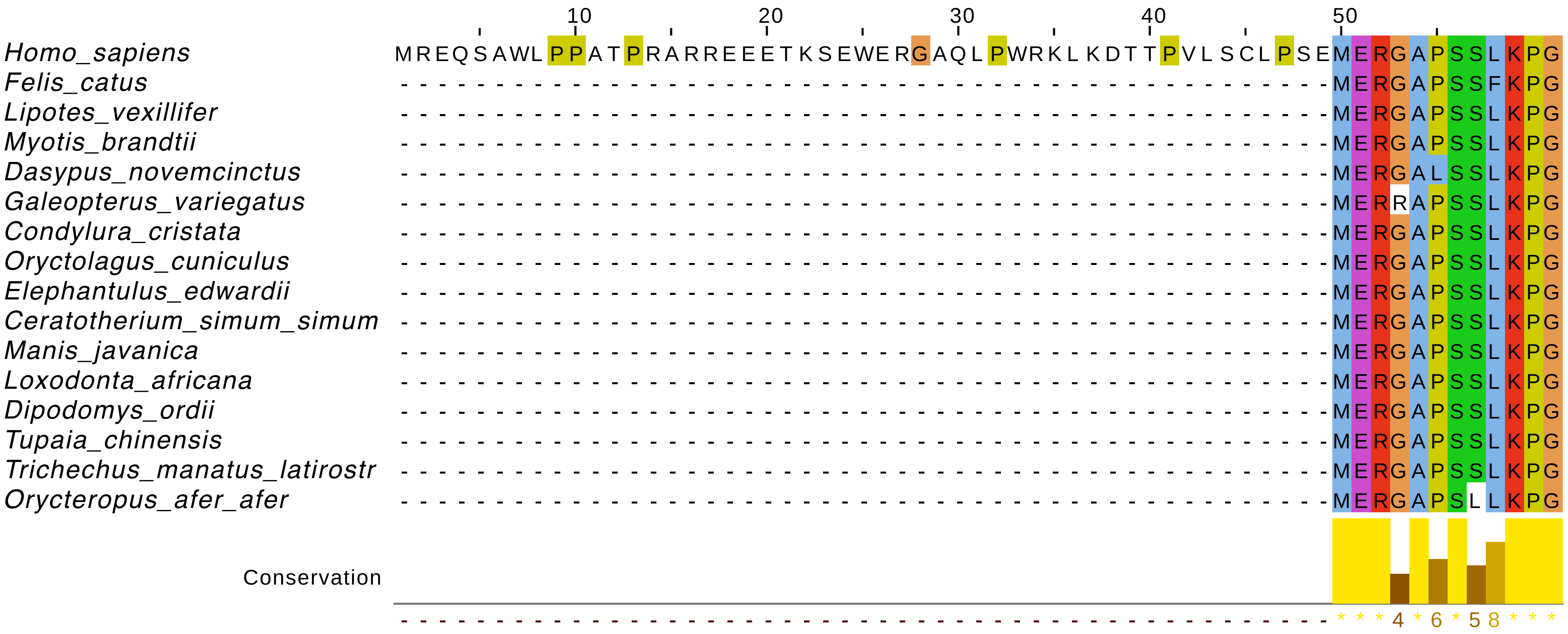

HG0531

ENSG00000146834 (MEPCE)

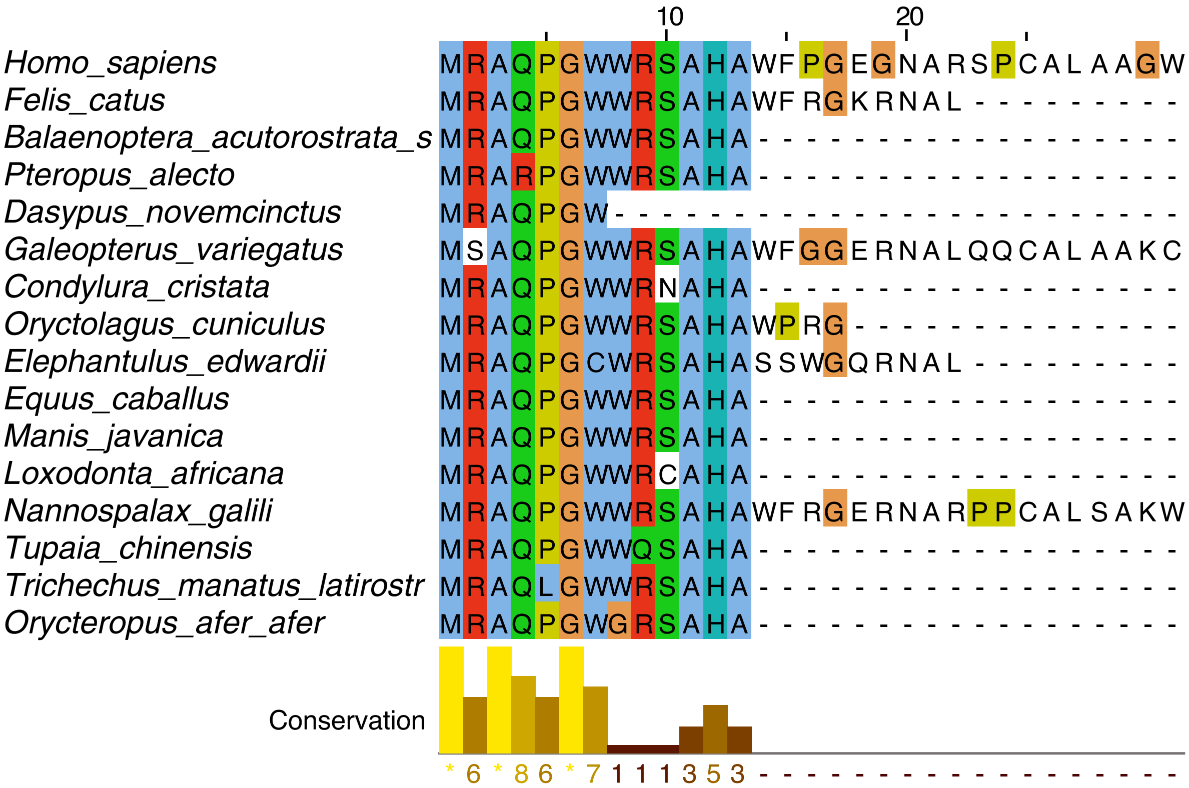

HG0532

ENSG00000105221 (AKT2)

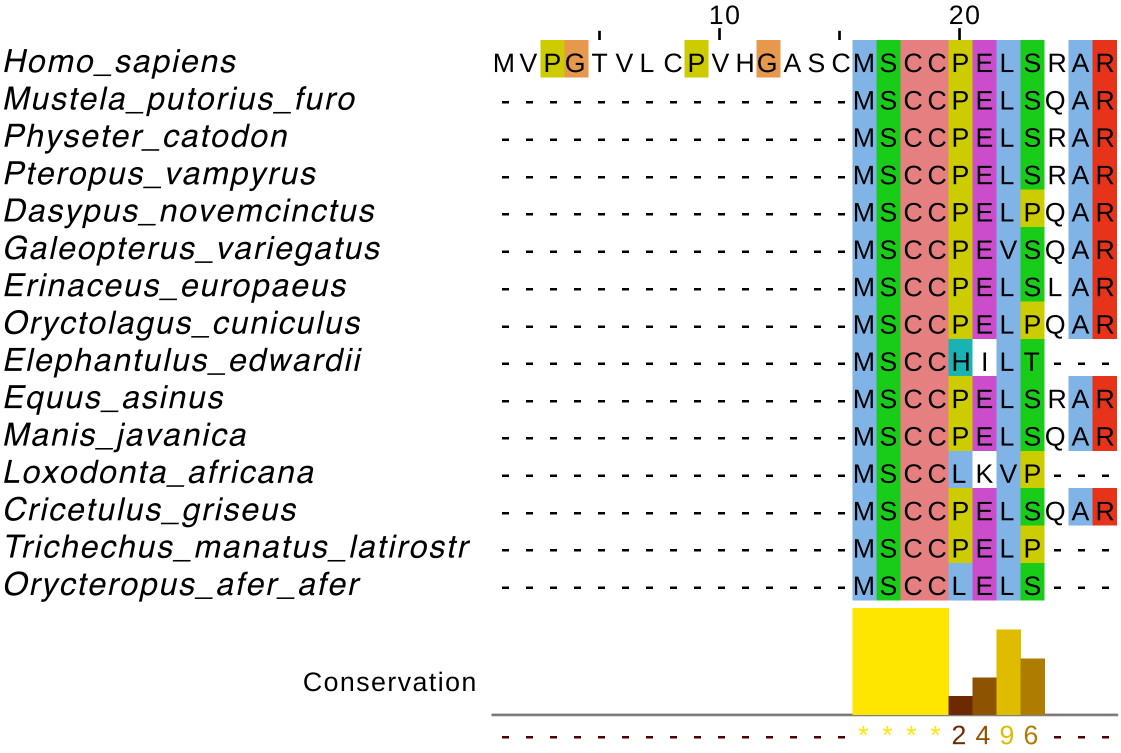

HG0533

ENSG00000114209 (PDCD10)

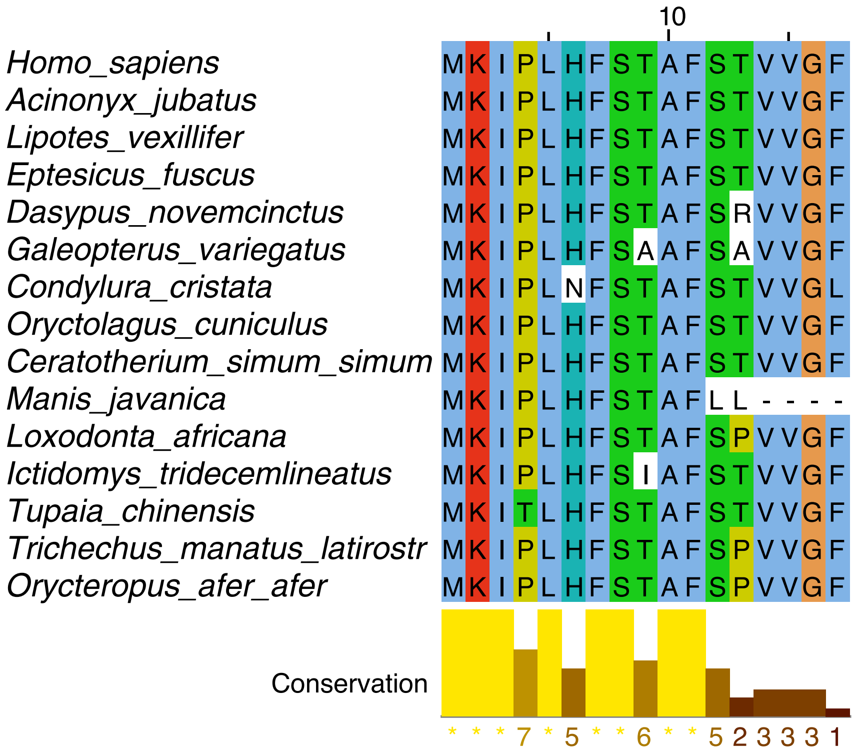

HG0534

ENSG00000117408 (IPO13)

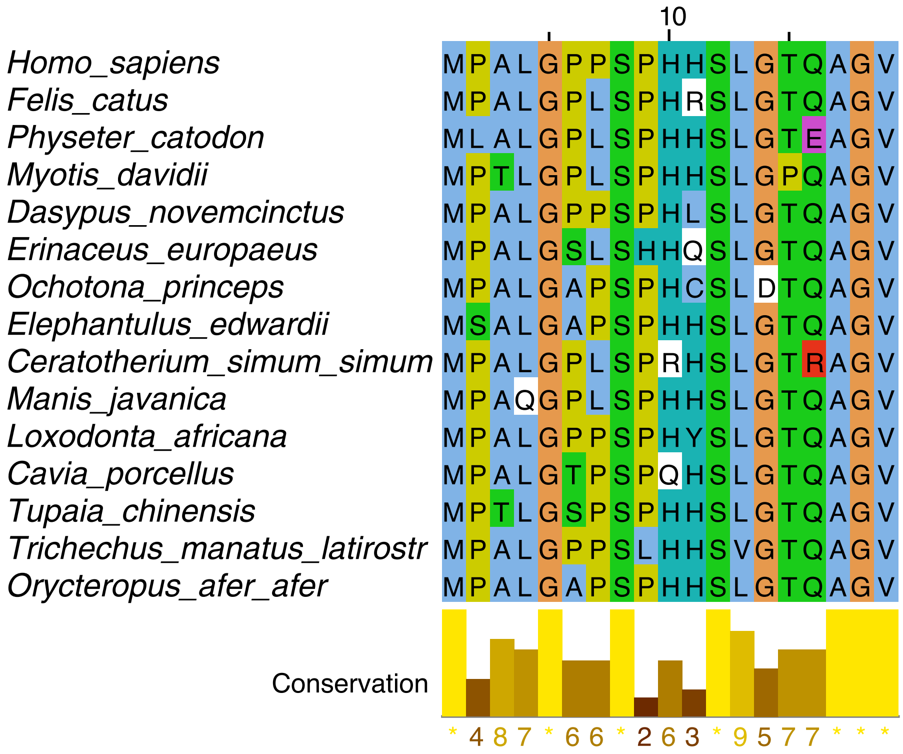

HG0535

ENSG00000118407 (FILIP1)

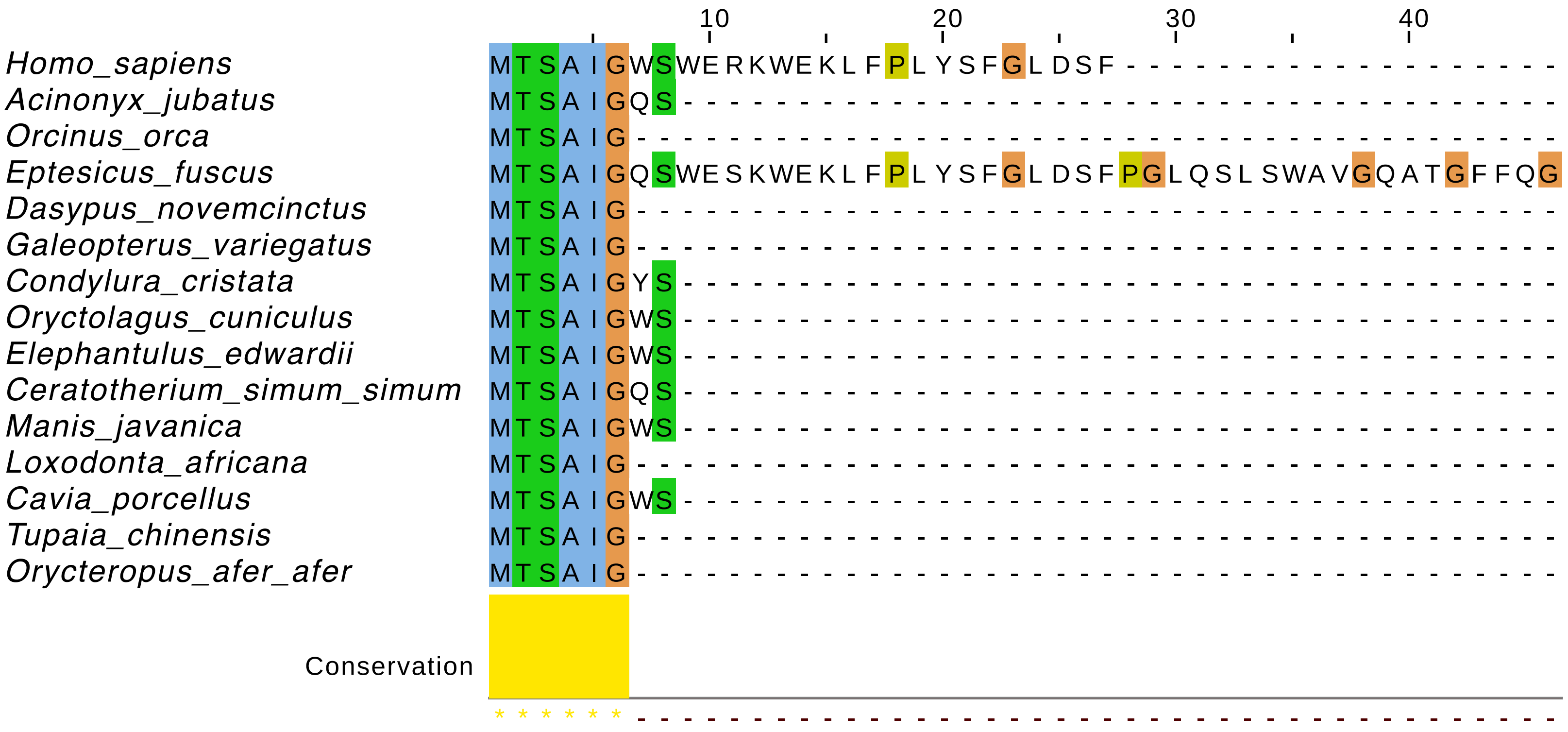

HG0536

ENSG00000181751 (C5orf30)

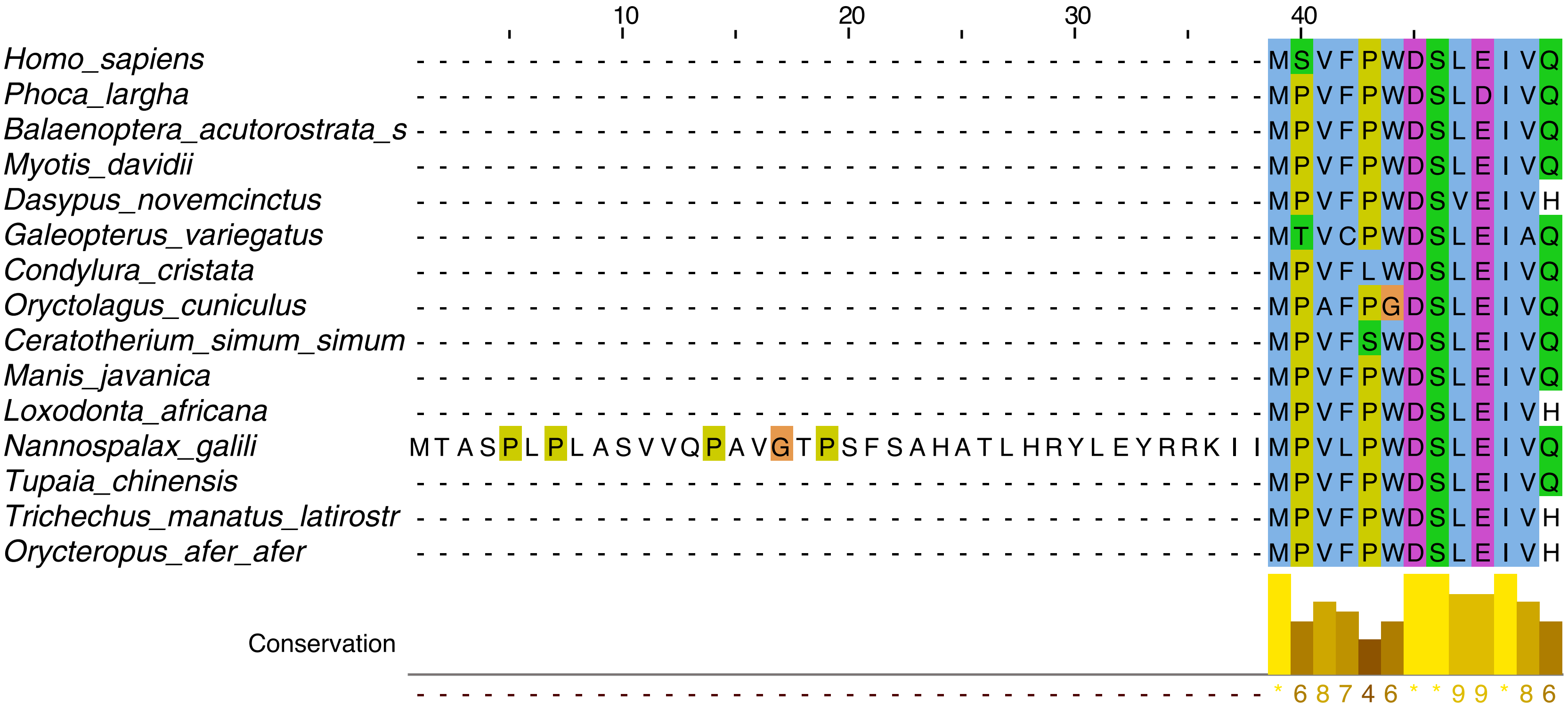

HG0537

ENSG00000184481 (FOXO4)

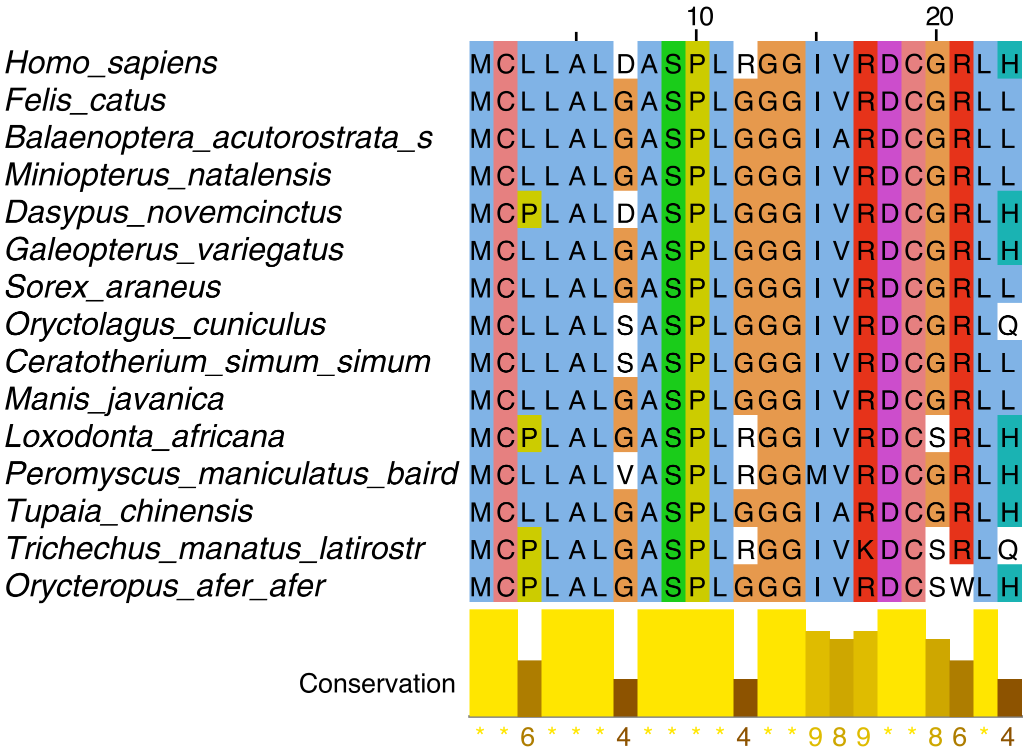

HG0538

ENSG00000006116 (CACNG3)

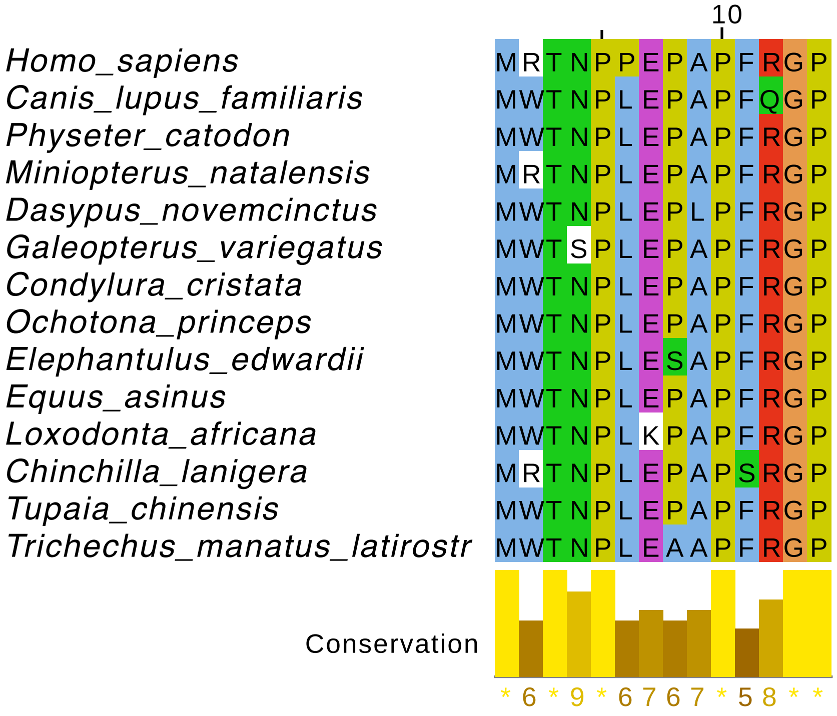

HG0539

ENSG00000115966 (ATF2)

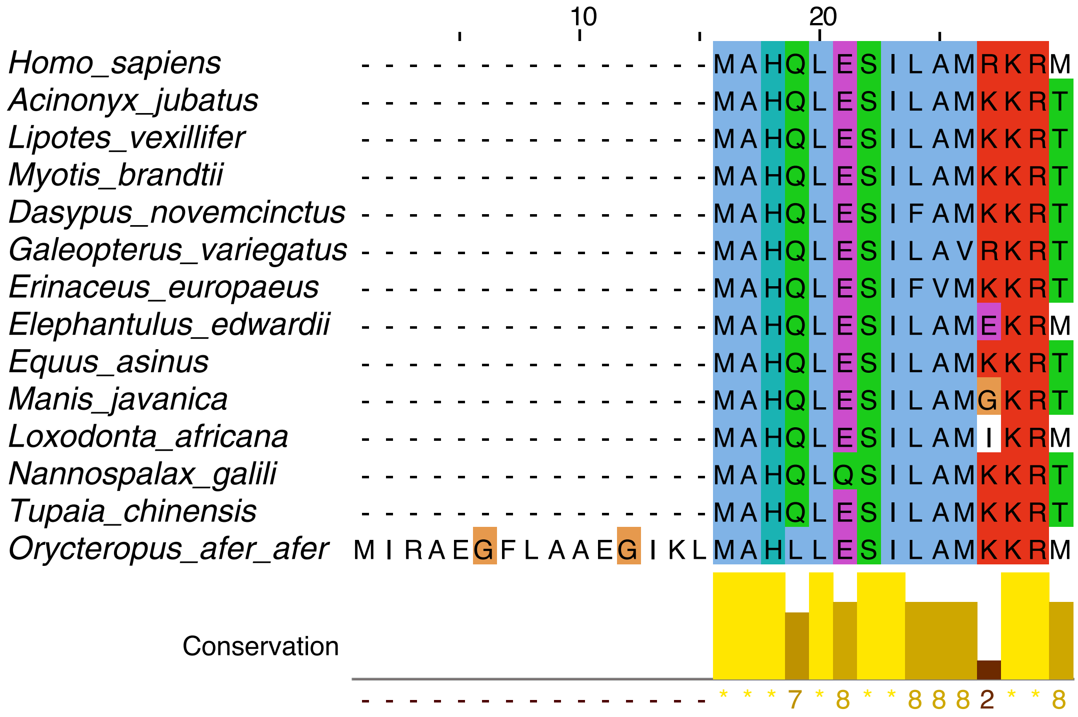

HG0540

ENSG00000120519 (SLC10A7)

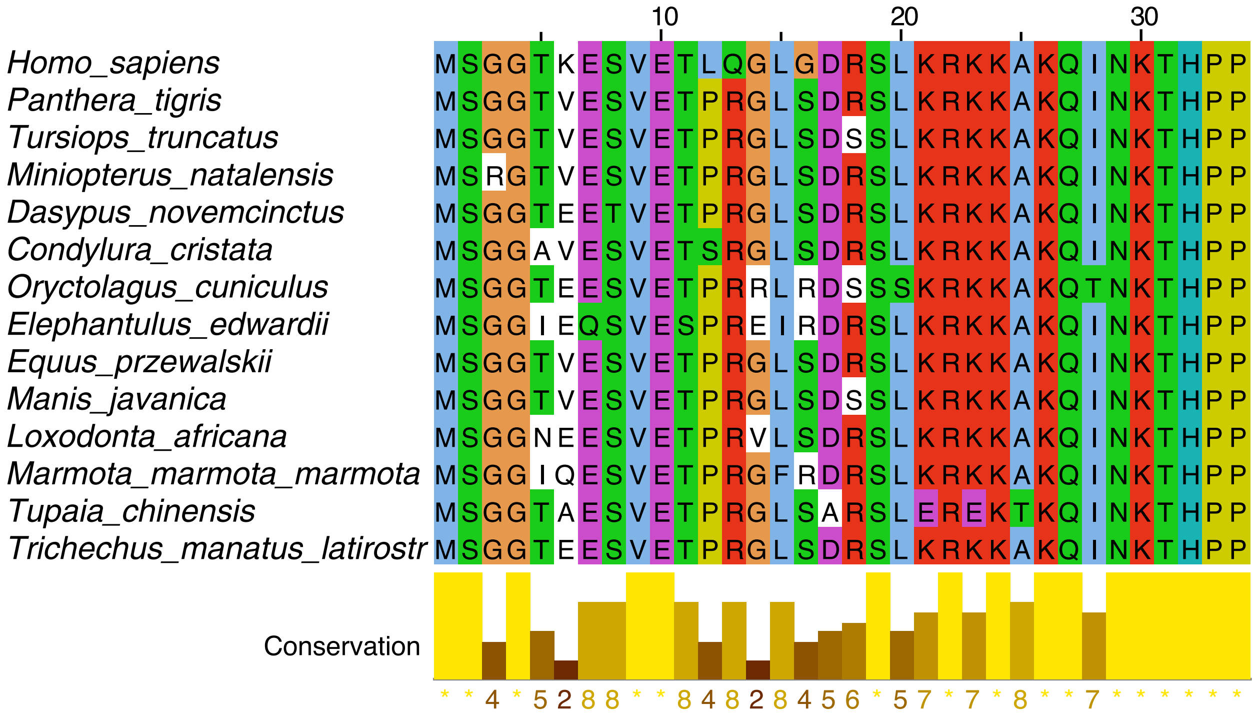

HG0541.1

ENSG00000122584 (NXPH1)

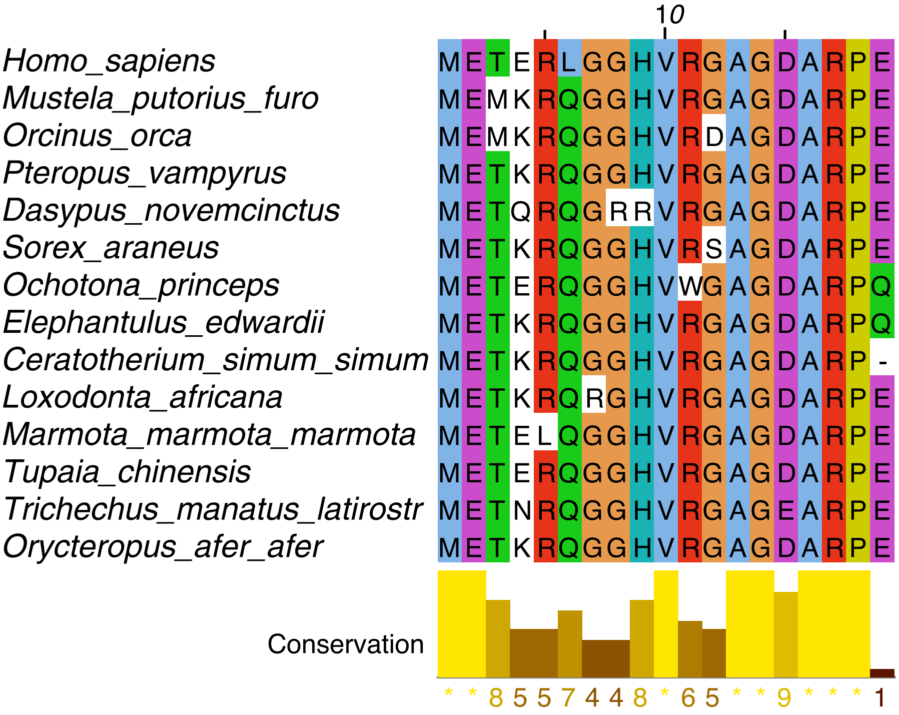

HG0541.2

ENSG00000122584 (NXPH1)

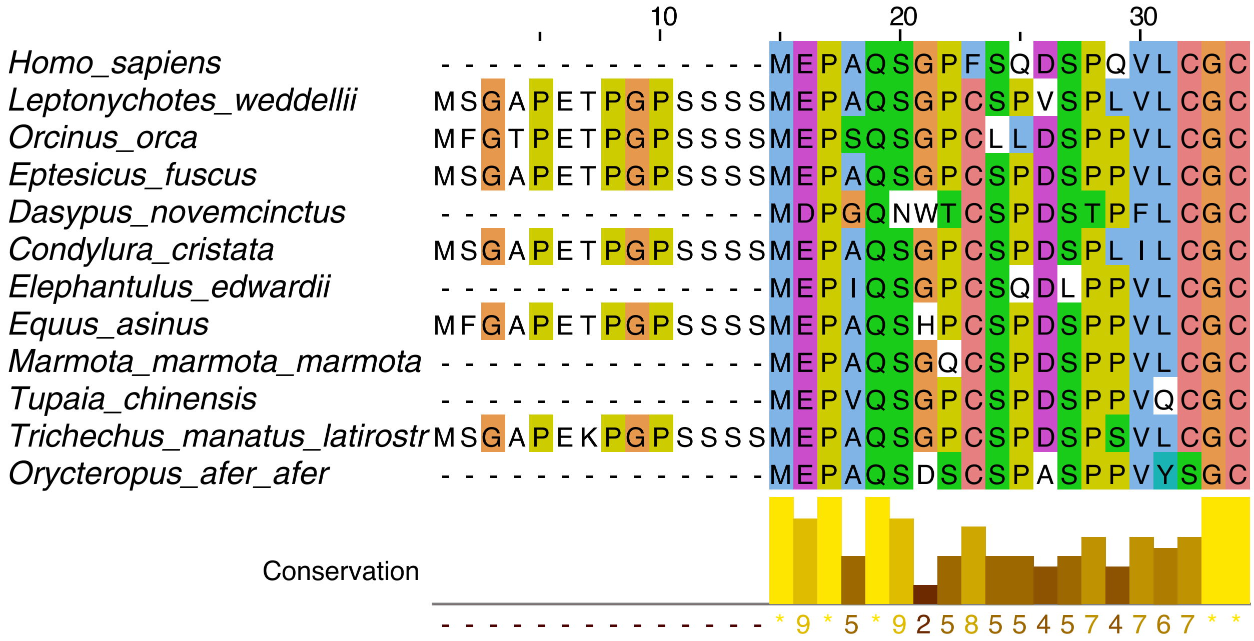

HG0542

ENSG00000126581 (BECN1)

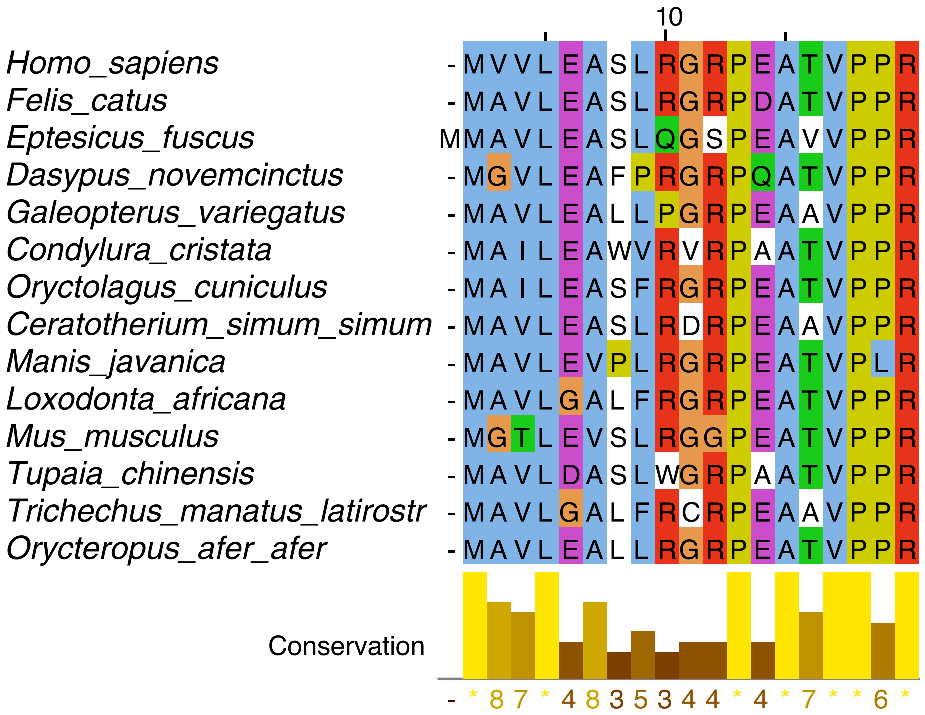

HG0543

ENSG00000131931 (THAP1)

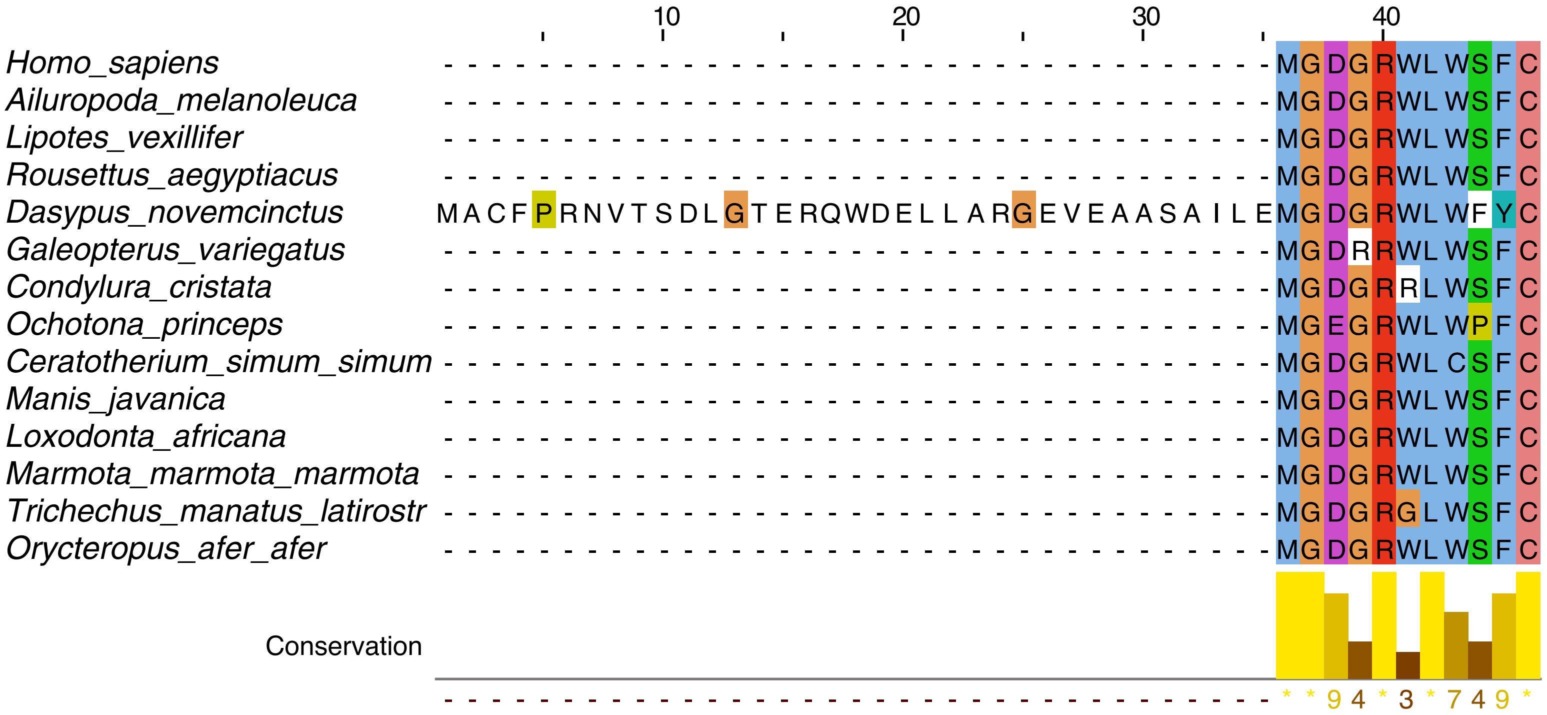

HG0544

ENSG00000136802 (LRRC8A)

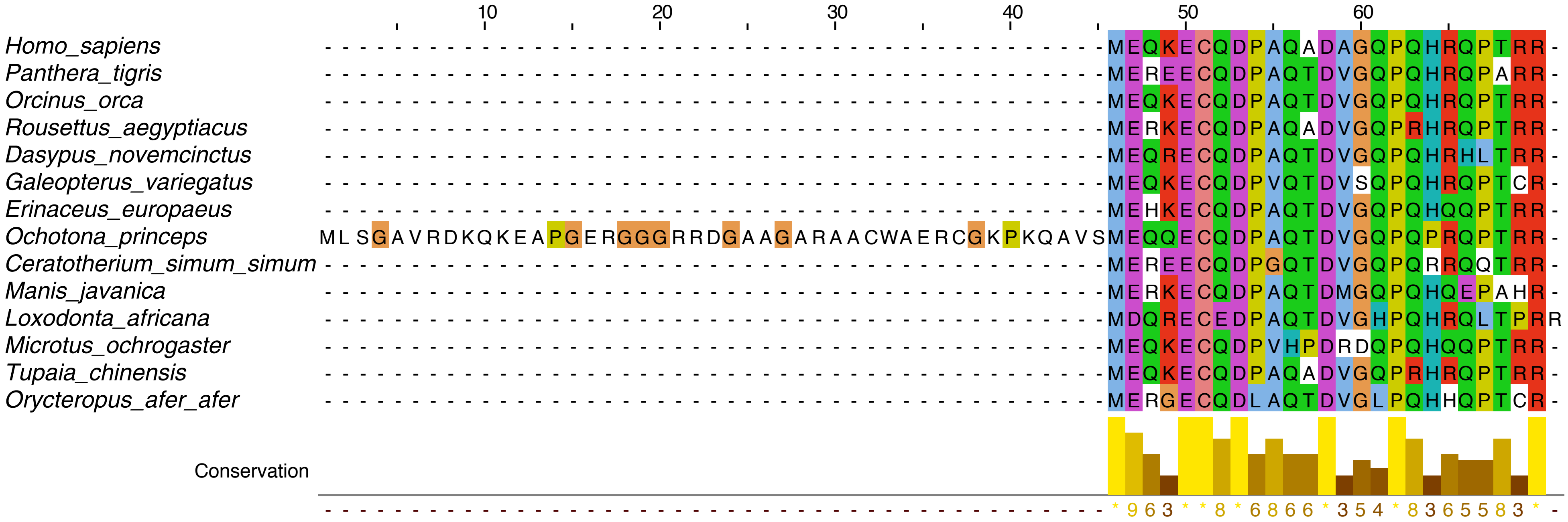

HG0545

ENSG00000142784 (WDTC1)

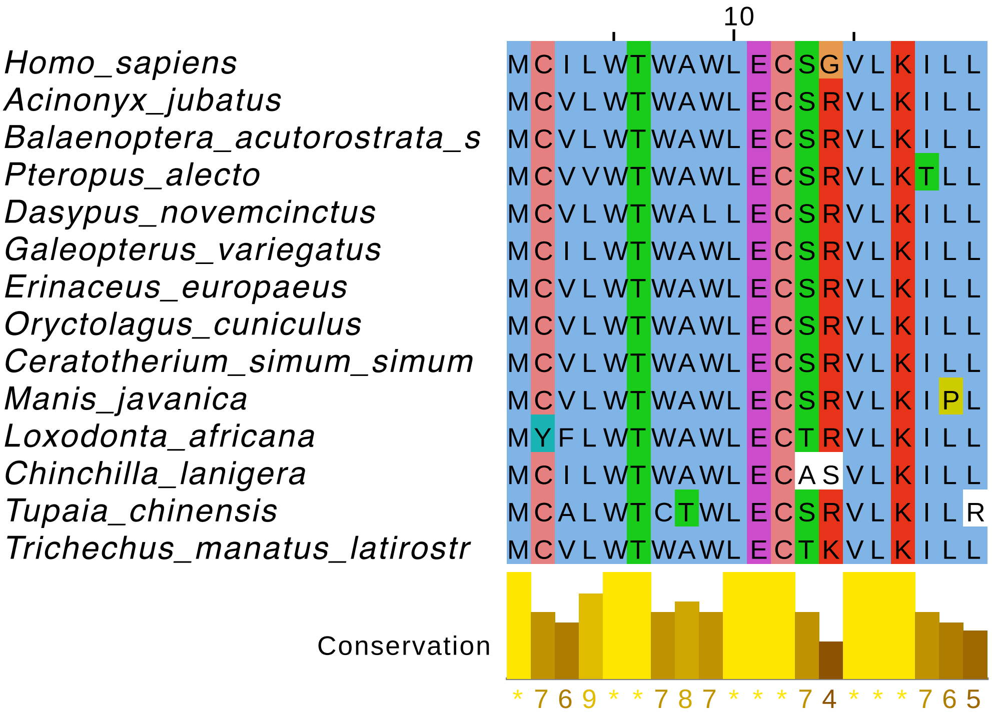

HG0546

ENSG00000148143 (ZNF462)

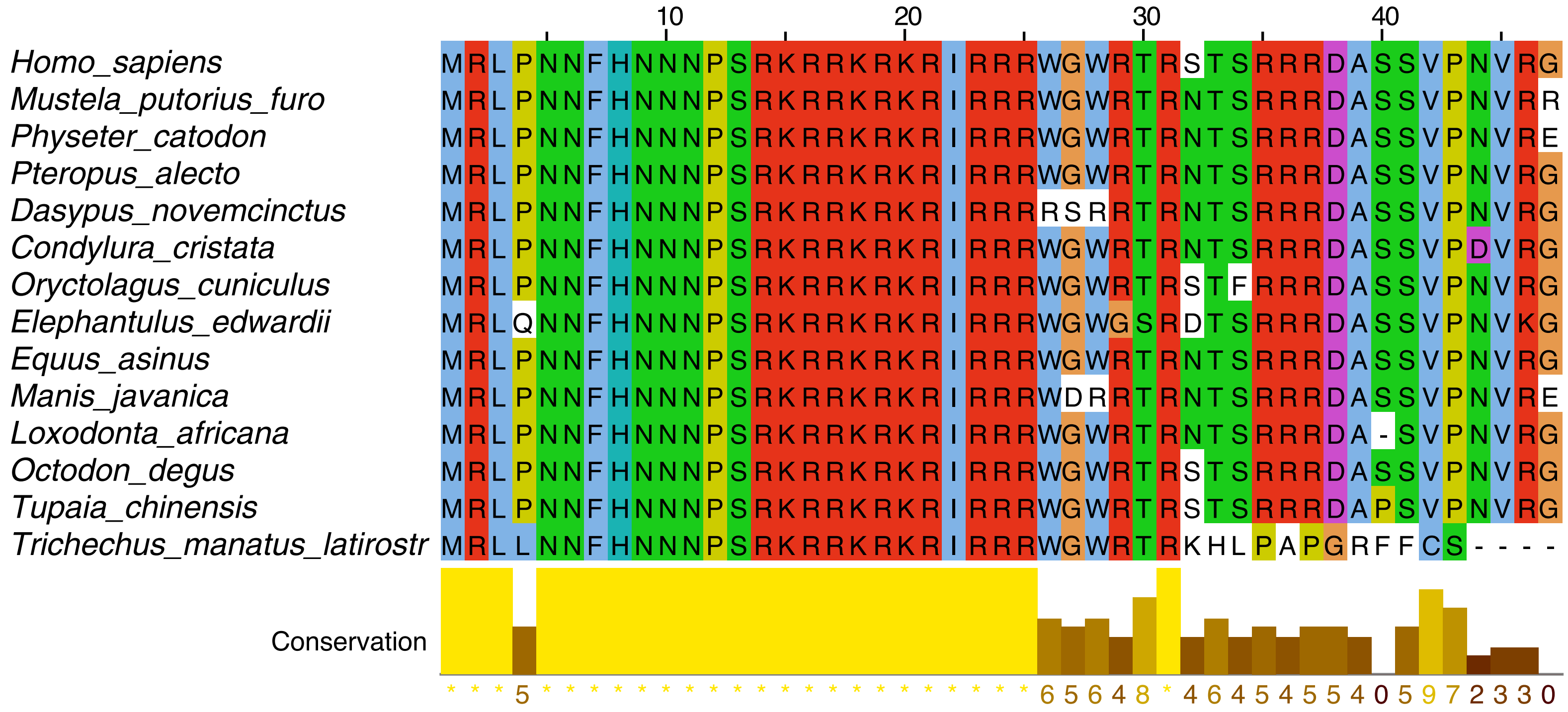

HG0547

ENSG00000149596 (JPH2)

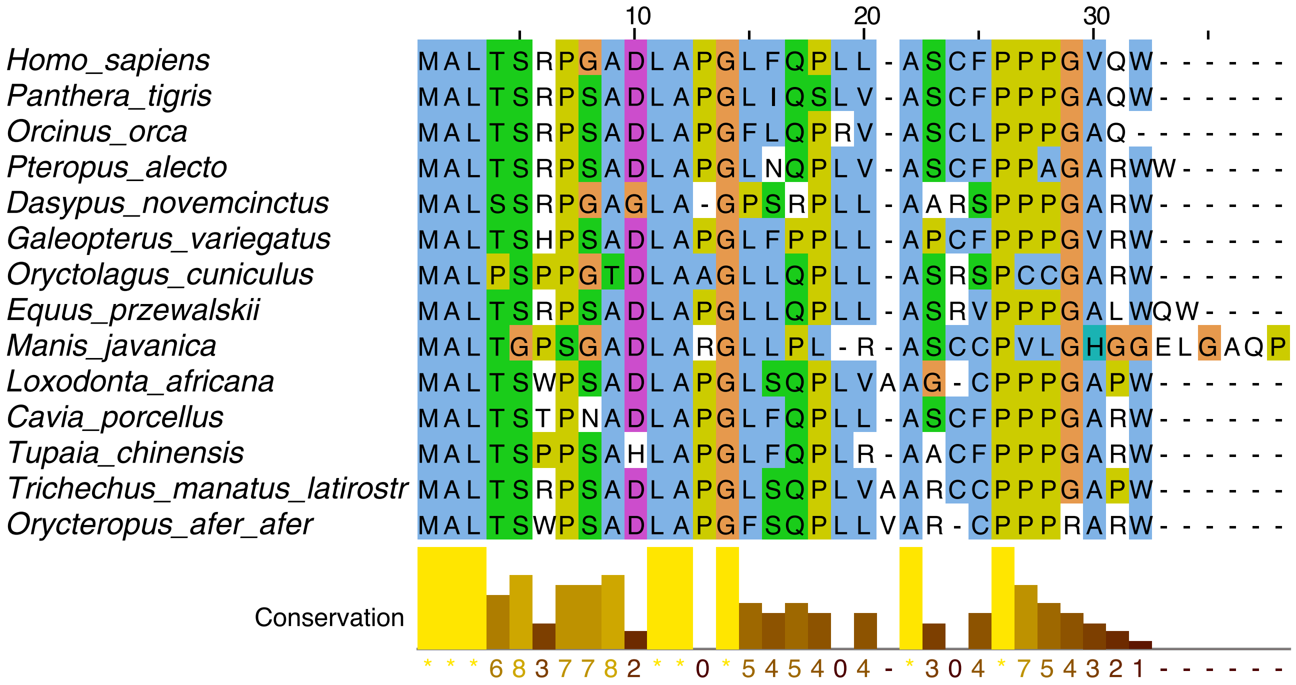

HG0548

ENSG00000155744 (FAM126B)

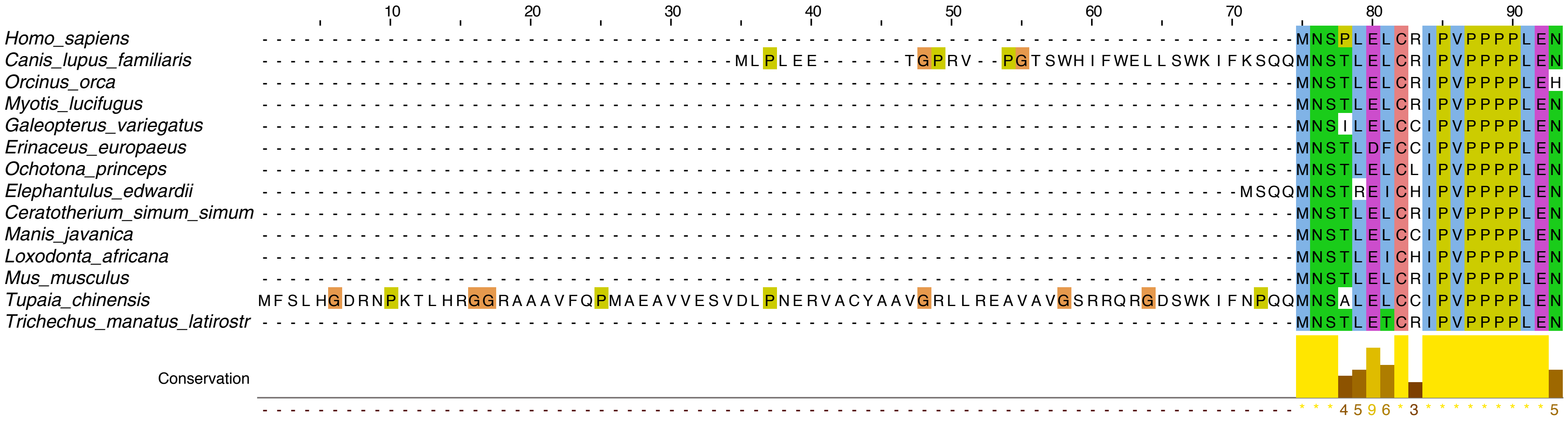

HG0549

ENSG00000156256 (USP16)

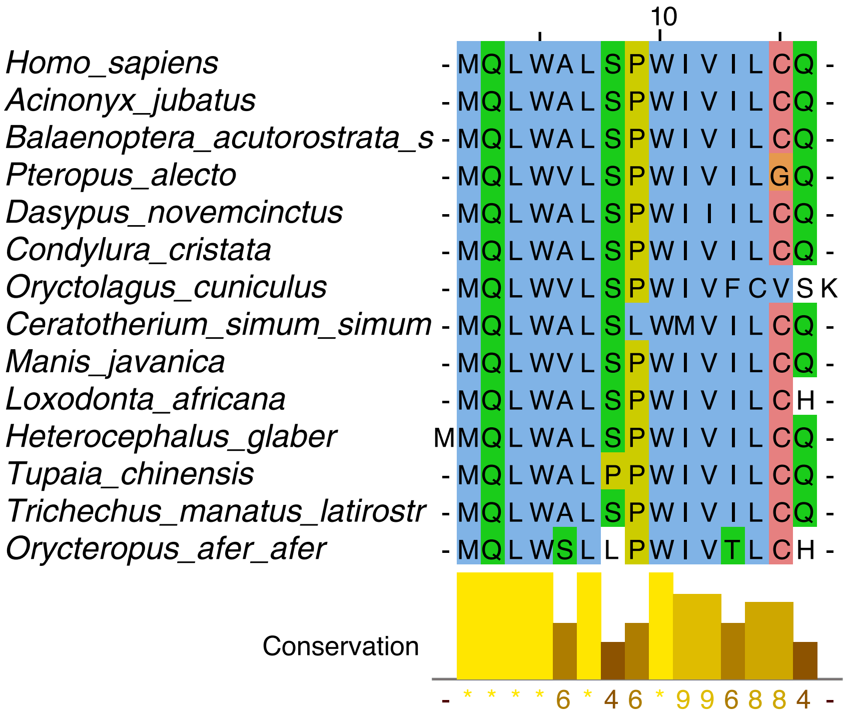

HG0550.1

ENSG00000156650 (KAT6B)

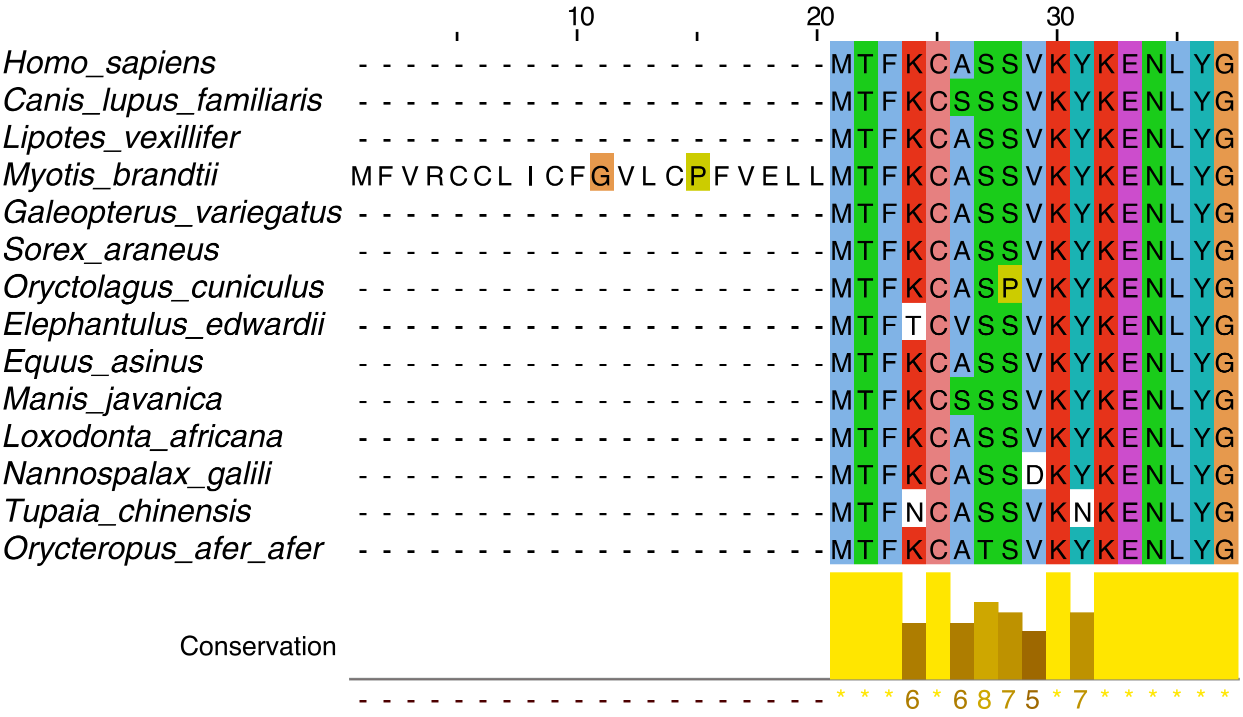

HG0550.2

ENSG00000156650 (KAT6B)

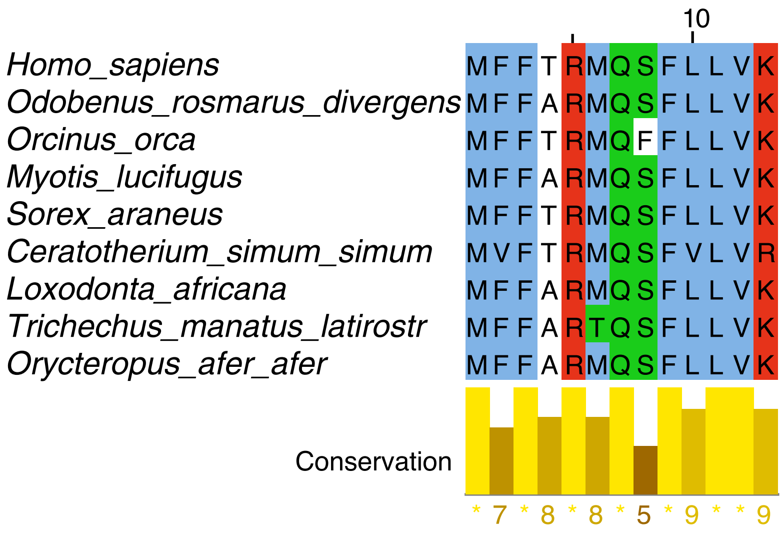

HG0551

ENSG00000162951 (LRRTM1)

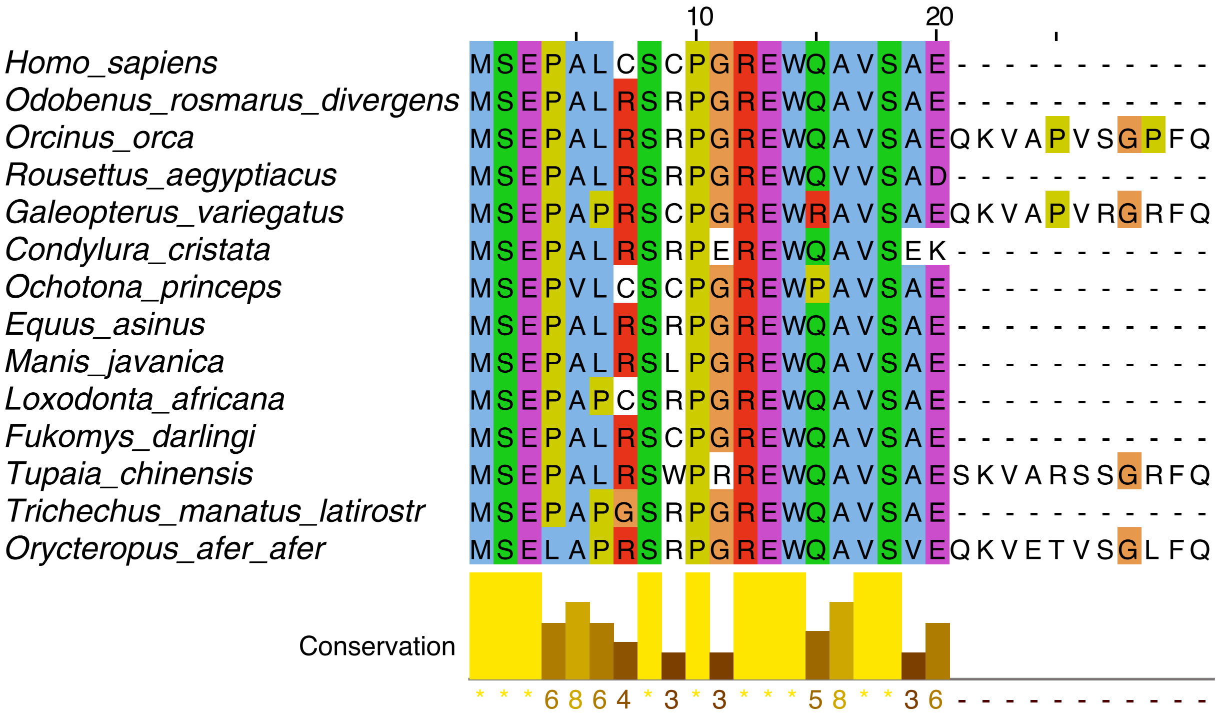

HG0552

ENSG00000169641 (LUZP1)

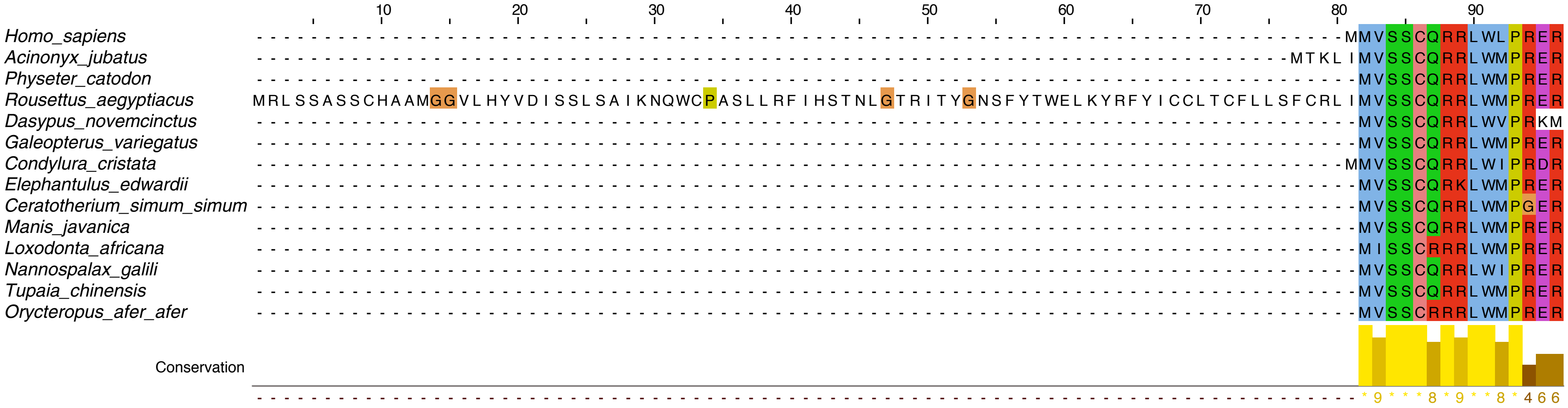

HG0553

ENSG00000183475 (ASB7)

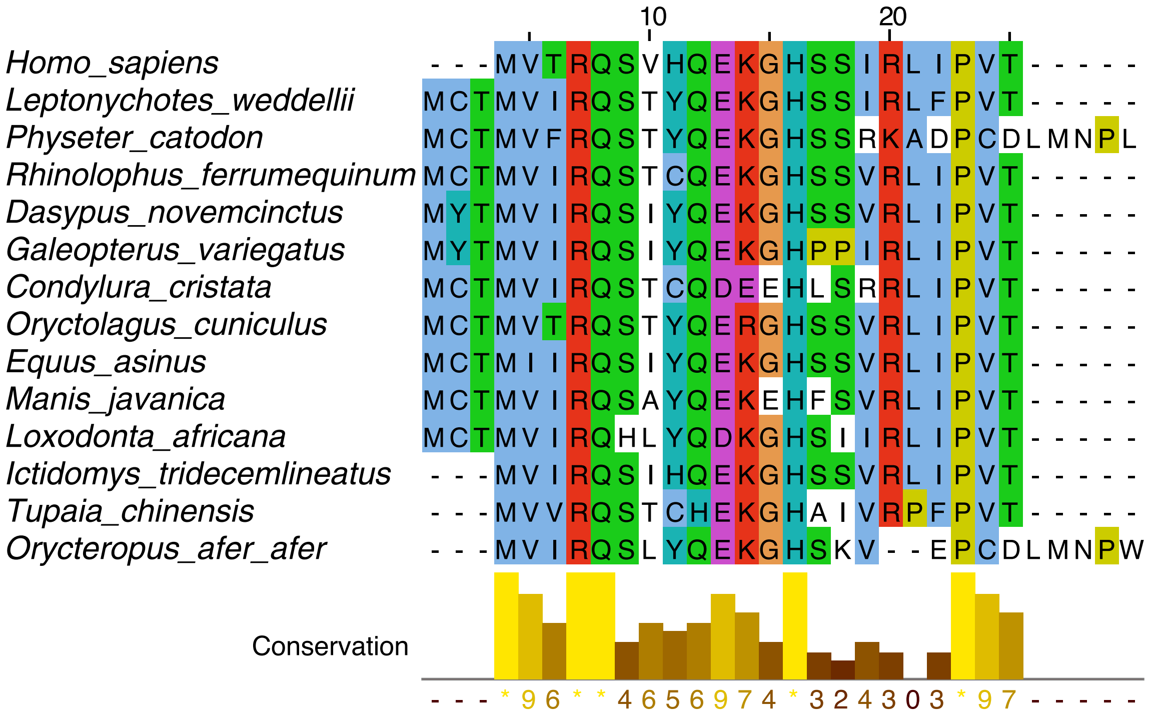

HG0554

ENSG00000196914 (ARHGEF12)

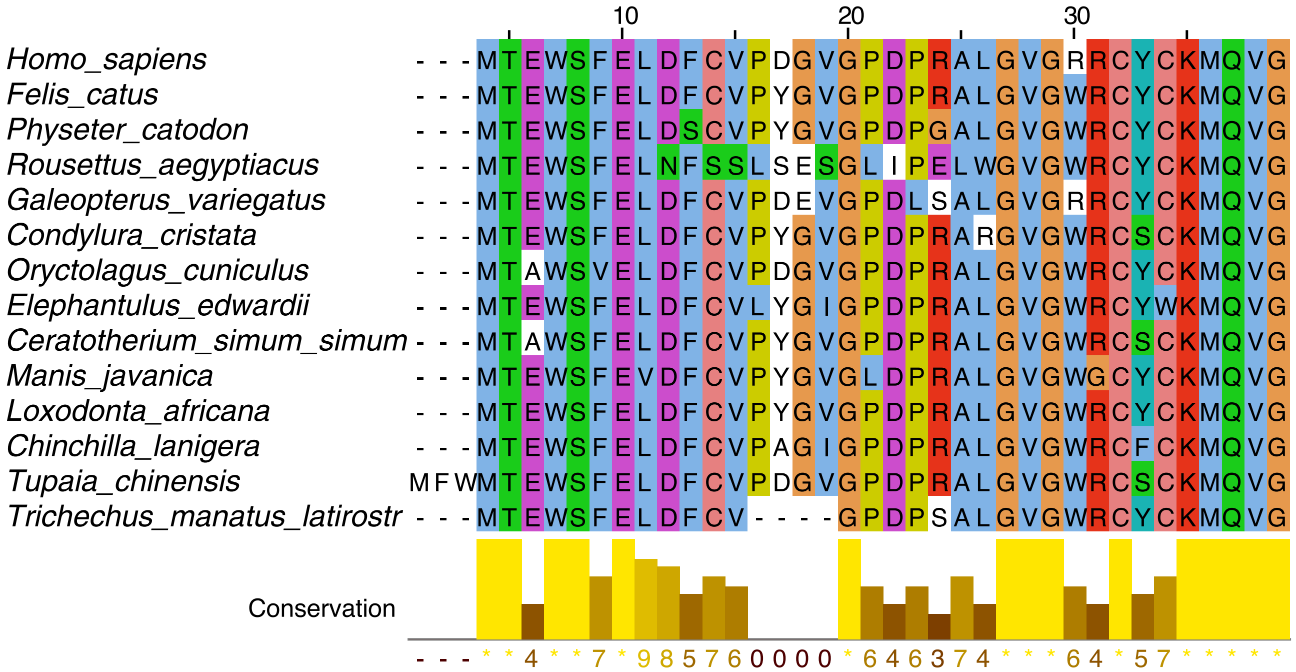

HG0555

ENSG00000204310 (AGPAT1)

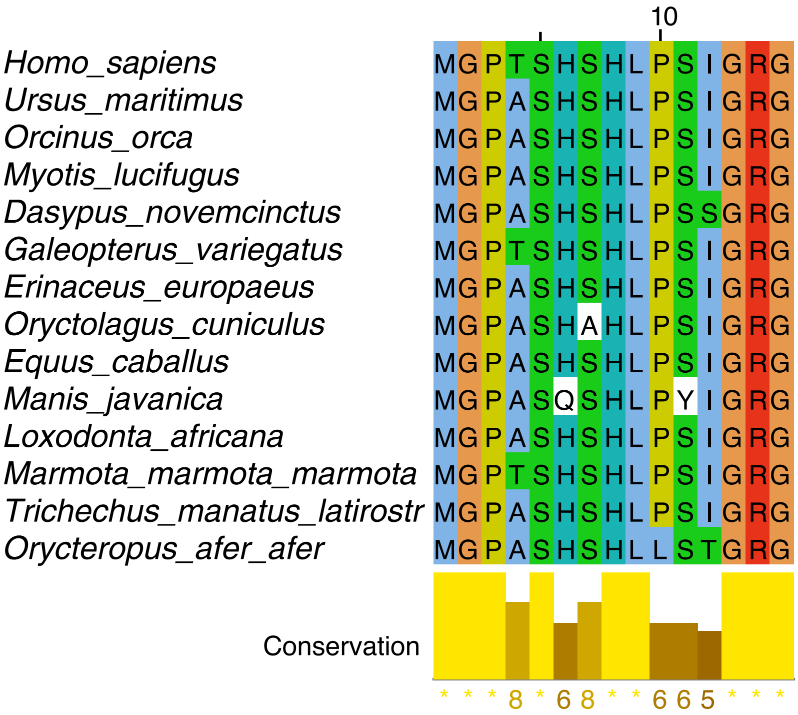

HG0556

ENSG00000273841 (TAF9)

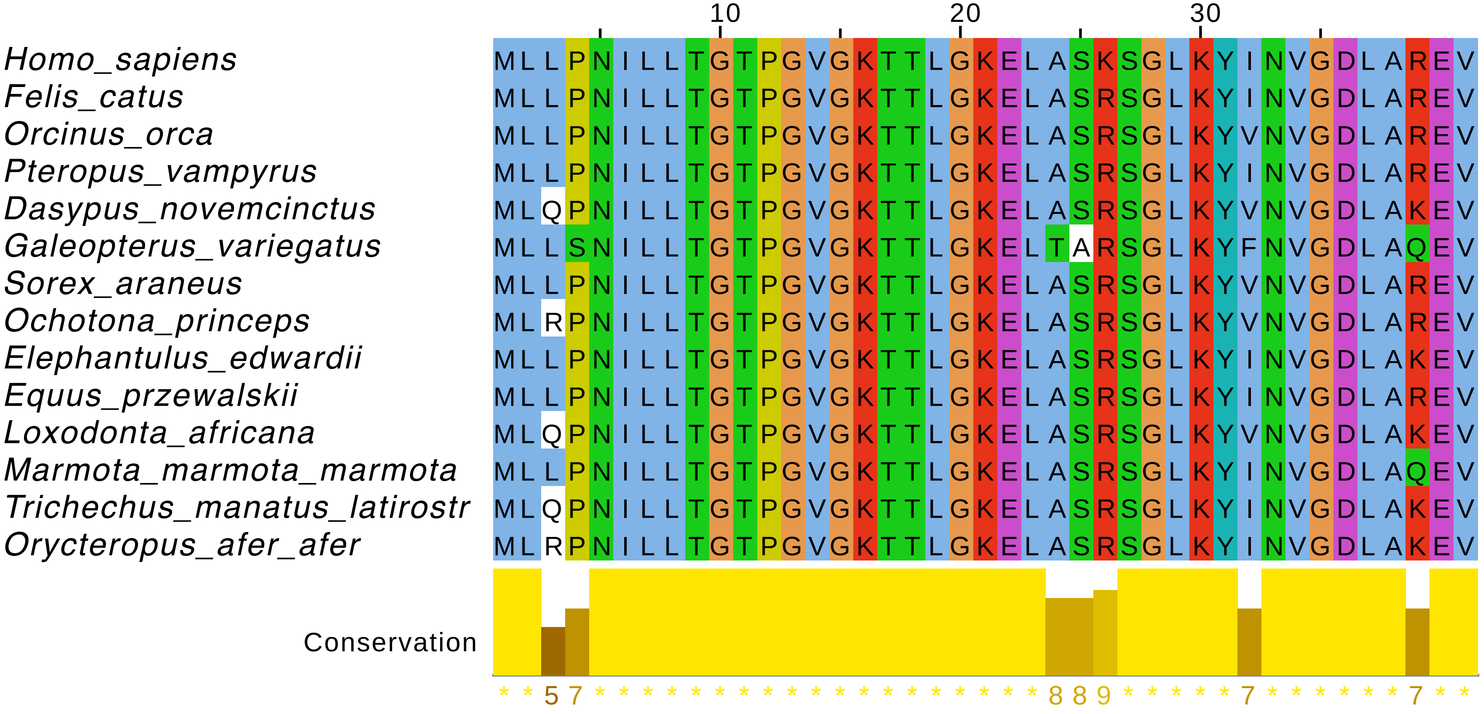

HG0557

ENSG00000012232 (EXTL3)

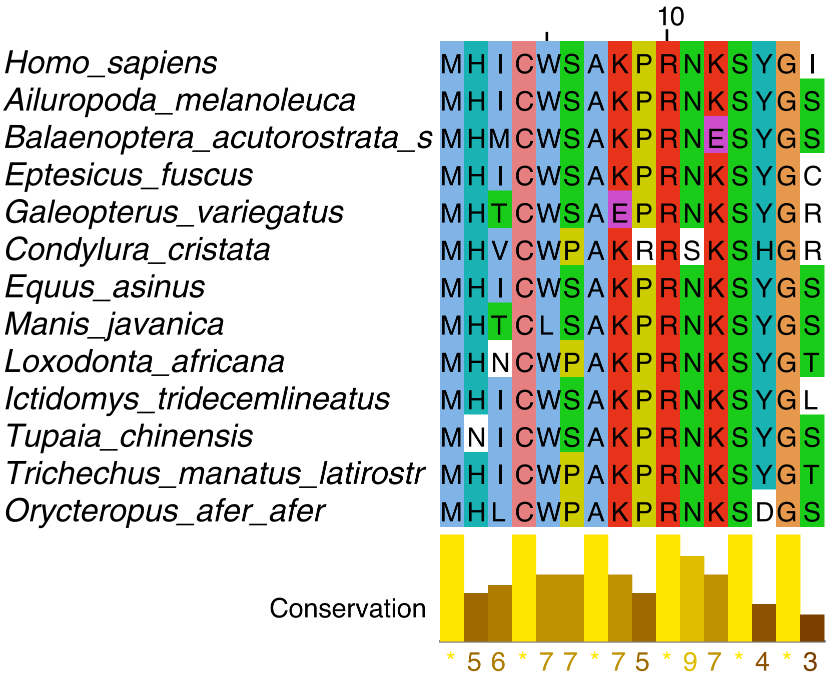

HG0558

ENSG00000029363 (BCLAF1)

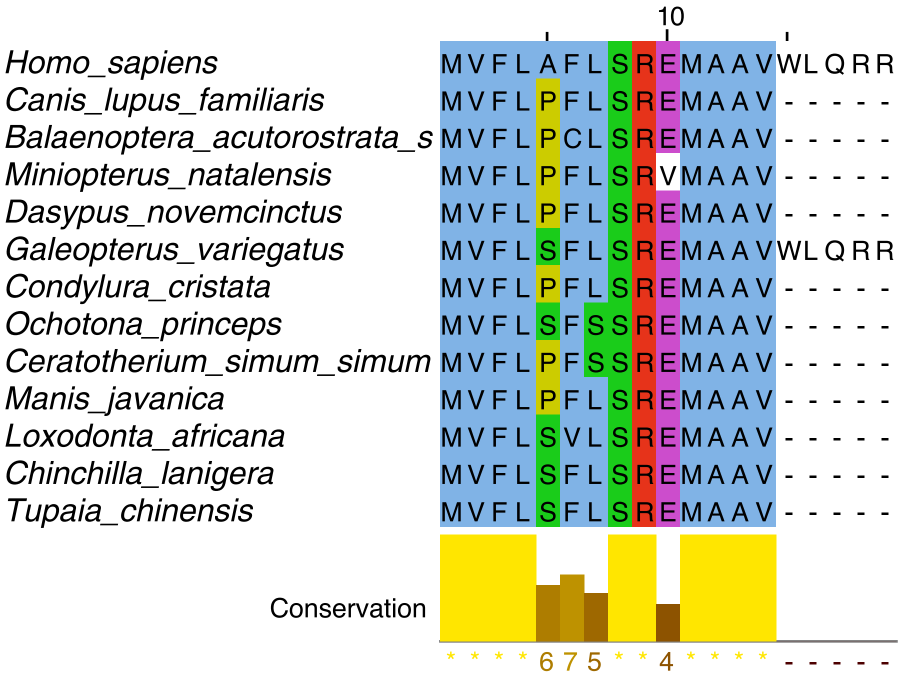

HG0559

ENSG00000078747 (ITCH)

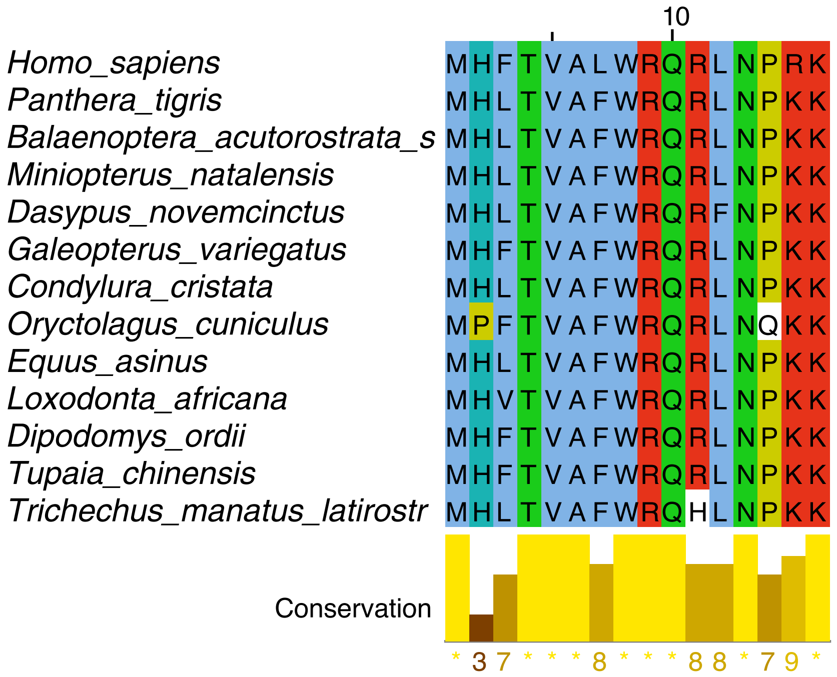

HG0560.1

ENSG00000092051 (JPH4)

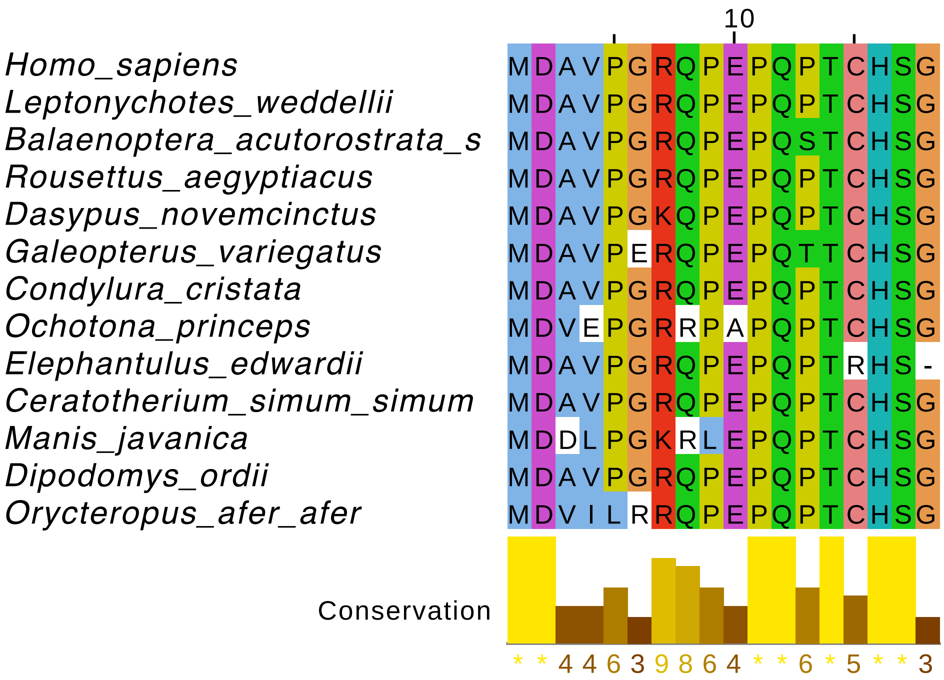

HG0560.2

ENSG00000092051 (JPH4)

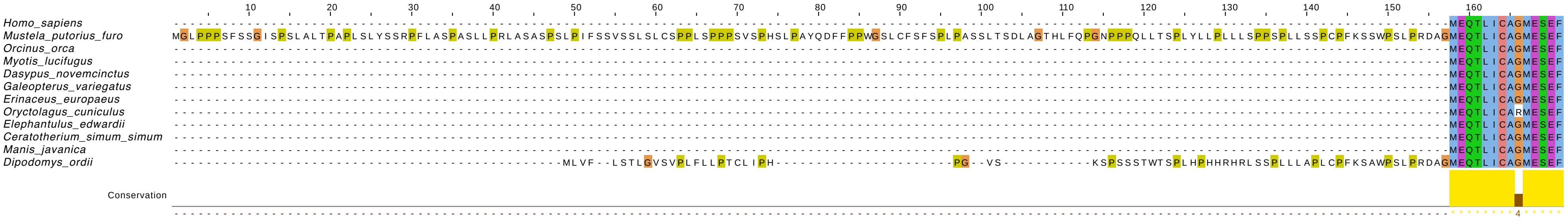

HG0561

ENSG00000100393 (EP300)

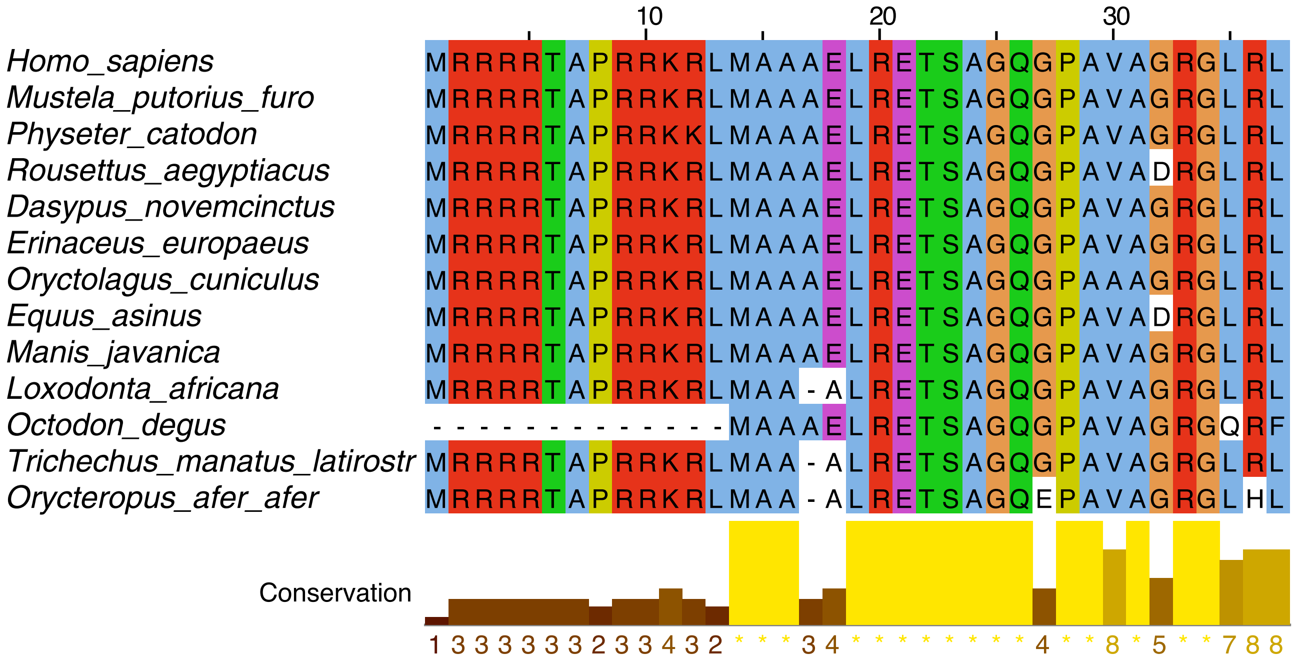

HG0562

ENSG00000105821 (DNAJC2)

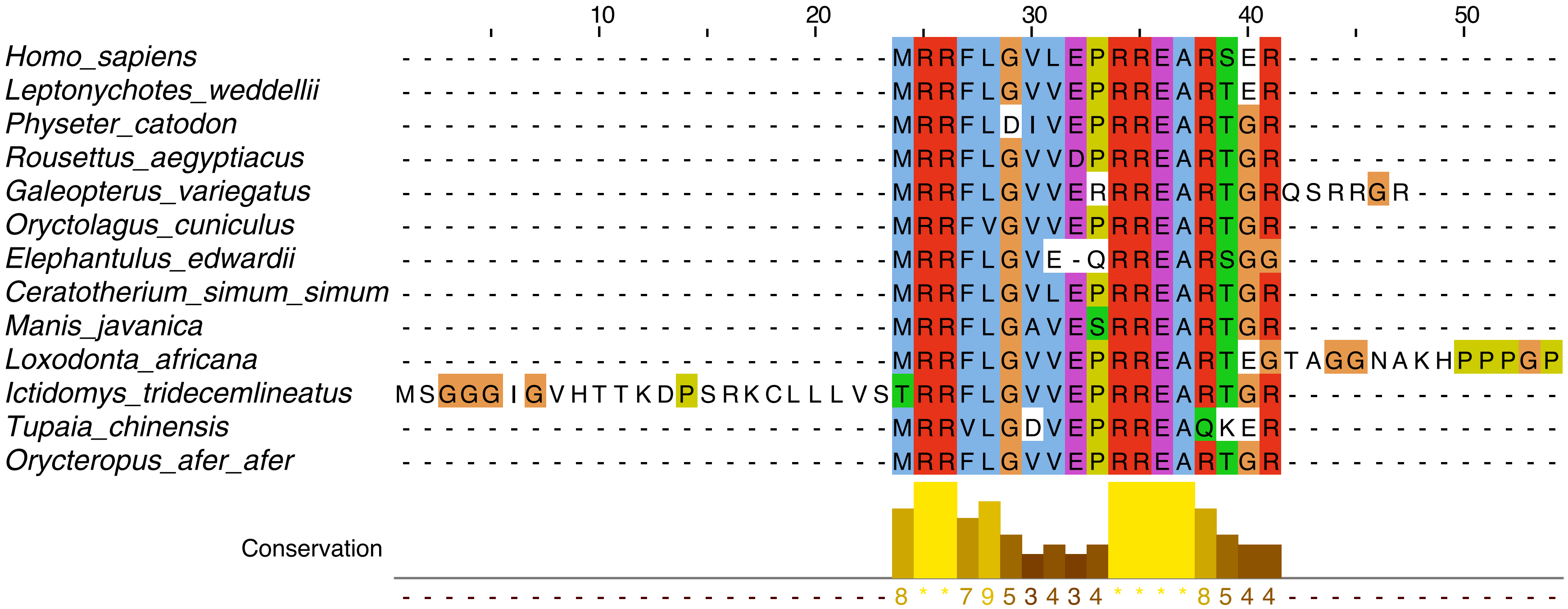

HG0563

ENSG00000107249 (GLIS3)

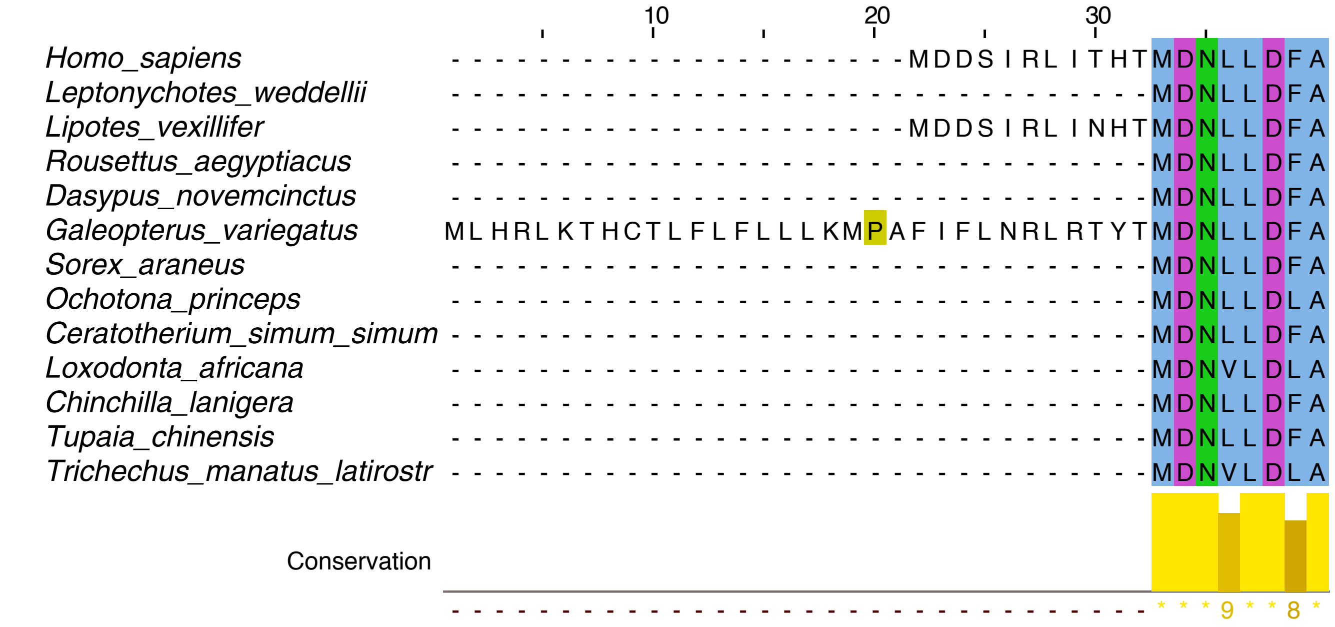

HG0564

ENSG00000109171 (SLAIN2)

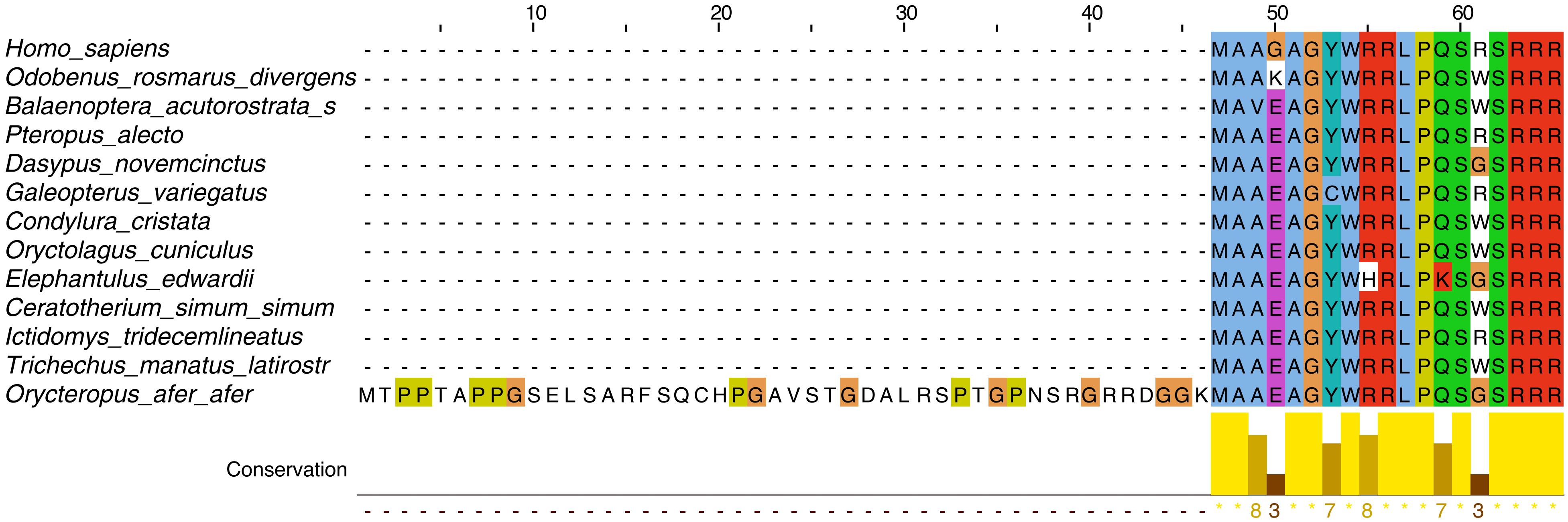

HG0565

ENSG00000120533 (ENY2)

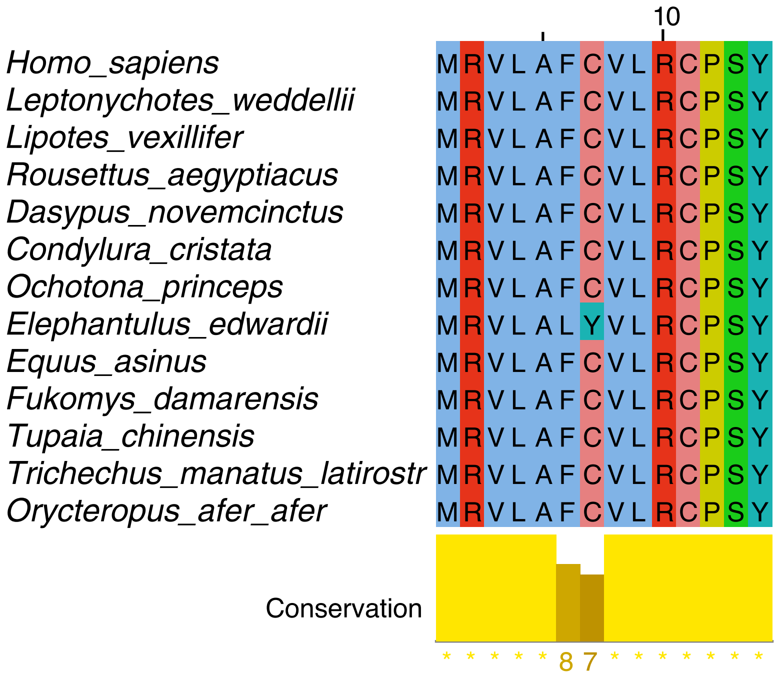

HG0566

ENSG00000124496 (TRERF1)

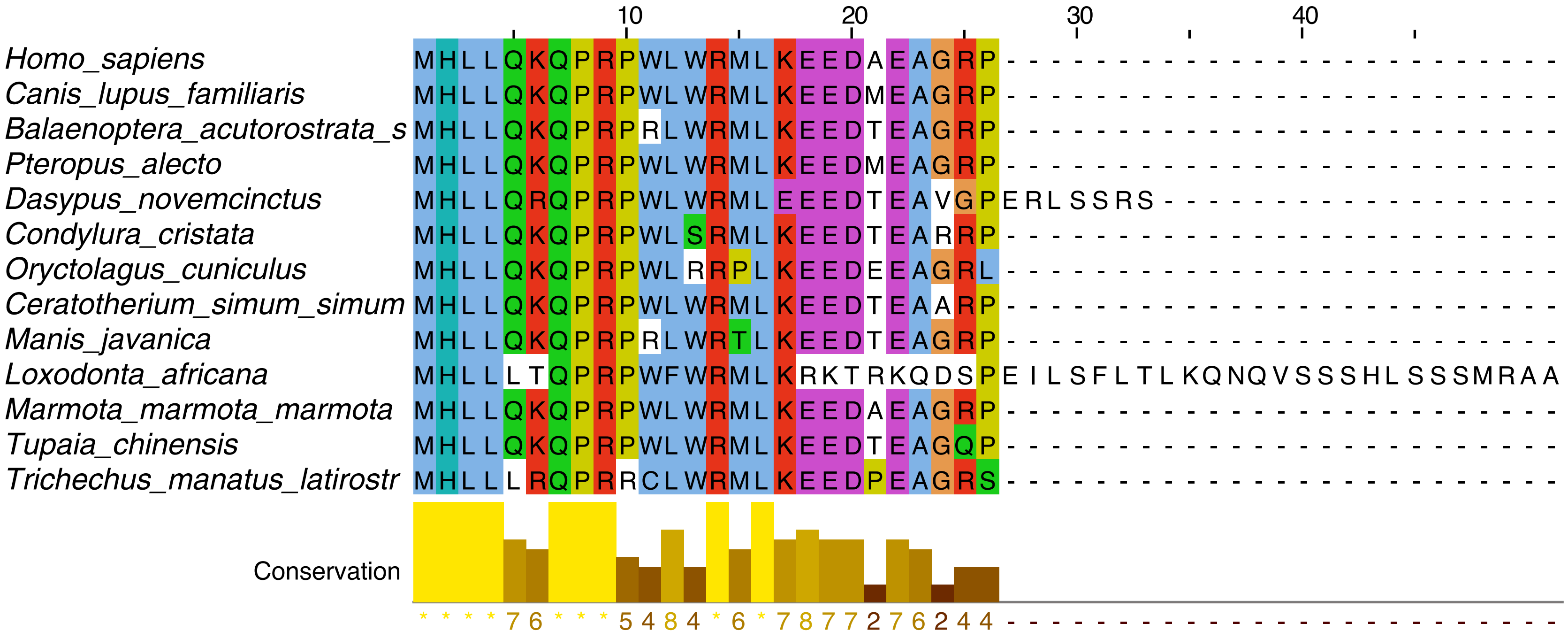

HG0567

ENSG00000132155 (RAF1)

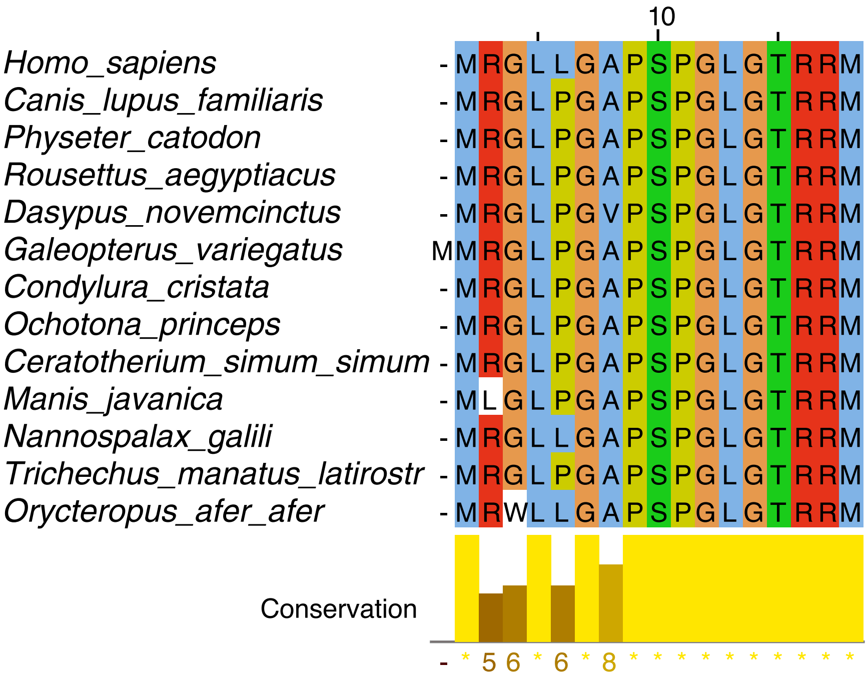

HG0568.1

ENSG00000138271 (GPR87)

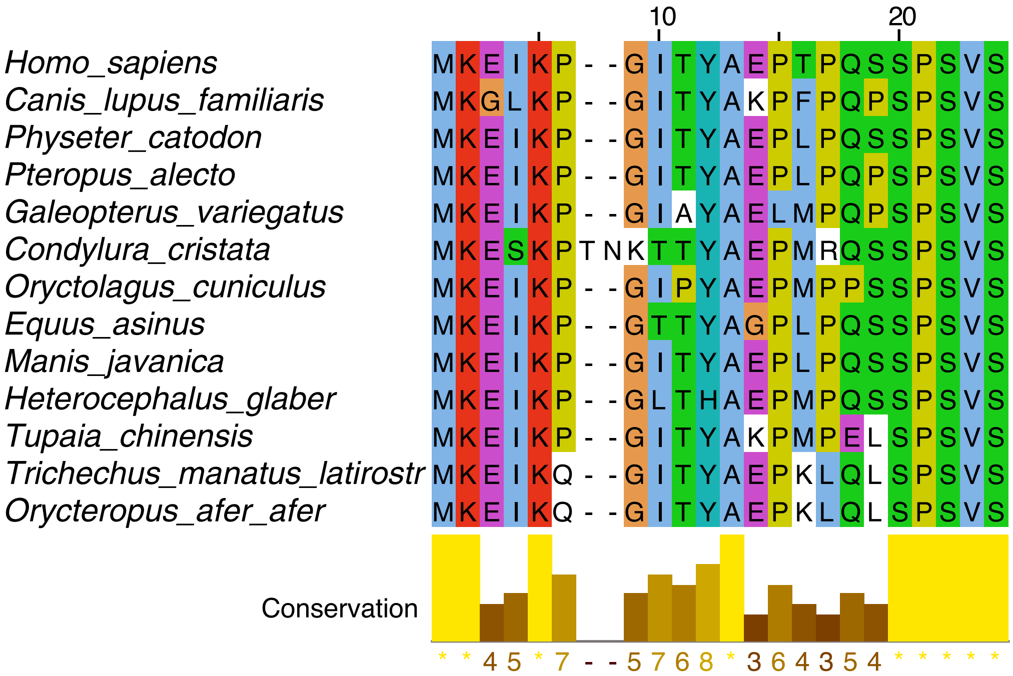

HG0568.2

ENSG00000138271 (GPR87)

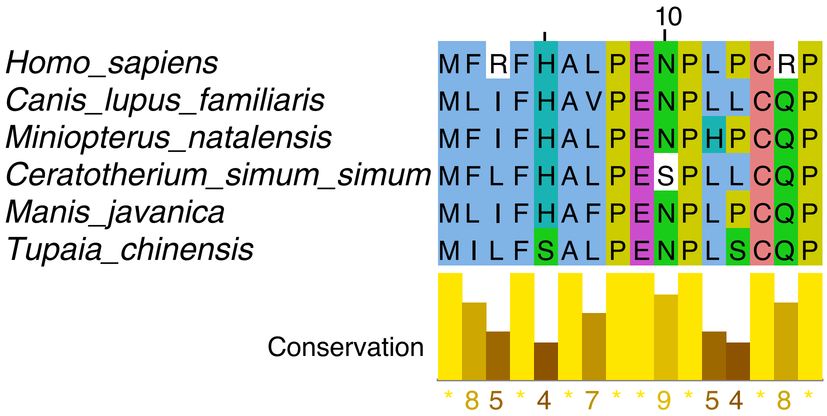

HG0569

ENSG00000139651 (ZNF740)

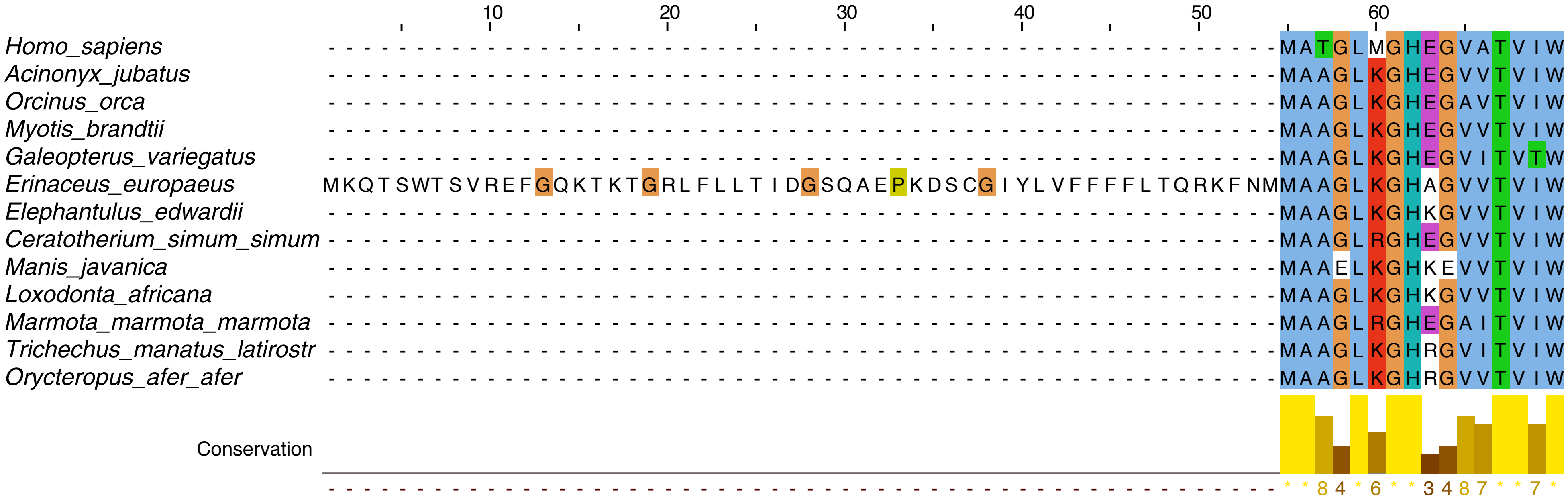

HG0570

ENSG00000152214 (RIT2)

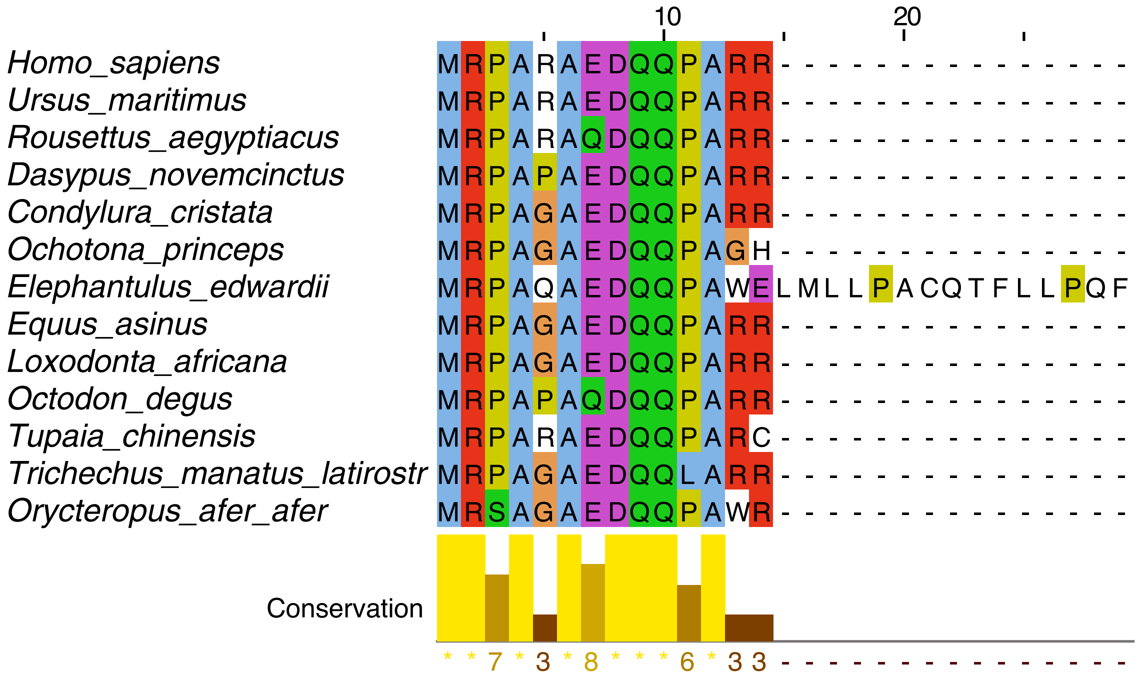

HG0571

ENSG00000153266 (FEZF2)

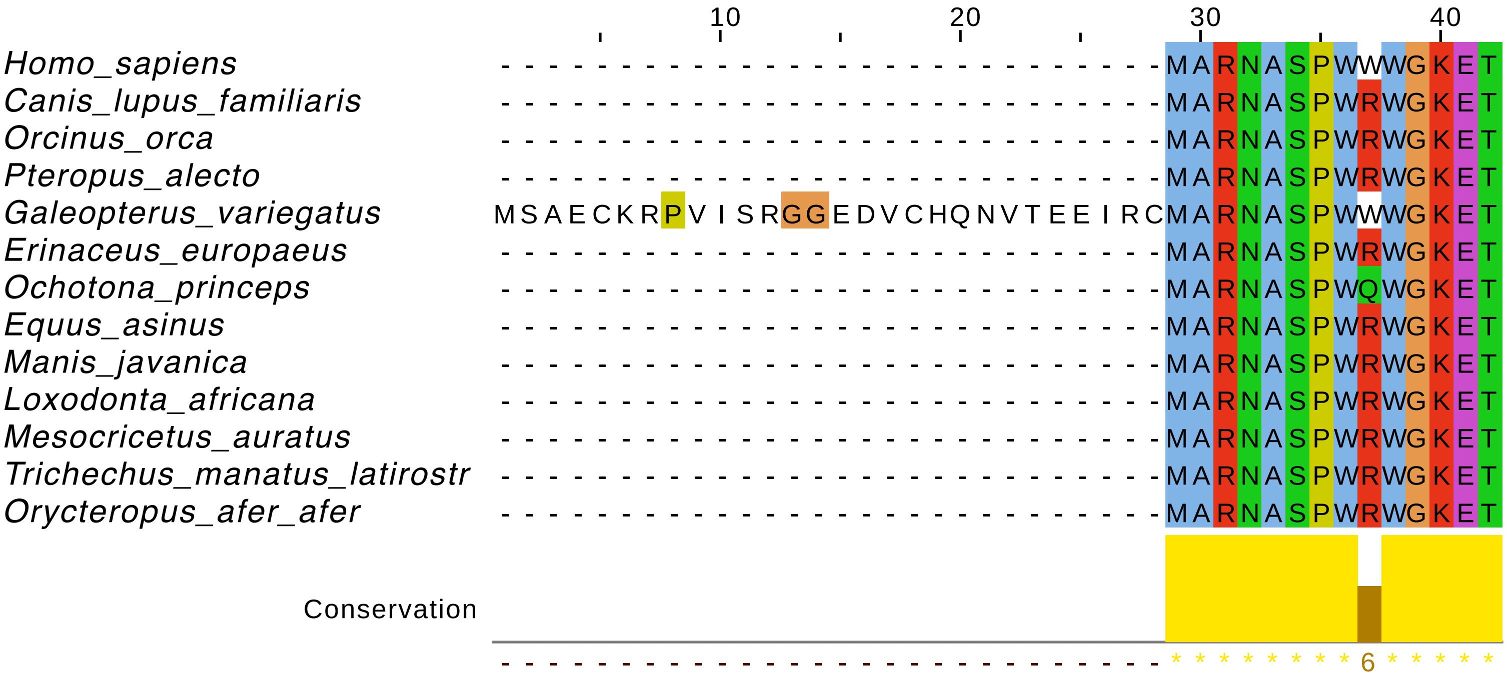

HG0572

ENSG00000158161 (EYA3)

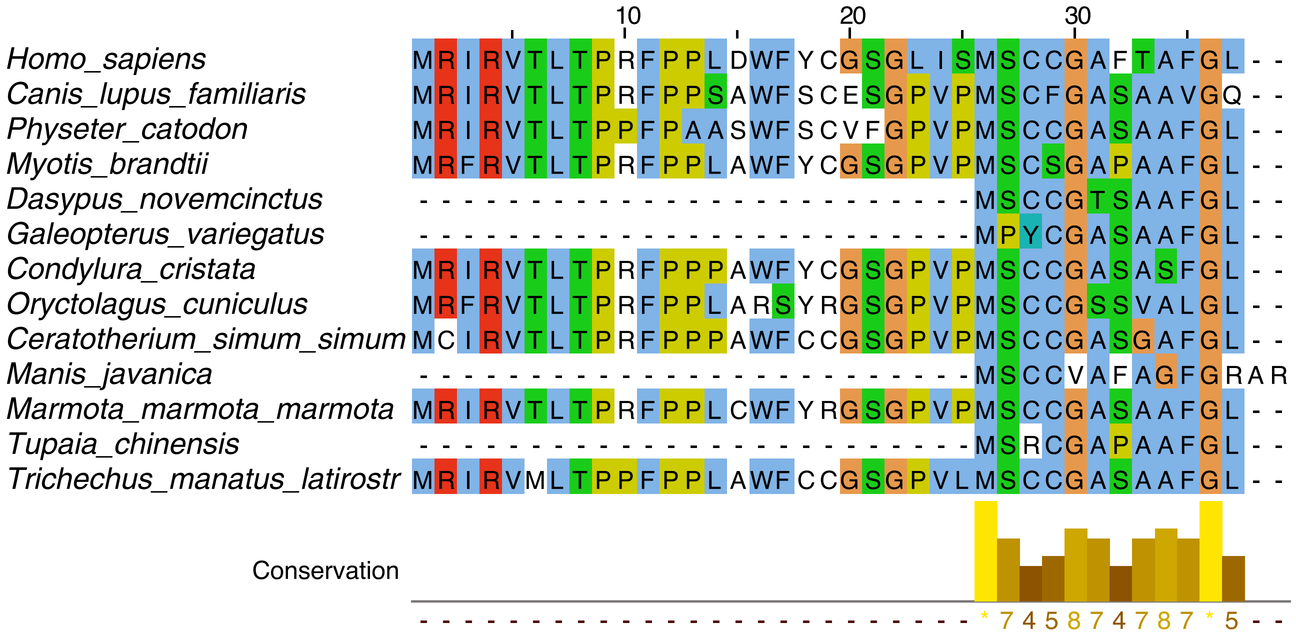

HG0573.1

ENSG00000162526 (TSSK3)

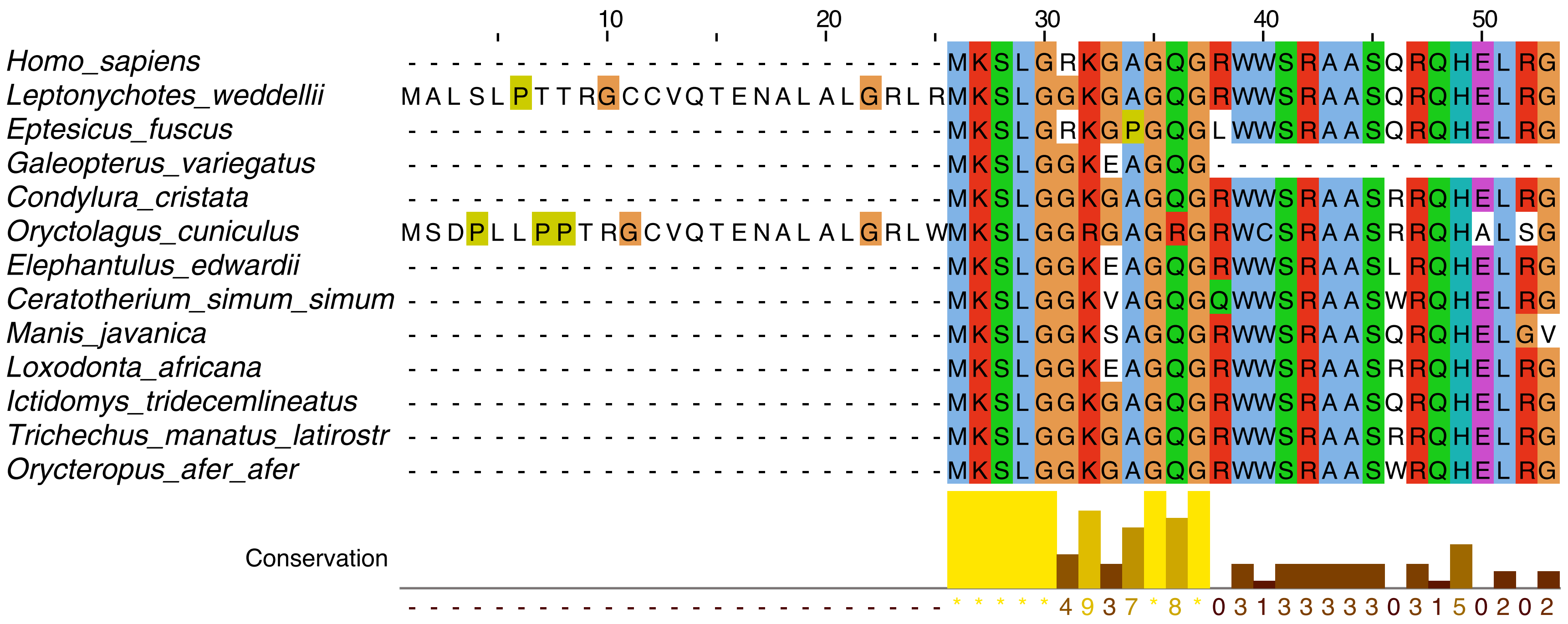

HG0573.2

ENSG00000206203 (TSSK2)

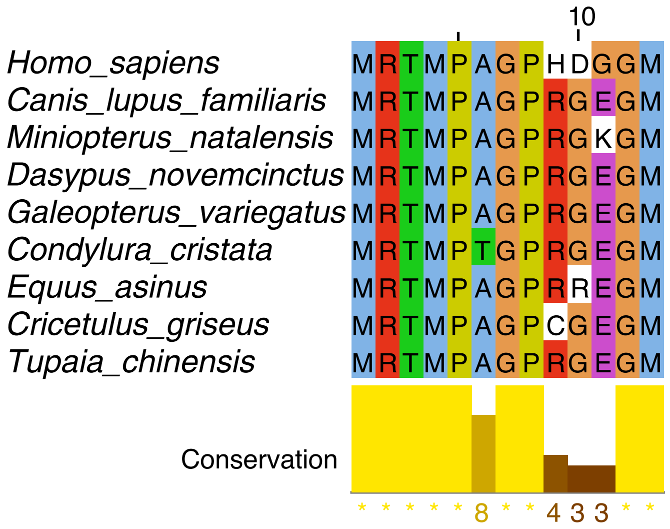

HG0574

ENSG00000164663 (USP49)

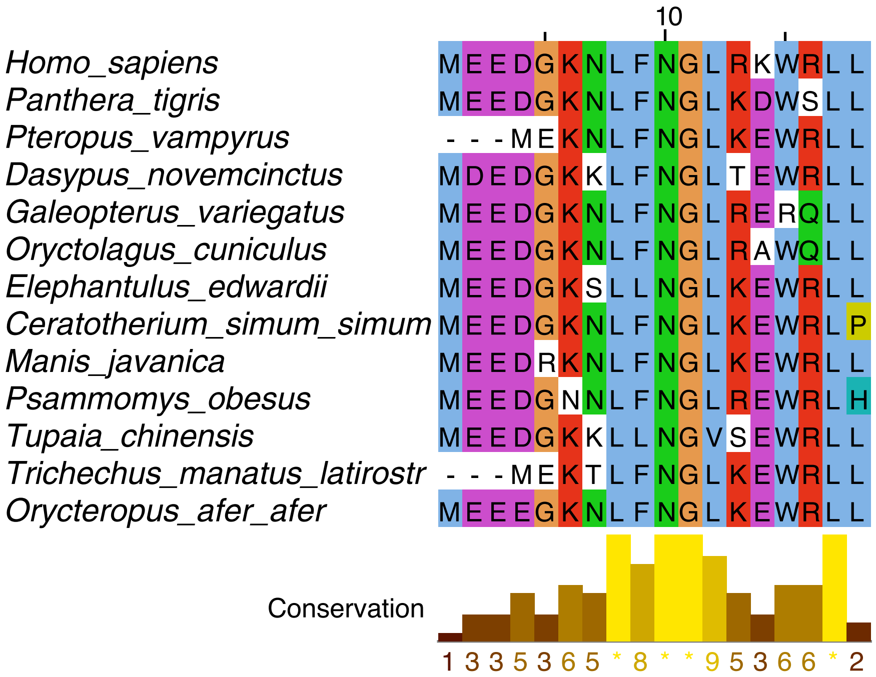

HG0575

ENSG00000164663 (USP49)

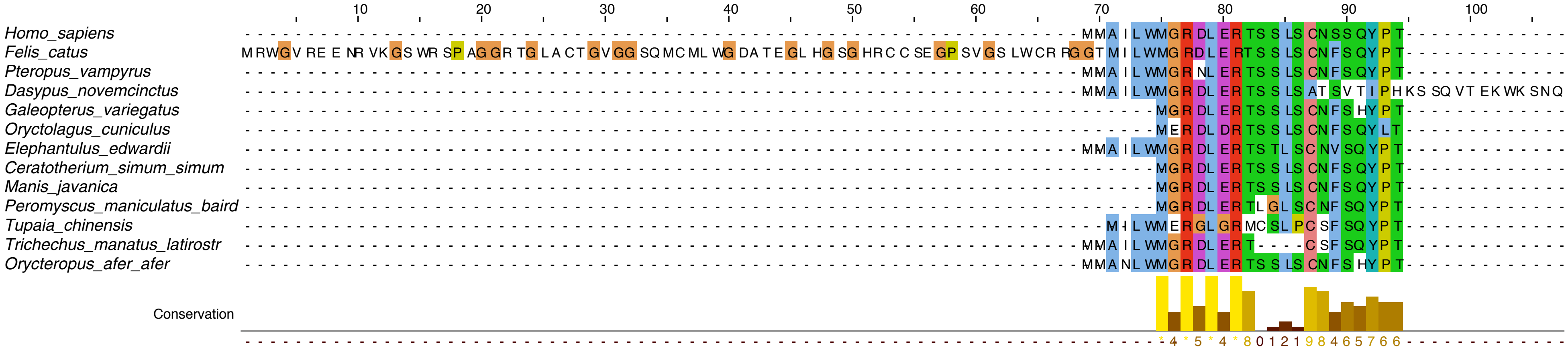

HG0576

ENSG00000175029 (CTBP2)

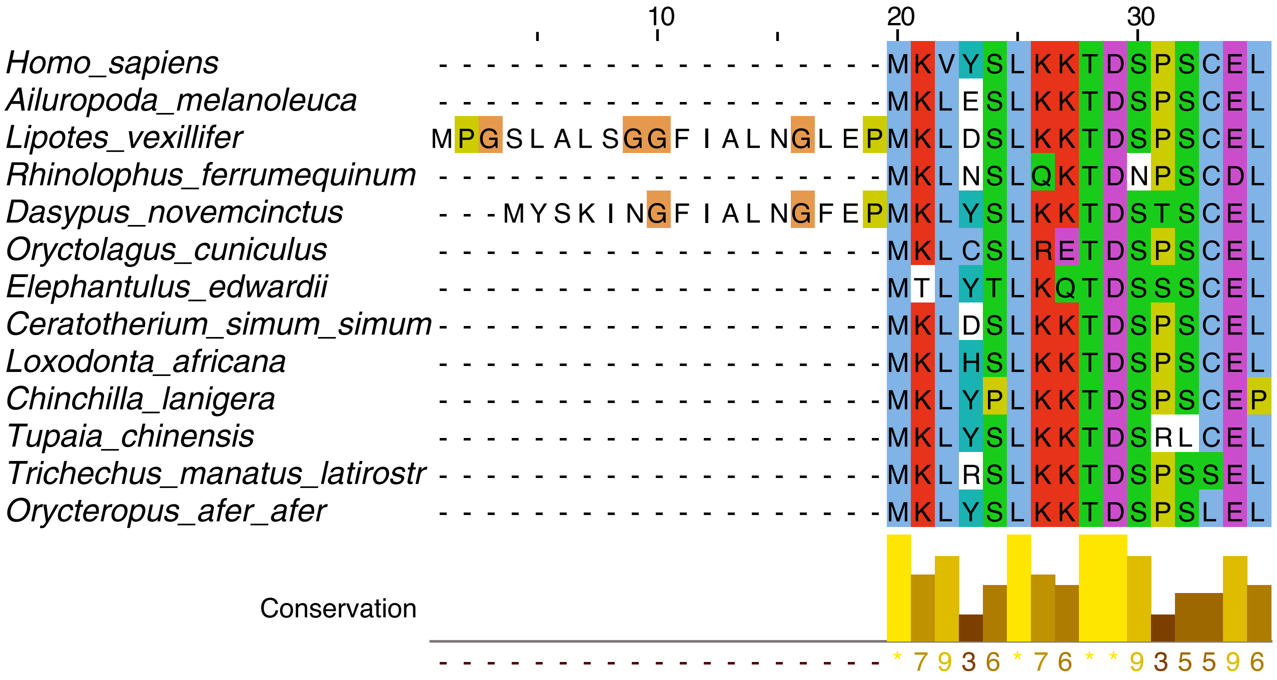

HG0577

ENSG00000250510 (GPR162)

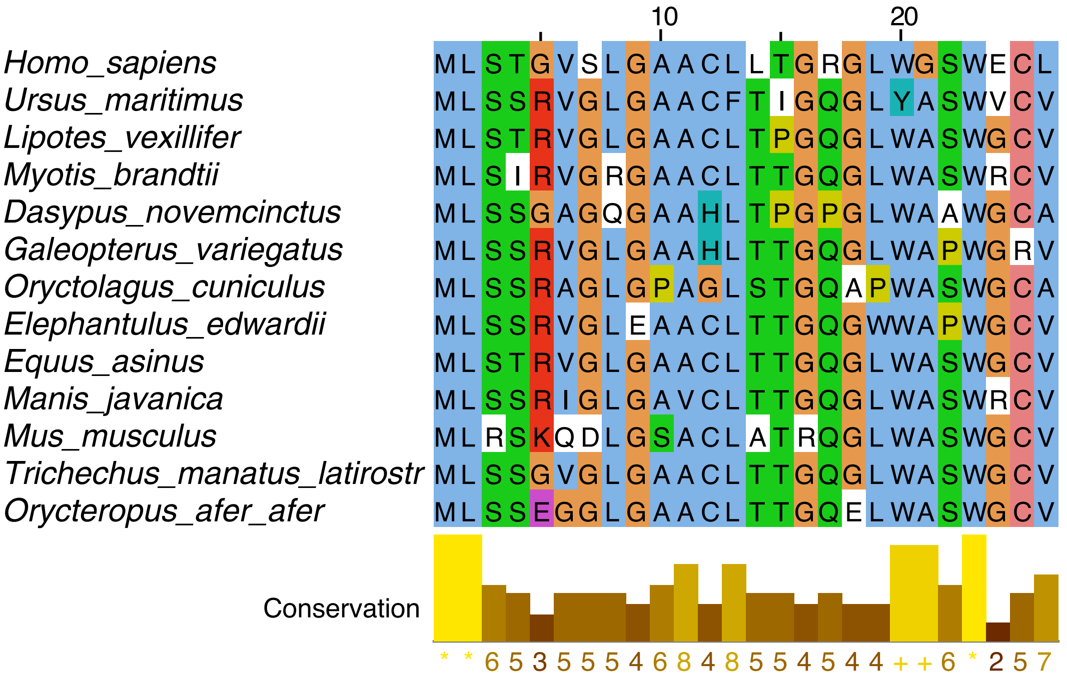

HG0578

ENSG00000004897 (CDC27)

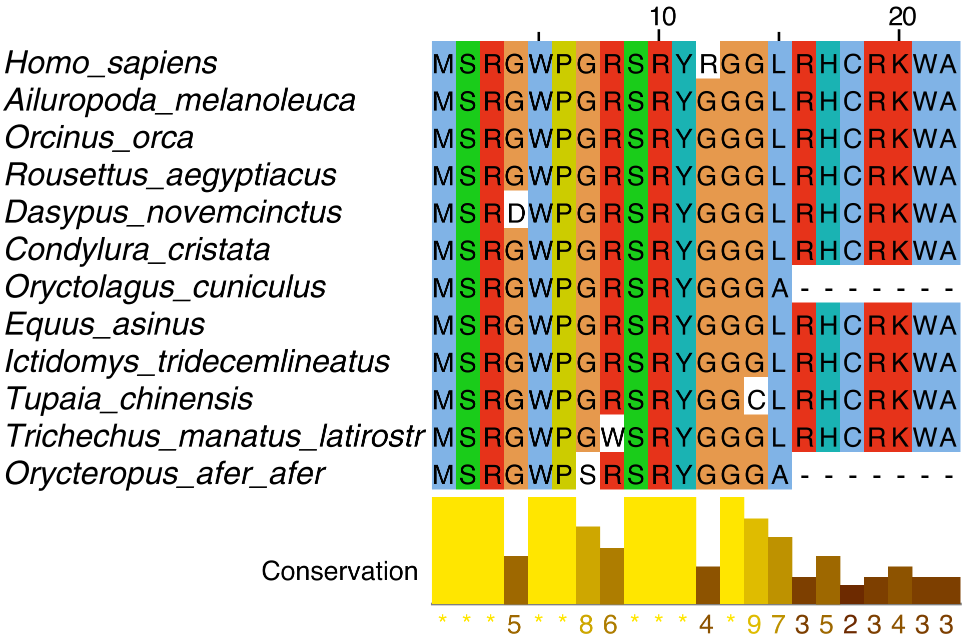

HG0579

ENSG00000010818 (HIVEP2)

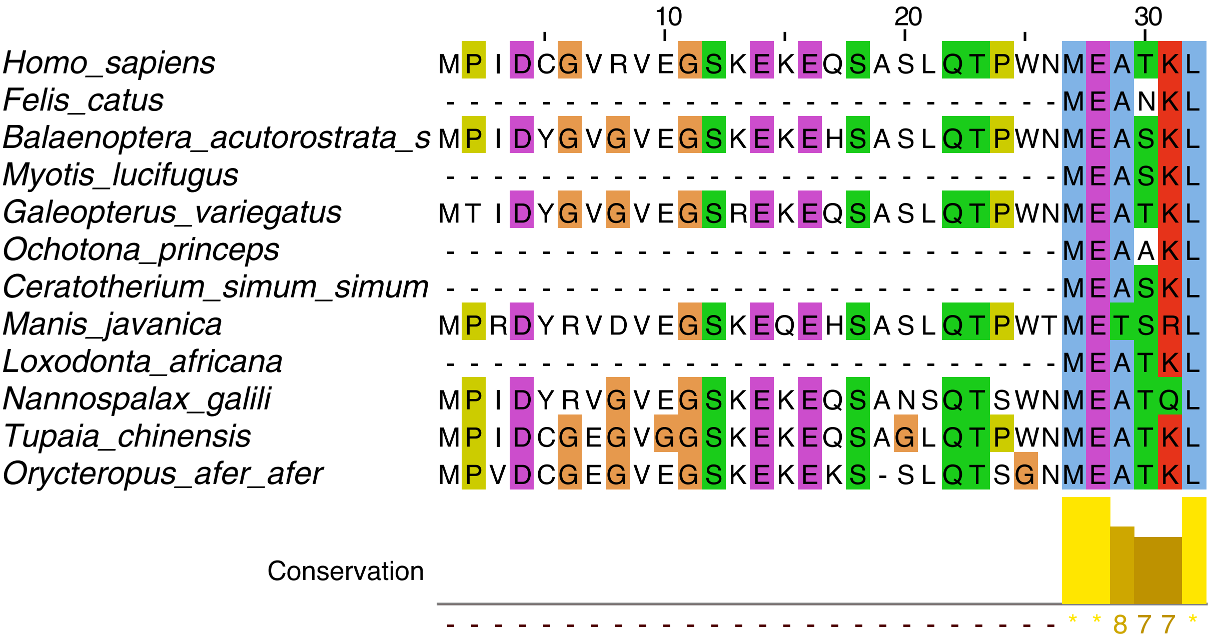

HG0580

ENSG00000073711 (PPP2R3A)

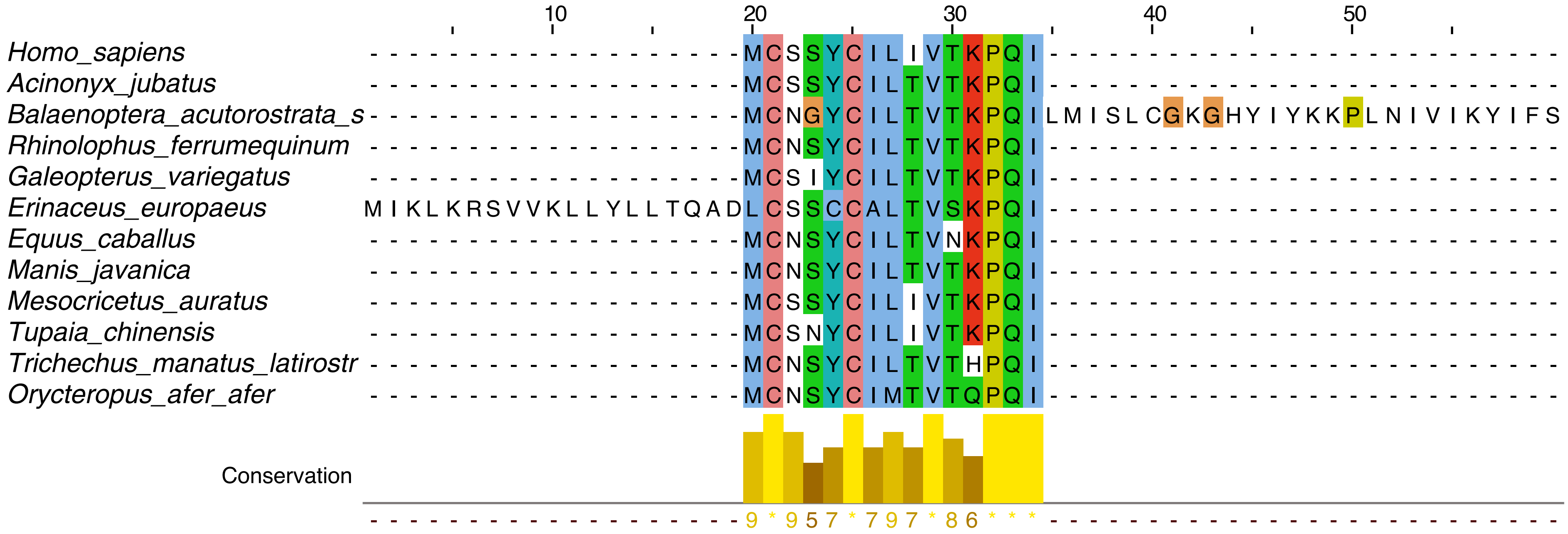

HG0581

ENSG00000087074 (PPP1R15A)

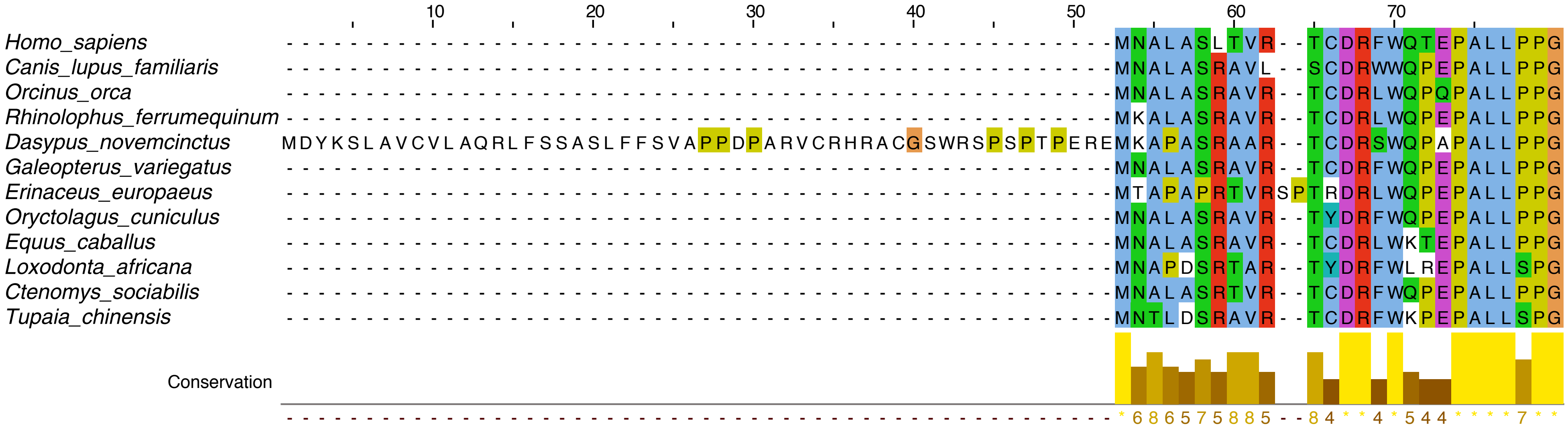

HG0582

ENSG00000087338 (GMCL1)

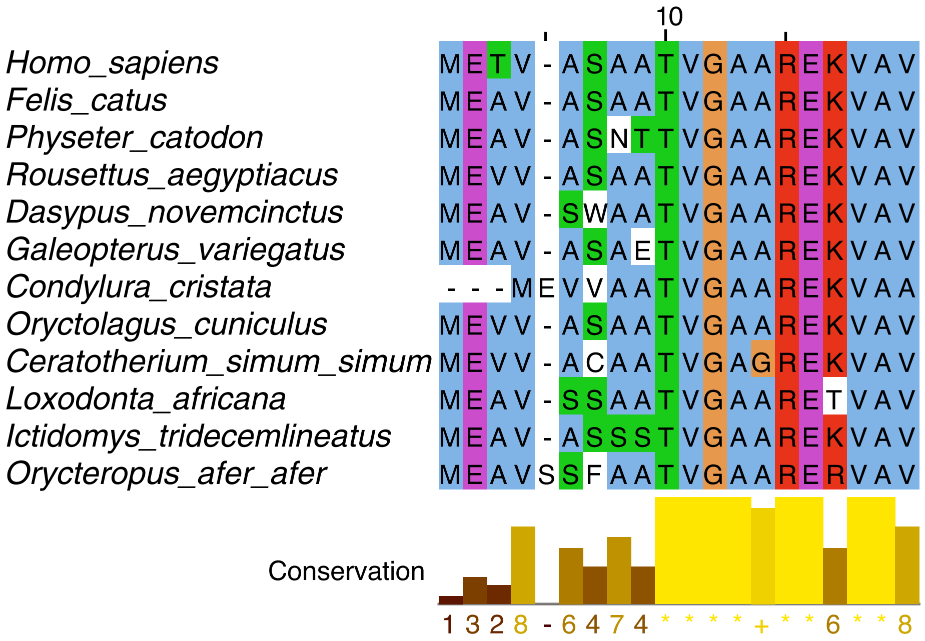

HG0583

ENSG00000087502 (ERGIC2)

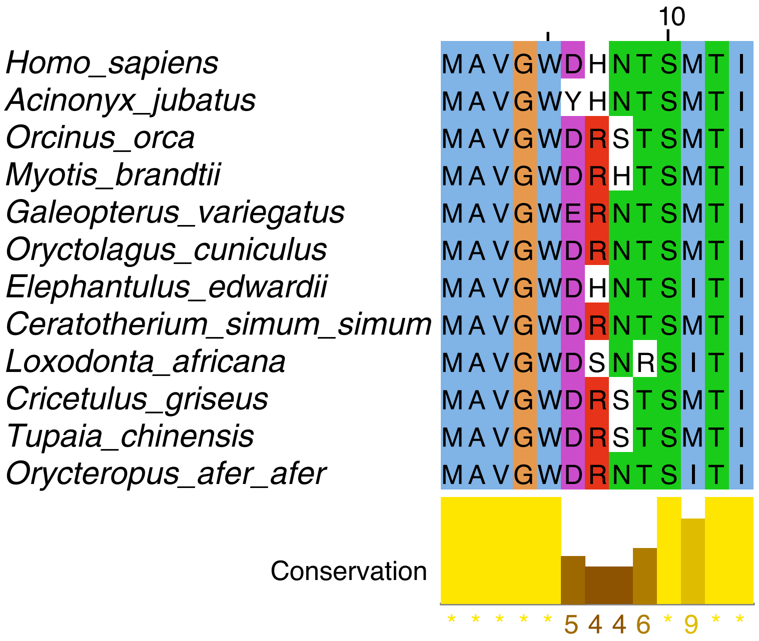

HG0584

ENSG00000092051 (JPH4)

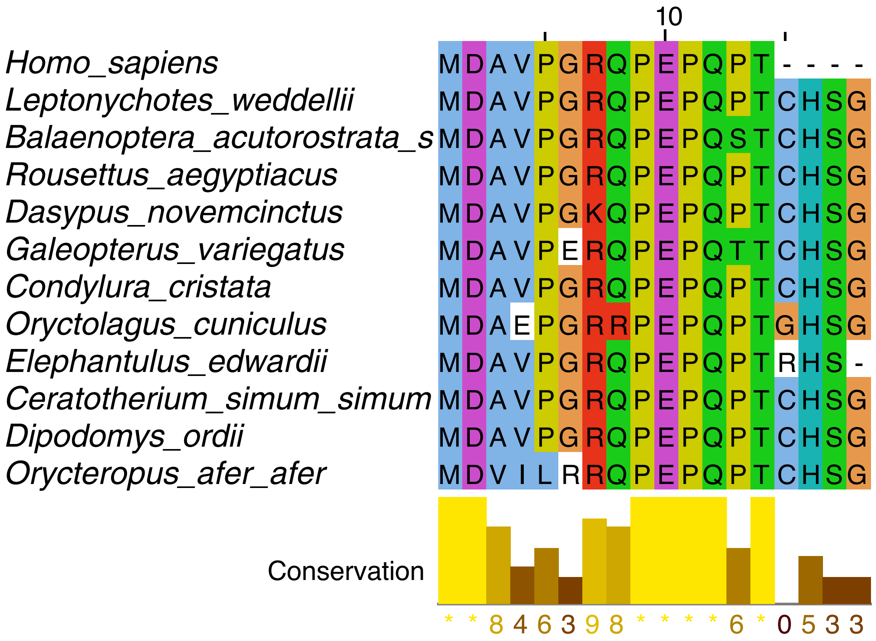

HG0585

ENSG00000106038 (EVX1)

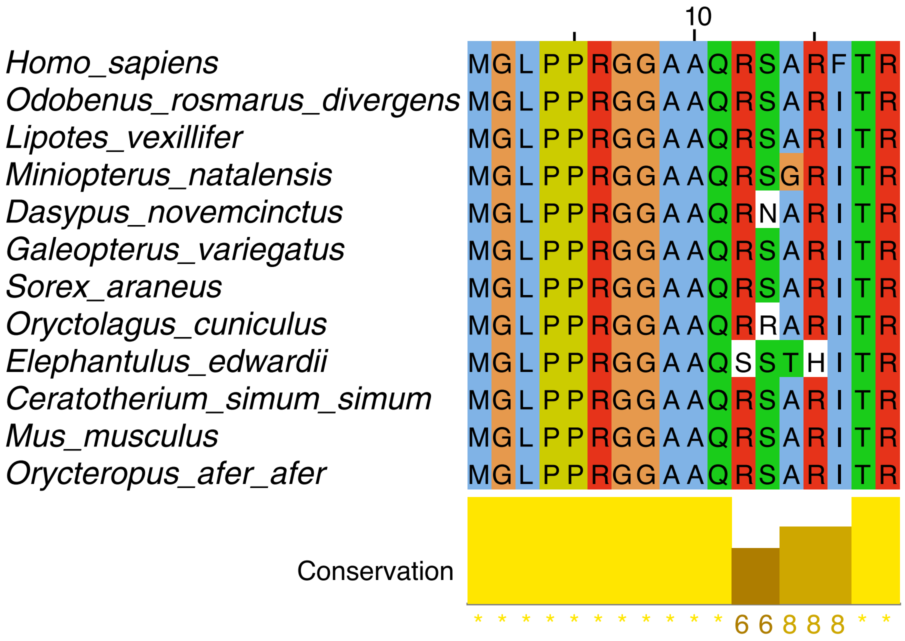

HG0586.1

ENSG00000108375 (RNF43)

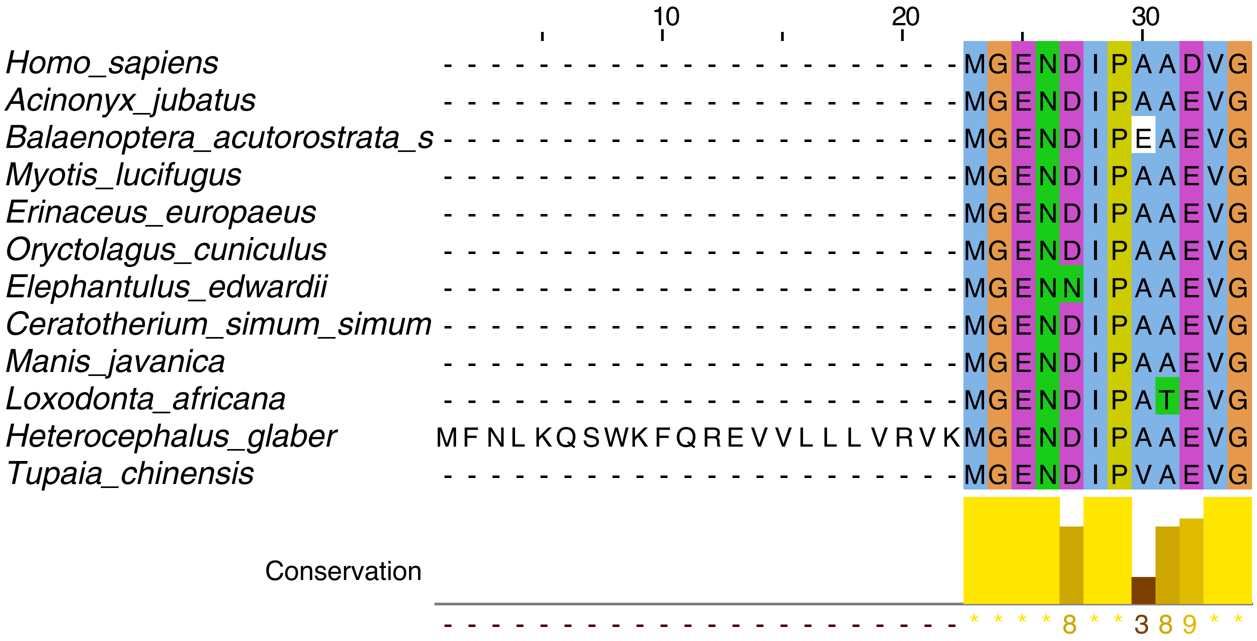

HG0586.2

ENSG00000108375 (RNF43)

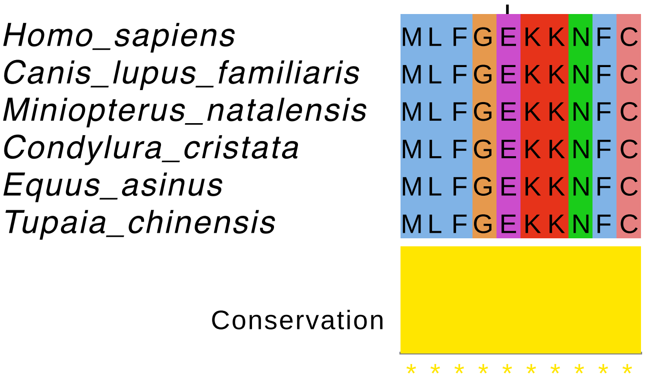

HG0587

ENSG00000119138 (KLF9)

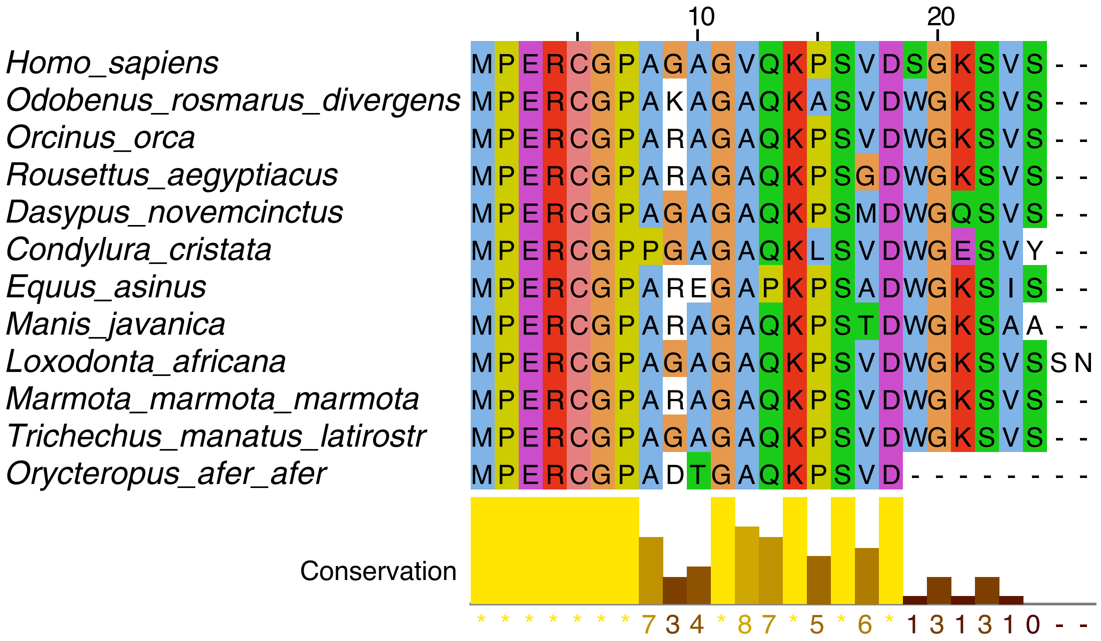

HG0588

ENSG00000120162 (MOB3B)

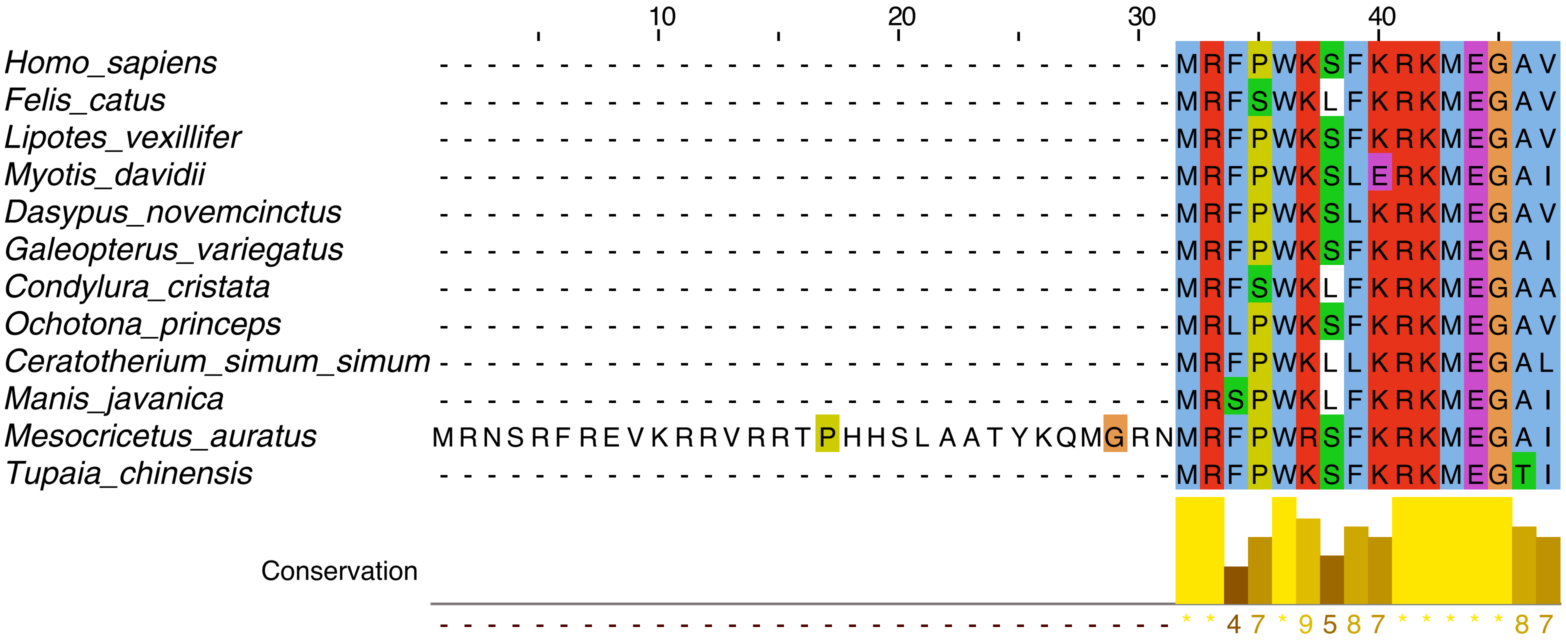

HG0589.1

ENSG00000121871 (SLITRK3)

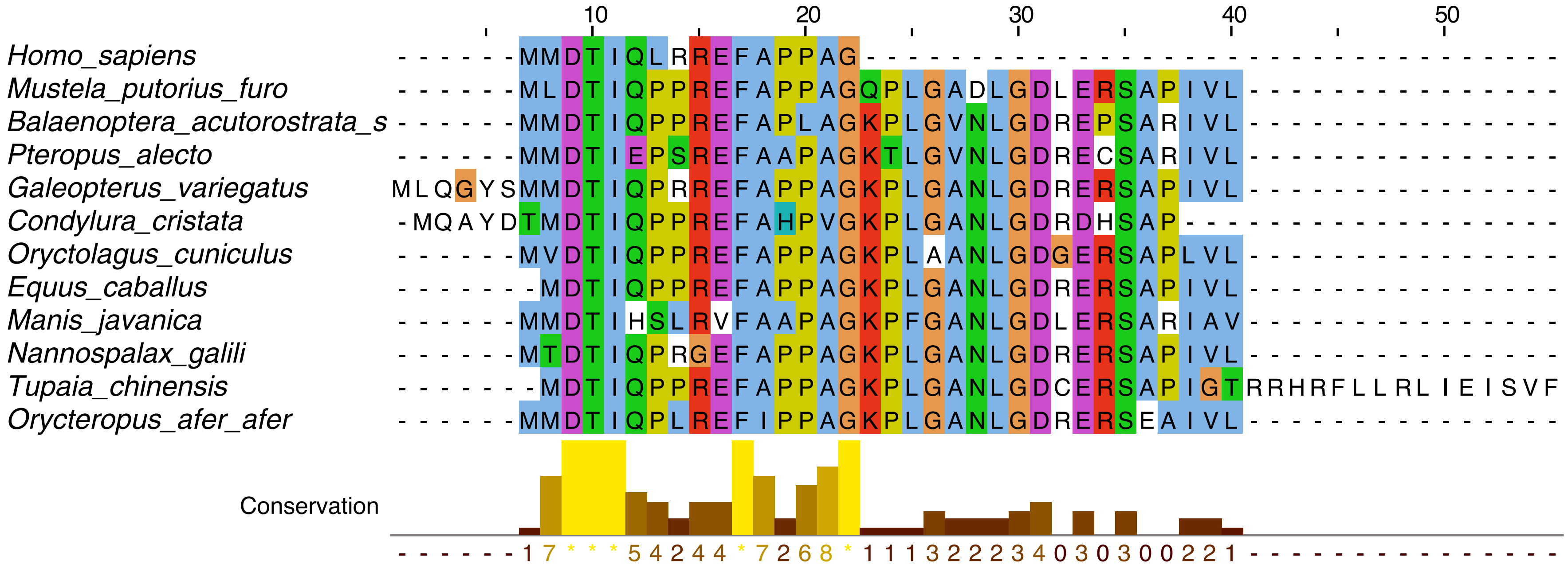

HG0589.2

ENSG00000121871 (SLITRK3)

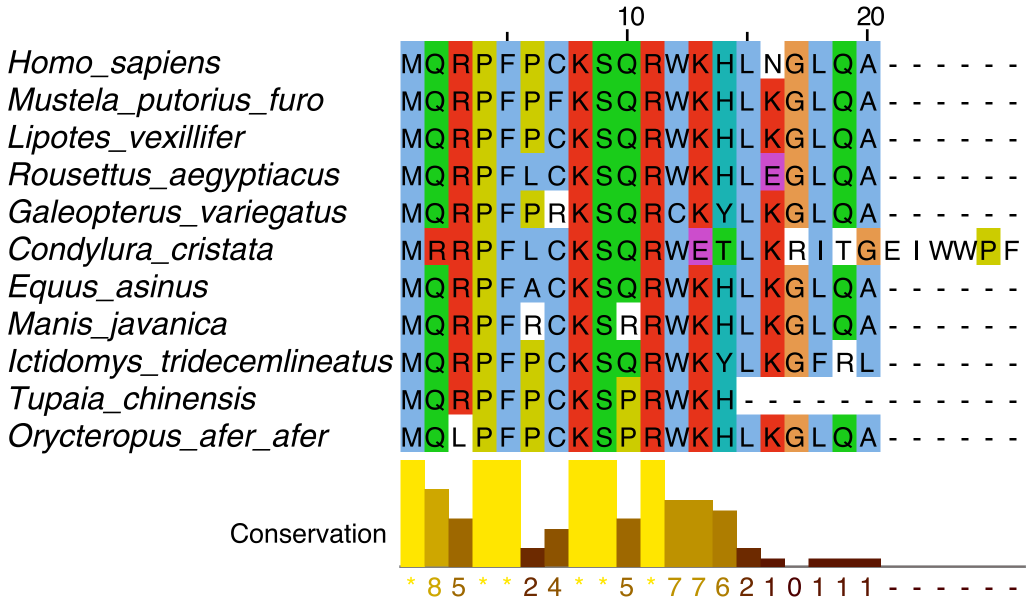

HG0589.3

ENSG00000121871 (SLITRK3)

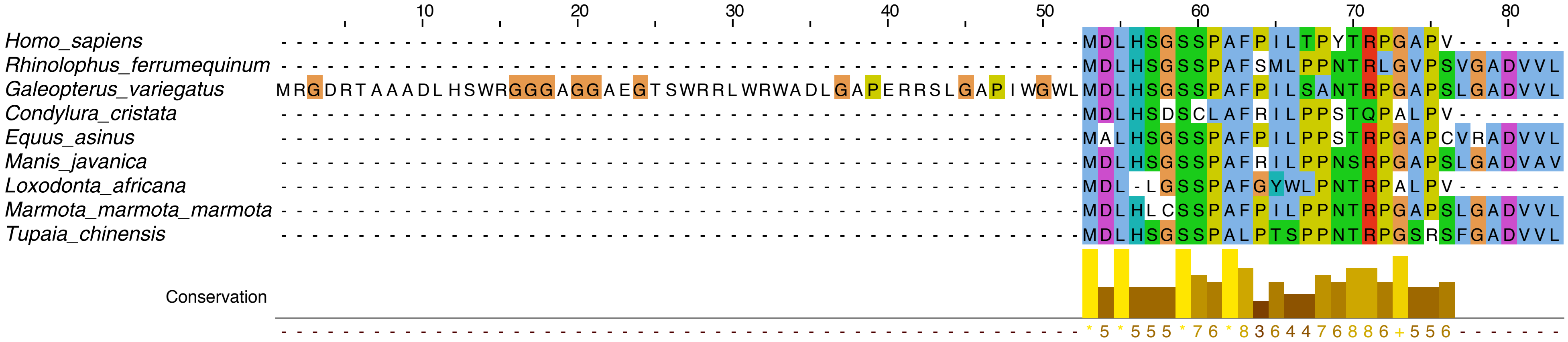

HG0590

ENSG00000122557 (HERPUD2)

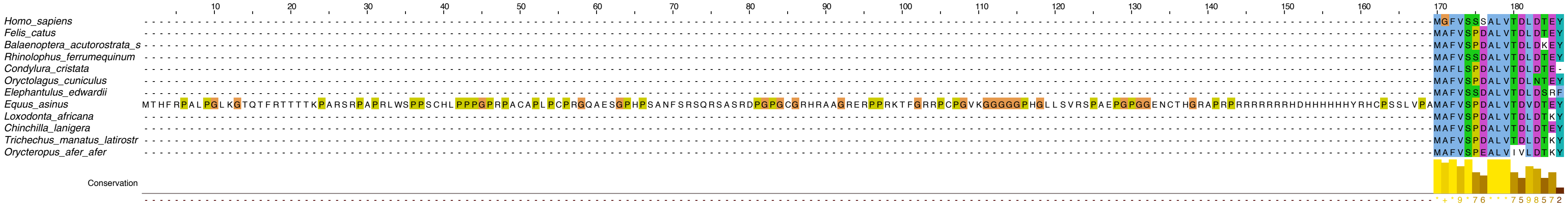

HG0591.1

ENSG00000127124 (HIVEP3)

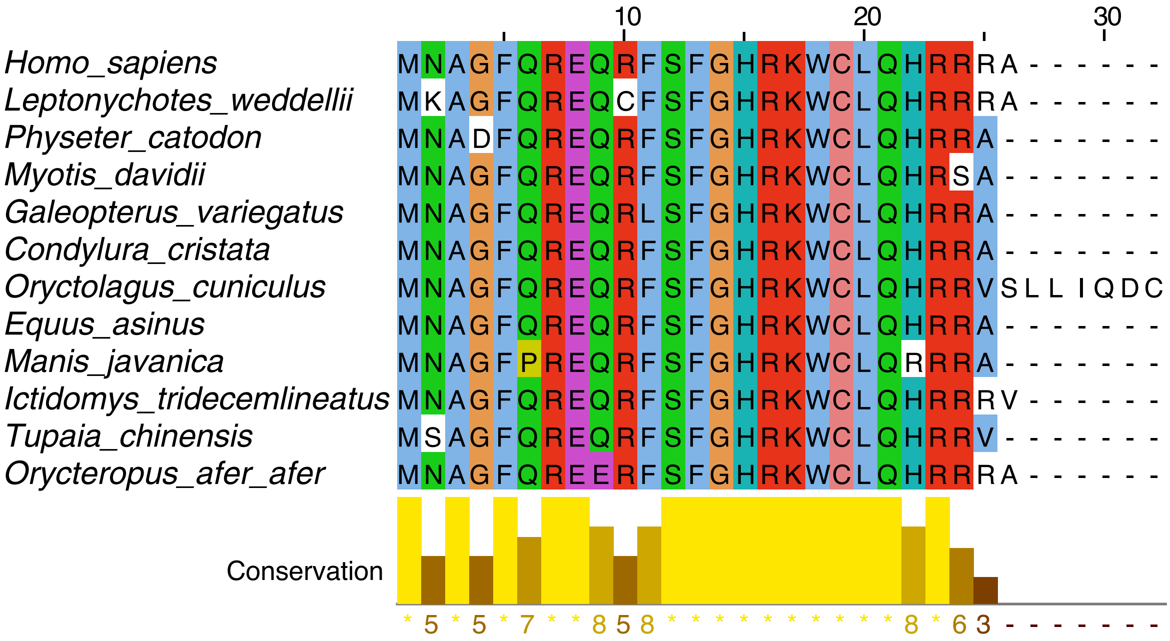

HG0591.2

ENSG00000127124 (HIVEP3)

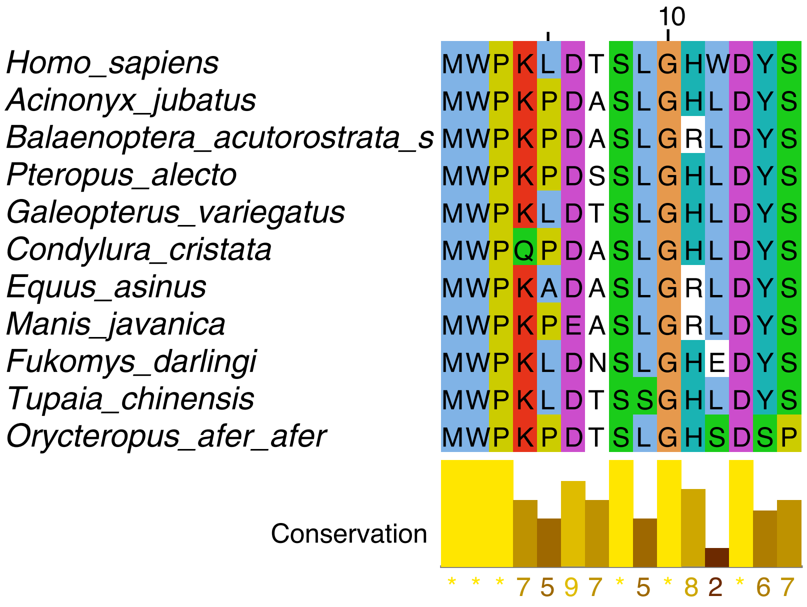

HG0591.3

ENSG00000127124 (HIVEP3)

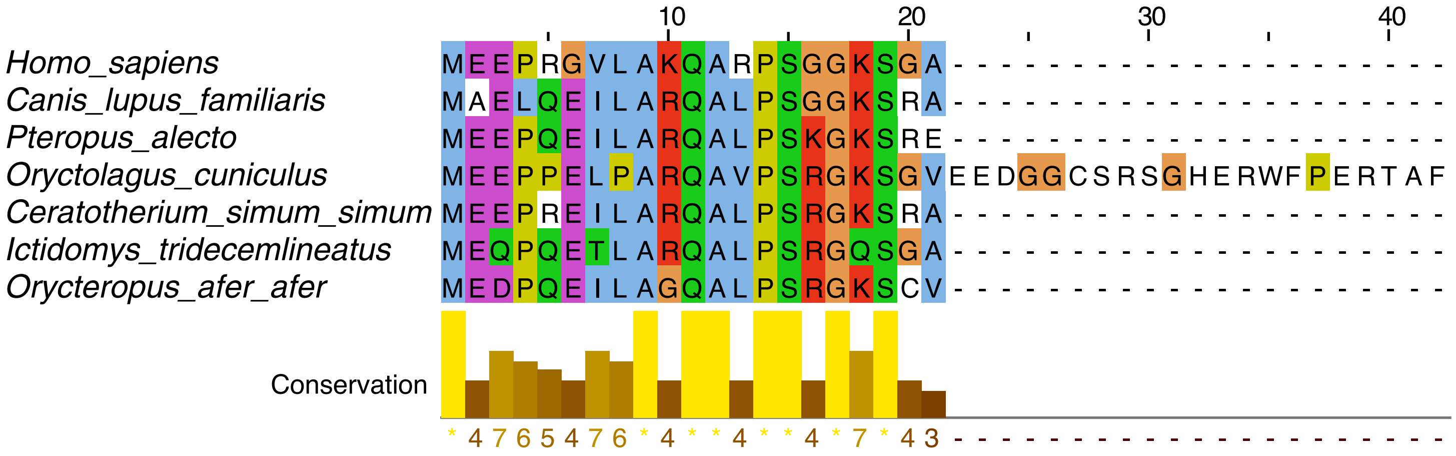

HG0591.4

ENSG00000127124 (HIVEP3)

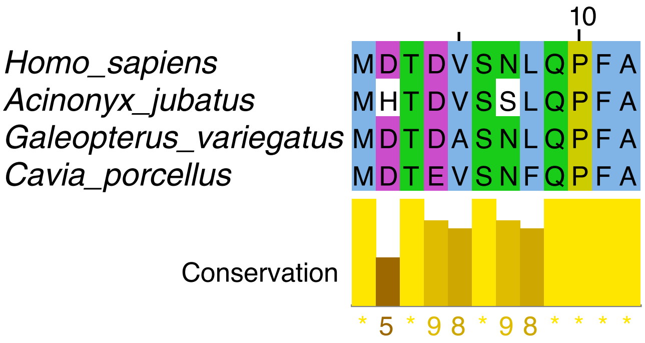

HG0592

ENSG00000133401 (PDZD2)

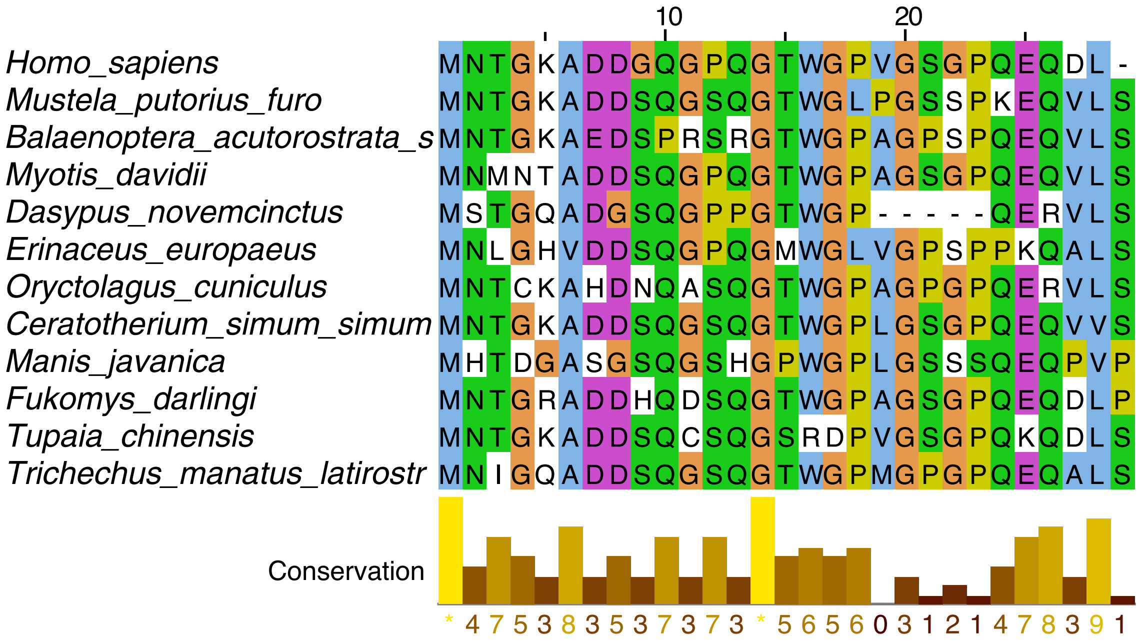

HG0593

ENSG00000135048 (TMEM2)

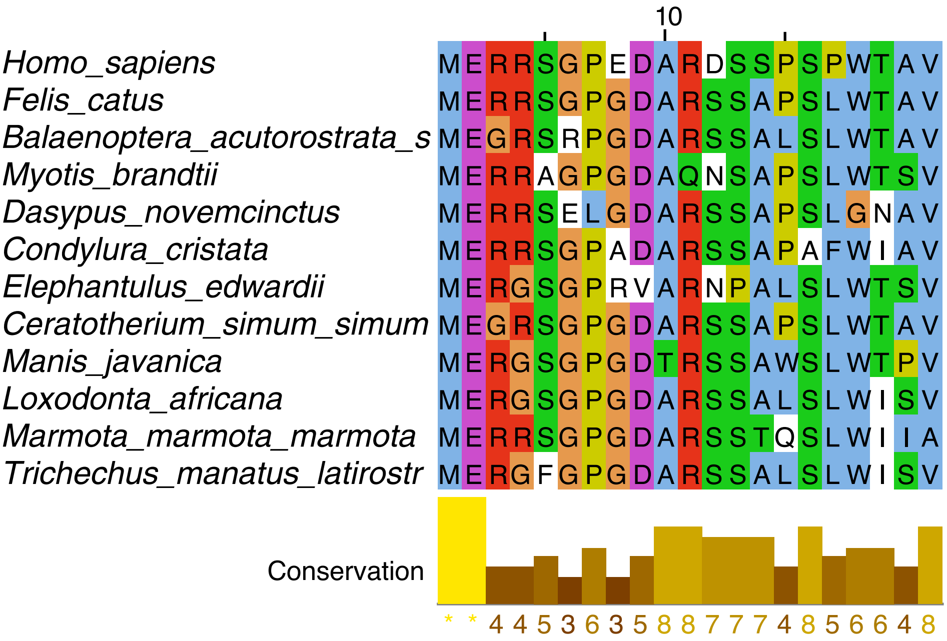

HG0594

ENSG00000136169 (SETDB2)

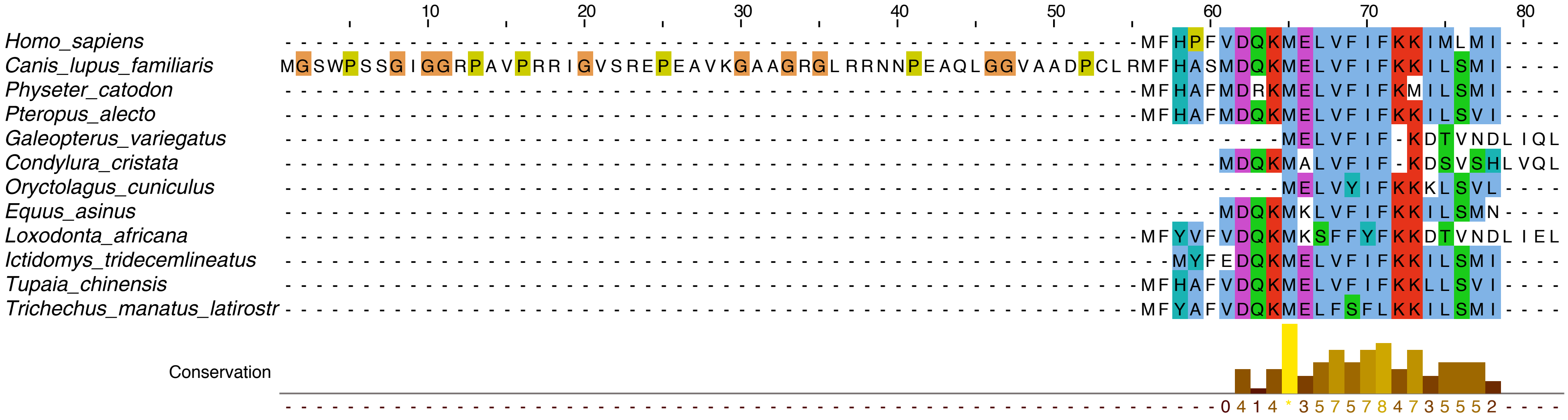

HG0595

ENSG00000141098 (GFOD2)

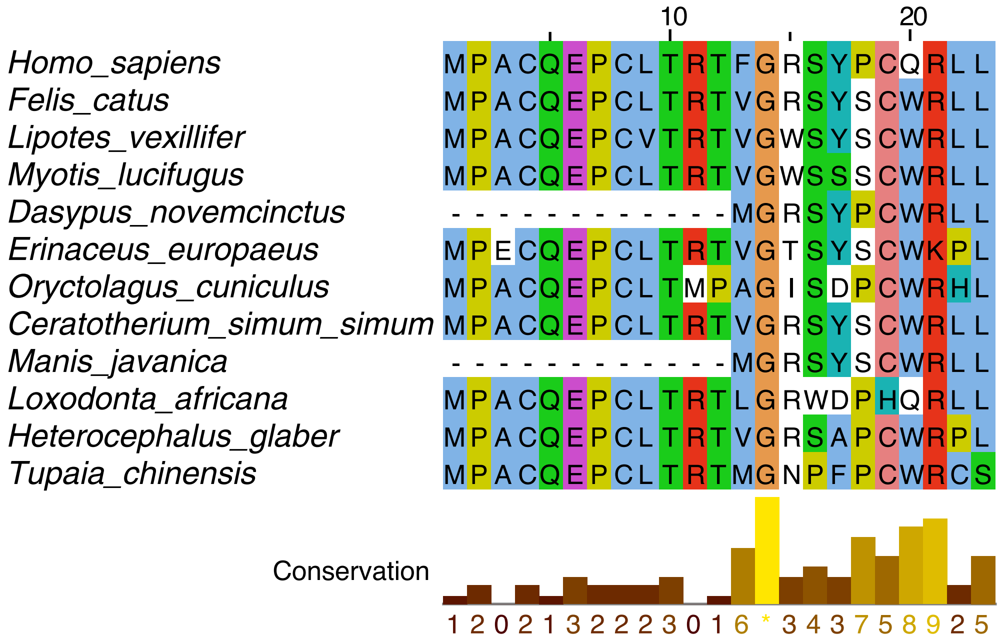

HG0596

ENSG00000157483 (MYO1E)

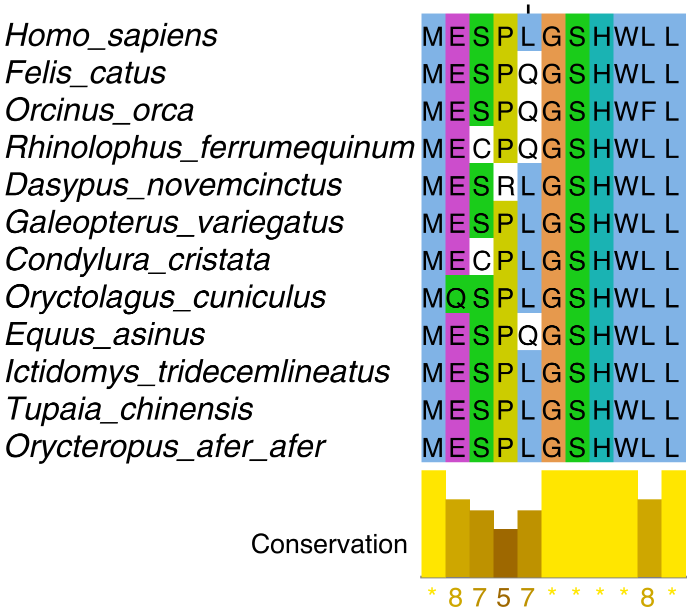

HG0597

ENSG00000163032 (VSNL1)

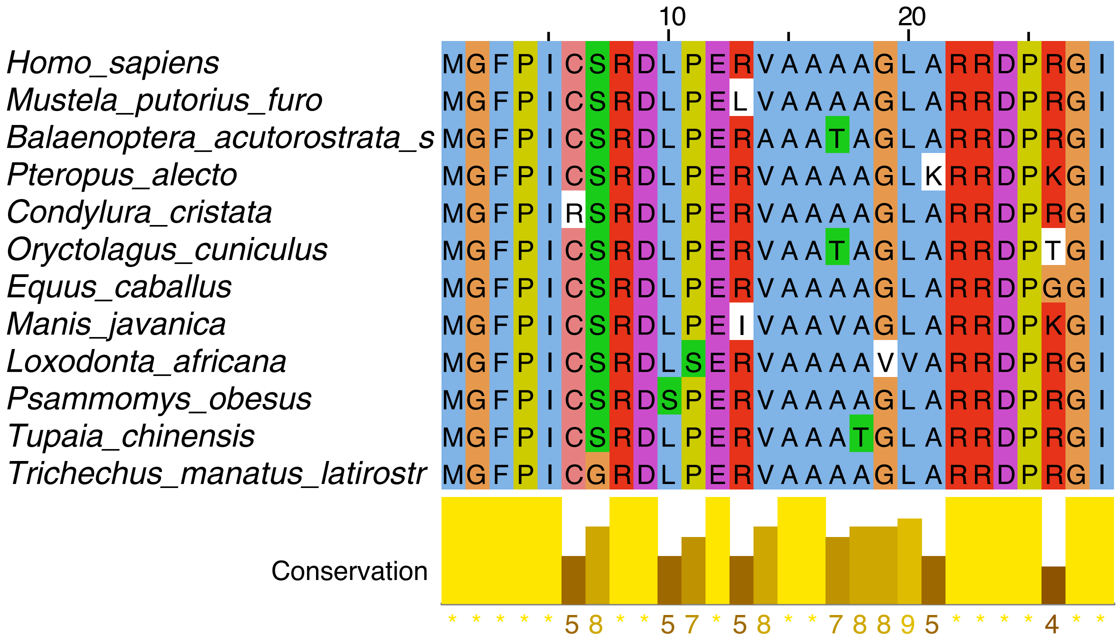

HG0598

ENSG00000163788 (SNRK)

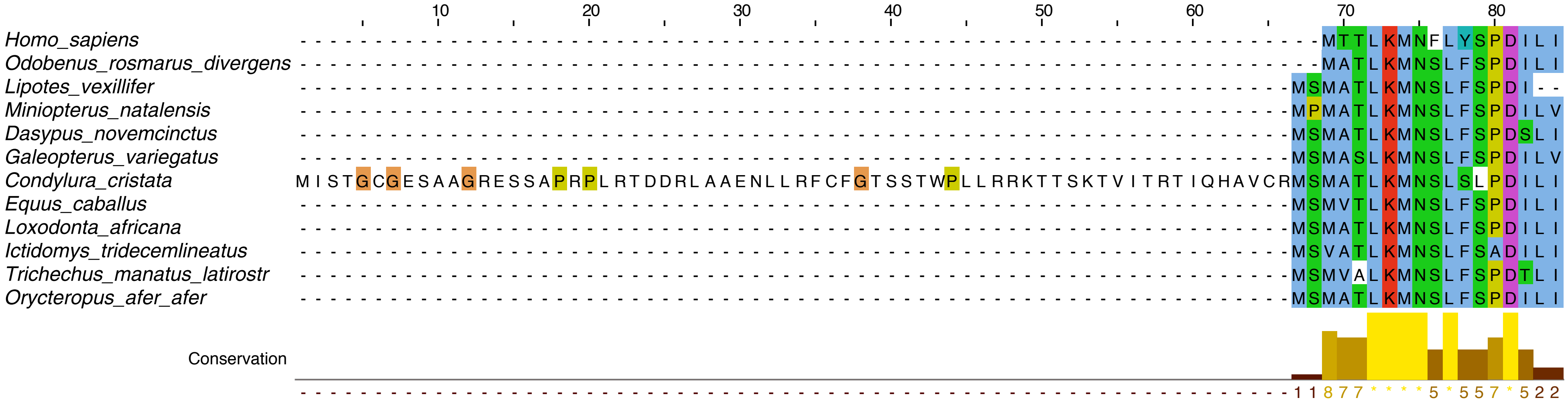

HG0599

ENSG00000164930 (FZD6)

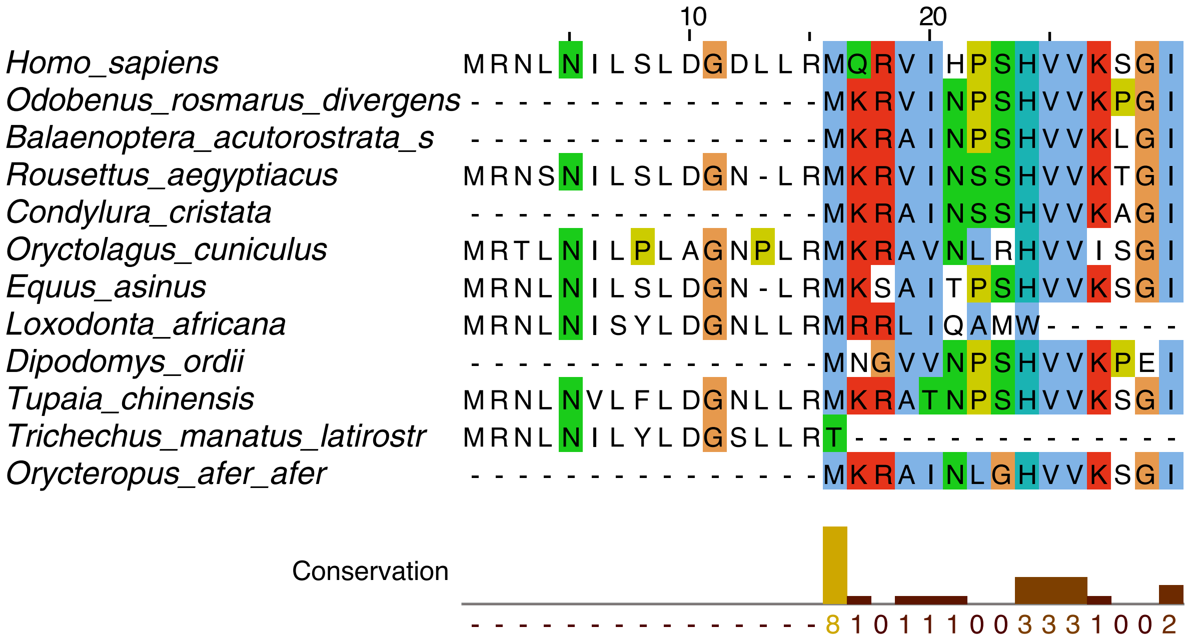

HG0600

ENSG00000173065 (FAM222B)

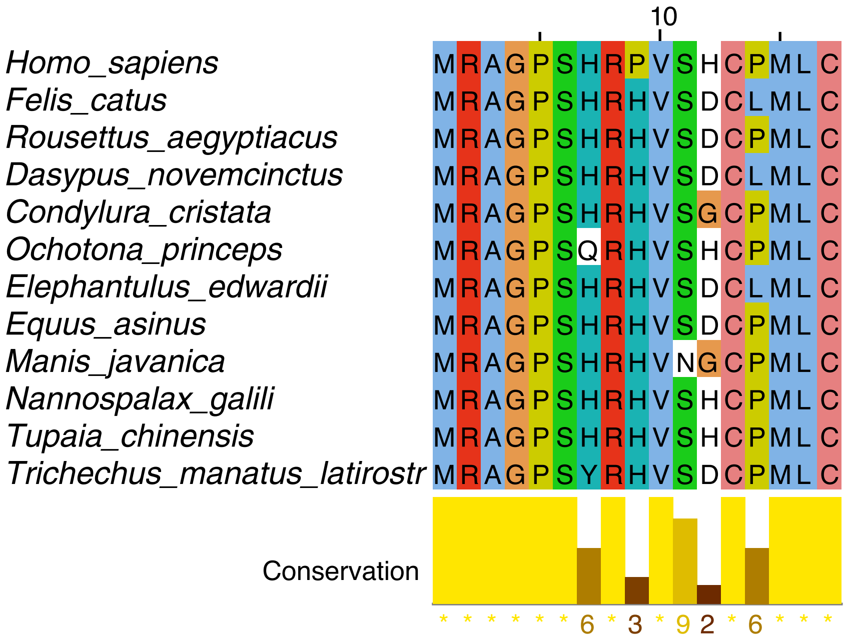

HG0601

ENSG00000175893 (ZDHHC21)

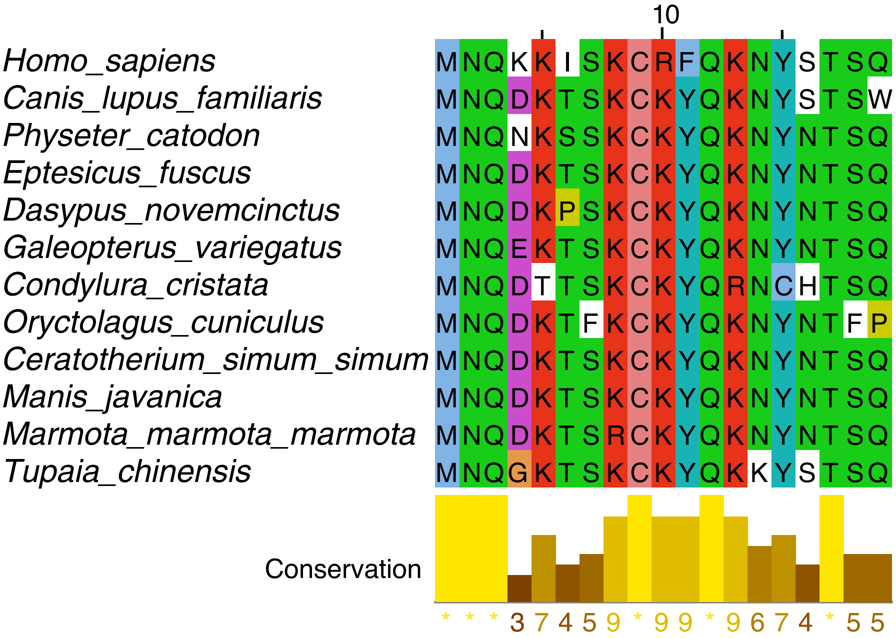

HG0602

ENSG00000179195 (ZNF664)

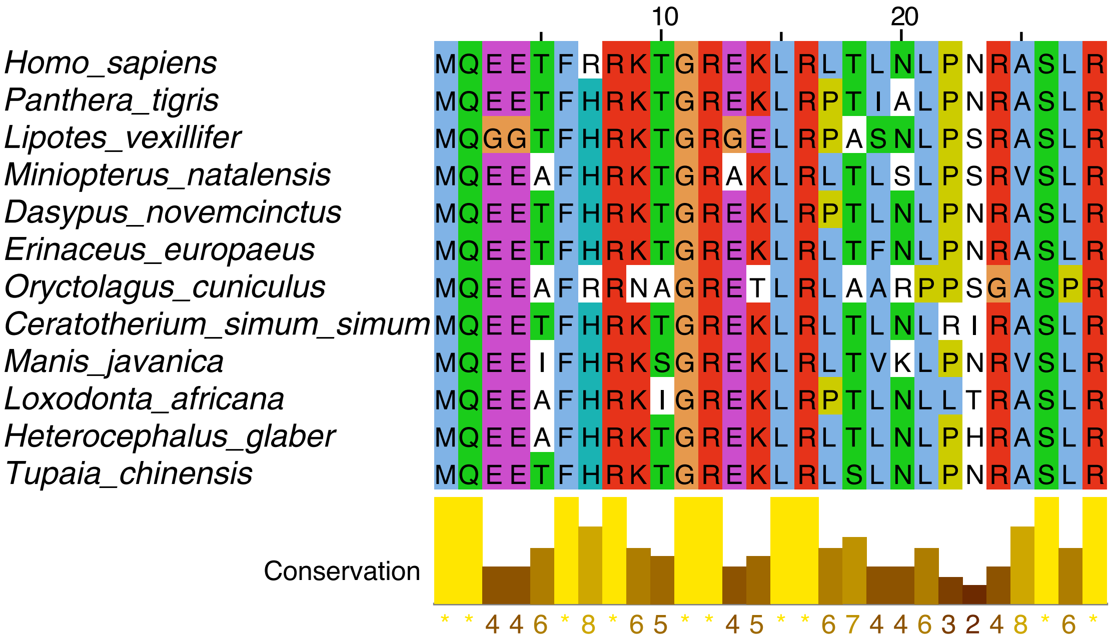

HG0603

ENSG00000197329 (PELI1)

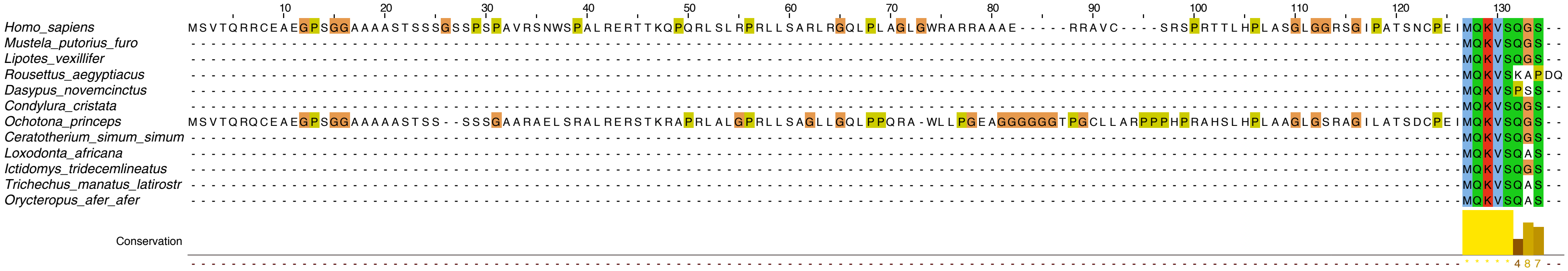

HG0604.1

ENSG00000003147 (ICA1)

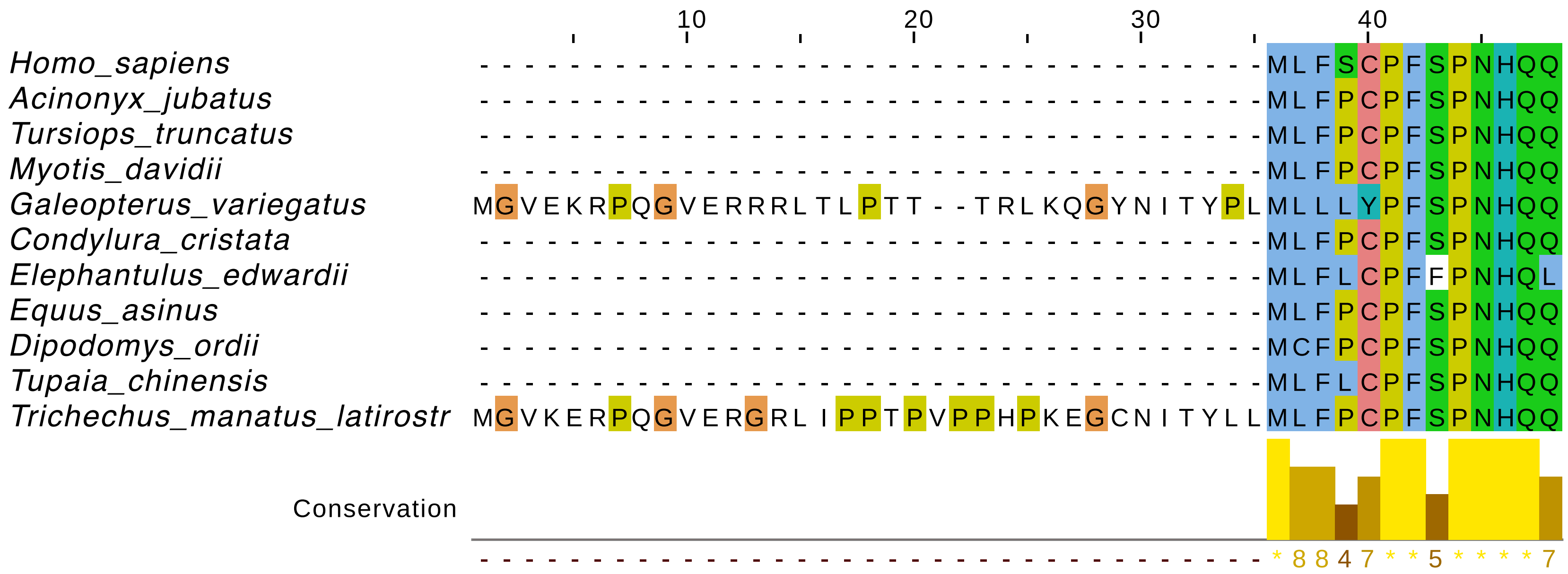

HG0604.2

ENSG00000003147 (ICA1)

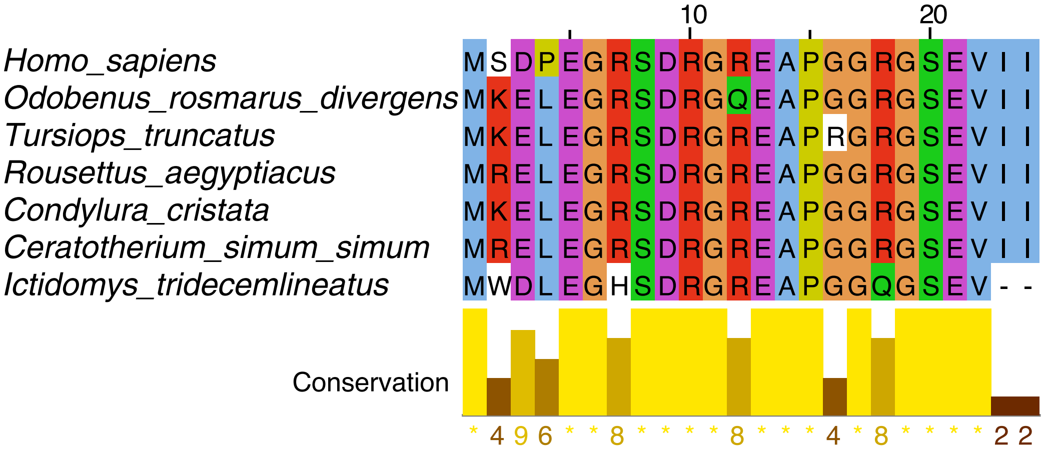

HG0604.3

ENSG00000003147 (ICA1)

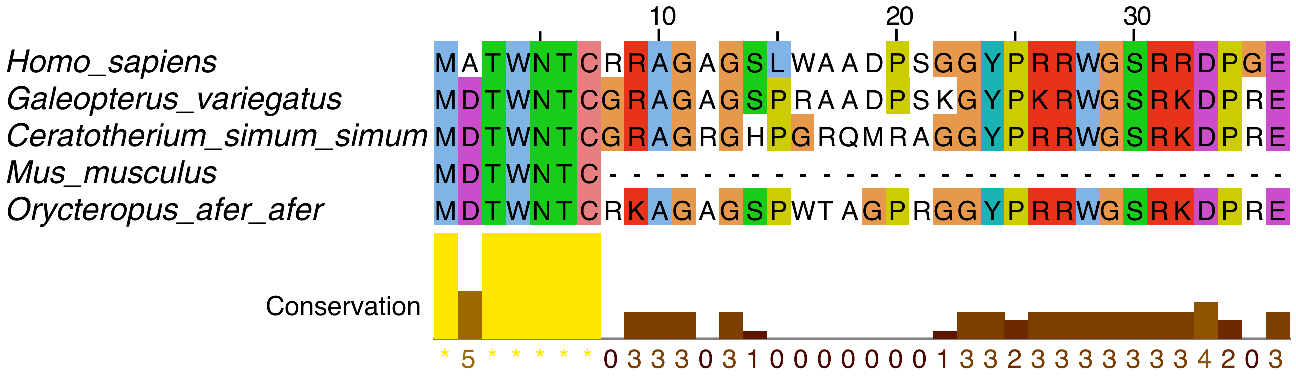

HG0605

ENSG00000053254 (FOXN3)

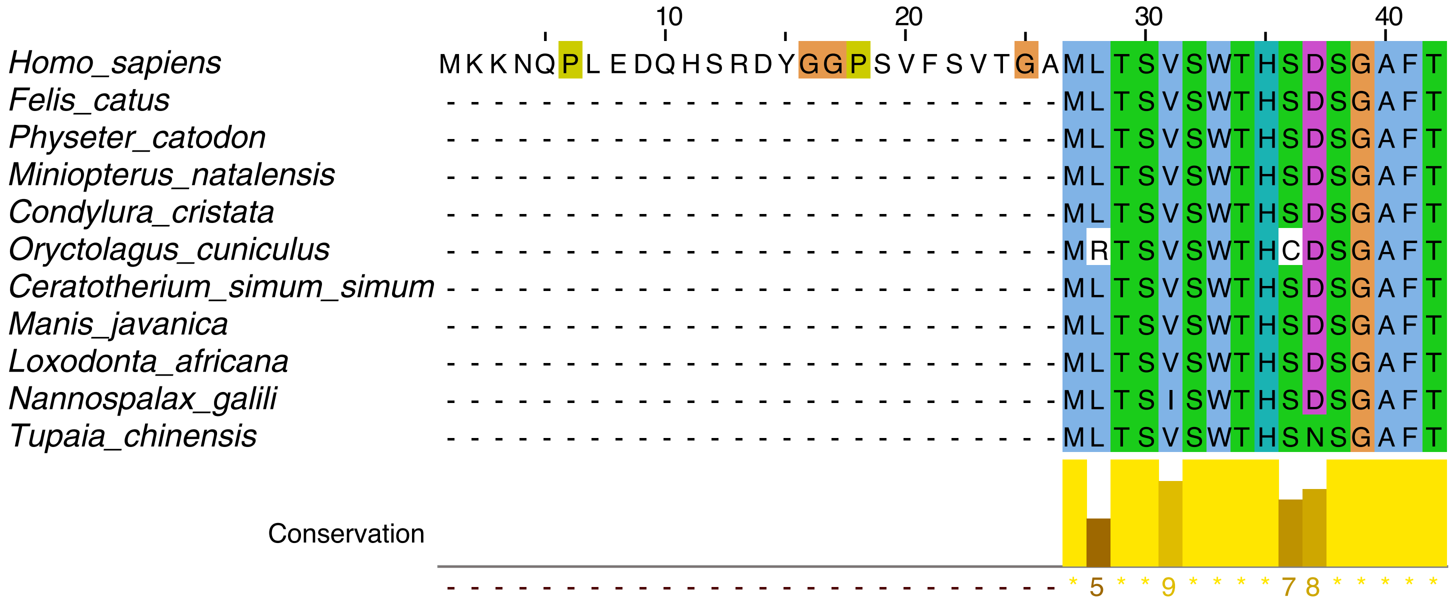

HG0606

ENSG00000055130 (CUL1)

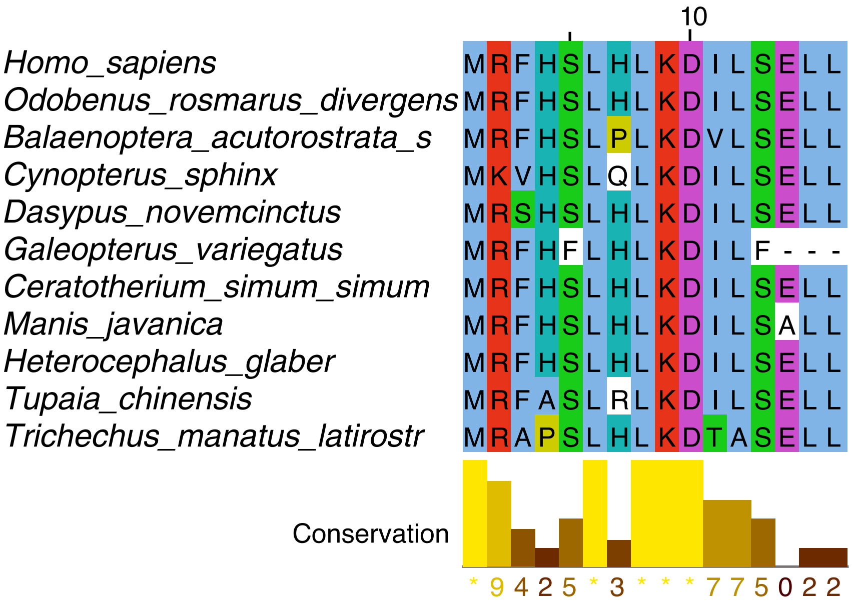

HG0607

ENSG00000074657 (ZNF532)

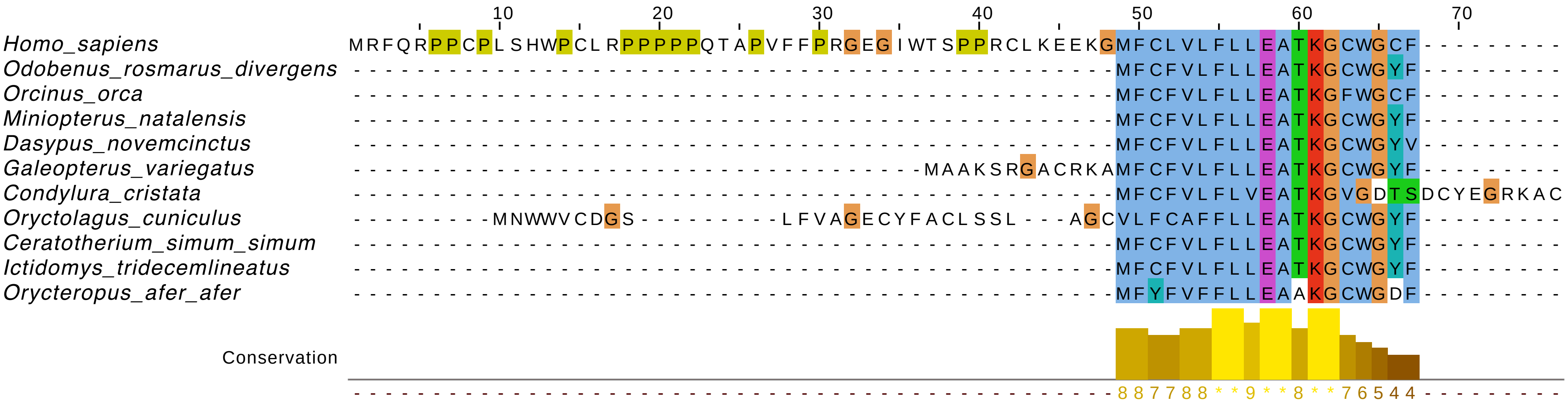

HG0608

ENSG00000084731 (KIF3C)

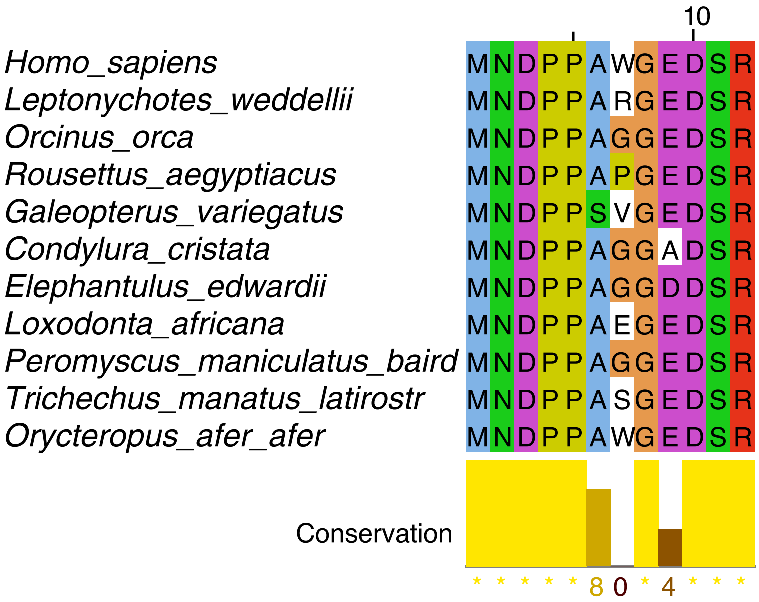

HG0609

ENSG00000095539 (SEMA4G)

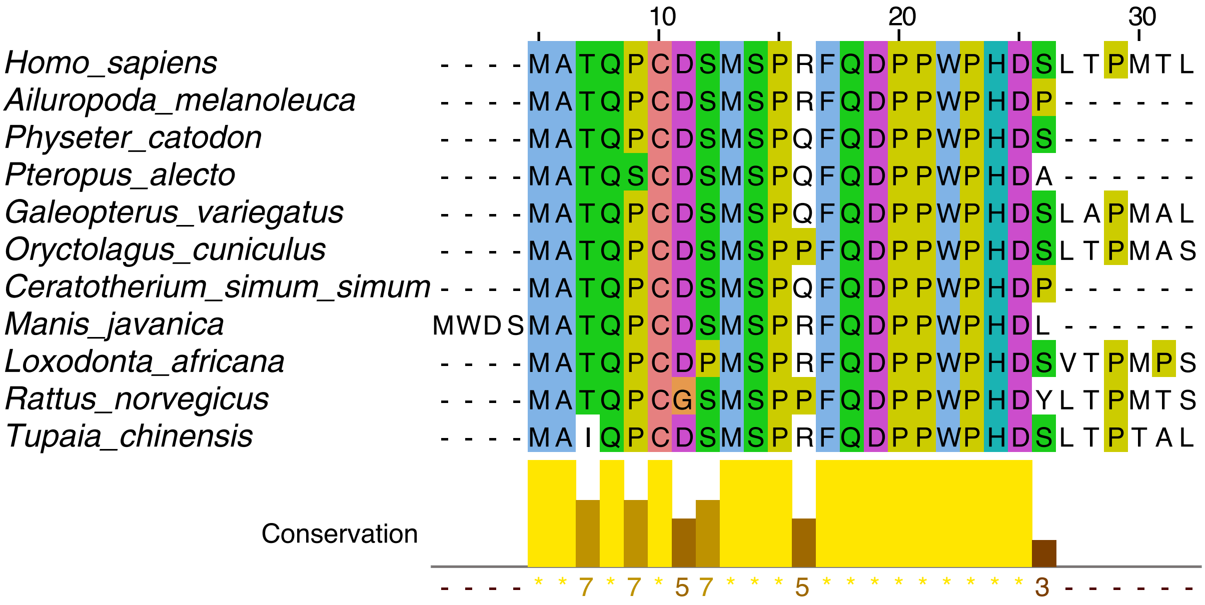

HG0610

ENSG00000106069 (CHN2)

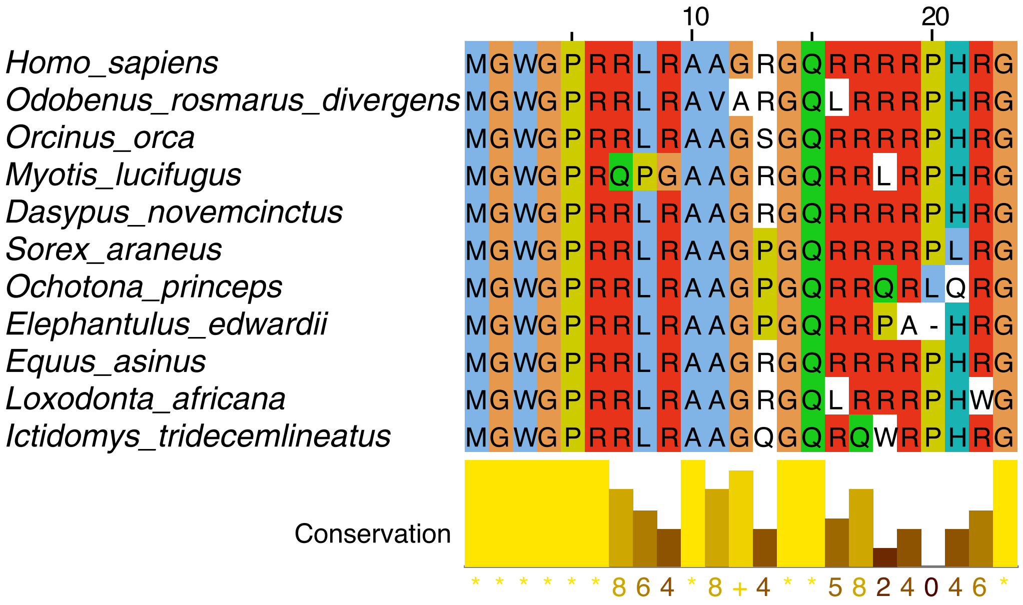

HG0611

ENSG00000108309 (RUNDC3A)

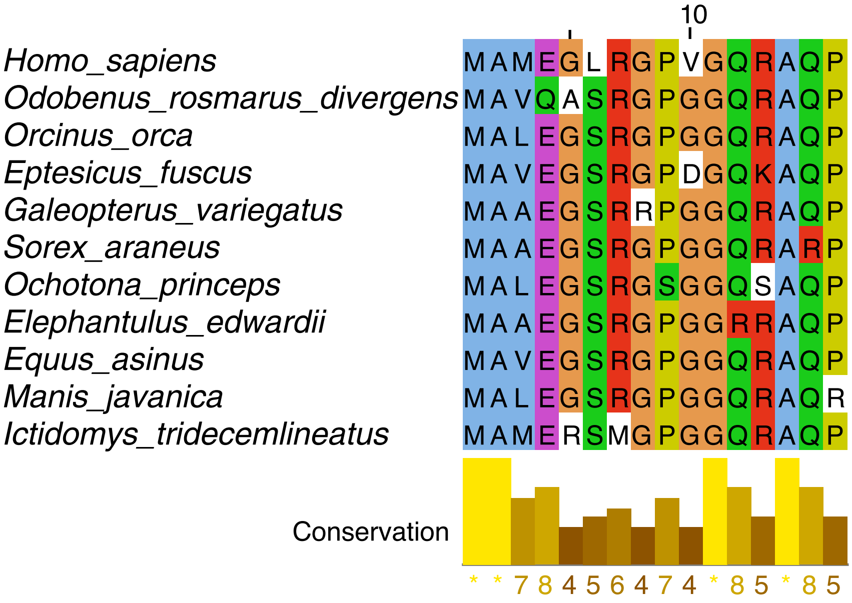

HG0612.1

ENSG00000110422 (HIPK3)

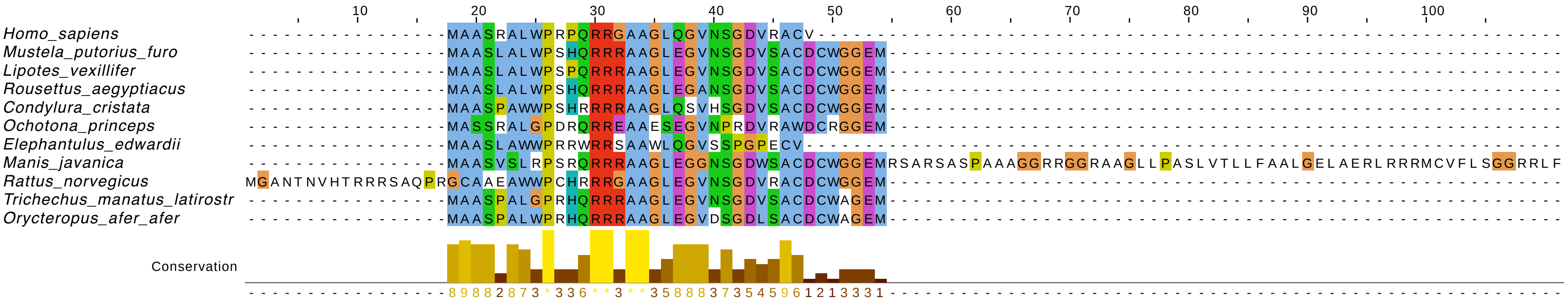

HG0612.2

ENSG00000110422 (HIPK3)

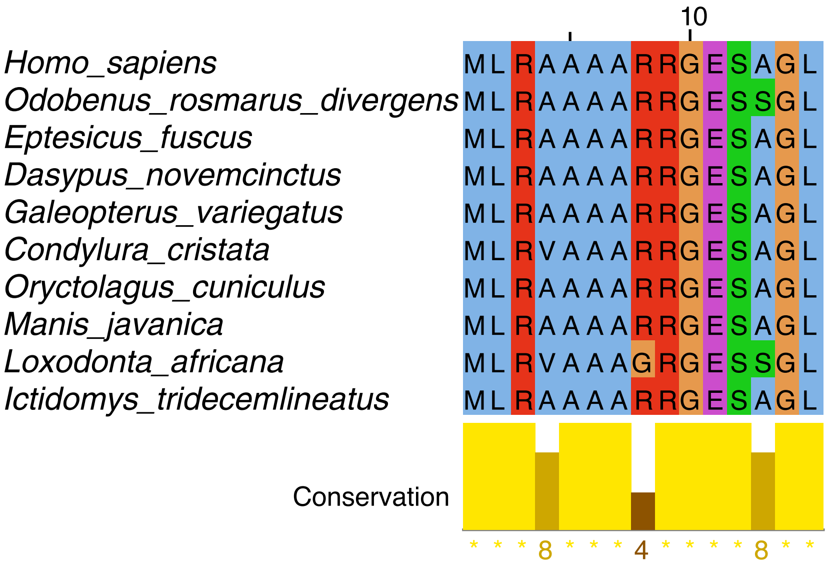

HG0612.3

ENSG00000110422 (HIPK3)

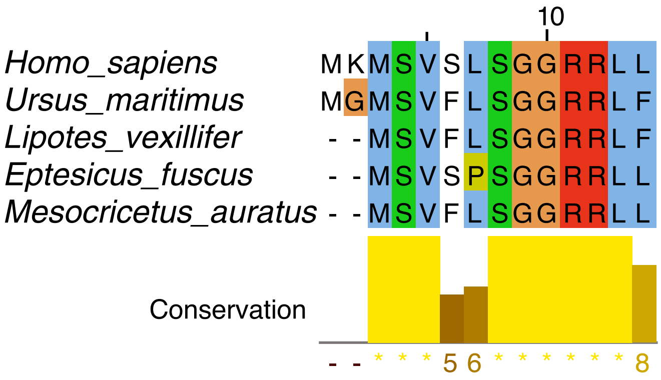

HG0613

ENSG00000114861 (FOXP1)

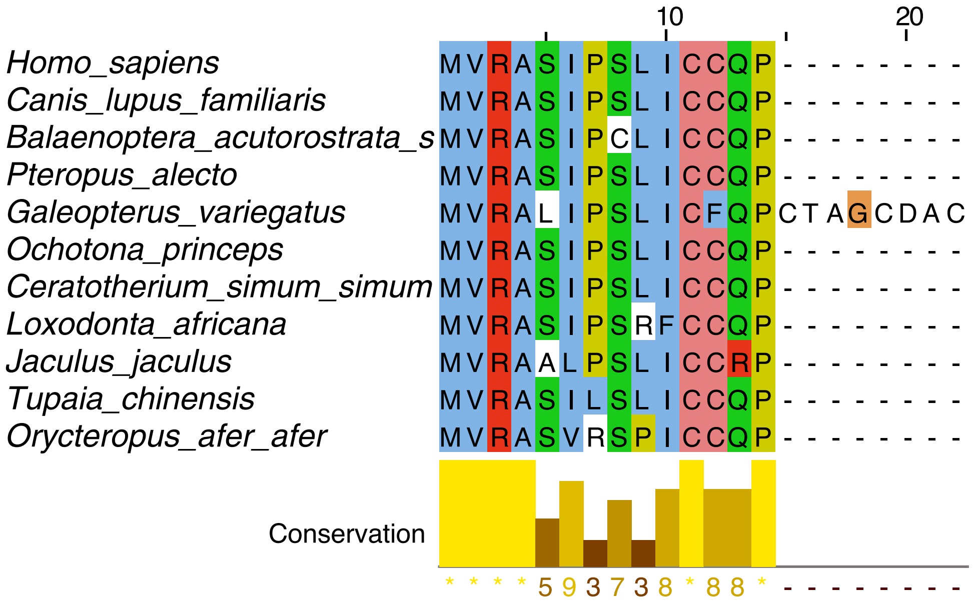

HG0614

ENSG00000122203 (KIAA1191)

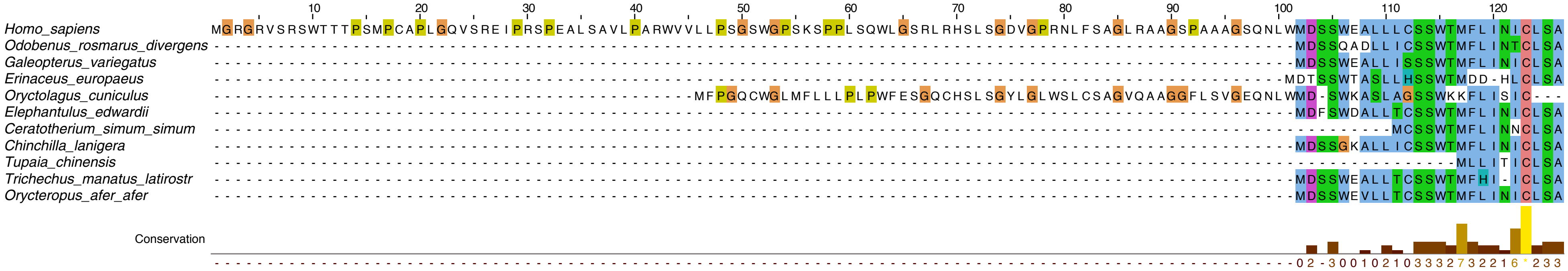

HG0615

ENSG00000128191 (DGCR8)

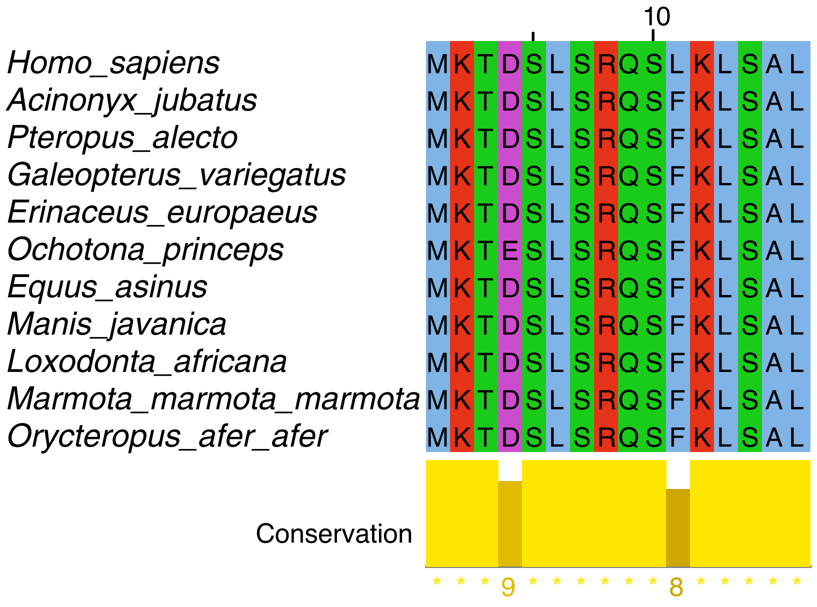

HG0616

ENSG00000128739 (SNRPN)

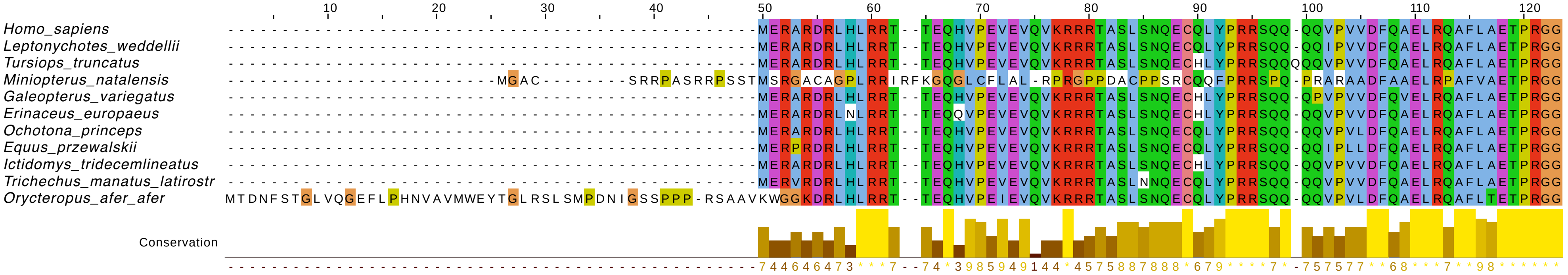

HG0617

ENSG00000135164 (DMTF1)

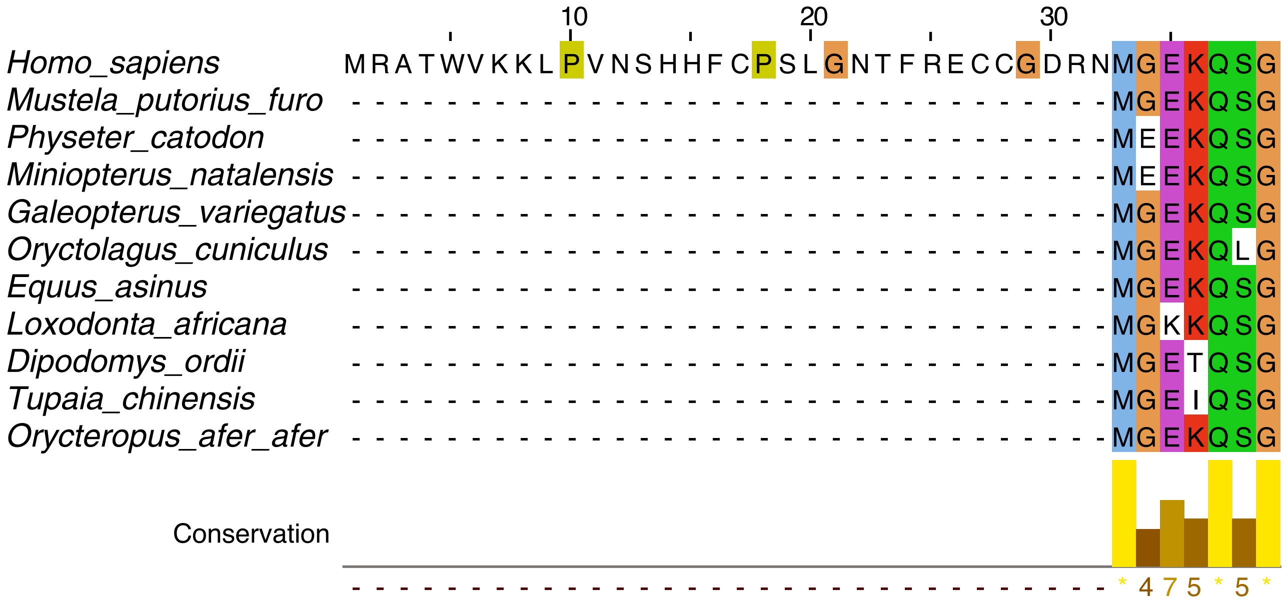

HG0618

ENSG00000137817 (PARP6)

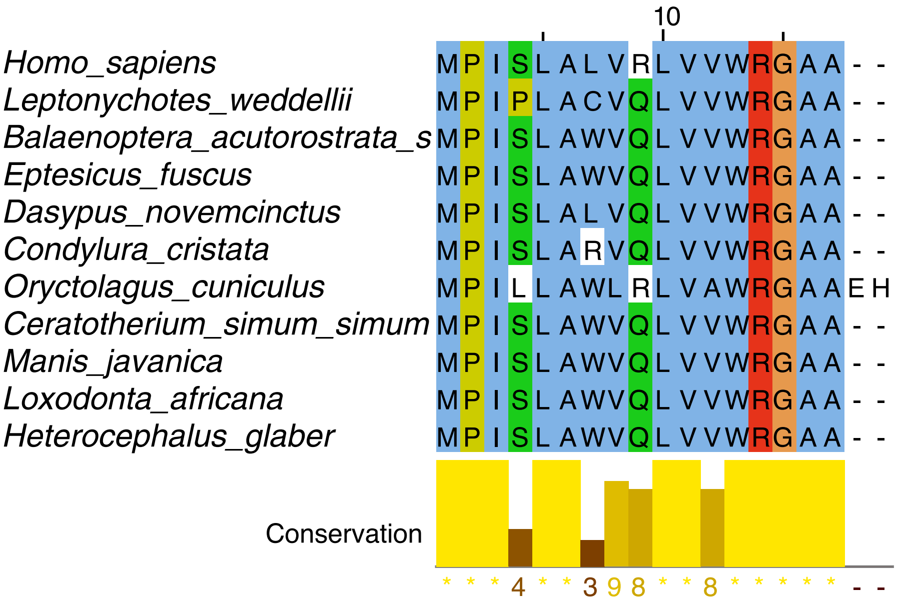

HG0619

ENSG00000138593 (SECISBP2L)

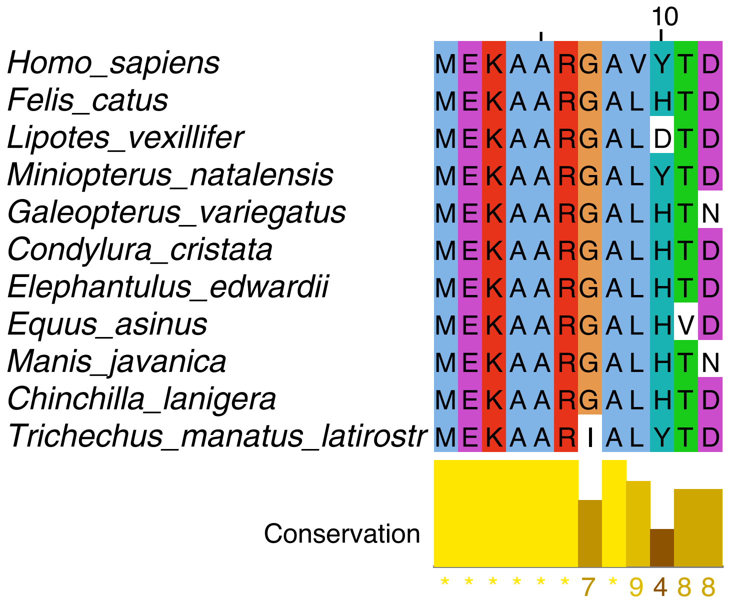

HG0620

ENSG00000145555 (MYO10)

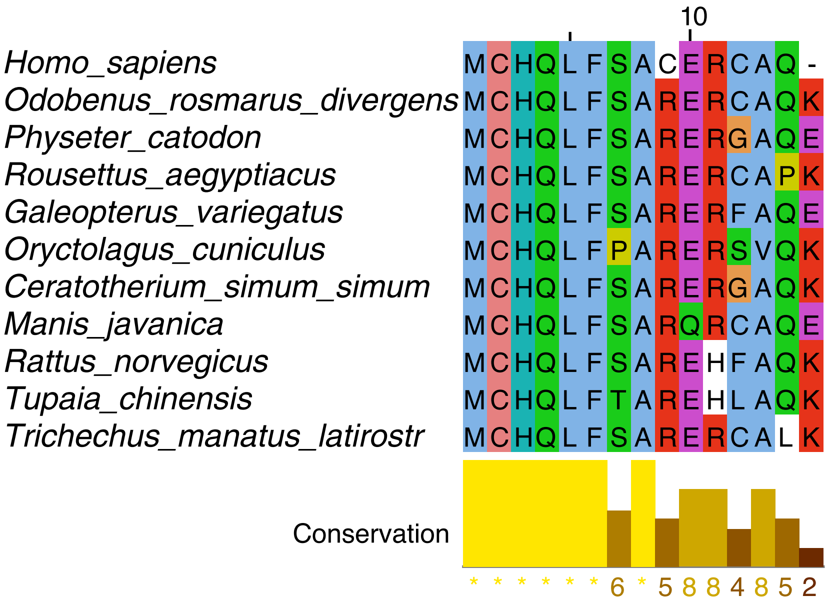

HG0621.1

ENSG00000146352 (CLVS2)

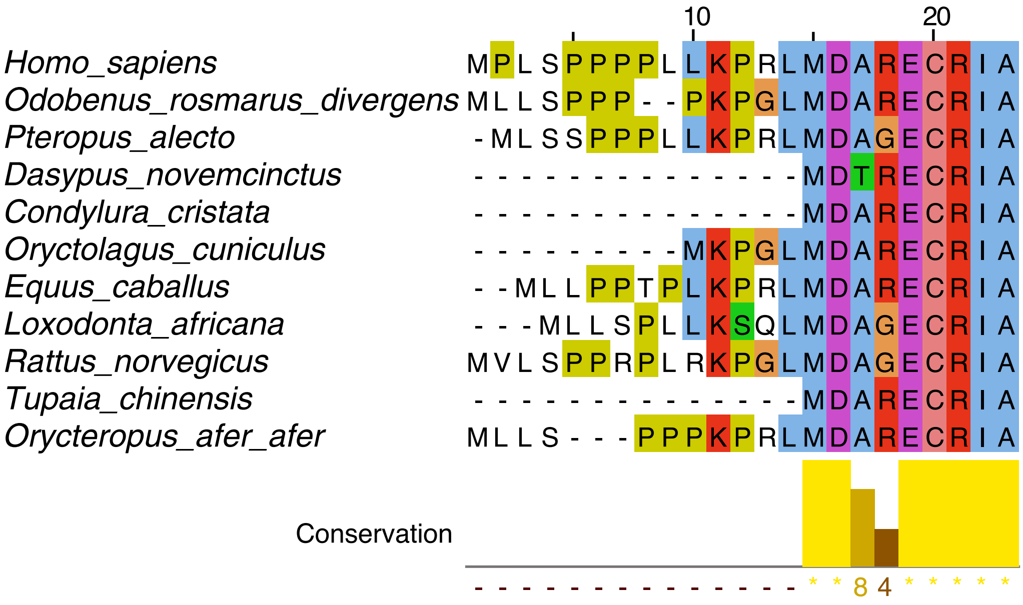

HG0621.2

ENSG00000146352 (CLVS2)

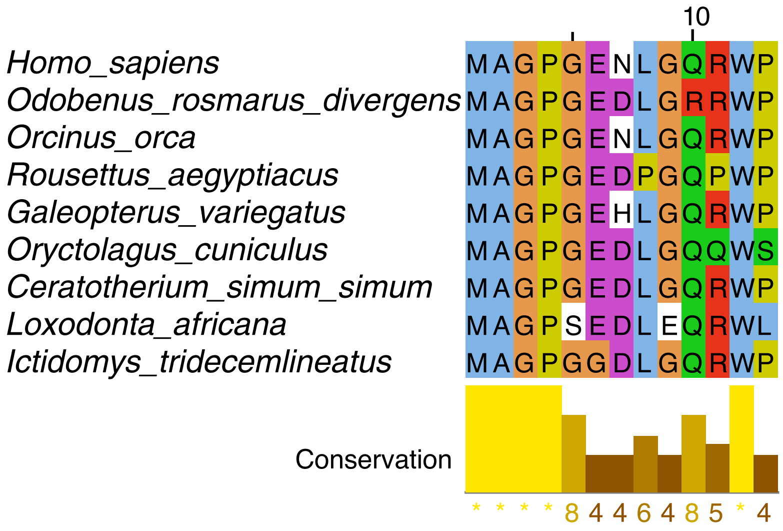

HG0621.3

ENSG00000146352 (CLVS2)

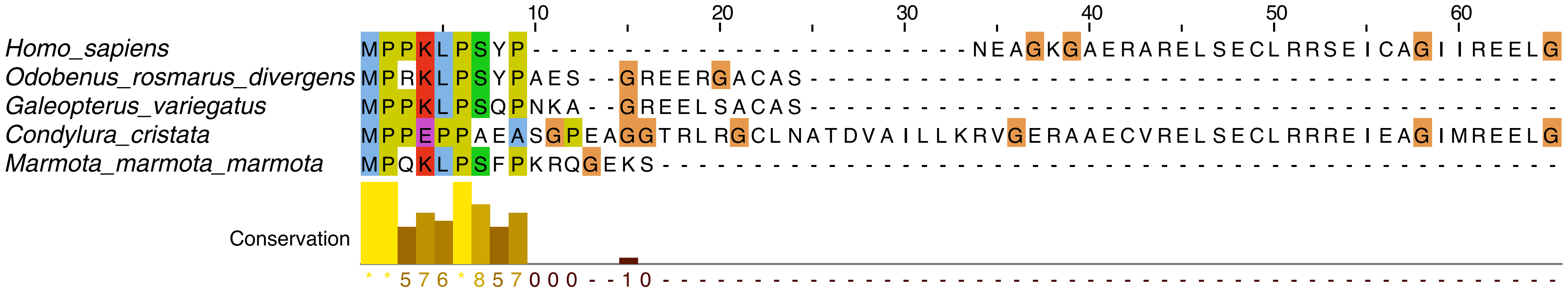

HG0622

ENSG00000148677 (ANKRD1)

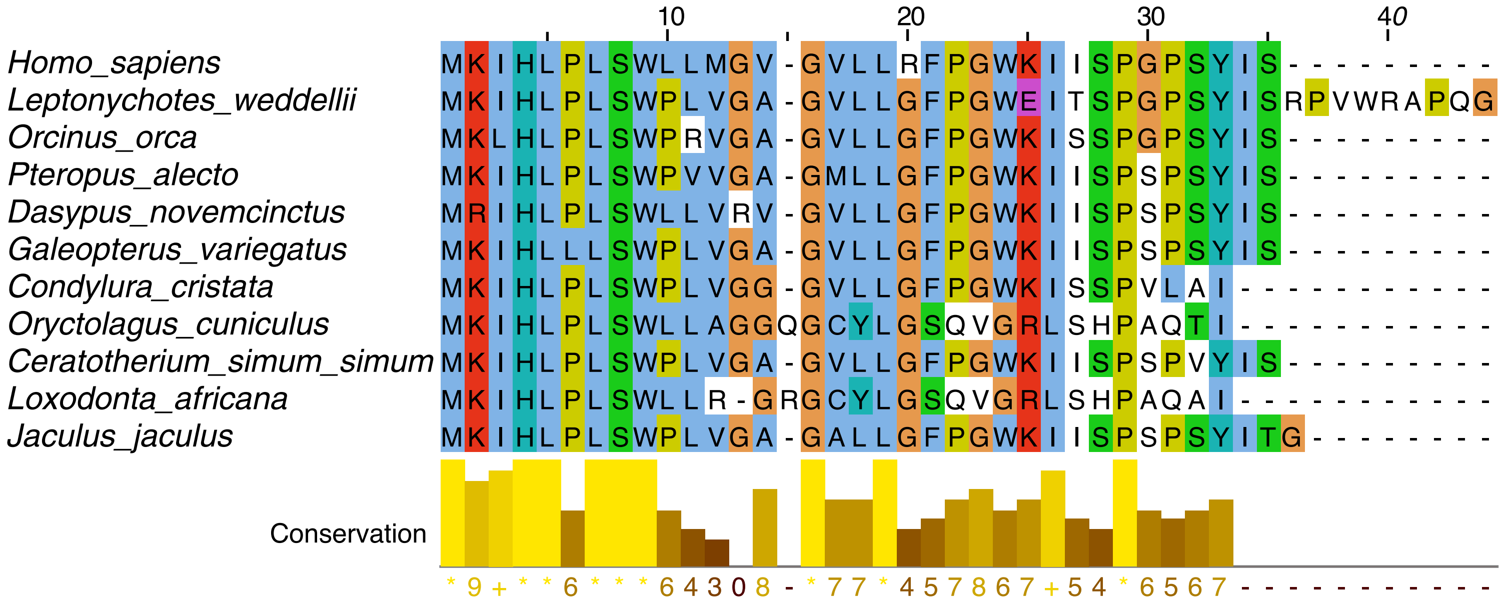

HG0623

ENSG00000151422 (FER)

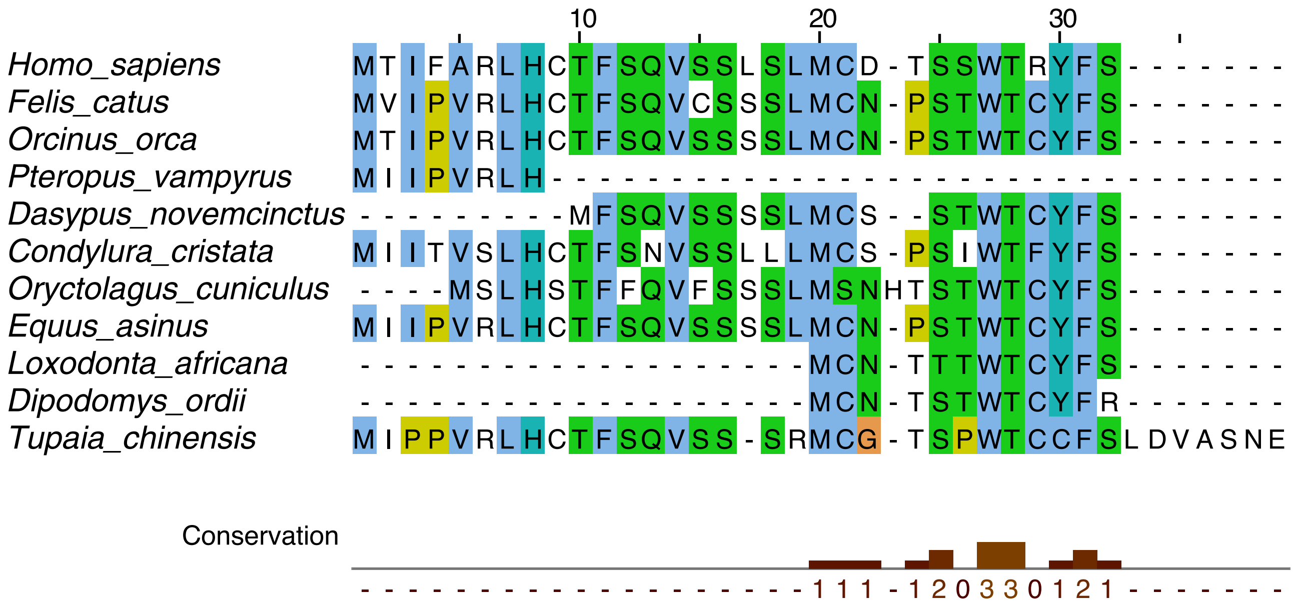

HG0624

ENSG00000156853 (ZNF689)

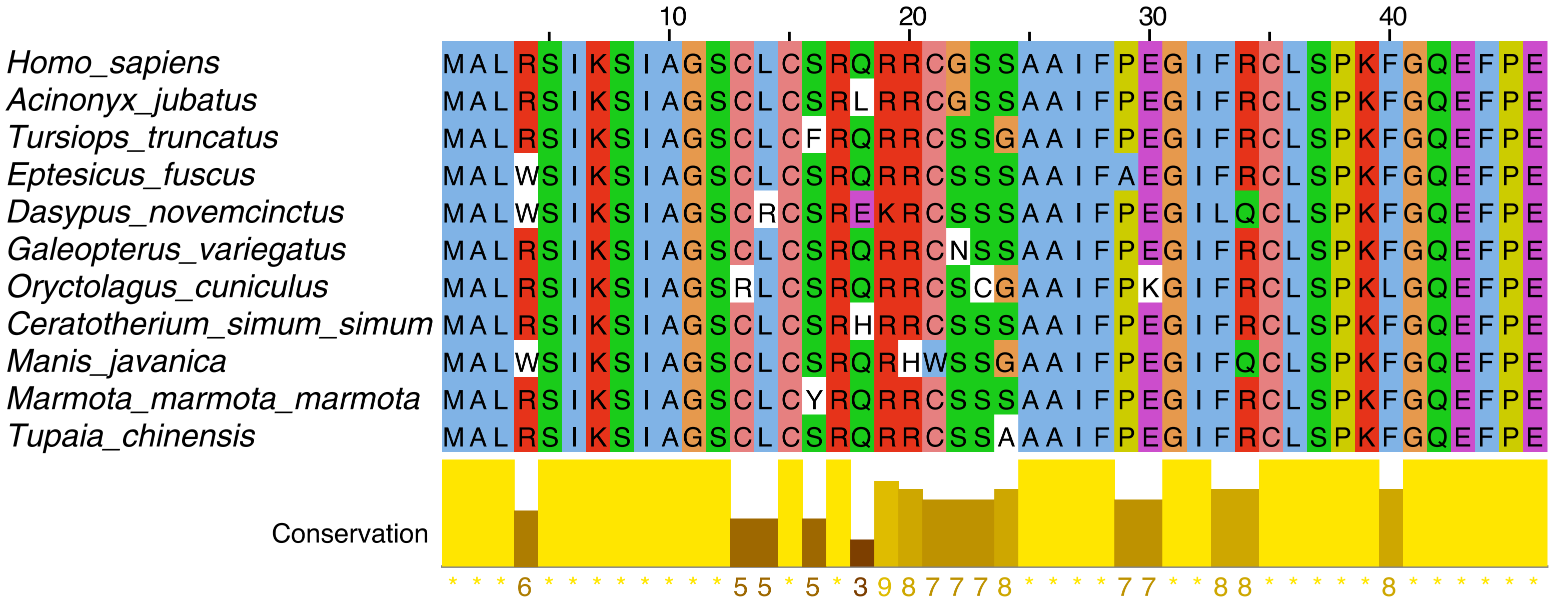

HG0625

ENSG00000164050 (PLXNB1)

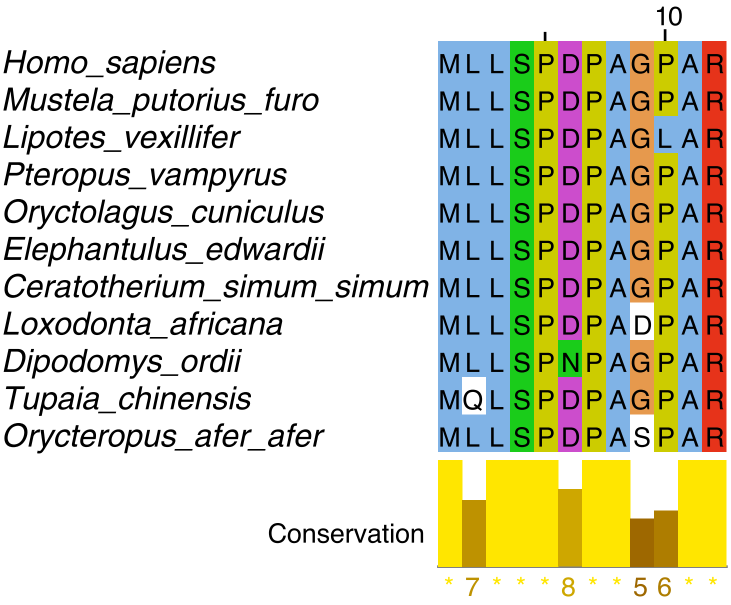

HG0626

ENSG00000168939 (SPRY3)

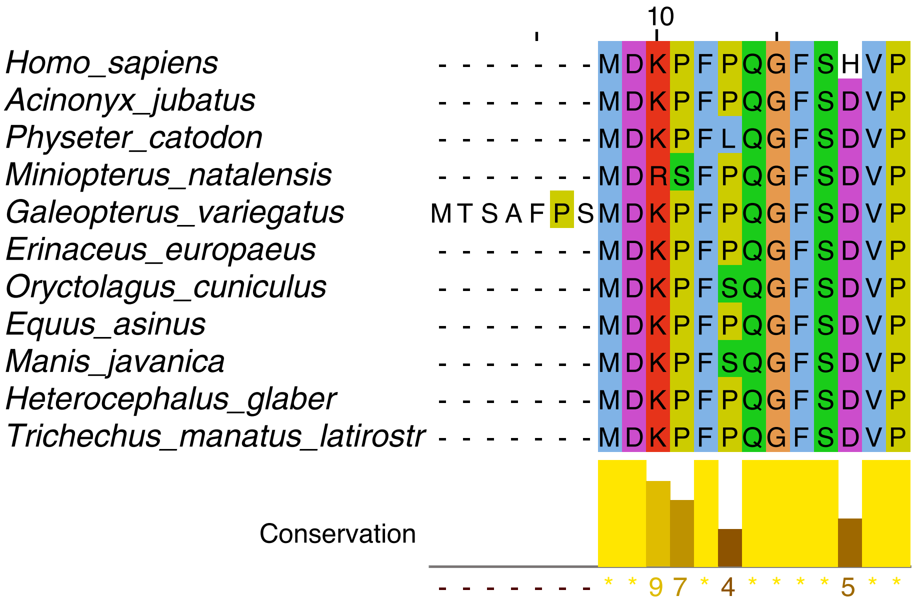

HG0627

ENSG00000169760 (NLGN1)

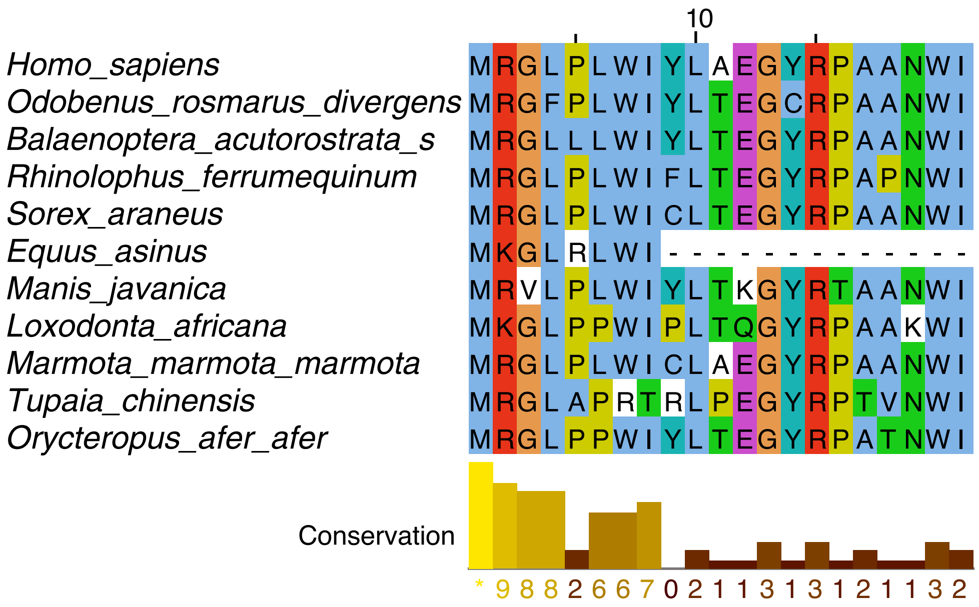

HG0628

ENSG00000170677 (SOCS6)

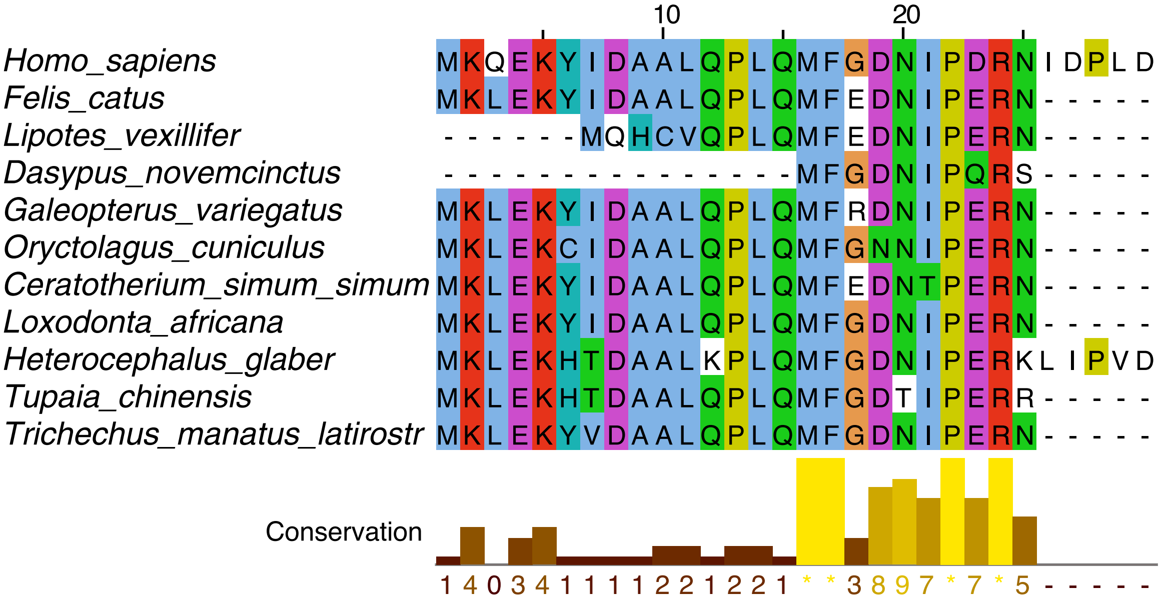

HG0629

ENSG00000170961 (HAS2)

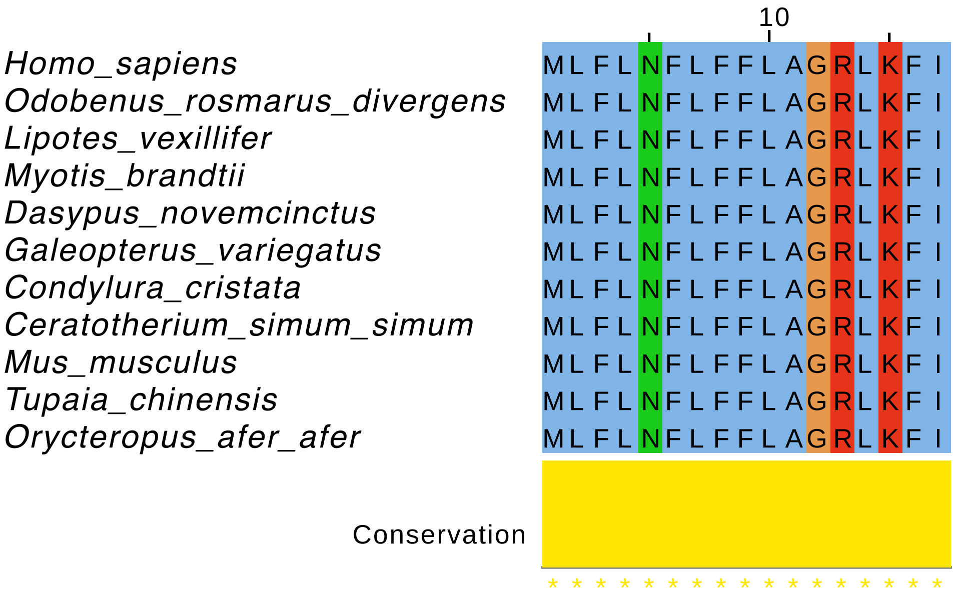

HG0630

ENSG00000171552 (BCL2L1)

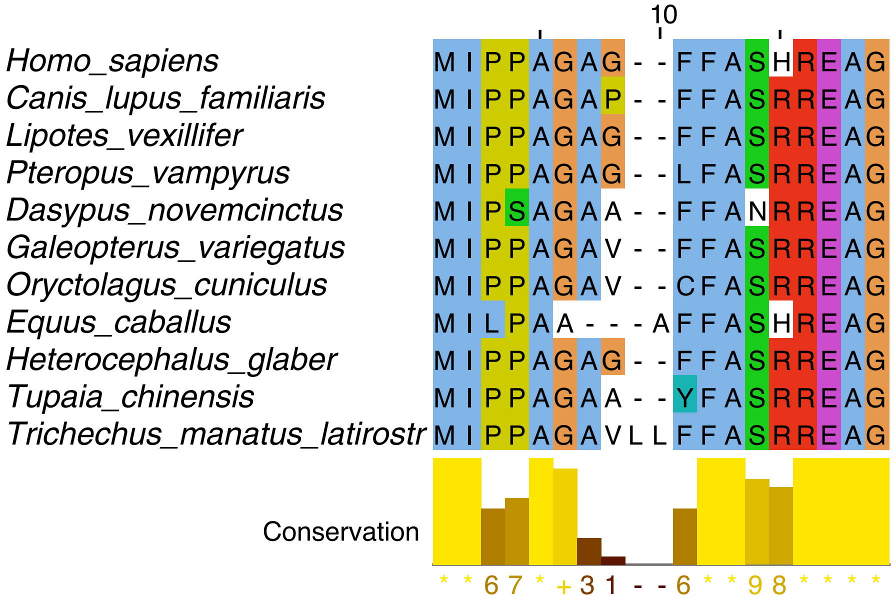

HG0631

ENSG00000173548 (SNX33)

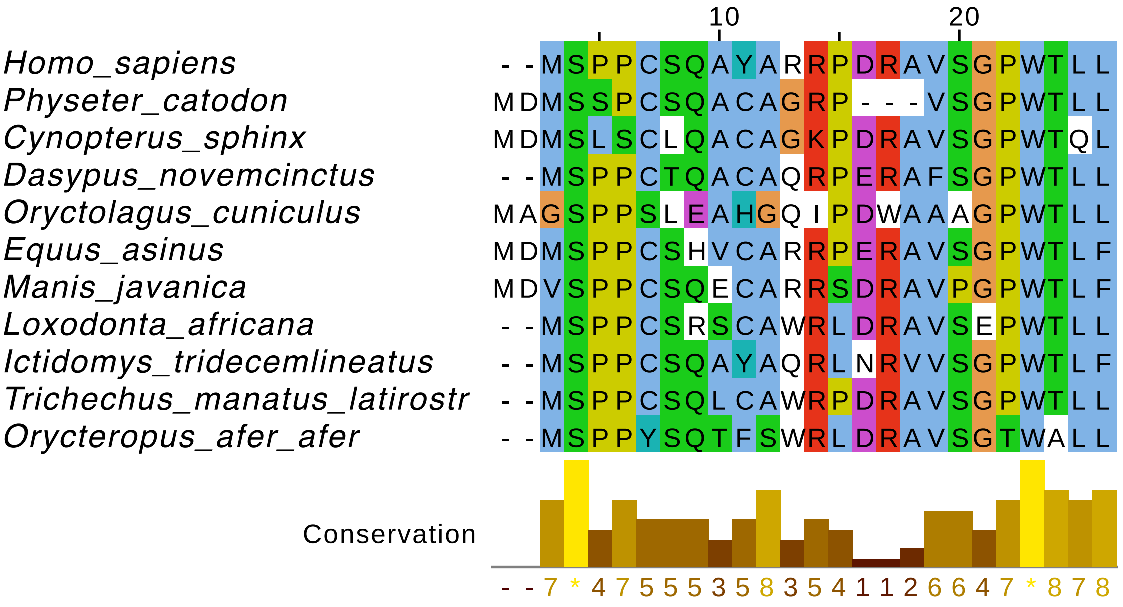

HG0632.1

ENSG00000181315 (ZNF322)

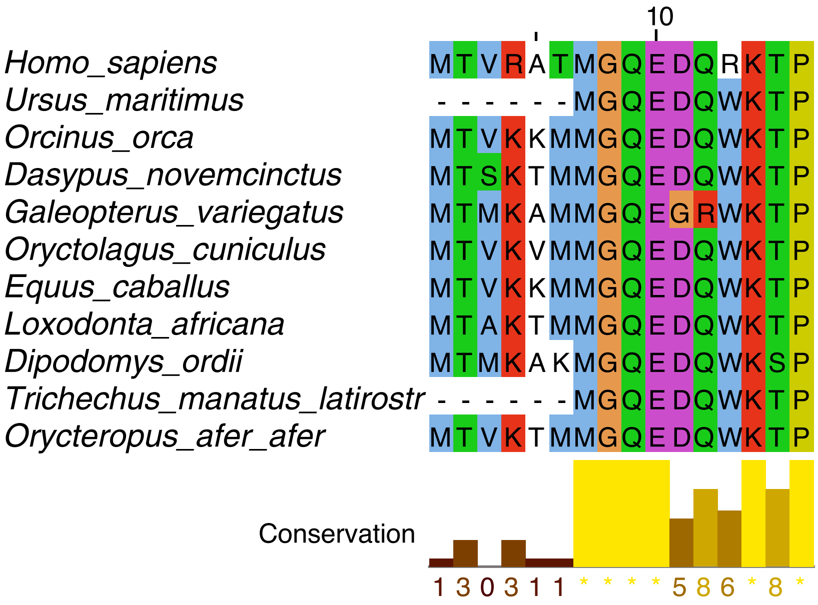

HG0632.2

ENSG00000181315 (ZNF322)

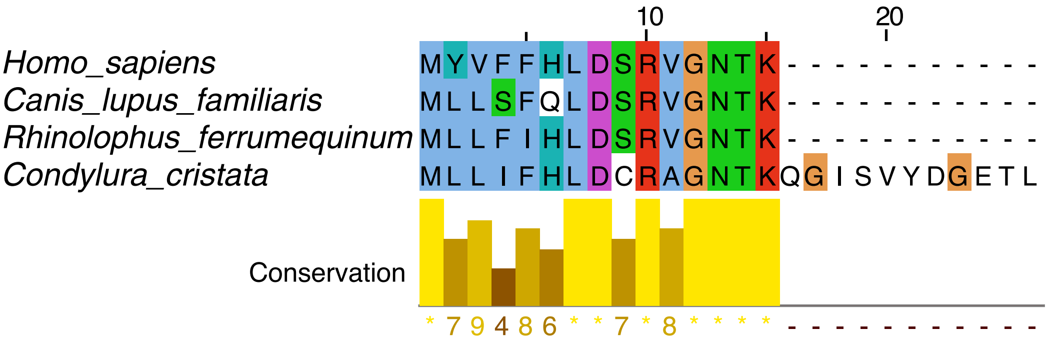

HG0633

ENSG00000186174 (BCL9L)

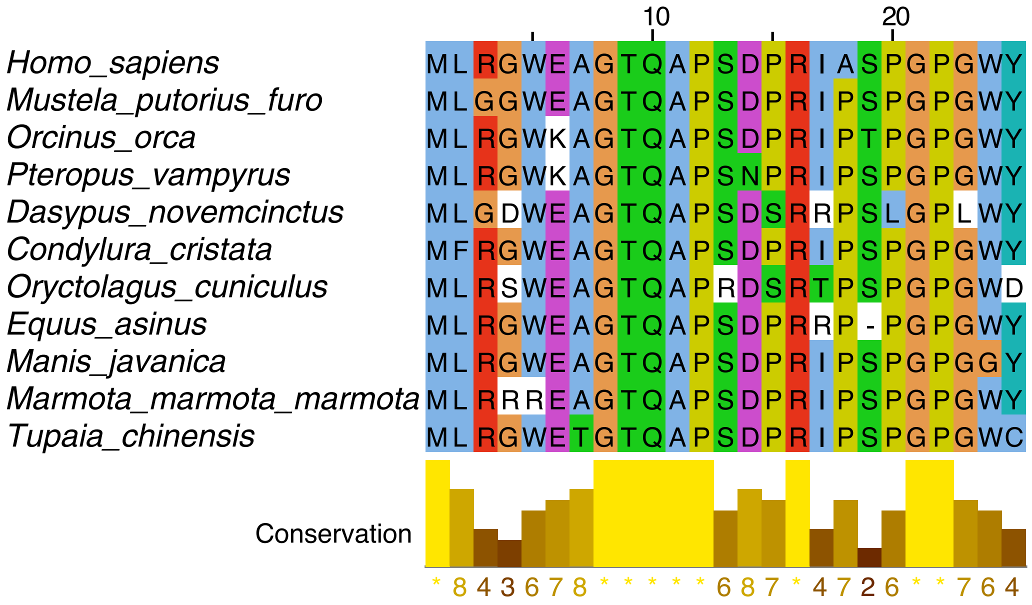

HG0634

ENSG00000198026 (ZNF335)

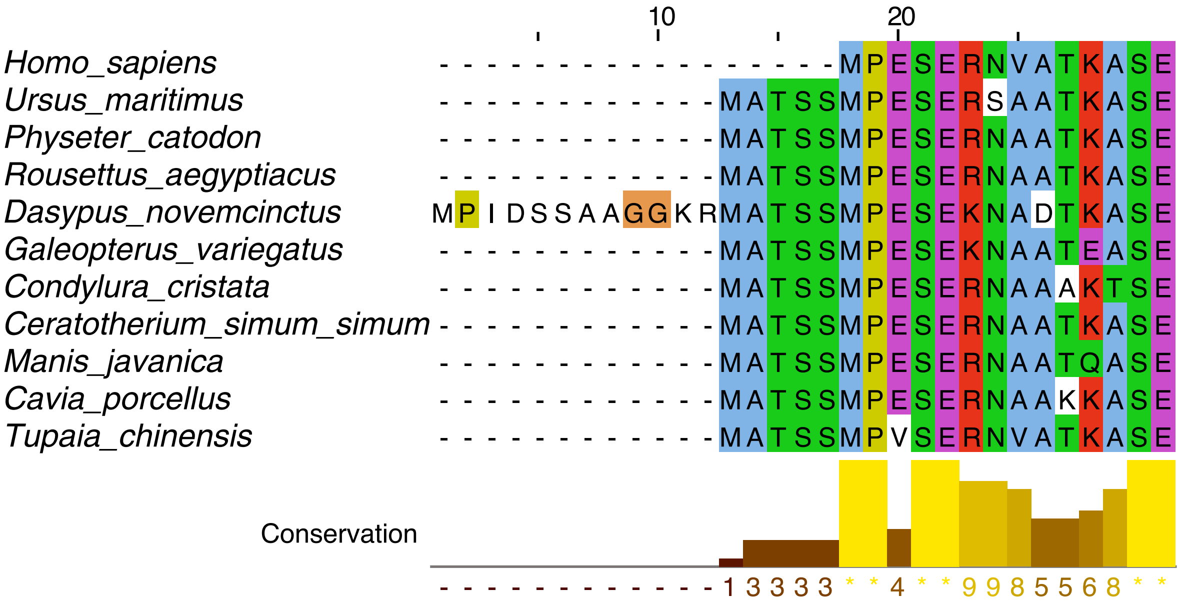

HG0635

ENSG00000259332 (ST20-MTHFS)

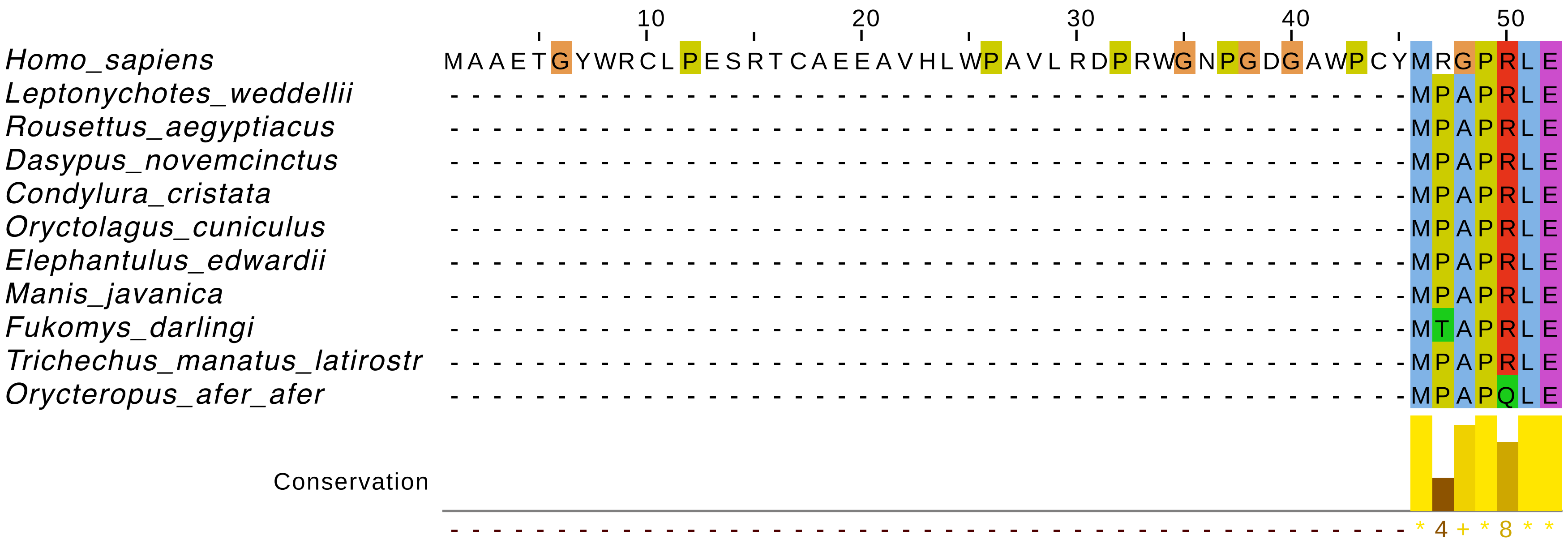

HG0636

ENSG00000002587 (HS3ST1)

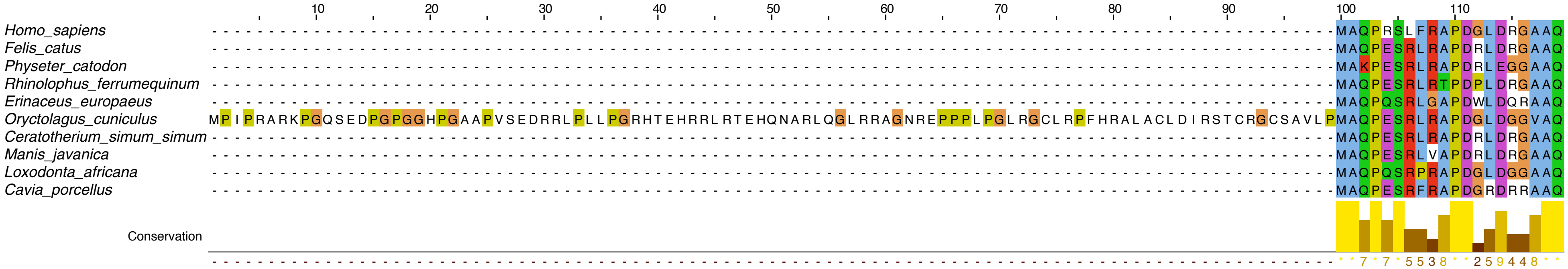

HG0637

ENSG00000005102 (MEOX1)

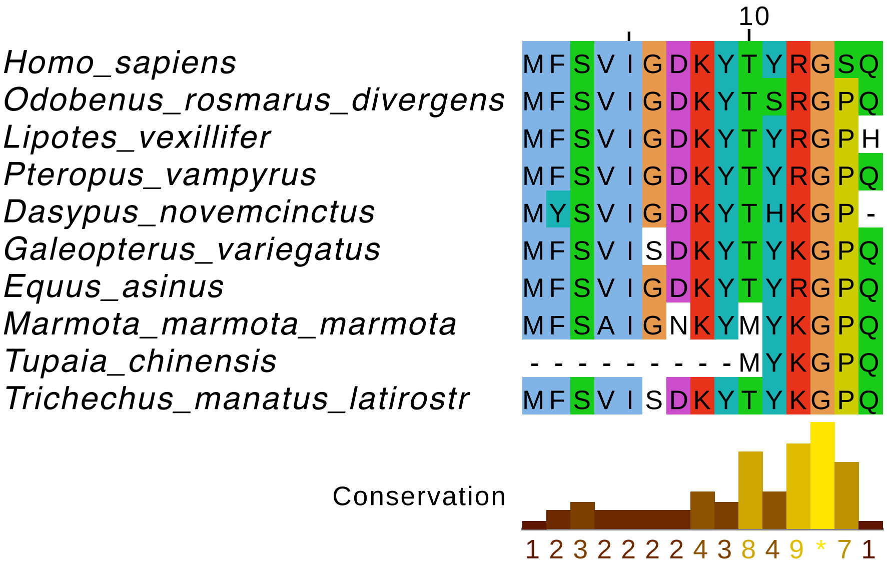

HG0638

ENSG00000013561 (RNF14)

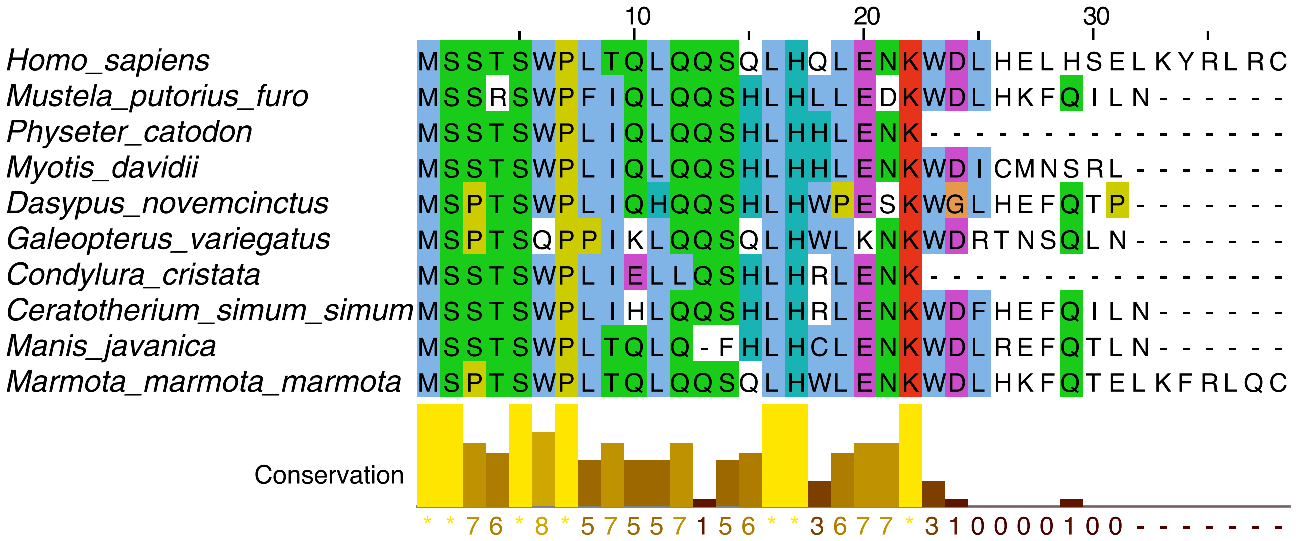

HG0639

ENSG00000018236 (CNTN1)

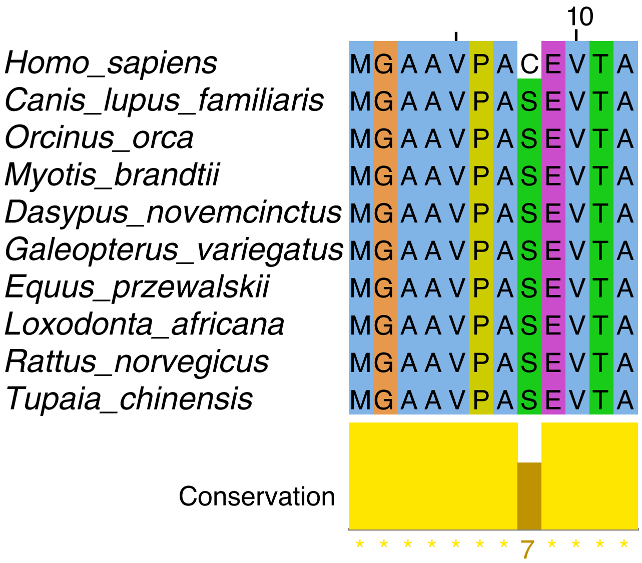

HG0640

ENSG00000062194 (GPBP1)

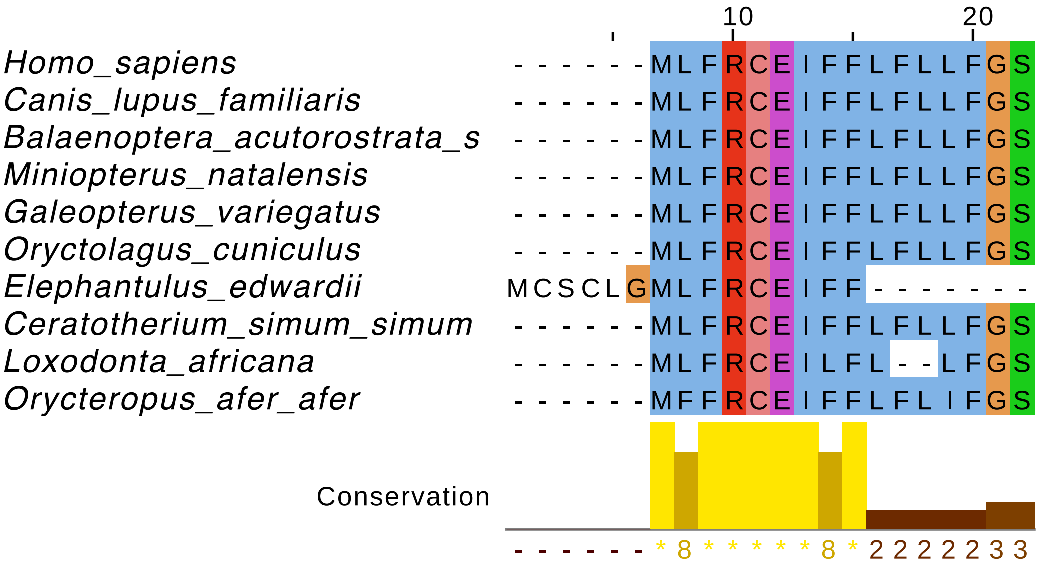

HG0641

ENSG00000100359 (SGSM3)

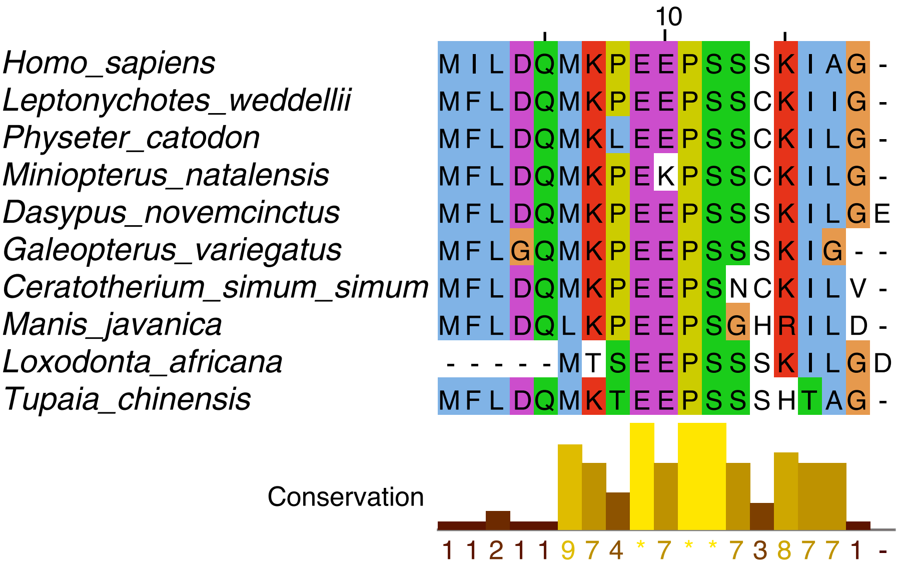

HG0642

ENSG00000100815 (TRIP11)

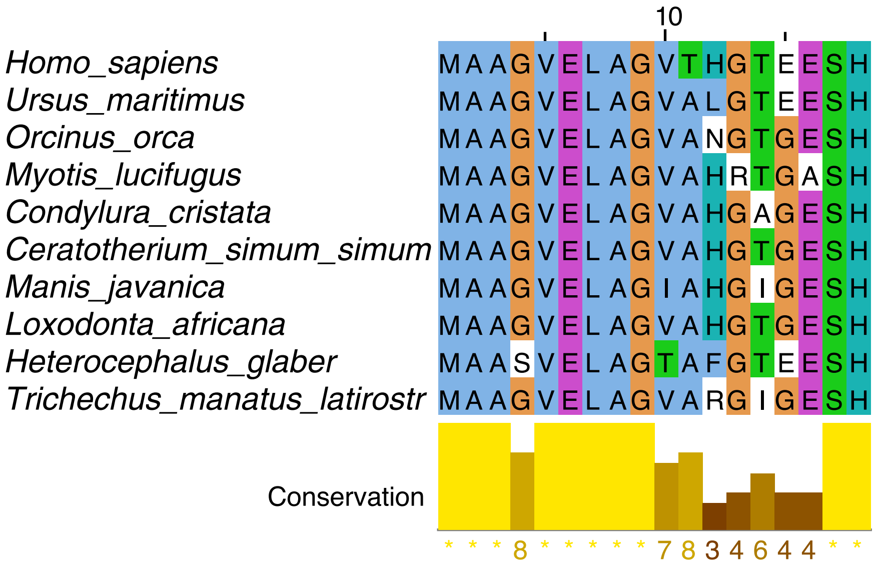

HG0643

ENSG00000103653 (CSK)

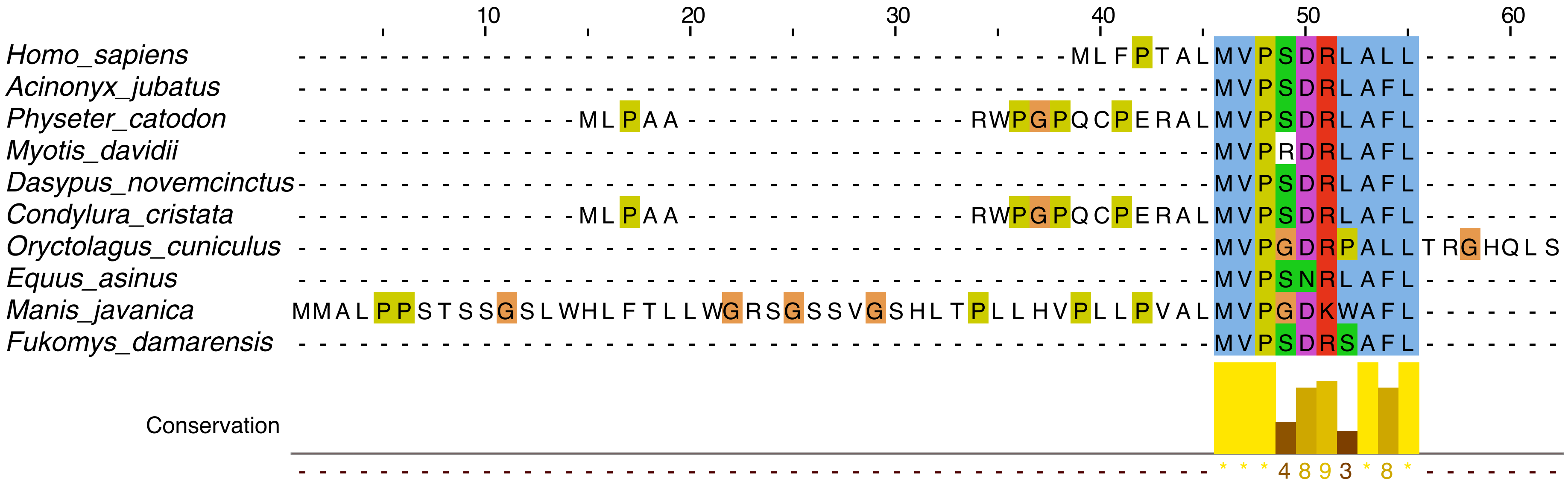

HG0644

ENSG00000104299 (INTS9)

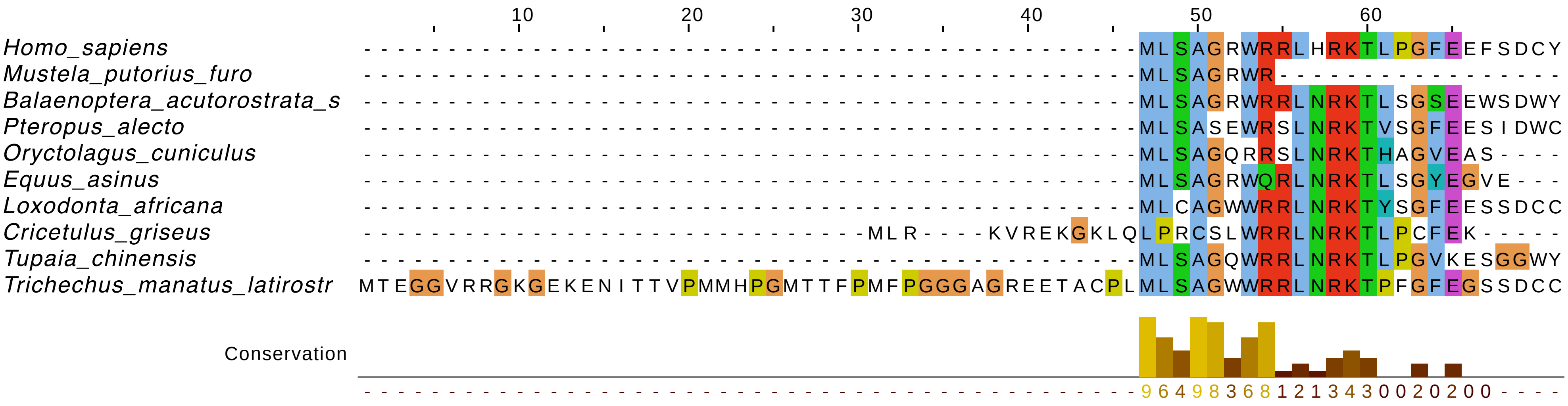

HG0645

ENSG00000107282 (APBA1)

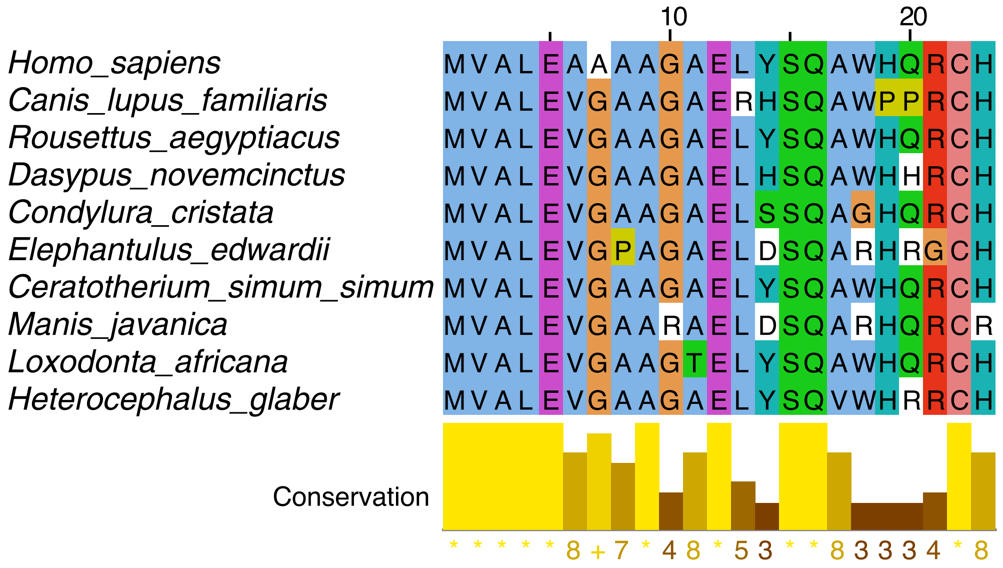

HG0646

ENSG00000108312 (UBTF)

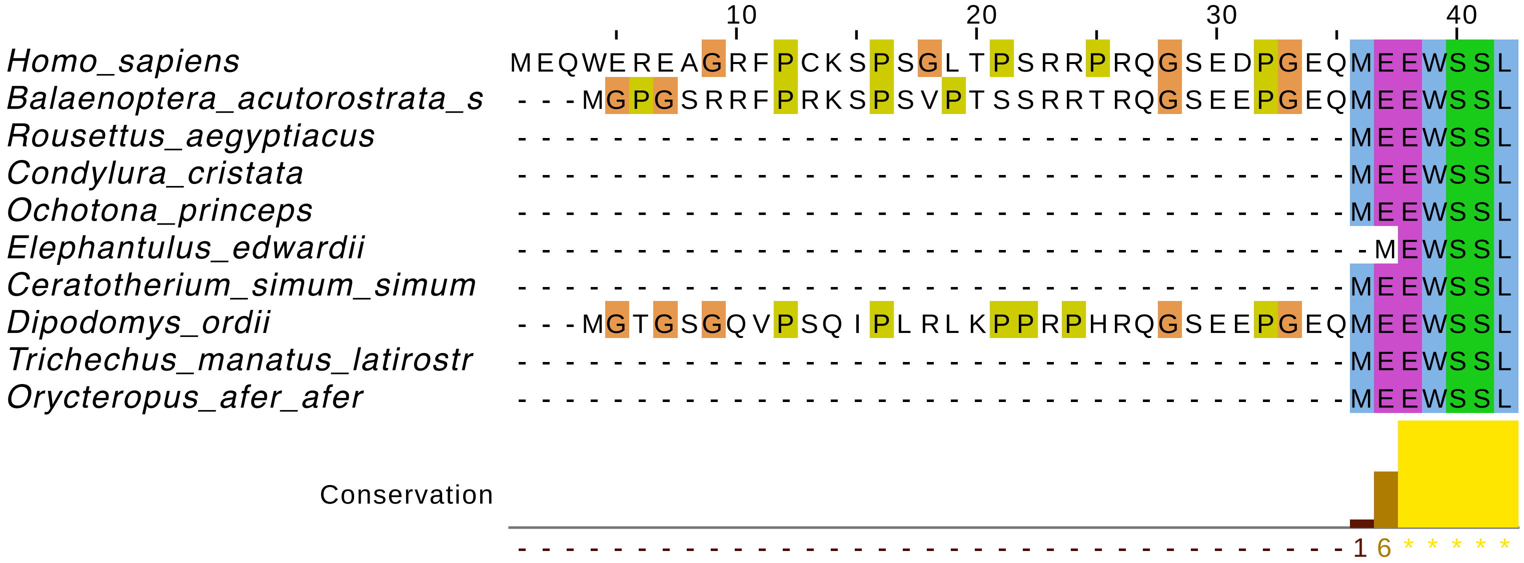

HG0647

ENSG00000108395 (TRIM37)

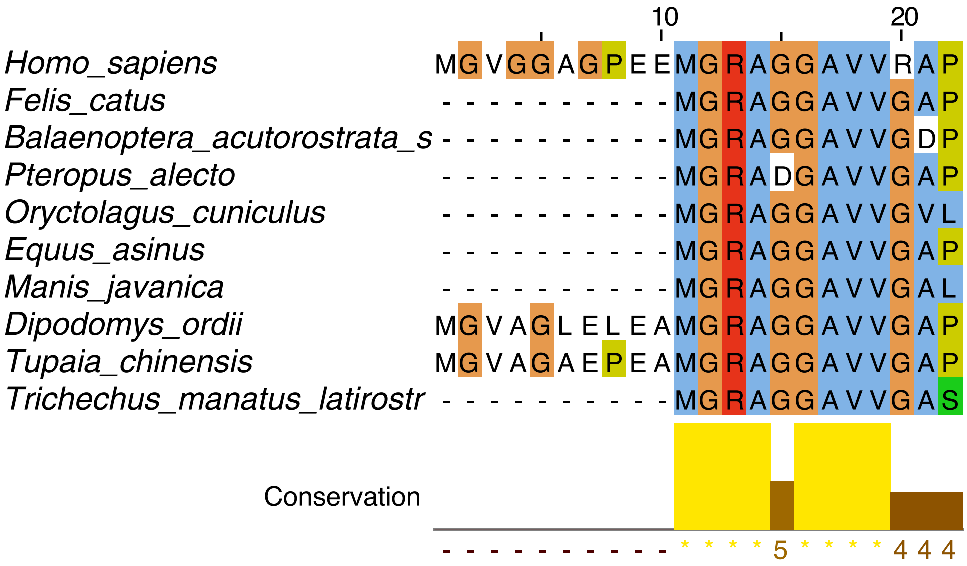

HG0648

ENSG00000114331 (ACAP2)

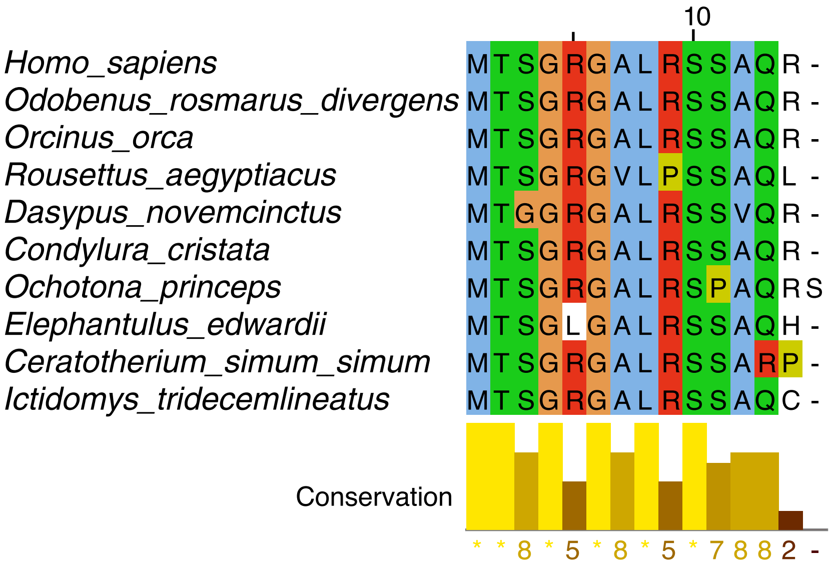

HG0649.1

ENSG00000114439 (BBX)

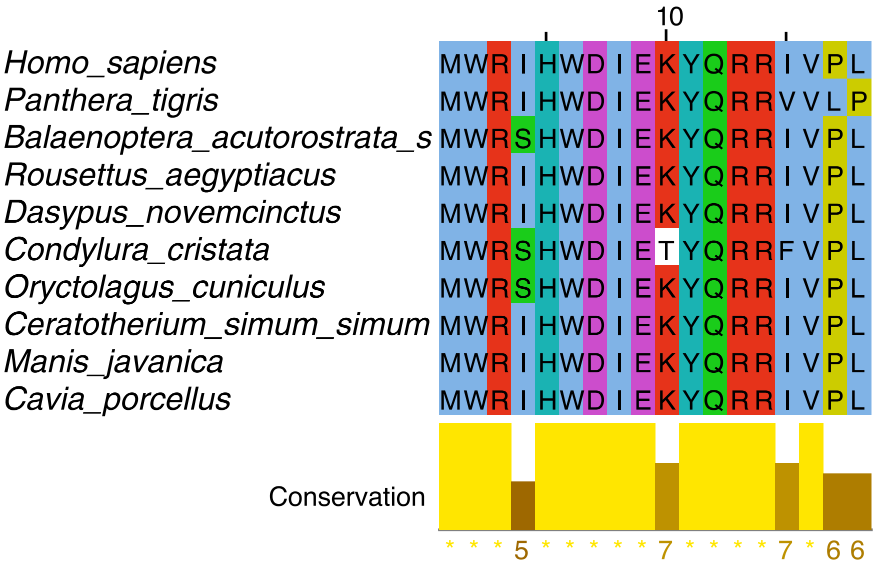

HG0649.2

ENSG00000114439 (BBX)

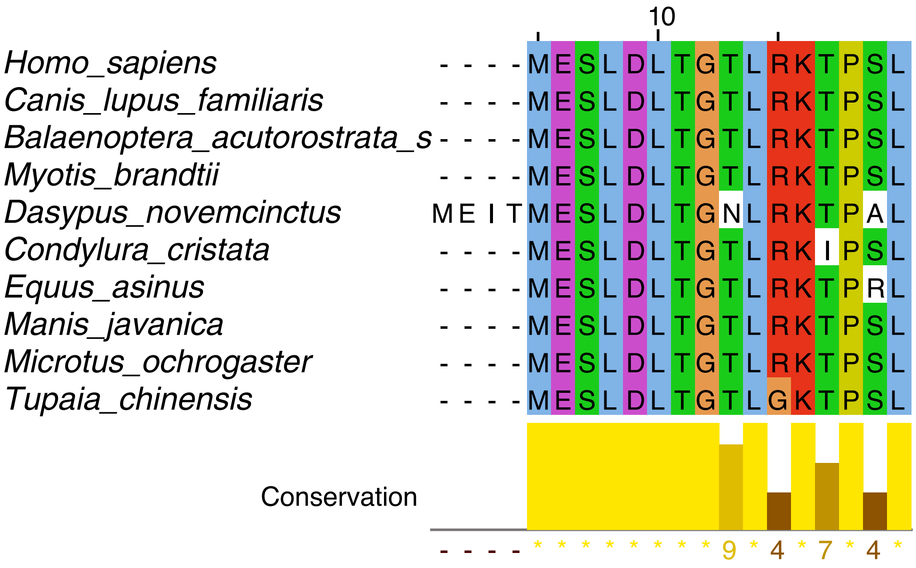

HG0650

ENSG00000115226 (FNDC4)

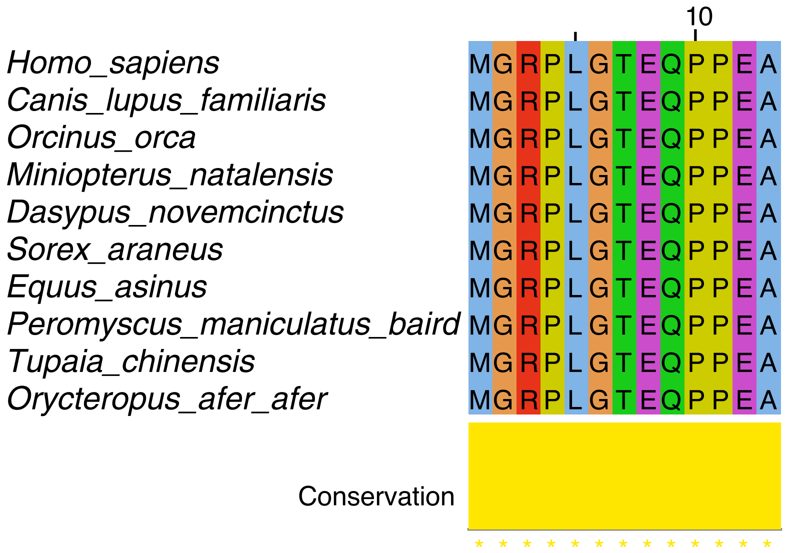

HG0651

ENSG00000115935 (WIPF1)

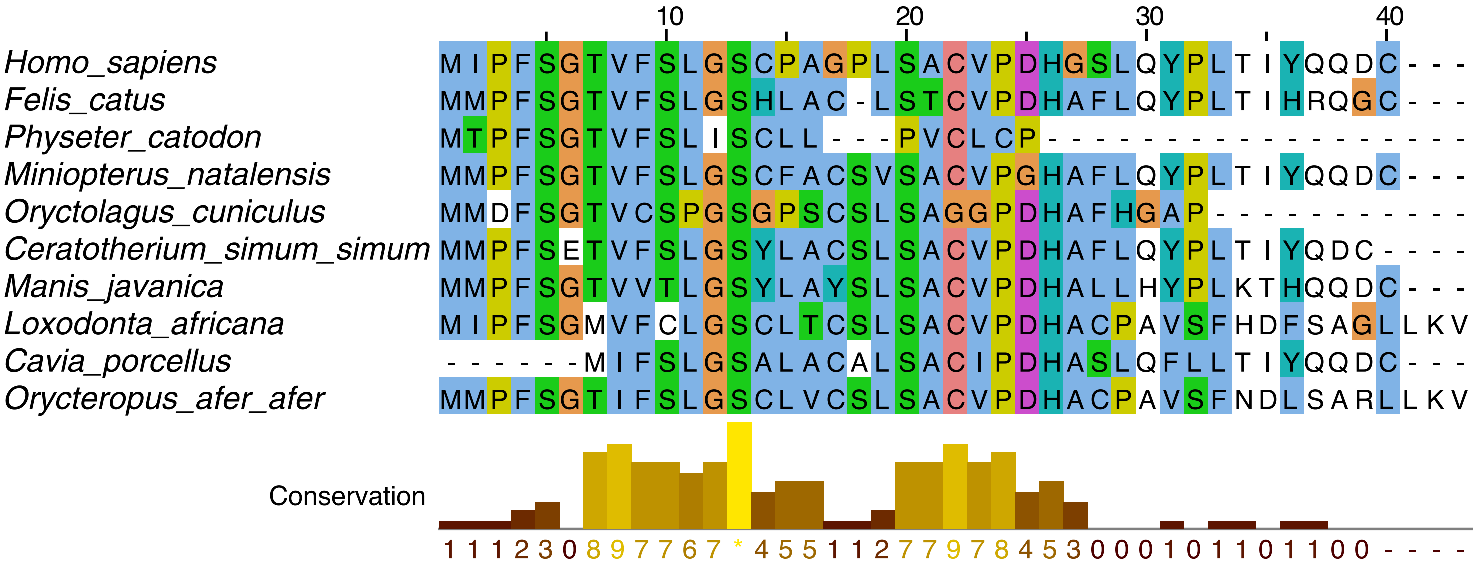

HG0652

ENSG00000118515 (SGK1)

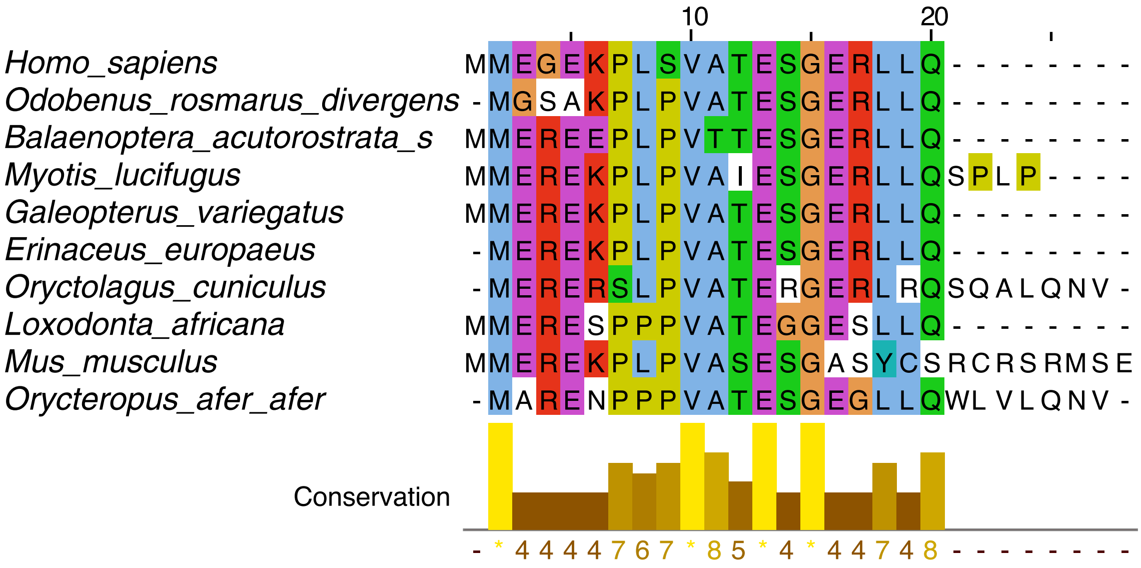

HG0653

ENSG00000120334 (CENPL)

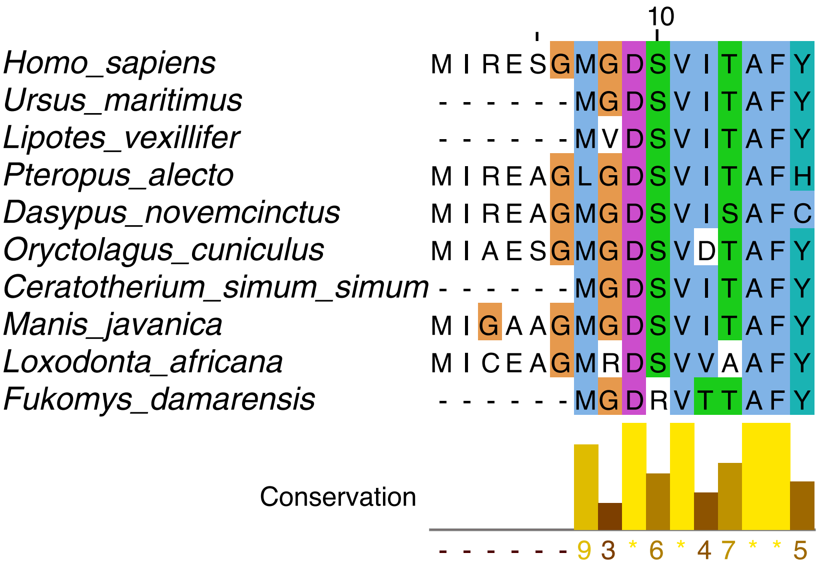

HG0654

ENSG00000133794 (ARNTL)

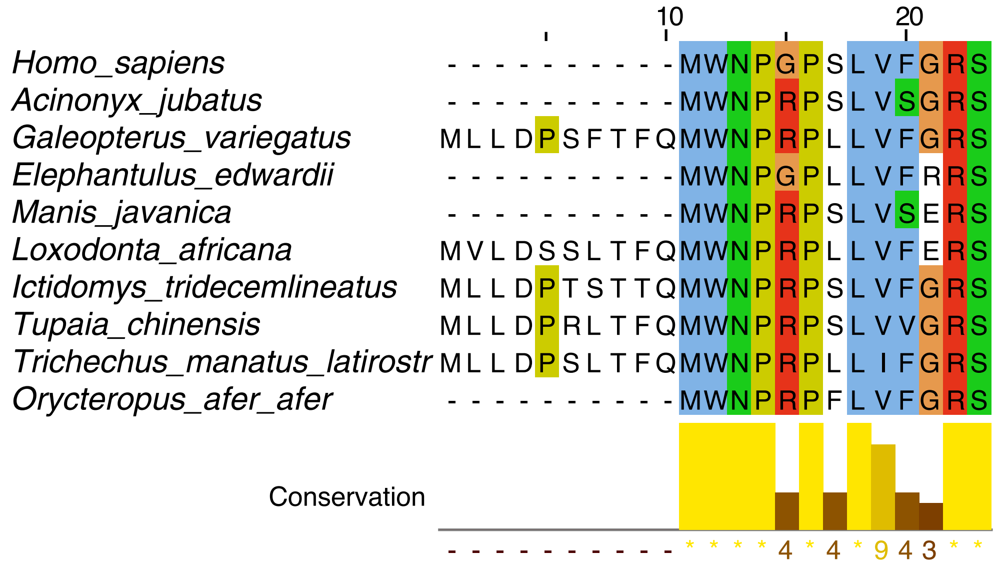

HG0655

ENSG00000143061 (IGSF3)

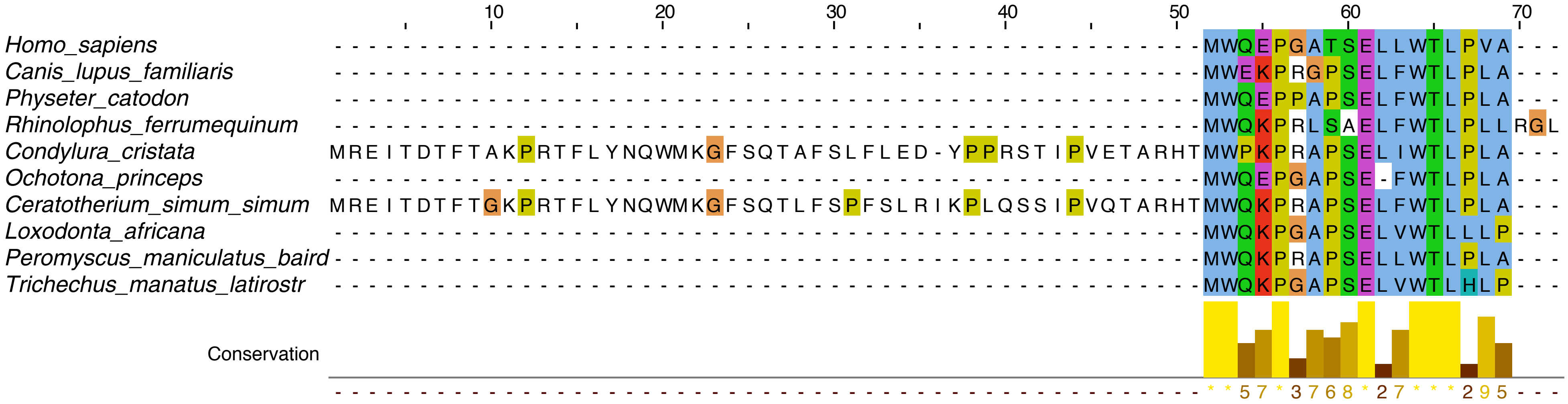

HG0656

ENSG00000150776 (C11orf57)

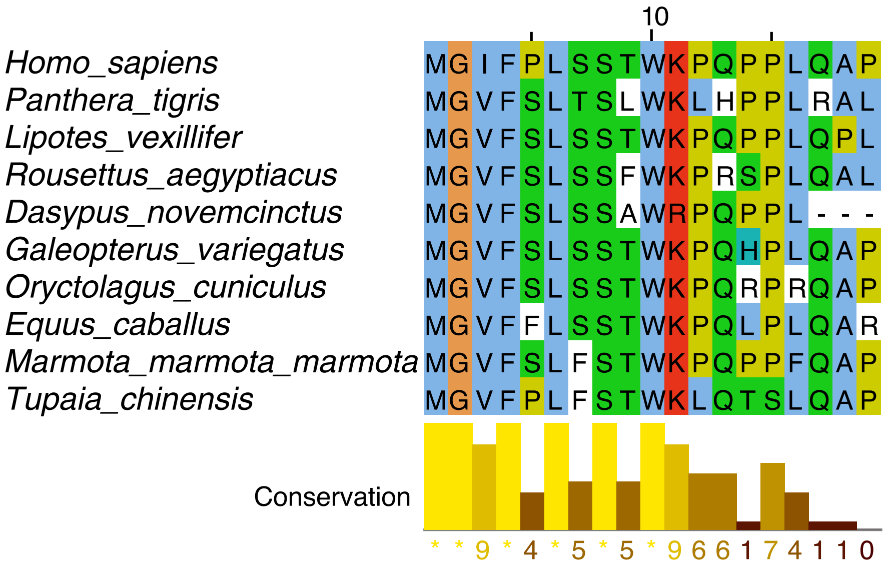

HG0657

ENSG00000151276 (MAGI1)

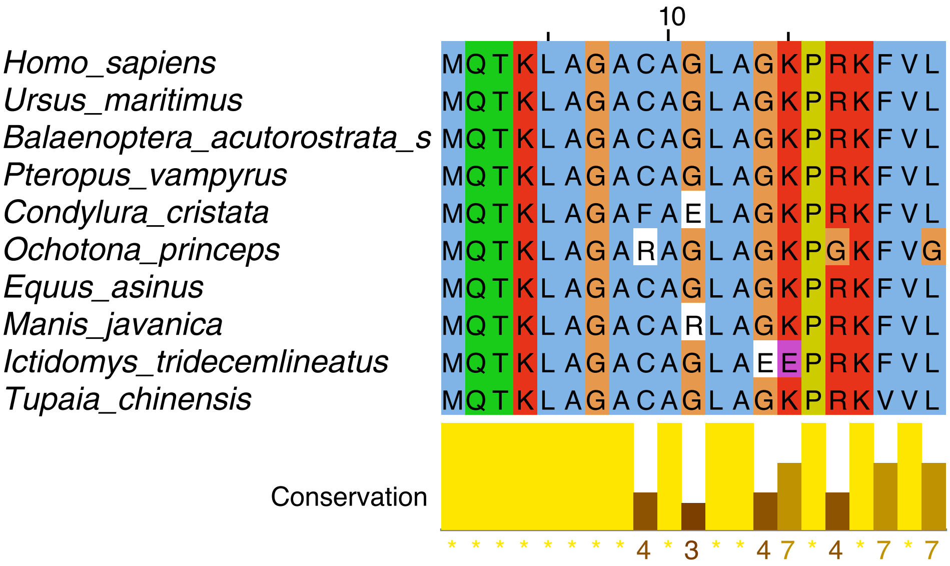

HG0658

ENSG00000153786 (ZDHHC7)

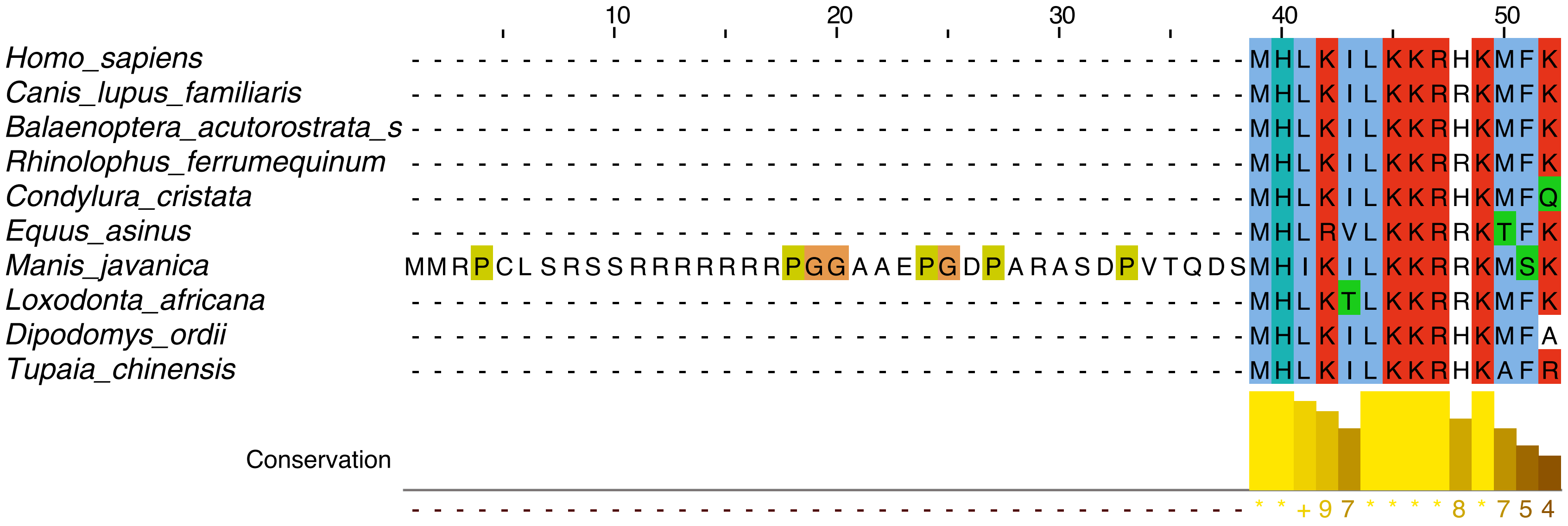

HG0659.1

ENSG00000158856 (DMTN)

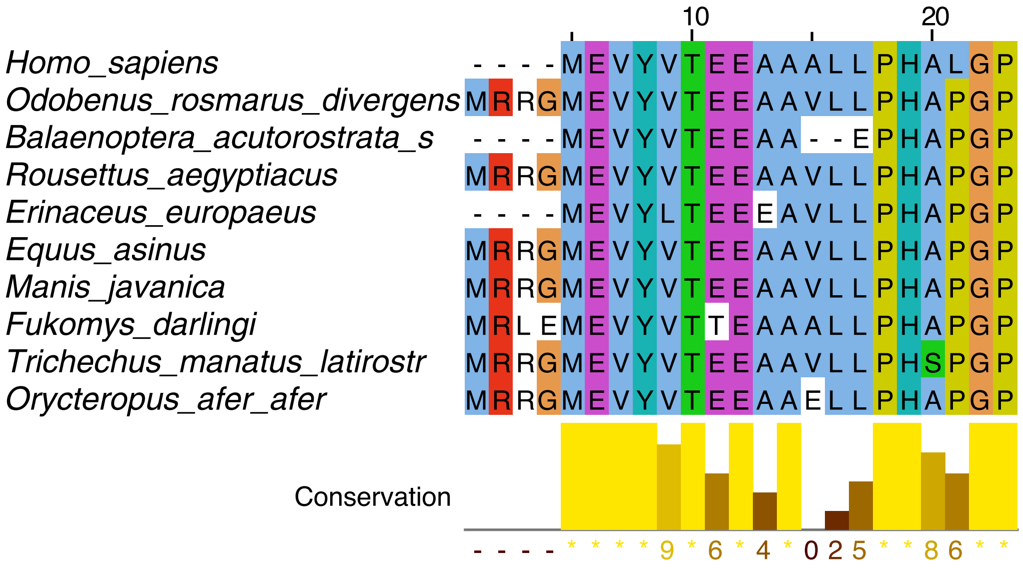

HG0659.2

ENSG00000158856 (DMTN)

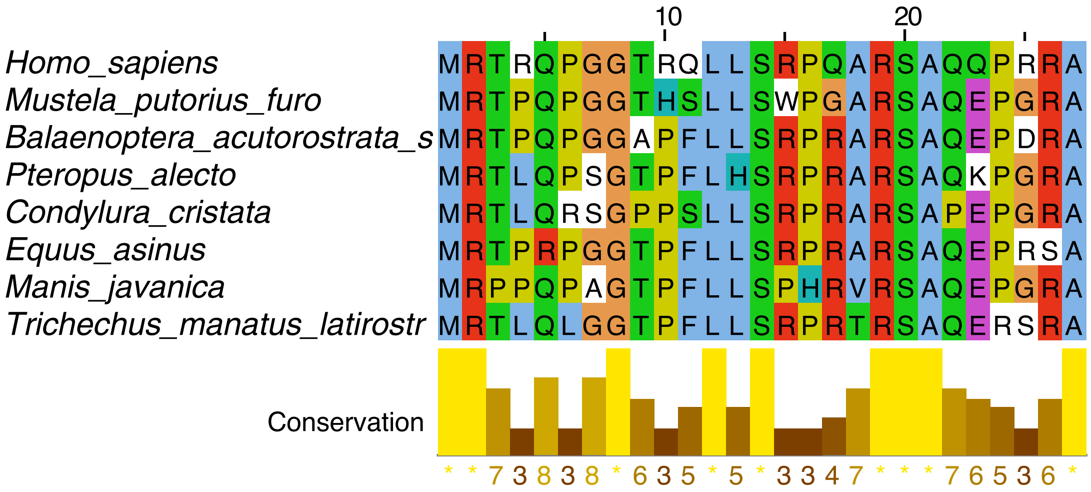

HG0660

ENSG00000165861 (ZFYVE1)

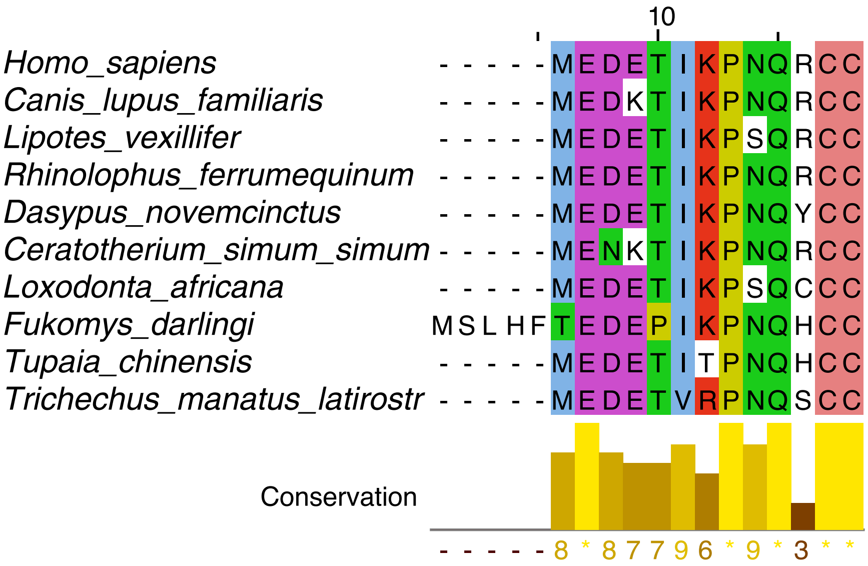

HG0661

ENSG00000166845 (C18orf54)

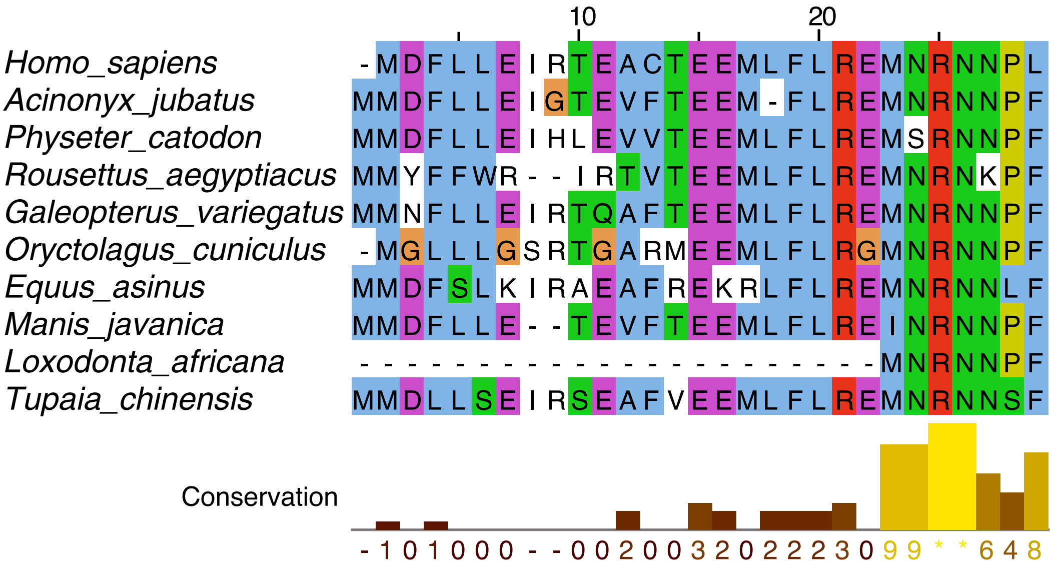

HG0662

ENSG00000166897 (ELFN2)

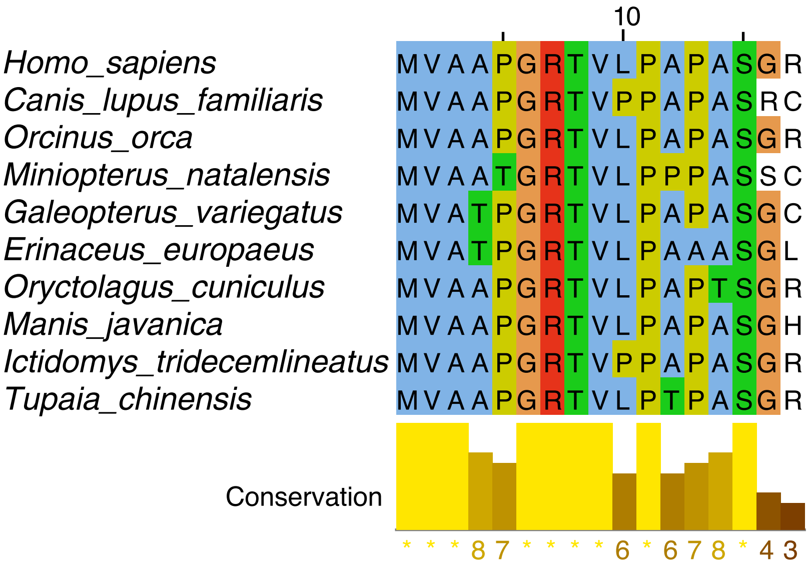

HG0663

ENSG00000168615 (ADAM9)

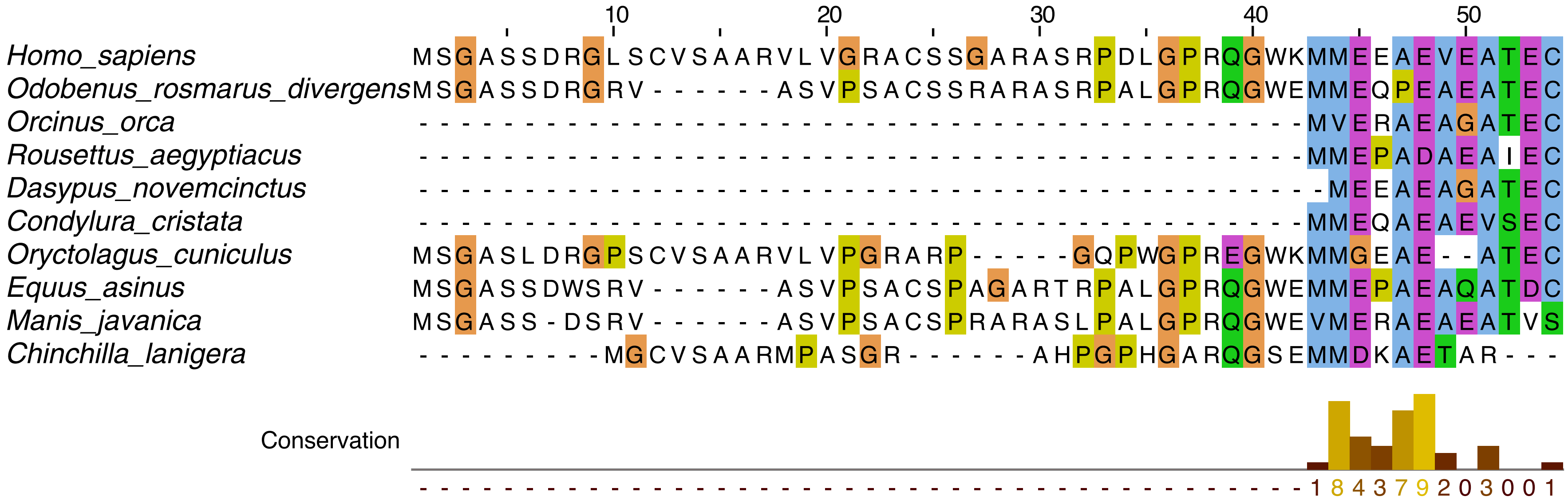

HG0664

ENSG00000169085

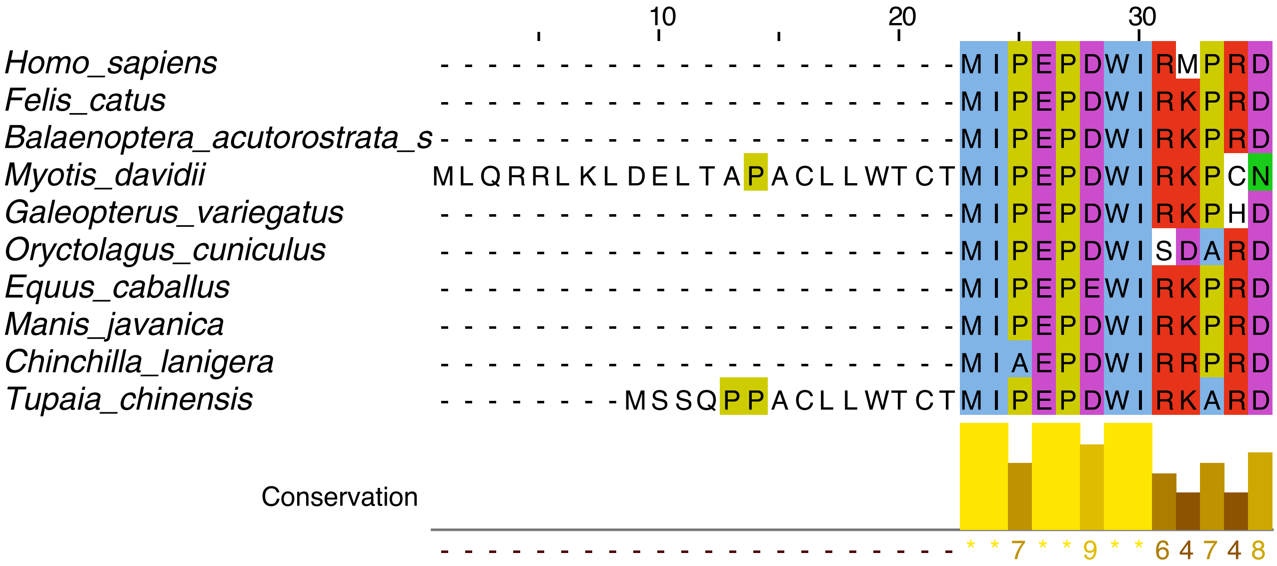

HG0665

ENSG00000169515 (CCDC8)

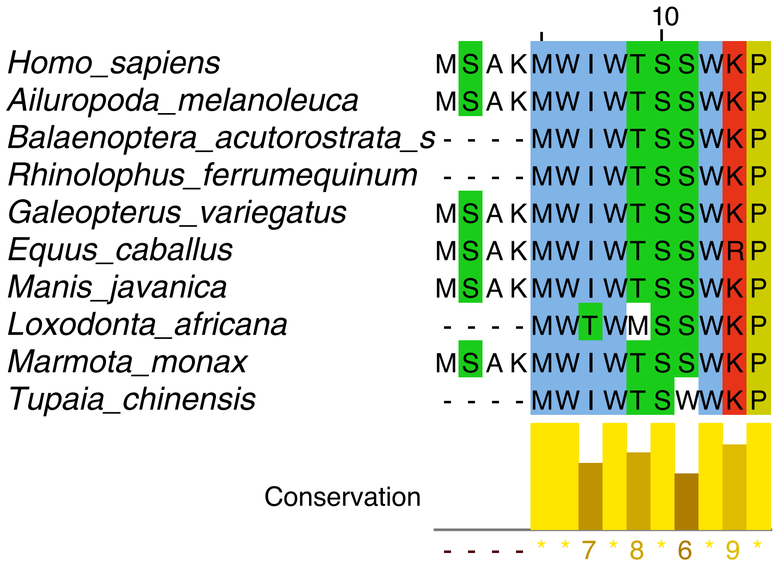

HG0666

ENSG00000171943 (SRGAP2C)

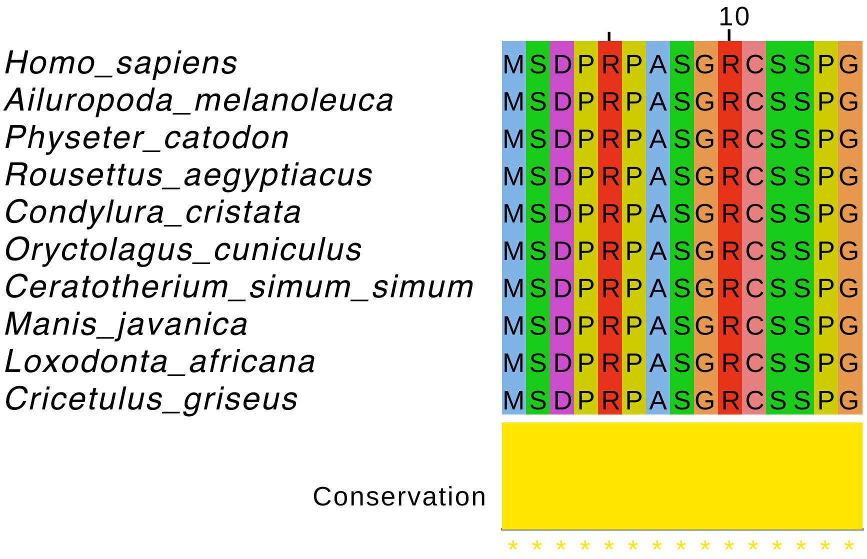

HG0667.1

ENSG00000182700 (IGIP)

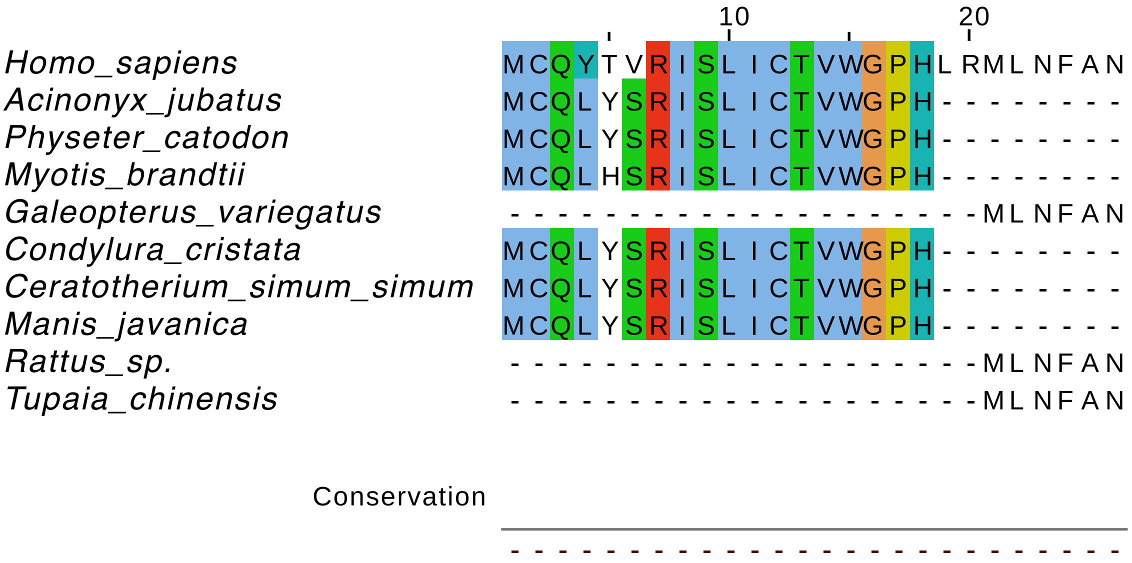

HG0667.2

ENSG00000182700 (IGIP)

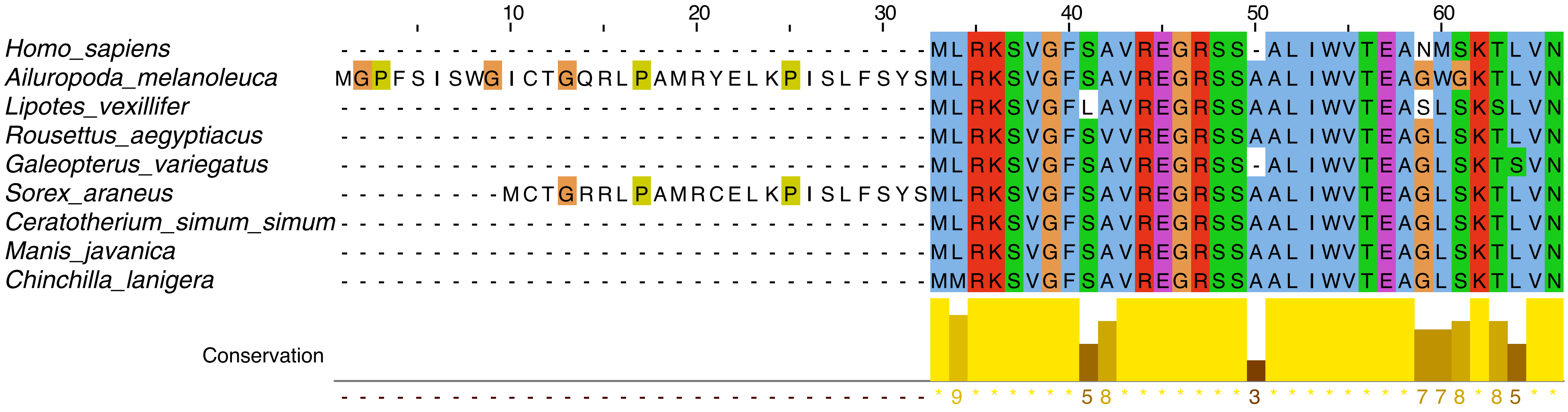

HG0668

ENSG00000182944 (EWSR1)

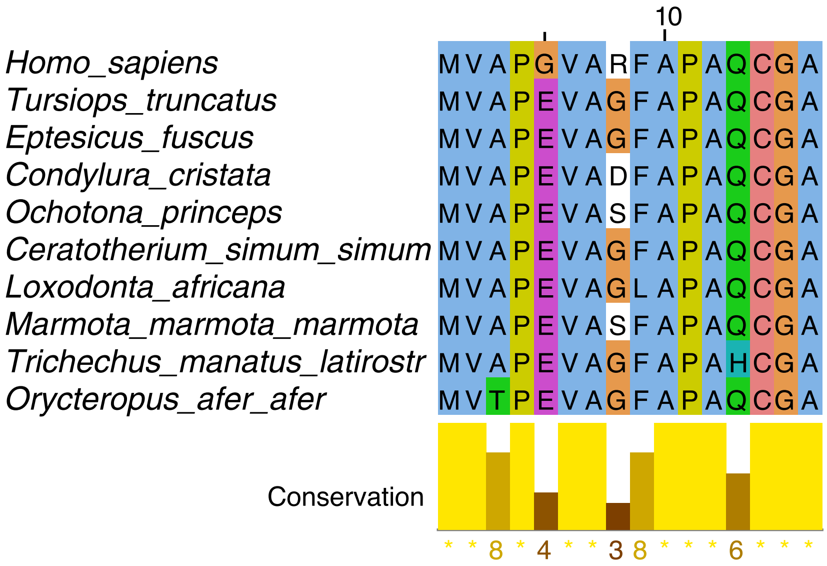

HG0669

ENSG00000183876 (ARSI)

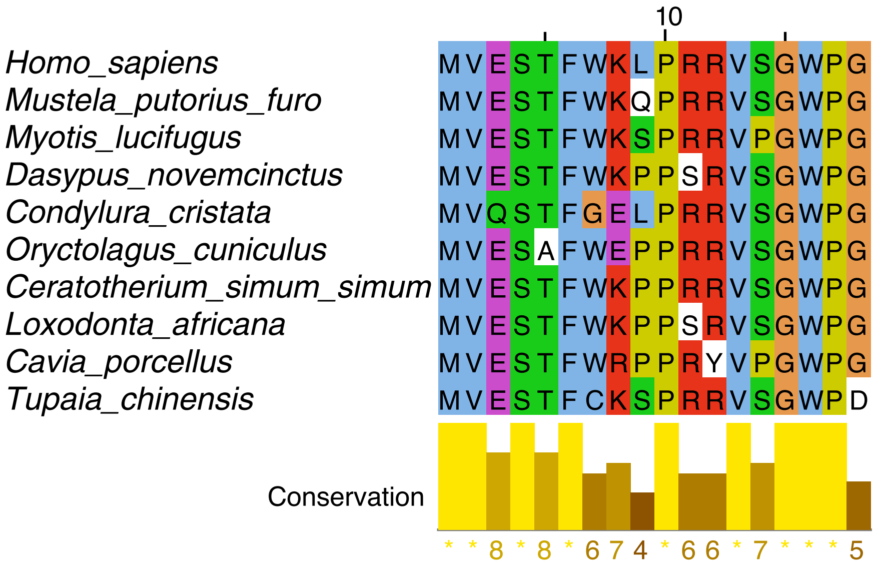

HG0670

ENSG00000187164 (SHTN1)

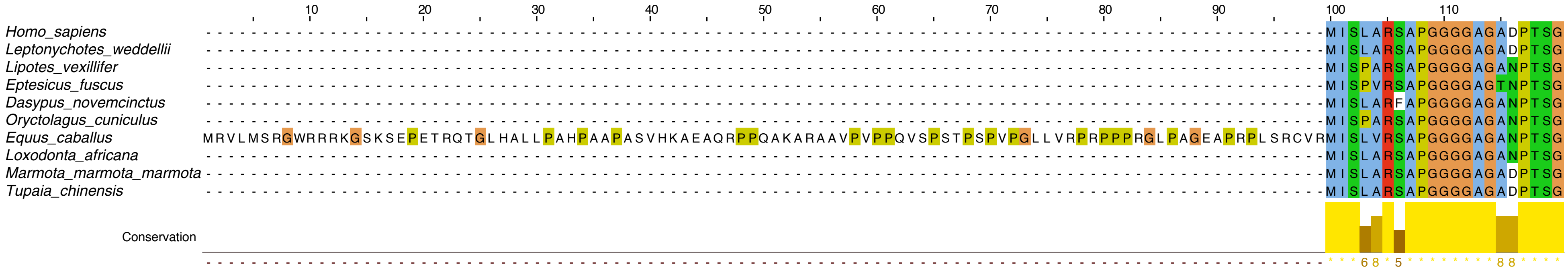

HG0671

ENSG00000189184 (PCDH18)

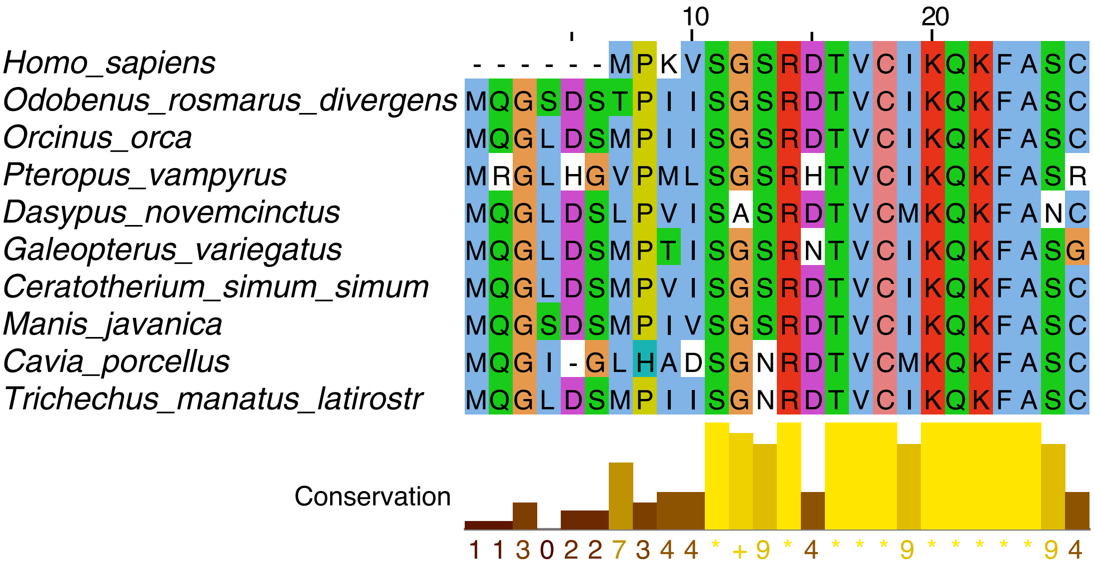

HG0672

ENSG00000196369 (SRGAP2B)

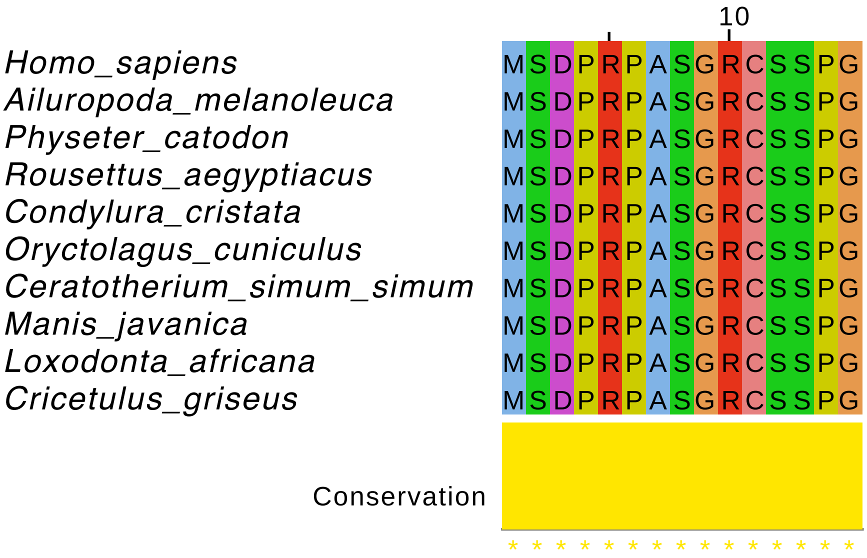

HG0673

ENSG00000266028 (SRGAP2)

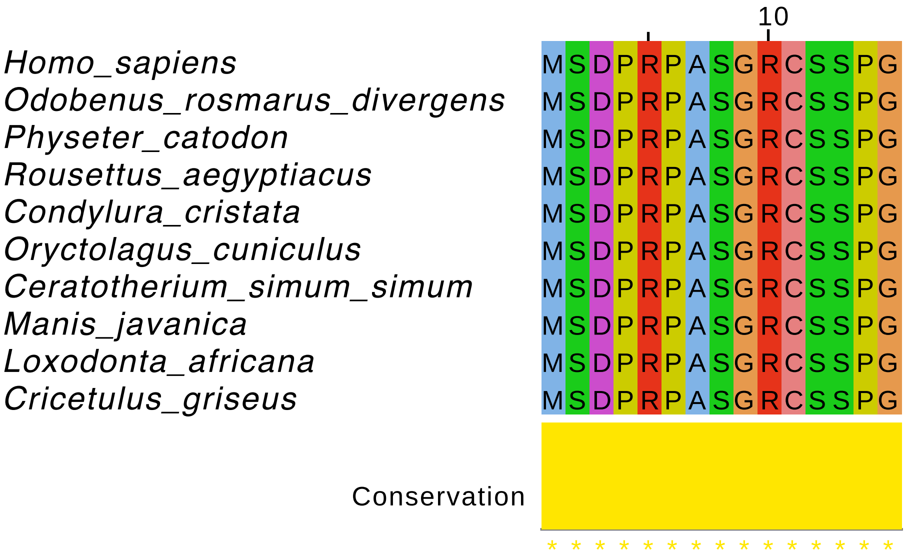

HG0674.1

ENSG00000006576 (PHTF2)

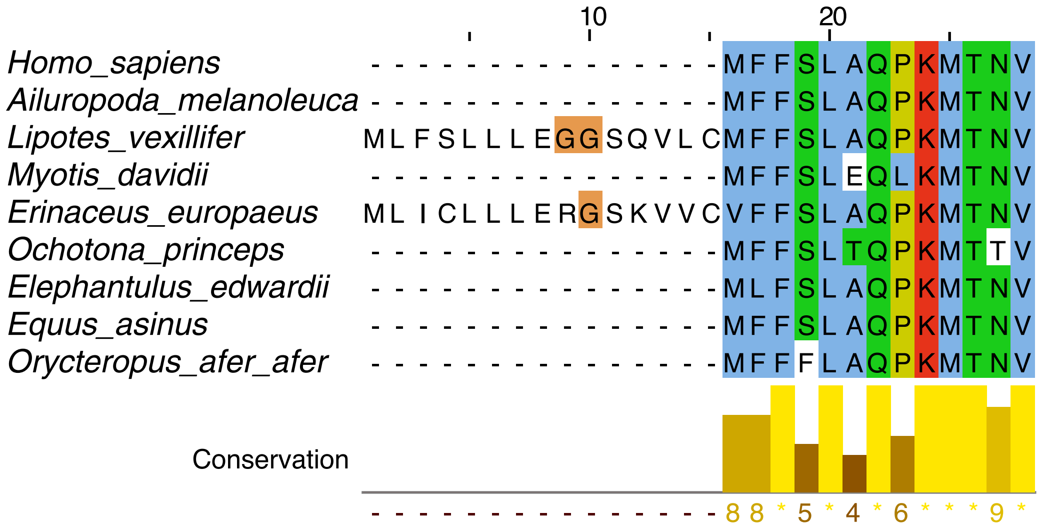

HG0674.2

ENSG00000116793 (PHTF1)

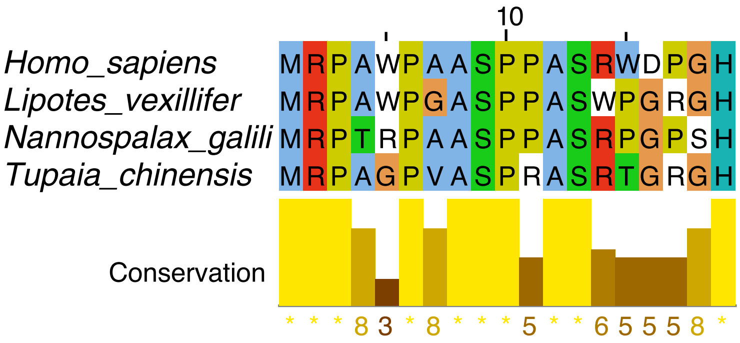

HG0675

ENSG00000048991 (R3HDM1)

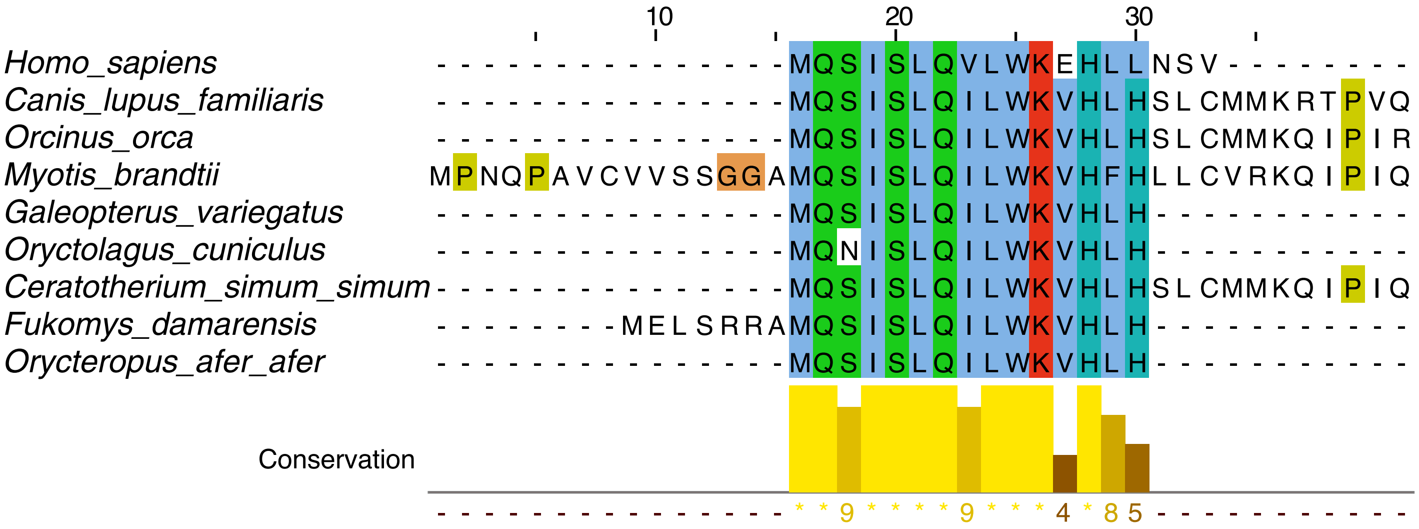

HG0676

ENSG00000058673 (ZC3H11A)

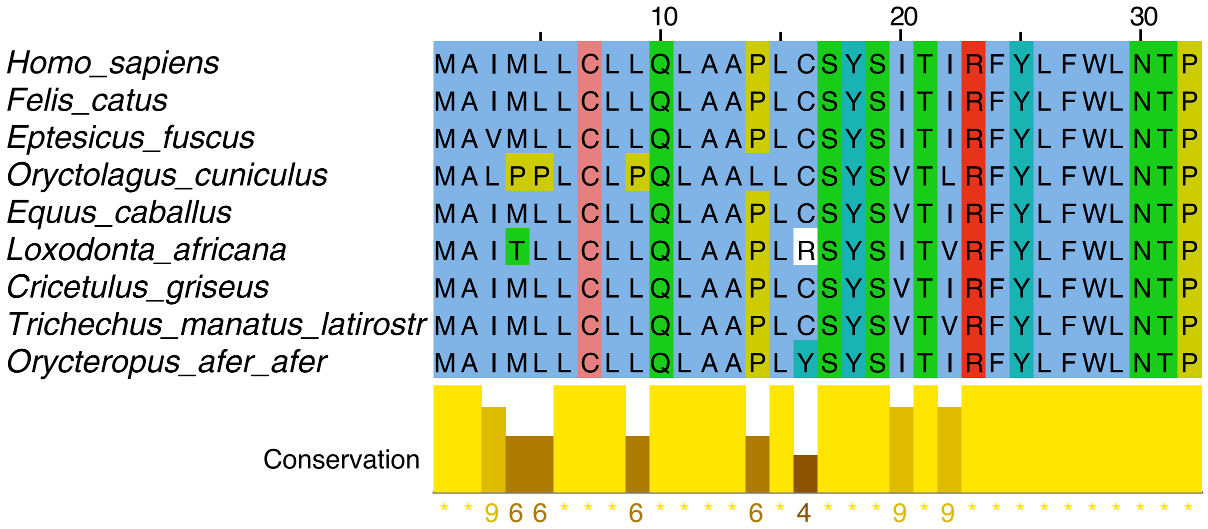

HG0677

ENSG00000064989 (CALCRL)

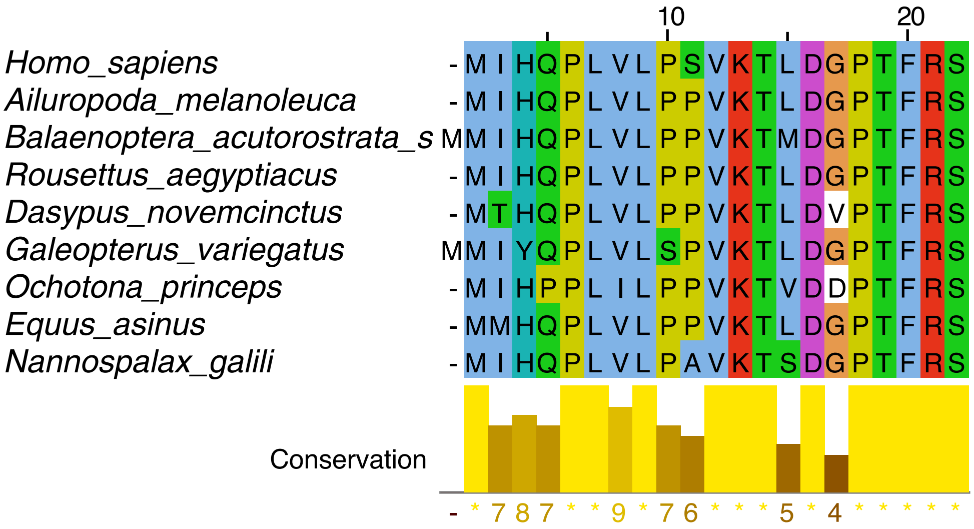

HG0678.1

ENSG00000090776 (EFNB1)

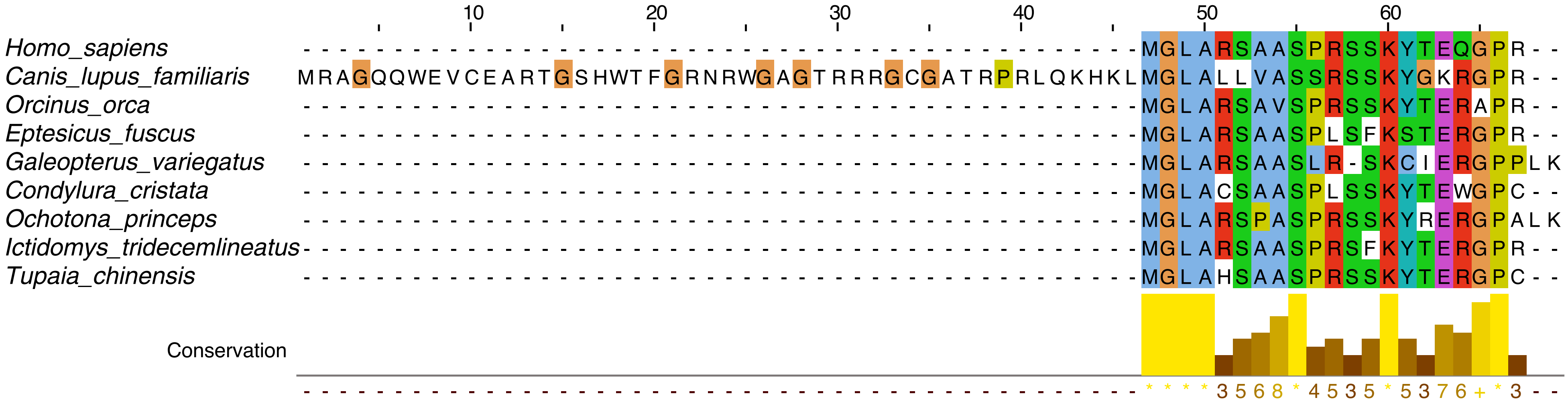

HG0678.2

ENSG00000108947 (EFNB3)

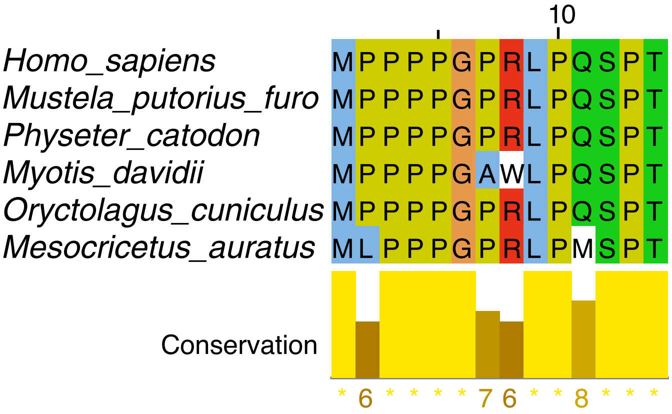

HG0679

ENSG00000100281 (HMGXB4)

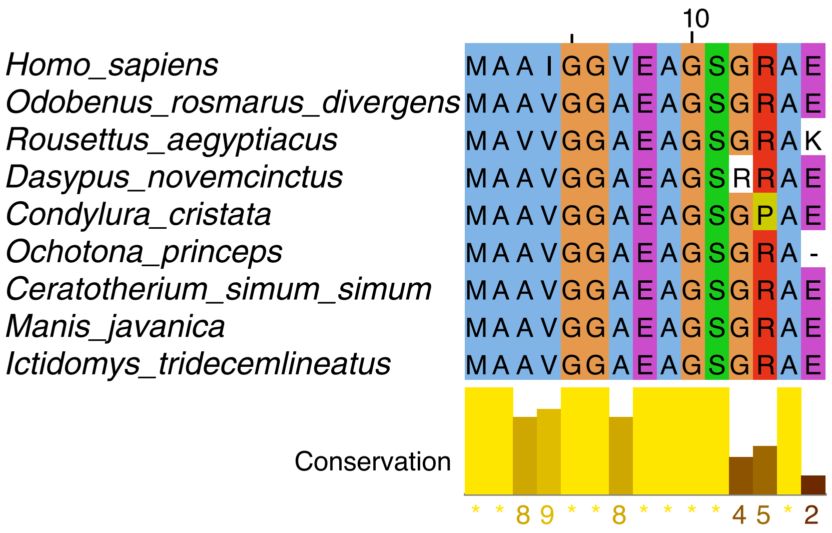

HG0680

ENSG00000100678 (SLC8A3)

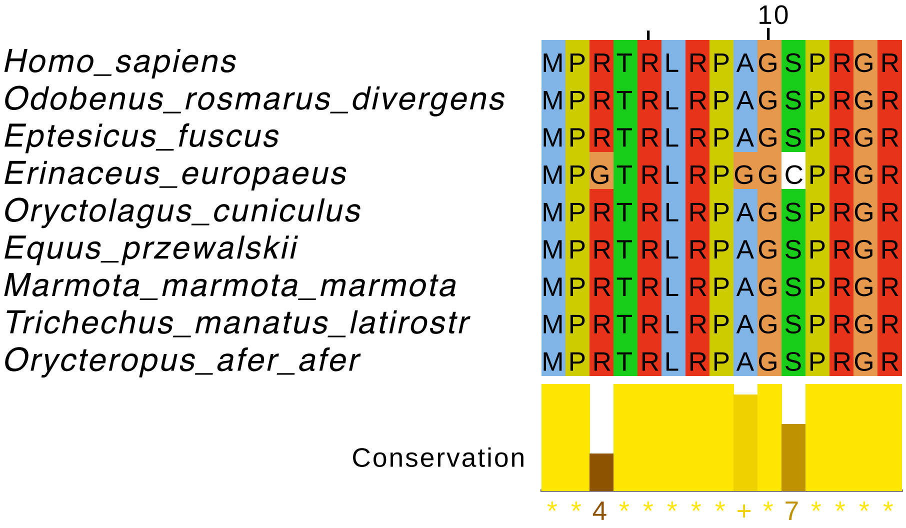

HG0681

ENSG00000101349 (PAK5)

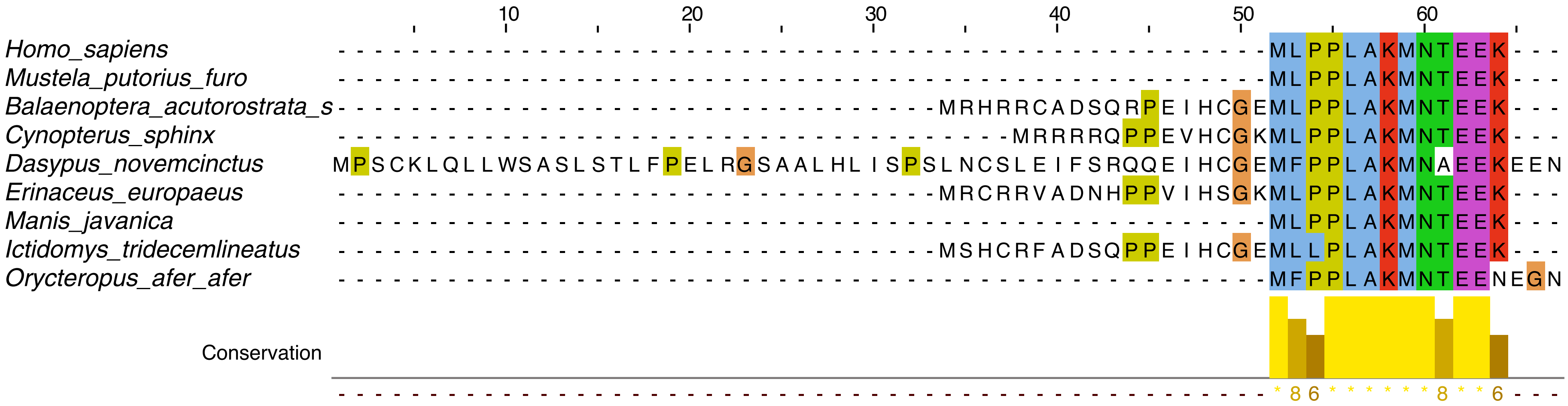

HG0682

ENSG00000105829 (BET1)

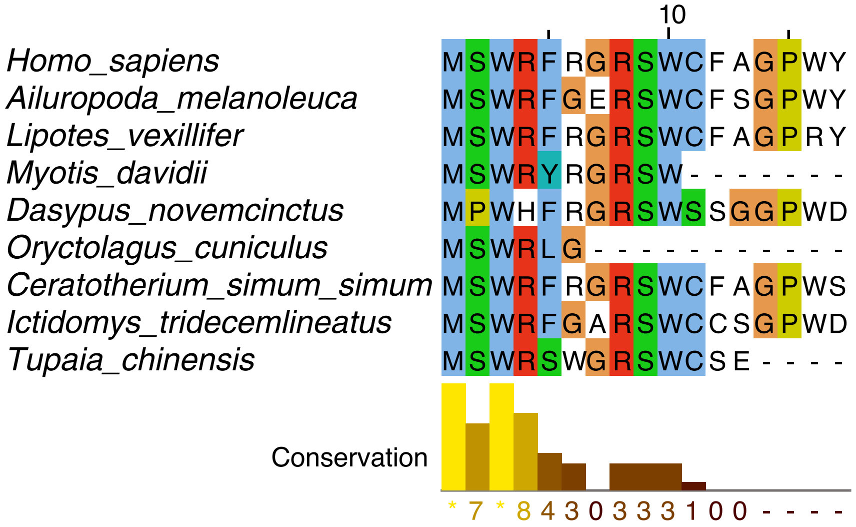

HG0683

ENSG00000111837 (MAK)

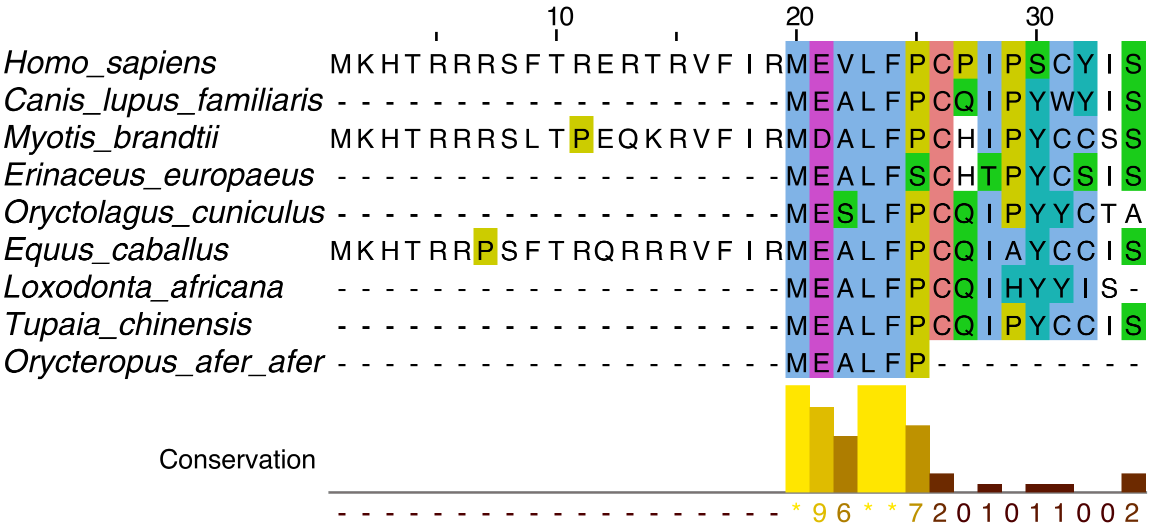

HG0684

ENSG00000113916 (BCL6)

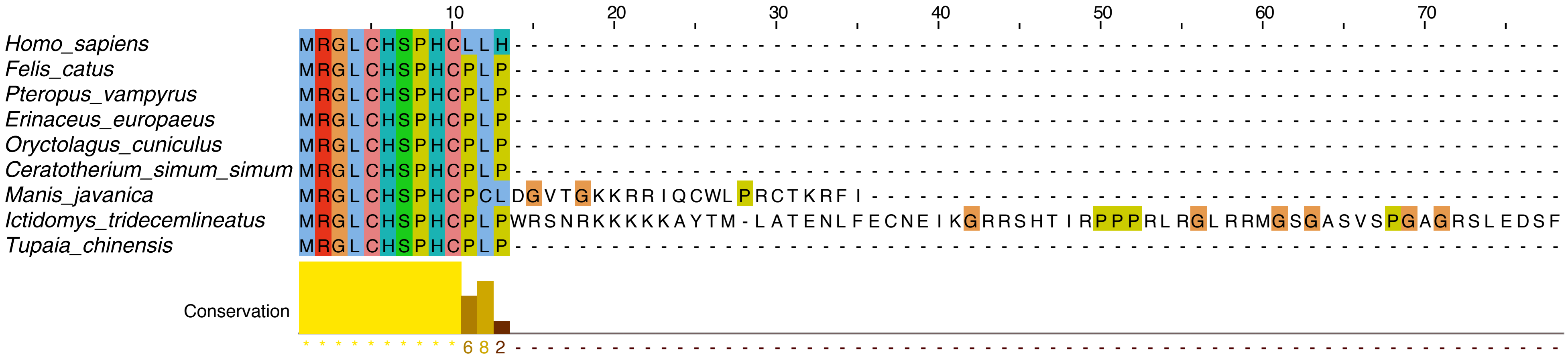

HG0685

ENSG00000117262 (GPR89A)

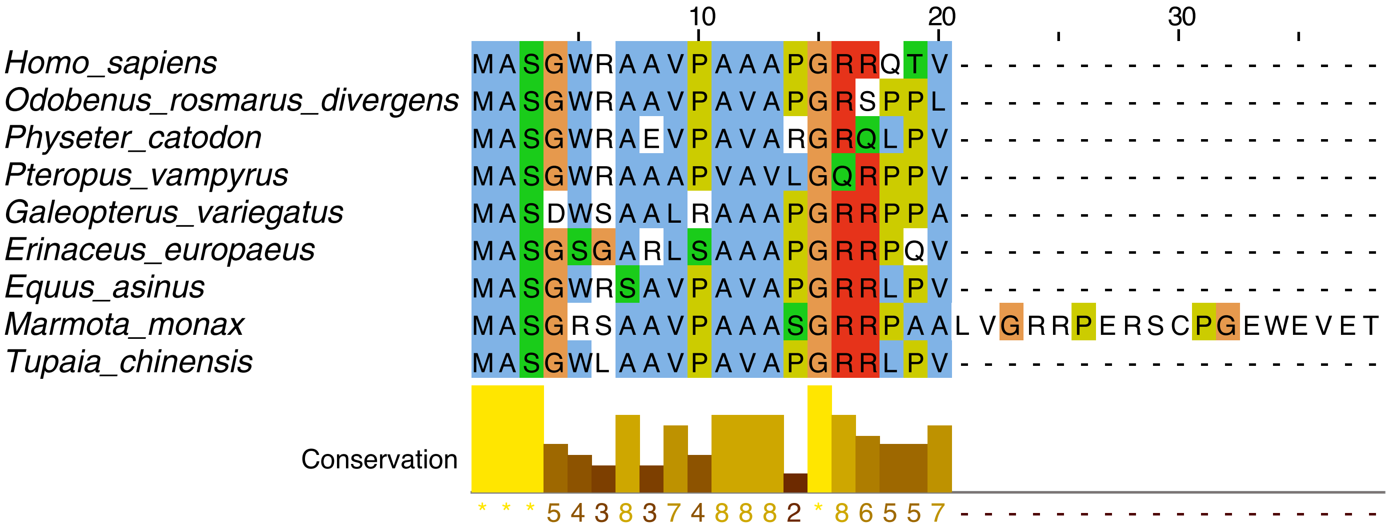

HG0686

ENSG00000118985 (ELL2)

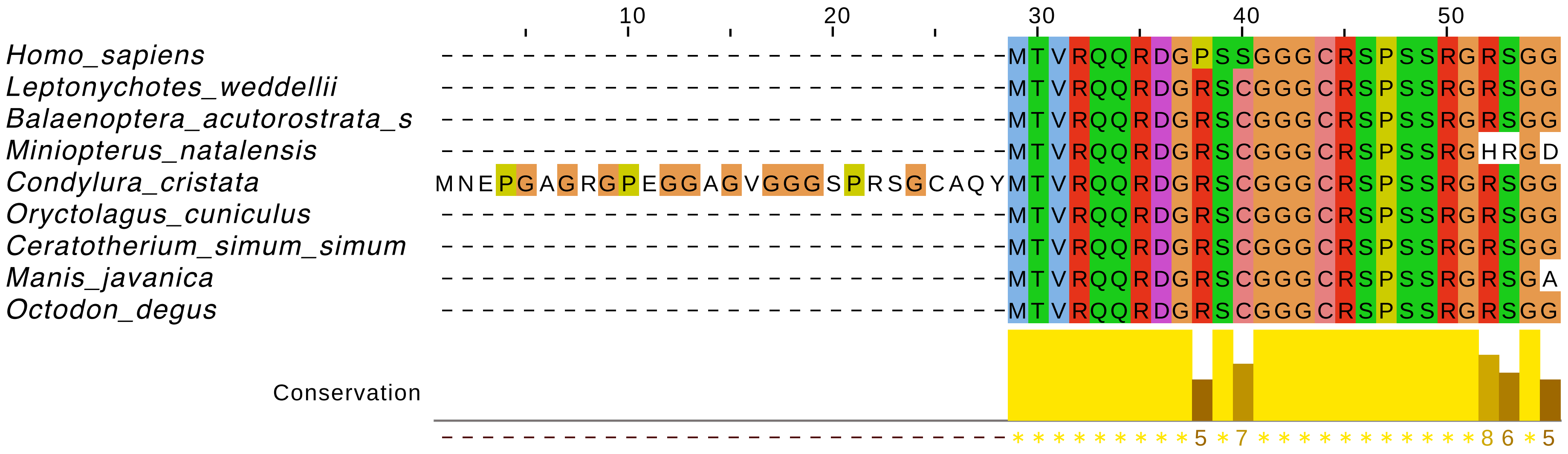

HG0687.1

ENSG00000120549 (KIAA1217)

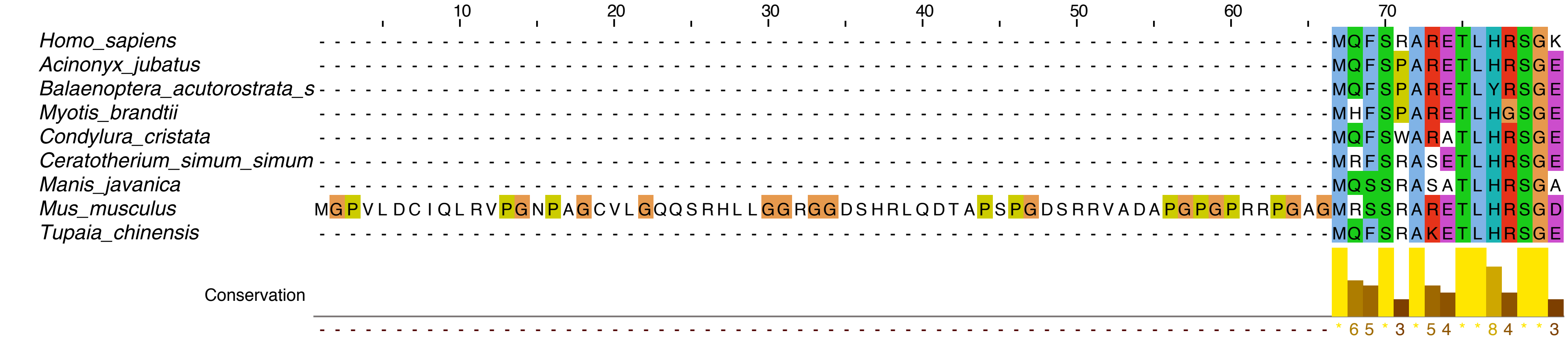

HG0687.2

ENSG00000120549 (KIAA1217)

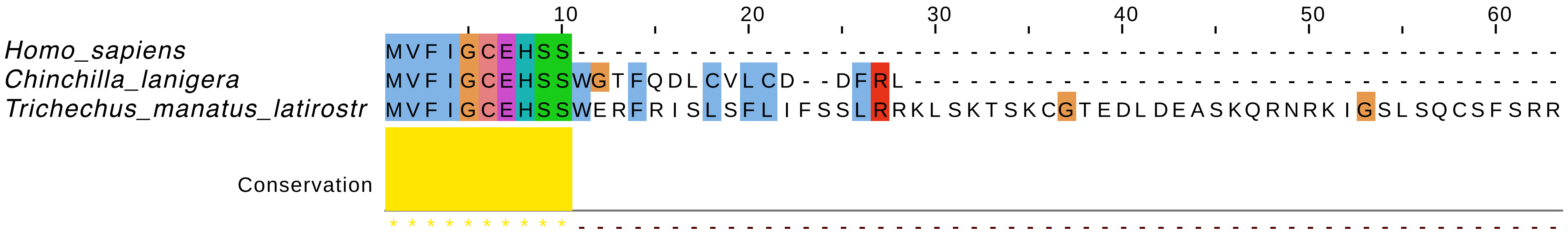

HG0688

ENSG00000122420 (PTGFR)

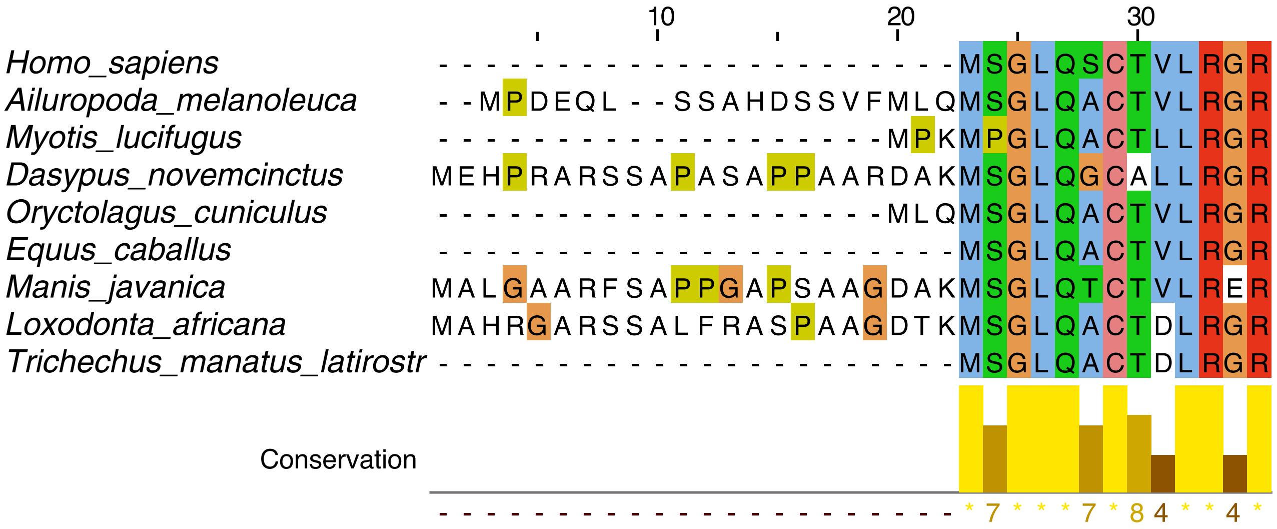

HG0689

ENSG00000123352 (SPATS2)

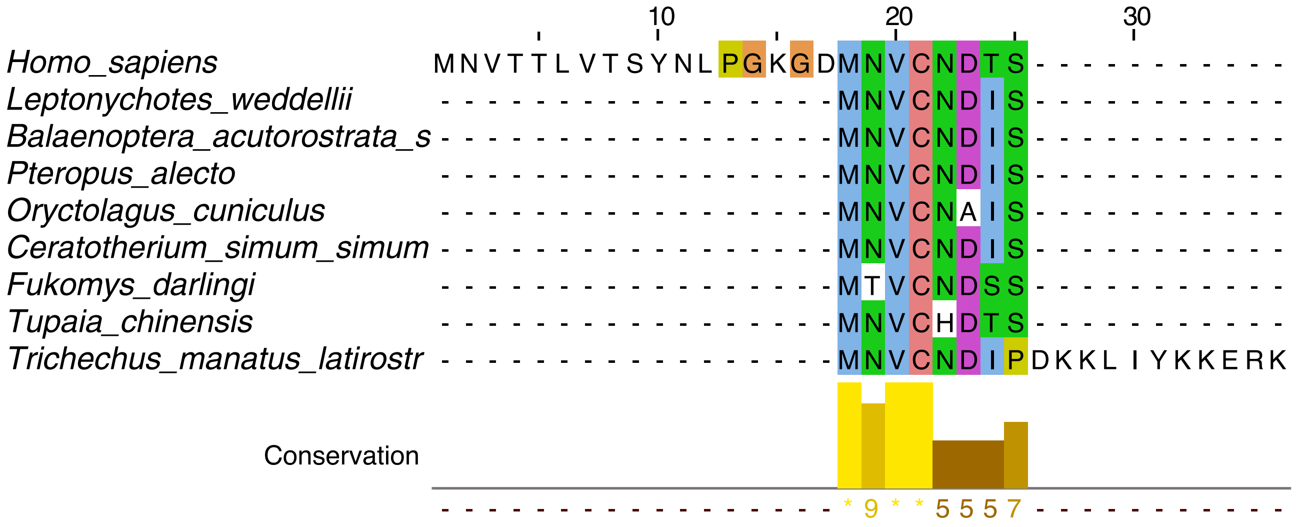

HG0690

ENSG00000125931 (CITED1)

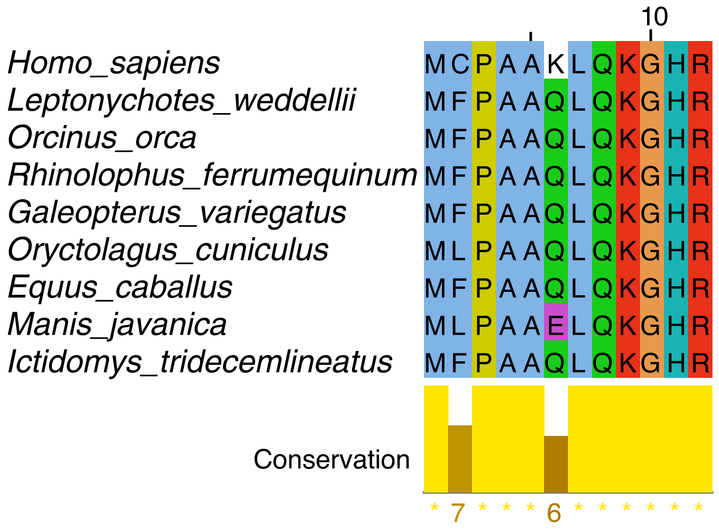

HG0691

ENSG00000129535 (NRL)

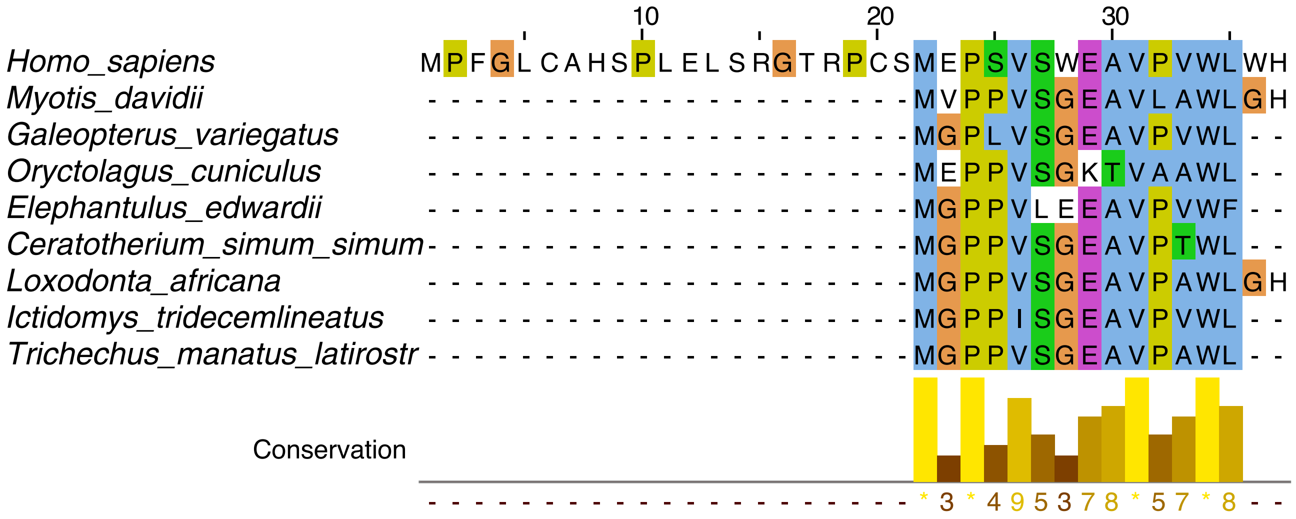

HG0692.1

ENSG00000130338 (TULP4)

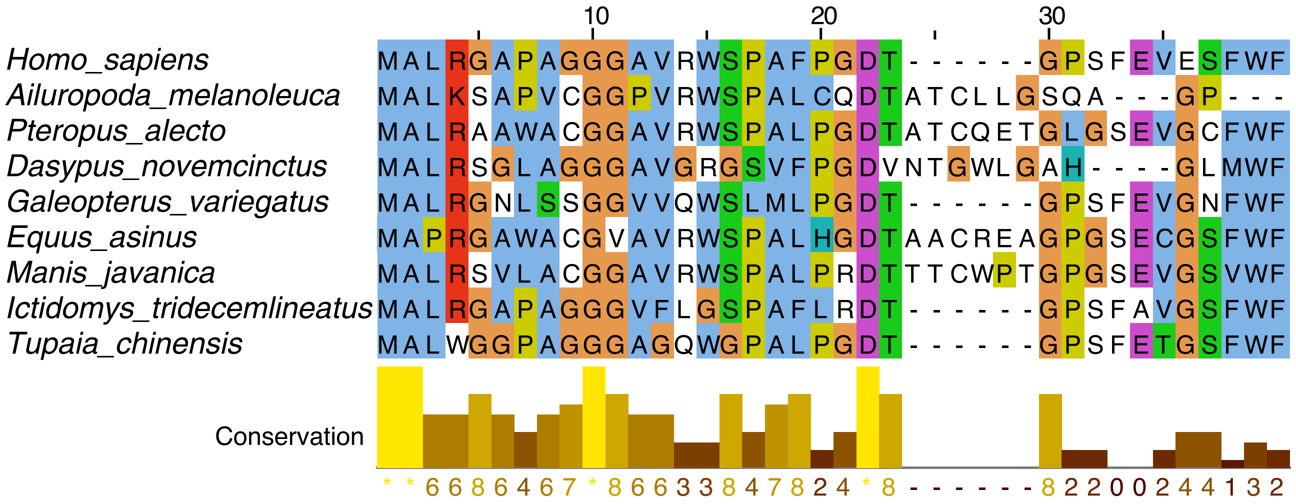

HG0692.2

ENSG00000130338 (TULP4)

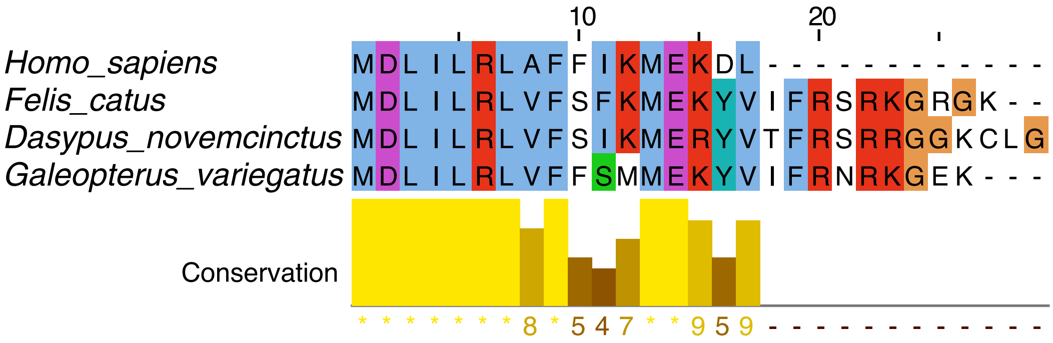

HG0693

ENSG00000131653 (TRAF7)

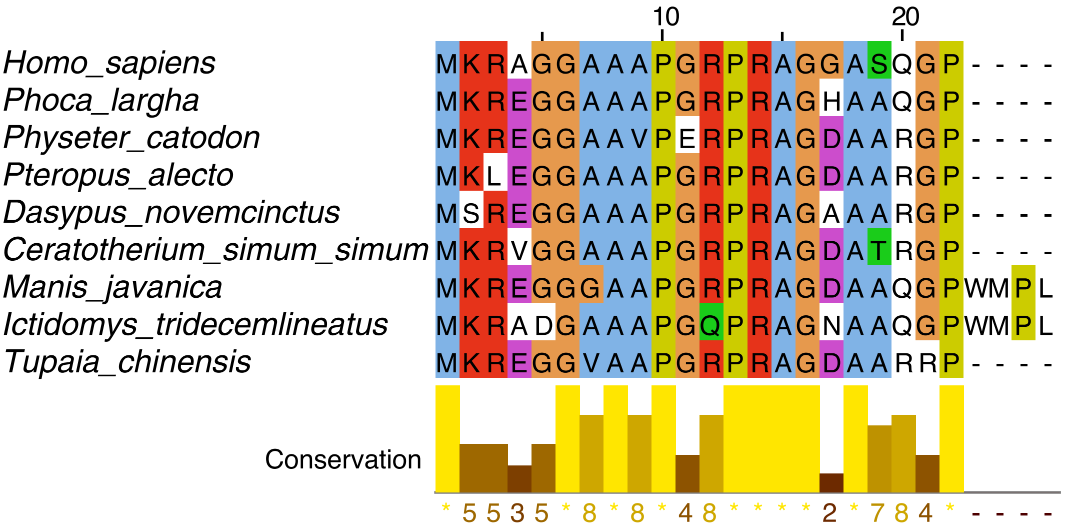

HG0694

ENSG00000133265 (HSPBP1)

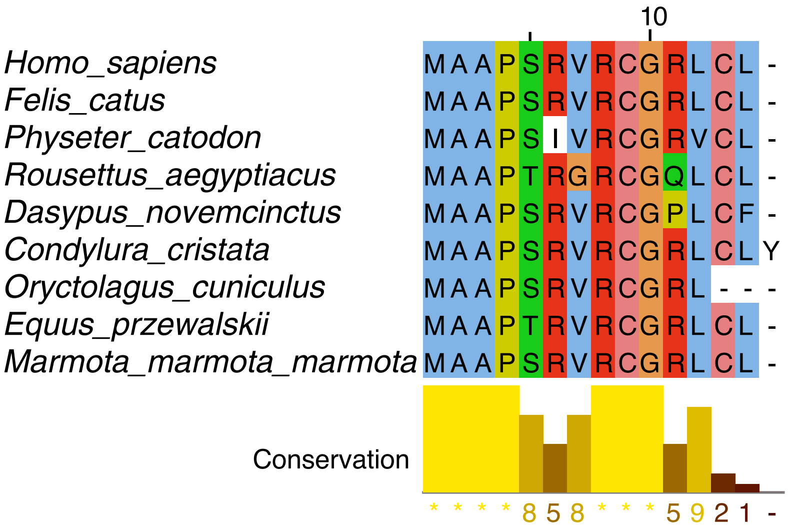

HG0695

ENSG00000133704 (IPO8)

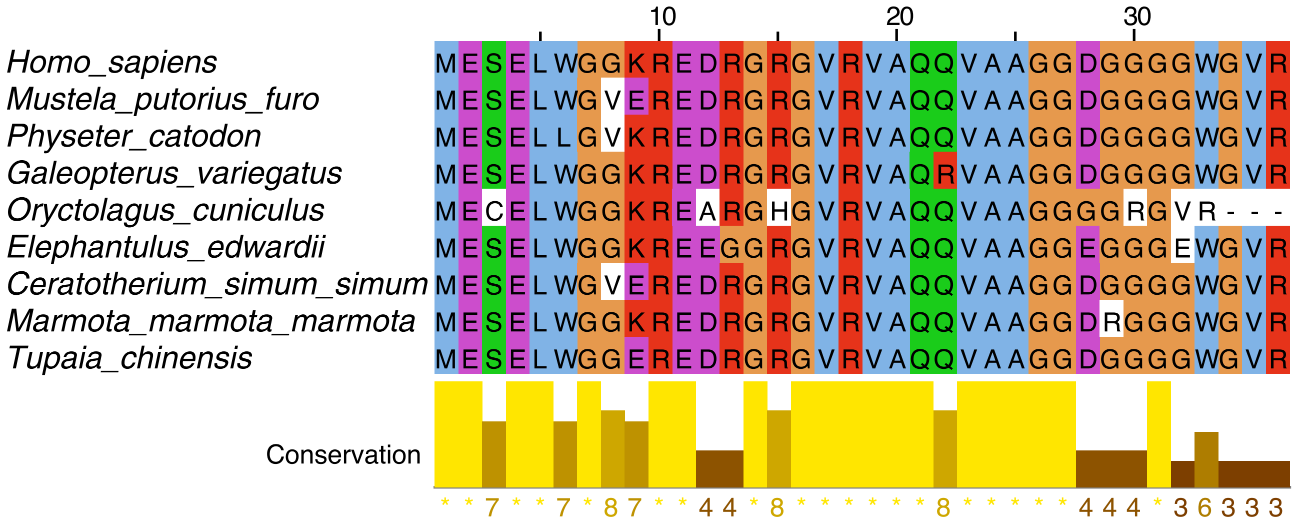

HG0696

ENSG00000138380 (CARF)

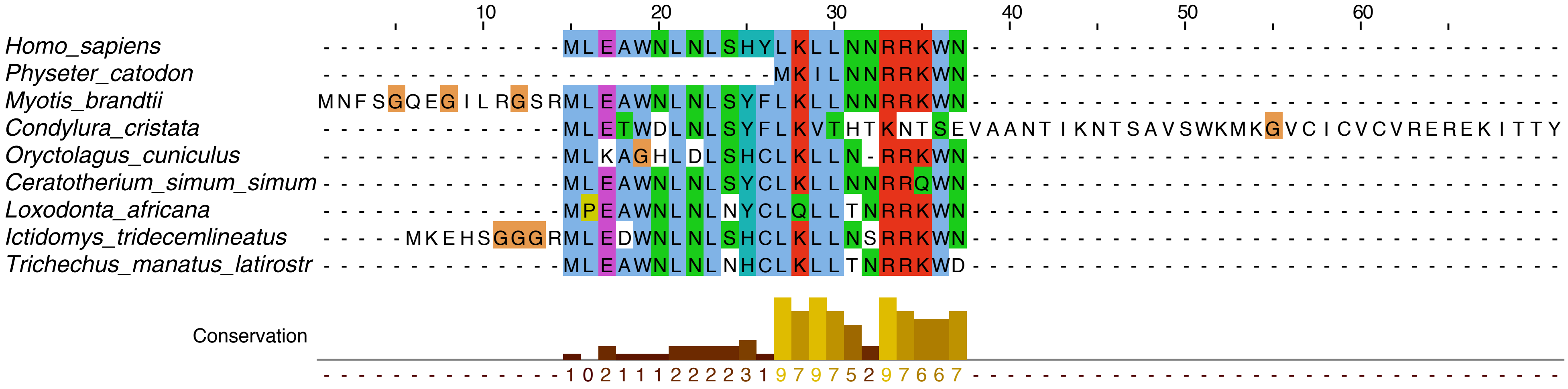

HG0697

ENSG00000143344 (RGL1)

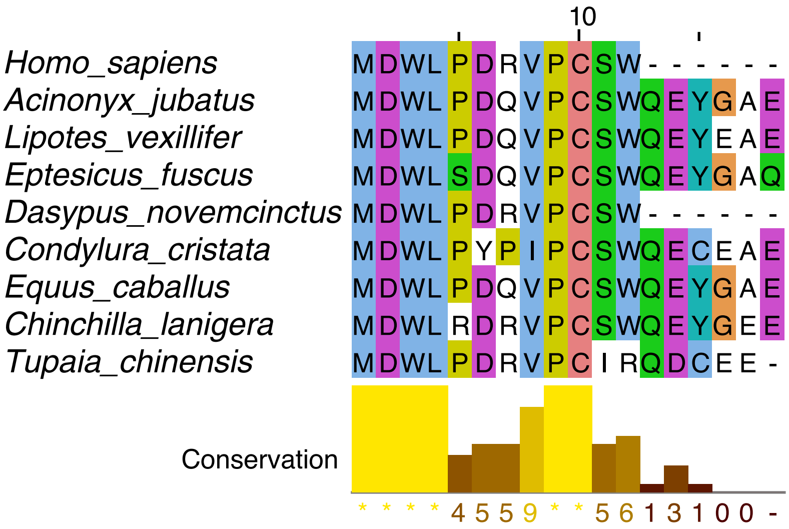

HG0698

ENSG00000157554 (ERG)

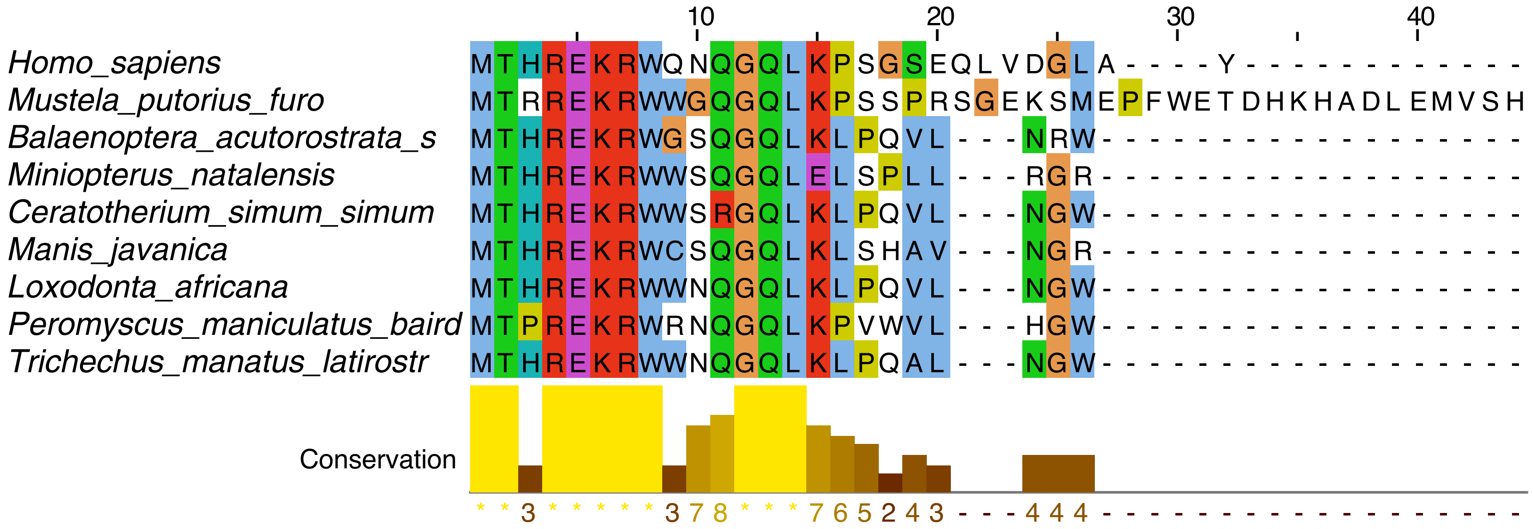

HG0699

ENSG00000160785 (SLC25A44)

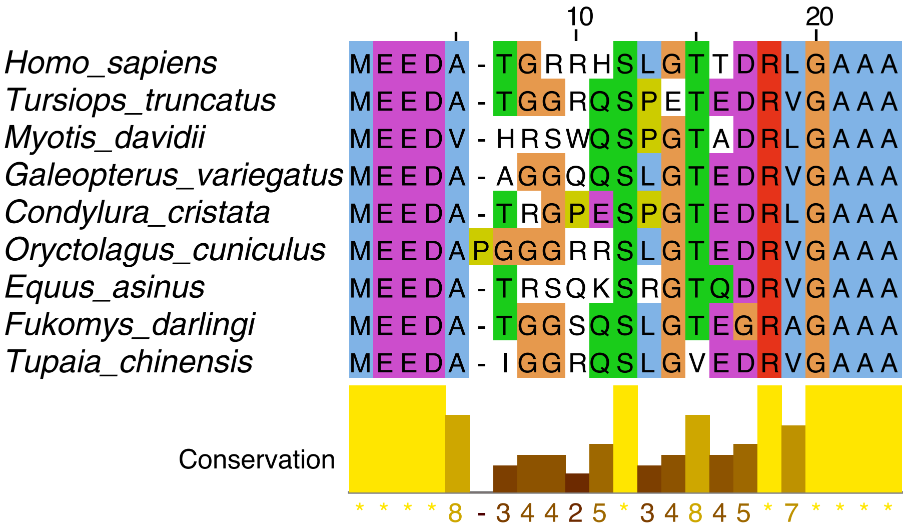

HG0700

ENSG00000165156 (ZHX1)

```
                                    10        20        30        40        50        60
                                     |         |         |         |         |         |
Homo_sapiens                 MQDG-GGVALRGAAAPAGSGASRLRDQRARRLWAAGGNWSAESAAGTGKEKTDCCFGPVGNDGLS
Odobenus_rosmarus_divergens  --------------------------------------------------MADRCFRPVGNDGLS
Lipotes_vexillifer           MQDG-GGGALRGTAAPAGDGR--------------------------------------------
Myotis_ricketti              --------------------------------------------------MADQCFGPAGNDGLS
Erinaceus_europaeus          MQDG-GGRALRGAAAPAGHGR--------------------------------------------
Ceratotherium_simum_simum    --------------------------------------------------MADRYFGPVGNDGLS
Manis_javanica               --------------------------------------------------MADRYFGPVGNDGLS
Ictidomys_tridecemlineatus   MQDG-GGGVLRGAAAPAGED---------------------------------------------
Trichechus_manatus_latirostr MQDGDGGGALRGAADPAGESR--------------------------------------------

Conservation
                             -----------------------------------------------------------------
```

HG0701

ENSG00000166292 (TMEM100)

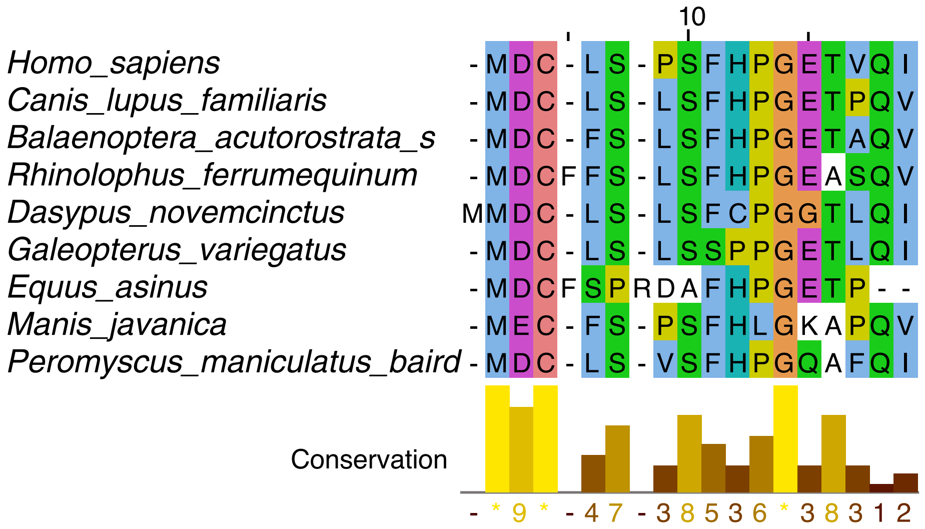

HG0702

ENSG00000167377 (ZNF23)

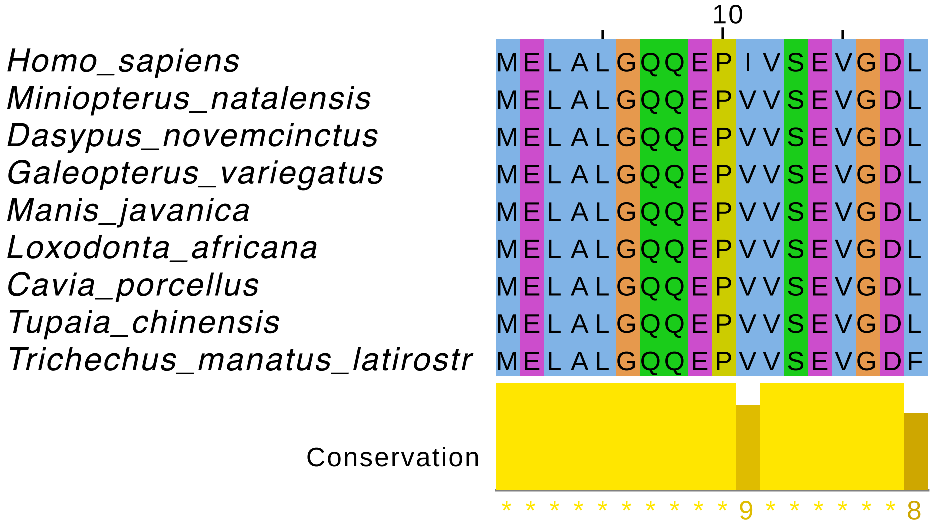

HG0703

ENSG00000171862 (PTEN)

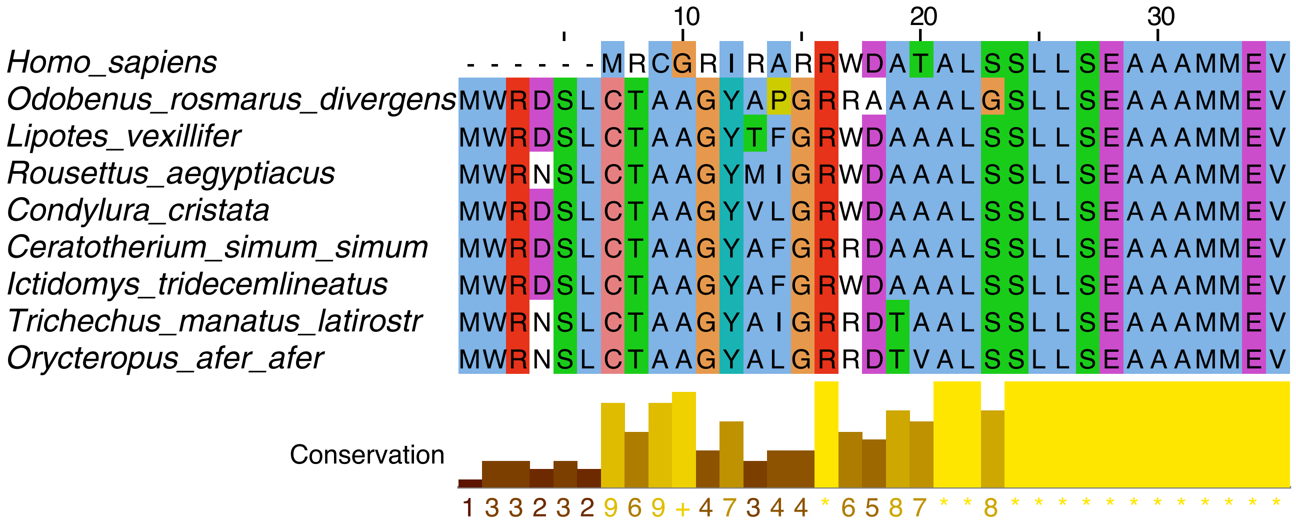

HG0704.1

ENSG00000173598 (NUDT4)

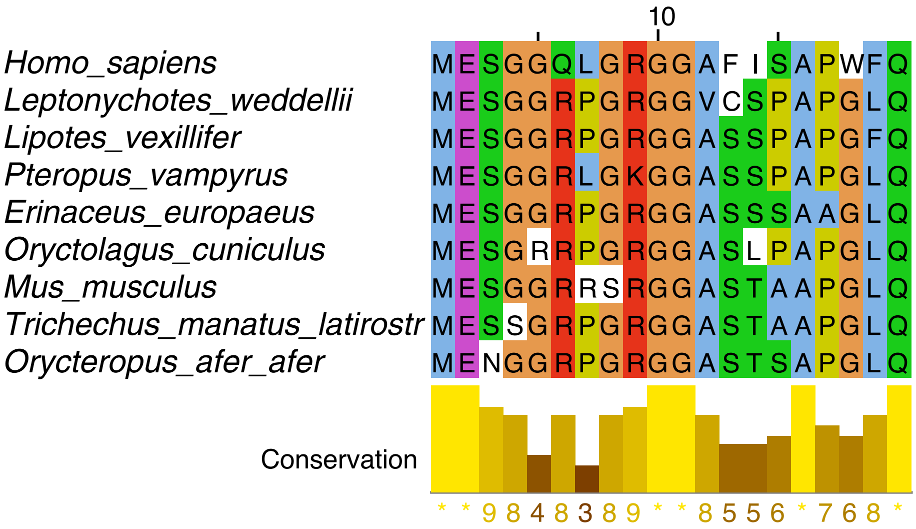

HG0704.2

ENSG00000272325 (NUDT3)

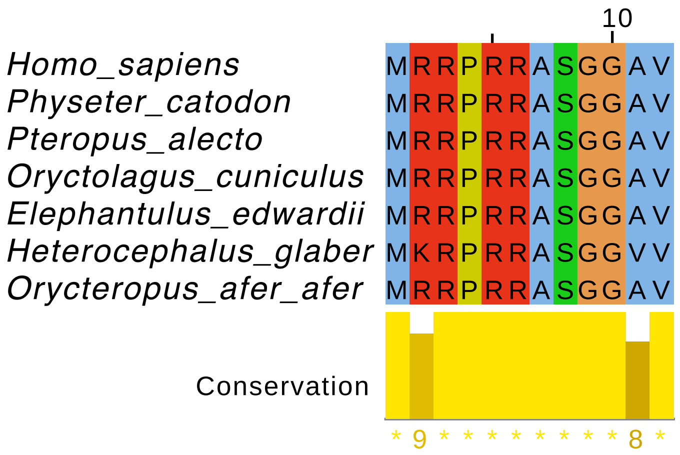

HG0705.1

ENSG00000175224 (ATG13)

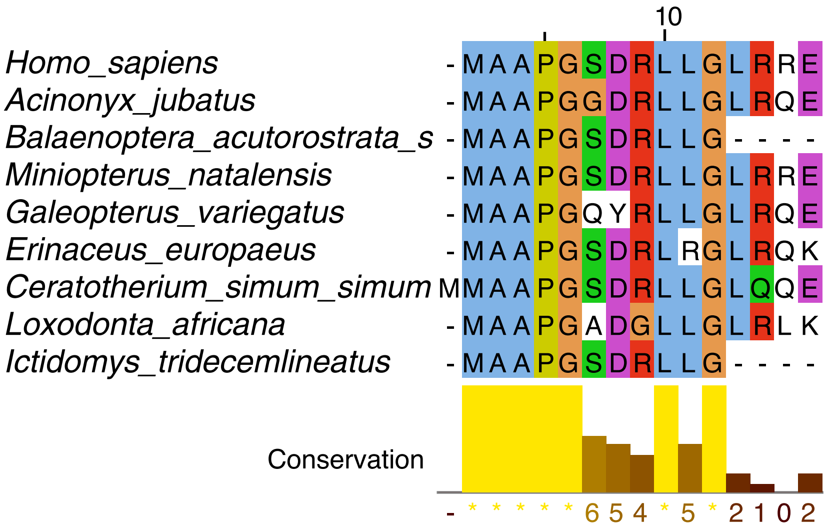

HG0705.2

ENSG00000175224 (ATG13)

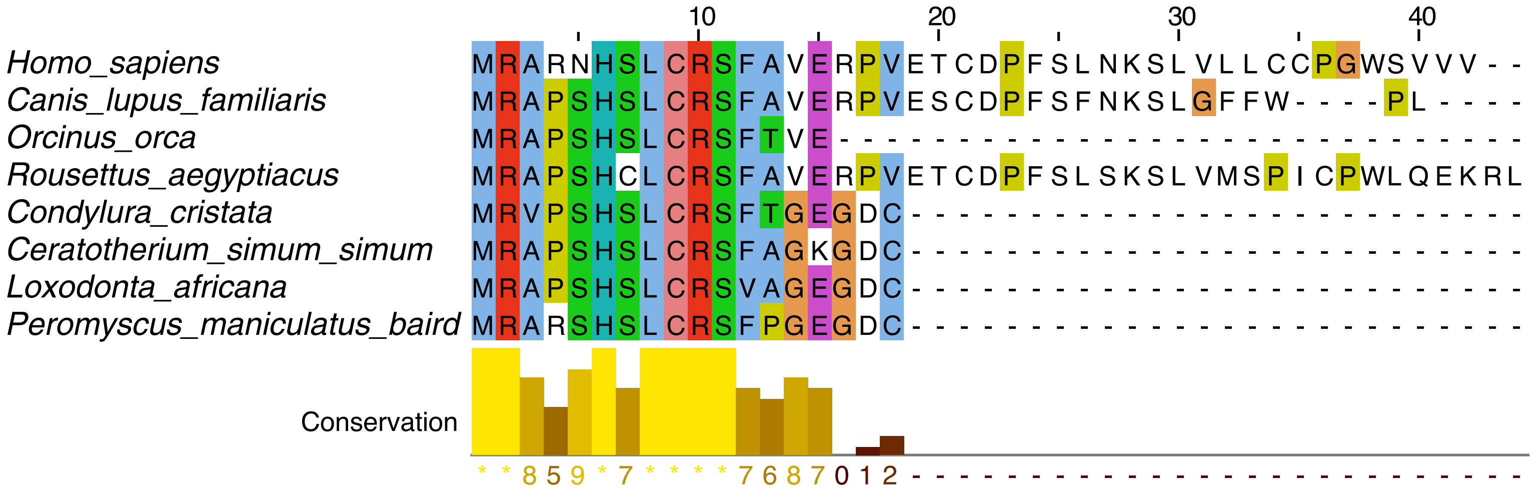

HG0706

ENSG00000178498 (DTX3)

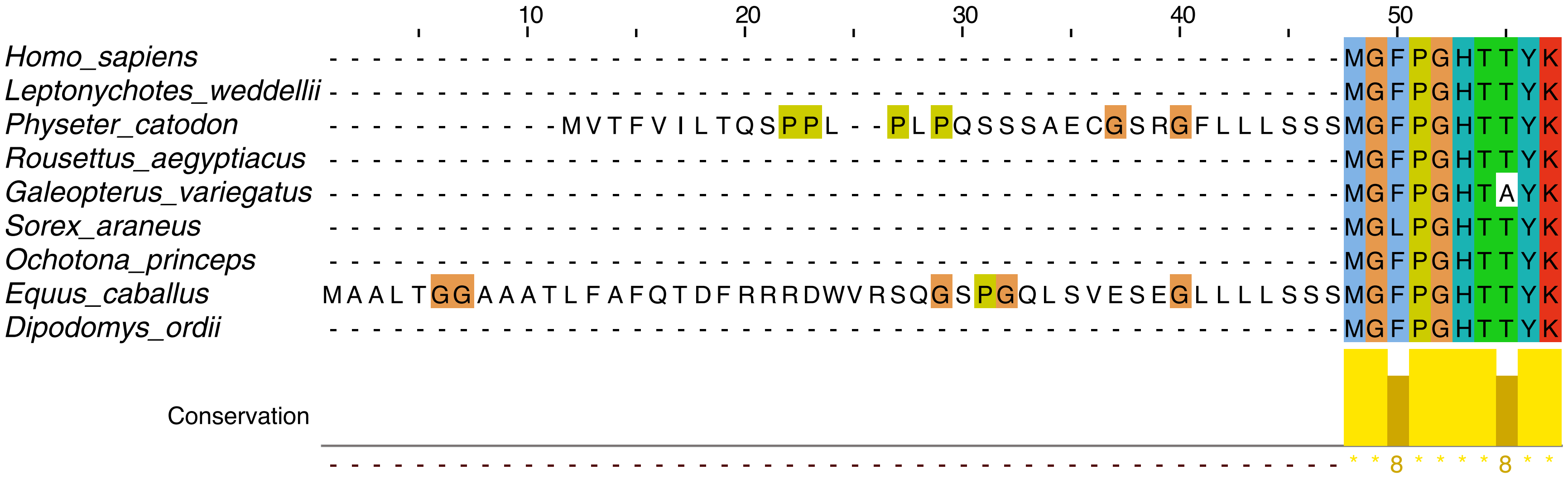

HG0707

ENSG00000179796 (LRRC3B)

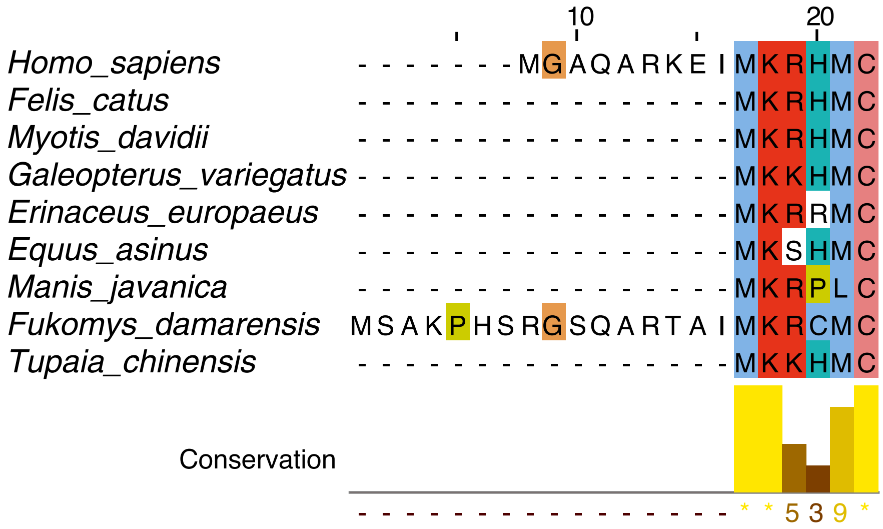

HG0708

ENSG00000184613 (NELL2)

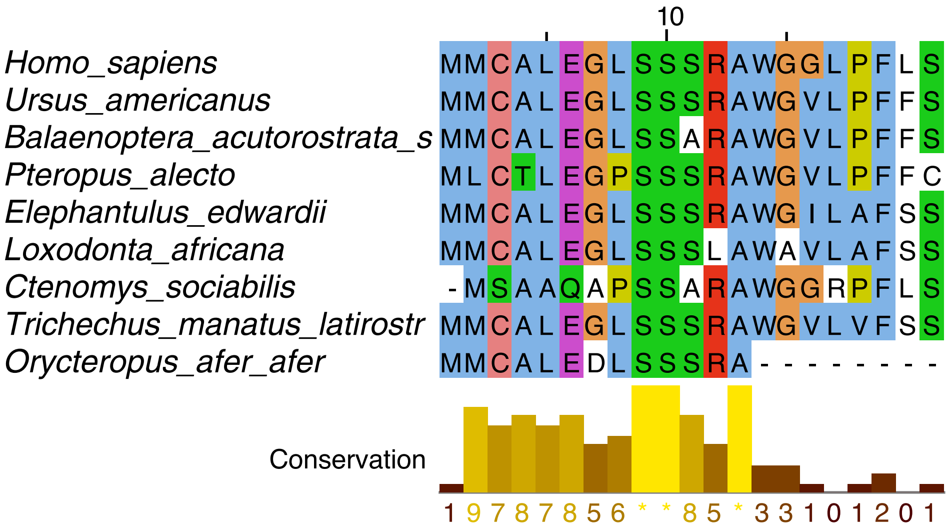

HG0709

ENSG00000186439 (TRDN)

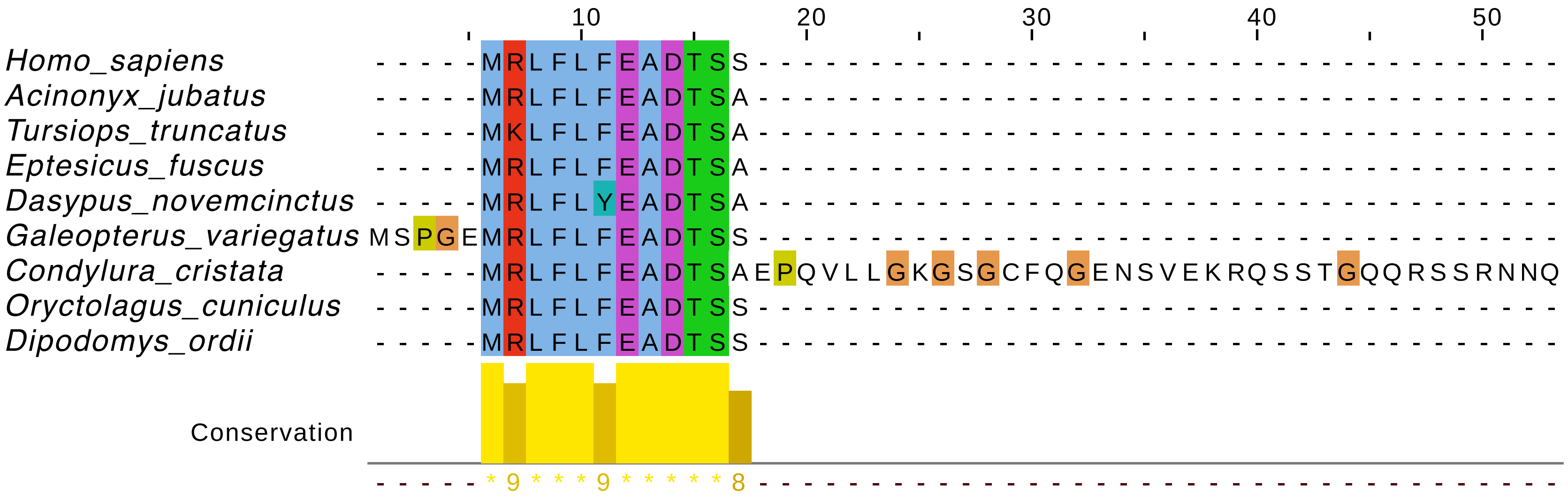

HG0710

ENSG00000196505 (GDAP2)

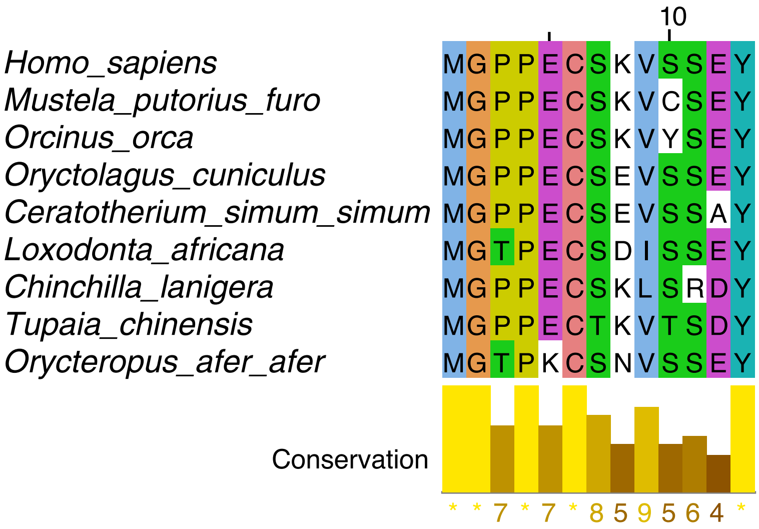

HG0711

ENSG00000198300 (PEG3)

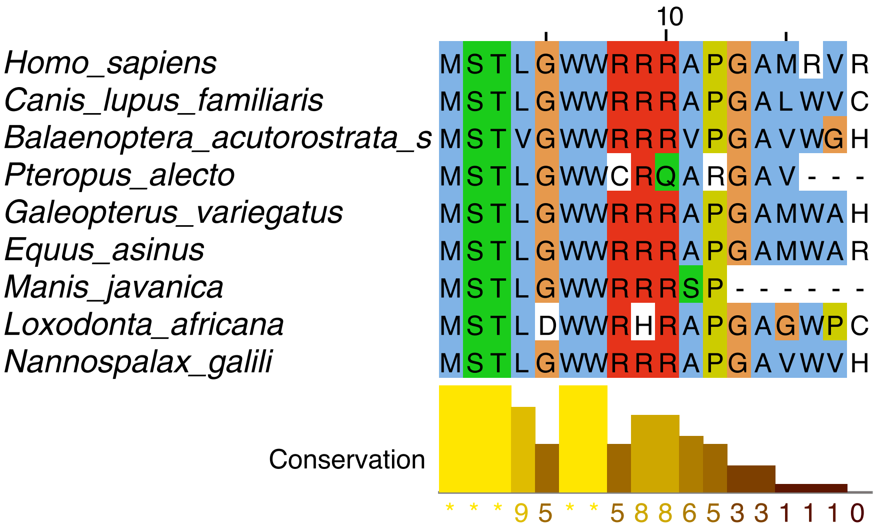

HG0712

ENSG00000213949 (ITGA1)

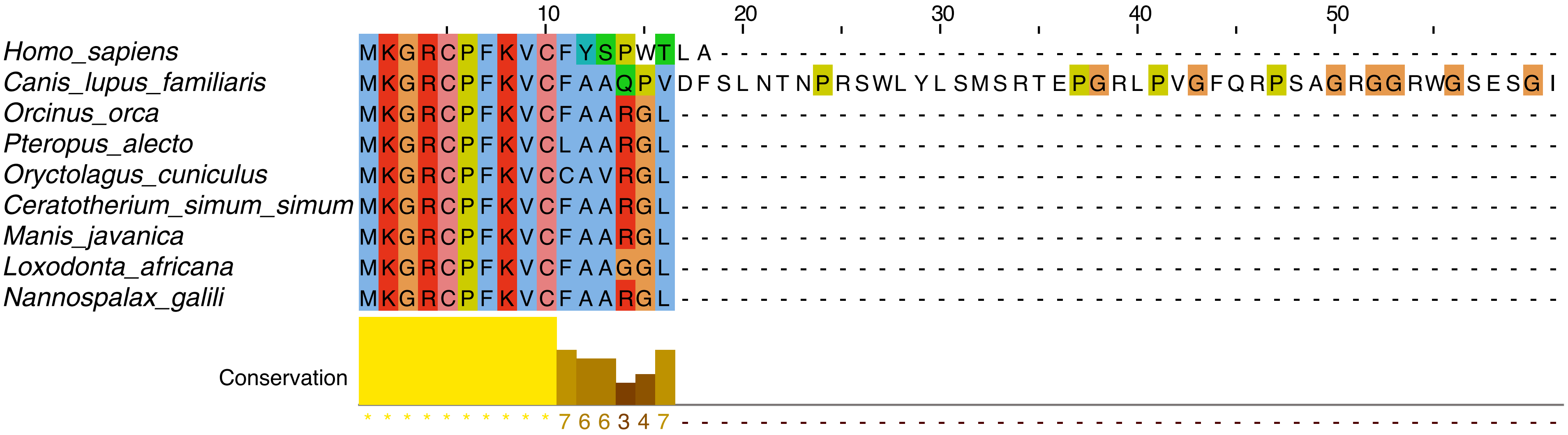

HG0713

ENSG00000008513 (ST3GAL1)

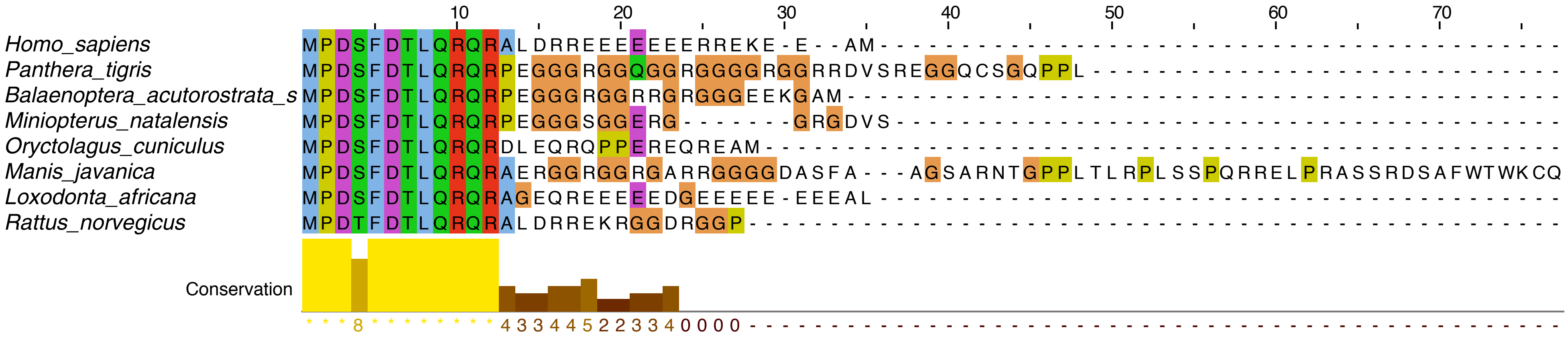

HG0714

ENSG00000051825 (MPHOSPH9)

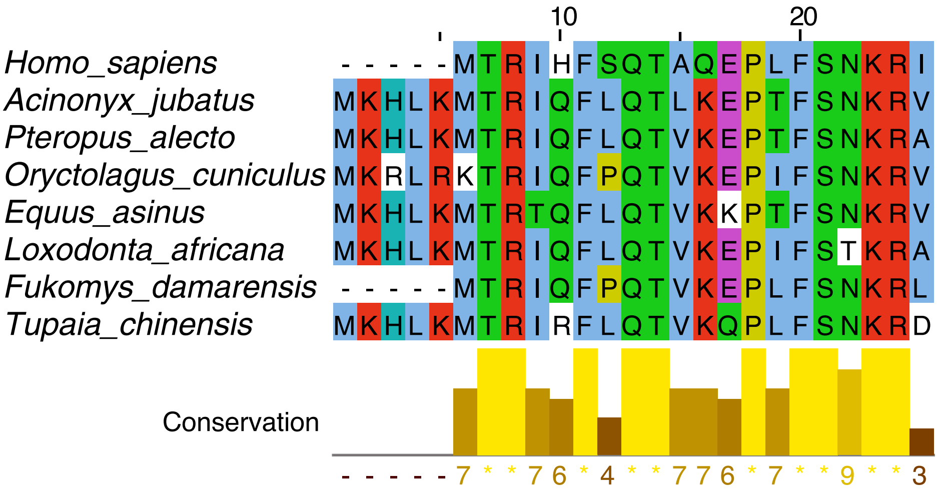

HG0715

ENSG00000071246 (VASH1)

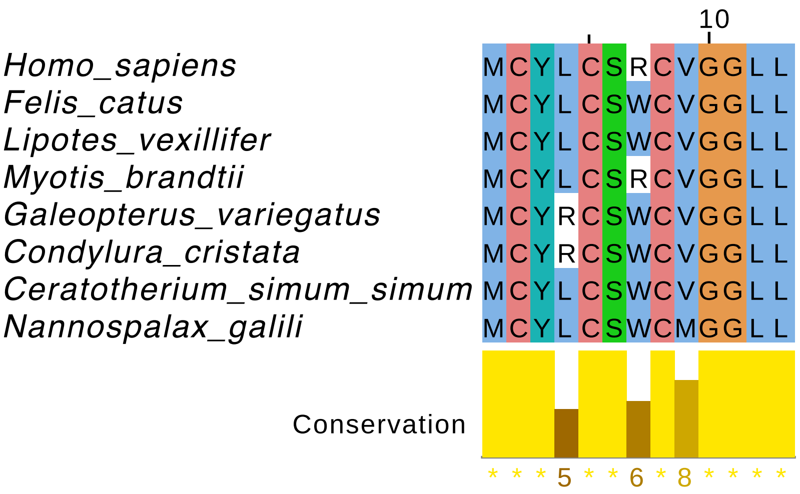

HG0716

ENSG00000075413 (MARK3)

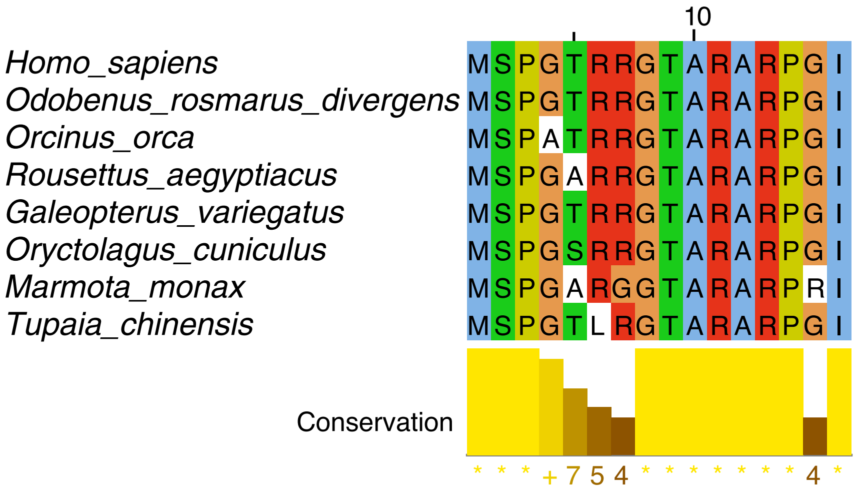

HG0717

ENSG00000083857 (FAT1)

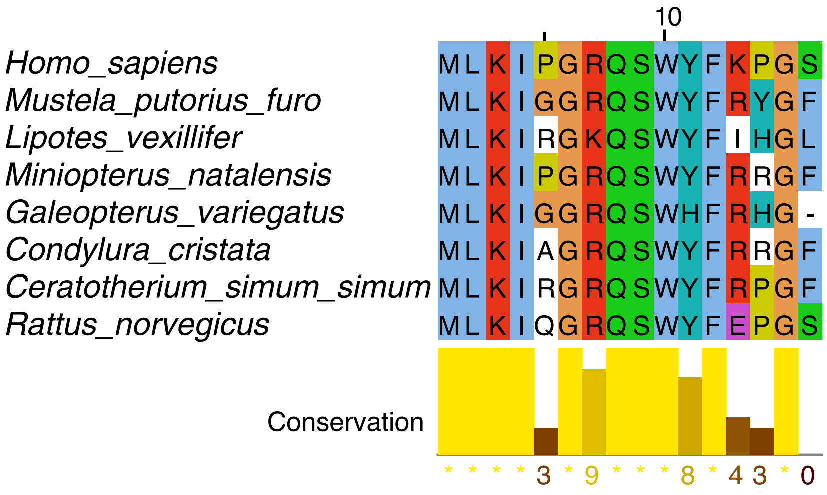

HG0718

ENSG00000085274 (MYNN)

```
                                   10        20        30        40        50        60        70        80        90       100       110
Homo_sapiens                  -------------------------------MMGEDKQLQAPARSAQLASG-ERPAVLCCRASAQSPGRA--------PDADCRHQNARDEEDRRSAAS------------------
Acinonyx_jubatus              -------------------------------MMGETKKLQAPACSAQSGSR-KED--FR----AVLPDLGPEREMLTIGRPECLE--------------------------------
Rousettus_aegyptiacus         MISLNSKIPPRPRAPVHKAAVPTRGAPKKGRMLGEDKQLQAPACSAQFGSS-KE--ALRCSSSRPRPRMRAERETPTVGRPECRE--------------------------------
Galeopterus_variegatus        --------------------------------MGEDANYK-PQRSARKPAPGRRPAAHRHPASAQNPGLAPMLS---------AFQNAQNEEDRRSAVSQPPGLVSMAPAIAGTVAC
Ceratotherium_simum_simum     MVES------R----RTQSSCSHSWGPEEGEGDGRRQATTSPSMQCASWAPGKRSAAQRCAASASNGGRARDADFQ------------------------PSKMLRMRK-TGGAL--
Manis_javanica                -------------------------------MMGKEERLQAPACSAQCGGWKERPAAFRCPASAQKAGAA-----------------------------------------------
Ictidomys_tridecemlineatus    -------------------------------MTGEDKQLQAPACSAQRGL----PRVWRCRLPGLSPESG-----------------------------------------------
Trichechus_manatus_latirostr  -------------------------------MMGEDEQLQAPA--------------------------------------------------------------------------
```

Conservation

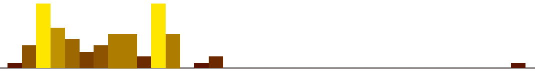

```
-------------------------------14*7534662*60120000-------00----0001000-----------------------------------------------
```

HG0719

ENSG00000085998 (POMGNT1)

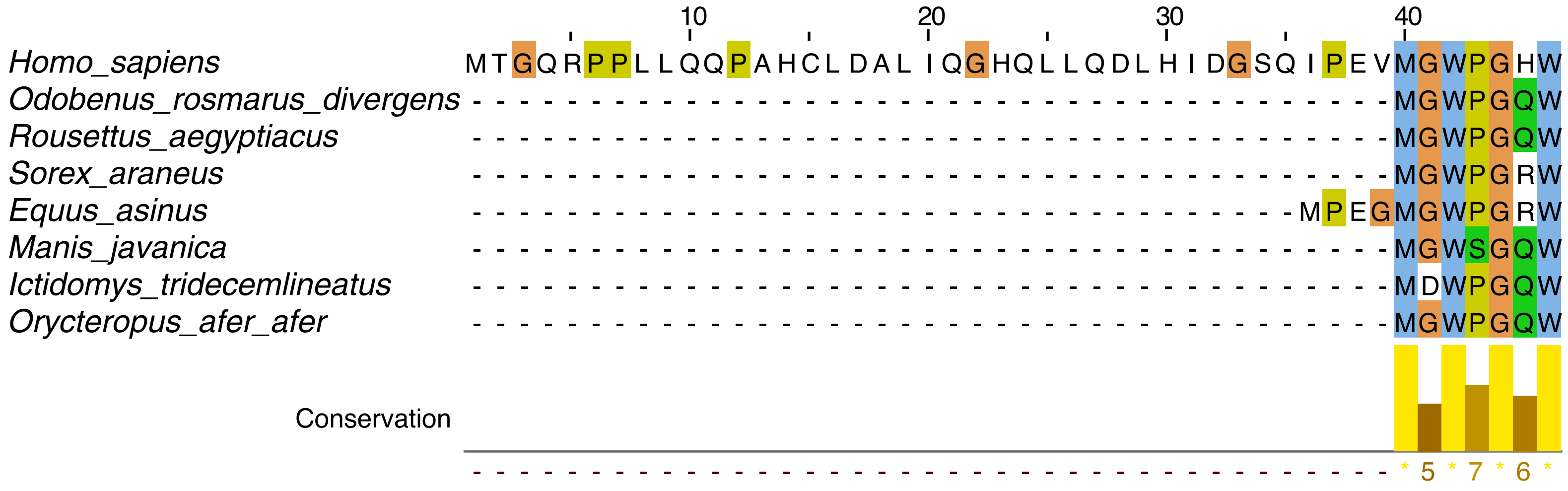

HG0720

ENSG00000087053 (MTMR2)

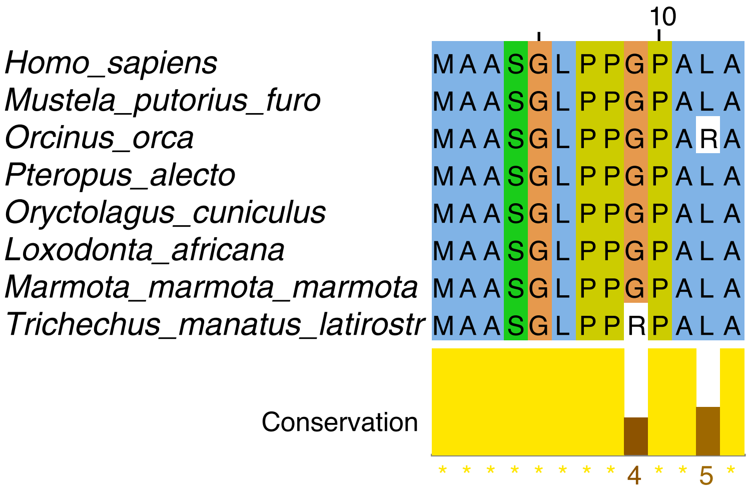

HG0721

ENSG00000095951 (HIVEP1)

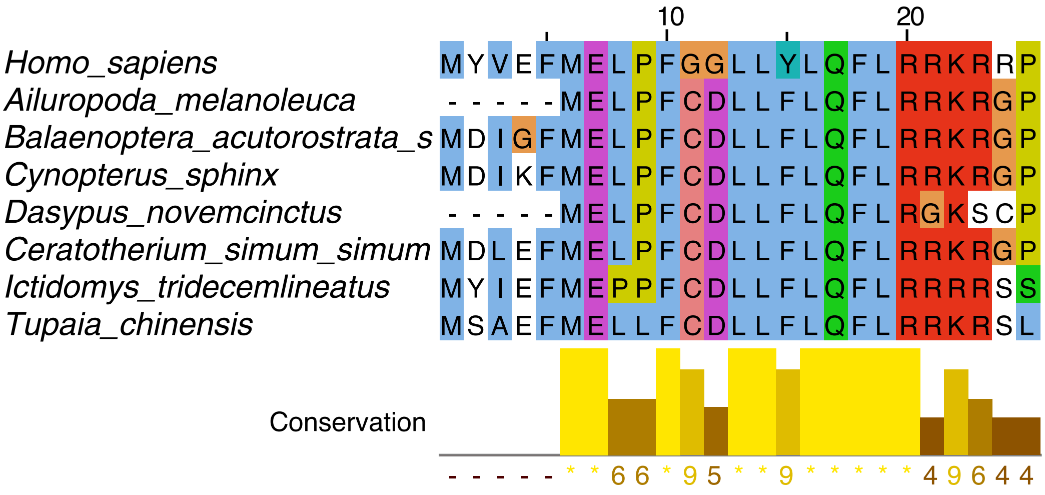

HG0722

ENSG00000101445 (PPP1R16B)

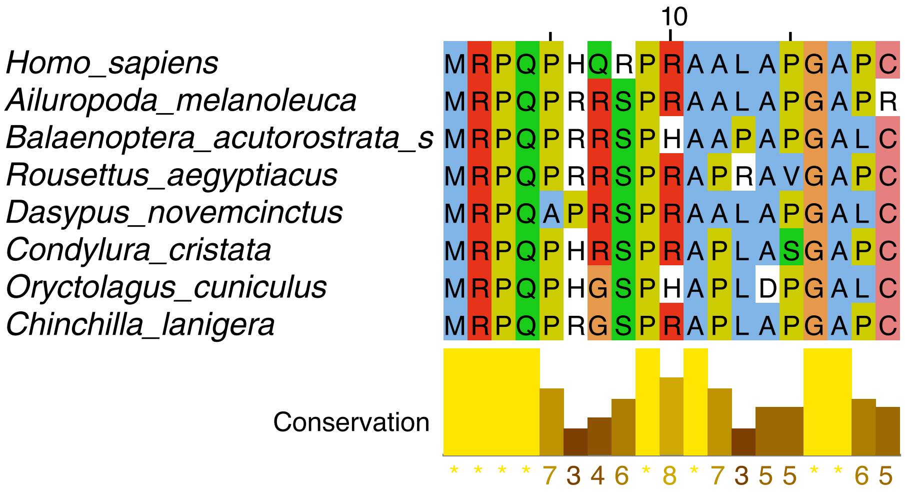

HG0723

ENSG00000102974 (CTCF)

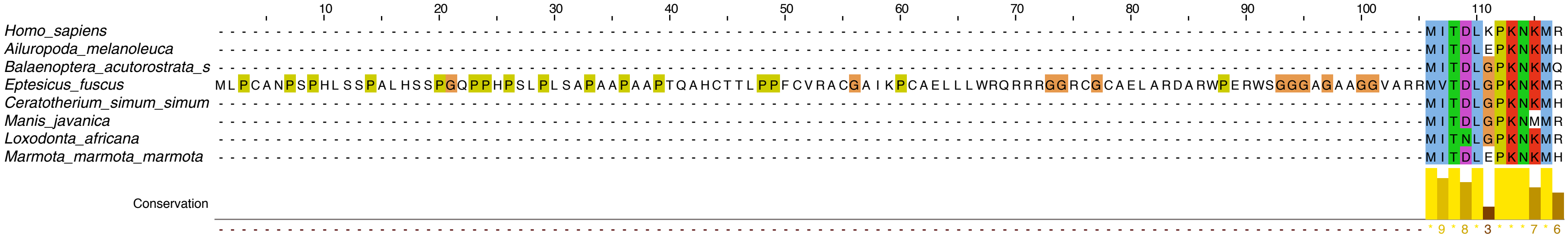

HG0724

ENSG00000104783 (KCNN4)

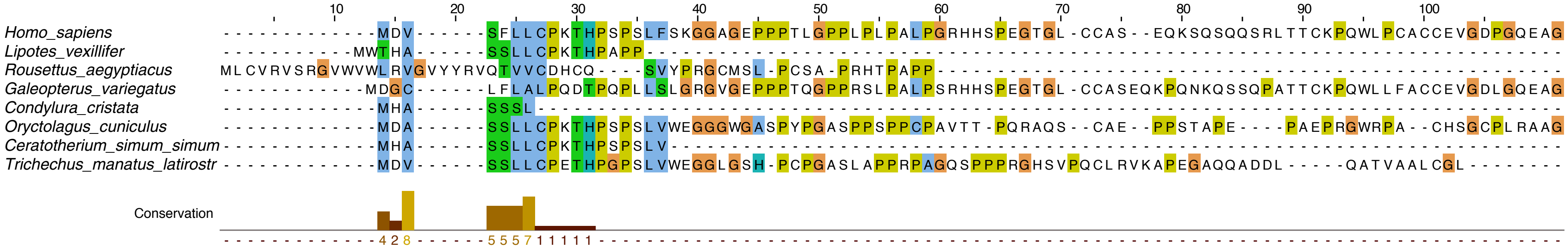

HG0725

ENSG00000112218 (GPR63)

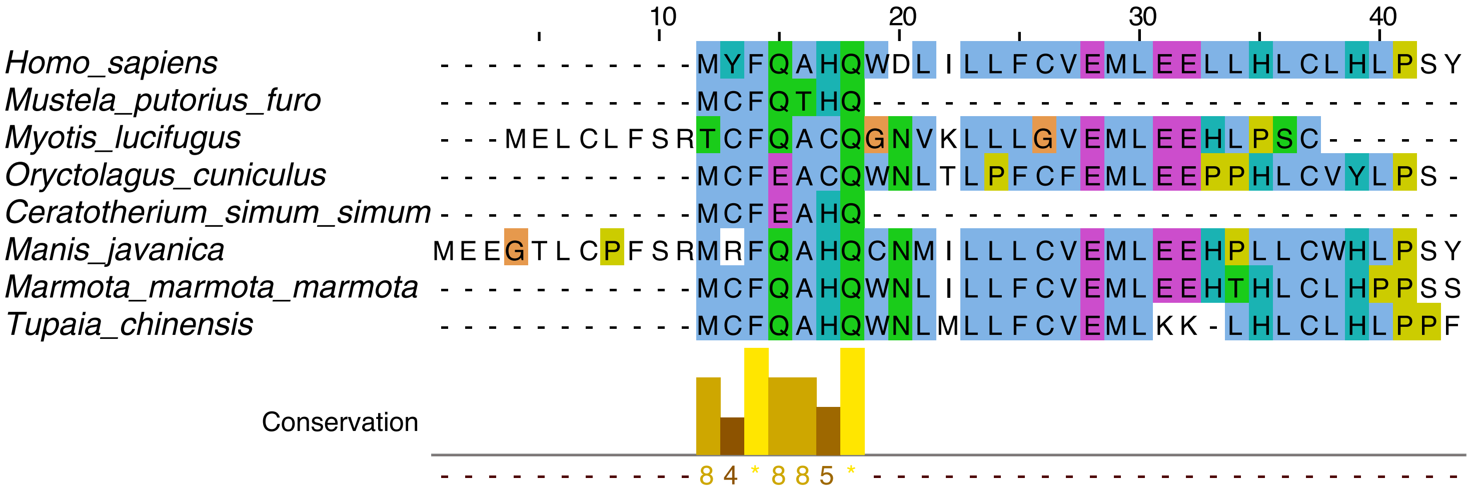

HG0726.1

ENSG00000115504 (EHBP1)

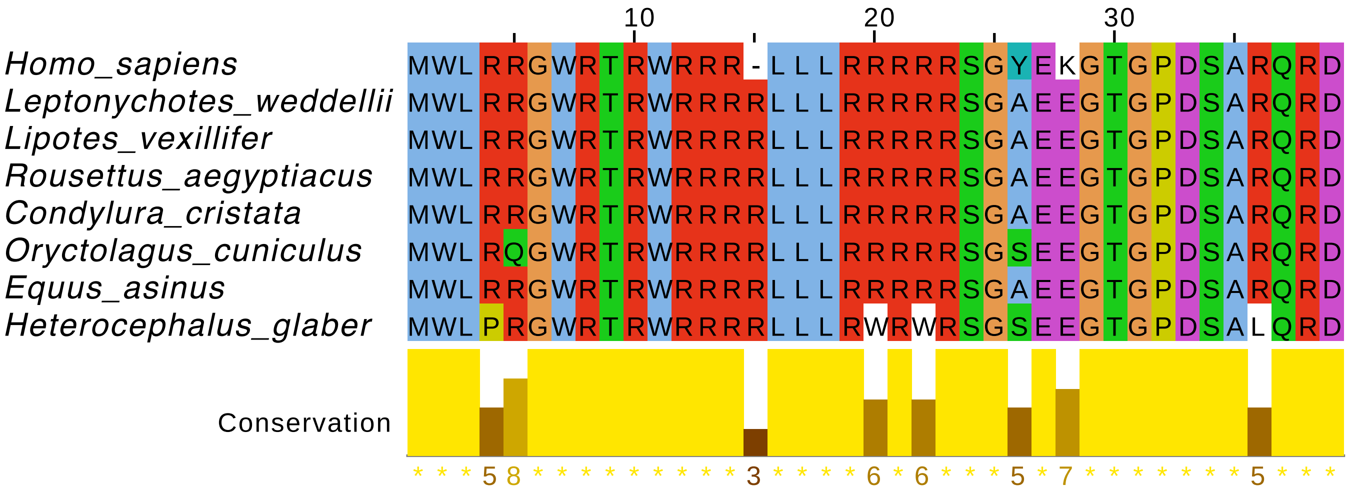

HG0726.2

ENSG00000115504 (EHBP1)

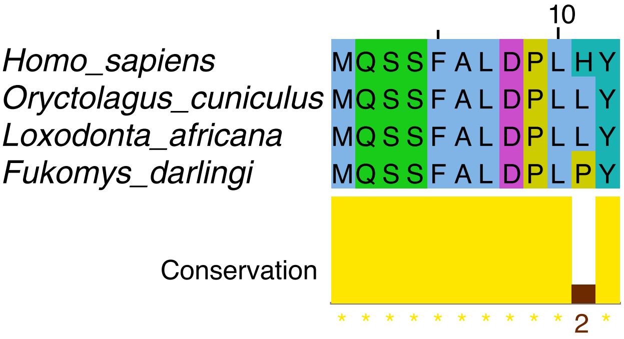

HG0727

ENSG00000115825 (PRKD3)

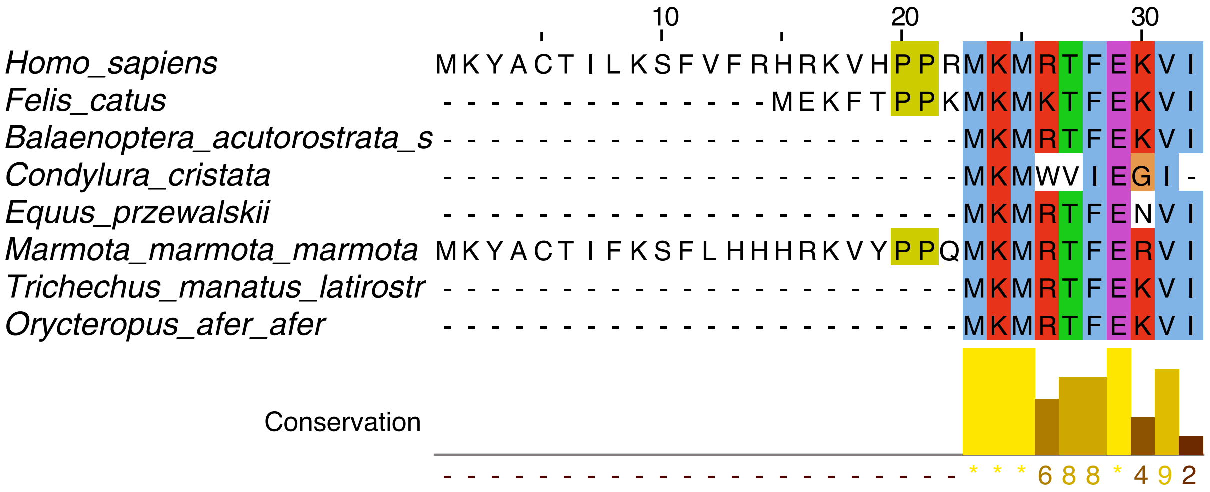

HG0728

ENSG00000116991 (SIPA1L2)

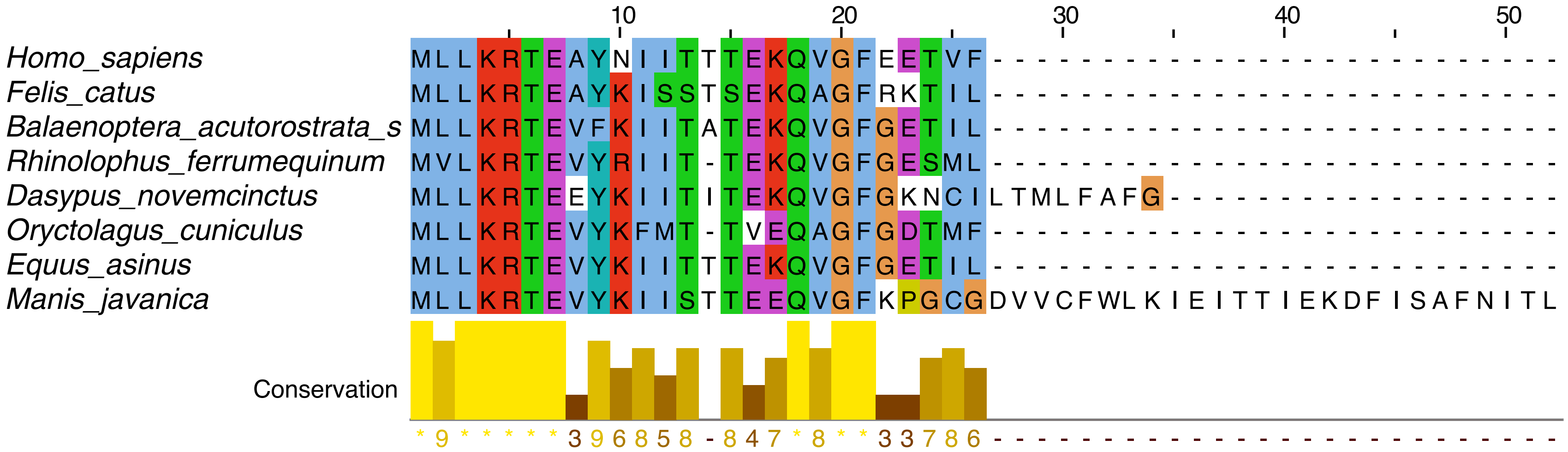

HG0729

ENSG00000119383 (PTPA)

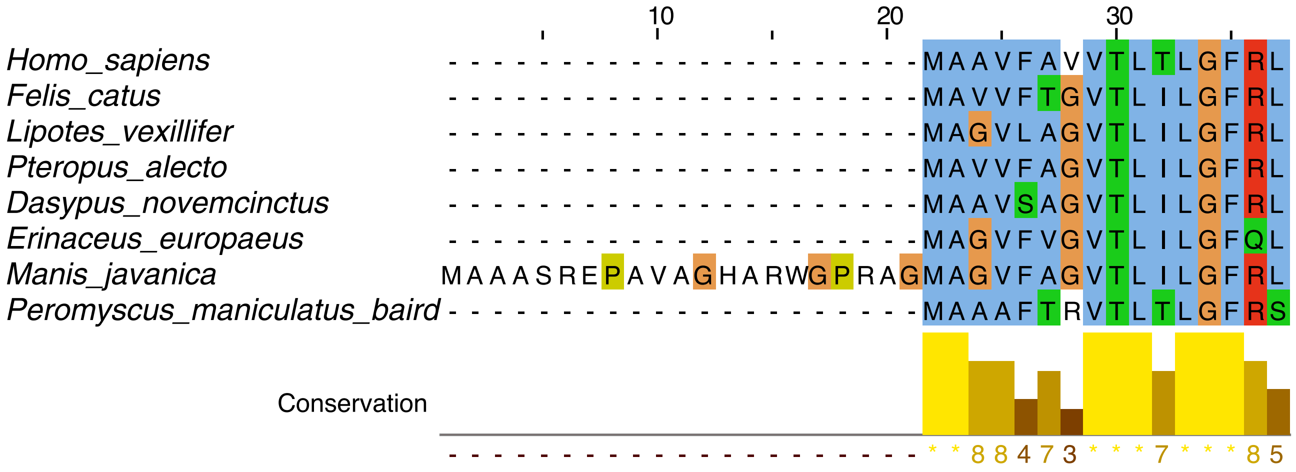

HG0730

ENSG00000125170 (DOK4)

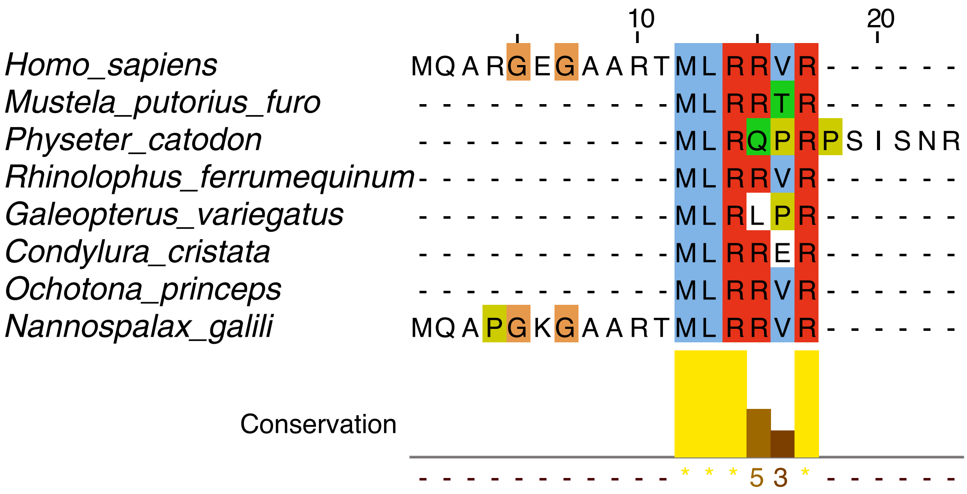

HG0731

ENSG00000129255 (MPDU1)

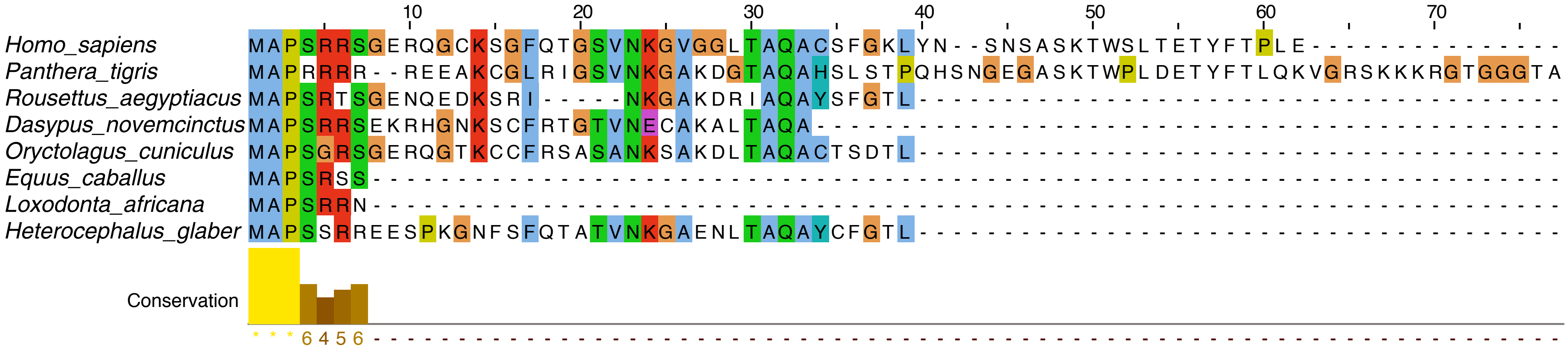

HG0732

ENSG00000135914 (HTR2B)

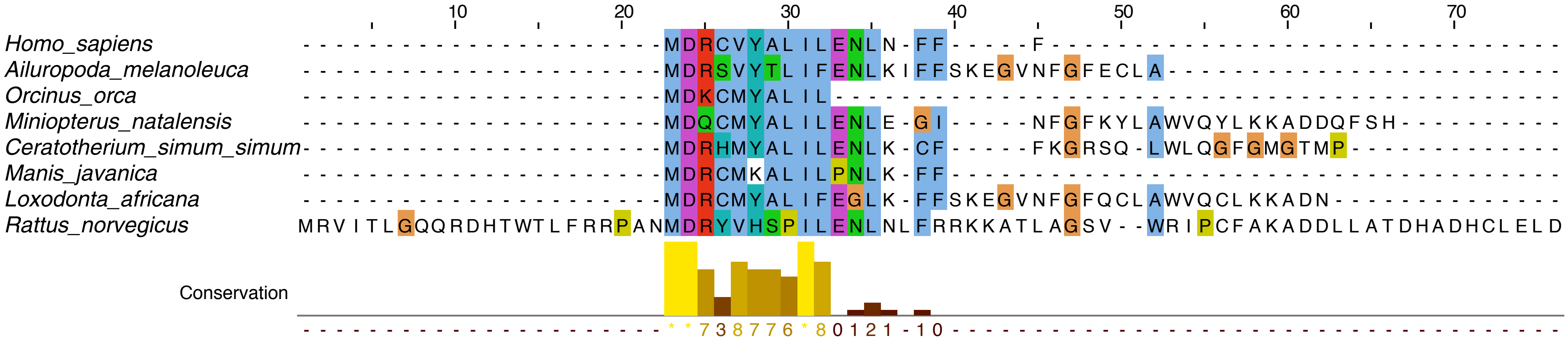

HG0733

ENSG00000136247 (ZDHHC4)

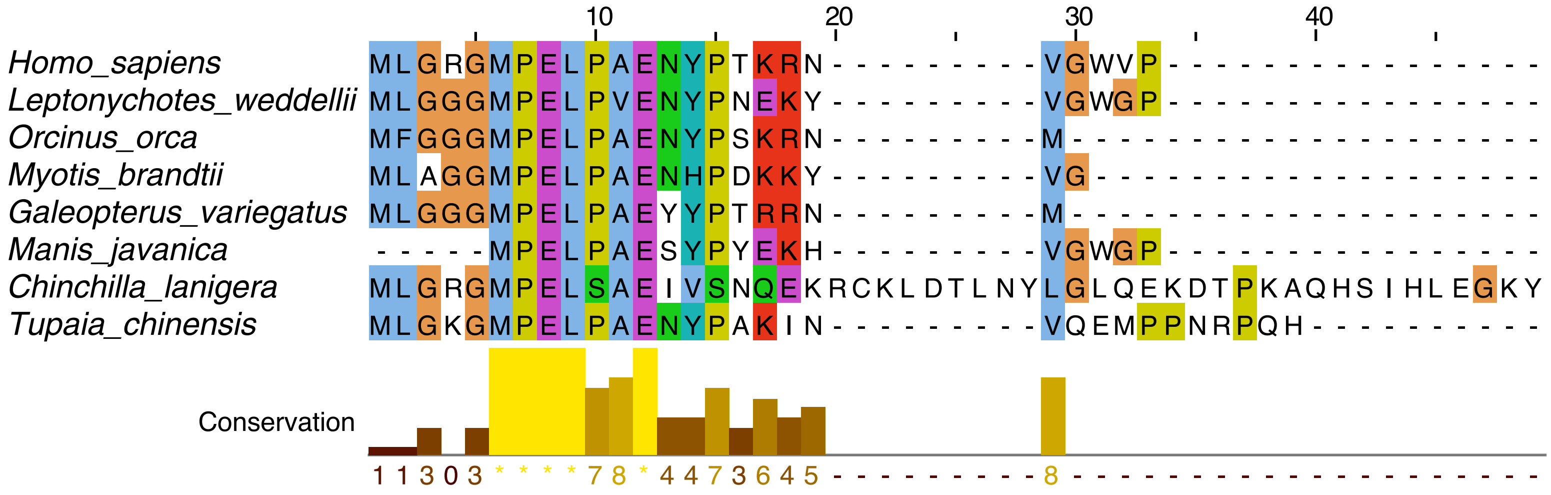

HG0734

ENSG00000139318 (DUSP6)

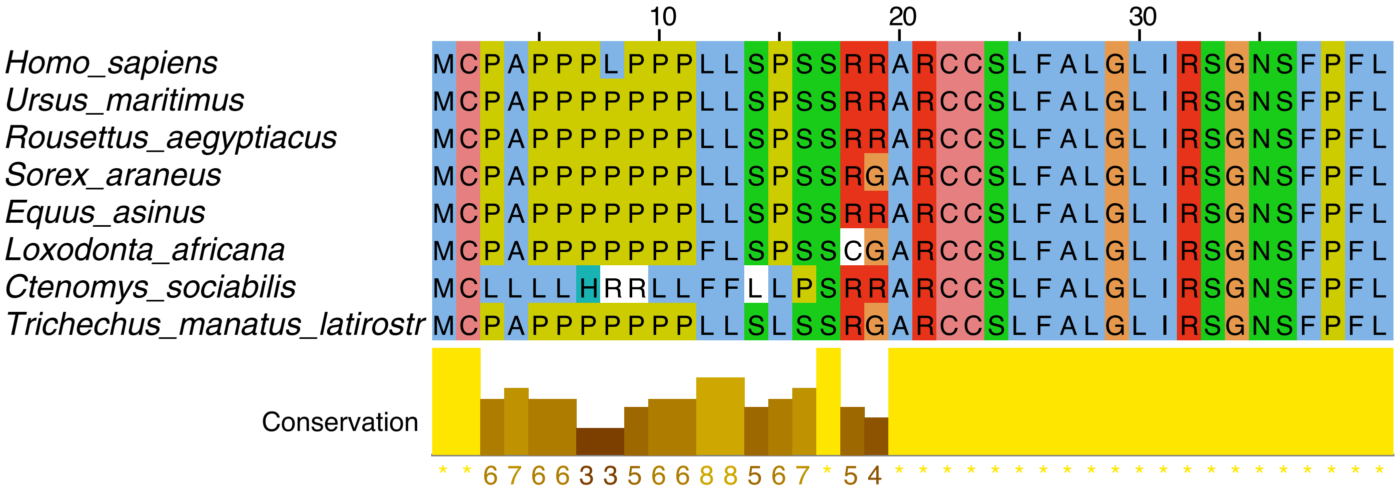

HG0735

ENSG00000139579 (NABP2)

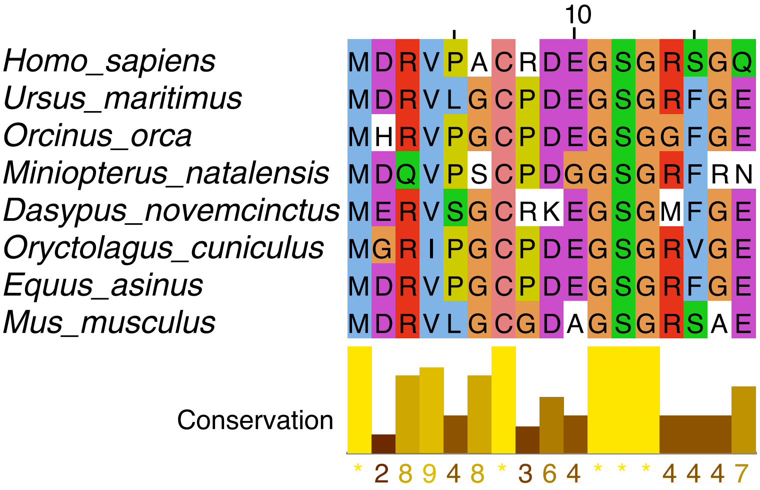

HG0736

ENSG00000139826 (ABHD13)

```
                                  10        20        30        40        50        60        70
Homo_sapiens              MWEPAGAFAGDTRAGAGSGATARSRSSGLDRGAGSGPWAGGG-------------------------------------
Felis_catus               MWEPAGTFAGDTERGPEVEEPR-----AAPQELRARPWRRRRPLGWRRTMRSDGSGAGGRCRAATGSVSAAHCAACEQV
Physeter_catodon          MWEPAGVFAGYTERGPEVGEPR-----AAPQELRATLWRRRRPLGWRRTMRSDGSGAGGRFCAAPGSESAADCAACEQV
Pteropus_alecto           MWEPAVAFAGDTEFGQEVEESR-----PAPQDLRARLWQQRWLFCWKRTMRSDGSGTAGRCCAALGCVFATDCAACEQI
Dasypus_novemcinctus      MWDPAGVFAGDTRA-----------------------------------------------------------------
Galeopterus_variegatus    MCEPAGAFAGDTQAGAGSGAADG-RAAGAPG------------------------------------------------
Equus_asinus              MWESAGVFAGDTKRGPEVEEQT-----AAPQELRVRPWRRRRPLGWRRTMRNDGSGAGGSCCAAPGSVSAADCAACEQV
Marmota_marmota_marmota   MWEPAGTFAGDTPALAGSRAAKS-RAAGSPG------------------------------------------------
Conservation              *797*87***5*3310010000-----1010------------------------------------------------
```

HG0737

ENSG00000141639 (MAPK4)

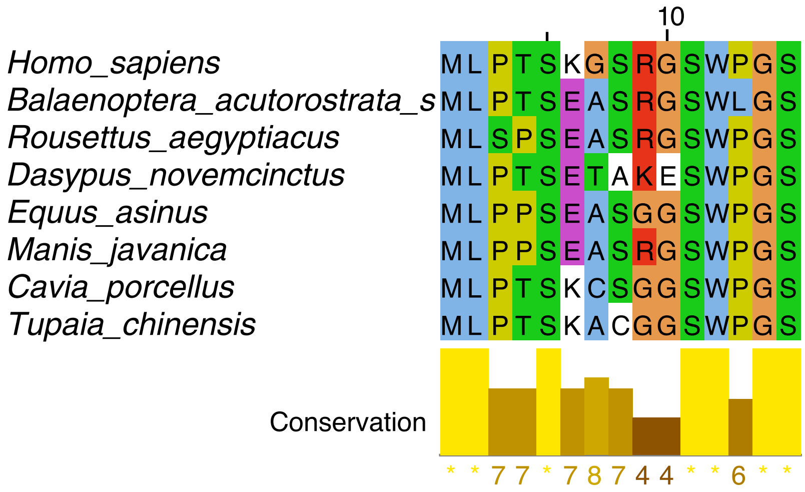

HG0738

ENSG00000143393 (PI4KB)

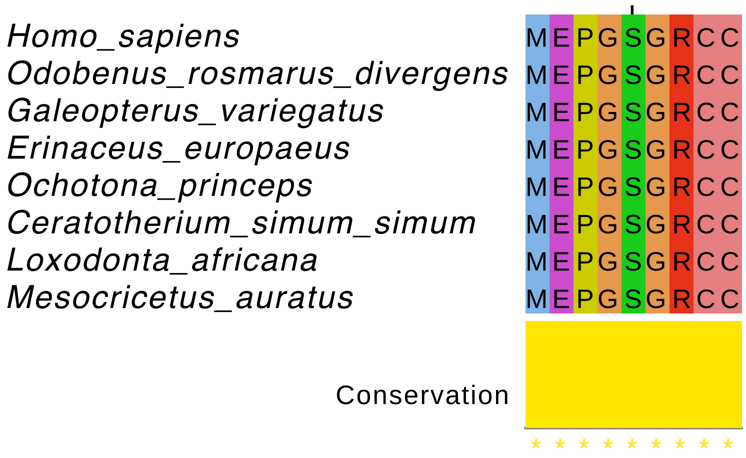

HG0739

ENSG00000144331 (ZNF385B)

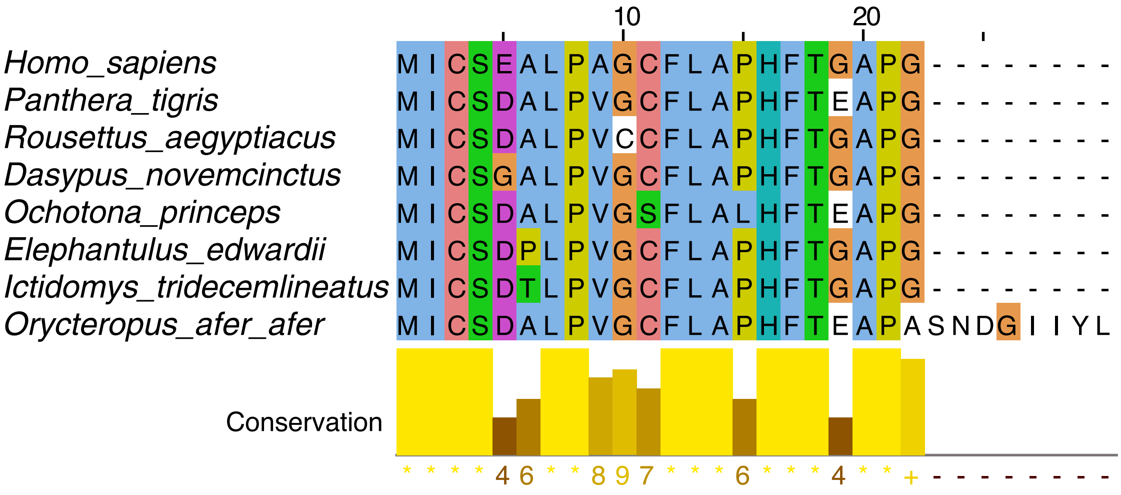

HG0740

ENSG00000144331 (ZNF385B)

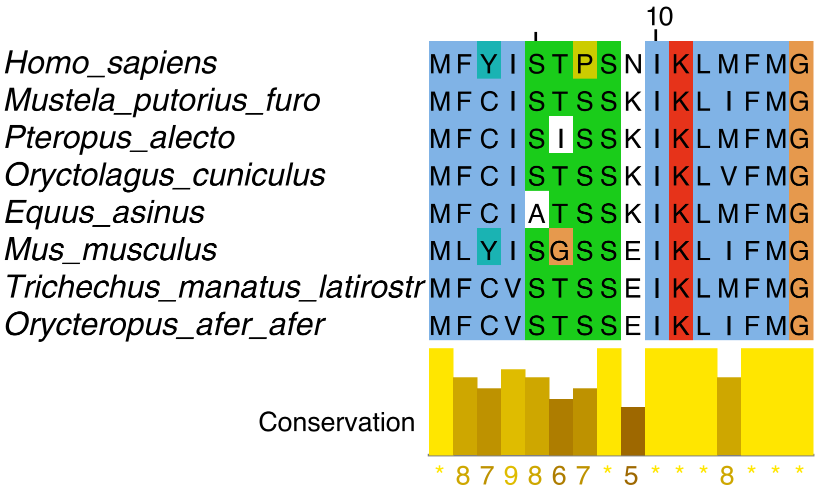

HG0741

ENSG00000144834 (TAGLN3)

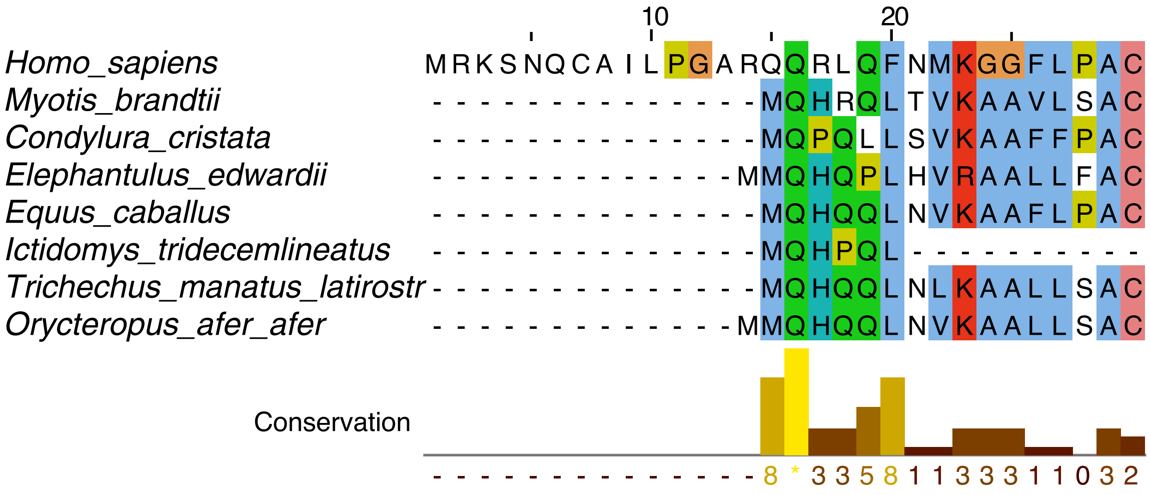

HG0742

ENSG00000154556 (SORBS2)

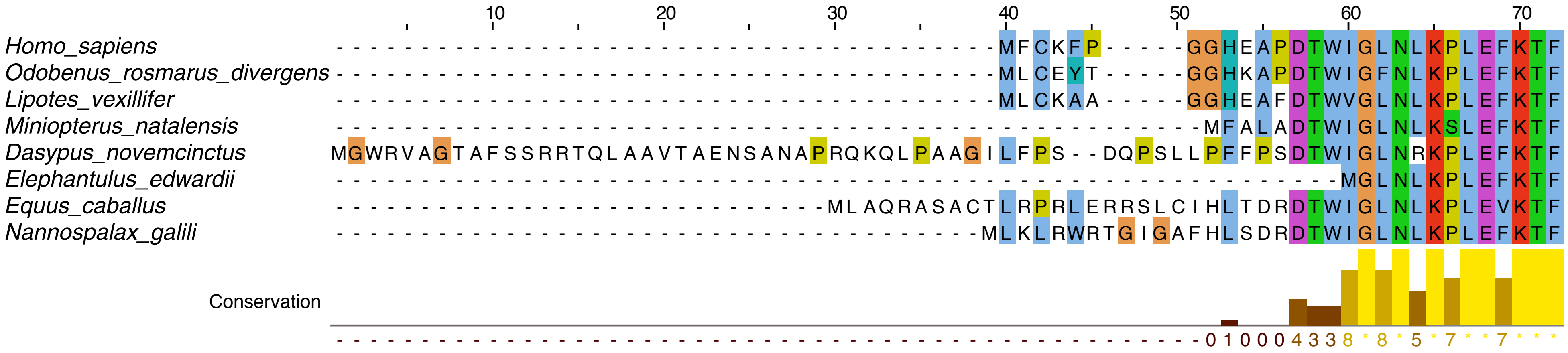

HG0743

ENSG00000154814 (OXNAD1)

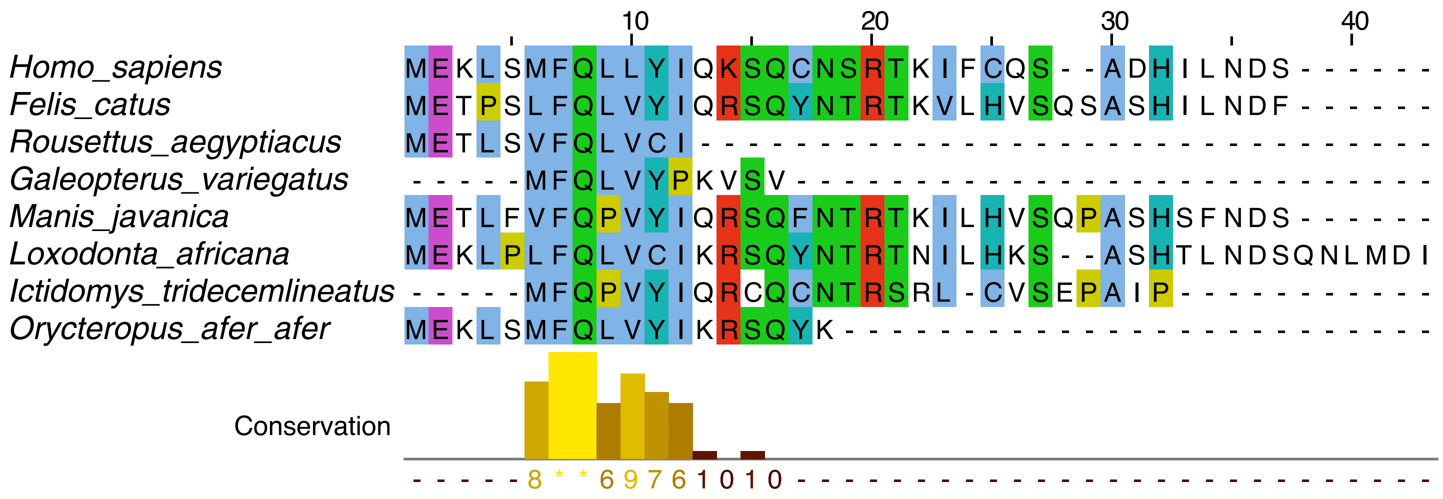

HG0744

ENSG00000163053 (SLC16A14)

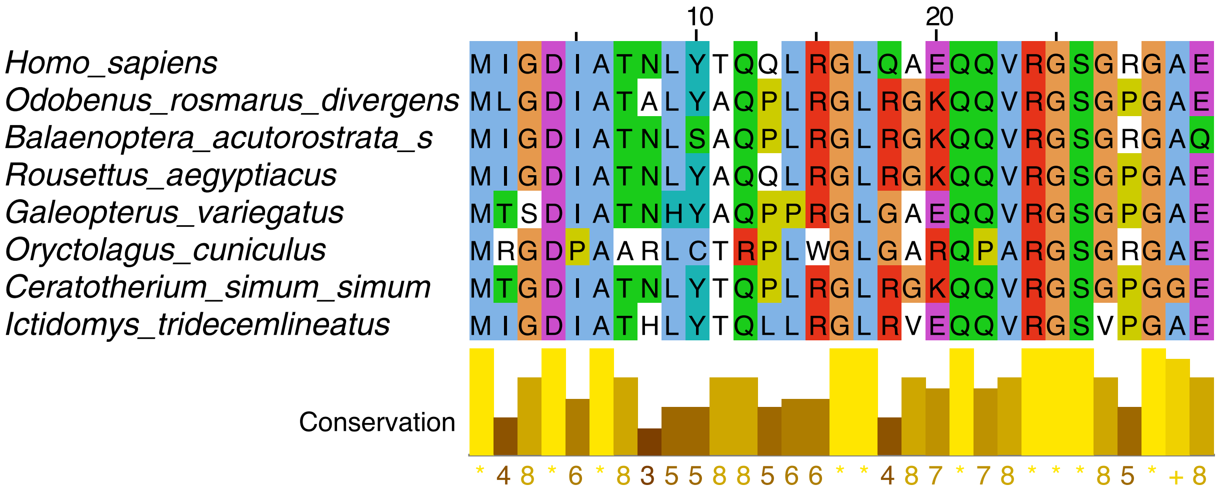

HG0745

ENSG00000163145 (C1QTNF7)

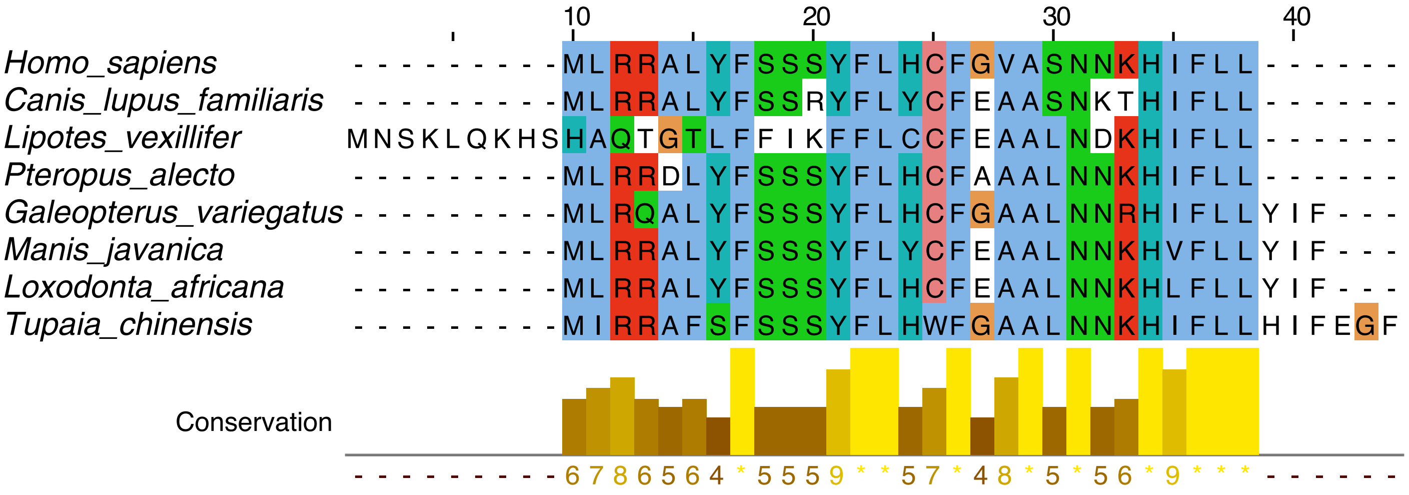

HG0746

ENSG00000164741 (DLC1)

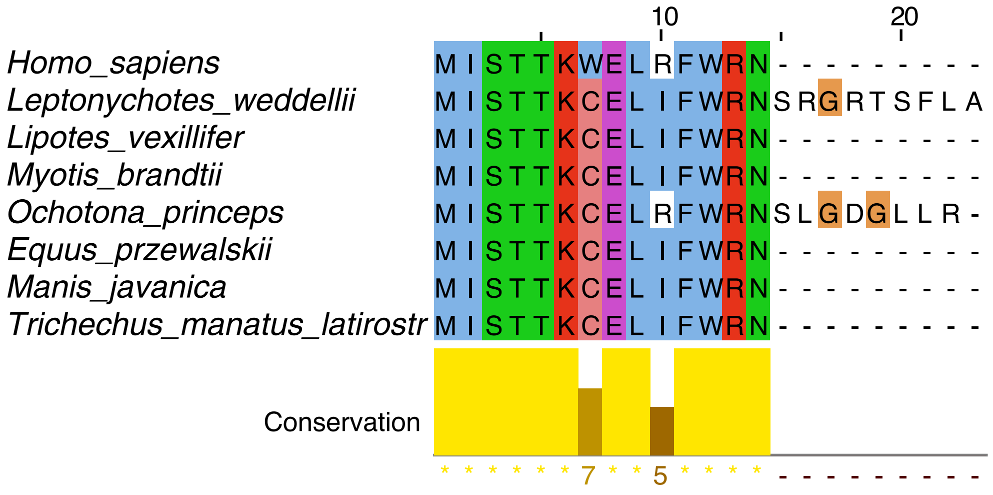

HG0747

ENSG00000168036 (CTNNB1)

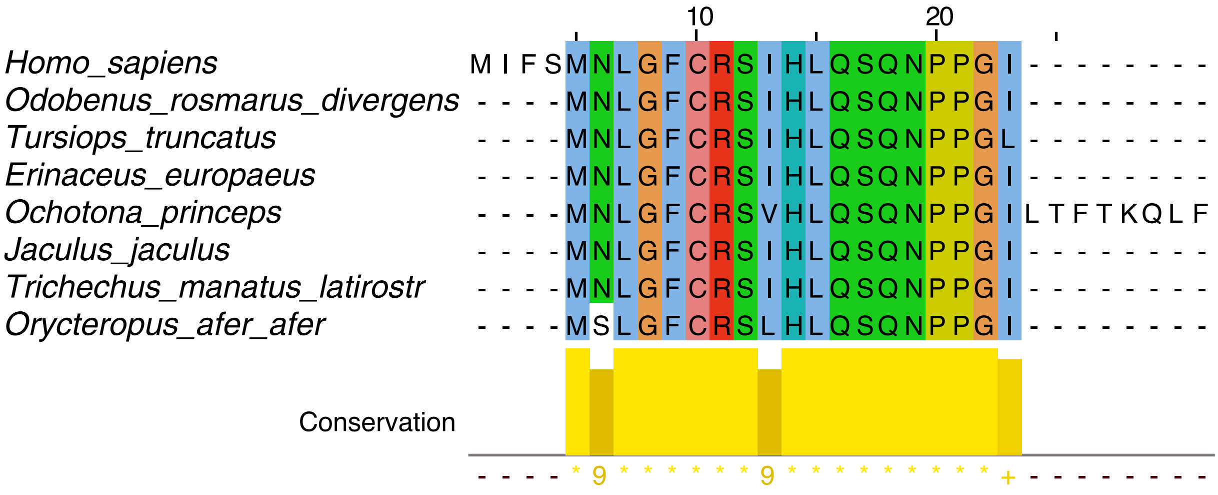

HG0748

ENSG00000168830 (HTR1E)

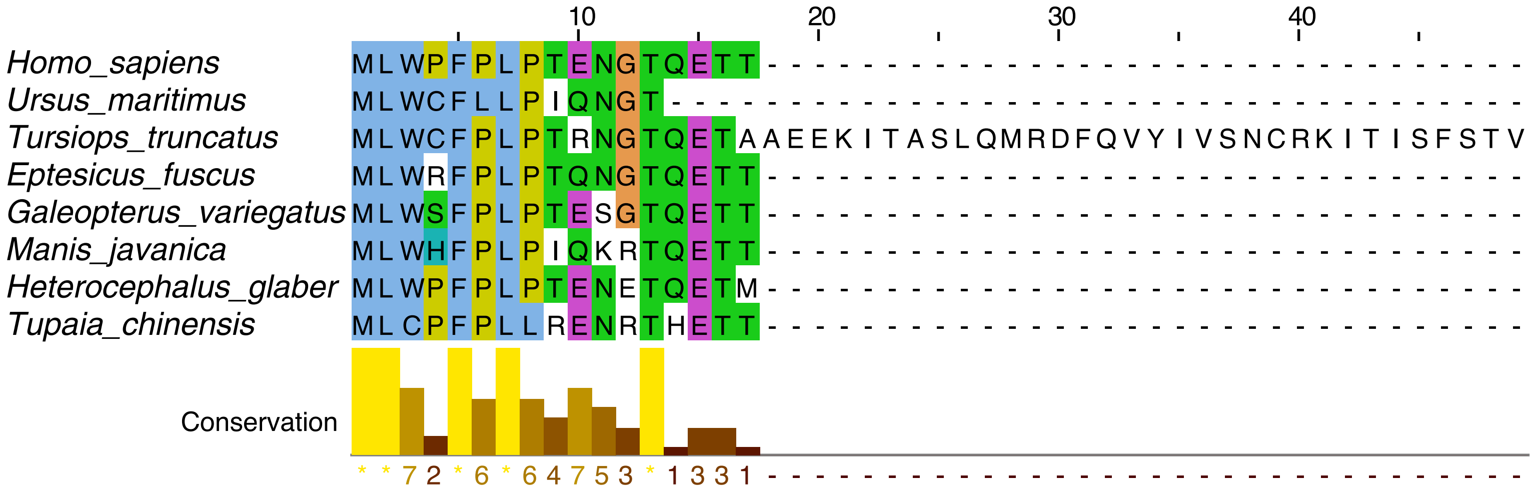

HG0749

ENSG00000169760 (NLGN1)

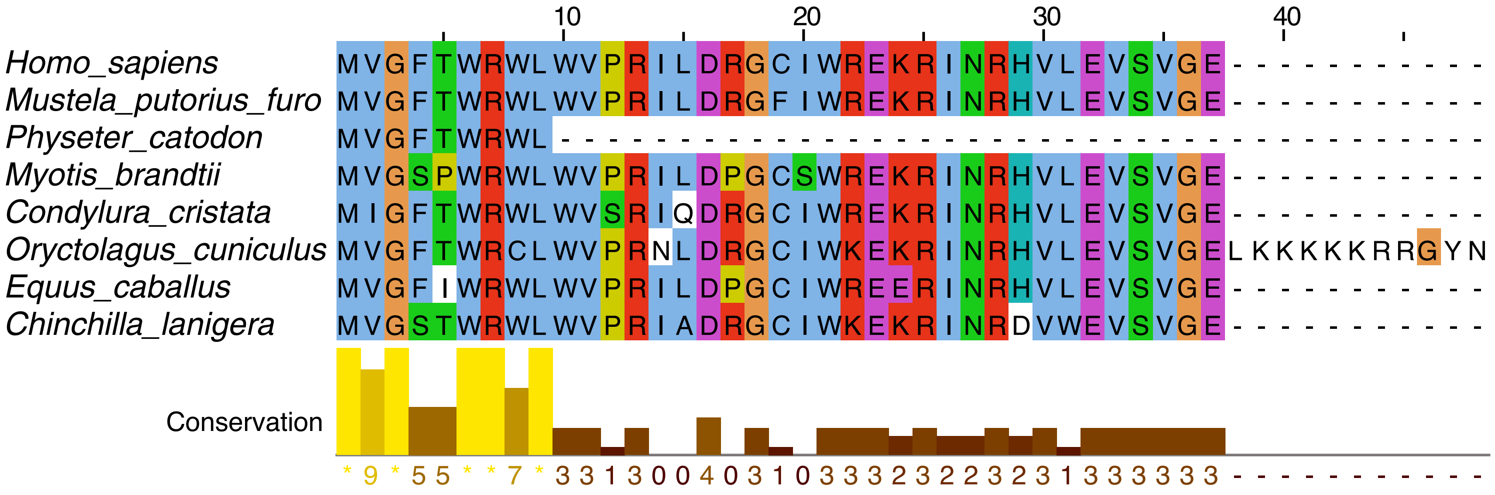

HG0750

ENSG00000170881 (RNF139)

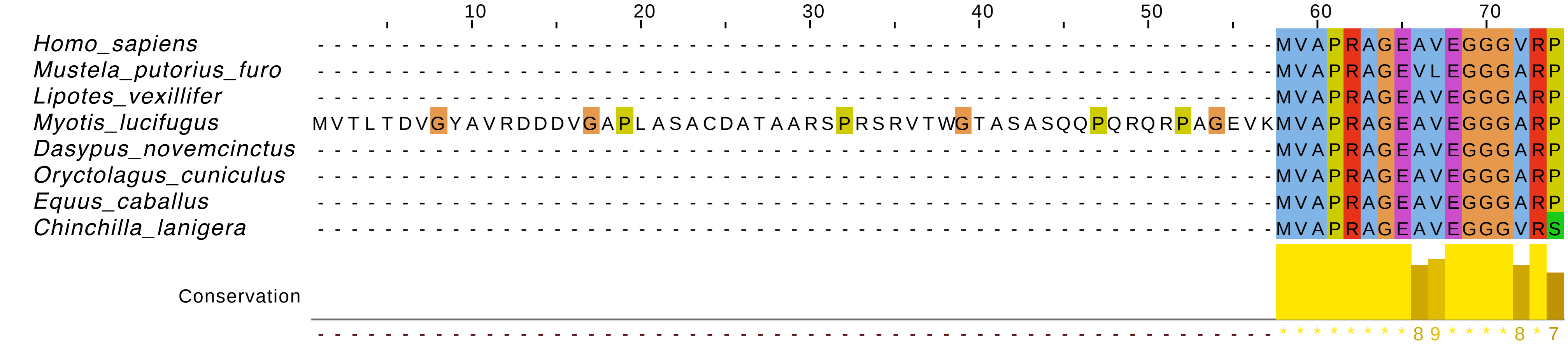

HG0751

ENSG00000171791 (BCL2)

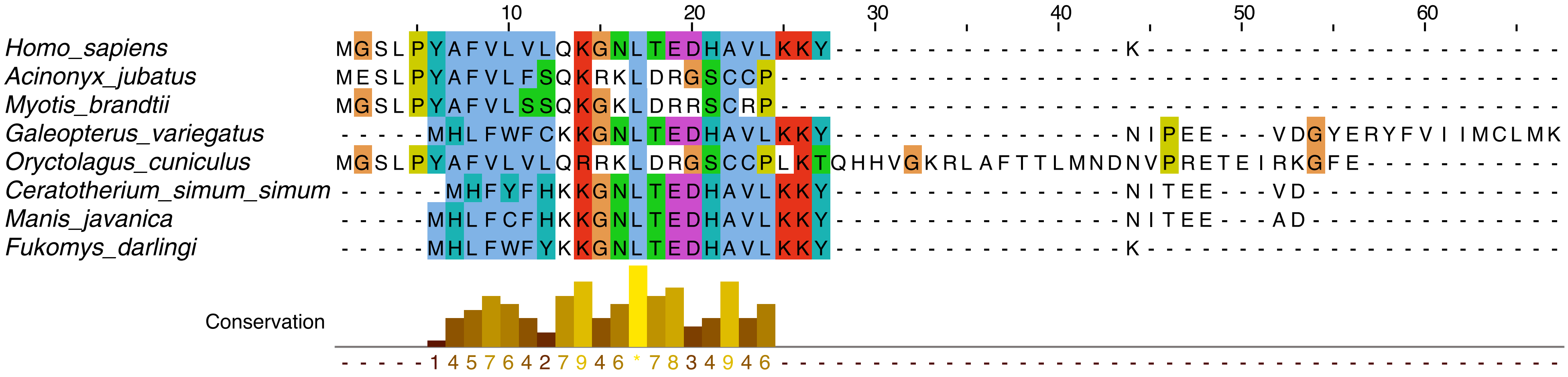

HG0752

ENSG00000172269 (DPAGT1)

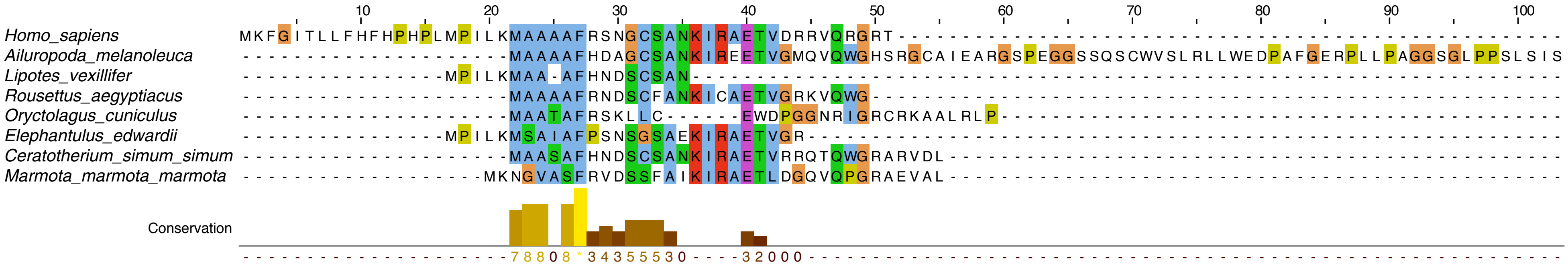

HG0753

ENSG00000173406 (DAB1)

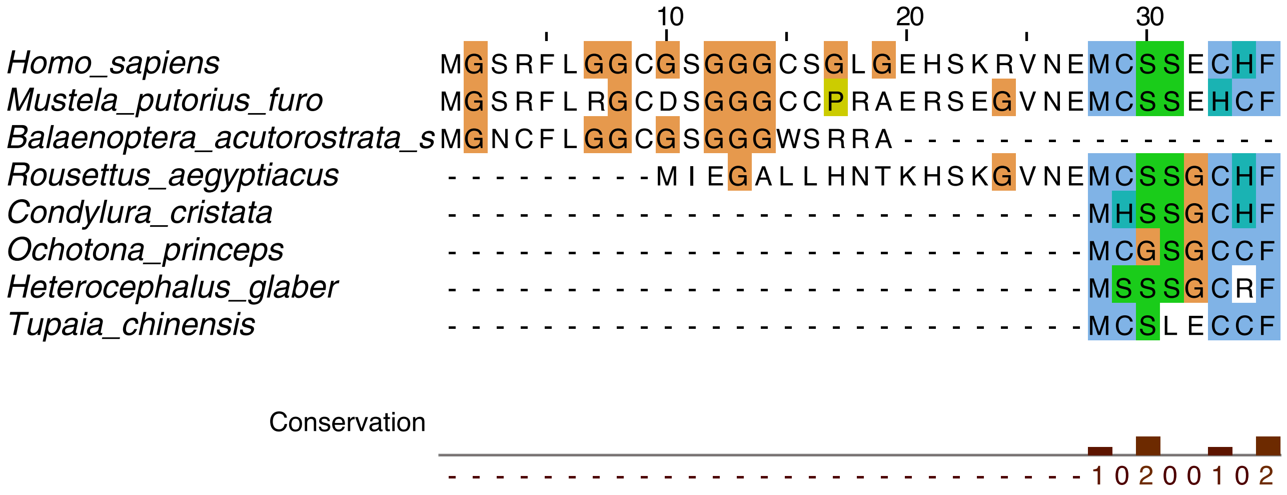

HG0754

ENSG00000175182 (FAM131A)

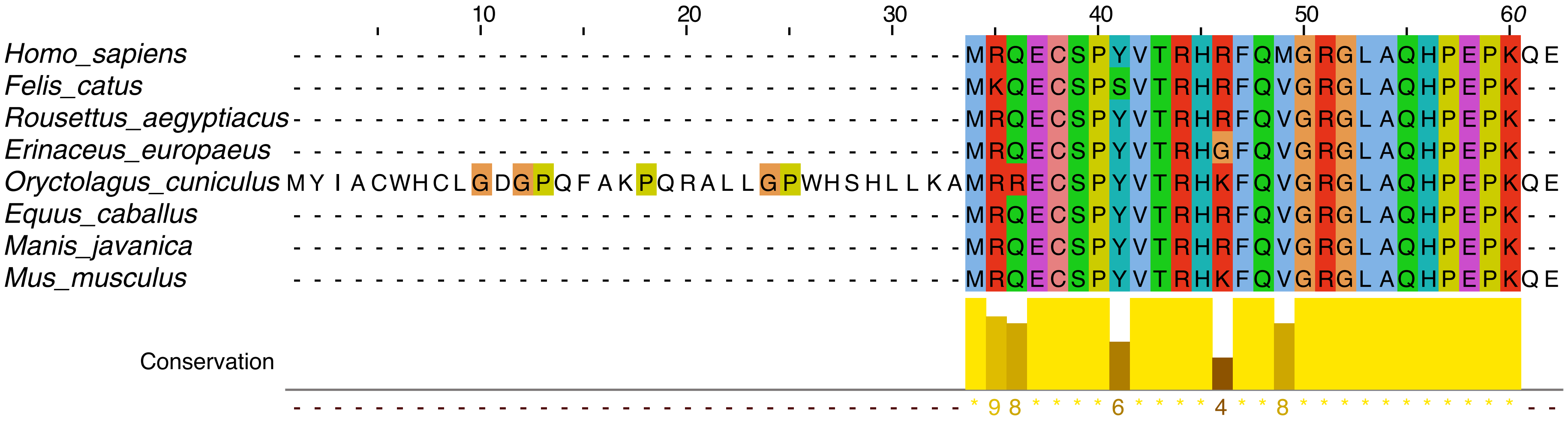

HG0755

ENSG00000176597 (B3GNT5)

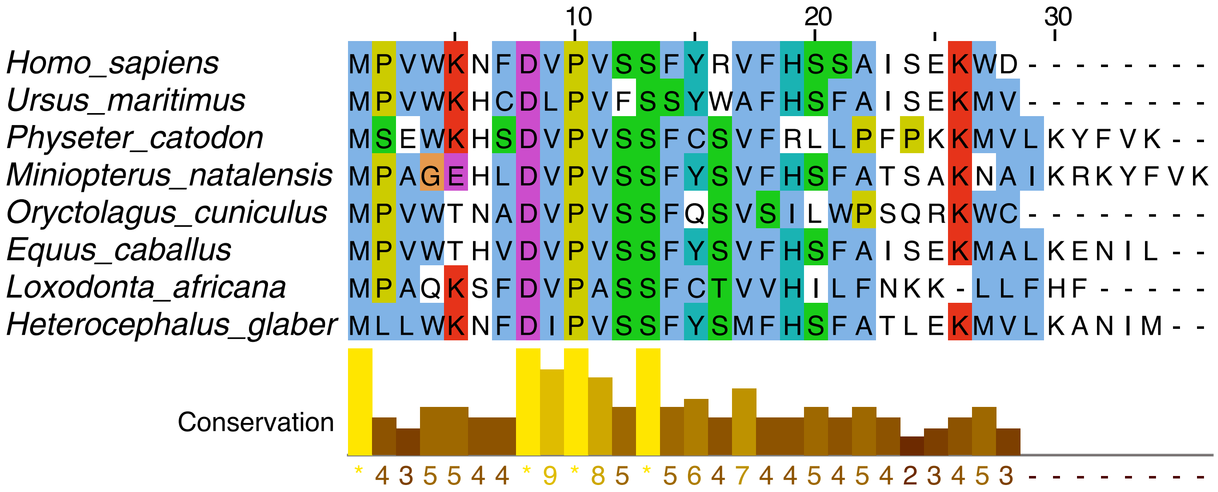

HG0756

ENSG00000177051 (FBXO46)

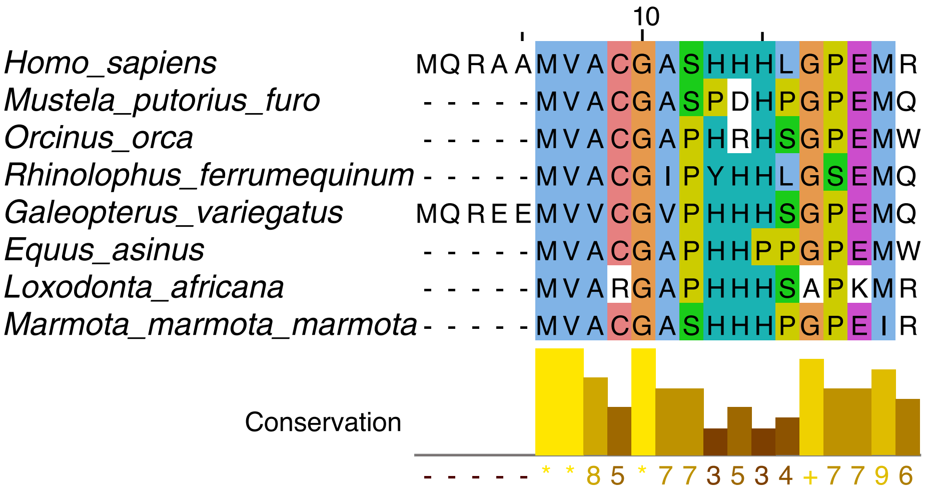

HG0757.1

ENSG00000177301 (KCNA2)

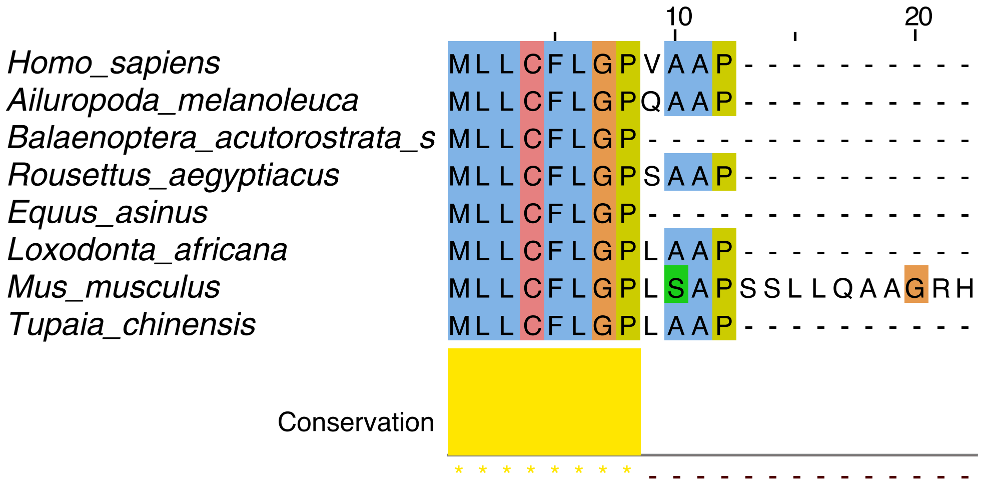

HG0757.2

ENSG00000182255 (KCNA4)

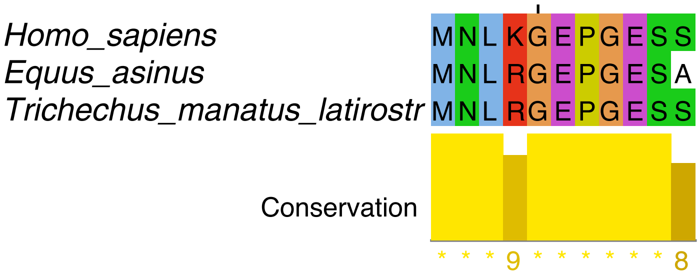

HG0758

ENSG00000179299 (NSUN7)

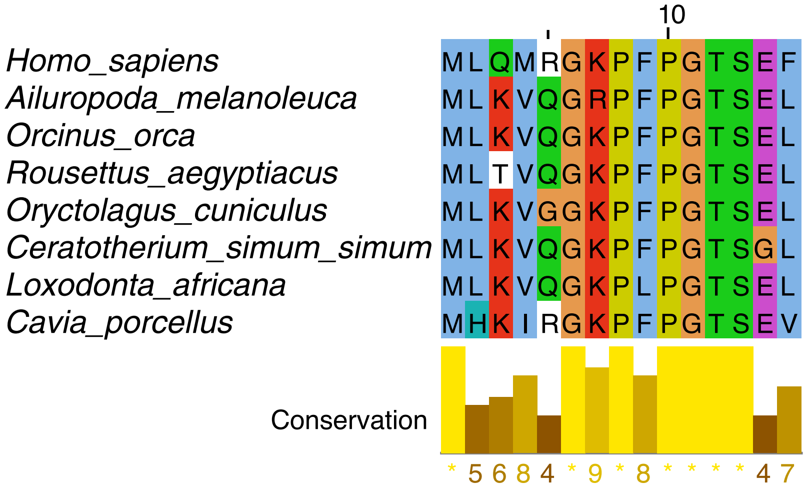

HG0759

ENSG00000180929 (GPR62)

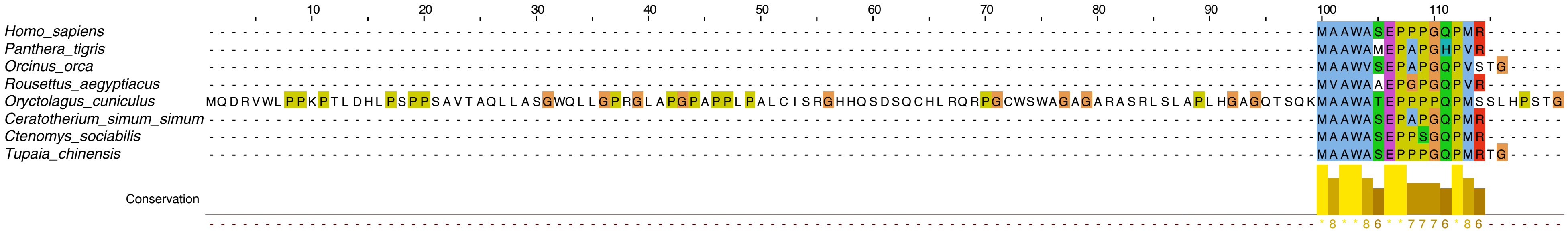

HG0760

ENSG00000182463 (TSHZ2)

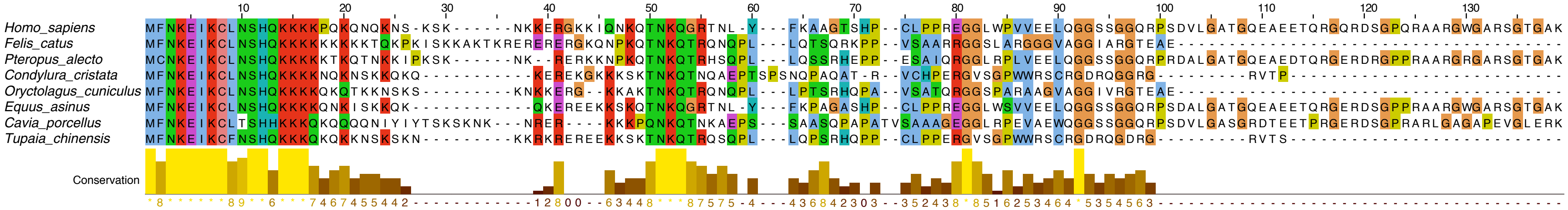

HG0761

ENSG00000185985 (SLITRK2)

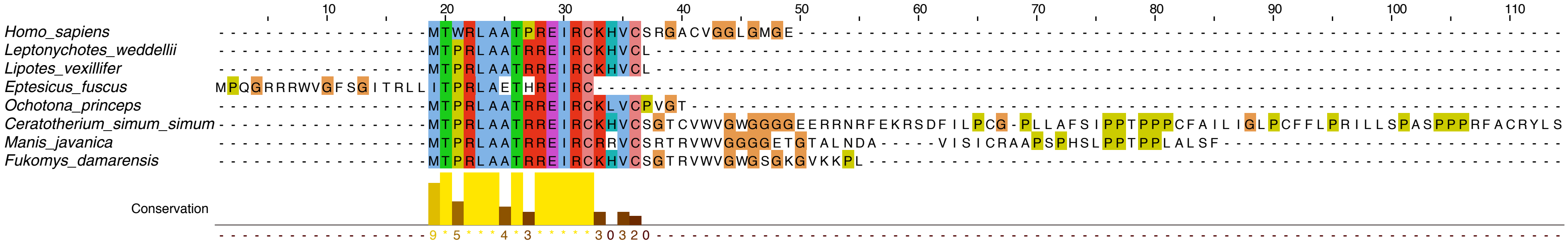

HG0762

ENSG00000187147 (RNF220)

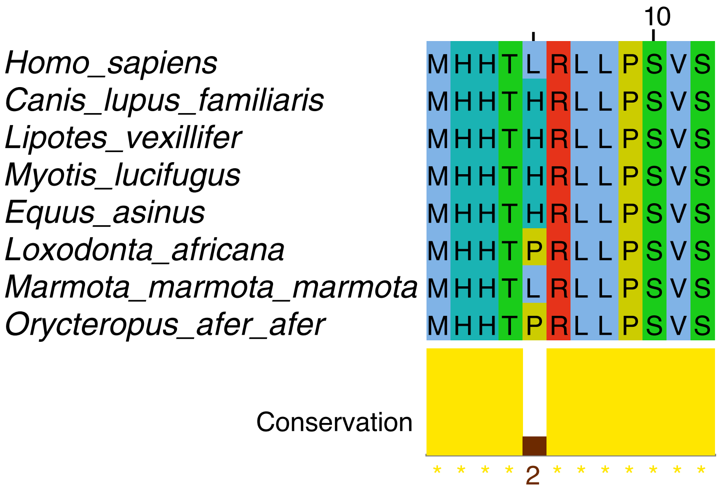

HG0763

ENSG00000188050 (RNF133)

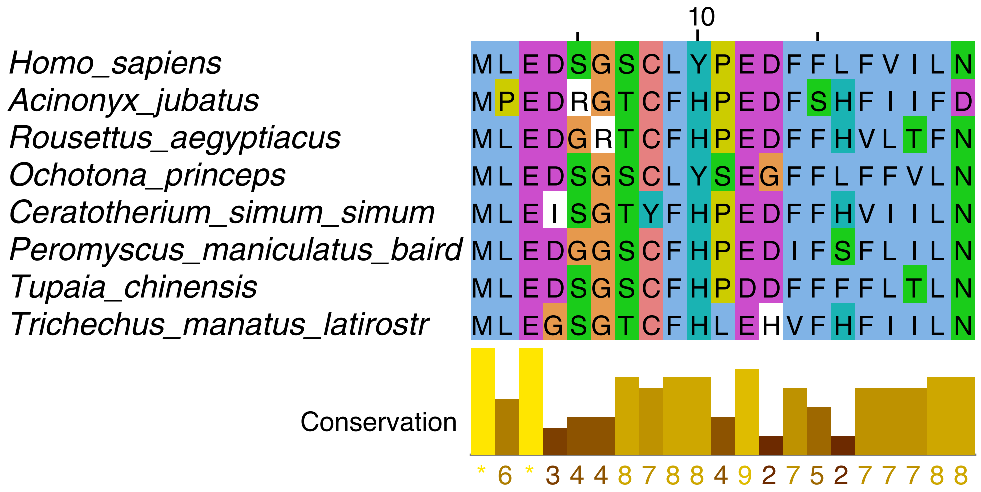

HG0764

ENSG00000188725 (SMIM15)

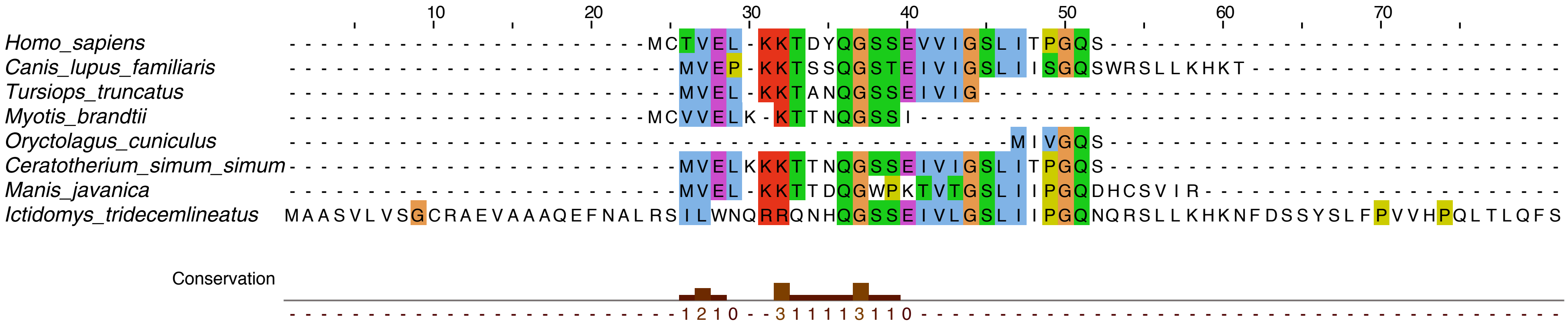

HG0765

ENSG00000196123

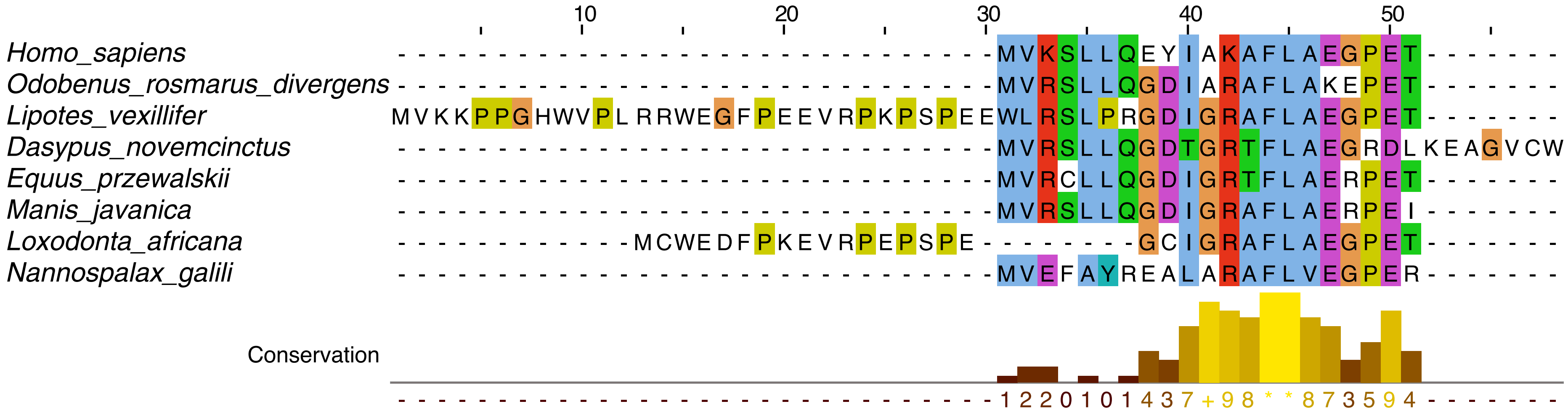

HG0766

ENSG00000196189 (SEMA4A)

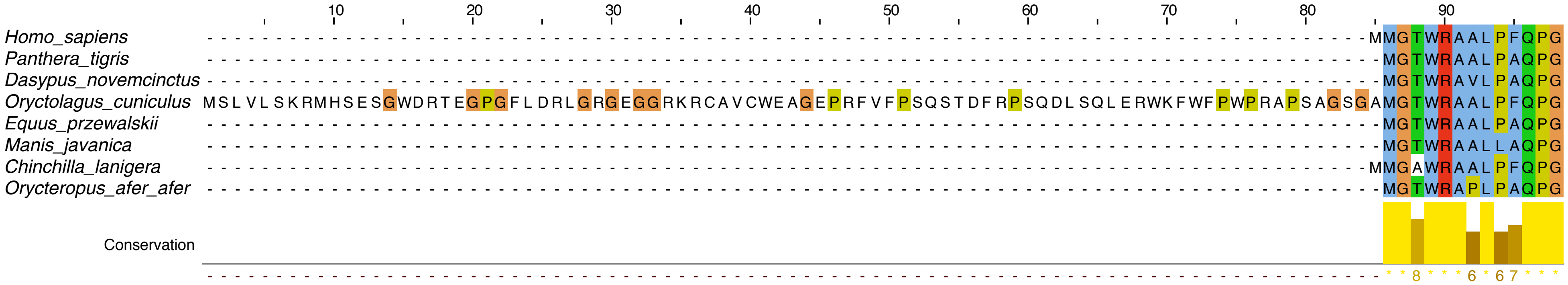

HG0767.1

ENSG00000197555 (SIPA1L1)

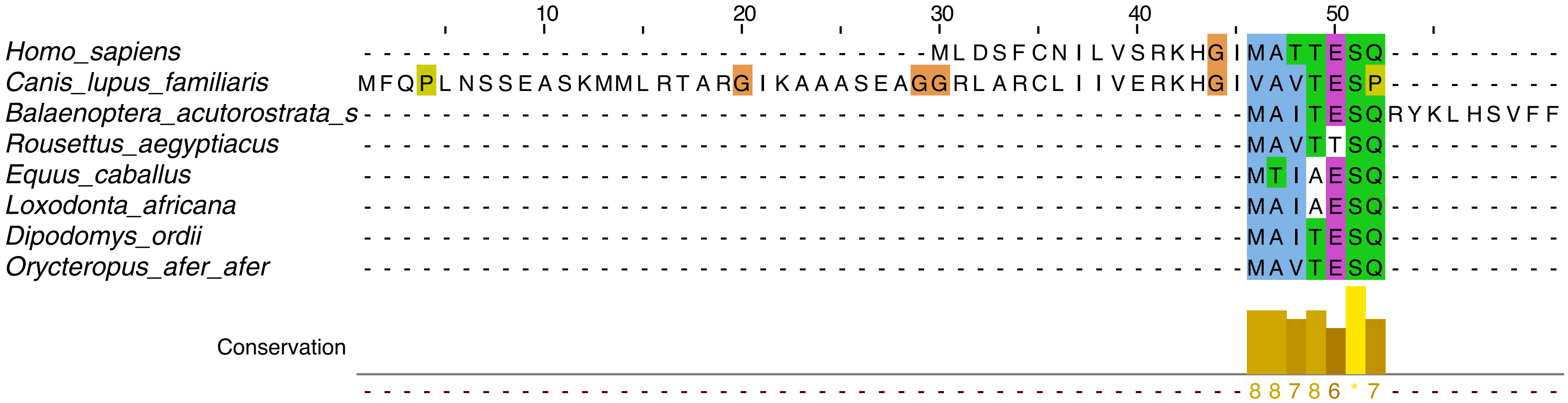

HG0767.2

ENSG00000197555 (SIPA1L1)

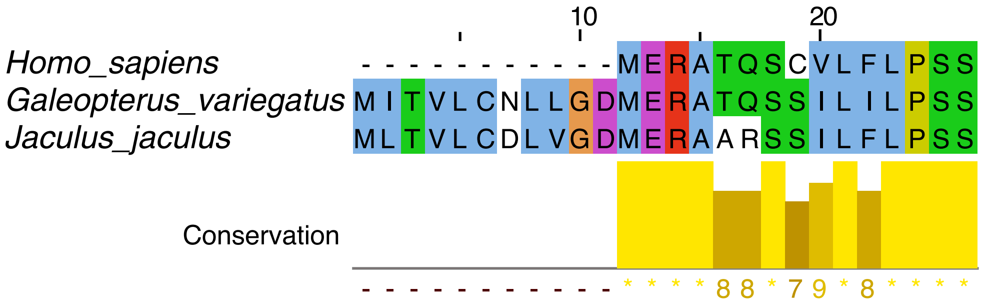

HG0768

ENSG00000213023 (SYT3)

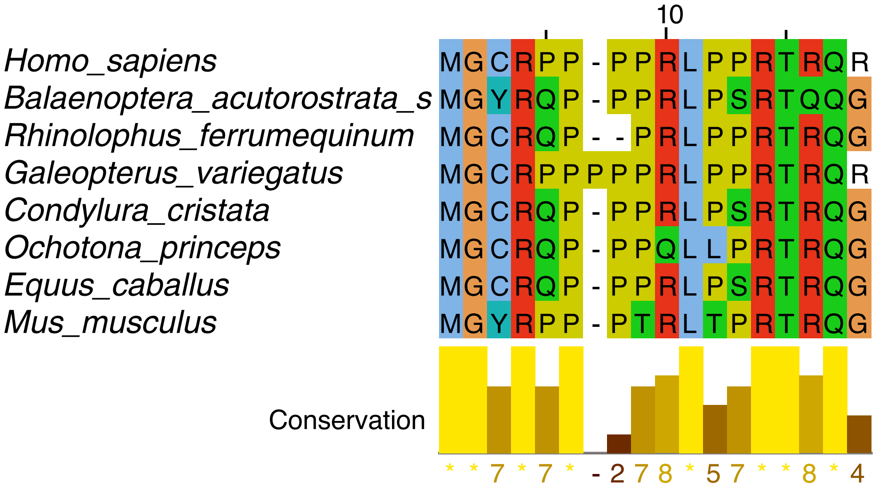

HG0769.1

ENSG00000280165 (PCDH20)

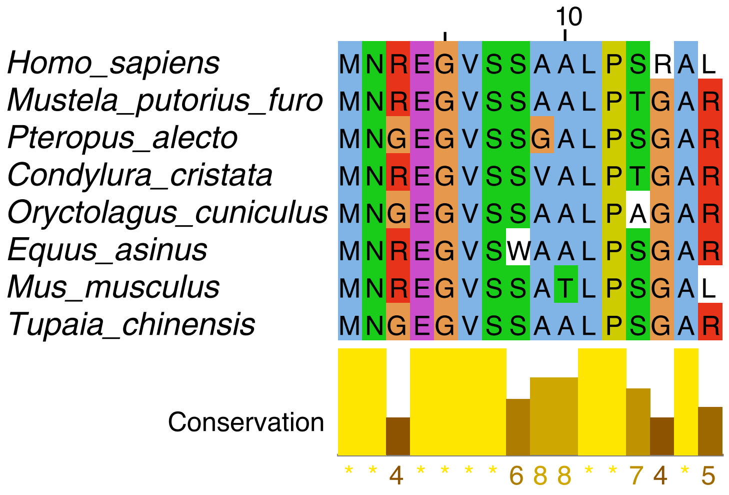

HG0769.2

ENSG00000197991

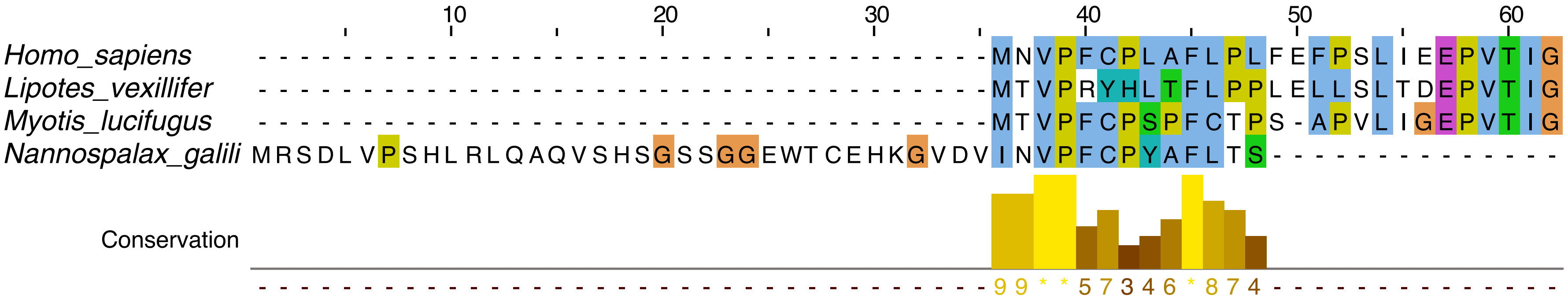

HG0770

ENSG00000010165 (METTL13)

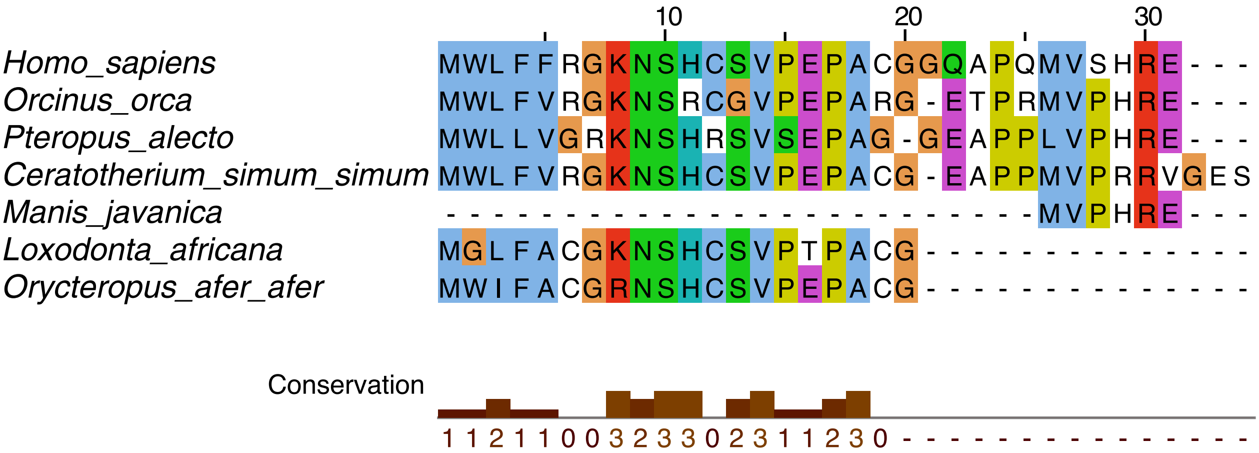

HG0771

ENSG00000013306 (SLC25A39)

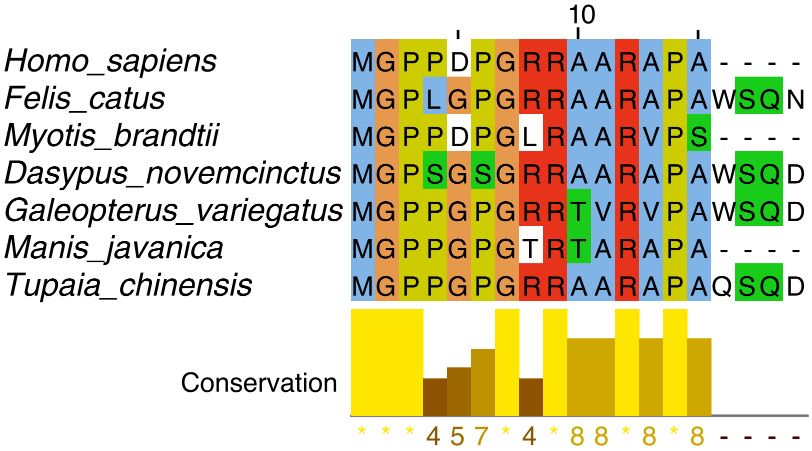

HG0772

ENSG00000064309 (CDON)

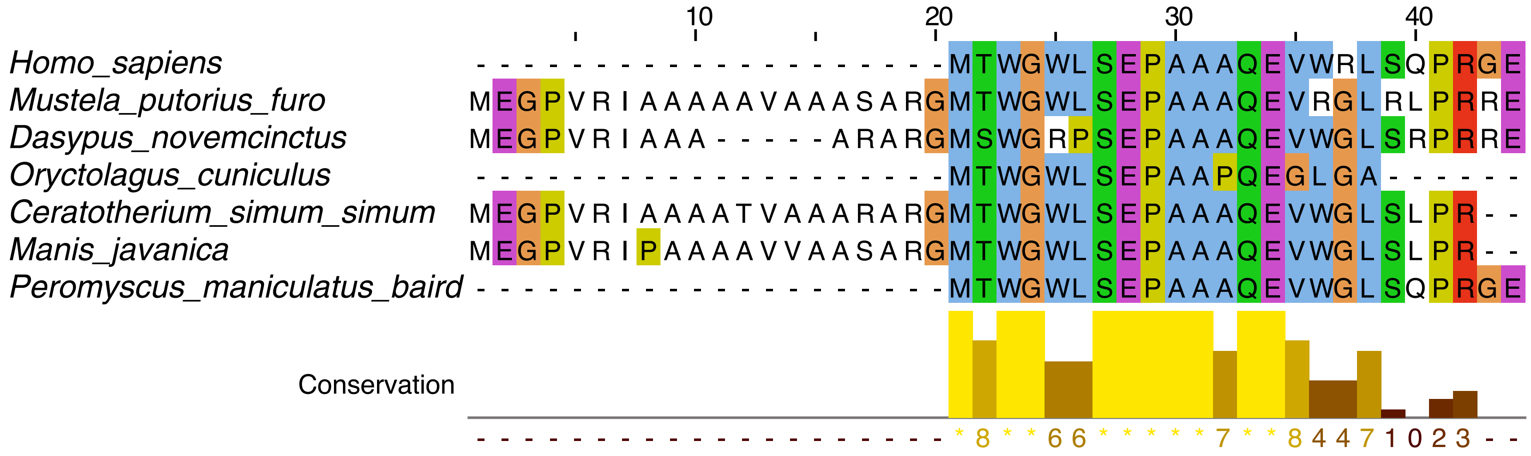

HG0773

ENSG00000065548 (ZC3H15)

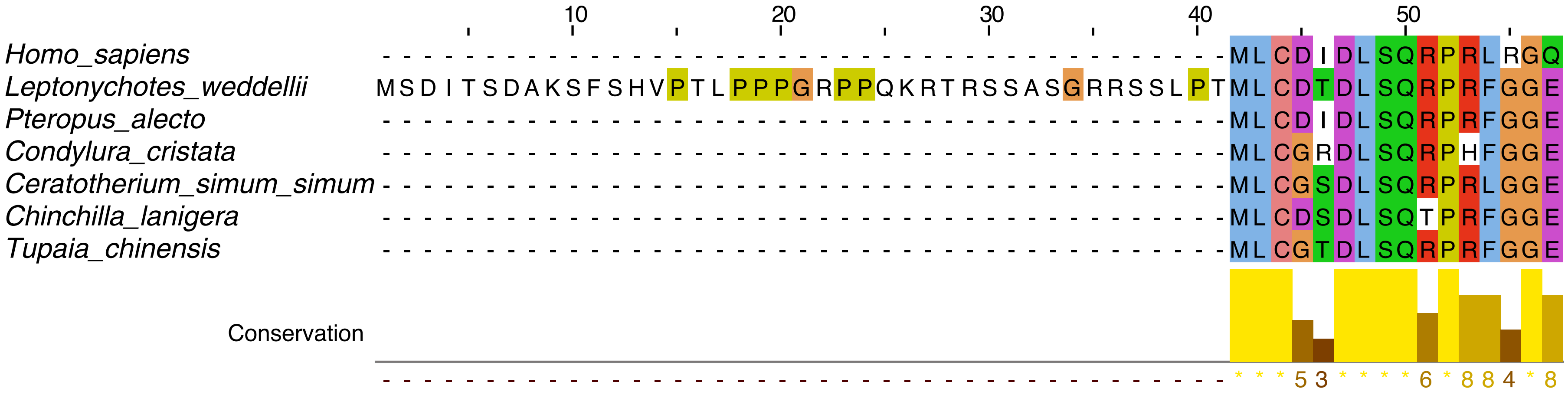

HG0774

ENSG00000066933 (MYO9A)

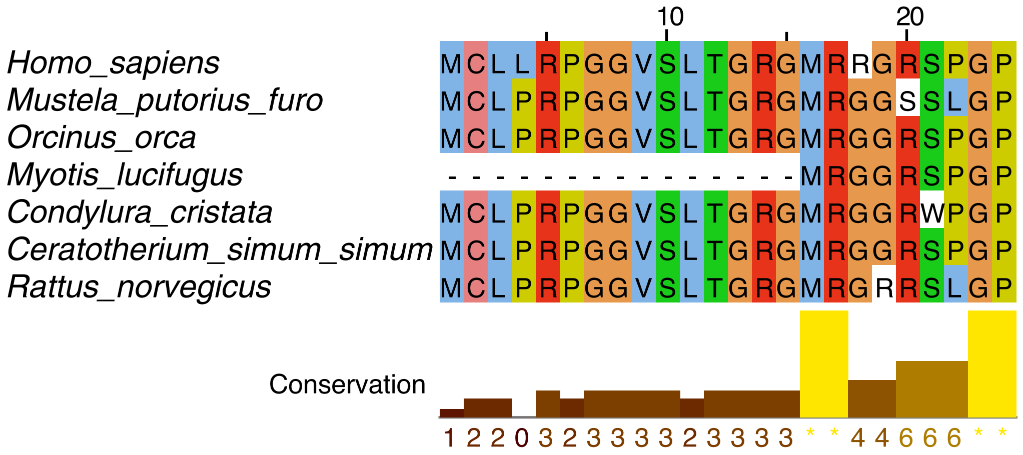

HG0775

ENSG00000068001 (HYAL2)

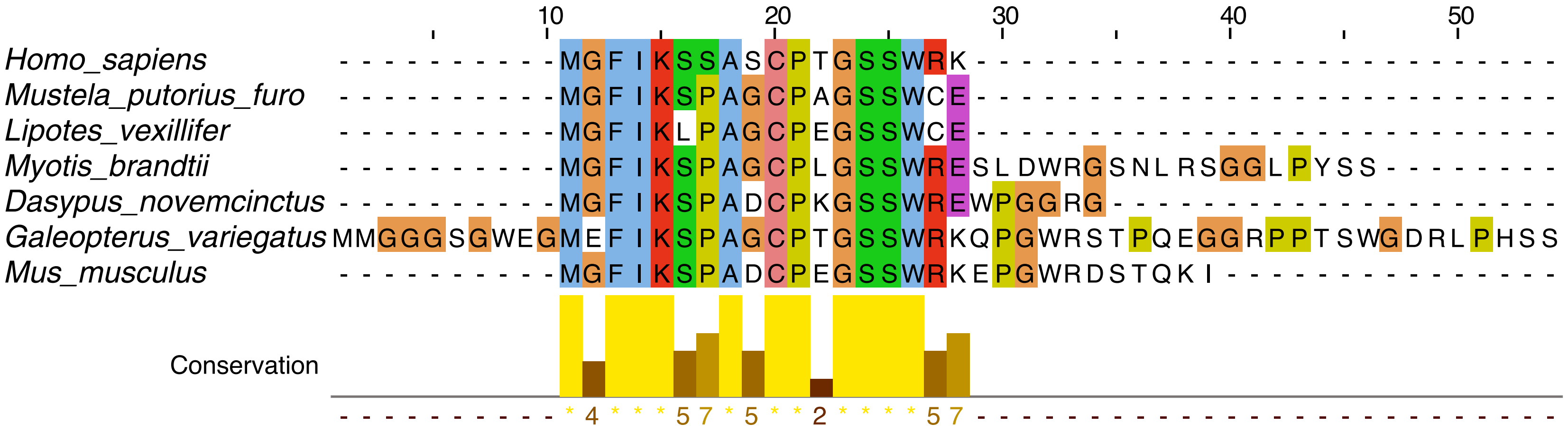

HG0776

ENSG00000082014 (SMARCD3)

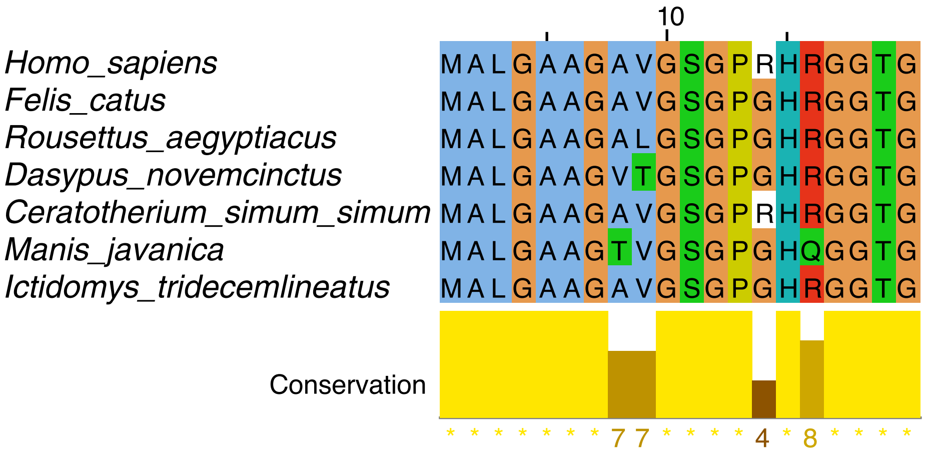

HG0777

ENSG00000085644 (ZNF213)

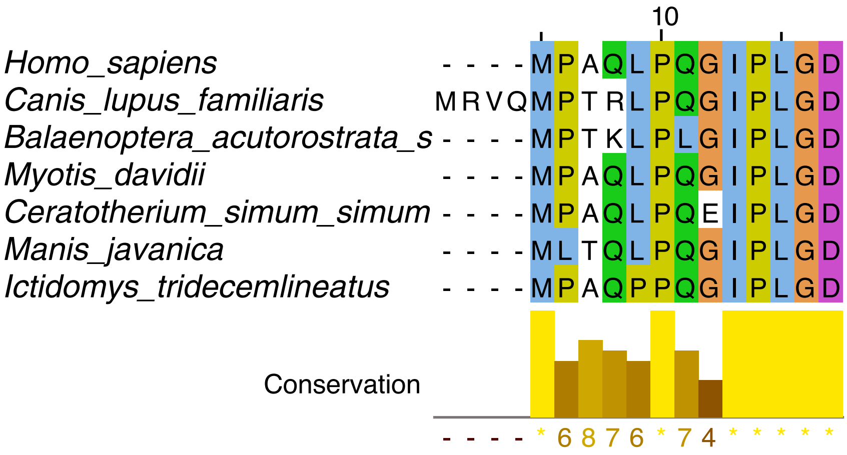

HG0778

ENSG00000089116 (LHX5)

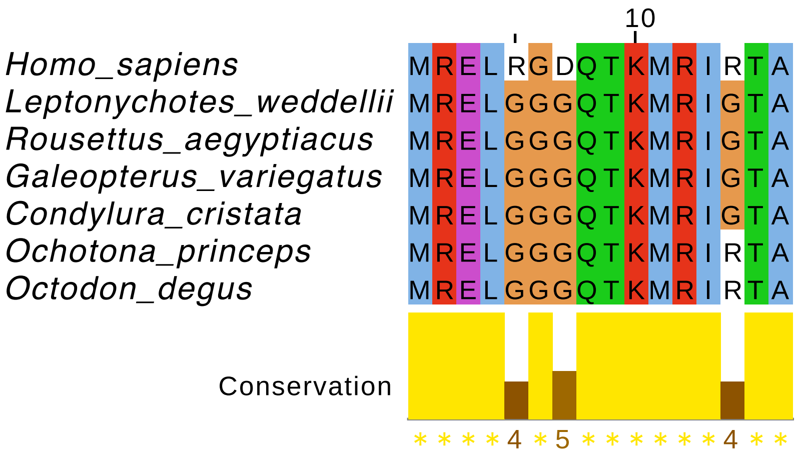

HG0779

ENSG00000091140 (DLD)

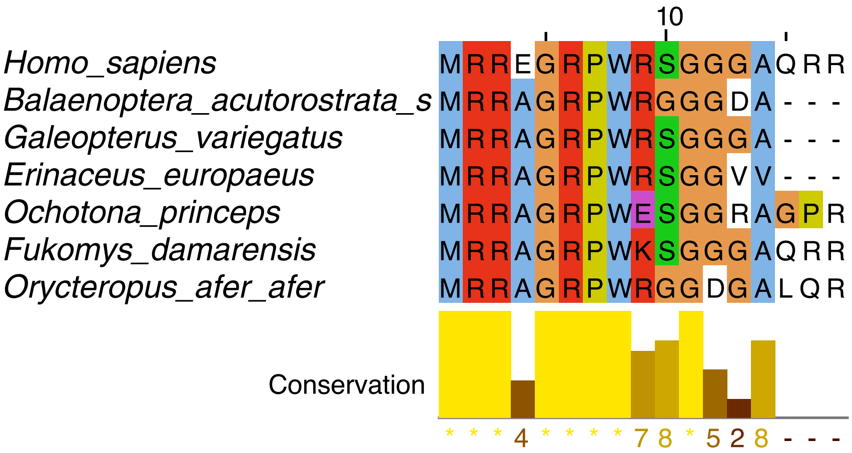

HG0780

ENSG00000092199 (HNRNPC)

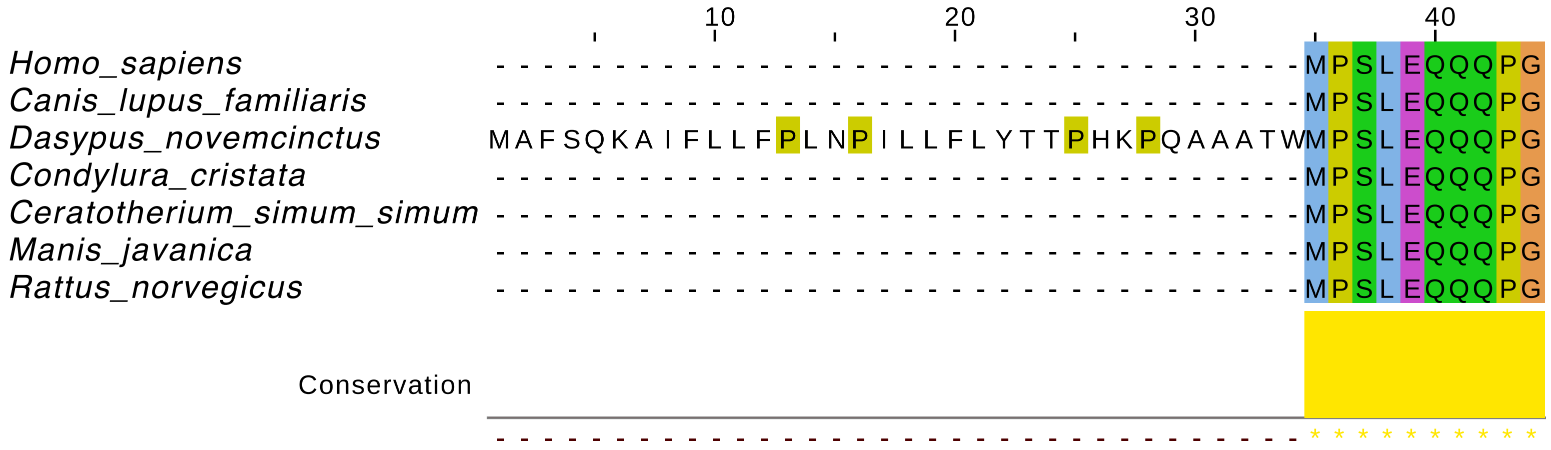

HG0781

ENSG00000100099 (HPS4)

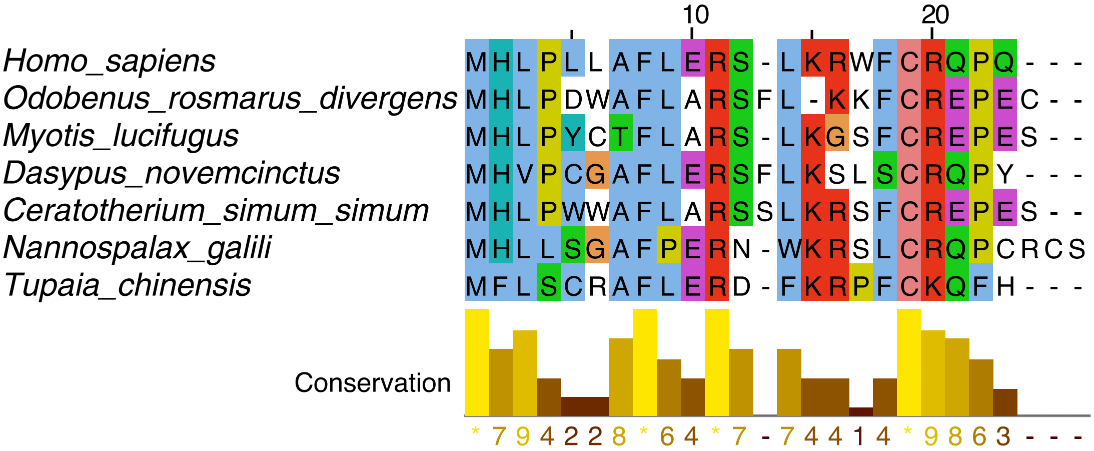

HG0782

ENSG00000100368 (CSF2RB)

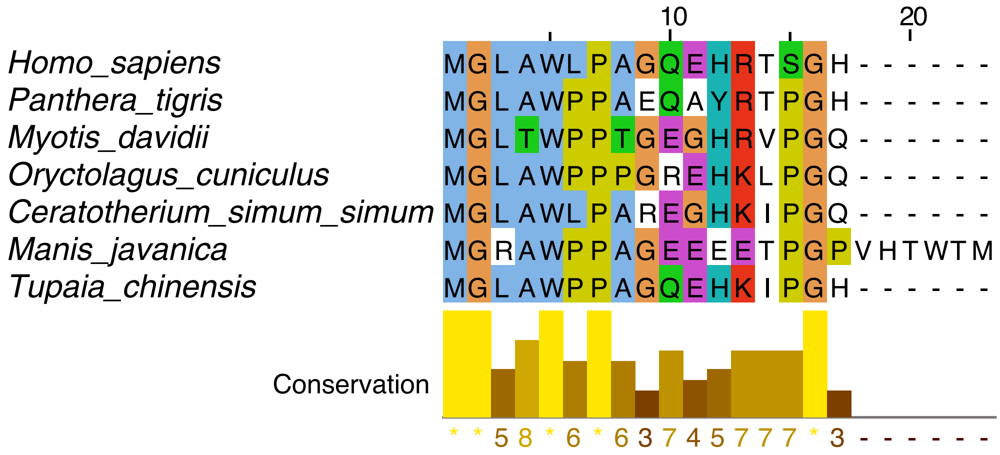

HG0783

ENSG00000105053 (VRK3)

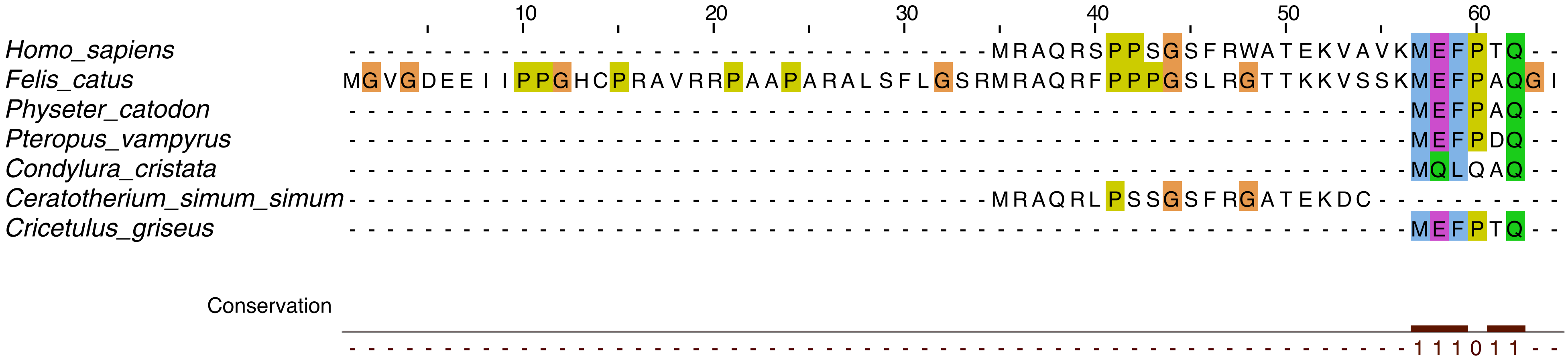

HG0784

ENSG00000105851 (PIK3CG)

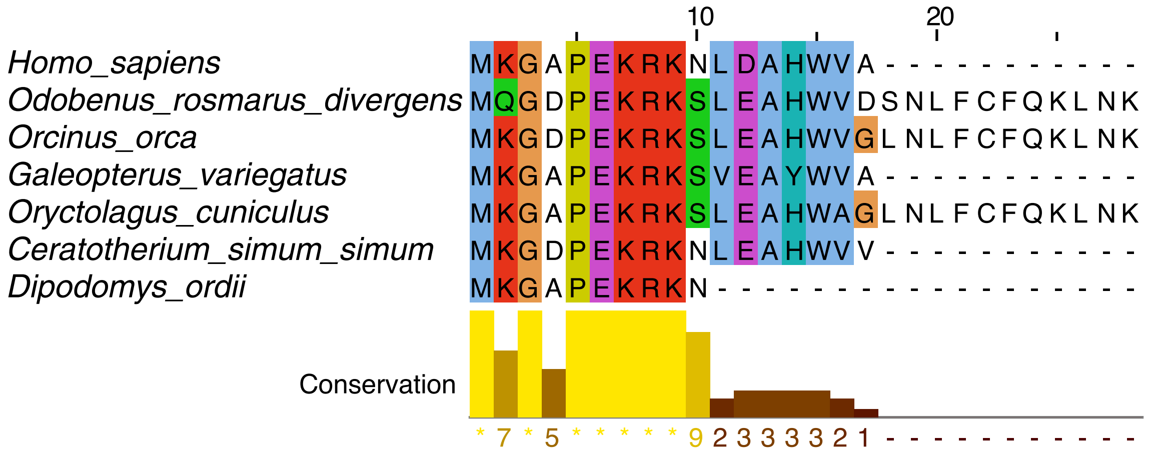

HG0785

ENSG00000107242 (PIP5K1B)

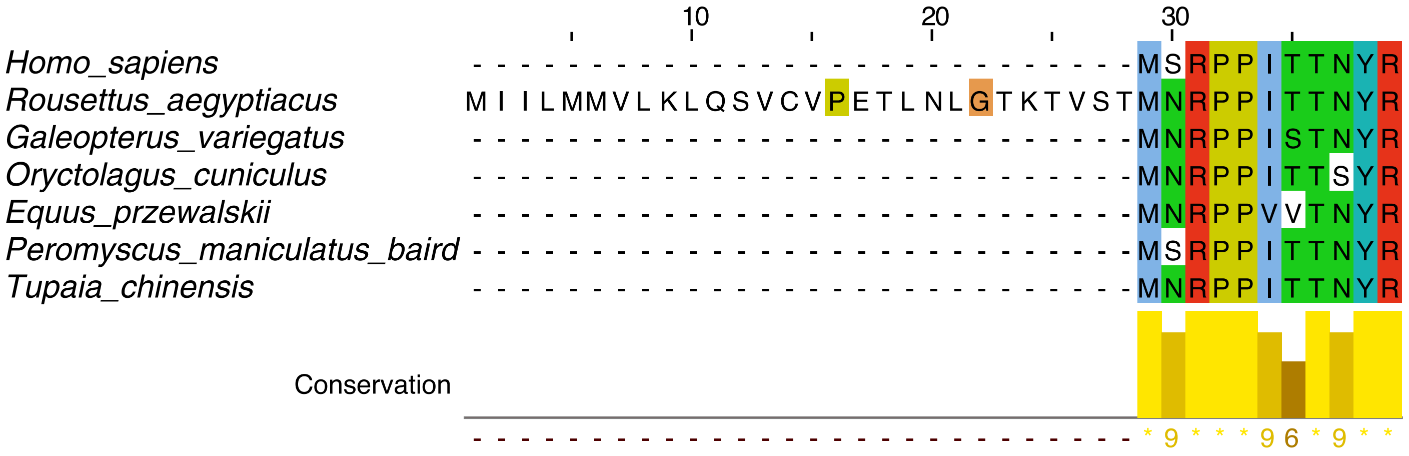

HG0786

ENSG00000107862 (GBF1)

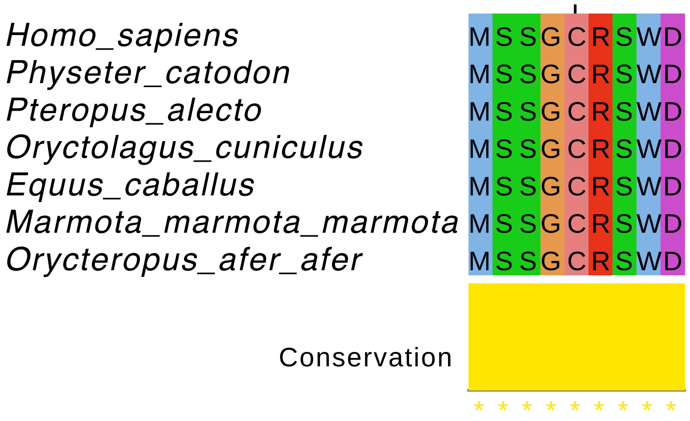

HG0787

ENSG00000108592 (FTSJ3)

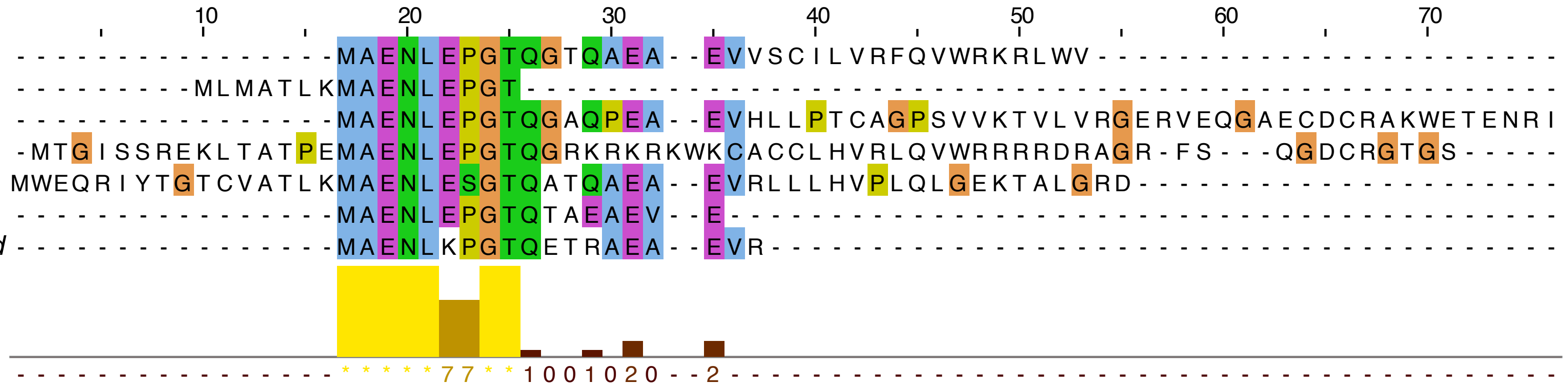

HG0788

ENSG00000113739 (STC2)

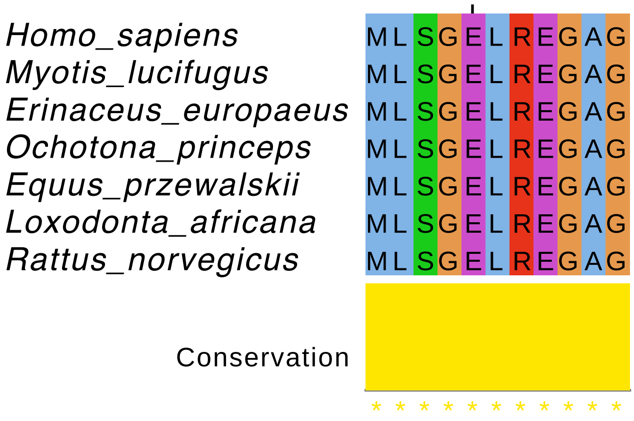

HG0789

ENSG00000115355 (CCDC88A)

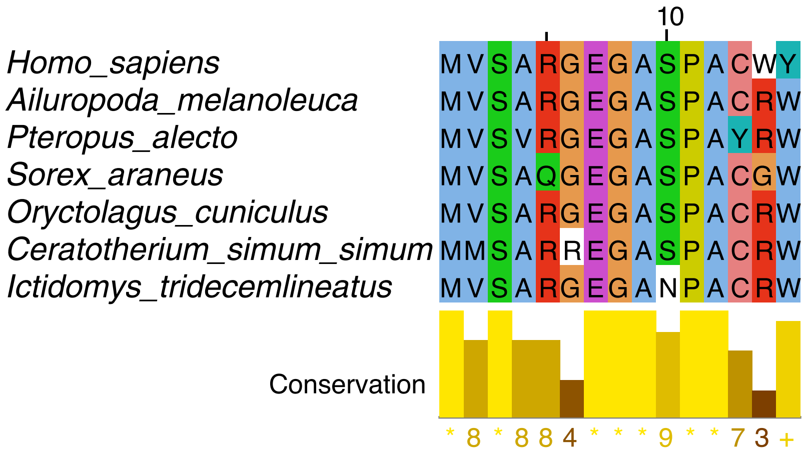

HG0790.1

ENSG00000115568 (ZNF142)

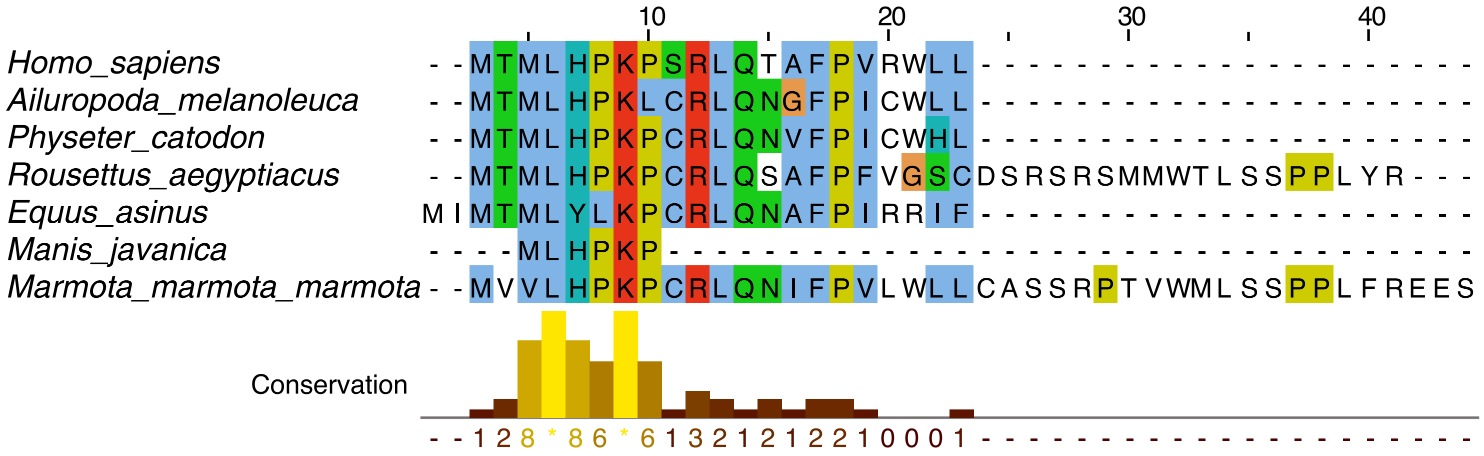

HG0790.2

ENSG00000115568 (ZNF142)

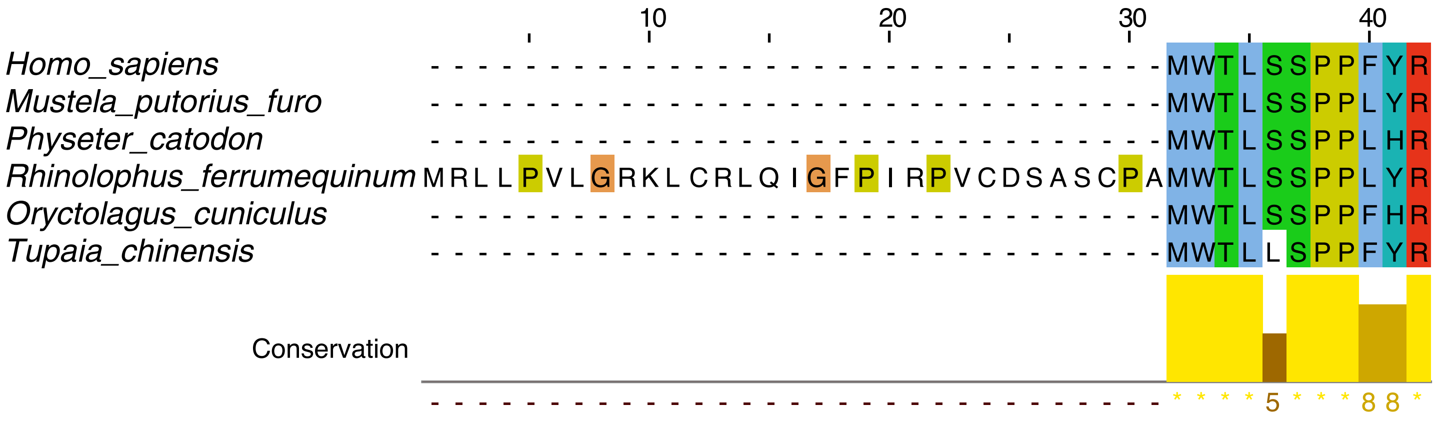

HG0791

ENSG00000131323 (TRAF3)

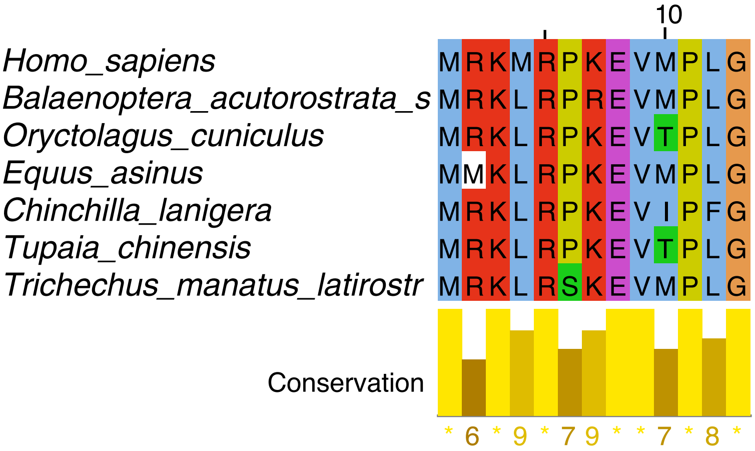

HG0792

ENSG00000132424 (PNISR)

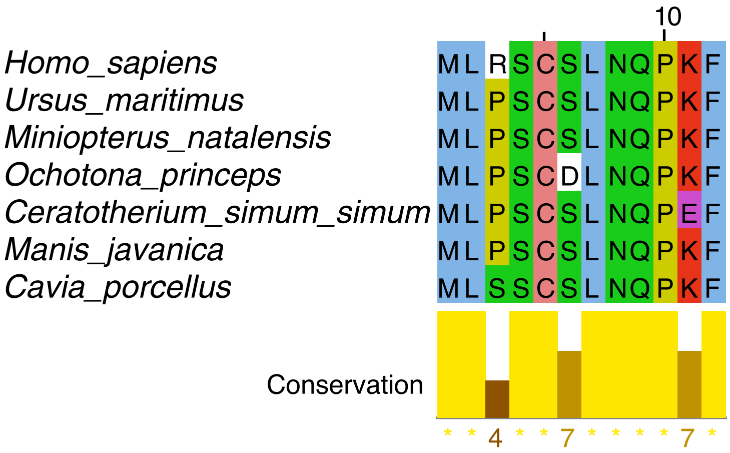

HG0793

ENSG00000132698 (RAB25)

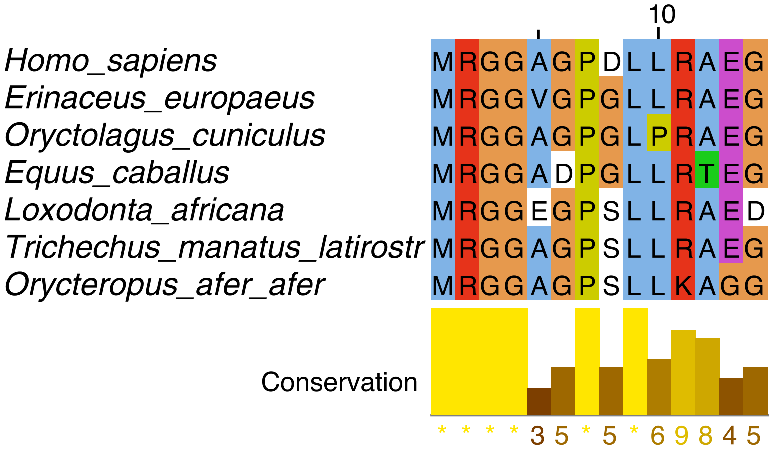

HG0794.1

ENSG00000135218 (CD36)

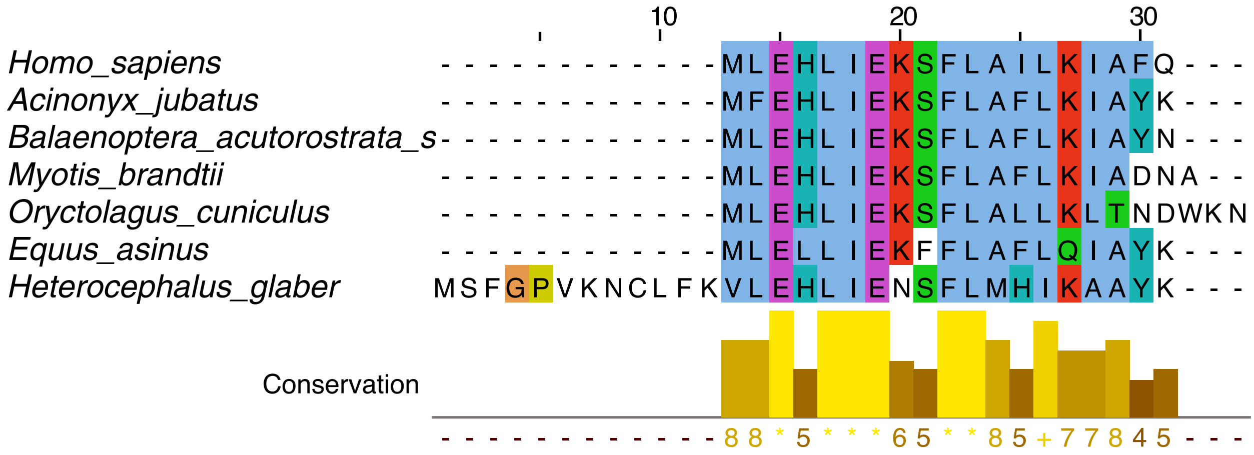

HG0794.2

ENSG00000135218 (CD36)

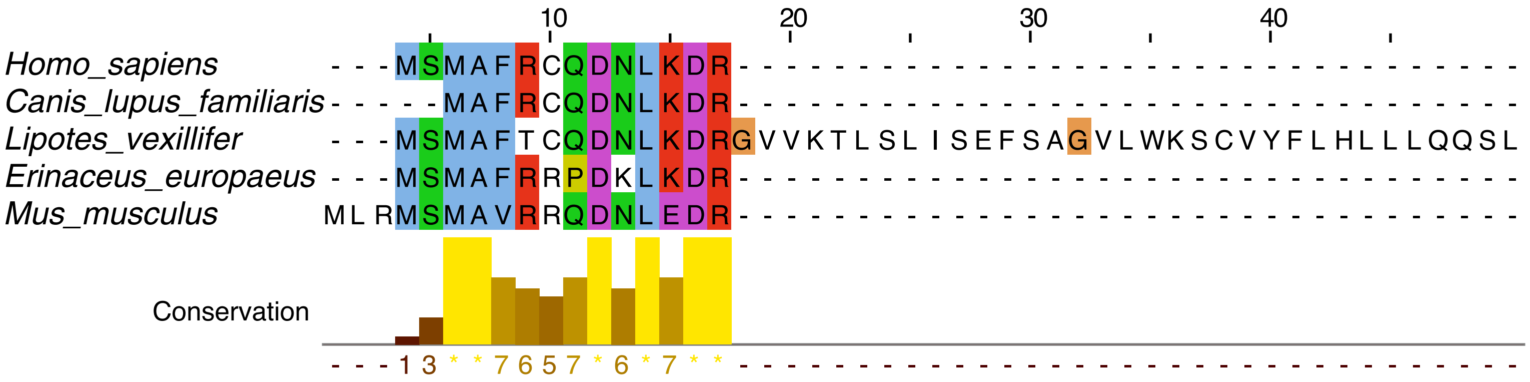

HG0795

ENSG00000137601 (NEK1)

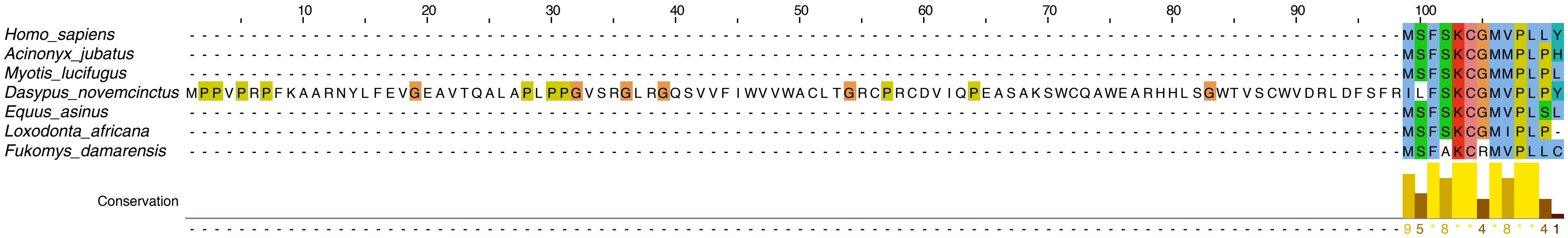

HG0796

ENSG00000138074 (SLC5A6)

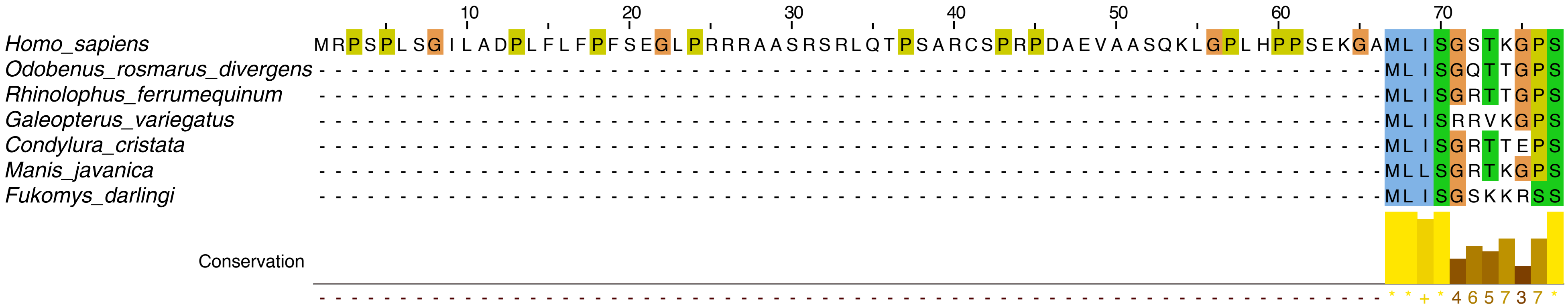

HG0797

ENSG00000140526 (ABHD2)

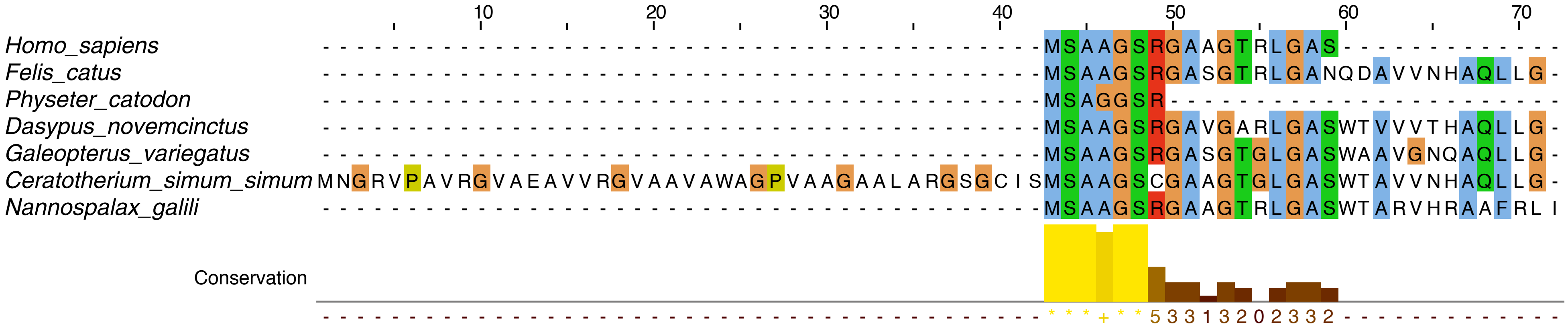

HG0798.1

ENSG00000142949 (PTPRF)

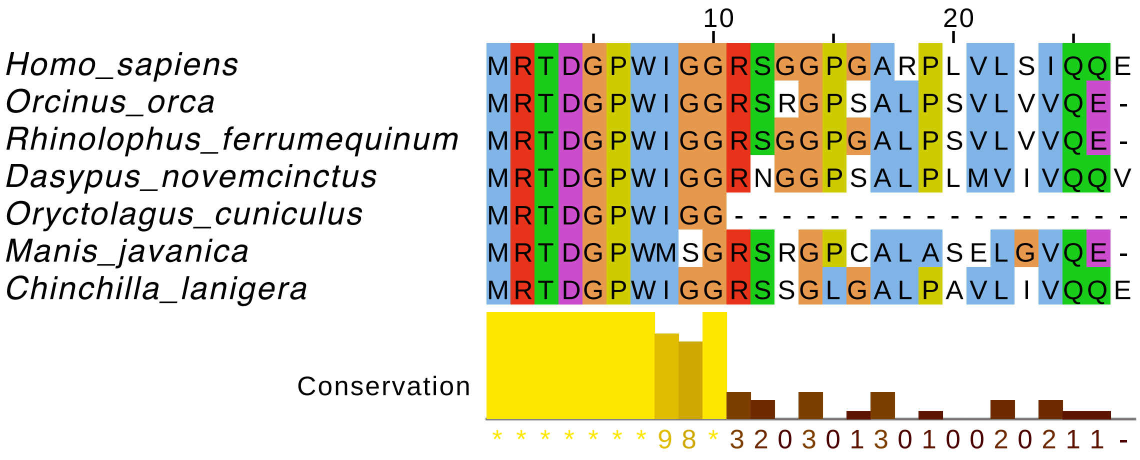

HG0798.2

ENSG00000153707 (PTPRD)

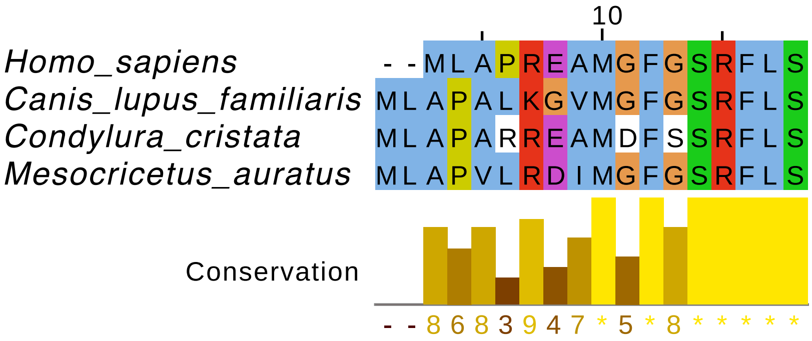

HG0799

ENSG00000143398 (PIP5K1A)

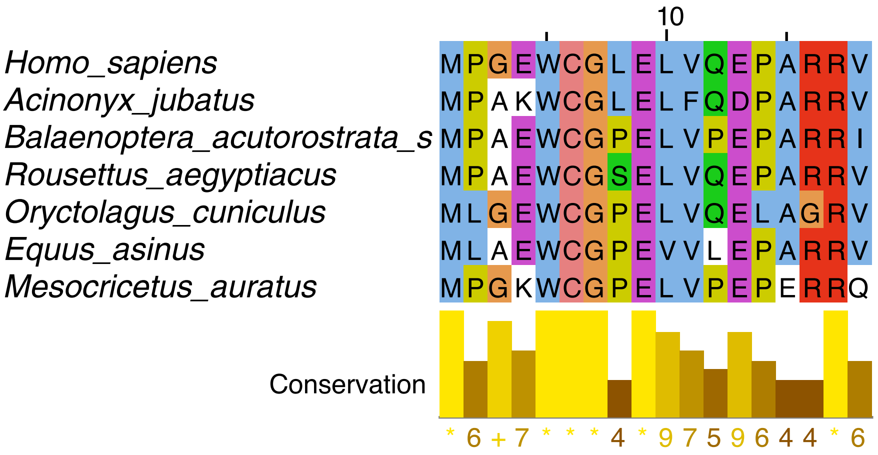

HG0800

ENSG00000143612 (C1orf43)

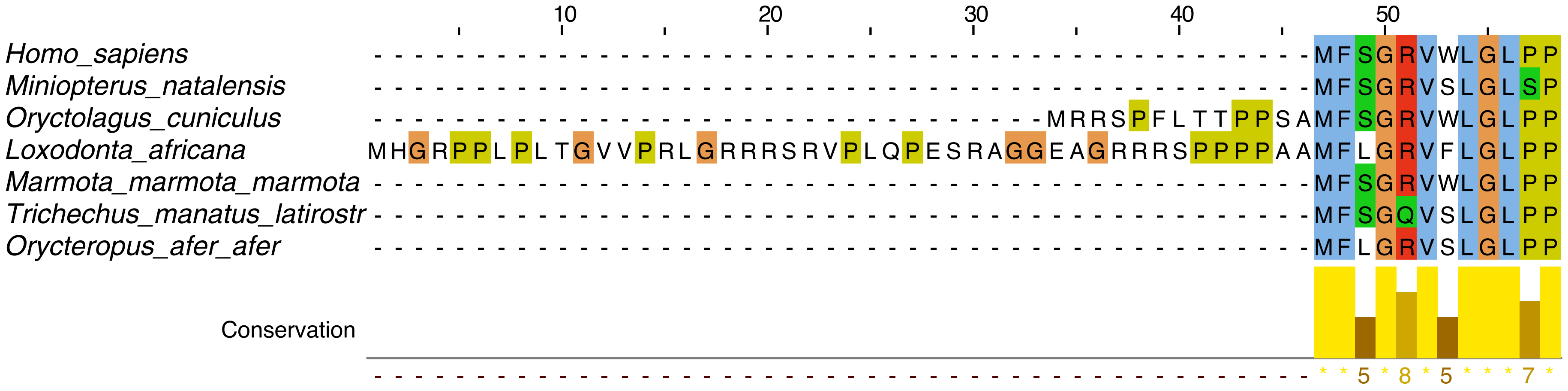

HG0801

ENSG00000144278 (GALNT13)

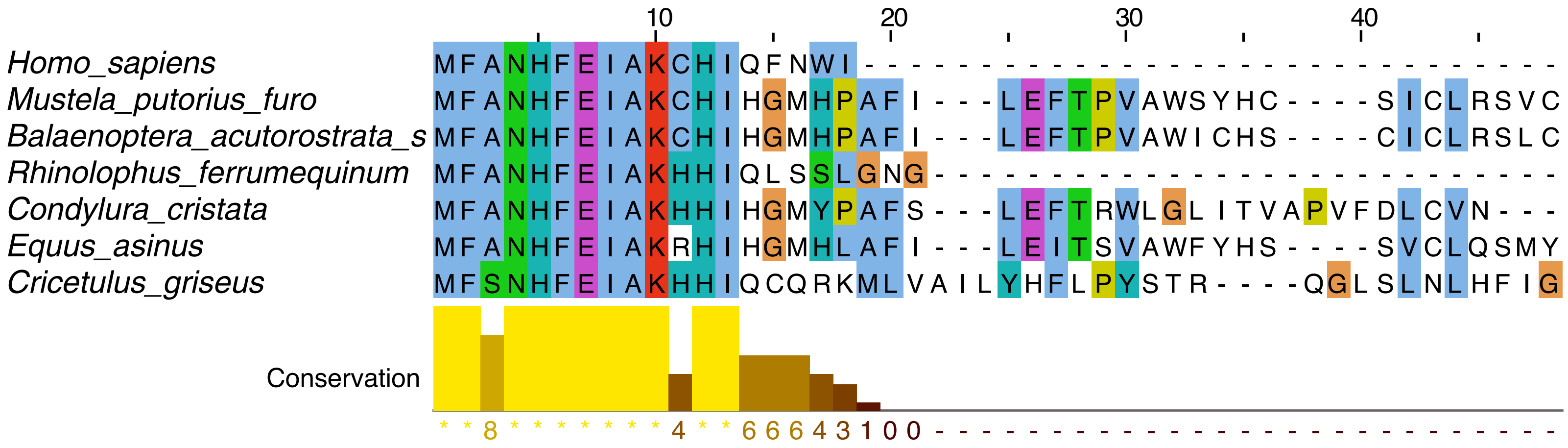

HG0802

ENSG00000144283 (PKP4)

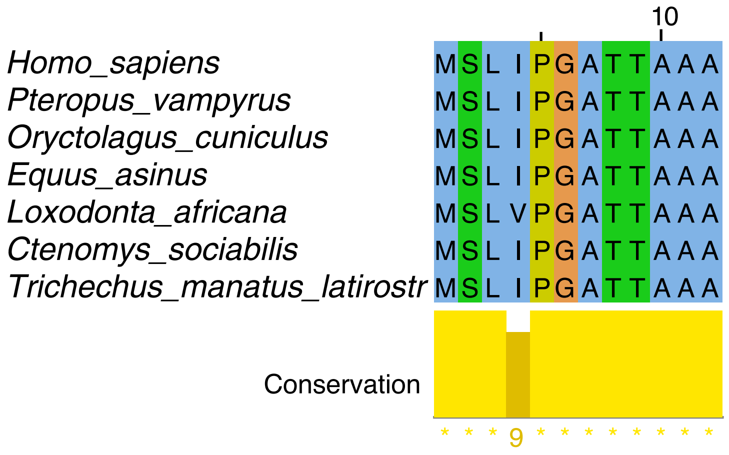

HG0803

ENSG00000144802 (NFKBIZ)

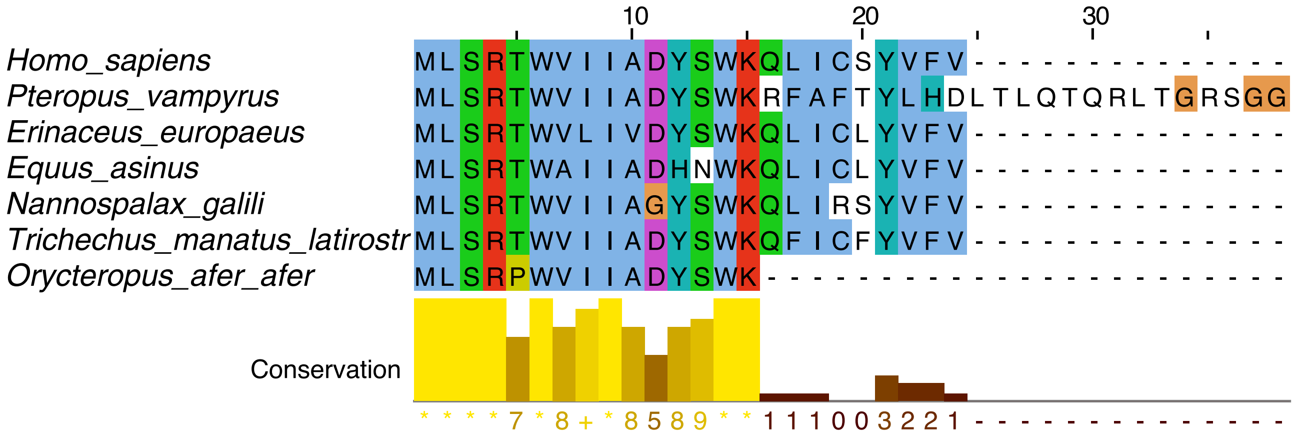

HG0804

ENSG00000144815 (NXPE3)

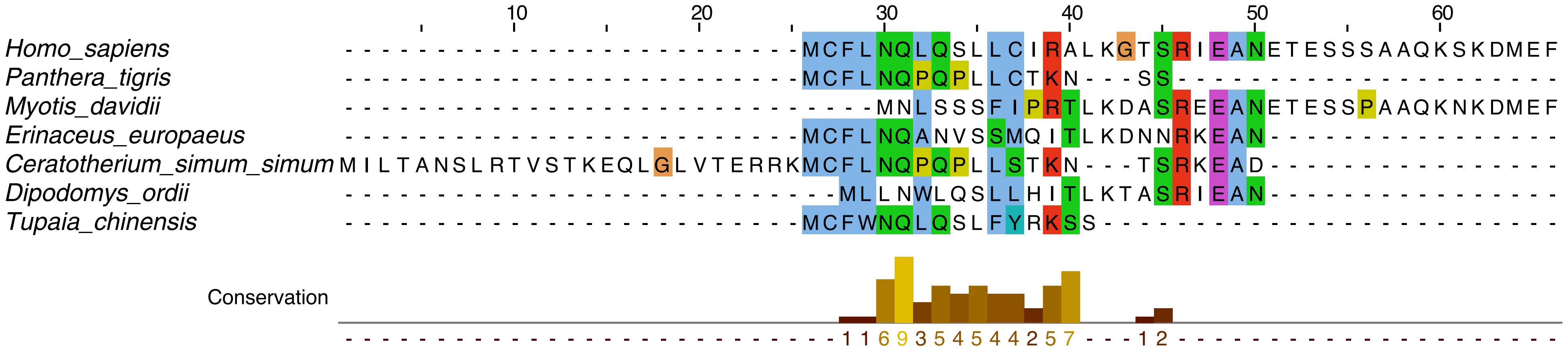

HG0805.1

ENSG00000145936 (KCNMB1)

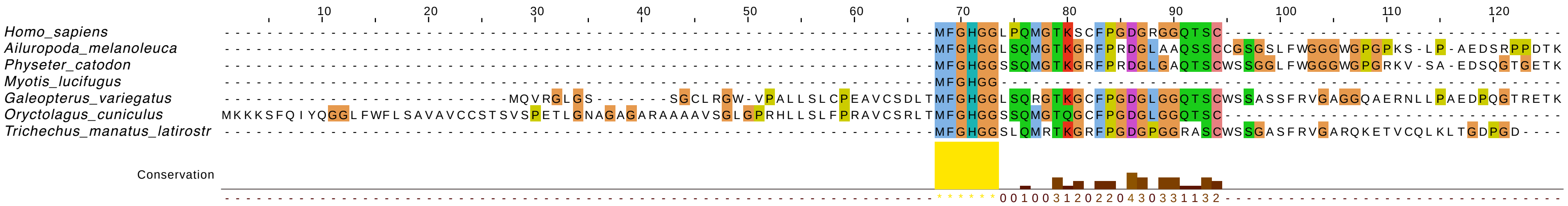

HG0805.2

ENSG00000145936 (KCNMB1)

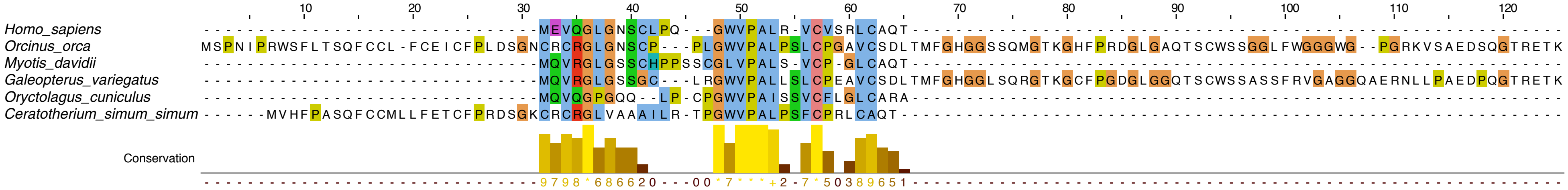

HG0806

ENSG00000147481 (SNTG1)

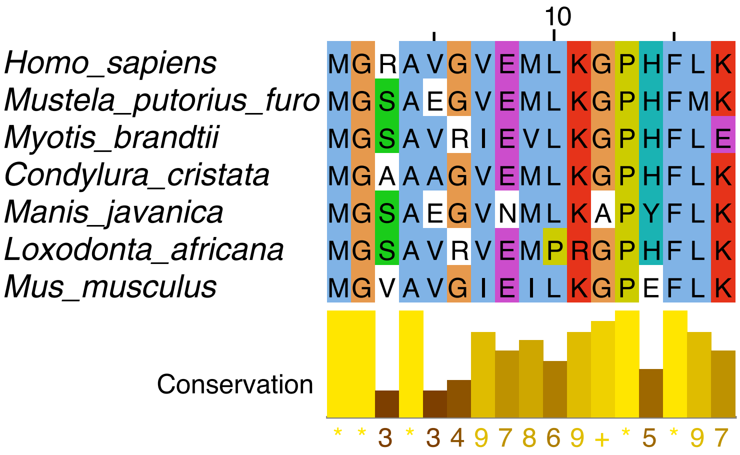

HG0807.1

ENSG00000147488 (ST18)

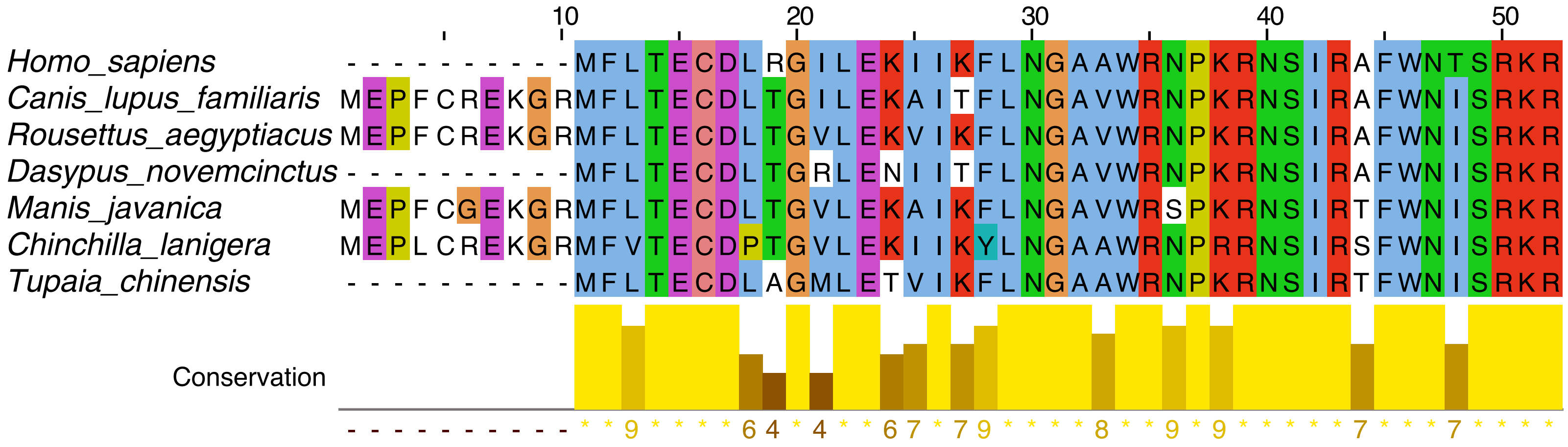

HG0807.2

ENSG00000147488 (ST18)

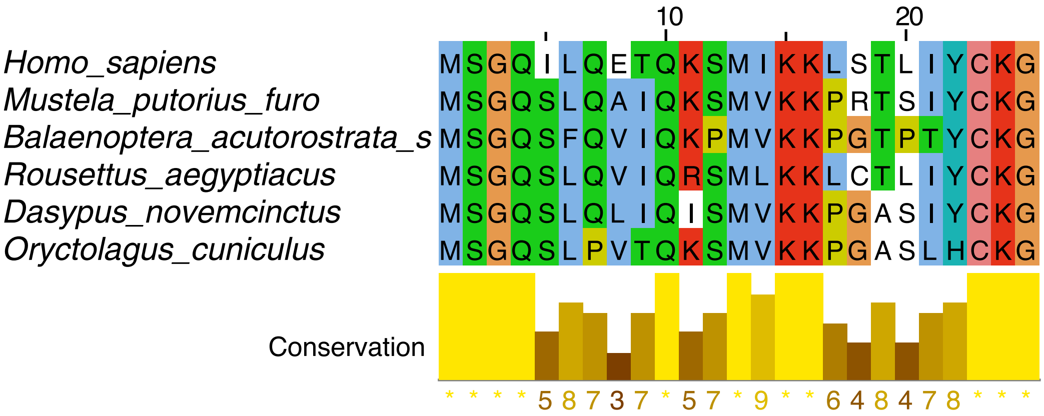

HG0807.3

ENSG00000186487 (MYT1L)

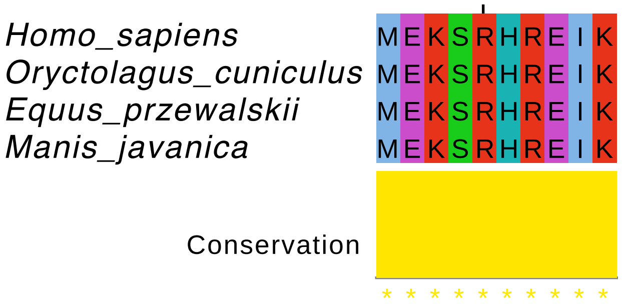

HG0808

ENSG00000149269 (PAK1)

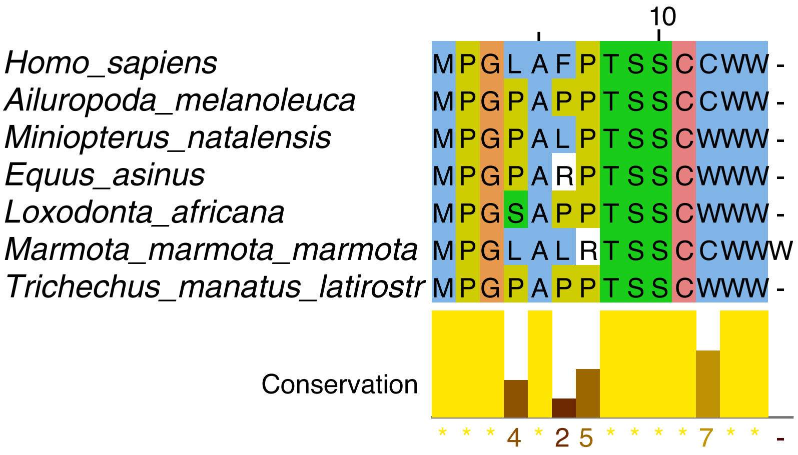

HG0809

ENSG00000151287 (TEX30)

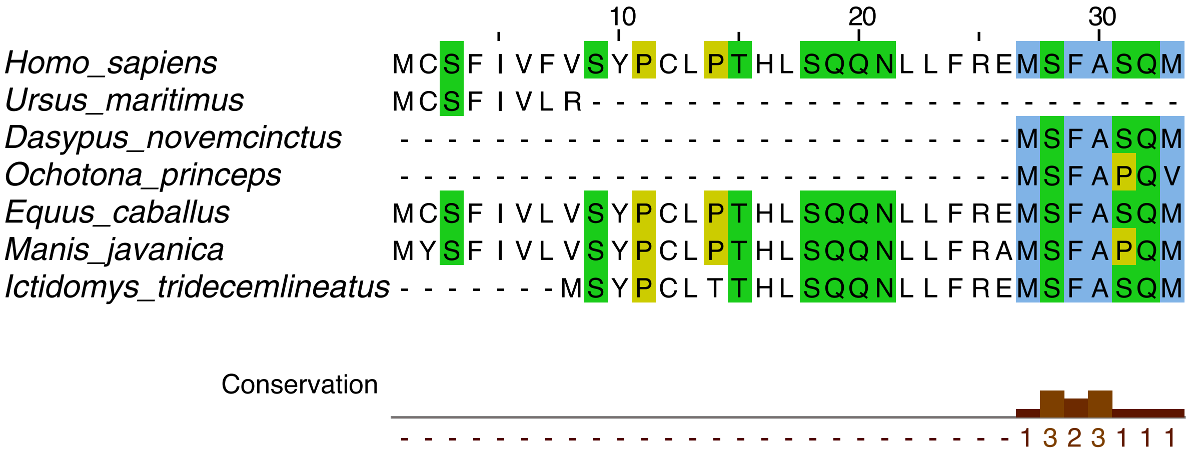

HG0810

ENSG00000156504 (FAM122B)

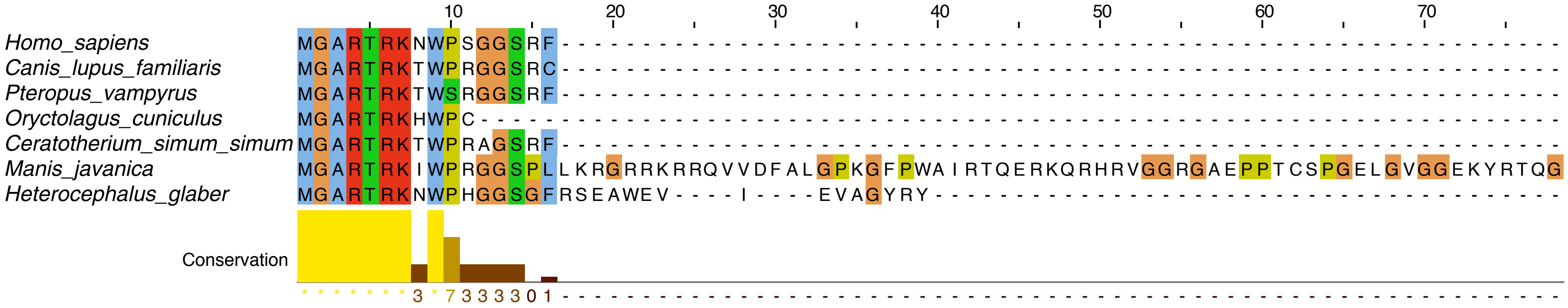

HG0811

ENSG00000157350 (ST3GAL2)

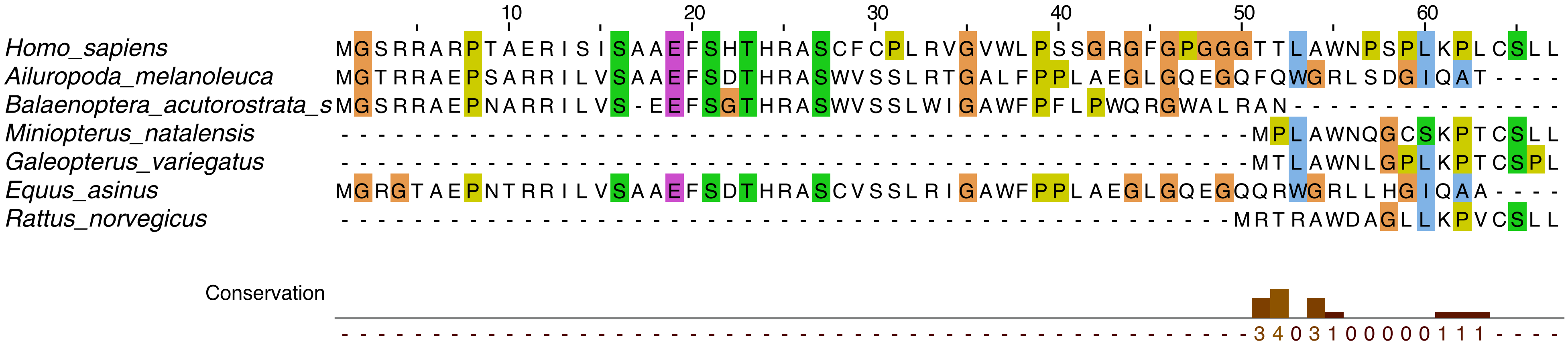

HG0812

ENSG00000157368 (IL34)

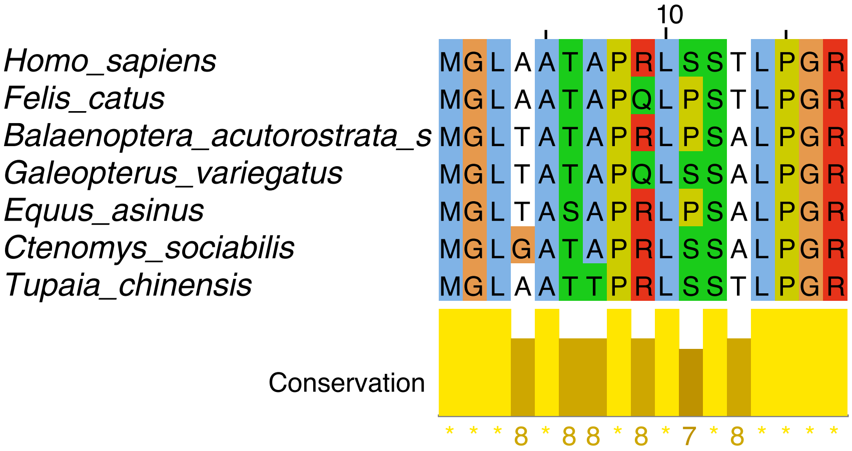

HG0813

ENSG00000160199 (PKNOX1)

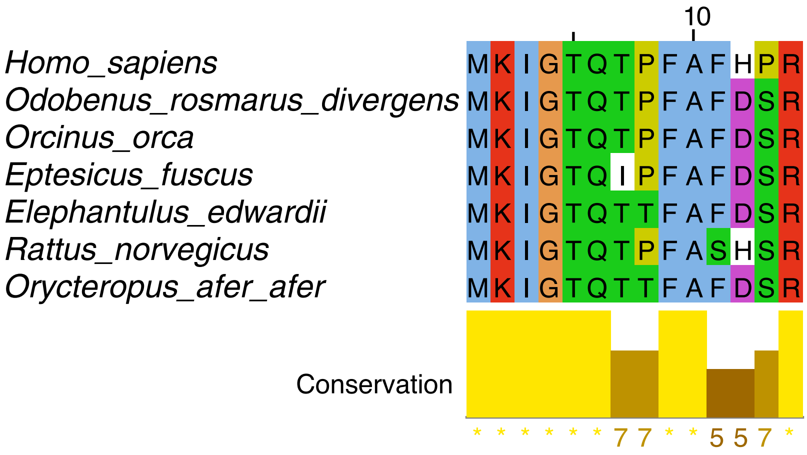

HG0814

ENSG00000163071 (SPATA18)

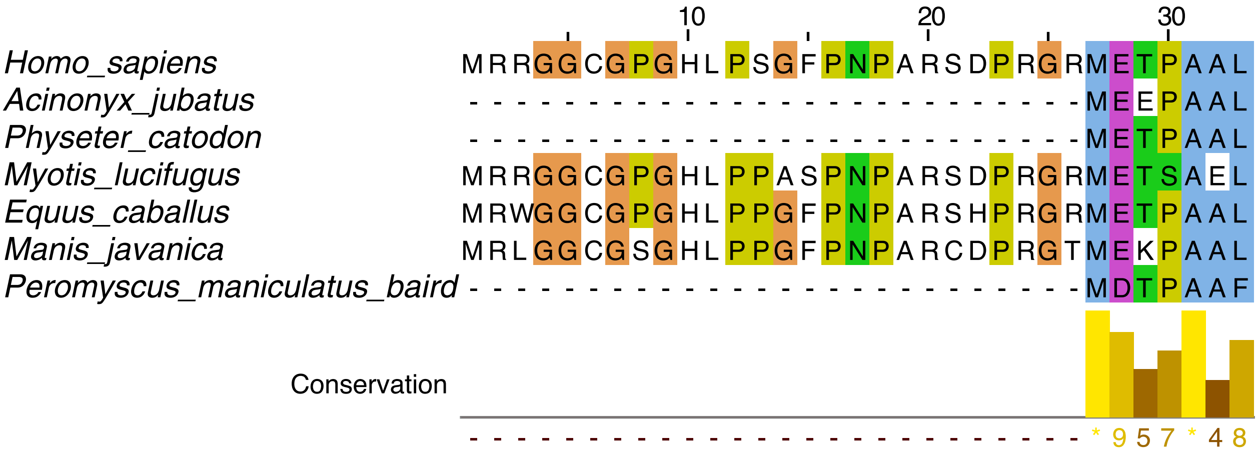

HG0815

ENSG00000163072 (NOSTRIN)

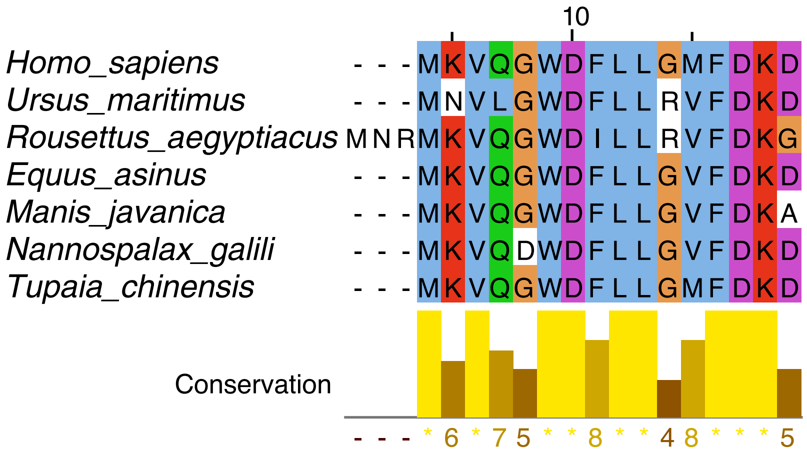

HG0816

ENSG00000163794 (UCN)

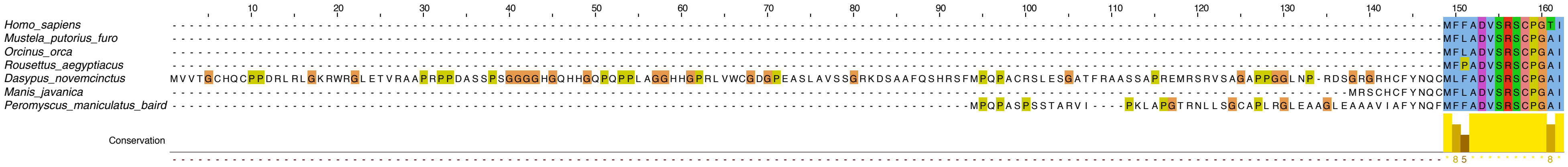

HG0817

ENSG00000163888 (CAMK2N2)

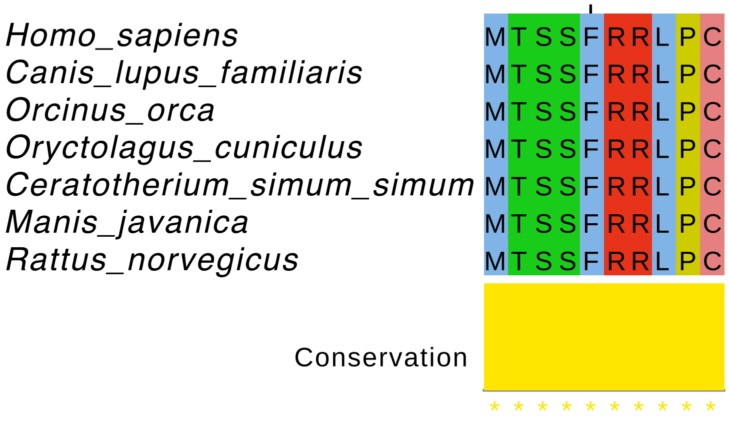

HG0818

ENSG00000164125 (FAM198B)

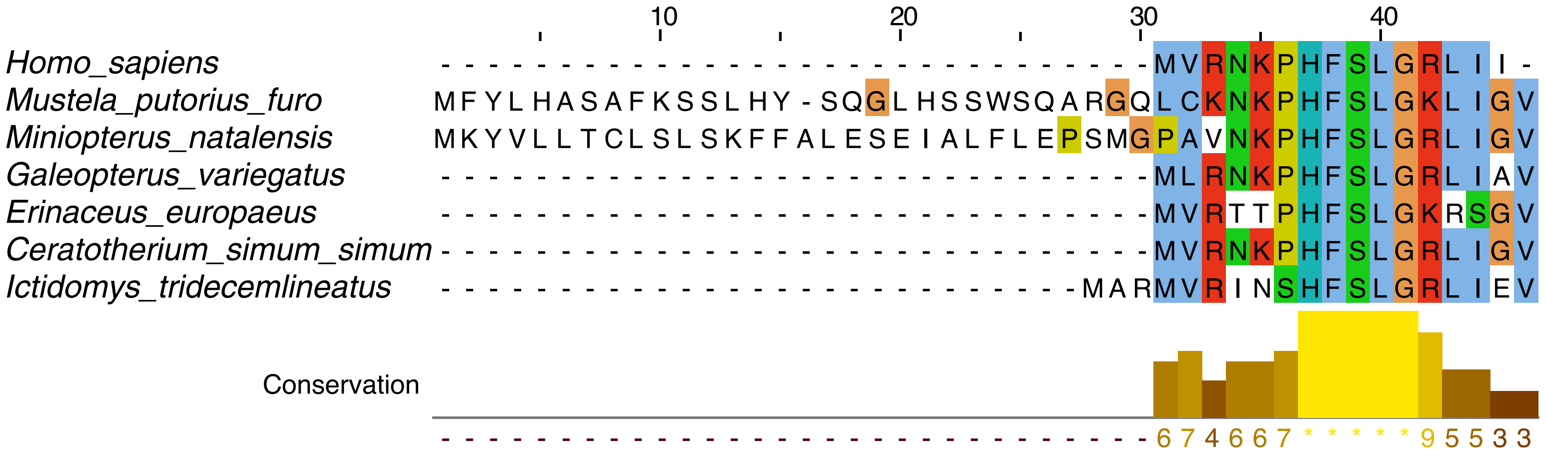

HG0819

ENSG00000164188 (RANBP3L)

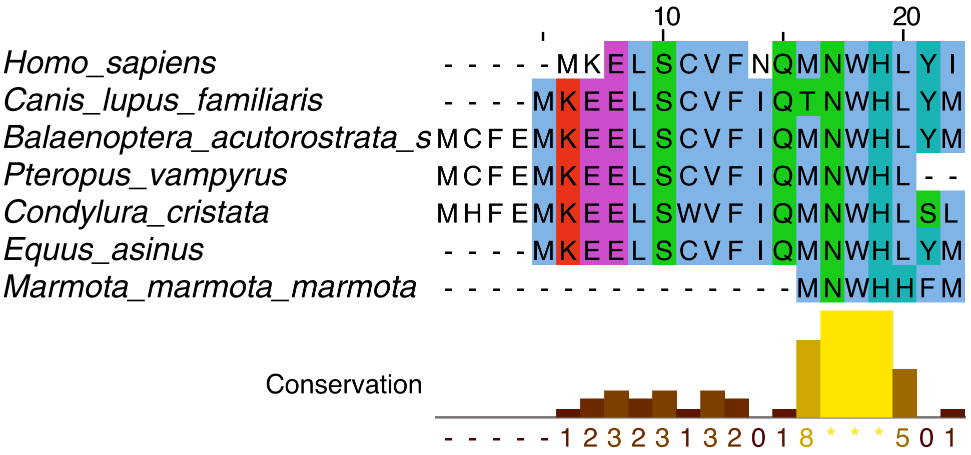

HG0820

ENSG00000164946 (FREM1)

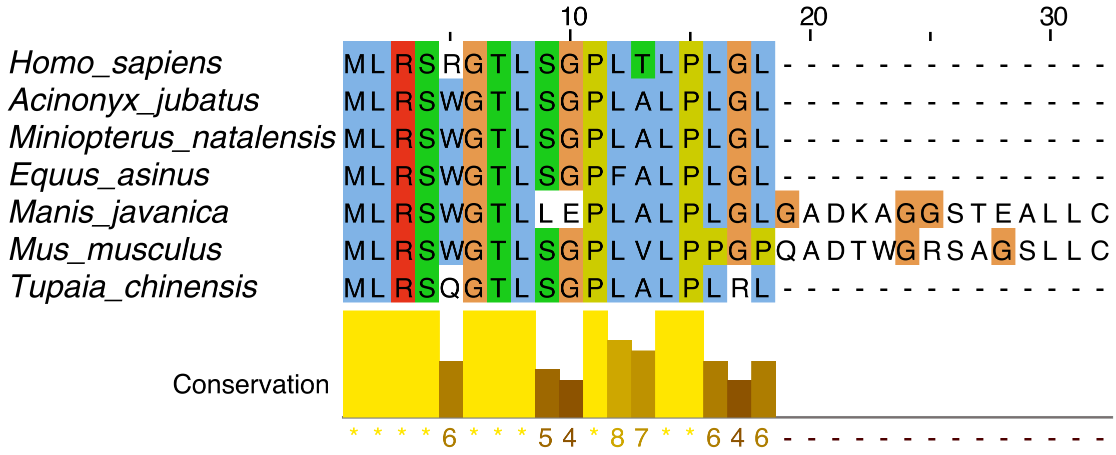

HG0821

ENSG00000165300 (SLITRK5)

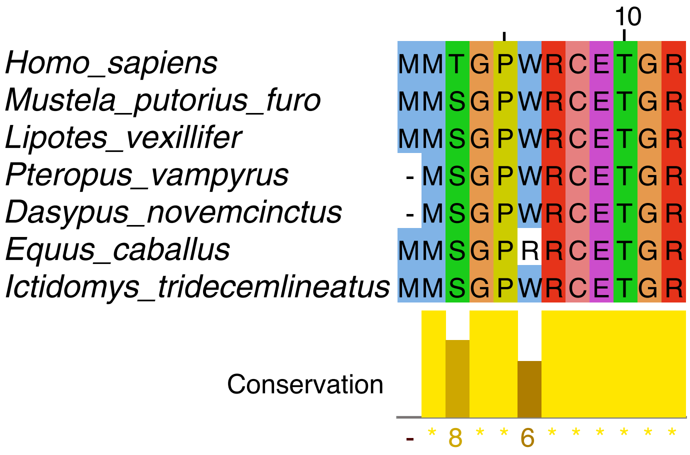

HG0822

ENSG00000166170 (BAG5)

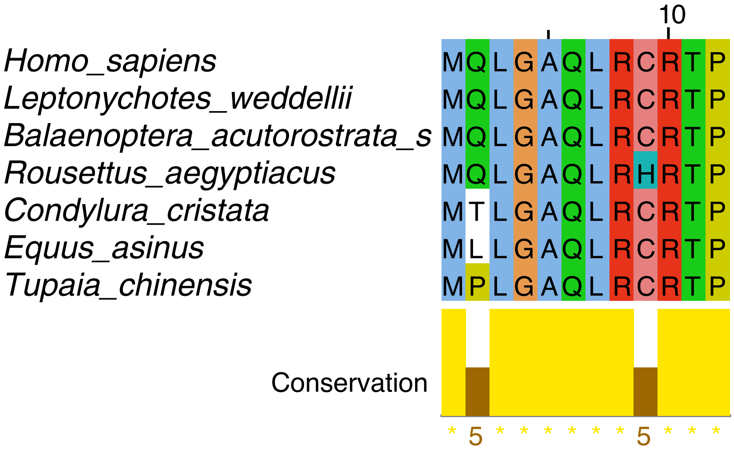

HG0823

ENSG00000166268 (MYRFL)

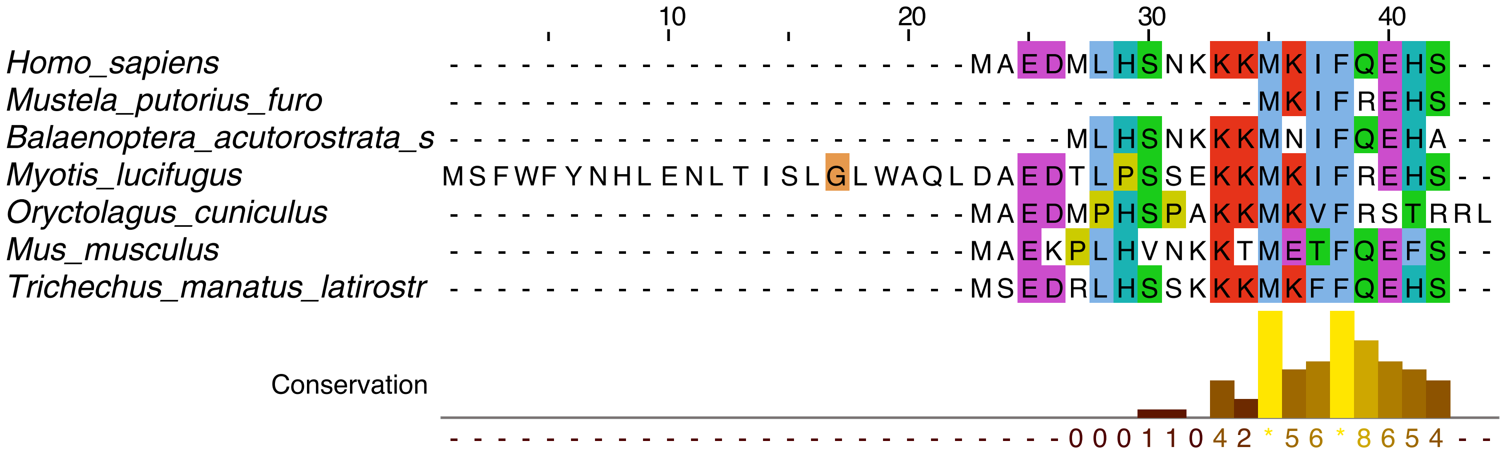

HG0824

ENSG00000167100

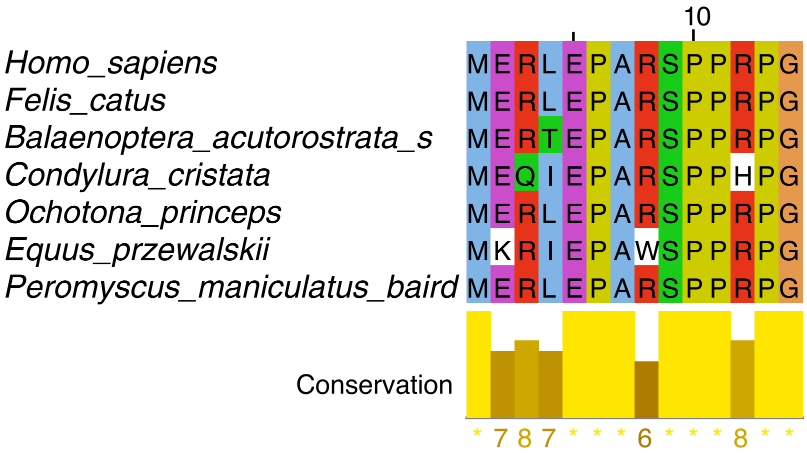

HG0825

ENSG00000169180 (XPO6)

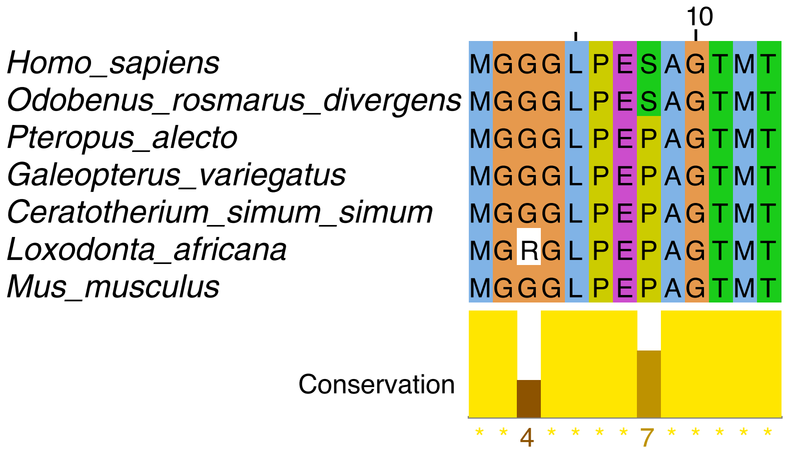

HG0826

ENSG00000169851 (PCDH7)

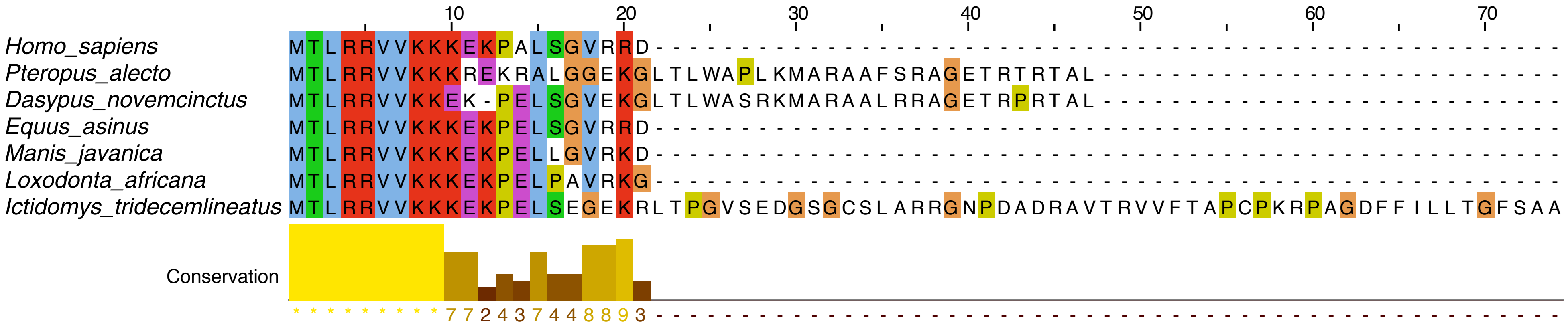

HG0827

ENSG00000170365 (SMAD1)

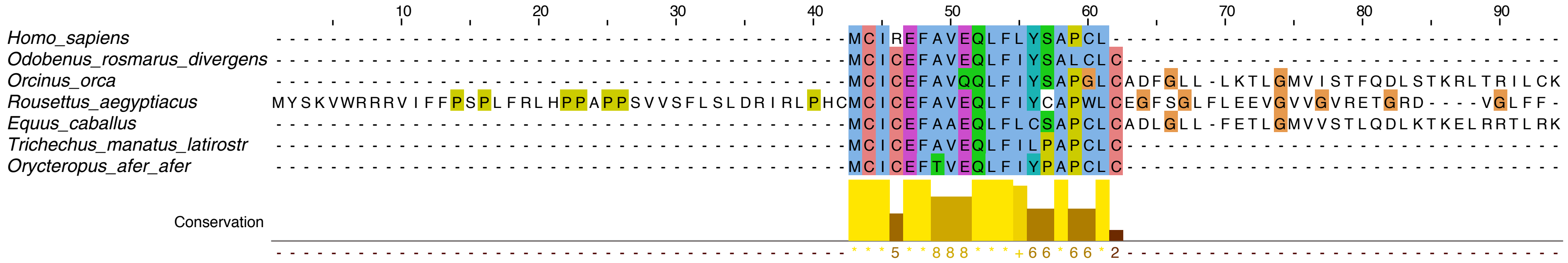

HG0828

ENSG00000170390 (DCLK2)

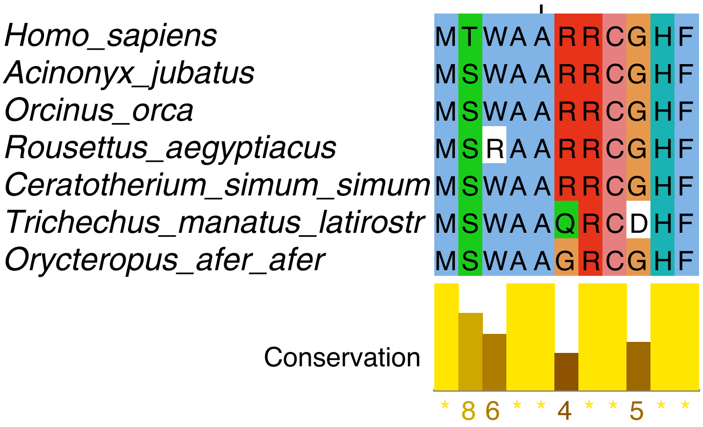

HG0829

ENSG00000171502 (COL24A1)

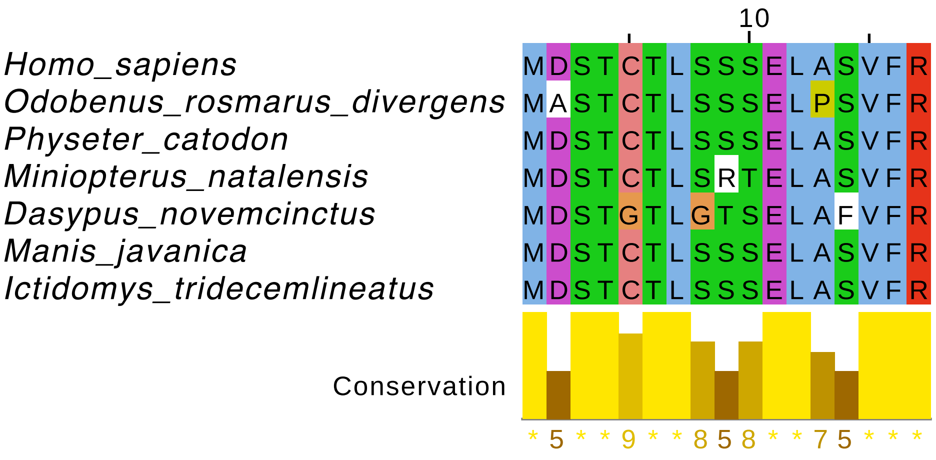

HG0830

ENSG00000171631 (P2RY6)

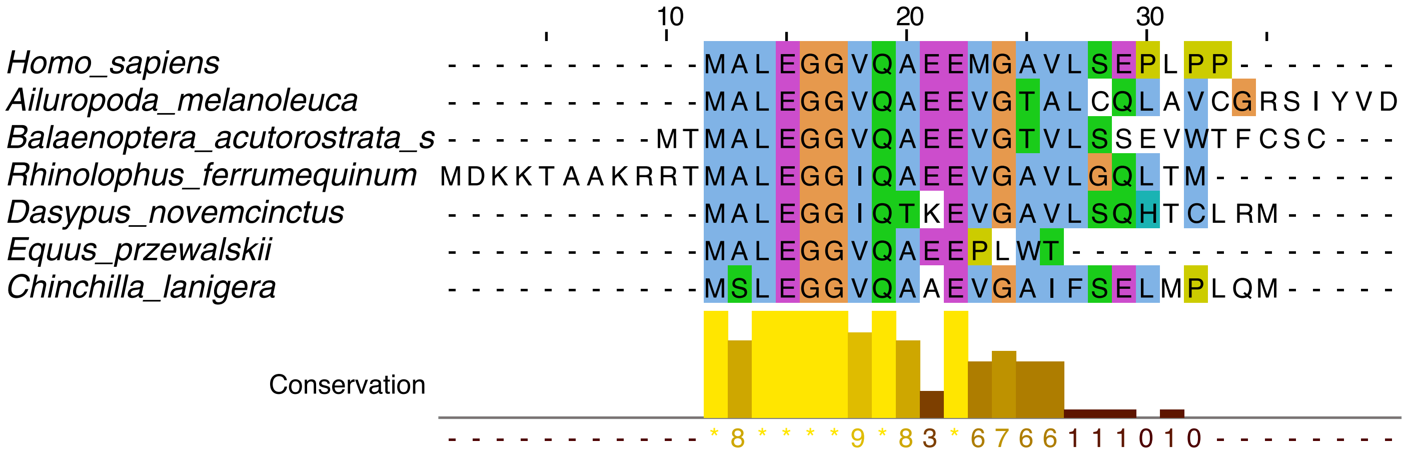

HG0831.1

ENSG00000174482 (LINGO2)

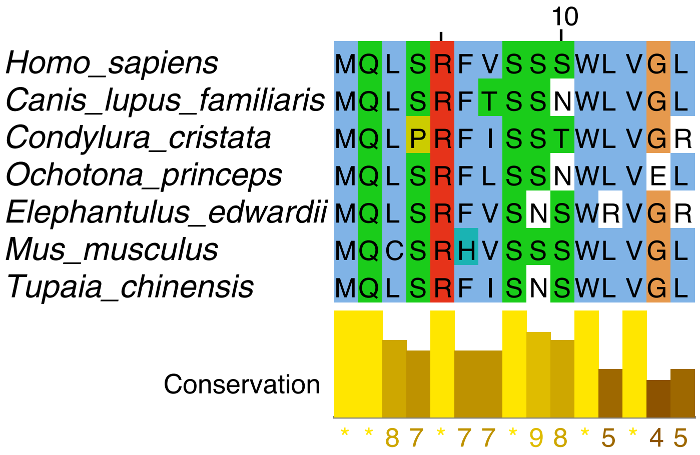

HG0831.2

ENSG00000174482 (LINGO2)

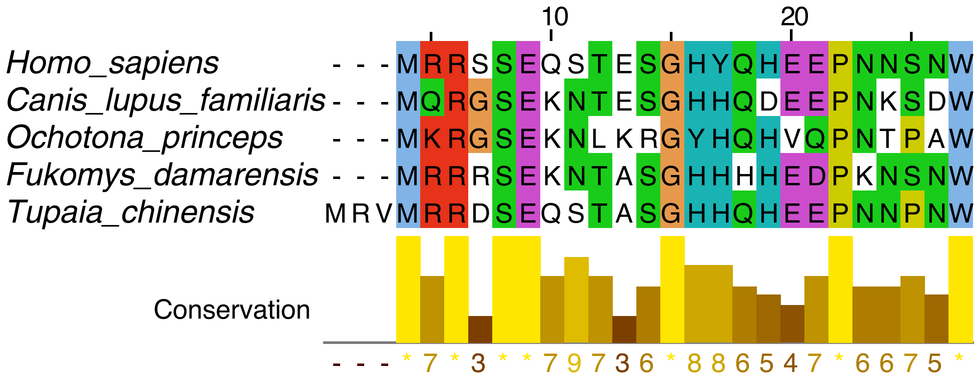

HG0832

ENSG00000175048 (ZDHHC14)

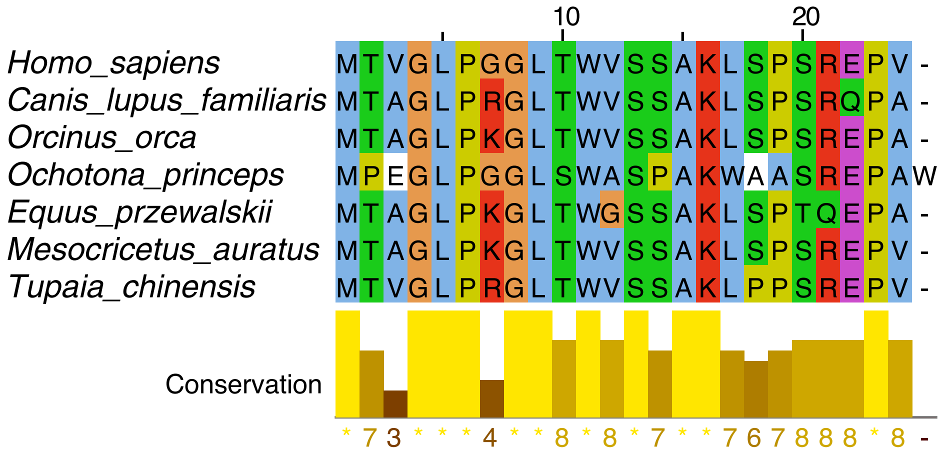

HG0833

ENSG00000176641 (RNF152)

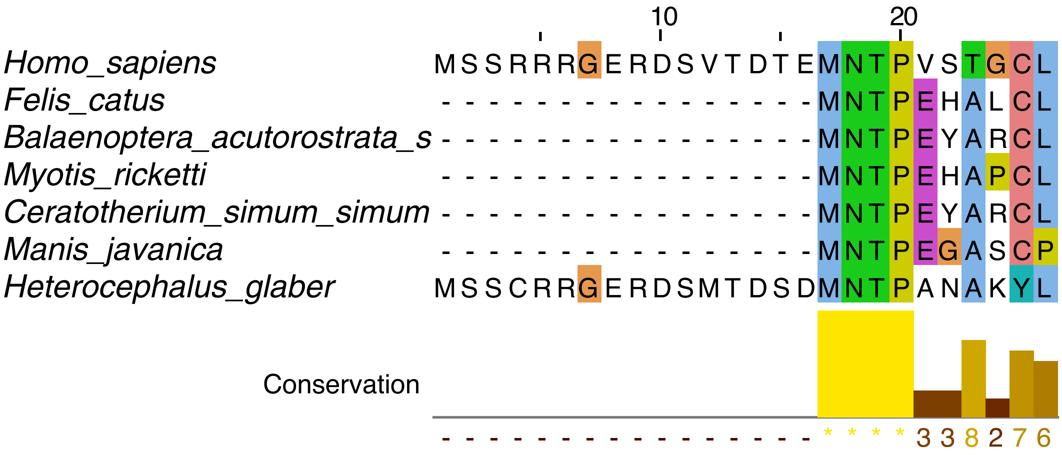

HG0834

ENSG00000182489 (XKRX)

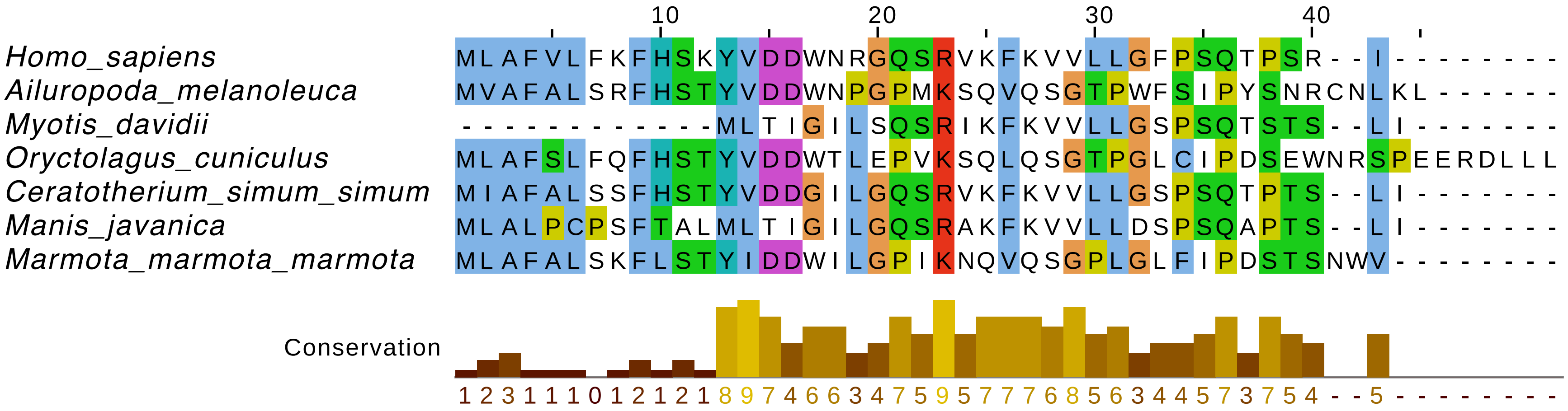

HG0835

ENSG00000182901 (RGS7)

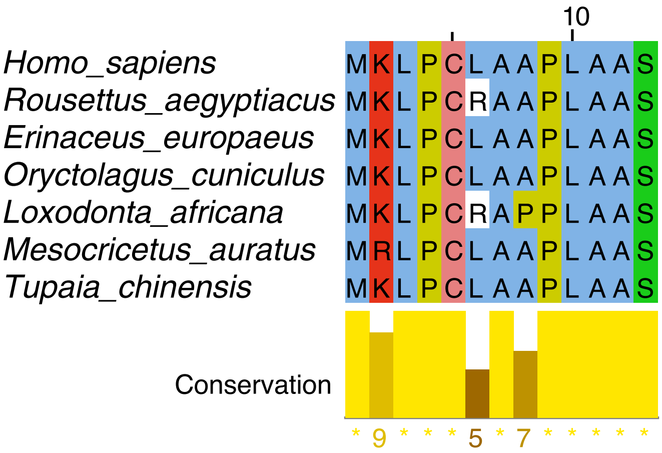

HG0836

ENSG00000197461 (PDGFA)

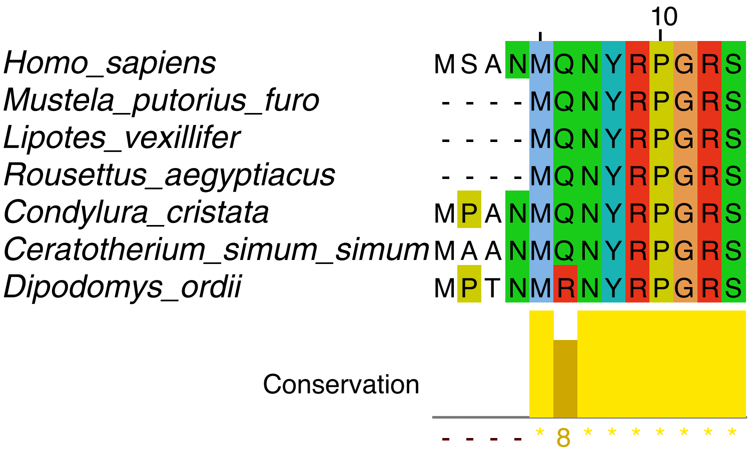

HG0837

ENSG00000198690 (FAN1)

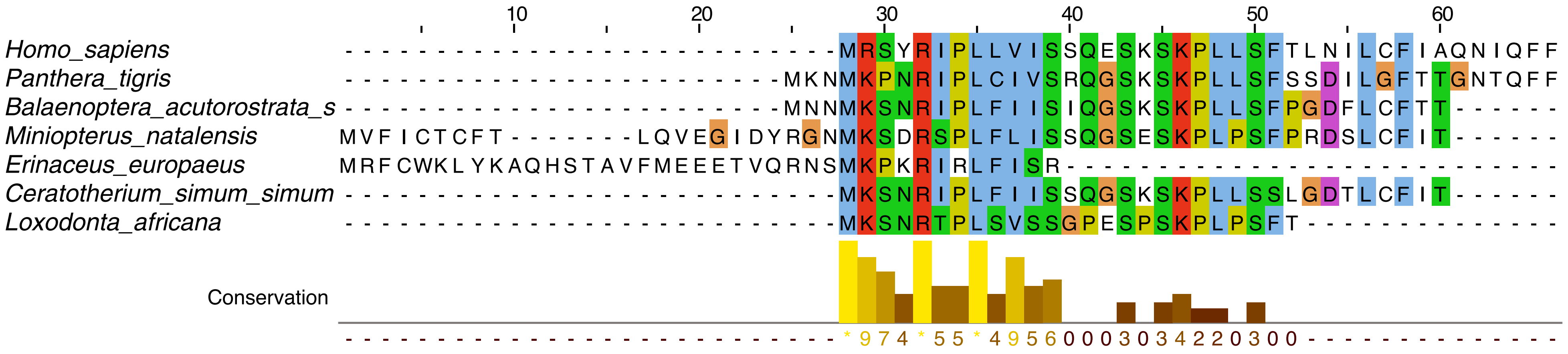

HG0838

ENSG00000198707 (CEP290)

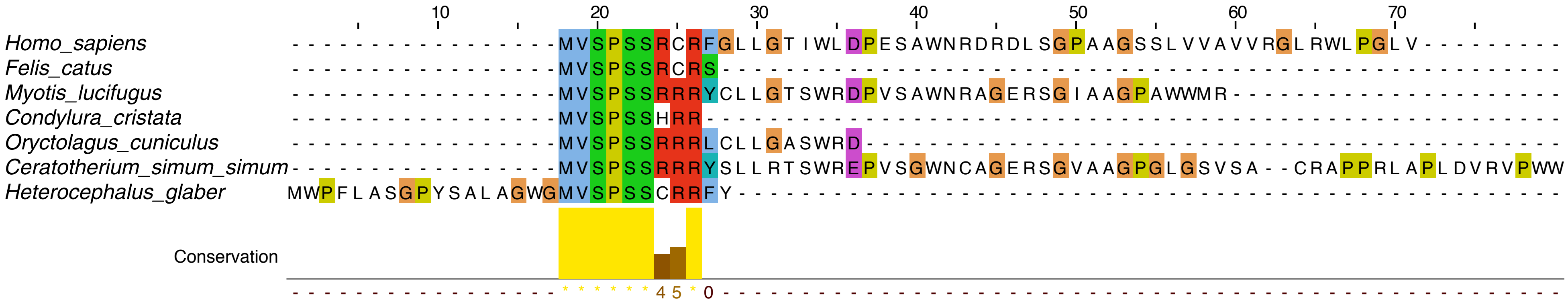

HG0839

ENSG00000198791 (CNOT7)

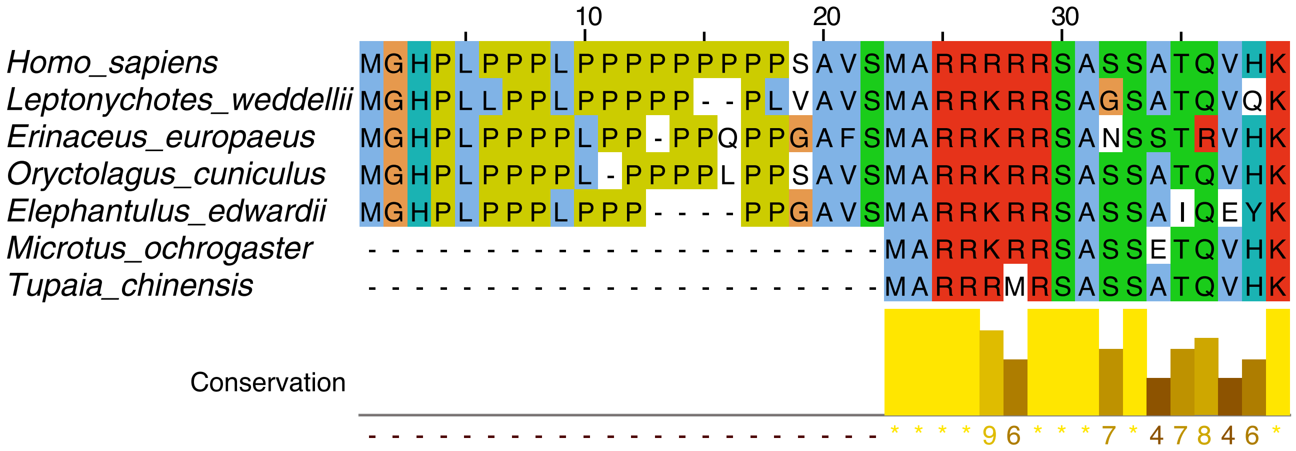

HG0840

ENSG00000278195 (SSTR3)

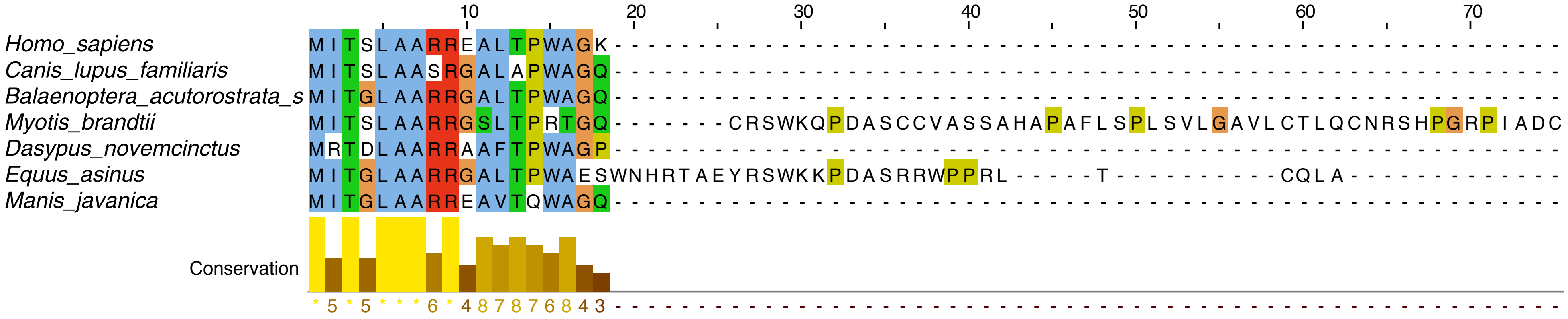

HG0841

ENSG00000278259 (MYO19)

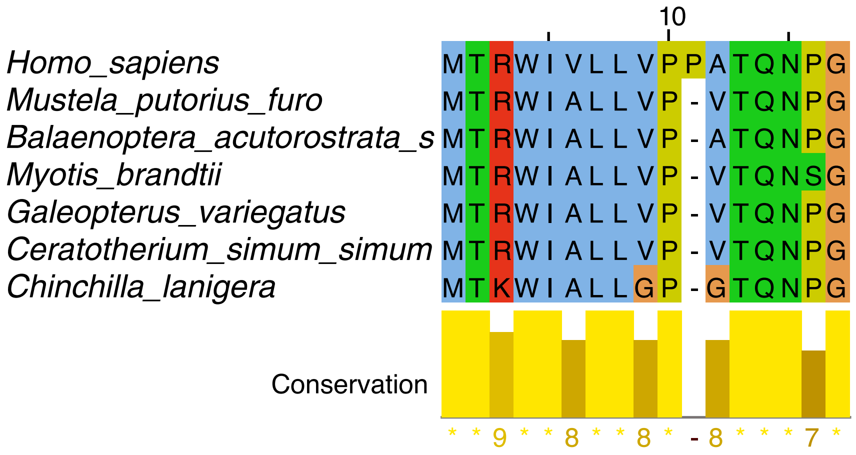

HG0842

ENSG00000002746 (HECW1)

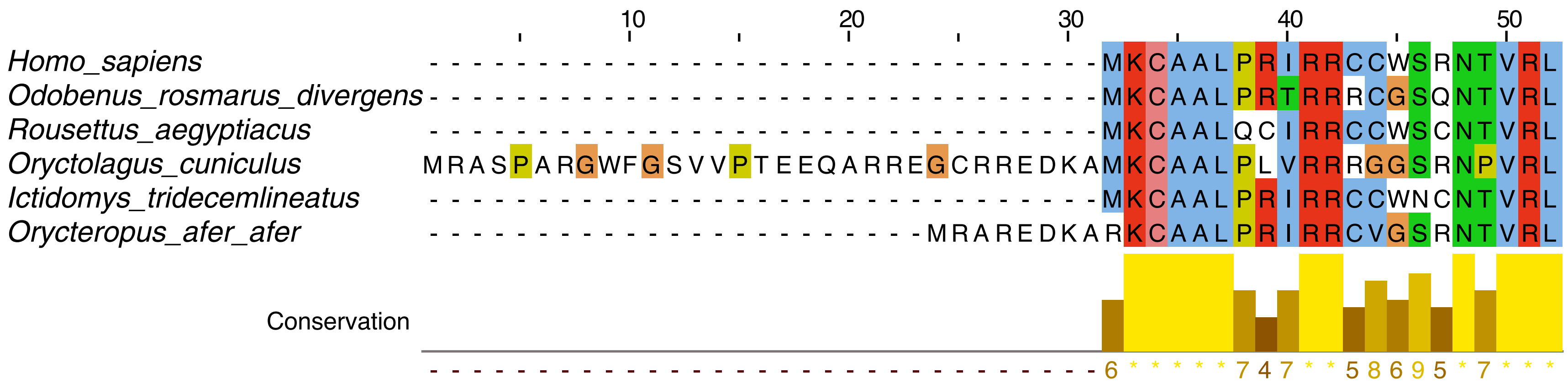

HG0843

ENSG00000043093 (DCUN1D1)

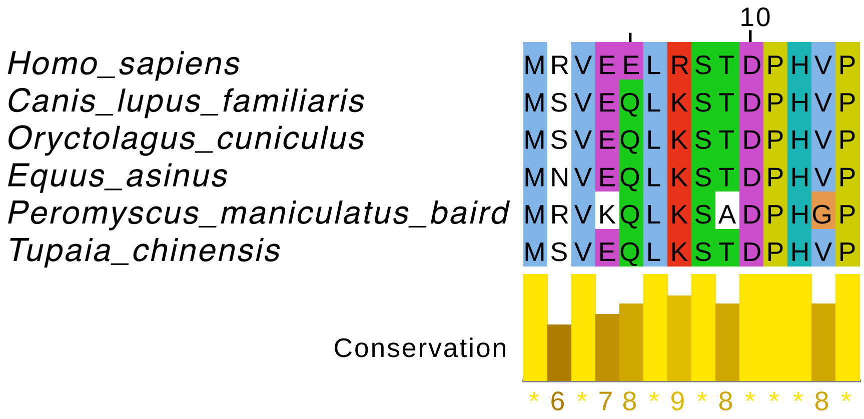

HG0844

ENSG00000091009 (RBM27)

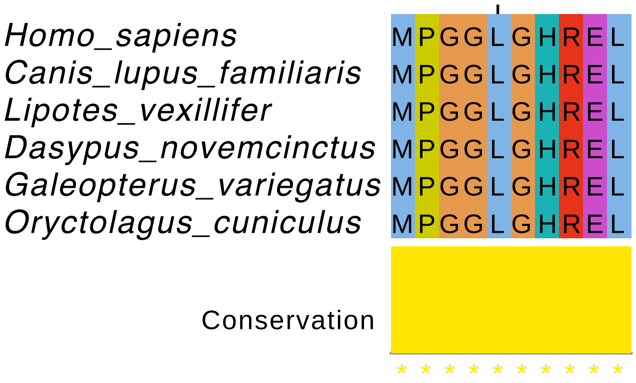

HG0845

ENSG00000099260 (PALMD)

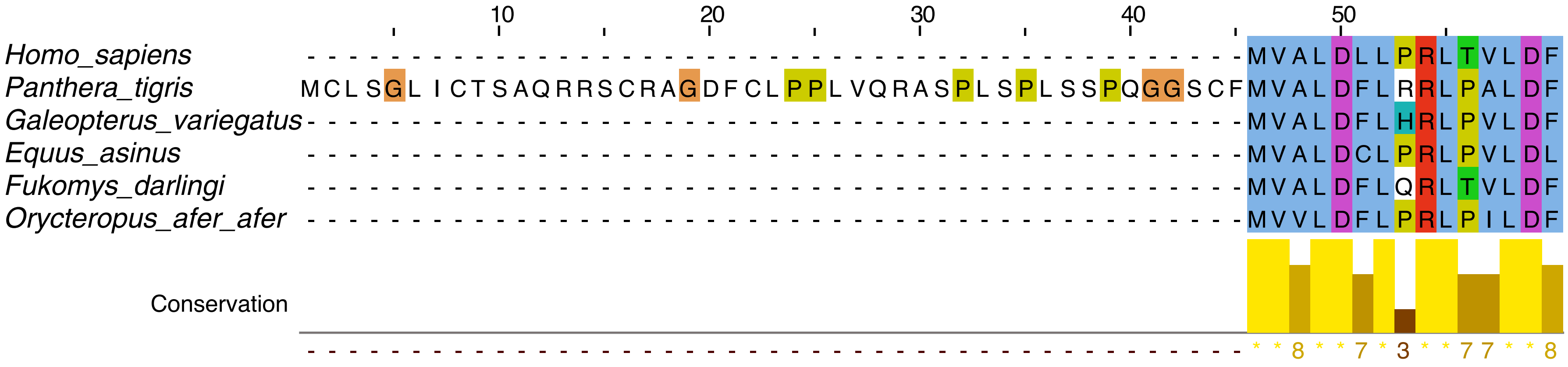

HG0846

ENSG00000100852 (ARHGAP5)

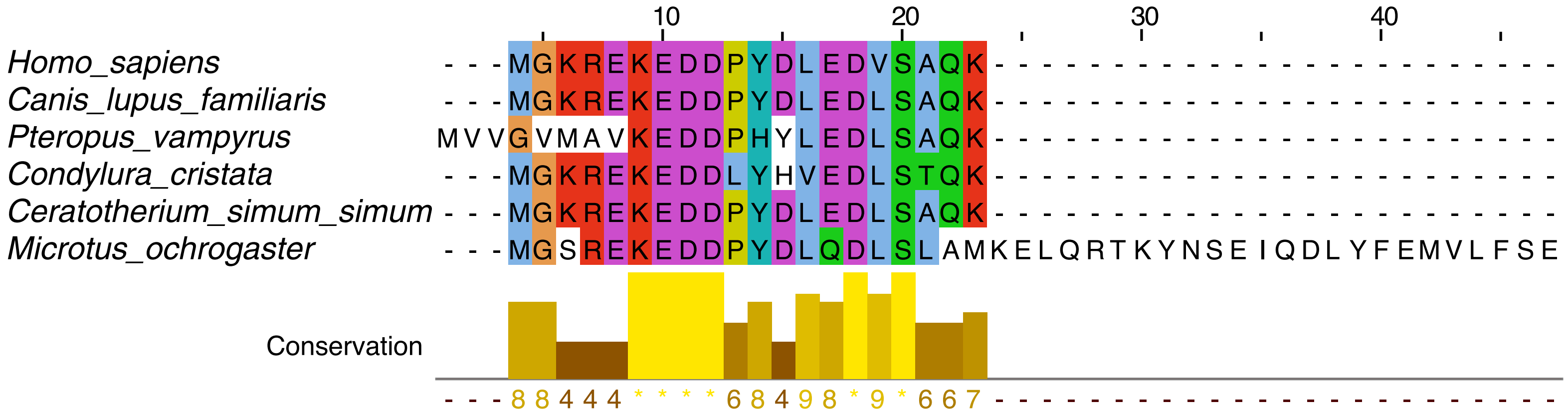

HG0847

ENSG00000106086 (PLEKHA8)

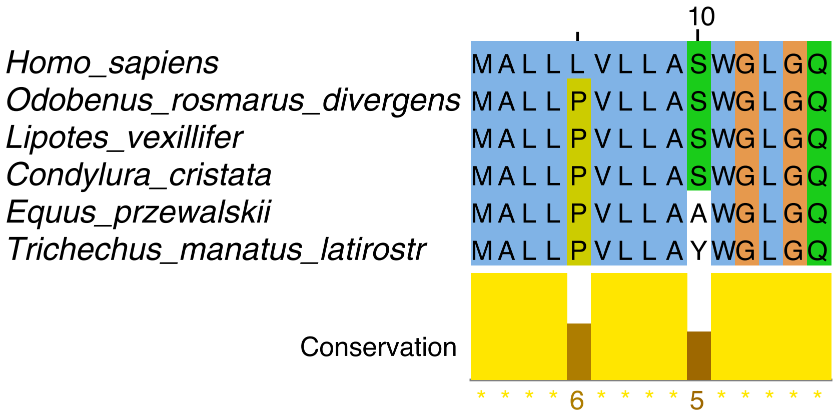

HG0848

ENSG00000111817 (DSE)

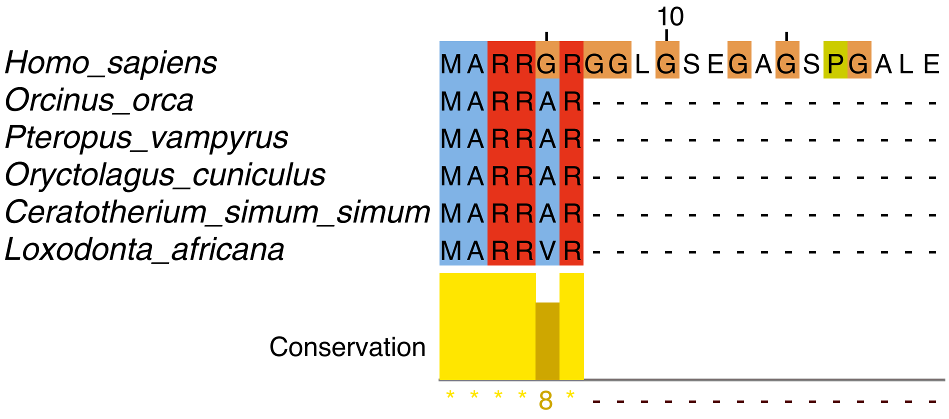

HG0849

ENSG00000111846 (GCNT2)

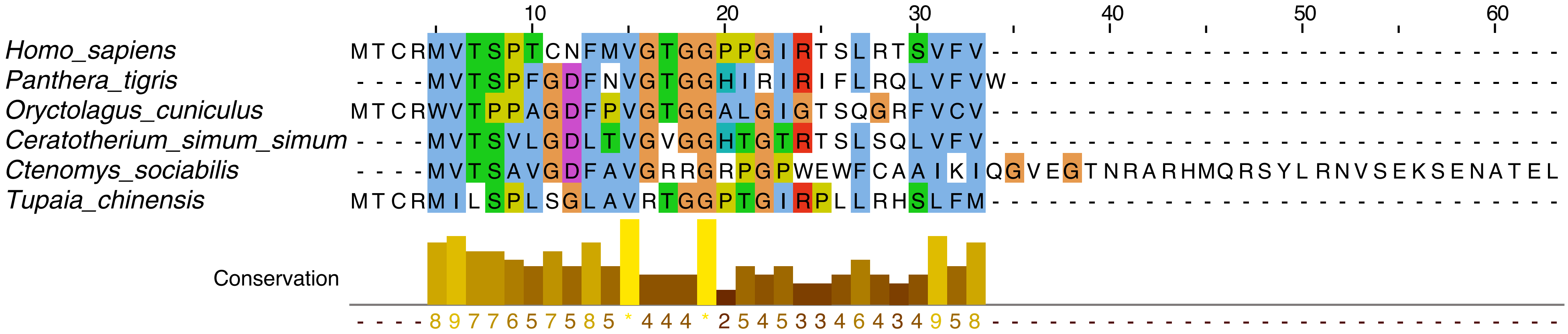

HG0850

ENSG00000113916 (BCL6)

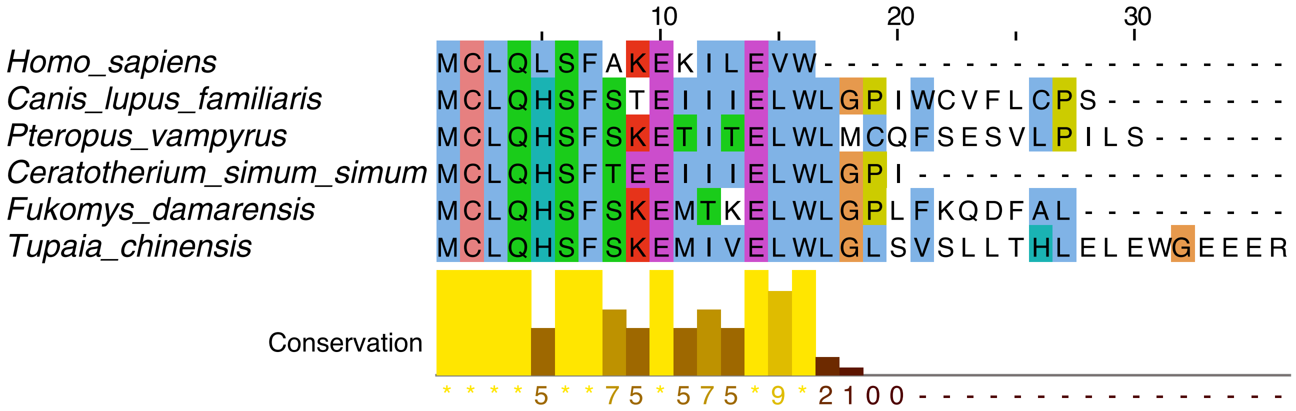

HG0851

ENSG00000114279 (FGF12)

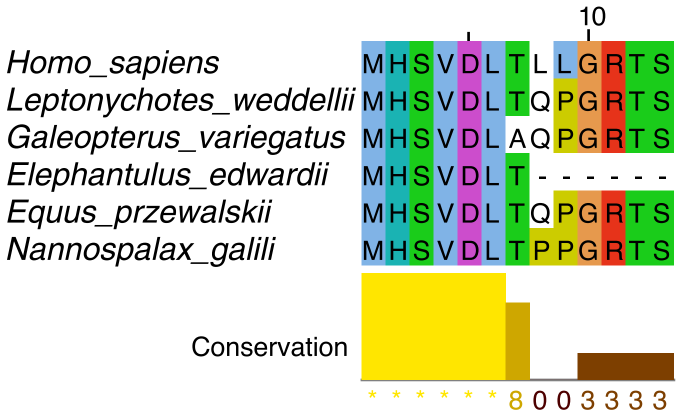

HG0852

ENSG00000114942 (EEF1B2)

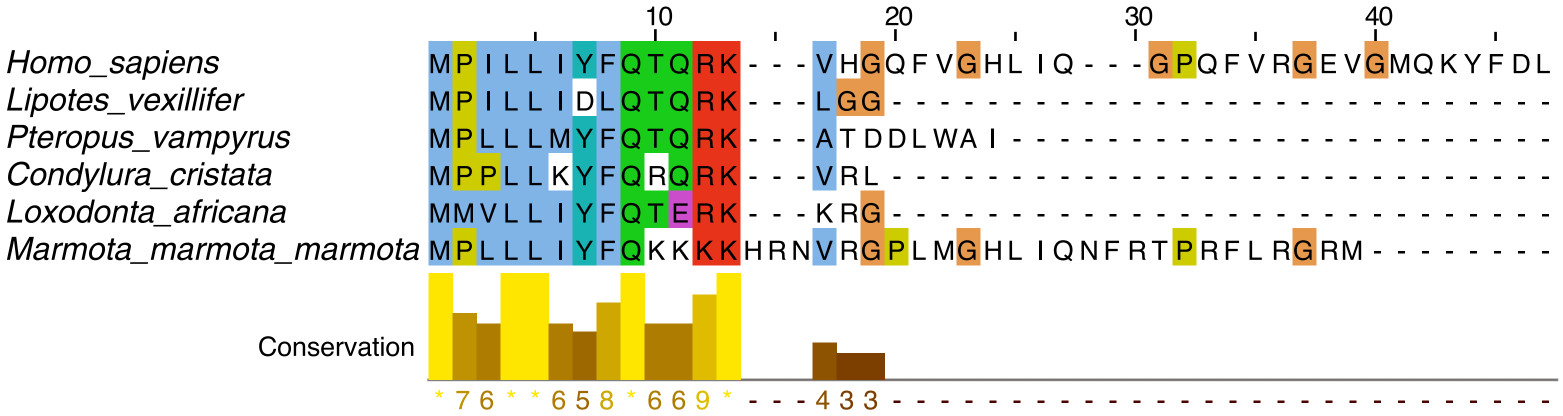

HG0853

ENSG00000121807 (CCR2)

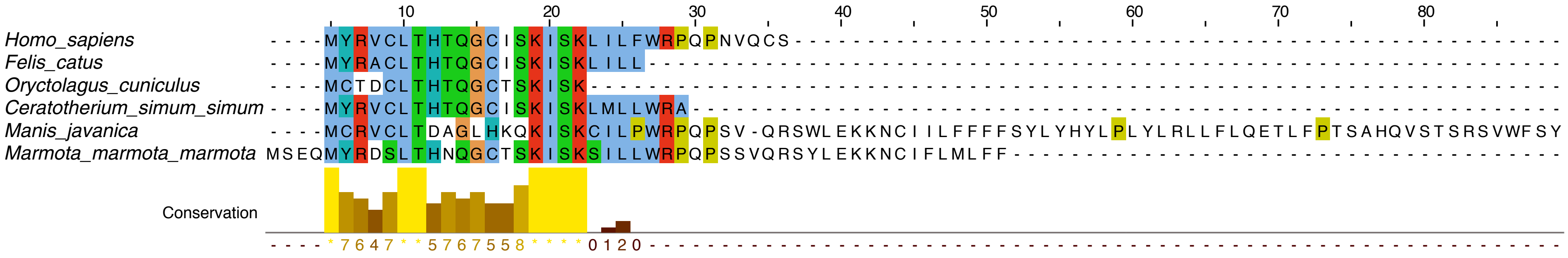

HG0854

ENSG00000127249 (ATP13A4)

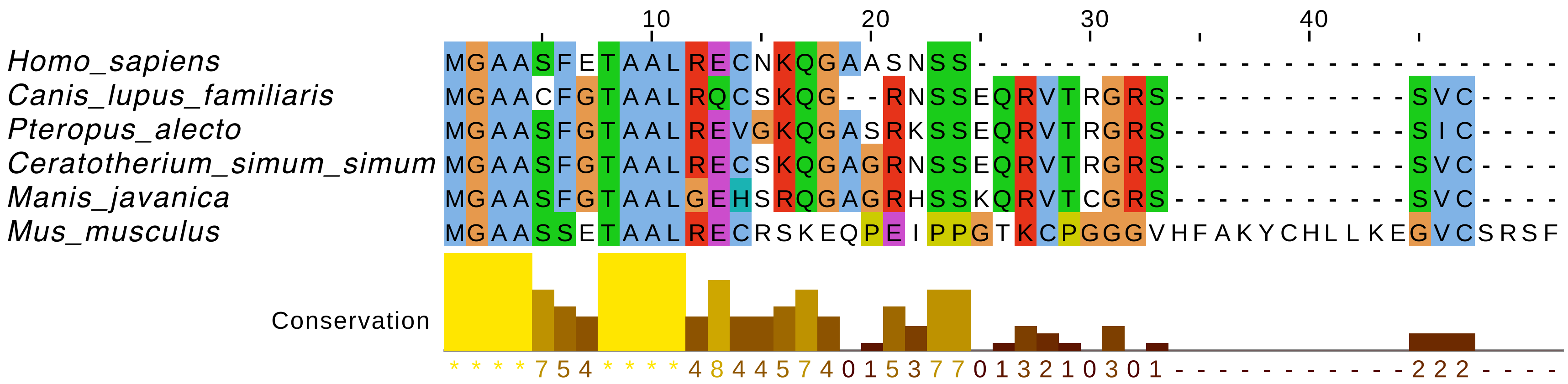

HG0855

ENSG00000127804 (METTL16)

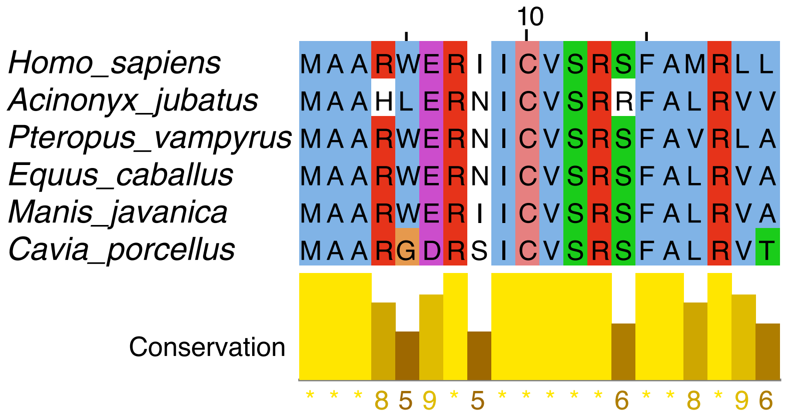

HG0856

ENSG00000130055 (GDPD2)

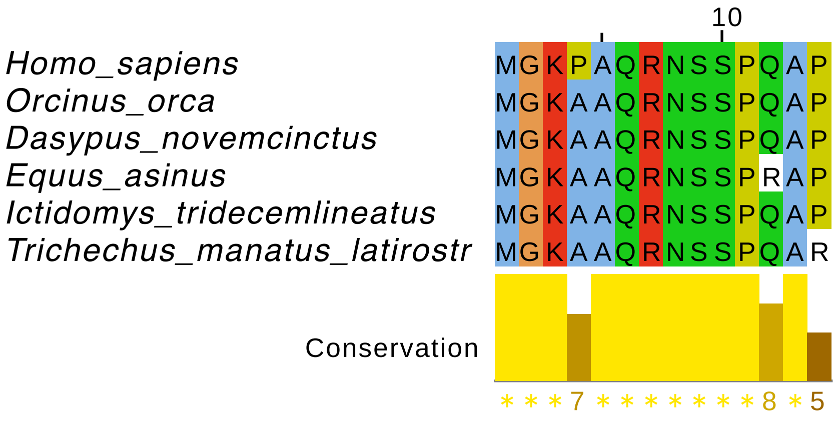

HG0857

ENSG00000130347 (RTN4IP1)

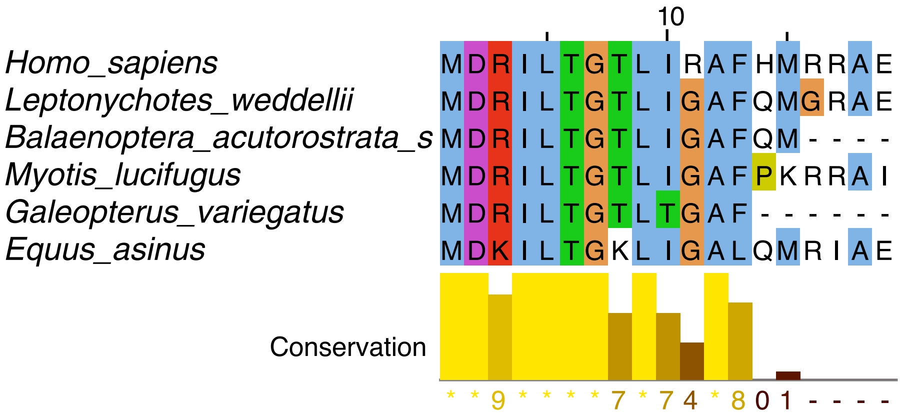

HG0858

ENSG00000132702 (HAPLN2)

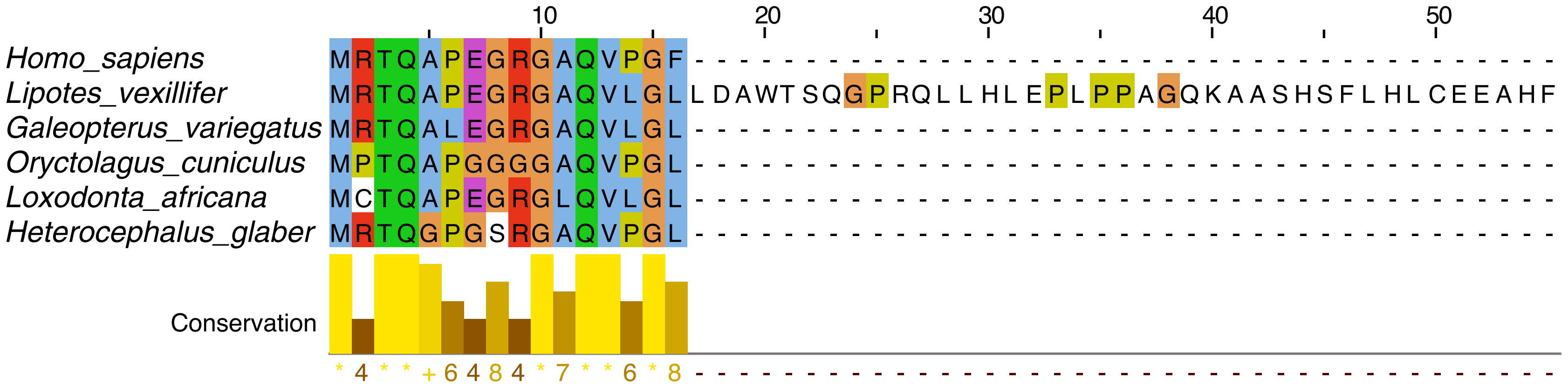

HG0859

ENSG00000132842 (AP3B1)

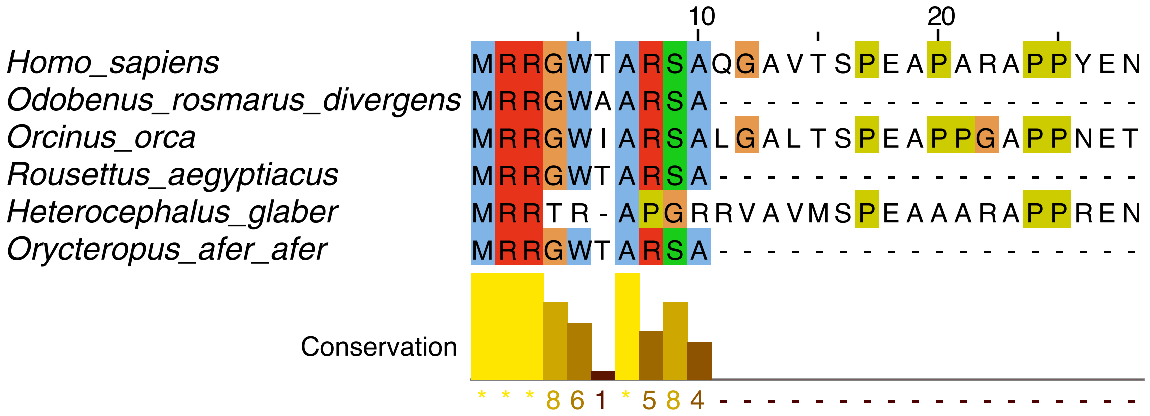

HG0860

ENSG00000133056 (PIK3C2B)

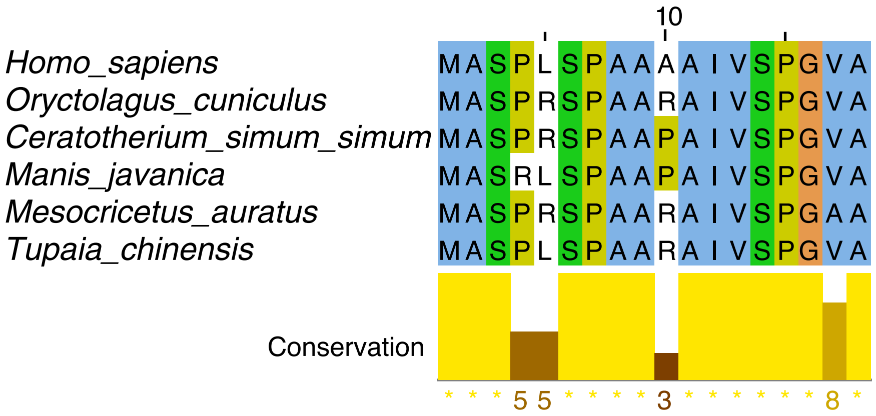

HG0861

ENSG00000134001 (EIF2S1)

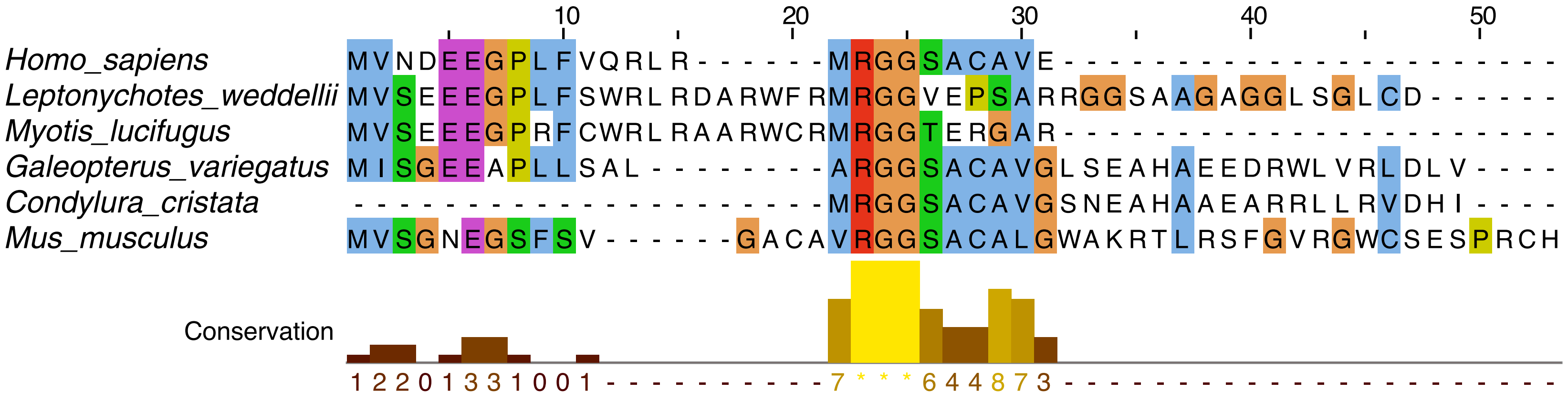

HG0862

ENSG00000134245 (WNT2B)

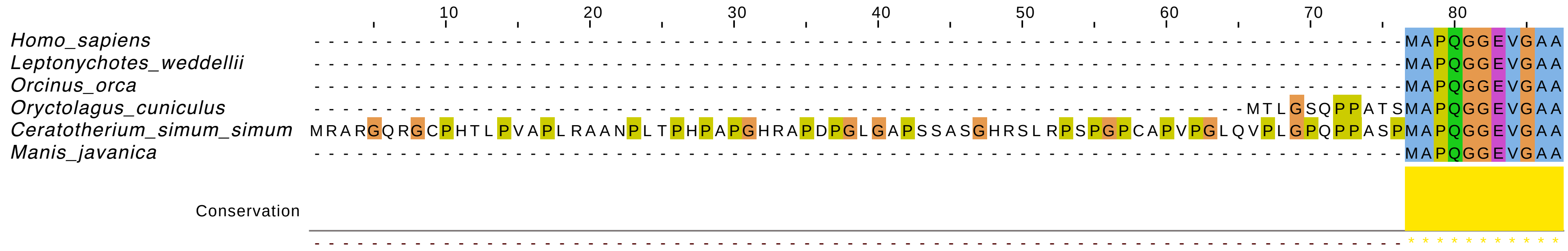

HG0863

ENSG00000134313 (KIDINS220)

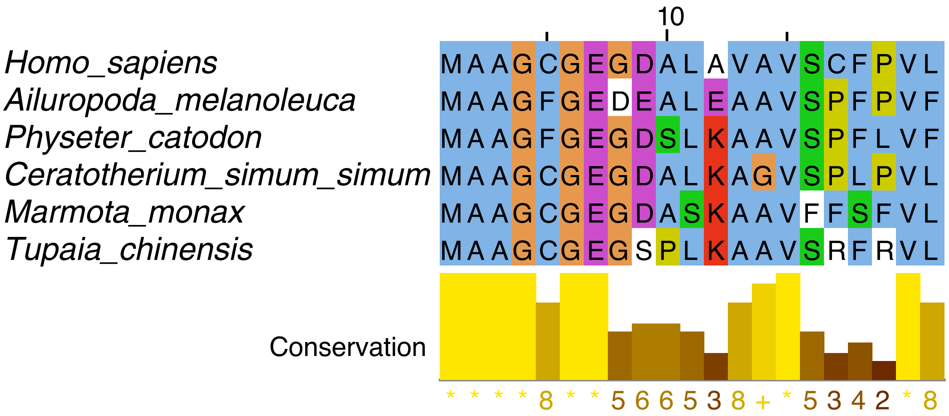

HG0864

ENSG00000134533 (RERG)

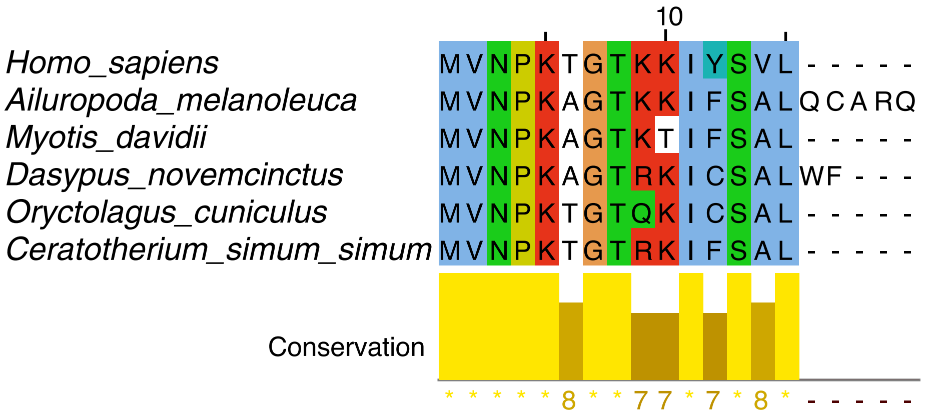

HG0865

ENSG00000135540 (NHSL1)

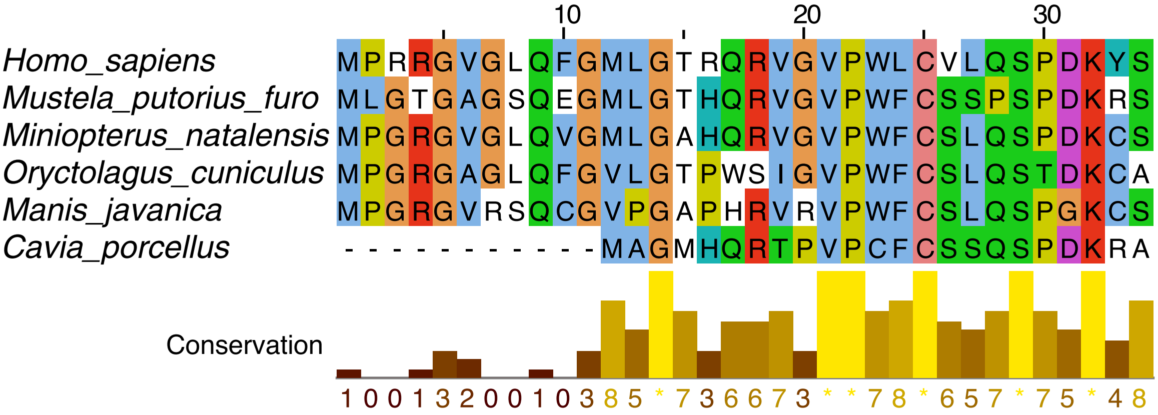

HG0866

ENSG00000136011 (STAB2)

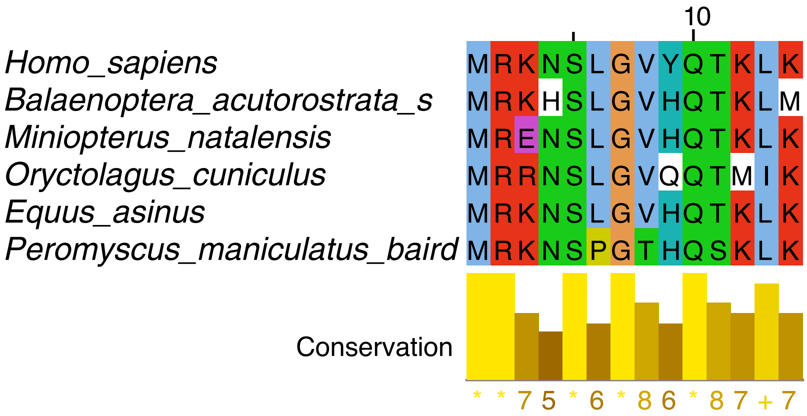

HG0867

ENSG00000137203 (TFAP2A)

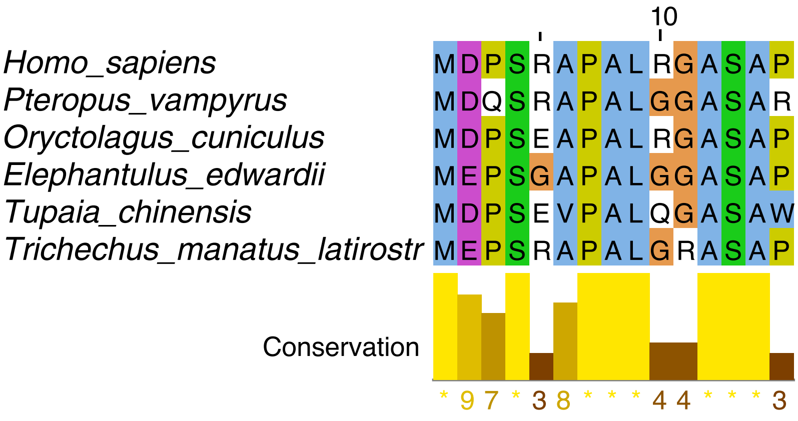

HG0868

ENSG00000137843 (PAK6)

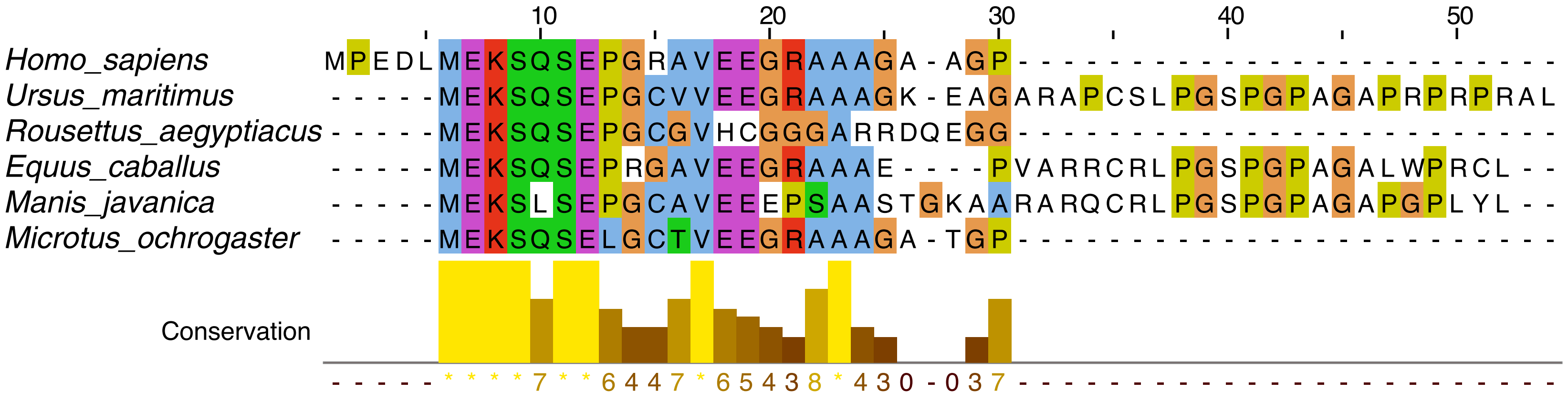

HG0869

ENSG00000139910 (NOVA1)

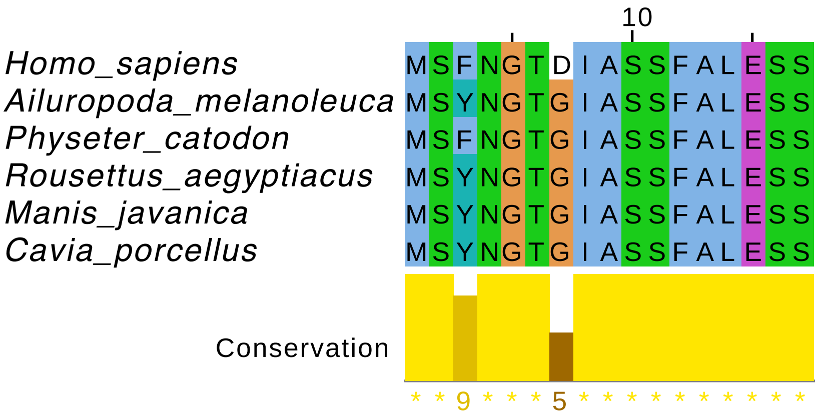

HG0870

ENSG00000140993 (TIGD7)

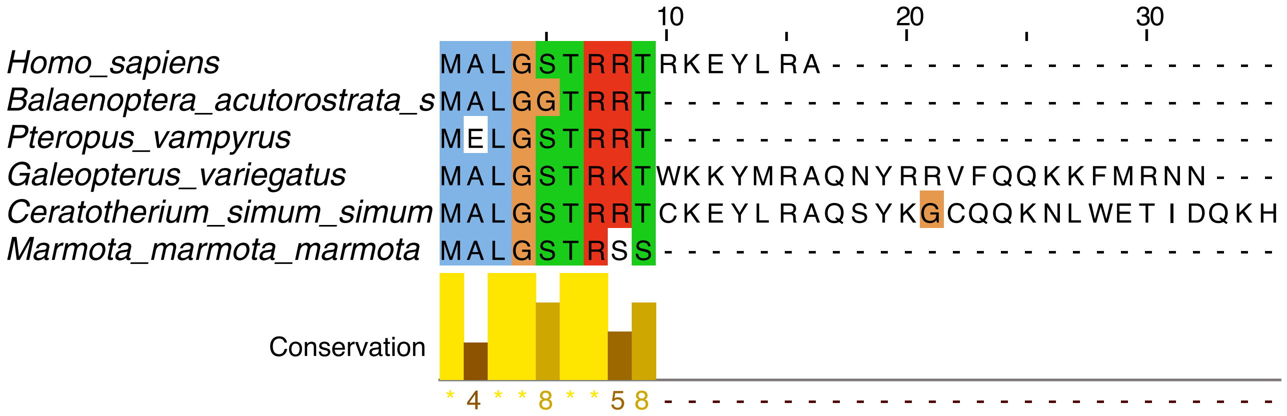

HG0871

ENSG00000141127 (PRPSAP2)

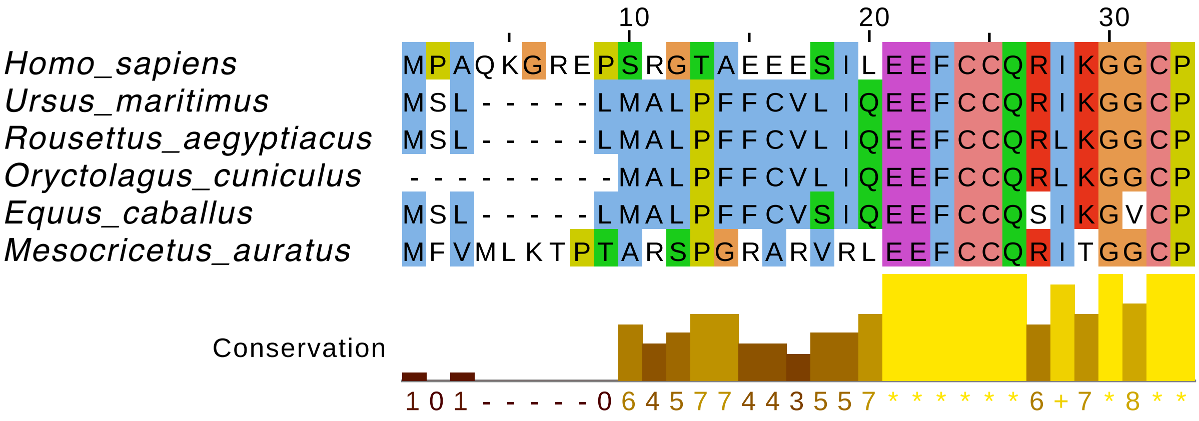

HG0872

ENSG00000142583 (SLC2A5)

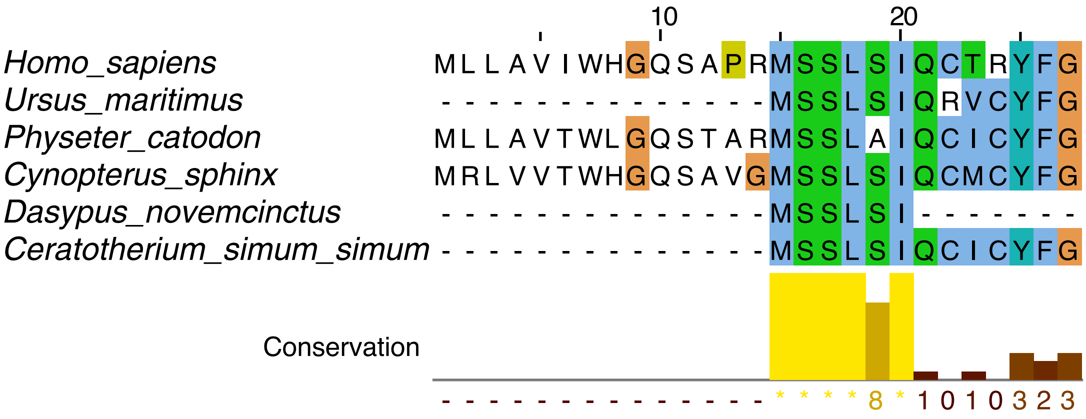

HG0873

ENSG00000142875 (PRKACB)

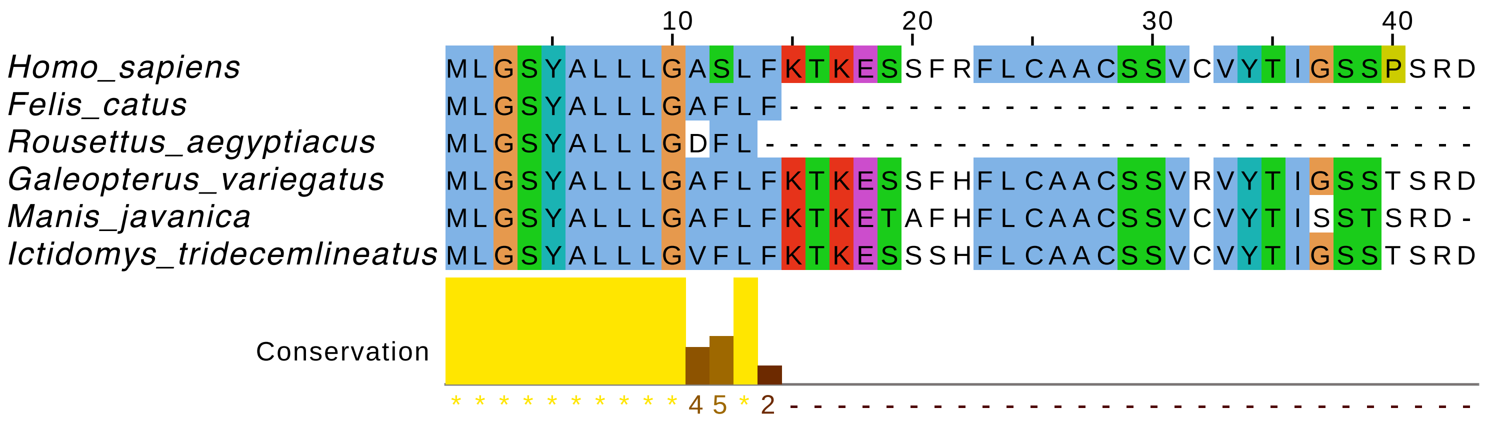

HG0874

ENSG00000143515 (ATP8B2)

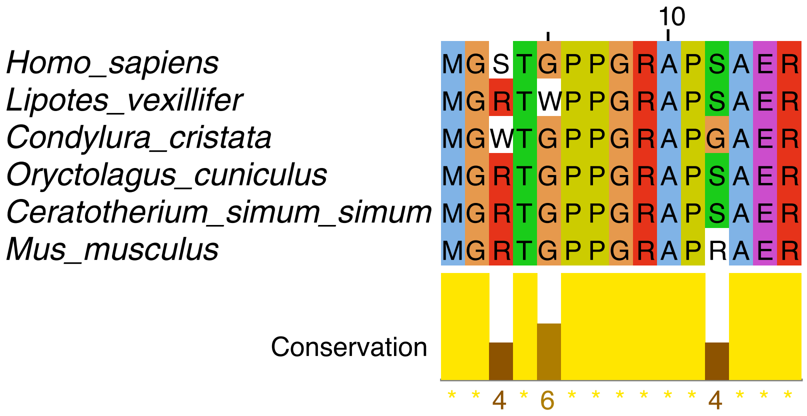

HG0875

ENSG00000145284 (SCD5)

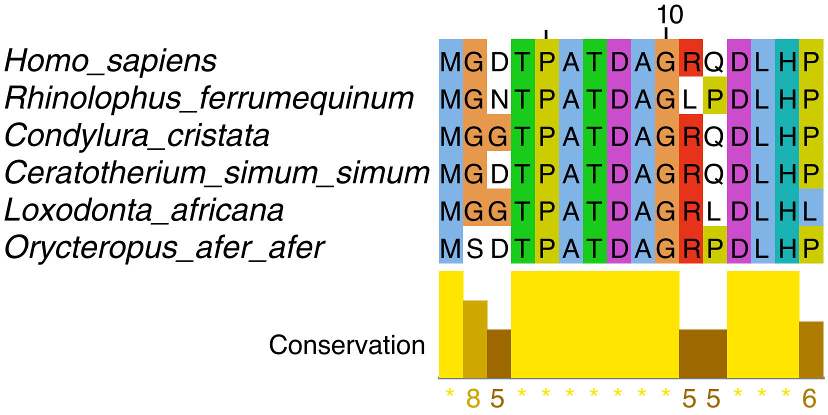

HG0876

ENSG00000145908 (ZNF300)

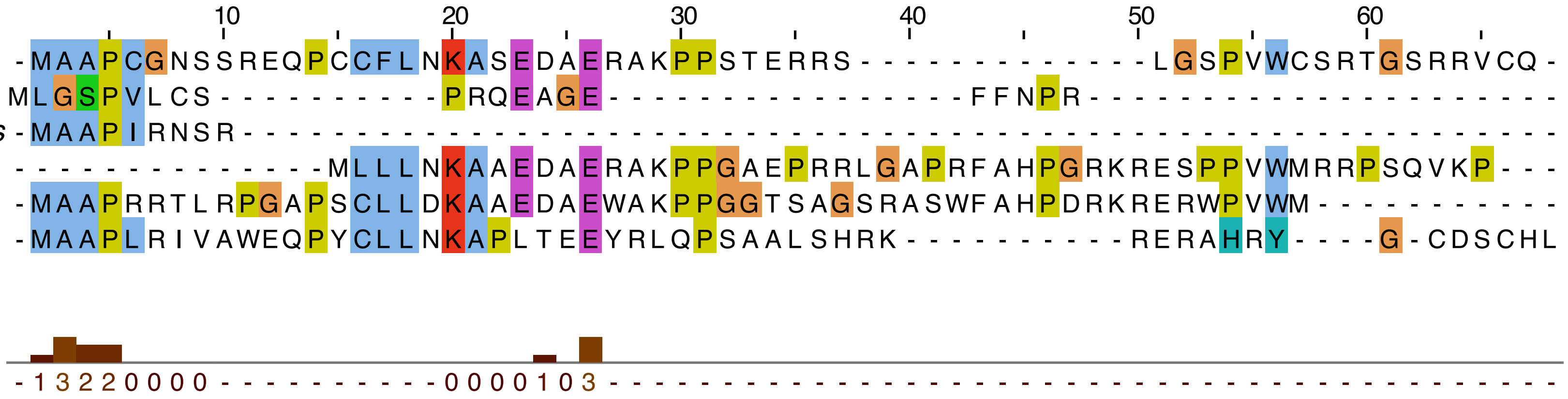

HG0877

ENSG00000146426 (TIAM2)

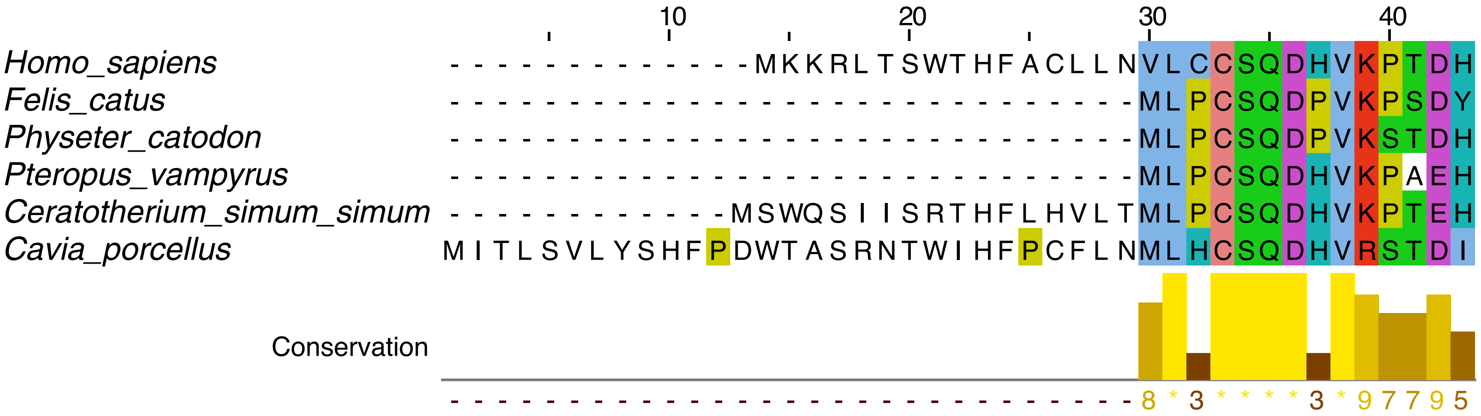

HG0878

ENSG00000146802 (TMEM168)

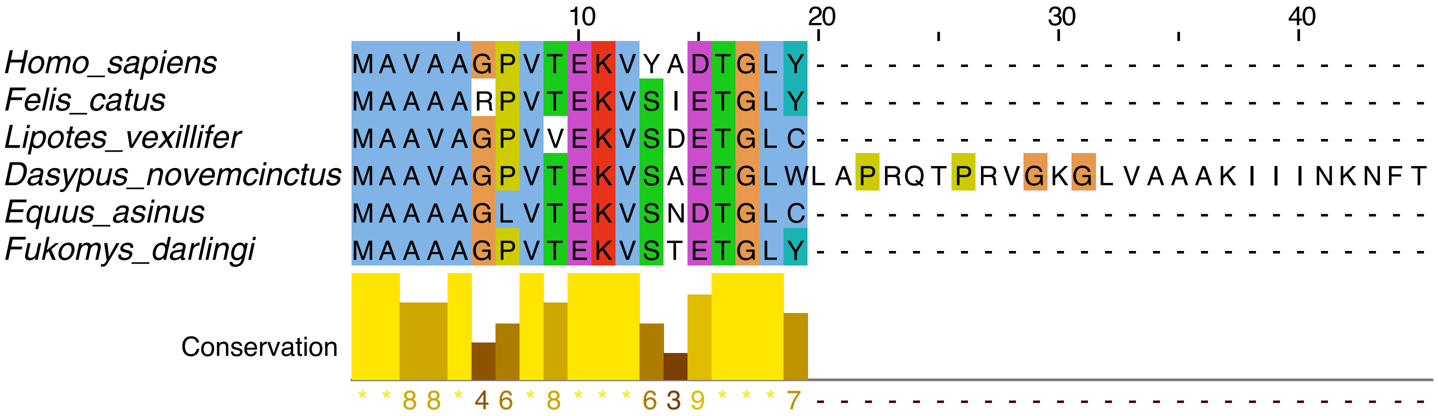

HG0879

ENSG00000147145 (LPAR4)

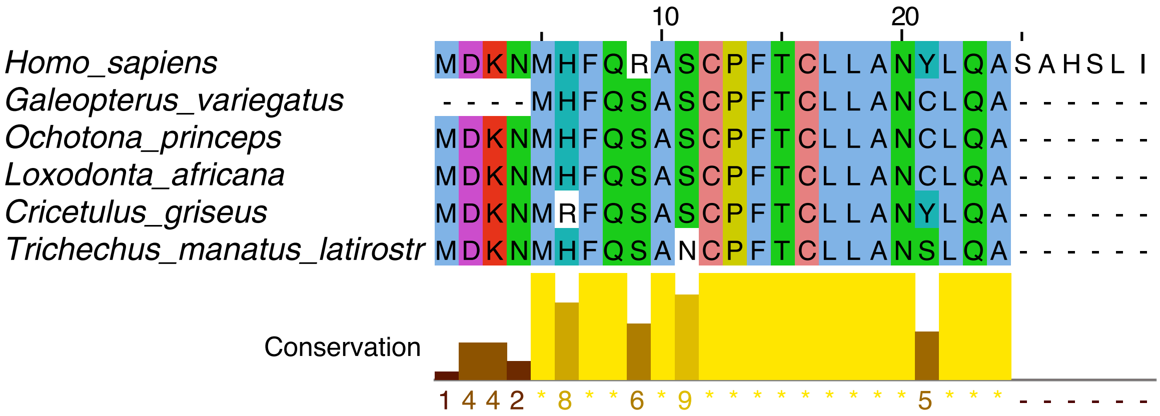

HG0880

ENSG00000151789 (ZNF385D)

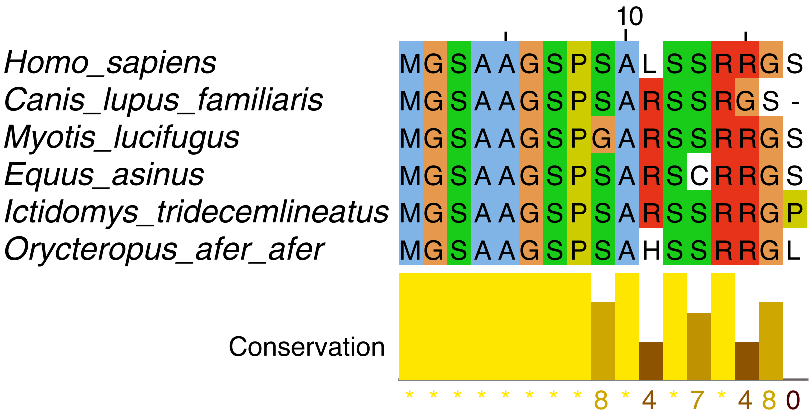

HG0881

ENSG00000153822 (KCNJ16)

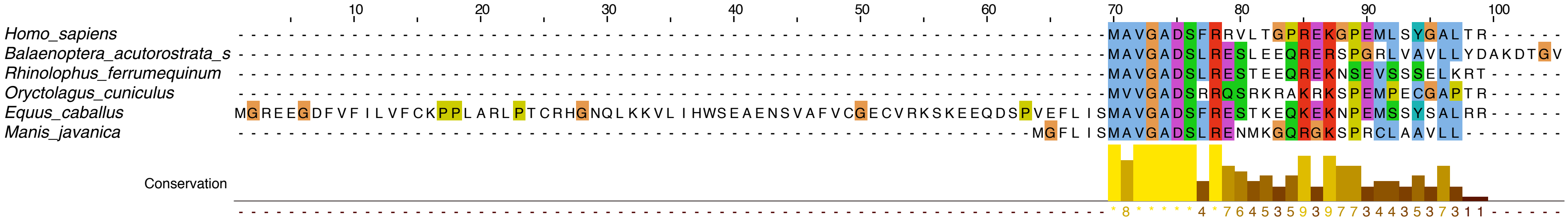

HG0882.1

ENSG00000154832 (CXXC1)

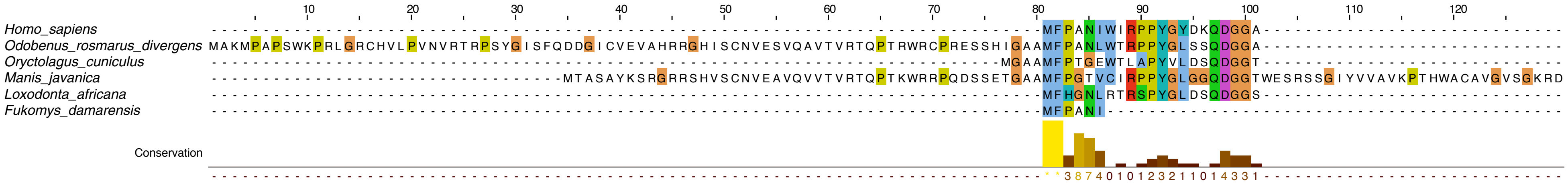

HG0882.2

ENSG00000154832 (CXXC1)

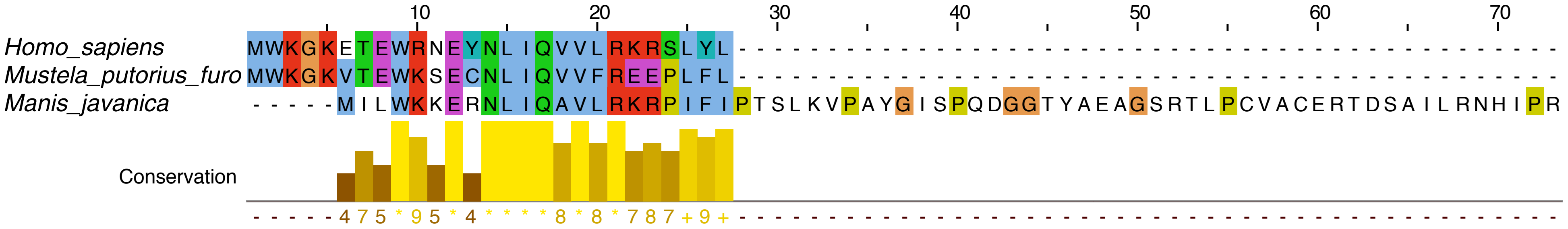

HG0883

ENSG00000156011 (PSD3)

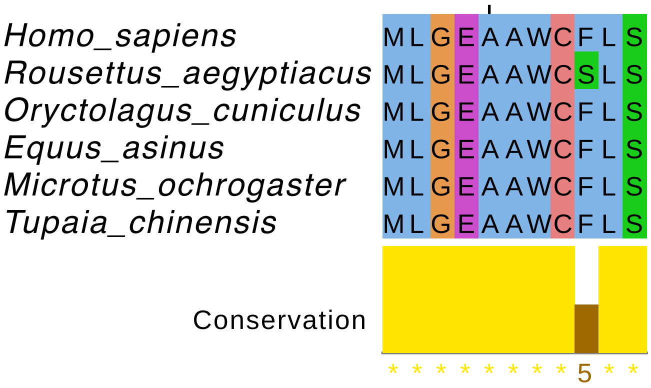

HG0884

ENSG00000156463 (SH3RF2)

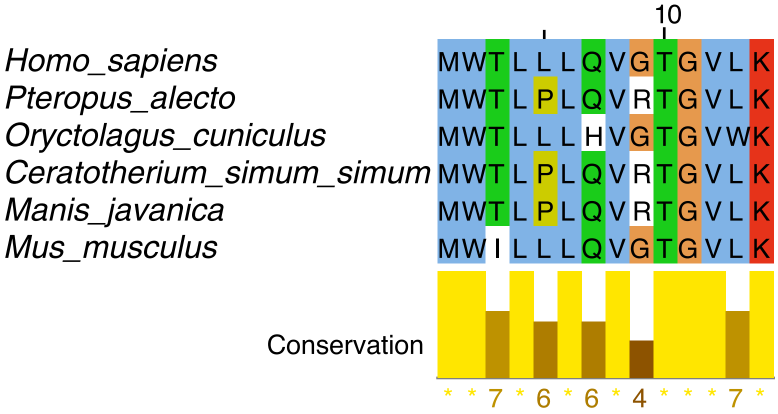

HG0885.1

ENSG00000157502 (MUM1L1)

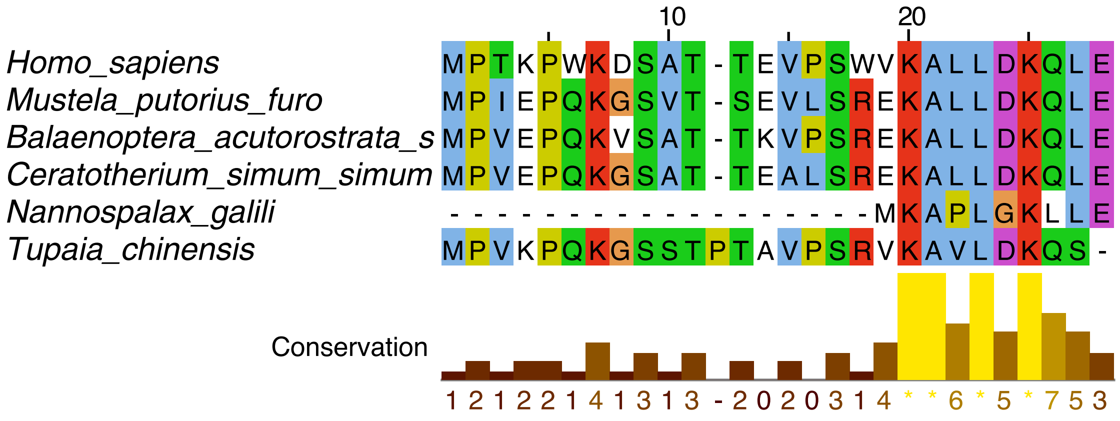

HG0885.2

ENSG00000157502 (MUM1L1)

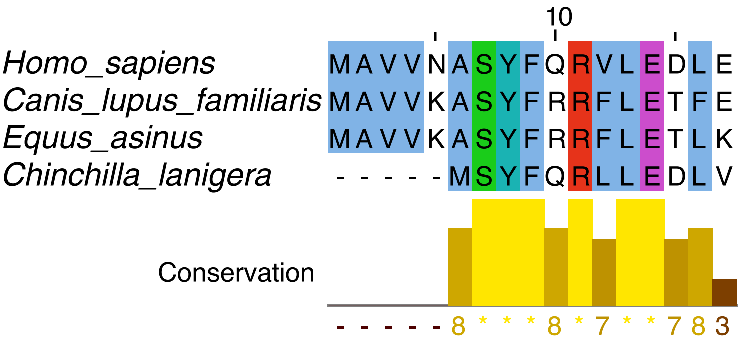

HG0886

ENSG00000158850 (B4GALT3)

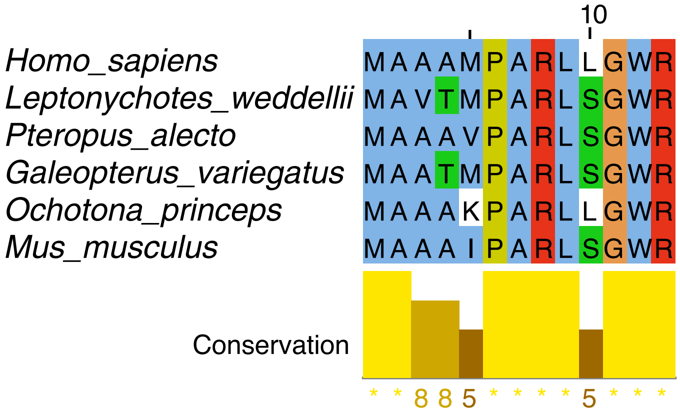

HG0887

ENSG00000160959

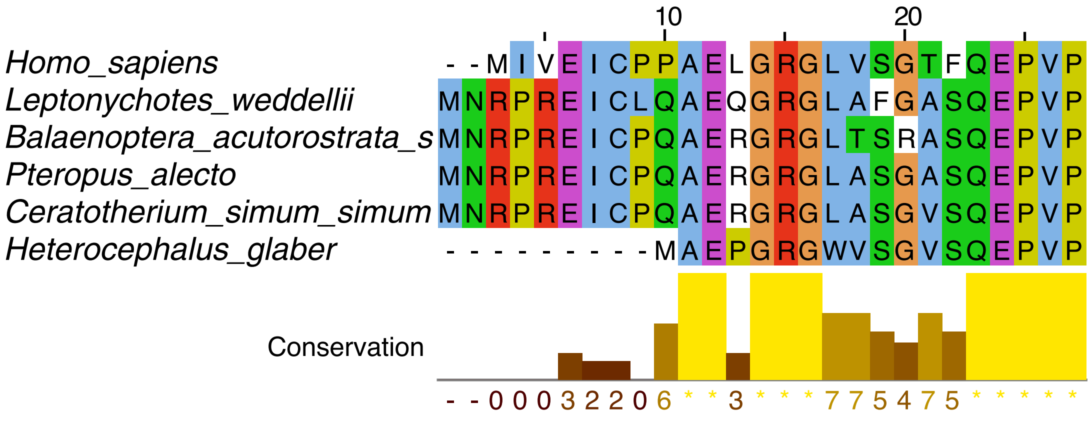

HG0888

ENSG00000164023 (SGMS2)

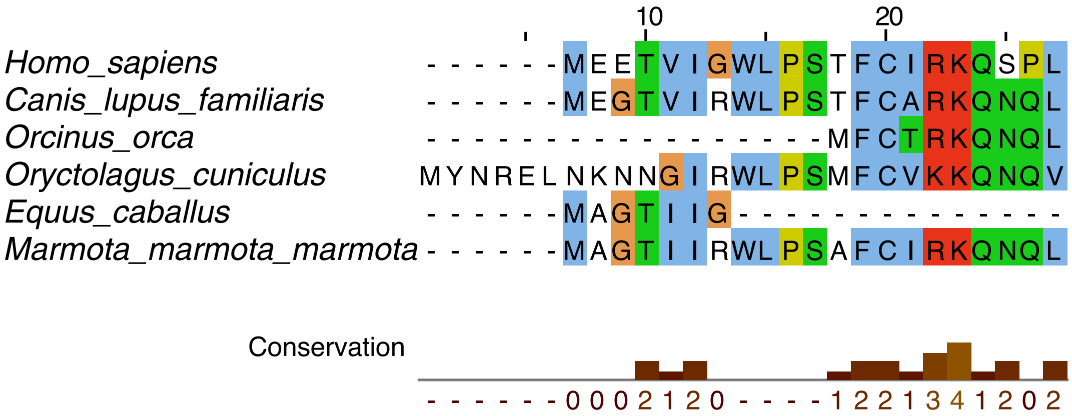

HG0889

ENSG00000164091 (WDR82)

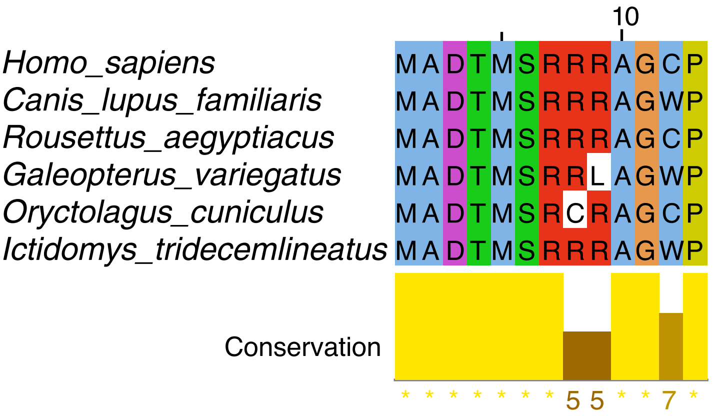

HG0890

ENSG00000165813

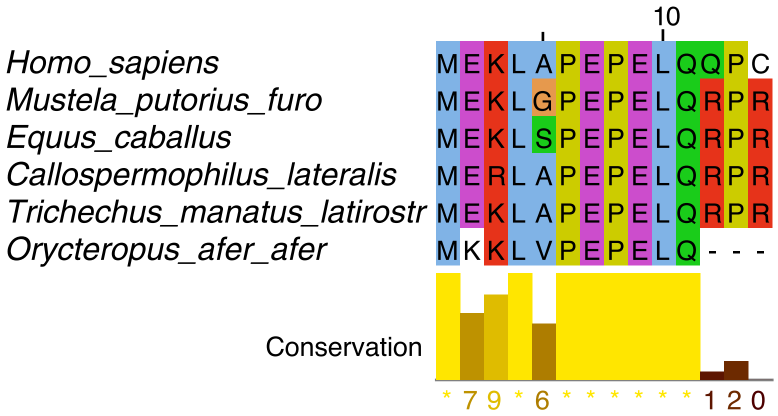

HG0891

ENSG00000166211 (SPIC)

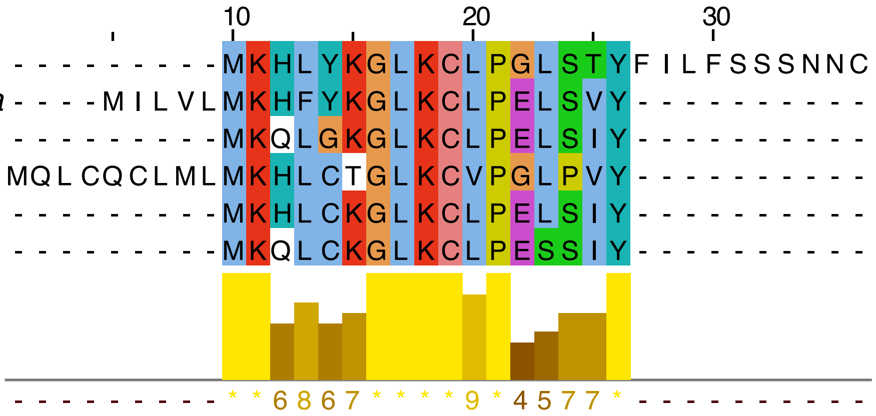

HG0892

ENSG00000168418 (KCNG4)

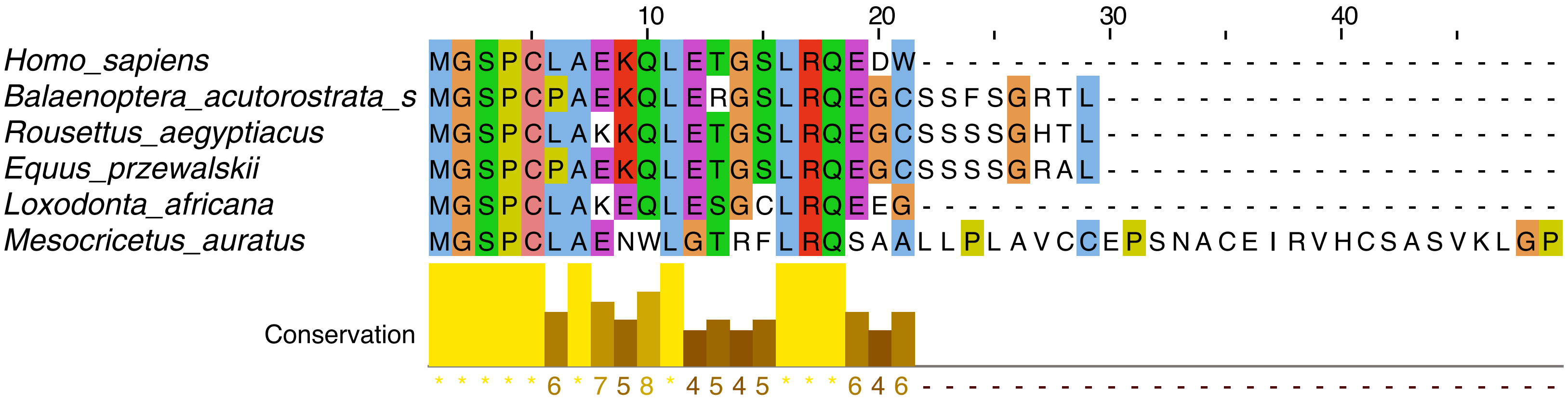

HG0893

ENSG00000168769 (TET2)

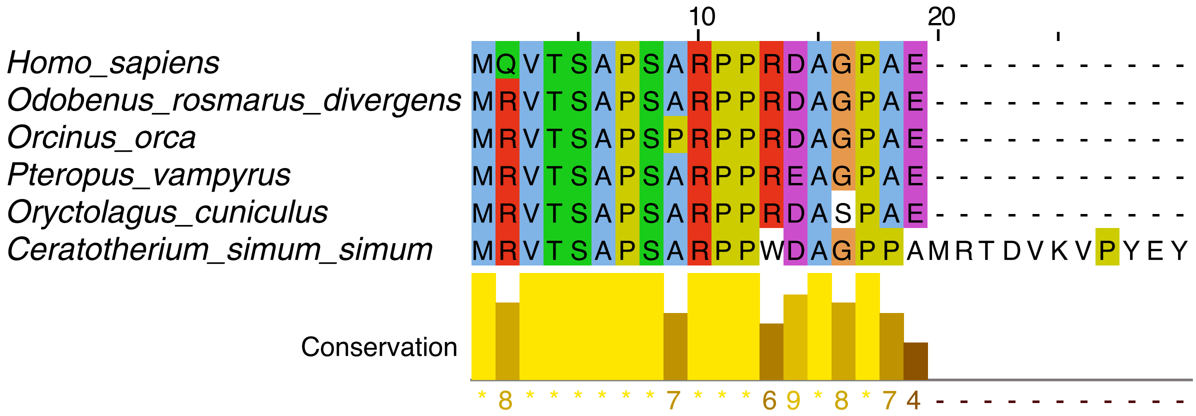

HG0894.1

ENSG00000169282 (KCNAB1)

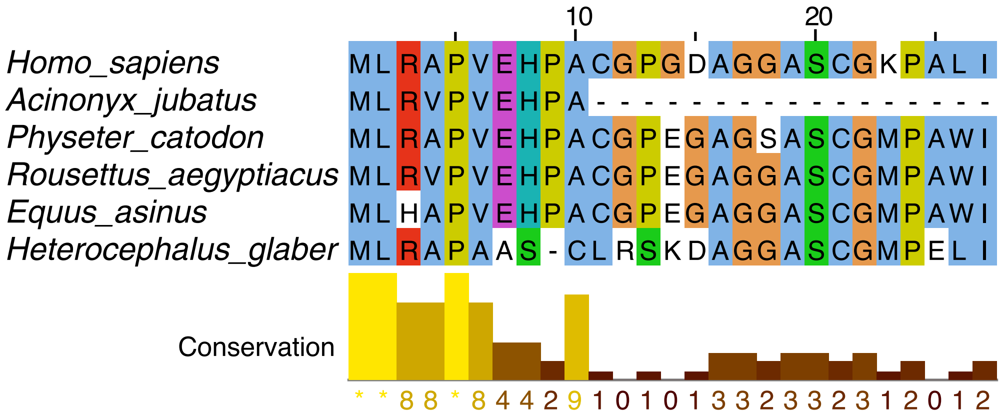

HG0894.2

ENSG00000069424 (KCNAB2)

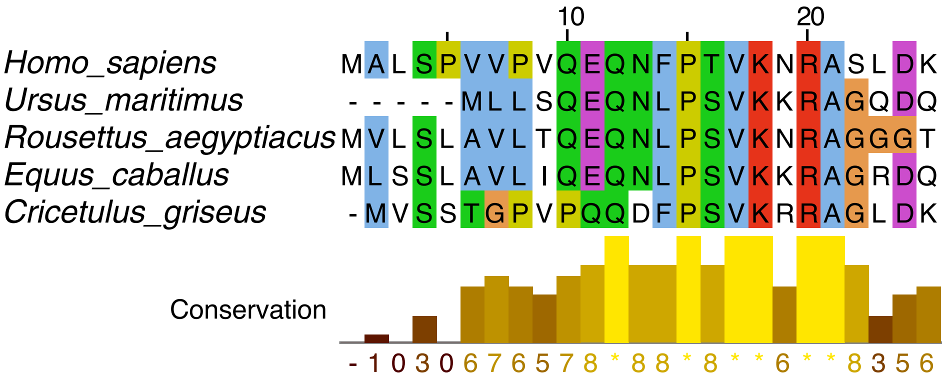

HG0895

ENSG00000170035 (UBE2E3)

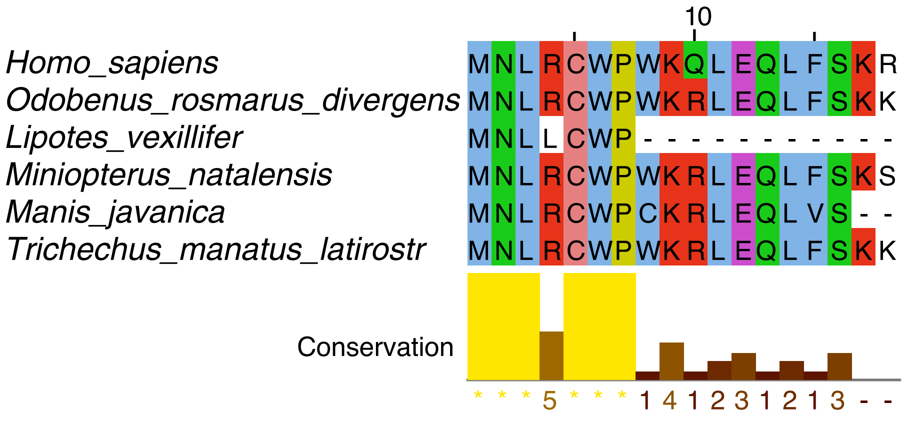

HG0896

ENSG00000170365 (SMAD1)

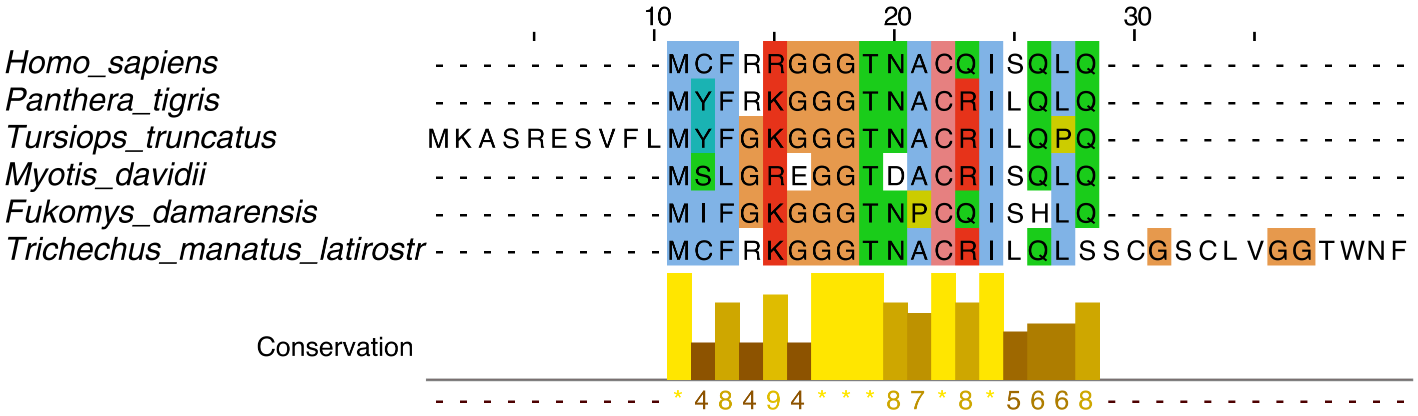

HG0897

ENSG00000171435 (KSR2)

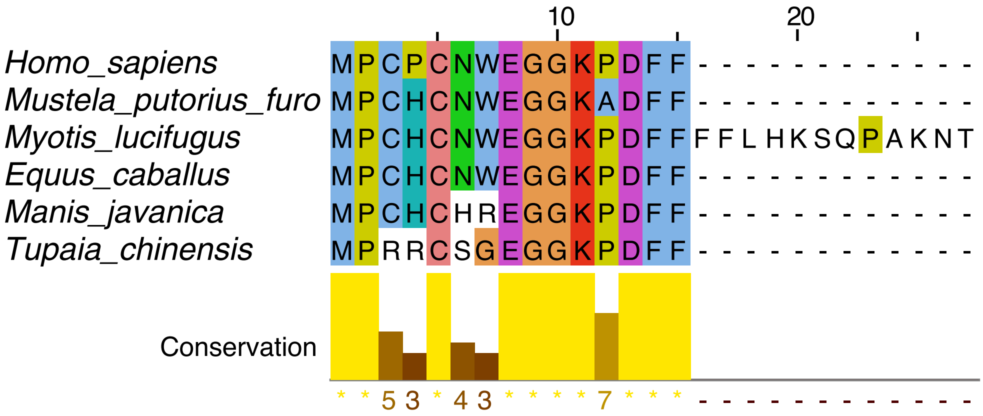

HG0898

ENSG00000171729 (TMEM51)

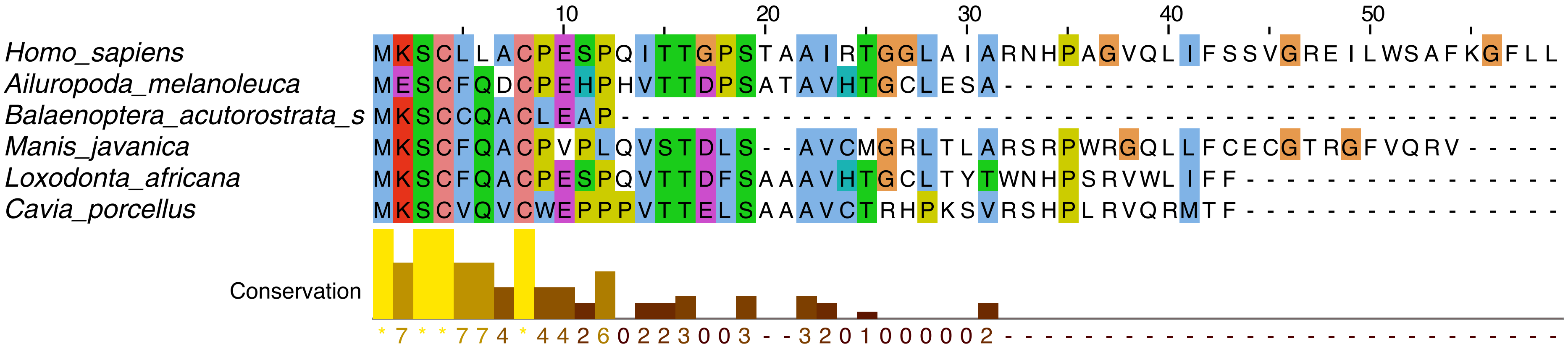

HG0899

ENSG00000173320 (STOX2)

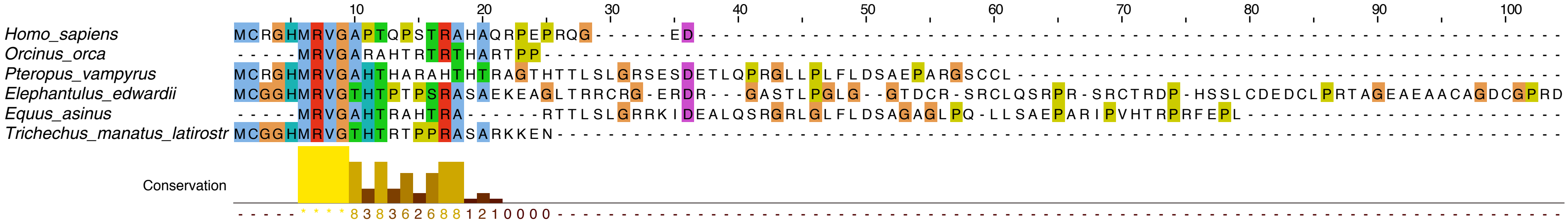

HG0900

ENSG00000173517 (PEAK1)

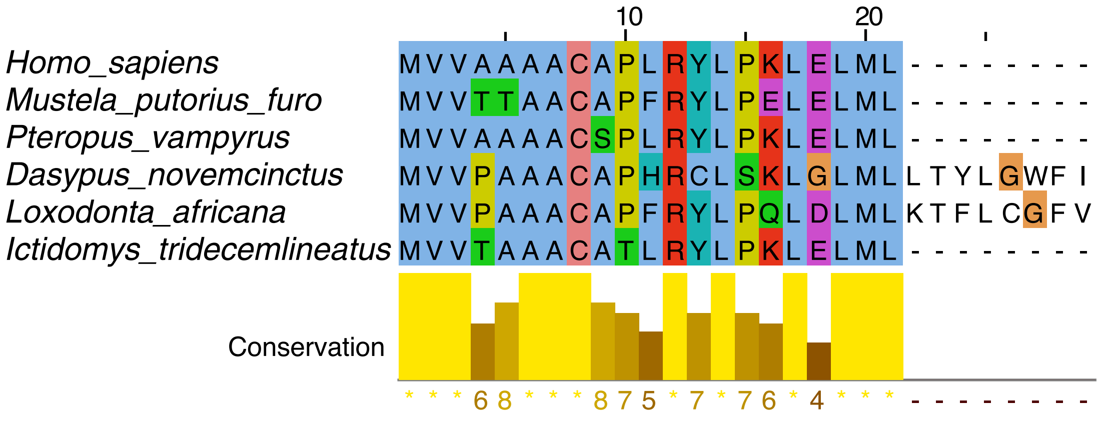

HG0901

ENSG00000175697 (GPR156)

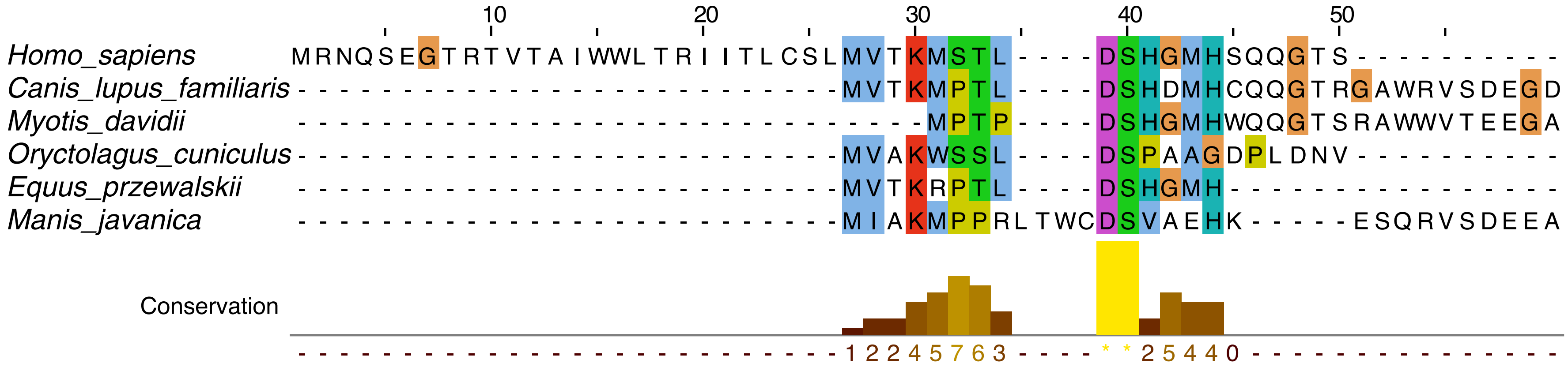

HG0902

ENSG00000177182 (CLVS1)

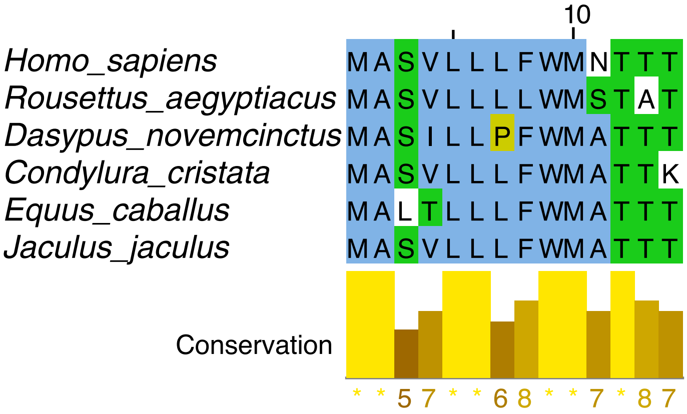

HG0903

ENSG00000180616 (SSTR2)

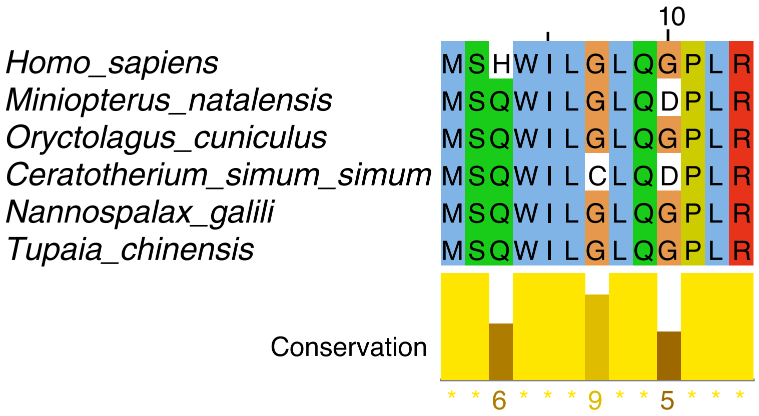

HG0904

ENSG00000182329

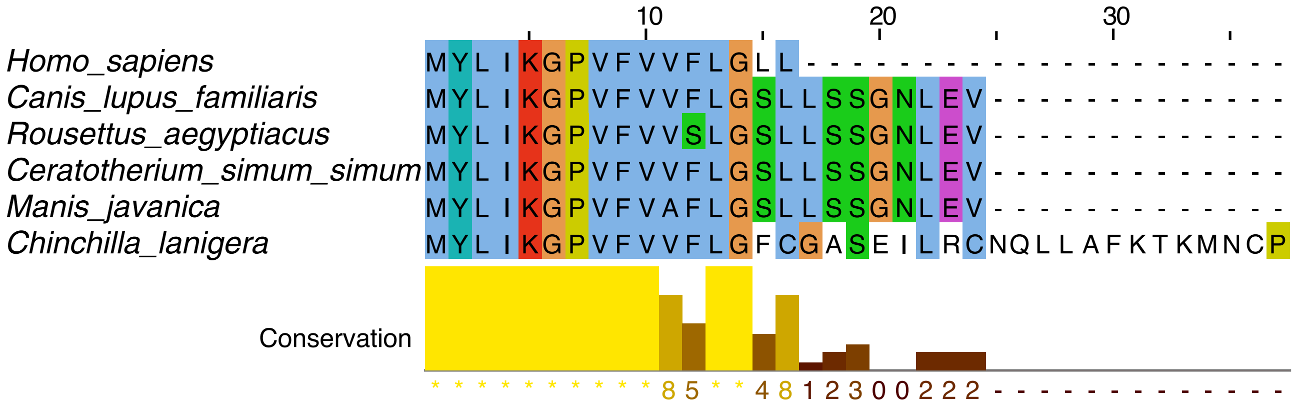

HG0905

ENSG00000183337 (BCOR)

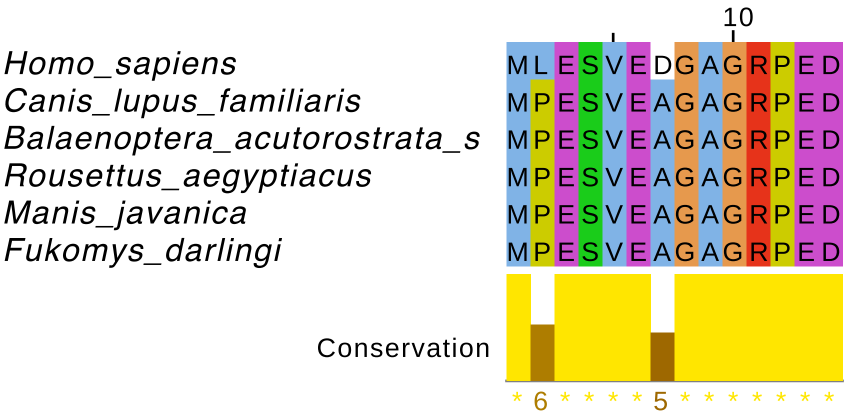

HG0906

ENSG00000183773 (AIFM3)

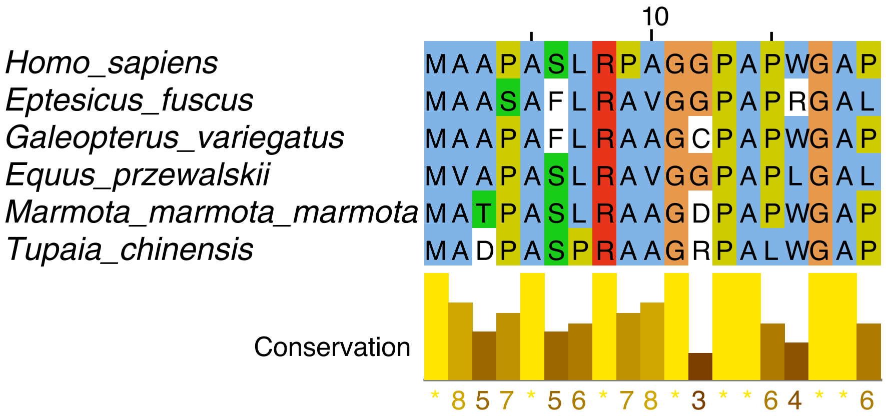

HG0907

ENSG00000186862 (PDZD7)

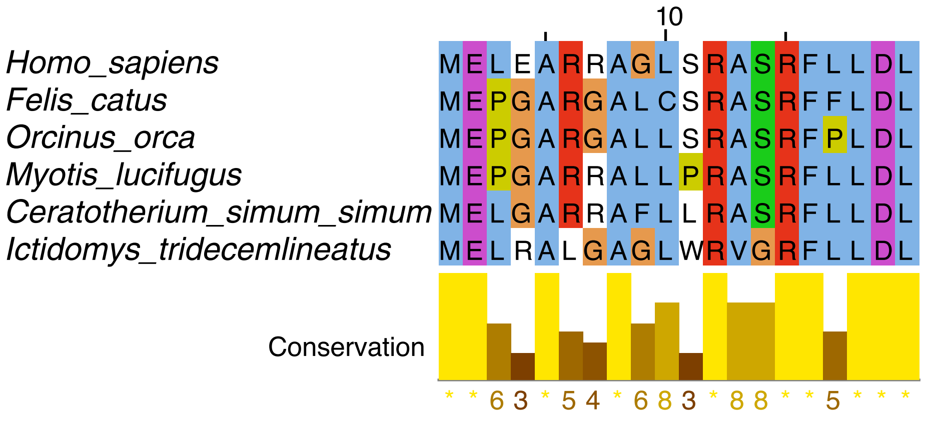

HG0908.1

ENSG00000188554 (NBR1)

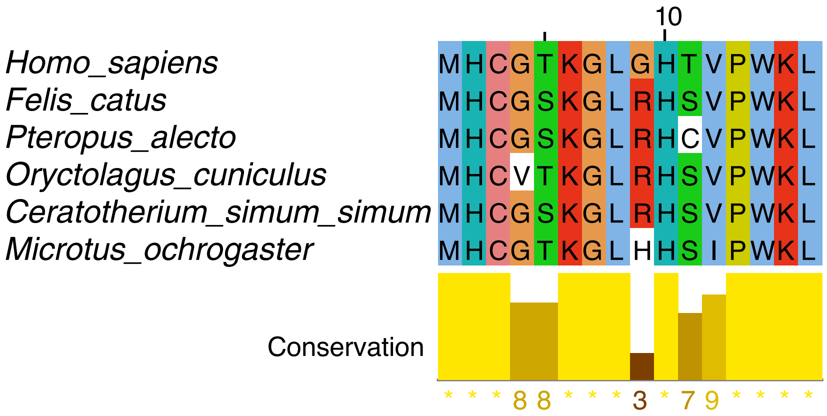

HG0908.2

ENSG00000188554 (NBR1)

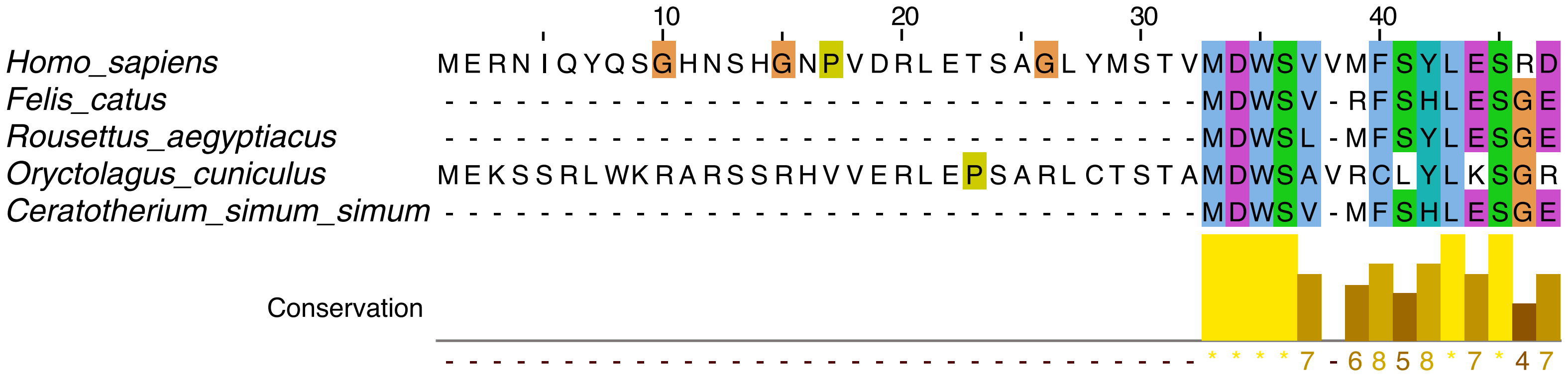

HG0909

ENSG00000189079 (ARID2)

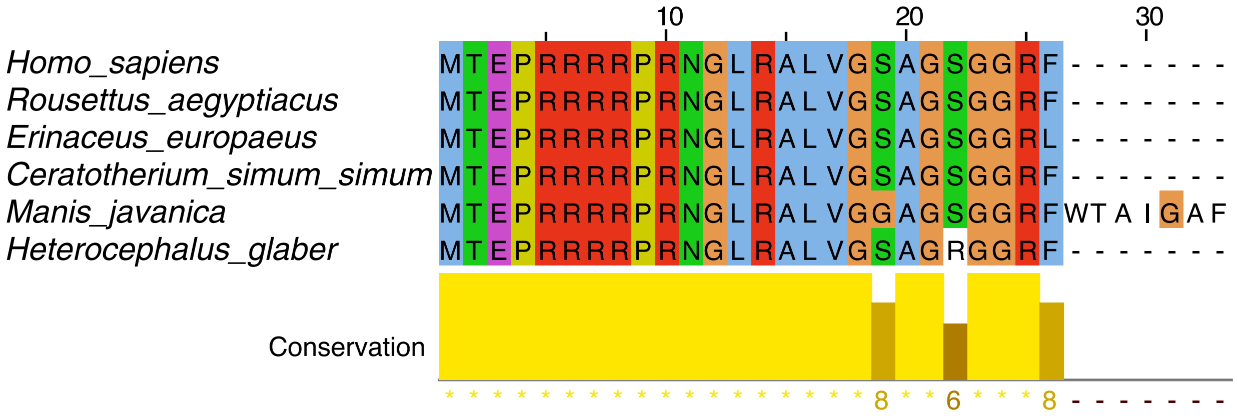

HG0910

ENSG00000197935 (ZNF311)

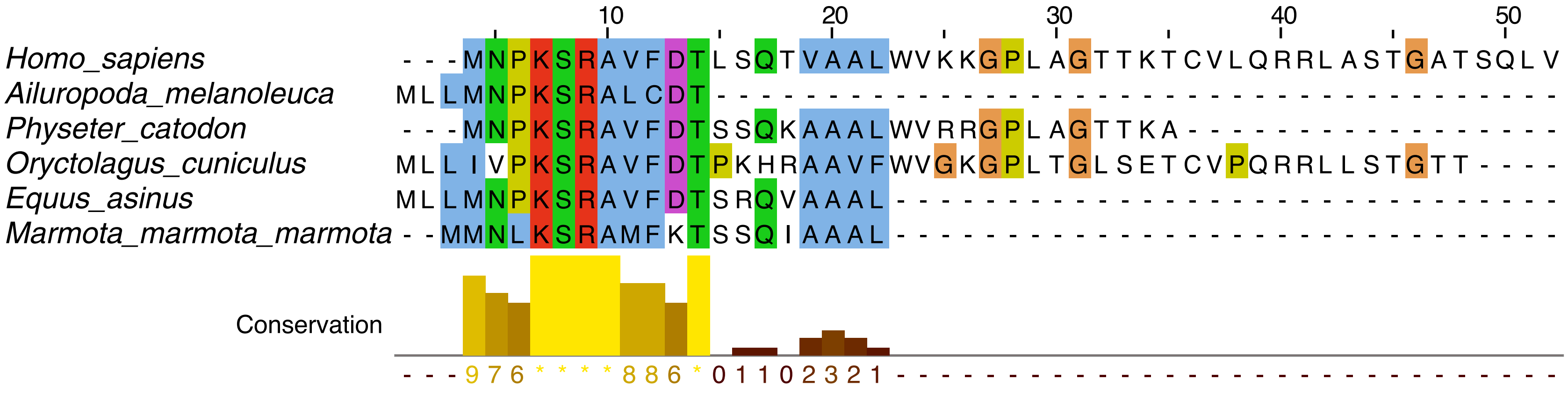

HG0911

ENSG00000198673 (FAM19A2)

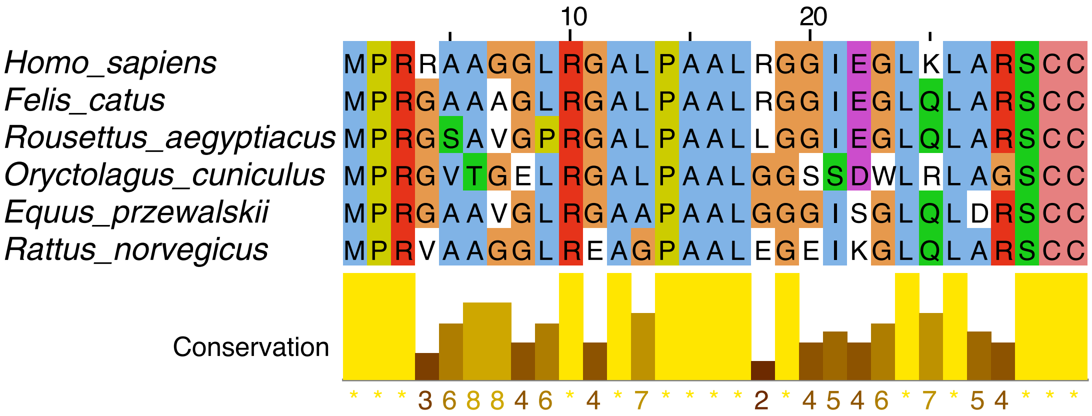

HG0912

ENSG00000198822 (GRM3)

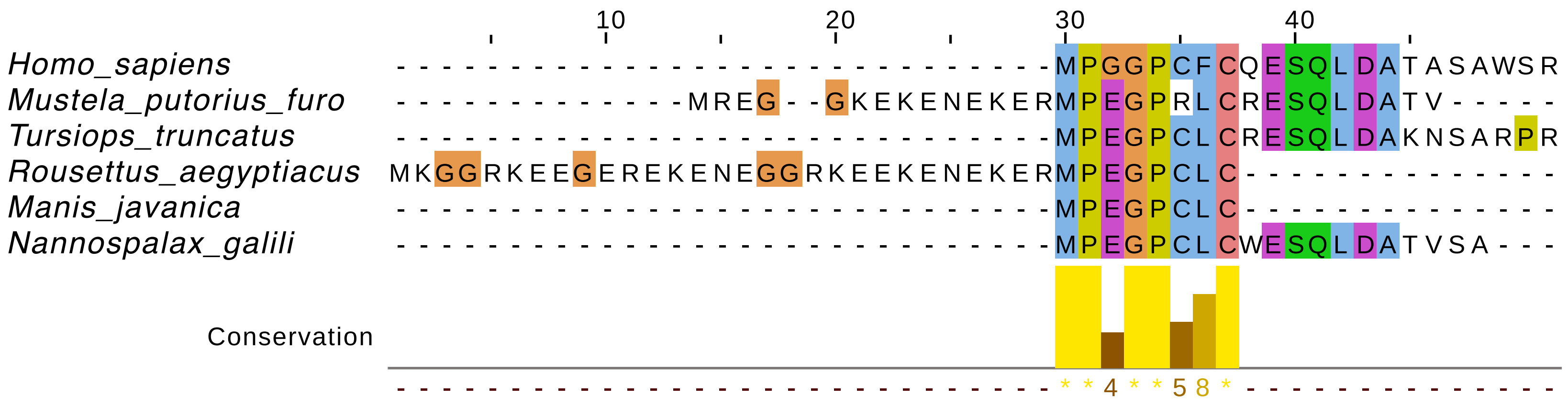

HG0913.1

ENSG00000204599 (TRIM39)

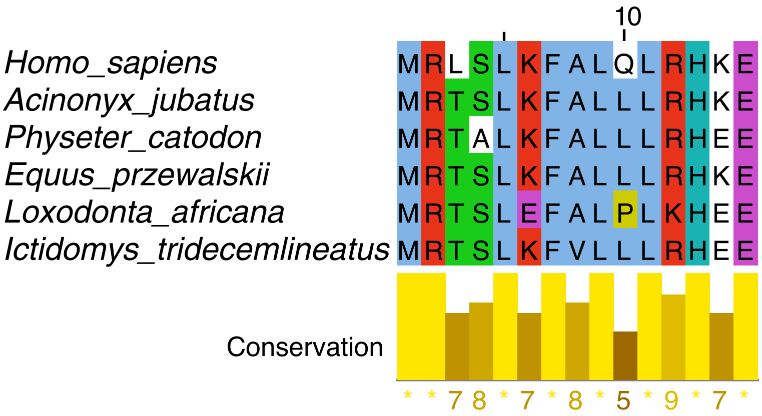

HG0913.2

ENSG00000204599 (TRIM39)

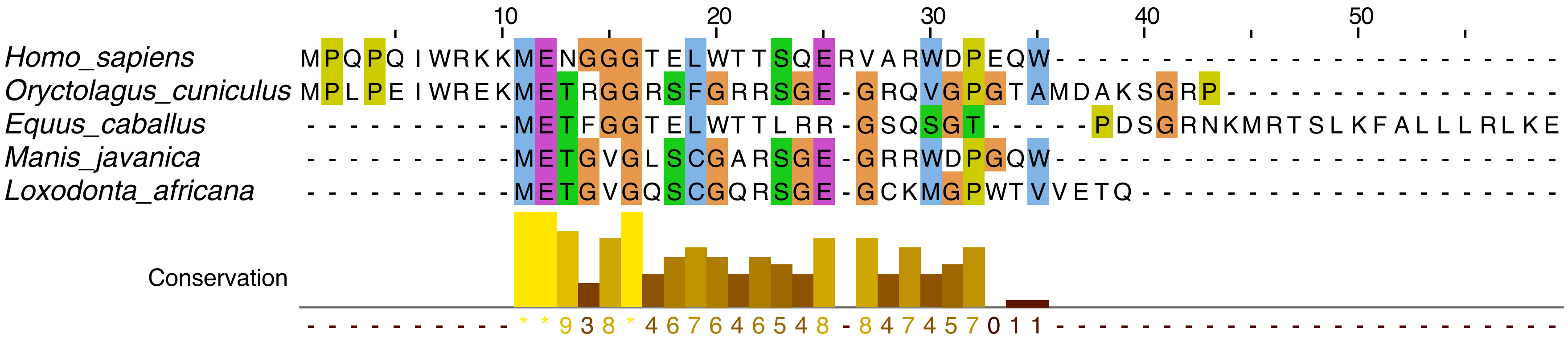

HG0914

ENSG00000227877 (MRLN)

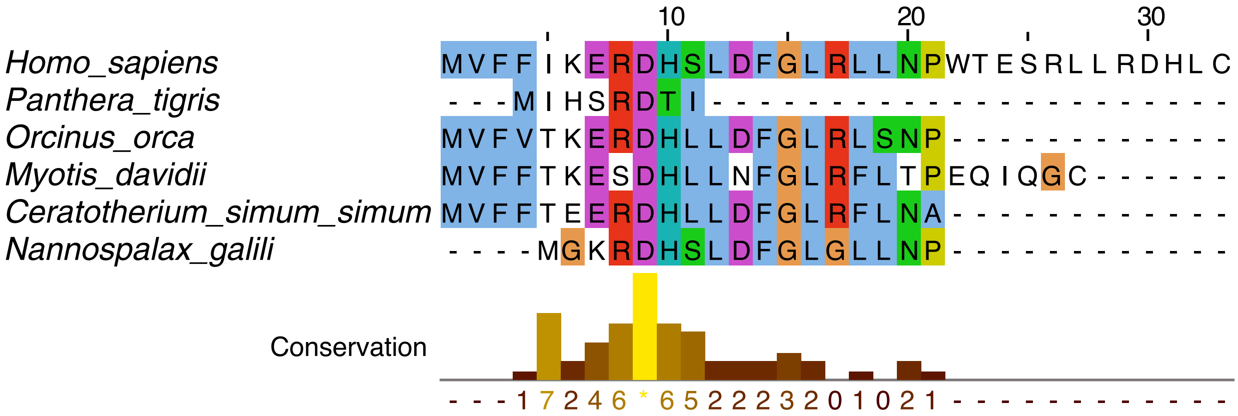

HG0915

ENSG00000254469 (AP002495.2)

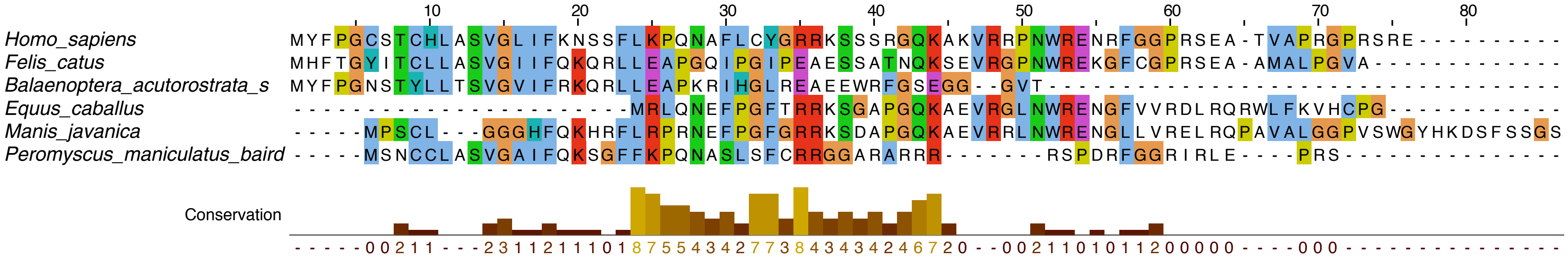

HG0916

ENSG00000020633 (RUNX3)

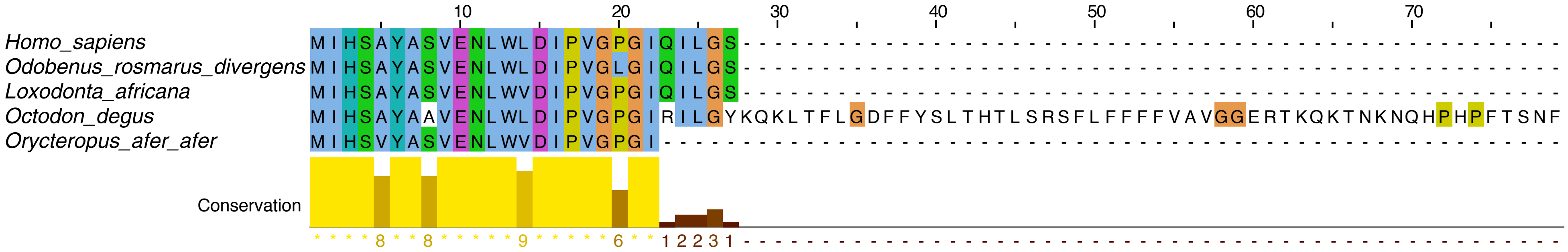

HG0917

ENSG00000042980 (ADAM28)

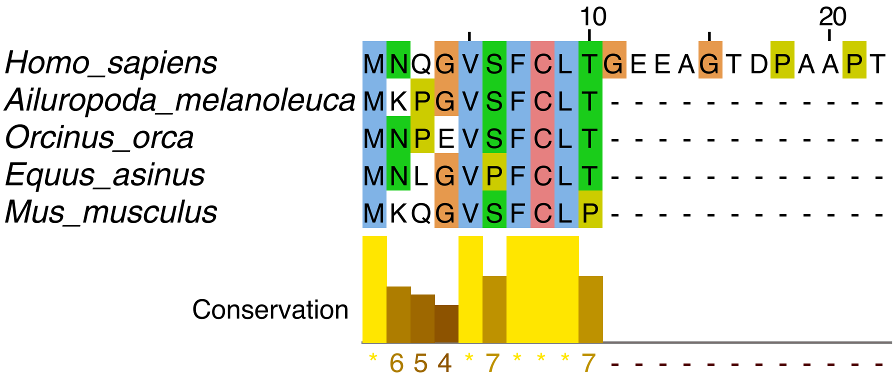

HG0918

ENSG00000047648 (ARHGAP6)

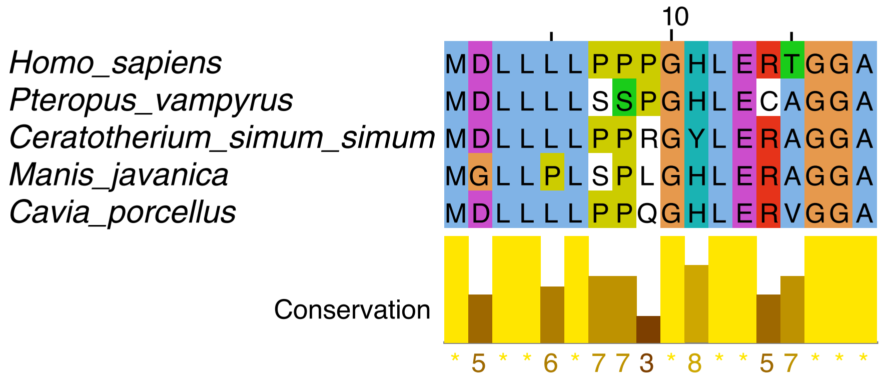

HG0919

ENSG00000051009 (FAM160A2)

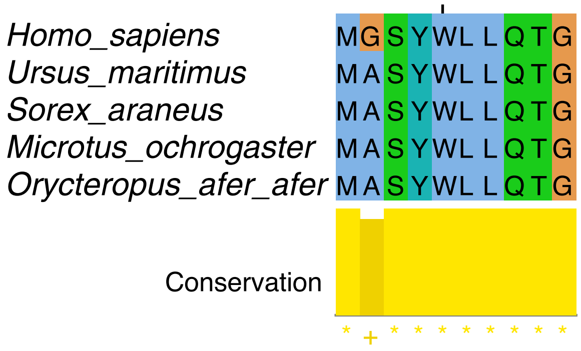

HG0920

ENSG00000051382 (PIK3CB)

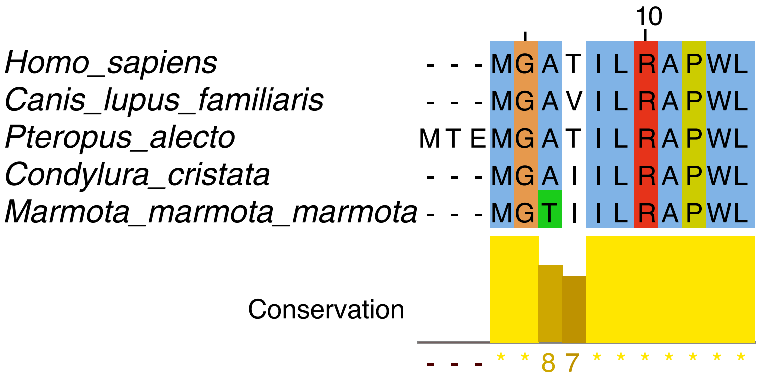

HG0921

ENSG00000058799 (YIPF1)

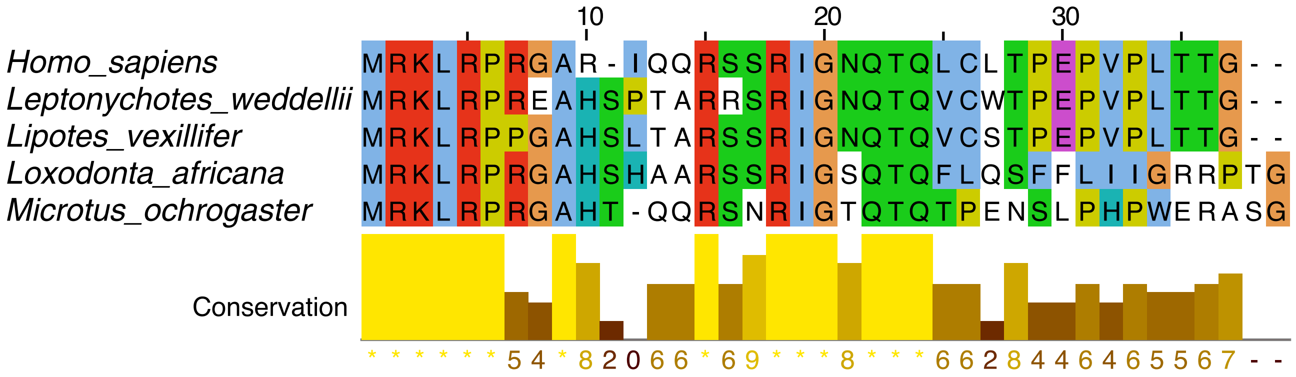

HG0922

ENSG00000079841 (RIMS1)

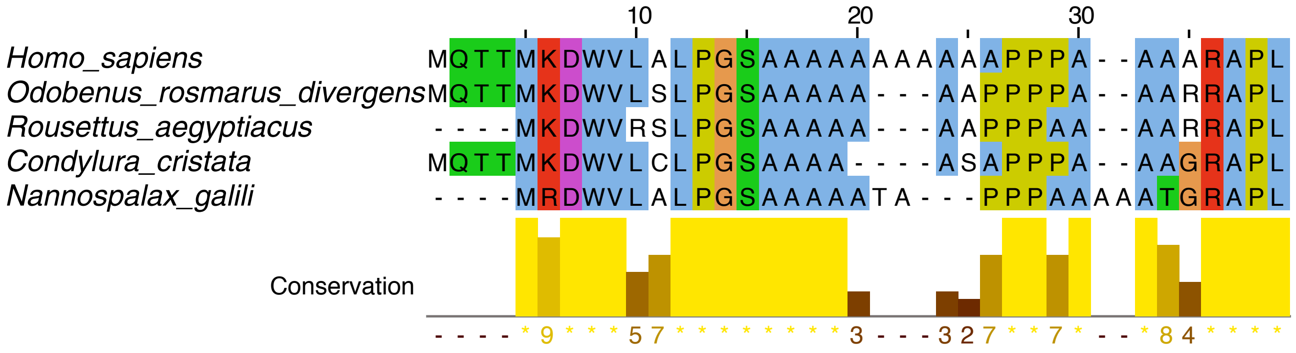

HG0923

ENSG00000081138 (CDH7)

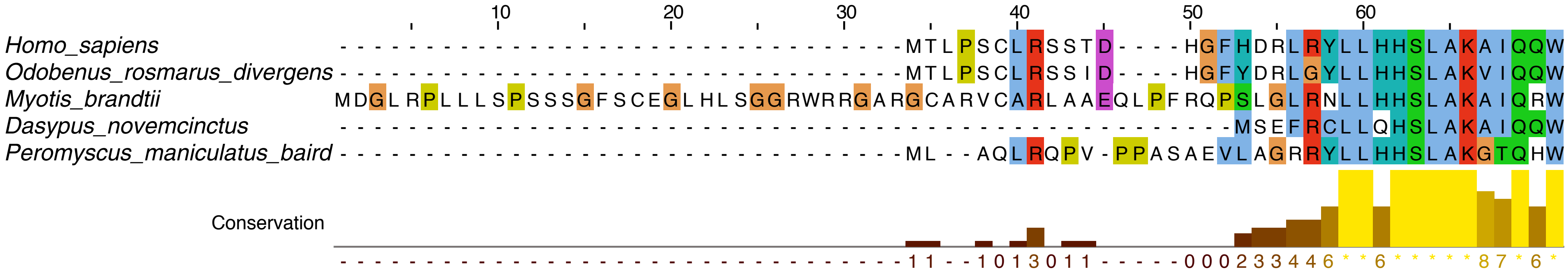

HG0924

ENSG00000081307 (UBA5)

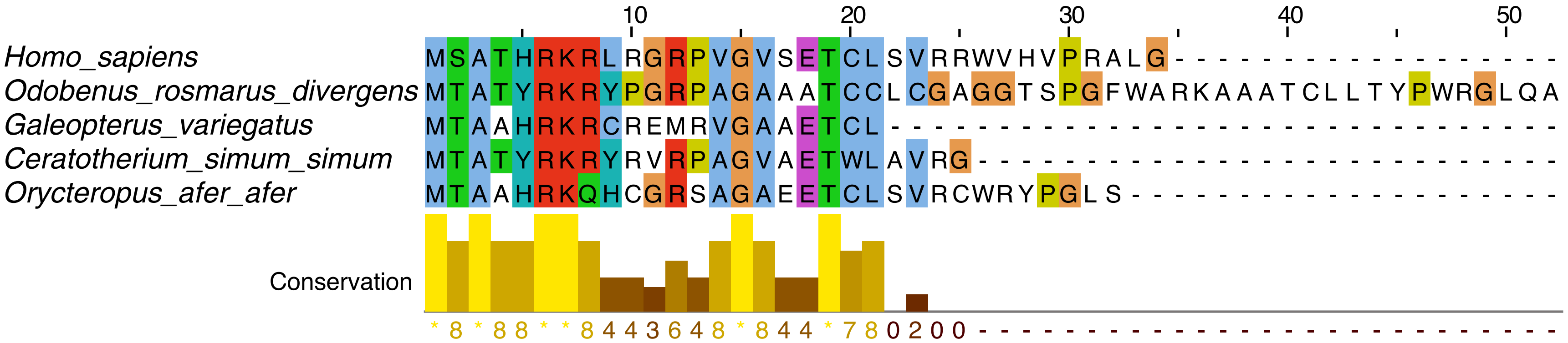

HG0925

ENSG00000088766 (CRLS1)

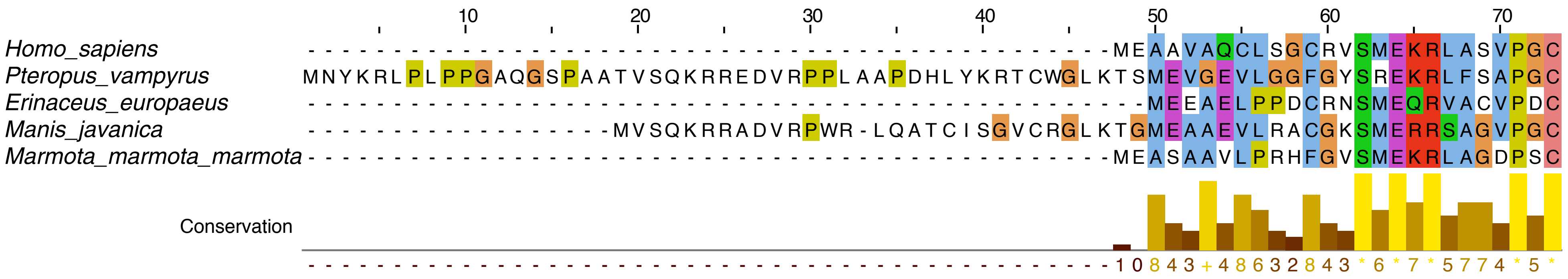

HG0926

ENSG00000095397 (WHRN)

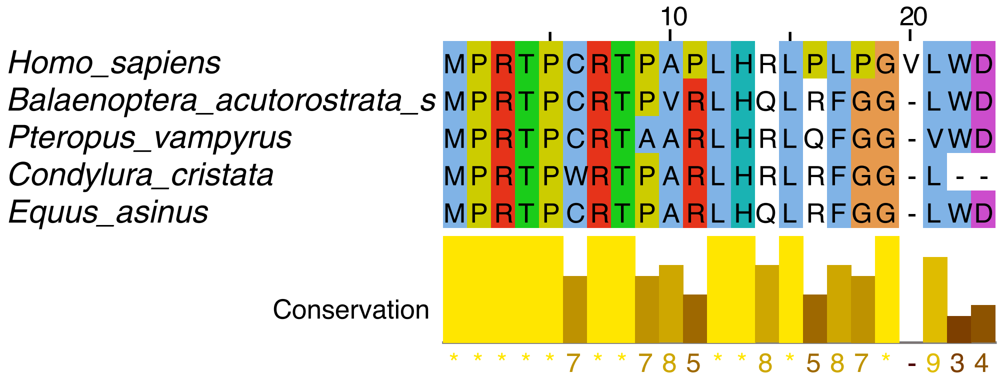

HG0927

ENSG00000095951 (HIVEP1)

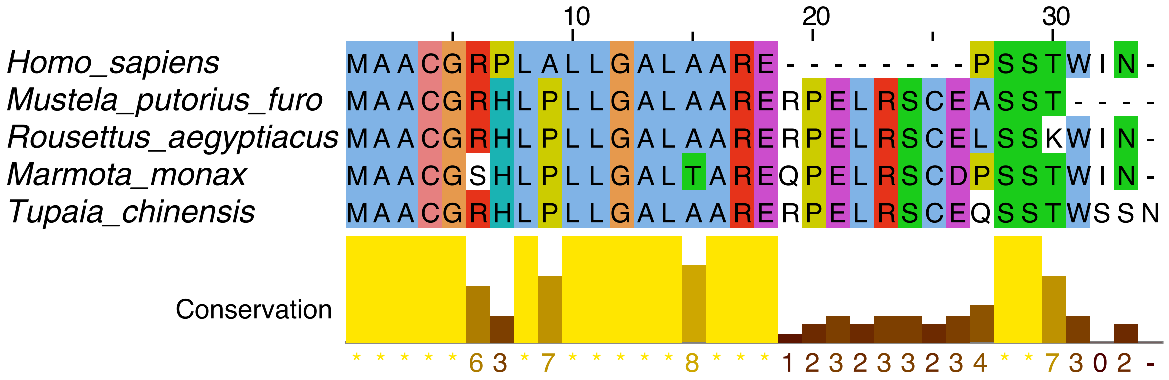

HG0928

ENSG00000102057 (KCND1)

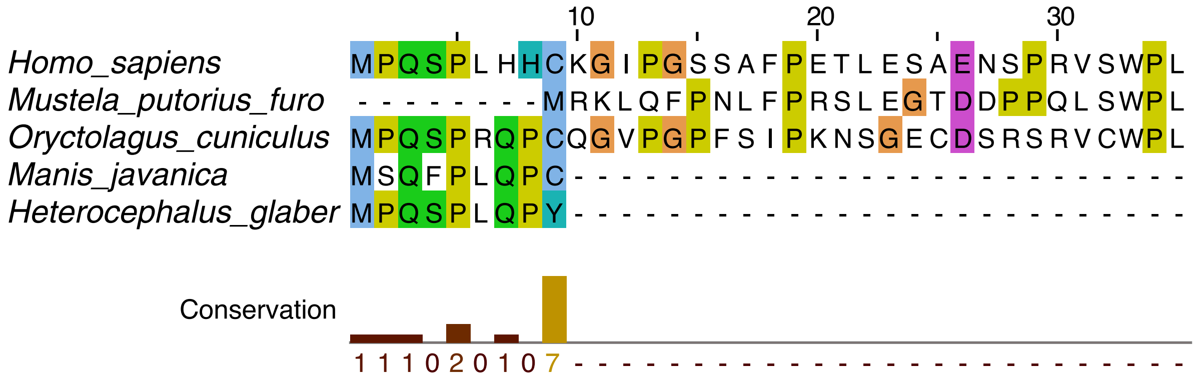

HG0929

ENSG00000102239 (BRS3)

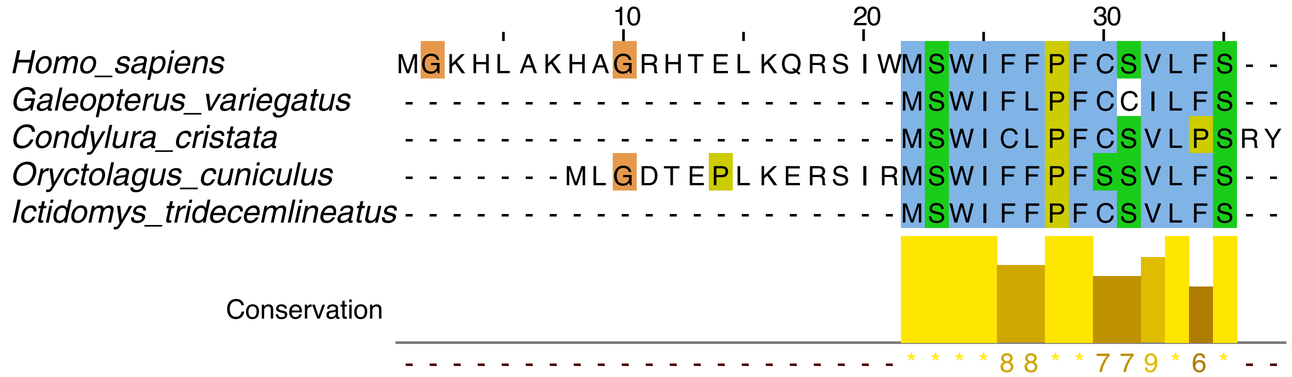

HG0930

ENSG00000102524 (TNFSF13B)

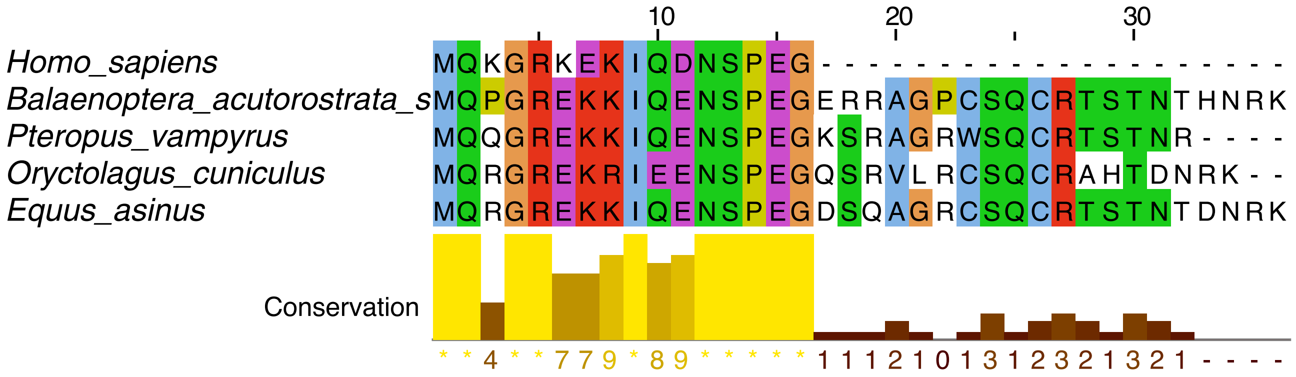

HG0931

ENSG00000104951 (IL4I1)

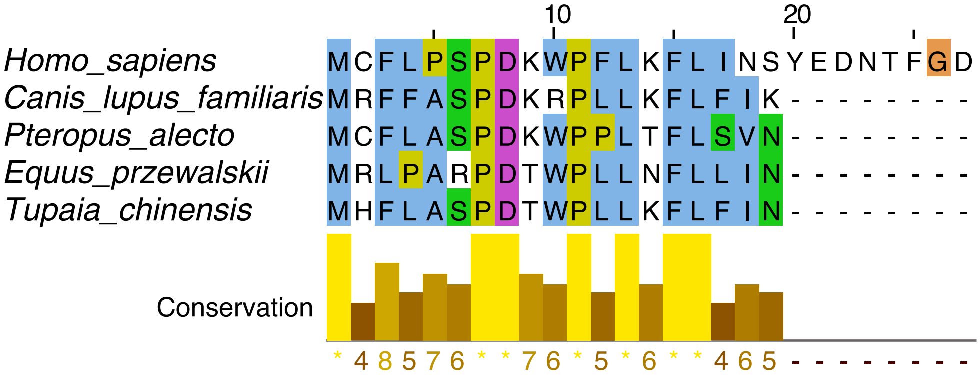

HG0932

ENSG00000105997 (HOXA3)

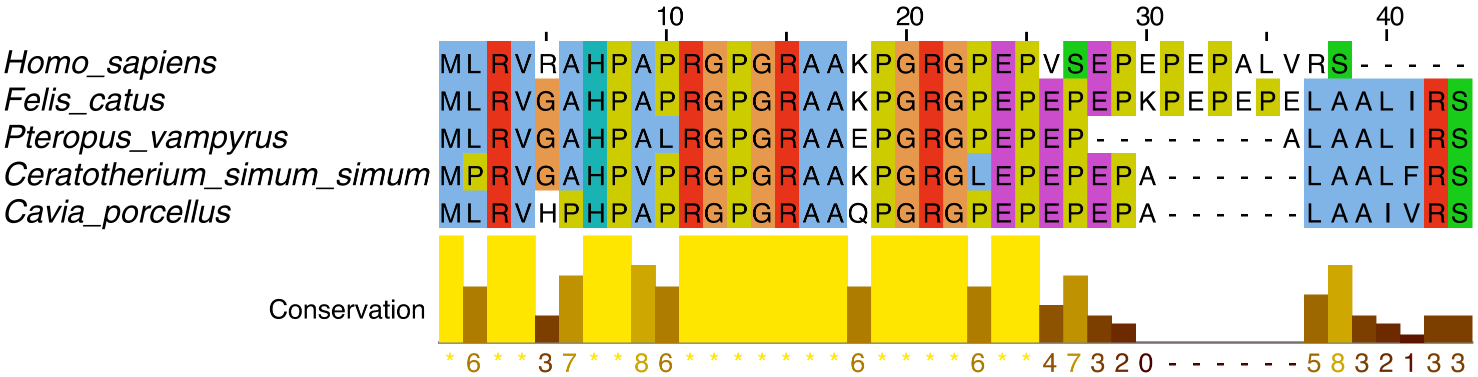

HG0933

ENSG00000106330 (MOSPD3)

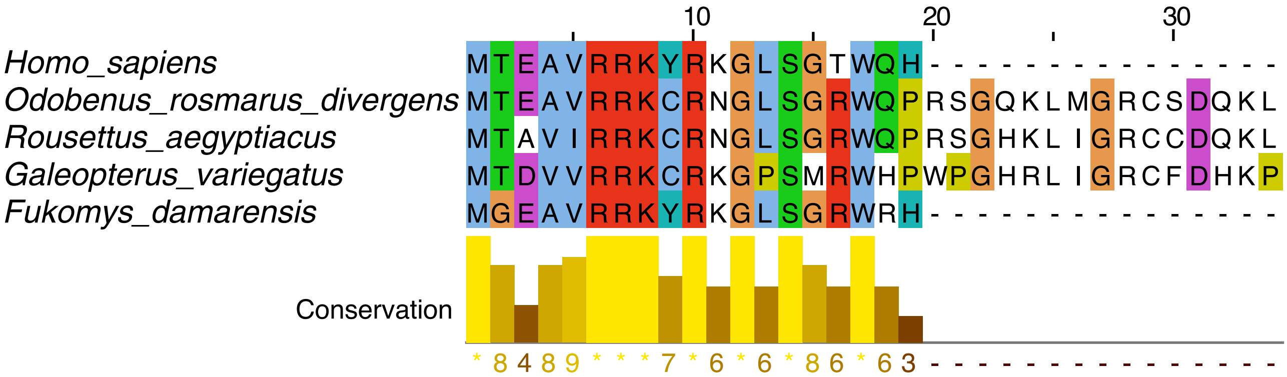

HG0934

ENSG00000107443 (CCNJ)

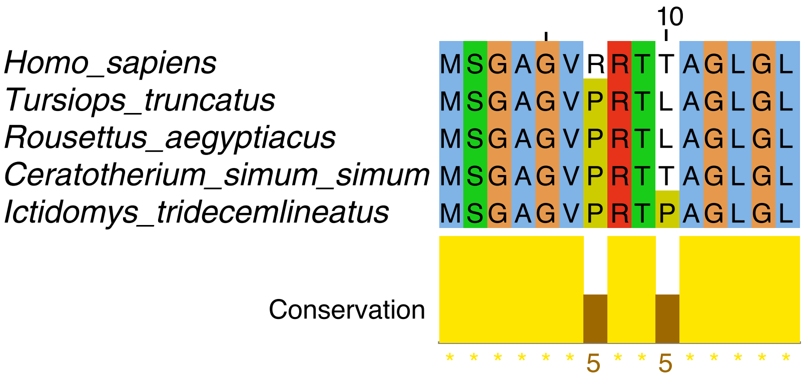

HG0935

ENSG00000108518 (PFN1)

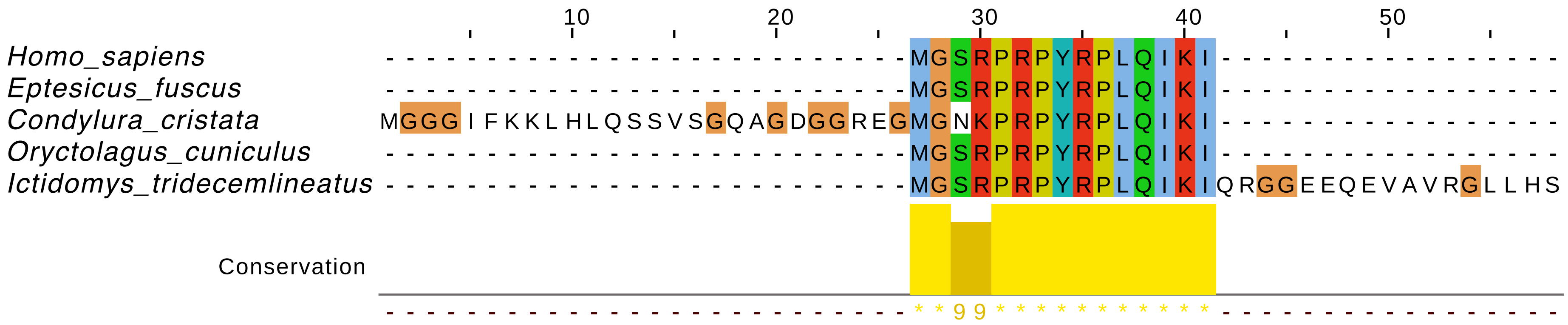

HG0936

ENSG00000108799 (EZH1)

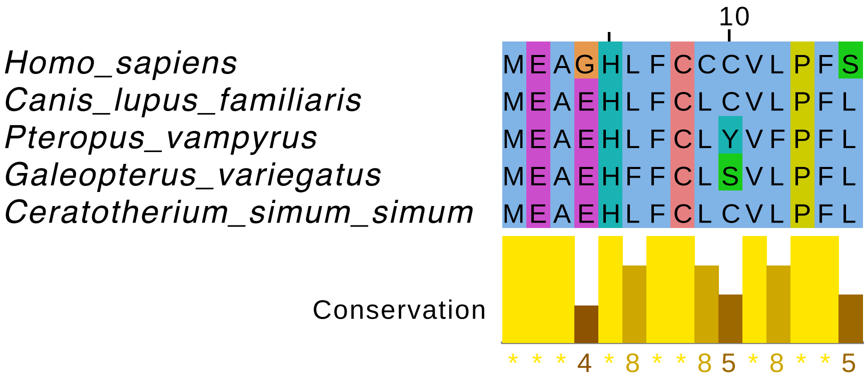

HG0937

ENSG00000109846 (CRYAB)

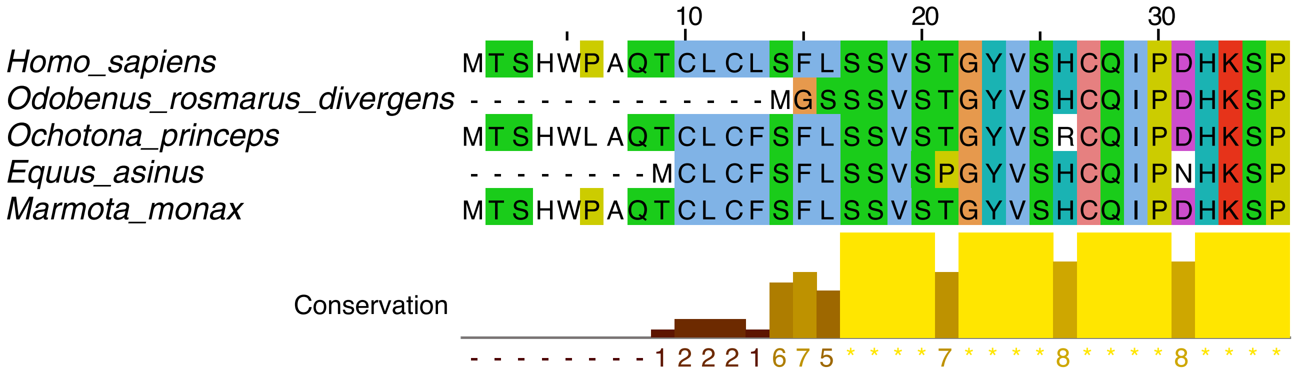

HG0938

ENSG00000111665 (CDCA3)

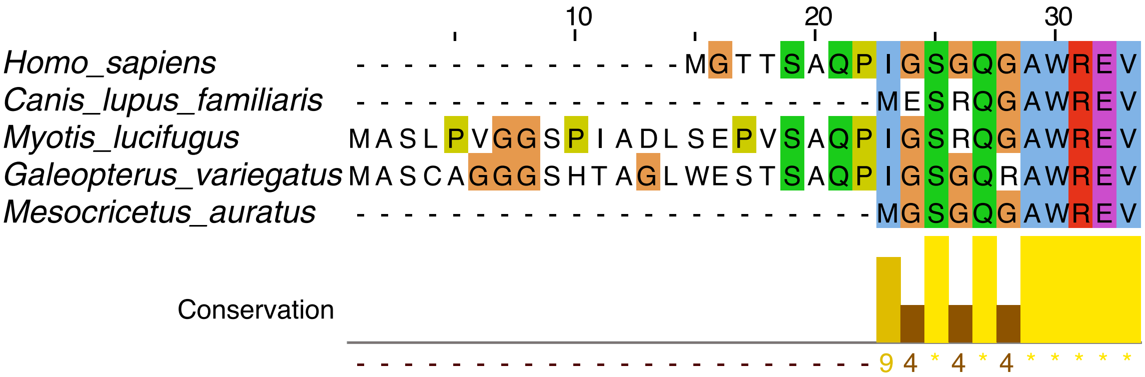

HG0939

ENSG00000111674 (ENO2)

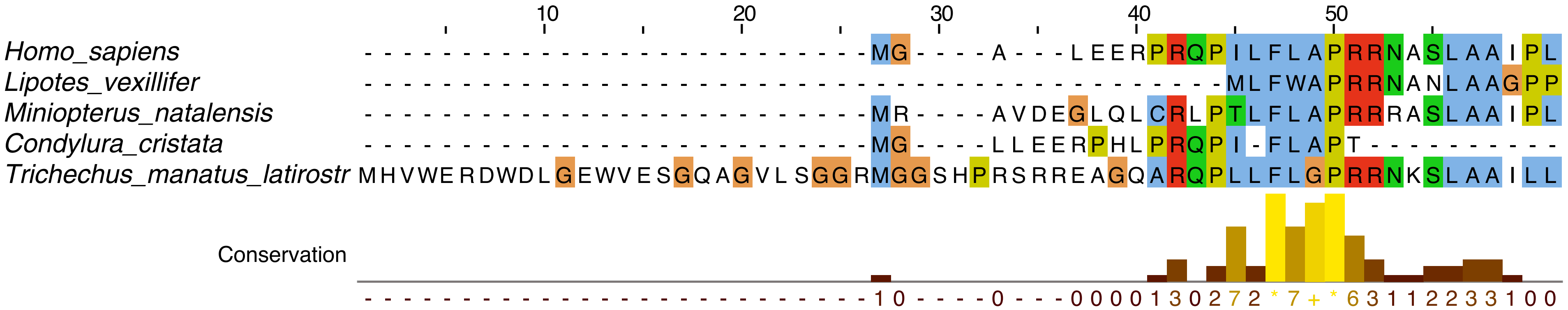

HG0940

ENSG00000115556 (PLCD4)

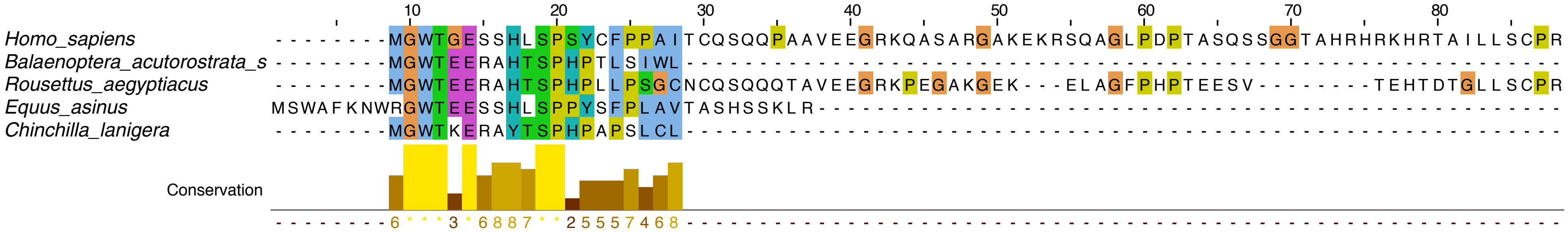

HG0941

ENSG00000116285 (ERRFI1)

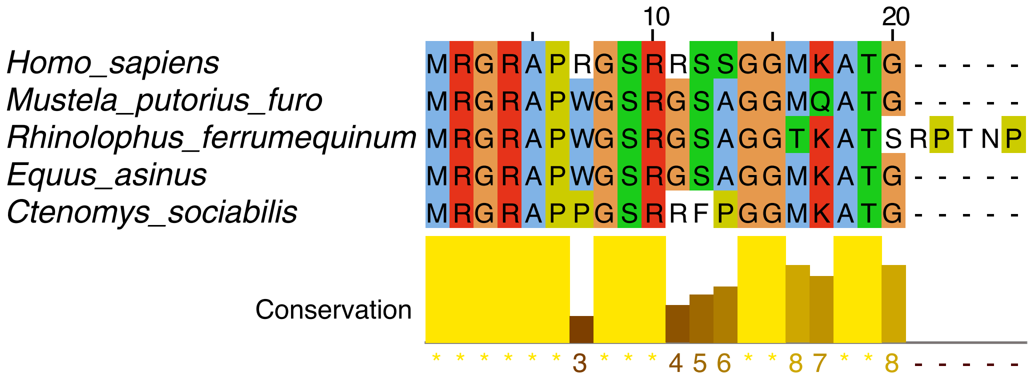

HG0942

ENSG00000117477

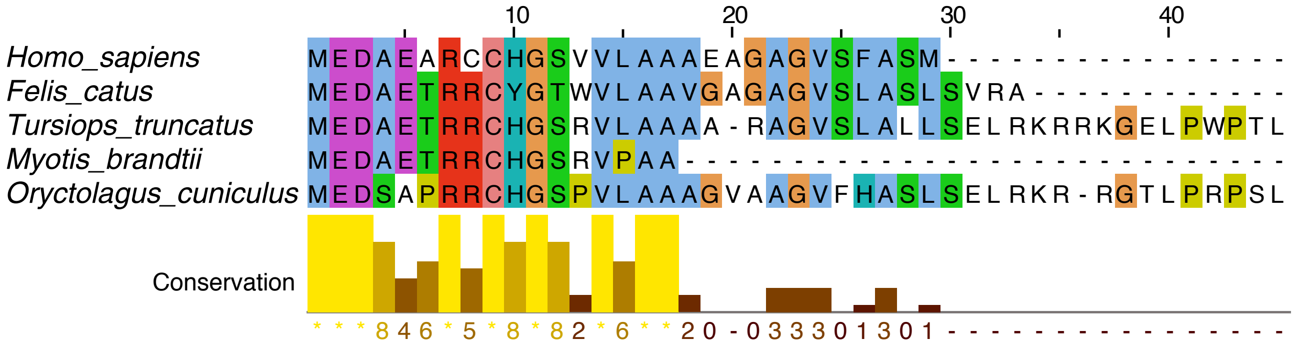

HG0943

ENSG00000120690 (ELF1)

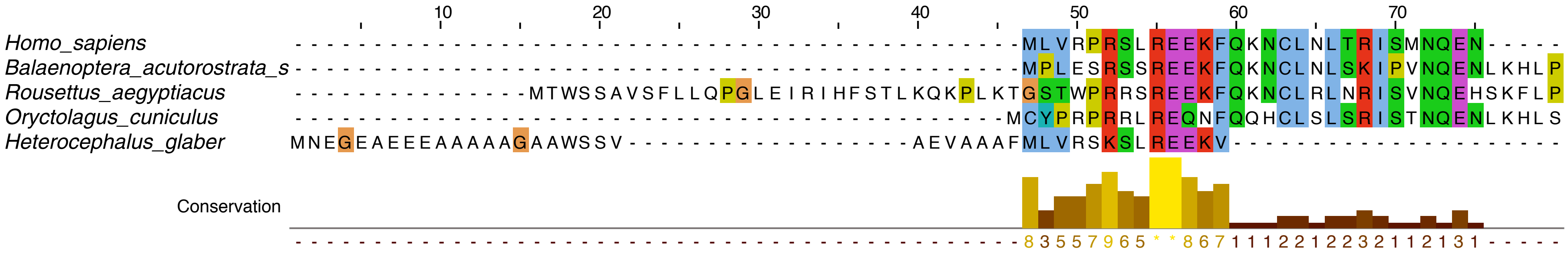

HG0944

ENSG00000120833 (SOCS2)

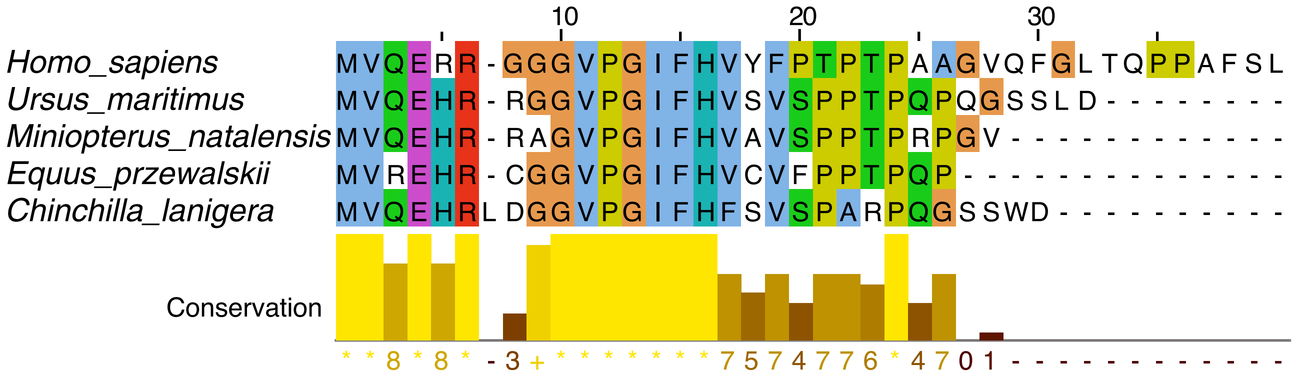

HG0945

ENSG00000124782 (RREB1)

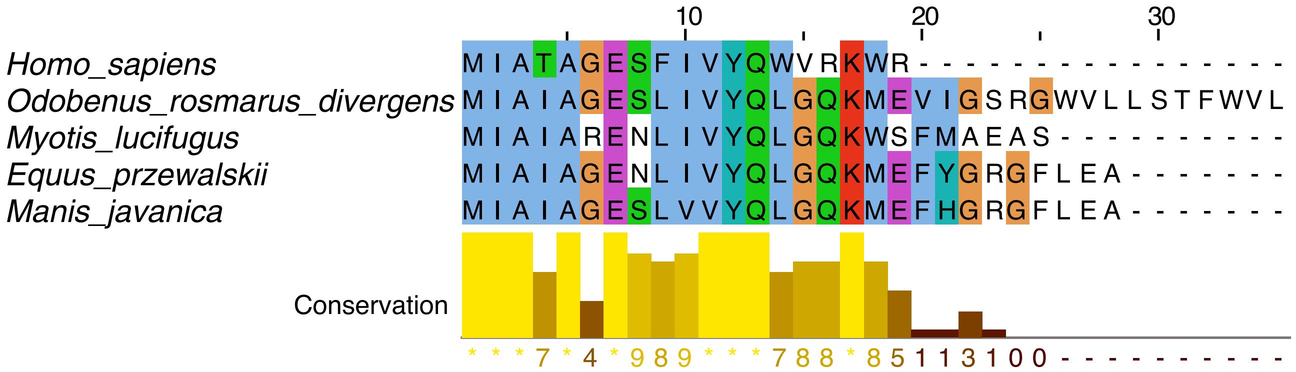

HG0946.1

ENSG00000127324 (TSPAN8)

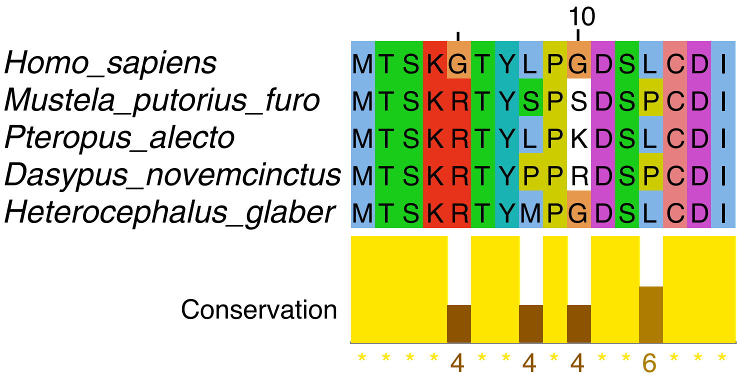

HG0946.2

ENSG00000127324 (TSPAN8)

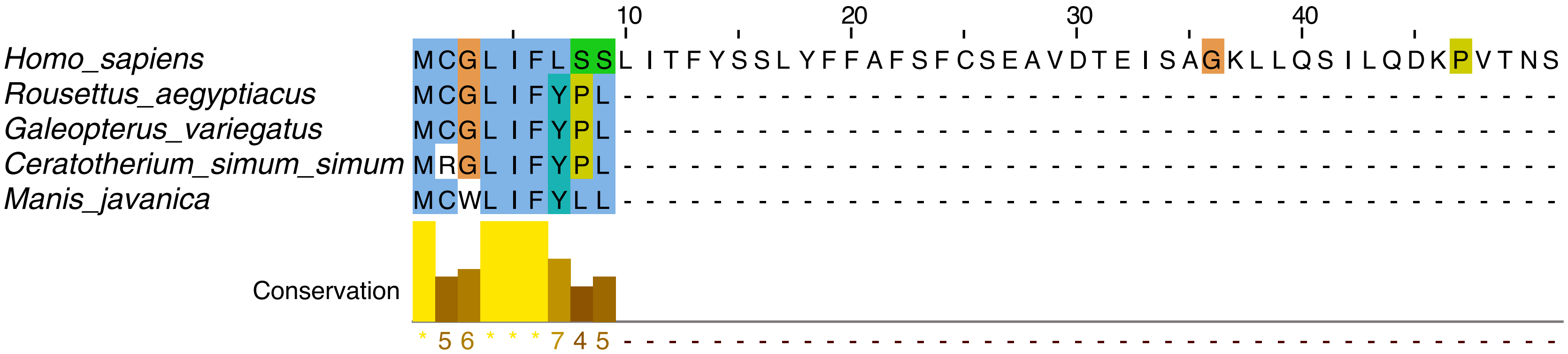

HG0947

ENSG00000129226 (CD68)

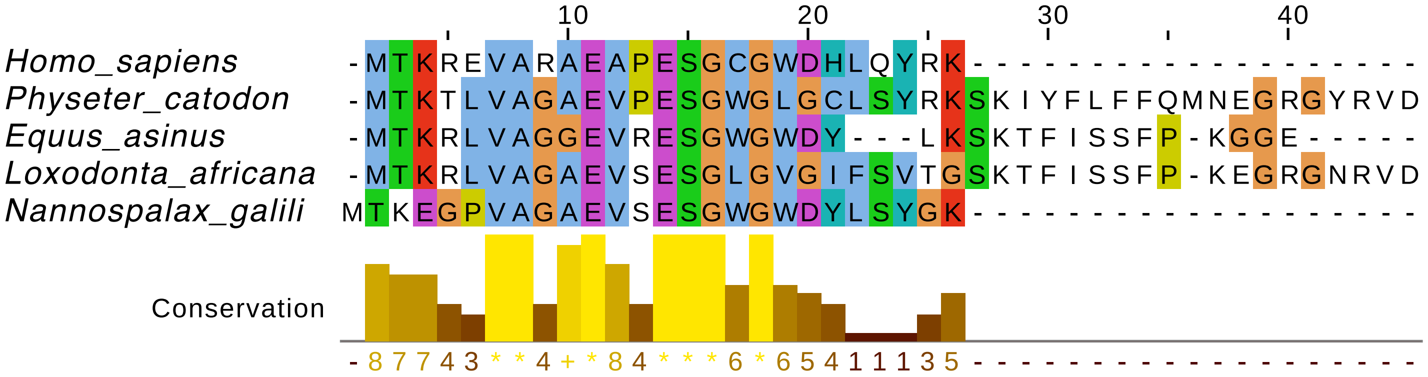

HG0948

ENSG00000129595 (EPB41L4A)

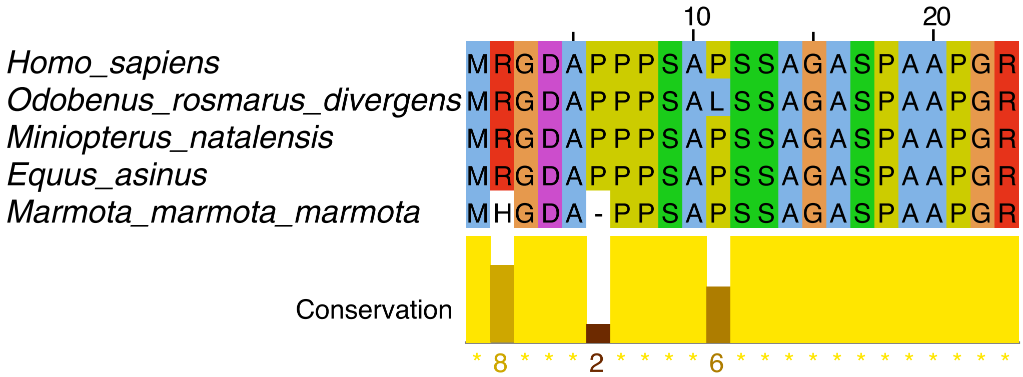

HG0949

ENSG00000131381 (RBSN)

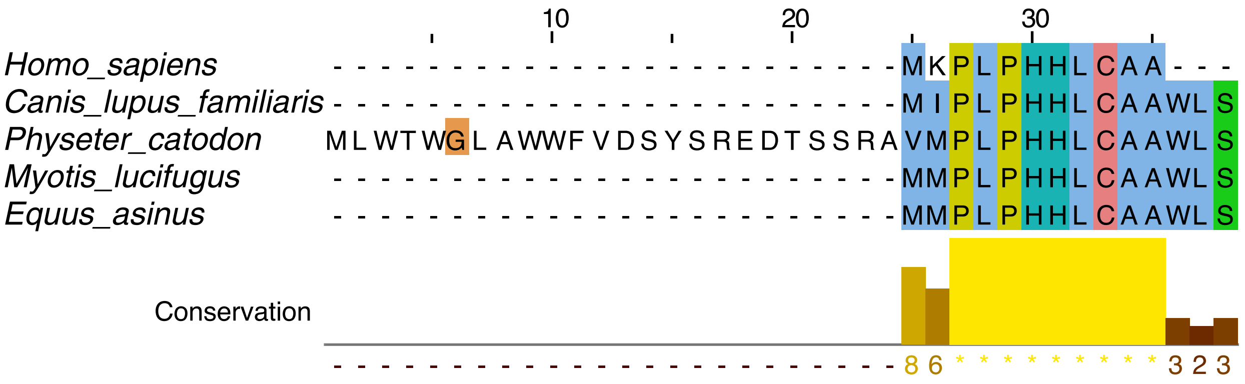

HG0950

ENSG00000132964 (CDK8)

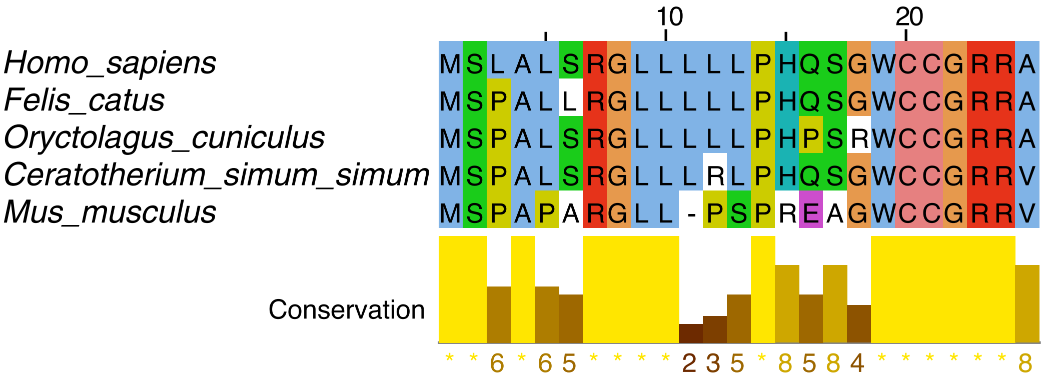

HG0951

ENSG00000134061 (CD180)

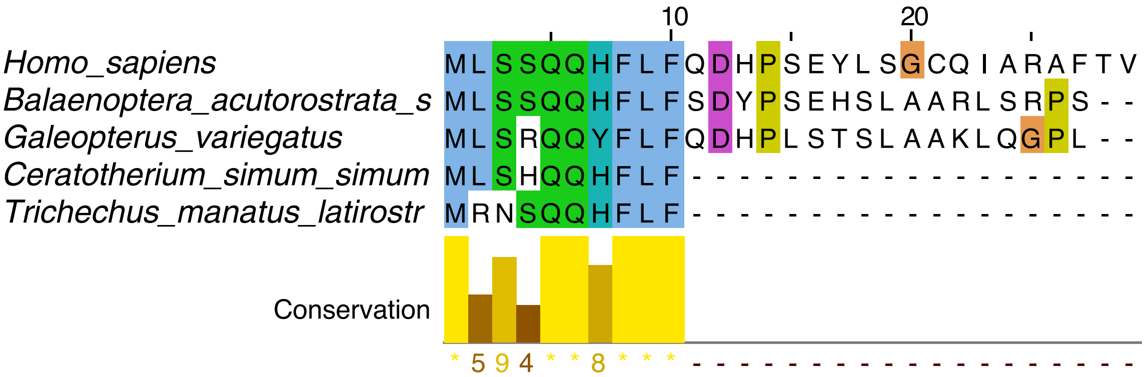

HG0952

ENSG00000134453 (RBM17)

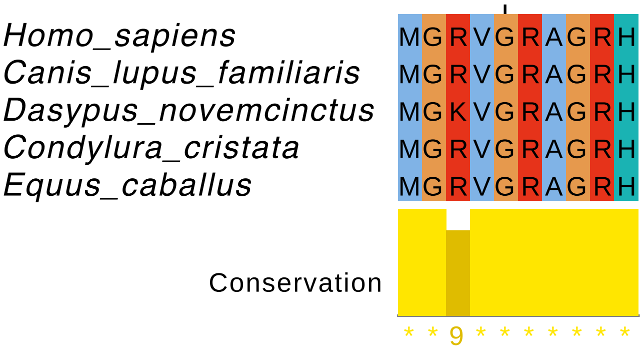

HG0953

ENSG00000134815 (DHX34)

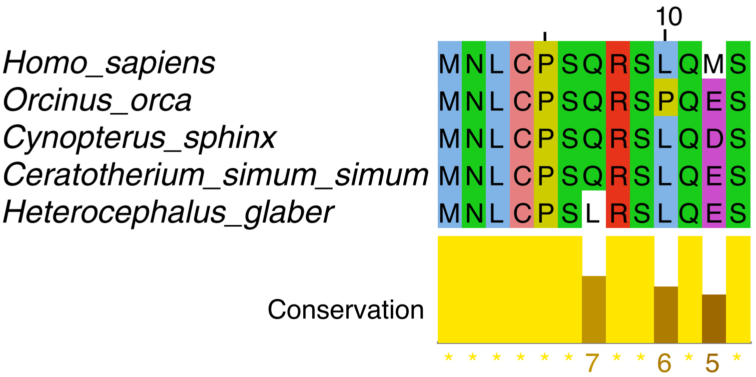

HG0954

ENSG00000134874 (DZIP1)

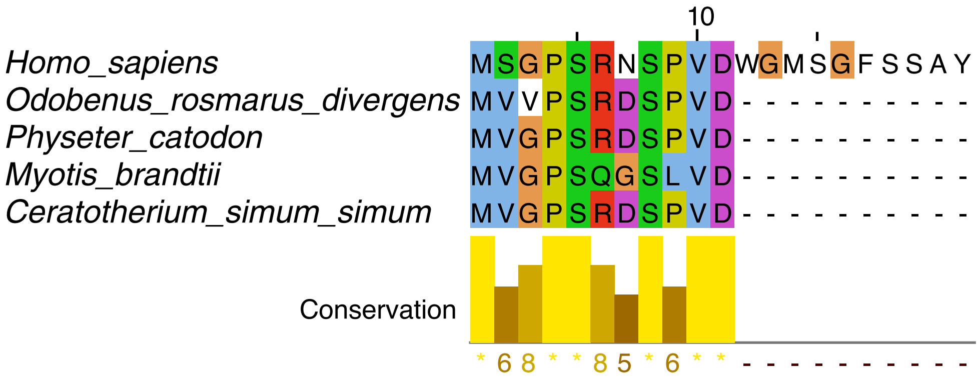

HG0955

ENSG00000134982 (APC)

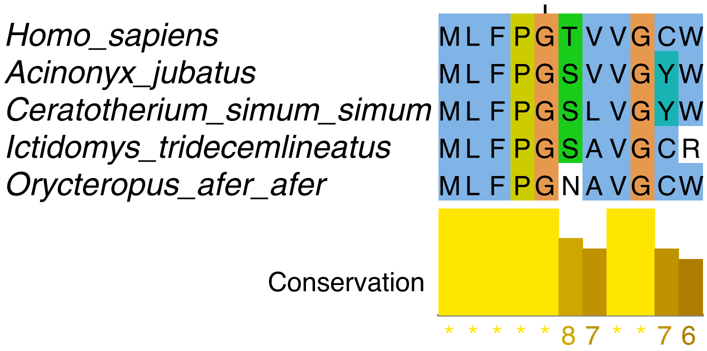

HG0956

ENSG00000136235 (GPNMB)

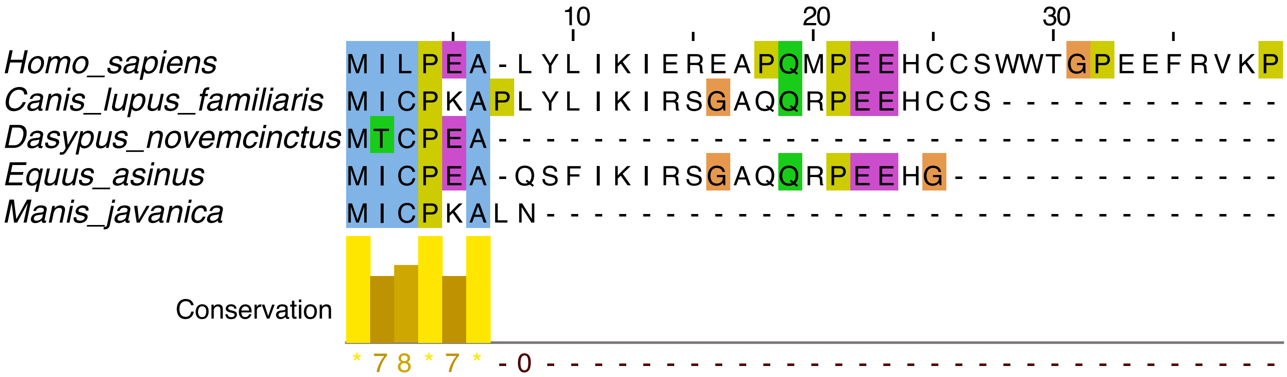

HG0957

ENSG00000136874 (STX17)

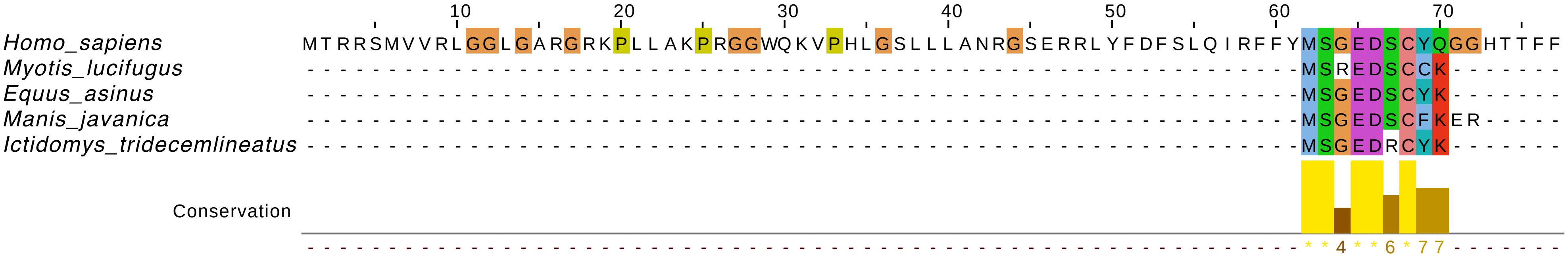

HG0958

ENSG00000137497 (NUMA1)

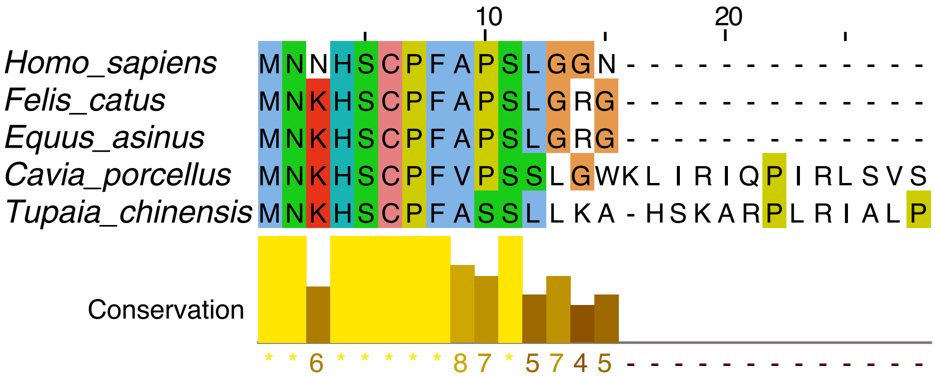

HG0959

ENSG00000137558 (PI15)

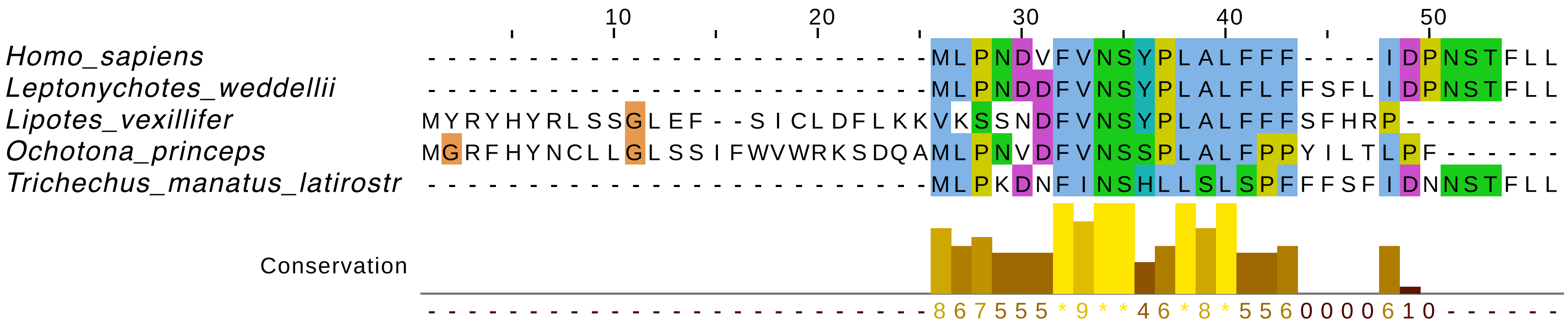

HG0960

ENSG00000140297 (GCNT3)

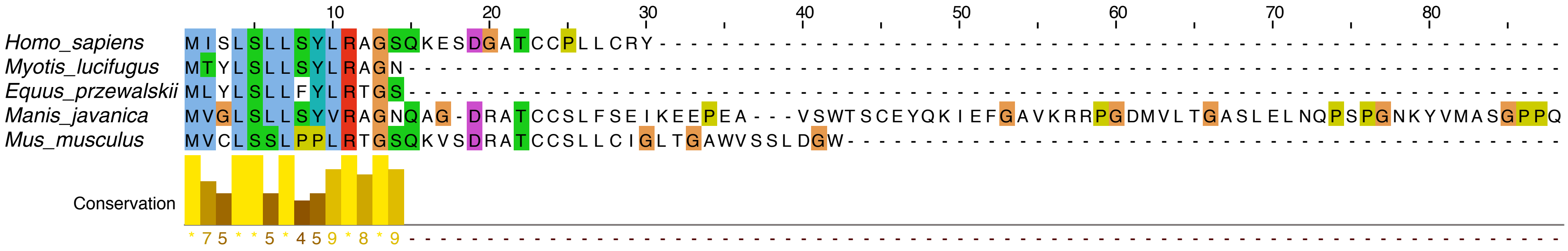

HG0961

ENSG00000140853 (NLRC5)

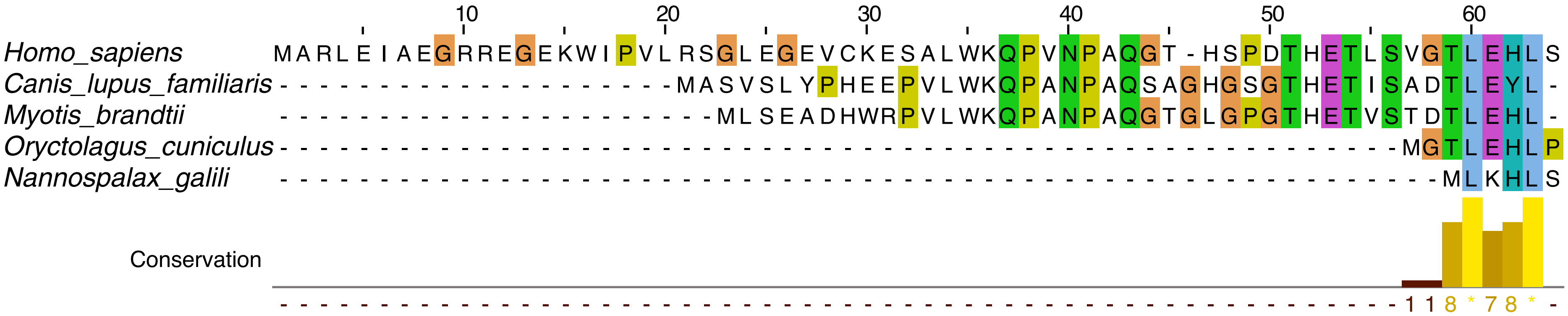

HG0962.1

ENSG00000143319 (ISG20L2)

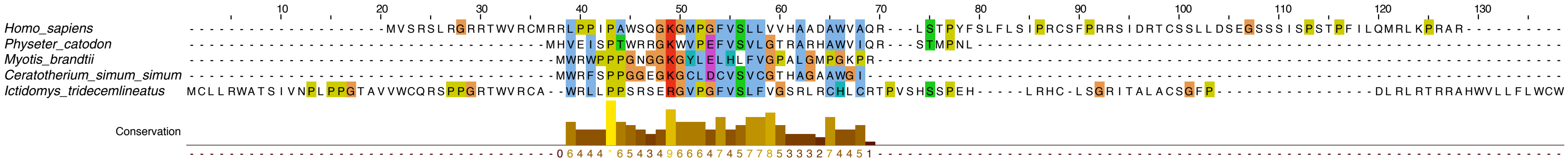

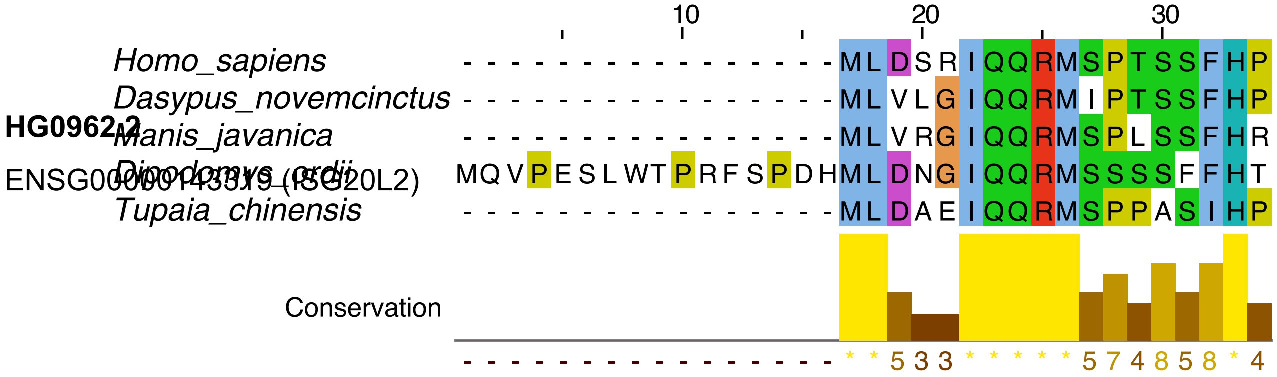
HG0962.2
ENSG00000143319 (ISG20L2)
Homo_sapiens
Dasypus_novemcinctus
Manis_javanica
Dipodomys_ordii
Tupaia_chinensis
Conservation
10
20
30

HG0963

ENSG00000143867 (OSR1)

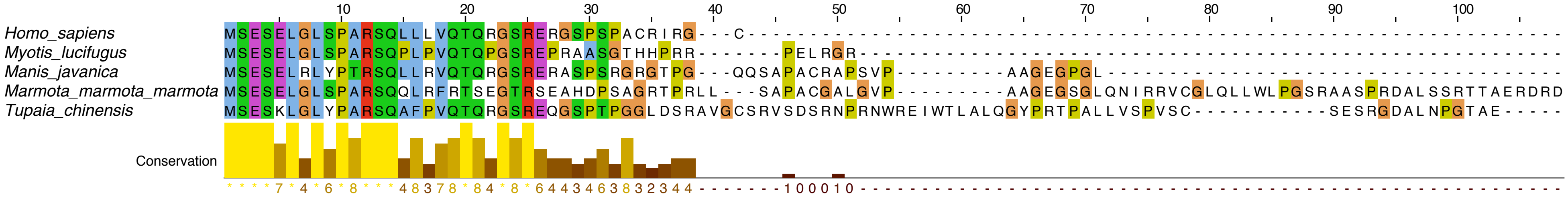

HG0964

ENSG00000145246 (ATP10D)

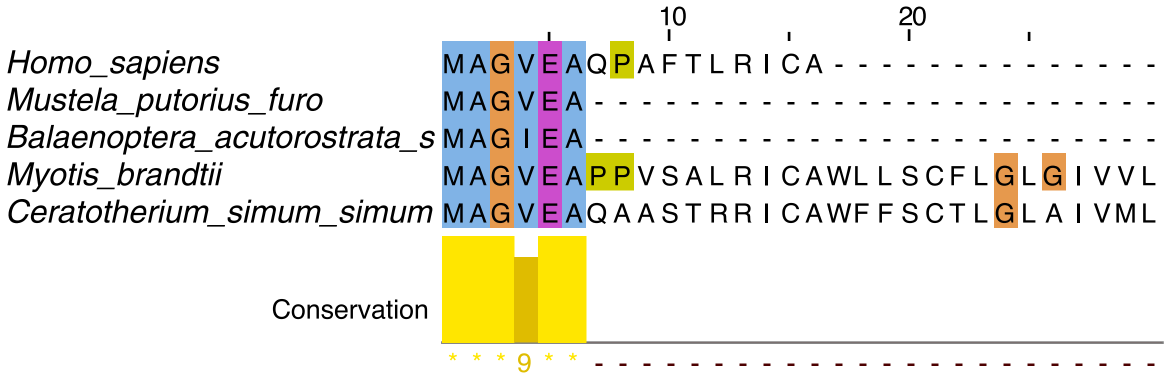

HG0965

ENSG00000146006 (LRRTM2)

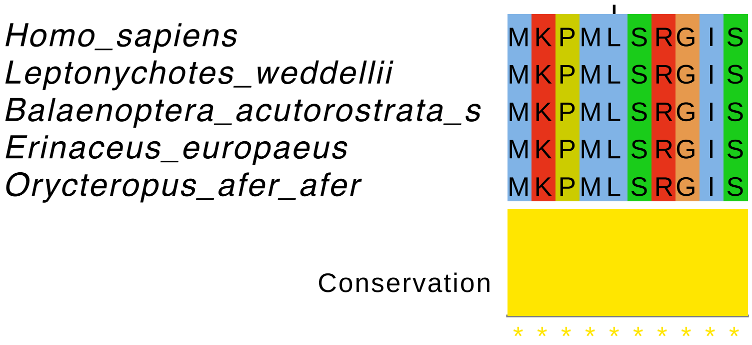

HG0966

ENSG00000148356 (LRSAM1)

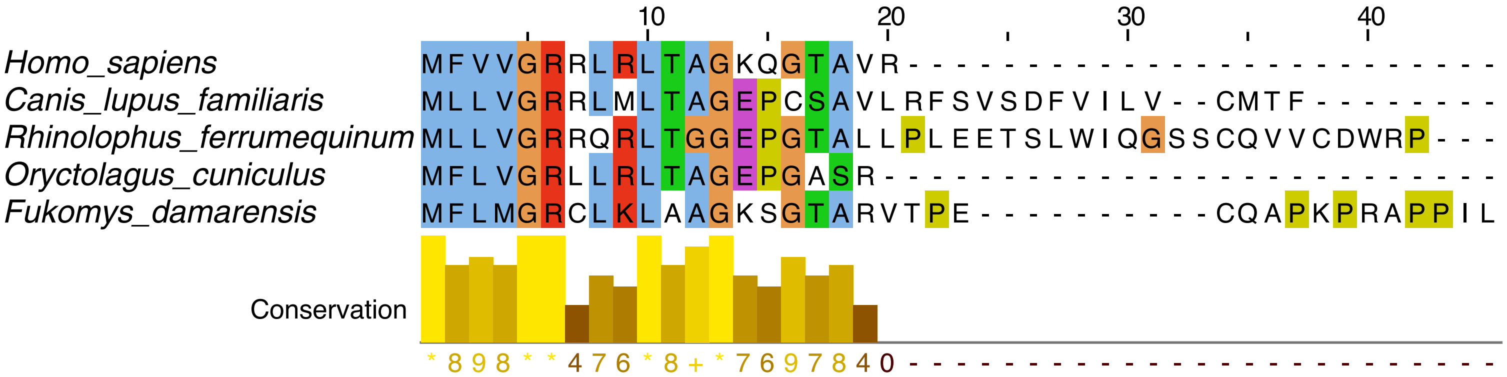

HG0967

ENSG00000149925 (ALDOA)

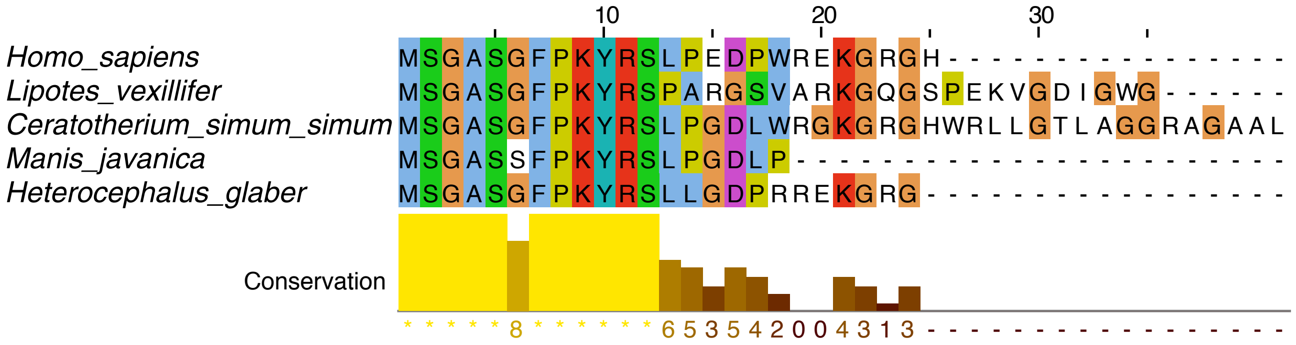

HG0968

ENSG00000154319 (FAM167A)

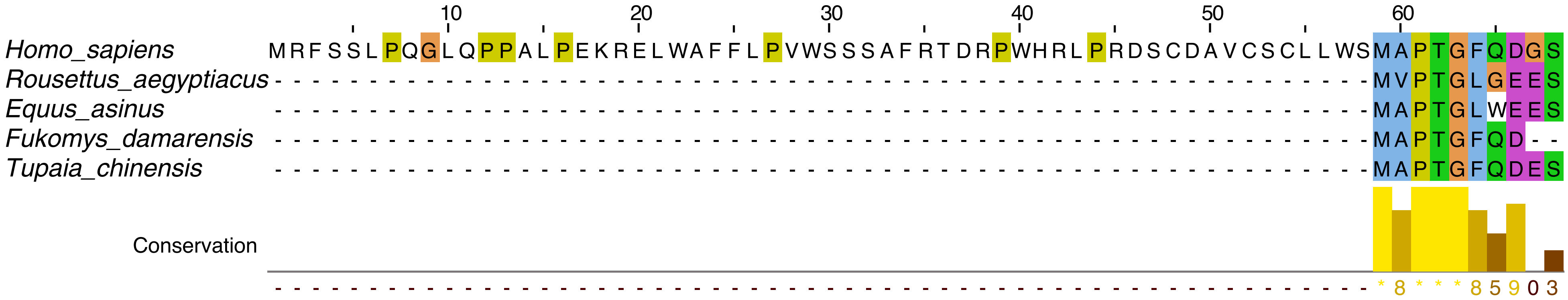

HG0969

ENSG00000154645 (CHODL)

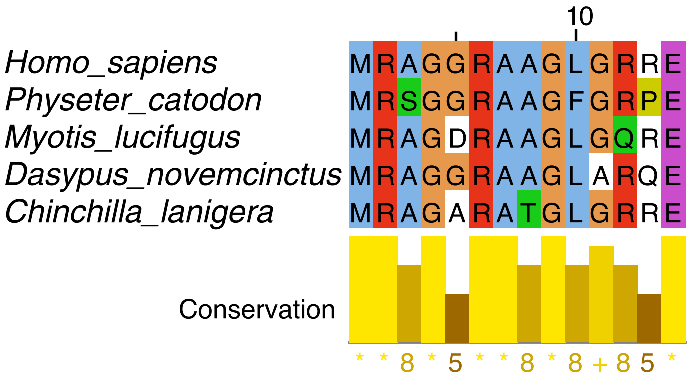

HG0970

ENSG00000154727 (GABPA)

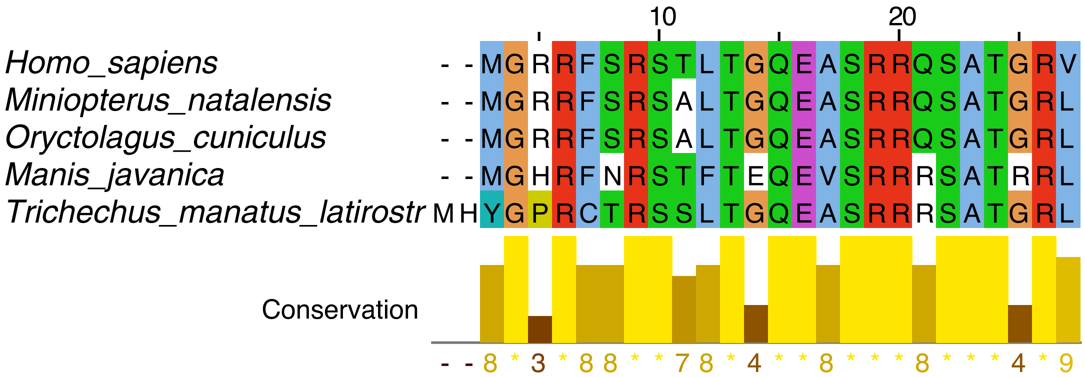

HG0971

ENSG00000155592 (ZKSCAN2)

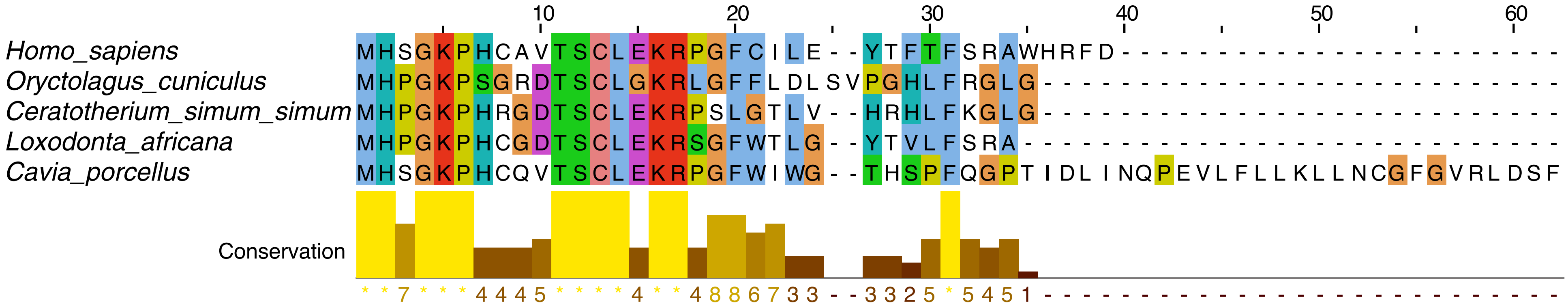

HG0972

ENSG00000155926 (SLA)

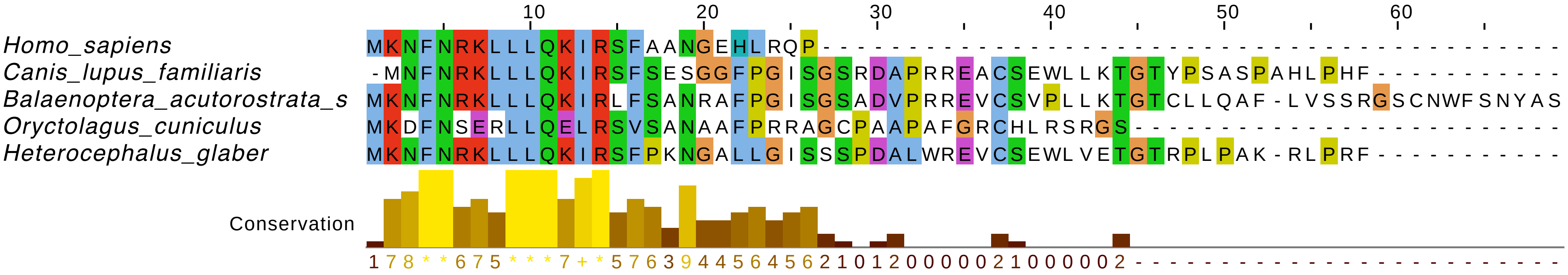

HG0973

ENSG00000156535 (CD109)

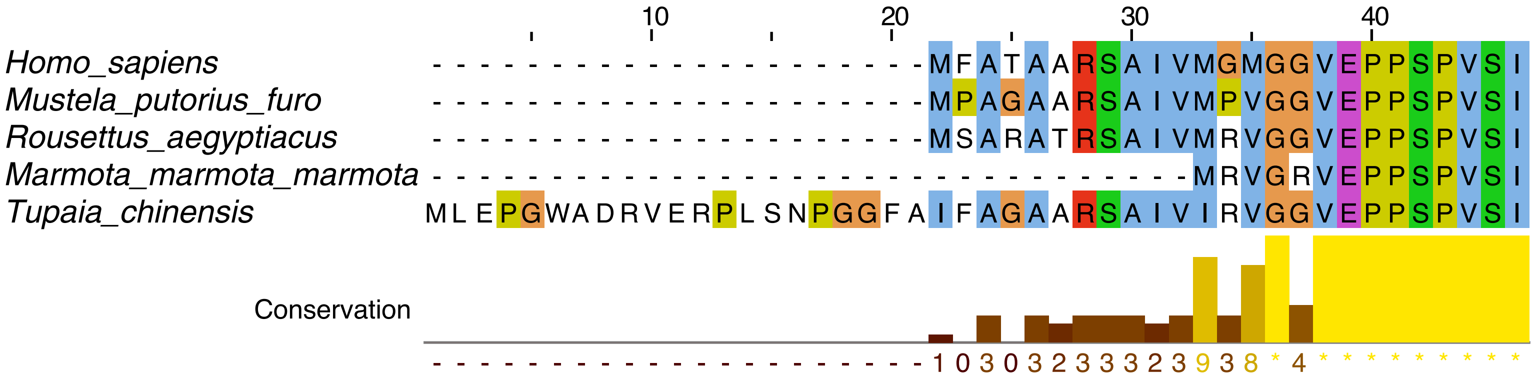

HG0974

ENSG00000157578 (LCA5L)

```
                                 10        20        30        40        50        60        70        80        90        100
Homo_sapiens               MQTNDHPYADHFEYLQPCFCHCLCRKATCFKKKSSWSAY--ERVGCFLQ-----------LIFPKQTIPPAISGFLKYILSLKRDQNLSKKYYRGKLINTTV
Mustela_putorius_furo      -----------------------------------------------------------------------MMVFLKYILSLKRDQNMSKNTALETS-----
Miniopterus_natalensis     ---------------------------------------------------------------------------------------MSKNTTLGTS-----
Equus_przewalskii          ----------------------------------MWEFRLFVPQSFFFGFLERHQKRQVEMETDGHPCADHFASFLKYILSVKRDQNMSKNTTLETS-----
Dipodomys_ordii            -------------------------------------MVVQFPFLCYF---------------------GALRCFLKYIF----------------------

Conservation
                           -----------------------------------------------------------------------101224321-------1341210020-----
```

HG0975.1

ENSG00000182158 (CREB3L2)

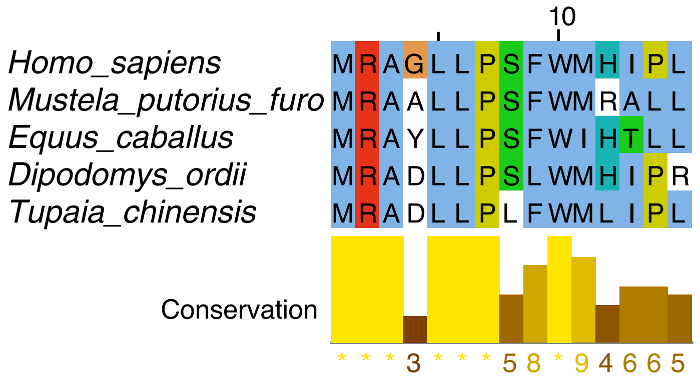

HG0975.2

ENSG00000157613 (CREB3L1)

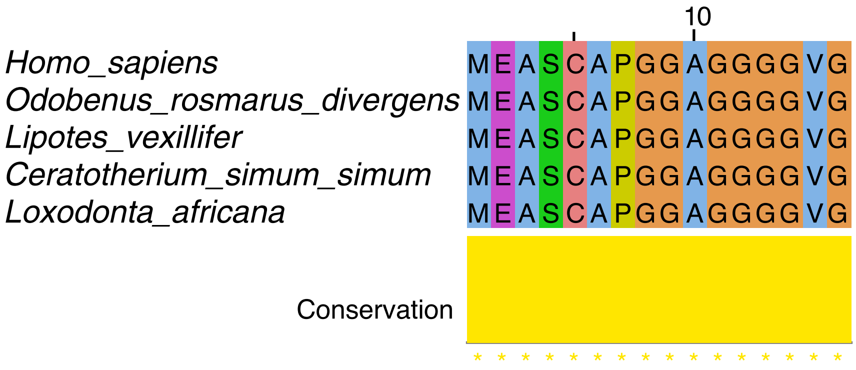

HG0976

ENSG00000158008 (EXTL1)

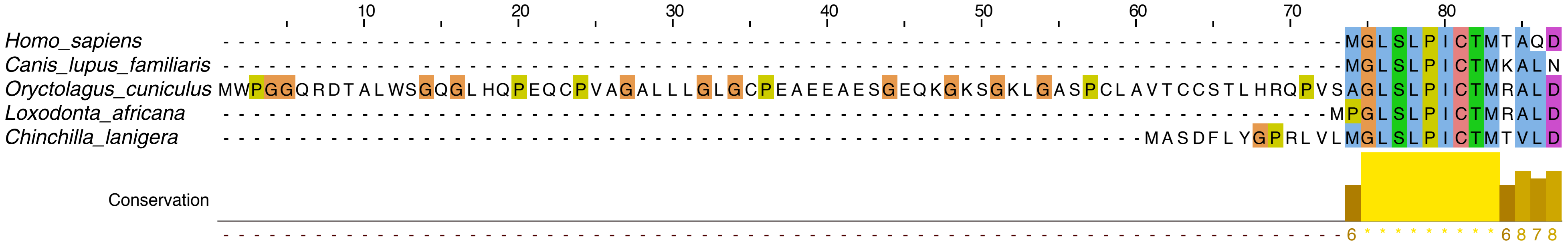

HG0977

ENSG00000158321 (AUTS2)

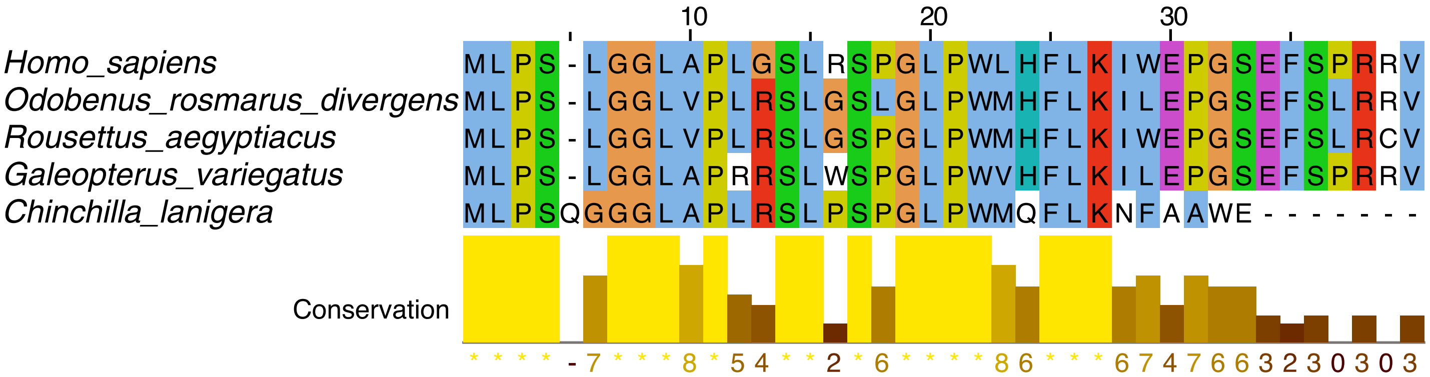

HG0978

ENSG00000159023 (EPB41)

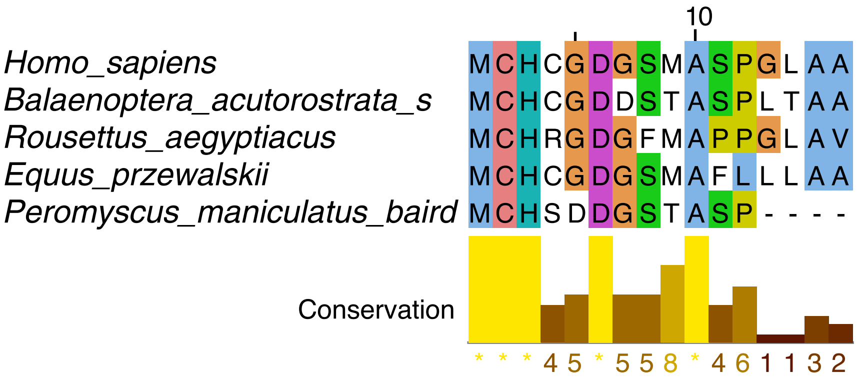

HG0979

ENSG00000159202 (UBE2Z)

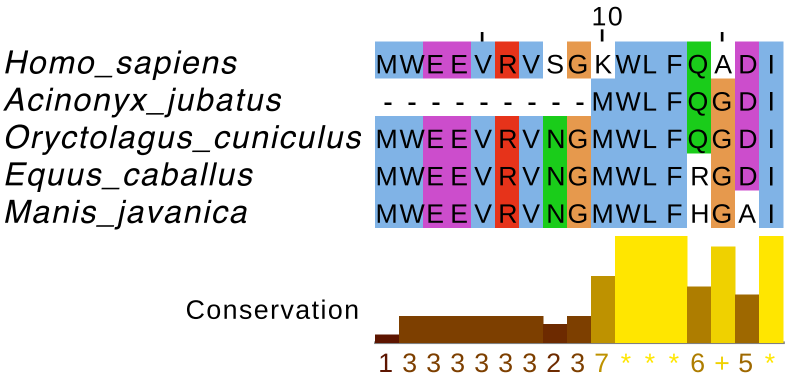

HG0980

ENSG00000162670 (BRINP3)

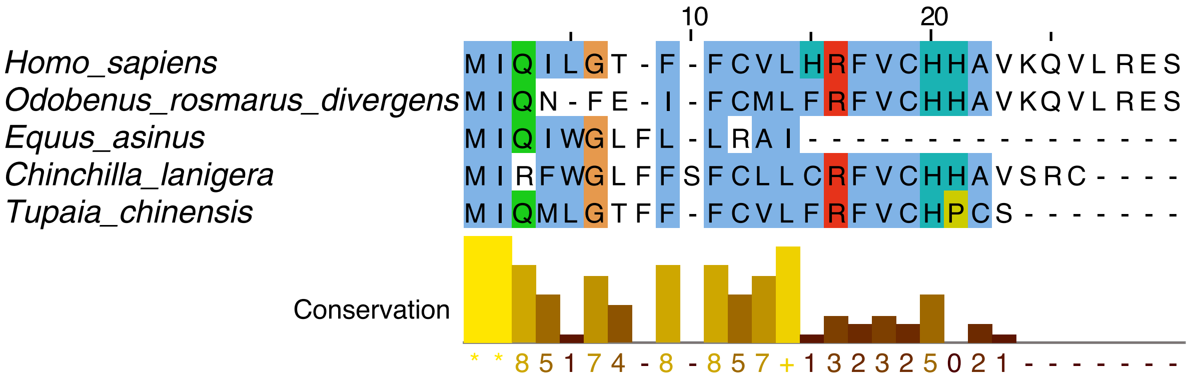

HG0981

ENSG00000163629 (PTPN13)

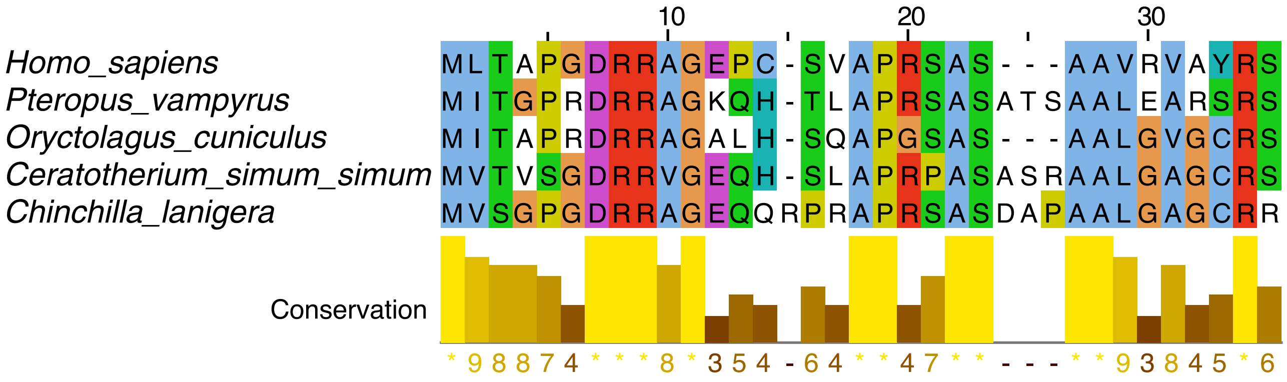

HG0982

ENSG00000164116 (GUCY1A3)

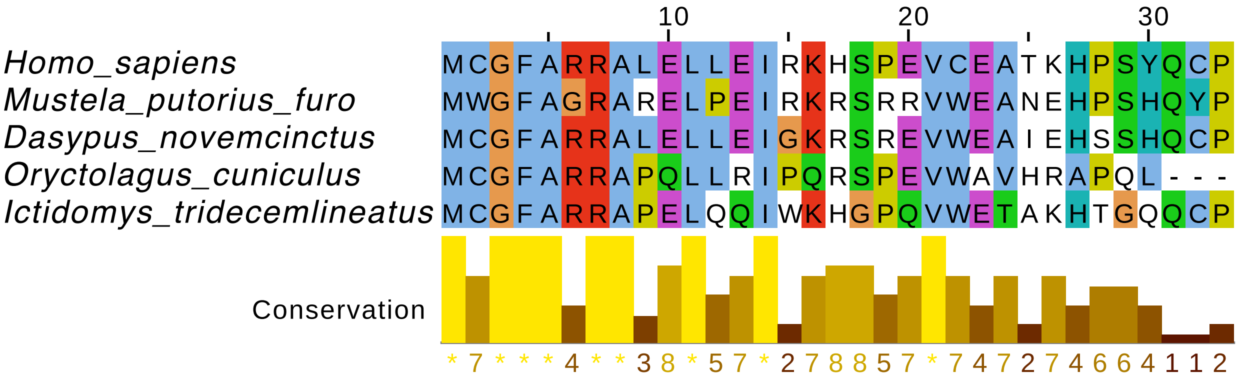

HG0983

ENSG00000164128 (NPY1R)

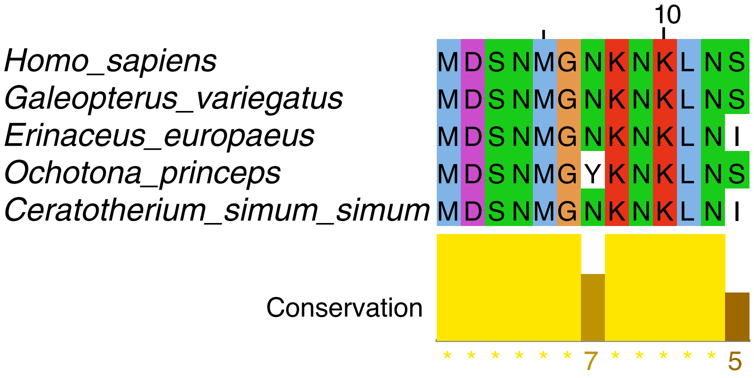

HG0984

ENSG00000164185 (ZNF474)

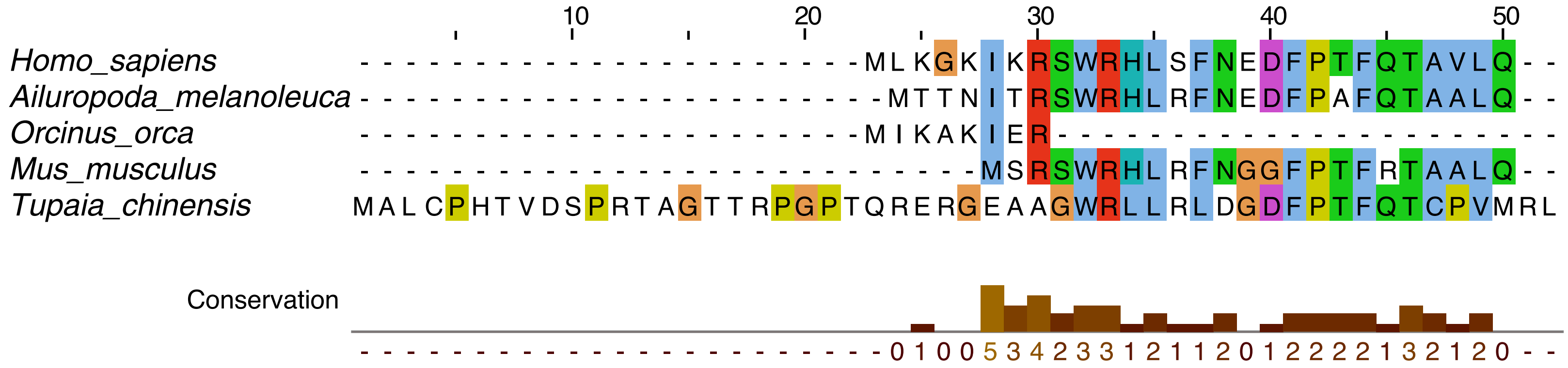

HG0985

ENSG00000164604 (GPR85)

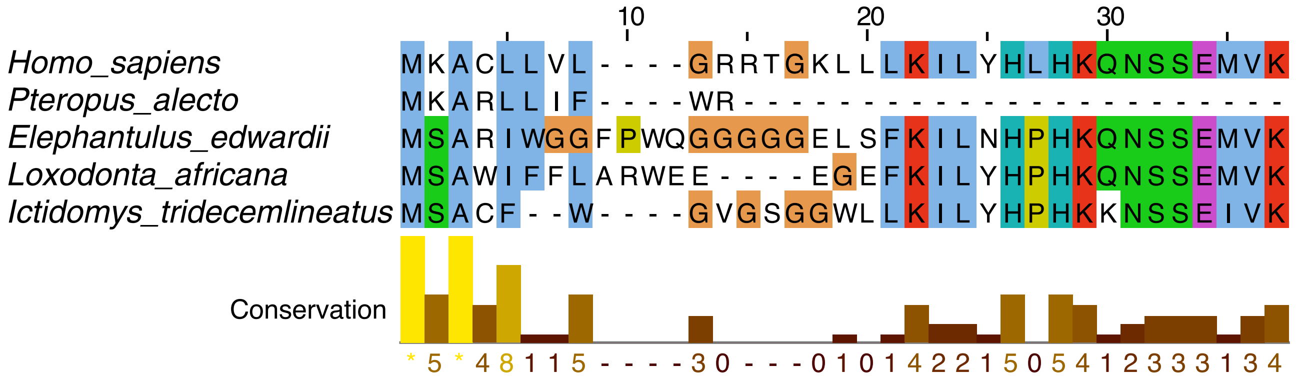

HG0986

ENSG00000164663 (USP49)

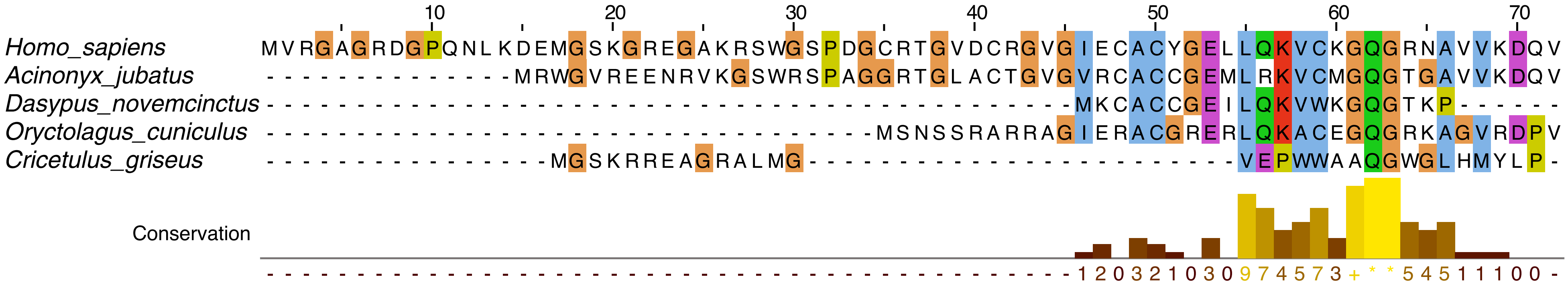

HG0987

ENSG00000164932 (CTHRC1)

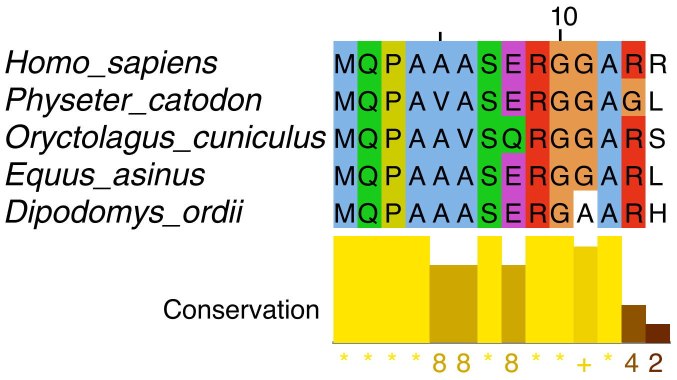

HG0988

ENSG00000166130 (IKBIP)

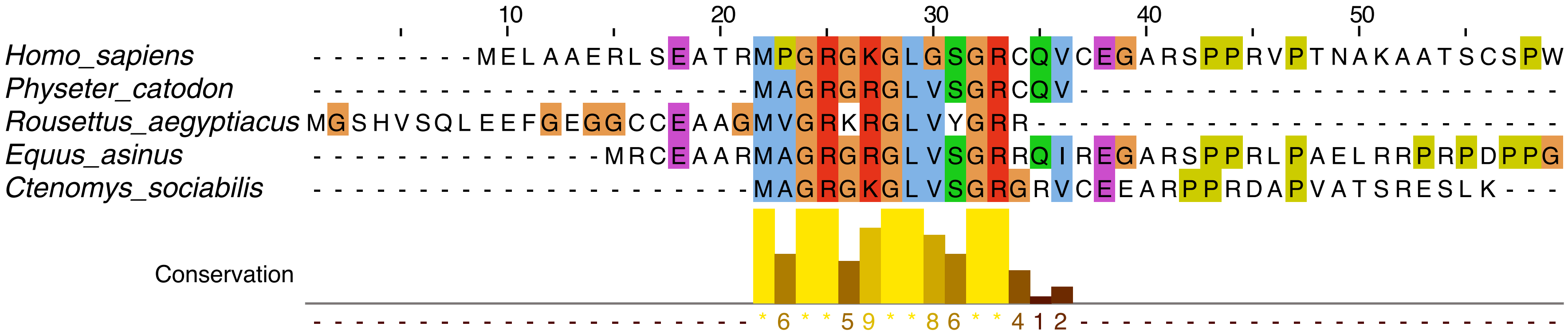

HG0989

ENSG00000166603 (MC4R)

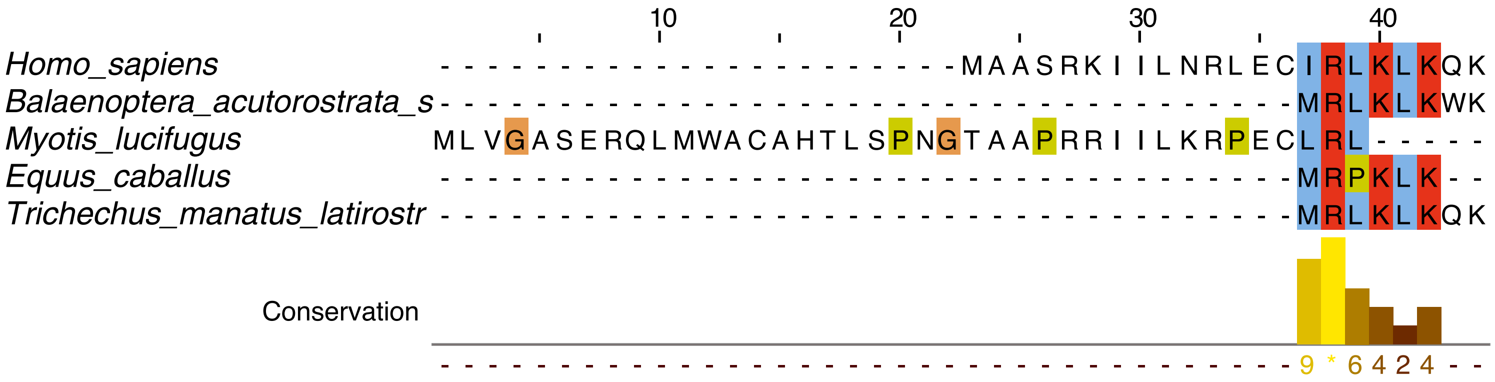

HG0990

ENSG00000166866 (MYO1A)

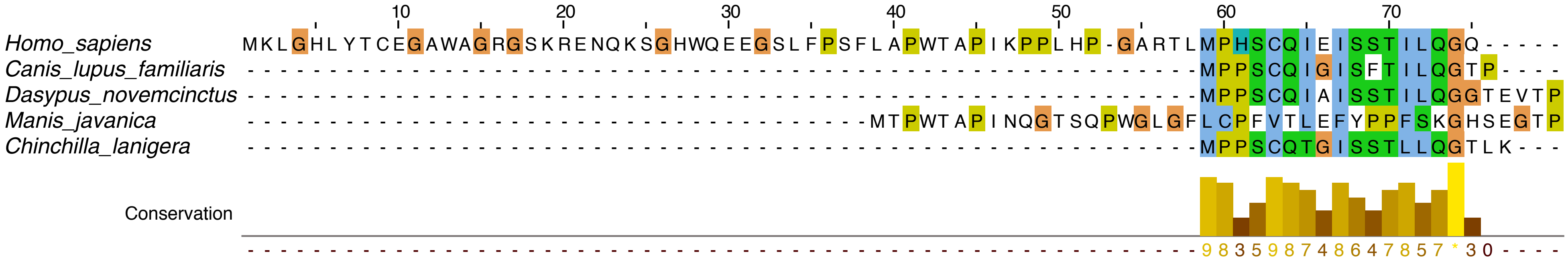

HG0991

ENSG00000167654 (ATCAY)

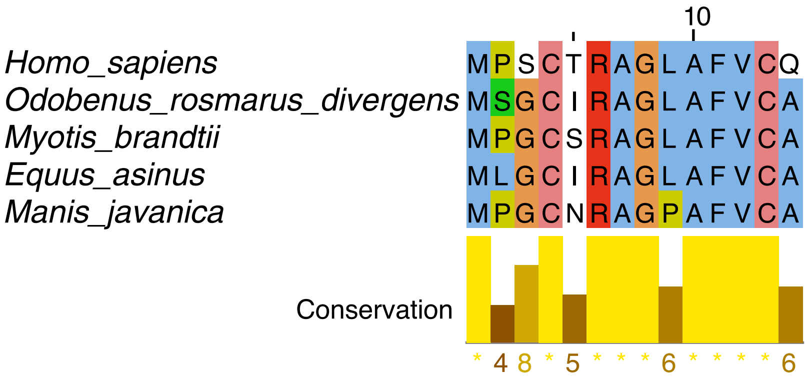

HG0992

ENSG00000168000 (BSCL2)

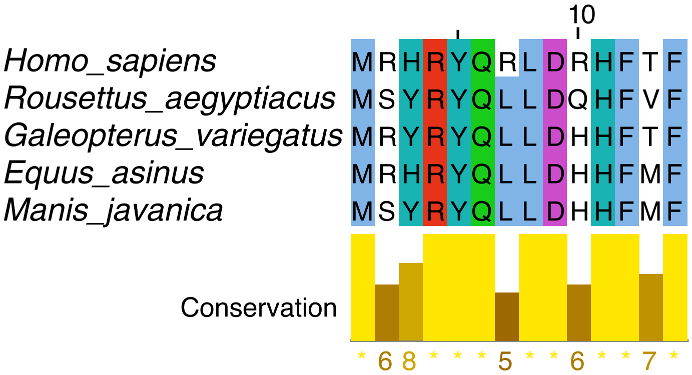

HG0993

ENSG00000169306 (IL1RAPL1)

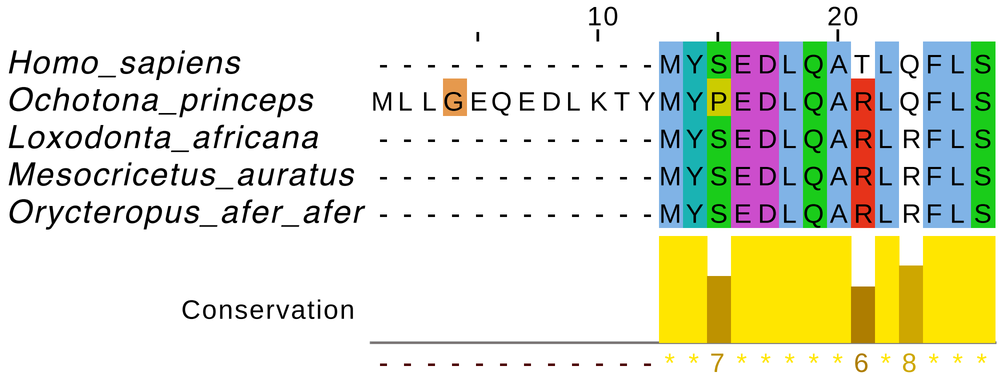

HG0994

ENSG00000170852 (KBTBD2)

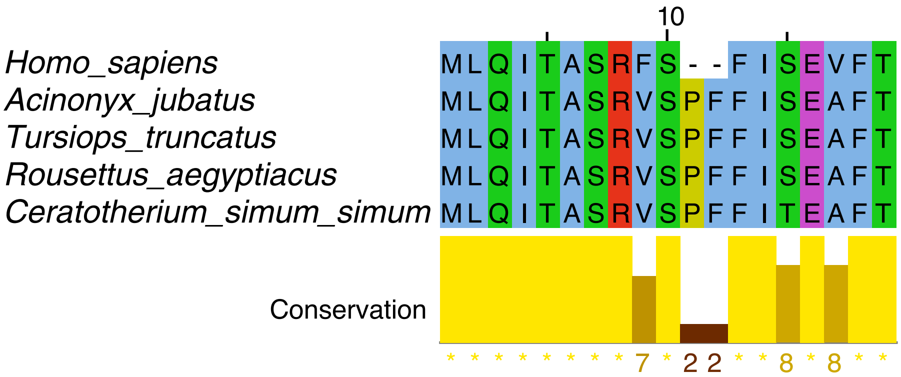

HG0995

ENSG00000171307 (ZDHHC16)

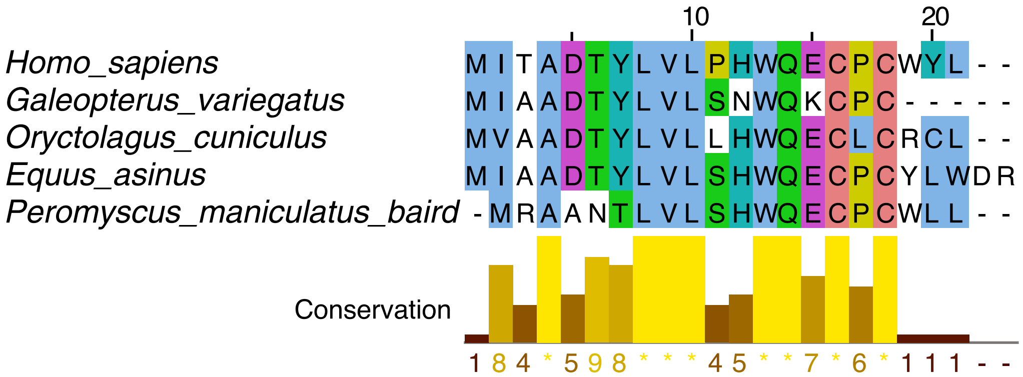

HG0996

ENSG00000173890 (GPR160)

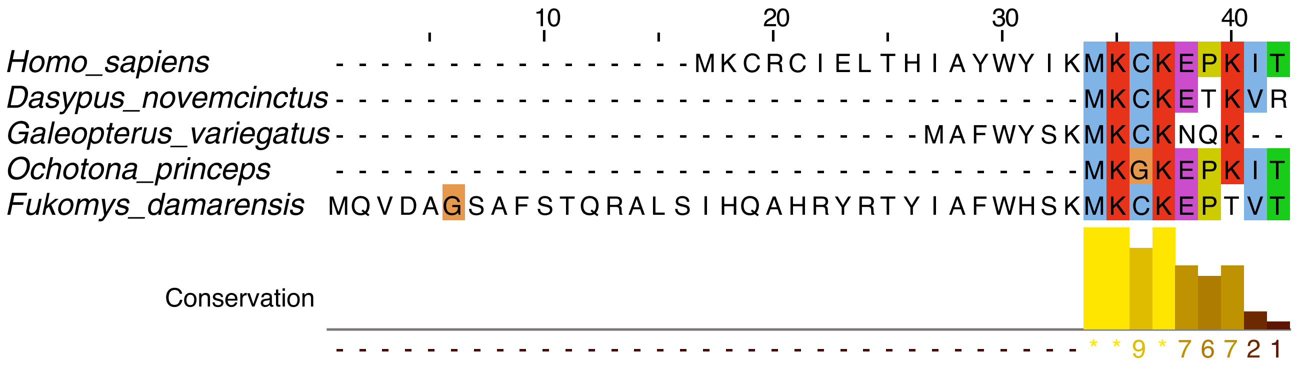

HG0997

ENSG00000177728 (TMEM94)

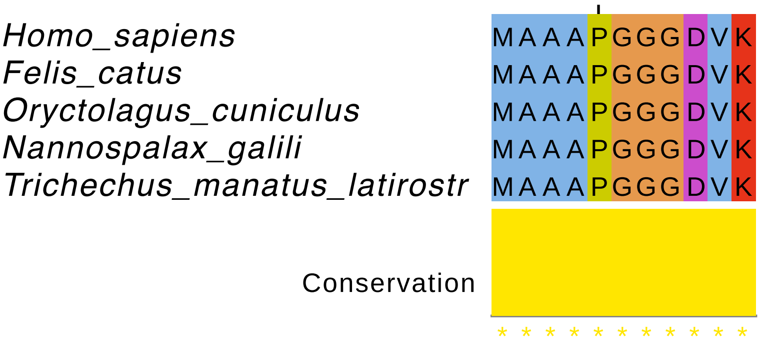

HG0998

ENSG00000178172 (SPINK6)

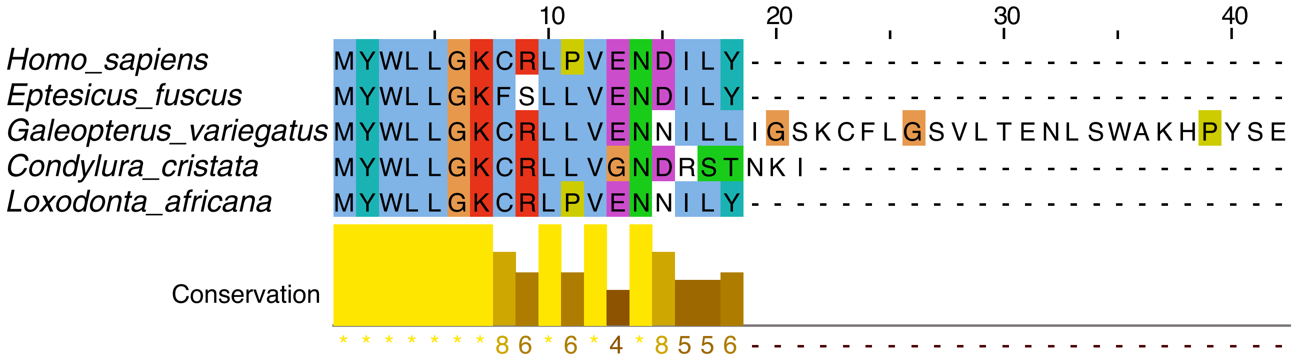

HG0999

ENSG00000178401 (DNAJC22)

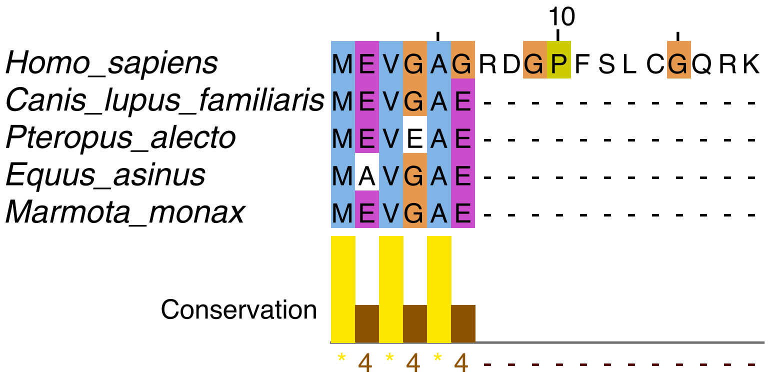

HG1000

ENSG00000179083 (FAM133A)

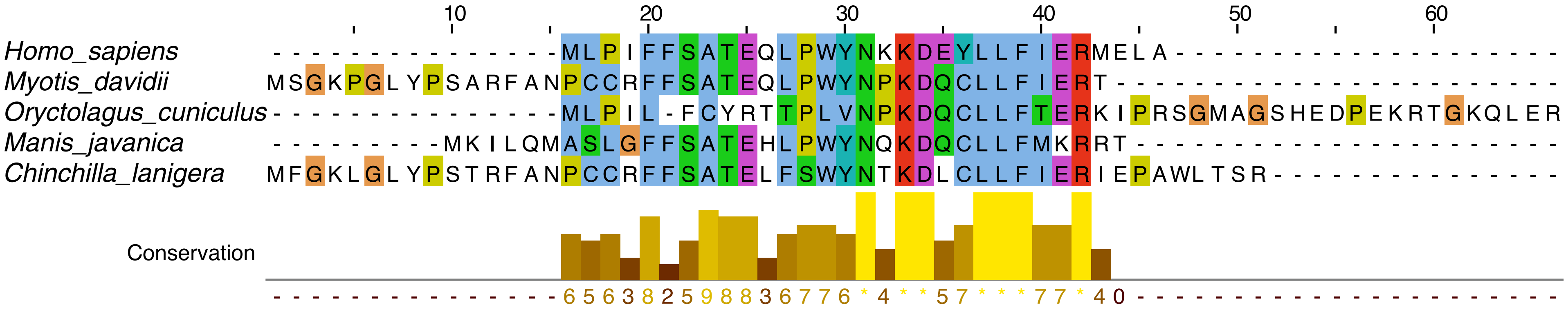

HG1001

ENSG00000179761 (PIPOX)

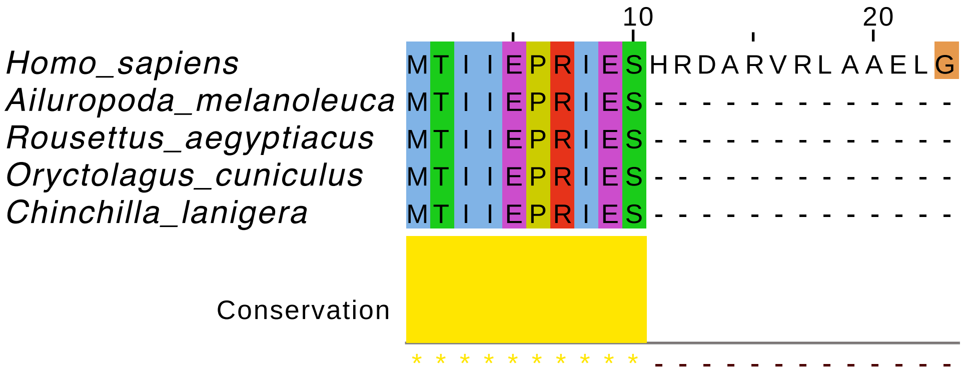

HG1002

ENSG00000181027 (FKRP)

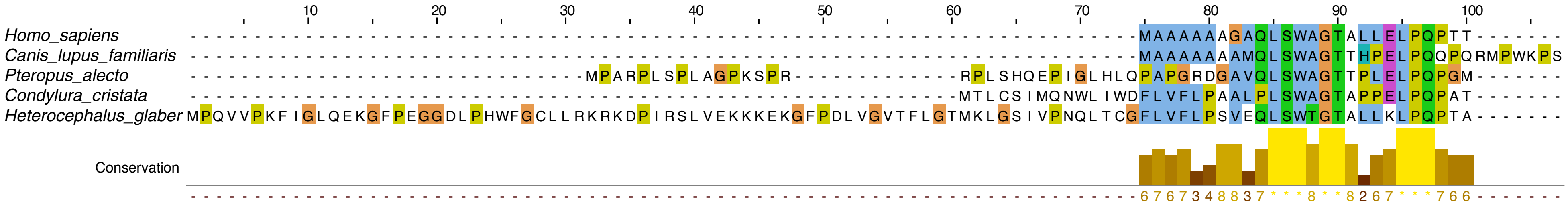

HG1003

ENSG00000184867 (ARMCX2)

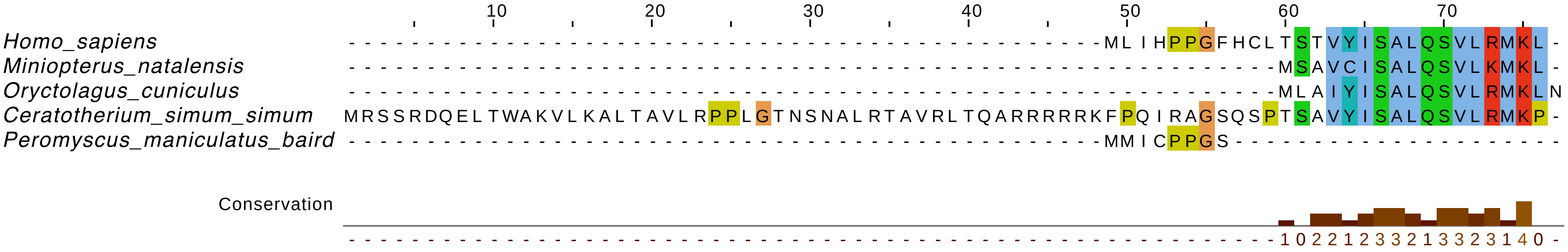

HG1004

ENSG00000185875 (THNSL1)

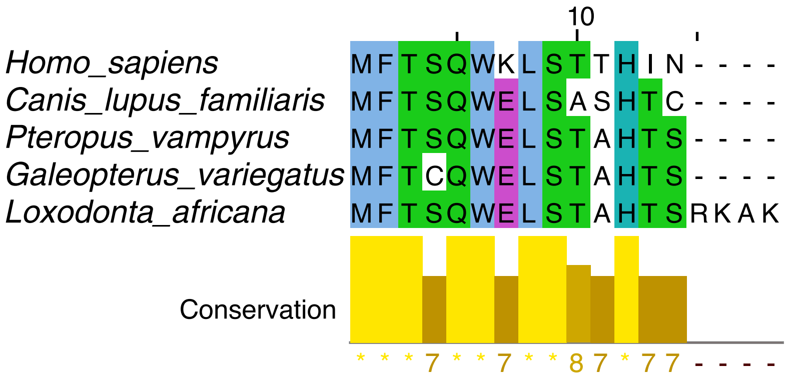

HG1005

ENSG00000186472 (PCLO)

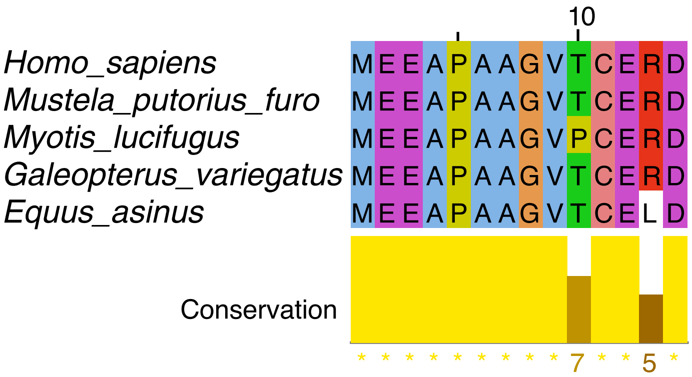

HG1006.1

ENSG00000188227 (ZNF793)

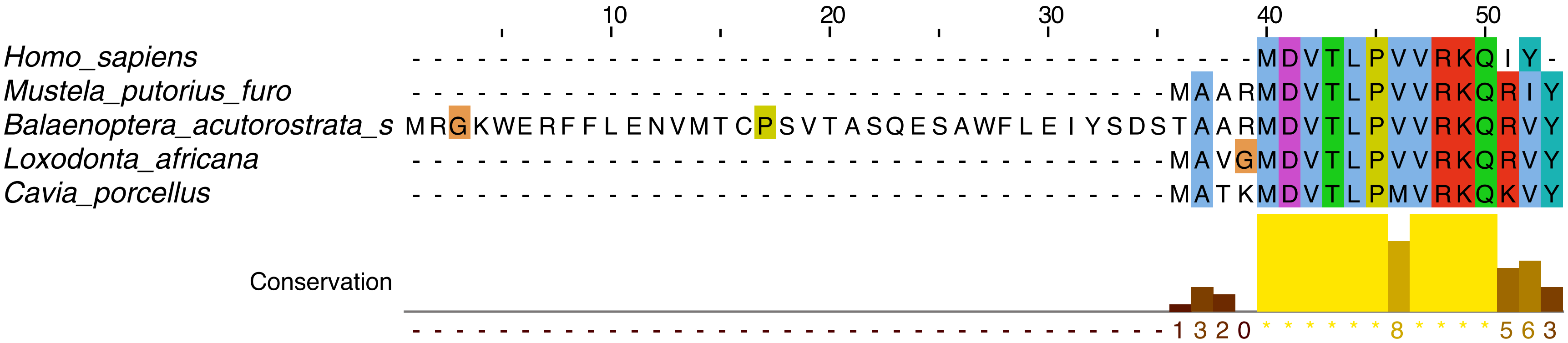

HG1006.2

ENSG00000188227 (ZNF793)

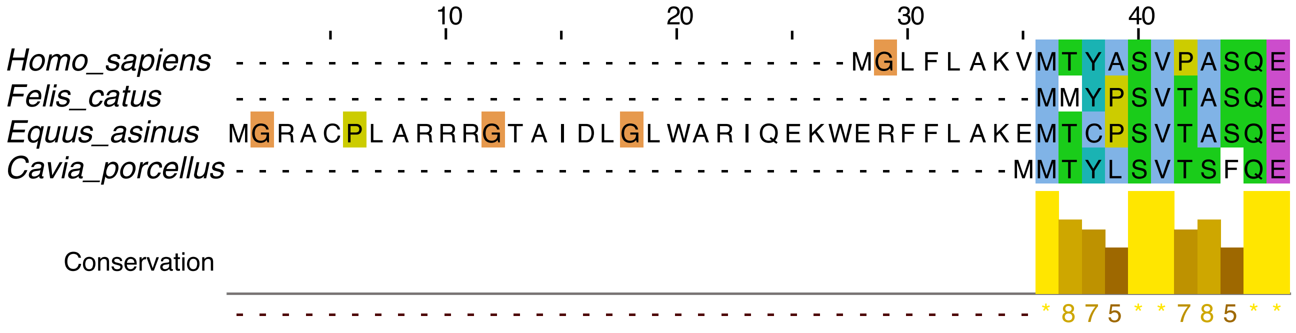

HG1007

ENSG00000188910 (GJB3)

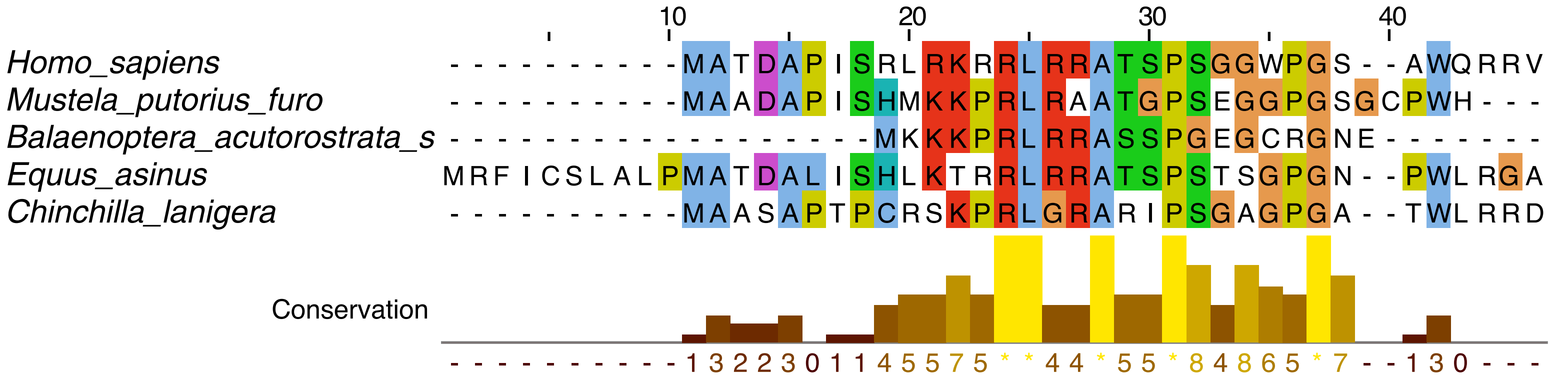

HG1008

ENSG00000198252 (STYX)

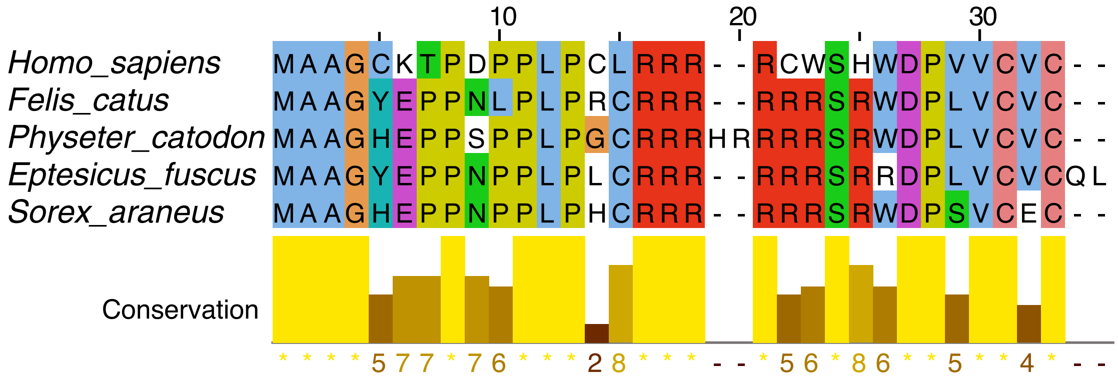

HG1009

ENSG00000198909 (MAP3K3)

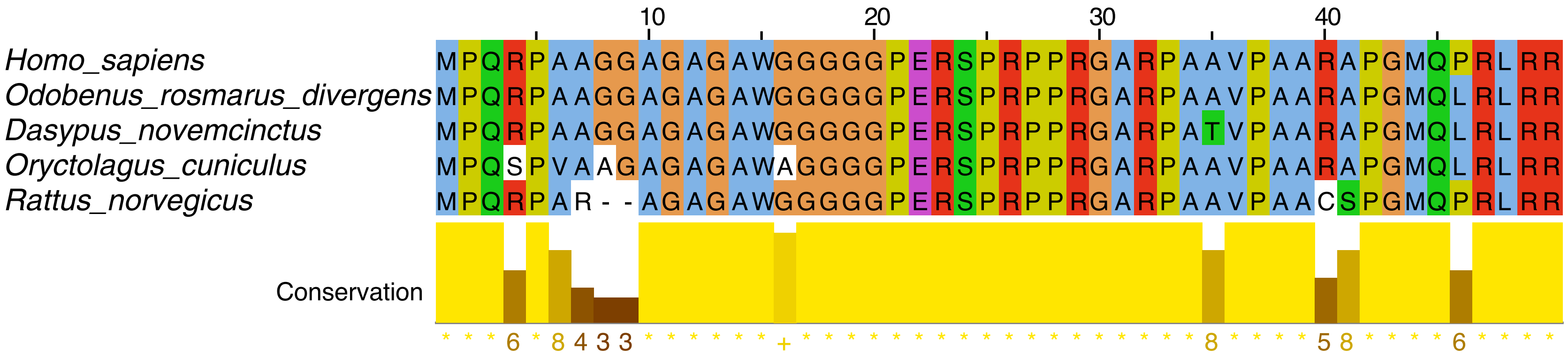

HG1010

ENSG00000203740 (METTL11B)

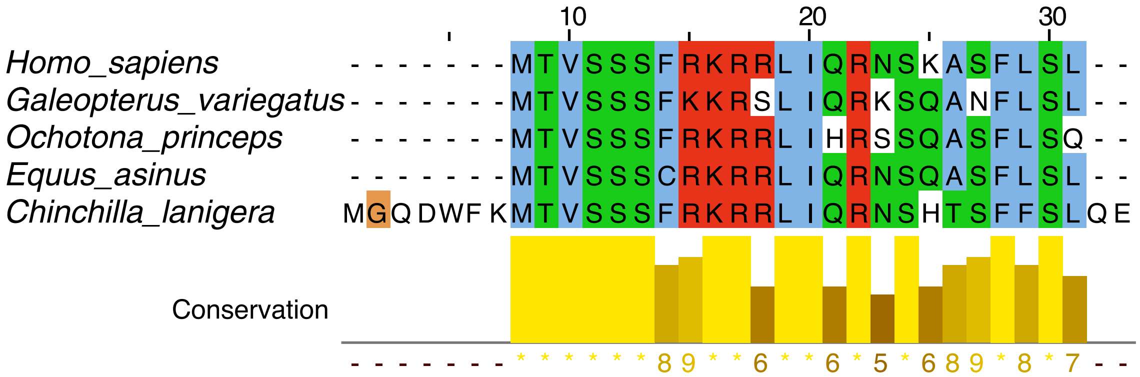

HG1011

ENSG00000204439 (C6orf47)

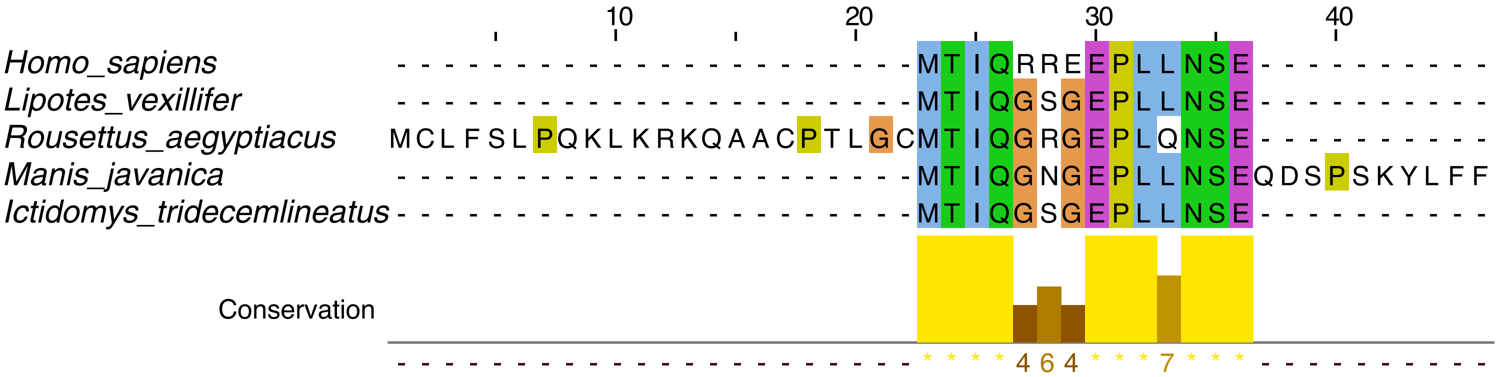

HG1012

ENSG00000213694 (S1PR3)

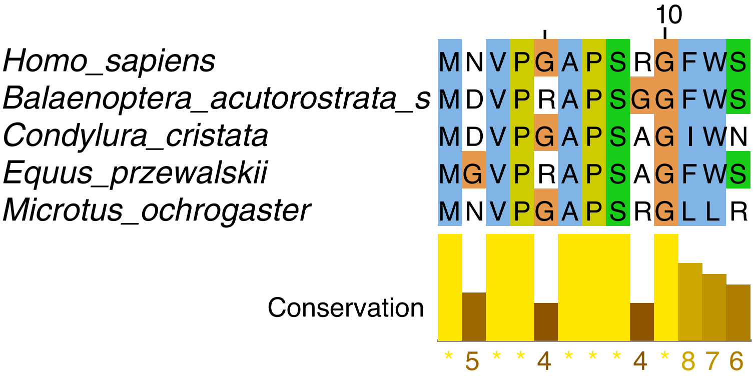

HG1013

ENSG00000214022 (REPIN1)

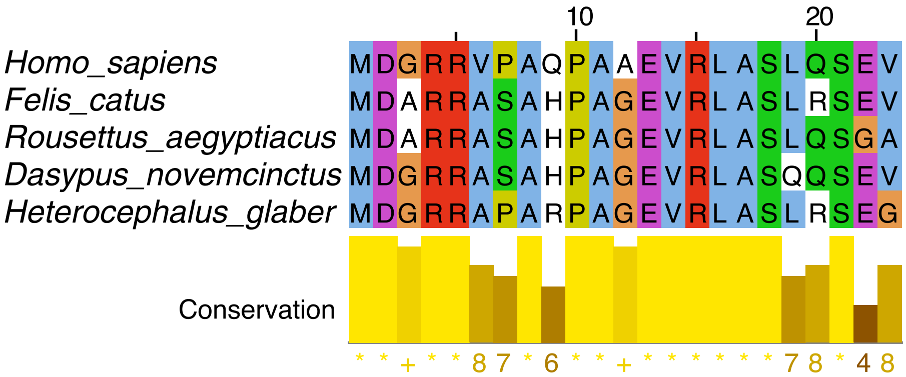

HG1014.1

ENSG00000214595 (EML6)

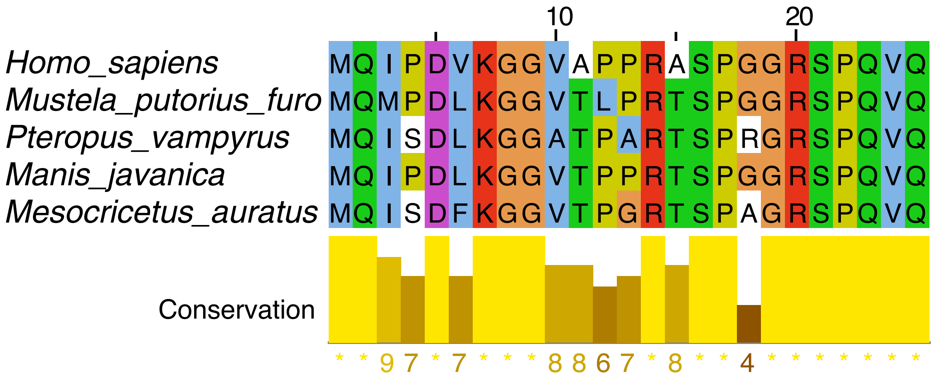

HG1014.2

ENSG00000214595 (EML6)

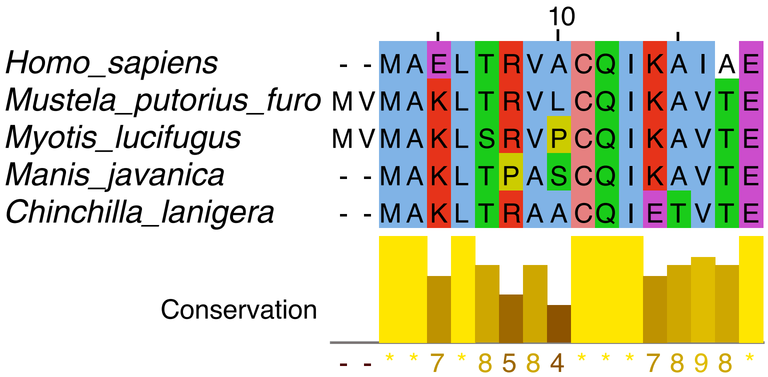

HG1015

ENSG00000240720 (LRRD1)

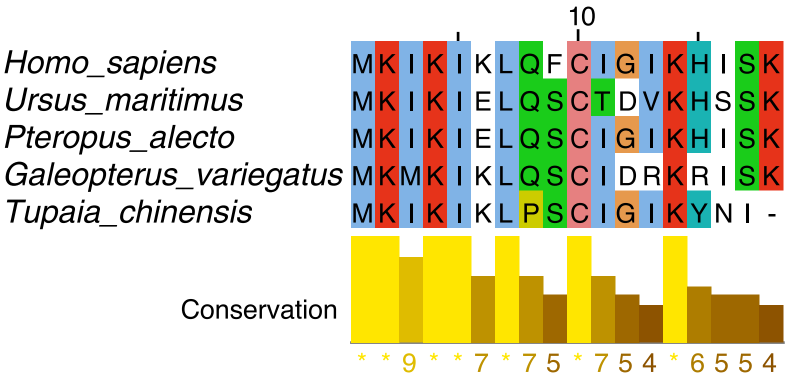

HG1016

ENSG00000254004 (ZNF260)

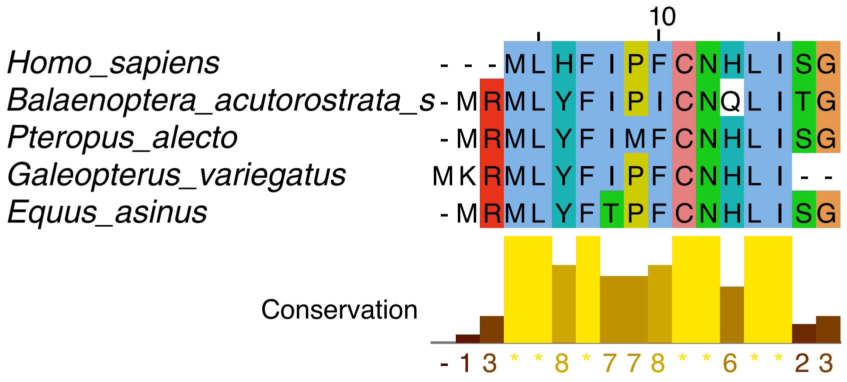

HG1017

ENSG00000255154 (HTD2)

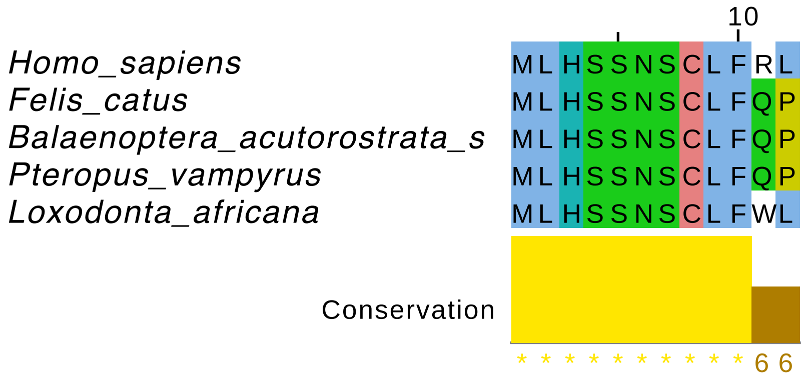

HG1018

ENSG00000259120 (SMIM6)

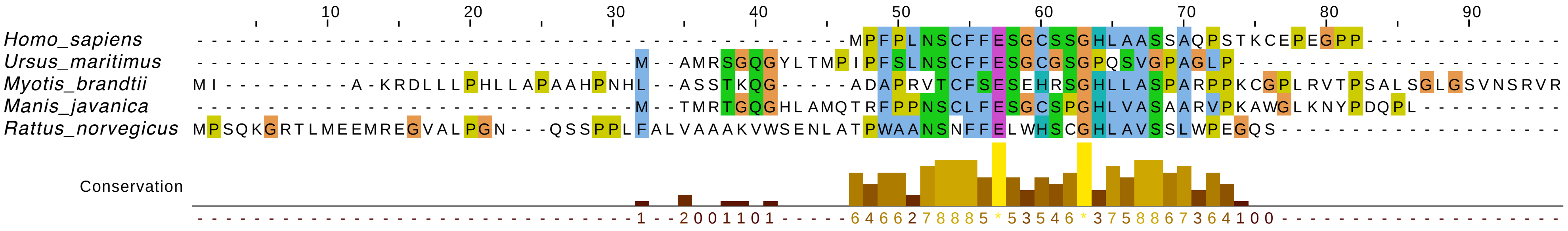

HG1019

ENSG00000019995 (ZRANB1)

```
                                       10        20        30        40
Homo_sapiens                    MASLKLNISISGFPLISSFLDQTSL-YLLPISFAFQSSVLLNPRV---
Odobenus_rosmarus_divergens     ----------------------MSL-YLLLISFAF-------------
Lipotes_vexillifer              MTWLRLNISVRGFLI---------------------------------
Orycteropus_afer_afer           MSWLKLNISIGGLPILTNPFPLKTFSTDLPLSLAYYLPCTAKYNFITS

Conservation
                                ------------------------------------------------
```

HG1020

ENSG00000023902 (PLEKHO1)

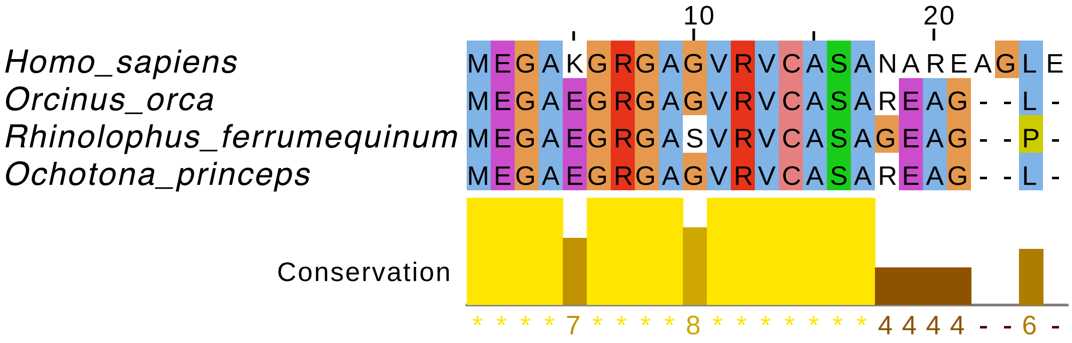

HG1021

ENSG00000064692 (SNCAIP)

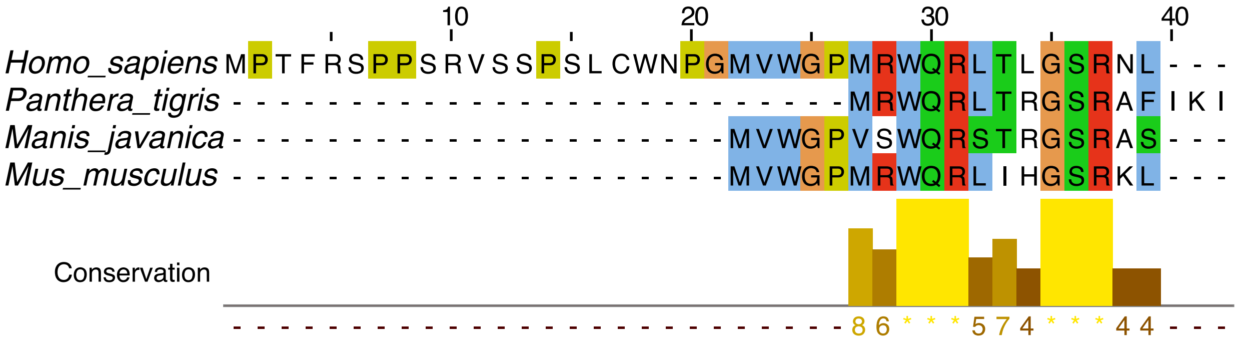

HG1022

ENSG00000065357 (DGKA)

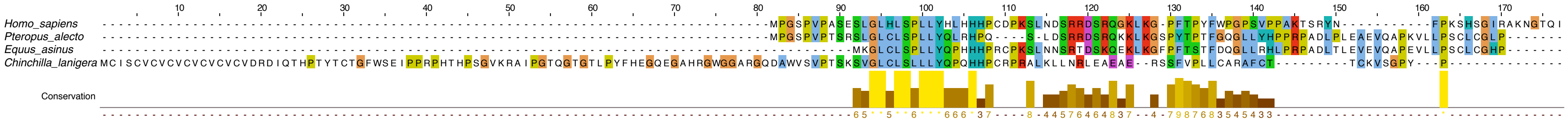

HG1023

ENSG00000068001 (HYAL2)

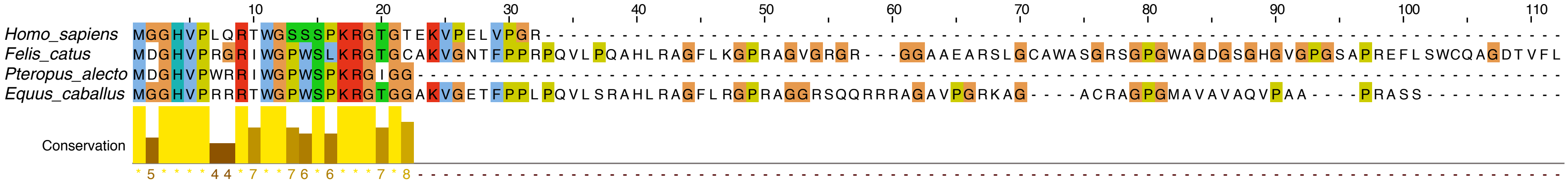

HG1024

ENSG00000073464 (CLCN4)

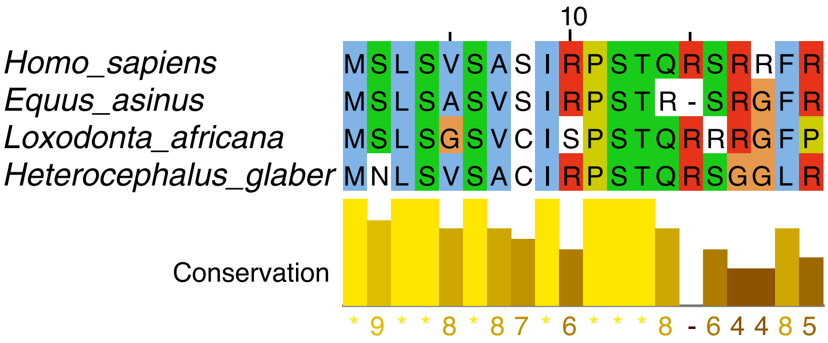

HG1025

ENSG00000074621 (SLC24A1)

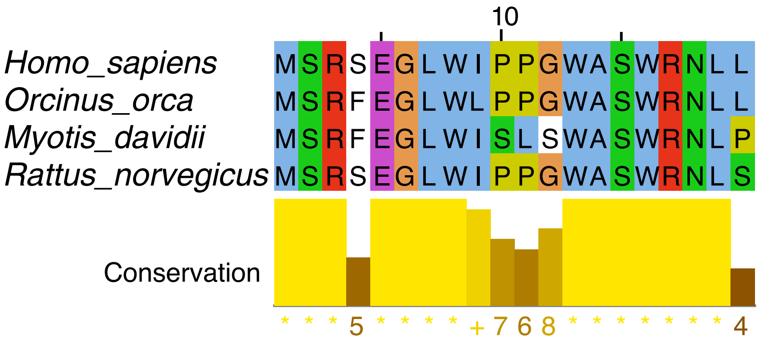

HG1026.1

ENSG00000075035 (WSCD2)

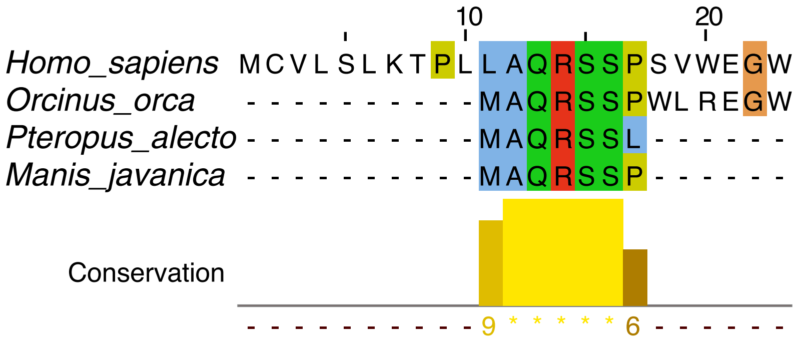

HG1026.2

ENSG00000179314 (WSCD1)

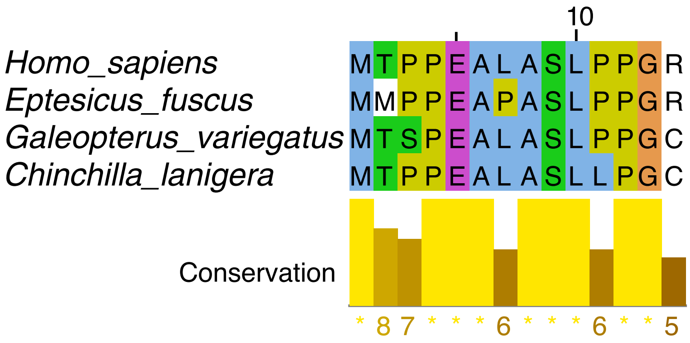

HG1026.3

ENSG00000179314 (WSCD1)

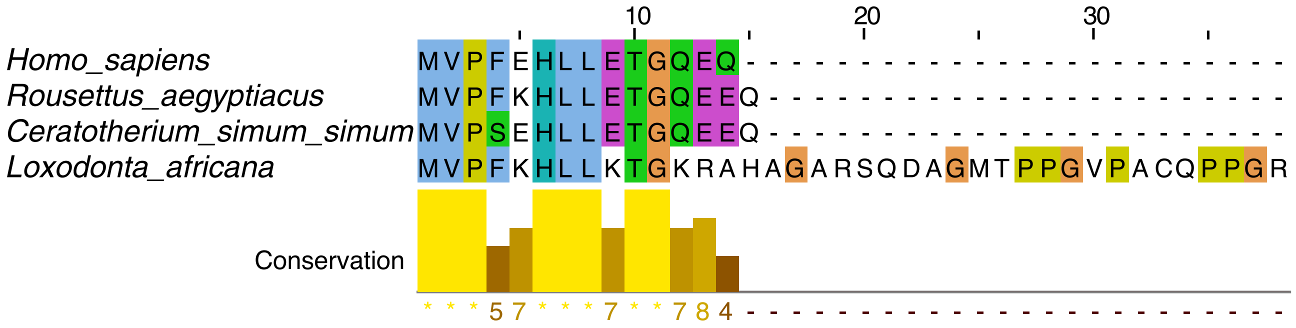

HG1027

ENSG00000075223 (SEMA3C)

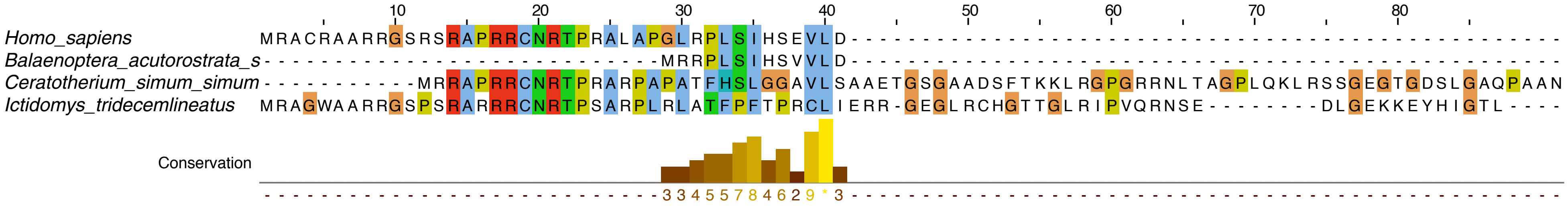

HG1028

ENSG00000076356 (PLXNA2)

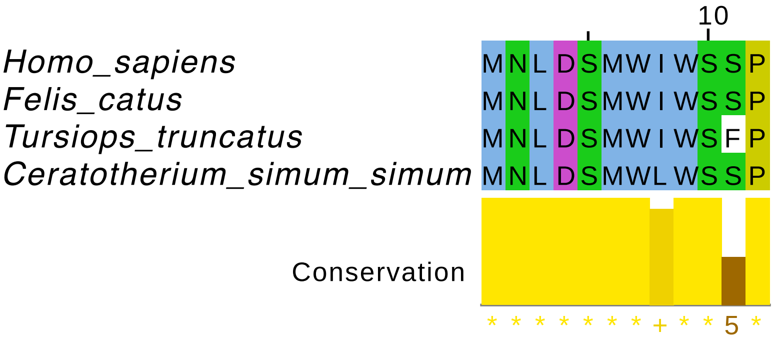

HG1029

ENSG00000079482 (OPHN1)

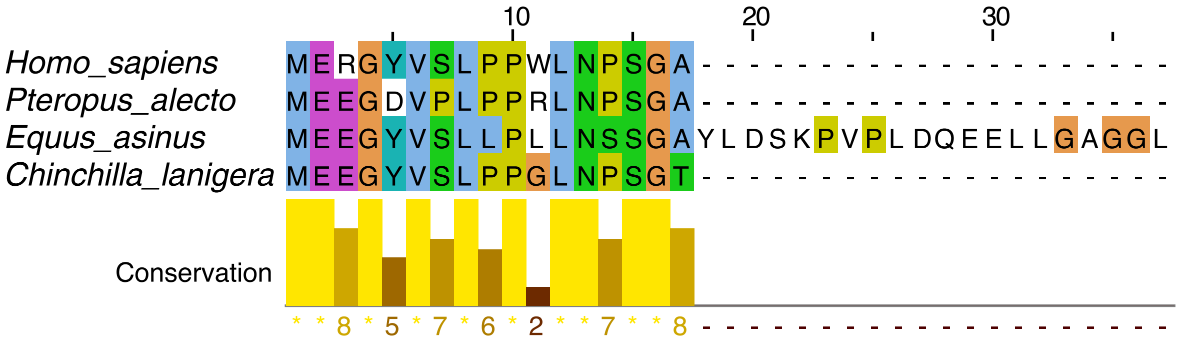

HG1030

ENSG00000082175 (PGR)

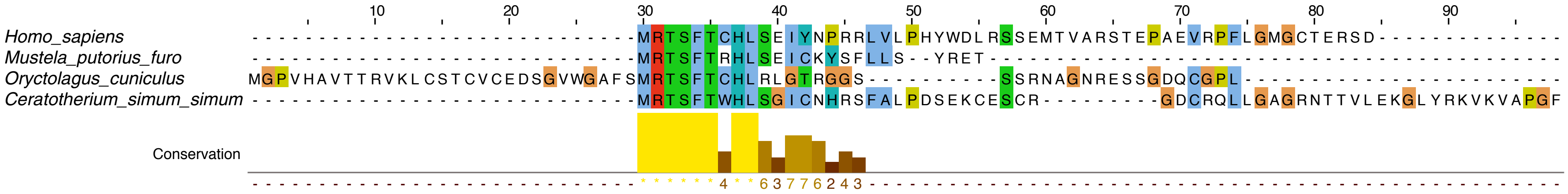

HG1031

ENSG00000082269 (FAM135A)

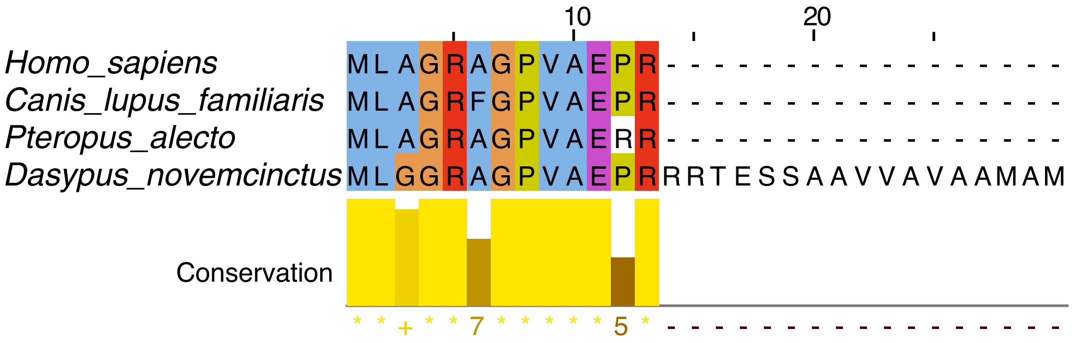

HG1032

ENSG00000084453 (SLCO1A2)

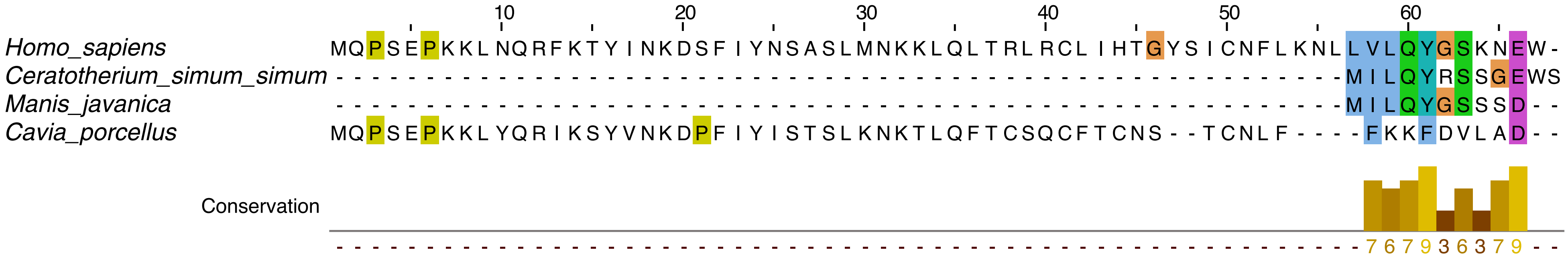

HG1033

ENSG00000089225 (TBX5)

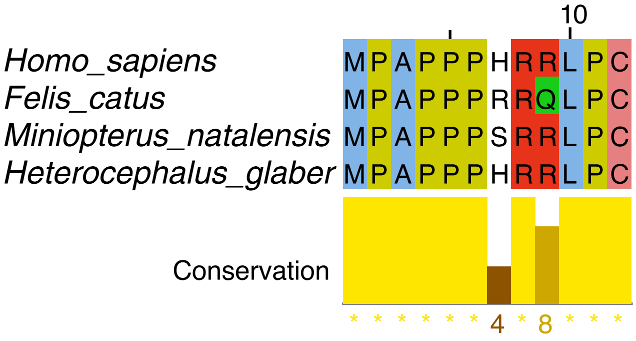

HG1034

ENSG00000090487 (SPG21)

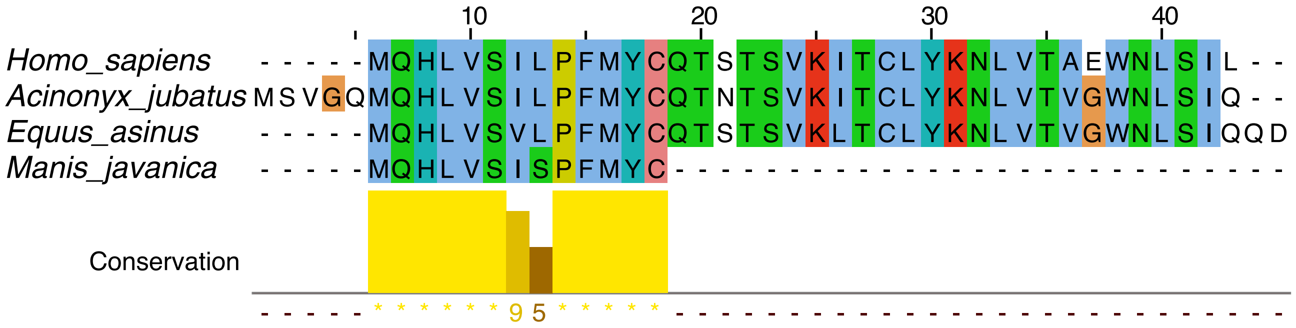

HG1035

ENSG00000093000 (NUP50)

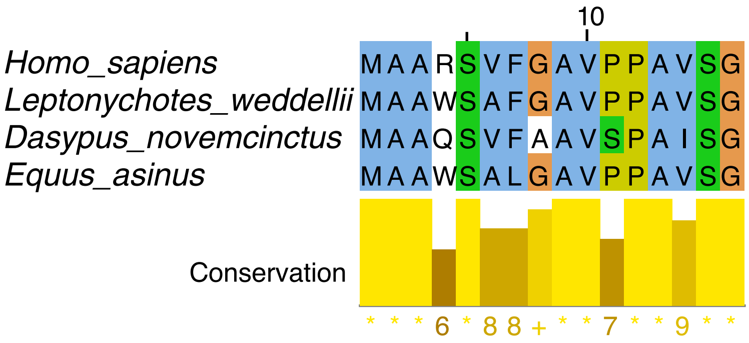

HG1036

ENSG00000100813 (ACIN1)

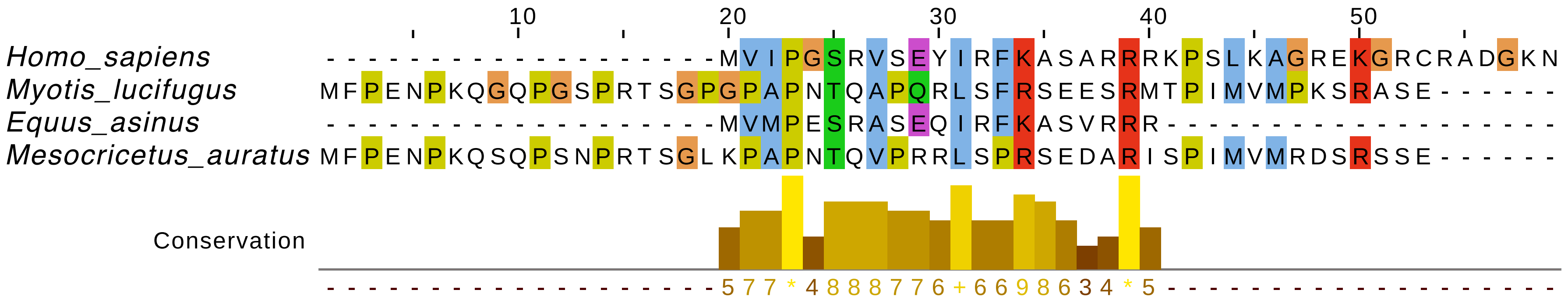

HG1037

ENSG00000102710 (SUPT20H)

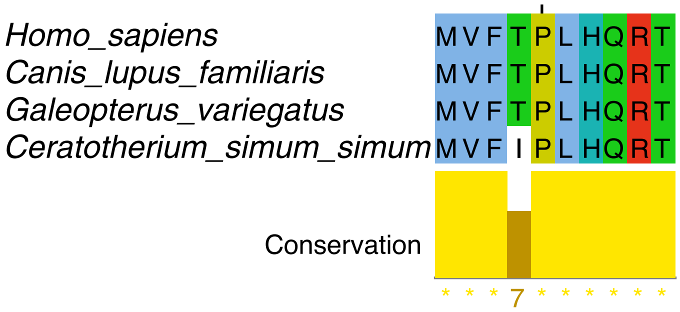

HG1038

ENSG00000104331 (IMPAD1)

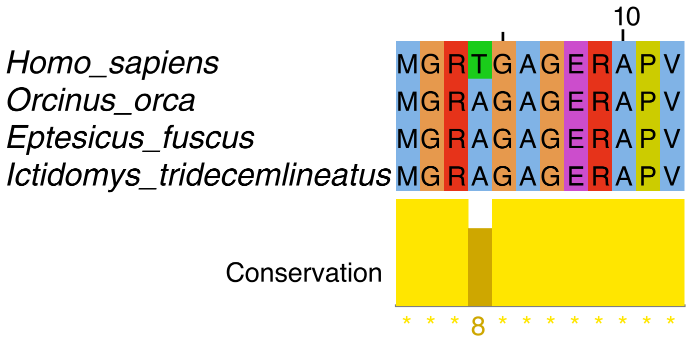

HG1039

ENSG00000104804 (TULP2)

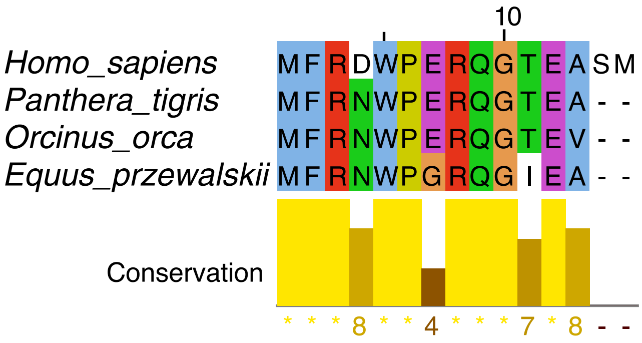

HG1040

ENSG00000105223 (PLD3)

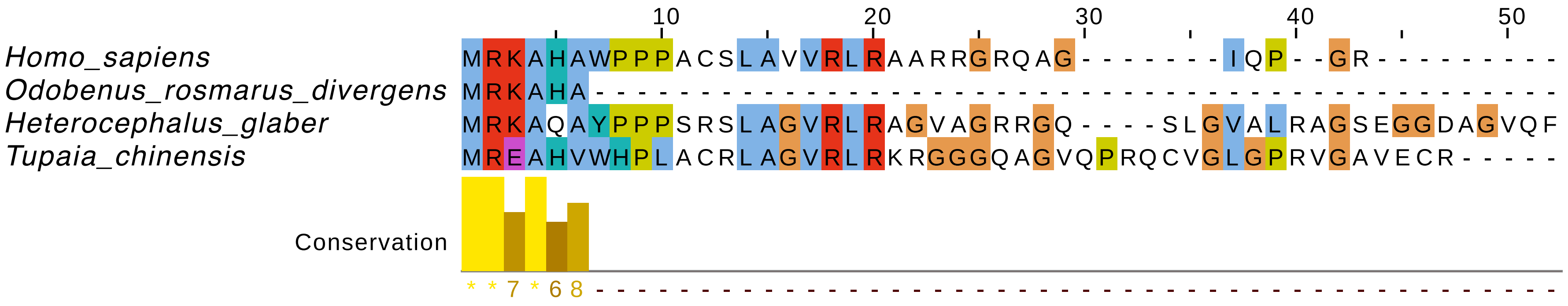

HG1041

ENSG00000106536 (POU6F2)

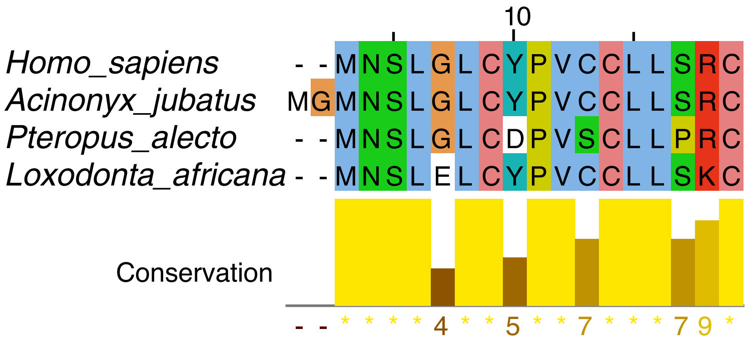

HG1042

ENSG00000107249 (GLIS3)

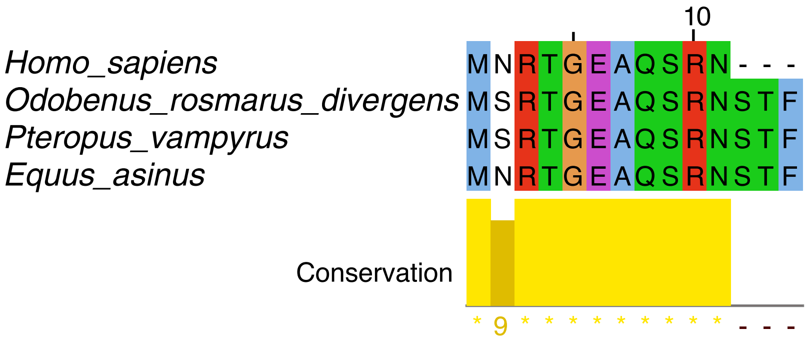

HG1043

ENSG00000107736 (CDH23)

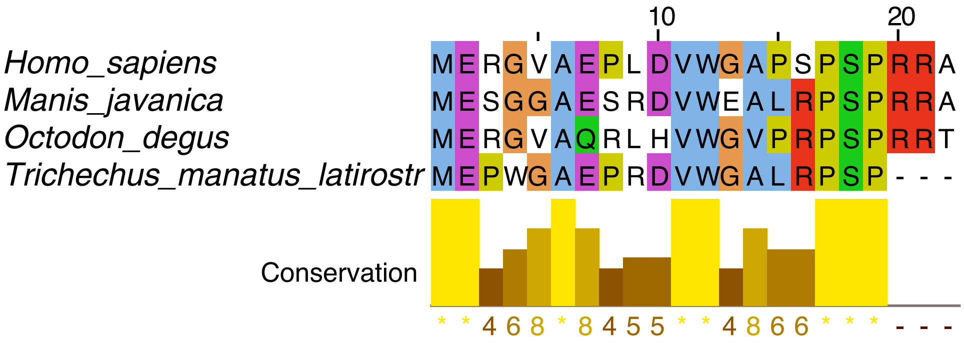

HG1044

ENSG00000109381 (ELF2)

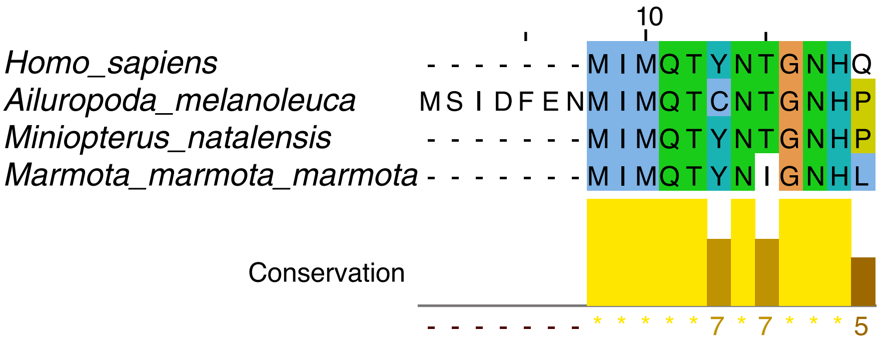

HG1045

ENSG00000110925 (CSRNP2)

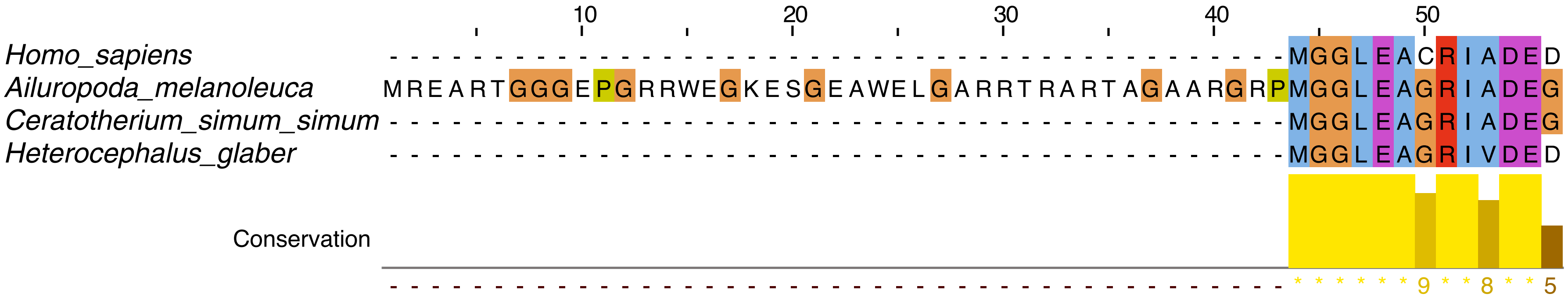

HG1046

ENSG00000112701 (SENP6)

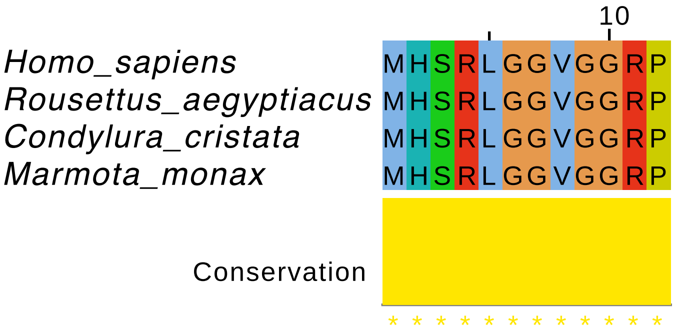

HG1047

ENSG00000112769 (LAMA4)

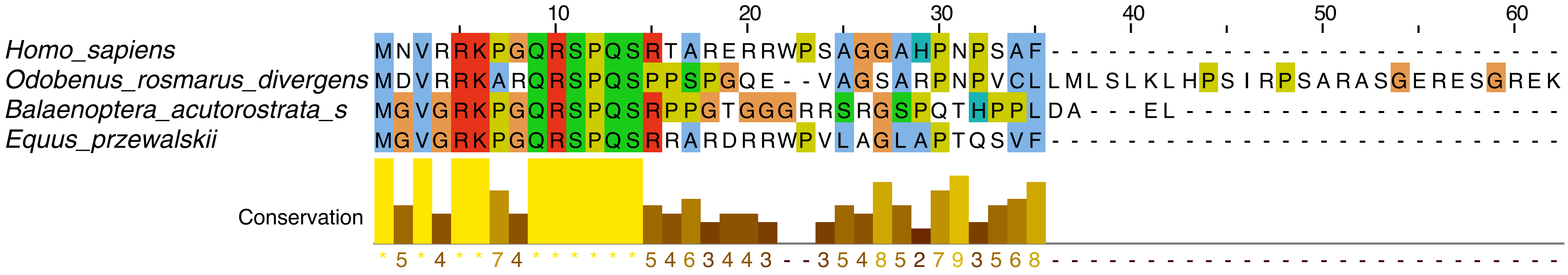

HG1048

ENSG00000115295 (CLIP4)

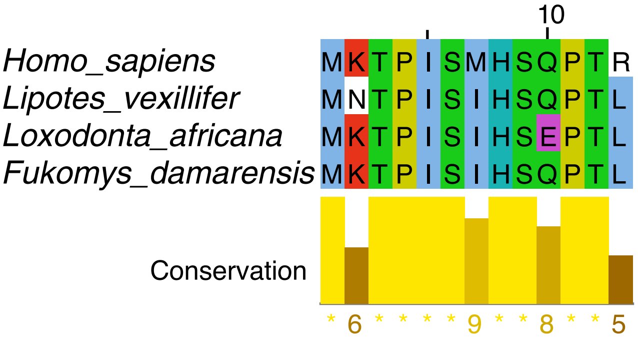

HG1049

ENSG00000116212

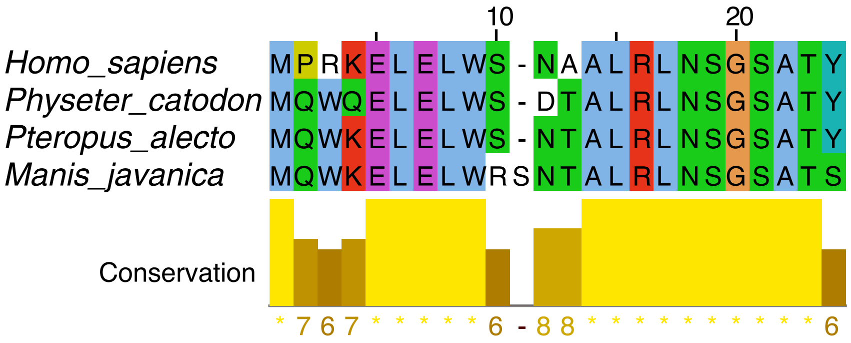

HG1050

ENSG00000116489 (CAPZA1)

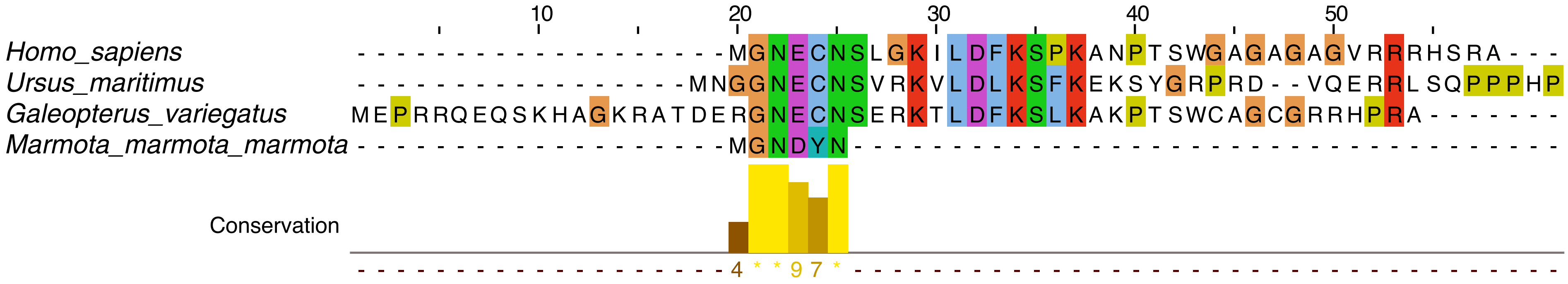

HG1051

ENSG00000116852 (KIF21B)

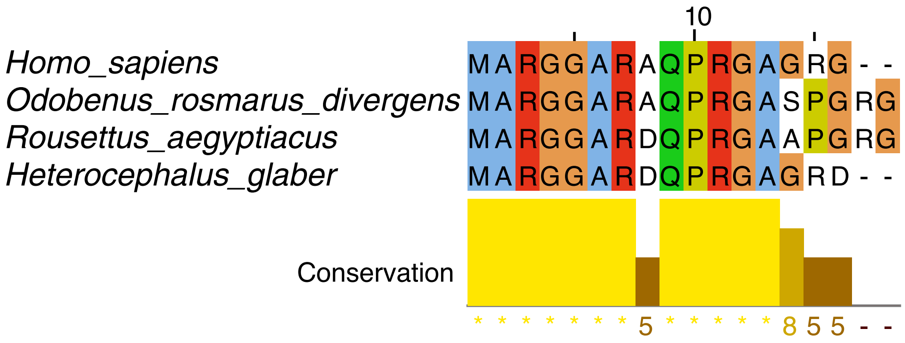

HG1052

ENSG00000118640 (VAMP8)

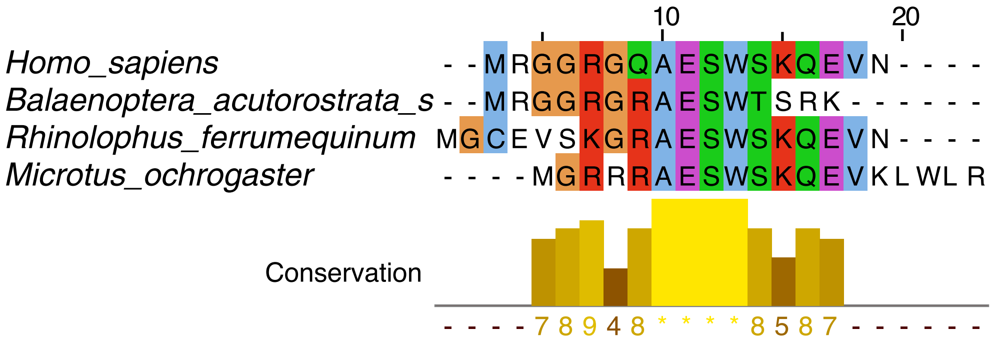

HG1053

ENSG00000119522 (DENND1A)

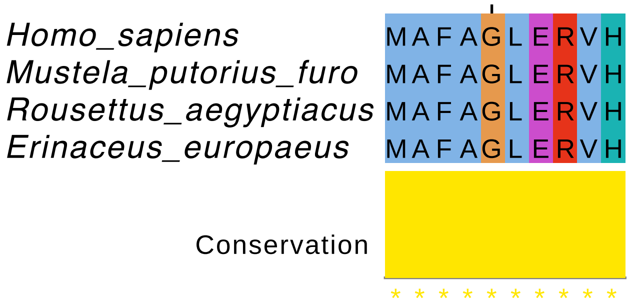

HG1054

ENSG00000120832 (MTERF2)

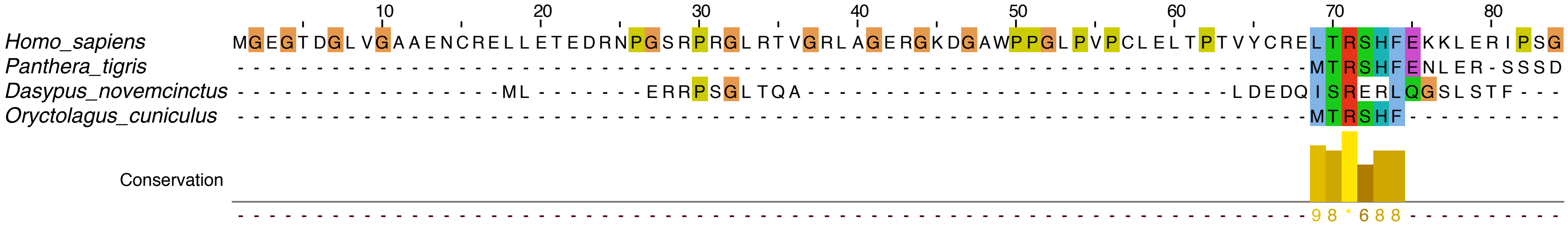

HG1055

ENSG00000122299 (ZC3H7A)

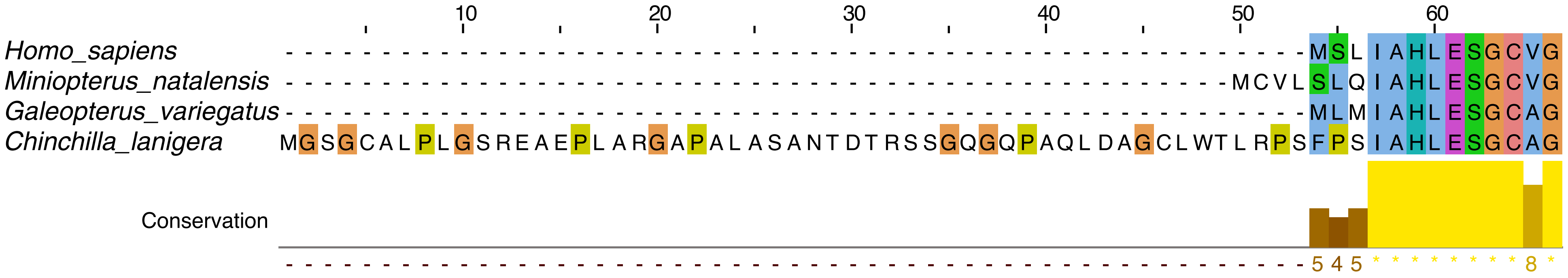

HG1056

ENSG00000128271 (ADORA2A)

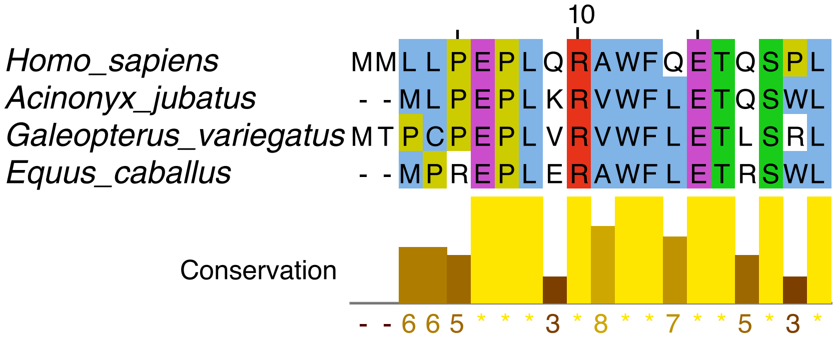

HG1057

ENSG00000131097 (HIGD1B)

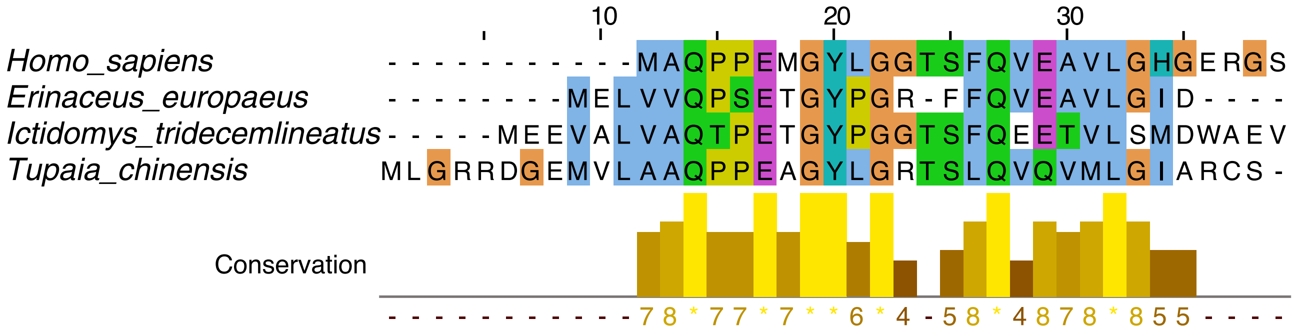

HG1058

ENSG00000131386 (GALNT15)

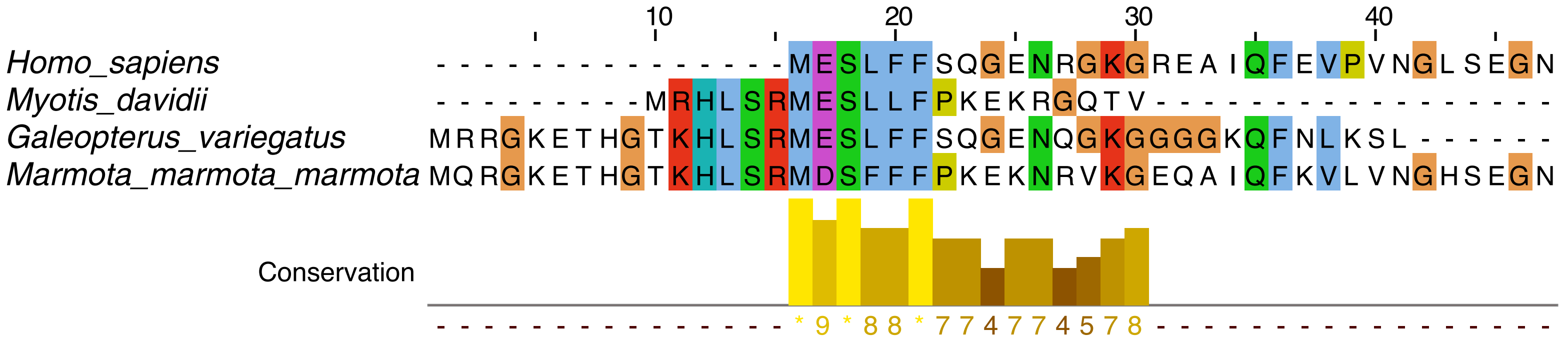

HG1059.1

ENSG00000132970 (WASF3)

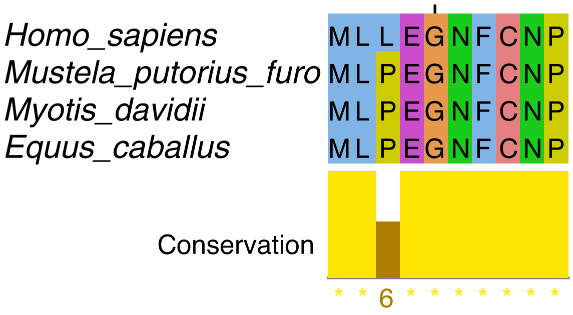

HG1059.2

ENSG00000112290 (WASF1)

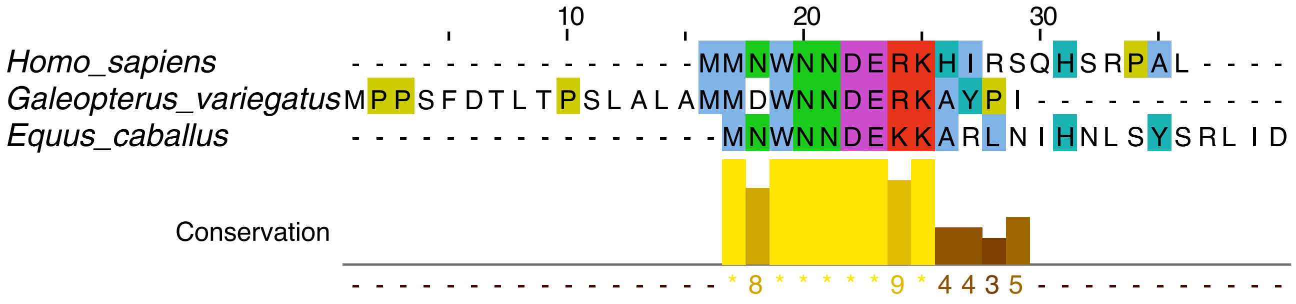

HG1060

ENSG00000134242 (PTPN22)

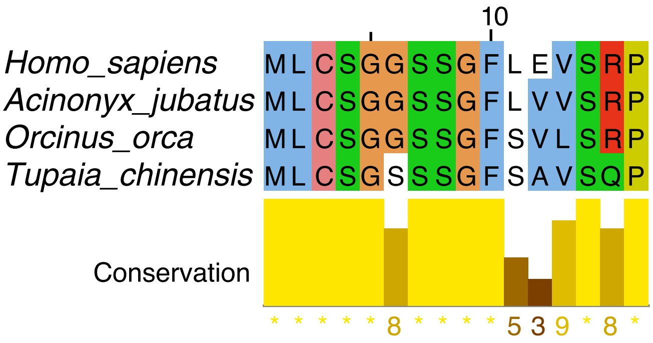

HG1061

ENSG00000134802 (SLC43A3)

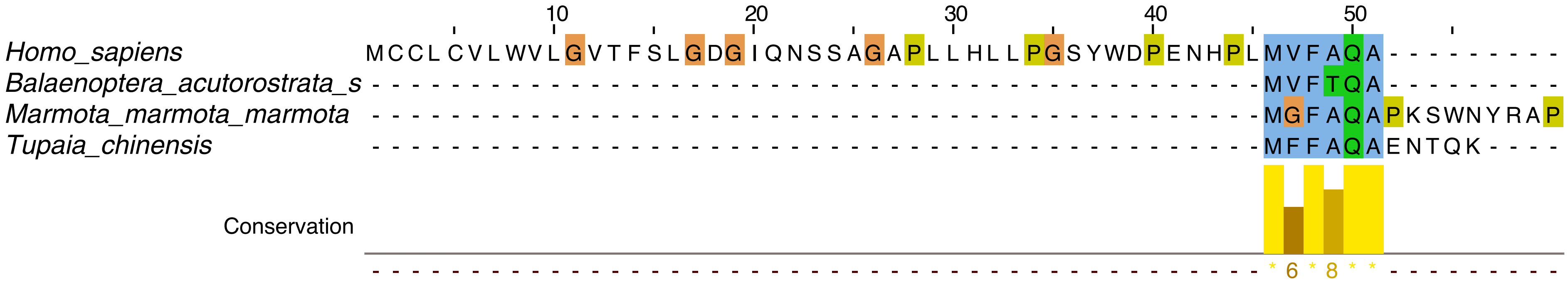

HG1062

ENSG00000137337 (MDC1)

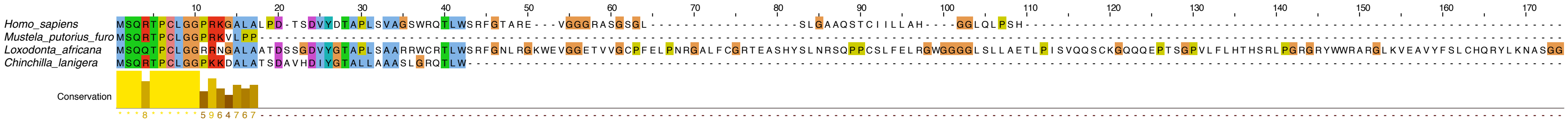

HG1063

ENSG00000138162 (TACC2)

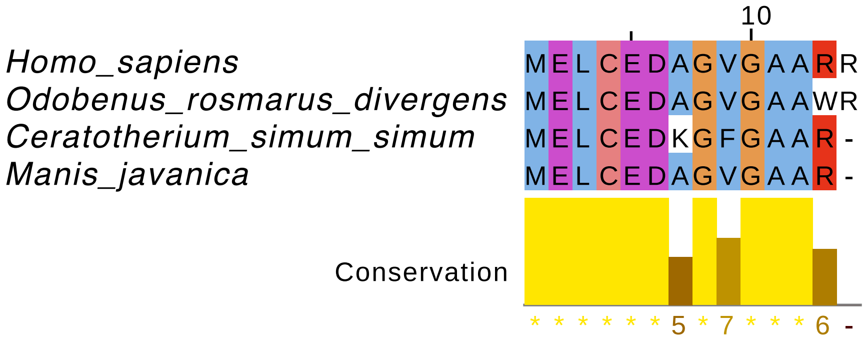

HG1064

ENSG00000139734 (DIAPH3)

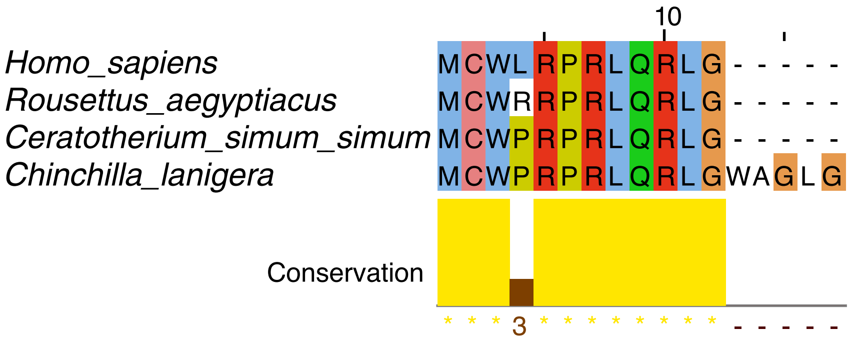

HG1065

ENSG00000140030 (GPR65)

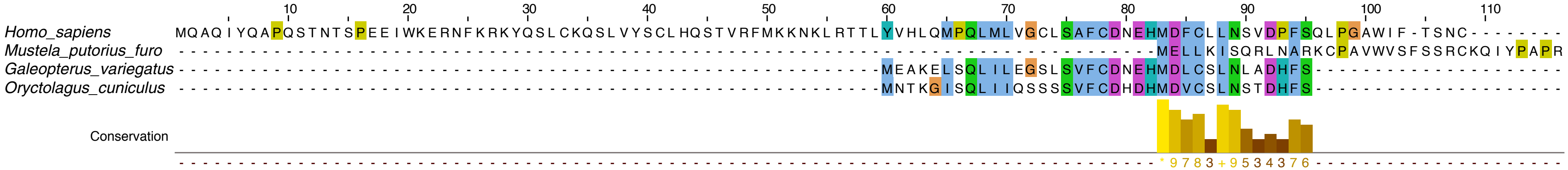

HG1066

ENSG00000140553 (UNC45A)

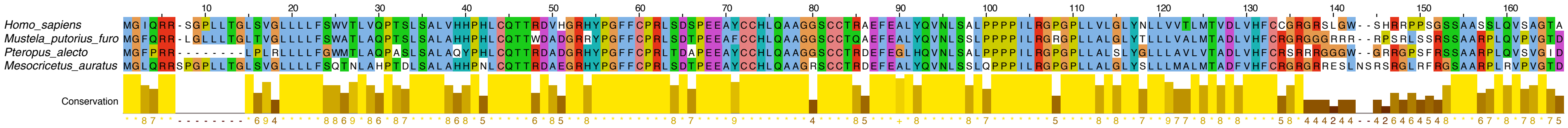

HG1067

ENSG00000143340 (FAM163A)

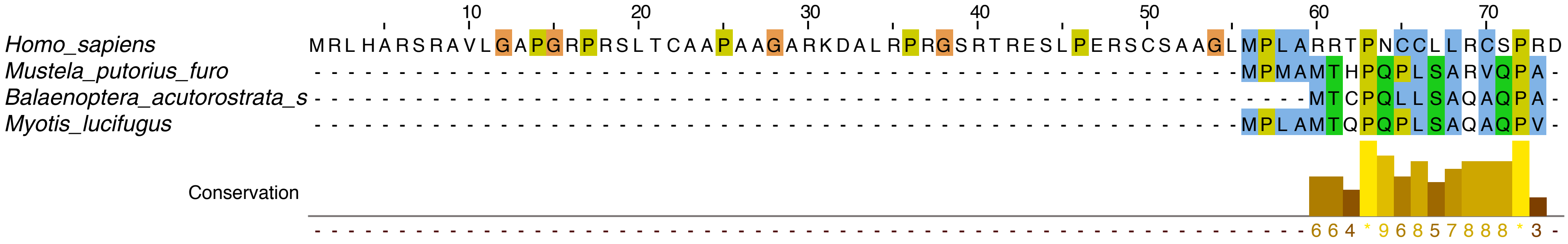

HG1068

ENSG00000144791 (LIMD1)

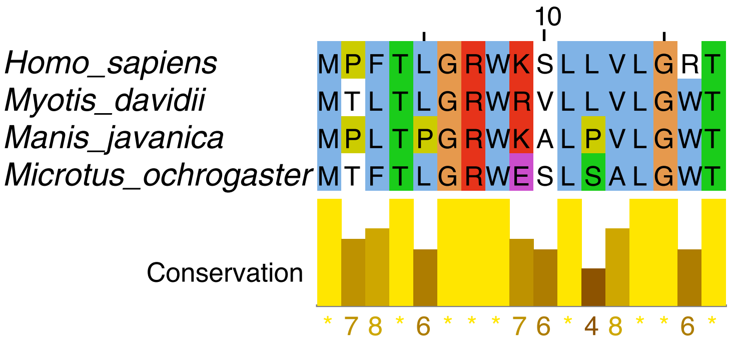

HG1069

ENSG00000146414 (SHPRH)

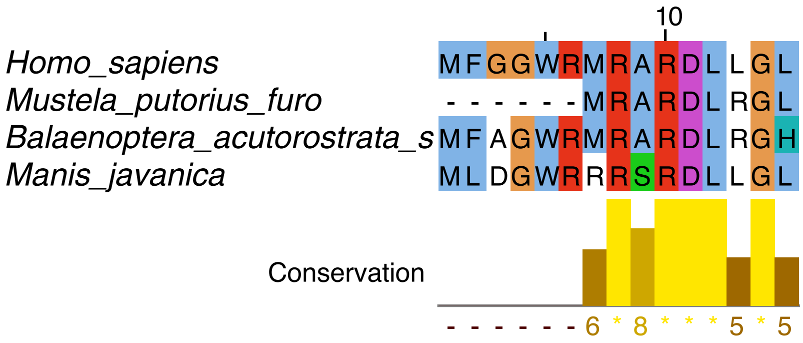

HG1070

ENSG00000147459 (DOCK5)

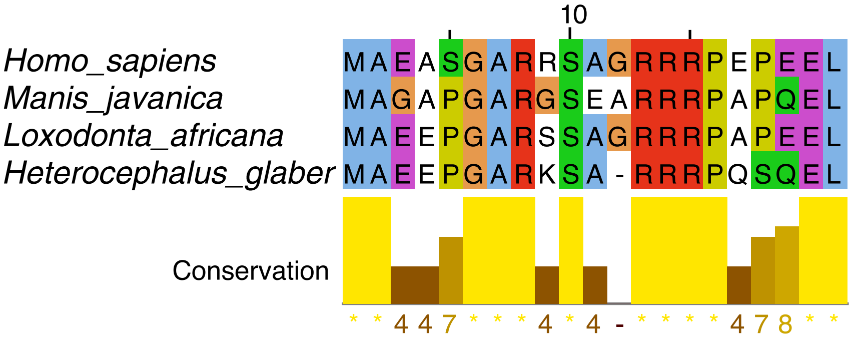

HG1071

ENSG00000147573 (TRIM55)

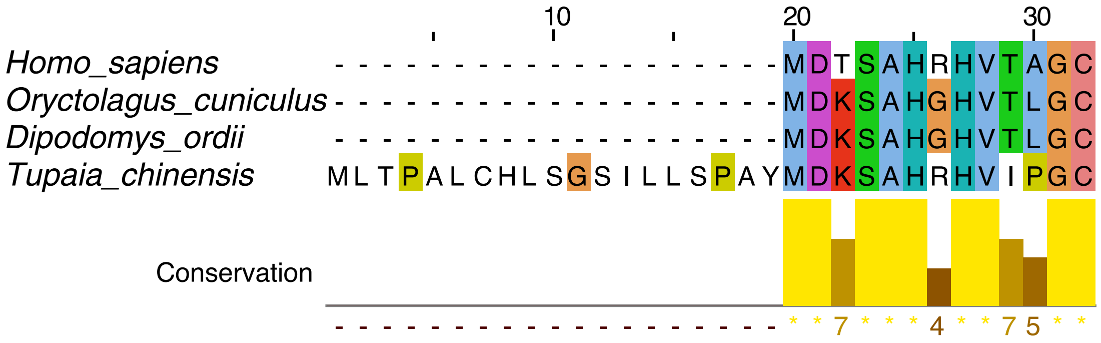

HG1072

ENSG00000147874 (HAUS6)

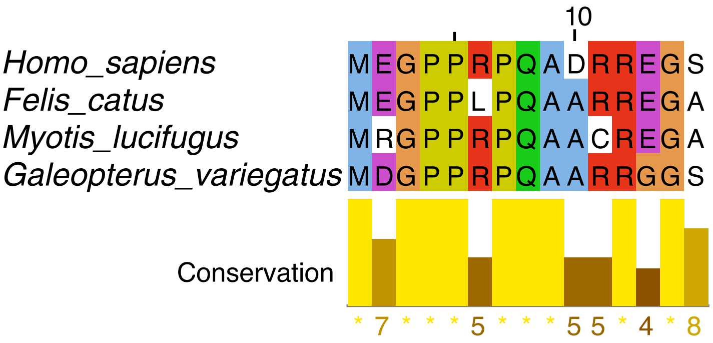

HG1073

ENSG00000149187 (CELF1)

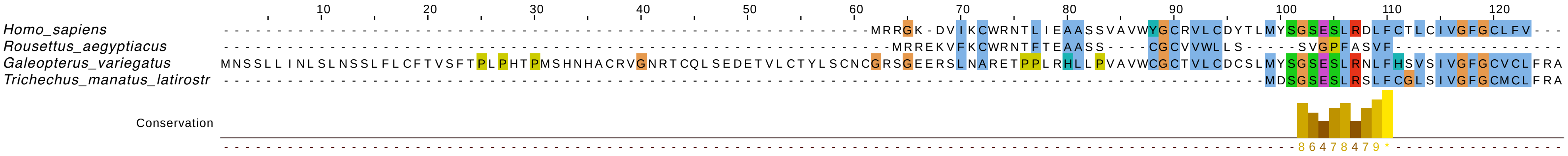

HG1074

ENSG00000150275 (PCDH15)

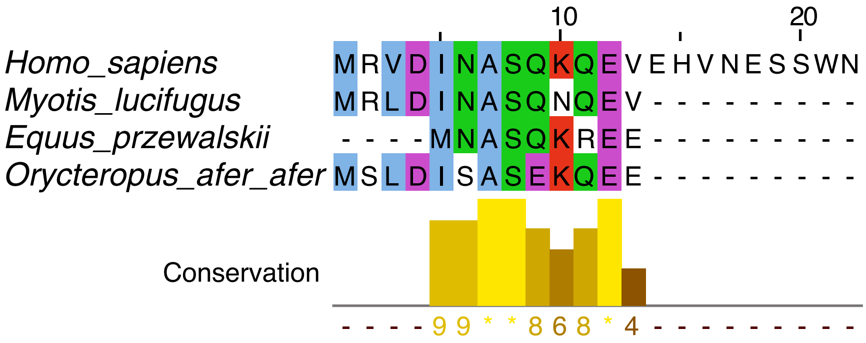

HG1075

ENSG00000150347 (ARID5B)

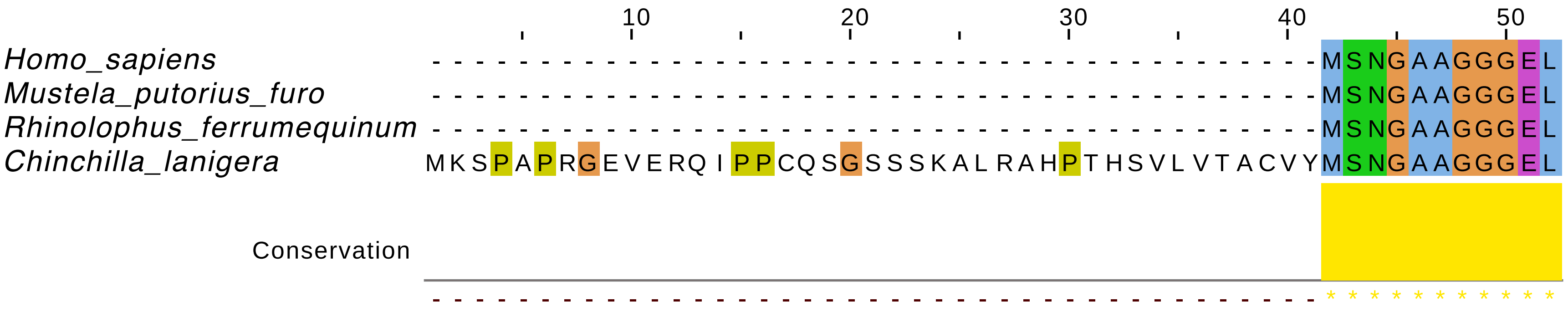

HG1076

ENSG00000150967 (ABCB9)

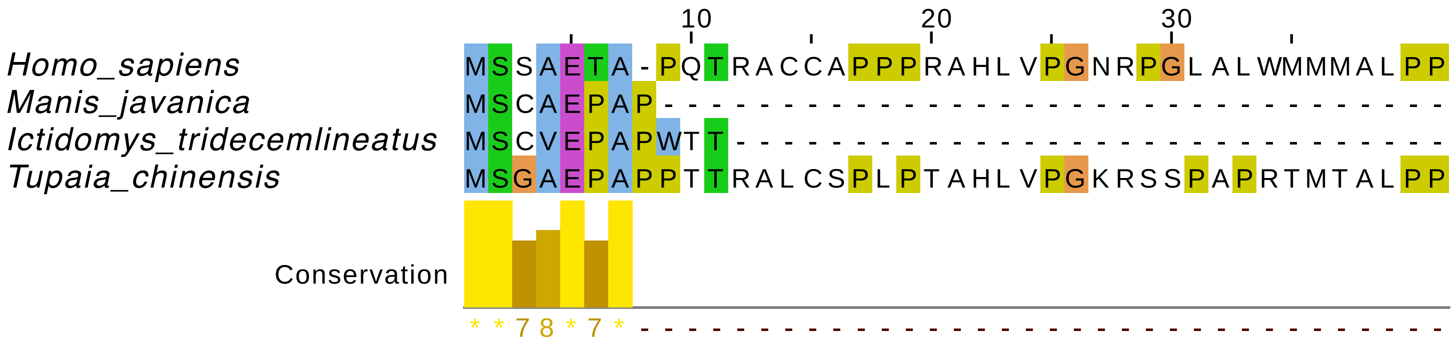

HG1077

ENSG00000151474 (FRMD4A)

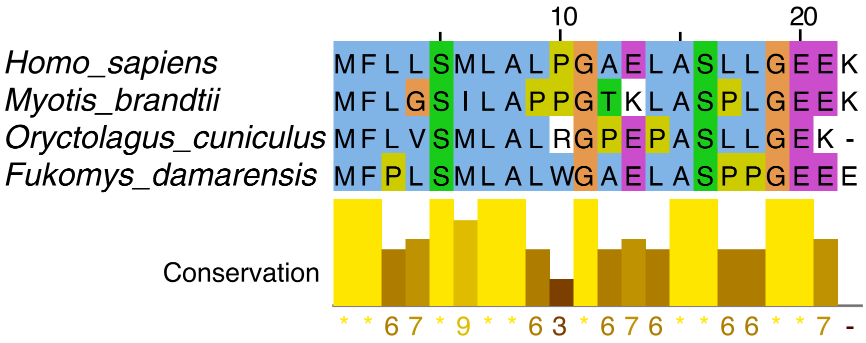

HG1078

ENSG00000162607 (USP1)

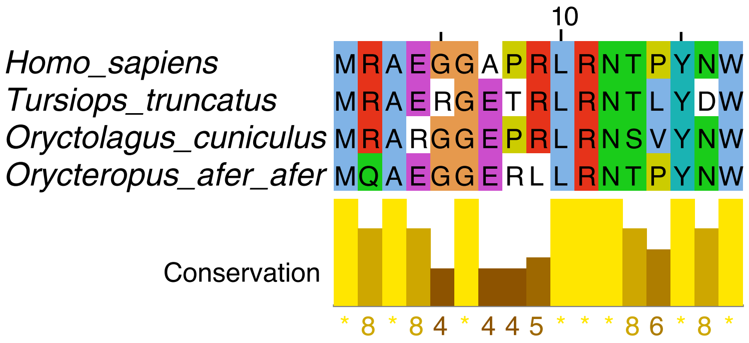

HG1079

ENSG00000162975 (KCNF1)

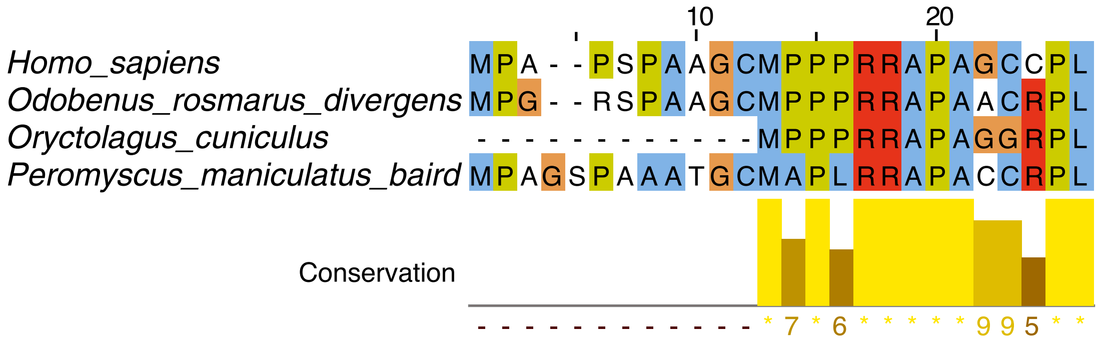

HG1080

ENSG00000164168 (TMEM184C)

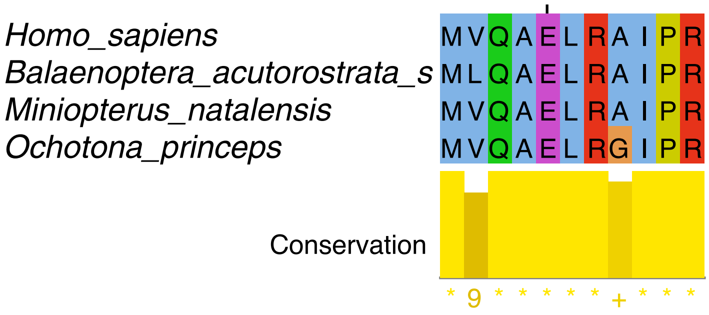

HG1081

ENSG00000165392 (WRN)

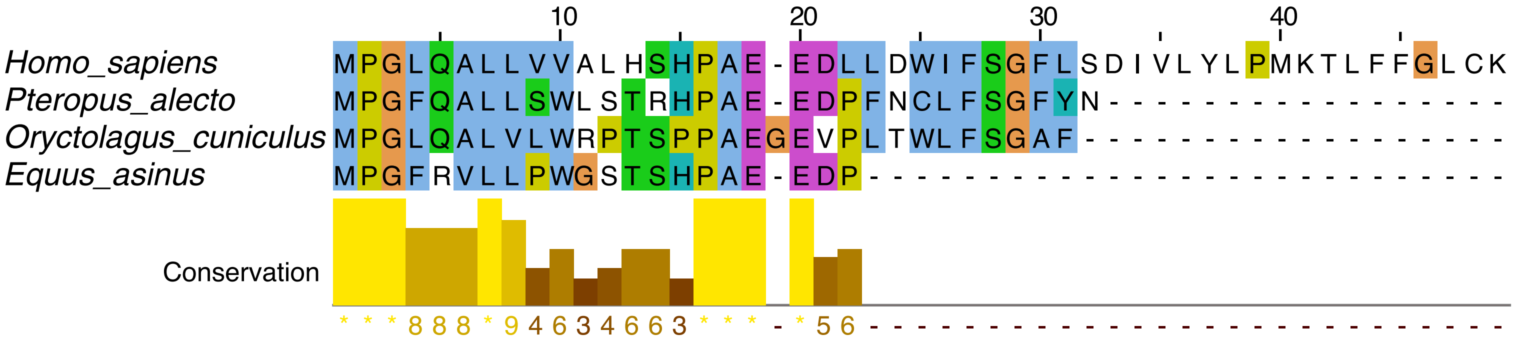

HG1082

ENSG00000165434 (PGM2L1)

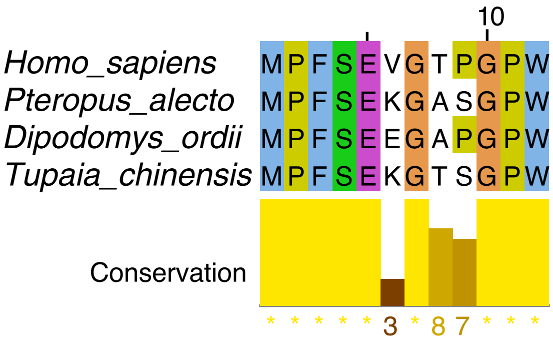

HG1083

ENSG00000165804 (ZNF219)

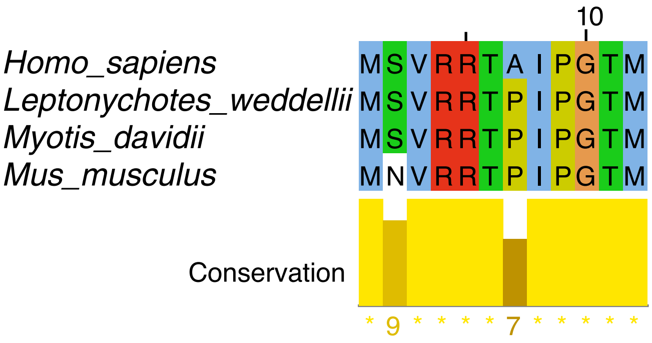

HG1084

ENSG00000165923 (AGBL2)

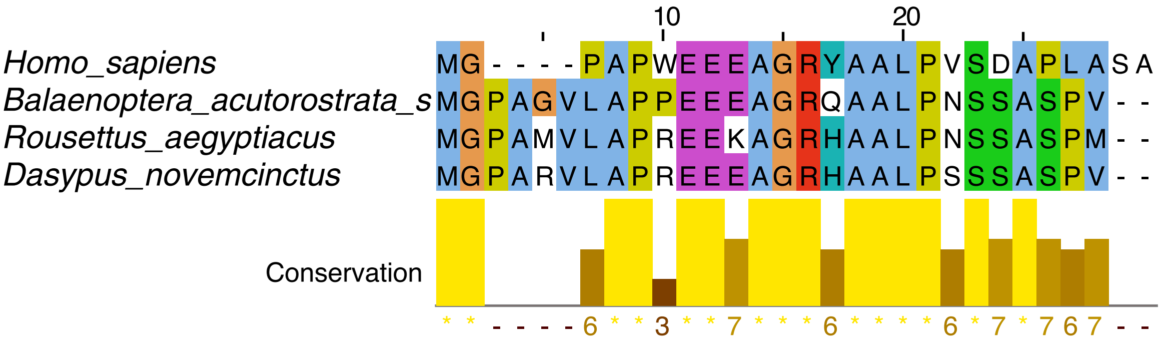

HG1085

ENSG00000166532 (RIMKLB)

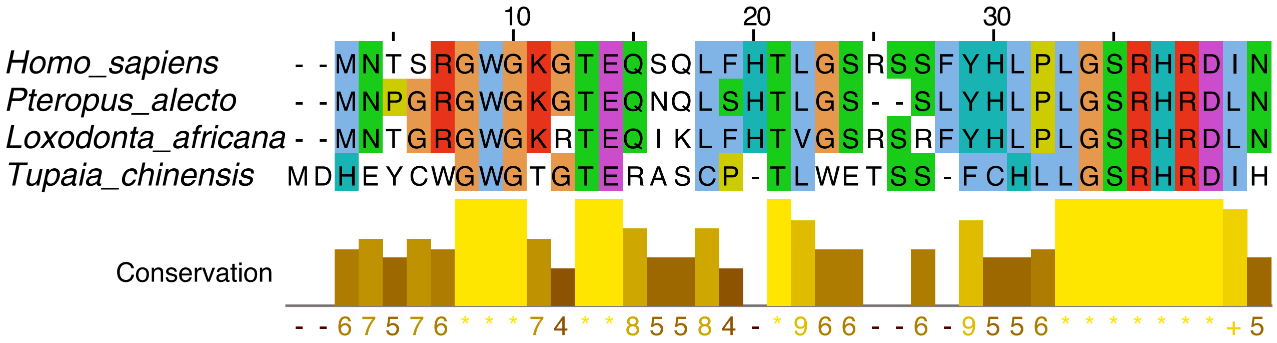

HG1086

ENSG00000167377 (ZNF23)

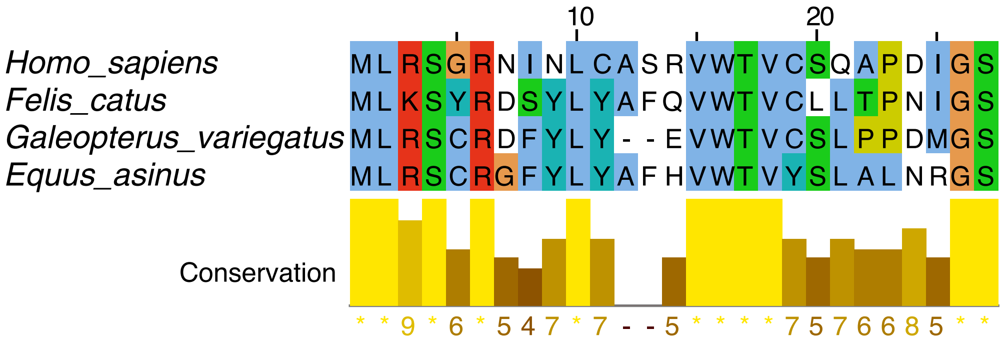

HG1087

ENSG00000168675 (LDLRAD4)

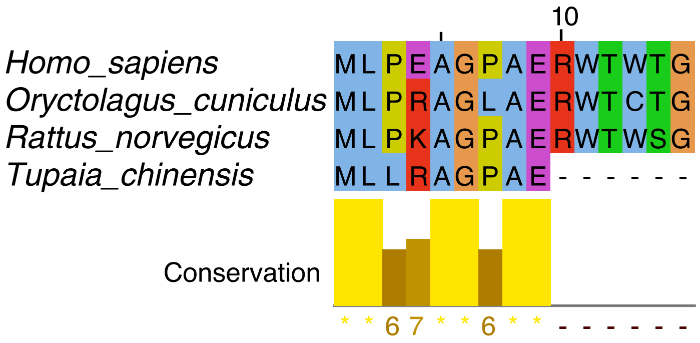

HG1088

ENSG00000169740 (ZNF32)

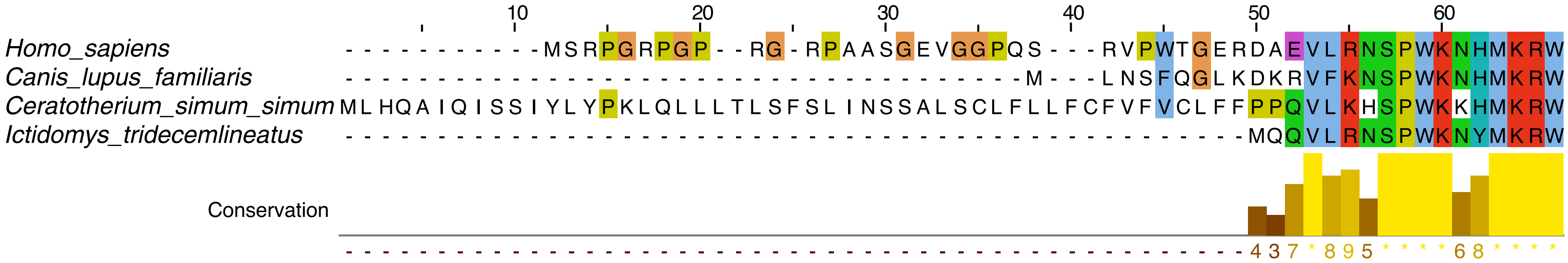

HG1089

ENSG00000169813 (HNRNPF)

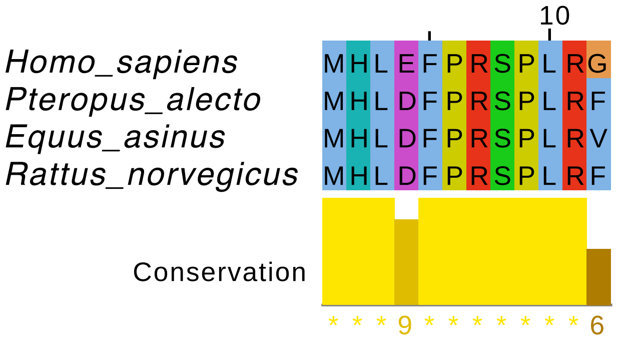

HG1090.1

ENSG00000170458 (CD14)

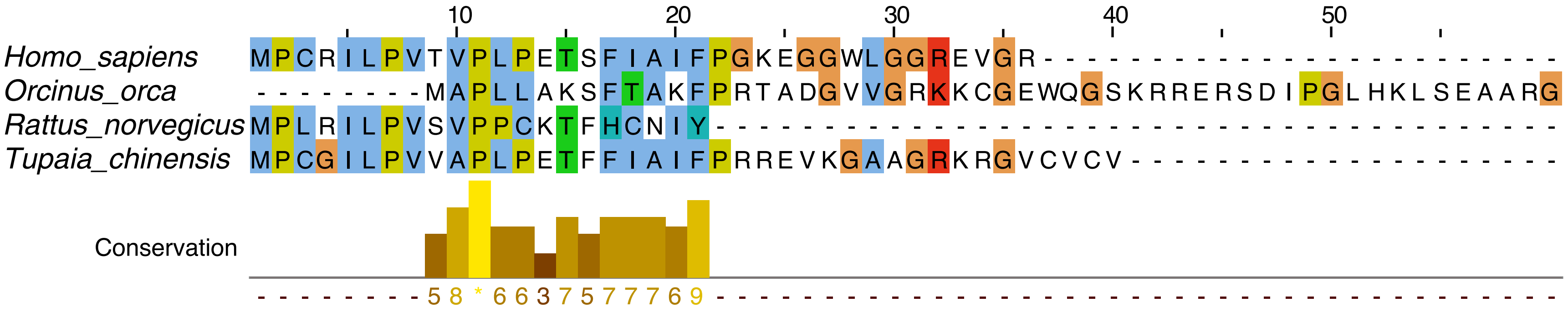

HG1090.2

ENSG00000170458 (CD14)

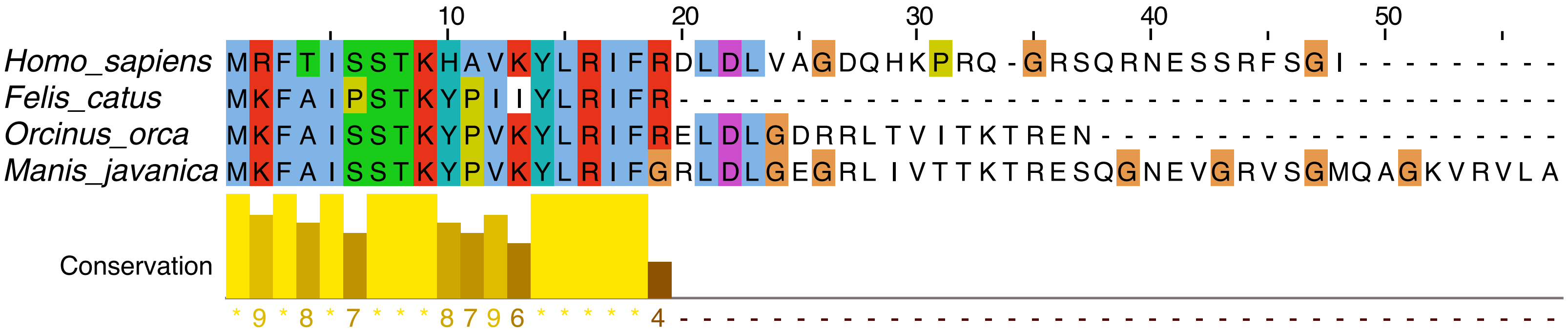

HG1091

ENSG00000172183 (ISG20)

```
                                   10        20        30        40        50        60        70        80        90       100       110
Homo_sapiens                ----------------------MRHLRRGRAGAKEGHPDMEPASSVSPDSARS--------------------------------------------------------------
Orcinus_orca                ----------------------------------MGHTDKEPASLVNSDSGH---------------------------------------------------------------
Myotis_lucifugus            -----------------------------MGGAEAGQRDMEPVRSASPDSGAC-------------RPSCISRQEMATCSLENEPETEAAPGQRGQDSPLEVRGDSPLTATDLGS
Ceratotherium_simum_simum   MFILQAGRRVRKERKMLQSTPSTASSEEGRAGAKAGHPDMEPASFVSPDSGHRALHLPAAPGDGAAQPTCLAFRP----------------------------------------

Conservation                ----------------------------------4*64*7**864897**+3---------------------------------------------------------------
```

HG1092

ENSG00000173327 (MAP3K11)

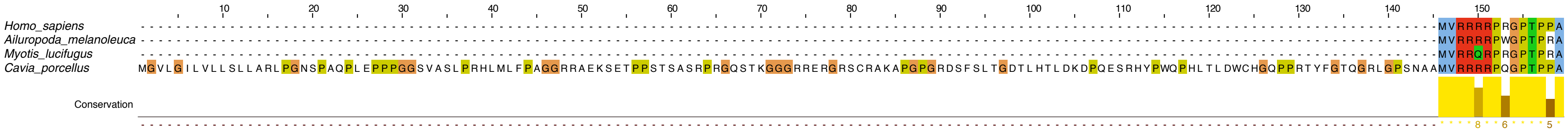

HG1093

ENSG00000174371 (EXO1)

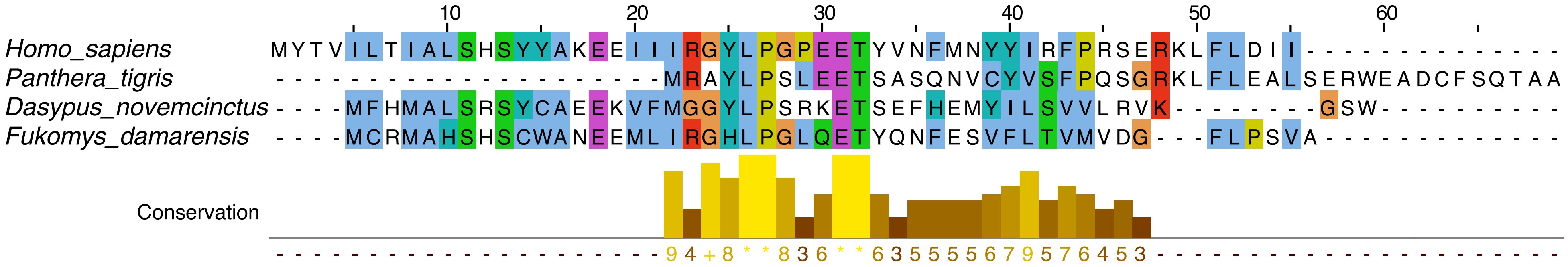

HG1094

ENSG00000177917 (ARL6IP6)

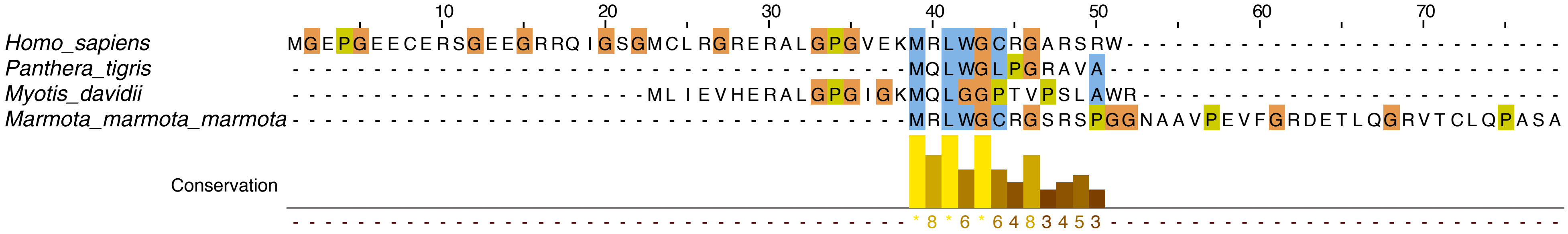

HG1095

ENSG00000179921 (GPBAR1)

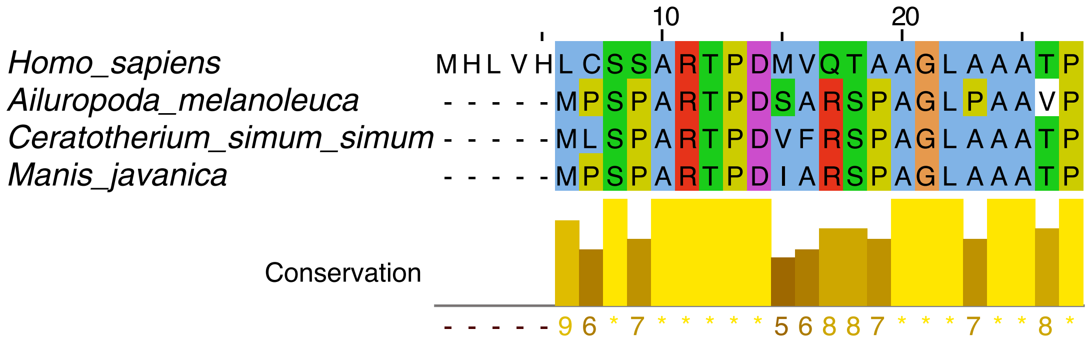

HG1096

ENSG00000181544 (FANCB)

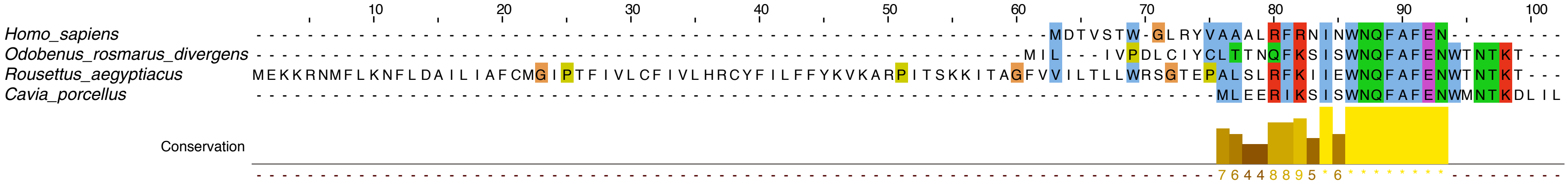

HG1097

ENSG00000182481 (KPNA2)

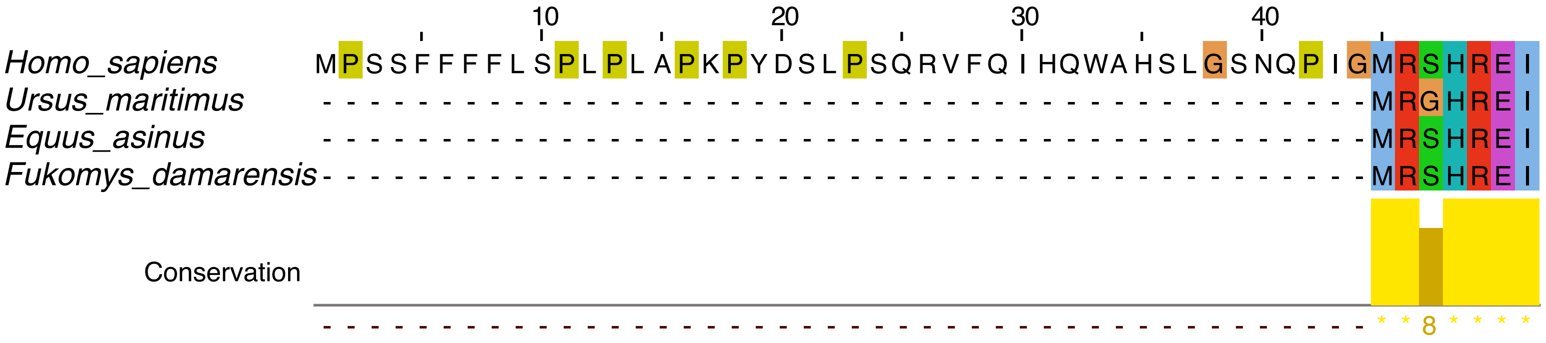

HG1098

ENSG00000183775 (KCTD16)

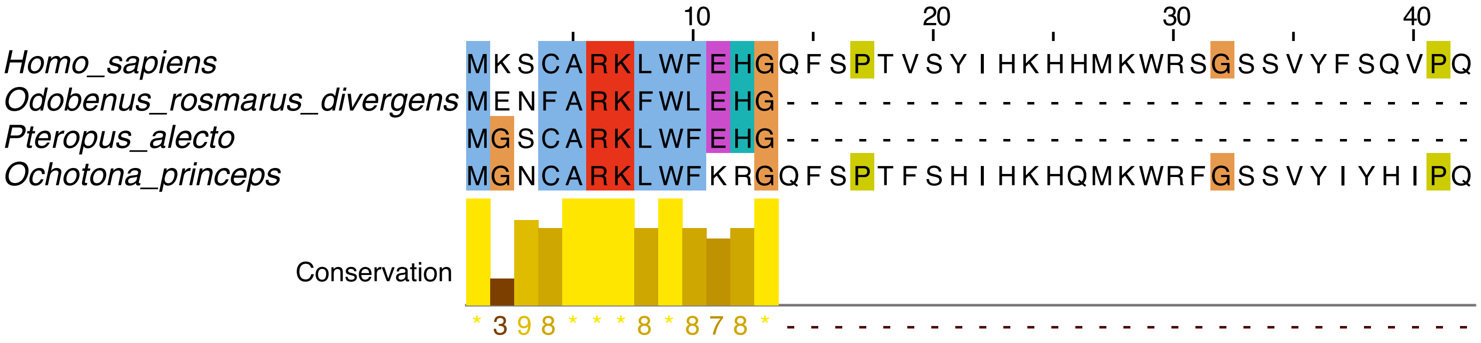

HG1099

ENSG00000186334 (SLC36A3)

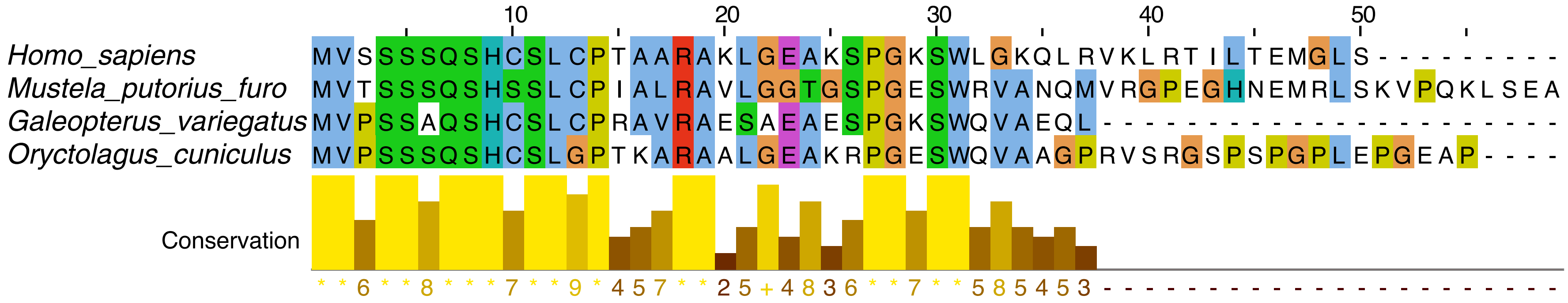

HG1100

ENSG00000187815 (ZFP69)

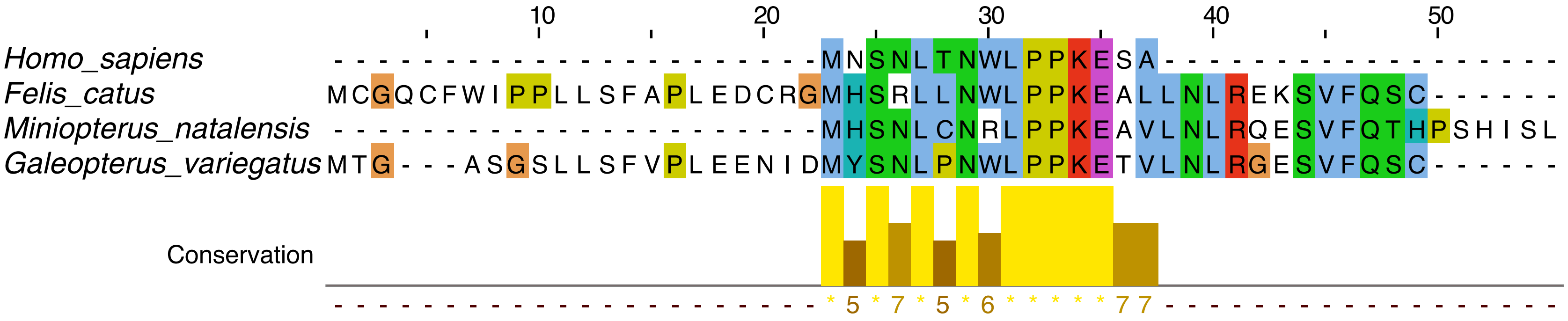

HG1101

ENSG00000189334 (S100A14)

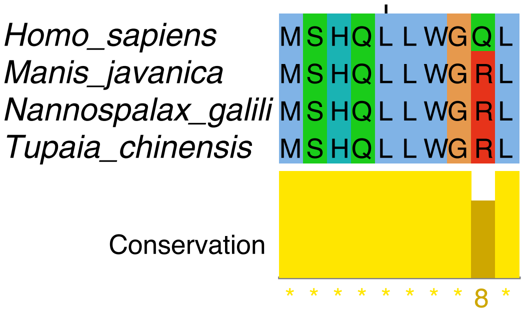

HG1102

ENSG00000196704 (AMZ2)

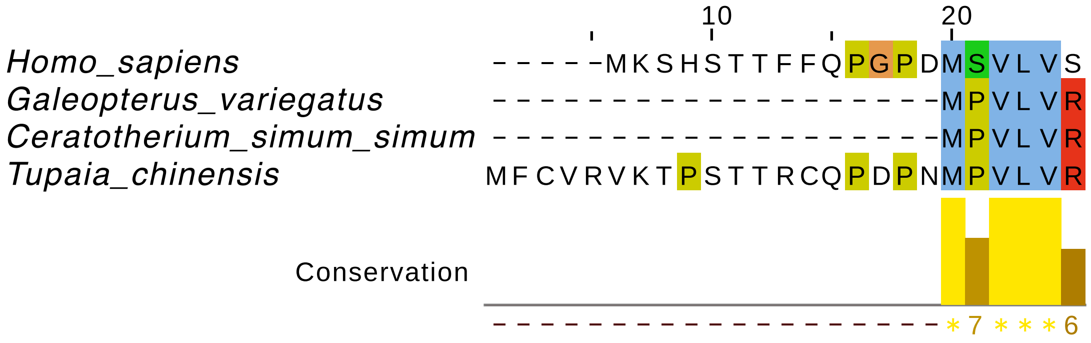

HG1103

ENSG00000197620 (CXorf40A)

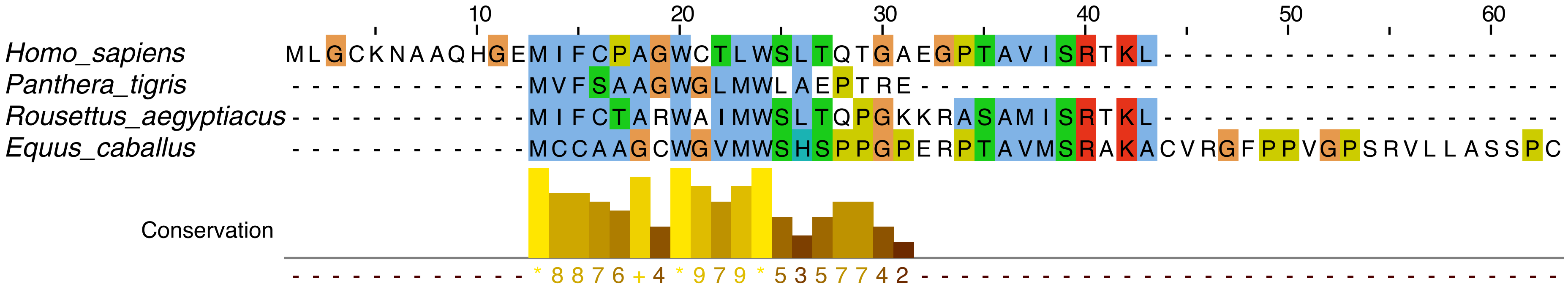

HG1104

ENSG00000205155 (PSENEN)

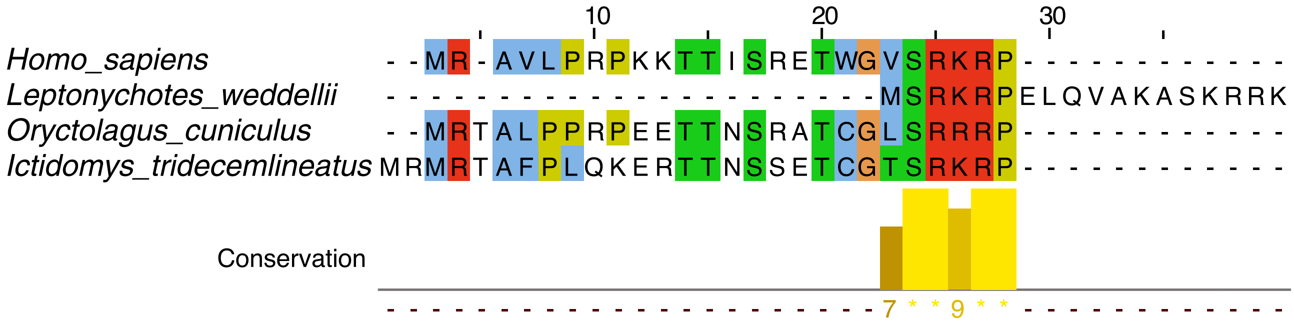

HG1105

ENSG00000213654 (GPSM3)

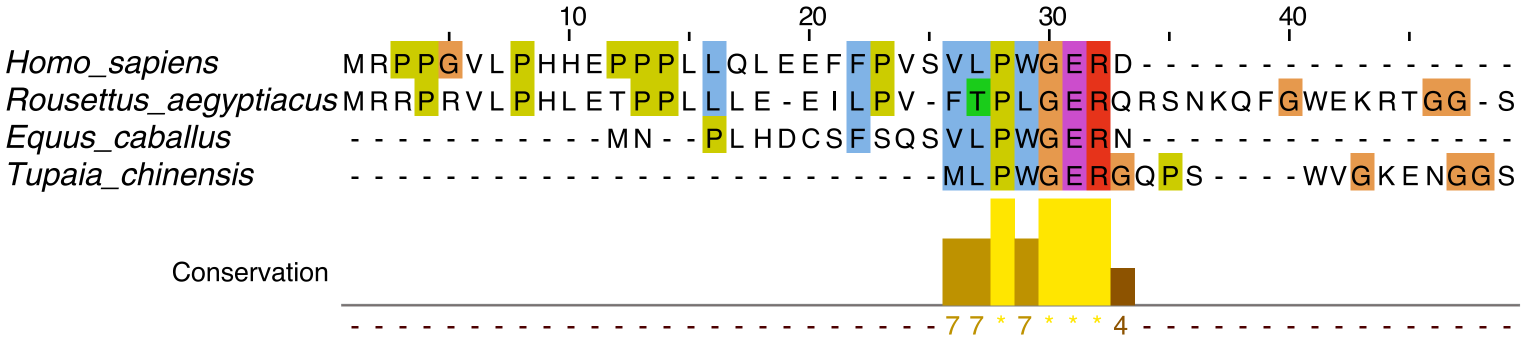

HG1106

ENSG00000215021 (PHB2)

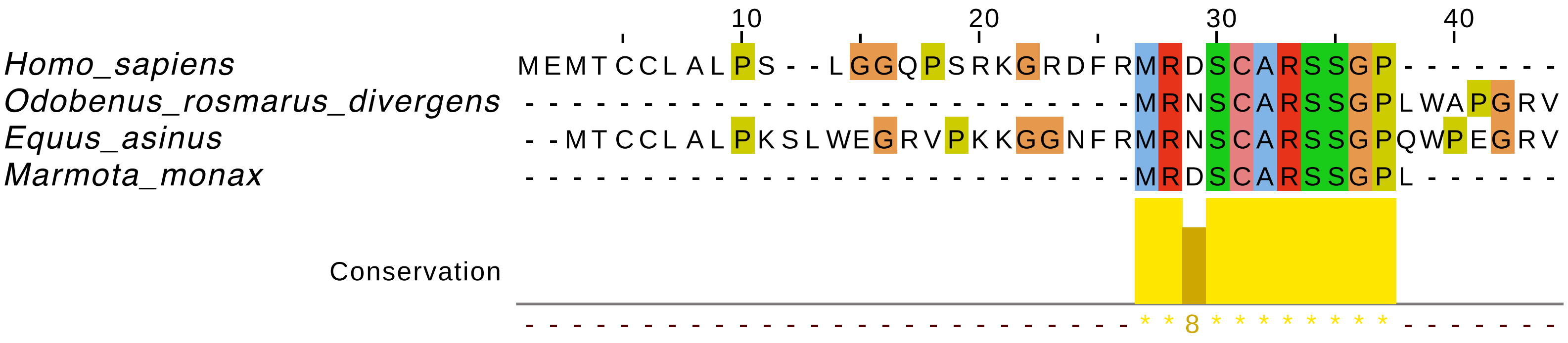

HG1107

ENSG00000217930 (PAM16)

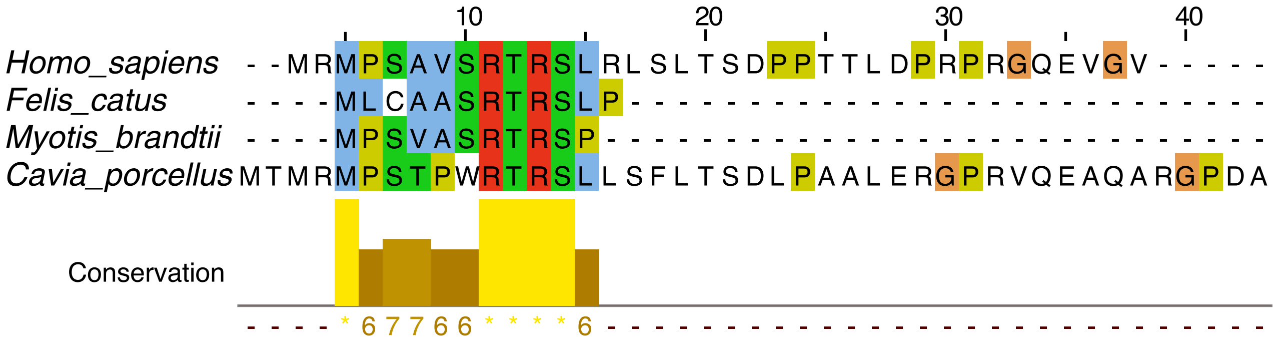

HG1108

ENSG00000251247 (ZNF345)

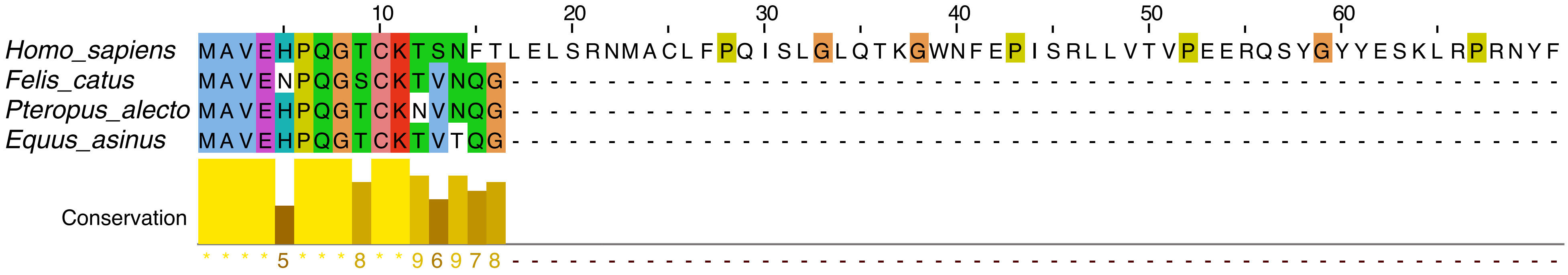

HG1109

ENSG00000278311 (GGNBP2)

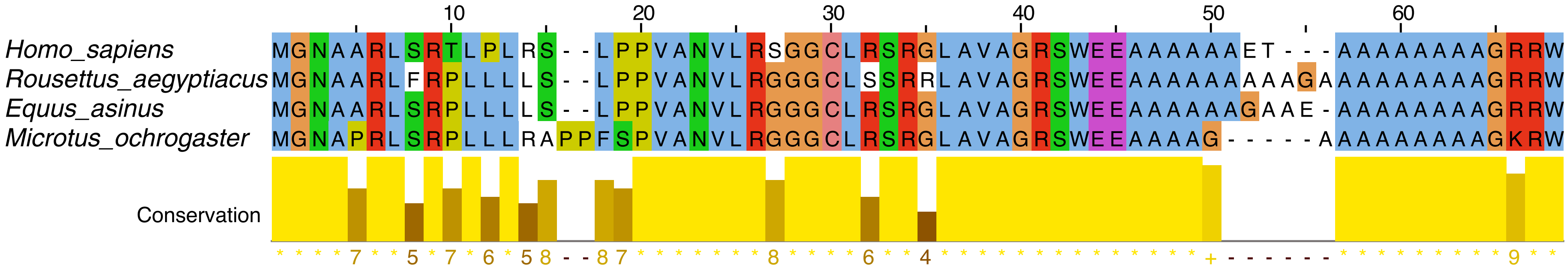

HG1110

ENSG00000034152 (MAP2K3)

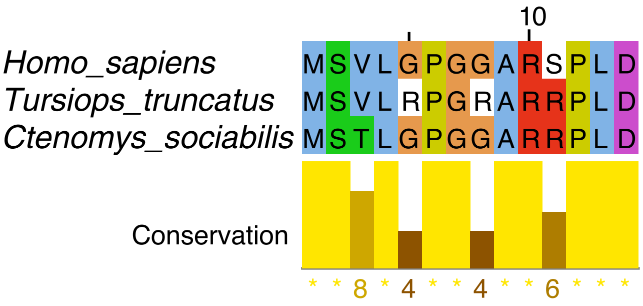

HG1111

ENSG00000040531 (CTNS)

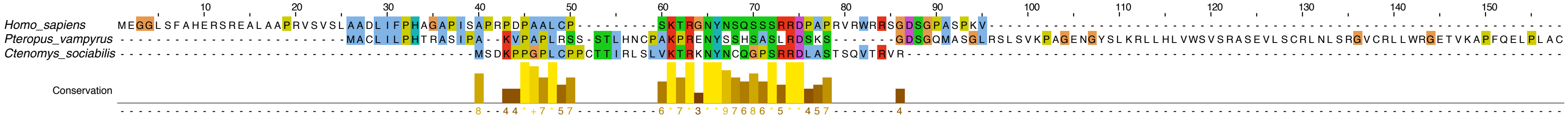

HG1112

ENSG00000068885 (IFT80)

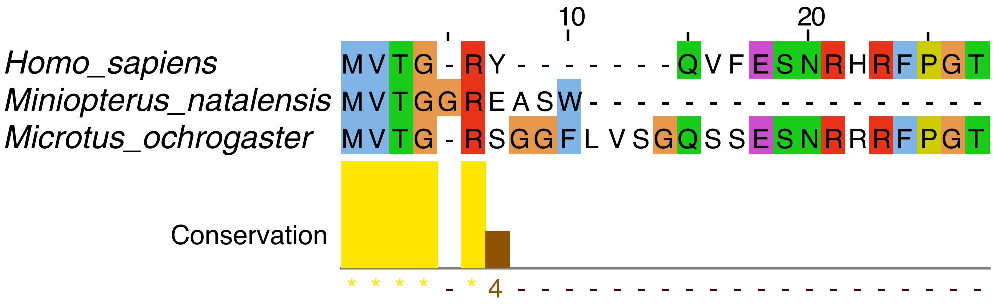

HG1113

ENSG00000072201 (LNX1)

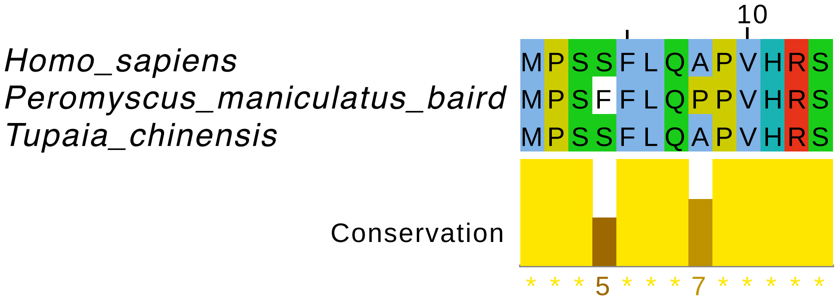

HG1114

ENSG00000073737 (DHRS9)

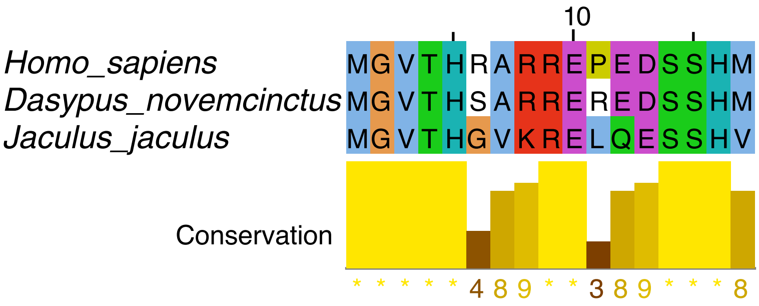

HG1115

ENSG00000074219 (TEAD2)

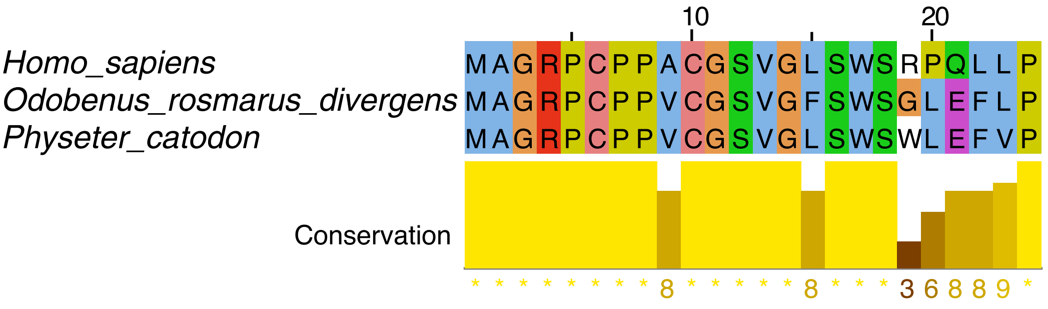

HG1116

ENSG00000084090 (STARD7)

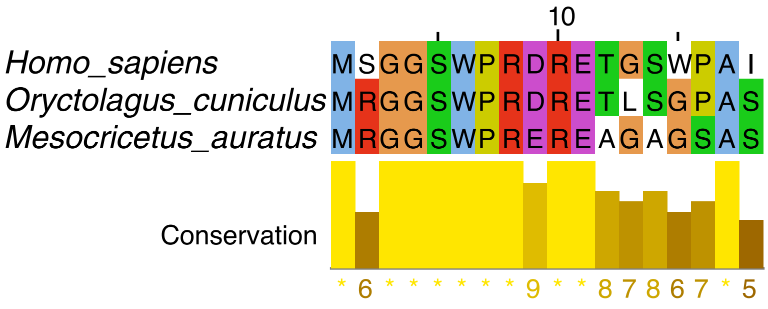

HG1117

ENSG00000089902 (RCOR1)

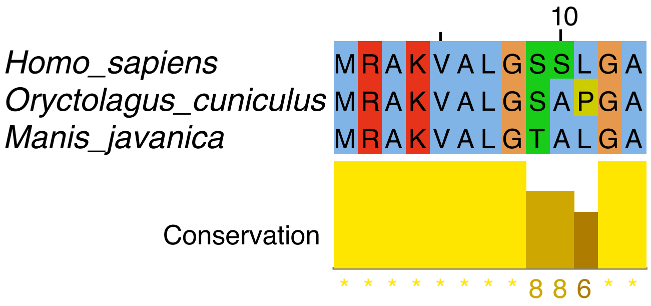

HG1118

ENSG00000090975 (PITPNM2)

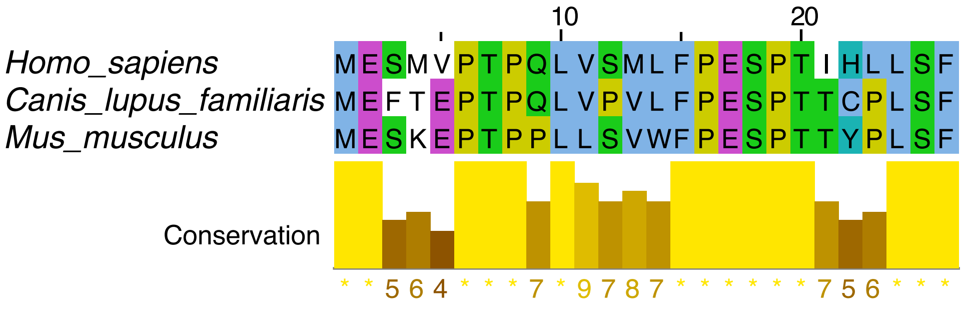

HG1119

ENSG00000092020 (PPP2R3C)

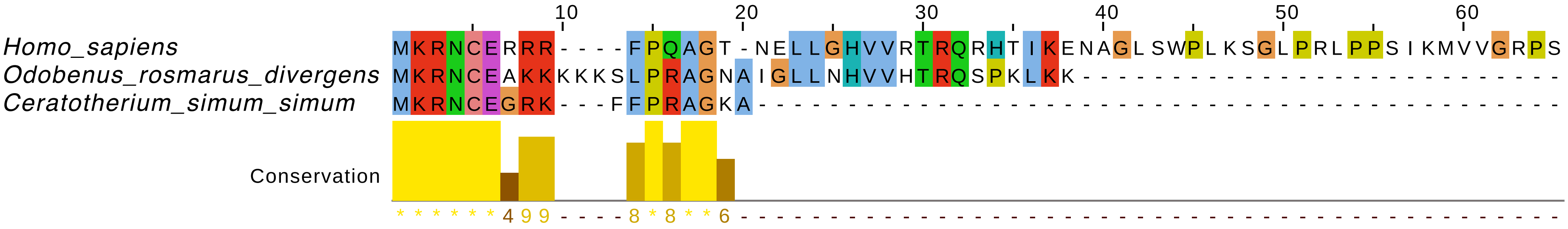

HG1120

ENSG00000095585 (BLNK)

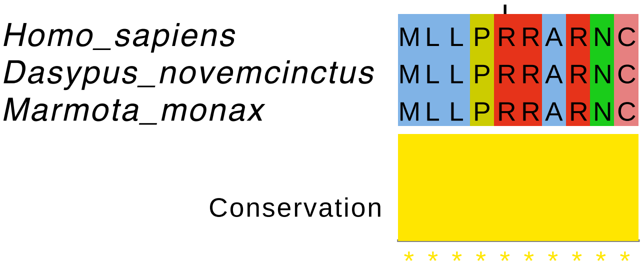

HG1121

ENSG00000100350 (FOXRED2)

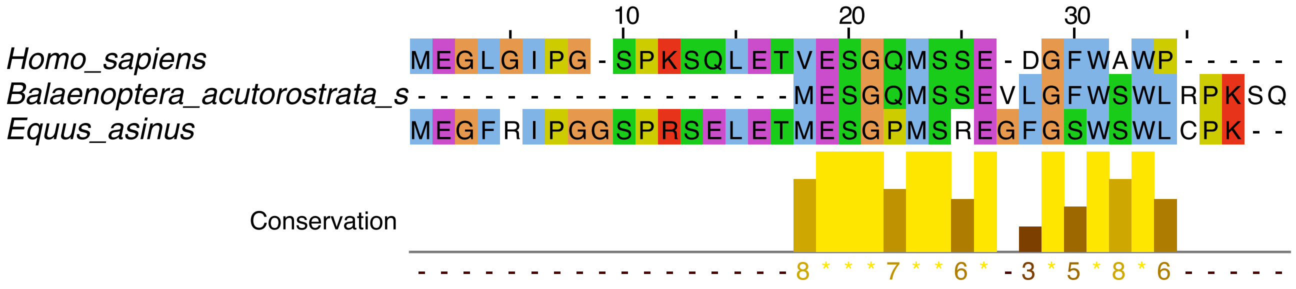

HG1122

ENSG00000100410 (PHF5A)

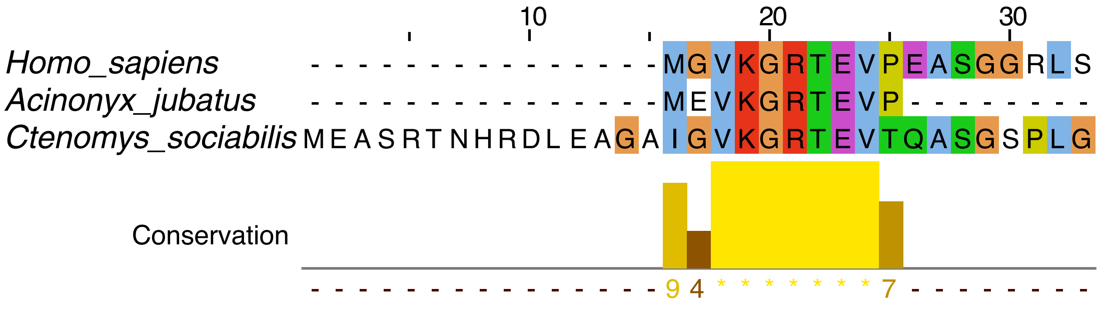

HG1123

ENSG00000100884 (CPNE6)

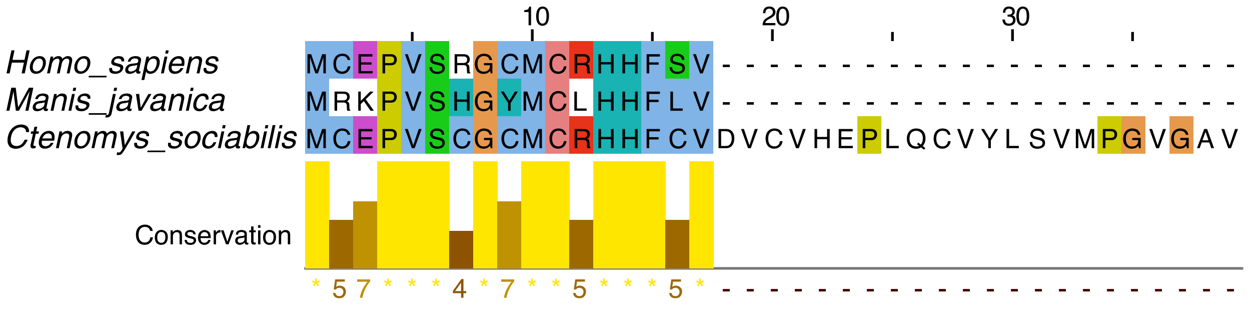

HG1124

ENSG00000100934 (SEC23A)

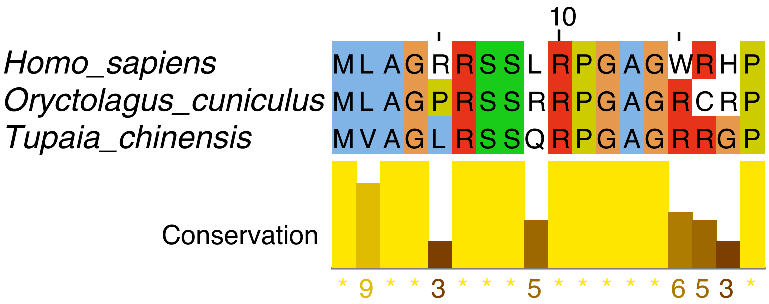

HG1125

ENSG00000101574 (METTL4)

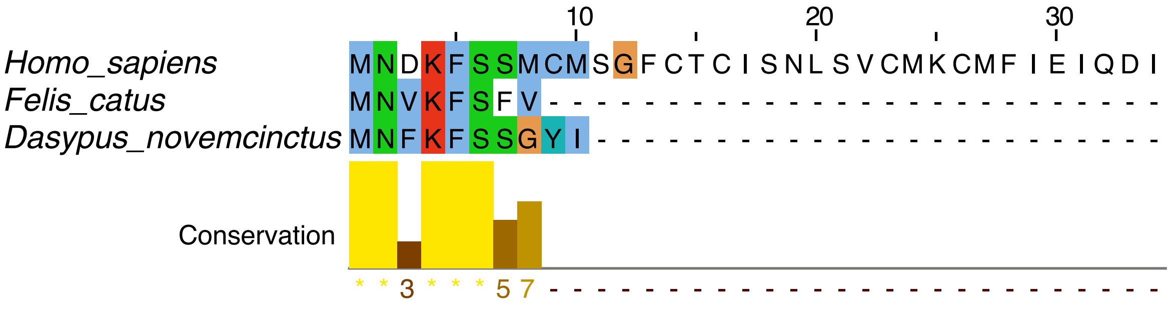

HG1126

ENSG00000101890 (GUCY2F)

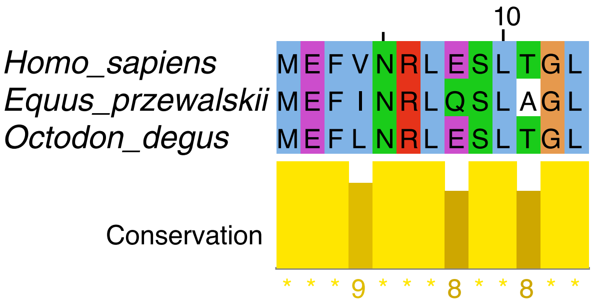

HG1127

ENSG00000103064 (SLC7A6)

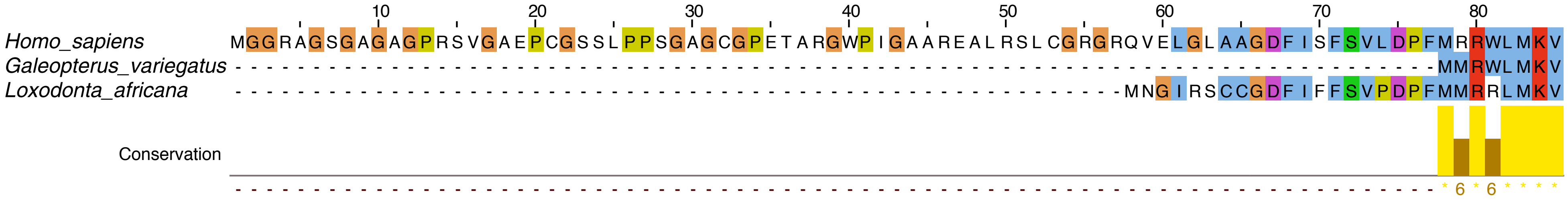

HG1128

ENSG00000103852 (TTC23)

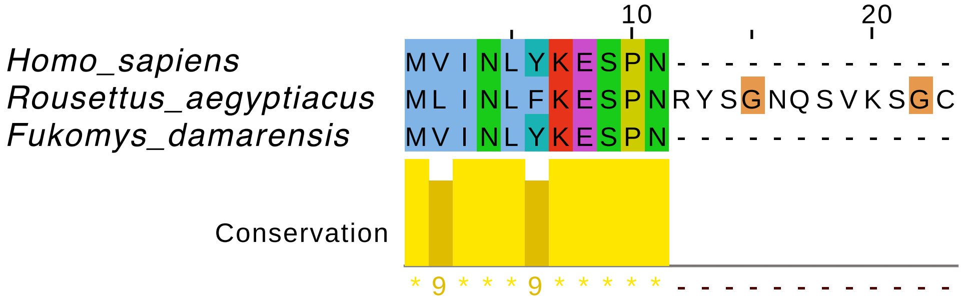

HG1129

ENSG00000104432 (IL7)

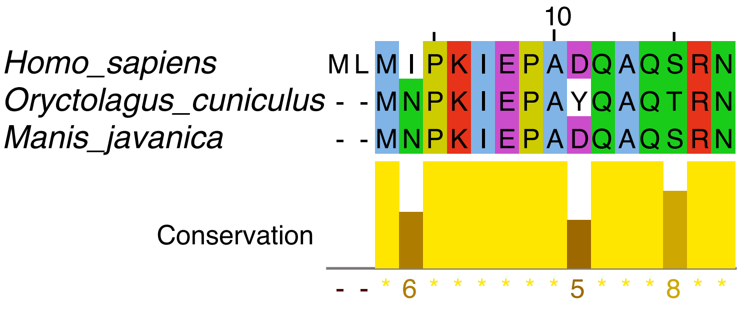

HG1130

ENSG00000105559 (PLEKHA4)

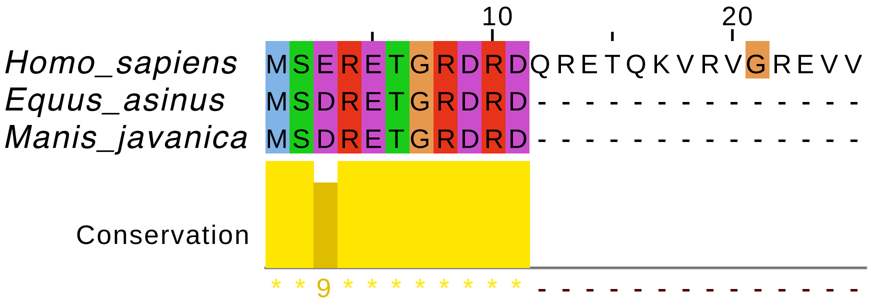

HG1131

ENSG00000106780 (MEGF9)

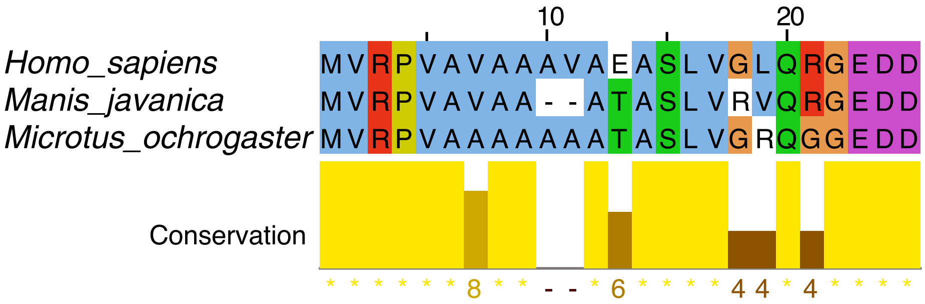

HG1132

ENSG00000109771 (LRP2BP)

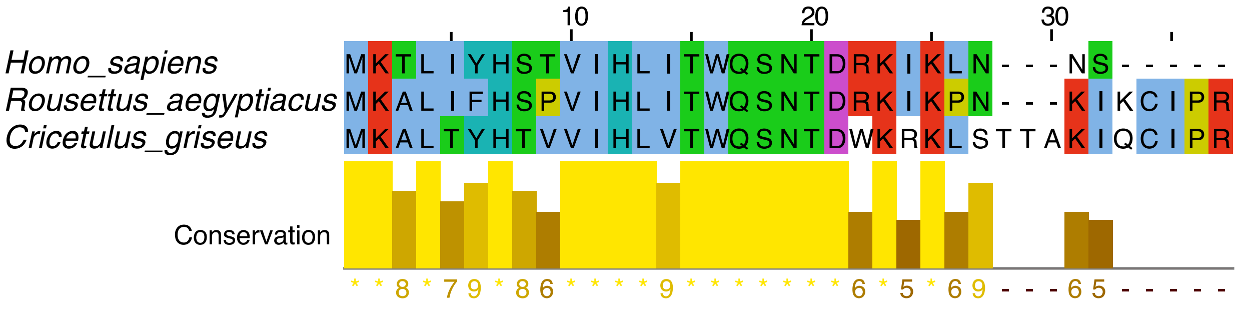

HG1133

ENSG00000111328 (CDK2AP1)

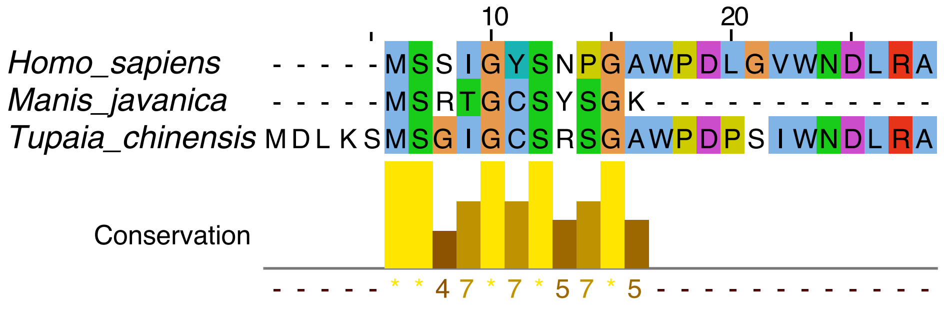

HG1134

ENSG00000112984 (KIF20A)

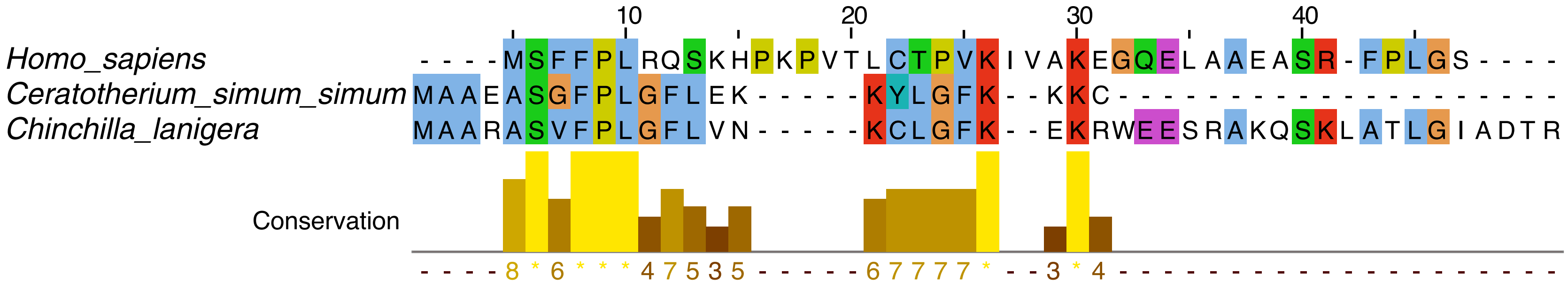

HG1135

ENSG00000113597 (TRAPPC13)

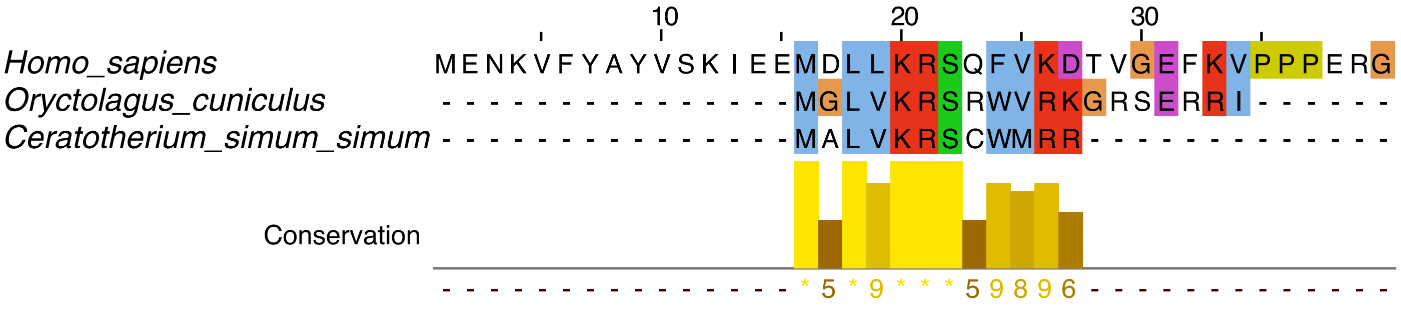

HG1136

ENSG00000113749 (HRH2)

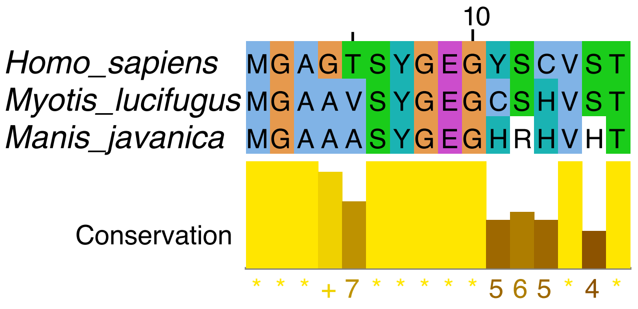

HG1137

ENSG00000115307 (AUP1)

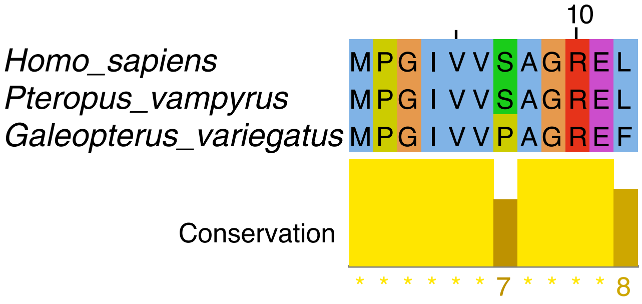

HG1138

ENSG00000116871 (MAP7D1)

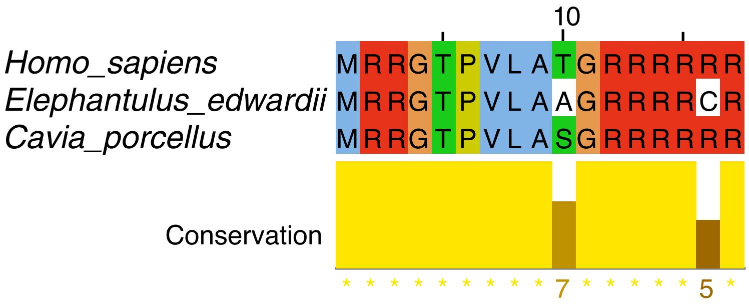

HG1139.1

ENSG00000118308 (LRMP)

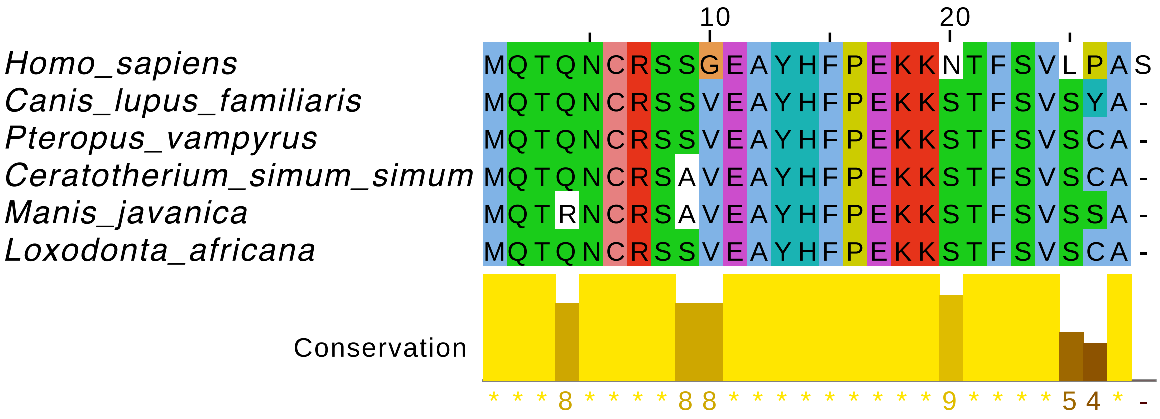

HG1139.2

ENSG00000118308 (LRMP)

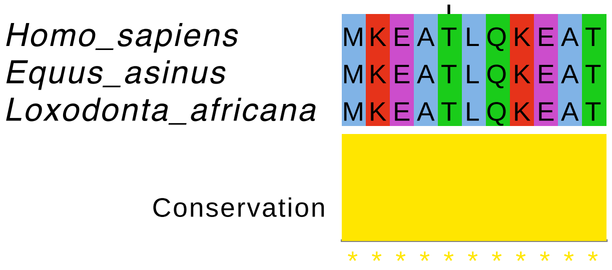

HG1140

ENSG00000118495 (PLAGL1)

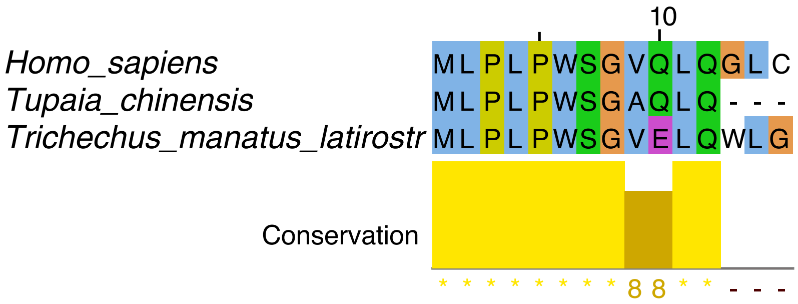

HG1141

ENSG00000121964 (GTDC1)

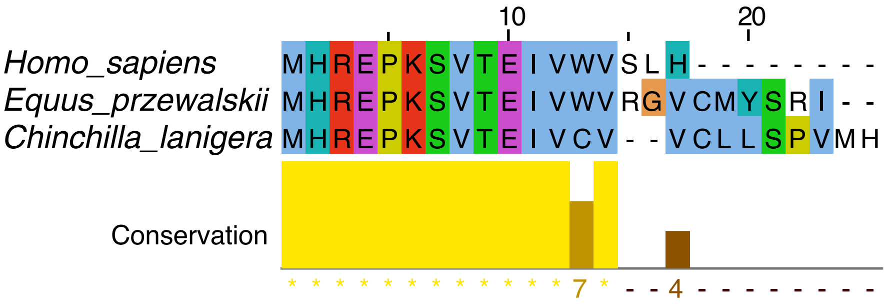

HG1142

ENSG00000124613 (ZNF391)

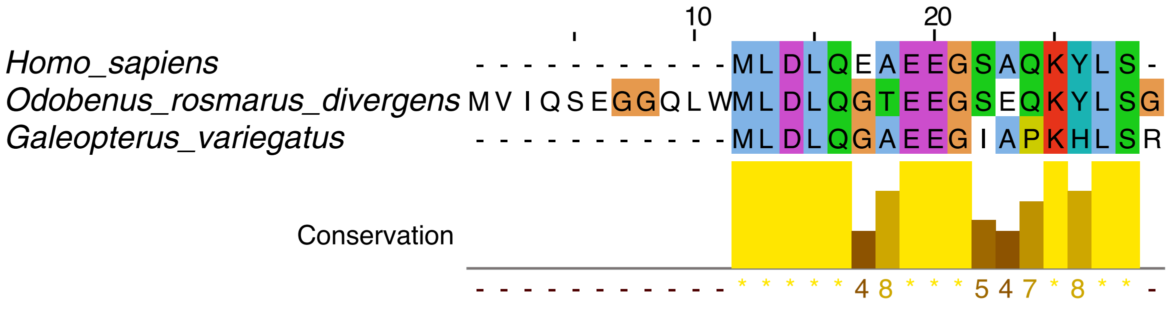

HG1143

ENSG00000125245 (GPR18)

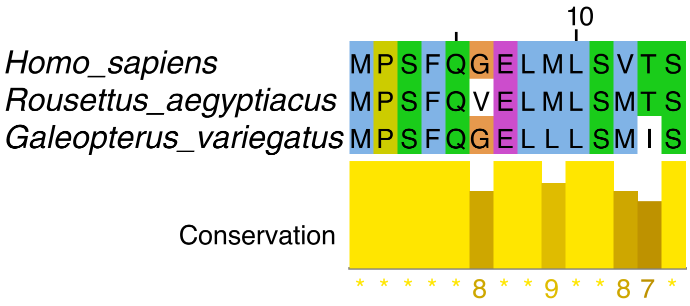

HG1144

ENSG00000128203 (ASPHD2)

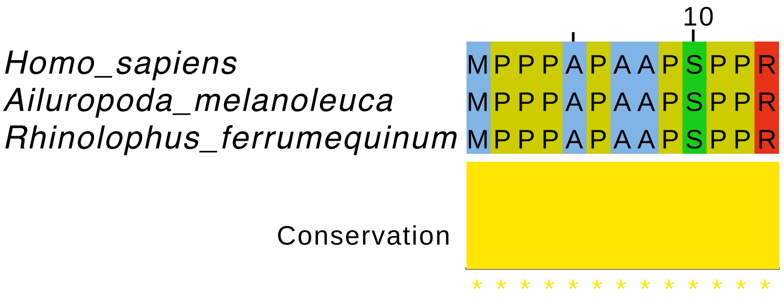

HG1145

ENSG00000128585 (MKLN1)

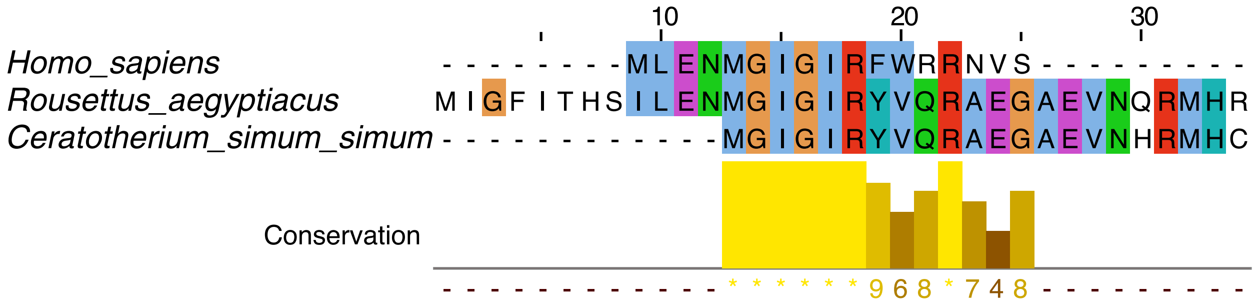

HG1146

ENSG00000130413 (STK33)

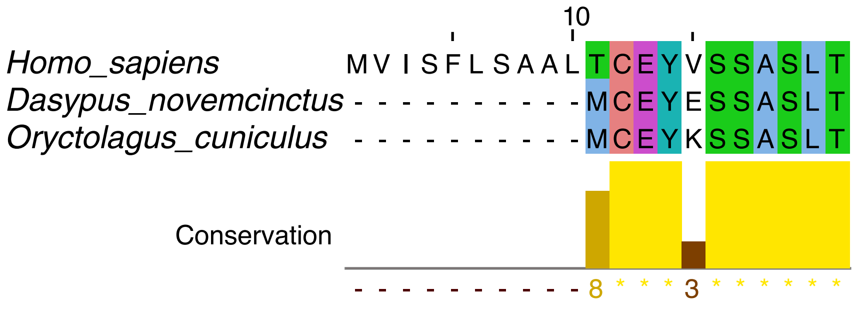

HG1147

ENSG00000131781 (FMO5)

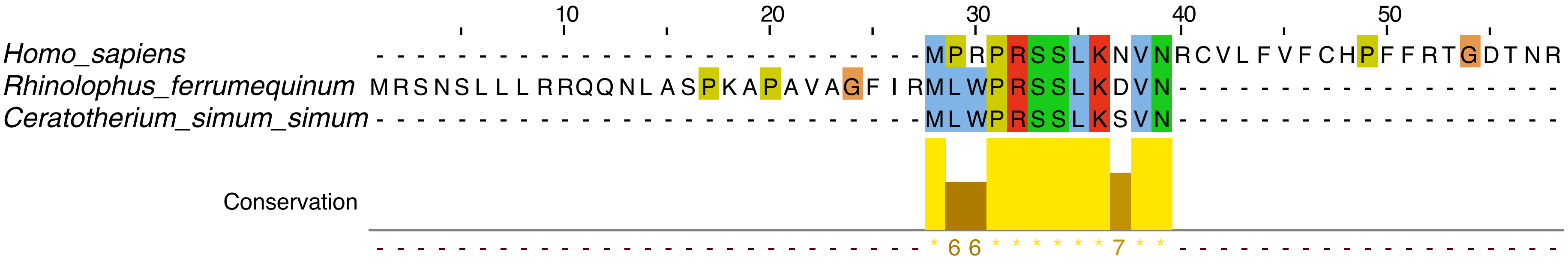

HG1148

ENSG00000132938 (MTUS2)

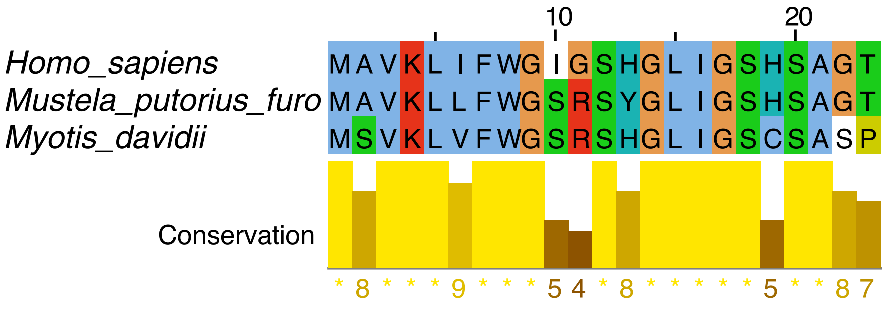

HG1149

ENSG00000132975 (GPR12)

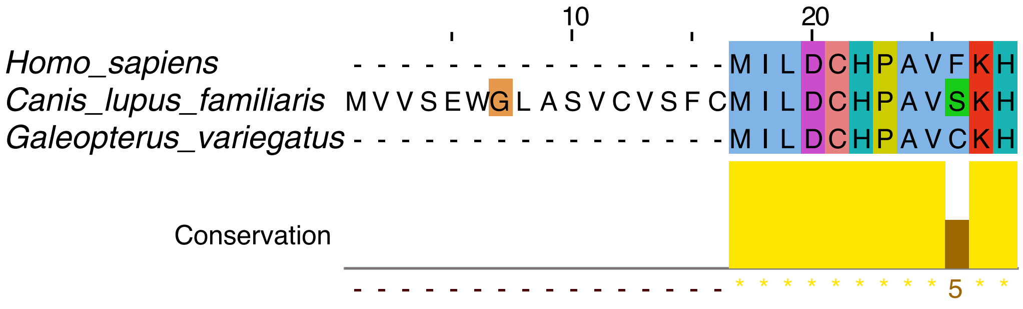

HG1150

ENSG00000135299 (ANKRD6)

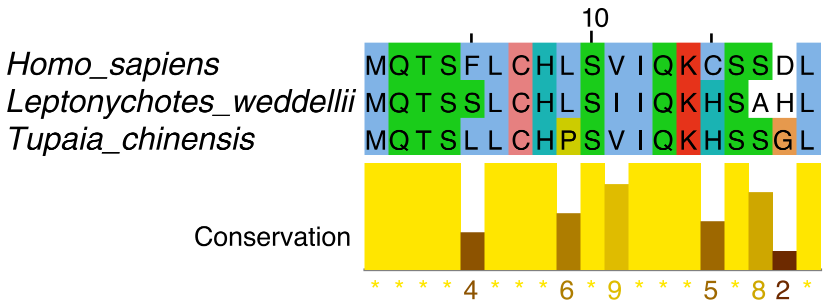

HG1151

ENSG00000137168 (PPIL1)

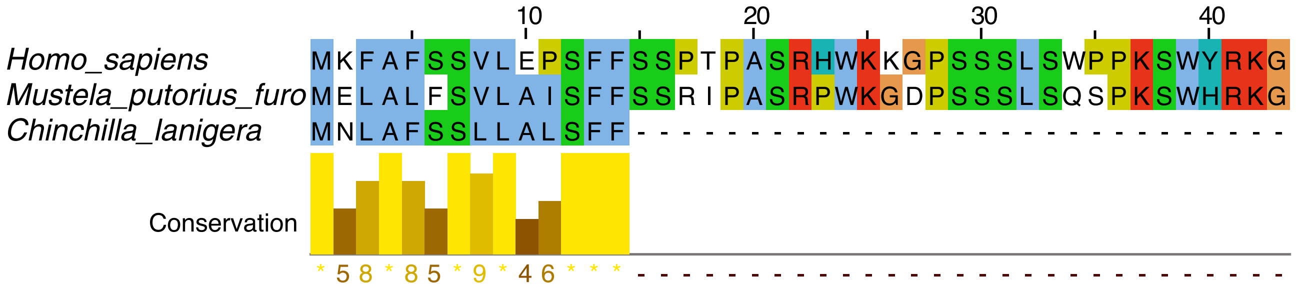

HG1152

ENSG00000138658 (ZGRF1)

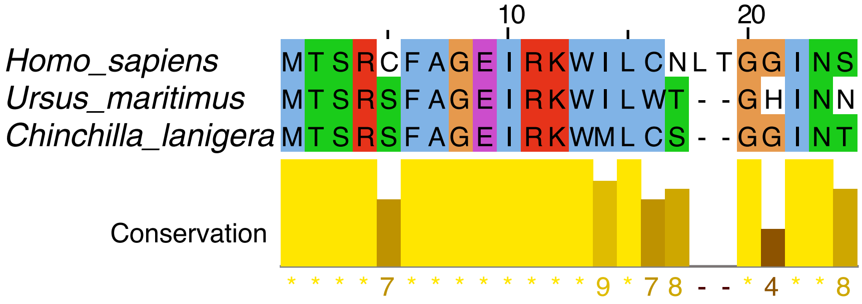

HG1153

ENSG00000138738 (PRDM5)

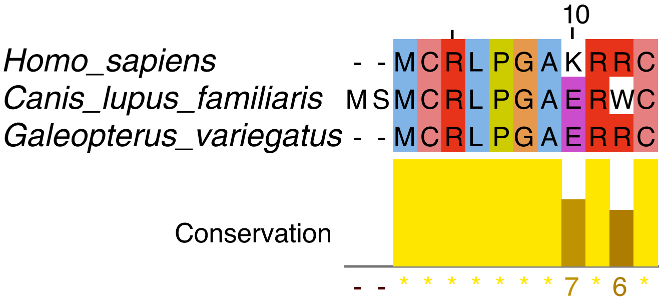

HG1154

ENSG00000139725 (RHOF)

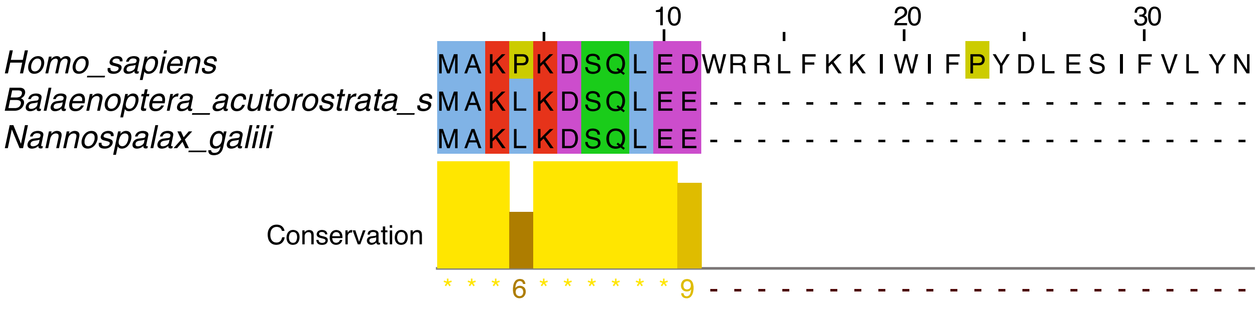

HG1155

ENSG00000141668 (CBLN2)

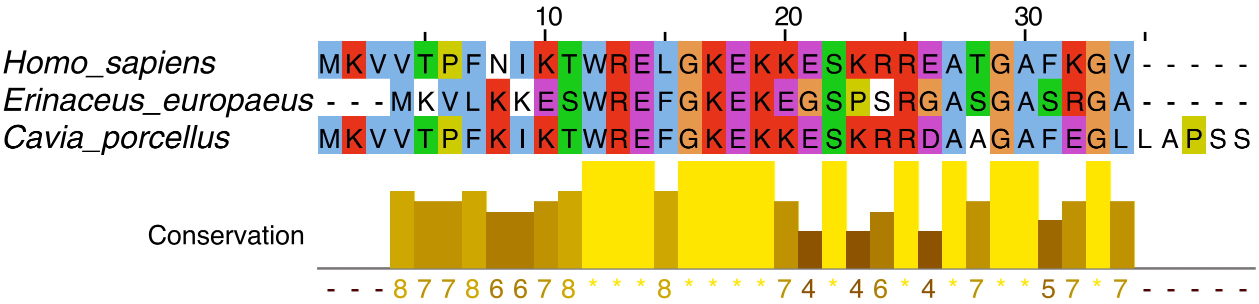

HG1156

ENSG00000144741 (SLC25A26)

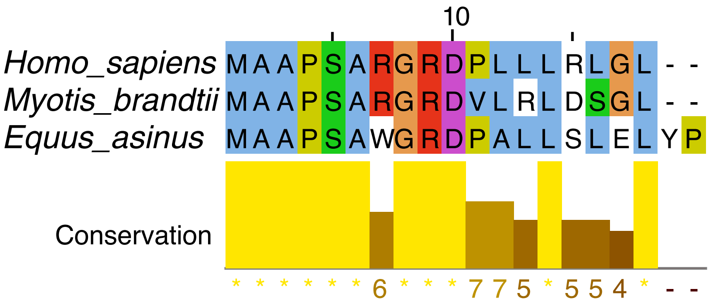

HG1157

ENSG00000148123 (PLPPR1)

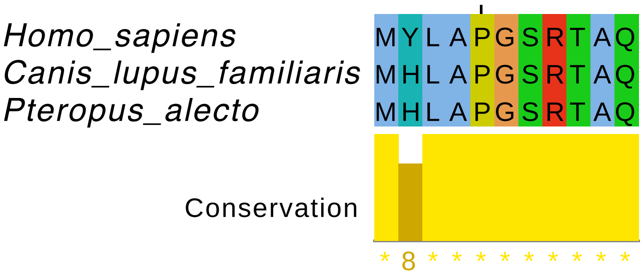

HG1158

ENSG00000149571 (KIRREL3)

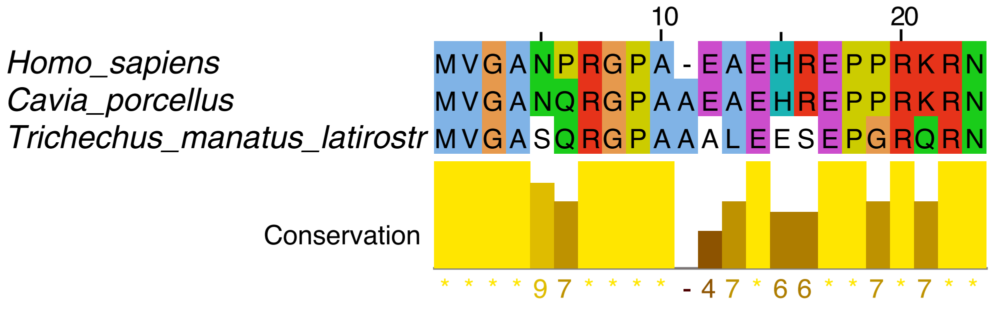

HG1159

ENSG00000152822 (GRM1)

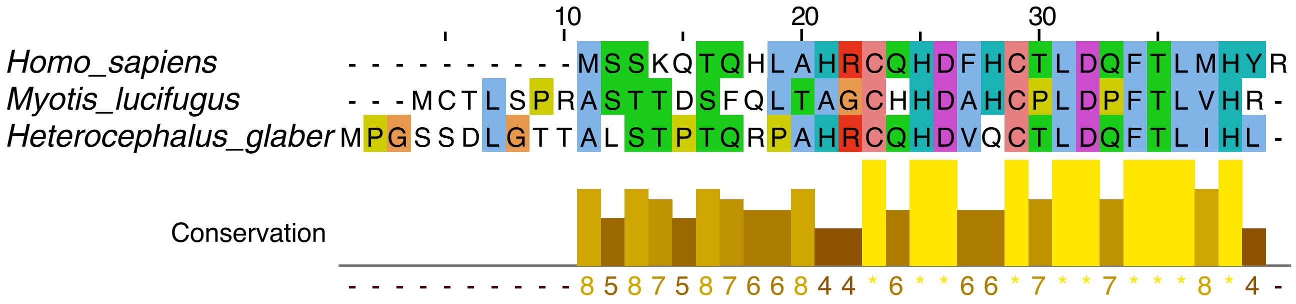

HG1160

ENSG00000154162 (CDH12)

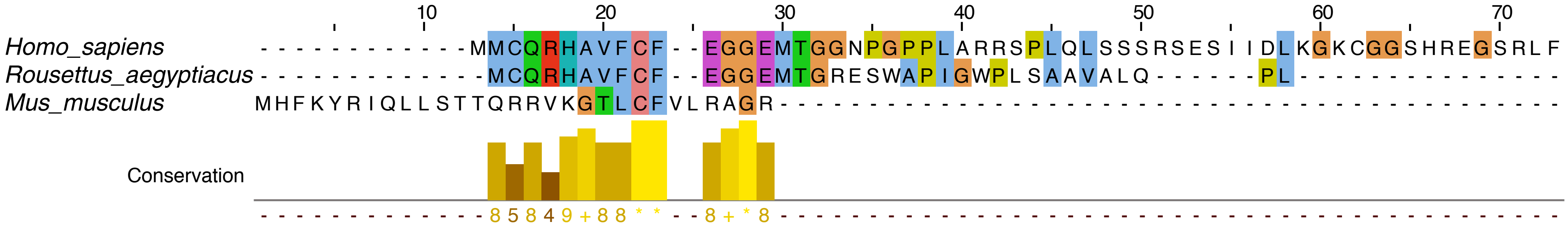

HG1161

ENSG00000154227 (CERS3)

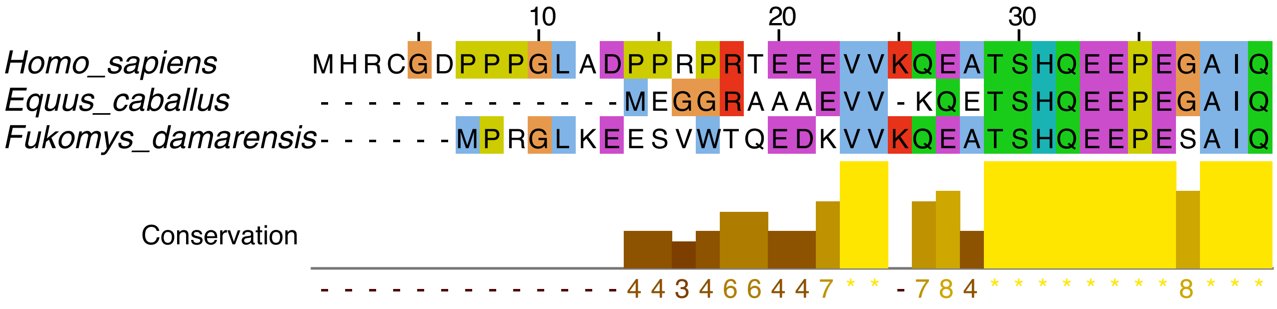

HG1162

ENSG00000155380 (SLC16A1)

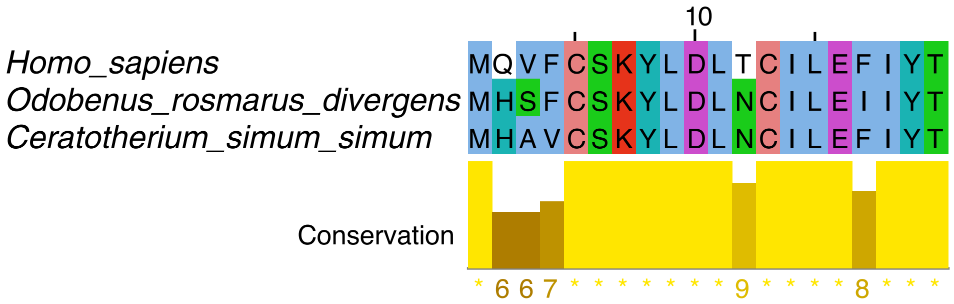

HG1163

ENSG00000157637 (SLC38A10)

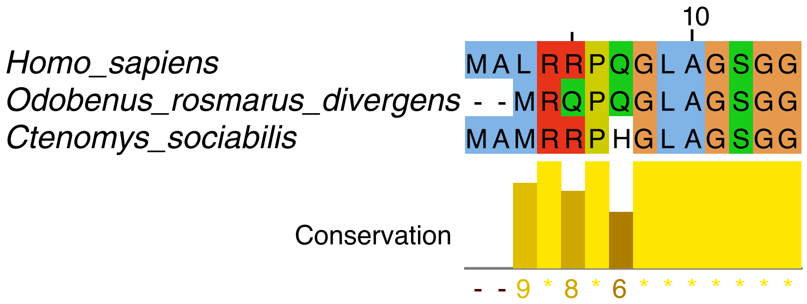

HG1164

ENSG00000162639 (HENMT1)

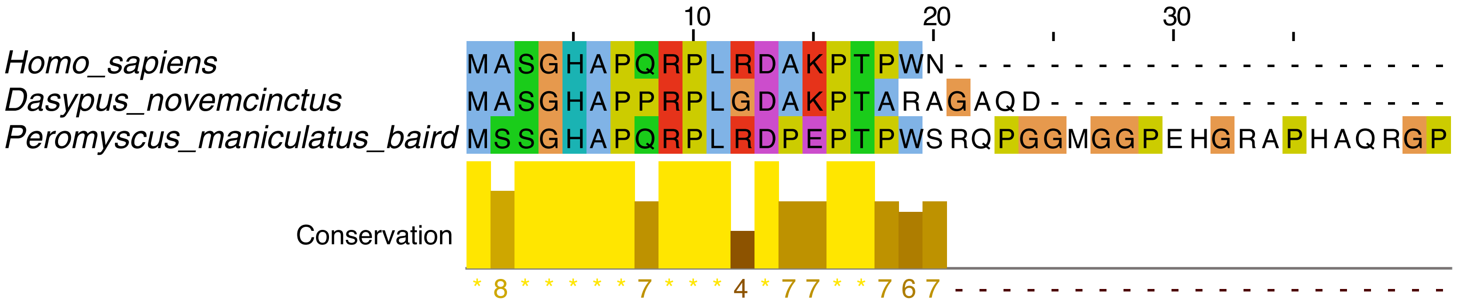

HG1165

ENSG00000162694 (EXTL2)

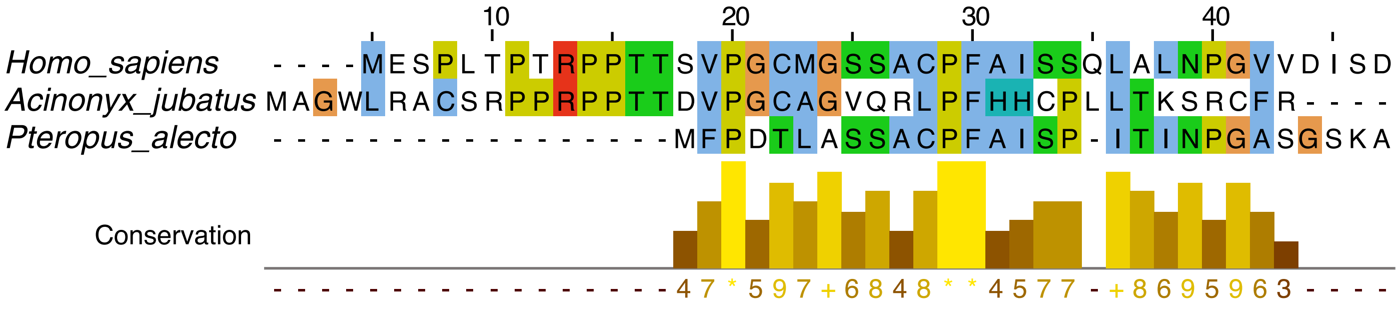

HG1166

ENSG00000165071 (TMEM71)

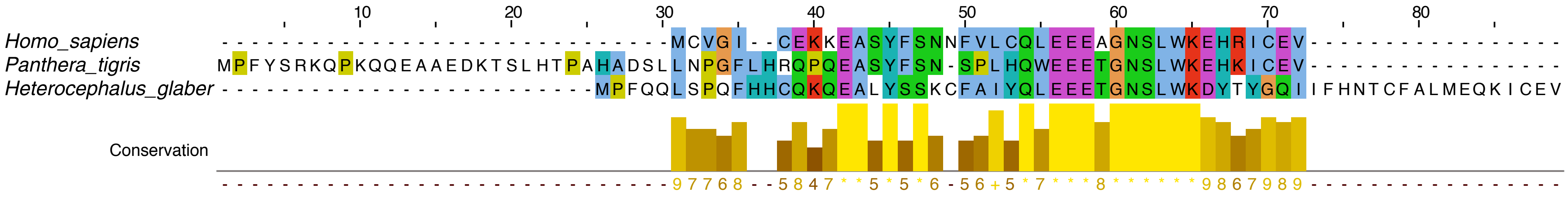

HG1167

ENSG00000165934 (CPSF2)

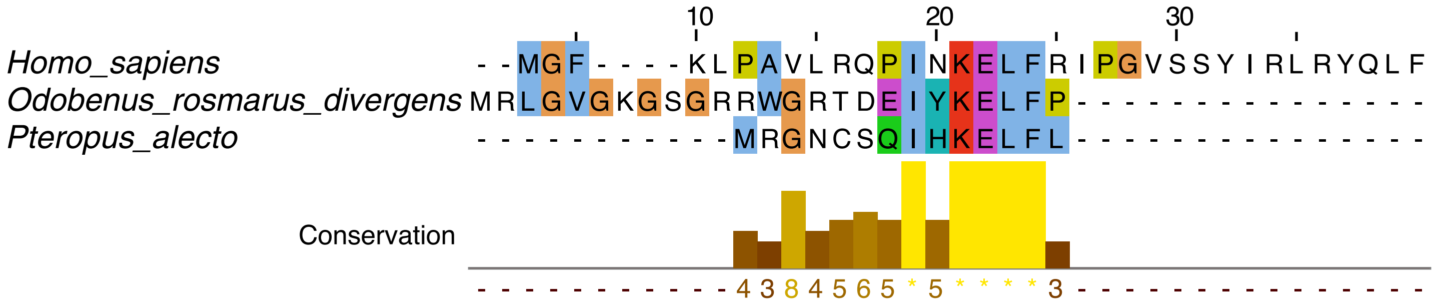

HG1168

ENSG00000166669 (ATF7IP2)

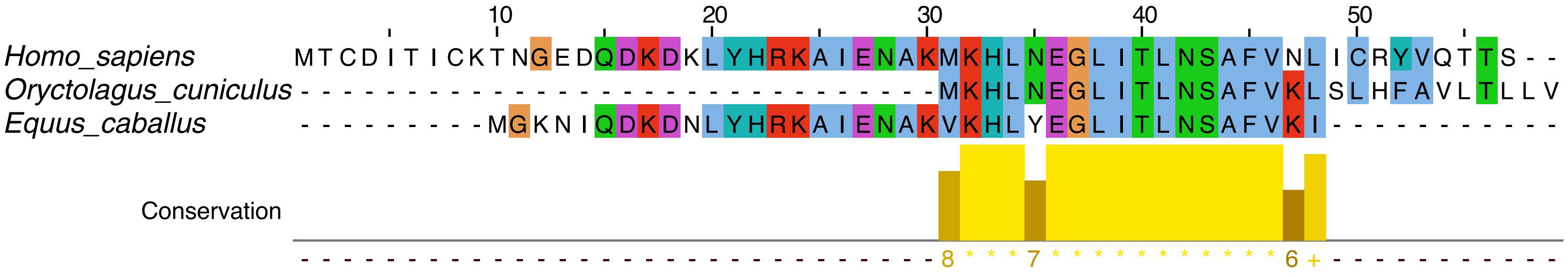

HG1169

ENSG00000167637 (ZNF283)

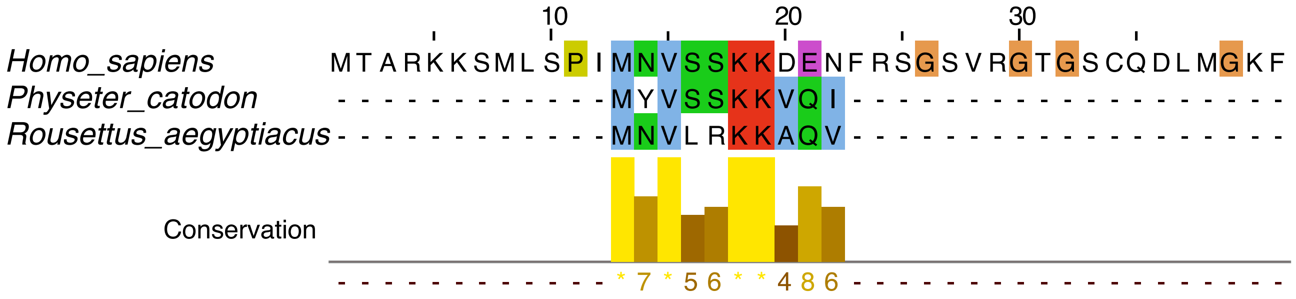

HG1170

ENSG00000167858 (TEKT1)

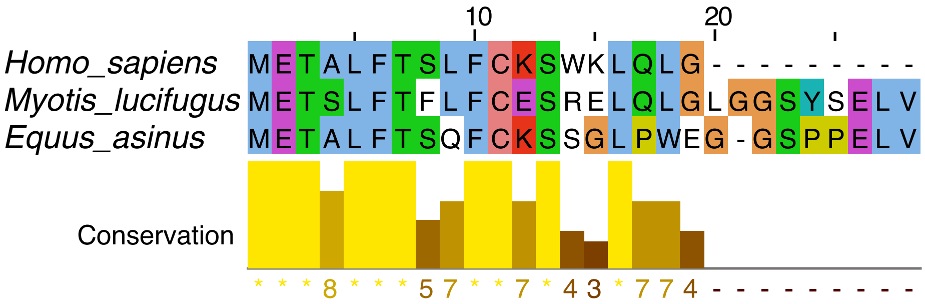

HG1171

ENSG00000167968 (DNASE1L2)

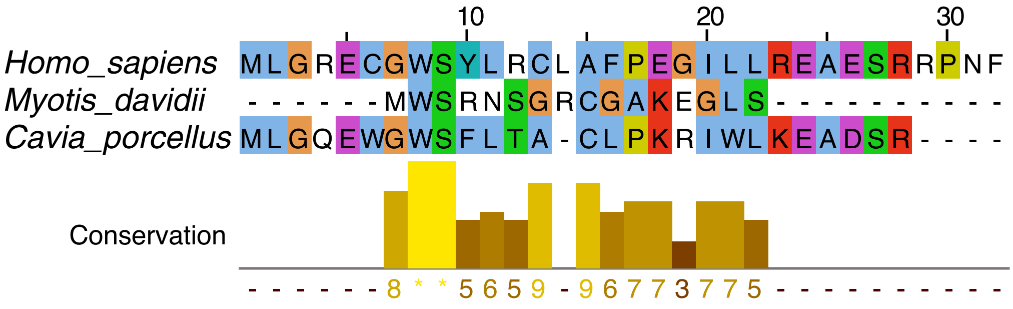

HG1172

ENSG00000170381 (SEMA3E)

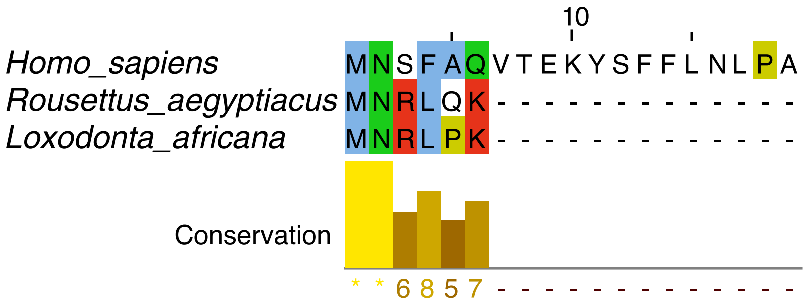

HG1173

ENSG00000170464 (DNAJC18)

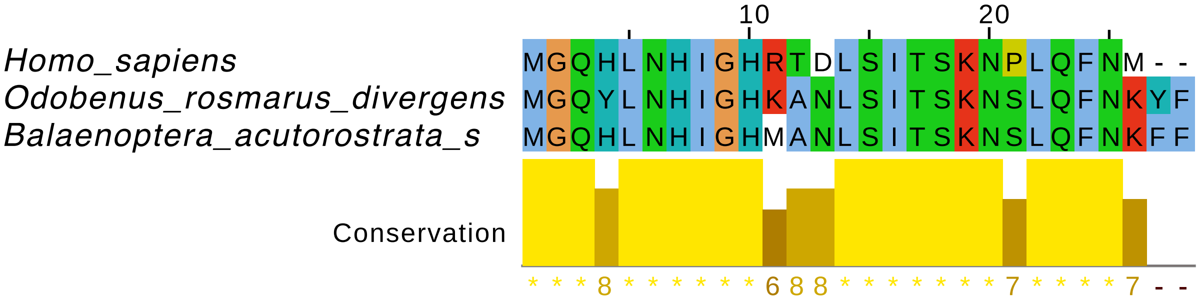

HG1174

ENSG00000173918 (C1QTNF1)

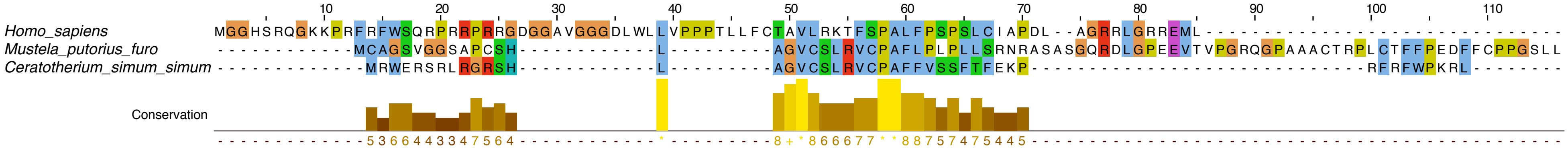

HG1175

ENSG00000174473 (GALNTL6)

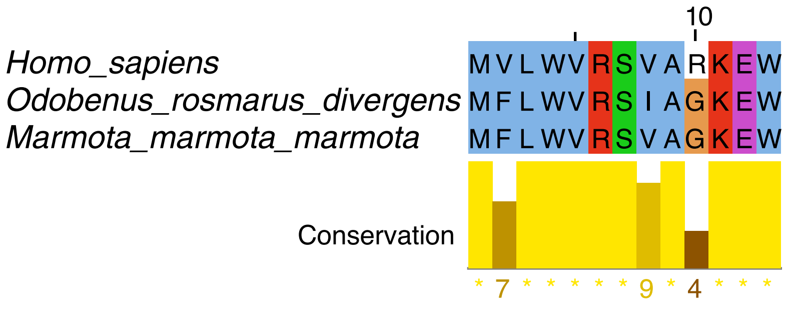

HG1176

ENSG00000176472 (ZNF575)

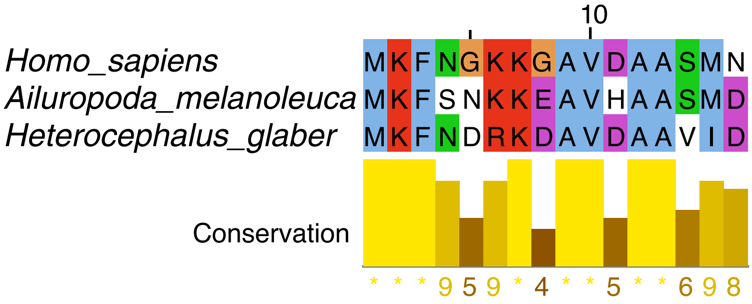

HG1177

ENSG00000178295 (GEN1)

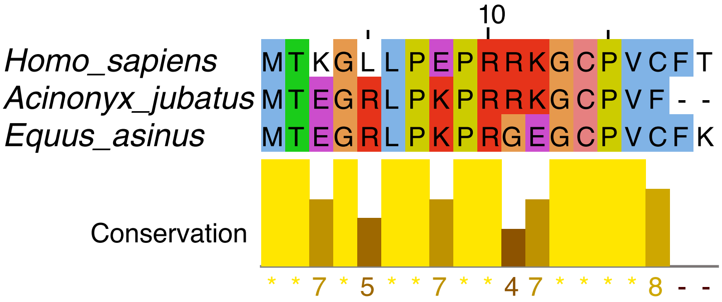

HG1178

ENSG00000182670 (TTC3)

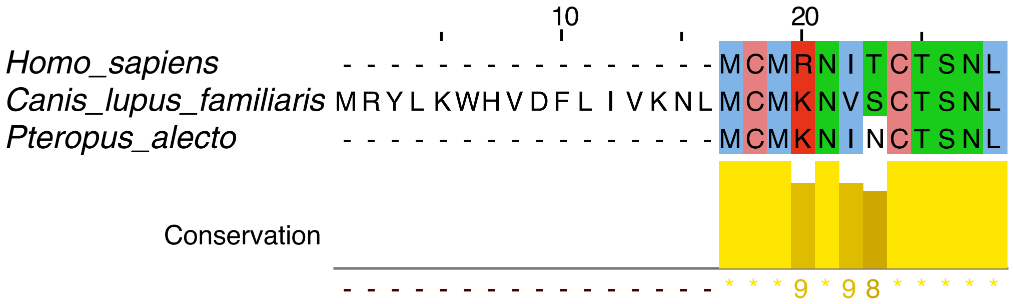

HG1179

ENSG00000185933 (CALHM1)

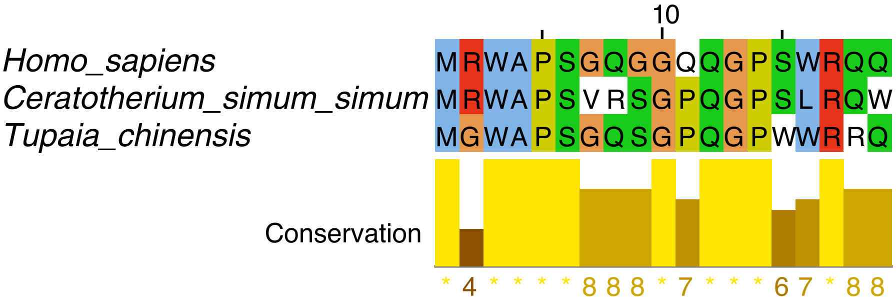

HG1180

ENSG00000188674

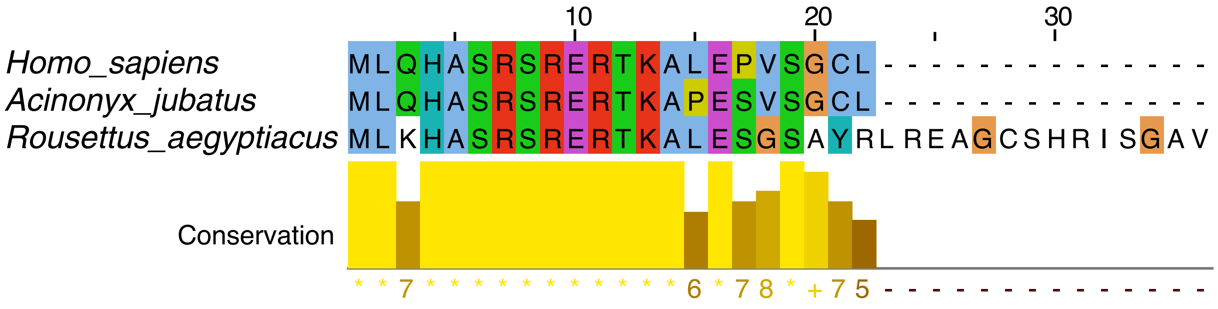

HG1181

ENSG00000189058 (APOD)

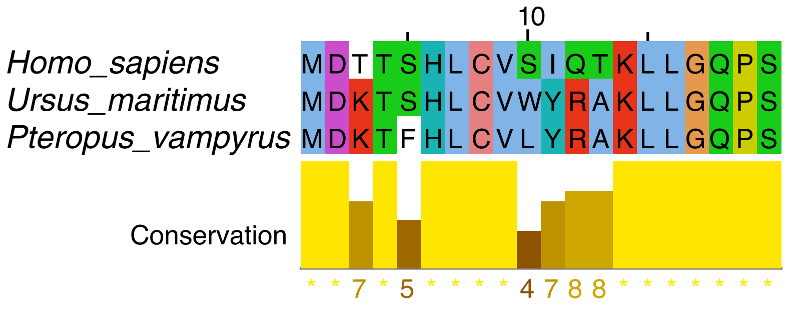

HG1182

ENSG00000196155 (PLEKHG4)

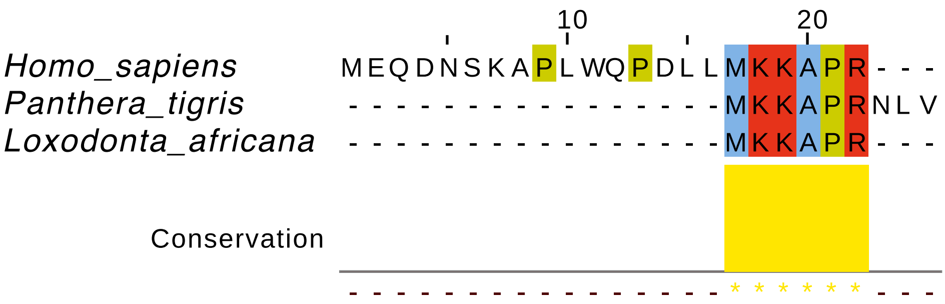

HG1183

ENSG00000198133 (TMEM229B)

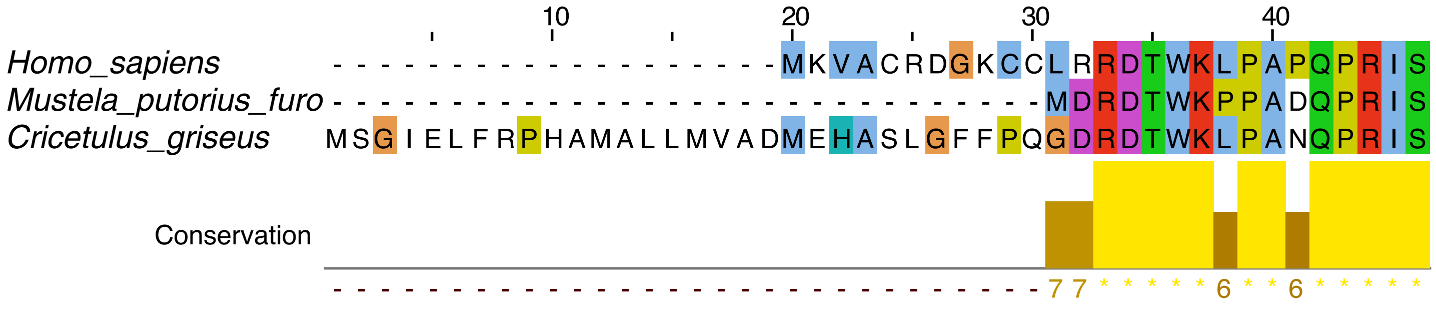

HG1184

ENSG00000198894 (CIPC)

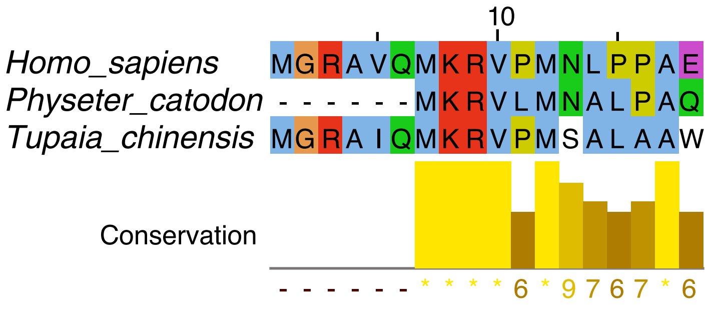

HG1185

ENSG00000198920 (KIAA0753)

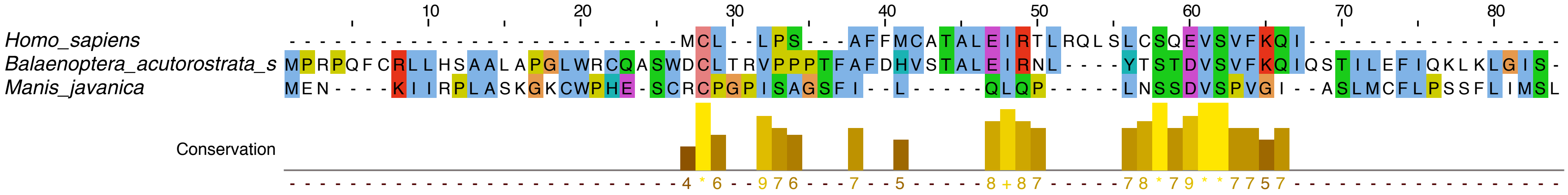

HG1186

ENSG00000204923

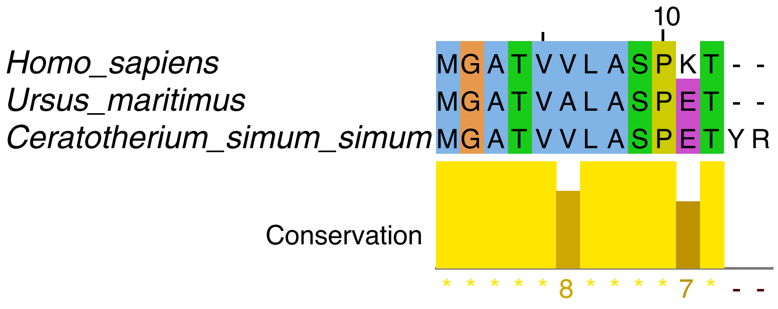

HG1187

ENSG00000213762 (ZNF134)

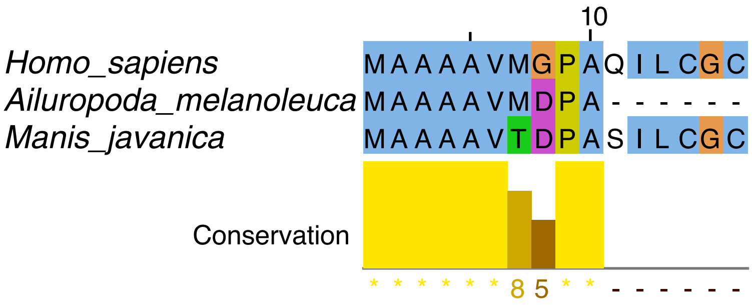

HG1188

ENSG00000214415 (GNAT3)

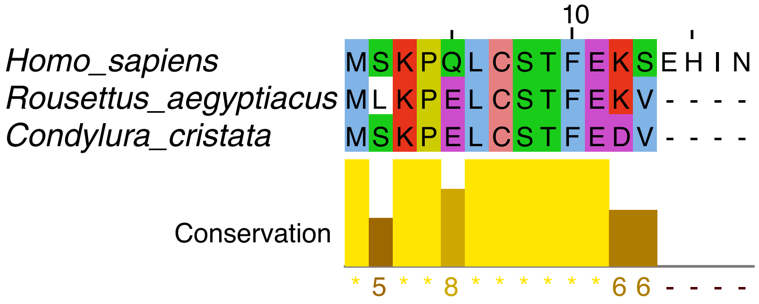

HG1189

ENSG00000279909

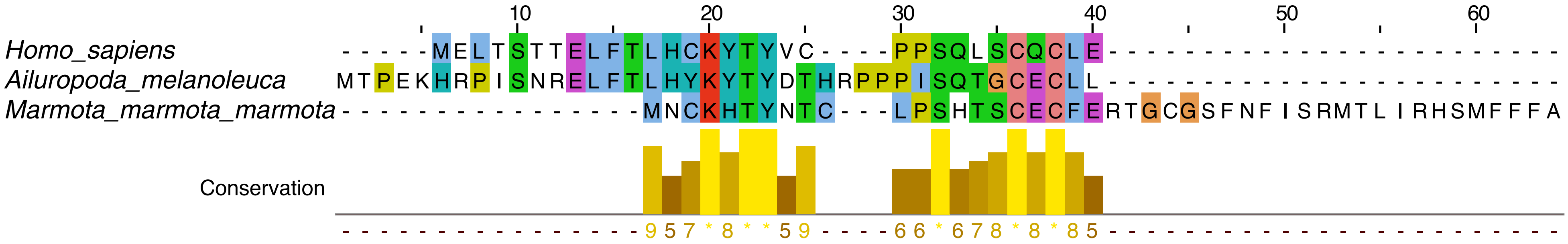

HG1190

ENSG00000123684 (LPGAT1)

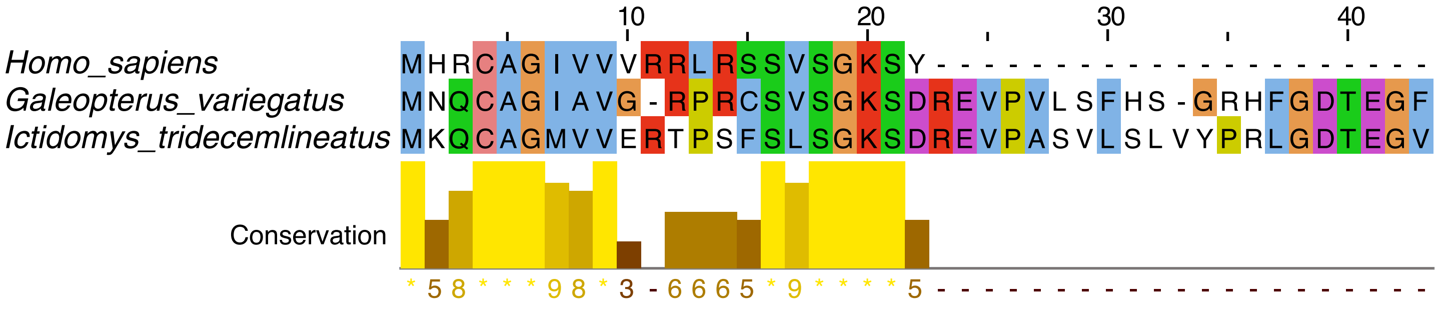

HG1191

ENSG00000164331 (ANKRA2)

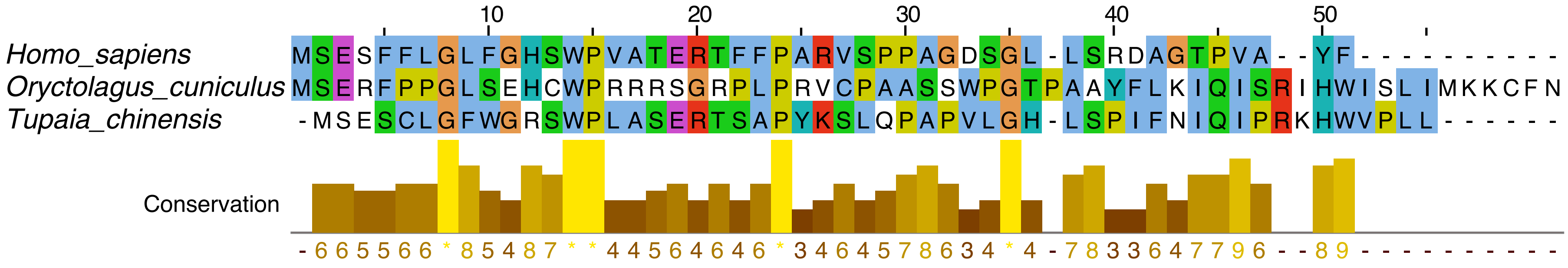

HG1192

ENSG00000168758 (SEMA4C)

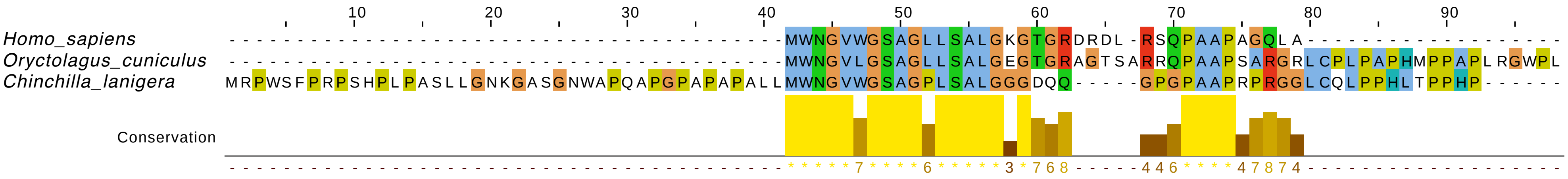

HG1193

ENSG00000177706 (FAM20C)

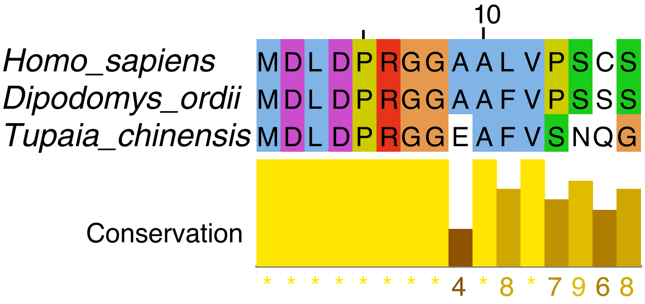

HG1194

ENSG00000214694 (ARHGEF33)

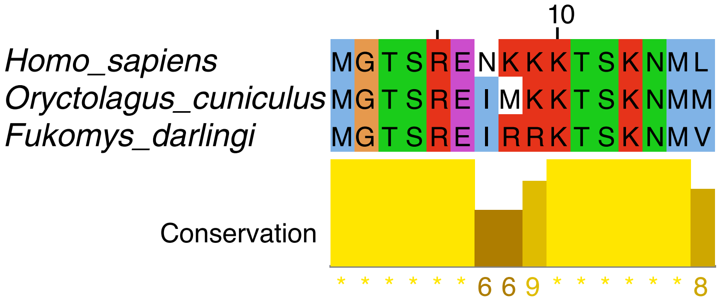

Supplementary Figure S4. Alignments of the extracted CPuORF sequences by ESUCA. The amino acid sequences of the extracted CPuORFs and their homologous putative uORFs were aligned using Clustal Ω ver. 1.2.2 and displayed using Jalview ver. 2.10.2. For each alignment, a putative uORF sequence was selected from each order in which uORF-tBLASTn and mORF-tBLASTn hits were found. In the alignments of the ENSG00000112245 (HG0001), ENSDARG00000003077 (HG0002), ENSGALG00000041706 (HG0004.1), ENSG00000031003 (HG0004.1), ENSGALG00000009013 (HG0010.1), ENSG00000189266 (HG0018), ENSDARG00000087059 (HG0026), ENSG00000172995 (HG0043), ENSDARG00000061687 (HG0044), ENSG00000139329 (HG0056), ENSG00000280987 (HG0057), ENSDARG00000018060 (HG0059.1), ENSDARG00000040008 (HG0076), ENSG00000164600 (HG0076), ENSG00000204406 (HG0079), ENSGALG00000016176 (HG0105), ENSG00000181690 (HG0125.2), ENSG00000017427 (HG0135), ENSG00000180530 (HG0170), ENSG00000177311 (HG0171.2), ENSG00000023445 (HG0262.1), ENSG00000204256 (HG0274.1), ENSG00000100239 (HG0275.1), ENSG00000175928 (HG0279.1), ENSGALG00000007717 (HG0403.1), ENSGALG00000026736 (HG0436), ENSG00000180008 (HG0460), ENSG00000166444 (HG0484), ENSG00000144460 (HG0504), ENSG00000177853 (HG0506.1), ENSG00000126860 (HG0513), ENSG00000273841 (HG0556), ENSG00000010818 (HG0579), ENSG00000164930 (HG0599), ENSG00000003147 (HG0604.2), ENSG00000074657 (HG0607), ENSG00000122203 (HG0614), ENSG00000259332 (HG0635), ENSG00000100359 (HG0641), ENSG00000166845 (HG0661), ENSG00000111837 (HG0683), ENSG00000085998 (HG0719), ENSG00000104783 (HG0724), ENSG00000115825 (HG0727), ENSG00000163794 (HG0816), ENSG00000127324 (HG0946.2), ENSG00000136235 (HG0956), ENSG00000154319 (HG0968), ENSG00000065357 (HG1022), ENSG00000082175 (HG1030), ENSG00000084453 (HG1032), ENSG00000140030 (HG1065), ENSG00000166532 (HG1085), ENSG00000174371 (HG1093), ENSG00000177917 (HG1094), ENSG00000092020 (HG1119), ENSG00000101574 (HG1125) CPuORFs, some putative uORF sequences were manually removed. After manual removal, K_a/K_s analysis was conducted using the putative uORF sequences shown in the alignments.

Supplementary Figure S5

```
HG0001 ENSDARG00000006242 ptp4a1

>Danio_rerio
-------------------------------------------------------------------MIKLQSSMSKHI-P--FCGVLGHTFMEF-LKGSGDYCQ--AQHGVYAEK-----
>Acipenser_sinensis
-------------------------------------------------------------------MIKLQSSMSKHIPQ--FCGVLGHTFMEF-LKGSGDYCQ--AQHEVYADK-----
>Actinia_tenebrosa
--------------------------------------------------------------------------MWT-TNI--CYDHCGHSLMEF-LIDSGDWCQ--AQHN----------
>Acanthogorgia_aspera
--------------------------------------------------------------------------MSR-AYCLFGFRSRFEKFMEYFLKDSGEFCQ--AQHE----------
>Amia_calva
-------------------------------------------------------------------MIKLQSSMSKHIPQ--FCGVLGHTFMEF-LKGSGDYCQ--AQHDVYADK-----
>Channa_argus
MS-------------------QQTEEAPP----QQQFQISDENSSC-HIEEEQKGDLTVVSALPPLSLRSHGISMFILLFG--FCVDLSRTYMAC-V--SGEFCQ--AQHLTSVEK-----
>Kaupichthys_hyoproroides
-------------------------------------------------------------------MIKLQSSMSKHIPQ--FCGVLGHTFMEF-LKDSGDYCQ--AQHEVYADK-----
>Anser_cygnoides
--------------------------------------------------------------------------------------------MEF-LKGSGDYCQ--AQHDLYADK-----
>Podocoryna_carnea
--------------------------------------------------------------------------------------------MEF-LRGSGDYCF--AQHRT---------
>Xenopus_tropicalis
MLFTGHGEEPQGRRSGPRQLSPDHRALRL---GDSSLL-YAPSPPAPLLFHRLFL--------GTGCMIKIQSSMSKHS-P--FCGVLGHTFMEF-LKGSGDYCQ--AQHELYADK-----
>Chaetura_pelagica
--------------------------------------------------------------------------------------------MEF-LKGSGDYCQ--AQHDLYADK-----
>Apteryx_australis_mantelli
--------------------------------------------------------------------------------------------MEF-LKGSGDYCQ--AQHDLYADK-----
>Stegodyphus_lineatus
---------------------------------------------------------------------MLDASMC-IDIA--VNQIQTFLLMEV-LVGSDEYCR--AQHK----------
>Meinertellus_cundinamarcensi
--------------------------------------------------------------------------MCR-DLV--FLGETGLTYMEF-QLDSGEYCQ--AQHK----------
>Scapharca_broughtonii
--------------------------------------------------------------------------MFR-KIL--FFGEFGHTFMEF-VTNSGDYCQ--AQHV----------
>Porichthys_notatus
-------------------------------------------------------------------MIKLQSSMSKHIPQ--FCGVLGHTFMEF-LKGRGDYCQ--AQHAADADE-----
>Oryzias_melastigma
-------------------------------------------------------------------MIKLQSSMSKHIPQ--FCGVLGHTFMEF-LKGSGDYCQ--AQHAAYADE-----
>Tripterygion_delaisi
--------------------------------------------------------------------------MFSLLFG--FCVDLSRTYMAC-V--SGEFCQ--AQHLTSVEK-----
>Buceros_rhinoceros_silvestri
--------------------------------------------------------------------------------------------MEF-LKGSGDYCQ--AQHDLYADK-----
>Caprimulgus_carolinensis
--------------------------------------------------------------------------------------------MEF-LKGSGDYCQ--AQHDLYADK-----
>Scyliorhinus_torazame
--------------------------------------------------------------------------------------------MEF-LKGSGDYCQ--AQHAVYADK-----
>Cariama_cristata
--------------------------------------------------------------------------------------------MEF-LKGSGDYCQ--AQHDLYADK-----
>Canis_lupus_familiaris
-------------------------------------------------------------------MIRPQSSMSKHI-P--FCGVLGHTFMEF-LKGSGDYCQ--AQHDLYADK-----
>Alatina_alata
---------------------------------------------------------------------MRRPCLC-T-------CFCGKTFMEF-LKDSGDWCQ--SQHK----------
>Hynobius_retardatus
MRAPELGRSEARAQCGGGSVAADTATLPPRSPAARPVW-YTARRACP--PATRAR--------GTGRMIQPRSSMSNHSPQ--FCGVLGHTFMEF-LKGSGDYCQ--AQHDLYADK-----
>Micropterus_floridanus
--------------------------------------------------------------------------------------------MEF-VRRSGDYCH--AQHI----------
>Balaenoptera_acutorostrata_s
--------------------------------------------------------------------------------------------MEF-LKGSGDYCQ--AQHDLYADK-----
>Astyanax_mexicanus
-------------------------------------------------------------------MVKLQSSMSKHIPQ--FCGVLGHTFMEF-LKGSGDYCQ--AQHGVYADK-----
>Calidris_pugnax
--------------------------------------------------------------------------------------------MEF-LKGSGDYCQ--AQHDLYADK-----
>Callorhinchus_milii
--------------------------------------------------------------------------------------------MEF-LKGSGDYCQ--AQHDVYADK-----
>Miniopterus_natalensis
-------------------------------------------------------------------MIRPQSSMSKHI-P--FCGVLGHTFMEF-LKGSGDYCQ--AQHDLYADK-----
>Haplochromis_burtoni
-------------------------------------------------------------------MIKLQSSMSKHIPQ--FCGVLGHTFMEF-LKGSGDYCQ--AQHAAYADE-----
>Pterocles_gutturalis
--------------------------------------------------------------------------------------------MEF-LKGSGDYCQ--AQHDLYADK-----
>Eucidaris_tribuloides
--------------------------------------------------------------------------MCK-HTE--VLAVINKPFMAL-VLDSGEFCQ--SQHK----------
>Dasypus_novemcinctus
-------------------------------------------------------------------MIRPQSSMSKHI-P--FCGVLGHTFMEF-LKGSGDYCQ--AQHDLYADK-----
>Alosa_alosa
-------------------------------------------------------------------MIKLQSSMSKHIPQ--FCGVLGHTFMEF-LKGSGDYCQ--AQHGVYADE-----
>Latimeria_menadoensis
-------------------------------------------------------------------MIKIQSSMSKHIPQ--FCGVLGHTFMEF-LKGSGDYCQ--AQHDLYADK-----
>Pogonus_chalceus
--------------------------------------------------------------------------MCM-ALV--FLGEKVFAFMEF-QLDSAEYCQ--AQHK----------
>Colius_striatus
--------------------------------------------------------------------------------------------MEF-LKGSGDYCQ--AQHDLYADK-----
>Columba_livia
--------------------------------------------------------------------------------------------MEF-LKGSGDYCQ--AQHDLYADK-----
>Leptosomus_discolor
--------------------------------------------------------------------------------------------MEF-LKGSGDYCQ--AQHDLYADK-----
>Ricordea_yuma
--------------------------------------------------------------------------MWR-GNP--VYGNRAHTFMEF-IIDSGDWCQ--AQHE----------
>Alligator_mississippiensis
-------------------------------------------------------------------MIRLQSSMSNRR-P--FCGVLGHTFMEF-LKGSGDYCQ--AQHDLYADK-----
>Cuculus_canorus
--------------------------------------------------------------------------------------------MEF-LKGSGDYCQ--AQHDLYADK-----
>Poecilia_formosa
-------------------------------------------------------------------MIKLQSSMSKHIP---FCGVLGRTFMEF-LKGSGDYCQ--AQHAAYADE-----
>Sarcophilus_harrisii
--------------------------------------------------------------------------------------------MEF-LKGSGDYCQ--AQHDLYADK-----
>Macrobrachium_nipponense
--------------------------------------------------------------------MKFKYTRS-NNAQ--LSSGRGYTLMEL-LKGSGEFCK--AQYHIK--------
>Forficula_auricularia
--------------------------------------------------------------------------------------------MEF-RLDSAEYCQ--AQHK----------
>Galeopterus_variegatus
--------------------------------------------------------------------------------------------MEF-LKGSGDYCQ--AQHDLYADK-----
>Monodelphis_domestica
-------------------------------------------------------------------MIRPQSSMSKHI-P--FCGVLGHTFMEF-LKGSGDYCQ--AQHDLYADK-----
>Occasjapyx_japonicus
--------------------------------------------------------------------------MCR-DLV--CLGKIGLTFMEF-FLDSGEYCQ--AQHK----------
>Macropus_eugenii
----------------------------------------------------------------------------MKAKQ--FCGVLGHTFMEF-LKGSGDYCQ--AQHDLYADK-----
>Arachnocampa_luminosa
--------------------------------------------------------------------------------------------MEF-HLDSAEYCQ--AQHK----------
>Eurylophella_sp._AD-2013
--------------------------------------------------------------------------MCR-DLV--FLGEIGLTYMEF-QLDSGEYCQ--AQHK----------
>Esox_lucius
-------------------------------------------------------------------MIKLQSSMSKHIP---FCGVLGHTFMEF-LKGSGDYCQ--AQHVAAYADE----
>Meganyctiphanes_norvegica
--------------------------------------------------------------------MKFKYCGN-NNTQ--LSCGRGYTLMEL-LKGSGEFCK--AQYHIK--------
>Haliaeetus_leucocephalus
-------------------------------------------------------------------MIRLQSSMSNHIPQ--FCGVLGHTFMEF-LKGSGDYCQ--AQHDLYADK-----
>Coturnix_japonica
-------------------------------------------------------------------MIRLQSSMSNHIP---FCGVLGHTFMEF-LKGSGDYCQ--AQHDLYADK-----
>Gavia_stellata
--------------------------------------------------------------------------------------------MEF-LKGSGDYCQ--AQHDLYADK-----
>Acanthogobius_hasta
-------------------------------------------------------------------MIKLQSSMSKHIPQ--FCGVLGHTFMEF-LKGSGDYCQ--AQHVVDADE-----
>Eurypyga_helias
--------------------------------------------------------------------------------------------MEF-LKGSGDYCQ--AQHDLYADK-----
>Apteronotus_leptorhynchus
--------------------------------------------------------------------------------------------MAF-LQRAGDYCH--AQHWCC--------
>Okanagana_villosa
--------------------------------------------------------------------------MCM-AIV--FLGETWLTFMEF-HLDSAEYCQ--AQHK----------
>Megachile_rotundata
--------------------------------------------------------------------------------------------MEF-QLDSAEYCQ--AQHK----------
>Erinaceus_europaeus
-------------------------------------------------------------------MIKPQSSMSKHIP---FCGVLGHTFMEF-LKGSGDYCQ--AQHDLYADK-----
>Oryctolagus_cuniculus
-------------------------------------------------------------------MIRPQSSMSKHI-P--FCGVLGHTFMEF-LKGSGDYCQ--AQHDLYADK-----
>Rhodinia_newara
--------------------------------------------------------------------------------------------MEF-HLDSAEYCQ--AQHK----------
>Protopterus_aethiopicus
-------------------------------------------------------------------MIKLQSSMSKHIPQ--FCGVIGHTFMEF-LKGSGDYCQ--AQHALYADK-----
>Lingula_anatina
-------------------------------------------------------------------------------MQ--FFGEFGHTFMEF-VTNSGEYCQ--AQHK----------
>Elephantulus_edwardii
-------------------------------------------------------------------MIRPQSSMSKHIPQ--FCGVLGHTFMEF-LKGSGDYCQ--AQHDLYADK-----
>Metallyticus_splendidus
--------------------------------------------------------------------------MCM-ALV--FLGETVLTYMEF-QLDSGEYCQ--AQHK----------
>Boreus_hyemalis
--------------------------------------------------------------------------------------------MEF-QLDSAEYCQ--AQHK----------
>Ornithorhynchus_anatinus
-------------------------------------------------------------------MIATHSSMSKHS-P--FCGVLGHTFMEF-LKDSGDYCQ--AQHDLYADK-----
>Cypridininae_sp._BMR-2011
-------------------------------------------------------------MEDSETSLLYKYRLCM-RPQ--VLAGVCCSFMEF-VLADGEYCQ--AQHK----------
>Perna_viridis
--------------------------------------------------------------------------MCR-NIL--FFGVLGHTFMEF-VTNSGDYCQ--AQHV----------
>Eptatretus_burgeri
--------------------------------------------------------------------------MNRHNAQ--FCGVLGRTFMEF-VKGRGEYCE--TQHIALADK-----
>Conwentzia_psociformis
---------------------------------------------------------------------------MV-NFN--SFSERVLTYMEF-QLDSAEYCQ--AQHK----------
>Triops_newberryi
--------------------------------------------------------------------------MCT-TGQ--RWVEWRHKFLAV-LLGSGDYCE--AQHK----------
>Hapalochlaena_maculosa
--------------------------------------------MC-RNILDLLGGI--VEKTDSPSTLAWPHHLCR-SIK--FFGEFGHTFMEF-VTNSGDFCQ--AQHV----------
>Epiophlebia_superstes
--------------------------------------------------------------------------------------------MEF-QLDSGEYCQ--AQHK----------
>Opisthocomus_hoazin
--------------------------------------------------------------------------------------------MEF-LKGSGDYCQ--AQHDLYADK-----
>Stenobothrus_lineatus
MV-------------------V------E----ASAVALADEGRTAGRHVMRLERAS--SPSSMVDSQAAGGGRLCA-HQQ--FRGEIVLTFMEF-QLDSGEYCQ--AQHK----------
>Plecoglossus_altivelis
--------------------------------------------------------------------------------------------MEF-LQGSGDYCH--AQHPSI--------
>Scleropages_formosus
--------------------------------------------------------------------------MCGLLFR--FCVDLGRPFMAF-VSVSGDFCQ--AQHLASADK-----
>Crassostrea_gigas
------------------------------------------MRDP-RGPVTVRGKF--VTGFV---LCSSLLNCWQ-HTE--FRSNFGHTYMEF-VTNSGDYCQ--AQHV----------
>Endeis_spinosa
--------------------------------------------------------------------------------------------MEF-LKESGDYCYCRAQHV----------
>Zonotrichia_albicollis
-------------------------------------------------------------------MIRLQSSMSNHI-P--FCGVLGHTFMEF-LKGSGDYCQ--AQHDLYADK-----
>Pecten_maximus
--------------------------------------------------------------------------------------------MEF-VTNSGDYCQ--AQHAL---------
>Pelecanus_crispus
--------------------------------------------------------------------------------------------MEF-LKGSGDYCQ--AQHDLYADK-----
>Sebastes_caurinus
-------------------------------------------------------------------MIKLQSSMSKHIP---FCGVLGHTFMEF-LKGSGDYCQ--AQHAAYADE-----
>Equus_przewalskii
-------------------------------------------------------------------MIRPQSSMSKHSPQ--FCGVLGHTFMEF-LKGSGDYCQ--AQHDLYADK-----
>Peruphasma_schultei
--------------------------------------------------------------------------------------------MEF-LPNSGEYCQ--AQHK----------
>Menopon_gallinae
--------------------------------------------------------------------MNFRFSGCM-ALK--NFHSTSSRLMEF-FLDSAEYCQ--AQHTK---------
>Glycera_dibranchiata
-------------------------------------------------------------ME------QCHPVLKT-HSK--FFGVLGHTFMEF-LCDSGEYCQ--AQHV----------
>Leuctra_sp._AD-2013
--------------------------------------------------------------------------MCM-ALV--LVGEIVLTYMEF-QLDSGEYCQ--AQHRK---------
>Cynoglossus_semilaevis
--------------------------------------------------------------------------------------------MEF-LKGSGDYCQ--AQHAAYADE-----
>Gorilla_gorilla
-------------------------------------------------------------------MIRPQSSMSKHIPQ--FCGVLGHTFMEF-LKGSGDYCQ--AQHDLYADK-----
>Loxodonta_africana
-------------------------------------------------------------------MIRPQSSMSKHI-P--FCGVLGHTFMEF-LKGSGDYCQ--AQHDLYADK-----
>Fulmarus_glacialis
--------------------------------------------------------------------------------------------MEF-LKGSGDYCQ--AQHDLYADK-----
>Nestor_notabilis
--------------------------------------------------------------------------------------------MEF-LKGSGDYCQ--AQHDLYADK-----
>Pinctada_martensii
--------------------------------------------------------------------------MCR-NIV--FFGEYGHTFMEF-VTNSGDYCQ--AQHK----------
>Leucoraja_erinacea
--------------------------------------------------------------------------MRKHIPQ--FCGVLGHTFMEF-LKGSGDYCQ--AQHDVYADK-----
>Inocellia_crassicornis
--------------------------------------------------------------------------------------------MEF-QLDSAEYCQ--AQHK----------
>Rhopilema_esculentum
-------------------------------------------------------------------------MNS-YHLL--LSKYFGKPFMEF-LKDSAEWCK--AQHVE---------
>Rattus_norvegicus
-------------------------------------------------------------------MIRPQSSMSRHI-P--FCGVLGHTFMEF-LKGSGDYCQ--AQHDLYADK-----
>Salmo_salar
-------------------------------------------------------------------MIKLQSSMSKHIPQ--FCGVLGHTFMEF-LKGSGDYCQ--AQHAAAYADE----
>Acropora_digitifera
--------------------------------------------------------------------------MWR-FSL--TFGSRAHTFMEF-IIDSGDWCQ--AQHE----------
>Tityus_serrulatus
--------------------------------------------------------------------------------------------MEY-LKTTGEYCR--AQHK----------
>Aurelia_aurita
-------------------------------------------------------------------------MTS-YHLQ--L-KCFGETFMEF-LKDSAEWCK--AQHVE---------
>Lepisosteus_oculatus
-------------------------------------------------------------------MIKLQSSMSKHIPQ--FCGVLGHTFMEF-LKGSGDYCQ--AQHGVYADK-----
>Sepia_pharaonis
--------------------------------------------MC-RNILDLLGGI--VKKTDSPSTLAWPHHLWR-LVK--FFGEFGHTFMEF-VTNSGDFCQ--AQHV----------
>Sepioloidea_lineolata
--------------------------------------------------------------------------------------------MEF-VTNSGDFCQ--AQHV----------
>Ictalurus_punctatus
-------------------------------------------------------------------MVKLQSSMSKHIPQ--FCGVLGHTFMEF-LKGSGDYCQ--AQHGVYADK-----
>Oropsylla_silantiewi
--------------------------------------------------------------------------------------------MEF-QLDSAEYCQ--AQHK----------
>Trichechus_manatus_latirostr
-------------------------------------------------------------------MIRPQSSMSKHIPQ--FCGVLGHTFMEF-LKGSGDYCQ--AQHDLYADK-----
>Aptenodytes_forsteri
--------------------------------------------------------------------------------------------MEF-LKGSGDYCQ--AQHDLYADK-----
>Streblospio_benedicti
--------------------------------------------------------------------------MCR-IIL--FFGQIGYTFMEF-LCNSGEYCQ--AQHVAIPSVLSRLH
>Squalus_acanthias
--------------------------------------------------------------------------MNRLVTQ--FSGISGCSFMEF-LKGSRDYCQ--AQHGLLADK-----
>Python_bivittatus
-------------------------------------------------------------------MIRLQSSMSNHI-P--FCGVLGHTFMEF-LKGSGDYCQ--AQHGLYADK-----
>Stylops_melittae
--------------------------------------------------------------------------MCI-GIL--NLGNKVVEYMEF-HFGSSEYCK--AQHK----------
>Tyto_alba
--------------------------------------------------------------------------------------------MEF-LKGSGDYCQ--AQHDLYADK-----
>Struthio_camelus_australis
--------------------------------------------------------------------------------------------MEF-LKGSGDYCQ--AQHDLYADK-----
>Deroceras_reticulatum
--------------------------------------------------------------------------MSS-FVL--FVGEFGHTFMEF-VTNSEDFCQ--AQHYRLVE------
>Pelodiscus_sinensis
-------------------------------------------------------------------MIRLQSSMSNHI-P--FCGVLGHTFMEF-LKGSGDYCQ--AQHDLYADK-----
>Takifugu_rubripes
-------------------------------------------------------------------MIKLQSSMSKHIPQ--FCGVLGHTFMEF-LKGSGDYCQ--AQHAVYADE-----
>Watasenia_scintillans
--------------------------------------------------------------------------------------------MEF-VTNSGDFCQ--AQHV----------
>Gynaikothrips_ficorum
----------------------------------------------MVHCAKFNGEN--SISMVIRRVIFDNKWPSV-GYQ--TCGEILPTFMEF-HLDSAEYCQ--AQHK----------
>Tinamus_guttatus
--------------------------------------------------------------------------------------------MEF-LKGSGDYCQ--AQHDLYADK-----
>Tetronarce_californica
--------------------------------------------------------------------------------------------MEF-VRNSSGYCP--AQHSIFADK-----
>Hydroptila_sp._AD-2013
--------------------------------------------------------------------------------------------MEF-RLDSGEYCQ--AQHK----------
>Calypte_anna
--------------------------------------------------------------------------------------------MEF-LKGSGDYCQ--AQHDLYADK-----
>Apaloderma_vittatum
--------------------------------------------------------------------------------------------MEF-LKGSGDYCQ--AQHDLYADK-----
>Orycteropus_afer_afer
-------------------------------------------------------------------MIRPQSSMSKHIPQ--FCGVLGHTFMEF-LKGSGDYCQ--AQHDLYADK-----
>Ruditapes_philippinarum
--------------------------------------------------------------------------MNR-IIL--FFGELGHTFMEF-LTNSGEYCL--AQHVHV--------
>Limulus_polyphemus
--------------------------------------------------------------------------------------------MEF-LKTAGEYCQ--SQHK----------
>Palythoa_caribaeorum
--------------------------------------------------------------------------MR--RDY--LAYDRGRTLMEF-LIDSGDWCQ--AQHE----------
>Thermobia_domestica
--------------------------------------------------------------------------------------------MEF-QLDSGEYCQ--AQHK----------
```

```
HG0001 ENSDARG00000035676 ptp4a2b

>Danio_rerio
---------------------------------------------------------------------MVGPFFCFRVDSDHTYMAFVTVCREFCQAQHLTFADK-
>Acipenser_sinensis
-------------------------------------------------------------M-IKLQSSMSKHIPQFCGVLGHTFMEFLKGSGDYCQAQHEVYADK-
>Amia_calva
-------------------------------------------------------------M-IKLQSSMSKHIPQFCGVLGHTFMEFLKGSGDYCQAQHDVYADK-
>Channa_argus
MS-QQTEEAPPQQ---QFQISDENSSCHIEEEQKGDLTV-VSALPPLS-------------L-RSHGISMFILLFGFCVDLSRTYMACV--SGEFCQAQHLTSVEK-
>Kaupichthys_hyoproroides
-------------------------------------------------------------M-IKLQSSMSKHIPQFCGVLGHTFMEFLKDSGDYCQAQHEVYADK-
>Anas_platyrhynchos
-------------------------------------------------------------------------------------MAILSVRADFCQAQHSILADK-
>Podocoryna_carnea
---------------------------------------------------------------------MNSLLSCFCVDLGRTFMAFLSVSGDFCQAQHLAFADK-
>Xenopus_tropicalis
MLFTGHGEEPQGRRSGPRQLSPDHRA-----LRLGDSSLLYAPSPPAPLLFHR-LFLGTGCM-IKIQSSMSKH-SPFCGVLGHTFMEFLKGSGDYCQAQHELYADK-
>Chaetura_pelagica
-------------------------------------------------------------------------------------MEFLKGSGDYCQAQHDLYADK-
>Apteryx_australis_mantelli
-------------------------------------------------------------------------------------MAILSVRADFCQAQHSTLADK-
>Scapharca_broughtonii
----------------------------------------------------------------------MFRKILFFGEFGHTFMEFVTNSGDYCQAQHV------
>Menidia_menidia
---------------------------------------------------------------------MFNLLFGFCVDVSRSYMACV--TGEFCQAQHLTSAEK-
>Porichthys_notatus
-------------------------------------------------------------M-IKLQSSMSKHIPQFCGVLGHTFMEFLKGRGDYCQAQHAADADE-
>Oryzias_latipes
---------------------------------------------------------------------MFSLFFGFCVDSSRTYMACV--TGEFCQAQHLTSAEK-
>Tripterygion_delaisi
-----------------------------------------------MVCLHPGA---------VTFSCIHSMNPSY--QHSYTCMAFARLGGEFCQAQHLLIIVDK
>Buceros_rhinoceros_silvestri
-------------------------------------------------------------------------------------MEFLKGSGDYCQAQHDLYADK-
>Caprimulgus_carolinensis
-------------------------------------------------------------------------------------MEFLKGSGDYCQAQHDLYADK-
>Trachinotus_blochii
-----------------------------------------------MVCLHPGA---------VTFSCVRSMNHLLSSHHSCTCMAFARLGGEFCQAQHLLLIVDK
>Scyliorhinus_torazame
-------------------------------------------------------------------------------------MEFLKGSGDYCQAQHAVYADK-
>Cariama_cristata
-------------------------------------------------------------------------------------MAILSVRADFCQAQHSILADK-
>Mustela_putorius_furo
-------------------------------------------------------------------------------------MAILSVRADFCQAQHSVFADK-
>Hynobius_chinensis
-------------------------------------------------------------M-IQPRSSMSNHSPQFCGVLGHTFMEFLKGSGDYCQAQHDLYADK-
>Micropterus_floridanus
-----------------------------------------------MVCLHPGA---------VTFSCVRSMNHLLSSHQSCTCMAFVKLGGEFCQAQHLLLIVDK
>Orcinus_orca
-------------------------------------------------------------------MPSPQLASVFRWNIRCTFMAILSVRADFCQAQHSVSADK-
>Astyanax_mexicanus
---------------------------------------------------------------------MFSLFFCFCVDLGRTFMAFV--SGDFCQAQHLSFENK-
>Calidris_pugnax
-------------------------------------------------------------------------------------MAILSVRADFCQAQHSILADK-
>Callorhinchus_milii
-------------------------------------------------------------------------------------MEFLKGSGDYCQAQHDVYADK-
>Rousettus_aegyptiacus
-------------------------------------------------------------------MPSPQLASVFRWNIRCTFMAILSVRADFCQAQHSVFADK-
>Haplochromis_burtoni
-------------------------------------------------------------------------------------MACV--SGEFCQAQHLTSVEK-
>Pterocles_gutturalis
-------------------------------------------------------------------------------------MEFLKGSGDYCQAQHDLYADK-
>Dasypus_novemcinctus
-------------------------------------------------------------------MPSPQLASVFRWNIRFTFMAILSVRADFCQAQHSVFADK-
>Clupea_harengus
---------------------------------------------------------------------MFNLLFCFCVELGRTYMAFVSVSGDFCQAQHLAFVDK-
>Latimeria_chalumnae
-------------------------------------------------------------M-IKIQSSMSKHIPQFCGVLGHTFMEFLKGSGDYCQAQHDLYADK-
>Colius_striatus
-------------------------------------------------------------------------------------MEFLKGSGDYCQAQHDLYADK-
>Columba_livia
-------------------------------------------------------------------------------------MAILSVRADFCQAQHSTLADK-
>Merops_nubicus
-------------------------------------------------------------------------------------MAILSVRADFCQAQHSPLADK-
>Corynactis_australis
----------------------------------------------------------------------MWRGNRVYGNRAHTFMEFIIDSGDWCQAQHE------
>Alligator_sinensis
-------------------------------------------------------------------------------------MAILSVRADFCQAQHSTLADK-
>Cuculus_canorus
-------------------------------------------------------------------------------------MEFLKGSGDYCQAQHDLYADK-
>Austrofundulus_limnaeus
---------------------------------------------------------------------MFSLLFGFCVDFNRTYMACV--TGEFCQAQHLTSVEK-
>Sarcophilus_harrisii
-------------------------------------------------------------------------------------MEFLKGSGDYCQAQHDLYADK-
>Galeopterus_variegatus
-------------------------------------------------------------------MPSPQLASVFRWNIRCTFMAILSVRADFCQAQHSVFADK-
>Monodelphis_domestica
-------------------------------------------------------------------------------------MAILSVRADFCQAQHSAFADK-
>Macropus_eugenii
-----------------------------------------------------------------------MKAKQFCGVLGHTFMEFLKGSGDYCQAQHDLYADK-
>Esox_lucius
-------------------------------------------------------------------------------------MAYVSVSGDFCQAQHLAFVDK-
>Haliaeetus_leucocephalus
------------------------------------------------------------------------MPRVFCWNIRRTFMAILSVRADFCQAQHSILADK-
>Asterias_amurensis
----------------------------------------------------------------------MSHLTGISSVPVSSYMAFVAISGEYCRSQHK------
>Coturnix_japonica
------------------------------------------------------------------------MPGVFWWNSRRTFMAILSVRAEFCQAQHSALADK-
>Gavia_stellata
-------------------------------------------------------------------------------------MAILSVRADFCQAQHSILADK-
>Acanthogobius_hasta
-------------------------------------------------------------M-IKLQSSMSKHIPQFCGVLGHTFMEFLKGSGDYCQAQHVVDADE-
>Chlamydotis_macqueenii
-------------------------------------------------------------------------------------MAILSVRADFCQAQHRILADK-
>Apteronotus_leptorhynchus
-------------------------------------------------------------------------------------MAFV--SGDFCQAQHLAFVNK-
>Glossoscolex_paulistus
----------------------------------------------------------------------MYTIASLSKASGHHLMALVYLCGEFCQAQHV------
>Cerapachys_biroi
-------------------------------------------------------------------------------------MEFQLDSAEYCQAQHK------
>Sorex_araneus
-------------------------------------------------------------------------------------MAILSVRADFCQAQHSVFADK-
>Ochotona_princeps
-------------------------------------------------------------------------------------MEFLKGSGDYCQAQHDLYADK-
>Protopterus_aethiopicus
-------------------------------------------------------------M-IKLQSSMSKHIPQFCGVIGHTFMEFLKGSGDYCQAQHALYADK-
>Lingula_anatina
-------------------------------------------------------------------------------------MEFVTNSGEYCQAQHK------
>Elephantulus_edwardii
-------------------------------------------------------------M-IRPQSSMSKHIPQFCGVLGHTFMEFLKGSGDYCQAQHDLYADK-
>Ornithorhynchus_anatinus
-------------------------------------------------------------M-IATHSSMSKHSPQFCGVLGHTFMEFLKDSGDYCQAQHDLYADK-
>Tauraco_erythrolophus
-------------------------------------------------------------------------------------MAILSVRADFCQAQHSILADK-
>Perna_viridis
----------------------------------------------------------------------MCRNILFFGVLGHTFMEFVTNSGDYCQAQHV------
>Eptatretus_burgeri
---------------------------------------------------------------------MNRHNAQFCGVLGRTFMEFVKGRGEYCETQHIALADK-
>Octopus_bimaculoides
-------------------------------------------------------------------------------------MEFVTNSGDFCQAQHV------
>Opisthocomus_hoazin
-------------------------------------------------------------------------------------MEFLKGSGDYCQAQHDLYADK-
>Scleropages_formosus
---------------------------------------------------------------------MCGLLFRFCVDLGRPFMAFVSVSGDFCQAQHLASADK-
>Crassostrea_gigas
-----------------------------------------MRDPRGPVTVRGKFVTGF---VLCSSLLNCWQHTEFRSNFGHTYMEFVTNSGDYCQAQHV------
>Corvus_brachyrhynchos
-------------------------------------------------------------------------------------MAILSVRADFCQAQHSTLAAK-
>Pecten_maximus
-------------------------------------------------------------------------------------MEFVTNSGDYCQAQHAL-----
>Egretta_garzetta
-------------------------------------------------------------------------------------MAILSVRADFCQAQHSILADK-
>Lateolabrax_maculatus
-----------------------------------------------MVCLHPGA---------VTSSCVRSMNHLLGSHRGRTCMAFARLGGEFCQAQHLLLIVDK
>Notothenia_coriiceps
-------------------------------------------------------------------------------------MACV--SGEFCQAQHLTSVEK-
>Ceratotherium_simum_simum
-------------------------------------------------------------------------------------MAILSVRADFCQAQHSVFADK-
>Perinereis_aibuhitensis
---------------------------------------------------------------MSSEAGVTTKLHQFFGELGHTFMEFLCNSGEYCQAQHV------
>Picoides_pubescens
-------------------------------------------------------------------------------------MAILSVRADFCQAQHSILADK-
>Platichthys_flesus
---------------------------------------------------------------------MFSLLFNFCVDLSHTYMACV--SGEFCQAQHLTSVEK-
>Homo_sapiens
-------------------------------------------------------------------MPSPQLASVFRWNIRCTFMAILSVRADFCQAQHSIFADK-
>Loxodonta_africana
-------------------------------------------------------------------------------------MAILSVRADFCQAQHSIFADK-
>Fulmarus_glacialis
-------------------------------------------------------------------------------------MAILSVRADFCQAQHSILADK-
>Nestor_notabilis
-------------------------------------------------------------------------------------MAILSVRAEFCQAQHSILADK-
>Leucoraja_erinacea
---------------------------------------------------------------------MRKHIPQFCGVLGHTFMEFLKGSGDYCQAQHDVYADK-
>Peromyscus_maniculatus_baird
-------------------------------------------------------------------MPSPQLASVFRWNIRCTFMAILSVRADFCQAQHSVFADK-
>Oncorhynchus_mykiss
---------------------------------------------------------------------MSSLLFCFCVDLDHTYMAYVSVSGDFCQAQHLAFVDK-
>Tupaia_chinensis
-------------------------------------------------------------------------------------MAILSVRADFCQAQHSVFADK-
>Ctenactis_echinata
----------------------------------------------------------------------MWRFNLVYGSRAHTFMEFIIDSGDWCQAQHE------
>Lepisosteus_oculatus
---------------------------------------------------------------------MSSLPFCFCVDLVRTFMAFLSVSGDFCQAQHLAFADK-
>Sepia_pharaonis
-------------------------------------------MCRNILDLLGGIVKKTDSPSTLAWPHHLWRLVKFFGEFGHTFMEFVTNSGDFCQAQHV------
>Sepioloidea_lineolata
-------------------------------------------------------------------------------------MEFVTNSGDFCQAQHV------
>Trichomycterus_areolatus
---------------------------------------------------------------------MFSLFFCFRADLSRTFMAFV--SGDFCHAQHLTIVNK-
>Trichechus_manatus_latirostr
-------------------------------------------------------------------MPSSQLASVFRWNIRCTFMAILSVRADFCQAQHSIFADK-
>Aptenodytes_forsteri
-------------------------------------------------------------------------------------MAILSVRADFCQAQHSILADK-
>Squalus_acanthias
---------------------------------------------------------------------MNRLVTQFSGISGCSFMEFLKGSRDYCQAQHGLLADK-
>Micrurus_fulvius
------------------------------------------------------------------------MLPVFHLNPSHTFMAILSVRADFCRAQHSTLAEK-
>Tyto_alba
-------------------------------------------------------------------------------------MEFLKGSGDYCQAQHDLYADK-
>Struthio_camelus_australis
-------------------------------------------------------------------------------------MAILSVRADFCQAQHSTLADK-
>Deroceras_reticulatum
----------------------------------------------------------------------MSSFVLFVGEFGHTFMEFVTNSEDFCQAQHYRLVE--
>Chrysemys_picta
-------------------------------------------------------------------------------------MAILSIRADFCQAQHSTLADK-
>Takifugu_rubripes
---------------------------------------------------------------------MFSLLFGFSVDLSHTFMACV--SGEFCQAQHLTSAEK-
>Watasenia_scintillans
-------------------------------------------------------------------------------------MEFVTNSGDFCQAQHV------
>Tinamus_guttatus
------------------------------------------------------------------------MPRVFCWNIRRTFMAILRVRADFCQAQHSTLADK-
>Tetronarce_californica
-------------------------------------------------------------------------------------MEFVRNSSGYCPAQHSIFADK-
>Calypte_anna
-------------------------------------------------------------------------------------MEFLKGSGDYCQAQHDLYADK-
>Apaloderma_vittatum
-------------------------------------------------------------------------------------MEFLKGSGDYCQAQHDLYADK-
>Orycteropus_afer_afer
-------------------------------------------------------------M-IRPQSSMSKHIPQFCGVLGHTFMEFLKGSGDYCQAQHDLYADK-
>Eurhomalea_rufa
-------------------------------------------------------------------------------------MEFLTNSGEYCLAQHVHV----
>Limulus_polyphemus
-------------------------------------------------------------MSSTNDSAKVPLLLPFEVRRHHTLMEFLKTAGEYCQSQHK------
```

HG0001 ENSDARG00000039997 ptp4a3

>Danio_rerio
---MAFLQRTGDYCHAQHLHI--------
>Acipenser_sinensis
--MIKLQSSMSKHIPQFCGVLGHTFMEFLKGSGDYCQAQHEVYADK-----
>Metridium_senile
--MWTGIAYRHSREYTLMEFLTNSGDWCQAQHH----------
>Amia_calva
--MIKLQSSMSKHIPQFCGVLGHTFMEFLKGSGDYCQAQHDVYADK-----
>Kaupichthys_hyoproroides
--MIKLQSSMSKHIPQFCGVLGHTFMEFLKDSGDYCQAQHEVYADK-----
>Anser_cygnoides
---MEFLKGSGDYCQAQHDLYADK-----
>Podocoryna_carnea
---MEFLRGSGDYCFAQHRT---------
>Xenopus_laevis
--MIKIHSSMSKHS-PFCGVLGYTFMEFLKGSGDYCQAQHALYADK-----
>Chaetura_pelagica
---MEFLKGSGDYCQAQHDLYADK-----
>Apteryx_australis_mantelli
---MEFLKGSGDYCQAQHDLYADK-----
>Scapharca_broughtonii
--MFRKILFFGEFGHTFMEFVTNSGDYCQAQHV----------
>Porichthys_notatus
--MIKLQSSMSKHIPQFCGVLGHTFMEFLKGRGDYCQAQHAADADE-----
>Oryzias_latipes
---MEFVRQSGDYCHAQHI----------
>Buceros_rhinoceros_silvestri
---MEFLKGSGDYCQAQHDLYADK-----
>Caprimulgus_carolinensis
---MEFLKGSGDYCQAQHDLYADK-----
>Scyliorhinus_torazame
---MEFLKGSGDYCQAQHAVYADK-----
>Cariama_cristata
---MEFLKGSGDYCQAQHDLYADK-----
>Canis_lupus_familiaris
--MIRPQSSMSKHI-PFCGVLGHTFMEFLKGSGDYCQAQHDLYADK-----
>Hynobius_retardatus
MRAPELGRSEARAQCGGGSVAADTATLPPRSPAARPVWYTARRACPPAT----------RARGT--GRMIQPRSSMSNHSPQFCGVLGHTFMEFLKGSGDYCQAQHDLYADK-----
>Micropterus_floridanus
---MEFVRRSGDYCHAQHI----------
>Physeter_catodon
---MEFLKGSGDYCQAQHDLYADK-----
>Pygocentrus_nattereri
---MAFLQRAGDYCHAQHFQS--------
>Charadrius_vociferus
---MEFLKGSGDYCQAQHDLYADK-----
>Callorhinchus_milii
---MEFLKGSGDYCQAQHDVYADK-----
>Miniopterus_natalensis
--MIRPQSSMSKHI-PFCGVLGHTFMEFLKGSGDYCQAQHDLYADK-----
>Neolamprologus_brichardi
---MEFLKGSGDYCQAQHAAYADE-----
>Pterocles_gutturalis
---MEFLKGSGDYCQAQHDLYADK-----
>Dasypus_novemcinctus
---MEFLKGSGDYCQAQHDLYADK-----
>Clupea_harengus
---MAFLQRAGDYCHAQHPRV--------
>Latimeria_menadoensis
--MIKIQSSMSKHIPQFCGVLGHTFMEFLKGSGDYCQAQHDLYADK-----
>Colius_striatus
---MEFLKGSGDYCQAQHDLYADK-----
>Columba_livia
---MEFLKGSGDYCQAQHDLYADK-----
>Leptosomus_discolor
---MEFLKGSGDYCQAQHDLYADK-----
>Alligator_sinensis
---MEFLKGSGDYCQAQHDLYADK-----
>Cuculus_canorus
---MEFLKGSGDYCQAQHDLYADK-----
>Fundulus_heteroclitus
--MIKLQSSMSKHIP-FCGVLGRTFMEFLKGSGDYCQAQHAAYADE-----
>Sarcophilus_harrisii
---MEFLKGSGDYCQAQHDLYADK-----
>Galeopterus_variegatus
---MEFLKGSGDYCQAQHDLYADK-----
>Monodelphis_domestica
---MEFLKGSGDYCQAQHDLYADK-----
>Macropus_eugenii
---MKAKQFCGVLGHTFMEFLKGSGDYCQAQHDLYADK-----
>Esox_lucius
---MAFLQRTGDYCHAQHPRA--------
>Haliaeetus_leucocephalus
--MIRLQSSMSNHIPQFCGVLGHTFMEFLKGSGDYCQAQHDLYADK-----
>Leptasterias_sp._AR-2014
---MSHS---------TGISSVPVSMAFVSISGEYCRSQHK----------
>Meleagris_gallopavo
---MEFLKGSGDYCQAQHDLYADK-----
>Gavia_stellata
---MEFLKGSGDYCQAQHDLYADK-----
>Acanthogobius_hasta
--MIKLQSSMSKHIPQFCGVLGHTFMEFLKGSGDYCQAQHVVDADE-----
>Balearica_regulorum_gibberic
---MEFLKGSGDYCQAQHDLYADK-----
>Apteronotus_leptorhynchus
---MAFLQRAGDYCHAQHWCC--------
>Condylura_cristata
--MIRPQSSMSKHI-PFCGVLGHTFMEFLKGSGDYCQAQHDLYADK-----
>Oryctolagus_cuniculus
--MIRPQSSMSKHI-PFCGVLGHTFMEFLKGSGDYCQAQHDLYADK-----
>Protopterus_aethiopicus
--MIKLQSSMSKHIPQFCGVIGHTFMEFLKGSGDYCQAQHALYADK-----
>Lingula_anatina
--MQFFGEFGHTFMEFVTNSGEYCQAQHK----------
>Elephantulus_edwardii
--MIRPQSSMSKHIPQFCGVLGHTFMEFLKGSGDYCQAQHDLYADK-----
>Ornithorhynchus_anatinus
--MIATHSSMSKHS-PFCGVLGHTFMEFLKDSGDYCQAQHDLYADK-----
>Perna_viridis
--MCRNILFFGVLGHTFMEFVTNSGDYCQAQHV----------
>Opisthocomus_hoazin
---MEFLKGSGDYCQAQHDLYADK-----
>Plecoglossus_altivelis
---MEFLQGSGDYCHAQHPSI--------
>Scleropages_formosus
---MCGLLFRFCVDLGRPFMAFVSVSGDFCQAQHLASADK-----
>Crassostrea_gigas
-------------------------MRDPRGP------------------------VTVR-GKFVTGFVLCSSLLNCWQHTEFRSNFGHTYMEFVTNSGDYCQAQHV----------
>Endeis_spinosa
---MEFLKESGDYCYCRAQHV--------
>Lepidothrix_coronata
--MIRLQSSMSNHIPQFCGVLGHTFMEFLKGSGDYCQAQHDLYADK-----
>Pecten_maximus
---MEFVTNSGDYCQAQHAL---------
>Egretta_garzetta
---MEFLKGSGDYCQAQHDLYADK-----
>Sebastes_caurinus
--MIKLQSSMSKHIP-FCGVLGHTFMEFLKGSGDYCQAQHAAYADE-----
>Equus_przewalskii
--MIRPQSSMSKHSPQFCGVLGHTFMEFLKGSGDYCQAQHDLYADK-----
>Extatosoma_tiaratum
---MHMEFLLSSGEYCQAQHK----------
>Perinereis_aibuhitensis
---MSSEAGVTTKLHQFFGELGHTFMEFLCNSGEYCQAQHV----------
>Cynoglossus_semilaevis
---MEFLKGSGDYCQAQHAAYADE-----
>Gorilla_gorilla
--MIRPQSSMSKHIPQFCGVLGHTFMEFLKGSGDYCQAQHDLYADK-----
>Loxodonta_africana
--MIRPQSSMSKHI-PFCGVLGHTFMEFLKGSGDYCQAQHDLYADK-----
>Fulmarus_glacialis
---MEFLKGSGDYCQAQHDLYADK-----
>Melopsittacus_undulatus
---MEFLKGSGDYCQAQHDLYADK-----
>Leucoraja_erinacea
---MRKHIPQFCGVLGHTFMEFLKGSGDYCQAQHDVYADK-----
>Rattus_norvegicus
--MIRPQSSMSRHIP-FCGVLGHTFMEFLKGSGDYCQAQHDLYADK-----
>Salmo_salar
---MAFLQRTGDYCHAQHPRA--------
>Tityus_serrulatus
---MEYLKTTGEYCRAQHK----------
>Lepisosteus_oculatus
------------MACEG-----------------SFSWFRAETVISVSSSGSLLRVIPLRDTDF--IRSRKQHDTKVRAAKTSCTVTGGSLMEFLRGSGDYCHAQHHSV--------
>Sepia_pharaonis
-------------MCRN--------ILDLLGG------------------------IVKK-TD---SPSTLAWPHHLWRLVKFFGEFGHTFMEFVTNSGDFCQAQHV----------
>Ictalurus_punctatus
---MLMEFLQGSGDYCHAQHRNA--------
>Trichechus_manatus_latirostr
--MIRPQSSMSKHIPQFCGVLGHTFMEFLKGSGDYCQAQHDLYADK-----
>Aptenodytes_forsteri
---MEFLKGSGDYCQAQHDLYADK-----
>Streblospio_benedicti
--MCRIILFFGQIGYTFMEFLCNSGEYCQAQHVAIPSVLSRLH
>Crotalus_horridus
--MIRLQSSMSNHIPQFCGVLGHTFMEFLKGSGDYCQAQHGLYADK-----
>Tyto_alba
---MEFLKGSGDYCQAQHDLYADK-----
>Struthio_camelus_australis
---MEFLKGSGDYCQAQHDLYADK-----
>Chrysemys_picta
--MIRLQSSMSNHLPQFCGVLGHTFMEFLKGSGDYCQAQHDLYADK-----
>Takifugu_rubripes
--MIKLQSSMSKHIPQFCGVLGHTFMEFLKGSGDYCQAQHAVYADE-----
>Tinamus_guttatus
---MEFLKGSGDYCQAQHDLYADK-----
>Calypte_anna
---MEFLKGSGDYCQAQHDLYADK-----
>Apaloderma_vittatum
---MEFLKGSGDYCQAQHDLYADK-----
>Orycteropus_afer_afer
--MIRPQSSMSKHIPQFCGVLGHTFMEFLKGSGDYCQAQHDLYADK-----
>Ruditapes_philippinarum
--MNRIILFFGELGHTFMEFLTNSGEYCLAQHVHV--------
>Limulus_polyphemus
---MEFLKTAGEYCQSQHK----------
>Palythoa_caribaeorum
---MRRDYLAYDRGRTLMEFLIDSGDWCQAQHE----------

HG0001 ENSDARG00000054814 PTP4A3

```
>Danio_rerio
----------------------------------------------------------------------------------MEFWQGSGDYCHAQHHTA--------
>Acipenser_sinensis
-----------------------------------------------------------MIKLQSSMSKHIPQFCGVLGHTFMEFLKGSGDYCQAQHEVYADK-----
>Metridium_senile
---------------------------------------------------------MWTG----------IAYRHSREYTLMEFLTNSGDWCQAQHH----------
>Amia_calva
----------------------------------------------------------------------------------MESSRGRGDYCHAQHPTA--------
>Kaupichthys_hyoproroides
-----------------------------------------------------------MIKLQSSMSKHIPQFCGVLGHTFMEFLKDSGDYCQAQHEVYADK-----
>Anser_cygnoides
----------------------------------------------------------------------------------MEFLKGSGDYCQAQHDLYADK-----
>Podocoryna_carnea
----------------------------------------------------------------------------------MEFLRGSGDYCFAQHRT---------
>Xenopus_tropicalis
MLFTGHGEEPQGRRSGPRQLSPDHR-ALRL---GDSSLLYAPSPPAPLLFHRLFLGTGCMIKIQSSMSKHS-PFCGVLGHTFMEFLKGSGDYCQAQHELYADK-----
>Chaetura_pelagica
----------------------------------------------------------------------------------MEFLKGSGDYCQAQHDLYADK-----
>Apteryx_australis_mantelli
----------------------------------------------------------------------------------MEFLKGSGDYCQAQHDLYADK-----
>Stegodyphus_mimosarum
---------------------------------------------------M-LDASMCID----------IAVNQIQTFLLMEVLVGSDEYCRAQHK----------
>Machilis_hrabei
---------------------------------------------------------MCKDLVFLG----------ETALTYMEFQLDSGEYCQAQHK----------
>Scapharca_broughtonii
---------------------------------------------------------MFRK----------ILFFGEFGHTFMEFVTNSGDYCQAQHV----------
>Porichthys_notatus
-----------------------------------------------------------MIKLQSSMSKHIPQFCGVLGHTFMEFLKGRGDYCQAQHAADADE-----
>Oryzias_latipes
-----------------------------------------------------------MIKLQSSMSKHIPQFCGVLGHTFMEFLKGSGDYCQAQHAAYADE-----
>Buceros_rhinoceros_silvestri
----------------------------------------------------------------------------------MEFLKGSGDYCQAQHDLYADK-----
>Caprimulgus_carolinensis
----------------------------------------------------------------------------------MEFLKGSGDYCQAQHDLYADK-----
>Scyliorhinus_torazame
----------------------------------------------------------------------------------MEFLKGSGDYCQAQHAVYADK-----
>Cariama_cristata
----------------------------------------------------------------------------------MEFLKGSGDYCQAQHDLYADK-----
>Mustela_putorius_furo
-----------------------------------------------------------MIRPQSSMSKHIPQFCGVLGHTFMEFLKGSGDYCQAQHDLYADK-----
>Alatina_alata
---------------------------------------------------M-RRPCLCT---------------CFCGKTFMEFLKDSGDWCQSQHK----------
>Hynobius_retardatus
MRAPELGRSEARAQCGGGSVAADTA-TLPPRSPAARPVWYTARRACP--PATRARGTGRMIQPRSSMSNHSPQFCGVLGHTFMEFLKGSGDYCQAQHDLYADK-----
>Micropterus_salmoides
----------------------------------------------------------------------------------MEFVRRSGDYCHAQHI----------
>Orcinus_orca
----------------------------------------------------------------------------------MEFLKGSGDYCQAQHDLYADK-----
>Astyanax_mexicanus
----------------------------------------------------------------------------------MEFLQGSGDYCHAQHRNA--------
>Calidris_pugnax
----------------------------------------------------------------------------------MEFLKGSGDYCQAQHDLYADK-----
>Callorhinchus_milii
----------------------------------------------------------------------------------MEFLKGSGDYCQAQHDVYADK-----
>Eptesicus_fuscus
-----------------------------------------------------------MIRPQSSMSKHIPQFCGVLGHTFMEFLKGSGDYCQAQHDLYADK-----
>Neolamprologus_brichardi
----------------------------------------------------------------------------------MEFLKGSGDYCQAQHAAYADE-----
>Pterocles_gutturalis
----------------------------------------------------------------------------------MEFLKGSGDYCQAQHDLYADK-----
>Dasypus_novemcinctus
-----------------------------------------------------------MIRPQSSMSKHI-PFCGVLGHTFMEFLKGSGDYCQAQHDLYADK-----
>Alosa_alosa
-----------------------------------------------------------MIKLQSSMSKHIPQFCGVLGHTFMEFLKGSGDYCQAQHGVYADE-----
>Latimeria_chalumnae
-----------------------------------------------------------MIKIQSSMSKHIPQFCGVLGHTFMEFLKGSGDYCQAQHDLYADK-----
>Colius_striatus
----------------------------------------------------------------------------------MEFLKGSGDYCQAQHDLYADK-----
>Columba_livia
----------------------------------------------------------------------------------MEFLKGSGDYCQAQHDLYADK-----
>Leptosomus_discolor
----------------------------------------------------------------------------------MEFLKGSGDYCQAQHDLYADK-----
>Alligator_mississippiensis
-----------------------------------------------------------MIRLQSSMSNRR-PFCGVLGHTFMEFLKGSGDYCQAQHDLYADK-----
>Cuculus_canorus
----------------------------------------------------------------------------------MEFLKGSGDYCQAQHDLYADK-----
>Cyprinodon_variegatus
-----------------------------------------------------------MIKLQSSMSKHIP-FCGVLGRTFMEFLKGSGDYCQAQHAAYADE-----
>Sarcophilus_harrisii
----------------------------------------------------------------------------------MEFLKGSGDYCQAQHDLYADK-----
>Cancer_borealis
--------------------------------------------------MK-FKYCGDNN----------AQLSSGRGYTLMELLKGSGEFCKAQYHIK--------
>Galeopterus_variegatus
----------------------------------------------------------------------------------MEFLKGSGDYCQAQHDLYADK-----
>Monodelphis_domestica
-----------------------------------------------------------MIRPQSSMSKHI-PFCGVLGHTFMEFLKGSGDYCQAQHDLYADK-----
>Occasjapyx_japonicus
---------------------------------------------------------MCRDLVCLG----------KIGLTFMEFFLDSGEYCQAQHK----------
>Macropus_eugenii
--------------------------------------------------------------------MKAKQFCGVLGHTFMEFLKGSGDYCQAQHDLYADK-----
>Rhagoletis_zephyria
------------------------------------------------------------MVVTRKQISDRPKELARPVPTSMEFHLDSAEYCQAQHK----------
>Isonychia_bicolor
---------------------------------------------------------MCRDLVFLG----------EIGLTYMEFQLDSGEYCQAQHK----------
>Esox_lucius
------------MASRGSLNWLRAETAICVRDS-GRLRVALPNRAPGLILTGTLL---------------NIKLCVPAGRSLMEFFQGSGDYCHAQHPSA--------
>Meganyctiphanes_norvegica
--------------------------------------------------MK-FKYCGNNN----------TQLSCGRGYTLMELLKGSGEFCKAQYHIK--------
>Haliaeetus_leucocephalus
-----------------------------------------------------------MIRLQSSMSNHIPQFCGVLGHTFMEFLKGSGDYCQAQHDLYADK-----
>Meleagris_gallopavo
----------------------------------------------------------------------------------MEFLKGSGDYCQAQHDLYADK-----
>Gavia_stellata
----------------------------------------------------------------------------------MEFLKGSGDYCQAQHDLYADK-----
>Acanthogobius_hasta
-----------------------------------------------------------MIKLQSSMSKHIPQFCGVLGHTFMEFLKGSGDYCQAQHVVDADE-----
>Balearica_regulorum_gibberic
----------------------------------------------------------------------------------MEFLKGSGDYCQAQHDLYADK-----
>Apteronotus_leptorhynchus
----------------------------------------------------------------------------------MAFLQRAGDYCHAQHWCC--------
>Trialeurodes_vaporariorum
---------------------------------------------------------MCVVVVNVRE---------LCCLPLMEFQLDSSEYCQAQHK----------
>Dinoponera_quadriceps
----------------------------------------------------------------------------------MEFQLDSAEYCQAQHK----------
>Condylura_cristata
-----------------------------------------------------------MIRPQSSMSKHIP-FCGVLGHTFMEFLKGSGDYCQAQHDLYADK-----
>Oryctolagus_cuniculus
-----------------------------------------------------------MIRPQSSMSKHIP-FCGVLGHTFMEFLKGSGDYCQAQHDLYADK-----
>Rhodinia_newara
----------------------------------------------------------------------------------MEFHLDSAEYCQAQHK----------
>Protopterus_aethiopicus
-----------------------------------------------------------MIKLQSSMSKHIPQFCGVIGHTFMEFLKGSGDYCQAQHALYADK-----
>Lingula_anatina
----------------------------------------------------------------------------------MEFVTNSGEYCQAQHK----------
>Elephantulus_edwardii
-----------------------------------------------------------MIRPQSSMSKHIPQFCGVLGHTFMEFLKGSGDYCQAQHDLYADK-----
>Metallyticus_splendidus
---------------------------------------------------------MCMALVFLG----------ETVLTYMEFQLDSGEYCQAQHK----------
>Ornithorhynchus_anatinus
-----------------------------------------------------------MIATHSSMSKHS-PFCGVLGHTFMEFLKDSGDYCQAQHDLYADK-----
>Perna_viridis
---------------------------------------------------------MCRN----------ILFFGVLGHTFMEFVTNSGDYCQAQHV----------
>Eptatretus_burgeri
---------------------------------------------------------MNR---------HNAQFCGVLGRTFMEFVKGRGEYCETQHIALADK-----
>Hapalochlaena_maculosa
-------------MCRNILDLLGGI---VEK----------TDSP---STLA-WPHHLCRS----------IKFFGEFGHTFMEFVTNSGDFCQAQHV----------
>Epiophlebia_superstes
----------------------------------------------------------------------------------MEFQLDSGEYCQAQHK----------
>Opisthocomus_hoazin
----------------------------------------------------------------------------------MEFLKGSGDYCQAQHDLYADK-----
>Prosarthria_teretrirostris
---------------------------------------------------------MCMAIVSRG----------EIVLTYMEFQLDSGEYCQAQHK----------
>Plecoglossus_altivelis
----------------------------------------------------------------------------------MEFLQGSGDYCHAQHPSI--------
>Crassostrea_gigas
-------------MCNNVLLMRDPRGPVTVR----------GKFVTGFVLCS-SLLNCWQH----------TEFRSNFGHTYMEFVTNSGDYCQAQHV----------
>Pseudopodoces_humilis
-----------------------------------------------------------MIRLQSSMSNHIPQFCGVLGHTFMEFLKGSGDYCQAQHDLYADK-----
>Pecten_maximus
----------------------------------------------------------------------------------MEFVTNSGDYCQAQHAL---------
>Egretta_garzetta
----------------------------------------------------------------------------------MEFLKGSGDYCQAQHDLYADK-----
>Sebastes_caurinus
-----------------------------------------------------------MIKLQSSMSKHIP-FCGVLGHTFMEFLKGSGDYCQAQHAAYADE-----
>Equus_przewalskii
-----------------------------------------------------------MIRPQSSMSKHSPQFCGVLGHTFMEFLKGSGDYCQAQHDLYADK-----
>Extatosoma_tiaratum
--------------------------------------------------------------------------------MHMEFLLSSGEYCQAQHK----------
>Menopon_gallinae
--------------------------------------------------MN-FRFSGCMAL----------KNFHSTSSRLMEFFLDSAEYCQAQHTK---------
>Perinereis_aibuhitensis
-------------------------------------------------MSS-EAGVTTKL----------HQFFGELGHTFMEFLCNSGEYCQAQHV----------
>Leuctra_sp._AD-2013
---------------------------------------------------------MCMALVLVG----------EIVLTYMEFQLDSGEYCQAQHRK---------
>Cynoglossus_semilaevis
----------------------------------------------------------------------------------MEFLKGSGDYCQAQHAAYADE-----
>Homo_sapiens
----------------------------------------------------------------------------------MEFLKGSGDYCQAQHDLYADK-----
>Loxodonta_africana
-----------------------------------------------------------MIRPQSSMSKHI-PFCGVLGHTFMEFLKGSGDYCQAQHDLYADK-----
>Fulmarus_glacialis
----------------------------------------------------------------------------------MEFLKGSGDYCQAQHDLYADK-----
>Melopsittacus_undulatus
----------------------------------------------------------------------------------MEFLKGSGDYCQAQHDLYADK-----
>Ectopsocus_briggsi
----------------------------------------------------------------------------------MEFFLDSAEYCQAQHRK---------
>Leucoraja_erinacea
------------------------------------------------------------------MRKHIPQFCGVLGHTFMEFLKGSGDYCQAQHDVYADK-----
>Mus_musculus
-----------------------------------------------------------MIRPQSSMSRHIPQFCGVLGHTFMEFLKGSGDYCQAQHDLYADK-----
>Salmo_salar
----------------------------------------------------------------------------------MEFLQGSGDYCHAQHLSA--------
>Porites_astreoides
-------------------------------------------------------------------MWSSNLEFGSRAHTFMEFIIDSGDWCQAQHE----------
>Tityus_serrulatus
----------------------------------------------------------------------------------MEYLKTTGEYCRAQHK----------
>Lepisosteus_oculatus
------------MACEGSFSWFRAETVISVSSS-GSLLRVIPLRDTDFIRSRKQHDTKVR---------AAKTSCTVTGGSLMEFLRGSGDYCHAQHHSV--------
>Sepia_pharaonis
-------------MCRNILDLLGGI---VKK----------TDSP---STLA-WPHHLWRL----------VKFFGEFGHTFMEFVTNSGDFCQAQHV----------
>Sepioloidea_lineolata
----------------------------------------------------------------------------------MEFVTNSGDFCQAQHV----------
>Ictalurus_punctatus
--------------------------------------------------------------------------------MLMEFLQGSGDYCHAQHRNA--------
>Trichechus_manatus_latirostr
-----------------------------------------------------------MIRPQSSMSKHIPQFCGVLGHTFMEFLKGSGDYCQAQHDLYADK-----
>Aptenodytes_forsteri
----------------------------------------------------------------------------------MEFLKGSGDYCQAQHDLYADK-----
>Streblospio_benedicti
---------------------------------------------------------MCRI----------ILFFGQIGYTFMEFLCNSGEYCQAQHVAIPSVLSRLH
>Squalus_acanthias
---------------------------------------------------------MNR---------LVTQFSGISGCSFMEFLKGSRDYCQAQHGLLADK-----
>Crotalus_horridus
-----------------------------------------------------------MIRLQSSMSNHIPQFCGVLGHTFMEFLKGSGDYCQAQHGLYADK-----
>Tyto_alba
----------------------------------------------------------------------------------MEFLKGSGDYCQAQHDLYADK-----
>Struthio_camelus_australis
----------------------------------------------------------------------------------MEFLKGSGDYCQAQHDLYADK-----
>Chrysemys_picta
-----------------------------------------------------------MIRLQSSMSNHLPQFCGVLGHTFMEFLKGSGDYCQAQHDLYADK-----
>Takifugu_rubripes
-----------------------------------------------------------MIKLQSSMSKHIPQFCGVLGHTFMEFLKGSGDYCQAQHAVYADE-----
>Watasenia_scintillans
----------------------------------------------------------------------------------MEFVTNSGDFCQAQHV----------
>Tinamus_guttatus
----------------------------------------------------------------------------------MEFLKGSGDYCQAQHDLYADK-----
>Hydroptila_sp._AD-2013
----------------------------------------------------------------------------------MEFRLDSGEYCQAQHK----------
>Calypte_anna
----------------------------------------------------------------------------------MEFLKGSGDYCQAQHDLYADK-----
>Apaloderma_vittatum
----------------------------------------------------------------------------------MEFLKGSGDYCQAQHDLYADK-----
>Orycteropus_afer_afer
-----------------------------------------------------------MIRPQSSMSKHIPQFCGVLGHTFMEFLKGSGDYCQAQHDLYADK-----
>Ruditapes_philippinarum
---------------------------------------------------------MNRI----------ILFFGELGHTFMEFLTNSGEYCLAQHVHV--------
>Limulus_polyphemus
----------------------------------------------------------------------------------MEFLKTAGEYCQSQHK----------
>Palythoa_caribaeorum
--------------------------------------------------------------------MRRDYLAYDRGRTLMEFLIDSGDWCQAQHE----------
>Tricholepidion_gertschi
----------------------------------------------------------------------------------MEFQLDSGEYCQAQHK----------
```

HG0001 ENSDARG00000087443 ptp4a2a

>Danio_rerio
--MQSSQLVFC-S-LIEGCTFMAFVSVSGDFCQAQHLVIV-DK----
>Acipenser_sinensis
--MIKLQS--SMSKHIPQFCGVLGHTFMEFLKGSGDYCQAQHEVYA-DK----
>Metridium_senile
---MWTGIA-YRHSREYTLMEFLTNSGDWCQAQHH----------
>Amia_calva
--MIKLQS--SMSKHIPQFCGVLGHTFMEFLKGSGDYCQAQHDVYA-DK----
>Hyalella_azteca
---MKLKY-C--GGNCNQL-VLSGRGYSLMELLQASGEFCKAQYTVLLPHD----
>Channa_argus
-------------------MSQQTEEAPPQQQF---QISDENSSCHIEEEQKGDLTVVSAL------PPLSLRSHGISMFILLFG-FCVDLSRTYMACV--SGEFCQAQHLTSV-EK----
>Kaupichthys_hyoproroides
--MIKLQS--SMSKHIPQFCGVLGHTFMEFLKDSGDYCQAQHEVYA-DK----
>Anser_cygnoides
---MAILSVRADFCQAQHSILA-DK----
>Podocoryna_carnea
--MNSLLSC-FCVDLGRTFMAFLSVSGDFCQAQHLAFA-DK----
>Xenopus_laevis
--MIKIHS--SMSKHS-PFCGVLGYTFMEFLKGSGDYCQAQHALYA-DK----
>Chaetura_pelagica
---MEFLKGSGDYCQAQHDLYA-DK----
>Apteryx_australis_mantelli
---MEFLKGSGDYCQAQHDLYA-DK----
>Meinertellus_cundinamarcensi
---MCRDLV-FLGETGLTYMEFQLDSGEYCQAQHK----------
>Scapharca_broughtonii
---MFRK-ILFFGEFGHTFMEFVTNSGDYCQAQHV----------
>Menidia_menidia
--MFNLLFG-FCVDVSRSYMACV--TGEFCQAQHLTSA-EK----
>Porichthys_notatus
--MIKLQS--SMSKHIPQFCGVLGHTFMEFLKGRGDYCQAQHAADA-DE----
>Oryzias_melastigma
--MIKLQS--SMSKHIPQFCGVLGHTFMEFLKGSGDYCQAQHAAYA-DE----
>Tripterygion_delaisi
---MVCL------HPGAVTFSC--IHSMNPS-Y--QHSYTCMAFARLGGEFCQAQHLLIIVDK----
>Buceros_rhinoceros_silvestri
---MEFLKGSGDYCQAQHDLYA-DK----
>Caprimulgus_carolinensis
---MEFLKGSGDYCQAQHDLYA-DK----
>Trachinotus_blochii
---MVCL------HPGAVTFSC--VRSMNHL-LSSHHSCTCMAFARLGGEFCQAQHLLLIVDK----
>Scyliorhinus_torazame
---MEFLKGSGDYCQAQHAVYA-DK----
>Cariama_cristata
---MAILSVRADFCQAQHSILA-DK----
>Canis_lupus_familiaris
---MAILSVRADFCQAQHSVFA-DK----
>Alatina_alata
---MRRPCL-CTCFCGKTFMEFLKDSGDWCQSQHK----------
>Hynobius_retardatus
MRAPELGRSEARAQCGGGSVAADTATLPPRSPAARPVWYTARRACPPATRARG--------------TGRMIQPRS--SMSNHSPQFCGVLGHTFMEFLKGSGDYCQAQHDLYA-DK----
>Micropterus_floridanus
---MVCL------HPGAVTFSC--VRSMNHL-LSSHQSCTCMAFVKLGGEFCQAQHLLLIVDK----
>Orcinus_orca
--MPSPQLASV-FRWNIRCTFMAILSVRADFCQAQHSVSA-DK----
>Astyanax_mexicanus
--MRRSRLF-FTHTESCTFMAFV--SGDFCQAQHLVIV-DK----
>Calidris_pugnax
---MPRV-SRWNIRRTFMAILSVRADFCQAQHSILA-DK----
>Callorhinchus_milii
---MEFLKGSRDYCQAQHGVLA-DK----
>Rousettus_aegyptiacus
--MPSPQLASV-FRWNIRCTFMAILSVRADFCQAQHSVFA-DK----
>Oreochromis_niloticus
---MVCL------HPGAVTFSC--VRSMNHL-LSSHHGCTCMAFARLGGEFCQAQHLLLIVDK----
>Pterocles_gutturalis
---MEFLKGSGDYCQAQHDLYA-DK----
>Eucidaris_tribuloides
---MCKHTE-VLAVINKPFMALVLDSGEFCQSQHK----------
>Dasypus_novemcinctus
---MEFLKGSGDYCQAQHDLYA-DK----
>Clupea_harengus
--MRNS-YRVF-FTPDESCTFMAFVSVSGDFCQAQHLVIV-DK----
>Latimeria_chalumnae
--MIKIQS--SMSKHIPQFCGVLGHTFMEFLKGSGDYCQAQHDLYA-DK----
>Colius_striatus
---MEFLKGSGDYCQAQHDLYA-DK----
>Columba_livia
---MEFLKGSGDYCQAQHDLYA-DK----
>Leptosomus_discolor
---MEFLKGSGDYCQAQHDLYA-DK----
>Corynactis_australis
---MWRGNR-VYGNRAHTFMEFIIDSGDWCQAQHE----------
>Alligator_mississippiensis
--MIRLQS--SMSNRR-PFCGVLGHTFMEFLKGSGDYCQAQHDLYA-DK----
>Cuculus_canorus
---MEFLKGSGDYCQAQHDLYA-DK----
>Poeciliopsis_prolifica
---MAFARLGGAFCQAQHLLLIVDK----
>Sarcophilus_harrisii
---MEFLKGSGDYCQAQHDLYA-DK----
>Sclerodactyla_briareus
---MYDRRKPSISERFYSTSAVDSPNNITPMY--RFNTA---SQLHTSLSWMAFLGAFGEFCRSQHT----------
>Galeopterus_variegatus
--MPSPQLASV-FRWNIRCTFMAILSVRADFCQAQHSVFA-DK----
>Monodelphis_domestica
--MIRPQS--SMSKHI-PFCGVLGHTFMEFLKGSGDYCQAQHDLYA-DK----
>Occasjapyx_japonicus
---MCRDLV-CLGKIGLTFMEFFLDSGEYCQAQHK----------
>Macropus_eugenii
---MKAKQFCGVLGHTFMEFLKGSGDYCQAQHDLYA-DK----
>Evechinus_chloroticus
---MCKPAE-VSAISNLAFMASVLDSGEYCQSQHK----------
>Ephemera_danica
---MEFQLDSGEYCQAQHK----------
>Esox_lucius
---MAYVSVSGDFCQAQHLAFV-DK----
>Aquila_chrysaetos_canadensis
---MPRV-FCWNIRRTFMAILSVRADFCQAQHSILA-DK----
>Asterias_amurensis
---MSHLTG-ISSVPVSSYMAFVAISGEYCRSQHK----------
>Coturnix_japonica
--MIRLQS--SMSNHI-PFCGVLGHTFMEFLKGSGDYCQAQHDLYA-DK----
>Gavia_stellata
---MAILSVRADFCQAQHSILA-DK----
>Acanthogobius_hasta
--MIKLQS--SMSKHIPQFCGVLGHTFMEFLKGSGDYCQAQHVVDA-DE----
>Chlamydotis_macqueenii
---MAILSVRADFCQAQHRILA-DK----
>Apteronotus_leptorhynchus
---MAFV--SGDFCQAQHLAFV-NK----
>Glossoscolex_paulistus
---MYTIAS-LSKASGHHLMALVYLCGEFCQAQHV----------
>Planococcus_citri
---MDIV-TKSALCFTFMEFLLDSSEYCQAQHK----------
>Sorex_araneus
---MAILSVRADFCQAQHSVFA-DK----
>Oryctolagus_cuniculus
--MIRPQS--SMSKHI-PFCGVLGHTFMEFLKGSGDYCQAQHDLYA-DK----
>Protopterus_aethiopicus
--MIKLQS--SMSKHIPQFCGVIGHTFMEFLKGSGDYCQAQHALYA-DK----
>Lingula_anatina
---MEFVTNSGEYCQAQHK----------
>Elephantulus_edwardii
--MIRPQS--SMSKHIPQFCGVLGHTFMEFLKGSGDYCQAQHDLYA-DK----
>Metallyticus_splendidus
---MCMALV-FLGETVLTYMEFQLDSGEYCQAQHK----------
>Ornithorhynchus_anatinus
--MPSPRLASA-FRWNIRCTFMAILSVRADFCQAQHSVCA-DK----
>Tauraco_erythrolophus
---MAILSVRADFCQAQHSILA-DK----
>Cypridininae_sp._BMR-2011
--MEDSETSLLYKY--RLCMRPQ-VLAGVCCSFMEFVLADGEYCQAQHK----------
>Perna_viridis
---MCRN-ILFFGVLGHTFMEFVTNSGDYCQAQHV----------
>Eptatretus_burgeri
--MNRHNAQ-FCGVLGRTFMEFVKGRGEYCETQHIALA-DK----
>Octopus_bimaculoides
---MEFVTNSGDFCQAQHV----------
>Epiophlebia_superstes
---MEFQLDSGEYCQAQHK----------
>Opisthocomus_hoazin
---MEFLKGSGDYCQAQHDLYA-DK----
>Prosarthria_teretrirostris
---MCMAIV-SRGEIVLTYMEFQLDSGEYCQAQHK----------
>Plecoglossus_altivelis
---MEFLQGSGDYCHAQHPSI--------
>Scleropages_formosus
---MAFVSVSGDFCQAQHLASA-DK----
>Crassostrea_gigas
---MRDPRGPVTVRGKF------VTGFVLCSSLLNCWQHTE-FRSNFGHTYMEFVTNSGDYCQAQHV----------
>Parus_major
--MIRLQS--SMSNHI-PFCGVLGHTFMEFLKGSGDYCQAQHDLYA-DK----
>Pecten_maximus
---MEFVTNSGDYCQAQHAL---------
>Egretta_garzetta
---MAILSVRADFCQAQHSILA-DK----
>Lateolabrax_maculatus
---MVCL------HPGAVTSSC--VRSMNHL-LGSHRGRTCMAFARLGGEFCQAQHLLLIVDK----
>Sebastes_caurinus
--MIKLQS--SMSKHIP-FCGVLGHTFMEFLKGSGDYCQAQHAAYA-DE----
>Ceratotherium_simum_simum
---MAILSVRADFCQAQHSVFA-DK----
>Perinereis_aibuhitensis
---MSSE-----AG--VTTKLHQ-FFGELGHTFMEFLCNSGEYCQAQHV----------
>Picoides_pubescens
---MAILSVRADFCQAQHSILA-DK----
>Perla_marginata
---MEFQLDSGEYCQAQHK----------
>Cynoglossus_semilaevis
---MVCP------HHRAVTTPC--VRSMNNL-LSSHDSCTCMAFARVGGEFCQAQHLLLIVDK----
>Cercocebus_atys
--MPSPQLASV-FRWNIRCTFMAILSVRADFCQAQHSVFA-DK----
>Loxodonta_africana
--MIRPQS--SMSKHI-PFCGVLGHTFMEFLKGSGDYCQAQHDLYA-DK----
>Fulmarus_glacialis
---MAILSVRADFCQAQHSILA-DK----
>Nestor_notabilis
---MAILSVRAEFCQAQHSILA-DK----
>Leucoraja_erinacea
---MRKHIPQFCGVLGHTFMEFLKGSGDYCQAQHDVYA-DK----
>Rhopilema_esculentum
--MNSYHLL-LSKYFGKPFMEFLKDSAEWCKAQHVE---------
>Heterocephalus_glaber
--MPSPQLASV-FRWNIRCTFMAILSVRADFCQAQHSVFA-DK----
>Salmo_salar
--MFNLLFC-FCVDLGHTYMAYVSVSGDFCQAQHLAFV-DK----
>Tupaia_chinensis
---MAILSVRADFCQAQHSVFA-DK----
>Porites_astreoides
---MWSSNL-EFGSRAHTFMEFIIDSGDWCQAQHE----------
>Aurelia_aurita
--MTSYHLQ-L-KCFGETFMEFLKDSAEWCKAQHVE---------
>Lepisosteus_oculatus
--MSSLPFC-FCVDLVRTFMAFLSVSGDFCQAQHLAFA-DK----
>Sepia_pharaonis
---MCRNILDLLGGIVKKTD-------SPSTLAWPH--HLWRLVK-FFGEFGHTFMEFVTNSGDFCQAQHV----------
>Sepioloidea_lineolata
---MEFVTNSGDFCQAQHV----------
>Ictalurus_punctatus
--MQSSRHF-FPCTESCTFMAFVSVSGDFCQAQHLVIV-DK----
>Trichechus_manatus_latirostr
--MPSSQLASV-FRWNIRCTFMAILSVRADFCQAQHSIFA-DK----
>Aptenodytes_forsteri
---MAILSVRADFCQAQHSILA-DK----
>Echinaster_spinulosus
---MCQLTAILNTISVSTLMAFAAVSGEYCRSQHK----------
>Streblospio_benedicti
---MCRIIL-FFGQIGYTFMEFLCNSGEYCQAQHVAIPSVLSRLH
>Squalus_acanthias
---MNRLVTQFSGISGCSFMEFLKGSRDYCQAQHGLLA-DK----
>Python_bivittatus
--MIRLQS--SMSNHI-PFCGVLGHTFMEFLKGSGDYCQAQHGLYA-DK----
>Tyto_alba
---MEFLKGSGDYCQAQHDLYA-DK----
>Struthio_camelus_australis
---MAILSVRADFCQAQHSTLA-DK----
>Deroceras_reticulatum
---MSSFVL-FVGEFGHTFMEFVTNSEDFCQAQHYRLVE------
>Sphaerechinus_granularis
---MCKPAE-DSAISNQAFMATVLDSGEYCQSQHK----------
>Pelodiscus_sinensis
--MIRLQS--SMSNHI-PFCGVLGHTFMEFLKGSGDYCQAQHDLYA-DK----
>Takifugu_rubripes
--MIKLQS--SMSKHIPQFCGVLGHTFMEFLKGSGDYCQAQHAVYA-DE----
>Watasenia_scintillans
---MEFVTNSGDFCQAQHV----------
>Tinamus_guttatus
---MPRV-FCWNIRRTFMAILRVRADFCQAQHSTLA-DK----
>Tetronarce_californica
---MEFVRNSSGYCPAQHSIFA-DK----
>Hydroptila_sp._AD-2013
---MEFRLDSGEYCQAQHK----------
>Calypte_anna
---MEFLKGSGDYCQAQHDLYA-DK----
>Apaloderma_vittatum
---MEFLKGSGDYCQAQHDLYA-DK----
>Orycteropus_afer_afer
--MIRPQS--SMSKHIPQFCGVLGHTFMEFLKGSGDYCQAQHDLYA-DK----
>Ruditapes_philippinarum
---MNRI-ILFFGELGHTFMEFLTNSGEYCLAQHVHV--------
>Limulus_polyphemus
--MSSTNDS----A--KVPLLLP-FEVRRHHTLMEFLKTAGEYCQSQHK----------
>Protopalythoa_variabilis
--MRRDY-LAYDRGRTLMEFLIDSGDWCQAQHE----------
>Thermobia_domestica
---MEFQLDSGEYCQAQHK----------

HG0001 ENSG00000112245 PTP4A1

>Homo_sapiens
---MIRPQSSMSKHIPQF--CGVLGHTFMEF-LKGSGDYCQAQHDL-YADK-----
>Acipenser_sinensis
---MIKLQSSMSKHIPQF--CGVLGHTFMEF-LKGSGDYCQAQHEV-YADK-----
>Actinia_tenebrosa
---MWTTNIC--YDHCGHSLMEF-LIDSGDWCQAQHN----------
>Acanthogorgia_aspera
---MSRAYCLFGFRSRFEKFMEYFLKDSGEFCQAQHE----------
>Amia_calva
---MIKLQSSMSKHIPQF--CGVLGHTFMEF-LKGSGDYCQAQHDV-YADK-----
>Channa_argus
-----------------MSQQTEEAPPQQQF---QISDENSSC-HIEEEQKGDLTVVSALPPLSLRSH-GISMFILLFGF--CVDLSRTYMAC---VSGEFCQAQHLT-SVEK-----
>Kaupichthys_hyoproroides
---MIKLQSSMSKHIPQF--CGVLGHTFMEF-LKDSGDYCQAQHEV-YADK-----
>Anser_cygnoides
--MEF-LKGSGDYCQAQHDL-YADK-----
>Podocoryna_carnea
--MEF-LRGSGDYCFAQHRT----------
>Megophrys_sangzhiensis
---MIKPQSSMSKHSPQF--CGVLGHTFMEF-LKGSGDYCQAQHDL-YADK-----
>Chaetura_pelagica
--MEF-LKGSGDYCQAQHDL-YADK-----
>Apteryx_australis_mantelli
--MEF-LKGSGDYCQAQHDL-YADK-----
>Nephilengys_cruentata
---MLD-ASMC--IDLAV--NQIHPFSLMEI-LVGSDEYCRAQHK----------
>Lepismachilis_y-signata
---MCKDLVF--LGETALTYMEF-QLDSGEYCQAQHK----------
>Scapharca_broughtonii
---MFRKILF--FGEFGHTFMEF-VTNSGDYCQAQHV----------
>Porichthys_notatus
---MIKLQSSMSKHIPQF--CGVLGHTFMEF-LKGRGDYCQAQHAA-DADE-----
>Oryzias_melastigma
---MIKLQSSMSKHIPQF--CGVLGHTFMEF-LKGSGDYCQAQHAA-YADE-----
>Tripterygion_delaisi
--MFSLLFGF--CVDLSRTYMAC---VSGEFCQAQHLT-SVEK-----
>Buceros_rhinoceros_silvestri
--MEF-LKGSGDYCQAQHDL-YADK-----
>Caprimulgus_carolinensis
--MEF-LKGSGDYCQAQHDL-YADK-----
>Scyliorhinus_torazame
--MEF-LKGSGDYCQAQHAV-YADK-----
>Cariama_cristata
--MEF-LKGSGDYCQAQHDL-YADK-----
>Leptonychotes_weddellii
---MIRPQSSMSKHIPQF--CGVLGHTFMEF-LKGSGDYCQAQHDL-YADK-----
>Alatina_alata
---MRR-PCLC---------TCFCGKTFMEF-LKDSGDWCQSQHK----------
>Hynobius_retardatus
MRAPELGRSEARAQCGGGSVAADTATLPPRSPAARPVWYTARRAC-PPATRARG----------TGRMIQPRSSMSNHSPQF--CGVLGHTFMEF-LKGSGDYCQAQHDL-YADK-----
>Micropterus_salmoides
--MEF-VRRSGDYCHAQHI----------
>Physeter_catodon
--MEF-LKGSGDYCQAQHDL-YADK-----
>Astyanax_mexicanus
---MVKLQSSMSKHIPQF--CGVLGHTFMEF-LKGSGDYCQAQHGV-YADK-----
>Calidris_pugnax
--MEF-LKGSGDYCQAQHDL-YADK-----
>Callorhinchus_milii
--MEF-LKGSGDYCQAQHDV-YADK-----
>Eptesicus_fuscus
---MIRPQSSMSKHIPQF--CGVLGHTFMEF-LKGSGDYCQAQHDL-YADK-----
>Oreochromis_niloticus
---MIKLQSSMSKHIPQF--CGVLGHTFMEF-LKGSGDYCQAQHAA-YADE-----
>Pterocles_gutturalis
--MEF-LKGSGDYCQAQHDL-YADK-----
>Eucidaris_tribuloides
---MCKHTEV--LAVINKPFMAL-VLDSGEFCQSQHK----------
>Dasypus_novemcinctus
---MIRPQSSMSKHIPQF--CGVLGHTFMEF-LKGSGDYCQAQHDL-YADK-----
>Alosa_alosa
---MIKLQSSMSKHIPQF--CGVLGHTFMEF-LKGSGDYCQAQHGV-YADE-----
>Latimeria_menadoensis
---MIKIQSSMSKHIPQF--CGVLGHTFMEF-LKGSGDYCQAQHDL-YADK-----
>Gyrinus_marinus
---MCMAIVW--PGETVLTFMEF-QLDSAEYCQAQHK----------
>Colius_striatus
--MEF-LKGSGDYCQAQHDL-YADK-----
>Columba_livia
--MEF-LKGSGDYCQAQHDL-YADK-----
>Leptosomus_discolor
--MEF-LKGSGDYCQAQHDL-YADK-----
>Ricordea_yuma
---MWRGNPV--YGNRAHTFMEF-IIDSGDWCQAQHE----------
>Alligator_mississippiensis
---MIRLQSSMSNRRPQF--CGVLGHTFMEF-LKGSGDYCQAQHDL-YADK-----
>Cuculus_canorus
--MEF-LKGSGDYCQAQHDL-YADK-----
>Cyprinus_carpio
---MIKLQSSMSKHIPQF--CGVLGHTFMEF-LKGSGDYCQAQHGV-YAEK-----
>Fundulus_grandis
---MIKLQSSMSKHIPQF--CGVLGRTFMEF-LKGSGDYCQAQHAA-YADE-----
>Sarcophilus_harrisii
--MEF-LKGSGDYCQAQHDL-YADK-----
>Cancer_borealis
--MK-FKYCGDNNAQL--SSGRGYTLMEL-LKGSGEFCKAQYHI-K--------
>Galeopterus_variegatus
--MEF-LKGSGDYCQAQHDL-YADK-----
>Monodelphis_domestica
---MIRPQSSMSKHIPQF--CGVLGHTFMEF-LKGSGDYCQAQHDL-YADK-----
>Occasjapyx_japonicus
---MCRDLVC--LGKIGLTFMEF-FLDSGEYCQAQHK----------
>Macropus_eugenii
--MKAKQF--CGVLGHTFMEF-LKGSGDYCQAQHDL-YADK-----
>Chironomus_riparius
---MDIVL--LRQLALTSMEF-KLDSAEYCQAQHK----------
>Isonychia_bicolor
---MCRDLVF--LGEIGLTYMEF-QLDSGEYCQAQHK----------
>Esox_lucius
---MIKLQSSMSKHIPQF--CGVLGHTFMEF-LKGSGDYCQAQHVAAYADE-----
>Meganyctiphanes_norvegica
--MK-FKYCGNNNTQL--SCGRGYTLMEL-LKGSGEFCKAQYHI-K--------
>Haliaeetus_leucocephalus
---MIRLQSSMSNHIPQF--CGVLGHTFMEF-LKGSGDYCQAQHDL-YADK-----
>Marthasterias_glacialis
---MSHLTGI--CSVPVSSYMAF-VAISGEYCRSQHK----------
>Coturnix_japonica
---MIRLQSSMSNHIPQF--CGVLGHTFMEF-LKGSGDYCQAQHDL-YADK-----
>Gavia_stellata
--MEF-LKGSGDYCQAQHDL-YADK-----
>Acanthogobius_hasta
---MIKLQSSMSKHIPQF--CGVLGHTFMEF-LKGSGDYCQAQHVV-DADE-----
>Eurypyga_helias
--MEF-LKGSGDYCQAQHDL-YADK-----
>Apteronotus_leptorhynchus
--MAF-LQRAGDYCHAQHWC-C--------
>Planococcus_citri
---MDIVT--KSALCFTFMEF-LLDSSEYCQAQHK----------
>Orussus_abietinus
--MEF-QLDSAEYCQAQHK----------
>Condylura_cristata
---MIRPQSSMSKHIPQF--CGVLGHTFMEF-LKGSGDYCQAQHDL-YADK-----
>Ornithodoros_turicata
--MKSDAAGT--NGEPSHSWMEVSLGPSGEYCLAQHK----------
>Ochotona_princeps
---MIRPQSSMSKHIPQF--CGVLGHTFMEF-LKGSGDYCQAQHDL-YADK-----
>Antheraea_yamamai
--MEF-RLDSAEYCQAQHK----------
>Protopterus_aethiopicus
---MIKLQSSMSKHIPQF--CGVIGHTFMEF-LKGSGDYCQAQHAL-YADK-----
>Lingula_anatina
---MQF--FGEFGHTFMEF-VTNSGEYCQAQHK----------
>Elephantulus_edwardii
---MIRPQSSMSKHIPQF--CGVLGHTFMEF-LKGSGDYCQAQHDL-YADK-----
>Metallyticus_splendidus
---MCMALVF--LGETVLTYMEF-QLDSGEYCQAQHK----------
>Nannochorista_philpotti
---MCMAIVV--FPTVALRYMEF-HLDSAEYCQAQHK----------
>Ornithorhynchus_anatinus
---MIATHSSMSKHSPQF--CGVLGHTFMEF-LKDSGDYCQAQHDL-YADK-----
>Cypridininae_sp._BMR-2011
--MED---SETSLL-YKYRLCMRPQV--LAGVCCSFMEF-VLADGEYCQAQHK----------
>Perna_viridis
---MCRNILF--FGVLGHTFMEF-VTNSGDYCQAQHV----------
>Eptatretus_burgeri
--MNRHNAQF--CGVLGRTFMEF-VKGRGEYCETQHIA-LADK-----
>Osmylus_fulvicephalus
---MCMARVF--LGETVLTYMEF-QLDSAEYCQAQHK----------
>Triops_newberryi
---MCTTGQR--WVEWRHKFLAV-LLGSGDYCEAQHK----------
>Hapalochlaena_maculosa
---MC-RNILDLLGGIVEKTDSPST---LA-WPHHLCRSIKF--FGEFGHTFMEF-VTNSGDFCQAQHV----------
>Epiophlebia_superstes
--MEF-QLDSGEYCQAQHK----------
>Opisthocomus_hoazin
--MEF-LKGSGDYCQAQHDL-YADK-----
>Stenobothrus_lineatus
-------------------MV------VEASAV---ALADEGRTAGRHVMRLERASSPSSM---VDSQAA-GGGRLCAHQQF--RGEIVLTFMEF-QLDSGEYCQAQHK----------
>Plecoglossus_altivelis
--MEF-LQGSGDYCHAQHPSI---------
>Scleropages_formosus
--MCGLLFRF--CVDLGRPFMAF-VSVSGDFCQAQHLA-SADK-----
>Crassostrea_gigas
---MRDP-RGPVTVRGKFVTGFV---L---CS-SLLNCWQHTEF--RSNFGHTYMEF-VTNSGDYCQAQHV----------
>Parus_major
---MIRLQSSMSNHIPQF--CGVLGHTFMEF-LKGSGDYCQAQHDL-YADK-----
>Pecten_maximus
--MEF-VTNSGDYCQAQHAL---------
>Pelecanus_crispus
--MEF-LKGSGDYCQAQHDL-YADK-----
>Sebastes_caurinus
---MIKLQSSMSKHIP-F--CGVLGHTFMEF-LKGSGDYCQAQHAA-YADE-----
>Equus_przewalskii
---MIRPQSSMSKHSPQF--CGVLGHTFMEF-LKGSGDYCQAQHDL-YADK-----
>Peruphasma_schultei
--MEF-LPNSGEYCQAQHK----------
>Menopon_gallinae
--MN-FRFSGCMALKN--FHSTSSRLMEF-FLDSAEYCQAQHTK---------
>Perinereis_aibuhitensis
--M---SS-EAGVTTKLHQF--FGELGHTFMEF-LCNSGEYCQAQHV----------
>Perla_marginata
--MEF-QLDSGEYCQAQHK----------
>Cynoglossus_semilaevis
--MEF-LKGSGDYCQAQHAA-YADE-----
>Loxodonta_africana
---MIRPQSSMSKHIPQF--CGVLGHTFMEF-LKGSGDYCQAQHDL-YADK-----
>Fulmarus_glacialis
--MEF-LKGSGDYCQAQHDL-YADK-----
>Nestor_notabilis
--MEF-LKGSGDYCQAQHDL-YADK-----
>Ectopsocus_briggsi
--MEF-FLDSAEYCQAQHRK---------
>Leucoraja_erinacea
--MRKHIPQF--CGVLGHTFMEF-LKGSGDYCQAQHDV-YADK-----
>Inocellia_crassicornis
--MEF-QLDSAEYCQAQHK----------
>Rhopilema_esculentum
--MNSYHLLL--SKYFGKPFMEF-LKDSAEWCKAQHVE---------
>Mesocricetus_auratus
---MIRPQSSMSRHIPQF--CGVLGHTFMEF-LKGSGDYCQAQHDL-YADK-----
>Salmo_salar
---MIKLQSSMSKHIPQF--CGVLGHTFMEF-LKGSGDYCQAQHAAAYADE-----
>Acropora_digitifera
---MWRFSLT--FGSRAHTFMEF-IIDSGDWCQAQHE----------
>Tityus_serrulatus
--MEY-LKTTGEYCRAQHK----------
>Aurelia_aurita
--MTSYHLQL---KCFGETFMEF-LKDSAEWCKAQHVE---------
>Lepisosteus_oculatus
---MIKLQSSMSKHIPQF--CGVLGHTFMEF-LKGSGDYCQAQHGV-YADK-----
>Sepia_pharaonis
---MC-RNILDLLGGIVKKTDSPST---LA-WPHHLWRLVKF--FGEFGHTFMEF-VTNSGDFCQAQHV----------
>Sepioloidea_lineolata
--MEF-VTNSGDFCQAQHV----------
>Ictalurus_punctatus
---MVKLQSSMSKHIPQF--CGVLGHTFMEF-LKGSGDYCQAQHGV-YADK-----
>Oropsylla_silantiewi
--MEF-QLDSAEYCQAQHK----------
>Trichechus_manatus_latirostr
---MIRPQSSMSKHIPQF--CGVLGHTFMEF-LKGSGDYCQAQHDL-YADK-----
>Aptenodytes_forsteri
--MEF-LKGSGDYCQAQHDL-YADK-----
>Streblospio_benedicti
---MCRIILF--FGQIGYTFMEF-LCNSGEYCQAQHVA-IPSVLSRLH
>Squalus_acanthias
--MNRLVTQF--SGISGCSFMEF-LKGSRDYCQAQHGL-LADK-----
>Crotalus_horridus
---MIRLQSSMSNHIPQF--CGVLGHTFMEF-LKGSGDYCQAQHGL-YADK-----
>Stylops_melittae
---MCIGILN--LGNKVVEYMEF-HFGSSEYCKAQHK----------
>Tyto_alba
--MEF-LKGSGDYCQAQHDL-YADK-----
>Struthio_camelus_australis
--MEF-LKGSGDYCQAQHDL-YADK-----
>Deroceras_reticulatum
---MSSFVLF--VGEFGHTFMEF-VTNSEDFCQAQHYR-LVE------
>Pelodiscus_sinensis
---MIRLQSSMSNHIPQF--CGVLGHTFMEF-LKGSGDYCQAQHDL-YADK-----
>Takifugu_rubripes
---MIKLQSSMSKHIPQF--CGVLGHTFMEF-LKGSGDYCQAQHAV-YADE-----
>Watasenia_scintillans
--MEF-VTNSGDFCQAQHV----------
>Gynaikothrips_ficorum
---MVHCAKFNGENSISMV---IRRVIF-DNKWPSVGYQT--CGEILPTFMEF-HLDSAEYCQAQHK----------
>Tinamus_guttatus
--MEF-LKGSGDYCQAQHDL-YADK-----
>Tetronarce_californica
--MEF-VRNSSGYCPAQHSI-FADK-----
>Hydroptila_sp._AD-2013
--MEF-RLDSGEYCQAQHK----------
>Calypte_anna
--MEF-LKGSGDYCQAQHDL-YADK-----
>Apaloderma_vittatum
--MEF-LKGSGDYCQAQHDL-YADK-----
>Orycteropus_afer_afer
---MIRPQSSMSKHIPQF--CGVLGHTFMEF-LKGSGDYCQAQHDL-YADK-----
>Eurhomalea_rufa
--MEF-LTNSGEYCLAQHVH-V--------
>Limulus_polyphemus
--MEF-LKTAGEYCQSQHK----------
>Palythoa_caribaeorum
---MRRDY-L--AYDRGRTLMEF-LIDSGDWCQAQHE----------
>Thermobia_domestica
--MEF-QLDSGEYCQAQHK----------

HG0001 ENSG00000184007 PTP4A2

```
>Homo_sapiens
-----------------------MAILSVRADFCQAQHSIFADK
>Acipenser_sinensis
MIKLQSSMSKHIPQFCGVLGHTFMEFLKGSGDYCQAQHEVYADK
>Amia_calva
MIKLQSSMSKHIPQFCGVLGHTFMEFLKGSGDYCQAQHDVYADK
>Kaupichthys_hyoproroides
MIKLQSSMSKHIPQFCGVLGHTFMEFLKDSGDYCQAQHEVYADK
>Anser_cygnoides
-----------------------MAILSVRADFCQAQHSILADK
>Podocoryna_carnea
-------MNSLLSCFCVDLGRTFMAFLSVSGDFCQAQHLAFADK
>Leptobrachium_boringii
------MNSSPVTDSFWNIRLTFMAIRRVRTDYCQAQHSVGADK
>Chaetura_pelagica
-----------------------MEFLKGSGDYCQAQHDLYADK
>Apteryx_australis_mantelli
-----------------------MAILSVRADFCQAQHSTLADK
>Porichthys_notatus
MIKLQSSMSKHIPQFCGVLGHTFMEFLKGRGDYCQAQHAADADE
>Oryzias_latipes
MIKLQSSMSKHIPQFCGVLGHTFMEFLKGSGDYCQAQHAAYADE
>Buceros_rhinoceros_silvestri
-----------------------MEFLKGSGDYCQAQHDLYADK
>Caprimulgus_carolinensis
-----------------------MEFLKGSGDYCQAQHDLYADK
>Scyliorhinus_torazame
-----------------------MEFLKGSGDYCQAQHAVYADK
>Cariama_cristata
-----------------------MAILSVRADFCQAQHSILADK
>Acinonyx_jubatus
-----------------------MAILSVRADFCQAQHSVFADK
>Hynobius_retardatus
-----MTGPSRRTVSCRNIRLTFMAIVSVRADFCQAQHCPVAGK
>Orcinus_orca
-----MPSPQLASVFRWNIRCTFMAILSVRADFCQAQHSVSADK
>Astyanax_mexicanus
MVKLQSSMSKHIPQFCGVLGHTFMEFLKGSGDYCQAQHGVYADK
>Calidris_pugnax
----------MPRVSRWNIRRTFMAILSVRADFCQAQHSILADK
>Callorhinchus_milii
-----------------------MEFLKGSGDYCQAQHDVYADK
>Myotis_davidii
-----------------------MAILSVRADFCQAQHSVFADK
>Oreochromis_niloticus
MIKLQSSMSKHIPQFCGVLGHTFMEFLKGSGDYCQAQHAAYADE
>Pterocles_gutturalis
-----------------------MEFLKGSGDYCQAQHDLYADK
>Dasypus_novemcinctus
-----MPSPQLASVFRWNIRFTFMAILSVRADFCQAQHSVFADK
>Clupea_harengus
------MRNSYRVFFTPDESCTFMAFVSVSGDFCQAQHLVIVDK
>Latimeria_chalumnae
------------------------MAECVRADFCQAQHGILAEK
>Colius_striatus
-----------------------MEFLKGSGDYCQAQHDLYADK
>Columba_livia
-----------------------MAILSVRADFCQAQHSTLADK
>Merops_nubicus
-----------------------MAILSVRADFCQAQHSPLADK
>Alligator_sinensis
-----------------------MAILSVRADFCQAQHSTLADK
>Cuculus_canorus
-----------------------MEFLKGSGDYCQAQHDLYADK
>Sinocyclocheilus_anshuiensis
-----------------------MAFVSVSGDFCQAQHLVIVDK
>Kryptolebias_marmoratus
MIELHSSMSKHIPQFCGVLGHTFMEFLKGSGDYCQAQHAAYADE
>Sarcophilus_harrisii
-----------------------MEFLKGSGDYCQAQHDLYADK
>Galeopterus_variegatus
-----MPSPQLASVFRWNIRCTFMAILSVRADFCQAQHSVFADK
>Monodelphis_domestica
-----------------------MAILSVRADFCQAQHSAFADK
>Macropus_eugenii
---------MKAKQFCGVLGHTFMEFLKGSGDYCQAQHDLYADK
>Esox_lucius
-------MFSLLFYFCVELGHPYMAYVSVSGDFCQAQHLAFVDK
>Haliaeetus_leucocephalus
----------MPRVFCWNIRRTFMAILSVRADFCQAQHSILADK
>Numida_meleagris
----------MPGVFWWKIRRTFMAILSVRADFCQAQHSALADK
>Gavia_stellata
-----------------------MAILSVRADFCQAQHSILADK
>Acanthogobius_hasta
MIKLQSSMSKHIPQFCGVLGHTFMEFLKGSGDYCQAQHVVDADE
>Chlamydotis_macqueenii
-----------------------MAILSVRADFCQAQHRILADK
>Apteronotus_leptorhynchus
-------------------------MAFVSGDFCQAQHLAFVNK
>Condylura_cristata
-----MPSPQLASVFRWNIRCTFMAILSVRADFCQAQHSVFADK
>Ochotona_princeps
-----------------------MEFLKGSGDYCQAQHDLYADK
>Protopterus_aethiopicus
MIKLQSSMSKHIPQFCGVIGHTFMEFLKGSGDYCQAQHALYADK
>Elephantulus_edwardii
MIRPQSSMSKHIPQFCGVLGHTFMEFLKGSGDYCQAQHDLYADK
>Ornithorhynchus_anatinus
-----MPSPRLASAFRWNIRCTFMAILSVRADFCQAQHSVCADK
>Tauraco_erythrolophus
-----------------------MAILSVRADFCQAQHSILADK
>Opisthocomus_hoazin
-----------------------MEFLKGSGDYCQAQHDLYADK
>Scleropages_formosus
-----------------------MAFVSVSGDFCQAQHLASADK
>Corvus_cornix_cornix
-----------------------MAILSVRADFCQAQHSTLAAK
>Egretta_garzetta
-----------------------MAILSVRADFCQAQHSILADK
>Sebastes_caurinus
MIKLQSSMSKHIP-FCGVLGHTFMEFLKGSGDYCQAQHAAYADE
>Ceratotherium_simum_simum
-----MPSPQLASVFRWNIRCTFMAILSVRADFCQAQHSVFADK
>Picoides_pubescens
-----------------------MAILSVRADFCQAQHSILADK
>Cynoglossus_semilaevis
-----------------------MEFLKGSGDYCQAQHAAYADE
>Loxodonta_africana
-----------------------MAILSVRADFCQAQHSIFADK
>Fulmarus_glacialis
-----------------------MAILSVRADFCQAQHSILADK
>Nestor_notabilis
-----------------------MAILSVRAEFCQAQHSILADK
>Leucoraja_erinacea
-------MRKHIPQFCGVLGHTFMEFLKGSGDYCQAQHDVYADK
>Microtus_ochrogaster
---------MREKVFRWNIRCTFMAILSVRADFCQAQHSIFADK
>Coregonus_maraena
-------MFNLLFCFCVDLGHTYMAYVSVSGDFCQAQHLAFVDK
>Tupaia_chinensis
-----------------------MAILSVRADFCQAQHSVFADK
>Lepisosteus_oculatus
-------MSSLPFCFCVDLVRTFMAFLSVSGDFCQAQHLAFADK
>Trichomycterus_areolatus
-----------------------MAFVSVSGDFCQAQHLVIVDK
>Trichechus_manatus_latirostr
-----MPSSQLASVFRWNIRCTFMAILSVRADFCQAQHSIFADK
>Aptenodytes_forsteri
-----------------------MAILSVRADFCQAQHSILADK
>Squalus_acanthias
-------MNRLVTQFSGISGCSFMEFLKGSRDYCQAQHGLLADK
>Micrurus_fulvius
----------MLPVFHLNPSHTFMAILSVRADFCRAQHSTLAEK
>Tyto_alba
-----------------------MEFLKGSGDYCQAQHDLYADK
>Struthio_camelus_australis
-----------------------MAILSVRADFCQAQHSTLADK
>Chrysemys_picta
-----------------------MAILSIRADFCQAQHSTLADK
>Takifugu_rubripes
MIKLQSSMSKHIPQFCGVLGHTFMEFLKGSGDYCQAQHAVYADE
>Tinamus_guttatus
----------MPRVFCWNIRRTFMAILRVRADFCQAQHSTLADK
>Tetronarce_californica
-----------------------MEFVRNSSGYCPAQHSIFADK
>Calypte_anna
-----------------------MEFLKGSGDYCQAQHDLYADK
>Apaloderma_vittatum
-----------------------MAILSVRADFCQAQHSILADK
>Orycteropus_afer_afer
MIRPQSSMSKHIPQFCGVLGHTFMEFLKGSGDYCQAQHDLYADK
```

HG0001 ENSGALG00000003265 PTP4A2

>Gallus_gallus
---MPGVFCWNLRRTFMAILSVRAEFCQAQHSALADK-----
>Acipenser_sinensis
---MIKLQSSMSKHIPQFCGVLGHTFMEFLKGSGDYCQAQHEVYADK-----
>Amia_calva
---MIKLQSSMSKHIPQFCGVLGHTFMEFLKGSGDYCQAQHDVYADK-----
>Channa_argus
MSQQTEEAPPQQQFQISDENSSCHIEEEQKGDLTVVSALPPLSLRSHGISMFILLFGFCVDLSRTYMACV--SGEFCQAQHLTSVEK-----
>Kaupichthys_hyoproroides
---MIKLQSSMSKHIPQFCGVLGHTFMEFLKDSGDYCQAQHEVYADK-----
>Anser_cygnoides
--MAILSVRADFCQAQHSILADK-----
>Podocoryna_carnea
--MNSLLSCFCVDLGRTFMAFLSVSGDFCQAQHLAFADK-----
>Pelophylax_nigromaculatus
---MNSSPGSSWNIRRTFMAIRRVSTEYCQAQHSVCADK-----
>Chaetura_pelagica
--MEFLKGSGDYCQAQHDLYADK-----
>Apteryx_australis_mantelli
--MAILSVRADFCQAQHSTLADK-----
>Menidia_menidia
--MFNLLFGFCVDVSRSYMACV--TGEFCQAQHLTSAEK-----
>Porichthys_notatus
---MIKLQSSMSKHIPQFCGVLGHTFMEFLKGRGDYCQAQHAADADE-----
>Oryzias_latipes
---MIKLQSSMSKHIPQFCGVLGHTFMEFLKGSGDYCQAQHAAYADE-----
>Tripterygion_delaisi
--MFSLLFGFCVDLSRTYMACV--SGEFCQAQHLTSVEK-----
>Buceros_rhinoceros_silvestri
--MEFLKGSGDYCQAQHDLYADK-----
>Caprimulgus_carolinensis
--MEFLKGSGDYCQAQHDLYADK-----
>Scyliorhinus_torazame
--MEFLKGSGDYCQAQHAVYADK-----
>Cariama_cristata
--MAILSVRADFCQAQHSILADK-----
>Acinonyx_jubatus
--MAILSVRADFCQAQHSVFADK-----
>Hynobius_chinensis
--MTGPSRRTVSCWNIRLTFMAIVSVRADFCQAQHCPVAGK-----
>Orcinus_orca
--MPSPQLASVFRWNIRCTFMAILSVRADFCQAQHSVSADK-----
>Pygocentrus_nattereri
--MFSLFFCFCVDLGRTFMAFV--SGDFCQAQHLAFENK-----
>Calidris_pugnax
---MPRVSRWNIRRTFMAILSVRADFCQAQHSILADK-----
>Callorhinchus_milii
--MEFLKGSGDYCQAQHDVYADK-----
>Miniopterus_natalensis
--MPSPQLASVFRWNIRCTFMAILSVRADFCQAQHSVFADK-----
>Neolamprologus_brichardi
---MIKLQSSMSKHIPQFCGVLGHTFMEFLKGSGDYCQAQHAAYADE-----
>Pterocles_gutturalis
--MEFLKGSGDYCQAQHDLYADK-----
>Eucidaris_tribuloides
---MCKHTEVLAVINKPFMALVLDSGEFCQSQHK----------
>Dasypus_novemcinctus
--MPSPQLASVFRWNIRFTFMAILSVRADFCQAQHSVFADK-----
>Clupea_harengus
--MFNLLFCFCVELGRTYMAFVSVSGDFCQAQHLAFVDK-----
>Latimeria_chalumnae
---MIKIQSSMSKHIPQFCGVLGHTFMEFLKGSGDYCQAQHDLYADK-----
>Colius_striatus
--MEFLKGSGDYCQAQHDLYADK-----
>Columba_livia
--MAILSVRADFCQAQHSTLADK-----
>Merops_nubicus
--MAILSVRADFCQAQHSPLADK-----
>Alligator_sinensis
--MAILSVRADFCQAQHSTLADK-----
>Cuculus_canorus
--MEFLKGSGDYCQAQHDLYADK-----
>Sinocyclocheilus_anshuiensis
--MFGPFFHFCVDLDHTYMAFVTICGEFCQAQHLTFVDK-----
>Fundulus_heteroclitus
---MIKLQSSMSKHIP-FCGVLGRTFMEFLKGSGDYCQAQHAAYADE-----
>Sarcophilus_harrisii
--MEFLKGSGDYCQAQHDLYADK-----
>Galeopterus_variegatus
--MPSPQLASVFRWNIRCTFMAILSVRADFCQAQHSVFADK-----
>Monodelphis_domestica
--MAILSVRADFCQAQHSAFADK-----
>Macropus_eugenii
--MKAKQFCGVLGHTFMEFLKGSGDYCQAQHDLYADK-----
>Esox_lucius
--MFSLLFYFCVELGHPYMAYVSVSGDFCQAQHLAFVDK-----
>Aquila_chrysaetos_canadensis
---MPRVFCWNIRRTFMAILSVRADFCQAQHSILADK-----
>Gavia_stellata
--MAILSVRADFCQAQHSILADK-----
>Acanthogobius_hasta
---MIKLQSSMSKHIPQFCGVLGHTFMEFLKGSGDYCQAQHVVDADE-----
>Chlamydotis_macqueenii
--MAILSVRADFCQAQHRILADK-----
>Apteronotus_leptorhynchus
--MAFV--SGDFCQAQHLAFVNK-----
>Xenophysella_greensladeae
---MCMATVFLGDACATFMEFHLDSAEYCQAQHK----------
>Sorex_araneus
--MAILSVRADFCQAQHSVFADK-----
>Ochotona_princeps
--MEFLKGSGDYCQAQHDLYADK-----
>Protopterus_aethiopicus
---MIKLQSSMSKHIPQFCGVIGHTFMEFLKGSGDYCQAQHALYADK-----
>Elephantulus_edwardii
---MIRPQSSMSKHIPQFCGVLGHTFMEFLKGSGDYCQAQHDLYADK-----
>Ornithorhynchus_anatinus
--MPSPRLASAFRWNIRCTFMAILSVRADFCQAQHSVCADK-----
>Tauraco_erythrolophus
--MAILSVRADFCQAQHSILADK-----
>Eptatretus_burgeri
--MNRHNAQFCGVLGRTFMEFVKGRGEYCETQHIALADK-----
>Octopus_bimaculoides
--MEFVTNSGDFCQAQHV----------
>Opisthocomus_hoazin
--MEFLKGSGDYCQAQHDLYADK-----
>Scleropages_formosus
--MAFVSVSGDFCQAQHLASADK-----
>Zonotrichia_albicollis
---MPTVFCWRILGTFMAILSVRAEFCQAQHSTLAAK-----
>Pecten_maximus
--MEFVTNSGDYCQAQHAL---------
>Egretta_garzetta
--MAILSVRADFCQAQHSILADK-----
>Lateolabrax_maculatus
-------------------------MVCL---------H--PGAVTSS-CVRSMNHLLGSHRGRTCMAFARLGGEFCQAQHLLLIVDK----
>Notothenia_coriiceps
--MACV--SGEFCQAQHLTSVEK-----
>Equus_asinus
--MPSPQLASVFRWNIRCTFMAILSVRADFCQAQHSVFADK-----
>Perinereis_aibuhitensis
---MSSEA-GVTTKLHQFFGELGHTFMEFLCNSGEYCQAQHV----------
>Picoides_pubescens
--MAILSVRADFCQAQHSILADK-----
>Cynoglossus_semilaevis
--MEFLKGSGDYCQAQHAAYADE-----
>Macaca_nemestrina
--MAILSVRADFCQAQHSVFADK-----
>Loxodonta_africana
--MAILSVRADFCQAQHSIFADK-----
>Fulmarus_glacialis
--MAILSVRADFCQAQHSILADK-----
>Nestor_notabilis
--MAILSVRAEFCQAQHSILADK-----
>Leucoraja_erinacea
--MRKHIPQFCGVLGHTFMEFLKGSGDYCQAQHDVYADK-----
>Fukomys_damarensis
--MAILSVRADFCQAQHSVFADK-----
>Salmo_salar
--MFSLLFCFCVDLDHTYMAYVSVSGDFCQAQHLAFVDK-----
>Tupaia_chinensis
--MAILSVRADFCQAQHSVFADK-----
>Lepisosteus_oculatus
--MSSLPFCFCVDLVRTFMAFLSVSGDFCQAQHLAFADK-----
>Sepia_pharaonis
---------------------MCRNILDLLGGIVKKTDS--PSTLAWP-HHLWRLVKFFGEFGHTFMEFVTNSGDFCQAQHV----------
>Ictalurus_punctatus
---MVKLQSSMSKHIPQFCGVLGHTFMEFLKGSGDYCQAQHGVYADK-----
>Trichechus_manatus_latirostr
--MPSSQLASVFRWNIRCTFMAILSVRADFCQAQHSIFADK-----
>Aptenodytes_forsteri
--MAILSVRADFCQAQHSILADK-----
>Streblospio_benedicti
---MCRIILFFGQIGYTFMEFLCNSGEYCQAQHVAIPSVLSRLH
>Squalus_acanthias
--MNRLVTQFSGISGCSFMEFLKGSRDYCQAQHGLLADK-----
>Gekko_japonicus
---MRKAACEVFLWNPRHTFMAILSIRADFCRAQHSTLADK-----
>Tyto_alba
--MEFLKGSGDYCQAQHDLYADK-----
>Struthio_camelus_australis
--MAILSVRADFCQAQHSTLADK-----
>Deroceras_reticulatum
---MSSFVLFVGEFGHTFMEFVTNSEDFCQAQHYRLVE------
>Chrysemys_picta
--MAILSIRADFCQAQHSTLADK-----
>Takifugu_rubripes
---MIKLQSSMSKHIPQFCGVLGHTFMEFLKGSGDYCQAQHAVYADE-----
>Watasenia_scintillans
--MEFVTNSGDFCQAQHV----------
>Tinamus_guttatus
---MPRVFCWNIRRTFMAILRVRADFCQAQHSTLADK-----
>Tetronarce_californica
--MEFVRNSSGYCPAQHSIFADK-----
>Calypte_anna
--MEFLKGSGDYCQAQHDLYADK-----
>Apaloderma_vittatum
--MAILSVRADFCQAQHSILADK-----
>Orycteropus_afer_afer
---MIRPQSSMSKHIPQFCGVLGHTFMEFLKGSGDYCQAQHDLYADK-----

HG0001 ENSGALG00000016271 PTP4A1

```
>Gallus_gallus
--------------------------------------------------------------------------MIRLQSSMS--NHIPQFCGVLGHTFME-FLKGSGDYCQAQHDL-YADK----
>Acipenser_sinensis
--------------------------------------------------------------------------MIKLQSSMS--KHIPQFCGVLGHTFME-FLKGSGDYCQAQHEV-YADK----
>Actinia_tenebrosa
----------------------------------------------------------------------------MWTTNIC--------YDHCGHSLME-FLIDSGDWCQAQHN----------
>Acanthogorgia_aspera
----------------------------------------------------------------------MSRAYCLFG------------FRSRFEKFMEYFLKDSGEFCQAQHE----------
>Amia_calva
--------------------------------------------------------------------------MIKLQSSMS--KHIPQFCGVLGHTFME-FLKGSGDYCQAQHDV-YADK----
>Channa_argus
---------------------MSQQTEEAPPQQQFQISDENSSCHIEEE----------QKGDLTVVSALPPLSLRSHGISMF--ILLFGFCVDLSRTYMA-C--VSGEFCQAQHLTSVEK-----
>Kaupichthys_hyoproroides
--------------------------------------------------------------------------MIKLQSSMS--KHIPQFCGVLGHTFME-FLKDSGDYCQAQHEV-YADK----
>Anser_cygnoides
---------------------------------------------------------------------------------------------------ME-FLKGSGDYCQAQHDL-YADK----
>Podocoryna_carnea
---------------------------------------------------------------------------------------------------ME-FLRGSGDYCFAQHRT---------
>Pseudacris_regilla
MLFTGHG----GEVCERRSGPHPARH-RSPPHRALATGF--SRLLYAPSPPAPRPHHRFFLGT---G------CMIKLQSSMS--KHSPQFCGVLGHTFME-FLKGSGDYCQAQHDL-YADK----
>Chaetura_pelagica
---------------------------------------------------------------------------------------------------ME-FLKGSGDYCQAQHDL-YADK----
>Apteryx_australis_mantelli
---------------------------------------------------------------------------------------------------ME-FLKGSGDYCQAQHDL-YADK----
>Nephilengys_cruentata
----------------------------------------------------------------------------MLDASMC--IDLAV-NQIHPFSLME-ILVGSDEYCRAQHK----------
>Meinertellus_cundinamarcensi
---------------------------------------------------------------------------------MC--RDLVF-LGETGLTYME-FQLDSGEYCQAQHK----------
>Scapharca_broughtonii
---------------------------------------------------------------------------------MF--RKILF-FGEFGHTFME-FVTNSGDYCQAQHV----------
>Menidia_menidia
---------------------------------------------------------------------------------MF--NLLFGFCVDVSRSYMA-C--VTGEFCQAQHLTSAEK-----
>Porichthys_notatus
--------------------------------------------------------------------------MIKLQSSMS--KHIPQFCGVLGHTFME-FLKGRGDYCQAQHAA-DADE----
>Oryzias_melastigma
--------------------------------------------------------------------------MIKLQSSMS--KHIPQFCGVLGHTFME-FLKGSGDYCQAQHAA-YADE----
>Tripterygion_delaisi
---------------------------------------------------------------------------------MF--SLLFGFCVDLSRTYMA-C--VSGEFCQAQHLTSVEK-----
>Buceros_rhinoceros_silvestri
---------------------------------------------------------------------------------------------------ME-FLKGSGDYCQAQHDL-YADK----
>Caprimulgus_carolinensis
---------------------------------------------------------------------------------------------------ME-FLKGSGDYCQAQHDL-YADK----
>Scyliorhinus_torazame
---------------------------------------------------------------------------------------------------ME-FLKGSGDYCQAQHAV-YADK----
>Cariama_cristata
---------------------------------------------------------------------------------------------------ME-FLKGSGDYCQAQHDL-YADK----
>Mustela_putorius_furo
--------------------------------------------------------------------------MIRPQSSMS--KHIPQFCGVLGHTFME-FLKGSGDYCQAQHDL-YADK----
>Alatina_alata
------------------------------------------------------------------------------MRRPC--LC----TCFCGKTFME-FLKDSGDWCQSQHK----------
>Hynobius_retardatus
MRAPELGRSEARAQCGG--GSVAADTATLPPRSPAA-----RPVWYTARR--ACPPATRARGT---G------RMIQPRSSMS--NHSPQFCGVLGHTFME-FLKGSGDYCQAQHDL-YADK----
>Micropterus_floridanus
---------------------------------------------------------------------------------------------------ME-FVRRSGDYCHAQHI----------
>Orcinus_orca
---------------------------------------------------------------------------------------------------ME-FLKGSGDYCQAQHDL-YADK----
>Astyanax_mexicanus
--------------------------------------------------------------------------MVKLQSSMS--KHIPQFCGVLGHTFME-FLKGSGDYCQAQHGV-YADK----
>Calidris_pugnax
---------------------------------------------------------------------------------------------------ME-FLKGSGDYCQAQHDL-YADK----
>Callorhinchus_milii
---------------------------------------------------------------------------------------------------ME-FLKGSGDYCQAQHDV-YADK----
>Eptesicus_fuscus
--------------------------------------------------------------------------MIRPQSSMS--KHIPQFCGVLGHTFME-FLKGSGDYCQAQHDL-YADK----
>Maylandia_zebra
--------------------------------------------------------------------------MIKLQSSMS--KHIPQFCGVLGHTFME-FLKGSGDYCQAQHAA-YADE----
>Pterocles_gutturalis
---------------------------------------------------------------------------------------------------ME-FLKGSGDYCQAQHDL-YADK----
>Eucidaris_tribuloides
---------------------------------------------------------------------------------MC--KHTEV-LAVINKPFMA-LVLDSGEFCQSQHK----------
>Dasypus_novemcinctus
--------------------------------------------------------------------------MIRPQSSMS--KHIPQFCGVLGHTFME-FLKGSGDYCQAQHDL-YADK----
>Alosa_alosa
--------------------------------------------------------------------------MIKLQSSMS--KHIPQFCGVLGHTFME-FLKGSGDYCQAQHGV-YADE----
>Latimeria_chalumnae
--------------------------------------------------------------------------MIKIQSSMS--KHIPQFCGVLGHTFME-FLKGSGDYCQAQHDL-YADK----
>Gyrinus_marinus
---------------------------------------------------------------------------------MC--MAIVW-PGETVLTFME-FQLDSAEYCQAQHK----------
>Colius_striatus
---------------------------------------------------------------------------------------------------ME-FLKGSGDYCQAQHDL-YADK----
>Columba_livia
---------------------------------------------------------------------------------------------------ME-FLKGSGDYCQAQHDL-YADK----
>Leptosomus_discolor
---------------------------------------------------------------------------------------------------ME-FLKGSGDYCQAQHDL-YADK----
>Ricordea_yuma
----------------------------------------------------------------------------MWRGNPV--------YGNRAHTFME-FIIDSGDWCQAQHE----------
>Alligator_mississippiensis
--------------------------------------------------------------------------MIRLQSSMS--NRRPQFCGVLGHTFME-FLKGSGDYCQAQHDL-YADK----
>Cuculus_canorus
---------------------------------------------------------------------------------------------------ME-FLKGSGDYCQAQHDL-YADK----
>Cyprinus_carpio
--------------------------------------------------------------------------MIKLQSSMS--KHIPQFCGVLGHTFME-FLKGSGDYCQAQHGV-YAEK----
>Fundulus_heteroclitus
--------------------------------------------------------------------------MIKLQSSMS--KHIPQFCGVLGRTFME-FLKGSGDYCQAQHAA-YADE----
>Sarcophilus_harrisii
---------------------------------------------------------------------------------------------------ME-FLKGSGDYCQAQHDL-YADK----
>Carcinus_maenas
-----------------------------------------------------------------------------MKFKYCGNNNAQL-SSGRGYTLME-LLKGSGEFCKAQYHIK--------
>Galeopterus_variegatus
---------------------------------------------------------------------------------------------------ME-FLKGSGDYCQAQHDL-YADK----
>Monodelphis_domestica
--------------------------------------------------------------------------MIRPQSSMS--KHIPQFCGVLGHTFME-FLKGSGDYCQAQHDL-YADK----
>Occasjapyx_japonicus
---------------------------------------------------------------------------------MC--RDLVC-LGKIGLTFME-FFLDSGEYCQAQHK----------
>Macropus_eugenii
-------------------------------------------------------------------------------------MKAKQFCGVLGHTFME-FLKGSGDYCQAQHDL-YADK----
>Chironomus_riparius
-------------------------------------------------------------------------------------MDIVL-LRQLALTSME-FKLDSAEYCQAQHK----------
>Isonychia_bicolor
---------------------------------------------------------------------------------MC--RDLVF-LGEIGLTYME-FQLDSGEYCQAQHK----------
>Esox_lucius
--------------------------------------------------------------------------MIKLQSSMS--KHIPQFCGVLGHTFME-FLKGSGDYCQAQHVAAYADE----
>Meganyctiphanes_norvegica
-----------------------------------------------------------------------------MKFKYCGNNNTQL-SCGRGYTLME-LLKGSGEFCKAQYHIK--------
>Haliaeetus_leucocephalus
--------------------------------------------------------------------------MIRLQSSMS--NHIPQFCGVLGHTFME-FLKGSGDYCQAQHDL-YADK----
>Marthasterias_glacialis
---------------------------------------------------------------------------------MS---HLTGICSVPVSSYMA-FVAISGEYCRSQHK----------
>Gavia_stellata
---------------------------------------------------------------------------------------------------ME-FLKGSGDYCQAQHDL-YADK----
>Acanthogobius_hasta
--------------------------------------------------------------------------MIKLQSSMS--KHIPQFCGVLGHTFME-FLKGSGDYCQAQHVV-DADE----
>Eurypyga_helias
---------------------------------------------------------------------------------------------------ME-FLKGSGDYCQAQHDL-YADK----
>Apteronotus_leptorhynchus
---------------------------------------------------------------------------------------------------MA-FLQRAGDYCHAQHWCC--------
>Planococcus_citri
-------------------------------------------------------------------------------------MDIVT-KSALCFTFME-FLLDSSEYCQAQHK----------
>Lasius_neglectus
---------------------------------------------------------------------------------------------------ME-FQLDSAEYCQAQHK----------
>Condylura_cristata
--------------------------------------------------------------------------MIRPQSSMS--KHIPQFCGVLGHTFME-FLKGSGDYCQAQHDL-YADK----
>Ornithodoros_turicata
-----------------------------------------------------------------------------MKSDAAG-----T-NGEPSHSWMEVSLGPSGEYCLAQHK----------
>Ochotona_princeps
--------------------------------------------------------------------------MIRPQSSMS--KHIPQFCGVLGHTFME-FLKGSGDYCQAQHDL-YADK----
>Antheraea_yamamai
---------------------------------------------------------------------------------------------------ME-FRLDSAEYCQAQHK----------
>Protopterus_aethiopicus
--------------------------------------------------------------------------MIKLQSSMS--KHIPQFCGVIGHTFME-FLKGSGDYCQAQHAL-YADK----
>Lingula_anatina
---------------------------------------------------------------------------------------MQF-FGEFGHTFME-FVTNSGEYCQAQHK----------
>Elephantulus_edwardii
--------------------------------------------------------------------------MIRPQSSMS--KHIPQFCGVLGHTFME-FLKGSGDYCQAQHDL-YADK----
>Metallyticus_splendidus
---------------------------------------------------------------------------------MC--MALVF-LGETVLTYME-FQLDSGEYCQAQHK----------
>Nannochorista_philpotti
---------------------------------------------------------------------------------MC--MAIVV-FPTVALRYME-FHLDSAEYCQAQHK----------
>Ornithorhynchus_anatinus
--------------------------------------------------------------------------MIATHSSMS--KHSPQFCGVLGHTFME-FLKDSGDYCQAQHDL-YADK----
>Cypridininae_sp._BMR-2011
--------------------------------------------------------------------MEDSETSLLYKYRLC--MRPQV-LAGVCCSFME-FVLADGEYCQAQHK----------
>Perna_viridis
---------------------------------------------------------------------------------MC--RNILF-FGVLGHTFME-FVTNSGDYCQAQHV----------
>Eptatretus_burgeri
--------------------------------------------------------------------------------MNR--HNAQF-CGVLGRTFME-FVKGRGEYCETQHIALADK-----
>Osmylus_fulvicephalus
---------------------------------------------------------------------------------MC--MARVF-LGETVLTYME-FQLDSAEYCQAQHK----------
>Triops_newberryi
---------------------------------------------------------------------------------MC--TTGQR-WVEWRHKFLA-VLLGSGDYCEAQHK----------
>Hapalochlaena_maculosa
----------------------------------------------------MCRNILDLLGGIVEK--TDSPSTLAWPHHLC--RSIKF-FGEFGHTFME-FVTNSGDFCQAQHV----------
>Epiophlebia_superstes
---------------------------------------------------------------------------------------------------ME-FQLDSGEYCQAQHK----------
>Opisthocomus_hoazin
---------------------------------------------------------------------------------------------------ME-FLKGSGDYCQAQHDL-YADK----
>Stenobothrus_lineatus
------------------------------------MVVEASAVALADEGRTAGRHVMRLERA--SSPSSMVDSQAAGGGRLC--AHQQF-RGEIVLTFME-FQLDSGEYCQAQHK----------
>Plecoglossus_altivelis
---------------------------------------------------------------------------------------------------ME-FLQGSGDYCHAQHPSI--------
>Scleropages_formosus
---------------------------------------------------------------------------------MC--GLLFRFCVDLGRPFMA-FVSVSGDFCQAQHLASADK-----
>Crassostrea_gigas
---------------------------------------------MR-----DPRGPVTVRGKFVTGFVLCSSLLNCW-------QHTEF-RSNFGHTYME-FVTNSGDYCQAQHV----------
>Endeis_spinosa
---------------------------------------------------------------------------------------------------ME-FLKESGDYCYCRAQHV--------
>Lepidothrix_coronata
--------------------------------------------------------------------------MIRLQSSMS--NHIPQFCGVLGHTFME-FLKGSGDYCQAQHDL-YADK----
>Pecten_maximus
---------------------------------------------------------------------------------------------------ME-FVTNSGDYCQAQHAL---------
>Pelecanus_crispus
---------------------------------------------------------------------------------------------------ME-FLKGSGDYCQAQHDL-YADK----
>Sebastes_caurinus
--------------------------------------------------------------------------MIKLQSSMS--KHIP-FCGVLGHTFME-FLKGSGDYCQAQHAA-YADE----
>Equus_przewalskii
--------------------------------------------------------------------------MIRPQSSMS--KHSPQFCGVLGHTFME-FLKGSGDYCQAQHDL-YADK----
>Peruphasma_schultei
---------------------------------------------------------------------------------------------------ME-FLPNSGEYCQAQHK----------
>Menopon_gallinae
---------------------------------------------------------------------------MNFRFSGC--MALKN-FHSTSSRLME-FFLDSAEYCQAQHTK---------
>Perinereis_aibuhitensis
--------------------------------------------------------------------------MSSEAGVTT--KLHQF-FGELGHTFME-FLCNSGEYCQAQHV----------
>Perla_marginata
---------------------------------------------------------------------------------------------------ME-FQLDSGEYCQAQHK----------
>Cynoglossus_semilaevis
---------------------------------------------------------------------------------------------------ME-FLKGSGDYCQAQHAA-YADE----
>Gorilla_gorilla
--------------------------------------------------------------------------MIRPQSSMS--KHIPQFCGVLGHTFME-FLKGSGDYCQAQHDL-YADK----
>Loxodonta_africana
--------------------------------------------------------------------------MIRPQSSMS--KHIPQFCGVLGHTFME-FLKGSGDYCQAQHDL-YADK----
>Fulmarus_glacialis
---------------------------------------------------------------------------------------------------ME-FLKGSGDYCQAQHDL-YADK----
>Nestor_notabilis
---------------------------------------------------------------------------------------------------ME-FLKGSGDYCQAQHDL-YADK----
>Ectopsocus_briggsi
---------------------------------------------------------------------------------------------------ME-FFLDSAEYCQAQHRK---------
>Leucoraja_erinacea
---------------------------------------------------------------------------------MR--KHIPQFCGVLGHTFME-FLKGSGDYCQAQHDV-YADK----
>Inocellia_crassicornis
---------------------------------------------------------------------------------------------------ME-FQLDSAEYCQAQHK----------
>Rhopilema_esculentum
----------------------------------------------------------------------------------MNSYHLLL-SKYFGKPFME-FLKDSAEWCKAQHVE---------
>Heterocephalus_glaber
--------------------------------------------------------------------------MIRPQSSMS--RHIPQFCGVLGHTFME-FLKGSGDYCQAQHDL-YADK----
>Salmo_salar
--------------------------------------------------------------------------MIKLQSSMS--KHIPQFCGVLGHTFME-FLKGSGDYCQAQHAAAYADE----
>Acropora_digitifera
----------------------------------------------------------------------------MWRFSLT--------FGSRAHTFME-FIIDSGDWCQAQHE----------
>Tityus_serrulatus
---------------------------------------------------------------------------------------------------ME-YLKTTGEYCRAQHK----------
>Aurelia_aurita
----------------------------------------------------------------------------------MTSYHLQL--KCFGETFME-FLKDSAEWCKAQHVE---------
>Lepisosteus_oculatus
--------------------------------------------------------------------------MIKLQSSMS--KHIPQFCGVLGHTFME-FLKGSGDYCQAQHGV-YADK----
>Sepia_pharaonis
----------------------------------------------------MCRNILDLLGGIVKK--TDSPSTLAWPHHLW--RLVKF-FGEFGHTFME-FVTNSGDFCQAQHV----------
>Sepioloidea_lineolata
---------------------------------------------------------------------------------------------------ME-FVTNSGDFCQAQHV----------
>Ictalurus_punctatus
--------------------------------------------------------------------------MVKLQSSMS--KHIPQFCGVLGHTFME-FLKGSGDYCQAQHGV-YADK----
>Oropsylla_silantiewi
---------------------------------------------------------------------------------------------------ME-FQLDSAEYCQAQHK----------
>Trichechus_manatus_latirostr
--------------------------------------------------------------------------MIRPQSSMS--KHIPQFCGVLGHTFME-FLKGSGDYCQAQHDL-YADK----
>Aptenodytes_forsteri
---------------------------------------------------------------------------------------------------ME-FLKGSGDYCQAQHDL-YADK----
>Streblospio_benedicti
---------------------------------------------------------------------------------MC--RIILF-FGQIGYTFME-FLCNSGEYCQAQHVAIPSVLSRLH
>Squalus_acanthias
---------------------------------------------------------------------------------MN--RLVTQFSGISGCSFME-FLKGSRDYCQAQHGLLAD-K----
>Takydromus_sexlineatus
--------------------------------------------------------------------------MIRLQSSMS--NHIPQFCGVLGHTFME-FLKGSGDYCQAQHGL-YADK----
>Stylops_melittae
---------------------------------------------------------------------------------MC--IGILN-LGNKVVEYME-FHFGSSEYCKAQHK----------
>Tyto_alba
---------------------------------------------------------------------------------------------------ME-FLKGSGDYCQAQHDL-YADK----
>Struthio_camelus_australis
---------------------------------------------------------------------------------------------------ME-FLKGSGDYCQAQHDL-YADK----
>Deroceras_reticulatum
---------------------------------------------------------------------------------MS--SFVLF-VGEFGHTFME-FVTNSEDFCQAQHYRLVE------
>Pelodiscus_sinensis
--------------------------------------------------------------------------MIRLQSSMS--NHIPQFCGVLGHTFME-FLKGSGDYCQAQHDL-YADK----
>Takifugu_rubripes
--------------------------------------------------------------------------MIKLQSSMS--KHIPQFCGVLGHTFME-FLKGSGDYCQAQHAV-YADE----
>Watasenia_scintillans
---------------------------------------------------------------------------------------------------ME-FVTNSGDFCQAQHV----------
>Gynaikothrips_ficorum
-----------------------------------------------------MVHCAKFNGE--NSISMVIRRVIFDNKWPS--VGYQT-CGEILPTFME-FHLDSAEYCQAQHK----------
>Tinamus_guttatus
---------------------------------------------------------------------------------------------------ME-FLKGSGDYCQAQHDL-YADK----
>Tetronarce_californica
---------------------------------------------------------------------------------------------------ME-FVRNSSGYCPAQHSIFADK-----
>Annulipalpia_sp._AD-2013
---------------------------------------------------------------------------------MC--LAERLEACSLATTLME-FRLDSSEYCQAQHK----------
>Calypte_anna
---------------------------------------------------------------------------------------------------ME-FLKGSGDYCQAQHDL-YADK----
>Apaloderma_vittatum
---------------------------------------------------------------------------------------------------ME-FLKGSGDYCQAQHDL-YADK----
>Orycteropus_afer_afer
--------------------------------------------------------------------------MIRPQSSMS--KHIPQFCGVLGHTFME-FLKGSGDYCQAQHDL-YADK----
>Ruditapes_philippinarum
---------------------------------------------------------------------------------MN--RIILF-FGELGHTFME-FLTNSGEYCLAQHVHV--------
>Limulus_polyphemus
----------------------------------------------------------------------MSST--NDSAKVP--LLLPF-EVRRHHTLME-FLKTAGEYCQSQHK----------
>Palythoa_caribaeorum
-------------------------------------------------------------------------------------MRRDYLAYDRGRTLME-FLIDSGDWCQAQHE----------
>Thermobia_domestica
---------------------------------------------------------------------------------------------------ME-FQLDSGEYCQAQHK----------
```

HG0001 FBgn0024734 PRL-1

```
>Drosophila_melanogaster
-------------------------------------------MCM--------------------AIV--L-VRPVPTSME-FHLDSAEYCQAQHK------
>Acipenser_sinensis
--------------------------------------------------------MIKLQSSMSKHI-PQFCGVLGHTFME-FLKGSGDYCQAQHEVYADK-
>Exaiptasia_pallida
-----------------------------------------MW-----------------------RGL--ALGRRDYSLME-FLIDSGDWCQAQHYK-----
>Acanthogorgia_aspera
---------------------------------------------------------------MSRAYCLFGFRSRFEKFMEYFLKDSGEFCQAQHE------
>Amia_calva
--------------------------------------------------------MIKLQSSMSKHI-PQFCGVLGHTFME-FLKGSGDYCQAQHDVYADK-
>Anser_cygnoides
--------------------------------------------------------------------------------ME-FLKGSGDYCQAQHDLYADK-
>Xenopus_tropicalis
----------------------------------MYVRFDTRAQRPA------DNGMPTQGGVRDWAA-RQFCGVLGHTFME-FLKGSGDYCQAQHELYADK-
>Chaetura_pelagica
--------------------------------------------------------------------------------ME-FLKGSGDYCQAQHDLYADK-
>Apteryx_australis_mantelli
--------------------------------------------------------------------------------ME-FLKGSGDYCQAQHDLYADK-
>Nephilengys_cruentata
--------------------------------------MLDASMCID---------------L-----A--VNQIHPFSLME-ILVGSDEYCRAQHK------
>Meinertellus_cundinamarcensi
-------------------------------------------MCR--------------------DLV--FLGETGLTYME-FQLDSGEYCQAQHK------
>Scapharca_broughtonii
-----------------------------------------MFRKI-----------------------L-FFGEFGHTFME-FVTNSGDYCQAQHV------
>Buceros_rhinoceros_silvestri
--------------------------------------------------------------------------------ME-FLKGSGDYCQAQHDLYADK-
>Caprimulgus_carolinensis
--------------------------------------------------------------------------------ME-FLKGSGDYCQAQHDLYADK-
>Scyliorhinus_torazame
--------------------------------------------------------------------------------ME-FLKGSGDYCQAQHAVYADK-
>Cariama_cristata
--------------------------------------------------------------------------------ME-FLKGSGDYCQAQHDLYADK-
>Mustela_putorius_furo
--------------------------------------------------------MIRPQSSMSKHI-PQFCGVLGHTFME-FLKGSGDYCQAQHDLYADK-
>Alatina_alata
------------------------------------------MRRP-----------------------CLCTCFCGKTFME-FLKDSGDWCQSQHK------
>Hynobius_retardatus
MRAPELGRSEARAQCGGGSVAADTATLPPRSPAARPVWYTARRACPPATRARGTGRMIQPRSSMSNHS-PQFCGVLGHTFME-FLKGSGDYCQAQHDLYADK-
>Orcinus_orca
--------------------------------------------------------------------------------ME-FLKGSGDYCQAQHDLYADK-
>Astyanax_mexicanus
--------------------------------------------------------MVKLQSSMSKHI-PQFCGVLGHTFME-FLKGSGDYCQAQHGVYADK-
>Charadrius_vociferus
--------------------------------------------------------------------------------ME-FLKGSGDYCQAQHDLYADK-
>Callorhinchus_milii
--------------------------------------------------------------------------------ME-FLKGSGDYCQAQHDVYADK-
>Myotis_lucifugus
--------------------------------------------------------MIRPQSSMSNHI-P-FCGVLGHTFME-FLKGSGDYCQAQHDLYADK-
>Oreochromis_niloticus
--------------------------------------------------------MIKLQSSMSKHI-PQFCGVLGHTFME-FLKGSGDYCQAQHAAYADE-
>Pterocles_gutturalis
--------------------------------------------------------------------------------ME-FLKGSGDYCQAQHDLYADK-
>Eucidaris_tribuloides
-------------------------------------------MCKH---------------T-----E--VLAVINKPFMA-LVLDSGEFCQSQHK------
>Dasypus_novemcinctus
--------------------------------------------------------MIRPQSSMSKHI-P-FCGVLGHTFME-FLKGSGDYCQAQHDLYADK-
>Latimeria_chalumnae
--------------------------------------------------------MIKIQSSMSKHI-PQFCGVLGHTFME-FLKGSGDYCQAQHDLYADK-
>Dastarcus_helophoroides
-------------------------------------------MCM--------------------AIV--YFGELIPSFME-FHLDSAEYCQAQHK------
>Colius_striatus
--------------------------------------------------------------------------------ME-FLKGSGDYCQAQHDLYADK-
>Columba_livia
--------------------------------------------------------------------------------ME-FLKGSGDYCQAQHDLYADK-
>Leptosomus_discolor
--------------------------------------------------------------------------------ME-FLKGSGDYCQAQHDLYADK-
>Corynactis_australis
-----------------------------------------MWRGN-----------------------R-VYGNRAHTFME-FIIDSGDWCQAQHE------
>Alligator_mississippiensis
--------------------------------------------------------MIRLQSSMSNRR--PFCGVLGHTFME-FLKGSGDYCQAQHDLYADK-
>Cuculus_canorus
--------------------------------------------------------------------------------ME-FLKGSGDYCQAQHDLYADK-
>Sinocyclocheilus_grahami
--------------------------------------------------------MIKLQSSMSKHI-PQFCGVLGHTFME-FLKDSGDYCQAQHGVYAEK-
>Austrofundulus_limnaeus
--------------------------------------------------------------------------------MA-FARLGGEFCQAQHLLLIVDK
>Sarcophilus_harrisii
--------------------------------------------------------------------------------ME-FLKGSGDYCQAQHDLYADK-
>Forficula_auricularia
--------------------------------------------------------------------------------ME-FRLDSAEYCQAQHK------
>Galeopterus_variegatus
--------------------------------------------------------------------------------ME-FLKGSGDYCQAQHDLYADK-
>Monodelphis_domestica
--------------------------------------------------------MIRPQSSMSKHI--PFCGVLGHTFME-FLKGSGDYCQAQHDLYADK-
>Occasjapyx_japonicus
-------------------------------------------MCR--------------------DLV--CLGKIGLTFME-FFLDSGEYCQAQHK------
>Macropus_eugenii
-----------------------------------------------------------------MKA-KQFCGVLGHTFME-FLKGSGDYCQAQHDLYADK-
>Evechinus_chloroticus
-------------------------------------------MCKP---------------A-----E--VSAISNLAFMA-SVLDSGEYCQSQHK------
>Eurylophella_sp._AD-2013
-------------------------------------------MCR--------------------DLV--FLGEIGLTYME-FQLDSGEYCQAQHK------
>Esox_lucius
--------------------------------------------------------MIKLQSSMSKHI-P-FCGVLGHTFME-FLKGSGDYCQAQHVAAYADE
>Falco_cherrug
--------------------------------------------------------------------------------ME-FLKGSGDYCQAQHDLYADK-
>Leptasterias_sp._AR-2014
-------------------------------------------MSH-----------------------S-TGISSVPVSMA-FVSISGEYCRSQHK------
>Meleagris_gallopavo
--------------------------------------------------------------------------------ME-FLKGSGDYCQAQHDLYADK-
>Gavia_stellata
--------------------------------------------------------------------------------ME-FLKGSGDYCQAQHDLYADK-
>Acanthogobius_hasta
--------------------------------------------------------MIKLQSSMSKHI-PQFCGVLGHTFME-FLKGSGDYCQAQHVVDADE-
>Balearica_regulorum_gibberic
--------------------------------------------------------------------------------ME-FLKGSGDYCQAQHDLYADK-
>Okanagana_villosa
-------------------------------------------MCM--------------------AIV--FLGETWLTFME-FHLDSAEYCQAQHK------
>Leptopilina_boulardi
-------------------------------------------MCM--------------------AIL--FPDECILTYME-FQLDSAEYCQAQHK------
>Condylura_cristata
--------------------------------------------------------MIRPQSSMSKHI--PFCGVLGHTFME-FLKGSGDYCQAQHDLYADK-
>Ochotona_princeps
--------------------------------------------------------------------------------ME-FLKGSGDYCQAQHDLYADK-
>Azaleodes_micronipha
-------------------------------------------MCM--------------------AAV--LEVSSLATLME-FRLDSAEYCQAQHK------
>Lingula_anatina
--------------------------------------------------------------------------------ME-FVTNSGEYCQAQHK------
>Elephantulus_edwardii
--------------------------------------------------------MIRPQSSMSKHI-PQFCGVLGHTFME-FLKGSGDYCQAQHDLYADK-
>Metallyticus_splendidus
-------------------------------------------MCM--------------------ALV--FLGETVLTYME-FQLDSGEYCQAQHK------
>Nannochorista_philpotti
-------------------------------------------MCM--------------------AIV--VFPTVALRYME-FHLDSAEYCQAQHK------
>Ornithorhynchus_anatinus
--------------------------------------------------------MIATHSSMSKH--SPFCGVLGHTFME-FLKDSGDYCQAQHDLYADK-
>Cypridininae_sp._BMR-2011
---------------------MED----SETS---LLYKYRLCMRP-----------------------Q-VLAGVCCSFME-FVLADGEYCQAQHK------
>Perna_viridis
-----------------------------------------MCRNI-----------------------L-FFGVLGHTFME-FVTNSGDYCQAQHV------
>Osmylus_fulvicephalus
-------------------------------------------MCM--------------------ARV--FLGETVLTYME-FQLDSAEYCQAQHK------
>Epiophlebia_superstes
--------------------------------------------------------------------------------ME-FQLDSGEYCQAQHK------
>Opisthocomus_hoazin
--------------------------------------------------------------------------------ME-FLKGSGDYCQAQHDLYADK-
>Prosarthria_teretrirostris
-------------------------------------------MCM--------------------AIV--SRGEIVLTYME-FQLDSGEYCQAQHK------
>Crassostrea_gigas
MRDPR-----GPVTVRGKFVTGF-------VL---CSSLLNCWQHT-----------------------E-FRSNFGHTYME-FVTNSGDYCQAQHV------
>Sturnus_vulgaris
--------------------------------------------------------MIRLQSSMSNHI-PQFCGVLGHTFME-FLKGSGDYCQAQHDLYADK-
>Pecten_maximus
--------------------------------------------------------------------------------ME-FVTNSGDYCQAQHAL-----
>Egretta_garzetta
--------------------------------------------------------------------------------ME-FLKGSGDYCQAQHDLYADK-
>Ceratotherium_simum_simum
--------------------------------------------------------------------------------ME-FLKGSGDYCQAQHDLYADK-
>Medauroidea_extradentata
-------------------------------------------MCM--------------------ALV--FTAGVAVRHME-FLLDSGEYCQAQHK------
>Menopon_gallinae
-------------------------------------MNFRFSGCM--------------------ALK--NFHSTSSRLME-FFLDSAEYCQAQHTK-----
>Glycera_dibranchiata
-----------------------------------------MEQCHP---------------VLKTHSK--FFGVLGHTFME-FLCDSGEYCQAQHV------
>Leuctra_sp._AD-2013
-------------------------------------------MCM--------------------ALV--LVGEIVLTYME-FQLDSGEYCQAQHRK-----
>Homo_sapiens
--------------------------------------------------------------------------------ME-FLKGSGDYCQAQHDLYADK-
>Loxodonta_africana
--------------------------------------------------------MIRPQSSMSKHI-P-FCGVLGHTFME-FLKGSGDYCQAQHDLYADK-
>Fulmarus_glacialis
--------------------------------------------------------------------------------ME-FLKGSGDYCQAQHDLYADK-
>Melopsittacus_undulatus
--------------------------------------------------------------------------------ME-FLKGSGDYCQAQHDLYADK-
>Ectopsocus_briggsi
--------------------------------------------------------------------------------ME-FFLDSAEYCQAQHRK-----
>Leucoraja_erinacea
---------------------------------------------------------------MRKHI-PQFCGVLGHTFME-FLKGSGDYCQAQHDVYADK-
>Inocellia_crassicornis
--------------------------------------------------------------------------------ME-FQLDSAEYCQAQHK------
>Rhopilema_esculentum
----------------------------------------------------------------MNSYHLLLSKYFGKPFME-FLKDSAEWCKAQHVE-----
>Rattus_norvegicus
--------------------------------------------------------MIRPQSSMSRHI-P-FCGVLGHTFME-FLKGSGDYCQAQHDLYADK-
>Ctenactis_echinata
-----------------------------------------MWRFN-----------------------L-VYGSRAHTFME-FIIDSGDWCQAQHE------
>Aurelia_aurita
----------------------------------------------------------------MTSYHLQL-KCFGETFME-FLKDSAEWCKAQHVE-----
>Lepisosteus_oculatus
--------------------------------------------------------MIKLQSSMSKHI-PQFCGVLGHTFME-FLKGSGDYCQAQHGVYADK-
>Sepia_pharaonis
--MCR-----NILDLLGGIVKKTD----SPST---LAWPHHLWRLV-----------------------K-FFGEFGHTFME-FVTNSGDFCQAQHV------
>Sepioloidea_lineolata
--------------------------------------------------------------------------------ME-FVTNSGDFCQAQHV------
>Trichomycterus_areolatus
--------------------------------------------------------------------------------ME-FLQGSGDYCHAQHRDA----
>Oropsylla_silantiewi
--------------------------------------------------------------------------------ME-FQLDSAEYCQAQHK------
>Trichechus_manatus_latirostr
--------------------------------------------------------MIRPQSSMSKHI-PQFCGVLGHTFME-FLKGSGDYCQAQHDLYADK-
>Aptenodytes_forsteri
--------------------------------------------------------------------------------ME-FLKGSGDYCQAQHDLYADK-
>Henricia_sp._AR-2014
-----------------------------------------MCQLT-----------------------AILNTISVPTLMA-FAGISGEYCRSQHK------
>Crotalus_horridus
--------------------------------------------------------MIRLQSSMSNHI-PQFCGVLGHTFME-FLKGSGDYCQAQHGLYADK-
>Stylops_melittae
-------------------------------------------MCI--------------------GIL--NLGNKVVEYME-FHFGSSEYCKAQHK------
>Tyto_alba
--------------------------------------------------------------------------------ME-FLKGSGDYCQAQHDLYADK-
>Struthio_camelus_australis
--------------------------------------------------------------------------------ME-FLKGSGDYCQAQHDLYADK-
>Sphaerechinus_granularis
-------------------------------------------MCKP---------------A-----E--DSAISNQAFMA-TVLDSGEYCQSQHK------
>Chelonia_mydas
--------------------------------------------------------------------------------ME-FLKGSGDYCQAQHDLYADK-
>Watasenia_scintillans
--------------------------------------------------------------------------------ME-FVTNSGDFCQAQHV------
>Gynaikothrips_ficorum
MVHC-------------AKFNGEN----SISMVIRRVIFDNKWPSV--------------------GYQ--TCGEILPTFME-FHLDSAEYCQAQHK------
>Tinamus_guttatus
--------------------------------------------------------------------------------ME-FLKGSGDYCQAQHDLYADK-
>Hydroptila_sp._AD-2013
--------------------------------------------------------------------------------ME-FRLDSGEYCQAQHK------
>Calypte_anna
--------------------------------------------------------------------------------ME-FLKGSGDYCQAQHDLYADK-
>Apaloderma_vittatum
--------------------------------------------------------------------------------ME-FLKGSGDYCQAQHDLYADK-
>Orycteropus_afer_afer
--------------------------------------------------------MIRPQSSMSKHI-PQFCGVLGHTFME-FLKGSGDYCQAQHDLYADK-
>Eurhomalea_rufa
--------------------------------------------------------------------------------ME-FLTNSGEYCLAQHVHV----
>Limulus_polyphemus
--------------------------------------------------------------------------------ME-FLKTAGEYCQSQHK------
>Protopalythoa_variabilis
---------------------------------------------------------------MR-RDY--LAYDRGRTLME-FLIDSGDWCQAQHE------
>Tricholepidion_gertschi
--------------------------------------------------------------------------------ME-FQLDSGEYCQAQHK------
```

HG0002 ENSDARG00000003077

>Danio_rerio
---MAANSVYDFTVETL----EE-----KSVSLDIFRGKVLLIMNVATF---
>Acipenser_sinensis
---MSLKMAGGIKKFYDFSAKIL----NT----GEALNFSKFKGKVVLIENVASL---
>Anemonia_viridis
------------------------------MLRTVFQFSS--------------------V----SR----PVS----RLIK--------SQIRSMASVHDFTVQ-N----LQ----GEPVSLSKYKGKVILIENVASL---
>Talitrus_saltator
---------------------MK---PILALLATAGLANA--------------------------L----YNPDNATECC------------AVDGSLFDFKLKNL----TE----DGSYDLSKLRGKVVLVVNVATF---
>Channa_striata
---MAGNVTGFYEITAKLL----S-----GETFSFSTLKGKVVLLENVASL---
>Anguilla_anguilla
---MAGNIKKFYDFSLKLL----S-----GDVLNLSSLKGKVVLIENVASL---
>Hydractinia_symbiolongicarpu
---MNSSARMASFYDFSVKTL----K-----GETKSMSDYKGKVVLIENTATL---
>Microhyla_fissipes
---MVMCRVMAVRSVFEFSARLL----S-----GEAVSLERYRGKVLLIENVASL---
>Nephilengys_cruentata
------------------MGWLG---LAFATLACGVIL----------------------------G----SAAKVPSRSCF--------RPGQSDLTVYNFTLSDL----LQ----TRNISLSEYQDKILLIVNVATY---
>Meinertellus_cundinamarcensi
--------------MGAWSPWMA---ALSVLVAY-------------------------------VG----ATDVVKSRPCY--------HNRRANTTIYEFTAQDM----MK----TKNISLSDYRGKVVLIVNVATY---
>Scapharca_broughtonii
---MAGRASQGALLLATV-LG----IV----DATLPRKAVCT--------QPKNATATIYDYSLKEI----YG----VKTLNMSMFQGQVVLFVNVATY---
>Argulus_siamensis
---------------------------MNRRIADKSLLTT--------------LLLCLLA----GW----SRADLPSVG----------CRGNFPGTIHHYNVTAL----DG----QSLIDFKLFKNQVLLIVNVATY---
>Menidia_menidia
---MENKSVYDFSVETL----DG-----HQVPLRRFRGKVLLIVNVATF---
>Opsanus_beta
---MAAVRKVKRFYDLTAKLL----S-----GETLTFSALKGKVVLIENVASL---
>Oryzias_latipes
---MGEKSVYDFFATSL----DG-----QQIPLGTFRNKVLLIVNVATF---
>Cryptocercus_wrighti
---MITIEKRPCTILGTNMHL-------------AGILAL-------------LLLTLHGS----HG----KVERVASVQCD--------NEDLIENSIYNYTLPDL----WE----VQNVSLSDFRGKVVLIVNVATY---
>Tripterygion_delaisi
---MSTIYDFTSKLL----T-----GETFSFSSLKGKVVLIENVASL---
>Calanus_helgolandicus
---MPAFYDIRAKKI----G-----GEVVSMADYKGKVVLIENTASL---
>Arenicola_marina
---MAESKEK--------DWWKKAGSIYEFSAQDI----DG-----NDTSLEKYKGHVCLIVNVATK---
>Trachinotus_blochii
---MAGNLKRFYGLTAKLL----S-----GETFSFPALRGKVVLIENVASL---
>Acinonyx_jubatus
---MCAAPLAAA-ALAAAAPRSVYSFSARPL----AG----GEPMSLGSLRGKVLLIENVASL---
>Hynobius_retardatus
---MAYVAKSFYDLKATNL----E-----GETVDFNIFRGRVVLIENVASL---
>Micropterus_floridanus
--MATTLYNFTAKLL----T-----GETFNFSSLQGKVVLIENVASL---
>Balaenoptera_acutorostrata_s
------------------------MTPVARLLKGGAS----------------RTPCSSSP-----R--LSPVSLGTHTMCAAQRSAA-ALAAAAPRSVYAFSARPL----AG----GEPVNLGSLRGKVLLIENVASL---
>Pygocentrus_nattereri
--MANVKSFYDITAKTL----T-----GEEFKFSTLQGKVVLIENVASL---
>Callorhinchus_milii
--MANSRKFYGFSTKLL----N-----GQTLNFSKFKGKVVLIENVASL---
>Rhinolophus_ferrumequinum
--------MGRGRGGGAAGKVLSPHDPIARLLKGGAA----------------RDPFCRVP-----S--ATPS--ETSAMCAAQLVAA-ARAAAAPRSVYDFSARPL----AG----GDPVNLGSLRGKVLLIENVASL---
>Haplochromis_burtoni
--MATSFYDLTAKLL----T-----GETFNFSSLKGKVVLIENVASL---
>Dasypus_novemcinctus
---MCAAALSAA---ALAAQRSVHAFSARPL----AG----GEPVSLGSLRGKVLLIENVASL---
>Clupea_harengus
--MAAIKTLYDISAKLL----T-----GELLNFSSLKGKVILIENVASL---
>Latimeria_chalumnae
---MAVGMKKFYDFSAKIL----NT----GELLSFSKFKGKVVLLENVASL---
>Rhodactis_indosinensis
-----------------MYLWYV---FVAFCLYWGNIGGT--------------------------E----RQK----SRSL--------CREEHKKSFYDFSMK-N----LD----GELVSMSDFRGHVVMAVNVATF---
>Alligator_mississippiensis
------------MNWGRLAKRAL-----------------------LCG----------------AVAARGLG-RSMCAQVD--------EDWRSAKSIYYFHAKDI----DG-----NDVSLEKYQGQVCIITNVASK---
>Echinococcus_multilocularis
---MCASR------LSAAAQSTVFSFSARPL----AG----GEPVSLGSLRGKVLLIENVASL---
>Cyprinodon_variegatus
---MEAKSIYDFSVETL----E-----GENVPLSVYRGKVLLIVNVATF---
>Sarcophilus_harrisii
---MC---------AAAAVPRSVHAFSARLL----GS----GELVSLASLRGKVLLIENVASL---
>Procambarus_clarkii
------------------MLW-------------AGLVSLAAVG---------V-------------V----AQDQVASRACF--------HHHSDGGNIYDFEEMDL----LE----TRNISLADFRGKVLMIVNVATY---
>Sclerodactyla_briareus
---MSSKARTIYSFSAKAL----EG----GGIVDMARYRGKVLLIENVASL---
>Galeopterus_variegatus
---MCATR------LAAVAHSSVHAFSARPL----AG----GEPVSLGSLQGKVLLIENVASL---
>Occasjapyx_japonicus
--------------MRVDGRGVS---PTPRNP-------------LAGGVAALVLLGCMPWSS----WAASPPDQVPSRPCY--------HNLSLGGDVYDFSEEDL----DR----ASNVSLADFRGKVLLVVNVATY---
>Evechinus_chloroticus
---------MVSRVLHRISRAVP---QFG--R------------TACGVGAILSVGVYFYASRNSPFVTTMA-AGAGKNDP--------NWWKTAQNIYAFSANDI----DG-----NEVSLEKYKGHVCLIVNVASS---
>Ciona_intestinalis
--MHQKGAVPAVLLVLG----TL----AVA-TSIENS---------TCFHNTFSVYDHSFPTL----SG----E-TVNLGEFRGNVSMVINVATY---
>Esox_lucius
----MYVRYC-YKMWR-INPVLV-----------------------VFG------------------TLASCG-LLAMSHPT--------EDWQKATSIYDFSAKDI----DG-----NVVSLEKYRGDVVIIVNVASK---
>Gadus_morhua
--MASKFFEITAKRL----T-----GEVLNFSSLKGKVVLIQNVASL---
>Glomeris_pustulata
--MNTSCCTSFYEFSAKDI----Q-----GKDVDFSKYKGKVVLIENVASL---
>Acanthogobius_hasta
---MASAEFYKFSPKLL----S-----GDTLNFSSLQGKVVLIENVASL---
>Sipunculus_nudus
----MLTGYHSGRLLTKLNEG---------V-------------LACA---------------LTGCVNRFS-AMAEAKDK--------DSWKKAQSIYEFTVKDI----DG-----NEVSLEKYKGHVCLIVNVASK---
>Chanos_chanos
--MAESKTLYDFSTKSL----T-----GEVFNFSALKGKVVLIENVASL---
>Galloisiana_yuasai
--------MAPS------------------FLGLGGILAV-------------FLLALAGS----DA----LVKRVPKVPCQ--------DHREINGSIHDYTIQRF----WD----SCNISLSDYQGKVVLLANVATY---
>Apteronotus_leptorhynchus
--MAKSFYDIKAKTL----E-----GAEFAFSSLQGKVVLIENVASL---
>Glossoscolex_paulistus
---MADARPK--------DWWKKAASIYDFTVKDI----DG-----NEVSLEKYRGHVCLIVNVATK---
>Cimex_lectularius
---MMKAQIFLAFATLFVG----SGLATSTVARVAKRKC---------LDAGDDANVYNFSFMDL----SG----KTNVSLGDFKGKMILVVNVATY---
>Oscarella_carmela
--MSAIKSFFQLAARSL----T-----GELVAFSRYKGKVLLVENVATA---
>Lasius_turcicus
---MAATSIYDFKANDI----DG-----HEVSLEKYKGHVVLICNVACK---
>Erinaceus_europaeus
---MCAAVRSVHAFTARPL----SG----GDALDLGSLRGKVLLIENVASL---
>Armadillidium_vulgare
--------------------------MIRYLLLLGTL----------------------LG----PS----MGESYDSVNC------------TAEEDIYQFSAKLL----DG----SATVNFSDYRGKVFVIYNVATY---
>Ixodes_persulcatus
--MATSAS--------DGWKTAASIYDFDAVDI----DG-----NKVSLSKYRGHVALIVNVATK---
>Oryctolagus_cuniculus
---MCAAR-------MAAAAQSVYSFSAHPL----AG----GEPVNLGSLRGKVLLIENVASL---
>Sarsinebalia_urgorrii
--MAFYSFTAKLL----S-----GEAVSMSRYKGKVILIENTASL---
>Clytia_hemisphaerica
MAKSILFDLKVSTLLGIIVTVVA---MLA--I------------YKA------FTGYLFPT--LS-AKETAN-TNLLHSNP--------QSPEKASSIYDFSALDI----DG-----NMVNLSKYNGLVTYIVNVASE---
>Lingula_anatina
--MARSPVKIRNFFDFSAKLL----S-----GEDINMSRYKGKVILVENVASL---
>Lithobius_forficatus
--MSFYDLAAKNL----V-----GQQVSFSKYQGKVVLIENVASL---
>Lutjanus_erythropterus
---MARNVKRFYDLTAKLL----S-----GEMFSFSALKGKVVLIENVASL---
>Macrostomum_lignano
--M---------ASSAETSTVYDFEAVDI----DG----N-NVSMDKYKGRVLIVVNVACK---
>Ornithorhynchus_anatinus
---MCASPNSVYGFSGRLL----RS----GEPFSLAALRGKVLLIENVASL---
>Mytilus_californianus
-----------------ME---------------GF-VDILTI----------VLATLWSV----SG----QDLSSRQVACY--------HHDDNLHSVYNFSMPDI----HG----EKTIDFSHYRGKVLLLVNVASF---
>Triops_newberryi
------------------------------------MASRLLLP---------VLLF---V----AG----VRSDTHVGSTL--------CNSSTTGTIYPFSQKRI----LG----GEVVNFSDFEGKVVLIVNVATY---
>Hapalochlaena_maculosa
MVEPLTTSMEPSKLMFLSHLLAS---FLSICLCEGFSITTGLTEEMTTRQNH-VHSHGHSH----GH----LHRSTNFLTCY--------QSDNHSTSLYDYKSYDI----HG----VEEIPLSVYRNKVLLIMNVASF---
>Calopteryx_splendens
--------------MGEGKHWLA---PLLCLVSLGLG-GV--------------------------D----AGDRVASRMCS---NFSPNSVKSPRNGVYNFSIPDV----YG----RRNISLSEYQGKVLLIVNVATY---
>Ophiocoma_echinata
---------MFASVLRVLSHTVS---KYP--P-------------------SLPLGF--A---GAAVVTKSL-CTMAEGGP--------SAPEKASSIYEFTVKDI----DG-----NDVSLEKYKGHVCLIVNVASK---
>Clonorchis_sinensis
--MAS---------CQTKPEETIYNFTVKSI----DG----E-SICLSQYAGFVCLVVNVASK---
>Tetrix_subulata
---------------------------MEALASFVFLVT--------------LLSPCALA----AS----KVERVSSLPCS--------TDYTDGDTIFNFTAEDI----HK----QRNISLSKFRGKVVLIANVATF---
>Osmerus_mordax
---MSGTIKTFYDFTAKLL----S-----GNILKLSSLQGKVVLVENVASL---
>Scleropages_formosus
--MAGEKTFYDFSAALV----T-----GELFSFSSLRGKVVLIENVASL---
>Crassostrea_gigas
--------------------------MAGACCSLAFLAFLGV-------------------------I----CRVSGTEVVCN--------QPPSSAESVYSFTAPDI----YQ----TKNVSLSKYRGKVLVITNLASF---
>Endeis_spinosa
--MIVNTAAF---
>Pecten_maximus
---MASVVNV--------RSPVKIQNFFDFHATTL----EG-----EKINFSTYRGKVILVMNVASL---
>Lateolabrax_japonicus
--MAASFYDFTSKLL----T-----GETFDFSALKGKVVLVENVASL---
>Sebastes_rastrelliger
---MTMAGNVKRFYDLTAKLL----S-----GKSLSFSALKGKVVLIENVASL---
>Ceratotherium_simum_simum
---MFAA---HL---ASVAPRSVYAFSARPL----AG----GELVSLGSLRGKVLLIENVASL---
>Laternula_elliptica
---MAVQRGQLLVAAL-VG----LL----APAHGARVQCT--------MPTNFTDTVYNYNAMNA----YE----NQTIDLSQYRGKVLAIMNVATY---
>Manis_javanica
---MC----------ASAALRSVYAFSARPL----AG----GEPVSLGSLRGKVLLIENVASL---
>Spadella_cephaloptera
---MVRLTTSLLKSFYDLSAKTLACGGYT----ASNVTFDAFKGKAVLIENVASL---
>Glycera_dibranchiata
------------------------------MIRDGGVLLL-------------PLLVASI----SL----AVDCLQMSTC------------SPEESIYDHYLN-D----LD----GRPVSLEKYAGNVLLIINTATF---
>Paralichthys_olivaceus
---MAGNAKRFYELTAKLL----S-----GESFSFAALKGKVVLVENVASL---
>Rhinopithecus_bieti
---MCAAG------LAAAAAQSVYAFSARPL----AG----GESVSLGSLRGKVLLIENVASL---
>Loxodonta_africana
---MCAAP---V---AAAALRSVHAFSARPL----AG----GEPVNLGSLRGKVLLIENVASL---
>Pinctada_maxima
---MYSVCDERTDGKTLYSMDTKDL----D-----GGVKELSDYRGAVTMVINVATF---
>Fukomys_darlingi
---MCAAR------LAEAAQATVYSFSARPL----AG----EEPLSLGSLRGKVLLIENVASL---
>Owenia_fusiformis
-------------------------------M------------TMGLLTSALLLLCGFL-------APGLAHNEAKTACY--------HSRRNNASIYDYRVETL----SKYFPDKDIVNLSDYRGKVVLIVNVATY---
>Oncorhynchus_kisutch
--MACVKSIYDFSAETL----NG-----QSVPLSIYRGKVLLIVNLATF---
>Tupaia_chinensis
---MCAAQA--------AAVAQATVYXARPL----GA----EEPMSLGSLRGKVLLIENVASL---
>Porites_astreoides
-------------------MWVV---NLAYLLATAFVASAMTIT---------QTLTTTSP----RK----FHKSNNLLTCY--------QSDNHTTSVYDFVMKDV----YG----EKTIPLSIYKGKVLMIVNVASF---
>Thunnus_thynnus
---MAVRRFYDLTVKLL----S-----GETFSLSALKGKVVLIENVASL---
>Tityus_serrulatus
---MAKRSFYEFSAKLL----N-----GETLNFSQYRGKVILIENVASL---
>Lepisosteus_oculatus
---MAGGMKKFYDFSMNLL----ST----GEAYNFSTLKGKVVLIENVASL---
>Sepia_pharaonis
---MQ-N----LD----GNVVSLKKFKGKYLMIVNVATF---
>Euprymna_scolopes
---MSRSPATPTGRKSFFDFSAKTL----A-----GENIDFSRFKGKVVLVENVASL---
>Ictalurus_furcatus
--MAKSFYDLSVKTL----T-----GEELKLSCLQGKVVLIENVASL---
>Caligus_rogercresseyi
--MENSFYDFKVEKL-----N----GSTMHLNTLKGKIVLIENTASL---
>Trichechus_manatus_latirostr
---MCAALGADVAALAAAVPRSVHAFSARRL----AG----GEPVSLGSLRGKVLLIENVASL---
>Pagrus_major
---MAKFYDLTAKLL----T-----GETFKFSSLRGKVVLIENVASL---
>Henricia_sp._AR-2014
-------------MRGIGTA--------------ALAVAL-------------ILLPGY-------G----SALGLLESRC------------AGNTSIYQHTFTTL----NG----SQEITLSQYKGKILLVVNVASF---
>Streblospio_benedicti
---MAARSIFQFTAKLI----N-----GQEVNLNSFKGKVVLVENVASL---
>Squalus_acanthias
---MSAQP--------ADWQKATSIFEFDAKDI----DG-----NEVSLEKYKGFVSIITNVASK---
>Protobothrops_mucrosquamatus
--MTTPIAKSFYDLSATDL----Q-----GEKVDFNHFRGRVVLIENVASF---
>Schistosoma_mansoni
--MS--------SSHKSWNSIYEFTVKDI----NG-----VDVSLEKYRGHVCLIVNVACK---
>Nesiohelix_samarangae
--------MGPW---------------------------------STTGPGGIMIAFCGLF----AA----ASAGVIMLDCK--------QPVSDKTTLYDFTLMDV----HR----VKNISLSDYRGKVVLIVNVATY---
>Symphylella_vulgaris
----------------------------MKSQ-------------MLVHL-AFIVLFCHLASA----L------KLDFTNC---------TAPEANQDIYKFVAEDL----NR----TKNVSLSEFRGKVLLIINVATFCDSTTQYKGLRTLQTEFGSENFSVLAFPCNHLACKTSSQCH
>Monopterus_albus
-------------------MNLI-----------------------QST------------------VLFSFL-LQAMSGLT--------EDWQRATSIYDFSATDI----DG-----NVVSLEKYRGNVVIITNVASK---
>Alvinella_pompejana
---MATA-SEEKSEKH--------DDWKKATSIYDFTCKDI----DG-----NEVSLEKYRGHVCLIVNVATN---
>Chelonia_mydas
---MGLQ-FTYSCAQA--------DDWRAAKSIYEFHAKDI----DG-----SDVSLEKYRGFVCIITNVASK---
>Takifugu_rubripes
---MAGSVKKFYELTAKLL----S-----GEVLSFSALRGKVVLIENVASL---
>Watasenia_scintillans
--MARTPATAKTFFDFSAKTL----A-----GENVDFSRYKGKVILVENVASL---
>Clio_pyramidata
--------MG-R---------------------------------TSVNSGGLLACLCGLV----VA----AAGSAVMLDCH--------QPANDPKSIYDFTLMDI----HH----QKNVSLSDYRGKVVLIVNVATY---
>Phagocata_gracilis
--MVVNTACK---
>Orycteropus_afer_afer
---MCAAPLTAAAARAAAAPRSVYAFSARPL----AG----GDPVSLGSLRGKVLLIENVASL---
>Villosa_lienosa
---MAVRSHVKPQNFFDFTAKTL----K-----GQDVNLSNYKGKVILVENVASL---
>Mastigoproctus_giganteus
--MFASKSFYDFAAKTL----Q-----GETIEFSLYKDRVVLIQNVASL---
>Patiria_pectinifera
------------------------------MA-------------TAGLWMPASAVLMILWSV-----AAAAG-GTLESIC---------RADGAASTVHQYSLHPL----DE---TSPPVPLSDFAGKILLLINVATY---
>Sinonovacula_constricta
---MAVPAGQFVLLSMA-G----LL----ALASGTRVSC---------VPGTPAKTVYDFSAMSI----YE----NETISFDRYKGKVLAIINLATF---
>Limulus_polyphemus
--MSIAKSFYDLAAKSL----T-----GEIINFSKYEGKVVLIENVASL---
>Palythoa_caribaeorum
-----MEAFEASRLKS------------LALILFGFVVFA---------------------------Y----ATDR-PKQVSD--------CQNRQKDSFHDFAALDL----H-----EEYIPFKRYEGKVVLVVNVATF---
>Thermobia_domestica
---MG----NK----MP---WKSNK---------LLTDIPNSLYDLSAIDI----DG----Q-KVGLSKFAGKCVMVVNVASK---

HG0003 ENSGALG00000014906 MOCS2

>Gallus_gallus
------------------------MSCQVTVLYFARSAELVGLRSES-V--SVPQRIT--SL-Q-----------LW---EEIVKIHPRLA--VIRD---QVVFAVRQEYVLLGD--QLLVLQTGDEVAIIPPISGG
>Acipenser_sinensis
------------------------MTTEVVVLYFAKSAELAEVRSEN-I--TVSHQVT--SL-Q-----------LW---QELVRKHPRLA--AIRD---QVVLAVRQEYVSLSD--ELLILHPGDEVAVIPPLSGG
>Adineta_ricciae
-----------------------MSTIQVAIIYFGRLRELVGISSEK-I--DFDSAIS--YTQQ----------SIL---DRIIEHRPILKSFLVTN---TFRLAVNQQYLLEGD---LIQFSKESEVALLPPFGGG
>Acanthogorgia_aspera
--MLALNEIYIDLE---ETVNLRSTDKIAVIPPLSGG
>Amia_calva
------------------------MNTEVVVLYFAKSAEIADVRSET-I--SVPQEIT--SL-Q-----------LW---QELVNRHPSLS--VLRD---QVVLAVRQEYVALGE--QLLSLQSGDEVAVIPPLSGG
>Talitrus_saltator
------------------MTQYGLEIVPVKIVFFASARELSGCSEVE-IDLSTPTT-----AKK-----------LF---SEVLNKHPELQ--KLEK---SVMVAVNQEYIDD-DT-NQVSLRAGDEVAFIPPISGG
>Podocoryna_carnea
--------------------MEESFKVPVSILFFAKSRELVGKTSDT-I--YLEQKTY---LKK-----------LL---EEIIHAYPSLK--NIGN---NFIIAYNQKYLTE-SE-DCITLKASDEIAIIPPISGG
>Megophrys_sangzhiensis
------------------------MTCEVVVLYFAKSSELTGVRSEI-I--NVPQEIT---SQN-----------LW---EEIVRFHPRLC--TIKD---QVVFAVRQEYVAI-GD-ELLSLRPGDEIAVIPPISGG
>Milnesium_tardigradum
------------------------MEISVKVLFFAKAKDLVGTGQGI-L--TLKEDIL--SLGS-----------LL---SNLYSQFPALL--PLQN---QVVLSLNQKYIDADDLQRPVSINIGDEIAVLPPISGG
>Apteryx_australis_mantelli
-------------------------MYDVLEVYWK--QNCVSLRSID-L--SCPRLCT--SL-LFKEISWLNANTLFRNLFVLLPIPLRLA--VIRD---QVVFAVQQEYVLLGD--QLLVLHPGDEVAIIPPISGG
>Machilis_hrabei
--------------------MAALCESEIRLLFFAKSRELAGKSEAS-I--CVPSLVH---VQD-----------LL---DLIVRE-FSLD--LIKN---NLILAHNQEYLDRE---QSVNIRTGDEIAVIPPLSGG
>Scapharca_broughtonii
--------------------MATPEEVQVTLLFFAKSRELSGLKTSQ-L--KTPTQVN---FQQ-----------LL---GLIVTAFPSLE--IISE---NIVLSLNEDYIDK--D-SSVNLNSGDEIAVIPPISGG
>Oryzias_latipes
------------------------MTAQVSVIYFGQSAELTGLKGEE-L--VSVPTPI--SSRE-----------LW---ELLQQRHPRLA--VLRG---HVVLAVAQQYIPIAE--QLVVLRDGDEVAVVPPLSGG
>Prorhinotermes_simplex
--------------------MADVSVVEIQVLFFAKARELCGQKQSC-L--LIP-SVT--SYNN-----------LL---DEIVTK-FSLE--SIRD---NLLLAINEEYTTSE---SEVQLKEGDEVAVIPPLSGG
>Eurytemora_affinis
-----------------------MADVDVKLILFAKARELVGASEVN-V--RFPQELS---SEH-----------LK---SKIIQSFPVLD--KLGG---TWLLALNEAYIDT----DRVTLNTGDTLAVIPPISGG
>Alatina_alata
---------------------MSNITVTVEVRFFAKARELAGIQKTH-V--RTRERIT---AHE-----------LI---SQIIDAFPKLD--VIRQ---CFLLAHNQEYCSL--D-QELNISDGDEIAVIPPISGG
>Hynobius_chinensis
------------------------MNSEVIVLYFAKSAELTGVRSET-L--SVPQQIT--PS-Q-----------LW---DEIVNRQPRLA--AIRD---NVVLAVRQEYVILGD--QLLTLQPGDEVAVIPPISGG
>Siniperca_chuatsi
------------------------MTAQVSVLYFAQSVELTGVKEEE-L--VAVPTPI--SSQD-----------LW---RLLLQRHPRLS--LLQD---QVVLAVRQQYVAIGD--QPVTLGDGDEVAVVPPLSGG
>Astyanax_mexicanus
------------------------MKSEVLVLYFAKSAELTGIKSET-I--SVQSELS--TL-Q-----------LW---QELEKLHPKLS--AVRD---QVVLAVRQEYVSLGD--QRVILRDGDEVAIIPPLSGG
>Callorhinchus_milii
------------------------MTAEVSVLYFARSAELSGVRSET-L--SVPQQLT--SM-Q-----------LW---EKIVNKHPSLI--AIRD---HVLLAVRQEYILLGD--QILFLQPGDEIAVIPPLSGG
>Oreochromis_niloticus
------------------------MTAQVCVLYFAKSVELTGVKEEK-T--VAVPTPI--SSHD-----------LW---KLLVQRHPRLC--TLQD---GVVLAVRQEYVPIGD--QVLTLGDGDEVAVVPPLSGG
>Alosa_alosa
------------MFGSA-VLQAPFMISEVSVLYFAKGAELTGVKSET-V--KLPSELT--TI-Q-----------LW---LELEKRHPKLC--VLRE---HVVLAVRQEYVDLCD--QTVSLHDGDEVAVIPPLSGG
>Latimeria_chalumnae
------------------------MTAQISVLYFAKSAELAGIRSEV-I--TVAQQIT--SQ-Q-----------LW---QEIVNRHPRLA--AIHD---HVILAVHQEYILLGD--QFLLLQPGDEVAIIPPISGG
>Priacma_serrata
------------------------MSVTVTILFFAKAREIVGKNSSS-L--EVPPALT--CT-D-----------LL---NTIVNE-YSLH--VIRN---NIILAVNEEFCAGN---DRLSLKTGDEIAVIPPLSGG
>Corynactis_australis
--------------MEH-INNDTTGKVEVTVLFFAKSRELVEQRSAE-V--LLSKRLS---GRE-----------LI---ASIVDNFPSLS--VIQD---NLVVAVNQSYVEK--E-SEIEFQGGEEVAIIPPISGG
>Cuculus_canorus
-------------------MTAELPLVQVSVLYFARSAELAGLRRET-L--RVPRQVT--AL-Q-----------LW---EEIVSLHPRLA--VIRD---QVVFAVRQEYVLLGD--QLLVLQPGDEVAIIPPISGG
>Ctenopharyngodon_idella
------------------------MNTEVSVLYFAKSAELTGLKAEN-I--TVPSNIT--SL-Q-----------LW---QDLENRHPRLS--VLRD---QVVLAVRQEYVPLGA--QPLTLRQGDEVAIIPPLSGG
>Fundulus_heteroclitus
------------------------MSAQVTVLYFAKSAELTGLKEEP-V--AVPTPIS--S-GD-----------LW---ALLLRKQPRLV--ALQG---HVILAVRQRYVAIGE--QQVTLEDGDEVAVVPPLSGG
>Petrolisthes_cinctipes
---------------------MTEREVSVSVLFFAGARDLAGVERSA-I--TLPVVTT--P-EQ-----------LL---ARVVRSFGELD--IIRD---SVIISLNQEYCVPG---QELVLSAEDEVAIIPPISGG
>Sclerodactyla_briareus
------------------------MPVDVTVQYYAISRDITGTREDR-L--TVPTHVT--L-KQ-----------LF---GCLTERHPGLK--QIET---NCLLAINGDWLTEES--GVVELKPGDEVAVIPPLSGG
>Forficula_auricularia
----------------M-NQDLNCDQVTVEVLFFAKARELSGVNKAI-I--QTPSLTN--S-EE-----------LL---DSLINR-FNLQ--QISS---CLVLAVDENWVESG----PLKLNSGSIIAVIPPLSGG
>Occasjapyx_japonicus
-----------------------MQTTELRVLFFASARELAKCNAAV-LRLDSAQ--Q--SGES-----------LL---EQVVLSFPDLQ--RVRG---SLMLALNQNYVQL--D-QRLTVSSGDELAVIPPISGG
>Sarcophaga_crassipalpis
-------------------MNSELPVINIHILFFAKARELSGLSESN-HQISTPKIIA---S-D-----------LL---EQIC-STYNLS--VIRD---NLIIAINESYCEN-LN-TELQLQEGDEIAIIPPISGG
>Echiniscus_testudo
--------------------------MQITVLLFARMRDLAGGRSEILI--DVPADCENGSV---------SSKRLL---QLIIAQCPRLK--EIK----HYQLAVNRQYVDLNSD-NLVQLGPTSEIALIPPVSGG
>Ciona_intestinalis
----------------------MGKNVQVNIKFFAKSRELVGIESEQ-L--ILPQDVT--SF-Q-----------LI---ETINSKYPRLS--DLQG---CFTLAVNGEYIKTDES-YSILLTDQSEIAVIPPLSGG
>Baetis_sp._AB-2009
----------------------MGQPIVVKVLLFASAREISGERESE-I--QVQQEET--Y-DT-----------LF---RKIVQR-FKLE--KLQN---SLILAINEDYPDAEN--TQLNLKPGDHIAVIPPLSGG
>Esox_lucius
------------------------MNTEVTVLYFAKSAELTGIKSET-I--RVPSELT--TL-D-----------LW---LDLVSRHPRLS--TVRE---SVVLAVRQEYVAVGE--LRVSLREGDEVAVIPPLSGG
>Leptasterias_sp._AR-2014
------------------MASICADVVEINVLFFAKSRELTGVKEGH-L--QVKTNLK--PE-E-----------LL---LQILSQYPQLS--IIQD---TVVLSVNQEYIAKGD--GTLNLQPGDEVAVIPPISGG
>Gadus_morhua
------------------------MTARVTVLYFAKSAELTGHRSEV-V--PLPAATS--SC-Q-----------LW---ALLLELHPSLA--ALQD---QVVLAVRQQYVALGD--QALLLADGDEVAVVPPLSGG
>Chlamydotis_macqueenii
--MLLGD--QLLVLQPGDEVAVIPPISGG
>Galloisiana_yuasai
--------------------MDLNLKISVNVLFFAKARELCGKKESK-L--QVVPSIS--Y-NL-----------LL---DHIIKE-FSLE--SIKG---NFILAINEEYTTTD---RELDLKEGDEIAIIPPLSGG
>Tigriopus_californicus
------------------------MSVSIKVLLFAKARELAGTHELT-L--NVVPNLS---ASD-----------LV---TQIEAQCPDLA--ILRG---SFVLALNQDYVDH--N-QVLDLKSGDELAVIPPISGG
>Acanthosoma_haemorrhoidale
----------------------MDCNVKIKILFFAKALELSGTKESF-L--SVSPNIS---TPL-----------LV---NKIV-DLFGLH--SISN---SILIAKNEEYVIKDGQ-EPLNLQEGDEIAIIPPISGG
>Myrmica_ruginodis
-------------------MDETATPIQATVLFFAKARELTGCKECK-L--CVPRKLS--SA-D-----------LL---DKIIHA-FELG--SIRN---QIILAVNEEFVVPD---SILVLSEKDKIAVIPPLSGG
>Rhipicephalus_appendiculatus
----------------------MRPEVPVRLLLFAKARELAGTSEAS-L--VVPAVLR--DVEA-----------LK---EVIFDAFPKLA--SLQR---SAVIAVNESYVDS--G-VEVTLRQGDEVAFIPPISGG
>Dyseriocrania_subpurpurella
--------------------MDDLQLVQVKVLFFAQSREVSGRREAS-I--NLPSKLS--CA-E-----------LL---DIFVDN-FSLS--AIKN---NILIAKNEELCCED---NLLEIKTGDEFAVIPPLSGG
>Lutjanus_erythropterus
------------------------MTAQVCVLYFAKSVELTGAKEEQ-I--VAVPTPI--SSQD-----------LW---KLLLQRHPRLS--VLQD---QVVLAVRQQYVAIGD--QVVTLGDGDEVAVVPPLSGG
>Mantis_religiosa
------------------MDVSSQQRVQIKVLFFAKARELSGIKESD-F--ISETPTS--Y-EL-----------LL---QQIVDK-YSLS--AIKS---SVVLAINEEYCSPS---EHFKLNEGDEVAVIPPLSGG
>Nannochorista_philpotti
----------------------MTEEVEVKLLFFANARDLAGINETL-L--LTRSKIL--CS-E-----------LL---RDITIN-YSLE--AIKN---NILLAVNEEYCESPN--TELNLKAGDEIAIIPPLSGG
>Dermanyssus_gallinae
-------------------------MVRVKLLFFAKARELAGTAERS-V--DVAERVF--SLDQ-----------LK---ELIAQLEPSVK--TLLD---TSILAVDLNYVVDSV----EIGTRTGEIAVIPPVSGG
>Ornithorhynchus_anatinus
-----------------------MVLSPVTVLYFAKSAELAGVRSET-I--SVPQEIK--SL-Q-----------LW---EEIESRHPRLA--DVRD---QVIFAVRQEYVQLGD--QVLLLQPGDEIAIIPPISGG
>Perna_viridis
---------------------MANNKVQVSLLFFAKSRELSGVSNSQ-I--KVSSQCS---FFQ-----------LF---ETIVQQYPSLS--VISE---NIILSLNEEYISR--D-GSITVQEGDEIAVIPPISGG
>Osmylus_fulvicephalus
------------------------MSVEVRILFFAKAKDFAQKSEST-L--NIPNTLT--F-KE-----------LL---TIIVDN-YNLQ--AIRN---TINISLNANFCDRD---ATLKFNTGDEIAVIPPLSGG
>Hapalochlaena_maculosa
--------------MDH-SFAGNKDPVKINVLFFAKSRELVGQNKSE-V--SITSRSV--TTAQ-----------LT---SFLLETFPRLK--PLKQ---SMIIALNEEYLSGD---ETIHLSNGDEIAVIPPLSGG
>Epiophlebia_superstes
--------------------MGDKDFVTVKVLFFAKARELSGIKFGQ-LKVNAAP--S---YTF-----------LL---EEIV-TKFSLE--KIRN---NILLAINEEYSGS--E-LQINLKDGDEIAVIPPISGG
>Opisthorchis_felineus
----------------------MKQAVRARVILFGEAKDLTGFQSAL-I--LVPVTCT--A-KE-----------LY---DLILGHFPKLK--LIEK---SLLLTLDFQYIDIEDNNTLLHISDSSELGVLPPLGGG
>Stenobothrus_lineatus
--------------------MDNQKKIEVKILFFAKARELAGCSESS-L--KVASHIS---YEE-----------LC---TLIV-ENFSLA--AIKK---NFILAVNEEYLSS--E-STLDFKEGDEIAVIPPISGG
>Osmerus_mordax
----------------------MTTKVSVLVLYFAKSADLTGLKSEV-I--HVQPELT--TL-A-----------LW---QELVNRHSRLS--AVRE---QIVLAVSQEYVTIGC--QLVSLKNGDEVAVIPPLSGG
>Scleropages_formosus
------------------------MNTEILVLYFAKSAELTGLRSEN-V--RVPPGLT--SL-Q-----------LW---EELVTRHPRLS--MLRT---QVVLAVREEYVPLGD--QPVRLQEGDEVAVIPPLSGG
>Crassostrea_gigas
----------------------MEGDVTINLLFFAKSRELVGSGSGE-L--VLTANTN---FDT-----------LK---QALLLKYPSLQ--VIAE---NIILSLNEEYIDD--N-QQLTLREGDEVAVIPPISGG
>Hypsibius_dujardini
--------------------------MDVNLKFLALAKDLAGTGDAT-I--RITAEESPFTVGS-----------FL---AKLIRTFPRLG--EIKD---QLILAHNLEYLDLQNPSASFELKEGDEIAVLPPVSGG
>Taeniopygia_guttata
------------------------MRCQVSVLYFARSAELAGLRCET-L--SVPQEIT--SL-Q-----------LW---EEIVKVHPRLA--LIRD---QVVFAVRQEYVLLGD--QLLVLQPGDEVAIIPPISGG
>Pecten_maximus
-----------------------MADVEVSVLFFAKSRELAGKKTAQ-IIVESPTN-----LQQ-----------LI---STIVSQFPSLQ--IIAD---NLVVSINEDYLQA--G-QEVHLNTGDEVAVIPPISGG
>Dissostichus_mawsoni
------------------------MTAQVTVLYFAKSAELSGLKEEE-L--VAVPTPI--SSQD-----------LW---SLLLQRHPRLC--VLQH---QVVLAVRQQYVAIGD--ELVALGDGDEVAVVPPLSGG
>Equus_asinus
----------------------MVPRCQVEVLYFAKSAEIAGIRSET-I--SVPQEIK--AL-Q-----------LW---NEIETRHPGMS--
>Timema_cristinae
-------------------MMTNNLKVAVKVLFFAKARELSGWKESD-L--EVECSLT--Y-EC-----------LL---DTLVKE-FSLE--LVQN---HLILAVNQEYKSAG---QTIVLEAGDEIAVIPPLSGG
>Menopon_gallinae
--------------------MNDSSHVSVKLLFFAKARELTGKNEAI-V--DVDRNVK--G-AD-----------LL---DFLVSK-FELD--CIRK---NVILALNGDYVDLE---CDLELNSADELAIIPPLSGG
>Perinereis_aibuhitensis
------------------------MAVQVKILFFAKSKEIAQTNESE-L--ALPNSIS---LAQ-----------LK---EVLCQQYNGLS--AIIE---NCIISLNQNYVEE----GDLNLNSGDEIAVVPPISGG
>Fasciola_hepatica
----------------------MSTVISVSVLFFGEAREVVKLHRVC-L--SLPTTCT--P-AE-----------LK---REILKHFPQLE--LLSS---SLGLALDLQYLDCSSETDRITLSDLSEVSVFPPLSGG
>Colobus_angolensis_palliatus
----------------------MVPLCQAEVLYFAKSVEITGVRSET-I--SVPQEIK--AL-Q-----------LW---KEIETRHPGLA--DVRN---QILFAVHQEYVELGD--QLLLLQPGDEIAVIPH----
>Acerentomon_sp._AD-2013
----------------------MAYDVSVNVLFFAKAKELTNLSEVL-L--VLKTETT---GNE-----------IL---SALEKKFPVLS--RIKE---IIHLAVNEAYCED-LS-CSITLNSGDTVAVITPISGG
>Melopsittacus_undulatus
------------------------MRCQVSVLYFARSAELAGLRRET-L--SVPRQIT--AI-Q-----------LW---EEIVKVHPRLA--VIRD---QVVFAVRQEYVLLGD--QFLVLQPGDEVAIIPPISGG
>Liposcelis_bostrychophila
--------------MID-GFDEEATQVSIKILFFALVKDLVGKNEDR-I--YVPSTIV--G-SA-----------LL---DLIIKK-YNLE--TVKN---NVILAVNEDWVDLD---SLLILKDSDEVAVIPPLSGG
>Xanthostigma_xanthostigma
------------------------MSVEVKILFFASASTISNLSESI-L--NVSGQIA---YDD-----------LL---ELIV-EKYSLQ--NIKQ---NILLAHNESICEK--N-QTLNLSDGDEIAVVPPFSGG
>Rhopilema_esculentum
--------------------MDAVNEVSVRLLFFAKSRDIVGLHEAH-I--DISSVVT---GQE-----------LL---RAIVTKFPGLH--ILRE---NLLLSCNQEYLES--D-QVVELAGGEEVAVIPPISGG
>Marmota_marmota_marmota
----------------------MVPRCQVEILYFAKSAEITGVRSET-I--SVPQEIK--AL-Q-----------LW---HEIETRHPGLA--DVRN---QVIFAVRQEYVELGD--QLLLLHPGDEIAIIPPISGG
>Coregonus_clupeaformis
------------------------MNTEVSVFYFAKSAELTGLKSET-I--RVPSELT--TL-E-----------LW---QDLVSRHPRLS--AVRD---SVVLAVRQEYVALGD--QCVSLREGDEVAVIPPLSGG
>Favia_lizardensis
--------------MDH-FSNDTTEQVEVTVLFFAKSRELVEKRSAQ-I--SVGKQIT---GTD-----------LI---HYILNNFPSLA--VIQE---NLVIAVNQSYIEK--D-SEITFEGGEEVAVIPPISGG
>Sepioloidea_lineolata
---MLPPLSGG
>Ictalurus_punctatus
------------------------MNTEVTVLYFAKSAELTGVKSEI-I--RVNSELT--SL-Q-----------LW---QELEHKHPKLS--AVRE---QVVLAVRREYVSVGE--QVVRLRHGDEVAVIPPLSGG
>Ceratophyllus_gallinae
---------------------MGNGEVSIKVIFFAKSRELSGVSETS-L--KVNSNIS--C-GE-----------LI---QLICAI-HGLQ--SIQN---NIIIALNEVYYQDLA--AILTLKTGDEVAIIPPLSGG
>Streblospio_benedicti
--------MLLFLIMLT-VLNILKLLYLVKVLFFAKSRELVGHSDSY-I--NLPLICL---ESQ-----------IR---TSIVENNPSLS--PISD---SFVLALNQEYIGE--G-EEVHISEGDEIAVIPPISGG
>Squalus_acanthias
------------------------MIAQVSVLYFARSAELAGVRSET-L--SVPQQLT--SL-Q-----------LW---QEIVKRHPRKQRENLREIQPRSVLNIFYHPVL-------------------------
>Takydromus_sexlineatus
------------------------MSCEVVVLYFARSAELAGVRTET-I--SVPQQIT--SL-Q-----------LW---EEIVRRHSRLT--AIQD---QVVFAVRQEYVLLGD--QLVVLNPGDEVAVIPPISGG
>Stylops_melittae
-----------------------MNTVTIKVLFFAKSRQLSKRKEGH-I--TLPAEIA---YDD-----------LL---TLIC-NQFQLE--EIKK---TIFLALNETYLQS--N-TLVHLHSDDIVAVIPPISSG
>Schistosoma_mansoni
-----------------------MTICNKKVLLFGEASELVGCKEIT-I--QFPLSCT--N-KT-----------LK---DIIIEQLVKLE--PLKD---SLTVAVNWKYTDINTSLNQISIEEIEELAILPPISGG
>Alvinella_pompejana
MKAYVICKPKYDHYDDKNFCNDTNCILKVTVFFFAKGRELVGKKEDQ-I--DIAPHNS--GT-A-----------LY---KLVVDRYPCLK--PIQE---NFALALNEEYIDKTD--EEIILSNGDQLAEIPPISGG
>Pelodiscus_sinensis
------------------------MSCEVVALYFARSAELAGVRSET-I--SVPQQIT--SL-Q-----------LW---EEIVKRHPRLA--AIQD---QVVFAVCQEYVLLGD--QLLVLQSGDEVAIIPPISGG
>Hydroptila_sp._AD-2013
---------------------MEVSKVVVKVLFFAASREIVGAEERE-L--EVPARSS--Y-SS-----------LL---HLLVHT-FKLD--IIKD---NFIVAKNEELCLDNG--HEIVFEKNDEIAIIPPLSGG
>Villosa_lienosa
---MQLHQGDEIAVIPPISGG
>Sinonovacula_constricta
----------------------MAAANVIQVLFFAKSRELTGIKQTN-L--LVNSSLT---AHE-----------LL---KKIISEFPGLQ--VLAD---TLILSLNEEYLALEDP-KILNLQEGDTVAVIPPISGG
>Limulus_polyphemus
----------------------MSSGVKVKLLFFAKARELVGVTETG-L--VVPNGLY--TQKK-----------LL---SIIVETFPVLC--SIQG---NFIVALNEECMDQS---QSIELKEGDQLAVIPPLSGG
>Palythoa_caribaeorum
--------------MEL-ANDAGCEKVQVTVLFFAKCRELVGTSSVQ-I--YLARRLS---GSK-----------LI---ETLLNLFPSLS--EIER---NLIVAVNQEYIEK--E-SNIELFGNDEIAVIPPISGG
>Thermobia_domestica
--------------------MAESEKVIVKLLFFAKARELSGHKDIT-I--NLPSITT---YSI-----------LV---SLIA-KEFSLE--PIQE---NLILALNQEYIES--D-RDISLKNGDEIAIIPPISGG

HG0003 FBgn0039280 Mocs2

>Drosophila_melanogaster
-------------------MNADGPVVNVHVLFFAKSRELANTPR-STVEVPTE---ITA--T-ELLDHLVSK-FGLTS--IR---DNLILAHNESYIDNLS--DRILFKEGDELAIIPPLSGG
>Acipenser_sinensis
------------------------MTTEVVVLYFAKSAELAEVRS-ENITVSHQ---VTS--L-QLWQELVRKHPRLAA--IR---DQVVLAVRQEYVSLSD--ELLILHPGDEVAVIPPLSGG
>Anemonia_viridis
----------------MDNKYNGTEDVPVILLFFAKSRELTECAS-KEVQLPRL---VTR--E-DLISSIIDIFPSLVQ--IE---KTIVVAVNQEYLDTGK---KVELRKGDTVAIIPPLSGG
>Adineta_ricciae
-----------------------MSTIQVAIIYFGRLRELVGISS-EKIDFDSA---ISYTQQ-SILDRIIEHRPILKSFLVT---NTFRLAVNQQYLLEGD---LIQFSKESEVALLPPFGGG
>Acanthogorgia_aspera
---MLALNEIYIDLEE---TVNLRSTDKIAVIPPLSGG
>Amia_calva
------------------------MNTEVVVLYFAKSAEIADVRS-ETISVPQE---ITS--L-QLWQELVNRHPSLSV--LR---DQVVLAVRQEYVALGE--QLLSLQSGDEVAVIPPLSGG
>Talitrus_saltator
------------------MTQYGLEIVPVKIVFFASARELSGCSE-VEIDLSTP---TTA--K-KLFSEVLNKHPELQK--LE---KSVMVAVNQEYIDDD--TNQVSLRAGDEVAFIPPISGG
>Hydractinia_symbiolongicarpu
---------------------MEDCSVEVNILFFAKSKELTGKSV-DVVNLKKS---NCL--D-NLLKEIIHTYPALNI--LK---GRFIIAHNQAYLRDT--KEVVILETGDEIAIIPPISGG
>Xenopus_tropicalis
------------------------MTCEVSVLYFAKSSELAGVRS-ETLTVSQE---ITS--Q-QLWDQLVHSHPSLCA--IR---DQVVLAVRQEYVPIG--NELLTLNTGDEIAVIPPISGG
>Milnesium_tardigradum
------------------------MEISVKVLFFAKAKDLVGTGQ-GILTLKED---ILS-LG-SLLSNLYSQFPALLP--LQ---NQVVLSLNQKYIDADDLQRPVSINIGDEIAVLPPISGG
>Machilis_hrabei
--------------------MAALCESEIRLLFFAKSRELAGKSE-ASICVPSL---VHV--Q-DLLDLIVRE-FSLDL--IK---NNLILAHNQEYLDREQ---SVNIRTGDEIAVIPPLSGG
>Scapharca_broughtonii
--------------------MATPEEVQVTLLFFAKSRELSGLKT-SQLKTPTQ---VNF--Q-QLLGLIVTAFPSLEI--IS---ENIVLSLNEDYIDKD---SSVNLNSGDEIAVIPPISGG
>Argulus_siamensis
-----------------------MSLITVSVLFFAKARELVGAHQ-TTITLPQS---LIG--H-KLVALILDYYEALKP--IE---KNFILALNENYIELD---EEISLSEGDVIAVIPPISGG
>Oryzias_latipes
------------------------MTAQVSVIYFGQSAELTGLKGEELVSVPTP---ISS--R-ELWELLQQRHPRLAV--LR---GHVVLAVAQQYIPIAE--QLVVLRDGDEVAVVPPLSGG
>Prorhinotermes_simplex
--------------------MADVSVVEIQVLFFAKARELCGQKQ-SCLLIPSV----TS-YN-NLLDEIVTK-FSLES--IR---DNLLLAINEEYTTSES---EVQLKEGDEVAVIPPLSGG
>Tripterygion_delaisi
------------------------MSARVVVLYFAQSAELAGLKEEEIVAVPSP---ISS--L-DLWNLLVQRHPRLGV--LR---GHVVLAVRQQYVAIGD--EQVALGDGDEVAVVPPLSGG
>Eurytemora_affinis
-----------------------MADVDVKLILFAKARELVGASE-VNVRFPQE---LSS--E-HLKSKIIQSFPVLDK--LG---GTWLLALNEAYIDTD----RVTLNTGDTLAVIPPISGG
>Seriola_quinqueradiata
------------------------MTAQVCVLYFAKSVELTGVKQEDIVAVPTP---ISS--R-DLWSLLLQRHPRLSV--LQ---DQVVLAVRQHYVAIGD--QVVTLGDGDEVAVVPPLRGG
>Alatina_alata
---------------------MSNITVTVEVRFFAKARELAGIQK-THVRTRER---ITA--H-ELISQIIDAFPKLDV--IR---QCFLLAHNQEYCSLD---QELNISDGDEIAVIPPISGG
>Hynobius_retardatus
------------------------MNSEVIVLYFAKSAELTGVRS-ERLSVPQQ---ITS--S-QLWDEIVNRQPRLAA--IR---DNVVLAVRQEYVVLGD--QLLTLQPGDEVAVIPPISGG
>Siniperca_chuatsi
------------------------MTAQVSVLYFAQSVELTGVKEEELVAVPTP---ISS--Q-DLWRLLLQRHPRLSL--LQ---DQVVLAVRQQYVAIGD--QPVTLGDGDEVAVVPPLSGG
>Astyanax_mexicanus
------------------------MKSEVLVLYFAKSAELTGIKS-ETISVQSE---LST--L-QLWQELEKLHPKLSA--VR---DQVVLAVRQEYVSLGD--QRVILRDGDEVAIIPPLSGG
>Callorhinchus_milii
------------------------MTAEVSVLYFARSAELSGVRS-ETLSVPQQ---LTS--M-QLWEKIVNKHPSLIA--IR---DHVLLAVRQEYILLGD--QILFLQPGDEIAVIPPLSGG
>Oreochromis_niloticus
------------------------MTAQVCVLYFAKSVELTGVKEEKTVAVPTP---ISS--H-DLWKLLVQRHPRLCT--LQ---DGVVLAVRQEYVPIGD--QVLTLGDGDEVAVVPPLSGG
>Alosa_alosa
-------------MFGSAVLQAPFMISEVSVLYFAKGAELTGVKS-ETVKLPSE---LTT--I-QLWLELEKRHPKLCV--LR---EHVVLAVRQEYVDLCD--QTVSLHDGDEVAVIPPLSGG
>Latimeria_chalumnae
------------------------MTAQISVLYFAKSAELAGIRS-EVITVAQQ---ITS--Q-QLWQEIVNRHPRLAA--IH---DHVILAVHQEYILLGD--QFLLLQPGDEVAIIPPISGG
>Priacma_serrata
------------------------MSVTVTILFFAKAREIVGKNS-SSLEVPPA---LTC--T-DLLNTIVNE-YSLHV--IR---NNIILAVNEEFCAGND---RLSLKTGDEIAVIPPLSGG
>Ricordea_yuma
---------------MEYTNHVSTDKVEINVLFFAKSRELVEQRS-AKVLLAKR---LSG--R-ELIASLVDSFPSLTV--IR---DNLVVAVNQSYVEKET---EIEFEGGEEVAIIPPISGG
>Cuculus_canorus
-------------------MTAELPLVQVSVLYFARSAELAGLRR-ETLRVPRQ---VTA--L-QLWEEIVSLHPRLAV--IR---DQVVFAVRQEYVLLGD--QLLVLQPGDEVAIIPPISGG
>Ctenopharyngodon_idella
------------------------MNTEVSVLYFAKSAELTGLKA-ENITVPSN---ITS--L-QLWQDLENRHPRLSV--LR---DQVVLAVRQEYVPLGA--QPLTLRQGDEVAIIPPLSGG
>Fundulus_grandis
------------------------MSAQVTVLYFAKSSELTGLKE-EPVAVPTP---ISS--G-DLWALLLRKQPRLVA--LQ---GHVILAVRQRYVAIGE--QQVTLEDGDEVAVVPPLSGG
>Macrobrachium_nipponense
---------------------MTESEVSVNILFFASARDLAGVDR-SILTVPVN---TTP--E-ELLSQVVRSFGEISS--IK---DSVILSHNQQYCEPG---QGLTLSTGDEVAIIPPISGG
>Sclerodactyla_briareus
------------------------MPVDVTVQYYAISRDITGTRE-DRLTVPTH---VTL--K-QLFGCLTERHPGLKQ--IE---TNCLLAINGDWLTEES--GVVELKPGDEVAVIPPLSGG
>Forficula_auricularia
-----------------MNQDLNCDQVTVEVLFFAKARELSGVNK-AIIQTPSL---TNS--E-ELLDSLINR-FNLQQ--IS---SCLVLAVDENWVESGP----LKLNSGSIIAVIPPLSGG
>Occasjapyx_japonicus
-----------------------MQTTELRVLFFASARELAKCNA-AVLRLDSA---QQS--GESLLEQVVLSFPDLQR--VR---GSLMLALNQNYVQLD---QRLTVSSGDELAVIPPISGG
>Echiniscus_testudo
--------------------------MQITVLLFARMRDLAGGRSEILIDVPADCENGSVSSK-RLLQLIIAQCPRLKE--IK----HYQLAVNRQYVDLNS-DNLVQLGPTSEIALIPPVSGG
>Evechinus_chloroticus
------------------MADDLAGKVEVTVLFFAKSRDLAGTKE-ASLQIQSD---IKG--E-VLLNVISEKFPRLDA--IR---DSIVLCINQEYISSES---SYHLSPGDEVAVIPPLSGG
>Ciona_intestinalis
----------------------MGKNVQVNIKFFAKSRELVGIES-EQLILPQD---VTS--F-QLIETINSKYPRLSD--LQ---GCFTLAVNGEYIKTDE-SYSILLTDQSEIAVIPPLSGG
>Baetis_sp._AB-2009
----------------------MGQPIVVKVLLFASAREISGERE-SEIQVQQE---ETY--D-TLFRKIVQR-FKLEK--LQ---NSLILAINEDYPDAEN--TQLNLKPGDHIAVIPPLSGG
>Esox_lucius
------------------------MNTEVTVLYFAKSAELTGIKS-ETIRVPSE---LTT--L-DLWLDLVSRHPRLST--VR---ESVVLAVRQEYVAVGE--LRVSLREGDEVAVIPPLSGG
>Asterias_rubens
------------------MASICADVVEINLLFFAKSRELAGVKE-GHLQVQTN---LKP--E-ELLLTIVNHYPQLSV--IQ---DTVVLSLNQEYIAKG--DDTLNLRPGDEVAVIPPISGG
>Gadus_morhua
------------------------MTARVTVLYFAKSAELTGHRS-EVVPLPAA---TSS--C-QLWALLLELHPSLAA--LQ---DQVVLAVRQQYVALGD--QALLLADGDEVAVVPPLSGG
>Gallus_gallus
------------------------MSCQVTVLYFARSAELVGLRS-ESVSVPQR---ITS--L-QLWEEIVKIHPRLAV--IR---DQVVFAVRQEYVLLGD--QLLVLQTGDEVAIIPPISGG
>Chlamydotis_macqueenii
---MLLGD--QLLVLQPGDEVAVIPPISGG
>Galloisiana_yuasai
--------------------MDLNLKISVNVLFFAKARELCGKKE-SKLQVVPS---ISY--N-LLLDHIIKE-FSLES--IK---GNFILAINEEYTTTDR---ELDLKEGDEIAIIPPLSGG
>Tigriopus_californicus
------------------------MSVSIKVLLFAKARELAGTHE-LTLNVVPN---LSA--S-DLVTQIEAQCPDLAI--LR---GSFVLALNQDYVDHN---QVLDLKSGDELAVIPPISGG
>Trialeurodes_vaporariorum
------------------------MNVSVEVLLFGRAKELAGEEK-VRVQVPSS---SSR--S-HLLKNLVTR-FSLQA--IE---SILILAINEEYVLESD---EIKLQDGDRVAIIPPLSGG
>Diadromus_collaris
-----------------MTALMAPNPRNIRVVFFAKARELAGLSE-STIDVDGI---LSC--D-QLFARLIDK-FGLDC--IR---DNLILAINEQFVERGA---TLDLKEGDEIAVIPPLSGG
>Ornithodoros_turicata
----------------------MLSNVAIRVLFFAKARELLGKSE-VTLLIPNN---VQD--EKALKDFIFSSFPLLSV--LV---KCTILAVNELYLEPG---ASVDLKEGDEIAVIPPISGG
>Helicoverpa_armigera
--------------------MEIENDVTVKLLFFARSKELAGTKE-STIRLPSK---ISY--S-KLLNIICDN-YNLQT--IK---DNILLAKNEEVCDEDI---DIDIKEKDSIAVIPPISGG
>Lutjanus_erythropterus
------------------------MTAQVCVLYFAKSVELTGAKEEQIVAVPTP---ISS--Q-DLWKLLLQRHPRLSV--LQ---DQVVLAVRQQYVAIGD--QVVTLGDGDEVAVVPPLSGG
>Metallyticus_splendidus
------------------MDGNGQHKVRIKVLFFAKARELSGTRE-SSLISDSV---ISY--E-SLLQQIIET-YSLSS--IK---SSVVLAINEEYCSAAT---NVMLNEGDEVAVIPPLSGG
>Boreus_hyemalis
-----------------------MGTAKIKIIFFAKSRDLSGKTE-DTFSVPTN---ITC--S-ELLSLISSN-YGLDA--IK---DNVLLALNEEYCDDLN--AQLILKTGDEIAVIPPLSGG
>Dermanyssus_gallinae
-------------------------MVRVKLLFFAKARELAGTAE-RSVDVAER----VFSLD-QLKELIAQLEPSVKT--LL---DTSILAVDLNYVVDSV---EIG-TRTGEIAVIPPVSGG
>Ornithorhynchus_anatinus
-----------------------MVLSPVTVLYFAKSAELAGVRS-ETISVPQE---IKS--L-QLWEEIESRHPRLAD--VR---DQVIFAVRQEYVQLGD--QVLLLQPGDEIAIIPPISGG
>Perna_viridis
---------------------MANNKVQVSLLFFAKSRELSGVSN-SQIKVSSQ---CSF--F-QLFETIVQQYPSLSV--IS---ENIILSLNEEYISRD---GSITVQEGDEIAVIPPISGG
>Osmylus_fulvicephalus
------------------------MSVEVRILFFAKAKDFAQKSE-STLNIPNT---LTF--K-ELLTIIVDN-YNLQA--IR---NTINISLNANFCDRDA---TLKFNTGDEIAVIPPLSGG
>Hapalochlaena_maculosa
---------------MDHSFAGNKDPVKINVLFFAKSRELVGQNK-SEVSITSR---SVT-TA-QLTSFLLETFPRLKP--LK---QSMIIALNEEYLSGDE---TIHLSNGDEIAVIPPLSGG
>Epiophlebia_superstes
--------------------MGDKDFVTVKVLFFAKARELSGIKF-GQLKVNAA---PSY--T-FLLEEIVTKF-SLEK--IR---NNILLAINEEYSGSE---LQINLKDGDEIAVIPPISGG
>Opisthorchis_felineus
----------------------MKQAVRARVILFGEAKDLTGFQS-ALILVPVT---CTA--K-ELYDLILGHFPKLKL--IE---KSLLLTLDFQYIDIEDNNTLLHISDSSELGVLPPLGGG
>Ceuthophilus_sp._AD-2013
--------------------MADGHQVDVKILLFAKARELSGVKE-SNLEITSS---ISY--S-SLFDILVKT-FSLDA--IK---GNLILAINQEYSSADS---ILNLKAGDEIAVIPPLSGG
>Osmerus_mordax
----------------------MTTKVSVLVLYFAKSADLTGLKS-EVIHVQPE---LTT--L-ALWQELVNRHSRLSA--VR---EQIVLAVSQEYVTIGC--QLVSLKNGDEVAVIPPLSGG
>Crassostrea_gigas
----------------------MEGDVTINLLFFAKSRELVGSGS-GELVLTAN---TNF--D-TLKQALLLKYPSLQV--IA---ENIILSLNEEYIDDN---QQLTLREGDEVAVIPPISGG
>Hypsibius_dujardini
--------------------------MDVNLKFLALAKDLAGTGD-ATIRITAEESPFTV--G-SFLAKLIRTFPRLGE--IK---DQLILAHNLEYLDLQNPSASFELKEGDEIAVLPPVSGG
>Taeniopygia_guttata
------------------------MRCQVSVLYFARSAELAGLRC-ETLSVPQE---ITS--L-QLWEEIVKVHPRLAL--IR---DQVVFAVRQEYVLLGD--QLLVLQPGDEVAIIPPISGG
>Pecten_maximus
-----------------------MADVEVSVLFFAKSRELAGKKT-AQIIVESP---TNL--Q-QLISTIVSQFPSLQI--IA---DNLVVSINEDYLQAG---QEVHLNTGDEVAVIPPISGG
>Dissostichus_mawsoni
------------------------MTAQVTVLYFAKSAELSGLKEEELVAVPTP---ISS--Q-DLWSLLLQRHPRLCV--LQ---HQVVLAVRQQYVAIGD--ELVALGDGDEVAVVPPLSGG
>Equus_asinus
----------------------MVPRCQVEVLYFAKSAEIAGIRS-ETISVPQE---IKA--L-QLWNEIETRHPGMS--
>Sipyloidea_sipylus
--------------------MTEGNKVVIKVLFFAKARELSGKTE-STILIEPK---TTY--S-KVLDHIAKE-FSLEL--IK---NNVILAVNQSYASADQ---TLELKTGDELAVIPPLSGG
>Menopon_gallinae
--------------------MNDSSHVSVKLLFFAKARELTGKNE-AIVDVDRN---VKG--A-DLLDFLVSK-FELDC--IR---KNVILALNGDYVDLEC---DLELNSADELAIIPPLSGG
>Fasciola_hepatica
----------------------MSTVISVSVLFFGEAREVVKLHR-VCLSLPTT---CTP--A-ELKREILKHFPQLEL--LS---SSLGLALDLQYLDCSSETDRITLSDLSEVSVFPPLSGG
>Colobus_angolensis_palliatus
----------------------MVPLCQAEVLYFAKSVEITGVRS-ETISVPQE---IKA--L-QLWKEIETRHPGLAD--VR---NQILFAVHQEYVELGD--QLLLLQPGDEIAVIPH----
>Acerentomon_sp._AD-2013
----------------------MAYDVSVNVLFFAKAKELTNLSE-VLLVLKTE---TTG--N-EILSALEKKFPVLSR--IK---EIIHLAVNEAYCEDL--SCSITLNSGDTVAVITPISGG
>Melopsittacus_undulatus
------------------------MRCQVSVLYFARSAELAGLRR-ETLSVPRQ---ITA--I-QLWEEIVKVHPRLAV--IR---DQVVFAVRQEYVLLGD--QFLVLQPGDEVAIIPPISGG
>Liposcelis_bostrychophila
---------------MIDGFDEEATQVSIKILFFALVKDLVGKNE-DRIYVPST---IVG--S-ALLDLIIKK-YNLET--VK---NNVILAVNEDWVDLDS---LLILKDSDEVAVIPPLSGG
>Inocellia_crassicornis
------------------------MSVEVKLLFFASARTISNLNE-SVLKVPDE---ISY--K-DLLGLIVEKY-SLQK--IR---HSVLLAHNESICDKN---QILSLSEGDEVAVIPPFSGG
>Rhopilema_esculentum
--------------------MDAVNEVSVRLLFFAKSRDIVGLHE-AHIDISSV---VTG--Q-ELLRAIVTKFPGLHI--LR---ENLLLSCNQEYLESD---QVVELAGGEEVAVIPPISGG
>Marmota_marmota_marmota
----------------------MVPRCQVEILYFAKSAEITGVRS-ETISVPQE---IKA--L-QLWHEIETRHPGLAD--VR---NQVIFAVRQEYVELGD--QLLLLHPGDEIAIIPPISGG
>Coregonus_clupeaformis
------------------------MNTEVSVFYFAKSAELTGLKS-ETIRVPSE---LTT--L-ELWQDLVSRHPRLSA--VR---DSVVLAVRQEYVALGD--QCVSLREGDEVAVIPPLSGG
>Favia_lizardensis
---------------MDHFSNDTTEQVEVTVLFFAKSRELVEKRS-AQISVGKQ---ITG--T-DLIHYILNNFPSLAV--IQ---ENLVIAVNQSYIEKDS---EITFEGGEEVAVIPPISGG
>Sepioloidea_lineolata
--MLPPLSGG
>Trichomycterus_areolatus
----------------------MNPEVQVLVLYFAKSSELSGVKS-ETISVQSE---LNS--L-KLWQQLELRHPKLSV--IR---EQVVLAVRREYVALGE--HRVHLQNGDEVAVIPPLSGG
>Oropsylla_silantiewi
---------------------MEVGQVSIKVKFFAKARELSGVSE-TLLKVNSN---FSC--G-ELVQLICIK-YGLQS--IQ---SNLIIALNETYCKDLA--AILDLKTGDEIAVIPPLSGG
>Streblospio_benedicti
---------MLLFLIMLTVLNILKLLYLVKVLFFAKSRELVGHSD-SYINLPLI---CLE--S-QIRTSIVENNPSLSP--IS---DSFVLALNQEYIGEG---EEVHISEGDEIAVIPPISGG
>Squalus_acanthias
------------------------MIAQVSVLYFARSAELAGVRS-ETLSVPQQ---LTS--L-QLWQEIVKRHPRKQRENLREIQPRSVLNIF--YH--------------------PVL---
>Atractaspis_aterrima
------------------------MSCEVTVLYFARSAELAGLRT-ETISVPQQ---ITS--L-QLWREIVKRHSSLAD--IE---DQVIFAICQEYVPLGD--QQFILHTGDEVAIIPPLSGG
>Stylops_melittae
-----------------------MNTVTIKVLFFAKSRQLSKRKE-GHITLPAE---IAY--D-DLLTLICNQF-QLEE--IK---KTIFLALNETYLQSN---TLVHLHSDDIVAVIPPISSG
>Trichobilharzia_regenti
-----------------------MSVCSKKVLLFGEASELVGCKE-IHLPFPKS---CTQ--K-ALKNIVIDQLAELEP--LK---DCITLAVNWCYVDTEDSINSISVEEIEEIAVLPPISGG
>Lytechinus_variegatus
------------MAEDAVSDSIAGEKVEVTVLFFAKSRDLTGTKE-ANIRVQSK---INT--G-SLLNVLSEKYPRLSA--IQ---DSIVLCINQEYISAEG---SFHLSPGDEIAVIPPLSGG
>Alvinella_pompejana
MKAYVICKPKYDHYDDKNFCNDTNCILKVTVFFFAKGRELVGKKE-DQIDIAPH---NSG--T-ALYKLVVDRYPCLKP--IQ---ENFALALNEEYIDKTD--EEIILSNGDQLAEIPPISGG
>Trachemys_scripta
------------------------MSCEVVALYFARSAELAGVRS-EIISVPQQ---LTS--L-QLWEEIVKRHPRLAA--IQ---DQVVFAVRQEYVLLGD--QLLVLQSGDEVAIIPPISGG
>Hydroptila_sp._AD-2013
---------------------MEVSKVVVKVLFFAASREIVGAEE-RELEVPAR---SSY--S-SLLHLLVHT-FKLDI--IK---DNFIVAKNEELCLDNG--HEIVFEKNDEIAIIPPLSGG
>Villosa_lienosa
--MQLHQGDEIAVIPPISGG
>Meretrix_meretrix
---------------------MDDDKVEVTVLFFAKSRELAGTRE-TTLQTFKE---ITA--A-GLLNQIISQYNSLDI--LR---ESIVLSLNEEYLSLTD-SEQLTLRSGDTVAVIPPISGG
>Limulus_polyphemus
----------------------MSSGVKVKLLFFAKARELVGVTE-TGLVVPNG---LYT-QK-KLLSIIVETFPVLCS--IQ---GNFIVALNEECMDQSQ---SIELKEGDQLAVIPPLSGG
>Protopalythoa_variabilis
---------------MELANDAGCEKVQVTVLFFAKCRELVGTSS-VQIYLARR---LSG--S-KLIETLLNLFPSLSE--IE---RNLIVAVNQEYIEKE---SNIELFGNDEIAVIPPISGG
>Tricholepidion_gertschi
----------------MGVFLMELSLITVKVLFFAKARELSGKVE-DNFQIVCS---LSY--P-ELLSQIVKE-FCLEP--IE---RNIILAINEEYIGTEE---KLCLKEGDEIAVIPPLSGG

HG0004.1 ENSDARG00000076779 fam13b

>Danio_rerio
---MRNYRLIFCHNLKQP
>Acipenser_sinensis
---MRNYRLIFCHNLKQP
>Amia_calva
---MRNYRLIFCHNLKQP
>Channa_punctata
---MRNYRLIFCHNLKQP
>Anas_platyrhynchos
---MRNYRLIFCHNLKQP
>Xenopus_laevis
MVTSCSDCGPLAVNAFKCIHKILTERDCWGQRELKGLSKRKPQQNQS-YRHTAEELIELWISSMRNYRLIFCHNLKQP
>Chaetura_pelagica
---MRNYRLIFCHNLKQP
>Menidia_menidia
---MRNYRLIFCHNLKQP
>Porichthys_notatus
---MRNYRLIFCHNLKQP
>Oryzias_latipes
---MRNYRLIFCHNLKQP
>Tripterygion_delaisi
---MRNYRLIFCHNLKQP
>Buceros_rhinoceros_silvestri
---MRNYRLIFCHNLKQP
>Caprimulgus_carolinensis
---MRNYRLIFCHNLKQP
>Cariama_cristata
---MRNYRLIFCHNLKQP
>Canis_lupus_familiaris
---MRNYRLIFCHNLKQP
>Hynobius_retardatus
---MRNYRLIFCHNLKQP
>Micropterus_floridanus
---MRNYRLIFCHNLKQP
>Balaenoptera_acutorostrata_s
---MRNYRLIFCHNLKQP
>Pygocentrus_nattereri
---MRNYRLIFCHNLKQP
>Calidris_pugnax
---MRNYRLIFCHNLKQP
>Callorhinchus_milii
---MRNYRLIFCHNLKQP
>Pteropus_vampyrus
---MRNYRLIFCHNLKQP
>Maylandia_zebra
---MRNYRLIFCHNLKQP
>Pterocles_gutturalis
---MRNYRLIFCHNLKQP
>Clupea_harengus
--------------------------------------MRKPFQ-------LSEVLHGICGLSMRNYRLIFCHNLKQP
>Latimeria_chalumnae
---MRNYRLIFCHNLKQP
>Colius_striatus
---MRNYRLIFCHNLKQP
>Columba_livia
---MRNYRLIFCHNLKQP
>Merops_nubicus
---MRNYRLIFCHNLKQP
>Alligator_mississippiensis
---MRNYRLIFCHNLKQP
>Cuculus_canorus
---MRNYRLIFCHNLKQP
>Kryptolebias_marmoratus
---MRNYRLIFCHNLKQP
>Sarcophilus_harrisii
---MRNYRLIFCHNLKQP
>Galeopterus_variegatus
---MRNYRLIFCHNLKQP
>Monodelphis_domestica
---MRNYRLIFCHNLKQP
>Anopheles_funestus
---MLNYRLVSTHFLKQP
>Esox_lucius
---MRNYRLIFCHNLKQP
>Falco_cherrug
---MRNYRLIFCHNLKQP
>Gallus_gallus
---MRNYRLIFCHNLKQP
>Gavia_stellata
---MRNYRLIFCHNLKQP
>Balearica_regulorum_gibberic
---MRNYRLIFCHNLKQP
>Erinaceus_europaeus
---MRNYRLIFCHNLKQP
>Oryctolagus_cuniculus
---MRNYRLIFCHNLKQP
>Eudarcia_simulatricella
---MLNYRLVSTHFLKQP
>Tauraco_erythrolophus
---MRNYRLIFCHNLKQP
>Opisthocomus_hoazin
---MRNYRLIFCHNLKQP
>Plecoglossus_altivelis
--MLFAPVVFDLLTSEILNRICGLSMRNYRLIFCHNLKQP
>Scleropages_formosus
---MRNYRLIFCHNLKQP
>Lepidothrix_coronata
---MRNYRLIFCHNLKQP
>Nipponia_nippon
---MRNYRLIFCHNLKQP
>Trematomus_bernacchii
---MRNYRLIFCHNLKQP
>Ceratotherium_simum_simum
---MRNYRLIFCHNLKQP
>Manis_javanica
---MRNYRLIFCHNLKQP
>Glycera_dibranchiata
---MHGYRLILCRTLKQP
>Picoides_pubescens
---MRNYRLIFCHNLKQP
>Platichthys_stellatus
---MRNYRLIFCHNLKQP
>Homo_sapiens
---MRNYRLIFCHNLKQP
>Loxodonta_africana
---MRNYRLIFCHNLKQP
>Fulmarus_glacialis
---MRNYRLIFCHNLKQP
>Nestor_notabilis
---MRNYRLIFCHNLKQP
>Mus_musculus
---MRNYRLIFCHNLKQP
>Salmo_salar
---MRNYRLIFCHNLKQP
>Tupaia_chinensis
---MRNYRLIFCHNLKQP
>Lepisosteus_oculatus
---MRNYRLIFCHNLKQP
>Ameiurus_nebulosus
---MTVFCLTTDVLYRICGPSMRNYRLIFCHNLKQP
>Trichechus_manatus_latirostr
---MRNYRLIFCHNLKQP
>Aptenodytes_forsteri
---MRNYRLIFCHNLKQP
>Crotalus_horridus
---MRNYRLIFCHNLKQP
>Tyto_alba
---MRNYRLIFCHNLKQP
>Struthio_camelus_australis
---MRNYRLIFCHNLKQP
>Chelonia_mydas
---MRNYRLIFCHNLKQP
>Takifugu_rubripes
---MRNYRLIFCHNLKQP
>Tinamus_guttatus
---MRNYRLIFCHNLKQP
>Calypte_anna
---MRNYRLIFCHNLKQP
>Apaloderma_vittatum
---MRNYRLIFCHNLKQP
>Orycteropus_afer_afer
---MRNYRLIFCHNLKQP

HG0004.1 ENSG00000031003 FAM13B

>Homo_sapiens
---MRNYRLIFCHNLKQP
>Acipenser_sinensis
---MRNYRLIFCHNLKQP
>Amia_calva
---MRNYRLIFCHNLKQP
>Channa_punctata
---MRNYRLIFCHNLKQP
>Anas_platyrhynchos
---MRNYRLIFCHNLKQP
>Xenopus_laevis
MVTSCSDCGPLAVNAFKCIHKILTERDCWGQRELKGLSKRKPQQNQS-YRHTAEELIELWISSMRNYRLIFCHNLKQP
>Chaetura_pelagica
---MRNYRLIFCHNLKQP
>Menidia_menidia
---MRNYRLIFCHNLKQP
>Porichthys_notatus
---MRNYRLIFCHNLKQP
>Oryzias_latipes
---MRNYRLIFCHNLKQP
>Tripterygion_delaisi
---MRNYRLIFCHNLKQP
>Buceros_rhinoceros_silvestri
---MRNYRLIFCHNLKQP
>Caprimulgus_carolinensis
---MRNYRLIFCHNLKQP
>Cariama_cristata
---MRNYRLIFCHNLKQP
>Canis_lupus_familiaris
---MRNYRLIFCHNLKQP
>Hynobius_retardatus
---MRNYRLIFCHNLKQP
>Micropterus_floridanus
---MRNYRLIFCHNLKQP
>Balaenoptera_acutorostrata_s
---MRNYRLIFCHNLKQP
>Pygocentrus_nattereri
---MRNYRLIFCHNLKQP
>Calidris_pugnax
---MRNYRLIFCHNLKQP
>Callorhinchus_milii
---MRNYRLIFCHNLKQP
>Pteropus_vampyrus
---MRNYRLIFCHNLKQP
>Maylandia_zebra
---MRNYRLIFCHNLKQP
>Pterocles_gutturalis
---MRNYRLIFCHNLKQP
>Clupea_harengus
--------------------------------------MRKPFQ-------LSEVLHGICGLSMRNYRLIFCHNLKQP
>Latimeria_chalumnae
---MRNYRLIFCHNLKQP
>Colius_striatus
---MRNYRLIFCHNLKQP
>Columba_livia
---MRNYRLIFCHNLKQP
>Merops_nubicus
---MRNYRLIFCHNLKQP
>Alligator_mississippiensis
---MRNYRLIFCHNLKQP
>Cuculus_canorus
---MRNYRLIFCHNLKQP
>Danio_rerio
---MRNYRLIFCHNLKQP
>Kryptolebias_marmoratus
---MRNYRLIFCHNLKQP
>Sarcophilus_harrisii
---MRNYRLIFCHNLKQP
>Galeopterus_variegatus
---MRNYRLIFCHNLKQP
>Monodelphis_domestica
---MRNYRLIFCHNLKQP
>Esox_lucius
---MRNYRLIFCHNLKQP
>Falco_cherrug
---MRNYRLIFCHNLKQP
>Gallus_gallus
---MRNYRLIFCHNLKQP
>Gavia_stellata
---MRNYRLIFCHNLKQP
>Balearica_regulorum_gibberic
---MRNYRLIFCHNLKQP
>Erinaceus_europaeus
---MRNYRLIFCHNLKQP
>Oryctolagus_cuniculus
---MRNYRLIFCHNLKQP
>Tauraco_erythrolophus
---MRNYRLIFCHNLKQP
>Opisthocomus_hoazin
---MRNYRLIFCHNLKQP
>Plecoglossus_altivelis
--MLFAPVVFDLLTSEILNRICGLSMRNYRLIFCHNLKQP
>Scleropages_formosus
---MRNYRLIFCHNLKQP
>Lepidothrix_coronata
---MRNYRLIFCHNLKQP
>Nipponia_nippon
---MRNYRLIFCHNLKQP
>Trematomus_bernacchii
---MRNYRLIFCHNLKQP
>Ceratotherium_simum_simum
---MRNYRLIFCHNLKQP
>Manis_javanica
---MRNYRLIFCHNLKQP
>Picoides_pubescens
---MRNYRLIFCHNLKQP
>Platichthys_stellatus
---MRNYRLIFCHNLKQP
>Loxodonta_africana
---MRNYRLIFCHNLKQP
>Fulmarus_glacialis
---MRNYRLIFCHNLKQP
>Nestor_notabilis
---MRNYRLIFCHNLKQP
>Mus_musculus
---MRNYRLIFCHNLKQP
>Salmo_salar
---MRNYRLIFCHNLKQP
>Tupaia_chinensis
---MRNYRLIFCHNLKQP
>Lepisosteus_oculatus
---MRNYRLIFCHNLKQP
>Ictalurus_punctatus
---MRNYRLIFCHNLKQP
>Trichechus_manatus_latirostr
---MRNYRLIFCHNLKQP
>Aptenodytes_forsteri
---MRNYRLIFCHNLKQP
>Crotalus_horridus
---MRNYRLIFCHNLKQP
>Tyto_alba
---MRNYRLIFCHNLKQP
>Struthio_camelus_australis
---MRNYRLIFCHNLKQP
>Chelonia_mydas
---MRNYRLIFCHNLKQP
>Takifugu_rubripes
---MRNYRLIFCHNLKQP
>Tinamus_guttatus
---MRNYRLIFCHNLKQP
>Calypte_anna
---MRNYRLIFCHNLKQP
>Apaloderma_vittatum
---MRNYRLIFCHNLKQP
>Orycteropus_afer_afer
---MRNYRLIFCHNLKQP

HG0004.1 ENSGALG00000041706

>Gallus_gallus
---MRNYRLIFCHNLKQP
>Acipenser_sinensis
---MRNYRLIFCHNLKQP
>Amia_calva
---MRNYRLIFCHNLKQP
>Channa_punctata
---MRNYRLIFCHNLKQP
>Anas_platyrhynchos
---MRNYRLIFCHNLKQP
>Xenopus_laevis
MVTSCSDCGPLAVNAFKCIHKILTERDCWGQRELKGLSKRKPQQNQS-YRHTAEELIELWISSMRNYRLIFCHNLKQP
>Chaetura_pelagica
---MRNYRLIFCHNLKQP
>Menidia_menidia
---MRNYRLIFCHNLKQP
>Porichthys_notatus
---MRNYRLIFCHNLKQP
>Oryzias_latipes
---MRNYRLIFCHNLKQP
>Tripterygion_delaisi
---MRNYRLIFCHNLKQP
>Buceros_rhinoceros_silvestri
---MRNYRLIFCHNLKQP
>Caprimulgus_carolinensis
---MRNYRLIFCHNLKQP
>Cariama_cristata
---MRNYRLIFCHNLKQP
>Canis_lupus_familiaris
---MRNYRLIFCHNLKQP
>Hynobius_retardatus
---MRNYRLIFCHNLKQP
>Micropterus_floridanus
---MRNYRLIFCHNLKQP
>Balaenoptera_acutorostrata_s
---MRNYRLIFCHNLKQP
>Pygocentrus_nattereri
---MRNYRLIFCHNLKQP
>Calidris_pugnax
---MRNYRLIFCHNLKQP
>Callorhinchus_milii
---MRNYRLIFCHNLKQP
>Pteropus_vampyrus
---MRNYRLIFCHNLKQP
>Maylandia_zebra
---MRNYRLIFCHNLKQP
>Pterocles_gutturalis
---MRNYRLIFCHNLKQP
>Clupea_harengus
--------------------------------------MRKPFQ-------LSEVLHGICGLSMRNYRLIFCHNLKQP
>Latimeria_chalumnae
---MRNYRLIFCHNLKQP
>Colius_striatus
---MRNYRLIFCHNLKQP
>Columba_livia
---MRNYRLIFCHNLKQP
>Merops_nubicus
---MRNYRLIFCHNLKQP
>Alligator_mississippiensis
---MRNYRLIFCHNLKQP
>Cuculus_canorus
---MRNYRLIFCHNLKQP
>Danio_rerio
---MRNYRLIFCHNLKQP
>Kryptolebias_marmoratus
---MRNYRLIFCHNLKQP
>Sarcophilus_harrisii
---MRNYRLIFCHNLKQP
>Galeopterus_variegatus
---MRNYRLIFCHNLKQP
>Monodelphis_domestica
---MRNYRLIFCHNLKQP
>Esox_lucius
---MRNYRLIFCHNLKQP
>Falco_cherrug
---MRNYRLIFCHNLKQP
>Gavia_stellata
---MRNYRLIFCHNLKQP
>Balearica_regulorum_gibberic
---MRNYRLIFCHNLKQP
>Erinaceus_europaeus
---MRNYRLIFCHNLKQP
>Oryctolagus_cuniculus
---MRNYRLIFCHNLKQP
>Tauraco_erythrolophus
---MRNYRLIFCHNLKQP
>Opisthocomus_hoazin
---MRNYRLIFCHNLKQP
>Plecoglossus_altivelis
--MLFAPVVFDLLTSEILNRICGLSMRNYRLIFCHNLKQP
>Scleropages_formosus
---MRNYRLIFCHNLKQP
>Lepidothrix_coronata
---MRNYRLIFCHNLKQP
>Nipponia_nippon
---MRNYRLIFCHNLKQP
>Trematomus_bernacchii
---MRNYRLIFCHNLKQP
>Ceratotherium_simum_simum
---MRNYRLIFCHNLKQP
>Manis_javanica
---MRNYRLIFCHNLKQP
>Picoides_pubescens
---MRNYRLIFCHNLKQP
>Platichthys_stellatus
---MRNYRLIFCHNLKQP
>Homo_sapiens
---MRNYRLIFCHNLKQP
>Loxodonta_africana
---MRNYRLIFCHNLKQP
>Fulmarus_glacialis
---MRNYRLIFCHNLKQP
>Nestor_notabilis
---MRNYRLIFCHNLKQP
>Mus_musculus
---MRNYRLIFCHNLKQP
>Salmo_salar
---MRNYRLIFCHNLKQP
>Tupaia_chinensis
---MRNYRLIFCHNLKQP
>Lepisosteus_oculatus
---MRNYRLIFCHNLKQP
>Ictalurus_punctatus
---MRNYRLIFCHNLKQP
>Trichechus_manatus_latirostr
---MRNYRLIFCHNLKQP
>Aptenodytes_forsteri
---MRNYRLIFCHNLKQP
>Crotalus_horridus
---MRNYRLIFCHNLKQP
>Tyto_alba
---MRNYRLIFCHNLKQP
>Struthio_camelus_australis
---MRNYRLIFCHNLKQP
>Chelonia_mydas
---MRNYRLIFCHNLKQP
>Takifugu_rubripes
---MRNYRLIFCHNLKQP
>Tinamus_guttatus
---MRNYRLIFCHNLKQP
>Calypte_anna
---MRNYRLIFCHNLKQP
>Apaloderma_vittatum
---MRNYRLIFCHNLKQP
>Orycteropus_afer_afer
---MRNYRLIFCHNLKQP

HG0004.1 FBgn0028494

>Drosophila_melanogaster
MLRYRLVSTHFLKQP
>Steatoda_grossa
MLSLRLVSCRFLKQP
>Prorhinotermes_simplex
MLTLRLVSTHFLKQP
>Anoplophora_glabripennis
MLKFRLVSTHFLKQP
>Eurylophella_sp._AD-2013
MLTLRLVSTHFLKQP
>Cimex_lectularius
MLTLRLVSTHFLKQP
>Leptopilina_clavipes
MLKLRLVSTHFLKQP
>Eudarcia_simulatricella
MLNYRLVSTHFLKQP
>Metallyticus_splendidus
MLTLRLVSTHFLKQP
>Corydalus_cornutus
MLTLRLVSTHFLKQP
>Conwentzia_psociformis
MLTLRLVSTHFLKQP
>Medauroidea_extradentata
MLTLRLVSTHFLKQP
>Tityus_serrulatus
MLSLRLVSCRFLKQP
>Oropsylla_silantiewi
MLTYRLVSTHFLKQP
>Annulipalpia_sp._AD-2013
MLTYRLVSTHFLKQP
>Limulus_polyphemus
MLSMRLVSCRFLKQP
>Zorotypus_caudelli
MLTLRLVSTHFLKQP

HG0004.2 ENSG00000138640 FAM13A

```
>Homo_sapiens
---------MLEAALRLPWGLCIGSCFSHQRICAEWNFSILLSF
>Mustela_putorius_furo
MRSKTSLPKMLGAALRLP--------------------------
>Physeter_catodon
---------MLGAALRSPWGLRIWSRVSHRSISTEWNSSVLLSF
>Eptesicus_fuscus
---------MLGAALRLRWGLRIGSCFSNQSIPTGWNFSLLLSF
>Loxodonta_africana
MRSTASLPKLLGTALHLPWGLWIGSRFLNQSISTEWNFSILLLF
```

HG0005 ENSDARG00000011055 fbxo9

>Danio_rerio
---MAAVWRARKFA-RCVVRFS-DRDL----
>Acipenser_sinensis
---MAAVWRARNFA-RCVVRFS-DRELN---
>Channa_argus
---MAAFLRARKYV-CCVVRFS-DREL----
>Kaupichthys_hyoproroides
---MAAVWRARNFS-RCVVRFS-DRELY---
>Podocoryna_carnea
---MAAVRRARNFA-RCVVCFS-DRELN---
>Xenopus_laevis
---MAAVRRSRSDS-RCVLRFS-DRELC---
>Apteryx_australis_mantelli
---MAAVRRARSFS-RCVVRFS-DRELC---
>Stegodyphus_mimosarum
---MAAVLIARRYV-RCVIRFNSDRELID--
>Menidia_menidia
---MAAFLRARKYV-RCVVRFS-DREL----
>Opsanus_beta
---MAAFLRARKYV-RCVVRFS-DREL----
>Oryzias_latipes
---MAAFLRARKYV-RCVVRFS-DRDL----
>Prorhinotermes_simplex
---MAAVIRAKKCF-RSVVRYS-DRESLP--
>Calanus_finmarchicus
---MAAVWMAKCFT-RSVVRFS-DRDS----
>Trachinotus_blochii
---MAAFLRARKYV-RCVVRFS-DREL----
>Panthera_tigris
---MAAVRRARSYC-RCLVRFS-DRELC---
>Hynobius_retardatus
---MAATRRARAHP-RGVIRFS-DRDLY---
>Micropterus_floridanus
---MAAFLRARKYV-CCVVRFS-DREL----
>Pygocentrus_nattereri
---MAAVWRARKFA-RCVVRFS-DRDL----
>Miniopterus_natalensis
---MAAVRRARSYS-RCLVRFS-DRELC---
>Ophthalmotilapia_ventralis
---MAAFLRARKYV-RCVVRFS-DREL----
>Clupea_harengus
---MAAVWRARKFP-RSVVRFS-DREL----
>Latimeria_chalumnae
---MAAVWRARNFI-GCVVRFS-DRELC---
>Alligator_mississippiensis
---MAAVRRARSDS-RCVVRFS-DRELC---
>Fundulus_heteroclitus
---MAAFLRARKYV-RCVVRFS-DREL----
>Sarcophilus_harrisii
---MAAVRRARSHC-RCLVRFS-DRELC---
>Homarus_americanus
---MAAVVRSRIYF-SCVVRFS-DRESIA--
>Sclerodactyla_briareus
---MAAVARFNRYH-QNIVRYS-DRD-----
>Macropus_eugenii
---MAAVRRARSHC-RCLVRFS-DRELC---
>Haploembia_palaui
---MAAVLQAKGFF-ASVIKYS-DREFMP--
>Ephemera_danica
---MAAVQRAAKLAGQLVVRFS-DRELN---
>Esox_lucius
---MAAFLRYLQFP-RCVVRFS-DRDL----
>Meganyctiphanes_norvegica
---MAAVARSYMYF-ASVVRFS-DRECIA--
>Falco_cherrug
---MAAVRRARSYS-RCVVRFS-DRELC---
>Leptasterias_sp._AR-2014
---MAAFLRFLEFS-NCIVRFS-DRDCKA--
>Coturnix_japonica
---MAAVRRARSYS-RCVVRFS-DRELC---
>Gillichthys_mirabilis
---MAAFLRARKYV-SCVVRFS-DREL----
>Chanos_chanos
---MAAVWRSRKFA-RCVVRFS-DRDL----
>Euschistus_heros
---MATNMAAVYVCLRLG-EIILRFS-DRDSKI--
>Apis_dorsata
---MAAVLLARLYF-RCVVRFS-DREYKG--
>Erinaceus_europaeus
---MAAVRRARSYC-RCLVRFS-DRELC---
>Rhipicephalus_appendiculatus
---MASGPLVGNYKSMAAVVKARRYR-NCVLRYNSDRECIA--
>Ochotona_princeps
---MAAVRRARSYC-RCLVRFS-DRELC---
>Lutjanus_erythropterus
---MAAFLRARKYV-RCVVRFS-DREL----
>Calopteryx_splendens
---MAAVLRAKELS-TCVVRYS-DRESSA--
>Ophiocoma_echinata
---MAAVNKSLLYF-SCFVRFS-DRDWLA--
>Locusta_migratoria
---MAAVFMARKYF-ESVIRFS-DRESVP--
>Plecoglossus_altivelis
---MAAFQRSRKFV-RSVVRFS-DRDLVSLI
>Scleropages_formosus
---MAAVWRARNFA-RCVVRFS-DRELN---
>Junco_hyemalis
---MAAVRRARRDS-RCVVRFS-DRELC---
>Lateolabrax_japonicus
---MAAFLRARKYV-RCVVRFS-DREL----
>Trematomus_bernacchii
---MAAFLRARKYV-RCVIRFS-DRDL----
>Equus_asinus
---------------------------------------MLGALAQVPGQKGAAPQRRLGSPAGPILRCSHSLRPRLAAGAPPLRESPAA--APARPSLAPCPAKMAAVRRARSYC-RCLVRFS-DRELC---
>Medauroidea_extradentata
---MAAALKARKFI-GSVIRYS-DRESLP--
>Perla_marginata
--MAKKYF-KSVIRFT-DRECLP--
>Cynoglossus_semilaevis
---MAAFLRARKYV-RCVVRFS-DREL----
>Microcebus_murinus
MVAKKYVRILAQTPCGTAKGLVRKIPRPSPQHPPRRASNRPGSSPSSRRIGGAAPLRRPGSPIGPTRRCSHGPRPRLAAGVPLLRESPAAAAAPARPDLAPCPAKMAAVRRARSYC-RCLVRFS-DRELC---
>Acerentomon_sp._AD-2013
--MF-ECVIRFT-DREIYG--
>Melopsittacus_undulatus
---MCLCCSRPACPSLAGKMAAVRRARSYS-RCVVRFS-DRELC---
>Ectopsocus_briggsi
---MAAVLNAKFYF-GLVIRYS-DRELIR--
>Leucoraja_erinacea
---MAAVWRARNFL-DYVVRFS-DRELQ---
>Cavia_porcellus
---MAAVRRARSYC-RCLVRFS-DRELC---
>Salvelinus_alpinus
---MAAFLRSLKFP-RCVVRFS-DRDL----
>Tupaia_chinensis
---MAAVRRARSYC-RCLVRFS-DRELC---
>Tityus_serrulatus
---MAAVIKGRRYF-GCVIRFNSDREYVA--
>Lepisosteus_oculatus
---MAAMWRARNFA-RCVVRFS-DRELY---
>Trichomycterus_areolatus
--MYMRETPGTSGRLGMSVTLLYK--ADLQRPCRLDSTQTLSVMAAVWRARKFS-RCVVRFS-DRDL----
>Sparus_aurata
---MAAFLRARKYV-RCVVRFS-DREL----
>Gekko_japonicus
---MTQTN----------------------THTGSRAHDLALSHLLALSRVGKMAAVRRARSDS-RCVVRFS-DRELC---
>Pelodiscus_sinensis
---MAAVRRARSYS-RCVVRFS-DRELC---
>Takifugu_rubripes
---MAAFLRARKFV-RSVVRFS-DREL----
>Patiria_miniata
---MYCFGRPSADRRRNMAAFLRFLEFS-KCITRFS-DRDCEA--
>Zorotypus_caudelli
---MAAVWMARKYY-ESIIRFT-DREFFP--
>Thermobia_domestica
--MTKMAAVLRARKYC-GSVIRYS-DRESFS--

HG0005 ENSGALG00000016320

```
>Gallus_gallus
-------------------------------------------------------------------------------MAAVRRARSYS-RCVVRFSD-RELC---
>Acipenser_sinensis
-------------------------------------------------------------------------------MAAVWRARNFA-RCVVRFSD-RELN---
>Channa_argus
-------------------------------------------------------------------------------MAAFLRARKYV-CCVVRFSD-REL----
>Kaupichthys_hyoproroides
-------------------------------------------------------------------------------MAAVWRARNFS-RCVVRFSD-RELY---
>Podocoryna_carnea
-------------------------------------------------------------------------------MAAVRRARNFA-RCVVCFSD-RELN---
>Xenopus_tropicalis
-------------------------------------------------------------------------------MAAVRRSRSDS-RCVLRFSD-RELC---
>Apteryx_australis_mantelli
-------------------------------------------------------------------------------MAAVRRARSFS-RCVVRFSD-RELC---
>Stegodyphus_mimosarum
-------------------------------------------------------------------------------MAAVLIARRYV-RCVIRFNSDRELID--
>Apostichopus_japonicus
-------------------------------------------------------------------------------MAAVGIANRYP-GSVTRYSD-RELVD--
>Menidia_menidia
-------------------------------------------------------------------------------MAAFLRARKYV-RCVVRFSD-REL----
>Opsanus_beta
-------------------------------------------------------------------------------MAAFLRARKYV-RCVVRFSD-REL----
>Oryzias_melastigma
-------------------------------------------------------------------------------MAAFLRARKYV-RCVVRFSD-REL----
>Prorhinotermes_simplex
-------------------------------------------------------------------------------MAAVIRAKKCF-RSVVRYSD-RESLP--
>Trachinotus_blochii
-------------------------------------------------------------------------------MAAFLRARKYV-RCVVRFSD-REL----
>Panthera_tigris
-------------------------------------------------------------------------------MAAVRRARSYC-RCLVRFSD-RELC---
>Micropterus_floridanus
-------------------------------------------------------------------------------MAAFLRARKYV-CCVVRFSD-REL----
>Pygocentrus_nattereri
-------------------------------------------------------------------------------MAAVWRARKFA-RCVVRFSD-RDL----
>Miniopterus_natalensis
-------------------------------------------------------------------------------MAAVRRARSYS-RCLVRFSD-RELC---
>Ophthalmotilapia_ventralis
-------------------------------------------------------------------------------MAAFLRARKYV-RCVVRFSD-REL----
>Clupea_harengus
-------------------------------------------------------------------------------MAAVWRARKFP-RSVVRFSD-REL----
>Latimeria_chalumnae
-------------------------------------------------------------------------------MAAVWRARNFI-GCVVRFSD-RELC---
>Alligator_mississippiensis
-------------------------------------------------------------------------------MAAVRRARSDS-RCVVRFSD-RELC---
>Sinocyclocheilus_grahami
-------------------------------------------------------------------------------MAAVWRARKFA-RCVVRFSD-RDL----
>Fundulus_heteroclitus
-------------------------------------------------------------------------------MAAFLRARKYV-RCVVRFSD-REL----
>Sarcophilus_harrisii
-------------------------------------------------------------------------------MAAVRRARSHC-RCLVRFSD-RELC---
>Homarus_americanus
-------------------------------------------------------------------------------MAAVVRSRIYF-SCVVRFSD-RESIA--
>Sclerodactyla_briareus
-------------------------------------------------------------------------------MAAVARFNRYH-QNIVRYSD-RD-----
>Macropus_eugenii
-------------------------------------------------------------------------------MAAVRRARSHC-RCLVRFSD-RELC---
>Haploembia_palaui
-------------------------------------------------------------------------------MAAVLQAKGFF-ASVIKYSD-REFMP--
>Ephemera_danica
-------------------------------------------------------------------------------MAAVQRAAKLAGQLVVRFSD-RELN---
>Esox_lucius
-------------------------------------------------------------------------------MAAFLRYLQFP-RCVVRFSD-RDL----
>Meganyctiphanes_norvegica
-------------------------------------------------------------------------------MAAVARSYMYF-ASVVRFSD-RECIA--
>Falco_cherrug
-------------------------------------------------------------------------------MAAVRRARSYS-RCVVRFSD-RELC---
>Leptasterias_sp._AR-2014
-------------------------------------------------------------------------------MAAFLRFLEFS-NCIVRFSD-RDCKA--
>Gillichthys_mirabilis
-------------------------------------------------------------------------------MAAFLRARKYV-SCVVRFSD-REL----
>Chanos_chanos
-------------------------------------------------------------------------------MAAVWRSRKFA-RCVVRFSD-RDL----
>Grylloblatta_bifratrilecta
-------------------------------------------------------------------------------MAADLRCKKYS-TPVIRYSD-RELLP--
>Apis_cerana
-------------------------------------------------------------------------------MAAVLLARLYF-RCVVRFSD-REYKG--
>Erinaceus_europaeus
-------------------------------------------------------------------------------MAAVRRARSYC-RCLVRFSD-RELC---
>Ixodes_persulcatus
-------------------------------------------------------------------------------MAAAVKARRYR-NCVLRFNSDRECIA--
>Ochotona_princeps
-------------------------------------------------------------------------------MAAVRRARSYC-RCLVRFSD-RELC---
>Lutjanus_erythropterus
-------------------------------------------------------------------------------MAAFLRARKYV-RCVVRFSD-REL----
>Calopteryx_splendens
-------------------------------------------------------------------------------MAAVLRAKELS-TCVVRYSD-RESSA--
>Ophiocoma_echinata
-------------------------------------------------------------------------------MAAVNKSLLYF-SCFVRFSD-RDWLA--
>Locusta_migratoria
-------------------------------------------------------------------------------MAAVFMARKYF-ESVIRFSD-RESVP--
>Osmerus_mordax
-------------------------------------------------------------------------------MAAFQRYRKFV-RSVVRFSD-RDLVSVI
>Scleropages_formosus
-------------------------------------------------------------------------------MAAVWRARNFA-RCVVRFSD-RELN---
>Corvus_cornix_cornix
-------------------------------------------------------------------------------MAAVRRARRDS-RCVVRFSD-RELC---
>Lateolabrax_japonicus
-------------------------------------------------------------------------------MAAFLRARKYV-RCVVRFSD-REL----
>Sebastes_caurinus
-------------------------------------------------------------------------------MAAFLRARKYV-RCVVRFSD-REL----
>Ceratotherium_simum_simum
MRAAAAAAAAALRMLGASAQVPVAERAAPQPRPGSPAGPTLRCSHSPRPRLAAGAPSLRESPAA--APARPSLAPSPAKMAAVRRARSYC-RCLVRFSD-RELC---
>Ramulus_artemis
-------------------------------------------------------------------------------MAAILKARKYI-GSVIRYSD-RESLP--
>Menopon_gallinae
-------------------------------------------------------------------------------MAALLSAKRHF-GLVIRFSD-REFQG--
>Perla_marginata
------------------------------------------------------------------------------------MAKKYF-KSVIRFTD-RECLP--
>Cynoglossus_semilaevis
-------------------------------------------------------------------------------MAAFLRARKYV-RCVVRFSD-REL----
>Papio_anubis
-------------MLGASAQVCAAEGAAPQRRPGSPVGLTLRCSHNSLLRLATGVPPLPESPAAAATPARPDLAPCPAKMAAVRRARSYC-RCLVRFSD-RELC---
>Acerentomon_sp._AD-2013
----------------------------------------------------------------------------------------MF-ECVIRFTD-REIYG--
>Melopsittacus_undulatus
---------------------------------------------------------------MCLCCSRPACPSLAGKMAAVRRARSYS-RCVVRFSD-RELC---
>Ectopsocus_briggsi
-------------------------------------------------------------------------------MAAVLNAKFYF-GLVIRYSD-RELIR--
>Leucoraja_erinacea
-------------------------------------------------------------------------------MAAVWRARNFL-DYVVRFSD-RELQ---
>Microtus_ochrogaster
-------------------------------------------------------------------------------MAAVRRARSYC-RCVVRFSD-RELC---
>Salvelinus_alpinus
-------------------------------------------------------------------------------MAAFLRSLKFP-RCVVRFSD-RDL----
>Tupaia_chinensis
-------------------------------------------------------------------------------MAAVRRARSYC-RCLVRFSD-RELC---
>Tityus_serrulatus
-------------------------------------------------------------------------------MAAVIKGRRYF-GCVIRFNSDREYVA--
>Lepisosteus_oculatus
-------------------------------------------------------------------------------MAAMWRARNFA-RCVVRFSD-RELY---
>Trichomycterus_areolatus
--------------------------MYMRETPGTS------------GRLGMSVTLLY--KADLQRPCRLDSTQTLSVMAAVWRARKFS-RCVVRFSD-RDL----
>Sparus_aurata
-------------------------------------------------------------------------------MAAFLRARKYV-RCVVRFSD-REL----
>Echinaster_spinulosus
-------------------------------------------------------------------------------MAAFLRFLEFA-NCIVRFSD-RDWKA--
>Streblospio_benedicti
-------------------------------------------------------------------------------MAAFLRYRMFS-SCVICFSW-RELTV--
>Gekko_japonicus
--------------------------------------MT-------------QTNTHTGSRAHDLALSHLLALSRVGKMAAVRRARSDS-RCVVRFSD-RELC---
>Chelonia_mydas
-------------------------------------------------------------------------------MAAVRRARSYS-RCVVRFSD-RELC---
>Takifugu_rubripes
-------------------------------------------------------------------------------MAAFLRARKFV-RSVVRFSD-REL----
>Thermobia_domestica
----------------------------------------------------------------------------MTKMAAVLRARKYC-GSVIRYSD-RESFS--
```

HG0006 FBgn0029971 CG18624

```
>Drosophila_melanogaster
---------------------------------MGSGIVVQLARKYGAVIFFPTVAVGSIYADWSHTRQWKRQQLQLAHRAQLKD--QR---------
>Echiuropus_macronychus
------------------------------------MGVLEIVRRFRVVVIFPAFVTTCIWADYKHTAEWKKRNFLERDQLRDRES-LRN--------
>Machilis_hrabei
--------------------------------MALWSSFRQFWSRYCVVIIFPTACVTSIYADYSHTQKWKAQQLRIQEDKLKAE-NWS---------
>Blaberus_atropos
-------------------------------------MAVSQLFRRYWIVVLFPTVTAGSIFADLIHTKQWKEQKAQLQTAAELEE--TL---------
>Hynobius_chinensis
---------------------------------MGQLSNIGVFWRRYRVSILFPAFSIGTIYADYAHTQKYKKSLADFQRKQQLQ--------------
>Trypoxylus_dichotomus
-----------------------------------MSFKIGKLVNKYWVVTLLPTFVASTIYADYTHTQEWKKSLNKLN--------------------
>Litopenaeus_vannamei
--------------------------------MAPRLTALG-FLRKYKVVIFFPLVTFSAITADILHTRKWKAEKAAAALAESA---------------
>Apachyus_charteceus
------------------------MVLLSIPVISVYMSSIINFCRKYSLSILFPTLSLSIIIADFNHSRKWKKSLKNE---------------------
>Daphnia_pulex
-------------------------------------MSVLAFWRRYRVSIIFPVLSFSFIYADWSYTRRWKQENLKKLAEAVDPE--VIKA-------
>Campodea_augens
MADS----------RIVTSKLPLDAPPVDPTSFSARCKRANFLWRYYRASVLFPTVTVGLILLDYNHTQKWKREQKELSEKQAEK--------------
>Haploembia_palaui
----------------------MDK-------PPSVLRRTGYLIRKYFIVVALPTALGYAVIADYLHTQKWKREQAALQNKPSM---------------
>Isonychia_bicolor
-------------------------------------MAVGYYIRRYGISVLFPTFVALSIFADYNHTRRWKQQHKLLTSQESNLS-------------
>Onuphis_iridescens
---------------------------------MSGLAYYKYLIRRNFVSVIFPVGLGLVIYADWSRTQKYKASQK-----------------------
>Galloisiana_yuasai
-------------------------------MASGKLSTINKLWKTYRVVIFFPTFATAAIVADLVHTRNWKVQKASLEAKENN---------------
>Amynthas_koreanus
----------------------------------MQLSFAYAVFKRNFVSVIFPVCTVWAIYADWSRTRRYKAAKAALPSTHFQS--------------
>Tigriopus_californicus
-----------------------------------MFKQGLALFRKYAVSVIVPTVVFGSIAWDFNRTQRWKRNQIEFDESQKIANQ------------
>Adelphocoris_lineolatus
-----------------------------------MALPIRSFVRKYGPSIVFPVAAFSAIYADYSHTQNWKKEQLLKAKTSELL--------------
>Lineus_viridis
-----------------------MEKE-----VVSAPVKVLRFAKRNFVSVIFPVVAAWAIYSDYSRTQRFKAKKARELLVTGAVQKELIKDSF-----
>Tenthredo_koehleri
-------------------------------MAAPGKVSIGKLISRYAAVVFFPTFSATTIFLDWNHTRKWKIAESERVSALNSELQ------------
>Armadillidium_vulgare
--------------------------------MEGKTHPIIKFWKRAGRSIIFSTVSISLITADYLHTRKWKKELETKYENFYV---------------
>Dermacentor_variabilis
----------------------MAATGTA----LEKVRPLLKVCRRYAATVLFPTISGWLIYADYSRTQRWKKSKASAE--------------------
>Triodia_sylvina
-------------------------------------MAIGKFLRRYGVVIFFPTFAATSIYLDWSHTKKWKQQNKLFVNSKEIV--------------
>Mantis_religiosa
------------------------------MEV-SRLAQFRRFWKTYWVVITLPVVTAGSIFADLNHTRKWKKEKRELAKADILKR--TLSEEQ-----
>Tanzaniophasma_sp._AD-2013
-------------------------------MAVGILSSARNFWRRYCVVILFPTFTITTIAADLIHTRNWKAKTVAAEVLDKTVSEDTVYS-------
>Boreus_hyemalis
-------------------------------MALGKASAIRHFWKKYSVVIFFPTFTLATIYADYSHTQQWKAQQGSTK--------------------
>Sialis_lutaria
--------------------------------MAVRLSTIGSFWRKYRVVIIFPVITISSIAADYQHTQKWKASQTNQ---------------------
>Mytilus_galloprovincialis
----------------------MAGS--------RTISKILRIVRRHSLTVIFPVLCFGAIYLDYSRTQEYKRQRKLDH--------------------
>Xibalbanus_tulumensis
-------------------------------MDVTLVGRVFRFLRFYKFVIIFPVACGGAIFADYNHTRKWKEEKARLEALAYQAEN-PENIVQ-----
>Euroleon_nostras
------------------------------MSVGRKLKTVYGYWRYYRIVILFPTFTIGTISADLLHTKHWKEQQLALENKSKSEF--D----------
>Triops_newberryi
----------------------------------MALSALRAFWRRYNVVIVFPTLTAASIYADYSHTQQYKAAKALAASRFSELLSE-----------
>Epiophlebia_superstes
---------------------------------MAKGNAAVKFLKKYGISIFFPSATVAAIYADLSHTKKWKALQVQVAKVSQE---------------
>Phalangium_opilio
----------------------------------MNFKIALRTLKRYKVVILFPTITIWSIYADYTRTLRYKATKLQILQRQTVL--------------
>Hypochthonius_rufulus
----------------------MANESPSLNHNKLWYERLVKEFRRYRRTILFPTVLLGLVYLDFSHTQKWKKQKQLSEENQSQIVLN-----------
>Stenobothrus_lineatus
-----------------------------------MLKATGKFLRRYGVVIFFPTVVTAAIITDLSYTRQWKRLKAEKLRQQELLTD------------
>Phyllium_philippinicum
--------------------------------MEVSKLVAAKNLVKKYFWVVFFPTFAAVTISADWQHTRKWLKKAALAEEQSKQA--VLAEDQSSNYQ
>Glycera_tridactyla
---------------------------------MSPAAGPLRLLKVNLVSITFPTVVAWAIYADWSRTRRFKAQKALERVKEHY---------------
>Cosmioperla_kuna
------------------------------MAAVKEVSAIGKFLRRYKVVIFFPTFTAATIFADLRHTRNWKAQETAQESTVKYTAEKLL---------
>Acerentomon_sp._AD-2013
MVHQTTTPHGSSSASKYGKQPTLVATQAEQDANLSFFRRALKTAWKYSLVVTFPSIAFAAIYADYSRTQKWKAEQARLKETE-----------------
>Ectopsocus_briggsi
------------------------------------MANVRYLLRRYSVVVIFPTLTAWSIYADLSHTRKWKAQEEARKAKEE----------------
>Inocellia_crassicornis
-------------------------------MAVQKVGFIRAFWRKYGVVITFPTLSFSAILADYTHTYKWKKQQAALLQELKDPE--LLKS-------
>Trichomycterus_areolatus
------------------------------MAVAKTPSALGLFWRKYRVSIIFPVLSVSAIFADYSRTSSWKKISKFER--------------------
>Oropsylla_silantiewi
---------------------------------MAYLASFRQLWKRYSIVVLFPSFALGSIYADWSNTQKWKQNTKKQ---------------------
>Gluvia_dorsalis
-------------------------------MASGSARGILYQLWRYKYVILFPTGTALSIYADYSHTKQWEALNKNKE--------------------
>Stylops_melittae
---------------------------------MIKIRFRLRSITRHISTALFPLVAAGLIWADYSATQNWKARKGLREAK------------------
>Alvinella_pompejana
----------------------MEGGT-----ALNRFQTLVAKLRRNFITIIFPVLTTWAIYADWSHTQKYKAAKAAALLQASEESQSIG---------
>Gynaikothrips_ficorum
------------------------------------MVKVANIVKRYFLTGFFPVLFVTPIYLDWSRTQRYKAEKLKESGGSS----------------
>Rhyacophila_fasciata
-------------------------------------MGLGKLVKRYSVVVLFPTVTVTSIVADLRHTYLWKKQKAELLKQ------------------
>Zorotypus_caudelli
---------------------------------MAVLKTARELFSRYFISVLFPTIAISSIVADYKHTQKWKAKQQAIIQSS-----------------
>Atelura_formicaria
----------------------------------MAAGGVGLFWRRYRVVIIFPTIVAACISADYAHTREWKAQQAAELKSRESVH-------------
```

HG0007 FBgn0034372 Gint3

>Drosophila_melanogaster
--------------------------------------MSFNFYVESFNS---T-------L--------QSIAR---QVEQFAGVALTAAVD------LRGNLE-------------SLTG---------DFRWEN-GPIAFTDLQATLLKVLIVLLLGTCVLAGYSWSIYGKVITEKFVRPST--LK-EI--EELKLSVAKLKLPKEHSPRI-
>Stegodyphus_lineatus
--MKSL-------------------DII--SNLITPSGVTE-------------YLYSVFPEDIPDA-AISLVTSFNTVDEFQLKILRVSLCILLINLFLIWCAWRKYGEKITERFMK-PA--SSKSL--EDLKREATELKLPKEHTPRI-
>Machilis_hrabei
MS---------S-------------------------VIDFE-------------------------SETSSLFE---ALHQLFQELTGFICETF-GLSLEVSQEE---------FKSYFWS---------VYHSII-DWLSFDDFQWKLLQAFLIILFTNIVLIYIAWRVYGERIADRFMYPES--VR-AI--EELKASVQRLKLPREHTPRI-
>Argulus_siamensis
--MFLD-----FD---------------------NLYLLLYDLY---IQ-LVEKLYE-ELYTKNDFRLRTIQCFSFIICVNIILIIIAWKIYGPRIAEKFMKPVP--TV--V--EQLSKDIASLKLPPEYSPRV-
>Cryptocercus_wrighti
--MSWKIYGKRISEKFMRPAT--CK-TI--EELKRSVSSLKLPKEHSPRV-
>Meloe_violaceus
MN---------A-------------------------FLNDFSTNSSLFD-----------VIYTYLTTINIYF-----------------IDTL-SLLRDHPKH---------------KDLLSVF------DNLP-ELLDFNEFQLTLLKTFLIVLFANLILIFIAWTIYGKRICDRFMKPAT--NK-AI--EELKASVSKLKLPKEHTPRI-
>Eucyclops_serrulatus
MNIWESLTNFTSSISTLILEDNIQPGRETDLLTDLFNFDSSIFSIESLYS-----------VANSFQNTLVGIFNSTCEISSVFGQVAGSIL----NEATTGNIS-----------SA-------VSLTFLESEKLF-DWFNFTEFQVKLLREFSIILLGNIVLVIFAWKIYGPRISSKFSVSPSRGSRQVI--EDLRLSMSELQLPNEHDYKLK
>Apachyus_charteceus
--MKPAT--SK-TI--EDLKKSVSLLKLPKEHSPRV-
>Daphnia_magna
----------------M--------------------VDEKSFGDQQSTLWVEL-------FVEYFVNLIYVLVT---SVIHLTEVL----V------------E-----YG----EHFLQSIFLLLISLSETIVAI-FDLAFSDFQIKLLQVFFCILAIHILAICVAWKIYSERITQRFLKPSL--YEISS--EQLKTAVSELKLPAEHTPRW-
>Occasjapyx_japonicus
--MKPAS--LR-AV--EELRSSVAELRLPQEHSPRI-
>Aposthonia_japonica
MI---------P-------------------------LFGNYF-NSTVLD-----------ATTYYVFKLFALA-----------------NEVV-GISSSKTFDDS-------ESS--FTIQI---------FDLG-TWFAFDDFQITLLKVFLSFLCVNLICIYISWKIYGRRISEKFMKPAT--PK-AI--EELKKSVFLLKLPKEHSPRV-
>Isonychia_bicolor
MS---------S-------------------------LSGDVSANST------------------VIDSIFQYL----------GKFCLLLQQAL-GLGPVVPLPEPLQDL----PHLPTTDILTDFIEW----------FAFDDFQFKLLKGFLVVLTFNIILIFIAWRFYGRNISEKFMRPAT--SK-AI--QDLKNSVSQLKLPSEHTPRI-
>Galloisiana_yuasai
MT---------S-------------------------INEDDSNSLSFIN-----------VITNYIYEISTVI-----------------YTAF-GFTSNIKVENN-------IPLHFIIECY---------YNII-NWLAFDEFQIKLVKGFTFILFANILFIYMSWKVYGKRISEKFMRPAT--SK-AI--EELKKSVSLLKLPKEHSPRI-
>Graphocephala_atropunctata
MT---------S-------------------------NVDEQLDSSSLWN-----------LLYFYCNQIYFAI-----------------FSNL-NITSNFTTSTLTSFIGNEEHSLNILSYL---------RYIA-SLLPYDEFQIELLKLFCAIILTNICLVAIAWKVYGKYICQRFMKPAT--PK-VI--EELKKSVSQLKLPKQHSPRI-
>Tenthredo_koehleri
MS---------S---SV--------------------CNDVAPGSGSLLD-----------IINKFACDLIGLV-----------------FSTI-GITSNINSSNLDDDTSSEHHRHTIIQFY---------EEFS-AWLAFDEFQLQLFKVFAFLLVSNVALIYVAWHVYGARISNRFMKPAT--SA-AV--EELKKSVSRLKLPKEHSPRV-
>Rhipicephalus_annulatus
--MFWF---------------SASQK--EEEAESPLW-----WTSSLLTGPLVEAY---------EQLDTWCLGCDHFERSVAQALLVVFAINVVLILCVWRRCGDQIADQFLNLAT--SSSQL--EEFQKEASSLELPEEHSPRI-
>Enteucha_acetosae
MS---------A-------------------------FFETYIINSTLFE-----------VLSLTVNDSFGYF----------SEL---VIRCF-ESV----------------KVLEISNKLEKICENTFSKEFL-SSLEFDDFQFLLLKTLLIIFFATFLLICFVWKLYGKRISERFMKPAT--SA-AI--QELKDSVSKLKLPKEHTPRI-
>Empusa_pennata
--MKPAT--SK-AI--EDLKKSVSLLKLPKEHSPRV-
>Panorpa_vulgaris
---MNILFIYFAWNFYKDDIWEYFMRPTS--VD-ED--AELQKRIELLNLPREHTPRI-
>Sialis_lutaria
MT---------S-------------------------YITDNLFNTSYYT---D-------VILKYSEEVNNIYL---KVIQLFSEHETAIHP------L---IL-------------FYQS---------VVDFAF-SLVHFNEFQISILKYLLLVLIINLFLICIYWKLYGKAIGERFMKPAT--SR-AI--EELKASVDRLKLPKVHTPRI-
>Euroleon_nostras
--MRPVT--SR-AI--EELKASVARLKLPKVHSPRI-
>Triops_newberryi
MK-----------------------------------FWLPNFDGVSLWSWWESAVDSVSDLSENVKSSVFPWIP-----VSLFSLFWF-LVES-----VTS-----------------AWGSL-----TYNLSVVG-ESFSFDDFQTKFLQALFVVLFCNIGLTLLAWRVYGKKITERFMQTSS--DV-LK--EQLKTAVSELKLPNEHTPRL-
>Calopteryx_splendens
MT---------S-------------------------FNDALFSNSTLLD-----------VAHYFIHEWSNIL-----------------LELL-GIGSEHEIEKAD--------SNTLLHYC---------EDLI-QWFAFDEFQTTLLKGFAIILSANIVLICISWKVYGKRISERFMRPPS--TK-AI--EELRKSVSQLKLPKEHSPRV-
>Nothrus_palustris
---MIDTLLFWLLIILVSNLLLIVIFWNIYGKRIDVLITRKEE--KQQIL--DQLRREAFELKLPKEHSPRI-
>Ceuthophilus_sp._AD-2013
MT---------S-------------------------VAEDAI-NSSFVD-----------VIRVYVFELFTAV-----------------TKIF-GLASNIPLQDT-------TSTRLFFECY---------NSVI-SWLAFDDFQVKLLKGFVFILCANVVLIFISWKVYGKKISERFMKPPT--SK-AI--EELKKSVSLLKLPKEHSPRV-
>Crassostrea_gigas
--MSSESLSS-----------A---TTQEAL------------------VIVDYISG--V----------------GKVIWDFL-------VDAFLL-LRHEQYDVQSKLLVVLIFWLFFNLFLIGIAWQYYGGQNICEKLNRSKEK----------------------------
>Ramulus_artemis
MT---------S-------------------------VNGDIWFNFSFLE-----------PVLHCICELYKSL-----------------NEYL-ADSLNFNYSAP---------AYSRVETL---------VVLG-SWVTFDDFQINLLKGFILFFFINILLIFISWKYYGKKISEKFMGPVT--PK-AI--EELKKSVSLLKLPREHSPRV-
>Menopon_gallinae
MD-----------------------------------QDSSTLGQTTFLD-----------NLYACFSKIFQEA-----------------YELL-SSVIQSEI----------LSYFEGVSFN---------LNVL-QYFAFDELQIKLLKGFLLIIVFNSVLIWITWRIYGKRISNRFMKTAS--SK-TI--EDLKKSASMLKLPKEHNPRV-
>Platynereis_dumerilii
MS--ERAP----I--D-------------------SKIAEFNSTEAEGNTW-------------VDFG---YD---L-CKEFGKFWDDKLEFRVKLLVVLIICLAVNLNLIHCAWRKYGNKITERFMKSGS--SRASS--EN--KEVPEQRLHRE------
>Leuctra_sp._AD-2013
--MRPPT--SK-SI--EELKKSVSLLKLPKEHSPRV-
>Acerentomon_sp._AD-2013
--MRPAS--LK-VI--EELKRSIAELKLPREHSPKI-
>Liposcelis_bostrychophila
--MRTAS--HK-AI--EDLKKSISMLKLPKEHSPRV-
>Xanthostigma_xanthostigma
MT---------S-------------------------FSYLDFVNASYND------------INYYAYEVYDYIL---QILNLFVFWKDCST----------------------------EH---------YLSLYQ-SFIVFNEFQVNILKSLLIIFLCINLLIFIYWKIYGHQICERFMRPAT--SR-AI--EELKASVAKLKLPKVHSPRI-
>Tityus_serrulatus
--MV--SHLF----------------------Y--FT-DI--I-YEYLVIPFINIEEFEWKLLRSLLVFLLINLVFITVAWNKYGNKISERFMK-PV--ANKIL--EDLKKEATELKLPKEHTPRI-
>Trichomycterus_areolatus
--MRPAT--SK-AI--QELKESVSRLKLPKEHTPRI-
>Archaeopsylla_erinacei
--MSLSNYNFTYFNTVYDQL------EYLYTVLWENLIKTLYSTES----------------EVSFFDPI--------QETFE-YLINFDEYQLTLLKVLFVLICLNLLFIYVAWNYYKDVIWDCSIRPTT--LD-ED--VELIERNAKLNLPRVHSPRL-
>Streblospio_benedicti
--MTEQEL----QNEK---------------VIHADTLQTL-------DLE-------------SLKET--------FCL-IVTDFWGKSDFQIKLFCILTVWLLINLVLIKIAWSTYGERLSEMFSGLTSRQTSRQTSEERVVEGVRAENIQRE------
>Stylops_melittae
---MV-NLQTNLTWVF------N-EFKQLFVSEI---------LYLE-SLTSLSNLQIRIVQVFTLLTILNLTLILIVWNIYGQRISNRFIKVAT--SK-EI--EELKLSVSKLKLPKEHSPRF-
>Frankliniella_cephalica
MS---------T-------------------------FTEESLFNTSAL------------LMNSLVSSISNTV-----------------LYAI-GLTSNVTEGC----------SHLSALPL---------FDWQ-ALFSYDDFQVVLLKVFLFILAFNLVSICIAWKIYGKNIYETFMKPVT--PK-II--EDLKKSVSRLNLPKEHSPRV-
>Annulipalpia_sp._AD-2013
--MSWW-------------RAFSEE-----G--LPLP----------LSLPSQESLSEFVQYFVPEIPL-GSVQFDDFHALLLKTFLVILFTTLFLIFVSWKLYGKKISERFMKPAT--SK-AI--QELKESVSRLKLPKEHTPRI-
>Limulus_polyphemus
MS--QAVDAA-NQSEL-------------------EKTENSTNADPQNITW-------------LTGSLLGSAREGL-ELWISDNFSDIDEFQWNILKTLLTFLCINLTLICLAWKKYGGKITERFMK-PD--SSKAL--DDLRKEATELKLPKEHCPRI-
>Zorotypus_caudelli
--MFICIAWKIYGEQISERFIKPAN--AK-FI--EDLKKSVSQLKLPKEHSPRV-
>Thermobia_domestica
--MK-PA--STKSF--EDLAKGASELKLPKEHSPRV-

HG0008 ENSG00000175567 UCP2

```
>Homo_sapiens
--------------------------------------MTIRCFVSHPFSMENQGDR-AMIATGSFEERDTFREA-----
>Acipenser_sinensis
--------------------------------------MLVQRFVFGRLSMEQRGNL--IVTESNFSERDTFREEC----
>Podocoryna_carnea
--------------------------------------MLVQRFVFGRLSMEPRGNL--IITESNFSERDTFREEC----
>Megophrys_sangzhiensis
--------------------------------------MFIQHLVLHQFSMESKGYR-IFTASGKCDGRDAFREA-----
>Oryzias_latipes
--------------------------------------MILGLSTSEISSMEKQGQT--VKCAANFSQRDTFRES-----
>Acinonyx_jubatus
--------------------------------------MTIRCFVSQPFSMENQGDR-AMIATGSFEEWDAFREA-----
>Hynobius_retardatus
--------------------------------------MITRTAGSKPSPMEDQGYR-----ERKFDMRDTFRET-----
>Micropterus_floridanus
--------------------------------------MILRLITSEKSSMEKQGNQ--FIAGTKFSQRDTFRES-----
>Orcinus_orca
MCLEQMALSLLGCGPLRSISLSKLSSF---ALTCRTHSMTIRCFISQPFSMENQRDQ-AMIATGSFEEWDAFREA-----
>Astyanax_mexicanus
--------------------------------------MFFRCSFSERFSMEDRGNE--IIVESHFNQRDAFRES-----
>Miniopterus_natalensis
--------------------------------------MTFRCFVSRPFSMENQQDR-AMIATGSFEEWDAFREA-----
>Clupea_harengus
--------------------------------------MLLYCLVLERFSMEEQGNV--IIVESNFNQRDTFRES-----
>Latimeria_chalumnae
--------------------------------------MIIRYSDSAEFSMDQ-EKT--SIVKNNFNERDIFRES-----
>Alligator_sinensis
--------------------------------------MVIRCPACKRAPMERNGHR-MVVASSKCDGRDAFRES-----
>Sinocyclocheilus_grahami
--------------------------------------MFLICTGSNRFSMEERGNK--IQVEGHFNQRDTFRES-----
>Kryptolebias_marmoratus
---------MIKPDYLKKYYLLFFFLFLLKCLFSRSWSMILRLKVSERSSMEKQGNQ--FIGGTRFNQRDTFRES-----
>Sminthopsis_crassicaudata
--------------------------------------MIIRCSVSQQFSMENQGKC-TMVARSNFEERDTFREA-----
>Galeopterus_variegatus
--------------------------------------MTFGCFVSQPFSMENQGDR-AMIATGSFEERDAFREA-----
>Monodelphis_domestica
--------------------------------------MIIRCSVSQQFSMENQAKW-NMVAVSNFEERDTFREA-----
>Trichosurus_vulpecula
--------------------------------------MIIRCSVSQQLSMENQGKW-AMVAVSNFEQRDTFREA-----
>Esox_lucius
--------------------------------------MFLGHIVFERCSMEEQGNQ--IIVESNFDQRDAFRES-----
>Acanthogobius_hasta
--------------------------------------------------MENKRTQ--IIAGTKFSQRDAFREA-----
>Chanos_chanos
---------------------------------------MFMCPVFKHFSMEEQGNE--IIAESTFNQRDTFRES-----
>Condylura_cristata
--------------------------------------MTVRGFVSQPFFMENQRDQ-AMLTADSFEEWDAFREA-----
>Oryctolagus_cuniculus
--------------------------------------MNFRCFVSQPFSMENPGDR-GMIATGGFEERDAFREA-----
>Elephantulus_edwardii
--------------------------------------MTIRYSVSQPFSMENQKDQMIMIATGSFKEWDVFREA-----
>Scleropages_formosus
--------------------------------------MFLKCYTLKRVSMEDQVNE--IIAESQFSERDTFREA-----
>Lateolabrax_japonicus
--------------------------------------MILRLITSKRSSMEKQGYQ--FIAGTKFSQRDTFRES-----
>Sebastes_caurinus
--------------------------------------MILKLITSESCSMERQGNQ--CIAGTKFSQRDAFRES-----
>Equus_asinus
--------------------------------------MTIRCFVSQPFSMENQGDR-AMIATGSFEERDAFREA-----
>Manis_javanica
--------------------------------------MTIRCFVSQPFSMENQRDL-AMIATGSFEEWDAFKEA-----
>Loxodonta_africana
--------------------------------------MTIRCSVSQPFSMENQRDQ-IMIATGSFEEWDVFREA-----
>Leucoraja_ocellata
--------------------------------------MII-WRFISELSMEQRQSQ--TVVKRGYNERDTFRESSPARL
>Psammomys_obesus
--------------------------------------MIFKCFVSKPFSMENQGDR-AMIATGSFEERDTFREA-----
>Oncorhynchus_tshawytscha
--------------------------------------MFLRYIVFERFSMEEKGNQ--IIVESNFDQRDAFRES-----
>Tupaia_chinensis
--------------------------------------MTIRCSVSQPFSMENQGDW-AMIATGSFKEWDVFREA-----
>Lepisosteus_oculatus
--------------------------------------MLFTSLVLGIFSMEQQGTQ--IIVESNFNERDTFRES-----
>Ictalurus_punctatus
--------------------------------------MFLKCPFSELFSMEEQGNE--IIVDSHFNQRDAFREA-----
>Trichechus_manatus_latirostr
--------------------------------------MTIRCSVSQPFSMENQRDQ-IMIATGSFEEWDVFREA-----
>Squalus_acanthias
--------------------------------------MII-RRFISELSME-RQSQ--IIVKKSYNERDTFRES-----
>Gekko_japonicus
--------------------------------------MIIRCRLSAAFSMASKGSR-AAVALSNFDERDTFRES-----
>Chrysemys_picta
--------------------------------------MILKCLVSKRVSMENQGDQ-IVVALSKFDERDTFRES-----
>Orycteropus_afer_afer
--------------------------------------MTIRCFVSQPFSMENQRDHQIMIATGSFEEWDVFREA-----
```

HG0009 FBgn0050100 CG30100

>Drosophila_melanogaster
-----MKMVQPSRQQILRLYKHLIRYGNHLKLTDKNYFLGRVRHEFRDNRSLTNPVEVEFSFKRGETLLKKGRIL
>Talitrus_saltator
-----------------------------MELSDRRFFEKRIRREFTKQRNS--PEELDTAVQRAEAFLEKGRLL
>Latrodectus_geometricus
--------MTVSRQKVLSLYKELLKYSKNLKYTDKSYYLNRLKNEFLENRNLQNASEINFQIQRGKNFLEKKRLL
>Meinertellus_cundinamarcensi
-------MQIPNKQQVLCLYKSLLRYGETLRLTDKQYFRTRVRSEFQKNKSSDDSKEIVFQYEKGLKVLNSKRIL
>Blattella_germanica
-------MSNPTAREVLKLYRDILKYGRQLQFTDKNYFGQRIRNEFKAGKDLKNQEDINFHFQRGEALLKNKRVV
>Calanus_finmarchicus
------------MSQATLLYKSLLRYGRTLQMTDKDYFYRRVRFEFEKNSVLLEKQEVQFQLDKARQFLKNKRLV
>Photinus_pyralis
--------MHPNAKDILKLYKNLLRYGQELQYTDKNYFARRIKNEFRRNRDITDEADIQFNYKRGISLLEKRAVV
>Apachyus_charteceus
-----MASTLPNTRQILSLYKNLMRYGQKLKFTNQSYFYERVRREFENNKTIPED-KREFYYKKGLSLLEKKRVV
>Daphnia_pulex
MNTIFIIMNVPNKTQIFELFRSCIRYSGQLKLSDKRLVSKRIKSEFHKNKTLSNPEEICKAYEKGQSFLSKRRLL
>Occasjapyx_japonicus
---MAAYMGVPSPVQVLRLYRSLVRYGKELKYTDRDFYQRRIRNEFERHRLLTKDEDIRFQYEKGLAFISRKRII
>Haploembia_palaui
-------MSSPSASEVLRLYRELLRFGRQLQFTDQKYFLRRIKTQFQENKHLEDKEQIEFQFNKGQSLLKRKRVV
>Baetis_sp._AD-2013
--------MSVARSEVLNLYRSILKYGKDIKLTDKDYFRKRIRQEFRSNQKVTNAEEIQFHVERAKAFLANKRVV
>Glomeris_pustulata
--------MTIQRQSVLKLYRELLRYGQTLQYTDKKFYFRRIRKEFEQNRSLTAPADIQYYYEKGLTFLTNKRLI
>Galloisiana_yuasai
-------MTNIGGRQIIKLYRDLLRYGKRLEFTDINYFNRRIKTEFLKNKNLASLDEIQFQFERGISLLKNKRVV
>Tigriopus_californicus
---------MSQRSEALRLYKSLLRYGQSLRLTDSAYLHRRCTAEFRTPRST--PAQIERALAQARALLSQRRLL
>Cercopis_vulnerata
-------MNKPTVKQIVQLYKDLLRYGNNLKLTDKEYYIDRIHLEFRKNQSLDNPKDIQFCFDRGLALLKNKRVV
>Argochrysis_armilla
-----------MRSAILRLYKEILRYGETLKYTDKTYFRERIRTTFRNNKDLTNEKDIDFQFKKGQVFLEKRRVV
>Rhipicephalus_appendiculatus
------MSKPPSKQAVLSLYRSLVRYGHRLELSDKNYYFKRLREEFEARKTLTEPKELQFYFNKGKAFLERKRLI
>Papilio_machaon
--------MSITRSEILNLYKKLMQYSQSLNLTDVQYFRTRVSNEFRRNKTLTKPEDISFAYKKGEALLRKGSVL
>Mantis_religiosa
-------MSIPSPRQVLQLYKSILRYGQELKFTDKEYFYYRINQEFKNNKDIKRTEDIIFNFQRGQALLKNKRVV
>Panorpa_vulgaris
-------MSVPNSSHILRLYKDLLRYGQSLQLTDKLYFRSKIVNEFKKNKTIENHKDISFNYEKGKSLLKKGRVV
>Dermanyssus_gallinae
---MGPVSGAPSVQAMRSLYKQLARYGQQLVLTDRRWFERQLRNEFAKPRVT--AEEQNFAYRKGQRFLELKRLV
>Osmylus_fulvicephalus
-------MLIPNNGQVRNLYRHILRYGQTLRYTDKIYFYNRVRNEFKQNKSLTDPADIVFNFKKGEELLRKKTVC
>Triops_newberryi
-------MSGPSPYQVLKLYRSLLRYSSTLQYTDKSFYIKRIRSEFKKKKDLTNEEEIRKAFEKGLSFLSKKRIV
>Calopteryx_splendens
-------MCQPSPSQVLSLYRSLLRYGNQLKYTDKKYFIKRIRKEFESNRNLIP-KERGFQYERGLVLLHNKRVI
>Hypochthonius_rufulus
--------MSVSREHILRLYKNILRYSNSLVYTDKSYYLQRVKREFRENKQLTDDSDIRFYYEKGITFLNKKRLI
>Prosarthria_teretrirostris
-------MNAPTAHEVLRLYRKLLQYGKQLKLTDKKYFFDRIRKEFKTNKTLTNQEDVTFQFKRGLALLENKRVV
>Ramulus_artemis
-------MNTPARVQVLRLYRDLLRYGQNLQLTDKDYFCKRVREEFRNNRVLENKNDLLFHFNKGESLLKTKRVV
>Menopon_gallinae
-------MCVPTTREVLTLYKRLLRYGRQLKYTDQDYYSKRIKQEFQSNKDVKGN-EAIFHYEKGLQLLKLKRVV
>Perla_marginata
-------MSGPTAKQVIQLYRDLLRYSKHLKFTDKNFFTKRVRKEFKTNKRLSNEEDIQFHYQKGLILLKNKSVI
>Polydesmus_angustus
-----MSASVVGKSQILHLYRQLLRYGRTLTYTDKNYYYGRIRAEFEKHREAEL-KTQLFQYKKGKALLENGRLI
>Acerentomon_sp._AD-2013
--MCWQQMGIPTNGQILKLYRSLLRYGRTLQYTDKDFFKRKVQQEFHRNGAITAPNEIKFQYEKGISFLSRKRLV
>Liposcelis_bostrychophila
-------MSAPSRTQVLRLYKDILRYGEELTYTNKTYFKNRVKNEFLSAKSAKDNKEIDNLYQKGVALLMRRRVV
>Xanthostigma_xanthostigma
-------MEVPSSTQVLRLYRQLIRYGQKLQYTDKNYYYKRVIAEFKRNRNLEDPKEIKFQFEKGQSLLKRKSVI
>Trichomycterus_areolatus
---------MPSRSEILKLYKQLLRYGQQLRLTDKSYYQERINQEFKQHKNLASNKDITFNFEKGKKLLEKGLVM
>Ctenocephalides_felis
-------MNTPSAAQVLRLYKNLLRYGQNLQYTDKDYFCNRIRKEFRNNKTCSRTEDLIFNFEVCI---------
>Stylops_melittae
-----------MRLKVLNLYKSLMRYGKSLRLTDNEYFLERIRSEFRRNKDLYSNQEIDYHFKKGETLLKKKLLV
>Symphylella_vulgaris
--------MNPTRVQILSLYKTLLRYADNLKYSDTNYLKQRVRVEFSQHRSLTAKKDIEFYFKRGKSFLERKRLV
>Frankliniella_cephalica
------MSTLINSNQVLQLYRSLIRYGQSLKLTDQKYYLKRVRKEFRANKDLQDPEKIEFMYKKGVSLLDRKRLI
>Philopotamus_ludificatus
---------MPSKREILKLYKQLLRYGQQLQLTDKTYFQQRIVNEFKKNKDLSKSDDITFNFKKGQQLLNKGLVL
>Zorotypus_caudelli
--------MNPGKPGILILYKSLIRYGKNLKYTDKKYFIERIRQEFKNRKELENEEDIKFAFQRGVSVLNNKRII
>Atelura_formicaria
-------MVAPSSKQVLQLYRNLMRYGRQLKYTGKDYYSKRVREEFKRNKSLENDEKKIFHFEKGQALLSQKRIV

HG0010.1 ENSG00000125863 MKKS

>Homo_sapiens
---MSLRNLWRDYKVLVVMVPLVGLIHLGWYRIKSSPVFQIPK---N-DDIPEQDSLGLSNLQ-KSQIQG-----K--------
>Amia_calva
---MGLSSVWKNYKVLIVMGTSLGLIHWGWYSIKANPIFHKSE---T-NYFPEPGIVSFVSEP-QHQPKG-----K--------
>Micruropus_glaber
--MVFRWGRVIESLKTNVVLVVMVPAIVVIHLGWRKLQDNPNLVDPNMKRE-----IPPVEGFSYLKNKIVEELFTTTDK--------
>Anas_platyrhynchos
---MGLKTFWKSYKVLIVMGTGLGLVHWGWFHIKSSPIFQVKT---E-DFVPEPAIVAYVMQK-DNKNKE-----K--------
>Xenopus_laevis
---MGLGNLWKNYKVLIVMGTGLGLVHWGWYNIKSNPAFQPAK---EDSLEPEPAISAEKNTM----KKG-----K--------
>Apteryx_australis_mantelli
---MGLKTFWKNYKVLIVMGPSLGLVHWGWFHIKSSPIFQVKT---EENFVPEPGIVAYMMRN-EHKNKE-----KQQ------
>Mastotermes_darwiniensis
---MSGKHGGFFNRNAVTVVMIPALVLMHWGWYKLQDVEVLVKKEEKKE-----LPVVVGARYLVHILEEKLQSFRGGSQSSGEK-
>Acinonyx_jubatus
---MSFQNFWRDYKVLVVMVPLVGLIHLGWHRIKSSPVFQMPN---K-DDISEPDSPVLGRPQ-KSHIQG-----K--------
>Hynobius_retardatus
---MGLKNLWKNYKVIIVMGTGLSLMHWGWYHVKTNPLFHPKR---D-EFVPEPSIVTYVLQG-NQQTKA-----K--------
>Physeter_catodon
---MSFQKFWRDYKVLVVMVPLVGLIHLGWHRIKSSPVFQIPN---K-DDLPEPDSLALTSPE-KSHIPG-----K--------
>Calidris_pugnax
---MGLKTIWKDYKVLIVMGTSLGLVHWGWFYVKSSPIFQVKT---E-DIVPEPGIVAYVMQG-DHKSKE-----K--------
>Miniopterus_natalensis
---MSFQSFWRDYKVLVVMVPLIGLIHLGWHRIKSSPVFQVPS---K-DNVPEPDSLALASLQ-KDHIQK-----K--------
>Echinarachnius_parma
---MQPNPPKKNFFMRNRVLFVGLISLTFIHVGWFHIANDPVFNRDAVSGQASR--KLESVPAYIKS-KTGSASAE---KSS------
>Latimeria_chalumnae
---MGLKSIWSNYKVLIVVGTGLGLIHWGWYSIKSNPVFHKNR---E-DYAPEPGIATSIMQQ-DKQPKG-----KQQ------
>Columba_livia
---MGLKTIWKDYKVLIVMGTGLGLVHWGWFYMKSSPIFQVKT---E-KFVPEPGIVAYVMQS-DQKNKE-----K--------
>Alligator_sinensis
---MGLKTIWKNYKVLIVMGPSLVLIHWGWYNIQSNPIFKMKR---E-DLDQEPGIVTHVMQQ-DSKNQG-----K--------
>Callinectes_sapidus
--MGTGMGRAREFLRRNIVLVVMFPILVGVHFGWQKIQDVEMFVPKQQRID-----LPVIEGARYLREGVEKKLGLKKEE--------
>Sclerodactyla_briareus
---MTKPSFIRRYRVLIVAFAGLSLIHLGWYRLSKNPDFTGIAVGAE------PEIKPSYLKQ-KS-GKG-----TSA------
>Monodelphis_domestica
---MSLKTFWRDYKVLLVMIPSIGLIHIGWKSIKSNPIFQTAN--ANEDSIPEPGIVAHMIPQ-KNQNQV-----K--------
>Strongylocentrotus_purpuratu
---MNPPKKNFFVRNKVLFVGLISLSLIHVGWFHIANDPVFNKGEK----KT--VSDLLPSYIKS-KALKAPAE---KSN------
>Meganyctiphanes_norvegica
--MMWGRVSEFAKRNLVMIIMVPTLFGLHLGWLKIQDTEAFVPKHQKME-----LPAVTAAARLQEEIKRKVKGDNNDQ-------
>Aquila_chrysaetos_canadensis
---MGLKTIWKDYKVLIVMGTSLGLVHWGWFYIKSSPIFQVKT---E-DFVPEPGIVAYVMQS-DHKNKE-----K--------
>Marthasterias_glacialis
--MSFFARYRVLTVAAVGLTMVHFGWYYIASDPVFNPDK------K--VLGITPEYFKS-KQAQQKKA---AVEGVTKSQ
>Numida_meleagris
--MMSLKTLWKDYKVLIVMGTSLGLVHWSWFHIKSSPLFQVKT---E-EIVPEPGIVAYVMQR-DRKNKE-----K--------
>Meccus_pallidipennis
---MNNNKDKFFKKHLVMIVFIPLIGLIHYSWHLLQQNEILVKNRKHNE-----LPIITIFKRLTQGRESD---------------
>Oryctolagus_cuniculus
---MNLQKFWRDYKVLVVMVPLVGLIHLGWHRIKSSPIFQVPN---K-DNIPEPDSLALASLP-KGPPQG-----K--------
>Ornithorhynchus_anatinus
---MSLKTIWRDYKVLIVMVPSIGLIHLGWHSIKNSPIFQIPN---K-DTIPEPAIVTHVMQQ-EASKKG-----K--------
>Octopus_bimaculoides
---MLKQLKAFAKNYTVLLVMIPSVVALHVGWYYMQFNPDLVEPEKQEI-------RILGKIFKK-T--PKE--------------
>Ceuthophilus_sp._AD-2013
---MLDKGKMQGFINRNLVSIVMVPGLILLHWGWTRIQDIEVFVKKEEKKN-----LPVFLGVHYIQE----KFLKWKEGDGVHPDE-
>Scleropages_formosus
---MGLGNVWRQYRVLIVMSASLGLIHWGWFQLKGHPALHGKQQREDDEYIPEPAIVSYVASG-RQSG-G-----K--------
>Lepidothrix_coronata
---MGLKTIWKDYKVLIVMGTGLGLVHWGWFYIKSSPIFQVKT---E-DYVPEPGIVTYVMQS-DQKNKE-----K--------
>Ceratotherium_simum_simum
---MSFQNFWRDYKVLVVMVPLVGLIHLGWHRIKSSPVFQIPN---K-DKVPEPDNLALASSQ-KSHIQE-----K--------
>Manis_javanica
---MSFQTFWRDYKVLVVMVPLVGLIHLGWHRIKSSPVFQIPN---K-DDGSEPDSLVLASPQ-KKHIQG-----K--------
>Glycera_dibranchiata
--MMSVAAFLRKNIVLVVCIPSLALIHYGWYNLQFKDNFIPQDQRHI-----RIA--GIDIDP---EKR-RTTTSQE-------
>Leuctra_sp._AD-2013
--MTGASSKFAGFINRNLVAIVMVPVLIGIHWGWNRIQNVEVFVTKEEKKD-----LPIILGARYLEQKIEEKVATWKDGDKAVTKE-
>Chinchilla_lanigera
---MSFQNFWRDYKVLLVMVPLIGLIHVGWHRIKSSPVFRVPT---K-DDIPEPHSLGLASPP-KSQIQG-----K--------
>Tupaia_chinensis
MRRVARGAKVPGNRGGSGRRFGLWGLLSVAAQGGAAAGGQVHQCCITMSFQNFWRDYKVLVVMVPLVGLIHLGWHRIKSSPVFQIPS---K-DDISEPDSLALASSQ-KSQIQG-----K--------
>Aptenodytes_forsteri
---MGLKTIWKDYKVLIVMGTSLGLVHWGWFYIKSSPIFQVKT---E-DFVPEPGIVAYVMRS-DHKNKE-----K--------
>Henricia_sp._AR-2014
--MSFFSRYRVLTIGVVGLAAIHAGWFYIAHNPTFNQEK------E--VVNITPEYYKS-KRSKLGAT---TASSDAKNI
>Takydromus_sexlineatus
---MGIKTIWKNYKVLIVMVPSLGLIHWGWYNIKSNPMFQPKR---E-DTVPVPGIVTYALQQ-DNKSKG-----K--------
>Pelodiscus_sinensis
-------------------------MILQYSHGCVC----RTGVDASMSLKTIWKNYKVLIVMGTSLGLIHWGWYKIKSNSIFQTKR---E-DYVPEPGIVTYVMQH-ESQNKG-----K--------
>Zorotypus_caudelli
---MGQKGRLWEFVNRNIVTLVMVPGLVLIHWGWLRLQENDIFVPPQDRKE-----FVIFRSFRDMKTNLEERYRSIVDGNSKD----

HG0010.1 ENSGALG00000009013 MKKS

>Gallus_gallus
--MMSLKTLWKDYKVLIVMGISLGLVHWSWFHIKSSPLFQVK---TE-EIVPEPGIVTYVMQSDHKNKEK----------
>Amia_calva
---MGLSSVWKNYKVLIVMGTSLGLIHWGWYSIKANPIFHKS---ET-NYFPEPGIVSFVSEPQHQPKGK----------
>Anas_platyrhynchos
---MGLKTFWKSYKVLIVMGTGLGLVHWGWFHIKSSPIFQVK---TE-DFVPEPAIVAYVMQKDNKNKEK----------
>Xenopus_tropicalis
---MGLGNLWKNYKVLIVMGTGLGLVHWGWYNIKSNPIFQPE---REDTLEPEPAITGYAVEKNTAKKAK----------
>Apteryx_australis_mantelli
---------------------------------------MAGRCQFTMGLKTFWKNYKVLIVMGPSLGLVHWGWFHIKSSPIFQVK---TEENFVPEPGIVAYMMRNEHKNKEKQQ--------
>Canis_lupus_familiaris
---MSFQNFWKDYKVLIVMVPLVGLIHMGWHRIKSSPVFQMPN----KDNVSEPENPELGCPPKNHIQGK----------
>Hynobius_retardatus
---MGLKNLWKNYKVIIVMGTGLSLMHWGWYHVKTNPLFHPK---RD-EFVPEPSIVTYVLQGNQQTKAK----------
>Orcinus_orca
---MSFQKFWRDYKVLIVMVPLVGLIHLGWHRIKSSPVFQIPN----KDDLPEPGRVAVTSPEKNHLPGK----------
>Calidris_pugnax
---MGLKTIWKDYKVLIVMGTSLGLVHWGWFYVKSSPIFQVK---TE-DIVPEPGIVAYVMQGDHKSKEK----------
>Miniopterus_natalensis
---MSFQSFWRDYKVLVVMVPLIGLIHLGWHRIKSSPVFQVPS----KDNVPEPDSLALASLQKDHIQKK----------
>Echinarachnius_parma
---MQPNPPKKNFFMRNRVLFVGLISLTFIHVGWFHIANDPVFNRDAVSGQ--ASRKLESVPAYIKSKTGSASAEKSS------
>Latimeria_chalumnae
---MGLKSIWSNYKVLIVVGTGLGLIHWGWYSIKSNPVFHKN---RE-DYAPEPGIATSIMQQDKQPKGKQQ--------
>Columba_livia
---MGLKTIWKDYKVLIVMGTGLGLVHWGWFYMKSSPIFQVK---TE-KFVPEPGIVAYVMQSDQKNKEK----------
>Alligator_mississippiensis
---MGLKTIWKNYKVLIVMGPSLVLIHWGWYNIQSNPIFKMK---RE-DLDQEPGIVTHVMQQDSKNQGK----------
>Sclerodactyla_briareus
---MTKPSFIRRYRVLIVAFAGLSLIHLGWYRLSKNPDFTGIAV------GAEPEIKPSYLKQKSGKGTSA---------
>Monodelphis_domestica
---MSLKTFWRDYKVLLVMIPSIGLIHIGWKSIKSNPIFQTANA--NEDSIPEPGIVAHMIPQKNQNQVK----------
>Strongylocentrotus_purpuratu
---MNPPKKNFFVRNKVLFVGLISLSLIHVGWFHIANDPVFNKGEK------KTVSDLLPSYIKSKALKAPAEKSN------
>Aquila_chrysaetos_canadensis
---MGLKTIWKDYKVLIVMGTSLGLVHWGWFYIKSSPIFQVK---TE-DFVPEPGIVAYVMQSDHKNKEK----------
>Marthasterias_glacialis
--MSFFARYRVLTVAAVGLTMVHFGWYYIASDPVFNPDK--------KVLGITPEYFKSKQAQQKKAAVEGVTKSQ
>Oryctolagus_cuniculus
---MNLQKFWRDYKVLVVMVPLVGLIHLGWHRIKSSPIFQVPN----KDNIPEPDSLALASLPKGPPQGK----------
>Ornithorhynchus_anatinus
---MSLKTIWRDYKVLIVMVPSIGLIHLGWHSIKNSPIFQIPN----KDTIPEPAIVTHVMQQEASKKGK----------
>Octopus_bimaculoides
---MLKQLKAFAKNYTVLLVMIPSVVALHVGWYYMQFNPDLVEPEK-------QEIRILGKIF-KKTPKE------------
>Laupala_kohalensis
--MISGKIRKFVNRNLVMIVMIPSLIFVHWGWLQIQQKDIFVKEEE---RKGLPIIGMAKIVMKKIMP-AEKEDSTPTQPE-
>Scleropages_formosus
---MGLGNVWRQYRVLIVMSASLGLIHWGWFQLKGHPALHGKQQREDDEYIPEPAIVSYVASGRQSGGK-----------
>Manacus_vitellinus
---MGLKTIWKDYKVLIVMGTGLGLVHWGWFYIKSSPLFQVK---TE-DYVPEPGIVTYVMQSDQKNKEK----------
>Ceratotherium_simum_simum
---MSFQNFWRDYKVLVVMVPLVGLIHLGWHRIKSSPVFQIPN----KDKVPEPDNLALASSQKSHIQEK----------
>Manis_javanica
---MSFQTFWRDYKVLVVMVPLVGLIHLGWHRIKSSPVFQIPN----KDDGSEPDSLVLASPQKKHIQGK----------
>Glycera_dibranchiata
--MMSVAAFLRKNIVLVVCIPSLALIHYGWYNLQFKDNFIPQDQ-------RHIRIAGIDIDPEKRRTTTSQE-------
>Nomascus_leucogenys
---MSLRNLWKDYKVLVVMVPLVGLIHLGWYRIKSSPVFQIPR----NDDIPEQDSLGLSNLQKSQIQGK----------
>Fukomys_darlingi
---MSLQKFWRDYKVLVVMVPLIGLIHLGWHRIKSSPIFQIPT----KDNIPEPDSPALASPPKRQIQGK----------
>Tupaia_chinensis
MRRVARGAKVPGNRGGSGRRFGLWGLLSVAAQGGAAAGGQVHQCCITMSFQNFWRDYKVLVVMVPLVGLIHLGWHRIKSSPVFQIPS----KDDISEPDSLALASSQKSQIQGK----------
>Sepia_pharaonis
---MASRLTAFLKEYKVLLIMLPAVAGIHYVWYKMQKSPVFMEGEK-------TKI--FGKDLVSDSPK-------------
>Aptenodytes_forsteri
---MGLKTIWKDYKVLIVMGTSLGLVHWGWFYIKSSPIFQVK---TE-DFVPEPGIVAYVMRSDHKNKEK----------
>Henricia_sp._AR-2014
--MSFFSRYRVLTIGVVGLAAIHAGWFYIAHNPTFNQEK--------EVVNITPEYYKSKRSKLGATTASSDAKNI
>Takydromus_sexlineatus
---MGIKTIWKNYKVLIVMVPSLGLIHWGWYNIKSNPMFQPK---RE-DTVPVPGIVTYALQQDNKSKGK----------
>Pelodiscus_sinensis
---MSLKTIWKNYKVLIVMGTSLGLIHWGWYKIKSNSIFQTK---RE-DYVPEPGIVTYVMQHESQNKGK----------

HG0010.2 ENSG00000125863 MKKS

>Homo_sapiens
--MKNTSWIRKNWLLVAGISFIGVHLGTYFLQRSAKQSVKFQSQSKQKSIEE----
>Amia_calva
--MRNRSWLRKNWLLIAGLSFLGIHLGTYVMQKTMKNSVRSDLQ--LKRKDASE--
>Anser_cygnoides
--MRKASWSRKNLLLVAGLSFIGVHFGTMLVNFVAKKSVRSHSETKKDDRSE----
>Xenopus_tropicalis
---MIKKQTWLRKNWLWVAGISFLGIHFGTYFIQKLAKQSAKSNLQLKESKNSE----
>Apteryx_australis_mantelli
---MKTSWSRKNFLLVAGLSLIGVHVGSMLVNLVAKKSVQSHSEIKKDRRE-----
>Ursus_maritimus
---MLKEEMKNTSWIRKNWLLVAGVSFIGVHLGTYFIQRAAKQSVKSQSQSRGKQQNIEE--
>Ambystoma_mexicanum
--MKSKSWLRKNWLLVAGISFVGLHIGTYIIQKVAKTSARSGSELKQQKDYDE---
>Physeter_catodon
--MKNSNWIRKNWLLVAGISFTGIHLGTYFIQRAAKQSVKSQPEGKQKSLNNELK-
>Calidris_pugnax
--MRKTSWSRKNFLLLAGLSVIAVHFGSMLVNFVAKKSARSHSEAKKEDRRE----
>Miniopterus_natalensis
--MKNTSWIRKNWLLVAGVSFIGVHLGTYFIQRAAKQSVKSQSGDKQK--SIRE--
>Dasypus_novemcinctus
---MKISWIKKNWLLVAGISFLGVHLGTYIIQRTAKQSVKSQSGVKPKHIEE----
>Latimeria_chalumnae
--MKNHSWMRKNWLLVAGLSFLGIHLGTYIMQKSVKNSTKSTENTVLKAKDI----
>Columba_livia
--MRKTTWNRKNLLLVAGLSVIGVHFGSMLVNFVAKKSARSHSEAKKEDHRE----
>Alligator_mississippiensis
--MKKTSWLRKNWLLVAGLSFLGVHIGTYLIQRAAKNSVKSTVEIKHKNLDE----
>Sarcophilus_harrisii
--MKQTSWIRRNWLLVAGLTFIGVHFSTYFLQIAAKHSAKSQIEYRQKNIKEIK--
>Galeopterus_variegatus
--MKNTSWIRKNWLLVAGVSFIGVHLGTYFIQRTAKQSVKSQSGNK----------
>Monodelphis_domestica
--MKQTSWIRKNWLLVAGLTFIGVHLGTYFIQVAAKHSAKSQSGSKEKNIKEMK--
>Aquila_chrysaetos_canadensis
--MKKASWSRKNFLLVAGLSLIGVHFGSMLVNFVAKKSVRSHSEAKKGDRHE----
>Asterias_rubens
--MKLPPWVARNKLLSVGIALLSIHLTIYIVQKTALATKSTKRLR-GMDMEQTERK
>Meleagris_gallopavo
--MRKASWSKKNFLLVAGLSLIGVHLGTMLVNFVAKKSARSHSETKRDDHRE----
>Condylura_cristata
--MKNTSWIRKNWLLVAGVSFIGVHLGTYFLQWSAKQSVKSQSIGKQKNIEE----
>Oryctolagus_cuniculus
--MRNTSWIRKNWLLVAGVSFVGVHLGTYFIQRAAKQSVKSQSGSKQKSTEE----
>Ornithorhynchus_anatinus
--MRKSSWIRRNWLLVAGLSFIGVHLGTYIIQITAKNSAKSVLETKKKSIQE----
>Scleropages_formosus
--MTMKNQSWLRKNWLWVAAASFLGIHVGTWLMQKAMKSSAHSQTRTKQKS--VDE--
>Ficedula_albicollis
--MKKTTWNRKNFLLVAGLSLIGVHLGSMVVNYVAKKSAQSQSEAKRSS-------
>Ceratotherium_simum_simum
--MKNISWIRKNWLLVAGVSFIGVHLGTYFIQRTAKQSVKYQSGGNNVLLNNEVK-
>Manis_javanica
--MKNTSWIRKNWLLIAGVSFIGVHLGTYFMQRAAKQSVKSQSRNKQENIEE----
>Melopsittacus_undulatus
--MRKANWNRKNFLLVVGLSVIGVHFGSMLVNFVAKKSAHSHSEAKKDRHE-----
>Marmota_marmota_marmota
--MKSTSWIRKNWLLVAGVSFIGVHLGTYFIQRAAKQSIKSQSVGKQKSIEE----
>Tupaia_chinensis
MRRVARGAKVPGNRGGSGRRFGLWGLLSVAAQGGAAAGGQKEATLNLSLILKEDMRNTSWIRKNWLLVAGVSFIGVHLGTYIIQRTAKHSVKSQSGDKQKNIEE----
>Lepisosteus_oculatus
--MSNRSWLQKNWLLVAGLSFLGIHLGTYLLQKTVKSSTRSDLA--LKQKDVK---
>Pygoscelis_adeliae
--MRKASWSRKNFLLVAGLSLIGFHFGSMLVNFVAKKSVRSHSEAKKEDHHE----
>Echinaster_spinulosus
--MKLPVWVANNKLLSIGIALATLHLTIYTIQKMAHSTSKSKRSLTGPPQHSRE--
>Takydromus_sexlineatus
--MKKISWIRKNWLLVAGLSFVGVHLGTYFIQRAAKSSAKSGLNSKSKGLDE----
>Struthio_camelus_australis
--MLQKEEMKTSWSRKNFLLVAGLSLIGVHIGSTLVNLVAKKSVHSHSEIKKDRRE-----
>Pelodiscus_sinensis
--MRKTSWIRKNWLLVAGFSFLGVHLGTYFIQRAAKNSAKSALEVKQKNLEE----
>Patiria_miniata
--MKLPVWVAKHKLLSVGIALLTIHLTIYTVQKTALSTSKTRRSLTGPSPPADKED

HG0010.2 ENSGALG00000009013 MKKS

```
>Gallus_gallus
-----MRKASWSKKNFLLVAGLSLIGVHFGTMLVNFVAKKSARSHSETK--RDNHRE---
>Amia_calva
-----MRNRSWLRKNWLLIAGLSFLGIHLGTYVMQKTMKNSVRSDLQ--LKRKDASE---
>Anser_cygnoides
-----MRKASWSRKNLLLVAGLSFIGVHFGTMLVNFVAKKSVRSHSETK--KDDRSE---
>Xenopus_tropicalis
----MIKKQTWLRKNWLWVAGISFLGIHFGTYFIQKLAKQSAKSNLQLK--ESKNSE---
>Apteryx_australis_mantelli
------MKTSWSRKNFLLVAGLSLIGVHVGSMLVNLVAKKSVQSHSEIK--KDRRE----
>Mustela_putorius_furo
-----MKNISWIRKNWLLVAGVSFIGVHLGTYFIQRAAKQSVKSQSTGK--QESIKE---
>Ambystoma_mexicanum
-----MKSKSWLRKNWLLVAGISFVGLHIGTYIIQKVAKTSARSGSELK--QQKDYDE--
>Balaenoptera_acutorostrata_s
-----MKNSNWIRKNWLLVAGISFTGIHLGTYLIQRAAKQSVKSQSEGK--QKSLNNELK
>Calidris_pugnax
-----MRKTSWSRKNFLLLAGLSVIAVHFGSMLVNFVAKKSARSHSEAK--KEDRRE---
>Eptesicus_fuscus
-----MKTTSWIRKNWLLVAGVSFIGVHLGTYVIQWAAKQSVKSQSGNK--QKSIKE---
>Dasypus_novemcinctus
------MKISWIKKNWLLVAGISFLGVHLGTYIIQRTAKQSVKSQSGVK--PKHIEE---
>Latimeria_chalumnae
-----MKNHSWMRKNWLLVAGLSFLGIHLGTYIMQKSVKNSTKSTENTVLKAKDI-----
>Columba_livia
-----MRKTTWNRKNLLLVAGLSVIGVHFGSMLVNFVAKKSARSHSEAK--KEDHRE---
>Alligator_mississippiensis
-----MKKTSWLRKNWLLVAGLSFLGVHIGTYLIQRAAKNSVKSTVEIK--HKNLDE---
>Sarcophilus_harrisii
-----MKQTSWIRRNWLLVAGLTFIGVHFSTYFLQIAAKHSAKSQIEYR--QKNIKEIK-
>Galeopterus_variegatus
-----MKNTSWIRKNWLLVAGVSFIGVHLGTYFIQRTAKQSVKSQSGNK-----------
>Monodelphis_domestica
-----MKQTSWIRKNWLLVAGLTFIGVHLGTYFIQVAAKHSAKSQSGSK--EKNIKEMK-
>Aquila_chrysaetos_canadensis
-----MKKASWSRKNFLLVAGLSLIGVHFGSMLVNFVAKKSVRSHSEAK--KGDRHE---
>Condylura_cristata
-----MKNTSWIRKNWLLVAGVSFIGVHLGTYFLQWSAKQSVKSQSIGK--QKNIEE---
>Oryctolagus_cuniculus
-----MRNTSWIRKNWLLVAGVSFVGVHLGTYFIQRAAKQSVKSQSGSK--QKSTEE---
>Ornithorhynchus_anatinus
-----MRKSSWIRRNWLLVAGLSFIGVHLGTYIIQITAKNSAKSVLETK--KKSIQE---
>Scleropages_formosus
---MTMKNQSWLRKNWLWVAAASFLGIHVGTWLMQKAMKSSAHSQTRTKQKS--VDE---
>Corvus_brachyrhynchos
-----MKKTTWSRKNFLLVAGLSLIGVHLGSMLVNFVAKKSAQSHSEAK--RDRHE----
>Equus_asinus
-----MKNISWIRKNWLLVAGVSFLGVHLGTYFIQRAAKQSVKSQSGGN--KVLLNDEVK
>Manis_javanica
-----MKNTSWIRKNWLLIAGVSFIGVHLGTYFMQRAAKQSVKSQSRNK--QENIEE---
>Microcebus_murinus
-----MKNTSWVRKNWLLVAGLSLIGVHLGTYFIQRAAKQSVKSQSGAK--QKSIEE---
>Melopsittacus_undulatus
-----MRKANWNRKNFLLVVGLSVIGVHFGSMLVNFVAKKSAHSHSEAK--KDRHE----
>Dipodomys_ordii
-----MKNPSWIRKNWLLVAGVSFIGVHLGTYFMQRIAKQSVRSQSADK--QRNVEE---
>Tupaia_chinensis
-----MRNTSWIRKNWLLVAGVSFIGVHLGTYIIQRTAKHSVKSQSGDK--QKNIEE---
>Lepisosteus_oculatus
-----MSNRSWLQKNWLLVAGLSFLGIHLGTYLLQKTVKSSTRSDLA--LKQKDVK----
>Pygoscelis_adeliae
-----MRKASWSRKNFLLVAGLSLIGFHFGSMLVNFVAKKSVRSHSEAK--KEDHHE---
>Anolis_carolinensis
-----MKKISWIRKNWLLVAGLTFVGVHLGTYLVQRAAKSSAKSGLDIK--SKGLDE---
>Struthio_camelus_australis
MLQKEEMKTSWSRKNFLLVAGLSLIGVHIGSTLVNLVAKKSVHSHSEIK--KDRRE----
>Trachemys_scripta
-----MRKTSWIKKNWLLVAGLSFLGVHLGTYFIQRAAKNSAKSALEIK--QKDLGE---
```

HG0010.3 ENSG00000125863 MKKS

>Homo_sapiens
------------MLISFINIELFDFIATMLHIHTLIPKE-----------------
>Canis_lupus_familiaris
------MSLSHYMLISFINLELFSFIATLLHVQDFHS-------------------
>Miniopterus_natalensis
------MSLNHYMLISFINMKLFNFITTMLRIQTVIPEEYFFKMLNRKLWLTK---
>Dasypus_novemcinctus
-----------------MNMKLFNFIPTMLHVQTFILRVIFLNVRTHDNVIYAKCV
>Galeopterus_variegatus
------MSLNHFVLINFINIELFNFIDTMLHA------------------------
>Condylura_cristata
------MSLNLYILITFINMELFNFIATYLDFCS----------------------
>Oryctolagus_cuniculus
MWNHQHVIKSLSVSISFIIIEPFHFLASVLHIQTFTPKE-----------------
>Equus_caballus
------MSLNHYMLISFVNIELFNFMATMLHIQTFIPEE-----------------
>Manis_javanica
-------------------MEPFNFIAIVPHIPIFIP-------------------
>Fukomys_damarensis
------MLLSHYVLISFRNTESYNFIATTLNIPEEYFKKYFKTHGHK---------
>Tupaia_chinensis
------MSLNHYILISFTNIELFNFIAMMLHVALSVLQNNFFKC------------

HG0011 FBgn0027360 Tim10

>Drosophila_melanogaster
--MRVRERMRKANQGDYDSSAASDRRFDY---------
>Euphrynichus_bacillifer
-------MVSKLVKINIAWAVIATIGISSFWLAKSSVDKNRYEAMKVRERMAKANVGEYEPSYRKFTS------------
>Stegodyphus_tentoriicola
------MALSKLTKINIIGATLAISGILSFFVVKKLVTIQRVEAMKARERMAKSNVGEYEVKRKFA--------------
>Machilis_hrabei
---MQLMITSKVAKIGISWLLITGAGVGGFVWTKRLVDKERYESMKIRERIRQSNFGEYKPSSRKFTG------------
>Prorhinotermes_simplex
------MPLGNVTKIAVGWAVVTAAGLYSFVLSKQSVDKHRYEAMKVRERIRRSNEGEYDSGPRKFTG------------
>Hynobius_chinensis
------MRLGKLGKIGVGWTILVVVGVSGFTYSKTSVDKKRYDNMRVRERMKKSNEGLYEATERFVGGETNKSQ------
>Dascillus_cervinus
------MLLNKTAKIGIGWGFVVSAGIYGFYLSKENITKKRYEHMKVRERMRQANFGEYEPSPRKFD-------------
>Scylla_paramamosain
------MLTSKWARIAVGWSAVIVIGVGGFVFTKQTIEQQRRESMKVRERMRNANTGDYESKRNFTG-------------
>Forficula_auricularia
-----MLILGTHAKVALGWSLIVATGIYSFVLAKKSIEKNRYENMKVRERMRNSNIGEYISTEKRKF-------------
>Aposthonia_japonica
---MSIMSLGHVQKIAIGWTLVISGSLYAIVWARDSINKSRYEVMKSRERMKRSNIGDYEVQDRKY--------------
>Isonychia_bicolor
------MPLTKVTKVGIGWAIITFAGISGFILSKKSIEKRRYEHMQVRERMRRSNYGEYAQSERKF--------------
>Meganyctiphanes_norvegica
------MFRTKTAKIVVGWAAAISCGVGGFILTKISVEDQRRDAMKVRERMRNANTGEYEAKRTFTG-------------
>Grylloblatta_bifratrilecta
MYNVSIMPLGNHAKIGIGWTIIIVSGLYSFVLSKRSIDKHRYDAMKVRERMRKSNAGDYGETARTFTG------------
>Meccus_pallidipennis
------MPMNVIHKICISWSLIVAAGIYSFVLSKRSIERNRYENMKVRERMRNANKGNYIASERKFTG------------
>Cephus_cinctus
------MRLNNHSKIAIGWLVVISTGLYGFVLSKQRVEVHRYESMKARERMRQAPQGDLSTRVQSST-------------
>Ixodes_persulcatus
------MRVGTVRRITIAWALVTVAGIGSFVYAKKLVDKQRYEAMKVRERMKNCNTGDYEPQRKFTF-------------
>Biston_suppressaria
------MPLGKFGKITLSWLAVTVTSLSLFVVAKDHINGQRLENMRVRERMRKSNQGEYEDVYRKL--------------
>Lithobius_forficatus
--MKIRQRMRKSNSGEYEQSPRHSD-------------
>Mantis_religiosa
--MKVRERMRKANYGEYETTRKFSG-------------
>Tanzaniophasma_sp._AD-2013
-----MMQPGSIKKVVIGWTVVTIVGLYGFVLSKKSVDKNRYEAMKVRERMRKANEGSYETERKFTG-------------
>Nannochorista_philpotti
-----MLQLTKATKIGICWSVITVSSLFGFVKSKEHIDKRRYENMKVRERMRKSNCGDYDKSTSRYI-------------
>Corydalinae_sp._KMRSPBM-2012
--MKVRERMRKSNFGEYKESNRKFNG------------
>Metaseiulus_occidentalis
-----MLWKSPIFKICVGWGIAVVGGLGAFVVARDKIIAQRYEAMKSRERMRNANAGSYEQGNRVFRKDGTTEKIQNFKF
>Xibalbanus_tulumensis
------MALGNVAKVAVGWTIVISAGVGAFVLAKISIDKNRFASMKARERIRKANEGDYVPVRKYTT-------------
>Conwentzia_psociformis
------MKLGKVAKIGIGWTIITTASLYAFYLSKQSIDNQRYEHMKARERMRNANIGDYKSSSRNF--------------
>Triops_newberryi
--------MNRVTKVNIGWAILISVGIGSFVVAKNLVDTQRLEHMRSRERMRNANKGEYEPSERKFVD------------
>Epiophlebia_superstes
------MVLNNAGKIAIGWIVVITVGISSFVLSKQSIEQKRLENMRVRERMRMANSGDYPESYRKFNG------------
>Prosarthria_teretrirostris
------MPLNNVAKIGISWAFIVAASLYTFVLSKRYVDNHRYESMKARERMRKANSGDYEPSPRKFTG------------
>Phyllium_philippinicum
--MKVRQRIRDANKGVYESSERKFD-------------
>Cosmioperla_kuna
---MSIMPLGKYAKIGIGWSFITAAGLYGFVLSKRSVDKRRYDSMKVRERMRNSNYGKYDTSARKFTG------------
>Ectopsocus_briggsi
----MPIKLGRDAKVAIGWAVVVVGGLYSFVLSKQSIDRKRYDNMKIRERMRNANVGEYEPSPRSFH-------------
>Raphidia_ariadne
------MVLGNSSKILIGWSVITFAGIYAFVLSKRSIEAKRYEHMKIRERMRKSNVGDYQITGRYSKSS-----------
>Tityus_serrulatus
------MPLNTLAKIGIGWVVLSIAGIGGFFVAKNSVNKHRYESMKVRERMRKANQGPYKPSPRFSR-------------
>Trichomycterus_areolatus
------MKLGNVGKVGVGWTIFVILGISGFAASKNSVDKKRYDSMKVRERMRKANEGEYQIPETRRFEA-----------
>Ceratophyllus_gallinae
-----MVAIGKFAKIGLSWAALTFASISAFVLSKQHIDAQRYENMKIRERIRNSNTGEYEKSYRHFD-------------
>Hydroptila_sp._AD-2013
------MKLSNFAKITISWAFLGVAGIGSFILSKNSVVHRRREDMVIRERMRNSNVGEYEASSRKF--------------
>Tricholepidion_gertschi
------MPVSKVAKICIGWAIASTAGVSGFIFCKHIIEKQRHESMKVRERMRDSNKGEYEPSYRKFTE------------

HG0012 FBgn0064116 CG33713

```
>Drosophila_melanogaster
MATAAAVAKVGKSVHRIFVGNLPWTVGHQELRGYFREFGRVVSA--NVIFDKRTGCSKGYGFVSFNSLTALEKIENEQKHILEGNYLNIQKS-------
>Boeckaxelia_carpenterii
--------MSMRSFSKIFTGNLPWSVGNRELGQYFSQFGRVLSA--QVHFDKKLGKSKSYGFVTFKDRQSAESALSHQNHILEGNILRVDRTENF----
>Machilis_hrabei
--------MAARNLQKIFVGNLPWTVGQRELRNYFSEFGHVVTA--NVVFDKNTGMSRGYGFVQFGTRDGYNSASNKQQHFLEGNYLNIQPASN-----
>Blaberus_atropos
--------MATKAIRKIFVGNLPWTVSHRELRNYFSEFGHVISA--NVIFDRNTGMSRGYGFVVFSTPGGFENASNKQSHILEGSKITIHPASN-----
>Gyrinus_marinus
------MAVTARSLYRLFVGNLPWTISNTELKLYFSKFGHVYNA--SVVFDKNTGLSRNYGFVTFSNQNGFDSATNVQTHKLEGNILKVQPAASS----
>Macrobrachium_rosenbergii
--------MAARSIHKLFIGNLPWTVGHRELRQHFSQFGYVITA--NVIFDRSKGMSKGFGFVQFGNRNAYEAALNKSPQFLEGNTLNVQPLHP-----
>Forficula_auricularia
------MANLIRKGPKIFVGNLPWTVGHFDLEKYFSKFGPVVHS--KVIFNKNTGLSKGYGFVEFDSKESIPKVLG-QKHILEGKLISTEININSN---
>Campodea_augens
---------MASSRLKLFVGNIPWTICHQELRQYFSEFGHVITS--NVMFDRNNGLSKGFGFITFSSQEAFNKAINKQTHFLEGNYLNIQPASTT----
>Aposthonia_japonica
--------MASRAIRKLFVSNLPWTVGHRELKTYFSEFGHVVSA--YVVFDRNTGLTRGYGFVVFGSPESYTNAVDYQNHFLEGNVLQVQNSAN-----
>Eurylophella_sp._AD-2013
--------MAARSIQKLFVGNLPWTVGNRELRQYFSEFGHVVSA--NVVFDKNTGMSRGYGFIMFGNQNGYSNATNKQHHVLEGNQITVQSSAN-----
>Meganyctiphanes_norvegica
--------MAARNLNKIFVNNIPWTIGHQELRQHFAQFGFVTSA--NVVFDRSTGMSRGYGFVQFANRQGVEAASAKSLQFLEGNMVNIRDANSK----
>Galloisiana_yuasai
--------MASRAIRKLFVGNLPWTISHRELKTYFSEFGHVVSA--NVIFDRNTGMSRGYGFIIFSTQGGYDSATNKQSHLLEGNQLTVQVASN-----
>Xenophysella_greensladeae
-----MASAAARSAQKLFVGNLPWTISSRELRQYFSEFGHVSSA--IVVFDRDTGLSRGYGFITFGTSGAYDSVARQQKHVLEGNDLTIQPAST-----
>Tenthredo_koehleri
-----MASKVGIIGNKLYVGNLPWTIANKQLKEYFSQFGHISRA--IVVFDKNSGMSKGFGFVTFSTGKSINTVQNKSVHMLEGHTLNIGSVDVSQQTE
>Metaphatus_ochraceus
------MASSGRALNRLFIGNLPWTVGHRQLREYFSQFGPVQSA--RVIFDKSTGLSKGYGFIEFATKSAAADATNKQVHTVEGHNILVQPQNN-----
>Mantis_religiosa
--------MAARALHKLFVGNLPWTIGHRELRTYFSEFGNVVSA--NVVFDKNTGMSRGYGFVIFGTKGGYESASKKQSHILEGHTLIVQPASN-----
>Tanzaniophasma_sp._AD-2013
----MAANATGRAIRKLFVSNLPWTVGHRELKTYFSEFGHVVSA--HVVFDRNTGMSRGYGFVIFSTQGGLDSATIKQNHLLEGNTLAVQVAA------
>Boreus_hyemalis
-----MASSVRNALHKLFVGNLPWTVGHRELRTYFSEFGPVASA--NVIYDRSSGVSRGYGFVVFSGRSGFENATNKQTHALEGHTLTIQPASN-----
>Corydalus_cornutus
--MATLPRQSINYVKRLFVGNLPWTVSHKELQHYFSKFGSVNTAGTRVVFDKKTGFSKKFGFVQFNNRNSIDDVI-KQTHTLEGNVLTVHETNQ-----
>Chrysopa_pallens
-MGIRKVATAAKALNRVFVGNLPWTISNRELKQYFSQFGLVSQV--NVIFDKKNGFSKGYGFVQFAKKESIQLLTETPNHNLEGNVLTIQPATF-----
>Triops_newberryi
--------MASRSAGRLFVSNLAWTVGSRELRQYFSQFGAVAQA--NVIFDKNTGMSRNYAFVVFATREGYNKALTHPTHTVEGQTVNIQPTGNTTE--
>Cordulegaster_boltonii
-------MAGRLVARKLFVGNLPWTVGHQELRQYFSEFGHVASA--NVVFNRETGISRGYGFVVFGNKSGIAAAMDRGNHQLEGNQITIQMASN-----
>Stenobothrus_lineatus
--------MAARAAYKLFVGNLPWTVGHRELRMYFSEFGPVASA--VVIFDKNTGLSRGYGFVLLGNKSGFSKVTNKQTHVLEGNQITVQAASH-----
>Ramulus_artemis
--------MASKVMKKLFVGNLPWTVGSRELKNYFSEFGHLVSA--NVVFDRNTGISRGYGFVIFSAQRGLESVLEKQSHILDGSTMSVQAATN-----
>Menopon_gallinae
-----MSLLNAPIRHKFLIKNLPWTVGDKQLTSYFVRFGGVVKS--CVEVDTKTGFSKGFGYVFFSRFL--SGDINSQPHILHNEVLEVIPVNL-----
>Leuctra_sp._AD-2013
-------MAARNAVNKLFVGNLPWTIGHEELFRYFSEFGSISSA--RVIFDNVTGMSKGYGFVMFNSKGALDNALGKQNHKLEGNKINIQRSTD-----
>Acerentomon_sp._AD-2013
---------MSSKITTVFVAYLSWSVSRLELGRRFSEFGKVLAA--DVVFDKETGFSKRYGFVRFADRHSAERALQTRNVFIDGKLAVVKPSGSGGSA-
>Liposcelis_bostrychophila
------MASRVVRSPKLVIFNLPWSVGPKELRQHFAQFGAVTYA--DVKFDRATGFSKGFGFIEFGAPEDTHKALEAPVHKLEGNLLNIRRSENE----
>Xanthostigma_xanthostigma
------MARFPRALNRLFVGNIPWTVGNKELRKYFSKFGLVNSV--NVMFDKQTGFSKNYGFVYYSMKDGFDNALNSKPHELEGSTLIVEPVSNPNGN-
>Trichomycterus_areolatus
-----MASTSVRALNRLFVGNLPWTVSHRELTQYFSSFGIVKSA--KVVFDRSSGLSRGYGFITFASRTGYDTAVNKPQHRLEGSVLTVNEASQ-----
>Ceratophyllus_gallinae
------MASTARSLNKLFVANLPWTIGHRELRKYFAEFGHVSTA--NVIFDKSTGISKGFGFVFFSAKGGLEKALNQQVHNLEGRVLNVKPSNN-----
>Gynaikothrips_ficorum
-----------MSSYLIKVSKLPWTVGAKELSAYCSSFGPVHSV--RVPVDYSTGFQYGFGWVYFANADSKSRFLE-KKHYLEGHQLIVDGSRSKIE--
>Rhyacophila_fasciata
-----MASQVGRSVSKLFVGNLPWTIGHKELGEYFSRFGRVQVA--KVVFDRSTGVSRGYGFVEFSSKTAYENAINQQMHSLEGKVLSVNPSSA-----
>Zorotypus_caudelli
--------MAARAVNKFFIGNLPWTVGPKELRNYFSEFGAVTSA--NVLFDKNTGFSKGFGFITFGSNENSEAIKNKQTFVLEGNNLVVEPAE------
>Atelura_formicaria
--------MAARAVNKLFVANLPWTVGHLELRQYFAQFGHVQSA--HVVFDKKTGMSRGYGFIQYATQDACNSATDKRNHVLEGNQLAVQISAAPK---
```

HG0013 FBgn0261381 mtTFB1

>Drosophila_melanogaster
------------------MSASEQGPKIKYGE--SAPKLDKAQLQFMKLIEEQNLDRVQKLKRIRRNNLLTAGALGVSVLAIYGYSIFSVQQEKFLDDFEEPKKVSS-----
>Artemia_franciscana
------------------MAGNDQMPKVDLAK--DRPKFSEAQLDHMRLVEKLNALRVTELQRLKRNNYITAGVIATCVLGIYGYSMFAVKQEKFLDELE------------
>Meinertellus_cundinamarcensi
------------------------MQKVDLEK--DKPKLSRAQLDYMKLLEKQNAERVMKLQRIRRNNIITGSLLGVGVLSIYFYSIFAVKQEKFLDDFDAPKKISASSS--
>Argulus_siamensis
------------------MANDNVMPKLNLQN---DPQLKQIQLEYIKKIERENLIRVNRMKKLKMKNVYAGCGLLGFVLGVYTYTLYSVKQETFLDDLEDPSHV-------
>Mastotermes_darwiniensis
------------------MGKNEQMPKVDLVK--DRANISRTTLDYMKIVERENLRRVQALQKLRQKNVRTGLMLGAGVLGIYLYTIYSVRQEKFLDDFDEPATTSN-----
>Hynobius_chinensis
--MYAVKQEKFLDDFEEPAKEELTQKQL
>Dastarcus_helophoroides
-----------------MMSEQPKAP----KL--DYSKLKEAELKFVKLVEKQNFERVQKLKAIRKRNLITAGAVGAAVLGIYGYTLFAVHQESFLDDFDEPAKTVQ-----
>Forficula_auricularia
-------------------MSSNQMPKVDFVK--DKELLKNTAVDYIKHIEKLNIDRAKKYIRIRRNNIITGCLLGLGITSIFAFTIIGVKQEKFLDDFEEPAKISS-----
>Campodea_augens
-----------------MAESAEKMPKLDLQK--DKPQLHQAQLYYMQMIENKNLERVQKLQKLRKRNLATAGILGIAVVSIFSYSMLAVKQEKFLDEVFDETEAKATLKQ-
>Haploembia_palaui
------------------MADSEQMPKVDFAK--DKAELKHTAIDYVKLINKVNEERALKFQRIRKSNIITGCVLGFAALGIYSYTIYAVKQERFLDDFEEPERINIAN---
>Eurylophella_sp._AD-2013
------------------------MPKVDFVK--DKANLNPASLDYIRLIERQNRERVLKLQKLRKVNLWTAGILGVSVFSIYFYSMFAVKQETFLDDFNEPERGSNPN---
>Grylloblatta_bifratrilecta
------------------MGDSDQMPKVDFVK--DKDKLKFTAVKYVEILEKKNLERVQKLLRTRRNNNITGWLLGGGVFGIYLYSMFAVQQEKFLDDFNEPEKTED-----
>Anoplocnemis_curvipes
------------------MGDADQMPKLNLAK----DKVDKNVLDYIRLIEEQNRSRVEKLRRIRRNNLLTAFGLGIGVLSIYGYSIFAVRQETFLDDFERPAIKE------
>Neodiprion_sertifer
-------------------MADEQMPKIHEGE--NFRKLTLVEQEYMRLIEERNLERVTRLKRMQRNNRYTGLALGALVLGIYGYSMYAVRQENFLDDFEEPVKTTE-----
>Acanthopteroctetes_unifascia
------------------MGGNDVMPKINLDK-EKPEKLKKFELEYMKVINQKNLERVQKLERIRRNNILTGSILGVGVLGIYFYSMYAVKQESFLDDFNEPEKIIRNN---
>Empusa_pennata
-------------------MDKDQMQKVDLVK--DKANISRVVVDYMKLMEKENLERVSRLKRLRKRNNLTGLLLGGAVFGVYLYTILSVKQEKFLDDFEPPELVEKN----
>Tanzaniophasma_sp._AD-2013
--MFAVQQENFLDDFEEPAKNVE-----
>Boreus_hyemalis
------------------MSGKDVMPKLNLSD-KSQQKLQPTELDFMKIIEKQNLARVQKLKRIRRNNLITAGILATGVFGIYGYSIFAVQQEKFLDDFKEPEKLTTNN---
>Corydalus_cornutus
------------------MDNNEQMPKVNLKS----SNIRQSDIDFMKVIEKQNIERVRKLQRMKRNNLITAGLLGSGVFGIYLYSMLAVKQETFLDDFNEPAKVVE-----
>Conwentzia_psociformis
-----------------------------MLD-EEKKKLTPTQLRFMQEIEQKNLDRVQKLQRIRKNNILVGCSIGLSVLAIYGYSIYSVQQEKFLDDFNPPSKVQS-----
>Triops_cancriformis
-------------------MASDQMPKVDLVK--DATKLRQPQLDYMKLIEKQNLERVTKLKRIRKNNLLTGAALFASVFGIYVYSIAAVKQEKFLDDLETPSSKPSH----
>Cordulegaster_boltonii
---------------MAGKENSNEMPKVDFEK--DRGQLGRAELGYIRQIEEQTRTRAERLKKMRRGNLITGFALTAGVLGIYIYSIVAVKQEKFLDDFEPPKLEPKQ----
>Schistocerca_gregaria
-------------------MENKQDPK-------NKEFISKSMAEYMKLIEKQNLERVQKLQKTRRNNLITAGILGAAVTGIYFYSMYSVKQETFLDDFEEPKKISQ-----
>Timema_cristinae
------------------MADKESMPKVDFEK--DKAKFKNTAVDYIKLIEKLNLERVQKLQRIRRNNIITGCVLGGSVLGIYAYSMYSVKQETFLDDFDEPATTSN-----
>Menopon_gallinae
------------------MTSSEDMPRADLSD----EKLKN-AQAYAKYLEHKNYMRSLYGKRILRRKRFMAGFCCAFVVGVYCFTLFRVQQEAFLDDFEEPPRVNRDEKN-
>Perla_marginata
------------------MGDNERMPKVDFTK--DKENIKLSVIDYMKLVEKQNLERVEKLRRIQRRNLITGCILGVGVLGIYGYSMYAVKQEKFLDDFDEPAKTEAKLS--
>Acerentomon_sp._AD-2013
--------------MALTEKPGDVMPEVDLKK--DAADINQVHLDYMKLLEAKNTKRAHRYVRMIKKNKLLGSGLIFGVASIYVYSMYAVKQEKFLDDFEHPDANIPPPQ--
>Liposcelis_bostrychophila
------------------MARENEMPEVDFKD-IDKNKKLRQAYIYAKQLEERNVFRALIGARNLKWSRIIGITCGLFAAGVYSLTINRVQQETFLEDWEEPEIPGRS----
>Inocellia_crassicornis
------------------MSGSDQMPKVSFGT----SKINKTELDFMKFIEKQNLDRVEKLKILRRKNVITGCVLGAGILSIYAYSILSVKQEKFLDDFEEPKKVYAETQ--
>Ceratophyllus_gallinae
------------------MADNKLVQKPSISD--EPRKLSKVELDFMRIIEKENLARVEKLKRVRRNNLITGTLLTGSVFGIYLYTILSVKQEKFLDDFNEPAKISD-----
>Caligus_rogercresseyi
MSVNAGLLRALRAPSRNFVRGAKTMKQVNVDDSETRAKLGSIQMDLIHRAEKMNSRRATQFSKYRRHDVVVGGVCFSMAIAIYLYTIFAIKQEKFLDDFDMPDPMLPVQSKE
>Stylops_melittae
----------------MQVSG----AKVNPKD----KEFTPEQLELFKQFDNDKLRRLNRIRLRNSNNKKIGLALGFTVAAFYAYSMVAVQQEHFLDDFDKPEIVQD-----
>Gynaikothrips_ficorum
------------------MDKNEAMVKVDLSK----AQVDPTTLEHMKIMEKYNLARVQQMKRIKRKSNFMSLGLVSLAVGTYLYSMYAVRQEGFLDDFEEPKLKTK-----
>Platycentropus_radiatus
------------------MDSTEQKGKI--VS-TSKPAFSKGQIDYMKVVENLNLERVQKLKRIKRNNFITGSVLGGIVLGIYAYSIFSVKQEHFLDDFIEPTTVHKET---
>Atelura_formicaria
------------------MGDSERMPKVNLGK----DHMKQTELDYMKLVEKQNLERVQKLKTLRRRNVITGSLLGLAVVGIYGYSIYAVKQERFLDDFDEPQKTYEQK---

HG0014 FBgn0260392 CG42518

>Drosophila_melanogaster
MMDLSPNNQIEDRKPILTADGLVQTSNSPFEPTISQETQTSNG--IGGQCHLTVDQLDIEILPIIYDIVRCVEKDPLEN-AVKLRESQDCNHKIFELQKRFESAREQIRQLPGIDFNKEEQQQRLELLRNQLKLKQQLIRKYKDTEF--
>Echinogammarus_veneris
--MSITGATVAAEDIDLDLLPLIYQYLRCLEKEQN--QTDLNRVALDATQKLTELNNKITLAREQVPKLAGVEYSPADQLQKLSALRAQLTLKKQLLAKYKSK-ASADG-------VTTRD----------------------------------
>Reticulitermes_flavipes
-----------------------------------MDSSEQNG--AALTCKLTVDDVDIEFLPLIYEIIRSVERDPH-DTSQKTRESQDTSQKVLELQKKLDQARNQIRRLPGVEHSKEEQLQKLETLRKQLQLKKELLLKYRN-MCTFDIPKS---
>Agrilus_planipennis
----------------------------------MDSNNESN---SDLQKRMTVDDLNIEILPVFYEIIKSVERDHHDN-TAKTRESQDCSQKVLELQKRFDQARSQISLLPGIDYSKEQQLQHLEALKTQLHLKQELLYKYRF-MYPFQMQK--
>Petrolisthes_cinctipes
--MSSPVVAAEDVDVDFLPVIYQYLRCLEKEQ----TDLGRVSQESSQKVLELQRKIQTAREQVRKLPGVEYSREEQLRRLEALRKQLALKKRLLIKYKSK-SEATH--
>Apachyus_charteceus
-----------------------------------MDISEST-S-QSLIQNLTVDDLDVEFLPIIYDIIRSVERDPL-DPSQKNRESQDSSQKVLELQKKLDQARNQVKQLQGIEYSKAEQLKKVEILRKQLQLKRDLLNKYRT-MCTFDIPK--
>Daphnia_pulex
--MATQLVEGGDFDLLSTIYDIIKSVEKEQQ-DNAQKQKDSQEAGQKVLELQRKLDHAREVVKKLPGIELSKDEQLLQIDLLRRQLTLKRDLLNKYRL-ITSFDREAQSEFQIHQH-----------------------------------
>Campodea_augens
---MAASNPIEQIDVEFLPTIYEIIRNIERDSG-DTTQKTRDLQEAGQKTLELQRKLDQAREQIQKFPGIEYNKEEQLRRLETLRKQLTLKRELLLKYKN-MCSFEVPKA---
>Haploembia_palaui
----------------------------------MEEQQNLNGS-VAASTQLTVQDLDLEFLPLIYEILRSIEKDPH-DSSQKVRESQDISQKILELQKKLDTARSQVQQLPGVEYSKEEQLQKLETLRKQLQLKKDLLLKYRH-MCSFEVPK--
>Baetis_sp._AD-2013
-----------------------------------MDSAEQP---TVTNGHLSAEEVDVELLPLIYELMRGIERESI-ETAQKPRESQDTSSKVLDLHKKLNLLREQIRKLPGIEYNREDQLKQLDHLRKQLRLKRELLLKYRN-MCSFEMPKA---
>Tigriopus_californicus
---MRTGIYGLSGIENSPKEQLKQLEILREQLRMKKKLIHKYKE-LNLKVFGLSGTY--
>Cuerna_arida
---------------------------MDVH---AINNDQQE-QTPTDYKPLTVENLDVDFLPLIYDIIRSVERDPH-DNAQKSSQSQDISQKVIELHNKLEQAKKQVRRLPGVEYSKEEQLEKLETLRKQLRLKRELLQKYRN-MYLLDSPKP---
>Tenthredo_koehleri
----------------------------------MMEVVEIAE-ETPARSQFTVDDLDIEILPLVYEIIRSVEKDPH-DNTQKAKESQDTSHKILDLQKKLDSARSQIKRLPGIEYSKEEQLQKLETLRRQLRLKQELLLKYRN-MCTFDVPKV---
>Armadillidium_vulgare
--MSSHTIAAEEVDVDFLPLIYQYLRCLEKDQ----NDLNRISVESSQKILEIQKKIDIAREQVQRLPGVEYSKEEQLQRLEALRKQLSLKKKLLPKYKSK-AQPEQ-------SH-------------------------------------
>Plutella_xylostella
----------------------------------MENNTDHAE--QNSCAPLSVQDVDVDFLPIVYEIIRSVERDLHDN-SAKVRESQDCSQKVLELQKKFDAARAQILRLPGIDYNKQDQLKQFEILRTQLKLKRELLQKYRNNMCPF--
>Empusa_pennata
-----------------------------MF---SEDQNGSAG--SALPAQLTVDDVDVEFLPLIYEIIKSVERDPH-DTSQKTRESQDTSQKVLELQKKLDQARNQIRRLPGIEYSKEEQIQKLETLRKQLQLKKELLLKYRH-MCTFDIPKST--
>Tanzaniophasma_sp._AD-2013
-----------------------------------MDVGEQNG--TSMPTQLTVDDVDVEFLPLVYEIIRSVERDPH-DTSQKTRESQDTSQKVLELQKKLDQARSQISRLPGVQYSKEEQLMKLETLHKQLQLKKELLLKYRH-MCSFDIPKS---
>Panorpa_vulgaris
-----------------------------ME---PMVTDQSE---LKNNTTLTVDQVEIDILPTIYDIIRSVERDPHDS-AGKLRESQDCATKVLELQKKLLKARNQIKQLPGIDLNPEEQLRKVDTLRNQLKLKEELLHKYRN-MYSFDSSKI---
>Corydalus_cornutus
-----------------------------ME---VSEQNDSN---NRSQEPLTVEEVDIEILPIIYDIIRSIERDPHDN-AAKARESQDCSQKVLELQKKLEFSRDQIRRLTGINCSREEQIAKLTSLRTQLKLKQDLLRKYRE-MVSLESTKS---
>Pseudomallada_prasinus
-----------------------------ME---VSESNELN---NRNSTQLNIEELDFEILPVIYEIIRSVERDPHDN-AAKARESQDCSQKVLELQKKLDQARAQIRRLPGIEFSREQQLQRLEALKTQLRLKQELLQKYRH-MCSLDSIKA---
>Triops_newberryi
---MTATSEEVDIEFLPLIYDIIKSFERDPV-DNTQKLKDSQETYQKVLELQKKLDHAREQIKKLPGVEFSKEEQLEQLDLLRQQLTLKRELLLKYRN-LGPFDLPK--
>Epiophlebia_superstes
-----------------------------------MDSEEQKT--SLSPSQLSAEEVDVEILPLIYEIIRSIERDPH-DATQKPRESQDTAQKILELQKKLDQAREQVKRLPGVDLSREEQLKRLEFLRTQLRLKRELLLKYRT-TCTFEIPSNPTGNSNSFSSPSLSSSTQQQQQINTNSLPTSNSQPIVNNTGKA
>Locusta_migratoria
-----------------------------------MESIEQNGS-TATPSSLTVDDVDVDFLPLIYDIIRSVERDPH-DSTQKTRESQDISQKILELHKKFDEARSQIGRLPGVEYSKDEQLRKLETFRKQLQLKKDLLRKYRE-MCTFEIPK--
>Phyllium_philippinicum
-------------------MEQKNATDSCEH---DMEGCEQNS--STHASELSVKDVDVEFLPLIYDIIRGVEKDPQ-DTSQKSRDSQDTSQKVLDLHKKLDQARNQIRRLTGIEYSKEEQLKKLETLRKQLQLKKDLLLKYRH-MCSFDVPK--
>Cosmioperla_kuna
-----------------------------ME---ASDQSNQEG--SSIPTYLTVNDVDVEFLPLIYEIIRSVEKDPH-DISQKTRESQDTSHKVLELQKKLDQARSQIQRLPGVEYSKEEQLLKLETLRKQLHLKRELLQKYRN-MCSFEIPKV---
>Acerentomon_franzi
--MADVETDEIDTDFLPLIYDIIRSIEREPTGDLSAKPKEGPDANQKILDLNKKLEQAREQIRQFPGIECNKEEQLRRFDALKQQLTLKEQLLAKYRT-LCSFELQ---
>Ectopsocus_briggsi
---------------MAEQTALSNAVKIEME---NLPNGNST-TDLVANNSTNNKEFDFEFLPLIYEVIKSVGPDQ--DPTGKGRESQDASIKILELERKLNEARQQILVLPGIEHSKEEQLRQLELCRKQVILKQKLIAKYRS-FCNFSLAD--
>Xanthostigma_xanthostigma
-----------------------------ME---VTD--QSE---TLNCKQLSVEELDIDILPIVYEIIRSIERDHHDN-TAKTREAQDCSQKVIELQKKLEQARSQIRQLPGIEYSREQQLQKLEALRTQLKLKQELLQKYRH-MCTLDFNKS---
>Trichomycterus_areolatus
-----------------------------ME------SVEEGE--GEGVGVVAESPLQLELLPALYDIVRSVEREVSEGGANKARESQDCSLKVLELHRQLEAARQQVRSLAGISQTKREQLRQFEILRTQLFLKRKLLKKYRA-MCLTESSFK---
>Oropsylla_silantiewi
-----------------------------ME---SMNTELSEQ--QTNANPLTVDQVEIDILPTIYDIIRSVERDPHDN-AGKARESQDCSTKVLELQKKLDKIRTQVALLPGIDYNKEEQLQQLDNLRKQLKLKQELLHKYRT-MYSFDSMKI---
>Mengenilla_moldrzyki
-----------------------------MD------ISN-----TEIEQGDSAEQITSEILPAVYEVIRSLEKDSYD----ISFKIHDCSQKILHLKDKFDRARQLIEDFPGIRYSIDDNKALMKSLRQQLKLNHELIQKYRN-IRDFGTTKP---
>Platycentropus_radiatus
-----------------------------MD---VINNDLNEP--QNNFSPLTVQDVDVDILPIVYDIIRSVERDVHDN-SAKARESQDCSLKVLELQKKLDSARSQIKRLPGIDYNKQEQLRQFEILRRQLHLKRELLQKYRN-MCSFDAPFK---
>Zorotypus_caudelli
-----------------------------------MDSNDQPAVPTVINNPATVDDIDVDFLPLIYEIIRSVERDPH-DTSQKSRESVDTSQKVLELQKKLDLARSQVRLLPGVELSKSEQLAHLDTLYKQLQLKRDLLHKYRN-MCSFDISKVQ--
>Atelura_formicaria
-----------------------------ME---SVEQNPVPTV-APSPSTITVDDADVEFLPVIYEILRSIERDPH-DSTQKSRESQDTSQKVLELQKKLDQAREQIVKLPGIEYSKEDQLKKLDTLRKQLRLKQELLLKYRN-MCTFELPKV---

HG0015 ENSDARG00000007377 odc1

```
>Danio_rerio
MRFKNSCLREV----HTGPGLEAYLKGFNF-TGEPPWLSDIC
>Acipenser_sinensis
MLFKNAPFKKVYNGFSEGLGLALYLKEFNF-TGEPPW-----
>Amia_calva
MLFKNTRFSKAHNGFLEGPDFAFYLKVFNF-KGEPPWL----
>Channa_striata
MLFKNHHSKKSVTAYSSGPGLESYLKGFNF-TGEPPWLLCT-
>Podocoryna_carnea
MLFKNAPFKKVYNGFSVGLGLALYLKEFNF-TGEPPW-----
>Xenopus_laevis
MLFKNTNSRTAQSGSIESVGLVLYLPGFYF-TGEPPWE----
>Basilichthys_microlepidotus
MLFKNPHSRKSHSGCSTGPGLESYLKGFNF-TGEPPWLLCT-
>Porichthys_notatus
MLVKTHHCRTAHSDYSAGPGLESYLKGVNF-TGDPPWILCT-
>Oryzias_latipes
MLFQNLHSRKSYRGSSTGPSIESYLKGFNFFTGEPPWPQRLD
>Hynobius_chinensis
MLFKNSCSTTSYHGSSGGLRLSLYLPGFYF-TGEPPWQQS--
>Micropterus_floridanus
MLFKNHPLRKTHTGYSAGPGLESYLKGFNF-TGEPPWLLCT-
>Astyanax_mexicanus
MLFKNTRLRKAHTGY-AGPGLEAYLKGFNF-TGEPPWVL---
>Callorhinchus_milii
MFYKNNQIRKLYNGYSEGLRFLLYLKVFSF-VGEPPWRRS--
>Haplochromis_burtoni
MLFKNLHSRKSHTGYSAGPGLESYLKGFNF-TGEPPWLLCT-
>Alosa_alosa
MLLKNNHLRTLHT-YSEGNGLESYLKGFNF-TGEPPWLLYT-
>Latimeria_chalumnae
MLFKN-YISKLYNGCTEGSGLALYRKGFDF-AGAPPWEHG--
>Poecilia_reticulata
MLFKNLHSRKAHSSCSTGPGLELYLKGFNF-TGEPPWLLCT-
>Esox_lucius
MLFKNNRFRKAHTGYSAGPGLESYLKGFNF-TGEPPWLLCT-
>Gadus_morhua
MLFKINHFRKVHTGYSAGRGLESYLKGFNF-TGEPPWLPLT-
>Acanthogobius_hasta
MLFKNLHLRRS----ETGPGLEAYLKGFNF-TGEPPWLLCT-
>Apteronotus_leptorhynchus
MLFKTTCLRKAHTGYSAGPGLEVYLKGFNF-TGEPPWVI---
>Erinaceus_europaeus
MRFKGPRLRNCHTSSGC-------CTSFEV-TGDPP------
>Lutjanus_erythropterus
MLFKNHRSRQAHTGYSAGPGLESYLKGFNF-TGEPPWLLCT-
>Osmerus_mordax
MLFKTNCFRKAHTGYSAGPGLESYLKGFNF-TGEPPWLLCT-
>Scleropages_formosus
MLFKNSRLRKAHFRYSTGCGLESYLKGFNF-TGEPPWLLCT-
>Lateolabrax_japonicus
MLFKNNHSRKTHT--GAGPGLESYLKGFNF-TGEPPWLLCT-
>Perca_flavescens
MLFKNNHSRKTHT----GPGLECYLKGFNF-TGEPPWLLCT-
>Paralichthys_olivaceus
MLFKNFNSRRSHTGYSTGPGLESYLKGFNF-TGEPPWLLCT-
>Leucoraja_erinacea
MFFKNERVKRVFHGYTESPVLTLYLRGFNS-VGKPPWRTS--
>Salmo_salar
MLFKNNRFRKAHTGYSAGPGLESYLKGFNF-TGEPPWLLCT-
>Lepisosteus_oculatus
MLFKNAHFRKADIGFLEGPGLASYLKGFNF-TGEPPWLSG--
>Trichomycterus_areolatus
MLFKTSRLSKAH----TGPGLEVYLKGFNF-TGEPPWGL---
>Squalus_acanthias
MFFKNEQARKVYNGYVESPDLALYLRGFNS-VGKPPWRDS--
>Takifugu_rubripes
MLFQNPRFRKTNTGYSAGPGLESYLKGFNF-TGEPPWALYS-
```

HG0016 ENSGALG00000010399 SELENOT

>Gallus_gallus
---MAYATGPLLKFQICVS
>Acipenser_sinensis
--------------MAKIWLSFSVLFLGVFYLY-S-AVADNG--APGKKLKMQFATGPQLKFQICVS
>Kaupichthys_hyoproroides
--------------MGRRWLSFCLLFLGVF-FLSCA-SADNGG---VKKLKMQFATGPLLKFQICIS
>Podocoryna_carnea
--------------MAKRWLSFSLLFLGVFYLY-S-ALADNG--APGKKLKMQFSTGPRLKFQICVS
>Xenopus_laevis
---MQYTAGPLLKFQICVS
>Acinonyx_jubatus
--------------MRF--LLVLL--VAASAVIRSDASANLGG-VPSKRLKMQYATGPLLKFQICVS
>Ambystoma_mexicanum
---MHYTSGPLLRFQICVS
>Tursiops_truncatus
------------------MRLLLFLLVAASAVVRSDASANLGG-VPGKRLKMQYATGPLLKFQICVS
>Eptesicus_fuscus
------------------MRLLLFLLVAASALVRSDASANLGG-VPSKRLKMQYATGPLLKFQICVS
>Dasypus_novemcinctus
-------------------MRLLLLLLAASAVVRSEASANLGG-LPSKRLKMQYATGPLLKFQICVS
>Alligator_mississippiensis
---MAYATGPLLKFQICVS
>Misgurnus_anguillicaudatus
----------------MRWLPLSVLILWAC-SLYS-VSADNSG---LKKMKMQFATGPLLKFQICVS
>Galeopterus_variegatus
------------------MRLLLLLLVAASAMVRSEASANRGG-VPSKRLKMQYATGPLLKFQICVS
>Monodelphis_domestica
------------------MRFLLLL-LMAAAALQGEASADTGG-VPGKRLKMQYATGPLLKFQICVS
>Trichosurus_vulpecula
------------------MRFLLLL-LMAAAALRGEASADTGG-VPGKRLKMQYATGPLLKFQICVS
>Aegypius_monachus
--------------MRAAVLLLLLLALAAGPGGG---SAEQGG-LPAKKLRMAYATGPLLKFQICVS
>Gadus_morhua
-------------MASSRWLRFSLLFLGVF-SLYCATSADNSG---VKKMKMQFATGPLLKFQICIS
>Condylura_cristata
------------------MRLLLLLLVAASAVVGTDASANLGG-VPSKRLKMQYATGPLLKFQICVS
>Oryctolagus_cuniculus
------------------MRLLLLLLVAASAVARSEASANLGG-VPSKRLKMQYATGPLLKFQICVS
>Pseudopodoces_humilis
--------MRAAGLRRLLLLLLLLAGLAAAPGGGGAAAAEQGG-LPAKKLRMAYATGPLLKFQICVS
>Trematomus_bernacchii
----------------MKWLQFSLL-VGLS-SLLCASSGDNSG---VKKMKMQFANGPLLKFQICIS
>Ceratotherium_simum_simum
------------------MRLLVLLLVAASAVVRSDASANLGG-VPGKRLKMQYATGPLLKFQICVS
>Manis_javanica
------------------MRLLLLLLVAASAMIRSDASANLGG-MPSKRLKMQYATGPLLKFQICVS
>Platichthys_stellatus
--------------MEMRWPRFLLLALGAF-SLCCATSGDISG---VKKMKMQFATGPLLKFQICIS
>Aotus_nancymaae
------------------MRLLLLLLVAASAVVRSEASANLGG-VPSKRLKMQYATGPLLKFQICVS
>Loxodonta_africana
------------------MRLLLLLLMAASAVARSEASANLGG-MPSKRLKMQYATGPLLKFQICVS
>Melopsittacus_undulatus
----------------MRAVLLLLLALAARPAGG---SAEQGG-LPAKKLRMAYATGPLLKFQICVS
>Marmota_marmota_marmota
MKPNINFEFWVVNREITRKRLLLLLLVAASAVARSEASANLGG-VPSKRLKMQYATGPLLKFQICVS
>Oncorhynchus_mykiss
---MKMQFATGPLLKFQICIS
>Tupaia_chinensis
------------------MRLLLLFLVAASAVVRSEASANLGG-VPSKRLKMQYATGPLLKFQICVS
>Trichechus_manatus_latirostr
------------------MRFLLLLLMAVSAVVRSEASANLGG-VPSKRLKMQYATGPLLKFQICVS
>Gekko_japonicus
-------------MKRALALLL-LLSV-----GLDWASAQPGGLPPAKKLRMAYATGPLLKFQICVS
>Mauremys_reevesii
---MAYTTGPLLKFQICVS
>Orycteropus_afer_afer
------------------MRLLLLLLMAASAVVRSEASANLGG-VPNKRLKMQYASGPLLKFQICVS

HG0017 ENSDARG00000043154 ucp2

>Danio_rerio
--MFFICTSSQRFSMEERGNKI-QVESHFNQRDAFRES-----
>Acipenser_sinensis
--MLVQRFVFGRLSMEQRGNLI-VTESNFSERDTFREEC----
>Podocoryna_carnea
--MLVQRFVFGRLSMEPRGNLI-ITESNFSERDTFREEC----
>Oryzias_latipes
--MILGLSTSEISSMEKQGQTV-KCAANFSQRDTFRES-----
>Micropterus_floridanus
--MILRLITSEKSSMEKQGNQF-IAGTKFSQRDTFRES-----
>Astyanax_mexicanus
--MFFRCSFSERFSMEDRGNEI-IVESHFNQRDAFRES-----
>Callorhinchus_milii
--MII-QRFISGFSMEQPETQI-IVKKSFNERDIFWES-----
>Neolamprologus_brichardi
--MIIRLI-AVKKSMEKQEKLF-IAGTKFSQRDAFRES-----
>Alosa_alosa
--MLLYCLVLERFSMEDQGNVI-IVESNFNQRDTFRES-----
>Latimeria_menadoensis
--MIIRYSDSAEFSMDQE-KTS-IVKNNFNERDIFRES-----
>Kryptolebias_marmoratus
-----------------MIKPDYLKKYYLLFFFLFLLK--------CLFSRSWSMILRLKVSERSSMEKQGNQF-IGGTRFNQRDTFRES-----
>Sminthopsis_crassicaudata
--MIIRCSVSQQFSMENQGKCTMVARSNFEERDTFREA-----
>Galeopterus_variegatus
--MTFGCFVSQPFSMENQGDRAMIATGSFEERDAFREA-----
>Monodelphis_domestica
--MIIRCSVSQQFSMENQAKWNMVAVSNFEERDTFREA-----
>Trichosurus_vulpecula
--MIIRCSVSQQLSMENQGKWAMVAVSNFEQRDTFREA-----
>Esox_lucius
--MFLGHIVFERCSMEEQGNQI-IVESNFDQRDAFRES-----
>Acanthogobius_hasta
--MENKRTQI-IAGTKFSQRDAFREA-----
>Chanos_chanos
---MFMCPVFKHFSMEEQGNEI-IAESTFNQRDTFRES-----
>Oryctolagus_cuniculus
--MNFRCFVSQPFSMENPGDRGMIATGGFEERDAFREA-----
>Osmerus_mordax
--MFFGYTVFEACSMEE-GKQI-IGESNFDQRDTFRES-----
>Scleropages_formosus
--MFLKCYTLKRVSMEDQVNEI-IAESQFSERDTFREA-----
>Lateolabrax_japonicus
--MILRLITSKRSSMEKQGYQF-IAGTKFSQRDTFRES-----
>Trematomus_bernacchii
--MILRLNTSEKSSMEKQGNLF-KAGTTFNQRDTFRES-----
>Equus_asinus
--MTIRCFVSQPFSMENQGDRAMIATGSFEERDAFREA-----
>Propithecus_coquereli
--MTIRCFVSQPFSMENQGNRAMIATGSFEEWDAFREA-----
>Leucoraja_ocellata
--MII-WRFISELSMEQRQSQT-VVKRGYNERDTFRESSPARL
>Ctenomys_sociabilis
MQAPPPALKSSASRADRIASPAAALFGHRHRWCPLTADCQRPLSRVLPAAGTRSMIFKCLVSQSFSMENQGDQAMIATGNFEERDTFREA-----
>Salmo_salar
--MFFGYVVFERFSMEEQGNQI-IIESNFDQRDAFRES-----
>Lepisosteus_oculatus
--MLFTSLVLGIFSMEQQGTQI-IVESNFNERDTFRES-----
>Ictalurus_furcatus
--MFLKCPFSELFSMEERGNEI-IVESHFNQRDAFREA-----
>Squalus_acanthias
--MII-RRFISELSME-RQSQI-IVKKSYNERDTFRES-----
>Gekko_japonicus
--MIIRCRLSAAFSMASKGSRAAVALSNFDERDTFRES-----
>Chrysemys_picta
--MILKCLVSKRVSMENQGDQIVVALSKFDERDTFRES-----

HG0018 ENSDARG00000053291 pnrc2

>Danio_rerio
---------------------------------------MFNSVPRQNN--AHWHMKTSDLA------GHTCAARSR-S-TTSRTWSPIGEEKLKSEKNHA--LNFFTTER-
>Acipenser_sinensis
---------------------------------------MYDSSTRFN--TNFRHMKTKTTA------GLTRSERSGEI-PTSPTWSQTGEKVSRKEEANK--SCYFTITKR
>Amia_calva
---------------------------------------MFGSAARPD--TAVRHMSAPSPA------GPTRATRSGFI-STSRTWSPVGEEKLKRPD-------FCTTKR-
>Channa_striata
---------------------------------------MFNSAPRENN--AHRHMKTSDFA------GHKRAARSRQK-TISRTWGPIGGERLKRD-LCF--VDFQTTKR-
>Anguilla_japonica
---------------------------------------MFGSASRQNC-SYFWHMKTPKLA------GLRRTERSGPT-PTSRTWSPIGEEKLRVEEVRT--RYFLTTKR-
>Podocoryna_carnea
---------------------------------------MYDSSTRFN--TNFRHMKTKTTA------GLRRSERSGEI-PTSPTWSQTGDKVSRKEEVNK--PCFFTITKR
>Basilichthys_microlepidotus
---------------------------------------MFSSASRENN--ALWHMRTSDLA------GHTSAARSRQK-ATSRTWGPIGGERLKRD-LCF--DNFLTTKR-
>Opsanus_beta
---------------------------------------MFSSAPREND--ANWHMKTSHCT-----------HKGRQK-AASRTWGPTGGERLKRN-LCSV-----TTKR-
>Oryzias_melastigma
---------------------------------------MFSSVPRENI--AHWHMKTSDLA------GQKSAVRSRQK-ATSRTWGPTGGERLKKD-LCF--VNIPPTKR-
>Tripterygion_delaisi
---------------------------------------MFYSAPRDIN--AHCHMKTSDLA------GHNTSAARSRLRTTSRTWGPVGGERLKRD-LCSVFTT----KR-
>Trachinotus_blochii
---------------------------------------MFSSAPRENN--AHWHMKTSDLA------GHKSAARSRQK-TTSRTWGPIGGERLKID-LCF--INFLTTKR-
>Mustela_putorius_furo
---------------------------------------MLASAPRLNS--ADWPMKTSVLR------QRKGSSRKQH--LLSWAWQQGRGQVVEILQSE------KQTER-
>Micropterus_dolomieu
---------------------------------------MFSPAPRENN--ALWHMKTTDLA------GNKIAARSRQR-TTSRTWGPIGGERLKRD-HCF--FNFLTTKR-
>Pygocentrus_nattereri
---------------------------------------MFNSASRQNI--ARWHMKTSDTV------GLKRAVRSRA--ATSRTWSPIGEEKLRNKDIYAVIFN---TKR-
>Miniopterus_natalensis
---------------------------------------MLASAPRLNS--ADWPMKTSVLR------QRKGSVRKQH--LLSWAWQQGIGQVVEILQSE------KQTER-
>Haplochromis_burtoni
---------------------------------------MFSSASRENN--AYWHMKTSDPA------GHKCAARGRQK-TTSRTWGPIGGERLKRD-VYS--VNFLTTKR-
>Alosa_alosa
---------------------------------------MFSSVTRQYT--AHWHMKTSDLA------GHKCAARSRFS-TTSRTWSPVGEEKLRSEIIHV--LNFLTTKR-
>Poecilia_mexicana
---------------------------------------MFSSASRQNN--DHWHMKTSDLA------GHTSAARSRQR-TTSRTWGPIGGERLKQD-LIV--FSLTTKR--
>Esox_lucius
---------------------------------------MFSSASLENN--AHWHMKTSDLA------GLKSAARSRSK-TTLRTWSPIGEEKLRNH-LCT--FNILITKR-
>Acanthogobius_hasta
---------------------------------------MFSSVPRQNI--ALWHMRTSVLA------GHKSAVRSRR--TTSRTWGPIGGVRLKRA-HCNT---FLTTKR-
>Chanos_chanos
---MKTSGLA------GRKCAARSR-T-TTSRTWSPIGEEKLRSKDIDE--LNLVTTKR-
>Plecoglossus_altivelis
MNTSFTRQPDQYLTKGFYHPSQKKSRFSSSRSTVFRGELMFSSGTRENI--ADRHMKTADLA------GHKSAARSRSK-TTLRTWSPIGEEKLRKKALCS--FHFLTTKR-
>Lateolabrax_japonicus
---------------------------------------MFSSAPRENN--APWHMKTSDLA------GHRSAARSRQR-TTSRTWGPIGGERLKRD-HCF--VDFLTTKR-
>Sebastes_caurinus
---------------------------------------MFSSVPRENNSTTHWHMKTSDLA------GHRSTARSRQKITTSRTWGPIGGERLKGD-LSFFYFNFLTTKR-
>Cynoglossus_semilaevis
---------------------------------------MFSSAARVNN--AHWHMKTSDPA------GHISAVRSRQK-TTSRTWGPIGGERLKED-SCS--TNFLTTKR-
>Microcebus_murinus
---------------------------------------MLASAPRLNS--ADWPMKTSVLT------QRKGSFRKQH--LLSWAWQQGRGQVVEILQSE------KQTER-
>Ictidomys_tridecemlineatus
---------------------------------------MLASAPRLNS--ADWPMKTSVLR------QRKGSVRKQH--LLSWAWQQGRGQVVEILQSE------KQTER-
>Oncorhynchus_tshawytscha
---------------------------------------MFYSASREND--AHWHMTTPDLA------GPKSAARSRPK-TTSRTWSPIGKEKLRRKYLCT--FNFLTTKR-
>Lepisosteus_oculatus
---------------------------------------MFGSASRYS--PDFRHMKTPNLA------GLTPSERSGLR-PTSRTWSPVGEEKLRGKESNR--LCFSTAKR-
>Ictalurus_punctatus
---------------------------------------MFSSASRQNA--AFWHMNTINTITTINIELNTCAVRSRR--TTSRTWRQISDETVRRRNLCT--GNFTTTTER
>Trichechus_manatus_latirostr
---------------------------------------MLASAPRLNS--ADWPMKTSVFR------QRKGSVRKQH--LLSWAWQQGRGQVLEILQSE------KQTER-
>Takifugu_rubripes
---------------------------------------MFSSVPRQNN--ALWHMKTLGLA------GHRSAVRSRSR-AAPRTRSPVEDPSKIGS------AHFATTKR-

HG0018 ENSG00000189266 PNRC2

>Homo_sapiens
---------------MLASAPRLNSADRPMKTSVLRQRKGSVRKQHLLSWAWQQGRGQVVEILQSEKQTER
>Anas_platyrhynchos
MQLDFFPWSFSAENPMLGSAPWLNTADHPMKTSIPSQRKESSERHHLLFQAWKQERGQELDGVESEKATER
>Chaetura_pelagica
---------------MLGSATWLDTADQPMKTSIPSQRKESSKRQHLLFQAWKQERGQELESVESEKTTER
>Apteryx_australis_mantelli
---------------MLGSATWLKAAYQPMKTSIPSQRKESSKRQHLRFQAWKQERGQELESVESEKTTER
>Buceros_rhinoceros_silvestri
---------------MLGSAAWLKSADQPMNTSVRSQRQESGKRQHLLFQAWKQERGHELESVGSEKRTER
>Caprimulgus_carolinensis
---------------MLGSATWLNTADQPMKTSIPSQRKESSKRQHLLFQAWKQERGQEWESVESEKTTER
>Cariama_cristata
---------------MLGSATWLNTAHQPMKTSIPSQRKESSKRQHLLFQAWKQERGQELESVESEKTTER
>Odobenus_rosmarus_divergens
---------------MLASAPRLNSADRPMKTSVLRQRKGSGRKQHLLSWAWQQGRGQVVEILQSEKQTER
>Orcinus_orca
---------------MLASAPRLNSADRPMKTSVLRQRKGSIRKQHLLSWAWQQGRGQVVEILQSEKQTER
>Charadrius_vociferus
---------------MLGSATWLNTADQPMKTSIPSQRKESSKRQHLLFQAWKQERGQELESVESEKTTER
>Eptesicus_fuscus
---------------MLASAPRLNSADWPMKTSVLRQRRGSVRKQHLLSWAWQQGRGQVVEILQSEKQTER
>Pterocles_gutturalis
---------------MLGSATWLNTADQPMKTSIPSQRKESSKRQHLLFQAWKQERGQELESVESEKTTER
>Dasypus_novemcinctus
---------------MLTSAPRLNSADQPMKTSVFRQRKGSVRKQHLLTWAWQQGRGQVVEILQSEKQTER
>Colius_striatus
---------------MLGSAPWLNTAEQPMKTSIPSQRKESSERQHLLFQAWKQERGQELESVESEKTTER
>Columba_livia
---------------MLGSAPWLNTAHQPMKTSIPGQRKESSERQHLLFQAWKQERGQELERAESEKTTER
>Merops_nubicus
---------------MLGSATWLNTADQPMKTSIPSQRKESSKRQHLLFQAWKQERGQELESVESEKTTER
>Alligator_mississippiensis
---------------MWGSASWLDTADRPMKTSIPSQRKRSGKRQHLLFQAWQQKRGREVAAVESEKKTER
>Cuculus_canorus
---------------MLGSAPWLNTADQPMKTYICSPRKESSERQHLLFQAWKQERGQELESVESEKTTER
>Sarcophilus_harrisii
---------------------MLNSADQPMKTSILRQRNRSGRKQHLLSWAWQQGRRQVVEILQSAKQTER
>Galeopterus_variegatus
---------------MLASAPRLNSTDWPMKTSVLRQRKGSTRRQHLLSWAWQQGRGQVVEILQSEKQTER
>Monodelphis_domestica
---------------------MLNSVDQPMKTSILRERNGSGRKQYLLSWAWQQGRRQVMEILQSAKRTER
>Macropus_eugenii
---------------------MLNSADQPMKTSILRQRNRSGRKQHLLSWAWQQGRRQVVEILQSAKQTER
>Falco_cherrug
---------------MLASATWLNTADQPMKTSIPSQRKESSKRQHLLFQAWKQERGQELESVESEKTTER
>Gallus_gallus
---------------MLGSAAWLNTADRPMKTSVPSQRKESSERQHLLFQAWKQERGQELESVESEKTTER
>Gavia_stellata
---------------MLGSATWLNTADQPMKTSIPSQRKESSKRQHLLFQAWKQERGQELESVESEKTTER
>Balearica_regulorum_gibberic
---------------MLGSATWLNTADQPMKTSIPSQRKESSKRQHLLFQAWKQERGQELESVESEKTTER
>Condylura_cristata
---------------MLASAPRLNSADRPMKTYVLRQRKGSVRKQHLLSWAWQQGRGQVVEILQSEKQTER
>Oryctolagus_cuniculus
---------------MLASAPRLNSADRPMNTSVLRQRRGSARKQHLGPWAWQRGRGQAVGTLHSGQQTER
>Ornithorhynchus_anatinus
---------------MLGSASWLNTADQPMKTSTRRQRERSGGKQHLLSQARQQGRGQAVEILWSEKQTER
>Tauraco_erythrolophus
---------------MLGSATWLNTADRPMKTSIPSQRKESSKRQHLLFQAWKQERGQELETVESEKTTER
>Opisthocomus_hoazin
---------------MLGSAPWLNTADQPMKTSIPSQRKESSKRQHLLFQAWKQERGQELESVQSEKTTER
>Sturnus_vulgaris
---------------MLGSAPWLKTADQPMKTSIPSQRKESSTRQHLFFQAWKQERGQELESVESEKTTER
>Phaethon_lepturus
---------------MLGSATWLNTADQPMKTSIPSQRKESSKRQHLLFQAWKQERGQALESVESEKTTER
>Ceratotherium_simum_simum
---------------MLASAPRLNSADRPMKTSVLRQRKGSVRKQHLLSWAWQQGRGQVVEILQSEKQTER
>Manis_javanica
---------------MLASAPRLNSADRPMKTSVLRQRKGSVRKQHLLSWAWQQGRGQVLEILRSAKQTER
>Picoides_pubescens
---------------MLGSAPWLNTADQPMKTSIPSQRKESSKRQHLLFQAWKQERGRELESVESEKTTER
>Loxodonta_africana
---------------MLASAPRLNSADRPMKTSVFRQRKGSVRKQHLLAWALQQGRGQVVEILRSEKQTER
>Fulmarus_glacialis
---------------MLGSATWLNTADQPMKTSIPSQRKESSKRQHLLFQAWKQERGQELESVESEKTTER
>Melopsittacus_undulatus
---------------MLGSAPRLNTADQPMNTSVLSQRKESSKRQHLRFQAWKQERGQGLESGESEKTTER
>Ictidomys_tridecemlineatus
---------------MLASAPRLNSADWPMKTSVLRQRKGSVRKQHLLSWAWQQGRGQVVEILQSEKQTER
>Tupaia_chinensis
---------------MLASAPRLNSADRPMKTYALRQRKGSVRKQLLLPWAWQPGRGQVVEILQSEKQTER
>Trichechus_manatus_latirostr
---------------MLASAPRLNSADWPMKTSVFRQRKGSVRKQHLLSWAWQQGRGQVLEILQSEKQTER
>Aptenodytes_forsteri
---------------MLGSATWLNTADQPMKTSIPSQRKESSKRQHLLFQAWKQERGQELESVESEKTSER
>Gekko_japonicus
---------------MLGSASWLKTADRPMRTTIPKQRKGSNRSQHLFFQVWQQERGQEVETVDSEKKTER
>Tyto_alba
---------------MWGSATWLNTANQPMKTSIPSQRKESSKRQHLLFQAWKQERGQELESVESEKTTER
>Struthio_camelus_australis
---------------MLGSATWLNAADQPMKTSIPSQRQESSKRQHLFFQAWTQERGQELESVESEKTTER
>Trachemys_scripta
---------------MLGSASWLNTADQPMKTSILSQRKGSNRRQHLLFQAWQQERGREVEAGESEKKTER
>Tinamus_guttatus
---------------MLGSATWLNAADQPMKTSIPSQRKESSKRQHLLFQAWKQERGQDLESVESEKTSER
>Calypte_anna
---------------MLGSAAWLNTADQPMKTSIPSQRKESSKRQHLLFQAWKQERGHELESVESEKTTER
>Apaloderma_vittatum
---------------MLGSAAWLKTADQPMKTSIPSQRKESSKRQHLLFQAWKQERGQELESVESEKTTER
>Orycteropus_afer_afer
---------------MLASAPRLNSADRPMKTSVLRQRRGLVRKQHLLSWAWQQGRRQVVEILQSEKQTER

HG0018 ENSGALG00000004122 PNRC2

>Gallus_gallus
--MLGSAAWLNTADRPMKTSVPSQRKESSERQHLLFQAWKQERGQELESVESEKTTER
>Anas_platyrhynchos
-------------------------------------MQLDFFPWSFSAENPMLGSAPWLNTADHPMKTSIPSQRKESSERHHLLFQAWKQERGQELDGVESEKATER
>Chaetura_pelagica
--MLGSATWLDTADQPMKTSIPSQRKESSKRQHLLFQAWKQERGQELESVESEKTTER
>Apteryx_australis_mantelli
MCFQKRVQERHGGACDARGWLDGEGRLRSAGVSRGPVIWLRLLPWSLCAENPMLGSATWLKAAYQPMKTSIPSQRKESSKRQHLRFQAWKQERGQELESVESEKTTER
>Buceros_rhinoceros_silvestri
--MLGSAAWLKSADQPMNTSVRSQRQESGKRQHLLFQAWKQERGHELESVGSEKRTER
>Caprimulgus_carolinensis
--MLGSATWLNTADQPMKTSIPSQRKESSKRQHLLFQAWKQERGQEWESVESEKTTER
>Cariama_cristata
--MLGSATWLNTAHQPMKTSIPSQRKESSKRQHLLFQAWKQERGQELESVESEKTTER
>Odobenus_rosmarus_divergens
--MLASAPRLNSADRPMKTSVLRQRKGSGRKQHLLSWAWQQGRGQVVEILQSEKQTER
>Balaenoptera_acutorostrata_s
--MLASAPRLNSADRPMKTSVLRQRKGSIRKQHLLSWAWQQGRGQVVEILQSEKQTER
>Charadrius_vociferus
--MLGSATWLNTADQPMKTSIPSQRKESSKRQHLLFQAWKQERGQELESVESEKTTER
>Eptesicus_fuscus
--MLASAPRLNSADWPMKTSVLRQRRGSVRKQHLLSWAWQQGRGQVVEILQSEKQTER
>Pterocles_gutturalis
--MLGSATWLNTADQPMKTSIPSQRKESSKRQHLLFQAWKQERGQELESVESEKTTER
>Dasypus_novemcinctus
--MLTSAPRLNSADQPMKTSVFRQRKGSVRKQHLLTWAWQQGRGQVVEILQSEKQTER
>Colius_striatus
--MLGSAPWLNTAEQPMKTSIPSQRKESSERQHLLFQAWKQERGQELESVESEKTTER
>Columba_livia
--MLGSAPWLNTAHQPMKTSIPGQRKESSERQHLLFQAWKQERGQELERAESEKTTER
>Merops_nubicus
--MLGSATWLNTADQPMKTSIPSQRKESSKRQHLLFQAWKQERGQELESVESEKTTER
>Alligator_mississippiensis
--MWGSASWLDTADRPMKTSIPSQRKRSGKRQHLLFQAWQQKRGREVAAVESEKKTER
>Cuculus_canorus
--MLGSAPWLNTADQPMKTYICSPRKESSERQHLLFQAWKQERGQELESVESEKTTER
>Sarcophilus_harrisii
--MLNSADQPMKTSILRQRNRSGRKQHLLSWAWQQGRRQVVEILQSAKQTER
>Galeopterus_variegatus
--MLASAPRLNSTDWPMKTSVLRQRKGSTRRQHLLSWAWQQGRGQVVEILQSEKQTER
>Monodelphis_domestica
--MLNSVDQPMKTSILRQRNGSGRKQYLLSWAWQQGRRQVVEILQSAKRTER
>Macropus_eugenii
--MLNSADQPMKTSILRQRNRSGRKQHLLSWAWQQGRRQVVEILQSAKQTER
>Aquila_chrysaetos_canadensis
--MLGSATWLNTADQPMKTSIPSQRKESSKRQHLLFQAWKQERGQELESVESEKTTER
>Gavia_stellata
--MLGSATWLNTADQPMKTSIPSQRKESSKRQHLLFQAWKQERGQELESVESEKTTER
>Mesitornis_unicolor
--MLGSAAWLNTADQPMKTSVPSQRKESSERQHLLFQAWKQERGQGLESVESEKTTER
>Condylura_cristata
--MLASAPRLNSADRPMKTYVLRQRKGSVRKQHLLSWAWQQGRGQVVEILQSEKQTER
>Oryctolagus_cuniculus
--MLASAPRLNSADRPMNTSVLRQRRGSARKQHLGPWAWQRGRGQAVGTLHSGQQTER
>Ornithorhynchus_anatinus
--MLGSASWLNTADQPMKTSTRRQRERSGGKQHLLSQARQQGRGQAVEILWSEKQTER
>Tauraco_erythrolophus
--MLGSATWLNTADRPMKTSIPSQRKESSKRQHLLFQAWKQERGQELETVESEKTTER
>Opisthocomus_hoazin
--MLGSAPWLNTADQPMKTSIPSQRKESSKRQHLLFQAWKQERGQELESVQSEKTTER
>Serinus_canaria
--MLGSAPWLKTAHQPMKTSIPSQRKESSTRQHLFFQAWKQERGQELESVESEKTTER
>Phalacrocorax_carbo
--MLGSATWLNTADQPMKTSVPSQRKESSKRQHLLFQAWKQERGQELESVESEKTTER
>Ceratotherium_simum_simum
--MLASAPRLNSADRPMKTSVLRQRKGSVRKQHLLSWAWQQGRGQVVEILQSEKQTER
>Manis_javanica
--MLASAPRLNSADRPMKTSVLRQRKGSVRKQHLLSWAWQQGRGQVLEILRSAKQTER
>Picoides_pubescens
--MLGSAPWLNTADQPMKTSIPSQRKESSKRQHLLFQAWKQERGRELESVESEKTTER
>Nomascus_leucogenys
--MLASAPWLNSADRPMKTSVLRQRKGSVRKQHLLSWAWQQGRGQVVEILQSEKQTER
>Loxodonta_africana
--MLASAPRLNSADRPMKTSVFRQRKGSVRKQHLLAWALQQGRGQVVEILRSEKQTER
>Fulmarus_glacialis
--MLGSATWLNTADQPMKTSIPSQRKESSKRQHLLFQAWKQERGQELESVESEKTTER
>Nestor_notabilis
--MLGSAPWLNTADRPMNTSVPSQRKESSKRQHLRFQAWKQERGQDLESGESEKTTER
>Ictidomys_tridecemlineatus
--MLASAPRLNSADWPMKTSVLRQRKGSVRKQHLLSWAWQQGRGQVVEILQSEKQTER
>Tupaia_chinensis
--MLASAPRLNSADRPMKTYALRQRKGSVRKQLLLPWAWQPGRGQVVEILQSEKQTER
>Trichechus_manatus_latirostr
--MLASAPRLNSADWPMKTSVFRQRKGSVRKQHLLSWAWQQGRGQVLEILQSEKQTER
>Aptenodytes_forsteri
--MLGSATWLNTADQPMKTSIPSQRKESSKRQHLLFQAWKQERGQELESVESEKTSER
>Gekko_japonicus
--MLGSASWLKTADRPMRTTIPKQRKGSNRSQHLFFQVWQQERGQEVETVDSEKKTER
>Tyto_alba
--MWGSATWLNTANQPMKTSIPSQRKESSKRQHLLFQAWKQERGQELESVESEKTTER
>Struthio_camelus_australis
--MLGSATWLNAADQPMKTSIPSQRQESSKRQHLFFQAWTQERGQELESVESEKTTER
>Trachemys_scripta
--MLGSASWLNTADQPMKTSILSQRKGSNRRQHLLFQAWQQERGREVEAGESEKKTER
>Tinamus_guttatus
--MLGSATWLNAADQPMKTSIPSQRKESSKRQHLLFQAWKQERGQDLESVESEKTSER
>Calypte_anna
--MLGSAAWLNTADQPMKTSIPSQRKESSKRQHLLFQAWKQERGHELESVESEKTTER
>Apaloderma_vittatum
--MLGSAAWLKTADQPMKTSIPSQRKESSKRQHLLFQAWKQERGQELESVESEKTTER
>Orycteropus_afer_afer
--MLASAPRLNSADRPMKTSVLRQRRGLVRKQHLLSWAWQQGRRQVVEILQSEKQTER

HG0019 ENSG00000178397 FAM220A

```
>Homo_sapiens
-----MAA----ALSGLAVRLSRSAAARSYGVFCKGLTRTLLIFFDLAWRLRINFPYLYIVASMMLNVRLQVHIEIH--
>Canis_lupus_familiaris
-----MAA----ALSGLAVRLSRSAAARSYGVFCKGLTRTLLIFFDLAWRLRINFPYLYIVASMMLNVRLQVHIEIH--
>Hynobius_retardatus
------MA----ALAVLAARVSQSAAVRSYCVFSKGLSRTLLIFFDMAWRLRINFPYLYVIASMMLNVRLQVHIEIH--
>Balaenoptera_acutorostrata_s
---------------------------------------------------------------MMLNVRLQVHIEIH--
>Pteropus_vampyrus
-----MAA----ALSGLALRLSRSAAARSYGVFCKGLTRTLLIFFDLAWRLRINFPYLYIVASMMLNVRLQVHIEIH--
>Echinarachnius_parma
MAATYSSPWLLSQYQERRERRLKLAALQTVQP-FPLLTRTLNFFFNLAYQCRLRIPTIIFVGLLAFDVRLQLQINVVQI
>Latimeria_menadoensis
------MA----GLTALVVRFSGSAAARSYGVFSKNLTRTLLIFFDLAWRLRLRFPYLYIITSLMLNVRLQVHIEIH--
>Sarcophilus_harrisii
-----MAA----ALSALALRLSRSAAARSYGVFCKGLTRTLLIFFDLAWRLRINFPYLYIVASMMLNVRLQVHIEIH--
>Monodelphis_domestica
-----MAA----ALSALALRLSRSAAARSYGVFCKGLTRTLLIFFDLAWRLRINFPYLYIVASMMLNVRLQVHIEIH--
>Arachnocampa_luminosa
----------------MIFNYLAKSLLNIYPSVSGRLSPLMLFCFNFAWRAFTLFPYMFLATTMATGFQIRVEIEFE--
>Strongylocentrotus_purpuratu
-----MAPWMIYSRFQRHHERKLTAVIRTPPP-FPLLTRTLSFFFNLAYQCRMRIPTIVFVGLLAFDVRLQLQINVVQI
>Marthasterias_glacialis
---------MFKMLGMMRDPDSMIQVGASMPV-RPFLSRTLTFFFDMAFRCRFKLPSIVAFVLFAFDIQLQLQINVNH-
>Condylura_cristata
---------------------------------------------------------------MMLNVRLQVHIEIH--
>Oryctolagus_cuniculus
---------------------------------------------------------------MMLNVRLQVHIEIH--
>Lingula_anatina
------------MLE---NYIP-------QRMGPSTLSDVCMFFFKLAWQSRIRLPVILTVVFMILDTRLQLEININININ
>Crassostrea_gigas
--------------------MVLQTLNQSYASFARSLSRILLFFFRFAWQTKFSLPYLLNSTMLALDFRVRVVILL---
>Equus_asinus
-----MAA----ALSGLAVRLSRSAAARSYGVFCKGLTRTLLIFFDLAWRLRINFPYLYIVASMMLNVRLQVHIEIH--
>Manis_javanica
---------------------------------------------------------------MMLNVRLQVHIEIH--
>Brachionus_manjavacas
----------------MIFNYLAKSLLNIYPSVSGRLSPLMLFCFNFAWRAFTLFPYMFLATTMATGFQIRVEIEFE--
>Heterocephalus_glaber
-----MAA----VLSGLAVRLSRSAAARSYGVFCKGLTRTLLIFFDLAWRLRINFPYLYIVASMMLNVRLQVHIEIH--
>Tupaia_chinensis
---------------------------------------------------------------MMLNVRLQVHIEIH--
>Echinaster_spinulosus
-------------MLRISDPDSFIQVCTVLPV-RNVMSRTLTFFFDMAFRCRFKLPSIVAFVLFAFDIQLQLQINVNH-
>Takydromus_sexlineatus
MAVTAAAT----LLSALVARFSQSAAARSYGVFCKGLTRTLLIFFDLAWKLRINFPYLYIVASMMLNVRLQVHIEIH--
>Sphaerechinus_granularis
----MMAPWLMYLRYQRSQQRKVQ----TVSP-FPLLTRTLSFFFNLAYQCRMRIPTIVFVGLLAFDVRLQLQINVVQI
>Pelodiscus_sinensis
-----MAA----DLSALAARLSQSAAARSYGVFCKGLTRTLLIFFDLAWKLRINFPYLYIVASMMLNVRLQVHIEIH--
>Villosa_lienosa
------------------MLKLFSALWSTYSNFTRHLTPILMFFFRFAWLSRFELPGILTTVFLALNIRVTVEIHM---
```

HG0020 FBgn0035436 CG12016

>Drosophila_melanogaster
--MASNAQLGKIILISAIAVFFYYFFWVAVLPFMLIDEGNPIRLFFPPLKYAFIVPTVFGVIFLGGIAAFSFYHIWSLRVKRD-----
>Meinertellus_cundinamarcensi
--MISDAAAGRLAIVVGSSIFSYYTLWVIVLPFVDREHA--VRDFFPDVTYALVLPNILGVVFIGSLVYYACASLLKERCTS------
>Macrobrachium_nipponense
---MYGHDRVIGKCVLSVTGVALLYYLTWVVCLPFADDDSS--IRLLFPDPLVALIVPTIVFVFLITLTGFYIGHSLYVDYTTTSISS--
>Forficula_auricularia
---MSDPIYGKLIVISSVIVLIYFSCWVFLTPFADATDY--FFKVFPSLRYALIIPAVSGLIFVGIISLFILCSVI------------
>Daphnia_pulex
--MAVGHDVLIGKILLFLCVIFVLYYTFWTICLPLLEPNFF--AHQYFPDSFYAIGVPLSVLSVMVTVLSVYALWLMKFQ----------
>Aposthonia_japonica
---MEDATLGKVILFCCGLFFIYYSMWVLVLPFVDENHV--IRRFFPNVFHAIFTPAVLGLSFISSLIFFTIYAMTFEQS--------
>Eurylophella_sp._AD-2013
--MVHTRTVGQLILLISSLLLLYYTLWVVALPFADSDHF--LVQLFPSRRYAIIVPAISGLTFIGSLAVFTVWSMYSC----------
>Meganyctiphanes_norvegica
---MLGCDQLIGKCVLCVFLSLSLYYIVWVVGLPFTDEDSF--LRALFPDPIYAIVGPTLVFVALLLLNGVYMLKAYFKVQKLKAQ----
>Grylloblatta_bifratrilecta
---MVVTDATCGKAILILTLLPFCYYTVWVLILPFTDAEDV--LNAVFPSNRYALVVPALAGLCFVGGLVAFTLYSLAS-----------
>Xenophysella_greensladeae
--MGALTDSTLGALLLTGASVFLLYYTLWVILSPFYEP-----ISSLFPPIRYALLVPAFTGLTFISSLIFFTVWALLSS----------
>Sceliphron_caementarium
---MFDSDREKGKLILIVTIILFLYYTIWIIGLPFIDDDRL---RSLFCSHDFALVVPAVSGLCFIGGLVLFTLYHVRPYLLSDRASKIQ
>Asellus_aquaticus
---MYGKDYMLGKVLLISSIVFVTYYLSWTILLPFVKDDSV--IRIFFPGPLLAILIPTSLFLFLLVTTFGYLLYTVAVNKKELL-----
>Pararge_aegeria
---MSFTLYHIYPYL---------
>Metallyticus_splendidus
---MNDSALGKILLIASCLFFCYYTTWVIVLPFSDRDHI--INILFPPVQHALLVPACMGVAFIGSLIIYTVYNIKQ-----------
>Tanzaniophasma_sp._AD-2013
---MNDSTLGTFVVIFSVLFFMYYTFWVLVLPFVEQDHV--IHTIFTSVDFALMVPAVAGLFFIGGLVIFTLFALRH-----------
>Euroleon_nostras
---MKDVVLGRTLLIFSGIFVLYYTFWVVVLPLLPLADDNTFLLFFPDVTYALLLPAILGTLFIGSLSAFTLYHVLPHLKLKKK----
>Calopteryx_splendens
---MGISDAQLGKIILVLSASLFVYYTLWLWVPPFISPSSFNTFPTVFPPTRYALILPAISGLLFIGGLLVFTIAALQNA----------
>Gryllotalpa_sp._AD-2013
--MLSDATLGKIILFSSCSFFIYYTAWVMFLPFADNIQV--IKS-FPPIWIALLVPAIMGLLFVGALMMYTVHAIRRK----------
>Ramulus_artemis
MILLCLLKKILRSLKKSILSVEHCLLHAFFLKYQQLFNFINCDTEYVIMVSDSTVGKLVLTATCIFFTYYSFWVLVLPFTDSEDK--INSFFPSVWYALVIPAVMGLIFIGSLVVFTLCAVETRGRS-------
>Cosmioperla_kuna
---MSDALIGKVILIASCTFFCYYTAWTLLLPFADGDST--INSLFPSIWYALFVPAYMGLAFIGSLMVFTYIALRT-----------
>Liposcelis_bostrychophila
---MLAKLILALAVSFFSWYTFCVIALPFFEPTHL--IVSYFPGPRYTLIIPALFGLTFFGSLLIFTIAVLWKEKP--------
>Trichomycterus_areolatus
---MILPDRILGKSIVLITTFAFVYLVIWLTLLPFTDFEKDHWLYKGFLDREYALILCISWGFIFIGSLMLFTLYQIYPYL---------
>Thrips_palmi
---MNDKTVGSLLIALSASFSFYYSVWVLGLPFFDSDHF--IHSLFPAVEFALLVPALMGLTFIGSLIIFCIIELRKAGAASK-----
>Hydroptila_sp._AD-2013
---MIISDSNLGKLIVLAAVSIFTYLVIWLTFLPFTDFDEDHWLYKGFLPREYALIFCIGWGFLFIGSLMGFTLYHLYPFLK--------
>Thermobia_domestica
---MTIADETLGKLLLIGSLLFTLYYTFWIIILPFVDPDHI--LHDLFPPVTYGVMTPAVMGLCFIGSLFVFTVVCFKSEK---------

HG0021.1 ENSDARG00000068708 ifrd1

```
>Danio_rerio
MYRSRSKSNTGIAFNTFLFTVPLEYTQTKRHYSKNWSCIG
>Amia_calva
MCRFRSQTFTGIAFYSYLLTVPLDGFHLERQYSKNWSSIG
>Channa_punctata
MYRCSQQSFSRIAFDCFLSTVQREYFHFTRQYNKSWSCIG
>Anguilla_japonica
MYRFRPQIFTGTAFNSYLLTLLLKGFYLKRHCTRNWSSIG
>Podocoryna_carnea
MCRFCSPTFTRIAFYSSLLAVPFEDFQLMRHYSKNCLSVG
>Menidia_menidia
MYRLGQQTSFKIAFDFFLSTVPREHFHFTRQYNKSRSCIG
>Opsanus_beta
MYRLGLQLIAKITFTSIFCTVPREYSYFTRQYSKSWSCIG
>Oryzias_melastigma
MYRLGQQFISKIAFDYFLSTVPREHFTFTRQYNKSWSCIG
>Tripterygion_delaisi
MHRLGQQSFSRIAFNSFLPTVPREYFLFTRQYNKGWSCIG
>Astyanax_mexicanus
MYRFRSQALIGIAFNSLLFTVPLDDIQFKRRYSKNWSCIG
>Neolamprologus_brichardi
MYRLGPQSISKIAFDFFLSTVPREYFHFTRQYNKGWSCIG
>Clupea_harengus
MYRLRSKSFTGIALNTFFAAVPLEEIYFRRQYSKNWSSIG
>Fundulus_heteroclitus
MYRLGRQSISKIAFNFFLSTVPRERFHFTRQYNKSWSCIG
>Esox_lucius
MYRFRTQIFSRTAFNSFLTTVPREDCQFMRQFNKNWSCIG
>Gadus_morhua
----------------------------MRQYNKGWSCIG
>Acanthogobius_hasta
MYRLAQQVFSKIALISSLPAVPREYFHFTRQYNKGWSCIG
>Apteronotus_leptorhynchus
MYRFRLQAFIGIAFYSLPFTVALEDGKFKRHYSKNWSCIG
>Plecoglossus_altivelis
MYRLGSQSFSRIAFNSFLSTVPREDLHFIRQYNKNWSCIG
>Perca_flavescens
MYRLGQQCIPKIAFNSFLSAVPREYFHFTRHYNKSWSCIG
>Cynoglossus_semilaevis
MYRLGQQFISKIAFNSFLSTVPREYFHFTRRCNKNWSCIG
>Coregonus_clupeaformis
MYRLRPQSFSRIAFNSFLSTVPREDCQFMRQFNKNWSCIG
>Lepisosteus_oculatus
MCRFRLQTFTGIAFYSYLSTVPCGDFHLERQYSKNWSSIG
>Ictalurus_punctatus
MYRSRSQAFIGIAFNPLLFTVPFEDFQFKRHYSKNWSCIG
>Sparus_aurata
MYRLGQQTFSKIAFDSFLSTKPREYFHFTRQYSKSCSCIG
>Takifugu_rubripes
MYRLGQLTISKIAFNSFLSAVPREYFHFTRQYNKSWSCIG
```

```
HG0021.1 ENSG00000006652 IFRD1

>Homo_sapiens
------------------------------------------MYRFRSQLFTGISAAATAHSYP---RRFSTLLLAEDSPLSRPPHRR-TS--KKCSSIG
>Xenopus_laevis
------------------------------------------MYRLHSQAFTGVSV----TSTF---RHFYPIPIEDS---KLIPERR-FS--KKCSSIG
>Apteryx_australis_mantelli
------------------------------------------MHRFRSRAFTGISAFATDSS-FACFRQSPPLPRSGK----SASQRR-CSKKRSCSSIG
>Leptonychotes_weddellii
------------------------------------------MYRFRPQLFTGISAAATAHSYP---RRFSPLLLAEDSPLSRPPHRR-TS--KKCSSIG
>Ambystoma_mexicanum
------------------------------------------MYRFRSQALIGFPWSRRRCSPA---RRCSPLPLGDL---NLIPERR-FS--KKCSSIG
>Physeter_catodon
------------------------------------------MYRFRSQLFTGISAAATAHSYP---RRFSPLLLVENSPLSRPPHRR-TS--KKCSSIG
>Rhinolophus_ferrumequinum
------------------------------------------MYRFRSQLFTGISAAATARSHP---RRSSPLLLAEASPLSRPPHRP-TS--KKCSSIG
>Dasypus_novemcinctus
------------------------------------------MCRFRSQLFTGISATATTHSYP---RRFSPLLLVEDSPLSRPPHRR-TS--KKCSSIG
>Latimeria_menadoensis
------------------------------------------MIRSGSQTVTRIA------SCY---HHLLAVPF---GDLHLVPERR-YS--KKCSSIG
>Sarcophilus_harrisii
------------------------------------------MYRFRSQLFTGISAAAIAHSSP---RRFSPLLLAEDLSLSQPPHRR-TS--KNCSSIG
>Galeopterus_variegatus
MCRDWLVRVRVPPTCGDDRRRSCYLRRRGLDRPALAPALERNMYRFRSQLFTGISAAATAHSYP---RRFSPLLLAEDSPLSRPPHRR-TS--KKCSSIG
>Monodelphis_domestica
------------------------------------------MYRFRSQLFTGISAAAIAYSSP---RRFSPLLLAEDLSLSHPPHRR-TS--KKCSSIG
>Coturnix_japonica
------------------------------------------MHRLRSRAFTGISAIAAGSS-FACLRQSPPLPRPEK----SASQRL-WNKKRSCSSIG
>Sorex_araneus
------------------------------------------MYRFRSQLFTGISAAATAHSHP---RRFSPLLLAEDSPLCRPPHRR-TS--KKCSSIG
>Ochotona_princeps
------------------------------------------MYRFRSQLFTGISAAATAHSYP---RRFSPLLLAQDSPLSRPTHRR-TS--KKCSSIG
>Elephantulus_edwardii
------------------------------------------MHRFRSQLFTGISAAATAHSHP---RRFSPLLLAEDSPLSLQPHRR-TS--KKCSSIG
>Equus_przewalskii
------------------------------------------MYRFRLQLFTGISAAATAHSYP---RRFSPLLLAEDSPLSRLPHRR-TS--KKCSSIG
>Manis_javanica
------------------------------------------MCRFRSQLLTGISAAATTHFYP---RRCSPLLLAEDSPFSRPPHRR-TS--KKCSSIG
>Loxodonta_africana
------------------------------------------MYRFRSQLFTGISAAATAHSHP---RRFSPLLLVEYSPLSRLPHRR-TS--KKCSSIG
>Marmota_monax
------------------------------------------MYRFRSQLFTGISAAATAHFYP---RRFSPLLLAEDSPLSRPPHRR-TS--RKCSSIG
>Tupaia_chinensis
------------------------------------------MYRFRSQLFTGISAAATAHSYP---RRFSPLLLAEDSSLSRPPHRR-TS--KKCSSIG
>Trichechus_manatus_latirostr
------------------------------------------MYRFRSQLFTEISAAATAHSHP---RRFSPLLLVEDSLLNRLPHRR-TS--KKCSSIG
>Anolis_carolinensis
------------------------------------------MYRFRSRASTGISAAATALPIIARLPQSHPLPRSGK----PASQRRFYSKKRSCSSIG
>Struthio_camelus_australis
------------------------------------------MHRFRSRAFTGISAFATDSS-FACFRQSPPLPRSGK----SASQRR-CSKKRSCSSIG
>Chelonia_mydas
------------------------------------------MHRFCSRAFTGISTATTALS-FACLRQSPPLPRSGK----SAPQRR-CSKKRSCSSIG
```

HG0021.1 ENSGALG00000009448 IFRD1

```
>Gallus_gallus
-----------------------------------------------------MHRLRSRAFTGISAIAASSSFACLRQSPPLPRPEKS----ASQRRW-NKKRSCSSIG
>Xenopus_laevis
-----------------------------------------------------MYRLHSQAFTGVSVTS----T--FRHFYPIPIED---SKLIPERRF---SKKCSSIG
>Apteryx_australis_mantelli
-----------------------------------------------------MHRFRSRAFTGISAFATDSSFACFRQSPPLPRSGKS----ASQRRC-SKKRSCSSIG
>Canis_lupus_familiaris
-----------------------------------------------------MYRFRSQLFTGISAAATAHSY--PRRFSPLLLAEDSPLSRPPHRRT---SKKCSSIG
>Hynobius_chinensis
-----------------------------------------------------MHRFRSQALTGVSSRC-------PPHTAPLPLGD---LHLIPERRF---SKKCSSIG
>Lipotes_vexillifer
-----------------------------------------------------MYRFRSQLFTGISAAATAHSY--PRRFSPVLLVENSPLSRPPHRRT---SKKCSSIG
>Calidris_pugnax
-----------------------------------------------------MHRLRSRAFTGISAFAAGSSSACFRQSPPLPRPEKS----ASLTRW-NKKRSCSSIG
>Rousettus_aegyptiacus
-----------------------------------------------------MCRFRSQLFTGISAAAAARSH--PRRSSPLSLATASPLSRPPHRRA---SRKCSSIG
>Dasypus_novemcinctus
-----------------------------------------------------MCRFRSQLFTGISATATTHSY--PRRFSPLLLVEDSPLSRPPHRRT---SKKCSSIG
>Alligator_mississippiensis
-----------------------------------------------------MHRFRSRASTGISAAS-ASSIACRRQSPPLPRPTKP----AAHRRC-SKKRSCFSIG
>Cuculus_canorus
-----------------------------------------------------MHRLRSRASTGISAFAADSSSSCFRQSPPLPRPGKS----TSQKRW-NKKRSCSSIG
>Sarcophilus_harrisii
-----------------------------------------------------MYRFRSQLFTGISAAAIAHSS--PRRFSPLLLAEDLSLSQPPHRRT---SKNCSSIG
>Galeopterus_variegatus
-----------MCRDWLVRVRVPPTCGDDRRRSCYLRRRGLDRPALAPALERNMYRFRSQLFTGISAAATAHSY--PRRFSPLLLAEDSPLSRPPHRRT---SKKCSSIG
>Monodelphis_domestica
-----------------------------------------------------MYRFRSQLFTGISAAAIAYSS--PRRFSPLLLAEDLSLSHPPHRRT---SKKCSSIG
>Falco_peregrinus
-----------------------------------------------------MHRLRSRASTGISAFAAGSSSACFRQSPPLPRFGKS----ASQRRW-SKKRSCSSIG
>Sorex_araneus
-----------------------------------------------------MYRFRSQLFTGISAAATAHSH--PRRFSPLLLAEDSPLCRPPHRRT---SKKCSSIG
>Ochotona_princeps
-----------------------------------------------------MYRFRSQLFTGISAAATAHSY--PRRFSPLLLAQDSPLSRPTHRRT---SKKCSSIG
>Elephantulus_edwardii
-----------------------------------------------------MHRFRSQLFTGISAAATAHSH--PRRFSPLLLAEDSPLSLQPHRRT---SKKCSSIG
>Junco_hyemalis
-----------------------------------------------------MHRLRSRASTGISAFAAGSPSACSRQSPPLPRPEKS----ASQRRW-SKKRSCSSIG
>Ceratotherium_simum_simum
MSRAKWDSGGECYGYWLGRVRVPPTDGDDRRRSCYLRRRGLDRPALAPALKRNMYRFRLQLFTGISAAATARSY--PRRFSPLLLAEDSPLSRRPHRRT---SKKCSSIG
>Picoides_pubescens
-----------------------------------------------------MHRLRSRASTGISAFAACLSSVCFRQSPPLPRTGKS----TSQRRW-SKKPSCSSIG
>Otolemur_garnettii
-----------------------------------------------------MYRFRSQLFTGISATATAHSY--PRRFSPLLLTEDSPLSRPPHRRT---SKKCSSIG
>Melopsittacus_undulatus
-----------------------------------------------------MHRLRSRASTGISAFAASSSSACFRQSPPLPRTAKS----TSQRRW-SKKRSCSSIG
>Marmota_monax
-----------------------------------------------------MYRFRSQLFTGISAAATAHFY--PRRFSPLLLAEDSPLSRPPHRRT---SRKCSSIG
>Tupaia_chinensis
-----------------------------------------------------MYRFRSQLFTGISAAATAHSY--PRRFSPLLLAEDSSLSRPPHRRT---SKKCSSIG
>Takydromus_sexlineatus
-----------------------------------------------------MYRFRSRASTGISAAATALPIACFPQSHPLPRSGKP----ASQRRFYNKKRSCSSIG
>Struthio_camelus_australis
-----------------------------------------------------MHRFRSRAFTGISAFATDSSFACFRQSPPLPRSGKS----ASQRRC-SKKRSCSSIG
>Trachemys_scripta
-----------------------------------------------------MHRFCSRAFTGISTATTDSSFACLRQSPPLPRSGKS----APQRRC-SKKRSCSSIG
```

HG0021.2 ENSDARG00000036811 ifrd2

>Danio_rerio
MVESGAATCTILRPKHFQKHLPSQPGIRHRLPGDNHR
>Acipenser_sinensis
MVEFTRATCSDIQ--LLQTACLSQPRIRYILPGDNHR
>Menidia_menidia
MVDLATVACSELRPKHFEKYLPSQPGVRYRLPGDNHR
>Oryzias_latipes
MVDLAKGACSKLRHKHFETYLTSQLSVRYRLPGDNHR
>Trachinotus_blochii
MVDFATAACSKLRPKHFKKYLQSQPGSRYRLPGDNHR
>Pygocentrus_nattereri
MVESAAATCTILRPKHFEKSLPSQPGIRYRLPGDKHR
>Oreochromis_niloticus
MVDLATTACSKLRPKRFEKYLPSHPSIRYRLPGDNHR
>Alosa_alosa
MVESAAATCTIPRPKLSEKYLSSQPGIRYWLPGDNHR
>Cyprinodon_variegatus
MVDLATAACSKLRPKHFEKYLPSQPGVRYRLPGDNHR
>Esox_lucius
MVEFGTATCSILLPKHFEKYCISQPGIRYRLPGDNHR
>Gadus_morhua
MVDFATATCSKLRPNYLLISLSSQPGIKYRLPGYNQR
>Acanthogobius_hasta
MVISETAACSKLRPKHCPRTSLSQPGIRYRLPGDNHR
>Plecoglossus_altivelis
MVEFATATCSILRPKHFEKYLPSQPGIRYMLPGDNHR
>Scleropages_formosus
MVEFAAATCAILRPKHFENYIPSQPGIRYWLPGDNHR
>Lateolabrax_japonicus
MVDFATTSCSELRPKHFGKDLLSQPGIRFRLPGDTHR
>Trematomus_bernacchii
MVDLATTACSLLRPKHFGKTFISPPGIRYRLPGDNHR
>Cynoglossus_semilaevis
MVDFVTAACSKLRPKLSEKISLSQPGIRYRLPGDNHR
>Salmo_salar
MVEFETVMCSILCPKHFEKYFLSQPGIRYRLPGDNHR
>Lepisosteus_oculatus
MVDLGAAACTILRPKRSEKYLPSQPGIRYRLPGDNHR
>Ictalurus_punctatus
MVEAAAATCTNLRPKHFGKYLPSQPGIRYWLPGDKHR
>Takifugu_rubripes
MVDLATAACSKLRPKHFEKYLLSQPGSRYRLPGDNHR

HG0022 FBgn0061359 CG33671

```
>Drosophila_melanogaster
MSKYDSKYLEEKLKRELQTEYVSVTDESDGCGGKFSAVIVSPAFS-GKTLLQKHRLVNSTLAEELKEIHAFSQKSYTPEEWEKVKAQ---
>Talitrus_saltator
MSEYTAEYIKAKLEKELDSTHVSVVDESDGCGGKFSAIIVSSAFQ-GKPLLQRHRLVNSTLAEELKQIHAFSMKTYTPEQWEQQQSKEQ-
>Meinertellus_cundinamarcensi
-MVFTEQYIREKLVKELEAVHVEVIDESDGCGAKFNVLIVSFLFE-GKPLLQRHRLVNSILEEELKTIHAFSQKTLTPEQWEKQKSS---
>Prorhinotermes_simplex
-MSYTADYIKSKLLKELEAVYVEVQDESDGCGAKFSVVIVSDKFD-GKPLLQRHRLVNSILEEELKSIHAFSQKTLTVQQWEKQKSS---
>Leptinotarsa_decemlineata
-MTYTEEHLRNKLIKELEASHVEVIDESDGCGGKFSCVIVSEVFK-GKPLLQRHRLVNSVLSEELKSIHAFSQKTYTPEQWEQQK-----
>Farfantepenaeus_aztecus
MTTYTEDYIRDKLTKELDAKHVSVEDMSDGCGAKFNVVVVSPKFE-GKPLLQRHRLVNGALEEELKTIHAFSMKTLTPEQWEKQGQ----
>Meganyctiphanes_norvegica
MTSYTEEYIKEKLTRELEASHVAVEDMSDGCGAKFSVVIVSPKFS-GKPLLARHRLVNSTLEEELKEIHAFSMKTLTPEQWQQQQQ----
>Acyrthosiphon_pisum
-MTINEDLLKSKLTVALDASYVSVNDDSDGCGAKFSVVVVSDKFN-GKTLLQRHRMVNEVLAEELKEIHAFSQKTLTTEQWNNLNKV---
>Ganaspis_sp._G1
-MPYTAEHIRNKLITELKAEHVEVVDESDGCGGKFSAIIVSEQFK-GKPLLQRHRLVNAALAEELKDIHAFSQKTLTPEQWEKEQS----
>Triodia_sylvina
-MVYTEEYLKEKLVRGLDAVHVEVVDESDGCGGKFSVVVVSDKFQ-GKPLLARHRLVNSVLQEELKEIHAFTQKTYTIEQWSNQTNAVNE
>Mantis_religiosa
-MPLTEEYIKNKLMKELEATHVEVQDESDGCGAKYNVLIVSDKFE-GKPLLQRHRLVNAVLEEELKLVHAFSQRTLTPAQWEEQKAA---
>Boreus_hyemalis
-MKYTEEYIREKLIKELNAEHVEVTDDSDGCGGKFSAIVVASIFQ-GKALLARHKLVNAALAEELKTIHAFSQKTFTPQQWQEKNTS---
>Corydalus_cornutus
-MSVTEEQIKQKLLRELDATHVKVVDESDGCGAKFSVVIVSDKFV-GKSLLQRHRLVNTTLQEELKNIHAFSQKTFTIDQWEKENSSN--
>Conwentzia_psociformis
-MSYTEGHIKDKLTKELDAVHCEVIDESDGCGGKFSAIIVSDKFN-GKPLLQRHRLVNAVLQEELKSIHAFSQKTFTREQWEAAKKDSH-
>Cordulegaster_boltonii
MSVYTTNYIKEKLRTGVEATHVEVQDDSDGCGAKFSVLVVSPKFE-GLPLLQRHRLVNSALAEELPNIHAFSQKTFTPKQWDDQKNKDAN
>Teleogryllus_commodus
-MSYTEEYVKNKLLKELEATHVEVEDLSDGCGAKFNAIIVSSKFE-GKPLLQRHRLVNSILSEELKSIHAFSQKTMTPKEWEKQNSQ---
>Gasterosteus_aculeatus
------------------------------------MLGNTSKIKSRRQYKLITLQINSILEEELKTIHALTLKTLTPHQWETRQK----
>Menopon_gallinae
-MPYTKDYIQSKLEKELDPVYIKVEDESDGCGAKFSAIIVSPKFQ-GKPLLQRQRLVNSILSEELKDIHAFSQRTLTPEQWEKENS----
>Perla_marginata
-MPYTENDIKTKLIKELDATHVDVRDDSDGCGAKFSVVIVSEKFE-GKPLLARHRLVNSVLEEELKEIHAFSQKTLTPQQWDQQKSA---
>Liposcelis_bostrychophila
-MPYTEDYIQNKLQKELDAIYVKVEDQSDGCGAKFSAIIVSPKFE-GKPLLQRHRLVNSVLEEELKDIHAFSQRTLTPQQWEQQNSS---
>Ctenocephalides_felis
-MSYTKQYLEEKLKTQLQAEHVEVVDLSDGCGDKFSAVIVSSVFE-GKPLLARHKLVNSTLAEELKTIHAFSQKPLTPEQWKAQQ-----
>Thrips_palmi
-MSISVDHIKDKLSKELQPVHIEVVDESDGCGGKFNVIVVSDKFE-GKPLLQRHRLVNSILEEELKVIHAFSQKTLTPEQWAKQSSQ---
>Hydroptila_sp._AD-2013
--MVSEEEIRSKLLTQLEAAHVEVTDESDGCGGKFSAVIVSDKFI-GKPLLARHRLVNAALEEELKQIHAFSQKTFTVEQWEKSKAL---
>Thermobia_domestica
-MAYTEDYIKDKLIKELEAIHVEVEDISDGCGAKFNAVIVSPKFE-GKPLLQRHRLVNSVLEEELKTIHAFSQKTLTPEQWEKQKQT---
```

HG0023.1 ENSG00000175197 DDIT3

>Homo_sapiens
-MLKMSGW----QRQ-----------SQNQSWNLRRECSRRKCIFIHHHT
>Xenopus_laevis
-MINMSPKSLNVQPQTSS---RCRFIRQKRTDPTWRKCWRRKTIFIHHHT
>Ursus_americanus
-MLKMSGW----QPQ-----------SQNQSRNLRRECSRRKCIFIHHHT
>Hynobius_retardatus
-MVNMSLRSPSE-PSTAARPDPPRALQRPQAGCRRRKCWRTKTIFIHHHT
>Tursiops_truncatus
-MLKMSGW----QRQ-----------SQNQSRNLRRECSRRKCIFIHHHT
>Rousettus_aegyptiacus
-MLKMSGW----QRQ-----------SQNQSRKLRRECSRRKCIFIHHHT
>Dasypus_novemcinctus
MMLKMSGW----QRQ-----------SQSQSRNLRRECSRRKCIFIHHHT
>Alligator_mississippiensis
MQARMSR--------------GQRNRS-GDERWPRRKSSRRKCIFIHHHT
>Sarcophilus_harrisii
MMMKMTWW----QRQ-----------SQNQSRDPRRECSRRKSIFIHHHT
>Galeopterus_variegatus
-MLKMSGW----QRQ-----------SQNQSRNLRRECSRRKCIFIHHHT
>Haliaeetus_leucocephalus
MRSRMSG--------------GQRSGWSRGEPGPPRKASRRKCVLVDHHT
>Oryctolagus_cuniculus
---------------------------MANICVCSPQCSRRKCIFIHHHT
>Eogystia_hippophaecolus
-MLKMSGW----QRQ-----------SQNNSRNLRRECSRRKCIFIHHHT
>Parus_major
MRTRMNR--------------GQRPERGRGEPGPPRKASRRKCVLADHHT
>Ceratotherium_simum_simum
-MLKMSGW----QPQ-----------SQNQSRNLRRECSRRKCIFIHHHT
>Manis_javanica
-MLKMSGW----QRQ-----------SQNQSWNPRRECSRRKCIFIHHHT
>Loxodonta_africana
-MLKMSGW----QRQ-----------SQSQSRNLRRKCSRRKCIFIHHHT
>Chinchilla_lanigera
-MLKMSRW----QRQ-----------SQSQSWNLRRECSRRKCIFIHHHT
>Trichechus_manatus_latirostr
-MLKMNGW----QRQ-----------SQNQSRNLRRKCSRRKCIFIHHHT
>Trachemys_scripta
MRARMSR--------------RQRGSRGQGEQGPRRKSSRRKCVFIHHHT

HG0023.2 ENSDARG00000059836 ddit3

```
>Danio_rerio
MVNMSDQPSLQKHTQTLNQKQPRKRNNKKKRSYWDKISPTTHIQT
>Amia_calva
MVNMSYHQSTPENQNTANPRQSRKRNNKRKRICWGTPSPPTHRQT
>Kaupichthys_hyoproroides
MVNMSYHQSQQKYIHTPTPRQTRKRNNKRKRICWDKLSPTAHRQT
>Oryzias_melastigma
MVNMSYQQFLLKYLHS-SSRQSRRRANKRKRNHWDKLSPTHFQT-
>Pygocentrus_nattereri
MVNMSYQQPQQKYIHTPSPRETRKRHNKRKRNFWDKISPTTHNHT
>Haplochromis_burtoni
MVNMSYHQFLLKYLHS-NSRQSRRRANKRKRNYWDKLSPTNCQT-
>Alosa_alosa
MVNMCCHQSQLKYIHTPNPRQSRKRHNKRKRNCWDTLSPTTHRQT
>Austrofundulus_limnaeus
MVNMSDHQFLLKYLLS-ASRQSRRRANKRKRNYWDNISPTIFQT-
>Esox_lucius
MVNMSYHQSQLKYIHTPNPRQTRKRNNKRKRTCWDKLSPTPNCQT
>Apteronotus_leptorhynchus
MVNMSYHQSQQKYTHTPNPRQLRRRHNKRKRNFWDKISPTTHNHT
>Scleropages_formosus
MVNMSYQQSQLKYIHTPNPRQSKKRNNKRKRTFWDKLSPTTHQQT
>Notothenia_coriiceps
MVNMSYHQFLLKYPHS-SSRQSRRRTNKRKRDCWDKLSPTNWQT-
>Oncorhynchus_mykiss
MVNMSYHQSQLKYIHTPNPRQSRKRNNKRKRTCWDKLSPTTNCQT
>Lepisosteus_oculatus
MVNMSYHHSTPENKNSPNHRQSRKRNNKRKRIVSDTPTPPNHRQT
>Trichomycterus_areolatus
MVNMNYQQSQQKHIHSPSLRQSRKRHNKRKRNYWDTISPTTHNHT
```

HG0024.1 ENSDARG00000093406

```
>Danio_rerio
MGPPRCRSRRLLPERTEDFGRFVSLS
>Acipenser_sinensis
MGPPGSRNCCVTFERMDDFGWLVSLS
>Amia_calva
MGPPGSKPRCVSFERMEDFGWFVNLS
>Channa_argus
MGPPGDRTQHMSTERTEDSSWIVDLS
>Podocoryna_carnea
MGPPGSRNCCVTFERMEDFGWLVSLS
>Menidia_menidia
MGPPGDRTCHMTPERTEDSRWIVDLS
>Oryzias_latipes
MGSPGDKTGSMSTERTEDFSWIVDLS
>Astyanax_mexicanus
MGPPRGKSSRLLTERRENFSWFVHLS
>Haplochromis_burtoni
MGPPGDRKLHMSTERTEDLSWIVDLS
>Alosa_alosa
MGPPRSKSRRISPERTEDFCWFVTLS
>Kryptolebias_marmoratus
MGPPGDRTRHMSTKRTEDSRWIVDLS
>Esox_lucius
MGPPGDKPRRVSSERTEDFIWFVNLS
>Plecoglossus_altivelis
MGPPGDKPRRVSTERTEDFSWFVNLS
>Trematomus_bernacchii
MGPPGDRTGHMSTERTEDFCWIVDLS
>Cynoglossus_semilaevis
MGPPGDRRRHLLTERTEDFSMIVDLS
>Salmo_salar
MGPPGDNPRRVSSERTEDFSWFVNLS
>Lepisosteus_oculatus
MGPPGSKPRRVSFERMEDFGWFVNLS
>Ictalurus_punctatus
MGPPRSKSSRLLSERRENLNWFVSLS
>Takifugu_rubripes
MGPPADRTCYMSTERTEDFGWIVDLS
```

```
HG0024.2 ENSG00000112308 C6orf62

>Homo_sapiens
MDFRD-WSGVSCVLLHT
>Mustela_putorius_furo
MDFKD-WTGVSCVLLHT
>Tursiops_truncatus
MDFKD-WTDVSCVLLHT
>Pteropus_vampyrus
MDFKD-WTGVTCVLLHT
>Dasypus_novemcinctus
MDFKD-WTGVSCVLLHT
>Sarcophilus_harrisii
MDLKD-WAGVSCVLLYT
>Galeopterus_variegatus
MDFKD-WTGVSCILLHT
>Monodelphis_domestica
MDLKD-WAGVSCVLLYT
>Erinaceus_europaeus
MDFKD-WTDVSCVLLHT
>Oryctolagus_cuniculus
MDFED-WTGVVLSHT--
>Elephantulus_edwardii
MGFKDF-----------
>Phalacrocorax_carbo
---MD-WAGASCFLLYT
>Equus_asinus
MDSKD-WTGVSCVLLHT
>Manis_javanica
MDFKD-RTGVSFVLLHP
>Loxodonta_africana
MNFKDFWTGVSCVLLHT
>Dipodomys_ordii
MDFKD-WTGVSCVLLHT
>Orycteropus_afer_afer
MDFKDFWTGVSCVLLHT
```

HG0024.2 ENSGALG00000013628 C6orf62

```
>Gallus_gallus
MDWACASCFLLYT
>Anser_cygnoides
MDWACASCFLLYT
>Apteryx_australis_mantelli
MDWACASCFLLYT
>Buceros_rhinoceros_silvestri
MDWACASCFLLYT
>Caprimulgus_carolinensis
MDWACASCFLLYT
>Cariama_cristata
MDWACASCFLLYT
>Calidris_pugnax
MDWACASCFLLYT
>Pterocles_gutturalis
MDWACASCFLLYT
>Colius_striatus
MDWACASCFLLYT
>Columba_livia
MDWACASCFLLYT
>Leptosomus_discolor
MDWACASCFLLYT
>Alligator_sinensis
MDWACASCFLLYT
>Aquila_chrysaetos_canadensis
MDWACASCFLLYT
>Gavia_stellata
MDWACASCFLLYT
>Balearica_regulorum_gibberic
MDWACASCFLLYT
>Tauraco_erythrolophus
MDWACASCFLLYA
>Opisthocomus_hoazin
MDWVCASCSLSYT
>Taeniopygia_guttata
MDWACASCFLLYT
>Pelecanus_crispus
MDWACASCFLLYT
>Picoides_pubescens
MDWACASCFLLYT
>Fulmarus_glacialis
MDWACASCFLLYT
>Melopsittacus_undulatus
MDWACASCFLLYT
>Aptenodytes_forsteri
MDWACASCFLLYT
>Tyto_alba
MDWACASCFLLYT
>Struthio_camelus_australis
MDWACASCFLLYT
>Chrysemys_picta
MDWACASCFLLYT
>Tinamus_guttatus
MDWDCASCFLLFT
```

HG0024.3 ENSGALG00000013628 C6orf62

>Gallus_gallus
---------MAGQLLILFLDPSNTTFP-
>Anser_cygnoides
MVKPVCCPAMAGQLL-LFLDPSDTTFS-
>Apteryx_australis_mantelli
---------MVGQLLILFLDPSNKAFS-
>Buceros_rhinoceros_silvestri
---------MAGQLFSLFLDPSNTTFS-
>Caprimulgus_carolinensis
---------MAGQLLTLVFDPSDTTSS-
>Cariama_cristata
---------MAGQLLRLFLDPSDTTTFS
>Calidris_pugnax
---------MAGQLLTLFLDPSNTTFS-
>Pterocles_gutturalis
---------MAGQLLTLFRDPSDTTFS-
>Colius_striatus
---------MAGQLLILFRGPSDTTVS-
>Columba_livia
---------MAGQLLTLFLGPSDTTFS-
>Leptosomus_discolor
---------MTGQLLTLFLDPSDTTFS-
>Alligator_mississippiensis
---------MVGQRLILFLDPFNMTVS-
>Cuculus_canorus
---------MAGQLLTLFLDPSNTMFS-
>Falco_cherrug
---------MAGQLLTLFLDPSNTTFS-
>Gavia_stellata
---------MAGQLLSLFLDPSDTTFS-
>Chlamydotis_macqueenii
---------MAGQLLTLFLDPSDTTFS-
>Tauraco_erythrolophus
---------MAGQLLTLFLDPSDTTFS-
>Opisthocomus_hoazin
---------MAGRLLTLFLDPSNATFS-
>Corvus_cornix_cornix
---------MAGQLLSLFLDPSNTTFS-
>Pelecanus_crispus
---------MAGQLLTLFLDPSNTTFS-
>Picoides_pubescens
---------MAEQLLSWFLDPSDTTFS-
>Fulmarus_glacialis
---------MAGQLLTLFLDPSDTTFS-
>Nestor_notabilis
---------MAGQLLTLFLDPSERPFS-
>Aptenodytes_forsteri
---------MAGQLLTLFLDPSDTTLS-
>Anolis_carolinensis
---------MVGRLLILFYDPSNKTVS-
>Tyto_alba
---------MAGQLLTLFLDPSDTTFS-
>Struthio_camelus_australis
---------MVGQLLILFLDPSNTTFS-
>Chelonia_mydas
---------MVGQLHSLFLDPSNMIAS-
>Tinamus_guttatus
---------MVGQLLILFLDPSNTTFS-

HG0025 ENSDARG00000060054 epc1b

>Danio_rerio
---MRTLIGPAGNIRGSDRFMLHLEL
>Anguilla_anguilla
---MRTKTGKVVNFRGSDRFMLHLEL
>Podocoryna_carnea
---MITQAGTVLNFRGSDRFMLNLEL
>Oryzias_latipes
---MKAPLGTVVNIRRSDRFILQLEL
>Pygocentrus_nattereri
---MTALIGSVVNIRRSDRFMLHLEL
>Haplochromis_burtoni
---MKAPLGTVVNIRRSDRFILHLEL
>Clupea_harengus
---MRTKICTVVNRRGSDRYMLHLSL
>Kryptolebias_marmoratus
---MRTRSRPAVNLSRSDRFMLHLLL
>Esox_lucius
---MRTQISTVVHLRRSDRFMLHLVL
>Acanthogobius_hasta
MRPGLAAIGRGAVGAGPGAERSGSLLNVRHVVHGALKRRGAARSGASHKHSPPESDQSRPSPPRRRPTHFHSPTVDPDPPHTSMKAPLGSAGDLCTSDRFMSHLEL
>Apteronotus_leptorhynchus
---MTTLIGSVVNIRGSDRFMLHLEL
>Scleropages_formosus
---MRTLTGTVLNSRGSDRFILHLEL
>Notothenia_coriiceps
---MKAPLGTVVNIRRSDRFILHLEL
>Salvelinus_alpinus
---MKAQLETDVNLRRSDRFMLHLEF
>Lepisosteus_oculatus
---MRAQTGTVVNFRGSDRFMLHLEI
>Takifugu_rubripes
---MKALLGTVVNIRRSDRFILHLEL

HG0026 ENSDARG00000087059 fam213ab

>Danio_rerio
-----------MELV----TRAVGSVGLTV--IEA-LRSF-TELFLTQPVC-ATLTQLADTDLRTLD-----------GD-DRVFKARELWESSGAVIMAVRRPG
>Oxyacanthus_curtus
----------MSLVPEMLGSWGWLLTGGTLA-GIITYCNLPPRFWFGHVSKSNDIELLRDAPLQRLL-----------PAEGSVMTGQQLWKDTGAVVMAIRRPG
>Scapharca_broughtonii
----------MALIS----PTLLGIAGLTA--GAAILLNL-PASWLFSTKK-STLSYLESAKLQTLH-----------SSNNRNFSARELWKDKPAVIMAVRRPG
>Micropterus_dolomieu
MTFILEGELYNM--------WY-GALAFVATLMVVVALAN-TDLFLTKSAP-ASLEELGATDLQTTT-----------GD-GKTVKGQLLWERSGAVILAVRRPG
>Rhodactis_indosinensis
-----------MA-------WFLGAGGAVA--IAG-IVLA-HWDWYSNSAR-PDLAYLSSIKLKALN-----------GT-NKDVMASELWRESGAVIMVVRRPG
>Strongylocentrotus_purpuratu
------------------MALLWGAAAAAV--GG-LLVAN-MDFWLPRGGAATTEAYLAGAVLQLLGETKLQAQLSLVDEAVRTFKAKDLWKDTGAVVLAVRRPG
>Meganyctiphanes_norvegica
--MLLDGGLSIMALSTMLPNWPWLVTGALT--GFVAYCNL-PAYWLNAPQK-ATTEYLSGAPLMKLD-----------GS-GGQILSNELWQKNGALVMAVRRPG
>Perna_viridis
-----------MVFS----KALLGVAGFTA--GAAILINL-PTRFFMASEK-ATLSYLTTAKLQTID-----------EA-KKNFSASDLWKNNGIVMMAVRRPG
>Octopus_bimaculoides
--MAEK-ATLAYLQGATLKTLG-----------DD-PKEFGAGELWKNNGAIIMVVRRPG
>Saccostrea_glomerata
-----------MGIS----PVLVGFAGLTA--GIAVIGNL-PVTWFTTVEK-ATLKYLEKGKLQTIE-----------AS-PRIFSVKDLWKDHGVVIMAVRRPG
>Pecten_maximus
-----------MGVT----KALLGMAGVTA--MAATLCNV-PVSWFMSSER-ATLNYLENARVQTLD-----------SQ-PKNITASELWKKNGVVMMAVRRPG
>Anoplopoma_fimbria
--------------M----MEIIAAVGGFI--TDV-MNFF-TDKFLTPPLK-ATLKHLEEAELKTLK-----------GE-TRTLKGRSLWERSGAVIMAVRRPG
>Montastraea_faveolata
-----------MA-------WFLGAGGVAA--LTG-VIIA-NWDWYKNSPR-PDLAYLSSIKLKALD-----------GT-GKEVMASELWSKAGAVIMVVRRPG
>Sepia_officinalis
--MAEK-ATVGYLAGATLKTLG-----------DD-PKEFGAAELWRKSGAVIMVVRRPG
>Sepioloidea_lineolata
--MAEK-ATVGYLAGATLKTLG-----------DD-PKEFGAAELWKNTGAVIMVVRRPG
>Doryteuthis_pealeii
--MAEK-ATVGYLAGATLKTLG-----------DD-PKEFGAAELWKKTGAVIMVVRRPG

HG0027 ENSG00000077254 USP33

>Homo_sapiens
---------------------------MERNNRYQPLQKHAKL------------------
>Channa_argus
MLREFVTTRPALEELMEEALIWGGGVGRGRKNRHQPLQKHTKVERPLAL------------
>Millepora_alcicornis
---------------------------MERKNWYQQLQKHTKI------------------
>Latrodectus_hesperus
---------------------------MERNNWYQPLQKHTKL------------------
>Hynobius_retardatus
MLREFITTRPALQELLKEALN------MERKDHYQPLQKHTEVHRPVTL------------
>Chironomus_riparius
MLRDFVTTRPALQELLKEALN------MERNNWYQPLQKHTKCKDHRHYEETASTNGKITS

HG0028.1 ENSDARG00000032103 mapk6

>Danio_rerio
---MEDSFDCDCGELEPPDH
>Channa_argus
---MEDSFDCDCGELEPPDH
>Kaupichthys_hyoproroides
---MEDCFDCDCGELEPPDH
>Anas_platyrhynchos
---MDGSFDCDCGELEPPDH
>Microhyla_fissipes
---MDASFDCDCGELEPPDH
>Chaetura_pelagica
---MDGSFDCDCGELEPPDH
>Apteryx_australis_mantelli
-------------------------------------MYWDIDL----------------FSLLFRLSRNKMDGSFDCDCGELEPPDH
>Porichthys_notatus
MELYLADTDTWSRIRHRPDCNHTHRETDKELLDRTDPFLLSADSSTPSLMDSYSLHQDGSDGFWPRLYSSKMEDSFDCDCGELEPPDH
>Oryzias_latipes
---MEDSFDCDCGELEPPDH
>Buceros_rhinoceros_silvestri
---MDGSFDCDCGELEPPDH
>Caprimulgus_carolinensis
---MDGSFDCDCGELEPPDH
>Trachinotus_blochii
---MEDSFDCDCGELEPPDH
>Cariama_cristata
---MDGSFDCDCGELEPPDH
>Canis_lupus_familiaris
---MDGSFDCDCGELEPPDH
>Hynobius_retardatus
---MDSSFDCDCGELEPPDH
>Micropterus_floridanus
---MEDSFDCDCGELEPPDH
>Tursiops_truncatus
---MDGSFDCDCGELEPPDH
>Pygocentrus_nattereri
---MEDSFDCDCGELEPPDH
>Charadrius_vociferus
---MDGSFDCDCGELEPPDH
>Callorhinchus_milii
---MDDSFDCDCGELEPPDH
>Rousettus_aegyptiacus
---------------------MPLKVS-LD----INFFLWVQDDLLRE---KINSGQ-NGAQTASGLYRSKMDGSFDCDCGELEPPDH
>Haplochromis_burtoni
---MLYPSKMEDSFDCDCGELEPPDH
>Pterocles_gutturalis
---MDGSFDCDCGELEPPDH
>Dasypus_novemcinctus
---MDGSFDCDCGELEPPDH
>Clupea_harengus
---MEDSFDCDCGELEPPDH
>Latimeria_chalumnae
---MDDSFDCDCGELEPPDH
>Colius_striatus
---MDGSFDCDCGELEPPDH
>Columba_livia
---MDGSFDCDCGELEPPDH
>Leptosomus_discolor
---MDGSFDCDCGELEPPDH
>Alligator_mississippiensis
---MDGSFDCDCGELEPPDH
>Cuculus_canorus
---MDGSFDCDCGELEPPDH
>Kryptolebias_marmoratus
---MEDSFDCDCGELEPPDH
>Sarcophilus_harrisii
---MDGSFDCDCGELEPPDH
>Galeopterus_variegatus
---MDGSFDCDCGELEPPDH
>Monodelphis_domestica
---MDGSFDCDCGELEPPDH
>Esox_lucius
---MEDSFDCDCGELEPPDC
>Haliaeetus_albicilla
---MDGSFDCDCGELEPPDH
>Gallus_gallus
---MDGSFDCDCGELEPPDH
>Gavia_stellata
---MDGSFDCDCGELEPPDH
>Acanthogobius_hasta
---MEDSFDCDCGELEPPDH
>Balearica_regulorum_gibberic
---MDGSFDCDCGELEPPDH
>Apteronotus_leptorhynchus
---MEDSFDCDCGELEPPDQ
>Condylura_cristata
---MDGSFDCDCGELEPPDH
>Oryctolagus_cuniculus
---MDGSFDCDCGELEPPDH
>Elephantulus_edwardii
---MDGSFDCDCGELEPPDH
>Ornithorhynchus_anatinus
---MDGSFDCDCGELEPPDQ
>Tauraco_erythrolophus
---MDGSFDCDCGELEPPDH
>Opisthocomus_hoazin
---MDGSFDCDCGELEPPDH
>Scleropages_formosus
---MEDSFDCDCGELEPPDH
>Lepidothrix_coronata
---MDGSFDCDCGELEPPDH
>Egretta_garzetta
---MDGSFDCDCGELEPPDH
>Notothenia_coriiceps
---MEDSFDCDCGELEPPDH
>Equus_asinus
----MAATAA-KREPRRRRCRQPSRRPEPT----LGAV----R--GPR---TPSMSR-RSPVSVCVLYQSKMDGSFDCDCGELEPPDH
>Manis_javanica
---MDGSFDCDCGELEPPDH
>Picoides_pubescens
---MDGSFDCDCGELEPPDH
>Cynoglossus_semilaevis
---MEDSFDCDCGELEPPDH
>Aotus_nancymaae
----MAATAA-KREPRRRRCRQHSRRPEPT----LGAVR------GLR---TLSMNR-RSPVSVCVLYQSKMDGSFDCDCGELEPPDH
>Loxodonta_africana
---MDGSFDCDCGEVEPPDH
>Fulmarus_glacialis
---MDGSFDCDCGELEPPDH
>Melopsittacus_undulatus
---MDGSFDCDCGELEPPDH
>Mus_musculus
----MAATAA-KREPRKRRCRQHSRRPEPT----LRAVR------GPC---TLSMSR-RSP--VSVLYQSKMDGSFDCDCGELEPPDH
>Oncorhynchus_kisutch
---MEDSFDCDCGELEPPDC
>Tupaia_chinensis
---MDGSFDCDCGELEPPDH
>Lepisosteus_oculatus
---MEDSFDCDCGELEPPDH
>Ictalurus_punctatus
---MEDSFDCDCGELEPPDH
>Trichechus_manatus_latirostr
---MDGSFDCDCGELEPPDH
>Pygoscelis_adeliae
---MDGSFDCDCGELEPPDH
>Squalus_acanthias
---MDDSFDCDCGELEPPDH
>Hypsiglena_sp._JMG-2014
---MDGSFDCDCGELEPPDH
>Tyto_alba
---MDGSFDCDCGELEPPDH
>Struthio_camelus_australis
---MDGSFDCDCGELEPPDH
>Pelodiscus_sinensis
--MMAGTVLRLSRNKMDGSFDCDCGELEPPDH
>Takifugu_rubripes
---MEDSFDCDCGELEPPDH
>Tinamus_guttatus
---MDGSFDCDCGELEPPDH
>Calypte_anna
---MDGSFDCDCGELEPPDH
>Apaloderma_vittatum
---MDGSFDCDCGELEPPDH
>Orycteropus_afer_afer
---MDGSFDCDCGELEPPDH

HG0028.1 ENSG00000069956 MAPK6

>Homo_sapiens
---MDGSFDCDCGELEPPDH
>Channa_argus
---MEDSFDCDCGELEPPDH
>Kaupichthys_hyoproroides
---MEDCFDCDCGELEPPDH
>Anas_platyrhynchos
---MDGSFDCDCGELEPPDH
>Xenopus_laevis
---MDGSFDCDCGELEPPDH
>Chaetura_pelagica
---MDGSFDCDCGELEPPDH
>Apteryx_australis_mantelli
---MYWDIDLFSLLFRLSRNKMDGSFDCDCGELEPPDH
>Porichthys_notatus
---------------MELYLADTDTWSRIRHRPDCNHTHRETDKELL----DRTDPFL-----------LSADSSTPSLMDSYSLHQDGSD--GFWPRLYSSKMEDSFDCDCGELEPPDH
>Oryzias_latipes
---MEDSFDCDCGELEPPDH
>Buceros_rhinoceros_silvestri
---MDGSFDCDCGELEPPDH
>Caprimulgus_carolinensis
---MDGSFDCDCGELEPPDH
>Trachinotus_blochii
---MEDSFDCDCGELEPPDH
>Cariama_cristata
---MDGSFDCDCGELEPPDH
>Ailuropoda_melanoleuca
---MDGSFDCDCGELEPPDH
>Hynobius_retardatus
---MDSSFDCDCGELEPPDH
>Micropterus_floridanus
---MEDSFDCDCGELEPPDH
>Tursiops_truncatus
---MDGSFDCDCGELEPPDH
>Pygocentrus_nattereri
---MEDSFDCDCGELEPPDH
>Charadrius_vociferus
---MDGSFDCDCGELEPPDH
>Callorhinchus_milii
---MDDSFDCDCGELEPPDH
>Miniopterus_natalensis
---MDGSFDCDCGELEPPDH
>Haplochromis_burtoni
---MLYPSKMEDSFDCDCGELEPPDH
>Pterocles_gutturalis
---MDGSFDCDCGELEPPDH
>Dasypus_novemcinctus
---MDGSFDCDCGELEPPDH
>Clupea_harengus
---MEDSFDCDCGELEPPDH
>Latimeria_chalumnae
---MDDSFDCDCGELEPPDH
>Colius_striatus
---MDGSFDCDCGELEPPDH
>Columba_livia
---MDGSFDCDCGELEPPDH
>Leptosomus_discolor
---MDGSFDCDCGELEPPDH
>Alligator_mississippiensis
---MDGSFDCDCGELEPPDH
>Cuculus_canorus
---MDGSFDCDCGELEPPDH
>Sinocyclocheilus_rhinocerous
MTPVRRLTKWLSAGRPSFPSHTTATWLNH-PQPLCLQTQTGTRANPLHMCYEHCGVFYSLQEKQESMTSDSVDTSELGFMNNHTQETLPKR--AVMSVVSQCKMEDSFDCDCGELEPPDH
>Poecilia_formosa
--MTKSKTSHGNS--TGYHRLYPSKMEDSFDCDCGELEPPDH
>Sarcophilus_harrisii
---MDGSFDCDCGELEPPDH
>Galeopterus_variegatus
---MDGSFDCDCGELEPPDH
>Monodelphis_domestica
---MDGSFDCDCGELEPPDH
>Esox_lucius
---MEDSFDCDCGELEPPDC
>Haliaeetus_albicilla
---MDGSFDCDCGELEPPDH
>Coturnix_japonica
---MDGSFDCDCGELEPPDH
>Gavia_stellata
---MDGSFDCDCGELEPPDH
>Acanthogobius_hasta
---MEDSFDCDCGELEPPDH
>Balearica_regulorum_gibberic
---MDGSFDCDCGELEPPDH
>Apteronotus_leptorhynchus
---MEDSFDCDCGELEPPDQ
>Condylura_cristata
---MDGSFDCDCGELEPPDH
>Oryctolagus_cuniculus
---MDGSFDCDCGELEPPDH
>Elephantulus_edwardii
---MDGSFDCDCGELEPPDH
>Ornithorhynchus_anatinus
---MDGSFDCDCGELEPPDQ
>Tauraco_erythrolophus
---MDGSFDCDCGELEPPDH
>Opisthocomus_hoazin
---MDGSFDCDCGELEPPDH
>Scleropages_formosus
---MEDSFDCDCGELEPPDH
>Manacus_vitellinus
---MDGSFDCDCGELEPPDH
>Egretta_garzetta
---MDGSFDCDCGELEPPDH
>Notothenia_coriiceps
---MEDSFDCDCGELEPPDH
>Equus_asinus
-----------------------------------MAATAAKREPRRRRCR----------------QPSRRPEPTLGAVRGPRTPSMSRRSPVSVCVLYQSKMDGSFDCDCGELEPPDH
>Manis_javanica
---MDGSFDCDCGELEPPDH
>Picoides_pubescens
---MDGSFDCDCGELEPPDH
>Platichthys_stellatus
---MEDSFDCDCGELEPPDH
>Loxodonta_africana
---MDGSFDCDCGEVEPPDH
>Fulmarus_glacialis
---MDGSFDCDCGELEPPDH
>Melopsittacus_undulatus
---MDGSFDCDCGELEPPDH
>Mus_musculus
-----------------------------------MAATAAKREPRKRRCR----------------QHSRRPEPTLRAVRGPCTLSMSRRSPVS--VLYQSKMDGSFDCDCGELEPPDH
>Oncorhynchus_kisutch
---MEDSFDCDCGELEPPDC
>Tupaia_chinensis
---MDGSFDCDCGELEPPDH
>Lepisosteus_oculatus
---MEDSFDCDCGELEPPDH
>Ictalurus_punctatus
---MEDSFDCDCGELEPPDH
>Trichechus_manatus_latirostr
---MDGSFDCDCGELEPPDH
>Aptenodytes_forsteri
---MDGSFDCDCGELEPPDH
>Squalus_acanthias
---MDDSFDCDCGELEPPDH
>Python_bivittatus
---MVCF----------------QTLR-------SQHG-----IQATTSHLQEGFSQNKMDGSFDCDCGELEPPDH
>Tyto_alba
---MDGSFDCDCGELEPPDH
>Struthio_camelus_australis
---MDGSFDCDCGELEPPDH
>Chrysemys_picta
---MDGSFDCDCGELEPPDH
>Takifugu_rubripes
---MEDSFDCDCGELEPPDH
>Tinamus_guttatus
---MDGSFDCDCGELEPPDH
>Calypte_anna
---MDGSFDCDCGELEPPDH
>Apaloderma_vittatum
---MDGSFDCDCGELEPPDH
>Orycteropus_afer_afer
---MDGSFDCDCGELEPPDH

HG0028.1 ENSGALG00000031448 MAPK6

>Gallus_gallus
---MDGSFDCDCGELEPPDH
>Channa_argus
---MEDSFDCDCGELEPPDH
>Kaupichthys_hyoproroides
---MEDCFDCDCGELEPPDH
>Anas_platyrhynchos
---MDGSFDCDCGELEPPDH
>Xenopus_laevis
---MDGSFDCDCGELEPPDH
>Chaetura_pelagica
---MDGSFDCDCGELEPPDH
>Apteryx_australis_mantelli
---MYWDIDLFSLLFRLSRNKMDGSFDCDCGELEPPDH
>Porichthys_notatus
---------------MELYLADTDTWSRIRHRPDCNHTHRETDKELL----DRTDPFL-----------LSADSSTPSLMDSYSLHQDGS--DGFWPRLYSSKMEDSFDCDCGELEPPDH
>Oryzias_latipes
---MEDSFDCDCGELEPPDH
>Buceros_rhinoceros_silvestri
---MDGSFDCDCGELEPPDH
>Caprimulgus_carolinensis
---MDGSFDCDCGELEPPDH
>Trachinotus_blochii
---MEDSFDCDCGELEPPDH
>Cariama_cristata
---MDGSFDCDCGELEPPDH
>Ailuropoda_melanoleuca
---MDGSFDCDCGELEPPDH
>Hynobius_retardatus
---MDSSFDCDCGELEPPDH
>Micropterus_floridanus
---MEDSFDCDCGELEPPDH
>Tursiops_truncatus
---MDGSFDCDCGELEPPDH
>Pygocentrus_nattereri
---MEDSFDCDCGELEPPDH
>Charadrius_vociferus
---MDGSFDCDCGELEPPDH
>Callorhinchus_milii
---MDDSFDCDCGELEPPDH
>Miniopterus_natalensis
---MDGSFDCDCGELEPPDH
>Haplochromis_burtoni
---MLYPSKMEDSFDCDCGELEPPDH
>Pterocles_gutturalis
---MDGSFDCDCGELEPPDH
>Dasypus_novemcinctus
---MDGSFDCDCGELEPPDH
>Clupea_harengus
---MEDSFDCDCGELEPPDH
>Latimeria_chalumnae
---MDDSFDCDCGELEPPDH
>Colius_striatus
---MDGSFDCDCGELEPPDH
>Columba_livia
---MDGSFDCDCGELEPPDH
>Leptosomus_discolor
---MDGSFDCDCGELEPPDH
>Alligator_mississippiensis
---MDGSFDCDCGELEPPDH
>Cuculus_canorus
---MDGSFDCDCGELEPPDH
>Sinocyclocheilus_rhinocerous
MTPVRRLTKWLSAGRPSFPSHTTATWLNH-PQPLCLQTQTGTRANPLHMCYEHCGVFYSLQEKQESMTSDSVDTSELGFMNNHTQETLPK--RAVMSVVSQCKMEDSFDCDCGELEPPDH
>Poecilia_formosa
--MTKSKTSHGN--STGYHRLYPSKMEDSFDCDCGELEPPDH
>Sarcophilus_harrisii
---MDGSFDCDCGELEPPDH
>Galeopterus_variegatus
---MDGSFDCDCGELEPPDH
>Monodelphis_domestica
---MDGSFDCDCGELEPPDH
>Esox_lucius
---MEDSFDCDCGELEPPDC
>Haliaeetus_albicilla
---MDGSFDCDCGELEPPDH
>Gavia_stellata
---MDGSFDCDCGELEPPDH
>Acanthogobius_hasta
---MEDSFDCDCGELEPPDH
>Balearica_regulorum_gibberic
---MDGSFDCDCGELEPPDH
>Apteronotus_leptorhynchus
---MEDSFDCDCGELEPPDQ
>Condylura_cristata
---MDGSFDCDCGELEPPDH
>Oryctolagus_cuniculus
---MDGSFDCDCGELEPPDH
>Elephantulus_edwardii
---MDGSFDCDCGELEPPDH
>Ornithorhynchus_anatinus
---MDGSFDCDCGELEPPDQ
>Tauraco_erythrolophus
---MDGSFDCDCGELEPPDH
>Opisthocomus_hoazin
---MDGSFDCDCGELEPPDH
>Scleropages_formosus
---MEDSFDCDCGELEPPDH
>Manacus_vitellinus
---MDGSFDCDCGELEPPDH
>Egretta_garzetta
---MDGSFDCDCGELEPPDH
>Notothenia_coriiceps
---MEDSFDCDCGELEPPDH
>Equus_asinus
-----------------------------------MAATAAKREPRRRRCRQPS----------------RRPEPTLGAVRGPRTPSMSRRSPVSVCVLYQSKMDGSFDCDCGELEPPDH
>Manis_javanica
---MDGSFDCDCGELEPPDH
>Picoides_pubescens
---MDGSFDCDCGELEPPDH
>Platichthys_stellatus
---MEDSFDCDCGELEPPDH
>Aotus_nancymaae
-----------------------------------MAATAAKREPRRRRCRQHS----------------RRPEPTLGAVRGLRTLSMNRRSPVSVCVLYQSKMDGSFDCDCGELEPPDH
>Loxodonta_africana
---MDGSFDCDCGEVEPPDH
>Fulmarus_glacialis
---MDGSFDCDCGELEPPDH
>Melopsittacus_undulatus
---MDGSFDCDCGELEPPDH
>Mus_musculus
-----------------------------------MAATAAKREPRKRRCRQHS----------------RRPEPTLRAVRGPCTLSMSRRSPVS--VLYQSKMDGSFDCDCGELEPPDH
>Oncorhynchus_kisutch
---MEDSFDCDCGELEPPDC
>Tupaia_chinensis
---MDGSFDCDCGELEPPDH
>Lepisosteus_oculatus
---MEDSFDCDCGELEPPDH
>Ictalurus_punctatus
---MEDSFDCDCGELEPPDH
>Trichechus_manatus_latirostr
---MDGSFDCDCGELEPPDH
>Aptenodytes_forsteri
---MDGSFDCDCGELEPPDH
>Squalus_acanthias
---MDDSFDCDCGELEPPDH
>Python_bivittatus
---MVCFQT-----------------------LRSQHGI-----QATTSHLQEGFSQNKMDGSFDCDCGELEPPDH
>Tyto_alba
---MDGSFDCDCGELEPPDH
>Struthio_camelus_australis
---MDGSFDCDCGELEPPDH
>Chrysemys_picta
---MDGSFDCDCGELEPPDH
>Takifugu_rubripes
---MEDSFDCDCGELEPPDH
>Tinamus_guttatus
---MDGSFDCDCGELEPPDH
>Calypte_anna
---MDGSFDCDCGELEPPDH
>Apaloderma_vittatum
---MDGSFDCDCGELEPPDH
>Orycteropus_afer_afer
---MDGSFDCDCGELEPPDH

HG0028.2 ENSG00000069956 MAPK6

```
>Homo_sapiens
MFIEACCVHIYSHFC--------
>Anser_cygnoides
MLIEAYYVHIYSHFC--------
>Rhacophorus_omeimontis
MMIEAYILHIYSHFCSMSTCFAH
>Chaetura_pelagica
MLIEAYYVHIYSHFC--------
>Apteryx_australis_mantelli
MLIEAYYVHIYSHFC--------
>Buceros_rhinoceros_silvestri
MLIEAYYVHIYSHFC--------
>Caprimulgus_carolinensis
MLIEAYYVHIYSHFC--------
>Cariama_cristata
MLIEAYYVHIYSHFC--------
>Acinonyx_jubatus
MFIEACCVHIYSHFC--------
>Balaenoptera_acutorostrata_s
MFIEACCVHIYSHFC--------
>Calidris_pugnax
MLIEAYYVHIYSHFC--------
>Eptesicus_fuscus
MFIEACCVHIYSHFC--------
>Pterocles_gutturalis
MLIEAYYVHIYSHFC--------
>Dasypus_novemcinctus
MFIEACCVHIYSHFC--------
>Colius_striatus
MLIEAYYVHIYSHFC--------
>Columba_livia
MLIEAYYVHIYSHFC--------
>Leptosomus_discolor
MLIEAYYVHIYSHFC--------
>Alligator_sinensis
MLIEAYYVHIYSYFC--------
>Cuculus_canorus
MLIEAYYVHIYSHFC--------
>Sarcophilus_harrisii
MFREAYCVHIYSLFC--------
>Galeopterus_variegatus
MFIEACCVHIYSHFC--------
>Aquila_chrysaetos_canadensis
MLIEAYYVHIYSHFC--------
>Coturnix_japonica
MLIEAYYVHIYSHFC--------
>Gavia_stellata
MLIEAYYVHIYSHFC--------
>Chlamydotis_macqueenii
MFIEAYYVHIYSHFC--------
>Condylura_cristata
MFIEACCVHIYSHFC--------
>Oryctolagus_cuniculus
MFIEACCVHIYSHFC--------
>Elephantulus_edwardii
MSIEACCVHIYSHFC--------
>Ornithorhynchus_anatinus
MLIEACFVHIYSHFC--------
>Tauraco_erythrolophus
MLKEAYYVHIYSHFC--------
>Opisthocomus_hoazin
MLIETYYVHIYSHFC--------
>Acanthisitta_chloris
MLIEAYYVHIYSHFC--------
>Egretta_garzetta
MLIEAYYVHIYSHFC--------
>Ceratotherium_simum_simum
MFIEACCVHIYSHFC--------
>Picoides_pubescens
MLIEAYYVHIYSHFC--------
>Loxodonta_africana
MFIEACCVHIYSHFC--------
>Fulmarus_glacialis
MLIEAYYVHIYSHFC--------
>Melopsittacus_undulatus
MLIEAYYVHIYSHFC--------
>Marmota_marmota_marmota
MFIEACCVHIYSHFC--------
>Tupaia_chinensis
MFLEACCVHIYSHFC--------
>Trichechus_manatus_latirostr
MFIEARCVHIYSRFC--------
>Pygoscelis_adeliae
MLIEAYYVHIYSHFC--------
>Takydromus_sexlineatus
MLIEAYYVHIYSHFC--------
>Tyto_alba
MLIEAYYVHIYSHFC--------
>Struthio_camelus_australis
MLIEAYYVHIYSHFC--------
>Pelodiscus_sinensis
MLLEAHYVHIYSHFC--------
>Tinamus_guttatus
MLIEAYYVHIYSHFC--------
>Calypte_anna
MLIEAYYVHIYSHFC--------
>Apaloderma_vittatum
MLIEAYYVHIYSHFC--------
>Orycteropus_afer_afer
MFIEACCVHIYSHFC--------
```

HG0029 ENSDARG00000007523 kmt2e

>Danio_rerio
--MHLDVCNECCG------------------
>Amia_calva
--MHLDVCNEHCGWHAPVFWIQMLRTQ----
>Channa_punctata
--MHLDVCNDCCGWHASVFWTPILWTQ----
>Xenopus_tropicalis
--MHLDVCNEHCGWHAPVFWIPMQRTP----
>Apteryx_australis_mantelli
--MGLWMHLDVCNEHCGWHAPVFWIPMHRTP----
>Menidia_menidia
--MHLDVCNECCGWHASVFWTPFLWTQLDKN
>Porichthys_notatus
--MHLDVCNECCGWHASVFWTSILWTQ----
>Oryzias_melastigma
--MHLDVCNDCCGWHASVFWTPFLWTQ----
>Canis_lupus_familiaris
--MGLWMHLDVCNEHCGWHAPVFWIPMHRTP----
>Hynobius_retardatus
--MHLDVCNEHCGCYASVLWPPMHRTP----
>Micropterus_dolomieu
--MHLDVCNDCCGWHASVFWTPILWTQ----
>Orcinus_orca
--MGLWMHLDVCNEHCGWHAPVFWIPMHRTP----
>Pygocentrus_nattereri
--MHLDVCNECCGWHASVFWIV-LWTQ----
>Charadrius_vociferus
--MHLDVCNEHCGWHAPVFWIPMHRTP----
>Callorhinchus_milii
--MHLDVCNEHCGWHAPVFWMPMYRTP----
>Myotis_brandtii
--MGLWMHLDVCNEHCGWHAPVFWIPMHRTP----
>Maylandia_zebra
--MHLDVCNECCGWHASVFWTPFLWTQ----
>Dasypus_novemcinctus
--MGLWMHLDVCNEHCGWHAPVFWIPMHRTP----
>Alosa_alosa
--MHLDVCNECCGWHASVYWIPLLWTQ----
>Latimeria_chalumnae
--MHLDVCNEHCGWHAPVFWIPMHRTP----
>Columba_livia
--MHLDVCNEHCGWHAPVFWIPMHRTP----
>Cuculus_canorus
--MHLDVCNEHCGWHAPVFWIPMHRTP----
>Poecilia_formosa
--MHLDVCNDCCGWHASVFRTPFLWTQ----
>Esox_lucius
--MHLDVCNECCG------------------
>Falco_cherrug
--MHLDVCNEHCGWHAPVFWIPMHRTP----
>Gadus_morhua
--MHLDVCNECCG------------------
>Acanthogobius_hasta
--MHLDVCNDCCGWYASVFRTAILWTQ----
>Chlamydotis_macqueenii
--MHLDVCNEHCGWHAPVFWIPMHRTP----
>Apteronotus_leptorhynchus
--MHLDVCNESCGWHASVFWIAVLWTQ----
>Condylura_cristata
--MDLWMHLDVCNEHCGWHAPVFWIPMHRTP----
>Ochotona_princeps
--MHLDVCNEHCGWHAPVFWIPMHRTP----
>Elephantulus_edwardii
--MGLWMHLDVCNEHCGWHAPVFWIPMHRTP----
>Ornithorhynchus_anatinus
--MHLDVCNEHCGWHAPVFWIPMHRTP----
>Plecoglossus_altivelis
--MHLDVCNECCGWHASVFWTPMLWTQ----
>Scleropages_formosus
MAWQDGLGLYHGGPLSLAAFAHLRAHRGNFKEDPQYEAASRPVGSTQGQAKWMHLDVCNECCGWYASVFWIPTLWTL----
>Manacus_vitellinus
--MHLDVCNEHCGWHAPVFWIPMHRTP----
>Lateolabrax_japonicus
--MHLDVCNDCCGWHASVFWTPILWTQ----
>Sebastes_caurinus
--MHLDVCNDCCGWHASVFWTPILWTQ----
>Ceratotherium_simum_simum
--MGLWMHLDVCNEHCGWHAPVFWIPMHRTP----
>Manis_javanica
--MGLWMHLDVCNEHCGWHAPVFWIPMHRTP----
>Picoides_pubescens
--MHLDVCNEHCGWHAPVFWIPMHRTP----
>Platichthys_stellatus
--MHLDVCNDCCGWHASVFWTPILWTQ----
>Aotus_nancymaae
--MGLWMHLDVCNEHCGWHAPVFWIPMHRTP----
>Loxodonta_africana
--MHLDVCNEHCGWHAPVFWIPMHRTP----
>Leucoraja_erinacea
--MHLDVCNEHCGWHAPVFWMPMCRTP----
>Mesocricetus_auratus
--MHLDVCNEHCGWHAPVFWIPMHRTP----
>Salmo_salar
--MHLDVCNECCGWHASVFWTPMLWTQ----
>Tupaia_chinensis
--MGLWMHLDVCNEHCGWHAPVFWIPMHRTP----
>Lepisosteus_oculatus
--MHLDVCNEHCGWHAPVFWIPMLRTQ----
>Trichechus_manatus_latirostr
--MDLWMHLDVCNEHCGWHAPVFWIPMHRTP----
>Aptenodytes_forsteri
--MHLDVCNEHCGWHAPVFWIPMHRTP----
>Squalus_acanthias
--MHLDVCNEHCGWHAPVFWMPMYRTP----
>Thamnophis_sirtalis
--MSLWMHLDVCNEHCGWHAPVFWIPMHRNP----
>Struthio_camelus_australis
--MHLDVCNEHCGWHAPVFWIPMHRTP----
>Chelonia_mydas
--MHLDVCNEHCGWHAPVFWIPMHRTP----
>Takifugu_rubripes
--MHLDVCNDCCGWHASVFWTPILWTQ----
>Tinamus_guttatus
--MHLDVCNEHCGWHAPVFWISMHRTP----
>Calypte_anna
--MHLDVCNEHCGWHAPVFWIPMHRTP----
>Apaloderma_vittatum
--MHLDVCNEHCGWHAPVFWIPMHRTP----
>Orycteropus_afer_afer
--MGFWMHLDVCNEHCGWHAPVFWIPMHRTP----

HG0029 ENSG00000005483 KMT2E

>Homo_sapiens
---------------------------MGLWMHLDVCNEHCGWHAPVFWIPMHRTP----
>Acipenser_sinensis
-------------------------------MHLDVCNEHFGWHASVFWIPMLRTP----
>Amia_calva
-------------------------------MHLDVCNEHCGWHAPVFWIQMLRTQ----
>Channa_punctata
-------------------------------MHLDVCNDCCGWHASVFWTPILWTQ----
>Anser_cygnoides
-------------------------------MHLDVCNEHCGWHAPVFWIPMHRTL----
>Xenopus_tropicalis
-------------------------------MHLDVCNEHCGWHAPVFWIPMQRTP----
>Chaetura_pelagica
-------------------------------MHLDVCNEHCGWHAPVFWIPMHRTP----
>Apteryx_australis_mantelli
---------------------------MGLWMHLDVCNEHCGWHAPVFWIPMHRTP----
>Menidia_menidia
-------------------------------MHLDVCNECCGWHASVFWTPFLWTQLDKN
>Porichthys_notatus
-------------------------------MHLDVCNECCGWHASVFWTSILWTQ----
>Oryzias_latipes
-------------------------------MHLDVCNDCCGWHASVFWTPFLWTQ----
>Buceros_rhinoceros_silvestri
-------------------------------MHLDVCNEHCGWHAPVFWIPMHRTP----
>Caprimulgus_carolinensis
-------------------------------MHLDVCNEHCGWHAPVFWIPMHRTP----
>Cariama_cristata
-------------------------------MHLDVCNEHCGWHAPVFWIPMHRTP----
>Mustela_putorius_furo
MLYRSILDSRFCEDIYSFLDLRFARHLMGLWMHLDVCNEHCGWHAPVFWIPMHRTP----
>Hynobius_retardatus
-------------------------------MHLDVCNEHCGCYASVLWPPMHRTP----
>Micropterus_dolomieu
-------------------------------MHLDVCNDCCGWHASVFWTPILWTQ----
>Orcinus_orca
---------------------------MGLWMHLDVCNEHCGWHAPVFWIPMHRTP----
>Pygocentrus_nattereri
-------------------------------MHLDVCNECCGWHASVFWIV-LWTQ----
>Calidris_pugnax
-------------------------------MHLDVCNEHCGWHAPVFWIPMHRTP----
>Callorhinchus_milii
-------------------------------MHLDVCNEHCGWHAPVFWMPMYRTP----
>Myotis_brandtii
---------------------------MGLWMHLDVCNEHCGWHAPVFWIPMHRTP----
>Haplochromis_burtoni
-------------------------------MHLDVCNECCGWHASVFWTPFLWTQ----
>Pterocles_gutturalis
-------------------------------MHLDVCNEHCGWHAPVFWIPMHRTP----
>Dasypus_novemcinctus
---------------------------MGLWMHLDVCNEHCGWHAPVFWIPMHRTP----
>Clupea_harengus
-------------------------------MHLDVCNECCGWHASVFWIPLLWTQ----
>Latimeria_chalumnae
-------------------------------MHLDVCNEHCGWHAPVFWIPMHRTP----
>Columba_livia
-------------------------------MHLDVCNEHCGWHAPVFWIPMHRTP----
>Merops_nubicus
-------------------------------MHLDVCNEHCGWHAPVFWIPMHRTP----
>Alligator_mississippiensis
-------------------------------MHLDVCNEHCGWHAPVFWIPMHRTP----
>Cuculus_canorus
-------------------------------MHLDVCNEHCGWHAPVFWIPMHRTP----
>Sinocyclocheilus_angustiporu
-------------------------------MHLDVCNECCG------------------
>Austrofundulus_limnaeus
-------------------------------MHLDVCNDCCGWHASVFWTPFLWTQ----
>Sarcophilus_harrisii
-------------------------------MHLDVCNEHCGWHAPVFWIPMHRTP----
>Galeopterus_variegatus
---------------------------MGLWMHLDVCNEHCGWHAPVFWIPMHRTP----
>Monodelphis_domestica
-------------------------------MHLDVCNEHCGWHAPVFWIPMHRTP----
>Esox_lucius
-------------------------------MHLDVCNECCG------------------
>Falco_cherrug
-------------------------------MHLDVCNEHCGWHAPVFWIPMHRTP----
>Gadus_morhua
-------------------------------MHLDVCNECCG------------------
>Coturnix_japonica
-------------------------------MHLDVCNEHCGWHAPVFWIPMHRTL----
>Acanthogobius_hasta
-------------------------------MHLDVCNDCCGWYASVFRTAILWTQ----
>Chlamydotis_macqueenii
-------------------------------MHLDVCNEHCGWHAPVFWIPMHRTP----
>Apteronotus_leptorhynchus
-------------------------------MHLDVCNESCGWHASVFWIAVLWTQ----
>Erinaceus_europaeus
---------------------------MGLWMHLDVCNEHCGWHAPVFWIPMHRTP----
>Ochotona_princeps
-------------------------------MHLDVCNEHCGWHAPVFWIPMHRTP----
>Elephantulus_edwardii
---------------------------MGLWMHLDVCNEHCGWHAPVFWIPMHRTP----
>Ornithorhynchus_anatinus
-------------------------------MHLDVCNEHCGWHAPVFWIPMHRTP----
>Plecoglossus_altivelis
-------------------------------MHLDVCNECCGWHASVFWTPMLWTQ----
>Scleropages_formosus
-------------------------------MHLDVCNECCGWYASVFWIPTLWTL----
>Parus_major
-----------------------MHLFMGLWMHLDVCNEHCGWHAPVFWIPMHRTP----
>Nipponia_nippon
-------------------------------MHLDVCNEHCGWHAPVFWIPMHRTP----
>Lateolabrax_japonicus
-------------------------------MHLDVCNDCCGWHASVFWTPILWTQ----
>Sebastes_caurinus
-------------------------------MHLDVCNDCCGWHASVFWTPILWTQ----
>Ceratotherium_simum_simum
---------------------------MGLWMHLDVCNEHCGWHAPVFWIPMHRTP----
>Manis_javanica
---------------------------MGLWMHLDVCNEHCGWHAPVFWIPMHRTP----
>Picoides_pubescens
-------------------------------MHLDVCNEHCGWHAPVFWIPMHRTP----
>Platichthys_stellatus
-------------------------------MHLDVCNDCCGWHASVFWTPILWTQ----
>Loxodonta_africana
---------------------------MGLWMHLDVCNEHCGWHAPVFWIPMHRTP----
>Leucoraja_erinacea
-------------------------------MHLDVCNEHCGWHAPVFWMPMCRTP----
>Mesocricetus_auratus
---------------------------MGLWMHLDVCNEHCGWHAPVFWIPMHRTP----
>Salmo_salar
-------------------------------MHLDVCNECCGWHASVFWTPMLWTQ----
>Tupaia_chinensis
---------------------------MGLWMHLDVCNEHCGWHAPVFWIPMHRTP----
>Lepisosteus_oculatus
-------------------------------MHLDVCNEHCGWHAPVFWIPMLRTQ----
>Trichomycterus_areolatus
-------------------------------MHLDVCHACSGWYASVFGIAQLWTH----
>Trichechus_manatus_latirostr
---------------------------MDLWMHLDVCNEHCGWHAPVFWIPMHRTP----
>Aptenodytes_forsteri
-------------------------------MHLDVCNEHCGWHAPVFWIPMHRTP----
>Squalus_acanthias
-------------------------------MHLDVCNEHCGWHAPVFWMPMYRTP----
>Thamnophis_sirtalis
---------------------------MSLWMHLDVCNEHCGWHAPVFWIPMHRNP----
>Tyto_alba
-------------------------------MHLDVCNEHCGWHAPVFWIPMHRTP----
>Struthio_camelus_australis
-------------------------------MHLDVCNEHCGWHAPVFWIPMHRTP----
>Chelonia_mydas
-------------------------------MHLDVCNEHCGWHAPVFWIPMHRTP----
>Takifugu_rubripes
-------------------------------MHLDVCNDCCGWHASVFWTPILWTQ----
>Tinamus_guttatus
-------------------------------MHLDVCNEHCGWHAPVFWISMHRTP----
>Calypte_anna
-------------------------------MHLDVCNEHCGWHAPVFWIPMHRTP----
>Apaloderma_vittatum
-------------------------------MHLDVCNEHCGWHAPVFWIPMHRTP----
>Orycteropus_afer_afer
---------------------------MGFWMHLDVCNEHCGWHAPVFWIPMHRTP----

HG0029 ENSG00000168137

>Homo_sapiens
---MSITCWMRLCSSPPLSEWSVSINWTP
>Anas_platyrhynchos
---MSVTCWPRLCGSSPPSERPVAALWTP
>Xenopus_tropicalis
---MSVKCLSRLCGSPP-SVWSVAAIPTP
>Apteryx_australis_mantelli
---MSVTCWPRLCGSSPPSERPVAALWTP
>Menidia_menidia
---MTVSGAVRLCGAPP-LEWSAAGVWTL
>Buceros_rhinoceros_silvestri
---MSVTCWPRLCGSSPPSERPVAALWTP
>Caprimulgus_carolinensis
---MSVTCWPRLCGSSPPSERPVAALWTP
>Cariama_cristata
---MSVTCWPRLCGSSPPSERPVAALWTP
>Canis_lupus_familiaris
---MSITCWLRLCSSPPLSEWPVSINWTP
>Physeter_catodon
---MSITCWLRLCSSPPLSEWPVSDNWTP
>Charadrius_vociferus
---MSVTCWPRLCGSSPPSERPVAALWTP
>Pteropus_vampyrus
---MSITCWLRLCSSPPLSEWPVSINWTP
>Pterocles_gutturalis
---MSVTCWPRLCGSSPPSERPVAALWTP
>Dasypus_novemcinctus
---MSITCCLRLCSAPPLSEWSVSLNWIP
>Latimeria_chalumnae
---MSMKCWSRHCGSPP-LVWSVAAIWTP
>Colius_striatus
---MSVTCWPRLCGSSPPSERPVAVLWTP
>Columba_livia
---MSVTCWPRLCGSSPPSERPVAALWTP
>Leptosomus_discolor
---MSVTCWPRLCGSSPPSERPVAALWTP
>Alligator_mississippiensis
---MSVTCWPRLCGSSPPLERPVATLWTP
>Poecilia_mexicana
---MTVSRGLRLDGAPL-LEWSVAISWTL
>Sarcophilus_harrisii
---MSLTCWLRLCGSPPLSEWPVSINWTP
>Monodelphis_domestica
---MSLTCWSRLCGSAPLSEWPVSINWIP
>Aquila_chrysaetos_canadensis
---MSVTCWPRLCGSSPPSERPVAALWTP
>Gallus_gallus
---MSVTCWPRLCGSSPPSERPVAALWTP
>Gavia_stellata
---MSVTCWPRLCGASPPSGRPVAALWTP
>Mesitornis_unicolor
---MSVTCWPRLCGSSPPSERPVAALWTP
>Condylura_cristata
---MSITCWLRLCSSPPLSEWPVSINWTP
>Oryctolagus_cuniculus
---MSVTCWPRLCSSPPLSEWSVSINWTP
>Ornithorhynchus_anatinus
------------------------MLTRSSESAAVM-------------------IF--RRRWQRGYW-DFTSTGQYKGFLMSVTCWLMLCGSPPLSEWSVSIKWTP
>Opisthocomus_hoazin
---MSVTCWPRLCGSPPPPERPVAALWTP
>Plecoglossus_altivelis
---MSVSRRLRLCGAPP-LEWSVAAVRTP
>Zonotrichia_albicollis
---MSVTCWPRLCGSSPPSERPVAALWTP
>Egretta_garzetta
---MSVTCWPRLCGSSPPSERPVAALWTP
>Equus_asinus
---MSITCWLRLCSSPPLSEWPVSINWTP
>Manis_javanica
---MSIICWLRLCSSPPLSEWPVSINWTP
>Picoides_pubescens
---MSVTCWPRLCGSSPPSERPVAALWTP
>Loxodonta_africana
---MSVTWWSRLCSSPPLSEWSVSINWTP
>Fulmarus_glacialis
---MSVTCWPRLCGSSPPSERPVAALWTP
>Melopsittacus_undulatus
---MSVTCWPRLCGSSPPSERPVAALWTP
>Cricetulus_griseus
--MAGFLMSITCWLRLCSSPPLSEWSVSINWTP
>Tupaia_chinensis
---MSITCWLRLRSSPPLSEWSVSVNWTP
>Trichechus_manatus_latirostr
---MSITCWLRLCSSPPLSEWSVSINWTP
>Aptenodytes_forsteri
---MSVTCWPRLCGSSPPSERPVAALWTP
>Anolis_carolinensis
---MSVTCWPRLCGCSPPLELPVATLWTP
>Struthio_camelus_australis
---MSVTCWPRLCGSSPPSERPVAALWTP
>Trachemys_scripta
MGRGSGRVRIAPGASAGAPRALSAVSARPVEEAQPVVLRGRGRGCRRRRGSRSRLLPRKRRGAEPGLVARGARATGRPGFPMSVTCWPRLCGSSPPLERPVATFWTP
>Tinamus_guttatus
---MSVTCWPRLCGSSPPSERPVAALWTP
>Calypte_anna
---MSVTCWPRLCGSSPPSERPLAALWTP
>Apaloderma_vittatum
---MSVTCWPGLCGSSPPSERPVAALWTP
>Orycteropus_afer_afer
---MSISCWLRLCSLPPLPEWSISINWTP

HG0029 ENSGALG00000008167

>Gallus_gallus
-------------------------------MHLDVCNEHCGWHAPVFWIPMHRTL----
>Acipenser_sinensis
-------------------------------MHLDVCNEHFGWHASVFWIPMLRTP----
>Amia_calva
-------------------------------MHLDVCNEHCGWHAPVFWIQMLRTQ----
>Channa_punctata
-------------------------------MHLDVCNDCCGWHASVFWTPILWTQ----
>Anser_cygnoides
-------------------------------MHLDVCNEHCGWHAPVFWIPMHRTL----
>Xenopus_tropicalis
-------------------------------MHLDVCNEHCGWHAPVFWIPMQRTP----
>Chaetura_pelagica
-------------------------------MHLDVCNEHCGWHAPVFWIPMHRTP----
>Apteryx_australis_mantelli
---------------------------MGLWMHLDVCNEHCGWHAPVFWIPMHRTP----
>Menidia_menidia
-------------------------------MHLDVCNECCGWHASVFWTPFLWTQLDKN
>Porichthys_notatus
-------------------------------MHLDVCNECCGWHASVFWTSILWTQ----
>Oryzias_latipes
-------------------------------MHLDVCNDCCGWHASVFWTPFLWTQ----
>Buceros_rhinoceros_silvestri
-------------------------------MHLDVCNEHCGWHAPVFWIPMHRTP----
>Caprimulgus_carolinensis
-------------------------------MHLDVCNEHCGWHAPVFWIPMHRTP----
>Cariama_cristata
-------------------------------MHLDVCNEHCGWHAPVFWIPMHRTP----
>Mustela_putorius_furo
MLYRSILDSRFCEDIYSFLDLRFARHLMGLWMHLDVCNEHCGWHAPVFWIPMHRTP----
>Ambystoma_mexicanum
-------------------------------MHLDVCNEHCGWNASVFWIPMQRTP----
>Micropterus_dolomieu
-------------------------------MHLDVCNDCCGWHASVFWTPILWTQ----
>Orcinus_orca
---------------------------MGLWMHLDVCNEHCGWHAPVFWIPMHRTP----
>Pygocentrus_nattereri
-------------------------------MHLDVCNECCGWHASVFWIV-LWTQ----
>Calidris_pugnax
-------------------------------MHLDVCNEHCGWHAPVFWIPMHRTP----
>Callorhinchus_milii
-------------------------------MHLDVCNEHCGWHAPVFWMPMYRTP----
>Myotis_brandtii
---------------------------MGLWMHLDVCNEHCGWHAPVFWIPMHRTP----
>Haplochromis_burtoni
-------------------------------MHLDVCNECCGWHASVFWTPFLWTQ----
>Pterocles_gutturalis
-------------------------------MHLDVCNEHCGWHAPVFWIPMHRTP----
>Dasypus_novemcinctus
---------------------------MGLWMHLDVCNEHCGWHAPVFWIPMHRTP----
>Clupea_harengus
-------------------------------MHLDVCNECCGWHASVFWIPLLWTQ----
>Latimeria_chalumnae
-------------------------------MHLDVCNEHCGWHAPVFWIPMHRTP----
>Columba_livia
-------------------------------MHLDVCNEHCGWHAPVFWIPMHRTP----
>Merops_nubicus
-------------------------------MHLDVCNEHCGWHAPVFWIPMHRTP----
>Alligator_mississippiensis
-------------------------------MHLDVCNEHCGWHAPVFWIPMHRTP----
>Cuculus_canorus
-------------------------------MHLDVCNEHCGWHAPVFWIPMHRTP----
>Sinocyclocheilus_grahami
-------------------------------MHLDVCNECCG------------------
>Austrofundulus_limnaeus
-------------------------------MHLDVCNDCCGWHASVFWTPFLWTQ----
>Sarcophilus_harrisii
-------------------------------MHLDVCNEHCGWHAPVFWIPMHRTP----
>Galeopterus_variegatus
-------------------------------MHLDVCNEHCGWHAPVFWIPMHRTP----
>Monodelphis_domestica
-------------------------------MHLDVCNEHCGWHAPVFWIPMHRTP----
>Esox_lucius
-------------------------------MHLDVCNECCG------------------
>Falco_cherrug
-------------------------------MHLDVCNEHCGWHAPVFWIPMHRTP----
>Gadus_morhua
-------------------------------MHLDVCNECCG------------------
>Acanthogobius_hasta
-------------------------------MHLDVCNDCCGWYASVFRTAILWTQ----
>Chlamydotis_macqueenii
-------------------------------MHLDVCNEHCGWHAPVFWIPMHRTP----
>Apteronotus_leptorhynchus
-------------------------------MHLDVCNESCGWHASVFWIAVLWTQ----
>Condylura_cristata
---------------------------MDLWMHLDVCNEHCGWHAPVFWIPMHRTP----
>Ochotona_princeps
-------------------------------MHLDVCNEHCGWHAPVFWIPMHRTP----
>Elephantulus_edwardii
---------------------------MGLWMHLDVCNEHCGWHAPVFWIPMHRTP----
>Ornithorhynchus_anatinus
-------------------------------MHLDVCNEHCGWHAPVFWIPMHRTP----
>Plecoglossus_altivelis
-------------------------------MHLDVCNECCGWHASVFWTPMLWTQ----
>Scleropages_formosus
-------------------------------MHLDVCNECCGWYASVFWIPTLWTL----
>Manacus_vitellinus
-------------------------------MHLDVCNEHCGWHAPVFWIPMHRTP----
>Nipponia_nippon
-------------------------------MHLDVCNEHCGWHAPVFWIPMHRTP----
>Lateolabrax_japonicus
-------------------------------MHLDVCNDCCGWHASVFWTPILWTQ----
>Sebastes_caurinus
-------------------------------MHLDVCNDCCGWHASVFWTPILWTQ----
>Ceratotherium_simum_simum
---------------------------MGLWMHLDVCNEHCGWHAPVFWIPMHRTP----
>Manis_javanica
---------------------------MGLWMHLDVCNEHCGWHAPVFWIPMHRTP----
>Picoides_pubescens
-------------------------------MHLDVCNEHCGWHAPVFWIPMHRTP----
>Platichthys_stellatus
-------------------------------MHLDVCNDCCGWHASVFWTPILWTQ----
>Pan_paniscus
-------------------------------MHLDVCNEHCGWHAPVFWIPMHRTP----
>Loxodonta_africana
-------------------------------MHLDVCNEHCGWHAPVFWIPMHRTP----
>Leucoraja_erinacea
-------------------------------MHLDVCNEHCGWHAPVFWMPMCRTP----
>Mesocricetus_auratus
---------------------------MGLWMHLDVCNEHCGWHAPVFWIPMHRTP----
>Salmo_salar
-------------------------------MHLDVCNECCGWHASVFWTPMLWTQ----
>Tupaia_chinensis
---------------------------MGLWMHLDVCNEHCGWHAPVFWIPMHRTP----
>Lepisosteus_oculatus
-------------------------------MHLDVCNEHCGWHAPVFWIPMLRTQ----
>Ictalurus_punctatus
-------------------------------MHLDVCHACSGWYASVFGIARLCSQ----
>Trichechus_manatus_latirostr
---------------------------MDLWMHLDVCNEHCGWHAPVFWIPMHRTP----
>Aptenodytes_forsteri
-------------------------------MHLDVCNEHCGWHAPVFWIPMHRTP----
>Squalus_acanthias
-------------------------------MHLDVCNEHCGWHAPVFWMPMYRTP----
>Thamnophis_sirtalis
---------------------------MSLWMHLDVCNEHCGWHAPVFWIPMHRNP----
>Tyto_alba
-------------------------------MHLDVCNEHCGWHAPVFWIPMHRTP----
>Struthio_camelus_australis
-------------------------------MHLDVCNEHCGWHAPVFWIPMHRTP----
>Chelonia_mydas
-------------------------------MHLDVCNEHCGWHAPVFWIPMHRTP----
>Takifugu_rubripes
-------------------------------MHLDVCNDCCGWHASVFWTPILWTQ----
>Tinamus_guttatus
-------------------------------MHLDVCNEHCGWHAPVFWISMHRTP----
>Calypte_anna
-------------------------------MHLDVCNEHCGWHAPVFWIPMHRTP----
>Apaloderma_vittatum
-------------------------------MHLDVCNEHCGWHAPVFWIPMHRTP----
>Orycteropus_afer_afer
---------------------------MGFWMHLDVCNEHCGWHAPVFWIPMHRTP----

```
HG0030.1 ENSG00000100664 EIF5

>Homo_sapiens
-----------------------------------------------------------MSSLLLQTEDTKKLQSKISSSY-
>Acipenser_sinensis
MSLFYENGEYPDDRSDGWERAVA--LVPVWRKLGLQARSLQPQWRKLFHVRDRVHLQSFMSSALLQTEDTKKLQAKIFLSY-
>Kaupichthys_hyoproroides
-----------------------------------------------------------MSSTLLQTEDTKKLQSKIFLSC-
>Anser_cygnoides
-----------------------------------------------------------MSSRLLQTEDTKKLQSKISSSY-
>Megophrys_sangzhiensis
-----------------------------------------------------------MSSSLLQTEDTKKLQSKIFSSY-
>Chaetura_pelagica
-----------------------------------------------------------MSSRLLQTEDTKKLQSKISSSY-
>Apteryx_australis_mantelli
-----------------------------------------------------------MSSRLLQTEDTKKLQSKISSSY-
>Porichthys_notatus
-----------------------------------------------------------MSSLLFQTEDPEKSPTTILLSY-
>Oryzias_latipes
-----------------------------------------------------------MSSPFFQTEDPKKSPTKILLPY-
>Buceros_rhinoceros_silvestri
-----------------------------------------------------------MSSRLLQTEDTKKLQSKISSSY-
>Caprimulgus_carolinensis
-----------------------------------------------------------MSSRLLQTEDTKKLQSKISSSY-
>Seriola_quinqueradiata
-----------------------------------------------------------MSSPLFQTEDPEKSPTKILLSY-
>Cariama_cristata
-----------------------------------------------------------MSSRLLQTEDTKKLQSKISSSY-
>Mustela_putorius_furo
-----------------------------------------------------------MSSLLLQTEDTKKLQSKISSSY-
>Hynobius_chinensis
-----------------------------------------------------------MSSPLLQTEDTKKLLSQIFSSY-
>Siniperca_chuatsi
-----------------------------------------------------------MSSPLFQTEDPEKSPTKILLSY-
>Lipotes_vexillifer
-----------------------------------------------------------MSSLLLQTEDTKKLQSKISSSY-
>Astyanax_mexicanus
-----------------------------------------------------------MSSLLLQTEDTKKSPSKIFLSC-
>Calidris_pugnax
-----------------------------------------------------------MSSRLLQTEDTKKLQSKISSSY-
>Callorhinchus_milii
-----------------------------------------------------------MSSILLQTEDTKKFNSKIFSLYS
>Miniopterus_natalensis
-----------------------------------------------------------MSSLLLQTEDTKKLQSKISSSYR
>Haplochromis_burtoni
-----------------------------------------------------------MSSPLFQTEDPEKSPTKILLSY-
>Pterocles_gutturalis
-----------------------------------------------------------MSSRLLQTEDTKKLQSKISSSY-
>Dasypus_novemcinctus
-----------------------------------------------------------MSSLLLQTEDTKKLQSKISSSY-
>Latimeria_chalumnae
-----------------------------------------------------------MSSILLQAEDTKKLQSKIFSSY-
>Colius_striatus
-----------------------------------------------------------MSSRLLQTEDTKKLQSKISSSY-
>Columba_livia
-----------------------------------------------------------MSSRLLQTEDTKKLQSKISSSY-
>Merops_nubicus
-----------------------------------------------------------MSSRLLQTEDTKKLQSKISSSY-
>Alligator_mississippiensis
-----------------------------------------------------------MSSRLLQTEDTKKLQSKISSSY-
>Cuculus_canorus
-----------------------------------------------------------MSSRLLQTEDTKKLQSKISSSY-
>Ctenopharyngodon_idella
MSLFCENGEYLDDRSYGWESAIVQLTEPVWGELSQRARSLEPQWPYPFHVRDKVLLQSFMSSPLLQTEDTNNCQFKIFSSC-
>Fundulus_heteroclitus
-----------------------------------------------------------MSSPLFQTEDPEKSPTKILLSY-
>Sarcophilus_harrisii
-----------------------------------------------------------MSSLLLQTEDTKKLQSKISSSY-
>Galeopterus_variegatus
-----------------------------------------------------------MSSLLLQTEDTKKLQSKISSSY-
>Monodelphis_domestica
-----------------------------------------------------------MSSLLLQTEDTKKLQSKISSSY-
>Esox_lucius
-----------------------------------------------------------MSPPLCQTEDTKNLQSKIFLSY-
>Aquila_chrysaetos_canadensis
-----------------------------------------------------------MSSRLLQTEDTKKLQSKISSSY-
>Gallus_gallus
-----------------------------------------------------------MSSRLLQTEDTKKLQSKISSSY-
>Gavia_stellata
-----------------------------------------------------------MSSRLLQTEDTKKLQSKVSSSY-
>Chanos_chanos
-----------------------------------------------------------MSSSLLQTEDTKNRQSRIFSSC-
>Chlamydotis_macqueenii
-----------------------------------------------------------MSSRLLQTEDTKKLQSKISSSY-
>Apteronotus_leptorhynchus
-----------------------------------------------------------MSSLLLQTEDTPKSPSKIFLFC-
>Condylura_cristata
-----------------------------------------------------------MSSLLLQTEDTKKLQSKISSSY-
>Oryctolagus_cuniculus
-----------------------------------------------------------MSSLLLQTEDTKKLQSKISSSY-
>Elephantulus_edwardii
-----------------------------------------------------------MSSLLLQTEDTKKL---------
>Ornithorhynchus_anatinus
-----------------------------------------------------------MSSLLLQTEDTKKLQSKISSPY-
>Tauraco_erythrolophus
-----------------------------------------------------------MSSRLLQTEDTKKLQSKISSSY-
>Opisthocomus_hoazin
-----------------------------------------------------------MSSRLLQTEDTKKLQSKISSSY-
>Osmerus_mordax
MSLFYENGEYPDDRSDGWDRAVAQLLEPVWGKLSLQARSLQPQWQDLFHVRARLLLQLFMSSPLGQTEDTKNCQSKIFLSY-
>Scleropages_formosus
-MFGYLSLAYLATSPVFKLQAVAQLLEPVWDTRNLQARKPQPRGQNLFHVRDRVLLQPFMSSTLLQTEDTKKSPSKIFLPY-
>Acanthisitta_chloris
-----------------------------------------------------------MSSSLLQTEDTKKLQSKISSPY-
>Nipponia_nippon
-----------------------------------------------------------MSSRLLQTEDTKKLQSKISSSY-
>Notothenia_coriiceps
-----------------------------------------------------------MSSPLFQTEDPKKSPTKILLSY-
>Ceratotherium_simum_simum
-----------------------------------------------------------MSSLLLQTEDTKKLQSKISSSY-
>Manis_javanica
-----------------------------------------------------------MSSLLLQTEDTKKLQSKISSSY-
>Picoides_pubescens
-----------------------------------------------------------MSSRLLQTEDTKKLQSKISSSY-
>Loxodonta_africana
-----------------------------------------------------------MSSLLLQTEDTKKLQSKISSSY-
>Fulmarus_glacialis
-----------------------------------------------------------MSSRLLQTEDTKKLQSKISSSY-
>Melopsittacus_undulatus
-----------------------------------------------------------MSSRLLQTEDTKKLQSKISSSY-
>Leucoraja_erinacea
-----------------------------------------------------------MSSILLQTEDTKKFHSKIFSLHS
>Jaculus_jaculus
-----------------------------------------------------------MSSLLLQTEDTKKLQSKISSSY-
>Salmo_salar
MSLFCENGEYPDDRSDGWERAVAQLLEPVWGELNLQARSLQPHWQDLFHVRARTLFQSFMSSHLRQTEDTKNLQSKIFLSY-
>Tupaia_chinensis
-----------------------------------------------------------MSSLLLQTEDTKKLQSKISSSY-
>Lepisosteus_oculatus
MSLFYENGEYPDDRSDGWERAVAQLLEPVWGKLSLQARSLQPQWRKLFHVRDRVHLQSFMSSTLLQTEDTKKLQSKIFLSC-
>Ictalurus_furcatus
-----------------------------------------------------------MSSRLLQTEDTQKSPSRIFLFC-
>Trichechus_manatus_latirostr
-----------------------------------------------------------MSSLLLQTEDTKKLQSKISSSY-
>Aptenodytes_forsteri
-----------------------------------------------------------MSSRLLQTEDTKKLQSKISSSY-
>Gekko_japonicus
-----------------------------------------------------------MSSRLLQTEDTKKLQSKISASY-
>Tyto_alba
-----------------------------------------------------------MSSRLLQTEDTKKLQSKISSSY-
>Struthio_camelus_australis
-----------------------------------------------------------MSSRLLQTEDTKKLQSKISSSY-
>Chrysemys_picta
-----------------------------------------------------------MSSRLLQTEDTKKLQSKISSSY-
>Tinamus_guttatus
-----------------------------------------------------------MSSRLLQTEDTKKLQSKISSSY-
>Calypte_anna
-----------------------------------------------------------MSSRLLQTEDTKKLQSKISSSY-
>Apaloderma_vittatum
-----------------------------------------------------------MSSRLLQTEDTKKLQSKISSSY-
>Orycteropus_afer_afer
-----------------------------------------------------------MSSLLLQTEDTKNLQSKISSSY-
```

HG0030.1 ENSGALG00000011464 EIF5

>Gallus_gallus
---MSSRLLQTEDTKKLQSKISSSY-
>Acipenser_sinensis
MSLFYENGEYPDDRSDGWERAVA--LVPVWRKLGLQARSLQPQWRKLFHVRDRVHLQSFMSSALLQTEDTKKLQAKIFLSY-
>Kaupichthys_hyoproroides
---MSSTLLQTEDTKKLQSKIFLSC-
>Anas_platyrhynchos
---MSSRLLQTEDTKKLQSKISSSY-
>Leptobrachium_boringii
---MSSSLLQTEDTKKLQSKIFSSY-
>Chaetura_pelagica
---MSSRLLQTEDTKKLQSKISSSY-
>Apteryx_australis_mantelli
---MSSRLLQTEDTKKLQSKISSSY-
>Oryzias_latipes
---MSSPFFQTEDPKKSPTKILLPY-
>Buceros_rhinoceros_silvestri
---MSSRLLQTEDTKKLQSKISSSY-
>Caprimulgus_carolinensis
---MSSRLLQTEDTKKLQSKISSSY-
>Seriola_quinqueradiata
---MSSPLFQTEDPEKSPTKILLSY-
>Cariama_cristata
---MSSRLLQTEDTKKLQSKISSSY-
>Mustela_putorius_furo
---MSSLLLQTEDTKKLQSKISSSY-
>Hynobius_chinensis
---MSSPLLQTEDTKKLLSQIFSSY-
>Siniperca_chuatsi
---MSSPLFQTEDPEKSPTKILLSY-
>Lipotes_vexillifer
---MSSLLLQTEDTKKLQSKISSSY-
>Astyanax_mexicanus
---MSSLLLQTEDTKKSPSKIFLSC-
>Calidris_pugnax
---MSSRLLQTEDTKKLQSKISSSY-
>Callorhinchus_milii
---MSSILLQTEDTKKFNSKIFSLYS
>Miniopterus_natalensis
---MSSLLLQTEDTKKLQSKISSSYR
>Oreochromis_niloticus
---MSSPLFQTEDPEKSPTKILLSY-
>Pterocles_gutturalis
---MSSRLLQTEDTKKLQSKISSSY-
>Dasypus_novemcinctus
---MSSLLLQTEDTKKLQSKISSSY-
>Latimeria_chalumnae
---MSSILLQAEDTKKLQSKIFSSY-
>Colius_striatus
---MSSRLLQTEDTKKLQSKISSSY-
>Columba_livia
---MSSRLLQTEDTKKLQSKISSSY-
>Merops_nubicus
---MSSRLLQTEDTKKLQSKISSSY-
>Alligator_sinensis
---MSSRLLQTEDTKKLQSKISSSY-
>Cuculus_canorus
---MSSRLLQTEDTKKLQSKISSSY-
>Danio_rerio
MSLFCENGDYLDDRSYGWESAIVQLPEPVWGELSQRARSLEPQWPYPFHVRDKVFLQSFMSSPLLQTEDTKNCQFKIFSSC-
>Cyprinodon_variegatus
---MSSPLFQTEDPEKSPTKILLSY-
>Sarcophilus_harrisii
---MSSLLLQTEDTKKLQSKISSSY-
>Galeopterus_variegatus
---MSSLLLQTEDTKKLQSKISSSY-
>Monodelphis_domestica
---MSSLLLQTEDTKKLQSKISSSY-
>Esox_lucius
---MSPPLCQTEDTKNLQSKIFLSY-
>Aquila_chrysaetos_canadensis
---MSSRLLQTEDTKKLQSKISSSY-
>Gavia_stellata
---MSSRLLQTEDTKKLQSKVSSSY-
>Chanos_chanos
---MSSSLLQTEDTKNRQSRIFSSC-
>Chlamydotis_macqueenii
---MSSRLLQTEDTKKLQSKISSSY-
>Apteronotus_leptorhynchus
---MSSLLLQTEDTPKSPSKIFLFC-
>Condylura_cristata
---MSSLLLQTEDTKKLQSKISSSY-
>Oryctolagus_cuniculus
---MSSLLLQTEDTKKLQSKISSSY-
>Elephantulus_edwardii
---MSSLLLQTEDTKKL---------
>Ornithorhynchus_anatinus
---MSSLLLQTEDTKKLQSKISSPY-
>Tauraco_erythrolophus
---MSSRLLQTEDTKKLQSKISSSY-
>Opisthocomus_hoazin
---MSSRLLQTEDTKKLQSKISSSY-
>Plecoglossus_altivelis
---MSSPLGQTEDTKNCQSKIFLSY-
>Scleropages_formosus
-MFGYLSLAYLATSPVFKLQAVAQLLEPVWDTRNLQARKPQPRGQNLFHVRDRVLLQPFMSSTLLQTEDTKKSPSKIFLPY-
>Serinus_canaria
---MSSRLLQTEDTKKLQSKISSSY-
>Nipponia_nippon
---MSSRLLQTEDTKKLQSKISSSY-
>Notothenia_coriiceps
---MSSPLFQTEDPKKSPTKILLSY-
>Equus_asinus
---MSSLLLQTEDTKKLQSKISSSY-
>Manis_javanica
---MSSLLLQTEDTKKLQSKISSSY-
>Picoides_pubescens
---MSSRLLQTEDTKKLQSKISSSY-
>Macaca_nemestrina
---MSSLLLQTEDTKKLQSKISSFY-
>Loxodonta_africana
---MSSLLLQTEDTKKLQSKISSSY-
>Fulmarus_glacialis
---MSSRLLQTEDTKKLQSKISSSY-
>Melopsittacus_undulatus
---MSSRLLQTEDTKKLQSKISSSY-
>Leucoraja_erinacea
---MSSILLQTEDTKKFHSKIFSLHS
>Jaculus_jaculus
---MSSLLLQTEDTKKLQSKISSSY-
>Salmo_salar
MSLFCENGEYPDDRSDGWERAVAQLLEPVWGELNLQARSLQPHWQDLFHVRARTLFQSFMSSHLRQTEDTKNLQSKIFLSY-
>Tupaia_chinensis
---MSSLLLQTEDTKKLQSKISSSY-
>Lepisosteus_oculatus
MSLFYENGEYPDDRSDGWERAVAQLLEPVWGKLSLQARSLQPQWRKLFHVRDRVHLQSFMSSTLLQTEDTKKLQSKIFLSC-
>Ictalurus_punctatus
---MSSRLLQTEDTQKSPSRIFLFC-
>Trichechus_manatus_latirostr
---MSSLLLQTEDTKKLQSKISSSY-
>Aptenodytes_forsteri
---MSSRLLQTEDTKKLQSKISSSY-
>Gekko_japonicus
---MSSRLLQTEDTKKLQSKISASY-
>Tyto_alba
---MSSRLLQTEDTKKLQSKISSSY-
>Struthio_camelus_australis
---MSSRLLQTEDTKKLQSKISSSY-
>Chrysemys_picta
---MSSRLLQTEDTKKLQSKISSSY-
>Tinamus_guttatus
---MSSRLLQTEDTKKLQSKISSSY-
>Calypte_anna
---MSSRLLQTEDTKKLQSKISSSY-
>Apaloderma_vittatum
---MSSRLLQTEDTKKLQSKISSSY-
>Orycteropus_afer_afer
---MSSLLLQTEDTKNLQSKISSSY-

HG0030.2 FBgn0030719 eIF5

>Drosophila_melanogaster
--MNSKGRTKNHRTSSNRHRKLQPFRYT-
>Hyalella_azteca
---MIP-EGQ--FSRVV-----------------YDYPRACVSPTPLHRMCAHNNDSPINIRNRQFSSNRHRKLQPFRTS-
>Meinertellus_cundinamarcensi
---MTVLSWKEP----------------------LLARGGS------GIDHTS-REHHPASQGPAVDRCMTS-PKR---------------------------------------QHPL------SYRTSKFSSNRHRKLQPFRET-
>Argulus_siamensis
---MNLYSWNDL----------------------LLSPGNP------ASFRKQ-LVA---SCGPDPSGCMFA-AKK---------------------------------------YEPL------SYRKTKFSSNRHRKLQPFRS--
>Coptotermes_gestroi
---MIS-SKR---------------------------------------QEPI------SYRTSKFSSNRHRKLQPFRET-
>Agrilus_planipennis
--MT-SQR---------------------------------------QEL------HCNRTTKSSSNRHRKLQPFRET-
>Callinectes_sapidus
---MILFSWHK--------------------GHISPGNCILSVEPFGGLLT-----------------------QS--------CMCACASDTPLDIRNRQFSSNRHRKLQPFRTS-
>Apachyus_charteceus
--MTLLSICRET----------------------TLSRRKS------GSFHI--ALEP---IRGLDSRCMSS-SKQ---------------------------------------QERI--NHRPVYRTSKFSTNRHRKLQPFRET-
>Occasjapyx_japonicus
---MIF-FQR---------------------------------------QEPI------SFRTSKFSSNRHRKLQPFRET-
>Haploembia_palaui
---MTLNTCRET----------------------TLTRGKS------GSFHL--SLEP---FRGLDSTCMIS-SKR---------------------------------------QEPI------TYRTSKFSSNRHRKLQPFRET-
>Isonychia_bicolor
---MTFHRCNEI----------------------SLSRGKS------GRLHI--ALEP---IFGLDPRCMSS-SKW---------------------------------------QNPI------SCRTSKFSSNRHRKLQPFRET-
>Meganyctiphanes_norvegica
---MILISWHK--------------------GHISPGHSILLVEPLGGLLT-----------------------KS--------CMCACASDTPLDIRNRQFSSNRHRKLQPFRTS-
>Galloisiana_yuasai
--MTLTSTCRET----------------------TLSRRKS------GSFHI--SLEP---IRGLDSRCMIS-SKR---------------------------------------QEPI------SYRTSKFSTNRHRKLQPFRET-
>Tupiocoris_notatus
---MIS-SKL---------------------------------------QWPV------SYRTSKFSSNRHRKSQPFRET-
>Copidosoma_floridanum
MTFLRQRETSRISSRGKGTVYNTGGGGGSTSSLRRTSRAV-DGLLSSRRTIASTTTSPSDVKITGKQRNNINVLSSGSKEECGQCQYRRCSPLFL--SSSCSAPAACMII-EGRGRLSSRSSSSSSSCCGCASHKQQLQDEDDHCQNAA---LRVHLNRLLLVDHRTAKFSSNRHRKLQPFRET-
>Eudarcia_simulatricella
---MTLA-RSTETSIQ----------------CGEKSCCIE------------------------RVQR----AADCARS-----CMTLYGPRTTGRRTSKFSSNRHRKLQPFRET-
>Metallyticus_splendidus
--MTLLPTCRET----------------------TLSRGKS------GSFHI--ALEP---VRGLDSRCMIS-SKR---------------------------------------QEPI------SYRTSKFSSNRHRKLQPFRET-
>Tanzaniophasma_sp._AD-2013
---MTLTLTR--------IQR----------FSRKFSGCFYI-SPKPSFG-----------------------------LEI---SRMILSKWQELLVHRTKFSSNRHRKSQPFREC-
>Panorpa_vulgaris
---MTLERYRETS-VLRGVPR------SPTALSPRGTKCMI--PPRRHLSYRTSKFSSNRHRKLQPFRET-
>Corydalus_cornutus
---MTLAGCIET----------------------LLSRRKP------GITQYS-VLEP---VLGIDTRCMIS-SKF---------------------------------------QEPI------SYRISKFSTNRHRKLQPFRET-
>Pseudomallada_prasinus
-MRQRRAETFRISSRGKGSVCSKGSGGRTLSRHRRNNSTTIISYIRCNRSL---------------------RWCDGSR------LSTWCSEIIED---TRYGPAACMII-ENYPPLPTRSRHNSN--------------------QDT---TQHFLNHLVLPKHRTSKFSSNRHRKLQPFRET-
>Calopteryx_splendens
--MT-LLSSKGI----------------------SLSRGRS------GFFSI--ALEP---ILGLDSRCMIS-SKR---------------------------------------QEPI------SYRTSKFSSNRHRKLQPFRET-
>Gryllotalpa_sp._AD-2013
--MTLLPTCRET----------------------TLTRGKS------GSFHI--ALAP---TRGLDSRCMIS-SKR---------------------------------------QEPI------SYRTSKFSSNRHRKLQPFRET-
>Clitarchus_hookeri
---MTLLTCRET----------------------ILSRGKS------GSFHI--AEEP---ARGQDSRCMIS-TKR---------------------------------------QEPI------CYRTSKFSSNRHRKLQPFRET-
>Menopon_gallinae
---MTLKHFKE---ASLTRRKCRPNLSP----GRGQF--RCMISHKGQPSYRKSKLSTNRHRKLQPFRET-
>Leuctra_sp._AD-2013
--MTLLPTCRET----------------------ILTRGKS------GSFHR--ALEP---VRGLDSRCMIS-YKR---------------------------------------RDPI------SHRISKFSTNRHRKLQPFRET-
>Polydesmus_angustus
---MTLISLK-------------ELLL----LRGLNCCCKLT-D-----------------------PHWESVLSRC--------MFSTEKFKPVSHRTPKFQSNRHRKLQPFRS--
>Acerentomon_sp._AD-2013
---MTLINSKEEKRSKDGSGKLYIRLQ--TFCGPGFINCMNLYQPHS-------------------------------SASPLSALCCT----EPSKSHYSSHSNQHRKLLPFREES
>Liposcelis_bostrychophila
---MTLTHFKE---ASLTRRKCRPNLSP----GRGQF--RCMISHKGQPSYRKSKLSTNRHRKLQPFRET-
>Inocellia_crassicornis
---MTLVNHTET----------------------LLSRGKS------GSSRYS-ALEP---ALGIDTRCMSS-SKR---------------------------------------QGPI------SYRISKYSTSRHRKLQPFRET-
>Ctenocephalides_felis
---MTLERYRETS-VLRGVDR------SACAPSSHSAKCMT--PTRRRHCYRQSKFSSNRHRKLQPFRET-
>Thrips_palmi
---MTFRSCKETW---------------------TVSRGRS------GSSPI--AQEP---GRGPGGRCMIS-YQP---------------------------------------HGPI------SHRTTKFSSNRHRKSQPFRET-
>Zorotypus_caudelli
--MTLLPLCRE-----------------------TLTRRKS------GNFHI--ALVP---TGGLDSGCMIS-SKR---------------------------------------KQPI------CYRTSKFSSNRHRKLQPFRET-
>Tricholepidion_gertschi
---MTLLSWKEI----------------------SLSRGKS------GSFHI--TLEP---ILGLDTRCMIS-SKR---------------------------------------QEPI------SYRTSKFSSNRHRKLQPFRET-

HG0031 ENSDARG00000000540 asnsd1

>Danio_rerio
---MSSKNIQRT-E-----PGASEERAQKEELNQKIKEQKIVVDELSNLKKNRRVYTQQPNSNIFFLADKGETLSSCKQDLDNMRKEYQDI-------------
>Acipenser_sinensis
---MST-K-SGQDN-----TSEV-NTKQKEKLNKKIKEQQVLVDELSNLKKNRKVYIQQPNSNIFFLGDKSETLSESKEMLDNMKKEYQDL-------------
>Amia_calva
---MSNKS-QAKDD-----QING-KAKQKEELNKKMKEQKVVIDELSNLKKNRKVYVQQQNSNIFFLEDKSETLATCKKSLDHMKKEYQDL-------------
>Channa_striata
---MKKELQDM-------------
>Anser_cygnoides
--------------------------------------MAARRGEEEEKKEEEEG------EGESAVLRPRDQLSRRIKEQKVIIDELSNLKKNRKVYKQQPNSNIFFLVDRTETLSQCKIVLDELKKAHQEMENSEKTKIKK---
>Polypedates_megacephalus
---MSDVG-VVLGE-----DCRSWTDKQKEEFYAKFREQNVVVDELSNLKKDRKVYRQQPNSSIYFLADRAKTLSDCKKTLDDLKHTHQLLENSENIQDTD---
>Porichthys_notatus
---MSSKL-HDIDA-----SFG--GTPSIEVLNRKISEQKVVLDELSNLKKNRKVYIQQQNSNIFFLADRKQTLSSCKKELDNLKRDLQDS-------------
>Oryzias_latipes
---MKMSSKK-YNNSD-----NSQE-QTTYKEELSKKIKEQKVVVDELSNLKKNRKVYIQQRNSNIFFLADRSQTLSTSRKELDYLKKELQDI-------------
>Trachinotus_blochii
---MSSKN-RDNDD-----NFEG-QSALKEELNKKIKEQKVVVDELSNLKKNRKVYIQQRNSNIFFLGDRSQTLGSCKKELDNMKKELQDM-------------
>Cariama_cristata
--MLSQCKNTLDELKKAHQEMENSENTKVKK---
>Phoca_largha
---MPSRGSRPEDGAGLF--SADHSTLHKEDLSSKIKEQKIVVDELSNLKKNRKVYRQQQNSNIFFLADRTEMLSESKNILDELKKEYQEIENSEKTKIKK---
>Hynobius_retardatus
---MSGTKPKN------KEDGSDSAAWTKEDLDTKIREQTVVVDELSNLKPNRKVYTQQPNSNIFFLADRTATLSESKKTLDDLKTAHQELEKSQKARVK----
>Micropterus_salmoides
--MNKMSSKI-RDNDD-----NCEG-RCALKEELNKKIKEQRVVVDELSNLKKNRKVYIQQRNSNIFFLADRSQTLASCKREMDNMKKDLQDM-------------
>Lipotes_vexillifer
---MPSRGARPEDGSGLV--PTDNSTQHKEDLSSKIKEQKIVVDELSNLKKNRKVYRQQQNSDIFFLADRTEMLSESKNTLDELRKEYQEIENSEKTKIKK---
>Astyanax_mexicanus
---MSIRSSEQPED-----PAQE-ERPTREELNTKIKEQKVLVDELSNLKKNRKVYLQQPNSNIFFLSDKGETLSSCKKELDKMKKDYQDM-------------
>Calidris_pugnax
---MSGRAARQEEEEEKEKREESAKARHKEELSRRIKEQKVVVDELSNLKKNRKVYRQQPNSNIFFLVDRTETLSQCKNTLDELKKAHQEMENSEKTKVKK---
>Callorhinchus_milii
--MEAAETRAAPGGEE-----RETRDQATRRQELHNKIKEQNVAINELSNLKETRRVYKQQPNSNIFFLTDKTTALTECKRAFDELKKEHQDLENPDKMMK-----
>Myotis_lucifugus
---MEMPAREARPEDGAGRA-------APGREDLSSRIKEQKIVVDELSNLKKNRTVYRQQQNSNIFFLADRTELLSESKNILDELKKEYQEIENSEKTKTKK---
>Oreochromis_niloticus
---MSSKG-CSSDE-----QFEG-QAAVKEELNKKIKEQKVLVDELCNLKKNRKVYIQQRNSNIFFVGDRSQTLTSCKKELDYMKKELQDI-------------
>Clupea_harengus
---MTTKRNLPAAN-----TVQ-DETPQKEELSKKIKEQKILVDELSNLKKNRRVYLQQPNSNIFFLTDRGQMLSTSKKELDNLKKDYQDM-------------
>Latimeria_chalumnae
---MSGRV-QGEDN-----ESQ-LRTSHKEELHKKIKEQKVLVDELSNLKKNRKVYQQQPNSNIFFLADRSKTLSDGKKKLDDMKREYQEL-------------
>Columba_livia
---MSGRPPRQEE------GGDSAKLRHKDELSRKIKEQKVVVDELSNLKKNRKVYRQQPNSNIFFLVDRTETLSQCKNTLDELKKAHQEMENSEKTKIKK---
>Alligator_sinensis
MRRERARVRNHPAAWVGGASCAEGVWLGGAEVRSQSKGAARLVSGMSGRAPRR--------DEEEARLRRKEELNGKIKEQKIVVDELSNLKKNRKVYRQQPNSNIFFLADRTETLSQCKNKLDELKKAHQEIENSEKSKIKK---
>Kryptolebias_marmoratus
---MSLKS-PDSSD-----NSAE-QSALKEELNKKIKEQKIVLDELSNLKKNRRVFIQQRNSNIFFLADRSQTLSSCKNDLDVLKKELQDV-------------
>Sarcophilus_harrisii
---MPGRGTRVEDGPEPA--ISAKLPLNKEELNSKIKEQKVVVDELSNLKKNRKVYKQQPNSNIFFLADRTEMLSESKNTLDELKKIHQEIENTEKNKSKK---
>Monodelphis_domestica
---MPGKGTRREDATEPG--TFASLPLNKEELNSKIKEQKVVVDELSNLKKNRKVYKQQPNSNIFFLSDRTEMLSESKNTLDELKKVHQEIENTEKNKIKK---
>Trichosurus_vulpecula
--MLSESKNTLDELKKIHQEIENTEKNKIKK---
>Esox_lucius
---MSV-----KNE-----IFDEEESALKVELNKKIKEQKVVVDELSNLKKSRRVYVQQPNSNIFFLADRGQTLSSCKRARQHEEGSPKYVIF-----------
>Aquila_chrysaetos_canadensis
---MSGRAPRQEEG-------ESAKLRRKEELSRRIKEQKVVVDELSNLKKNRKVYRQQPNSNIFFLVDRTETLSQCKNTLDELKKAHQEMENSEKAKIKK---
>Gadus_morhua
---MTSN--SREDE-----ISEA-HSAIKEELNKKIKEQKIVVDELSNLKKNRKVYVQQRNSNIFFLADKGQMLGTNKKELDILKKELQDM-------------
>Numida_meleagris
--MSRREEEEGAGVCRKEELSRRIKEQKVVVDELSNLKKNRKVYKQQPNSNIFFLVDRTETLSQCKNTLDQLKKAHQELENSEKTKVKK---
>Acanthogobius_hasta
--MS-RNTED-----NNEG-LSALKEEMNKKIREQKVVLDELSNLKKGRKVYTQQRNSNIFFLAERSQTLSACKKELDVMKKELQDI-------------
>Condylura_cristata
---MPSQ--GPEEGARLV--SSDNSSLIKEDLSSKIKEQKIVVDELSNLKKNRKVYRQQQNSNVFFLADRTEMLSESKNILDELKKEYQEIENLDKTQIKK---
>Oryctolagus_cuniculus
---MPGRGVHPEDSAVPV--PAGGRTLHKEDLCNKIKEQKVVVDELSNLKKNRRVYRQQQNSNIFFLADRTEMLSESKNILDELKKEYQEIENSEKSSIKK---
>Lutjanus_erythropterus
---MKKELQDM-------------
>Ornithorhynchus_anatinus
--MLSESKNTLDELKKTHQEMDSLEKTKAKK---
>Plecoglossus_altivelis
---MSSKCQLDLEN-----LFAGQESKRKEELNKKINEQKVVVDELNNLKKNRKVYSQQPNSNIFFLADRQQCLSSYKNKVDNMKKELLDI-------------
>Scleropages_formosus
---MSVRE-QEKEG-----ASLG-DTSLKEELNKKIKEQKVVVDELSNLKKNRRVYVQQPNSNIFFLADKAETLGSCRRTLDNMKKEYQDL-------------
>Pseudopodoces_humilis
---MSGRPPRQEDG-------ESAALRHKEELSRRIKEQKIVVDELSNLKKNRKVYKQQPNSNIFFLADRTEMLSQCKNTLDELKKAHQELENSEKAKIKK---
>Lateolabrax_japonicus
---MSS-K-SDNSD-----NFDG-QSAAKEELNTKIKEQKVVVDELSNLKKNRKVYIQQRNSNIFFLADRGQTLGSCKKELDNMKKELLDM-------------
>Anoplopoma_fimbria
---MSSKI-HSNED-----NCEG-TSALKEELNIKIKEQKVVVDELSNLKKNRKVYVQQRNSNIFFLADRGQTLGSCKKELDNMKKHLQDM-------------
>Ceratotherium_simum_simum
---MPSRGTRPEDGAGLV--PTDDSAPHKEDLSNKIKEQKIVVDELSNLKKNRKVYRQQQNSNIFFLADRTEMLSESKNILDELKKEYQEIENSEKTKIKK---
>Manis_javanica
---MPSGGSRPEDGAAPV--PTENSILHKEDLSSKIKEQKIVVDELSNLKKNRKVYRQQQNSNIFFLADRTEMLSESKNILDELKKEYQEIENSEKTKIKK---
>Hippoglossus_hippoglossus
---MSSKG-RDNED-----SFEW-QSALKEELNKKIKEQKVVVDELSNLKKNRNVYIQQRNSNIFFLADRGQTLGSCKKELDNMKKELQDM-------------
>Pongo_abelii
---MPSRGTRPEDSSVLI--PTDNSTPHKEDLSSKIKEQKIVVDELSNLKKNRKVYRQQQNSNIFFLADRTEMLSESKNILDELKKEYQEIENLDKTKIKK---
>Loxodonta_africana
---MEMPSSRAHPEDSAELV--PSDNSTPHKEELNSKIKEQKIVVDELSNLKKNRKVYKQQQNSNIFFLADRTEMLSESKNILDELKKEYQEIENLEKTKIKK---
>Callospermophilus_lateralis
---------------------------------------MLRGVEMPIRGTRSDDSAVLF--STDYSTAQKEELSNKIKEQKIVVDELSNLKKNRKVYRQQQNSNIFFLSDRTEMLSENKNILDELKKEYQDIENSEKTKIKK---
>Oncorhynchus_mykiss
---MSAKNQREN-E-----NIDEEESTLKEELNKKIKEQKIVFDELSNLKKNRRVYVQQPNSNIFFLADRGQTLASCKKELDNMKKDM----------------
>Tupaia_chinensis
---MEMPSRGARLEDSAVRG--PTDDSTPHREDLHSKIKEQKIVVDELSNLKKNRRVYKQQQNSNIFFLADRAELLSESKNVLDELKKEYQEIENSEKTKIKK---
>Lepisosteus_oculatus
---MSNKN-QVKDD-----VVQA-KAKQKEELNKKIREQKIVIDELSNLKKTRRVYVQQPNSHIFFLANKAETLSASKNSLENMKKEYQDV-------------
>Ictalurus_punctatus
--MSTN-----LVHE-ERATKEELNKQIKEQKVVIDELSNLKKNRKVFVQQPNSNIFFLADKGEMLSTCKKDLDKMKKEYQDM-------------
>Trichechus_manatus_latirostr
---MEMPSSRAHPEDSTELV--PSDNSTPHREDLSSKIKEQKIVVDELSNLKKNRKVYKQQQNSNIFFLADRTEMLSESKNTLDELKKEYQEIENLEKTKIKKSSS
>Squalus_acanthias
--MEGTSD-----KVQSSEADRKEELNKQMKEQNVVIDELSNLKKSRRVYKQQPNSNIFFSADRTQTLSECKCALEEMKKEYQDLENSEKMKK-----
>Thamnophis_sirtalis
-------------MIISATGKEPGLLLT------------TQQEQMSS-------QALLREGGEGSKLPDKEELNRKIKEQKIVVDEISNLKKNRKVYRQQPNSNIFFLADKTQTLSECKITLDELKKAHQEIENPEKTKTKK---
>Tyto_alba
---MLDELKKAHQEMENSEKTKSKK---
>Trachemys_scripta
---MSGREPRR--------EEEGAKPHQKEELNRKIKEQKVVVDELSNLKKNRKVYKQQPNSNIFFLVDRTETLSECKNTLDDLKRAHQEIENSEKTKIRK---
>Takifugu_rubripes
---MAFND-----HFDE-QSTLKEELNRKLKEQKVVVDELSNLKKNRKVYTQQRNSNIFFLADRAETLSSNKKKLDFMKKEVQNMKD-----------
>Orycteropus_afer_afer
--MLSESKNILDELKKEYQEIENSEKTKIKK---

HG0031 ENSG00000138381 ASNSD1

>Homo_sapiens
---MPSRGTRPE---DSSVLIPTDNSTPHKEDLSSKIKEQKIVVDELSNLKKNRTEQKCCLRARIYWMN------------------------------------
>Acipenser_sinensis
--M----STK---SGQD-NTSEVNTKQKEKLNKKIKEQQVLVDELSNLKKNRKVYIQQPNSNIFFLGDKSETLSESKEMLDNMKKEYQDL-------------
>Amia_calva
---MS----NKS---QAKD-DQINGKAKQKEELNKKMKEQKVVIDELSNLKKNRKVYVQQQNSNIFFLEDKSETLATCKKSLDHMKKEYQDL-------------
>Channa_argus
---MT----SKT---QDNDINVEGQ-CGLKEELNRKIKEQKVAVDELSNLKKNRKVYIQQRNSNIFFLADRSQTLGSCKKELDNMKKELQDM-------------
>Anser_cygnoides
---MAARRGEEEEKKEEEEGEGESAVLRPRDQLSRRIKEQKVIIDELSNLKKNRKVYKQQPNSNIFFLVDRTETLSQCKIVLDELKKAHQEMENSEKTKIKK---
>Nanorana_parkeri
---MSD---------IVAGEDCRGWTDKQKEDFYTKFREQKVVIDELTNLKKDRKVYRQQPNSNIFFVADKVKTLSECKKTLADLEHTYQILENSEKLQDKK---
>Porichthys_notatus
---MS----SKL---HDID-ASFGG-TPSIEVLNRKISEQKVVLDELSNLKKNRKVYIQQQNSNIFFLADRKQTLSSCKKELDNLKRDLQDS-------------
>Oryzias_latipes
---MK--MS----SKK---YNNSDNSQEQ-TTYKEELSKKIKEQKVVVDELSNLKKNRKVYIQQRNSNIFFLADRSQTLSTSRKELDYLKKELQDI-------------
>Trachinotus_blochii
---MS----SKN---RDNDDNFEGQ-SALKEELNKKIKEQKVVVDELSNLKKNRKVYIQQRNSNIFFLGDRSQTLGSCKKELDNMKKELQDM-------------
>Acinonyx_jubatus
---MPSRGSQPE---DGAALSSADNSTPHKEDLSSKIKEQKIVVDELSNLKKNRKVYRQQQNSNIFFLADRTEMLSESKNILDELKKEYQEIENSEKTKIKK---
>Hynobius_chinensis
---MSGTKPKNK-------EAGSDSAAWTKEDLDTKIREQTVVVDELSNLKPNRKVYTQQPNSNIFFLADRTATLSESKKTLDDLKTARQELEKSQEVRVK----
>Micropterus_salmoides
--MNK--MS----SKI---RDNDDNCEGR-CALKEELNKKIKEQRVVVDELSNLKKNRKVYIQQRNSNIFFLADRSQTLASCKREMDNMKKDLQDM-------------
>Orcinus_orca
---MPSRGARPE---DGSGLVPTDNSTQHKEDLSSKIKEQKIVVDELSNLKKNRI----------HWMS------------------------------------
>Astyanax_mexicanus
---MS----IRSS--EQPE-DPAQEERPTREELNTKIKEQKVLVDELSNLKKNRKVYLQQPNSNIFFLSDKGETLSSCKKELDKMKKDYQDM-------------
>Calidris_pugnax
---MSGRAA-RQEEEEEKEKREESAKARHKEELSRRIKEQKVVVDELSNLKKNRKVYRQQPNSNIFFLVDRTETLSQCKNTLDELKKAHQEMENSEKTKVKK---
>Callorhinchus_milii
---MEAAETRAAP--GGEE-RETRDQATRRQELHNKIKEQNVAINELSNLKETRRVYKQQPNSNIFFLTDKTTALTECKRAFDELKKEHQDLENPDKMMK-----
>Rousettus_aegyptiacus
---MEMPFCETRPE---NGAELVPTDNSAPHKVDLSSKIKEQKVVVDELSNLKKNRI----------YWMS------------------------------------
>Neolamprologus_brichardi
---MS----SKS---YSNDEQFEGQ-AAVKEELNKKIKEQKVLVDELCNLKKNRKVYIQQRNSNIFFVGERSQTLTSCKKELDYMKKELQDI-------------
>Clupea_harengus
---MT----TKRN--LPAANTVQDE-TPQKEELSKKIKEQKILVDELSNLKKNRRVYLQQPNSNIFFLTDRGQMLSTSKKELDNLKKDYQDM-------------
>Latimeria_chalumnae
---MS----GRV---QGED-NESQLRTSHKEELHKKIKEQKVLVDELSNLKKNRKVYQQQPNSNIFFLADRSKTLSDGKKKLDDMKREYQEL-------------
>Columba_livia
---MSGRPP-RQEE------GGDSAKLRHKDELSRKIKEQKVVVDELSNLKKNRKVYRQQPNSNIFFLVDRTETLSQCKNTLDELKKAHQEMENSEKTKIKK---
>Alligator_sinensis
MRRERARVRNHPAAWVGGASCAEGVWLGGAEVRSQSKGAARLVSG--MSGRAP-RRD--------EEEARLRRKEELNGKIKEQKIVVDELSNLKKNRKVYRQQPNSNIFFLADRTETLSQCKNKLDELKKAHQEIENSEKSKIKK---
>Danio_rerio
---MS----SKNI--QRTE-PGASEERAQKEELNQKIKEQKIVVDELSNLKKNRRVYTQQPNSNIFFLADKGETLSSCKQDLDNMRKEYQDI-------------
>Kryptolebias_marmoratus
---MS----LKS---PDSSDNSAEQ-SALKEELNKKIKEQKIVLDELSNLKKNRRVFIQQRNSNIFFLADRSQTLSSCKNDLDVLKKELQDV-------------
>Sarcophilus_harrisii
---MPGRGTRVE---DGPEPAISAKLPLNKEELNSKIKEQKVVVDELSNLKKNRTEQKCSLKVKTH---------------------------------------
>Monodelphis_domestica
---MPGKGTRRE---DATEPGTFASLPLNKEELNSKIKEQKVVVDELSNLKKNRIEQKCSLKAKIH---------------------------------------
>Esox_lucius
---MS----------VKNEIFDEEE-SALKVELNKKIKEQKVVVDELSNLKKSRRVYVQQPNSNIFFLADRGQTLSSCKRARQHEEGSPKYVIF-----------
>Aquila_chrysaetos_canadensis
---MSGRAP-RQEEG-------ESAKLRRKEELSRRIKEQKVVVDELSNLKKNRKVYRQQPNSNIFFLVDRTETLSQCKNTLDELKKAHQEMENSEKAKIKK---
>Meleagris_gallopavo
---MSRREEEESAELGHKEELSRRIKEQKVVVDELSNLKKNRKVYRQQPNSNIFFLVDRTETLSQCKNTLDQLKKAHQELENSDKTKVKK---
>Acanthogobius_hasta
--MS---RNTEDNNEGL-SALKEEMNKKIREQKVVLDELSNLKKGRKVYTQQRNSNIFFLAERSQTLSACKKELDVMKKELQDI-------------
>Condylura_cristata
---MPSQ--GPE---EGARLVSSDNSSLIKEDLSSKIKEQKIVVDELSNLKKNRKVYRQQQNSNVFFLADRTEMLSESKNILDELKKEYQEIENLDKTQIKK---
>Oryctolagus_cuniculus
---MPGRGVHPE---DSAVPVPAGGRTLHKEDLCNKIKEQKVVVDELSNLKKNRRVYRQQQNSNIFFLADRTEMLSESKNILDELKKEYQEIENSEKSSIKK---
>Plecoglossus_altivelis
---MS----SKCQ--LDLENLFAGQESKRKEELNKKINEQKVVVDELNNLKKNRKVYSQQPNSNIFFLADRQQCLSSYKNKVDNMKKELLDI-------------
>Scleropages_formosus
---MS----VRE---QEKE-GASLGDTSLKEELNKKIKEQKVVVDELSNLKKNRRVYVQQPNSNIFFLADKAETLGSCRRTLDNMKKEYQDL-------------
>Zonotrichia_albicollis
---MSGRPP-RQE-------DGDSAALRHKEELSRRIKEQKVVVDELSNLKKNRKVYKQQPNSNIFFLADRTEMLSQSKNTLDELKKAHQELENSEKAKIKK---
>Lateolabrax_japonicus
--M----SSK---SDNSDNFDGQ-SAAKEELNTKIKEQKVVVDELSNLKKNRKVYIQQRNSNIFFLADRGQTLGSCKKELDNMKKELLDM-------------
>Perca_flavescens
---MS----SKS---RDNDDNFEGQ-SAHKEEMNRKIKEQKIVVDELSNLKKNRKVYIQQRNSNIFFLADRSQTLGLCKKELDNMKKDLQDM-------------
>Ceratotherium_simum_simum
---MPSRGTRPE---DGAGLVPTDDSAPHKEDLSNKIKEQKIVVDELSNLKKNRKVYRQQQNSNIFFLADRTEMLSESKNILDELKKEYQEIENSEKTKIKK---
>Manis_javanica
---MPSGGSRPE---DGAAPVPTENSILHKEDLSSKIKEQKIVVDELSNLKKNRKVYRQQQNSNIFFLADRTEMLSESKILFPSLHRYIG---------------
>Hippoglossus_hippoglossus
---MS----SKG---RDNEDSFEWQ-SALKEELNKKIKEQKVVVDELSNLKKNRNVYIQQRNSNIFFLADRGQTLGSCKKELDNMKKELQDM-------------
>Loxodonta_africana
---MEMPSSRAHPE---DSAELVPSDNSTPHKEELNSKIKEQKIVVDELSNLKKNRTEQKCFLKAKIYWMN------------------------------------
>Ictidomys_tridecemlineatus
---MLRGVEMPIRGTRSD---DSAVSVSTDNSTAHKEELSNKIKEQKIVVDELSNLKKNRKVYRQQQNSNIFFLADRTEMLSENKNILDELKKEYQDIENSEKTKIKK---
>Oncorhynchus_mykiss
---MS----AKNQ--RENENIDEEE-STLKEELNKKIKEQKIVFDELSNLKKNRRVYVQQPNSNIFFLADRGQTLASCKKELDNMKKDM----------------
>Tupaia_chinensis
---MEMPSRGARLE---DSAVRGPTDDSTPHREDLHSKIKEQKIVVDELSNLKKNRRVYKQQQNSNIFFLADRAELLSESKNVLDELKKEYQEIENSEKTKIKK---
>Lepisosteus_oculatus
---MS----NKN---QVKD-DVVQAKAKQKEELNKKIREQKIVIDELSNLKKTRRVYVQQPNSHIFFLANKAETLSASKNSLENMKKEYQDV-------------
>Ictalurus_punctatus
--MST-NLVHEERATKEELNKQIKEQKVVIDELSNLKKNRKVFVQQPNSNIFFLADKGEMLSTCKKDLDKMKKEYQDM-------------
>Trichechus_manatus_latirostr
---MEMPSSRAHPE---DSTELVPSDNSTPHREDLSSKIKEQKIVVDELSNLKKNRKVYKQQQNSNIFFLADRTEMLSESKNTLDELKKEYQEIENLEKTKIKKSSS
>Squalus_acanthias
---ME--GTSD-KVQSSEADRKEELNKQMKEQNVVIDELSNLKKSRRVYKQQPNSNIFFSADRTQTLSECKCALEEMKKEYQDLENSEKMKK-----
>Protobothrops_mucrosquamatus
---MSSPA--------LLGEGGEESKLPDKEELNRKIKEQKIVVDELSNLKKNRKVYRQQPNSNIFFLADRTQTLSECRITLDELKKAHQEIENSEKTKTKK---
>Trachemys_scripta
---MSGREP-RRE--------EEGAKPHQKEELNRKIKEQKVVVDELSNLKKNRKVYKQQPNSNIFFLVDRTETLSECKNTLDDLKRAHQEIENSEKTKIRK---
>Takifugu_rubripes
---MAFNDHFDEQ-STLKEELNRKLKEQKVVVDELSNLKKNRKVYTQQRNSNIFFLADRAETLSSNKKKLDFMKKEVQNMKD-----------

HG0031 ENSGALG00000041666

>Gallus_gallus
--MSRREEEETAELGYKGELSRRIKEQKVVVDELSNLKKNRKVYRQQPNSNIFFLVDRTEALSQCKNTLDQLKKAHQELENSEKTKVKK---
>Acipenser_sinensis
---MSTK-SGQD------NTS-EVNTKQKEKLNKKIKEQQVLVDELSNLKKNRKVYIQQPNSNIFFLGDKSETLSESKEMLDNMKKEYQDL-------------
>Amia_calva
---MSNKSQAKD------DQI-NGKAKQKEELNKKMKEQKVVIDELSNLKKNRKVYVQQQNSNIFFLEDKSETLATCKKSLDHMKKEYQDL-------------
>Channa_striata
---MKKELQDM-------------
>Anas_platyrhynchos
--MENSEKTKIKK---
>Pelophylax_nigromaculatus
---MTDV------EVEKREDCRAWTDKQKEEFYTKFREQKVVVDELSNLKQDRKVYRQQPNSNIFFLADKAKTLSECKKTLDDLKKTSQILENSEKIQDQK---
>Porichthys_notatus
---MSSKLHDID--------ASFGGTPSIEVLNRKISEQKVVLDELSNLKKNRKVYIQQQNSNIFFLADRKQTLSSCKKELDNLKRDLQDS-------------
>Oryzias_latipes
---MKMSSKKYNNS------DNS-QEQTTYKEELSKKIKEQKVVVDELSNLKKNRKVYIQQRNSNIFFLADRSQTLSTSRKELDYLKKELQDI-------------
>Caprimulgus_carolinensis
--MENSEKTKIKK---
>Trachinotus_blochii
---MSSKNRDND------DNF-EGQSALKEELNKKIKEQKVVVDELSNLKKNRKVYIQQRNSNIFFLGDRSQTLGSCKKELDNMKKELQDM-------------
>Cariama_cristata
--MLSQCKNTLDELKKAHQEMENSENTKVKK---
>Acinonyx_jubatus
---MPSRGSQPED--GAALSSADNSTPHKEDLSSKIKEQKIVVDELSNLKKNRKVYRQQQNSNIFFLADRTEMLSESKNILDELKKEYQEIENSEKTKIKK---
>Hynobius_retardatus
---MSGTKPK------NKEDGSDSAAWTKEDLDTKIREQTVVVDELSNLKPNRKVYTQQPNSNIFFLADRTATLSESKKTLDDLKTAHQELEKSQKARVK----
>Micropterus_salmoides
--MNKMSSKIRDND------DNC-EGRCALKEELNKKIKEQRVVVDELSNLKKNRKVYIQQRNSNIFFLADRSQTLASCKREMDNMKKDLQDM-------------
>Lipotes_vexillifer
---MPSRGARPED--GSGLVPTDNSTQHKEDLSSKIKEQKIVVDELSNLKKNRKVYRQQQNSDIFFLADRTEMLSESKNTLDELRKEYQEIENSEKTKIKK---
>Pygocentrus_nattereri
---MSVRSSEQT------DDLAQDERSAREELNRRIKEQKVVIDELSNLKKNRKVYIQQPNSNIFFLTDKGETLSSCKKELDKMKRHYQDM-------------
>Calidris_pugnax
---MSGRAARQEEEEEKEKREESAKARHKEELSRRIKEQKVVVDELSNLKKNRKVYRQQPNSNIFFLVDRTETLSQCKNTLDELKKAHQEMENSEKTKVKK---
>Callorhinchus_milii
--MEAAETRAAPGG-----EERETRDQATRRQELHNKIKEQNVAINELSNLKETRRVYKQQPNSNIFFLTDKTTALTECKRAFDELKKEHQDLENPDKMMK-----
>Myotis_lucifugus
---MEMPAREARPED--GAGRA-----APGREDLSSRIKEQKIVVDELSNLKKNRTVYRQQQNSNIFFLADRTELLSESKNILDELKKEYQEIENSEKTKTKK---
>Oreochromis_niloticus
---MSSKGCSSD------EQF-EGQAAVKEELNKKIKEQKVLVDELCNLKKNRKVYIQQRNSNIFFVGDRSQTLTSCKKELDYMKKELQDI-------------
>Pterocles_gutturalis
--MENSEKTKIKK---
>Alosa_alosa
--------------------------MCGIQVDNIS---YIVLFLLTMTTKRNLPA------ADTVQEETTQKEELSKQIKEQNILVDELSNLKKNRRVYLQQPNSNIFFLTDREQMLSTCKKDLDNLKKEYQAM-------------
>Latimeria_chalumnae
---MKREYQEL-------------
>Columba_livia
---MSGRPPR------QEEGGDSAKLRHKDELSRKIKEQKVVVDELSNLKKNRKVYRQQPNSNIFFLVDRTETLSQCKNTLDELKKAHQEMENSEKTKIKK---
>Alligator_sinensis
MRRERARVRNHPAAWVGGASCAEGVWLGGAEVRSQSKGAARLVSG--MSGRAPR------RD--EEEARLRRKEELNGKIKEQKIVVDELSNLKKNRKVYRQQPNSNIFFLADRTETLSQCKNKLDELKKAHQEIENSEKSKIKK---
>Cuculus_canorus
--MENSQKTEIKK---
>Pimephales_promelas
---MSSKNIHQP------EPGTQEELAQKEELNKKIKEQKILVDELSNLKKNRRVYTQQPNSNIFFLADKGESLSSCKKDLDNMRKEYQDI-------------
>Poecilia_formosa
---MSQTNSGSS------DNV-SKYSALKEELNRKVKEQKIIVDELSNLKKNRKVYIQQRNSNVFFLADRSQTLSSCKKQLDYMKKEQQDL-------------
>Sarcophilus_harrisii
---MPGRGTRVED--GPEPAISAKLPLNKEELNSKIKEQKVVVDELSNLKKNRKVYKQQPNSNIFFLADRTEMLSESKNTLDELKKIHQEIENTEKNKSKK---
>Monodelphis_domestica
---MPGKGTRRED--ATEPGTFASLPLNKEELNSKIKEQKVVVDELSNLKKNRKVYKQQPNSNIFFLSDRTEMLSESKNTLDELKKVHQEIENTEKNKIKK---
>Trichosurus_vulpecula
--MLSESKNTLDELKKIHQEIENTEKNKIKK---
>Esox_lucius
---MSVKN----------EIFDEEESALKVELNKKIKEQKVVVDELSNLKKSRRVYVQQPNSNIFFLADRGQTLSSCKRARQHEEGSPKYVIF-----------
>Aquila_chrysaetos_canadensis
---MSGRAPR------QEE-GESAKLRRKEELSRRIKEQKVVVDELSNLKKNRKVYRQQPNSNIFFLVDRTETLSQCKNTLDELKKAHQEMENSEKAKIKK---
>Gadus_morhua
---MTSNS-RED------EIS-EAHSAIKEELNKKIKEQKIVVDELSNLKKNRKVYVQQRNSNIFFLADKGQMLGTNKKELDILKKELQDM-------------
>Gavia_stellata
--MENSEKTKIKK---
>Acanthogobius_hasta
--MSRNTE------DNN-EGLSALKEEMNKKIREQKVVLDELSNLKKGRKVYTQQRNSNIFFLAERSQTLSACKKELDVMKKELQDI-------------
>Balearica_regulorum_gibberic
--MENSEKTKIKK---
>Condylura_cristata
---MPSQ--GPEE--GARLVSSDNSSLIKEDLSSKIKEQKIVVDELSNLKKNRKVYRQQQNSNVFFLADRTEMLSESKNILDELKKEYQEIENLDKTQIKK---
>Oryctolagus_cuniculus
---MPGRGVHPED--SAVPVPAGGRTLHKEDLCNKIKEQKVVVDELSNLKKNRRVYRQQQNSNIFFLADRTEMLSESKNILDELKKEYQEIENSEKSSIKK---
>Lutjanus_erythropterus
---MKKELQDM-------------
>Ornithorhynchus_anatinus
--MLSESKNTLDELKKTHQEMDSLEKTKAKK---
>Plecoglossus_altivelis
---MSSKCQLDL-----ENLFAGQESKRKEELNKKINEQKVVVDELNNLKKNRKVYSQQPNSNIFFLADRQQCLSSYKNKVDNMKKELLDI-------------
>Scleropages_formosus
---MSVREQEK-------EGASLGDTSLKEELNKKIKEQKVVVDELSNLKKNRRVYVQQPNSNIFFLADKAETLGSCRRTLDNMKKEYQDL-------------
>Taeniopygia_guttata
--MSGGPA------RQEDGESAALRHKEELSRRIKEQKVVVDELSNLKKNRKVYKQQPNSNIFFLADRTEMLSQCKNTLDELKKAHQELENSEKAKIKK---
>Nipponia_nippon
--MENSEKTRNKK---
>Lateolabrax_japonicus
---MSSK-SDNS------DNF-DGQSAAKEELNTKIKEQKVVVDELSNLKKNRKVYIQQRNSNIFFLADRGQTLGSCKKELDNMKKELLDM-------------
>Perca_flavescens
---MSSKSRDND------DNF-EGQSAHKEEMNRKIKEQKIVVDELSNLKKNRKVYIQQRNSNIFFLADRSQTLGLCKKELDNMKKDLQDM-------------
>Ceratotherium_simum_simum
---MPSRGTRPED--GAGLVPTDDSAPHKEDLSNKIKEQKIVVDELSNLKKNRKVYRQQQNSNIFFLADRTEMLSESKNILDELKKEYQEIENSEKTKIKK---
>Manis_javanica
---MPSGGSRPED--GAAPVPTENSILHKEDLSSKIKEQKIVVDELSNLKKNRKVYRQQQNSNIFFLADRTEMLSESKNILDELKKEYQEIENSEKTKIKK---
>Picoides_pubescens
--MENPEKTKIKK---
>Hippoglossus_hippoglossus
---MSSKGRDNE------DSF-EWQSALKEELNKKIKEQKVVVDELSNLKKNRNVYIQQRNSNIFFLADRGQTLGSCKKELDNMKKELQDM-------------
>Microcebus_murinus
---MPSRATHPED--SAVLVSTDESTPYKGDLSNKIKEQKIVVDELSNLKKNRKVYKQQQNSNIFFLADRTEMLSQSKNTLDELKKEYQEIENSEKTKIKK---
>Loxodonta_africana
---MEMPSSRAHPED--SAELVPSDNSTPHKEELNSKIKEQKIVVDELSNLKKNRKVYKQQQNSNIFFLADRTEMLSESKNILDELKKEYQEIENLEKTKIKK---
>Fulmarus_glacialis
--MENSEKTKIKK---
>Melopsittacus_undulatus
--MENSEKTKIEK---
>Callospermophilus_lateralis
---MLRGVEMPIRGTRSDD--SAVLFSTDYSTAQKEELSNKIKEQKIVVDELSNLKKNRKVYRQQQNSNIFFLSDRTEMLSENKNILDELKKEYQDIENSEKTKIKK---
>Oncorhynchus_mykiss
---MSAKNQREN------ENIDEEESTLKEELNKKIKEQKIVFDELSNLKKNRRVYVQQPNSNIFFLADRGQTLASCKKELDNMKKDM----------------
>Tupaia_chinensis
---MEMPSRGARLED--SAVRGPTDDSTPHREDLHSKIKEQKIVVDELSNLKKNRRVYKQQQNSNIFFLADRAELLSESKNVLDELKKEYQEIENSEKTKIKK---
>Lepisosteus_oculatus
---MSNKNQVKD-------DVVQAKAKQKEELNKKIREQKIVIDELSNLKKTRRVYVQQPNSHIFFLANKAETLSASKNSLENMKKEYQDV-------------
>Ictalurus_punctatus
---MST------NLV-HEERATKEELNKQIKEQKVVIDELSNLKKNRKVFVQQPNSNIFFLADKGEMLSTCKKDLDKMKKEYQDM-------------
>Trichechus_manatus_latirostr
---MEMPSSRAHPED--STELVPSDNSTPHREDLSSKIKEQKIVVDELSNLKKNRKVYKQQQNSNIFFLADRTEMLSESKNTLDELKKEYQEIENLEKTKIKKSSS
>Aptenodytes_forsteri
--MENSEKTKIKK---
>Squalus_acanthias
---ME----G-T-----SDKVQSSEADRKEELNKQMKEQNVVIDELSNLKKSRRVYKQQPNSNIFFSADRTQTLSECKCALEEMKKEYQDLENSEKMKK-----
>Anolis_carolinensis
---MSSQMPQ------REEEGEGAKLGDKEELSRKIKEQKVIVDELSNLKKNRKVYRQQQNSNIFFLADRTQTLSECKNTLDELKKAHQEMENSEKTGIKK---
>Tyto_alba
---MLDELKKAHQEMENSEKTKSKK---
>Chelonia_mydas
---MSGREPR------REE--EGAKLHQKDELTRKIKEQKVVVDELSNLKKNRKVYKQQPNSNIFFLVDRTEMLSECKNTLDDLKRAHQEIENSEKTKIRK---
>Takifugu_rubripes
---MAF-----NDHF-DEQSTLKEELNRKLKEQKVVVDELSNLKKNRKVYTQQRNSNIFFLADRAETLSSNKKKLDFMKKEVQNMKD-----------
>Calypte_anna
--MENSDKTKVKK---
>Orycteropus_afer_afer
--MLSESKNILDELKKEYQEIENSEKTKIKK---

HG0032 ENSDARG00000104981 SLC45A4

>Danio_rerio
MGILRCFPSGFSRPSPLGLPS--TLY-----------STASIQIMKLVLTEWQLIWIFTVSSRQ
>Acipenser_sinensis
MGINRGAPTCVA-FPSLCCQP--TLF-----------STCSIAIMKLVLTEWQLIWIL-NCSRQ
>Amia_calva
MGIHRGAPTCVA-RPLLHCSP--SLF-----------STSSSEIMKLVLTEWQLIWIFTNCSRQ
>Channa_argus
MGIFRGAPPTAAACPPPPSH------CTPTHSIIQPSIQPFLYKMTLELTEWSSIWIFSACNRQ
>Pseudacris_regilla
MGINSASPVCVA-FPACSRKT--ENF-----------TTSSIEIMKLVLTEWQL-WIFNSNSQ-
>Chaetura_pelagica
--MKLVLTEWQL-WIFNSNSQ-
>Oryzias_melastigma
MGIFRGAPPAVVTCPPPSRP-PCSLLPAPPQTVIEPSIQPPQHRMTLELTEWSSIRIFTACNRQ
>Mustela_putorius_furo
MGSNGGPPSCVS-FPPLCSRT--NNF-----------STASITMMELVLTEWQL-WIFNSNSQ-
>Hynobius_retardatus
MGINSGAPLCVA-FTTRCSQT--NHF-----------TTSSIEIMKLVLTEWQL-WIFNSSSQ-
>Pygocentrus_nattereri
MGIHRRAPTCVS-CPPACRLP--SLY-----------NTSSIEIMKLVLTEWQMIWIFTVSSRQ
>Calidris_pugnax
MGINSGAPLCVA-FPPLCSKN--NNF-----------STSSIIIMKLVLTEWQL-WIFNSNSQ-
>Callorhinchus_milii
MGSNSGAPPCVA-VPALYYKQ---NL-----------NTSSIKIMKLVLTEWQLIWIFNSNRQ-
>Myotis_brandtii
MGSNGGAPSRVS-APPLRSPR--NHA-----------CTPSVMMMKLVLTEWQL-WICNSHSQ-
>Haplochromis_burtoni
MGIFRGAPPTVAAFPPPPTPSPRDLLIAPTHSVIQPSIQPSIQKMTLELTEWSSIWIFTACNRQ
>Pterocles_gutturalis
--MKLVLTEWQL-WIFNSNSQ-
>Dasypus_novemcinctus
MGIHSGPPFCVT-SPPLCSKK--NNF-----------STSSIIMMKLVLTEWQL-WIFNSNSQ-
>Alosa_alosa
MGIHRGAPTCAV-CPPPRRLP--SLY-----------RTSSIQIMKLVLTEWQSIWIFTNFSRQ
>Latimeria_chalumnae
MGIHGGPPLCVA-FPPLCSKK--NIF-----------NTSSIKIMKLILTEWQLLWIFINSSRQ
>Colius_striatus
--MKLVLTEWQL-WIFNSNSQ-
>Columba_livia
MGINSGAPLCVA-FPPLCSKN--NNF-----------STSSIIIMKLVLTEWQL-WIFNSNSQ-
>Leptosomus_discolor
--MKLVLTEWQL-WIFNSNSQ-
>Alligator_mississippiensis
MGINSGAPLCVA-FPPLCNKK---NF-----------STSSIIIMKLVLTEWQL-WIFNSNSQ-
>Cuculus_canorus
--MKLVLTEWQL-WIFNSNSQ-
>Poecilia_formosa
MGIFAGAPPAIATRPSPPS--HCSLSPVPTHTI----IQPPRHKMTLELTEWSSIWIFTACNRQ
>Sarcophilus_harrisii
MGINSGPPFCDT-FSPLCSKK--NNF-----------STSSIIMMKLVLTEWQL-WIFNSNSQ-
>Monodelphis_domestica
MGINSGPPFSDT-FPLLCSKK--NNF-----------STSSIIMMKLVLTEWQL-WIFNSNSQ-
>Esox_lucius
MGIHRGALPCFASCPPPRCLP--SLF-----------CTSSTQIMKLVLTEWQLIWIFTDSSRQ
>Aquila_chrysaetos_canadensis
MGINSGAPLCVA-FPPLCSKN--NNF-----------STSSIIIMKLVLTEWQL-WIFNSNSQ-
>Coturnix_japonica
MGINSGAPLCVA-CPPLCSKN--NNF-----------STSSIIIMKLVLTEWQL-WIFNSNSQ-
>Eurypyga_helias
--MKLVLTEWQL-WIFNSNSQ-
>Condylura_cristata
MGSNRGPPLCVA-LPPLRGTE--SSF-----------STASAIMMKLVLTEWQL-WIFNSDSQ-
>Oryctolagus_cuniculus
MGISRAPAVSVT-VPPLHSKK--RAC-----------STCSVMMMKLVLTEWQL-WIFNSNSQ-
>Opisthocomus_hoazin
--MKLVLTEWQL-WIFNSNSQ-
>Scleropages_formosus
MGIYRGAPTCVA-SPPLHRLP--PLF-----------STSSTEIMKLVLTEWQLIWIFTDHSRQ
>Acanthisitta_chloris
MGINSGAPLCVA-FPPLCSTN--NNF-----------STSSIIIMKLVLTEWQL-WIFNTNSQ-
>Phaethon_lepturus
MGINSGAPLCVA-FPPLCSKN--NNF-----------STSSIIIMKLVLTEWQL-WIFNSNSQ-
>Equus_asinus
MGSNGGPPFCVT-FPPLCSKK--NNF-----------STSSIIMMKLVLTEWQL-WIFNSNSQ-
>Manis_javanica
MGSNGGPPFCVT-FPPLCSKK--SNF-----------STTSVIMMKLVLTEWQL-WIFNSNSQ-
>Picoides_pubescens
--MKLVLTEWQL-WIFNSNSQ-
>Platichthys_stellatus
MGIFRGAPPTIAACPPPAPS-PR----APTLSV----IQPSIHKMTLELTEWSSIWIFTACNRQ
>Macaca_fascicularis
MGITSGTPVCVT-FPPLCSKK--IHF-----------STSSVIMMKLVLTEWQL-WIFNSNSQ-
>Loxodonta_africana
MGINSGLSFCVT-IPPLYSKK--NNF-----------STSSVIMMKLVLTEWQL-WIFNSNSQ-
>Melopsittacus_undulatus
--MKLVLTEWQL-WIFNSNSQ-
>Cavia_porcellus
MGIHRGLPVFVT-FPPLSSKR--SDC-----------SASSVMMMKLVLTEWHL-WIFNSNLQ-
>Oncorhynchus_mykiss
MGIHRGALTCVASCPPPCRLP--SLF-----------CTSSTHIMKLVLTEWQLIWIFTDSSRQ
>Tupaia_chinensis
MGSVSAAAVGAA-SKPLCSKS--HNR-----------STSSVKMMKLVLTEWQL-WICNSHSQ-
>Lepisosteus_oculatus
MGIFRGAPTGVA-SPPLRRLP--SLF-----------RTSSTEIMKLVLTEWQLIWIFINCSRQ
>Ictalurus_punctatus
MGIHRRAPSCVS-CPAARCLP--SLF-----------NTSSTEIMKLVLTEWQMIWIFTVSNRQ
>Aptenodytes_forsteri
--MKLVLTEWQL-WIFNSNSQ-
>Anolis_carolinensis
MGITSGAAVCVT-IPPLCSKQ--NNF-----------STSSIIIMKLVLTEWQL-WIFNSNSQ-
>Tyto_alba
MGINSGAPLCVA-FPPLCSKN--NNF-----------STSSIIIMKLVLTEWQL-WIFNSNSQ-
>Struthio_camelus_australis
MGINSGAPLCVA-FPPLCSKK--NNF-----------STSSIIIMKLVLTEWQL-WIFNSNSQ-
>Chrysemys_picta
MGINSGAPFCVA-FPPLCSKN--NNF-----------STLSIIIMKLVLTEWQL-WIFNSNSQ-
>Takifugu_rubripes
MGIIRGAPPNCTACPSPPSP-PSGLYFTPTHSDIQPTILPSIPKMTLELTEWSSIWIFTAYNRQ
>Tinamus_guttatus
MGINSGAPLCVA-FPPLCSKK--NNF-----------STSSIIIMKLVLTEWQL-WIFNSNSQ-
>Calypte_anna
--MKLVLTEWQL-WIFNSNSQ-

HG0033 ENSG00000112144 ICK

>Homo_sapiens
--MYLGDSHVLLNTLCWCCHRKIWLHSGEDCYHCRTEPLRP---------
>Anser_cygnoides
--MIPATVHTVLSALYLCCHRKNWLHSEEK-HKKH---------------
>Apteryx_australis_mantelli
--------------------------MQLKLPEDGGLSDQRLSLENMIPATIHTVLSALYLCCHRKNWLHSEEK-HKNH---------------
>Menidia_menidia
--MSPPA---LPTV-FCCCHRKIWLHSATGRERICFPPN-----------
>Oryzias_melastigma
--MTPPA---LPPG-FCCCHRKIWLHSAAGEEHVGFEVLSKDHVDG----
>Buceros_rhinoceros_silvestri
--MIPATVHTVLSALYLCCHRKNWLHLEEKQTKR----------------
>Caprimulgus_carolinensis
--MIPATIHSVLSALYLCCHRKNWLHSEEK-HQKH---------------
>Cariama_cristata
--MIPATVHTVLSALYLCCHRKNWLQSEEK-HKKH---------------
>Odobenus_rosmarus_divergens
--MYLGDSHVLLNTLCWCCHRKIWLHSGEACRLCRTTPPRP---------
>Physeter_catodon
--MYLGDSHVIWNTLCWCCHRKIWLHSGEVCYGCRTDPPRP---------
>Charadrius_vociferus
--MIPATVHTVLSALYLCCHRKNWLHSEEK-HKKH---------------
>Pteropus_vampyrus
--MYLGDSHVVLNTLCWCCHRKIWLHSGEACCHCRTEPPRP---------
>Pterocles_gutturalis
--MIPATVHTVLSALYLCCHRKNWLHSEEK-YEKHKKR------------
>Colius_striatus
--MIPATVHTVLSALYLCCHRKDWLHSEEK-H------------------
>Columba_livia
--MIPATVHTVLSALYLCCHRKNWLHSEKK-H------------------
>Merops_nubicus
--MIPATVHTVLSALYLCCHRKNWLHSEEK-HRKHQGLKCACS-------
>Alligator_mississippiensis
--MILAAFHGVLSALYLCCHRKNWLHSEEEHREVYPKD------------
>Cuculus_canorus
--MIPATVHTVLSALYLCCHRKNWLHSEEK-HKKHEGLKCACS-------
>Xiphophorus_maculatus
--MNTPA---QPNV-FCCCHRKIWLHSAVEGEHKCFSS------------
>Sarcophilus_harrisii
--MYLRDCHDISNTLCWCCHRKVWLHSEENCFHDRREQSQD---------
>Galeopterus_variegatus
--MYLGDSHVLLNALCWCCHRKIWLHSGEACCHCRTEPPRP---------
>Monodelphis_domestica
--MYLRHCPAIPNTLCWCWHRKVWLHSGEDCCSHERKGQSQD--------
>Esox_lucius
--MSPPA---VPVV-HCCCHRKIWLHSTAARGLPFVFSSSP---------
>Aquila_chrysaetos_canadensis
--MIPATVHTVLSALYLCCHRKNWLHSEEK-HKKH---------------
>Gallus_gallus
-------------------------------------MYQRLSLENMIPATVDTVLSALCLCCHRKNWLQSEKKH-------------------
>Gavia_stellata
--MIPATVHTVLSALYLCCHRKNWLHSEEK-HKNH---------------
>Eurypyga_helias
--MIPATVHTILSALYLCCHRKNWLHSEEE-HKKH---------------
>Erinaceus_europaeus
--MYLGDSHVLLNTLCWCCHRKIWLHSGEACCHCRTEPPQP---------
>Oryctolagus_cuniculus
--MYLGDSHVLLTTLCWCCHRKIWLHSGEACYHCRTEPPQP---------
>Ornithorhynchus_anatinus
--MRLGDHHVVSDSLFLCCHRKIWLRSGERRVLGN---------------
>Tauraco_erythrolophus
--MIPATVHTVLSALYLCCHRKNWLHSEEK-HKKH---------------
>Plecoglossus_altivelis
--MNPST---LPAV-HCCCHRKIWLHSAAGGGHLSLFLLVPAHPHLQFAE
>Parus_major
MSRARPALGRSCSAGRRGRGAAIPGLSAGRAGAGRHVRDQRLSLENMIPATVHTVLSALYLCCHRKNWLHSEEKKHRKRQGLKCACS-------
>Egretta_garzetta
--MIPATVHIVLSALYLCCHRKNWLHSEEK-H------------------
>Lateolabrax_japonicus
--MSPPA---LPVVQLCCCHRKIWLHSAARGISRQ---------------
>Ceratotherium_simum_simum
--MYLGDSHVLLNTLCWCCHRKIWLHSGEACCHCRTEPPRP---------
>Manis_javanica
--MYLGDSGVVLNTLGWCCHRKIWLHSGEACFCCGTAPPRL---------
>Picoides_pubescens
--MIPATVDTVLSALYLCCHRKNWLHSEKK-HRKH---------------
>Cynoglossus_semilaevis
--MSPPA---WPSV-SCCCHRKIWLHSAAGHHHSDAFNS-----------
>Loxodonta_africana
---------------------------MEQTSLSTVFISRRLIFENMYLGDSCVVLNPLCWCCHRKVWLHAGEACYRCRTEPPQP---------
>Fulmarus_glacialis
--MIPATVHTVLSALYLCCHRKNRLHSEEK-RKKH---------------
>Nestor_notabilis
--MIPATVHTVLSALYLCCHRKNWLHSEEK-HKKH---------------
>Mus_musculus
--MYLGDNHVLLNTLCWCCHRKIWLQSGEACYHCRTEPPRP---------
>Oncorhynchus_nerka
--MSPTA---VPVV-HCCCHRKIWLHSTDAGGFKPATSVFLLAIVI----
>Tupaia_chinensis
--MYLGDSRVLLNTLCWCCHRKIWLHSGEACDHGRTEPPRP---------
>Trichechus_manatus_latirostr
--MYLRDSHVVLNTLCWCCHRKVWLHSGEACYHCRTEPPQP---------
>Pygoscelis_adeliae
--MIPATVHTVLSALYLCCHRKNWLHSEEK-HKKH---------------
>Anolis_carolinensis
--MSPENFHAALEILCLCCHRKNWLHSEEKCFSCS---------------
>Tyto_alba
--MIPATVHTVLSALYLCCHRKNWLHSEKK-Y------------------
>Struthio_camelus_australis
--------------------MYMHVICNRDDLQLNSLSHQRLRLENMIPA-VHTVLSALYLCCHRKNWLHSEEKHKKH----------------
>Chrysemys_picta
--MTLVAIHAVLSTLYLCCHRKNWLHSEEKHVSCREAFTKD---------
>Calypte_anna
--MIPATVHTVLSALYLCCHRKNWLHSEEK-LKKH---------------
>Apaloderma_vittatum
--MIPATVHTVLSALYLCCHRKNWLHSEEKPRNTKD--------------
>Orycteropus_afer_afer
--MYLGDRHV-LNTLCWCCHRKIWLHSGGACYHCRTEPPQP---------

HG0033 ENSGALG00000016321 ICK

>Gallus_gallus
--------------------------MIPATVDTVLSALCLCCHRKNWLQSEKKH-----------
>Anas_platyrhynchos
----------MRVWSGQADQRLSLENMIPATVDTVLSALYLCCHRKNWLQSEEQHKNSGH------
>Apteryx_australis_mantelli
--MQL--KLPEDGGLSGNDQRLSLENMIPATIHTVLSALYLCCHRKNWLHSEEKHKNH--------
>Buceros_rhinoceros_silvestri
--------------------------MIPATVHTVLSALYLCCHRKNWLHLEEKQTKR--------
>Caprimulgus_carolinensis
--------------------------MIPATIHSVLSALYLCCHRKNWLHSEEKHQKH--------
>Cariama_cristata
--------------------------MIPATVHTVLSALYLCCHRKNWLQSEEKHKKH--------
>Ailuropoda_melanoleuca
--------------------------MYLGDSHVLLNTLCWCCHRKIWLHSGEACRLCRTAPPRP-
>Balaenoptera_acutorostrata_s
--------------------------MYLGDSHVLLSTLCWCCHRKIWLHSGEVCCGCRTDPPRP-
>Charadrius_vociferus
--------------------------MIPATVHTVLSALYLCCHRKNWLHSEEKHKKH--------
>Pteropus_alecto
--------------------------MYLGDSHVVLNTLCWCCHRKIWLHSGEACCHCRTEPPRP-
>Pterocles_gutturalis
--------------------------MIPATVHTVLSALYLCCHRKNWLHSEEKYEKHKKR-----
>Latimeria_menadoensis
--------------------------MSTVDLPVLLCYMYCCCHRKGWLHSKK-------------
>Colius_striatus
--------------------------MIPATVHTVLSALYLCCHRKDWLHSEEKH-----------
>Columba_livia
--------------------------MIPATVHTVLSALYLCCHRKNWLHSEKKH-----------
>Leptosomus_discolor
--------------------------MIPATVHTVLSALYLCCHRKNWLYSEKKH-----------
>Alligator_mississippiensis
--------------------------MILAAFHGVLSALYLCCHRKNWLHSEEEHREVYPKD----
>Cuculus_canorus
--------------------------MIPATVHTVLSALYLCCHRKNWLHSEEKHKKHEGLKCACS
>Sarcophilus_harrisii
--------------------------MYLRDCHDISNTLCWCCHRKVWLHSEENCFHDRREQSQD-
>Galeopterus_variegatus
--------------------------MYLGDSHVLLNALCWCCHRKIWLHSGEACCHCRTEPPRP-
>Haliaeetus_leucocephalus
--------------------------MIPATVHTVLSALYLCCHRKNWLHSEEKH-----------
>Gavia_stellata
--------------------------MIPATVHTVLSALYLCCHRKNWLHSEEKHKNH--------
>Balearica_regulorum_gibberic
--------------------------MIPATVHTVLSALYLCCHRKNWLHSEEKHKKH--------
>Erinaceus_europaeus
--------------------------MYLGDSHVLLNTLCWCCHRKIWLHSGEACCHCRTEPPQP-
>Oryctolagus_cuniculus
--------------------------MYLGDSHVLLTTLCWCCHRKIWLHSGEACYHCRTEPPQP-
>Tauraco_erythrolophus
--------------------------MIPATVHTVLSALYLCCHRKNWLHSEEKHKKH--------
>Opisthocomus_hoazin
--------------------------MIHATVLTVLSALYLCCHRKNWLHLEEKHRKHYGLKCACS
>Lepidothrix_coronata
--------------------------MIPATVHTVLSALYLCCHRKNWLQSKEKHKKH--------
>Nipponia_nippon
--------------------------MIPATVHTVLSALYLCCHRKNWLHSEEKHKKH--------
>Equus_asinus
--------------------------MYLGDSHVLLNTLCWCCHRKIWLHSGEACCRCRTGPPRP-
>Manis_javanica
--------------------------MYLGDSGVVLNTLGWCCHRKIWLHSGEACFCCGTAPPRL-
>Picoides_pubescens
--------------------------MIPATVDTVLSALYLCCHRKNWLHSEKKHRKH--------
>Homo_sapiens
--MG-YSFFPRSCGSLLPGRRLIFGNMYLGDSHVLLNTLCWCCHRKIWLHSGEDCYHCRTEPLRP-
>Loxodonta_africana
-------MEQTSLSTVFISRRLIFENMYLGDSCVVLNPLCWCCHRKVWLHAGEACYRCRTEPPQP-
>Fulmarus_glacialis
--------------------------MIPATVHTVLSALYLCCHRKNRLHSEEKRKKH--------
>Nestor_notabilis
--------------------------MIPATVHTVLSALYLCCHRKNWLHSEEKHKKH--------
>Cricetulus_griseus
--------------------------MYLGDNHVLLNTLCWCCHRKIWLQSGEACYCCRTDPPRP-
>Tupaia_chinensis
--------------------------MYLGDSRVLLNTLCWCCHRKIWLHSGEACDHGRTEPPRP-
>Trichechus_manatus_latirostr
--------------------------MYLRDSHVVLNTLCWCCHRKVWLHSGEACYHCRTEPPQP-
>Aptenodytes_forsteri
--------------------------MIPATVHTVLSALYLCCHRKNWLHSEEKHKKH--------
>Anolis_carolinensis
--------------------------MSPENFHAALEILCLCCHRKNWLHSEEKCFSCS-------
>Tyto_alba
--------------------------MIPATVHTVLSALYLCCHRKNWLHSEKKY-----------
>Struthio_camelus_australis
MYMHVICNRDDLQLNSLSHQRLRLENMIPA-VHTVLSALYLCCHRKNWLHSEEKHKKH--------
>Chrysemys_picta
--------------------------MTLVAIHAVLSTLYLCCHRKNWLHSEEKHVSCREAFTKD-
>Calypte_anna
--------------------------MIPATVHTVLSALYLCCHRKNWLHSEEKLKKH--------
>Apaloderma_vittatum
--------------------------MIPATVHTVLSALYLCCHRKNWLHSEEKPRNTKD------
>Orycteropus_afer_afer
--------------------------MYLGDRHV-LNTLCWCCHRKIWLHSGGACYHCRTEPPQP-

HG0034 ENSDARG00000060991 mief1

```
>Danio_rerio
------------------------------MGGWSRSAVLQLYRALLRAGQHLQYTDRDYYRRVVTREFRRCQT-LSAPAEREDALKRGQFFLNSGLGDLV
>Acipenser_sinensis
------------------------------MGGWSRSAVLQLYRALLRAGRPLQHTDRNYYRAALSREFRRCQG-LREEGARELALKRGLYFLHSGLGGLV
>Channa_punctata
MLHVQLYSLAVNRVPTSRCQGVPPLHVWSLMGGWSRCAVLELYRALLRAGRYLQYTDRNYYRRAVAREFRRCQD-LTIPQDKEEALKRGQFFLSSRLGGLM
>Anser_cygnoides
------------------------------MAAWSREAVLSLYRALLRRGRALRYTDRDFYRAAVRSEFRRQRG-LQRPEDRERQLEKGQAFLRSGLGGLI
>Rhacophorus_omeimontis
------------------------------MAGWSREAVLKLYRALLREGRKLQYTDRDYYSSYIRREFRKNQN-LANLEEKEKQLAKGQFFLQTKLGGLV
>Basilichthys_microlepidotus
------------------------------MGSWSRSAVLELYRALLRAGRHLQYTDRNYYRRAVAREFRRCQT-LTVPEDKEEALKRGQFFLNSRLGGLM
>Porichthys_notatus
------------------------------MGGWSRSAVLELYRALLRAGRRLQYTDRNYYYRVVALEFRRSQA-LTAPDQKEAALKRGQFFLSSKLGGLM
>Oryzias_melastigma
------------------------------MGSWSRVAVLELYRALLRAGRHLQYTDRNYYRQAVAREFRRCQA-LTVPEDKEEALKRGQFFLNSRLGGLM
>Tripterygion_delaisi
------------------------------MGSWSRGAVLELYRALLRAGRQLQHTERGYYRRAVAREFRRCQT-LTLPQDKEDALRRGQFFLSSRLGGLM
>Trachinotus_blochii
------------------------------MGGWSRSAVLELYRALLRAGRHLQYTDRNYYRRAVAREFRRCQA-LTVPADKEDALKRGQFFLNSRLGGLM
>Ailuropoda_melanoleuca
------------------------------MAPWSREAVLSLYRALLRQGRELRYTDRDFYLASIRREFRKNQK-LEDPEAREKQLEKGLVFLHSKLGGII
>Ambystoma_mexicanum
------------------------------MAAWSRTAVLNLYRTLLREGRHLKYTDRDFYFATVRREFRRNQD-LTEPDEKARQLEKGQYFLQTRLGGLV
>Micropterus_floridanus
------------------------------MGGWSRSAVLELYRALLRAGRHLQYTDRNYYRRAVAREFRRCQA-LTVPEDKEDALRRGQFFLRSRLGGLM
>Orcinus_orca
------------------------------MAPWSREAVLSLYRALLRQGRELRYTDRDFYFASIRREFRKNQK-LEDPEARERQLEKGLVFLHSKLGGII
>Astyanax_mexicanus
------------------------------MGGWSRTAVLELYRALLRAGRHLQYTDRNYYRRAVTREFRRCQA-LSTPGEKEDALRRGQFFLTSRLGGLV
>Calidris_pugnax
------------------------------MAAWSRQAVLTLYRALLRQGRGLRYTDQDFYLASIRREFRKNQG-LQQLEEKERQLEKGQAFLQSKLGGLV
>Callorhinchus_milii
------------------------------MATWSRTAVLNLYRALLRAGQHLRYTDREYYYSYVKREFRKNQH-LTEEQQRTQQLKKGIYFLNNKLGGLV
>Pteropus_vampyrus
------------------------------MAPWSREAVLSLYRALLRQGRELRYTDRDFYLASIRREFRKNQK-LEDPEARERQLEKGLVFLHSKLGGII
>Haplochromis_burtoni
------------------------------MGSWSRSAVLELYRALLRAGRHLQYTDRNYYRRAVAREFRRCQA-LTVPEDKEEALKRGRFFLSSRLGGLM
>Alosa_alosa
------------------------------MGGWSRSAVLELYRALLRAGRHLQYTDRNYYRRAVAREFRRCQA-LSAPQEKEDALKRGQFFLNSRLGGLV
>Latimeria_chalumnae
------------------------------MAGWSRTAVLNLYRALLREGRRLRYTDRDYYYNYVKREFRKNQG-LSQPEDREKQLEKGMFFLQNKLGGLI
>Alligator_mississippiensis
------------------------------MAAWSRQAVLSLYRTLLRQGRRLRFTDRDFYLATIRREFRKNKQ-LEQLEDKERQLEKGQAFLHSKLGGLV
>Cyprinodon_variegatus
------------------------------MGSWSRGAVLELYRALLRAGRHLQYTDRNYYRRAVTREFRRCQA-LTVPEDKEEALKRGQFFLSSRLGGLM
>Sarcophilus_harrisii
------------------------------MAEPGRGAVLGLYRTLLRRARELRLTDRDFYVETIRREFRRPLPPGAGPDAVQRQLEKGRAFLRGERGRLV
>Galeopterus_variegatus
------------------------------MAPWSREAVLSLYRALLRQGRELRYTDRDFYLAFIRREFRKNQK-LEDPEARERQLEKGLVFLHSKLGGII
>Monodelphis_domestica
------------------------------MAEPGRGAVLGLYRALLRRARGLRYTDRDFYVEAVRREFRRPLPPGVGPEAVQRQLDKGRAFLRGERGRLV
>Esox_lucius
------------------------------MGGWSRSAVLDLYRALLRAGRHLQYTDRDYYRRAVAHEFRRCQT-LTAPSDREDALKRGQFFLHSRLGGLM
>Numida_meleagris
------------------------------MAAWSRQAVLTLYRALLREGRGLRYTDRDFYLASIRREFRKNQE-LQRLEDKERQLEKGQAFLQSKLGGLV
>Acanthogobius_hasta
------------------------------MGGWSRSAVLELYRALLRAGRHLQYTDRNYYRRAVTREFRRCQA-LSGSQEKEDALKRGQFFLNSKLGGLM
>Apteronotus_leptorhynchus
------------------------------MGGWSRSAVLELYRALLRAGRHLQYTDRSYYQRAVTREFRRCQA-LNAPEEKEDALNRGRFFLSSRLGGLV
>Condylura_cristata
------------------------------MAPWSRSAVLSLYRALLRQGRELRYSDRDFYLASIRREFRKNQK-LEDPEAREKQLEKGLVFLHSKLGGLV
>Ochotona_princeps
------------------------------MAPWSRQAVLSLYRALLRQGRELRYTDRDFYLASIRREFRKNQK-LEEPEGRERQLEKGLVFLRSKLGGLV
>Plecoglossus_altivelis
------------------------------MGGWSRSAVLDLYRALLRAGRHLQHTDRNYYRRAVAREFRRCQA-LSAPSDKEDALKRGQFFLSSRLGGLV
>Scleropages_formosus
------------------------------MGGWSKTAVLELYRALLRAGRHLQYTDRNYYRHTLAREFRRCQA-LTNPQAREEALKRGQFFLRSRLGGLV
>Zonotrichia_albicollis
------------------------------MAAWSREAVLTLYRALLRRGRGLRYTDRDFYLASIRREFRRNQG-LQRLEDKERQLEKGQAFLRSSLGGLV
>Lateolabrax_japonicus
------------------------------MGGWSRSAVLELYRALLRAGRHLQYTDRNYYRRAVAREFRRCQA-LTIPEDKEDALKRGQFFLSSRLGGLM
>Anoplopoma_fimbria
------------------------------MGGWSRSAVLELYRALLRAGRHLQYTDRNYYRRAVAHEFRRCQA-LTIPEDKEEALRRGQFFLNSRLGGLM
>Ceratotherium_simum_simum
------------------------------MAPWSREAVLSLYRALLRQGRELRYTDRDFYLASIRREFRKNQK-LEDPEARERQLEKGLVFLHSKLGGLI
>Cynoglossus_semilaevis
------------------------------MGGWSRSAVLGLYKALLRAGRNLQYTDRNYYRRAVAREFRRCQA-LTIPEDKEEALKRGQFFLSSRLGGLM
>Pongo_abelii
------------------------------MAPWSREAVLSLYRALLRQGRQLRYTDRDFYFASIRREFRKNQK-LEDPVARERQLEKGLVFLNGKLGRII
>Leucoraja_erinacea
------------------------------MAAWSRVAVLNLYRSLLREGRRLRYTDREFYYTFIKREFRKNQH-LSGEEEKAQQLEKGLYFLESKIGGLV
>Jaculus_jaculus
------------------------------MAPWSREAVLSLYRALLRQGRELRYTDRDFYFASVRREFRKNQK-LQDPVAREKQLEKGLVFLHSKLGGLV
>Salvelinus_alpinus
------------------------------MGGWSRSAVLELYRALLRAGRHLQYTDRNYYRRAVAGEFRRCQA-LTVPSDKEDALKRGQFFLSSRLGGLM
>Lepisosteus_oculatus
------------------------------MGGWSRTAVLELYRALLRAGHHLQHTDRNYYRRAVAREFRRCQG-LTAPGDKERALQRGQFFLSSRLGGLV
>Ictalurus_punctatus
------------------------------MGGWSRGAVLELYRALLRAGRHLQYTDRNYYRRTIAREFRRCQS-LSEPREKEEALKRGQFFLNSRLGGLI
>Trichechus_manatus_latirostr
------------------------------MAPWSREAVLSLYRTLLRRGRELRYTDRDFYLASIRREFRKNQK-LEDPEARERQLEKGLFFLHSKLGGIV
>Squalus_acanthias
------------------------------MATWSRVAVLNLYRALLREGRNLQYTDREYYYTFIKREFRKNQR-LTGEEEKVKQLEKGIYFLKSKLGGLV
>Crotalus_adamanteus
------------------------------MATWSREAVLNLYRALLRQGRGLRYTDRDFYLSSIRKEFRKNQH-LTSPEDKERHLKKGQTFLHSKLGGLI
>Chrysemys_picta
------------------------------MAAWSREAVLNLYRALLRQGRGLRYTDRDFYLASIRREFRKNQQ-LERLEDRERQLEKGQAFLHSRLGGLV
>Takifugu_rubripes
------------------------------MGGWSRSAVLELYRALLRAGRHLQYTDRNYYRHAVVREFRRCQA-LTAPEEKEEALKRGQFFLKSRLGGLM
>Orycteropus_afer_afer
------------------------------MAPWSRAAVLSLYRTLLRQGRQLRYTDRDFYFASIRQEFRKNQK-LEDPAARERQLEKGLFFLHSKLGGII
```

HG0034 ENSG00000100335 MIEF1

```
>Homo_sapiens
------------------------------MAPWSREAVLSLYRALLRQGRQLRYTDRDFYFASIRREFRKNQK-LEDAEARERQLEKGLVFLNGKLGRII
>Acipenser_sinensis
------------------------------MGGWSRSAVLQLYRALLRAGRPLQHTDRNYYRAALSREFRRCQG-LREEGARELALKRGLYFLHSGLGGLV
>Channa_punctata
MLHVQLYSLAVNRVPTSRCQGVPPLHVWSLMGGWSRCAVLELYRALLRAGRYLQYTDRNYYRRAVAREFRRCQD-LTIPQDKEEALKRGQFFLSSRLGGLM
>Anser_cygnoides
------------------------------MAAWSREAVLSLYRALLRRGRALRYTDRDFYRAAVRSEFRRQRG-LQRPEDRERQLEKGQAFLRSGLGGLI
>Xenopus_tropicalis
------------------------------MAPWSREAVLSLYRALLRQGRQLRYTDRDFYFASIRREFRKNQK-LEDAEARERQLEKGLVFLNGKLGRII
>Basilichthys_microlepidotus
------------------------------MGSWSRSAVLELYRALLRAGRHLQYTDRNYYRRAVAREFRRCQT-LTVPEDKEEALKRGQFFLNSRLGGLM
>Porichthys_notatus
------------------------------MGGWSRSAVLELYRALLRAGRRLQYTDRNYYYRVVALEFRRSQA-LTAPDQKEAALKRGQFFLSSKLGGLM
>Oryzias_latipes
------------------------------MGSWSRVAVLELYRALLRAGRHLQYTDRNYYRQAVAREFRRCQA-LTVAEDKEEALKRGQFFLNSRLGGLM
>Tripterygion_delaisi
------------------------------MGSWSRGAVLELYRALLRAGRQLQHTERGYYRRAVAREFRRCQT-LTLPQDKEDALRRGQFFLSSRLGGLM
>Trachinotus_blochii
------------------------------MGGWSRSAVLELYRALLRAGRHLQYTDRNYYRRAVAREFRRCQA-LTVPADKEDALKRGQFFLNSRLGGLM
>Ursus_maritimus
------------------------------MAPWSREAVLSLYRALLRQGRELRYTDRDFYLASIRREFRKNQK-LEDPEAREKQLEKGLVFLHSKLGGII
>Hynobius_chinensis
------------------------------MAAWSRQAVLSLYRALLRGGRRLQYTDRDFYFASVRREFRRQQG-LTRPEDKERQLEKGLFFLRTQLGGLL
>Micropterus_floridanus
------------------------------MGGWSRSAVLELYRALLRAGRHLQYTDRNYYRRAVAREFRRCQA-LTVPEDKEDALRRGQFFLRSRLGGLM
>Orcinus_orca
------------------------------MAPWSREAVLSLYRALLRQGRELRYTDRDFYFASIRREFRKNQK-LEDPEARERQLEKGLVFLHSKLGGII
>Pygocentrus_nattereri
------------------------------MGGWSRSAVLELYRALLRAGRQLQYTDRNYYQRAVTQEFRRCQA-LSAPGEKEDALKRGQFFLSSRLGGLV
>Calidris_pugnax
------------------------------MAAWSRQAVLTLYRALLRQGRGLRYTDQDFYLASIRREFRKNQG-LQQLEEKERQLEKGQAFLQSKLGGLV
>Callorhinchus_milii
------------------------------MATWSRTAVLNLYRALLRAGQHLRYTDREYYYSYVKREFRKNQH-LTEEQQRTQQLKKGIYFLNNKLGGLV
>Pteropus_vampyrus
------------------------------MAPWSREAVLSLYRALLRQGRELRYTDRDFYLASIRREFRKNQK-LEDPEARERQLEKGLVFLHSKLGGII
>Haplochromis_burtoni
------------------------------MGSWSRSAVLELYRALLRAGRHLQYTDRNYYRRAVAREFRRCQA-LTVPEDKEEALKRGRFFLSSRLGGLM
>Alosa_alosa
------------------------------MGGWSRSAVLELYRALLRAGRHLQYTDRNYYRRAVAREFRRCQA-LSAPQEKEDALKRGQFFLNSRLGGLV
>Latimeria_chalumnae
------------------------------MAGWSRTAVLNLYRALLREGRRLRYTDRDYYYNYVKREFRKNQG-LSQPEDREKQLEKGMFFLQNKLGGLI
>Alligator_mississippiensis
------------------------------MAAWSRQAVLSLYRTLLRQGRRLRFTDRDFYLATIRREFRKNKQ-LEQLEDKERQLEKGQAFLHSKLGGLV
>Sinocyclocheilus_rhinocerous
------------------------------MGSWSRSAVLQLYRALLRAGQHLQYTDRDYYRRAVTREFRRCQT-LSAPAEREDALKKGQFFLNSGLGRLV
>Kryptolebias_marmoratus
------------------------------MGSWSRSAVLELYRALLRAGRRLQYTDRNYYRWAVAREFRRCQA-LTGAEDKEEALKRGRFFLRSGLGGLM
>Sarcophilus_harrisii
------------------------------MAEPGRGAVLGLYRTLLRRARELRLTDRDFYVETIRREFRRPLPPGAGPDAVQRQLEKGRAFLRGERGRLV
>Galeopterus_variegatus
------------------------------MAPWSREAVLSLYRALLRQGRELRYTDRDFYLAFIRREFRKNQK-LEDPEARERQLEKGLVFLHSKLGGII
>Monodelphis_domestica
------------------------------MAEPGRGAVLGLYRALLRRARGLRYTDRDFYVEAVRREFRRPLPPGVGPEAVQRQLDKGRAFLRGERGRLV
>Esox_lucius
------------------------------MGGWSRSAVLDLYRALLRAGRHLQYTDRDYYRRAVAHEFRRCQT-LTAPSDREDALKRGQFFLHSRLGGLM
>Numida_meleagris
------------------------------MAAWSRQAVLTLYRALLREGRGLRYTDRDFYLASIRREFRKNQE-LQRLEDKERQLEKGQAFLQSKLGGLV
>Acanthogobius_hasta
------------------------------MGGWSRSAVLELYRALLRAGRHLQYTDRNYYRRAVTREFRRCQA-LSGSQEKEDALKRGQFFLNSKLGGLM
>Apteronotus_leptorhynchus
------------------------------MGGWSRSAVLELYRALLRAGRHLQYTDRSYYQRAVTREFRRCQA-LNAPEEKEDALNRGRFFLSSRLGGLV
>Erinaceus_europaeus
------------------------------MAPWSREAVLSLYRALLRQGRELRYTDRDFYLASIRREFRKNQK-LEDPAAREKQLEKGLVFLHSKLGGII
>Ochotona_princeps
------------------------------MAPWSRQAVLSLYRALLRQGRELRYTDRDFYLASIRREFRKNQK-LEEPEGRERQLEKGLVFLRSKLGGLV
>Plecoglossus_altivelis
------------------------------MGGWSRSAVLDLYRALLRAGRHLQHTDRNYYRRAVAREFRRCQA-LSAPSDKEDALKRGQFFLSSRLGGLV
>Scleropages_formosus
------------------------------MGGWSKTAVLELYRALLRAGRHLQYTDRNYYRHTLAREFRRCQA-LTNPQAREEALKRGQFFLRSRLGGLV
>Sturnus_vulgaris
------------------------------MAAWSREAVLSLYRALLRRGRGLRYTDRDFYLASIRREFRRNQG-LQRLEDKERQLEKGQAFLQNRLGGLV
>Lateolabrax_japonicus
------------------------------MGGWSRSAVLELYRALLRAGRHLQYTDRNYYRRAVAREFRRCQA-LTIPEDKEDALKRGQFFLSSRLGGLM
>Trematomus_bernacchii
------------------------------MGGWSRGAVLELYRALLRAGRQLQYTDRNYYRRAVAHEFRRCQD-LKKAQDKEEALKRGQFFLDSRLGGLM
>Equus_asinus
------------------------------MAPWSREAVLSLYRALLRQGRELRYTDRDFYLASIRREFRKNQK-LEDPEARERQLEKGLVFLHSKLGGII
>Cynoglossus_semilaevis
------------------------------MGGWSRSAVLGLYKALLRAGRNLQYTDRNYYRRAVAREFRRCQA-LTIPEDKEEALKRGQFFLSSRLGGLM
>Leucoraja_erinacea
------------------------------MAAWSRVAVLNLYRSLLREGRRLRYTDREFYYTFIKREFRKNQH-LSGEEEKAQQLEKGLYFLESKIGGLV
>Psammomys_obesus
------------------------------MAPWSREAVLSLYRALLRQGRELRYTDRDFYFASIRREFRKNQK-LEDLEAREKQLEKGLVFLHSKLGGLI
>Salmo_salar
------------------------------MGGWSRSAVLELYRALLRAGRHLQYTDRNYYRRAVAGEFRRCQA-LTVSRDKEAALKRGQFFLSSRLGGLM
>Lepisosteus_oculatus
------------------------------MGGWSRTAVLELYRALLRAGHHLQHTDRNYYRRAVAREFRRCQG-LTAPGDKERALQRGQFFLSSRLGGLV
>Trichomycterus_areolatus
------------------------------MGGWSRGAVLQLYRALLRAGRNLQYTDRDYYRRAIAREFRRHQA-LTEPQEKEEALRRGQFFLSSRLGGLV
>Trichechus_manatus_latirostr
------------------------------MAPWSREAVLSLYRTLLRRGRELRYTDRDFYLASIRREFRKNQK-LEDPEARERQLEKGLFFLHSKLGGIV
>Squalus_acanthias
------------------------------MATWSRVAVLNLYRALLREGRNLQYTDREYYYTFIKREFRKNQR-LTGEEEKVKQLEKGIYFLKSKLGGLV
>Gekko_japonicus
------------------------------MAAWTREAVLNLYRSLLRQGRGLRYTDRDFYFTSIRKEFRKNQQ-LERLEDKERQLEKGQAFLRSKLGGLV
>Chrysemys_picta
------------------------------MAAWSREAVLNLYRALLRQGRGLRYTDRDFYLASIRREFRKNQQ-LERLEDRERQLEKGQAFLHSRLGGLV
>Takifugu_rubripes
------------------------------MGGWSRSAVLELYRALLRAGRHLQYTDRNYYRHAVVREFRRCQA-LTAPEEKEEALKRGQFFLKSRLGGLM
>Orycteropus_afer_afer
------------------------------MAPWSRAAVLSLYRTLLRQGRQLRYTDRDFYFASIRQEFRKNQK-LEDPAARERQLEKGLFFLHSKLGGII
```

HG0035 ENSG00000197147 LRRC8B

>Homo_sapiens
MKTNTEMVCLRNFKRCRPPD
>Anser_cygnoides
MKTNVKMACPRNFKRCRPPD
>Leptobrachium_boringii
MKSNAEMSRIKHFKRCRPPD
>Chaetura_pelagica
MKTNVKMACPRNFKRCRPPD
>Buceros_rhinoceros_silvestri
MKTNVKMACPRNFKRCRPPD
>Caprimulgus_carolinensis
MKTNVKMACPRNFKRCRPPD
>Cariama_cristata
MKTNVKMACPRNFKRCRPPD
>Acinonyx_jubatus
MKTNTEMVCLRNFKRCRPPD
>Ambystoma_mexicanum
------MQCTRKFKRCRPPD
>Physeter_catodon
MKTITEMVCLRNFKRCRPPD
>Charadrius_vociferus
MKTNVKMACPRNFKRCRPPD
>Myotis_ricketti
MKTNTEMVFLRNFKRCRPPD
>Pterocles_gutturalis
MKTNVKMACPRNFKRCRPPD
>Dasypus_novemcinctus
MKTNTEMVCLRNFKRCRPPD
>Colius_striatus
MKTNVKMACPRNFKRCRPPD
>Columba_livia
MKTNVKMACPRNFKRCRPPD
>Merops_nubicus
MKTNVKMACPRNFKRCRPPD
>Alligator_mississippiensis
MKANAKMACPRNFKRCRPPD
>Cuculus_canorus
MKANVKMACPRNFKRCRPPD
>Sarcophilus_harrisii
MKTKAEMVCLRNFKRCRPPD
>Galeopterus_variegatus
MKTNTEMVCLRNFKRCRPPD
>Monodelphis_domestica
MKTKAEMVCLRNFKRCRPPD
>Macropus_eugenii
MKTKAEMVCLRNFKRCRPPD
>Aquila_chrysaetos_canadensis
MKTNVKMACPRNFKRCRPPD
>Coturnix_japonica
MKTNVKMACPRNFKRCRPPD
>Gavia_stellata
MKTNVKMACPRNFKRCRPPD
>Balearica_regulorum_gibberic
MKTNVKMACPRNFKRCRPPD
>Condylura_cristata
MKTNTEMVCLRNFKRCRPPD
>Oryctolagus_cuniculus
MKKNTEMVCLRNFKRCRPPD
>Elephantulus_edwardii
MKTNTEMVCLRNFKRCRPPD
>Tauraco_erythrolophus
MKTNVKMACPRNFKRCRPPD
>Opisthocomus_hoazin
MKTNVKMACPRNFKKCRPPD
>Acanthis_flammea
MKTNVKMACPRNFKRCRPPD
>Egretta_garzetta
MKTNVKMACPRNFKRCRPPD
>Ceratotherium_simum_simum
MKTNTEMVCLRNFKRCRPPD
>Manis_javanica
MKTNTEMVCLRNFKRCRPPD
>Picoides_pubescens
MKTNVKMACPRNFKRCRPPD
>Loxodonta_africana
MKTNTEMVCLRNFKRCRPPD
>Melopsittacus_undulatus
MKTNVKMACPRNFKRCRPPD
>Nannospalax_galili
MKTNSEMVCLRNFKRCRPPD
>Tupaia_chinensis
MKTNTDMVCLRNFKRCRPPD
>Trichechus_manatus_latirostr
MKTNTEMVCLRNFKRCRPPD
>Aptenodytes_forsteri
MKTNVKMACPRNFKRCRPPD
>Gekko_japonicus
MKTVVKMACLRNFKRCRPPD
>Tyto_alba
MKTNVKMACPRNFKRCRPPD
>Struthio_camelus_australis
MKTNAKMACPRNFKRCRPPD
>Chrysemys_picta
MKINAKMACPRNFKRCRPPD
>Tinamus_guttatus
MKTNAKMACPRNFKRCRPPD
>Calypte_anna
MKTNVKMACPRNFKRCRPPD
>Apaloderma_vittatum
MKTNVKMACPRNFKRCRPPD
>Orycteropus_afer_afer
MKTNTEMVCLRNFKRCRPPD

HG0035 ENSGALG00000006167 LRRC8B

>Gallus_gallus
----------------------MKTNVKMACPRNFKRCRPPD
>Anser_cygnoides
----------------------MKTNVKMACPRNFKRCRPPD
>Leptobrachium_boringii
----------------------MKSNAEMSRIKHFKRCRPPD
>Chaetura_pelagica
----------------------MKTNVKMACPRNFKRCRPPD
>Buceros_rhinoceros_silvestri
----------------------MKTNVKMACPRNFKRCRPPD
>Caprimulgus_carolinensis
----------------------MKTNVKMACPRNFKRCRPPD
>Cariama_cristata
----------------------MKTNVKMACPRNFKRCRPPD
>Felis_catus
--------------MLAAHKCRMKTNTEMVCLRNFKRCRPPD
>Ambystoma_mexicanum
----------------------------MQCTRKFKRCRPPD
>Physeter_catodon
----------------------MKTITEMVCLRNFKRCRPPD
>Charadrius_vociferus
----------------------MKTNVKMACPRNFKRCRPPD
>Myotis_ricketti
----------------------MKTNTEMVFLRNFKRCRPPD
>Pterocles_gutturalis
----------------------MKTNVKMACPRNFKRCRPPD
>Dasypus_novemcinctus
----------------------MKTNTEMVCLRNFKRCRPPD
>Colius_striatus
----------------------MKTNVKMACPRNFKRCRPPD
>Columba_livia
----------------------MKTNVKMACPRNFKRCRPPD
>Merops_nubicus
----------------------MKTNVKMACPRNFKRCRPPD
>Alligator_mississippiensis
----------------------MKANAKMACPRNFKRCRPPD
>Cuculus_canorus
----------------------MKANVKMACPRNFKRCRPPD
>Sarcophilus_harrisii
----------------------MKTKAEMVCLRNFKRCRPPD
>Galeopterus_variegatus
----------------------MKTNTEMVCLRNFKRCRPPD
>Monodelphis_domestica
----------------------MKTKAEMVCLRNFKRCRPPD
>Macropus_eugenii
----------------------MKTKAEMVCLRNFKRCRPPD
>Aquila_chrysaetos_canadensis
----------------------MKTNVKMACPRNFKRCRPPD
>Gavia_stellata
----------------------MKTNVKMACPRNFKRCRPPD
>Balearica_regulorum_gibberic
----------------------MKTNVKMACPRNFKRCRPPD
>Condylura_cristata
----------------------MKTNTEMVCLRNFKRCRPPD
>Oryctolagus_cuniculus
----------------------MKKNTEMVCLRNFKRCRPPD
>Elephantulus_edwardii
----------------------MKTNTEMVCLRNFKRCRPPD
>Tauraco_erythrolophus
----------------------MKTNVKMACPRNFKRCRPPD
>Opisthocomus_hoazin
----------------------MKTNVKMACPRNFKKCRPPD
>Acanthis_flammea
----------------------MKTNVKMACPRNFKRCRPPD
>Egretta_garzetta
----------------------MKTNVKMACPRNFKRCRPPD
>Ceratotherium_simum_simum
----------------------MKTNTEMVCLRNFKRCRPPD
>Manis_javanica
----------------------MKTNTEMVCLRNFKRCRPPD
>Picoides_pubescens
----------------------MKTNVKMACPRNFKRCRPPD
>Homo_sapiens
----------------------MKTNTEMVCLRNFKRCRPPD
>Loxodonta_africana
----------------------MKTNTEMVCLRNFKRCRPPD
>Melopsittacus_undulatus
----------------------MKTNVKMACPRNFKRCRPPD
>Nannospalax_galili
----------------------MKTNSEMVCLRNFKRCRPPD
>Tupaia_chinensis
----------------------MKTNTDMVCLRNFKRCRPPD
>Trichechus_manatus_latirostr
----------------------MKTNTEMVCLRNFKRCRPPD
>Aptenodytes_forsteri
----------------------MKTNVKMACPRNFKRCRPPD
>Anolis_carolinensis
----------------------MKTLVRMACPRNFKRCRPPD
>Tyto_alba
----------------------MKTNVKMACPRNFKRCRPPD
>Struthio_camelus_australis
----------------------MKTNAKMACPRNFKRCRPPD
>Chelonia_mydas
MRVLFWKREPTELESHLKCLSRMKINAKMACPRNFKRCRPPD
>Tinamus_guttatus
----------------------MKTNAKMACPRNFKRCRPPD
>Calypte_anna
----------------------MKTNVKMACPRNFKRCRPPD
>Apaloderma_vittatum
----------------------MKTNVKMACPRNFKRCRPPD
>Orycteropus_afer_afer
----------------------MKTNTEMVCLRNFKRCRPPD

HG0036 ENSG00000155096 AZIN1

```
>Homo_sapiens
MIGGFHLPTCPFCCIF
>Anser_cygnoides
MIVGFHLLTCPFCCII
>Chaetura_pelagica
MIVGFHLLTCPFCCII
>Apteryx_australis_mantelli
MIVGFHLLTCPFCCII
>Buceros_rhinoceros_silvestri
MIVGFHLLTYPFCCII
>Caprimulgus_carolinensis
MIVGFHLLTCPFCCII
>Cariama_cristata
MIVGFHLLTCPFCCII
>Canis_lupus_familiaris
MIGGFHLLTCPFCCIF
>Ambystoma_mexicanum
MIVGFHLLTCSICCIF
>Lipotes_vexillifer
MIGGFHLLTCPFCCIF
>Calidris_pugnax
MIVGFHLLTCPFCCII
>Rousettus_aegyptiacus
MIGGFHLLTCPFCCIF
>Pterocles_gutturalis
MIVGFHLLTCPFCCII
>Dasypus_novemcinctus
MIGGFHLLTCPFCCIF
>Columba_livia
MIVGFHLLTCPFCCII
>Leptosomus_discolor
MIVGFHLLTCPFCCII
>Alligator_mississippiensis
MIVGFHLLTCPFCCII
>Cuculus_canorus
MIVGFHLLTCPFCCII
>Sarcophilus_harrisii
MIGGFHLLTCPFCCIF
>Galeopterus_variegatus
MIGGFHLLTCPFCCIF
>Monodelphis_domestica
MIGGFHLLTCPFCCIF
>Macropus_eugenii
MIGGFHLLTRPFCCIF
>Aquila_chrysaetos_canadensis
MIVGFHLLTCPFCCII
>Coturnix_japonica
MIVGFHLLTCPFCCII
>Mesitornis_unicolor
MIVGFHLLTCPFCCII
>Condylura_cristata
MIGGFHLLTCPFCCIF
>Elephantulus_edwardii
MIGGFHLLTCPFCCIF
>Tauraco_erythrolophus
MIVGFHLLTCPFCCII
>Opisthocomus_hoazin
MIVGFHLLTCPFCCII
>Ficedula_albicollis
MIVGFHLLTCPFCCII
>Phalacrocorax_carbo
MIVGFHLLTCPFCCII
>Equus_przewalskii
MIGGFHLLTCPFCCIF
>Manis_javanica
MIGGFHLLTCPFCCIF
>Picoides_pubescens
MIVGFHLLTCPFCCII
>Loxodonta_africana
MIGGFHLLTCPFCCIF
>Fulmarus_glacialis
MIVGFHLLTCPFCCII
>Melopsittacus_undulatus
MIVGFHLLTCPFCCII
>Cricetulus_griseus
MIGGFHLLTCPFCCIF
>Tupaia_chinensis
MIGGFHLLTCPFCCIF
>Trichechus_manatus_latirostr
MIGGFHLLTCPFCCIF
>Aptenodytes_forsteri
MIVGFHLLTCPFCCII
>Boiga_irregularis
MIVGFHLLTCPFCCII
>Tyto_alba
MIVGFHLLTCPFCCII
>Struthio_camelus_australis
MIVGFHLLTCPFCCII
>Chelonia_mydas
MIVGFHLLTCPFCCII
>Tinamus_guttatus
MIVGFHLLTCPFCCII
>Calypte_anna
MIVGFHLLTCPFCCII
>Apaloderma_vittatum
MIVGFHLLTCPFCCII
>Orycteropus_afer_afer
MIGGFHLLTCPFCCIF
```

HG0036 ENSGALG00000029295

>Gallus_gallus
MIVGFHLLTCPFCCII
>Anser_cygnoides
MIVGFHLLTCPFCCII
>Chaetura_pelagica
MIVGFHLLTCPFCCII
>Apteryx_australis_mantelli
MIVGFHLLTCPFCCII
>Buceros_rhinoceros_silvestri
MIVGFHLLTYPFCCII
>Caprimulgus_carolinensis
MIVGFHLLTCPFCCII
>Cariama_cristata
MIVGFHLLTCPFCCII
>Canis_lupus_familiaris
MIGGFHLLTCPFCCIF
>Hynobius_chinensis
MIVVFHLLTCPICCIL
>Lipotes_vexillifer
MIGGFHLLTCPFCCIF
>Calidris_pugnax
MIVGFHLLTCPFCCII
>Eptesicus_fuscus
MIGGFHLLTCPFCCIF
>Pterocles_gutturalis
MIVGFHLLTCPFCCII
>Dasypus_novemcinctus
MIGGFHLLTCPFCCIF
>Columba_livia
MIVGFHLLTCPFCCII
>Leptosomus_discolor
MIVGFHLLTCPFCCII
>Alligator_mississippiensis
MIVGFHLLTCPFCCII
>Cuculus_canorus
MIVGFHLLTCPFCCII
>Sarcophilus_harrisii
MIGGFHLLTCPFCCIF
>Galeopterus_variegatus
MIGGFHLLTCPFCCIF
>Monodelphis_domestica
MIGGFHLLTCPFCCIF
>Macropus_eugenii
MIGGFHLLTRPFCCIF
>Aquila_chrysaetos_canadensis
MIVGFHLLTCPFCCII
>Balearica_regulorum_gibberic
MIVGFHLLTCPFCCII
>Condylura_cristata
MIGGFHLLTCPFCCIF
>Elephantulus_edwardii
MIGGFHLLTCPFCCIF
>Tauraco_erythrolophus
MIVGFHLLTCPFCCII
>Opisthocomus_hoazin
MIVGFHLLTCPFCCII
>Ficedula_albicollis
MIVGFHLLTCPFCCII
>Phalacrocorax_carbo
MIVGFHLLTCPFCCII
>Ceratotherium_simum_simum
MIGGFHLLTCPFCCIF
>Manis_javanica
MIGGFHLLTCPFCCIF
>Picoides_pubescens
MIVGFHLLTCPFCCII
>Aotus_nancymaae
MIGGFHLPTCPFCCIF
>Loxodonta_africana
MIGGFHLLTCPFCCIF
>Fulmarus_glacialis
MIVGFHLLTCPFCCII
>Melopsittacus_undulatus
MIVGFHLLTCPFCCII
>Jaculus_jaculus
MIGGFHLLTCPFCCIF
>Tupaia_chinensis
MIGGFHLLTCPFCCIF
>Trichechus_manatus_latirostr
MIGGFHLLTCPFCCIF
>Aptenodytes_forsteri
MIVGFHLLTCPFCCII
>Boiga_irregularis
MIVGFHLLTCPFCCII
>Tyto_alba
MIVGFHLLTCPFCCII
>Struthio_camelus_australis
MIVGFHLLTCPFCCII
>Chelonia_mydas
MIVGFHLLTCPFCCII
>Tinamus_guttatus
MIVGFHLLTCPFCCII
>Calypte_anna
MIVGFHLLTCPFCCII
>Apaloderma_vittatum
MIVGFHLLTCPFCCII
>Orycteropus_afer_afer
MIGGFHLLTCPFCCIF

HG0037 ENSG00000140092 FBLN5

>Homo_sapiens
MKCSFLTFLLQLV
>Anser_cygnoides
MKCSFLTFLLQLV
>Xenopus_laevis
MKCSFLTFLQQLV
>Chaetura_pelagica
MKCSFLTFLLQLV
>Apteryx_australis_mantelli
MKCSFLTFLLQLV
>Buceros_rhinoceros_silvestri
MKCSFLTFLLQLV
>Caprimulgus_carolinensis
MKCSFLTFLLQLV
>Cariama_cristata
MKCSFLTFLLQLV
>Felis_catus
MKCSFLKFLLQLV
>Hynobius_retardatus
MKCSFLTFLLQLV
>Balaenoptera_acutorostrata_s
MKCSFLTFLLQLV
>Calidris_pugnax
MKCSFLTFLLQLV
>Myotis_brandtii
MKCSFLTFLLQLV
>Pterocles_gutturalis
MKCSFLTFLLQLV
>Dasypus_novemcinctus
MKCSFLAFLLQLV
>Colius_striatus
MKCSFLTFLLQLV
>Columba_livia
MKCSFLTFLLQLV
>Leptosomus_discolor
MKCSFLTFLLQLV
>Cuculus_canorus
MKCSFLTFLLQLV
>Monodelphis_domestica
MKCSFLTFLLQLV
>Trichosurus_vulpecula
MKCSFLTFLLQLV
>Aquila_chrysaetos_canadensis
MKCSFLTFLLQLV
>Coturnix_japonica
MKCSFLTFLLQLV
>Gavia_stellata
MKCSFLTFLLQLV
>Chlamydotis_macqueenii
MKCSFLTFLLQLV
>Condylura_cristata
MKCSFLTFLLQLV
>Oryctolagus_cuniculus
MKCSFLTFLLQLV
>Ornithorhynchus_anatinus
MKCSFLTFLLQLV
>Tauraco_erythrolophus
MKCSFLTFLLQLV
>Opisthocomus_hoazin
MKCSFLTFLLQLV
>Corvus_brachyrhynchos
MKCSFLTFLLQLV
>Egretta_garzetta
MKCSFLTFLLQLV
>Ceratotherium_simum_simum
MKCSFLTFLLQLV
>Picoides_pubescens
MKCSFLTFLLQLV
>Loxodonta_africana
MKCSFLTFLLQLV
>Fulmarus_glacialis
MKCSFLTFLLQLV
>Nestor_notabilis
MKCSFLTFLLQLV
>Chinchilla_lanigera
MKCSFLTFLLQLV
>Tupaia_chinensis
MKCSFLTFLLQLV
>Trichechus_manatus_latirostr
MKCSFLTFLLQLV
>Aptenodytes_forsteri
MKCSFLTFLLQLV
>Tyto_alba
MKCSFLTFLLQLV
>Struthio_camelus_australis
MKCSFLTFLLQLV
>Pelodiscus_sinensis
MKCSFLTFLLQLV
>Tinamus_guttatus
MKCSFLTFLLQLV
>Calypte_anna
MKCSFLTFLLQLV
>Apaloderma_vittatum
MKCSFLTFLLQLV

HG0037 ENSGALG00000010747 FBLN5

>Gallus_gallus
MKCSFLTFLLQLV
>Anser_cygnoides
MKCSFLTFLLQLV
>Xenopus_laevis
MKCSFLTFLQQLV
>Chaetura_pelagica
MKCSFLTFLLQLV
>Apteryx_australis_mantelli
MKCSFLTFLLQLV
>Buceros_rhinoceros_silvestri
MKCSFLTFLLQLV
>Caprimulgus_carolinensis
MKCSFLTFLLQLV
>Cariama_cristata
MKCSFLTFLLQLV
>Felis_catus
MKCSFLKFLLQLV
>Hynobius_retardatus
MKCSFLTFLLQLV
>Balaenoptera_acutorostrata_s
MKCSFLTFLLQLV
>Calidris_pugnax
MKCSFLTFLLQLV
>Myotis_brandtii
MKCSFLTFLLQLV
>Pterocles_gutturalis
MKCSFLTFLLQLV
>Dasypus_novemcinctus
MKCSFLAFLLQLV
>Colius_striatus
MKCSFLTFLLQLV
>Columba_livia
MKCSFLTFLLQLV
>Leptosomus_discolor
MKCSFLTFLLQLV
>Cuculus_canorus
MKCSFLTFLLQLV
>Monodelphis_domestica
MKCSFLTFLLQLV
>Trichosurus_vulpecula
MKCSFLTFLLQLV
>Aquila_chrysaetos_canadensis
MKCSFLTFLLQLV
>Gavia_stellata
MKCSFLTFLLQLV
>Chlamydotis_macqueenii
MKCSFLTFLLQLV
>Condylura_cristata
MKCSFLTFLLQLV
>Oryctolagus_cuniculus
MKCSFLTFLLQLV
>Ornithorhynchus_anatinus
MKCSFLTFLLQLV
>Tauraco_erythrolophus
MKCSFLTFLLQLV
>Opisthocomus_hoazin
MKCSFLTFLLQLV
>Corvus_brachyrhynchos
MKCSFLTFLLQLV
>Egretta_garzetta
MKCSFLTFLLQLV
>Ceratotherium_simum_simum
MKCSFLTFLLQLV
>Picoides_pubescens
MKCSFLTFLLQLV
>Aotus_nancymaae
MKCSFLTFLLQLV
>Loxodonta_africana
MKCSFLTFLLQLV
>Fulmarus_glacialis
MKCSFLTFLLQLV
>Nestor_notabilis
MKCSFLTFLLQLV
>Chinchilla_lanigera
MKCSFLTFLLQLV
>Tupaia_chinensis
MKCSFLTFLLQLV
>Trichechus_manatus_latirostr
MKCSFLTFLLQLV
>Aptenodytes_forsteri
MKCSFLTFLLQLV
>Tyto_alba
MKCSFLTFLLQLV
>Struthio_camelus_australis
MKCSFLTFLLQLV
>Pelodiscus_sinensis
MKCSFLTFLLQLV
>Tinamus_guttatus
MKCSFLTFLLQLV
>Calypte_anna
MKCSFLTFLLQLV
>Apaloderma_vittatum
MKCSFLTFLLQLV

HG0038 ENSGALG00000006571

>Gallus_gallus
---MSVTCWPRLCGSSPPSERPVAALWTP
>Anas_platyrhynchos
---MSVTCWPRLCGSSPPSERPVAALWTP
>Xenopus_tropicalis
---MSVKCLSRLCGSPP-SVWSVAAIPTP
>Apteryx_australis_mantelli
---MSVTCWPRLCGSSPPSERPVAALWTP
>Buceros_rhinoceros_silvestri
---MSVTCWPRLCGSSPPSERPVAALWTP
>Caprimulgus_carolinensis
---MSVTCWPRLCGSSPPSERPVAALWTP
>Cariama_cristata
---MSVTCWPRLCGSSPPSERPVAALWTP
>Ailuropoda_melanoleuca
---MSITCWLRLCSSPPLSEWPVSINWTP
>Orcinus_orca
---MSITCWLRLCSSPPLSEWPVSDNWTP
>Charadrius_vociferus
---MSVTCWPRLCGSSPPSERPVAALWTP
>Rousettus_aegyptiacus
---MSITCWLRLCGSPPLSEWPVSINWTP
>Pterocles_gutturalis
---MSVTCWPRLCGSSPPSERPVAALWTP
>Dasypus_novemcinctus
---MSITCCLRLCSAPPLSEWSVSLNWIP
>Latimeria_chalumnae
---MSMKCWSRHCGSPP-LVWSVAAIWTP
>Colius_striatus
---MSVTCWPRLCGSSPPSERPVAVLWTP
>Columba_livia
---MSVTCWPRLCGSSPPSERPVAALWTP
>Merops_nubicus
---MSVTCWPRLCGSSPPSERPVAALWTP
>Alligator_mississippiensis
---MSVTCWPRLCGSSPPLERPVATLWTP
>Sarcophilus_harrisii
---MSLTCWLRLCGSPPLSEWPVSINWTP
>Monodelphis_domestica
---MSLTCWSRLCGSAPLSEWPVSINWIP
>Aquila_chrysaetos_canadensis
---MSVTCWPRLCGSSPPSERPVAALWTP
>Gavia_stellata
---MSVTCWPRLCGASPPSGRPVAALWTP
>Chlamydotis_macqueenii
---MSVTCWPRLCGSSPPSERPVAALWTP
>Condylura_cristata
---MSITCWLRLCSSPPLSEWPVSINWTP
>Oryctolagus_cuniculus
---MSVTCWPRLCSSPPLSEWSVSINWTP
>Ornithorhynchus_anatinus
---MLTRSSESAAVMIFRRRW-QRGYWDFTSTGQYKGFLMSVTCWLMLCGSPPLSEWSVSIKWTP
>Opisthocomus_hoazin
---MSVTCWPRLCGSPPPPERPVAALWTP
>Ficedula_albicollis
---MSVTCWPRLCGSSPPSERPVAALWTP
>Nipponia_nippon
---MSVTCWPRLCGSSPPSERPVAALWTP
>Equus_asinus
---MSITCWLRLCSSPPLSEWPVSINWTP
>Manis_javanica
---MSIICWLRLCSSPPLSEWPVSINWTP
>Picoides_pubescens
---MSVTCWPRLCGSSPPSERPVAALWTP
>Callithrix_jacchus
-------------------------------MCSSMLFF-SILICVSTFSRSSELL-----ADNF-GGLHIQKSIKVTDARFLMSVTCWMRLCSSPPLSEWSVSIKWTP
>Loxodonta_africana
---MSVTWWSRLCSSPPLSEWSVSINWTP
>Fulmarus_glacialis
---MSVTCWPRLCGSSPPSERPVAALWTP
>Melopsittacus_undulatus
---MSVTCWPRLCGSSPPSERPVAALWTP
>Fukomys_darlingi
---MSVTCWLRICGSPPLSEWSVSISWTP
>Tupaia_chinensis
---MSITCWLRLRSSPPLSEWSVSVNWTP
>Trichechus_manatus_latirostr
---MSITCWLRLCSSPPLSEWSVSINWTP
>Aptenodytes_forsteri
---MSVTCWPRLCGSSPPSERPVAALWTP
>Anolis_carolinensis
---MSVTCWPRLCGCSPPLELPVATLWTP
>Struthio_camelus_australis
---MSVTCWPRLCGSSPPSERPVAALWTP
>Trachemys_scripta
MGRGSGRVRIAPGASAGAPRALSAVSARPVEEAQPVVLRGRGRGCRR--RRGSRSRLLPRKRRGAEPGLVARGARATGRPGFPMSVTCWPRLCGSSPPLERPVATFWTP
>Tinamus_guttatus
---MSVTCWPRLCGSSPPSERPVAALWTP
>Calypte_anna
---MSVTCWPRLCGSSPPSERPLAALWTP
>Apaloderma_vittatum
---MSVTCWPGLCGSSPPSERPVAALWTP
>Orycteropus_afer_afer
---MSISCWLRLCSLPPLPEWSISINWTP

HG0039.1 ENSG00000077092 RARB

>Homo_sapiens
MIIWINYRLLAGLSVIIHDSGLGKRPTAYVPKKGQSLMELGGLFY-AICLHT--
>Acipenser_sinensis
-------------------------------------MELGPLLH-AISLYT--
>Amia_calva
MIIWINYRLLAVLSVIIHDSSLGKRPRSSVPKRGQSLMELGPLLH-AISLHT--
>Anser_cygnoides
MIIWINYRLLAVLSVIIHDSGLGKRPTAYVPKKGQSLMELGVLLC-AIFLHT--
>Xenopus_tropicalis
MIIWINYRLLAVLSVIIHDSGLGKRPTAYVPKKGAEFDGVGCTFSIAICCAPKG
>Chaetura_pelagica
MIIWINYRLLAVLSVIIHDSGLGKRPTAYVPKKGQSLMELGVLSC-AIFLHT--
>Apteryx_australis_mantelli
MIIWINYRLLAVLSVIIHDSGLGKRPTAYVPKKGQSLMELGVLLC-AIFLLT--
>Buceros_rhinoceros_silvestri
MIIWINYRLLAVLSVIIHDSGLGKRPTAYVPKKGQSLMELGVLLC-AIFLHT--
>Ursus_maritimus
MIIWINYRLLAGLSVIIHDSGLGKRPTAYVPKKGQSLMELGGLFY-AICLHT--
>Orcinus_orca
MIIWINYRLLAGLSVIIHDSRLGKRPTAYVPKRGQSLMELGGLFY-AICLHT--
>Calidris_pugnax
MIIWINYRLLAVLSVIIHDSGLGKRPTAYVPKKGQSLMELGVLLC-AIFLHT--
>Callorhinchus_milii
MIIWINYRGF--
>Eptesicus_fuscus
MIIWINYRLLAGLSVIIHDSGLGKRPTAYVPKKGQSLMELGGLFY-AICLHT--
>Dasypus_novemcinctus
MIIWINYRLLAGVSVIIHDSGPAGSPRACVPKKGQSLMELGGLVLDAICLRT--
>Latimeria_chalumnae
MIIWINYRLLAVLSVIIHDSGLGKRPRAYVPKKRGRVSWSWVYFYMPFSTTPRG
>Colius_striatus
-------------------------------------MELGVLLC-AIFLHT--
>Columba_livia
MIIWINYRLLAVLSVIIHDSGLGKRPTAYVPKKGQSLMELGVLLC-AIFLHT--
>Cuculus_canorus
-------------------------------------MELGVLLC-AIFLHT--
>Galeopterus_variegatus
MIIWINYRLLAGLSVIIHDSGLGKRPTAYVPKKGQSLMELGGLFY-AICLYT--
>Falco_cherrug
MIIWINYRLLAVLSVIIHDSGLGKRPTAYVPKKGQSLMELGVLLC-AIFLHT--
>Coturnix_japonica
MIIWINYRLLAVLSVIIHDSGLGKRPTAYVPKKGQSLMELGVLLC-AIFLHT--
>Gavia_stellata
MIIWINYRLLAVLSVIIHDSGLGKRPTAYVPKKGQSLMELGVLLC-AIFLHT--
>Mesitornis_unicolor
MIIWINYRLLAVLSVIIHDSGLGKRPTAYVPKKGQSLMELGVLLC-AIFLHT--
>Apteronotus_leptorhynchus
MITWIVSKLLATLSVIIRDSGLGKGARNMSNQR---------------------
>Condylura_cristata
MIIWINYRLLAGLSVIIHDSGLGKRPTAYVPKKGQSLMELGGLFY-AICLHT--
>Oryctolagus_cuniculus
MIIWINYRLLAGLSVIIHDSGLGKEPTAYVPKKGQSLMELGGLFY-AICLHT--
>Elephantulus_edwardii
MIIWINYRLLAGLSVIIHDSGLGKRPTAYVPKRGQSLMELGGLFP-AVCLRT--
>Opisthocomus_hoazin
-------------------------------------MELGLLLR-AIFLRA--
>Geospiza_fortis
MIIWINYRLLAVLSVIIHDSGLGKRPTAYVPKKGQSLMELGVLLC-AIFLHT--
>Nipponia_nippon
MIIWINYRLLAVLSVIIHDSGLGKRPTAYVPKKGQSLMELGVLLC-AIFLHT--
>Ceratotherium_simum_simum
MIIWINYRLLAGLSVIIHDSGLGKRPTAYVPKKGQSLMELGGLFY-AICLHT--
>Manis_javanica
MIIWINYRLLAGLSVIIHDSGLGKRPTAYVPKKGQSLMELGGLFY-AICLHT--
>Picoides_pubescens
MIIWINYRLLAVLSVIIHDSGLGKRPTAYVPKKGQSLMELGVLLC-AIFLHT--
>Loxodonta_africana
MTIWINYRLLAGLSVIIHDSGLGKRPTAYVPKKGQSLMELGGLFY-AICLRT--
>Melopsittacus_undulatus
MIIWINYRLLAVLSVIIHDSGLGKRPTAYVPKKGQSLMELGVLLC-AIFLYT--
>Callospermophilus_lateralis
MIIWINYRLLAGLSVIIHDSGLGKRPTAYVPKKGQSLMELGGLFY-AICLHT--
>Salmo_salar
-------------------------------------MELCALMF-AIIIHT--
>Tupaia_chinensis
MIIWINYRLLAGLSVIIHDSGLGKSPTAYVPKKGQSLMELGGLFY-AICLLT--
>Lepisosteus_oculatus
MIIWINYRLLVVLSVIIHDSSLGKRPRSYVPKKGQSLMELVPLLH-AISLHT--
>Trichechus_manatus_latirostr
MIIWINYRLLAGLSVIIHDSGLGKRPTAYVPKKGQSLMELGGLFY-AICLRT--
>Pygoscelis_adeliae
MIIWINYRLLAVLSVIIHDSGLGKRPTAYVPKKGQSLMELGVLLC-AIFLHT--
>Squalus_acanthias
MIIWINYRLLAVLSVIIHDSGLGKRPTAQRAKKRGEEKI---------------
>Struthio_camelus_australis
MIIWINYRLLAVLSVIIHDSGLGKRPTAYVPKKGQSLMELGVLLC-AIFLHT--
>Trachemys_scripta
MIIWINYRLLAVLSVIIHDSGLGKRRTAYVPKKGQSLMELGVLLY-AIFLHT--
>Tinamus_guttatus
MIIWINYRLLAVLSVIIHDSGLGKRPTAYVPKKGQSLMELGVLLC-AIFLHT--
>Calypte_anna
MIIWINYRLLAVLSVIIHDSGLGKRPTAYVPKKGQSLMELGVLLC-AIFLHT--
>Orycteropus_afer_afer
MIIWINYRLLAGLSVIIHDSGLGKRPTAYVPKKGQSLMELGGLFY-AICLRT--

HG0039.2 ENSG00000077092 RARB

>Homo_sapiens
MPRTRAIRAGFVWAPSG
>Odobenus_rosmarus_divergens
MPRTRASRAGFVWAPSG
>Orcinus_orca
MPRTRASRAGLVWAPSG
>Rousettus_aegyptiacus
MPRTRASRAGLVWAPSG
>Galeopterus_variegatus
MPRTRAIRAGFVWAPSG
>Condylura_cristata
MPRTRASRAGFVWAPSG
>Ochotona_princeps
MPRTRATQAGSLWAPSG
>Elephantulus_edwardii
MRRTRASRAGAVWAPSG
>Equus_przewalskii
MPRTRASRAGFVWAPSG
>Marmota_marmota_marmota
MPRTRASRAGFVWAPSG
>Tupaia_chinensis
MPRTRASRAGLVWAPSG
>Trichechus_manatus_latirostr
MPRTRAIRAGFVWAPSG
>Orycteropus_afer_afer
MPRTQTIRAGFVWAPSG

HG0040.1 ENSG00000164190 NIPBL

>Homo_sapiens
--MGSNDSWHLN
>Anser_cygnoides
--MGSNDSWHLN
>Xenopus_laevis
--MGSNDSWHFN
>Chaetura_pelagica
--MGSNDSWHLN
>Apteryx_australis_mantelli
--MGSNDSWHLN
>Buceros_rhinoceros_silvestri
--MGSNDSWHLN
>Caprimulgus_carolinensis
--MGSNDSWHLN
>Cariama_cristata
--MGSNDSWHLN
>Felis_catus
--MGSNDSWHLN
>Balaenoptera_acutorostrata_s
--MGSNDSWHLN
>Calidris_pugnax
--MGSNDSWHLN
>Miniopterus_natalensis
--MGSNDSWHLN
>Pterocles_gutturalis
--MGSNDSWHLN
>Dasypus_novemcinctus
--MHKYSVWEMGSNDSWHLN
>Columba_livia
--MGSNDSWHLN
>Leptosomus_discolor
--MGSNDSWHLN
>Cuculus_canorus
--MGSNDSWHLN
>Sarcophilus_harrisii
MVDNWRNWCISRGKQIPRSISVSSAGASFGLLGSIIPPKQKVCIYTYRCIIISKLCQIFLKWHIHLVARRSPENPIESRGYPKSGHPPAAFPKSGEKPGHRSHPISFILHPSGPLRFSRERGRRLEGKPPNAFLWSPSREARRRPKLRSSHFPHSRLGLGLPTDSNADSPSVWEMGSNDSWHLS
>Galeopterus_variegatus
--MESNDSWHLN
>Monodelphis_domestica
--MGSNDSWHLS
>Haliaeetus_leucocephalus
--MGSNDSWHMN
>Coturnix_japonica
--MGSNDSWHLN
>Gavia_stellata
--MGSNDSWHLN
>Chlamydotis_macqueenii
--MGSNDSWHLN
>Condylura_cristata
--MGSNDSWHLN
>Oryctolagus_cuniculus
--MGSNDSWHLN
>Elephantulus_edwardii
--MGSNDSWHLN
>Ornithorhynchus_anatinus
--MGSNDSWHLN
>Tauraco_erythrolophus
--MGSNDSWHLN
>Opisthocomus_hoazin
--MGSNDSWHLN
>Lepidothrix_coronata
--MGSNDSWHLN
>Nipponia_nippon
--MGSNDSWHLN
>Equus_przewalskii
--MGSNDSWHLN
>Picoides_pubescens
--MGSNDSWHLN
>Loxodonta_africana
--MGSNDSWHLN
>Melopsittacus_undulatus
--MGSNDSWHLN
>Peromyscus_maniculatus_baird
--MGSNDSWHLN
>Tupaia_chinensis
--MGSNDSWHLN
>Aptenodytes_forsteri
--MGSNDSWHLN
>Gekko_japonicus
--MGSNDSWHLN
>Tyto_alba
--MGSNDSWHLN
>Pelodiscus_sinensis
--MGSNDSWHLN
>Tinamus_guttatus
--MGSNDSWHLN
>Calypte_anna
--MGSNDSWHLN
>Apaloderma_vittatum
--MGSNDSWHLN
>Orycteropus_afer_afer
--MGSNDSWHLN

HG0040.2 ENSG00000164190 NIPBL

```
>Homo_sapiens
MFPALRRRKRSRSHPPSRPGL--------------------------
>Apteryx_australis_mantelli
MFAALRR-RRKRSQPPSRPGLLLLYYLPSSSCSAAAAAAQQRRRLL-
>Mustela_putorius_furo
MFPALRRRKRSRSHPPSRPGL--------------------------
>Physeter_catodon
MFPALRRRKRSRSHPPSRPGL--------------------------
>Miniopterus_natalensis
MFPALRRRKRSRSHPPSRPGL--------------------------
>Alligator_mississippiensis
MFAALRR-RRKRSQPPSRPGLLLLYYLPSSSCSAAAAAAQQRRRLL-
>Galeopterus_variegatus
MFPALRRRKRSRSHPPSRPGL--------------------------
>Aquila_chrysaetos_canadensis
MFAALRR-RRKRSQPPSRPGLLLLYYLPSSSCSAAAAAAQRRRRRLL
>Coturnix_japonica
MFAALRR-RRKRSQPPSRPGLLLLYYLPSSSCSAAAAAAQRRRRLL-
>Condylura_cristata
MFPALRRRKRSRSHPPSRPGL--------------------------
>Oryctolagus_cuniculus
MFPALRRRKRSRSHPPSRPGL--------------------------
>Elephantulus_edwardii
MFPALRSRKRSRSHPPSRPGL--------------------------
>Ficedula_albicollis
MFAALRR-RRKRSQPPSRPGLLLLYYLPSSSCSAAAAAQRRRRLL--
>Equus_caballus
MFPALRRRKRSRSHPPSRPGL--------------------------
>Loxodonta_africana
MFPALRRRKRSRSHPPSRPGL--------------------------
>Melopsittacus_undulatus
MFAALRR-RRKRSQPPSRPGLLLLYYLPSSSCSAAAAVAQRRRRLL-
>Chinchilla_lanigera
MFPALRRRKRSRSHPPSRPGL--------------------------
>Trichechus_manatus_latirostr
MFPALRRRKRSRSHPPSWPGL--------------------------
>Anolis_carolinensis
MFAALRR-RRKRSQPPSRPGLLLLYYLPSSSSSAAQRRIL-------
>Pelodiscus_sinensis
MFAALRR-RRKRSQPPSRPGLLLLYYLPSSSCSAAAAAQQRRRLL--
>Orycteropus_afer_afer
MFPALRRRKRSRSHPPSRPGL--------------------------
```

HG0041 ENSDARG00000061352 bcl11aa

```
>Danio_rerio
----------------------------------------------------------------------------------------------------------MTAIPQSIFPAP-SLYYF
>Acipenser_sinensis
----------------------------------------------------------------------------------------------------------MTAIPQSIFPAP-SLYYF
>Anser_cygnoides
----------------------------------------------------------------------------------------------------------MTAIPQFIFPAP-SLYYF
>Pelophylax_nigromaculatus
----------------------------------------------------------------------------------------------------------MTAVPQFIFPAP-SLYYF
>Chaetura_pelagica
----------------------------------------------------------------------------------------------------------MTAIPQFIFPAP-SLYYF
>Apteryx_australis_mantelli
----------------------------------------------------------------------------------------------------------MTAIPQFIFPAP-SLYYF
>Oryzias_melastigma
------------------------MSGVRPQPDVQVRRERHVRALELAQLEGGGGEGEGKGGFCHFFP-FSLSIHLSLILSLSLFSL------------LSFLKKAMTAIPRSIFASAQSLYYF
>Tripterygion_delaisi
----------------------------------------------------------------------------------------------------------MTAIPRSIFASAPSLYYF
>Mustela_putorius_furo
----------------------------------------------------------------------------------------------------------MTALPQFIFPAP-SLYYF
>Lipotes_vexillifer
----------------------------------------------------------------------------------------------------------MTALPQFIFPAP-SLYYF
>Pygocentrus_nattereri
MSVHWFCYFHSLLPLPGFALPMFCMSVSIHLLTFKFAGTSRP-HL----------NLQLRGAFAIFS-SLS----------------------------LSFLKKAMTAIPQSIFPAP-SFYYF
>Calidris_pugnax
---------------------MFCMSLSIQAPDVQVRRDVTS-AL----------ELAAQGGFCHFFH-LSLSLLLSLPLSPS-PSLSLFFFPC-----TKKKKKAMTAIPQFIFPAP-SLYYF
>Rousettus_aegyptiacus
----------------------------------------------------------------------------------------------------------MTALPQFIFPAP-SLYYF
>Dasypus_novemcinctus
----------------------------------------------------------------------------------------------------------MTALPQFIFPAP-SLYYF
>Alosa_alosa
----------------------------------------------------------------------------------------------------------MTAIPQSIFPAP-SLYYF
>Latimeria_chalumnae
----------------------------------------------------------------------------------------------------------MTAIPQSIFPAP-SLYYF
>Alligator_mississippiensis
----------------------------------------------------------------------------------------------------------MTAIPQFIFPAP-SLYYF
>Sarcophilus_harrisii
----------------------------------------------------------------------------------------------------------MTALPQFIFPAP-SLYYF
>Galeopterus_variegatus
----------------------------------------------------------------------------------------------------------MTALPQFIFPAP-SLYYF
>Esox_lucius
----------------------------------------------------------------------------------------------------------MTAIPQSIFPAP-SLYYF
>Haliaeetus_leucocephalus
---------------------MFCMSLSIQAPDVQVRRDVTS-AL----------ELAAQGGFCHFFH-LSLSFSLSLSLPL--PLSLSFFFPL-----HKKKKKAMTAIPQFIFPAP-SLYYF
>Gallus_gallus
------------------------MSLSIQAPDVQVRGDVTS-AL----------ELAAQGGFCHFFH-LALFL-PSFPLSPS--LSPSLFFPR-----TQKKKKAMTAIPQFIFPAP-SLYYF
>Erinaceus_europaeus
----------------------------------------------------------------------------------------------------------MTALPQFIFPAP-SLYYF
>Ochotona_princeps
----------------------------------------------------------------------------------------------------------MTALPQFIFPAP-SLYYF
>Elephantulus_edwardii
----------------------------------------------------------------------------------------------------------MTALPQFIFPAP-SLYYF
>Ornithorhynchus_anatinus
----------------------------------------------------------------------------------------------------------MTALPQFIFPAP-SLYYF
>Scleropages_formosus
----------------------------------------------------------------------------------------------------------MSAVPQSIFSAP-SLHYP
>Geospiza_fortis
------------------------MSLSIQAPDVQVRRDVTS-AL----------ELAAQGGFCHFFH-LSLSPSLSPSLSLP-LPLSLFFLAQKKKKKKKKKKKAMTAIPQFIFPAP-SLYYF
>Egretta_garzetta
----------------------------------------------------------------------------------------------------------MTAIPQFIFPAP-SLYYF
>Ceratotherium_simum_simum
----------------------------------------------------------------------------------------------------------MTALPQFIFPAP-SLYYF
>Manis_javanica
----------------------------------------------------------------------------------------------------------MTALPQFIFPAP-SLYYF
>Callithrix_jacchus
MCVHWCELPLPSSPSLLLALPMFCVSLSIQTPDVQVRRDVTS-AL----------ELAAQGGFCHFFSSLSLSLSLSPPLSLFSLSLSFFFFFF-----LLKKKKAMTALPQFIFPAP-SLYYF
>Loxodonta_africana
----------------------------------------------------------------------------------------------------------MTALPQFIFPAP-SLYYF
>Melopsittacus_undulatus
------------------------MSLSIQAPDVQVRRDVTS-AL----------ELAAQGGFCHFFH-LSLSFSPSLSLSLS----SFFFFPFSL--HKKKKKKAMTAIPQFIFPAP-SLYYF
>Mus_musculus
----------------------------------------------------------------------------------------------------------MTALPQFIFPAP-SLYYF
>Salmo_salar
----------------------------------------------------------------------------------------------------------MTAIPQSIFPAP-SLYYF
>Lepisosteus_oculatus
----------------------------------------------------------------------------------------------------------MTAIPQSIFPAP-SLYYF
>Ictalurus_punctatus
----------------------------------------------------------------------------------------------------------MTAIPQSIFSAP-SLYYF
>Trichechus_manatus_latirostr
----------------------------------------------------------------------------------------------------------MTALPQFIFPAP-SLYYF
>Thamnophis_sirtalis
---------------------MFCMSLSIQPLDVQVRRDVTS-AL----------ELAAQGGFCHFFH-LSLSLSLLSPPTVS---------PSLAQKGKKEKEKAMTAIPQFIFPAP-SLYYF
>Pelodiscus_sinensis
----------------------------------------------------------------------------------------------------------MTAIPQFIFPAP-SLYYF
>Calypte_anna
----------------------------------------------------------------------------------------------------------MTAIPQFIFPAP-SLYYF
>Orycteropus_afer_afer
----------------------------------------------------------------------------------------------------------MTALPQFIFPAP-SLYYF
```

HG0041 ENSG00000119866 BCL11A

```
>Homo_sapiens
-------------------------------------------------------------------MTALPQFIFP-APSLYYF
>Acipenser_sinensis
-------------------------------------------------------------------MTAIPQSIFP-APSLYYF
>Anser_cygnoides
-------------------------------------------------------------------MTAIPQFIFP-APSLYYF
>Pelophylax_nigromaculatus
-------------------------------------------------------------------MTAVPQFIFP-APSLYYF
>Chaetura_pelagica
-------------------------------------------------------------------MTAIPQFIFP-APSLYYF
>Apteryx_australis_mantelli
-------------------------------------------------------------------MTAIPQFIFP-APSLYYF
>Tripterygion_delaisi
------------------------------------------------------------------MTAIPRSIFASAPSLYYF
>Canis_lupus_familiaris
-------------------------------------------------------------------MTALPQFIFP-APSLYYF
>Lipotes_vexillifer
-------------------------------------------------------------------MTALPQFIFP-APSLYYF
>Astyanax_mexicanus
-------------------------------------------------------------------MTAIPQFIFP-APSLYYF
>Calidris_pugnax
MFCMSLSIQAPDVQVRRDVTSALELAAQGGFCHFFHLSLSLLLSLPLSPSPSLSLFFFPCTKKKKKAMTAIPQFIFP-APSLYYF
>Rousettus_aegyptiacus
-------------------------------------------------------------------MTALPQFIFP-APSLYYF
>Dasypus_novemcinctus
-------------------------------------------------------------------MTALPQFIFP-APSLYYF
>Alosa_alosa
-------------------------------------------------------------------MTAIPQSIFP-APSLYYF
>Latimeria_chalumnae
-------------------------------------------------------------------MTAIPQSIFP-APSLYYF
>Alligator_mississippiensis
-------------------------------------------------------------------MTAIPQFIFP-APSLYYF
>Sinocyclocheilus_rhinocerous
-------------------------------------------------------------------MTAIPQSIFP-APSLYYF
>Sarcophilus_harrisii
-------------------------------------------------------------------MTALPQFIFP-APSLYYF
>Galeopterus_variegatus
-------------------------------------------------------------------MTALPQFIFP-APSLYYF
>Esox_lucius
-------------------------------------------------------------------MTAIPQSIFP-APSLYYF
>Haliaeetus_leucocephalus
MFCMSLSIQAPDVQVRRDVTSALELAAQGGFCHFFHLSLSFSLSLSLPL-PLSLSFFFPLHKKKKKAMTAIPQFIFP-APSLYYF
>Gallus_gallus
---MSLSIQAPDVQVRGDVTSALELAAQGGFCHFFHLALFL-PSFPLSPS-LSPSLFFPRTQKKKKAMTAIPQFIFP-APSLYYF
>Erinaceus_europaeus
-------------------------------------------------------------------MTALPQFIFP-APSLYYF
>Ochotona_princeps
-------------------------------------------------------------------MTALPQFIFP-APSLYYF
>Elephantulus_edwardii
-------------------------------------------------------------------MTALPQFIFP-APSLYYF
>Ornithorhynchus_anatinus
-------------------------------------------------------------------MTALPQFIFP-APSLYYF
>Scleropages_formosus
-------------------------------------------------------------------MSAVPQSIFS-APSLHYP
>Sturnus_vulgaris
-------------------------------------------------------------------MTAIPQFIFP-APSLYYF
>Egretta_garzetta
-------------------------------------------------------------------MTAIPQFIFP-APSLYYF
>Ceratotherium_simum_simum
-------------------------------------------------------------------MTALPQFIFP-APSLYYF
>Manis_javanica
-------------------------------------------------------------------MTALPQFIFP-APSLYYF
>Loxodonta_africana
-------------------------------------------------------------------MTALPQFIFP-APSLYYF
>Melopsittacus_undulatus
---MSLSIQAPDVQVRRDVTSALELAAQGGFCHFFHLSLSFSPSLSLSLSSFFFFPFSLHKKKKKKAMTAIPQFIFP-APSLYYF
>Dipodomys_ordii
-------------------------------------------------------------------MTALPQFIFP-APSLYYF
>Salmo_salar
-------------------------------------------------------------------MTAIPQSIFP-APSLYYF
>Lepisosteus_oculatus
-------------------------------------------------------------------MTAIPQSIFP-APSLYYF
>Ictalurus_punctatus
-------------------------------------------------------------------MTAIPQSIFS-APSLYYF
>Trichechus_manatus_latirostr
-------------------------------------------------------------------MTALPQFIFP-APSLYYF
>Thamnophis_sirtalis
MFCMSLSIQPLDVQVRRDVTSALELAAQGGFCHFFHLSLSLSLLSPPT---VSPSLAQKGKKEKEKAMTAIPQFIFP-APSLYYF
>Pelodiscus_sinensis
-------------------------------------------------------------------MTAIPQFIFP-APSLYYF
>Calypte_anna
-------------------------------------------------------------------MTAIPQFIFP-APSLYYF
>Orycteropus_afer_afer
-------------------------------------------------------------------MTALPQFIFP-APSLYYF
```

HG0041 ENSGALG00000034048

>Gallus_gallus
--MTAIPQFIFP-APSLYYF
>Acipenser_sinensis
--MTAIPQSIFP-APSLYYF
>Anser_cygnoides
--MTAIPQFIFP-APSLYYF
>Pelophylax_nigromaculatus
--MTAVPQFIFP-APSLYYF
>Chaetura_pelagica
--MTAIPQFIFP-APSLYYF
>Apteryx_australis_mantelli
--MTAIPQFIFP-APSLYYF
>Tripterygion_delaisi
--MTAIPRSIFASAPSLYYF
>Mustela_putorius_furo
--MTALPQFIFP-APSLYYF
>Lipotes_vexillifer
--MTALPQFIFP-APSLYYF
>Astyanax_mexicanus
--MTAIPQFIFP-APSLYYF
>Calidris_pugnax
MFCMSLSIQAPDVQVRRDVTSALELAAQGGFCHFFHL-SLSLLLSLPLSP-SPSLSLFFFPCTKKKKAMTAIPQFIFP-APSLYYF
>Rousettus_aegyptiacus
--MTALPQFIFP-APSLYYF
>Dasypus_novemcinctus
--MTALPQFIFP-APSLYYF
>Alosa_alosa
--MTAIPQSIFP-APSLYYF
>Latimeria_chalumnae
--MTAIPQSIFP-APSLYYF
>Alligator_sinensis
--MTAIPQFIFP-APSLYYF
>Catla_catla
--MTAIPQSIFP-APSLYYF
>Sarcophilus_harrisii
--MTALPQFIFP-APSLYYF
>Galeopterus_variegatus
--MTALPQFIFP-APSLYYF
>Esox_lucius
--MTAIPQSIFP-APSLYYF
>Haliaeetus_leucocephalus
MFCMSLSIQAPDVQVRRDVTSALELAAQGGFCHFFHL-SLSFSLSLSLPL--PLSLSFFFPLHKKKKKAMTAIPQFIFP-APSLYYF
>Erinaceus_europaeus
--MTALPQFIFP-APSLYYF
>Ochotona_princeps
--MTALPQFIFP-APSLYYF
>Elephantulus_edwardii
--MTALPQFIFP-APSLYYF
>Ornithorhynchus_anatinus
--MTALPQFIFP-APSLYYF
>Scleropages_formosus
--MSAVPQSIFS-APSLHYP
>Sturnus_vulgaris
--MTAIPQFIFP-APSLYYF
>Egretta_garzetta
--MTAIPQFIFP-APSLYYF
>Ceratotherium_simum_simum
--MTALPQFIFP-APSLYYF
>Manis_javanica
--MTALPQFIFP-APSLYYF
>Callithrix_jacchus
MFCVSLSIQTPDVQVRRDVTSALELAAQGGFCHFFSSLSLSLSLSPPLSLFSLSLSFFFFFFLLKKKKAMTALPQFIFP-APSLYYF
>Loxodonta_africana
--MTALPQFIFP-APSLYYF
>Melopsittacus_undulatus
---MSLSIQAPDVQVRRDVTSALELAAQGGFCHFFHL-SLSFSPSLSLSL-SSFFFFPFSLHKKKKKKAMTAIPQFIFP-APSLYYF
>Mus_musculus
--MTALPQFIFP-APSLYYF
>Salmo_salar
--MTAIPQSIFP-APSLYYF
>Lepisosteus_oculatus
--MTAIPQSIFP-APSLYYF
>Ictalurus_punctatus
--MTAIPQSIFS-APSLYYF
>Trichechus_manatus_latirostr
--MTALPQFIFP-APSLYYF
>Gekko_japonicus
--MTAIPQFIFP-APSLYYF
>Chrysemys_picta
--MTAIPQFIFP-APSLYYF
>Calypte_anna
--MTAIPQFIFP-APSLYYF
>Orycteropus_afer_afer
--MTALPQFIFP-APSLYYF

HG0042.1 ENSG00000140937 CDH11

>Homo_sapiens
----MGG-VCYRNWQQVSNG
>Anas_platyrhynchos
----MCG-ICYVNWQQVSSG
>Chaetura_pelagica
----MCG-ICYENWQRVSSG
>Apteryx_australis_mantelli
----MCG-ICYVSWQQVSSG
>Buceros_rhinoceros_silvestri
----MCG-ICYINWQQVSSG
>Caprimulgus_carolinensis
----MCG-ICYINWQQVSSG
>Acinonyx_jubatus
----MGG-VCYRNWQQVSNG
>Balaenoptera_acutorostrata_s
MTNQMGGVLRYRNWQQVSNG
>Charadrius_vociferus
----MCG-ICYINWQQVSSG
>Eptesicus_fuscus
----MGG-VCYRNWQQVSNG
>Pterocles_gutturalis
----MCG-ICYINWQQVSSG
>Dasypus_novemcinctus
----MGE-LSYRNWQQVSNG
>Columba_livia
----MCG-ICYINWQQVSSG
>Leptosomus_discolor
----MCG-ICYINWQQVSSG
>Alligator_mississippiensis
----MGG-ICYINWQQVTNG
>Galeopterus_variegatus
----MGR-VCYRNWQQVSNG
>Monodelphis_domestica
----MGG-VCYRNWQQVSNG
>Falco_cherrug
----MCG-ICYINWQQVSSG
>Coturnix_japonica
----MCG-ICYINWQRVSSG
>Gavia_stellata
----MCG-ICYINWQQVSSG
>Chlamydotis_macqueenii
----MCG-ICYINWQQVSSG
>Sorex_araneus
----MGG-VCYRNWQQVSNG
>Oryctolagus_cuniculus
----MGG-VCYRNWQQVSNG
>Tauraco_erythrolophus
----MCG-ICYINWQQVSSG
>Opisthocomus_hoazin
----MCG-ICYINWQQVSSG
>Pseudopodoces_humilis
----MCG-ICYINWQQVSSG
>Phaethon_lepturus
----MCG-ICYINWQQVSNG
>Ceratotherium_simum_simum
----MGG-VCYRNWQQVSNG
>Manis_javanica
----MGG-VCYRNWQQVSNG
>Picoides_pubescens
----MCG-ICYINWQQVPSG
>Loxodonta_africana
----MGG-VYYRNWQQVSNG
>Fulmarus_glacialis
----MCG-ICYINWQQVSSG
>Melopsittacus_undulatus
----MCG-ICYINWQQVSSG
>Heterocephalus_glaber
----MGG-VCYRNWQQVSNG
>Tupaia_chinensis
----MGG-LCYRNWQQVSNG
>Aptenodytes_forsteri
----MSG-ICYITWQQVSSG
>Takydromus_sexlineatus
----MGG-ICYFNWQWVSSG
>Struthio_camelus_australis
----MCG-ICYRSWQQVSSG
>Chelonia_mydas
----MGG-ICYINWQQVSSG
>Tinamus_guttatus
----MCG-ICYISWQQVSSG
>Calypte_anna
----MCG-ICYINWQQVSSG
>Apaloderma_vittatum
----MCG-TCYINWQQVSSG
>Orycteropus_afer_afer
MTNQMGR-VCYRNWQQVSNG

HG0042.2 ENSGALG00000039985

>Gallus_gallus
MHSRCGGSVCAEHFI
>Anas_platyrhynchos
MHSMCGCNLCAEHFI
>Charadrius_vociferus
MCLMCGGILCAEHFI
>Columba_livia
MCLMCGGILCAEHFI
>Falco_cherrug
MCLMCGGILCAEHFI
>Balearica_regulorum_gibberic
MCLMCGGILCAEHFI
>Opisthocomus_hoazin
MCLMCGGLLCAEHFI
>Phalacrocorax_carbo
MCLMCGGILCAEHFI
>Fulmarus_glacialis
MCSVCGGILCAEHFI
>Melopsittacus_undulatus
MHLMCGGILCAQHFI
>Aptenodytes_forsteri
MCLMCGGILCAEHFI
>Struthio_camelus_australis
MHFMCGAILCAEHFI
>Tinamus_guttatus
MHFMCGGILCAERFI
>Apaloderma_vittatum
MCLMCGGILCAEHFI

HG0042.3 ENSG00000140937 CDH11

>Homo_sapiens
MPRGPLAAAADL
>Leptonychotes_weddellii
MPRGPLAAAADL
>Orcinus_orca
MPRGPLAAAADL
>Rousettus_aegyptiacus
MPRGPLAAAADL
>Manis_javanica
MPRGPLAAAADL
>Loxodonta_africana
MPRGPLAAAADL
>Octodon_degus
MPRGPLAAAADL
>Orycteropus_afer_afer
MPRGPLAAAADL

HG0042.4 ENSG00000140937 CDH11

>Homo_sapiens
MSSTSRDIPSQERSA
>Mustela_putorius_furo
MYSVSRDIPFQERSA
>Pteropus_vampyrus
MCSASRDIPFQERSA
>Condylura_cristata
MLSASRDIPLQDRSA
>Ceratotherium_simum_simum
MCSASRDIPLQERSA
>Octodon_degus
MCSESKDIPLQERSA

HG0042.5 ENSG00000140937 CDH11

>Homo_sapiens
------------------------------MQKSLCFQLSGPV-TFLRVVIC
>Leptonychotes_weddellii
MGPGLGSGLTPLRSSCARDRRPVARAHPPGPQKSLYFRLSGPV-TLVPVVIC
>Eptesicus_fuscus
--MIIPVAVC
>Erinaceus_europaeus
MGPGLGPGVPPLRSP--RDRPPEARAHPLGPQKSFPFWLSGPE-AFSPVGIC
>Peromyscus_maniculatus_baird
------------------------------MTKSLCFWLCGPVKTFISVVIC

HG0043 ENSG00000172995 ARPP21

```
>Homo_sapiens
--------------------------------------------MLFNEGASASLSNKRSMREDRAVHGYGYWTRIFGKVD-DVSHCVRESHGDGHSELLMGTWDSSCL--------------
>Anas_platyrhynchos
MLIQHSHCAVQFNYCYFPAFIYRLSQRGEHTHIQGSFLCARCVLAPLLI------LLSLRFQVCAVLFQFIPLLLCHIQVDDDLSNSLKNQHGDWHAELLMGTWDSSCH--------------
>Chaetura_pelagica
----------------------------------------------------------------------------------------------------MGTWDSGCH--------------
>Buceros_rhinoceros_silvestri
----------------------------------------------------------------------------------------------------MGTWDSRCH--------------
>Caprimulgus_carolinensis
----------------------------------------------------------------------------------------------------MGTWDSRCH--------------
>Cariama_cristata
----------------------------------------------------------------------------------------------------MGTWDSRCR--------------
>Mustela_putorius_furo
-------------------------------MLHWGVLCCLFL-MLFNEGASASLRNKRSMMEDRAVHGFGYWRRIYEKVD-DVSNRVRESHGDGHSELLMGTWDSSCL--------------
>Hynobius_retardatus
----------------------------------------------------------------------------------------------------MGTWDSHCH--------------
>Lipotes_vexillifer
----------------------------------------------------------------------------------------------------MGTWDSRCL--------------
>Callorhinchus_milii
----------------------------------------------------------------------------------------------------MRTWDSLCYCSAVLISQVNSIR-
>Rousettus_aegyptiacus
-----------------------------------------------MKEQAPHLANKRSMMEDRAVRGYGYWRRIFGKVD-DVSNCVRESHGHGRSDLLMGTWDSSFL--------------
>Pterocles_gutturalis
----------------------------------------------------------------------------------------------------MGTWDSRRH--------------
>Dasypus_novemcinctus
----------------------------------------------------------------------------------------------------MGTWDSSCR--------------
>Latimeria_chalumnae
----------------------------------------------------------------------------------------------------MGTWDSRCH--------------
>Colius_striatus
----------------------------------------------------------------------------------------------------MGTWDSHCH--------------
>Columba_livia
----------------------------------------------------------------------------------------------------MGTWDSRCHLSLAWT--------
>Leptosomus_discolor
----------------------------------------------------------------------------------------------------MGTWDSHCH--------------
>Alligator_sinensis
----------------------------------------------------------------------------------------------------MGTWDAHCLS-------------
>Cuculus_canorus
----------------------------------------------------------------------------------------------------MGTWDSRCH--------------
>Galeopterus_variegatus
-------------------------------MLHRGVLCSLFL-MLFNEGASASLSNKRSMREDRAVRGYGYWRTIFGKVDDDVSKCVKESHGDGHSELLKGTWDSSCL--------------
>Aquila_chrysaetos_canadensis
-----------------------------------------------------------------MKHSLPSPPFLRRQVDYDPSNSLKDQHGDWHSELLMGTWDSRCH--------------
>Gallus_gallus
----------------------------------------------------------------------------------------------------MGTWDSSCR--------------
>Balearica_regulorum_gibberic
----------------------------------------------------------------------------------------------------MGTWDSRCH--------------
>Erinaceus_europaeus
----------------------------------------------------------------------------------------------------MGTWDSSCL--------------
>Oryctolagus_cuniculus
----------------------------------------------------------------------------------------------------MGTWDSSCL--------------
>Elephantulus_edwardii
----------------------------------------------------------------------------------------------------MGTWDSGHP--------------
>Tauraco_erythrolophus
----------------------------------------------------------------------------------------------------MRTWDSRCH--------------
>Opisthocomus_hoazin
----------------------------------------------------------------------------------------------------MGTWDSRCH--------------
>Parus_major
----------------------------------------------------------------------------------------------------MGTWDSRCR--------------
>Egretta_garzetta
----------------------------------------------------------------------------------------------------MGTWDSRCH--------------
>Equus_asinus
-------------------------------MLHWGVLCSLFL-MLFNEGASASLSNKRSMMEDRAVRGYGYWRRISGKVD-DVSNCVRESHGDGRSELLMGTWDSSCL--------------
>Manis_javanica
-------------------------------------------------------------------------------MVDDVSHCVRKPHGDEHSELLVGTWDPSCLCSLVWILLVHDSRR
>Picoides_pubescens
----------------------------------------------------------------------------------------------------MGTWDSRCH--------------
>Loxodonta_africana
----------------------------------------------------------------------------------------------------MGTWDSSCL--------------
>Fulmarus_glacialis
----------------------------------------------------------------------------------------------------MGTWDSRCH--------------
>Nestor_notabilis
----------------------------------------------------------------------------------------------------MGTWDSRCH--------------
>Heterocephalus_glaber
-------------------------------MLRWGVLCSLFL-MPFNERASTSLSNKRRMRKDSSVHGYGSWRRIFGKVDDDVSKCVREPHGDGHSALLMGTWDSSCF--------------
>Pygoscelis_adeliae
----------------------------------------------------------------------------------------------------MGTWDSRCH--------------
>Python_bivittatus
----------------------------------------------------------------------------------------------------MGSWDSHCH--------------
>Tyto_alba
----------------------------------------------------------------------------------------------------MGTWDSRCH--------------
>Tinamus_guttatus
----------------------------------------------------------------------------------------------------MGTWDSHCR--------------
>Calypte_anna
----------------------------------------------------------------------------------------------------MGTWDSRCH--------------
>Orycteropus_afer_afer
----------------------------------------------------------------------------------------------------MGTWDSGCL--------------
```

HG0044 ENSDARG00000061687 atxn1a

>Danio_rerio
--MIPHELHGPLSRS-----RCDFNCL-----------RLKSVSIRP----
>Acipenser_sinensis
--MIPHEVPGSLSDS-----RCDFQCI-----------RLRSVTSDTEASQ
>Amia_calva
--MIPHEVHGSLSES-----RRDFPSL-----------RLRSMLVRH----
>Anser_cygnoides
--MIPHEVTGSLAEI-----KCDFPFI-----------RLKSEPARQ----
>Rhacophorus_dennysi
--MIPHEVVGVPSGI-----KCDFPFT-----------RLKSVSVRQ----
>Apteryx_australis_mantelli
--MIPHEVTGSLSEI-----KCDFPFI-----------RLKSKPARQ----
>Oryzias_latipes
--MIPHEVHGAHFRS-----WSNSIFS-----------CVKPGAGQQ----
>Canis_lupus_familiaris
--MIPHEEPGSPTEI-----KCDFPCI-----------RLKPEPARQ----
>Hynobius_retardatus
--MIPHEVSGSSSGI-----KCDFLSL-----------RLKPIPARQ----
>Balaenoptera_acutorostrata_s
--MIPHEEPGSPTEI-----KCDFPFI-----------RLKAEPARE----
>Pygocentrus_nattereri
--MIPHEPQAQLFCG-----RHVFSCF-----------RLRSASIQP----
>Calidris_pugnax
--MIPHEVTGSLAEI-----KCDFPFI-----------RLKSEPARQ----
>Miniopterus_natalensis
--MIPHEEPGSPTEI-----KCDFPFI-----------RLKSEPARQ----
>Dasypus_novemcinctus
--MIPHEAPGAPAAV-----PCDFPCS-----------RLKSQPARQ----
>Latimeria_chalumnae
--MIPHEVPGSPSEN-----QCDFTFTF----------RLKSEQKRH----
>Columba_livia
--MIPHEVTGSLAEI-----KCDFPFI-----------RLKSEPARQ----
>Alligator_mississippiensis
--MIPHEVTGSPSEI-----KCDFPFI-----------RLKSEPARQ----
>Sarcophilus_harrisii
--MIPHEVSGSPSEI-----KCDFPFT-----------RLKPELARQ----
>Monodelphis_domestica
--MIPHEVSGSPSEI-----KCDFPFT-----------RLKSELARQ----
>Aquila_chrysaetos_canadensis
--MIPHEVTGSLAEI-----KCDFPFI-----------RLKSEPARQ----
>Coturnix_japonica
--MIPHEVTGSLAEI-----KCDFPFI-----------RLKSEPARQ----
>Eurypyga_helias
--MIPHEVTGSLAEI-----KCDFPFI-----------RLKSEPARQ----
>Condylura_cristata
--MIPHEGPGSPTEI-----KCDFPFI-----------RLKSEPARQ----
>Oryctolagus_cuniculus
--MIPHEEPGSPTEI-----KCDFPFI-----------RLKSEPASQ----
>Opisthocomus_hoazin
--MIPHEVTGSLAEI-----KCDFPFI-----------RLKSEPARQ----
>Scleropages_formosus
--MIPHEVCGPLTNS-----RRCLPFF-----------RLRPAPIRR----
>Lepidothrix_coronata
--MIPHEVTGSLAEI-----KCDFPFI-----------RLKSEPDRQ----
>Egretta_garzetta
--MIPHEVTGSLAEI-----KCDFPFI-----------RLKSEPARQ----
>Equus_asinus
--MIPHEEPGSPTDI-----QCDFPFI-----------RLKSEPARQ----
>Manis_javanica
--MIPHEGPGSPTEI-----KCDFPFI-----------RLKSEPARQ----
>Cercocebus_atys
--MIPHEEPGSPTEI-----KCDFPFI-----------RLKSEPARQ----
>Loxodonta_africana
--MIPHEEPGSPTEI-----KCDFPFI-----------RLKPEPASQ----
>Melopsittacus_undulatus
--MIPHEVTGSLAEI-----KCDFPFI-----------RLKSEPARQ----
>Ictidomys_tridecemlineatus
--MIPHEEPGSPTEI-----KCDFPFI-----------RLKSEPARQ----
>Salmo_salar
MQQEKA----TPEGYSRR---LLQ--KATPDTLVPAAIQEASSQREMIPHEVDGRLSSSFHGNGRCDSRCSHGNSRCAPLCFSLSQSQASDRR--
>Tupaia_chinensis
MLFPGADVSISLPSFPLSSLPFAPSLPSSSFSQKPELLSADCTHGEMVPHEEPGSPTEI-----KCDFSFI-----------RLKAESARQ----
>Lepisosteus_oculatus
--MIPHEVHGSHTDS-----RRDFPCI-----------RLRSVPVRH----
>Ictalurus_punctatus
--MIPHELWQVLSHS-----RHVFCCF-----------QLRSVSIQP----
>Aptenodytes_forsteri
--MIPHEVTGSLAEI-----KCDFPFI-----------RLKSEPARQ----
>Anolis_carolinensis
-----------------------------------MRCLQRFVLTEMIPHEVSGSASQI-----KCDFPLS-----------RLKPEPARQ----
>Struthio_camelus_australis
MLF-------------SSLKPFVQWSSSTKMAPNPEELSKGCAHGEMIPHEVTGSLSEI-----KCDFPFI-----------RLKSEPARQ----
>Pelodiscus_sinensis
--MIPHEVTGSLSEI-----KCDFPFI-----------RLKSEPARQ----
>Tinamus_guttatus
--MIPHEVTGSLSDI-----KCDFPFI-----------RLKPEAARQ----

HG0044 ENSG00000124788 ATXN1

```
>Homo_sapiens
----------------------------------------------MIPHEEPGSPTEIKCDFPFI-RLKSEPSRQ----
>Acipenser_sinensis
----------------------------------------------MIPHEVPGSLSDSRCDFQCI-RLRSVTSDTEASQ
>Amia_calva
----------------------------------------------MIPHEVHGSLSESRRDFPSL-RLRSMLVRH----
>Anser_cygnoides
----------------------------------------------MIPHEVTGSLAEIKCDFPFI-RLKSEPARQ----
>Pelophylax_nigromaculatus
----------------------------------------------MIPHEVVGVPSGIKCDFPFT-RLKSVSVRQ----
>Apteryx_australis_mantelli
----------------------------------------------MIPHEVTGSLSEIKCDFPFI-RLKSKPARQ----
>Felis_catus
----------------------------------------------MIPHEEPGSPTEIKCDFPCI-RLKPEPARQ----
>Hynobius_retardatus
----------------------------------------------MIPHEVSGSSSGIKCDFLSL-RLKPIPARQ----
>Balaenoptera_acutorostrata_s
----------------------------------------------MIPHEEPGSPTEIKCDFPFI-RLKAEPARE----
>Calidris_pugnax
----------------------------------------------MIPHEVTGSLAEIKCDFPFI-RLKSEPARQ----
>Pteropus_vampyrus
----------------------------------------------MIPHEEPGSPTEIKCDFPFI-RLKSEPAKQ----
>Dasypus_novemcinctus
----------------------------------------------MIPHEAPGAPAAVPCDFPCS-RLKSQPARQ----
>Latimeria_chalumnae
----------------------------------------------MIPHEVPGSPSENQCDFTFTFRLKSEQKRH----
>Columba_livia
----------------------------------------------MIPHEVTGSLAEIKCDFPFI-RLKSEPARQ----
>Alligator_mississippiensis
--------------------MQDSQEAEQGEMENPEQLLEDCAHKEMIPHEVTGSPSEIKCDFPFI-RLKSEPARQ----
>Sinocyclocheilus_anshuiensis
-------------------------MAS-LSTKVLPAVLETHSQREMIPHELHGPLSHSRCDFHCL-RLKSVSIRP----
>Sarcophilus_harrisii
----------------------------------------------MIPHEVSGSPSEIKCDFPFT-RLKPELARQ----
>Monodelphis_domestica
----------------------------------------------MIPHEVSGSPSEIKCDFPFT-RLKSELARQ----
>Aquila_chrysaetos_canadensis
----------------------------------------------MIPHEVTGSLAEIKCDFPFI-RLKSEPARQ----
>Coturnix_japonica
----------------------------------------------MIPHEVTGSLAEIKCDFPFI-RLKSEPARQ----
>Eurypyga_helias
----------------------------------------------MIPHEVTGSLAEIKCDFPFI-RLKSEPARQ----
>Condylura_cristata
----------------------------------------------MIPHEGPGSPTEIKCDFPFI-RLKSEPARQ----
>Oryctolagus_cuniculus
----------------------------------------------MIPHEEPGSPTEIKCDFPFI-RLKSEPASQ----
>Opisthocomus_hoazin
----------------------------------------------MIPHEVTGSLAEIKCDFPFI-RLKSEPARQ----
>Scleropages_formosus
----------------------------------------------MIPHEVCGPLTNSRRCLPFF-RLRPAPIRR----
>Corvus_brachyrhynchos
----------------------------------------------MIPHEVTGSPAEIKCDFPFI-RLKSEPARQ----
>Egretta_garzetta
----------------------------------------------MIPHEVTGSLAEIKCDFPFI-RLKSEPARQ----
>Equus_asinus
----------------------------------------------MIPHEEPGSPTDIQCDFPFI-RLKSEPARQ----
>Manis_javanica
----------------------------------------------MIPHEGPGSPTEIKCDFPFI-RLKSEPARQ----
>Loxodonta_africana
----------------------------------------------MIPHEEPGSPTEIKCDFPFI-RLKPEPASQ----
>Melopsittacus_undulatus
----------------------------------------------MIPHEVTGSLAEIKCDFPFI-RLKSEPARQ----
>Marmota_monax
----------------------------------------------MIPHEEPGSPTEIKCDFPFI-RLKSEPARQ----
>Tupaia_chinensis
MLFPGADVSISLPSFPLSSLPFAPSLPSSSFSQKPELLSADCTHGEMVPHEEPGSPTEIKCDFSFI-RLKAESARQ----
>Lepisosteus_oculatus
----------------------------------------------MIPHEVHGSHTDSRRDFPCI-RLRSVPVRH----
>Aptenodytes_forsteri
----------------------------------------------MIPHEVTGSLAEIKCDFPFI-RLKSEPARQ----
>Thamnophis_sirtalis
------------------------------------MLVEDCGHKEMIPHEVSGSTQEIKCDFPLG-RLKSEPARQ----
>Struthio_camelus_australis
----------------------------------------------MIPHEVTGSLSEIKCDFPFI-RLKSEPARQ----
>Chrysemys_picta
-----------------------------MVLWNPEQLLEHCAHREMIPHEVTGSPSEIKCDFPFI-RLKSEPARQ----
>Tinamus_guttatus
----------------------------------------------MIPHEVTGSLSDIKCDFPFI-RLKPEAARQ----
```

HG0045.1 ENSG00000083168 KAT6A

>Homo_sapiens
MVNWFPASENLVFHGVKLTQHQDKG
>Anser_cygnoides
MVSCFPSSENLFSHRVKLVQHRDKG
>Apteryx_australis_mantelli
MVNCFPSSENLFSHGVKLVQHRDKG
>Buceros_rhinoceros_silvestri
MVSWFLSFENLFSHRVKLVQHRDKG
>Canis_lupus_familiaris
MVNWFPASENVVFHGVKLTQHQDKG
>Physeter_catodon
MVNWFPASGNVVFHGVKLTQHQDKG
>Charadrius_vociferus
MVSWFPSFENLFSHCVKLVQHRDKG
>Callorhinchus_milii
MVNCFLSRRNLFLLCVELVQHRDKG
>Eptesicus_fuscus
MVNWFPASENVVFHGVKLTQHQDKG
>Latimeria_chalumnae
MVNWFTSSENLFSHCVEFVQHQDKG
>Colius_striatus
MVSCFPSLENLFSHRVTLVQHRDKG
>Columba_livia
MVSWFLSFENLFSHRVKLVQHQDKG
>Leptosomus_discolor
MVSWFPSFENLFSHRVKLVQHRDKG
>Alligator_sinensis
MVSWLLSSENLFSHGVKLVQHRDKG
>Cuculus_canorus
MVSWFPSFENLFSHRVKLVQHRDKG
>Sarcophilus_harrisii
MVNWFPTSENLFFHGVKHIQHQDKG
>Galeopterus_variegatus
MVDWFPASENLIFRGVKLIQHQDKG
>Monodelphis_domestica
MVNWFPASENLVFHGVKLIQHQDKG
>Aquila_chrysaetos_canadensis
MVSWFPSFENLFSHCVKLVQHRDKG
>Coturnix_japonica
MVSCFPSSENLFSHCVKLVQHQDKG
>Balearica_regulorum_gibberic
MVSWFPSFENLFSHRVKLVQHRDKG
>Erinaceus_europaeus
MVNWFPVSENVVFHGVKLTQHQDKG
>Oryctolagus_cuniculus
MVSWFPASESRVSHGVGLTQHQDKG
>Elephantulus_edwardii
MVNWFPGSENLVFHGVKFTQHQDKG
>Tauraco_erythrolophus
MVSWFPSFENLFSHCVKLVQHRDKG
>Pseudopodoces_humilis
MVSWFPSFEDLLSHHLKLVQHQDKG
>Phaethon_lepturus
MVSWFPSFENLFSHCVKLVQHQDKG
>Equus_asinus
MVNWFPASEKVVFHGVKLTQHQDKG
>Manis_javanica
MVSWFPASENVVFHGVKLTEHQDKG
>Loxodonta_africana
MVNWFPASENLVFHGVKLTQHQDKG
>Fulmarus_glacialis
MVSWFPSFENLFSHRVKLVQHRDKG
>Melopsittacus_undulatus
MVSWFLSLENLFSHRVRLVQHRDKG
>Ctenomys_sociabilis
MVNWFPSSENLVFHGVKLTQHQDKG
>Tupaia_chinensis
MVNWFPASENLVFHGVKFTQHQDKG
>Trichechus_manatus_latirostr
MVNWFPASENLVFHGVKLSQHQDKG
>Aptenodytes_forsteri
MVSWFPSFENLFSHRVKLFQHRDKG
>Anolis_carolinensis
MVNWSPFSDNLFSYCVKLLQHRDKG
>Tyto_alba
MVSWFSSFENLFSHCVKLVQLRDKG
>Struthio_camelus_australis
MVNWFPSSENLFSHGVKLVQHRDKG
>Chelonia_mydas
MVNWFPSSKNLFSHGVKLIQHRDKG
>Tinamus_guttatus
MVNCFPSSENLFSHGVKLIQHRDKG

HG0045.2 ENSG00000083168 KAT6A

```
>Homo_sapiens
MAAVSVWGISAVRPGAPGSRLSFLPA---
>Mustela_putorius_furo
MAAVSVWGISAVRPGAPGSRFSSPPA---
>Balaenoptera_acutorostrata_s
MAAVSVWGISAVRPGAPLTRRSSPPA---
>Rousettus_aegyptiacus
MAAVSVWGISAVRPGAPGSRLSSPPA---
>Haliaeetus_leucocephalus
MAAVSVSGFAAVRPLPLGSRLSQASA---
>Gallus_gallus
MAAVSVSGFAAVRPLLLGSRLSQSSV---
>Ochotona_princeps
MAAVSVWGISAVRLGAPGSRLSFPPA---
>Elephantulus_edwardii
MAAVSVWGISAVRPGAPGSRLSSPPV---
>Pseudopodoces_humilis
MAAVSVSGFAAVRPSPPGSRRSRASA---
>Ceratotherium_simum_simum
MAAVSVWGISAVRPGAPGSRLCFPPA---
>Ctenomys_sociabilis
MAAVSVWGISAVRPGAPGSRLSPPPDSFY
>Gekko_japonicus
MAAVSVWVFAAVRPQAPGLRLS-------
>Chrysemys_picta
MAAVSVWGFAAVRPPARGLRLSQAPI---
```

HG0046 ENSDARG00000018817 bdnf

>Danio_rerio
MMTSNKTHYLPASVGVCGY
>Anser_cygnoides
MMTSNKTHYLPASVGETGF
>Nanorana_parkeri
MMTSNKRHYLPASVGETGF
>Chaetura_pelagica
MMTSNKTHYLPASVGETGF
>Canis_lupus_familiaris
MMTSNKTHYLPASVGETGF
>Lipotes_vexillifer
MMTSNKTHYLPASVGETGF
>Calidris_pugnax
MMTSNKTHYLPASVGETGF
>Miniopterus_natalensis
MMTSNKTHYLPASVGETGF
>Haplochromis_burtoni
MMTSNKTHYLPASVGVSGY
>Columba_livia
MMTSNKTHYLPASVGETGF
>Cuculus_canorus
MMTSNKTHYLPASVGETGF
>Austrofundulus_limnaeus
MMTSNKTHYLPASVGVSGY
>Galeopterus_variegatus
MMTSNKTHYLPASVGETRF
>Monodelphis_domestica
MMTSNKTHYLPASVGETGF
>Aquila_chrysaetos_canadensis
MMTSNKTHYLPASVGETGF
>Coturnix_japonica
MMTSNKTHYLPASVGETRF
>Condylura_cristata
MMTSNKTHYLPASVGETGF
>Ochotona_princeps
MMTSNKTHYLPASVGETRF
>Elephantulus_edwardii
MMTSNKTHYLPASVGETGF
>Opisthocomus_hoazin
MMTSNKTHYLPASVRETGF
>Corvus_brachyrhynchos
MMTSNKTHYLPASVGETGF
>Egretta_garzetta
MMTSNKTHYLPASVGETGF
>Notothenia_coriiceps
MMTSNKTHYLPASVGVSGY
>Ceratotherium_simum_simum
MMTSNKTHYLPASVGETGF
>Manis_javanica
MMTSNKTHYLPASVGETGF
>Cynoglossus_semilaevis
MMTSNKTHYLPASVGVSGY
>Aotus_nancymaae
MMTSNKTHYLPASVGETRF
>Loxodonta_africana
MMTSNKTHYLPASVGETGF
>Melopsittacus_undulatus
MMTSNKTHYLPASVGETGF
>Dipodomys_ordii
MMTSNKTHYLPASVGETGF
>Salmo_salar
MMTSNKTHYLPASVGVSGY
>Lepisosteus_oculatus
MMTSNKTHYLPASVGESGF
>Trichechus_manatus_latirostr
MMTSNKTHYLPASVGETGF
>Aptenodytes_forsteri
MMTSNKTHYLPASVGETGF
>Anolis_carolinensis
MMTSNETHSLPASGGETGL
>Struthio_camelus_australis
MMTSNKTHYLPASVGETGF
>Chelonia_mydas
MMTSNKTHYLPASVGETGF
>Tinamus_guttatus
MMTSNKTHYLPASVGETGF
>Calypte_anna
MMTSNKTHYLPASVGETGF
>Orycteropus_afer_afer
MMTSNKTHYLPASVGETRF

HG0046 ENSG00000176697 BDNF

>Homo_sapiens
MMTSNKTHYLPASVGETRF
>Anser_cygnoides
MMTSNKTHYLPASVGETGF
>Nanorana_parkeri
MMTSNKRHYLPASVGETGF
>Chaetura_pelagica
MMTSNKTHYLPASVGETGF
>Canis_lupus_familiaris
MMTSNKTHYLPASVGETGF
>Lipotes_vexillifer
MMTSNKTHYLPASVGETGF
>Calidris_pugnax
MMTSNKTHYLPASVGETGF
>Eptesicus_fuscus
MMTSNKTHYLPASVGETGF
>Haplochromis_burtoni
MMTSNKTHYLPASVGVSGY
>Columba_livia
MMTSNKTHYLPASVGETGF
>Cuculus_canorus
MMTSNKTHYLPASVGETGF
>Danio_rerio
MMTSNKTHYLPASVGVCGY
>Austrofundulus_limnaeus
MMTSNKTHYLPASVGVSGY
>Galeopterus_variegatus
MMTSNKTHYLPASVGETRF
>Monodelphis_domestica
MMTSNKTHYLPASVGETGF
>Aquila_chrysaetos_canadensis
MMTSNKTHYLPASVGETGF
>Gallus_gallus
MMTSNKTHYLPASVGETRF
>Condylura_cristata
MMTSNKTHYLPASVGETGF
>Ochotona_princeps
MMTSNKTHYLPASVGETRF
>Elephantulus_edwardii
MMTSNKTHYLPASVGETGF
>Opisthocomus_hoazin
MMTSNKTHYLPASVRETGF
>Acanthis_flammea
MMTSNKTHYLPASVGETGF
>Egretta_garzetta
MMTSNKTHYLPASVGETGF
>Notothenia_coriiceps
MMTSNKTHYLPASVGVSGY
>Ceratotherium_simum_simum
MMTSNKTHYLPASVGETGF
>Manis_javanica
MMTSNKTHYLPASVGETGF
>Cynoglossus_semilaevis
MMTSNKTHYLPASVGVSGY
>Loxodonta_africana
MMTSNKTHYLPASVGETGF
>Melopsittacus_undulatus
MMTSNKTHYLPASVGETGF
>Rattus_norvegicus
MMTSNKTHYLPASVGETRF
>Salmo_salar
MMTSNKTHYLPASVGVSGY
>Lepisosteus_oculatus
MMTSNKTHYLPASVGESGF
>Trichechus_manatus_latirostr
MMTSNKTHYLPASVGETGF
>Aptenodytes_forsteri
MMTSNKTHYLPASVGETGF
>Anolis_carolinensis
MMTSNETHSLPASGGETGL
>Struthio_camelus_australis
MMTSNKTHYLPASVGETGF
>Chelonia_mydas
MMTSNKTHYLPASVGETGF
>Tinamus_guttatus
MMTSNKTHYLPASVGETGF
>Calypte_anna
MMTSNKTHYLPASVGETGF
>Orycteropus_afer_afer
MMTSNKTHYLPASVGETRF

HG0047 ENSGALG00000015422 NRG1

>Gallus_gallus
MLLLLSLLP------LPLLL----QILLLLLLPHDNC-FPRLSRYQPQMLKVRVLPFALGYWFT----
>Anser_cygnoides
MLLLLSLLP------LPLLL----QILLLLLLPHDNC-FPRLSRYQPQMLKGDVLSLPQKSWNCLRGV
>Xenopus_laevis
MVLLLSLLP--------RLLLFPLLLAFLLLLLHDSY-FLP-NRYHLQMLQVREQQFTLGYWFT----
>Chaetura_pelagica
MLLLLSLLP------LP--L----QILLLLLLLHDNC-FPRLSRYQPQMLKVRVLPFALGYWFT----
>Buceros_rhinoceros_silvestri
--MLKVRVMPFALGYWFT----
>Caprimulgus_carolinensis
MLLLLSLLP------LPLLL----QILLLLLLPLDNC-FPRLSRYQPQMLKVRVLPFALGYWFT----
>Mustela_putorius_furo
MLLLLSLLPLLPPPLLPPP-PPPLVLLLLLLLLHDSC-FLHLSRHQLQMLEVRNMPFSLGYWFT----
>Hynobius_retardatus
MRWLLSLLP------LRL---------PVLLSPHV-TSFLQRSGCPSPVLQVRSLHTALGYWFT----
>Physeter_catodon
--MLEVRNMPFSLGYWFT----
>Calidris_pugnax
MLLLLSLLP------LPLLL----QILLLLLLPHDNC-FPRLSRYQPQMLKVRILPFALGYWFT----
>Eptesicus_fuscus
MLLLLSLLPLLPPPLLPPP-PPALVLLLLLLLLHDSC-FLHLSRYQLQMLEVRNMPFSLGYWFT----
>Pterocles_gutturalis
--MLKVRILPFALGYWFT----
>Dasypus_novemcinctus
------MLGLL--SPPLPPPPPPLVLLLLAPLPHDSC-FRHLSGQQLQGLEGSPLPFSLGSWFT----
>Columba_livia
MLLLLSLLP------LPLLL----QILLLLLLPHDSC-FPRLSRYQPQTLKVRVLPFALGYWFT----
>Leptosomus_discolor
--MLKVRVLAFALGYWFT----
>Cuculus_canorus
MLLLLSLLP------LPLLL----QILLLLLLPHDNC-FPHLSRYQPQMLKVRVLPFALGYWFT----
>Galeopterus_variegatus
--MLEVRNMPFTLGYWFT----
>Haliaeetus_leucocephalus
MLLLLSLLP------LPLLL----QILLLLLLPHDNC-FPRLSRYQPQMLKVRVLPFALGYWFT----
>Gavia_stellata
MLLLLSLLP------LPLLL----QILLLLLLPHDNC-FPRLSRYQPQMLKVRVLPFALGYWFT----
>Balearica_regulorum_gibberic
--MLKVRVLPFALGYWFT----
>Condylura_cristata
MLLLLSLLPLLPPPLLPPP-PPPLVLLLLLLLLHDSC-FLHLSRQQLQMLEVRNIPFSLGYWFT----
>Oryctolagus_cuniculus
MLLLLSLLPLLPPPLLP---PPPLLLFLLLLLLHDSC-FLHLSRHQLQMLGVRNLPFSLGYWFT----
>Elephantulus_edwardii
MLLLLSLLPLLPPPLLPPP-PPPLVLLWLLLLLHDSC-FLHLSRQQLQMLEVRNLPFSLGYWFT----
>Ornithorhynchus_anatinus
MLLLLSLLYLLLLL-LLLL-LLPLLLLLLLLLLRDSC-FLHLSRHQPQMLEVRNLPFTLGYWFT----
>Opisthocomus_hoazin
MLLLLSLLP------LPLLL----QILLLLLLPHDNC-FPRLSRYQPQTLKVRVLPFALGYWFT----
>Parus_major
MLLLLSLLP------LPLLL----EILLLLLLPHDNC-FPRLSRYQPQMLKVRVLPFALGYWFT----
>Egretta_garzetta
MLLLLSLLP------LPLML----QILLLLLLPHDNC-FPRLSRYQPQMLKVRVLPFALGYWFT----
>Ceratotherium_simum_simum
MLLLLSLLPLLPPPLLPPP-PPPLILLLLLLLLHDSC-FLHLSRHQLQMLEVRNVPFSLGYWFT----
>Manis_javanica
ML--------VLPAPLPLLPP-PPVLPLLLLLPHDSG-FLHLSRPQLQTLEVRNMPFRLGYWFT----
>Mandrillus_leucophaeus
MLLLLSLLPLLPPPLLPPP-PPALVLLLLLLLLHDSC-FLHLSRHQLQMLEVRNMPFSLGYWFT----
>Loxodonta_africana
MLLLLSLLPLLPPPLLPPP-PPPLVLLLLLLLLHDSC-FLHLSRQQLQMLEVRNLPFSLGYWFT----
>Melopsittacus_undulatus
MLLLLSLLP------LPPLL----QILLLLLLPHDNC-FPRLSRYQPQMLKVRVLPFALGYWFT----
>Chinchilla_lanigera
MLLLLSLLPLLPPLLLPPPPPPPLVLLLLLLLLHDSC-FLHLSRHQLQMLEVRNVPFSLGYWFT----
>Trichechus_manatus_latirostr
MLLLLSLLPLLPPPL---LPPPPLVLLLLLLLLHDSC-FLHLSRQQLQMLEVRNLPFSLGYWFT----
>Pygoscelis_adeliae
MLLLLSLLP------LPLLL----QILLLLLLPHDNC-FPRLSRYQPQMLKVRVLPFALGYWFT----
>Anolis_carolinensis
MLVLLSLLL------LLPLPPLPFLLLFLLLLLHDGCVFLHLSSHQPQMLKVRYQPFTLGCWFT----
>Chrysemys_picta
MLLLLSLLP------LPLLPPLLLFLILLLLLLHDNC-FLHLSRHQPQMLKVRSQPFALGYWFT----
>Calypte_anna
MLLLLSLLP------LPLLL----QILLLLLLPHDNC-FPRLSRYQPQMLKVRVLPFALGYWFT----
>Apaloderma_vittatum
--MLKVRVLPFALGYWFT----
>Orycteropus_afer_afer
MLLLLSLLPLLPPPLLPP-PPPPLVLLLLLLLLHDSC-FLHLSRQQLQMLEVRNLPFSLGYWFT----

HG0048 ENSG00000177565 TBL1XR1

>Homo_sapiens
--MGYILCCDLMV
>Anser_cygnoides
--MGCVLCCDLMV
>Chaetura_pelagica
--MGCVLCCDLMV
>Apteryx_australis_mantelli
--MGCVLCCDLMV
>Cariama_cristata
--MGCVLCCDLMV
>Acinonyx_jubatus
--MGYILCCDLMV
>Lipotes_vexillifer
--MGYILCCDLMV
>Charadrius_vociferus
--MGCVLCCDLMV
>Myotis_brandtii
--MGYILCCDLMV
>Colius_striatus
--MGCVLCCDLMV
>Columba_livia
--MGCVLCCDLMV
>Leptosomus_discolor
--MGCVLCCDLMV
>Alligator_sinensis
--MGYILRCDLMV
>Cuculus_canorus
--MGCVLCCDLMV
>Sarcophilus_harrisii
--MGYILCCDLMV
>Galeopterus_variegatus
--MGYILCCDLMV
>Monodelphis_domestica
--MGYILCCDLMV
>Aquila_chrysaetos_canadensis
--MGCVLCCDLMV
>Coturnix_japonica
MTFSCSLFGSAWKGRQTCYLRMRHSASPLCGKRHLKGKLLHINALPMGCVLCCDLMV
>Balearica_regulorum_gibberic
--MGCVLCCDLMV
>Condylura_cristata
--MGYILCCDLMV
>Oryctolagus_cuniculus
---------------------MHQQQQLVIGGTGYSVAHSHINALPMGYILCCDLMV
>Tauraco_erythrolophus
--MGCVLCCDLMV
>Opisthocomus_hoazin
--MGCVLCCDLMV
>Corvus_cornix_cornix
--MGCVLCCDLMV
>Phaethon_lepturus
--MGCVLCCDLMV
>Ceratotherium_simum_simum
--MGYILCCDLMV
>Picoides_pubescens
--MGCVLCCDLMV
>Fulmarus_glacialis
--MGCVLCCDLMV
>Melopsittacus_undulatus
--MGCVLCCDLMV
>Cricetulus_griseus
--MGYILCCDLMV
>Tupaia_chinensis
--MGHVLCCGLMV
>Trichechus_manatus_latirostr
--MGYILCCDLMV
>Pygoscelis_adeliae
--MGCVLCCDLMV
>Anolis_carolinensis
--MGYILCRDLMV
>Tyto_alba
--MGCVLCCDLMV
>Struthio_camelus_australis
--MGCVLCCDLMV
>Chelonia_mydas
--MGYILCRDLMV
>Orycteropus_afer_afer
--MGYILCCDLMV

HG0048 ENSGALG00000039182

>Gallus_gallus
------------------------MGCVLCCDLMV
>Anser_cygnoides
------------------------MGCVLCCDLMV
>Chaetura_pelagica
------------------------MGCVLCCDLMV
>Apteryx_australis_mantelli
------------------------MGCVLCCDLMV
>Cariama_cristata
------------------------MGCVLCCDLMV
>Ailuropoda_melanoleuca
------------------------MGYILCCDLMV
>Lipotes_vexillifer
------------------------MGYILCCDLMV
>Calidris_pugnax
------------------------MGCVLCCDLMV
>Rousettus_aegyptiacus
------------------------MGYILCCDLMV
>Colius_striatus
------------------------MGCVLCCDLMV
>Columba_livia
------------------------MGCVLCCDLMV
>Leptosomus_discolor
------------------------MGCVLCCDLMV
>Cuculus_canorus
------------------------MGCVLCCDLMV
>Sarcophilus_harrisii
------------------------MGYILCCDLMV
>Galeopterus_variegatus
------------------------MGYILCCDLMV
>Monodelphis_domestica
------------------------MGYILCCDLMV
>Aquila_chrysaetos_canadensis
------------------------MGCVLCCDLMV
>Balearica_regulorum_gibberic
------------------------MGCVLCCDLMV
>Condylura_cristata
------------------------MGYILCCDLMV
>Oryctolagus_cuniculus
MHQQQLVIGGTGYSVAHSHINALPMGYILCCDLMV
>Tauraco_erythrolophus
------------------------MGCVLCCDLMV
>Opisthocomus_hoazin
------------------------MGCVLCCDLMV
>Acanthisitta_chloris
------------------------MGCVLCCDLMV
>Egretta_garzetta
------------------------MGCVLCCDLMV
>Equus_asinus
------------------------MGFILCCDLMV
>Picoides_pubescens
------------------------MGCVLCCDLMV
>Cercocebus_atys
------------------------MGYILCCDLMV
>Fulmarus_glacialis
------------------------MGCVLCCDLMV
>Melopsittacus_undulatus
------------------------MGCVLCCDLMV
>Chinchilla_lanigera
------------------------MGYVLCCDLMV
>Tupaia_chinensis
------------------------MGHVLCCGLMV
>Trichechus_manatus_latirostr
------------------------MGYILCCDLMV
>Aptenodytes_forsteri
------------------------MGCVLCCDLMV
>Gekko_japonicus
------------------------MGCILRCDLMV
>Tyto_alba
------------------------MGCVLCCDLMV
>Struthio_camelus_australis
------------------------MGCVLCCDLMV
>Apaloderma_vittatum
------------------------MDCVLCCDLMV
>Orycteropus_afer_afer
------------------------MGYILCCDLMV

HG0049 ENSDARG00000044485 sall4

```
>Danio_rerio
---------------MINRNALLIIIITLTCAFLLEGLISPNLENF----------------
>Channa_argus
MCEPLDWSQSAVRGTVINRNALLIIIITLTCAFLLEGLIFRGEGFVEEAEEN----------
>Anser_cygnoides
---------------MINRNALLIIIITLTCAFRPMGVISQL--------------------
>Xenopus_laevis
------------MGGSI---------QQLLEFFFFFGGGERE--------------------
>Oryzias_latipes
MCERSGLEPISCQGLVINRNALLIIIITLTCAFPTRGANFPSRRICRRRAGKLSATLCS---
>Cyprinodon_variegatus
MCEPLDRSQSAVREPAINRNALLIIIITLTCAFLLEREGG----------------------
>Coturnix_japonica
---------------MINRNALLIIIITLTCAFRPMGVISQL--------------------
>Plecoglossus_altivelis
---------------MINRRALLII-ITGTCAVDGL--DFRNFELIFLV--IFYSFLADV--
>Ficedula_albicollis
---------------MINRNALLIIIITLTCAFRPKFPNSRNLL------------------
>Cebus_capucinus_imitator
-----------------------MSRIAGTCAFLLKGGNFRTPGIRCVK-------------
>Rattus_norvegicus
---------------MINRNALLIIIITGTCAFLPKGGKIS---------------------
>Lepisosteus_oculatus
---------------MINRNALLIIIITLTCAFLLEGLISQL--------------------
>Ictalurus_punctatus
---------------MINRNALLIIIITLTCAFLLEGLISADLENLLRRRSKACYYLPQMVV
>Chrysemys_picta
---------------MINRNALLIIIITLTCAFRPRGVISQL--------------------
```

HG0049 ENSGALG00000039238

>Gallus_gallus
MINRNALLIIIIIAGHMRGARRTGEAGGGRLDFRNFE-----KIFF--------
>Acipenser_sinensis
MINRNALLIIIIIEGHMRGGWARFP-----------------------------
>Anser_cygnoides
---------------MRGARRIGEVGGGRLDFRNFE-----KIFF--------
>Xenopus_tropicalis
MINRNALLIIIIMAGHMRAGWGSLPAIGSTFWE---------------------
>Chaetura_pelagica
MINRNALLIIIIIAGHMRGARRIGEAGGGRLDFRNFE-----KIFF--------
>Apteryx_australis_mantelli
MINRNALLIIIIIAGHMRGARRIGEVGGGRLDFRNFE-----KIFF--------
>Menidia_menidia
MINRRALLII--ITGHMRGGWARFT-----------------------------
>Oryzias_latipes
MINRRALLII--ITGHMRGGWARFT-----------------------------
>Charadrius_vociferus
MINRNALLIIIIIAGHMRGARRIGEAGGGRLDFRNFE-----KIFF--------
>Pteropus_vampyrus
MINRNALLIIIIIAGHMRAAPGAGEPGRGRAGRRAGGAAAAAAARFP-------
>Haplochromis_burtoni
MINRRALLII--ITGHMRGGCSRFT-----------------------------
>Alosa_alosa
MINRKALLII--ITGHMRGGWARFS-----------------------------
>Columba_livia
MINRNALLIIIIIAGHMRGARRIGEAGGGRLDFRNFE-----KIFF--------
>Cuculus_canorus
MINRNALLIIIIIAGHMRGARRIGEAGGGGSISVILR-----RFFFSNFLFCPS
>Ctenopharyngodon_idella
MINREALLII--ITGHMRGGWARFP-----------------------------
>Kryptolebias_marmoratus
MINRTALLII--ITGHMRGGWGSIY---EIFEEVL-------------------
>Monodelphis_domestica
MINRNALLIIIIIAGHMRAARRAGESGRGAGRREGGATA---AARFP-------
>Esox_lucius
MINRQALLII--ITGHMRGGWARFP-----------------------------
>Falco_cherrug
MINRNALLIIIIIAGHMRGARRIGEAGGGEARFP--------------------
>Chanos_chanos
MINRKALLII--ITGHMRGGWARFP-----------------------------
>Apteronotus_leptorhynchus
MINREALLII--ITGHMRGGWTRYP-----------------------------
>Plecoglossus_altivelis
MINRRALLII--ITGTCAVDGLDFRNFELIFLVIFYSF-------LADV-----
>Scleropages_formosus
---------------MRAGWAPLGRALS----------EKVCGNFP-------
>Corvus_brachyrhynchos
MINRNALLIIIIIAGHMRGARRIGEAGGGRLDFRNFE-----KIFF--------
>Egretta_garzetta
MINRNALLIIIIIAGHMRGARRIGEAGGGRLDFRNFE-----KIFF--------
>Trematomus_bernacchii
MINRRALLII--ITGHMRGGWARFT-----------------------------
>Picoides_pubescens
MINRNALLIIIIIAGHMRGARRIGEAGGGRLDFRNFE-----KIFF--------
>Cynoglossus_semilaevis
MINRTALLII--ITGHMRGGWARFF---T-------------------------
>Cebus_capucinus_imitator
MINRNALLIIIIIEGHMRAAPGAGEPGRGRAGRRAGGAAAAAAARFP-------
>Melopsittacus_undulatus
MINRNALLIIIIIAGHMRGARRTGEAGGGGSISVILR-----RFFFSNFLFCPS
>Peromyscus_maniculatus_baird
MINRNALLIIIIIAGHMRAVPGAGEPGRGRAGRRAGGAAAA-AARFP-------
>Salvelinus_alpinus
MINRKALLII--ITGHMRGGWTRFS-----------------------------
>Ictalurus_punctatus
MINRNALLIIIIITLTCAFLLEGLISADLENLL----RRRSKACYYLPQMVV---
>Aptenodytes_forsteri
MINRNALLIIIIIAGHMRGARRIGEAGGGEARFP--------------------
>Struthio_camelus_australis
MINRNALLIIIIIAGHMRGARRIGEAGGGRLDFRNFE-----KIFF--------
>Chelonia_mydas
MINRNALLIIIIIAGHMRGAAWLGE--GGGSISVILR-----RFFFSNFLFCPS
>Tinamus_guttatus
MINRNALLIIIIIAGHMRGARRTGAVGGGEARFP--------------------
>Calypte_anna
MINRNALLIIIIIAGHMRGARRIGEAGGGRLDFRNFE-----KIFF--------

HG0050.1 ENSG00000074054 CLASP1

>Homo_sapiens
------------------------------MVHCCHSPDCIFETQALS--NLQRT--KRQPPRYVCWEGV----IVTAL------
>Amia_calva
------------------------------MLHCCHTPACISILQPLC--DLQRI--SGQPLG----------------------
>Anser_cygnoides
------------------------------MVHCCHSQNCIFETQTLS--NLHRT--KRRPPASVRGGICRTVCPLAFLLVLCF-
>Rhacophorus_dennysi
------------------------------MVNCCHSQDCIFDGQALR--KLFQTSDDH--EEVIC-------------------
>Apteryx_australis_mantelli
------------------------------MVHCCHSRNCIFETQTLS--NLHRT--KRRPPAYVCRRIFWTCCPLAFLLVLYF-
>Ailuropoda_melanoleuca
------------------------------MVHCCHSPDCIFETQALS--NFQRT--KRQPPGYVYWEGV----TLTAL------
>Hynobius_retardatus
------------------------------MLNCFHSPHCILGTQAHSIRNLHRT--KRRPRGYDC--------PLAFIVVVLDW
>Tursiops_truncatus
------------------------------MVHCCHSPDCISETQALS--NLQQT--KRPPPGDACWEGV----TPTAL------
>Pygocentrus_nattereri
------------------------------MVHCCHSQACIPKAQPLA--SLRCT------------------------------
>Calidris_pugnax
------------------------------MVHCCHSQNCIFETQTLS--NLHRT--KRRPPAYVYRGIFRTYCPLAFLLVLYF-
>Callorhinchus_milii
------------------------------MLHCCRSQDCICSPRPLD--NLQET--DRRPLG----------------------
>Rousettus_aegyptiacus
------------------------------MVHCCHSPDCIFETQALS--NLYRT--NRQPPGYVYWEGV----TLTAL------
>Dasypus_novemcinctus
------------------------------MVHCCHSPDCIFETQALS--NLWRARATRQPPGHVYQEGSS--------------
>Columba_livia
------------------------------MVHCCHSQNCIFETQTLS--NLHRT--KRRPPAYVYRGIFRTYCPLAFLLVLYF-
>Alligator_mississippiensis
------------------------------MVHCWHSQNCIFETQAFS--NLHRT--KRQPTGFICRGIFPTCCPLASFSLVVYL
>Sarcophilus_harrisii
------------------------------MVHCCHSPDCIFETKALS--NLYQT--QRQPPGSLPSGRFSLTAL----------
>Galeopterus_variegatus
------------------------------MVHCCHSPDCIFETQALS--NLQRT--KRQPPRYVYWEGV----ILTAL------
>Monodelphis_domestica
------------------------------MVHCCHSPDCISETKALS--NLCQT--KRQPPGSLPSKEIQPDCPLAFFGLCCI-
>Aquila_chrysaetos_canadensis
------------------------------MVHCCHSQNCIFETQTLS--NLHRT--KRQPPAYVYRGIFRTYCPLAFLLVLYF-
>Coturnix_japonica
------------------------------MVHCCHSQNCIFETQALS--NLH--------------------------------
>Condylura_cristata
------------------------------MVHCCHSPDCIFETQTLS--NLQRT--KRQPPGSFYLEGV----TLTAL------
>Oryctolagus_cuniculus
------------------------------MVHCCHSPDCIFETQALS--NLQQT--KRQLPRYGYWEGV----ILTAL------
>Elephantulus_edwardii
MYDILLPLTSSSSRYLNWTFNIQPAALQTHMVHCCHSPDCIFETQALG--NSQQT--KRQLLGSVYREGV----VLTAL------
>Taeniopygia_guttata
------------------------------MVHCCHSQNCIFETQALS--NLHRR--KRRPPAYICRGSFWPCCPLAFWLLLYF-
>Ceratotherium_simum_simum
------------------------------MVHCCHSPDCIFETQALS--NLQQT--KRQPPRYVYREGV----TLTAP------
>Manis_javanica
------------------------------MVHCCHSLDCIFEAQALS--NLQQT--KRQPPGHVYWEGV----TLTAL------
>Loxodonta_africana
------------------------------MVHCCHSLDCIFETQTLS--NLLQT--KR--------------------------
>Dipodomys_ordii
------------------------------MVHCCHSPDCIFETQALS--NLQRT--KRPPPGLGYREGV----ILTAL------
>Tupaia_chinensis
------------------------------MVHCCHSPDCIFETQALS--NLQRT--KRQPPGYIYREGV----ILTAL------
>Lepisosteus_oculatus
------------------------------MVHCFHSQACISVLQPLS--NLQHI------------------------------
>Trichechus_manatus_latirostr
------------------------------MVHCCHSPDCIFETQTLS--NLQQT--KRQPPGSLYQEGV----ILTAL------
>Aptenodytes_forsteri
------------------------------MVHCCHSQNCIFETQTLS--NLH--------------------------------
>Squalus_acanthias
------------------------------MVHCCRSPDRICSPELLS--NLQQA--DQRPLG----------------------
>Anolis_carolinensis
------------------------------MVHCCHSQNHIFEIQALG--NLHQT--KRRPRGSS-RRTAQTCRPLASLLPVLLF
>Struthio_camelus_australis
------------------------------MVHCCHSQNCIFETQTLS--NLHRT--KRQPPEYVCRGIFWTCCPLAFLLVLYF-
>Chelonia_mydas
------------------------------MVHCCHSQNCIFETQALS--NLHRT--KRRPTGYIYSGVSQTYCPLALFSPVLYF
>Tinamus_guttatus
------------------------------MVHCCHSQNCIFEIQTLS--NLHRT--KRRPPAYSCRGIF---------------
>Orycteropus_afer_afer
------------------------------MVHCCHSPDYIFETQALS--NLQQT--KRQPPGSVYQEGN----ILTAL------

HG0050.2 ENSG00000074054 CLASP1

>Homo_sapiens
MKYFRPSS-RTSLCHSLLYLLLVI
>Acinonyx_jubatus
MKNFRPKS-RTSLCHSLLYLLLVI
>Tursiops_truncatus
MKYSRPRR-RTSLCHSLPSPLPVI
>Myotis_brandtii
MKYFRPNT-RTSLCHSLLYRLLVI
>Dasypus_novemcinctus
MKYFRPNS-VASLCHSLLYRLLVI
>Galeopterus_variegatus
MKYFRPNS-RTSLCHSLLYLLLVI
>Condylura_cristata
MKYFRANS-RTSLCHSLLYLLLVI
>Oryctolagus_cuniculus
MKYFKPSSRRTSLCHSLLYLLLVI
>Elephantulus_edwardii
MKYFRPLC-MTSFCHSLHLLLDI-
>Ceratotherium_simum_simum
MKYFRPNS-RTSLCHSLLYLQLVI
>Fukomys_darlingi
MKYFRPSS-RTSLCHSLLYLLLVI
>Orycteropus_afer_afer
MKYFRHNS-IASLCHSLLYLLLDI

HG0051 ENSG00000116679 IVNS1ABP

```
>Homo_sapiens
------------MNVSISSEMNQIMMHHYHRRNSCL
>Anas_platyrhynchos
------------------------MMHDYHRRNSCL
>Apteryx_australis_mantelli
-------------------------MHDYHRRNSCL
>Ailuropoda_melanoleuca
------------MSFSISSEMNQIMMHDYHRRNSCL
>Hynobius_chinensis
------------MSLTVCAQRNQIMIHDYHKRNSCL
>Physeter_catodon
------------MSFSISSEMNQIMMHDYHRRSSCL
>Callorhinchus_milii
------------------------MMQHHHSRNSCL
>Rousettus_aegyptiacus
------------MSFSISSEMNQIMMHDYHRRNSCL
>Dasypus_novemcinctus
------------------------MMHDYHRRNSCL
>Latimeria_menadoensis
----------MRMNFIVYSTRNQIMMQDYHSRKQSL
>Alligator_sinensis
------------MSSLVRADRHQFMMHYYHRRNSCL
>Sarcophilus_harrisii
------------MNFFVVSQRNQIIMHDYHRRNSCL
>Galeopterus_variegatus
------------------------MMHHYHRRNSCL
>Macropus_eugenii
------------MNFFVLSQRNQIMMHDYHRRNSCL
>Aquila_chrysaetos_canadensis
------------MNSIVYAERHQIMMHDYHRRNSCL
>Numida_meleagris
------------MSCIVRAERQKIMMHDYHRRNSCL
>Erinaceus_europaeus
------------MSFPISSEMNQIMMHDYHRRNSCL
>Oryctolagus_cuniculus
MRSWRTANSDEKHGTQRSPGVQEIMMHHSHRRNSCL
>Elephantulus_edwardii
------------MNFSISTERNQIMMHDYHRRNSCL
>Ornithorhynchus_anatinus
------------------------MMHDYHRRNSCL
>Pseudopodoces_humilis
------------MFTAVRAQRHQVMMHDYHRRNSCL
>Ceratotherium_simum_simum
------------MSFSISSEMNQIMMHDYHRRNSCL
>Manis_javanica
------------MSVSISTEMNQIIMHDYHRRNSCL
>Loxodonta_africana
------------MNFSVSAERSQTMMQDYHRRNSCL
>Heterocephalus_glaber
------------MSFSISSEMNQIMMHRYHRRNSCL
>Tupaia_chinensis
------------MNFSISSEMNQIMMYHYHRRNWCL
>Trichechus_manatus_latirostr
------------MNFSISTERNQIMMHDYHRRNSCL
>Aptenodytes_forsteri
------------------------MMHDYHRRNSCL
>Anolis_carolinensis
------------MSAVVCGERDQSMMNDYHRRNSCL
>Struthio_camelus_australis
------------------------MMHDYHRRNSCL
>Chrysemys_picta
------------MNGVVCAERHQIMMHDYHRRNSCL
>Tinamus_guttatus
------------------------MMHDYHRRNSCL
>Calypte_anna
------------------------MMHDYHRRNSCL
>Orycteropus_afer_afer
------------MNFSTSTERNQIMMHDYHRRNSCL
```

HG0051 ENSGALG00000037162

>Gallus_gallus
------------MSCIIRAERQKIMMHDYHRRNSCL
>Acipenser_sinensis
------------MSTVVYSHRNQVIMRDYHRRTTFL
>Anas_platyrhynchos
------------------------MMHDYHRRNSCL
>Apteryx_australis_mantelli
-------------------------MHDYHRRNSCL
>Ailuropoda_melanoleuca
------------MSFSISSEMNQIMMHDYHRRNSCL
>Cynops_pyrrhogaster
------------MSLIVCAERNQIMIHDYHKRSSCL
>Physeter_catodon
------------MSFSISSEMNQIMMHDYHRRSSCL
>Rousettus_aegyptiacus
------------MSFSISSEMNQIMMHDYHRRNSCL
>Dasypus_novemcinctus
------------------------MMHDYHRRNSCL
>Latimeria_chalumnae
----------MRMNSIVYSTRNQIMMQDYHSRKQSL
>Alligator_sinensis
------------MSSLVRADRHQFMMHYYHRRNSCL
>Rutilus_rutilus
------------MSTIVNRQPDQVIMHDQHRKGLSL
>Sarcophilus_harrisii
------------MNFFVVSQRNQIIMHDYHRRNSCL
>Galeopterus_variegatus
------------------------MMHHYHRRNSCL
>Macropus_eugenii
------------MNFFVLSQRNQIMMHDYHRRNSCL
>Aquila_chrysaetos_canadensis
------------MNSIVYAERHQIMMHDYHRRNSCL
>Erinaceus_europaeus
------------MSFPISSEMNQIMMHDYHRRNSCL
>Oryctolagus_cuniculus
MRSWRTANSDEKHGTQRSPGVQEIMMHHSHRRNSCL
>Elephantulus_edwardii
------------MNFSISTERNQIMMHDYHRRNSCL
>Ornithorhynchus_anatinus
------------------------MMHDYHRRNSCL
>Sturnus_vulgaris
------------MFSTVRAQRHQVMMHDYHRRNSCL
>Ceratotherium_simum_simum
------------MSFSISSEMNQIMMHDYHRRNSCL
>Manis_javanica
------------MSVSISTEMNQIIMHDYHRRNSCL
>Pan_paniscus
------------MNVSISSEMNQIMMHHYHRRNSCL
>Loxodonta_africana
------------MNFSVSAERSQTMMQDYHRRNSCL
>Mus_musculus
------------MNFAISSEMTQIMMHDYHRRNSCL
>Salmo_salar
------------MSTTTRCQKPQVIMHDQHRKSLTL
>Tupaia_chinensis
------------MNFSISSEMNQIMMYHYHRRNWCL
>Trichechus_manatus_latirostr
------------MNFSISTERNQIMMHDYHRRNSCL
>Aptenodytes_forsteri
------------------------MMHDYHRRNSCL
>Squalus_acanthias
------------MIYIVRFVPRKDNMQDHHNRNSCL
>Anolis_carolinensis
------------MSAVVCGERDQSMMNDYHRRNSCL
>Struthio_camelus_australis
------------------------MMHDYHRRNSCL
>Pelodiscus_sinensis
------------MSGVVCAERHQIMMHDYHRRNSCL
>Tinamus_guttatus
------------------------MMHDYHRRNSCL
>Calypte_anna
------------------------MMHDYHRRNSCL
>Orycteropus_afer_afer
------------MNFSTSTERNQIMMHDYHRRNSCL

HG0052 ENSG00000178235 SLITRK1

>Homo_sapiens
MNLNCVCGEQDGRCYFVMRSGMNCSL
>Anser_cygnoides
-----------------MKSGMNCSV
>Apteryx_australis_mantelli
MNWNCFRCEEDGCCYFVMRSGMNRSV
>Buceros_rhinoceros_silvestri
MNWNCFPCEEDGCCYFVMRSGMNCSV
>Caprimulgus_carolinensis
MNWNSFPCEEDGCCYFVMRSGMNCSV
>Ailuropoda_melanoleuca
MNLNCVCGEQDGRCYFVMRSGMNCSL
>Lipotes_vexillifer
MNLNCVCGEQDGRCYFVMRSGMNCSL
>Calidris_pugnax
MNWNCFPCEEDGCCYFVMRSGMNCSV
>Miniopterus_natalensis
MNLNCVCGEQDGCCYFVMRSG-----
>Pterocles_gutturalis
MNWNCFPCEEDGCCYFVMRSGMNSSV
>Columba_livia
MNWNSFPWEEDGCCYFVMRSGMHCWV
>Leptosomus_discolor
MNWNCFPREEDGCCYFVMRSGMNCSV
>Galeopterus_variegatus
MNLNCVCGEQDGRCYFVMRSGMNCSL
>Monodelphis_domestica
MNLNCVCGKQDDCCYFVMKSGMNCSV
>Falco_cherrug
MNWNCFPCEKDGCCYFVMRSGMNCSV
>Coturnix_japonica
MNWNCFPREEDGCCYFVMRSGMNCSV
>Balearica_regulorum_gibberic
MNWNCFPFEEDGCCYFVMRSGMNCSV
>Erinaceus_europaeus
MNLNCVCGEQDGRCYFVMRSGMNCSL
>Oryctolagus_cuniculus
MNLNCVCGEQDGCCYFVMRSGMNCSL
>Elephantulus_edwardii
MNLDCVCGEQDGRCYFVMKSGMNCSL
>Tauraco_erythrolophus
-----------------MRSGMNCSV
>Lepidothrix_coronata
MNWNCFPCEEDGCCYFVMRSGMNCSV
>Egretta_garzetta
MNWNCFPCEEDGCCYFVMRSGMNCSV
>Ceratotherium_simum_simum
MNLNCVCGEQDGRCYFVMRSGMNCSL
>Manis_javanica
-----------------MRSGMNCSL
>Picoides_pubescens
-----------------MRSGMNCSV
>Loxodonta_africana
-----------------MRSGMNCSL
>Melopsittacus_undulatus
MNWNCFPCEEDGCCYFVTRSGMNCSV
>Jaculus_jaculus
MNLNCVCGEQDGGCYFVMRSGMNCSL
>Tupaia_chinensis
MNLNCVCGEQDGRCYFVMRSGMNCSL
>Trichechus_manatus_latirostr
-----------------MRSGMNCSL
>Aptenodytes_forsteri
MNWNCFPCEEDGCCYFVMRSGMNRSV
>Struthio_camelus_australis
MNWNCFRCEEDDCCYFVMRSGMNRSV
>Chrysemys_picta
MNWNCFRCEDDGCCYFVMTSGMNCSV
>Tinamus_guttatus
MDWNCFRYEEDGGCYFVMRSGMNCSV
>Apaloderma_vittatum
MNWNCFPCKEDGCCYFVMRSGMNCSV
>Orycteropus_afer_afer
MNLNCVCGEQDGRCYFVMRSGMNCSL

HG0053.1 ENSG00000184564 SLITRK6

>Homo_sapiens
---MVNYMQKSQHSCEVCREF
>Anser_cygnoides
-------MQKSQHSCEVCCEF
>Chaetura_pelagica
-------MQKSQHSSEVCCEF
>Buceros_rhinoceros_silvestri
MLQTVNYMQKSQHSCEVCCEF
>Caprimulgus_carolinensis
-------MQKSQHSCEVCCEF
>Cariama_cristata
-------MQKSQHSCEVCCEF
>Acinonyx_jubatus
---MVNYMQKSQHSCEVCREF
>Charadrius_vociferus
-------MQKKQRSCEVCCEF
>Eptesicus_fuscus
---MVNYMQKSQHSCEVCPEF
>Pterocles_gutturalis
-------MQKSQHSCEVCCEF
>Colius_striatus
-------MQKSQHSCEVCSEF
>Columba_livia
-------MQKTQHSCEVCCEF
>Merops_nubicus
-------MQKSQHSCEVCCEF
>Cuculus_canorus
-------MQKSQHCCEVCCEF
>Falco_cherrug
-------MQKSQHSCEVCCEF
>Gallus_gallus
-------MRKSQHSCEVCCEF
>Gavia_stellata
-------MQKSQHSCEVCCEF
>Mesitornis_unicolor
-------MQKSQRSCEVCREF
>Condylura_cristata
---MVNYMQKTQHSCEVCCEF
>Ornithorhynchus_anatinus
---MVNYMQKSQHSCEVCCEF
>Tauraco_erythrolophus
-------MQKSQHSYEVCCEC
>Opisthocomus_hoazin
-------MQKGQHSCEVCFEY
>Acanthisitta_chloris
-------MQKSQPSCEVCCEF
>Pelecanus_crispus
-------MQKSQHSCEVCCEF
>Equus_caballus
---MVNYMQKSQHSCEVCCEF
>Manis_javanica
---MVNYMQKSQHSCEVCCEF
>Picoides_pubescens
-------MQKSQHSCEVCCEF
>Loxodonta_africana
---MVNYMQKSQRSCEVCREF
>Fulmarus_glacialis
-------MQKSQHSCEVCCEF
>Nestor_notabilis
-------MQISQHSCEVCCEF
>Marmota_marmota_marmota
---MVNYMQKSQHSCEVCCEF
>Trichechus_manatus_latirostr
---MVNYMQKSQHSCEVCREF
>Aptenodytes_forsteri
-------MQKSQHSCEVCCEF
>Chelonia_mydas
---MVNYMQKSQHRWEVCCEF
>Calypte_anna
-------MQKSQHSCEVCCEF
>Apaloderma_vittatum
-------MQTTQHSCEVCCEF
>Orycteropus_afer_afer
---MVNYMQKSQHSCEVCREF

HG0053.2 ENSG00000184564 SLITRK6

```
>Homo_sapiens
----------MLSILSLTRGFNFMKLWDLTKSDI------------
>Canis_lupus_familiaris
-----------------------MKQWDLPKSDI------------
>Physeter_catodon
----------MLSILSLTRGFNFIKRWDLPKSDI------------
>Rousettus_aegyptiacus
----------MLSILGLTRGFHFIK---------------------
>Galeopterus_variegatus
----------MLSVLSLTRGFNFMKLWDLPKSDI------------
>Condylura_cristata
----------MLSILSLNRGFNFMKQRNCQKVIFQKKMHSGWCFLF
>Oryctolagus_cuniculus
----------MLSIFSWTRSLNFLMLGNTPQSDI------------
>Equus_asinus
MHSARNIFNEMLSILSLTKGFNFMKQWDLPKSDI------------
>Manis_javanica
----------MLSILSLTRGFNFMKQWDLPKSDII-----------
>Loxodonta_africana
MHSARNIFSEMLSILSLIGAVNFRKLWDLPKSDI------------
>Fukomys_darlingi
----------MLSILSLTRDFNFPKLWDLPKSDI------------
>Tupaia_chinensis
----------MLSIVSLTRGFNFMKLWDLPKSDI------------
>Orycteropus_afer_afer
----------MLSILSLTGVFNFKKLWDMPKSDI------------
```

HG0054 ENSG00000124479 NDP

```
>Homo_sapiens
-------MTTRKLQPDPALSLNGTGS------
>Anser_cygnoides
-------MTAKRLKPDFSLSWRGTGSHDLEFF
>Leptonychotes_weddellii
-------MTTRKLQPDPALCLKGTGS------
>Balaenoptera_acutorostrata_s
-------MTTRKLQPDPALSLKGTGA------
>Miniopterus_natalensis
-------MTTRKLQPDPALSLEGTGS------
>Dasypus_novemcinctus
-------MITRKLQSDPALSLRGTRS------
>Galeopterus_variegatus
-------MTTRKLQPDPALSLKGTGS------
>Meleagris_gallopavo
-------MTTKRLKPDFSLSWRGTGSQYLVFF
>Gavia_stellata
-------MTAKRLKPDFLLSWKGTGCQYLEFF
>Sorex_araneus
-------MTARKLQPDPALSLEGTGS------
>Oryctolagus_cuniculus
-------MTTRKLQPDPALSLKGSGA------
>Ceratotherium_simum_simum
-------MTTRKLQPDPALSLKGTGS------
>Loxodonta_africana
-------MTTRKLQPDPALSLKGTGS------
>Ctenomys_sociabilis
-------MTTTKLQPDPALALKGTGS------
>Tupaia_chinensis
-------MTTRKLQPDPTLSLKGTGS------
>Trichechus_manatus_latirostr
-------MTTRKLQPDPALSLKGTGS------
>Python_bivittatus
MSNPKPAMTTWRLKTDPLLSWQGTGPQSLEGF
>Orycteropus_afer_afer
-------MTTRKLQPDPTLSLKGTGS------
```

HG0054 ENSGALG00000016222 NDP

>Gallus_gallus
-----------MTAKRLKPDFSLSWRGTGSQYLEFF
>Anser_cygnoides
-----------MTAKRLKPDFSLSWRGTGSHDLEFF
>Apteryx_australis_mantelli
-----------MTAKRPKPDFSPSWKETGSQYLEFF
>Cariama_cristata
-----------MTAKRLKPDFSPSWKGTGCQYLEFF
>Felis_catus
-----------MTTRKLRPDRALCWEGTGS------
>Balaenoptera_acutorostrata_s
-----------MTTRKLQPDPALSLKGTGA------
>Calidris_pugnax
-----------MTAKRLKPDFSPSWKGTGCQYLEFF
>Pteropus_vampyrus
-----------MTTRKLQPDPALSLKGTGS------
>Pterocles_gutturalis
-----------MTAKRLKPDFSPSWKGTGCQYLEFF
>Colius_striatus
-----------MTSKRLKPVFSPSWEGTGGQYLEFC
>Leptosomus_discolor
-----------MTAKRLKPDFSPSWKGTGCQCLEFF
>Cuculus_canorus
-----------MPAKRLKPGFSPSWKGTDCQYLEFF
>Galeopterus_variegatus
-----------MTTRKLQPDPALSLKGTGS------
>Haliaeetus_albicilla
-----------MTAKRLKPDFSPSWRGTGCQYLEFF
>Gavia_stellata
-----------MTAKRLKPDFLLSWKGTGCQYLEFF
>Balearica_regulorum_gibberic
-----------MTAKRLKPDFSPSWKGTGCQYLEFF
>Sorex_araneus
-----------MTARKLQPDPALSLEGTGS------
>Oryctolagus_cuniculus
-----------MTTRKLQPDPALSLKGSGA------
>Tauraco_erythrolophus
-----------MTAKRLKPDFSPSWKGTGCQDLEFF
>Opisthocomus_hoazin
-----------MTAKRLKPDFSPSWKGTGCPYLEFF
>Nipponia_nippon
MISVNSNSRRRMTAKRLKPDFSPSWKGTGCQYLEFF
>Ceratotherium_simum_simum
-----------MTTRKLQPDPALSLKGTGS------
>Aotus_nancymaae
-----------MTTRKLQPDPALSLKGTGS------
>Loxodonta_africana
-----------MTTRKLQPDPALSLKGTGS------
>Fulmarus_glacialis
-----------MTAKRLKPDFSPSWKGTGCQYLEFF
>Nestor_notabilis
-----------MTAKALKPDFSPSWKGTGCHYLEFF
>Heterocephalus_glaber
-----------MTTKKLRPDPALTLKGTGS------
>Tupaia_chinensis
-----------MTTRKLQPDPTLSLKGTGS------
>Trichechus_manatus_latirostr
-----------MTTRKLQPDPALSLKGTGS------
>Pygoscelis_adeliae
-----------MTAKRLKPDFSSSWKGTGCQYLEFF
>Gekko_japonicus
----MSTPKLAMTTQRLKTDLSLSSKGTGPQSLECF
>Struthio_camelus_australis
-----------MTAKRLKPDFSPSWKGTGSRYLEFF
>Chrysemys_picta
-----------MTTKRLKPGFSSSLKGTESQYLEFF
>Tinamus_guttatus
-----------MTVKRTNPDFSLSWKGMGSQYLEFF
>Calypte_anna
-----------MTAKRLKPDSSPSWKGTDCQVLEFF
>Orycteropus_afer_afer
-----------MTTRKLQPDPTLSLKGTGS------

HG0055.1 ENSGALG00000010533 ELAVL4

>Gallus_gallus
MLHIVFQRVSCHDLIKRGGGCVCCSA---
>Anser_cygnoides
MLHIVFQRVSCHDLIKRGGGLCVLQRLVYVDF-VASLGSRAVRRNVSFYITLSLLPTR---------
>Chaetura_pelagica
MLHIVFQRVSCHDLIKRGGGLYVLQRLVYVDF-VASLGSSAVRRNVSFYITLSLLFDSVRGCKGPSL
>Apteryx_australis_mantelli
MLHIVFQRVSCHDLIKRGGGLYVLQRLVYVDF-VASLGSGTVRRNVSFYITFFLLLTL---------
>Caprimulgus_carolinensis
MLHIVFQRVSCHDLIKRGGGLYVLQRLVYVDF-VASLGSSAVRRNVSFYITLFLLLTL---------
>Canis_lupus_familiaris
MLHLVFQRVSCHDLIKKRRRLVCVAVPGVLSCCFLPWAAAL--------------------------
>Hynobius_retardatus
MLHIVFQRVSCHDLIKRGGGLYVLQRIVYVDL-LHRWAAVL--------------------------
>Lipotes_vexillifer
MLHLVFQRVSCHDLIKKRRRLVCVAVPGVLSCCFLLWAAAL--------------------------
>Calidris_pugnax
MLHIVFQRVSCHDLIKRGGGLYVLQRLVYVDF-VASLGSSAVRRNVSFYITLFLLLTL---------
>Myotis_lucifugus
MLHLVFQRVSCHDLIKKRRRLVCVAVPGVLSCCFLLWAAAL--------------------------
>Dasypus_novemcinctus
MLHLVFQRVSCHDLIKKRRRLVCVAVPGVLSCCFLLWAAAL--------------------------
>Cuculus_canorus
MLHIVFQRVSCHDLIKRGGGVYVLQCLVYVDF-VASLGSSAVRRNVSFYITLFLLSTL---------
>Galeopterus_variegatus
MLHLVFQRVSCHDLIKKRRRLVCVAVPGVLSCCFLLWAAAL--------------------------
>Aquila_chrysaetos_canadensis
MLHIVFQRVSCHDLIKRGGGLYVLQRLVYVDF-VASLGSSAVRRNVSFYITLFLLLTL---------
>Balearica_regulorum_gibberic
MLHIVFQRVSCHDLIKRGGGLYVLQRLVYVDF-VASLGSSAVRRNVSFYITLFLLLTL---------
>Condylura_cristata
MLHLVFQRVSCHDLIKKRRRLVCVAVPGVLSCCFLLWAAAL--------------------------
>Oryctolagus_cuniculus
MLHLVFQRVSCHDLIKKRRRLVCVAVPGVLSCCFLLWAAAL--------------------------
>Elephantulus_edwardii
MLHLVFQRVSCHDLIKKRRRLVCVAVPGVLNCCFLLWAAAL--------------------------
>Opisthocomus_hoazin
MLHIVFQRVSCHDLIKRGGGLYVLQRLVYVDF-VASLGSSAVRRNVSFYITLFLLLTL---------
>Corvus_brachyrhynchos
MLHIVFQRVSCRDLIKRGGGLYVLQCLVYVDF-VASLGSSAVRRNVSFYITLFLLLTL---------
>Egretta_garzetta
MLHIVFQRVSCHDLIKRGGGLYVLQRLVYVDF-VASLGSSAVRRNVSFYITLFLLLTL---------
>Ceratotherium_simum_simum
MLHLVFQRVSCHDLIKKRRRLVCVAVPGVLSCCFLLWAAAL--------------------------
>Manis_javanica
MLHLVFQRVSCHDLIKKRRRLVCVAVPGVLSCCFLLWAAAL--------------------------
>Aotus_nancymaae
MLHLVFQRVSCHDLIKKRRRLVCVAVPGVLSCCFLLWAAAL--------------------------
>Loxodonta_africana
MLHLVFQRVSCHDLIKKRRRLVCVAVPGVLSCCFLLWAAAL--------------------------
>Fulmarus_glacialis
MLHIVFQRVSCHDLIKRGGGLYVLQRLVYVDF-VASLGSSAVRRNVSFYITLFLLLTL---------
>Melopsittacus_undulatus
MLHIVFQRVSCRDLIKRGGGLYVLQCLVYVDF-VASLGSSAVRRNVSFYITLFLLSTL---------
>Dipodomys_ordii
MLHLVFQRVSCHDLIKRGGGLYV--
>Trichechus_manatus_latirostr
MLHLVFQRVSCHDLIKKRRRLVCVAVPGVLSCCFLLWAAAL--------------------------
>Aptenodytes_forsteri
MLHIVFQRVSCHDLIKRGGGLYVLQRLVYVDF-VASLGSSAVRRNVSFYITLFLLLTL---------
>Anolis_carolinensis
MLHIVFQRVSCHDLIKRGGGLYVYQRLVYADL-LHRWAAVL--------------------------
>Tyto_alba
MLHIVFQRVSCHDLIKRGGGLYVLQRLVYVDF-VASLGSSAVRRNVSFYITLFLLLTL---------
>Struthio_camelus_australis
MLHIVFQRVSCHDLIKRGGGLYVLQRLVYVDF-VASLGSSTVRRNVSFYITFFLLLTL---------
>Chelonia_mydas
MLHIVFQRVSCHDLIKRGGGLYVLQRLVYVDF-VASLGSSTVRRNVSFYITFFLLLTL---------
>Tinamus_guttatus
MLHIVFQRVSCHDLIKRGGGSCVLRRLVYVDF-VASLGSGTVRRNVSFYITLSLLPTL---------
>Orycteropus_afer_afer
MLHLVFQRVSCHDLIKKRRRLVCVAVPGVLSCCFLLWAAAL--------------------------

HG0055.2 ENSGALG00000010533 ELAVL4

>Gallus_gallus
-------MSILLHRWAAAL
>Anser_cygnoides
-------MSILLHRWAAAL
>Chaetura_pelagica
MCCSAWYMSILLHRWAAAL
>Apteryx_australis_mantelli
-------MSILLHRWAAVL
>Caprimulgus_carolinensis
-------MSILLHRWAAAL
>Calidris_pugnax
-------MSILLHRWAAAL
>Cuculus_canorus
-------MSILLHRWAAAL
>Aquila_chrysaetos_canadensis
-------MSILLHRWAAAL
>Balearica_regulorum_gibberic
-------MSILLHRWAAAL
>Opisthocomus_hoazin
-------MSILLHRWAAAL
>Corvus_brachyrhynchos
-------MSILLHRWAAAL
>Egretta_garzetta
-------MSILLHRWAAAL
>Fulmarus_glacialis
-------MSILLHRWAAAL
>Melopsittacus_undulatus
-------MSILLHRWAAAL
>Aptenodytes_forsteri
-------MSILLHRWAAAL
>Tyto_alba
-------MSILLHRWAAAL
>Struthio_camelus_australis
-------MSILLHRWAAAL
>Pelodiscus_sinensis
-------MSILLHRWAAVL
>Tinamus_guttatus
-------MSILLHRWAAVL

HG0056 ENSG00000139329 LUM

```
>Homo_sapiens
----------------MSWAVLSPHVHLHFVREQCSHAT--PQDPHNDITPFRDWRDWAG-SPHPPSALVSLRIWQPVPS----------------
>Ursus_maritimus
----------------MSGAVLAPHVHLHFVREQNAHMP--PCK----IPTMT-------------------------------------------
>Hynobius_chinensis
----------------MGWPFLS--IHLHFVREQYFHMP--HHK----FTMMT-------------------------------------------
>Orcinus_orca
-------------------------------------MP--HHK----IPTMT-------------------------------------------
>Calidris_pugnax
MSTSIKNNRWVVLVQNMVSTCT--------SLESCIFGHATPQDHHDDIAASENSVTRAFF--FFFFLFVTERIWQTFTS----------------
>Myotis_ricketti
----------------MYWAVLAHQSTCTSLESRAFTCH--TTRSHNDITPFRVWRDWAG-SPPFSSCITKNLAASSALTEFTAYIGGCLSILHLL
>Haliaeetus_leucocephalus
----------------MGCPCTKYGIHLHFIRESCIFVHATPQDHHDDIAASENSVTRAFF--FF-FSLVTQRIWQTFTS----------------
>Mesitornis_unicolor
----------------MVSTCT--------SLESYIFVHATPQDHHDDIAASQNSVTRAFF--F---SLVTQRIWQTLTS----------------
>Ficedula_albicollis
----------------MVSTCT--------SLESCIFVHATPQDHHDDIAASENSVTRAFF--FF-SPLVTQRIWQSFTS----------------
>Ceratotherium_simum_simum
----------------MSWADFAHHVHLHFVREQSVHMP--HHK----IPTMT-------------------------------------------
>Picoides_pubescens
----------------MGSTCT--------SLELHFFVHATPQDPHDDIAASHNSLTRAFLSFFFFPPAVTQRIWQIFTS----------------
>Loxodonta_africana
----------------MSRAVLSLHVHLHFVRELSFYTP--HHE----IPTMK-------------------------------------------
>Nestor_notabilis
----------------MVSTCT--------SLESCIFVHATPQDHHDDIAASENSVTRAFF--F---SLVTQRIWQTCTS----------------
>Marmota_marmota_marmota
----------------MSWAVVAQHVHLHFVREQSVNMP--HHK----SPTMT-------------------------------------------
>Tupaia_chinensis
----------------MSRAVLVHHVHLHFVREQCVHMP--HHK----LPTMT-------------------------------------------
>Pygoscelis_adeliae
----------------MGCPCTKYGIHLHFIRELHFCTCHTTRSP---------------------------------------------------
>Python_bivittatus
----------MGRIDGLSFTIFS--LHLHLIRQQQFHIP--HAR----IP----------------------------------------------
>Orycteropus_afer_afer
----------------MSWAVLALHVHLHFVREQSFHMP--HHK----IPTMK-------------------------------------------
```

HG0056 ENSGALG00000011271 LUM

>Gallus_gallus
MGCPCTECGIHLHFIKRAAFSYMPHHKITMMT
>Anser_cygnoides
----------------------MPHHKITMMT
>Apteryx_australis_mantelli
----------------------MPHHKITMMT
>Caprimulgus_carolinensis
----------------------MPHHKITMMT
>Cariama_cristata
----------------------MPHHKITMMT
>Hynobius_chinensis
MGWPF--LSIHLHFVRE-QYFHMPHHKFTMMT
>Charadrius_vociferus
----------------------MPHHKITMMT
>Myotis_ricketti
----------------------MPHHKIPQ--
>Pterocles_gutturalis
----------------------MPHHKITMMT
>Latimeria_chalumnae
---------MHLHLVKE-LCFFIPHHKLIMMT
>Colius_striatus
MDCPYTKYGIHLHFIRE-LHFCSCHTTISMMT
>Columba_livia
----------------------MPHHKITMMT
>Merops_nubicus
----------------------MPHHKITMMT
>Alligator_sinensis
----------------------MPHHKITMMT
>Haliaeetus_leucocephalus
MVS-------TCTSLERAAFLYMPHHRITMMT
>Gavia_stellata
----------------------MPHHKITMMT
>Chlamydotis_macqueenii
MGCPCTKCGIHLHFIRE-LHFRTCYTTNSP--
>Condylura_cristata
----------------------MPHHKIPTMT
>Ficedula_albicollis
MGCPCTKYGIHLHFIRE-LHFCTCHTTRSP--
>Nipponia_nippon
MGCPCTKYGIHLHFIRD-LHFSTCHTTRSP--
>Equus_przewalskii
----------------------MPHHKIPTMT
>Picoides_pubescens
MGCPYTKYGIHLHFIRA-AFFCPCHTTRSP--
>Propithecus_coquereli
MNWAVLAPHVHLHFVRE-QSVHMPHHKIPTMT
>Loxodonta_africana
MSRAVLSLHVHLHFVRE-LSFYTPHHEIPTMK
>Fulmarus_glacialis
----------------------MPHHKITMMT
>Nestor_notabilis
----------------------MPHHKITMMT
>Tupaia_chinensis
MSRAVLVHHVHLHFVRE-QCVHMPHHKLPTMT
>Pygoscelis_adeliae
----------------------MPHHKITMMT
>Gekko_japonicus
----------------------MPHHQNPMMT
>Tyto_alba
----------------------MPHHKITMMT
>Struthio_camelus_australis
----------------------MPHHKITMMT
>Pelodiscus_sinensis
MGCPCTVYTVHLHFIRE-QHLYIPHHKIIMMT
>Tinamus_guttatus
MGCPCTKYGIHLHFITE-LHFCTYHTTRSL--
>Apaloderma_vittatum
----------------------MPHHKITMMT
>Orycteropus_afer_afer
MSWAVLALHVHLHFVRE-QSFHMPHHKIPTMK

HG0057 ENSG00000280987 MATR3

>Homo_sapiens
---------------------------MLGAQWRRNQPSRAAESCLLVLSLKKILQSLLILFLGVTIFEAKLT
>Anas_platyrhynchos
--------------------------------MTK--TACFSTELSGVSQLEEDLQSLLILFLGVTIFEVKFA
>Apteryx_australis_mantelli
--------------------------------MTK--TACFSTELSGVSQLEEDLQSLLILFLGVTIFEVKFA
>Buceros_rhinoceros_silvestri
----------------MMHK--LNCICLKGL-MTK--TACFSTELSGVSQLEEDLQSLLILFLGVTLLEVKFA
>Caprimulgus_carolinensis
--------------------------------MTK--TACFSTELSGVSQLEEDLQSLLILFLGVTIFELKFA
>Cariama_cristata
--------------------------------MTK--PACFSTELSGVSQLEEDLQSLLILFLGVTIFEVKFA
>Odobenus_rosmarus_divergens
---------------------------MLGAQWRRNQPPRAAESCLLVLSLKKILQSLLILFLGVTIF-----
>Calidris_pugnax
--------------------------------MTK--TACFSTELSGVSQLEEDLQSLLILFLGVTVFEVKFA
>Miniopterus_natalensis
---------------------------MLGAQWRRNQPPRAAESCLLVLSLKKILQSLLILFLGVTIFEAQLT
>Pterocles_gutturalis
--------------------------------MTK--TACFSTELSGVSQLEEDLQSLLILFLGVTIFEVKFA
>Colius_striatus
--------------------------------MTK--TACFSTELSGVSQLEEDLQSLLILFLGVTVVEVKFA
>Columba_livia
--------------------------------MTK--TACFSTELSGVSQLEEDLQSLLILFLGVTIFEVKFA
>Merops_nubicus
--------------------------------MTK--PACFSTELSGVSQLEEDLQSLLILFLGVTIFEVKFA
>Cuculus_canorus
--------------------------------MTK--TACFSTELSGVSQLEEDLQSLLILFLGVTILK----
>Monodelphis_domestica
---------------------------MLGTQW--
>Gallus_gallus
MLAVQGRPPRCRFALLFFTPLPEHXIAPRGPGARS--HRRXRAELSGVSQLEEDLQSLLILFLGVTIFEVKFA
>Gavia_stellata
--------------------------------MTK--TACFSTELSGVSQLEEDLQSLLILFLGVTIFEVKFA
>Chlamydotis_macqueenii
--------------------------------MTK--TACFSTELSGVSQLEEDLQSLLILFLGVTIFEVKFA
>Condylura_cristata
---------------------------MLGAQWRRNQPPRAAESCLLVLSLKKILQSLLILFLGVTIF-----
>Oryctolagus_cuniculus
---------------------------MLGAQWRRRQSPRAAESCLLVLSLKKILQSLLILFLGVTIF-----
>Tauraco_erythrolophus
--------------------------------MTK--TACFSTELSGVSQLEEDLQSLLILFLGVTVLK----
>Opisthocomus_hoazin
--------------------------------MTK--TACFSTELSGVSQLEEDLQSLLILFLGVTVF-----
>Acanthisitta_chloris
--------------------------------MTK--TACFSTELSGVSQLEEDLQSLLILFLGVTVFEVKFA
>Egretta_garzetta
--------------------------------MTK--TACFSTELSGVSQLEEDLQSLLILFLGVTIFEVKFA
>Ceratotherium_simum_simum
---------------------------MLGAQWRRKQPLRAAESCLLFLSLKKILQSLLILFLGVTIF-----
>Picoides_pubescens
--------------------------------MTK--TACFSTELSGVSQLDEDLQSLLILFLGVTIFEVKFA
>Loxodonta_africana
---------------------------MLGAQGRRSQPPRAAESCLLVLSLKKILQSLLILFLGVTIF-----
>Fulmarus_glacialis
--------------------------------MTK--TACFSTELSGVSQLEEDLRSLLILFLGVTIFEVKFA
>Melopsittacus_undulatus
--------------------------------MTK--PACFSTELSGVSQLEEDLQSLLILFLGVTIFEVKFA
>Jaculus_jaculus
-----------------MRA-SRSAAAMLGAQWRRRQPRRAAESCLLVLSLKKILQSLLILFLGVTIF-----
>Trichechus_manatus_latirostr
---------------------------MLGAQWRRSQPPRAAESCLLVLSLKKILQSLLILFLGVTIF-----
>Aptenodytes_forsteri
--------------------------------MTK--TACFSTELSGVSQLEEDLQSLLILFLGVTIFEVKFA
>Tyto_alba
--------------------------------MTK--TACFSTELSGVSQLEEDLQSLLILFLGVTIFEVKFA
>Struthio_camelus_australis
--------------------------------MTK--TACFSTELSGVSQLEEDLQSLLILFLGVTIFEVKFA
>Tinamus_guttatus
--------------------------------MTK--TACFSTELSGVSQLEEDLQSLLILFLGVTIFEVKFA

HG0058 ENSG00000180332 KCTD4

>Homo_sapiens
--------------------------------MLPAFPFHHQAGAE------
>Anser_cygnoides
--------------------------------MLAAFPFQHRAGAEERCF--
>Xenopus_laevis
MQIVFFPAVLVISCCLHSSYATYCKSELRAHAMLAAFPFHHRGGAG------
>Apteryx_australis_mantelli
--------------------------------MPAAFPFQHRAGAE------
>Caprimulgus_carolinensis
--------------------------------MRAAFPFQHRAGAE------
>Cariama_cristata
--------------------------------MLAAFPFQHRAGAE------
>Ailuropoda_melanoleuca
--------------------------------MLPAFPFHHQAGAE------
>Charadrius_vociferus
--------------------------------MLAAFPFQHRAGAE------
>Miniopterus_natalensis
--------------------------------MLPAFPFHHQAGAE------
>Colius_striatus
--------------------------------MIAAFPFQHRAGAE------
>Columba_livia
--------------------------------MLAAFPFQHRAGAE------
>Leptosomus_discolor
--------------------------------MLAAFPFQHRAGAE------
>Galeopterus_variegatus
--------------------------------MLPAFPFHHQAGAE------
>Haliaeetus_albicilla
--------------------------------MLAAFPFQHRAGAE------
>Gavia_stellata
--------------------------------MLAAFPFQHRAGAE------
>Chlamydotis_macqueenii
--------------------------------MLAAFPFQHRAGAE------
>Erinaceus_europaeus
--------------------------------MLPAFPFHHQAGAE------
>Ochotona_princeps
--------------------------------MLPAFPFPRQAGTE------
>Tauraco_erythrolophus
--------------------------------MLAAFPF-------------
>Opisthocomus_hoazin
--------------------------------MLAAFPFQHRAGAE------
>Manacus_vitellinus
--------------------------------MLAAFPFQHRSGAE------
>Egretta_garzetta
--------------------------------MLAAFPFQHRAGAE------
>Ceratotherium_simum_simum
--------------------------------MLPAFPFHHQAGAE------
>Manis_javanica
--------------------------------MLPAFSFHHQAGLNKGVFVW
>Fulmarus_glacialis
--------------------------------MLAAFPFQHRAGAE------
>Nestor_notabilis
--------------------------------MLAAFPFQHRAGAE------
>Ictidomys_tridecemlineatus
--------------------------------MLPAFPFHRQAGTE------
>Tupaia_chinensis
--------------------------------MLPAFPFHHQAGAE------
>Trichechus_manatus_latirostr
--------------------------------MLPAFPFHHQAGTE------
>Pygoscelis_adeliae
--------------------------------MLAAFPFQHRAGAE------
>Tyto_alba
--------------------------------MLAAFPFQHRAGAE------
>Trachemys_scripta
--------------------------------MLAALPFHHRAGAE------
>Calypte_anna
--------------------------------MLSAFPFQHRAGAE------
>Apaloderma_vittatum
--------------------------------MLAAFPFQHRAGAE------
>Orycteropus_afer_afer
--------------------------------MLPVFPFHQQAGAE------

HG0059.1 ENSDARG00000018060 pik3r2

>Danio_rerio
----------MLHSLCSIIWLNRGFKLRKVACTANTGQV---------------------------
>Acipenser_sinensis
--------MLVKLSLCSIVWLNRGFKRRGVLLTMGLGHH---------------------------
>Amia_calva
--------MLPTSSLCSIVWLNRGFKLRKVLSS-NTGQN---------------------------
>Channa_punctata
--------MLPTLSLCSIIWLNRGIKLRKVHRSTNTGQV---------------------------
>Anas_platyrhynchos
MPSRERWIMVVSFSLCSIIWLNRGFKLRKRGCT---------------------------------
>Xenopus_tropicalis
MIPEKSWIMFVSLFLCSIIWLNRGFKLRITGT----------------------------------
>Menidia_menidia
--------MLPTLSLCSIIWLNRGIKLRKVHRFTNTDQV---------------------------
>Tripterygion_delaisi
--------MLPTRSLCSIIWLNRGIKLRKVHQSANTGQV---------------------------
>Cariama_cristata
MPAHQCWIMFVSFSLCSIIWLNRGFKLRKRGCT---------------------------------
>Hynobius_chinensis
--------MIVAPSLCSIIWLNRGFKLRKGGT----------------------------------
>Micropterus_salmoides
--------MLPTLSLCSIIWLNRGIKLRKVHQFTNTGQV---------------------------
>Calidris_pugnax
MPARQCWIMFVSFSLCSIIWLNRGFKLRKRGCT---------------------------------
>Haplochromis_burtoni
--------MLPTISLCSIIWLNRGIKLRKVHQSTNTGQV---------------------------
>Clupea_harengus
--------MFPFLSLCSIIWLNRGFKLRKVLCVANTGQI---------------------------
>Latimeria_menadoensis
MVVVKCWSML-ALSLCSIIWLNRGLKLRKGVPLQDCALLPMWTEELDITSASKIELPVIWGRGPPI
>Columba_livia
MPAHKCWIMFVSFSLCSIIWLNRGFKLRKRGCT---------------------------------
>Poeciliopsis_prolifica
--------MLPTLSLCSIIWLNRGIKLRKVQQSSNTGQL---------------------------
>Monodelphis_domestica
---------MVRRALCSIVWLSRGFKLRKSG-----------------------------------
>Esox_lucius
--------MLPTLSLCSIVWLNRGIKLRKVLHCANTGQL---------------------------
>Aquila_chrysaetos_canadensis
MPAHKCWIMFVSFSLCSIIWLNRGFKLRKRGCT---------------------------------
>Coturnix_japonica
MPTRECWIMFVSFSLCSIIWLNRGFKLRKRGCT---------------------------------
>Lutjanus_erythropterus
--------MLPTLSLCSIIWLNRGIKLRKVHQATNTGQV---------------------------
>Scleropages_formosus
--------MLPTLSLCSIVWLNRGIKLRKVLYSANTGQF---------------------------
>Lepidothrix_coronata
MPAHQCWIMFVSFSLCSIIWLNRGFKLRKRGCT---------------------------------
>Lateolabrax_japonicus
--------MLPTLSLCSIIWLNRGIKLRKVHQSTNTGQV---------------------------
>Sebastes_caurinus
--------MLPTLSLCSIIWLNRGIKLRKVHQPTNTGQV---------------------------
>Cynoglossus_semilaevis
--------MLPSLSLCSIIWLNRGIKLRKLHLSTNTGQI---------------------------
>Salmo_salar
--------MLPTLSLCSIIWLNRGIKLRKVLHCANTGQL---------------------------
>Lepisosteus_oculatus
--------MLPTLSLCSIVWLNRGFKLRKALLS-NTGQH---------------------------
>Aptenodytes_forsteri
MPAHQCWIMFVSFSLCSIIWLNRGFKLRKRGCT---------------------------------
>Gekko_japonicus
--------MLLTFSLCSIIWLNRGFKLRERGCT---------------------------------
>Struthio_camelus_australis
MPARNCWIMFVSFSLCSIIWLNRGFKLRKRGCT---------------------------------
>Chrysemys_picta
MPSHECWIMFVAFSLCSIIWLNRGFKLRKRGCTSACGSLSVCGAILTRLR--STWLVQRW------
>Takifugu_rubripes
--------MLPTLSLCSIIWLNRGFKLRKVHQSSNTGQV---------------------------

HG0059.1 ENSGALG00000003428

>Gallus_gallus
MPTHECWIMFVSFSLCSIIWLNRGFKLRKR-GCT--------------------------------
>Acipenser_sinensis
--------MLVKLSLCSIVWLNRGFKRRGVLLTMGLGHH---------------------------
>Amia_calva
--------MLPTSSLCSIVWLNRGFKLRKVLSSNTG-QN---------------------------
>Channa_argus
--------MLPTLSLCSIIWLNRGIKLRKMYRSTNTGQV---------------------------
>Anas_platyrhynchos
MPSRERWIMVVSFSLCSIIWLNRGFKLRKR-GCT--------------------------------
>Megophrys_sangzhiensis
MVPVKSWIMFVSLFLCSIIWLNRGFKLRIR-GT---------------------------------
>Menidia_menidia
--------MLPTLSLCSIIWLNRGIKLRKVHRFTNTDQV---------------------------
>Ambystoma_mexicanum
MCGGKCWIMFVALSLCSIIWLNRGFKLRKG-GT---------------------------------
>Micropterus_salmoides
--------MLPTLSLCSIIWLNRGIKLRKVHQFTNTGQV---------------------------
>Haplochromis_burtoni
--------MLPTISLCSIIWLNRGIKLRKVHQSTNTGQV---------------------------
>Clupea_harengus
--------MFPFLSLCSIIWLNRGFKLRKVLCVANTGQI---------------------------
>Latimeria_chalumnae
MVVVKCWSML-ALSLCSIIWLNRGLKLRKGVPLQDCALLSMWTEELDITSASKIELPVIWGRGPPI
>Danio_rerio
----------MLHSLCSIIWLNRGFKLRKVACTANTGQV---------------------------
>Poecilia_mexicana
--------MLPMLSLCSIIWLNRGIKLRKVQQSTTLVSSEAAATTV-IT-AATL------------
>Monodelphis_domestica
---------MVRRALCSIVWLSRGFKLRKS-G----------------------------------
>Esox_lucius
--------MLPTLSLCSIVWLNRGIKLRKVLHCANTGQL---------------------------
>Falco_cherrug
MPTHKCWIMFVSFSLCSIIWLNRGFKLRKR-GCT--------------------------------
>Lutjanus_erythropterus
--------MLPTLSLCSIIWLNRGIKLRKVHQATNTGQV---------------------------
>Scleropages_formosus
--------MLPTLSLCSIVWLNRGIKLRKVLYSANTGQF---------------------------
>Lateolabrax_japonicus
--------MLPTLSLCSIIWLNRGIKLRKVHQSTNTGQV---------------------------
>Sebastes_caurinus
--------MLPTLSLCSIIWLNRGIKLRKVHQPTNTGQV---------------------------
>Platichthys_stellatus
--------MLPTISLCSIIWLNRGIKLRKSHQSTNTGQV---------------------------
>Oncorhynchus_mykiss
--------MLPTLSLCSIIWLNRGIKLRKVLHSANTGLL---------------------------
>Lepisosteus_oculatus
--------MLPTLSLCSIVWLNRGFKLRKALLSNTG-QH---------------------------
>Crotalus_adamanteus
--------MLVTFCLCFIIWLNRGFKLRERRGCT--------------------------------
>Takifugu_rubripes
--------MLPTLSLCSIIWLNRGFKLRKVHQSSNTGQV---------------------------

HG0059.2 ENSG00000145675 PIK3R1

```
>Homo_sapiens
--------------------------MDSVTKVSERIGPRCESQPGFKVLTSC
>Canis_lupus_familiaris
MDLQGGNHWEFGEARVRSDTVQIRRQMDSVTRVSERIGPRCENQPGGQVLTSC
>Balaenoptera_acutorostrata_s
--------------------------MDSVTRASERIGPRRESQPRVNVLTSC
>Myotis_brandtii
--------------------------MDSVTRVSERIGPRCESQPGVQVLTSC
>Dasypus_novemcinctus
--------------------------MDSVTRASERIGPRCESQPGGQVLTSR
>Erinaceus_europaeus
--------------------------MDSVTGVSERIGPRCENQPGVKMLTSC
>Oryctolagus_cuniculus
--------------------------MDSVARASERIGPGRERQPRVWVLTSC
>Equus_asinus
--------------------------MDSVTGVSERIGPRCESQPGVKVLASC
>Manis_javanica
--------------------------MDSVTRVSERIGPRCESQSEVQVLTSC
>Loxodonta_africana
--------------------------MDSVTRASERIGPRGESQPGVKC----
>Mus_musculus
--------------------------MDSVTGTSERIGPRCESQPGVKVLTSC
>Tupaia_chinensis
--------------------------MDSVTRASERIGPRCESRPGVRVLTSC
```

HG0059.3 ENSG00000105647 PIK3R2

>Homo_sapiens
MVAACTLGSLVWCSQQSRQPWAPMALRVRA-------
>Felis_catus
MVTACALGPLVWRSRKSRQPRAPMAPRVRE-------
>Balaenoptera_acutorostrata_s
MVTACALGPLAWRSWKSRQPREPMAPHVRE-------
>Rousettus_aegyptiacus
MVTACALGSLVWRSRKSRQPWAPMAPRVRE-------
>Condylura_cristata
MVTSSALGPLVWRSWKSRQSRAPMAPHVKE-------
>Equus_asinus
MVTACVLGPLIWRSWKRRQPWEPMAAHVRE-------
>Manis_javanica
MVTACALGPLVWRSWKSRQPRAPMAHHVRE-------
>Chinchilla_lanigera
MVAALTLCSLVWRSWKSWQPRVPMALYHRRMANPVAK

HG0059.4 ENSG00000145675 PIK3R1

>Homo_sapiens
MDSRMASHLSS
>Dasypus_novemcinctus
MDSRMASHLSS
>Equus_asinus
MDSRMANHLSS
>Loxodonta_africana
MDSRMASHLSS
>Nannospalax_galili
MDSRMASHLSS
>Tupaia_chinensis
MDSRMASHLSS

HG0059.5 ENSDARG00000038524 pik3r1

>Danio_rerio
---MGRVCMKHLEGAPNHWTISFT
>Astyanax_mexicanus
---MGKLCVKHREGALDHWRISFT
>Alosa_alosa
---MGSLCLKHHAGAMEHWPINMT
>Apteronotus_leptorhynchus
MLNCIFKLTPLVSCDSYRKERFSRADFDGFLFNLAIYQSRGCFGQSTGADFSQMGTLCLKHREGAQEHWRIRLT
>Ictalurus_punctatus
---METLCLKHHEAPLDHWRISFT

HG0060.1 ENSG00000049192 ADAMTS6

>Homo_sapiens
--------------------------------MNPDIEESAGCWIKVALKQWNIQ
>Anser_cygnoides
--------------------------------MTADLEESTGCWKRIPVKKWNVQ
>Chaetura_pelagica
--------------------------------MTADVEEGTGCWKRIPVKKWNI-
>Buceros_rhinoceros_silvestri
--------------------------------MTADIEESTGCWKRILVKKWNNQ
>Caprimulgus_carolinensis
--------------------------------MTADIEESTGCWKRIPVKKWNIQ
>Cariama_cristata
--------------------------------MTTDIEESTGYWKRIPVKKWNIQ
>Odobenus_rosmarus_divergens
-----------------MKILHHSFFPFGLKKMNPDIEESTGCWKKVPVKQWNI-
>Lipotes_vexillifer
-----------------MKILPSLSFSFWTEKMNPDTEESTGCWKKIPVKQWNI-
>Charadrius_vociferus
--------------------------------MTADIEESTGCWKRIPVKMWNIQ
>Pteropus_alecto
MLKNGHTLVTNQPNALLMKILPSLIFSFWIEKMNPDIEESTGCWKKVPVKQWTD-
>Dasypus_novemcinctus
----------------------------MKTKMNQDTEESMGCWTKVPVKQRNI-
>Columba_livia
--------------------------------MTADIEESTGCWKRIPVKRWNIQ
>Leptosomus_discolor
--------------------------------MTADIEEGTGCWKRIPVEKWNVQ
>Sarcophilus_harrisii
--------------------------------MNPERGESTGCWKRAPAKKWNI-
>Galeopterus_variegatus
--------------------------------MNPDLEESMGCWKKVLVKQWNT-
>Monodelphis_domestica
--------------------------------MNPERGESTGCWKRAPAKKWNI-
>Aquila_chrysaetos_canadensis
--------------------------------MTADIEESTGCWKRIPVKKWNIQ
>Gallus_gallus
--------------------------------MTADIEE--SCWKRVPVKKWNIQ
>Eurypyga_helias
--------------------------------MTADLEENTGCWKRIPVKKWNVQ
>Sorex_araneus
--------------------------------MNPVIEESMGCWKKVSIIQWNI-
>Oryctolagus_cuniculus
--------------------------------MNPDIEESTGCWKKLLVKQWNI-
>Tauraco_erythrolophus
--------------------------------MTADIEESTGCWKRIPVKKWNIQ
>Opisthocomus_hoazin
--------------------------------MTADIEEGTGFWKRIPVKKWNIQ
>Nipponia_nippon
--------------------------------MTADIEESTGCWKRIPFKKWNIQ
>Ceratotherium_simum_simum
-----------------MKILPSLFFSFWIEKMNPDIEESMGCWKKVPVKQ----
>Manis_javanica
--------------------------------MNPHLEESMGCWKKVPAKQWNI-
>Loxodonta_africana
--------------------------------MNPDTEESMGCWEKVPVKQWNIY
>Fulmarus_glacialis
--------------------------------MTADIEESTGCWKRIPVKKWNIQ
>Ictidomys_tridecemlineatus
--------------------------------MNPDIEESAGCWKKEPVKQWNI-
>Tupaia_chinensis
--------------------------------MNPDMDDSMGFWKKVPVKQWKM-
>Pygoscelis_adeliae
--------------------------------MTADIEESTGCWKRIPVKKWNLQ
>Struthio_camelus_australis
--------------------------------MTADIEESTGCRKRIPVKKWNIQ
>Chrysemys_picta
--------------------------------MTADIEESTGCWKRVPVKKWNIQ
>Orycteropus_afer_afer
--------------------------------MNPDTEESTEYWEKIPVKQWNI-

HG0060.1 ENSGALG00000014751

```
>Gallus_gallus
--------------------------------MTADIEE--SCWKRVPVKKWNIQ
>Anser_cygnoides
--------------------------------MTADLEESTGCWKRIPVKKWNVQ
>Chaetura_pelagica
--------------------------------MTADVEEGTGCWKRIPVKKWNI-
>Buceros_rhinoceros_silvestri
--------------------------------MTADIEESTGCWKRILVKKWNNQ
>Caprimulgus_carolinensis
--------------------------------MTADIEESTGCWKRIPVKKWNIQ
>Cariama_cristata
--------------------------------MTTDIEESTGYWKRIPVKKWNIQ
>Odobenus_rosmarus_divergens
-----------------MKILHHSFFPFGLKKMNPDIEESTGCWKKVPVKQWNI-
>Lipotes_vexillifer
-----------------MKILPSLSFSFWTEKMNPDTEESTGCWKKIPVKQWNI-
>Charadrius_vociferus
--------------------------------MTADIEESTGCWKRIPVKMWNIQ
>Pteropus_alecto
MLKNGHTLVTNQPNALLMKILPSLIFSFWIEKMNPDIEESTGCWKKVPVKQWTD-
>Dasypus_novemcinctus
----------------------------MKTKMNQDTEESMGCWTKVPVKQRNI-
>Columba_livia
--------------------------------MTADIEESTGCWKRIPVKRWNIQ
>Leptosomus_discolor
--------------------------------MTADIEEGTGCWKRIPVEKWNVQ
>Sarcophilus_harrisii
--------------------------------MNPERGESTGCWKRAPAKKWNI-
>Galeopterus_variegatus
--------------------------------MNPDLEESMGCWKKVLVKQWNT-
>Monodelphis_domestica
--------------------------------MNPERGESTGCWKRAPAKKWNI-
>Aquila_chrysaetos_canadensis
--------------------------------MTADIEESTGCWKRIPVKKWNIQ
>Eurypyga_helias
--------------------------------MTADLEENTGCWKRIPVKKWNVQ
>Oryctolagus_cuniculus
--------------------------------MNPDIEESTGCWKKLLVKQWNI-
>Tauraco_erythrolophus
--------------------------------MTADIEESTGCWKRIPVKKWNIQ
>Opisthocomus_hoazin
--------------------------------MTADIEEGTGFWKRIPVKKWNIQ
>Nipponia_nippon
--------------------------------MTADIEESTGCWKRIPFKKWNIQ
>Equus_asinus
-----------------MKILPSLFFSFWIEKMNPDIEESMRCWKKVPVKQWNI-
>Manis_javanica
--------------------------------MNPHLEESMGCWKKVPAKQWNI-
>Cercocebus_atys
-----------------MKTFTIIIFSFWIEKMNPDIEESAGCWIKVPLKQWNIQ
>Loxodonta_africana
--------------------------------MNPDTEESMGCWEKVPVKQWNIY
>Fulmarus_glacialis
--------------------------------MTADIEESTGCWKRIPVKKWNIQ
>Ictidomys_tridecemlineatus
--------------------------------MNPDIEESAGCWKKEPVKQWNI-
>Pygoscelis_adeliae
--------------------------------MTADIEESTGCWKRIPVKKWNLQ
>Struthio_camelus_australis
--------------------------------MTADIEESTGCRKRIPVKKWNIQ
>Chrysemys_picta
--------------------------------MTADIEESTGCWKRVPVKKWNIQ
>Orycteropus_afer_afer
--------------------------------MNPDTEESTEYWEKIPVKQWNI-
```

HG0060.2 ENSG00000049192 ADAMTS6

>Homo_sapiens
MLRFWYGIDSLAHR
>Anas_platyrhynchos
MVRFWSGVSSIAHR
>Chaetura_pelagica
MVRFWSGVNSVSHR
>Canis_lupus_familiaris
MLRFWYGIDSLAHR
>Physeter_catodon
MLRFWHGIDSLAHR
>Charadrius_vociferus
MVQFWSGVNSVAHR
>Dasypus_novemcinctus
MLRFWYGVDSLAHR
>Columba_livia
MVRYWSGANSIAHR
>Leptosomus_discolor
MVRFWSGVNFIAHR
>Galeopterus_variegatus
MLRLWYDIDSLTHR
>Aquila_chrysaetos_canadensis
MVRFWSGVNSVAHR
>Gallus_gallus
MVQFWSGVNPIAHR
>Eurypyga_helias
MVRFWSGVNSVAHR
>Sorex_araneus
MLRFWYDIDSLAHR
>Oryctolagus_cuniculus
MLRFWYGIDSLAHR
>Tauraco_erythrolophus
MVPFWSGVNSIAHR
>Opisthocomus_hoazin
MVRFWSGVNSIAHR
>Acanthisitta_chloris
MVRFWSGVNSIAHR
>Phaethon_lepturus
MVRFWSGVNSLAHR
>Ceratotherium_simum_simum
MLRFWYGIDSLAHR
>Manis_javanica
MLRFWYGIDSLAHR
>Loxodonta_africana
MLRFWYGIDSLAHR
>Fulmarus_glacialis
MVRFWSGVNSTAHR
>Nannospalax_galili
MLRFWYGIDSLAHR
>Tupaia_chinensis
MLRFGYDSDSLAHR
>Pygoscelis_adeliae
MVRFWSGVNSVAHR
>Thamnophis_sirtalis
MVQFWSGVKAIAHR
>Struthio_camelus_australis
MVRFWSGVKSIAHR
>Chrysemys_picta
MVRFWSGVNSIAHR
>Orycteropus_afer_afer
MLRFWHGVDSLAHR

HG0060.2 ENSGALG00000014751

>Gallus_gallus
MVQFWSGVNPIAHR
>Anser_cygnoides
MVRFWSGVSSIAHR
>Chaetura_pelagica
MVRFWSGVNSVSHR
>Buceros_rhinoceros_silvestri
MVLFWSGVNFIAHR
>Caprimulgus_carolinensis
MVRFWSGVNSIARR
>Cariama_cristata
MVRFWSGVNSVAHK
>Charadrius_vociferus
MVQFWSGVNSVAHR
>Dasypus_novemcinctus
MLRFWYGVDSLAHR
>Columba_livia
MVRYWSGANSIAHR
>Leptosomus_discolor
MVRFWSGVNFIAHR
>Aquila_chrysaetos_canadensis
MVRFWSGVNSVAHR
>Eurypyga_helias
MVRFWSGVNSVAHR
>Tauraco_erythrolophus
MVPFWSGVNSIAHR
>Opisthocomus_hoazin
MVRFWSGVNSIAHR
>Manacus_vitellinus
MVQFWSGVNSIAHK
>Nipponia_nippon
MVRFWSGVNSIAHR
>Microcebus_murinus
MLRFWYGIDSIAHR
>Loxodonta_africana
MLRFWYGIDSLAHR
>Fulmarus_glacialis
MVRFWSGVNSTAHR
>Chinchilla_lanigera
MLRFWYGINSLAHR
>Pygoscelis_adeliae
MVRFWSGVNSVAHR
>Thamnophis_sirtalis
MVQFWSGVKAIAHR
>Struthio_camelus_australis
MVRFWSGVKSIAHR
>Chrysemys_picta
MVRFWSGVNSIAHR
>Orycteropus_afer_afer
MLRFWHGVDSLAHR

HG0061.1 ENSG00000166398

```
>Homo_sapiens
----MVTCLMEMPFLKLRFAELHYFYT
>Anas_platyrhynchos
-----------MPFVKPMYTVLHCYCT
>Xenopus_laevis
-----------MPFTKPVFADFIYYLT
>Caprimulgus_carolinensis
-----------MPFVKLMYTVLHCYCT
>Felis_catus
----MVTCLMEMPFLKLRFAELHYFYT
>Hynobius_retardatus
-----------MPFTKPSYPVPHYYT-
>Physeter_catodon
----MVTCLMEMPFLKLRFAELHYFYT
>Cynopterus_sphinx
----MVTCLMEMPFLKLIFAELHYFYT
>Columba_livia
-----------MPFVKPMYAVLHCYCT
>Alligator_mississippiensis
-----------MPFMKPAYAVLHYYYT
>Sarcophilus_harrisii
-----------MPFMELLFAVLYCYYT
>Galeopterus_variegatus
----MATCLMEMPFLKLGLAELHYFYT
>Monodelphis_domestica
-----------MPFMELLFAVLHYYYT
>Condylura_cristata
----MVTCLMKMPFLKLRFAELHYFYT
>Oryctolagus_cuniculus
----MVICLLEMSFLKLTFAEVHYFYT
>Phaethon_lepturus
-----------MPFVKPMATVLHCYYT
>Ceratotherium_simum_simum
----MVICLMEMPFLKLRFAELHYFYT
>Manis_javanica
----MVTFLMEMPFLKLRFAELHYFYT
>Melopsittacus_undulatus
-----------MPFVKPTYTVLHFYCT
>Ctenomys_sociabilis
MFDHMVTCLMEMPFLKLQFAELHYFYT
>Tupaia_chinensis
---------MEMPFLKHRFAELQYFYT
>Tyto_alba
-----------MPFVKLMYMVLHCYCT
>Struthio_camelus_australis
-----------MPFVKPMYAVLHCYCT
>Pelodiscus_sinensis
-----------MPFLKRMYTVLHCYYT
>Calypte_anna
-----------MPFVKPMYTVLHCYCT
```

HG0061.1 ENSGALG00000004949

>Gallus_gallus
MPFVKPMYTVLHCYCT----
>Anser_cygnoides
MPFVKPMYTVLHCYCT----
>Leptobrachium_boringii
MPFTKPMYVALLCYLT----
>Chaetura_pelagica
MPFVKPMYTVLHCYCT----
>Apteryx_australis_mantelli
MPFVKPMYTVLHCYCT----
>Buceros_rhinoceros_silvestri
MPFVEPMYTVLHCYCT----
>Caprimulgus_carolinensis
MPFVKLMYTVLHCYCT----
>Cariama_cristata
MPFVKPMYTVLHCYCT----
>Charadrius_vociferus
MPFVKPMYTVLHCYCTKLYI
>Pterocles_gutturalis
MPFVKPMYTVLHCYCT----
>Colius_striatus
MPFVKPMYTVHHCYCT----
>Columba_livia
MPFVKPMYAVLHCYCT----
>Merops_nubicus
MPFVKPMYTVLHCYCT----
>Alligator_sinensis
MPFMKPAYAVLHYYYT----
>Cuculus_canorus
MPFVKPMYTVLHCYCT----
>Falco_cherrug
MPFVKPMYTVLHCYCT----
>Gavia_stellata
MPFVKPMYTVLHCYCT----
>Balearica_regulorum_gibberic
MPFVKPMYTVLHCYCT----
>Ornithorhynchus_anatinus
MPFVKPGFTVLHRYHT----
>Tauraco_erythrolophus
MPFVKPMYTVLHCYCT----
>Opisthocomus_hoazin
MPFVKRMCAVLDCYCTELYI
>Corvus_brachyrhynchos
MPFVKPMYTVLHCYCT----
>Egretta_garzetta
MPFVKPMYTVLHCYCT----
>Picoides_pubescens
MPFVTPMYTAPHRRCT----
>Fulmarus_glacialis
MPFVKPMYTVLHCYCT----
>Nestor_notabilis
MPFVKPVYTVLHCYCT----
>Aptenodytes_forsteri
MPFVKPMYTVLHCYCT----
>Tyto_alba
MPFVKLMYMVLHCYCT----
>Struthio_camelus_australis
MPFVKPMYAVLHCYCT----
>Pelodiscus_sinensis
MPFLKRMYTVLHCYYT----
>Tinamus_guttatus
MPFVKPMYTVLHCYCT----
>Calypte_anna
MPFVKPMYTVLHCYCT----
>Apaloderma_vittatum
MPFVKPMYTVLHCYCT----

HG0061.2 ENSGALG00000004949

```
>Gallus_gallus
--------------------------------------------------------MLQRKQLPQQTRKGEEGLQ----------------------------------
>Anas_platyrhynchos
--------------------------------------------------------MLQRKQLPQQTRKGEEGLQ----------------------------------
>Calidris_pugnax
--------------------------------------------------------MLQRKQLPQQRRKGGKGKKACNNK-HSNF------------------------
>Columba_livia
--------------------------------------------------------MLQRKQLPQQTRKGEEGLQ----------------------------------
>Sarcophilus_harrisii
--------------------------------------------------------MLQRKQLPQKTKGKEEAAEQLEITNTPNF------------------------
>Monodelphis_domestica
--------------------------------------------------------MLQRKQLPQKTEGKEEAPWQLEITNTPVFNNLTCEKRRHFIFFSCSIKKKHYR
>Aquila_chrysaetos_canadensis
--------------------------------------------------------MLQRKQLPQQTRKGEEGLQ----------------------------------
>Corvus_brachyrhynchos
--------------------------------------------------------MLQRKQLPQQTGKGEEGLQ----------------------------------
>Aptenodytes_forsteri
--------------------------------------------------------MLQRKQLPQQTRKGEEGLQ----------------------------------
>Struthio_camelus_australis
MNLLLLKYSKPVNSLESLLTEHRSCGNDLEALQIHLCIFVPSLRLRANAIWKHHFLTLQRKQLPQQTRKGEEGLQ----------------------------------
```

HG0061.3 ENSG00000166398

>Homo_sapiens
MLQGNRYLSKQKEWRRRLTTNA-I------------------------------
>Pteropus_alecto
MLQGNRYLSKQKERRRRLITNA-IF-----------------------------
>Galeopterus_variegatus
MLQGNCYLSKQKERRRRLIITNGIFKKTC--FRKTLEE-I--------------
>Condylura_cristata
MLQGNRYLSKQKERRRLIIYNMPFF-----------------------------
>Oryctolagus_cuniculus
MLQGNHYLSKQKERSRRLKITNDIFTKKQTCFQKTLEEIWTFLASGRTFVSKWI
>Ceratotherium_simum_simum
MLQGSHYLNKQKERRRRLIITNAIFKKKLG-FRKY-------------------
>Nannospalax_galili
MLQGNRYLSKEKERRRRRTVTDAM-FKENL--FSENPTGDL--------------
>Tupaia_chinensis
MLQGNRYLSKQKERRRRLTTTNAIFKKQTNSFPENIEE-I--------------

HG0062.1 ENSDARG00000075569 gpbp1l1

>Danio_rerio
MPPSTDGRHWT
>Pygocentrus_nattereri
MPRSTDGRHWT
>Clupea_harengus
MPRSTEGRHWT
>Poecilia_formosa
MPPSTDQKHWT
>Apteronotus_leptorhynchus
MPRSTDGRHWT
>Salmo_salar
MPRSTDGRHWT
>Trichomycterus_areolatus
MPRSTDGRHWT

HG0062.1 ENSGALG00000010276 GPBP1L1

>Gallus_gallus
MPHFIEGRHWT
>Anser_cygnoides
MPHFIEGRHWT
>Chaetura_pelagica
MPHFIEGRHWT
>Apteryx_australis_mantelli
MPHFIEGRHWT
>Buceros_rhinoceros_silvestri
MPHFIEGRHWT
>Caprimulgus_carolinensis
MPHFVEGRHWT
>Cariama_cristata
MPHFIEGRHWT
>Physeter_catodon
MPHLVEKRHWT
>Charadrius_vociferus
MPHFIEGRHWT
>Pterocles_gutturalis
MPHFIEGRHWT
>Colius_striatus
MPHFIEGRHWT
>Columba_livia
MPHFIEGRHWT
>Leptosomus_discolor
MPHFIEGRHWT
>Alligator_mississippiensis
MPHFIEGRHWT
>Cuculus_canorus
MPHFIEGRHWT
>Falco_cherrug
MPHFIEGRHWT
>Eurypyga_helias
MPHFIEGRHWT
>Tauraco_erythrolophus
MPHFIEGRHWT
>Opisthocomus_hoazin
MPHFVEGRHWT
>Geospiza_fortis
MPHFIEGRHWT
>Egretta_garzetta
MPHFIEGRHWT
>Picoides_pubescens
MPHFIEGRHWT
>Fulmarus_glacialis
MPHFIEGRHWT
>Melopsittacus_undulatus
MPHFIEGRHWT
>Oncorhynchus_mykiss
MPHSTDGRHWT
>Aptenodytes_forsteri
MPHFIEGRHWT
>Gekko_japonicus
MPRFIEGRHWT
>Tyto_alba
MPHFIEGRHWT
>Struthio_camelus_australis
MPHFIEGRHWT
>Pelodiscus_sinensis
MPHFIEGRHWT
>Tinamus_guttatus
MPHFIERRHWT
>Calypte_anna
MPHFVEGRHWT
>Apaloderma_vittatum
MPHFIEGRHWT

HG0062.2 ENSGALG00000010276 GPBP1L1

>Gallus_gallus
MSCPADLGVSVQY
>Anser_cygnoides
MNYPAELGVSVQY
>Caprimulgus_carolinensis
MNYPADLSVSVQY
>Charadrius_vociferus
MNYPADLSVSVQY
>Pterocles_gutturalis
MNYPADLSVSVQY
>Colius_striatus
MSYPADLGVSAQY
>Columba_livia
MNDPADLSVSVQY
>Cuculus_canorus
MNYPADLSVSVQY
>Haliaeetus_leucocephalus
MNYPADLSVSVQY
>Gavia_stellata
MNYPADLSVSVQY
>Chlamydotis_macqueenii
MNYPADLSVSVQY
>Opisthocomus_hoazin
MNYPADLSVSVQY
>Acanthisitta_chloris
MNCPAALSVSVQY
>Phaethon_lepturus
MNYPADLSVSVQY
>Fulmarus_glacialis
MNYPADLSVSVQY
>Melopsittacus_undulatus
MNYPADLGVSVQY
>Pygoscelis_adeliae
MNYPADLSVSVQY
>Tyto_alba
MNYPADLSVSVQY
>Struthio_camelus_australis
MNYPADLGASVQY
>Tinamus_guttatus
MNYPADLGASVQY
>Calypte_anna
MNYPADLSVSVQY

HG0062.3 ENSG00000159592 GPBP1L1

>Homo_sapiens
--MSYLIKQHHSAITQGS
>Leptonychotes_weddellii
MSMYYLFKQHHSAITQGS
>Balaenoptera_acutorostrata_s
MSMYYLFKQHHRAITQGS
>Rousettus_aegyptiacus
--MYYLFKQHHSAITQGS
>Dasypus_novemcinctus
MSMYYPFKQHHSTITQGS
>Galeopterus_variegatus
MSMYYLFKQHHSAITQGS
>Condylura_cristata
MSLNYLFKQHHSDITQGS
>Oryctolagus_cuniculus
MPMYYPFKQQHSAITQGS
>Ceratotherium_simum_simum
MSMYYLFKQHHSAITQGS
>Loxodonta_africana
MSMHYLFKQHHSAITQRS
>Marmota_marmota_marmota
MSMYYLFKQHHSAITQGS
>Trichechus_manatus_latirostr
MSMYYLFKQHHSTITQGS
>Orycteropus_afer_afer
MSMYYLFKQHHSAITQRS

HG0063.1 ENSG00000162630 B3GALT2

>Homo_sapiens
------------------MCACSIASFLLK---
>Anas_platyrhynchos
------------------MCACSIASFLLK---
>Chaetura_pelagica
------------------MCACSIASFLLK---
>Buceros_rhinoceros_silvestri
------------------MCACSIASFLLK---
>Caprimulgus_carolinensis
------------------MCACSRASFLLK---
>Cariama_cristata
------------------MCACSIASFLLK---
>Mustela_putorius_furo
------------------MCACSIASFLLK---
>Charadrius_vociferus
------------------MCACSIASFLLK---
>Pterocles_gutturalis
---MGYREQQPQEADGWGMCACSIASFLLK---
>Dasypus_novemcinctus
------------------MCACSIASFWLK---
>Colius_striatus
------------------MCACSIASFLLK---
>Columba_livia
------------------MCACSIASFLLK---
>Cuculus_canorus
------------------MCACSIASILLK---
>Galeopterus_variegatus
------------------MCACSIASFLLK---
>Monodelphis_domestica
------------------MCACSIASFLLK---
>Macropus_eugenii
------------------MCACSIASFLLK---
>Aquila_chrysaetos_canadensis
------------------MCACSIASFLLK---
>Gallus_gallus
------------------MCACSIASFLLK---
>Gavia_stellata
------------------MCACSIASFLLK---
>Mesitornis_unicolor
------------------MCACSIASFLLK---
>Condylura_cristata
------------------MCACSIASFLLK---
>Ornithorhynchus_anatinus
MDGSDTGEEPSREAERWGMCACSMASF--
>Tauraco_erythrolophus
------------------MCACSIASFLLK---
>Opisthocomus_hoazin
------------------MCACSIASFLLK---
>Phalacrocorax_carbo
MDRLDTVKKQSQEAGCWGMCACSIASFLLK---
>Ceratotherium_simum_simum
------------------MCACSIASFLLK---
>Manis_javanica
------------------MCACSIASLLLK---
>Loxodonta_africana
------------------MCACSLTSFLLK---
>Fulmarus_glacialis
------------------MCACSIASFLLK---
>Nannospalax_galili
------------------MCACSIASFLLK---
>Aptenodytes_forsteri
------------------MCACSIASFLLK---
>Chelonia_mydas
------------------MCACSIASFLLK---
>Apaloderma_vittatum
------------------MCACSIASFLPKRWRAKTIFWGYNPRTINGLTKEVSHLQTTTQHKQRKMNTKE

HG0063.1 ENSGALG00000002477

```
>Gallus_gallus
------------------MCACSIASFLLK-----------------------------------------
>Anas_platyrhynchos
------------------MCACSIASFLLK-----------------------------------------
>Chaetura_pelagica
------------------MCACSIASFLLK-----------------------------------------
>Buceros_rhinoceros_silvestri
------------------MCACSIASFLLK-----------------------------------------
>Caprimulgus_carolinensis
------------------MCACSRASFLLK-----------------------------------------
>Cariama_cristata
------------------MCACSIASFLLK-----------------------------------------
>Mustela_putorius_furo
------------------MCACSIASFLLK-----------------------------------------
>Charadrius_vociferus
------------------MCACSIASFLLK-----------------------------------------
>Pterocles_gutturalis
---MGYREQQPQEADGWGMCACSIASFLLK-----------------------------------------
>Dasypus_novemcinctus
------------------MCACSIASFWLK-----------------------------------------
>Colius_striatus
------------------MCACSIASFLLK-----------------------------------------
>Columba_livia
------------------MCACSIASFLLK-----------------------------------------
>Cuculus_canorus
------------------MCACSIASILLK-----------------------------------------
>Galeopterus_variegatus
------------------MCACSIASFLLK-----------------------------------------
>Monodelphis_domestica
------------------MCACSIASFLLK-----------------------------------------
>Macropus_eugenii
------------------MCACSIASFLLK-----------------------------------------
>Aquila_chrysaetos_canadensis
------------------MCACSIASFLLK-----------------------------------------
>Gavia_stellata
------------------MCACSIASFLLK-----------------------------------------
>Mesitornis_unicolor
------------------MCACSIASFLLK-----------------------------------------
>Condylura_cristata
------------------MCACSIASFLLK-----------------------------------------
>Ornithorhynchus_anatinus
MDGSDTGEEPSREAERWGMCACSMASF--------------------------------------------
>Tauraco_erythrolophus
------------------MCACSIASFLLK-----------------------------------------
>Opisthocomus_hoazin
------------------MCACSIASFLLK-----------------------------------------
>Phalacrocorax_carbo
MDRLDTVKKQSQEAGCWGMCACSIASFLLK-----------------------------------------
>Ceratotherium_simum_simum
------------------MCACSIASFLLK-----------------------------------------
>Manis_javanica
------------------MCACSIASLLLK-----------------------------------------
>Cercocebus_atys
------------------MCACSIASFLLK-----------------------------------------
>Loxodonta_africana
------------------MCACSLTSFLLK-----------------------------------------
>Fulmarus_glacialis
------------------MCACSIASFLLK-----------------------------------------
>Nannospalax_galili
------------------MCACSIASFLLK-----------------------------------------
>Aptenodytes_forsteri
------------------MCACSIASFLLK-----------------------------------------
>Chelonia_mydas
------------------MCACSIASFLLK-----------------------------------------
>Apaloderma_vittatum
------------------MCACSIASFLPKRWRAKTIFWGYNPRTINGLTKEVSHLQTTTQHKQRKMNTKE
```

HG0063.2 ENSG00000162630 B3GALT2

>Homo_sapiens
MD-TETRTQHCIVQPGK---
>Anser_cygnoides
MDTEEKRTQHCIVRQGKHQM
>Canis_lupus_familiaris
MD-TEKRIQHCIVRPGKQQM
>Balaenoptera_acutorostrata_s
MD-TEKRIQHCIVRPGKQQM
>Rousettus_aegyptiacus
MD-TEKRTQHCIVRPGKQQM
>Dasypus_novemcinctus
MD-TEKRTQHCIVQPGKQQM
>Galeopterus_variegatus
MD-TEKRTQHCIVQPGKQQM
>Monodelphis_domestica
MD-TEKRIQHCIVRQGKYKM
>Macropus_eugenii
MD-TEKRIQHCIVRQGKYEM
>Aquila_chrysaetos_canadensis
MDTGEKRTQHCIVRQGKHQM
>Gallus_gallus
MDTGERRTQHCIVRQGKHQM
>Erinaceus_europaeus
MD-TQKRIQHCIVRPGKQQM
>Oryctolagus_cuniculus
MD-TEKRTQHCIVQPGKQQM
>Lepidothrix_coronata
MDTGERRTQHCIVRHGKHQM
>Ceratotherium_simum_simum
MD-TEKRTQHCIVRPGKQQM
>Manis_javanica
MD-TEKRIQHCIVRPGKQQM
>Loxodonta_africana
MD-TEKRIKHCIVRPGKQQT
>Dipodomys_ordii
MD-TEKRTQHCIVQPGKQQM
>Tupaia_chinensis
MD-TGKRTQHRIVQPGKQQL
>Trichechus_manatus_latirostr
MD-TEKRIQHCIVQPGKQQM
>Gekko_japonicus
MD-TEKRTQHCIVQQGKHQL
>Chelonia_mydas
MD-TEKRIQHCIVRQGKCQM
>Orycteropus_afer_afer
MD-TEKRIQHCIVQPGKQQM

HG0064.1 ENSG00000102678 FGF9

>Homo_sapiens
MVILISNQMEKLRIFFPYY-GRMG-----
>Anas_platyrhynchos
MVILILNQMEKLRIFFFITVGWAEDLIAC
>Chaetura_pelagica
MVILILNQMEKLRIFFFITVGWAEDRIAC
>Canis_lupus_familiaris
MVILISNQMEKLRIFFPYY-GRMG-----
>Balaenoptera_acutorostrata_s
MVILISNQMEKLRIFFPYY-GRMG-----
>Charadrius_vociferus
MVILILNQMEKLRIFFFITVGWAEDRIAC
>Rousettus_aegyptiacus
MVIPIWNQMEKLRIFSLMTASWDEDLPVC
>Dasypus_novemcinctus
MVILISNPMEKLRIFFLITVGWDEDLTAC
>Leptosomus_discolor
MVILILNQMEKLRIFFFITVGWAEDRIAC
>Cuculus_canorus
MVILTLNQMEKLRIFFFITVGWAEDRIAC
>Galeopterus_variegatus
MVILISNQMEKLQIFFLITVGWDEDLPAY
>Macropus_eugenii
MVILISNQMEKLRIFFLITVGWDEDLTAC
>Aquila_chrysaetos_canadensis
MVILILNQMEKLRIFFFITVGWAEDRIAC
>Coturnix_japonica
MVILFLNQMEKLRIFFLYY-GWMG-----
>Eurypyga_helias
MVILILNQMEKLRIFFLITVGWAEDRIAC
>Condylura_cristata
MVILISNQMEKLRIFFPYY-GRMG-----
>Oryctolagus_cuniculus
MVILISKQMEKLRIFFPYY-GRMG-----
>Elephantulus_edwardii
MVILISNQMEKLRIFFLIMAGWDEDLTAC
>Opisthocomus_hoazin
MVILVLNQMEKLRIFFLDY-GWMG-----
>Lepidothrix_coronata
MIILILNQMEKLRIFFLYY-SWMG-----
>Egretta_garzetta
MVILILNQMEKLRIFFFITVGWAEDRIAC
>Equus_przewalskii
MVILISNQMEELRIFFPYY-GRMG-----
>Manis_javanica
MVILISNQMEKLRIFFLITVGWDEDLSAC
>Picoides_pubescens
MVILILNQMEKLRIFFLITVGWAEDRIAC
>Loxodonta_africana
MVILISSQMEKLRIFFLITVGWDEDLTAC
>Peromyscus_maniculatus_baird
MVILISKQMEKLRIFFPYY-GRMG-----
>Tupaia_chinensis
MVILISNPMEKLRIFFPYY-GRMG-----
>Aptenodytes_forsteri
MVILILNQMEKLRIFFFITVGWAEDRIAC
>Struthio_camelus_australis
MVILILNQMEKLRIFFFITVGWAEDLIAC
>Chelonia_mydas
MVILILNQMEKLRIFFLIAVGWAEDLTAC
>Tinamus_guttatus
MVFLILNQMEKLRIFFLYY-SWMG-----
>Calypte_anna
MVILILNQMEKLRIFFFITVGWAEDRIAC
>Orycteropus_afer_afer
MVILISNQMDKLQIFFLITVGWDEDFTAC

HG0064.2 ENSG00000113578 FGF1

>Homo_sapiens
MVCGFTQFFSWGFSELHSYQKGSRRERTSGSNSLRSRGIRASPPFLGGHSLLMVNGNSLPPAAACLQLSW---------------------------
>Panthera_tigris
--MENGNSLPPAAACLQHSR---------------------------
>Myotis_brandtii
--MANGNSLPAAAAGLPCSW---------------------------
>Galeopterus_variegatus
--MVNGNSLLPAAACLRCSWRGSVDTAPEKKLLRAKAKSSKSATTSS
>Condylura_cristata
--MENGNSLPPAAASPQRSW---------------------------
>Ceratotherium_simum_simum
--MENGNSLPPAAACLQRSC---------------------------
>Nannospalax_galili
--MVNGNSLPQSL--LAASW---------------------------
>Orycteropus_afer_afer
--MGNGNSLPPAPACLK------------------------------

HG0065 ENSGALG00000029927 STRBP

>Gallus_gallus
--MKRFCQAKQLLSKTPRRREKEKERES-----
>Anser_cygnoides
--MKRFCQAKQRLAKTPRRREKEKGREKLKH--
>Chaetura_pelagica
--MKRFCQAKQCLAKTPRRREKEKGRERELKH-
>Apteryx_australis_mantelli
--MKRFCQAKQCLAKTPRRREKEKGRGRELKH-
>Buceros_rhinoceros_silvestri
--MKRFCQTKQCLAKTPRRREKEKGRERELKH-
>Caprimulgus_carolinensis
--MKRFCQAKQCLAKTPRRREKEKGRERELKH-
>Cariama_cristata
--MKRFCQAKQCLAKTPRRREKEKGRERELKH-
>Balaenoptera_acutorostrata_s
--MRRYCHRKQCLVKTTRKREKDXGRRS-----
>Charadrius_vociferus
--MKRFCQAKQCLAKTPRRREKEKGRERELKH-
>Colius_striatus
--MKRFYQAKQCLAKTPRRREKEKGRERELKR-
>Columba_livia
--MKRFCQTKQGLAKTPRRREKEKGRESELKH-
>Merops_nubicus
--MKRFCQAKQCLAKTPRRREKEKGREKELKH-
>Alligator_mississippiensis
--MRRFCQAKQCLAKTPRRREKEKGEGERS---
>Cuculus_canorus
--MKRFCQAKQCLAKTPRRREKEKRRRRELKH-
>Monodelphis_domestica
--MRRFCLTKQGPLKTTRKREKERGKS------
>Aquila_chrysaetos_canadensis
--MKRFCQAKQCLAKTPRRREKEKGRERELKH-
>Gavia_stellata
--MKRFCQAKQCLAKTPRRREKEKGRERELKH-
>Balearica_regulorum_gibberic
--MKRFCQAKQCLAKTPRRREKEKGRERELKH-
>Condylura_cristata
--MRRYCHTKQCLVKTPRKRGKDHRKKKLKTL-
>Tauraco_erythrolophus
--MKRFCQAKQCLAKTPRRREKEKGRERELKH-
>Zonotrichia_albicollis
MEHVCHVGFPVFLIPEAGIARWLTNLPGISSNWHGKVVTCVWAHHKEVAFHSDICLLRYKWISDCPIWKLGCFDQWFASCAASSTSGINTPS--LLISSCFSLFAGELEKVTP--RAPWPYELLSWQGTERLMKRFCQAKQCLAKTPRRREKEKGRERELQH-
>Phaethon_lepturus
--MKRFCQAKQCLAKTPRRREKEKGRERELKH-
>Picoides_pubescens
--MKRFCQAKQCLAKTPRRREKEKGRERELKH-
>Otolemur_garnettii
--MRRYCHTKQCLVKTTRKREKDREKS------
>Fulmarus_glacialis
--MKRFCQAKQCLAKTPRRREKEKGRERELKH-
>Melopsittacus_undulatus
--MKRFCQAKQCLAKTPRRREKEKGRERELKH-
>Aptenodytes_forsteri
--MKRFCQAKQCLAKTPRRREKEKGREKELKH-
>Anolis_carolinensis
---------------------------------MGARGAVTWGPERRLA-------------------------------------AVELPERSERRDGEDAAAAGVLQSRIPGASVPLLRDLLSRQETERHMRSSWQAKQSLAKTPRRREKEKADRRRS---
>Struthio_camelus_australis
--MKRFCQAKQCLAKTPRRREKEKGRGKELKH-
>Chrysemys_picta
--MRRFCQAKTVPSKDTEEEGKGEGGGGAELRL
>Tinamus_guttatus
--MKRFCQTKQCLAKTPRRREKEKGRGRDLKH-
>Calypte_anna
--MKRLCQAKQCLAKTPRRREKEKGREKELKH-
>Apaloderma_vittatum
--MKRFCQAKQCLAKTPRRREKEKGWERELKH-

HG0066 ENSG00000112182 BACH2

>Homo_sapiens
-----MDCQSLLISPFAQFPAS
>Anser_cygnoides
-----MDCCSLLISPLILFPAS
>Mustela_putorius_furo
-----MDCWSLLISPFAQFPAS
>Orcinus_orca
-----MDCRSLLFSPFAQFPAS
>Charadrius_vociferus
-----MDCCSLLISPLILFPAS
>Eptesicus_fuscus
-----MDCCSLLISPFAQFPAS
>Dasypus_novemcinctus
-----MDCCSLLISPFAQFSAS
>Columba_livia
-----MDCCSLLISPLILFPAS
>Alligator_mississippiensis
-----MDCCSLLISPFTQFPAS
>Cuculus_canorus
-----MDCCSLLISPLIPFPAS
>Sarcophilus_harrisii
-----MDCCNLLISPFIQFPAS
>Monodelphis_domestica
-----MDCCSLFISPFIQFPAS
>Macropus_eugenii
-----MDCCSLLISPFIQFPAS
>Falco_cherrug
-----MDCCSLLISPLILFPAS
>Gallus_gallus
-----MDCCSLLISPLILFPAS
>Mesitornis_unicolor
-----MDCCSLLISPVILFPAS
>Condylura_cristata
-----MDCCSFLISPFAQCPAS
>Oryctolagus_cuniculus
-----MDCCSLLISPFAQSPAS
>Elephantulus_edwardii
-----MDCCSLLISPFAQFPAS
>Opisthocomus_hoazin
-----MDCCSLLISPLILFPAS
>Pseudopodoces_humilis
-----MDCSSLLISPLILFPAS
>Egretta_garzetta
-----MDCCSLLISPLILFPAS
>Equus_asinus
-----MDCCSLLISPFAQFPAS
>Manis_javanica
-----MDCCSHLISPFAQFPAS
>Melopsittacus_undulatus
-----MDCCSLLLSPLILFPAS
>Heterocephalus_glaber
-----MDCCSLLISPFAQFPAS
>Trichechus_manatus_latirostr
-----MDCCSLLISPFAQFPAS
>Aptenodytes_forsteri
-----MDCCSLLISPLILFPAS
>Gekko_japonicus
-----MDCCKLLISPFVHFLVS
>Struthio_camelus_australis
-----MDCCSLLISPLILFPAS
>Chrysemys_picta
MVHILMDCCSLLISPFIQFPAS
>Tinamus_guttatus
-----MDCCSLFISPLIPFPAS
>Orycteropus_afer_afer
-----MDCCSILISPFAQFPAS

HG0067 ENSG00000166862 CACNG2

```
>Homo_sapiens
METGDQNFRKRD-------------------------------------------
>Channa_punctata
METGDQNFRKKENLNGYFDFFPVFIAFCLLSLPVWRKPANYLINSLSLDFLTPSG
>Anser_cygnoides
METGDQNFRKRD-------------------------------------------
>Microhyla_fissipes
METEDQNFRKRD-------------------------------------------
>Oryzias_latipes
METGDRNFRKKEKRSC---------------------------------------
>Canis_lupus_familiaris
METGDQNFRKRD-------------------------------------------
>Balaenoptera_acutorostrata_s
METGDQNFRKRD-------------------------------------------
>Astyanax_mexicanus
MESGDQNFRKKEN------------------------------------------
>Calidris_pugnax
METGDQNFRKRD-------------------------------------------
>Rousettus_aegyptiacus
METGDQNFRKRD-------------------------------------------
>Maylandia_zebra
METGDQNFRKKENLSG---------------------------------------
>Dasypus_novemcinctus
METGDQNFRKRD-------------------------------------------
>Alosa_alosa
METGDQNFRKKEN------------------------------------------
>Sinocyclocheilus_anshuiensis
METGDQNFRKKDI------------------------------------------
>Poecilia_mexicana
METGDQNFRKKENLSG---------------------------------------
>Galeopterus_variegatus
METGDQNFRKRD-------------------------------------------
>Monodelphis_domestica
METGDQNFRKRD-------------------------------------------
>Aquila_chrysaetos_canadensis
METGDQNFRKRD-------------------------------------------
>Meleagris_gallopavo
METGDQNFRKRD-------------------------------------------
>Apteronotus_leptorhynchus
METGDQNFRKKDIYQLFILFNFRFHFSVY--------------------------
>Zonotrichia_albicollis
METGDQNFRKRD-------------------------------------------
>Ceratotherium_simum_simum
METGDQNFRKRD-------------------------------------------
>Manis_javanica
METGDQNFRKRD-------------------------------------------
>Melopsittacus_undulatus
METGDQNFRKRD-------------------------------------------
>Nannospalax_galili
METGDQNFRKRD-------------------------------------------
>Salmo_salar
METGDQNFRKKEI------------------------------------------
>Tupaia_chinensis
METGDQNFRKRD-------------------------------------------
>Ictalurus_punctatus
METGDQNFRKKLN------------------------------------------
>Thamnophis_sirtalis
METGDQNFRKRD-------------------------------------------
>Struthio_camelus_australis
METGDQNFRKRD-------------------------------------------
>Chelonia_mydas
METGDQNFRKRD-------------------------------------------
>Takifugu_rubripes
METGDQNFRKKENPCVSFGFSF---SSCVPTRTI---------------------
>Tinamus_guttatus
METGDQNFRKRD-------------------------------------------
```

HG0068 ENSDARG00000071235

>Danio_rerio
--MRFFRL-TFKCFVDCF
>Amia_calva
--MRFFRL-TFKCFVDCF
>Xenopus_tropicalis
--MRFFRL-TFKCFVDCF
>Menidia_menidia
--MRFFRL-TFKCFVDCF
>Porichthys_notatus
--MRFFRL-TFKCFVDCF
>Oryzias_melastigma
--MRFFRL-TFKCFVDCF
>Hynobius_chinensis
--MRFFRL-TFKCFVDGF
>Micropterus_floridanus
--MRFFRL-TFKCFVDCF
>Astyanax_mexicanus
--MRFFRL-TFKCFVDCF
>Rousettus_aegyptiacus
--MRFFRL-TFKCFVDCF
>Haplochromis_burtoni
--MRFFRL-TFKCFVDCF
>Clupea_harengus
--MRFFRL-TFKCFVDCF
>Latimeria_chalumnae
MLLPYPSVQCRELSQTRSAAREILSYRDSKCSQEGISPAAWLPSFYPYFPLLLFAHMRYFRL-TFKCFVDCF
>Cyprinodon_variegatus
--MRFFRL-TFKCFVDCF
>Monodelphis_domestica
--MRFFRL-TFKCFVDCF
>Ciona_intestinalis
--MRYFRLFTFKCFIDCF
>Esox_lucius
--MRFFRL-TFKCFVDCF
>Gallus_gallus
--MRFFRL-TFKCFVDCF
>Erinaceus_europaeus
--MRFFRL-TFKCFVDCF
>Scleropages_formosus
--MRFFRL-TFKCFVDCF
>Notothenia_coriiceps
--MRFFRL-TFKCFVDCF
>Platichthys_stellatus
--MRFFRL-SFKCFVDCF
>Papio_anubis
--MRFFRL-TFKCFVDCF
>Leucoraja_erinacea
--MRFFRL-TFKCFVDCF
>Psammomys_obesus
--MRFFRL-TFKCFVDCF
>Salmo_salar
--MRFFRL-SFKCFVDCF
>Lepisosteus_oculatus
--MRFFRL-TFKCFVDCF
>Macropisthodon_rudis
--MRFFRL-TFKCFVDCF
>Botryllus_schlosseri
--MHYFKLTTFKCFIDCF
>Tetronarce_californica
--MRFFRL-TFKCFVDCF

HG0068 ENSG00000130821 SLC6A8

>Homo_sapiens
--MRFFRL-TFKCFVDCF
>Xenopus_tropicalis
--MRFFRL-TFKCFVDCF
>Menidia_menidia
--MRFFRL-TFKCFVDCF
>Porichthys_notatus
--MRFFRL-TFKCFVDCF
>Oryzias_latipes
--MRFFRL-TFKCFVDCF
>Tripterygion_delaisi
--MRFFRL-TFKCFVDCF
>Hynobius_chinensis
--MRFFRL-TFKCFVDGF
>Micropterus_floridanus
--MRFFRL-TFKCFVDCF
>Pygocentrus_nattereri
--MRFFRL-TFKCFVDCF
>Rousettus_aegyptiacus
--MRFFRL-TFKCFVDCF
>Haplochromis_burtoni
--MRFFRL-TFKCFVDCF
>Clupea_harengus
--MRFFRL-TFKCFVDCF
>Latimeria_chalumnae
MLLPYPSVQCRELSQTRSAAREILSYRDSKCSQEGISPAAWLPSFYPYFPLLLFAHMRYFRL-TFKCFVDCF
>Danio_rerio
--MRFFRL-TFKCFVDCF
>Cyprinodon_variegatus
--MRFFRL-TFKCFVDCF
>Monodelphis_domestica
--MRFFRL-TFKCFVDCF
>Ciona_intestinalis
--MRYFRLFTFKCFIDCF
>Esox_lucius
--MRFFRL-TFKCFVDCF
>Gallus_gallus
--MRFFRL-TFKCFVDCF
>Erinaceus_europaeus
--MRFFRL-TFKCFVDCF
>Notothenia_coriiceps
--MRFFRL-TFKCFVDCF
>Platichthys_stellatus
--MRFFRL-SFKCFVDCF
>Leucoraja_erinacea
--MRFFRL-TFKCFVDCF
>Psammomys_obesus
--MRFFRL-TFKCFVDCF
>Salmo_salar
--MRFFRL-SFKCFVDCF
>Lepisosteus_oculatus
--MRFFRL-TFKCFVDCF
>Macropisthodon_rudis
--MRFFRL-TFKCFVDCF
>Botryllus_schlosseri
--MHYFKLTTFKCFIDCF
>Tetronarce_californica
--MRFFRL-TFKCFVDCF

HG0068 ENSGALG00000041419

>Gallus_gallus
--MRFFRL-TFKCFVDCF
>Amia_calva
--MRFFRL-TFKCFVDCF
>Xenopus_tropicalis
--MRFFRL-TFKCFVDCF
>Menidia_menidia
--MRFFRL-TFKCFVDCF
>Porichthys_notatus
--MRFFRL-TFKCFVDCF
>Oryzias_latipes
--MRFFRL-TFKCFVDCF
>Tripterygion_delaisi
--MRFFRL-TFKCFVDCF
>Hynobius_chinensis
--MRFFRL-TFKCFVDGF
>Micropterus_floridanus
--MRFFRL-TFKCFVDCF
>Pygocentrus_nattereri
--MRFFRL-TFKCFVDCF
>Rousettus_aegyptiacus
--MRFFRL-TFKCFVDCF
>Haplochromis_burtoni
--MRFFRL-TFKCFVDCF
>Clupea_harengus
--MRFFRL-TFKCFVDCF
>Latimeria_chalumnae
MLLPYPSVQCRELSQTRSAAREILSYRDSKCSQEGISPAAWLPSFYPYFPLLLFAHMRYFRL-TFKCFVDCF
>Danio_rerio
--MRFFRL-TFKCFVDCF
>Cyprinodon_variegatus
--MRFFRL-TFKCFVDCF
>Monodelphis_domestica
--MRFFRL-TFKCFVDCF
>Ciona_intestinalis
--MRYFRLFTFKCFIDCF
>Esox_lucius
--MRFFRL-TFKCFVDCF
>Erinaceus_europaeus
--MRFFRL-TFKCFVDCF
>Scleropages_formosus
--MRFFRL-TFKCFVDCF
>Taeniopygia_guttata
--MRFFRL-TFKCFVDCF
>Notothenia_coriiceps
--MRFFRL-TFKCFVDCF
>Platichthys_stellatus
--MRFFRL-SFKCFVDCF
>Cebus_capucinus_imitator
--MRFFRL-TFKCFVDCF
>Leucoraja_erinacea
--MRFFRL-TFKCFVDCF
>Mus_musculus
--MRFFRL-TFKCFVDCF
>Salmo_salar
--MRFFRL-SFKCFVDCF
>Lepisosteus_oculatus
--MRFFRL-TFKCFVDCF
>Macropisthodon_rudis
--MRFFRL-TFKCFVDCF
>Botryllus_schlosseri
--MHYFKLTTFKCFIDCF
>Tetronarce_californica
--MRFFRL-TFKCFVDCF

HG0069 ENSG00000100697 DICER1

>Homo_sapiens
-MSKCSSDKSNTEISNIKT
>Apteryx_australis_mantelli
MMSKCSSGSINTEVAEIKT
>Cariama_cristata
MMSKCSSGSINTETAEIKT
>Ailuropoda_melanoleuca
-MSKCSSDNSNTEISNIKT
>Balaenoptera_acutorostrata_s
-MSKCSSDNSNTEISNIKT
>Charadrius_vociferus
MMSKCSSGSINTETAEIKT
>Pteropus_alecto
-MSKCSSDNSNTEISNIKT
>Pterocles_gutturalis
MMSKCSSGSINTETAEIKT
>Dasypus_novemcinctus
-MSKCSSDNSNTEISNIKT
>Columba_livia
MMSKCSSGSINTETAEIKT
>Alligator_sinensis
MMSKCSSENTNTKVSEIKT
>Galeopterus_variegatus
-MSKCSSDNSNTEISNIKT
>Aquila_chrysaetos_canadensis
MMSKCSSGSINTETAEIKT
>Gavia_stellata
MMSKCSSGSINTETAEIKT
>Mesitornis_unicolor
MMSKCSSGSVNTETAEIKT
>Erinaceus_europaeus
MMSKCSSDNSNTEIANIKT
>Elephantulus_edwardii
-MSKCGSDSSNTDIADIKT
>Ornithorhynchus_anatinus
MMSKCSSGNINTEVSEIKT
>Ficedula_albicollis
MMSKCSSGGINTETAEIKT
>Phaethon_lepturus
MMSKCSSGSINTEAAEIKT
>Ceratotherium_simum_simum
-MSKCSSDNSNTEISNIKT
>Loxodonta_africana
-MSKCSSDNSNAEISNIKT
>Melopsittacus_undulatus
MMSKCSSGSINTETAEIKT
>Ictidomys_tridecemlineatus
-MSKCSSDSSNTEISNIKT
>Trichechus_manatus_latirostr
-MSKCSSDNSNTEISNIKT
>Aptenodytes_forsteri
MMSKCSSGSINTETAEIKT
>Tyto_alba
MMSKCSSGSINTETAEIKT
>Struthio_camelus_australis
MMSKCNSGSINTEVAEIKT
>Chrysemys_picta
MMSKCSSKNTNTEISEIKT
>Tinamus_guttatus
MMSKCSSGNINTEVAEIKT
>Apaloderma_vittatum
MMSKCSSGSINTETAEIKT
>Orycteropus_afer_afer
-MSGCSSDGSNTEASHIKT

HG0069 ENSGALG00000010999 DICER1

>Gallus_gallus
MMSKCSLESISTEAAEIKT--------------------------
>Anser_cygnoides
MMSKCSLESISTEAAEIKT--------------------------
>Chaetura_pelagica
MMSKCSLGSIKTETAEIKTAVLEQKCNERNTG-------------
>Apteryx_australis_mantelli
MMSKCSSGSINTEVAEIKT--------------------------
>Buceros_rhinoceros_silvestri
MMSKCSSGSINTATAEIKT--------------------------
>Cariama_cristata
MMSKCSSGSINTETAEIKT--------------------------
>Calidris_pugnax
MMSKCSLGSINTETAEIKT--------------------------
>Pterocles_gutturalis
MMSKCSSGSINTETAEIKT--------------------------
>Colius_striatus
MMSKCSLGSINTETAEIKMAVLEQKCNERNTG-------------
>Columba_livia
MMSKCSSGSINTETAEIKT--------------------------
>Merops_nubicus
MMSKCSLGSINTETAEIKT--------------------------
>Alligator_sinensis
MMSKCSSENTNTKVSEIKT--------------------------
>Cuculus_canorus
MMSRCSLGSINSETAEIKTAVLKQKCK------------------
>Aquila_chrysaetos_canadensis
MMSKCSSGSINTETAEIKT--------------------------
>Gavia_stellata
MMSKCSSGSINTETAEIKT--------------------------
>Balearica_regulorum_gibberic
MMSKCSSGSINTETAEIKTTVLEQKCNERNTG-------------
>Erinaceus_europaeus
MMSKCSSDNSNTEIANIKT--------------------------
>Ornithorhynchus_anatinus
MMSKCSSGNINTEVSEIKT--------------------------
>Opisthocomus_hoazin
MMSKCSLGIINTETAEIKT--------------------------
>Acanthisitta_chloris
MMSKCSLGSINTETAEIKTAVLEQKCNERNTG-------------
>Phaethon_lepturus
MMSKCSSGSINTEAAEIKT--------------------------
>Picoides_pubescens
MMSKCSSGSINTETAKIKTAVLEQKCNERNTG-------------
>Fulmarus_glacialis
MMSKCSSGSINTETTEIKTAVLEQKCNERNTG-------------
>Melopsittacus_undulatus
MMSKCSSGSINTETAEIKT--------------------------
>Jaculus_jaculus
-MSKCSSDNSSTEVADIKT--------------------------
>Aptenodytes_forsteri
MMSKCSSGSINTETAEIKT--------------------------
>Tyto_alba
MMSKCSSGSINTETAEIKT--------------------------
>Struthio_camelus_australis
MMSKCNSGSINTEVAEIKT--------------------------
>Pelodiscus_sinensis
MMSKCSSKNTNTEVSEIKTQVRKYPSRTPAVNNTTWVIEEALQRQ
>Tinamus_guttatus
MMSKCSSGNINTEVAEIKT--------------------------
>Calypte_anna
MMSKCSLESINTETAEIKTAVLEQKCNERNTG-------------
>Apaloderma_vittatum
MMSKCSSGSINTETAEIKT--------------------------

HG0070 ENSDARG00000040926 nr2f2

>Danio_rerio
MDDCKCPWTWPLRARLLFGLLQF----------------------------
>Amia_calva
MADCRQPWTWPLSARLLFSLSSHQV--------------------------
>Oryzias_latipes
MADCKRPWTWPLSARQRLSSPTHQF--------------------------
>Tripterygion_delaisi
MADCKRPWTWPLRARQRLSSPLLHHF-------------------------
>Mustela_putorius_furo
MADCGLPWTWPPDSRLSSSSSSSSSSS-------YS------NSSAHQL--
>Orcinus_orca
MADCGLPWTWPPDSRLSSSTSSSSSSS-------SSS---SNSSAAHQL--
>Pygocentrus_nattereri
MADCRRPWTWPLRAGPRFGSPSPHF--------------------------
>Haplochromis_burtoni
MADCKRPWTWPLSARQRLSSPSHQF--------------------------
>Clupea_harengus
MDECKRPWTWPSRARLRFGSSSVLTIC--S--------------ATF----
>Latimeria_chalumnae
MADCELPWTWPLDTRLFFNSTVRSGLNIFE--------------AIFLLFA
>Austrofundulus_limnaeus
MADCKRPWTWPLSARQPLRSPSHQF--------------------------
>Esox_lucius
MADCKRPWTWPLSARLRFSSLSHPF--------------------------
>Acanthogobius_hasta
MADCTRPWTWPLSAGQRLGSPSHHF--------------------------
>Osmerus_mordax
MADCKRPWTWPLRERQRLSSPSHQF--------------------------
>Scleropages_formosus
MADCRRIWTWPLSHRTTFNT-------------------------------
>Lepidothrix_coronata
MADCELPWTWPPDTRLSSFNYSAADQL------------------------
>Notothenia_coriiceps
MADCKRPWTWPLSARQRLSSPSHQF--------------------------
>Platichthys_stellatus
MADCKRPWTWPLSARQRLSSPSHQF--------------------------
>Cercocebus_atys
MADCGLPWTWPPDSRLSSSSTSSFTTTSSSSSSSSSSSSSANSSAAHQL--
>Loxodonta_africana
MADCGLPWTWPPDSRLSSFSSSSSS-S-------SSF---SNSSAAHQL--
>Nannospalax_galili
MADCGLPWTWPPDSRLSSSSSSISSFS-------SSSSFSSSNSAAH----
>Salmo_salar
MADCKRPWTWPLSARLRFSSPSHQF--------------------------
>Lepisosteus_oculatus
MADCRQRWTWPLNAQLLSSSSTHQV--------------------------
>Ictalurus_punctatus
MDDCRRPWTWPLRAGLRFGSSCCQF--------------------------
>Trichechus_manatus_latirostr
MADCGLPWTWPPDSRLSSFSSSSFS-S-------S-----SNSSAAHQL--
>Takifugu_rubripes
MADCKRPWTWPLSARQRLGSPSHQF--------------------------

HG0070 ENSDARG00000052695 nr2f1a

>Danio_rerio
MADCNLPWNWPPDNCIS--------------------------------
>Acipenser_sinensis
MAGCILPWNWPPDTCIS--------------------------------
>Xenopus_laevis
MADCILPWIWPPDTYKP--------------------------------
>Menidia_menidia
MADCNLPWNWPPDTCIS--------------------------------
>Scyliorhinus_torazame
MADCVLPWIWPPDTCVP--------------------------------
>Mustela_putorius_furo
MADCGLPWTWPPDSRLSSSSSSSS----------SSSYS---NSSAHQL
>Ambystoma_mexicanum
MAACGLPWTWPPDTRRP--------------------------------
>Orcinus_orca
MADCGLPWTWPPDSRLSSSTSSSS----------SSSSSSSNSSAAHQL
>Pygocentrus_nattereri
MADCNLPWNWPPDSCIS--------------------------------
>Haplochromis_burtoni
MADCNLPWNWPPDTCIS--------------------------------
>Alosa_alosa
MADCNFPWNWPPDTCIS--------------------------------
>Latimeria_chalumnae
MADCVLPWTWPPDTCIS--------------------------------
>Cyprinodon_variegatus
MADCNLPWNWPPDTCIS--------------------------------
>Esox_lucius
MADCNLPWNWPPDACIS--------------------------------
>Gadus_morhua
MAVCNLPWNWPPDTCTS--------------------------------
>Acanthogobius_hasta
MAGCVLPWTWPPDTCVS--------------------------------
>Apteronotus_leptorhynchus
MADCNLPWNWSPDNCIS--------------------------------
>Osmerus_mordax
MADCNIPWNWPPDTCIS--------------------------------
>Lepidothrix_coronata
MADCELPWTWPPDTRLSSFNYSAADQL----------------------
>Perca_flavescens
MAGCNLPWNWPPDTCIS--------------------------------
>Manis_javanica
MADCGLRWTWPPDSRLSSSASSSA--------SSSNSSA-------PQV
>Cynoglossus_semilaevis
MADCNLPWNWPPDTCIS--------------------------------
>Cercocebus_atys
MADCGLPWTWPPDSRLSSSSTSSFTTTSSSSSSSSSSSSSANSSAAHQL
>Loxodonta_africana
MADCGLPWTWPPDSRLSSFSSSSS----------S-SSSFSNSSAAHQL
>Mesocricetus_auratus
MADCGLPWTWPPDSRLSFSSSSSS--------SSSSSFSSSNSSAAH--
>Oncorhynchus_mykiss
MANCNLPWNWPPDTCIS--------------------------------
>Lepisosteus_oculatus
MADCNLPWNWPPDTCIS--------------------------------
>Trichomycterus_areolatus
MADCELPWNWPPDNCIS--------------------------------
>Trichechus_manatus_latirostr
MADCGLPWTWPPDSRLSSFSSSSF----------S-SS--SNSSAAHQL
>Takydromus_sexlineatus
MADCGLLWIWPPDTSFSSCNSAWLPEREGGGDL----------------
>Takifugu_rubripes
MADCNLPWNWPPDTCIS--------------------------------

HG0070 ENSG00000175745 NR2F1

>Homo_sapiens
MAGCVRPWTWPPDTRAP-------------------------------------
>Acipenser_sinensis
MAGCILPWNWPPDTCIS-------------------------------------
>Xenopus_laevis
MADCILPWIWPPDTYKP-------------------------------------
>Menidia_menidia
MADCNLPWNWPPDTCIS-------------------------------------
>Oryzias_latipes
MADCKRPWTWPLSARQRLSSPTHQF-----------------------------
>Scyliorhinus_torazame
MADCVLPWIWPPDTCVP-------------------------------------
>Canis_lupus_familiaris
MADCGLPWTWPPDSRLSSSSSSSSSSSSSSSSSSSSSSSSSSSSSYSNSSAHQL
>Hynobius_retardatus
MAGCALPWTWPPDTSEP-------------------------------------
>Orcinus_orca
MADCGLPWTWPPDSRLSSSTSSSSSSSSSSSNS---------------SAAHQL
>Pygocentrus_nattereri
MADCNLPWNWPPDSCIS-------------------------------------
>Haplochromis_burtoni
MADCNLPWNWPPDTCIS-------------------------------------
>Clupea_harengus
MAGCNIPWNWPPDTCIS-------------------------------------
>Latimeria_chalumnae
MADCVLPWTWPPDTCIS-------------------------------------
>Catostomus_commersonii
MAGCNLPWNWPPDNCIS-------------------------------------
>Austrofundulus_limnaeus
MADCKRPWTWPLSARQPLRSPSHQF-----------------------------
>Esox_lucius
MADCKRPWTWPLSARLRFSSLSHPF-----------------------------
>Gadus_morhua
MAVCNLPWNWPPDTCTS-------------------------------------
>Acanthogobius_hasta
MAGCVLPWTWPPDTCVS-------------------------------------
>Osmerus_mordax
MADCNIPWNWPPDTCIS-------------------------------------
>Lepidothrix_coronata
MADCELPWTWPPDTRLSSFNYSAADQL---------------------------
>Perca_flavescens
MAGCNLPWNWPPDTCIS-------------------------------------
>Manis_javanica
MADCGLRWTWPPDSRLSSSASSSASSSNSSAPQV--------------------
>Platichthys_stellatus
MAGCNIPWNWPPDTCIS-------------------------------------
>Loxodonta_africana
MADCGLPWTWPPDSRLSSFSSSSSS-SSSFSNS---------------SAAHQL
>Nannospalax_galili
MADCGLPWTWPPDSRLSSSSSSISSFSSSSSFSSSN------------SAAH--
>Salmo_salar
MANCNLPWNWPPDTCIS-------------------------------------
>Lepisosteus_oculatus
MADCNLPWNWPPDTCIS-------------------------------------
>Trichomycterus_areolatus
MADCELPWNWPPDNCIS-------------------------------------
>Trichechus_manatus_latirostr
MADCGLPWTWPPDSRLSSFSSSSFS-SS--SNS---------------SAAHQL
>Takifugu_rubripes
MADCNLPWNWPPDTCIS-------------------------------------

HG0070 ENSG00000185551 NR2F2

>Homo_sapiens
MADCGLPWTWPPDSRLSSSSTSSFTTTSSSSSSSSSSSANSSAAHQL
>Menidia_menidia
MADCNLPWNWPPDTCIS------------------------------
>Mustela_putorius_furo
MADCGLPWTWPPDSRLSSSSSSSS--------SSSYS---NSSAHQL
>Ambystoma_mexicanum
MAACGLPWTWPPDTRRP------------------------------
>Orcinus_orca
MADCGLPWTWPPDSRLSSSTSSSS--------SSSSSSSNSSAAHQL
>Pygocentrus_nattereri
MADCNLPWNWPPDSCIS------------------------------
>Haplochromis_burtoni
MADCNLPWNWPPDTCIS------------------------------
>Latimeria_chalumnae
MADCVLPWTWPPDTCIS------------------------------
>Ctenopharyngodon_idella
MADCNLPWNWPPDNCIS------------------------------
>Fundulus_grandis
MADCNLPWNWPPDTCTS------------------------------
>Esox_lucius
MADCNLPWNWPPDACIS------------------------------
>Osmerus_mordax
MADCNIPWNWPPDTCIS------------------------------
>Lepidothrix_coronata
MADCELPWTWPPDTRLSSFNYSAADQL--------------------
>Manis_javanica
MADCGLRWTWPPDSRLSSSASSSASSSNSSAP-------------QV
>Cynoglossus_semilaevis
MADCNLPWNWPPDTCIS------------------------------
>Loxodonta_africana
MADCGLPWTWPPDSRLSSFSSSSS--------S-SSSFSNSSAAHQL
>Mesocricetus_auratus
MADCGLPWTWPPDSRLSFSSSSSSSSS------SSFSSSNSSAAH--
>Lepisosteus_oculatus
MADCNLPWNWPPDTCIS------------------------------
>Trichomycterus_areolatus
MADCELPWNWPPDNCIS------------------------------
>Trichechus_manatus_latirostr
MADCGLPWTWPPDSRLSSFSSSSF--------S-SS--SNSSAAHQL
>Squalus_acanthias
MADCVLPWSWPPDSRLFSADRV-------------------------
>Takydromus_sexlineatus
MADCGLLWIWPPDTSFSSCNSAWLPEREGGGDL--------------
>Takifugu_rubripes
MADCNLPWNWPPDTCIS------------------------------

HG0071.1 ENSDARG00000098240 meis2a

>Danio_rerio
MRHDSLVTCRLLMGRL
>Acipenser_sinensis
MRHDSLVTCGLLMGRL
>Oryzias_latipes
MRHDSLVTCRLLMGRL
>Odobenus_rosmarus_divergens
MKHDSSVTCGLLTGRS
>Lipotes_vexillifer
MKHDSSVTCGLLTGRS
>Pygocentrus_nattereri
MRHDSLVTCTLLMGRL
>Myotis_brandtii
MKHDSSVTCGLLTGRS
>Lipochromis_sp._matumbi_hunt
MRHDSLVTCRLLMGRL
>Clupea_harengus
MRHDSLVTCRLLMGRL
>Latimeria_chalumnae
MRHDSLVTCGLLTGRS
>Alligator_sinensis
MRHDSLVTCGLLTGRS
>Kryptolebias_marmoratus
MRHDSLVTCRLLMGRL
>Esox_lucius
MRHDSLVTCRLLMGRL
>Condylura_cristata
MKHDSSVTCGLLTGRS
>Ochotona_princeps
MKHDSSVTCGLLTGRS
>Elephantulus_edwardii
MKHDSSVTCGLLTGRS
>Zonotrichia_albicollis
MRHDSLVTCGLLTGRS
>Notothenia_coriiceps
MRHDSLVTCRLLMGRL
>Ceratotherium_simum_simum
MKHDSSVTCGLLTGRS
>Cynoglossus_semilaevis
MRHDSLVTCRLLMGRL
>Colobus_angolensis_palliatus
MKHDSSVTCGLLTGRS
>Loxodonta_africana
MKHDSSVTCGLLTGRS
>Jaculus_jaculus
MKHDSSVTCGLLTGRS
>Salmo_salar
MRHDSLVTCRLLMGRL
>Lepisosteus_oculatus
MRHDSLVTCGLLMGRL
>Ictalurus_punctatus
MRHDSLVTCRLLMGRL
>Python_bivittatus
MRHDSLVTCGLLTGRS
>Struthio_camelus_australis
MRHDSLVTCGLLTGRS
>Takifugu_rubripes
MRHDSLVTCRLLMGRL
>Orycteropus_afer_afer
MKHDSSVTCGLLTGRS

HG0071.1 ENSG00000134138 MEIS2

>Homo_sapiens
MKHDSSVTCGLLTGRS
>Acipenser_sinensis
MRHDSLVTCGLLMGRL
>Oryzias_latipes
MRHDSLVTCRLLMGRL
>Odobenus_rosmarus_divergens
MKHDSSVTCGLLTGRS
>Lipotes_vexillifer
MKHDSSVTCGLLTGRS
>Astyanax_mexicanus
MRHDSLVTCTLLMGRL
>Myotis_davidii
MKHDSSVTCGLLTGRS
>Haplochromis_burtoni
MRHDSLVTCRLLMGRL
>Clupea_harengus
MRHDSLVTCRLLMGRL
>Latimeria_chalumnae
MRHDSLVTCGLLTGRS
>Alligator_sinensis
MRHDSLVTCGLLTGRS
>Danio_rerio
MRHDSLVTCRLLMGRL
>Poecilia_reticulata
MRHDSLVTCRLLMGRL
>Esox_lucius
MRHDSLVTCRLLMGRL
>Erinaceus_europaeus
MKHDSSVTCGLLTGRS
>Ochotona_princeps
MKHDSSVTCGLLTGRS
>Elephantulus_edwardii
MKHDSSVTCGLLTGRS
>Zonotrichia_albicollis
MRHDSLVTCGLLTGRS
>Notothenia_coriiceps
MRHDSLVTCRLLMGRL
>Ceratotherium_simum_simum
MKHDSSVTCGLLTGRS
>Cynoglossus_semilaevis
MRHDSLVTCRLLMGRL
>Loxodonta_africana
MKHDSSVTCGLLTGRS
>Jaculus_jaculus
MKHDSSVTCGLLTGRS
>Salmo_salar
MRHDSLVTCRLLMGRL
>Lepisosteus_oculatus
MRHDSLVTCGLLMGRL
>Ictalurus_punctatus
MRHDSLVTCRLLMGRL
>Python_bivittatus
MRHDSLVTCGLLTGRS
>Struthio_camelus_australis
MRHDSLVTCGLLTGRS
>Takifugu_rubripes
MRHDSLVTCRLLMGRL
>Tetronarce_californica
MRDDSFVTSGLLTGRS
>Orycteropus_afer_afer
MKHDSSVTCGLLTGRS

HG0071.2 ENSG00000143995 MEIS1

>Homo_sapiens
MLSDSGALLQVP
>Panthera_tigris
MLSDSGALLQVP
>Balaenoptera_acutorostrata_s
MLSDSGALLQVP
>Myotis_davidii
MLSDSGALLQVP
>Galeopterus_variegatus
MLSDSGALLQVP
>Condylura_cristata
MLSDSGALLQVP
>Ochotona_princeps
MLSDSGGLLQVP
>Equus_caballus
MLSDSGALLQVP
>Manis_javanica
MLSDSGALLQVP
>Loxodonta_africana
MLSDSGALLQVP
>Mus_musculus
MLNDAGALLQVP
>Tupaia_chinensis
MLSDSGALLQVP

HG0072 ENSG00000104435 STMN2

>Homo_sapiens
---MGGADREGLISISNCILVLCTPAPLADQYLMLGDSDSAGVMSGDLGSQSA----
>Chaetura_pelagica
---------------------------------MPRDSGSAGVMSGDL---------
>Odobenus_rosmarus_divergens
---------------------------------MLGDSDSAGVMSGDLGSQSA----
>Hynobius_retardatus
---MSGDLWSQSAGAEP
>Tursiops_truncatus
---------------------------------MLGDSDSAGVMSGDLGSQSA----
>Charadrius_vociferus
---------------------------------MPGDSGSAGVMSGDL---------
>Myotis_davidii
---------------------------------MLGGSDSARVMSGDLGSQSARASG
>Dasypus_novemcinctus
---MSGDLGSQSA----
>Columba_livia
---------------------------------MPRDSGSAGVMSGDL---------
>Cuculus_canorus
---------------------------------MPRDSGSAGVMSGDL---------
>Galeopterus_variegatus
---MGGAAREGLISISNCILVLCTPAPLADQYLMLGDSDSAGVMSGDLGSQSA----
>Falco_cherrug
---------------------------------MPGDSGSAGVMSGDL---------
>Gallus_gallus
---------------------------------MPGDSGSAGVMSGDL---------
>Condylura_cristata
---------------------------------MLGDSDSAGVVSGDLGSQSA----
>Ochotona_princeps
---MGGAAREGLISISNCIVVLCTPAPLADQYLMLRDSDSAGVMSGDLGSQSA----
>Opisthocomus_hoazin
---------------------------------MPGGSGSAGVMSGDL---------
>Sturnus_vulgaris
---------------------------------MPGDSGSAGVMSGDLSSQSAWVEE
>Egretta_garzetta
---------------------------------MPGDSGSAGVMSGDL---------
>Ceratotherium_simum_simum
---------------------------------MLGDSDSAGVMSGDLGSQSA----
>Picoides_pubescens
---------------------------------MPGDSGSAGVMSGDL---------
>Loxodonta_africana
---MGGAAREGLISISNCILVLCTLAPLADQYLMLGDSDSAGVMSGDLGSQSA----
>Melopsittacus_undulatus
---------------------------------MPGDSGSAGVMSGDL---------
>Marmota_marmota_marmota
---MGGAAREGLISISNCILVLCTPAPLADQYSMLGDSDSAGVMSGDLGSQSA----
>Tupaia_chinensis
---------------------------------MLGDSDSAGVMSGDLGSQSA----
>Aptenodytes_forsteri
---------------------------------MPGDSGSAGVMSGDL---------
>Squalus_acanthias
MFHWKKRGGPILICISNCILALCTLASLVDQYLMPGDPENAPVMSRDLFSQSALLGR
>Anolis_carolinensis
---------------------------------MLADSGSAGVMSGELWSQSAWLEE
>Struthio_camelus_australis
---------------------------------MPGDSGSAGVMSGDL---------
>Chelonia_mydas
---------------------------------MLGDSSSAGVMSGDL---------
>Tinamus_guttatus
---------------------------------MPGDSGSAGVMSGDL---------
>Calypte_anna
---------------------------------MPGDSGSAGVMSGDL---------

HG0072 ENSGALG00000039690 STMN2

>Gallus_gallus
------------------------------MPGDSGSAGVMSGDL---------
>Chaetura_pelagica
------------------------------MPRDSGSAGVMSGDL---------
>Canis_lupus_familiaris
------------------------------MLGDSDSAGVMSGDLGSQSA----
>Lipotes_vexillifer
------------------------------MLGDSDSAGVMSGDLGSQSA----
>Charadrius_vociferus
------------------------------MPGDSGSAGVMSGDL---------
>Cynopterus_sphinx
------------------------------MPGDSDSAGVMSGDLGSQSAWVSE
>Columba_livia
------------------------------MPRDSGSAGVMSGDL---------
>Cuculus_canorus
------------------------------MPRDSGSAGVMSGDL---------
>Galeopterus_variegatus
MGGAAREGLISISNCILVLCTPAPLADQYLMLGDSDSAGVMSGDLGSQSA----
>Haliaeetus_leucocephalus
------------------------------MPGDSGSAGVMSGDL---------
>Opisthocomus_hoazin
------------------------------MPGGSGSAGVMSGDL---------
>Pseudopodoces_humilis
------------------------------MPGDSGSAGVMSGDLSSQSAWVEE
>Egretta_garzetta
------------------------------MPGDSGSAGVMSGDL---------
>Ceratotherium_simum_simum
------------------------------MLGDSDSAGVMSGDLGSQSA----
>Picoides_pubescens
------------------------------MPGDSGSAGVMSGDL---------
>Cebus_capucinus_imitator
MGGAAREGLITISNCILVLCTPALLADQYLMLGDSDSAGVMSGDLGSQSA----
>Loxodonta_africana
MGGAAREGLISISNCILVLCTLAPLADQYLMLGDSDSAGVMSGDLGSQSA----
>Melopsittacus_undulatus
------------------------------MPGDSGSAGVMSGDL---------
>Ictidomys_tridecemlineatus
------------------------------MLGDSDSAGVMSGDLGSQSA----
>Tupaia_chinensis
------------------------------MLGDSDSAGVMSGDLGSQSA----
>Aptenodytes_forsteri
------------------------------MPGDSGSAGVMSGDL---------
>Anolis_carolinensis
------------------------------MLADSGSAGVMSGELWSQSAWLEE
>Struthio_camelus_australis
------------------------------MPGDSGSAGVMSGDL---------
>Chelonia_mydas
------------------------------MLGDSSSAGVMSGDL---------
>Tinamus_guttatus
------------------------------MPGDSGSAGVMSGDL---------
>Calypte_anna
------------------------------MPGDSGSAGVMSGDL---------

HG0073 ENSDARG00000041708 csrp2bp

>Danio_rerio
------------------------------MSTASKVVLGLSIVLTISTVAGVHIKQNWDRQRLREGVVRDLERLERKRENLRALEEQIQLTRELVTERNRRQAEASETHDS--------------
>Amia_calva
------------------------------MSTTSKVVLGVSLALTLGTVAGVHIKQSLDRERLREGVYRDLERQARKKENLRQLEEQIALTRELEKERQRRAAGTQGS-----------------
>Channa_striata
------------------------------MSTTSKVVLGLSVSLTLTTVAGVHLKQALDRQRLHEGVVQDLERLERKKENLRLLQEQRVLTRHLEEERQRREAELHPQDHN--------------
>Xenopus_laevis
------------------------------MSTISKVVLGISVVLSVGTVVGVHVKQRLDRERLREGVLQDLERQSRKQENLRLLEEQIHLTKRLEAEREKILLTEGSSKF---------------
>Porichthys_notatus
------------------------------MSRVSKVVLGLSVVLTLGTVTGVHLLQARDRQRLHDGVLRDLERVQRKKDNLRRLQDQKVLSRRLEAERQRREAELQTTGHTHTD-----------
>Tripterygion_delaisi
------------------------------MSTTSKVVLGVSVVLSLSIVAGVHLKQDWDRQRLHEGVIRDLERLAKKKENLRLLEEQRILTIQLEEERQQREAQLSSQES---------------
>Odobenus_rosmarus_divergens
------------------------------MSRSSKAVLGLSVVLTAATVAGVHLKQRQDRQRLHDGVIRDIERQNRKKENIRLLGEQIILTEQLEAEREKMLLAKGSQKT---------------
>Hynobius_chinensis
------------------------------MSAASKVVLGVSVLASVATVAGVHLKQKLDRERLREGVFRDIERQHRKQENLRLLEEQIVLTRQLEVEREKMLMTKGSQKS---------------
>Pygocentrus_nattereri
------------------------------MSTASKVVLGVSVVLTVSTVVGVHVKQNWDRQRLREGVLRDLERLERKRENLRALEEQIQLTRELVAEQAYQQAEAARGS----------------
>Calidris_pugnax
---MEERDKALMAKGSQQP---------------
>Callorhinchus_milii
------------------------------MSAVSKVVLGVSVAVTAGTVAGVHLRQQLDREKLREGVFRDLERQEHKKKNLRLLEEQIILTNQLEADRVASESIQKSEKHKSDF-----------
>Rhinolophus_ferrumequinum
------------------------------MSRTSKVVLGLSVVLTVATVAGVHLKQRQDQQRLRDGVIRDIERQNRKKENIRLLGEQIILTEQLEAEREKTLLAKDLKKH---------------
>Maylandia_zebra
------------------------------MSAVSKVVLGVSVVLTVSTVAAVHLKQAWDRERLREGVVRDLERLERRKENLRMLEEQRLLTRQLEEERRRREARLHPQDT---------------
>Alosa_alosa
------------------------------MSTASKVVLGLSVVLTISTVAGVHIKQNWDRQRLHEGVLRDLERVERKRENLRLLEEQIQLTQELETERQRREASAASGATQSP------------
>Merops_nubicus
---MEERDKALMEKGSQQS---------------
>Cuculus_canorus
--MSRKKQRLHDGVLRDLERQHQKKENIRLLEEQIALTKQLVEERDKGLMSKGSQES---------------
>Nothobranchius_furzeri
MNIADESSVVGIWLT---------EVRPNVMSAASKVVLGVSVLLTLGTVAAVHLNQAWDRERLREGVVQDLERVERKKENLRLLEEQRILTRHLEEERRRGEERLQSQDT---------------
>Numida_meleagris
------------------------------MSRSSRAVLVVTALLSAATIAAVHVQQRRERERLHSGVLRDLERQNQKKENIRLLEEQIALTKQLTEERDKMLMEKGSQQS---------------
>Acanthogobius_hasta
------------------------------MSRASRAVLGVSVVLTLSTVAGVHLKQAWDRERLHQGVVRDLERLQRKKENLQLLEEQRELTRILQQDRDQGRDQGQGRDQGQDQGRGQGQGQGRD
>Apteronotus_leptorhynchus
------------------------------MTVVSKVVLGISVVLTIGSVVGVHIKQNWDRERLREGVLRDLERLERKRKNLQDLEDQIQLTKVLVAERERRETQTANSAQGS-------------
>Plecoglossus_altivelis
------------------------------MSKTSKLVLGVSVVLTLSTVAGVHLKQTWDRERLHQGVLRDLERMERKKENLRVLEEQRTLTKELEAERDRRTTALQSPDQPHT------------
>Zonotrichia_albicollis
------------------------------MSRGSRLALAVSALLSAAIVAAVHIQQRRELERLRSGVVRDLERQKQKKENVRLLEEQIALTKRLIEERDKALMEKRSQQP---------------
>Gasterosteus_aculeatus
------------------------------MTTVSKVVLGVSVVLTLSTVAAVHLNQSWDRQRLHQGVVRDLERLERKKENLRLLEEQRILTSQLEEERLRREAERRPQDH---------------
>Ceratotherium_simum_simum
------------------------------MSRSSKVVLGLSVVLTAATVAGVHLKQRQDQQRLRDGVIRDIERQNRKKENIRLLGEQIILTEQLEAEREKMVLAKGSQKT---------------
>Papio_hamadryas
------------------------------MSRTSKVVLGLSVLLTAATLAGVHVKQQWDRQRLRDGVIRDIERQIRKKENIRLLGEQIILTEQLEAEREKTLLAKGSQKS---------------
>Mus_musculus
------------------------------MSRSSKAVLGLSVLLTAATVAGVHLKQRQDRQRLRDGVIRDIERQNQKKENIRLLGEQIILTKQLEAEREKMLEKGSQN-T---------------
>Salmo_salar
------------------------------MSTTSKVVLGVSVILTVSTVAGVHLKQNWDQERLHAGVLRDIERLERKRENLRVLEQQRSLTKELKAERDRRESGQQGSDQPQE------------
>Ictalurus_punctatus
------------------------------MSTASKVVLGLSVVLTVGTVAGVHIKQNWDRQRLREGVLRDLERVERKLENQRALEEQIRLTTELVAHREQQEAGETAGSN---------------
>Takydromus_sexlineatus
------------------------------MSAASKAVLAASVAVSGAVVAAVHLQQSRVRERLREGVLRDFERQNRKQENLRLLEQQIALTEQLEMERSKMLMGKESPH----------------
>Trachemys_scripta
----------MHWPLELCGSRSANGPVGEGMSTASKVALGISVLLSAGTVAAVHIQQRRVKERLHEGVIRDLERQSRKLENIRLLQEQIILTTQLEAERDKMLMEKGSQQS---------------
>Takifugu_rubripes
------MLSVSSWLA---------KTNRNVMSRASKVVLGVSVVLTLSTVTAVHLKQTWDRQRLHDGVLRDLERLEKKKENLRLLEEQRVLTRQLEYERQGRET----------------------

HG0074 ENSDARG00000078624 arhgef9b

>Danio_rerio
---MTLDVQTVAVFTVIAILLLVNVILIFFMGSS
>Maylandia_zebra
---MALDVQTCAVFTVIAVLVLVNVLLMFILGTR
>Fundulus_heteroclitus
---MALDVQTCAVFTVIAVLVLVNVLLMFILGTR
>Esox_lucius
-------------------------------MVDAIWTVKFKQPRSRAEQTSNANLPSSRQSTHRR-GSGVLLALQHCTMALDVQTFVVFTVIAVLVLVNVLLMFILGTR
>Cynoglossus_semilaevis
---MALDVQTCVVFTVIAFLVLVNVLLMFILGTR
>Salmo_salar
MSCNSACRVKTRSVAWEAVFPAVFFFSICSQMVDAVWTVKFKQSRSRPEQSSSARLPSSGQAAHRHRSSRIYLALQHYTMALDVQTFVVFTVIAVLVLVNVLLMFILGTR

HG0074 ENSG00000131089 ARHGEF9

>Homo_sapiens
------MDSLTEQ----RLTS-PNLPAPH--------LEHYSVLHCTMTLDVQTVVVFAVIVVLLLVNVILMFFLGTR
>Anser_cygnoides
------MDSLTEQ----RLTS-PNLPAPH--------LEHYSVLHCTMTLDVQTVVVFAVIVVLLLVNVILMFFLGTR
>Nanorana_parkeri
------MDSFAEQ----RLTP-QNLPAPR--------LDHYNVLHCTMTLDVQTVVVFAVIVVLLLVNVILMFFLGTR
>Apteryx_australis_mantelli
------MDSLTEQ----RLTS-PNLPAPH--------LEHYSVLHCTMTLDVQTVVVFAVIVVLLLVNVILMFFLGTR
>Oryzias_latipes
-------------------MARGGGGAGR--WSRQRMGFLNTVLRCNLPPETQKVVVFFIMMLLIIINVVLMFLLAFQ
>Panthera_tigris
------MDSLTEQ----RLTS-PNLPAPH--------LEHYSVLHCTMTLDVQTVVVFAVIVVLLLVNVILMFFLGTR
>Hynobius_retardatus
------MDSFTEQ----RLTT-QNLQAPH--------LEQYSVLHCTMTLDVQTVVVFAVIVVLLLVNVILMFFLGTR
>Orcinus_orca
------MDSLTEQ----RLTS-PNLPAPH--------LEHYSVLHCTMTLDVQTVVVFAVIVVLLLVNVILMFFLGTR
>Calidris_pugnax
------MDSLTEQ----RLTS-PNLPAPH--------LEHYSVLHCTMTLDVQTVVVFAVIVVLLLVNVILMFFLGTR
>Myotis_brandtii
------MDSLTEQ----RLTS-PNLPAPH--------LEHYSVLHCTMTLDVQTVVVFAVIVVLLLVNVILMFFLGTR
>Neolamprologus_brichardi
--------------------MARGGGSGR--WSRQRMGFFNTMLRCNLPPETQKVVVFLIMMLLIIINVVLMFLLAFQ
>Danio_rerio
---MTLDVQTVAVFTVIAILLLVNVILIFFMGSS
>Fundulus_heteroclitus
--------------------MARGNGSGR--WSRQKMGFFNTLLRCNLPPDTQKVVVFVIMMLLIIINVVLMFLLAFQ
>Monodelphis_domestica
--------------------MA---KGPD--CSRPQWSILHTFLRCNMNPETQRVVVFVILLFLIIINVVLMFLLAFR
>Macropus_eugenii
---MTLDVQTIVVFAVIVVLLLVNVILMFFLGTR
>Esox_lucius
MVDAIWTVKFKQPRSRAEQTSNANLPSSRQSTHRRGSGVLLALQHCTMALDVQTFVVFTVIAVLVLVNVLLMFILGTR
>Aquila_chrysaetos_canadensis
------MDSLTEQ----RLTS-PNLPAPH--------LEHYSVLHCTMTLDVQTVVVFAVIVVLLLVNVILMFFLGTR
>Meleagris_gallopavo
------MDSLTEQ----RLTS-PNLPAPH--------LEHYSVLHCTMTLDVQTVVVFAVIVVLLLVNVILMFFLGTR
>Condylura_cristata
------MDSLTEQ----RLTS-PNLPAPH--------LEHYSVLHCTMTLDVQTVVVFAVIVVLLLVNVILMFFLGTR
>Ochotona_princeps
------MDSLTEQ----RLTS-PNLPAPH--------LEHYSVLHCTMTLDVQTVVVFAVIVVLLLVNVILMFFLGTR
>Elephantulus_edwardii
------MDSLTEQ----RLTS-PNLPAPH--------LEHYSVLHCTMTLDVQTVVVFAVIVVLLLVNVILMFFLGTR
>Pseudopodoces_humilis
------MDSLTEQ----RLTS-PNLPAPH--------LEHYSVLHCTMTLDVQTVVVFAVIVVLLLVNVILMFFLGTR
>Ceratotherium_simum_simum
------MDSLTEQ----RLTS-PNLPAPH--------LEHYSVLHCTMTLDVQTVVVFAVIVVLLLVNVILMFFLGTR
>Cynoglossus_semilaevis
---MALDVQTCVVFTVIAFLVLVNVLLMFILGTR
>Cricetulus_griseus
------MDSLTEQ----RLTS-PNLPAPH--------LEHYSVLHCTMTLDVQTVVVFAVIVVLLLVNVILMFFLGTR
>Salmo_salar
--------------------MARGG-GAH--RARQRLGYLNTLLRCNLHPETQKVVVFMIMMLLVIINVVLMFLLAF-
>Lepisosteus_oculatus
------MDSYTEP----RLTSENNLPAQH--------LEYYSLLHCTMTLDVQTVVVFTVIVVLVLVNVILMFFLGSR
>Trichechus_manatus_latirostr
------MDSLTEQ----RLTS-PNLPAPH--------LEHYSVLHCTMTLDVQTVVVFAVIVVLLLVNVILMFFLGTR
>Struthio_camelus_australis
------MDSLTEQ----RLTS-PNLPAPH--------LEHYSVLHCTMTLDVQTVVVFAVIVVLLLVNVILMFFLGTR
>Pelodiscus_sinensis
------MDSLTEQ----RLTS-PNLPAPH--------LEHYSVLHCTMTLDVQTVVVFAVIVVLLLVNVILMFFLGTR

HG0075 ENSG00000165699 TSC1

>Homo_sapiens
---MKDTRLTALETEVPVVARTVW-----------
>Anas_platyrhynchos
---MKGVWLTALQTEAQRIHRTVW-----------
>Apteryx_australis_mantelli
---MKGVWLIAVETEARLIRRTAW-----------
>Leptonychotes_weddellii
---MKDTRLTALETEVPIVPRTVW-----------
>Balaenoptera_acutorostrata_s
MATCQGCAVGKRWKPANPELPLLCLGSHFIVRLVLTDACFFSSVTMKDTRLTALETEVPIVPRTVW-----------
>Calidris_pugnax
---MKGVWLTALQTEARLVHRTVW-----------
>Miniopterus_natalensis
---MKDTRLTALETELPIVPRTVW-----------
>Dasypus_novemcinctus
---MKDTKLTALESEVSFVPRTVW-----------
>Columba_livia
---MKGVWLTALQTEAQLIHRTVW-----------
>Alligator_mississippiensis
---MKGARLTALKTEALLVHRTVW-----------
>Sarcophilus_harrisii
---MKDTRLRALETEVLIVHRTVW-----------
>Galeopterus_variegatus
---MKDSRLTALETEVPIIPRTAW-----------
>Monodelphis_domestica
---MKDTRLTALETEVLIVHRTVW-----------
>Haliaeetus_leucocephalus
---MKGVWLTALQTEAQLVHRTVW-----------
>Coturnix_japonica
---MKGVWLTALQTEAQGIHRTVW-----------
>Condylura_cristata
---MRDTRLTALETDVPVVPRTGWSRR--------
>Oryctolagus_cuniculus
---MRDNKLAALGTEAPVVHRAVYSWPQ-------
>Elephantulus_edwardii
---MKDTRLTALETEVSVVPRTV------------
>Ornithorhynchus_anatinus
---MKVSRLTTLETEGPIIYRTVW-----------
>Parus_major
---MKGVWLTALQTEAQVIHRTVW-----------
>Ceratotherium_simum_simum
---MKDTRLTALETEVPVVPRTVW-----------
>Manis_javanica
---MKDTRLTALETEVPVVPRTV------------
>Loxodonta_africana
---MKDTRLTALETEISIVPRAVL-----------
>Mesocricetus_auratus
---MKDTRLTALETDVPTVPRTVW-----------
>Tupaia_chinensis
---MRDTGLTALETEISIVPRTVW-----------
>Trichechus_manatus_latirostr
---MKDTRLTALETEVSIVPRAVW-----------
>Aptenodytes_forsteri
---MKGVWLTALQTEARLVHRTVW-----------
>Struthio_camelus_australis
----------------------------------MDVQTRMKFLIMKGVWLIALETEAQLIHRTVW-----------
>Chrysemys_picta
---MKGARLTALETEVLLVHRTVW-----------
>Orycteropus_afer_afer
---MKETRLTALETEVSIVPQSSLVVAPMKSLWSL

HG0076 ENSDARG00000040008 neurod6a

>Danio_rerio
MGTSSGSWHQCHFS--
>Anser_cygnoides
MGTSSGSWHQCHFLEINEIVQIY--TDLDSRRRCDTQTEKIWKAKNEN-----
>Microhyla_fissipes
MGTSSGSWHQCHFF--
>Canis_lupus_familiaris
MGTSSGSWHQCHFLEINEIVQIY--TDLETPGDDATLSLKRFGRYK--MKTDY
>Hynobius_retardatus
MGTSSGSWHQCHFLEINEIVQLH--TDPDSWRRRGIQTEQTWKPKKWKLIID-
>Lipotes_vexillifer
MGTSSGSWHQCHFLEINEIVQIH--TDLETSGDDATLSLKRCGRSK--MKTDY
>Calidris_pugnax
MGTSSGSWHQCHFLEINEIVQIH--TDLDSRRRCDIQTEKIWKAKNEN-----
>Pteropus_alecto
MGTSSGSWHQCHFLEINEIVQIY--TDLETPGDDATLSLKRFGRSK--MKTDY
>Sarcophilus_harrisii
MGTSSGSWHQCHFLEINEIVQRSIPRSRDSRRRCDIRTEKIWKTKK-------
>Galeopterus_variegatus
MGTSSGSWHQCHFLEINEIVQIY--TDLETPGDDATLSLKRFGRPK--MKTDY
>Macropus_eugenii
MGTSSGSWHQCHFLEINEIVQRSIPRSRDSRRRCDIRTEKIWKTKK-------
>Aquila_chrysaetos_canadensis
MGTSSGSWHQCHFLEINEIVQIY--TDLDSRRRCDIQTEKIWKAKNEN-----
>Gallus_gallus
MGTSSGSWHQCHFLEINEIVQIY--TDLGSRRRCDTQTEKIWKAKNEN-----
>Condylura_cristata
MGTSSGSWHQCHFLEINEIVQIY--TDLETPGDDATLSLKRFGRSK--MKTDY
>Ochotona_princeps
MGTSSGSWHQCHFLEINEIVQIY--TDLETPGDDATFSLKRFGRPK--MKTDY
>Elephantulus_edwardii
MGTSSGSWHQCHFLEINEIVQIY--TDLETRGDDATLSLKRFGRPK--MRTDY
>Ficedula_albicollis
MGTSSGSWHQCHFLEINEIVQIY--TDLDSSRRCDIQTEKIWKAKNEN-----
>Ceratotherium_simum_simum
MGTSSGSWHQCHFLEINEIVQIY--TDLETPGDDATLSLKRFGRSK--MKTDY
>Pongo_abelii
MGTSSGSWHQCHFLEINEIVQIY--TDLETPGDDATLSLKRFGRPK--MKTDY
>Loxodonta_africana
MGTSSGSWHQCHFLEINEIVQIH--TDLETPGDDATLSLKRFGRPK--MRTDY
>Peromyscus_maniculatus_baird
MGTSSGSWHQCHFLEINEIVQIH--TDLEAPGDDATLSLKRFGRSK--MKTDY
>Tupaia_chinensis
MGTSSGSWHQCHFLEINEIVQIH--TDLETPGDDATLSLKRFGRPK--MKTDY
>Trichechus_manatus_latirostr
MGTSSGSWHQCHFLEINEIVQIY--TDLETPGDDATLSLKRFGRPK--MRTDY
>Anolis_carolinensis
MGTSSGSWHQCHFLEINEIVQIY--TDLDSRRRCDIQIEKIWKAKK-------
>Struthio_camelus_australis
MGTSSGSWHQCHFLEINEIVQIH--TDLDSRRRCDIQTEKIWKAKNEN-----
>Chrysemys_picta
MGTSSGSWHQCHFLEINEIVQRY--TDLDSRRRCDIQTEKIWKAKNEN-----
>Tinamus_guttatus
MGTSSGSWHQCHFLEINEIVQIH--TDLGSRRRCDIQTEKIWKAKNEN-----
>Calypte_anna
MGTSSGSWHQCHFLEINEIVQIY--TDLDSRRRCDIQTEKIWKAKNEN-----
>Orycteropus_afer_afer
MGTSSGSWHQCHFLEINEIVQIY--TDLETSGDDATLSLKRFGRPK--MRTDY

HG0076 ENSG00000164600 NEUROD6

>Homo_sapiens
---------------MGTSSGSWHQCHFLEINEIVQIY--TDLETPGDDATLSLKRFG--RPKMKTDY
>Anser_cygnoides
---------------MGTSSGSWHQCHFLEINEIVQI--YTDLDSRRRCDTQTEKIWKAKNEN-----
>Microhyla_fissipes
MHHVVLCPVTSQADGMGTSSGSWHQCHFF---------------------------------------
>Panthera_tigris
---------------MGTSSGSWHQCHFLEINEIVQIY--TDLETPGDDATLSLKRFG--RYKMKTDY
>Hynobius_retardatus
---------------MGTSSGSWHQCHFLEINEIVQL--HTDPDSWRRRGIQTEQTWKPKKWKLIID-
>Orcinus_orca
---------------MGTSSGSWHQCHFLEINEIVQIH--TDLETPGDDATLSLKRCG--RSKMKTDY
>Calidris_pugnax
---------------MGTSSGSWHQCHFLEINEIVQI--HTDLDSRRRCDIQTEKIWKAKNEN-----
>Pteropus_alecto
---------------MGTSSGSWHQCHFLEINEIVQIY--TDLETPGDDATLSLKRFG--RSKMKTDY
>Sarcophilus_harrisii
---------------MGTSSGSWHQCHFLEINEIVQRSIPRSRDSRRRCDIRTEKIWKTKK-------
>Galeopterus_variegatus
---------------MGTSSGSWHQCHFLEINEIVQIY--TDLETPGDDATLSLKRFG--RPKMKTDY
>Macropus_eugenii
---------------MGTSSGSWHQCHFLEINEIVQRSIPRSRDSRRRCDIRTEKIWKTKK-------
>Aquila_chrysaetos_canadensis
---------------MGTSSGSWHQCHFLEINEIVQI--YTDLDSRRRCDIQTEKIWKAKNEN-----
>Gallus_gallus
---------------MGTSSGSWHQCHFLEINEIVQI--YTDLGSRRRCDTQTEKIWKAKNEN-----
>Condylura_cristata
---------------MGTSSGSWHQCHFLEINEIVQIY--TDLETPGDDATLSLKRFG--RSKMKTDY
>Ochotona_princeps
---------------MGTSSGSWHQCHFLEINEIVQIY--TDLETPGDDATFSLKRFG--RPKMKTDY
>Elephantulus_edwardii
---------------MGTSSGSWHQCHFLEINEIVQIY--TDLETRGDDATLSLKRFG--RPKMRTDY
>Scleropages_formosus
---------------MGTSSGSWHQCHCSKREEQGETERK--------DGPGTQRVYGT---------
>Lepidothrix_coronata
---------------MGTSSGSWHQCHFLEINEIVQI--YTDLDSRRRCDIQTEKIWKAKNEN-----
>Ceratotherium_simum_simum
---------------MGTSSGSWHQCHFLEINEIVQIY--TDLETPGDDATLSLKRFG--RSKMKTDY
>Loxodonta_africana
---------------MGTSSGSWHQCHFLEINEIVQIH--TDLETPGDDATLSLKRFG--RPKMRTDY
>Ictidomys_tridecemlineatus
---------------MGTSSGSWHQCHFLEINEIVQIY--TDLEAPGDDATLSLKRFG--RPKMKTDY
>Tupaia_chinensis
---------------MGTSSGSWHQCHFLEINEIVQIH--TDLETPGDDATLSLKRFG--RPKMKTDY
>Trichechus_manatus_latirostr
---------------MGTSSGSWHQCHFLEINEIVQIY--TDLETPGDDATLSLKRFG--RPKMRTDY
>Anolis_carolinensis
---------------MGTSSGSWHQCHFLEINEIVQI--YTDLDSRRRCDIQIEKIWKAKK-------
>Struthio_camelus_australis
---------------MGTSSGSWHQCHFLEINEIVQI--HTDLDSRRRCDIQTEKIWKAKNEN-----
>Chrysemys_picta
---------------MGTSSGSWHQCHFLEINEIVQR--YTDLDSRRRCDIQTEKIWKAKNEN-----
>Tinamus_guttatus
---------------MGTSSGSWHQCHFLEINEIVQI--HTDLGSRRRCDIQTEKIWKAKNEN-----
>Calypte_anna
---------------MGTSSGSWHQCHFLEINEIVQI--YTDLDSRRRCDIQTEKIWKAKNEN-----
>Orycteropus_afer_afer
---------------MGTSSGSWHQCHFLEINEIVQIY--TDLETSGDDATLSLKRFG--RPKMRTDY

HG0077.1 ENSGALG00000005074

```
>Gallus_gallus
------------------MIP-QPDPTTKEKKIVHLRLGRHCN
>Chaetura_pelagica
MLQRFDLTSSSSPEAAVAMIP-QPDPTTKDKRIVRLKLGRRCN
>Apteryx_australis_mantelli
------------------MIP-QPDPTTKEKRIVRLKLGRHCN
>Buceros_rhinoceros_silvestri
------------------MIP-QPDPTTKEKRIVRLKLGRRCN
>Caprimulgus_carolinensis
------------------MIP-QPDPTTKEKRIVCLKLGRRCN
>Cariama_cristata
------------------MIP-QPDPTTKEKRIVCLKLGRRCN
>Charadrius_vociferus
------------------MIP-QPDPTTKEKRIVHLKLGRRCN
>Pterocles_gutturalis
------------------MIP-QPDPTTKEKRIVCLKLGRHCN
>Colius_striatus
------------------MTP-QPDPTTKEKRIVRLKLGHRCN
>Columba_livia
------------------MIP-QPDPTTKEKRIVRLKLGRHCN
>Leptosomus_discolor
------------------MIP-QPDPTTKEKRVVRLKLGRRCN
>Alligator_mississippiensis
------------------MIP-QPDPTTSERRASYFEAEQSLN
>Cuculus_canorus
------------------MIP-QPDPTTKEKRIVRLKLGRRCN
>Falco_cherrug
------------------MIP-QPDPTTKEKRIVRLKLGRRCN
>Gavia_stellata
------------------MIP-QPDPTTKEKRIVRLKLGRRCN
>Chlamydotis_macqueenii
------------------MIP-QPDPTTKEKRIVCLKLGRRCN
>Opisthocomus_hoazin
------------------MIP-QPDPTTKEKRIVCLKLGRRCN
>Manacus_vitellinus
------------------MIP-QPDPTTKEKRIVRLKLGRHCN
>Phaethon_lepturus
------------------MIP-QPDPTTKEKRIVHLKLGRRCN
>Picoides_pubescens
------------------MIP-QPDPTTKEKRVVCLKLGRCCN
>Fulmarus_glacialis
------------------MIP-QPDPTTKEKRIVRLKLGRRCN
>Melopsittacus_undulatus
------------------MIP-QPDPTTKEKRIVCLKLGRRCN
>Pygoscelis_adeliae
------------------MIP-QPDPTTKEKRIVHLKLGRRCN
>Tyto_alba
------------------MIP-QPDPTTKEKRIVHLKLGRHCN
>Struthio_camelus_australis
------------------MIP-QPDPTTKEKRIVHLKLGRHCN
>Chrysemys_picta
MFQRFTLSSSSSPEACQGNDSTARSDHQREKRIVHLKLGRHCN
>Tinamus_guttatus
------------------MTP-QPDPSTKEKRIVRLKQGRHCN
>Calypte_anna
------------------MIP-QPDPTTKEKRIVHLKLGHPCN
>Apaloderma_vittatum
------------------MIP-QPDPTTKEKRIVCLKLGHRCN
```

HG0077.2 ENSGALG00000005074

>Gallus_gallus
MPSGGVLLKLGHCNL
>Caprimulgus_carolinensis
MLSGGVVLKTEHCSL
>Calidris_pugnax
MLSGGVVLKTEHCNL
>Pterocles_gutturalis
MLSGGVVLKTEHCSL
>Columba_livia
MLSGGVVLKTEHCNL
>Aquila_chrysaetos_canadensis
MLSGGVVLKTEHCSL
>Manacus_vitellinus
MLSGGVVLKTEHCSL
>Nipponia_nippon
MLSGGVVLKTEHCSL
>Nestor_notabilis
MLSGGVVLKTEHCSL

HG0078.1 ENSGALG00000016633 HMBOX1

>Gallus_gallus
MVNNTEDPQEAL
>Anas_platyrhynchos
MVNNTEDPQEAL
>Chaetura_pelagica
MVNNTEDPQEAL
>Apteryx_australis_mantelli
MVNNTEDPQEAL
>Buceros_rhinoceros_silvestri
MVNNTEDPQEAL
>Cariama_cristata
MVNNTEDPQEAL
>Charadrius_vociferus
MVNNTEDPQEAL
>Pterocles_gutturalis
MVNNTEDPREAL
>Colius_striatus
MVNNTEDLQEAL
>Leptosomus_discolor
MVNNTEDPQEAL
>Alligator_mississippiensis
MVNNTEDPHEAF
>Cuculus_canorus
MVNNTEDPQEAL
>Falco_cherrug
MVNNTEDPQEAL
>Gavia_stellata
MVNNTEDPQEAL
>Chlamydotis_macqueenii
MVNNTEDPQEAL
>Tauraco_erythrolophus
MVNNTEDPQEAL
>Opisthocomus_hoazin
MVNNTEDPQEAL
>Zonotrichia_albicollis
MVNNTEDPQEAL
>Egretta_garzetta
MVNNTEDPQEAL
>Picoides_pubescens
MRNNTEDPQEAL
>Fulmarus_glacialis
MVNNTEDPREAL
>Melopsittacus_undulatus
MVNNTEDPQEAL
>Aptenodytes_forsteri
MVNNTEDPQEAL
>Tyto_alba
MVNNTEDPQEAL
>Struthio_camelus_australis
MVNNTEDPQEAL
>Pelodiscus_sinensis
MVNNAEDPQEAF
>Tinamus_guttatus
MVNNTEDPYEAL
>Calypte_anna
MVNNTEDPQEAL
>Apaloderma_vittatum
MVNNTEDPQEAL

HG0078.2 ENSG00000147421 HMBOX1

>Homo_sapiens
MVDNADHLWKGY
>Felis_catus
MINNADHL----
>Pteropus_alecto
MVNNADHL----
>Dasypus_novemcinctus
MINYANHLWKGY
>Erinaceus_europaeus
MVDNTDHLWKGY
>Oryctolagus_cuniculus
MINHIDHLWKGY
>Equus_asinus
MVNNADHL----
>Loxodonta_africana
MVNIADHLWKGY
>Heterocephalus_glaber
MVNNADHLWKGY
>Trichechus_manatus_latirostr
MVNNADHLWKGY
>Orycteropus_afer_afer
MINNADHLWKGY

HG0079 ENSG00000204406 MBD5

>Homo_sapiens
MSTIFWREKRYSLIGTRKDIEHLEINCLHYTAESHTTLVMFSSLLSRRLHYWNFEVMKTSDEPTCNTLDSDWKIKRKFKECGL---
>Anas_platyrhynchos
--MKETQE---ESLDNERKIKRKQKECGI---
>Xenopus_laevis
---MKSPGKHCLNLSTDWKIRRKQEECDK---
>Odobenus_rosmarus_divergens
--MKTSDEPTYNILDSDWKMKKKFKECGL---
>Ambystoma_mexicanum
------------MV-SSPEAGSSDLPSL-YTEE--PSSSEFVSLETERLCNQDLGIMRIIQEKASEVFVMDWKIKRKLKESCF---
>Orcinus_orca
------------------------------------MLCFY-QFLCRRLHYWNFEDMKTSDEPTCNILDSDWKMKRKFKECGL---
>Calidris_pugnax
--MKETQE---DNLDNERKIKRKQKECGI---
>Eptesicus_fuscus
MSTVFWREKRYSHIGIHKDIEQLEICCLHYAAGSHSTCYVF-KFLSRRLQYWNFEDMNTSDEPTCNILDSDWKMKRKFKECGL---
>Dasypus_novemcinctus
--MKTSNEPTCNALDSDWKIKRKYKECDL---
>Columba_livia
--MKETQE---DNLDHEWKIKRKQKECGI---
>Alligator_sinensis
--MKESQE---ETLDNEWKIKRKQKECGI---
>Galeopterus_variegatus
--MKTSDEPTCNTLDSDWKIKRKFKECGL---
>Monodelphis_domestica
--MNKSEERIYNTLNIEKKI--KLKECGI---
>Falco_cherrug
--MKETQE---DNLDNERKIKRKQKECGI---
>Gallus_gallus
--MKGTQE---ENLDNERKIKRKQKECGI---
>Condylura_cristata
--MKTSEEPTCNILNSVWKMKRKFKECGL---
>Oryctolagus_cuniculus
--MKTSDEPTCNTLDSDWKIKRKFKECGL---
>Manacus_vitellinus
--MKESQE---DNLDSEQKIKSKQKECGMQRV
>Equus_przewalskii
--MKTSDEPACNILNSDWKMKRKLKECGL---
>Manis_javanica
--MKTSDEPTCSILNSDWKMKRKFKECGL---
>Loxodonta_africana
--MKTSEEPACNTLNSDWKIERKFKECGQ---
>Tupaia_chinensis
--MKTSDEPTCNTLDSDWKIK--FKGCGL---
>Trichechus_manatus_latirostr
--MKTSDEPTCNTLDSDWKIERKFKECGQ---
>Aptenodytes_forsteri
--MKETQE---DNLDNERKIKRKQKECGI---
>Python_bivittatus
--MRKQKECGI---
>Struthio_camelus_australis
--MKESQE---ENLDNEWKIKRKQKECGI---
>Chrysemys_picta
--MKESQEDTLSTLDNEWKIKRKQKECGI---
>Tinamus_guttatus
--MKESQE---ENLDNEWKIKRKQKECGI---
>Orycteropus_afer_afer
--MKTSDEPTCNTLDSDWKIERKFKECGQ---

HG0080.1 ENSG00000179603 GRM8

```
>Homo_sapiens
---------------------------MGPDGSLQKVQPQVVPFLLWQE
>Anser_cygnoides
---------------------MVLYYLLGTDGSLQKLQFQMVNLIL---
>Canis_lupus_familiaris
---------------------------MGPDGSLQKVQPLVVPLLLWQE
>Orcinus_orca
---------------------------MGPDGSLQKVQPQVVPLLLWQE
>Calidris_pugnax
---------------------MILYYLLGTDGSLQKVQLQMVNLIL---
>Myotis_lucifugus
MEIKLNLRGLTVTFQSRKLAEAHRAFLRGPDGSLQKVRPQVVPLLLWQE
>Dasypus_novemcinctus
---------------------------MGPDGSLQKVQPQVVPLLLWRE
>Galeopterus_variegatus
---------------------------MGPDGSLQKEQPQVVPLLLWRE
>Monodelphis_domestica
---------------------------MGPDGSLQKV------------
>Aquila_chrysaetos_canadensis
---------------------MVLYYLLGTDGSLQKVQRQMVNLIL---
>Coturnix_japonica
---------------------MVLYHLLGTDGSLQKVQFQMVNLSL---
>Sorex_araneus
---------------------------MGPDGRLQTVEPQVVLFLLWQE
>Oryctolagus_cuniculus
---------------------------MGPDGSLQKVQPQAVPLLLWQE
>Elephantulus_edwardii
---------------------------MGPNGGLQQTQPQVVLLLLWQE
>Ornithorhynchus_anatinus
---------------------------MGPDDTRWKVRLQTVPLLLQQE
>Lepidothrix_coronata
---------------------MVLYHLLGTDGSLQKVQLQMVSLIL---
>Equus_asinus
---------------------------MGPDGSLQKVQPQVVPLLLWQE
>Manis_javanica
---------------------------MGPDGSLQKVQALVVPLLLRQK
>Loxodonta_africana
---------------------------MGPDGSLQKVQPQVVPLFLWQE
>Mus_musculus
---------------------------MGPDGSLQKVQPQVVPLLLWQE
>Tupaia_chinensis
---------------------------MGPDGSLQKVQAQVVPFLLWQE
>Trichechus_manatus_latirostr
---------------------------MGPDGSLRKVQPQVVPLFLWQE
>Anolis_carolinensis
--------------------MVLYSASLGTDGSLQKGKLQIVHFLL---
>Struthio_camelus_australis
---------------------MVLYYLLGTDGSLQKVQLQMVNLIL---
>Trachemys_scripta
----------------------------------------MVHLLF---
>Tinamus_guttatus
---------------------MVLYYLLGTDGSLQKVQLQMVNFTL---
>Calypte_anna
----------------------------------------MVNLIL---
>Orycteropus_afer_afer
---------------------------MGPDGSLQKVQPQVVPILLWQE
```

HG0080.1 ENSGALG00000039403

```
>Gallus_gallus
--------------MVLY-HLLGTDGSLQKVQFQMVNLNL---
>Anser_cygnoides
--------------MVLY-YLLGTDGSLQKLQFQMVNLIL---
>Leptonychotes_weddellii
---------------------MGPDGSLQKVQPLVVPLLLWQE
>Orcinus_orca
---------------------MGPDGSLQKVQPQVVPLLLWQE
>Calidris_pugnax
--------------MILY-YLLGTDGSLQKVQLQMVNLIL---
>Dasypus_novemcinctus
---------------------MGPDGSLQKVQPQVVPLLLWRE
>Galeopterus_variegatus
---------------------MGPDGSLQKEQPQVVPLLLWRE
>Aquila_chrysaetos_canadensis
--------------MVLY-YLLGTDGSLQKVQRQMVNLIL---
>Oryctolagus_cuniculus
---------------------MGPDGSLQKVQPQAVPLLLWQE
>Lepidothrix_coronata
--------------MVLY-HLLGTDGSLQKVQLQMVSLIL---
>Equus_asinus
---------------------MGPDGSLQKVQPQVVPLLLWQE
>Aotus_nancymaae
MGIHCDVQIKKTCCCPQSLFPMGPDGSLQKVQPQVVPLLLWQE
>Loxodonta_africana
---------------------MGPDGSLQKVQPQVVPLFLWQE
>Fukomys_damarensis
---------------------MGPDGSLQKVQPQVVPLPLCQQ
>Tupaia_chinensis
---------------------MGPDGSLQKVQAQVVPFLLWQE
>Trichechus_manatus_latirostr
---------------------MGPDGSLRKVQPQVVPLFLWQE
>Anolis_carolinensis
--------------MVLYSASLGTDGSLQKGKLQIVHFLL---
>Struthio_camelus_australis
--------------MVLY-YLLGTDGSLQKVQLQMVNLIL---
>Tinamus_guttatus
--------------MVLY-YLLGTDGSLQKVQLQMVNFTL---
>Calypte_anna
----------------------------------MVNLIL---
>Orycteropus_afer_afer
---------------------MGPDGSLQKVQPQVVPILLWQE
```

HG0080.2 ENSG00000179603 GRM8

>Homo_sapiens
--MTRNNSATRLISRT---------------
>Odobenus_rosmarus_divergens
--MTRNNSATGLTSRT---------------
>Myotis_brandtii
--MPRNNSAAGLISRTWIVDLIPTSEPRRLF
>Condylura_cristata
--MTRNNSATGLTSRT---------------
>Oryctolagus_cuniculus
--MTRNNSATGLTSRT---------------
>Chinchilla_lanigera
--MTRNNSATGLTSRT---------------
>Tupaia_chinensis
--MTRNNSATGLTSRT---------------
>Trichechus_manatus_latirostr
MGDPRVTGRGQARLGCSDGADRGRILEHSFSDCLPQVTSWTCPGGTRTCPAEMTRNNSATGLTSRT---------------

HG0080.3 ENSG00000179603 GRM8

>Homo_sapiens
---MEETWRLLGQLSPLSPEISHKCGRCARLTRRRRRRRRR--------------------------------
>Mustela_putorius_furo
---MEDTWRRPGQLSPLSPETSHKCGRGACLARRRRRR-----------------------------------
>Erinaceus_europaeus
---MEATWGWPAQLCPLGPQISHKCGRGAGQARRRRRHGSAGFL--TAPSAGSRE------------------
>Oryctolagus_cuniculus
---MEETWRRLGQLSPLNPESSHKCGRGARLARGGGGGGGGADMELRAPQRASSPRPLRGAGNNSATGLTSRT
>Mus_musculus
MVGAKRLVIPGAIPAAAPRAAWGEWPRAEGREAPGVDQSRPPVGVFVIHMEETRRWLGRLSPLSPETSHKCGRGARLSRWPRRRQR---------------------------------

HG0081.1 ENSDARG00000028228 zbtb18

>Danio_rerio
MMSEQLTGCHPPVFSSNAQTGS---
>Anser_cygnoides
MMSEQLTACHPSAFYSNTQTGS---
>Xenopus_laevis
MMSEQLTACHPSAFYSNTQTGS---
>Apteryx_australis_mantelli
MMSEQLTACHPSAFYSNTQTGS---
>Canis_lupus_familiaris
MMSEQLTACHPSAFYSNTQTGS---
>Balaenoptera_acutorostrata_s
MMSEQLTACHPSAFYSNTQTGS---
>Astyanax_mexicanus
MMSEQLTGCHPPVFSSTAQTGS---
>Calidris_pugnax
MMSEQLTACHPSAFYSNTQTGS---
>Miniopterus_natalensis
MMSEQLTACHPSAFYSNTQTGS---
>Dasypus_novemcinctus
MMSEQLTACHPSAFYSNTQTGS----------------SCVR-SCAGGGI--------------------SFLSNIIS-----------------------TLHLSS------------------------
>Columba_livia
MMSEQLTACHPSAFYSNTQTGS---
>Kryptolebias_marmoratus
MMSEQLTGCHPPHLLLHRLHPSPVLYCESQRQPGRVSALCVCVCVCVRVFAAADQRQLEGGGGRRGGGGRWYLVPPPTSPPLCICPSQFEQRGFFLFVCLFSLPLSLSFFFFFAYLPSSSALLFYPLIDSE
>Monodelphis_domestica
MMSEQLTACHPSAFYSNTQTGS---
>Esox_lucius
MMSEQLTGCHPPVFYSNTQTGS---
>Coturnix_japonica
MMSEQLTACHPSAFYSNTQTGS---
>Erinaceus_europaeus
MMSEQLTACHPSAFYSNTQTGS---
>Oryctolagus_cuniculus
MMSEQLTACHPSAFYSNTQTGS---
>Corvus_brachyrhynchos
MMSEQLTACHPSAFYSNTQTGS---
>Ceratotherium_simum_simum
MMSEQLTACHPSAFYSNTQTGS---
>Manis_javanica
MMSEQLTACHPSAFYSNTQTGS---
>Cercocebus_atys
MMSEQLTACHPSAFYSNTQTGS---
>Cricetulus_griseus
MMSEQLTACHPSAFYSNTQTGS---
>Salmo_salar
MMSEQLTGCHPPVFYSNAQTGS---
>Tupaia_chinensis
MMSEQLTACHPSAFYSNTQTGS---
>Ictalurus_punctatus
MMSEQLTGCHPPVFSSAAQTAS---
>Anolis_carolinensis
MMSEQLTACHPSAFYSNTQTGS----------------ECVCRSVCKEGI--------------------SFLSNIIS-----------------------TLHLSLS-----------------------
>Chelonia_mydas
MMSEQLTACHPSAFYSNTQTGS---
>Tinamus_guttatus
MMSEQLTACHPSAFYSNTQTGS---

HG0081.2 ENSDARG00000028228 zbtb18

```
>Danio_rerio
MIVPIVSYFFFPPPLSVVQRVSKMNDGNGAASEP
>Astyanax_mexicanus
-----------------------MNDSNGAASEP
>Esox_lucius
-----------------------MNDGNGEGSEP
```

HG0082 ENSDARG00000062379 slc35a4

>Danio_rerio
--------------------------------MAEDKDPVTKLKDLVHLKDQLEEIQKKVENEVQAGVPQGGSLLASPFLKGFLAGYVVSRLRSSAVLGVALGTLSGIFAAQNYQVPNIEATLRDILNSLKKRPGE
>Acipenser_sinensis
--------------------------------MADDKDALSKLKDLAHLKNQLEDIQKRVEEEVQAGIPQGGSLLASPFLKGFLAGYVVAKLRSSAVMGVLIGTCTGIYAAQSFDVPNIEKTVKDYFNSIKKGPN-
>Amia_calva
--------------------------------MAGDKDPLSKLKDLAQLKDQLEDIQKRVENEVQAGIPQGGSLLASPFLKGFLAGYVVARLRSSAVLGVVVGTCTGIYAAQNFDVPNIEKSVKDYYNSMKKRPN-
>Odorrana_margaretae
MVAISTCSRPQSVSCSPAVWLSVR--LPWLDNMA-DDKGTPKLKDLAFLKDQLEGLQRKLENEVQAGVSQGGSFLSSPFLKGFLAGYIISKLRASAVLGFAVGAFTGIYAAQAYAVPNVEKTIKDYLGSLRKGPD-
>Porichthys_notatus
--------------------------------MANDKDPLKQLKDLTQLRNQLQEIQRQVENELSVGIPQGSTVWGSPFLKGFLAGYVVAKLRSSAVIGVLLGTVTGMYVAQNYQVPIIDKTFKEYMNHVKNAPK-
>Oryzias_melastigma
--MTIREYMNSLKKGPK-
>Trachinotus_blochii
--------------------------------MADDKDPLKQLKDLTQLRNQLEEIQRRVENEVSVGIPQGGSVLGSPFLKGFLAGYVVAKLRSSAILGVLLGTITGMYAAQNYQVPNIERTVKEYLNNMKKGPK-
>Ursus_maritimus
--------------------------------MADDKDSLPKLKDLAFLKNQLERLQQRVEDEVSSGVSQDGSLMSSPFLKGFLAGYVVAKLRASAVLGFAVGTCTGIYAAQAYAVPNVEKTLRDYLRSLRKGPD-
>Micropterus_floridanus
---MVAKLRSSAIAGVLLGTITGMYVAQSYQVPNIERTVRDYLNNMKKGPK-
>Pteropus_vampyrus
-----------MAGALDIRWEGCACATSSTGKMADDKDSLPKLKDLAFLKNQLEHLQQRVEDEVNSGVGQDGSLLSSPFLKGFLAGYVVAKLRASAVLGFAVGTCTGIYAAQTYAVPNVEKTLRDYLRSLRKGPD-
>Maylandia_zebra
--------------------------------MADDKDPLKQLRDLTQLKNQLDEIQRRVEHEVSAGIPQGGSVLGSPFLKGFLAGYVVARLRSSAFLGVLLGTVTGMYAAQNYQVPNIERTFKEYMNNLKKGPK-
>Dasypus_novemcinctus
--------------------------------MADDKDSLPKLKDLAFLKNQLERLQRRVEDEVNSGVGQDGSLLSSPFLKGFLAGYVVAKLRASAVMGFVVGTCTGIYIAQAYAVPNVEKTLRDYLRSLRKGPD-
>Latimeria_chalumnae
--------------------------------MADDKDTLRKLKDLAQLKDQLEDIQKRVEEEVNAGIPQGGSLLASPFLKGFLTGYVVARLRSSALLGVVVGTCTGIYAAQNYKVPNIENTVKDYISSLKKGSN-
>Fundulus_grandis
--------------------------------MADNKDPLRQLKDLTQLKNQLEAIQRRVENEVSSGIPQGSSVLGSPFLKGFLAGYVVAKLRSSAFLGVLLGTVTGIYAAQNYQVPNVERTVKDYMSTLRKGPK-
>Sarcophilus_harrisii
---MADDKEGSFLSSPFLKGFLAGYVVAKLRASAVMGFAVGLCTGIYAAQSYAVPNVEKTLQDYLRSLRKGPD-
>Esox_lucius
--------------------------------MADDKDPFKQMKDLNQLKNQLESIQKRVESEFATGIPQGGSVLGSPFLKGFLAGYVVAKLRSSAIIGVVLGTFTGIYAAQNYQVPNIERTLKDYMSLFKKGP--
>Gallus_gallus
--------------------------------MADDKDSLPKLKDLALLKGQLESLQRRVEDEVHAGVGQGGSLLASPFLKGFLAGYLVAKLRFSAVLGFAVGTCTGIYAAQNYAVPDVEKTVRDYLSALRKGGE-
>Acanthis_flammea
--------------------------------MADDKDSLPKLKDLAFLKGQLESLQRRVEDEVQAGVGQDGSLLASPLLKGFLAGYLVAKLRFSAVLGFVAGTCTGIYAAQNYAVPNVEKTIRDYVNSLKKGRD-
>Lateolabrax_japonicus
---MYAAQNYQVPNIERTMKDCMNNLRKGPK-
>Trematomus_bernacchii
--------------------------------MAGDKDPLKQLKDLTKLKSQLEEIQRRVENEVTLGIPQGGSVLGSPFLKGFLAGYAVAKLRSSAFLGLLVGTATGIYAAQNYPVPNIERTVKDYINSLKKGPK-
>Equus_asinus
--------------------------------MADDKDSLPKLKDLAFLKDQLESLQRRVEDEVNSGVGQDGSLLSSPFLKGFLAGYVVAKLRASAVLGFAVGTCTGIYAAQAYAVPNVEKTLRDYLRSLRKGPD-
>Manis_javanica
--------------------------------MADDKDSLPKLKDLAFLKNQLERLQQRVEDEVNSGVSQDGSLLSSPFLKGFLAGYVVAKLRASAVLGFAVGTCTGIYAAQAYAVPNVEKTLRDYLRSLRKGPD-
>Platichthys_stellatus
--------------------------------MADDKDPLKQLRDLTHLKSQLEEIQRRVESEASLGIPQGGSVLGSPLLKGFLAGYVVAKLRSSAILGVLLGTLTGIYAAQSYQVPNIERTVKDYMNTFKKGPK-
>Nomascus_leucogenys
--------------------------------MADDKDSLPKLKDLAFLKNQLERLQRRVEDEVSSGVDQDGSLLSSPFLKGFLAGYVVAKLRASAVLGFAVGTCTGIYAAQAYAVPNVEKTLRDYLQSLRKGPD-
>Marmota_monax
--------------------------------MADDKDSLPKLKDLAFLKNQLERLQQRVEDEVSSGVGQDGSLLSSPFLKGFLAGYVVAKLRASAVLGFAVGTFTGIYAAQAYAVPNVEKTLRDYFRSLRKGPD-
>Salmo_salar
--------------------------------MAGDKDPFKQMKDLNQLKNQLEDIQKRVETEFAAGIPQGGSVLASPFLKGFLAGYVVAKLRSSAIIGVLLGTFTGIYAAQNYQVPNIESTLKDYMSLFRKGPK-
>Trichomycterus_areolatus
--------------------------------MANDKDPLSQVKDLALLKDQLADIQKRVENEVQAGIPQGGSLLASPFLKGFLAGYVVARLRASAVLGVALGTLTGIYAAQNYEVPNIERTIKDYLNSLKKGPGN
>Gekko_japonicus
--------------------------------MADDKDSLPKLKDLAFLKDQLANLQQRVENEVQTGVGQEGSLLASPFLKGFLAGYVVAKLRSSAVLGFLVGTCSGVYVAQTYAIPNVEKTIKEFFSSFRKGPD-

HG0082 ENSG00000176087 SLC35A4

>Homo_sapiens
-------------------MADDKDSLPKLKDLAFLKNQLESLQRRVEDEVNSGVGQDGSLLSSPFLKGFLAGYVVAKLRASAVLGFAVGTCTGIYAAQAYAVPNVEKTLRDYLQLLRKGPD-
>Acipenser_sinensis
-------------------MADDKDALSKLKDLAHLKNQLEDIQKRVEEEVQAGIPQGGSLLASPFLKGFLAGYVVAKLRSSAVMGVLIGTCTGIYAAQSFDVPNIEKTVKDYFNSIKKGPN-
>Amia_calva
-------------------MAGDKDPLSKLKDLAQLKDQLEDIQKRVENEVQAGIPQGGSLLASPFLKGFLAGYVVARLRSSAVLGVVVGTCTGIYAAQNFDVPNIEKSVKDYYNSMKKRPN-
>Pelophylax_nigromaculatus
-------------------MADD-KGSPKLKDLAFLKNQLEGLQRKLENEVQAGVSQEGSFLSSPFLKGFLAGYIVSKLRASAVLGFAVGAFTGIYAAQAYAVPNVEKTIKDYLGSLRKGPD-
>Porichthys_notatus
-------------------MANDKDPLKQLKDLTQLRNQLQEIQRQVENELSVGIPQGSTVWGSPFLKGFLAGYVVAKLRSSAVIGVLLGTVTGMYVAQNYQVPIIDKTFKEYMNHVKNAPK-
>Oryzias_melastigma
---MTIREYMNSLKKGPK-
>Trachinotus_blochii
-------------------MADDKDPLKQLKDLTQLRNQLEEIQRRVENEVSVGIPQGGSVLGSPFLKGFLAGYVVAKLRSSAILGVLLGTITGMYAAQNYQVPNIERTVKEYLNNMKKGPK-
>Ursus_maritimus
-------------------MADDKDSLPKLKDLAFLKNQLERLQQRVEDEVSSGVSQDGSLMSSPFLKGFLAGYVVAKLRASAVLGFAVGTCTGIYAAQAYAVPNVEKTLRDYLRSLRKGPD-
>Micropterus_floridanus
--MVAKLRSSAIAGVLLGTITGMYVAQSYQVPNIERTVRDYLNNMKKGPK-
>Rhinolophus_ferrumequinum
-------------------MADDKDSLPKLKDLAFLKNQLERLQQRVEDEVNSGMGQDGSLLSSPFLKGFLAGYVVAKLRASAVLGFAVGTCTGIYAAQAYAVPNVEKTLRDYLRSLRKGPD-
>Maylandia_zebra
-------------------MADDKDPLKQLRDLTQLKNQLDEIQRRVEHEVSAGIPQGGSVLGSPFLKGFLAGYVVARLRSSAFLGVLLGTVTGMYAAQNYQVPNIERTFKEYMNNLKKGPK-
>Dasypus_novemcinctus
-------------------MADDKDSLPKLKDLAFLKNQLERLQRRVEDEVNSGVGQDGSLLSSPFLKGFLAGYVVAKLRASAVMGFVVGTCTGIYIAQAYAVPNVEKTLRDYLRSLRKGPD-
>Latimeria_chalumnae
-------------------MADDKDTLRKLKDLAQLKDQLEDIQKRVEEEVNAGIPQGGSLLASPFLKGFLTGYVVARLRSSALLGVVVGTCTGIYAAQNYKVPNIENTVKDYISSLKKGSN-
>Pimephales_promelas
--MRSSAILGVALGTLTGIYAVQNYQVPNIEQTVRDFLNSLKKGPRD
>Fundulus_grandis
-------------------MADNKDPLRQLKDLTQLKNQLEAIQRRVENEVSSGIPQGSSVLGSPFLKGFLAGYVVAKLRSSAFLGVLLGTVTGIYAAQNYQVPNVERTVKDYMSTLRKGPK-
>Sarcophilus_harrisii
--MADDKEGSFLSSPFLKGFLAGYVVAKLRASAVMGFAVGLCTGIYAAQSYAVPNVEKTLQDYLRSLRKGPD-
>Esox_lucius
-------------------MADDKDPFKQMKDLNQLKNQLESIQKRVESEFATGIPQGGSVLGSPFLKGFLAGYVVAKLRSSAIIGVVLGTFTGIYAAQNYQVPNIERTLKDYMSLFKKGP--
>Gallus_gallus
-------------------MADDKDSLPKLKDLALLKGQLESLQRRVEDEVHAGVGQGGSLLASPFLKGFLAGYLVAKLRFSAVLGFAVGTCTGIYAAQNYAVPDVEKTVRDYLSALRKGGE-
>Acanthis_flammea
-------------------MADDKDSLPKLKDLAFLKGQLESLQRRVEDEVQAGVGQDGSLLASPLLKGFLAGYLVAKLRFSAVLGFVAGTCTGIYAAQNYAVPNVEKTIRDYVNSLKKGRD-
>Lateolabrax_japonicus
--MYAAQNYQVPNIERTMKDCMNNLRKGPK-
>Trematomus_bernacchii
-------------------MAGDKDPLKQLKDLTKLKSQLEEIQRRVENEVTLGIPQGGSVLGSPFLKGFLAGYAVAKLRSSAFLGLLVGTATGIYAAQNYPVPNIERTVKDYINSLKKGPK-
>Equus_asinus
-------------------MADDKDSLPKLKDLAFLKDQLESLQRRVEDEVNSGVGQDGSLLSSPFLKGFLAGYVVAKLRASAVLGFAVGTCTGIYAAQAYAVPNVEKTLRDYLRSLRKGPD-
>Manis_javanica
-------------------MADDKDSLPKLKDLAFLKNQLERLQQRVEDEVNSGVSQDGSLLSSPFLKGFLAGYVVAKLRASAVLGFAVGTCTGIYAAQAYAVPNVEKTLRDYLRSLRKGPD-
>Cynoglossus_semilaevis
--------MSRIPSTPPSKMANDKDPLKQLKDLTQLKNQLEEIQRRVENEISAGIPQGGSVLGSPFLKGFLAGYVVAKLRSSAILGLVIGTCTGIYAAQNYQVPNIEQTVKEYMRNIKKGPK-
>Heterocephalus_glaber
-------------------MADDKDSLPKIKDLAFLKNQLERLQQRVENEVNSGVGQDGSLLSSPFLKGFLAGYVVAKLRASAVLGFAVGTCTGIYAAQAYALPNVEKTLRDYFQSLRKGPD-
>Salmo_salar
MLLSTPGIRVDHRAFNRAKMADDKDPFKQMKNLNQLKNQLEEIQKRVESEFAEGIPQGGSVLASPFLKGFLAGYMVAKLRSSAVIGVVLGTFTGIYAAQNYQVPNIESTLKDYMSLFRKGPK-
>Ictalurus_punctatus
-------------------MANGKDPLTQLKDLAQLKDQLEDIQKRVENEVQIGIPQGGSLLASPFLKGFLAGYVVAKLRASAVLGVALGTLTGIYTAQTYQVPNIETTIKDYLNSLKKGPNN
>Gekko_japonicus
-------------------MADDKDSLPKLKDLAFLKDQLANLQQRVENEVQTGVGQEGSLLASPFLKGFLAGYVVAKLRSSAVLGFLVGTCSGVYVAQTYAIPNVEKTIKEFFSSFRKGPD-

HG0083.1 ENSGALG00000008039 MFSD13A

>Gallus_gallus
MDWPRQRCNRCCEKTSHLKWR
>Anser_cygnoides
MDWPQQRCSRCWGKTSHLKWR
>Chaetura_pelagica
MDWLQQRHSRCWEKTSHLKWR
>Apteryx_australis_mantelli
MDWPQQRSSRCWEKTSHLKWR
>Buceros_rhinoceros_silvestri
MDWPRQRCSRCWEKTSHPKWR
>Cariama_cristata
MDWPQQRCSRCWEKTSHLKWR
>Charadrius_vociferus
MDWPQQRCSRCWEKTSHLKWR
>Pterocles_gutturalis
MDWLQQGCSRCWEKTSHLKWR
>Colius_striatus
MDWPQQRCRRCWEKTSHVKWR
>Columba_livia
MDWPQQRCSRCWDETSHLKWR
>Merops_nubicus
MDWPQQRCSRCWEKTSHLKWR
>Cuculus_canorus
MDLPQQRCSRCWEKTSHLKWR
>Falco_cherrug
MDWPQQRCSRCWEKTSHLTWR
>Gavia_stellata
MDWPQKICSRCWEKTSHLKWR
>Eurypyga_helias
MDWPQQRCSRCWEKTSHLKWR
>Tauraco_erythrolophus
MDWSQQRCSRCWEKTFHLKWR
>Opisthocomus_hoazin
MDWPQQRCGRCWEKASHLKWR
>Sturnus_vulgaris
MDWPQQRCSRCWEKTSHLKWR
>Egretta_garzetta
MDWPQQICSRCWEKTSHLKWR
>Picoides_pubescens
MDQLQQRCSWCWEKTSHVKWR
>Fulmarus_glacialis
MDWPQQISSRCWEKTSHVKWR
>Nestor_notabilis
MDWPQKRCRRCWDKTSHLKWR
>Aptenodytes_forsteri
MDWPQQICSRCWEKTSHLKWR
>Tyto_alba
MDWPQQRCSRCWEKTSHLKWR
>Struthio_camelus_australis
MDWPQQRSSRCWEKTSHLKWR
>Chelonia_mydas
MDWPPTKVNRCCGKVSHLKWR
>Calypte_anna
MDWPQQRCSRCWEKTSHLKWR
>Apaloderma_vittatum
MDWPQQRCNRCWEKISHLKWR

HG0083.2 ENSG00000138111 MFSD13A

>Homo_sapiens
MGRPPVAMSPPRCSWG
>Ailuropoda_melanoleuca
MARLLLDMSAPRCSWG
>Balaenoptera_acutorostrata_s
MARLPVAMSAPQYSWG
>Dasypus_novemcinctus
MARLPVAMSPPQCSCG
>Ceratotherium_simum_simum
MARLLVAMSAPRCFWG
>Cavia_porcellus
MARPPVISSPPRCSWG

HG0084.1 ENSGALG00000009207 TRIM2

>Gallus_gallus
MMFYSFGLLRNCSSIPARILSSALH-
>Anser_cygnoides
MMFCSFGLLRNCSSIPARIWSSALH-
>Chaetura_pelagica
MMVCSFGLLRNCSSIPARILSSALH-
>Apteryx_australis_mantelli
MMFYSFGLLRNCSSVPARILSSALH-
>Buceros_rhinoceros_silvestri
MMVCSFGLLRNCSSSLQGFYPQHCID
>Caprimulgus_carolinensis
MMVCSFGFLRNCSSVPARILSSALH-
>Cariama_cristata
MMVCSFGLLRNCSSVPARILSSALH-
>Calidris_pugnax
MMVYSFGLLRNCSSVPARILSSALH-
>Pterocles_gutturalis
MMVCSFGFLRNYSSVPARILSSALH-
>Colius_striatus
MMVCSFGLLRNCSSVPARILSSALH-
>Merops_nubicus
MMVCSFGLLRNCSSVPARILSSALH-
>Cuculus_canorus
MMVCSFGLLRNCSSVPARILSSARH-
>Aquila_chrysaetos_canadensis
MMVCSFGLLRNCSSVPARILSSALH-
>Gavia_stellata
MMVCSFGLLRNCSSVPARILSSALH-
>Balearica_regulorum_gibberic
MMVCSFGLLRNCSSVPARILSSALH-
>Tauraco_erythrolophus
MMVCSLGLLRNCSSVPARILSSALH-
>Opisthocomus_hoazin
MMVCSFGLLRNCSSVPARILSSALH-
>Lepidothrix_coronata
MMVCSFGLLRNCSSVPARILSSALH-
>Pelecanus_crispus
MMVCSFGLLRNCSSVPARILSSALH-
>Picoides_pubescens
MMVCSFGLLRNCSSVPARILSSALH-
>Fulmarus_glacialis
MMVCSFGLLRNCSSVPARILSSALH-
>Melopsittacus_undulatus
MMVCSFGLLRNCSSVPARILSSALH-
>Tyto_alba
MMVCSFGLLRNCSSVPARILSSALH-
>Struthio_camelus_australis
MMICSFGLLRNCSSVPARILSSALH-
>Chelonia_mydas
-MFCSFGLLRNCSSIPERILYAALL-
>Tinamus_guttatus
MMFCSPGLLRNCSSVPARILSSALY-
>Calypte_anna
MMVCSLGLLWNCSSVPARILSSALH-
>Apaloderma_vittatum
MMVCSFGLLRNCSSVSARILSSALH-

HG0084.2 ENSG00000154447 SH3RF1

>Homo_sapiens
MSHFKGCTQL
>Canis_lupus_familiaris
MSHFKGCTEL
>Equus_asinus
MNHFKGCTQL
>Nannospalax_galili
MNHFKGCTQL
>Orycteropus_afer_afer
MSHYKGCTQL

HG0085 ENSG00000107779 BMPR1A

>Homo_sapiens
MHQFNTVLEFMRWKHRSKLFGENQKYSFI------------------------------
>Polypedates_megacephalus
MHQFDTVLELMNWKYG---
>Chaetura_pelagica
MHFHNITFELMKWKYR---
>Odobenus_rosmarus_divergens
MHQFNTVLEFMRWKHRSKLFGENWKYSFI------------------------------
>Orcinus_orca
MHQFNTVLEFMRWKHRSKLFGENWKYSFI------------------------------
>Calidris_pugnax
MHFHNTTFELMKWKYR---
>Rousettus_aegyptiacus
MHQFNTVLEFMRWKHRSKLFGENWKSSFI------------------------------
>Dasypus_novemcinctus
MHQFNTVLEFMRWKHRSKLFGENWKYNFI------------------------------
>Alligator_mississippiensis
MHCYNIILELMKWKYR---
>Monodelphis_domestica
MCHFNIALELMKWKYRSKQFGENRKSSFIQQTLLRKQETAVEVGIIIKLLQAEFH----
>Falco_cherrug
MHFHNITFELMKWKYR---
>Gallus_gallus
MRFHNTTFELMKWKYR---
>Gavia_stellata
MHFHNITFELMKWKYR---
>Chlamydotis_macqueenii
MHCHSITFELMKWKYR---
>Erinaceus_europaeus
MHQFNTVLEFMRWKHRSKVFGENWKYSFI------------------------------
>Oryctolagus_cuniculus
MHPFNTVLEFMRWKHRSKPFRENWKCSSVWAHL--------------------------
>Ornithorhynchus_anatinus
MHFFNTVLELMKWKYRSKQFEEIWKYSLARPFSKESGNCSWSSS--------NY-----
>Opisthocomus_hoazin
MHFHNITFELMKWKYR---
>Parus_major
MHFHNTTFELMKWKYR---
>Equus_asinus
MHQFNTVLEFMRWKHRSKLFGENWKYSFI------------------------------
>Manis_javanica
MHQFNTVLEFMRWKHRSKLFGENWKHSFI------------------------------
>Picoides_pubescens
MHFHNTTFELMKWKYR---
>Loxodonta_africana
MHQFNTVLEFMRWKHRSKLFGENWKHSFI------------------------------
>Melopsittacus_undulatus
MHFHNITFELMKWKYR---
>Marmota_monax
MHQLNTVLEFMRWKHRSKLFGENWKYSFIATSL-RSEEKAIRGGVIVKWLWPSTRNLIE
>Tupaia_chinensis
MHPFNTVLEFMRWKHRSKPRGEDLKYGFI------------------------------
>Trichechus_manatus_latirostr
MHQFNTVLEFMRWKHRSKLFGENWKYSFI------------------------------
>Tinamus_guttatus
MHFHNTTFELMKWKYR---

HG0085 ENSGALG00000002003 BMPR1A

```
>Gallus_gallus
------MRFHNTTFELMKWKYR---------------------------------------
>Anas_platyrhynchos
MEIDRNMHFHSITFELMKWKYR---------------------------------------
>Chaetura_pelagica
------MHFHNITFELMKWKYR---------------------------------------
>Calidris_pugnax
------MHFHNTTFELMKWKYR---------------------------------------
>Alligator_mississippiensis
------MHCYNIILELMKWKYR---------------------------------------
>Monodelphis_domestica
------MCHFNIALELMKWKYRSKQFGENRKSSFIQQTLLRKQETAVEVGIIIKLLQAEFH
>Falco_cherrug
------MHFHNITFELMKWKYR---------------------------------------
>Gavia_stellata
------MHFHNITFELMKWKYR---------------------------------------
>Balearica_regulorum_gibberic
------MHFHNITFELMKWKYR---------------------------------------
>Ornithorhynchus_anatinus
------MHFFNTVLELMKWKYRSKQFEEIWKYSLARPFSKESGNCSWSSS--------NY-
>Opisthocomus_hoazin
------MHFHNITFELMKWKYR---------------------------------------
>Parus_major
------MHFHNTTFELMKWKYR---------------------------------------
>Picoides_pubescens
------MHFHNTTFELMKWKYR---------------------------------------
>Melopsittacus_undulatus
------MHFHNITFELMKWKYR---------------------------------------
>Struthio_camelus_australis
------MQFHNITFELMKWKYR---------------------------------------
>Chrysemys_picta
------MHCYNIILELMKWKYR---------------------------------------
>Tinamus_guttatus
------MHFHNTTFELMKWKYR---------------------------------------
```

HG0086 ENSGALG00000009835 XPOT

>Gallus_gallus
MLVATSELWGGSRHRRDGGVTGKMIVISTLEEVGKPVSRSRKRRV-
>Anser_cygnoides
-----------------------MIVGSPLEEVGKPVSRSKKPIV-
>Rana_clamitans
-----------------------MLVDSAVPERGKPIVRNRELEV-
>Chaetura_pelagica
-----------------------MIVASPLEEVGKPVRSRKQRI--
>Apteryx_australis_mantelli
-----------------------MIVVSPLEEVGKPVRNRKQRV--
>Buceros_rhinoceros_silvestri
-----------------------MIVVSPLEEVGKPVRSRKQRV--
>Caprimulgus_carolinensis
-----------------------MIVVSPLKEVGKPVESRKRRVQQ
>Cariama_cristata
-----------------------MIVVSPLEEVGKPVRSRKQRV--
>Hynobius_chinensis
-----------------------MFVVYAEGDGGEPSKENRKLEL-
>Charadrius_vociferus
-----------------------MIVVSLLEEVGKPVRSRKQRV--
>Colius_striatus
-----------------------MIVVSPVEEVGKPVRSRKQKV--
>Columba_livia
-----------------------MIVGSPLEEVGKPVRSRKQRV--
>Merops_nubicus
-----------------------MIVVSPLEEVGKPVRSRKQRA--
>Cuculus_canorus
-----------------------MIVVSLLEEVGKPVRSRKRSV--
>Haliaeetus_leucocephalus
-----------------------MIVVSSLEEVGKPVRSRKQRV--
>Gavia_stellata
-----------------------MIVVFLLEEVGKPVRSRKQRV--
>Mesitornis_unicolor
-----------------------MIVVSPLEEVGKPVRNRKQRV--
>Tauraco_erythrolophus
-----------------------MVVVSPLEEVGKPVGSRKQRV--
>Opisthocomus_hoazin
-----------------------MIVASPLEEVGKPVTCRKQRVQQ
>Manacus_vitellinus
-----------------------MIVVSPLEEVGKPVRSRKQRV--
>Phaethon_lepturus
-----------------------MVVVSPLEEVGKPVRSRKQRV--
>Fulmarus_glacialis
-----------------------MIVVSPLEEVGKPVRSRKQRVYQ
>Melopsittacus_undulatus
-----------------------MMVVPPLEEVGRPVRSRKQRV--
>Pygoscelis_adeliae
-----------------------MIVVSPLEEVGKPVRSRKQRV--
>Tyto_alba
-----------------------MFVVSPLEEVGKPVRSRKQRV--
>Struthio_camelus_australis
-----------------------MIVVSPLEEVGKAVRNRKQRV--
>Chrysemys_picta
-----------------------MFVVSTIGEGGKPVKNRKQKV--
>Apaloderma_vittatum
-----------------------MIVVSPLEEVGKPVRSRRQKAEQ

HG0087 ENSG00000164548 TRA2A

>Homo_sapiens
-------------------------MWKINQDKRRLMKSIKDQKIKIEWGKKLNRRLVR---------
>Xenopus_tropicalis
-----MSDVEGRVNVREEMEEFFPRILKIKQDKRRLMKSFKDQKKSIEWGNFLNRRLGR---------
>Canis_lupus_familiaris
MSDVEENNFEGRVNVREEIEEFFPRMWKINQDKRRLMKSIKDQKIKIEWGKKLNRRLVR---------
>Orcinus_orca
MSDVEENNFEGRVNVREEIEEFFPRMWKINQDKRRLMKSIKDQKIKIEWGKKLNRRLVR---------
>Calidris_pugnax
MSDVEENNFEGRVNVREEIEEFFPRMWKINQDKRRLMKSIKDQKIKIEWGKKLNRRLVR---------
>Myotis_brandtii
MSDVEENNFEGRVNVREEIEEFFPRMWKINQDKRRLMKSIKDQKIKIEWGKKLNRRLVR---------
>Haplochromis_burtoni
------------------------------------MKTYTEQNIELEREEVYRKNGVR---------
>Dasypus_novemcinctus
MSDVEENNFEGRVNVREEIEEFFPRMWKINQDKRRLMKSIKDQKIKIEWGKKLNRRLVR---------
>Latimeria_chalumnae
MSDVEENTFEGRVNVREEIEEFYPRMWKNNQDKRRLMKNVKDQKIKIEWGKKLNRRNVR---------
>Columba_livia
MSDVEENNFEGRVNVREEIEEFFPRMWKINQDKRRLMKSIKDQKIKIEWGKKLNRRLVR---------
>Sarcophilus_harrisii
-------------------------MWKINQDKRRLMKSIKDQKIKIEWGKKLNRRLVR---------
>Aquila_chrysaetos_canadensis
MSDVEENNFEGRVNVREEIEEFFPRMWKINQDKRRLMKSIKDQKIKIEWGKKLNRRLVR---------
>Coturnix_japonica
MSDVEENNFEGRVNVREEIEEFFPRMWKINQDKRRLMKSIKDQKIKIEWGKKLNRRLVR---------
>Condylura_cristata
MSDIEENNFEGRVNVREEIEEFFPRMWKINQDKRRLMKSIKDQKIKIEWGKKLNRRLVR---------
>Ochotona_princeps
MSDVEENNFEGRVNVREEIEEYFPRMWKINQDKRRLMKSIKDQKIKIEWGKKLNRRLVR---------
>Scleropages_formosus
MSDIEDGNFGGRVIGRDGFEEFYPRMWKNIQDKRRLMKTCTDQKI--RWEGKNERKNGQIEALALCHV
>Parus_major
MSDVEENNFEGRVNVREEIEEFFPRMWKINQDKRRLMKSIKDQKIKIEWGKKLNRRLVR---------
>Lateolabrax_japonicus
------------------------------------MKTCTEQNIEVEREEM----------------
>Anoplopoma_fimbria
------------------------------------MKTCTEQNIEVEREEM----------------
>Equus_asinus
MSDVEENNFEGRVNVREEIEEFFPRMWKINQDKRRLMKSIKDQKIKIEWGKKLNRRLVR---------
>Manis_javanica
MSDIEENNFEGRVNVREEIEEFFPRMWKINQDKRRLMKSIKDQKIKIEWGKKLNRRLVR---------
>Cynoglossus_semilaevis
------------------------------------MKTCTEQYIEVQRVENVTEEWCQIEALVV---
>Marmota_marmota_marmota
MSDVEENNFEGRVNVREEIEEFFPRMWKINQDKRRLMKSIKDQKIKIEWGKKLNRRLVR---------
>Trichechus_manatus_latirostr
-------------------------MWKINQDKRRLMKSIKDQKIKIEWGKKLNRRLVR---------
>Pygoscelis_adeliae
MSDVEENNFEGRVNVREEIEEFFPRMWKINQDKRRLMKSIKDQKIKIEWGKKLNRRLVR---------
>Struthio_camelus_australis
-------------------------MWKINQDKRRLMKSIKDQKIKIEWGKKLNRRLVR---------
>Pelodiscus_sinensis
-------------------------MWKINQDKRRLMKSIKDQKIKIEWGKKLNRRLVR---------
>Tinamus_guttatus
-------------------------MWKINQDKRRLMKSIKDQKIKIEWGKKLNRRLVR---------

HG0088 ENSG00000273079 GRIN2B

>Homo_sapiens
---MQARRRDWTF----PTCSLP
>Anas_platyrhynchos
---MQARRGDWTF----PTCSLP
>Chaetura_pelagica
---MQARRGDWTF----PTCSLP
>Apteryx_australis_mantelli
---MQARRGDWTF----PTCSLP
>Canis_lupus_familiaris
---MPARRGDWTF----PTCSLP
>Orcinus_orca
---MLFLLFILRLSYVDMQARRHDWIY----PTCSLP
>Charadrius_vociferus
---MQARRGDWTF----PTCSLP
>Eptesicus_fuscus
---MQARRGAWTF----PTCSLP
>Columba_livia
---MQARRGDWTF----PTCSLP
>Cuculus_canorus
---MQARRGDWTF----PTCSLP
>Haliaeetus_leucocephalus
---MQARRGDWTF----PTCSLP
>Coturnix_japonica
---MQARRGAWTF----PTCSLP
>Condylura_cristata
---MQARREDWTF----STCLLP
>Ochotona_princeps
---MILRSFILCLPYVDMQARRGDCTFPTSFPTCSLP
>Elephantulus_edwardii
---MIFLSFTLRLSYVDMQARRGDWTS----PTCSLP
>Opisthocomus_hoazin
---MQARRGDWTF----PTCSLP
>Manacus_vitellinus
---MQARRGDWTF----PTCSLP
>Nipponia_nippon
---MQARRGDWTF----PTCSLP
>Manis_javanica
---MQARRGDWTP----PPCSLP
>Ctenomys_sociabilis
---MQARRGDWTF----PTCSLP
>Tupaia_chinensis
---MISRSFILRLSYVDMKARRGDWTS----PTCPLP
>Trichechus_manatus_latirostr
---MIFLSFILRLSYVDMQARRGDWTF----PTCSLP
>Aptenodytes_forsteri
---MQARRGDWTF----PTCSLP
>Thamnophis_sirtalis
MISLDPAYFDFPLYWIRFILEGDILLCLCTTSRRKIASVEASCKKAVSMCIWHRPFPICSPNLILLYVDMQARRGDWIF----PTCSLP
>Struthio_camelus_australis
---MQARRGDWTF----PTCSLP
>Chrysemys_picta
---MQARREVWIV----PTCSLL
>Tinamus_guttatus
---MQARRGDWTF----PTCSLP
>Orycteropus_afer_afer
---MIFLSFTLRLSYVDMQARRGDWTF----PTCSLP

HG0089.1 ENSG00000092421 SEMA6A

>Homo_sapiens
MTKACEGESAA--RGPERSPR----
>Chaetura_pelagica
MTKDCKGET--GSSFPERSP-----
>Canis_lupus_familiaris
MTKACRGESAA--RGPERSPR----
>Hynobius_retardatus
MTKDCQGESAAGSRFPERSP-----
>Balaenoptera_acutorostrata_s
MTKACEGESAA--RGPERSPR----
>Calidris_pugnax
MTKDCKGES--GSSFPERSP-----
>Miniopterus_natalensis
MTKACEGESAA--RGPERSPR----
>Columba_livia
MTKDCKGES--GSSFPERSP-----
>Cuculus_canorus
MTKDCQGES--GSSFPERSP-----
>Galeopterus_variegatus
MTKACEGENTA--RGPKRAPR----
>Monodelphis_domestica
MTKDCEGESGP--RSPERSPI----
>Falco_cherrug
MTKDCKGES--GSSFPERSP-----
>Coturnix_japonica
MTKDCKGES--GSSFPERSP-----
>Sorex_araneus
MTKACKGESAA--RGPERSPSIMDY
>Oryctolagus_cuniculus
MTKACGGESAA--RGPERAPR----
>Lepidothrix_coronata
MTKDCKGES--GSSFPERSPKM-DY
>Egretta_garzetta
MTKDCKGES--GSSFPERSP-----
>Ceratotherium_simum_simum
MTKACEGESAA--RGPERSPR----
>Manis_javanica
MTKACEAESAA--RGPESSPR----
>Picoides_pubescens
MTKDCKGES--GSSFPERSP-----
>Loxodonta_africana
MTKACEGESAA--RSPERSPSIMDY
>Heterocephalus_glaber
MTKACKAESAA--RGPEKSPR----
>Trichechus_manatus_latirostr
MTKACEGESAA--RGPERSPR----
>Python_bivittatus
MTKDCKGES--GSSFAERSP-----
>Struthio_camelus_australis
MTKDCKGES--GSSFPERSP-----
>Chrysemys_picta
MTKDCKGES--GSSFPERSP-----
>Orycteropus_afer_afer
MTKACKGESEA--RCPEKSPR----

HG0089.1 ENSGALG00000002294 SEMA6A

>Gallus_gallus
MTKDCKGES--GSSFPERSP-------------------
>Nanorana_parkeri
MTKDCQRVP-TGSAFPERSLSV---DY------------
>Chaetura_pelagica
MTKDCKGET--GSSFPERSP-------------------
>Odobenus_rosmarus_divergens
MTKACKGESAA--CGPERSPR------------------
>Hynobius_retardatus
MTKDCQGESAAGSRFPERSP-------------------
>Lipotes_vexillifer
MTKACEGESAA--RGPERSPR------------------
>Calidris_pugnax
MTKDCKGES--GSSFPERSP-------------------
>Miniopterus_natalensis
MTKACEGESAA--RGPERSPR------------------
>Columba_livia
MTKDCKGES--GSSFPERSP-------------------
>Alligator_mississippiensis
MTKDCTGERGCGV--PRAVPGHGLLNGTLPQLGLSSGCR
>Cuculus_canorus
MTKDCQGES--GSSFPERSP-------------------
>Monodelphis_domestica
MTKDCEGESGP--RSPERSPI------------------
>Falco_cherrug
MTKDCKGES--GSSFPERSP-------------------
>Sorex_araneus
MTKACKGESAA--RGPERSPSI--MDY------------
>Oryctolagus_cuniculus
MTKACGGESAA--RGPERAPR------------------
>Ficedula_albicollis
MTKDCKGES--GSSFPERSPKM---DY------------
>Egretta_garzetta
MTKDCKGES--GSSFPERSP-------------------
>Equus_caballus
MTKACKGESTA--RGPERSPR------------------
>Picoides_pubescens
MTKDCKGES--GSSFPERSP-------------------
>Cebus_capucinus_imitator
MTKACEGESAA--RGPERSPR------------------
>Loxodonta_africana
MTKACEGESAA--RSPERSPSI--MDY------------
>Heterocephalus_glaber
MTKACKAESAA--RGPEKSPR------------------
>Trichechus_manatus_latirostr
MTKACEGESAA--RGPERSPR------------------
>Takydromus_sexlineatus
MTKDCKGES--GSSFAERSP-------------------
>Struthio_camelus_australis
MTKDCKGES--GSSFPERSP-------------------
>Chrysemys_picta
MTKDCKGES--GSSFPERSP-------------------
>Orycteropus_afer_afer
MTKACKGESEA--RCPEKSPR------------------

HG0089.2 ENSG00000092421 SEMA6A

>Homo_sapiens
MNTSRLHPRK
>Balaenoptera_acutorostrata_s
MNTARLHPRK
>Rhinolophus_ferrumequinum
MNTERLHPRK
>Galeopterus_variegatus
MNTARLHPRK
>Sorex_araneus
MNTARLHPRK
>Oryctolagus_cuniculus
MNTARLHPRK
>Equus_asinus
MNTARLHPRK
>Manis_javanica
MNTARLHPRK
>Jaculus_jaculus
MNTARLHPRK
>Trichechus_manatus_latirostr
MNTARLHPRK

HG0089.3 ENSGALG00000002294 SEMA6A

>Gallus_gallus
MNKLHLQFRK
>Chaetura_pelagica
MNKLHLQFRK
>Calidris_pugnax
MNKLHLQFRK
>Columba_livia
MNKLHLQFRK
>Falco_cherrug
MNKLHLQFRK
>Egretta_garzetta
MNKLHLQFRK
>Picoides_pubescens
MNKLHLQFRK
>Struthio_camelus_australis
MNKLHLQFRK

HG0090.1 ENSG00000172765 TMCC1

>Homo_sapiens
MKIGMKPSSHSCMRSHC
>Mustela_putorius_furo
----MKPPSHSCMCSYC
>Balaenoptera_acutorostrata_s
----MKPSSQSCMCSYC
>Miniopterus_natalensis
----MKPSSHSCMCSYC
>Dasypus_novemcinctus
----MKPPSYSCMCSHC
>Sorex_araneus
----MKPSSHSCMCSCW
>Ceratotherium_simum_simum
----MKPPSHSCMCSYC
>Loxodonta_africana
MKIGMKPPSHSSMCSHC
>Chinchilla_lanigera
----MKPPSHSCMHSHC
>Tupaia_chinensis
MKIGMKPSSHCCMCSHY
>Trichechus_manatus_latirostr
----MKPPSHSCMCSNC

HG0090.1 ENSGALG00000008487 TMCC1

```
>Gallus_gallus
-MEISMRSPWHSCMCSHS
>Anser_cygnoides
-MEISMRSPWHSCMCSHS
>Apteryx_australis_mantelli
-MEISMRSPWHNCMCSH-
>Buceros_rhinoceros_silvestri
-MEISMRSPWHSCMCSHS
>Mustela_putorius_furo
-----MKPPSHSCMCSYC
>Calidris_pugnax
-MEISMRSPWHSCMCSHS
>Eptesicus_fuscus
-----MKPPSHSCMCSYY
>Merops_nubicus
-MEISMRSPWHSCMCSHS
>Alligator_mississippiensis
-----MRSPWHCCMRSHS
>Cuculus_canorus
-----MRSPWHSCMCSHS
>Sarcophilus_harrisii
MEEISMRPSSHSCMCRHS
>Aquila_chrysaetos_canadensis
-MEISMRSPWHSCMCSHS
>Mesitornis_unicolor
-MEISMRSPWHSCMCSRS
>Condylura_cristata
-----MKPPSHSCMCSYW
>Tauraco_erythrolophus
-----MRSPWHSCMCSHS
>Opisthocomus_hoazin
-MEISMRSPWHSCMCSHS
>Geospiza_fortis
MMEISMRSPWHSCMCSHS
>Phaethon_lepturus
-MEISMRSPWHSCMCSHS
>Ceratotherium_simum_simum
-----MKPPSHSCMCSYC
>Picoides_pubescens
-MEISMRSPWHSCMCSHS
>Nestor_notabilis
-----MRSPWHSCMYSHS
>Trichechus_manatus_latirostr
-----MKPPSHSCMCSNC
>Aptenodytes_forsteri
-----MRSPWHSCMCSHC
>Takydromus_sexlineatus
-----MRPPWHSCMCIHS
>Tyto_alba
-MEISMRSPWHSCMCSHS
>Struthio_camelus_australis
-MEISMRSPWHSCMCSHS
>Chrysemys_picta
-MEISMRPPWHSCMCSRS
```

HG0090.2 ENSDARG00000060954 tmcc1b

```
>Danio_rerio
--------------------------MTSMTLRQTTDACTLSEST----------
>Channa_argus
-----------------------------MTLSQVTDACPL--------------
>Basilichthys_microlepidotus
--------------------------MTSMTLSQATDACPLKRPN----------
>Oryzias_melastigma
--------------------------MTSMTLSQATDACPLKGSD----------
>Trachinotus_blochii
--------------------------MTSMTLSQATDACPLKRPN----------
>Micropterus_floridanus
--------------------------MTSMTLSQATDACALKRPN----------
>Pygocentrus_nattereri
---------MDRAIPELRARGGQAGLMTSMTLSQSADACTLAEST----------
>Maylandia_zebra
--------------------------MTSMTLSQATDACPLKRPN----------
>Austrofundulus_limnaeus
--------------------------MTSMTLSQATDACPLKRPN----------
>Esox_lucius
--------------------------MTSMTLSQSADACTGSK------------
>Gadus_morhua
--------------------------MTSMTLRQTTDACTLV-------------
>Apteronotus_leptorhynchus
---------------------------MSMTLSQSADACTLTEST----------
>Plecoglossus_altivelis
--------------------------MTSMTLSQSSDACTLAVPI----------
>Lateolabrax_japonicus
--------------------------MTSMTLSQATDACPLKRPN----------
>Notothenia_coriiceps
--------------------------MTSMTLSQATDACPLKRPN----------
>Ictalurus_punctatus
MNGHSAQSSQGSPIPELRARGGRAGPMTSMTLSQSTDACTLTESTRVPSPLLPGF
>Takifugu_rubripes
--------------------------MTSMTLSQATDACPHKGH-----------
```

HG0090.3 ENSGALG00000008487 TMCC1

```
>Gallus_gallus
----------------MLSFVQWRDCCMSAMLKGK
>Anas_platyrhynchos
---------------------------MSAMLKGK
>Apteryx_australis_mantelli
---------------------------MSAMLKDN
>Charadrius_vociferus
-------MQRDELASAILSFVQWRDCCMSAMLKGK
>Merops_nubicus
---------------------------MSAMLKGK
>Cuculus_canorus
---------------------------MSTMLKGK
>Aquila_chrysaetos_canadensis
---------------------------MSAMLKGK
>Tauraco_erythrolophus
---------------------------MSAMLKGK
>Opisthocomus_hoazin
---------------------------MSAMLKGK
>Ficedula_albicollis
MSCARCSCVRVLTAFTPVSSVQWRDCCESAMLKGK
>Nipponia_nippon
---------------------------MSAMLKGK
>Nestor_notabilis
---------------------------MSAMLKGK
>Tyto_alba
---------------------------MSAMLKGK
>Struthio_camelus_australis
---------------------------MPAMLKDA
```

HG0091.1 ENSG00000172209 GPR22

>Homo_sapiens
-----------------------------------MAHYRHIFSLRSFSKRCL---
>Nanorana_parkeri
-----------------------------------MAQHKHIFSLRSFSGEICSTCFPVGFAYK----------------------------------
>Chaetura_pelagica
-----------------------------------MAQYRHKFSLRSFFEDMLITLFPFYSANK----------------------------------
>Caprimulgus_carolinensis
-----------------------------------MAQYRHKFSLRSFFEDMLITLFLSLLLC-----------------------------------
>Odobenus_rosmarus_divergens
-----------------------------------MAQYRHIFSLRSFLKRCL---
>Hynobius_retardatus
-----------------------------------MAQHKHIFSLRSFSEEMLMALFPWYSAYK----------------------------------
>Orcinus_orca
MYFQMLIDSTVGRVRDCSSLQAALHVPYISSTAETMAQYRHTFSLRSFSKRCL---
>Calidris_pugnax
-----------------------------------MAHYRHKFSLRSFLEDMLITLFPFYSANK----------------------------------
>Rousettus_aegyptiacus
-----------------------------------MAQYRHIFSLRSFQRDVYEPLPQVL--------------------------------------
>Pterocles_gutturalis
-----------------------------------MAHYRHKFSLRSFLEDMLITLFPFYSANK----------------------------------
>Colius_striatus
-----------------------------------MAQYRHKFSLRSFFEDMLITIFPFYSANK----------------------------------
>Merops_nubicus
-----------------------------------MAQYRHKFSLRSFLEDMFITLFPFYSANK----------------------------------
>Galeopterus_variegatus
-----------------------------------MAQYRHIFSLRSFLKRCL---
>Aquila_chrysaetos_canadensis
-----------------------------------MAQYRHKFSLRSFFEDMLITLFPFYSANK----------------------------------
>Coturnix_japonica
-----------------------------------MAHYRHKFSLRSFFEDVLFTLFPFFSANK----------------------------------
>Erinaceus_europaeus
-----------------------------------MAQYRHIFSLRSFRRDVYDPLPPILY-------------------------------------
>Oryctolagus_cuniculus
-----------------------------------MAQHRHIFSLRSFLKRCL---
>Tauraco_erythrolophus
-----------------------------------MAQYRHKFSLRSFFEDMLITLFKNSANK-----------------------------------
>Zonotrichia_albicollis
-----------------------------------MAQYRHKFSLRSFFEDMLITLFPFYSANKVNKMNPSNLELLHLHREFTKSLVEGMQQLIQFYY
>Phaethon_lepturus
-----------------------------------MAQYRHKFSLRSFFEEMLITLFSFYSANK----------------------------------
>Ceratotherium_simum_simum
-----------------------------------MAQYRHIFSLRSFSKRCL---
>Manis_javanica
-----------------------------------MAQYRHIFSLRSFPKRCL---
>Melopsittacus_undulatus
-----------------------------------MAQYRHKFSLRSFFEDMLITLFPFYSANK----------------------------------
>Ictidomys_tridecemlineatus
-----------------------------------MAQYRHIFSLRSFLKRCL---
>Aptenodytes_forsteri
-----------------------------------MAQYRHKFSLRSFFEDMLITLFPFYSANK----------------------------------
>Calypte_anna
-----------------------------------MAHYRHKFSLRSFFEDMLITLLPFYSANK----------------------------------
>Apaloderma_vittatum
-----------------------------------MAQYRHKFSLRSFFGDTLITLFPFYSANK----------------------------------

HG0091.2 ENSG00000172209 GPR22

>Homo_sapiens
--------------MSKYILEATNVMFPFLFSHILENKEILSCTPSKGNIIPII----------------------------
>Canis_lupus_familiaris
--------------MSKYILEARNRMFPFLFSHVLENKETLSYIPSEGNIIPIIEEVSLGRDFLRQNKHKYFVLVFRNPSAN
>Orcinus_orca
--------------MSKYILEARNLMF-LLFSHVLENKETSSHILSKGNNSYHLRKFLSVVTF-------------------
>Pteropus_alecto
--------------MSKYILGARNLMFPFLFSHVLENKKTL---
>Dasypus_novemcinctus
--------------MAKYIFETRNLMYPFLFSRILENKETL---
>Galeopterus_variegatus
--------------MSKYILEARNLMFPFLFSHILENKETLSYIPSKGNIISII----------------------------
>Monodelphis_domestica
-------MFSCNKGMFKYISKARNQMSQFLFSLILENKNTQPHSPQKGNIINVT----------------------------
>Oryctolagus_cuniculus
--------------MSKYILEARKLVFPFLFSHILENRDNTIYSLKGKSNSYHLRKFLLVTTF-------------------
>Ornithorhynchus_anatinus
--------------MLKYISEARDRLSGFLFPAILENKNTRPHQARG-NRINIS----------------------------
>Ceratotherium_simum_simum
MRTAIETDRFVTKGCLNIFWKLETLCFLFCYHYVLENKETPSYKPSKGNIILII----------------------------
>Manis_javanica
--------------MSKYILEARNLMFPFLFSNVLGNRHFHIFPQREM----------------------------------
>Heterocephalus_glaber
--------------MSKYILEVRNLMFPFLFSHILENKETLS--
>Chrysemys_picta
--------------MPKYIFEARNWMSLFLFSFILENKNMLP--

HG0091.3 ENSG00000172209 GPR22

>Homo_sapiens
-------------MYPLLMKTNKIKQQMVFIKRTWPGFII-----
>Odobenus_rosmarus_divergens
-----------MTMYPLLMKTNQIKEQMVFIKRTWTGFII-----
>Lipotes_vexillifer
-------------MYPLLMKTNQIK--------------------
>Pteropus_alecto
-------------MYPLLMKTNQIK--------------------
>Galeopterus_variegatus
MTNGLFHLSFITNLYLLLMKTNQIKEQMVFIKKNMDWIYNIKQVR
>Monodelphis_domestica
--MDIFSLNIMICYVSFLMKISQIK--------------------
>Erinaceus_europaeus
-------------MYLLLMKTSQIKQQMAFIKRT-----------
>Oryctolagus_cuniculus
-------------MYPLLMKTNQIK--------------------
>Ornithorhynchus_anatinus
-------------MYPPLMKINQIKWQMVSLKSTRTGFYSLMQLM
>Ceratotherium_simum_simum
-------------MYPVLMRTNQIKYQMAFIKRTWTGFII-----
>Manis_javanica
-------------MYLLLMKTNQIK--------------------
>Heterocephalus_glaber
-------------MYPLLMKTNQIK--------------------

HG0092.1 ENSGALG00000009966 FRS2

>Gallus_gallus
--------------------------------------MISSVTRQHDTLKYDRSPDAATEAGNINGQI
>Anser_cygnoides
--------------MVIKQLLSCSAAVC----LHVLGQMISSVTRQHDTLKYDRTPDAATEAGNINGQI
>Xenopus_laevis
--MRHVILV------------------
>Apteryx_australis_mantelli
------------MLHGAKCLMLMTLGWK----VTFLDQMITSVTRQHDTLKYDRSPDAAAEAGNINGQI
>Buceros_rhinoceros_silvestri
MI-SSVTRQHDTLSMTEALMLQQRLGILMAESRDHMVF-------------------------------
>Charadrius_vociferus
MI-SSVTRQHDALSMTEALMLQQRLGILMAKSRDHMVF-------------------------------
>Pteropus_alecto
MRGIWFKLQYREFRLDIKNFQKIKYQILKR-------FVMSSNKKKHGILKYD----------------
>Dasypus_novemcinctus
--------------MQLKLFKQIGTKLHLFFSEESSTRLSQQT--KHVILKYD----------------
>Latimeria_menadoensis
---MKYERGPDVARGIRNLNGQI
>Colius_striatus
MI-SSMTRQHDTLSMTEALMLQQRLGILMAKSRDLMVF-------------------------------
>Columba_livia
--------------------------------------MISSVTRHHDTLKYDRSPDAAAEPGNINGQI
>Leptosomus_discolor
MI-SSVTRQHDTLSMTEALMLQQRLGILMAKSRDHMVF-------------------------------
>Alligator_mississippiensis
------------MSPFDDC---SDMGACI---ECILDPVVAHVKRQHDILKYDRSPDAATEARNINGQI
>Cuculus_canorus
MI-SSVTRQHDTSSMTEALMLQQRLGILMAKSRDHMVF-------------------------------
>Sarcophilus_harrisii
--------------MQLLLFSHIRRKLHLIFSKEKFARLCKQA--KYGILKYD----------------
>Aquila_chrysaetos_canadensis
MI-SSVTRQHDTLSMTEALMLQQRLGILMAKSRDHMVF-------------------------------
>Gavia_stellata
MI-SSVTRQHDPLSMTEALMLQQRLGILMAKSRDHMVF-------------------------------
>Balearica_regulorum_gibberic
--------------------------------------MISSVTRQHDTLKYDRSPDAAAEAGNINGQI
>Condylura_cristata
--------------MQLWLFNQIRAKLYLFFSEDNSTRISHHTEKSHGILKYD----------------
>Tauraco_erythrolophus
MI-SSVTRQHDTLSMTEALMLQQRLGILMAKCRDHMVF-------------------------------
>Opisthocomus_hoazin
--------------------------------------MISSVTRQHDTLKYDRSPDAAAEAGNINGQI
>Zonotrichia_albicollis
MI-SSVTRQHDTLSMTEALMLQQRLGILMAKSRDQMVF-------------------------------
>Phaethon_lepturus
--------------------------------------MISSVTRQHDTLKYDRSPDAAAEAGNINGQI
>Fulmarus_glacialis
MI-SSVTRQYDTLSMTEALMLQQRLGILMARSRDHMVF-------------------------------
>Aptenodytes_forsteri
MI-SSVTRQHDTLSMTEALMLQQRLGILMAKSRDHMVF-------------------------------
>Pelodiscus_sinensis
--------------------------------------MFVYVRRQHAILKYDRSPDAATEARNINGQI
>Calypte_anna
MI-SSVARQHDTLSMTEALMLQQRLGILMAKSRDPMVF-------------------------------

HG0092.2 ENSG00000166225 FRS2

>Homo_sapiens
------MKQSICHKVRCSCGMSTSLEQNCICFSQKRIPQG--------------------------------
>Panthera_tigris
------MKQSVCHKVRCSCGFSTRLEQNCICFSQKRIPQG--------------------------------
>Orcinus_orca
------MKQSICHKVRCSCGFSTRLGQNCIYFSQKRIPQEKTKMRGIWFKLQYKEFRLDIKSFQKIKYQILK
>Rousettus_aegyptiacus
------MKQSICHKVRCSCGFSTRLEQNCICFSQKRIPQGARQVYCIKQRFEDS------------------
>Dasypus_novemcinctus
------MKQSICHKVRCS--
>Monodelphis_domestica
------MKLFICDEVKCSRGFLVISEGNCI--
>Condylura_cristata
------MKQSICHKLRCNCGFSTRLEQNCISFSQKIIPQG--------------------------------
>Oryctolagus_cuniculus
------MKQPICHKVRCSSGFSTRLEQNCICFSQKRTPQG--------------------------------
>Ceratotherium_simum_simum
------MKQSICPKVRCSCGFSTRLEQNCIYFSQKRIPQG--------------------------------
>Manis_javanica
----MQSDETICHKVRCSCGFSTRLDQNCVSFSQRRILQG--------------------------------
>Loxodonta_africana
------MKQTICHKVKCSCGFSTRLEQNCICFSQKRIPQG--------------------------------
>Fukomys_damarensis
------MKQSICHKIRCSCGFSTRLEQNYICFSQKRIPQG--------------------------------
>Tupaia_chinensis
MPGYQVMKQSIWYKVRCSCGYSTRLEQNYICFSQKRILQG--------------------------------

HG0093 ENSG00000169554 ZEB2

```
>Homo_sapiens
MLFLLTISGQNYKQDFAAPCLN-------------
>Anas_platyrhynchos
MLLLLTISGQNYNQDFAESACC-------------
>Chaetura_pelagica
MLFLLTISGQNYKQDLAEPACC-------------
>Apteryx_australis_mantelli
MLFLLTISGQNYKQDFAEPACC-------------
>Odobenus_rosmarus_divergens
MLFLLTISGQNYKQDFADRACVLPKQGAESMRTAI
>Lipotes_vexillifer
MLFLLTISGQNYKQDFADRACVLPKQGAESMRTAI
>Myotis_brandtii
MLFLLTISGQNYKQDFADRACVLPEQGAESMRTAI
>Dasypus_novemcinctus
MLFLLTISGQNYKQDFADRACVLPKQGAESMRTAI
>Columba_livia
MLFLLTISGQNYKQDFAEPACC-------------
>Alligator_sinensis
MLFLLPISGQNYNQDFAESACC-------------
>Galeopterus_variegatus
MLFLLTISGQNYKQDFTDRACVLPKQGAESMRTAI
>Falco_peregrinus
MLFLLTISGQNYKQDFAEPACC-------------
>Coturnix_japonica
MLFLLTISGQNYKQDFAESACC-------------
>Erinaceus_europaeus
MLFLLTISGQNYKQDFTDRACVLPEPGAESMRAAI
>Oryctolagus_cuniculus
MLFLLTISGQNYKQDFADRACVLPKQGAESMRTAI
>Parus_major
MLFLLTISGQNYKQDFAEPACCCCCRRRCC-----
>Egretta_garzetta
MLFLLTISGQNYKQDFAEPACC-------------
>Ceratotherium_simum_simum
MLFLLTISGQNYKQDFADRACVLPKQGAESMRTAI
>Loxodonta_africana
MLFLLTISGQNYKQDFADRACVLPKQGAESMRTAI
>Peromyscus_maniculatus_baird
MLFLLTISGQNYKQDFAARCLN-------------
>Trichechus_manatus_latirostr
MLFLLTISGQNYKQDFADRACVLPKQGAESMRTAI
>Pygoscelis_adeliae
MLFLLTISGQNYKQDFAESACC-------------
>Thamnophis_sirtalis
MLLLLFTAGQNYKQDFAESACC-------------
>Struthio_camelus_australis
MLFLLTISGQNYRQDFAEPACC-------------
>Chelonia_mydas
MLFLLTISGQNYKQHFAGRVCVLLNQGVESMRTAI
>Orycteropus_afer_afer
MLFLLTISGQNYKQDFADRACVLPKQGAESMRTAI
```

HG0093 ENSGALG00000040465 Sip1

>Gallus_gallus
MLFLLTISGQNYKQDFAESACC------------
>Anas_platyrhynchos
MLLLLTISGQNYNQDFAESACC------------
>Chaetura_pelagica
MLFLLTISGQNYKQDLAEPACC------------
>Apteryx_australis_mantelli
MLFLLTISGQNYKQDFAEPACC------------
>Odobenus_rosmarus_divergens
MLFLLTISGQNYKQDFADRACVLPKQGAESMRTAI
>Lipotes_vexillifer
MLFLLTISGQNYKQDFADRACVLPKQGAESMRTAI
>Myotis_brandtii
MLFLLTISGQNYKQDFADRACVLPEQGAESMRTAI
>Dasypus_novemcinctus
MLFLLTISGQNYKQDFADRACVLPKQGAESMRTAI
>Latimeria_chalumnae
MSLLLVIASPN------------------------
>Columba_livia
MLFLLTISGQNYKQDFAEPACC------------
>Alligator_mississippiensis
MLFLLPISGQNYNQDFAESACC------------
>Galeopterus_variegatus
MLFLLTISGQNYKQDFTDRACVLPKQGAESMRTAI
>Falco_cherrug
MLFLLTISGQNYKQDFAEPACC------------
>Condylura_cristata
MLFLLTISGQSYKQDFADRACVLPKQGAESMRTAI
>Oryctolagus_cuniculus
MLFLLTISGQNYKQDFADRACVLPKQGAESMRTAI
>Parus_major
MLFLLTISGQNYKQDFAEPACCCCCRRRCC-----
>Egretta_garzetta
MLFLLTISGQNYKQDFAEPACC------------
>Ceratotherium_simum_simum
MLFLLTISGQNYKQDFADRACVLPKQGAESMRTAI
>Otolemur_garnettii
MLFLLTISGQNYKQDFADRACVLPKQGAESMRTAI
>Loxodonta_africana
MLFLLTISGQNYKQDFADRACVLPKQGAESMRTAI
>Peromyscus_maniculatus_baird
MLFLLTISGQNYKQDFADRACVLPKQGAESMRTAI
>Trichechus_manatus_latirostr
MLFLLTISGQNYKQDFADRACVLPKQGAESMRTAI
>Pygoscelis_adeliae
MLFLLTISGQNYKQDFAESACC------------
>Python_bivittatus
MLLLLFTAGQNYKQDFAESACC------------
>Struthio_camelus_australis
MLFLLTISGQNYRQDFAEPACC------------
>Chelonia_mydas
MLFLLTISGQNYKQHFAESACC------------
>Orycteropus_afer_afer
MLFLLTISGQNYKQDFADRACVLPKQGAESMRTAI

HG0094.1 ENSG00000112175 BMP5

>Homo_sapiens
---------------------MQQKLLTTVDNWKSEFQLS
>Anser_cygnoides
-----MPRSADK--ILVFCSINAEKTITTVENWKSEFQLS
>Apteryx_australis_mantelli
-----MSRSADK--ILVFCSISAEKTITTVENWKSEFQLS
>Mustela_putorius_furo
---------------------MQQKLLTTVDNWKSKFQLS
>Balaenoptera_acutorostrata_s
---------------------MQQKLLTTVDNWKSEFQLS
>Calidris_pugnax
-----MPRSADK--ILVFCSINAEKTITTVENWKSEFQLS
>Pteropus_alecto
---------------------MQQKLLTTVDNWKSEFQPS
>Columba_livia
-----MPRSADK--ILVFCSINAEKTITTVENWKSEFQLS
>Alligator_mississippiensis
-----MPRSADK--VLFFCSINAEKTITTVENWKSEFQLS
>Monodelphis_domestica
-----MHRSFDQIILAVFCYINAVQTITTVDNWKSEFQLS
>Aquila_chrysaetos_canadensis
-----MPRSADK--ILVFCSINAEKTITTVENWKSEFQLS
>Coturnix_japonica
-----MPRSADK--ILVFCSINAEKTITTVENWKSEFQLS
>Sorex_araneus
---------------------MQQKLLTTVDNWKSEFQLS
>Ochotona_princeps
---------------------MKQKLLTTVDNWRSEFQLS
>Elephantulus_edwardii
---------------------MQQKLLTTVDNWKFEFQLS
>Corvus_brachyrhynchos
-----MPRSADK--ILVFCSINAEKTITTVENWKSEFQLS
>Ceratotherium_simum_simum
---------------------MQQKLLTTVDNWKSEFQLS
>Manis_javanica
---------------------MQQKLLTTVDNWKSEFQLS
>Loxodonta_africana
---------------------MQQKLLTTVDNWKSKFQLS
>Marmota_marmota_marmota
---------------------MQQKLLATVDNWKSEFQLS
>Trichechus_manatus_latirostr
---------------------MQQKLLTTVDNWKSKFQLS
>Aptenodytes_forsteri
-----MPRSADK--ILVFCSINAEKTITTVENWKSEFQLS
>Anolis_carolinensis
MLCTGMPASTNQ--VLVFCSIHVEKTVMTVENWKSEFQLS
>Struthio_camelus_australis
-----MPRSADK--ILVFCSINAEKTITTVENWKSEFQLS
>Pelodiscus_sinensis
-----MPRSADK--ILVFCSINAEKAITTVENWKSEFQLS
>Tinamus_guttatus
-----MSRSADK--ILVFCSINAEKTITTVENWKSEFQLS
>Orycteropus_afer_afer
---------------------MQQKLLTTVDNWKYEFQLS

HG0094.2 ENSG00000112175 BMP5

>Homo_sapiens
MDLQEGFQVNSGKHIYLNSTT--
>Ailuropoda_melanoleuca
MDLQEGFQVNSGKHIYLNSTTLSLILH--
>Pteropus_vampyrus
MDLQEGFQVNTGKHIYLNSTTLSLILH--
>Dasypus_novemcinctus
MDLQEGFQVNSGKHIYLNSTTLSLILH--
>Galeopterus_variegatus
MDLQEGFQVNSGKHIYLNSTTLSLILH--
>Monodelphis_domestica
MDFREGLQVNSGKHIYFTSTILSFILH--
>Sorex_araneus
MDLKEGFQVNSGKHIYLNSRTLNLVLH--
>Oryctolagus_cuniculus
MDLQEGFQVNSGEHIYLNSTTLSVILH--
>Ceratotherium_simum_simum
MDLQEGFQVNSGKHIYLNSTTLSLILH--
>Manis_javanica
MDLQEGFQGNSGKHIYLNSTTLSLILHQDDTKDVKVITKLPDKYISQHQGADEHRSVIQKSGSVLERTQGLRRTQEQVKITLEIQFIRRSTLLNSNTKGLIIINSYRNA
>Loxodonta_africana
MDLQEGFQVNSGKHIYLNSRTLSLILH--
>Ictidomys_tridecemlineatus
MDLQEGFQVNSGKHIYLNSTTLSLILH--
>Trichechus_manatus_latirostr
MDLQEGFQINSGKHIYLNSRTLSLISH--
>Chelonia_mydas
MDLQKFFQVNSGKHMN---
>Orycteropus_afer_afer
MDLQEGFQVNSGKHIYLNSTTLSLTLH--

HG0095 ENSDARG00000013708 usp9

>Danio_rerio
MEERSSLILGYAMVSATYPVPQVVLSLSEVCRFSVA-----PEE----KLISSGVPAT
>Amia_calva
------------MVSAKHPVLQVVLSYTDVCWFSVI-----VEE----AIISSLTTTT
>Channa_argus
------------MVSAAHPDLQVLVSLSCVFG--FSSRCSTAEVA----VSSLLACAP
>Microhyla_fissipes
------------MVSAKRSVFEVELCKADICRLAF------LEG----TIISSLVTAT
>Menidia_menidia
------------MVSAAHPDLQVLVSLSRAFG--LSSLCSTAEVA----VISLLVSAP
>Porichthys_notatus
------------MVSAAHPDLQVLVSLSRVLG--LDSLCSTAELA----VISLLASAP
>Oryzias_melastigma
------------MVSATHPDLQVLVSLSCVFG--LSSLCSTAEVA----VISLLASAP
>Ambystoma_mexicanum
------------MVSAKCGVHEVVLCQPHTCWYYVY-----IKG----SPISSLTLAT
>Pygocentrus_nattereri
------------MVSAAHPVLQVVLSLSDVCRFSIS-----PEE----AVIFSCTPAT
>Callorhinchus_milii
------------MVSARQPELEVVLLCTDLNWFSIF-----TEE----AIHSSLATTT
>Haplochromis_burtoni
------------MVSAAHPDLQVLVSLSRVFR--LSSLCSTAEVA----VISLLASAP
>Dasypus_novemcinctus
------------MVSARWFILDLELFKTDICWYFVF-----YKW----TIISFFKTTT
>Alosa_alosa
------------MVSAIPPVLQVVLSLSRACRLSVS-----LEE----AVISPRAPAT
>Latimeria_chalumnae
------------MLSAKQSVLEVVLLYTDTCWFAIF-----TEG----VIISSLKTTT
>Poeciliopsis_prolifica
------------MVSAVHPDLQVLATLSRVFR--LSFLRCTAEVA----VISLLASAP
>Sarcophilus_harrisii
------------MVSARWFVLNLELFKTDICWYFIF-----YKR----TLISPLTTTT
>Esox_lucius
------------MVSAARPDLQVVLSLSLAPRPQCSSLRSVSEVAEAPPVISLLASAP
>Gadus_morhua
------------MVSAAHPDLQVLVSLSCVFG--LVYLCSTAEAA----VIPLVASAP
>Apteronotus_leptorhynchus
------------MVTATQPKPQVLLSCLLALWIRPA-----ALQ----AVISRLAPTT
>Ornithorhynchus_anatinus
------------MVAATWLALELELFKTDTCRYCIV-----SKR----TIIPSLPTTT
>Scleropages_formosus
------------MVAASRPALQLVLSHTDVCWFSVT-----TDE----ALISSPAIAT
>Trematomus_bernacchii
------------MVSAAHPDLQVLVSLSRAFG--LSSLCSTAEVA----VISLLASAP
>Platichthys_stellatus
------------MVSAAHPDLQVLVSLSRVFG--LSSLCSTAEVA----VISLLASAP
>Macaca_nemestrina
------------MVSARWFILELDLFKTDKCWYFIF-----YKW----TIISFLKTTT
>Oncorhynchus_mykiss
------------MVSAARPDLQVVLSLSLVPRPLLSSLCSSSEVAEAPPVISLVVSAA
>Lepisosteus_oculatus
------------MVSAKHPVLQVVLSSTDACWFSII-----VEE----AIISSLTTTT
>Ictalurus_punctatus
------------MVS------ASALALSAVCYFSIS-----PEE----AVISSRAPVT

HG0096 ENSGALG00000014186 MPPED1

>Gallus_gallus
-------------------------------MVFSCRCRKIADMIA
>Anas_platyrhynchos
-------------------------------MVFFHQCRKIADMIA
>Chaetura_pelagica
-------------------------------MVFFRGCRKIADMIA
>Apteryx_australis_mantelli
-------------------------------MVFFCRCRKIADMIA
>Buceros_rhinoceros_silvestri
-------------------------------MVFFHRSRKIAEMIA
>Caprimulgus_carolinensis
-------------------------------MVFFCRCRKIADMIA
>Cariama_cristata
-------------------------------MVFFHRCRKIADMIA
>Calidris_pugnax
MLVVSQPLMQEENPRLKGFSEGSVLIRSGDVMVFFRRCRKIADMIA
>Pterocles_gutturalis
-------------------------------MVFFHRCRKIAAMIA
>Colius_striatus
-------------------------------MVFFHRCRKIADMIA
>Columba_livia
-------------------------------MVFFHRCRKIADMIA
>Leptosomus_discolor
-------------------------------MVFFRRCRKIADMIA
>Cuculus_canorus
-------------------------------MVVFRRCRKIADMIA
>Haliaeetus_albicilla
-------------------------------MVFSRRCRKIADMIA
>Gavia_stellata
-------------------------------MVFFHRCRKIADMIA
>Mesitornis_unicolor
-------------------------------MVFFHRCRKIADMIA
>Tauraco_erythrolophus
-------------------------------MVFSRRCRKIADMIA
>Opisthocomus_hoazin
-------------------------------MVFFRRCRKIADMIA
>Acanthisitta_chloris
-------------------------------MVFFHRCRKIADMIA
>Nipponia_nippon
-------------------------------MVFFRRCRKIADMIA
>Fulmarus_glacialis
-------------------------------MVFFHRCRKIADMIA
>Nestor_notabilis
-------------------------------MVFFRRCRKIADMIA
>Aptenodytes_forsteri
-------------------------------MVFFHRCRKIADMIA
>Tyto_alba
-------------------------------MVFFRRCRKIADMIA
>Struthio_camelus_australis
-------------------------------MVFFRRCRKIADMIA
>Chrysemys_picta
-------------------------------MVFFCRCRKIAQMII
>Tinamus_guttatus
-------------------------------MVFFCRYRKIADMIA

HG0097 ENSGALG00000037496

>Gallus_gallus
MELQPRAGAADTPSAFLRSSHHERGRFHLVKMAVWDRCNL
>Ailuropoda_melanoleuca
-------------------------------MAVRDRCNL
>Orcinus_orca
-------------------------------MAVRDRCNL
>Calidris_pugnax
-------------------------------MAVWDRCNL
>Rousettus_aegyptiacus
-------------------------------MAVRDRCNL
>Dasypus_novemcinctus
-------------------------------MAVRGRCHL
>Columba_livia
-------------------------------MAVWDRCNL
>Alligator_mississippiensis
-------------------------------MAVGDRCNL
>Sarcophilus_harrisii
-------------------------------MAVRDHCNL
>Galeopterus_variegatus
-------------------------------MAVRDRCNL
>Monodelphis_domestica
-------------------------------MAVRDHCNL
>Falco_peregrinus
-------------------------------MAVWDRCNL
>Erinaceus_europaeus
-------------------------------MAVRDRCNL
>Elephantulus_edwardii
-------------------------------MAVRDRCNL
>Lepidothrix_coronata
-------------------------------MAVWDRCNL
>Nipponia_nippon
-------------------------------MAVWDRCNL
>Ceratotherium_simum_simum
-------------------------------MAVRDRCNL
>Manis_javanica
-------------------------------MAVRDRCNL
>Pongo_abelii
-------------------------------MAVRDRCNL
>Loxodonta_africana
-------------------------------MAVRDRCNL
>Melopsittacus_undulatus
-------------------------------MAVRDRCNL
>Microtus_ochrogaster
-------------------------------MAVCDRCNL
>Trichechus_manatus_latirostr
-------------------------------MAVRDRCNL
>Aptenodytes_forsteri
-------------------------------MAVWDRCNL
>Gekko_japonicus
-------------------------------MAVWDRCNL
>Pelodiscus_sinensis
-------------------------------MAVWDHCNL
>Calypte_anna
-------------------------------MAVWDRYNL

HG0098 ENSG00000198739 LRRTM3

>Homo_sapiens
MQKRNIHEDPIT
>Anser_cygnoides
MQKRNIHEDPIT
>Panthera_tigris
MQKRNIHEDPIT
>Tursiops_truncatus
MQKRNIHEDPIT
>Calidris_pugnax
MQKRNIHEDPIT
>Pteropus_alecto
MQKRNIHEDPIT
>Dasypus_novemcinctus
MQKRNIHEDPIT
>Galeopterus_variegatus
MQKRNIHEDPIT
>Monodelphis_domestica
MQKRNIHEDPIT
>Aquila_chrysaetos_canadensis
MQKHNIHEDPIT
>Coturnix_japonica
MQKRNIHEDPIT
>Condylura_cristata
MQKRNIHEDPIT
>Oryctolagus_cuniculus
MQKRNIHEDPIT
>Elephantulus_edwardii
MQKRNIHEDPIT
>Ornithorhynchus_anatinus
MQKRNIHEDPIT
>Lepidothrix_coronata
MQKRNIHEDPIT
>Ceratotherium_simum_simum
MQKRNIHEDPIT
>Manis_javanica
MQKRNIHEDPIT
>Loxodonta_africana
MQKRNIHEDPIT
>Marmota_marmota_marmota
MQKRNIHEDPIT
>Tupaia_chinensis
MQKRNIHEDPIT
>Trichechus_manatus_latirostr
MQKRNIHEDPIT
>Anolis_carolinensis
MQKHIIHEDPIT
>Struthio_camelus_australis
MQKHNIHEDPIT
>Chrysemys_picta
MQNRNIHEDPIT
>Tinamus_guttatus
MQKRNIHEDPIT
>Orycteropus_afer_afer
MQKRNIHEDPIT

HG0099.1 ENSG00000168575 SLC20A2

```
>Homo_sapiens
--------------------------------------------MRKT---NGSEKVYRIIGGDITAFPDREEKYGMCFTAD---------
>Anas_platyrhynchos
----------------------------------------------------------------------------MCFTADREQANEQYL
>Mustela_putorius_furo
MFLTAPLLTQLLLCIIFELLSSTAIAGSPLMNRVLFHVDGELVATRRN---GKGYRNQLQLGGDITAFPDWEEKYGMCFTAD---------
>Balaenoptera_acutorostrata_s
----------------------------------------------------------------------------MCFTAD---------
>Miniopterus_natalensis
--------------------------------------------MRENPSFSMSSVEIKEPGGDITAFPDREEKYGMCFTAD---------
>Dasypus_novemcinctus
----------------------------------------------------------------------------MCFTAD---------
>Cuculus_canorus
----------------------------------------------------------------------------MCFTADRRQPNEQYL
>Sarcophilus_harrisii
----------------------------------------------------------------------------MCFTAD---------
>Galeopterus_variegatus
----------------------------------------------------------------------------MCFNAD---------
>Monodelphis_domestica
------------MCQK------------KLIIEQV--QSLDFLQPRKKDACKRGY----------HSVSSLREKYGMCFTAD---------
>Haliaeetus_albicilla
----------------------------------------------------------------------------MCFTADRRQPNEQYL
>Condylura_cristata
----------------------------------------------------------------------------MCFTAD---------
>Oryctolagus_cuniculus
----------------------------------------------------------------------------MHFPAD---------
>Elephantulus_edwardii
----------------------------------------------------------------------------MCFTTDCTQPNELD-
>Ornithorhynchus_anatinus
----------------------------------------------------------------------------MCFTAD---------
>Sturnus_vulgaris
----------------------------------------------------------------------------MCFTADRRQPNEQYL
>Equus_asinus
----------------------------------------------------------------------------MCFTAD---------
>Manis_javanica
----------------------------------------------------------------------------MCFTPD---------
>Loxodonta_africana
----------------------------------------------------------------------------MCFTAD---------
>Cavia_porcellus
----------------------------------------------------------------------------MCFTAD---------
>Tupaia_chinensis
-----------MGCSILELIRPLTG-----------TDSCRHVHPQAP---PSSDVGSCMAGGDITAFPGWEEKYGMCFPAD---------
>Trichechus_manatus_latirostr
----------------------------------------------------------------------------MCFTAD---------
>Struthio_camelus_australis
----------------------------------------------MPSCAGGSFIPGLLVTG-HHSCTSLGAKYGMCFTADRTQPNEQYL
>Chrysemys_picta
-------------------------------------------------------------MTGHCSCTSFEAKYGMCFTAD---------
>Tinamus_guttatus
----------------------------------------------------------------------------MCFTADRMQPNEQYL
>Orycteropus_afer_afer
----------------------------------------------------------------------------MCFTADRTQPNELY-
```

HG0099.2 ENSG00000144136 SLC20A1

>Homo_sapiens
MNLRPFSSPPWNSAPCF
>Leptonychotes_weddellii
MNLRPFSSPPWNS-PCF
>Tursiops_truncatus
MNLRPFSSPPWNSAPCF
>Condylura_cristata
MNLRPFSSPPWNSAPC-
>Oryctolagus_cuniculus
MNLRPFSSPPWNFAPCF
>Equus_przewalskii
MNLRPFSSQPWNSAPCF
>Manis_javanica
MNRRPCSSKPWNSAPLL
>Rattus_norvegicus
MNLRPFSNPPWNSAPCF
>Tupaia_chinensis
MNLRPFSSPPWNSAPCF
>Trichechus_manatus_latirostr
MNLRPCSSLPWNSAPCF
>Orycteropus_afer_afer
MNLRPCFSRPWNSAPCF

HG0099.3 ENSG00000168575 SLC20A2

```
>Homo_sapiens
-----------------------------------MMLCIVHQGTPLGRISH-------
>Leptonychotes_weddellii
-----------------------------------MMLCIVQQSTPLGQISH-------
>Eptesicus_fuscus
-----------------------------------MMLCIVHQSTPLGQISH-------
>Galeopterus_variegatus
MPVAKKSLLFLFVSTCKNHLKEDRVIGPNSIISIIVMLCIVH-----------------
>Erinaceus_europaeus
MPVVKKLALFSLLVLAKTTYKKLVVLGLNSVICIIMMLCIVHQNTPLSQISH-------
>Elephantulus_edwardii
MPVVKGAYYSIFVSTCKSNLKEVSAVGLTSIISIIVMLCIVHQSTPLCQISH-------
>Ceratotherium_simum_simum
MPVVKKSLLFLFVSTCKNNLKEVSIIGLKSIISIIMMLCIVHRARPLARFPIKRTPTLS
>Heterocephalus_glaber
-------------------------------MGIIMMLCIVHQGTPLGHISH-------
>Tupaia_chinensis
MPVVKELTL--SVSTCKINLKEVSIIGLHSVISIIVMLCIVHQGTPLGQISH-------
>Orycteropus_afer_afer
-----------------------------------MMLCIVHQSTPLCQISH-------
```

HG0099.4 ENSDARG00000010641 slc20a1b

```
>Danio_rerio
MDVRCICLIVQAKTNRNRSSLFRG
>Trachinotus_blochii
MAVHSLCLIAQDPKKSNRSSIITG
>Astyanax_mexicanus
MDVRSVCLITQTKTHCSSTSLFRG
>Clupea_harengus
----MGCLIHQSQTNCSKSSLLRG
>Apteronotus_leptorhynchus
MDVCSVCLIAQTKTHCSRYSLFRG
>Lutjanus_erythropterus
MAVCSLCLIAQHQSNSNRSSIITG
>Sebastes_caurinus
MAVCSICLISEHQRNRNRSSLITG
>Platichthys_stellatus
MAVMSLCLIAQRSKNSNRSSLILG
>Trichomycterus_areolatus
MDVRSVCLIGQTKTHCSSTSLFRG
```

HG0100.1 ENSDARG00000104148 crebbpb

>Danio_rerio
--MRVLGVGLSWGEWRWRSGALLSRV----
>Acipenser_sinensis
--MWVLGVELSWGEWRWRGAVATFLRFFVA
>Channa_punctata
--MWVLGVGLSWGEWRWRSGALLHLF----
>Menidia_menidia
---MIIMWVLGVGLSWGEWRWRSGALLHFF----
>Porichthys_notatus
--MWVLGVGLSWVDLRRRSVALLPFF----
>Oryzias_latipes
MKTFDVTQNGYVVEHLAIARLPKCCSVVPDFLNGRFETRPVHGDRCSWGYLMTIMWVLGVGLTWGEWRWRSGALLHLF----
>Trachinotus_blochii
--MWVLGVGLSWGEWRWRSGALLHLF----
>Micropterus_floridanus
--MLGHLTIIMWVLGVGLSWGEWRWRSGALLHLF----
>Pygocentrus_nattereri
--MWVLGVGLSWGEWRWRRAALLLLF----
>Maylandia_zebra
--MWVLGVGLSWGEWRWRSGALLHLF----
>Alosa_alosa
--MIMWVLGVGLSWGEWRWRSGALLLLF----
>Nothobranchius_furzeri
MKKFDVTQNGYFEH-LELRLYPRNAEIHLNIPDRHLKARFLHGDPFISGYLMIIMWVLGVGLSWGEWRWRSGALLHLF----
>Esox_lucius
--MWVLGVGLSWGDCQWRCVALLPFF----
>Acanthogobius_hasta
--MIMWVLGVGLSWGEWRWRSGALLHLF----
>Apteronotus_leptorhynchus
--MMVFGVGLSWGEWQWRSGALLLVL----
>Lutjanus_erythropterus
--MWVLGVGLSWGEWRWRSGALLHLF----
>Plecoglossus_altivelis
--MWVLGVGLSWGEWRWRSGALLHLF----
>Scleropages_formosus
--MWVLGVGLSWGERRWRSGALLLLF----
>Lateolabrax_japonicus
--MWVLGVGLSWGEWRWRSGALLHLF----
>Trematomus_bernacchii
--MWVLGVGLSWGEWRWRSGALLHLF----
>Cynoglossus_semilaevis
-----------------------------MLSNCRAEVGSLHGEYFIVRYLTIIMWVLGVGLSWGEWRWRSGALLHLF----
>Salvelinus_alpinus
--MWVLVVGLSWVEWRWRSGALLHLF----
>Lepisosteus_oculatus
--MWVLGVGLSWGEWFWRSGAL--------
>Trichomycterus_areolatus
--MSVIGVDLSWVEWQWRSGALLLVF----
>Takifugu_rubripes
MNTFDVTQNDFFTH-LDITFFLGNAAVHLTSLDCHPEVRFLHGEYFILGYLTMIMWVLGVGLSWGEWRWRSGALLHLF----

HG0100.1 ENSDARG00000104609 crebbpa

>Danio_rerio
--MIMWVLGVGLSWGEWWWRRGALLLLF----
>Acipenser_sinensis
--MWVLGVELSWGEWRWRGAVATFLRFFVA
>Channa_punctata
--MWVLGVGLSWGEWRWRSGALLHLF----
>Menidia_menidia
---MIIMWVLGVGLSWGEWRWRSGALLHFF----
>Porichthys_notatus
--MWVLGVGLSWVDLRRRSVALLPFF----
>Oryzias_latipes
MKTFDVTQNGYVVEHLAIARLPKCCSVVPDFLNGRFETRPVHGDRCSWGYLMTIMWVLGVGLTWGEWRWRSGALLHLF----
>Trachinotus_blochii
--MWVLGVGLSWGEWRWRSGALLHLF----
>Micropterus_floridanus
--MLGHLTIIMWVLGVGLSWGEWRWRSGALLHLF----
>Pygocentrus_nattereri
--MWVLGVGLSWGEWRWRRAALLLLF----
>Maylandia_zebra
--MWVLGVGLSWGEWRWRSGALLHLF----
>Clupea_harengus
--MIMWVLGVGLSWGEWRWRSGALLLLF----
>Latimeria_chalumnae
---MKCVLTVGFLSAIMWVLGVVLSGGDRWWRRRGALLLLH---
>Nothobranchius_furzeri
MKKFDVTQNGYFEH-LELRLYPRNAEIHLNIPDRHLKARFLHGDPFISGYLMIIMWVLGVGLSWGEWRWRSGALLHLF----
>Esox_lucius
--MWVLGVGLSWGDCQWRCVALLPFF----
>Acanthogobius_hasta
--MIMWVLGVGLSWGEWRWRSGALLHLF----
>Apteronotus_leptorhynchus
--MMVFGVGLSWGEWQWRSGALLLVL----
>Lutjanus_erythropterus
--MWVLGVGLSWGEWRWRSGALLHLF----
>Plecoglossus_altivelis
--MWVLGVGLSWGEWRWRSGALLHLF----
>Scleropages_formosus
--MWVLGVGLSWGERRWRSGALLLLF----
>Lateolabrax_japonicus
--MWVLGVGLSWGEWRWRSGALLHLF----
>Sebastes_caurinus
--MWVLGVGLSWGEWRWRSGALLHLF----
>Cynoglossus_semilaevis
-----------------------------MLSNCRAEVGSLHGEYFIVRYLTIIMWVLGVGLSWGEWRWRSGALLHLF----
>Oncorhynchus_kisutch
--MWVLVVGLSWVEWRWRSGALLHLF----
>Lepisosteus_oculatus
--MWVLGVGLSWGEWFWRSGAL--------
>Trichomycterus_areolatus
--MSVIGVDLSWVEWQWRSGALLLVF----
>Takifugu_rubripes
MNTFDVTQNDFFTH-LDITFFLGNAAVHLTSLDCHPEVRFLHGEYFILGYLTMIMWVLGVGLSWGEWRWRSGALLHLF----

HG0100.2 ENSDARG00000061108 ep300b

>Danio_rerio
---MAEKTDASSEDKSRENCS--VAEEAT
>Channa_punctata
---MADRTECSIEDGSWDNFC--VAEEAT
>Micropterus_floridanus
---MADRTECSTEDGSWDSFC--VAEEAT
>Pygocentrus_nattereri
---MAVKMDPALEERSRHQVS--VAHEAT
>Pundamilia_nyererei
---MPGRTEYSPEDRSWDGFC--VAEEAT
>Clupea_harengus
MRRTPERRYRGAFGLHSLSCFPSSLTRYDALAQRNRTELLISPPTMAEKTDTAMEDKSWDNLP--VAEEAT
>Esox_lucius
---MAEKTDAAMEDRSWDNVS--VTEEAK
>Apteronotus_leptorhynchus
---MAENTDTAIEDKSRDNVF--VADEAT
>Plecoglossus_altivelis
---MAEKTDATMEDSSWDNFS--VAEEAT
>Scleropages_formosus
---MAEKTDAPAEDRSWDNLNFTVAEEAT
>Notothenia_coriiceps
---MAERTESSTEDGSWDSFC--VAEEAT
>Salmo_salar
---MAEKTDAAMEDRSWDNFS--AAKEAK

HG0101 ENSG00000099250 NRP1

>Homo_sapiens
MARAVAPGRGTSVGKGRGSRS
>Apteryx_australis_mantelli
MVGAVAPAEGTSVGKGRGSLS
>Odobenus_rosmarus_divergens
MARAVAPGRGTSVGKERGNRS
>Orcinus_orca
MARAVAPGRGTSVGKERGNRS
>Calidris_pugnax
MAGAAAPGEGTSVGKGRGSRS
>Rousettus_aegyptiacus
MARAVAPGRGTSVGKERGNRS
>Dasypus_novemcinctus
MARAVAPGRGTSVGKGRGNRS
>Monodelphis_domestica
MARAAALGSGTSVGKERGNRS
>Aquila_chrysaetos_canadensis
MAGAAAPGEGTSVGKGRGSRS
>Coturnix_japonica
MAGAAALGAGTSVGKGRGSRS
>Condylura_cristata
MARAVAPGRGTSVGKERGNRS
>Oryctolagus_cuniculus
MARAVAPGRGTSVGKGRGSRS
>Elephantulus_edwardii
MARAVAPGRGTSVGKGRGNRS
>Ornithorhynchus_anatinus
MARAASSGRGTSVGREIGNRS
>Geospiza_fortis
MARAAAPGEGTSVGKGRGSRS
>Ceratotherium_simum_simum
MARAVAPGRGTSVGKERGNRS
>Manis_javanica
MARAVAP--------------
>Loxodonta_africana
MARAVALGRGTSVGKERGNRS
>Melopsittacus_undulatus
MAGAAAPGEETSVGKGKGNRS
>Microtus_ochrogaster
MARAVAPGRGTSVGKGRGNRS
>Tupaia_chinensis
MARAVAPGRGTSVGKGRGNRS
>Lepisosteus_oculatus
-MATPAPGRGTSLGKERGNQS
>Aptenodytes_forsteri
--MAGAPGEGTSVGKGRGSRS
>Anolis_carolinensis
MAKAVTPGKGTSVGKGRGNWS
>Struthio_camelus_australis
MVGAVAPGEGTSVGKGRGSRS
>Pelodiscus_sinensis
MAGAATPGKGATVGKGRGNQS

HG0102 ENSG00000182263 FIGN

>Homo_sapiens
-----------------------------MISNHRHLHLTLASIHQQT----HRIDATLTYRVLSPRVQVK
>Acipenser_sinensis
-----------------------------MISNRRHPHLTRAPIHQQT----HRIDVTLTSEAALPHTQA-
>Anser_cygnoides
MLAPCKAAVLGLKFQHGRGCSQSSLQKLGMISNHRHLHLTRAPIHQQT----HRIDATLTYRVLVPNLQVK
>Apteryx_australis_mantelli
-----------------------------MISNHRHLHLTRAPIHQQT----RRIDATLTYRVLVPNLQVK
>Acinonyx_jubatus
-----------------------------MISNHRHLHLTLASIHQQT----HRIDATLTYRVFAPRVQVK
>Physeter_catodon
-----------------------------MISNHRHLHLTLASIHQQT----HRIDATLTYRVFAPRVQVK
>Calidris_pugnax
-----------------------------MISNHRHLHLTRAPIHQQT----HRIDATLTYRVLVPNLQVK
>Rousettus_aegyptiacus
-----------------------------MISNHRHLHLTLASIHQQT----HRIDATLTYRLFAPRVQVK
>Dasypus_novemcinctus
-----------------------------MISNHRHLHLTRASIHQQT----HRIDATLTYRVLARRVQVK
>Alligator_mississippiensis
MLAPCKAAVLGLKFQHGRGCSQSSLQKLGMISNHRHLHLTRAPIHQQT----HRIDAILTYRVLIPNLQVK
>Galeopterus_variegatus
-----------------------------MISNHRHLHLTLASIHQQT----HRIDATLTYRVLAPRVQVK
>Gallus_gallus
MLAPCKAAVLGLKFQHGRGCSQSSLQKLGMISNHRHLHLTRAPIHQQT----HRIDAALTYRVLEPNLQVK
>Apteronotus_leptorhynchus
-----------------------------MVSNRMHLRLTRTTIHQRT----NRIDVALTSEVILSYTQV-
>Ochotona_princeps
-----------------------------MISNHKHLRLTLASIHQQT----QRIDATLTYRVLAPRVQVK
>Ornithorhynchus_anatinus
-----------------------------MISNHRHLHLTRAPIHQQT----HRIDATLTYRVPGPRIQVK
>Scleropages_formosus
-----------------------------MVSNGKHPRLTRAAVHQKKKIQHSGIDVTLTCEAALSHTQV-
>Manacus_vitellinus
-----------------------------MISNHRHLHLTRAPIHQQT----HRIDATLTYRVLVPNLQVK
>Equus_asinus
-----------------------------MISNHRHLHLTLASIHQQT----HRIDATLTYRVFAPRVQVK
>Manis_javanica
-----------------------------MISNHRHLHLTRASIHQQT----HRIDATLTYRVFAPRVQVK
>Ictidomys_tridecemlineatus
-----------------------------MISNHRHLHLTLASIHQQT----HRIDATLTYRVLAPRVQVK
>Tupaia_chinensis
-----------------------------MISNHRHLHLTLASIHQQT----HRIDATLTYRVLAPRVQVK
>Lepisosteus_oculatus
-----------------------------MISNRRHPRLTLAPIHQQT----YRIDVTLTSEAALPHIQA-
>Trichechus_manatus_latirostr
-----------------------------MISNHRHLHLTRASIHQQT----HRIDATLTYRVLPPRVQVK
>Struthio_camelus_australis
-----------------------------MISNHRHLHLTRAPIHQQT----PRIDATLTYRVLVPNLQVK
>Chrysemys_picta
MLAPCKAAVLGLKFQHGRGCSQSSLQKLGMISNHRHLHLTRAPIHQQT----QRIDATLTYRVLVPNLQVK
>Tinamus_guttatus
-----------------------------MISNHRHLHLTRAPVHQQT----RRIDATLTYRVLLPNLQVK

HG0103 ENSG00000165197 VEGFD

>Homo_sapiens
---MPFETIFSSCLMSTA-
>Canis_lupus_familiaris
---MPFETVFSSCLMSTAW
>Orcinus_orca
---MPFEPVCSSCLRSTAW
>Rousettus_aegyptiacus
---MPFETVFSSCLMSTSW
>Dasypus_novemcinctus
---MPFDTIFSSCLTSTA-
>Sorex_araneus
---MPFETVFSSCLMSTAW
>Oryctolagus_cuniculus
---MPFETIFSACLMSTA-
>Equus_asinus
---MPFETVFSSCLMSSAW
>Manis_javanica
---MPFETVSSSCLMSTVW
>Loxodonta_africana
---MPFEAIFSICLMSAAC
>Octodon_degus
---MPFETVFSSCLMSTA-
>Tupaia_chinensis
---MPFATIFSSCLMSTV-
>Trichechus_manatus_latirostr
MRTFYIFFFSLSPPPTRLCKNVPCLIEVISLDFDHNYLFFHVRSWGFQLSGEEMPFEAIFSICLMSTAC

HG0103 ENSGALG00000016558 VEGFD

```
>Gallus_gallus
----------------MHFKRDASSCMTTTA
>Anser_cygnoides
MTEKLESFLVCIGDREMHFKRDASSCVTTTA
>Chaetura_pelagica
----------------MHFETDTSSCMTTTA
>Apteryx_australis_mantelli
----------------MHFKTATSSCMTTTV
>Buceros_rhinoceros_silvestri
----------------MHFETDASICMTTTA
>Cariama_cristata
----------------MHFETDASSCMTTTA
>Charadrius_vociferus
----------------MHFETDASSCMTTTA
>Pterocles_gutturalis
----------------MHFETDASSCMTTTA
>Colius_striatus
----------------MHFKTDVSSCMTTTA
>Columba_livia
----------------MHFETDTSSYMTTTA
>Merops_nubicus
----------------MHFETDASSCMTTTE
>Alligator_mississippiensis
----------------MHFKTDSSSCTISTA
>Cuculus_canorus
----------------MHFETDASSCVMTTA
>Aquila_chrysaetos_canadensis
----------------MHFETDASSCMTTTA
>Mesitornis_unicolor
----------------MHFKTDASSCMTTTA
>Ornithorhynchus_anatinus
----------------MHFERDSSSCTISTA
>Tauraco_erythrolophus
----------------MHFKTDACSCMTTTA
>Opisthocomus_hoazin
----------------MHFKTDASSCMMTTA
>Lepidothrix_coronata
----------------MHFETDASSCMTTTA
>Egretta_garzetta
----------------MHFETDASSCMTTTA
>Melopsittacus_undulatus
----------------MHFETDASSCMTTSA
>Aptenodytes_forsteri
----------------MHFETDASSCMTTTA
>Struthio_camelus_australis
----------------MHFETDASSCMTITA
>Chelonia_mydas
----------------MHFETEPSSCMISTA
>Apaloderma_vittatum
----------------MHFKTDASSCMTLTA
```

HG0104.1 ENSGALG00000038097 EI24

>Gallus_gallus
---MMREAWVRLFPSPCS
>Anas_platyrhynchos
---MMREAWVRLFPSPCS
>Chaetura_pelagica
--MREVWDRLFPSPCS
>Cariama_cristata
--MREVWVRLFPSPCS
>Charadrius_vociferus
--MREVWVRLFPSPCS
>Colius_striatus
--MREVWVLLFPSPCS
>Columba_livia
--MREVRVRLFPSPRS
>Merops_nubicus
--MREVWGRLFPSPCS
>Sarcophilus_harrisii
--MRDFGVLLFPSPCS
>Monodelphis_domestica
--------------------------MGNPR----------PREGKLCVSRLFMMRDFGILLFPSPCS
>Trichosurus_vulpecula
--MRDFGILLFPSPCS
>Haliaeetus_leucocephalus
--MREVWVRLFPSPCS
>Gavia_stellata
--MREVWVRLFPSPCS
>Balearica_regulorum_gibberic
--MREVWVRLFPSPCS
>Tauraco_erythrolophus
--MREVWAQLFPSPRS
>Sturnus_vulgaris
MVVPSGKPVRAGNPVGNHQGVTELVIPGQPTVSSSAMALCVLSPQAPSVSHLSRMREAWVRLSPSPCS
>Phalacrocorax_carbo
--MREVWVRLFPSPCS
>Fulmarus_glacialis
--MREVWVRLFPSPCS
>Melopsittacus_undulatus
--MREVWVRLFPSPCS
>Aptenodytes_forsteri
---------------------------------MPAAVRCFLSPQAPSVSHLSRMREVWVRLFPSPCS
>Anolis_carolinensis
--MRQGWVHFFPSPCS
>Tyto_alba
--MREVWVRFFPSPCS
>Chelonia_mydas
---MMTEVWALLFPSPCS
>Calypte_anna
--MREVWVGLSPSPCS
>Apaloderma_vittatum
--MREVWVRLFPSPCS

HG0104.2 ENSG00000149547 EI24

```
>Homo_sapiens
---------MLVLK-------------QKSSLQSLSKNKSRPRSFMMRDLGTLLSPVCS-
>Leptonychotes_weddellii
---------------------------------------------MMKDFETLLPPLCSW
>Lipotes_vexillifer
---------------------------------------------MMRHLGTLLSPLCS-
>Miniopterus_natalensis
---------------------------------------------MMKEFGMLLYPLCSW
>Condylura_cristata
---------------------------------------------MMKVLGALLSPLCNW
>Oryctolagus_cuniculus
MPSAGSVAPALGAPRRSERRARRSRAAGGGRGTGPSRGGRGAGPFMMRDLGAFLSPLCSR
>Ceratotherium_simum_simum
---------------------------------------------MMKDFGTLLSPPCSW
>Manis_javanica
---------------------------------------------MMKDFGMLLPLLCSW
```

HG0105 ENSG00000135298 ADGRB3

```
>Homo_sapiens
------------------------------------------------MGNGDWDRRGVSPSLLLGRVPVLVS--
>Anser_cygnoides
MWLDLVDPEPKLLFSLGGVCVCRAGVFSPRFLSSAAFFNLGFEPEPGKMGNGASDRRGISPSLLLGRVPVVLVF-
>Chaetura_pelagica
------------------------------------------------MGNGASDRRGISPSLLLGRVPVVLVF-
>Odobenus_rosmarus_divergens
------------------------------------------------MGNGDWDRRGVSPSLLLGRVPVLVS--
>Lipotes_vexillifer
------------------------------------------------MGNGTWDRRGVSPSLLLGRVPVLVS--
>Pteropus_alecto
------------------------------------------------MGNGDWDRRGVSPSLLLGRVPVLVS--
>Dasypus_novemcinctus
------------------------------------------------MGNGDWDRRGVSPSLLLGRVPVLVS--
>Galeopterus_variegatus
------------------------------------------------MGNGDWDRRGVSPSLLLGRVPVLVS--
>Monodelphis_domestica
------------------------------------------------MGNGDSARRGVSPSLLLGRVPVLGS--
>Aquila_chrysaetos_canadensis
------------------------------------------------MGNGASDRRGISPSLLLGRVPVVLVF-
>Coturnix_japonica
------------------------------------------------MGNGASDRRGISPSLVSGRVPVVLEV-
>Condylura_cristata
------------------------------------------------MGNGDWDRRGVSPSLLLGRVPVLVS--
>Oryctolagus_cuniculus
------------------------------------------------MGNGDWERRGVSPSLLLGRVPVLVS--
>Parus_major
------------------------------------------------MGNGASVRRGISPSLLLGRVPVVLVF-
>Nipponia_nippon
------------------------------------------------MGNGASDRRGISPSLLLGRVPVVLVF-
>Equus_caballus
------------------------------------------------MGNGDWDRRGVSPSLLLGRVPVLVS--
>Manis_javanica
------------------------------------------------MGNGAWDRRGVSPSLLPGRVPALVS--
>Loxodonta_africana
------------------------------------------------MGNGDGARRGVSPSLLLGRVAVLVS--
>Ictidomys_tridecemlineatus
------------------------------------------------MGNGDWDRRGVSPSLLLGRVPVLVS--
>Trichechus_manatus_latirostr
------------------------------------------------MGNGDWDRRGVSPSLLLGRVPVLVS--
>Pygoscelis_adeliae
------------------------------------------------MGNGASDRRGISPSLLLGRVPVVLVF-
>Thamnophis_sirtalis
------------------------------------------------MGNDASARRGMSPCLFLGHVLVVLGSS
>Struthio_camelus_australis
MWLDLVDPEPKLLFFLGGVYVWRAGVFSLRFSSSAAFFNLGFEPEPGKMGNGASDRRGISPSLLSGRVPVVLVF-
>Chrysemys_picta
------------------------------------------------MGNGASDRRGISPSLLLGRVPVVLVF-
>Calypte_anna
MWLDLVDPESKLLFFLCGVCVWRAGVFSLRFLSSTAFLNLGFEPEPGKMGNGASDRRGISPSLLLGRVPVVLVF-
```

HG0105 ENSGALG00000016176

>Gallus_gallus
MWLDLADPEPELLFFRGAVGVREACGGYLAAFFSLLLPFFFFFFFFLVGFEPEPGKMGNGASDRRGISPSLVSGRVPVVLEV-
>Anser_cygnoides
MWLDLVDPEPKLLFSLGGVCVCRAG------VFS--PRFLSSAAFFNLGFEPEPGKMGNGASDRRGISPSLLLGRVPVVLVF-
>Chaetura_pelagica
--MGNGASDRRGISPSLLLGRVPVVLVF-
>Leptonychotes_weddellii
--MGNGDWDRRGVSPSLLLGRVPVLVS--
>Orcinus_orca
--MGNGAWDRRGVSPSLLLGRVPVLVS--
>Dasypus_novemcinctus
--MGNGDWDRRGVSPSLLLGRVPVLVS--
>Galeopterus_variegatus
--MGNGDWDRRGVSPSLLLGRVPVLVS--
>Monodelphis_domestica
--MGNGDSARRGVSPSLLLGRVPVLGS--
>Aquila_chrysaetos_canadensis
--MGNGASDRRGISPSLLLGRVPVVLVF-
>Condylura_cristata
--MGNGDWDRRGVSPSLLLGRVPVLVS--
>Ochotona_princeps
--MGNGGWERRGISRSLLLGRVPVLVS--
>Lepidothrix_coronata
MWLDLVDPEPKLLFFLGGVCVWRAG------IFS--LRFLSSAAFFNLGFEPEPGKMGNGASVRRGISPSLLLGRVPVVLVF-
>Nipponia_nippon
--MGNGASDRRGISPSLLLGRVPVVLVF-
>Manis_javanica
--MGNGAWDRRGVSPSLLPGRVPALVS--
>Microcebus_murinus
--MGNGDWDRRGVSPSLLLGRVPVLVS--
>Loxodonta_africana
--MGNGDGARRGVSPSLLLGRVAVLVS--
>Ictidomys_tridecemlineatus
--MGNGDWDRRGVSPSLLLGRVPVLVS--
>Trichechus_manatus_latirostr
--MGNGDWDRRGVSPSLLLGRVPVLVS--
>Pygoscelis_adeliae
--MGNGASDRRGISPSLLLGRVPVVLVF-
>Thamnophis_sirtalis
--MGNDASARRGMSPCLFLGHVLVVLGSS
>Struthio_camelus_australis
MWLDLVDPEPKLLFFLGGVYVWRAG------VFS--LRFSSSAAFFNLGFEPEPGKMGNGASDRRGISPSLLSGRVPVVLVF-
>Pelodiscus_sinensis
--MGNGASDRRGISPSLVLGRVPVVLVF-
>Calypte_anna
MWLDLVDPESKLLFFLCGVCVWRAG------VFS--LRFLSSTAFLNLGFEPEPGKMGNGASDRRGISPSLLLGRVPVVLVF-

HG0106 ENSG00000147548 NSD3

>Homo_sapiens
--MEQTEGPMRERES
>Pseudacris_regilla
--MEQTEGPMRERES
>Canis_lupus_familiaris
--MEQTEGPMRERES
>Hynobius_retardatus
--MDQTEGPMRERES
>Balaenoptera_acutorostrata_s
--MEQSEGPMRERES
>Calidris_pugnax
--MEQTEGPMRRRES
>Miniopterus_natalensis
--MEQTEGPMRERES
>Latimeria_chalumnae
--MEQTEGPMRKRES
>Sarcophilus_harrisii
--MEQTEGPMRERES
>Galeopterus_variegatus
--MEQTEGPMRERES
>Aegypius_monachus
MRRGALRAPLGARRKGSHLPARRGRRRPRRRPLSPLGWVDAAGAVRMEQTEGPMRKRES
>Gallus_gallus
--MEQTEGPMRKRES
>Condylura_cristata
--MEQTEGPMRERES
>Oryctolagus_cuniculus
--MEQTEGPMRERES
>Elephantulus_edwardii
--MEQTEVPMREREN
>Ornithorhynchus_anatinus
MRLRPAPAPS------PSAPGSSCGPTLLRAPVSLLGSHRHGRGVRMEQTEGPMRERES
>Lepidothrix_coronata
--MEVTEGPMRKRES
>Ceratotherium_simum_simum
--MEQTEGPMRERES
>Manis_javanica
--MEQTEGPMRERES
>Ictidomys_tridecemlineatus
--MEQTEGPMRERES
>Lepisosteus_oculatus
--MEHAEGPVRERES
>Trichechus_manatus_latirostr
--MEQTEGPMREREN
>Takydromus_sexlineatus
--MEQTDGPMRKRES
>Chrysemys_picta
--MEQTDGPMRERES
>Orycteropus_afer_afer
--MEQTEGPMREREN

HG0107.1 ENSG00000058668 ATP2B4

>Homo_sapiens
MHILFTPSSGQVGLS
>Chaetura_pelagica
MHILFTPLSGEVGLG
>Ailuropoda_melanoleuca
MHILFTPPSGQVGLS
>Balaenoptera_acutorostrata_s
MHILFTPPSGQVGLS
>Charadrius_vociferus
MHILFTPLSGEVGLG
>Callorhinchus_milii
MHFLTTPSSGEVGLS
>Rhinolophus_ferrumequinum
MHILFTPPSGQVGLS
>Dasypus_novemcinctus
MHILFILQSGQVGLS
>Latimeria_chalumnae
MHFLFTPLSGEVGLG
>Columba_livia
MHILFTPLSGEVGLG
>Alligator_mississippiensis
MHILFTPLSGEVGLG
>Galeopterus_variegatus
MHILFTPSSGQVGLS
>Aquila_chrysaetos_canadensis
MHILFTPLSGEVGLG
>Gallus_gallus
MHILFTPSSGGLGLG
>Condylura_cristata
MHILFTPPSGQVGLS
>Ornithorhynchus_anatinus
MHILFTPRSREVGLG
>Lepidothrix_coronata
MHILFTPLSGEVGLG
>Equus_asinus
MHILFTPPSGQVGLS
>Nestor_notabilis
MHILFTPLSGEVGLG
>Ictidomys_tridecemlineatus
MHILFTPSSGQVSLN
>Tupaia_chinensis
MFILFIPPSGQVGLS
>Pygoscelis_adeliae
MHILFTPLSGEVGLG
>Gekko_japonicus
MCILFTPSSEEVGLG
>Chrysemys_picta
MHILFTPLSGEVGLS

HG0107.1 ENSGALG00000003601 ATP2B4

>Gallus_gallus
MHILFTPSSGGLGLG
>Anser_cygnoides
MRILFTPASGGEGLG
>Chaetura_pelagica
MHILFTPLSGEVGLG
>Ailuropoda_melanoleuca
MHILFTPPSGQVGLS
>Balaenoptera_acutorostrata_s
MHILFTPPSGQVGLS
>Calidris_pugnax
MHILFTPLSGEVGLG
>Rhinolophus_ferrumequinum
MHILFTPPSGQVGLS
>Latimeria_chalumnae
MHFLFTPLSGEVGLG
>Columba_livia
MHILFTPLSGEVGLG
>Alligator_sinensis
MHILFTPLSGEVGLG
>Galeopterus_variegatus
MHILFTPSSGQVGLS
>Aquila_chrysaetos_canadensis
MHILFTPLSGEVGLG
>Condylura_cristata
MHILFTPPSGQVGLS
>Ornithorhynchus_anatinus
MHILFTPRSREVGLG
>Ficedula_albicollis
MHILFTPSSPQLGLG
>Equus_asinus
MHILFTPPSGQVGLS
>Cebus_capucinus_imitator
MHILFTPSSGQVGLS
>Nestor_notabilis
MHILFTPLSGEVGLG
>Ictidomys_tridecemlineatus
MHILFTPSSGQVSLN
>Pygoscelis_adeliae
MHILFTPLSGEVGLG
>Gekko_japonicus
MCILFTPSSEEVGLG
>Chrysemys_picta
MHILFTPLSGEVGLS

```
HG0107.2 ENSG00000070961 ATP2B1

>Homo_sapiens
-------------------------------------------MIDMEKLVMGQRSRYFSGKCCC
>Odobenus_rosmarus_divergens
-------------------------------------------MIDMKKLVMGQRSRYFSGKCC-
>Lipotes_vexillifer
-------------------------------------------MTDRKKLVMGQRSRYFSGKCC-
>Pteropus_vampyrus
-------------------------------------------MIDMRKLIMGQRSRYFSGKCC-
>Dasypus_novemcinctus
MRREPSPRSRLLVSVTGPRPFFSFLFLSPSENGSCYRARYVHLMIDMKKLVMGQRSRHFLGKSCC
>Galeopterus_variegatus
---------------------------------------------------MGQRSRYFSGKCCY
>Monodelphis_domestica
---------------------------------------------------MGQSSRDLHGKCC-
>Condylura_cristata
-------------------------------------------MIDVKKLVMGQRSRYFSGKCC-
>Oryctolagus_cuniculus
-------------------------------------------MIEMKKQVMGQRSRYFSGKCCC
>Elephantulus_edwardii
-------------------------------------------MIDRKKLVMGQRSRYFSGKCCC
>Ceratotherium_simum_simum
-------------------------------------------MIDRKKLVMGQRSRYFSGKCC-
>Manis_javanica
-------------------------------------------MIDTKKLVMGQRSRYFSGKCC-
>Loxodonta_africana
----------------------------------------MHLMIDMKKLVMGQRSRYFSGKCCC
>Cavia_porcellus
-------------------------------------------MIDMKKQVMGQRSRYFSGKCCC
>Tupaia_chinensis
-------------------------------------------MIDMKKLVMGQKSRYFSEKCCY
>Orycteropus_afer_afer
----------------------------------------MHLMIDMKKLVMGHMSRYFSGKCCC
```

HG0107.2 ENSGALG00000030550

```
>Gallus_gallus
----------MGRPQRYLSKMGLLLG
>Anas_platyrhynchos
----------MGRPQRYLSRRGLLLG
>Caprimulgus_carolinensis
----------MGQAERYLSRWGLLLR
>Calidris_pugnax
----------MGQPQRYLSRCGLLLG
>Colius_striatus
----------MGQPQRYLSRRGLLLE
>Columba_livia
----------MGQPQRYLSRRGLLLG
>Merops_nubicus
----------MGPPQRYLSRRGLLLG
>Cuculus_canorus
----------MGQPQRYLSRRGLLLG
>Aquila_chrysaetos_canadensis
----------MGQPQRYLSRRGLLLG
>Eurypyga_helias
----------MGQPQRYLSRRDLLLG
>Tauraco_erythrolophus
----------MGQPQRYLSRRDLLLG
>Opisthocomus_hoazin
----------MGQPQRYLSRRGLLLG
>Corvus_brachyrhynchos
----------MGQPQRYLSRRGLLLG
>Nipponia_nippon
----------MGQPQRYLSRRGLLLG
>Fulmarus_glacialis
----------MGQPQRYLSRRGLLLG
>Nestor_notabilis
----------MGQPQRFLSRRGFLLG
>Aptenodytes_forsteri
----------MGQPQRYLSRQGLLLG
>Tyto_alba
----------MGQPQRYLSRRGLLLG
>Struthio_camelus_australis
MPFGLGTELTMGQHQRYISRWGLLLG
>Tinamus_guttatus
MPFGLGTELAMGQHQRYLSRRGLLLG
>Calypte_anna
----------MGQPERFLSRRGLLLG
>Apaloderma_vittatum
----------MGQPQRYLSRRGLLLG
```

HG0107.3 ENSDARG00000063433 atp2b2

```
>Danio_rerio
MGNHVWSDAGLQPW
>Acipenser_sinensis
MGNCIWSEAGLQP-
>Channa_argus
MGSHTWSRSGLQPW
>Menidia_menidia
MGSHTWSGSGLQPW
>Porichthys_notatus
MGSHTWSMSGLQPW
>Oryzias_latipes
MGSHTWSGSGRQPW
>Micropterus_salmoides
MGNHTWSGSGLQPW
>Pygocentrus_nattereri
MGNHVWSDAGLQPW
>Haplochromis_burtoni
MGSHTWSGSGLQPW
>Austrofundulus_limnaeus
MGSHTWSGSGLQPW
>Lateolabrax_japonicus
MGSHTWSGSGLQPW
>Platichthys_stellatus
MGSHTWSGSGLQPW
>Lepisosteus_oculatus
MGNYIWSGAGLQP-
>Ictalurus_punctatus
MGNAVWSDAALQPR
```

HG0107.3 ENSG00000157087 ATP2B2

```
>Homo_sapiens
-----------MPVPGSSMGPTEWRLLGLQP
>Canis_lupus_familiaris
-----------MPAPGSSMGPTEWRLLGLQP
>Lipotes_vexillifer
-----------MPAPGSSMGPAEWRLLGLQP
>Myotis_davidii
-----------MPAPGSSMGPTEWRLLGLDP
>Dasypus_novemcinctus
-----------MSAPGSSMGPAEWRLLGLQP
>Condylura_cristata
------------------MGPTEWRLLGLQP
>Oryctolagus_cuniculus
-----------MPALGSSMGPTEWRLLGLQP
>Equus_asinus
-----------MQAPGSSMGPTEWRLLGLQP
>Manis_javanica
MILGATTPDHVDASSGSSMGPTEWRLLGLQP
>Nannospalax_galili
-----------MPAPGSSMGPTEWRLLGLQP
>Tupaia_chinensis
------------------MGPTGWRLLGLQP
>Trichechus_manatus_latirostr
-----------MPALGSSMGATEWRLLGLQP
>Orycteropus_afer_afer
-----------MPALGNSMGATEWRLLGLQP
```

HG0107.4 ENSG00000070961 ATP2B1

>Homo_sapiens
MSVWFKKDFHT
>Mustela_putorius_furo
MSVWFKKDFHI
>Balaenoptera_acutorostrata_s
MSVWFKKDFHI
>Rousettus_aegyptiacus
MSVWFKKDFHI
>Galeopterus_variegatus
MSVWFKKDFHI
>Condylura_cristata
MSVWLKKDFHI
>Ceratotherium_simum_simum
MSVWFKKDFHI
>Loxodonta_africana
MSVWFKEDFHI
>Mus_musculus
MSVWFKEDFHI
>Tupaia_chinensis
MSVWFKKDFHI
>Trichechus_manatus_latirostr
MSVWFKEDFHI

HG0108.1 ENSGALG00000007396 TAOK3

>Gallus_gallus
-------------------MKEETDWDLKAPTSS
>Anas_platyrhynchos
-------------------MKEEIDWELKARASS
>Apteryx_australis_mantelli
-------------------MKEEIDWESKARTSS
>Buceros_rhinoceros_silvestri
-------------------MKEETDWKSKAQTGS
>Cariama_cristata
-------------------MKEEIVWKLKARTSS
>Calidris_pugnax
-------------------MKEEIDWELKARTSS
>Colius_striatus
-------------------MKEEIDWKLKAQTSS
>Columba_livia
-------------------MKEETDWKLKARTGS
>Merops_nubicus
-------------------MKEEIDWELKAQTS-
>Alligator_mississippiensis
-------------------MKAETDWEFKARTRS
>Cuculus_canorus
-------------------MKEESDWELKARTSS
>Falco_cherrug
-------------------MKEEIDWELKARTSS
>Gavia_stellata
-------------------MKEEIDWELKVRTSS
>Eurypyga_helias
-------------------MKEETDWELKARASS
>Tauraco_erythrolophus
-------------------MKEEIDWKLKARTSS
>Opisthocomus_hoazin
-------------------MKEEVDWELKGRTSS
>Corvus_cornix_cornix
-------------------MKEEIGWDLRAQTSS
>Phaethon_lepturus
-------------------MKEEIDWELKARTSS
>Nestor_notabilis
-------------------MKEETDWELKAQTSL
>Aptenodytes_forsteri
-------------------MKEEIDWELKARTSS
>Takydromus_sexlineatus
MLIAIVTQILNCYLQTPLSMREETDWELEAGTSS
>Tyto_alba
-------------------MKEEIGWKLKARTSS
>Pelodiscus_sinensis
-------------------MREETDWEFKARTRS
>Tinamus_guttatus
-------------------MKEEVDWESKARTSS

HG0108.2 ENSG00000135090 TAOK3

```
>Homo_sapiens
----------------MNLSNMEYFVPHTKRY
>Acinonyx_jubatus
MQKTEVLKEDKGFAAKMNLSNTVYFVPFTKRY
>Balaenoptera_acutorostrata_s
----------------MSLSNTVYFVPYTKRY
>Eptesicus_fuscus
----------------MNLSNAVYFVPYTKRY
>Condylura_cristata
----------------MNLSNTVHFVPYTKRC
>Oryctolagus_cuniculus
----------------MNLSNPVHFLPHTKRY
>Equus_asinus
----------------MNLSNTVYFVPYTKRY
>Manis_javanica
----------------MNLRNTVYFVPYTKRY
>Marmota_marmota_marmota
----------------MNLSNTAHFVPHTKRY
>Tupaia_chinensis
----------------MNLSNT-NFVPHTKRY
>Trichechus_manatus_latirostr
----------------MNLSNTVYFVPCTKRC
```

HG0108.3 ENSDARG00000098304 taok1b

>Danio_rerio
MSNQRGLTCRSRTAR
>Pygocentrus_nattereri
MSHQRGLARRSRTAR
>Ictalurus_punctatus
MSHHRGLACRSRTAR

HG0109.1 ENSG00000048540 LMO3

>Homo_sapiens
MNGSHRQTSDCSHLQAFLKGEGCVLFPGYSGEALQSSHCAY
>Anser_cygnoides
----------------------MLLFDLSQGEELQSSHCAY
>Chaetura_pelagica
----------------------MLLFDLSQGEELQSSHCAY
>Ursus_maritimus
----------------------MLLFDLSQGEALQSSHCAF
>Charadrius_vociferus
----------------------MLLFDLFQGEELQSSHCAY
>Myotis_lucifugus
----------------------MLLFDLSQGEALQSSHCAF
>Columba_livia
----------------------MLLFDLSQGEELQSSHCAY
>Alligator_sinensis
----------------------MLLFDLSQGEELQSSHCAY
>Cuculus_canorus
----------------------MLLFDLSQGEELQSSHCAY
>Galeopterus_variegatus
----------------------MLLFDLSQGEALQSSHCAY
>Aquila_chrysaetos_canadensis
----------------------MLLFDLFQGEELQSSHCAY
>Coturnix_japonica
----------------------MLLFDLSQGEELQSSHCAY
>Condylura_cristata
----------------------MLLFDLSQGEALQSSHCAY
>Lepidothrix_coronata
----------------------MLLFDLFQGEELQSSHCAY
>Nipponia_nippon
----------------------MLLFDLFQGEELQSSHCAY
>Equus_asinus
----------------------MLLFDLSQGEALQSSHCAF
>Picoides_pubescens
----------------------MLLFDLSQGEELQSSHCAY
>Melopsittacus_undulatus
----------------------MLLFDLFQGEELQSSHCAY
>Marmota_marmota_marmota
----------------------MLLFDLSQGEALQSSHCAY
>Struthio_camelus_australis
----------------------MLLFDLSQGEELQSSHCAY
>Chelonia_mydas
----------------------MLLFDLSQGEELQSSHCAY
>Tinamus_guttatus
----------------------MLLFDLSQGEELQSSHCAY
>Calypte_anna
----------------------MLLFDLSQGEELQSSHCAY
>Orycteropus_afer_afer
----------------------MLLFDLSQGEALQSSHCAY

HG0109.2 ENSG00000048540 LMO3

```
>Homo_sapiens
MQHIFYG----DYSDCCYEIQVRFMIFFILNMICYANAACDSLIKSICYQEAKRGRWDFRITGRVREACGFVIRHH--FAHCEGL----RV-CALSCLAVLIPSPTPCRHS------DPLPKWLHNSRSSAV----------
>Mustela_putorius_furo
------------------------MIFFILNMICYANAACDSLIKSICYHEAKRGRWDFRITGRVREACGFVIRHH--FAHCHTARGSVCALCLLWPPSSLVPSPTPCRHS------DPLPKWLHNSRSSAV----------
>Hynobius_retardatus
-------------------------------MIWYANAACDSLIKSICYQPAKRDHWDYRITGRVREACGFVIRHH--LHTAATCEGLAACLCLCVCVPE------------------------------------------
>Tursiops_truncatus
------------------------MIFFILNMICYANAACDSLIKSICYHDAKRGRWDFRITGRVREACGFVIRHH--FAHCHTARGSVCAPCLVWPPSSFPPPPAPPLRP------A--PKVASQ----------------
>Myotis_brandtii
MQHIFIGWGEKNFPQSCYEIQLRFMIFFILNMICYANAACDSLIKSICYREAQRGRWDFRIMGRVREACGFVIRHR--FAHRHPARGL--SVCAASRLAALVPSPTPCRHS------DPLPKWLHNSRSSSGVGCAPGPPSS
>Galeopterus_variegatus
------------------------MIFFILNMICYANAACDSLIKSICYQEAKRGLWDFRITGRVREACGFVIRHY--FAHCHTARGSVCV-CAVSCLAVLLPSPPPCRHS------DPLPKWLHNTRSSAV----------
>Oryctolagus_cuniculus
------------------------MIFFILNMICYANAACDSLIKSICYQEAKGGRWDFRITGRVREACGFVIRHR--FAHRHTARGSVCAPCLVWQPSPFPH-PLPPLRP------A--PKVASQ----------------
>Loxodonta_africana
----------------------------------------------------------------------MWLCHSPSFCTLPHCEGL--RVCAVSRLAVLIPSPTLRRHS------DPLPKWLHNSRSSAV----------
>Ctenomys_sociabilis
------------------------MTFFILNMICYANAACDSLIKSICYQEAKKGRWDFRIAGRVREACGFVIRHH--FAHCHAARGSVCALCLVRPPSSLPPPPASTPTLSQSGFTVVDPQWWCRLRTRSTLEPN------
>Trichechus_manatus_latirostr
----------------------------------------------------------------------MWLCRSPSFCTLPPCEGL--RVCAVSLLAVLIPSPTLRRHS------DPLPKWLHNSRSSAV----------
>Orycteropus_afer_afer
----------------------------------------------------------------------MWLCHSPSFCTLPHCEGL--LVRALSRLAVLFPSPTPRRH--------------------------------
```

HG0109.3 ENSG00000048540 LMO3

```
>Homo_sapiens
-----------MYACLDGPEKPNLRNLPMYRTSQLRGSH---------------------------------
>Canis_lupus_familiaris
-----------MCACLDGPEKPNLRNLPMYRTNQLKGFLLIRRL----------------------------
>Orcinus_orca
-----------MCACLDRPEKPSLRELPMYRTNPLKGFLLICRL----------------------------
>Rousettus_aegyptiacus
-----------MCACLDGPEKPNLRNLPMYRTNQLKGFLLICRL----------------------------
>Dasypus_novemcinctus
-----------MCACSDGPAKPNLRKLPMYRTNQLKGVLLICRL----------------------------
>Galeopterus_variegatus
------MG-EVRVRLLRGPEQPNLRNLPMHRPNQLKGFPLICRLSVNFVIFQEKRYKAATVLTERPPPISLN
>Monodelphis_domestica
MSKDLFIQMRCMCACISIRERQKLRNLLIYRSNQLKGVVLTC------------------------------
>Oryctolagus_cuniculus
-----------MCACIDGPGKPNLRNLPMHRTSQRKGFSLICRL----------------------------
>Elephantulus_edwardii
----------------------------MYRTNQLKGVSLICRL----------------------------
>Equus_asinus
-----------MCSCLDGPEKPNLRNLPMYRTNQLKGSY---------------------------------
>Manis_javanica
------MGEVHMCLLR-PARKAKHRKLPMYRTNQLKGFLLICRL----------------------------
>Cavia_porcellus
----------------------------MFRTNQLKGFSLICRL----------------------------
>Tupaia_chinensis
--------MRCMCACLDGPEKPNLRISAYVQNKSAE-GVLINLPSVNFVLFQEKRYKAASVLTEQPPPVSLN
>Trichechus_manatus_latirostr
-----------MCACLNRPAKPNLRNLPMYRTNQLKGVLLFCRL----------------------------
>Orycteropus_afer_afer
----------------------------MYRTNQLKGVLLICRL----------------------------
```

HG0110.1 ENSG00000091656 ZFHX4

>Homo_sapiens
MPPTFCSSVFIFTQ
>Anas_platyrhynchos
MPPTSCSIIFIFTQ
>Chaetura_pelagica
MPPTSCSIIFIFTQ
>Canis_lupus_familiaris
MPPTFCFSVFIFTQ
>Orcinus_orca
MPPTFCSSVFIFTQ
>Myotis_brandtii
MPPTFCSSVFIFTQ
>Columba_livia
MPPTSCSIIFIFTQ
>Cuculus_canorus
MPPTSCSIIFIFTQ
>Falco_cherrug
MPPTSCSIIFIFTQ
>Gallus_gallus
MPPTSCSIIFIFTQ
>Condylura_cristata
MPPTFCSSVFIFTQ
>Ochotona_princeps
MPPTFCSSVFIFTQ
>Opisthocomus_hoazin
MPPTSCSVIFIFTQ
>Corvus_cornix_cornix
MPPTSCSIIFIFTQ
>Egretta_garzetta
MPPTSCSIIFIFTQ
>Ceratotherium_simum_simum
MPPTFCSSVFIFTQ
>Loxodonta_africana
MPPTFCSSVFIFTQ
>Melopsittacus_undulatus
MPPTSCSIIFIFTQ
>Ictidomys_tridecemlineatus
MPPTFCSSVFIFTQ
>Tupaia_chinensis
MPPTFCSSVFIFTQ
>Aptenodytes_forsteri
MPPTSCSIIFIFTQ
>Struthio_camelus_australis
MPPTSCSIIFIFTQ
>Calypte_anna
MPPTSCSIIFIFTQ

HG0110.2 ENSDARG00000073944

>Danio_rerio
---MRSVLIPAI-FCTKVISRNKDRTDFFSLSPPSSN---------RSQGASVVCSTT----------------------KKQTLL--------NALRNTEIPRLTSAA---
>Oryzias_latipes
MKHMRTELITAI-FCRKLISRNKDPKSFFFSITRSHFHLFLAR------------S--------------DTRLIGCFASKLFLYC-------L--
>Odobenus_rosmarus_divergens
MKHMRTELITAI-FCRKLISRIKIQRLFF-LFT-HQLPLPARPLRPLQPP-----PPPPPPPPPGRS--RRRHRLLCIPITA--
>Ambystoma_mexicanum
MKHMRTALITAI-FCRKLISRIKIQRVSWLRCSGSVQQQQQPPLSGQRRGLGAACTGDRGDPAPGAASAKGRGPGGCVQRASQGAGD------PRAAEDPEQPV----PGTGAQPWPRTRSPRRASGVQRTGHTQGTAAPYPGTLRPRRTPPAAGGCRGRAGEEGGRAGAAAAAAAMERGRRRRCCRAGLPAAPLLSPPSAA
>Astyanax_mexicanus
MKHMRTELITAIFFCRKLISRNKDPKSFFSLFN-HQISLSPLPLSL--
>Neolamprologus_brichardi
---MRTELITAI-FCRKLISRNKDPKSFFFSIIRSHFHLFLAP------------S--------------NTRLIACFASKLFLYC-------L--
>Latimeria_chalumnae
MKHMRTELITAI-FCRKLISRIKIQRLFF-LFN-HQISLSPLPLSLQ------------------------HRHIACFASKLFVTFSLLLFFANSLPNNNALPILMQTDPKELPSWDL--
>Nothobranchius_furzeri
MKHMRTELITAI-FCRKLISRNKDPKSFFFSIIRSHFHLFLAP------------S--------------NTRLIACFASKLFLYC-------L--
>Notothenia_coriiceps
MKHMRTELITAI-FCRKLISRNKDPKSFFFSIIRSHFHLFLAP------------S--------------NTRLIACFASKLFLYC-------L--
>Salmo_salar
MKHMRTELITAI-FCRKLISRNKDPKSFF-LFN-HQISLSPLPRSL--
>Lepisosteus_oculatus
MKHMRTELITAI-FCRKLISRNKDPKSFFSFQS-PDLTFTSSSLPLTQDT-----SPALHP------------NYFYIPFTTNTS----FFYTPQQQ---
>Takifugu_rubripes
MKHMRTELITAI-FCRKLISRNKDPKSFFFSITRSHFHLFLAR------------S--------------NTRLIACFASKLFLYR-------L--

HG0111 ENSGALG00000034944

>Gallus_gallus
MEVISWGGPRALWARVLLSDHWLPPE
>Anser_cygnoides
MEVISWGGPRAPWARVLLSDHWLPPA
>Caprimulgus_carolinensis
MEVISWGGPRALWARVLLSDHWLPPA
>Calidris_pugnax
MEVISWGGPRALWARVLLSDHWLPPA
>Pterocles_gutturalis
MEVISWGGPRALWARVLLSDHWLPPA
>Leptosomus_discolor
MEVISWGGPRALWARVLLSDHWLPPA
>Alligator_sinensis
MEVIIQGGPRALWAQVLLSDHWLPPA
>Cuculus_canorus
MEVISWGGPRALWAGVLLSDHWLPTA
>Aquila_chrysaetos_canadensis
MEVISWGGPRALWARVLLSDHWLPPA
>Gavia_stellata
MEVISWGGPRALWARVLLSDHWLPPA
>Balearica_regulorum_gibberic
MEVISWGGPRALWARVLLSDHWLPPA
>Tauraco_erythrolophus
MEVISWGGPRALWARVLLSDHWLPPA
>Opisthocomus_hoazin
MEVISWGGPRALWARVLLSDHWLPPA
>Lepidothrix_coronata
MEVISWGGPRALWARVLLSDHWLPPA
>Egretta_garzetta
MEVISWGGPRALWARVLLSDHWLPPA
>Melopsittacus_undulatus
MEVISWGGPRALWARVLLGDHWLPPA
>Aptenodytes_forsteri
MEVISWGGPRALWARVLLSDHWLPPA
>Protobothrops_mucrosquamatus
-----MGGLRVLWVRVLLNNHWLIAT
>Struthio_camelus_australis
MEVISWGGPRALWARVLLSDHWLPPA
>Chrysemys_picta
MEVISWGGPRALWARVLLSYHWLPPA
>Tinamus_guttatus
MEGMSWGGPRALWARVLLSDHWLPPA
>Calypte_anna
MEVISWGGPRALWAGVLLSDHWLPPA
>Apaloderma_vittatum
MEVISWGGPRALWARVLLGDHWLPPA

HG0112 ENSG00000105997 HOXA3

>Homo_sapiens
--MKRSARGGHWRRSVT
>Anser_cygnoides
MC--KDT-ASWLSAKKMYMLLVENEERTAKYPMADFRRLR---------------------------------SARRGHWRKSAT
>Xenopus_laevis
---MEMN-AGFLVPGVISDPSCFNRSDQGGHWRRNAT
>Ailuropoda_melanoleuca
---M----PRVGDN-HWIS-LHLSRSARGGHWRRSVT
>Calidris_pugnax
--M-DFILRSARRGHWRKSAT
>Eptesicus_fuscus
----------------MPRLLVEIECRAAQPHMADCHQLRAPGHRKRRFPER----SGVGDNWGFRL-KYLSRSARGSHWRRSVT
>Columba_livia
--M-DFILRSARRGHWRKSAT
>Poecilia_reticulata
------M-GIHVLTSRVEQPLFGHLTFPFRPPLTDRRPSRPPGRTASPCVTE-IRPSNFR--PSLGNTLSCLRFTRGGHWRRGVT
>Esox_lucius
--------------------------------MEDKQVVKTAVYETLVAQRHEIYQNKSLPYYC----VHETRFTQGGHWRRGVT
>Coturnix_japonica
--M-DFILRSARSGHWRSSAT
>Erinaceus_europaeus
---M----CRR------GS-CGNPRSARGSHWRRSIT
>Scleropages_formosus
----------------MRKLHISHE--------LCGMELEHPKHVKKRLAGQDFSLNKVITIYDLGEKFPPLRSTRGGHWRRGVT
>Sturnus_vulgaris
--M-DFILRSARRGHWRKSAT
>Notothenia_coriiceps
-----------------------MEKKQDLDVMADGE-------------EHEIQPSKFRASHALQDTLNCLRFTRGGHWRRGVT
>Equus_asinus
--M-EFILRSARGGHWRRSVT
>Cynoglossus_semilaevis
--MRKYCSV-----LCVRRQRKRFTRGGHWRRGVT
>Chinchilla_lanigera
--M-EFILRSARGGHWRRSVT
>Salmo_salar
--------------------------------MA----------VDEILKRYEFCLNKSHPSYSLHEILPCHRFTRGGHWRRGVT
>Pygoscelis_adeliae
--M-DFILRSARRGHWRKSAT
>Anolis_carolinensis
MCSCRKTQSLGCALMKMYTFWEENPARREKIHMAGCHHPSSPVHWKIRFKDS-LSLEII-----LPGVGNARRSTRRGHWRRNVT
>Struthio_camelus_australis
MC--KDT-ASWLSAKKMYTLSVEIEPRTAKYPMADYHHLREPVHRKTPICLADRSLEQFS----PTL-SDFKRSARRGHWRKSAT
>Takifugu_rubripes
------------------------MKREADLGMA-------------ECTMHEIQPSKFTPSLGARKTINCLRFTRGGHWRRGVT
>Tinamus_guttatus
--M-DFILRSARRGHWRKSAT

HG0113 ENSDARG00000099727 ssbp2

```
>Danio_rerio
-------------------MGGIAPAVAMVTGLCPGRL
>Acipenser_sinensis
-------------------MGSDAPCVAMVTGLCPGRL
>Polypedates_megacephalus
----------------------------MVTGLFPGRL
>Porichthys_notatus
-------MERGRSRTAGHPMRVAAACVAMVTGLCPGRL
>Oryzias_latipes
-------------------MEYDAACVTMVTGLCPGRL
>Canis_lupus_familiaris
-------------------MVGVAAWVAMGTGLFPGRL
>Orcinus_orca
----------------------------MGTGLFPGRL
>Haplochromis_burtoni
-------------------MGDDAACVTMVTGLCPGRL
>Dasypus_novemcinctus
----------------------------MGTGLFPGRL
>Alosa_alosa
MSVRGVTKQNGRFFCARCPMGAIGPAVTMVTGLCPGRL
>Latimeria_menadoensis
----------------------------MVTGLFPGRL
>Austrofundulus_limnaeus
-------------------MEDAAACVTMVTGLCPGRL
>Falco_peregrinus
-------------------MGCVCGSVSMVTGLFPGRL
>Sorex_araneus
----------------------------MGTGLFPGRL
>Ornithorhynchus_anatinus
-------------------MAGVAAWVAMGTGLFPGRL
>Trematomus_bernacchii
----------------------------MVTGLCPRRL
>Ceratotherium_simum_simum
----------------------------MGTGLFPGRL
>Platichthys_stellatus
-------MASGRSRPAGHPMGGDAACVAMVTGPCPGRL
>Callithrix_jacchus
----------------------------MGTGLFPGRL
>Microtus_ochrogaster
-------------------MVGVAAWVAMGTGLFPGRL
>Tupaia_chinensis
----------------------------MGTGLFPGRL
>Pelodiscus_sinensis
-------------------MGCVCGSVSMVTGLFPGRL
```

HG0113 ENSGALG00000023089 SSBP2

>Gallus_gallus
------------------------MGCVCGGVAMATGLFPGRL
>Odorrana_margaretae
---------------------------------MVTGLFPGRL
>Canis_lupus_familiaris
------------------------MVGVAAWVAMGTGLFPGRL
>Hynobius_retardatus
-------------MGGPPACAPSNRRRRCMRVAMATGLFQGRL
>Orcinus_orca
---------------------------------MGTGLFPGRL
>Pteropus_vampyrus
---------------------------------MGTGLFPGRL
>Dasypus_novemcinctus
---------------------------------MGTGLFPGRL
>Latimeria_chalumnae
---------------------------------MVTGLFPGRL
>Monodelphis_domestica
---------------------------------MGTGLFPGRL
>Falco_peregrinus
------------------------MGCVCGSVSMVTGLFPGRL
>Erinaceus_europaeus
MEGEACPSRALPGRRGRGLRAGRPIVGVAAWVAMGTGLFPGRL
>Ochotona_princeps
---------------------------------MGTGLFPGRL
>Ornithorhynchus_anatinus
------------------------MAGVAAWVAMGTGLFPGRL
>Ceratotherium_simum_simum
---------------------------------MGTGLFPGRL
>Cercocebus_atys
---------------------------------MGTGLFPGRL
>Marmota_marmota_marmota
---------------------------------MGTGLFPGRL
>Tupaia_chinensis
---------------------------------MGTGLFPGRL
>Anolis_carolinensis
------------------------MARVCGGVSMATGLFPGRL
>Pelodiscus_sinensis
------------------------MGCVCGSVSMVTGLFPGRL

HG0114.1 ENSGALG00000003701 SGMS1

>Gallus_gallus
--MKPRRSLLM-LWSRSDCCPL
>Anser_cygnoides
--MKPRKSFLM-HWSHSDCCPI
>Apteryx_australis_mantelli
------------------------------MLSPACGCSRQGLIVGQT---GSSDKMKKPRRSLLLPWCHSGCCPV
>Cariama_cristata
--MKPRRSFPT-RWNRSDCCPI
>Calidris_pugnax
MNRVLWVEKHLQEEECQHSYEVTSFLCLPQHLDARIRQLRQGLAYCQT---ASLDKMKPRRSFLM-RWSRSDCCPI
>Pterocles_gutturalis
--MKPRRSFLM-RWSRSDCCPI
>Columba_livia
--MKPRRSFAT-CQSRADRCPI
>Merops_nubicus
--MKPRRSFPTRRWSRSDCCPI
>Cuculus_canorus
--MKPRRSFLT-CWSHLDCCPI
>Haliaeetus_albicilla
--MKPRRSFPT-RWSRSDCCPV
>Gavia_stellata
--MKPRRSFPT-RWSRSDCCPI
>Balearica_regulorum_gibberic
--MKPRRSFPT-RWSRSDCCPI
>Opisthocomus_hoazin
--MKPRRSFLT-HWSHSDCCPV
>Pseudopodoces_humilis
--MKPRRSFPP-RWSRSDCCPL
>Nipponia_nippon
--MKPRRSFPM-RWSRSDCCPI
>Fulmarus_glacialis
--MKPRRSFLT-RWSRSDCCPI
>Nestor_notabilis
--MKPRRSFPT-HWSRSDCCPI
>Aptenodytes_forsteri
--MKPRRSFPT-RWSCSDCCPI
>Tyto_alba
--MKPRRSFPM-CWSRSDCCPI
>Chrysemys_picta
------------------MYKVFRNL---------------YLSTCQTGFCCTSDRMNKPKRGFLLYWKNSDCCLV
>Calypte_anna
--MKPRKSFPV-HWSRSDCCPI
>Apaloderma_vittatum
--MKPRRSFVT-RQNRSDCCPV

HG0114.2 ENSG00000198964 SGMS1

>Homo_sapiens
MTRMFLNNPRKNPAQK
>Felis_catus
MTRIFLNNQRKTPGAP
>Myotis_davidii
MTRIFLSNPRKNLVEK
>Galeopterus_variegatus
MTRIFLNNPRKNPVQK
>Condylura_cristata
MTRIFLNNQRKNLVQK
>Equus_asinus
MTRIFLNNQRKNLAQK
>Fukomys_damarensis
MTRIFLHNLRKNPVQK

HG0115.1 ENSGALG00000010976 CCDC126

```
>Gallus_gallus
MESRPAK-PRRCVQDDPARRLLTL------------
>Anas_platyrhynchos
MESRPTK-LRRCVQDDPARRLFTL------------
>Apteryx_australis_mantelli
MESRPTKTWKRCIQDDLAGGLFTL------------
>Buceros_rhinoceros_silvestri
--------MRRCIQDDPARRFFIS------------
>Caprimulgus_carolinensis
--------MRRCIQDDSARRLFNS------------
>Cariama_cristata
--------MRRCIQDDPARRFFTS------------
>Charadrius_vociferus
--------MRRCIQDDPARRFFTS------------
>Colius_striatus
--------MRRCIQDDPDRRLFTSQKFGFV------
>Columba_livia
--------MRRCIQDDPSRRLFTS------------
>Leptosomus_discolor
--------MRSCIQDDPARRFFTS------------
>Alligator_mississippiensis
MGSRLSIIWRECVQDGPSRRFVTL------------
>Aquila_chrysaetos_canadensis
--------MRRCIQDDPARRFFTS------------
>Gavia_stellata
--------MRRCIQDDPARRFFTSY-----------
>Balearica_regulorum_gibberic
--------MRRCIQDDPARRFFTSQKSYFCLICNII
>Tauraco_erythrolophus
--------MRRCIQDDPATRFFTSQKS---------
>Opisthocomus_hoazin
--------MRRCIQDDPARR----------------
>Lepidothrix_coronata
--------MRRCIQDDPTRRFFTS------------
>Phaethon_lepturus
--------MRRCIQDDPARRFFTS------------
>Melopsittacus_undulatus
--------MRRCIQDD--------------------
>Tyto_alba
---------MRCNQDDPARRFFTSQKSYFCLICNII
>Struthio_camelus_australis
MESRPTKTWKRCIQDDQARGFFTL------------
>Apaloderma_vittatum
--------MRRYIQDDPARRFFTS------------
```

HG0115.2 ENSG00000169193 CCDC126

```
>Homo_sapiens
MSANRVNRVDTTLLKMKNIQY
>Ursus_maritimus
---------------MKNIQY
>Pteropus_alecto
---------------MKNIQY
>Galeopterus_variegatus
---------------MENIKY
>Oryctolagus_cuniculus
---------------MKNIQY
>Marmota_marmota_marmota
---------------MKIIPY
>Tupaia_chinensis
---------------MKDIQY
```

HG0116 ENSG00000010270 STARD3NL

>Homo_sapiens
-------------MCLLFRDGEVGKRLL
>Ursus_maritimus
MRSPTSFQCLGWDAGTGFRDGEVGERLL
>Myotis_ricketti
------------MRPLLSRDGEVGKRLL
>Condylura_cristata
------------MRPFLSRDGEIGKRLL
>Ceratotherium_simum_simum
------------MRSLLSRDGEVGKRLL
>Marmota_marmota_marmota
------------MRCLLSRDGEVGKRFL

HG0116 ENSGALG00000033038

>Gallus_gallus
--MDQHSSDGETGKGLL
>Apteryx_australis_mantelli
--MDQPSSDGETGRRLL
>Buceros_rhinoceros_silvestri
--MDQPSSDGETGRPPL
>Caprimulgus_carolinensis
--MDQSSSDGETGRRPL
>Cariama_cristata
--MDQPSSDGETGRRPL
>Calidris_pugnax
--MDQPSSDGETGRRPL
>Pterocles_gutturalis
--MDQPSSDGETGRRPL
>Colius_striatus
--MDQPSSDGETGRRPL
>Columba_livia
--MDQPSSDGETGRRPL
>Leptosomus_discolor
--MDQPSSDGETGRRPL
>Alligator_mississippiensis
--MDQPSSDGEAGRRLL
>Cuculus_canorus
--MDQPSRDGETARRLL
>Aquila_chrysaetos_canadensis
--MDQPSSDGETGRRPL
>Gavia_stellata
--MDQPSSDGETGRRLL
>Balearica_regulorum_gibberic
--MDQPSSDGETGRRPL
>Opisthocomus_hoazin
--MDQPSSDGETGRRQL
>Lepidothrix_coronata
--MDQPSSDGETGRRPL
>Egretta_garzetta
--MDQPSSDGETGRRPL
>Tyto_alba
--MDQHSSDGETGRLPL
>Struthio_camelus_australis
MGKDDIAVPSDVPLTQKIDILLLTSFFFLLLLACKLSPALLSTFFQYFQKNIVEHVCESCVTVQLLLLGCHESIAIWGKVWLKETRKRSSSCMDQPSSDGETGRRLP
>Chrysemys_picta
--MDQPASDGEAGKRLL
>Tinamus_guttatus
--MDQPSSDGETGRRLL

HG0117.1 ENSG00000140945 CDH13

>Homo_sapiens
MLSAAACMNENAAGRF
>Anser_cygnoides
MLSAAECMNEKASGTF
>Ursus_maritimus
MLSAAKCMNENAAGRF
>Lipotes_vexillifer
MLSAAACMNENAARRF
>Calidris_pugnax
MLSAAECMNEKASGTF
>Rhinolophus_ferrumequinum
MLSAAACMNENAAGRF
>Dasypus_novemcinctus
MLSAAACMNENAAGRF
>Columba_livia
MLSAAECMNEKASGTF
>Numida_meleagris
MLSAAECMNEKASGTF
>Condylura_cristata
MLSAAACMNENAAGRF
>Oryctolagus_cuniculus
MLSAAACMNENAAGRC
>Sturnus_vulgaris
MLSAAECMNEKASGTF
>Ceratotherium_simum_simum
MLSAAACMNENAAGRF
>Manis_javanica
MLSAAKCMNENAAGRV
>Loxodonta_africana
MLSAAACVNENAAGRF
>Ctenomys_sociabilis
MLSAAACMNENAAGRF
>Tupaia_chinensis
MLSAAACMNENAAGRF
>Trichechus_manatus_latirostr
MLSAAASVNENAAGLF
>Aptenodytes_forsteri
MLSAAECMNEKASGTF
>Anolis_carolinensis
MLSAAECMKEKASGVF
>Struthio_camelus_australis
MLSAAECMNEKASGTF
>Pelodiscus_sinensis
MLSAAECMNEKASGTF

HG0117.2 ENSG00000140945 CDH13

>Homo_sapiens
MPPPRGSLLARCSPRICHAKRGSVRKESVL
>Dasypus_novemcinctus
------------------------MKESVL
>Condylura_cristata
----------MYSPHICHAERGSFMKESVL
>Oryctolagus_cuniculus
------------------------MKESVL
>Mus_musculus
------------------------MKESVL
>Tupaia_chinensis
------------------------MKESVL

HG0118.1 ENSG00000183662 FAM19A1

>Homo_sapiens
--MTRFPPLKPWLLF
>Anas_platyrhynchos
-----------------------------------MLGDDLKVKMRRFPPLKPWPLF
>Mustela_putorius_furo
--MTRFLPLKPWLLF
>Hynobius_retardatus
--MTRFPPLESWLLF
>Lipotes_vexillifer
--MTRFPALKPWFLF
>Pteropus_vampyrus
--MTRFPPLKPWLLF
>Monodelphis_domestica
--MTRFPPLKPWLLF
>Aquila_chrysaetos_canadensis
-----------------------------------MLGDDLKVKMRRFPPLKPWPLF
>Erinaceus_europaeus
--MTRFPPLKPWLLF
>Oryctolagus_cuniculus
--MTRFPPLKPWLLF
>Elephantulus_edwardii
--MTRFPPLKPWLLF
>Parus_major
-----------------------------------MLGNDLKVKMRRFPPLKPWPLF
>Equus_asinus
--MTRFPPLKPWLLF
>Manis_javanica
--MTRFPPIKPWLLL
>Loxodonta_africana
MHWSGDRSVTDYKLNLVRKLHIISPSLQRSRQAEVTPGDDLKVKMTRFPPLKAWLLF
>Fulmarus_glacialis
-----------------------------------MLGDDLKVKMRRFPPLKPWPLF
>Nannospalax_galili
--MTRFPPLKPWLLF
>Tupaia_chinensis
--MTRFPPLKPWLLF
>Trichechus_manatus_latirostr
--MTRFPPLKPWLLF
>Struthio_camelus_australis
--MRRFPPLKPWPLF
>Chrysemys_picta
--MTRFPPLKPWPLF
>Orycteropus_afer_afer
--MTRFPPLKPWLLF

HG0118.2 ENSG00000183662 FAM19A1

>Homo_sapiens
MHWSGDGPSATIN---
>Lipotes_vexillifer
MHWSGDGPSLTIN---
>Pteropus_alecto
MHWNGDGPSLTIN---
>Condylura_cristata
MHWRGDGPSLTIN---
>Ochotona_princeps
MHWSGDGPAVAQLIPIWRLHILSPPLQKSLQAEVTPGDYLKVKMTRFLLLKPWLLF
>Elephantulus_edwardii
MHWSGGAPSLTIN---
>Tupaia_chinensis
MHWSGNGPSVTIN---
>Orycteropus_afer_afer
MHWSGDGPSLTIN---

HG0119 ENSGALG00000043570 SGK3

>Gallus_gallus
------------------------MMEFTFSQEELRILDQYR
>Anser_cygnoides
------------------------MMEFRFSQEERRISDQHR
>Chaetura_pelagica
------------------------MMEFTFSEEKLRVVDQYR
>Buceros_rhinoceros_silvestri
------------------------MMEFTFSREKLRLLGQHR
>Caprimulgus_carolinensis
------------------------MMEFTFSQEKLRILDQYR
>Cariama_cristata
------------------------MMGFTFSPEKLCILDQYR
>Calidris_pugnax
------------------------MMEFTFSQEKLRILDQDR
>Pterocles_gutturalis
------------------------MMEFTFPREKLRILDQYH
>Leptosomus_discolor
------------------------MMEFTFSEEKLHISDQYR
>Alligator_mississippiensis
------------------------MMESRFSQDKLHLLDQYR
>Aquila_chrysaetos_canadensis
MDNLTVLLNTKYVKYGGRGGREDYMMEFTFSQEKLRILDQYR
>Gavia_stellata
-------------------------MEFTFFQEKLRILDQYR
>Chlamydotis_macqueenii
------------------------MMEFTFSQEKLRILDQYR
>Tauraco_erythrolophus
------------------------MMEFTFSQEKLRILDQYR
>Ficedula_albicollis
------------------------MMEFTFSQEKLRILDQYR
>Pelecanus_crispus
------------------------MMEFTFSQEKLRILDQYH
>Fulmarus_glacialis
------------------------MMEFTFSQEKLRILDQYR
>Pygoscelis_adeliae
---------------MLSCRDSDYMMEFTFSQEKLRILDQYR
>Takydromus_sexlineatus
------------------------MMEFKFTQERLHVLDQYC
>Struthio_camelus_australis
------------------------MMELTFFQEKLHILDQYR
>Chrysemys_picta
------------------------MMEFKFSQEKLHILDQYR
>Apaloderma_vittatum
------------------------MMEFTFSQEKLRIKDQYH

HG0120 ENSG00000022355 GABRA1

>Homo_sapiens
MKSPPISHPVCPGTYYYCLGKEKSFVV------------------------------
>Mustela_putorius_furo
MKSPPISHPVCPGTYYYCLGKEKSFVV------------------------------
>Calidris_pugnax
MKSQPISHPVYPGTYYYCLGKEKAFVV------------------------------
>Rhinolophus_ferrumequinum
MKSPPISHPVCPGTYYYCLGKEKSFVV------------------------------
>Dasypus_novemcinctus
MKSPPISHPVCPGTYYYCLGKEKSFVVSLQIGSWEANLGVKSSAEEHAESMMAQTE-
>Galeopterus_variegatus
MKSPPISHPVCPGTYYYCLGREKSFVV------------------------------
>Monodelphis_domestica
MKSLPISHPVYPGTYYYCLGKERSFVVYLQTEYWEEIWV------------------
>Macropus_eugenii
MKSLPISHPVYPGTYYYCLGKEKSFVVYLQTEYWEEIWV------------------
>Esox_lucius
MKSQPISHPVYPGTYYYCPGKVKLHLAAICVFRV--DFLVTNLLGT-EKSLTPHTNE
>Condylura_cristata
MKSPPISHPVCPGTYYYCLGKEKSFVV------------------------------
>Ochotona_princeps
MKSPPISHPVCPGTYYYCLGKEKSFVV------------------------------
>Scleropages_formosus
MKSQPIPHPVYSGTHYYCLG-------------------------------------
>Zonotrichia_albicollis
MKSQPISHPVYPGTYYYCLGKEKAFVV------------------------------
>Ceratotherium_simum_simum
MKSPPISHPVCPGTYYYCLGKEKSFVV------------------------------
>Manis_javanica
MKSPPISHPVCPGTYYYCLGKEKSFVV------------------------------
>Cavia_porcellus
MKSPPISHPVCPGTYYYCLGKEKSFVV------------------------------
>Salmo_salar
MKSQPISHPVYPGTYYYCPGKVKLYLAAISVFTV--DFVVR----------------
>Tupaia_chinensis
MKSPPISHPVCPGTYYYCLGKEKSFVV------------------------------
>Lepisosteus_oculatus
MKSQPISHPVYPGTYYYCLGKENLYLAAISVFRV--DFAVRLPIYR-DR--------
>Trichechus_manatus_latirostr
MKSPPISHPVCPGTYYYCLGKEISFVV------------------------------
>Thamnophis_sirtalis
MKSQPISHPVYPGTYYYCLGKVQSFLL------------------------------
>Chrysemys_picta
MKSQPISHPVYPGTYYYCLGKEKAFVV------------------------------

HG0121 ENSG00000122786 CALD1

>Homo_sapiens
--------MCSLSMDFWEGPRKGGLEFY---
>Anas_platyrhynchos
--------MYSLSIDFWEGPRKGCSFIE---
>Apteryx_australis_mantelli
--------MYSLSIDFWEGPRKGCSFIE---
>Canis_lupus_familiaris
--------MCSLSTDFWEGPRKGGLEFY---
>Balaenoptera_acutorostrata_s
MPTVNIGDMCSLSMDFWEGLRKGGLEFY---
>Calidris_pugnax
--------MYSLSIDFWEGPRKSCSFIE---
>Dasypus_novemcinctus
--------MCSLSMDFWEGLRKGGLQFY---
>Columba_livia
--------MYSLSIDFWEGPRKSCSFIE---
>Galeopterus_variegatus
--------MCSLSMDFWEGPRKGGLEFY---
>Haliaeetus_leucocephalus
--------MYSLSIDFWEGPRKSCSFIE---
>Coturnix_japonica
--------MYSLSIDFWEGPKQGCSFIE---
>Condylura_cristata
--------MCSLSMGFWEELRKGRAVWSLVE
>Oryctolagus_cuniculus
--------MCSLSMDFWEGPRKGGLQFY---
>Corvus_brachyrhynchos
--------MYSLSIDFWEGPRKSCTFIE---
>Nipponia_nippon
--------MYSLSIDCWEGPRKSCSFIE---
>Equus_asinus
--------MCSLSMDFWEGLRKGGLEFY---
>Manis_javanica
-------------MDFWEGLREGGLEFY---
>Loxodonta_africana
--------MCSLSVDCWERLRKGGLQFIE--
>Nannospalax_galili
--------MCSLSMDFWEGPRKGGLQFY---
>Tupaia_chinensis
--------MCSLSLDFWEGPRKGGLEFY---
>Aptenodytes_forsteri
--------MYSLSIDFWEGPRKSCSFIE---
>Struthio_camelus_australis
--------MYSLSIDFWEGPRKGCSFIE---

HG0122 ENSG00000169925 BRD3

>Homo_sapiens
-----------------------------MLSCDRPSSCLEEAGLQPGSL---------PLCPD-HPHIPKAVAWLGPEMGRRAFSGSVEAARASPSRCCDRK-
>Felis_catus
---MGRRASERSTVEARASPSRCCDGQ-
>Balaenoptera_acutorostrata_s
---MGCRTSAGSTAEARASPSCCCRDGQ
>Calidris_pugnax
---MGCQSRSGSIKEERKSPSRCCRDSE
>Pteropus_alecto
---MGCHASAGSTEEARAPPSRCCDGQ-
>Cuculus_canorus
---MGCQSRSGNIKEERKSPSRCCCDSE
>Sarcophilus_harrisii
---MGCQSYSGSIKKERESPSCCCDSK-
>Galeopterus_variegatus
MQMPRRQTCRAGTTASCLPDWLRASPCHSLLPSQTPEL-LLLVMIEPRSVPCSRAPAPHAVCGSAEVWGAAGVAQLGPEMGCHTSPGSIGAARASRSRCCDRK-
>Monodelphis_domestica
---MGCQSYSGSIKKERESPSCCCDSK-
>Macropus_eugenii
---MGCQSYSGSIKKERESPSCCCDSK-
>Balearica_regulorum_gibberic
---MGCQSHSGSIKEERKSPSCCCCDRE
>Erinaceus_europaeus
---MGCHAPKGSTEHISTSLSRCCDN--
>Oryctolagus_cuniculus
---MGRRASLGSVDAARAPARRRCCGRK
>Ornithorhynchus_anatinus
---MGCQSYSGSIKQVKESPSCCCDDSE
>Equus_caballus
---MGRRASRGSTEELRASPSRCCDGQ-
>Manis_javanica
---MGCHASAGSTEETRASLSRCCDGQ-
>Loxodonta_africana
---MGCRAASGGVEGERAPAGCGCDRK-
>Ictidomys_tridecemlineatus
---MGCQASSGSTEGAGAFWSCSCDGK-
>Tupaia_chinensis
---MGCRGPSGSIAAARAPASPCRDGK-
>Trichechus_manatus_latirostr
---MGCRAASGSLEGARASAGRGCDGK-
>Chelonia_mydas
---MGCQSHSGSIKEDRESPSCCCDSK-
>Orycteropus_afer_afer
---MGCRASSGIVEGTWASLGRGCDE--

HG0123 ENSG00000173276 ZBTB21

```
>Homo_sapiens
MTSSLVWDDVKFKMDGITAAAAAAARASRV-
>Amia_calva
-------------MDGITTATRSTEF-----
>Channa_argus
MTSSLVWDDVKFKMDGITTSAQKSTQH----
>Xenopus_laevis
MTSSVVWDDVKFKMDGITAAAARQRLAE---
>Menidia_menidia
-------------MDGITISSQKFTQH----
>Oryzias_latipes
-------------MDGITTKTQKSCTTVNKV
>Hynobius_retardatus
-------------MDGITAAARAGQF-----
>Myotis_lucifugus
MTSSLVWDDVKFKMDGITAAAAAAARASRV-
>Oreochromis_niloticus
MTSSLVWDDVKFKMDGITTSAEKSTQH----
>Pimephales_promelas
MTSSLEWDDVKYKMDGITTASCSTQL-----
>Fundulus_heteroclitus
MTSSLVWDDVKFKMDGITTSRLKATQH----
>Galeopterus_variegatus
MTSSLVWDDVKFKMDGITAAAAAVARASRV-
>Esox_lucius
-------------MDGITTTTHSTEF-----
>Coturnix_japonica
MTSSLVWDDVKFKMDGITAAAAARAAEP---
>Lateolabrax_japonicus
MTSSLVWDDVKFKMDGITTSAQKSTQH----
>Trematomus_bernacchii
----MVWDDVKFKMDGITTSAQKSTQQH---
>Platichthys_stellatus
MTSSLVWDDVKFKMDGITTSAQKAPQQ----
>Heterocephalus_glaber
MTSSLVWDDVKFKMDGITAAAAAAARASRV-
>Salmo_salar
MTSSLE-------------------------
>Thamnophis_sirtalis
MTSSLVWDDVKFKMDGITAVAAARAGES---
>Chrysemys_picta
MTSSLVWDDVKFKMDGITAVAAARAGES---
>Takifugu_rubripes
MTSSLVWDDVKFKMDGITTAA----------
```

HG0124.1 ENSDARG00000031763 smad6b

```
>Danio_rerio
------------------MQYSIVPSKLMLWLCHRYTVLGCARAARHSANI-NVARAQK-----
>Acipenser_sinensis
MHFDFNVAVCNCVTLAIYLARYCICPNLMLQSGCKYTAFGCAIVARHLQKL-HVLRRPWN----
>Channa_argus
----------------------------MLRLCHQCLSVGCARIARHFTDI-NVLQRP------
>Porichthys_notatus
--MQQNSLFFILLLFLFPPSLPLFSRSLMVRLGHRCSSLGCARVARRSTHI-NVLQRP------
>Eptesicus_fuscus
----------------------------MLQPCGAPSTTGCARSARRPAAVLHVGLRPAQPAPR
>Oreochromis_niloticus
----------------------------MLRFCHQCSSLGCACTARHFSVI-NVLQRPEKCL--
>Dasypus_novemcinctus
----------------------------MLQPCGAPSTTGCARSARHPTAELHVGLRPAQPAPR
>Alosa_alosa
------------------MQHYIVASNLMLCFSHQYSAFGCACVARQSTNI-NVVQRA------
>Esox_lucius
------------------MQYTTVNYILMLRVGHKSTTQGCACSARRSQNL-DVVRRP------
>Apteronotus_leptorhynchus
------------------MLHNIVPSK-LMWFYHQHRVLGCACIAQQSANI-NVRSQKLF----
>Oryctolagus_cuniculus
----------------------------MLQPCGAPSTTGCARSARRSAAELHVGLRPAPR---
>Lateolabrax_japonicus
----------------------------MLRLCHQCSSVGCACIARHFTDI-NVLQRP------
>Notothenia_coriiceps
----------------------------MLRFCHQCSSVGCAGIARHFTDI-NVLQRPEKYL--
>Equus_asinus
----------------------------MLQPCGAPSTTGCARSARRPAAELHVGLRPA-----
>Cynoglossus_semilaevis
----------------------------MLRLCHQCSSLGCACAARRFTYI-NVLQRR------
>Callithrix_jacchus
----------------------------MLQPCGAPSTTGCARSARRSAAVLHVGLRPAQPAPR
>Microtus_ochrogaster
----------------------------MLQPCGAPSTTGCARSARRPAAELHVGLRPAQPAPR
>Oncorhynchus_kisutch
------------------MQHTTVNYILMLRVSHKSTTPGCACSARRLQNI-NVVRRP------
>Lepisosteus_oculatus
----------------------------MLQFCCKYTALSCAFIARHLRKM-NVWRRP------
>Ictalurus_punctatus
MHFVFSVALHFIDALYILMLPNNVPFNPMLWFCYRPTVLCCARAAQRSANL-NVAQAQKQL---
>Takifugu_rubripes
----------------------------MLRLCHQCSSVSCAWSARHFTHI-NVLQRP------
```

HG0124.2 ENSDARG00000053209 smad6a

```
>Danio_rerio
MMGINQWLHCSPQG----PRVCVSEEERE---------------------------------------------------------------------
>Pygocentrus_nattereri
------MRECVRECDCVYVWDCVRISRRRKRGRETAQGNNRRSPLILSAARNERIRRVAGARAFTLPVEGSFVRACFFAPLHEASFCAVRADWNVNSS
>Latimeria_chalumnae
MMGGSQ--------------------------------------------------------------------------------------------
>Scleropages_formosus
MMGGSQWLLSYPRG----PRVCVSGGGKKRREKR-----GKANPRVLSEQI-----------------------------------------------
>Salmo_salar
MMGINQWLQSFPGA----P-VCVRASVSEKNSRK----------------------------------------------------------------
```

HG0125.1 ENSDARG00000051926 plag1

```
>Danio_rerio
MRKKLLKIREIVESQEKYRRKDVNAPAGLMSLLC
>Channa_argus
MRKKLLKIRKIVESQEKLNRKDVKASGALESGRC
>Xenopus_laevis
MGKKLLKNRKGI----------------------
>Menidia_menidia
MRKKLLKIRKIVESPEKFNRKDVEASGALKYRQC
>Oryzias_melastigma
MRKKFVKIRKTVESPEKHSRKDVKATRAFESRQC
>Mustela_putorius_furo
MRKKLLEIPRAV----------------------
>Pygocentrus_nattereri
MRKKLLKIRRTVESPEKRPRKDVKAPTGLVSLWC
>Myotis_lucifugus
MRKKLLEIPRAV----------------------
>Maylandia_zebra
MRKKLLKIRKIVESEEKLNRKDDKASGTPVSRQC
>Clupea_harengus
MRKKLLKIRKIVETQEKHRRKDVNASADVGSLRC
>Poecilia_reticulata
MRKKLQKIRKIVESQEKVHRKDVEAPRAP-----
>Esox_lucius
MRKKLLKIRKIVESQEKLHRKDVKAPVAPESWRC
>Lateolabrax_japonicus
MRKKLLKIRKIVESQEKLNRKNVKAPGAPESGQC
>Notothenia_coriiceps
MRKKLLKIRKIVESEEKPNRKNVKATGAPESGRC
>Manis_javanica
MRKKLLEIPRAV----------------------
>Platichthys_stellatus
MRKKLLKIRKIVESEEKLNRKDVKASGAPESGRC
>Rhinopithecus_bieti
MRKKLLEIPRAV----------------------
>Rattus_norvegicus
MRKKLLEIPRAV----------------------
>Lepisosteus_oculatus
MRKKLLKIRKILQS--------------------
>Trichomycterus_areolatus
MRKNLLKIRKIVESQEKGSRKDAKAPKCLVSLWC
>Takifugu_rubripes
MRKKLIKILKLVESQEKLNRKDAEAPRAPGSSWC
```

HG0125.2 ENSG00000181690 PLAG1

```
>Homo_sapiens
MLKPRDWPKWEGLDSTLFHEESMGLAKIKV
>Mustela_putorius_furo
MLKPRDWPKWEGLDSALFHEESMGLAKIKV
>Lipotes_vexillifer
MLKPRDWPKWEGLDSALFHEESMELAKIKV
>Myotis_davidii
MLKPRDWPKWEGLDSALFHEESVGLAKIKV
>Dasypus_novemcinctus
----------------------MGLAKIKV
>Galeopterus_variegatus
MLKPRDWPKWEGLDSTLFHEESMELAKIKV
>Erinaceus_europaeus
MLKPRDWPKWEGLDSTLLHEESMELAKIKV
>Ochotona_princeps
MLKPRDWPKWEGLDSTLFHEELMGLAKIKI
>Elephantulus_edwardii
MVKPRDWPKWEGLDSTLFHKD---------
>Equus_caballus
MLKPRDWPKWEGLNSALFHEESMGLAKIKV
>Manis_javanica
MLKPRDWSKWERLDSALFYEESMGLAKIKV
>Loxodonta_africana
MVKPRDWPKWEGLDSALFHKESVGLAKIKV
>Jaculus_jaculus
MLKPRDWPKWEGLDSTLFHEELMGLAKIRV
>Tupaia_chinensis
MLKPRDWPKWEGLDSTLFHEESMGLAKI--
>Trichechus_manatus_latirostr
MVKLCDWPKWEGLDSALFHKESMGLAKIKV
>Anolis_carolinensis
--------------------MSVELVKIKT
>Orycteropus_afer_afer
MVKPRDWPKWEELDSALFHKESMGLAKIKV
```

HG0126.1 ENSGALG00000010899 G6PC2

>Gallus_gallus
MNCEKIQANMFQLFIRRIA---------------------------
>Anser_cygnoides
MNCEKIQANMFQLFIERIA---------------------------
>Chaetura_pelagica
---------MFQLFIQRIS---------------------------
>Apteryx_australis_mantelli
MNCEKIQANMFQLFIQRIA---------------------------
>Buceros_rhinoceros_silvestri
MDCEKIRANMFQLFTQRIA---------------------------
>Caprimulgus_carolinensis
---------MFQLFIQRIA---------------------------
>Merops_nubicus
---------MFQLFIQRTA---------------------------
>Cuculus_canorus
MNCEKNQANMFQLFIQRIS---------------------------
>Haliaeetus_albicilla
MNCEKIQANMFQLFIQRIA---------------------------
>Gavia_stellata
MNCEKIQANMFQLFIQRIA---------------------------
>Chlamydotis_macqueenii
MNCEKIQANMFQLFIQRIA---------------------------
>Opisthocomus_hoazin
MNCEKIQANMFQLFIQRIAPVTHCASTKLSPQFAAAQHVLHLVSSN
>Acanthisitta_chloris
MNCDKIQANMFQLFIQSIA---------------------------
>Phalacrocorax_carbo
MNCEKIQANMFQLFIQRIA---------------------------
>Picoides_pubescens
MNCEKIQANMFQLFIQRIA---------------------------
>Fulmarus_glacialis
MNCEKIQANMFQLCIQRIA---------------------------
>Nestor_notabilis
MNCEKIQANMFQLFIERIA---------------------------
>Aptenodytes_forsteri
MNCEKIQANMFQLFIQRIA---------------------------
>Tyto_alba
MNCEKIQANMFQLFIQRIA---------------------------
>Struthio_camelus_australis
MNCEKIQANMFQLFIQRIA---------------------------
>Chrysemys_picta
MNCVKIQANIFQLFIQRVA---------------------------

HG0126.2 ENSGALG00000010899 G6PC2

```
>Gallus_gallus
MCFPRDSEPRVPGMAGYATLIQYKAQPSGWY----------------
>Anas_platyrhynchos
MCFPGDLEPRVPGMAGYTPPIRYKGNVNHLLSLFNSSLPWLSFSLET
>Apteryx_australis_mantelli
MCFLGDLESHVPGMAGYTTLIQYKAQSSGWY----------------
>Buceros_rhinoceros_silvestri
MCFPGDLEPRVPGMAG-------------------------------
>Caprimulgus_carolinensis
-------------MAGYTTPIQYKAQPSDWY----------------
>Cuculus_canorus
MCFPGDLEPRVPGMSGYTMAIQYKAQPSGWY----------------
>Haliaeetus_albicilla
-------------MAGYTTPIQYKAQPSGWY----------------
>Gavia_stellata
MCFPGDLEPRVPGMAGYTTPIQYKAQPSGWY----------------
>Opisthocomus_hoazin
MCFPGDLETRVPGMAGYTTAIQYKAQPSGWY----------------
>Corvus_cornix_cornix
MCFPRDLEPRVSGMAGYTTPI--------------------------
>Phalacrocorax_carbo
MCFPGDLEPRVPGMAGCTTLIQYKAQPSGWY----------------
>Fulmarus_glacialis
MCFPGDLEPRVPGMAGYTTPIQYKAQPSGWY----------------
>Aptenodytes_forsteri
-------------MAGYTTLIQYKAQPSGWY----------------
>Tyto_alba
-------------MAGCTTPIQYKAQPSGWY----------------
>Struthio_camelus_australis
MCFLRDLESRVPGMAGTPL----------------------------
>Tinamus_guttatus
MCFPGDLESHVPGMAGYTTLIQYKGNVNHFLSFFNSSLPWFSFSLI-
```

HG0127.1 ENSGALG00000029709

>Gallus_gallus
MQNQSTH-EEKSPLRGNVRGIAVGDNLLHSPTP-----
>Anas_platyrhynchos
MQNPSAH-EEKSPLHGNVRGIALGDNLLHSAAP-----
>Buceros_rhinoceros_silvestri
MQNSSTH-EEKSPL--VVMSEE----------------
>Caprimulgus_carolinensis
MQNPST--------------------------------
>Cariama_cristata
MQNPSTH-EEPSHG--NVRGIAIGDNLLHSATL-----
>Calidris_pugnax
MQNPSTH-EEKSPL--IVMLEE----------------
>Colius_striatus
MQNPSTH-EEKSSL--VVMLEE----------------
>Columba_livia
-----------MISRGNVRGIAAGDNPLHSAAL-----
>Falco_cherrug
-----------MISRGNVRGIAVGDNPLHSATVQDKHR
>Gavia_stellata
MQNPSTH-EENSPL--VVMLEE----------------
>Eurypyga_helias
MQNPSTH-EEKSPL--VVMLEE----------------
>Corvus_brachyrhynchos
-----ME-DFEGERHGNVRGIAVGDNLLHSATL-----
>Pelecanus_crispus
MQNPSTH-EEKSPL--VVMLEE----------------
>Picoides_pubescens
MQNPSSH-EEKSPLL--VTLEE----------------
>Fulmarus_glacialis
MQNPSTH-EEESPL--VVMLEE----------------
>Pygoscelis_adeliae
MQNPSTH-EEKSPL--VVMLEE----------------
>Tyto_alba
MQNPSTH-EEKSPL--VVMLEE----------------
>Struthio_camelus_australis
MQNLFTQ-EERS-----CLGIATGDNLLHSDPLQEKHH
>Trachemys_scripta
MQNHTVSQEENSPHYA----------------------
>Tinamus_guttatus
MQNPSTQ-EEKSPL------------------------
>Apaloderma_vittatum
MQNPSTH-EEKSPL--VIMLKE----------------

HG0127.2 ENSG00000109452 INPP4B

>Homo_sapiens
-------------------------------MVLPGKRSSVFYEKLDKGFTGGIVLEICS
>Canis_lupus_familiaris
-------------------------------MVLPGKRSVVFYEKSDQGFQRGI------
>Myotis_brandtii
-------------------------------MVLPGKRSALSYEKSDQEFQSGISGL---
>Dasypus_novemcinctus
-------------------------------MVLPGKKSAVFYEKSSQEL----------
>Condylura_cristata
--------------------------MEPKEMVLPGRRFAVFYEKTDQEL----------
>Oryctolagus_cuniculus
-------------------------------MVLPGKRSVL-YEKSDQEP----------
>Fukomys_damarensis
MKYNLTRNCTIVTLRIFHLLKVEIRHQEPKEMVLPGKRSAVFYEKSDQES----------
>Tupaia_chinensis
-------------------------------MVLPGKRAAVFSEKSDQEP----------

HG0128 ENSDARG00000091029 phox2bb

>Danio_rerio
--MRTICSRDLGLGGSLGGFS
>Anas_platyrhynchos
--MRTICSRDLRQGIS-----
>Apteryx_australis_mantelli
--MRTICSRDLRQGIS-----
>Oryzias_latipes
--MRTICSRDLGLGGS-----
>Mustela_putorius_furo
--MRTICSRDLRQGIS-----
>Rousettus_aegyptiacus
MQESMIGRLDSSVPQCQPNRRMSYCHVKSASNKTNRFAIVQVERAKFIMRTICSRDLRQGIS-----
>Haplochromis_burtoni
--MRTICSRDLGLGGS-----
>Austrofundulus_limnaeus
--MRTICSRDLGLGGS-----
>Monodelphis_domestica
--MRTICSRDLRQGFS-----
>Gallus_gallus
MQESAIGRLDSSVPQCQPNRLMSYCHVKSASNKTNRFAIVQVERAKFIMRTICSRDLRQGIS-----
>Oryctolagus_cuniculus
MQESMIGRLDSSVPQCQPNRRMSYCHVKSASNKTNRFAIVQVERAKFIMRTICSRDLRQGIS-----
>Corvus_brachyrhynchos
MQESAIGRLDSSVPQCQPNRLMSYCHVKSASNKTNRFAIVQVERAKFIMRTICSRDLRQGIS-----
>Ceratotherium_simum_simum
--MRTICSRDLRQGIS-----
>Cercocebus_atys
MQESMIGRLDSSVPQCQPNRRMSYCHVKSASNKTNRFAIVQVERAKFIMRTICSRDLRQGIS-----
>Microtus_ochrogaster
MQESMIGRLDSSVPQCQPNRRMSYCHVKSASNKTNRFAIVQVERAKFIMRTICSRDLRQGISQEAFS
>Salmo_salar
--MRTICSRDLRLGGS-----
>Lepisosteus_oculatus
--MRTICSRDLRQGCS-----
>Pelodiscus_sinensis
---------------------MSYCHVKSASNKTNRFAIVQVERAKFIMRTICSRDLRQGIS-----

HG0128 ENSG00000109132 PHOX2B

>Homo_sapiens
MQESMIGRLDSSVPQCQPNRRMSYCHVKSASNKTNRFAIVQVERAKFIMRTICSRDLRQGIS-----
>Anas_platyrhynchos
--MRTICSRDLRQGIS-----
>Xenopus_tropicalis
---------------------MSYCHVKSTSNKTNRFAIVQVERAKFIMRTLCSGDPRRGSS-----
>Apteryx_australis_mantelli
--MRTICSRDLRQGIS-----
>Oryzias_latipes
--MRTICSRDLGLGGS-----
>Mustela_putorius_furo
--MRTICSRDLRQGIS-----
>Hynobius_retardatus
---------------------MSYCHVKSASPETNRPAIVQVQRAQCTSGAAML-------------
>Rousettus_aegyptiacus
MQESMIGRLDSSVPQCQPNRRMSYCHVKSASNKTNRFAIVQVERAKFIMRTICSRDLRQGIS-----
>Haplochromis_burtoni
--MRTICSRDLGLGGS-----
>Rutilus_rutilus
--MRTICSRDLGLGGSLGGFS
>Poecilia_formosa
--MRTICSRDLGLGGS-----
>Monodelphis_domestica
--MRTICSRDLRQGFS-----
>Meleagris_gallopavo
MQESAIGRLDSSVPQCQPNRLMSYCHVKSASNKTNRFAIVQVERAKFIMRTICSRDLRQGIS-----
>Apteronotus_leptorhynchus
--MRTICSRDLGLGGSLGGF-
>Oryctolagus_cuniculus
MQESMIGRLDSSVPQCQPNRRMSYCHVKSASNKTNRFAIVQVERAKFIMRTICSRDLRQGIS-----
>Corvus_brachyrhynchos
MQESAIGRLDSSVPQCQPNRLMSYCHVKSASNKTNRFAIVQVERAKFIMRTICSRDLRQGIS-----
>Ceratotherium_simum_simum
--MRTICSRDLRQGIS-----
>Microtus_ochrogaster
MQESMIGRLDSSVPQCQPNRRMSYCHVKSASNKTNRFAIVQVERAKFIMRTICSRDLRQGISQEAFS
>Salmo_salar
--MRTICSRDLRLGGS-----
>Lepisosteus_oculatus
--MRTICSRDLRQGCS-----
>Pelodiscus_sinensis
---------------------MSYCHVKSASNKTNRFAIVQVERAKFIMRTICSRDLRQGIS-----

HG0129 ENSDARG00000099437

>Danio_rerio
--MPAVFLLLRSLVVRLFGSRLAASGVQLLRRILTTATGHLGTVLRNIWERISSQQSKEAILGCVLCLLNMHKKVDN------
>Amia_calva
--MHKKVDN------
>Channa_argus
--MPAVFILLRSLVIRLLSSRLAGSVAQFLRRSLSTGIAHLGTALRHVWDCVRSQESKEAILGCVLCILNMHKKVEN------
>Xenopus_laevis
--MPVPFLLIRTLLVRAAGSRLAVSGARQLTKGARWARARLLILLQRLWDRLTSEETRQALLSCVLCLLNLKHKSNT------
>Menidia_menidia
--MPAVFILLRSLVIRLLSSRLAGSLAQFLRRSLSTGAAHLGTALRHIWDRVRSQESKEAILGCVLCILNMHKKVEN------
>Porichthys_notatus
--MHKKVEN------
>Oryzias_melastigma
--MHKKVEN------
>Ambystoma_mexicanum
--MPLPFLLLRTLLIRLAGSRLAASGVQFLRKRLAWAGARLAPLLRRVWRRATSEESRQAVLGCVLCVLNLRKRVDGE-----
>Astyanax_mexicanus
--MHKKVDN------
>Oreochromis_niloticus
--MPAVFILLRSLVIRLLSSRLAGSVAQYLRRILSTGATHLGTALRHIWDRVRSQESKEAILGCVLCILNMHKKVEN------
>Alosa_alosa
--MPAVFLLLRSLVIRLLSSKLAGSAAQFLRRSLSAAVSHLGTALRHIWDRVRSQESKEAILGCVLCILNMHKKVDN------
>Latimeria_chalumnae
--MPALFLLLRTLFIRLLGSQLAVSAGLFLRRSLAAAGGRLAALLRRVWDRIRSEESRQVALSCVLCILNLHKKVDE------
>Nothobranchius_furzeri
--MPAVFILLRSLVIRLLSSRLAGSLAQFLRRSLTTGAAHLGTALRHIWERVRSQESKEAVLGCVLCILNMHKKVEN------
>Esox_lucius
--MPAVLVLLRA----LLSSRLTGSLVQFLRRTLSSTFGHVGAALRYVWDRISSQESKEAILGCVLCLLNMHKKVDEPEAESR
>Apteronotus_leptorhynchus
--MQKKVDN------
>Lutjanus_erythropterus
--MHKKVEN------
>Sebastes_caurinus
--MPAVFILLRSLVIRLLSSRLAGSVAQFLRRTLSTGAAHLGTALRHVWDRIRSQESKEAVLGCVLCILNMHKKVET------
>Platichthys_stellatus
---MPVFILLRSLVIRLLSSKLAGSLAQYLRRGLSAGAAHLGTALRYVWDRIRSQESKEAVLGCVLCVLGMAKKVEK------
>Salmo_salar
MQERENPCKRERIPVREREREKSKTRSRSQRGGDFIPVLFLSFLSVFLLLISLCVSGCGSSIMPAVFLFLL---RTLLSSRMIGSLLQFLRRTLSSTFSHVGAALRYVWDRISSQESKEAILGCVLCLLNMHKKVDQPAAESH
>Lepisosteus_oculatus
--MPAFFLLLRSLLIRLLSSKLAGSAAQFLRRSLSATASHIGSLLRHIWDRIRSQESREAILSCVLCILNMHKKVDN------
>Trachemys_scripta
--MPAPLLLLPLLR-------------TLLARLLLLARKRLLPLLRRLGARLGSRESREAVLTCLLCILNLRKKADD------

HG0130 ENSGALG00000002993

>Gallus_gallus
MSCWEIRRIFFSLVEEEPCPGG-WLH-GL-------F-----WRAKEAKGSCLSRTQR----ELGHVLSAHL-GNTVCKSRRKIAGIIQTAILIFVIFFRRGMHLA-------------------------------
>Anas_platyrhynchos
MSCWEIWWIFYSLVEEEPCAGG-RLR-AL-------F-----WRAKEGKGSRLSRTER----ELGHVLSAHL-GNTGCKSERKIAGIVQTAILIFVIFFRRGMHLA-------------------------------
>Panthera_tigris
MSSWGARRIGVSFLQEEPGAGGFRWLPPPLL---GLFPPLVRGGEETGDRGDLRGS-------EASDMCQHIWGAHPSSPRGDLPEQFKLRY---
>Orcinus_orca
MTSWGSRRIGVSFLQEEPRAGGFRWLPPPLL---VLFPPLV-QGVETGEQGDFPGS-------EASDMCQHIWGTHPSSPRGDLLEQFKLRY---
>Calidris_pugnax
MSCWEIWWIFFSLVEEEPGAGS-WLR-GL-------F-----WRAKEGKGSHLSRGER----ELGHVLSAHL-GNTVCKSERKFAGIVQTAILIFLIFFRRGMHLA-------------------------------
>Pteropus_alecto
MSSWGARRIGVSFLQKEPGAGGFRWLPPPLL---VLFPPLRRG-AETGERGHLRGS-------EASDMCQHIWGAHPSSPRGDLPEQFKLRY---
>Columba_livia
MSCWEIWWIFFSLVEEEPGAGS-WLR-GL-------F-----WRAKEGKGYRLSHAES----SDMC--CQHIWGIQSASPKGNLLGSFK-------VRY--------------------------------------
>Aquila_chrysaetos_canadensis
MSCWEIWWIFFSLVEEEPGAGG-WLR-GL-------F-----WRVKEGKGSRLSRAER----ELGHVLSAHL-GNTVCKSERKFAGIVQTAILIFVIFFRRGMHLA-------------------------------
>Chlamydotis_macqueenii
MSRWEIWWIFFLLVEEEPGAGS-WLR-GV-------F-----WRAKEGKGSRLSRAER----ELGHVLSAHL-GNTVCKSERKFAGIVQTAILIFVIFFRCGMHLA-------------------------------
>Condylura_cristata
MSSWGARRIGVSFLQEEPRAGGFRWLLPPLL---VLFPPLVLG-AETGERGDLRSS-------EASDMCQHIWGAHQSSPRGDLPEQFKLRY---
>Oryctolagus_cuniculus
MSSWGARRIGVSFLQERAQRGWLQVAGAAAA--------AARADSAAGPRSRDAGAGRPRRQRGIGHVSAHL-GRTSVEPEGSFAGTLQTAILILGVTVPGRLSGGSASVRPLESARACVCVCRVGPPLPPPCFPVE
>Elephantulus_edwardii
MSSWGARRIGVSLVPEEPSAGGFRWLPPPPL---LVLFPALGRRAETGERGDLQGG-------EASDMCQHIWGAQPSSPRGDLPEQFKLRY---
>Lepidothrix_coronata
MSCWEIWWIFFSLVEEEPGAGS-WLR-GL-------F-----WRAKEGKGSRLSRAER----EPGHVLSAHL-GNTVCKSERKFAGIVQTAILIFVIFFRRGMHLA-------------------------------
>Ceratotherium_simum_simum
MSSWGARRIGVSFLPEEPSAGGFRWLPPPLL---VLFPPLVRG-GETGERGDLRGS-------EASDMCQHIWGAHPSSPRGDLPEQFKLRY---
>Carlito_syrichta
MSSWGARRIGVSFLQEEPSAGGFRWLPPPSL---LVLSPPRVRGAETRERGDLRGS-------EASDMCQHIWGAHPSSPRGDLPEQFKLRY---
>Loxodonta_africana
MSSWGARRIGVSLVQEEPSAGGFRWLPPPLL---VLFPPHV-RRAETGEWGDLLGS-------EASDMCQHIWGAHLSSPRGDLLEQFKLRY---
>Microtus_ochrogaster
MSSWGARRIGVSVLQEEPRAGGFRWLTPPPPRSPQVLFPPLVRGAKTQEQGDLRGK-------EASDMCQHIWGAHPKSPRGDLPKQFKLRY---
>Trichechus_manatus_latirostr
MSSWGDRQIGVSLVQEEPIAGGFRWLLPPLL---VLFPPLV-RRAETGERGDVLGS-------EASDMCQHTWGAHPSSPRGDLPEQFKLQY---
>Chrysemys_picta
MSCWEIWWIFVSLLEEEPRAGGCRLL-GL-------L-----WRAKEGKGSRLSRAER----ELGHVLSAHL-GNTVCKSERRFAGIVQSAILIFVISFRRGMHLA-------------------------------
>Calypte_anna
MSCWEIWWIFFSLVEEEPGAGS-WLR-GL-------F-----WRLKEGKGSRLSRAGR----ELGHVLSAHL-GNTVCKSQRKFAGIVQTAILIFVIFFRRGMHLV-------------------------------
>Orycteropus_afer_afer
MSSWGARRIVVSLVKEEASTGSFRWLLPPPPL--LVLCSPLVRRAETGERGDLLGS-------EASDMCQHICGARPSSPRGDLPEQFELRY---

HG0131 ENSGALG00000013135 GALNT1

```
>Gallus_gallus
------------------------------------MAVNLQRRYR-TWSLKLD---
>Anas_platyrhynchos
------------------------------------MAVNLQRRYR-TWSLKLD---
>Apteryx_australis_mantelli
------------------MLYSNAVGSHHQCLWTIQMAVNIQRRYR-TWSLKLD---
>Buceros_rhinoceros_silvestri
------------------------------------MAVNLQRRYR-TWSLKLD---
>Charadrius_vociferus
------------------------------------MAVNLQRRYR-TVCLKLD---
>Colius_striatus
------------------------------------MAVNLQRRYR-TWSVKLD---
>Columba_livia
--MSEWPRPVCYSMVPTTGALQQCIGILPPVLVDLQMAVNLQRRYRRTWSLKLD---
>Leptosomus_discolor
MFMSEWPRPVCS-MVPTTGALQQCIGILPPVLVDHPDAVNLQRRYRT-WSLKLD---
>Alligator_mississippiensis
------------------------------------MAVNLQKRYRTWSQTGLEQSC
>Cuculus_canorus
------------------------------------MAVNLQRRYR-TWCLKLD---
>Aquila_chrysaetos_canadensis
------------------------------------MAVNLQRRYR-TWSLKLD---
>Mesitornis_unicolor
------------------------------------MAVNLQRRYR-T---------
>Tauraco_erythrolophus
------------------------------------MAVNLQRRYR-TWCLKLD---
>Opisthocomus_hoazin
------------------------------------MAVNLQRRYR-TWSLKLD---
>Acanthisitta_chloris
------------------------------------MAVNLQRRYR-TWSLKLD---
>Phaethon_lepturus
-------------MVPTTGAPRQCIGILPPVLVDHPDAVNLQRRYRT-WSLKLD---
>Melopsittacus_undulatus
------------------------------------MAINLQKRYRT-WCLKLD---
>Pygoscelis_adeliae
------------------------------------MAVNLQRRYR-TWSLKLD---
>Struthio_camelus_australis
--MSEWPRPIWILPQ---VLYSNAVGSRHQCWWTIQMAVNIQRRYR-TWSLKLD---
>Chelonia_mydas
------------------------------------MAVNLQKRYRT-WSRKLD---
>Tinamus_guttatus
--------------------------------------MAIQRRYRM-WCLKLD---
```

HG0132 ENSG00000151067 CACNA1C

```
>Homo_sapiens
----MRYSFTRGGSSG-KEQFLGFDAIMGIR
>Anas_platyrhynchos
----MHCNFTKGGSSG-KDQFLVFDAIMGIR
>Xenopus_laevis
----MQSFSAKGGSRG-TDQTRGFDAIMGIR
>Panthera_tigris
----MHYNFTKGGSSG-KEQFLVFDAIMGIR
>Hynobius_retardatus
----MHCYPTRGDCSGEKEQFLGFDSIMGIR
>Tursiops_truncatus
----MHYNFTEGGSSG-KDQFLVFDAIMGIR
>Calidris_pugnax
----MHCNFTKGGSSG-KDQFLVFDAIMGIR
>Pteropus_vampyrus
----MHYNFTQGGSSG-KDQFLVFDAIMGIR
>Dasypus_novemcinctus
----MHYNFTQGGSSG-KDQFSVFDAIMGIR
>Galeopterus_variegatus
----MHDSLSEGGGSG-RQQFAVFDAIMGIR
>Gallus_gallus
----MHCNFTKGGSSG-KDQFLVFDAIMGIR
>Condylura_cristata
----MHCNLTKGGGRG-KAQFLVFDAIMGIR
>Oryctolagus_cuniculus
----MHCKLTRGGSSG-KEQFLVFDAIMGIR
>Scleropages_formosus
MYRGMRCSVLLGGSSE--CLFLFFNSIMGIR
>Lepidothrix_coronata
----MHCNFTKGGSSG-KDQFLVFDAIMGIR
>Ceratotherium_simum_simum
----MHYNSTQGGSSG-KDQFLVFDAIMGIR
>Manis_javanica
----MYYNFTKGGSSG-KEQFWVFDAIMGIR
>Chinchilla_lanigera
----MHYNLTKGGSSG-KEQFLVFDAIMGIR
>Anolis_carolinensis
----MLCNCTKGGSSG-KDQILGFDAIMGIR
>Struthio_camelus_australis
----MHCNFTKGGSSG-KDQFLVFDAIMGIR
>Tinamus_guttatus
----MHCNLTKGGSSG-KEQFLVFDAIMGIR
```

HG0133.1 ENSDARG00000051886 ankrd11

>Danio_rerio
------MESTVDDDAMEA
>Acipenser_sinensis
------MESTVDDDAMEA
>Channa_striata
------MESTVDDDAMAA
>Menidia_menidia
------MESTVDDDAMAA
>Porichthys_notatus
------MESTVDDDAMAA
>Oryzias_melastigma
------MESTVDDDAMAA
>Astyanax_mexicanus
------MESTVDDDAMEA
>Haplochromis_burtoni
------MESTVDDDAMAA
>Alosa_alosa
------MESTVDDDAMAA
>Kryptolebias_marmoratus
------MESTVDDDAMAA
>Esox_lucius
------MESTVDDDAIEA
>Acanthogobius_hasta
------MESTVDDDAMAA
>Scleropages_formosus
------MESTVDDDAMEA
>Anoplopoma_fimbria
------MESTVDDDAMAA
>Platichthys_stellatus
------MESTVDDDAMAA
>Salmo_salar
------MESTVDDDAMEA
>Lepisosteus_oculatus
------MESTVDDDAMEP
>Ictalurus_punctatus
------MESTVDDDAMEA
>Squalus_acanthias
MPWWRAMESTVDDDAMDA
>Takifugu_rubripes
------MESTVDDDAMAA

HG0133.1 ENSG00000167522 ANKRD11

>Homo_sapiens
MESQTVDDEAPAV
>Canis_lupus_familiaris
MESQTVDDEAMAV
>Miniopterus_natalensis
MESQTVDDEAMAV
>Ceratotherium_simum_simum
MESQTVDDEAMAV
>Loxodonta_africana
MESQTVDDEAMAV
>Chinchilla_lanigera
MESQTVDDEAMAV

HG0133.2 ENSDARG00000051886 ankrd11

>Danio_rerio
MGKTSIGREYQV
>Pygocentrus_nattereri
MGKSSIGREYQA
>Esox_lucius
MGKTSIGQEYQG

```
HG0133.3 ENSG00000167522 ANKRD11

>Homo_sapiens
-MGSARA--------PPLSRRGAAQPSEHEYIQPCSGTHLHWI------
>Rousettus_aegyptiacus
MAGPNGEGQIVNRTEVEIIYLTKLYSLVSEYTQHCSGHTLYWI------
>Psammomys_obesus
-MGSARA--------PPPFRRGAASPSEPEHTV-----LLEFIPSPEQH
>Pelodiscus_sinensis
--------------------------MEEEQIHSCFGTHLYWI------
```

HG0134.1 ENSGALG00000041267 BMI1

>Gallus_gallus
MAAWLTFIFC
>Odobenus_rosmarus_divergens
MAAWLTFIFC
>Ambystoma_mexicanum
MAAWLTFIFC
>Lipotes_vexillifer
MAAWLTFIFC
>Astyanax_mexicanus
MAAWVAFIFC
>Calidris_pugnax
MAAWLTFIFC
>Latimeria_chalumnae
MAAWLTFIFC
>Alligator_mississippiensis
MAAWLTFIFC
>Danio_rerio
MAAWLVFIFC
>Sarcophilus_harrisii
MAAWLTFIFC
>Ochotona_princeps
MAAWLAFIFC
>Corvus_brachyrhynchos
MAAWLTFIFC
>Ceratotherium_simum_simum
MAAWLTFIFC
>Manis_javanica
MAAWLTFIFC
>Loxodonta_africana
MAAWLTFIFC
>Chinchilla_lanigera
MAAWLTFIFC
>Pygoscelis_adeliae
MAAWLTFIFC
>Anolis_carolinensis
MAAWLTFIFC
>Chelonia_mydas
MAAWLTFIFC
>Orycteropus_afer_afer
MAAWLTFIFC

HG0134.2 ENSG00000168283 BMI1

>Homo_sapiens
MRGRDRGETMGMWAREPRSGLAAPPSPAE
>Odobenus_rosmarus_divergens
---------MGMLAREPRSGLETPPSPAE
>Orcinus_orca
---------MGMLAREPRSGLETPPSPAE
>Pteropus_vampyrus
---------MGMLAREPRSGLETPPSPAE
>Ochotona_princeps
---------MGMLARAPRSGLETPPGPAE
>Ceratotherium_simum_simum
---------MGLLAREPRSGLETPPSPAE
>Loxodonta_africana
---------MGRLAREPRSGLETPPSPAE
>Dipodomys_ordii
---------MGMWAREPRSGLETPPRPAE
>Orycteropus_afer_afer
---------MGLLAREPQSGLETPPSPAE

HG0135 ENSDARG00000094132 igf1

>Danio_rerio
MLPQLFPVENVSVM
>Acipenser_sinensis
MFPQLFPVENVCVM
>Amia_calva
MFLQLFPVENVCVM
>Xenopus_laevis
MLPQLFPVYSVCVM
>Menidia_menidia
MLPQLFPVENVCVM
>Oryzias_melastigma
MLPQLFPVENVCVM
>Austrofundulus_limnaeus
MLPQLFPVENVCVM
>Meleagris_gallopavo
MLPQLFPVYSVCVM
>Parus_major
MLPQLFPVYSVCVM
>Mesocricetus_auratus
MLPQLFPVYSVCVL
>Oncorhynchus_kisutch
MLPQLFPVENVCVM
>Lepisosteus_oculatus
MFPQLFPVENVCVM

HG0135 ENSG00000017427 IGF1

>Homo_sapiens
MFPQLFPVYSVCVL
>Acipenser_sinensis
MFPQLFPVENVCVM
>Amia_calva
MFLQLFPVENVCVM
>Xenopus_laevis
MLPQLFPVYSVCVM
>Menidia_menidia
MLPQLFPVENVCVM
>Oryzias_melastigma
MLPQLFPVENVCVM
>Ailuropoda_melanoleuca
MFPQLFPVYSVCVL
>Myotis_brandtii
MFPQLFPVYSVCVL
>Dasypus_novemcinctus
MFPQLFPVYSVCVL
>Austrofundulus_limnaeus
MLPQLFPVENVCVM
>Monodelphis_domestica
MFPQLFPVYSVCVL
>Meleagris_gallopavo
MLPQLFPVYSVCVM
>Erinaceus_europaeus
MFPQLFPVYSVCVL
>Oryctolagus_cuniculus
MFPQLFPVYSVCVL
>Parus_major
MLPQLFPVYSVCVM
>Equus_caballus
MFPQLFPVYSVCVL
>Nannospalax_galili
MFPQLFPVYSVCVL
>Oncorhynchus_kisutch
MLPQLFPVENVCVM
>Tupaia_chinensis
MFPQLFPVYSVCVL
>Lepisosteus_oculatus
MFPQLFPVENVCVM

HG0136.1 ENSG00000125845 BMP2

>Homo_sapiens
--MWTLFQWTCPRV---LLRRTAVS
>Felis_catus
--MWRLFQWTCPRV---LLRRSAVS
>Physeter_catodon
--MWRLFQWTCPRV---LLRRSAVS
>Eptesicus_fuscus
--MWRLFQWTCPRV---LLRRSAVS
>Dasypus_novemcinctus
--MRRRSRWTCPRV---LPRRSAAS
>Latimeria_chalumnae
--MWILFQWTCSSV---LLSQSAAS
>Galeopterus_variegatus
--MWRLFQWTCPRV---LLRRTAVS
>Monodelphis_domestica
--MWRLFQWTCPRV---LLSQSAVS
>Condylura_cristata
--MWRFFQWTCPRV---LLRRSAVS
>Oryctolagus_cuniculus
--MWRLFQWTCPRG---LLRRTAVS
>Ornithorhynchus_anatinus
MRKKTHREQRRLPQTPAPPPSPSSSRSPSRSPWSPPRPAPDPPAAPVSSARDFSMWRLFRWTCPRV---LLRQSAVS
>Ceratotherium_simum_simum
--MWRLFQWTCPRV---LLRRSAVS
>Manis_javanica
--MWRLFQWTCPRV---LLRRSAVS
>Loxodonta_africana
--MWRLFQWTCPRV---LLRRSAVS
>Dipodomys_ordii
--MWRLFQWTCPRV---LLRRTAVS
>Tupaia_chinensis
--MWRLFQWTCHRV---LLRRTAVS
>Lepisosteus_oculatus
--MWKLFQWTCQRV---LLSRSPLS
>Trichechus_manatus_latirostr
--MWRLFQWTCLRV---LLRRSAVS
>Trachemys_scripta
--MWSPFQWTCPSAPPLLLRRSAGS
>Orycteropus_afer_afer
--MWRLFQWTCPRV---LLRRSAVS

HG0136.2 ENSG00000125845 BMP2

>Homo_sapiens
MRSTYCRRSGAWDALAEV
>Oryctolagus_cuniculus
MPSTSCRGSGARDVPAEV
>Ceratotherium_simum_simum
MPITSCRSSGAWDAPAEV
>Marmota_monax
MPSTSCRGSGAWDVMVEV

HG0137.1 ENSG00000181722 ZBTB20

>Homo_sapiens
---------------MNICTRPKPWSNLPEE
>Amia_calva
---------------MKICCRPHTWTDLPEE
>Anser_cygnoides
---------------MNVCTRSKAWSNLPEE
>Panthera_tigris
---------------MNICTRPKPWSNLPEE
>Balaenoptera_acutorostrata_s
---------------MNICTRPKPWSDLPEE
>Rousettus_aegyptiacus
---------------MNICTRPKPWSDLPEE
>Dasypus_novemcinctus
---------------MNIFTRPKRWSDLPEE
>Latimeria_menadoensis
---------------MKIRVRPKRWTDLPEK
>Gallus_gallus
---------------MNVCTKSKAWSNLPEE
>Erinaceus_europaeus
---------------MNISTRPKSWSDLPEE
>Oryctolagus_cuniculus
---------------MNICTRPQPWSDLPEE
>Elephantulus_edwardii
---------------MNICTRPKPWSDLPEE
>Lepidothrix_coronata
---------------MNVCTRSKAWSNLPEE
>Equus_asinus
---------------MNICTRPKPWSDLPEE
>Manis_javanica
---------------MNICTRPQPWSDLPEE
>Cavia_porcellus
MSLFLLCRSSLFPAPMNICTRPKPWSDLPEE
>Tupaia_chinensis
---------------MNICTRPKPWSDLPEE
>Trichechus_manatus_latirostr
---------------MNICTRPKPWSDLPEE
>Pelodiscus_sinensis
---------------MNICTRAKAWSDLPEE
>Orycteropus_afer_afer
---------------MNICTRPKPWSNLPEE

HG0137.2 ENSG00000181722 ZBTB20

```
>Homo_sapiens
----------------MSWKCRNLSLPSRKNL-------------------------
>Mustela_putorius_furo
MRSLNSFHLLLTVQSSMSWKCRNLSLPSRKTVAGTFPPRFERKRIGWNKTLPSIWST
>Lipotes_vexillifer
----------------MSWKCRNLSLPSRKIP-------------------------
>Rousettus_aegyptiacus
MRSLNSFHLLLTVQSSMSWKCRNLSLPSRKTVTGTFPPRFERKRIGWNKTLLSIWST
>Erinaceus_europaeus
----------------MSWKCRNLSLPSRKIP-------------------------
>Elephantulus_edwardii
----------------MSWKCRNLSLPSRQIL-------------------------
>Equus_przewalskii
MRSLNSFHLLLTVQSSMSWKCRNLSLPSRKTVAGTFPPRFERKRIGWNKTLLSIWST
>Cricetulus_griseus
MSSQNSFHLLLTVQSSMSWKCRNLSLPSRKTLWNTVNSTN-----------------
>Tupaia_chinensis
MRSLSSFHLLLTVQSSMSWKCRNLSLPSRKIL-------------------------
```

HG0138.1 ENSG00000185070 FLRT2

>Homo_sapiens
MPRDPRCCRASVFPFHLPDPVGFG
>Anser_cygnoides
MP--EPSCSATFSPFNLPDPVGFG
>Felis_catus
MPREPRCCRASFFPFHLPDPVGFG
>Lipotes_vexillifer
MPREPRCCRASFLPFHLPDPVGFG
>Calidris_pugnax
MP--EPSCSATFSPFNLPDPVGFG
>Myotis_davidii
MPREPRCCRASFFPFHLPDPVGFG
>Dasypus_novemcinctus
MPREPRCCRASFFPFHLPDPVGFG
>Monodelphis_domestica
MQQEPSYYCTSFFSFHLPDPVGFG
>Oryctolagus_cuniculus
MPREPRCYRAGFFPFHLPDPVGFG
>Corvus_brachyrhynchos
MP--EPSCSATFSPFNLPDPVGFG
>Equus_asinus
MPREPGCCRASFFPFHLPDPVGFG
>Loxodonta_africana
MPREPRYCHASFFPFHLPDPVGFG
>Chinchilla_lanigera
MPREPHCCRASFFPFHLPDPVGFG
>Tupaia_chinensis
MPREPRSCRASFFPFHLPDPVGFG
>Trichechus_manatus_latirostr
MPRKPRYCLASFFPFHLPDPVGFG
>Crotalus_adamanteus
------MPGLSCFALHLPDPVGFG
>Struthio_camelus_australis
MP--EPSCSATFSPFNLPDPVGFG
>Pelodiscus_sinensis
MQ--EASASASVSPFHLPDPAGFG
>Tinamus_guttatus
MP--EPSSSAAFSPLNLPDPVGFG
>Orycteropus_afer_afer
MPREPRCCRASFFPFHLPDPVGFG

HG0138.2 ENSG00000185070 FLRT2

>Homo_sapiens
---MGRRDLGGDP
>Odobenus_rosmarus_divergens
MFETCRCPLRVSHLPLSPQRNERTEITSVATISAPRRCAASARGASLALGSGCLAAARRQHCACRRVHPAPGSGWRFSTMGRRDLGGDP
>Orcinus_orca
---MGRRDLGGDP
>Pteropus_alecto
---MGRRDLGGDP
>Dasypus_novemcinctus
---MGRRDLGGDP
>Oryctolagus_cuniculus
---MGRRDLGGDP
>Equus_asinus
---MGRRDLGGDP
>Loxodonta_africana
---MGRRDLGGDP
>Cricetulus_griseus
---MGRRDLGGDP

HG0139 ENSGALG00000006379 SHH

>Gallus_gallus
MCSVISDLKAAIPAPVLLGIGG-----------
>Anas_platyrhynchos
MCSVISDLKPAIPAPVLLGIGG-----------
>Apteryx_australis_mantelli
MCSVISDLKAAIPAPVLIGIGG-----------
>Charadrius_vociferus
MCSVISDLKPAIPAPVLLGIGG-----------
>Myotis_brandtii
MCSVISDQQPAAPAPVWVGIGRESPALVAGRVL
>Latimeria_chalumnae
MCSVINDLKPVISAPTLIGIAG-----------
>Columba_livia
MCSVISDLTPAIPAPVLLGIGG-----------
>Falco_cherrug
MCSVISDLKPAIPAPVLLGIGG-----------
>Oryctolagus_cuniculus
MCSVISDRQPAIRAPVWVGIGGESPAAAAGKVI
>Opisthocomus_hoazin
MCSVISDLKPAIPAPVLLGIGG-----------
>Parus_major
MCSVISDLKPAIPAPVLLGIGG-----------
>Nipponia_nippon
MCSVISDLKPAIPAPVLLGIGG-----------
>Ceratotherium_simum_simum
MCSVISDQQPAVPAPVWVGISGESPAAAAGKVI
>Manis_javanica
MCSVISAQQPAEPAPVWVGIAGQSPAAAAGKVV
>Chlorocebus_sabaeus
MCSVISGRQPAIPAPVWVGSGGECPAAAAGKVI
>Fukomys_damarensis
MCSAISDRQPAIAAPVWAEIGGEYTAAAAGKVI
>Lepisosteus_oculatus
MYSVINEQKADISAPASVGIGG-----------
>Pygoscelis_adeliae
MCSVISDLKPAIPAPVLLGIGG-----------
>Chrysemys_picta
MCSVISDLKPAIPTPVLLGIGG-----------
>Tinamus_guttatus
MCSVISDLKAAIPAPVLLGIGG-----------

HG0140 ENSG00000009307 CSDE1

>Homo_sapiens
--MKCNRTDFTT
>Apteryx_australis_mantelli
--MKCNRTEFTT
>Canis_lupus_familiaris
--MKCNRTDFTT
>Physeter_catodon
--MKCNRTDFTT
>Myotis_davidii
--MKCNRTDFTT
>Dasypus_novemcinctus
--MKCNRTDFTT
>Alligator_mississippiensis
--MKCNRTEFTT
>Sarcophilus_harrisii
--MKCNRTDFTT
>Galeopterus_variegatus
--MKCNRTDFTT
>Monodelphis_domestica
--MKCNRTDFTT
>Sorex_araneus
--MKCNRTDFTT
>Oryctolagus_cuniculus
--MKCNRTDFTT
>Ceratotherium_simum_simum
--MKCNRTDFTT
>Manis_javanica
MSMKCNRTDFTT
>Loxodonta_africana
--MKCNRTDFTT
>Jaculus_jaculus
--MKCNRTDFTT
>Trichechus_manatus_latirostr
--MKCNRTDFTT
>Python_bivittatus
--MKCNRTEFTT
>Chrysemys_picta
--MKCNRTEFTT
>Orycteropus_afer_afer
--MKCNRTDFTT

HG0141.1 ENSGALG00000010435 RASGRP3

>Gallus_gallus
MFVLNTSAEPSTIFLEA-QLRMQPTIK-----------
>Anser_cygnoides
MFVLNTSAEPSTIFLEERKLRMQPTIK-----------
>Charadrius_vociferus
MFVLNTSAEPSTIFLEV-ELRMQPTIK-----------
>Pterocles_gutturalis
MFVLNTSAEPSTIFLE-VQLRMQPTIK-----------
>Columba_livia
MFVLNTSAEPSTIFLEG-RLRMQPTIK-----------
>Alligator_mississippiensis
---------------------MQPAIKR----------
>Cuculus_canorus
MFVLNTSAEPSTIFLE-VQLRMQPTIK-----------
>Aquila_chrysaetos_canadensis
MFVLNTSAEPSTIFP-----------------------
>Eurypyga_helias
MFVLNTSAEPSTIFLE-VKLRMQPKIK-----------
>Tauraco_erythrolophus
MFVLNTSAEPSTIFLE-VQLRMQPTIK-----------
>Opisthocomus_hoazin
---------------------MQPTIK-----------
>Zonotrichia_albicollis
MFVLNTSAEPSTIFIE-VQLRMQPTK------------
>Egretta_garzetta
MFVLNTSAEPSTIFLE-VQLRMQPTIK-----------
>Picoides_pubescens
MFVLNTSAEPSTISPE-VQLRMQPTIK-----------
>Python_bivittatus
MFVLNTSAKLSTFFEASQR--MQLTVIEWGPSCFSLLW
>Struthio_camelus_australis
MFVLNTSAEPSTLFCRPACL------------------
>Chrysemys_picta
MFVLNTSAEPSTIFHEA---------------------
>Tinamus_guttatus
MFVLNTSAEPSTIFPE-GQLRMQPTIK-----------
>Apaloderma_vittatum
---------------------MQPTIK-----------

HG0141.2 ENSG00000152689 RASGRP3

>Homo_sapiens
--MIMEMVYLMLKMSLVF
>Canis_lupus_familiaris
----MEMVYLMLKMCLVF
>Balaenoptera_acutorostrata_s
--MIMEMVYMMLKMPLVF
>Rousettus_aegyptiacus
--MIMEIVCLMLKMSLVF
>Dasypus_novemcinctus
----MKMVYLMLKMSLVF
>Erinaceus_europaeus
----MEMVCLMWKMSLLF
>Ochotona_princeps
MYMIMEMVYLMLKMSLVF
>Equus_przewalskii
--MIIEMVCLMLKMSLVF
>Loxodonta_africana
--MIMEMVYLMLKMSIVF
>Tupaia_chinensis
----MEMAYLMLKMSLVF
>Orycteropus_afer_afer
--MIMEMVYLMPKMSTVF

HG0141.3 ENSG00000152689 RASGRP3

>Homo_sapiens
MLMRWLSQLRGYEVVCE---
>Mustela_putorius_furo
--MKWLSQLRGYEA-CELGE
>Equus_asinus
MLTKWLSQLRGYEKVCKLRE
>Manis_javanica
MLMKWLNQLRG---------
>Tupaia_chinensis
MLMRWLSQLRG---------

HG0142.1 ENSG00000204217 BMPR2

>Homo_sapiens
MFSPRPGYFFDIVKLRGK---
>Apteryx_australis_mantelli
MFPPKPVCFFDIVKLRGK---
>Odobenus_rosmarus_divergens
MFSPRPGYFFDIVKLRGK---
>Hynobius_retardatus
MFHPKPGCFFDIVKLRGK---
>Lipotes_vexillifer
MFSPRPGYFFDIVKLRGK---
>Pteropus_alecto
MFSPRPGYFFDIVKLRGK---
>Monodelphis_domestica
MFPSKSGFFFDIVKLRGKSSW
>Aquila_chrysaetos_canadensis
MFPSQPGSFFDIVKLRGK---
>Condylura_cristata
MFSPRPGYFFDIVKLRGK---
>Oryctolagus_cuniculus
MFSPRPGYFFDIVKLRGK---
>Elephantulus_edwardii
MFSPRPGSFFDIVKLRGE---
>Acanthis_flammea
MFPSQPGAFFDVVKLRGK---
>Loxodonta_africana
MFSLRPGSFFDIVKLRGE---
>Jaculus_jaculus
MFSPRPGYFFDIVKLRGK---
>Trichechus_manatus_latirostr
MFSPRPGYFFDIVKLRGE---
>Pygoscelis_adeliae
MFPSPPGCFFDIVKPRRK---
>Struthio_camelus_australis
MFAPTPGCFFDIVKLRGK---
>Chelonia_mydas
MFHAKPGCFFDIVKLRGK---
>Orycteropus_afer_afer
MFSPRPGYFFHIVKLRGE---

HG0142.2 ENSG00000204217 BMPR2

>Homo_sapiens
--------MKALQLGPL-ISHLSFQTVL
>Acinonyx_jubatus
--------MKALQLGPL-ICHLSFQTAL
>Lipotes_vexillifer
--------MKALQLGPL-ICHLSFQTAL
>Miniopterus_natalensis
--------MKALQLGPL-ICHLSFQTAL
>Monodelphis_domestica
MKGIFCSGVRALQTGPLSICHLSFQTAL
>Condylura_cristata
--------MNALQLGPR-ICHLSFQTAL
>Oryctolagus_cuniculus
--------MKALPLRPL-PCHLSFQTAL
>Elephantulus_edwardii
--------MNALQLGLL-ICHLSFQTAL
>Ictidomys_tridecemlineatus
--------MKALQLGPL-ICHLSFQTAL
>Trichechus_manatus_latirostr
--------MKALQLGPL-ICHLSFQTAL
>Orycteropus_afer_afer
--------MKALQLGPL-ICHLSFQTAL

HG0143 ENSG00000101958 GLRA2

>Homo_sapiens
MMPRTGTFSFFSA-NCTKP-NLFLIFKETRFLPNFESGQ
>Mustela_putorius_furo
MMPRTGTFCFFSA-NCTNP-NLFLIFKETRFLPDFESGQ
>Orcinus_orca
MMPSTGTFCFFSA-NCTSP-NLFLIFKETRFLPDFESGQ
>Rousettus_aegyptiacus
-MPRTGTFCFFSA-NCTNP-NLFLIFKETRFLPDFEFGQ
>Dasypus_novemcinctus
MMPRTGTFCFFSA-NCTNP-NLFLIFKEIRFLPDFQSGQ
>Galeopterus_variegatus
MMPRTGTFCFFSV-NCTNP-NLFLIFKETRFLPDFESGR
>Monodelphis_domestica
MIPRTGTFYFFSP-NYTNP-HLFLIFKAIRLLTDFESGQ
>Sorex_araneus
MMPRTGTFCFFSA-NCTNP-NLFLIFKETRFLPNFEPGQ
>Oryctolagus_cuniculus
MMPRTGTFCFFSA-NCSIP-NLFLIFKETRFLPDFESGQ
>Elephantulus_edwardii
MMPRSGTFCFFLV-NCTNP-NLFLVFKEIRFLPDFESGQ
>Equus_caballus
MMPRTGTFCFFSA-NCANP-NLFLIFKETRLLSDFESGQ
>Manis_javanica
MMPRTGTFCFFSA-NCTNR-NLFLIFKETRFLTDFESGQ
>Loxodonta_africana
MMPRTGTFCFFSV-NCTNP-NLFLVFKEIRFLPDFESGQ
>Peromyscus_maniculatus_baird
MMPRTGTFCFFSA-NCTTP-NLFLIFKETRFLPDFESGQ
>Tupaia_chinensis
MMPKTGTFCFFSA-N------------------------
>Trichechus_manatus_latirostr
MMPRTGTFCFFSV-NCTNP-NLFLVFKEIRFLPDFESGQ
>Anolis_carolinensis
MIPRTGTFDFLPSPRHADEGLLFLILKNFKLLLDLGI--
>Chelonia_mydas
MIPRTGTSYFLPP-ICRHPDLLFLIFKKIKLLLDLGI--
>Orycteropus_afer_afer
MMPKTGTFCFFLV-NCTNP-NLFLVFKEIRFLPDFGSGQ

HG0144 ENSG00000117114 ADGRL2

>Homo_sapiens
MMQTDGFDEAGHL
>Acinonyx_jubatus
MMQTAGFDEAGHL
>Ambystoma_mexicanum
-MQTDGFDEVGNA
>Balaenoptera_acutorostrata_s
MMQTAGFDEAGHL
>Pteropus_alecto
MMQIAGFDEAGHI
>Alligator_mississippiensis
-MQTTGFDDAGHL
>Galeopterus_variegatus
MMQTAGFDEAGHL
>Monodelphis_domestica
MMYTAGFNEAGHL
>Sorex_araneus
MMQTAVFDEAGHL
>Oryctolagus_cuniculus
MMQTAGFDEAGHL
>Elephantulus_edwardii
MMQTAGFDEAGHI
>Equus_caballus
MMQTPGFDEAGHL
>Manis_javanica
MMQTAGFDEAGHL
>Loxodonta_africana
MMQTAGFDEAGHL
>Ictidomys_tridecemlineatus
MMQTAGFDEAGHL
>Tupaia_chinensis
MMQTAGFDEAGHL
>Trichechus_manatus_latirostr
MMQTAGFDEAGHL
>Chrysemys_picta
MIQTTGFNDAGHL
>Orycteropus_afer_afer
MMQTAGFNEAGHL

HG0145 ENSG00000162599 NFIA

```
>Homo_sapiens
MIPYGMDRTTCCPTCHCKCFLN
>Canis_lupus_familiaris
MIPYGMDRTTCCPTCHCKCFLN
>Pygocentrus_nattereri
MIPYGMERATCCPTCQCKCFLN
>Charadrius_vociferus
MIPYGMDRATCCPTCQCKCFLN
>Alosa_alosa
MIPYGMERATCCPTCQCKCFLN
>Cuculus_canorus
MIPYGMDRATCCPTCQCKCFLN
>Sinocyclocheilus_anshuiensis
MIPYGMEWATCCPTCQCKCFLN
>Monodelphis_domestica
MIPYGMDRTTCCPTCHRKCFLN
>Haliaeetus_leucocephalus
MIPYGMDRATCCPTCQCKCFLN
>Apteronotus_leptorhynchus
MIPYGMERATCCPTCQCKCFLN
>Sturnus_vulgaris
MIPYGMDRATCCPTCQCKCFLN
>Nipponia_nippon
MIPYGMDRATCCPTCQCKCFLN
>Lateolabrax_maculatus
MIPYGME-ATCCPTCQCKCFLN
>Manis_javanica
MIPYGMDRTTCCPTCHCKCFLN
>Melopsittacus_undulatus
MIPYGMDRATCCPTCQCKCFLN
>Mus_musculus
MIPYGMDRTTCCPTCHCKCFLN
>Salmo_salar
MIPYGMERATCCPTCQCKCFLN
>Lepisosteus_oculatus
MIPYGMDWATCCPTCQCKCFLN
>Ameiurus_nebulosus
-----MERATCCPTCQCKCFLN
```

HG0146.1 ENSG00000163681 SLMAP

>Homo_sapiens
MLQ---CCNVSATKGEKRPRSQTKHKNWRVTHLLGYLQSPNCVP-GVFLAEAARCLSLFFPEKKMD
>Anas_platyrhynchos
-----------------------------MTHLLGFETFYPSFKEPLLLFEGIRCLFLFFQRRMD-
>Apteryx_australis_mantelli
-----------------------------MTHLVGFKTSYPSFKEPLLLFEGIRCLLLFFQRRMD-
>Panthera_tigris
MLQ---CCNISATEGEKRPRSQTKHKNWCVTHLLGYLQSLNCLP-GVFLAGADRCLSLFFPGKRMD
>Calidris_pugnax
-----------------------------MTHLLGFKTFYPSFKELLLLFEGIRCLFLFFQRRMD-
>Pteropus_alecto
MLQ---CCNISATEGEKRPRSQTKHKNWCVTHLLGYLQSSNCLP-GVFLAEAVQCLSLFFPRKKMD
>Galeopterus_variegatus
MLQ---CCNVSATKGEKRPRSQTKHKNWCVTHLLGYLQSPNCLP-GVFLAEAA--------------
>Haliaeetus_albicilla
-----------------------------MTHLLGFKTFYPSFKEPLLLFEGIRCLFLFFQRRMD-
>Meleagris_gallopavo
-----------------------------MTHLPGFKTFSPSVEEPFLLSEEIRCLFLFFQRRMD-
>Condylura_cristata
MLQ---CCNISATKGEKRPRSQTKRKNWRVTHLLGYLQSPNCLP-GLFLAEAVRCLNLFFLGKKMD
>Oryctolagus_cuniculus
MLQ---CCNVSASEGAPRPRSHTEPRNRRVTHLLGDLQPQNCLP-GVFLAEAARCLSLSFPGKMD-
>Pseudopodoces_humilis
-----------------------------MTHLLGFKTLYPSFPGPLLLSEGIRCLFLFFQRRMD-
>Equus_asinus
MLQ---CCNISATEGERRPRSQTKHKNWFVTHLLGYLQSPNCLP-GVFLAEAVRCLSLFFPGKRMD
>Manis_javanica
MLQ---GCNISATEGERRPRSQTKPANCCVTHLCGYLQSPNCLP-GVL-AETVRCLSLFFPGKKMD
>Marmota_marmota_marmota
MLQ---CCNVSATKGEKRPRSQTKHKNWCVTHLLGYLQSPNCLP-GVFLAEAARCLSLFFPGKMD-
>Tupaia_chinensis
MLQ---CCNVSATEGEKRPRSQTKHKNWCVTHLLGYLQSPNCLP-GVFLAEAARCSSLFFPGKKMD
>Pygoscelis_adeliae
-----------------------------MTHLLGFKTFCPSFKEPLLLFEGIRCLFLFFRRRMD-
>Struthio_camelus_australis
MLHRSGFWSVLFTEGGKKKAVEIPNKSICMTHLVGFKTS---------------------------

HG0146.2 ENSG00000163681 SLMAP

>Homo_sapiens
MLRQCAVCVNLKFWVG
>Felis_catus
MLRQCAVCFNLKFWGG
>Pteropus_alecto
MLRQCAFCFNLKFWGG
>Galeopterus_variegatus
MLRQCVVCFNLEFWGG
>Condylura_cristata
MLRQCAVCFDLKFWGG
>Ceratotherium_simum_simum
MLRQCTVCFNLKFWGG
>Manis_javanica
MLRQCAVCFNLKFGGG
>Marmota_marmota_marmota
MLRQCAVCFNLKFWGG
>Tupaia_chinensis
MLRQCAVCVNLQFWGG

HG0146.3 ENSG00000163681 SLMAP

>Homo_sapiens
MAAGAGKVGKFPASSGAA
>Felis_catus
MAAGAGKVGKFPALSGTA
>Rousettus_aegyptiacus
MAAGAGKVGKFSASSGTA
>Condylura_cristata
MAAGAGKVGKFPASSDMV
>Oryctolagus_cuniculus
MAAGAGKVGKFPASSGTA
>Mus_musculus
MAANAGKVGKFPASSATA

HG0147 ENSG00000108984 MAP2K6

>Homo_sapiens
MKIARLQLASLLQN
>Acinonyx_jubatus
MKIARLQLASLLQN
>Orcinus_orca
MKIARLQLASLLQN
>Eptesicus_fuscus
MKIARLQLASLLQN
>Dasypus_novemcinctus
MKIARLQLASLLQN
>Macropus_eugenii
MKTARLQLESLLQN
>Meleagris_gallopavo
MKIERLQFASLLQN
>Erinaceus_europaeus
MKIARLQLASLLQN
>Oryctolagus_cuniculus
MKIARLQLASLLQN
>Elephantulus_edwardii
MKIARLQLASLLQN
>Equus_asinus
MKIARLQLASLLQN
>Manis_javanica
MKIARLQLASLLQN
>Loxodonta_africana
MKIARLQLASLLQN
>Nannospalax_galili
MKIARLQLASLLQN
>Trichechus_manatus_latirostr
MKIARLQLASLLQN
>Gekko_japonicus
MKIERLQFASLLQN
>Struthio_camelus_australis
MKIERLQFASLLQN
>Pelodiscus_sinensis
MKIGRLQFASLLQN

HG0147 ENSGALG00000004370 MAP2K6

>Gallus_gallus
MKIERLQFASLLQN
>Acinonyx_jubatus
MKIARLQLASLLQN
>Orcinus_orca
MKIARLQLASLLQN
>Eptesicus_fuscus
MKIARLQLASLLQN
>Dasypus_novemcinctus
MKIARLQLASLLQN
>Latimeria_chalumnae
MKITSLHFASLLQN
>Erinaceus_europaeus
MKIARLQLASLLQN
>Ochotona_princeps
MKIARLQLASLLQN
>Elephantulus_edwardii
MKIARLQLASLLQN
>Equus_asinus
MKIARLQLASLLQN
>Manis_javanica
MKIARLQLASLLQN
>Otolemur_garnettii
MKIARLQLASLLQN
>Loxodonta_africana
MKIARLQLASLLQN
>Dipodomys_ordii
MKIARLQFASLLQN
>Trichechus_manatus_latirostr
MKIARLQLASLLQN
>Gekko_japonicus
MKIERLQFASLLQN
>Struthio_camelus_australis
MKIERLQFASLLQN
>Trachemys_scripta
MKIERLQFASLLQN

HG0148.1 ENSGALG00000011855 RHOJ

>Gallus_gallus
MLEIRWKMEWI
>Apteryx_australis_mantelli
MLEMKWKMEWI
>Caprimulgus_carolinensis
MLEMKWKMEWI
>Charadrius_vociferus
MLEMKWKMEWI
>Columba_livia
MLEMKWKMEWI
>Merops_nubicus
MLEMRWKMEWI
>Cuculus_canorus
MLEMKWKMEWI
>Falco_cherrug
MLEMKWKMEWI
>Gavia_stellata
MLEMKWKMEWI
>Eurypyga_helias
MLEMKWKMEWI
>Opisthocomus_hoazin
MLEMKWKMEWI
>Corvus_brachyrhynchos
MLEMKWKMEWI
>Nipponia_nippon
MLEMKWKMEWI
>Picoides_pubescens
MLEMKWKMEWI
>Melopsittacus_undulatus
MLEMKWKMEWI
>Tyto_alba
MLEMKWKMEWI
>Pelodiscus_sinensis
MLEMKWKMEWI
>Calypte_anna
MLEMKWKMEWI

HG0148.2 ENSGALG00000011855 RHOJ

```
>Gallus_gallus
-----------MILWTALVIVRSSCDSLETLV
>Anas_platyrhynchos
-----------MILLTALVIVRSGCDSLETLV
>Apteryx_australis_mantelli
--MYIFNLFTSMILLTALVIVRSGCDSLETLV
>Caprimulgus_carolinensis
-----------MILLTDLVIVRSGCDSLETLV
>Cariama_cristata
-----------MILLTDLVIVRSGCDSLETLV
>Calidris_pugnax
MIFLSVNLFTSMILLTDLVIVKSGCDSLETLV
>Columba_livia
-----------MILLTDLAIVRSGCDSLETLV
>Merops_nubicus
-----------MLLLTDLVIVRSGCDSLETLV
>Cuculus_canorus
-----------MILLTDLVIVRSGCDSLETLV
>Aquila_chrysaetos_canadensis
-----------MILLTDLVIVRSGCDSLETLV
>Gavia_stellata
-----------MILLTDLVIVRSGCDSLETLV
>Mesitornis_unicolor
-----------MILSTDLVIVRSGCDSLETLV
>Opisthocomus_hoazin
-----------MILLTDLVIVRSGCDSLETLV
>Manacus_vitellinus
-----------MILLTDLVIVRSGCDSLETLL
>Nipponia_nippon
-----------MILLTDLVIVRSGCDSLETLV
>Picoides_pubescens
-----------MILLTDLVIVRSGCDSLETLV
>Melopsittacus_undulatus
-----------MILFTDLVIVRSGCDSLETLV
>Aptenodytes_forsteri
-----------MILLTDLVIVRSGFDSLETLV
>Tyto_alba
-----------MILLTDLVIVRSGCDSLETLV
>Struthio_camelus_australis
-----------MILLTALVIVRSGCDSLETLV
>Calypte_anna
-----------MILWTDLVIVRSGCDSLEALV
```

HG0149.1 ENSGALG00000014645 MEF2C

>Gallus_gallus
MILTIKRVYVQERMQGTVN
>Anas_platyrhynchos
MILTIKRVYVQERMQGTVN
>Calidris_pugnax
-------------MQGTVN
>Columba_livia
MILTIKRVYV-ERMQGTVN
>Alligator_mississippiensis
MILRIKKEYVQERMQGTVN
>Cuculus_canorus
-------------MQGTVN
>Monodelphis_domestica
----MKFLFLQERMQGSVN
>Aquila_chrysaetos_canadensis
MILTIKRVYVQERMQGTVN
>Balearica_regulorum_gibberic
-------------MQGTVN
>Ornithorhynchus_anatinus
-------MYVQERMQGSVN
>Lepidothrix_coronata
MILTIKRVYVQERMQRTVN
>Phaethon_lepturus
-------------MQGTVN
>Otolemur_garnettii
-------------MQGFGN
>Fulmarus_glacialis
-------------MQGTVN
>Aptenodytes_forsteri
MILTIKRVYVQEQMQGTVN
>Anolis_carolinensis
MILNKRKGYVQERMPGSVN
>Chrysemys_picta
MILTIKRVYVQERMQGSVN
>Tinamus_guttatus
-------------MQGTVN

HG0149.2 ENSG00000081189 MEF2C

>Homo_sapiens
MWNILKDNIKVNECRNLGTELCKC
>Ursus_maritimus
----------MNECRNLGTEQCKC
>Orcinus_orca
----------MNECRNLGTEQCKC
>Rousettus_aegyptiacus
----------MNECRNLGTEQCKC
>Dasypus_novemcinctus
----------MNECRNLRTELCKC
>Condylura_cristata
----------MNECRNLGTEQCKC
>Ochotona_princeps
----------MNECRNLGTELCKC
>Ceratotherium_simum_simum
----------MNECRNLGTELCKC
>Manis_javanica
----------MNECRNSGTEQGKC
>Loxodonta_africana
----------MNECRNLGTELCKC
>Heterocephalus_glaber
----------MNECRNLGTELCKC
>Tupaia_chinensis
----------MNECRNLGTELCKC
>Trichechus_manatus_latirostr
----------MNECGNLGTELCKC
>Orycteropus_afer_afer
----------MNECRNLGTELCKC

HG0150.1 ENSGALG00000032289 IKZF5

```
>Gallus_gallus
---------MAAAAEGGGRGAGRRRRHQQMEHKLT
>Anser_cygnoides
MGRESFGSFVCGALRFNAFLLEKMRRHQQMEHKLT
>Cariama_cristata
-----------------------------MEHKLT
>Charadrius_vociferus
-----------------------------MEHKLT
>Pterocles_gutturalis
-----------------------------MEHKLT
>Merops_nubicus
-----------------------------MEHKLT
>Alligator_mississippiensis
-----------------------------MEHKLT
>Haliaeetus_leucocephalus
-----------------------------MEYKLT
>Gavia_stellata
-----------------------------MEHKLT
>Mesitornis_unicolor
-----------------------------MEHKLT
>Tauraco_erythrolophus
-----------------------------MEHKLT
>Opisthocomus_hoazin
-----------------------------MEHKLT
>Parus_major
-----------------------------MEHKLT
>Egretta_garzetta
-----------------------------MEHKLT
>Fulmarus_glacialis
-----------------------------MEHKLT
>Nestor_notabilis
-----------------------------MEHKLT
>Aptenodytes_forsteri
-----------------------------MEHKLT
>Chelonia_mydas
-----------------------------MEHKLT
```

HG0150.2 ENSG00000095574 IKZF5

```
>Homo_sapiens
MAFWSPLLYLRSKFQLLYISSMLEWQEKGGTDLG-------------
>Mustela_putorius_furo
MAFWSPLVCLQSKFRLLYISSMLEWQEKGGTDLG-------------
>Orcinus_orca
MAFWSPLVYLQSKFRLLYISSMLEWQEKGGTDLG-------------
>Pteropus_alecto
MAFWSPLVCLQSKFHLLYISSMLEWQEKGGTDLG-------------
>Dasypus_novemcinctus
MAFWSPMVCLQSKFRLLYISSMLEWQEKGGADLGEKLFFLGITNCKL
>Sarcophilus_harrisii
---------------------MLEWKEKGGIDLG-------------
>Galeopterus_variegatus
MAFWSPLACHQSEFRLLHISSMLQWQEKGT-DLG-------------
>Monodelphis_domestica
MAFWSQLMYFQRGFQLLYISSMLEWKEKSGIDLG-------------
>Condylura_cristata
MAFWSPWVCLQSKFR--------------------------------
>Oryctolagus_cuniculus
---------------------MLEWQEKGT-DLG-------------
>Ornithorhynchus_anatinus
MACWSPLAHRQSGFLILGYSSMLEREERSGTDLG-------------
>Equus_asinus
MAFWSPLVCLQSKFLLLYISSMLEWQDKGGTDLG-------------
>Manis_javanica
---------------------MLEWQEKGS-DLG-------------
>Loxodonta_africana
MAFWSPWLCLQSKFPLLFISSMVEWQEKGGTDLG-------------
>Marmota_marmota_marmota
MAFWSPLVCLQNKFQILYISSMLEWQEKGGTDLG-------------
>Tupaia_chinensis
MAFWSPVVYLQNKFQLLYISSMLEWQEKGITDLG-------------
```

HG0151 ENSG00000006468 ETV1

>Homo_sapiens
MFLLNLEGSFCSLSPSVAFSPVRCKA--SLVD----R----HC-------
>Acinonyx_jubatus
MFLLNLEGIFCSRSPSVAFSPVRCKA--SLVD----R----HC-------
>Balaenoptera_acutorostrata_s
MFLLKLEGIFCSRSPSVAFSPVRCKA--SLVD----R----HC-------
>Miniopterus_natalensis
MFLLNLEGIFCSSSPSVAFSLKAILFPSRLIEVQIPRKSPAKCSKYKTLS
>Dasypus_novemcinctus
MVLLNLEGIFCSRSPSVAFSPVRCKA--SLVD----R----HC-------
>Alligator_mississippiensis
MFLLNLEGIFCSRSPCVTFRL---------LAVQILRKAADQMPRK----
>Galeopterus_variegatus
MFLLNLEGIFCSCSPSVAFSPVRCKA--SLVD----R----HC-------
>Haliaeetus_leucocephalus
MFLLNLEGIFCSRPPRVAFRL---------IGVQIPRKSPDQMPSK----
>Condylura_cristata
MFLLNLEGFFCSRSPSVAFRL---------IDVQIPRKSPA---------
>Pseudopodoces_humilis
MFLLNLEGIFCSRSPRVAFRL---------IGVQIPRKSPDQMPSK----
>Ceratotherium_simum_simum
MFLLNLEGIFCSRSPSVAFSPVRCKA--SLVD----R----HC-------
>Manis_javanica
MFLLNLKGFFCSSSPSVAFSPVRCKA--SLVD----R----HC-------
>Loxodonta_africana
MFLLNLEGIFCSRSPSVAFSPVRCKA--SLVD----R----HC-------
>Chinchilla_lanigera
MFLLNLEGIFCSRSPSVAFSPVRCKA--SLVD----R----HC-------
>Tupaia_chinensis
MFLLNLEGIVCSRSPSVAFSPVRCKA--SLAD----R----HC-------
>Struthio_camelus_australis
MFLLNLEGIFCSRSPGVAFRL---------IGVQIPKKSPDQMPSK----
>Chelonia_mydas
MFLLNLEGIFCSRSPCVTFRL---------IGEQIPRKSSGQMPRK----
>Orycteropus_afer_afer
MFLPNLEGIFCSSSPTVAF---------RLIEVQIPRKSPAKCSKYKILS

HG0151 ENSGALG00000035504 ER81

```
>Gallus_gallus
MFLLNLEGNFLQPIAARGLQADWSPDPEEISRSNAH-
>Anas_platyrhynchos
MFLLNLEGIFSQPTAARGLQADWRPDPEEISRSNAQ-
>Rousettus_aegyptiacus
MFLLNLEGIFLQQFA----------------------
>Mesitornis_unicolor
MFLLNLEGIFLQPIAAPGLQADWSPDPEEISRSNAQ-
>Manacus_vitellinus
MFLLNLEGIFLQPIAARGLQADWSPDPEEISRSNAQ-
>Nomascus_leucogenys
MFLLNLEGSFCSLSPSVAF----SPVRCKASLVDRHC
>Struthio_camelus_australis
MIA----WSW-LAKTSLWEKADWSPDPEEISRSNAQ-
```

HG0152 ENSDARG00000015536 sox6

```
>Danio_rerio
--------MLHDGKCFEYLRHGRLH--------------------------------------------------------
>Megophrys_sangzhiensis
--------MLHDGKCLEYLQPWLLTFTAKCDSPPAPPNQVTAQSPKAPLALQTYANDLDSGDLPAAHPYLLWGSLGFLIHE
>Menidia_menidia
--------MLHDGKCFEYLRHGCAH--------------------------------------------------------
>Felis_catus
--------MLHDGKCFEYLRHGRSH--------------------------------------------------------
>Pygocentrus_nattereri
--------MLRDGKCFEYLRHGRSH--------------------------------------------------------
>Miniopterus_natalensis
--------MLHDGKCFEYLRHGRSH--------------------------------------------------------
>Haplochromis_burtoni
--------MLHDGKCFEYLRHGCAH--------------------------------------------------------
>Latimeria_chalumnae
--------MLHDGKCFEYLRHGRSH--------------------------------------------------------
>Fundulus_heteroclitus
--------MLHDGKCFEYLRHGCAH--------------------------------------------------------
>Equus_asinus
--------MLHDGKCFEYLRHGRSH--------------------------------------------------------
>Platichthys_stellatus
--------MLHDGKCFEYLRHGCAH--------------------------------------------------------
>Cebus_capucinus_imitator
--------MLHDGKCFEYLRHGRSH--------------------------------------------------------
>Peromyscus_maniculatus_baird
--------MLHDGKCFEYLRHGRSH--------------------------------------------------------
>Salmo_salar
--------MLHDGKCFEYLRHGRSH--------------------------------------------------------
>Lepisosteus_oculatus
-------MMLHDGKCFEYLRHGR----------------------------------------------------------
>Ictalurus_punctatus
MPLATHFLMLHDGKCFECLRHGRAH--------------------------------------------------------
>Pelodiscus_sinensis
--------MLHDGKCFEYLRHGRSH--------------------------------------------------------
>Takifugu_rubripes
--------MLHDGKCFEYLRHGCAH--------------------------------------------------------
```

HG0153 ENSGALG00000003136 IKZF2

>Gallus_gallus
MEEVPAGLPLATAPLPQFP-----------
>Odobenus_rosmarus_divergens
MEEVKTGLPLATDPLP--------------
>Ambystoma_mexicanum
MEEVRAGLPLATAAALP-------------
>Orcinus_orca
MEEVKTGLPLATDPLP--------------
>Rousettus_aegyptiacus
MEEVKTGLPLATDPLP--------------
>Galeopterus_variegatus
MEEVKTGLPLATDPLP--------------
>Erinaceus_europaeus
MEEVKTGLPLATDPLP--------------
>Oryctolagus_cuniculus
MEEVKTGLPLATDPLP--------------
>Elephantulus_edwardii
MEEVKTGLPLATDPLP--------------
>Ceratotherium_simum_simum
MEEVKTGLPLATDPLP--------------
>Manis_javanica
MEEVKTGLPLATEPLP--------------
>Macaca_mulatta
MEEVKTGLPLATDPLP--------------
>Loxodonta_africana
MEEVKTGLPLATEPLP--------------
>Mus_musculus
MEEVKTGLPLATDPLP--------------
>Tupaia_chinensis
MEEVKTGLPLATDPLP--------------
>Trichechus_manatus_latirostr
MEEVKTGLPLATDPLP--------------
>Chrysemys_picta
MEEVKTGLPLATDPLLTVSITNCSAQTHKH
>Orycteropus_afer_afer
MEEVKTGLPLATDPLP--------------

HG0154 ENSG00000128573 FOXP2

```
>Homo_sapiens
MPSVWDVIGQRCTHSSVNTAVNSCLMVALTVS--
>Leptobrachium_boringii
MPSVWDLISQRCIHSSVNTAVNSYWMVTVTAS--
>Mustela_putorius_furo
MPSVWDVIGQRCTHSSVNTAVNSCLMVALTVS--
>Hynobius_retardatus
MPSVWDVIGQRCIHSSVNTAVNSCLKVALTVS--
>Calidris_pugnax
-------------------------MVALTVS--
>Pteropus_alecto
MPSVWDVIGQRCTHSSVNTAVNSCLMVALTVS--
>Kryptolebias_marmoratus
--------------------------MSLTVSAS
>Galeopterus_variegatus
MPSVWDVIGQRCTHSSVNTAVNSCLMVALTVS--
>Monodelphis_domestica
MPSVWDVIGQRCTHSSVNTAVNSCLMVALTVS--
>Condylura_cristata
MPSVWDVIGQRCTHSSVNTAVNSCLMVALTVS--
>Oryctolagus_cuniculus
MPSVWDVIGQRCTHSSVNTAVNSCLMVALTVS--
>Lepidothrix_coronata
MPSVWDVIGQRCTHSSVNTAVNSCLMVALTVS--
>Mus_musculus
MPSVWDVIGQRCTHSSVNTAVNSCLMVALTVS--
>Salmo_salar
-MGLGREQAEVLSHSSVNTAGNTFLMMSLTVSVS
>Lepisosteus_oculatus
MPSVWDVIGQRCTHSSVNTAVNSCLIMALTVS--
>Ictalurus_punctatus
-------------------------MMALTVSVS
>Anolis_carolinensis
MPSVWDVIGQRCSRRSVNTAVNSCLMVALTVS--
>Pelodiscus_sinensis
MPSVWDVIGQRCTHSSVNTAVNSCLMVALTVS--
```

HG0155 ENSG00000136535 TBR1

>Homo_sapiens
MAGAVSSAAQAARSKVSLF
>Anser_cygnoides
MAEAVSSSPQAARSKVSLS
>Canis_lupus_familiaris
MAGAVSSAAQAARSKVSLF
>Orcinus_orca
MAGAVSSAAQAARSKVSLF
>Pteropus_vampyrus
MAGAVSSAAQAARSKVSLF
>Sarcophilus_harrisii
MAGAVSSAAQAARSKVSLF
>Monodelphis_domestica
MAEAVSSAAQAARSKVSLF
>Aquila_chrysaetos_canadensis
MAEAVSSSPQAARSKVSLS
>Gallus_gallus
MAGAVSSSPQAARSKVSLS
>Condylura_cristata
MAGAVSSAAQAARSKVSLF
>Ochotona_princeps
MAGAVSSAAQAVRSKVSLF
>Elephantulus_edwardii
MAGAVSSAAQAARSKVSLF
>Ceratotherium_simum_simum
MAGAVSSAAQAARSKVSLF
>Ictidomys_tridecemlineatus
MAGAVSSAAQAARSKVSLF
>Trichechus_manatus_latirostr
MAGAVSSAAQAARSKVSLF
>Struthio_camelus_australis
MAEAVSSSPQAARSKVSLS
>Tinamus_guttatus
MAGAVSSSPQAARSKVSLS
>Orycteropus_afer_afer
MARAVSSAAQAARSKVSLF

HG0156 ENSG00000156113 KCNMA1

>Homo_sapiens
MAVDGCLGWRWRREELPRAPAPAARC
>Apteryx_australis_mantelli
MAGDGFFGGRWRREELSEAPARPARC
>Odobenus_rosmarus_divergens
MAVDGCSGWRWRREELPRAPAPAARC
>Orcinus_orca
MAVDGCSGWRWRREELPRAPAPAARC
>Pteropus_vampyrus
MAVDGCSGWRWRREELPRAPAPAARC
>Macropus_eugenii
MAVDGCLGWRWRREELPGAPAPPAHC
>Aquila_chrysaetos_canadensis
MAGDGFFGGRWRREELSEAPARPARC
>Gallus_gallus
MAGDGLFGGRWRREELSAAPARPARC
>Condylura_cristata
MAVDGCSGWRWRREELPRAPAPAARC
>Zonotrichia_albicollis
MAGDGLFGGRWRREELSEAPARPARC
>Ceratotherium_simum_simum
MAVDGCSGWRWRREELPRAPAPAARC
>Manis_javanica
MAVDGCSGWRWRREELPRAPAPAARC
>Loxodonta_africana
MAVDGCSGWRWRREELPRAPAPAARC
>Melopsittacus_undulatus
MAGDGVFGGRWRREELSEAPARPARC
>Microtus_ochrogaster
MAVDGCSGWRWRREELPRAPAPAARC
>Trichechus_manatus_latirostr
MAVDGCSGWRWRREELPRAPAPAARC
>Chrysemys_picta
MAGEGCFGGRWRWGELSGAPARPARC
>Orycteropus_afer_afer
MAVDGCSGWRWRREELPRAPAPAARC

HG0157 ENSG00000173926 MARCH3

```
>Homo_sapiens
MCPGPLGREQPPTGGNLALRTAAGTSVCYRDSARTMEILYECGSH
>Phoca_largha
-----------------------------------MEILYECGSH
>Balaenoptera_acutorostrata_s
-----------------------------------MEILYECGSH
>Pteropus_alecto
-----------------------------------MEILYECGSH
>Dasypus_novemcinctus
-----------------------------------MEILYECGSH
>Galeopterus_variegatus
-----------------------------------MEILYECGSH
>Condylura_cristata
MCPGALGPEQPPVGATWDSVPAPGASVCYLDSARRMEIL------
>Oryctolagus_cuniculus
-----------------------------------MEILHERGSH
>Elephantulus_edwardii
-----------------------------------MEILYECGSH
>Ceratotherium_simum_simum
-----------------------------------MEILYECGSH
>Manis_javanica
-----------------------------------MEILYECGSH
>Loxodonta_africana
-----------------------------------MAILYECGSH
>Heterocephalus_glaber
MCPASLGREQPPVRDNLGPRTCGRASVGDPESAGRMEILYQCGSH
>Tupaia_chinensis
MCPGPLGSEQPPIGATWDSGPAAGASVCDRDTARKMEILYECGSH
>Trichechus_manatus_latirostr
-----------------------------------MEILYECGSH
>Gekko_japonicus
-----------------------------------MEILYECGSH
>Pelodiscus_sinensis
-----------------------------------MEILYECGSH
>Orycteropus_afer_afer
-----------------------------------MEILYECGSH
```

HG0158 ENSG00000198939 ZFP2

>Homo_sapiens
---MTVYGEVMLENYRNPVSLGNHLSKPNGTSQLERE-LSLMQKEPPE--GGFRD---
---FLGYCCQI--NLTFSPL
IILSQSRGESID--

>Acinonyx_jubatus
--MAARPLPLGTQFQESVTFKDVSADFTWEEWLRLDSAQGNLYGKVMLENYRDLVSLGHRLSKPNVSSQLELEELSQVTKELPAC--LSPG---
---LLF---SRFSSM-LL----
-----PD---

>Lipotes_vexillifer
--MLGNYSNLVSLGHQLSKPNVISQLD---

>Rousettus_aegyptiacus
--MAPGLLTTRDQALMAFQDIAVAFSEKEWKLLSPAQRTLYRDVMLENYSHLVSLGIAFSKPQLITQLEQEDEPWKDKSECLL-DLCPG---
---ENIQCNDE----------FKW--

>Dasypus_novemcinctus
--MTKAQGLLSFEDVAVVFTWQEWQLLDPAQKDLYKDVMLENYNNLLSVGYQATKPDSLFKLEQGEPPWMVEGKIQSWPYPAHSMAGT-L-AFIQNSAVTN---
---HSTALGKKSYGCNEDAVKA---------------------------CKPLVTVPERTHAGKKPHECCDYKRAFASKSLLIPHQT-PIEERLYGCSQCQKAFITKSALIYHQKTYHREGESYGCNKCGKAFPWE
SKLIL--HQRIHSGERPFECKVCGKAFMVRTHLTVHERTHTGEKPYECSDCKNAFSKKAQLLIHQRIHTGERPYGC
SECQQAFIQKSDLTNHKKIHLVGRKSHGCSECGKVFPWKSKLLVHQRTHSGERPYRCNACDKAFTSKAHLIVHQRLHTGEKPYECCDCRKAFSTKAHLIIHQRTHTGERPYECKECQQAFIQKSGLTNHLQTCHPRVKLYGCHECGKVLSCKSTLIIHQRTHTGEKPF----KCNVCDKAFTAKSYLSVHQRIHTGEKPYECSECKKAFTTL
STLIGHQRTHTGERPYECNECQKAFFRKSALTNHQQTQHTGGNVGI---
>Sarcophilus_harrisii
--MLENYRNLFFLGLPVSKPEVISQLEQGEVPWIAEKEATF-RIHTG---
---EKP---YACNECTKAFCDG
SSLTK--HQRIHTGEKPYKCSDCGKSFSDSSSLA--

>Galeopterus_variegatus
--MLENYTNLVSLGYSASKPDVITLLEQGKEPCMVVKDVTG-GWCPG---
---LFSRHKTK----------TLSPEKDIYEISLSK--

>Monodelphis_domestica
--MASVPLPT-GPCQESVTFQDVAVEFTWEEWRYLNPSQKKLYKDVMLENYQSLISLGFEVSKPDVIHQLERKETSWMPEADVSQ-SSYPG---
---MKEFTGEKPYECSECGNVFSSRTQLTMHQRIHTRKFLYECKECGKACHCNSELIRHQRIHT-GEKPYECTVCGKAFSMN
GNLIV--HQRIHTGEKPFECSECGKSFRQRTHLYVHQTVHTQEISHECSECGKAFMRKTQLAVHQRIHTGGRPYGC
SVCGKDFFCSSDLIKHQKIHI-IEKSYECSECGKVFRSKIQLTVHQRIHPGKSLYECKECGKEFHCNSNLIKHQRIHTEEKPYQCNDCGKAFRRKAHLTLHQRIHTGDILYGCQECEKTFLYISDLTRHQR-IHTEEKPYECAVCVKAFRSKTQLSVHQRIHTGEKPY----ECTECGKFFRQRSHLYIHQRIHTVDILYECKECGKAFPYN
SDLIQHQKVHTGEKPHECTVCTKAFRSKTQLTVHQRIH-TGEKPYECTECGKFFRQRTHLYIHQRIHTGEILYECKDYEKAFPYKLELIQH----------
>Trichosurus_vulpecula
---MLESVTFNDVAVDFTQEEWKQPDPAQRSLYRDVLLENYRNFISLGQQVSKPHMISQLEQGEEPWMVESIIQK-GICPE---
---FSFPLPME---------------QKTFAFSIPFRL---G
G---

>Sorex_araneus
---MTHPASRSAPGTVAFEDVAVYFTRLEWSCLFPEQRTPYRDVMLENYRHLASLGFLASKPALVSLLEQGREPGALILQ--
----

>Ochotona_princeps
--MLENYRNVASVGCQLFKPGLISWLEEELESMIGERGVLQ-GVTTH---
---VQSNAGEKLYECKECGKAFITSSRLSEHLRSHTGEKPYGCYECGKAFASSSYLTAHLRTHT-GEKPFVCTTCGKAFTRS
CYLRV--HMRTHTGEKPYECQECGKAFTGRSWLTIHLRTHTGEKPYECKECEKAFTSFALLNEHIKTHTGEKPFVC
NICTKSFRNSS-----------------------------CLKTHFRIHTGIKPYQCKDCGKAFSGRAGFTKHVLTHTGEKPYECKECGKTFRTSSGLTEHVRIHTGEKPFECYQCGKAMAHSSSLVAHLR-THTGEKPFECNMCEKAFTSSSYLRVHMRSHTGEKPY----VCKDCG----------------------------KAFIAH
SGLSQHLRTHTGEKPNECKQ---
>Equus_asinus
--MLENYRNLVSLGFPFSKPGIIS--

>Manis_javanica
--MHNEFLQTVSKGSVMFRDVSINFCQEEWECLDSGQMNLYKEVMLENFSNLVSVGLSNSKPAVISLLEQGKEPWMVERKLTR-SLCSD---
---LVSMCETK----------ILSLKKRHFSQVISTHE--------DMPTFIQPFLTPHQKAIN-EEKVCECKICGKAFNQN
SQLTQ--YQRIHSAEKNYECKECGKAFI--

>Loxodonta_africana
--MIEAQESLAFEDVAVKFTWEEWQLLDPAQKNLYWDVMLENFSNLVSMGYQVTTPDALSRLEREELPWNILDEINSQNISEIRKVVDHLPEHLRNESRVNRLERCQEHNTWENIFHQRRSHFSLRQNHNIFDLDEESLKSNLTLIDQSRSYEVKN
SAEFNRDEKTFLHASHEQFHTEIKFPQSQKSNSTKSKIIKHQNT-------HKIEKPHVCSECGKAFIRKSWLISHQIIHSGERPHRGYSCGEAFSRKFKLTEHHQRKHGGEEFLECMGRGKAFLKKSQLNTHRKIHTDEKWYKCSECGKGFTWKRNLISHQRIHTEEKPYMCSECGKGFIRKGNLIIHQRIHT-GEKPYVCGQCGKGFMWK
GNLVL--HQRIHTGEKSYICIECGKGFIKKGDFIIHQRTHTGEKPYACSECGKGFSQKGNLIIHQRIHSGEKPYIC
AECGKGFIQKRDFIIHQRIHT-GEKPYACGECGKDFIRKQYLIIHQRIHSGEKPYVCTDCGKRFIQKRDFVIHQRTHTGEKPYVCGQCGKGFIRKGHLIIHQRIHSGEKPYICTKCGKGFIQKRKLIVHQR-SHSGEKPYICTECGKGFIQKRDYDIHQRIHTGEKLY----ICRECGKAFTRKGNLTIHQRIHTGEKPYICSECGKGFIQK
GNLIIHQRIHSGEKPYICTECGKGFNQKGSLITHQRIH-TGEKPYVCGKCGKGFRKKSHLITHQRFHTXKSHLACSICGQSYSQESSFIEHQKIHTVEKPF
>Marmota_marmota_marmota
--MHLDSAQRNLYGKVMLENYRNLVSLGHQLSKPLVISQLEQEELNLKNKELPGC--ASPD---
---FLG---YYCQTN-LLFSPL
SILSKPRRERID--

>Tupaia_chinensis
MKSLPLVTCRSRPPHRMMGTALPPSFCACAAPPTPPSYSGNSSSCGFSCACPADLGMGSVFLKEFVSFEDVAVGFTWEEWQDLDHAQRTLYRDVMLETYSNLVSLGHCITKPELILKMEQGAEPWMVEEPPNQ-SLPDVQKVDD----------LNETSEKSQDKTLWQLVITNNNIST--KERIELEKTFSLSLNHVLKLITNNENYIGMI
PEEFSVCQNMLLSSNFDELHAQEE-----PDLNPTGKFLRHPEHFSQLPKIPVGQQPFEYSGQEKAFHREAVLLTQKRIHM------YGACKYAYAKAF--------------------------DNSALIAQERTQEGRKPFEYDVYGNIFYKNCDLTKHPIIHTGEKPYRFSEFEKSFIKKSYLTKHQRTQT-REKPYGCNKCRKFFHEK
SDLIV--HQKTHIGEKPHECNECKKSFYRKSDLTVHQRTHTGEKPYECNECRKSFNQKSNLSRHQRIHTGEKPYEC
NRCKKSFCQKSDLTFHQRTHT-GERPHECKECRKSFYKKSDLTVHKRTHTGEKPYECNVCRKTFYQKSKLTVHQRTHTGEKPFECNECRKSFNRKADLTVHQRTHTGEKPYECNECRKSFYQKSVLTVHQR-THTGEKPYECNNCRKAFCQKSHLSRHQKTHTGGKPYRKTFSCNVCGKMFYEKSKFTKHQKIHTGEKPYKCKECEKSFISK
SKVIRHERTHTGEKPYRCNKCEKSFRQKSNLTVHQRIH-TGGKSYKCFECGKTFGQKSTLIVHHRIHTGRNPMNVKNVGNLST------------------
>Trichechus_manatus_latirostr
--MLENYRILVSLGLCFSKPSVILLLEQGKEPWMVKRELTE-GLCSG---
---E--

>Gekko_japonicus
---MLSPVTFEDVAVYFTEGQVALLDPNQRALYREVMMENYENMASLGFLFIKPALISQLEQGEELWVPESLDWD-AAETL---
---DTSGEGSR---

HG0159.1 ENSG00000182197 EXT1

>Homo_sapiens
MQENYSPLLARPGCEIFSPALSRALCNPKMKDRRGES
>Acinonyx_jubatus
MQENYSPLLARPG------------------------
>Balaenoptera_acutorostrata_s
MQENYSPLLARPGCEIFSPALSRLLCNPKMKDRRGES
>Pteropus_vampyrus
MQENYSPLLARPGCEIFSPALSRPLCNPKMKDRRGES
>Dasypus_novemcinctus
MQENSSPLLARPGCEVFSPALSRPLCNPKMKDRRGES
>Sarcophilus_harrisii
-----------------------------MKDRRGES
>Galeopterus_variegatus
-----------------------------MKDRRGES
>Monodelphis_domestica
-----------------------------MKDRRGES
>Condylura_cristata
MQENYSLLVARPGCEIFCPALSRPLCNPKMKDRRGES
>Oryctolagus_cuniculus
-----------------------------MKDRRGES
>Ceratotherium_simum_simum
MQENYSPLLARPGCEIFSLALSRPLCNPKMKDRRGES
>Manis_javanica
MRGNYSPLLARPGCKIFSPALSRPLCNPKMKDRRGES
>Loxodonta_africana
MQENYCPLLARPGCEIFRPALSRPLCNPKMKDRRGES
>Marmota_marmota_marmota
MQENYSPLLARPGCEIFSPGLSRALCNPKMKDRRGES
>Trichechus_manatus_latirostr
MQENYRQLLARPGCEIFRPALSRPLCNPKMKDRRGES
>Takydromus_sexlineatus
-----------------------------MKDRRGES
>Chelonia_mydas
-----------------------------MKDRRGES

HG0159.2 ENSG00000182197 EXT1

>Homo_sapiens
MQGFVRLRRPSCKMH
>Ailuropoda_melanoleuca
MQGFVRLRRPSCKMH
>Balaenoptera_acutorostrata_s
MQGFVRLRRPSCKMH
>Pteropus_alecto
MQGFVRLRRPSCKMH
>Dasypus_novemcinctus
MQGFVRLRRSSCRMH
>Galeopterus_variegatus
MQGFVRLRRPSCKMH
>Monodelphis_domestica
MQGFVRLRRSSWKM-
>Sorex_araneus
MQGFVRLRRPSCKMH
>Oryctolagus_cuniculus
MQGFVRLRRPSCRMH
>Ceratotherium_simum_simum
MQGFVRLRRPSCKMH
>Manis_javanica
MQGFVRLRRPGCKMH
>Loxodonta_africana
MQGFVRLPRSGCEMH
>Cricetulus_griseus
MQGFVRLRRPSCKMH
>Trichechus_manatus_latirostr
MQGFVRLRRSGCKMH
>Takydromus_sexlineatus
MQGFVRLRRSGWKM-
>Pelodiscus_sinensis
MQGFVRLRRESWKM-

HG0160 ENSDARG00000060089 btaf1

```
>Danio_rerio
MAPHIKPAAWLLFCDLQNYFSPFVRLKYRFRGSA--EG
>Amia_calva
MAPHIKPASWVLSSEQQKYPLFLIRMKYLSARSS--GG
>Channa_punctata
MAPHIKTVFWVAFNVQQNNSLHLIRLKYLSARTS--EG
>Megophrys_sangzhiensis
MAPHIKPGVWLLRCHRQISSL--IRFKRLLGSCRGPDG
>Menidia_menidia
MAPHIKTVFWVGLVAQQNNSLILIRLKYLSASSS--EG
>Oryzias_melastigma
MAPHIKTVFWIALYVEQKRALILIRLKYLSAKTS--EG
>Micropterus_salmoides
MAPHIKTVFWVALNVLQNNSLHLIRLKYLSSKSS--EG
>Astyanax_mexicanus
MAPRIKPASWLLFCDQQNYFSLLIRLKYLFGRSA--EG
>Haplochromis_burtoni
MAPHIKTVFWVVLNVQQNNSLNLIRLKYLSAKSS--EG
>Alosa_alosa
MAPHIKPASWLLFCDQQNYFFLLIRLKYLSGRSS--EG
>Latimeria_chalumnae
MAPHIKSGFWVLSSNHQIFSLNLLNDQSMFRCCP--EG
>Kryptolebias_marmoratus
MAPHIKTVFWVSPNEQRNKSLFLVRLKYLSADSS--EG
>Esox_lucius
MAPHIKSASWVPLGEQQNYSLYLIRLKYLSGSSS--EG
>Notothenia_coriiceps
MAPHIKTVFWVALGLLQNNSLHLIRLRYLSSKSS--EG
>Salmo_salar
MAPHIKSASWVPFSEHKNYSLNLIRLKYLSGSSS--EG
>Lepisosteus_oculatus
MAPHIKPDSWVLFSEQQNYCLFLIRIKYLSGCSS--AG
>Ictalurus_punctatus
MAPHIKSASRLLFCEQQNYFSLLIRVKYLFGTSA--EG
```

HG0161 ENSGALG00000017191

>Gallus_gallus
MCWLFFVPYTFPNS
>Anas_platyrhynchos
MCWLLFVLYTFPNS
>Caprimulgus_carolinensis
MCWLLFVP------
>Cariama_cristata
MCWLLFVQ------
>Charadrius_vociferus
MCWLLFVL------
>Pterocles_gutturalis
MSWLLFVLQTFPNS
>Columba_livia
MCWLLFVL------
>Leptosomus_discolor
MCWLLFVL------
>Haliaeetus_leucocephalus
MCWLLFVL------
>Gavia_stellata
MCWLLFVL------
>Chlamydotis_macqueenii
MCWLLFVL------
>Opisthocomus_hoazin
MCWLLFALQTFPNS
>Zonotrichia_albicollis
MCWLPFVLQTFPNS
>Fulmarus_glacialis
MCWLLFVL------
>Aptenodytes_forsteri
MCWLLFVL------
>Tyto_alba
MCWLLFVL------
>Chrysemys_picta
MCWLRFVLYTFPNT

HG0162 ENSG00000138347 MYPN

>Homo_sapiens
----MFHRHMVYTAQNNLGPVS--
>Anas_platyrhynchos
----MLRRHMVYAAQNNLGPAN--
>Xenopus_laevis
----MLYRHMVHRAQNNTQPASDKCCCCFSGDRLTQLHTIIFPKFQDNPCSLSSAFTKIGLFHFHPHPWTISIEETAVRAGH
>Canis_lupus_familiaris
MPGGMFHRHMVYTAQNNLGPVS--
>Lipotes_vexillifer
----MFHRHMVYTAQNNLGPVS--
>Eptesicus_fuscus
----MFDRHMVYTAQNNLGPVS--
>Gallus_gallus
----MLCRHMVYAAQNNLGPAN--
>Erinaceus_europaeus
----MFHRHMVYTAQNNLGPVS--
>Oryctolagus_cuniculus
----MFHRHMVYTAQNNLGPAS--
>Parus_major
----MLRGHMVYTAQNNLGPAN--
>Equus_asinus
----MFHRHMVYTAQNNLGPAS--
>Manis_javanica
----MFHRHMVYTAQNNLGPVS--
>Loxodonta_africana
----MFHRHMVYTAQNNLGPVS--
>Ictidomys_tridecemlineatus
----MFHRHMVYTAQNNLGPVS--
>Pygoscelis_adeliae
---------MVYAAQNNLGPAN--
>Anolis_carolinensis
----MFHKHMVYVSQNNLGPAN--
>Orycteropus_afer_afer
----MFHRHMVYTAQNNLGPVS--

HG0163 ENSG00000146285 SCML4

```
>Homo_sapiens
MFAAGAQGCWRLTVSY-----------------------------------------
>Xenopus_tropicalis
MFAAGERSCWRLSVC------------------------------------------
>Ailuropoda_melanoleuca
MFAAGEQGCWRLTVSY-----------------------------------------
>Hynobius_retardatus
MFAAGESGCWRLTVC------------------------------------------
>Orcinus_orca
MFAAGEQGCWRLTVSY-----------------------------------------
>Rousettus_aegyptiacus
MFAAGEQGCWRLTVSY-----------------------------------------
>Dasypus_novemcinctus
MFAVGEQGCWRQTVSS-----------------------------------------
>Gallus_gallus
MFAAGERSCWRLARC------------------------------------------
>Sorex_araneus
MFATGELGCWRLTVSSSLPSGRLASAGLGLHVLKVFSLLKQHSWLGGEPARALVNKL
>Elephantulus_edwardii
MFAAGEQGCWRLTVNDSHTSGKQVLQFSVCMCRKSSRFRVNIPDLGKNLSRALVNKP
>Corvus_brachyrhynchos
MFAAGELSCWRLTLC------------------------------------------
>Ceratotherium_simum_simum
MFAAGEQGCWRLTVSS-----------------------------------------
>Manis_javanica
MFAAGEQGCCRLTVSC-----------------------------------------
>Loxodonta_africana
MFAAGEQGCWRLTVSDSHTSGRQVLQYSVCMCRKSSVF-------------------
>Ictidomys_tridecemlineatus
MFAAGEQGCWRLTVSY-----------------------------------------
>Anolis_carolinensis
MFAAGEQSCWRCTVR------------------------------------------
>Chrysemys_picta
MFAAGELSCWRLTVC------------------------------------------
```

HG0164 ENSG00000151292 CSNK1G3

```
>Homo_sapiens
MLTLPIWYSYLVMYLFSQYPVSVCLS
>Xenopus_laevis
------------MFPFSQHPI-----
>Felis_catus
------------MYLFSQYPISVCLS
>Lipotes_vexillifer
MLTLPTWYSYLVMYLFSQYPISVWLS
>Eptesicus_fuscus
MLTLPAWYSYLVMYLFSQYPISVCLS
>Dasypus_novemcinctus
MLTLPTWYSYLVMYLFSQHPISVCLS
>Alligator_sinensis
------------MFPFSHYPI-----
>Galeopterus_variegatus
MLTLPTWCSYLVMYLFSQYPISVCLS
>Erinaceus_europaeus
MLTLPTWYSYLVMYLFSQYPISVCLS
>Oryctolagus_cuniculus
MLTLPTWCSTLVMYLLSQYPISVCWS
>Ceratotherium_simum_simum
MLTLPTWYSYLVMYLFSQYPISVCLS
>Manis_javanica
------------MYLFSQYPISVCLS
>Loxodonta_africana
MLTLRTWYSYLVMYLFSEYPISACLS
>Octodon_degus
MLTLPGWYSYLVMYLFSQYPVSVCLS
>Tupaia_chinensis
MLTLPSWHSDLVMYLFPQYLISVCLS
>Trichechus_manatus_latirostr
MLTLSTWYSYLVMYLFLQYPISACLS
>Orycteropus_afer_afer
MLTLSTWYSYLVMYLFSQYPISVCLS
```

HG0165 ENSG00000177508 IRX3

>Homo_sapiens
MSIRARSPGNLRQSVRT
>Canis_lupus_familiaris
MSIRARSPGNLRQSVRT
>Orcinus_orca
MSIRARSPGNLRQSVRT
>Pygocentrus_nattereri
MSIRARSPNNLRQSVRT
>Callorhinchus_milii
MSIRPSTPNNLHESVRT
>Latimeria_chalumnae
MSIRAVSPNNLKQSVRT
>Catostomus_commersonii
MSITKRSSNNLKQSVRT
>Esox_lucius
MSIRARSPNNLRQSVRT
>Gallus_gallus
MSIRARSPNNLRQSVRT
>Erinaceus_europaeus
MSIRAQSPGNRGQSVRT
>Scleropages_formosus
MSIRAKSPNNLRQSLRT
>Parus_major
MSIRAQSPNNLRQSVRT
>Ceratotherium_simum_simum
MSIRARSPGNLRQSVRT
>Heterocephalus_glaber
MSIRARSPGNLRQSVRT
>Lepisosteus_oculatus
MSIRAKSPNNLRQSVRT
>Trichechus_manatus_latirostr
MSIRARSPGNLRQSVRT
>Trachemys_scripta
MSIRTQSPNNLRQSVRT

HG0166.1 ENSG00000148948 LRRC4C

```
>Homo_sapiens
---------------------------MKLQRRWREGDRI
>Anas_platyrhynchos
---------------------------MKMQRSWREGKRI
>Felis_catus
---------------------------MKLQRRWREGDRI
>Rousettus_aegyptiacus
---------------------------MKLQRRWREGDRI
>Dasypus_novemcinctus
---------------------------MKLQRRWRKGDRI
>Aquila_chrysaetos_canadensis
---------------------------MKMQRSWREGKRI
>Gallus_gallus
---------------------------MKMQRSWREGKRI
>Condylura_cristata
---------------------------MKLQRR-------
>Ochotona_princeps
---------------------------MKLQRRRREGDRI
>Corvus_brachyrhynchos
---------------------------MKLQRSWREGKRI
>Equus_przewalskii
---------------------------MKLQRRCGEGDRI
>Microtus_ochrogaster
---------------------------MKSQRRWREGDRI
>Trichechus_manatus_latirostr
---------------------------MKLQRRWREGDRI
>Struthio_camelus_australis
MDLFLTAGGNLEFFPLPPSLYSIYCEIMKMQRSWREGKRI
>Pelodiscus_sinensis
---------------------------MKMQRSWREGKRI
>Orycteropus_afer_afer
---------------------------MKLQRRWREGDRI
```

HG0166.2 ENSG00000148948 LRRC4C

>Homo_sapiens
MQDAAFSWNRTQWIN------------
>Leptonychotes_weddellii
MRDAALSWNRTQWIN------------
>Balaenoptera_acutorostrata_s
MRDAALSWNRTQWIN------------
>Rousettus_aegyptiacus
MRDAALSWNRTQWIN------------
>Dasypus_novemcinctus
MRDAALSWNRTQWIN------------
>Sorex_araneus
MQDAAPSGERTQWIN------------
>Elephantulus_edwardii
MRDAALSQKRTQWRN------------
>Equus_przewalskii
MQDAALSWNRTQWIN------------
>Microtus_ochrogaster
MRDAALSWSRTQRIN------------
>Trichechus_manatus_latirostr
MRDAALSWNRTQWINCAREKEERSFFL
>Orycteropus_afer_afer
MRDAAFSWNRTQWINCAREKEGRSFFF

HG0166.3 ENSG00000148948 LRRC4C

>Homo_sapiens
MIGNEEPRIHKEKSMFIFLYKGESEPRRYFWNEKFGAFLVK--
>Acinonyx_jubatus
--------------MFVFLYKGESKPRRYFWN---
>Balaenoptera_acutorostrata_s
--------------MFIFFCKGESKPRRYFWNEKFGAFFSKVKT---------------------------------------
>Miniopterus_natalensis
MISNGEPTSHKEKVCSLFFYTGESQPRIYFWNEKFGAFFFFFFFFRRVKDLI-------------------------------
>Dasypus_novemcinctus
--------------MFVFLYKGESKPRRLFYFFIFGTKSLGLY--
>Condylura_cristata
MIGNGERRIHKEKVCS-FFSKGESKPRR---
>Oryctolagus_cuniculus
--------------MFVFLCKGESKPRRYFWNEKFGAFFRKVKDLRRWCFLFSFEFPTRGEEINNTSAKKFQRRKVDRGRMRH
>Equus_przewalskii
--------------MFIFLSKGESEPRRYFCNEKFGAFLGK--
>Nannospalax_galili
--------------MFLFLYKEESTPRRYFWNEKFGAFFRKVKT---------------------------------------

HG0166.4 ENSG00000148948 LRRC4C

>Homo_sapiens
MEGDWGFCRAGSREPGRWR
>Leptonychotes_weddellii
MEGDWGFCRAGNREPGRWR
>Dasypus_novemcinctus
MEGEKGFRSAASREPGRWR
>Condylura_cristata
MEGDPGCCRAGSPEPGRWR
>Ochotona_princeps
MEGDWGFCCVASREPGRWR
>Peromyscus_maniculatus_baird
MEGDLGFRGTESREPGLWH
>Trichechus_manatus_latirostr
MEGDWGFCRAGSREPGRWR
>Orycteropus_afer_afer
MEGDQCFCRAGSREPGRWR

HG0167 ENSDARG00000104005 slc4a3

```
>Danio_rerio
----------------------MPPHRLTGNPAVKEKG
>Menidia_menidia
----------------------MPPQRLPGTPAVKEKG
>Porichthys_notatus
----------------------MPPQRLPGTPAVKEKG
>Oryzias_latipes
----------------------MPSQRLPGSPAEKEKG
>Micropterus_floridanus
----------------------MPPKRLPGSPTVKEKG
>Pygocentrus_nattereri
----------------------MPPHRLTGNPTVKEKG
>Haplochromis_burtoni
----------------------MPPQRLPGSPAVKEKG
>Alosa_alosa
----------------------MPPHRLTGSPTVKEKG
>Latimeria_chalumnae
----------------------MPPHRILGRATVKEKG
>Fundulus_heteroclitus
----------------------MPPQRLPGSPVVKEKG
>Apteronotus_leptorhynchus
----------------------MPPHRLTGNPTVKEKG
>Scleropages_formosus
MQRESRSAGCSELRAQRACAAVMPPHRLTGTPTVKGNG
>Platichthys_stellatus
----------------------MPPQGLRGPPAVKEKG
>Salvelinus_alpinus
----------------------MPPHRLPGTPTVKEKG
>Lepisosteus_oculatus
----------------------MPPHSFTGIPTVKEKG
>Ictalurus_punctatus
-------------------MQVMPPHRLTGNPTVKEKG
```

HG0168 ENSGALG00000006997 BRINP1

>Gallus_gallus
MPAIWIKEGDFLSEHEKDC
>Anas_platyrhynchos
MPAIWIKEGDFLSEHEKDC
>Hynobius_chinensis
MPEIWSRDWAFLSEHEKDC
>Calidris_pugnax
MPAIWIKEGDFLSEHEKDC
>Columba_livia
MPAIWIKEGDFLSEHEKDC
>Alligator_mississippiensis
MPASRIQGRDFLSEHEKDC
>Cuculus_canorus
MPAIWIKEEDFLSEHEKDC
>Monodelphis_domestica
MPAIWITEGDLKAEYEKDC
>Haliaeetus_leucocephalus
MPAIWIKEGDFLSEHEKDC
>Ornithorhynchus_anatinus
MPAIWIREGDLTAEHEKGC
>Lepidothrix_coronata
MPAIWIKEGDFLSEHEKDC
>Picoides_pubescens
MPAICIKEGDFLSEHEKDC
>Melopsittacus_undulatus
MPAIWIKEGDFLSEHEKDC
>Aptenodytes_forsteri
MPAIWIKEGDFLSEHEKDC
>Pelodiscus_sinensis
MPAIWIKERDFLSEHEKDC
>Calypte_anna
MPAIWIKEGYFLSEHEKDC

HG0169 ENSG00000114933 INO80D

>Homo_sapiens
MVGKLSFRRAEEQLSGVMSSQCL
>Anas_platyrhynchos
MVGKLLFWRAEEPLSDVMSSRCL
>Chaetura_pelagica
MVGKLLFWRAEEPLSDVMSSQCL
>Hynobius_retardatus
MVGKLLFWRAEELLSGVMSSQCL
>Physeter_catodon
MVGKLSFRRAEELLSGVMSSQCL
>Calidris_pugnax
MVGKLLFWRAEEPLSDVMSSQCL
>Pteropus_vampyrus
MVGKLSFRRAEERLSGVMSSQCL
>Columba_livia
MVGKLLFWRAEEPLSDVMYSQCL
>Monodelphis_domestica
MVGKLSFRRAEERLSGVMSSQCR
>Aquila_chrysaetos_canadensis
MVGKLLFWRAEEPLSDVMSSQCL
>Gallus_gallus
MVGKLLFWRAEEPLCDVMSSRCL
>Serinus_canaria
MVGKLLFWRAEEPLSDVMSSQCL
>Egretta_garzetta
MVGKLLFWRAEEPLSDVMSSQCL
>Picoides_pubescens
MVGKLLFWRAEDPLSDVMSSQCL
>Melopsittacus_undulatus
MVGKLLFWRAEEPLSDVMSSQCL
>Aptenodytes_forsteri
MVGKLLFWRAEEPLSDVMSSQCL

HG0170 ENSG00000180530 NRIP1

>Homo_sapiens
MKALRSAEQIDYSGVPV----LGTTAVRERTLIFAFNGEQKMKK--------EKEYI---H------
>Anas_platyrhynchos
MKALLSAEQIDCYSVPV----LGTTAVKERNSMCLMEMEKDEKQTGFMEKGEEGKYLMELHYLRSVF
>Canis_lupus_familiaris
MKALLSAEQIDCSGVPV----LGTTAVKERTPLLAFNWKQKMKK--------EKQCI---H------
>Balaenoptera_acutorostrata_s
--------------------------------MFAFIVEQKVKK--------EKQYI---R------
>Rhinolophus_ferrumequinum
---MKK--------EKQDI---H------
>Dasypus_novemcinctus
---MKK--------EKQYI---H------
>Galeopterus_variegatus
---MKE--------EKHYI---H------
>Oryctolagus_cuniculus
---MKT--------AKQYI---H------
>Equus_asinus
----MTSERLFNSPLNFLSQKSETQILEIEELMFAFNGQQKMKK--------EKRYI---H------
>Manis_javanica
---MKK--------EKQYI---H------
>Loxodonta_africana
---MKK--------EKQYI---H------
>Ictidomys_tridecemlineatus
---MKM--------EKQYI---H------
>Tupaia_chinensis
---MKK--------ENQCI---H------
>Trichechus_manatus_latirostr
---MKK--------EKQYI---H------
>Anolis_carolinensis
MKALLSAEQIDCYSVPV----LGTTAVKERLLALTLHINHLEGE--------EKKHI---NCVQSSV
>Orycteropus_afer_afer
---MKK--------VKQYI---H------

HG0171.1 ENSG00000177311 ZBTB38

>Homo_sapiens
---MYS--------KEKRTLAMVQLNQDSEADMQCHLQH
>Acinonyx_jubatus
--MQCHLQH
>Balaenoptera_acutorostrata_s
--MQCHLQH
>Myotis_davidii
--MQCHFQH
>Dasypus_novemcinctus
--MQCHLQH
>Columba_livia
--------MM---------------SECHCATVAKKSLRGERRERKKTRSFASSNNMRCAYTLNQDSVADTQCHLQH
>Galeopterus_variegatus
--MQCHLQH
>Monodelphis_domestica
--MYSSLLEFTVLNQDSVADMQCHFQH
>Condylura_cristata
--MQCHLQH
>Corvus_cornix_cornix
--MQCHLQH
>Equus_asinus
--MQCHLQH
>Manis_javanica
MPTRAHRAMMWGWGRWFHHWVRLGATVSVRATFISGVLVGERCA--------LKRRGLVVVQLNQDSEADMQCHLQH
>Heterocephalus_glaber
--MQCHLQH
>Struthio_camelus_australis
--MQCHLQH
>Tinamus_guttatus
--MQCHLQH

HG0171.2 ENSG00000177311 ZBTB38

>Homo_sapiens
MRNFFFQSCFPVDKFSSVITVHSSCSAYDFCVYVTIASKTCEQEDREISWSPSLYIKMAECLVMLALH-----
>Felis_catus
---MAECLVMLALH-----
>Pteropus_vampyrus
---MAECLVMLALH-----
>Galeopterus_variegatus
---MAECLVMLALH-----
>Monodelphis_domestica
---MAECAAMLVRH-----
>Erinaceus_europaeus
---MAECPVMLALH-----
>Sturnus_vulgaris
---MAGCIAMLTLL-----
>Equus_asinus
MRNFFFQSCFPVDKFSSRDHSTLKLFCVRFCVCVTIASKTCEQEDGEISWSPSLYIKMAECLVMLALH-----
>Manis_javanica
----------------------------MICVCVTIASKTCEQEDGEISWSPSLYIKMAECHASTALSDTYRF
>Trichechus_manatus_latirostr
---MAECLVMLALH-----
>Tinamus_guttatus
---MAGCIATLTLH-----

HG0171.3 ENSG00000177311 ZBTB38

>Homo_sapiens
MLAVLQKAFRAADLDVEEMEDSEPRISK
>Acinonyx_jubatus
------------------MEDSEPRISK
>Physeter_catodon
------------------MEDSEPRISK
>Myotis_davidii
------------------MEDSEPRISK
>Galeopterus_variegatus
------------------MEDKEPRISK
>Erinaceus_europaeus
------------------MEDSEPRISM
>Equus_asinus
------------------MEDSEPRISK
>Manis_javanica
------------------MEDSEPRISK
>Heterocephalus_glaber
------------------MEDSEPRISK
>Trichechus_manatus_latirostr
------------------MEDSESRISK

HG0171.4 ENSG00000177311 ZBTB38

>Homo_sapiens
MGDIIRKICGADHKK---------------------------------
>Acinonyx_jubatus
MGDIIRKICGADLNKK--------------------------------
>Balaenoptera_acutorostrata_s
MGDIIRKICGADLDKK--------------------------------
>Rousettus_aegyptiacus
MGDIIRKICGADFNKK--------------------------------
>Condylura_cristata
MRDVIRKICGADLDEK--------------------------------
>Equus_asinus
MGDIIRKICGAALNK---------------------------------
>Manis_javanica
MGDIIRKICGADLNKK--------------------------------
>Heterocephalus_glaber
MSDIIRKICGADLNRVKNPEQESVCDSWAQGVGSVWASEEQLIRIRLS

HG0172.1 ENSG00000196782 MAML3

>Homo_sapiens
MQLSSQSTLRTRGGINTKTFLWKENAISK---LAVFRCGVE--------------------------------------
>Mustela_putorius_furo
MQLSSQSTLRTRGGINTKTFLWKENAISK---LAVFRCGVE--------------------------------------
>Balaenoptera_acutorostrata_s
MQLSSQSTLRTRGGINTKTFFVEGKCNKQACRFPM-RCGVSVCRACPHWRHMLVCVHGVCFSVRREKMLANHRKSPGIY
>Myotis_brandtii
MQLSSQSTLRTRGGINTKTFLWKENAISK---LAVFRCGVE--------------------------------------
>Dasypus_novemcinctus
MQLNSQSTLRTRGGINTKTFLWKENAISK---LAVF---
>Monodelphis_domestica
MQPRSQSTLRTRGGINTKTFLWKENAIRK---LAVF---
>Gallus_gallus
MQPGSASTLRARRGTSAQTFL--
>Condylura_cristata
MQLSSQSTLRTRGGINTKTFLWKENAISR---LAVFRCGVE--------------------------------------
>Oryctolagus_cuniculus
MQLSSQSTLRTRGGINTKTFLWKENAISK---LAVLRCAVE--------------------------------------
>Ficedula_albicollis
MQLSSQSTLRTRCGINTKTFFQKENTISK---RLCLSFDAAECE-----------------------------------
>Ceratotherium_simum_simum
MQLSSQSTLRTRGGINTKTFLWKENAISK---LAVFRCGVE--------------------------------------
>Manis_javanica
MQLSSQSTLRTRGGINTKTFLWKENAISK---LAVFRCGVE--------------------------------------
>Mus_musculus
MQLSSQSTLRTRGGINTKTFLWKENAISK---LAVFRCGVE--------------------------------------
>Tupaia_chinensis
MQLSSQSTLRTRGGINTKTFLWKENAISK---LAVFRCGVE--------------------------------------
>Pelodiscus_sinensis
MQLSSQSTLSTRCGINTKTFFLEGKYNKKAALPVFWCCGV---------------------------------------

HG0172.2 ENSG00000196782 MAML3

>Homo_sapiens
MDYKKKASKERISP-----------------
>Mustela_putorius_furo
MDYKKKAGEERISP-----------------
>Balaenoptera_acutorostrata_s
MDYKKKAGEERIFP-----------------
>Myotis_brandtii
MDYKKKAGEERISP-----------------
>Dasypus_novemcinctus
MDYKKKAGEERSTP-----------------
>Condylura_cristata
MDYKKKAGDERIAP-----------------
>Oryctolagus_cuniculus
MDYKKKAGEERRSP-----------------
>Ceratotherium_simum_simum
MDYKKKAGKERISP-----------------
>Nannospalax_galili
MDYKKKAGEERIAP-----------------
>Tupaia_chinensis
MDYKKKAGEERLSPGVAIRFAAGSVCCDSFC

HG0173 ENSG00000101746 NOL4

>Homo_sapiens
---------------------------------------MIELACHRHWYLS
>Anser_cygnoides
---------------------------------------MIELACHRHWYLS
>Lipotes_vexillifer
MQERLALTVQRREFRGSQTNPWVHLERKVGPRLRDAVSAMIELACHRHWYLS
>Calidris_pugnax
---------------------------------------MIELACHRHWYLS
>Pteropus_alecto
---------------------------------------MIELAFHRHWYLS
>Aquila_chrysaetos_canadensis
---------------------------------------MIELACHRHWYLS
>Sorex_araneus
---------------------------------------MIELACHRYWYLS
>Oryctolagus_cuniculus
---------------------------------------MIELVRHRHWYLS
>Elephantulus_edwardii
---------------------------------------MIELACHRHWYLS
>Corvus_cornix_cornix
---------------------------------------MIELACHRHWYLS
>Loxodonta_africana
---------------------------------------MIELACHRHWYLS
>Fukomys_damarensis
---------------------------------------MIELACHRHWYLS
>Tupaia_chinensis
---------------------------------------MIELACHRHWYLS
>Trichechus_manatus_latirostr
---------------------------------------MIELACHRHWYLS
>Chrysemys_picta
---------------------------------------MIELACHRHWYLS

HG0174 ENSG00000144355 DLX1

>Homo_sapiens
--------MRLTRGRAASPALVQRSPSASDQSPVIMQDSGPISSASS
>Anas_platyrhynchos
-----------------------------------MQDGGPISSAVS
>Mustela_putorius_furo
-----------------------------------MQDSGPISSASS
>Rousettus_aegyptiacus
-----------------------------------MQGSGPISSASS
>Galeopterus_variegatus
-----------------------------------MQDSGPISSASS
>Monodelphis_domestica
MITRRGSGTHLSQVLLASLPLVQPSQSTSDQSQAIMQDGGPIRSASS
>Coturnix_japonica
-----------------------------------MQDGEPISSAGS
>Ochotona_princeps
-----------------------------------MQDGGPISSASS
>Ornithorhynchus_anatinus
-----------------------------------MQDGGPIRSASS
>Zonotrichia_albicollis
-----------------------------------MQDGGPISSAVS
>Equus_przewalskii
---------------------------------MIMQDSGPISSASS
>Loxodonta_africana
-----------------------------------MQDSGPISSANL
>Octodon_degus
-----------------------------------MQDGGPISSASS
>Tupaia_chinensis
-----------------------------------MQDSGPISSASS
>Chrysemys_picta
-----------------------------------MQDGGPISSASS

HG0175 ENSG00000151320 AKAP6

>Homo_sapiens
MCCKHQKKTVGSGDAVLERSEV---
>Anas_platyrhynchos
MCCKHQKRMI---------------
>Felis_catus
MCCKHQKETVGAGDAVLERSKV---
>Balaenoptera_acutorostrata_s
MCCKHQKKAVGAGDAVLERSKA---
>Calidris_pugnax
MCRKHQKRMI---------------
>Rousettus_aegyptiacus
MCCKHQKKTVGAGDAVLERSKV---
>Dasypus_novemcinctus
MCRKHQKRTVGAGDAVLERSKV---
>Monodelphis_domestica
MCRKHQKRMVRAGDALLERSMV---
>Condylura_cristata
MCCKHQKRTVRAGDAVLERSKI---
>Parus_major
MCRKHQKRMI---------------
>Equus_caballus
MCCKHQKKTVGPGDAVLERSKV---
>Manis_javanica
MCCKHQKQTVGAGDELPEEGLGHLR
>Fukomys_darlingi
MCCKHQKKTVGAGDAVLERSKV---
>Struthio_camelus_australis
MCRKHQKGTI---------------
>Pelodiscus_sinensis
MCRKHQKRMV---------------

HG0176 ENSG00000170962 PDGFD

>Homo_sapiens
---MQEVWGELGD
>Apteryx_australis_mantelli
---MQEVWGKLGE
>Physeter_catodon
---MQEVWGELGD
>Pteropus_alecto
---MQEVWGELGD
>Galeopterus_variegatus
---MQEVWGELGE
>Monodelphis_domestica
---MQEVWGELGD
>Macropus_eugenii
MLGLDQDRHRHIWILLQLPLLATGGAVGRKSTCCSSSPTGLTNFSMQEVWGELGD
>Haliaeetus_leucocephalus
---MQEVWGQLGE
>Oryctolagus_cuniculus
---MQEVWGELGD
>Ceratotherium_simum_simum
---MQEVWGELSD
>Loxodonta_africana
---MQEVWGELGD
>Heterocephalus_glaber
---MQEVWGKLSD
>Tupaia_chinensis
---MQEVWGELGD
>Trichechus_manatus_latirostr
---MQEVWGELGD
>Orycteropus_afer_afer
---MQEVWGELGD

HG0177 ENSG00000171843 MLLT3

>Homo_sapiens
MLRNHLLYPGAA--AAAAAFAFGAEFNKRASEQASAGGKRQRMSAIYPPLLGALSFIAASS
>Felis_catus
--MSAIYPPLLGALSFIAASS
>Orcinus_orca
--MSAIYPPLLGALSFIAASS
>Myotis_brandtii
--MSAIYPLLLGALSFIAASS
>Dasypus_novemcinctus
--MSAIYPPLLGALSFIAASS
>Alligator_mississippiensis
MCRAHALTAALQALELELELLLGAQFNKSERK-RQGEKERKRMSAISPPLLGALPVIAASS
>Galeopterus_variegatus
--MSAIYPPLLGALSFIAASS
>Monodelphis_domestica
--MSAIYPLLLGALSFISASS
>Condylura_cristata
--MSAIYPPLLGALSFIAASS
>Oryctolagus_cuniculus
--MSAIYPPLPGALSFIAASS
>Elephantulus_edwardii
--MSAIYPPLPGALSFIAASS
>Equus_caballus
MLRNLILWPWSCCCCCCCFCFWG--------------------------------------
>Ictidomys_tridecemlineatus
--MTAIYPPFLGALSFIAASS
>Pelodiscus_sinensis
--MSAIYPPLPCAPSVTSASS
>Orycteropus_afer_afer
--MSAIYPLLLGALSFIAASP

HG0178.1 ENSG00000131634 TMEM204

>Homo_sapiens
------------------------------MGPE-ASSFSTGLALLQVQEGG
>Odobenus_rosmarus_divergens
--------------------------------MGREAGCSSGLALLQVQEGG
>Balaenoptera_acutorostrata_s
--------------------------------MGSSEGFSSGLALLQVQEGG
>Pteropus_vampyrus
--------------------------------MGQAAAFSSGLALFQVQEGG
>Galeopterus_variegatus
------------------------------MGLTAATSFNTGLALLQVQEGG
>Monodelphis_domestica
------------------------------MGLS-AAWFCTGLALLKVQAGG
>Trichosurus_vulpecula
------------------------------MGLS-AAWFSTGLALLKVQAGG
>Oryctolagus_cuniculus
------------------------------MGLE-AAGFSTGLALLQVQEGG
>Ceratotherium_simum_simum
--------------------------------MGRAGGFSSGLALLQVQEGG
>Ctenomys_sociabilis
MFVASIASGSQKDGILTTLDSCWCPGFFPNMGLA-ATGFSTGLALLQVQEGG
>Tupaia_chinensis
------------------------------MGFT-AAAFSTGLALLQVQEAG

HG0178.1 ENSGALG00000029047 TMEM204

```
>Gallus_gallus
------------------------------MDLHVFQFITGLALLIKVQEGG
>Anser_cygnoides
------------------------------MEVPIFQFITGLALLIKVQEGG
>Apteryx_australis_mantelli
------------------------------MDLPIFQFITGLALLIKVQEGG
>Calidris_pugnax
------------------------------MDLPILQFSTGLALLIKVQEGG
>Callorhinchus_milii
------------------------------MDLCAHQLLTAASPVKKAQEGG
>Columba_livia
------------------------------MDLPVIQFITGLALLIKVQEGG
>Alligator_mississippiensis
------------------------------MDLPVLQFITGLALLIKVQEGG
>Aquila_chrysaetos_canadensis
------------------------------MDLPIVQFITGLALLIKVQEGG
>Corvus_brachyrhynchos
------------------------------MDLPVIQFTTGLALLIKVQEGG
>Egretta_garzetta
------------------------------MDLPVVQFITGLALLIKVQEGG
>Fukomys_damarensis
MFVASLASGSQKDGILRTLDSRRGPCFFPEMDLAATGFSTGLALLQV-QQGG
>Aptenodytes_forsteri
------------------------------MDLPIVQFITGLALLIKVQEGG
>Struthio_camelus_australis
------------------------------MDLPVFQFITGLALLIKVQEGG
>Chelonia_mydas
------------------------------MDLPVLQFITRLALLIQVQEGG
```

HG0178.2 ENSGALG00000029047 TMEM204

>Gallus_gallus
--MFDCRGSGSVCCSRLRPEAHQNSHKNSHFL--

>Anser_cygnoides
--MFDCCGSGSVCCSRLRPEAHQKQPQKQPFPLSWGK---GGGRQT-----FSSQQ----------------------PVLWS-------------
LPLAVRRSETDTA-EQ-----IPQD--T------PNFH--SSLG-------------------------
>Apteryx_australis_mantelli
--MFDCRGSGSVCCSRLRPEAHQKQPQKQQFPLSGETEREGGKKTL-----FLTAV----------------------SALQS-------------
PPLAALMFREGHSGAN-----SSRDSKLSEFLWDENIR--VEFGLALR---MTILSVCGIF--------
>Calidris_pugnax
--MFDCRGSGSVCCSRLRPEAHQNQPQKQPFPLRGEVG---GPFPR-----SSC--
----SSIFREGHGTAN-----SSRYFKLAELLWDASIH--IQLGLALR---E-----------------
>Aquila_chrysaetos_canadensis
--MFDCCGSGSVCCSRLRPEAHQKQPQKQPFPLRGEVG---GAFSS---Q-QWLFSGPCTRSSD-----------LQRGTRQS-------------
KLLEILQTCRAPLG---
>Lepidothrix_coronata
--MFDCRGSGSVCCSRLRPEAHQKQPQKQPFPLGGEVG---GPFPH-----CSGCSPPGTHSSD-----------LRRGTRQS-------------
SPLEILQAC----------------------------R--APLG-------------------------
>Microcebus_murinus
MSTASLGPVSQHRSLPVSTAMGETAGLPCAWDPWPLLWDRKVGRGQQRGWPGAGPQ---KSP---QAFPIRKHILLVSGCGGRAAICCSWLRPPAHQNSLKNNHFLSAERRGELELEHTHTSLQRALCFPGPAIRCGAPGKGPGSRGAAAKPALLRARPGAGLPRCRLPP
ELLSVFCRCRGRGTRSLLGPLLPRTTA-RSRLWIRAGAPVSSPGWASQQLAAAQARPCSRCRKEDKAGP
>Mus_musculus
--------------------MSVLAGIPD----------YSYGTG---RWGGAASGGLEQASEVSPGLSHQEAHPVSVWLCGRAAICCSWLRPPAHQKQPQKQPFPLC--
----
>Struthio_camelus_australis
--MFDCRGSGSVCCSRLRPEAHQKQPQKQPFPLS--

HG0179 ENSDARG00000036549 agpat3

>Danio_rerio
-------MLSHIERLP-CYNRLR----DTAVAQYE---REKLEKVAGFPPTGSVVLSEGPCKRGL
>Opsanus_beta
--------------------MLN----QSAVMIFTGICGALVVMAMAYYIYWSVVLAEGPCKRGL
>Oryzias_latipes
--------------------MLN----QSAVMIFTGICGAMVLMAMAYYVYWSVILADGPCKRGL
>Trachinotus_blochii
---MCMSVVLAEGPCKRGL
>Pygocentrus_nattereri
--MTAGALQHNGSVVLSEGPCKRGL
>Neolamprologus_brichardi
--------------------MLN----QSAVMIFTGICGAMVFMAMAYYVYWSVVLAEGPCKRGL
>Clupea_harengus
--------------------MHT----Q-SLLHWSKSVS-QIFFKPKKGPVRSVVLSEGPCKRGL
>Fundulus_heteroclitus
--------------------MLN----QSAVMIFTGICGAMVFMAMAYYVYWSVVLAEGPCKRGL
>Esox_lucius
--------------------MLS----QSTVMIFSGICGAFVVLAMGYYVYWTIVLSEGPCRRGL
>Scleropages_formosus
-------MLC------GCYQLRCGVFRGIVAVRWTGS-GLVPGGQVSWKRTRSVILSEGPCKRGL
>Ficedula_albicollis
---------------------------MEDCLLWEG----------LHPGTGTIILSDGPCSRF-
>Salmo_salar
MLKNKYVYGSGVQRHQCCFSVELK---TNALDLFGNV------NRRQDPEIRSVVLADGPCKRGL
>Lepisosteus_oculatus
-----------------------MLANQTSVLIFTGICAALLILAVAYYTYWSVVLSEGPCKRY-
>Pelodiscus_sinensis
-----------------------MRANGKDMETTQ-----NSKEHLGSPAVMTVILSEGPCKAY-

HG0179 ENSG00000160216 AGPAT3

>Homo_sapiens
-------MLRWVSVASPEFLLNEVCRVIWTAVLSEGPCTRS-
>Opsanus_beta
-MLNQSAVMIFTGICGALVVMAMAYYIYWSVVLAEGPCKRGL
>Canis_lupus_familiaris
------------------MSCFSARGRIRTAVLSEGPCTRS-
>Astyanax_mexicanus
-MLNQGAVMIFTGICGALVVMAVAYYAYWSVVLSEGPCKRGL
>Myotis_lucifugus
-MLASYSTCLFSGVCAALVAVALTYYLYWTAVLSEGPCSRY-
>Haplochromis_burtoni
-MLNQSAVMIFTGICGAMVFMAMAYYVYWSVVLAEGPCKRGL
>Danio_rerio
-MLNQGAVMIFTGVCGALVVMAVAYYAYWSVVLSEGPCKRGL
>Fundulus_heteroclitus
-MLNQSAVMIFTGICGAMVFMAMAYYVYWSVVLAEGPCKRGL
>Monodelphis_domestica
-MLDSYSTSLFSAVCATLFVMALAYYLYWTSVLSVGPCTKS-
>Macropus_eugenii
-MLANYSVCLFSAICANLFVMALAYYLYWTSVLSEGPCTHY-
>Esox_lucius
-MLSQSTVMIFSGICGAFVVLAMGYYVYWTIVLSEGPCRRGL
>Equus_asinus
----------MVGSTSP-------ELWPRTAVLSEGPCTRY-
>Rattus_norvegicus
-MLASSSTCLFSGVCVALLAVALAYYFYWTAVLSEGPCGRP-
>Lepisosteus_oculatus
MLANQTSVLIFTGICAALLILAVAYYTYWSVVLSEGPCKRY-

HG0180.1 ENSG00000112246 SIM1

```
>Homo_sapiens
--MSIFRGPGFAAGSRRKG-----WLLRGPTGI
>Anas_platyrhynchos
--MTLFRGAMLSATSRRKGQKYLIWLLRGAASF
>Odobenus_rosmarus_divergens
--MSIFWGPGFASPNRRKG-----WLLRGPAGI
>Orcinus_orca
--MSISREPGFAARNRRKG--------------
>Myotis_brandtii
--MSLFQGPGFAARNRRKG-----WLLRGPAGI
>Monodelphis_domestica
--MSVFRVLVFAAWNRREA-----WFLDGPIDI
>Sorex_araneus
--MSVFRAPGCAAWNRKKG-----WPRRAPAGL
>Oryctolagus_cuniculus
--MSTFRAPRFAAQNGRKG-----WLLRGPAGI
>Equus_asinus
MIMSIFRGPGFAARNRRKG-----WLLGDLAGI
>Manis_javanica
-MMSTFREPGYEAPNRRKG-----WLLRSPAGI
>Loxodonta_africana
--MSIFRGPGFAARNRRKG-----WLLWGASGI
>Heterocephalus_glaber
--MSIFQGTGFAAENRRKR-----WLLRGSASI
>Tupaia_chinensis
--MSIFRGPGCASPNRKKN-----WLPWGLAGI
>Trichechus_manatus_latirostr
--MSIFRGPGFAARNRRKV-----WLLRGPAGI
```

HG0180.2 ENSG00000112246 SIM1

>Homo_sapiens
---MRTVKRWAPPKPLPLVGTEGP
>Mustela_putorius_furo
MVGMSTIKRWAPLHPLPPAGTEVA
>Pteropus_alecto
---MNTIKRRASPHPLPPAGTEGR
>Loxodonta_africana
---MSAIKRWAPPHPLPPVGTEAA
>Tupaia_chinensis
---MIAIRRWAPAHPLPPAGTEVA

HG0181.1 ENSG00000143776 CDC42BPA

>Homo_sapiens
----------MHSVFFLPF--------L-FFFFPGSELLMQIGLYSLMMQPDSFQDYVARVEF
>Ailuropoda_melanoleuca
---------------------------------------MRIGPDSLMMQPDSLRDCVARVEF
>Pteropus_vampyrus
---------------------------------------MQIGLNSLMMQPDSFQDYVARIEF
>Galeopterus_variegatus
---------------------------------------MQIGLYSFMMQPDSFQD-------
>Monodelphis_domestica
MHYSSVGGGNLHLGFRLPLVTYFVFLLLFSIHPSKSELLMPSGLYSLMMQRDSFQDCVA----
>Coturnix_japonica
-------------------------------------MRPEWGFYTLMMQWDSFQGCVA----
>Condylura_cristata
---------------------------------------MHLGLNSLVMQPDSFQDYVARVEF
>Ochotona_princeps
---------------------------------------MQMGLYSLMMQPDSFQDYVARVEF
>Equus_asinus
---------------------------------------MQIGLNSLMMQPDSFQDYVARVEF
>Loxodonta_africana
---------------------------------------MRIGLYSLMMQPASFQDYVA----
>Mesocricetus_auratus
---------------------------------------MPRGLYSLMMQPDSFQDYVARIEF
>Tupaia_chinensis
---------------------------------------MQIRLYSLMMQPDSFQDYVARVEF
>Thamnophis_sirtalis
---------------------------------------MEWGLYTLMMQKDSFRDWVS----
>Chrysemys_picta
-------------------------------------MQTEWGFYTLMMQWDSFQDYVA----

HG0181.2 ENSG00000143776 CDC42BPA

>Homo_sapiens
MYMGEIEDASVK
>Balaenoptera_acutorostrata_s
MYMCEIEDASVK
>Rhinolophus_ferrumequinum
MYMCEIEDASVK
>Erinaceus_europaeus
MYMCEIEDASVK
>Ochotona_princeps
MYMGEIEDASVK
>Ceratotherium_simum_simum
MYMCEIEDASVK
>Cricetulus_griseus
MFMGEIEDASVK
>Tupaia_chinensis
MFMGEIEDASVK
>Trichechus_manatus_latirostr
MYMCEIEDASVK

HG0182 ENSGALG00000031991

>Gallus_gallus
MDVNQPKQEHLLALKGPVSH---------------------
>Ailuropoda_melanoleuca
MEVNPPKQEHLLALKGPVSL---------------------
>Lipotes_vexillifer
MEVNPPKQEHLLALKGPVSL---------------------
>Dasypus_novemcinctus
MEVNPPKQEHLLALKGPVFL---------------------
>Sarcophilus_harrisii
MEVNPPKQEHLLALKGPVSSFSSVQNVERRTRKSNKF----
>Trichosurus_vulpecula
MEVNPPKQEHLLALKGTVSSFSSVQNVERKTRKSYNYEFKF
>Oryctolagus_cuniculus
MEVNPPKQEHLLALKGPVSL---------------------
>Sturnus_vulgaris
MDVNQPKQEHLLALKGPVFH---------------------
>Manis_javanica
MEVNPPKQEHLLALKGPVSL---------------------
>Papio_anubis
MEVNPPKQEHLLALKGPVSL---------------------
>Cricetulus_griseus
MEVNPPKQEHLLALKGPVSSDLARETVL-------------
>Tupaia_chinensis
MEVNPPKQEHLLALKGPVSL---------------------
>Aptenodytes_forsteri
MDVNQPKQEHLLALKGPVIH---------------------
>Protobothrops_mucrosquamatus
MDVTQSKQEHLFALKCPVLSYTAHRIRFF------------

HG0183 ENSG00000011465 DCN

>Homo_sapiens
-----------------------MW---VSAGLCSLRETLFFNCAME------------------------------------
>Leptonychotes_weddellii
MGRKKADKLSGCNTRDISLPYDLMW---VSAGLCSLRETLFFNCAME------------------------------------
>Lipotes_vexillifer
-----------------------MW---VSAGLCSLRETLFFNCAME------------------------------------
>Rousettus_aegyptiacus
-----------------------MW---VSAG---
>Dasypus_novemcinctus
-----------------------MW---VSAGLCSLRETFIF---
>Galeopterus_variegatus
-----------------------MW---VSAGLCSLRETLFFNCAME------------------------------------
>Erinaceus_europaeus
-----------------------MW---VSAGLSSLRETVFFLTAMQ------------------------------------
>Elephantulus_edwardii
-----------------------MW---VSAGLCSLRETFFFFSVCYGVEAGGFQPSDSHRAAPNPLLLSTPANSFAWAEYLV
>Manis_javanica
-----------------------MW---VSAGLCSLRETLFFNCAME------------------------------------
>Loxodonta_africana
-----------------------MW---VSAGLCSLRETLFFNCAME------------------------------------
>Dipodomys_ordii
-----------------------MW---VSAGLCSLRETLFFNCAME------------------------------------
>Protobothrops_mucrosquamatus
----------------------MMYCGCQQPGVFSLRKTLLFNCAME------------------------------------
>Pelodiscus_sinensis
-----------------------MW---VSAWIVFIEETLFFNCAVE------------------------------------
>Orycteropus_afer_afer
-----------------------MW---VSAGLCSLRETLFFNCAME------------------------------------

HG0184 ENSG00000136542 GALNT5

>Homo_sapiens
---MLCYEMPRRQRLTSGRN-
>Odobenus_rosmarus_divergens
--MPRRQRLTSSRS-
>Orcinus_orca
---MLCCEMPRRQRLTSGRS-
>Pteropus_alecto
---MLCCEMPRRQRLTSGRS-
>Dasypus_novemcinctus
---MLCCEMPRRQRLTSSRS-
>Monodelphis_domestica
---MVCCEMPRRQRLTSSSRS
>Condylura_cristata
---MLCYEMPRRQTDKG----
>Oryctolagus_cuniculus
MRSGVELQRADRERGGEKKHQPQPAPPFLGLVGRWTRVHRENTSDRLNYLVAGIACSWNDLRAVFTRVQPGPAEQAPMLYCEMPRRQRLTSSRS-
>Equus_caballus
---MLCYEMPRRQRLTSGRS-
>Manis_javanica
---MLCCEMPRRQRLTSGRS-
>Loxodonta_africana
---MLCCEMPRRQRLTSSRS-
>Ictidomys_tridecemlineatus
---MRCCEMPRRQRLTGGRS-
>Pelodiscus_sinensis
---MPCCKMPRRQRLTGSRI-
>Orycteropus_afer_afer
---MLCCEMPRRQRLTSSRS-

HG0185 ENSG00000143033 MTF2

>Homo_sapiens
MHRQSAGNQNGEGLY---------------------------------------
>Canis_lupus_familiaris
MHRQSAGNQNGEGL--
>Lipotes_vexillifer
MHRQSAGNQNGEGL--
>Miniopterus_natalensis
MHRQSAGNQNGEGL--
>Galeopterus_variegatus
MHRQSAGNQNGEGL--
>Erinaceus_europaeus
MHRQSAGNQNGEGL--
>Ochotona_princeps
MHRQSAGNQNGEGL--
>Equus_asinus
MHRQSAGNQNGEGL--
>Loxodonta_africana
MHRQSAGNQNGEGLY---------------------------------------
>Heterocephalus_glaber
MHRQSAGNQNGEGL--
>Tupaia_chinensis
MHRQSAGNQNGEGL--
>Trichomycterus_areolatus
MHRDSAGNQNGETKSRKIFV------GSNWNNS---RLLTCDSFF---------
>Trichechus_manatus_latirostr
MHRQSAGNQNGEGLYGSGLCLRLVYERPLYPNPSPQDFGLCVRLCGVPGGAGAQ
>Orycteropus_afer_afer
MHRQSAGNQNGDGLY---------------------------------------

HG0186 ENSG00000167552 TUBA1A

>Homo_sapiens
---MASLGRLGVWTNRKRPRPIPMVTELYIGSRIRLCAAGSLTSTA----
>Mustela_putorius_furo
---MVTELYIGSRIRPCAAGSLTSTA----
>Orcinus_orca
---MVTELYIGSRIRPCAAGSLTSTA----
>Rousettus_aegyptiacus
---MVTELYIGSRIRPCAAGSLTSTA----
>Sarcophilus_harrisii
---MVTELYIGSCIGPCAVSSLTSTS----
>Sorex_araneus
---MVTESYLGSRIRPCAAGSLTSTA----
>Ochotona_princeps
MDARAAGFCSLSLSEGCRKVYGYWAGGGGGTHYLIPCRLHQSAPSLHGSPGADAGVWTNRNRPRPIPMATGLYIGSLRRPHRRFSYIDRLRVAL
>Equus_asinus
------------------------------------MSLAPITTLLSWLLWADRGVWTNRKRPQPIPMVTELYIGSRIRPCAAGSLTSTA----
>Manis_javanica
---MVTELYIGSCILPCTAGFLTSTA----
>Loxodonta_africana
---MVTELYIGSCIRPCAAGSLTSTA----
>Marmota_marmota_marmota
---MVTELYIGSRIRLCAAGSLTSTA----
>Trichechus_manatus_latirostr
------------------------------------MSLAPITTLLSWLLWADRGVWTNRKRPRPIPMVTELYIGSRIRPCAAGSLTSTA----
>Python_bivittatus
---MVTGLYIGSRVILCATGSLTSTS----
>Orycteropus_afer_afer
---MVTELYIGSRIRSSAAGSLTSTA----

HG0187.1 ENSGALG00000004058 GPR146

```
>Gallus_gallus
MDKMFLPAST-------GQQGTAYQKSW
>Anser_cygnoides
MGKMLLPAIK-------KLQGGAYQESW
>Caprimulgus_carolinensis
MGKMFLPAIK-------EQQGCAYQKSW
>Pterocles_gutturalis
---MFLPAIT-------EHQGSAYQKSW
>Alligator_mississippiensis
MSKMLLQAIKKH---SFKQQGSAYQKSW
>Aquila_chrysaetos_canadensis
MGKMFLPTIK-------GQQGTAYQKSW
>Gavia_stellata
MGKMFLPAIE-------EQ---------
>Lepidothrix_coronata
---MFLPAVK-------GQQGRAYQKSW
>Fulmarus_glacialis
MGKMFLPAIK-------EQQGSAYQKSW
>Aptenodytes_forsteri
MGKMFLPAIK-------EQQGSAYQKSW
>Takydromus_sexlineatus
---MFLPAVN-------LRKGSAYQESW
>Struthio_camelus_australis
MGKMFLPAVRKH---SLKQQGSAYQKSW
>Chrysemys_picta
---MFLPAIKKPICYSLKQQGRAYRKSW
```

HG0187.2 ENSDARG00000059610 gpr146

>Danio_rerio
MSMEIQQNPMPP
>Pygocentrus_nattereri
MSVEIQQNPMPP
>Esox_lucius
MSVEVQQNPMPP
>Chanos_chanos
MSMEVQQNPMPP
>Apteronotus_leptorhynchus
MSMEIKQNPMPP
>Oncorhynchus_kisutch
MSVEVQQNPMPP
>Trichomycterus_areolatus
MSVEIKQDPMPP

HG0187.3 ENSGALG00000004058 GPR146

```
>Gallus_gallus
MCMYVQGKSHVNTDSVP-------------
>Anas_platyrhynchos
MCMYVQGKSHVNTDSVPRTGLVQPSAGVF-
>Caprimulgus_carolinensis
MCMYVQGKSHVYTEFSATTEGSAAASAVFF
>Pterocles_gutturalis
MRMYVQGKSHVYTEFGATTQGDAAVSAVFS
>Haliaeetus_leucocephalus
MCIYVQGKSRVYTKFGAATEGSAAASAVFF
>Fulmarus_glacialis
MCMYVQGKSHVYTEFGATTEGSAATSAVLF
>Pygoscelis_adeliae
MCMYVQGKSRVYTEFGATTEASAAASAVFF
```

HG0188 ENSG00000157625 TAB3

>Homo_sapiens
------------MTVVLEDHYFWKAG
>Felis_catus
MTLSVLKRTDQKMTAVLEDHYYWKAG
>Lipotes_vexillifer
------------MTAVLEDHYYWKTD
>Pteropus_vampyrus
------------MTVVLEDYYYWKAG
>Dasypus_novemcinctus
------------MTVVLKDRYYWKAG
>Sarcophilus_harrisii
------------MTETLEHHFYWKAN
>Galeopterus_variegatus
MTLTVLRSTDQKMTVVLEDHYFWKAH
>Erinaceus_europaeus
------------MTVVLEDHYYWKAG
>Oryctolagus_cuniculus
------------MTVVLEVHYFWKAG
>Ceratotherium_simum_simum
------------MTAVLEDHYYWKAG
>Manis_javanica
------------MTAVLEDHYYWKAG
>Loxodonta_africana
------------MTAVFEDHCYWKAS
>Fukomys_darlingi
------------MTVVLEDHYFWKAS
>Tupaia_chinensis
MTWTVLRSTDQKMTMVLEDHYFWKAG
>Trichechus_manatus_latirostr
------------MTAVLEDHCYWKAS

HG0188 ENSGALG00000016284 TAB3

>Gallus_gallus
--------------------MPEASEDHNHWKVC
>Anas_platyrhynchos
--------------------MPEASEDHNHWKAC
>Pelophylax_nigromaculatus
--------------------MQEGSEDHNHWRAL
>Apteryx_australis_mantelli
-----------------MQKMPEASEDHNHWKAY
>Merops_nubicus
--------------------MPEASEDHNHWKAC
>Alligator_mississippiensis
MVLPPEP------SP-PRTKMPEASEDHNHWKAC
>Aquila_chrysaetos_canadensis
--------------------MPEASEDHNHWKAC
>Gavia_stellata
--------------------MPEASEDHNHWKAC
>Parus_major
-MQPPEPQPQPEPSPQRSTKMPEASEDHNHWKAC
>Gekko_japonicus
-MLLLE------PV-PPRATMPEASEDHNHWEAC
>Struthio_camelus_australis
--------------------MPEASEDHNHWKAY
>Chrysemys_picta
--------------------MPEASEDHIHWKTY
>Tinamus_guttatus
--------------------MPEASEDHNHWKAY

HG0189 ENSDARG00000001807 tnfrsf21

>Danio_rerio
--------MGIIAFVFQNQALIPYYLKPVHSGAQTIKRREGTEFVH---------
>Amia_calva
--------MGIIPC-LQNQALIPYYLKPVHFATLTLKNCRWKKDQC---------
>Trachinotus_blochii
--------MGITTAFFLNQALIPYYLKPVHLETQTIKHCS---------------
>Micropterus_salmoides
--------MGITTAFFLNQALIPYYLKPVHLETRTIKRCS---------------
>Oreochromis_niloticus
-MPPSVVAMGINTASSVKQTLTLHYLKPVHLKTQSIKHCSGDCHCSEPGSASLLR
>Latimeria_chalumnae
--------MGYRPTGFRNQALLPYYLKPVHLVKVIFTNWAESYF-----------
>Esox_lucius
--------MGIKPF-FQNQALIPYYLKPVHFETQTIKNCRLKEHQQ---------
>Lutjanus_erythropterus
--------MGISTAFLLKPALIPYYLKPVHLETRTIKHCS---------------
>Trematomus_bernacchii
--------MGITTAFFLNQALIPYYLKPVHLETRTIKHCS---------------
>Leucoraja_ocellata
--------MGINAADIDDQSLLPYYLKPVDLVKAAARCLQRWSFLYQ--------
>Salmo_salar
--------MGIIPF-FQNQALIPYYLKPVHFETQTIKTAG---------------
>Lepisosteus_oculatus
MNSHSGIAMGIIPF-FQNRALIPYYLKPVHFDTMTLKDWRWNKYQY---------
>Squalus_acanthias
--------MGINAADIDDQSLLPYYLKPVDLVKVAARFLQWWNRWCQ--------

HG0190 ENSGALG00000006949 CHD2

>Gallus_gallus
MSFFPRTFLY
>Anser_cygnoides
MSFFPRTFLY
>Apteryx_australis_mantelli
MSFFPRTFLY
>Cariama_cristata
MSFFPRTFLY
>Cuculus_canorus
MSFFPRTFLY
>Monodelphis_domestica
MSFFPRTFLY
>Aquila_chrysaetos_canadensis
MSFFPRTFLY
>Gavia_stellata
MSFFPRTFLY
>Chlamydotis_macqueenii
MSFFPRTFLY
>Manacus_vitellinus
MSFFPRTFLY
>Egretta_garzetta
MSFFPRTFLY
>Pygoscelis_adeliae
MSFFPRTFLY
>Struthio_camelus_australis
MSFFPRTFLY

HG0191 ENSGALG00000036097

```
>Gallus_gallus
MNKRKNPFWTVFLNQASPSISRTF
>Anas_platyrhynchos
MNKQKNPFWTVFLNEASPSISGTY
>Apteryx_australis_mantelli
MNKQKKTFWTVFLNQASPSILGTY
>Calidris_pugnax
MNKQKNPFWTVFLNQASPSVSGTY
>Alligator_mississippiensis
MDRLKKKFWTVFINQASPSLLETY
>Monodelphis_domestica
MDKLKNSFWIVFPNETSPSSFSAQ
>Aquila_chrysaetos_canadensis
MSKQKNPFWTVFLNQASPSVLGTY
>Ornithorhynchus_anatinus
MDKLKKPLWTVFLPEASLSLLGCH
>Lepidothrix_coronata
MNKRKTPIWTVFLNQASPSVSGTY
>Anolis_carolinensis
MDRIKKPFWTVFLNQFFPSPMEAC
>Struthio_camelus_australis
MNKQKKTFWTVFLNQASPSILGTY
>Pelodiscus_sinensis
MDILKKTFWIVFLSQASPSFLGTY
>Tinamus_guttatus
MNKQKKTFWTVFLNQASPSILGTY
```

HG0192 ENSG00000125107 CNOT1

>Homo_sapiens
MQKLPRSCQAISQLSIQKETYHSKDLRSCMPGNGMKTKCAMFQNRVRPLVKKN
>Felis_catus
--MFQNRVIPLVKKN
>Lipotes_vexillifer
--MFQNRVRPLVKN-
>Myotis_brandtii
--MFQNRVRPLVKKN
>Dasypus_novemcinctus
--MFQNRVRLLVKKN
>Sarcophilus_harrisii
--MFQNRIRSLVKKN
>Monodelphis_domestica
--MFQNRIRSLVKKN
>Condylura_cristata
--MFQNRVRPLVKKN
>Ceratotherium_simum_simum
--MFQNRLRPLVKKN
>Loxodonta_africana
--MFQNRVRPLVKKN
>Ictidomys_tridecemlineatus
--MFQNRVRPLVKKN
>Trichechus_manatus_latirostr
--MFQNRVRPLVKKN
>Pelodiscus_sinensis
--MFQNRIRPLGKKS

HG0193 ENSG00000134853 PDGFRA

>Homo_sapiens
MISFRRMDYNIESITKRGF--------
>Anser_cygnoides
MISFGRMDYNIESITGRGL--------
>Ursus_maritimus
MISFRRMDYNIESITKRGF--------
>Ambystoma_mexicanum
MIRLGRMGYNIESITRRAFLGRLQSGN
>Lipotes_vexillifer
MISFRRMDYNIESITKRGF--------
>Myotis_ricketti
MISFRRMDYNIESITKRGF--------
>Dasypus_novemcinctus
MISFRRMDYNIESITKRGF--------
>Monodelphis_domestica
MISF-RIDFNIESITKRGV--------
>Oryctolagus_cuniculus
MISFRRMDYNIESITKRGF--------
>Lepidothrix_coronata
MISFGRMDYNIESITERGL--------
>Equus_asinus
MISFRRMDYNIESITKSGF--------
>Fukomys_damarensis
MISFRRMDYNIESITKSGFCAHYS---
>Pelodiscus_sinensis
MISFGRMDSNIESITRLGL--------

HG0194 ENSG00000143507 DUSP10

```
>Homo_sapiens
--------MCESIAEEERQMVEEYTYL---
>Ailuropoda_melanoleuca
--------MGESLAEAERQMVEGYTYL---
>Balaenoptera_acutorostrata_s
--------MCESTAEAERQMVEGYTYL---
>Sarcophilus_harrisii
MKLSEWGLDAPSIATVKRQMVEGYTYL---
>Monodelphis_domestica
MKLSEWGFNVQSIATVKRQMVEGYTYL---
>Macropus_eugenii
--------MYKSIATVKRQMVEGYTYL---
>Sorex_araneus
-------------------MVEGYTY----
>Elephantulus_edwardii
-------------------MVKGYYFIKLF
>Equus_asinus
--------MRESIAEAERQMVEGYTYL---
>Marmota_marmota_marmota
--------MCQSIAEAERQMVEGYTYL---
>Tupaia_chinensis
--------MYETVAQEERQMVEGSTYL---
>Trichechus_manatus_latirostr
--------MCESIAEEERQMVEGYTHL---
>Chrysemys_picta
-------------------MLKGNTYL---
```

HG0195 ENSG00000165966 PDZRN4

>Homo_sapiens
MKNSSQVWRVIPFQFTCLSVLGKLEDLLSLSAS
>Odobenus_rosmarus_divergens
MKNSSQVWRVIPFQFTCLSVLGKLEDLLSLSAS
>Lipotes_vexillifer
MKNSSQVWRVIPFQFTCLSVLGKLEDLLSLSAC
>Pteropus_alecto
MKNSSQVWRVIPFQFTCLSVLGKLEDLLSLSAS
>Latimeria_chalumnae
MKNWSQEWREIPCQCHYLSVLGKLESFALVC--
>Gallus_gallus
MKNSSQVWRVIPCQCICLSVLGK----------
>Erinaceus_europaeus
MKNSSQVWRVIPFQFTCLSVLGELEDLLSLSAA
>Oryctolagus_cuniculus
MKNSSRDWRVIHFQLPCRSVLGTLEDQLPRSAS
>Elephantulus_edwardii
MKNSSQVWRVIPFQFTCLSVLGKLEDLLSLSAS
>Manis_javanica
MKTGGQVWRVLPFRLACLPVPGKLEDSRARSAP
>Loxodonta_africana
MKNSSQVWRVIPFQFTCLSVLGKLEDLLSLSAS
>Ictidomys_tridecemlineatus
MKNSSQVWRVIPFQFTCLSVLGKLEDLLSLSAS
>Trichechus_manatus_latirostr
MKNSSQVWRVIPFQFTCLSVLGKLEDLLSLSAS

HG0196 ENSG00000189403 HMGB1

>Homo_sapiens
MLQSGESEE-AASGSRSHSHCSTLSSIETAPGQVRAGRALGDSVPR----
>Megophrys_sangzhiensis
MLQIGERVKRLRIDPASHSHCSTLSSIDTALETS----------------
>Odobenus_rosmarus_divergens
MLQSGESEE-AASGSRSHSHCSTLSSIETAPGQVRARRALGDSVPR----
>Pteropus_vampyrus
MLQSGESEE-AASGSRSHSHCSTLSSIETAPGQVRAGRALGDSVLAEEK-
>Dasypus_novemcinctus
MLQSGESEE-AASGSRSHSHCSTLSSIETAPGKCEPGGRWATRSLADEK-
>Galeopterus_variegatus
MLQSGESEE-AASGSRSHSHCSTLSSIETAPGQVRAGRALGDSVPR----
>Macropus_eugenii
MLQNGESEE-AASGSRSHSHCSTLSSIETAPGQVRAGRALGYSVRR----
>Condylura_cristata
MLQSGESEE-AASGSRSHSHCSTLSSIETAPGQVRAGRALGDSVPR----
>Ochotona_princeps
MLQSGESEE-AASGSRSHSHCSTLSSIETAPGQARAGRALGDSVPRGGK-
>Equus_przewalskii
MLQSGESEE-AASGSRSHSHCSTLSSIETAPGQVRAGRALGDSVPR----
>Manis_javanica
MLQSGESEE-AASGSRSHSHCSTLSSIETAPGQVRGRRAPGDSVPR----
>Ictidomys_tridecemlineatus
MLQSGESEE-AASGSRSHSHCSTLSSIETAPGQVRAGRALGDSVPR----
>Trichechus_manatus_latirostr
MLQSGESEE-AASGSRSHSHCSTLSSIETAPGQAEPDGTGRLCASRTKNY

HG0197 ENSG00000196132 MYT1

>Homo_sapiens
MIAFRDVILKEDRQ
>Odobenus_rosmarus_divergens
MIAFRDVILKEDRQ
>Balaenoptera_acutorostrata_s
MIAFRDVILKEDRQ
>Myotis_davidii
MIAFRDVILKEDRQ
>Galeopterus_variegatus
MIAFRDVILKEDRQ
>Monodelphis_domestica
MIAFRDVILKGERQ
>Coturnix_japonica
MIGFRDVILQRGRQ
>Oryctolagus_cuniculus
MIAFRDVILKEDRQ
>Equus_asinus
MIAFRDVILKEDRQ
>Manis_javanica
MIAFRDVILKEDRQ
>Chinchilla_lanigera
MIAFRDVILKEDRQ
>Tupaia_chinensis
MIAFRDVILKEDRQ
>Pelodiscus_sinensis
MIAFRDVMLKRARQ

HG0198.1 ENSDARG00000018688 elk3

>Danio_rerio
------------------------MCVCVRIRVCAR-VPECRMVARGGKACLHRRAERLGNRARREEVSRLAVCLLQLPAHRERERKSE-----------------------------
>Calidris_pugnax
---------------------MLQPC-------C---ASVARARGEGRKSLFTQTANRLGNNA-GEEVSRLAV---
>Latimeria_menadoensis
-------------------MCVLYYC-------N---VEHQWAGREGRKSLFTQTAKCLGNNA-GKEVSRLSV---
>Fundulus_heteroclitus
MCGGFEANEASLKASQRSRRCEFKDCRSFRQHVCRQRVGPLLAAEEGRKSVFTQTASCLGNRR-GEEVSRLCVCLLQLPALRKKKPRIRFQRSTTVQTRSQHGKTGERTGFDVKSFAE
>Monodelphis_domestica
------------------------MCVCVCVCVCVC-VCVC----------------------VVTVTILLCVCLILQPC--------------------------------------
>Coturnix_japonica
---------------------MLQPC-------R---TSVARARGEGRKSLFTQTANRLGNNA-GKEVSRLSV---
>Ornithorhynchus_anatinus
---------------------MLQPC-------C---TSAARERGAGRKSLFTQTANRLGNNA-VKEVSRLSV---
>Manacus_vitellinus
---------------------MLQPC-------C---TSTARARGAGRKRLFTQTANRPGNSA-GEEVSRAAV---
>Nannospalax_galili
------------------------MRASEGSRGT---QPRPRRTARGGKACLHRLHTAWGIMQ---
>Lepisosteus_oculatus
-------------------MCVIIER-------C---WTSVGRGRRGEESLFTQTANRLGNNA-GKEVSRLSV---
>Ictalurus_punctatus
----------------MGTDSGYSITVCVRVCVG---PRWAGREERGGKACLHRRPSAWGTE--
>Python_bivittatus
------------------------MCLMLQQCCL---HISGQRGEKGGKACLHRLQTAWGIMQERK--

HG0198.2 ENSG00000111145 ELK3

>Homo_sapiens
MESRGLSPGSS
>Mustela_putorius_furo
MESRGLSPGSS
>Lipotes_vexillifer
MESRGLSPGSS
>Rousettus_aegyptiacus
MESRGLSPGSS
>Dasypus_novemcinctus
MESRGVSPGSS
>Galeopterus_variegatus
MESRGLSPGSS
>Ochotona_princeps
MESRGLSPGSS
>Elephantulus_edwardii
MESRGVSPGSS
>Ceratotherium_simum_simum
MESRGLSPGSS
>Manis_javanica
MESRGLSPGSS
>Microtus_ochrogaster
MESRGLSPGSS
>Tupaia_chinensis
MESRGLSPGSS
>Trichechus_manatus_latirostr
MESRGVSPGSS

HG0199 ENSGALG00000007989 ADNP

>Gallus_gallus
MLRQKGNEQDMTLLLCPGFGLTQ
>Anas_platyrhynchos
----------MMLLLCPGFGLTQ
>Apteryx_australis_mantelli
MLRQKGKEQDMILLLCPGFGLTQ
>Calidris_pugnax
MLRQKGKEQDMILLLCPGFGLTQ
>Columba_livia
-----MLNQDMILLLCPGFGLTQ
>Alligator_mississippiensis
----------MSLLLCPGFGLTQ
>Aquila_chrysaetos_canadensis
MLRQKGKEQDMILLLCPGFGLTQ
>Manacus_vitellinus
MLRQKGKEQDMILLLCPGWGLPQ
>Aptenodytes_forsteri
MLRQKGKEQDMILLLCPGFGLTQ
>Struthio_camelus_australis
----------MILLLCPGFGLTQ
>Chrysemys_picta
----------MSLLLCPGFGLTQ
>Tinamus_guttatus
----------MILLLCPGFGLTQ

HG0200 ENSGALG00000012015 NDST4

>Gallus_gallus
MHCNGHMNVSGEESAPLRLIVIMAL
>Anser_cygnoides
MYCNGHMNISGGESAPLHLLIIMAL
>Apteryx_australis_mantelli
MHCNGHMNNSVGESAPLRLLVTMAL
>Mustela_putorius_furo
MVCNGYMNLWGVIFAPSHTLFTMAL
>Hynobius_retardatus
MHCNGHMNCWGAAPAQSCTLFTMSL
>Calidris_pugnax
MHCNGHMNVLGGESAPPHLLIPMAL
>Lepidothrix_coronata
MLCNGHMNVSEGESAPLQLLIPMAL
>Nannospalax_galili
MVCNGYMNLWEVESAPSHALATMAL
>Thamnophis_sirtalis
MNCNGHMNIWRVVFIPSRGLFMMAL
>Struthio_camelus_australis
MYCNGHMNNSVGESAPLRLLLTMAL
>Chrysemys_picta
MHCNGHMNILGVVPAPFFVLSTMAL
>Tinamus_guttatus
MHCNGHMNSSGPIST--HLLVTMAL

HG0201 ENSGALG00000015256 PAM

```
>Gallus_gallus
--------------------MVCQKLEDACRAHNNVVMN
>Anser_cygnoides
--------------------MVCQKLEDACRAHNNEVVN
>Calidris_pugnax
--------------------MVCQKLEDARRAHNNEVVN
>Columba_livia
--------------------MVCQKLEDACRAHNNEVVN
>Alligator_mississippiensis
--------------------MVCQKL-------------
>Sarcophilus_harrisii
MIRDNIASSTFLIYRASKTDGLSSAVGDACRAHDNEVVN
>Falco_cherrug
--------------------MVCQKLEDACGAHNNEVVN
>Pseudopodoces_humilis
--------------------MVCQKLEDACRAHNNEVVK
>Aptenodytes_forsteri
--------------------MVCQKLEDAHRAHNNEAVN
>Takydromus_sexlineatus
--------------------MVCQKL-------------
>Struthio_camelus_australis
--------------------MVCQKL-------------
>Trachemys_scripta
--------------------MVCQKL-------------
```

HG0202 ENSG00000124766 SOX4

>Homo_sapiens
--MRSDLELLIARGDYYCIGFQANGKPGGGVWHEEALVTAADWLQPRL---
>Channa_punctata
--MRSDTRLLIARADYY--
>Oryzias_melastigma
--MRSDTRLLIAHADYYCVSFSTNE--
>Mustela_putorius_furo
--MRSDLELLIARGDYYCIGFQANGKAGGGAWHEEALVTAADWPRPRL---
>Ambystoma_mexicanum
MLLAGSHPCTVCTPPLPPLHPGSGYTAHSPNPLEHIRTPSQINGSLPAPPKRFTLNPKISASPRVPFFEKFALWLGMRSALEPFIARGDYYCIGCRANGGGRGRDRACGNVGY-SD---
>Pteropus_vampyrus
--MRSDLELLIARGDYYCIGFQANGKPRGGAWHEEALVTAADWPWPRL---
>Pimephales_promelas
--MRSDPKLLIARADYY--
>Austrofundulus_limnaeus
--MRSDTRLLIARADYY--
>Scleropages_formosus
--MRSDITLLIARAHDY--
>Ceratotherium_simum_simum
--MRSDMELLIARGDYYCIGFQANGKPGGGAWHEEALVTAADWPRPRL---
>Cavia_porcellus
--MRSDLELLIARGDYYCIGFQANRKGGGGAWHEEALVTAADWLRPKDKEARGGIGVCSKLQQEKQCVRERGLSHSQVSSSCTLFKSLH
>Chrysemys_picta
--MRSDLELLIARGDYYCIGFQANSSRRGGVWHEEALDTASDWPGRGL---

HG0203 ENSG00000128606 LRRC17

>Homo_sapiens
------------MSL----WHP-FPKFSLGTPRTPAGSHCSRTALVKITQTWISKEYFHCSVCNTK
>Xenopus_laevis
------------MSL----WHP-FPEPRARIPSTPAAHTEYL----IITG----------------
>Ursus_maritimus
------------MSL----WHP-FPKFRD-SHRTSAGSHHS-------------------------
>Physeter_catodon
------------MRLEREAETR-RPRVSKGLHRTSAGSHCS-------------------------
>Rousettus_aegyptiacus
------------MSL----WHP-FPKFRDSTTPQLDLQALHLH-----------------------
>Dasypus_novemcinctus
------------MSL----WHP-FPKFSDS--TDPEGSQCSLTAQVTIAQTWISKKYIHCFICHIK
>Condylura_cristata
MVSA-----GMAPCEACEGWIRGRPHSLVWTTCTSAGSHCS-------------------------
>Oryctolagus_cuniculus
------------MSL----WHP-FPKLRTSTEPSWVPLFL----------NYVS------------
>Equus_caballus
------------MSL----WHP-FPKFRE-LHGTSAGSHCSSTALVKLTHTRISKEYFHCSICHTK
>Manis_javanica
MLPRLIGQDQFLLITWPQSWLR--KIPEMGLHCTSAVSHCS-------------------------
>Marmota_monax
------------MSL----WHP-FPKFRNSESPSWVSLFL----------NCI-------------
>Tupaia_chinensis
------------MSL----WHP-FPKFRDS--TCWVALFL----------SCIS------------

HG0204 ENSG00000162599 NFIA

>Homo_sapiens
MPLTPAYSRHV
>Felis_catus
MPLTPAYSRHV
>Myotis_lucifugus
MPLTPAYSRHV
>Galeopterus_variegatus
MPLTPAYSRHV
>Condylura_cristata
MPLTPAYSRHV
>Ochotona_princeps
MPLTPAYSRHV
>Elephantulus_edwardii
MPLTPAYSRHV
>Loxodonta_africana
MPLTPAYSRHV
>Nannospalax_galili
MPLTPAYSRHV
>Thamnophis_sirtalis
MPLTPGYSRHV
>Chrysemys_picta
MPLTPGYSRHV
>Orycteropus_afer_afer
MPLTPAYSRHV

HG0205 ENSG00000184611 KCNH7

>Homo_sapiens
MGREFWFQKRMLCHKERLARWPGL
>Leptonychotes_weddellii
MGRDFWFQKRMLCHKERLARWPGL
>Physeter_catodon
MGRDFWFQKRMLCHKERLARWPGL
>Rousettus_aegyptiacus
MGRDFWFQKRMLCYKERLARWPGL
>Monodelphis_domestica
----------MICHKEKLAQWSGL
>Erinaceus_europaeus
MGRDFWFQKKMLCHKERLARWPGL
>Ochotona_princeps
MGRAFWFQMRMLCQPERLARWPGL
>Equus_caballus
MGRDFWFQKRMLCHKERLARWPGL
>Loxodonta_africana
MGRDFWFPKRMLCHKERLARWPGL
>Marmota_marmota_marmota
MGRDFWFQKRMLCHKERLARWPGL
>Trichechus_manatus_latirostr
MGRDFWFQKRMLCHKERLARWPGL
>Pelodiscus_sinensis
MGRDAVIQRRMICHKG--------

HG0206.1 ENSGALG00000002069

>Gallus_gallus
MVGAIPRQPRRFWGVPGWQVSSASCANDRPLWDGEIDPEWSGILLQREPHTGGLTWTS
>Anser_cygnoides
MVGAIPRQPRRFWGVPGWQVSSASCANDSPRRDGEVDPEHGGVLLQRDAALQHIP---
>Calidris_pugnax
MVGAIPRQPRRFWGVPRWQVSSANCANDRPLRDKEVNPEQSRVLLQRDAALQCTP---
>Columba_livia
MVGAIPRQPRRFWGVPDWQVSSASCANDRPLWDRETDPEQSRVLLQRDDALQSTL---
>Alligator_mississippiensis
MVGAIPRQPRRFWGVPGWQVSPASCANDRVLRGHQGYLARRGGRLRSDAVLQRAP---
>Falco_cherrug
MVGAIPRQPRRFWGVPGWQVSSASCANDRLLRDREVNPEQSGVLLQRDAVLHRTL---
>Manacus_vitellinus
MVGAIPRQPRRFWGVPDWQVSSAGCANDRLLWDREVNPEISRVLLQRDAVLQTTS---
>Pygoscelis_adeliae
MVGAIPRQPRRFWGVPGWQVSSASCANGRPLRDPEVNPEESGVLLQRDAALQCTP---
>Boiga_irregularis
MVGAIPRQPRRFWGVPGWQVSPASFTSHRRLLQQEKTPARGRDPVQWDVAVQPTS---
>Struthio_camelus_australis
MVGAIPRQPRRFWGVPGWQVSSASCANDYPLQDGEPNLEPCGVLLQRDAALQRTP---
>Chrysemys_picta
MVGAIPRQPRRFWGVPGWQVSPASCANDRLLLGQESCSARGG----------------

HG0206.2 ENSG00000005238 FAM214B

```
>Homo_sapiens
--------------------------------MGLVLLSSGAARNGGSHLASAAGMDQTAS
>Canis_lupus_familiaris
-------------------------------------------------------MDQTAS
>Lipotes_vexillifer
-------------------------------------------------------MDQTAS
>Myotis_davidii
-------------------------------------------------------MDQTTS
>Sarcophilus_harrisii
-------------------------------------------------------MGQTTS
>Galeopterus_variegatus
-------------------------------------------------------MDQTAS
>Monodelphis_domestica
MEPPAREGED-------TFSTKPYSPRGVLSSHMESHLSQGAARDGGSDRASGVGMGQTTS
>Condylura_cristata
-------------------------------------------------------MDQTAS
>Ochotona_princeps
-------------------------------------------------------MDQTAS
>Elephantulus_edwardii
-------------------------------------------------------MDQTAN
>Ceratotherium_simum_simum
MEGVVWGGRGGRGGRWGCWGAGRGGRPHQLVGNGLVQLSPGATRNGGSHLASAAGMDQTAS
>Manis_javanica
-------------------------------------------------------MDQTAS
>Marmota_marmota_marmota
-------------------------------------------------------MDQTAS
>Tupaia_chinensis
-------------------------------------------------------MDQTAS
```

HG0207.1 ENSGALG00000007121 TNFSF8

>Gallus_gallus
-----------------------------------MSQDDGENPARNRDGLRPEPDQGEIQTSTLTAI
>Anser_cygnoides
--------------------------------------MKGENPARNRDGLQPEPDQGEIQSSTLRAI
>Canis_lupus_familiaris
--------------------------------------MQGENPSWNRDGLRTESDEER---------
>Pteropus_vampyrus
MKNEPLPHPCCCLSPHAPREEFLRKAAREGFFPSHMIQDAGENPSWNRDGPRTESDEER---------
>Sarcophilus_harrisii
--------------------------------------MEGENPTWNRDGL-----------------
>Monodelphis_domestica
--------------------------------------MKGENPAWNRDGL-----------------
>Trichosurus_vulpecula
--------------------------------------MKGENPAWNRDGL-----------------
>Parus_major
--------------------------------------MKGENPARSRDGLQLEPDRGEPQSSAWRAI
>Ceratotherium_simum_simum
-----------------------------------MIQDAGENPSWNRDGPRTESDEER---------
>Pan_paniscus
MKNEPLPHPCCRLSPHAPREEFLRQAAGEGFFPSHMIQDSGENPSWNRDGPRTESDEER---------
>Chrysemys_picta
--------------------------------------MKGENPARSGDGLRMRI-------------

HG0207.2 ENSG00000106952 TNFSF8

```
>Homo_sapiens
----------MGPELNQMKRDKV------
>Ailuropoda_melanoleuca
----------MGPEVNQMRRDKVCCGKAI
>Physeter_catodon
----------MGPEPNQMRRDKV------
>Rousettus_aegyptiacus
MQGRILLGTEMGPEPNQMRRDKV------
>Dasypus_novemcinctus
MQGENPSWNRDGPEPTQMRRDKV------
>Galeopterus_variegatus
----------MGPELNQMKTDKV------
>Sorex_araneus
----------MGPEPNQMRRDKV------
>Oryctolagus_cuniculus
----------MSPEPSQMKRDKV------
>Ceratotherium_simum_simum
MQGRILLGTEMGPEPNQMRRDKV------
>Manis_javanica
----------MGPEPNQMRRDKV------
>Loxodonta_africana
----------MGPEPTQMRRDKV------
>Marmota_marmota_marmota
----------MGPEPDQMRRDKV------
```

HG0208 ENSGALG00000013272 PRMT8

>Gallus_gallus
MPSWSLHAQVQFSWCFSPV
>Anser_cygnoides
MPSWSLHAQVRFSWCFSPV
>Chaetura_pelagica
MPSWSLHAQVQFSWCFSPI
>Charadrius_vociferus
MPGWSLHAQVQFSWCFSPV
>Aquila_chrysaetos_canadensis
MPSWSLHAQVQFSWCFSPV
>Mesitornis_unicolor
MPSWSLHAQVQFSWCSSPV
>Zonotrichia_albicollis
MPSWSLHAQVQFSWCFSPV
>Nipponia_nippon
MPSWSLHAQVQISWCFSPV
>Struthio_camelus_australis
MPSWSLYAQVQFSSCFSPI
>Chrysemys_picta
MPRWSLHAQVQFSWGFFAF
>Calypte_anna
MPSWSLYAQVQFSWCFSPV

HG0209 ENSG00000101040 ZMYND8

```
>Homo_sapiens
MHPQSPKSASLFSLPNCGKMRRLLEHLLAWLKRK-----------
>Canis_lupus_familiaris
-------------------MRRLLEHLLAWPKRK-----------
>Rousettus_aegyptiacus
-------------------MRRLLEHVG-----------------
>Dasypus_novemcinctus
MHPQSPKSASLFSLPNCGKMRRLLEHLLAWLKRK-----------
>Oryctolagus_cuniculus
-------------------MRRLLEHLLAWLKRK-----------
>Elephantulus_edwardii
MHPQSPHSASLFSLPNSGKMRRLLEHLVAWLKRK-----------
>Ceratotherium_simum_simum
-------------------MRRLLEHLLAWLKRK-----------
>Loxodonta_africana
-------------------MRRLLEHLLAWLKRK-----------
>Chinchilla_lanigera
MHPQSPKSASLFSLPNCGKMRRLLEHWLAWLKRK-----------
>Trichechus_manatus_latirostr
MHPQSPKSASLFSLPNCGKMRRLLEHLLAAFLPKVFSFSRLLGAK
>Chrysemys_picta
-------------------MRWLLEQLLG----------------
```

HG0210 ENSG00000155849 ELMO1

>Homo_sapiens
--------------MPCRCKMCLWPAGGRKRK
>Felis_catus
--------------MPCRCKMCLWPAGGRKRK
>Balaenoptera_acutorostrata_s
--------------MPCRCKMCLWPAGGRKRK
>Rhinolophus_ferrumequinum
--------------MPRSCKTCLWPAGGRKRK
>Dasypus_novemcinctus
--------------MPCRCKMCLWPAGGRKRK
>Condylura_cristata
--------------------MCLWPAGGRKRK
>Oryctolagus_cuniculus
--------------MPCRCKMCLWPAGGRKRK
>Equus_asinus
--------------MPCRCKMCLWPAGGRKRK
>Manis_javanica
--------------------MCLWPAGGRKRK
>Nannospalax_galili
--------------MPCRCKMSLRPAGGRKRK
>Anolis_carolinensis
MAEARLLSSVPRREPPCRCKSRLQPAGGRKRK

HG0211 ENSG00000176204 LRRTM4

>Homo_sapiens
MDKPSLHSF
>Mustela_putorius_furo
MDKPSLHSF
>Rousettus_aegyptiacus
MDKPSLHSF
>Condylura_cristata
MDKPSLHSF
>Oryctolagus_cuniculus
MDKPSLHSF
>Lepidothrix_coronata
MDKPSLHSF
>Manis_javanica
MDKPSLHSF
>Loxodonta_africana
MDKPSLHSF
>Heterocephalus_glaber
MDKPSLHSF
>Tupaia_chinensis
MDKPSLHSF
>Trichechus_manatus_latirostr
MDKPSLHSF

HG0212 ENSDARG00000100244 ebf3a

>Danio_rerio
MLPSILQRRAKGPSSSSPVSGNTSTVIIYAFICLLEPEVVDSAVSRSLF----------
>Hynobius_retardatus
------------------------MVIIFSFICLLEQESLARSARFRSVLKLF------
>Alosa_alosa
MLPSILQ-DAKGPSSTSPVSGNTSMVIIFSFICLLEPERLARSGRFSSVLKLF------
>Latimeria_chalumnae
------------------------MVIIFSFICLLEQESLARSGRFSSVLKLF------
>Esox_lucius
MLLSILQ-DAKGPSSTSPVSGNTSMVIIFFVYLSCGAGAPCQKWSIQQCPEAFLML---
>Apteronotus_leptorhynchus
MLPSILQ-DAKGPSSTSPVSGNTSMVIIFSFICLLEPERLARSGRFSSVLKLF------
>Lepisosteus_oculatus
MLPSILQ-DAKGPSSTSPVSGNTSMVIIFSFICLLEQERLARSGRFSSVLKVFDAVVVG
>Anolis_carolinensis
------------------------MVIIFSFICLLEQSRPALPEVVDSAVS--------
>Pelodiscus_sinensis
------------------------MVIIFSFICLLEQESLARSGRFSSVLKLF------
>Tetronarce_californica
------------------------MVIIFRLFVFWSRRTLSE--VVDSAVS--------

HG0213.1 ENSGALG00000001946 RSPO1

>Gallus_gallus
MGLDPSSCAPYGDEGRTMLPAT
>Anser_cygnoides
MGLDPSSCAPYGDEGRTMLPAT
>Xenopus_laevis
MGLDPNSCGPYKDESRTMLPVT
>Apteryx_australis_mantelli
MGLDPRSCAPYGVDGRIMLPAT
>Hynobius_retardatus
MGLHLSTFDPYGDENRAMLPAT
>Calidris_pugnax
MGLEPSPCAPYGDEGRAMLPAT
>Lepidothrix_coronata
MGPRPSSCAPHRDEGRAMLPAT
>Struthio_camelus_australis
MGLDPHSCAPYGVDGRIMLPAT
>Pelodiscus_sinensis
MGLDPSSSAPYGVEGRLMLPAT

HG0213.2 ENSGALG00000001946 RSPO1

>Gallus_gallus
--------------------------MGMESTALRPGFINVQIALI
>Anser_cygnoides
--------------------------MGMESTALRPGFINVQIALI
>Calidris_pugnax
--------------------------MGMESTALRPGFINVQIALI
>Lepidothrix_coronata
MQEFALDAFTKLDTLVIIIIKSPDPVDGDGVYGLRPGFINVQIALI
>Struthio_camelus_australis
--------------------------MGMESTALKPGFINVQIALI
>Chelonia_mydas
--------------------------MGMESAALWAGVINVPIALI

```
HGO214.1 ENSGALG00000012917 CDH6

>Gallus_gallus
METNQIESPLGSGCLHAGVKKCCRSSGSPSERRGG
>Anas_platyrhynchos
METNQIESPFGFSSLAA------------------
>Ambystoma_mexicanum
METNQIESPLGFACLPASVKSDVGRASAS------
>Calidris_pugnax
METNQTESPFGFSSLPA------------------
>Alligator_sinensis
METNQIESPLGFSSLHARVKSDAGREGAPLRCQ--
>Monodelphis_domestica
METNQIESPFGFSFPACLS----------------
>Acanthis_flammea
METNQTESPFGSCSLPA------------------
>Struthio_camelus_australis
METNQIESPFGCSSLPA------------------
>Chrysemys_picta
METNQIESPLGFGSLHARVKSDAGRERAPLRCE--
```

HG0214.2 ENSG00000040731 CDH10

>Homo_sapiens
---MNKTLMKCWKPVETQ--
>Mustela_putorius_furo
---MSKTLMRCWKPVETQ--
>Miniopterus_natalensis
MLRESWSCIEVEAGGENNAGNTQKRDVVTLSPGLSGLAPTSFIPRLRKKAFGNMSKTLMKCWKPVETQ--
>Galeopterus_variegatus
---MYKTLMRCWKPVEMQ--
>Monodelphis_domestica
---------------------------------------MLLPSPEGESKIGNMNKILINCWKPIGYPTY
>Oryctolagus_cuniculus
---MNKPSMKCWKPVETQ--
>Equus_caballus
---MSEALMKCWKPVETQ--
>Cavia_porcellus
---MNKALMKCWKPAETQ--
>Tupaia_chinensis
---MYKTLRNCWKPVETQ--

HG0215 ENSDARG00000079280 dhrsx

>Danio_rerio
MQSLISPVTKAILVALFIFAILLILYVILWYICRDVDCDHGI
>Channa_argus
MQSLISPVTKAILVALFIFAILLILYVILWYICRDVDCDHGI
>Pygocentrus_nattereri
MQSLISPVTKAVLVALFIFAILLILYVILWYICRDVDCDHGI
>Maylandia_zebra
MQSFISPVTKAILVALFIFAILLILYVILWYICRDVDCDHGI
>Poecilia_latipinna
MQSLISPVTKAILVALFIFAILLILYVILWYICRDVDCDHGI
>Esox_lucius
MQSFLSPFNKAILVALFIFAILLILYVILWYICRDVDCDHGI
>Platichthys_stellatus
MQSIISPVTKAILVALFIFAILLILYVILWYICRDVDCDHGI

HG0215 ENSGALG00000016681 DHRSX

>Gallus_gallus
----MQSLISPVTKAILVALFIFAILLILYVILWYICRDVDCDHGI
>Astyanax_mexicanus
----MQSLISPVTKAVLVALFIFAILLILYVILWYICRDVDCDHGI
>Maylandia_zebra
----MQSFISPVTKAILVALFIFAILLILYVILWYICRDVDCDHGI
>Sinocyclocheilus_rhinocerous
----MQSLISPVTKAILVALFIFAILLILYVILWYICRDVDCDHGI
>Austrofundulus_limnaeus
----MQSLISPVTKAILVALFIFAILLILYVILWYICRDVDCDHGI
>Esox_lucius
MSSVMQSFISPVTKAILVALFIFAILLILYVILWYICRDVDCDHGI
>Zonotrichia_albicollis
----MQSLISPVTKAILVALFIFAILLILYVILWYICRDVDCDHGI
>Platichthys_stellatus
----MQSIISPVTKAILVALFIFAILLILYVILWYICRDVDCDHGI
>Salmo_salar
MSSVMQSFLSPVNKAILVALFIFAILLILYVILWYICRDVDCDHGI

HG0216.1 ENSGALG00000020922

```
>Gallus_gallus
-------------------------------------MICGSVQLELSE
>Anas_platyrhynchos
-------------------------------------MICGSVQLELSE
>Calidris_pugnax
-------------------------------------MICGWVQLELSE
>Columba_livia
-------------------------------------MICGWVQLELSE
>Alligator_mississippiensis
-------------------------------------MICGSVQLELTE
>Zonotrichia_albicollis
MPGAEILGWFCLTKVEGTREPRREVDSSCQDERASLEMICGSVQLELSE
>Aptenodytes_forsteri
-------------------------------------MICGSVQLELSE
>Takydromus_sexlineatus
-------------------------------------MVCGSVKLELTK
>Chrysemys_picta
-------------------------------------MICGSVQLELTE
```

HG0216.2 ENSG00000116667 C1orf21

>Homo_sapiens
------------------------------MICSSARLKLTE
>Canis_lupus_familiaris
------------------------------MICSSARLKLTE
>Orcinus_orca
------------------------------MLCSSARLKLTE
>Eptesicus_fuscus
------------------------------MICSSARLKLSE
>Dasypus_novemcinctus
------------------------------MICCSARLKLTE
>Sarcophilus_harrisii
MSFYLMFSLILVDFFFLFFKKQKYVPVSVEMICSSARLELTE
>Monodelphis_domestica
------------------------------MICSSARLELTE
>Macropus_eugenii
------------------------------MICSSPRLELTE
>Condylura_cristata
------------------------------MICSSARLKLTE
>Elephantulus_edwardii
------------------------------MICSSARLKLTE
>Ceratotherium_simum_simum
------------------------------MICSSARLKLTE
>Manis_javanica
------------------------------MICSSARLKLTE
>Loxodonta_africana
------------------------------MICSSARLKLTE
>Ictidomys_tridecemlineatus
------------------------------MICSSARLKLTE
>Tupaia_chinensis
------------------------------MICSSARLKLTE
>Trichechus_manatus_latirostr
------------------------------MICSSARLKLTE

HG0217.1 ENSG00000150394 CDH8

>Homo_sapiens
---MNFQVVDLPLRSAQ
>Odobenus_rosmarus_divergens
---MNFQVVDLPLRSAQ
>Orcinus_orca
---MNFQVVDLPLRSAQ
>Galeopterus_variegatus
MFLSSASALMLELGPPRAARTARKGASSPGGSDGGGALGVLGSEERKVVDPLGRGKGSHRASATAKGEGGRGRGGGTAYMNFQVIDLPLRSAQ
>Gallus_gallus
---MNFQALDLPPRSAQ
>Loxodonta_africana
---MNFQVVDLPLRSAQ
>Ctenomys_sociabilis
---MNFQVVDLPLRSAQ
>Trichechus_manatus_latirostr
---MNFQVVDLPLRSAQ
>Orycteropus_afer_afer
---MNFQVVDLPLRSAQ

HG0217.2 ENSGALG00000005319 CDH8

>Gallus_gallus
MRRKSGESCLFTGTGEWLFYLTLSIRV
>Chaetura_pelagica
MKRKSGGSCLFTGTG------------
>Apteryx_australis_mantelli
MKRKSGESCLFTGTG------------
>Buceros_rhinoceros_silvestri
MKRKSGKACLFTGTG------------
>Calidris_pugnax
MRRKSGESCLFTGTG------------
>Pterocles_gutturalis
MTRRSGESCLFAGTG------------
>Columba_livia
MKRKSAKSCLFPGTG------------
>Falco_cherrug
MKRKSGESCLFTGTG------------
>Gavia_stellata
MKRQSGESCLFTGTG------------
>Chlamydotis_macqueenii
MKRKSGESCLFAGTG------------
>Sturnus_vulgaris
MRRKSGAPCLFPATG------------
>Egretta_garzetta
MKRKSGESCLFTGTG------------
>Picoides_pubescens
MKRKSGKSCLFTGTG------------
>Nestor_notabilis
MKRKPGESCVFTGTG------------
>Pygoscelis_adeliae
MKRKSGESCLFAGSG------------
>Struthio_camelus_australis
MKRKSGESCLFTGTG------------
>Tinamus_guttatus
MKRKAGESCLFTGTG------------
>Calypte_anna
MKRKSGESCLFRGTG------------

HG0218 ENSDARG00000100149 axin2

```
>Danio_rerio
--------------------------------------MDERSSPADLRERGDYS--
>Channa_argus
---------------------------------MRGDGQPKRSSPADIRE-------
>Xenopus_laevis
MQL-------QKWGALAESSGCTEW-----STLHTHEGRGRRGSPADLRGRADYRGI
>Haplochromis_burtoni
---------------------------------MREAGQPERSPPADIRE-------
>Cyprinodon_variegatus
---------------------------------MREDGQPERSPPADIRE-------
>Gadus_morhua
MRIRALDTPTVDYGMFLCGYQTGVFFEELTHGSMRENGQTERSSPADIRE-------
>Lutjanus_erythropterus
---------------------------------MREDGQPERSSPADIRE-------
>Notothenia_coriiceps
---------------------------------MREDGQPERSSPADIRE-------
>Cynoglossus_semilaevis
---------------------------------MGEDGQPERSSPADI---------
```

HG0219 ENSDARG00000104082 trps1

```
>Danio_rerio
MAHAKAPPSFRLQRLLQFN-QQ----------------------
>Pygocentrus_nattereri
---------------MQFN-QQVLL-------------------
>Clupea_harengus
MAYAKAPPSFRLQRLLQFN-QQ----------------------
>Sarcophilus_harrisii
MALATAPPSFGLLRLLLPINQQIYRSALKMSSVSSVHLLRQLH-
>Monodelphis_domestica
MALATAPPSFGLLRLLLPINQQIYRSALKMSSVSSVHLLRQLH-
>Esox_lucius
MAYAKAPPSFRLQRLLQFN-QQRAVAGR--KTS-----------
>Apteronotus_leptorhynchus
MVHAKAPPSFRLQRQLQFN-QQPGLAV-----------------
>Salmo_salar
MAYAKAPPSFRLQRLLQFN-QQREKHCD--SCCGGTQDCYYL--
>Trichomycterus_areolatus
---------------MLFN-QQAGLSSDCAKQCDGPWTLIQNHR
```

HG0220 ENSGALG00000008418 LARGE2

>Gallus_gallus
-------------MLGPAGLGLPT
>Anser_cygnoides
-------------MLGPAGLGLPT
>Calidris_pugnax
-------------MLGPVGLGLPT
>Aquila_chrysaetos_canadensis
MPSGGVAPVPGSRMLGPAGLGLPT
>Acanthis_flammea
-------------MPGPAGLGLPT
>Aptenodytes_forsteri
-------------MLGPAGLGLPT
>Struthio_camelus_australis
-------------MLGPAGLGLPT
>Pelodiscus_sinensis
-------------MMGPAGLDLPT
>Tinamus_guttatus
-------------MLGPAGLGLPT

HGO221 ENSGALG00000010859 EPGN

>Gallus_gallus
--MPTGNRKLGFQEKTRSQC
>Canis_lupus_familiaris
MFDSYPGMLLPGLQISELSFVGLLGRWLATLELVQVCPGNGAFPEFRFFFVVLSTFTYTIMPTGNRKPVFQKKT----
>Calidris_pugnax
--MPTGNRKPGFQKKI----
>Galeopterus_variegatus
--MIMPTGNRKPVFPKKT----
>Erinaceus_europaeus
--MIMPTGNRKPGFQKKA----
>Oryctolagus_cuniculus
--MPTGNRKPVFQKKT----
>Lepidothrix_coronata
--MPTGNRKPGFQKKT----
>Homo_sapiens
--MIMPTGNRKPVFQKKT----
>Struthio_camelus_australis
--MPTGNRKLEFQKKS----

HG0222 ENSGALG00000011564 RUFY3

>Gallus_gallus
MQKPGFLWLLRINSL---
>Anser_cygnoides
MRKSGFLWLLRINSLQHI
>Alligator_sinensis
MQRARFLWMLRIHCL---
>Aquila_chrysaetos_canadensis
MQKPGFLWLL--------
>Corvus_brachyrhynchos
--------MLRISSL---
>Pygoscelis_adeliae
MQKPGFLWSLRINSL---
>Struthio_camelus_australis
MQKPGFL-----------
>Pelodiscus_sinensis
MQWPGFL-----------
>Calypte_anna
MQKPGFLWWLRIGSLQHS

HG0223 ENSG00000091656 ZFHX4

>Homo_sapiens
--MDGRVRERLRLPLPFRVLLRLRLRLGCDWDATVTRASIASIESNAKTQPGSLTGWMK
>Anas_platyrhynchos
--MDGRGSEKPL--QPCLLLVRLRQRLGCDWDATVTWASIASIESNAKTQPGSLTGWMK
>Monodelphis_domestica
--MDGRGRERLQMPLPFRLLVRLRQRLGCDWDTTVTWASIASIESNAKTQPGSLTGWMK
>Coturnix_japonica
MVYFFFFFFFQLCFSWIFSGSILGSGAGVFKKIICSIIFLVWWWMDGRGSEKPL--QPCLLLVRLRQRLGCDWDATVTWASIASIESNAKTQPGSLTGWMK
>Oryctolagus_cuniculus
--MDGRVRERLRLPLPFRVLLRLRLRLGCDWDATVTRASIASIESNAKTQPGSLTGWMK
>Ficedula_albicollis
--MDGRGSEKPL--QPCLLLVRLRQRLGCDWDATVTWASIASIESNAKTQPGSLTGWMK
>Ceratotherium_simum_simum
--MDGRVRERLRLPLPFLVLLRLRLRLGCDWDTTVTRASIASIESNAKTQPGSLTGWMK
>Rattus_norvegicus
---MDGVRERLRLPLPFRLVLRLRLRLGCDWDATVTQASIASIESNAKTQPGSLTGWMK
>Tinamus_guttatus
--MDGRGSEKPL--QPCLLLVRLRQRLGCDWDATVTWASIASIESNAKTQPGSLTGWMK

HG0224 ENSG00000118007 STAG1

>Homo_sapiens
MPPDGGLGLQLR
>Ailuropoda_melanoleuca
MPPDGGLGLQLR
>Lipotes_vexillifer
MPPDGGLGLQLR
>Rousettus_aegyptiacus
MPPDGGLGLQLR
>Gallus_gallus
MPPDGGLGLRLR
>Condylura_cristata
MPPDGGLGLQLR
>Ochotona_princeps
MPPDGGLGLQLR
>Equus_asinus
MPPDGGLGLQLR
>Mus_musculus
MPPDGGLGLQLR

HG0225 ENSG00000150051 MKX

```
>Homo_sapiens
MFAGWDRQSGNMRRPRYSCSRAARAGLGK-----------
>Amia_calva
MFAGRAA---------------------------------
>Mustela_putorius_furo
MFAGWAGQSRNMRRPRYSCSRAARARLGK-----------
>Pteropus_alecto
MFAGWARQSGNMRRPRYSCSRAARARLGK-----------
>Oreochromis_niloticus
MFAVSGCVLGNMSRVRYSESRAARRRPLDKAEEETAVRAV
>Poecilia_latipinna
MFAGSASVQGNMSRRRYSESRAAG----------------
>Manis_javanica
MFAGWAGQSGNMRRPQHSCSRAARAPPGK-----------
>Ictidomys_tridecemlineatus
MFAGWARQSGNMRRPRYSCSRAARARLGK-----------
>Salmo_salar
MFAGWGSVLGNMRRVTYSVSRAENRRPLNKAERRPRFVWF
```

HG0226.1 ENSG00000180592

```
>Homo_sapiens
MERFIYRLYYLLWEGRWQYKGMLISGRFWGEGVEY-QFLLHHLSGVSNQRWL--------------------
>Mustela_putorius_furo
MERFIYRLYYLLWEGRWQYKGMLISGRFWGEGVEY-QFLLHHLSGVSNQRWL--------------------
>Miniopterus_natalensis
MERFIYRLYYLLWEGRWQYKGMLISGRFWGEGVEY-QFLLHHLSGVSNQRWL--------------------
>Coturnix_japonica
---------------------MLISGRFLGGRGGISIFIASPVRSVQSALALGELIDEGVSGRAHFTLSFYL
>Ceratotherium_simum_simum
MERFIYRLYYLLWEGRWQYKGMLISGRFWGEGVEY-QFLLHHLSGVSNQRWL--------------------
>Manis_javanica
MERFIYRLYYLLWEGRWQYKGMLISGRFWGEGVEY-QFLLHHLSGVSNQRWL--------------------
>Cavia_porcellus
MERFIYRLYYLLWEGRWQYKGMLISGRFWGEGVEY-QFLLHHLSGVSNQRWL--------------------
>Salmo_salar
--------------------MYLTNGGGGAQGVEY-PFLLHHLSVASNRIRV--------------------
```

HG0226.2 ENSG00000180592

>Homo_sapiens
MAEQPIESADMVNKF-IKETPWL-------------LKNSTLYIWICTSGKAQKYPFILLITRMCLFLEEQGNT
>Mustela_putorius_furo
---MCLFLEEQGNI
>Manis_javanica
---MCLFLEEQGNM
>Chinchilla_lanigera
----------MSESFFFGRGEYLLVRPEQGKVELLNLKKEIIVHFFFL--EGSEISVYSLTTRMCLFLEEEGNI

HG0226.3 ENSG00000180592

```
>Homo_sapiens
----------------------------------MRLGGPLLSFGNILDILENDTKICLNLTKKRLNHIDFHPK
>Mustela_putorius_furo
MPGLHLCCSCKHRTVKKILPNFAKEPTSHLGILIMQLGRPLLSFGNILDILGNDTKICFNLTKKRLNNIDIHPK
>Chinchilla_lanigera
MPGLHLCCSCKHRTVKKVLPNSAKQPTKRLGTLVIQVGRLLLSFGNILDSLENDSKLCFNLTKKRLNHTDIHPK
```

HG0227.1 ENSGALG00000031929

```
>Gallus_gallus
-MTALNNWWHFGGYGWSSVY-
>Anser_cygnoides
-MTALNNWWHFGGYHWIPVY-
>Ambystoma_mexicanum
MMTTFNNSWQFGGFHWIRAY-
>Calidris_pugnax
-MTALNNWWQFGGFGWISVYS
>Aquila_chrysaetos_canadensis
-MTALNNRWRFGGFRWISVC-
>Lepidothrix_coronata
-MTALNNWWQFGGFRWISV--
>Struthio_camelus_australis
-MTALNNWWHFGRFHWIPVY-
>Chrysemys_picta
MMTAFNNWWQFGGFHWISVC-
```

HG0227.2 ENSG00000275163

>Homo_sapiens
--------------MGKVSEEE-PFLP--FVAISTGLQGTSFLLGFYVCFTHCLSFLCLRHFHE----
>Anser_cygnoides
--------------MEKVSNWECSLLPCLSVAISAGLLGVAFLLGFCVCFTYCFPLPFGQEPFYDQAR
>Coturnix_japonica
MRSLQQNCFVTDVSMEKVSNWECSLFPCLSVAISAGL-------------------------------
>Lepidothrix_coronata
--------------MEKVSNWECSLLPSLAVAISAGLQGVAFLLGFCVCFTYCFPLPFGQELFYDLAK

HG0228 ENSDARG00000087887 phf21b

>Danio_rerio
MSLTASKEARTCCAARVV-LAIVIVAFQNTSLTPQITRIASEVEEKCS
>Astyanax_mexicanus
MSLVASKEARTCSAA---------------------------------
>Alosa_alosa
MSPVPSKEARTCFAA---------------------------------
>Latimeria_chalumnae
MSLVPSKEARTCCAAGVVVLSIVIVGFF--------------------
>Apteronotus_leptorhynchus
MSLVPSKEARTCFAA---------------------------------
>Scleropages_formosus
MSPIGSKETRTCFGD---------------------------------
>Salmo_salar
MSPLHSKEARTCFAD---------------------------------
>Lepisosteus_oculatus
MSPIPSKEARTCFAD---------------------------------

HG0229 ENSGALG00000005474

>Gallus_gallus
MCIHFWWTLPFLGV-------------
>Apteryx_australis_mantelli
MCMHFGWTLPFLAV-------------
>Calidris_pugnax
MCIHFGWTLPLLCI-------------
>Haliaeetus_leucocephalus
MCIHFEWTLPFLGI-------------
>Lepidothrix_coronata
MCIRFGWTLPFLGI-------------
>Phalacrocorax_carbo
MCIHFGWTLPLLLI-------------
>Pygoscelis_adeliae
MCIHFGWTLPLLCI-------------
>Pelodiscus_sinensis
MCAHFRWTLPCLGFSDLVLNSPEADIY

HG0230 ENSGALG00000011007 OSBPL3

>Gallus_gallus
--MQIMLLECIWVRDEFCITLRSLPIHLLDYLLMTF
>Anser_cygnoides
--MQIMPLECI-------------------------
>Calidris_pugnax
MVAVDLPFLPSSSCWLWGAAAGGHGGGARWAVARPAAGERLRNVHGIQIVPLDCIWVKDESRVSLRSLPTHFLNYLLMVF
>Aquila_chrysaetos_canadensis
--MQIMPLDCIWIKDEFCISFRSLPTHFLNCFLMVF
>Sturnus_vulgaris
-----------------------MGEECSIAVKRQQFSPPLGSFHGIQIMPLDCIWVKDEFSISLRSLPTHFPDYLLMVF
>Pygoscelis_adeliae
--MQIMPLDCIWVKDEFCVSLRSLPTHFLNYLFMVF
>Anolis_carolinensis
---MLLECIWEHNDFCLPDPRLPVPLMVLQSD--
>Chrysemys_picta
---MPLECIWVNNDSCLSRPCLPIHFLNSLWMVF

HG0231 ENSG00000150636

>Homo_sapiens
MQEILLFTHVAATETL-EQPSKPRGS-----
>Anas_platyrhynchos
MQEILLFTRLCNTEKQ-EEPRKPRG------
>Canis_lupus_familiaris
MQEILLFTHVSVTLSR---------------
>Dasypus_novemcinctus
MQEILLFTHVSVTLRR---------------
>Monodelphis_domestica
MQEIQLFTHVSVTLRSKSSPASPEGALGRCL
>Pseudopodoces_humilis
MQEILLFTHVSVTLRSKRSPAN---------
>Struthio_camelus_australis
MQEILLFTHVFVTLRSKSSPASPEGVKLD--
>Pelodiscus_sinensis
MQE-ILFTHVSAALSSKNSPASPKGVKSH--

HG0232 ENSG00000180875 GREM2

>Homo_sapiens
-------------------------------MGVCCSLAAVELKAAAGDQT
>Alligator_mississippiensis
-------------------------------MGICSSLAKEELKAAAGYI-
>Monodelphis_domestica
-------------------------------MGICCSPAKAELNPAEGDQT
>Oryctolagus_cuniculus
-------------------------------MGVCWSPPAVELKAAAGDQT
>Equus_caballus
MQRREESELSPGLLPHLQGCQPGLHTRPALLMGVCWRPAAVQLSAAAGDQT
>Ictidomys_tridecemlineatus
-------------------------------MGFCWSLAAVELKAAAGDQT
>Tupaia_chinensis
-------------------------------MGIGWSPAAIELQAAAGDQT
>Pelodiscus_sinensis
-------------------------------MGICSNLVEAELKAAVGDHT

HG0233 ENSG00000206432 TMEM200C

```
>Homo_sapiens
MSGGEEGSEAGCGMRDAEDLTLLGNSWKWLPPQRRRGLR-AWGGRSPPRGC------------------------
>Mustela_putorius_furo
-------------MRAAEDLTLLGNSWKWLPPQPRRGLR-TWGGRSPPLGC------------------------
>Balaenoptera_acutorostrata_s
-------------MQDAEDLTLLGNSWKWLPPRHRRGLR-IPGGRSPPLGC------------------------
>Rousettus_aegyptiacus
-------------MQDAEDLTLLGNSWKWLPPQHRRGLR-AWGGRSPPRGC------------------------
>Monodelphis_domestica
-------------MKDGEGITLLGNSSPWLLPQAWRRGSENITGATSPAGSPGILSRGEMRDAGRLHLRATVHSG
>Oryctolagus_cuniculus
-------------MQDAADLTLLGNSWKWLPPLRRRGLR-ARGGRSPPRGC------------------------
>Equus_asinus
-------------MQDAEDLTLLGNSRKWLPPQHQRGLR-TRGGRSPPRGC------------------------
>Chrysemys_picta
-------------MKDGEGTTLLGNSLEWLCPGAPRGLTS--SGRSLPLSLLGL---------------------
```

HG0234.1 ENSGALG00000011250 ASIC4

>Gallus_gallus
MLTCLELLSIPWILLAHDA
>Calidris_pugnax
MLTCLELLSIPWILLAHDA
>Columba_livia
MLTCLELLSIPWILLAHDA
>Aquila_chrysaetos_canadensis
MLTCLELLSIPWILLAHDA
>Lepidothrix_coronata
MLTCLELLSIPWILLAHDA
>Thamnophis_sirtalis
MLTCLELLSIPWILLAHDA
>Tinamus_guttatus
MLTCLELLSIPWILLAHDA

```
HG0234.2 ENSG00000108684 ASIC2

>Homo_sapiens
---MVNMGHYALVNRLRAAQRLVLSIFSLALA-FNLVSSGVCRGEDFISIG
>Mustela_putorius_furo
MPGMLHAGHYARVNRLRAAERLV----------------------------
>Monodelphis_domestica
---MLNTGHYALVNRLGAAERLVLSILSCLLLILSLVVCVCVQGKDFISIG
>Condylura_cristata
---MLNTGHYALVNRLRAAERLVLSIFSLALA-FNLVSSGVCRGEDFISIG
>Rattus_norvegicus
---MLNTGHYALVNRLRAAQRLVLSIFSLALA-FNLVSSGVCRGEDFISIG
```

HG0235.1 ENSGALG00000035927

```
>Gallus_gallus
MLATSESLGNFTVGALRAVLEL-----TAKEARRGQTMGCPVLGLLHEQTPRAPLGCPAKRA
>Anser_cygnoides
MFPTSESPGNFTVEASRCAQSWSSHPKGARKGQQGRTMGYVVLGLLREKPANVPLGFPAKRA
>Calidris_pugnax
-------------------------------------MGYLVLGLLRDKPASVPLRFPAKRA
>Aquila_chrysaetos_canadensis
MLPTSESPGNFTMEASNCAQSWS---------------------------------------
>Sturnus_vulgaris
MLPTSESPGNFTMEASHCSQSWS---------------------------------------
>Struthio_camelus_australis
MLPTSESPGNFKLEVSHCAQSWS---------------------------------------
>Pelodiscus_sinensis
MFPTSDAPGNFTVETSDCAQFFS---------------------------------------
```

HG0235.2 ENSG00000101638 ST8SIA5

>Homo_sapiens
MPPAPAAPTRNFAPRSPPPA--------------------
>Mustela_putorius_furo
MPPAPSPPGGNFAPPSPPPG--------------------
>Myotis_lucifugus
---MPATPAGNFAPRSPPPPDCLVGLVPP-PPPPPTGPRA
>Ceratotherium_simum_simum
MLPAPAGPATNFARRSPPPD--------------------
>Ictidomys_tridecemlineatus
MPPARAAPAGNFALRSPPPAGPRCETPVRPRAAGVSEPRA

HG0236 ENSGALG00000004437 LHFPL2

```
>Gallus_gallus
MVAAGAGQGEPGGARAGGGGGSGAEPQRTGGAGERALLAGGIARLLKWLTKAKNRGHWCVMSSSGQSPRPSPPVRSVMCQYEQ
>Anas_platyrhynchos
------------------------------------------------------------MSNSGQSLQPALPARSVMCQYEQ
>Calidris_pugnax
------------------------------------------------------------MSNSGQSLRPPPPACSVMCQYEY
>Alligator_mississippiensis
------------------------------------------------------------MSRSGQSLGPPPHYCNVMCQYEY
>Aquila_chrysaetos_canadensis
------------------------------------------------------------MSKSGQSLRPPPPACSIMCQYEY
>Manacus_vitellinus
------------------------------------MKNTSIARHLKWLTKPKDLEQWCVMSNSAQSFQPPPPACSVICQYEY
>Struthio_camelus_australis
-----------------------------------------------------------------------------MCQYEH
```

HG0237 ENSGALG00000012123 PAX6

```
>Gallus_gallus
---------MCGVISGF-EKR-APARPHFPLWLRRREEVFCWKMMTEVRLC--
>Cyprinodon_variegatus
MEKTTPYFLSTQFFLSFSSSL-LLPWLCFPLWLWHRGEVFIWGMMTEVGSR--
>Esox_lucius
---------MCGLISVFWRPVSPLSPHRFPLWLWRREKSLRGADDDRGRLMGK
>Callithrix_jacchus
-------------------------------------------MMTEVRLR--
>Salmo_salar
---------MCRVISRF-EKL-ALALPRFPLCTAE------------------
>Lepisosteus_oculatus
-------------------------------------------MMTEVRLC--
>Chrysemys_picta
---------MCGVISGF-EKR-ALAQPHFPLWLRRREEVFCWKMMTEVRLC--
```

HG0238 ENSG00000049618 ARID1B

```
>Homo_sapiens
------------------------------------------MLGPIGSASRVFTHIRAR-EEK-R---KVSFKPELKTFTMKAHSRSRPRA--
>Channa_argus
--------------------------------MPCWATRAP------GDAAAYYREPGWRRGGGG-KEKKVSFKPGRETFNMKLH---------
>Latimeria_chalumnae
MKKLWSMSLQGLHASTPFHIFQALCSNKREVDNSCWSSLSHAMLGQRGSGRHVFTHIRVR-EEKKK---KVSFKPEQKTFNMKSHSRSRHRHRQ
>Kryptolebias_marmoratus
----------------------------------------------------------MEKRRRG-ERKKVSFKPGRETFNMKPH---------
>Esox_lucius
--------------------------------------------------------MHLPISGLE-ERKKVSFKPGRQTSNMKPH---------
>Parus_major
------------------------------------------MLGQRGSGRRVFTHIRVR-EERRRKKKKFHLNLNKKTFNMKSHSGNGHGP--
>Lepisosteus_oculatus
--------------------------------------------------------MHLPISGLERKGRKVSFKTWTKTFNMKSHRGSRQSQ--
```

HG0239 ENSG00000149256 TENM4

>Homo_sapiens
MEPGRLKLPCVQIKIAYFKDYLKDYS
>Mustela_putorius_furo
MEPGRLKLPCVQIKIAYLKNYSKDYS
>Physeter_catodon
MEPGRLKLPCVQIKIAYFKDYLKDYS
>Pteropus_alecto
MEPGRLKLPCVQIKIAYFKDYLKDYS
>Condylura_cristata
MEPGRLKLPCVQIKIAYFKDYLKDYS
>Chinchilla_lanigera
MEPGRLKLPCVQIKIAYFKDYLKDYS
>Anolis_carolinensis
MESGKLQSAYDQVKTAYFKAHLMDYS

HG0240 ENSG00000166342 NETO1

>Homo_sapiens
MTDPFFLPSMPQRKIPKGWSEERCRWTSSRGGCSDLLRGASETGD
>Calidris_pugnax
MTDPLIPSSMHQ-KKPKGWKSLGS---ESCRKCFHLLLVLYETGD
>Myotis_lucifugus
MTDPFLLPPMPQRKIPQGWTEEGCRSSSRRARLPQPAAR---GG-
>Falco_peregrinus
MTDPLIPSSMHQ-KKPKGWKSLGS---ESCRKCFDLLLVLYETGD
>Pseudopodoces_humilis
MTDPLIPSSMHQ-KKPKGWKSLGS---ESCRKCFDLLLVLYETGD
>Mesocricetus_auratus
MTDPCLLPSMPQQKTPKGWREKSCGWTSSRRRCSDLVLLVDETGD
>Chrysemys_picta
MTDPLILPSMHQ-KNPKVWETLGL---KSYRKCFDRLLVLYETGD

HG0241 ENSG00000198561 CTNND1

>Homo_sapiens
--ME-----VSGWLCSLLPASSLLWWLGCFFHDFLNLDWAVLCVKPISCDLLLTV-----------------
>Balaenoptera_acutorostrata_s
---MSAP-LK-----VSQICSSLLPASSLLWWLGCFSHDILNLDWAVLC-----------------------------
>Coturnix_japonica
---------MGAVTQP-----------TACPRAEAV-RSEG-RTGGRPTAAAGPSRRYPACRSHPKYELTACLFALCGESSLPPASSLWWLGYFSHDSLRLDLAVPCLCQSHPSFLL-------------------
>Oryctolagus_cuniculus
---MIVLNLDWAVLCVKSISYDILTVV-----------------
>Lepidothrix_coronata
---------MS-----------------------A---------VGSPGLAAQP------------APMGQRLAPKSPSALFPPASSLWWLGCFSHDSLRLDLAVPVPLPIKPELPPLTVKRGAPAPSGSLFLPSP
>Ictidomys_tridecemlineatus
---MIFLNLDWAVLCVKTINYDPSLNSVK---------------
>Python_bivittatus
MIRKDVAEQLGPWHSPKEKVLFFLSVALFPPRPDSFLNLQGSEANLLPEAAAKGTVRFPERSARG----------TKAQAFFLLLPCCWWLGCFSHDFLHLDQAVP------------------------------

HG0242.1 ENSGALG00000007112 LINS1

```
>Gallus_gallus
---------MSRGNLEEECLL--ACLFLLLDKNNFKTDLQQSEIIGEDLQRSVIY
>Apteryx_australis_mantelli
-----------------------------MDKNNFETNLQ---------------
>Columba_livia
-----------------------MLLFLLLDKKNFEADLH---------------
>Haliaeetus_albicilla
----MVEEVRSLGNIEEKCLI--VLLFLLLDKNNFKADLQRP-------------
>Sturnus_vulgaris
-----MRSLHLPRNLEEKCPV--VLLFLLLDESNWEAALQ---------------
>Pelodiscus_sinensis
MSLSSRGFCIIRAILQKECLYRATNPFLLLDRSNFKSDP----------------
```

HG0242.2 ENSG00000140471 LINS1

>Homo_sapiens
-----MREEVPACTFCELRWPRAGICVPS---NGGVKRVRLSQ---------------IPGFTEETS
>Mustela_putorius_furo
-----MRRKRSACAFPELRWPGG-WCCSF---NGGVKAGSPNPDLRQKLQERRPACVIKQSFPEETS
>Manis_javanica
MWPLRMREEGPTCTFRELRLPGGGSCALEWRFEGRVRFAR---SLAGK-------------------

HG0243.1 ENSG00000163697 APBB2

>Homo_sapiens
---MGSLLSRQLIFQIITYFLHHC
>Physeter_catodon
------MALSQDTFQIITYFLHHC
>Equus_asinus
MVPRGSVSSRQLIFQVITYFLHLC
>Manis_javanica
---------MQLVFQIITYFLHHC
>Chinchilla_lanigera
MVPGGSLFSRQLILQIITYFRHHC
>Struthio_camelus_australis
----------MKRFQIIIYFLVHC

HG0243.2 ENSG00000163697 APBB2

>Homo_sapiens
MLWSDLPSRGTETFAGWKIHKRKLL
>Felis_catus
MLWSDLPSRGTETFAGWKIHKRKLL
>Balaenoptera_acutorostrata_s
MLWSALPSRGTETFAGWKIHKRKLL
>Rousettus_aegyptiacus
MLWSDLPSRGTETFAGWKIHKRKLL
>Dasypus_novemcinctus
MLWLDLPSRGTEIFAGWKIHKRKLL
>Galeopterus_variegatus
MLWSDLPSRGTETFAGWKIHKRKLL
>Condylura_cristata
MLGSDLPSCGTEAFAGWKIHKRKLL
>Ceratotherium_simum_simum
MLWSDLPSRGTETFAGWKIHKRKLL
>Manis_javanica
MLWSDLPSRGTETFAGWKIHKRKLL
>Loxodonta_africana
MLWSDLPSRGRETFAGWKIHKRKLL
>Fukomys_damarensis
MLWSDLPSRGTETFAGWKIHKRKLL
>Tupaia_chinensis
MLWSDLPSCCTETFAGWKIHKRKFL

HG0244 ENSGALG00000010837 ASB5

>Gallus_gallus
MLTGTLRSLKAVVMKQPKYQHFSRIDPSEQLMLMVVF-
>Xenopus_tropicalis
MLTGTIRSLNAVVIKHPKITFRKKPYSMSDI-------
>Calidris_pugnax
MRTGTLRSLKAVVMKQPKCWCFSRIDPSQQLMIRAVF-
>Columba_livia
-------------------------------MIRVVF-
>Tauraco_erythrolophus
------------------------------MMIRVVF-
>Saimiri_boliviensis
-------------MKQPKDQLFRSTTDESQAHLRLGSN

HG0245 ENSG00000091831 ESR1

>Homo_sapiens
--MQPHTPFYLPYLGYSVV--LPRVILCTHYVFLANEEGESNRKRDKQRYIGVWHGAHKAAH-------------------
>Mustela_putorius_furo
MRKL---RAG-----------TILLLAKHSSSYRYTLTPPPPFSPDVSSQSPQLLNIAVCRWQHGQPHSPFYLPYLSYRVA--LPRVILCTHYILPANEESESNRKRDKQR--------------------------------
>Pteropus_alecto
--MYTLHGPANEKSASSERHKQR--------------------------------
>Monodelphis_domestica
-----------------------------MSSC---LF-PSLQRRLLSSPAVAKYSLAVCGWLHRQPHTQFYLPYLGYRID--LPRVIFCTHYIFLENMKSESNRGKR-----------------------------------
>Rattus_norvegicus
MAVLEGTPWGVFFFFFPGEETEGFLDSKCSSSYTRSL--SARLALDASYLRPLMLNIAVCGWLHMQSYTPSYLPYLSYRYSYCPSRVILCTHHAVPAN-KTESNRKGDKQRQIGAWHRYTGNTSGKKERKETSLWLSLHLLQT
>Thamnophis_sirtalis
--MKMNKTRSLDKRR--------------------------------

HG0246 ENSG00000162676 GFI1

>Homo_sapiens
MSKPLGLGVKVSSGLG-L--------------------SKP---SE---
>Mustela_putorius_furo
MSKPLGLGVKVFSALLGVFIPKCFHNKLFRGSGAGFALRTP---RPLAGLQAGGTT--R-R--
>Rousettus_aegyptiacus
MSKPLGLGVKVSA--
>Ochotona_princeps
MSKPLGLGVKISAGPG-L--------------------RKL---WP---
>Parus_major
MSKPLGLGLKVFSAPLGVFIPEEFSTTSYF---
>Rattus_norvegicus
MSKPLGLGVKVFSASLGVFILKCFHNKLFRGPEAQVSLCGRGGQWPGSSPAAPGFGDSRLRSGSDACPSRRPGCVAHGQRVQELCELPARSARQLPSICVLNL

HG0247.1 ENSDARG00000014746 rbfox1

>Danio_rerio
MLALGRCCRDWD----
>Apteryx_australis_mantelli
MLALGRCCMAWD----
>Calidris_pugnax
MLALGRCCMAWD----
>Corvus_brachyrhynchos
MLALGRCCMAWDQSSC
>Chrysemys_picta
MLALGRCCMAWD----

HG0247.2 ENSG00000100320 RBFOX2

>Homo_sapiens
MGGSWRKKEGERLPERKRKERKKH
>Canis_lupus_familiaris
MGGSWREKEGERLPERKRKERKKR
>Orcinus_orca
MGGSWRKKEGERLPERKRKERKKH
>Myotis_brandtii
MGGSWRTKEGERLPERKRKERKKH
>Dasypus_novemcinctus
MGGSWRKKEGERLPERKRKERKKH
>Galeopterus_variegatus
MGGSWRK-EGERLPERKRKERKKH
>Macropus_eugenii
MGGSWRKKEGERLPERKRKERKKH
>Condylura_cristata
MGGSWRKKEGERLPERKRKERKKH
>Ceratotherium_simum_simum
MGGSWRRKEGERLPERKRKERKKH
>Manis_javanica
MGGSWRKKEGERLPERKRKERKKH
>Cricetulus_griseus
MGGSWRKKEGERLPERKRKERKKH
>Tupaia_chinensis
MGGSWRKKEGERLPERKRKERKKH

HG0248.1 ENSGALG00000004570 NCOA3

>Gallus_gallus
MVSDSGDPVKSILCLNAC
>Anser_cygnoides
MTSDSEGPMKNMLCLNVC
>Aquila_chrysaetos_canadensis
MTSDSEGPLKNMLCLNVC
>Manacus_vitellinus
MTSDSEGPLKNRLCLNVC
>Chrysemys_picta
MITGPDGPLKSLLCLNVC

HG0248.2 ENSG00000124151 NCOA3

```
>Homo_sapiens
---------MAAGGSKYLLDGGLRDQ
>Leptonychotes_weddellii
---------MAAGGSKYLLNSGVTDQ
>Orcinus_orca
---------MAAGGSKYLLNSGIRDQ
>Myotis_lucifugus
---------MAAGGSKYLLNSGIRDQ
>Galeopterus_variegatus
MASKSEHADISTAGSKYLMNSGLRDQ
>Condylura_cristata
---------MAAGGSKYLLNSGVRDQ
>Oryctolagus_cuniculus
---------MAAGGSKYLLNSEHRDQ
>Equus_asinus
---------MAAGGSKYLLNSGVRDQ
>Manis_javanica
---------MAAGGSEYLLNSGVRDQ
>Loxodonta_africana
---------MAAVGSKHLLNSGPRDQ
>Nannospalax_galili
---------MAAGGSKYLLNSGLRDN
```

HG0249 ENSDARG00000062550 rc3h1a

>Danio_rerio
--------MLCFSHYVLNAHNARKYLRTVIFGRALSVPVEPLGLTAVSFYAKRT
>Pygocentrus_nattereri
---------------MLRVHNA--------------------------------
>Apteronotus_leptorhynchus
MNRFR-HILLCIQHCVLNVHSA--------------------------------
>Scleropages_formosus
----------------MNVYNVRIY-----------------------------
>Gekko_japonicus
MNTWSSRDKGYISHYVLTVCTYRRYLKTIFFPKNLI-PVESVL-----------

HG0250 ENSGALG00000014861

```
>Gallus_gallus
-------------MNWKHLDLCRALNQILV-----------------
>Anser_cygnoides
-------------MNWKHLGLFRAVNQLFV-----------------
>Haliaeetus_leucocephalus
MKENYALKSQLFKMNWKHPDLFRVVNQLFV-----------------
>Manis_javanica
-------------MNWKHIDLCRRMPCECSPRRRSLAQKAAGRHSLG
>Aptenodytes_forsteri
-------------MNWKRLDLFRAVNQLFV-----------------
```

HG0251 ENSG00000185053 SGCZ

>Homo_sapiens
MMLTREAAFFLGMQQFAGF
>Mustela_putorius_furo
MMLSREAAFFLGMQQFAGF
>Rousettus_aegyptiacus
MMLSREAAFFLGMQQFAGF
>Chinchilla_lanigera
MMLSREAAFFLGMQQFAGF
>Chrysemys_picta
-MLSSEAVFFLGMQQFAGF

HG0252.1 ENSGALG00000009645 ESRRG

>Gallus_gallus
MSLHGVVDFPSKETGFGFFKIPDDSPD
>Alligator_mississippiensis
MSLHGVVDFPSQETGFGFLQIPDDSPD
>Sturnus_vulgaris
MSLHGVVDFPSKETGFGFFKIPDDSPD
>Microcebus_murinus
MFLHGGVL-------------------

HG0252.2 ENSG00000119715 ESRRB

```
>Homo_sapiens
---------------------------MVVRLFFFFFFFCQLPVISITESGSIYRLG--VLCP-----------------------------
>Myotis_davidii
------------------------------MVVGIFFFFCQLPVISITESDLFIGWEYCVL--SH--KETQTGDCQLVLEELSSESSATGST
>Corvus_brachyrhynchos
MPTGDCGGLLPGHSAELKAQLLLPCLRPGYFFFFFFFFFCQLPVISITELGSIYRLGLGVLCPSHKERQRHRGDCRLVQAEPKDI-------
>Mus_musculus
------------------------------MVVGFFFLFCQLPVISITESGSIYSLR--VLCP-----------------------------
```

HG0252.3 ENSG00000196482 ESRRG

>Homo_sapiens
MCNVPAWGFCRLS-----------------------
>Mustela_putorius_furo
MCNVPAWGFCRLS-----------------------
>Orcinus_orca
MCNVPAWGFCRLSQPKRPASASLKSPTTHLLNLLQF
>Monodelphis_domestica
MCKVPAWGFGGVGCRLS-------------------
>Manis_javanica
MCNVPAWGFCRLS-----------------------
>Mus_musculus
MCDVPAWGVCRLS-----------------------

HG0253.1 ENSGALG00000015184

>Gallus_gallus
MYQNVNDEALLNLVKGVTSAERAVGAARSGGAGRRAGQGRVGQGGPGRAGHGAPSSAAAASRSLDRQRRARKPNTNKAARTVPSSSSLSLFLIF
>Lipotes_vexillifer
MYQNVND---
>Pteropus_vampyrus
MYQNVND---
>Anolis_carolinensis
MYQNVNDPPLLNLVKGVTSAESVVRAVPSGGAVPGLPLLLLLPRG--RD---

HG0253.2 ENSG00000140332 TLE3

>Homo_sapiens
MSAQSQALTRSTQPREGGRPLPAEP
>Odobenus_rosmarus_divergens
MSAQPRALTRSTQWGEGGRPQPAEP
>Orcinus_orca
MSAQPPALTRSTQRGEGGRPQPAEP
>Rousettus_aegyptiacus
MSAQSPALTCSTQPREGGRLQPAEP
>Oryctolagus_cuniculus
MSAQPPALTRSTQPREGGRPKPAEP
>Elephantulus_edwardii
MSSQSPALIRQTQPREGYRPQPADP
>Equus_caballus
MSAQPPALTRSTQPGEGGRPQPAEP
>Manis_javanica
MSARSQALTRTTQPGEGGRPQPTEP
>Jaculus_jaculus
MSAQPRALTLSTQPWEGGRPQPAEP
>Trichechus_manatus_latirostr
MSAQSPALTRSTQPGEGGRPQPAEP
>Orycteropus_afer_afer
MSAQSPALTRSTQPEEGGRLQLAEP

HG0253.3 ENSG00000140332 TLE3

>Homo_sapiens
MPGRLICTY
>Odobenus_rosmarus_divergens
MPGRLICTY
>Equus_caballus
MPGRLICTY
>Manis_javanica
MPGRLICTY

HGO254.1 ENSGALG00000035919

>Gallus_gallus
MLDGACGCQTQPEAAADGR-R--------DPSCAPPVPPRS--------------------------
>Aquila_chrysaetos_canadensis
MLDGACGCQTQPEAAAAAGYRDIPLRVSRGPFPAPPAPSASTFAATDWAWVVSVLRYMKHYCCGEVY
>Pseudopodoces_humilis
MLDGACGCQAQPAAAGHRDTGTPR---------PPRARSDEDFFSQI--------------C-----
>Trachemys_scripta
MLAGACGCQSQPEAAAAAGQPDPR-----APLPAPRRRRQQVMVSCVLHLFL---EERK--------

HG0254.2 ENSG00000072134 EPN2

>Homo_sapiens
MTIQGSYSSLITETEPS
>Galeopterus_variegatus
MTVQGSCSKSITETEPS
>Loxodonta_africana
MIVQGCSSRLITETDPS
>Nannospalax_galili
MTIQGSCSIWITETEPS
>Orycteropus_afer_afer
MIVQGSSSWLITETDPS

HG0255 ENSGALG00000012139 ARL14EP

```
>Gallus_gallus
-------------MSATGSAAVGGAARKPGAGVPAQRRRKVHGKGDLHRNH
>Alligator_mississippiensis
-------------MAASSTAAVVGGTRESGSGAAGQGRKKAYGKGDPHRNY
>Haliaeetus_leucocephalus
MVSRRVGGGEGRLMAATSSAAVAEGTRKSGAGVPAQRRKKVHGKGDLHRNY
>Trachemys_scripta
-------------MAATSTTALVGGTRESGSGVAAQGRKKAYGKGDPHRNY
```

HG0256 ENSGALG00000039901

>Gallus_gallus
MAATTTISLRHSQPCNRFPRDVPELLEFKPPKGPRSTQKQCP
>Calidris_pugnax
MAATTTISLRNSQPCNKFPTNVPELLEFKPP-----------
>Alligator_mississippiensis
MAATTTISLRNSHPCNTFPRMCLNSWSSNRLKGPTTAQTQYS
>Haliaeetus_leucocephalus
MAATTTISLRNSQPCNKFPTNVPELLEFKPP-----------

HG0257 ENSG00000198791 CNOT7

>Homo_sapiens
--MLSPVMRRPLVSLRGFWDCSREREGFEISLPKNQQE---LCL-----------------------------
>Physeter_catodon
--MLPPVMRGPLMPLRGFWNCSRERWRKAVFN-YRVFP---LCVCVFSEEGYKGRGPGSFHCVCVCVCVCVCV
>Galeopterus_variegatus
--------------------------MPLCCPLVSLGACSTEMLPPVMRRLWVSFRGFWNCSKERGGGKHC--
>Corvus_brachyrhynchos
MGQVRRDHSGSPGPTSLLEQGHPGAYSTRLCPDSSGMSSLRQTPKPFWAVRSRSLHGFWSCSSSCSGFPLKLSVHQFLPIISCPIVWHHQDEPGTRPLGIHTH----------

HG0258.1 ENSGALG00000016797 FHL2

>Gallus_gallus
MSNCLITKYIVWVIQ-------
>Columba_livia
MPDCLTTKHIVWAIQ-------
>Pelodiscus_sinensis
MPDCLITKYIVWVIQCSNKPLY

HG0258.2 ENSGALG00000016797 FHL2

>Gallus_gallus
MDVTLCPWSRR
>Chaetura_pelagica
MDVTPCPWSRR
>Apteryx_australis_mantelli
MDVTPCPWSRR
>Cariama_cristata
MDVTPCPWSRR
>Calidris_pugnax
MDVTPCPWSRR
>Colius_striatus
MDVTPCPWSRR
>Columba_livia
MDVTPCPWSRR
>Cuculus_canorus
MDVTPCPWSRR
>Mesitornis_unicolor
MDVTPCPWSRR
>Opisthocomus_hoazin
MDVTPCPWSRR
>Acanthisitta_chloris
MDVTPCPWSRR
>Nipponia_nippon
MDVTPCPWSRR
>Aptenodytes_forsteri
MDVTPCPWSRR
>Struthio_camelus_australis
MDVTPCPWSRR

HG0259 ENSGALG00000009936 AP4S1

>Gallus_gallus
MLKSKMAAPGKGAQFRP--------
>Gekko_japonicus
MLESKMAAPGKAVQPREPRGGCAE
>Pelodiscus_sinensis
-----MAAPGKGAQFGP--------

HG0260.1 ENSGALG00000016890 MBNL2

>Gallus_gallus
MLLSMIFFSQKSTSGT
>Anas_platyrhynchos
MLLSMVVFSQKSTSGT
>Apteryx_australis_mantelli
MLLSMVFFSQKSTSGT
>Buceros_rhinoceros_silvestri
MQLSMVFPSQKSTTGT
>Calidris_pugnax
MRLSMVFPSQKSTSGT
>Columba_livia
MRLSMVFPSQKSTSGT
>Merops_nubicus
MRLSMVFPSQKSTSGT
>Cuculus_canorus
MQLSMFFPSQKSTSGT
>Aquila_chrysaetos_canadensis
MRLSMFFPSQKSTSGT
>Mesitornis_unicolor
MRLSMVFPSQKSTSGT
>Tauraco_erythrolophus
MQLSMVFPSQKSASGT
>Opisthocomus_hoazin
MRLSMVFRSQKSTSGT
>Taeniopygia_guttata
MRLSMCFPSQKSTSGT
>Egretta_garzetta
MQLSMVFPSQKSTSGT
>Fulmarus_glacialis
MQLSMVFPSQKSTSGT
>Nestor_notabilis
MRLSMVFPSQKSTSGT
>Aptenodytes_forsteri
MRLSMVFPSQKSTSGT
>Struthio_camelus_australis
MLLSMVFFSRKSTSGT
>Tinamus_guttatus
MLLSMFVFSQKSTSGT
>Apaloderma_vittatum
MRLSMVFPSQKSTSGT

HG0260.2 ENSG00000139793 MBNL2

```
>Homo_sapiens
MSDCLSIHSLIILFLGLDFTKQFITHLQTYMWEFSQQ
>Mustela_putorius_furo
------------------------------MWGFSQQ
>Lipotes_vexillifer
MSDYLPINSLLTLFPGLGFFKQFVTYLQTYMWDFSQQ
>Pteropus_alecto
MSDCLPIII-SSLLLGLDFIKQFITYLQTYMWDFSQ-
>Dasypus_novemcinctus
MSDCLPINSLI-LFCGLDFIKQFIIYLKTYMWDFSQQ
>Galeopterus_variegatus
MSDCLPINSLITLLPGLDFIKRFITYLQTYMWEFSQQ
>Condylura_cristata
MSDRLLINSLLTLSPGLDFIKQFITYLQTYMWDFPPQ
>Oryctolagus_cuniculus
------------------------------MWEFSRQ
>Elephantulus_edwardii
------------------------------MWDLSQH
>Equus_asinus
MSDCPPINSLLTLFPGLDFIKQFITYLQTYMWDFSQ-
>Manis_javanica
MSDCLPINSLFTLLPRLNFIKTFIPYLQTYMWDFSQQ
>Loxodonta_africana
------------------------------MWDFSQQ
>Chinchilla_lanigera
MSDCLPIHSLSTLSPGLDFIKQLITYLQTYMWEFSQQ
>Tupaia_chinensis
MSDCPLINSLITLFPRLDFIKQFITYLQTYMWEFSQQ
>Trichechus_manatus_latirostr
------------------------------MWDCSQQ
```

HG0260.3 ENSG00000152601 MBNL1

>Homo_sapiens
MTPTMPWALGRQWGRL
>Odobenus_rosmarus_divergens
MTPTMPWALGRQSGCL
>Lipotes_vexillifer
MTPTMPWALARQSGCL
>Pteropus_alecto
----MPWALGRQSGCL
>Condylura_cristata
----MPWAPGRQSGCL
>Manis_javanica
----MPWALGRQSGRL
>Heterocephalus_glaber
MTPTMPWALGRQSGCL
>Tupaia_chinensis
MTPTMPWALGRLSGCL
>Orycteropus_afer_afer
MTPTMPWVLCLQSGCL

HG0260.4 ENSG00000152601 MBNL1

>Homo_sapiens
MSCGAHNAPMTRS
>Odobenus_rosmarus_divergens
MSCGAHNAPVTGN
>Lipotes_vexillifer
MSCGAHNAPVTGN
>Pteropus_alecto
MSCGAHNAPVTGN
>Dasypus_novemcinctus
MSCGAHNAPVTRN
>Condylura_cristata
MSSGAHNAPVTRN
>Tupaia_chinensis
MSCGAHNAAVTRN
>Orycteropus_afer_afer
MSCGAHNAPVTRN

HG0260.5 ENSG00000152601 MBNL1

>Homo_sapiens
MFFPPVSPPGSKTVWCILRRQSHQIAGF-------------------------
>Odobenus_rosmarus_divergens
MFFPPVSHPGSKTVWCILKWQPHQILTVFCRSDSHLQPNLAGFLTKSPIRRPL
>Pteropus_vampyrus
MFFPPASHPGSKTVWCICKEAAPSDLTVLS-----------------------
>Orycteropus_afer_afer
MFFPPVLPPGSKTMWCILRRQPHQIPGFYSNLI--LTPS--------------

HG0260.6 ENSG00000152601 MBNL1

>Homo_sapiens
--MGLRFICSGDNMAYLPWNIPVFQK--
>Odobenus_rosmarus_divergens
MIWGCGVFCSNNNMT---
>Orcinus_orca
-------------MTYLPWTITVFKKMNILC---
>Pteropus_vampyrus
-------------MTYLPWNSPVFQKMVFYFLKRGPLLSINHIMQSWGERGFYISNIIYKLDGKLVCECLCLCDNQFY

HG0261.1 ENSGALG00000005867 MARCH8

>Gallus_gallus
MSAAFKPPSCK-
>Anas_platyrhynchos
MSAAFKPPSCK-
>Buceros_rhinoceros_silvestri
MRAAFKPPSCKS
>Charadrius_vociferus
MSAAFKPPSCK-
>Columba_livia
MSAAFKPPSCK-
>Haliaeetus_leucocephalus
MSTAFKPPSCK-
>Eurypyga_helias
MSTAFKPPSCK-
>Corvus_cornix_cornix
MSTAFKPPSCK-
>Nipponia_nippon
MSAAFKPPSCK-
>Picoides_pubescens
MSTAFKPPSCKQ
>Pygoscelis_adeliae
MSAAFKPPSCK-
>Apaloderma_vittatum
MSIAFKPPSCK-

HG0261.2 ENSGALG00000009520 MARCH1

```
>Gallus_gallus
----------------MEYLTQRQEMKPGSNVKGHVKLLHSRNLLKDCFT
>Anser_cygnoides
MGFRRCRYFHCRKRNMRLPQDENCMEMNPGSNVKGHVKLCHSRNLLKDCFA
>Calidris_pugnax
-------------------------MNPGRNVKGHVKLCHSRSLLKDCFT
>Zonotrichia_albicollis
-------------------------MNPGSNVKGHVKLCHSRNFLKDCFT
>Struthio_camelus_australis
-------------------------MNPRSRLKGHVKLCHSRNLLKDCFT
```

HG0261.3 ENSG00000145416 MARCH1

>Homo_sapiens
MPAPRDAGRTQPGGRLSYFTLRR-------------
>Pteropus_alecto
MPAPRDAGYAQPGGCLSDFALRR-------------
>Monodelphis_domestica
MPEPRDMGTVQTCGRLSDFTLRWKCTQG--------
>Nannospalax_galili
MPAPRDAGCAQPGGRLNHFILPRKCTQEEF------
>Tupaia_chinensis
MPASGAAGCAQPGGRLSHFTPRGFQQRLSESVKSTA

HG0261.4 ENSG00000165406 MARCH8

>Homo_sapiens
MLEAGQGGSAASSEPT
>Ailuropoda_melanoleuca
MLRAGWGGPAASSAPT
>Pteropus_vampyrus
MLQAGPGGLAASSAPT

HG0262.1 ENSG00000023445 BIRC3

```
>Homo_sapiens
MFLLPPGNKALKSAVEKPNVCEIQETMLANHW-----------------------
>Canis_lupus_familiaris
---MPPGNKALKVWSRGPSVGGPDMD-----PREGTHHDLFVGRC----------
>Balaenoptera_acutorostrata_s
MFLLPPGNKALKSAVESPNICGTQEACLQNTSRKKNHGDLFIGYY----------
>Dasypus_novemcinctus
MFLLPPGNKVLESAVENPNICASQQTMLAKSLVEKHA-VYWLSLVLLKYLFTNAR
>Oryctolagus_cuniculus
MFLPPPGHRVLKSAVRKPQHL----------------------------------
>Equus_asinus
MFLLPPGNK-LKSAVESANLCGTQETMLANHCLENQD-DLFVGHC----------
>Cavia_porcellus
MFLLLPGNQVLKSAPRLQILVEKAL------------------------------
```

HG0262.2 ENSG00000023445 BIRC3

>Homo_sapiens
-------------------MSLILNLICGNKGKVILAGAYC
>Ailuropoda_melanoleuca
MNRGRVTKSSHRSCNCKVTITLNLNVTEGHEGEVILAGTYC
>Balaenoptera_acutorostrata_s
---------------------------------MIVAGTYC
>Dasypus_novemcinctus
---------------------------------MTVAGAYC
>Cavia_porcellus
-------------------MILILNLICGSKE-VITMEAYC

HG0262.3 ENSGALG00000017186

>Gallus_gallus
MHSTRKAHNVTVAQEKVLFDIPVFVNG--VDQLLH------
>Haliaeetus_albicilla
-----------MIQGEVLFDMLSSIVRKGRGSFCTESSCVS
>Fulmarus_glacialis
-----------MIQGKVLFDMLSSIVKMGWGSFCTESSCVN

HG0262.4 ENSG00000023445 BIRC3

>Homo_sapiens
MNTKKIFITMCKIFGQGKEY
>Rousettus_aegyptiacus
---------MCKLFGQ----
>Equus_asinus
MNTKKVFITMCKVFEQ----

HG0263.1 ENSG00000169826 CSGALNACT2

>Homo_sapiens
MEILILFLIFDNFLLKV
>Leptonychotes_weddellii
MEILILFLIFDNLLLKV
>Physeter_catodon
MEILILFLLFYNLSQKV
>Pteropus_alecto
MEILVLFLIFDNLLLKV
>Equus_asinus
MEILILFLIFDNLLLKV
>Manis_javanica
MEILILFSVFDNLLLKV
>Microtus_ochrogaster
MEILIFFLIFDNFLLKL

HG0263.2 ENSGALG00000002543 CSGALNACT2

>Gallus_gallus
--------------------------------MANVGTCDLWLCNDT
>Calidris_pugnax
------------------------------MVMDDIGTSDLSLCNDT
>Sturnus_vulgaris
------------------------------MVMDDIGTSDLHLCNDT
>Struthio_camelus_australis
MSPFPFNGFLFSLGWDILNKSFGVNHAYKLMVMDDVGTSNLYLCNDT

HG0263.3 ENSGALG00000002543 CSGALNACT2

>Gallus_gallus
MIPGWCLPFCSPSAVFFVWDKKS
>Calidris_pugnax
MTPWWCLPSSSSSAGFFVWGRKS
>Corvus_brachyrhynchos
MIPCWCLPSSSPSAGFSCLG---
>Pygoscelis_adeliae
MTPWWCLPPSSPSAGFFVWGRKS

HG0263.4 ENSG00000147408 CSGALNACT1

>Homo_sapiens
MDASSSGGPKRMLPVV
>Balaenoptera_acutorostrata_s
MDESSSGTPGRVLPVI
>Cynopterus_sphinx
MDESSSGGPKRMLPVV
>Ceratotherium_simum_simum
MDESSSRGFRRMLPVV

HG0264.1 ENSG00000151458 ANKRD50

>Homo_sapiens
MELCDSLKFRKSVAELWNQAFLPQFNN--
>Acinonyx_jubatus
MELCDPSRFRKSIATLWNQAIFHQFNN--
>Lipotes_vexillifer
MEFCDPSRYRKSVGQGLEPGSFPSVQQFI
>Rousettus_aegyptiacus
MELCDPSRYRKTVAELWNRAIFYQFNP--
>Dasypus_novemcinctus
MALCDLPKHRKSVTKF-------------
>Sarcophilus_harrisii
MELYDLLSHMKSIIEFWDQTSFHQINN--
>Galeopterus_variegatus
MQLCDSSRCRKSVAELWNQAFFPSVQQLN
>Oryctolagus_cuniculus
MELCDLSSYWKGVTKLWS-----------
>Equus_asinus
MELCDPSRYRKRVAKLWNQATFHQFNN--
>Manis_javanica
MELCDPSRYRKSVAKFWNQAIFYQFNS--
>Loxodonta_africana
MELCDPSRHRKSVTEFWN-----------
>Fukomys_darlingi
MELYGPSRYSKSVAELWHQSCFHQFNN--
>Trichechus_manatus_latirostr
MELCDPSRHRKSVTELWN-----------
>Orycteropus_afer_afer
MKLCDPSKHKKSVTELWNKAFFPSVKQ--

HG0264.2 ENSG00000151458 ANKRD50

>Homo_sapiens
MYCLISVLETSTIT----------
>Mustela_putorius_furo
MYCLICTLETSTIT----------
>Orcinus_orca
MYCLICILETSTIT----------
>Myotis_lucifugus
MYCLICILEGSTIT----------
>Sarcophilus_harrisii
MYCLISILETSTISINSRCSHGVI
>Galeopterus_variegatus
MYCLIYILEISTIT----------
>Ceratotherium_simum_simum
MYCLICIFETSTVT----------
>Manis_javanica
MYCLIHILETSTVT----------
>Cavia_porcellus
MYCLIYVLETSTIT----------
>Tupaia_chinensis
MYCLICILETSTIT----------
>Orycteropus_afer_afer
MYCLICVLETFTIT----------

HG0264.3 ENSGALG00000011831 ANKRD50

>Gallus_gallus
MDSFFWIYIRRD
>Anser_cygnoides
MDSFFWIYIQRD
>Calidris_pugnax
MDSFFWMYIQRD
>Zonotrichia_albicollis
MDSFFCVYIQRD
>Aptenodytes_forsteri
MDSFFWMYIQRD

HG0264.4 ENSG00000151458 ANKRD50

>Homo_sapiens
MERPVLMYPDSSK---
>Physeter_catodon
------MYPDSSKC--
>Manis_javanica
------MYPDSSKC--
>Trichechus_manatus_latirostr
------MYPDSSKCRR

HG0265.1 ENSG00000144285 SCN1A

>Homo_sapiens
MELFLQINTSGVMEAGGS
>Mustela_putorius_furo
MELFLQINTSGTMEAGGS
>Rhinolophus_ferrumequinum
MELFLQINTSGAMEAGGS
>Condylura_cristata
MQLFLQINTSGAMEAGRS
>Oryctolagus_cuniculus
MGLFLLINTSGAMEAEGS
>Equus_asinus
MELLLQINTSGAMEAGGS
>Manis_javanica
MELFLQINTSGAMEAGGS
>Heterocephalus_glaber
MELFLQINTSGAMEAGGS

HG0265.2 ENSG00000196876 SCN8A

>Homo_sapiens
MAPTAVPPAASSAPLQSGRASRAPR
>Odobenus_rosmarus_divergens
MAPTAVPPAAASAPLQSGRASRAPR
>Orcinus_orca
MAPTAVPPAASSAPQQSGRASRAPR
>Oryctolagus_cuniculus
MAPTAVPPAAASAPLQSGRASRAPR
>Elephantulus_edwardii
MAPTAVPPAAASVPLQSGRASRAPR
>Equus_asinus
MAPTAVPPAAASAPLQSGRASRAPR
>Mus_musculus
MAPTAVPPAAASAPLQSGRASRAPR

HG0265.3 ENSDARG00000018032 scn8ab

>Danio_rerio
MATSAVSPGSFKHP----------
>Astyanax_mexicanus
MATSAVSPGSLRHP----------
>Austrofundulus_limnaeus
MATAAVTPDSFLHPQFTDSCRDAC
>Apteronotus_leptorhynchus
MATSAVSPGSFRHP----------
>Salmo_salar
MATTAVSPESFRHLQSNNAFLGSL
>Lepisosteus_oculatus
MATTAVSPESFRHLQSSDAVLDFC

HG0266.1 ENSG00000184185 KCNJ12

>Homo_sapiens
--MAATTNSALKTVPCPLSTIT-
>Odobenus_rosmarus_divergens
---------------------MELICAPSQGLLATDSSPAGVWEMAAAADTALKTATCPLSTVT-
>Orcinus_orca
--MAASASAALKTVTCLVSTVT-
>Rhinolophus_ferrumequinum
MQPARRRPWPGTAARSSEDAERAAPGGLTEGLLATDLSPIGAWEMAAATNAALKTVTCPLSTVT-
>Galeopterus_variegatus
--MAAAANSALKTVTCPLSTIT-
>Oryctolagus_cuniculus
---------------------------------MIGSSPADAWEMAVTAHSALKTVKCSLSTIT-
>Equus_przewalskii
--MAATANAALKTATCPLSLLRR
>Fukomys_damarensis
--MAAAANSALKTVTCPLSTIT-
>Tupaia_chinensis
--MAAAANSALKTVTCLLSTVT-

HG0266.1 ENSG00000260458 KCNJ18

>Homo_sapiens
---MAATTNSALKTVPCLLSTIT
>Canis_lupus_familiaris
MWQPRAPTPPLPGRTVPRAPGRHLVIFRGLNSHHGLLWPSRGGAGGSIGRGVQRPGFLCPSCHSLQCSFVTLGKSLLCSEPLFPCRVELILPTSQGLLVTDSSPAGVQEMEAAADTALKTATCLLSTVT
>Orcinus_orca
---MAASASAALKTVTCLVSTVT
>Rhinolophus_ferrumequinum
-----------------------------MQPARRRPWPGTAARSS---------------------------------E---DAERAAPGGLTEGLLATDLSPIGAWEMAAATNAALKTVTCPLSTVT
>Galeopterus_variegatus
---MAAAANSALKTVTCPLSTIT
>Condylura_cristata
-----------------------------MRPARRRPGPGTAAR------------------------------------SSEDAECAAPGALTEGLPVTSSFAAGTWEMAASASAALKTAACLLSTVT
>Oryctolagus_cuniculus
--MIGSSPADAWEMAVTAHSALKTVKCSLSTIT
>Fukomys_damarensis
---MAAAANSALKTVTCPLSTIT
>Tupaia_chinensis
---MAAAANSALKTVTCLLSTVT

HG0266.2 ENSGALG00000001181 KCNJ5

>Gallus_gallus
MFCHAGQREGRRRRPRDQKTTTACW
>Calidris_pugnax
MFSPNRQREEGRGQPRDPKTSPALW
>Aquila_chrysaetos_canadensis
MFSNNRQREEGRRQPRDQKTTPAIW
>Parus_major
MFAASRQREGGRRQPGAPRTIPAPW
>Struthio_camelus_australis
MFCNNRQREEGEREPRDKKTTPALW

HG0267 ENSDARG00000028173 slc4a2a

>Danio_rerio
MLPESSQLSLSKREKG
>Pygocentrus_nattereri
MLPESSQLSLSEREKG
>Apteronotus_leptorhynchus
MLPKNCQLSLSEKEKG
>Scleropages_formosus
MLPKSTPLSLSEKEKG
>Lepisosteus_oculatus
MLPKSSPLSLSEKEKG
>Trichomycterus_areolatus
MLPRTTQLSLSKREKG

HG0267 ENSDARG00000052330 slc4a2b

>Danio_rerio
MLPVNSCLSLSEKEKG
>Trachinotus_blochii
MLP-KSCLSPSEKEKG
>Pygocentrus_nattereri
MLPESSQLSLSEREKG
>Pundamilia_nyererei
MLP-KSCLSPSEKEKG
>Alosa_alosa
MLPISSCLSPSEKEKG
>Kryptolebias_marmoratus
MLP-KSCLSPSEKEKG
>Apteronotus_leptorhynchus
MLPKNCQLSLSEKEKG
>Plecoglossus_altivelis
MLPKRSCLSPSEKEKG
>Scleropages_formosus
MLPKSTPLSLSEKEKG
>Sebastes_caurinus
MLP-KSCLSPSEKEKG
>Platichthys_stellatus
MLP-KSCLSPSEKEKG
>Lepisosteus_oculatus
MLPKSSPLSLSEKEKG

HG0267 ENSG00000164889 SLC4A2

>Homo_sapiens
------------------------MPPPRCPESRSDSEKG
>Panthera_tigris
------------------------MPPPCCPESRSDSEKG
>Balaenoptera_acutorostrata_s
------------------------MPPPCCPESRSYSEKG
>Myotis_brandtii
MVSRSQRPRGHLCTGSHFSPGHLVMPPPCCPESRSDSEKG
>Galeopterus_variegatus
------------------------MPPPCCPESRSDSEKG
>Oryctolagus_cuniculus
------------------------MPPPCCSESRSDSEKG
>Equus_asinus
------------------------MPPPCCPESRSDSEKG
>Manis_javanica
------------------------MPPPCCLESRSDSEKG
>Loxodonta_africana
------------------------MPPPCCPESRSDSEKG
>Mus_musculus
------------------------MPPPCCPESRSDSEKG
>Trichechus_manatus_latirostr
------------------------MPPPCCPESRSDSEKG
>Orycteropus_afer_afer
------------------------MPPPCCPESLSDSEKG

HG0268.1 ENSDARG00000011703 clocka

>Danio_rerio
MSTSRLKVLSLQLRP
>Pygocentrus_nattereri
MSTSRLKLLSLPLQP
>Clupea_harengus
MSTSRLKVVSLPLRP
>Lepisosteus_oculatus
MSTSRLKCDSLRLRP
>Trichomycterus_areolatus
MSTSRLKLLSVPLQP
>Takifugu_rubripes
MSTSRLKVLSPPLRP

HG0268.2 ENSG00000134852 CLOCK

>Homo_sapiens
----------------------MVGHIEEYLEPIVS
>Panthera_tigris
----------------------MVSHIEEYIEPIVF
>Myotis_lucifugus
-------MP-PYVTLSDRILRLMVSHIEEYIEPIVF
>Galeopterus_variegatus
----------------------MIGHTEEYLEPIVF
>Equus_asinus
MNPGASFMPPNSHHILRRILRLMVSHIEEYIEPVVF
>Heterocephalus_glaber
----------------------MVGHIEEALEPIVF

HG0268.3 ENSG00000134852 CLOCK

```
>Homo_sapiens
------------------------------MITRRQLVLRRSTNVYYKTKT
>Physeter_catodon
--------------------------------------MRRSTNVYYKTKT
>Pteropus_alecto
-------------------------------MLIPQIKKRRSTNVYHKTKT
>Loxodonta_africana
------------MKETFDV-RDKDQTKRENTKCGKWRPYRRSTNVYHKTKT
>Heterocephalus_glaber
MTRTAEFVSGKPAAASFSRKINSMQSSPILPLCRPATILRRSTNVYHKTKT
```

HG0269.1 ENSGALG00000028685 SEMA5A

>Gallus_gallus
MVTERCFSPMSARRVSS
>Anser_cygnoides
MVTKRCFSHMSARRVAG
>Columba_livia
MVTKRCFSHMSARRVAS
>Corvus_brachyrhynchos
MVTKRCFSHMSARRVDS
>Aptenodytes_forsteri
MVTKRCFSHMSARRVVS
>Struthio_camelus_australis
MVTKRCFSHMSARRTAS

HG0269.2 ENSDARG00000061471 sema5ba

>Danio_rerio
MLVMPLCIPEAHLGS
>Salmo_salar
--MTSCCIPEAHLGS
>Ictalurus_punctatus
-MSLWKCIPEAHLGS

HG0269.3 ENSGALG00000011686 SEMA5B

>Gallus_gallus
MPLTQTEITKDFPWCLTSNMTLQ
>Lepidothrix_coronata
---------------MTLNMTLQ
>Aptenodytes_forsteri
---------------MTSNMTLQ

HG0270.1 ENSGALG00000015147 ALDH1A1

>Gallus_gallus
MVNVTVFKMFHKCRSRITIFPSCFLFFFFSFLFFFFFIS--SLARMSE----LD--------
>Calidris_pugnax
MVNLTGFKMFCKCRSRITIFPCF-LFFFF----FFFSIS--SLARMSE----LD--------
>Pterocles_gutturalis
MVNLTGFKMFRKYRSRITIFPCF-LFLFF-----FFLIS--SLARMSE----LD--------
>Columba_livia
MVNLTGFKTFHKCRSRITIFSCFLFFFF---------LFVFSFHHWQESVNWIDMEVIEEEK
>Aquila_chrysaetos_canadensis
MVNLTGFKMFCKCRSRITIFPHF-LFFFLS-FFFFFFIS--SLARMSE----LD--------
>Gavia_stellata
MVNLTGFKMFRKCRSRITIFPCF-LFFFF--FFFFFLIS--SLGRMSE----LD--------
>Chlamydotis_macqueenii
MVNLTGFKMFRKSRSRITIFPCFLFFFF---------FFFFSFHHWQE--------------
>Opisthocomus_hoazin
MINLTGFKMPHKCRSRITNFPCFLFFLF-SFFHFIIGKN--------E--------------
>Manacus_vitellinus
MVNLTGFKMYRKCRSGITIFPCFLFFPF-FFFHFIIGKN--------E--------------
>Phaethon_lepturus
MVNLTGFKMFRKCRSRITIFPCF-LFFFF-----FFFIS--SLARMSE----LD--------
>Melopsittacus_undulatus
MVIITGFKMFCKCRSRITILPSLFFLFFFLFCLW----------------------------
>Aptenodytes_forsteri
MVNLTGFKMFRKCRFKITIFLCFLFFFF------FIFFSFFSFHHWQE--------------
>Tyto_alba
MVNLTGFKMFHKCRSRITIFPCFLFFFF-SFLFFFFFFFFFFFPHWQE--------------
>Apaloderma_vittatum
MVNLTGFKMFCKRRSRITIFPFS-F------FFFFFFIS--SLARMSE----LD--------

HG0270.2 ENSGALG00000015147 ALDH1A1

>Gallus_gallus
MISFLFLALPPPSLSLISLT
>Calidris_pugnax
MISFLFLALPPPSLSH----
>Pterocles_gutturalis
MISFLFLALPPPSLSH----
>Columba_livia
MISFLSLALPPPSLSH----
>Falco_cherrug
MISFLFLALPPPSLSLISLT
>Gavia_stellata
MISFLFLALPPPSLSH----
>Chlamydotis_macqueenii
MISFLFLALLPPSLSH----
>Opisthocomus_hoazin
MISFLFLALPPP-LSLISLT
>Lepidothrix_coronata
MISFLFLALPPPSLSLV---
>Nipponia_nippon
MISFLFLALPPPSLSH----
>Fulmarus_glacialis
MISFLFLALPPPSLSH----
>Aptenodytes_forsteri
MISFLFLAVPPPSLSLISLT
>Tyto_alba
MMSFLFLALPPPSLSH----

HG0270.3 ENSG00000128918 ALDH1A2

>Homo_sapiens
MEAAPGVLGRMDQPPAPRPSRPIRRAPA
>Leptonychotes_weddellii
----------MDQPPAPRPSRPIRKAPA
>Lipotes_vexillifer
----------MDQPPAPRPSRPIRRAPA
>Eptesicus_fuscus
----------MDQPPRPGPHVQ------
>Dasypus_novemcinctus
----------MDQPPAPRPSRPIRRAPA
>Monodelphis_domestica
----------MDQPPLPLPSCPIRRDPA
>Macropus_eugenii
----------MDQPPLPLPSCPIRRDPA
>Condylura_cristata
----------MDQPPAPRASRPIRRAPA
>Elephantulus_edwardii
----------MDQPPTPRPSRPIRRAPA
>Ceratotherium_simum_simum
----------MDQPPAPRPSRPIRRAPA
>Ictidomys_tridecemlineatus
----------MDQPPAPRPSRPIRRVPA

HG0271.1 ENSGALG00000004064 GPER1

>Gallus_gallus
MELELCHLPTCTKENC-
>Caprimulgus_carolinensis
MELKFCYLPACMKKLC-
>Cariama_cristata
MELKFYYLPTCTKKHC-
>Calidris_pugnax
MELKFCRLPTCPEKRCR
>Pterocles_gutturalis
MELKFYYLPTCMKKHC-
>Merops_nubicus
MELKFYYLPPCTKKHC-
>Haliaeetus_albicilla
MELKFYHLPTCVKKHC-
>Gavia_stellata
MELKFYYLPTCMKKHC-
>Eurypyga_helias
MELKFYYLPTCMKKHC-
>Pelecanus_crispus
MELKFYYLPTCMKKHC-
>Fulmarus_glacialis
MELKFYYLPTCMKKHC-
>Apaloderma_vittatum
MEIKFYYLPTCMKKHC-

HG0271.2 ENSG00000164850 GPER1

>Homo_sapiens
MPGSLQGESRFFLKMDSPFKTELWEPFGKS
>Orcinus_orca
MPGSLPRASPFLLKRDFLLKNGFWEPCGKS
>Equus_asinus
MSGSLQRAAPFFLKMDLLFKTDFCGPWGKS
>Mesocricetus_auratus
MPGSLPRESPFFVKMQLLLKTDFCDPFSKS
>Tupaia_chinensis
MLDSLQRESPFFLTMDSWFKTDFCDFFGNS

HG0271.3 ENSDARG00000074661 gper1

>Danio_rerio
MLMDWRKQDCAGLNTCEKQSVQKKKKKQDP
>Pygocentrus_nattereri
MPVDWRRQGSAGIKTCEEQSVQKNQERHNP
>Salmo_salar
MHMDWMRQASTGIRTCEKQTAQKKRKKQSP
>Ictalurus_punctatus
MLLDWRRQSSTGIKMCKEQTVQKKHSL---

HG0272.1 FBgn0034649 CG9865

```
>Drosophila_melanogaster
------MSDTRKDPCKPNACRIQACLTKNHYQEDKCLDVLEAMRQCCLKWHKES--LCCSGIDLEKTYLPDASKEKNATGKSNK--------------------------
>Boeckaxelia_potanini
MDREYNSVLKPRDPCKPKACAIQRCLERTHYNVVLCRPFYEQLTECCRQFAARS--DACGSEEQPPASALDREPVA----------------------------------
>Parasteatoda_tepidariorum
--------MQKKEPCQKYACELQKCLKENNFQQEHCLSVIKSLLQCCEKWNFKS--KCCEGFIESSDMVPDL--------------------------------------
>Meinertellus_cundinamarcensi
---------MKKDPCKIFACEIQRCLQENKFQESACQAAINALKKCCKKWREDS--LVCSGIKIDDELNKDIPQK-----------------------------------
>Mastotermes_darwiniensis
---------MKKDPCKPHACSIQKCLKENNYQEASCQEFIELLKECCRKWSSAS--ISCSGIDTQEAPSQTPEDKKQ---------------------------------
>Carabus_granulatus
-------MPVTKDPCKATACKIQKCLKENNYQESACADVLEEMKECCRKWAKVS--LCCSGIKIEDKAGSSSFNQSVK--------------------------------
>Carcinus_maenas
---------MKKDPCQRQACDIQDCLQRNKYQEAACEIQINALQECCRKWHEES--GCCAGVKLKGTVTSDAALKTKPSSQKSNVK------------------------
>Forficula_auricularia
-----MPRIDPKNPCMHYSCDYNKCLTETNYKEDKCKYYLDQLRNCCKKFAHLNS-LSCEGIKFDPPKENDHTETVTK--------------------------------
>Occasjapyx_japonicus
------MPAKPNDPCKPLACEIQRCLVANDFLESKCEEEVKRMLACCDLWGETSGGRSCSGFWINRPGEAPRKRQEAKK-------------------------------
>Aposthonia_japonica
--------MPKKDPCEPHACKIQKCLTENNFQEQKCKAVIDDMINCCKKWGGES--LCCSGFLSKGNKTDSSKEGSSAVTNEGKPSSET---------------------
>Eurylophella_sp._AD-2013
---------MKGDPCQLQACAIQKCLQVNNYKEAACPKEFEEMRKCCEKWGQLS--GCCSGFKDPELISSENRQGDKVASK-----------------------------
>Galloisiana_yuasai
----------MKDPCKPYACKIQKCLKEHNYQENACKDVIEEMVICCRKWGDIS--LCCSGIDTGKTNGSSGSKTVSGKT------------------------------
>Pachypsylla_venusta
-------MSKKKDPCKVHACAIQKCLKENDFQESKCLQYFEYMRQCCVKFHADS--ICCSGIDINKSYLKDNEKSSPSKTDHIERSTLVAKISSYYSSRSNNNLFERFFQ
>Brachycistis_timberlakei
--------MTTTDPCKPYACKLQKCLKDNVYQPSRCEEVIEEIRQCCIKHSAKS--IVCEGIDTSKQYEHNTVDYKKATK------------------------------
>Ixodes_persulcatus
--------MKAVDPCQKEACAIQKCLQEHNYQEDACVKFIKEMLKCCEKWREMS--GCCSGFLKDEASKKAKGELKSKT-------------------------------
>Antheraea_yamamai
---------MSKDPCKRFACDIQRCLVENNYQEVRCASVFEAMRRCCLKFKPSS--LVCEGYDLEPRKFAPVTDRAKK--------------------------------
>Mantis_religiosa
---------MKKDPCKPYACEIQKCLKEHNYQIEPCQKFVEYLRDCCRKWGDVS--ISCSGIDTRTSPNKASDKE-----------------------------------
>Panorpa_vulgaris
------MSNVPKDPCKPSACKIQQCLQEHNFQEQSCIDVIEQMRLCCVKWEGKS--FVCNGINTAEPADLAISPKHTKHS----RY------------------------
>Dermanyssus_gallinae
--------MPKGDPCQKQACDIQKCLQANNYQEALCVEAFRAMDLCCEKWGLQS--GCCTGFIKEQQLKKKIANESTKTPISFN--------------------------
>Pseudomallada_prasinus
------MPKPYIDPCKEFACRIQKCLRENNFQEHRCKQVIEELRQCCVKWKSES--ISCSGIDINSPKEEQ---------------------------------------
>Triops_newberryi
------------------------------------------MKECCSKWQERS--QSCAGFLVPEIEKKKNEPLS----------------------------------
>Calopteryx_splendens
------------------------------------------MQKCCDKWFDES--LCCSGFKKPSES------------------------------------------
>Stenobothrus_lineatus
---------MRKDPCKPIACKIQKCLTENRFQEPACQRVLEEMKECCRKWAHIS--LCCSGIDTSDKPKD----------------------------------------
>Extatosoma_tiaratum
----------MKDPCKPQACKIQKCLTENNYQEAACKDVIKEMVECCRKWADVS--LCCSGINTKETTANSPEEI-----------------------------------
>Menopon_gallinae
--------MTKKDPCKSLACALQKCLKENDFQEERCKKVLDDIRTCCKKFKEIS--VVCSGIDVSEKIK-----------------------------------------
>Cosmioperla_kuna
--------MKKRDPCKAHACRIQKCLAENNFQESACQAVLEDMRSCCRKFKNVS--LCCSGIDIGDTPTLETASEPKK--------------------------------
>Ectopsocus_briggsi
-------MPKKVEPCKPLACALQKCLTENNYQEAACKEAISALKDCCAKWGETSP-VVCSGFNK----------------------------------------------
>Xanthostigma_xanthostigma
----------MKDPCKLTACKIQKCLQENHFQEDACADVLEEMRQCCLKWYKES--LCCSGIRLDEPKIKEKKSINQSKTN-----------------------------
>Ctenocephalides_felis
--------MRSKDPCKANACMIQKCLSENNFQESACTDVIELMRLCCVKWGTKS--FVCNGIKTEKETSSTSSTVENN----KIPYKLDHKS------------------
>Thrips_palmi
------MSKRSKDPCKPFACAIQDCLNAKNFQESACVDVIENLNECCRHWKHKS--LVCSGMLIGPAEVEKSDVKSDGTSGVEVRKA-----------------------
>Hydroptila_sp._AD-2013
--------MKQKDPCKATACLIQKCLRERGFQEERCADVLEEMRQCCLKWHKVS--LCCSGIKLDPPKIEKETK------------------------------------
>Zorotypus_caudelli
-------MKKKKDPCELYACMLQKCLTENNFRESCCIAVLEQLRECCRIWGNAS--ICCSGFDNSPKAEKEPENKEPESSAKK---------------------------
>Thermobia_domestica
--------MKGGDPCQKQACDIQKCLKIHNYQVSACKDFFEAMKKCCEKWGDLS--GCCSGFKKERELEEAQKTK-----------------------------------
```

HG0272.2 FBgn0034649 CG9865

```
>Drosophila_melanogaster
---MESVRKANQRIRNYPVLLSKCADKATAYAVCVSRD--------LNVQHKICDTEFKEFLSCIRKTALEMKTKL
>Oxyacanthus_curtus
---MESVRKARERMKRFPMLLKECHDPATSYARCVALK--------ENVLKDDCSQEFQMFKNCLASAAKKMGTKM
>Machilis_hrabei
---MESVRKAKVRLQMYPKLLAQCGREGSAYAQCVITI--------ENLKKGDCSKEFQDFRVCIQKAACKLNTRL
>Argulus_siamensis
---MDSVKNAQLKLSKYTQSIVKCNQEASMYAQCVLVQ--------DDLKKHACSEEFKKLVICINKVIGKK----
>Periplaneta_americana
---MESVLKAKQRLRRYPELLVQCTGPATAYAMCVLAK--------ESVRHAECDREFQEFQLCLQKAARHLGTRL
>Euwallacea_fornicatus
---MESVSKAKQRFRQYPVIFAKCSREASVYATCVLKQ--------DNIKKNDCAEEFSGFRTCLRKAAVSLKTKI
>Farfantepenaeus_aztecus
---MESVKKAQNRLRNYPKLVANCAPVATEYARCVALK--------GNVLKGDCAQEFEAFKRCILESAKKMKTKI
>Occasjapyx_japonicus
---MESVKRARSRFQMYPKLLAECGGPAAAYAKCVATH--------EDLPKGACEKEFQGLKECLQAAAKKMGTRL
>Aposthonia_japonica
---MESVRKAKVRMRRYPELLKTCSQPAAAYAKCVAKS--------GNIRRHECEELFIKFMSCMKTAAKNLGSRL
>Eurylophella_sp._AD-2013
---MESVSRARARLRMFPRFLAECAAEGTSYAQCVAQQ--------DNPQRGACAKEFEQFSRCVRGAAQKHGTRI
>Meganyctiphanes_norvegica
---MESVKRARNRLKAYPKLAAQCTGVATEYARCVALK--------ENVMKGDCNAEFEAFKKCLMDSAKRMNTRL
>Grylloblatta_bifratrilecta
---MDSVNKGRQRLQRYPGLLVECNQPAITYATCVVSK--------DNVKKAECEKEFKLLLECLHKAAKKRGTRL
>Kerria_lacca
---MDSVSKAKNRIHKYPVLFIECRNEGKLYAACIASK--------QDIQFNACVNEFRNFKNCLMKSAIKLGTKL
>Pseudomasaris_vespoides
---MEAVKNAKDRLRKYPLLLAECHDAATKYAVCVLAK--------KNLQKNDCEAEFHEFKICLTKAAAKHRTKL
>Metaphatus_ochraceus
---MESVQKARDRLAKYPLIFGKCGKQATLYARCVCLK--------DNVKKDDCAPEFKEFMSCLKTTAKDMKVRF
>Mantis_religiosa
---MESVRKAKQRLRHYPKLLAECHGPATAYASCVLAK--------ENVKRGDCEQEFQALRKCLHQAAKHIGSRL
>Boreus_hyemalis
MQVMDSVRSARQRLSQYPKFLAECTQSATQYAMCVCRD--------TNVQKDQCINEFNAFKQCFQKAALKAKVKL
>Chrysopa_pallens
---MESVKKAHKRFRNYPVLLTECRNEASEYAACVLNS--------GGIKYKECAKEFTNFKNCIQKAAVKHKTKL
>Triops_newberryi
---MEPLRKARTRLHNYPLVIAECGPQATSYGRCIALKL-------DDLKKDTCVKEFEAFKGCLQQASKKLKTKL
>Epiophlebia_superstes
---MEAVNRAKGRMRRFPRLLSLCTAEGNAYAVCILAAHESGGPRGTGLTKGQCDAEFQALKLCVSEAARKAGTRI
>Stenobothrus_lineatus
---MEAVQAARSRLRKYPLLLSKCSTEASLYATCVLSR--------DNIKHSECEQEFRKLTTCLQKAAQKAGTRL
>Medauroidea_extradentata
---MDSVRKAHSRLQRYPELLRRCSKEATAYAGCVIGK--------ENVRKWDCDEEFKQLMSCLRSAAYKAKIRL
>Menopon_gallinae
MGKVEVLKMAKTRLRKYPDLFAECRTEGAAYAACVVKQ--------ENVTRNSCAAEFQNFKNCAMSAAKRLSTRL
>Cosmioperla_kuna
---MESVRRARARLRRYPELFAVCHAPATKYAACVVNK--------DNIKHNECGKEFLEFKQCLEQAAHKCGTRL
>Ectopsocus_briggsi
---MEAVQRSRARLARYPFHLTACAIEGGAYATCVLEK--------ENLTKDACVKEFRALVKCMKVSAAKSGSRL
>Xanthostigma_xanthostigma
---MESVKLARTRFRKYPLLLTECHNFAAAYASCVLVK--------ENIQQNDCAKEFEAFKNCLKKTALKHKTRL
>Oropsylla_silantiewi
---MDSVRKARARFSQYPSLLGECATQATTYASCVCRD--------LNVEKDRCLKEFTELQKCVKKAASKAKVRI
>Gynaikothrips_ficorum
---MESVKRAKLRLSRYPALLAECREPAKQYAACVSRQ--------DNVVKGSCQTEFVRFKGCLLKASAKMATRM
>Annulipalpia_sp._AD-2013
---MESVKKAHTRLRQYPVLLAKCGESASAYAKCVIRL--------DTVDKNDCSKEFNSFKSCLMKAAKDMKTKM
>Zorotypus_caudelli
---MDALTKARNRLKKYPQHLKECTEPAAAYASCIVRK--------DNLKKGDCEKEFKELKKCLLLVAEKRKTKL
>Atelura_formicaria
---MESVKKARGRIRKYPQLLAGCAKEGTKYAKCVMDN--------ANVQKGCCTKEFEELKTCLIKAAKKQGTRI
```

HG0272.3 ENSG00000143315 PIGM

```
>Homo_sapiens
MPDVIVCSRFGWQPGGWEMRRPPAAKNRREG
>Rhinolophus_ferrumequinum
------------------MRRQPEGRSRS--
>Ceratotherium_simum_simum
------------------MRRPPEARNRRED
>Manis_javanica
------------------MRGPPEARNAS--
```

HG0273.1 ENSGALG00000003293 MBTPS1

>Gallus_gallus
MSLFIRLYLKRQ
>Anas_platyrhynchos
MSLFIRLYLKRP
>Chaetura_pelagica
MSLFIRLYLKRP
>Apteryx_australis_mantelli
MSLFIRLYLKRP
>Cariama_cristata
MSLFIRLYLKRP
>Calidris_pugnax
MSLFIRLYLKRP
>Merops_nubicus
MSLFIRLYLKRP
>Cuculus_canorus
MSLFIRLYLKRP
>Aquila_chrysaetos_canadensis
MSLFIRLYLKRP
>Gavia_stellata
MSLFIRLYLKRP
>Eurypyga_helias
MSLFIRLYLKRP
>Opisthocomus_hoazin
MSLFIRLYLKRP
>Nipponia_nippon
MSLFTRLYLKRQ
>Fulmarus_glacialis
MSLFIRLYLKRP
>Aptenodytes_forsteri
MSLFIRLYLKRP
>Struthio_camelus_australis
MSLFIRLYLKRP
>Tinamus_guttatus
MSLFIRLYLKRP

HG0273.2 ENSG00000140943 MBTPS1

>Homo_sapiens
MECPGVFAAEAPLSRARRP--
>Felis_catus
METRGVFAAEAPLSRARGTRA
>Lipotes_vexillifer
MEILGVFAAEAPLSRAGRTRA
>Rhinolophus_ferrumequinum
METRGVFAAEAPLSRAHRTRA
>Condylura_cristata
MESRGVFAAETPLS-------
>Equus_asinus
MESRGVFAAEAPLSRARRTRA
>Manis_javanica
MEARGVLAAEAPLSPARRN--
>Psammomys_obesus
METRGVFAAEAPFSRARQLRA
>Trichechus_manatus_latirostr
METRGVLSVEARLSRARRTGA

HG0273.3 ENSG00000140943 MBTPS1

>Homo_sapiens
MLQLLAETKIHF
>Chinchilla_lanigera
MLQLLAETKIHF
>Tupaia_chinensis
MLQLLAETKIHF

HG0274.1 ENSG00000204256 BRD2

>Homo_sapiens
MTGPSGQYRPPRSPWSSPFSGPAQSSESVHPLYSALKRISKMEAAAP
>Ailuropoda_melanoleuca
MTGPGGQYRPHRSPRRSPFSGPAQSSESVHPLYSALKRISKMEAAAP
>Orcinus_orca
MTGPGGQYRPHRSPRSSPFSSPAQSSESVHPLYSALKRISKMEAAAP
>Myotis_davidii
MTVPGGQYRPHRSPRSSPFSGPAQSSESVHPLYSALKRISKMEAAAP
>Dasypus_novemcinctus
MTGPGGQY---------------------------------------
>Galeopterus_variegatus
MTGPCGQCRPHRSPRSSPFSGPAQSSESVHPLYSALKRISKMEAAAP
>Condylura_cristata
---MEAAAP
>Oryctolagus_cuniculus
MTGPGGQYRPYRSPRSSPFSGPAQSSESVHPLYSALKRISKMEAASP
>Elephantulus_edwardii
MTRPGGQYRPHRSPRSSPFPGPAQSSESIHPLYSALKRISKMEAAAP
>Equus_caballus
MTGPGGQYRPHRSPRSSPFSGPAQSSESIHPLYSALKRISKMEAAAP
>Manis_javanica
MIGPGGQYRPHRSPRSFPFSGPAQSSESVHPLYSALERISKMVAAAP
>Loxodonta_africana
MTDTGGQYRPHRSPRSSPFPGPAQSSESIHPLYSALKRISKMEAAAP
>Trichechus_manatus_latirostr
MTGPGGQYRPHRSPRSSPFPGPAQSSESVRPLYSALKRISKMEAAAP
>Orycteropus_afer_afer
MTGPGGQYRPHRSPRSSPFLGPAQSSESIHPLYSALKRISKMEAAAP

HG0274.2 ENSDARG00000100129

```
>Danio_rerio
----------------------------MLLRNKSYPKICKDNATGAARSESNKVQHIHLRLAHSDRSSRSV
>Astyanax_mexicanus
---------------------------MLLRCNKSDPKICEENAAGAARTESNKTC----------------
>Ictalurus_punctatus
MGGQTHTHTHTHTHTHTQTTESSQPCIMPLRCNKKLPENLHGNATGAAWSESNKVVLTL-RY----------
```

HG0275.1 ENSG00000100239 PPP6R2

>Homo_sapiens
--MKKIFLKLHILQEKNHKCFSVLPEYMI-------------------------
>Canis_lupus_familiaris
--MKKSFLKLHIVQEKNPKCFHILFELRIKKFP---------------------
>Balaenoptera_acutorostrata_s
--MKKFFLKLHILQEKIPNVFIFCLNTKFKQGVSTEVRRLRRKCASPCSGLLTG
>Rousettus_aegyptiacus
MCVRSAAGRPGFASASALAATRASQEPPRRPARGAGEEIINLILNEKSFLKVAYTPRKNPKCFHILPEYKI-------------------------
>Ceratotherium_simum_simum
--MKKFFLKLHVLQEKNRSFLNILPEFKI-------------------------
>Manis_javanica
MCVRPTPGRPGFASASVLASTRASEEPPQRPARGAGEEIINLLLNEKIILKVTYSPRKNPKCFYILPENKI-------------------------
>Cavia_porcellus
--MKKRILKVGYTPRKKPKCFSVLPDYKI-------------------------

HG0275.2 ENSDARG00000045540 ppp6r2a

>Danio_rerio
MLGHSVPSAFCHSNACRPVEEV
>Pygocentrus_nattereri
MPGRSVLSAFCHSNACLLVEEV
>Ictalurus_punctatus
-----MPSAVCHSNACLLVEKV

HG0276.1 ENSGALG00000008912

>Gallus_gallus
MDSSSSITVTWNIQHHM
>Anas_platyrhynchos
MDLSLSVTVTWNIQHCL
>Calidris_pugnax
MDSSLHVTATWSIQHHL
>Manacus_vitellinus
MDSSLSVTVTWSIQQHL
>Pygoscelis_adeliae
MDSSLSVTVTWSTQHHL

HG0276.2 ENSG00000073734 ABCB11

>Homo_sapiens
MMKTEVGKGCETF
>Equus_asinus
-MKTEVGKGCKTL
>Trichechus_manatus_latirostr
MMKTEVGKGCKTL

HG0277.1 ENSG00000102531 FNDC3A

>Homo_sapiens
MAAAAFVVPRVKQKAGATRRGSEERG
>Felis_catus
MAAAAFVVPRVKQKAGATRRGSEERG
>Orcinus_orca
MAAAAFVVPRVKQKAGATRRGSEERG
>Pteropus_vampyrus
MAAAAFVVPRVKQKAGATRRGSEERG
>Monodelphis_domestica
MAAVGFVISRVKQKEGATGRLGEERG
>Condylura_cristata
MAAAAFVVPRVKQKAGAARRGSEERG
>Ochotona_princeps
MAAAAFVVPRVKQKAGATRRGSEERG
>Elephantulus_edwardii
MAAAAFVVPRVKQKAGATRRGSGERG
>Ceratotherium_simum_simum
MAAAAFVVPRVKQKAGATRRGSEERG
>Octodon_degus
MAAAAFVVPRVKQKAGATRRGSEERG
>Trichechus_manatus_latirostr
MAAAAFVVPRVKQKAGATRRGSEERG
>Orycteropus_afer_afer
MAAAAFVVPRVKQKAGATRRGSEERG

HG0277.2 ENSGALG00000004169

>Gallus_gallus
MAERSVGGRQRRG
>Calidris_pugnax
MAERSVGGRQRRG
>Haliaeetus_leucocephalus
MAERSVGGRQRRG

HG0278.1 ENSG00000119946 CNNM1

>Homo_sapiens
---MPGHLQPPAGTSCPGGV----------------
>Myotis_lucifugus
MGNFPGQWGRRGAGRELLPGRSSLRCCLLLKQEALLQAKLSLGCSSDIPECVRVMHRAVMRGHFQPPAGTSCPDRVRTPTASSPRAQGACTW
>Dasypus_novemcinctus
---MLCAVMRGHLQPSSGTSCPG-----------------
>Condylura_cristata
---MRGHLWPPAGTTCPGRV----------------
>Oryctolagus_cuniculus
---MRGHLQPPAPTPCPGRVCAPTASSPRAQGARTW
>Equus_caballus
----------------------------------MLQGKLSPDCNSDTTKCVWVMQSAVMRGHLQPPAKTSCPGRV----------------

```
HG0278.2 ENSDARG00000078733 cnnm2b

>Danio_rerio
-------------------------------------------------------------------------------MHKSRRRAAEMKP--DWL
>Channa_argus
MYIKKSRVESCRSFYMRCTCFGISFVEEAADAPPTSQRRFPVGRRGRSSRGHQGRHELARKPALASRQCRTRSGNEQVPMHKSRQPSAEMKADSDWL
>Alosa_alosa
-------------------------------------------------------------------------------MHKLKRRTAEMKADSDWL
>Lateolabrax_japonicus
-------------------------------------------------------------------------------MHKSRQPSAEMKADSDWL
```

HG0279.1 ENSG00000175928 LRRN1

>Homo_sapiens
MFTFSARCPTYHNIVFTFC
>Leptonychotes_weddellii
MFTFSACCPTYHYLVFNFC
>Balaenoptera_acutorostrata_s
MFTFSARCPSYHYTVFTFC
>Rhinolophus_ferrumequinum
MFTFSARCPT---------
>Galeopterus_variegatus
MFTFSACCPTCHRTVVTFC
>Ceratotherium_simum_simum
MFTFSACCATYHYIVFTCC
>Manis_javanica
MFTFLVCCPTYHQTVSAFC
>Nannospalax_galili
MFTFSACCST---------
>Orycteropus_afer_afer
MLAFPACCPTHHYIGFTFC

HG0279.2 ENSGALG00000035099 Lrrn1

```
>Gallus_gallus
MSAPPLRGGEERLRG-QRPPGCRNVFRHGFVEDPGTQRSS
>Anas_platyrhynchos
---------MTRLRTKANGTIYRNIFRHRFAEDPGTQRSG
>Calidris_pugnax
-----MLSGTP--NSRLCSRAVRNLFRHRFAEDSGTQRNS
```

HG0280.1 ENSG00000156299 TIAM1

```
>Homo_sapiens
----------------MAHLEILTKPLLFPMGFLAHSHRLR
>Acinonyx_jubatus
------------------------------MGFLAPPHRLR
>Oryctolagus_cuniculus
MLCILVPQLGPEQPAWMAPLEILTKLLLFCMGFSAHYPR--
>Equus_caballus
------------------------------MGFLAHYHRFR
>Ictidomys_tridecemlineatus
----------------MAPLEILTKPLLFGMGFLAHSSRLR
>Tupaia_chinensis
----------------MAYLKILTQLLLSEMGILAHSHRLR
```

HG0280.2 FBgn0085447 sif

>Drosophila_melanogaster
MGAKESVEAAAKMNADGWGHVERSSRYHNHRH-NQRSQSLNRGQALSQ------ADVSGMW
>Rhynchophorus_ferrugineus
MGAKGSVEAA-KINAQNWSNFEHNHSKQFVGH-HQRSQSLNRGVREQWSGRGADLGGSDMW
>Trichoplusia_ni
MGAKESVEAA-KFSAQNWNNFEHAAVKHPYRHGTQRSQSLNRGHSLSR------TD--GTW
>Chrysopa_pallens
MGAKGSVEAA-KYNAQQWNSMEGSRHRH-----SQRSHSLNRGHSLSR------AP--DMW
>Oropsylla_silantiewi
MGAKGSVEAA----KENWEHIERGR--HHHRL-HQRSQSLNRGHSLSR------ADTAGMW

HG0281.1 ENSGALG00000006766 DEPDC5

>Gallus_gallus
MCRMSSTVFTRNS
>Anas_platyrhynchos
---MSSTVFTQSS
>Apteryx_australis_mantelli
---MSSTVFAQSS
>Buceros_rhinoceros_silvestri
---MTSTVFTQSS
>Calidris_pugnax
MCTMSSTVFTQSS
>Colius_striatus
---MSSTVFAQSS
>Aquila_chrysaetos_canadensis
---MSSTVFTQSS
>Chlamydotis_macqueenii
MCKMSSTVFTQGS
>Pelecanus_crispus
---MSSTVFTQSS
>Picoides_pubescens
---MSSNVFTQSS
>Fulmarus_glacialis
---MSSTVFTQSS
>Melopsittacus_undulatus
---MSSTVFTQSS
>Calypte_anna
---MSSTIFTQSS

HG0281.2 ENSG00000100150 DEPDC5

>Homo_sapiens
MTSLPQAWNS
>Leptonychotes_weddellii
MTSLPQAWNS
>Balaenoptera_acutorostrata_s
MISLPQAWNS
>Miniopterus_natalensis
MTSLPQAWNS
>Dasypus_novemcinctus
MTSLPLAWNS
>Galeopterus_variegatus
MTSLPQAWNS
>Condylura_cristata
MTSLPQAWNS
>Oryctolagus_cuniculus
MTSLPQAWNS
>Equus_przewalskii
MTSLPQAWNS
>Manis_javanica
MTSHPQAWNS

HG0282.1 ENSDARG00000061328 cdon

```
>Danio_rerio
----------------------------------MEGPVRICHRENVFRC
>Channa_argus
---------------------MFFPSARTSSPCLMEGPVRICHQKNAFCC
>Trachinotus_blochii
----------------------------------MEGPVRICHQKNASCC
>Micropterus_floridanus
----------------------------------MEGPVRICLQKNAFCC
>Pygocentrus_nattereri
----------------------------------MEGPVRICCQKNAFCC
>Neolamprologus_brichardi
---------------------MSLKPSRTLSPFLMEGPVRICQQKNAFCC
>Esox_lucius
----------------------------------MEGPVRICHQKNELFF
>Lateolabrax_maculatus
MWVEWNSSLTPPPPPPPPAVFMFLPSSRTLSPCLMEGPVRICLQKNAFCC
>Sebastes_caurinus
---------------------MLLPSSRKHSPCLMEGPVRICLQKNAFCC
>Cynoglossus_semilaevis
---------------------MSRPLSYTFCPCLMEGPVRICHQKNASCC
>Oncorhynchus_kisutch
----------------------------------MEGPVRICHQKNELFC
>Trichomycterus_areolatus
----------------------------------MEGPVRICCYRNAFCC
```

HG0282.2 ENSG00000144857 BOC

>Homo_sapiens
MKSCRHLYRLRVAARK
>Condylura_cristata
MKSCQHSHRLRVAAVK
>Loxodonta_africana
MKSCQHSHRRRVAATK

HG0283.1 ENSGALG00000010270 AREL1

>Gallus_gallus
MQVPGVPFSVTAFSMASLC
>Anser_cygnoides
MQIPGVSFSVIIFSMASSC
>Apteryx_australis_mantelli
MQVPGVSLSVMNFSLASSC
>Charadrius_vociferus
MQVPGVSLSVIIFSMDSSC
>Colius_striatus
MQVPGN-------------
>Leptosomus_discolor
MHVPGISLSVIIFSMAASC
>Haliaeetus_leucocephalus
MQVPGVSPSVIIFSMASSC
>Mesitornis_unicolor
MQVPRVSLSVIIFSMASSC
>Opisthocomus_hoazin
MQAPGVSPSVIIFSMASSC
>Phaethon_lepturus
MQVPGVSLSVIIFSMASSC
>Picoides_pubescens
MQVPGVSLSVIIFSMTSSC
>Fulmarus_glacialis
MQVPGVSLSVTIFSMASSC
>Struthio_camelus_australis
MRVPGVSLSVMNFSMASSC
>Apaloderma_vittatum
MQIPGVSLSVIIFSMASSC

HG0283.2 ENSG00000119682 AREL1

```
>Homo_sapiens
MDRPFLLTLWRFSFMWKLEDKVKGAKSYLLW-
>Panthera_tigris
MDRHFPLTLQQFSFLWKLEDKVNGARSYLLW-
>Balaenoptera_acutorostrata_s
MDRHFLLTLQLFSFMWKLEDKVKVAKSYLLW-
>Pteropus_alecto
MDRHFLLTLQLFSLMWKLKTR-----------
>Dasypus_novemcinctus
MDRPILLTLWLFSSMWKLEDKVKGGKSYLFW-
>Galeopterus_variegatus
MDRPFLLTLRLFSFMWKLEDKAKGAKSYLLW-
>Condylura_cristata
MDRHFLLTLQLFSFMWNLEDKVTATKSTCSGE
>Oryctolagus_cuniculus
MDNDLPMTLWLFSFMWKLEDKVKEARDYLLW-
>Equus_asinus
MDRHFLLTLWLFSFLWKLEDNVKGAKNYPLW-
>Manis_javanica
MDRCFPSTLQLFSSMWKLEDKVKGAPEYLLC-
>Dipodomys_ordii
MDRPFLVTLWLFFFMWKLEDKVKGAKSYLLR-
>Tupaia_chinensis
MDRGFLLTLQLFSFMWQTRWKTR---------
>Trichechus_manatus_latirostr
MDRPFLLTLQLFSPMWKLEDRVKGAEVICFGE
>Orycteropus_afer_afer
MDRPFLLTLQLFSSMWKLEDKVEGARSDLLW-
```

HG0284.1 ENSG00000007168 PAFAH1B1

>Homo_sapiens
--MGVKDGRGPAEAAVQRSGGMNLTC
>Leptonychotes_weddellii
--MGVKDGRGPAEAAVQRSGGMNPIC
>Physeter_catodon
--MGVKDGRGPAEAAVQRSGGMNLTC
>Miniopterus_natalensis
--MGVKDGRGPAEAAVQRSGGMNLTC
>Galeopterus_variegatus
--MGVKDGRGPAEAAVQRSGGMNLTC
>Erinaceus_europaeus
--MGVKDGRGPAEAAVQRSGGMNLAC
>Ochotona_princeps
--MGVKDGRGPAEAAVQRSGGMNLTC
>Ceratotherium_simum_simum
--MGVKDGRGPAESAVQRSGGMNLTC
>Mesocricetus_auratus
MPCFLSLLSGNSDAAWLFVFVVMVTFTLSLSLSLTPSLPPSLPPSFLPSLSLPLSRRWMGVKDGRGPAEAAVQRSGGMNLTC
>Tupaia_chinensis
--MGVKDGRGPAEAAGQRSGGMNLTC
>Trichechus_manatus_latirostr
--MGVKDGRGPAEAAVQRSGGMNLTC
>Orycteropus_afer_afer
--MGVKDGRGPAEAAVQRSGGMNLTC

HG0284.2 ENSGALG00000005834 PAFAH1B1

```
>Gallus_gallus
MPPGFSVGFPIWRNICALSHCGKLNCGS
>Anser_cygnoides
MPLGFPIGFHIWRNISDLSHSVAD----
>Haliaeetus_albicilla
MPPDFLLDFTCGKIL-------------
>Melopsittacus_undulatus
MPPDFLSGFTSGKLL-------------
```

HGO285.1 ENSGALG00000012156 DPP10

>Gallus_gallus
MLRGRPRAAGQEEHGGGADTGLGFPPAGAVAPRLPPAFLFLYFSSSSAAAPLRGEVGVMRLAPREGCPGPQRQ
>Calidris_pugnax
--MRLAPREGCPGPQRQ
>Acanthis_flammea
--MRLAPREGCPGPQQQ

HG0285.2 ENSG00000175497 DPP10

>Homo_sapiens
-----------------MNQVNALFRAK
>Loxodonta_africana
MELSLAFCYISPRALRMMNQVNALFRAK
>Ctenomys_sociabilis
-----------------MNQVNALFRAK

HG0286.1 ENSG00000116698 SMG7

>Homo_sapiens
MSLQSAQYLRF--------------
>Leptonychotes_weddellii
MSLQSAQYLRF--------------
>Lipotes_vexillifer
MSLQSAQYLRF--------------
>Eptesicus_fuscus
MSLQSAQYLRF--------------
>Erinaceus_europaeus
MSLQSAQYLRF--------------
>Elephantulus_edwardii
MSLQSAQYLRF--------------
>Heterocephalus_glaber
MSLQSAQYLRFMDSKWKAMGFYMLD
>Trichechus_manatus_latirostr
MSLQSAQYLRF--------------
>Orycteropus_afer_afer
MSLQSAQYLRF--------------

HG0286.2 ENSDARG00000060767 smg7

>Danio_rerio
-MDNMAPGVDLACESVVV
>Pygocentrus_nattereri
MMDNMAPGVDLASGSVVV
>Apteronotus_leptorhynchus
----MAPGVDLASGPVVV
>Trichomycterus_areolatus
-MDNMAPGIDLAPEPAVV

HG0287.1 ENSG00000170325 PRDM10

>Homo_sapiens
MLQTDLSVLHEHTCGRQMTVLYPVLL
>Acinonyx_jubatus
-----------------MTVPHPVLF
>Balaenoptera_acutorostrata_s
-----------------MTVLHPELL
>Rousettus_aegyptiacus
-----------------MTVLHPMLL
>Oryctolagus_cuniculus
-----------------MTALSPVLL
>Equus_asinus
-----------------MTVLHPLLP
>Loxodonta_africana
-----------------MSVVCP---
>Heterocephalus_glaber
MLQTDPSVFHAHACAGQMTVPHPVLL
>Tupaia_chinensis
-MLRSPSVLHEHVCGGQMTALLPVLL

HG0287.2 ENSDARG00000104251 prdm10

```
>Danio_rerio
MQGSLLCSYHSEGRPTSTFS----------------------------
>Amia_calva
MQCGHLCSYHSEVRPTWPFPLKTQISPPISEPKPKSRRRGT-------
>Channa_punctata
MQCGHLCSYHSEVRTTCTFSRKCKKTRLCFVIF---FVRQLLLVLLLI
>Oryzias_latipes
MQCSRLCSYHSEVRTACTFFAKCKITQNSRATSSTTLVQTIIFS----
>Pygocentrus_nattereri
MQCGHLCSYHSEVRPTSTILLK--------------------------
>Plecoglossus_altivelis
MQRSLLCSYHSEELCDNKVTGGEESS----------------------
>Cynoglossus_semilaevis
MQRDHLCSYHSEVRTTYRESAGDSCY----------------------
```

HG0288.1 ENSGALG00000006655 MAP3K13

>Gallus_gallus
MIRYWLPASRLNCPVL--
>Apteryx_australis_mantelli
MIRYWVHMLHLNCVVLIL
>Calidris_pugnax
MIRYWVRVSHLNCVVLIL
>Falco_cherrug
MIRYWLHVSHLNCVVLIL
>Aptenodytes_forsteri
MIRYWVCVSHLNCVVLIL

HG0288.2 ENSG00000073803 MAP3K13

>Homo_sapiens
-----------------------MYCGIQI--LALWERFWSPLLSQNSVPKIF
>Mustela_putorius_furo
MERAKDRAELVGLPVFLVSTYQLLRCAVAVRGRTHTHRFWSPLLHPNSVPLIL
>Rhinolophus_ferrumequinum
------------------------------MCRNSSLLFWSPLLRQNSIRVIL
>Mus_musculus
------------------------------------MPFWSPLLHQTSVAAIF
>Tupaia_chinensis
---MHQNSLPATL

HG0289 ENSG00000072415 MPP5

>Homo_sapiens
MDFILQNQENWLIGKIDL
>Odobenus_rosmarus_divergens
MDFILQNQEIGFIGEIDL
>Balaenoptera_acutorostrata_s
MDFILQNQEIGFIGKIEL
>Miniopterus_natalensis
MDFILQNQEIGFIGKIDL
>Dasypus_novemcinctus
MDFILQNQENWLYRKF--
>Galeopterus_variegatus
MDSILQNQENWLYRKN--
>Condylura_cristata
MDFILQNQEIGFIGKFEL
>Ceratotherium_simum_simum
MDFILQNQEIGFIGKIDL
>Manis_javanica
MDFMLQNQEIGFLGKIDL
>Loxodonta_africana
MDFILQNQENWLYRKN--
>Nannospalax_galili
MDFILQNQENWLYRKI--
>Tupaia_chinensis
MDFILQNQEHWLYRKN--
>Trichechus_manatus_latirostr
MDFILQNQENWLYRKN--
>Orycteropus_afer_afer
MDFILQNQENWLYRKIDL

HG0289 ENSGALG00000009581 MPP5

>Gallus_gallus
--------MDFTQQRNENWPNISISI---
>Anser_cygnoides
--------MDFTQHHNKNWPNSISV----
>Chaetura_pelagica
--------MDFTQHHNENWPHSSISV---
>Apteryx_australis_mantelli
--------MDFIPHHNKNWPYSSICV---
>Buceros_rhinoceros_silvestri
--------MDFTQHHNENWPNSSISVCGE
>Caprimulgus_carolinensis
--------MDFTQHHNENWPNRSISV---
>Cariama_cristata
--------MDFTQHRNKNWPNSSISV---
>Calidris_pugnax
--------MDFTQHHNENWPNISISV---
>Pterocles_gutturalis
--------MDFTQHHNENWPSSSISV---
>Colius_striatus
--------MDSTQHHNKNCPNSSISV---
>Columba_livia
--------MDFTQHRNKNWPSSSISV---
>Leptosomus_discolor
--------MDFMQRHNENWPGSSISV---
>Cuculus_canorus
--------MDFTQHHNENWPNSSISV---
>Aquila_chrysaetos_canadensis
--------MDFTQHHNENWPNSSISV---
>Gavia_stellata
--------MDFTQHHNKNWPSSSISV---
>Mesitornis_unicolor
--------MDFMQHHNENWPNSSISV---
>Tauraco_erythrolophus
--------MDFTQHHNENCPNSSISV---
>Opisthocomus_hoazin
--------MDSTQHHNKNWPNSTISV---
>Lepidothrix_coronata
-------MMDFTQHHNKNWPNSSICV---
>Phalacrocorax_carbo
--------MDFTQHRNENWPNSSISV---
>Picoides_pubescens
--------MDFTHHHNENWLNSSIFA---
>Fulmarus_glacialis
--------MDSTQQHNKNWPNSSISV---
>Melopsittacus_undulatus
--------MDFTQCHSENWPNNSISI---
>Aptenodytes_forsteri
--------MDFTQHHNKNWPNSSISV---
>Tyto_alba
--------MDFTQHHNENWPNSSISD---
>Struthio_camelus_australis
--------MDFMPHHNENWPYSSICV---
>Tinamus_guttatus
--------MDFIPHHNENWPFSTICV---
>Calypte_anna
MGNIFQVVMDFRQHQNESWPSSSISA---
>Apaloderma_vittatum
--------MDFTQHPNENWPNSSFSV---

HG0290.1 ENSG00000116539 ASH1L

>Homo_sapiens
MLVSSFGDPCFLWNTVL
>Acinonyx_jubatus
MLVASFGDPCFLWNTVL
>Balaenoptera_acutorostrata_s
MLVASFGDPCFLWNTVL
>Rhinolophus_ferrumequinum
MLVAHFGDPCFLWNTVL
>Dasypus_novemcinctus
MLVASFGDPCFLWNTVL
>Sarcophilus_harrisii
MLVASFGEQPFLFNTTF
>Monodelphis_domestica
MLVASFGEQPFLFKTVL
>Sorex_araneus
MLVASFGDPCFLWNRVL
>Ochotona_princeps
MLVPSFGDPCFLWNTIL
>Ceratotherium_simum_simum
MLVASFGRPCFLWNTVL
>Manis_javanica
MLVASFGDPCFLWNTVL
>Loxodonta_africana
MLVASLGDLCFLWNIVL
>Dipodomys_ordii
MLVASFGKSCFLWNTVL
>Tupaia_chinensis
MLVSSFGDPCFLWNTVL
>Trichechus_manatus_latirostr
MLVASLGGLCFLWNVVL
>Orycteropus_afer_afer
MLVASLGDLCFLWNIVL

HG0290.2 ENSDARG00000070981 ash1l

>Danio_rerio
MEARLTREARSDEPELH
>Astyanax_mexicanus
MEARLTPQARSDSPELD
>Haplochromis_burtoni
MEARLTREARADTSRPS
>Austrofundulus_limnaeus
MEARLTREARADTSRPS
>Notothenia_coriiceps
MEARLTREARADTSRPS
>Cynoglossus_semilaevis
MEARLTREARADTSRPS

HG0291.1 ENSGALG00000009173 GFRA1

```
>Gallus_gallus
---------------MDRNLESLHLGGFGISTC
>Anser_cygnoides
---------------MDVNLESLDLGGFGISTR
>Apteryx_australis_mantelli
---------------MDLNLESLDLGGCGISTC
>Buceros_rhinoceros_silvestri
---------------MDVNLESLDLGGFGISTC
>Calidris_pugnax
---------------MDLNSESLDLGGFGTSTC
>Pterocles_gutturalis
---------------MDLNLESLDLGGFGISTC
>Colius_striatus
MFFPFSFFFFSPVNSMDLNLESLDLGGFGISTC
>Leptosomus_discolor
---------------MDLNLESLDLGGFGISTC
>Cuculus_canorus
---------------MDLNLESLDLGGFGISTC
>Balearica_regulorum_gibberic
---------------MDLNLESLDLGGFGISTC
>Opisthocomus_hoazin
---------------MDLNLEPSDLGGFGISTC
>Lepidothrix_coronata
---------------MDVNLEPLDLGGFGISTC
>Pelecanus_crispus
---------------MDLNLESLDLGGFGISTC
>Calypte_anna
---------------MDRNLESSNVGGSRISTC
>Apaloderma_vittatum
---------------MDVNLESLDLDSFGISTC
```

HG0291.2 ENSG00000151892 GFRA1

>Homo_sapiens
MELNFGRPEQHSCPGIAAR
>Mustela_putorius_furo
MELNFGRPEQRGCPGIAVC
>Lipotes_vexillifer
MELNFGRPEQRGRPGIAAR
>Galeopterus_variegatus
MELNFGRPEERSRPGTAAC
>Condylura_cristata
MELNFGRPEQRGCPGIAAC
>Ochotona_princeps
MELNFGRPEEHDLPGIAAC
>Equus_przewalskii
MELNFGRPEQRGSPGIAAC
>Rattus_norvegicus
MELNFEWPEERSRPGIAAR
>Trichechus_manatus_latirostr
MELNFGRPEQRGRPSFAAC
>Orycteropus_afer_afer
MELNFGRREQRGRPSIAAC

HG0292.1 ENSGALG00000006502 PCGF5

>Gallus_gallus
MPGKVPLRRAAPQLS
>Chaetura_pelagica
MPGKVELHRAAPQLL
>Buceros_rhinoceros_silvestri
MPGEVHLHRAAPQLL
>Caprimulgus_carolinensis
MPGEVQLHRAAPQLL
>Cariama_cristata
MPGEVQLHRAAPQL-
>Calidris_pugnax
MPGEVQPRRAAPQLS
>Leptosomus_discolor
MPGEVQLPRAAPQLS
>Falco_cherrug
MPGEVQLHRAAPQLP
>Balearica_regulorum_gibberic
MPGEVQLHRAAPQLL
>Ficedula_albicollis
MPGEVQLHRAAPQLL
>Nipponia_nippon
MPGEVQLHRAAPQLL
>Aptenodytes_forsteri
MPGEVQLRRAAPQLL

HG0292.2 ENSG00000180628 PCGF5

>Homo_sapiens
MALSPLRRRWDRLGEDAAEETNSQDMGKRNHQKE
>Felis_catus
---------------------MVSDMGKQNHQKE
>Lipotes_vexillifer
-------------------------MGKQNHQKE
>Pteropus_vampyrus
-------------------------MGKRNHQKE
>Dasypus_novemcinctus
-------------------------MGKQNHQKE

HG0293 ENSG00000152127 MGAT5

>Homo_sapiens
----MEVRKGNQLTQEPE
>Acinonyx_jubatus
----MEMRKGNQLTQEPE
>Orcinus_orca
----METRKGNRLTQEPE
>Pteropus_alecto
----MEARKGNWLTQEPE
>Sarcophilus_harrisii
----MELKEGNRLTQEPE
>Galeopterus_variegatus
----MGVKKGNRLTQEPE
>Monodelphis_domestica
----MEMKEGNWLTQEPE
>Condylura_cristata
----MEARKGNRLTQEPE
>Ochotona_princeps
----MEVRKGSLLTQEPE
>Ceratotherium_simum_simum
----MEVSKGNRLTQEPE
>Loxodonta_africana
----MELRKGHRLTQEPE
>Chinchilla_lanigera
MKQRMEARKGNQLTQEPE
>Tupaia_chinensis
----MDVRKGNWLTQEPE
>Orycteropus_afer_afer
----MELRKGHQLTQEPE

HG0293 ENSGALG00000012187 MGAT5

>Gallus_gallus
MLRKAHWPTNRMELKYGYMLTKESE
>Anser_cygnoides
-----------MELRYRYTLTKESE
>Chaetura_pelagica
-----------MELKYGYTLTKELE
>Buceros_rhinoceros_silvestri
-----------MELKYGYTLTKELE
>Caprimulgus_carolinensis
-----------MELKYRYVLTKELE
>Cariama_cristata
-----------MELKYGYTLTKELE
>Calidris_pugnax
-----------MELKYGHTLTKELE
>Haliaeetus_albicilla
-----------MELKYGYTLTKELE
>Gavia_stellata
-----------MELKYGYTLTKELE
>Eurypyga_helias
-----------MELKYGYMLTKELE
>Sturnus_vulgaris
-----------MELKYGYVLTKEWE
>Pelecanus_crispus
-----------MELKYGYMLTKELE
>Fulmarus_glacialis
-----------MDLKYGYTLTKELE
>Nestor_notabilis
-----------MELKHGYMLTKELE
>Tinamus_guttatus
-----------MEVKYNYMLTQESD
>Calypte_anna
-----------MELKHGYTLTKELE
>Apaloderma_vittatum
-----------MELKYGYTLTEELE

HG0294.1 ENSGALG00000017036 LHFP

```
>Gallus_gallus
---------MMGRNILLAFRIRASPSVRICQG
>Anser_cygnoides
---------MMGGNIPLAVRIRASPSARICQG
>Apteryx_australis_mantelli
---------MMGENIIMAVKIQASPRARIYQG
>Calidris_pugnax
---------MMGGNIPPAVRIQASPSARICQG
>Columba_livia
---------MMGGNIPPAVRIRASPSVRICQG
>Aquila_chrysaetos_canadensis
MGQKRIFDFMMGGNIPLAVRIRASPSARICQG
>Zonotrichia_albicollis
---------MMGGNIPLALRIRASPSVRICQG
>Struthio_camelus_australis
---------MMGENIFMAVRIQASPGARIYQG
>Tinamus_guttatus
---------MMGENISMAVKIRVSPGARIYQG
```

HG0294.2 ENSG00000183722 LHFP

```
>Homo_sapiens
MFPPEKDSKLCVRPGRGREGGRRPPAMDHHL----------
>Myotis_ricketti
MFRPGKTASCVCAPARG-EGGRRPPAMDHHPYYGGPEVEAQ
>Galeopterus_variegatus
MFPPARESKLCVRPGPGREGGRRPPAMDHRSYYRGWEVDAQ
>Oryctolagus_cuniculus
-----MFRQVVCAPGTHREGGRRPPTMDHRSYHREWEVDAQ
>Dipodomys_ordii
MCVSGKDLKLCARPGPGRKGGCRPPAPRPHPTTLSHG----
>Tupaia_chinensis
MFPPGNDSKLCARSGPGREGGRRPPAMDHHPYYREWEVDTQ
```

HG0295.1 ENSG00000166888 STAT6

>Homo_sapiens
MYVCALSYFSFWWWWWKGGGASRASLELAGQSYRPMGPGSAR
>Ailuropoda_melanoleuca
-----------------------------------MGPGSAR
>Balaenoptera_acutorostrata_s
-----------------------------------MGPGSAR
>Pteropus_alecto
-----------------------------------MGPRSAR
>Sarcophilus_harrisii
-----------------------------------MGPGSVH
>Galeopterus_variegatus
---------------------MQVSPELAGQSYRPMGPGSAH
>Monodelphis_domestica
-----------------------------------MGPGSVH
>Erinaceus_europaeus
-----------------------------------MGPGSAH
>Ceratotherium_simum_simum
-----------------------------------MGPGSAR
>Manis_javanica
-----------------------------------MGPGSAC
>Loxodonta_africana
-----------------------------------MGPGSAR
>Nannospalax_galili
-----------------------------------MGPGSAR
>Tupaia_chinensis
-----------------------------------MGPGSAR
>Trichechus_manatus_latirostr
-----------------------------------MGPGSAR
>Orycteropus_afer_afer
-----------------------------------MGPGSAC

HG0295.2 ENSGALG00000003282 STAT5B

```
>Gallus_gallus
-----------------------------------------------------------MRAGVPSLCP-----------------------QLRCAGGGRKQPEHSAARLRRQLTFWKEIAALSSFL
>Odobenus_rosmarus_divergens
------------------------------------------------------------------------------------------------------------MVYRRQLTFEEEIAVLCSFL
>Orcinus_orca
------------------------------------------------------------------------------------------------------------MVYQRQLTFEEEIAALGSFL
>Pteropus_alecto
------------------------------------------------------------------------------------------------------------MVYQRQLTFEEEIAALCSFL
>Elephantulus_edwardii
------------------------------------------------------------------------------------------------------------MVYRRQLTFEEEIAALCSFL
>Ceratotherium_simum_simum
MRKLRPWFGISRARDFPALPPPSSGHSCA--S-----GPPQSRAAGRWDSRGSPPSGQSPRPAPPSLATPGPAAAAAAHSPGSQRPRAEAGRERQRLGGVLAKARALCMVYRRQLTFEEEIAALCSFL
>Chlorocebus_sabaeus
MRKLRPRFGISRARDFPAPPTNHIPPAIL--TRPGSGTLPRSRSRGPAGTPGGPRRRGSPHARRRPALPPAGTAAAVAHSQGSRRPRAEAGRERAGLGGVLARARALCMVYRRQLTFEEEIAALRSFL
>Cricetulus_griseus
------------------------------------------------------------------------------------------------------------MVYRRQLTFEEEIAALCSFL
>Trichechus_manatus_latirostr
MRKLRPWFGISRARGFPTLPLSPQRPLQRLLPATISGRFRGAAGKGWLGLPGDPAVGAAPRAAPPRLAARGPATAAAGHSPGSQQPRAEAGRELGSREGELGRARALCMVYRRQLTFEEEIAALRSFL
```

HG0296 ENSGALG00000006912 CPEB3

```
>Gallus_gallus
MQIARGRRWRRRRVLGKTGTRISFSLQGESSEFRCKRKETWRNCCISLPYKHISHLD
>Mustela_putorius_furo
MQITG-GGW----CLGKNRNQNQFLIAG-----------------------------
>Rousettus_aegyptiacus
MQINR-GGW----CLGKNRNQNQFLIAG-----------------------------
>Monodelphis_domestica
MQITG-GGW----CLEKTGTRISFSLQGERSEFR-----------------------
>Papio_anubis
MQITG-GGW----CLGKNRNQNQFLIAGRTL--------------------------
>Mesocricetus_auratus
MQITG-GGW----CLGKNRNQNQFLIAG-----------------------------
```

HG0297 ENSGALG00000031684

>Gallus_gallus
MCFCFVLQRFFWRLIFTF
>Lipotes_vexillifer
MCFCFVL-----------
>Rousettus_aegyptiacus
MCFCFVL-----------
>Oryctolagus_cuniculus
MCFCFVL-----------
>Elephantulus_edwardii
MCFCFVL-----------
>Cebus_capucinus_imitator
MCFCFVL-----------
>Jaculus_jaculus
MCFCFVL-----------

HG0298 ENSGALG00000042308

>Gallus_gallus
MESIVWECG
>Acinonyx_jubatus
MESIVWECG
>Myotis_davidii
MESVVWECG
>Cercocebus_atys
MESVVWECG

HG0299 ENSGALG00000040465 Sip1

>Gallus_gallus
-------------------------------------MLGKEKEKNVIRRGVTRQSVPRFVFPGVAERDQS---
>Notothenia_coriiceps
MLTFGSP-----QLSPPVKIGGVRFR--GLLLCVTRQRKGGGKKKNVIRRGVTRQSLPRFVSWSGCKTSVVNLP
>Platichthys_stellatus
MLTFGSP-----QLSPPVKIGGVRFR--GLLLCVTWQRKGGGKKKNVIRRGVTRQSLPRFVSWSGCKTSVVNLP
>Takifugu_rubripes
MFNPEDAHVWLSSIIPSREDRWRAFQGASPPLCST-AKKRRWKEKNVIRRGVTRQSLPRFVSWSGCKTSVVNLP

HG0300 FBgn0031668 CG31917

>Drosophila_melanogaster
------------------------MVNVMKGVLVECDPAMKQFLLHLDEKLALGRKFIIQDLDENHLFISTDIVEVLQARVDDLMDRISFPLHDKDA---------
>Eulimnogammarus_cruentus
------------------------MVNVIKGILIKCDPVMKQLLLHLDETLALGSKFVLANLDDCHLFVAQDVYEQLKVRVDELYEKVYNNYSASNPKSFE-----
>Latrodectus_tredecimguttatus
------------------------MVYVMKGILVECDPAMKQFLLHLDETNAFKKKFIIEDLDETHLFIAPDMLEILQTRIDDLMDQISFPLAEK-----------
>Machilis_hrabei
------------------------MVNVKKGILVECDQAMKQFLLHLDETLGLGQKFIVQDLDETHVFIAPEILETLQSRIDDLMDQISFPLADKGM---------
>Argulus_siamensis
------------------------MVNFLKGILVECDPAMKQFLLHLDETFALGKKFIIQDLDDNHLFISSDIIETLKARIDDLMDQISFPLAEKGA---------
>Mastotermes_darwiniensis
---------MKYIQYL-RRKSTEIMVNVMKGVLIECDQAMKQFLLHLDETMALGRKFIIQDLDETHLFISADILETLQSRVDDLMDQISFPLADRGL---------
>Sitophilus_oryzae
------------------------MVNVMKGVLVRCDNAMKQFLLHLDETLKLGRKFIIQDLDENHLFISADILDTLQAKIDDLMDQISFPLADKGN---------
>Eriocheir_sinensis
------------------------MVNVMKGILIKCDPVMKQFLLHLDETMKLGSKFVLVDLDECHLFVQPEVYNSLKNLIDEQYEKVYYNQKSSLE---------
>Forficula_auricularia
------------------------MVNVMKGILIECDHAMKQFLLHLDETSALGKRFIIQDLDETHLFISSDILETLQARVDDLMDKISFPLADRGL---------
>Daphnia_magna
---------------------------------------MKQLLLHLDETHALGNKFIVQDLDETHLFIAPEYLEVLQTRIDDLMDRISFPLAERETD--------
>Occasjapyx_japonicus
------------------------MVNVMKGILVECDPAMKQFLLHLDETSALGRKFIIQDLDETHIFISADIIEVLQTQIDDLMDQISFPLADKNN---------
>Haploembia_palaui
------------------------MVHVTKGILIECDHAMKQFLLHLDETLALGKKFILQDLDETHLFISADILTTLQARIDDLMDQISFPLADKGF---------
>Eurylophella_sp._AD-2013
------------------------MVNVTKGSLITCDPAMKQFLLHLDDTLALGKKFIIQDLDETHLFVSSDIVEILRNRVDDLMDQISFTDKQQQ----------
>Meganyctiphanes_norvegica
------------------------MVNVMKGLLIKCDPVMKQLILHLDETLGLGSKFVLVDLDECHLFVQSEVYKTLKTRIDELYEKVYYNKSTSDSSWQASRYYE
>Grylloblatta_bifratrilecta
------------------------MVNVMKGILIECDQAMKQFLLHLDETTALGRKFIIQDLDETHLFISADILETLQARVDDLMDQISFPLADKGL---------
>Cuerna_arida
------------------------MVNVMKGILVECDHAMKQFLLHLDETVALGRKFIIQDLDETHLFISADILDVLQARVDDLMDQISFPLAEKGL---------
>Copidosoma_floridanum
------------------------MVNVMKGVLVECDPAMKQFLLHLDETLALGRKFILQDLDETHLFISADIVETLQGRVDDLMDQLSIPVHEKGL---------
>Asellus_aquaticus
------------------------MVQVQKGILIKCDPVMKQFLLHLDEKQTLGQRFVLVDLDDCHLFVQSDIYNSLKVRIDELYEKIYYNISGSNQQKNFE----
>Antheraea_assama
------------------------MVNVMKGVLVECDPAMKQFLLHLDETLALGRKFILQDLDETHLFISADIVETLQARVDDLMDQLSIPVHDKGL---------
>Mantis_religiosa
------------------------MVNVMKGVLVECDQAMKQFLLHLDDKLALGRKFIIQDLDDTHLFISADILDALKARVDDLMDQISFPLADKGF---------
>Tanzaniophasma_sp._AD-2013
---------------------------------------MKQFLLHLDDTTALGRKFILQDLDETHLFIAADILETLQARVDDLMDQISFPLADKGL---------
>Boreus_hyemalis
------------------------MVNVMKGVLVECDQAMKQFLLHLDETLALGRKFIIQDLDETHVFISSDIIETLQARVDDLMDQISFPLHDKGL---------
>Corydalinae_sp._KMRSPBM-2012
------------------------MVNVMKGILVQCDQPMKQFLLHLDETLKLGRRFIIQDLDETHLFISADVIEILQAKIDDLMDQISFPLADKGN---------
>Cypridininae_sp._BMR-2011
------------------------MVNALKGILVECDPAMKQFLLHLDEKEILGRRFIIQDLDETKLFISSDILDTLQAKIDDLMDKISFPITET-----------
>Osmylus_fulvicephalus
------------------------MVNVMKGVLVQCDQAMKQFLLHLDETLKLGRRFIIHDLDETHLFISADVIETLQAKIDDLMDQISFPLSEKGY---------
>Triops_newberryi
------------------------MVNVVKGILVECDPAMKQFLLHLDETNALGRKFILQDLDDTHVFISTEILEVLQARIDDLMDQISFPLHEKDP---------
>Epiophlebia_superstes
------------------------MVNVMKGILVECDHAMKQFLLHLDDTVALGRKFIIQDLDETHLFISADILGVLRARIDDLMDQISFPLADKGY---------
>Platynothrus_peltifer
---------------------------------------MKQFLLHLDETNALGTRFIIQDLDANHLFISSDILDKLKNSIDDLMDQISFVDVN------------
>Prosarthria_teretrirostris
------------------------MVNVMKGVLVECDQAMKQFLLHLDETLALGKKFIIQDLDETHLFISADIVETLQARVDDLMDQISFPLADRGL---------
>Timema_cristinae
------------------------MVNVMKGILVECDQAMKQFLLHLDETIALGRKFIIQDLDETHLFISADIVETLQARVDDLMDQISFPLSDKGL---------
>Menopon_gallinae
----------------------------MKGILVECDQAMKQFLLHLDETLALGRKFIIQDLDETHLFVSADVLNILQTKVDDLIDEISAPITEK-----------
>Leuctra_sp._AD-2013
------------------------MVNVLKGVLIGCDQAMKQFLLHLDETNALGRKFIIQDLDETHLFISADILGTLQAKIDDLMDQISFPLADKGL---------
>Polydesmus_angustus
------------------------MVNVMKGVLIECDPAMKQFLVYLDDTNALKRKFIIRDLDDTHLFVSVDIIPILQTKIDDLMDQISFPLAEK-----------
>Acerentomon_sp._AD-2013
------------------------MVNVVKGVLVECDPAMKQFLLQLDETNALNRKFIIQDLDETHLFISSDILETLQEKIDDLMDQISFPLSEK-----------
>Liposcelis_bostrychophila
------------------------MVNVTKGILVECDPAMKQFLLHLDETSALGGKFIIQDLDDTHLFIAAEYLSILRSKVDDLMDQISAPITDK-----------
>Inocellia_crassicornis
------------------------MVNVMKGVLIECDQAMKQFLLHLDESLKLGRRFIIQDLDDTHLFVSADVIEILQTKIDDLMDQISFPIAEKAQ---------
>Ceratophyllus_gallinae
------------------------MVNLKKGILIECDQAMKQFLLHLDETQALGRKFIIEDLDETHVFISADILETLQARIDDLMDQISFPLHEKGL---------
>Archispirostreptus_gigas
------------------------MVNVMKGVLIECDPAMKQFLLHLDETNALGKHFIIRDLDETHLFITADSLETLQGKIDDLMDQISFPLSEKGI---------
>Stylops_melittae
MSLKLVKYRQKFFDNIRPHRNILLYVNVVKGVLIKCDPAIKQFLIVLDETLKLGRRFIVQDLDETHLFVSSDIVDTLQTKLDDILDQINFTVVEKS----------
>Thrips_palmi
------------------------MVNVVKGVLVECDQAMKQFLLHLDETMALGCRFIIQNLDDTHLFISADILTTLQSRIDDLMDQVSFPLVEKDA---------
>Platycentropus_radiatus
------------------------MVNVMKGVLVECDQAMKQFLLHLDETLALGRKFILQDLDETHVFISTDIVETLQARIDDLMDQINFPLHEKGQ---------
>Zorotypus_gurneyi
------------------------MVNVMKGILVECDHAMKQFLLHLDETIALGRKFIIQDLDETHLFISADILETLQARVDDLMDQISFPLAEK-----------
>Atelura_formicaria
------------------------MVNVTKGVLVSCDRAMKQFLLHLDETLALGRKFIVHDLDETHLFISADILETLQARVDDLMDQISFPLADKY----------

HG0301 FBgn0036856 CG9666

```
>Drosophila_melanogaster
----------------------------------MAFP---TTSAQQA-ETNRKILEEIQTKKQLLAGGIINLGLSPPNQMPA--PQLLGQPTTVNPDFQAGV--------GIATNATSTARSAFNPTSSTTLGFFIPQDSYFGNSFIPVLPRLEPLPSPATTPTTPNAP
PSHSISK
>Machilis_hrabei
----------------------------MTLDFDMAFQ--PANNKQEM-Q-NRKILQELQLKKQLLLKEGAAPTSPL---STP--PLS---PN-----PQGFPLLP-----SEAHMLPSSQRTALQCAHSQSPGYFITQDSSFGNIILPVLPRFEQNSK-----------
-------
>Periplaneta_americana
----------------------------------MAFQ--PPSARQEL-A-NRKILEEIQLKKQQILKQGVAPSLNSPTL-TP--SLSAVGGG-----VNLPVSMS-----PEAHMMSSSQRAALQHAHSQSFGYFVTQDSSFGNLILPVLPRFDIK-------------
-------
>Pogonus_chalceus
----------------------------------MAFT--QTNAQREI-T-SRKILEELHLKKQMLLKQGAVTSLNNTPAP--------ILPLVSPQTGTIPFTGAPAAALLGEAQTTSAQRTALHSAHSQSFGFFITQDSSFGNLILPVIPRFDNK-------------
-------
>Procambarus_clarkii
----------------------------------------------------------------MILKQGVSQALNAPATSQQ--PT-G------------------WSSGTDGHIAGNSQRSNLLQANSSSFGYFIPQDSSFGNFILPVLPRLEPK-------------
-------
>Apachyus_charteceus
----------------------------------MAFQ--PPSARQEI-A-NRKILEELQLKKQILLKQGATSTLNTQLTPNV--PVSGTTPSFVSCGLNRGPLVSASSPMSSEPYMTAAQRTA----LQQSYGYFVTQDSLYGNSILPVLPRFESK-------------
-------
>Campodea_augens
----------------------------------MAFQ--PVNNRQDL-Q-NRKILEELQMKKQMLKQGAAPVSLNSTTSPSG--M------GTVTAANSAFSHL-----PPEMQMLQHNQRNALQLANSQSFGHFVTQDSAFGNTLLPVLPRLETPKP-----------
-------
>Haploembia_palaui
----------------------------------MAYQ--PPTARQDV-ATSRKILEELQLKKQLLLKQGVQSTLTSTSLSSL--SMPAT------------DTAN-----ITANMMSSAQRAALNHAHSTSFGYFISQDSSFGNIILPVLPRHESK-------------
-------
>Isonychia_bicolor
----------------------------------MAFQ--PPSNRQEL-Q-NKKILEELHLKKQLLLKQGAVTALNSSNFQS----------S-----PSVPTYGS-----SDTHMMSNNQRTQLQIANTQSYGYFVPQESSFGNFILPVLPRFDTPPSK----------
-------
>Meganyctiphanes_norvegica
----------------------------------MASQ-APGRGAADS-NTQRKILEDLQLKKQMILKQGASQALNAPAPQQT--AT-G------------------WSSGTDGHIAGNSQRQV-QQTTNQSFGYFVTQDSSFGNFILPVLPRLEPTK------------
-------
>Galloisiana_yuasai
----------------------------------MALQ--PTNVRQEL-A-NRKILEELQLKKQLLLKQGAAPSLINTPTITT--SLSMT--A-----GTGPLSMS-----PEAHMLSSNQRAALQHAHAQSFGFFVSQDSSFGNLIIPVLPRFDVAK------------
-------
>Tigriopus_californicus
MAANPSHHTPNSTSGGASGPAVSAGTAAAATGGSLAFG---NPSSGKI-DQQRKILEELERQKKQLAKGNLGPSAANASPAPNHTPNSGG--------PASGTLSKAMEQGDVPSILTQAQKAAMELANKSSFGYFIPQDSAFGNSILPVVPRLQPK-------------
-------
>Clastoptera_arizonana
----------------------------------MDFR--QSQSAREL-A-NRKILEELALKKQMLLKQGAQPLGST---------P--ISPMPV-----TPATNIPANIAPDVHVMSSTTRAALAHANSQSFGFFVPQDSSFGNLILPVLPRLEK--------------
-------
>Leptopilina_clavipes
----------------------------------MAFS--QTN-SREL-Q-NRKILEELQLKKQMLLKQGVTTNLGSSLTSTAGS-----------------ATTVPATQTSDGVTISASQRAALHNAHSNSYGFFVTQDSSFGNLILPVLPRFDNK-------------
-------
>Bombyx_mori
----------------------------------MAFP--QPNAQREL-T-NRKILEDLQLKKQMLLKQGAVAPLTAA---LSQPNP--VSQLPMCSIPPV-SATAGFPQLPEANVINTSHRAALQHANATSCGYFVSQDSSFGNQILPVLPRFENK-------------
-------
>Mantis_religiosa
----------------------------------MAFQ--PPSARQEI-A-NRKILEEIQLKKQLLKQGVVTQNLS-----TP--IAPLVGAG-----VNVPMTVS-----PEAHMLSSSQRAAFQHALLQSFGYFIPQDSSFGNLILPVLPRFDK--------------
-------
>Tanzaniophasma_sp._AD-2013
----------------------------------MAFQ--P-ASRQEI-A-NRKILEELQLKKQLLLKQGVTPTLNTPAL-SQ--SIPGVSPS-----PILPTSLP-----SDAHMMTSSQRVALQHAHQMSFGYFVTQDSSFGNLILPVLPRFDTK-------------
-------
>Nannochorista_philpotti
----------------------------------MAFP--Q-TNTQD----HRKILEELQAKKQQLLKGGAATLTTTPQLPN-------------------ILTTPVANQTIDTHAINSTQRNALNNANSTSFGYFVPTDSSFGNPILPVLPRFDPKV------------
-------
>Corydalus_cornutus
----------------------------------MAFP--QPNAQRDL-A-ARKILEELQLKKQLLLKQGVQATTTANSVTT----N--LSTLPTTTGAPNSTGGP---LSTDSHSMSSAQRAALHAAHTQSFGYFVTQDSSFGNLILPVLPRYDTK-------------
-------
>Euroleon_nostras
----------------------------------MAFP--QPN-QREL-A-NRKILEELQLKKQILLKQGVQATANTNNLTT----P--LGPSVITSGTVINQTGP---GTPQEHNLTSAQRAALHSAHTQSFGYFIPQDSAFGNLILPVLPRFDVK-------------
-------
>Triops_newberryi
----------------------------------MSFQ--PAPGKQEL-A-SRKILEEIQMQKKLLKQGGPLPTPTTVNP-VP--LASQ------------TGQVSAPTANPELQSLNPTQRAALQHAHAHSFGYYVTQESAYGNLILPVIPRLELVAPSK---------
-------
>Calopteryx_splendens
----------------------------------MAFQ--PPSSRQEI-Q-NRKILEELQLKKQLLLKQGAASALNTTPIPPP--PMPV---G-----NTMPL-PT-----GEQHLVSSTQRSALQFAHTHSFGFFVTQDSLFGNLILPVLPRFDMK-------------
-------
>Ceuthophilus_sp._AD-2013
----------------------------------MAFQ--PPSARQEL-Q-NRKILEELQLKKQILLKQGVAPTLSI----SP--SLSV---G-----GSNAVAMT-----PEAHMMSSSQRAALQHAHTQSFGFFVSQDSLFGNLILPVLPRFDK--------------
-------
>Peruphasma_schultei
----------------------------------MAYQ--PPSVRQEM-A-TRKILEELQLKKQMLLKQGVAPTLSSQNLSA----------------IPMSGGLT-----PDAHMMSSAQRTALQNANSQSCGYFITQDSSFGNLILPVLPRFDSK-------------
-------
>Menopon_gallinae
----------------------------------MAFQ---PSTRPDIQNPQKKILEELNLRKQLLKIG-TAQTLNPSALNTP---I--------------GVPLINSNPVSTDNQMSTTARAALQHANSTSFGYFVPQESSFGNNIIAVLPRFNESK------------
-------
>Perla_marginata
----------------------------------MAFQ--APNLRQEL-A-NRKILEELQLKKQMLLKQGVTQATLNTSIPSS--IPIG---M-----PASGLGVP-----AELHLVSPAQRQALQNANSQSFGVFVCQDSSFGNLILPVLPRFEVKPSL----------
-------
>Acerentomon_sp._AD-2013
----------------------------------MAFQQTTPNSRPDL-QQSKKILEELQMKKQQLLKGVNSTNQSSCP------PA-----------STAPISG-----TPDVHMMSPSQRSALQNANATSYGFFIAQDSSFGNLILPVLPRYDNK-------------
-------
>Ectopsocus_briggsi
----------------------------------MAFQ---SNSAGDFPNPQRKILQELNLKKQLLKSG-TAQTLNPSALNAP---I--------------GLPSLNP-PVAPENQMSNIARSALNSANTTSFGYFVAQESSFGNNILAVLPRFPEAK------------
-------
>Xanthostigma_xanthostigma
----------------------------------MAFP--QPNAQREL-A-NRKILEELQLKKQLLLKQGVQASTTTVASNIS---P--IGASTLPAPGPTVQSGA---PSMDTHNLSSAQRAALHSAHTQSFGFFIAQDSSFGNLILPVLPRFDTK-------------
-------
>Ceratophyllus_gallinae
----------------------------------MALP--PTNAQQD----HRKILEELQQKKQQLLKGGAPTLNTTLPTSN-------------------IPNPQGIIPGNDMHMINSTQRIALRNANSTSFGYFVPTDSSFGNQILPVLPRIDAE-------------
-------
>Gynaikothrips_ficorum
----------------------------------MSFQ--PTN-SRKD-P-NRKILEELQQKKQMLLKSGAGVVSPPSVP-----------------------------NLASEPQANKVQCMVMGAAIAQSFAYYIPQESNFGNPILPVIPRFDKN-------------
-------
>Annulipalpia_sp._AD-2013
----------------------------------MAFN--QPNAQREL-T-NRKILEELQLKKQILLKQGPAPNLTGSSVSLTSAPT--PAQLPVSTIPPI-SATTILPQISDTHLINTSHRAALNNANATSCGFFVPQDSSFGNQILPVLPRFDSK-------------
-------
>Zorotypus_gurneyi
----------------------------------MAFQ--PPSSRQEQ-A-NRKLLEELQMKKQLLLKQGVSQTLTVPSITP-------LSVS-----TPVISGLT-----PEAHMMSPAQRVALQHAHSQSFGYFITQDSSFGNQILPVLPRFDSK-------------
-------
>Thermobia_domestica
MFRLLNHLRIERW-NG--------KENLGIRVCMMAFQ--APNARQEI-Q-NRKILEEIQMKKQLLLKQGAAPNLNSPPNLTP--TN--IPVT-----GPMPMYMS-----PEAHMMSSSQRTALQHAQCQSFGYFITQDSSFGNLILPVLPRFDNK-------------
-------
```

>Drosophila_melanogaster
-------MDSDSTVTSLEENTENFC----TNPTRDILAEGITNLFKPTIERLDERVASTIQLQAELRGQLDALAAQLRDIEKAQ-SQIPEFADKVKELLNVKHKVTVISNVLVTSQERLTGLHKLIEKEQRRRQALLDSALSTNIS----------------------
>Oxyacanthus_flavus
------MADSDSVSTSVGDQTEDLV---SP--TRDALVDGIMGMLKPCLDQLDDRVRQTRASQSELQQQLEGLQQQLTSVQQKT-QCPLHLDQYIKKLQNAKRRVVVVNNILQTTQERLNKLNNQMIKENTRRQALLDPSMTAS------------------------
>Machilis_hrabei
-----MEVDTESTNTSLDDKTEDFG----ENPTRDTLAEGIMCVIKPTIDQLDERVKVTRISQVELKQQIEALAEELKKIADAQ-QCPLDLDLYVKKLLNAKRRVTVVSNVLQNAQDRLNKIHQLVAREGSRRRALLEPSPVTTPGSGP-GT---------SRQVNPI
>Cryptocercus_wrighti
-----MDLDTESTTTSIDDKTEDFC----ENPTRDTLAEGLMCLLKPTIDQLDERVRATRISQVELKQQIEGLAEELKKISESQ-QCPLDLDLYVRKLINAKLKVTVVSNILQTAQDRLNKVHQQIAREQSRRRALLEPSPITTPLEAT-NA---------SIQ----
>Pogonus_chalceus
-----MDVDSESTATSIDENTENFC----DNPTRDTLAEGLMGLIKPTVDQLDERVRATRISQLELKQCIESLSEELKKVSETQ-QTVVDLDAYVKKLINAKHRVTVLSNVLQNAQDRLNKVHQSIERETIKRKAFLESTPGEATTSTTSSVI---------------
>Astacus_astacus
------MADTESSCTSVDDRTDDLV---SP--TRDALVDGIVGLLKPCLDQLDDRVRATRVSQSELHQQLEGLAAELSRVQEQT-RCPLQLDAYIKKLQNAKRRVVVVNNILQNTQDRLNKLHSQVNKENARRRALLETTSPLPITPSTSLHA-P-------------
>Occasjapyx_japonicus
-----MEVDTASTTTSLEDKTEDFGFGDGDNPTRDSLAEGLVALLKPTLDQLDERVKNTKISQVELRQQIEALTEELRGIAGEQ-QCPIDLDHYVKKLINARRRVTVVSTVLQTTQERLNKLHQLVQRETGRRRPLLEPSPGAPPSASALPFQ-P-------------
>Baetis_sp._AD-2013
---MCLLKPTIDSLDERVRATRISQVELRQQIDGLADEIKKISDEQ-QCPLDLESYVKKLLNAKRRVTVVSNILQVAQERLNKVHQLVERESARRKALTEPSPVISPSAHP-IE---------TNPSIQ-
>Meganyctiphanes_norvegica
MIQSFNMADTESTCTSVDDRTEDLV---SP--TRDALVDGIVSLLKPCLDQLDERVRTTRLSQSELHQQLEGLAVELSRVKDQV-ECPLQLDSYIKKLQNAKRRVVVVNNILQNTQDRLNKLHSQVNKETTRRRALLETTST-ASTPSTSLHA-P-------------
>Galloisiana_yuasai
-----MDLDTESTTTSIDDKTEDFG----ENPTRDTLAEGLICLLKPTIDQLDERVRATRISQVELKQKIEGLSDELKKISGEQ-QCPLDLDLYVKKLANAKQKVTVVSNVLQAVQERLNKVLIQIDKDEARRRALLEPSPVISPLDVE-ST---------STQ----
>Clastoptera_arizonana
-----MDVETGSTNTSLDDKTEDFC----ENPTRDTLAEGLMCMLKPTIDQLDERVKVTRISQLELKQQIEGLSEELKKISAEQ-SSPLDWEAYANKLTNAKHKITVVSNILQAAQERLYKVSQQINKETARRQALLKQSSPTSSTDSKPLS---------SVQ----
>Ganaspis_sp._G1
-----MDLDTASDNTSLDDKTEDFC----DNPTRDALTEGLMSLLKPTVDQLDERIRSTRISQIELKQQIDSLNEELVKISESL-QQPLELDSYLKKLVNAKQKVTIVSNILQTTQDRLNKIHQAIERESAKKRALLDHSSSYNDDETQKSAK-ERETGHSSVDVQQ-
>Asellus_aquaticus
------MADSDSTCTSVNDRTEDLI---SPKPTRDALVDGIIGLLKPCLDQLDEKVRSTRLSQSELQQQLETLSTQLNRVQDQV-QCPLQLDSYIKKLQNAKRRVVIVNNVLQSTQDRLNKLHGQVGKETSRRKALLESQPSSSTALI--------------------
>Triodia_sylvina
-----MDADTESTTTSAEENTGNLC----DNPTRDTLAEGILGLLKPTVDQLDERVRATRISQVELKQQIDTLTEELKKINQAL-SNHPDIESYVKKLITCKHKVTVVLNVLQTSQERLNEIRRMISLEEGVRRAVLPALEAQSEPEPSTSATLISPEGS--IN----
>Mantis_religiosa
---MCLLKPTIDQLDERVRATRISQVELRQQIEGLAEELKKISEAQ-QCPLDLDVYVRKLINAKLKVTVVSNILQTAQERLNKIHQQIARETSRRKALMEPSPITTPLEAG-NP---------SIQ----
>Tanzaniophasma_sp._AD-2013
---MCLLKPTIDQLDERVRATRISQVELKQQIEGLSEELRKISDGQ-QCPLDLDVYVKKLLNAKQKVTVVSNILQTAQERLNKVHIQIAKETARRRALLEPSPISTPLEVS-NT---------TVQ----
>Nannochorista_philpotti
-----MDADSESTATSLDENTENFC----DNPTRDTLSEGLMGILKPTIDQLDERVRATRVSQLELKQQIDRVSEDVRKILDNK-KPPAELDDYVKKLVNTKLKVTVITNILQSVQERLNKIHSQIDRETARREAQLSSQTEPYDSEVASGSN-Q-------LQNNLN
>Corydalus_cornutus
------MDDTESTATSIDENTENFC----ENPTRDTLAEGLMGLLKPTIDQLDERVRAMRISQSELKQQIEILHEELRKIAELQ-QYDLDLDVYVKKLVTAKHKVTIVLNILQCAQDRLNKIHAGIEKETIRRKALLEKLPTPVQSPPPVPQTSSDDLGH--FGG---
>Chrysopa_pallens
------MEDTESTATSIDENTENSC----DNPIRDTLAEGLMGLLKPTIVQLNERANAVRISQSELKQQIETLQEEIKKISELQ-QSDVDIDSYVKKLMTAKHKVVAVSTVLQNAQDRLNKIHSGIEKETNRRKIVLEKSNTTLQEPSSSSIS---------------
>Triops_newberryi
-----MDSEVISNDTSLDDKIEDFG----DYPTRDILAEGIVNLLRPTVEGLDQSIRDTKQSQAELRTQIEALTSALNVVAENH-SCPIDLEPYIKKLLDSKRRITVISSVLQTSQDRLVKVHHLAQKDIARRKSSLDTQASVPAP----------------------
>Calopteryx_splendens
-----MEQDNDNKVSASVSKTNSFG----ENPTGDALAEGLICLFKPTIDLLDERITATRNSQLELKDKIEILAEELRKISELH-KCPLDLELYVKKLAGAKRKVTVVSNVLQNTQERLSKVQVQVAREAQRRRALLEPSPAMSPVSVESNA---------SLQ----
>Prosarthria_teretrirostris
-----MELDTESTTTSIDDKTEDFC----ENPTRDTLAEGLMCLLKPTVDQLDERVRATRITQVELKQQIEGLAEELRKLSATQ-QCPLDLDLYVKKLMNAKQKVTVVSNILQTAQDRLNKVHLLIEKESSRRRTLLEPSPSTTPLEAT-NP---------SIQ----
>Medauroidea_extradentata
---MCLLKPTIDQLDDRVRAVRISQVELKQQIDGLSEELKKIAESQ-SCPLELDAYVRKLMNAKLKVTVVSNILQTAQERLNKVHANIAKEAAKRKALLEPSQKTTPLEVS-QP---------SIQ----
>Menopon_gallinae
-----MEVDTESTNTSVEDKTEDFC----DNPTRDALTEGLMRLFKPTIDQLDEKVRTIRISQVELKQQIESLSDELKKINESSTDKEIDLDVYVKKLMTVKHKVTVVSNILQNSEERLLKLQQNISKDSTSRKALLESTSVTQVSVTEPDD--------SSVQ----
>Leuctra_sp._AD-2013
-----MDLDDASTTTSIDDKTEDFC----ENPIRDTLTEGLMCLLKPTIDQLDERVRATRISQVELKQQIEGLSDELRKISDAQ-QCPLNLDLYVRKLTNAKLKVTVVSNILQTAQERLNKVHQMIAREAAKRRSLLEPSPISSPLETV-NP---------SIQ----
>Acerentomon_sp._AD-2013
-----MEIDTDSRNTSLDDKTEDYD----DNPTRDALVDGMISLLKPAIDQIDERVKATRLSQLELGEQIGSLAEEIKRVSEEQ-NCPLQLDQYVQKLQNAKKRITVVSNVLQSVQDRLNQLHNIATQELTRQQSLTEVEAPGQSSEDTQ------------------
>Liposcelis_bostrychophila
---MRLFKPTIDQLDDKVRTIRISQVELKQQIESLSEELKRISESNVDKELDLDVYVKKLMNVKHKVTVVSNILQNSEERLLKLQQNISKDSTSHKALLETTETSQATQAEPDS--------SAIQ----
>Inocellia_crassicornis
------MDDTESTATSIDENTENFC----DNPTRDTLAEGLMGLLKPTVDQLDERVRATRISQCELKQQIEGLSEELKKISEAQ-QTNLELDAYVKKLINSKQKVILVSNILQAAQDRLNKIHLAIDKESAKRKALLEKTPAGSTSTLETSQTNR-------------
>Oropsylla_silantiewi
-----MEPDSESTNTSLDENTENFC----DYPTRDTLSEGLMGILKPTVDQLEERVQATKASQIALKSQIDEVSNEISKILSNR-KPPLELDEYLKKLVNTKNKITVVTNILQATQERLNRIHISIEKETQRRESLLATNISLPS---TSGSQ-----------INL-
>Gynaikothrips_ficorum
-----MEGDTASTNTSNDDKTDDYC----DNPTRDTLADGLMCLLKPTVDQLDERVRATRISQNEVKQHLDNLSEELKKISDAQ-QCPIDLDLYVQKLNNAKQKITIVSTFLNSAQDRLSKLSQQISREQSRRKALIEPSQSTLFDASS-GS---------VI-----
>Platycentropus_radiatus
-----MDGDTESTATSVEENTENFC----DNPTRDTLVEGLMGLLKPTVDKLDESVRATRISQVELKQQIDGLADELKKISDSQ-SNFLDLEEYVKKLMNSKRKITAISTILQSTQERLDKVHHLVSREAARKQALLESSEQSASTSGFNDV--SQ-EGN--IN----
>Atelura_formicaria
-----MDVDTESTNTSIDDKTEDFG----ENPTRDTLAEGFMCLLKPTIDQLDERVRATRISQVELRQQIDGLAEELRKISEAQ-HCPLDLDVYVKKLLDAKRRVTVVSNILQNAQDRLNKVHQLVARESVRRRALMEPSPVTTPSTAL-PE---------AKSSQQ-

HG0303 FBgn0260464

>Drosophila_melanogaster
MHTDL-SSHLHTPACNKLIEELQACHEN-NAFAKFVGVCNSIDDKVVKCLKGERIARSAANRAKARERQAKYKEKLLQQESN---------
>Hyalellopsis_costata
MHPDL-SPHLHTPECNEIIQKLFNCHKE-HSLLKFLGTCNDLDHQVWTCLKAERVERRALNRFKAEQRYQQLKEHMKSS------------
>Machilis_hrabei
MHPDL-STHLHTDECNQLIQLLKDCHDR-NIFNKFLGVCNGEDNAVVKCLRKEREENRRKNLEKSREKQRKISQARAAQQ-----------
>Mastotermes_darwiniensis
MHTDL-STHLHTEECNKLIELLKDCHKE-HSFGKFLGYCNDADREVIKCLRKEREQNRKANQQKAQLMQDKVRTSYPE-------------
>Gyrinus_marinus
MHTDL-SPHLHSKECNELIKQLQTCHTE-FPIGKFFGYCNSVDAAMVRCLKAERIERSKLNREKARERQEQIKRIL---------------
>Astacus_leptodactylus
MHPDL-SPHLHSDECNELISKLHACHKE-NKFLKFVGICNDIDSAVWKCMKAERLQRRAKNRALGEEQRKKVFAKLDEGYSWKKM------
>Forficula_auricularia
MHPDL-SPHLHGETCLKIIEAYKTCLIE-HPFGKFINYCGKLDAELNRCLKEERETRRRQNFEKSSYYKDMLKKGDQEK------------
>Daphnia_pulex
MHSNL-SPHLHTPECNQLIQLLLACHKE-NPVGRFVGFCNKQDTLMVACLRKEREANRSVNAEKSKIKQEEIRRRLKEQESI---------
>Occasjapyx_japonicus
MHPDL-SAHLHTPECNVLIEALKACHSE-HKFAKFVGYCNEADSAVLRCLRDERENRRKANFEKSAEKRRRLRNAADQARSE---------
>Isonychia_bicolor
MHPDL-SSHLHTEKCNLLITDLKACHSQ-NKFGRFLGFCNDLDKELVKCLREEREQNRKKNFDKSRAKQKAVLERL---------------
>Meganyctiphanes_norvegica
MHPDL-SPHLHLEECNILIAKLKDCHNE-NKFAKFMGACNDIDKAVNKCLKKEREIKRNANRAAGKEKREATLKKIEEGHSWKEL------
>Galloisiana_yuasai
MHPDL-SAHLHTTECNILITKLKECHEE-NKFRKFLGICNDIDTKLAACLRQERADNRKANQEKARQTQE----KLKRVALKAKSQNL---
>Cercopis_vulnerata
MHPDL-SSHLHTPECNELITQLLECHKN-HNFLRYFGHCTHLDNVMLKCLKRERLTRRAANAAKAVEKNKQVRERMRELYN----------
>Fopius_arisanus
MSDTYSRLPLHQGACNELYYQVQECEKSHHALSKIFGKCSDVHVALTKCLKQNKLNLAAANREQSRIRQENARKKLKAEKNSNKQ------
>Biston_suppressaria
MHTDL-SAHLHTDECNVLIKKLQDCHSE-HSFLKFVGYCNDFDYEMRKCLKGERLRRQKANLDESRKRHAEIRARIVAQEQAKH-------
>Mantis_religiosa
MHPDL-SPHLHTEECQNLINKFHECHKN-YPYKKFLGVCNDLDKAVNKCLKNERIERRRLNQLKARELQEKAKKSKPSA------------
>Tanzaniophasma_sp._AD-2013
MHPDL-SPNLHSAECNILVTKLRECHQE-HKFGRFLGLCNDADLELVRCLRKEREANRLNNPNWSRERQKNICESKASN------------
>Nannochorista_philpotti
MHPDL-SPHLHTEECNIIVQQLKECHNE-HQFRKFVGFCNSIDSKLIKCFKAERENNRRRNKELADTRRETTYRLMEAAKE----------
>Corydalus_cornutus
MHTDL-SPHLHTEECNYIINLLKACHTD-NPFKRFLGVCNDIDRDLIKCLRKEREQNRKLNFEKSREKQERLKQTQKLT------------
>Euroleon_nostras
MHPDL-SPHLHTPECNELIQQLHECHRD-HPLRKFVGYCNDIDRVLLKCLKRERQERSRLNRQKAFEKQERIKRLLSEQEQKS--------
>Triops_newberryi
MHSDL-SAHLHSPECNALIELLLKCHKE-NPVRKFTGYCNSQDTAVTVCLKKEREQNRKLNADKSAQRQKAIQERLRQAQQEKAQS-----
>Cordulegaster_boltonii
MHPDL-SPHLHTEECNILITKLKTCHSE-NRFAKFMGICNDIDRELLDCLRKERNDRRKANFEKSREKRQIVQEKLKQEKVK---------
>Ceuthophilus_sp._AD-2013
MHPDL-SPHLHTDECNVLIRKLHECHRV-NNIGKFFGYCNDVDNELVKCLRKERDIRRKSNLENSRARQEKYKDILKVQKEGQS-------
>Peruphasma_schultei
MSKNV-LKHNYSGNCTGLLEKLTECRED-NKYTKFFGVCNDAFKELDKCLQKEYEDRRTENHAKARALQERKLGKQSQQQT----------
>Perla_marginata
MHPDL-SPHLHTEECNVLIKKLKDCHEQ-NTFGKFLGFCNSADNELLKCLRREREIRRKANFDKAKEFQAKIRQPIPQV------------
>Acerentomon_sp._AD-2013
MHTNL-APHLHTDECNELIQKLFQCRLD-HPVLRFVGYCNDLDILMTKCLKRERLERRRISIEQSTKSRLERLELRRQERQKETESANQQA
>Inocellia_crassicornis
MHSDL-SPHLHSEDCNKVIAELHKCHAD-HPFKKFIGHCNDIDRALVKCLKKERLERTRINYEKSIIRQKEIRERMKNMS-----------
>Ceratophyllus_gallinae
MHSDL-STHLHSEECNAIVQRLTACRQE-NSFKKFLGACNDIDRELVKCFRKDREARRKKNKEASDLRYNKKLNI----------------
>Rhyacophila_fasciata
MHTDL-SPQLHTEECNKIITALKTCHEQ-HAFRKFIGFCNSIDDQMIKCLKEERLRRRAANIKASAEKHAAILKRIEQNTNA---------
>Zorotypus_gurneyi
MHTDL-SRHLHTEECNILIQKLQECHTG-HPFAKFIGFCNDFDSEMRRCLKKERLERKRINLEQAVARRERMNKAIHADVNKN--------
>Tricholepidion_gertschi
MHPDL-SAHLHTDECNILISKLKECHKE-YKFRRFVGACNDIDHELTKCLRAEREANRKVNLERARRQQEVVRQKMREV------------

HG0304 FBgn0260467 CG7071

>Drosophila_melanogaster
---------------MKIMISQVGRELYKVPLRILDHRVFVNGEIEAFLENFEVRRNDSEVEKLFQVTETVGSLKYDLSRCSATGRGSGAE-NLAQLDTEVSHLLDGVNALLAKA-KV-ERTASTQLQEARLAREQRRAEFLTNLEHGYRRIENSFEEKEEEIAELYSDL
QLKLNIAK-------
>Hyalella_azteca
--------------------MAITSEVEEVFSRLFDHRPFLKGEISFFKREFEEKRGDREVEELFRALELTTEIKQAQVEK---VVEASDA-NLPRTIADIQVALRMCHTSLDSD--SRTSRLSSEIERQREDRQQRLAVAKAEVEAKLASINAAYDLKEKELREKFAKL
DSSNTCDS-------
>Machilis_hrabei
--MCGRIAEKE--D-SRRSVDTLEENRVSREHEWETFVTDMAEKCSKIDQTFGNKGDEMKEYYSEL
EKKLHLDQ-------
>Mastotermes_darwiniensis
-------------------MAAVVKDVGEIWTRLFDHRPFLSGEIKFFLKEFEEKRSDREVERLFQILERATEIKETQLDR---VKLASDV-HLPTLNANLEVAVGMCNRILEKE--E-EHQSDTALTARREIRKAEWESFIEDMTQKCSKVDATFQEKEEELKEFYSDL
EKKLHIGK-------
>Calanus_finmarchicus
---------------MSANNRSGFEDITEVHSRLFNHRPFLQGEVKHFVREFEEKRGDREVENIFSCLERVSELRDLGLPG---LLSSAGSNSSTSLVANLEVAESMLGRILEQG--E-TGEVEKTLVASREAREKEWTNFLKEVQIRCKKVDQSFKEQEDSIRKKYQDL
ESGS-----------
>Pogonus_chalceus
-------------------MSQLSKDIGEIWNRLFDHRAFLNGEINFVLKEFEQKRLDKEVEYLFRLLEQITDIKDTQIVR---LRQVGNT-SLPSINKNLDFALNACKRIEDIE--L-KYKEDTSLALAEEERKLRWEKFVDDMSHKCSRVDNTFDEKEEELHEFYTDL
ERKLQIAK-------
>Eucyclops_serrulatus
---------------------MVSKDVFGVYSRLFNHRPFLQGEARHFVKEFEEKRNDREVENIFNTLETVTELKDCQIDK---VKGQCEL-NLTTLSANLLVANSMCSKILDQE--R-TSDFDQALESSRQVREKEWSQFLSDIQNKCEKIDDAYKQKEKDLRLHYQKL
ENVFLQD--------
>Farfantepenaeus_aztecus
-------------------MASAASDVNEVFSRLFDHRPFLKGEIEYFKKEFEEKRGDREVEQLFRSLELITEIKEGQIEK---IVNSSDD-NLPRTIADIQVALHMLEDTIDTE--KKFNS-EELLAKKRAERKSKLTATQQEVQEKLNFLENNYLEKEQALRAQFESL
EKSAGF---------
>Forficula_auricularia
--MCNRIVEKE--Q-LTRKAITLQKHRESRRAEWEKFIGDMTTTCSKVDQTFLEKEDELRHFYSDL
EDKLHIAN-------
>Occasjapyx_japonicus
--MATRISACD--T-QFDSESILCERRVAREQEWERFLQDTSEKSVRVDNAFRDKELQLRGYYSLL
EDKMRP---------
>Baetis_sp._AD-2013
-------------------MSSIPKDVGEIWSRLFDHKPFVQGEVKFFLKEFEEKREDREVEKVFQLLEQVADIKDVQIEK---TIKSCGN-QLSILNSNLEVATGMCDSILSRE--E-ENSVDVALMVKRESRKAEWEAFIRDMGEKCARVDKTFKEREEEIREDYQEL
ERKLHLDKTPE----
>Meganyctiphanes_norvegica
-------------------MAAAVGDVNEVFSRLFDHRPFLKGEIEYFRKEFEEKRGDREVEQLFRCLELTTEIKEAQIDK---VVGSSDD-NLPRTIADIQVALHMCEDTIQVE--KKFAT-EELLSKKRTERQIRLQSAQADVSEKLALLQNTFQDKELELRRQFESL
EREAGYS--------
>Galloisiana_yuasai
--MCNRILEKE--E-VYRSDTAIFAKREIRKTEWQNFIEGMTHKCVFVDQTFQEKEDQIAEFYSDL
EQKLHLEK-------
>Graphocephala_atropunctata
-------------------MNNIIRDCGEIWNRLFDHRPFLNGEILYFQEEFEVKRSDREITKLFEVLEHVTEIRGSQIEK---VKSLCET-NLPDYQTKLSALLSSCDAILDDE--N-KYDIDTNLEVNREIRKSDWERFQSEMQVKYSNVDETFNDKEKELLEFYSDL
EKKLHINNTTISRKE
>Solenopsis_invicta
-------------------MASITKDTGEIWSRLFDHRPFVQGEITFFLREFQEKRADREVERLFKILEYSTELKESQLDR---TEQLGDC-HLPSLKANVDVALSMCNLVLQKE--E-NFDSDSVLNENRLARRTEWEKFINDMSDKCQRVDQTFQEKENEIQEFYVDL
EKKLHITP-------
>Eogystia_hippophaecolus
-------------------MTELSREIAEVWSRLFDHRPFLNGEIKFMLKEFEEKRNDREVENLFVILEKLTDIKDTQVDR---IKRTSE--SLPVLSEKLEQALQLSEKVEKDFLEN-HKTIEEKRLANQSKRQKEWDQFIDDMNFKCQRIDNAFEEKEEELRDLYADL
KHKLNIAK-------
>Tanzaniophasma_sp._AD-2013
--MTEKCAKVDATFEEKEQELREFYTDL
ERKLHIGK-------
>Panorpa_vulgaris
-------------------MTEIIKDVGEIWSRLFDHRPFLNGEIKFFLREFEEKRSDHEVENLFSTLEIATDIKDTDISR---TTLAADS-HLPVLIENIQAAVTLCDKIVDLG--H-SEDLTKQLEENREKRKAEWVLFVDDMTHKCSRIDNTFEEKEEELKDFYSDL
ERKLHIHK-------
>Corydalus_cornutus
------------------MSAEYLKDIGVIWTRLFDHRPFLNGEINFALKEFEEKRSDKEVEQLFTLLEHAADINDSQIKR---FKTAVDT-EIPQLKNQIESILKVTDNILEQA--E-RYKQDETLEINNRARKKEWEEFVEDMTYKCRQVDNTFEEKEEELQEFYADL
EKKLIRQ--------
>Osmylus_fulvicephalus
-------------------MSDIFKDIGNLWTRLFDHRSFLHGEKQFFLREFEEKRTDTEVKQLFNILETITDIKDTEIDR---MKLIANN-HLPSVEINLLLAFDLVDGILQNE--S-NDAQKEKLIQNRTLRDVSLKEFLKDIDFKKERVDRTFEEKEVEILELYLNL
ERKLRLSN-------
>Triops_newberryi
--MSALDNLIKEKELSYRQYYASL
EEQLKAK--------
>Epiophlebia_superstes
--MCNRILEKE--E-EYCPDRALVNNRERRKAEWEAFVEDMTQKCSKVEITFGEKEKEMREFYEQL
GQKLHID--------
>Stenobothrus_lineatus
-------------------MAAIIKDTGELWTRLFDHRPFLNGEIKFVLREFEEKRSDGEVEKLFKILECVSEIKDTQIER---VKLASDI-HLPNLNGNLEVAVSMCNRILEKE--Q-QHRLDTTLEAKREIRKAEWENFIEDMAQKCTKVDTTFQEKEQELKEFYLDL
EKKLHITK-------
>Ramulus_artemis
--MSCRILDKE--Q-QHLADNSLEARRELRKAEWHDFITDMTDKCFKVDRTFEEKERELSDFYVDL
ERKLHLDQ-------
>Cosmioperla_kuna
--MCNRILEND--E-RHRSDTTLVAKRELRKVEWEKFIEDMTQKCSRVDKTFEEKEEELKEFYADL
EKKLHLNN-------
>Inocellia_crassicornis
--------------MSEVVKDTIICDIAGIWTRLFDHRPFLHGEIQFMLKEFQEKRSDSEIENLFKILECGTDIKDTQTDR---IKHRINN-NLPELKTKIDEALNICNDINQQE--D-LYKSDKTLAVNRITRKKEWELFIEDMTYKCSKVDNTFEEKEGELQEFYTDL
ERKLHITK-------
>Ctenocephalides_felis
-------------------MTEIFKDINETWMRLFDHRPFLNGEINYMQQEFEGKRKDIEIEHLFSTLQVSTEIVETHIPR---AKICSES-YLPSIKSSVDKALEVSESVFSED--F-EKSVKEALLEKRAKREAGWESFLEDVNQRYQSIDLTFEEKEQELNEFYSDL
NRKLHLHT-------
>Lepeophtheirus_salmonis
MPGNSAITAGGILEYSGKRTMSVAKDIFDIHDRLFNHRPFLQGEIRHFIKEFEEKREDREVHNLFNVLEKVTELRESEIDK---IITQCDG-IFPNLSANLLVAQSMSNKILDQG--R-TREIEQALENSKDTREKEWTSFMSEIQDTCAKIDDAYKRKEEDITTQYSKL
EESIEKKN-------
>Frankliniella_cephalica
--MCNRMIKSE--E-EHLADNSLEAKRALRKADWENFISDMAGRCSRVDSTFQEKEEELREFYEDL
EAKLHVVK-------
>Rhyacophila_fasciata
-------------------MSDLTKDCGKIWMRLFDHRPFLNGEIKFMLREFEEKRSDREVTNLFSALENATEIKDNQVER---MKESAAL-QLPELATHLVKALSAYEDIEGYK--T-ELSEDTTLEKRREIRKKEWARFTEEMSDTYTRIDNAFEQKEEELKDFYADL
EHKLNITTK------
>Atelura_formicaria
--MCSRIIEKE--E-EHCIDTALLDNRLSRKAEWELFIGDMTDRCSKVDVTFEDKEGELREYYADL
EKKLRTEQ-------

HG0305 FBgn0050290 Ppcdc

```
>Drosophila_melanogaster
MTSQIYSQKKFVPTPPEKGSFPLDHEGLCKKQFLLYASCLRKNAQDTSQCRQDAQNYLACRMDNNLMEKTEWSKLGFHDQSTKTDQKEPEVQKQ
>Asprogammarus_rhodophthalmus
MGSMTLGTRSFQPTPPAKGSFPLDHEGECKTFMIKYMECMHQTGGNNSECREKARDYLSCRMEKGLMAREEWSKLGYADLASSAARDGSE----
>Meinertellus_cundinamarcensi
MTTMTFGQKSFKPTPPDKGSFPLDHEGECKRTMLKYMLCLRENNNDNSQCRVQAKDYLQCRMDNNLMAKEDWKTLGLGDIVPEK----------
>Prorhinotermes_simplex
MSSVTFSQKKFTPIPPDKGSFPLDHEGQCKKAMVKYMICLKTNKNDSSPCREDAMAYLDCRMSNHLMAKEEWSKLGFGDVKTKTSEK-------
>Dastarcus_helophoroides
MSAATFSQQRFVPTPPEKGSFPLDHEGLCKKFMVKYMGCLRSNKNNNSACREEARAYLACRIDNNLMTKEEWSKLGFSDSEPKEQH--------
>Astacus_leptodactylus
MASMTFSQKSFTPTPPQKGSFPLDHEGECKKFMIKYMLCLRENQNENNKCREESREYLGCRMEKGLMAKEEWSKLGYSDLTTSAHHTS------
>Forficula_auricularia
MSAMTFSQKQFIPTAPDKGSFPLDHEGICKKVMLKYMICLNENDNKNSLCRQEAKDYLSCRMNNNLMAKEEWETLGYKDEKN------------
>Daphnia_pulex
MASNTFGQKNFTPTGPLKGSFPLDHDGECKINMLRYMICLRENSNQSSECRQLSKEYLDCRMQKQLMVREEWTKLGFGDISEENKQ--------
>Occasjapyx_japonicus
MSSTTFGQKTFTPTPPEKGSFPLDHEGLCRRFMLRYLLCIERQSHDNSACRGEARDYLECRMDRQLMAREDWGKLGFSDSSAAPPAEKP-----
>Aposthonia_japonica
MSSMTFGQKTFTPTPPDKGSFPLDHEGLCKRQMLKYMICLREYDNDGAKCRAESKDYLSCRMENNLMAKEDWSKLGFDDNVKMKNL--------
>Meganyctiphanes_norvegica
MSSMTFGQKSFTPTPPQKGSFPLDHEGECKKFMIKYMLCLRESKNENSLCRQQSKDYLECRMEKGLMAKEEWTKLGLADVGSAEPSNSEQ----
>Grylloblatta_bifratrilecta
MSSVTFGQKSFTPTPPDKGSFPLDHQGQCKKEMLKYMICLRRNRNDNSACREESMRYLGCRMDNNLMAREEWSKLGFSDLELKPEKNN------
>Halyomorpha_halys
MSSLTFGQKKFTPTPPEKGSFPLDHDGQCKRTMLKYMICLRNTNSDSSKCRQEAKEYLACRMDNNLMAKEDWSKLGFKEEEVKQEN--------
>Psyttalia_concolor
MSSYTFSQKIFTPIPPQKGSFPLDHEGYCKLPMIKYMRCLHTNKNDNTKCRDDAREYLGCRMENNLMSREEWSKLGFHDDDKIEEGEKPKNT--
>Armadillidium_vulgare
MSSMTFGQKLFKPIPPEEGSFPLDHEGLCKKSMINYMQCLSKYNNNNSECRLEAKEYLDCRMRNNLMASEEWSKLGFSDKDKQQSEVTEVKDN-
>Metallyticus_splendidus
MSTMTFGQKQFAPVPPQKGSFPLDHEGQCKKAMLKYMICLNANSNDNSSCRAEAKVYLQCRMDNNLMAKEEWSKLGLSDVEAKLQKE-------
>Boreus_hyemalis
MSSMTFGQKTFIPTAPDKGSFPLDHEGLCKKLMLKYMHCLRMSKNDNSLCRQEAKEYLGCRMDNELMAKEDWSKLGFKESDVVK----------
>Chrysopa_pallens
MSTMTFGQKSFTPTPPDKGSFPLDHEGICKETMYNYMRCLRLNKNNNSACRIEAKDYLACRMDNNLMSKEEWSKLGFQNQNKSTINK-------
>Gryllotalpa_sp._AD-2013
MSSMTFGQKTFTPVPPEKGSFPLDHEGVCRTLMLKYMLCLRRNDNDNSQCREEAKSYLGCRMDNHLMAKEEWSKLGFKDLDMKDDSKEN-----
>Medauroidea_extradentata
MSSVTFGQKSFTPTPPDKGSFPLDHEGQCKREMLKYLLCLRANNNDNSACRREAKTYLGCRMDNNLMAKEEWSKLGFSDLNLDSKGSDKSL---
>Menopon_gallinae
---MAYQGKVFKPVPPMKGSFPLDHDRSCRPEMLKYMKCLMENKYDSEFCRTEIKTYLECRMDNNLMAKESWEALGLKDIAESNSEYSS-----
>Cosmioperla_kuna
MSSMTFGQKSFKPTAPDKGSFPLDHEGQCKHEMIKYMLCLSTNNNNNSACREEAKSYLGCRMDNQLMAKEDWSKLGFDDLRSDSNRVEH-----
>Acerentomon_sp._AD-2013
MGTRSFQQKPLQSIPPEKGSFPLDHENECKRSMLEYMICLQQNKNDNSACRMKAKGYLKCRMDNGLMAIEDWPSLGFKDGNVEASVSAKL----
>Ectopsocus_briggsi
MSSMTFNQKKFTPTAPDKGSFPLDHENLCKQQMMKYMQCLRVSKADNSKCRLESKDYLACRMEHQLMTKEPWSKLGLEDLEDQKTVQ-------
>Inocellia_crassicornis
MSAMNF-QRTFIPTPPDKGSFPLDHEGLCKDQMIRYMTCLRQNRNDNSCCRIEAKDYLGCRMDNNLMLKEDWSKLGYSDNKNNVKKD-------
>Ceratophyllus_gallinae
MSSMTFSQKSFTPTAPQKGSFPLDHEGQCKKFMIKYMSCLRQHKNDNSSCRNEAQAYLGCRMDNELMAKEDFSKLGFNSNEINK----------
>Caligus_rogercresseyi
MASNVFGAKNFKPVAPEKGSFPLDYEGKCKKEYLKFMICLKDNKYVVENCREESKNYLVCRMDHGLMAKETLPNLGFKEEENKKADK-------
>Mengenilla_moldrzyki
MSNPLLRQKVSKPIPPQKGSFPLDHNAICKKEMLKYIICLRNNENGGSNCRELAKSYLKCRMDNNLMASEEWSHLGF-----------------
>Frankliniella_cephalica
--MAQFAKSRFVPTPPEKGSFPLDHDGVCKDLMRKYMTCLSSNKQDNTKCRKEIKTYLDCRMNNNLMAREEWKLLGLEDVGAKSEVVQK-----
>Zorotypus_gurneyi
MSSATFGKKIFTPTPPDKGSFPLDHEGQCKRVMLQYMSCLTKNNNDNSFCRLEAKNYLACRMENNLMAKEEWSKLGFQD---------------
```

HG0306 FBgn0259726

>Drosophila_melanogaster
--------MSIPQ--------------YRPPK------E--------IQDLLDQ---KEQQ----------QANQRRRTAVLPK---RTWIQRNPRLFQISFITGSLLIFFSKPLFDCFIADPLPPPENKVPPHKR----------------------------------
----
>Micruropus_glaber
-----------------------MSNAKTTGRRSSVPP---LHGQ--KYDWLYQKPALKVRTQEHFDAEEYLKRRIEVPKTVSNPWWLKFYNRNPRRFFITFCVVTCGTFYSRFLYDAFVVPTLYIVSSLKEIVFQPPVPAIEPLQLASVTTNSTCDSVQPSRTEPTKRV
SESASATAQTAKPEAVASETSK
>Machilis_hrabei
---MAIK----YGSG-------RVSPHLPK---RTLMQKYPRIISGLLITTGLLVFFSKPLYDAFCGDTVTVETLRARKAAKSQSQ------------------------------
----
>Reticulitermes_flavipes
---MK----------QQPPFVRQKVLKD---RNWIQRNPRTFVCLFTASSLLVLFSRPLYDIFVREYPPVPVDPQLPK------------------------------------
----
>Calanus_finmarchicus
MADRGWHSPGHPS--------------YKKDRVGQTDPS--------RGTSLDE---IKRRSKQKNADKRFNSPFAFHEPKYNK---VNLINRYPRLFGVAFTTVCVLLLYSKPIYDATLREPTQYELDRANFLKRRME--VGGWWDNPFWKR---SSDKPE-----K--
----
>Carabus_granulatus
---MNDNR----------------RTVIRK---RSWLQRHPKIFFWGTVSSSLLIFFSRPIYDIFIRTDFIPPPTPKEKSKQ----------------------------------
----
>Farfantepenaeus_aztecus
--------MSKPVKSPYPRAKIDLPGSYRSGPTGSQRPGRETEGSNSRSRLLQQQDPSHYR----YNPESVKS---NKPEVSRF---RRWIYRNPRRFQIIGLGIGLTIFFSRPLYDIFLREHIEGPLPVKQK-------------------------------------
----
>Occasjapyx_japonicus
---MPGP---------RPTPVVR---RGTYNLPK---RSWRQRNPRAFTGVFVGTCLLVLFSRPLYDAFLRDEGSPSAPRPN--------------------------------------
----
>Eurylophella_sp._AD-2013
--MNRPKPP---------KK----P---HYLNQIGK---RSWVQRNPRAFVIIFTTTCLTIFYSRPLYDIFFREYPEGHIPKNYIFQKKEE-------------------------------
----
>Grylloblatta_bifratrilecta
---MK----------QQPIVRRKLILKD---SPWYQRNPRLFVGLFTTGCLLVLFSRPLYDVFLRPAPTGPAPHPSKR------------------------------------
----
>Meccus_pallidipennis
---MWKE----------KVETPKFRKTSKD---LPWVNRNPRIFIILTTAFALSIFFSKPAYDLMYAERVPHPSLSRSLDAGKGQ-------------------------------
----
>Chyphotes_mellipes
--------------------------------MTALDPCKK----------------FACKLQQCLKDNVYQP--SRCEGVIEE-------LRQCCIKNSTHSVVCEGIDISKPYDHKTVDYKTAIR---
----
>Palaephatus_luteolus
--MDQRRRSKVLGK---RSWMYRYPKTFQITFTTLGLGIFFSKPIYDIFIRKYEDFDYTEPPTTLSQRRN------------------------------
----
>Mantis_religiosa
---MN----------PKP-FIRRKVLKD---RSWIYRYPRAFVGITTAASMLIFFSRPLYDAFVRDLPEAPPEPRVPRLKPSHF------------------------------
----
>Tanzaniophasma_sp._AD-2013
---MK----------EQPIVRRK-VLKD---SPWYIRNPRTFVGIFTTSCLLVLFSRPLYDAFLRDYTSSPPPAAPRSLK----------------------------------
----
>Boreus_hyemalis
---MSVKRGPTMIH---RTWIQRNPRLFSGVFITGSLLILFSRPIYDMFFRTDFAPPRQSLYKK------------------------------------
----
>Corydalus_cornutus
---MSNRK----------------NPVMVK---HSWARRNPKLFSAIFVTTSLLIFWSRPIYDIFFRPVNEMAPPRKVKFD-----------------------------------
----
>Chrysopa_pallens
---MKE----------------RTVLKK---RSWAQRNPRAFFAIFATTSLLILYSRPIYDIFIREDYDTYKKRMEKERLSRQ--AAE--------------------------
----
>Triops_newberryi
---MDPSN---------RKVVWSRFGEKVK---LPWYRRKPILFQKIFISVSLLIFFSRPLYDIFLRSYIEGPNPIRDPYSINER--LGSRVVLK--------PKTPE-----EKS
E---------------------
>Cordulegaster_boltonii
--MNEFKPW---------KEKKFPP---PSRKLLKE---HSWIRRNPGIFSSVFTLGCLMILFSRPLYDAFLRDSVEGRQVLPSNSK-----------------------------------
----
>Schistocerca_gregaria
---MKES----------K-ESPVRRKVLKD---QPWIKRNPRAFAGLLTASFLLAFFSRPLYDIFLRKDNVVPLESPRIAK-----------------------------------
----
>Timema_cristinae
---MKEKLVRRQVLKD---RPFYQRNPRLFGGVFTTVCLLIYFSRPIYDIFFRPDFAPAPIDQIRAIRKKVSD-----------------------------
----
>Leuctra_sp._AD-2013
---MDRK----------PS-PVVRRQVLKD---LPWTRRNPRLFAGIFLTGSMLLLFSRPIYDIFIRDNPEQTPLSAN--------------------------------------
----
>Raphidia_ariadne
--MQKRNTILHK---PTFLQRNPRIIGFSLITLSMLMLFSKPIYDTFFAPNYKFPPKPKN--------------------------------------
----
>Oropsylla_silantiewi
---MNQGPRRRAIRKD---LPWAYRNPRKAANIIVIGSILTIFSKPIYDIFLQEPIDYTVKWVQKIKANIESKKGSE-------------------------
----
>Annulipalpia_sp._AD-2013
--------MSYPY--------------GKPPK------P--------VDPPI--------K----------RDPLKQRRPVLKN---QSFVYRYPGTFTGVMTFLGLAIFFSRPIYDVFFSEMFPDLADPILCDHP-TRGVKGKRYVAP--------EQQ---------Q

>Zorotypus_caudelli
---MN----------SEGPKIRKAVLKD---RSWIYRHPQLFIYSCATIGFLIIFHRPIYEMFNPLPPRIKTQ-LDEAKSKKNSVKISE-------------------------
----
>Atelura_formicaria
---MQ---PRQKPLKD---RSWVQRNPRTFTVTLTSTCLLILFSRPLYDAFLRPAEPPSVPMKK--------------------------------------
----

HG0307 FBgn0037822 CG14683

>Drosophila_melanogaster
-MSAQYQRFLKVLEKWPAEKSKV--GRDLGEQIRKQVTKLTSLEG---GATDKELDRQINSLERLSNNVYAKKYPRTFE-STATGLTAAQCSQVLSSEFLQYLNEDSKKKK---------------------
>Hyalella_azteca
MSGSSYHRFVELLKLWPVDKTKI-GGRDLGEYIQHRISKTFVQGVAT-SVDEEKCSAIVSSLTRLAKDTHKNKFPRTRE-SSALGLTKEECSGLLSNEFLALLHDREK-PFFNRFSNFPNKKT---------
>Machilis_hrabei
MASIYYKKFIEILGKWPLDSTKK--GRDFGEVIRKWVSGTFSHGEAS-SVDVKECEKVYVTLNSLANNKYRDTYKRTHS-TSATGLTRKECQDIVSTEFLQKLQEEENRGILRSVWSSMKSK----------
>Argulus_siamensis
--MLKQSDALSKMLNNSYKKMYPRKFK-STALGLDYETCKVANSNEFLKSMSK---------------------------
>Cryptocercus_wrighti
-MARSYRNFLQLLELWPVDSTKV--GRDLGQHIREQVKVAFKTGEVF-PGDETECNRIYASLKRLADNRYGQMYPREST-TCASGLLADECQAILSHQFLEELRESER-GFFSKLFHRKS------------
>Carabus_granulatus
-MAASYKRMLKLLEKWPVDKNKR--GQDLGEHLRKQVQAAFAARQF--DTNQEECDRKYLSLKRIAEDHHLKTHPRTLK-SSATGLTAEQCSQILSPEFLEALEREDQ-SVFRRIFSF-------GSKK---
>Litopenaeus_vannamei
MARNSYRNFLRLLEKWPLDESKK-GGRDLGEHLRIRVSEAFRLGDAT-PTDEVECQRVCASLNRLANNTYATRYPRKLT-STSTGVTIQECHQIASTEFLKAMEDSEK-GLLSGMFKQG-------------
>Forficula_auricularia
-MAHSYKIALQILEKWPICSSKK--GRDLGEFIRNQFNIGFKDQQIL-FGSEVECNRNLESIKRISENYHANKYKRTLN-SSSTGLTAEECEKSLSNEFLKHLNK---------------------------
>Occasjapyx_japonicus
MSGATYRNYLKLLEKWPVDANKK--GRDLGEHLRTRISEAFHRGAAT-QVDEADCASKLSSLTRLTVDHHAKQYPRRYE-SAATGLSADKCRQIVSTEFINQLLEEEK-GLVEKLLATKLEKKSKD------
>Eurylophella_sp._AD-2013
-MAQHYKNFLRILEKWPLDKSKG--ERDLGLIVRQRVKQAFPSGEST-PVNIDECDKTYGILKRLVDNHHLNLHKRKLE-SSSTGLSAEECRQVLSTEFLKELEKQDR-SLWSRLFNRNKE-----------
>Meganyctiphanes_norvegica
MSQKSYRTFLRLLENWPMDTTKT-GGRDLGEHIRIRVSEAFHKGETT-PVNETECMRIYSSLNRIASNTYFNQYPRSRN-STSTGLSGDICRQVISTEGLASMRVKEP-PTFEKKDN---------------
>Galloisiana_yuasai
-MAKTYRQFLILLEKWPIDSSKR--GRDLGEHLREEVKKAFTAGEVT-PVDEAKCSRYQASLQRLVNNHYGQLYRRSSS-SSASGLTAQECSDLVSSEFLDYLQKEEK-GFFARYFKRK-------------
>Clastoptera_arizonana
-MANHYRNFLRLLEKWPVDTSKS--SRDLGFFIREQLKVNFPQGEIARNIDEKKCNEAYNSLNRLADNYYFDKYQRKFH-SSATGLDAEQCKTVLSDEFLEYLKEENNQTIVSKVFGNKNKEQ---------
>Argochrysis_armilla
-MAGNYKNYLKLLELWPLDKSKA--GRDLGQHIRDQVKVAFSKGDVGNDINREQCNRYYQSLQRIVGNHYGKTYPRTLS-SSASGLTKEQCNEILSPEFNEYLKYYDQ-SFFEKVRLSLALIFTRSNKAKKS
>Danaus_plexippus
-MTSQYKKFTKLIAKWPIDNNKA--ERDLGKFIRDKVKAAFEGGNSK-NLDSELCTRQLYSLNKIADNHYRNKYKRIHD-SSATGLSSEECNLVLSSEVLQYLKEENK-GFFKNIFKKD-------------
>Boreus_hyemalis
-MAASYKKYLQLLEKWPIDKTKN--ARDLGLYLRQQVKLTFGGGVVS-RAEEEKCERQLHALNRLADNTYGKLFSRTST-STATGLSADQCRQILSTDFLELLQEEDK-GMMSKIMSKAKPKT---------
>Euroleon_nostras
-MSNSYRRFLNLLKDWPLDTTKQ--NRDLGQHLRDRIKVAFATGEQF-QGDQQKCDLEYLALRRLADNYYAKKYPRTLQ-SSAIGLSLKECKDIISNEFLESVKEDEK-SVLQKLFSKKSRVVAN-------
>Calopteryx_splendens
-MAGRYKLFLRLLESWPVDNSKK--GRDLGYHIRKKLLEAFPMGETS-SFNAEECEKVYLSLKKINDNYYGDKYKRHTN-VTSTGLTYEQCAGVTSTEFLTEVSAGRR------LFKLSQKR----------
>Stenobothrus_lineatus
-MANNYRKFVKLLESWPVDPSKT--ERDIGFYLRERVKKAFTAGELF-EGDQEKCAREHASLKRLADNVYGKLYKRTTS-SSATGLTAEECNSVLASEFLTLLQKENR-SIFSRLFRGK-------------
>Clitarchus_hookeri
-MANHYRNFLKLLEGWPIDASKK--GRDLGEHLRDQVKTAFNAGEVS-RIDKAKCEKIYMSLKKLADNHYGNLYHRTSS-SSAMGLTAEQCGSVMSTEFLKSLEEEEK-GFFKRIFKRD-------------
>Leuctra_sp._AD-2013
-MAGIYRDFLRILEKWPVDTTKK--GRDIGEYIRQQVKVAFPAGEVF-KGDEEHCRKSAASLKRLSENHYGELYKRTSQ-SSATGLTAEQCNGVLSSEFMEMVEESNK-SFLSKLFGRNKDS----------
>Acerentomon_sp._AD-2013
MSAPLYHKYMKLLERWPVDLSKK--GRDLGEFLRTKIFSEFKYGETS-NVDVLNCERKFEALNRLSADHYMTKYPRLYT-STSSGISVEQCKLVLSTETLKQLEYEDT-GVLKRTFWNLTSYKNKVDDSKK-
>Xanthostigma_xanthostigma
-MASNYRRFLELLKKWPLDKTKE--GRDLGQHIRDKVKVAFAEGDIS-QTNQEECDKQYNSLKRLSESYYANTYPRKVS-SSSTGLKSDQCTQILSNEFLELLEQEDR-SFVSKLFNRSKK-----------
>Oropsylla_silantiewi
-MASSYKTFMKILKQWPIDKTKK--DRDLGRYLRDSIKTVFTKGEVS-TVDEVKCSKQLEALDRIANNTNFVKYKREFA-SSATGLTSEQCNTVLSTDFLEYLEYENK-GIVTKLVTKPPAVES--------
>Caligus_rogercresseyi
-MSASSSRWLKLLQSWPSEPSKK--SRDLSQILLKRKSPSS--------------EAEVRALETLLSNKLALKYSRVYSEATATALGREDLKTLTSTDFLESSTAYANLSFWERIKLKFFSK----------
>Thrips_palmi
-MAQLYERFLKVLQKWPLNADYPPPLRSLPAHLRDQGKAAFSARGEF-SGDTLQCEKRLSSLERLTTNQIARAYPRTQQ-TGALGLTSDQCVFALSDSYQKSLETSATTSLFQRIVSSFRQGSGKEKA----
>Platycentropus_radiatus
-MTSQYRNFLKLLEKWPLDPSKK--GRDLGDRIRVCIKAAFNTEQGF-TGDLKECQQKYESLNRLISNQHGNLFKRTKA-HSSTGLTAQECNLVLSSDFLEYLQQEER-SIFANILPQKD------------

HG0308 FBgn0264743 CG44001

>Drosophila_melanogaster
MPYIIIRGNLASYS--HKYPWRVLVSGLKADDIEQLNKFSCGG---YSDESTIVYLVHPCRILSALEILGFRVVASSSTAVK------QDYNEYMWTMRKEFDEPEPLEAESVVRENLSNIGREAASLGNYHKVDSPE--------
>Meinertellus_cundinamarcensi
MPYAIVTGNLKQSEGAGRPPWQVSVAGLKDKEIAQLNRFQCLT----KDKYTVVFYQHPCIILTALEVIGYRVVSSCPAGEG----NENRSDEYVWTVRKDFSEPEPSDAEEENGI------------------------------
>Coptotermes_gestroi
MPYILVRGNLSSYG--KDYPWRVLVSGLKACDIEQLKRFASGG---YCDESTIVYLQHPCVVLTALEVLGYQVVASSSTSIK------QDYNEYMWTMRKEFSEPEPMVTKPTLKQ------------------------------
>Lepicerus_sp._AD-2013
MPYILVRGNLASYG--HKYPFRVLVSGLKAGDIDQLNRFASGG---YSDDYTIVYLQHPCVILTALEVLGYRVVASSSTSVK------QDYNEHMWTMRKEFAEPEPDPDADTETDV-TTLC-E----------------------
>Apachyus_charteceus
MPYILVRGNLASYG--HKHPWRVLVSGLKASDIEQLKRFASGG---YTDESTIVYLQHPCVILTALEVLDYRVVASSSTSVK------QDYNEYMWTMRKEFSEPAPLLTCKPTN-------------------------------
>Aposthonia_japonica
--MWTMRREFTEPDPTSKPGF---------------------------------
>Ephemera_danica
MPYILVRGTVTQHP-------CAHVSGLKASDIEQLKHFGSARKAVPADDLTVSYALHPCVVLTALEVLGYSVVSCTAGPTG-----PGGETQYLWTLHKDFPEPEPDSDLYNHH-------------------------------
>Grylloblatta_bifratrilecta
MPYIIVRGNLASYG--HRNPWRVLVSGLKAGDLEQLKGFSSGG---YSDDSTIVYCHHPCVVLTALEVLGYKVIASSSTSVK------QDYNEYMWTMRKEFPEPEPDSQTKQPVLT----CN-----------------------
>Lygus_lineolaris
MPYIIVRGNLASYD--NRYPWRVLVSGLKADDLEQLQRFNCGG---YCDNSTIVYLVHPTVILSALEVLGYQVVASSSTAVK------QDYNEYMWTMRKEFAEPEPTKEKEKTNSA-----------------------------
>Bombus_terrestris
MPYILVRGNLASYG--HRYPWRVLVSGLKASDIEQLSRFASGG---YCDDSTIVYLQHPCVILTALEVLGYKVVASSSTSVK------QDYNEYMWTMRKDFSEPEPEPVIKTGKLP-RR--------------------------
>Zygaena_fausta
MPYILIRGNLASYG--HKNPWRVLVSGLKAADIEQLKRFACGG---YSDDSTIVYLQHPCVILSALEVLGYKVVASSSTAVK------QDYNEYMWTMRKEFSEPEPSIIVEQDNIN-NYSK-EAMHLGNYQKDDEV---------
>Mantis_religiosa
--MWTMRKDFASPEPQLAKPTLKQ------------------------------
>Tanzaniophasma_sp._AD-2013
MPYILVRGNLASYG--DRNPWRVLVSGLKAAEIEQLKGFASGG---YCDNSTIVYLQHPCVVLTALEVLDYKVVASSSTSIK------QDYNEYMWTMRKEFSEPNPQTKL-----------------------------------
>Boreus_hyemalis
MPYILIRGNLASYS--HKYPWRVLVSGLKATDIEQLKRFACGG---YCDDSTIVYLQHPCVILTALEVLGYKVVASSSTSVK------QDYNEYMWTMRKEVFEPETCIAPSTVTTI-SPSVDERDNIANISKEALTTITPNKIDE
>Corydalus_cornutus
MPYILVRGHLTSYG--HRYTPRVLVSGLKANDIEQLARFPSGG---YSDDLTIVYLSHPCVILTALEVLGYKVIASSSTSVK------QDYNEYMWTMRKDFTEPEPVNVKEVAHDA-----------------------------
>Chrysopa_pallens
MPYILVRGHLTSYG--HKYTPRVLVSGLKAPDIEQLSRFPSGG---YADDLTIVYHTHPCVILTALEVLGYKVIASSSTSVK------QDYNEYMWTMRKDFAEPEPDPLSK----------------------------------
>Triops_newberryi
MPYVIVSGNLTPYK--SGTPWKVGISGLKGIELQELDRFILEG---QADPHTVVFKHHPIVLLNALEVLGYRVISSYSTDKPTINISQEPVPISVWTMRREFPEPEPSDDDSDSNHPTDASL------------------------
>Epiophlebia_superstes
MPYIIIRGNLASYS--NRNPWRVLVSGLKADDITQLKRFPSGG---YCDDSTIVYMQHPCVILTALEVIGYRVVASSSTSVK------QDYNEYMWTMRKEFTEPEPSVTSFDSGIT-EPLP------------------------
>Ceuthophilus_sp._AD-2013
MPYILVRGNLASYG--HRHPWRVLVSGLKATDIEQLKRFASGG---YSDDSTIVYLQHPCVILTALEVLGYKVVASSSTAVK------QDYNEYMWTMRGEFAEPEPLLSKPTSPTS-----------------------------
>Peruphasma_schultei
MPYILVRGNLASYG--HKHPWRVLVSGLKASDIDQLKGFASGG---YCDESTIVYLQHPCVILTALEVLDYRVVASSSTSVK------QDYNEYMWTMRKEFSEPEPHAAKPGY--------------------------------
>Leuctra_sp._AD-2013
MPYILVRGNLASYG--HKNPWRVLVSGLKAADIEQLKRFASGG---YCDNSTIVYLQHPCVILTALEVLDYKVVASSSTVIK------QDYNEYMWTMKKDFAEPEPQTVSNPE--------------------------------
>Ectopsocus_briggsi
MPYIIVRGNLASYG--HRDPFRVIVSGLKAQDIEQLKQYPCGG---YTDEVTIVYLAHPCIILNVLEVLGYRVIASSTTGVK------QDYNEYMWTMRKEFHEPEPLERN-----------------------------------
>Ctenocephalides_felis
--MWTMTKEFSEPDPTKCGESKHE------------------------------
>Thrips_palmi
MPYIIVRGNLASYD--NRYPWRVLVSGLKAGDIEQLSRFASGG---YCDDSTIVYLQHPCVILSALEVLGYHVVASSSTSVK------QDYNEYMWTMRKDFSEPEPVPAPITAKPS-A---------------------------
>Annulipalpia_sp._AD-2013
MPYILIRGNLASYG--HKNPWRVLVSGLQAADIEQLKKFACGG---FCDTSTIVYLQHPCVILSALEVLGFRVVASSSTAVK------QDYNEYMWTMRREFEEPEPSVFMERDNIT-NIT-KEAMNIADIQNIKANDL-------
>Thermobia_domestica
MPYVIVRGNLTALGNNHSQPWRVLVSGLKADDILNLKRFQCGD---YPDDFTIAFLQHPCVILTALEVLGYRVVSSSSSS----------NNEYMWTMRKEFSEPEPIPQNSESFD------------------------------

HG0309 FBgn0037689 CG8135

>Drosophila_melanogaster
------MSSVEAEVLQKIDLIEP-TTGKLFFE--HKTELLFCRPHLMPLKTQALERLEEMHRDTARQLQKKR----QQKKKSTEPTGSL-------------------------------
>Talitrus_saltator
--------------MSGLKEVLP-RKGLLYQE--ESYQLALCRPKLMPLKSITLEKLEAMQQQLQERSKEQL----QQQELSTQQQ----------------------------------
>Argulus_siamensis
---------MSEEDLKLLKEVIP-YQGVVFQE--DVPNFILCKPKLLPMKSVTLEKLEKMQKESQERVKNQQ----NEVQDANIDSS---------------------------------
>Cryptocercus_wrighti
--------MSEERKIPALKEYIP-YKGLLHHE--EIPEFLLCKPKLMPLKSVTLEKLENMQKEAEEKLKKQQ----MNEQRS--------------------------------------
>Oxysternon_conspicillatum
------------MSEGEIKPIVP-KTGQLLIE--NPEELILCKPRLIPLKSYTLEKLEKMQEEAIKTLRESR----EKTEG---------------------------------------
>Eucyclops_serrulatus
--MPKG-EVKVTVQSEPFREVLP-KRGLIFQE--SSPQLVLCKPKLLPLKSFTLEKLDKMQKEATEKAREQM----IEQQKQEDEDERLFGSVSENAETHAPESVSFE------------
>Farfantepenaeus_aztecus
-------MPEGSEAGQVLKEVLP-RKGLLYQE--DSSPLALCRPKLMPLKSVTLEKLEAMQKAAQEKGRQQQ----EEQ-LERQATAPA------NGSRI------F-------------
>Occasjapyx_japonicus
-MSSEGSSKTTAAPQQLFKEILP-RKGLLHQE--DIPTYALCKPKLMPLKSFTLEKLERVQREAQEKVLDRS----NQPEQN--------------------------------------
>Eurylophella_sp._AD-2013
----------MGDSKDSVKEILP-KKGLLFSE--DIASLILCKPKLMPLKTLTLEKLEKMQKEAEVRGREEH----EKEANESF------------------------------------
>Tigriopus_californicus
--------------------------------------MVLCKPKLLPLKSFTLQKLEAMQSEAKIKAQAQL----RPNEDLEHNDEDE-QRLAADGAAFISESVSFE------------
>Nilaparvata_lugens
-----M-ANLEGSSDSNIQEYLP-TTGLVHNE--EIKELIFCKPKLMPLKSVTLEKLERMQKEADQKLKEQQ----RLPTAAPLPTFQS-------------------------------
>Argochrysis_armilla
-----M-SQAADNISEEIDIVLP-KQGLLYKE--ESLDYILCKPKLMPLKSVTLEKLEKMQKDAEQKLKDTE----VTTNGYKENK----------------------------------
>Metaphatus_ochraceus
--MRFERMQQEASRQWKEKL----KSAKKN--------------------------------------
>Boreus_hyemalis
MSINDLNKNITLDSKTVPQYVLP-PYGEICLK--YESKLKLSHPHIMPLKSVTLEKLEKMQQEAFKQVQENA----KLKEDY--------------------------------------
>Corydalus_cornutus
--------MSNTLNEKNVKYVIPSNTGQLFFE--DTRQLVLCKPRLMALKSLTLEKLEKMQIDAEEKMKLHK----SNYS--
>Osmylus_fulvicephalus
--------MSSGSSTTPMRPVLPINTGQLFFE--DTKELILCKPRLMPLKSLTLEKLEEMQKLAEAEVRAHR----NSTVEEHK------------------------------------
>Triops_newberryi
-------MEESKNTPEVPQRILP-EHGLIHLE-CNNSSWSLCKPKLMPLKSVTLDKLELMQKAAQEKMQQMK----DSSSQS--------------------------------------
>Cordulegaster_boltonii
--------MSDGFKIDR--ENFSVPRGLIQYQ--DFPHFVLCKPMLMPLKPVNLRKLEEMQREILKKVKSQE----NAQHE---------------------------------------
>Teleogryllus_commodus
---------MSKDQTPVLKDFIP-NSGMIYHE--DLPQYILCKPKLMPLKSVTLEKLEKMQKDAEDKLKQQR----LEQINEGNVNNVP-------------------------------
>Sipyloidea_sipylus
---------MSSHNQPALKEFIP-RKGFVVPE--DETQYILCKPKLMPLKSVTLMKLEKMQRDANEKLKKQK----AEPQNDTC------------------------------------
>Menopon_gallinae
-------MSSSHDNDTDLKYTLP-TTGYIFEE--DVNRYVLCKPKLMPMKSITLEKLEKMQKEAEDKFKSHM----NSKCEE--------------------------------------
>Liposcelis_bostrychophila
---------MSTVNNENLKFTLP-TTGFIYEE--DISQYILCKPKLMPLKSITLEKLEKMQKEAEEKLKSQQ----AYTKKE--------------------------------------
>Caligus_rogercresseyi
---MQEESQARAALVLREEGQHLLRSTGPQGIP--------PQHDAEILSMTTQTHRGNKDEEE
>Frankliniella_cephalica
-----------MSEPANVKHVIL-STGVIQQQEKDIFQPTLCKPKLMPLKSVTLQRLEDMQKLAEEKLRQQQ----INSSTGESTQL---------------------------------

HG0310 FBgn0039339 CG5116

```
>Drosophila_melanogaster
-MNDIKRLKDQQREKHPGFDGYMDCMTRSLFTGLATFCLSFSGTYFAQKIVQ-SKIR--YPIKYNILISSLVATGVSYQITSTRTKACQAAWMAFEDKHSVLKEKTF----------
>Poekilogammarus_pictoides
MSDRKSRMVNQFGEEHPAIGSYLECVSRAYLTGLAGFSLSFACTYSVQRLTA-HRLP--YTANGQLLLAAIVASAVAYKVTANHVQHCHNFWMAAEDKHTALNPIQDKGDTQQL---
>Machilis_hrabei
-MNDLNKLKEEYGDKYPAFGSYTECMSRALLTGLSSFVISFSGVYIFQTLMK-KHLP--YGSKANVLISSGVASIVSYYITAKRTQACQSAWMAAEDKHTYLNPISERGNKKDT---
>Argulus_siamensis
-------------------------MKTNYFYGFGYFVIGLGCAEVIQKVVG-AEKY--YGKSIKIGTSLMAAVLVAFKFTLNGAKECSAILKDRSH--------------------
>Prorhinotermes_simplex
-MENINELKKQQRDIHPGFGSYLECMTRALFTGVAAFTLAFSGSFIGQSLLK-KHIP--YGKNTYILLSALLATGISYQVTTQRTKACQAAWMAAEDKHTYFKRGSQAEE-------
>Eucyclops_serrulatus
MLLAQEASSETKKDS--IPASQVECQTRSVMYGVATGTISFVGVYFAQRFLTRHYKG--ASVSLFLATSLFFGGFSGYMVTDAKLKDCAKASKKFKEFSSN----PNK---------
>Farfantepenaeus_aztecus
-MNNNTELINQYKDAFPAFGGYMECMSRTLLTGLSSFCLAFGGTYITQHLTN-KYLP--YGRKNHIIVSSLIACAVAYKVTSIRAEACQAGWMAAEDKHTYLNPISEREKTVDVEEE
>Occasjapyx_japonicus
-MQNMNEVVKKTEEKYPSFGSYTECMSRALLSGMSAFALAFSGTYLLQTALS-RHLP--YGGKGFVLVSSFVAVGASYRTTTTRTRACQRAWVRADGIKDDF---------------
>Isonychia_bicolor
-MNNVKQLKEQYSGEHRGFGIYLECMSRALLMGLSSFCIAFATSYIPQSLLK-KQLPTSYGVGGRILTSSVLAAGVSYYITSQRTKTCQAAWMAVEEKYTYLTPISVIENIESK---
>Meganyctiphanes_norvegica
-MQNTSDTIKQYKGAFPAFGSYMECMSRTFLTGLSAFSLGFGGTYVAQHLTA-KHLP--YDRKLHILFSSIIAVAVSYKVTSVRTNACQAAWMAAEDKHTFLNPISERPKTVDEEE-
>Cuerna_arida
MSRGKERLLELYGDDHPGFGSYLECMTRANFTGLATFTIAFSSTYIAQSILK-NKLP--YSSKFFILSSTAIAAYFSYKVSKARSQVCQAAWLAAEDKSTYFTELEKTSPTPVS---
>Tenthredo_koehleri
-MNSNDNSDTIISAKFPGYGSYLECMTRSLFTGLSTFAIVFSGAFFGQRFMH-KSTP--YSKKSTILISTSLAALASYYITSSYTVACKKSWARANGN-------------------
>Mantis_religiosa
-MENINELKRQQSEKHPGFGSYLECMTRTLFTGLASFTLAFSGSFIGQSLLK-NKLP--YGKNGSILISSLLAVGVSYQVTSQRTKACQAAWMASEDKHTYLKDIPAST--------
>Boreus_hyemalis
-MNDIKKLKLQQKDAHPGFGSYLECMTRALFTGLATFSITFSGIYFIQKSQS-KRLP--YTSNGCILVSAGFAVLASYYITASKTKICQAAWMAAEDKHTYLKELDKVS--------
>Triops_newberryi
-MNNIGELKERYTQIYPAFGSYTECMSRALLTGLSASIFGFSASYFAQYLLK-NRIP--YGLKGQVLTSSAIAILVSYKVTSDRSKACQKAWMAAEDKHTYLQDVEKPRSS------
>Calopteryx_splendens
-MNNINALKEQYKEVHPGFGSYLECMSRTLLTGLASFTLAFAGTYIGQSLLK-KSLP--YKKKSFVIISSLVAVAVSYRVTSVRAKACQAGWLAAEEKHTFFSENPTSDK-------
>Ceuthophilus_sp._AD-2013
--------------------------------------MVFASTYLSQKLLQ-KKLP--YGQNMFVLVSSLLAVGVSYQVTAHRTRNCQSGWMAAEDKCTYFTQNMPDE--------
>Extatosoma_tiaratum
-MNDINQLKREQSEKHPGFGSYLECSSRGLLTGLASFTLAFSATYISQTLLQ-NRLP--YSRNSIILCSACAAIAVSYHVTSRRMQACQAAWLAAEDKHTYLSGIGEAD--------
>Leuctra_sp._AD-2013
-MNDIKKLKEQHSEAHPGFGSYLECMTRSLFTGLAAFTLAFSGSFLAQSLLK-KHLP--YRMNGYILVSSGLAVATSYHITAKRTSACQAAWMAVEDKHTYLTDQPEQKN-------
>Inocellia_crassicornis
-MNDIKILKEQQRESHPAFGSYLECMTRALFTGLASFTIVFSSTFFTQKLLVNKIFP--YRLPRAILISSACAVLSSYQITATRTKSCQAAWMAAEDKYTYLTETK-----------
>Thermobia_domestica
-MSS-SNQRKGLSQNHHAHGSYLECMTRAALTGLSSFTIAFSAAYLSQYTFR-HRLPF-SSRRGPIICSLVVASAFTYYTTSRTLKECELASVPSEEKTTSFRIIQEKQ--------
```

HG0311 FBgn0263251 vnc

>Drosophila_melanogaster
------------------------MSLQTGLAFIFTILASVSIVAAIILLGWFLIWKAFLSKFRLVRELLGQEEPEDQQPLAWDHQNQQPHHHGRARKARRD---
>Cryptocercus_wrighti
--------------------------MKELLLAVFSIISSLFLVLILVCLGWYLVWRFFLSRFRFVRELLGGMTESTSVS-------DLKNGRSRIKKTRRE---
>Aleochara_curtula
--------------------------MKDIILAITTVISSLAIVLFLILLGWYIVWKLFLSRFRFVRELLGGGSPAGETP-------E---NKNRSKRSRRD---
>Forficula_auricularia
MS------IDLVPNEGNSVFTPDDMSIQKILAIIAYSIVSIIIVLAIVAFVWYLLWKFFLSKFRFVREFVGAIYETPTEN-------E-NTKSTKSRKSRTRRLD
>Aposthonia_japonica
--------------------------MKEFLLAVFSVISSLLLVLGLVGFGWFLVWKLFLSRFRFVRELLGGSSESSSVN-------DLKNGRSRIKKTRKD---
>Graminella_nigrifrons
---------------------------MEFVLAIFSFLSSFVVVIAIVCLGWYLVWKVFLSNFRFVRELLGGMSDTPPVE-------G-ETRKARIKKARRE---
>Pseudomasaris_vespoides
--------------------------MQEIVLAAFSVISSLLIVFGLVLLGWYLVWKFFLSRFRFVRELLGGMSESSSVN-------DLKNGRSRIKKVRRD---
>Tischeria_quercitella
--------------------------MKQTFLAIVALISSVALVCLFVLICWFILWKCCLSKFRFVRELLGSMSESPPAS-------EVRQ-PTRAKKLRKE---
>Mantis_religiosa
--------------------------MKEFFLAIFSIISSVFLVLVFVCLGWYFVWKLFLSRFRFVRELLGGMSESNSAS-------DLKDGRSRMKKMRRD---
>Bittacus_pilicornis
--MTESSSVN-------DLKNGRNRIKKVRLD---
>Sialis_lutaria
--MSETSSQT-------NLKNEKGRVKKVRRE---
>Osmylus_fulvicephalus
-------------------MSKNIMNVENMILMVLTIFVTFSTVIVFIFLGWFIVWKCCLSRFRFVKELLSEKQEEEVPV----------RKSSRSRRVHRE---
>Laupala_kohalensis
--------------------------MMELFLAVFSIISSVLLVLVLVFLGWYLVWKLFLSRFRFVRELLGAMSESASVN-------DLKNGRSRMKKTRRD---
>Medauroidea_extradentata
--------------------------MKDFLLAFVSTVVSVAIVVTLVIFGWYLVWKLFLSRFRFVRELLGGANDTPPGS-------E----GKRAKKVRRD---
>Leuctra_sp._AD-2013
---------------------------MEILLTIFSVLFSLALVVSLAGLGWYLVWKFFLSRFRFVRELLGGMSESQSVA-------DLKSGRSRIKKTRLD---
>Ectopsocus_briggsi
--------------------------MISAIFSIFSFLIAFVVVIGLVTFGWFLLWKYLLSRFRFVRDLFGDSRDSTSDS-------P-ESTRPRTRPNRKARID
>Xanthostigma_xanthostigma
--MNGNEHPP-------NQRKSSRSSKKVRRD---
>Ctenocephalides_felis
--------------------------MKDFLLACVSIISSVLIVLILVSAGWYIVWKLFLSRFRFVRELLGGMSESSTVV-------DLKNSRNKIKKVRRD---
>Frankliniella_cephalica
--------------------------MKEILLAVFSLFSSLTLVLALVALGWFLVWKFFLSRFRFVRDLLGSRSESRTVA-------ELKTGRSRAKKSRRE---
>Annulipalpia_sp._AD-2013
MCFTHSQKLGKCRNHFRPMFE--GSSTSSLVWAAALSAGLPILLVLLIATGWFLVWKCFLSKFRFVRELLGGNADQSPSA-------EPKDNRNRSKKVRRE---
>Zorotypus_caudelli
--------------------------MNEFLLAAFSIISSFILVLILVSVGWYLVWILFLSRFRFVRELLGNMSENQVSS-------K----KSRLE--------

HG0312 FBgn0038641 CG7708

```
>Drosophila_melanogaster
----------MCGLHFRTRNSPSQKTSLRWGDCCLA-------------------------------------------------
>Hyalella_azteca
----------MCGLGFRLKNTPSKKTSLRWGDCCGCY------------------------------------------------
>Parasteatoda_tepidariorum
----------MVSLRTRLRNFPSKKTSLRWGDSCVRFFP-FSLTFQ---------------------------------------
>Argulus_siamensis
---------MTCNLGFRTKNTPCQKTSLRWGDYC---------------------------------------------------
>Prorhinotermes_simplex
----------MCGLAFRTRNSPSQKTSLRWGDCCTNYSFSHNYPLH-HRQLHSLQSVQKLKTSAMRVFTAFALHQRFHHSVSLKI
>Calanus_finmarchicus
--------MAVCSLGYQTENTPSKKTSLRWGDDCRVYAVFSLHACK-QDR-----------------------------------
>Nicrophorus_vespilloides
----------MCALGFRTRNSPSQKTSLRWGDCCLAS------------------------------------------------
>Homarus_americanus
----------MCGLGFRLKNTPSKKTSLRWGDCCGRGVVKRSCSSRR---QEGI-------------------------------
>Tigriopus_californicus
--------MTTATLGFTTENSPSKRTSLRWGDDCLQIKQTHLDRSC---------------------------------------
>Aphis_glycines
----------MCSLGFKTRNSPSQKTSLRWGDCCPLF------------------------------------------------
>Polistes_dominula
----------MGGLAFRTRNSPSQKTSLRWGDCCPPNPNPPSSSALWKNRQRDMPPVPV--------------------------
>Ornithodoros_turicata
----------MVSPRYRLRNFPSKRTSLRWGDSCTRGLHRHLLSAQ---------------------------------------
>Tischeria_quercitella
----------MCGLRFRTRNSPSQKTSLRWGDCC---------------------------------------------------
>Varroa_jacobsoni
MPYMSPGQKSNGNNLVRHRNFPSKRTSLRWGDSCPSAYPQLPPLHCAASL---LGPF--------------------H-------
>Triops_newberryi
----------MCGLGFRTRNSPSQKTSLRWGDCCFSRNSTFVQSF----------------------------------------
>Nothrus_palustris
---------MFRLIRLRLRNFPSKKTSLRWGDSCGQNVQLKNY------------------------------------------
>Locusta_migratoria
--------MSSCGPAFRTRNSPSQKTSLRWGDSCAAAAAHL--------------------------------------------
>Liposcelis_bostrychophila
----------MCGLGFKTRNSPSQKTSLRWGDCCFNYNY----------------------------------------------
>Oropsylla_silantiewi
----------MCGLGFRTRNSPSQKTSLRWGDCC---------------------------------------------------
>Caligus_rogercresseyi
-----------MSVGYGTENSPSKRTSLRWGDDCCLLAINSED------------------------------------------
```

HG0313 FBgn0259725

```
>Drosophila_melanogaster
------MSDTRKDPCKPNACRIQACLTSKHRR-----------------------------------------------------
>Gmelinoides_fasciatus
MDREYNSVLKPKDPCKPKACAIQRCLERTRYNVVLCQPYYEKLTECCRQFAARSDA--CGSEEQPPAS-A---------------
>Meinertellus_cundinamarcensi
---------MKKDPCKIFACEIQRCLQENKFQESACQAAINALKKCCKKWREDSLV--CSGIKIDDELNKDIPQK----------
>Reticulitermes_flavipes
---------MKKDPCKHHACNIQKCLKENSYQEQSCQQFIELLRECCCKWGNASVS--CSGIDTRKTP-SQTVRSLACLA-----
>Lepicerus_sp._AD-2013
--------MSSRDPCKPFACNIQKCLKGKEIKRIICFITLF--IVYLRKKLPRAGL--SKGNNTNDKM-L---------------
>Occasjapyx_japonicus
------MPAKPNDPCKPLACEIQRCLVANDFLESKCEEEVKRMLACCDLWGETSGGRSCSGFWINRPGEAPRKRQEAKK------
>Clastoptera_arizonana
---------MKKDPCKKFACEIQKCLKENEFQEDKCKQYIEYLRNCCKTFGPQSTS--CSGIKTSSPPKVD--------------
>Lasius_neglectus
--------MTTVDPCKQFACKLQKCLTDNVYQPSRCQEIIEELRKCCIKHSSISTV--CDGINTSKRYEHNTVDYRKVLK-----
>Papilio_xuthus
----MEFLYMPADPCKKFACEIQRCLSENKFQEVRCARVFESMRQCCLKFKHISLV--CEGYRLEPREFAPVTDRPDKN------
>Tanzaniophasma_sp._AD-2013
----------MKDPCKPFACRIQKCLFVYTCHC----------------------------------------------------
>Nannochorista_philpotti
------MPLPSKDPCKLNACKIQKCLKEHNYQEDACLDIIEQMRLCCVKWGNKSYV--CNGIKTESKQPKYETSNESVKI-----
>Cordulegaster_boltonii
------MKRTKKDPCKVHACRIQQCLKENKFQEDNCKDIINEMQKCCDKWFYESLC--CSGFKKPLQS-----------------
>Tetrix_subulata
--------MKKKDPCKPIACKIQKCLVV---------------------------------------------------------
>Ramulus_artemis
---------MKKDPCKVQACKIQKCLAENNYQESACKEVIKEMVECCRKWSDVSLC--CSGINTKVST-ASSAEV----------
>Cosmioperla_kuna
--------MKKRDPCKAHACRIQKCLAENNFQESACQAVLEDMRSCCRKFKNVSLC--CSGIDIGDTP-TLETASEPKK------
>Inocellia_crassicornis
----------MKDPCKLTACKIQKCLKENRYQEDACAEVLEEMRQCCLKWHQVSLC--CSGIRLDEPKIKD-KKLSSKDDNN---
>Oropsylla_silantiewi
--------MRPKDPCKANACMIQKCLNENNFQESACGEVLELMRLCCVKWGDKSFV--CNGINLEKAENSTDNTKSTKLKPQQKS
>Rhyacophila_fasciata
----------MKDPCKASACKIQTCLKENGYQESRCADVLEAMRQCCLKWHKESLC--CSGIRLEPRPVLQTRQAETHSENTDKN
```

HG0314 FBgn0260468 CG7950

>Drosophila_melanogaster
-----------------------------------MSGYQYLDVKIKLRDPDSVTLTPVFFCGCIHSSLASIFGEIGGQTILEIVKFSSSQKRSILRVPENVLDRVRVAIALIGYYQEVPC-HFQVLSTSRKPLDFEESPEEFVAFN-
>Blattella_germanica
-----------------------------------MSGYYYLDVSLEFEGGSRPELNAVYLKNHILQALNTLFGEVGASTRVDILKYNPNTQRAVLRCPSASYVKLRSSLSLCGNYNAVAC-VYRIHNTSSCLLSLLGTSRTYKH---
>Anoplophora_glabripennis
-----------------------------------MSKYYYLDVSLQLAKTSENTITATYFKNNIVNAVNQLFGEVATAVNIDILKYNSTNQRAILRVPKTHYIKLRSSLTLCGTYEGEPC-AYRIHKASPLLLALQGDSRDYQH---
>Aposthonia_japonica
---MLSMKTLFGETGTCIDVDIIKYNEKSRRAVIRCPSSSYIKLRSALTLSGNYSDIPC-VYRVHNASSSPLSLLGNSRNYKH---
>Xenophysella_greensladeae
---MQALKSLFGEAGQSTCVDILKYDPSHKRAILRCPSYAYVKLRSALTLCGNCSGEEC-AFRIHCCSPSLLALTANSRTYSHR--
>Psyttalia_concolor
--------------------------------------MQYLDVSLDLPSNPSREISPVFLKSHVTQSLKQLFGEKGADCSIDILKYNSTTRRFILRCSSQDYVRLRASLTLASTYEEDNC-IYTVNRSSASPFGLLANGRDYNH---
>Nemophora_degeerella
-----------------------------------MTDYFYLDVSLELDTSDNVEISALYLKKHIIDSLNTLFGEVGASIPVDILKYEPTTKRSILRVPEEFYVKLYSSLTLCGKYQNILC-AYRVHKTTPSLLSLLGDSRTHKF---
>Mantis_religiosa
-----------------------------------MSDYYYLDVSLEIAGKEKQEVTAVYFKNHVMQSLRTLFGEVGGCIIVDVLKYNPEEQRAVLRVPASSYVKLHSSLTLCGEYENTVC-AYRVHNTSPCLLSFLGNSRTYHH---
>Panorpa_vulgaris
-----------------------------------MSNYYYLDVSLELSAA-KSKVSAVLFKKHVIESLQSLFGSISSATQIDILRYNREQIRAIIRVPEKYYVKLRSALTLSGGYEKFQC-AYTIHSSSPCLLSILADSRSYEH---
>Chrysopa_pallens
-----------------------------------MSNYHYLDLTLELPKGLKREISAIFFKKHVMQALNALFGEVGASTTVDLLKYESIPHRAIIRVPSVYYVKLRSSLTLAGDYEGIPC-AYRIHKSSTYLLNLLGDSRNYNHE--
>Prosarthria_teretrirostris
MGRLAKCPCRVLQIQRNVCVAVRRFDNSTNGF--IMSDYYYMDISLICSSPTEEKVSAVYFKDHVMQSLRTLFGEVGSSINVDVLKYDIEKQRAVLRCPSASYVKLHSSLTLSGDYCGSPC-VYKVHQTSAYLLSLLGNSRTYKHI--
>Menopon_gallinae
-----------------------------------MKGYYYLDVLIKFEQG-KISLDENKLKTIIVYSLNTLFGECGSAIEVDVLKYTQTDQRAILRCLADKYVKVRASLSLLNEYAGVQL-QLQVVKASASLLSLQSNSRTYKFA--
>Liposcelis_bostrychophila
-----------------------------------MSSYYYLDVQTKFHQGETIQLTPSEAKETIRKNLKELFGECGTLQSVEILKSYLKEGRFILRCSCSFYVKLRSSLIVCSSLG---N-NFKIIKTSPSLLSLLSKPWETDF---
>Raphidia_ariadne
---------------------MNIFCNYFDIFVILLNVYK----YKEFEKGINKEVTVAFFKKHVLSALNTLHGEVGASTTVDILKYDSTKRRAVLRIPISNYIQLRNALTLVGDYEGIPC-AYRIHNCSAYLLNLIGDSSLYIH---
>Stylops_melittae
-----------------------------------MSNYFYFDISLELEANSTFKPSINNINSHIQQSIKLLFGELGLPHEFHILKYNKAENRFIIRIPKDDFIKFHTALTLSDTFNHVPIITYRIHKITPFLL-FLSNSRTFEY---
>Megalurothrips_sjostedti
-----------------------------------MSKFFYLDVSLEFEDKQKSQVDAVAFKNYIWTAIRTLFGDFGASINVDLLKYNEKDTRGIIRCPAREYVKVRGSLTFCGKIQGTTC-RFLVHQSSSLLLSLCGNSRTYKHC--
>Hydroptila_sp._AD-2013
------------------------------------MTYHYLDVTLDVNIQSAFNITPSSFMKNVTNSVEELFGEIGGAIDIKLLKYNQEDKRAILRVQSNSFEKLYAALTLKGVYESFEC-AYRIHKTSQSLLALTSSSLTYTH---
>Zorotypus_gurneyi
-----------------------------------MNDYSYFDVSLEVADE-NISISKNSFKRHVLSSLNTLFGEVGESVDLKILKYNVQLHRGIIKCPAESAVKLHSALTLSGKFDKYYS-VYKVHKSSTSLLSLAANSRTYNHVVK

HG0315 FBgn0260234 CG42508

```
>Drosophila_melanogaster
MKPKKSAFANSSTGYGRNKHGKGHSGKDKVEGGGKGKSR--DEKD-KHKHEQKVDEKDDKDLNSGFGDYLRTPEAFEMMKLFVFANTIMLIVTMAWPHIKEQFYMVNQWLESFREEHQQ
>Leptinotarsa_decemlineata
MI-----------------------KSRQLKRTKNS-----LNKPIKDREKKSEADRIDSDDEQGFSKWIRSEEGLDNLKLFVLGNTLIMFIAISWPQIKEVLDSAYYLYVEFTERR--
>Aposthonia_japonica
MK---------------------------------------KLMRSNSAMPAATNENRRDNNEEGFTAWLRSPEGIKFMHTFVLANTFVVILTVAWPHMTEVVDIIKSYIFD-------
>Isonychia_bicolor
------------------------------------------------------------------------------MKLFVIANSIIMLTTVGWPHITKTWEILQELLSGEL-----
>Acanthocasuarina_muelleriana
MKNKAAKSGSTAYRGG-------TAYKKLAPSAESKKET--N--PRPKVKKAPAPLTDHINENEGFGGWLSSEEGIGTMKMFVMANSVMMLITVGWPYLYQLLNYLHDSADNTPVRLY-
>Polistes_canadensis
MKKRINNTDNKNTKN------------RSNEERGKQKND--DEKDNEKNTIERLEQNEDEYSNQGFGDWLRSNDGAEMMRLFVIANSLLLFVTMAWPNMKESFYFIRDYFMGEEDDY--
>Micropterix_calthella
MKPRN-----KKSQN------------KANFNTGAALHK--T--ESKLLAKENTEKFVDEDEEKGFGGWLRSTEGIEIMRLFVIANSVVAFLTLAWPQMQKAFGIFQELVAENF-----
>Empusa_pennata
MKKRKSPNTV------------------AATE-----------DKVKPKI---KVDDVSDEENEGFGGWLKSSEGVAYMRLFVLVNSLVVFMTMTWPHIVECYDIVYSYFTE-------
>Euroleon_nostras
MKKHGRKQKKV-TI--------NEKQKQLQEKEKAKEKE--NKKPSDTVDKKN--DIDSDDENQGFSVWLRSSEGIELIQLFVLSNSIVLFVTVAWPQMQEAINIIISFFKETFST---
>Triops_newberryi
MKCKTGNLKK--SK--------------SIEA-----------KDIA-TQPSDTASGGPEEAAGGWTGWLASPEGVGWMQFFVITNAIIILTTTGLPHISAVWDIVKSWF---------
>Prosarthria_teretrirostris
------------------------MKKRKQENNKAHTTN--KDTSSSELVDNP--DISDTETNQGFGNWLRSNEGMEYMRLFVLANSLVVFLTMAWPQMSQAFNTIGSYFTSD------
>Leuctra_sp._AD-2013
MKKKKAGKLT------------------SPPATEV--EK--NISKINTNIVAKQELNEDDEENQGFSDWLKSSEGVSYMRLFVIANSLMVFMTMTWPHLSSVFEIISSFFTDD------
>Ectopsocus_briggsi
MQ----------------------HQHRKQQKEGAAKRKVAKSVVNAAESRKPDVDPDPYDEDAGFGGWLQSGDGVTYLRLFVISNSLMLFLTLGWPHIKESIEIITSFFYD-------
>Hydroptila_sp._AD-2013
------------------------------------------------------------------------------MRLFVIGNSIVVFLTMTWPHMQSTAKIVREMIFGEEE----
>Zorotypus_gurneyi
------------------------------------------------------------------------------MKLFVISNSVVMVLTMLWPQISSAVEVIGSILNDQN-----
```

HG0316 FBgn0041164 armi

>Drosophila_melanogaster
--MSS---EASGSITIK--E-----EAS-----------TSGTATNIPVESSETEAKKPEIGADKAESQSETTEGGKAAETT
ENHLKRLQNLRKELSYLSETDWMYESLDKKAAQ----
>Homarus_americanus
MASGNSSGLGASLFRGALHGNTSNTNNLSGNSSTSNQQHHHHHHTAGHHSQQGGVTGSPHIHHPQQPQP--------------------PPPQ---PTLGPVTFSTEETSRNRGTP-----------GSPDHMSTV------------MAKLWF---DHNVESSLSSLKK
EEHDKRTRDLRKQLDYITETNWKYSPVEKYIGQQ---
>Meganyctiphanes_norvegica
-MASNSSQFGTSIFRGGGSNNNSQNSNNSINNNNNNNNNNNNNTTPQNQQLSGALQQA---SHPFQSQQTTPIQQQEQQQQQLQQAQPSGSSS---QVQYQPGIDNTPNDYADNRS-----------QSPDFMATL------------MAKLWF---DHNVESSLSSLRK
EENEKRMRDLRKQLDYINDTNWKYAPVEKFLGQQ---
>Graphocephala_atropunctata
---MVRVSPGDHGIMGPMN--------------------TIPK---TILSSPSQS--E-AQGSLNS-----------NLNNTSSAV------------LSRLKF---EEAIEKNSVHIEK
EAHLKRVKNLRKELDYLQNTAWKYPSIDHYIGQ----
>Argochrysis_armilla
---MNSSTLSP---STLGHTSSQRTSESQDSEIT-----------TSGTQTSKI------------LNRMKS---ELQEQKQQKRLLK
ELHLKRVQSLRKELDYLKATEWRYQPIDRYLDQSNRN
>Phymatopus_californicus
--MSSNQSISQ---TLLTPPGKI--E-SQST---------------DGPVAH---------DIDQALSHIRL---EHNIERSLRNCHK
EIHLKRLQSLRKELEFLSSTDWQFEDRDKGTSQY---
>Teleogryllus_commodus
---MSST--------------------GLPK---TVLSSPTQS--E-SQASELS-----------T--TSLNSE------------LVHLKF---EHSFDQCLLQMQR
DAHNKRMKLLRKELDYIQVTAWKYPVIDKYIGQ----
>Ramulus_artemis
---MSTS--------------------RLSK---TVLAPPGQG--E-GQSSELS-----------SSSTLTPNQ------------SNLLKF---EYVMDQVMSASPR
EQHEKRMKVLRKEVEYLQNTAWKYQPIDKFIGP----
>Leuctra_sp._AD-2013
---MSSS--------------------GLSK---AVLSSPGQR--E-TQNTEQN-----------NRPTSAGDP------------LGLLKV---DCSVDQSLAQLQR
DDHKKRMKVLRKELNNLQSTAWQYPPIDKFVGQ----
>Thrips_palmi
---MSSP--------------------NINR---SVLSTTRAPRKE-TGSSELM--------------NNQLSL------------LARYKV---EHAVDQARSQVLK
EAHQKRLKVLRKELDYLKNSNWMYQPVDKFIGQ----
>Hydroptila_sp._AD-2013
---MSSSPSTSPVPSVSTAPSPSPAP-SAHSESSAVAHVHPHAAGSSSTVTNIG-----RSFHEDILKQVKL---DSKFNLLAKQAIT
AEHKTRMKSLRKETEYLNTTAWQFESVDKF-------
>Atelura_formicaria
---MMA------------FSRFLF---EHTVDHSHAFVQR
DEHQKRLKALRKELDYLQNTAWKYQPIEKYIGQ----

HG0317 FBgn0050011 gem

```
>Drosophila_melanogaster
MEK--SEIRLQRMSNEYQS----------QS-SYMYL-----RTKML---LKIENTL--LRSHRQRETTGIKKLYNSFFVLF
>Nicrophorus_vespilloides
-----------------------------MD-ILCNH-----LQVKM---IKIDNTF--LQNHRQRETTGIKKLYNSFFVMF
>Trachymyrmex_zeteki
------------------MRCSQGVAMIFEDAFVVFS-----RFLSL---KSRGCTL--LETHRQRETTGIKKLYNSFFVMF
>Papilio_polytes
MEAIPSHQRTYLIGNRFTSKPFPGLVPHIQY-HLCRS-----NTNML---LKIENEL--LQSHRQRETTGIKKLYNSFFVLF
>Osmylus_fulvicephalus
------------------------MDSGI-R-CHYCL-----VQIKM---NRIENSL--LQSHRQRETTGLKKLYNSFFVLF
>Xanthostigma_xanthostigma
------------------------MDTR--Y-FAFYF-----IQDKM---NTIENVL--LQSHRQRETTGIKKLYNSFFVLF
>Ceratophyllus_gallinae
-------MELKLLQFDF--------------S-STRIF-----VNKML---LKIENSL--LQSHRQRETTGIKKLYNSFFVLF
>Stylops_melittae
-------MENKLFG---------KMLSFY-AQFLSYKIFIRNRQRLMQPLLYHQVLLITKHSNQHQKMIDIKCLYNSFFVMF
```

HG0318 FBgn0035293 CG5687

```
>Drosophila_melanogaster
MKYRN-NRGTILQIQFRTFLRYSAANLIPFES-
>Tribolium_castaneum
MKYRSRSNRQIIQIQFRSFLR------------
>Copidosoma_floridanum
MRYAK-KTSKIIQLQFRTFLRNSSNTGYY----
>Boreus_hyemalis
MKYRN-KSGQILQIQFRTFLIVESGKRS-----
>Corydalus_cornutus
MKYNC-SNRQIIQIQFRTFLLDKRIIDNRILIR
>Osmylus_fulvicephalus
MKYRNRSNRQIIQIQFRTFLSTDKHSR------
>Oropsylla_silantiewi
MKYRN-SCGLILQIQFRTFLLDTRIYDNRG---
```

HG0319 FBgn0026778 Rad1

```
>Drosophila_melanogaster
MA---SESENTTSYFVPHLVDQVFELYRSKAESNSLTKEERGQFFYDLSILLGQELVLRSLHLLDDHN-FSFFHAKNNRSVCVVEISK-GTEYFRLIPGVSFCKCEFFQCHVLQLPRGILYQDLPSGEPGILEDWSEESRVSYTCQHILALRLHQFLKHTGGKTTEKILK
KEDIKELRTDVFRD------
>Gyrinus_marinus
-----------------------------------------------MHSLYGQV-FVRALELLEKYN-FTIFQT-TDGLRKVLKVIGT-TEEYALFHNINFCPCRAFQYQVLS------------------------SKSSYTCKHVLAAKLAEI----SGKFKVETYT
SEQITEVLNNQIPDG-----
>Polyommatus_icarus
-------------------------------MRNSTTKQLSDQDLITLHSVFGPV-LQRALEILEKQPLLEVYTT-ANKSRTLLEVKGEKDLRYRIFPMVNFCPCQAFKHQVLE------------------------KRIQLTCKHILAGKIALL----LGKTKEHEVT
QEQYQMLLHSMFEQQQ----
>Panorpa_vulgaris
-------MSKEKSTFLPHT---LVELIAKVKKSYSANKSFSEEDFLSLHSIFGTV-LEKALEILDRYN-ITVYRT-SS-SRCLIQVASH-DTYYKLFQDVNYCSCQAFKFQVLG------------------------TQKQYTCKHVLAAILADI----TGKIITEQIS
DEQFNVLIESMVSL------
>Annulipalpia_sp._AD-2013
MSYDSRTKKLSTCKILPRI---AFDLLKKVQNESSESKKISDDNLLTLHSVFGNV-LERALDVNSKRNSVTVYTL-QNGSRSLIEVRGSNGQTYRLLPQVNYCQCPAFQNYALQ------------------------LKTELTCKHVLAAYVADV----TQSAVENIVN
REQFDLLFDSFVASDEFELL
```

HG0320 FBgn0040993

```
>Drosophila_melanogaster
-MKT---PDTKAKLLNNISLSKHLSSKKGGSRTSNCGNCLEFSVLTRLSSVP
>Cosmioperla_kuna
MNSRAFVYQETKRFLPTVRLSKHLNTRKG-SRRGNAGNRLELQVLTQLTHVP
>Frankliniella_cephalica
MNNR---ADTKAKLLNNLSLSKHLSSKQGASRTTNCGNRLEFALLTLRQNIP
```

HG0321 ENSDARG00000070426 chac1

```
>Danio_rerio
--MHQAKKSVLRDCTITTAKQLGSDTVRGIAVSQPIPLLLDRRKLTLAT---
>Channa_punctata
MAMHRARQNQLRDSIIATAKQLGSNTVRGIPASQPVPLLLDRRKLTLAS---
>Anguilla_japonica
--MHQAKQKIRRYSIAKTAKQLGT--TRGITASQPIPLLLDREK--------
>Basilichthys_microlepidotus
MAMHSARQNQPRNSIFATAKQLGSNTVRGIPASQPVPLLLDRRKLTLAT---
>Oryzias_melastigma
MAMHSARQTRPRASTRATAKQLGSGTVRGIPASQPVPLLLYRRRPQLAAAAS
>Tripterygion_delaisi
MAMHRARQTQPRPSISAAARQSASSTVRGIPANQPVPLLLDRRKITIAG---
>Seriola_quinqueradiata
--MHRARQNQLRNSIFATAKQLGSNTVRGIPASQPVPLLLDRRKLTLAS---
>Micropterus_salmoides
--MHSARQNQLRNSIFATAKQLGSNTVRGIPASQPVPLLLDRRKLTLAS---
>Pygocentrus_nattereri
--MHQAKQKHPRTSILTTAKQLGSSAVRGIAVSQPIPLLLDRRKLVLAS---
>Haplochromis_sp._redtail_she
MAMHSARQNKQRNSIFATAKQLGSNTVRGIPASQPVPLLLDRRKLTLAS---
>Clupea_harengus
--MHSARQN-VNLSIFTTAKQLGSNTVRGIAVSQPIPLLLDRRKQILAS---
>Xiphophorus_maculatus_x_Xiph
MAMHSARQNQPRNSIFATAKQLGSNTGRGIPASQPVPLLLDRRKLTLTTS--
>Esox_lucius
--MHRARKNRLRNSIFTTAKQLGSNTVRGITVSQPIPLLLDRRKLTLAS---
>Apteronotus_leptorhynchus
--MHQAKKNGLRNSIFTTAKQLGSNTVRGIAVSQPIPLLLDRRKLTLGS---
>Lutjanus_erythropterus
MAMHSARRNQLRNSIFATAKQLGSSTVRGIPASQPVPLLLDRRKLTLAS---
>Plecoglossus_altivelis
--MHLARQNRQRNSIFTTAKQLGSNTVRGISVSQPIPLLLDRRKLILAS---
>Scleropages_formosus
--MHRTRQ----TCITKTAKQFGSHT-----ASRPIALLLDRRKYSTP----
>Lateolabrax_maculatus
--MHRARQHRARDSISAAAKQLGSSAVRGIPASQPVPLLLDRRKLTLAS---
>Gasterosteus_aculeatus
--MHSARQNPLRNSIFATAKQLGSNTVRGIPASQPVPLLLDRRKLTLAS---
>Solea_solea
MAMHQAKKYQLRDSILATAKQLGSNTVRGIPVSQPVPLLLDRRKLTLAS---
>Oncorhynchus_nerka
--MHRAKQNGLRYSIFTTAKQLGSNSVRGITVSQPIPLLLDRRKLTLPS---
>Ictalurus_punctatus
--MHQAKQTDLRNSIFTTAKQLGSSTVRGIAVSQPIPLLLDRRKLALAG---
>Pagrus_major
MAMHSARQNKLRNSIFAPAKQSGSNTVRGIPASQPVPLLLDRRKLTLAS---
>Takifugu_rubripes
MAMHSARQH-HRISILATAKQLGSNTVRGIPASQPVPLLLDRRKLTLAS---
```

HG0322 ENSDARG00000070917 kitlga

>Danio_rerio
MMILDMYLWDEENPFPARRSKIK
>Channa_punctata
-----MCTQDDDSPFPPRRSKIK
>Anguilla_anguilla
-----MCARNYENPFPPVRSRIK
>Menidia_menidia
-----MCTQDDDNPFPHRRSKIK
>Opsanus_beta
-----MCTQDDENPFPPRRSKIK
>Oryzias_latipes
-----MCTQDDDNPFPPRRSKIK
>Tripterygion_delaisi
-----MCTQDEDNPFPTRRSKIK
>Micropterus_salmoides
-----MCTQDDDNPFPPRRSKIK
>Pygocentrus_nattereri
-MSLDMRKRDEENPFPARRSKIK
>Haplochromis_burtoni
-----MCTQDDDNPFPPRRSKIK
>Austrofundulus_limnaeus
-----MCLQDDENPFPLRRSKIK
>Esox_lucius
-----MCTRDDENPFPPRRSKIK
>Gadus_morhua
-----MCTRDDESLFPHRRSKIK
>Apteronotus_leptorhynchus
-MILDMCRRHEESSFPARRSKIK
>Scleropages_formosus
-----MCTRNDESPFPPQRSKIK
>Lateolabrax_japonicus
-----MCTQDDDNPFPPRRSKIK
>Sebastes_caurinus
-----MCTQDDDNPFPPRRSKIK
>Cynoglossus_semilaevis
-----MCTQDDDSPFPPRRSKIK
>Salmo_salar
-----MCTRDDENPFPPRRSKIK
>Ictalurus_punctatus
-MVLDMHERDEEKSFPARRSKIK
>Takifugu_rubripes
-----MCTQDDDNPFPPRRSKIK

HG0323 ENSDARG00000071197 usp40

>Danio_rerio
---MFIPVKSSYARV-SPLIVNRELGKTKMR
>Channa_punctata
---MFSPVKSSYFRV-SPAIVNRELGKTKMR
>Menidia_menidia
---MFSPVKSSYFRV-SPAIVNRELGKTKMR
>Porichthys_notatus
---MFSPVKSSYFRV-SPAIVNRELGKTKMR
>Oryzias_latipes
---MFSPVNSSYFRV-SPAIVNRELGKTKMR
>Trachinotus_blochii
---MFSPVKSSYFRV-SPAIVNRELGKTKMR
>Micropterus_floridanus
---MFSPVKSSYFRV-SPAIVNRELGKTKMR
>Haplochromis_burtoni
---MFSPVKSSYFRV-SPAIVNRELGKTKMR
>Clupea_harengus
MIAMVSPVKSVHSGV-NPMVVNRELGKTKMR
>Poecilia_mexicana
---MFSPVKSSYFRV-SPTIINRELGKTKMR
>Esox_lucius
---MFTPVKSSYFRV-SPTTINRELGKTKMR
>Acanthogobius_hasta
---MFSPIKSSYFRV-SPAIINTELGKTKMR
>Apteronotus_leptorhynchus
---MFSPVKSAYLRV-SPTTVNRELGKTKMR
>Lutjanus_erythropterus
---MFSPVKSSYFRV-SPAIVNRELGKTKMR
>Plecoglossus_altivelis
---MFSPVKSSYFRV-SPTTVNRELGKTKMR
>Scleropages_formosus
---MFSAVQSEYPRLTLPRAVNRELGKTKMR
>Lateolabrax_japonicus
---MFSPVKSSYFRV-SPAIVNRELGKTKMR
>Notothenia_coriiceps
---MFSPVKSSYFRV-SPAIVNRELGKTKMR
>Hippoglossus_hippoglossus
---MFSPVKSSYFRV-SPATVNRELGKTKMR
>Salmo_salar
---MFTPVKSSYFRV-SPTTINRELGKTKMR
>Takifugu_rubripes
---MFSPVKSSYFRV-SPTIVNRELGKTKMR

HG0324 ENSDARG00000094965 nfil3-5

>Danio_rerio
MTVVFVEFHLFLIFLTSLDT
>Channa_punctata
MSEVFVEFHLFLIFLANLDT
>Menidia_menidia
MSEVFVEFHLFLIFLANLDT
>Porichthys_notatus
MSEIIVEVRLFLIFLANVDT
>Oryzias_latipes
MSEVFVEFHLFLIFLANLDT
>Micropterus_floridanus
MSEVFVEFHLFLIFLANMDT
>Astyanax_mexicanus
MRDIFIEFHLFLIFLANLDT
>Oreochromis_niloticus
MSEVFVEFHLFLIFLANLDT
>Clupea_harengus
MKEVYVEFHLFLIFLSNFDT
>Austrofundulus_limnaeus
MSEVFVEFHLFLIFLANLDT
>Acanthogobius_hasta
MYKVILEFHLFLIFLANLDT
>Lutjanus_erythropterus
MRVANAELQLFFIFLANLDT
>Plecoglossus_altivelis
MCEVFVEFHLFLIFLANFDT
>Scleropages_formosus
MRKLYVEFHLLLIFLANLDT
>Lateolabrax_japonicus
MSEVFVEFHLFLIFLAYLDT
>Sebastes_caurinus
MSEVFVEFHLFLIFLANMDT
>Cynoglossus_semilaevis
MSKIFVEFHLFLIFLANLDT
>Oncorhynchus_mykiss
MRGINAEFHCFLIFLASLDT
>Lepisosteus_oculatus
MWEIFVESHLFLIFLANLDT
>Takifugu_rubripes
MSEVFVEFHLFLIFLANMDT

HG0325 ENSDARG00000079738 znf219

```
>Danio_rerio
----------------MYLIQVKRNHDVCCH
>Channa_argus
----------------MYLIQVKRNHDVSCH
>Menidia_menidia
-MLVYNPDFHFFSQLVMYLIQVKRNHDVSCH
>Porichthys_notatus
----------------MYLIPVKRNHDVSCH
>Oryzias_melastigma
----------------MYLIQVKRNHDVWCH
>Micropterus_salmoides
----------------MYLIQVKRNHDVSCH
>Pygocentrus_nattereri
----------------MYLIQVKRNHDVCCH
>Haplochromis_burtoni
----------------MYLIQVKRNHDVSCH
>Alosa_alosa
----------------MYLIQVKRNHDVSCH
>Austrofundulus_limnaeus
----------------MYLIQVKRNHDVSCH
>Esox_lucius
----------------MYLIQVKRNHDVSCH
>Apteronotus_leptorhynchus
----------------MYLIQVKRNHDVSCH
>Scleropages_formosus
----------------MYLIQVKRNHDVSCH
>Lateolabrax_japonicus
----------------MYLIQVKRNHDVSCH
>Sebastes_caurinus
----------------MYLIQVKRNHDVSCH
>Cynoglossus_semilaevis
-MLVYKPDFHFFPQLVMYLIQVKRNHDVSCH
>Salmo_salar
MLVYNNPDFHFFPQLVMYLIQVKRNHDVSCH
>Ictalurus_punctatus
----------------MYLIQAKRNHDVSCH
>Takifugu_rubripes
----------------MYLIQVKRNHDVSCH
```

HG0326 ENSDARG00000012848 arih2

>Danio_rerio
---------MSDGECDFFCFFRCC
>Channa_argus
---------MSDGECDFFCFFRCC
>Menidia_menidia
---------MSDGECDFFCFFRCC
>Oryzias_latipes
---------MSDGECDFFCFFRCC
>Tripterygion_delaisi
---------MSDGECDFFCFFRCC
>Micropterus_salmoides
---------MSDGECDFFCFFRCC
>Pygocentrus_nattereri
---------MSDGECDFFCFFRCC
>Oreochromis_niloticus
---------MSDGECDFFCFFRCC
>Austrofundulus_limnaeus
---------MSDGECDFFCFFRCC
>Acanthogobius_hasta
---------MSDGECDFFCFFRCC
>Apteronotus_leptorhynchus
---------MSDGECDFFCFFRCC
>Plecoglossus_altivelis
---------MSDGECDFFCFFRCC
>Scleropages_formosus
---------MSDGECDFFCFFRCC
>Lateolabrax_japonicus
---------MSDGECDFFCFFRCC
>Anoplopoma_fimbria
---------MSDGECDFFCFFRCC
>Cynoglossus_semilaevis
---------MSDGECDFFCFFRCC
>Ictalurus_punctatus
---------MSDGECDFFCFFRCC
>Takifugu_rubripes
MNIQSSAVQMSDGECDFFCFFRCC

HG0327 ENSDARG00000022203 gtf2ird1

>Danio_rerio
-------------MTFNFTGLQNKE-RGKGTHSSVKENREKTGQTHQIN-HTECFSTD---YRPFEIMTFSKVAVAFL-----------------------------------
>Amia_calva
-------------MGFNFTGLQNKE-RGKGTHSSAKEKRKKGKNR-AII-HRRTVHKAPTISKCFGLQCFGLRWPLYRDRGTN----RAVSIGLNGEEGCVAGLTADGI----
>Channa_punctata
MLLLSWAGISRRRMSFNFTGLQNKE-RGKGHPALLREKRNK---LRQNT-QIIDY----KPRERSQQCDFSDYGASAGGTATLWMGIVSVSDGLKRGINLFRD----------
>Menidia_menidia
MLVLSWAGISRRRMSFNFTGLQNKE-RGKGHPAL---
>Porichthys_notatus
MLLLSGAGISRRRMSFNFTGLQNKE-RGKGHPAQLKQRNKKKEKNLKGK-RDRL---
>Oryzias_latipes
-------------MSFNFTGLQNKE-KEKGTPCSAKTGKTGINWEKSKE--SETAVQ----GAMIRIRVFF------NGREL-------------------------------
>Tripterygion_delaisi
MLLLSWAGISRRRMSFNFTGLQNKE-RGKGHPALVKGAKKQ---E--
>Pygocentrus_nattereri
-------------MSFNFTGLQNKE-RGKGTLCSVKEKQKEKRTGKQD---
>Oreochromis_niloticus
MLLLSWAGISRRRMSFNFTGLQNKE-RGKGHPALLRGKNRHKLRKKKNT-QIADN----GTWARSRRGDDPDYDVLADGTATLWMGIVSVSDGLKRGNKPIPGLTAATRLFNR
>Fundulus_heteroclitus
MLLLRWAGISRRRMSFNFTGLQNKEKRGKGHPALLKGINKKQNSENKTG-ETPGL----QES-------KPSPGQ--------------------------------------
>Esox_lucius
-------------MSFNFTGLQNKE-RGKGTHSTVKENSKKRGTFK---
>Apteronotus_leptorhynchus
-------------MSFNFTGLQNKE-RGKGTLSSVIENRKKRTKIDKII-SPKIFKQATKKRNMLQIMRFHKLAIAFL-----------------------------------
>Scleropages_formosus
-------------MAFNFTGLQNKE-RGERGPCAALKKTERNGHKH---
>Cynoglossus_semilaevis
MLLLSWAGINRRRMSFNFTGLQNKE-RGKGHPALLKG--
>Salmo_salar
-------------MSFNFTGLATEQ--RNGTHSTVKENRKNGGNRKIISVIT-----------------FC--
>Lepisosteus_oculatus
-------------MSFNFTGLQNKD-GKGDTLLR---
>Ictalurus_punctatus
-------------MSFNFTGLQNKE-KGKGDTVPALKTRKKDGGRKKKRSKSRCNLTRKGRRTRSRLCGFCEVSVALF-----------------------------------
>Takifugu_rubripes
MLLLSWAGISRRRMSFNFTGLQNKE-RGKGTPDRVKGEKQE--

HG0328 ENSDARG00000099999 tcf3b

>Danio_rerio
----------MWMEVVKMSTRRMNSPQL
>Menidia_menidia
----------MWTEVVKMSTRRMKSSQL
>Oryzias_latipes
----------MWTEVVKMSTRRMKSSQL
>Tripterygion_delaisi
----------MWTEVVKMSTRRMKSSQL
>Astyanax_mexicanus
----------MWMEVVKMSTRRMNSSQL
>Neolamprologus_brichardi
----------MWTEVVKMSTRRMKSSQL
>Alosa_alosa
MRKDTLLLPTMWMEVVKMSTRRMKSLQL
>Kryptolebias_marmoratus
----------MWTEVVKMSTRRMKSSQL
>Esox_lucius
MRRD-TLLLTMWTEVVKMSTRRMKSSQL
>Gadus_morhua
----------MWTEVVKMSARRMKSSQL
>Apteronotus_leptorhynchus
MRRNTLLLLTMWMVVAKMSTRRMNSSQL
>Plecoglossus_altivelis
----------MCSEVVKMSSRRMKSAQL
>Scleropages_formosus
----------MWMEVVKMSSRRMK----
>Lateolabrax_japonicus
----------MWTEVVKMSTRRMKSSQL
>Notothenia_coriiceps
----------MWTEVVKMSTRRMKSSQL
>Cynoglossus_semilaevis
----------MWTEVVKMSTRRMKSSQL
>Oncorhynchus_mykiss
MRRDTLLLLTMWTEVVKMSTRRMKSS--
>Takifugu_rubripes
----------MWTEVVKMSTRRMKSSQL

HG0329 ENSDARG00000036442 atp11c

```
>Danio_rerio
MRMVREDSIQLIWKRCSFTAALHIARGNR-LESR--------------------------
>Channa_argus
-----MNRNQTKWKRCSVAAVFHIARTNARVETR--------------------------
>Menidia_menidia
MRMVRGDRNQTKWRRCSVAAAFHIARTNVRVESR--------------------------
>Oryzias_melastigma
MRMVRRYRSQTKWKRCSVAAAFHIARTNDRLESR--------------------------
>Tripterygion_delaisi
MRMVRRSRNQTKWKPCSGAAASPIARTRVGTR----------------------------
>Micropterus_floridanus
MRMVRRYRNQTKWKRCSVAAAFHIARTNVRVENR--------------------------
>Pygocentrus_nattereri
MQMVREERSQPEWKRCSFTAALHIARRNR-VENR--------------------------
>Maylandia_zebra
MRMVRRYRNQTKWKRCSVAAAFHIARTNVRVENR--------------------------
>Alosa_alosa
MRMVRGERLPRKWKQCSNTATFHVARTNWIE-NR--------------------------
>Austrofundulus_limnaeus
MRMVRRYRNQTKWKRCSVAPAFHIARXNVRVENR--------------------------
>Esox_lucius
MRMVRRDRIQTKWKQCSIAAAFHIARWTSRVENR--------------------------
>Gadus_morhua
MRMVRRARNQTKWKRCSLDAVPHIARRHVRVETR--------------------------
>Apteronotus_leptorhynchus
MRMIREQRSETKWKQCSFTAAFHIDWKHR-LGNR--------------------------
>Plecoglossus_altivelis
MRMVRRERIQTKWKQCSIAAAFHIARTRV--GNR--------------------------
>Notothenia_coriiceps
MRMVRRYRNQTKWKRCSDAAAFHIARTNVRVENR--------------------------
>Salmo_salar
MRMVRRDRIQTKWKQCSIAAAFHIAISTTRVENRELVNKYKGNVTYLSEKLKQSIVNWFK
>Takifugu_rubripes
MRMVPRSRNRAQWEACSVAVAFHIARYSARVESR--------------------------
```

HG0330 ENSDARG00000039392 wnk1b

>Danio_rerio
MDEVDLTVFVCH--HQCLPLSNCVLFFYVYKRTPPLLGSGPL-HCKSNC----
>Acipenser_sinensis
MDGVVWTVLVSQ--CLRLLFCDCILFFSNYKRTPPVQGPDPL-HCNCIHCPY-
>Amia_calva
MDGVDLMVLVCQ--HPCLIFCDSVLFFSIYKRTPPLQGPDPV-RCKILNFLC-
>Menidia_menidia
MDGVDLKRLEYQ--QQHPAFSCSVLSLSIFKRTPPILHHQSPNYICKDIL---
>Pygocentrus_nattereri
MDGVDLTVLVCH--HQRLLLCDCVLFFYIYKRTPPLLSSDHK-YCNSNCL---
>Clupea_harengus
MDGVDVTVLQCP--LQCLPVCDCVLFFSIYKRTRPIQGFGPQ-CCLYSRNCSC
>Kryptolebias_marmoratus
MDGVNLKILLYR--HQCSAFCCSVRSFSTYKRTPPIIHPQSLRYIYKDSL---
>Esox_lucius
MDGVDLTVLVCH--HQRLLLCDCFLFFSIYKRTPPLLGPTPPERCKSNRDCCC
>Acanthogobius_hasta
MEENYLK---SP--PRHCGLYNSDQSFSNYKRTPPILHIVNL-HCKSRNYKQ-
>Apteronotus_leptorhynchus
MDGVDLTVLVCR--HQCLLLCDCILFFYVYKRTPPLLGLCPT-YCISNCL---
>Plecoglossus_altivelis
MDEVDLKSLVCL--HPCPPFCDCVL-FSIYKRTPPVLHLHRE-PCLHSNWL--
>Scleropages_formosus
MGCVDLMVLVCQQQHQCLLFCDGVLFFSTYKRTPPLVGPDPV-WCKSIRYFC-
>Lateolabrax_japonicus
MDGVDLKRLVYQ--QQHSEFCCGVQSFTIYKRTPPLLHLQSLHYIYKDRL---
>Trematomus_bernacchii
MDGVDLKRLVYQ--HQHSVFFCGVQSFTIYKRTPPILYPQSLHYIYKDRQ---
>Oncorhynchus_mykiss
MDGVDLTVLVCH--HQRLLLCDCFLFFSIYKRTPPLLGPTTPERCKSYSNYCC
>Lepisosteus_oculatus
MDCVNLMVIVCL--HQHLIFCDIILFFFIYKRTPPLQGPDPL-HCKILSFHC-
>Trichomycterus_areolatus
MDGVDLTLLIYH--RQRLCLSDCILFYYAYKRTPPLLG--------------

HG0331 ENSDARG00000044899 tmem183a

>Danio_rerio
MYATLSVSSIMAEDPGGAFFNFTLILTDFSLPLL--ERWQG----
>Channa_punctata
----------MAEDPGGTFFNFSSIFLFFSLPQLAIETWQDRKPR
>Oryzias_melastigma
---------MMAEDSGGTFFNFYLIFLPFFVPKLVIETWLDL---
>Micropterus_floridanus
---------MMAEDPGGTFFNFSLIFFSFSLPPLAIETWQDSQAR
>Pygocentrus_nattereri
---------MMAEDPGGAYRIFSFIFLRFSPPLL--QTWQGAQSR
>Neolamprologus_brichardi
---------MMAEDPGGTFFNFSVIFLSFSLPQLAIETWQDSQSR
>Cyprinodon_variegatus
---------MMAEDPGGTFFNLSVIFLSFSLTRLAIEPWQDSQAR
>Esox_lucius
---------MMAEDPGGTLFNFSPTFFSFSLLLLVLETWQDTQP-
>Gadus_morhua
---------MMAEDPGGTFLKFTQFFISFYLGLLVLETWQHTQSR
>Scleropages_formosus
---------MMAEDPGGPYFNLSFIFLSFSLSLLVVPTWQEPQLR
>Lateolabrax_maculatus
---------MMAEDPGGTLFKFSLIFLSFSLPPLP----------
>Sebastes_caurinus
----------MAEDPGGTFSNLSFIFLSFSLPPLAIETWQDPQAR
>Scophthalmus_maximus
---------MMAEDPGGALFNFSIIFLSFSLPRLAIEPWQDSHPR
>Salmo_salar
---------MMAEDPGGTIFNFSPTFLFFSLPLLVIETWQDTQPR
>Lepisosteus_oculatus
----------MAEDPGGVDFNFSFISFRFSLSLLVVATWQEPQSR
>Ictalurus_punctatus
----------MAEDPGGAYFNFSLNFLCFLLPLL--EIWQGTQSR
>Takifugu_rubripes
---------MMAEDPGGTISNFSLFFLFFALPPLAIATWQDSQVC

HG0332 ENSDARG00000102893 atg7

```
>Danio_rerio
MKRNSVYRFLSVFSCLLVCVFLF-FWGFAFYSMVLAP--
>Amia_calva
MKRNGVYQFFSVFSCLVVFLFLFLFWFFVFYTMVMTP--
>Channa_argus
MKRNGAYRFLSVFSCFVVCAFLFLFWFFIFYSLVLAP--
>Menidia_menidia
MKRNGAYRFLSVFSCFIVCVFLFLFWFFVFYSMMHAP--
>Porichthys_notatus
MKRNGAYRFLSVFSCFVVCVFLFLFWFFVFYSLVLAP--
>Oryzias_melastigma
MKRNGAYRFLSVFSCFIVCAFLFLFWSFVFYSMLRAP--
>Pygocentrus_nattereri
MKRTGAYRFLSVFSCLLVCVFLFLFWFFVFYSMVLAP--
>Haplochromis_burtoni
MKRNGAYRFLSVFSCFIVCAFLFMFWFFVFYSMVLAP--
>Poecilia_formosa
MKRNGAYRFLSVFSCFVVCVFLFLFWFFVFYSMVLAP--
>Esox_lucius
-MRSGAYKFLTVFSCLIVCGFMFMFWFFVFYSFAP----
>Apteronotus_leptorhynchus
MKRDGAYRLLSVFSCLVIFVFLLFFWVFVFYSMVLAP--
>Plecoglossus_altivelis
-MKRGAYKFLSVFSCFVVCAFLFLFWLFIFYSMMLAP--
>Scleropages_formosus
MKKICAYRLLSVLSCVGICLFLFLFWFFVFYTLVMAP--
>Lateolabrax_japonicus
MKRNGAYKFLSVFSCFIVCAFLLLFWFFIFYSMVLAP--
>Platichthys_stellatus
MKRSGAYRFLSVLSFFAVCASLLLFWFIIFYTMMHVT--
>Trichomycterus_areolatus
MKRNGAYRFLSVFSCFAVCAFIFIFWFFVFYSMFLAPEN
>Takifugu_rubripes
MKRNGAYKFLSVFSCFIVCAFLFIFWFFIFYSMVHAP--
```

HG0333 ENSDARG00000042518 hipk2

>Danio_rerio
MQEIAAIL----SAAQDGRFLLSCEQCCRFIFCHE
>Porichthys_notatus
MRE--ATADTRSSLLQDGRFLLSFVQKARFLLLP-
>Astyanax_mexicanus
MQETAAIL----SAAQDGRFLLSSELKYLFIFCHE
>Oreochromis_niloticus
MQETAS---TLSSAFQDGRFLLSFEQKYRFIFCHE
>Kryptolebias_marmoratus
MQETAATLSSPSSLFQDGRFLLSFKQKARFIFCHE
>Esox_lucius
MQETATL-----SVAQDGRFLLSSEQKSRFIFCNE
>Acanthogobius_hasta
MQEIAATLSSP--VSQDGRFLLSFERKSRCIPLP-
>Plecoglossus_altivelis
MQETATL-----TVAQDGRFLLSSEISLYFLP---
>Scleropages_formosus
MQEEPLL-----SADQDGRFLLSSELRSPPIYLP-
>Notothenia_coriiceps
MQETAA---TLSSLFQDGRFSLSFEQKSRFIVLP-
>Cynoglossus_semilaevis
MQENTATL-----SSQDGRFLLSFEQNRRFIFCHV
>Oncorhynchus_kisutch
MQETATL-----SGAQDGRFLLSSEQISRFIFCHE
>Lepisosteus_oculatus
MQETATL-----TVAQDGRFLLSSKLKSRFIFLP-
>Trichomycterus_areolatus
MR----DQDRIVSAAQDGRFLLSSGLKDLLTVLQ-
>Takifugu_rubripes
-MNWASNAATLSSALQDGRFSLSSEKKPLFIFLP-

HG0334 ENSDARG00000075349

```
>Danio_rerio
-------------------------MTSLCGYHRNQGSTADG----------------------
>Anguilla_japonica
-------------------------MTSLHGYHRNQGPSANG----------------------
>Trachinotus_blochii
-------------------------MTSLLGFNKSQSSTADG----------------------
>Micropterus_floridanus
MRVKLFRQEKKQHGLLCFGSFLLCDMTSVLGYNRSQSSTAAGGRHLVRCVFLQQCKALPRQSPQ
>Astyanax_mexicanus
-------------------------MTSPSGYHRNRRSTADS----------------------
>Maylandia_zebra
-------------------------MTSRLGYKRSSSSTADG----------------------
>Alosa_alosa
-------------------------MTSPCGCHRNQGCPADG----------------------
>Esox_lucius
-------------------------MRSLHGFYRNQGSTVDG----------------------
>Acanthogobius_hasta
-------------------------MTSLIEYTRNQSSSSDG----------------------
>Apteronotus_leptorhynchus
-------------------------MTSPSSCHRNQGSTADS----------------------
>Plecoglossus_altivelis
-------------------------MTSLHGYHRNQGSTAHG----------------------
>Lateolabrax_japonicus
-------------------------MTSLLGSNKSQSSTADG----------------------
>Anoplopoma_fimbria
-------------------------MTSLFGYNKSQSSTADG----------------------
>Oncorhynchus_mykiss
-------------------------MTSLSGYHRNQGSTAEG----------------------
>Ictalurus_furcatus
-------------------------MTSSSGCHRNQGSTADS----------------------
```

HG0335 ENSDARG00000034700 vegfab

```
>Danio_rerio
MWIVIALEEKFDLLLNNVRGPASWILIFLDILKIIGNS
>Channa_striata
MWIATPLQDDKSLLRNNVRRCASWILVFLDILKIVGNS
>Porichthys_notatus
MWIAIALKENKDLLRNNVTRRTSWILVFLDILKVVGNS
>Oryzias_latipes
MWIATP-QESQDLPRNDVRRCASWILVFLDILKIIGNS
>Pygocentrus_nattereri
MWIVIPFEEILDLLQNNVRGFASWVLTFLDILKTVSNG
>Ophthalmotilapia_ventralis
MWIATPLQENKDLLRNNVRRCASWILVFLDILKIIGNS
>Poecilia_latipinna
-----MWRENKDLLRNDVRRCASWILAFLDILKIVGNS
>Gadus_morhua
MWIAIPLQENDSLLQNNVKRYASWILVFLDILKNIGNS
>Apteronotus_leptorhynchus
MWIVILFEENISLPENNVRGFATWVLTFLDILKIIGNS
>Notothenia_coriiceps
MWIAIPLQEDKGLLRNNVRRFASRILVFLDILKIIGNS
>Scophthalmus_maximus
MWIATALQENTGPLRNNVRRYASWIFVFLDILKSIGNS
>Oncorhynchus_mykiss
MWIAISFEENKGLLQNNVKRYASWILMFLDILKIIGNS
>Ictalurus_punctatus
MWIVRAFEQKLSLRRDHVSGFASWLLTFLDILKIIGNS
>Takifugu_rubripes
MRILISLQENKGRLRKNVTSCASWILVFLDILKIIGNS
```

HG0336 ENSDARG00000028676 nuak1b

>Danio_rerio
---MRVIYVQNAGVVAAFSPEIFCGRLK----QRNNGEIMTERATGKRTRHNGVGYVIME
>Channa_argus
---------------------------------MRSCTWAVDKMRVIFIQNAGFVAAFSPRRCESVMLAAFRTTDNVNEAAELTTARSCQS------SEE
>Menidia_menidia
---------------------------------MRSRTWAADKMRVIFIQNAGFVAAFSPFS--------------------------------------
>Oryzias_latipes
---------------------------------MRCSTWAVDKMRVIFIQNAGFVAAFSPFS--------------------------------------
>Pundamilia_nyererei
---------------------------------MRSFTWAVDKMRVIFIQNAGFVAAFSPFS--------------------------------------
>Clupea_harengus
---MRVIYIQNAGFVAAFSGGNDG------------------------------------
>Kryptolebias_marmoratus
---MRVLFTQNAGFVAAFSPLL--------------------------------------
>Esox_lucius
---MRVIFIQNAGFVAAFSGGNDAEGFAC-------------------------------
>Apteronotus_leptorhynchus
---MRVIYIQNAGCVAAFSGSNDG------------------------------------
>Notothenia_coriiceps
---------------------------------MRSCTWAVDKMRVIFIQNAGFVAAFSPFRCESVMLAAFRTTDKVNEP-ELTTARSCKS------TEE
>Cynoglossus_semilaevis
---------------------------------MRVYTWAKDKMRVIFIQNAGFVAAFSPRRCESVTLAAFRVTNTA-EEPELNTNKSCQS------AE-
>Salmo_salar
MAIFKTSELDISDHSASLKSTWAWSTCAIHRVQQLNDVAPEDKMRVIFIQNAGFVAAFSGGNDAEGFAC-------------------------------
>Takifugu_rubripes
----------------------------------MNGQPHLDKMRVIFIQNAGFVAAFSPFRRESVMSAAFRTTDKVTDLQ-SASAGSCQA------IKE

HG0337 ENSDARG00000054906

```
>Danio_rerio
------------------MFLHRADLSLRPRTKYRDSD-------------------
>Channa_argus
------------------MFLHRADLNLSPRTKRFPLVRLRVGFLLPERSWRQTGGV
>Micropterus_salmoides
------------------MFLHRADLNLSPRTKRFPLR-------------------
>Oreochromis_niloticus
------------------MCLHRAHLNLSPRTKRAPLR-------------------
>Alosa_alosa
------------------MCLHRLDSNLRPRTKYRGLH-------------------
>Esox_lucius
------------------MFYHRADLSLRPRTKYRDFH-------------------
>Lutjanus_erythropterus
------------------MCLHRADLNLSPPTKRFPLR-------------------
>Plecoglossus_altivelis
------------------MFLHRTDLSLRPRTKYRDFH-------------------
>Lateolabrax_maculatus
------------------MFLHRADLNLSLRTKRAPLI-------------------
>Salmo_salar
------------------MFHYRADLSLRPRTKYRDFH-------------------
>Ictalurus_punctatus
MIAGARSLGGSGQGRSCSMFHHRANLSLRPRTKYRAFD-------------------
>Sparus_aurata
------------------MFLHRADLSFSPRTKRVPLK-------------------
```

HG0338 ENSDARG00000000151 thraa

```
>Danio_rerio
MLTCRGSSPPQSAVIGCWMESR
>Channa_argus
-------------MIGCWMELR
>Pygocentrus_nattereri
-------------MIGCWMELR
>Neolamprologus_brichardi
-------------MIGCWMELR
>Alosa_alosa
-------------MIGCWMELR
>Poecilia_reticulata
-------------MIGCWMELR
>Esox_lucius
-------------MIGCWMELR
>Lateolabrax_japonicus
-------------MIGCWMELR
>Trematomus_bernacchii
-------------MIGCWMELR
>Salmo_salar
-------------MIGCWMELR
>Ictalurus_punctatus
-------------MIGCWMELR
```

HG0339 ENSDARG00000063144 shisa7b

>Danio_rerio
MNPDLFMPVQWAQPCCPVRTGPKPPFVLFCWVELNPEQRHSRKRVQHT---
>Menidia_menidia
MNPDIFMSVLRAEPRCPARTGPKPSFVLFCWAKLNSEQRHSREGIVRTQTR
>Oryzias_latipes
MNPDIFMSALWAEPRCPARTGPKPSFVLFCWAKLNSEQRHSRDGVST----
>Pygocentrus_nattereri
MNPTSFMSVQWAQSRCPARTGPKPPFVLFCWAELNPEQRHSRKRVQHT---
>Oreochromis_niloticus
MNPDSFMSVLWAEPRCPARTGPKPSFVLFCWAKLNSEQRHSREGIKRT---
>Alosa_alosa
MNLSQFMGFLWTEPCSPARDGPKPTFVLFCWAELSPEQRHLCKGATHT---
>Kryptolebias_marmoratus
MNPDIFMSLLWAEPRCPARTGPKPSFVLFCWAKLNSEQRHSREGIERT---
>Esox_lucius
MNPDIFMSVLWAEPRCPARTGPKPTIVLFCWAELNSEQRHSRKGIKHT---
>Lateolabrax_japonicus
MNPDIFMSVLWAEPRCPARTGPKPSFVLFCWAKLNSEQRHSHKGIERT---
>Platichthys_stellatus
MNPDIFMSVLWAEPRCPARTGPKPSFVLFCWAKLNSEQRHSHKGIERTFL-
>Salmo_salar
MNPDIFMSVLWAEPRCPARTGPKPTFVLFCWAELNSEQRHSRKGIKRT---

HG0340 ENSDARG00000017591 fat1a

>Danio_rerio
--MQRLGTYKLHEKPPNKGR--
>Channa_punctata
--MPRSGLHKLHAKPPNLRVYQ
>Menidia_menidia
--MPRFGLHKLHAKPPNIRD--
>Neolamprologus_brichardi
--MPRFGIHKLHAKPPNLRI--
>Poecilia_reticulata
--MRRFGLQKLHAKPPNLRDY-
>Esox_lucius
--MQTFGPYKLHEKPPNPRR--
>Anoplopoma_fimbria
--MPRFGLHKLHAKPPNIRD--
>Oncorhynchus_masou
--MQTFVPYKLHEKPPNPRR--
>Ictalurus_furcatus
MYMYKLGTYKLHEKPPNHGR--

HG0341 ENSDARG00000039430 msl2b

>Danio_rerio
----------------MMTRIWSYFVPWKRGKR
>Amia_calva
-----------------MMFIWAYFVPWKRGKR
>Anguilla_japonica
MSLLFFNYIQVKDVHLMMMLVWAYFVPWKRGKR
>Alosa_alosa
-----------------MMILWAHFVPWKRGKR
>Esox_lucius
-----------------MMIIWAYFVPWKRGKR
>Apteronotus_leptorhynchus
------------MDFYPMMIVYAYFLPWKREKR
>Plecoglossus_altivelis
-----------------MMLIWAYFVPWKRGKR
>Salmo_salar
-----------------MMIIWAYFVPWKRGKR
>Lepisosteus_oculatus
------------------MLIWAYFVPWKRGKR

HG0342 ENSDARG00000045801 fbxl14b

>Danio_rerio
------------------------------------MYDRLDPRVERRLLSKRQTGSSAA
>Amia_calva
MQSETSLLHDCGRKRRSDRPGLFLLYITGLVEVFLKMYDRLDPRGELEGLSQDKVGSAV-
>Alosa_alosa
------------------------------------MYDRLDPRVDRGRLSEHKMGSVA-
>Esox_lucius
------------------------------------MYDRLDPRRERRHVSKGIIGTVV-
>Apteronotus_leptorhynchus
------------------------------------MYDRLDPRVERRRLSRHKMGSAV-
>Scleropages_formosus
------------------------------------MYDRLDPRQEAEGSSEHTSGCAAV
>Salmo_salar
------------------------------------MYDRLDPRREPRRVSKGIIGSVV-
>Lepisosteus_oculatus
------------------------------------MYDRLDPRGEREGLSQDKVGSAV-
>Trichomycterus_areolatus
------------------------------------MYDRLDPRVERRRLSEHIMGSNAV

HG0343 ENSDARG00000057062

```
>Danio_rerio
---------------MDYSRTDANLGFPTELLP---
>Menidia_menidia
------------MPVMDYGRTSANLGWHS-------
>Porichthys_notatus
------------MSVMDYSRTDANLSRHSQSSIRLH
>Trachinotus_blochii
------------MPVMDYSRTDANLSWHSQNSIPPY
>Micropterus_floridanus
------------MQVMDYSRTDANLSWHSQNSIPPY
>Pygocentrus_nattereri
---------------MDYSRTYANLGFPAKLSP---
>Alosa_alosa
MKRVGYCKVPVQAQAMDYSRTDANLGFPAKLSS---
>Notothenia_coriiceps
------------MLVMDYSRTDANLSWHSQNSIPPN
>Ictalurus_punctatus
------------MPVMDYRRTDANLAFSAEVSP---
```

HG0344 ENSDARG00000032072 cdk16

>Danio_rerio
MAEDYWERAAALGYYYCC
>Acipenser_sinensis
MAEDYWESTAALGKH---
>Amia_calva
MAEDYWEQTAAPGHY---
>Astyanax_mexicanus
MAEDYWEHTAALGHC---
>Clupea_harengus
MAEDYWESTVALGYYC--
>Esox_lucius
MAEDYWESTVALGYC---
>Salmo_salar
MAEDYWESTLALGYFC--
>Lepisosteus_oculatus
MAEDYWELPAARGHY---

HG0345 ENSDARG00000035910 znf281b

>Danio_rerio
MEEANSCKGTLFWL
>Channa_punctata
MEEPSSCKRTMFWL
>Trachinotus_blochii
MEEPSSCGKTMFWL
>Micropterus_salmoides
MEEPSSCGRTMFWL
>Astyanax_mexicanus
MEEGNSCERTMFWH
>Maylandia_zebra
MEEPSSCGRTMFWL
>Notothenia_coriiceps
MEEPSSCGRTMFWL
>Ictalurus_punctatus
MEEGNSCRRTMFWQ

HG0346 ENSDARG00000041982 adamts6

>Danio_rerio
------------------------------MFVCTVAASASAGKQMDSSAQVPNIDLLLWNNMKGVVMDSCPAHG
>Channa_punctata
---MQMDRSRQVPNIELPVWSYT------------
>Oryzias_melastigma
---MDRSGQVSSIGLPVWSST------------
>Pygocentrus_nattereri
---MDSSRQPPNIDLLLWNYLK-----------
>Apteronotus_leptorhynchus
---MDRSRQAPHIGLLLWN--------------
>Lateolabrax_maculatus
---MQMDRSRQVSNIELPVWSYT------------
>Notothenia_coriiceps
---MQMDRSRQVSNIELPVWSYT------------
>Lepisosteus_oculatus
MRKILKRKGEEGSELTAELQCRRVLLSVSSSVGNASDNEDQNGTQMDSSRQTSSIDLLFWNYLKSFN--------

HG0347 ENSDARG00000063194

>Danio_rerio
----------------------MADLSKSKPGLK---
>Pygocentrus_nattereri
----------------------MADLSKSNPGLK---
>Oreochromis_niloticus
----------------------MADLSKSNPGLK---
>Poecilia_formosa
MGEEYEMDSVSQRANRVDDEVKMADLSKSNPGLK---
>Apteronotus_leptorhynchus
----------------------MADLSKSNPGLK---
>Cynoglossus_semilaevis
----------------------MADLSKSNPGLK---
>Salmo_salar
----------------------MADLSKSNPGLKIET
>Lepisosteus_oculatus
----------------------MADLSKSNPGLK---

HG0348 ENSDARG00000036139 lctla

>Danio_rerio
MSFLLAALDKLAYLLSLVAFCVSLSYSWLK
>Opsanus_beta
MSLILTVLDKLAYFLSLVAFCVSLSSSWMK
>Pygocentrus_nattereri
MSFLLAALDKLTYFLSLIAFCVSVSYSWLK
>Oreochromis_niloticus
MSLILTVLDKLAYFLSLIAFCVSVSYSWLK
>Fundulus_heteroclitus
MSLILTVLDKLSYFLSLVAFCVSASHGWLR
>Esox_lucius
MSLLLTVLDKLTYLLSLIAICVSASNSWLR
>Salmo_salar
MSLILTVLDKLTYFLSLIAFCVSVSYGWLK

HG0349 ENSDARG00000028699 crkl

```
>Danio_rerio
------------------------------MHQSWLTLVAACLVKRQVAVQ
>Channa_punctata
-MVVVSFFVFIF-LYFGREHLLTAQRRNHIMHQSWIKLVAACL--------
>Micropterus_dolomieu
------------------------------MLQSWIKPVAACL--------
>Alosa_alosa
------------------------------MHQSWLMLVPACL--------
>Apteronotus_leptorhynchus
------------------------------MQESWFMPVAACL--------
>Notothenia_coriiceps
MFVCRKLFCFSILAAQTITQPLTAEHRNHIMFQSWIKPVAACL--------
```

HG0350 ENSDARG00000036194 pitx2

>Danio_rerio
---MQVDIISHTCPCHSGSPCSRLKPRYLLPSRNGKKIFP
>Lipochromis_sp._matumbi_hunt
--MGGRNMVTWRSGERRSWIDSAQATAEMNSEWCS----VCMPLDLISPTCPCHSDSQAR------------------
>Poecilia_reticulata
MLLFGRGKISASPGINTLADISRFCELRLVGHIHMDAPVRENRGGGKAVTWRCGERRSWMGRAQATAEMSRERWS----VCKPLDLISPTCPCHSDSQPR------------------
>Esox_lucius
---MALDLISPTCPCHSDTPRS------------------
>Apteronotus_leptorhynchus
----------------------------------MSSLMTCQKEGGFCVTSGCRERLSWTAKEQGTAEMKSERCALCENVCMQVDIISPTCPCHSGFIAARGMKLR------------
>Platichthys_stellatus
--MNTERCG----VCMPLDLISPTCPCHSDSQQR------------------

HG0351 ENSDARG00000068096 atf5a

>Danio_rerio
MRTKPCFLWLNSQTSLSCTLSSGI--
>Pygocentrus_nattereri
MLTQPCFLWLNSQTTISCTINSAV--
>Esox_lucius
MLTQPCSLWLNSQTPPTHIVPSDYSD
>Apteronotus_leptorhynchus
MLTQPFLLWLNSQTTRSCTVNSAV--
>Salmo_salar
MLTQPCSLWLNSQTPPTHILRSD---
>Ictalurus_punctatus
MHTQPCFLWLNSLTTLTCITSSDS--

HG0352 ENSDARG00000070214 slc41a1

```
>Danio_rerio
MGNIIQVLPKFRQDLTQAWRRGTHLPLFLDL---------------
>Astyanax_mexicanus
MGNIICMLPKFRQDLKQARRGVMHLPLLLNL---------------
>Alosa_alosa
MGSIICMLPKYRQDLSRAWSTGMHLPFLSDL---------------
>Esox_lucius
MENILELLPKHRQGLTCAWGRAATHTCLRGRTCSLVAPSNLFFITT
>Salmo_salar
MENILELLPKHRQEGTHAWGRAATHTCLCRRKSSLVAPLNRFFITT
>Ictalurus_punctatus
MGNIICMLPKFRQDLMQSLERAKAPASFTQPVEREL----------
```

HG0353 ENSDARG00000078864 lats2

>Danio_rerio
-----------------------------------MANMHTRLGWKKHADVNPHMSLEEGECEP
>Astyanax_mexicanus
--------------------------------------MQTMPEWNQPTSVTPFMSWEEGECDP
>Alosa_alosa
-----------------------------------MAKMCTRLAWSKAVLLTPHKTWDEGERDP
>Esox_lucius
-----------------------------------MAQMGARMVWGKGTLVTPHMTWNKEECDP
>Salmo_salar
-----------------------------------MAQMGARTVWGKGTLVTPHMTWNKEECDP
>Trichomycterus_areolatus
MSFFKTTRVKQSINELSDGKVSSVTLLTTVRNISLMAMMQTRLEWNQPTCMNPFMSAKEGKCDP

HG0354 ENSDARG00000019897 gpat4

>Danio_rerio
MIIFPYMPMKSMFQRRLQKLRHSRGVQQSVFR
>Astyanax_mexicanus
--------MKALFQRRLQKLRL----------
>Esox_lucius
--------MKALFQRRLQKLRLYMD-------
>Apteronotus_leptorhynchus
--------MKTLFQRRLQKLRL----------
>Ictalurus_punctatus
--------MKALFQRRLQKLRL----------

HG0355 ENSDARG00000019930 tal1

>Danio_rerio
MDDPPQNGSMHTLTSTARRR---
>Acipenser_sinensis
MDGPTQNGSTQFLTSKYKR----
>Amia_calva
MDGPPQNGSTHGIISTHKR----
>Osmerus_mordax
MDDHRQNGSTHTPSTTFKQRLRT
>Salmo_salar
MDDPRQNGSTHTLSTTLRLW---

HG0356 ENSDARG00000020475 znrf1

>Danio_rerio
MIRPGKWAVASARHSCFSQT
>Alosa_alosa
MFRPGERAVASHRHSWRSQT
>Esox_lucius
MFRSGKWAVASVRHSCFSQT
>Salmo_salar
MFLSGKWAVASVIHSCFSQT
>Ictalurus_punctatus
MIRPRKRALASPGHSCFSQT

HG0357 ENSDARG00000037285 mipa

```
>Danio_rerio
MSAGHAHHLSIRVPLLNLS-----------------------------
>Oreochromis_niloticus
MSAGHAYRLSIHVLLLTTSGVGL-------------------------
>Cyprinodon_variegatus
MSAGHT------------------------------------------
>Esox_lucius
MSAGHAQPLSIHVPLLTTIRVSILMQRDRYSAPRRQHPGLYDKAKKRL
>Salmo_salar
MSAGHAQPLSINVPLLTTSRVGI-------------------------
```

HG0358 ENSDARG00000062506 zc3h18

```
>Danio_rerio
MAALTVSCRVYAFFR------------------
>Pygocentrus_nattereri
MAALTVSCGVYAFLR------------------
>Alosa_alosa
MAALTLSCRVYYFLR------------------
>Plecoglossus_altivelis
MAALSVSCRFFVFFNIADKSEIHSFTLESKKLK
>Ictalurus_punctatus
MAALTVPCRACAFLR------------------
```

HG0359 ENSDARG00000070348 hoxc10a

```
>Danio_rerio
----------------------MSASYCPYPLRHKGDCFESKEKQDK
>Astyanax_mexicanus
----------------------MSASYCPNPLRRKRDCFKT------
>Alosa_alosa
---------MLLIF---LPPQKMSASYCPYPSRKKRNCCKTKE----
>Apteronotus_leptorhynchus
----------------------MSASYCPYPLRHKRDCLKT------
>Ictalurus_punctatus
MTGRLESIIMLPIFSLPPTSQKMSASYCPYPFRHKRACFKT------
```

HG0360 ENSDARG00000070801 opa1

```
>Danio_rerio
MCPIWKDELNFILQRDPKLIYICH-
>Pygocentrus_nattereri
MPPIWKDELQFVLK-----------
>Clupea_harengus
MSTIWKDDLNCVFTFKLKKLYICQL
>Cyprinodon_variegatus
MCPVWKDELNCTRR-----------
>Anoplopoma_fimbria
MCTIWKDELNRA-------------
```

HG0361 ENSDARG00000077581 zzz3

>Danio_rerio
MCSKRFAEVRCPHTAAEERAPR---
>Oryzias_latipes
--MFIIGQTRCPHSAAEERAPG---
>Haplochromis_burtoni
----MSPAGRCPHLAAEERAPR---
>Apteronotus_leptorhynchus
MCSRRFAEVRWPHSAAEESTTMRSQ
>Ictalurus_punctatus
--------MRCPYSAEEESTRMRSQ

HG0362 ENSDARG00000078355 zc3h4

```
>Danio_rerio
MSFISHRNCRT-ENLRNVFNLFLF--------------------
>Esox_lucius
MSFILQRNCRT-ENHRTQFQPISLTTVSGELTTMN---------
>Apteronotus_leptorhynchus
MSFISHRNCKDGENFRNVSACFSFKLLMGINCYELKLFLKPINK
>Scleropages_formosus
MSFILQRSCRT-ENLRTHFNLSLFKQLMGN--------------
>Salmo_salar
MSFILQRNCRT-ENLRTQFQPISLTTVSGELTTMN---------
```

HG0363 ENSDARG00000095896 pou3f3b

```
>Danio_rerio
MRYNLPALKKFLQKGGGDQQTYS
>Alosa_alosa
MRHNLPALKKFLRKGGGLHQTYS
>Esox_lucius
MRYNLPARKK-------------
>Notothenia_coriiceps
MRYNLPARKKRL-----------
>Cynoglossus_semilaevis
MRYNLPARNR-------------
```

HG0364 ENSDARG00000004988 ppp3ca

>Danio_rerio
MPLHQPHLVTANTRRE
>Alosa_alosa
MPLHQPHLAPAKTYRE
>Esox_lucius
MPLHQQHLATAKTHRE
>Salmo_salar
MPLHQPHLATAKTHRE

HG0365 ENSDARG00000016132 keap1a

```
>Danio_rerio
-----MPTWLETESCYSGLLTRQKKHGSLSLNDNQLCLYFFMARGWV
>Pygocentrus_nattereri
MYHSVMPLWSEMDHCHDGLRTRQKKHGSLSPSDHQLCLYFFMTRGWV
>Apteronotus_leptorhynchus
-----MPIRSETVPCHHGLQTRQNKRSSLSPSDHQLCLYFLMTRGWV
>Trichomycterus_areolatus
-----MPIWSAQDPSHDGLRLRLKKPGSLSPSDHQLCLYYFMARGWI
```

HG0366 ENSDARG00000017803 gsk3b

>Danio_rerio
MELSGSERP-RISVLKLGNWKILIRIAG
>Pygocentrus_nattereri
MELAGAERP-RVSVSKPGNWKTLLRILG
>Apteronotus_leptorhynchus
MELRGTERP-WISVFKLGNWKILIRIVG
>Ictalurus_punctatus
MELRGAEVQWWISVFKSGDW--------

HG0367 ENSDARG00000034056 csnk1g2b

```
>Danio_rerio
-----MFTVLPRTDFKETMRSTRGRSLVGFH---------------------------------------------
>Astyanax_mexicanus
MKNVCLFAPFPKTDLEETMRSLRGRFAVAFWIQ-------------------------------------------
>Esox_lucius
-----MSAVLPKTDQKEAMRGSGGSCQAVLWIQLLGF---------------------------------------
>Ictalurus_punctatus
------------------MRSPTGRSALADQIQYQEACVCACICERVSECACMRAHAITSCLMHAVMCSTPQKEEP
```

HG0368 ENSDARG00000058606 sik1

>Danio_rerio
MTCKCKCKNHLCCGRRWKLFAS
>Clupea_harengus
MTCKSAVVNLFDCGRRWNLFVC
>Chanos_chanos
MTCKFVSKSDFSCGRRWKLFVY
>Apteronotus_leptorhynchus
MTYKYASENHFGCERRWNLLVL

HG0369 ENSDARG00000059276 hoxd4a

```
>Danio_rerio
------MKSLHLDWPSWSHGRLTLFS
>Pygocentrus_nattereri
MASSALIGFLHLDWPSWSHGCPTLFS
>Lateolabrax_japonicus
------MDQHHLYWPIRSHGSLTLFS
>Sebastes_caurinus
------MNQHHLHWPIWSHGSLTLFS
```

HG0370 ENSDARG00000062693 nrxn3b

>Danio_rerio
-----MWQNNRIRSNMENLCFNPERRLKGTSSVKHF--
>Pygocentrus_nattereri
MLSFTMQRYCRVKSNMEDLCYKPGSRFKRMSSGKQIGH
>Esox_lucius
-----MGHGCGSEAKMATVCFNPCSRLKGNSSNGSLYH
>Salmo_salar
---------------MVAVCFNPCSRLKGKSSDGPLYH

HG0371 ENSDARG00000062765 whsc1l1

```
>Danio_rerio
-------------------MEHAEAGPVRKRET
>Pygocentrus_nattereri
-------------------MEH-AEGPVRKRET
>Alosa_alosa
MIVHLCEQKQGSNIWHLFRMEH-AEGPVRKRET
>Scleropages_formosus
-------------------MEH-AEGPVRERET
```

HG0372 ENSDARG00000069467 igsf9bb

>Danio_rerio
MQGMSKRVSAADSASNDQGLG-
>Apteronotus_leptorhynchus
---MSKRISADDSASNVLGARL
>Lepisosteus_oculatus
---MSKRVLAGDCANTVQGARR
>Ameiurus_nebulosus
---MSERIPAAASASSIPGARL

HG0373 ENSDARG00000074611 wdr37

>Danio_rerio
MTVALRSAEAVINALGFLFEVFLFFITLSSRLRMCLLCLSFTALHGCFR---------
>Pygocentrus_nattereri
---------------------------------MRLLCLSFTALHCPVPDRKCAVGRH
>Apteronotus_leptorhynchus
----------------------------MIALKMCLLCLSFTAPHCPVPGGTSAEGRH
>Trichomycterus_areolatus
---------------------------------MCLLCFSCTALHCPVAGRKCAVGRH

```
HG0374 ENSDARG00000075147 lrrc38a

>Danio_rerio
MISWKIELRVFVAAFRCTSLCRLQSRRQGKMPSRQT------------
>Pygocentrus_nattereri
MISWKTAFRVFVAVVRSRSFFSL-------------------------
>Esox_lucius
MISWKTLFRVSLA-ARWPGLCRLHTKRKKDASARTLTPFFLLLPPPLP
>Apteronotus_leptorhynchus
MISWKVASHISVA-VRFWSFCRL-------------------------
```

HG0375 ENSDARG00000076171 znf827

>Danio_rerio
MGKDLFSSYP------
>Porichthys_notatus
MGKNLFSSYPQTRTRM
>Plecoglossus_altivelis
MGKNLFSSYP------
>Oncorhynchus_kisutch
MGKNLFSSYP------

HG0376 ENSDARG00000077229 ano8b

>Danio_rerio
MTHNNLHFTLTFI
>Alosa_alosa
MSHNNLHFTFTFI
>Apteronotus_leptorhynchus
MTHNNLHFTFTFI
>Lepisosteus_oculatus
MSHGNLHFTFTFI

HG0377 ENSDARG00000079549 cdc42ep1a

>Danio_rerio
MQPRFSGIGVMHIFSSHLSSPNLSGYEQRPMAKTGHS----------------------------------
>Astyanax_mexicanus
----------MHIFNSHPSQLEHFGHGQEPATKELCVFLMEKAFLRNISLSCSFFSFALRSPEPGIVLHEW
>Alosa_alosa
----------MHIFNSHFS--
>Ictalurus_punctatus
----------MSIFISHLSQWDHFGYGQRPVAKNLCVHTYGKFSLRNISLSCTFFSLTFLEKP--------

HG0378 ENSDARG00000080009 bahcc1b

```
>Danio_rerio
MIGTYFFGRQSQNCAECKT--------
>Apteronotus_leptorhynchus
MTGTYFFGRQIKNCAECETWAKLVTGL
>Scleropages_formosus
MIGSYFSGWGSQQYRECNT--------
>Trichomycterus_areolatus
MTGTYFFGRHTQNGAECKT--------
```

HG0379 ENSDARG00000104372 gnb1b

>Danio_rerio
MRGFDSHTIVFFGQCHINKGHLRLRTDQNVSRQ------
>Esox_lucius
MRGLEGYTVTFSSQCQKTQSLRQLRKGSSVPAAGERQKY
>Apteronotus_leptorhynchus
MRGFDSYTIVFFGQCHIKQGQLRLSTDQCVSRQQE----
>Ameiurus_nebulosus
MRGFDSNTVFSFGQCHIKHGQLRLNTDPCVSRQ------

HG0380 ENSDARG00000005350 dip2ba

>Danio_rerio
-------------MAEGVVLRMDCYSPFNSRFMP
>Plecoglossus_altivelis
-------------MAEGPIVRMDCLPDLNACSMS
>Trichomycterus_areolatus
MGSRDQRSASSHTMAEGAAVRMDCSSHFNTRFES

HG0381 ENSDARG00000006124 kdm5c

>Danio_rerio
---------------MACSSDRMNGG
>Astyanax_mexicanus
MGLEGLCLGCDEKPKMACSLDRMNGG
>Clupea_harengus
---------------MACSTDRMNGG

HG0382 ENSDARG00000017242

>Danio_rerio
MTEPFFTADACGGGLTCRC
>Alosa_alosa
MQEPFFRTNECGGG-----
>Apteronotus_leptorhynchus
MKQPLFTADECGGGLTCLC

```
HG0383 ENSDARG00000036501 rab39bb

>Danio_rerio
-------MMLLLSLPFHLHKTSKHFSIFQTWIKT-------
>Alosa_alosa
MRDESGYMMLLMLLPFHQ-QNSKHFFIFKTWSENNTSQYHN
>Platichthys_stellatus
MRDESGDMMLLLALPFHQ-QTSKHTQPAPT-----------
```

HG0384 ENSDARG00000054748 cuedc1b

>Danio_rerio
MQLNKQKKRRKGFLSLQWTRFFIV
>Pygocentrus_nattereri
MQLNKQKKRKKAFSFLSGLT----
>Apteronotus_leptorhynchus
MQLKKQKRRRKGLLLPPWTPTTLH

HG0385 ENSDARG00000059549 akap10

>Danio_rerio
MNEGNRESSGCCL
>Salmo_salar
MNEGTRESTGCCH
>Ictalurus_punctatus
MNEGTRESTRCCH

HG0386 ENSDARG00000062084 clcn1a

>Danio_rerio
MCEEETERLSVCVCMCPCHPRVGMLRQTGSLFLGQ
>Alosa_alosa
-----------------------MLRQTSGLFLGQ
>Salmo_salar
-----------------------MLRQTAGLFLGQ

HG0387 ENSDARG00000071150 cica

>Danio_rerio
MQERNRVEDIDRLGLTGNQED
>Astyanax_mexicanus
MREGNRVEDIDRLRLTENLED
>Apteronotus_leptorhynchus
MRVGNRVEVLDRLGHTGNLED

HG0388 ENSDARG00000077228 ntrk3a

>Danio_rerio
MQPVEEDSLGALIFEPFLH
>Alosa_alosa
MQPGDEDSIGVSIFQPFHL
>Salmo_salar
MQPREEDKPGVVIFEPFLI

HG0389 ENSDARG00000077361 bptf

>Danio_rerio
MAAASDLKRGAGRSF
>Alosa_alosa
MAAMSDPKRDAGRSF
>Apteronotus_leptorhynchus
MAAASDSKRDAGHSF

HG0390 ENSDARG00000090035 rtn4r

>Danio_rerio
MTERGT-TGRRCSPSRARTATVG
>Esox_lucius
MTERGS-TRRRCSPTRFSRKTSG
>Apteronotus_leptorhynchus
MTERGRINGRLCSPSRAFKATVG

HG0391 ENSDARG00000099634 elna

>Danio_rerio
MIHRTKGFALS
>Pygocentrus_nattereri
MIHRTEGFALS
>Trichomycterus_areolatus
MIHRTEGFAFS

HG0392 ENSDARG00000100003 glulb

```
>Danio_rerio
MRMRDKQPGLPYKTLWKYSHSYSDYLYFLIGLI
>Astyanax_mexicanus
--MREKQARKSSRSSWTHSQSGIDYLFLLKGFL
>Ictalurus_punctatus
--MREKEFVLSY---------------------
```

HG0393 ENSGALG00000002090 GPSM2

>Gallus_gallus
MELKGPQMKELERVSDSLRCV--
>Anser_cygnoides
MELKGPQMQELEHVSESVRCVWQ
>Chaetura_pelagica
-------MKELEHVSEGLRCV--
>Apteryx_australis_mantelli
MELKGPQMQELERVSESLRCV--
>Buceros_rhinoceros_silvestri
MELKGPQMKEREHVSEGLRCVWQ
>Caprimulgus_carolinensis
MELKGPQMKEREHISEGLRCV--
>Cariama_cristata
MELKGPQMKELEHVSEGLRCV--
>Calidris_pugnax
MELKGPQMKELEHVSEGLRCV--
>Pterocles_gutturalis
MELKGPQMKELEHVSEALR----
>Colius_striatus
MELKGPQMKELENVSEGLRCV--
>Columba_livia
-------MKELEHVSEGFRGV--
>Leptosomus_discolor
MELKGPQMKELEHVSEGLRRV--
>Cuculus_canorus
MELKGPQMKELEHVSEGFRCV--
>Aquila_chrysaetos_canadensis
MELKGPQMKELEHVSEGLRCV--
>Gavia_stellata
MELKGPQMKELEHISEGLRCV--
>Mesitornis_unicolor
MEPKGPQMKELERVTEGLRCV--
>Tauraco_erythrolophus
MELKGPQMKELEHVSEGLRCV--
>Opisthocomus_hoazin
MELKGPEMRKPEHVSEGV-----
>Ficedula_albicollis
MELKGPQMKERTRTSLTV-----
>Phaethon_lepturus
MELKGPQMKELEHVSEGLRCV--
>Picoides_pubescens
MDLKGPQMKELEHVAEGLRCV--
>Fulmarus_glacialis
MELKGPQMKELEHVSEGLRCV--
>Nestor_notabilis
MELKGPQMKELEHVSEGLRCV--
>Aptenodytes_forsteri
MELKGPQMKELGTPF--------
>Tyto_alba
MELKGPQMKELEHVTEGLRCV--
>Struthio_camelus_australis
MELKGPQMQELEHVSESLRCV--
>Tinamus_guttatus
MELKGPQMQELEHGAECLRCI--
>Calypte_anna
MEIRGPEMKEQEHISEDLRCV--
>Apaloderma_vittatum
-------MKELEHVSEGL-----

HG0394 ENSGALG00000002479 MAT1A

>Gallus_gallus
MRSPCMHTQIQAHADFTD----
>Anser_cygnoides
MHTPCVHAQIQAHAEFTR----
>Chaetura_pelagica
MHSPCMCTQMQAHADFTH----
>Apteryx_australis_mantelli
MHTPCTHTQIQAHADFTH----
>Caprimulgus_carolinensis
MHTPCMRTQMQAHADFTH----
>Cariama_cristata
MHAPCMRTQMQAHAEFTH----
>Charadrius_vociferus
MHTPCMRTQMQARADFTY----
>Pterocles_gutturalis
MHTPCMRTQMQAHADFTH----
>Colius_striatus
MHTPCMHAQMQAHAGFTH----
>Columba_livia
MHTPCMCTQMQAHADFTH----
>Merops_nubicus
---------MQAHAEFTHSNLS
>Aquila_chrysaetos_canadensis
MHAPCMHTQMQAHADFTH----
>Gavia_stellata
MHTACMRTQMQAHADFTH----
>Chlamydotis_macqueenii
MHAPCMRTQMQAHADFTR----
>Tauraco_erythrolophus
MHTPCMCTRMQAHADFTH----
>Manacus_vitellinus
MRSPCVHAQMQAYAAFTH----
>Egretta_garzetta
MHTPCMCTQMQAHADFTH----
>Fulmarus_glacialis
MHTPCMRTQMQAHADFTH----
>Nestor_notabilis
MHTPCMCTQMQAHAVFTH----
>Pygoscelis_adeliae
MHTPCMRTQMQAHADFTH----
>Tyto_alba
MHTPCMRTQMQAHADFTH----
>Struthio_camelus_australis
MHAPCTHT--------------
>Tinamus_guttatus
MHTACTHSQLQARADFTH----
>Calypte_anna
MHAPCMCTQMKAHADFIH----
>Apaloderma_vittatum
MHTPCVCTQMQTHADFTH----

HG0395 ENSGALG00000014936 ESCO1

>Gallus_gallus
MLLAHRDKLLWPKHRLPLVKT
>Anser_cygnoides
MLLAHLDKSLWPEHRLPLVKT
>Apteryx_australis_mantelli
MLLAHLDKLLWPKHRLPLVKT
>Buceros_rhinoceros_silvestri
MLLAHLDKLLWPQHPLHLVKT
>Caprimulgus_carolinensis
MLLAHLDELLWPKHRLPLVKT
>Cariama_cristata
MLLAHLDKLLWPKHRLPLVKT
>Calidris_pugnax
MLLAHLDKLLWPKHRLPLVKT
>Colius_striatus
MLLAHLAKLLWPKHRLPFVKT
>Columba_livia
MLLAHLDKLLWPKHRLPLVKT
>Merops_nubicus
MLLAHLDKLLWPKHRFPLVKT
>Aegypius_monachus
MLLAHLDKLLWPKHRLPLVKT
>Gavia_stellata
MLLAHLNKLLWPKHRLPLVKT
>Chlamydotis_macqueenii
MLLAHLDKLRWPKHRLPLVKT
>Tauraco_erythrolophus
MLLAHLDKLLWPKHRLPLVKT
>Opisthocomus_hoazin
MPLAHLDKLLWPKHRLPLVKT
>Geospiza_fortis
MLLSHVDKLLWPKHRLPLVKT
>Egretta_garzetta
MLLAHLDKLLWPKHRLPLVKT
>Fulmarus_glacialis
MLLAHLDKLLWPKHRLPLVKT
>Nestor_notabilis
MLLAHLDKLPWPKHRLPLVET
>Pygoscelis_adeliae
MLLAHLDKLLWPKHRLPLVKT
>Tyto_alba
MLLARLDKLLWPKHRLPLVKS
>Struthio_camelus_australis
MLLAHLDKLLWPKHRLPLVKT
>Tinamus_guttatus
MLLAHLDKLLWPKHRLPLVET

HG0396.1 ENSGALG00000034253

>Gallus_gallus
------------------MKDHCKEPWTFVVSYNTVIQRV
>Chaetura_pelagica
MQIKQRKKECQEWPSLISAKDHCRVPWILAVSYNVLIQRV
>Caprimulgus_carolinensis
------------------MKDHCREPWILVYKCT------
>Cariama_cristata
------------------MKDHYREPWILVVSYNILIQRV
>Charadrius_vociferus
------------------MKDHFREPWILVVSYNILIQRV
>Pterocles_gutturalis
------------------MKDHSREPWILGVSYNILIQRV
>Colius_striatus
------------------MKDHCREPWILVVSYNILIQRV
>Merops_nubicus
------------------MKDHCKEPWLLVVSYNVLIQRV
>Gavia_stellata
------------------MKDHCREPWILVVSYNILIQRV
>Balearica_regulorum_gibberic
------------------MKDHCREPWMLVVSCNILIQRV
>Tauraco_erythrolophus
------------------MKDHCREPWILVVSFNILIQRI
>Opisthocomus_hoazin
------------------MKDHCREPWILVYKCT------
>Pelecanus_crispus
------------------MKDHCREPWILVVSYNILIQRV
>Picoides_pubescens
------------------MKGHCREPWILVVS--------
>Fulmarus_glacialis
------------------MKDHCREPWILVVSYNILIQRV
>Nestor_notabilis
------------------MKDHCREPWILLVSCNILIQKV
>Aptenodytes_forsteri
------------------MKDHCREPWILVVSYNILIQRV
>Tyto_alba
------------------MKDQCRKSWILAVSYNILIQRV
>Struthio_camelus_australis
------------------MKDHCEEPWTFVYKCT------
>Tinamus_guttatus
---------------MVSMKDHCEELWTFVYKCT------
>Calypte_anna
------------------MKDCYREPWILVVSYNILIQSV
>Apaloderma_vittatum
------------------MKDHCREPWILVVSYNVLIQRV

HG0396.2 ENSGALG00000034253

>Gallus_gallus
---MQKKTNKGRKDVKHDLP
>Anas_platyrhynchos
MKTNLSWLLQLHQLSATSSERPAQTPQVVDQSSFSSSSHLGAQSFYVVLGLNTRLLKLFVLMRKWISQLSTNKTNKGKKDVKHGLP
>Calidris_pugnax
---MWKWISQLNANQTNKGKKDVKRGLP
>Pterocles_gutturalis
---MWKWISQLNANQTNKGKKDVEHGLP
>Columba_livia
---MWKRILQLNANQTNKGKKDVKRGLP
>Gavia_stellata
---MWKWISQLNANQTNKGKKDVKRGLP
>Nipponia_nippon
---MWKWISQLNANQTNKGKKDVKRGLP
>Fulmarus_glacialis
---MWKWISQLNANQTNKGKKDVKCGLP
>Pygoscelis_adeliae
MKTNPSWLLQLHQLSAT--RFVLMWKWISQLNANQTNKGKKDVKRGLP

HG0397 ENSGALG00000009438 SLC39A9

>Gallus_gallus
MAESPSWDLGVCTG
>Apteryx_australis_mantelli
MAEGLSWDLGVCTG
>Buceros_rhinoceros_silvestri
MAESPSWELGVCTG
>Cariama_cristata
MAESLSWELGVCTG
>Colius_striatus
MAESLCWELGVCTG
>Leptosomus_discolor
MAESLSWELGVCTG
>Cuculus_canorus
MAESLCWELGVCTG
>Falco_cherrug
MAESLSWELGVCTG
>Gavia_stellata
MAESLSWELGVCTG
>Eurypyga_helias
MAESLSWELGVCTG
>Tauraco_erythrolophus
MAESLSWELGVCTG
>Opisthocomus_hoazin
MAESLSWELGVCTS
>Corvus_cornix_cornix
MAESLSWELGVCTG
>Egretta_garzetta
MAESLSWELGVCTG
>Fulmarus_glacialis
MAESLSWELGVCTG
>Aptenodytes_forsteri
MAESLSWELGVCTG
>Tyto_alba
MAERLSWELGVCTG
>Struthio_camelus_australis
MAGGLSWDLGVCTG
>Apaloderma_vittatum
MAESRSWELGVCTG

HG0398 ENSGALG00000005369 BTBD10

>Gallus_gallus
------------MMEEKVRLARAALNY
>Chaetura_pelagica
------------MMEEKVRLADGAVNY
>Buceros_rhinoceros_silvestri
------------MMEEKVRLADGAVNY
>Caprimulgus_carolinensis
------------MMEEKVRLADGAVNY
>Cariama_cristata
------------MMEEKVRLADGAVNY
>Charadrius_vociferus
------------MMEEKVRLADGALNY
>Pterocles_gutturalis
------------MMEEKVRLADGGMNY
>Colius_striatus
------------MMEEKVRLADGAVNY
>Leptosomus_discolor
------------MMEEKVRLADGAVNY
>Aquila_chrysaetos_canadensis
------------MMEEKVRLADGAVNY
>Chlamydotis_macqueenii
------------MMEEKVRLADGAVNY
>Tauraco_erythrolophus
------------MMEEKVRLAGGAVNY
>Opisthocomus_hoazin
------------MMEEKVRLADGALNY
>Corvus_brachyrhynchos
------------MMEEKVRLADGAVKY
>Egretta_garzetta
------------MMEEKVRLADGAVNY
>Nestor_notabilis
------------MMEEKVRLADGAVNY
>Struthio_camelus_australis
MYSYNPLSLEGKMMEEKVRLADVAVNY
>Calypte_anna
------------MMEEKVRLADGAVNY

HG0399 ENSGALG00000032659

>Gallus_gallus
-------MTNHFCVCTTRIQNS------
>Anser_cygnoides
-------MTNHICLCTTRIQNS------
>Chaetura_pelagica
-------MTNNICLCTTRIQEFLNRRLV
>Caprimulgus_carolinensis
MIFKCIFMTNHICLCTTRIQNS------
>Cariama_cristata
-------MTNHTCLCTTQIRNS------
>Charadrius_vociferus
-------MTNHIC---------------
>Pterocles_gutturalis
----MHFMTNHICLCTTQIQNS------
>Columba_livia
-------MTDHICLCTTREQNS------
>Leptosomus_discolor
MIFKCIFMTNHICLCTTRIQNS------
>Falco_cherrug
-------MTNHICLCTTQIQN-------
>Gavia_stellata
-------MTNHICLCTTRIQNS------
>Mesitornis_unicolor
-------MTNHICLCTTRIQNS------
>Tauraco_erythrolophus
-------MPNHICLCTTRIQNS------
>Taeniopygia_guttata
-------MTNYICLCTTRIQNS------
>Pelecanus_crispus
-------MTNHICLCTTRIQNS------
>Fulmarus_glacialis
-------MTNHICLCTTRIQNS------
>Aptenodytes_forsteri
-------MTNHICLCTTRIQNS------
>Apaloderma_vittatum
-------MTNRICLCTTRIQNS------

HG0400 ENSGALG00000004261 AQP9

>Gallus_gallus
MISLAVQHEISYFSV
>Anser_cygnoides
MISLAVQDEISYFNV
>Apteryx_australis_mantelli
MISLAVQDEI-----
>Caprimulgus_carolinensis
MTSLAVQDEISYFNV
>Calidris_pugnax
MISLAVQDEISYFNV
>Columba_livia
MISLAVQDEISYFNV
>Cuculus_canorus
MISLAVQDEISYFNV
>Aquila_chrysaetos_canadensis
MISLAVQDEISYFNV
>Balearica_regulorum_gibberic
MISLAVQDEISYFNV
>Geospiza_fortis
MISLAVQDEISYFNV
>Egretta_garzetta
MISLAVQDEISYFNV
>Picoides_pubescens
MISLAAQDEISYFNV
>Fulmarus_glacialis
MISLAVQDQISYFNV
>Aptenodytes_forsteri
MISLAVQDEISYFNV
>Tyto_alba
MISLAVQDEISYFNV
>Struthio_camelus_australis
MISLAVQDEISYFNV
>Tinamus_guttatus
MISLALRDEISYFNV

HG0401 ENSGALG00000009057 TASP1

>Gallus_gallus
--MILLQNLFLNGSMLFASRRC
>Anas_platyrhynchos
MQMIPLQNLFLNGSMLFSNRRW
>Apteryx_australis_mantelli
--MIPLQSLFLNGSMLFSSRRW
>Charadrius_vociferus
--MIPLQGLFLNGRMLFSSRRW
>Leptosomus_discolor
--MIPLQSLFLNGSMPFSSRRW
>Falco_cherrug
--MILLQSLFLNGKVLFSSRRW
>Gavia_stellata
--MIPLQSLFLDGRMLFSSRRW
>Balearica_regulorum_gibberic
--MIPLQSLFLNGRMLFSSRRW
>Sturnus_vulgaris
--MISLQSLFLNGSVLLPSRRW
>Egretta_garzetta
--MIPLQSLFLNGRMLFSSRRW
>Fulmarus_glacialis
--MIPLQSLFLNGRMLFSSRRW
>Nestor_notabilis
--MILLQSLFLNGRVLFSSRRW
>Pygoscelis_adeliae
--MIPLQSLFLNGRMLFSSRRW
>Tyto_alba
--MIPLLSLFLNGRMLFSSRRW
>Struthio_camelus_australis
--MIPLQSLLLNGSMIFSSRRW
>Tinamus_guttatus
--MIPLQRLFLNGSMFFSSRRW
>Apaloderma_vittatum
--MIPLQSLFLNGRMLFSSRRW

HG0402 ENSGALG00000035584

>Gallus_gallus
MARCWKGREQPMLLI
>Anas_platyrhynchos
MARCWKGRERPMLLL
>Chaetura_pelagica
MAQCWKGREQPMLLF
>Calidris_pugnax
MARCWKGREQPMLLF
>Pterocles_gutturalis
MARCWKGREQPMLLF
>Columba_livia
MARCWKGREQPMLLF
>Leptosomus_discolor
MALCWKGREQPMLLF
>Falco_cherrug
MARCWKGREQPMLLF
>Chlamydotis_macqueenii
MARCWKGREQPMLLF
>Tauraco_erythrolophus
MARCWKGREQPMLLF
>Taeniopygia_guttata
MARCWKGREQPMLLF
>Pelecanus_crispus
MARCWKGREQPMLLF
>Fulmarus_glacialis
MARCWKGREQPMLLF
>Aptenodytes_forsteri
MARCWKGREQPMLLF
>Tyto_alba
MAQCWKGREQPMLLF
>Struthio_camelus_australis
MARCWKGREQPMLLF
>Calypte_anna
MARCWKGREQPMLLF

HG0403.1 ENSGALG00000007717 GCNT7

>Gallus_gallus

MNPPPNTNPFSRMLSGKEAFIQHEIQSIPITCMPN-------------------------

>Anser_cygnoides

------------MLSAEEAFFQHVIQCIPVRIMR--------------------------

>Chaetura_pelagica

------------MLSGKEEFFQHEIQCLPTI-----------------------------

>Cariama_cristata

MNLPPNIQLLSGMLSGKEAFFQHEIQCIPII-----------------------------

>Charadrius_vociferus

------------MLSGKEAFFQHEIQCIPII-----------------------------

>Pterocles_gutturalis

------------MLSGKEAFFHPEIQCIQII-----------------------------

>Columba_livia

------------MLSGKEAFFQHGIQCIPII-----------------------------

>Merops_nubicus

------------MLSGKEEFFQHEIQCIPII-----------------------------

>Aquila_chrysaetos_canadensis

------------MLSGKEAFFQREIQCLPII-----------------------------

>Gavia_stellata

------------MLSGKEAFFQHEIQCIPII-----------------------------

>Balearica_regulorum_gibberic

------------MLSGKEAFFQHEIQCIPII-----------------------------

>Tauraco_erythrolophus

------------MLSGKKAFFQHEIQCIPIL-----------------------------

>Lepidothrix_coronata

------------MLSGKEAFFQHEIQCIPIISMFN-------------------------

>Fulmarus_glacialis

------------MLSGKEAFFQHEIQCIPVI-----------------------------

>Tyto_alba

------------MLSGKEAFFQNEIQCIPII-----------------------------

>Tinamus_guttatus

------------MLSGKKAFFQHQIQCIPEFCLGSSTSKASRKRPHAVATGFRTSVLSCF

HG0403.2 ENSGALG00000007717 GCNT7

>Gallus_gallus

MLSIAGN-------QVMLMFNPFQLYEISTQATE

>Anas_platyrhynchos

MLSIAGSQMKP-----------------------

>Apteryx_australis_mantelli

MLSIAGSQMKP-----------------------

>Calidris_pugnax

MLSIAGSQMNSQMKTVMLMFNPFHLRQISPQATE

>Struthio_camelus_australis

MLSIAGSQMKP-----------------------

HG0404 ENSGALG00000008700 PDCD4

>Gallus_gallus
MEHSTRKDKTVLTR---
>Apteryx_australis_mantelli
MEHSTGKNKTVLTW---
>Buceros_rhinoceros_silvestri
MEHSARKNKSVLTG---
>Caprimulgus_carolinensis
MEHSTRKNKTVLT----
>Charadrius_vociferus
MEHSTRKNKTVLTR---
>Colius_striatus
MEHSTRKNKTVLTR---
>Leptosomus_discolor
MEHSTRKNKIVLTR---
>Haliaeetus_leucocephalus
MEHSIRKNKTVLTR---
>Gavia_stellata
MEHSPRKDKTVLTR---
>Chlamydotis_macqueenii
MEHSTRKNKTVLTRQFA
>Tauraco_erythrolophus
MEHSTRRSKTVLTR---
>Ficedula_albicollis
MEHSTRKDKTVLTR---
>Fulmarus_glacialis
MEHSARKNKTVLTR---
>Nestor_notabilis
MEHSTTKTKTVLTR---
>Aptenodytes_forsteri
MEHSTRKNNTVLTR---
>Tyto_alba
MEHSTRKNKTVITR---

HG0405.1 ENSGALG00000009185

>Gallus_gallus
--MLLSSLWAFLDLRFFS
>Anser_cygnoides
------------MW------NTLL-AYLRVRE--AVQEVLCRLYKYSVRQ-----AVISLSLLQISAACLGHMLLSRLRAFLDLRFFS
>Cariama_cristata
--MLLISLRAFLDLRFFS
>Charadrius_vociferus
MKERLARVGDLKSFSPSPLTHSQTEELHRHQEITLHQDARSR-YFLPCTQQVTSFEILFSPLKQISAACLGYMLLISLRAFLDLRFFS
>Pterocles_gutturalis
--MLLSSLQAFLDLRCFS
>Colius_striatus
--MLLIGLRAFLDLRFFS
>Leptosomus_discolor
--MLLISLRAFLDLRFFS
>Aquila_chrysaetos_canadensis
--MDNHGKISTACLGYMLLISLRAFLDLRFFS
>Gavia_stellata
--MSTACLGYMLLISLRAFLDLRFFS
>Chlamydotis_macqueenii
--MLLISLQAFLDLGFFS
>Opisthocomus_hoazin
--MSTACLGCMLLISLRAFLDLRFSS
>Acanthisitta_chloris
--MLLISLLASLDLRFFS
>Phalacrocorax_carbo
--MSTACLGYMLLISLQAFLDLRFSS
>Struthio_camelus_australis
--MLLSSLRAFLDLRFFS

HG0405.2 ENSGALG00000009185

>Gallus_gallus
---MPHPDIFSPELSKLPVL------
>Charadrius_vociferus
---MPDPGIFSPVLSKLPVSRSCSVL
>Pterocles_gutturalis
---MPHPGILCPVLSKLQVLRSCSVL
>Colius_striatus
---MPNPGIFFPVLSKLAVLRSCPGL
>Leptosomus_discolor
---MPDPGIFSPVLSKLPFLRSCSVL
>Haliaeetus_albicilla
MKERLARAGELKSFSPSPLVSQCKDRRTTETSEISTLAFLRMPDPGIFSPALSKLPVSRLCSVL
>Chlamydotis_macqueenii
---MPDPDIFYPVLSKLPVLRSCSVL
>Phalacrocorax_carbo
---MPDPGIFSPILSKLPVLRSGSVL
>Picoides_pubescens
---MLDPGIFSPVLSKLPVLRSCSVL
>Fulmarus_glacialis
---MPDPGIFSPVLSKLPVLRSCSVL
>Nestor_notabilis
---MPDPGIFSTVLSKLPVSRSCSVL
>Pygoscelis_adeliae
---MPDPGIFSPVLSKLPVLRSCSVL

HG0406 ENSGALG00000004935 LSM14A

>Gallus_gallus
MKAAAMLGASGRFWCGVC
>Chaetura_pelagica
-----MLGASGRFRCGVC
>Apteryx_australis_mantelli
MKAAAMLGASGCFWCCLC
>Calidris_pugnax
MKAAAMLGASGRFWCGLC
>Columba_livia
MKAAAMLGASGRFWCGVC
>Leptosomus_discolor
MKAAAMLGASGRFWCGVC
>Aquila_chrysaetos_canadensis
MKAAAMLGASGCFWCGVC
>Tauraco_erythrolophus
MKPSSMWRASGRFRCGVC
>Pseudopodoces_humilis
MKAAAMLGAAGRGPC---
>Nipponia_nippon
MKAAAMFGASGRFWCGVC
>Picoides_pubescens
MKAAAMLGGSGRFWCGVC
>Melopsittacus_undulatus
MTAAAMLGASGSFWCGVC
>Pygoscelis_adeliae
MKAAAMFGASGWFWCGVC
>Calypte_anna
MKAAAMLGASGRFRCGVC

HG0407 ENSGALG00000015027 JAK2

>Gallus_gallus
MERARVLRGSRQLALLRRAPAGYIMFYHCLCACFI
>Anas_platyrhynchos
------------------------MFYHSLCACFV
>Chaetura_pelagica
------------------------MFHHCLCTCFL
>Buceros_rhinoceros_silvestri
------------------------MFHHCLCACFV
>Cariama_cristata
------------------------MFQHCLCACFV
>Calidris_pugnax
------------------------MFHHCLCACFA
>Columba_livia
------------------------MFHHCLCACFV
>Cuculus_canorus
------------------------MFHHCLCACIV
>Aquila_chrysaetos_canadensis
-----------------------MMFHHCLCACFV
>Eurypyga_helias
------------------------MFHHCLCACFA
>Tauraco_erythrolophus
------------------------MFHHCVCACFV
>Lepidothrix_coronata
MGRERA--DAACAPGCTASAAGYIMSHHCLCACFV
>Egretta_garzetta
------------------------MFYHCLCACFV
>Nestor_notabilis
------------------------MFHHCSCACFV

HG0408 ENSGALG00000036255 WNT7B

>Gallus_gallus
MTGRSPEAAAAARTA-AAAHTLPREQH
>Anas_platyrhynchos
MTGRSPEAAAAARAA-AAAHTLHREQH
>Chaetura_pelagica
MTGRSPEAAAAARAA-AAAHTLHREQH
>Apteryx_australis_mantelli
MTGRSPEAAAAARAA-AAAHTLHREQH
>Charadrius_vociferus
MTGRSPEAAAAARAA-AAAHTLHREQH
>Columba_livia
MTGRSPEAAAAARTA-AAAHTLPREQH
>Cuculus_canorus
MTGRSPEAAAAARAAAAAAHTLHREQH
>Aquila_chrysaetos_canadensis
MTGRSPEAAAAARAA-AAAHTLHREQH
>Lepidothrix_coronata
MTGRSPEAAAAARAA-APAHTLHREQH
>Nipponia_nippon
MTGRSPEAAAAARAA-AAAHTLHREQH
>Melopsittacus_undulatus
MTGRSPEAAAAARAA-AAAHTLHREQH
>Aptenodytes_forsteri
MTGRSPEAAAAARAA-AAAHTLHREQH
>Struthio_camelus_australis
MTGRSPEAAAAARAA-AAAHTLHREQH
>Calypte_anna
MTGRSPEAAAAARAA-AAAHTLHREQH

HG0409 ENSGALG00000002315

>Gallus_gallus

MVSTPCEPPLQK---------

>Anser_cygnoides

MDSTSCEPPLQK---------

>Apteryx_australis_mantelli

MNSTSCEPPLQKATLAQKWNL

>Cariama_cristata

MDSTSCEPPLQK---------

>Pterocles_gutturalis

MNSTSCEPPLQK---------

>Columba_livia

MDSTSCEPPLQK---------

>Chlamydotis_macqueenii

MDSTSCEPPLQK---------

>Opisthocomus_hoazin

MDSTSCEPPLQK---------

>Zonotrichia_albicollis

MDSTSCEPPLQK---------

>Phalacrocorax_carbo

MDSTSCEPPLQK---------

>Fulmarus_glacialis

MDSTSCEPPLQK---------

>Melopsittacus_undulatus

MDSTSCEPPLQK---------

>Pygoscelis_adeliae

MDSTSCEPPLQK---------

HG0410 ENSGALG00000008148 MYBPC3

>Gallus_gallus

MGASTEWFFWGSQKPCSLICV

>Anas_platyrhynchos

MGASTKSFFRGSQKPWRLIPV

>Cariama_cristata

MGASTKCFFRGSEKPCSLIPK

>Calidris_pugnax

MGASTECFFRGSQKPCRLIPV

>Columba_livia

MEASTKCFFRGFQKPCSLIPV

>Leptosomus_discolor

MGASTKCFFRGSQKPCSFIPV

>Aquila_chrysaetos_canadensis

MGASTKCFFRGSQKPCSLIPV

>Eurypyga_helias

MGSSTECFFRGSQKPHILIPD

>Parus_major

MGASIERFFRGSQKPCSHIPV

>Phaethon_lepturus

MGASTGCFFRGSQKPCRLIPV

>Fulmarus_glacialis

MGASTECFFRGSQKPCSLISV

>Melopsittacus_undulatus

MGASTKCFFRGSQKPCTLIPV

>Aptenodytes_forsteri

MGASTECFFRGSQKPCSLIPV

HG0411 ENSGALG00000012166 SLC35F5

>Gallus_gallus

--------MAIPTLTLLKWRLSSL

>Anser_cygnoides

MAEKWFYKMTTPTLTLLKWRLSSL

>Cariama_cristata

--------MTAPTLTLLKWRLSSL

>Calidris_pugnax

--------MTAPNLTQLKWRLSSL

>Aquila_chrysaetos_canadensis

--------MTAPTLTLLKWRLSSL

>Gavia_stellata

--------MTAPTLTLLKWRLSSL

>Eurypyga_helias

--------MTAPTLTLLRWRLSSL

>Tauraco_erythrolophus

--------MTASTLTLLKWRLSSL

>Sturnus_vulgaris

--------MTVPTLRLLEWRLSSL

>Nipponia_nippon

--------MTPPTLRLLKWRLSSL

>Picoides_pubescens

--------MTAPTVILLKWRLSSL

>Fulmarus_glacialis

--------MTAPTLTSLKWRLSSL

>Aptenodytes_forsteri

--------MTAPTLTLLKWRLSSL

HG0412 ENSGALG00000003446 PRLR

>Gallus_gallus
-----------------------------MNERNKIFLCTHESFREPLM
>Chaetura_pelagica
-----------------------------MKERNKNLPCTHESFSEHLI
>Caprimulgus_carolinensis
-----------------------------MKERNKNLPCTHESFSEPLI
>Cariama_cristata
-----------------------------MKERNRNLPCTHQSFCKPLI
>Calidris_pugnax
-----------------------------MKERNKNLHRTHESFSEPLI
>Pterocles_gutturalis
-----------------------------MKERNKNLPRTHESFSEPLI
>Colius_striatus
-----------------------------MKERNKNLPCTHESFSEPLI
>Columba_livia
-----------------------------MKERNKNLPCTHETFSEPLI
>Haliaeetus_leucocephalus
-----------------------------MKEKNKNLPCTHESFSEPLI
>Chlamydotis_macqueenii
MTENLQGVSPMAAWDEDGGYIRSSTDLTFMKKRNKNLHNTHESFREPLI
>Lepidothrix_coronata
-----------------------------MKERNKKLPYIHESFSEPLI
>Nipponia_nippon
-----------------------------MKEKNKNLPHTHESFSEPLI

HG0413 ENSGALG00000005475

>Gallus_gallus

MRSCCWLLLDFVSG

>Anser_cygnoides

MQSCCWLLLDFVSG

>Charadrius_vociferus

MQSCCQLLLEFVSG

>Colius_striatus

MQSCCQLLLGFVSG

>Falco_cherrug

MQSCCQLLMDFVSG

>Gavia_stellata

MQSCCQLLLDFVSG

>Balearica_regulorum_gibberic

MQSCCQLLLDFVSG

>Opisthocomus_hoazin

MQSCCQCLLDFVFG

>Egretta_garzetta

MQSCCQLLLDFVSG

>Melopsittacus_undulatus

MQSCCQLLLDSVSG

>Struthio_camelus_australis

MQSCCQFLLDFFSG

>Tinamus_guttatus

MQSCCQFLLVFLSG

HG0414 ENSGALG00000032599

>Gallus_gallus
MMKALQWERAVVQG
>Anser_cygnoides
MMKAPQWEHAVVQG
>Chaetura_pelagica
MMKALQWDRAVVQG
>Buceros_rhinoceros_silvestri
MMKALQWERAVVQG
>Charadrius_vociferus
MMKALQWERAVVQG
>Columba_livia
MMKALQWERAVVQG
>Cuculus_canorus
MMKAPQWERAVVQG
>Aquila_chrysaetos_canadensis
MMKALQWERAVVQG
>Sturnus_vulgaris
MMKALQWERAVVQG
>Phaethon_lepturus
MMKALQWEHAVVQG
>Pygoscelis_adeliae
MMKALQWERAVVQG
>Calypte_anna
MMKALQWDRAVVQG

HG0415 ENSGALG00000034970

>Gallus_gallus
MLSRTVMTREASLAKD
>Anas_platyrhynchos
MLSATVMTREASPAKD
>Chaetura_pelagica
MLSTTVMKREAPLARD
>Cariama_cristata
MLSATVMKKESPLARD
>Charadrius_vociferus
MLSATVMKKEAPLARD
>Pterocles_gutturalis
MLSTTVMKREAPLARD
>Leptosomus_discolor
MLSTTVMKREAPLARD
>Cuculus_canorus
MLSRTVMKIEALLAKD
>Gavia_stellata
MLSTTVMKREATLDRD
>Balearica_regulorum_gibberic
MLSTTVMKREAALARD
>Pelecanus_crispus
MLSTTVMKREASLARD
>Fulmarus_glacialis
MLSTTVMKREAPLARD

HG0416 ENSGALG00000015874 TTK

>Gallus_gallus
MKDYISCRCWILGII
>Anas_platyrhynchos
MESYISCLCWVLGII
>Charadrius_vociferus
MESYISCLCWILEII
>Leptosomus_discolor
MESYVSCLCWILGII
>Gavia_stellata
MESYISCLCWILGII
>Eurypyga_helias
MENYLSCLCWILRII
>Egretta_garzetta
MENYISCLCWILGII
>Fulmarus_glacialis
MESYISCLCWILGII
>Tyto_alba
MESYVSCLCWILGII
>Struthio_camelus_australis
MENYVSCLCWISGNI

HG0417 ENSGALG00000001522 PHF20

>Gallus_gallus

----------------MDFSALTIPDGGKHVVGGELGE

>Anas_platyrhynchos

----------------MDFSGLTIPDGGMHIVGGELGE

>Apteryx_australis_mantelli

MAVVIHLQGLPIVVGTMDFSGVTIPDGGVHIVGGELGE

>Calidris_pugnax

----------------MDLSGLSIPDGGVQIVGGELGE

>Columba_livia

MAVVIHLRGLPGVVGTMDFSGLTIPAGGVHVVGGELGE

>Haliaeetus_albicilla

MAVFIHLQGLPIVVGTMDFSGLTIPDGGVHIVGGELGE

>Parus_major

MAVVIHLQGVPIAVGTMDFSGLTIPDGGVHIVGGELGE

>Pygoscelis_adeliae

MAVVIHLQGLLIVVGTMDFSGLTIPDGGMHIVGGELGE

>Struthio_camelus_australis

MAVVIHLQGLPIVVGTMDFSGLTIPDGGVHIVGGELGE

HG0418 ENSGALG00000010337 GPR149

>Gallus_gallus
-------------------MKNCIFSSTFCTSLLPDFSRA-----------
>Anser_cygnoides
-------------------MKNCIFSSTFGTSLLPDFSRACVVLVIGDA--
>Caprimulgus_carolinensis
-------------------MKNCIFFSTFSTSLLPDFS-------------
>Calidris_pugnax
-------------------MKNCIFFSTFDASLLPDFSRA-----------
>Haliaeetus_albicilla
-------------------MKNCIFFSTFDTSLLPEFSRA-----------
>Eurypyga_helias
MACYYLNSLNYPSFSAERIMKNCFLFSTFATSLLPDFSRARVVLVIKGTFT
>Tauraco_erythrolophus
-------------------MRNCIFSATFDTSLLPDFSRA-----------
>Phaethon_lepturus
-------------------MKNCIFFSIFDTSLLPDFSRA-----------
>Tyto_alba
-------------------MKNCIFFSTFGTSLLPDFLRA-----------

HG0419 ENSGALG00000012542 RASD2

>Gallus_gallus

MGGRPLEPCSPSQSC

>Anas_platyrhynchos

MGGRPLEPCGPSHSC

>Apteryx_australis_mantelli

MGDRPLQSCSPSQSC

>Calidris_pugnax

MGEHPLKPCSPSQSS

>Columba_livia

MGECPLEPCGPSQSC

>Haliaeetus_albicilla

MGERPLEPCGPSQSC

>Corvus_brachyrhynchos

MGERPLEPCGPSQSC

>Aptenodytes_forsteri

MGERPLEPCGPSQSC

>Tinamus_guttatus

MGDSPLHICSPSQSC

HG0420 ENSGALG00000000336 PPP1R12B

>Gallus_gallus

MPKIIHLNRQGLITRAANSLISLF

>Anser_cygnoides

MPKIIHPKQQGLITRAVNSLISLF

>Apteryx_australis_mantelli

MPKIIHPKQRGLITRAGNSLIGLF

>Calidris_pugnax

MPKIIHPKQQGLITRAVNSLISLF

>Aquila_chrysaetos_canadensis

MPKIIHPKQQGLITRAVNSLISLF

>Corvus_brachyrhynchos

MPKIIHLKQQGLITRAVNSLISLF

>Nipponia_nippon

MPKITHPKQQGLITRAVNSLISLF

>Calypte_anna

MPKIIHPKQQGLITRAVNSLISLF

HG0421 ENSGALG00000003492

>Gallus_gallus

---MEANLKRAKKGDLKVSVHRMEIERIRYVLSSYLRCRLAKIEKFFP
HVLEKEKSRAKGEPSILSPEEFAFAKEYMANTETYLKNVALKHMPPNLQKVALLKSVPKPNLDSFVFLRVLKRQENILVEPETDEQREYTIDLEEGSQHLIRYKTI
APLVASGAVQLI

>Anser_cygnoides

---MESNLKRAKAGDLKVSVHRMEIERIRYVLSSYLRCRLVKIEKFFP
HVLEKEKSRAEGEPSILSPEEFAFAKEYMANTETYLKNVALKHMPPNLQKVSLLKSVPKPNLDSFVFLRVLERQENILVEPETDEQREYTIDLEEGSQHLIRYKTI
APLVASGAVQLI

>Apteryx_australis_mantelli

--MEIERIRYVLSSYLRCRLVKIEKFFP
HVLEKEKSRAEGEPSILSPEEFAFAKEYMANTETYLRNVALKHMPPNLQKVSLLKSVPKPNLDSFVFLRVLERQENILVEPETDEQREYTIDLEEGSQHLIRYKTI
APLVASGAVQLI

>Charadrius_vociferus

---MEANLKRAKRGDLKVSVHRMEIERIRYVLSSYLRCRLVKIEKFFP
HVLEKEKSRAEGEPSILSPEEFAFAKEYMANTETYLKNVALKHMPPNLQKVSLLKSVPKPNLDSFVFLRVLERQENILVEPETDEQREYTIDLEEGSQHLIRYKTI
APLVASGAVQLI

>Falco_cherrug

---MEANLKRAKRGDLKVSIHRMEIERIRYVLSSYLRCRLVKIEKFFP
HVLEKEKSRTEGEPSILSPEEFAFAKEYMANTETYLKNVALKHMPPNLQKVSLLKSVPKPNLDSFVFLRVLERQENILVEPETDEQREYAIDLEEGSQHLIRYRTI
APLVASGAVQLI

>Geospiza_fortis

MAGAMAAAAGGDSDGESEELVLTPAQLIHSLEQAWLNEKFAPELLESKPEIIECVVEQLDHMEANLKRAKKGDLKVSVHHMEIERIRFVLSSYLRCRLVKIEKFFP
HILEKEKSRAEGEPSILSPEEFAFAKEYMANTETYLKNVALKHMPPNLQKVSLLKSVPKPNLDSFVFLRVLERQENILVEPETDEQREYTIDLEEGSQHLIRYRTV
APLVASGAVQLI

>Nipponia_nippon

MAAASGEGTAADSDGDSEELVLTPAQLIRSLEQAWLNEKFAPELLESKPEIIECVVEQLDHMEANLKRAKRGDLKVSVHRMEIERIRYVLSSYLRCRLVKIEKFFP
HVLEKEKSRAEGEPSILSPEEFAFAKEYMANTETYLKNVALKHMPPNLQKVSLLKSVPKPNLDSFVFLRVLERQENILVEPETDEQREYTIDLEEGSQHLIRYKTI
APLVASGAVQLI

>Struthio_camelus_australis

--MNEANLKRAKKGDLKVSIHHMEIERIRYVLSSYLRCRLVKIEKFFP
HVLEKEKSRAEGEPSILSPEEFAFAKEYMANTETYLKNVALKHMPPNLQKVSLLKSVPKPNLDSFVFLRVLERQENILVEPETDEQREYTIDLEEGSQHLIRYKTI
APLVASGAVQLI

HG0422 ENSGALG00000005772 BOK

>Gallus_gallus

--------------------------MKGKMKNRLWESSSWEL

>Anas_platyrhynchos

------------------------------MKDRLFENSCWKL

>Leptosomus_discolor

MLIDAASPKKHESNPKKKSLRGGKCNMKGKMKNWPFENSCWEM

>Aquila_chrysaetos_canadensis

--------------------------MKGKMKNWPFENSCWEL

>Gavia_stellata

--------------------------MRGKMKNWPFENSCWEL

>Balearica_regulorum_gibberic

--------------------------MRGKMKNWPFESSCWEL

>Fulmarus_glacialis

--------------------------MRGKMKNWSFESSCWDL

>Aptenodytes_forsteri

--------------------------MRGKMKNWPFENSCWEL

HG0423 ENSGALG00000006329

>Gallus_gallus

MLSKLVGKKGGHLVSVL

>Leptosomus_discolor

MLSKLVGKNSGHLVSVL

>Aquila_chrysaetos_canadensis

MLSKLVGKNSGHLVSVL

>Gavia_stellata

MLSKLVGKNSGHLVSVL

>Balearica_regulorum_gibberic

MLSKLVGKNSGHLVSVL

>Phaethon_lepturus

MLSKLVGKNSGHLVSVL

>Tyto_alba

MLSKLVGKNSGHLVSVL

>Tinamus_guttatus

MLSKLVGRNSGHLVSVL

HG0424 ENSGALG00000039553

>Gallus_gallus
MTIYLSCIRQRCCLQ
>Anser_cygnoides
MTIYLSCIRKRFCLQ
>Calidris_pugnax
MTIFLSCIRKRFCL-
>Cuculus_canorus
MTIFLSCIRKRFC--
>Haliaeetus_leucocephalus
MTIFLSCIRKRFCL-
>Acanthisitta_chloris
MTIFLSCIRKRFCL-
>Phalacrocorax_carbo
MTIFLSCIRRRFCL-
>Aptenodytes_forsteri
MTIFLSCIRKRVCL-

HG0425 ENSGALG00000043044

>Gallus_gallus
MIQKTVRSCDCKMSSFYLMLKGGKLKLQEL
>Caprimulgus_carolinensis
------------MSSFHLMLKGGKLELQE-
>Falco_cherrug
------------MSSFHSLLKGGKLKLQE-
>Mesitornis_unicolor
------------MSSFHLMLKGGKLKHQE-
>Tauraco_erythrolophus
------------------MLKEGKRKLQE-
>Opisthocomus_hoazin
------------MSSFQLMPKGGKLKLQE-
>Ficedula_albicollis
--------MDCEMNSFHLILKEGKLKLQK-
>Tyto_alba
------------MSSFHLMLKGGKLKLQE-

HG0426 ENSGALG00000002192 PTPRC

>Gallus_gallus
---MLLRKDTPDFQK
>Anser_cygnoides
---MLLHKDTPDFQK
>Calidris_pugnax
---MLFHKDTPDFQK
>Columba_livia
---MLFHKDTPDFQK
>Falco_cherrug
---MLFHKDTPDFQK
>Ficedula_albicollis
MPVELRAGHSSLTSGWRWRKKVLAAVAAGLVKRSLVPLMYRMKAEISGCPQRTNPFNQTNQRERHMLFHKDTPDFQK
>Aptenodytes_forsteri
---MLFHKDTPDFQK

HG0427 ENSGALG00000005550 ST6GAL1

>Gallus_gallus
MKVFLELFSASRGCCVWGYPALNPGQKSANSQK
>Anser_cygnoides
MKVFLELFRVSHNRCVLVYPALTPGRKSANSQK
>Calidris_pugnax
MKVFLECFGASQNCCVLGYSA------------
>Columba_livia
MKLFLELFSASQNCCVLRCPA------------
>Aquila_chrysaetos_canadensis
MKVFLELFSASQNCCVLRYPA------------
>Ficedula_albicollis
MKVFLELFNTSRSCCVLGYPA------------
>Aptenodytes_forsteri
MKVFLELFSASQNCCVLGYPA------------

HG0428 ENSGALG00000005976 TCEA2

>Gallus_gallus
MSHPVAGRFAAPLLK
>Charadrius_vociferus
MSVPVDGRFSTPLLK
>Leptosomus_discolor
MSVPVDGRFSAPLLK
>Haliaeetus_albicilla
MSVPVDGRFSTPLLK
>Gavia_stellata
MSVPVDGRFSTPLLK
>Pelecanus_crispus
MSVPVDGRFSTPLLK
>Fulmarus_glacialis
MSVPVDGRFSTPLLK

HG0429 ENSGALG00000006626 PDE6C

>Gallus_gallus

----MPLYSMYKVPFSY

>Anser_cygnoides

---------MYKVPFSY

>Columba_livia

MYYACELYNMYKVPFSY

>Gavia_stellata

MYYACELYNMYKVPFSY

>Opisthocomus_hoazin

MYYACELYNMYKVPFSY

>Pelecanus_crispus

MYYACELYNMYKVPFSY

>Tinamus_guttatus

---------MYKVPFSY

HG0430 ENSGALG00000015490

>Gallus_gallus

---MLSMGTSASDALTLLKTYFVAWLKRR

>Anser_cygnoides

---MLSTGTSASDARTLLKTYFVVWLKGR

>Apteryx_australis_mantelli

MKVYVLKELTCLRRSQWYQGNGAQQKEGSPKTTNLAVLGHRCKMLSMGTFASDAR-ILKIYFFAWLTGR

>Calidris_pugnax

-----------MGIAPYF-----------FLVNAVNRLIQRSKMLSVGTSASDARTLQKAYFVAWLKGR

>Columba_livia

---MLSLETSAYDAGTLLKTYFVAQLKGR

>Haliaeetus_albicilla

---------------------------------MKIFSLIRSKMLSMETSASDARTLLKTYFVAWLKGR

>Zonotrichia_albicollis

---MLSMGTSASDARTLLKTSFVAWFKGR

HG0431 ENSGALG00000026542 FAM217B

>Gallus_gallus

MPAEAESKDVVMFAFGTLCCVDRKEWSRWKIM

>Aquila_chrysaetos_canadensis

----------MMFAFDTLCLANPKDWSRLKIM

>Manacus_vitellinus

-----------MSAFDTLCLANPKDWSRLKIM

>Melopsittacus_undulatus

------MQEVVMFAFDTLCLTNPKDWS-----

>Aptenodytes_forsteri

-----------MFAFDTLCLTNPKDWSRLKIM

>Struthio_camelus_australis

-----------MPAFDALCLINPKHWSPLKVM

>Tinamus_guttatus

---------MVMFAFDTLCLVNPKHWSPLKIM

HG0432 ENSGALG00000040546

>Gallus_gallus
MLENFTSVLIVISEHPPKN----
>Anser_cygnoides
MLENFTSVLIVISEHPPKN----
>Calidris_pugnax
MLEYFTSVLIVISEHPQRTETLH
>Columba_livia
MLENFTSVLIVISEHPQRTETLH
>Haliaeetus_albicilla
MLENFTSVLIVISEHPQRTETLH
>Corvus_cornix_cornix
MLENSTSVLTVIREHPQRSAAFH
>Aptenodytes_forsteri
MLENFTSVLIVISEHPQRTETLH

HG0433 ENSGALG00000002952 NRG4

>Gallus_gallus
MQISGGVDSADCPVRV
>Calidris_pugnax
MQMRDYVDSADCPVRV
>Columba_livia
MQIRDYVDSADCPVHV
>Aquila_chrysaetos_canadensis
MQIRDYVDSADCPVHV
>Zonotrichia_albicollis
MRIRDYVDSADCPVHV
>Aptenodytes_forsteri
MQIRDYVDSADCPVHV

HG0434 ENSGALG00000008357 TPCN1

>Gallus_gallus

MGARTFAFWWLEDGHDGRFPGKRF

>Calidris_pugnax

MGTRPCIFLCLEDTPDGRLPGESF

>Columba_livia

MGTRTFIFLCLEDTPDGRLSRDTF

>Aquila_chrysaetos_canadensis

MGTHTFIFLCLEDTPDGRLSGDSF

>Pygoscelis_adeliae

MSTHTFIFLCLEDTPDGRLSGESF

>Struthio_camelus_australis

MGTRVFVRFCLEDTPDGRLPGGRF

HG0435 ENSGALG00000010778 CHRM3

>Gallus_gallus

--MAKSFSSVWYIQLQAG

>Anser_cygnoides

--MAKSFSSAWYDGLQVG

>Pterocles_gutturalis

--MAKSFISVWYNGLPAG

>Aquila_chrysaetos_canadensis

MVMAKSFISIWYNGLPVG

>Egretta_garzetta

MVMAKSFISVWYNGLPVG

>Aptenodytes_forsteri

MVMAKSFISVWYNGLPVG

HG0436 ENSGALG00000026736 OGN

>Gallus_gallus
MLKLSTAPCSRILPLTLGTECWQSIFYPMESTFKTVNLGKLSHCSNLDVIHCWIVHNFTLLSVCMGLTYA
>Anser_cygnoides
----------------------------MESIFKTVNLGRSIHCSICIS---------------------
>Apteryx_australis_mantelli
----------------------------MESTFKTVNL--------------------------------
>Calidris_pugnax
----------------------------MESTFKTVKL--------------------------------
>Columba_livia
----------------------------MESTFKTVKLRMSIHFNNS-----------------------
>Pygoscelis_adeliae
----------------------------MESTFKTVKL--------------------------------

HG0437 ENSGALG00000001728 NACC2

>Gallus_gallus
MRGAAPARRPGPPFSPGPRRPASAAAARRKTMGRTMCPTLDTPALNA
>Haliaeetus_leucocephalus
-----------------------------------MCPTLDIPALNV
>Parus_major
-----------------------------------MCPTLDIPALNV
>Melopsittacus_undulatus
-----------------------------------MCPTLDIPALNV
>Aptenodytes_forsteri
-----------------------------------MCPTLDIPALNV

HG0438 ENSGALG00000008545 SENP5

>Gallus_gallus
MWYTARCCVTCKERQFCT
>Anser_cygnoides
MWYTAWC-----------
>Falco_cherrug
MWYAGWCCLTCKERWFCL
>Corvus_brachyrhynchos
MWFAGRCCLTCKERWFCV
>Aptenodytes_forsteri
MWYAGWCCLTCKERWFCV

HG0439 ENSGALG00000009172 OSBPL6

>Gallus_gallus
MQKSIPCIFVHE
>Calidris_pugnax
MQKTIPCLFVHE
>Aquila_chrysaetos_canadensis
MQKTIPCLFVHE
>Taeniopygia_guttata
MQKTIPCIFVHE
>Pygoscelis_adeliae
MQKAIPCLFVHE

HG0440 ENSGALG00000010090

>Gallus_gallus
MMLGHLLLTTGFSKEIQHGLMC
>Anas_platyrhynchos
-MLGHLLLSTGFSKEIQHGLDG
>Calidris_pugnax
MMLEHL----------------
>Corvus_cornix_cornix
MILQHL----------------
>Aptenodytes_forsteri
MMLEHL----------------

HG0441 ENSGALG00000015069 B4GALT4

>Gallus_gallus

MSHRTTRSAAALPTCSFPSLL

>Apteryx_australis_mantelli

MNCKTRGSVAAIPTSGFPSLL

>Corvus_brachyrhynchos

MNFKTAGSVAALPTSSFPSLL

>Struthio_camelus_australis

MNCKTRGSVAAVPTSGFPSLL

>Tinamus_guttatus

MNCKTRGSVAAVPTSAFPSLL

HG0442 ENSGALG00000030250

>Gallus_gallus

-----------MTFTSMMN-QEKPVKSS

>Calidris_pugnax

---MKYFLVLDMTFSITMNPQEKPVKSS

>Aquila_chrysaetos_canadensis

MAVMKYFLVLDMTFTTTMN-QEKPIKSS

>Corvus_brachyrhynchos

-----------MTFSTTMN-QEKPVKSS

>Struthio_camelus_australis

-----------MTFTIRMN-QEKPAKSS

HG0443 ENSGALG00000039454

>Gallus_gallus
MGWVSNHVSQDSVLL------------------------------------
>Anas_platyrhynchos
MGWVSNHVSQDSLFCNDGLRRCFPGEVRAGGWEAKRDVLVGFVVVGMEVNN
>Calidris_pugnax
MGWVSNHVS--
>Struthio_camelus_australis
MGWVSNHVS--
>Tinamus_guttatus
MGWVSNHVS--

HG0444 ENSGALG00000000558 SLC1A6

>Gallus_gallus

--------MLYLPKEKILHPAFELRESRNKTTQNNPPR------RKRPTVLLPDH

>Aquila_chrysaetos_canadensis

--------MLYLSKEKILHPAFELKELKNETTQNNPPR------RKRPAALPA--

>Lepidothrix_coronata

MLQSTMRATVYKTRENILLPAFELKELKNETTQNNPPR------RKRPAALPA--

>Struthio_camelus_australis

--------MLYLSKEKILQPAFDLKELQNKPTQNNPQEIRRVFRPKRPAVLPA--

HG0445 ENSGALG00000004017

>Gallus_gallus
MVALPGVGLGQPPQQGGDESLYKQGRK
>Anas_platyrhynchos
MVVLLGVGLGQPPHQQGDKSLHKQRRK
>Acanthis_flammea
MVAVRRAGLGQPPEQQGETAPYERRRK
>Pygoscelis_adeliae
--MVSVRGLGQPLEQQGDKSPYDQRRK

HG0446 ENSGALG00000006807 PLSCR5

>Gallus_gallus

MLPNTSLCSPARSAWALPFCSHASYLSAAHMCV-ISMATWALEVEI

>Anser_cygnoides

------------------------------------MATWALEVEI

>Calidris_pugnax

-MPHSSL--PGSVHRLCHFCSHASYLSADRMCVMISMATWALEAEI

>Parus_major

---------------------------------MISTATWAFKAGV

HG0447 ENSGALG00000008784

>Gallus_gallus

MKLNFKECFNQKEN

>Anser_cygnoides

MKLILK--------

>Calidris_pugnax

MKLILKECFTQKEN

>Corvus_cornix_cornix

MKLALRECFNQKEN

HG0448 ENSGALG00000011762 UBA6

>Gallus_gallus

MRPGLPAVPAC

>Aquila_chrysaetos_canadensis

MNPGLPAIPAC

>Lepidothrix_coronata

MSPGLPAIPAC

>Aptenodytes_forsteri

MNPGLPAIPAC

HG0449 ENSGALG00000014106 PPFIBP1

>Gallus_gallus

MDQWNWLKRNGGDQH

>Apteryx_australis_mantelli

MGWWYWLKSDGGDQH

>Aquila_chrysaetos_canadensis

MDQWYLLKSDSGDQH

>Pseudopodoces_humilis

MDQWYMLKSNSGDQH

HG0450 ENSGALG00000015528 JAKMIP1

>Gallus_gallus

---------MRDKTHMADGRDFCERFG

>Anas_platyrhynchos

MGMNGKRKDMRNS--THNGRDLCERFY

>Calidris_pugnax

---------MRDR--SHDGRDLCERFC

>Aptenodytes_forsteri

---------MRDC--SHDGRDLCERFC

HG0451 ENSGALG00000019489 CHST9

>Gallus_gallus
MGL----------------CTD---FR----LYRSESGQVTEEDAGTVRTSEK
>Apteryx_australis_mantelli
--MLKTCEK
>Aquila_chrysaetos_canadensis
MGLLAGFQQRFRQMLSVSCCMNNTSFVWGKTEEVRVSGEDTEEDAGTLKTSEK
>Lepidothrix_coronata
-------------MLPMSCYLNNTAFVYSETEEVGVSGEDTEEDAGMLKTSEK

HG0452 ENSGALG00000004227 FAM20B

>Gallus_gallus
MLPWQSVFYSASYE
>Chaetura_pelagica
MLQWQYLFHSASYE
>Apteryx_australis_mantelli
MLQWQYLFYSASYE

HG0453 ENSGALG00000005995 SLC13A5

>Gallus_gallus
MQVLADSPLSARLCAGVKFPLC
>Calidris_pugnax
MRVLADSPLSARL---------
>Manacus_vitellinus
MQVLADSPLSARLCTGVKFPLC

HG0454 ENSGALG00000007186 TMEM268

>Gallus_gallus

MLPCQWRAESQGSRQWESE

>Anas_platyrhynchos

MPPCSWKAESRGSLQWECE

>Falco_cherrug

MSPCSRKAGSPGSPQWECE

HG0455 ENSGALG00000010718 THSD7A

>Gallus_gallus

MTAAASQRKITCSVSWSPLRTAVSLLADGKLLLTAEWKSVEQQPY

>Anas_platyrhynchos

MTAAASQRKITCSVSWSPLRTAVSLLADGKLQLTAEWKSVE----

>Zonotrichia_albicollis

MTAAASQSKITCSVSWSPLRTAVSLLADGKLL-------------

HG0456 ENSGALG00000033256

>Gallus_gallus
MHCSKLFLVFSSG
>Columba_livia
MHYSKLFLVSSSG
>Pseudopodoces_humilis
MHYSKLFLVSSSG

HG0457 ENSGALG00000039538 CLDND1

>Gallus_gallus
MEQLGPAGSWRCVLLAYCRTPVARCL
>Apteryx_australis_mantelli
MEQLGQIGSLHFMLLAYYRTQIYL--
>Gavia_stellata
MEQLGQIGSLHFMLSAYHGTQTTLCL

HG0458 ENSG00000060339 CCAR1

>Homo_sapiens
MWRRGAAWRKRGKLAHAPKADGFEMASMLAGTRLRSML-----
>Acinonyx_jubatus
MRRRGEAWRKRGKLAHAPKADGFEMASVLAGTRFRSLL-----
>Balaenoptera_acutorostrata_s
------------------------MASVLAGTGFRSLLLKTNK
>Miniopterus_natalensis
------------------------MASVLAGTRFRSLL-----
>Dasypus_novemcinctus
MGRKGEAWRKREKLAHAPKADGFEMASVLAGSRLRLLF-----
>Sarcophilus_harrisii
------------------------MASALAATRLRSPL-----
>Galeopterus_variegatus
------------------------MASVLTGTRLR--------
>Monodelphis_domestica
------------------------MASALAATRLRSFL-----
>Trichosurus_vulpecula
------------------------MASALAATRLRSLL-----
>Sorex_araneus
------------------------MASLLAGTRFRSLP-----
>Oryctolagus_cuniculus
------------------------MASVLAGTRLRTLL-----
>Elephantulus_edwardii
------------------------MASVLTGFRLRSLL-----
>Equus_caballus
------------------------MASVLAGTRFRSLL-----
>Manis_javanica
------------------------MASVLAGTRFRSLL-----
>Loxodonta_africana
------------------------MASLLTRSRLRSLL-----
>Fukomys_damarensis
------------------------MASVLAGTRFRSLL-----
>Tupaia_chinensis
------------------------MASALAGTRLR--------
>Trichechus_manatus_latirostr
------------------------MASVLTGIRFRSLL-----
>Orycteropus_afer_afer
------------------------MASVLAGTRLRSLL-----

HG0459.1 ENSG00000163848 ZNF148

>Homo_sapiens
MKGRNSTMISFSTLQHSFP
>Canis_lupus_familiaris
MKGRNSTMISFSTLQHSLP
>Lipotes_vexillifer
MKGRNSTMISFSTLQHSLP
>Eptesicus_fuscus
MKGRNSTMISFSTVQNSFP
>Dasypus_novemcinctus
MKGGNSTMISFSILQHFLS
>Sarcophilus_harrisii
-------MISFSTLQHPLP
>Galeopterus_variegatus
-------MISFSTLQRSLP
>Monodelphis_domestica
-------MISFSTLQHPLP
>Condylura_cristata
-------MISFSTLQHSLP
>Oryctolagus_cuniculus
MKGRNSTMISVSTLQHSLP
>Elephantulus_edwardii
-------MISFSTLQHSLP
>Ceratotherium_simum_simum
MKGRNSTMISFSTLQHSLP
>Manis_javanica
-------MISFSTVQHCLP
>Loxodonta_africana
-------MISFSTLQHFLP
>Marmota_marmota_marmota
-------MISFSTLQHSLP
>Tupaia_chinensis
MKGRNSAMISFSTLQHSLP
>Trichechus_manatus_latirostr
-------MISFSTLQHSLP
>Orycteropus_afer_afer
-------MISFSTLQHSLP

HG0459.2 ENSG00000163848 ZNF148

>Homo_sapiens
MGVCLTYLMEESDGISD----
>Acinonyx_jubatus
MGICLTYYIEDLDGISD----
>Orcinus_orca
MGICLTYFVEELDGIND----
>Myotis_brandtii
MGICLTYFIEESDGISGLRWY
>Dasypus_novemcinctus
MGICLTYFIEELECISD----
>Galeopterus_variegatus
MGICLTYFIEELNGISD----
>Erinaceus_europaeus
MGICLTYLTEELDGIND----
>Oryctolagus_cuniculus
MGICLVDLIEELDGVSD----
>Ceratotherium_simum_simum
MGICLTYFIEELDGVSD----
>Jaculus_jaculus
MGICLTYFIEEFDGISD----
>Tupaia_chinensis
MGICSTYFIEELDGISD----
>Trichechus_manatus_latirostr
MGICLLYFIEELDDISD----
>Orycteropus_afer_afer
MGICLTYFIEELDGISD----

HG0459.3 ENSG00000163848 ZNF148

>Homo_sapiens
MSASLLLWEEEEKEEE-EEEEEGEEEEKGAGVGAVAEAATAKVLPPPPFPSSQGP--
>Lipotes_vexillifer
MSASLLLWEEEEKEEE--EEEEGEEEEKGAGVGAVAEAATAKVLPPPPFPSS---
>Rousettus_aegyptiacus
MSASLLPWEEEEKEEEEEEEEEGEEEEKGAGVGAVAEAATAKVLPPPPFPSPQGP--
>Galeopterus_variegatus
MSASLLLWEEEEKEEE-EEEEEGEEEEKGAGVGAVAEAATAKVLLPPPFPSSQGP--
>Trichechus_manatus_latirostr
MSASLLLWEEEEKEE---EEEEGEEEEKGAGVGAVAEAATAKVLPPPPFPSSPGPSEGWSCRRLQSVTARLGARPRIKGGICSLEIQPSDLAGWAFACYTL
>Orycteropus_afer_afer
MSASLLLWEEEEKEE---EEEEGEEEEKGAGVGAVAEAATAKVLPPPPFPSSQGP--

HG0460 ENSG00000180008 SOCS4

>Homo_sapiens
MAMV----MAAVTRTTPVKLRSGRYIQKVPRRNFLLEKMKKQYL
>Ursus_maritimus
MAMV----MAAVTRTAPVKLRSERYIQRVPRRNFLLEKLKKQYL
>Balaenoptera_acutorostrata_s
MAVV----MAAVTRTAPVKLRSERYIQKVPRRNILPEKLKEQYL
>Eptesicus_fuscus
MAMV----MAAVTRTAPIKLRSERYIQKVPRRNFLPEKLKKQYL
>Dasypus_novemcinctus
-MAM----MAPVTRTASVKLCSGRYIQKVPRRNFLLEKLKKQYL
>Sarcophilus_harrisii
MFLLPGRASHIQRWQLISPLGYVRYFLKRPRRNFLLESLKNQHI
>Galeopterus_variegatus
MAMV----MAAVIWTAPAKLRSGRYIQKVSRRNFLQEKLKKPYL
>Monodelphis_domestica
--------MEAAPWTARVKLRCGRYFHKGLRRNFLLKSLKNQHL
>Macropus_eugenii
--------MEAAPWTARVRRRCGRYFHKGPRRNFLLESLKNQHM
>Condylura_cristata
--------MAAVTPTAPVKLRSGRYIQKVPRRTFLLEKLKKQYL
>Ornithorhynchus_anatinus
MA----ASGAPTPRAARVKRRSGRYFQKVPGRSSPPEKRKGGRF
>Ceratotherium_simum_simum
MAMV----MAAVPRAAPVKLRFGRYIQKVPRRNFLPEKLKKQYS
>Manis_javanica
MAMT----MAAVTRTAPIKLRSEKYIQNVPRRNFLPEKLKKQYL
>Loxodonta_africana
----------------------MRYMQKVPRRNFLPEKLKKQYL
>Nannospalax_galili
MEAV----MAAAPRTAPVKLHSGRFIQKVPRRNFLSEKLKKQYL
>Tupaia_chinensis
MAMV----MAAVTRTAPVKLRSGRYIQKVPRRNFLPEKLEKQYL
>Trichechus_manatus_latirostr
MAVV----MAAVTRTAIVELRSKRYIQKVPRRNFLPEKLKKQYL
>Orycteropus_afer_afer
MAMV----MAAVTQRAPVKLRSGRYIQKVPGRNYLPEKLKKQYL

HG0461 ENSG00000064419 TNPO3

>Homo_sapiens
MAPVRERPWPRQ
>Ursus_maritimus
MAPVRERPWPTQ
>Lipotes_vexillifer
MAPVRERPWPTK
>Miniopterus_natalensis
MAPVGERPWPTQ
>Dasypus_novemcinctus
MAPVRERPWPRQ
>Sarcophilus_harrisii
MAPARERPWPRQ
>Galeopterus_variegatus
MAPVRERPWPRQ
>Monodelphis_domestica
MAPARERPWPRQ
>Erinaceus_europaeus
MAPVRERPWPTQ
>Oryctolagus_cuniculus
MAPVRERPWPRQ
>Elephantulus_edwardii
MAPARERLWPRQ
>Equus_asinus
MAPVRERPWPTQ
>Manis_javanica
MAPVWERPWPTQ
>Loxodonta_africana
MAPVRERSWPRQ
>Dipodomys_ordii
MAPVRERPWPSQ
>Tupaia_chinensis
MAPVRERPWPRQ
>Trichechus_manatus_latirostr
MAPVRERPWPRQ

HG0462 ENSG00000125686 MED1

>Homo_sapiens
MAAASSTLLFLPPGTSD
>Acinonyx_jubatus
MAAAGSTLLFLPPGTSD
>Orcinus_orca
MAAAASTLLFLPPGTSD
>Pteropus_alecto
MAAAASTLLFLPPGTSD
>Dasypus_novemcinctus
MAAAASSLIFFPPGSSD
>Sarcophilus_harrisii
MAAAAGTLQLLPPAASD
>Monodelphis_domestica
MAAAAGTLQLLPPGASD
>Sorex_araneus
MAAAGSTLLFLPPGTTD
>Oryctolagus_cuniculus
MAAAAGTLLFLPPGTSD
>Elephantulus_edwardii
MAAAESTLLFLPPGTSD
>Ceratotherium_simum_simum
MAAAASTLLFLPPGTSD
>Manis_javanica
MAAAASTLLFLPPGTSD
>Loxodonta_africana
MAAAASTLHFLPPGTSD
>Marmota_marmota_marmota
MAAAASTLLFLPPGTSD
>Tupaia_chinensis
MAAAASTLLFLPPGTSD
>Trichechus_manatus_latirostr
MAAAASTLLFLPPGTSD
>Orycteropus_afer_afer
MAAVASTLPFLPPGTSE

HG0463 ENSG00000129473 BCL2L2

>Homo_sapiens
--------------MKGPSWGLLATSAVS
>Ailuropoda_melanoleuca
--------------MKGPSWGLLATSAGS
>Balaenoptera_acutorostrata_s
--------------MKGPSWGLLATSAGS
>Eptesicus_fuscus
--------------MKGPSWGLLATSAGS
>Dasypus_novemcinctus
--------------MKGPSWGLLATGAGS
>Sarcophilus_harrisii
--------------MKGLSWGLPATSAVS
>Galeopterus_variegatus
--------------MKGPSWGLLATSAGS
>Monodelphis_domestica
--------------MKGLSWGLPATSAVS
>Erinaceus_europaeus
-------------MMKGPSWGLLATSAGS
>Oryctolagus_cuniculus
--------------MKGPSWGLLATGAGS
>Elephantulus_edwardii
--------------MKGPSWGLLATSAGS
>Ceratotherium_simum_simum
--------------MKGPSWGLLATSAGS
>Manis_javanica
--------------MKGPSWGLFATSAGS
>Loxodonta_africana
MDSATQLGSTLAGRMKGPSWGLLATGAGS
>Dipodomys_ordii
--------------MKGPSWGLLATSAGS
>Trichechus_manatus_latirostr
--------------MKGPSWRLFATSAGS
>Orycteropus_afer_afer
--------------MKGPSWGLLATSAGS

HG0464 ENSG00000188215 DCUN1D3

>Homo_sapiens
---MLRYMGQRQRGHPIQRGPL
>Odobenus_rosmarus_divergens
---MVGGLDPPGRRRFQKGFD------------WRNLWSSCWLAPLADGMLRHMGQWQRGHLIQRGPL
>Physeter_catodon
---MRSPLRGHFWSMPPHLAVFLPLHCPPTMLHSAEGWLAPLADGMLRHVGQWQQGHPIQRGPL
>Pteropus_alecto
---MVGGLDPPGRRRFQKGFD------------WRNLWSSCRLAPLADGMLRFKGQWQRGHPVQRGPL
>Dasypus_novemcinctus
---MLRHMGQGQQGHPVQRGPL
>Sarcophilus_harrisii
---MLRHAGQHQRGCPIRRGPL
>Galeopterus_variegatus
---MLRYMGQWQRGHPIQRGPL
>Monodelphis_domestica
---MLRHAGHHQRGCPIRRGPL
>Macropus_eugenii
---MLRHAGQHQRGCPIRRRPL
>Condylura_cristata
---MVGDLDPPGRRRFQSGFD------------WRNLWSSCWLTPLADGMLRHMGQRQKGRPIQGGPL
>Oryctolagus_cuniculus
---MVGGLDPPGRRRFQKGFD------------WRNLWSSCWLVPLADGMLKYMGQRQRGHPFQRGPL
>Ceratotherium_simum_simum
MEGAGLRGGRRRWAADEEEAAAVVEEEAEAAGVGSLETRAGMVGGLDPPGRRRFQKGFD------------WRNLWSSCWLAPLADGMLRHMGQWQRGHPVQRGPL
>Manis_javanica
-----------------------------------MCVQGGRQKQLELRLKEESWRLIS-----VQRKLSRATVFDLSGWLAPLADGMLRHKGQWQRGHPIQRGPL
>Loxodonta_africana
---MVGGLDPPSRRRFQKGSD------------WRNLWSSCGLAPLADGMLRHTGQRQQGHLIPRGPL
>Chinchilla_lanigera
---MLRYMGQWQRGHPIQRGPL
>Tupaia_chinensis
---MSHEGGWLAPLADGMLTYMGQWQQGHLIQRGPL
>Trichechus_manatus_latirostr
---MVGGLDPPSRRRFQKGFD------------WRNLWSSCWLAPLADGMLRHMGQRQRGHLIPRGPL

HG0465 ENSG00000132153 DHX30

>Homo_sapiens

--MRNSKERIQLQFKSLQNLRLSLLL
>Mustela_putorius_furo
MLRRWEGAPCAALTSAVQTRSLRCPRGARAPTHSAPLPEVPLAPAAAAPHWARRRGLAAGWPPSGVSPRRPLRAPGGGPAGDPLRRGVGAGGGAGSAGAAGGTSAS
GPGRGRAAAGGSCSPAVVGGARGSCAVHRGLFHICNNRRRPSLVMRNSKERIQLQFKNLQNLRLSLLV
>Orcinus_orca

--MRNSKERIQLQFKSPQNLRLSLLL
>Rousettus_aegyptiacus

--MRNSKERIQLQFKSLQNLRLSLLL
>Dasypus_novemcinctus

--MRNSKERIQLQLKAYKI-------
>Sarcophilus_harrisii

--MKNRTERIYFPFKSRRNLRFSLLL
>Galeopterus_variegatus

--MRNSKDRIQLQFKSLQNLRLSLLS
>Condylura_cristata

--MRNSKETIQLQFKSLQNLRPSLLL
>Elephantulus_edwardii

--MRNSKERIQLQFKSLQNLRLSLLL
>Equus_caballus

--MRNSKERIQLQFRSLQDLRLSLLS
>Manis_javanica

--MRRSKERIQLQLKSLHDLGVSLLL
>Loxodonta_africana

--MRNSKERIQLQFRSLQNLKLSLLL
>Rattus_norvegicus

--MRNSKERIQLQFESLQNLSPSLLL
>Tupaia_chinensis

--MRNSKERTQLQFKSLHNLRLSLHL
>Trichechus_manatus_latirostr

---MRNNSKERIQLQFKSLQNLRSSLLL
>Orycteropus_afer_afer

--MRNSKERIQLQFKSLQNLRLSLLL

HG0466 ENSG00000139679 LPAR6

>Homo_sapiens
MFLLGISEKKEIFIKPSKDPDNLQINWRL
>Mustela_putorius_furo
MFLTEISKKKEIFIKPSKDTKNLHILWRL
>Lipotes_vexillifer
---MSLLEEKEIFIKSRKDPKDLQIHWRL
>Miniopterus_natalensis
MFLLEILEEKEIFIKPPKDPENLQIHWGF
>Dasypus_novemcinctus
MFLSEILEKKEILIKPPKDPKNLHISWRL
>Sarcophilus_harrisii
MFLLEISKGKEILMKPSKDPKHLQINKRS
>Galeopterus_variegatus
MFLLEISEKKEIFIKPPRDPNNLQINWRL
>Monodelphis_domestica
-------------MKPTKDHLHLQVNRRL
>Macropus_eugenii
MFLLEISKGKEILMKPSKDPKHLQIN---
>Erinaceus_europaeus
MFLLETSEKKEILIKPSKDPKNLQLHWRL
>Oryctolagus_cuniculus
MFHLEISEKTEIFLKPRLDPNNLQIN---
>Equus_przewalskii
MFLLEISEKEEIFIKPPKDPKNLQIRWRL
>Manis_javanica
MFLFEISEKKEIFIKSPKDPRNLHILWRL
>Loxodonta_africana
MFLLEIPEKKEIFIKPPKEPKNLQVNWRL
>Marmota_marmota_marmota
MFLSYILEKKEIFIKPPKDPNNLRINWML
>Orycteropus_afer_afer
MLLSEISEKKEILIKPPQELKNL------

HG0467.1 ENSG00000163320 CGGBP1

>Homo_sapiens
-----------------MTSWHHRENRVQAQKLFPHHHLLKN
>Mustela_putorius_furo
-----------------MTSRHRRENRVQAQKLFPHHHLLKN
>Tursiops_truncatus
-----------------MTSQHLRENRVQAQKLFPHHHLLKN
>Pteropus_vampyrus
-----------------MTSRHRRENRVQAQKLFPHHHLLKN
>Dasypus_novemcinctus
-----------------MTFRHRRENRVQAQKLFPHHHLLKN
>Sarcophilus_harrisii
MPNSTFPEYSGLGTGKGMTTRYASENRVQAQKPFLYHHLLRN
>Galeopterus_variegatus
-----------------MTSRHRRENRVQAQKPFPHHHFLKN
>Monodelphis_domestica
-----------------MTARYASENRVQAQKPFLYHHLLRN
>Condylura_cristata
-----------------MISRHRRENRVQAQKLFPHHHLLKN
>Oryctolagus_cuniculus
-----------------MTSWHLRENRVQAQKLFPHHHLLKN
>Equus_asinus
-----------------MTSWHRRENRVQAQKLFPHHHLLKN
>Manis_javanica
-----------------MTSWHRRENRVQAQKLFLHHHLLKN
>Loxodonta_africana
-----------------MTFRYRRENRVQAQKLFPHHHLLKN
>Nannospalax_galili
-----------------MTSRHRRENRVQAQKLFPHHHLLKN
>Tupaia_chinensis
-----------------MISRHRRENRVQAQKLFPHHHLLKN
>Trichechus_manatus_latirostr
-----------------MTFRYRRENRVQAQKLFPHHHLLKN

HG0467.2 ENSG00000163320 CGGBP1

>Homo_sapiens

MRLWFRSGDHSLLSIKTKKTGN

>Orcinus_orca

MRLWFCSGDPSLLGIKTKKTGN

>Miniopterus_natalensis

MRLWFCAGGPLLLSIKTKKTGN

>Oryctolagus_cuniculus

MRLWLCAGDHSLLSIKTKKTGN

>Ceratotherium_simum_simum

MRLWICAGDPSLLSIKKKKTGN

>Marmota_marmota_marmota

MCLRFCAGDPSLLSIKKKKTGN

>Tupaia_chinensis

-MLWFCAGDPSLLSIQTKKTGI

HG0468 ENSG00000187778 MCRS1

>Homo_sapiens
MLLPRVLETDLDRSPTLGVHSWENEGRRVRISSQPLSSPGQNPWMSLWDRSS------------
>Acinonyx_jubatus
--MSLWDPSS------------
>Orcinus_orca
--MNLWDSS-F-----------
>Myotis_brandtii
--MSLWDPSS------------
>Dasypus_novemcinctus
--MSPWDVSF------------
>Galeopterus_variegatus
--MSLWDPSS------------
>Macropus_eugenii
--MNLWDSS-YPFGLALRDW--
>Erinaceus_europaeus
--MSLWDPSS------------
>Oryctolagus_cuniculus
--MSLWDPSSWPRGLKPVALGL
>Equus_asinus
--MSLWDPSS------------
>Manis_javanica
--MSLWEPSS------------
>Loxodonta_africana
--MSLWDPSSWPCGLEPVALGL
>Ictidomys_tridecemlineatus
--MRLWDPSS------------
>Tupaia_chinensis
--MSLWDPSF------------
>Trichechus_manatus_latirostr
--MSLWDPSSWPCGLEPVALGL
>Orycteropus_afer_afer
--MSLCDPSF------------

HG0469 ENSG00000198369 SPRED2

>Homo_sapiens
MKGAKLPAAGQASR
>Ailuropoda_melanoleuca
MKGAKLPAAGQASR
>Physeter_catodon
MKGAKLPAAGQASR
>Eptesicus_fuscus
MKGAKLPAAGQASR
>Dasypus_novemcinctus
MKGAKLPAAGQASR
>Sarcophilus_harrisii
MKGTELPAAGHSSR
>Monodelphis_domestica
MKGTELPAAGHSSR
>Condylura_cristata
MKGAKLPAAGQASR
>Oryctolagus_cuniculus
MKGAKLPAAGQASR
>Ornithorhynchus_anatinus
MKGTKLPAAGHSSR
>Ceratotherium_simum_simum
MKGAKLPAAGQASR
>Manis_javanica
MKGAKLPAAGQASR
>Loxodonta_africana
MKGAKLPAAGQASR
>Cavia_porcellus
MKGAKLPAAGQASR
>Tupaia_chinensis
MKGAKLLAAGQASR
>Trichechus_manatus_latirostr
MKGAKLPAAGQASR

HG0470 ENSG00000224470 ATXN1L

>Homo_sapiens
----MWAPQPEAERGTGKLQRSPF
>Panthera_tigris
----MWAPQPAAERGAGEPQRSPF
>Balaenoptera_acutorostrata_s
----MWAPQPAAERGAGEPQRSPF
>Rousettus_aegyptiacus
----MWAPQPSAERGAGEPQRSPF
>Dasypus_novemcinctus
----MWPPQPAAERGAGKPQRSCF
>Sarcophilus_harrisii
MEAQMWVPQSAVEGGTRKSQRSLF
>Galeopterus_variegatus
----MWAPQPAAERGAGKPQRSPF
>Condylura_cristata
----MWAPQPAAERGAGEPQRSPF
>Ochotona_princeps
----MWAPQPAAERGAGKPQRSPF
>Elephantulus_edwardii
----MWAPRPTAARGAGKPQRSPF
>Equus_przewalskii
----MWAPQPAAERGAGEPQRSPF
>Manis_javanica
----MWAPQPAAERGAGEPQRSPF
>Loxodonta_africana
----MWAPQPAAERGAGKPQRSPF
>Heterocephalus_glaber
----MWAPQPTAERGAGKPQRSPF
>Trichechus_manatus_latirostr
----MWAPQPATERGAGKPQRSPF
>Orycteropus_afer_afer
----MWAPQPAAERVAGKPQRSPF

HG0471 ENSG00000023041 ZDHHC6

>Homo_sapiens
---MTIKEHTWTCGTWKCAHRKRKSIILLK
>Ursus_americanus
---MTIKEHTWTCGTWKCVHRKRKSIIF--
>Balaenoptera_acutorostrata_s
---MTIKEHTWTCGTWKCVHRKRKSVVL--
>Pteropus_alecto
---MSIKEHTWTCGTWKCVHRKRKAVIL--
>Dasypus_novemcinctus
---MTVKGDTWTHGTWKCVHRQRKSIIL--
>Galeopterus_variegatus
---MTIKEHTWTCGTWKCMHRKRKSIVL--
>Macropus_eugenii
---MSINEDTWTFDTWKCVYKKGTLQGF--
>Condylura_cristata
MLVFGEQPQPGDLDHVWYSRLHWRLSLEPCKIEFHTKRLDSPSYHKELCVRNAENGIICINIRQLEGMTAKEHTWTWGTWKYVHRKRKSIIL--
>Oryctolagus_cuniculus
---MTVKEHTWTCGTWKCVHRKRKSVIL--
>Elephantulus_edwardii
--MLNSEDDILFV--NILESMTVEEDMWTCGMWKCMHRKRKSVIL--
>Ceratotherium_simum_simum
---MTVKEHTWTCGTWKCVHRKRKSIIL--
>Loxodonta_africana
--MTIKEEDTWTCATWKCVHRKRTSINL--
>Chinchilla_lanigera
---MTIKEHTWTCGTWKCVHRKRKSIIL--
>Trichechus_manatus_latirostr
---MTVKEDTWTCGTWKCVHRKRKSISL--
>Orycteropus_afer_afer
---MTVQEDKWTWGTWKCVPRKRKPVIL--

HG0472 ENSG00000105991 HOXA1

>Homo_sapiens
--------MEEVRKLAQSRRASQDQVTQ
>Leptonychotes_weddellii
--------MEEVRKLARSPRASQDPVPQ
>Tursiops_truncatus
--------MEEVRKLARSPRASQDPVTQ
>Rousettus_aegyptiacus
--------MEEVRKLARSPRASQDQVTQ
>Monodelphis_domestica
--------MEEVRKLARSPRARQDPVTV
>Condylura_cristata
--------MEEVRKLARSSRASQDQVTQ
>Oryctolagus_cuniculus
--------MEEVRKLARSPRASQDPVTQ
>Elephantulus_edwardii
--------MEEVRKLARSSRASQQPATQ
>Equus_przewalskii
--------MEEVRKLARSPRASQDPVTQ
>Manis_javanica
MSFFFSGPMEEVRKLARSPRASQNPVTQ
>Loxodonta_africana
--------MEEVRKLARSPRASQDPVTQ
>Peromyscus_maniculatus_baird
--------MEEVRKLARSPRASQDPVTQ
>Tupaia_chinensis
--------MEEVRKLAQSPRASQDPVTQ
>Trichechus_manatus_latirostr
--------MEEVRKLARSRRASQDPVTQ
>Orycteropus_afer_afer
--------MEEVRKLARSPRASQDPVTQ

HG0473.1 ENSG00000138650 PCDH10

>Homo_sapiens
MRKGDACHPSCAKI---------------
>Odobenus_rosmarus_divergens
MRKGDACHPSCAKIEK-------------
>Lipotes_vexillifer
MRKGDACHPSCAKTEKL--G---------
>Pteropus_alecto
MRKGDACHPSCAKIEK-------------
>Dasypus_novemcinctus
MRKRDVCHPSCTKTEK-------------
>Galeopterus_variegatus
MRKWDACHPSCAKIEK-------------
>Monodelphis_domestica
MRSGDVCLPSCAKREKGEAGWWGVEP---
>Condylura_cristata
MRRGDACRPACAKVEERG-----------
>Ochotona_princeps
MRIGDAGHPSCAEIEK-------------
>Equus_asinus
MRKGDACHPSCAKIEK-------------
>Manis_javanica
MRKGDACHPSCAKIEK-------------
>Loxodonta_africana
MRKGDACHPSCAKIEK-------------
>Marmota_marmota_marmota
MRKGDVCHLSCAKIEKL--DWIEDSFKMK
>Tupaia_chinensis
MRKGDACHPSCAKIEK-------------
>Trichechus_manatus_latirostr
MRKGDACHPSCAKIEK-------------

HG0473.2 ENSG00000240184 PCDHGC3

>Homo_sapiens
MSDSAPSAQALTR
>Leptonychotes_weddellii
MSDSAPSAQALTR
>Orcinus_orca
MSDSEPSARALTR
>Myotis_davidii
MSDSAPSAQALTR
>Dasypus_novemcinctus
MSDSAPSARALTR
>Erinaceus_europaeus
MSDSAPSAQALNR
>Oryctolagus_cuniculus
MSDSAPSAQALSR
>Equus_przewalskii
MSDSAPSAQALTR
>Manis_javanica
MSDSAPSAPALTR
>Loxodonta_africana
MSDSAPSAQALSH
>Mus_musculus
MSDSAPSAQALTR
>Trichechus_manatus_latirostr
MSDSAPSAQALTR

HG0474 ENSG00000139083 ETV6

>Homo_sapiens
MTASGWP-WSLSGLGRGKESGKNLRTS-----
>Ailuropoda_melanoleuca
MTASGWP-WSASGLGRGKESGKKLRTS-----
>Balaenoptera_acutorostrata_s
MTASGRP-WSVSGLGRGKESGKKLRTS-----
>Pteropus_alecto
MTASGWP-WSVSGLGRGKESGKKLRTS-----
>Sarcophilus_harrisii
MIASGCLFGGFLGWGRGKESGKKLRTS-----
>Monodelphis_domestica
MIASGWLFGGFLGWGRGKESGKKLRTS-----
>Condylura_cristata
MTASGRP-WSASGLGRGKESGKKLRTS-----
>Oryctolagus_cuniculus
MTASGWP-RSRSGLGRGKESGKTLRTCCSPPL
>Elephantulus_edwardii
MTASGWP-WSISGLGRGKESGKNQRTS-----
>Equus_przewalskii
MTASGWP-WSVSGLGRGKESGKKLRTS-----
>Loxodonta_africana
MTASGWP-WSISGLGRGKESGKKLRTS-----
>Octodon_degus
MTASGCP-WSVSGLGRGKESGKKLRTS-----
>Tupaia_chinensis
MTASGWP-WSFSGLGRGKESGKKLRTS-----
>Trichechus_manatus_latirostr
MTASGWP-WSLSGLGRGKESEKN---------
>Orycteropus_afer_afer
MTASGWP-WSISGLGRGKESGKKLRTS-----

HG0475 ENSG00000109118 PHF12

>Homo_sapiens
MAAPASLPGRTGCRQCRL
>Canis_lupus_familiaris
MAAPASLPGRTSGRQRRL
>Orcinus_orca
MAAPASFPGRTSGRQHRL
>Rousettus_aegyptiacus
MAAPASLPSRTSSRQRRL
>Dasypus_novemcinctus
MAAPASLPGRTSGHQRRL
>Sarcophilus_harrisii
MAAPASLPGRAGGRQRRL
>Galeopterus_variegatus
MAAPANLPGPTSGRQRRL
>Erinaceus_europaeus
MAAPANLPGRTSGRQRRL
>Oryctolagus_cuniculus
MAAPASLPGRTSGRQRRL
>Elephantulus_edwardii
MAAPDSLPGRTSGRQRRL
>Ceratotherium_simum_simum
MAAPASLPGRTSGRQRRL
>Heterocephalus_glaber
MAAPASLPGRTSGRQHRL
>Trichechus_manatus_latirostr
MAAPASLPGRTSSRQRRL
>Orycteropus_afer_afer
MAAPASLPGRTSGLQRRL

HG0476 ENSG00000130939 UBE4B

>Homo_sapiens
MNNTWWGEGER-VGVEG-------------------------------
>Mustela_putorius_furo
MNNTWWGEGVR-VGVEG-------------------------------
>Orcinus_orca
MNNTWWGEGVR-VGVEG-------------------------------
>Pteropus_alecto
MNNTWWGEGVR-VGVEG-------------------------------
>Dasypus_novemcinctus
MNNTWWGEGVR-VGVEG-------------------------------
>Galeopterus_variegatus
MNNTWWGEGVR-VGVEGRRIYSSSESIAVLPLPPARAPAWRRGVAGLF
>Monodelphis_domestica
MNNPWWGQGGQRRAEG--------------------------------
>Condylura_cristata
MNNTWWGEGVR-VGVEG-------------------------------
>Ochotona_princeps
MNNTWWGEGVR-VGVEG-------------------------------
>Equus_przewalskii
MNNTWWGEGVR-LGVEG-------------------------------
>Ictidomys_tridecemlineatus
MNNTWWGEGVR-VGVEG-------------------------------
>Tupaia_chinensis
MNNTCWGEGVRRVGVEG-------------------------------
>Trichechus_manatus_latirostr
MNNTWWGEGVR-VEVEG-------------------------------
>Orycteropus_afer_afer
MNNTWWGEGVK-VRVER-------------------------------

HG0477 ENSG00000166925 TSC22D4

>Homo_sapiens
MSDPDVPRGSDVPAMWPPCSRGA
>Phoca_largha
MSDPDVPRGPDAPAMWPPCSRGA
>Balaenoptera_acutorostrata_s
MSDPDVPRGPDVPTMWPPCSRGA
>Rhinolophus_ferrumequinum
MSDSDVPRGPDVPAMWPPCSRGA
>Dasypus_novemcinctus
MSDPDVPRGPDAPAMWPPCSRGA
>Sarcophilus_harrisii
MSDPDVARGPDPTAIWPPSARGA
>Galeopterus_variegatus
MSDPDVARGPDAPTMWPPCSRGA
>Monodelphis_domestica
MLDPDVPRGPDPTAIWPPSARGA
>Condylura_cristata
MSDPDVPRGPDAPAMWPPCSRGA
>Oryctolagus_cuniculus
MSDPDVPRGPDAPAMWPPCSRGA
>Equus_caballus
MSDPDVPRGPDVPAMWPPCSRGA
>Manis_javanica
MSDPDIPRGPDAPTMWPPCSRGA
>Loxodonta_africana
MSDPDVPRGSDVPAMWPPCSRGA
>Cricetulus_griseus
MSDPDVPRGPDVPAMWPPCSRGA

HG0478 ENSG00000168214 RBPJ

>Homo_sapiens
MISKRKENDTDTLAEVF
>Acinonyx_jubatus
MISKGKENDTDTLAEVF
>Lipotes_vexillifer
MISKGKENDTDTLAEVF
>Myotis_brandtii
MISKGKENDTDTLAEVF
>Dasypus_novemcinctus
MISKGKENDTDTLAEVF
>Sarcophilus_harrisii
MISRGKEND--TLAEVF
>Galeopterus_variegatus
MISKGKENETDTLAEVF
>Monodelphis_domestica
MISKGKEND--TLAEVF
>Oryctolagus_cuniculus
MISKGKENDTDTLAEVF
>Ornithorhynchus_anatinus
MISKGKEND--TLAEVF
>Ceratotherium_simum_simum
MISKGKENDTDTLAEVF
>Manis_javanica
MISKGKENDTDTLAEVF
>Dipodomys_ordii
MISKEKENDTDILAEVF
>Orycteropus_afer_afer
MISKGKENDTDTLAEVF

>Homo_sapiens

--MAQPTASAQKLVRPIRAVCRILQIPESDPSNLRP
>Leptonychotes_weddellii

--MAQPTASAQKLVRPIRAVCRILQIPESDPSNLRP
>Lipotes_vexillifer

--MAQPTASAQKLVRPIRAVCRILQIPEPDPSNLRP
>Myotis_lucifugus
MRSRWHRRGVGGSPRGTGGGRPRRGRRITGSCFPQGAGGGNRRSSFLTLSCASGDGDQRSCCAPRCALPPLAALPRFAPSLVLSSGFQDRRPLPGTTPSPGPSAFP
PLGRLRPGARVPPARLPLSLRGHRSLAPARPSPRRSTESPRPMAQPTASAQKLVRPIRAVCRILQIPESDPSNLRP
>Sarcophilus_harrisii

--MAQPTTSAQKLVRPIRAVCRILQIPESDPSNLRP
>Monodelphis_domestica

--MAQPTTSAQKLVRPIRAVCRILQIPESDPSNLRP
>Condylura_cristata

--MAQPTASAQKLVRPIRAVCRILQIPESDPSNLRP
>Oryctolagus_cuniculus

--MAQPTASAQKLVRPIRAVCRILQIPESDPSNLRP
>Ceratotherium_simum_simum

--MAQPTASAQKLVRPIRAVCRILQIPESDPSNLRS
>Manis_javanica

--MAQPTASAQKLVRPIRAVCRILQIPESDPSNLRP
>Ictidomys_tridecemlineatus

--MAQPTASAQKLVRPIRAVCRILQIPESDPSNLRP
>Tupaia_chinensis

--MAQPTASAQKLVRPIRAVCRILQIPESDPSNLRP
>Trichechus_manatus_latirostr

--MAQPTASAQKLVRPIRAVCRILQIPESDPSNLRS
>Orycteropus_afer_afer

--MAQPTASAQKLVRPIRAVCRILQIPESDPSNLRP

HG0479.2 ENSG00000168453 HR

>Homo_sapiens
MAIRGPAALSAALYLH----
>Odobenus_rosmarus_divergens
MAMRGPAALSAALSLH----
>Lipotes_vexillifer
MAVRGPAALSAGHSLH----
>Myotis_lucifugus
MAIRGPAALPAALSLH----
>Monodelphis_domestica
MAIGGPAALSVPLGLH----
>Condylura_cristata
MAIRGPAALSAALSLH----
>Oryctolagus_cuniculus
MAIGGPEALSAALSLH----
>Ceratotherium_simum_simum
MAIRGPAALSAALSLH----
>Manis_javanica
MAIGGPAALSAALSLH----
>Ictidomys_tridecemlineatus
MAIRGPAALSAALSLH----
>Trichechus_manatus_latirostr
MAIRGPAALSAALFAELSLH
>Orycteropus_afer_afer
MAIRSPAALSAALSLH----

HG0480 ENSG00000198018 ENTPD7

>Homo_sapiens
MPGTRRPPSQGKRKRR
>Canis_lupus_familiaris
MPETRRPVSQGKKKRR
>Balaenoptera_acutorostrata_s
MTGTRRPPSQGKRKRR
>Myotis_davidii
MPGTRRPPSQGKRKRR
>Galeopterus_variegatus
MPGTRRPPSQGKRKRR
>Monodelphis_domestica
MPGTGKPPCQGKRKRR
>Condylura_cristata
MPGTRRPSAQGKRKRR
>Oryctolagus_cuniculus
MPGTRRPPSQGKRKRR
>Equus_asinus
MPGTRRPPSQGKRKRR
>Manis_javanica
MSGTRRPPSQGKRKMR
>Loxodonta_africana
MPGTRRPHSQGKRKRR
>Dipodomys_ordii
MPGTRRPPSQGRRKRR
>Tupaia_chinensis
MPGTRRPPSQGKRKRR
>Trichechus_manatus_latirostr
MPGTRRPHSQGKRKRR

HG0481 ENSG00000198963 RORB

>Homo_sapiens
MVFSAEQLFADHLLHSC
>Ursus_maritimus
MVFSAEQLFADHLLHSC
>Balaenoptera_acutorostrata_s
MVFSAEQLFADYLPHSC
>Rousettus_aegyptiacus
MVFSAEQLFADHLLHSC
>Monodelphis_domestica
MVFSAEQIFGCSL-HSC
>Condylura_cristata
MVFSAKQLFAFHLLHSC
>Elephantulus_edwardii
MVFSAEQLFTDHLLPSC
>Ceratotherium_simum_simum
MIFSAEQLFADHLLHSC
>Manis_javanica
MVLSVEQLFADHLLQCC
>Loxodonta_africana
MVFSAEQLFTDHLLHLC
>Peromyscus_maniculatus_baird
MLLSAEQLFADRLLPSC
>Tupaia_chinensis
MVFSAEQLFTDYLLHSC
>Trichechus_manatus_latirostr
MVSSAEQLFTNHLLHSC
>Orycteropus_afer_afer
MVFSAEQPFVDHLVHSC

HG0482 ENSG00000067167 TRAM1

>Homo_sapiens

-----------------------------MGSEAPASGCSGAVRSSQREAAASR---------------

>Odobenus_rosmarus_divergens

MLTPAVGSPSFFFFFFFFGRPRRLRTGCGMGSEAPASGSGGAVRSSQGEAAAAASR-------------

>Physeter_catodon

-----------------------------MGSEAPASGSGGAVRSSQGEAAAASR--------------

>Pteropus_alecto

-----------------------------MGSKAPASGSGGAVRSSQGEAAAASR--------------

>Dasypus_novemcinctus

-----------------------------MGSGAPASGCGWAVSSSQGEAGAAASQ-------------

>Galeopterus_variegatus

-----------------------------MGSEAPASGCGGAVRSSQGAAAAASR--------------

>Monodelphis_domestica

-----------------------------MGSEVPESGCGWAENSSQGEETAGAATAEATAAAAAASCP

>Condylura_cristata

-----------------------------MGSEAPANGSSGAVRSSQGEAAAAFSR-------------

>Oryctolagus_cuniculus

-----------------------------MGSEAPASGCGGAVRSGQGEAAAAAVSR------------

>Equus_asinus

-----------------------------MGSEAPASGGGGAVRSGRGEEAEAAARR------------

>Manis_javanica

-----------------------------MGSEAPASCSDGAVRSSQGEAAAVACR-------------

>Rattus_norvegicus

-----------------------------MGSEAPASGCGGAVRSTQREAAVAALR-------------

>Trichechus_manatus_latirostr

-----------------------------MGSEALASGCGGAVRSSQGEVAAAACR-------------

HG0483 ENSG00000164463 CREBRF

>Homo_sapiens
MERGRGPCSGTGPSP
>Ailuropoda_melanoleuca
MERGRGPCSGTGPSP
>Orcinus_orca
MERGRGPCSGTGPSP
>Rousettus_aegyptiacus
MERGRGPCSGTGPSP
>Dasypus_novemcinctus
MERGRGPCSGTGPSS
>Sarcophilus_harrisii
MERGRGPRTRTGPSP
>Galeopterus_variegatus
MERGRGPCSGTGPSP
>Macropus_eugenii
MERGRGPRSRTGPSP
>Oryctolagus_cuniculus
MERGRGPCSGTGPSP
>Equus_przewalskii
MERGRGPCSGTGPRP
>Manis_javanica
MERGRGPCSGTGPRP
>Ictidomys_tridecemlineatus
MERGRGPCSGTGPIP
>Trichechus_manatus_latirostr
MERGRGPCSGTAPSP

HG0484 ENSG00000166444 ST5

>Homo_sapiens
MNQLASISLTNHRKMRDCLGATTSTMSPCTG-
>Canis_lupus_familiaris
--------------MRDCLGTSNSTMSPCTG-
>Eptesicus_fuscus
--------------MRDCLGTSTSTMRPCTG-
>Galeopterus_variegatus
--------------MRDCLGTSTSTMGPCTG-
>Monodelphis_domestica
--------------MRDCPGSASSTMSTACTD
>Elephantulus_edwardii
--------------MRDYLSANTSTMSPCTG-
>Ceratotherium_simum_simum
--------------MRDCLSTSPSTMSPCTG-
>Manis_javanica
--------------MRDCLGTSTSTRNPCTG-
>Loxodonta_africana
--------------MRDCLGASTSTMSPCTG-
>Chinchilla_lanigera
--------------MRDCLGSSSSTMSPCTG-
>Tupaia_chinensis
--------------MQDCFGASSSAMSPCTG-
>Trichechus_manatus_latirostr
--------------MRDCLGASTSTMSPCTG-
>Orycteropus_afer_afer
--------------MRDCLGASTSIMSPCTG-

HG0485.1 ENSG00000182667 NTM

>Homo_sapiens
MSGDNYGEVILSHTLGFLVVSFSKTVDLNLLAQA
>Mustela_putorius_furo
MSRDNY----------------------------
>Orcinus_orca
MRRDNY----------------------------
>Pteropus_alecto
MSRDNY----------------------------
>Monodelphis_domestica
MSRDNY----------------------------
>Macropus_eugenii
MSRDNY----------------------------
>Erinaceus_europaeus
MSRDNY----------------------------
>Ochotona_princeps
MSGDNYGEVILSHTLGFLVVSFSETVDLNLRA--
>Equus_caballus
MSRDNY----------------------------
>Manis_javanica
MSRDNY----------------------------
>Microtus_ochrogaster
MSGDNYGEVRLSHALGFLVVSFSKTVDLNLLGRA
>Tupaia_chinensis
MSGDNYGEVALSHTLGFL-VSFSETVDLNLLA--
>Trichechus_manatus_latirostr
MSGDNY----------------------------

HG0485.2 ENSG00000183715 OPCML

>Homo_sapiens
MQEGASGSARLES
>Mustela_putorius_furo
MQEGASGSARLES
>Orcinus_orca
MQEGASGSARLES
>Ochotona_princeps
MQEGASGSARLES
>Equus_asinus
MQEGASGSARLES
>Mesocricetus_auratus
MQEGASGSARLES

HG0486 ENSG00000067900 ROCK1

>Homo_sapiens
MRLWASERLWVAVAPGPGSGE-RRSPQ
>Canis_lupus_familiaris
MRLWASERLWVAVAPGPGSGE-RRSPQ
>Lipotes_vexillifer
MRLWASERLWVAVAPGPGSGE-RRSPQ
>Myotis_brandtii
MRLWASERLWVALAPGPGSGE-RRSPQ
>Dasypus_novemcinctus
MRLWASERLWVAVAPSPGSGE-WRSPQ
>Sarcophilus_harrisii
MRLWDSVRLRVAVAPRARPGERRAAQ-
>Sorex_araneus
MRLWASERLWVAVAPGPGSPAERRSPQ
>Ochotona_princeps
MRLWACERLWVAVVPGPGSGEPRC-PQ
>Equus_asinus
MRLWASERLWVAVAPGPGSGE-RRSPQ
>Dipodomys_ordii
MRLWASGRLWVAVAPGPGSGERRWSPQ
>Tupaia_chinensis
MRLWASERLWVAVAPGPGSGE-RRSPQ
>Orycteropus_afer_afer
MRLWASERLWVAVAPGPGSGE-RRSPQ

HG0487 ENSG00000091831 ESR1

>Homo_sapiens
----------------------------------MEHFWKDVLDPAGWPAGF
>Mustela_putorius_furo
----------------------------------MEHFWKDALDPAGWPLGC
>Lipotes_vexillifer
----------------------------------MEHFWKDALDPAGWPVGC
>Rousettus_aegyptiacus
----------------------------------MEPFWKDALDPAGWPVGC
>Monodelphis_domestica
MNPCNFAHAYRATCTYTHILSFILTLSAFEFCISMEHFRKDALCPVGWPTGC
>Condylura_cristata
----------------------------------MEHFWKDALDPAGWPVCC
>Oryctolagus_cuniculus
----------------------------------MEHFWKDTLDPAGWPVGC
>Ceratotherium_simum_simum
----------------------------------MEHFWKDALDPAGWPAGC
>Manis_javanica
----------------------------------MDHFWKDALDPAGWRVGC
>Ictidomys_tridecemlineatus
----------------------------------MEHFWKDTLAPAGWPTGC
>Tupaia_chinensis
----------------------------------MEHFWKDDLDPAGWPVGC
>Trichechus_manatus_latirostr
----------------------------------MEHLWKDAFDPAGWPVGC

HG0488.1 ENSG00000116194 ANGPTL1

>Homo_sapiens
----------------------------------MWQTEQSHNAFSAGLKTAHRPELPLYYLKLHRWLVKFN----
>Canis_lupus_familiaris
----------------------------------MWQTEQSHNAFSAGLKTAHRPELPLYYLKLHRWLVKFNRLVL
>Balaenoptera_acutorostrata_s
----------------------------------MWQTEQSHNAFSAGLKTARRPGTSTILLKIT-----------
>Myotis_lucifugus
----------------------------------MWQTEQSHNAFSAGLKTAHRPELPLHYLRLHRWLVKFD----
>Dasypus_novemcinctus
----------------------------------MWQTEQSHNAFSAGLKTALRPELPLYYLKLHRWLVKFN----
>Sarcophilus_harrisii
----------------------------------MKQTEERHNTFSAGKKDISHMEICAVLSKII-----------
>Condylura_cristata
----------------------------------MWLTEQSHNAFSAGLKTTHRVDRRLCYLKLHRWLVKFN----
>Oryctolagus_cuniculus
----------------------------------MWQTEQSHNAFSAGLRTAHRPELLLYDLKLHRWLVKLN----
>Equus_przewalskii
MWAELESTEKERESLFKGTVYTIWVSCSWLLHFSMWQTEQNHNAFSAGLKTAHRPELPLYYLKLHRWLVKFN----
>Manis_javanica
----------------------------------MWQTEQSHSAFSAGLQIAHRPELPLYYLKLHRWLVKFN----
>Callospermophilus_lateralis
----------------------------------MWQTEQSHKAFSEGLDTAHRPELPLHYLKLHRWLVKFN----
>Tupaia_chinensis
MWAALESTEKERESLFKGTVYTGRVSCSSLLHFSMWQTAQGHSAFSAGLKTAHRPELHHTT---------------

HG0488.2 ENSG00000116194 ANGPTL1

>Homo_sapiens
MQLPLKSQMNKLLH
>Felis_catus
MQLPLKSQMNKLLH
>Balaenoptera_acutorostrata_s
MQLPLKSQTNKLLH
>Rousettus_aegyptiacus
MQLPLKSQMNKLLH
>Galeopterus_variegatus
--------MNKLLH
>Oryctolagus_cuniculus
MQLPLKSQMNKLLN
>Equus_caballus
MQLPLKSQTSKLLH
>Loxodonta_africana
MQLPLESQMNKLHF
>Marmota_marmota_marmota
MQLPLKSQMNKLLH
>Tupaia_chinensis
MQLPLKSQMKKLLH

>Homo_sapiens
--MLWEKPLPTCPLAWHCPL
>Odobenus_rosmarus_divergens
--MPWEKPLPTCPLAWRCPL
>Balaenoptera_acutorostrata_s
--MPWEKPLPICSLTWHCLL
>Myotis_brandtii
--MGGAPPTCLLAWHCPL
>Dasypus_novemcinctus
--MGEPLPSCPPTWHCPL
>Galeopterus_variegatus
--MPWEKPLPTCPLAWHCLF
>Monodelphis_domestica
--MASAWESPLPAYPLAWHCPL
>Condylura_cristata
--MPWEKPLPIRPLAWHCPL
>Equus_asinus
--MPWEKPLPTCPLAWHCLL
>Loxodonta_africana
--MPWEKLLSTHPPTWHCPL
>Heterocephalus_glaber
MLSAGLGTQGTQPQPREVKPGFLAPFHRGTAGPRLLSEQEQLMAWEKPLPTCPLSWHCPL
>Tupaia_chinensis
--MPWEKPLLICPLAWHCPL

HG0490.1 ENSG00000152413 HOMER1

>Homo_sapiens

MAPFILFGRMNRSCLAFSSSSPPLLCSES

>Odobenus_rosmarus_divergens

MAPFILLGRMNRSCPAFSSSSPPPLCSES

>Myotis_lucifugus

MAPFI-LGRMNRSCPAFSASSPPLLGSES

>Dasypus_novemcinctus

MAPFILLGRMNRSCPAFSSSSPPPLCS--

>Galeopterus_variegatus

MAPFILLERINRSCPAFSSSSTPPLCFES

>Monodelphis_domestica

MASFILFWRMNRSSPAFSSCSPPPLSSDS

>Sorex_araneus

MAPFILLGRMNRSCPVFSSSSPPPLCSES

>Manis_javanica

MVPFILLGRMNRSCPAFSSSSPPPLCSES

>Loxodonta_africana

MAPFILLGRVNRSCPVFSSFSLPPLCSES

>Dipodomys_ordii

MAPFLLLGRMNRSCLAFSSTSPPLR-SES

>Tupaia_chinensis

MAPFI-LGRMNRSCPAFSSSSSPPLSSES

>Orycteropus_afer_afer

MAPFILLGSMSRSCPVFSSSSLPPLCSES

HG0490.2 ENSG00000152413 HOMER1

>Homo_sapiens

MPPLFERFGIYKREIILPKRTN

>Acinonyx_jubatus

MPPLLERFGIYEREILLPKGTN

>Balaenoptera_acutorostrata_s

MPPLFEHFGISQREIILPKGTN

>Pteropus_vampyrus

MPPLFEHFGIYGREIILPKGTN

>Galeopterus_variegatus

MPPLFERFGVYQREIILPKRTN

>Condylura_cristata

MPPLFERFGVYEREIILPKGTN

>Ceratotherium_simum_simum

MPPLFERFGIYEGEIILPKGTN

>Manis_javanica

MSPLFERFDIYKREIILPKGTN

>Dipodomys_ordii

MPPLFERFGVYERELILPKRNN

>Tupaia_chinensis

MPPLFERFGIYEREIILPKRTN

HG0491 ENSG00000154114 TBCEL

>Homo_sapiens
-------MQDTGLRRPEWGTR-----------
>Mustela_putorius_furo
-------MQDTGLRRPEWGTRMPDIIRYRQLA
>Orcinus_orca
-------MQDTGLRRPEWGTR-----------
>Rousettus_aegyptiacus
MKAASGSMQDTGLLRAELGTR-----------
>Monodelphis_domestica
-------MQDTGLRRPELGTR-----------
>Condylura_cristata
-------MEDTGLRRPEWGTR-----------
>Ochotona_princeps
-------MQDTGLRRLDWGTR-----------
>Elephantulus_edwardii
-------MQDTWLRRSEWGPR-----------
>Ceratotherium_simum_simum
-------MQDTGLRRPEWGPR-----------
>Loxodonta_africana
-------MQDTGLGRPEWGTR-----------
>Ictidomys_tridecemlineatus
-------MQDTGLRRPEWGTR-----------
>Trichechus_manatus_latirostr
-------MQGTGLRPPELGTR-----------

HG0492 ENSG00000162670 BRINP3

>Homo_sapiens
-------------------------MEYLFESWMYFSHSLLAS------
>Mustela_putorius_furo
-------------------------MEYLFESWIYFSHSHLAS------
>Orcinus_orca
-------------------------MEYLLASWMYFSHSHLAS------
>Pteropus_alecto
-------------------------MEYLFECWMYFSHCHLAS------
>Galeopterus_variegatus
-------------------------MEYLFESWMYFPHGHLAS------
>Monodelphis_domestica
-------------------------MECLFDFWIHYSHSHLAS------
>Erinaceus_europaeus
-------------------------MDYLFESWMYFSHSHRAS------
>Equus_asinus
MPPGTADYFKVPPPCSYHCTLIWKIMEYLCESWMYISHSHLASWNFLWC
>Loxodonta_africana
------------------------MMEYLFESWTYFSHSHLAS------
>Heterocephalus_glaber
-------------------------MEYLFESWMYFSHGHLAS------
>Trichechus_manatus_latirostr
-------------------------MEYLFESWTYFSHSHLAS------
>Orycteropus_afer_afer
---------------------------------MYFSHSHLAY------

HG0493 ENSG00000164707 SLC13A4

>Homo_sapiens
MPPFIILSQFLFAGHKTMLINPT
>Canis_lupus_familiaris
MPPSITLPRCLSAGPRTMLSNPT
>Balaenoptera_acutorostrata_s
MPPFIILSRFLFAGHKTMLTNPT
>Rhinolophus_ferrumequinum
MSRFTILCRFLSAGHKTMLTNPT
>Dasypus_novemcinctus
MPPFTILSQYLFAGHKTMLINPT
>Galeopterus_variegatus
MPPFIILSQFLSAGHKTMLINPT
>Monodelphis_domestica
MPQFVILGQFPFPAQKTLLINPT
>Oryctolagus_cuniculus
MPPHRTLSQFLFAGHKTMLINPT
>Ceratotherium_simum_simum
MPPFIIFSHFLFAGHKTMLANPT
>Manis_javanica
-----------------MLTDPS
>Peromyscus_maniculatus_baird
-----------------MLLNPT
>Tupaia_chinensis
MPPFIILSQFLFAGHKTMLINPT

HG0494 ENSG00000184226 PCDH9

>Homo_sapiens
---MHAAVENIPELKKCNYVLCVLNAQKN---P---DPVGKRDEMWRE
>Mustela_putorius_furo
---MLDAVENTPELKKCNGMLC--VCVKYQKSP---DPVGNRDEMWRE
>Orcinus_orca
---MFDAVENTPELKKCNWMLCVR------------------------
>Rousettus_aegyptiacus
---MLDPVENIPELKKCNWMLCVLNTRRAQTSWVIETKCGEND-----
>Dasypus_novemcinctus
MRNFAGNCHLGEQPESPHHPNSDVSYQKLETSAVSISDGNRLLDAVGNTPALKKCSWMLCVC------------------------
>Monodelphis_domestica
---MQSTVENIPELKKCNWMLCVCSIQGAPIS------CG--------
>Condylura_cristata
---MLDAVENTPELKKCNWMVCVCVCVKYQKSP---DPMGNKDEMWRE
>Ceratotherium_simum_simum
---MLDAVENTPELKKCNWMLC--VC----------------------
>Loxodonta_africana
---MLDAVENTPELKKCNWMVCVCEIPE---EP---RPSG--------
>Nannospalax_galili
---MLDAVENTPELKKCNWMWCLLNTRS----P---DPVGNRDEMWRE
>Trichechus_manatus_latirostr
---MLDAVENSLELKKCNWMLCVC------------------------
>Orycteropus_afer_afer
---MLGAVENTPEVKKCNWVLCVC------------------------

HG0495.1 ENSG00000196730 DAPK1

>Homo_sapiens
----------------------MHEGATEAQERW
>Felis_catus
----------------------MHEGPTEAQERW
>Balaenoptera_acutorostrata_s
----------------------MHEGPTEAQERW
>Rousettus_aegyptiacus
----------------------MHEGPTEAQERW
>Galeopterus_variegatus
----------------------MHEGPTEAQERW
>Erinaceus_europaeus
----------------------MYEGPTEAQKRW
>Oryctolagus_cuniculus
MVGLRQRSGEIRGVLGARDWRPMHEGSTEAREPW
>Equus_asinus
----------------------MHEGPTEAQERW
>Loxodonta_africana
----------------------MHEGPTTAQERW
>Ictidomys_tridecemlineatus
MIGLRQRPERPGELRDAGDWRLMHEGPTSVQERW

HG0495.2 ENSG00000196730 DAPK1

>Homo_sapiens
-----------------------------------MVWEAELKCPGLW
>Mustela_putorius_furo
-----------------------------------MVWEAELKCAGLW
>Orcinus_orca
-----------------------------------MVWEAELKCAGLP
>Eptesicus_fuscus
-----------------------------------MVWEAELKCAGLW
>Dasypus_novemcinctus
MVGLRQRSGGTRGSPGRPGLETDARGARGGAGAAVMVWEAELKCAGLR
>Galeopterus_variegatus
-----------------------------------MVWEVEPKCPGLR
>Monodelphis_domestica
---------------------------------MVMVWEVELKSVGLW
>Condylura_cristata
-----------------------------------MVWDAELKCAGLW
>Equus_przewalskii
-----------------------------------MVWEAELKCAGLW
>Loxodonta_africana
-----------------------------------MVWEAELKCAGLW
>Nannospalax_galili
----------------------MHEGPAEAQEAVVMVWEAELKCPGLW
>Tupaia_chinensis
-----------------------------------MVWEEELKCARLW

HG0496 ENSG00000135913 USP37

>Homo_sapiens
MSSVPRRVLCLWHKDQNKGELLLHRVFR
>Canis_lupus_familiaris
MSSSPRRVHCLWHKDQNKGELLLHRVFQ
>Physeter_catodon
MSSSPRRAHCLWHKDQNKGELLLHRVFQ
>Pteropus_alecto
MSSSPRRVHCLWHKHQNKGELLLHRVFQ
>Monodelphis_domestica
MGSSPRRVLYLWHKHQNKDYWK------
>Oryctolagus_cuniculus
MSSFPRRVHCLWHKDQHKGELLLHRVFQ
>Equus_asinus
MSSFPRRVHCLWHKDQNKGELLLHRVFQ
>Manis_javanica
MSSSPRRVHCLWHKDQNKESFR------
>Loxodonta_africana
MSSSPRRVLCLWHKDQNKGELLLHRVFQ
>Octodon_degus
MSSFPRRVPCLWHKDQNKGELLLHRVFQ
>Trichechus_manatus_latirostr
MSSSPRRVLCLWHKDQNKGELLLHRVFQ

HG0497 ENSG00000147571 CRH

>Homo_sapiens
----------------------MIYAGAEAARNRAVKRASAY
>Mustela_putorius_furo
MGPGIKYTQAGQLALSPTDHGPMIYAGAEAARNRAVKRASAY
>Pteropus_alecto
----------------------MIYAGAEAARNRAVKRASAY
>Galeopterus_variegatus
----------------------MIYAGAEAARNRAVKRASAY
>Monodelphis_domestica
----------------------MIYSGADRARNLAVKKASAY
>Condylura_cristata
----------------------MIYAGAEAARNRAVKRASAY
>Oryctolagus_cuniculus
----------------------MIYAGAEAARNRAVKRASAY
>Equus_asinus
----------------------MIYAGAEAARNRAVKRASAY
>Loxodonta_africana
----------------------MIYAGAEAARNRAVKRASAY
>Peromyscus_maniculatus_baird
----------------------MIYAGAEAARNRAVKRASAY
>Tupaia_chinensis
----------------------MIYAGAEAARNRAVKRASAY

HG0498 ENSG00000005379 TSPOAP1

>Homo_sapiens
--------------------------------------MRQAAHRPRPP
>Mustela_putorius_furo
--------------------------------------MRQAAHRPRPP
>Balaenoptera_acutorostrata_s
--------------------------------------MRQAAHRPRPP
>Rousettus_aegyptiacus
--------------------------------------MRQAAHRPRPP
>Dasypus_novemcinctus
--------------------------------------MRQAAHRPRPP
>Monodelphis_domestica
MHTLMLGPSPIPRTLGHRELLGNPGFPRAEPLTSKGETMRQAAHRPRPP
>Equus_przewalskii
--------------------------------------MRQAAHRPRPP
>Manis_javanica
--------------------------------------MRQAAHRPRPP
>Mus_musculus
--------------------------------------MRQAAHRPRPP
>Tupaia_chinensis
--------------------------------------MRQAAHRPRPP

HG0499 ENSG00000011258 MBTD1

>Homo_sapiens

MLMLLISWGHPS

>Mustela_putorius_furo

MLMLLISWGHPS

>Orcinus_orca

MLMLLISWGHPS

>Myotis_lucifugus

MLMLLISWGHPS

>Dasypus_novemcinctus

MLMLLISWGHQS

>Sarcophilus_harrisii

MLMLLISWGHPN

>Oryctolagus_cuniculus

MLMLLISWGHPS

>Equus_caballus

MLMLLISWGHPS

>Manis_javanica

MLMLLISWGHPS

>Mus_musculus

MLMLLISWGHPS

HG0500 ENSG00000070778 PTPN21

>Homo_sapiens

MLPSSGHWRLRCFLMAAAAAG

>Ailuropoda_melanoleuca

MLPSPGLWGLSCFLMAAASAG

>Pteropus_vampyrus

MLPSPGLWRLSCILMEAAAAG

>Dasypus_novemcinctus

MLPSPGLWKRNCFLMAAAAAG

>Sarcophilus_harrisii

MIPSLRPWRIRCFLMAADAAG

>Monodelphis_domestica

MIPSPGPWRIRCFLMAADTAG

>Ceratotherium_simum_simum

MLPSPGLWRLRCFLMAAAAAG

>Manis_javanica

MLPSPGLWRLSCFLMAAAAAG

>Loxodonta_africana

MLPSPGLWRLDCFLMAAAAPG

>Rattus_norvegicus

MLPSLGRWRLGCFLMAAAAVG

HG0501 ENSG00000110400 NECTIN1

>Homo_sapiens

MTRATCGGHLPSL

>Mustela_putorius_furo

MTRATCGGHPPSL

>Pteropus_vampyrus

MTRATCGGHLPSF

>Sminthopsis_crassicaudata

MTRLTCGGHLPRL

>Monodelphis_domestica

MTRLTCGGHLPRL

>Condylura_cristata

MTRATCGGHLPSL

>Ochotona_princeps

MTRATCGGHLPSL

>Ceratotherium_simum_simum

MTRATCGGHLPSL

>Manis_javanica

MTRATCGGHLPSL

>Marmota_marmota_marmota

MTRATCGGHLPSL

HG0502 ENSG00000126882

>Homo_sapiens

MSEPHNYSLNYKLFLTQRISVIFVHCEEKKREPRAHEMQQNPYL

>Canis_lupus_familiaris

MSEQHDYPLTSRLSFF----------------------------

>Rhinolophus_ferrumequinum

-------------------------------------MQQNPYL

>Galeopterus_variegatus

-------------------------------------MQQSPRL

>Monodelphis_domestica

-------------------------------------MQQNPYL

>Erinaceus_europaeus

MSEQYDYLLTSKLFFF----------------------------

>Oryctolagus_cuniculus

-------------------------------------MQQNPYL

>Equus_caballus

-------------------------------------MQQNPYL

>Microtus_ochrogaster

MSEQHAYPLNYKLFFF----------------------------

>Orycteropus_afer_afer

-------------------------------------MQQNPYL

HG0503 ENSG00000141564 RPTOR

>Homo_sapiens
MGRSWLWLQQLRRVRDPQG
>Felis_catus
MGRSWLWLQQLRRVRDPQG
>Orcinus_orca
MGPSWLWLQQLRRVRDPQG
>Rhinolophus_ferrumequinum
MGRSWLWLQQLRRVRDPQG
>Galeopterus_variegatus
MGRSWLWLQQLRRVRDPQG
>Monodelphis_domestica
MGRSWLQLQQLGRMWGPQG
>Ceratotherium_simum_simum
MGRSWLWLQQLRRLRDPQG
>Manis_javanica
MGRSWLPLQQLRRVRDPQG
>Loxodonta_africana
MGRSWLCLQQLRRVRDPQG
>Ictidomys_tridecemlineatus
MGRSWLLPQQLRRVRDPQG

HG0504 ENSG00000144460 NYAP2

>Homo_sapiens

MQTDCRNREERLLLISEEYNQMRYISQLGDCFWSCNHLCSKVGKIIQFLPCWTWKERGCVFIPTH

>Mustela_putorius_furo

MQTDCRNREERLLLISEEYNQMRHLSQPG------------------------------------

>Myotis_lucifugus

MQTDCRNREERLLLISEEYNQMRHLSQPG------------------------------------

>Galeopterus_variegatus

---------------------MKYLSQPGDCFGSCNHLCSKVGKIIQFLPCWTWKERGCVFIPTH

>Condylura_cristata

MQTDCRNREERLLLISEEYNQMRHLSQPG------------------------------------

>Oryctolagus_cuniculus

---------------------MKYLSQPGDCFGSCNHLCSKVGKIIQFLLCWTWKERGCVFIPTH

>Manis_javanica

MQTDCTHREERLLLISEEYNQMRHLSQPG------------------------------------

>Peromyscus_maniculatus_baird

MQADCRSRGERLLLISEEHDQMRYLSQLGDCFGSCNHLCSKVGKIIQFFPCWTWKERGCALIPTH

>Tupaia_chinensis

---------------------MRHLSQPGDCFGSCNHLCSKVGKIILFLPCWTWKEQGCVFIPTH

HG0505 ENSG00000157470

>Homo_sapiens
MGSRGAPTPRADVNY
>Odobenus_rosmarus_divergens
MGSRGAPAPRADVNY
>Lipotes_vexillifer
MGSRGAPAPRADVNY
>Rhinolophus_ferrumequinum
MGSRGAPAPREDVNY
>Dasypus_novemcinctus
MGCRGAPAPRADVNY
>Monodelphis_domestica
MGSRIPPAPSADVNY
>Condylura_cristata
MGPRGAPDSRADVNY
>Oryctolagus_cuniculus
MGSRGAPGPRADVNY
>Manis_javanica
MGSRRAPAPCADVNY
>Rattus_norvegicus
MGSRGAPDPRADVNY

HG0506.1 ENSG00000177853 ZNF518A

>Homo_sapiens

MAAIRDSQAMGTTTDIISSFDHCS

>Ursus_maritimus

MAAIRDSQAMGTTTDIVSSFDHCS

>Physeter_catodon

MAAIRDSQAMGTTTDIISSFDHCS

>Miniopterus_natalensis

MAAIRDSQAMGTTTDIISSFDHCS

>Galeopterus_variegatus

MAAIRDSQAMGSTTDIISSFDHCS

>Trichosurus_vulpecula

MAAIGDSPAMELTPDFVSSFDQCS

>Oryctolagus_cuniculus

MAAIRDSQAMGTTTDI-ISFDHCS

>Equus_asinus

MAAIRDSQAMGTTTDI-ISFDHCS

>Manis_javanica

MAAIRDSQAMGTTTDTISSFDHCS

>Mesocricetus_auratus

MAAIWDSQAMGTTTDV-ISFDHCA

HG0506.2 ENSG00000177853 ZNF518A

>Homo_sapiens
MSLHSIVSCLNYR
>Mustela_putorius_furo
MSWHSIVSCLNYK
>Balaenoptera_acutorostrata_s
MSRHSIVSCSNYK
>Miniopterus_natalensis
MSQHNIVSCLNYK
>Dasypus_novemcinctus
MCQHSIVSCLNYE
>Galeopterus_variegatus
MSWHSIVSCLNYK
>Equus_asinus
MSRHSIVSCLNYK
>Manis_javanica
MSQHSIVSCLNYK
>Loxodonta_africana
MSQHNIVSCLNFK
>Marmota_marmota_marmota
MSRHSIISCLNYR

HG0507 ENSG00000060237 WNK1

>Homo_sapiens

MTAAPLLPPPARPPLAAGWMRTVRR

>Odobenus_rosmarus_divergens

MTAAPLLLQPSRPPLAAGWMRTVRR

>Rousettus_aegyptiacus

MTAAPLLLQPSRPPLAAGWMWTARR

>Monodelphis_domestica

MTAVPQLLVPARPPPAAGWMRSVRR

>Oryctolagus_cuniculus

MTAAPLLPQPSRPPLAAGWMRTVQR

>Loxodonta_africana

MTAAPLLLQLSWPPLAAGWMRTVRR

>Rattus_sp.

MTAALLLPQPYRPPLAAGWMRTVRR

>Trichechus_manatus_latirostr

MTAALLLLQPSWPTVAAGWMRTVRR

>Orycteropus_afer_afer

MTAAPLLLQSSWLPLAAGWMRTVRR

HG0508 ENSG00000061936 SFSWAP

>Homo_sapiens

MAAVLRLGTGCGVFD

>Acinonyx_jubatus

MAAVSSLGPGCGAFD

>Pteropus_alecto

MAAVSRLGPGCGACD

>Dasypus_novemcinctus

MAAVLRLVLGCGTFD

>Monodelphis_domestica

MAAVLRLRPGCGASD

>Oryctolagus_cuniculus

MAAVLRLGPGCGAFD

>Equus_asinus

MAAVLRLGPGCGAFD

>Manis_javanica

MAAMPRLGLGCGASD

>Marmota_monax

MAAVLRLRTGCGAFD

HG0509 ENSG00000068024 HDAC4

>Homo_sapiens
---------------------------MCWWEFGARWSYRFRGNFEPFRIT
>Canis_lupus_familiaris
MPRPAPRGRQDWGFPRLRANTPCRCTCMCPWNLGALWSCRLRGSCEPL---
>Balaenoptera_acutorostrata_s
---------------------------MCRWDSGALWSSRSCGSFEPF---
>Pteropus_alecto
---------------------------MCPWDFGALWSYCLRGNFEPS---
>Dasypus_novemcinctus
---------------------------MCWWNFGALWSYRLCGSIKPF---
>Galeopterus_variegatus
---------------------------MCWWNFGALCSYRLLGNFEPF---
>Ornithorhynchus_anatinus
---------------------------MYWWSIGALWSCGLCGNFDPF---
>Ceratotherium_simum_simum
---------------------------MCRWNF------------------
>Peromyscus_maniculatus_baird
---------------------------MCWWKFGAGWSYCLRGHFEPF---

HG0510 ENSG00000134769 DTNA

>Homo_sapiens
MVLKSSCCPFIEF
>Acinonyx_jubatus
MVLESSCCFFIEF
>Balaenoptera_acutorostrata_s
MVLKSSCCFFIEF
>Pteropus_alecto
MVLESSCCFFIEF
>Dasypus_novemcinctus
MVLESSCCCFIEF
>Monodelphis_domestica
MVLESSCCAFIEL
>Ceratotherium_simum_simum
MVLESSCCFFIEF
>Tupaia_chinensis
MVLESSCCFFIEF
>Orycteropus_afer_afer
MVLESSCCSFIEF

HG0511 ENSG00000143337 TOR1AIP1

>Homo_sapiens

MAETSSSPFSSSLVPPARRRERSTAADPAQALPLSQPSRHTMGPEAGLLHSSD----DAGGGPSDSQLPP

>Felis_catus

---MGPEAGLQHSSD----DAGGGPGDSQLPP

>Balaenoptera_acutorostrata_s

---MGPEAGLQHSSD----DAGGSPGDSQLPP

>Rhinolophus_ferrumequinum

---MGPEAGLQHSSD----DAGGSPGDSQLPP

>Dasypus_novemcinctus

---MGPEAGLQYSSD----DAGGSPGDSQLPP

>Sarcophilus_harrisii

---MGPEAAGQRGSGSGRSGGPTGPGDSQLPP

>Oryctolagus_cuniculus

---MGPGAASRHSSD----DAGGGPGDSQLPP

>Equus_asinus

---MGPEAGLRHSSD----DAGGGPGDSQLPP

>Marmota_marmota_marmota

---MGPEAGLRHSSD----DAGGGPGDSQLPP

HG0512.1 ENSG00000112319 EYA4

>Homo_sapiens
MSCFHQRHSAKGKLRHWKERE---
>Canis_lupus_familiaris
MSCLQQRHNAKGKLRRWKERE---
>Balaenoptera_acutorostrata_s
MSSFHQRHSVKGKLQHWKARE---
>Miniopterus_natalensis
MSCFHQRHSAKGKLRHWKERQ---
>Monodelphis_domestica
MSCFYQKNSREGKLRHQEGRRINK
>Equus_przewalskii
MSCFHQRHSAKGKLRHWKERE---
>Manis_javanica
MSCFRQRHSAEGRLRHWKERE---
>Mesocricetus_auratus
MSCFHQRHSAKGKLRHWKERE---

HG0512.2 ENSG00000104313 EYA1

>Homo_sapiens

------------MLSAAAVWWGRSAGRKAVTKQNGGS

>Leptonychotes_weddellii

------------MLSAAAVWWGKSAGLKAVTKQNGGS

>Physeter_catodon

--MVLFLCLGFSDAISCCCLEGKSAGLKAVTKQNGGS

>Pteropus_alecto

------------MLSAAAVWWGRSAGLKAVSPQHGGS

>Galeopterus_variegatus

------------MLSAAAVWWGRSAGRKAVAKQNGGS

>Oryctolagus_cuniculus

------------MLSAAAVRWGESAGRKAVTARSGGS

>Elephantulus_edwardii

MRLCCFFVSGSQMLSAAAVQWGKSAGLQAVTTQNGGS

>Equus_przewalskii

------------MLSAAAVEWGQSAGLKAVAKQNGGS

>Loxodonta_africana

------------MLSAAAV------------------

>Cavia_porcellus

------------MLSAAAVRWGKRAGRRAVPKQNSGS

HG0512.3 ENSG00000104313 EYA1

>Homo_sapiens
MPFTAAVSSFFSFG
>Panthera_tigris
MPFTAAVSSFFSFG
>Lipotes_vexillifer
MPFTAAVSSFFSFG
>Myotis_brandtii
MPFTAAVSSFFSFG
>Sorex_araneus
MPFTAAVFSFFSFG
>Elephantulus_edwardii
MPFTAAVSSFFSFG
>Ceratotherium_simum_simum
MPFTAAVSSFFSFG
>Loxodonta_africana
MPFTAAVSSFFSFG
>Ictidomys_tridecemlineatus
MPFTAAVSSFFSFG

HG0513 ENSG00000126860 EVI2A

>Homo_sapiens

MCFSCSGKEMCHLWFGFKSGKLAAHILFYCRFTLRLIFSKSILL-------------

>Odobenus_rosmarus_divergens

---------MCHLWFGFKSGKLAVHILFFF---------------------------

>Physeter_catodon

---------MCHLWLVES---------------------------------------

>Miniopterus_natalensis

MCFSCSEKEMCHLWFGLKSGKLAVHVLFCSRFTLTFIFLQVCSTWKLCLKTRKEVAY

>Manis_javanica

---------MCHLWFGFKTGKLAVHILFYCRFTFKVHDL------------------

>Loxodonta_africana

MCFSCSRKEMCHPSVVWL---------------------------------------

>Chinchilla_lanigera

MCLSCSGKEMCHLWFGFKSGKLAVRTLFCCRCTLHFVFSKSILL-------------

HG0514 ENSG00000147570 DNAJC5B

>Homo_sapiens

MKKRRDLKRDNQLAGKVTNEK

>Panthera_tigris

---MPPVQRDNQLADEVTNEK

>Physeter_catodon

---MTPVQRDNQLADRVTNQK

>Myotis_lucifugus

---MGPLQRDNQLADRVTNEK

>Ornithorhynchus_anatinus

MSSFRVFQRDNQLAARVTNDK

>Ceratotherium_simum_simum

---MPPVQRDNQLADRVTNEK

>Microtus_ochrogaster

---MPPVQRDNQLADKVTNEK

>Tupaia_chinensis

---MAHMQRDNQLAGRVTNEK

HG0515 ENSG00000170471 RALGAPB

>Homo_sapiens

MRAPPVASGAIWIVL

>Leptonychotes_weddellii

MRAPPVASGAIWIVL

>Balaenoptera_acutorostrata_s

MRAPPVASGAIWIVL

>Myotis_brandtii

MRAPPVASGAIWIVL

>Monodelphis_domestica

MRAGPLGAGTIWIVL

>Erinaceus_europaeus

MRAPPVASGAIWIVL

>Mesocricetus_auratus

MRAPPVASGAIWIVL

>Tupaia_chinensis

MRAPPVASGAIWIVL

HG0516 ENSG00000041515 MYO16

>Homo_sapiens

MRMSLK-LFAGSWNRASMQ-----

>Panthera_tigris

MRMSLK-LSAGSWNRASVR-----

>Rousettus_aegyptiacus

MRMSLK-LFA--------------

>Monodelphis_domestica

MRMSLKLLFARSWTRSCIQ-----

>Oryctolagus_cuniculus

MRMSGK-LLAGSWSGASVQ-----

>Manis_javanica

MRMSLK-LFAPSWNRASVQGCVLR

>Chinchilla_lanigera

MRMSLE-LFA--------------

HG0517 ENSG00000086717 PPEF1

>Homo_sapiens

MIDCVLSPQVRLKESTTSKVKI--

>Canis_lupus_familiaris

-MDCVLSLQVRLKDSATNKVKI--

>Monodelphis_domestica

-MDCVLSLLVRPKDSTMVKVEIWE

>Oryctolagus_cuniculus

-MDCVLSQQIRLRVSAANKVKI--

>Ceratotherium_simum_simum

-MDCVLSLQIRLKDTAASKMKI--

>Mus_musculus

-MDCALSLQVRMNDSATSEMKI--

>Tupaia_chinensis

MMDSVLNLQVQLKYSATSKAKI--

HG0518 ENSG00000111254 AKAP3

>Homo_sapiens

-----------MEGHRKKQDLELSKNIPASSLTL-------KVYSG----VFRGSLFQYYRDSLYYMAGGG------------------------------

>Canis_lupus_familiaris

MSLFSPNKGRYMEGWRRKQDLELSKNIPASSLTF-------KV--

>Pteropus_vampyrus

-----------MEGWVRKQDLELSKNIPASSLTL-------KLYSG----VFCGHYPS-TTEIPYITWQEGWVNLGIVKTKNN------------------

>Sarcophilus_harrisii

-----------MEGSGRKQDLELNKNITASLIDF-------KKCILESFVVHCSSAA----EVPD-----NYMAGGVGKLKNCEDYKTIQDPSLYLRICTQ

>Oryctolagus_cuniculus

-----------MEGSGRKQDLELSKDIPASSLTL-------KIYSG----VSCGSLLKCYRDS--------------------------------------

>Equus_asinus

-----------MEGWGRKQDLELSTNIFIDFQSIFWSLLWFTIPVLQRFHILHGRRG------------------G-------------------------

>Chinchilla_lanigera

-----------MEGWGRKQDLELSKNIPASSLTL-------KVYCG----IFCGSLVQSYRDSLYITWQEV------------------------------

HG0519 ENSG00000127074 RGS13

>Homo_sapiens

MVDCSTGYICQWNVSTYYTTNMRKK---

>Acinonyx_jubatus

MVDRSTGYICQWNFSTYHTTNMRKK---

>Pteropus_vampyrus

MVDCSSGYICQWNFSTCHTTNMRKK---

>Trichosurus_vulpecula

-MFCATRHICQWNVSTYHISQYEGKIYC

>Loxodonta_africana

MVDCCTGYICQRNVSTYTT-NMRKK---

>Cavia_porcellus

MVDSSTRYIC------------------

>Orycteropus_afer_afer

MVDCSSGYIC------------------

HG0520 ENSG00000197415 VEPH1

>Homo_sapiens

----MNNFRFTELSGFHSL-CPRS-LPESPAQSQTMQLSNCKGAFEE

>Panthera_tigris

----MNNFRFTELRGFHLL-CPRSPLPESRAQAQNL-----------

>Lipotes_vexillifer

----MNNFRFTELSRFHSL-CPWSPLPESLTQSQNRPLSSSKCAFEE

>Rousettus_aegyptiacus

MGSAMNNFRFTKLSGFCPLSVPRSPLPESPAQSQNIQLSNNKPAFEE

>Monodelphis_domestica

----MNNWRFPGLPDFS------------------------------

>Manis_javanica

----MNNFRFTELSGFHSL-CPRSQLPKSPTQSQHRQLSNSRHAFEE

>Tupaia_chinensis

----MNHSKFTELSGFHSL-CPRNLLPEFLTQSQNIPLSNRMGALEE

HG0521 ENSG00000038274 MAT2B

>Homo_sapiens
-----------------------MWRTARDSGIGIKMLI
>Myotis_lucifugus
-----------------------MLKTARVSGIGIKMLI
>Sarcophilus_harrisii
-----------------------MWETARDPETGI----
>Ceratotherium_simum_simum
-----------------------MWKTARDSGIGIKMLI
>Ictidomys_tridecemlineatus
MKHETSHWVFWKLPRGISCQLTALWKTARDSGIGIKMLI

HG0522 ENSG00000140396 NCOA2

>Homo_sapiens

MAAAASATASAAKVSADGSRHLTA

>Rousettus_aegyptiacus

MAAAASAAASAAKVSADGSRHLTA

>Monodelphis_domestica

MAAT--ASAPAGKVSAVGSRHLID

>Ochotona_princeps

MAAAASATATAAKVSADGSRHLTA

>Dipodomys_ordii

MAATVSAPAWAAKVSADGSRHLTA

HG0523 ENSG00000075213 SEMA3A

>Homo_sapiens

MGCSWYSYKVNMLLAAHIYP

>Monodelphis_domestica

MGCSLYSYKVNKLLTAYIYD

>Trichosurus_vulpecula

MGCSLYSYKVNKLLTAYIYD

>Mus_musculus

-----------MLLAAHVCA

HG0524 ENSG00000107929 LARP4B

>Homo_sapiens

MRTGRSVGDLESRG

>Macropus_eugenii

MRTGRSVGDLESRG

>Manis_javanica

MRTGRSVGATESRG

>Microtus_ochrogaster

MRTGRSVGDLESRG

HG0525 ENSG00000136167 LCP1

>Homo_sapiens

MTSPEIAVVQRKQSRGIELLQNARVSNIVSAAASYLTKVEEILYFQLRKLKIPPASDDQAPLDTEHCAFLLQVTSCLVTTHPGLTKLFCRSERRGSWSYTSTTKDS

FFPARSVT

>Canis_lupus_familiaris

---MICHSSHFLPC--DRTAGVDSCSADPRGGGFWSHASTTEDS

FSTRSVA-

>Ornithorhynchus_anatinus

--MTSCLVTT--------------------------

>Mus_musculus

--MLLERRKWK----------VASRFHCDLLSVTSCLVTTQPCLTRLLCRSEGLGFLTTQQCC---

----

HG0526 ENSG00000143569 UBAP2L

>Homo_sapiens

MRLEPERGERERARASEAWALPEYSTL

>Felis_catus

MRLEPERGERGRARASAAWAPPEYSTL

>Monodelphis_domestica

MRLGP-GGWREREGASAAWAWPEHSTL

>Chinchilla_lanigera

MRLQPEGGERERARASAAWALPEYSTL

HG0527 ENSG00000198382 UVRAG

>Homo_sapiens

MS----APGLSGVEGLHCGNMALP

>Monodelphis_domestica

MSALGLGSGRPGREVLHCGNMALP

>Oryctolagus_cuniculus

MS----GPGLCRVEGLHCGNMALP

>Chinchilla_lanigera

MS----APGLGPVEGLHCGNMALP

HG0528 ENSG00000204767

>Homo_sapiens

MGMFPWEHHVSSNKQTSRSFISPPVYLLWKEDAFKFPAVSPWKLMLWETKSCMF---

>Rousettus_aegyptiacus

MGMSPSGHHVSSKKPTRRSLISLPVYMLWKEDAFKFPVVSHWKSMLRERKPSVPEVAGFFWNLHFGSLALSSKWYFCCLFVQDSKRFPGSELPASLP

>Ornithorhynchus_anatinus

--MLLVDHHVSSEKQTYQSLIMLIIYT--

HG0529 ENSG00000112851 ERBIN

>Homo_sapiens
--------MLAQLRNIREINSTQCV
>Canis_lupus_familiaris
--------MLAQLRNIREINSTQCV
>Lipotes_vexillifer
--------MLAQLRNIREINSTQCV
>Pteropus_alecto
--------MLAQLRNSREINSAQCV
>Dasypus_novemcinctus
--------MLAQLRNIRQIDLLQCV
>Galeopterus_variegatus
--------MLAQLRNIREINSTQCV
>Sorex_araneus
--------MLAQLRNIRETNST---
>Oryctolagus_cuniculus
--------MLAQLRNTREINSTQCV
>Elephantulus_edwardii
--------MLAQLRNTSKINSALCV
>Equus_asinus
MREPLIHRMLAQLRNIREINSTQCV
>Manis_javanica
--------MLAQLRNIREINSTKSV
>Loxodonta_africana
--------MLAQLRNIREINSTQCV
>Cricetulus_griseus
--------MLAQLRNIREINSTQCF
>Tupaia_chinensis
--------MLAQLRSIREINSTKCV
>Trichechus_manatus_latirostr
--------MLAQLRNIREINSTQCV
>Orycteropus_afer_afer
--------MLAQLRNIIEINSTQSV

HG0530 ENSG00000128594 LRRC4

>Homo_sapiens
MREQSAWLPPATPRARREEETKSEWERGAQLPWRKLKDTTPVLSCLPSEMERGAPSSLKPG
>Felis_catus
---MERGAPSSFKPG
>Lipotes_vexillifer
---MERGAPSSLKPG
>Myotis_brandtii
---MERGAPSSLKPG
>Dasypus_novemcinctus
---MERGALSSLKPG
>Galeopterus_variegatus
---MERRAPSSLKPG
>Condylura_cristata
---MERGAPSSLKPG
>Oryctolagus_cuniculus
---MERGAPSSLKPG
>Elephantulus_edwardii
---MERGAPSSLKPG
>Ceratotherium_simum_simum
---MERGAPSSLKPG
>Manis_javanica
---MERGAPSSLKPG
>Loxodonta_africana
---MERGAPSSLKPG
>Dipodomys_ordii
---MERGAPSSLKPG
>Tupaia_chinensis
---MERGAPSSLKPG
>Trichechus_manatus_latirostr
---MERGAPSSLKPG
>Orycteropus_afer_afer
---MERGAPSLLKPG

HG0531 ENSG00000146834 MEPCE

>Homo_sapiens
MRAQPGWWRSAHAWFPGEGNARSPCALAAGW
>Felis_catus
MRAQPGWWRSAHAWFRGKRNAL---------
>Balaenoptera_acutorostrata_s
MRAQPGWWRSAHA------------------
>Pteropus_alecto
MRARPGWWRSAHA------------------
>Dasypus_novemcinctus
MRAQPGW------------------------
>Galeopterus_variegatus
MSAQPGWWRSAHAWFGGERNALQQCALAAKC
>Condylura_cristata
MRAQPGWWRNAHA------------------
>Oryctolagus_cuniculus
MRAQPGWWRSAHAWPRG--------------
>Elephantulus_edwardii
MRAQPGCWRSAHASSWGQRNAL---------
>Equus_caballus
MRAQPGWWRSAHA------------------
>Manis_javanica
MRAQPGWWRSAHA------------------
>Loxodonta_africana
MRAQPGWWRCAHA------------------
>Nannospalax_galili
MRAQPGWWRSAHAWFRGERNARPPCALSAKW
>Tupaia_chinensis
MRAQPGWWQSAHA------------------
>Trichechus_manatus_latirostr
MRAQLGWWRSAHA------------------
>Orycteropus_afer_afer
MRAQPGWGRSAHA------------------

HG0532 ENSG00000105221 AKT2

>Homo_sapiens
MVPGTVLCPVHGASCMSCCPELSRAR
>Mustela_putorius_furo
---------------MSCCPELSQAR
>Physeter_catodon
---------------MSCCPELSRAR
>Pteropus_vampyrus
---------------MSCCPELSRAR
>Dasypus_novemcinctus
---------------MSCCPELPQAR
>Galeopterus_variegatus
---------------MSCCPEVSQAR
>Erinaceus_europaeus
---------------MSCCPELSLAR
>Oryctolagus_cuniculus
---------------MSCCPELPQAR
>Elephantulus_edwardii
---------------MSCCHILT---
>Equus_asinus
---------------MSCCPELSRAR
>Manis_javanica
---------------MSCCPELSQAR
>Loxodonta_africana
---------------MSCCLKVP---
>Cricetulus_griseus
---------------MSCCPELSQAR
>Trichechus_manatus_latirostr
---------------MSCCPELP---
>Orycteropus_afer_afer
---------------MSCCLELS---

HG0533 ENSG00000114209 PDCD10

>Homo_sapiens
MKIPLHFSTAFSTVVGF
>Acinonyx_jubatus
MKIPLHFSTAFSTVVGF
>Lipotes_vexillifer
MKIPLHFSTAFSTVVGF
>Eptesicus_fuscus
MKIPLHFSTAFSTVVGF
>Dasypus_novemcinctus
MKIPLHFSTAFSRVVGF
>Galeopterus_variegatus
MKIPLHFSAAFSAVVGF
>Condylura_cristata
MKIPLNFSTAFSTVVGL
>Oryctolagus_cuniculus
MKIPLHFSTAFSTVVGF
>Ceratotherium_simum_simum
MKIPLHFSTAFSTVVGF
>Manis_javanica
MKIPLHFSTAFLL----
>Loxodonta_africana
MKIPLHFSTAFSPVVGF
>Ictidomys_tridecemlineatus
MKIPLHFSIAFSTVVGF
>Tupaia_chinensis
MKITLHFSTAFSTVVGF
>Trichechus_manatus_latirostr
MKIPLHFSTAFSPVVGF
>Orycteropus_afer_afer
MKIPLHFSTAFSPVVGF

HG0534 ENSG00000117408 IPO13

>Homo_sapiens
MPALGPPSPHHSLGTQAGV
>Felis_catus
MPALGPLSPHRSLGTQAGV
>Physeter_catodon
MLALGPLSPHHSLGTEAGV
>Myotis_davidii
MPTLGPLSPHHSLGPQAGV
>Dasypus_novemcinctus
MPALGPPSPHLSLGTQAGV
>Erinaceus_europaeus
MPALGSLSHHQSLGTQAGV
>Ochotona_princeps
MPALGAPSPHCSLDTQAGV
>Elephantulus_edwardii
MSALGAPSPHHSLGTQAGV
>Ceratotherium_simum_simum
MPALGPLSPRHSLGTRAGV
>Manis_javanica
MPAQGPLSPHHSLGTQAGV
>Loxodonta_africana
MPALGPPSPHYSLGTQAGV
>Cavia_porcellus
MPALGTPSPQHSLGTQAGV
>Tupaia_chinensis
MPTLGSPSPHHSLGTQAGV
>Trichechus_manatus_latirostr
MPALGPPSLHHSVGTQAGV
>Orycteropus_afer_afer
MPALGAPSPHHSLGTQAGV

HG0535 ENSG00000118407 FILIP1

>Homo_sapiens
MTSAIGWSWERKWEKLFPLYSFGLDSF-------------------
>Acinonyx_jubatus
MTSAIGQS--------------------------------------
>Orcinus_orca
MTSAIG--
>Eptesicus_fuscus
MTSAIGQSWESKWEKLFPLYSFGLDSFPGLQSLSWAVGQATGFFQG
>Dasypus_novemcinctus
MTSAIG--
>Galeopterus_variegatus
MTSAIG--
>Condylura_cristata
MTSAIGYS--------------------------------------
>Oryctolagus_cuniculus
MTSAIGWS--------------------------------------
>Elephantulus_edwardii
MTSAIGWS--------------------------------------
>Ceratotherium_simum_simum
MTSAIGQS--------------------------------------
>Manis_javanica
MTSAIGWS--------------------------------------
>Loxodonta_africana
MTSAIG--
>Cavia_porcellus
MTSAIGWS--------------------------------------
>Tupaia_chinensis
MTSAIG--
>Orycteropus_afer_afer
MTSAIG--

HG0536 ENSG00000181751 C5orf30

>Homo_sapiens
--------------------------------------MSVFPWDSLEIVQ
>Phoca_largha
--------------------------------------MPVFPWDSLDIVQ
>Balaenoptera_acutorostrata_s
--------------------------------------MPVFPWDSLEIVQ
>Myotis_davidii
--------------------------------------MPVFPWDSLEIVQ
>Dasypus_novemcinctus
--------------------------------------MPVFPWDSVEIVH
>Galeopterus_variegatus
--------------------------------------MTVCPWDSLEIAQ
>Condylura_cristata
--------------------------------------MPVFLWDSLEIVQ
>Oryctolagus_cuniculus
--------------------------------------MPAFPGDSLEIVQ
>Ceratotherium_simum_simum
--------------------------------------MPVFSWDSLEIVQ
>Manis_javanica
--------------------------------------MPVFPWDSLEIVQ
>Loxodonta_africana
--------------------------------------MPVFPWDSLEIVH
>Nannospalax_galili
MTASPLPLASVVQPAVGTPSFSAHATLHRYLEYRRKIIMPVLPWDSLEIVQ
>Tupaia_chinensis
--------------------------------------MPVFPWDSLEIVQ
>Trichechus_manatus_latirostr
--------------------------------------MPVFPWDSLEIVH
>Orycteropus_afer_afer
--------------------------------------MPVFPWDSLEIVH

HG0537 ENSG00000184481 FOXO4

>Homo_sapiens
MCLLALDASPLRGGIVRDCGRLH
>Felis_catus
MCLLALGASPLGGGIVRDCGRLL
>Balaenoptera_acutorostrata_s
MCLLALGASPLGGGIARDCGRLL
>Miniopterus_natalensis
MCLLALGASPLGGGIVRDCGRLL
>Dasypus_novemcinctus
MCPLALDASPLGGGIVRDCGRLH
>Galeopterus_variegatus
MCLLALGASPLGGGIVRDCGRLH
>Sorex_araneus
MCLLALGASPLGGGIVRDCGRLL
>Oryctolagus_cuniculus
MCLLALSASPLGGGIVRDCGRLQ
>Ceratotherium_simum_simum
MCLLALSASPLGGGIVRDCGRLL
>Manis_javanica
MCLLALGASPLGGGIVRDCGRLL
>Loxodonta_africana
MCPLALGASPLRGGIVRDCSRLH
>Peromyscus_maniculatus_baird
MCLLALVASPLRGGMVRDCGRLH
>Tupaia_chinensis
MCLLALGASPLGGGIARDCGRLH
>Trichechus_manatus_latirostr
MCPLALGASPLRGGIVKDCSRLQ
>Orycteropus_afer_afer
MCPLALGASPLGGGIVRDCSWLH

HG0538 ENSG00000006116 CACNG3

>Homo_sapiens
MRTNPPEPAPFRGP
>Canis_lupus_familiaris
MWTNPLEPAPFQGP
>Physeter_catodon
MWTNPLEPAPFRGP
>Miniopterus_natalensis
MRTNPLEPAPFRGP
>Dasypus_novemcinctus
MWTNPLEPLPFRGP
>Galeopterus_variegatus
MWTSPLEPAPFRGP
>Condylura_cristata
MWTNPLEPAPFRGP
>Ochotona_princeps
MWTNPLEPAPFRGP
>Elephantulus_edwardii
MWTNPLESAPFRGP
>Equus_asinus
MWTNPLEPAPFRGP
>Loxodonta_africana
MWTNPLKPAPFRGP
>Chinchilla_lanigera
MRTNPLEPAPSRGP
>Tupaia_chinensis
MWTNPLEPAPFRGP
>Trichechus_manatus_latirostr
MWTNPLEAAPFRGP

HG0539 ENSG00000115966 ATF2

>Homo_sapiens
---------------MAHQLESILAMRKRM
>Acinonyx_jubatus
---------------MAHQLESILAMKKRT
>Lipotes_vexillifer
---------------MAHQLESILAMKKRT
>Myotis_brandtii
---------------MAHQLESILAMKKRT
>Dasypus_novemcinctus
---------------MAHQLESIFAMKKRT
>Galeopterus_variegatus
---------------MAHQLESILAVRKRT
>Erinaceus_europaeus
---------------MAHQLESIFVMKKRT
>Elephantulus_edwardii
---------------MAHQLESILAMEKRM
>Equus_asinus
---------------MAHQLESILAMKKRT
>Manis_javanica
---------------MAHQLESILAMGKRT
>Loxodonta_africana
---------------MAHQLESILAMIKRM
>Nannospalax_galili
---------------MAHQLQSILAMKKRT
>Tupaia_chinensis
---------------MAHQLESILAMKKRT
>Orycteropus_afer_afer
MIRAEGFLAAEGIKLMAHLLESILAMKKRM

HG0540 ENSG00000120519 SLC10A7

>Homo_sapiens
MSGGTKESVETLQGLGDRSLKRKKAKQINKTHPP
>Panthera_tigris
MSGGTVESVETPRGLSDRSLKRKKAKQINKTHPP
>Tursiops_truncatus
MSGGTVESVETPRGLSDSSLKRKKAKQINKTHPP
>Miniopterus_natalensis
MSRGTVESVETPRGLSDRSLKRKKAKQINKTHPP
>Dasypus_novemcinctus
MSGGTEETVETPRGLSDRSLKRKKAKQINKTHPP
>Condylura_cristata
MSGGAVESVETSRGLSDRSLKRKKAKQINKTHPP
>Oryctolagus_cuniculus
MSGGTEESVETPRRLRDSSSKRKKAKQTNKTHPP
>Elephantulus_edwardii
MSGGIEQSVESPREIRDRSLKRKKAKQINKTHPP
>Equus_przewalskii
MSGGTVESVETPRGLSDRSLKRKKAKQINKTHPP
>Manis_javanica
MSGGTVESVETPRGLSDSSLKRKKAKQINKTHPP
>Loxodonta_africana
MSGGNEESVETPRVLSDRSLKRKKAKQINKTHPP
>Marmota_marmota_marmota
MSGGIQESVETPRGFRDRSLKRKKAKQINKTHPP
>Tupaia_chinensis
MSGGTAESVETPRGLSARSLEREKTKQINKTHPP
>Trichechus_manatus_latirostr
MSGGTEESVETPRGLSDRSLKRKKAKQINKTHPP

HG0541.1 ENSG00000122584 NXPH1

>Homo_sapiens

METERLGGHVRGAGDARPE

>Mustela_putorius_furo

MEMKRQGGHVRGAGDARPE

>Orcinus_orca

MEMKRQGGHVRDAGDARPE

>Pteropus_vampyrus

METKRQGGHVRGAGDARPE

>Dasypus_novemcinctus

METQRQGRRVRGAGDARPE

>Sorex_araneus

METKRQGGHVRSAGDARPE

>Ochotona_princeps

METERQGGHVWGAGDARPQ

>Elephantulus_edwardii

METKRQGGHVRGAGDARPQ

>Ceratotherium_simum_simum

METKRQGGHVRGAGDARP-

>Loxodonta_africana

METKRQRGHVRGAGDARPE

>Marmota_marmota_marmota

METELQGGHVRGAGDARPE

>Tupaia_chinensis

METERQGGHVRGAGDARPE

>Trichechus_manatus_latirostr

METNRQGGHVRGAGEARPE

>Orycteropus_afer_afer

METKRQGGHVRGAGDARPE

HG0541.2 ENSG00000122584 NXPH1

>Homo_sapiens

--------------MEPAQSGPFSQDSPQVLCGC

>Leptonychotes_weddellii

MSGAPETPGPSSSSMEPAQSGPCSPVSPLVLCGC

>Orcinus_orca

MFGTPETPGPSSSSMEPSQSGPCLLDSPPVLCGC

>Eptesicus_fuscus

MSGAPETPGPSSSSMEPAQSGPCSPDSPPVLCGC

>Dasypus_novemcinctus

--------------MDPGQNWTCSPDSTPFLCGC

>Condylura_cristata

MSGAPETPGPSSSSMEPAQSGPCSPDSPLILCGC

>Elephantulus_edwardii

--------------MEPIQSGPCSQDLPPVLCGC

>Equus_asinus

MFGAPETPGPSSSSMEPAQSHPCSPDSPPVLCGC

>Marmota_marmota_marmota

--------------MEPAQSGQCSPDSPPVLCGC

>Tupaia_chinensis

--------------MEPVQSGPCSPDSPPVQCGC

>Trichechus_manatus_latirostr

MSGAPEKPGPSSSSMEPAQSGPCSPDSPSVLCGC

>Orycteropus_afer_afer

--------------MEPAQSDSCSPASPPVYSGC

HG0542 ENSG00000126581 BECN1

>Homo_sapiens
-MVVLEASLRGRPEATVPPR
>Felis_catus
-MAVLEASLRGRPDATVPPR
>Eptesicus_fuscus
MMAVLEASLQGSPEAVVPPR
>Dasypus_novemcinctus
-MGVLEAFPRGRPQATVPPR
>Galeopterus_variegatus
-MAVLEALLPGRPEAAVPPR
>Condylura_cristata
-MAILEAWVRVRPAATVPPR
>Oryctolagus_cuniculus
-MAILEASFRGRPEATVPPR
>Ceratotherium_simum_simum
-MAVLEASLRDRPEAAVPPR
>Manis_javanica
-MAVLEVPLRGRPEATVPLR
>Loxodonta_africana
-MAVLGALFRGRPEATVPPR
>Mus_musculus
-MGTLEVSLRGGPEATVPPR
>Tupaia_chinensis
-MAVLDASLWGRPAATVPPR
>Trichechus_manatus_latirostr
-MAVLGALFRCRPEAAVPPR
>Orycteropus_afer_afer
-MAVLEALLRGRPEATVPPR

HG0543 ENSG00000131931 THAP1

>Homo_sapiens
-----------------------------------MGDGRWLWSFC
>Ailuropoda_melanoleuca
-----------------------------------MGDGRWLWSFC
>Lipotes_vexillifer
-----------------------------------MGDGRWLWSFC
>Rousettus_aegyptiacus
-----------------------------------MGDGRWLWSFC
>Dasypus_novemcinctus
MACFPRNVTSDLGTERQWDELLARGEVEAASAILEMGDGRWLWFYC
>Galeopterus_variegatus
-----------------------------------MGDRRWLWSFC
>Condylura_cristata
-----------------------------------MGDGRRLWSFC
>Ochotona_princeps
-----------------------------------MGEGRWLWPFC
>Ceratotherium_simum_simum
-----------------------------------MGDGRWLCSFC
>Manis_javanica
-----------------------------------MGDGRWLWSFC
>Loxodonta_africana
-----------------------------------MGDGRWLWSFC
>Marmota_marmota_marmota
-----------------------------------MGDGRWLWSFC
>Trichechus_manatus_latirostr
-----------------------------------MGDGRGLWSFC
>Orycteropus_afer_afer
-----------------------------------MGDGRWLWSFC

HG0544 ENSG00000136802 LRRC8A

>Homo_sapiens

---MEQKECQDPAQADAGQPQHRQPTRR-

>Panthera_tigris

---MEREECQDPAQTDVGQPQHRQPARR-

>Orcinus_orca

---MEQKECQDPAQTDVGQPQHRQPTRR-

>Rousettus_aegyptiacus

---MERKECQDPAQADVGQPRHRQPTRR-

>Dasypus_novemcinctus

---MEQRECQDPAQTDVGQPQHRHLTRR-

>Galeopterus_variegatus

---MEQKECQDPVQTDVSQPQHRQPTCR-

>Erinaceus_europaeus

---MEHKECQDPAQTDVGQPQHQQPTRR-

>Ochotona_princeps

MLSGAVRDKQKEAPGERGGGRRDGAAGARAACWAERCGKPKQAVSMEQQECQDPAQTDVGQPQPRQPTRR-

>Ceratotherium_simum_simum

---MEREECQDPGQTDVGQPQRRQQTRR-

>Manis_javanica

---MERKECQDPAQTDMGQPQHQEPAHR-

>Loxodonta_africana

---MDQRECEDPAQTDVGHPQHRQLTPRR

>Microtus_ochrogaster

---MEQKECQDPVHPDRDQPQHQQPTRR-

>Tupaia_chinensis

---MEQKECQDPAQADVGQPRHRQPTRR-

>Orycteropus_afer_afer

---MERGECQDLAQTDVGLPQHHQPTCR-

HG0545 ENSG00000142784 WDTC1

>Homo_sapiens
MCILWTWAWLECSGVLKILL
>Acinonyx_jubatus
MCVLWTWAWLECSRVLKILL
>Balaenoptera_acutorostrata_s
MCVLWTWAWLECSRVLKILL
>Pteropus_alecto
MCVVWTWAWLECSRVLKTLL
>Dasypus_novemcinctus
MCVLWTWALLECSRVLKILL
>Galeopterus_variegatus
MCILWTWAWLECSRVLKILL
>Erinaceus_europaeus
MCVLWTWAWLECSRVLKILL
>Oryctolagus_cuniculus
MCVLWTWAWLECSRVLKILL
>Ceratotherium_simum_simum
MCVLWTWAWLECSRVLKILL
>Manis_javanica
MCVLWTWAWLECSRVLKIPL
>Loxodonta_africana
MYFLWTWAWLECTRVLKILL
>Chinchilla_lanigera
MCILWTWAWLECASVLKILL
>Tupaia_chinensis
MCALWTCTWLECSRVLKILR
>Trichechus_manatus_latirostr
MCVLWTWAWLECTKVLKILL

HG0546 ENSG00000148143 ZNF462

>Homo_sapiens
MRLPNNFHNNNPSRKRRKRKRIRRRWGWRTRSTSRRRDASSVPNVRG
>Mustela_putorius_furo
MRLPNNFHNNNPSRKRRKRKRIRRRWGWRTRNTSRRRDASSVPNVRR
>Physeter_catodon
MRLPNNFHNNNPSRKRRKRKRIRRRWGWRTRNTSRRRDASSVPNVRE
>Pteropus_alecto
MRLPNNFHNNNPSRKRRKRKRIRRRWGWRTRNTSRRRDASSVPNVRG
>Dasypus_novemcinctus
MRLPNNFHNNNPSRKRRKRKRIRRRRSRRTRNTSRRRDASSVPNVRG
>Condylura_cristata
MRLPNNFHNNNPSRKRRKRKRIRRRWGWRTRNTSRRRDASSVPDVRG
>Oryctolagus_cuniculus
MRLPNNFHNNNPSRKRRKRKRIRRRWGWRTRSTFRRRDASSVPNVRG
>Elephantulus_edwardii
MRLQNNFHNNNPSRKRRKRKRIRRRWGWGSRDTSRRRDASSVPNVKG
>Equus_asinus
MRLPNNFHNNNPSRKRRKRKRIRRRWGWRTRNTSRRRDASSVPNVRG
>Manis_javanica
MRLPNNFHNNNPSRKRRKRKRIRRRWDRRTRNTSRRRDASSVPNVRE
>Loxodonta_africana
MRLPNNFHNNNPSRKRRKRKRIRRRWGWRTRNTSRRRDA-SVPNVRG
>Octodon_degus
MRLPNNFHNNNPSRKRRKRKRIRRRWGWRTRSTSRRRDASSVPNVRG
>Tupaia_chinensis
MRLPNNFHNNNPSRKRRKRKRIRRRWGWRTRSTSRRRDAPSVPNVRG
>Trichechus_manatus_latirostr
MRLLNNFHNNNPSRKRRKRKRIRRRWGWRTRKHLPAPGRFFCS----

HG0547 ENSG00000149596 JPH2

>Homo_sapiens
MALTSRPGADLAPGLFQPLL-ASCFPPPGVQW------
>Panthera_tigris
MALTSRPSADLAPGLIQSLV-ASCFPPPGAQW------
>Orcinus_orca
MALTSRPSADLAPGFLQPRV-ASCLPPPGAQ-------
>Pteropus_alecto
MALTSRPSADLAPGLNQPLV-ASCFPPAGARWW-----
>Dasypus_novemcinctus
MALSSRPGAGLA-GPSRPLL-AARSPPPGARW------
>Galeopterus_variegatus
MALTSHPSADLAPGLFPPLL-APCFPPPGVRW------
>Oryctolagus_cuniculus
MALPSPPGTDLAAGLLQPLL-ASRSPCCGARW------
>Equus_przewalskii
MALTSRPSADLAPGLLQPLL-ASRVPPPGALWQW----
>Manis_javanica
MALTGPSGADLARGLLPL-R-ASCCPVLGHGGELGAQP
>Loxodonta_africana
MALTSWPSADLAPGLSQPLVAAG-CPPPGAPW------
>Cavia_porcellus
MALTSTPNADLAPGLFQPLL-ASCFPPPGARW------
>Tupaia_chinensis
MALTSPPSAHLAPGLFQPLR-AACFPPPGARW------
>Trichechus_manatus_latirostr
MALTSRPSADLAPGLSQPLVAARCCPPPGAPW------
>Orycteropus_afer_afer
MALTSWPSADLAPGFSQPLLVAR-CPPPRARW------

HG0548 ENSG00000155744 FAM126B

>Homo_sapiens
--MNSPLELCRIPVPPPPLEN
>Canis_lupus_familiaris
----------------------------------MLPLEE------TGPRV--PGTSWHIFWELLSWKIFKSQQMNSTLELCRIPVPPPPLEN
>Orcinus_orca
--MNSTLELCRIPVPPPPLEH
>Myotis_lucifugus
--MNSTLELCRIPVPPPPLEN
>Galeopterus_variegatus
--MNSILELCCIPVPPPPLEN
>Erinaceus_europaeus
--MNSTLDFCCIPVPPPPLEN
>Ochotona_princeps
--MNSTLELCLIPVPPPPLEN
>Elephantulus_edwardii
--MSQQMNSTREICHIPVPPPPLEN
>Ceratotherium_simum_simum
--MNSTLELCRIPVPPPPLEN
>Manis_javanica
--MNSTLELCCIPVPPPPLEN
>Loxodonta_africana
--MNSTLEICHIPVPPPPLEN
>Mus_musculus
--MNSTLELCRIPVPPPPLEN
>Tupaia_chinensis
MFSLHGDRNPKTLHRGGRAAAVFQPMAEAVVESVDLPNERVACYAAVGRLLREAVAVGSRRQRGDSWKIFNPQQMNSALELCCIPVPPPPLEN
>Trichechus_manatus_latirostr
--MNSTLETCRIPVPPPPLEN

HG0549 ENSG00000156256 USP16

>Homo_sapiens
-MQLWALSPWIVILCQ-
>Acinonyx_jubatus
-MQLWALSPWIVILCQ-
>Balaenoptera_acutorostrata_s
-MQLWALSPWIVILCQ-
>Pteropus_alecto
-MQLWVLSPWIVILGQ-
>Dasypus_novemcinctus
-MQLWALSPWIIILCQ-
>Condylura_cristata
-MQLWALSPWIVILCQ-
>Oryctolagus_cuniculus
-MQLWVLSPWIVFCVSK
>Ceratotherium_simum_simum
-MQLWALSLWMVILCQ-
>Manis_javanica
-MQLWVLSPWIVILCQ-
>Loxodonta_africana
-MQLWALSPWIVILCH-
>Heterocephalus_glaber
MMQLWALSPWIVILCQ-
>Tupaia_chinensis
-MQLWALPPWIVILCQ-
>Trichechus_manatus_latirostr
-MQLWALSPWIVILCQ-
>Orycteropus_afer_afer
-MQLWSLLPWIVTLCH-

HG0550.1 ENSG00000156650 KAT6B

>Homo_sapiens
--------------------MTFKCASSVKYKENLYG
>Canis_lupus_familiaris
--------------------MTFKCSSSVKYKENLYG
>Lipotes_vexillifer
--------------------MTFKCASSVKYKENLYG
>Myotis_brandtii
MFVRCCLICFGVLCPFVELLMTFKCASSVKYKENLYG
>Galeopterus_variegatus
--------------------MTFKCASSVKYKENLYG
>Sorex_araneus
--------------------MTFKCASSVKYKENLYG
>Oryctolagus_cuniculus
--------------------MTFKCASPVKYKENLYG
>Elephantulus_edwardii
--------------------MTFTCVSSVKYKENLYG
>Equus_asinus
--------------------MTFKCASSVKYKENLYG
>Manis_javanica
--------------------MTFKCSSSVKYKENLYG
>Loxodonta_africana
--------------------MTFKCASSVKYKENLYG
>Nannospalax_galili
--------------------MTFKCASSDKYKENLYG
>Tupaia_chinensis
--------------------MTFNCASSVKNKENLYG
>Orycteropus_afer_afer
--------------------MTFKCATSVKYKENLYG

HG0550.2 ENSG00000156650 KAT6B

>Homo_sapiens
MFFTRMQSFLLVK
>Odobenus_rosmarus_divergens
MFFARMQSFLLVK
>Orcinus_orca
MFFTRMQFFLLVK
>Myotis_lucifugus
MFFARMQSFLLVK
>Sorex_araneus
MFFTRMQSFLLVK
>Ceratotherium_simum_simum
MVFTRMQSFVLVR
>Loxodonta_africana
MFFARMQSFLLVK
>Trichechus_manatus_latirostr
MFFARTQSFLLVK
>Orycteropus_afer_afer
MFFARMQSFLLVK

HG0551 ENSG00000162951 LRRTM1

>Homo_sapiens
MSEPALCSCPGREWQAVSAE-----------
>Odobenus_rosmarus_divergens
MSEPALRSRPGREWQAVSAE-----------
>Orcinus_orca
MSEPALRSRPGREWQAVSAEQKVAPVSGPFQ
>Rousettus_aegyptiacus
MSEPALRSRPGREWQVVSAD-----------
>Galeopterus_variegatus
MSEPAPRSCPGREWRAVSAEQKVAPVRGRFQ
>Condylura_cristata
MSEPALRSRPEREWQAVSEK-----------
>Ochotona_princeps
MSEPVLCSCPGREWPAVSAE-----------
>Equus_asinus
MSEPALRSRPGREWQAVSAE-----------
>Manis_javanica
MSEPALRSLPGREWQAVSAE-----------
>Loxodonta_africana
MSEPAPCSRPGREWQAVSAE-----------
>Fukomys_darlingi
MSEPALRSCPGREWQAVSAE-----------
>Tupaia_chinensis
MSEPALRSWPRREWQAVSAESKVARSSGRFQ
>Trichechus_manatus_latirostr
MSEPAPGSRPGREWQAVSAE-----------
>Orycteropus_afer_afer
MSELAPRSRPGREWQAVSVEQKVETVSGLFQ

HG0552 ENSG00000169641 LUZP1

>Homo_sapiens
--MMVSSCQRRLWLPRER
>Acinonyx_jubatus
--MTKLIMVSSCQRRLWMPRER
>Physeter_catodon
---MVSSCQRRLWMPRER
>Rousettus_aegyptiacus
MRLSSASSCHAAMGGVLHYVDISSLSAIKNQWCPASLLRFIHSTNLGTRITYGNSFYTWELKYRFYICCLTCFLLSFCRLIMVSSCQRRLWMPRER
>Dasypus_novemcinctus
---MVSSCQRRLWVPRKM
>Galeopterus_variegatus
---MVSSCQRRLWMPRER
>Condylura_cristata
--MMVSSCQRRLWIPRDR
>Elephantulus_edwardii
---MVSSCQRKLWMPRER
>Ceratotherium_simum_simum
---MVSSCQRRLWMPGER
>Manis_javanica
---MVSSCQRRLWMPRER
>Loxodonta_africana
---MISSCRRRLWMPRER
>Nannospalax_galili
---MVSSCQRRLWIPRER
>Tupaia_chinensis
---MVSSCQRRLWMPRER
>Orycteropus_afer_afer
---MVSSCRRRLWMPRER

HG0553 ENSG00000183475 ASB7

>Homo_sapiens
---MVTRQSVHQEKGHSSIRLIPVT-----
>Leptonychotes_weddellii
MCTMVIRQSTYQEKGHSSIRLFPVT-----
>Physeter_catodon
MCTMVFRQSTYQEKGHSSRKADPCDLMNPL
>Rhinolophus_ferrumequinum
MCTMVIRQSTCQEKGHSSVRLIPVT-----
>Dasypus_novemcinctus
MYTMVIRQSIYQEKGHSSVRLIPVT-----
>Galeopterus_variegatus
MYTMVIRQSIYQEKGHPPIRLIPVT-----
>Condylura_cristata
MCTMVIRQSTCQDEEHLSRRLIPVT-----
>Oryctolagus_cuniculus
MCTMVTRQSTYQERGHSSVRLIPVT-----
>Equus_asinus
MCTMIIRQSIYQEKGHSSVRLIPVT-----
>Manis_javanica
MCTMVIRQSAYQEKEHFSVRLIPVT-----
>Loxodonta_africana
MCTMVIRQHLYQDKGHSIIRLIPVT-----
>Ictidomys_tridecemlineatus
---MVIRQSIHQEKGHSSVRLIPVT-----
>Tupaia_chinensis
---MVVRQSTCHEKGHAIVRPFPVT-----
>Orycteropus_afer_afer
---MVIRQSLYQEKGHSKV--EPCDLMNPW

HG0554 ENSG00000196914 ARHGEF12

>Homo_sapiens
---MTEWSFELDFCVPDGVGPDPRALGVGRRCYCKMQVG
>Felis_catus
---MTEWSFELDFCVPYGVGPDPRALGVGWRCYCKMQVG
>Physeter_catodon
---MTEWSFELDSCVPYGVGPDPGALGVGWRCYCKMQVG
>Rousettus_aegyptiacus
---MTEWSFELNFSSLSESGLIPELWGVGWRCYCKMQVG
>Galeopterus_variegatus
---MTEWSFELDFCVPDEVGPDLSALGVGRRCYCKMQVG
>Condylura_cristata
---MTEWSFELDFCVPYGVGPDPRARGVGWRCSCKMQVG
>Oryctolagus_cuniculus
---MTAWSVELDFCVPDGVGPDPRALGVGWRCYCKMQVG
>Elephantulus_edwardii
---MTEWSFELDFCVLYGIGPDPRALGVGWRCYWKMQVG
>Ceratotherium_simum_simum
---MTAWSFELDFCVPYGVGPDPRALGVGWRCSCKMQVG
>Manis_javanica
---MTEWSFEVDFCVPYGVGLDPRALGVGWGCYCKMQVG
>Loxodonta_africana
---MTEWSFELDFCVPYGVGPDPRALGVGWRCYCKMQVG
>Chinchilla_lanigera
---MTEWSFELDFCVPAGIGPDPRALGVGWRCFCKMQVG
>Tupaia_chinensis
MFWMTEWSFELDFCVPDGVGPDPRALGVGWRCSCKMQVG
>Trichechus_manatus_latirostr
---MTEWSFELDFCV----GPDPSALGVGWRCYCKMQVG

HG0555 ENSG00000204310 AGPAT1

>Homo_sapiens
MGPTSHSHLPSIGRG
>Ursus_maritimus
MGPASHSHLPSIGRG
>Orcinus_orca
MGPASHSHLPSIGRG
>Myotis_lucifugus
MGPASHSHLPSIGRG
>Dasypus_novemcinctus
MGPASHSHLPSSGRG
>Galeopterus_variegatus
MGPTSHSHLPSIGRG
>Erinaceus_europaeus
MGPASHSHLPSIGRG
>Oryctolagus_cuniculus
MGPASHAHLPSIGRG
>Equus_caballus
MGPASHSHLPSIGRG
>Manis_javanica
MGPASQSHLPYIGRG
>Loxodonta_africana
MGPASHSHLPSIGRG
>Marmota_marmota_marmota
MGPTSHSHLPSIGRG
>Trichechus_manatus_latirostr
MGPASHSHLPSIGRG
>Orycteropus_afer_afer
MGPASHSHLLSTGRG

HG0556 ENSG00000273841 TAF9

>Homo_sapiens
MLLPNILLTGTPGVGKTTLGKELASKSGLKYINVGDLAREV
>Felis_catus
MLLPNILLTGTPGVGKTTLGKELASRSGLKYINVGDLAREV
>Orcinus_orca
MLLPNILLTGTPGVGKTTLGKELASRSGLKYVNVGDLAREV
>Pteropus_vampyrus
MLLPNILLTGTPGVGKTTLGKELASRSGLKYINVGDLAREV
>Dasypus_novemcinctus
MLQPNILLTGTPGVGKTTLGKELASRSGLKYVNVGDLAKEV
>Galeopterus_variegatus
MLLSNILLTGTPGVGKTTLGKELTARSGLKYFNVGDLAQEV
>Sorex_araneus
MLLPNILLTGTPGVGKTTLGKELASRSGLKYVNVGDLAREV
>Ochotona_princeps
MLRPNILLTGTPGVGKTTLGKELASRSGLKYVNVGDLAREV
>Elephantulus_edwardii
MLLPNILLTGTPGVGKTTLGKELASRSGLKYINVGDLAKEV
>Equus_przewalskii
MLLPNILLTGTPGVGKTTLGKELASRSGLKYINVGDLAREV
>Loxodonta_africana
MLQPNILLTGTPGVGKTTLGKELASRSGLKYVNVGDLAKEV
>Marmota_marmota_marmota
MLLPNILLTGTPGVGKTTLGKELASRSGLKYINVGDLAQEV
>Trichechus_manatus_latirostr
MLQPNILLTGTPGVGKTTLGKELASRSGLKYINVGDLAKEV
>Orycteropus_afer_afer
MLRPNILLTGTPGVGKTTLGKELASRSGLKYINVGDLAKEV

HG0557 ENSG00000012232 EXTL3

>Homo_sapiens
MHICWSAKPRNKSYGI
>Ailuropoda_melanoleuca
MHICWSAKPRNKSYGS
>Balaenoptera_acutorostrata_s
MHMCWSAKPRNESYGS
>Eptesicus_fuscus
MHICWSAKPRNKSYGC
>Galeopterus_variegatus
MHTCWSAEPRNKSYGR
>Condylura_cristata
MHVCWPAKRRSKSHGR
>Equus_asinus
MHICWSAKPRNKSYGS
>Manis_javanica
MHTCLSAKPRNKSYGS
>Loxodonta_africana
MHNCWPAKPRNKSYGT
>Ictidomys_tridecemlineatus
MHICWSAKPRNKSYGL
>Tupaia_chinensis
MNICWSAKPRNKSYGS
>Trichechus_manatus_latirostr
MHICWPAKPRNKSYGT
>Orycteropus_afer_afer
MHLCWPAKPRNKSDGS

HG0558 ENSG00000029363 BCLAF1

>Homo_sapiens

MVFLAFLSREMAAVWLQRR

>Canis_lupus_familiaris

MVFLPFLSREMAAV-----

>Balaenoptera_acutorostrata_s

MVFLPCLSREMAAV-----

>Miniopterus_natalensis

MVFLPFLSRVMAAV-----

>Dasypus_novemcinctus

MVFLPFLSREMAAV-----

>Galeopterus_variegatus

MVFLSFLSREMAAVWLQRR

>Condylura_cristata

MVFLPFLSREMAAV-----

>Ochotona_princeps

MVFLSFSSREMAAV-----

>Ceratotherium_simum_simum

MVFLPFSSREMAAV-----

>Manis_javanica

MVFLPFLSREMAAV-----

>Loxodonta_africana

MVFLSVLSREMAAV-----

>Chinchilla_lanigera

MVFLSFLSREMAAV-----

>Tupaia_chinensis

MVFLSFLSREMAAV-----

HG0559 ENSG00000078747 ITCH

>Homo_sapiens
MHFTVALWRQRLNPRK
>Panthera_tigris
MHLTVAFWRQRLNPKK
>Balaenoptera_acutorostrata_s
MHLTVAFWRQRLNPKK
>Miniopterus_natalensis
MHLTVAFWRQRLNPKK
>Dasypus_novemcinctus
MHLTVAFWRQRFNPKK
>Galeopterus_variegatus
MHFTVAFWRQRLNPKK
>Condylura_cristata
MHLTVAFWRQRLNPKK
>Oryctolagus_cuniculus
MPFTVAFWRQRLNQKK
>Equus_asinus
MHLTVAFWRQRLNPKK
>Loxodonta_africana
MHVTVAFWRQRLNPKK
>Dipodomys_ordii
MHFTVAFWRQRLNPKK
>Tupaia_chinensis
MHFTVAFWRQRLNPKK
>Trichechus_manatus_latirostr
MHLTVAFWRQHLNPKK

HG0560.1 ENSG00000092051 JPH4

>Homo_sapiens

MDAVPGRQPEPQPTCHSG

>Leptonychotes_weddellii

MDAVPGRQPEPQPTCHSG

>Balaenoptera_acutorostrata_s

MDAVPGRQPEPQSTCHSG

>Rousettus_aegyptiacus

MDAVPGRQPEPQPTCHSG

>Dasypus_novemcinctus

MDAVPGKQPEPQPTCHSG

>Galeopterus_variegatus

MDAVPERQPEPQTTCHSG

>Condylura_cristata

MDAVPGRQPEPQPTCHSG

>Ochotona_princeps

MDVEPGRRPAPQPTCHSG

>Elephantulus_edwardii

MDAVPGRQPEPQPTRHS-

>Ceratotherium_simum_simum

MDAVPGRQPEPQPTCHSG

>Manis_javanica

MDDLPGKRLEPQPTCHSG

>Dipodomys_ordii

MDAVPGRQPEPQPTCHSG

>Orycteropus_afer_afer

MDVILRRQPEPQPTCHSG

>Homo_sapiens

---MEQTLICAGMESEF
>Mustela_putorius_furo
MGLPPPSFSSGISPSLALTPAPLSLYSSRPFLASPASLLPRLASASPSLPIFSSVSSLSLCSPPLSPPPSVSPHSLPAYQDFFPPWGSLCFSFSPLPASSLTSDLA
GTHLFQPGNPPPQLLTSPLYLLPLLLSPPSPLLSSPCPFKSSWPSLPRDAGMEQTLICAGMESEF
>Orcinus_orca

---MEQTLICAGMESEF
>Myotis_lucifugus

---MEQTLICAGMESEF
>Dasypus_novemcinctus

---MEQTLICAGMESEF
>Galeopterus_variegatus

---MEQTLICAGMESEF
>Erinaceus_europaeus

---MEQTLICAGMESEF
>Oryctolagus_cuniculus

---MEQTLICARMESEF
>Elephantulus_edwardii

---MEQTLICAGMESEF
>Ceratotherium_simum_simum

---MEQTLICAGMESEF
>Manis_javanica

---MEQTLICAGMESEF
>Dipodomys_ordii
--MLVF--LSTLGVSVPLFLLPTCLIPH----------------------PG--VS----
-------KSPSSSTWTSPLHPHHRHRLSSPLLLAPLCPFKSAWPSLPRDAGMEQTLICAGMESEF

HG0561 ENSG00000100393 EP300

>Homo_sapiens
MRRRRTAPRRKRLMAAAELRETSAGQGPAVAGRGLRL
>Mustela_putorius_furo
MRRRRTAPRRKRLMAAAELRETSAGQGPAVAGRGLRL
>Physeter_catodon
MRRRRTAPRRKKLMAAAELRETSAGQGPAVAGRGLRL
>Rousettus_aegyptiacus
MRRRRTAPRRKRLMAAAELRETSAGQGPAVADRGLRL
>Dasypus_novemcinctus
MRRRRTAPRRKRLMAAAELRETSAGQGPAVAGRGLRL
>Erinaceus_europaeus
MRRRRTAPRRKRLMAAAELRETSAGQGPAVAGRGLRL
>Oryctolagus_cuniculus
MRRRRTAPRRKRLMAAAELRETSAGQGPAAAGRGLRL
>Equus_asinus
MRRRRTAPRRKRLMAAAELRETSAGQGPAVADRGLRL
>Manis_javanica
MRRRRTAPRRKRLMAAAELRETSAGQGPAVAGRGLRL
>Loxodonta_africana
MRRRRTAPRRKRLMAA-ALRETSAGQGPAVAGRGLRL
>Octodon_degus
-------------MAAAELRETSAGQGPAVAGRGQRF
>Trichechus_manatus_latirostr
MRRRRTAPRRKRLMAA-ALRETSAGQGPAVAGRGLRL
>Orycteropus_afer_afer
MRRRRTAPRRKRLMAA-ALRETSAGQEPAVAGRGLHL

HG0562 ENSG00000105821 DNAJC2

>Homo_sapiens
-----------------------MRRFLGVLEPRREARSER-------------
>Leptonychotes_weddellii
-----------------------MRRFLGVVEPRREARTER-------------
>Physeter_catodon
-----------------------MRRFLDIVEPRREARTGR-------------
>Rousettus_aegyptiacus
-----------------------MRRFLGVVDPRREARTGR-------------
>Galeopterus_variegatus
-----------------------MRRFLGVVERRREARTGRQSRRGR-------
>Oryctolagus_cuniculus
-----------------------MRRFVGVVEPRREARTGR-------------
>Elephantulus_edwardii
-----------------------MRRFLGVE-QRREARSGG-------------
>Ceratotherium_simum_simum
-----------------------MRRFLGVLEPRREARTGR-------------
>Manis_javanica
-----------------------MRRFLGAVESRREARTGR-------------
>Loxodonta_africana
-----------------------MRRFLGVVEPRREARTEGTAGGNAKHPPPGP
>Ictidomys_tridecemlineatus
MSGGGIGVHTTKDPSRKCLLLVSTRRFLGVVEPRREARTGR-------------
>Tupaia_chinensis
-----------------------MRRVLGDVEPRREAQKER-------------
>Orycteropus_afer_afer
-----------------------MRRFLGVVEPRREARTGR-------------

HG0563 ENSG00000107249 GLIS3

>Homo_sapiens
---------------------MDDSIRLITHTMDNLLDFA
>Leptonychotes_weddellii
--------------------------------MDNLLDFA
>Lipotes_vexillifer
---------------------MDDSIRLINHTMDNLLDFA
>Rousettus_aegyptiacus
--------------------------------MDNLLDFA
>Dasypus_novemcinctus
--------------------------------MDNLLDFA
>Galeopterus_variegatus
MLHRLKTHCTLFLFLLLKMPAFIFLNRLRTYTMDNLLDFA
>Sorex_araneus
--------------------------------MDNLLDFA
>Ochotona_princeps
--------------------------------MDNLLDLA
>Ceratotherium_simum_simum
--------------------------------MDNLLDFA
>Loxodonta_africana
--------------------------------MDNVLDLA
>Chinchilla_lanigera
--------------------------------MDNLLDFA
>Tupaia_chinensis
--------------------------------MDNLLDFA
>Trichechus_manatus_latirostr
--------------------------------MDNVLDLA

HG0564 ENSG00000109171 SLAIN2

>Homo_sapiens
--MAAGAGYWRRLPQSRSRRR
>Odobenus_rosmarus_divergens
--MAAKAGYWRRLPQSWSRRR
>Balaenoptera_acutorostrata_s
--MAVEAGYWRRLPQSWSRRR
>Pteropus_alecto
--MAAEAGYWRRLPQSRSRRR
>Dasypus_novemcinctus
--MAAEAGYWRRLPQSGSRRR
>Galeopterus_variegatus
--MAAEAGCWRRLPQSRSRRR
>Condylura_cristata
--MAAEAGYWRRLPQSWSRRR
>Oryctolagus_cuniculus
--MAAEAGYWRRLPQSWSRRR
>Elephantulus_edwardii
--MAAEAGYWHRLPKSGSRRR
>Ceratotherium_simum_simum
--MAAEAGYWRRLPQSWSRRR
>Ictidomys_tridecemlineatus
--MAAEAGYWRRLPQSRSRRR
>Trichechus_manatus_latirostr
--MAAEAGYWRRLPQSWSRRR
>Orycteropus_afer_afer
MTPPTAPPGSELSARFSQCHPGAVSTGDALRSPTGPNSRGRRDGGKMAAEAGYWRRLPQSGSRRR

HG0565 ENSG00000120533 ENY2

>Homo_sapiens
MRVLAFCVLRCPSY
>Leptonychotes_weddellii
MRVLAFCVLRCPSY
>Lipotes_vexillifer
MRVLAFCVLRCPSY
>Rousettus_aegyptiacus
MRVLAFCVLRCPSY
>Dasypus_novemcinctus
MRVLAFCVLRCPSY
>Condylura_cristata
MRVLAFCVLRCPSY
>Ochotona_princeps
MRVLAFCVLRCPSY
>Elephantulus_edwardii
MRVLALYVLRCPSY
>Equus_asinus
MRVLAFCVLRCPSY
>Fukomys_damarensis
MRVLAFCVLRCPSY
>Tupaia_chinensis
MRVLAFCVLRCPSY
>Trichechus_manatus_latirostr
MRVLAFCVLRCPSY
>Orycteropus_afer_afer
MRVLAFCVLRCPSY

HG0566 ENSG00000124496 TRERF1

>Homo_sapiens
MHLLQKQPRPWLWRMLKEEDAEAGRP------------------------
>Canis_lupus_familiaris
MHLLQKQPRPWLWRMLKEEDMEAGRP------------------------
>Balaenoptera_acutorostrata_s
MHLLQKQPRPRLWRMLKEEDTEAGRP------------------------
>Pteropus_alecto
MHLLQKQPRPWLWRMLKEEDMEAGRP------------------------
>Dasypus_novemcinctus
MHLLQRQPRPWLWRMLEEEDTEAVGPERLSSRS-----------------
>Condylura_cristata
MHLLQKQPRPWLSRMLKEEDTEARRP------------------------
>Oryctolagus_cuniculus
MHLLQKQPRPWLRRPLKEEDEEAGRL------------------------
>Ceratotherium_simum_simum
MHLLQKQPRPWLWRMLKEEDTEAARP------------------------
>Manis_javanica
MHLLQKQPRPRLWRTLKEEDTEAGRP------------------------
>Loxodonta_africana
MHLLLTQPRPWFWRMLKRKTRKQDSPEILSFLTLKQNQVSSSHLSSSMRAA
>Marmota_marmota_marmota
MHLLQKQPRPWLWRMLKEEDAEAGRP------------------------
>Tupaia_chinensis
MHLLQKQPRPWLWRMLKEEDTEAGQP------------------------
>Trichechus_manatus_latirostr
MHLLLRQPRRCLWRMLKEEDPEAGRS------------------------

HG0567 ENSG00000132155 RAF1

>Homo_sapiens

-MRGLLGAPSPGLGTRRM

>Canis_lupus_familiaris

-MRGLPGAPSPGLGTRRM

>Physeter_catodon

-MRGLPGAPSPGLGTRRM

>Rousettus_aegyptiacus

-MRGLPGAPSPGLGTRRM

>Dasypus_novemcinctus

-MRGLPGVPSPGLGTRRM

>Galeopterus_variegatus

MMRGLPGAPSPGLGTRRM

>Condylura_cristata

-MRGLPGAPSPGLGTRRM

>Ochotona_princeps

-MRGLPGAPSPGLGTRRM

>Ceratotherium_simum_simum

-MRGLPGAPSPGLGTRRM

>Manis_javanica

-MLGLPGAPSPGLGTRRM

>Nannospalax_galili

-MRGLLGAPSPGLGTRRM

>Trichechus_manatus_latirostr

-MRGLPGAPSPGLGTRRM

>Orycteropus_afer_afer

-MRWLLGAPSPGLGTRRM

HG0568.1 ENSG00000138271 GPR87

>Homo_sapiens

MKEIKP--GITYAEPTPQSSPSVS

>Canis_lupus_familiaris

MKGLKP--GITYAKPFPQPSPSVS

>Physeter_catodon

MKEIKP--GITYAEPLPQSSPSVS

>Pteropus_alecto

MKEIKP--GITYAEPLPQPSPSVS

>Galeopterus_variegatus

MKEIKP--GIAYAELMPQPSPSVS

>Condylura_cristata

MKESKPTNKTTYAEPMRQSSPSVS

>Oryctolagus_cuniculus

MKEIKP--GIPYAEPMPPSSPSVS

>Equus_asinus

MKEIKP--GTTYAGPLPQSSPSVS

>Manis_javanica

MKEIKP--GITYAEPLPQSSPSVS

>Heterocephalus_glaber

MKEIKP--GLTHAEPMPQSSPSVS

>Tupaia_chinensis

MKEIKP--GITYAKPMPELSPSVS

>Trichechus_manatus_latirostr

MKEIKQ--GITYAEPKLQLSPSVS

>Orycteropus_afer_afer

MKEIKQ--GITYAEPKLQLSPSVS

HG0568.2 ENSG00000138271 GPR87

>Homo_sapiens

MFRFHALPENPLPCRP

>Canis_lupus_familiaris

MLIFHAVPENPLLCQP

>Miniopterus_natalensis

MFIFHALPENPHPCQP

>Ceratotherium_simum_simum

MFLFHALPESPLLCQP

>Manis_javanica

MLIFHAFPENPLPCQP

>Tupaia_chinensis

MILFSALPENPLSCQP

HG0569 ENSG00000139651 ZNF740

>Homo_sapiens
--MATGLMGHEGVATVIW
>Acinonyx_jubatus
--MAAGLKGHEGVVTVIW
>Orcinus_orca
--MAAGLKGHEGAVTVIW
>Myotis_brandtii
--MAAGLKGHEGVVTVIW
>Galeopterus_variegatus
--MAAGLKGHEGVITVTW
>Erinaceus_europaeus
MKQTSWTSVREFGQKTKTGRLFLLTIDGSQAEPKDSCGIYLVFFFFLTQRKFNMMAAGLKGHAGVVTVIW
>Elephantulus_edwardii
--MAAGLKGHKGVVTVIW
>Ceratotherium_simum_simum
--MAAGLRGHEGVVTVIW
>Manis_javanica
--MAAELKGHKEVVTVIW
>Loxodonta_africana
--MAAGLKGHKGVVTVIW
>Marmota_marmota_marmota
--MAAGLRGHEGAITVIW
>Trichechus_manatus_latirostr
--MAAGLKGHRGVITVIW
>Orycteropus_afer_afer
--MAAGLKGHRGVVTVIW

HG0570 ENSG00000152214 RIT2

>Homo_sapiens
MRPARAEDQQPARR---------------
>Ursus_maritimus
MRPARAEDQQPARR---------------
>Rousettus_aegyptiacus
MRPARAQDQQPARR---------------
>Dasypus_novemcinctus
MRPAPAEDQQPARR---------------
>Condylura_cristata
MRPAGAEDQQPARR---------------
>Ochotona_princeps
MRPAGAEDQQPAGH---------------
>Elephantulus_edwardii
MRPAQAEDQQPAWELMLLPACQTFLLPQF
>Equus_asinus
MRPAGAEDQQPARR---------------
>Loxodonta_africana
MRPAGAEDQQPARR---------------
>Octodon_degus
MRPAPAQDQQPARR---------------
>Tupaia_chinensis
MRPARAEDQQPARC---------------
>Trichechus_manatus_latirostr
MRPAGAEDQQLARR---------------
>Orycteropus_afer_afer
MRSAGAEDQQPAWR---------------

HG0571 ENSG00000153266 FEZF2

>Homo_sapiens
----------------------------MARNASPWWWGKET
>Canis_lupus_familiaris
----------------------------MARNASPWRWGKET
>Orcinus_orca
----------------------------MARNASPWRWGKET
>Pteropus_alecto
----------------------------MARNASPWRWGKET
>Galeopterus_variegatus
MSAECKRPVISRGGEDVCHQNVTEEIRCMARNASPWWWGKET
>Erinaceus_europaeus
----------------------------MARNASPWRWGKET
>Ochotona_princeps
----------------------------MARNASPWQWGKET
>Equus_asinus
----------------------------MARNASPWRWGKET
>Manis_javanica
----------------------------MARNASPWRWGKET
>Loxodonta_africana
----------------------------MARNASPWRWGKET
>Mesocricetus_auratus
----------------------------MARNASPWRWGKET
>Trichechus_manatus_latirostr
----------------------------MARNASPWRWGKET
>Orycteropus_afer_afer
----------------------------MARNASPWRWGKET

HG0572 ENSG00000158161 EYA3

>Homo_sapiens
MRIRVTLTPRFPPLDWFYCGSGLISMSCCGAFTAFGL--
>Canis_lupus_familiaris
MRIRVTLTPRFPPSAWFSCESGPVPMSCFGASAAVGQ--
>Physeter_catodon
MRIRVTLTPPFPAASWFSCVFGPVPMSCCGASAAFGL--
>Myotis_brandtii
MRFRVTLTPRFPPLAWFYCGSGPVPMSCSGAPAAFGL--
>Dasypus_novemcinctus
-------------------------MSCCGTSAAFGL--
>Galeopterus_variegatus
-------------------------MPYCGASAAFGL--
>Condylura_cristata
MRIRVTLTPRFPPPAWFYCGSGPVPMSCCGASASFGL--
>Oryctolagus_cuniculus
MRFRVTLTPRFPPLARSYRGSGPVPMSCCGSSVALGL--
>Ceratotherium_simum_simum
MCIRVTLTPRFPPPAWFCCGSGPVPMSCCGASGAFGL--
>Manis_javanica
-------------------------MSCCVAFAGFGRAR
>Marmota_marmota_marmota
MRIRVTLTPRFPPLCWFYRGSGPVPMSCCGASAAFGL--
>Tupaia_chinensis
-------------------------MSRCGAPAAFGL--
>Trichechus_manatus_latirostr
MRIRVMLTPPFPPLAWFCCGSGPVLMSCCGASAAFGL--

HG0573.1 ENSG00000162526 TSSK3

>Homo_sapiens

-------------------------MKSLGRKGAGQGRWWSRAASQRQHELRG

>Leptonychotes_weddellii

MALSLPTTRGCCVQTENALALGRLRMKSLGGKGAGQGRWWSRAASQRQHELRG

>Eptesicus_fuscus

-------------------------MKSLGRKGPGQGLWWSRAASQRQHELRG

>Galeopterus_variegatus

-------------------------MKSLGGKEAGQG----------------

>Condylura_cristata

-------------------------MKSLGGKGAGQGRWWSRAASRRQHELRG

>Oryctolagus_cuniculus

MSDPLLPPTRGCVQTENALALGRLWMKSLGGRGAGRGRWCSRAASRRQHALSG

>Elephantulus_edwardii

-------------------------MKSLGGKEAGQGRWWSRAASLRQHELRG

>Ceratotherium_simum_simum

-------------------------MKSLGGKVAGQGQWWSRAASWRQHELRG

>Manis_javanica

-------------------------MKSLGGKSAGQGRWWSRAASQRQHELGV

>Loxodonta_africana

-------------------------MKSLGGKEAGQGRWWSRAASRRQHELRG

>Ictidomys_tridecemlineatus

-------------------------MKSLGGKGAGQGRWWSRAASQRQHELRG

>Trichechus_manatus_latirostr

-------------------------MKSLGGKGAGQGRWWSRAASRRQHELRG

>Orycteropus_afer_afer

-------------------------MKSLGGKGAGQGRWWSRAASWRQHELRG

HG0573.2 ENSG00000206203 TSSK2

>Homo_sapiens

MRTMPAGPHDGGM

>Canis_lupus_familiaris

MRTMPAGPRGEGM

>Miniopterus_natalensis

MRTMPAGPRGKGM

>Dasypus_novemcinctus

MRTMPAGPRGEGM

>Galeopterus_variegatus

MRTMPAGPRGEGM

>Condylura_cristata

MRTMPTGPRGEGM

>Equus_asinus

MRTMPAGPRREGM

>Cricetulus_griseus

MRTMPAGPCGEGM

>Tupaia_chinensis

MRTMPAGPRGEGM

HG0574 ENSG00000164663 USP49

>Homo_sapiens

MEEDGKNLFNGLRKWRLL

>Panthera_tigris

MEEDGKNLFNGLKDWSLL

>Pteropus_vampyrus

---MEKNLFNGLKEWRLL

>Dasypus_novemcinctus

MDEDGKKLFNGLTEWRLL

>Galeopterus_variegatus

MEEDGKNLFNGLRERQLL

>Oryctolagus_cuniculus

MEEDGKNLFNGLRAWQLL

>Elephantulus_edwardii

MEEDGKSLLNGLKEWRLL

>Ceratotherium_simum_simum

MEEDGKNLFNGLKEWRLP

>Manis_javanica

MEEDRKNLFNGLKEWRLL

>Psammomys_obesus

MEEDGNNLFNGLREWRLH

>Tupaia_chinensis

MEEDGKKLLNGVSEWRLL

>Trichechus_manatus_latirostr

---MEKTLFNGLKEWRLL

>Orycteropus_afer_afer

MEEEGKNLFNGLKEWRLL

>Homo_sapiens
--MMAILWMGRDLERTSSLSCNSSQYPT------------
-
>Felis_catus
MRWGVREENRVKGSWRSPAGGRTGLACTGVGGSQMCMLWGDATEGLHGSGHRCCSEGPSVGSLWCRRGGTMILWMGRDLERTSSLSCNFSQYPT------------
-
>Pteropus_vampyrus
--MMAILWMGRNLERTSSLSCNFSQYPT------------
-
>Dasypus_novemcinctus
--MMAILWMGRDLERTSSLSATSVTIPHKSSQVTEKWKSN
Q
>Galeopterus_variegatus
--MGRDLERTSSLSCNFSHYPT------------
-
>Oryctolagus_cuniculus
--MERDLDRTSSLSCNFSQYLT------------
-
>Elephantulus_edwardii
--MMAILWMGRDLERTSTLSCNVSQYPT------------
-
>Ceratotherium_simum_simum
--MGRDLERTSSLSCNFSQYPT------------
-
>Manis_javanica
--MGRDLERTSSLSCNFSQYPT------------
-
>Peromyscus_maniculatus_baird
--MGRDLERTLGLSCNFSQYPT------------
-
>Tupaia_chinensis
--MILWMERGLGRMCSLPCSFSQYPT------------
-
>Trichechus_manatus_latirostr
--MMAILWMGRDLERT----CSFSQYPT------------
-
>Orycteropus_afer_afer
--MMANLWMGRDLERTSSLSCNFSHYPT------------
-

HG0576 ENSG00000175029 CTBP2

>Homo_sapiens
-------------------MKVYSLKKTDSPSCEL
>Ailuropoda_melanoleuca
-------------------MKLESLKKTDSPSCEL
>Lipotes_vexillifer
MPGSLALSGGFIALNGLEPMKLDSLKKTDSPSCEL
>Rhinolophus_ferrumequinum
-------------------MKLNSLQKTDNPSCDL
>Dasypus_novemcinctus
---MYSKINGFIALNGFEPMKLYSLKKTDSTSCEL
>Oryctolagus_cuniculus
-------------------MKLCSLRETDSPSCEL
>Elephantulus_edwardii
-------------------MTLYTLKQTDSSSCEL
>Ceratotherium_simum_simum
-------------------MKLDSLKKTDSPSCEL
>Loxodonta_africana
-------------------MKLHSLKKTDSPSCEL
>Chinchilla_lanigera
-------------------MKLYPLKKTDSPSCEP
>Tupaia_chinensis
-------------------MKLYSLKKTDSRLCEL
>Trichechus_manatus_latirostr
-------------------MKLRSLKKTDSPSSEL
>Orycteropus_afer_afer
-------------------MKLYSLKKTDSPSLEL

HG0577 ENSG00000250510 GPR162

>Homo_sapiens

MLSTGVSLGAACLLTGRGLWGSWECL

>Ursus_maritimus

MLSSRVGLGAACFTIGQGLYASWVCV

>Lipotes_vexillifer

MLSTRVGLGAACLTPGQGLWASWGCV

>Myotis_brandtii

MLSIRVGRGAACLTTGQGLWASWRCV

>Dasypus_novemcinctus

MLSSGAGQGAAHLTPGPGLWAAWGCA

>Galeopterus_variegatus

MLSSRVGLGAAHLTTGQGLWAPWGRV

>Oryctolagus_cuniculus

MLSSRAGLGPAGLSTGQAPWASWGCA

>Elephantulus_edwardii

MLSSRVGLEAACLTTGQGWWAPWGCV

>Equus_asinus

MLSTRVGLGAACLTTGQGLWASWGCV

>Manis_javanica

MLSSRIGLGAVCLTTGQGLWASWRCV

>Mus_musculus

MLRSKQDLGSACLATRQGLWASWGCV

>Trichechus_manatus_latirostr

MLSSGVGLGAACLTTGQGLWASWGCV

>Orycteropus_afer_afer

MLSSEGGLGAACLTTGQELWASWGCV

HG0578 ENSG00000004897 CDC27

>Homo_sapiens
MSRGWPGRSRYRGGLRHCRKWA
>Ailuropoda_melanoleuca
MSRGWPGRSRYGGGLRHCRKWA
>Orcinus_orca
MSRGWPGRSRYGGGLRHCRKWA
>Rousettus_aegyptiacus
MSRGWPGRSRYGGGLRHCRKWA
>Dasypus_novemcinctus
MSRDWPGRSRYGGGLRHCRKWA
>Condylura_cristata
MSRGWPGRSRYGGGLRHCRKWA
>Oryctolagus_cuniculus
MSRGWPGRSRYGGGA-------
>Equus_asinus
MSRGWPGRSRYGGGLRHCRKWA
>Ictidomys_tridecemlineatus
MSRGWPGRSRYGGGLRHCRKWA
>Tupaia_chinensis
MSRGWPGRSRYGGCLRHCRKWA
>Trichechus_manatus_latirostr
MSRGWPGWSRYGGGLRHCRKWA
>Orycteropus_afer_afer
MSRGWPSRSRYGGGA-------

HG0579 ENSG00000010818 HIVEP2

>Homo_sapiens

MPIDCGVRVEGSKEKEQSASLQTPWNMEATKL

>Felis_catus

--------------------------MEANKL

>Balaenoptera_acutorostrata_s

MPIDYGVGVEGSKEKEHSASLQTPWNMEASKL

>Myotis_lucifugus

--------------------------MEASKL

>Galeopterus_variegatus

MTIDYGVGVEGSREKEQSASLQTPWNMEATKL

>Ochotona_princeps

--------------------------MEAAKL

>Ceratotherium_simum_simum

--------------------------MEASKL

>Manis_javanica

MPRDYRVDVEGSKEQEHSASLQTPWTMETSRL

>Loxodonta_africana

--------------------------MEATKL

>Nannospalax_galili

MPIDYRVGVEGSKEKEQSANSQTSWNMEATQL

>Tupaia_chinensis

MPIDCGEGVGGSKEKEQSAGLQTPWNMEATKL

>Orycteropus_afer_afer

MPVDCGEGVEGSKEKEKS-SLQTSGNMEATKL

HG0580 ENSG00000073711 PPP2R3A

>Homo_sapiens
-------------------MCSSYCILIVTKPQI--------------------------
>Acinonyx_jubatus
-------------------MCSSYCILTVTKPQI--------------------------
>Balaenoptera_acutorostrata_s
-------------------MCNGYCILTVTKPQILMISLCGKGHYIYKKPLNIVIKYIFS
>Rhinolophus_ferrumequinum
-------------------MCNSYCILTVTKPQI--------------------------
>Galeopterus_variegatus
-------------------MCSIYCILTVTKPQI--------------------------
>Erinaceus_europaeus
MIKLKRSVVKLLYLLTQADLCSSCCALTVSKPQI--------------------------
>Equus_caballus
-------------------MCNSYCILTVNKPQI--------------------------
>Manis_javanica
-------------------MCNSYCILTVTKPQI--------------------------
>Mesocricetus_auratus
-------------------MCSSYCILIVTKPQI--------------------------
>Tupaia_chinensis
-------------------MCSNYCILIVTKPQI--------------------------
>Trichechus_manatus_latirostr
-------------------MCNSYCILTVTHPQI--------------------------
>Orycteropus_afer_afer
-------------------MCNSYCIMTVTQPQI--------------------------

HG0581 ENSG00000087074 PPP1R15A

>Homo_sapiens
--MNALASLTVR--TCDRFWQTEPALLPPG
>Canis_lupus_familiaris
--MNALASRAVL--SCDRWWQPEPALLPPG
>Orcinus_orca
--MNALASRAVR--TCDRLWQPQPALLPPG
>Rhinolophus_ferrumequinum
--MKALASRAVR--TCDRLWQPEPALLPPG
>Dasypus_novemcinctus
MDYKSLAVCVLAQRLFSSASLFFSVAPPDPARVCRHRACGSWRSPSPTPEREMKAPASRAAR--TCDRSWQPAPALLPPG
>Galeopterus_variegatus
--MNALASRAVR--TCDRFWQPEPALLPPG
>Erinaceus_europaeus
--MTAPAPRTVRSPTRDRLWQPEPALLPPG
>Oryctolagus_cuniculus
--MNALASRAVR--TYDRFWQPEPALLPPG
>Equus_caballus
--MNALASRAVR--TCDRLWKTEPALLPPG
>Loxodonta_africana
--MNAPDSRTAR--TYDRFWLREPALLSPG
>Ctenomys_sociabilis
--MNALASRTVR--TCDRFWQPEPALLPPG
>Tupaia_chinensis
--MNTLDSRAVR--TCDRFWKPEPALLSPG

HG0582 ENSG00000087338 GMCL1

>Homo_sapiens

METV-ASAATVGAAREKVAV

>Felis_catus

MEAV-ASAATVGAAREKVAV

>Physeter_catodon

MEAV-ASNTTVGAAREKVAV

>Rousettus_aegyptiacus

MEVV-ASAATVGAAREKVAV

>Dasypus_novemcinctus

MEAV-SWAATVGAAREKVAV

>Galeopterus_variegatus

MEAV-ASAETVGAAREKVAV

>Condylura_cristata

---MEVVAATVGAAREKVAA

>Oryctolagus_cuniculus

MEVV-ASAATVGAAREKVAV

>Ceratotherium_simum_simum

MEVV-ACAATVGAGREKVAV

>Loxodonta_africana

MEAV-SSAATVGAARETVAV

>Ictidomys_tridecemlineatus

MEAV-ASSSTVGAAREKVAV

>Orycteropus_afer_afer

MEAVSSFAATVGAARERVAV

HG0583 ENSG00000087502 ERGIC2

>Homo_sapiens
MAVGWDHNTSMTI
>Acinonyx_jubatus
MAVGWYHNTSMTI
>Orcinus_orca
MAVGWDRSTSMTI
>Myotis_brandtii
MAVGWDRHTSMTI
>Galeopterus_variegatus
MAVGWERNTSMTI
>Oryctolagus_cuniculus
MAVGWDRNTSMTI
>Elephantulus_edwardii
MAVGWDHNTSITI
>Ceratotherium_simum_simum
MAVGWDRNTSMTI
>Loxodonta_africana
MAVGWDSNRSITI
>Cricetulus_griseus
MAVGWDRSTSMTI
>Tupaia_chinensis
MAVGWDRSTSMTI
>Orycteropus_afer_afer
MAVGWDRNTSITI

HG0584 ENSG00000092051 JPH4

>Homo_sapiens
MDAVPGRQPEPQPT----
>Leptonychotes_weddellii
MDAVPGRQPEPQPTCHSG
>Balaenoptera_acutorostrata_s
MDAVPGRQPEPQSTCHSG
>Rousettus_aegyptiacus
MDAVPGRQPEPQPTCHSG
>Dasypus_novemcinctus
MDAVPGKQPEPQPTCHSG
>Galeopterus_variegatus
MDAVPERQPEPQTTCHSG
>Condylura_cristata
MDAVPGRQPEPQPTCHSG
>Oryctolagus_cuniculus
MDAEPGRRPEPQPTGHSG
>Elephantulus_edwardii
MDAVPGRQPEPQPTRHS-
>Ceratotherium_simum_simum
MDAVPGRQPEPQPTCHSG
>Dipodomys_ordii
MDAVPGRQPEPQPTCHSG
>Orycteropus_afer_afer
MDVILRRQPEPQPTCHSG

HG0585 ENSG00000106038 EVX1

>Homo_sapiens
MGLPPRGGAAQRSARFTR
>Odobenus_rosmarus_divergens
MGLPPRGGAAQRSARITR
>Lipotes_vexillifer
MGLPPRGGAAQRSARITR
>Miniopterus_natalensis
MGLPPRGGAAQRSGRITR
>Dasypus_novemcinctus
MGLPPRGGAAQRNARITR
>Galeopterus_variegatus
MGLPPRGGAAQRSARITR
>Sorex_araneus
MGLPPRGGAAQRSARITR
>Oryctolagus_cuniculus
MGLPPRGGAAQRRARITR
>Elephantulus_edwardii
MGLPPRGGAAQSSTHITR
>Ceratotherium_simum_simum
MGLPPRGGAAQRSARITR
>Mus_musculus
MGLPPRGGAAQRSARITR
>Orycteropus_afer_afer
MGLPPRGGAAQRSARITR

HG0586.1 ENSG00000108375 RNF43

>Homo_sapiens
----------------------MGENDIPAADVG
>Acinonyx_jubatus
----------------------MGENDIPAAEVG
>Balaenoptera_acutorostrata_s
----------------------MGENDIPEAEVG
>Myotis_lucifugus
----------------------MGENDIPAAEVG
>Erinaceus_europaeus
----------------------MGENDIPAAEVG
>Oryctolagus_cuniculus
----------------------MGENDIPAAEVG
>Elephantulus_edwardii
----------------------MGENNIPAAEVG
>Ceratotherium_simum_simum
----------------------MGENDIPAAEVG
>Manis_javanica
----------------------MGENDIPAAEVG
>Loxodonta_africana
----------------------MGENDIPATEVG
>Heterocephalus_glaber
MFNLKQSWKFQREVVLLLVRVKMGENDIPAAEVG
>Tupaia_chinensis
----------------------MGENDIPVAEVG

HG0586.2 ENSG00000108375 RNF43

>Homo_sapiens

MLFGEKKNFC

>Canis_lupus_familiaris

MLFGEKKNFC

>Miniopterus_natalensis

MLFGEKKNFC

>Condylura_cristata

MLFGEKKNFC

>Equus_asinus

MLFGEKKNFC

>Tupaia_chinensis

MLFGEKKNFC

HG0587 ENSG00000119138 KLF9

>Homo_sapiens
MPERCGPAGAGVQKPSVDSGKSVS--
>Odobenus_rosmarus_divergens
MPERCGPAKAGAQKASVDWGKSVS--
>Orcinus_orca
MPERCGPARAGAQKPSVDWGKSVS--
>Rousettus_aegyptiacus
MPERCGPARAGAQKPSGDWGKSVS--
>Dasypus_novemcinctus
MPERCGPAGAGAQKPSMDWGQSVS--
>Condylura_cristata
MPERCGPPGAGAQKLSVDWGESVY--
>Equus_asinus
MPERCGPAREGAPKPSADWGKSIS--
>Manis_javanica
MPERCGPARAGAQKPSTDWGKSAA--
>Loxodonta_africana
MPERCGPAGAGAQKPSVDWGKSVSSN
>Marmota_marmota_marmota
MPERCGPARAGAQKPSVDWGKSVS--
>Trichechus_manatus_latirostr
MPERCGPAGAGAQKPSVDWGKSVS--
>Orycteropus_afer_afer
MPERCGPADTGAQKPSVD--------

HG0588 ENSG00000120162 MOB3B

>Homo_sapiens
-------------------------------MRFPWKSFKRKMEGAV
>Felis_catus
-------------------------------MRFSWKLFKRKMEGAV
>Lipotes_vexillifer
-------------------------------MRFPWKSFKRKMEGAV
>Myotis_davidii
-------------------------------MRFPWKSLERKMEGAI
>Dasypus_novemcinctus
-------------------------------MRFPWKSLKRKMEGAV
>Galeopterus_variegatus
-------------------------------MRFPWKSFKRKMEGAI
>Condylura_cristata
-------------------------------MRFSWKLFKRKMEGAA
>Ochotona_princeps
-------------------------------MRLPWKSFKRKMEGAV
>Ceratotherium_simum_simum
-------------------------------MRFPWKLLKRKMEGAL
>Manis_javanica
-------------------------------MRSPWKLFKRKMEGAI
>Mesocricetus_auratus
MRNSRFREVKRRVRRTPHHSLAATYKQMGRNMRFPWRSFKRKMEGAI
>Tupaia_chinensis
-------------------------------MRFPWKSFKRKMEGTI

HG0589.1 ENSG00000121871 SLITRK3

>Homo_sapiens
------MMDTIQLRREFAPPAG---------------------------------
>Mustela_putorius_furo
------MLDTIQPPREFAPPAGQPLGADLGDLERSAPIVL---------------
>Balaenoptera_acutorostrata_s
------MMDTIQPPREFAPLAGKPLGVNLGDREPSARIVL---------------
>Pteropus_alecto
------MMDTIEPSREFAAPAGKTLGVNLGDRECSARIVL---------------
>Galeopterus_variegatus
MLQGYSMMDTIQPRREFAPPAGKPLGANLGDRERSAPIVL---------------
>Condylura_cristata
-MQAYDTMDTIQPPREFAHPVGKPLGANLGDRDHSAP------------------
>Oryctolagus_cuniculus
------MVDTIQPPREFAPPAGKPLAANLGDGERSAPLVL---------------
>Equus_caballus
-------MDTIQPPREFAPPAGKPLGANLGDRERSAPIVL---------------
>Manis_javanica
------MMDTIHSLRVFAAPAGKPFGANLGDLERSARIAV---------------
>Nannospalax_galili
------MTDTIQPRGEFAPPAGKPLGANLGDRERSAPIVL---------------
>Tupaia_chinensis
-------MDTIQPPREFAPPAGKPLGANLGDCERSAPIGTRRHRFLLRLIEISVF
>Orycteropus_afer_afer
------MMDTIQPLREFIPPAGKPLGANLGDRERSEAIVL---------------

HG0589.2 ENSG00000121871 SLITRK3

>Homo_sapiens
MQRPFPCKSQRWKHLNGLQA------
>Mustela_putorius_furo
MQRPFPFKSQRWKHLKGLQA------
>Lipotes_vexillifer
MQRPFPCKSQRWKHLKGLQA------
>Rousettus_aegyptiacus
MQRPFLCKSQRWKHLEGLQA------
>Galeopterus_variegatus
MQRPFPRKSQRCKYLKGLQA------
>Condylura_cristata
MRRPFLCKSQRWETLKRITGEIWWPF
>Equus_asinus
MQRPFACKSQRWKHLKGLQA------
>Manis_javanica
MQRPFRCKSRRWKHLKGLQA------
>Ictidomys_tridecemlineatus
MQRPFPCKSQRWKYLKGFRL------
>Tupaia_chinensis
MQRPFPCKSPRWKH------------
>Orycteropus_afer_afer
MQLPFPCKSPRWKHLKGLQA------

HG0589.3 ENSG00000121871 SLITRK3

>Homo_sapiens
--MDLHSGSSPAFPILTPYTRPGAPV-------
>Rhinolophus_ferrumequinum
--MDLHSGSSPAFSMLPPNTRLGVPSVGADVVL
>Galeopterus_variegatus
MRGDRTAAADLHSWRGGGAGGAEGTSWRRLWRWADLGAPERRSLGAPIWGWLMDLHSGSSPAFPILSANTRPGAPSLGADVVL
>Condylura_cristata
--MDLHSDSCLAFRILPPSTQPALPV-------
>Equus_asinus
--MALHSGSSPAFPILPPSTRPGAPCVRADVVL
>Manis_javanica
--MDLHSGSSPAFRILPPNSRPGAPSLGADVAV
>Loxodonta_africana
--MDL-LGSSPAFGYWLPNTRPALPV-------
>Marmota_marmota_marmota
--MDLHLCSSPAFPILPPNTRPGAPSLGADVVL
>Tupaia_chinensis
--MDLHSGSSPALPTSPPNTRPGSRSFGADVVL

HG0590 ENSG00000122557 HERPUD2

>Homo_sapiens

---MGFVSSSALVTDLDTEY
>Felis_catus

---MAFVSPDALVTDLDTEY
>Balaenoptera_acutorostrata_s

---MAFVSPDALVTDLDKEY
>Rhinolophus_ferrumequinum

---MAFVSSDALVTDLDTEY
>Condylura_cristata

---MAFLSPDALVTDLDTE-
>Oryctolagus_cuniculus

---MAFVSPDALVTDLNTEY
>Elephantulus_edwardii

---MAFVSSDALVTDLDSRF
>Equus_asinus
MTHFRPALPGLKGTQTFRTTTTKPARSRPAPRLWSPPSCHLPPPGPRPACAPLPCPRGQAESGPHPSANFSRSQRSASRDPGPGCGRHRAAGRERPPRKTFGRRPC
PGVKGGGGGPHGLLSVRSPAEPGPGGENCTHGRAPRPRRRRRRRHDHHHHHHYRHCPSSLVPAMAFVSPDALVTDVDTEY
>Loxodonta_africana

---MAFVSPDALVTDLDTKY
>Chinchilla_lanigera

---MAFVSPDALVTDLDTEY
>Trichechus_manatus_latirostr

---MAFVSPDALVTDLDTKY
>Orycteropus_afer_afer

---MAFVSPEALVIVLDTKY

HG0591.1 ENSG00000127124 HIVEP3

>Homo_sapiens
MNAGFQREQRFSFGHRKWCLQHRRRA------
>Leptonychotes_weddellii
MKAGFQREQCFSFGHRKWCLQHRRRA------
>Physeter_catodon
MNADFQREQRFSFGHRKWCLQHRRA-------
>Myotis_davidii
MNAGFQREQRFSFGHRKWCLQHRSA-------
>Galeopterus_variegatus
MNAGFQREQRLSFGHRKWCLQHRRA-------
>Condylura_cristata
MNAGFQREQRFSFGHRKWCLQHRRA-------
>Oryctolagus_cuniculus
MNAGFQREQRFSFGHRKWCLQHRRVSLLIQDC
>Equus_asinus
MNAGFQREQRFSFGHRKWCLQHRRA-------
>Manis_javanica
MNAGFPREQRFSFGHRKWCLQRRRA-------
>Ictidomys_tridecemlineatus
MNAGFQREQRFSFGHRKWCLQHRRRV------
>Tupaia_chinensis
MSAGFQREQRFSFGHRKWCLQHRRV-------
>Orycteropus_afer_afer
MNAGFQREERFSFGHRKWCLQHRRRA------

HG0591.2 ENSG00000127124 HIVEP3

>Homo_sapiens
MWPKLDTSLGHWDYS
>Acinonyx_jubatus
MWPKPDASLGHLDYS
>Balaenoptera_acutorostrata_s
MWPKPDASLGRLDYS
>Pteropus_alecto
MWPKPDSSLGHLDYS
>Galeopterus_variegatus
MWPKLDTSLGHLDYS
>Condylura_cristata
MWPQPDASLGHLDYS
>Equus_asinus
MWPKADASLGRLDYS
>Manis_javanica
MWPKPEASLGRLDYS
>Fukomys_darlingi
MWPKLDNSLGHEDYS
>Tupaia_chinensis
MWPKLDTSSGHLDYS
>Orycteropus_afer_afer
MWPKPDTSLGHSDSP

HG0591.3 ENSG00000127124 HIVEP3

>Homo_sapiens

MEEPRGVLAKQARPSGGKSGA---------------------

>Canis_lupus_familiaris

MAELQEILARQALPSGGKSRA---------------------

>Pteropus_alecto

MEEPQEILARQALPSKGKSRE---------------------

>Oryctolagus_cuniculus

MEEPPELPARQAVPSRGKSGVEEDGGCSRSGHERWFPERTAF

>Ceratotherium_simum_simum

MEEPREILARQALPSRGKSRA---------------------

>Ictidomys_tridecemlineatus

MEQPQETLARQALPSRGQSGA---------------------

>Orycteropus_afer_afer

MEDPQEILAGQALPSRGKSCV---------------------

HG0591.4 ENSG00000127124 HIVEP3

>Homo_sapiens

MDTDVSNLQPFA

>Acinonyx_jubatus

MHTDVSSLQPFA

>Galeopterus_variegatus

MDTDASNLQPFA

>Cavia_porcellus

MDTEVSNFQPFA

HG0592 ENSG00000133401 PDZD2

>Homo_sapiens
MNTGKADDGQGPQGTWGPVGSGPQEQDL-
>Mustela_putorius_furo
MNTGKADDSQGSQGTWGLPGSSPKEQVLS
>Balaenoptera_acutorostrata_s
MNTGKAEDSPRSRGTWGPAGPSPQEQVLS
>Myotis_davidii
MNMNTADDSQGPQGTWGPAGSGPQEQVLS
>Dasypus_novemcinctus
MSTGQADGSQGPPGTWGP-----QERVLS
>Erinaceus_europaeus
MNLGHVDDSQGPQGMWGLVGPSPPKQALS
>Oryctolagus_cuniculus
MNTCKAHDNQASQGTWGPAGPGPQERVLS
>Ceratotherium_simum_simum
MNTGKADDSQGSQGTWGPLGSGPQEQVVS
>Manis_javanica
MHTDGASGSQGSHGPWGPLGSSSQEQPVP
>Fukomys_darlingi
MNTGRADDHQDSQGTWGPAGSGPQEQDLP
>Tupaia_chinensis
MNTGKADDSQCSQGSRDPVGSGPQKQDLS
>Trichechus_manatus_latirostr
MNIGQADDSQGSQGTWGPMGPGPQEQALS

HG0593 ENSG00000135048 TMEM2

>Homo_sapiens
MERRSGPEDARDSSPSPWTAV
>Felis_catus
MERRSGPGDARSSAPSLWTAV
>Balaenoptera_acutorostrata_s
MEGRSRPGDARSSALSLWTAV
>Myotis_brandtii
MERRAGPGDAQNSAPSLWTSV
>Dasypus_novemcinctus
MERRSELGDARSSAPSLGNAV
>Condylura_cristata
MERRSGPADARSSAPAFWIAV
>Elephantulus_edwardii
MERGSGPRVARNPALSLWTSV
>Ceratotherium_simum_simum
MEGRSGPGDARSSAPSLWTAV
>Manis_javanica
MERGSGPGDTRSSAWSLWTPV
>Loxodonta_africana
MERGSGPGDARSSALSLWISV
>Marmota_marmota_marmota
MERRSGPGDARSSTQSLWIIA
>Trichechus_manatus_latirostr
MERGFGPGDARSSALSLWISV

>Homo_sapiens
---MFHPFVDQKMELVFIFKKIMLMI----
>Canis_lupus_familiaris
MGSWPSSGIGGRPAVPRRIGVSREPEAVKGAAGRGLRRNNPEAQLGGVAADPCLRMFHASMDQKMELVFIFKKILSMI----
>Physeter_catodon
---MFHAFMDRKMELVFIFKMILSMI----
>Pteropus_alecto
---MFHAFMDQKMELVFIFKKILSVI----
>Galeopterus_variegatus
--MELVFIF-KDTVNDLIQL
>Condylura_cristata
--MDQKMALVFIF-KDSVSHLVQL
>Oryctolagus_cuniculus
--MELVYIFKKKLSVL----
>Equus_asinus
--MDQKMKLVFIFKKILSMN----
>Loxodonta_africana
---MFYVFVDQKMKSFFYFKKDTVNDLIEL
>Ictidomys_tridecemlineatus
---MYFEDQKMELVFIFKKILSMI----
>Tupaia_chinensis
---MFHAFVDQKMELVFIFKKLLSVI----
>Trichechus_manatus_latirostr
---MFYAFVDQKMELFSFLKKILSMI----

HG0595 ENSG00000141098 GFOD2

>Homo_sapiens
MPACQEPCLTRTFGRSYPCQRLL
>Felis_catus
MPACQEPCLTRTVGRSYSCWRLL
>Lipotes_vexillifer
MPACQEPCVTRTVGWSYSCWRLL
>Myotis_lucifugus
MPACQEPCLTRTVGWSSSCWRLL
>Dasypus_novemcinctus
------------MGRSYPCWRLL
>Erinaceus_europaeus
MPECQEPCLTRTVGTSYSCWKPL
>Oryctolagus_cuniculus
MPACQEPCLTMPAGISDPCWRHL
>Ceratotherium_simum_simum
MPACQEPCLTRTVGRSYSCWRLL
>Manis_javanica
------------MGRSYSCWRLL
>Loxodonta_africana
MPACQEPCLTRTLGRWDPHQRLL
>Heterocephalus_glaber
MPACQEPCLTRTVGRSAPCWRPL
>Tupaia_chinensis
MPACQEPCLTRTMGNPFPCWRCS

HG0596 ENSG00000157483 MYO1E

>Homo_sapiens
MESPLGSHWLL
>Felis_catus
MESPQGSHWLL
>Orcinus_orca
MESPQGSHWFL
>Rhinolophus_ferrumequinum
MECPQGSHWLL
>Dasypus_novemcinctus
MESRLGSHWLL
>Galeopterus_variegatus
MESPLGSHWLL
>Condylura_cristata
MECPLGSHWLL
>Oryctolagus_cuniculus
MQSPLGSHWLL
>Equus_asinus
MESPQGSHWLL
>Ictidomys_tridecemlineatus
MESPLGSHWLL
>Tupaia_chinensis
MESPLGSHWLL
>Orycteropus_afer_afer
MESPLGSHWLL

HG0597 ENSG00000163032 VSNL1

>Homo_sapiens
MGFPICSRDLPERVAAAAGLARRDPRGI
>Mustela_putorius_furo
MGFPICSRDLPELVAAAAGLARRDPRGI
>Balaenoptera_acutorostrata_s
MGFPICSRDLPERAAATAGLARRDPRGI
>Pteropus_alecto
MGFPICSRDLPERVAAAAGLKRRDPKGI
>Condylura_cristata
MGFPIRSRDLPERVAAAAGLARRDPRGI
>Oryctolagus_cuniculus
MGFPICSRDLPERVAATAGLARRDPTGI
>Equus_caballus
MGFPICSRDLPERVAAAAGLARRDPGGI
>Manis_javanica
MGFPICSRDLPEIVAAVAGLARRDPKGI
>Loxodonta_africana
MGFPICSRDLSERVAAAAVVARRDPRGI
>Psammomys_obesus
MGFPICSRDSPERVAAAAGLARRDPRGI
>Tupaia_chinensis
MGFPICSRDLPERVAAATGLARRDPRGI
>Trichechus_manatus_latirostr
MGFPICGRDLPERVAAAAGLARRDPRGI

HG0598 ENSG00000163788 SNRK

>Homo_sapiens
--MTTLKMNFLYSPDILI
>Odobenus_rosmarus_divergens
--MATLKMNSLFSPDILI
>Lipotes_vexillifer
--MSMATLKMNSLFSPDI--
>Miniopterus_natalensis
--MPMATLKMNSLFSPDILV
>Dasypus_novemcinctus
--MSMATLKMNSLFSPDSLI
>Galeopterus_variegatus
--MSMASLKMNSLFSPDILV
>Condylura_cristata
MISTGCGESAAGRESSAPRPLRTDDRLAAENLLRFCFGTSSTWPLLRRKTTSKTVITRTIQHAVCRMSMATLKMNSLSLPDILI
>Equus_caballus
--MSMVTLKMNSLFSPDILI
>Loxodonta_africana
--MSMATLKMNSLFSPDILI
>Ictidomys_tridecemlineatus
--MSVATLKMNSLFSADILI
>Trichechus_manatus_latirostr
--MSMVALKMNSLFSPDTLI
>Orycteropus_afer_afer
--MSMATLKMNSLFSPDILI

HG0599 ENSG00000164930 FZD6

>Homo_sapiens

MRNLNILSLDGDLLRMQRVIHPSHVVKSGI

>Odobenus_rosmarus_divergens

---------------MKRVINPSHVVKPGI

>Balaenoptera_acutorostrata_s

---------------MKRAINPSHVVKLGI

>Rousettus_aegyptiacus

MRNSNILSLDGN-LRMKRVINSSHVVKTGI

>Condylura_cristata

---------------MKRAINSSHVVKAGI

>Oryctolagus_cuniculus

MRTLNILPLAGNPLRMKRAVNLRHVVISGI

>Equus_asinus

MRNLNILSLDGN-LRMKSAITPSHVVKSGI

>Loxodonta_africana

MRNLNISYLDGNLLRMRRLIQAMW------

>Dipodomys_ordii

---------------MNGVVNPSHVVKPEI

>Tupaia_chinensis

MRNLNVLFLDGNLLRMKRATNPSHVVKSGI

>Trichechus_manatus_latirostr

MRNLNILYLDGSLLRT--------------

>Orycteropus_afer_afer

---------------MKRAINLGHVVKSGI

HG0600 ENSG00000173065 FAM222B

>Homo_sapiens

MRAGPSHRPVSHCPMLC

>Felis_catus

MRAGPSHRHVSDCLMLC

>Rousettus_aegyptiacus

MRAGPSHRHVSDCPMLC

>Dasypus_novemcinctus

MRAGPSHRHVSDCLMLC

>Condylura_cristata

MRAGPSHRHVSGCPMLC

>Ochotona_princeps

MRAGPSQRHVSHCPMLC

>Elephantulus_edwardii

MRAGPSHRHVSDCLMLC

>Equus_asinus

MRAGPSHRHVSDCPMLC

>Manis_javanica

MRAGPSHRHVNGCPMLC

>Nannospalax_galili

MRAGPSHRHVSHCPMLC

>Tupaia_chinensis

MRAGPSHRHVSHCPMLC

>Trichechus_manatus_latirostr

MRAGPSYRHVSDCPMLC

HG0601 ENSG00000175893 ZDHHC21

>Homo_sapiens

MNQKKISKCRFQKNYSTSQ

>Canis_lupus_familiaris

MNQDKTSKCKYQKNYSTSW

>Physeter_catodon

MNQNKSSKCKYQKNYNTSQ

>Eptesicus_fuscus

MNQDKTSKCKYQKNYNTSQ

>Dasypus_novemcinctus

MNQDKPSKCKYQKNYNTSQ

>Galeopterus_variegatus

MNQEKTSKCKYQKNYNTSQ

>Condylura_cristata

MNQDTTSKCKYQRNCHTSQ

>Oryctolagus_cuniculus

MNQDKTFKCKYQKNYNTFP

>Ceratotherium_simum_simum

MNQDKTSKCKYQKNYNTSQ

>Manis_javanica

MNQDKTSKCKYQKNYNTSQ

>Marmota_marmota_marmota

MNQDKTSRCKYQKNYNTSQ

>Tupaia_chinensis

MNQGKTSKCKYQKKYSTSQ

HG0602 ENSG00000179195 ZNF664

>Homo_sapiens
MQEETFRRKTGREKLRLTLNLPNRASLR
>Panthera_tigris
MQEETFHRKTGREKLRPTIALPNRASLR
>Lipotes_vexillifer
MQGGTFHRKTGRGELRPASNLPSRASLR
>Miniopterus_natalensis
MQEEAFHRKTGRAKLRLTLSLPSRVSLR
>Dasypus_novemcinctus
MQEETFHRKTGREKLRPTLNLPNRASLR
>Erinaceus_europaeus
MQEETFHRKTGREKLRLTFNLPNRASLR
>Oryctolagus_cuniculus
MQEEAFRRNAGRETLRLAARPPSGASPR
>Ceratotherium_simum_simum
MQEETFHRKTGREKLRLTLNLRIRASLR
>Manis_javanica
MQEEIFHRKSGREKLRLTVKLPNRVSLR
>Loxodonta_africana
MQEEAFHRKIGREKLRPTLNLLTRASLR
>Heterocephalus_glaber
MQEEAFHRKTGREKLRLTLNLPHRASLR
>Tupaia_chinensis
MQEETFHRKTGREKLRLSLNLPNRASLR

HG0603 ENSG00000197329 PELI1

>Homo_sapiens
MSVTQRRCEAEGPSGGAAAASTSSSGSSPSPAVRSNWSPALRERTTKQPQRLSLRPRLLSARLRGQLPLAGLGWRARRAAAE-----RRAVC----SRSPRTTLHP
LASGLGGRSGIPATSNCPEIMQKVSQGS--
>Mustela_putorius_furo

--------------------MQKVSQGS--
>Lipotes_vexillifer

--------------------MQKVSQGS--
>Rousettus_aegyptiacus

--------------------MQKVSKAPDQ
>Dasypus_novemcinctus

--------------------MQKVSPSS--
>Condylura_cristata

--------------------MQKVSQGS--
>Ochotona_princeps
MSVTQRQCEAEGPSGGAAAAASTSS--SSSGAARAELSRALRERSTKRAPRLALGPRLLSAGLLGQLPPQRA-WLLPGEAGGGGGGTPGCLLARPPPHPRAHSLHP
LAAGLGSRAGILATSDCPEIMQKVSQGS--
>Ceratotherium_simum_simum

--------------------MQKVSQGS--
>Loxodonta_africana

--------------------MQKVSQAS--
>Ictidomys_tridecemlineatus

--------------------MQKVSQGS--
>Trichechus_manatus_latirostr

--------------------MQKVSQAS--
>Orycteropus_afer_afer

--------------------MQKVSQAS--

HG0604.1 ENSG00000003147 ICA1

>Homo_sapiens
-----------------------------------MLFSCPFSPNHQQ
>Acinonyx_jubatus
-----------------------------------MLFPCPFSPNHQQ
>Tursiops_truncatus
-----------------------------------MLFPCPFSPNHQQ
>Myotis_davidii
-----------------------------------MLFPCPFSPNHQQ
>Galeopterus_variegatus
MGVEKRPQGVERRRLTLPTT--TRLKQGYNITYPLMLLLYPFSPNHQQ
>Condylura_cristata
-----------------------------------MLFPCPFSPNHQQ
>Elephantulus_edwardii
-----------------------------------MLFLCPFFPNHQL
>Equus_asinus
-----------------------------------MLFPCPFSPNHQQ
>Dipodomys_ordii
-----------------------------------MCFPCPFSPNHQQ
>Tupaia_chinensis
-----------------------------------MLFLCPFSPNHQQ
>Trichechus_manatus_latirostr
MGVKERPQGVERGRLIPPTPVPPHPKEGCNITYLLMLFPCPFSPNHQQ

HG0604.2 ENSG00000003147 ICA1

>Homo_sapiens

MSDPEGRSDRGREAPGGRGSEVII

>Odobenus_rosmarus_divergens

MKELEGRSDRGQEAPGGRGSEVII

>Tursiops_truncatus

MKELEGRSDRGREAPRGRGSEVII

>Rousettus_aegyptiacus

MRELEGRSDRGREAPGGRGSEVII

>Condylura_cristata

MKELEGRSDRGREAPGGRGSEVII

>Ceratotherium_simum_simum

MRELEGRSDRGREAPGGRGSEVII

>Ictidomys_tridecemlineatus

MWDLEGHSDRGREAPGGQGSEV--

HG0604.3 ENSG00000003147 ICA1

>Homo_sapiens

MATWNTCRRAGAGSLWAADPSGGYPRRWGSRRDPGE

>Galeopterus_variegatus

MDTWNTCGRAGAGSPRAADPSKGYPKRWGSRKDPRE

>Ceratotherium_simum_simum

MDTWNTCGRAGRGHPGRQMRAGGYPRRWGSRKDPRE

>Mus_musculus

MDTWNTC-----------------------------

>Orycteropus_afer_afer

MDTWNTCRKAGAGSPWTAGPRGGYPRRWGSRKDPRE

HG0605 ENSG00000053254 FOXN3

>Homo_sapiens
MKKNQPLEDQHSRDYGGPSVFSVTGAMLTSVSWTHSDSGAFT
>Felis_catus
--------------------------MLTSVSWTHSDSGAFT
>Physeter_catodon
--------------------------MLTSVSWTHSDSGAFT
>Miniopterus_natalensis
--------------------------MLTSVSWTHSDSGAFT
>Condylura_cristata
--------------------------MLTSVSWTHSDSGAFT
>Oryctolagus_cuniculus
--------------------------MRTSVSWTHCDSGAFT
>Ceratotherium_simum_simum
--------------------------MLTSVSWTHSDSGAFT
>Manis_javanica
--------------------------MLTSVSWTHSDSGAFT
>Loxodonta_africana
--------------------------MLTSVSWTHSDSGAFT
>Nannospalax_galili
--------------------------MLTSISWTHSDSGAFT
>Tupaia_chinensis
--------------------------MLTSVSWTHSNSGAFT

HG0606 ENSG00000055130 CUL1

>Homo_sapiens

MRFHSLHLKDILSELL

>Odobenus_rosmarus_divergens

MRFHSLHLKDILSELL

>Balaenoptera_acutorostrata_s

MRFHSLPLKDVLSELL

>Cynopterus_sphinx

MKVHSLQLKDILSELL

>Dasypus_novemcinctus

MRSHSLHLKDILSELL

>Galeopterus_variegatus

MRFHFLHLKDILF---

>Ceratotherium_simum_simum

MRFHSLHLKDILSELL

>Manis_javanica

MRFHSLHLKDILSALL

>Heterocephalus_glaber

MRFHSLHLKDILSELL

>Tupaia_chinensis

MRFASLRLKDILSELL

>Trichechus_manatus_latirostr

MRAPSLHLKDTASELL

HG0607 ENSG00000074657 ZNF532

>Homo_sapiens

MRFQRPPCPLSHWPCLRPPPPPQTAPVFFPRGEGIWTSPPRCLKEEKGMFCLVLFLLEATKGCWGCF---------

>Odobenus_rosmarus_divergens

--MFCFVLFLLEATKGCWGYF---------

>Orcinus_orca

--MFCFVLFLLEATKGFWGCF---------

>Miniopterus_natalensis

--MFCFVLFLLEATKGCWGYF---------

>Dasypus_novemcinctus

--MFCFVLFLLEATKGCWGYV---------

>Galeopterus_variegatus

------------------------------------MAAKSRGACRKAMFCFVLFLLEATKGCWGYF---------

>Condylura_cristata

--MFCFVLFLVEATKGVGDTSDCYEGRKAC

>Oryctolagus_cuniculus

---------MNWWVCDGS---------LFVAGECYFACLSSL---AGCVLFCAFFLLEATKGCWGYF---------

>Ceratotherium_simum_simum

--MFCFVLFLLEATKGCWGYF---------

>Ictidomys_tridecemlineatus

--MFCFVLFLLEATKGCWGYF---------

>Orycteropus_afer_afer

--MFYFVFFLLEAAKGCWGDF---------

HG0608 ENSG00000084731 KIF3C

>Homo_sapiens
MNDPPAWGEDSR
>Leptonychotes_weddellii
MNDPPARGEDSR
>Orcinus_orca
MNDPPAGGEDSR
>Rousettus_aegyptiacus
MNDPPAPGEDSR
>Galeopterus_variegatus
MNDPPSVGEDSR
>Condylura_cristata
MNDPPAGGADSR
>Elephantulus_edwardii
MNDPPAGGDDSR
>Loxodonta_africana
MNDPPAEGEDSR
>Peromyscus_maniculatus_baird
MNDPPAGGEDSR
>Trichechus_manatus_latirostr
MNDPPASGEDSR
>Orycteropus_afer_afer
MNDPPAWGEDSR

HG0609 ENSG00000095539 SEMA4G

>Homo_sapiens

----MATQPCDSMSPRFQDPPWPHDSLTPMTL

>Ailuropoda_melanoleuca

----MATQPCDSMSPRFQDPPWPHDP------

>Physeter_catodon

----MATQPCDSMSPQFQDPPWPHDS------

>Pteropus_alecto

----MATQSCDSMSPQFQDPPWPHDA------

>Galeopterus_variegatus

----MATQPCDSMSPQFQDPPWPHDSLAPMAL

>Oryctolagus_cuniculus

----MATQPCDSMSPPFQDPPWPHDSLTPMAS

>Ceratotherium_simum_simum

----MATQPCDSMSPQFQDPPWPHDP------

>Manis_javanica

MWDSMATQPCDSMSPRFQDPPWPHDL------

>Loxodonta_africana

----MATQPCDPMSPRFQDPPWPHDSVTPMPS

>Rattus_norvegicus

----MATQPCGSMSPPFQDPPWPHDYLTPMTS

>Tupaia_chinensis

----MAIQPCDSMSPRFQDPPWPHDSLTPTAL

HG0610 ENSG00000106069 CHN2

>Homo_sapiens
MGWGPRRLRAAGRGQRRRRPHRG
>Odobenus_rosmarus_divergens
MGWGPRRLRAVARGQLRRRPHRG
>Orcinus_orca
MGWGPRRLRAAGSGQRRRRPHRG
>Myotis_lucifugus
MGWGPRQPGAAGRGQRRLRPHRG
>Dasypus_novemcinctus
MGWGPRRLRAAGRGQRRRRPHRG
>Sorex_araneus
MGWGPRRLRAAGPGQRRRRPLRG
>Ochotona_princeps
MGWGPRRLRAAGPGQRRQRLQRG
>Elephantulus_edwardii
MGWGPRRLRAAGPGQRRPA-HRG
>Equus_asinus
MGWGPRRLRAAGRGQRRRRPHRG
>Loxodonta_africana
MGWGPRRLRAAGRGQLRRRPHWG
>Ictidomys_tridecemlineatus
MGWGPRRLRAAGQGQRQWRPHRG

HG0611 ENSG00000108309 RUNDC3A

>Homo_sapiens
MAMEGLRGPVGQRAQP
>Odobenus_rosmarus_divergens
MAVQASRGPGGQRAQP
>Orcinus_orca
MALEGSRGPGGQRAQP
>Eptesicus_fuscus
MAVEGSRGPDGQKAQP
>Galeopterus_variegatus
MAAEGSRRPGGQRAQP
>Sorex_araneus
MAAEGSRGPGGQRARP
>Ochotona_princeps
MALEGSRGSGGQSAQP
>Elephantulus_edwardii
MAAEGSRGPGGRRAQP
>Equus_asinus
MAVEGSRGPGGQRAQP
>Manis_javanica
MALEGSRGPGGQRAQR
>Ictidomys_tridecemlineatus
MAMERSMGPGGQRAQP

HG0612.1 ENSG00000110422 HIPK3

>Homo_sapiens
-----------------MAASRALWPRPQRRGAAGLQGVNSGDVRACV--

>Mustela_putorius_furo
-----------------MAASLALWPSHQRRRAAGLEGVNSGDVSACDCWGGEM--

>Lipotes_vexillifer
-----------------MAASLALWPSPQRRRAAGLEGVNSGDVSACDCWGGEM--

>Rousettus_aegyptiacus
-----------------MAASLALWPSHQRRRAAGLEGANSGDVSACDCWGGEM--

>Condylura_cristata
-----------------MAASPAWWPSHRRRRAAGLQSVHSGDVSACDCWGGEM--

>Ochotona_princeps
-----------------MASSRALGPDRQRREAAESEGVNPRDVRAWDCRGGEM--

>Elephantulus_edwardii
-----------------MAASLAWWPRRWRRSAAWLQGVSSPGPECV---

>Manis_javanica
-----------------MAASVSLRPSRQRRRAAGLEGGNSGDWSACDCWGGEMRSARSASPAAAGGRRGGRAAGLLPASLVTLLFAALGELAERLRRRMCVFLSG
GRRLF
>Rattus_norvegicus
MGANTNVHTRRRSAQPRGCAAEAWWPCHRRRGAAGLEGVNSGDVRACDCWGGEM--

>Trichechus_manatus_latirostr
-----------------MAASPALGPRHQRRRAAGLEGVNSGDVSACDCWAGEM--

>Orycteropus_afer_afer
-----------------MAASPALWPRHQRRRAAGLEGVDSGDLSACDCWAGEM--

HG0612.2 ENSG00000110422 HIPK3

>Homo_sapiens

MLRAAAARRGESAGL

>Odobenus_rosmarus_divergens

MLRAAAARRGESSGL

>Eptesicus_fuscus

MLRAAAARRGESAGL

>Dasypus_novemcinctus

MLRAAAARRGESAGL

>Galeopterus_variegatus

MLRAAAARRGESAGL

>Condylura_cristata

MLRVAAARRGESAGL

>Oryctolagus_cuniculus

MLRAAAARRGESAGL

>Manis_javanica

MLRAAAARRGESAGL

>Loxodonta_africana

MLRVAAAGRGESSGL

>Ictidomys_tridecemlineatus

MLRAAAARRGESAGL

HG0612.3 ENSG00000110422 HIPK3

>Homo_sapiens

MKMSVSLSGGRRLL

>Ursus_maritimus

MGMSVFLSGGRRLF

>Lipotes_vexillifer

--MSVFLSGGRRLF

>Eptesicus_fuscus

--MSVSPSGGRRLL

>Mesocricetus_auratus

--MSVFLSGGRRLL

HG0613 ENSG00000114861 FOXP1

>Homo_sapiens
MVRASIPSLICCQP--------
>Canis_lupus_familiaris
MVRASIPSLICCQP--------
>Balaenoptera_acutorostrata_s
MVRASIPCLICCQP--------
>Pteropus_alecto
MVRASIPSLICCQP--------
>Galeopterus_variegatus
MVRALIPSLICFQPCTAGCDAC
>Ochotona_princeps
MVRASIPSLICCQP--------
>Ceratotherium_simum_simum
MVRASIPSLICCQP--------
>Loxodonta_africana
MVRASIPSRFCCQP--------
>Jaculus_jaculus
MVRAALPSLICCRP--------
>Tupaia_chinensis
MVRASILSLICCQP--------
>Orycteropus_afer_afer
MVRASVRSPICCQP--------

HG0614 ENSG00000122203 KIAA1191

>Homo_sapiens
MGRGRVSRSWTTTPSMPCAPLGQVSREIPRSPEALSAVLPARWVVLLPSGSWGPSKSPPLSQWLGSRLRHSLSGDVGPRNLFSAGLRAAGSPAAAGSQNLWMDSSW
EALLLCSSWTMFLINICLSA
>Odobenus_rosmarus_divergens
---MDSSQ
ADLLICSSWTMFLINTCLSA
>Galeopterus_variegatus
---MDSSW
EALLISSSWTMFLINICLSA
>Erinaceus_europaeus
--MDTSSW
TASLLHSSWTMDD-HLCLSA
>Oryctolagus_cuniculus
---MFPGQCWGLMFLLLPLPWFESGQCHSLSGYLGLWSLCSAGVQAAGGFLSVGEQNLWMD-SW
KASLAGSSWKKFLISIC---
>Elephantulus_edwardii
---MDFSW
DALLTCSSWTMFLINICLSA
>Ceratotherium_simum_simum

----MCSSWTMFLINNCLSA
>Chinchilla_lanigera
---MDSSG
KALLICSSWTMFLINICLSA
>Tupaia_chinensis

----------MLLITICLSA
>Trichechus_manatus_latirostr
---MDSSW
EALLTCSSWTMFHI-ICLSA
>Orycteropus_afer_afer
---MDSSW
EVLLTCSSWTMFLINICLSA

HG0615 ENSG00000128191 DGCR8

>Homo_sapiens
MKTDSLSRQSLKLSAL
>Acinonyx_jubatus
MKTDSLSRQSFKLSAL
>Pteropus_alecto
MKTDSLSRQSFKLSAL
>Galeopterus_variegatus
MKTDSLSRQSFKLSAL
>Erinaceus_europaeus
MKTDSLSRQSFKLSAL
>Ochotona_princeps
MKTESLSRQSFKLSAL
>Equus_asinus
MKTDSLSRQSFKLSAL
>Manis_javanica
MKTDSLSRQSFKLSAL
>Loxodonta_africana
MKTDSLSRQSFKLSAL
>Marmota_marmota_marmota
MKTDSLSRQSFKLSAL
>Orycteropus_afer_afer
MKTDSLSRQSFKLSAL

>Homo_sapiens
---MERARDRLHLRRT--TEQHVPEVEVQVKRRRTASLSNQECQLYPRRSQQ-QQVPVVD
FQAELRQAFLAETPRGG
>Leptonychotes_weddellii
---MERARDRLHLRRT--TEQHVPEVEVQVKRRRTASLSNQECQLYPRRSQQ-QQIPVVD
FQAELRQAFLAETPRGG
>Tursiops_truncatus
---MERARDRLHLRRT--TEQHVPEVEVQVKRRRTASLSNQECHLYPRRSQQQQQVPVVD
FQAELRQAFLAETPRGG
>Miniopterus_natalensis
-------------------------MGAC--------SRRPASRRPSSTMSRGACAGPLRRIRFKGQGLCFLAL-RPRGPPDACPPSRCQQFPRRSPQ-PRARAAD
FAAELRPAFVAETPRGG
>Galeopterus_variegatus
---MERARDRLHLRRT--TEQHVPEVEVQVKRRRTASLSNQECHLYPRRSQQ-QPVPVVD
FQVELRQAFLAETPRGG
>Erinaceus_europaeus
---MERARDRLNLRRT--TEQQVPEVEVQVKRRRTASLSNQECHLYPRRSQQ-QQVPVVD
FQAELRQAFLAETPRGG
>Ochotona_princeps
---MERARDRLHLRRT--TEQHVPEVEVQVKRRRTASLSNQECQLYPRRSQQ-QQVPVLD
FQAELRQAFLAETPRGG
>Equus_przewalskii
---MERPRDRLHLRRT--TEQHVPEVEVQVKRRRTASLSNQECQLYPRRSQQ-QQIPLLD
FQAELRQAFLAETPRGG
>Ictidomys_tridecemlineatus
---MERARDRLHLRRT--TEQHVPEVEVQVKRRRTASLSNQECHLYPRRSQQ-QQVPVVD
FQAELRQAFLAETPRGG
>Trichechus_manatus_latirostr
---MERVRDRLHLRRT--TEQHVPEVEVQVKRRRTASLNNQECQLYPRRSQQ-QQVPVVD
FQAELRQAFLAETPRGG
>Orycteropus_afer_afer
MTDNFSTGLVQGEFLPHNVAVMWEYTGLRSLSMPDNIGSSPPP-RSAAVKWGGKDRLHLRRT--TEQHVPEIEVQVKRRRTASLSNQECQLYPRRSQQ-QQVPVLD
FQAELRQAFLTETPRGG

HG0617 ENSG00000135164 DMTF1

>Homo_sapiens
MRATWVKKLPVNSHHFCPSLGNTFRECCGDRNMGEKQSG
>Mustela_putorius_furo
--------------------------------MGEKQSG
>Physeter_catodon
--------------------------------MEEKQSG
>Miniopterus_natalensis
--------------------------------MEEKQSG
>Galeopterus_variegatus
--------------------------------MGEKQSG
>Oryctolagus_cuniculus
--------------------------------MGEKQLG
>Equus_asinus
--------------------------------MGEKQSG
>Loxodonta_africana
--------------------------------MGKKQSG
>Dipodomys_ordii
--------------------------------MGETQSG
>Tupaia_chinensis
--------------------------------MGEIQSG
>Orycteropus_afer_afer
--------------------------------MGEKQSG

HG0618 ENSG00000137817 PARP6

>Homo_sapiens
MPISLALVRLVVWRGAA--
>Leptonychotes_weddellii
MPIPLACVQLVVWRGAA--
>Balaenoptera_acutorostrata_s
MPISLAWVQLVVWRGAA--
>Eptesicus_fuscus
MPISLAWVQLVVWRGAA--
>Dasypus_novemcinctus
MPISLALVQLVVWRGAA--
>Condylura_cristata
MPISLARVQLVVWRGAA--
>Oryctolagus_cuniculus
MPILLAWLRLVAWRGAAEH
>Ceratotherium_simum_simum
MPISLAWVQLVVWRGAA--
>Manis_javanica
MPISLAWVQLVVWRGAA--
>Loxodonta_africana
MPISLAWVQLVVWRGAA--
>Heterocephalus_glaber
MPISLAWVQLVVWRGAA--

HG0619 ENSG00000138593 SECISBP2L

>Homo_sapiens
MEKAARGAVYTD
>Felis_catus
MEKAARGALHTD
>Lipotes_vexillifer
MEKAARGALDTD
>Miniopterus_natalensis
MEKAARGALYTD
>Galeopterus_variegatus
MEKAARGALHTN
>Condylura_cristata
MEKAARGALHTD
>Elephantulus_edwardii
MEKAARGALHTD
>Equus_asinus
MEKAARGALHVD
>Manis_javanica
MEKAARGALHTN
>Chinchilla_lanigera
MEKAARGALHTD
>Trichechus_manatus_latirostr
MEKAARIALYTD

HG0620 ENSG00000145555 MYO10

>Homo_sapiens
MCHQLFSACERCAQ-
>Odobenus_rosmarus_divergens
MCHQLFSARERCAQK
>Physeter_catodon
MCHQLFSARERGAQE
>Rousettus_aegyptiacus
MCHQLFSARERCAPK
>Galeopterus_variegatus
MCHQLFSARERFAQE
>Oryctolagus_cuniculus
MCHQLFPARERSVQK
>Ceratotherium_simum_simum
MCHQLFSARERGAQK
>Manis_javanica
MCHQLFSARQRCAQE
>Rattus_norvegicus
MCHQLFSAREHFAQK
>Tupaia_chinensis
MCHQLFTAREHLAQK
>Trichechus_manatus_latirostr
MCHQLFSARERCALK

HG0621.1 ENSG00000146352 CLVS2

>Homo_sapiens

MPLSPPPPLLKPRLMDARECRIA

>Odobenus_rosmarus_divergens

MLLSPPP--PKPGLMDARECRIA

>Pteropus_alecto

-MLSSPPPLLKPRLMDAGECRIA

>Dasypus_novemcinctus

--------------MDTRECRIA

>Condylura_cristata

--------------MDARECRIA

>Oryctolagus_cuniculus

---------MKPGLMDARECRIA

>Equus_caballus

--MLLPPTPLKPRLMDARECRIA

>Loxodonta_africana

---MLLSPLLKSQLMDAGECRIA

>Rattus_norvegicus

MVLSPPRPLRKPGLMDAGECRIA

>Tupaia_chinensis

--------------MDARECRIA

>Orycteropus_afer_afer

MLLS---PPPKPRLMDARECRIA

HG0621.2 ENSG00000146352 CLVS2

>Homo_sapiens

MAGPGENLGQRWP

>Odobenus_rosmarus_divergens

MAGPGEDLGRRWP

>Orcinus_orca

MAGPGENLGQRWP

>Rousettus_aegyptiacus

MAGPGEDPGQPWP

>Galeopterus_variegatus

MAGPGEHLGQRWP

>Oryctolagus_cuniculus

MAGPGEDLGQQWS

>Ceratotherium_simum_simum

MAGPGEDLGQRWP

>Loxodonta_africana

MAGPSEDLEQRWL

>Ictidomys_tridecemlineatus

MAGPGGDLGQRWP

HG0621.3 ENSG00000146352 CLVS2

>Homo_sapiens

MPPKLPSYP------------------------NEAGKGAERARELSECLRRSEICAGIIREELG

>Odobenus_rosmarus_divergens

MPRKLPSYPAES--GREERGACAS---

>Galeopterus_variegatus

MPPKLPSQPNKA--GREELSACAS---

>Condylura_cristata

MPPEPPAEASGPEAGGTRLRGCLNATDVAILLKRVGERAAECVRELSECLRRREIEAGIMREELG

>Marmota_marmota_marmota

MPQKLPSFPKRQGEKS---

HG0622 ENSG00000148677 ANKRD1

>Homo_sapiens

MKIHLPLSWLLMGV-GVLLRFPGWKIISPGPSYIS---------

>Leptonychotes_weddellii

MKIHLPLSWPLVGA-GVLLGFPGWEITSPGPSYISRPVWRAPQG

>Orcinus_orca

MKLHLPLSWPRVGA-GVLLGFPGWKISSPGPSYIS---------

>Pteropus_alecto

MKIHLPLSWPVVGA-GMLLGFPGWKIISPSPSYIS---------

>Dasypus_novemcinctus

MRIHLPLSWLLVRV-GVLLGFPGWKIISPSPSYIS---------

>Galeopterus_variegatus

MKIHLLLSWPLVGA-GVLLGFPGWKIISPSPSYIS---------

>Condylura_cristata

MKIHLPLSWPLVGG-GVLLGFPGWKISSPVLAI-----------

>Oryctolagus_cuniculus

MKIHLPLSWLLAGGQGCYLGSQVGRLSHPAQTI-----------

>Ceratotherium_simum_simum

MKIHLPLSWPLVGA-GVLLGFPGWKIISPSPVYIS---------

>Loxodonta_africana

MKIHLPLSWLLR-GRGCYLGSQVGRLSHPAQAI-----------

>Jaculus_jaculus

MKIHLPLSWPLVGA-GALLGFPGWKIISPSPSYITG--------

HG0623 ENSG00000151422 FER

>Homo_sapiens
MTIFARLHCTFSQVSSLSLMCD-TSSWTRYFS-------
>Felis_catus
MVIPVRLHCTFSQVCSSSLMCN-PSTWTCYFS-------
>Orcinus_orca
MTIPVRLHCTFSQVSSSSLMCN-PSTWTCYFS-------
>Pteropus_vampyrus
MIIPVRLH-------------------------------
>Dasypus_novemcinctus
---------MFSQVSSSSLMCS--STWTCYFS-------
>Condylura_cristata
MIITVSLHCTFSNVSSLLLMCS-PSIWTFYFS-------
>Oryctolagus_cuniculus
----MSLHSTFFQVFSSSLMSNHTSTWTCYFS-------
>Equus_asinus
MIIPVRLHCTFSQVSSSSLMCN-PSTWTCYFS-------
>Loxodonta_africana
-------------------MCN-TTTWTCYFS-------
>Dipodomys_ordii
-------------------MCN-TSTWTCYFR-------
>Tupaia_chinensis
MIPPVRLHCTFSQVSS-SRMCG-TSPWTCCFSLDVASNE

HG0624 ENSG00000156853 ZNF689

>Homo_sapiens
MALRSIKSIAGSCLCSRQRRCGSSAAIFPEGIFRCLSPKFGQEFPE
>Acinonyx_jubatus
MALRSIKSIAGSCLCSRLRRCGSSAAIFPEGIFRCLSPKFGQEFPE
>Tursiops_truncatus
MALRSIKSIAGSCLCFRQRRCSSGAAIFPEGIFRCLSPKFGQEFPE
>Eptesicus_fuscus
MALWSIKSIAGSCLCSRQRRCSSSAAIFAEGIFRCLSPKFGQEFPE
>Dasypus_novemcinctus
MALWSIKSIAGSCRCSREKRCSSSAAIFPEGILQCLSPKFGQEFPE
>Galeopterus_variegatus
MALRSIKSIAGSCLCSRQRRCNSSAAIFPEGIFRCLSPKFGQEFPE
>Oryctolagus_cuniculus
MALRSIKSIAGSRLCSRQRRCSCGAAIFPKGIFRCLSPKLGQEFPE
>Ceratotherium_simum_simum
MALRSIKSIAGSCLCSRHRRCSSSAAIFPEGIFRCLSPKFGQEFPE
>Manis_javanica
MALWSIKSIAGSCLCSRQRHWSSGAAIFPEGIFQCLSPKFGQEFPE
>Marmota_marmota_marmota
MALRSIKSIAGSCLCYRQRRCSSSAAIFPEGIFRCLSPKFGQEFPE
>Tupaia_chinensis
MALRSIKSIAGSCLCSRQRRCSSAAAIFPEGIFRCLSPKFGQEFPE

HG0625 ENSG00000164050 PLXNB1

>Homo_sapiens

MLLSPDPAGPAR

>Mustela_putorius_furo

MLLSPDPAGPAR

>Lipotes_vexillifer

MLLSPDPAGLAR

>Pteropus_vampyrus

MLLSPDPAGPAR

>Oryctolagus_cuniculus

MLLSPDPAGPAR

>Elephantulus_edwardii

MLLSPDPAGPAR

>Ceratotherium_simum_simum

MLLSPDPAGPAR

>Loxodonta_africana

MLLSPDPADPAR

>Dipodomys_ordii

MLLSPNPAGPAR

>Tupaia_chinensis

MQLSPDPAGPAR

>Orycteropus_afer_afer

MLLSPDPASPAR

HG0626 ENSG00000168939 SPRY3

>Homo_sapiens

-------MDKPFPQGFSHVP

>Acinonyx_jubatus

-------MDKPFPQGFSDVP

>Physeter_catodon

-------MDKPFLQGFSDVP

>Miniopterus_natalensis

-------MDRSFPQGFSDVP

>Galeopterus_variegatus

MTSAFPSMDKPFPQGFSDVP

>Erinaceus_europaeus

-------MDKPFPQGFSDVP

>Oryctolagus_cuniculus

-------MDKPFSQGFSDVP

>Equus_asinus

-------MDKPFPQGFSDVP

>Manis_javanica

-------MDKPFSQGFSDVP

>Heterocephalus_glaber

-------MDKPFPQGFSDVP

>Trichechus_manatus_latirostr

-------MDKPFPQGFSDVP

HG0627 ENSG00000169760 NLGN1

>Homo_sapiens
MRGLPLWIYLAEGYRPAANWI
>Odobenus_rosmarus_divergens
MRGFPLWIYLTEGCRPAANWI
>Balaenoptera_acutorostrata_s
MRGLLLWIYLTEGYRPAANWI
>Rhinolophus_ferrumequinum
MRGLPLWIFLTEGYRPAPNWI
>Sorex_araneus
MRGLPLWICLTEGYRPAANWI
>Equus_asinus
MKGLRLWI-------------
>Manis_javanica
MRVLPLWIYLTKGYRTAANWI
>Loxodonta_africana
MKGLPPWIPLTQGYRPAAKWI
>Marmota_marmota_marmota
MRGLPLWICLAEGYRPAANWI
>Tupaia_chinensis
MRGLAPRTRLPEGYRPTVNWI
>Orycteropus_afer_afer
MRGLPPWIYLTEGYRPATNWI

HG0628 ENSG00000170677 SOCS6

>Homo_sapiens

MKQEKYIDAALQPLQMFGDNIPDRNIDPLD

>Felis_catus

MKLEKYIDAALQPLQMFEDNIPERN-----

>Lipotes_vexillifer

------MQHCVQPLQMFEDNIPERN-----

>Dasypus_novemcinctus

---------------MFGDNIPQRS-----

>Galeopterus_variegatus

MKLEKYIDAALQPLQMFRDNIPERN-----

>Oryctolagus_cuniculus

MKLEKCIDAALQPLQMFGNNIPERN-----

>Ceratotherium_simum_simum

MKLEKYIDAALQPLQMFEDNTPERN-----

>Loxodonta_africana

MKLEKYIDAALQPLQMFGDNIPERN-----

>Heterocephalus_glaber

MKLEKHTDAALKPLQMFGDNIPERKLIPVD

>Tupaia_chinensis

MKLEKHTDAALQPLQMFGDTIPERR-----

>Trichechus_manatus_latirostr

MKLEKYVDAALQPLQMFGDNIPERN-----

HG0629 ENSG00000170961 HAS2

>Homo_sapiens
MLFLNFLFFLAGRLKFI
>Odobenus_rosmarus_divergens
MLFLNFLFFLAGRLKFI
>Lipotes_vexillifer
MLFLNFLFFLAGRLKFI
>Myotis_brandtii
MLFLNFLFFLAGRLKFI
>Dasypus_novemcinctus
MLFLNFLFFLAGRLKFI
>Galeopterus_variegatus
MLFLNFLFFLAGRLKFI
>Condylura_cristata
MLFLNFLFFLAGRLKFI
>Ceratotherium_simum_simum
MLFLNFLFFLAGRLKFI
>Mus_musculus
MLFLNFLFFLAGRLKFI
>Tupaia_chinensis
MLFLNFLFFLAGRLKFI
>Orycteropus_afer_afer
MLFLNFLFFLAGRLKFI

HG0630 ENSG00000171552 BCL2L1

>Homo_sapiens
MIPPAGAG--FFASHREAG
>Canis_lupus_familiaris
MIPPAGAP--FFASRREAG
>Lipotes_vexillifer
MIPPAGAG--FFASRREAG
>Pteropus_vampyrus
MIPPAGAG--LFASRREAG
>Dasypus_novemcinctus
MIPSAGAA--FFANRREAG
>Galeopterus_variegatus
MIPPAGAV--FFASRREAG
>Oryctolagus_cuniculus
MIPPAGAV--CFASRREAG
>Equus_caballus
MILPAA---AFFASHREAG
>Heterocephalus_glaber
MIPPAGAG--FFASRREAG
>Tupaia_chinensis
MIPPAGAA--YFASRREAG
>Trichechus_manatus_latirostr
MIPPAGAVLLFFASRREAG

HG0631 ENSG00000173548 SNX33

>Homo_sapiens

--MSPPCSQAYARRPDRAVSGPWTLL

>Physeter_catodon

MDMSSPCSQACAGRP---VSGPWTLL

>Cynopterus_sphinx

MDMSLSCLQACAGKPDRAVSGPWTQL

>Dasypus_novemcinctus

--MSPPCTQACAQRPERAFSGPWTLL

>Oryctolagus_cuniculus

MAGSPPSLEAHGQIPDWAAAGPWTLL

>Equus_asinus

MDMSPPCSHVCARRPERAVSGPWTLF

>Manis_javanica

MDVSPPCSQECARRSDRAVPGPWTLF

>Loxodonta_africana

--MSPPCSRSCAWRLDRAVSEPWTLL

>Ictidomys_tridecemlineatus

--MSPPCSQAYAQRLNRVVSGPWTLF

>Trichechus_manatus_latirostr

--MSPPCSQLCAWRPDRAVSGPWTLL

>Orycteropus_afer_afer

--MSPPYSQTFSWRLDRAVSGTWALL

HG0632.1 ENSG00000181315 ZNF322

>Homo_sapiens

MTVRATMGQEDQRKTP

>Ursus_maritimus

------MGQEDQWKTP

>Orcinus_orca

MTVKKMMGQEDQWKTP

>Dasypus_novemcinctus

MTSKTMMGQEDQWKTP

>Galeopterus_variegatus

MTMKAMMGQEGRWKTP

>Oryctolagus_cuniculus

MTVKVMMGQEDQWKTP

>Equus_caballus

MTVKKMMGQEDQWKTP

>Loxodonta_africana

MTAKTMMGQEDQWKTP

>Dipodomys_ordii

MTMKAKMGQEDQWKSP

>Trichechus_manatus_latirostr

------MGQEDQWKTP

>Orycteropus_afer_afer

MTVKTMMGQEDQWKTP

HG0632.2 ENSG00000181315 ZNF322

>Homo_sapiens

MYVFFHLDSRVGNTK-----------

>Canis_lupus_familiaris

MLLSFQLDSRVGNTK-----------

>Rhinolophus_ferrumequinum

MLLFIHLDSRVGNTK-----------

>Condylura_cristata

MLLIFHLDCRAGNTKQGISVYDGETL

HG0633 ENSG00000186174 BCL9L

>Homo_sapiens
MLRGWEAGTQAPSDPRIASPGPGWY
>Mustela_putorius_furo
MLGGWEAGTQAPSDPRIPSPGPGWY
>Orcinus_orca
MLRGWKAGTQAPSDPRIPTPGPGWY
>Pteropus_vampyrus
MLRGWKAGTQAPSNPRIPSPGPGWY
>Dasypus_novemcinctus
MLGDWEAGTQAPSDSRRPSLGPLWY
>Condylura_cristata
MFRGWEAGTQAPSDPRIPSPGPGWY
>Oryctolagus_cuniculus
MLRSWEAGTQAPRDSRTPSPGPGWD
>Equus_asinus
MLRGWEAGTQAPSDPRRP-PGPGWY
>Manis_javanica
MLRGWEAGTQAPSDPRIPSPGPGGY
>Marmota_marmota_marmota
MLRRREAGTQAPSDPRIPSPGPGWY
>Tupaia_chinensis
MLRGWETGTQAPSDPRIPSPGPGWC

HG0634 ENSG00000198026 ZNF335

>Homo_sapiens
-----------------MPESERNVATKASE
>Ursus_maritimus
------------MATSSMPESERSAATKASE
>Physeter_catodon
------------MATSSMPESERNAATKASE
>Rousettus_aegyptiacus
------------MATSSMPESERNAATKASE
>Dasypus_novemcinctus
MPIDSSAAGGKRMATSSMPESEKNADTKASE
>Galeopterus_variegatus
------------MATSSMPESEKNAATEASE
>Condylura_cristata
------------MATSSMPESERNAAAKTSE
>Ceratotherium_simum_simum
------------MATSSMPESERNAATKASE
>Manis_javanica
------------MATSSMPESERNAATQASE
>Cavia_porcellus
------------MATSSMPESERNAAKKASE
>Tupaia_chinensis
------------MATSSMPVSERNVATKASE

HG0635 ENSG00000259332 ST20-MTHFS

>Homo_sapiens
MAAETGYWRCLPESRTCAEEAVHLWPAVLRDPRWGNPGDGAWPCYMRGPRLE
>Leptonychotes_weddellii
---MPAPRLE
>Rousettus_aegyptiacus
---MPAPRLE
>Dasypus_novemcinctus
---MPAPRLE
>Condylura_cristata
---MPAPRLE
>Oryctolagus_cuniculus
---MPAPRLE
>Elephantulus_edwardii
---MPAPRLE
>Manis_javanica
---MPAPRLE
>Fukomys_darlingi
---MTAPRLE
>Trichechus_manatus_latirostr
---MPAPRLE
>Orycteropus_afer_afer
---MPAPQLE

HG0636 ENSG00000002587 HS3ST1

>Homo_sapiens

---MAQPRSL

FRAPDGLDRGAAQ

>Felis_catus

---MAQPESR

LRAPDRLDRGAAQ

>Physeter_catodon

---MAKPESR

LRAPDRLEGGAAQ

>Rhinolophus_ferrumequinum

---MAQPESR

LRTPDPLDRGAAQ

>Erinaceus_europaeus

---MAQPQSR

LGAPDWLDQRAAQ

>Oryctolagus_cuniculus

MPIPRARKPGQSEDPGPGGHPGAAPVSEDRRLPLLPGRHTEHRRLRTEHQNARLQGLRRAGNREPPPLPGLRGCLRPFHRALACLDIRSTCRGCSAVLPMAQPESR

LRAPDGLDGGVAQ

>Ceratotherium_simum_simum

---MAQPESR

LRAPDRLDRGAAQ

>Manis_javanica

---MAQPESR

LVAPDRLDRGAAQ

>Loxodonta_africana

---MAQPQSR

PRAPDGLDGGAAQ

>Cavia_porcellus

---MAQPESR

FRAPDGRDRRAAQ

HG0637 ENSG00000005102 MEOX1

>Homo_sapiens
MFSVIGDKYTYRGSQ
>Odobenus_rosmarus_divergens
MFSVIGDKYTSRGPQ
>Lipotes_vexillifer
MFSVIGDKYTYRGPH
>Pteropus_vampyrus
MFSVIGDKYTYRGPQ
>Dasypus_novemcinctus
MYSVIGDKYTHKGP-
>Galeopterus_variegatus
MFSVISDKYTYKGPQ
>Equus_asinus
MFSVIGDKYTYRGPQ
>Marmota_marmota_marmota
MFSAIGNKYMYKGPQ
>Tupaia_chinensis
---------MYKGPQ
>Trichechus_manatus_latirostr
MFSVISDKYTYKGPQ

HG0638 ENSG00000013561 RNF14

>Homo_sapiens

MSSTSWPLTQLQQSQLHQLENKWDLHELHSELKYRLRC

>Mustela_putorius_furo

MSSRSWPFIQLQQSHLHLLEDKWDLHKFQILN------

>Physeter_catodon

MSSTSWPLIQLQQSHLHHLENK----------------

>Myotis_davidii

MSSTSWPLIQLQQSHLHHLENKWDICMNSRL-------

>Dasypus_novemcinctus

MSPTSWPLIQHQQSHLHWPESKWGLHEFQTP-------

>Galeopterus_variegatus

MSPTSQPPIKLQQSQLHWLKNKWDRTNSQLN-------

>Condylura_cristata

MSSTSWPLIELLQSHLHRLENK----------------

>Ceratotherium_simum_simum

MSSTSWPLIHLQQSHLHRLENKWDFHEFQILN------

>Manis_javanica

MSSTSWPLTQLQ-FHLHCLENKWDLREFQTLN------

>Marmota_marmota_marmota

MSPTSWPLTQLQQSQLHWLENKWDLHKFQTELKFRLQC

HG0639 ENSG00000018236 CNTN1

>Homo_sapiens
MGAAVPACEVTA
>Canis_lupus_familiaris
MGAAVPASEVTA
>Orcinus_orca
MGAAVPASEVTA
>Myotis_brandtii
MGAAVPASEVTA
>Dasypus_novemcinctus
MGAAVPASEVTA
>Galeopterus_variegatus
MGAAVPASEVTA
>Equus_przewalskii
MGAAVPASEVTA
>Loxodonta_africana
MGAAVPASEVTA
>Rattus_norvegicus
MGAAVPASEVTA
>Tupaia_chinensis
MGAAVPASEVTA

HG0640 ENSG00000062194 GPBP1

>Homo_sapiens
------MLFRCEIFFLFLLFGS
>Canis_lupus_familiaris
------MLFRCEIFFLFLLFGS
>Balaenoptera_acutorostrata_s
------MLFRCEIFFLFLLFGS
>Miniopterus_natalensis
------MLFRCEIFFLFLLFGS
>Galeopterus_variegatus
------MLFRCEIFFLFLLFGS
>Oryctolagus_cuniculus
------MLFRCEIFFLFLLFGS
>Elephantulus_edwardii
MCSCLGMLFRCEIFF-------
>Ceratotherium_simum_simum
------MLFRCEIFFLFLLFGS
>Loxodonta_africana
------MLFRCEILFL--LFGS
>Orycteropus_afer_afer
------MFFRCEIFFLFLIFGS

HG0641 ENSG00000100359 SGSM3

>Homo_sapiens
MILDQMKPEEPSSSKIAG-
>Leptonychotes_weddellii
MFLDQMKPEEPSSCKIIG-
>Physeter_catodon
MFLDQMKLEEPSSCKILG-
>Miniopterus_natalensis
MFLDQMKPEKPSSCKILG-
>Dasypus_novemcinctus
MFLDQMKPEEPSSSKILGE
>Galeopterus_variegatus
MFLGQMKPEEPSSSKIG--
>Ceratotherium_simum_simum
MFLDQMKPEEPSNCKILV-
>Manis_javanica
MFLDQLKPEEPSGHRILD-
>Loxodonta_africana
-----MTSEEPSSSKILGD
>Tupaia_chinensis
MFLDQMKTEEPSSSHTAG-

HG0642 ENSG00000100815 TRIP11

>Homo_sapiens
MAAGVELAGVTHGTEESH
>Ursus_maritimus
MAAGVELAGVALGTEESH
>Orcinus_orca
MAAGVELAGVANGTGESH
>Myotis_lucifugus
MAAGVELAGVAHRTGASH
>Condylura_cristata
MAAGVELAGVAHGAGESH
>Ceratotherium_simum_simum
MAAGVELAGVAHGTGESH
>Manis_javanica
MAAGVELAGIAHGIGESH
>Loxodonta_africana
MAAGVELAGVAHGTGESH
>Heterocephalus_glaber
MAASVELAGTAFGTEESH
>Trichechus_manatus_latirostr
MAAGVELAGVARGIGESH

HG0643 ENSG00000103653 CSK

>Homo_sapiens
--------------------------------------MLFPTALMVPSDRLALL-------
>Acinonyx_jubatus
---MVPSDRLAFL-------
>Physeter_catodon
--------------MLPAA--------------RWPGPQCPERALMVPSDRLAFL-------
>Myotis_davidii
---MVPRDRLAFL-------
>Dasypus_novemcinctus
---MVPSDRLAFL-------
>Condylura_cristata
--------------MLPAA--------------RWPGPQCPERALMVPSDRLAFL-------
>Oryctolagus_cuniculus
---MVPGDRPALLTRGHQLS
>Equus_asinus
---MVPSNRLAFL-------
>Manis_javanica
MMALPPSTSSGSLWHLFTLLWGRSGSSVGSHLTPLLHVPLLPVALMVPGDKWAFL-------
>Fukomys_damarensis
---MVPSDRSAFL-------

HG0644 ENSG00000104299 INTS9

>Homo_sapiens
--MLSAGRWRRLHRKTLPGFEEFSDCY
>Mustela_putorius_furo
--MLSAGRWR-----------------
>Balaenoptera_acutorostrata_s
--MLSAGRWRRLNRKTLSGSEEWSDWY
>Pteropus_alecto
--MLSASEWRSLNRKTVSGFEESIDWC
>Oryctolagus_cuniculus
--MLSAGQRRSLNRKTHAGVEAS----
>Equus_asinus
--MLSAGRWQRLNRKTLSGYEGVE---
>Loxodonta_africana
--MLCAGWWRRLNRKTYSGFEESSDCC
>Cricetulus_griseus
------------------------------MLR----KVREKGKLQLPRCSLWRRLNRKTLPCFEK-----
>Tupaia_chinensis
--MLSAGQWRRLNRKTLPGVKESGGWY
>Trichechus_manatus_latirostr
MTEGGVRRGKGEKENITTVPMMHPGMTTFPMFPGGGAGREETACPLMLSAGWWRRLNRKTPFGFEGSSDCC

HG0645 ENSG00000107282 APBA1

>Homo_sapiens
MVALEAAAAGAELYSQAWHQRCH
>Canis_lupus_familiaris
MVALEVGAAGAERHSQAWPPRCH
>Rousettus_aegyptiacus
MVALEVGAAGAELYSQAWHQRCH
>Dasypus_novemcinctus
MVALEVGAAGAELHSQAWHHRCH
>Condylura_cristata
MVALEVGAAGAELSSQAGHQRCH
>Elephantulus_edwardii
MVALEVGPAGAELDSQARHRGCH
>Ceratotherium_simum_simum
MVALEVGAAGAELYSQAWHQRCH
>Manis_javanica
MVALEVGAARAELDSQARHQRCR
>Loxodonta_africana
MVALEVGAAGTELYSQAWHQRCH
>Heterocephalus_glaber
MVALEVGAAGAELYSQVWHRRCH

HG0646 ENSG00000108312 UBTF

>Homo_sapiens

MEQWEREAGRFPCKSPSGLTPSRRPRQGSEDPGEQMEEWSSL

>Balaenoptera_acutorostrata_s

---MGPGSRRFPRKSPSVPTSSRRTRQGSEEPGEQMEEWSSL

>Rousettus_aegyptiacus

-----------------------------------MEEWSSL

>Condylura_cristata

-----------------------------------MEEWSSL

>Ochotona_princeps

-----------------------------------MEEWSSL

>Elephantulus_edwardii

------------------------------------MEWSSL

>Ceratotherium_simum_simum

-----------------------------------MEEWSSL

>Dipodomys_ordii

---MGTGSGQVPSQIPLRLKPPRPHRQGSEEPGEQMEEWSSL

>Trichechus_manatus_latirostr

-----------------------------------MEEWSSL

>Orycteropus_afer_afer

-----------------------------------MEEWSSL

HG0647 ENSG00000108395 TRIM37

>Homo_sapiens

MGVGGAGPEEMGRAGGAVVRAP

>Felis_catus

----------MGRAGGAVVGAP

>Balaenoptera_acutorostrata_s

----------MGRAGGAVVGDP

>Pteropus_alecto

----------MGRADGAVVGAP

>Oryctolagus_cuniculus

----------MGRAGGAVVGVL

>Equus_asinus

----------MGRAGGAVVGAP

>Manis_javanica

----------MGRAGGAVVGAL

>Dipodomys_ordii

MGVAGLELEAMGRAGGAVVGAP

>Tupaia_chinensis

MGVAGAEPEAMGRAGGAVVGAP

>Trichechus_manatus_latirostr

----------MGRAGGAVVGAS

HG0648 ENSG00000114331 ACAP2

>Homo_sapiens
MTSGRGALRSSAQR-
>Odobenus_rosmarus_divergens
MTSGRGALRSSAQR-
>Orcinus_orca
MTSGRGALRSSAQR-
>Rousettus_aegyptiacus
MTSGRGVLPSSAQL-
>Dasypus_novemcinctus
MTGGRGALRSSVQR-
>Condylura_cristata
MTSGRGALRSSAQR-
>Ochotona_princeps
MTSGRGALRSPAQRS
>Elephantulus_edwardii
MTSGLGALRSSAQH-
>Ceratotherium_simum_simum
MTSGRGALRSSARP-
>Ictidomys_tridecemlineatus
MTSGRGALRSSAQC-

HG0649.1 ENSG00000114439 BBX

>Homo_sapiens

MWRIHWDIEKYQRRIVPL

>Panthera_tigris

MWRIHWDIEKYQRRVVLP

>Balaenoptera_acutorostrata_s

MWRSHWDIEKYQRRIVPL

>Rousettus_aegyptiacus

MWRIHWDIEKYQRRIVPL

>Dasypus_novemcinctus

MWRIHWDIEKYQRRIVPL

>Condylura_cristata

MWRSHWDIETYQRRFVPL

>Oryctolagus_cuniculus

MWRSHWDIEKYQRRIVPL

>Ceratotherium_simum_simum

MWRIHWDIEKYQRRIVPL

>Manis_javanica

MWRIHWDIEKYQRRIVPL

>Cavia_porcellus

MWRIHWDIEKYQRRIVPL

HG0649.2 ENSG00000114439 BBX

>Homo_sapiens
----MESLDLTGTLRKTPSL
>Canis_lupus_familiaris
----MESLDLTGTLRKTPSL
>Balaenoptera_acutorostrata_s
----MESLDLTGTLRKTPSL
>Myotis_brandtii
----MESLDLTGTLRKTPSL
>Dasypus_novemcinctus
MEITMESLDLTGNLRKTPAL
>Condylura_cristata
----MESLDLTGTLRKIPSL
>Equus_asinus
----MESLDLTGTLRKTPRL
>Manis_javanica
----MESLDLTGTLRKTPSL
>Microtus_ochrogaster
----MESLDLTGTLRKTPSL
>Tupaia_chinensis
----MESLDLTGTLGKTPSL

HG0650 ENSG00000115226 FNDC4

>Homo_sapiens

MGRPLGTEQPPEA

>Canis_lupus_familiaris

MGRPLGTEQPPEA

>Orcinus_orca

MGRPLGTEQPPEA

>Miniopterus_natalensis

MGRPLGTEQPPEA

>Dasypus_novemcinctus

MGRPLGTEQPPEA

>Sorex_araneus

MGRPLGTEQPPEA

>Equus_asinus

MGRPLGTEQPPEA

>Peromyscus_maniculatus_baird

MGRPLGTEQPPEA

>Tupaia_chinensis

MGRPLGTEQPPEA

>Orycteropus_afer_afer

MGRPLGTEQPPEA

HG0651 ENSG00000115935 WIPF1

>Homo_sapiens

MIPFSGTVFSLGSCPAGPLSACVPDHGSLQYPLTIYQQDC---

>Felis_catus

MMPFSGTVFSLGSHLAC-LSTCVPDHAFLQYPLTIHRQGC---

>Physeter_catodon

MTPFSGTVFSLISCLL---PVCLCP------------------

>Miniopterus_natalensis

MMPFSGTVFSLGSCFACSVSACVPGHAFLQYPLTIYQQDC---

>Oryctolagus_cuniculus

MMDFSGTVCSPGSGPSCSLSAGGPDHAFHGAP-----------

>Ceratotherium_simum_simum

MMPFSETVFSLGSYLACSLSACVPDHAFLQYPLTIYQDC----

>Manis_javanica

MMPFSGTVVTLGSYLAYSLSACVPDHALLHYPLKTHQQDC---

>Loxodonta_africana

MIPFSGMVFCLGSCLTCSLSACVPDHACPAVSFHDFSAGLLKV

>Cavia_porcellus

------MIFSLGSALACALSACIPDHASLQFLLTIYQQDC---

>Orycteropus_afer_afer

MMPFSGTIFSLGSCLVCSLSACVPDHACPAVSFNDLSARLLKV

HG0652 ENSG00000118515 SGK1

>Homo_sapiens
MMEGEKPLSVATESGERLLQ--------
>Odobenus_rosmarus_divergens
-MGSAKPLPVATESGERLLQ--------
>Balaenoptera_acutorostrata_s
MMEREEPLPVTTESGERLLQ--------
>Myotis_lucifugus
MMEREKPLPVAIESGERLLQSPLP----
>Galeopterus_variegatus
MMEREKPLPVATESGERLLQ--------
>Erinaceus_europaeus
-MEREKPLPVATESGERLLQ--------
>Oryctolagus_cuniculus
-MERERSLPVATERGERLRQSQALQNV-
>Loxodonta_africana
MMERESPPPVATEGGESLLQ--------
>Mus_musculus
MMEREKPLPVASESGASYCSRCRSRMSE
>Orycteropus_afer_afer
-MARENPPPVATESGEGLLQWLVLQNV-

HG0653 ENSG00000120334 CENPL

>Homo_sapiens

MIRESGMGDSVITAFY

>Ursus_maritimus

------MGDSVITAFY

>Lipotes_vexillifer

------MVDSVITAFY

>Pteropus_alecto

MIREAGLGDSVITAFH

>Dasypus_novemcinctus

MIREAGMGDSVISAFC

>Oryctolagus_cuniculus

MIAESGMGDSVDTAFY

>Ceratotherium_simum_simum

------MGDSVITAFY

>Manis_javanica

MIGAAGMGDSVITAFY

>Loxodonta_africana

MICEAGMRDSVVAAFY

>Fukomys_damarensis

------MGDRVTTAFY

HG0654 ENSG00000133794 ARNTL

>Homo_sapiens
----------MWNPGPSLVFGRS
>Acinonyx_jubatus
----------MWNPRPSLVSGRS
>Galeopterus_variegatus
MLLDPSFTFQMWNPRPLLVFGRS
>Elephantulus_edwardii
----------MWNPGPLLVFRRS
>Manis_javanica
----------MWNPRPSLVSERS
>Loxodonta_africana
MVLDSSLTFQMWNPRPLLVFERS
>Ictidomys_tridecemlineatus
MLLDPTSTTQMWNPRPSLVFGRS
>Tupaia_chinensis
MLLDPRLTFQMWNPRPSLVVGRS
>Trichechus_manatus_latirostr
MLLDPSLTFQMWNPRPLLIFGRS
>Orycteropus_afer_afer
----------MWNPRPFLVFGRS

HG0655 ENSG00000143061 IGSF3

>Homo_sapiens
---MWQEPGATSELLWTLPVA---
>Canis_lupus_familiaris
---MWEKPRGPSELFWTLPLA---
>Physeter_catodon
---MWQEPPAPSELFWTLPLA---
>Rhinolophus_ferrumequinum
---MWQKPRLSAELFWTLPLLRGL
>Condylura_cristata
MREITDTFTAKPRTFLYNQWMKGFSQTAFSLFLED-YPPRSTIPVETARHTMWPKPRAPSELIWTLPLA---
>Ochotona_princeps
---MWQEPGAPSE-FWTLPLA---
>Ceratotherium_simum_simum
MREITDTFTGKPRTFLYNQWMKGFSQTLFSPFSLRIKPLQSSIPVQTARHTMWQKPRAPSELFWTLPLA---
>Loxodonta_africana
---MWQKPGAPSELVWTLLLP---
>Peromyscus_maniculatus_baird
---MWQKPRAPSELLWTLPLA---
>Trichechus_manatus_latirostr
---MWQKPGAPSELVWTLHLP---

HG0656 ENSG00000150776 C11orf57

>Homo_sapiens

MGIFPLSSTWKPQPPLQAP

>Panthera_tigris

MGVFSLTSLWKLHPPLRAL

>Lipotes_vexillifer

MGVFSLSSTWKPQPPLQPL

>Rousettus_aegyptiacus

MGVFSLSSFWKPRSPLQAL

>Dasypus_novemcinctus

MGVFSLSSAWRPQPPL---

>Galeopterus_variegatus

MGVFSLSSTWKPQHPLQAP

>Oryctolagus_cuniculus

MGVFSLSSTWKPQRPRQAP

>Equus_caballus

MGVFFLSSTWKPQLPLQAR

>Marmota_marmota_marmota

MGVFSLFSTWKPQPPFQAP

>Tupaia_chinensis

MGVFPLFSTWKLQTSLQAP

HG0657 ENSG00000151276 MAGI1

>Homo_sapiens
MQTKLAGACAGLAGKPRKFVL
>Ursus_maritimus
MQTKLAGACAGLAGKPRKFVL
>Balaenoptera_acutorostrata_s
MQTKLAGACAGLAGKPRKFVL
>Pteropus_vampyrus
MQTKLAGACAGLAGKPRKFVL
>Condylura_cristata
MQTKLAGAFAELAGKPRKFVL
>Ochotona_princeps
MQTKLAGARAGLAGKPGKFVG
>Equus_asinus
MQTKLAGACAGLAGKPRKFVL
>Manis_javanica
MQTKLAGACARLAGKPRKFVL
>Ictidomys_tridecemlineatus
MQTKLAGACAGLAEEPRKFVL
>Tupaia_chinensis
MQTKLAGACAGLAGKPRKVVL

HG0658 ENSG00000153786 ZDHHC7

>Homo_sapiens
--------------------------------------MHLKILKKRHKMFK
>Canis_lupus_familiaris
--------------------------------------MHLKILKKRRKMFK
>Balaenoptera_acutorostrata_s
--------------------------------------MHLKILKKRHKMFK
>Rhinolophus_ferrumequinum
--------------------------------------MHLKILKKRRKMFK
>Condylura_cristata
--------------------------------------MHLKILKKRHKMFQ
>Equus_asinus
--------------------------------------MHLRVLKKRRKTFK
>Manis_javanica
MMRPCLSRSSRRRRRRRPGGAAEPGDPARASDPVTQDSMHIKILKKRRKMSK
>Loxodonta_africana
--------------------------------------MHLKTLKKRRKMFK
>Dipodomys_ordii
--------------------------------------MHLKILKKRHKMFA
>Tupaia_chinensis
--------------------------------------MHLKILKKRHKAFR

HG0659.1 ENSG00000158856 DMTN

>Homo_sapiens
----MEVYVTEEAAALLPHALGP
>Odobenus_rosmarus_divergens
MRRGMEVYVTEEAAVLLPHAPGP
>Balaenoptera_acutorostrata_s
----MEVYVTEEAA--EPHAPGP
>Rousettus_aegyptiacus
MRRGMEVYVTEEAAVLLPHAPGP
>Erinaceus_europaeus
----MEVYLTEEEAVLLPHAPGP
>Equus_asinus
MRRGMEVYVTEEAAVLLPHAPGP
>Manis_javanica
MRRGMEVYVTEEAAVLLPHAPGP
>Fukomys_darlingi
MRLEMEVYVTTEAAALLPHAPGP
>Trichechus_manatus_latirostr
MRRGMEVYVTEEAAVLLPHSPGP
>Orycteropus_afer_afer
MRRGMEVYVTEEAAELLPHAPGP

HG0659.2 ENSG00000158856 DMTN

>Homo_sapiens

MRTRQPGGTRQLLSRPQARSAQQPRRA

>Mustela_putorius_furo

MRTPQPGGTHSLLSWPGARSAQEPGRA

>Balaenoptera_acutorostrata_s

MRTPQPGGAPFLLSRPRARSAQEPDRA

>Pteropus_alecto

MRTLQPSGTPFLHSRPRARSAQKPGRA

>Condylura_cristata

MRTLQRSGPPSLLSRPRARSAPEPGRA

>Equus_asinus

MRTPRPGGTPFLLSRPRARSAQEPRSA

>Manis_javanica

MRPPQPAGTPFLLSPHRVRSAQEPGRA

>Trichechus_manatus_latirostr

MRTLQLGGTPFLLSRPRTRSAQERSRA

HG0660 ENSG00000165861 ZFYVE1

>Homo_sapiens
-----MEDETIKPNQRCC
>Canis_lupus_familiaris
-----MEDKTIKPNQRCC
>Lipotes_vexillifer
-----MEDETIKPSQRCC
>Rhinolophus_ferrumequinum
-----MEDETIKPNQRCC
>Dasypus_novemcinctus
-----MEDETIKPNQYCC
>Ceratotherium_simum_simum
-----MENKTIKPNQRCC
>Loxodonta_africana
-----MEDETIKPSQCCC
>Fukomys_darlingi
MSLHFTEDEPIKPNQHCC
>Tupaia_chinensis
-----MEDETITPNQHCC
>Trichechus_manatus_latirostr
-----MEDETVRPNQSCC

HG0661 ENSG00000166845 C18orf54

>Homo_sapiens

-MDFLLEIRTEACTEEMLFLREMNRNNPL

>Acinonyx_jubatus

MMDFLLEIGTEVFTEEM-FLREMNRNNPF

>Physeter_catodon

MMDFLLEIHLEVVTEEMLFLREMSRNNPF

>Rousettus_aegyptiacus

MMYFFWR--IRTVTEEMLFLREMNRNKPF

>Galeopterus_variegatus

MMNFLLEIRTQAFTEEMLFLREMNRNNPF

>Oryctolagus_cuniculus

-MGLLLGSRTGARMEEMLFLRGMNRNNPF

>Equus_asinus

MMDFSLKIRAEAFREKRLFLREMNRNNLF

>Manis_javanica

MMDFLLE--TEVFTEEMLFLREINRNNPF

>Loxodonta_africana

----------------------MNRNNPF

>Tupaia_chinensis

MMDLLSEIRSEAFVEEMLFLREMNRNNSF

HG0662 ENSG00000166897 ELFN2

>Homo_sapiens

MVAAPGRTVLPAPASGR

>Canis_lupus_familiaris

MVAAPGRTVPPAPASRC

>Orcinus_orca

MVAAPGRTVLPAPASGR

>Miniopterus_natalensis

MVAATGRTVLPPPASSC

>Galeopterus_variegatus

MVATPGRTVLPAPASGC

>Erinaceus_europaeus

MVATPGRTVLPAAASGL

>Oryctolagus_cuniculus

MVAAPGRTVLPAPTSGR

>Manis_javanica

MVAAPGRTVLPAPASGH

>Ictidomys_tridecemlineatus

MVAAPGRTVPPAPASGR

>Tupaia_chinensis

MVAAPGRTVLPTPASGR

HG0663 ENSG00000168615 ADAM9

>Homo_sapiens

MSGASSDRGLSCVSAARVLVGRACSSGARASRPDLGPRQGWKMMEEAEVEATEC

>Odobenus_rosmarus_divergens

MSGASSDRGRV------ASVPSACSSRARASRPALGPRQGWEMMEQPEAEATEC

>Orcinus_orca

--MVERAEAGATEC

>Rousettus_aegyptiacus

--MMEPADAEAIEC

>Dasypus_novemcinctus

---MEEAEAGATEC

>Condylura_cristata

--MMEQAEAEVSEC

>Oryctolagus_cuniculus

MSGASLDRGPSCVSAARVLVPGRARP-----GQPWGPREGWKMMGEAE--ATEC

>Equus_asinus

MSGASSDWSRV------ASVPSACSPAGARTRPALGPRQGWEMMEPAEAQATDC

>Manis_javanica

MSGASS-DSRV------ASVPSACSPRARASLPALGPRQGWEVMERAEAEATVS

>Chinchilla_lanigera

---------MGCVSAARMPASGR------AHPGPHGARQGSEMMDKAETAR---

HG0664 ENSG00000169085

>Homo_sapiens
----------------------MIPEPDWIRMPRD
>Felis_catus
----------------------MIPEPDWIRKPRD
>Balaenoptera_acutorostrata_s
----------------------MIPEPDWIRKPRD
>Myotis_davidii
MLQRRLKLDELTAPACLLWTCTMIPEPDWIRKPCN
>Galeopterus_variegatus
----------------------MIPEPDWIRKPHD
>Oryctolagus_cuniculus
----------------------MIPEPDWISDARD
>Equus_caballus
----------------------MIPEPEWIRKPRD
>Manis_javanica
----------------------MIPEPDWIRKPRD
>Chinchilla_lanigera
----------------------MIAEPDWIRRPRD
>Tupaia_chinensis
--------MSSQPPACLLWTCTMIPEPDWIRKARD

HG0665 ENSG00000169515 CCDC8

>Homo_sapiens
MSAKMWIWTSSWKP
>Ailuropoda_melanoleuca
MSAKMWIWTSSWKP
>Balaenoptera_acutorostrata_s
----MWIWTSSWKP
>Rhinolophus_ferrumequinum
----MWIWTSSWKP
>Galeopterus_variegatus
MSAKMWIWTSSWKP
>Equus_caballus
MSAKMWIWTSSWRP
>Manis_javanica
MSAKMWIWTSSWKP
>Loxodonta_africana
----MWTWMSSWKP
>Marmota_monax
MSAKMWIWTSSWKP
>Tupaia_chinensis
----MWIWTSWWKP

HG0666 ENSG00000171943 SRGAP2C

>Homo_sapiens
MSDPRPASGRCSSPG
>Ailuropoda_melanoleuca
MSDPRPASGRCSSPG
>Physeter_catodon
MSDPRPASGRCSSPG
>Rousettus_aegyptiacus
MSDPRPASGRCSSPG
>Condylura_cristata
MSDPRPASGRCSSPG
>Oryctolagus_cuniculus
MSDPRPASGRCSSPG
>Ceratotherium_simum_simum
MSDPRPASGRCSSPG
>Manis_javanica
MSDPRPASGRCSSPG
>Loxodonta_africana
MSDPRPASGRCSSPG
>Cricetulus_griseus
MSDPRPASGRCSSPG

HG0667.1 ENSG00000182700 IGIP

>Homo_sapiens

MCQYTVRISLICTVWGPHLRMLNFAN

>Acinonyx_jubatus

MCQLYSRISLICTVWGPH--------

>Physeter_catodon

MCQLYSRISLICTVWGPH--------

>Myotis_brandtii

MCQLHSRISLICTVWGPH--------

>Galeopterus_variegatus

--------------------MLNFAN

>Condylura_cristata

MCQLYSRISLICTVWGPH--------

>Ceratotherium_simum_simum

MCQLYSRISLICTVWGPH--------

>Manis_javanica

MCQLYSRISLICTVWGPH--------

>Rattus_sp.

--------------------MLNFAN

>Tupaia_chinensis

--------------------MLNFAN

HG0667.2 ENSG00000182700 IGIP

>Homo_sapiens
--------------------------------MLRKSVGFSAVREGRSS-ALIWVTEANMSKTLVN
>Ailuropoda_melanoleuca
MGPFSISWGICTGQRLPAMRYELKPISLFSYSMLRKSVGFSAVREGRSSAALIWVTEAGWGKTLVN
>Lipotes_vexillifer
--------------------------------MLRKSVGFLAVREGRSSAALIWVTEASLSKSLVN
>Rousettus_aegyptiacus
--------------------------------MLRKSVGFSVVREGRSSAALIWVTEAGLSKTLVN
>Galeopterus_variegatus
--------------------------------MLRKSVGFSAVREGRSS-ALIWVTEAGLSKTSVN
>Sorex_araneus
---------MCTGRRLPAMRCELKPISLFSYSMLRKSVGFSAVREGRSSAALIWVTEAGLSKTLVN
>Ceratotherium_simum_simum
--------------------------------MLRKSVGFSAVREGRSSAALIWVTEAGLSKTLVN
>Manis_javanica
--------------------------------MLRKSVGFSAVREGRSSAALIWVTEAGLSKTLVN
>Chinchilla_lanigera
--------------------------------MMRKSVGFSAVREGRSSAALIWVTEAGLSKTLVN

HG0668 ENSG00000182944 EWSR1

>Homo_sapiens
MVAPGVARFAPAQCGA
>Tursiops_truncatus
MVAPEVAGFAPAQCGA
>Eptesicus_fuscus
MVAPEVAGFAPAQCGA
>Condylura_cristata
MVAPEVADFAPAQCGA
>Ochotona_princeps
MVAPEVASFAPAQCGA
>Ceratotherium_simum_simum
MVAPEVAGFAPAQCGA
>Loxodonta_africana
MVAPEVAGLAPAQCGA
>Marmota_marmota_marmota
MVAPEVASFAPAQCGA
>Trichechus_manatus_latirostr
MVAPEVAGFAPAHCGA
>Orycteropus_afer_afer
MVTPEVAGFAPAQCGA

HG0669 ENSG00000183876 ARSI

>Homo_sapiens
MVESTFWKLPRRVSGWPG
>Mustela_putorius_furo
MVESTFWKQPRRVSGWPG
>Myotis_lucifugus
MVESTFWKSPRRVPGWPG
>Dasypus_novemcinctus
MVESTFWKPPSRVSGWPG
>Condylura_cristata
MVQSTFGELPRRVSGWPG
>Oryctolagus_cuniculus
MVESAFWEPPRRVSGWPG
>Ceratotherium_simum_simum
MVESTFWKPPRRVSGWPG
>Loxodonta_africana
MVESTFWKPPSRVSGWPG
>Cavia_porcellus
MVESTFWRPPRYVPGWPG
>Tupaia_chinensis
MVESTFCKSPRRVSGWPD

HG0670 ENSG00000187164 SHTN1

>Homo_sapiens
---MISLARS
APGGGGAGADPTSG
>Leptonychotes_weddellii
---MISLARS
APGGGGAGADPTSG
>Lipotes_vexillifer
---MISPARS
APGGGGAGANPTSG
>Eptesicus_fuscus
---MISPVRS
APGGGGAGTNPTSG
>Dasypus_novemcinctus
---MISLARF
APGGGGAGANPTSG
>Oryctolagus_cuniculus
---MISPARS
APGGGGAGANPTSG
>Equus_caballus
MRVLMSRGWRRRKGSKSEPETRQTGLHALLPAHPAAPASVHKAEAQRPPQAKARAAVPVPPQVSPSTPSPVPGLLVRPRPPPRGLPAGEAPRPLSRCVRMISLVRS
APGGGGAGANPTSG
>Loxodonta_africana
---MISLARS
APGGGGAGANPTSG
>Marmota_marmota_marmota
---MISLARS
APGGGGAGADPTSG
>Tupaia_chinensis
---MISLARS
APGGGGAGADPTSG

HG0671 ENSG00000189184 PCDH18

>Homo_sapiens

------MPKVSGSRDTVCIKQKFASC

>Odobenus_rosmarus_divergens

MQGSDSTPIISGSRDTVCIKQKFASC

>Orcinus_orca

MQGLDSMPIISGSRDTVCIKQKFASC

>Pteropus_vampyrus

MRGLHGVPMLSGSRHTVCIKQKFASR

>Dasypus_novemcinctus

MQGLDSLPVISASRDTVCMKQKFANC

>Galeopterus_variegatus

MQGLDSMPTISGSRNTVCIKQKFASG

>Ceratotherium_simum_simum

MQGLDSMPVISGSRDTVCIKQKFASC

>Manis_javanica

MQGSDSMPIVSGSRDTVCIKQKFASC

>Cavia_porcellus

MQGI-GLHADSGNRDTVCMKQKFASC

>Trichechus_manatus_latirostr

MQGLDSMPIISGNRDTVCIKQKFASC

HG0672 ENSG00000196369 SRGAP2B

>Homo_sapiens
MSDPRPASGRCSSPG
>Ailuropoda_melanoleuca
MSDPRPASGRCSSPG
>Physeter_catodon
MSDPRPASGRCSSPG
>Rousettus_aegyptiacus
MSDPRPASGRCSSPG
>Condylura_cristata
MSDPRPASGRCSSPG
>Oryctolagus_cuniculus
MSDPRPASGRCSSPG
>Ceratotherium_simum_simum
MSDPRPASGRCSSPG
>Manis_javanica
MSDPRPASGRCSSPG
>Loxodonta_africana
MSDPRPASGRCSSPG
>Cricetulus_griseus
MSDPRPASGRCSSPG

HG0673 ENSG00000266028 SRGAP2

>Homo_sapiens
MSDPRPASGRCSSPG
>Odobenus_rosmarus_divergens
MSDPRPASGRCSSPG
>Physeter_catodon
MSDPRPASGRCSSPG
>Rousettus_aegyptiacus
MSDPRPASGRCSSPG
>Condylura_cristata
MSDPRPASGRCSSPG
>Oryctolagus_cuniculus
MSDPRPASGRCSSPG
>Ceratotherium_simum_simum
MSDPRPASGRCSSPG
>Manis_javanica
MSDPRPASGRCSSPG
>Loxodonta_africana
MSDPRPASGRCSSPG
>Cricetulus_griseus
MSDPRPASGRCSSPG

HG0674.1 ENSG00000006576 PHTF2

>Homo_sapiens

---------------MFFSLAQPKMTNV

>Ailuropoda_melanoleuca

---------------MFFSLAQPKMTNV

>Lipotes_vexillifer

MLFSLLLEGGSQVLCMFFSLAQPKMTNV

>Myotis_davidii

---------------MFFSLEQLKMTNV

>Erinaceus_europaeus

MLICLLLERGSKVVCVFFSLAQPKMTNV

>Ochotona_princeps

---------------MFFSLTQPKMTTV

>Elephantulus_edwardii

---------------MLFSLAQPKMTNV

>Equus_asinus

---------------MFFSLAQPKMTNV

>Orycteropus_afer_afer

---------------MFFFLAQPKMTNV

HG0674.2 ENSG00000116793 PHTF1

>Homo_sapiens

MRPAWPAASPPASRWDPGH

>Lipotes_vexillifer

MRPAWPGASPPASWPGRGH

>Nannospalax_galili

MRPTRPAASPPASRPGPSH

>Tupaia_chinensis

MRPAGPVASPRASRTGRGH

HG0675 ENSG00000048991 R3HDM1

>Homo_sapiens
---------------MQSISLQVLWKEHLLNSV--------
>Canis_lupus_familiaris
---------------MQSISLQILWKVHLHSLCMMKRTPVQ
>Orcinus_orca
---------------MQSISLQILWKVHLHSLCMMKQIPIR
>Myotis_brandtii
MPNQPAVCVVSSGGAMQSISLQILWKVHFHLLCVRKQIPIQ
>Galeopterus_variegatus
---------------MQSISLQILWKVHLH-----------
>Oryctolagus_cuniculus
---------------MQNISLQILWKVHLH-----------
>Ceratotherium_simum_simum
---------------MQSISLQILWKVHLHSLCMMKQIPIQ
>Fukomys_damarensis
--------MELSRRAMQSISLQILWKVHLH-----------
>Orycteropus_afer_afer
---------------MQSISLQILWKVHLH-----------

HG0676 ENSG00000058673 ZC3H11A

>Homo_sapiens
MAIMLLCLLQLAAPLCSYSITIRFYLFWLNTP
>Felis_catus
MAIMLLCLLQLAAPLCSYSITIRFYLFWLNTP
>Eptesicus_fuscus
MAVMLLCLLQLAAPLCSYSITIRFYLFWLNTP
>Oryctolagus_cuniculus
MALPPLCLPQLAALLCSYSVTLRFYLFWLNTP
>Equus_caballus
MAIMLLCLLQLAAPLCSYSVTIRFYLFWLNTP
>Loxodonta_africana
MAITLLCLLQLAAPLRSYSITVRFYLFWLNTP
>Cricetulus_griseus
MAIMLLCLLQLAAPLCSYSVTIRFYLFWLNTP
>Trichechus_manatus_latirostr
MAIMLLCLLQLAAPLCSYSVTVRFYLFWLNTP
>Orycteropus_afer_afer
MAIMLLCLLQLAAPLYSYSITIRFYLFWLNTP

HG0677 ENSG00000064989 CALCRL

>Homo_sapiens
-MIHQPLVLPSVKTLDGPTFRS
>Ailuropoda_melanoleuca
-MIHQPLVLPPVKTLDGPTFRS
>Balaenoptera_acutorostrata_s
MMIHQPLVLPPVKTMDGPTFRS
>Rousettus_aegyptiacus
-MIHQPLVLPPVKTLDGPTFRS
>Dasypus_novemcinctus
-MTHQPLVLPPVKTLDVPTFRS
>Galeopterus_variegatus
MMIYQPLVLSPVKTLDGPTFRS
>Ochotona_princeps
-MIHPPLILPPVKTVDDPTFRS
>Equus_asinus
-MMHQPLVLPPVKTLDGPTFRS
>Nannospalax_galili
-MIHQPLVLPAVKTSDGPTFRS

HG0678.1 ENSG00000090776 EFNB1

>Homo_sapiens
--MGLARSAASPRSSKYTEQGPR--
>Canis_lupus_familiaris
MRAGQQWEVCEARTGSHWTFGRNRWGAGTRRRGCGATRPRLQKHKLMGLALLVASSRSSKYGKRGPR--
>Orcinus_orca
--MGLARSAVSPRSSKYTERAPR--
>Eptesicus_fuscus
--MGLARSAASPLSFKSTERGPR--
>Galeopterus_variegatus
--MGLARSAASLR-SKCIERGPPLK
>Condylura_cristata
--MGLACSAASPLSSKYTEWGPC--
>Ochotona_princeps
--MGLARSPASPRSSKYRERGPALK
>Ictidomys_tridecemlineatus
--MGLARSAASPRSFKYTERGPR--
>Tupaia_chinensis
--MGLAHSAASPRSSKYTERGPC--

HG0678.2 ENSG00000108947 EFNB3

>Homo_sapiens

MPPPPGPRLPQSPT

>Mustela_putorius_furo

MPPPPGPRLPQSPT

>Physeter_catodon

MPPPPGPRLPQSPT

>Myotis_davidii

MPPPPGAWLPQSPT

>Oryctolagus_cuniculus

MPPPPGPRLPQSPT

>Mesocricetus_auratus

MLPPPGPRLPMSPT

HG0679 ENSG00000100281 HMGXB4

>Homo_sapiens
MAAIGGVEAGSGRAE
>Odobenus_rosmarus_divergens
MAAVGGAEAGSGRAE
>Rousettus_aegyptiacus
MAVVGGAEAGSGRAK
>Dasypus_novemcinctus
MAAVGGAEAGSRRAE
>Condylura_cristata
MAAVGGAEAGSGPAE
>Ochotona_princeps
MAAVGGAEAGSGRA-
>Ceratotherium_simum_simum
MAAVGGAEAGSGRAE
>Manis_javanica
MAAVGGAEAGSGRAE
>Ictidomys_tridecemlineatus
MAAVGGAEAGSGRAE

HG0680 ENSG00000100678 SLC8A3

>Homo_sapiens
MPRTRLRPAGSPRGR
>Odobenus_rosmarus_divergens
MPRTRLRPAGSPRGR
>Eptesicus_fuscus
MPRTRLRPAGSPRGR
>Erinaceus_europaeus
MPGTRLRPGGCPRGR
>Oryctolagus_cuniculus
MPRTRLRPAGSPRGR
>Equus_przewalskii
MPRTRLRPAGSPRGR
>Marmota_marmota_marmota
MPRTRLRPAGSPRGR
>Trichechus_manatus_latirostr
MPRTRLRPAGSPRGR
>Orycteropus_afer_afer
MPRTRLRPAGSPRGR

HG0681 ENSG00000101349 PAK5

>Homo_sapiens
---MLPPLAKMNTEEK---
>Mustela_putorius_furo
---MLPPLAKMNTEEK---
>Balaenoptera_acutorostrata_s
---------------------------------MRHRRCADSQRPEIHCGEMLPPLAKMNTEEK---
>Cynopterus_sphinx
-------------------------------------MRRRRQPPEVHCGKMLPPLAKMNTEEK---
>Dasypus_novemcinctus
MPSCKLQLLWSASLSTLFPELRGSAALHLISPSLNCSLEIFSRQQEIHCGEMFPPLAKMNAEEKEEN
>Erinaceus_europaeus
---------------------------------MRCRRVADNHPPVIHSGKMLPPLAKMNTEEK---
>Manis_javanica
---MLPPLAKMNTEEK---
>Ictidomys_tridecemlineatus
---------------------------------MSHCRFADSQPPEIHCGEMLLPLAKMNTEEK---
>Orycteropus_afer_afer
---MFPPLAKMNTEENEGN

HG0682 ENSG00000105829 BET1

>Homo_sapiens

MSWRFRGRSWCFAGPWY

>Ailuropoda_melanoleuca

MSWRFGERSWCFSGPWY

>Lipotes_vexillifer

MSWRFRGRSWCFAGPRY

>Myotis_davidii

MSWRYRGRSW-------

>Dasypus_novemcinctus

MPWHFRGRSWSSGGPWD

>Oryctolagus_cuniculus

MSWRLG-----------

>Ceratotherium_simum_simum

MSWRFRGRSWCFAGPWS

>Ictidomys_tridecemlineatus

MSWRFGARSWCCSGPWD

>Tupaia_chinensis

MSWRSWGRSWCSE----

HG0683 ENSG00000111837 MAK

>Homo_sapiens
MKHTRRRSFTRERTRVFIRMEVLFPCPIPSCYIS
>Canis_lupus_familiaris
-------------------MEALFPCQIPYWYIS
>Myotis_brandtii
MKHTRRRSLTPEQKRVFIRMDALFPCHIPYCCSS
>Erinaceus_europaeus
-------------------MEALFSCHTPYCSIS
>Oryctolagus_cuniculus
-------------------MESLFPCQIPYYCTA
>Equus_caballus
MKHTRRPSFTRQRRRVFIRMEALFPCQIAYCCIS
>Loxodonta_africana
-------------------MEALFPCQIHYYIS-
>Tupaia_chinensis
-------------------MEALFPCQIPYCCIS
>Orycteropus_afer_afer
-------------------MEALFP---------

HG0684 ENSG00000113916 BCL6

>Homo_sapiens

MRGLCHSPHCLLH---

>Felis_catus

MRGLCHSPHCPLP---

>Pteropus_vampyrus

MRGLCHSPHCPLP---

>Erinaceus_europaeus

MRGLCHSPHCPLP---

>Oryctolagus_cuniculus

MRGLCHSPHCPLP---

>Ceratotherium_simum_simum

MRGLCHSPHCPLP---

>Manis_javanica

MRGLCHSPHCPCLDGVTGKKRRIQCWLPRCTKRFI---

>Ictidomys_tridecemlineatus

MRGLCHSPHCPLPWRSNRKKKKKAYTM-LATENLFECNEIKGRRSHTIRPPPRLRGLRRMGSGASVSPGAGRSLEDSF

>Tupaia_chinensis

MRGLCHSPHCPLP---

HG0685 ENSG00000117262 GPR89A

>Homo_sapiens
MASGWRAAVPAAAPGRRQTV------------------
>Odobenus_rosmarus_divergens
MASGWRAAVPAVAPGRSPPL------------------
>Physeter_catodon
MASGWRAEVPAVARGRQLPV------------------
>Pteropus_vampyrus
MASGWRAAAPVAVLGQRPPV------------------
>Galeopterus_variegatus
MASDWSAALRAAAPGRRPPA------------------
>Erinaceus_europaeus
MASGSGARLSAAAPGRRPQV------------------
>Equus_asinus
MASGWRSAVPAVAPGRRLPV------------------
>Marmota_monax
MASGRSAAVPAAASGRRPAALVGRRPERSCPGEWEVET
>Tupaia_chinensis
MASGWLAAVPAVAPGRRLPV------------------

HG0686 ENSG00000118985 ELL2

>Homo_sapiens
----------------------------MTVRQQRDGPSSGGGCRSPSSRGRSGG
>Leptonychotes_weddellii
----------------------------MTVRQQRDGRSCGGGCRSPSSRGRSGG
>Balaenoptera_acutorostrata_s
----------------------------MTVRQQRDGRSCGGGCRSPSSRGRSGG
>Miniopterus_natalensis
----------------------------MTVRQQRDGRSCGGGCRSPSSRGHRGD
>Condylura_cristata
MNEPGAGRGPEGGAGVGGGSPRSGCAQYMTVRQQRDGRSCGGGCRSPSSRGRSGG
>Oryctolagus_cuniculus
----------------------------MTVRQQRDGRSCGGGCRSPSSRGRSGG
>Ceratotherium_simum_simum
----------------------------MTVRQQRDGRSCGGGCRSPSSRGRSGG
>Manis_javanica
----------------------------MTVRQQRDGRSCGGGCRSPSSRGRSGA
>Octodon_degus
----------------------------MTVRQQRDGRSCGGGCRSPSSRGRSGG

HG0687.1 ENSG00000120549 KIAA1217

>Homo_sapiens
--MQFSRARETLHRSGK
>Acinonyx_jubatus
--MQFSPARETLHRSGE
>Balaenoptera_acutorostrata_s
--MQFSPARETLYRSGE
>Myotis_brandtii
--MHFSPARETLHGSGE
>Condylura_cristata
--MQFSWARATLHRSGE
>Ceratotherium_simum_simum
--MRFSRASETLHRSGE
>Manis_javanica
--MQSSRASATLHRSGA
>Mus_musculus
MGPVLDCIQLRVPGNPAGCVLGQQSRHLLGGRGGDSHRLQDTAPSPGDSRRVADAPGPGPRRPGAGMRSSRARETLHRSGD
>Tupaia_chinensis
--MQFSRAKETLHRSGE

HG0687.2 ENSG00000120549 KIAA1217

>Homo_sapiens

MVFIGCEHSS--

>Chinchilla_lanigera

MVFIGCEHSSWGTFQDLCVLCD--DFRL----------------------------------

>Trichechus_manatus_latirostr

MVFIGCEHSSWERFRISLSFLIFSSLRRKLSKTSKCGTEDLDEASKQRNRKIGSLSQCSFSRR

HG0688 ENSG00000122420 PTGFR

>Homo_sapiens
----------------------MSGLQSCTVLRGR
>Ailuropoda_melanoleuca
--MPDEQL--SSAHDSSVFMLQMSGLQACTVLRGR
>Myotis_lucifugus
-------------------MPKMPGLQACTLLRGR
>Dasypus_novemcinctus
MEHPRARSSAPASAPPAARDAKMSGLQGCALLRGR
>Oryctolagus_cuniculus
-------------------MLQMSGLQACTVLRGR
>Equus_caballus
----------------------MSGLQACTVLRGR
>Manis_javanica
MALGAARFSAPPGAPSAAGDAKMSGLQTCTVLRER
>Loxodonta_africana
MAHRGARSSALFRASPAAGDTKMSGLQACTDLRGR
>Trichechus_manatus_latirostr
----------------------MSGLQACTDLRGR

HG0689 ENSG00000123352 SPATS2

>Homo_sapiens
MNVTTLVTSYNLPGKGDMNVCNDTS-----------
>Leptonychotes_weddellii
-----------------MNVCNDIS-----------
>Balaenoptera_acutorostrata_s
-----------------MNVCNDIS-----------
>Pteropus_alecto
-----------------MNVCNDIS-----------
>Oryctolagus_cuniculus
-----------------MNVCNAIS-----------
>Ceratotherium_simum_simum
-----------------MNVCNDIS-----------
>Fukomys_darlingi
-----------------MTVCNDSS-----------
>Tupaia_chinensis
-----------------MNVCHDTS-----------
>Trichechus_manatus_latirostr
-----------------MNVCNDIPDKKLIYKKERK

HG0690 ENSG00000125931 CITED1

>Homo_sapiens
MCPAAKLQKGHR
>Leptonychotes_weddellii
MFPAAQLQKGHR
>Orcinus_orca
MFPAAQLQKGHR
>Rhinolophus_ferrumequinum
MFPAAQLQKGHR
>Galeopterus_variegatus
MFPAAQLQKGHR
>Oryctolagus_cuniculus
MLPAAQLQKGHR
>Equus_caballus
MFPAAQLQKGHR
>Manis_javanica
MLPAAELQKGHR
>Ictidomys_tridecemlineatus
MFPAAQLQKGHR

HG0691 ENSG00000129535 NRL

>Homo_sapiens
MPFGLCAHSPLELSRGTRPCSMEPSVSWEAVPVWLWH
>Myotis_davidii
---------------------MVPPVSGEAVLAWLGH
>Galeopterus_variegatus
---------------------MGPLVSGEAVPVWL--
>Oryctolagus_cuniculus
---------------------MEPPVSGKTVAAWL--
>Elephantulus_edwardii
---------------------MGPPVLEEAVPVWF--
>Ceratotherium_simum_simum
---------------------MGPPVSGEAVPTWL--
>Loxodonta_africana
---------------------MGPPVSGEAVPAWLGH
>Ictidomys_tridecemlineatus
---------------------MGPPISGEAVPVWL--
>Trichechus_manatus_latirostr
---------------------MGPPVSGEAVPAWL--

HG0692.1 ENSG00000130338 TULP4

>Homo_sapiens

MALRGAPAGGGAVRWSPAFPGDT------GPSFEVESFWF

>Ailuropoda_melanoleuca

MALKSAPVCGGPVRWSPALCQDTATCLLGSQA---GP---

>Pteropus_alecto

MALRAAWACGGAVRWSPALPGDTATCQETGLGSEVGCFWF

>Dasypus_novemcinctus

MALRSGLAGGGAVGRGSVFPGDVNTGWLGAH----GLMWF

>Galeopterus_variegatus

MALRGNLSSGGVVQWSLMLPGDT------GPSFEVGNFWF

>Equus_asinus

MAPRGAWACGVAVRWSPALHGDTAACREAGPGSECGSFWF

>Manis_javanica

MALRSVLACGGAVRWSPALPRDTTTCWPTGPGSEVGSVWF

>Ictidomys_tridecemlineatus

MALRGAPAGGGVFLGSPAFLRDT------GPSFAVGSFWF

>Tupaia_chinensis

MALWGGPAGGGAGQWGPALPGDT------GPSFETGSFWF

HG0692.2 ENSG00000130338 TULP4

>Homo_sapiens

MDLILRLAFFIKMEKDL------------

>Felis_catus

MDLILRLVFSFKMEKYVIFRSRKGRGK--

>Dasypus_novemcinctus

MDLILRLVFSIKMERYVTFRSRRGGKCLG

>Galeopterus_variegatus

MDLILRLVFFSMMEKYVIFRNRKGEK---

HG0693 ENSG00000131653 TRAF7

>Homo_sapiens
MKRAGGAAAPGRPRAGGASQGP----
>Phoca_largha
MKREGGAAAPGRPRAGHAAQGP----
>Physeter_catodon
MKREGGAAVPERPRAGDAARGP----
>Pteropus_alecto
MKLEGGAAAPGRPRAGDAARGP----
>Dasypus_novemcinctus
MSREGGAAAPGRPRAGAAARGP----
>Ceratotherium_simum_simum
MKRVGGAAAPGRPRAGDATRGP----
>Manis_javanica
MKREGGGAAPGRPRAGDAAQGPWMPL
>Ictidomys_tridecemlineatus
MKRADGAAAPGQPRAGNAAQGPWMPL
>Tupaia_chinensis
MKREGGVAAPGRPRAGDAARRP----

HG0694 ENSG00000133265 HSPBP1

>Homo_sapiens
MAAPSRVRCGRLCL-
>Felis_catus
MAAPSRVRCGRLCL-
>Physeter_catodon
MAAPSIVRCGRVCL-
>Rousettus_aegyptiacus
MAAPTRGRCGQLCL-
>Dasypus_novemcinctus
MAAPSRVRCGPLCF-
>Condylura_cristata
MAAPSRVRCGRLCLY
>Oryctolagus_cuniculus
MAAPSRVRCGRL---
>Equus_przewalskii
MAAPTRVRCGRLCL-
>Marmota_marmota_marmota
MAAPSRVRCGRLCL-

HG0695 ENSG00000133704 IPO8

>Homo_sapiens

MESELWGGKREDRGRGVRVAQQVAAGGDGGGGWGVR

>Mustela_putorius_furo

MESELWGVEREDRGRGVRVAQQVAAGGDGGGGWGVR

>Physeter_catodon

MESELLGVKREDRGRGVRVAQQVAAGGDGGGGWGVR

>Galeopterus_variegatus

MESELWGGKREDRGRGVRVAQRVAAGGDGGGGWGVR

>Oryctolagus_cuniculus

MECELWGGKREARGHGVRVAQQVAAGGGGRGVR---

>Elephantulus_edwardii

MESELWGGKREEGGRGVRVAQQVAAGGEGGGEWGVR

>Ceratotherium_simum_simum

MESELWGVEREDRGRGVRVAQQVAAGGDGGGGWGVR

>Marmota_marmota_marmota

MESELWGGKREDRGRGVRVAQQVAAGGDRGGGWGVR

>Tupaia_chinensis

MESELWGGEREDRGRGVRVAQQVAAGGDGGGGWGVR

HG0696 ENSG00000138380 CARF

>Homo_sapiens
--------------MLEAWNLNLSHYLKLLNNRRKWN----------------------------------
>Physeter_catodon
--------------------------MKILNNRRKWN----------------------------------
>Myotis_brandtii
MNFSGQEGILRGSRMLEAWNLNLSYFLKLLNNRRKWN----------------------------------
>Condylura_cristata
--------------MLETWDLNLSYFLKVTHTKNTSEVAANTIKNTSAVSWKMKGVCICVCVREREKITTY
>Oryctolagus_cuniculus
--------------MLKAGHLDLSHCLKLLN-RRKWN----------------------------------
>Ceratotherium_simum_simum
--------------MLEAWNLNLSYCLKLLNNRRQWN----------------------------------
>Loxodonta_africana
--------------MPEAWNLNLNYCLQLLTNRRKWN----------------------------------
>Ictidomys_tridecemlineatus
-----MKEHSGGGRMLEDWNLNLSHCLKLLNSRRKWN----------------------------------
>Trichechus_manatus_latirostr
--------------MLEAWNLNLNHCLKLLTNRRKWD----------------------------------

HG0697 ENSG00000143344 RGL1

>Homo_sapiens

MDWLPDRVPCSW------

>Acinonyx_jubatus

MDWLPDQVPCSWQEYGAE

>Lipotes_vexillifer

MDWLPDQVPCSWQEYEAE

>Eptesicus_fuscus

MDWLSDQVPCSWQEYGAQ

>Dasypus_novemcinctus

MDWLPDRVPCSW------

>Condylura_cristata

MDWLPYPIPCSWQECEAE

>Equus_caballus

MDWLPDQVPCSWQEYGAE

>Chinchilla_lanigera

MDWLRDRVPCSWQEYGEE

>Tupaia_chinensis

MDWLPDRVPCIRQDCEE-

HG0698 ENSG00000157554 ERG

>Homo_sapiens

MTHREKRWQNQGQLKPSGSEQLVDGLA----Y------------

>Mustela_putorius_furo

MTRREKRWWGQGQLKPSSPRSGEKSMEPFWETDHKHADLEMVSH

>Balaenoptera_acutorostrata_s

MTHREKRWGSQGQLKLPQVL---NRW------------------

>Miniopterus_natalensis

MTHREKRWWSQGQLELSPLL---RGR------------------

>Ceratotherium_simum_simum

MTHREKRWWSRGQLKLPQVL---NGW------------------

>Manis_javanica

MTHREKRWCSQGQLKLSHAV---NGR------------------

>Loxodonta_africana

MTHREKRWWNQGQLKLPQVL---NGW------------------

>Peromyscus_maniculatus_baird

MTPREKRWRNQGQLKPVWVL---HGW------------------

>Trichechus_manatus_latirostr

MTHREKRWWNQGQLKLPQAL---NGW------------------

HG0699 ENSG00000160785 SLC25A44

>Homo_sapiens

MEEDA-TGRRHSLGTTDRLGAAA

>Tursiops_truncatus

MEEDA-TGGRQSPETEDRVGAAA

>Myotis_davidii

MEEDV-HRSWQSPGTADRLGAAA

>Galeopterus_variegatus

MEEDA-AGGQQSLGTEDRVGAAA

>Condylura_cristata

MEEDA-TRGPESPGTEDRLGAAA

>Oryctolagus_cuniculus

MEEDAPGGGRRSLGTEDRVGAAA

>Equus_asinus

MEEDA-TRSQKSRGTQDRVGAAA

>Fukomys_darlingi

MEEDA-TGGSQSLGTEGRAGAAA

>Tupaia_chinensis

MEEDA-IGGRQSLGVEDRVGAAA

HG0700 ENSG00000165156 ZHX1

>Homo_sapiens

MQDG-GGVALRGAAAPAGSGASRLRDQRARRLWAAGGNWSAESAAGTGKEKTDCCFGPVGNDGLS

>Odobenus_rosmarus_divergens

--MADRCFRPVGNDGLS

>Lipotes_vexillifer

MQDG-GGGALRGTAAPAGDGR--

>Myotis_ricketti

--MADQCFGPAGNDGLS

>Erinaceus_europaeus

MQDG-GGRALRGAAAPAGHGR--

>Ceratotherium_simum_simum

--MADRYFGPVGNDGLS

>Manis_javanica

--MADRYFGPVGNDGLS

>Ictidomys_tridecemlineatus

MQDG-GGGVLRGAAAPAGED---

>Trichechus_manatus_latirostr

MQDGDGGGALRGAADPAGESR--

HG0701 ENSG00000166292 TMEM100

>Homo_sapiens
-MDC-LS-PSFHPGETVQI
>Canis_lupus_familiaris
-MDC-LS-LSFHPGETPQV
>Balaenoptera_acutorostrata_s
-MDC-FS-LSFHPGETAQV
>Rhinolophus_ferrumequinum
-MDCFFS-LSFHPGEASQV
>Dasypus_novemcinctus
MMDC-LS-LSFCPGGTLQI
>Galeopterus_variegatus
-MDC-LS-LSSPPGETLQI
>Equus_asinus
-MDCFSPRDAFHPGETP--
>Manis_javanica
-MEC-FS-PSFHLGKAPQV
>Peromyscus_maniculatus_baird
-MDC-LS-VSFHPGQAFQI

HG0702 ENSG00000167377 ZNF23

>Homo_sapiens
MELALGQQEPIVSEVGDL
>Miniopterus_natalensis
MELALGQQEPVVSEVGDL
>Dasypus_novemcinctus
MELALGQQEPVVSEVGDL
>Galeopterus_variegatus
MELALGQQEPVVSEVGDL
>Manis_javanica
MELALGQQEPVVSEVGDL
>Loxodonta_africana
MELALGQQEPVVSEVGDL
>Cavia_porcellus
MELALGQQEPVVSEVGDL
>Tupaia_chinensis
MELALGQQEPVVSEVGDL
>Trichechus_manatus_latirostr
MELALGQQEPVVSEVGDF

HG0703 ENSG00000171862 PTEN

>Homo_sapiens
------MRCGRIRARRWDATALSSLLSEAAAMMEV
>Odobenus_rosmarus_divergens
MWRDSLCTAAGYAPGRRAAAALGSLLSEAAAMMEV
>Lipotes_vexillifer
MWRDSLCTAAGYTFGRWDAAALSSLLSEAAAMMEV
>Rousettus_aegyptiacus
MWRNSLCTAAGYMIGRWDAAALSSLLSEAAAMMEV
>Condylura_cristata
MWRDSLCTAAGYVLGRWDAAALSSLLSEAAAMMEV
>Ceratotherium_simum_simum
MWRDSLCTAAGYAFGRRDAAALSSLLSEAAAMMEV
>Ictidomys_tridecemlineatus
MWRDSLCTAAGYAFGRWDAAALSSLLSEAAAMMEV
>Trichechus_manatus_latirostr
MWRNSLCTAAGYAIGRRDTAALSSLLSEAAAMMEV
>Orycteropus_afer_afer
MWRNSLCTAAGYALGRRDTVALSSLLSEAAAMMEV

HG0704.1 ENSG00000173598 NUDT4

>Homo_sapiens

MESGGQLGRGGAFISAPWFQ

>Leptonychotes_weddellii

MESGGRPGRGGVCSPAPGLQ

>Lipotes_vexillifer

MESGGRPGRGGASSPAPGFQ

>Pteropus_vampyrus

MESGGRLGKGGASSPAPGLQ

>Erinaceus_europaeus

MESGGRPGRGGASSSAAGLQ

>Oryctolagus_cuniculus

MESGRRPGRGGASLPAPGLQ

>Mus_musculus

MESGGRRSRGGASTAAPGLQ

>Trichechus_manatus_latirostr

MESSGRPGRGGASTAAPGLQ

>Orycteropus_afer_afer

MENGGRPGRGGASTSAPGLQ

HG0704.2 ENSG00000272325 NUDT3

>Homo_sapiens
MRRPRRASGGAV
>Physeter_catodon
MRRPRRASGGAV
>Pteropus_alecto
MRRPRRASGGAV
>Oryctolagus_cuniculus
MRRPRRASGGAV
>Elephantulus_edwardii
MRRPRRASGGAV
>Heterocephalus_glaber
MKRPRRASGGVV
>Orycteropus_afer_afer
MRRPRRASGGAV

HG0705.1 ENSG00000175224 ATG13

>Homo_sapiens

-MAAPGSDRLLGLRRE

>Acinonyx_jubatus

-MAAPGGDRLLGLRQE

>Balaenoptera_acutorostrata_s

-MAAPGSDRLLG----

>Miniopterus_natalensis

-MAAPGSDRLLGLRRE

>Galeopterus_variegatus

-MAAPGQYRLLGLRQE

>Erinaceus_europaeus

-MAAPGSDRLRGLRQK

>Ceratotherium_simum_simum

MMAAPGSDRLLGLQQE

>Loxodonta_africana

-MAAPGADGLLGLRLK

>Ictidomys_tridecemlineatus

-MAAPGSDRLLG----

HG0705.2 ENSG00000175224 ATG13

>Homo_sapiens

MRARNHSLCRSFAVERPVETCDPFSLNKSLVLLCCPGWSVVV--

>Canis_lupus_familiaris

MRAPSHSLCRSFAVERPVESCDPFSFNKSLGFFW----PL----

>Orcinus_orca

MRAPSHSLCRSFTVE-----------------------------

>Rousettus_aegyptiacus

MRAPSHCLCRSFAVERPVETCDPFSLSKSLVMSPICPWLQEKRL

>Condylura_cristata

MRVPSHSLCRSFTGEGDC--------------------------

>Ceratotherium_simum_simum

MRAPSHSLCRSFAGKGDC--------------------------

>Loxodonta_africana

MRAPSHSLCRSVAGEGDC--------------------------

>Peromyscus_maniculatus_baird

MRARSHSLCRSFPGEGDC--------------------------

HG0706 ENSG00000178498 DTX3

>Homo_sapiens
---MGFPGHTTYK
>Leptonychotes_weddellii
---MGFPGHTTYK
>Physeter_catodon
-----------MVTFVILTQSPPL--PLPQSSSAECGSRGFLLLSSSMGFPGHTTYK
>Rousettus_aegyptiacus
---MGFPGHTTYK
>Galeopterus_variegatus
---MGFPGHTAYK
>Sorex_araneus
---MGLPGHTTYK
>Ochotona_princeps
---MGFPGHTTYK
>Equus_caballus
MAALTGGAAATLFAFQTDFRRRDWVRSQGSPGQLSVESEGLLLLSSSMGFPGHTTYK
>Dipodomys_ordii
---MGFPGHTTYK

HG0707 ENSG00000179796 LRRC3B

>Homo_sapiens
-------MGAQARKEIMKRHMC
>Felis_catus
----------------MKRHMC
>Myotis_davidii
----------------MKRHMC
>Galeopterus_variegatus
----------------MKKHMC
>Erinaceus_europaeus
----------------MKRRMC
>Equus_asinus
----------------MKSHMC
>Manis_javanica
----------------MKRPLC
>Fukomys_damarensis
MSAKPHSRGSQARTAIMKRCMC
>Tupaia_chinensis
----------------MKKHMC

HG0708 ENSG00000184613 NELL2

>Homo_sapiens
MMCALEGLSSSRAWGGLPFLS
>Ursus_americanus
MMCALEGLSSSRAWGVLPFFS
>Balaenoptera_acutorostrata_s
MMCALEGLSSARAWGVLPFFS
>Pteropus_alecto
MLCTLEGPSSSRAWGVLPFFC
>Elephantulus_edwardii
MMCALEGLSSSRAWGILAFSS
>Loxodonta_africana
MMCALEGLSSSLAWAVLAFSS
>Ctenomys_sociabilis
-MSAAQAPSSARAWGGRPFLS
>Trichechus_manatus_latirostr
MMCALEGLSSSRAWGVLVFSS
>Orycteropus_afer_afer
MMCALEDLSSSRA--------

HG0709 ENSG00000186439 TRDN

>Homo_sapiens
-----MRLFLFEADTSS------------------------------------
>Acinonyx_jubatus
-----MRLFLFEADTSA------------------------------------
>Tursiops_truncatus
-----MKLFLFEADTSA------------------------------------
>Eptesicus_fuscus
-----MRLFLFEADTSA------------------------------------
>Dasypus_novemcinctus
-----MRLFLYEADTSA------------------------------------
>Galeopterus_variegatus
MSPGEMRLFLFEADTSS------------------------------------
>Condylura_cristata
-----MRLFLFEADTSAEPQVLLGKGSGCFQGENSVEKRQSSTGQQRSSRNNQ
>Oryctolagus_cuniculus
-----MRLFLFEADTSS------------------------------------
>Dipodomys_ordii
-----MRLFLFEADTSS------------------------------------

HG0710 ENSG00000196505 GDAP2

>Homo_sapiens
MGPPECSKVSSEY
>Mustela_putorius_furo
MGPPECSKVCSEY
>Orcinus_orca
MGPPECSKVYSEY
>Oryctolagus_cuniculus
MGPPECSEVSSEY
>Ceratotherium_simum_simum
MGPPECSEVSSAY
>Loxodonta_africana
MGTPECSDISSEY
>Chinchilla_lanigera
MGPPECSKLSRDY
>Tupaia_chinensis
MGPPECTKVTSDY
>Orycteropus_afer_afer
MGTPKCSNVSSEY

HG0711 ENSG00000198300 PEG3

>Homo_sapiens

MSTLGWWRRRAPGAMRVR

>Canis_lupus_familiaris

MSTLGWWRRRAPGALWVC

>Balaenoptera_acutorostrata_s

MSTVGWWRRRVPGAVWGH

>Pteropus_alecto

MSTLGWWCRQARGAV---

>Galeopterus_variegatus

MSTLGWWRRRAPGAMWAH

>Equus_asinus

MSTLGWWRRRAPGAMWAR

>Manis_javanica

MSTLGWWRRRSP------

>Loxodonta_africana

MSTLDWWRHRAPGAGWPC

>Nannospalax_galili

MSTLGWWRRRAPGAVWVH

HG0712 ENSG00000213949 ITGA1

>Homo_sapiens

MKGRCPFKVCFYSPWTLA--

>Canis_lupus_familiaris

MKGRCPFKVCFAAQPVDFSLNTNPRSWLYLSMSRTEPGRLPVGFQRPSAGRGGRWGSESGI

>Orcinus_orca

MKGRCPFKVCFAARGL--

>Pteropus_alecto

MKGRCPFKVCLAARGL--

>Oryctolagus_cuniculus

MKGRCPFKVCCAVRGL--

>Ceratotherium_simum_simum

MKGRCPFKVCFAARGL--

>Manis_javanica

MKGRCPFKVCFAARGL--

>Loxodonta_africana

MKGRCPFKVCFAAGGL--

>Nannospalax_galili

MKGRCPFKVCFAARGL--

HG0713 ENSG00000008513 ST3GAL1

>Homo_sapiens

MPDSFDTLQRQRALDRREEEEEEEERREKE-E--AM---

>Panthera_tigris

MPDSFDTLQRQRPEGGGRGGQGGRGGGGRGGRRDVSREGGQCSGQPPL-----------------------------

>Balaenoptera_acutorostrata_s

MPDSFDTLQRQRPEGGGRGGRRGRGGGEEKGAM--

>Miniopterus_natalensis

MPDSFDTLQRQRPEGGGSGGERG-------GRGDVS---

>Oryctolagus_cuniculus

MPDSFDTLQRQRDLEQRQPPEREQREAM---

>Manis_javanica

MPDSFDTLQRQRAERGGRGGRGARRGGGGDASFA---AGSARNTGPPLTLRPLSSPQRRELPRASSRDSAFWTWKCQ

>Loxodonta_africana

MPDSFDTLQRQRAGEQREEEEEDGEEEEE-EEEAL--

>Rattus_norvegicus

MPDTFDTLQRQRALDRREKRGGDRGGP--

HG0714 ENSG00000051825 MPHOSPH9

>Homo_sapiens

-----MTRIHFSQTAQEPLFSNKRI

>Acinonyx_jubatus

MKHLKMTRIQFLQTLKEPTFSNKRV

>Pteropus_alecto

MKHLKMTRIQFLQTVKEPTFSNKRA

>Oryctolagus_cuniculus

MKRLRKTRIQFPQTVKEPIFSNKRV

>Equus_asinus

MKHLKMTRTQFLQTVKKPTFSNKRV

>Loxodonta_africana

MKHLKMTRIQFLQTVKEPIFSTKRA

>Fukomys_damarensis

-----MTRIQFPQTVKEPLFSNKRL

>Tupaia_chinensis

MKHLKMTRIRFLQTVKQPLFSNKRD

HG0715 ENSG00000071246 VASH1

>Homo_sapiens
MCYLCSRCVGGLL
>Felis_catus
MCYLCSWCVGGLL
>Lipotes_vexillifer
MCYLCSWCVGGLL
>Myotis_brandtii
MCYLCSRCVGGLL
>Galeopterus_variegatus
MCYRCSWCVGGLL
>Condylura_cristata
MCYRCSWCVGGLL
>Ceratotherium_simum_simum
MCYLCSWCVGGLL
>Nannospalax_galili
MCYLCSWCMGGLL

HG0716 ENSG00000075413 MARK3

>Homo_sapiens

MSPGTRRGTARARPGI

>Odobenus_rosmarus_divergens

MSPGTRRGTARARPGI

>Orcinus_orca

MSPATRRGTARARPGI

>Rousettus_aegyptiacus

MSPGARRGTARARPGI

>Galeopterus_variegatus

MSPGTRRGTARARPGI

>Oryctolagus_cuniculus

MSPGSRRGTARARPGI

>Marmota_monax

MSPGARGGTARARPRI

>Tupaia_chinensis

MSPGTLRGTARARPGI

HG0717 ENSG00000083857 FAT1

>Homo_sapiens

MLKIPGRQSWYFKPGS

>Mustela_putorius_furo

MLKIGGRQSWYFRYGF

>Lipotes_vexillifer

MLKIRGKQSWYFIHGL

>Miniopterus_natalensis

MLKIPGRQSWYFRRGF

>Galeopterus_variegatus

MLKIGGRQSWHFRHG-

>Condylura_cristata

MLKIAGRQSWYFRRGF

>Ceratotherium_simum_simum

MLKIRGRQSWYFRPGF

>Rattus_norvegicus

MLKIQGRQSWYFEPGS

HG0718 ENSG00000085274 MYNN

>Homo_sapiens

-------------------------------MMGEDKQLQAPARSAQLASG-ERPAVLCCRASAQSPGRA--------PDADCRHQNARDEEDRRSAAS-------
----

>Acinonyx_jubatus

-------------------------------MMGETKKLQAPACSAQSGSR-KED--FR----AVLPDLGPEREMLTIGRPECLE---------------------
----

>Rousettus_aegyptiacus

MISLNSKIPPRPRAPVHKAAVPTRGAPKKGRMLGEDKQLQAPACSAQFGSS-KE--ALRCSSSRPRPRMRAERETPTVGRPECRE---------------------
----

>Galeopterus_variegatus

--------------------------------MGEDANYK-PQRSARKPAPGRRPAAHRHPASAQNPGLAPMLS---------AFQNAQNEEDRRSAVSQPPGLVS
MAPAIAGTVAC

>Ceratotherium_simum_simum

MVES------R----RTQSSCSHSWGPEEGEGDGRRQATTSPSMQCASWAPGKRSAAQRCAASASNGGRARDADFQ------------------------PSKMLR
MRK-TGGAL--

>Manis_javanica

-------------------------------MMGKEERLQAPACSAQCGGWKERPAAFRCPASAQKAGAA------------------------------------
----

>Ictidomys_tridecemlineatus

-------------------------------MTGEDKQLQAPACSAQRGL----PRVWRCRLPGLSPESG------------------------------------
----

>Trichechus_manatus_latirostr

-------------------------------MMGEDEQLQAPA---

HG0719 ENSG00000085998 POMGNT1

>Homo_sapiens
MTGQRPPLLQQPAHCLDALIQGHQLLQDLHIDGSQIPEVMGWPGHW
>Odobenus_rosmarus_divergens
---------------------------------------MGWPGQW
>Rousettus_aegyptiacus
---------------------------------------MGWPGQW
>Sorex_araneus
---------------------------------------MGWPGRW
>Equus_asinus
-----------------------------------MPEGMGWPGRW
>Manis_javanica
---------------------------------------MGWSGQW
>Ictidomys_tridecemlineatus
---------------------------------------MDWPGQW
>Orycteropus_afer_afer
---------------------------------------MGWPGQW

HG0720 ENSG00000087053 MTMR2

>Homo_sapiens
MAASGLPPGPALA
>Mustela_putorius_furo
MAASGLPPGPALA
>Orcinus_orca
MAASGLPPGPARA
>Pteropus_alecto
MAASGLPPGPALA
>Oryctolagus_cuniculus
MAASGLPPGPALA
>Loxodonta_africana
MAASGLPPGPALA
>Marmota_marmota_marmota
MAASGLPPGPALA
>Trichechus_manatus_latirostr
MAASGLPPRPALA

HG0721 ENSG00000095951 HIVEP1

>Homo_sapiens
MYVEFMELPFGGLLYLQFLRRKRRP
>Ailuropoda_melanoleuca
-----MELPFCDLLFLQFLRRKRGP
>Balaenoptera_acutorostrata_s
MDIGFMELPFCDLLFLQFLRRKRGP
>Cynopterus_sphinx
MDIKFMELPFCDLLFLQFLRRKRGP
>Dasypus_novemcinctus
-----MELPFCDLLFLQFLRGKSCP
>Ceratotherium_simum_simum
MDLEFMELPFCDLLFLQFLRRKRGP
>Ictidomys_tridecemlineatus
MYIEFMEPPFCDLLFLQFLRRRRSS
>Tupaia_chinensis
MSAEFMELLFCDLLFLQFLRRKRSL

HG0722 ENSG00000101445 PPP1R16B

>Homo_sapiens
MRPQPHQRPRAALAPGAPC
>Ailuropoda_melanoleuca
MRPQPRRSPRAALAPGAPR
>Balaenoptera_acutorostrata_s
MRPQPRRSPHAAPAPGALC
>Rousettus_aegyptiacus
MRPQPRRSPRAPRAVGAPC
>Dasypus_novemcinctus
MRPQAPRSPRAALAPGALC
>Condylura_cristata
MRPQPHRSPRAPLASGAPC
>Oryctolagus_cuniculus
MRPQPHGSPHAPLDPGALC
>Chinchilla_lanigera
MRPQPRGSPRAPLAPGAPC

>Homo_sapiens
---M
ITDLKPKNKMR
>Ailuropoda_melanoleuca
---M
ITDLEPKNKMH
>Balaenoptera_acutorostrata_s
---M
ITDLGPKNKMQ
>Eptesicus_fuscus
MLPCANPSPHLSSPALHSSPGQPPHPSLPLSAPAAPAAPTQAHCTTLPPFCVRACGAIKPCAELLLWRQRRRGGRCGCAELARDARWPERWSGGGAGAAGGVARRM
VTDLGPKNKMR
>Ceratotherium_simum_simum
---M
ITDLGPKNKMH
>Manis_javanica
---M
ITDLGPKNMMR
>Loxodonta_africana
---M
ITNLGPKNKMR
>Marmota_marmota_marmota
---M
ITDLEPKNKMH

>Homo_sapiens

-------------MDV------SFLLCPKTHPSPSLFSKGGAGEPPPTLGPPLPLPALPGRHHSPEGTGL-CCAS--EQKSQSQQSRLTTCKPQWLPCACCEVGDP

GQEAG

>Lipotes_vexillifer

-----------MWTHA------SSLLCPKTHPAPP---

>Rousettus_aegyptiacus

MLCVRVSRGVWVWLRVGVYYRVQTVVCDHCQ----SVYPRGCMSL-PCSA-PRHTPAPP---

-----

>Galeopterus_variegatus

------------MDGC------LFLALPQDTPQPLLSLGRGVGEPPPTQGPPRSLPALPSRHHSPEGTGL-CCASEQKPQNKQSSQPATTCKPQWLLFACCEVGDL

GQEAG

>Condylura_cristata

-------------MHA------SSSL--

>Oryctolagus_cuniculus

-------------MDA------SSLLCPKTHPSPSLVWEGGGWGASPYPGASPPSPPCPAVTT-PQRAQS--CAE--PPSTAPE----PAEPRGWRPA--CHSGCP

LRAAG

>Ceratotherium_simum_simum

-------------MHA------SSLLCPKTHPSPSLV---

>Trichechus_manatus_latirostr

-------------MDV------SSLLCPETHPGPSLVWEGGLGSH-PCPGASLAPPRPAGQSPPPRGHSVPQCLRVKAPEGAQQADDL-----QATVAALCGL---

-----

HG0725 ENSG00000112218 GPR63

>Homo_sapiens
-----------MYFQAHQWDLILLFCVEMLEELLHLCLHLPSY
>Mustela_putorius_furo
-----------MCFQTHQ-------------------------
>Myotis_lucifugus
---MELCLFSRTCFQACQGNVKLLLGVEMLEEHLPSC------
>Oryctolagus_cuniculus
-----------MCFEACQWNLTLPFCFEMLEEPPHLCVYLPS-
>Ceratotherium_simum_simum
-----------MCFEAHQ-------------------------
>Manis_javanica
MEEGTLCPFSRMRFQAHQCNMILLLCVEMLEEHPLLCWHLPSY
>Marmota_marmota_marmota
-----------MCFQAHQWNLILLFCVEMLEEHTHLCLHPPSS
>Tupaia_chinensis
-----------MCFQAHQWNLMLLFCVEMLKK-LHLCLHLPPF

HG0726.1 ENSG00000115504 EHBP1

>Homo_sapiens
MWLRRGWRTRWRRR-LLLRRRRRSGYEKGTGPDSARQRD
>Leptonychotes_weddellii
MWLRRGWRTRWRRRRLLLRRRRRSGAEEGTGPDSARQRD
>Lipotes_vexillifer
MWLRRGWRTRWRRRRLLLRRRRRSGAEEGTGPDSARQRD
>Rousettus_aegyptiacus
MWLRRGWRTRWRRRRLLLRRRRRSGAEEGTGPDSARQRD
>Condylura_cristata
MWLRRGWRTRWRRRRLLLRRRRRSGAEEGTGPDSARQRD
>Oryctolagus_cuniculus
MWLRQGWRTRWRRRRLLLRRRRRSGSEEGTGPDSARQRD
>Equus_asinus
MWLRRGWRTRWRRRRLLLRRRRRSGAEEGTGPDSARQRD
>Heterocephalus_glaber
MWLPRGWRTRWRRRRLLLRWRWRSGSEEGTGPDSALQRD

HG0726.2 ENSG00000115504 EHBP1

>Homo_sapiens
MQSSFALDPLHY
>Oryctolagus_cuniculus
MQSSFALDPLLY
>Loxodonta_africana
MQSSFALDPLLY
>Fukomys_darlingi
MQSSFALDPLPY

HG0727 ENSG00000115825 PRKD3

>Homo_sapiens
MKYACTILKSFVFRHRKVHPPRMKMRTFEKVI
>Felis_catus
--------------MEKFTPPKMKMKTFEKVI
>Balaenoptera_acutorostrata_s
----------------------MKMRTFEKVI
>Condylura_cristata
----------------------MKMWVIEGI-
>Equus_przewalskii
----------------------MKMRTFENVI
>Marmota_marmota_marmota
MKYACTIFKSFLHHHRKVYPPQMKMRTFERVI
>Trichechus_manatus_latirostr
----------------------MKMRTFEKVI
>Orycteropus_afer_afer
----------------------MKMRTFEKVI

HG0728 ENSG00000116991 SIPA1L2

>Homo_sapiens
MLLKRTEAYNIITTTEKQVGFEETVF--------------------------
>Felis_catus
MLLKRTEAYKISSTSEKQAGFRKTIL--------------------------
>Balaenoptera_acutorostrata_s
MLLKRTEVFKIITATEKQVGFGETIL--------------------------
>Rhinolophus_ferrumequinum
MVLKRTEVYRIIT-TEKQVGFGESML--------------------------
>Dasypus_novemcinctus
MLLKRTEEYKIITITEKQVGFGKNCILTMLFAFG------------------
>Oryctolagus_cuniculus
MLLKRTEVYKFMT-TVEQAGFGDTMF--------------------------
>Equus_asinus
MLLKRTEVYKIITTTEKQVGFGETIL--------------------------
>Manis_javanica
MLLKRTEVYKIISTTEEQVGFKPGCGDVVCFWLKIEITTIEKDFISAFNITL

HG0729 ENSG00000119383 PTPA

>Homo_sapiens
---------------------MAAVFAVVTLTLGFRL
>Felis_catus
---------------------MAVVFTGVTLILGFRL
>Lipotes_vexillifer
---------------------MAGVLAGVTLILGFRL
>Pteropus_alecto
---------------------MAVVFAGVTLILGFRL
>Dasypus_novemcinctus
---------------------MAAVSAGVTLILGFRL
>Erinaceus_europaeus
---------------------MAGVFVGVTLILGFQL
>Manis_javanica
MAAASREPAVAGHARWGPRAGMAGVFAGVTLILGFRL
>Peromyscus_maniculatus_baird
---------------------MAAAFTRVTLTLGFRS

HG0730 ENSG00000125170 DOK4

>Homo_sapiens
MQARGEGAARTMLRRVR------
>Mustela_putorius_furo
-----------MLRRTR------
>Physeter_catodon
-----------MLRQPRPSISNR
>Rhinolophus_ferrumequinum
-----------MLRRVR------
>Galeopterus_variegatus
-----------MLRLPR------
>Condylura_cristata
-----------MLRRER------
>Ochotona_princeps
-----------MLRRVR------
>Nannospalax_galili
MQAPGKGAARTMLRRVR------

HG0731 ENSG00000129255 MPDU1

>Homo_sapiens

MAPSRRSGERQGCKSGFQTGSVNKGVGGLTAQACSFGKLYN--SNSASKTWSLTETYFTPLE--------------

>Panthera_tigris

MAPRRRR--REEAKCGLRIGSVNKGAKDGTAQAHSLSTPQHSNGEGASKTWPLDETYFTLQKVGRSKKKRGTGGGTA

>Rousettus_aegyptiacus

MAPSRTSGENQEDKSRI-----NKGAKDRIAQAYSFGTL--------------------------------------

>Dasypus_novemcinctus

MAPSRRSEKRHGNKSCFRTGTVNECAKALTAQA--

>Oryctolagus_cuniculus

MAPSGRSGERQGTKCCFRSASANKSAKDLTAQACTSDTL--------------------------------------

>Equus_caballus

MAPSRSS--

>Loxodonta_africana

MAPSRRN--

>Heterocephalus_glaber

MAPSSRREESPKGNFSFQTATVNKGAENLTAQAYCFGTL--------------------------------------

HG0732 ENSG00000135914 HTR2B

>Homo_sapiens

----------------------MDRCVYALILENLN-FF-----F-------------------------------

>Ailuropoda_melanoleuca

----------------------MDRSVYTLIFENLKIFFSKEGVNFGFECLA------------------------

>Orcinus_orca

----------------------MDKCMYALIL--

>Miniopterus_natalensis

----------------------MDQCMYALILENLE-GI-----NFGFKYLAWVQYLKKADDQFSH----------

>Ceratotherium_simum_simum

----------------------MDRHMYALILENLK-CF-----FKGRSQ-LWLQGFGMGTMP-------------

>Manis_javanica

----------------------MDRCMKALILPNLK-FF-------------------------------------

>Loxodonta_africana

----------------------MDRCMYALIFEGLK-FFSKEGVNFGFQCLAWVQCLKKADN--------------

>Rattus_norvegicus

MRVITLGQQRDHTWTLFRRPANMDRYVHSPILENLNLFRRKKATLAGSV--WRIPCFAKADDLLATDHADHCLELD

HG0733 ENSG00000136247 ZDHHC4

>Homo_sapiens

MLGRGMPELPAENYPTKRN---------VGWVP---------------

>Leptonychotes_weddellii

MLGGGMPELPVENYPNEKY---------VGWGP---------------

>Orcinus_orca

MFGGGMPELPAENYPSKRN---------M-------------------

>Myotis_brandtii

MLAGGMPELPAENHPDKKY---------VG------------------

>Galeopterus_variegatus

MLGGGMPELPAEYYPTRRN---------M-------------------

>Manis_javanica

-----MPELPAESYPYEKH---------VGWGP---------------

>Chinchilla_lanigera

MLGRGMPELSAEIVSNQEKRCKLDTLNYLGLQEKDTPKAQHSIHLEGKY

>Tupaia_chinensis

MLGKGMPELPAENYPAKIN---------VQEMPPNRPQH----------

HG0734 ENSG00000139318 DUSP6

>Homo_sapiens

MCPAPPPLPPPLLSPSSRRARCCSLFALGLIRSGNSFPFL

>Ursus_maritimus

MCPAPPPPPPPLLSPSSRRARCCSLFALGLIRSGNSFPFL

>Rousettus_aegyptiacus

MCPAPPPPPPPLLSPSSRRARCCSLFALGLIRSGNSFPFL

>Sorex_araneus

MCPAPPPPPPPLLSPSSRGARCCSLFALGLIRSGNSFPFL

>Equus_asinus

MCPAPPPPPPPLLSPSSRRARCCSLFALGLIRSGNSFPFL

>Loxodonta_africana

MCPAPPPPPPPFLSPSSCGARCCSLFALGLIRSGNSFPFL

>Ctenomys_sociabilis

MCLLLLHRRLLFFLLPSRRARCCSLFALGLIRSGNSFPFL

>Trichechus_manatus_latirostr

MCPAPPPPPPPLLSLSSRGARCCSLFALGLIRSGNSFPFL

HG0735 ENSG00000139579 NABP2

>Homo_sapiens

MDRVPACRDEGSGRSGQ

>Ursus_maritimus

MDRVLGCPDEGSGRFGE

>Orcinus_orca

MHRVPGCPDEGSGGFGE

>Miniopterus_natalensis

MDQVPSCPDGGSGRFRN

>Dasypus_novemcinctus

MERVSGCRKEGSGMFGE

>Oryctolagus_cuniculus

MGRIPGCPDEGSGRVGE

>Equus_asinus

MDRVPGCPDEGSGRFGE

>Mus_musculus

MDRVLGCGDAGSGRSAE

HG0736 ENSG00000139826 ABHD13

>Homo_sapiens

MWEPAGAFAGDTRAGAGSGATARSRSSGLDRGAGSGPWAGGG-------------------------------------

>Felis_catus

MWEPAGTFAGDTERGPEVEEPR-----AAPQELRARPWRRRRPLGWRRTMRSDGSGAGGRCRAATGSVSAAHCAACEQV

>Physeter_catodon

MWEPAGVFAGYTERGPEVGEPR-----AAPQELRATLWRRRRPLGWRRTMRSDGSGAGGRFCAAPGSESAADCAACEQV

>Pteropus_alecto

MWEPAVAFAGDTEFGQEVEESR-----PAPQDLRARLWQQRWLFCWKRTMRSDGSGTAGRCCAALGCVFATDCAACEQI

>Dasypus_novemcinctus

MWDPAGVFAGDTRA---

>Galeopterus_variegatus

MCEPAGAFAGDTQAGAGSGAADG-RAAGAPG--

>Equus_asinus

MWESAGVFAGDTKRGPEVEEQT-----AAPQELRVRPWRRRRPLGWRRTMRNDGSGAGGSCCAAPGSVSAADCAACEQV

>Marmota_marmota_marmota

MWEPAGTFAGDTPALAGSRAAKS-RAAGSPG--

HG0737 ENSG00000141639 MAPK4

>Homo_sapiens

MLPTSKGSRGSWPGS

>Balaenoptera_acutorostrata_s

MLPTSEASRGSWLGS

>Rousettus_aegyptiacus

MLSPSEASRGSWPGS

>Dasypus_novemcinctus

MLPTSETAKESWPGS

>Equus_asinus

MLPPSEASGGSWPGS

>Manis_javanica

MLPPSEASRGSWPGS

>Cavia_porcellus

MLPTSKCSGGSWPGS

>Tupaia_chinensis

MLPTSKACGGSWPGS

HG0738 ENSG00000143393 PI4KB

>Homo_sapiens
MEPGSGRCC
>Odobenus_rosmarus_divergens
MEPGSGRCC
>Galeopterus_variegatus
MEPGSGRCC
>Erinaceus_europaeus
MEPGSGRCC
>Ochotona_princeps
MEPGSGRCC
>Ceratotherium_simum_simum
MEPGSGRCC
>Loxodonta_africana
MEPGSGRCC
>Mesocricetus_auratus
MEPGSGRCC

HG0739 ENSG00000144331 ZNF385B

>Homo_sapiens

MICSEALPAGCFLAPHFTGAPG--------

>Panthera_tigris

MICSDALPVGCFLAPHFTEAPG--------

>Rousettus_aegyptiacus

MICSDALPVCCFLAPHFTGAPG--------

>Dasypus_novemcinctus

MICSGALPVGCFLAPHFTGAPG--------

>Ochotona_princeps

MICSDALPVGSFLALHFTEAPG--------

>Elephantulus_edwardii

MICSDPLPVGCFLAPHFTGAPG--------

>Ictidomys_tridecemlineatus

MICSDTLPVGCFLAPHFTGAPG--------

>Orycteropus_afer_afer

MICSDALPVGCFLAPHFTEAPASNDGIIYL

HG0740 ENSG00000144331 ZNF385B

>Homo_sapiens

MFYISTPSNIKLMFMG

>Mustela_putorius_furo

MFCISTSSKIKLIFMG

>Pteropus_alecto

MFCISISSKIKLMFMG

>Oryctolagus_cuniculus

MFCISTSSKIKLVFMG

>Equus_asinus

MFCIATSSKIKLMFMG

>Mus_musculus

MLYISGSSEIKLIFMG

>Trichechus_manatus_latirostr

MFCVSTSSEIKLMFMG

>Orycteropus_afer_afer

MFCVSTSSEIKLIFMG

HG0741 ENSG00000144834 TAGLN3

>Homo_sapiens
MRKSNQCAILPGARQQRLQFNMKGGFLPAC
>Myotis_brandtii
--------------MQHRQLTVKAAVLSAC
>Condylura_cristata
--------------MQPQLLSVKAAFFPAC
>Elephantulus_edwardii
-------------MMQHQPLHVRAALLFAC
>Equus_caballus
--------------MQHQQLNVKAAFLPAC
>Ictidomys_tridecemlineatus
--------------MQHPQL----------
>Trichechus_manatus_latirostr
--------------MQHQQLNLKAALLSAC
>Orycteropus_afer_afer
-------------MMQHQQLNVKAALLSAC

HG0742 ENSG00000154556 SORBS2

>Homo_sapiens

---------------------------------------MFCKFP-----GGHEAPDTWIGLNLKPLEFKTF

>Odobenus_rosmarus_divergens

---------------------------------------MLCEYT-----GGHKAPDTWIGFNLKPLEFKTF

>Lipotes_vexillifer

---------------------------------------MLCKAA-----GGHEAFDTWVGLNLKPLEFKTF

>Miniopterus_natalensis

---MFALADTWIGLNLKSLEFKTF

>Dasypus_novemcinctus

MGWRVAGTAFSSRRTQLAAVTAENSANAPRQKQLPAAGILFPS--DQPSLLPFFPSDTWIGLNRKPLEFKTF

>Elephantulus_edwardii

---MGLNLKPLEFKTF

>Equus_caballus

-----------------------------MLAQRASACTLRPRLERRSLCIHLTDRDTWIGLNLKPLEVKTF

>Nannospalax_galili

--------------------------------------MLKLRWRTGIGAFHLSDRDTWIGLNLKPLEFKTF

HG0743 ENSG00000154814 OXNAD1

>Homo_sapiens

MEKLSMFQLLYIQKSQCNSRTKIFCQS--ADHILNDS------

>Felis_catus

METPSLFQLVYIQRSQYNTRTKVLHVSQSASHILNDF------

>Rousettus_aegyptiacus

METLSVFQLVCI-------------------------------

>Galeopterus_variegatus

-----MFQLVYPKVSV---------------------------

>Manis_javanica

METLFVFQPVYIQRSQFNTRTKILHVSQPASHSFNDS------

>Loxodonta_africana

MEKLPLFQLVCIKRSQYNTRTNILHKS--ASHTLNDSQNLMDI

>Ictidomys_tridecemlineatus

-----MFQPVYIQRCQCNTRSRL-CVSEPAIP-----------

>Orycteropus_afer_afer

MEKLSMFQLVYIKRSQYK-------------------------

HG0744 ENSG00000163053 SLC16A14

>Homo_sapiens

MIGDIATNLYTQQLRGLQAEQQVRGSGRGAE

>Odobenus_rosmarus_divergens

MLGDIATALYAQPLRGLRGKQQVRGSGPGAE

>Balaenoptera_acutorostrata_s

MIGDIATNLSAQPLRGLRGKQQVRGSGRGAQ

>Rousettus_aegyptiacus

MIGDIATNLYAQQLRGLRGKQQVRGSGPGAE

>Galeopterus_variegatus

MTSDIATNHYAQPPRGLGAEQQVRGSGPGAE

>Oryctolagus_cuniculus

MRGDPAARLCTRPLWGLGARQPARGSGRGAE

>Ceratotherium_simum_simum

MTGDIATNLYTQPLRGLRGKQQVRGSGPGGE

>Ictidomys_tridecemlineatus

MIGDIATHLYTQLLRGLRVEQQVRGSVPGAE

HG0745 ENSG00000163145 C1QTNF7

>Homo_sapiens

---------MLRRALYFSSSYFLHCFGVASNNKHIFLL------

>Canis_lupus_familiaris

---------MLRRALYFSSRYFLYCFEAASNKTHIFLL------

>Lipotes_vexillifer

MNSKLQKHSHAQTGTLFFIKFFLCCFEAALNDKHIFLL------

>Pteropus_alecto

---------MLRRDLYFSSSYFLHCFAAALNNKHIFLL------

>Galeopterus_variegatus

---------MLRQALYFSSSYFLHCFGAALNNRHIFLLYIF---

>Manis_javanica

---------MLRRALYFSSSYFLYCFEAALNNKHVFLLYIF---

>Loxodonta_africana

---------MLRRALYFSSSYFLHCFEAALNNKHLFLLYIF---

>Tupaia_chinensis

---------MIRRAFSFSSSYFLHWFGAALNNKHIFLLHIFEGF

HG0746 ENSG00000164741 DLC1

>Homo_sapiens

MISTTKWELRFWRN---------

>Leptonychotes_weddellii

MISTTKCELIFWRNSRGRTSFLA

>Lipotes_vexillifer

MISTTKCELIFWRN---------

>Myotis_brandtii

MISTTKCELIFWRN---------

>Ochotona_princeps

MISTTKCELRFWRNSLGDGLLR-

>Equus_przewalskii

MISTTKCELIFWRN---------

>Manis_javanica

MISTTKCELIFWRN---------

>Trichechus_manatus_latirostr

MISTTKCELIFWRN---------

HG0747 ENSG00000168036 CTNNB1

>Homo_sapiens

MIFSMNLGFCRSIHLQSQNPPGI--------

>Odobenus_rosmarus_divergens

----MNLGFCRSIHLQSQNPPGI--------

>Tursiops_truncatus

----MNLGFCRSIHLQSQNPPGL--------

>Erinaceus_europaeus

----MNLGFCRSIHLQSQNPPGI--------

>Ochotona_princeps

----MNLGFCRSVHLQSQNPPGILTFTKQLF

>Jaculus_jaculus

----MNLGFCRSIHLQSQNPPGI--------

>Trichechus_manatus_latirostr

----MNLGFCRSIHLQSQNPPGI--------

>Orycteropus_afer_afer

----MSLGFCRSLHLQSQNPPGI--------

HG0748 ENSG00000168830 HTR1E

>Homo_sapiens

MLWPFPLPTENGTQETT--------------------------------

>Ursus_maritimus

MLWCFLLPIQNGT------------------------------------

>Tursiops_truncatus

MLWCFPLPTRNGTQETAAEEKITASLQMRDFQVYIVSNCRKITISFSTV

>Eptesicus_fuscus

MLWRFPLPTQNGTQETT--------------------------------

>Galeopterus_variegatus

MLWSFPLPTESGTQETT--------------------------------

>Manis_javanica

MLWHFPLPIQKRTQETT--------------------------------

>Heterocephalus_glaber

MLWPFPLPTENETQETM--------------------------------

>Tupaia_chinensis

MLCPFPLLRENRTHETT--------------------------------

HG0749 ENSG00000169760 NLGN1

>Homo_sapiens

MVGFTWRWLWVPRILDRGCIWREKRINRHVLEVSVGE-----------

>Mustela_putorius_furo

MVGFTWRWLWVPRILDRGFIWREKRINRHVLEVSVGE-----------

>Physeter_catodon

MVGFTWRWL---------------------------------------

>Myotis_brandtii

MVGSPWRWLWVPRILDPGCSWREKRINRHVLEVSVGE-----------

>Condylura_cristata

MIGFTWRWLWVSRIQDRGCIWREKRINRHVLEVSVGE-----------

>Oryctolagus_cuniculus

MVGFTWRCLWVPRNLDRGCIWKEKRINRHVLEVSVGELKKKKKRRGYN

>Equus_caballus

MVGFIWRWLWVPRILDPGCIWREERINRHVLEVSVGE-----------

>Chinchilla_lanigera

MVGSTWRWLWVPRIADRGCIWKEKRINRDVWEVSVGE-----------

HG0750 ENSG00000170881 RNF139

>Homo_sapiens
---MVAPRAGEAVEGGGVRP
>Mustela_putorius_furo
---MVAPRAGEVLEGGGARP
>Lipotes_vexillifer
---MVAPRAGEAVEGGGARP
>Myotis_lucifugus
MVTLTDVGYAVRDDDVGAPLASACDATAARSPRSRVTWGTASASQQPQRQRPAGEVKMVAPRAGEAVEGGGARP
>Dasypus_novemcinctus
---MVAPRAGEAVEGGGARP
>Oryctolagus_cuniculus
---MVAPRAGEAVEGGGARP
>Equus_caballus
---MVAPRAGEAVEGGGARP
>Chinchilla_lanigera
---MVAPRAGEAVEGGGVRS

HG0751 ENSG00000171791 BCL2

>Homo_sapiens

MGSLPYAFVLVLQKGNLTEDHAVLKKY----------------K-----------------------

>Acinonyx_jubatus

MESLPYAFVLFSQKRKLDRGSCCP---

>Myotis_brandtii

MGSLPYAFVLSSQKGKLDRRSCRP---

>Galeopterus_variegatus

-----MHLFWFCKKGNLTEDHAVLKKY----------------NIPEE---VDGYERYFVIIMCLMK

>Oryctolagus_cuniculus

MGSLPYAFVLVLQRRKLDRGSCCPLKTQHHVGKRLAFTTLMNDNVPRETEIRKGFE-----------

>Ceratotherium_simum_simum

------MHFYFHKKGNLTEDHAVLKKY----------------NITEE---VD--------------

>Manis_javanica

-----MHLFCFHKKGNLTEDHAVLKKY----------------NITEE---AD--------------

>Fukomys_darlingi

-----MHLFWFYKKGNLTEDHAVLKKY----------------K-----------------------

HG0752 ENSG00000172269 DPAGT1

>Homo_sapiens

MKFGITLLFHFHPHPLMPILKMAAAAFRSNGCSANKIRAETVDRRVQRGRT--

>Ailuropoda_melanoleuca

---------------------MAAAAFHDAGCSANKIREETVGMQVQWGHSRGCAIEARGSPEGGSSQSCWVSLRLLWEDPAFGERPLLPAGGSGLPPSLSIS

>Lipotes_vexillifer

----------------MPILKMAA-AFHNDSCSAN--

>Rousettus_aegyptiacus

---------------------MAAAAFRNDSCFANKICAETVGRKVQWG--

>Oryctolagus_cuniculus

---------------------MAATAFRSKLLC------EWDPGGNRIGRCRKAALRLP--

>Elephantulus_edwardii

----------------MPILKMSAIAFPSNSGSAEKIRAETVGR---

>Ceratotherium_simum_simum

---------------------MAASAFHNDSCSANKIRAETVRRQTQWGRARVDL--

>Marmota_marmota_marmota

-------------------MKNGVASFRVDSSFAIKIRAETLDGQVQPGRAEVAL--

HG0753 ENSG00000173406 DAB1

>Homo_sapiens

MGSRFLGGCGSGGGCSGLGEHSKRVNEMCSSECHF

>Mustela_putorius_furo

MGSRFLRGCDSGGGCCPRAERSEGVNEMCSSEHCF

>Balaenoptera_acutorostrata_s

MGNCFLGGCGSGGGWSRRA----------------

>Rousettus_aegyptiacus

---------MIEGALLHNTKHSKGVNEMCSSGCHF

>Condylura_cristata

---------------------------MHSSGCHF

>Ochotona_princeps

---------------------------MCGSGCCF

>Heterocephalus_glaber

---------------------------MSSSGCRF

>Tupaia_chinensis

---------------------------MCSLECCF

HG0754 ENSG00000175182 FAM131A

>Homo_sapiens
---------------------------------MRQECSPYVTRHRFQMGRGLAQHPEPKQE
>Felis_catus
---------------------------------MKQECSPSVTRHRFQVGRGLAQHPEPK--
>Rousettus_aegyptiacus
---------------------------------MRQECSPYVTRHRFQVGRGLAQHPEPK--
>Erinaceus_europaeus
---------------------------------MRQECSPYVTRHGFQVGRGLAQHPEPK--
>Oryctolagus_cuniculus
MYIACWHCLGDGPQFAKPQRALLGPWHSHLLKAMRRECSPYVTRHKFQVGRGLAQHPEPKQE
>Equus_caballus
---------------------------------MRQECSPYVTRHRFQVGRGLAQHPEPK--
>Manis_javanica
---------------------------------MRQECSPYVTRHRFQVGRGLAQHPEPK--
>Mus_musculus
---------------------------------MRQECSPYVTRHKFQVGRGLAQHPEPKQE

HG0755 ENSG00000176597 B3GNT5

>Homo_sapiens

MPVWKNFDVPVSSFYRVFHSSAISEKWD--------

>Ursus_maritimus

MPVWKHCDLPVFSSYWAFHSFAISEKMV--------

>Physeter_catodon

MSEWKHSDVPVSSFCSVFRLLPFPKKMVLKYFVK--

>Miniopterus_natalensis

MPAGEHLDVPVSSFYSVFHSFATSAKNAIKRKYFVK

>Oryctolagus_cuniculus

MPVWTNADVPVSSFQSVSILWPSQRKWC--------

>Equus_caballus

MPVWTHVDVPVSSFYSVFHSFAISEKMALKENIL--

>Loxodonta_africana

MPAQKSFDVPASSFCTVVHILFNKK-LLFHF-----

>Heterocephalus_glaber

MLLWKNFDIPVSSFYSMFHSFATLEKMVLKANIM--

HG0756 ENSG00000177051 FBXO46

>Homo_sapiens

MQRAAMVACGASHHHLGPEMR

>Mustela_putorius_furo

-----MVACGASPDHPGPEMQ

>Orcinus_orca

-----MVACGAPHRHSGPEMW

>Rhinolophus_ferrumequinum

-----MVACGIPYHHLGSEMQ

>Galeopterus_variegatus

MQREEMVVCGVPHHHSGPEMQ

>Equus_asinus

-----MVACGAPHHPPGPEMW

>Loxodonta_africana

-----MVARGAPHHHSAPKMR

>Marmota_marmota_marmota

-----MVACGASHHHPGPEIR

HG0757.1 ENSG00000177301 KCNA2

>Homo_sapiens

MLLCFLGPVAAP----------

>Ailuropoda_melanoleuca

MLLCFLGPQAAP----------

>Balaenoptera_acutorostrata_s

MLLCFLGP--------------

>Rousettus_aegyptiacus

MLLCFLGPSAAP----------

>Equus_asinus

MLLCFLGP--------------

>Loxodonta_africana

MLLCFLGPLAAP----------

>Mus_musculus

MLLCFLGPLSAPSSLLQAAGRH

>Tupaia_chinensis

MLLCFLGPLAAP----------

HG0757.2 ENSG00000182255 KCNA4

>Homo_sapiens
MNLKGEPGESS
>Equus_asinus
MNLRGEPGESA
>Trichechus_manatus_latirostr
MNLRGEPGESS

HG0758 ENSG00000179299 NSUN7

>Homo_sapiens
MLQMRGKPFPGTSEF
>Ailuropoda_melanoleuca
MLKVQGRPFPGTSEL
>Orcinus_orca
MLKVQGKPFPGTSEL
>Rousettus_aegyptiacus
MLTVQGKPFPGTSEL
>Oryctolagus_cuniculus
MLKVGGKPFPGTSEL
>Ceratotherium_simum_simum
MLKVQGKPFPGTSGL
>Loxodonta_africana
MLKVQGKPLPGTSEL
>Cavia_porcellus
MHKIRGKPFPGTSEV

HG0759 ENSG00000180929 GPR62

>Homo_sapiens
---MAAWASE
PPPGQPMR-------
>Panthera_tigris
---MAAWAME
PAPGHPVR-------
>Orcinus_orca
---MAAWVSE
PAPGQPVSTG-----
>Rousettus_aegyptiacus
---MVAWAAE
PGPGQPVR-------
>Oryctolagus_cuniculus
MQDRVWLPPKPTLDHLPSPPSAVTAQLLASGWQLLGPRGLAPGPAPPLPALCISRGHHQSDSQCHLRQRPGCWSWAGAGARASRLSLAPLHGAGQTSQKMAAWATE
PPPPQPMSSLHPSTG
>Ceratotherium_simum_simum
---MAAWASE
PAPGQPMR-------
>Ctenomys_sociabilis
---MAAWASE
PPSGQPMR-------
>Tupaia_chinensis
---MAAWASE
PPPGQPMRTG-----

HG0760 ENSG00000182463 TSHZ2

>Homo_sapiens
MFNKEIKCLNSHQKKKKPQKQNQKNS-KSK------NKKERGKKIQNKQTNKQGRTNL-Y---FKAAGTSHP--CLPPREGGLWPVVEELQGGSSGGQRPSDVLGA
TGQEAEETQRGQRDSGPQRAARGWGARSGTGAK
>Felis_catus
MFNKEIKCLNSHQKKKKKKKKKTQKPKISKKAKTKRERERERGKQNPKQTNKQTRQNQPL---LQTSQRKPP--VSAARRGGSLARGGGVAGGIARGTEAE-----
----
>Pteropus_alecto
MCNKEIKCLNSHQKKKKKTKQTNKKIPKSK------NK--RERKKNPKQTNKQTRHSQPL---LQSSRHEPP--ESAIQRGGLRPLVEELQGGSSGGQRPRDALGA
TGQEAEDTQRGERDRGPPRAARGRGARSGTGAK
>Condylura_cristata
MFNKEIKCLNSHQKKKKNQKNSKKQKQ-----------KEREKGKKKSKTNKQTNQAEPTSPSNQPAQAT-R--VCHPERGVSGPWWRSCRGDRQGGRG-------
--RVTP---------------------------
>Oryctolagus_cuniculus
MFNKEIKCLNSHQKKKKQKQTKKNSKS---------KNKKERG--KKAKTNKQTRQNQPL---LPTSRHQPA--VSATQRGGSPARAAGVAGGIVRGTEAE-----
----
>Equus_asinus
MFNKEIKCLNSHQKKKKQNKISKKQK------------QKEREEKKSKQTNKQGRTNL-Y---FKPAGASHP--CLPPREGGLWSVVEELQGGSSGGQRPSDALGA
TGQEAEETQRGERDSGPPRAARGWGARSGTGAK
>Cavia_porcellus
MFNKEIKCLTSHHKKKQKQQQQNIYIYTSKSKNK---NRER----KKKPQNKQTNKAEPS---SAASQPAPATVSAAAGEGGLRPEVAEWQGGSSGGQRPSDVLGA
SGRDTEETPRGERDSGPRARLGAGAPEVGLERK
>Tupaia_chinensis
MFNKEIKCFNSHQKKKQKQKKNSKSKN---------KKRKREREEKKSKTNKQTRQSQPL---LQPSRHQPP--CLPPERGVSGPWWRSCRGDRQGDRG-------
--RVTS---------------------------

HG0761 ENSG00000185985 SLITRK2

>Homo_sapiens
------------------MTWRLAATPREIRCKHVCSRGACVGGLGMGE---

>Leptonychotes_weddellii
------------------MTPRLAATRREIRCKHVCL---

>Lipotes_vexillifer
------------------MTPRLAATRREIRCKHVCL---

>Eptesicus_fuscus
MPQGRRRWVGFSGITRLLITPRLAETHREIRC--

>Ochotona_princeps
------------------MTPRLAATRREIRCKLVCPVGT--

>Ceratotherium_simum_simum
------------------MTPRLAATRREIRCKHVCSGTCVWVGWGGGGEERRNRFEKRSDFILPCG-PLLAFSIPPTPPPCFAILIGLPCFFLPRILLSPASPPP
RFACRYLS
>Manis_javanica
------------------MTPRLAATRREIRCRRVCSRTRVWVGGGGETGTALNDA-----VISICRAAPSPHSLPPTPPLALSF---------------------
----
>Fukomys_damarensis
------------------MTPRLAATRREIRCKHVCSGTRVWVGWGSGKGVKKPL---

HG0762 ENSG00000187147 RNF220

>Homo_sapiens
MHHTLRLLPSVS
>Canis_lupus_familiaris
MHHTHRLLPSVS
>Lipotes_vexillifer
MHHTHRLLPSVS
>Myotis_lucifugus
MHHTHRLLPSVS
>Equus_asinus
MHHTHRLLPSVS
>Loxodonta_africana
MHHTPRLLPSVS
>Marmota_marmota_marmota
MHHTLRLLPSVS
>Orycteropus_afer_afer
MHHTPRLLPSVS

HG0763 ENSG00000188050 RNF133

>Homo_sapiens

MLEDSGSCLYPEDFFLFVILN

>Acinonyx_jubatus

MPEDRGTCFHPEDFSHFIIFD

>Rousettus_aegyptiacus

MLEDGRTCFHPEDFFHVLTFN

>Ochotona_princeps

MLEDSGSCLYSEGFFLFFVLN

>Ceratotherium_simum_simum

MLEISGTYFHPEDFFHVIILN

>Peromyscus_maniculatus_baird

MLEDGGSCFHPEDIFSFLILN

>Tupaia_chinensis

MLEDSGSCFHPDDFFFFLTLN

>Trichechus_manatus_latirostr

MLEGSGTCFHLEHVFHFIILN

HG0764 ENSG00000188725 SMIM15

>Homo_sapiens

-----------------------MCTVEL-KKTDYQGSSEVVIGSLITPGQS-----------------------------

>Canis_lupus_familiaris

-------------------------MVEP-KKTSSQGSTEIVIGSLIISGQSWRSLLKHKT--------------------

>Tursiops_truncatus

-------------------------MVEL-KKTANQGSSEIVIG-------------------------------------

>Myotis_brandtii

-----------------------MCVVELK-KTTNQGSSI---

>Oryctolagus_cuniculus

--MIVGQS-----------------------------

>Ceratotherium_simum_simum

-------------------------MVELKKKTTNQGSSEIVIGSLITPGQS-----------------------------

>Manis_javanica

-------------------------MVEL-KKTTDQGWPKTVTGSLIIPGQDHCSVIR-----------------------

>Ictidomys_tridecemlineatus

MAASVLVSGCRAEVAAAQEFNALRSILWNQRRQNHQGSSEIVLGSLIIPGQNQRSLLKHKNFDSSYSLFPVVHPQLTLQFS

HG0765 ENSG00000196123

>Homo_sapiens
------------------------------MVKSLLQEYIAKAFLAEGPET-------
>Odobenus_rosmarus_divergens
------------------------------MVRSLLQGDIARAFLAKEPET-------
>Lipotes_vexillifer
MVKKPPGHWVPLRRWEGFPEEVRPKPSPEEWLRSLPRGDIGRAFLAEGPET-------
>Dasypus_novemcinctus
------------------------------MVRSLLQGDTGRTFLAEGRDLKEAGVCW
>Equus_przewalskii
------------------------------MVRCLLQGDIGRTFLAERPET-------
>Manis_javanica
------------------------------MVRSLLQGDIGRAFLAERPEI-------
>Loxodonta_africana
------------MCWEDFPKEVRPEPSPE--------GCIGRAFLAEGPET-------
>Nannospalax_galili
------------------------------MVEFAYREALARAFLVEGPER-------

>Homo_sapiens
--MMGTWRAALPFQPG
>Panthera_tigris
---MGTWRAALPAQPG
>Dasypus_novemcinctus
---MGTWRAVLPAQPG
>Oryctolagus_cuniculus
MSLVLSKRMHSESGWDRTEGPGFLDRLGRGEGGRKRCAVCWEAGEPRFVFPSQSTDFRPSQDLSQLERWKFWFPWPRAPSAGSGAMGTWRAALPFQPG
>Equus_przewalskii
---MGTWRAALPAQPG
>Manis_javanica
---MGTWRAALLAQPG
>Chinchilla_lanigera
--MMGAWRAALPFQPG
>Orycteropus_afer_afer
---MGTWRAPLPAQPG

HG0767.1 ENSG00000197555 SIPA1L1

>Homo_sapiens
-----------------------------MLDSFCNILVSRKHGIMATTESQ---------
>Canis_lupus_familiaris
MFQPLNSSEASKMMLRTARGIKAAASEAGGRLARCLIIVERKHGIVAVTESP---------
>Balaenoptera_acutorostrata_s
---MAITESQRYKLHSVFF
>Rousettus_aegyptiacus
---MAVTTSQ---------
>Equus_caballus
---MTIAESQ---------
>Loxodonta_africana
---MAIAESQ---------
>Dipodomys_ordii
---MAITESQ---------
>Orycteropus_afer_afer
---MAVTESQ---------

HG0767.2 ENSG00000197555 SIPA1L1

>Homo_sapiens

-----------MERATQSCVLFLPSS

>Galeopterus_variegatus

MITVLCNLLGDMERATQSSILILPSS

>Jaculus_jaculus

MLTVLCDLVGDMERAARSSILFLPSS

HG0768 ENSG00000213023 SYT3

>Homo_sapiens

MGCRPP-PPRLPPRTRQR

>Balaenoptera_acutorostrata_s

MGYRQP-PPRLPSRTQQG

>Rhinolophus_ferrumequinum

MGCRQP--PRLPPRTRQG

>Galeopterus_variegatus

MGCRPPPPPRLPPRTRQR

>Condylura_cristata

MGCRQP-PPRLPSRTRQG

>Ochotona_princeps

MGCRQP-PPQLLPRTRQG

>Equus_caballus

MGCRQP-PPRLPSRTRQG

>Mus_musculus

MGYRPP-PTRLTPRTRQG

HG0769.1 ENSG00000280165 PCDH20

>Homo_sapiens

MNREGVSSAALPSRAL

>Mustela_putorius_furo

MNREGVSSAALPTGAR

>Pteropus_alecto

MNGEGVSSGALPSGAR

>Condylura_cristata

MNREGVSSVALPTGAR

>Oryctolagus_cuniculus

MNGEGVSSAALPAGAR

>Equus_asinus

MNREGVSWAALPSGAR

>Mus_musculus

MNREGVSSATLPSGAL

>Tupaia_chinensis

MNGEGVSSAALPSGAR

HG0769.2 ENSG00000197991

>Homo_sapiens

-----------------------------------MNVPFCPLAFLPLFEFPSLIEEPVTIG

>Lipotes_vexillifer

-----------------------------------MTVPRYHLTFLPPLELLSLTDEPVTIG

>Myotis_lucifugus

-----------------------------------MTVPFCPSPFCTPS-APVLIGEPVTIG

>Nannospalax_galili

MRSDLVPSHLRLQAQVSHSGSSGGEWTCEHKGVDVINVPFCPYAFLTS--------------

HG0770 ENSG00000010165 METTL13

>Homo_sapiens

MWLFFRGKNSHCSVPEPACGGQAPQMVSHRE---

>Orcinus_orca

MWLFVRGKNSRCGVPEPARG-ETPRMVPHRE---

>Pteropus_alecto

MWLLVGRKNSHRSVSEPAG-GEAPPLVPHRE---

>Ceratotherium_simum_simum

MWLFVRGKNSHCSVPEPACG-EAPPMVPRRVGES

>Manis_javanica

-------------------------MVPHRE---

>Loxodonta_africana

MGLFACGKNSHCSVPTPACG--------------

>Orycteropus_afer_afer

MWIFACGRNSHCSVPEPACG--------------

HG0771 ENSG00000013306 SLC25A39

>Homo_sapiens

MGPPDPGRRAARAPA----

>Felis_catus

MGPLGPGRRAARAPAWSQN

>Myotis_brandtii

MGPPDPGLRAARVPS----

>Dasypus_novemcinctus

MGPSGSGRRAARAPAWSQD

>Galeopterus_variegatus

MGPPGPGRRTVRVPAWSQD

>Manis_javanica

MGPPGPGTRTARAPA----

>Tupaia_chinensis

MGPPGPGRRAARAPAQSQD

HG0772 ENSG00000064309 CDON

>Homo_sapiens
--------------------MTWGWLSEPAAAQEVWRLSQPRGE
>Mustela_putorius_furo
MEGPVRIAAAAAVAAASARGMTWGWLSEPAAAQEVRGLRLPRRE
>Dasypus_novemcinctus
MEGPVRIAAA-----ARARGMSWGRPSEPAAAQEVWGLSRPRRE
>Oryctolagus_cuniculus
--------------------MTWGWLSEPAAPQEGLGA------
>Ceratotherium_simum_simum
MEGPVRIAAAATVAAARARGMTWGWLSEPAAAQEVWGLSLPR--
>Manis_javanica
MEGPVRIPAAAAVVAASARGMTWGWLSEPAAAQEVWGLSLPR--
>Peromyscus_maniculatus_baird
--------------------MTWGWLSEPAAAQEVWGLSQPRGE

HG0773 ENSG00000065548 ZC3H15

>Homo_sapiens
---MLCDIDLSQRPRLRGQ
>Leptonychotes_weddellii
MSDITSDAKSFSHVPTLPPPGRPPQKRTRSSASGRRSSLPTMLCDTDLSQRPRFGGE
>Pteropus_alecto
---MLCDIDLSQRPRFGGE
>Condylura_cristata
---MLCGRDLSQRPHFGGE
>Ceratotherium_simum_simum
---MLCGSDLSQRPRLGGE
>Chinchilla_lanigera
---MLCDSDLSQTPRFGGE
>Tupaia_chinensis
---MLCGTDLSQRPRFGGE

HG0774 ENSG00000066933 MYO9A

>Homo_sapiens

MCLLRPGGVSLTGRGMRRGRSPGP

>Mustela_putorius_furo

MCLPRPGGVSLTGRGMRGGSSLGP

>Orcinus_orca

MCLPRPGGVSLTGRGMRGGRSPGP

>Myotis_lucifugus

---------------MRGGRSPGP

>Condylura_cristata

MCLPRPGGVSLTGRGMRGGRWPGP

>Ceratotherium_simum_simum

MCLPRPGGVSLTGRGMRGGRSPGP

>Rattus_norvegicus

MCLPRPGGVSLTGRGMRGRRSLGP

HG0775 ENSG00000068001 HYAL2

>Homo_sapiens

----------MGFIKSSASCPTGSSWRK--------------------------

>Mustela_putorius_furo

----------MGFIKSPAGCPAGSSWCE--------------------------

>Lipotes_vexillifer

----------MGFIKLPAGCPEGSSWCE--------------------------

>Myotis_brandtii

----------MGFIKSPAGCPLGSSWRESLDWRGSNLRSGGLPYSS--------

>Dasypus_novemcinctus

----------MGFIKSPADCPKGSSWREWPGGRG--------------------

>Galeopterus_variegatus

MMGGGSGWEGMEFIKSPAGCPTGSSWRKQPGWRSTPQEGGRPPTSWGDRLPHSS

>Mus_musculus

----------MGFIKSPADCPEGSSWRKEPGWRDSTQKI---------------

HG0776 ENSG00000082014 SMARCD3

>Homo_sapiens

MALGAAGAVGSGPRHRGGTG

>Felis_catus

MALGAAGAVGSGPGHRGGTG

>Rousettus_aegyptiacus

MALGAAGALGSGPGHRGGTG

>Dasypus_novemcinctus

MALGAAGVTGSGPGHRGGTG

>Ceratotherium_simum_simum

MALGAAGAVGSGPRHRGGTG

>Manis_javanica

MALGAAGTVGSGPGHQGGTG

>Ictidomys_tridecemlineatus

MALGAAGAVGSGPGHRGGTG

HG0777 ENSG00000085644 ZNF213

>Homo_sapiens

----MPAQLPQGIPLGD

>Canis_lupus_familiaris

MRVQMPTRLPQGIPLGD

>Balaenoptera_acutorostrata_s

----MPTKLPLGIPLGD

>Myotis_davidii

----MPAQLPQGIPLGD

>Ceratotherium_simum_simum

----MPAQLPQEIPLGD

>Manis_javanica

----MLTQLPQGIPLGD

>Ictidomys_tridecemlineatus

----MPAQPPQGIPLGD

HG0778 ENSG00000089116 LHX5

>Homo_sapiens
MRELRGDQTKMRIRTA
>Leptonychotes_weddellii
MRELGGGQTKMRIGTA
>Rousettus_aegyptiacus
MRELGGGQTKMRIGTA
>Galeopterus_variegatus
MRELGGGQTKMRIGTA
>Condylura_cristata
MRELGGGQTKMRIGTA
>Ochotona_princeps
MRELGGGQTKMRIRTA
>Octodon_degus
MRELGGGQTKMRIRTA

HG0779 ENSG00000091140 DLD

>Homo_sapiens

MRREGRPWRSGGGAQRR

>Balaenoptera_acutorostrata_s

MRRAGRPWRGGGDA---

>Galeopterus_variegatus

MRRAGRPWRSGGGA---

>Erinaceus_europaeus

MRRAGRPWRSGGVV---

>Ochotona_princeps

MRRAGRPWESGGRAGPR

>Fukomys_damarensis

MRRAGRPWKSGGGAQRR

>Orycteropus_afer_afer

MRRAGRPWRGGDGALQR

HG0780 ENSG00000092199 HNRNPC

>Homo_sapiens
----------------------------------MPSLEQQQPG
>Canis_lupus_familiaris
----------------------------------MPSLEQQQPG
>Dasypus_novemcinctus
MAFSQKAIFLLFPLNPILLFLYTTPHKPQAAATWMPSLEQQQPG
>Condylura_cristata
----------------------------------MPSLEQQQPG
>Ceratotherium_simum_simum
----------------------------------MPSLEQQQPG
>Manis_javanica
----------------------------------MPSLEQQQPG
>Rattus_norvegicus
----------------------------------MPSLEQQQPG

HG0781 ENSG00000100099 HPS4

>Homo_sapiens
MHLPLLAFLERS-LKRWFCRQPQ---
>Odobenus_rosmarus_divergens
MHLPDWAFLARSFL-KKFCREPEC--
>Myotis_lucifugus
MHLPYCTFLARS-LKGSFCREPES--
>Dasypus_novemcinctus
MHVPCGAFLERSFLKSLSCRQPY---
>Ceratotherium_simum_simum
MHLPWWAFLARSSLKRSFCREPES--
>Nannospalax_galili
MHLLSGAFPERN-WKRSLCRQPCRCS
>Tupaia_chinensis
MFLSCRAFLERD-FKRPFCKQFH---

HG0782 ENSG00000100368 CSF2RB

>Homo_sapiens
MGLAWLPAGQEHRTSGH------
>Panthera_tigris
MGLAWPPAEQAYRTPGH------
>Myotis_davidii
MGLTWPPTGEGHRVPGQ------
>Oryctolagus_cuniculus
MGLAWPPPGREHKLPGQ------
>Ceratotherium_simum_simum
MGLAWLPAREGHKIPGQ------
>Manis_javanica
MGRAWPPAGEEEETPGPVHTWTM
>Tupaia_chinensis
MGLAWPPAGQEHKIPGH------

HG0783 ENSG00000105053 VRK3

>Homo_sapiens

----------------------------------MRAQRSPPSGSFRWATEKVAVKMEFPTQ--

>Felis_catus

MGVGDEEIIPPGHCPRAVRRPAAPARALSFLGSRMRAQRFPPPGSLRGTTKKVSSKMEFPAQGI

>Physeter_catodon

--MEFPAQ--

>Pteropus_vampyrus

--MEFPDQ--

>Condylura_cristata

--MQLQAQ--

>Ceratotherium_simum_simum

----------------------------------MRAQRLPSSGSFRGATEKDC----------

>Cricetulus_griseus

--MEFPTQ--

HG0784 ENSG00000105851 PIK3CG

>Homo_sapiens
MKGAPEKRKNLDAHWVA-----------
>Odobenus_rosmarus_divergens
MQGDPEKRKSLEAHWVDSNLFCFQKLNK
>Orcinus_orca
MKGDPEKRKSLEAHWVGLNLFCFQKLNK
>Galeopterus_variegatus
MKGAPEKRKSVEAYWVA-----------
>Oryctolagus_cuniculus
MKGAPEKRKSLEAHWAGLNLFCFQKLNK
>Ceratotherium_simum_simum
MKGDPEKRKNLEAHWVV-----------
>Dipodomys_ordii
MKGAPEKRKN------------------

HG0785 ENSG00000107242 PIP5K1B

>Homo_sapiens
----------------------------MSRPPITTNYR
>Rousettus_aegyptiacus
MIILMMVLKLQSVCVPETLNLGTKTVSTMNRPPITTNYR
>Galeopterus_variegatus
----------------------------MNRPPISTNYR
>Oryctolagus_cuniculus
----------------------------MNRPPITTSYR
>Equus_przewalskii
----------------------------MNRPPVVTNYR
>Peromyscus_maniculatus_baird
----------------------------MSRPPITTNYR
>Tupaia_chinensis
----------------------------MNRPPITTNYR

HG0786 ENSG00000107862 GBF1

>Homo_sapiens
MSSGCRSWD
>Physeter_catodon
MSSGCRSWD
>Pteropus_alecto
MSSGCRSWD
>Oryctolagus_cuniculus
MSSGCRSWD
>Equus_caballus
MSSGCRSWD
>Marmota_marmota_marmota
MSSGCRSWD
>Orycteropus_afer_afer
MSSGCRSWD

HG0787 ENSG00000108592 FTSJ3

>Homo_sapiens

----------------MAENLEPGTQGTQAEA--EVVSCILVRFQVWRKRLWV-----------------------

>Dasypus_novemcinctus

---------MLMATLKMAENLEPGT---

>Galeopterus_variegatus

----------------MAENLEPGTQGAQPEA--EVHLLPTCAGPSVVKTVLVRGERVEQGAECDCRAKWETENRI

>Oryctolagus_cuniculus

-MTGISSREKLTATPEMAENLEPGTQGRKRKRKWKCACCLHVRLQVWRRRRDRAGR-FS---QGDCRGTGS-----

>Equus_asinus

MWEQRIYTGTCVATLKMAENLESGTQATQAEA--EVRLLLHVPLQLGEKTALGRD---------------------

>Manis_javanica

----------------MAENLEPGTQTAEAEV--E---

>Peromyscus_maniculatus_baird

----------------MAENLKPGTQETRAEA--EVR---------------------------------------

HG0788 ENSG00000113739 STC2

>Homo_sapiens
MLSGELREGAG
>Myotis_lucifugus
MLSGELREGAG
>Erinaceus_europaeus
MLSGELREGAG
>Ochotona_princeps
MLSGELREGAG
>Equus_przewalskii
MLSGELREGAG
>Loxodonta_africana
MLSGELREGAG
>Rattus_norvegicus
MLSGELREGAG

HG0789 ENSG00000115355 CCDC88A

>Homo_sapiens

MVSARGEGASPACWY

>Ailuropoda_melanoleuca

MVSARGEGASPACRW

>Pteropus_alecto

MVSVRGEGASPAYRW

>Sorex_araneus

MVSAQGEGASPACGW

>Oryctolagus_cuniculus

MVSARGEGASPACRW

>Ceratotherium_simum_simum

MMSARREGASPACRW

>Ictidomys_tridecemlineatus

MVSARGEGANPACRW

HG0790.1 ENSG00000115568 ZNF142

>Homo_sapiens

--MTMLHPKPSRLQTAFPVRWLL---------------------

>Ailuropoda_melanoleuca

--MTMLHPKLCRLQNGFPICWLL---------------------

>Physeter_catodon

--MTMLHPKPCRLQNVFPICWHL---------------------

>Rousettus_aegyptiacus

--MTMLHPKPCRLQSAFPFVGSCDSRSRSMMWTLSSPPLYR---

>Equus_asinus

MIMTMLYLKPCRLQNAFPIRRIF---------------------

>Manis_javanica

----MLHPKP----------------------------------

>Marmota_marmota_marmota

--MVVLHPKPCRLQNIFPVLWLLCASSRPTVWMLSSPPLFREES

HG0790.2 ENSG00000115568 ZNF142

>Homo_sapiens
-------------------------------MWTLSSPPFYR
>Mustela_putorius_furo
-------------------------------MWTLSSPPLYR
>Physeter_catodon
-------------------------------MWTLSSPPLHR
>Rhinolophus_ferrumequinum
MRLLPVLGRKLCRLQIGFPIRPVCDSASCPAMWTLSSPPLYR
>Oryctolagus_cuniculus
-------------------------------MWTLSSPPFHR
>Tupaia_chinensis
-------------------------------MWTLLSPPFYR

HG0791 ENSG00000131323 TRAF3

>Homo_sapiens
MRKMRPKEVMPLG
>Balaenoptera_acutorostrata_s
MRKLRPREVMPLG
>Oryctolagus_cuniculus
MRKLRPKEVTPLG
>Equus_asinus
MMKLRPKEVMPLG
>Chinchilla_lanigera
MRKLRPKEVIPFG
>Tupaia_chinensis
MRKLRPKEVTPLG
>Trichechus_manatus_latirostr
MRKLRSKEVMPLG

HG0792 ENSG00000132424 PNISR

>Homo_sapiens
MLRSCSLNQPKF
>Ursus_maritimus
MLPSCSLNQPKF
>Miniopterus_natalensis
MLPSCSLNQPKF
>Ochotona_princeps
MLPSCDLNQPKF
>Ceratotherium_simum_simum
MLPSCSLNQPEF
>Manis_javanica
MLPSCSLNQPKF
>Cavia_porcellus
MLSSCSLNQPKF

HG0793 ENSG00000132698 RAB25

>Homo_sapiens

MRGGAGPDLLRAEG

>Erinaceus_europaeus

MRGGVGPGLLRAEG

>Oryctolagus_cuniculus

MRGGAGPGLPRAEG

>Equus_caballus

MRGGADPGLLRTEG

>Loxodonta_africana

MRGGEGPSLLRAED

>Trichechus_manatus_latirostr

MRGGAGPSLLRAEG

>Orycteropus_afer_afer

MRGGAGPSLLKAGG

HG0794.1 ENSG00000135218 CD36

>Homo_sapiens
------------MLEHLIEKSFLAILKIAFQ---
>Acinonyx_jubatus
------------MFEHLIEKSFLAFLKIAYK---
>Balaenoptera_acutorostrata_s
------------MLEHLIEKSFLAFLKIAYN---
>Myotis_brandtii
------------MLEHLIEKSFLAFLKIADNA--
>Oryctolagus_cuniculus
------------MLEHLIEKSFLALLKLTNDWKN
>Equus_asinus
------------MLELLIEKFFLAFLQIAYK---
>Heterocephalus_glaber
MSFGPVKNCLFKVLEHLIENSFLMHIKAAYK---

HG0794.2 ENSG00000135218 CD36

>Homo_sapiens

---MSMAFRCQDNLKDR---------------------------------

>Canis_lupus_familiaris

-----MAFRCQDNLKDR---------------------------------

>Lipotes_vexillifer

---MSMAFTCQDNLKDRGVVKTLSLISEFSAGVLWKSCVYFLHLLLQQSL

>Erinaceus_europaeus

---MSMAFRRPDKLKDR---------------------------------

>Mus_musculus

MLRMSMAVRRQDNLEDR---------------------------------

HG0795 ENSG00000137601 NEK1

>Homo_sapiens
--MSFSKCGM
VPLLY
>Acinonyx_jubatus
--MSFSKCGM
MPLPH
>Myotis_lucifugus
--MSFSKCGM
MPLPL
>Dasypus_novemcinctus
MPPVPRPFKAARNYLFEVGEAVTQALAPLPPGVSRGLRGQSVVFIWVVWACLTGRCPRCDVIQPEASAKSWCQAWEARHHLSGWTVSCWVDRLDFSFRILFSKCGM
VPLPY
>Equus_asinus
--MSFSKCGM
VPLSL
>Loxodonta_africana
--MSFSKCGM
IPLP-
>Fukomys_damarensis
--MSFAKCRM
VPLLC

HG0796 ENSG00000138074 SLC5A6

>Homo_sapiens
MRPSPLSGILADPLFLFPFSEGLPRRRAASRSRLQTPSARCSPRPDAEVAASQKLGPLHPPSEKGAMLISGSTKGPS
>Odobenus_rosmarus_divergens
--MLISGQTTGPS
>Rhinolophus_ferrumequinum
--MLISGRTTGPS
>Galeopterus_variegatus
--MLISRRVKGPS
>Condylura_cristata
--MLISGRTTEPS
>Manis_javanica
--MLLSGRTKGPS
>Fukomys_darlingi
--MLISGSKKRSS

HG0797 ENSG00000140526 ABHD2

>Homo_sapiens
--MSAAGSRGAAGTRLGAS-------------
>Felis_catus
--MSAAGSRGASGTRLGANQDAVVNHAQLLG-
>Physeter_catodon
--MSAGGSR-----------------------
>Dasypus_novemcinctus
--MSAAGSRGAVGARLGASWTVVVTHAQLLG-
>Galeopterus_variegatus
--MSAAGSRGASGTGLGASWAAVGNQAQLLG-
>Ceratotherium_simum_simum
MNGRVPAVRGVAEAVVRGVAAVAWAGPVAAGAALARGSGCISMSAAGSCGAAGTGLGASWTAVVNHAQLLG-
>Nannospalax_galili
--MSAAGSRGAAGTRLGASWTARVHRAAFRLI

HG0798.1 ENSG00000142949 PTPRF

>Homo_sapiens

MRTDGPWIGGRSGGPGARPLVLSIQQE

>Orcinus_orca

MRTDGPWIGGRSRGPSALPSVLVVQE-

>Rhinolophus_ferrumequinum

MRTDGPWIGGRSGGPGALPSVLVVQE-

>Dasypus_novemcinctus

MRTDGPWIGGRNGGPSALPLMVIVQQV

>Oryctolagus_cuniculus

MRTDGPWIGG-----------------

>Manis_javanica

MRTDGPWMSGRSRGPCALASELGVQE-

>Chinchilla_lanigera

MRTDGPWIGGRSSGLGALPAVLIVQQE

HG0798.2 ENSG00000153707 PTPRD

>Homo_sapiens
--MLAPREAMGFGSRFLS
>Canis_lupus_familiaris
MLAPALKGVMGFGSRFLS
>Condylura_cristata
MLAPARREAMDFSSRFLS
>Mesocricetus_auratus
MLAPVLRDIMGFGSRFLS

HG0799 ENSG00000143398 PIP5K1A

>Homo_sapiens

MPGEWCGLELVQEPARRV

>Acinonyx_jubatus

MPAKWCGLELFQDPARRV

>Balaenoptera_acutorostrata_s

MPAEWCGPELVPEPARRI

>Rousettus_aegyptiacus

MPAEWCGSELVQEPARRV

>Oryctolagus_cuniculus

MLGEWCGPELVQELAGRV

>Equus_asinus

MLAEWCGPEVVLEPARRV

>Mesocricetus_auratus

MPGKWCGPELVPEPERRQ

HG0800 ENSG00000143612 C1orf43

>Homo_sapiens
--MFSGRVWLGLPP
>Miniopterus_natalensis
--MFSGRVSLGLSP
>Oryctolagus_cuniculus
---------------------------------MRRSPFLTTPPSAMFSGRVWLGLPP
>Loxodonta_africana
MHGRPPLPLTGVVPRLGRRRSRVPLQPESRAGGEAGRRRSPPPPAAMFLGRVFLGLPP
>Marmota_marmota_marmota
--MFSGRVWLGLPP
>Trichechus_manatus_latirostr
--MFSGQVSLGLPP
>Orycteropus_afer_afer
--MFLGRVSLGLPP

HG0801 ENSG00000144278 GALNT13

>Homo_sapiens

MFANHFEIAKCHIQFNWI------------------------------

>Mustela_putorius_furo

MFANHFEIAKCHIHGMHPAFI---LEFTPVAWSYHC----SICLRSVC

>Balaenoptera_acutorostrata_s

MFANHFEIAKCHIHGMHPAFI---LEFTPVAWICHS----CICLRSLC

>Rhinolophus_ferrumequinum

MFANHFEIAKHHIQLSSLGNG---------------------------

>Condylura_cristata

MFANHFEIAKHHIHGMYPAFS---LEFTRWLGLITVAPVFDLCVN---

>Equus_asinus

MFANHFEIAKRHIHGMHLAFI---LEITSVAWFYHS----SVCLQSMY

>Cricetulus_griseus

MFSNHFEIAKHHIQCQRKMLVAILYHFLPYSTR----QGLSLNLHFIG

HG0802 ENSG00000144283 PKP4

>Homo_sapiens

MSLIPGATTAAA

>Pteropus_vampyrus

MSLIPGATTAAA

>Oryctolagus_cuniculus

MSLIPGATTAAA

>Equus_asinus

MSLIPGATTAAA

>Loxodonta_africana

MSLVPGATTAAA

>Ctenomys_sociabilis

MSLIPGATTAAA

>Trichechus_manatus_latirostr

MSLIPGATTAAA

HG0803 ENSG00000144802 NFKBIZ

>Homo_sapiens

MLSRTWVIIADYSWKQLICSYVFV--------------

>Pteropus_vampyrus

MLSRTWVIIADYSWKRFAFTYLHDLTLQTQRLTGRSGG

>Erinaceus_europaeus

MLSRTWVLIVDYSWKQLICLYVFV--------------

>Equus_asinus

MLSRTWAIIADHNWKQLICLYVFV--------------

>Nannospalax_galili

MLSRTWVIIAGYSWKQLIRSYVFV--------------

>Trichechus_manatus_latirostr

MLSRTWVIIADYSWKQFICFYVFV--------------

>Orycteropus_afer_afer

MLSRPWVIIADYSWK-----------------------

HG0804 ENSG00000144815 NXPE3

>Homo_sapiens

-------------------------MCFLNQLQSLLCIRALKGTSRIEANETESSSAAQKSKDMEF

>Panthera_tigris

-------------------------MCFLNQPQPLLCTKN---SS---------------------

>Myotis_davidii

-----------------------------MNLSSSFIPRTLKDASREEANETESSPAAQKNKDMEF

>Erinaceus_europaeus

-------------------------MCFLNQANVSSMQITLKDNNRKEAN----------------

>Ceratotherium_simum_simum

MILTANSLRTVSTKEQLGLVTERRKMCFLNQPQPLLSTKN---TSRKEAD----------------

>Dipodomys_ordii

---------------------------MLLNWLQSLLHITLKTASRIEAN----------------

>Tupaia_chinensis

-------------------------MCFWNQLQSLFYRKSS-------------------------

HG0805.1 ENSG00000145936 KCNMB1

>Homo_sapiens
---MFGHGGLPQMGTKSCFPGDGRGGQTSC------------

>Ailuropoda_melanoleuca
---MFGHGGLSQMGTKGRFPRDGLAAQSSCCGSGSLFWGGGW
GPGPKS-LP-AEDSRPPDTK

>Physeter_catodon
---MFGHGGSSQMGTKGRFPRDGLGAQTSCWSSGGLFWGGGW
GPGRKV-SA-EDSQGTGETK

>Myotis_lucifugus
---MFGHGG---------------------------------

>Galeopterus_variegatus
---------------------------MQVRGLGS-------SGCLRGW-VPALLSLCPEAVCSDLTMFGHGGLSQRGTKGCFPGDGLGGQTSCWSSASSFRVGAG
GQAERNLLPAEDPQGTRETK

>Oryctolagus_cuniculus
MKKKSFQIYQGGLFWFLSAVAVCCSTSVSPETLGNAGAGARAAAAVSGLGPRHLLSLFPRAVCSRLTMFGHGGSSQMGTQGCFPGDGLGGQTSC------------
----

>Trichechus_manatus_latirostr
---MFGHGGSLQMRTKGRFPGDGPGGRASCWSSGASFRVGAR
QKETVCQLKLTGDPGD----

HG0805.2 ENSG00000145936 KCNMB1

>Homo_sapiens
-------------------------------MEVQGLGNSCLPQ---GWVPALR-VCVSRLCAQT---
----
>Orcinus_orca
MSPNIPRWSFLTSQFCCL-FCEICFPLDSGNCRCRGLGNSCP---PLGWVPALPSLCPGAVCSDLTMFGHGGSSQMGTKGHFPRDGLGAQTSCWSSGGLFWGGGWG
--PGRKVSAEDSQGTRETK
>Myotis_davidii
-------------------------------MQVRGLGSSCHPPSSCGLVPALS-VCP-GLCAQT---
----
>Galeopterus_variegatus
-------------------------------MQVRGLGSSGC---LRGWVPALLSLCPEAVCSDLTMFGHGGLSQRGTKGCFPGDGLGGQTSCWSSASSFRVGAGG
QAERNLLPAEDPQGTRETK
>Oryctolagus_cuniculus
-------------------------------MQVQGPGQQ--LP-CPGWVPAISSVCFLGLCARA---
----
>Ceratotherium_simum_simum
------MVHFPASQFCCMLLFETCFPRDSGKCRCRGLVAAAILR-TPGWVPALPSFCPRLCAQT--
----

HG0806 ENSG00000147481 SNTG1

>Homo_sapiens

MGRAVGVEMLKGPHFLK

>Mustela_putorius_furo

MGSAEGVEMLKGPHFMK

>Myotis_brandtii

MGSAVRIEVLKGPHFLE

>Condylura_cristata

MGAAAGVEMLKGPHFLK

>Manis_javanica

MGSAEGVNMLKAPYFLK

>Loxodonta_africana

MGSAVRVEMPRGPHFLK

>Mus_musculus

MGVAVGIEILKGPEFLK

HG0807.1 ENSG00000147488 ST18

>Homo_sapiens

----------MFLTECDLRGILEKIIKFLNGAAWRNPKRNSIRAFWNTSRKR

>Canis_lupus_familiaris

MEPFCREKGRMFLTECDLTGILEKAITFLNGAVWRNPKRNSIRAFWNISRKR

>Rousettus_aegyptiacus

MEPFCREKGRMFLTECDLTGVLEKVIKFLNGAVWRNPKRNSIRAFWNISRKR

>Dasypus_novemcinctus

----------MFLTECDLTGRLENIITFLNGAVWRNPKRNSIRAFWNISRKR

>Manis_javanica

MEPFCGEKGRMFLTECDLTGVLEKAIKFLNGAVWRSPKRNSIRTFWNISRKR

>Chinchilla_lanigera

MEPLCREKGRMFVTECDPTGVLEKIIKYLNGAAWRNPRRNSIRSFWNISRKR

>Tupaia_chinensis

----------MFLTECDLAGMLETVIKFLNGAAWRNPKRNSIRTFWNISRKR

HG0807.2 ENSG00000147488 ST18

>Homo_sapiens

MSGQILQETQKSMIKKLSTLIYCKG

>Mustela_putorius_furo

MSGQSLQAIQKSMVKKPRTSIYCKG

>Balaenoptera_acutorostrata_s

MSGQSFQVIQKPMVKKPGTPTYCKG

>Rousettus_aegyptiacus

MSGQSLQVIQRSMLKKLCTLIYCKG

>Dasypus_novemcinctus

MSGQSLQLIQISMVKKPGASIYCKG

>Oryctolagus_cuniculus

MSGQSLPVTQKSMVKKPGASLHCKG

HG0807.3 ENSG00000186487 MYT1L

>Homo_sapiens
MEKSRHREIK
>Oryctolagus_cuniculus
MEKSRHREIK
>Equus_przewalskii
MEKSRHREIK
>Manis_javanica
MEKSRHREIK

HG0808 ENSG00000149269 PAK1

>Homo_sapiens
MPGLAFPTSSCCWW-
>Ailuropoda_melanoleuca
MPGPAPPTSSCCWW-
>Miniopterus_natalensis
MPGPALPTSSCWWW-
>Equus_asinus
MPGPARPTSSCWWW-
>Loxodonta_africana
MPGSAPPTSSCWWW-
>Marmota_marmota_marmota
MPGLALRTSSCCWWW
>Trichechus_manatus_latirostr
MPGPAPPTSSCWWW-

HG0809 ENSG00000151287 TEX30

>Homo_sapiens
MCSFIVFVSYPCLPTHLSQQNLLFREMSFASQM
>Ursus_maritimus
MCSFIVLR-------------------------
>Dasypus_novemcinctus
--------------------------MSFASQM
>Ochotona_princeps
--------------------------MSFAPQV
>Equus_caballus
MCSFIVLVSYPCLPTHLSQQNLLFREMSFASQM
>Manis_javanica
MYSFIVLVSYPCLPTHLSQQNLLFRAMSFAPQM
>Ictidomys_tridecemlineatus
-------MSYPCLTTHLSQQNLLFREMSFASQM

HG0810 ENSG00000156504 FAM122B

>Homo_sapiens

MGARTRKNWPSGGSRF--

>Canis_lupus_familiaris

MGARTRKTWPRGGSRC--

>Pteropus_vampyrus

MGARTRKTWSRGGSRF--

>Oryctolagus_cuniculus

MGARTRKHWPC---

>Ceratotherium_simum_simum

MGARTRKTWPRAGSRF--

>Manis_javanica

MGARTRKIWPRGGSPLLKRGRRKRRQVVDFALGPKGFPWAIRTQERKQRHRVGGRGAEPPTCSPGELGVGGEKYRTQG

>Heterocephalus_glaber

MGARTRKNWPHGGSGFRSEAWEV----I----EVAGYRY---------------------------------------

HG0811 ENSG00000157350 ST3GAL2

>Homo_sapiens

MGSRRARPTAERISISAAEFSHTHRASCFCPLRVGVWLPSSGRGFGPGGGTTLAWNPSPLKPLCSLL

>Ailuropoda_melanoleuca

MGTRRAEPSARRILVSAAEFSDTHRASWVSSLRTGALFPPLAEGLGQEGQFQWGRLSDGIQAT----

>Balaenoptera_acutorostrata_s

MGSRRAEPNARRILVS-EEFSGTHRASWVSSLWIGAWFPFLPWQRGWALRAN---------------

>Miniopterus_natalensis

--MPLAWNQGCSKPTCSLL

>Galeopterus_variegatus

--MTLAWNLGPLKPTCSPL

>Equus_asinus

MGRGTAEPNTRRILVSAAEFSDTHRASCVSSLRIGAWFPPLAEGLGQEGQQRWGRLLHGIQAA----

>Rattus_norvegicus

---MRTRAWDAGLLKPVCSLL

HG0812 ENSG00000157368 IL34

>Homo_sapiens
MGLAATAPRLSSTLPGR
>Felis_catus
MGLAATAPQLPSTLPGR
>Balaenoptera_acutorostrata_s
MGLTATAPRLPSALPGR
>Galeopterus_variegatus
MGLTATAPQLSSALPGR
>Equus_asinus
MGLTASAPRLPSALPGR
>Ctenomys_sociabilis
MGLGATAPRLSSALPGR
>Tupaia_chinensis
MGLAATTPRLSSTLPGR

HG0813 ENSG00000160199 PKNOX1

>Homo_sapiens
MKIGTQTPFAFHPR
>Odobenus_rosmarus_divergens
MKIGTQTPFAFDSR
>Orcinus_orca
MKIGTQTPFAFDSR
>Eptesicus_fuscus
MKIGTQIPFAFDSR
>Elephantulus_edwardii
MKIGTQTTFAFDSR
>Rattus_norvegicus
MKIGTQTPFASHSR
>Orycteropus_afer_afer
MKIGTQTTFAFDSR

HG0814 ENSG00000163071 SPATA18

>Homo_sapiens

MRRGGCGPGHLPSGFPNPARSDPRGRMETPAAL

>Acinonyx_jubatus

--------------------------MEEPAAL

>Physeter_catodon

--------------------------METPAAL

>Myotis_lucifugus

MRRGGCGPGHLPPASPNPARSDPRGRMETSAEL

>Equus_caballus

MRWGGCGPGHLPPGFPNPARSHPRGRMETPAAL

>Manis_javanica

MRLGGCGSGHLPPGFPNPARCDPRGTMEKPAAL

>Peromyscus_maniculatus_baird

--------------------------MDTPAAF

HG0815 ENSG00000163072 NOSTRIN

>Homo_sapiens

---MKVQGWDFLLGMFDKD

>Ursus_maritimus

---MNVLGWDFLLRVFDKD

>Rousettus_aegyptiacus

MNRMKVQGWDILLRVFDKG

>Equus_asinus

---MKVQGWDFLLGVFDKD

>Manis_javanica

---MKVQGWDFLLGVFDKA

>Nannospalax_galili

---MKVQDWDFLLGVFDKD

>Tupaia_chinensis

---MKVQGWDFLLGMFDKD

HG0816 ENSG00000163794 UCN

>Homo_sapiens

--MFFADVSRSCPGTI
>Mustela_putorius_furo

--MFLADVSRSCPGAI
>Orcinus_orca

--MFLADVSRSCPGAI
>Rousettus_aegyptiacus

--MFPADVSRSCPGAI
>Dasypus_novemcinctus
MVVTGCHQCPPDRLRLGKRWRGLETVRAAPRPPDASSPSGGGGHGQHHGQPQPPLAGGHHGPRLVWCGDGPEASLAVSSGRKDSAAFQSHRSFMPQPACRSLESGA
TFRAASSAPREMRSRVSAGAPPGGLNP-RDSGRGRHCFYNQCMLFADVSRSCPGAI
>Manis_javanica

-------------------------------MRSCHCFYNQCMFLADVSRSCPGAI
>Peromyscus_maniculatus_baird
---MPQPASPSSTARV
I----PKLAPGTRNLLSGCAPLRGLEAAGLEAAAVIAFYNQFMFFADVSRSCPGAI

HG0817 ENSG00000163888 CAMK2N2

>Homo_sapiens
MTSSFRRLPC
>Canis_lupus_familiaris
MTSSFRRLPC
>Orcinus_orca
MTSSFRRLPC
>Oryctolagus_cuniculus
MTSSFRRLPC
>Ceratotherium_simum_simum
MTSSFRRLPC
>Manis_javanica
MTSSFRRLPC
>Rattus_norvegicus
MTSSFRRLPC

HG0818 ENSG00000164125 FAM198B

>Homo_sapiens

------------------------------MVRNKPHFSLGRLII-

>Mustela_putorius_furo

MFYLHASAFKSSLHY-SQGLHSSWSQARGQLCKNKPHFSLGKLIGV

>Miniopterus_natalensis

MKYVLLTCLSLSKFFALESEIALFLEPSMGPAVNKPHFSLGRLIGV

>Galeopterus_variegatus

------------------------------MLRNKPHFSLGRLIAV

>Erinaceus_europaeus

------------------------------MVRTTPHFSLGKRSGV

>Ceratotherium_simum_simum

------------------------------MVRNKPHFSLGRLIGV

>Ictidomys_tridecemlineatus

---------------------------MARMVRINSHFSLGRLIEV

HG0819 ENSG00000164188 RANBP3L

>Homo_sapiens

-----MKELSCVFNQMNWHLYI

>Canis_lupus_familiaris

----MKEELSCVFIQTNWHLYM

>Balaenoptera_acutorostrata_s

MCFEMKEELSCVFIQMNWHLYM

>Pteropus_vampyrus

MCFEMKEELSCVFIQMNWHL--

>Condylura_cristata

MHFEMKEELSWVFIQMNWHLSL

>Equus_asinus

----MKEELSCVFIQMNWHLYM

>Marmota_marmota_marmota

---------------MNWHHFM

HG0820 ENSG00000164946 FREM1

>Homo_sapiens

MLRSRGTLSGPLTLPLGL--------------

>Acinonyx_jubatus

MLRSWGTLSGPLALPLGL--------------

>Miniopterus_natalensis

MLRSWGTLSGPLALPLGL--------------

>Equus_asinus

MLRSWGTLSGPFALPLGL--------------

>Manis_javanica

MLRSWGTLLEPLALPLGLGADKAGGSTEALLC

>Mus_musculus

MLRSWGTLSGPLVLPPGPQADTWGRSAGSLLC

>Tupaia_chinensis

MLRSQGTLSGPLALPLRL--------------

HG0821 ENSG00000165300 SLITRK5

>Homo_sapiens
MMTGPWRCETGR
>Mustela_putorius_furo
MMSGPWRCETGR
>Lipotes_vexillifer
MMSGPWRCETGR
>Pteropus_vampyrus
-MSGPWRCETGR
>Dasypus_novemcinctus
-MSGPWRCETGR
>Equus_caballus
MMSGPRRCETGR
>Ictidomys_tridecemlineatus
MMSGPWRCETGR

HG0822 ENSG00000166170 BAG5

>Homo_sapiens

MQLGAQLRCRTP

>Leptonychotes_weddellii

MQLGAQLRCRTP

>Balaenoptera_acutorostrata_s

MQLGAQLRCRTP

>Rousettus_aegyptiacus

MQLGAQLRHRTP

>Condylura_cristata

MTLGAQLRCRTP

>Equus_asinus

MLLGAQLRCRTP

>Tupaia_chinensis

MPLGAQLRCRTP

HG0823 ENSG00000166268 MYRFL

>Homo_sapiens
----------------------MAEDMLHSNKKKMKIFQEHS--
>Mustela_putorius_furo
----------------------------------MKIFREHS--
>Balaenoptera_acutorostrata_s
--------------------------MLHSNKKKMNIFQEHA--
>Myotis_lucifugus
MSFWFYNHLENLTISLGLWAQLDAEDTLPSSEKKMKIFREHS--
>Oryctolagus_cuniculus
----------------------MAEDMPHSPAKKMKVFRSTRRL
>Mus_musculus
----------------------MAEKPLHVNKKTMETFQEFS--
>Trichechus_manatus_latirostr
----------------------MSEDRLHSSKKKMKFFQEHS--

HG0824 ENSG00000167100

>Homo_sapiens
MERLEPARSPPRPG
>Felis_catus
MERLEPARSPPRPG
>Balaenoptera_acutorostrata_s
MERTEPARSPPRPG
>Condylura_cristata
MEQIEPARSPPHPG
>Ochotona_princeps
MERLEPARSPPRPG
>Equus_przewalskii
MKRIEPAWSPPRPG
>Peromyscus_maniculatus_baird
MERLEPARSPPRPG

HG0825 ENSG00000169180 XPO6

>Homo_sapiens

MGGGLPESAGTMT

>Odobenus_rosmarus_divergens

MGGGLPESAGTMT

>Pteropus_alecto

MGGGLPEPAGTMT

>Galeopterus_variegatus

MGGGLPEPAGTMT

>Ceratotherium_simum_simum

MGGGLPEPAGTMT

>Loxodonta_africana

MGRGLPEPAGTMT

>Mus_musculus

MGGGLPEPAGTMT

HG0826 ENSG00000169851 PCDH7

>Homo_sapiens

MTLRRVVKKKEKPALSGVRRD---

>Pteropus_alecto

MTLRRVVKKKREKRALGGEKGLTLWAPLKMARAAFSRAGETRTRTAL---------------------------

>Dasypus_novemcinctus

MTLRRVVKKEK-PELSGVEKGLTLWASRKMARAALRRAGETRPRTAL---------------------------

>Equus_asinus

MTLRRVVKKKEKPELSGVRRD---

>Manis_javanica

MTLRRVVKKKEKPELLGVRKD---

>Loxodonta_africana

MTLRRVVKKKEKPELPAVRKG---

>Ictidomys_tridecemlineatus

MTLRRVVKKKEKPELSEGEKRLTPGVSEDGSGCSLARRGNPDADRAVTRVVFTAPCPKRPAGDFFILLTGFSAA

HG0827 ENSG00000170365 SMAD1

>Homo_sapiens
--MCIREFAVEQLFLYSAPCL---------------------------------
>Odobenus_rosmarus_divergens
--MCICEFAVEQLFIYSALCLC--------------------------------
>Orcinus_orca
--MCICEFAVQQLFIYSAPGLCADFGLL-LKTLGMVISTFQDLSTKRLTRILCK
>Rousettus_aegyptiacus
MYSKVWRRRVIFFPSPLFRLHPPAPPSVVSFLSLDRIRLPHCMCICEFAVEQLFIYCAPWLCEGFSGLFLEEVGVVGVRETGRD----VGLFF-
>Equus_caballus
--MCICEFAAEQLFLCSAPCLCADLGLL-FETLGMVVSTLQDLKTKELRRTLRK
>Trichechus_manatus_latirostr
--MCICEFAVEQLFILPAPCLC--------------------------------
>Orycteropus_afer_afer
--MCICEFTVEQLFIYPAPCLC--------------------------------

HG0828 ENSG00000170390 DCLK2

>Homo_sapiens
MTWAARRCGHF
>Acinonyx_jubatus
MSWAARRCGHF
>Orcinus_orca
MSWAARRCGHF
>Rousettus_aegyptiacus
MSRAARRCGHF
>Ceratotherium_simum_simum
MSWAARRCGHF
>Trichechus_manatus_latirostr
MSWAAQRCDHF
>Orycteropus_afer_afer
MSWAAGRCGHF

HG0829 ENSG00000171502 COL24A1

>Homo_sapiens
MDSTCTLSSSELASVFR
>Odobenus_rosmarus_divergens
MASTCTLSSSELPSVFR
>Physeter_catodon
MDSTCTLSSSELASVFR
>Miniopterus_natalensis
MDSTCTLSRTELASVFR
>Dasypus_novemcinctus
MDSTGTLGTSELAFVFR
>Manis_javanica
MDSTCTLSSSELASVFR
>Ictidomys_tridecemlineatus
MDSTCTLSSSELASVFR

HG0830 ENSG00000171631 P2RY6

>Homo_sapiens

-----------MALEGGVQAEEMGAVLSEPLPP-------

>Ailuropoda_melanoleuca

-----------MALEGGVQAEEVGTALCQLAVCGRSIYVD

>Balaenoptera_acutorostrata_s

---------MTMALEGGVQAEEVGTVLSSEVWTFCSC---

>Rhinolophus_ferrumequinum

MDKKTAAKRRTMALEGGIQAEEVGAVLGQLTM--------

>Dasypus_novemcinctus

-----------MALEGGIQTKEVGAVLSQHTCLRM-----

>Equus_przewalskii

-----------MALEGGVQAEEPLWT--------------

>Chinchilla_lanigera

-----------MSLEGGVQAAEVGAIFSELMPLQM-----

HG0831.1 ENSG00000174482 LINGO2

>Homo_sapiens

MQLSRFVSSSWLVGL

>Canis_lupus_familiaris

MQLSRFTSSNWLVGL

>Condylura_cristata

MQLPRFISSTWLVGR

>Ochotona_princeps

MQLSRFLSSNWLVEL

>Elephantulus_edwardii

MQLSRFVSNSWRVGR

>Mus_musculus

MQCSRHVSSSWLVGL

>Tupaia_chinensis

MQLSRFISNSWLVGL

HG0831.2 ENSG00000174482 LINGO2

>Homo_sapiens

---MRRSSEQSTESGHYQHEEPNNSNW

>Canis_lupus_familiaris

---MQRGSEKNTESGHHQDEEPNKSDW

>Ochotona_princeps

---MKRGSEKNLKRGYHQHVQPNTPAW

>Fukomys_damarensis

---MRRRSEKNTASGHHHHEDPKNSNW

>Tupaia_chinensis

MRVMRRDSEQSTASGHHQHEEPNNPNW

HG0832 ENSG00000175048 ZDHHC14

>Homo_sapiens
MTVGLPGGLTWVSSAKLSPSREPV-
>Canis_lupus_familiaris
MTAGLPRGLTWVSSAKLSPSRQPA-
>Orcinus_orca
MTAGLPKGLTWVSSAKLSPSREPA-
>Ochotona_princeps
MPEGLPGGLSWASPAKWAASREPAW
>Equus_przewalskii
MTAGLPKGLTWGSSAKLSPTQEPA-
>Mesocricetus_auratus
MTAGLPKGLTWVSSAKLSPSREPV-
>Tupaia_chinensis
MTVGLPRGLTWVSSAKLPPSREPV-

HG0833 ENSG00000176641 RNF152

>Homo_sapiens
MSSRRRGERDSVTDTEMNTPVSTGCL
>Felis_catus
----------------MNTPEHALCL
>Balaenoptera_acutorostrata_s
----------------MNTPEYARCL
>Myotis_ricketti
----------------MNTPEHAPCL
>Ceratotherium_simum_simum
----------------MNTPEYARCL
>Manis_javanica
----------------MNTPEGASCP
>Heterocephalus_glaber
MSSCRRGERDSMTDSDMNTPANAKYL

HG0834 ENSG00000182489 XKRX

>Homo_sapiens

MLAFVLFKFHSKYVDDWNRGQSRVKFKVVLLGFPSQTPSR--I--------

>Ailuropoda_melanoleuca

MVAFALSRFHSTYVDDWNPGPMKSQVQSGTPWFSIPYSNRCNLKL------

>Myotis_davidii

------------MLTIGILSQSRIKFKVVLLGSPSQTSTS--LI-------

>Oryctolagus_cuniculus

MLAFSLFQFHSTYVDDWTLEPVKSQLQSGTPGLCIPDSEWNRSPEERDLLL

>Ceratotherium_simum_simum

MIAFALSSFHSTYVDDGILGQSRVKFKVVLLGSPSQTPTS--LI-------

>Manis_javanica

MLALPCPSFTALMLTIGILGQSRAKFKVVLLDSPSQAPTS--LI-------

>Marmota_marmota_marmota

MLAFALSKFLSTYIDDWILGPIKNQVQSGPLGLFIPDSTSNWV--------

HG0835 ENSG00000182901 RGS7

>Homo_sapiens

MKLPCLAAPLAAS

>Rousettus_aegyptiacus

MKLPCRAAPLAAS

>Erinaceus_europaeus

MKLPCLAAPLAAS

>Oryctolagus_cuniculus

MKLPCLAAPLAAS

>Loxodonta_africana

MKLPCRAPPLAAS

>Mesocricetus_auratus

MRLPCLAAPLAAS

>Tupaia_chinensis

MKLPCLAAPLAAS

HG0836 ENSG00000197461 PDGFA

>Homo_sapiens
MSANMQNYRPGRS
>Mustela_putorius_furo
----MQNYRPGRS
>Lipotes_vexillifer
----MQNYRPGRS
>Rousettus_aegyptiacus
----MQNYRPGRS
>Condylura_cristata
MPANMQNYRPGRS
>Ceratotherium_simum_simum
MAANMQNYRPGRS
>Dipodomys_ordii
MPTNMRNYRPGRS

HG0837 ENSG00000198690 FAN1

>Homo_sapiens

---------------------------MRSYRIPLLVISSQESKSKPLLSFTLNILCFIAQNIQFF

>Panthera_tigris

------------------------MKNMKPNRIPLCIVSRQGSKSKPLLSFSSDILGFTTGNTQFF

>Balaenoptera_acutorostrata_s

------------------------MNNMKSNRIPLFIISIQGSKSKPLLSFPGDFLCFTT------

>Miniopterus_natalensis

MVFICTCFT-------LQVEGIDYRGNMKSDRSPLFLISSQGSESKPLPSFPRDSLCFIT------

>Erinaceus_europaeus

MRFCWKLYKAQHSTAVFMEEETVQRNSMKPKRIRLFISR---------------------------

>Ceratotherium_simum_simum

---------------------------MKSNRIPLFIISSQGSKSKPLLSSLGDTLCFIT------

>Loxodonta_africana

---------------------------MKSNRTPLSVSSGPESPSKPLPSFT--------------

HG0838 ENSG00000198707 CEP290

>Homo_sapiens

-----------------MVSPSSRCRFGLLGTIWLDPESAWNRDRDLSGPAAGSSLVVAVVRGLRWLPGLV---------

>Felis_catus

-----------------MVSPSSRCRS---

>Myotis_lucifugus

-----------------MVSPSSRRRYCLLGTSWRDPVSAWNRAGERSGIAAGPAWWMR---------------------

>Condylura_cristata

-----------------MVSPSSHRR--

>Oryctolagus_cuniculus

-----------------MVSPSSRRRLCLLGASWRD--

>Ceratotherium_simum_simum

-----------------MVSPSSRRRYSLLRTSWREPVSGWNCAGERSGVAAGPGLGSVSA--CRAPPRLAPLDVRVPWW

>Heterocephalus_glaber

MWPFLASGPYSALAGWGMVSPSSCRRFY--

HG0839 ENSG00000198791 CNOT7

>Homo_sapiens
MGHPLPPPLPPPPPPPPPSAVSMARRRRRSASSATQVHK
>Leptonychotes_weddellii
MGHPLLPPLPPPPP--PLVAVSMARRKRRSAGSATQVQK
>Erinaceus_europaeus
MGHPLPPPPLPP-PPQPPGAFSMARRKRRSANSSTRVHK
>Oryctolagus_cuniculus
MGHPLPPPPL-PPPPLPPSAVSMARRKRRSASSATQVHK
>Elephantulus_edwardii
MGHPLPPPLPPP----PPGAVSMARRKRRSASSAIQEYK
>Microtus_ochrogaster
----------------------MARRKRRSASSETQVHK
>Tupaia_chinensis
----------------------MARRRMRSASSATQVHK

HG0840 ENSG00000278195 SSTR3

>Homo_sapiens

MITSLAARREALTPWAGK---

>Canis_lupus_familiaris

MITSLAASRGALAPWAGQ---

>Balaenoptera_acutorostrata_s

MITGLAARRGALTPWAGQ---

>Myotis_brandtii

MITSLAARRGSLTPRTGQ-------CRSWKQPDASCCVASSAHAPAFLSPLSVLGAVLCTLQCNRSHPGRPIADC

>Dasypus_novemcinctus

MRTDLAARRAAFTPWAGP---

>Equus_asinus

MITGLAARRGALTPWAESWNHRTAEYRSWKKPDASRRWPPRL-----T----------CQLA-------------

>Manis_javanica

MITGLAARREAVTQWAGQ---

HG0841 ENSG00000278259 MYO19

>Homo_sapiens

MTRWIVLLVPPATQNPG

>Mustela_putorius_furo

MTRWIALLVP-VTQNPG

>Balaenoptera_acutorostrata_s

MTRWIALLVP-ATQNPG

>Myotis_brandtii

MTRWIALLVP-VTQNSG

>Galeopterus_variegatus

MTRWIALLVP-VTQNPG

>Ceratotherium_simum_simum

MTRWIALLVP-VTQNPG

>Chinchilla_lanigera

MTKWIALLGP-GTQNPG

HG0842 ENSG00000002746 HECW1

>Homo_sapiens

-------------------------------MKCAALPRIRRCCWSRNTVRL

>Odobenus_rosmarus_divergens

-------------------------------MKCAALPRTRRRCGSQNTVRL

>Rousettus_aegyptiacus

-------------------------------MKCAALQCIRRCCWSCNTVRL

>Oryctolagus_cuniculus

MRASPARGWFGSVVPTEEQARREGCRREDKAMKCAALPLVRRRGGSRNPVRL

>Ictidomys_tridecemlineatus

-------------------------------MKCAALPRIRRCCWNCNTVRL

>Orycteropus_afer_afer

-----------------------MRAREDKARKCAALPRIRRCVGSRNTVRL

HG0843 ENSG00000043093 DCUN1D1

>Homo_sapiens

MRVEELRSTDPHVP

>Canis_lupus_familiaris

MSVEQLKSTDPHVP

>Oryctolagus_cuniculus

MSVEQLKSTDPHVP

>Equus_asinus

MNVEQLKSTDPHVP

>Peromyscus_maniculatus_baird

MRVKQLKSADPHGP

>Tupaia_chinensis

MSVEQLKSTDPHVP

HG0844 ENSG00000091009 RBM27

>Homo_sapiens
MPGGLGHREL
>Canis_lupus_familiaris
MPGGLGHREL
>Lipotes_vexillifer
MPGGLGHREL
>Dasypus_novemcinctus
MPGGLGHREL
>Galeopterus_variegatus
MPGGLGHREL
>Oryctolagus_cuniculus
MPGGLGHREL

HG0845 ENSG00000099260 PALMD

>Homo_sapiens

---MVALDLLPRLTVLDF

>Panthera_tigris

MCLSGLICTSAQRRSCRAGDFCLPPLVQRASPLSPLSSPQGGSCFMVALDFLRRLPALDF

>Galeopterus_variegatus

---MVALDFLHRLPVLDF

>Equus_asinus

---MVALDCLPRLPVLDL

>Fukomys_darlingi

---MVALDFLQRLTVLDF

>Orycteropus_afer_afer

---MVVLDFLPRLPILDF

HG0846 ENSG00000100852 ARHGAP5

>Homo_sapiens

---MGKREKEDDPYDLEDVSAQK------------------------

>Canis_lupus_familiaris

---MGKREKEDDPYDLEDLSAQK------------------------

>Pteropus_vampyrus

MVVGVMAVKEDDPHYLEDLSAQK------------------------

>Condylura_cristata

---MGKREKEDDLYHVEDLSTQK------------------------

>Ceratotherium_simum_simum

---MGKREKEDDPYDLEDLSAQK------------------------

>Microtus_ochrogaster

---MGSREKEDDPYDLQDLSLAMKELQRTKYNSEIQDLYFEMVLFSE

HG0847 ENSG00000106086 PLEKHA8

>Homo_sapiens
MALLLVLLASWGLGQ
>Odobenus_rosmarus_divergens
MALLPVLLASWGLGQ
>Lipotes_vexillifer
MALLPVLLASWGLGQ
>Condylura_cristata
MALLPVLLASWGLGQ
>Equus_przewalskii
MALLPVLLAAWGLGQ
>Trichechus_manatus_latirostr
MALLPVLLAYWGLGQ

HG0848 ENSG00000111817 DSE

>Homo_sapiens

MARRGRGGLGSEGAGSPGALE

>Orcinus_orca

MARRAR---------------

>Pteropus_vampyrus

MARRAR---------------

>Oryctolagus_cuniculus

MARRAR---------------

>Ceratotherium_simum_simum

MARRAR---------------

>Loxodonta_africana

MARRVR---------------

HG0849 ENSG00000111846 GCNT2

>Homo_sapiens

MTCRMVTSPTCNFMVGTGGPPGIRTSLRTSVFV------------------------------

>Panthera_tigris

----MVTSPFGDFNVGTGGHIRIRIFLRQLVFVW-----------------------------

>Oryctolagus_cuniculus

MTCRWVTPPAGDFPVGTGGALGIGTSQGRFVCV------------------------------

>Ceratotherium_simum_simum

----MVTSVLGDLTVGVGGHTGTRTSLSQLVFV------------------------------

>Ctenomys_sociabilis

----MVTSAVGDFAVGRRGRPGPWEWFCAAIKIQGVEGTNRARHMQRSYLRNVSEKSENATEL

>Tupaia_chinensis

MTCRMILSPLSGLAVRTGGPTGIRPLLRHSLFM------------------------------

HG0850 ENSG00000113916 BCL6

>Homo_sapiens

MCLQLSFAKEKILEVW--------------------

>Canis_lupus_familiaris

MCLQHSFSTEIIIELWLGPIWCVFLCPS--------

>Pteropus_vampyrus

MCLQHSFSKETITELWLMCQFSESVLPILS------

>Ceratotherium_simum_simum

MCLQHSFTEEIIIELWLGPI----------------

>Fukomys_damarensis

MCLQHSFSKEMTKELWLGPLFKQDFAL---------

>Tupaia_chinensis

MCLQHSFSKEMIVELWLGLSVSLLTHLELEWGEEER

HG0851 ENSG00000114279 FGF12

>Homo_sapiens
MHSVDLTLLGRTS
>Leptonychotes_weddellii
MHSVDLTQPGRTS
>Galeopterus_variegatus
MHSVDLAQPGRTS
>Elephantulus_edwardii
MHSVDLT------
>Equus_przewalskii
MHSVDLTQPGRTS
>Nannospalax_galili
MHSVDLTPPGRTS

HG0852 ENSG00000114942 EEF1B2

>Homo_sapiens

MPILLIYFQTQRK---VHGQFVGHLIQ---GPQFVRGEVGMQKYFDL

>Lipotes_vexillifer

MPILLIDLQTQRK---LGG----------------------------

>Pteropus_vampyrus

MPLLLMYFQTQRK---ATDDLWAI-----------------------

>Condylura_cristata

MPPLLKYFQRQRK---VRL----------------------------

>Loxodonta_africana

MMVLLIYFQTERK---KRG----------------------------

>Marmota_marmota_marmota

MPLLLIYFQKKKKHRNVRGPLMGHLIQNFRTPRFLRGRM--------

HG0853 ENSG00000121807 CCR2

>Homo_sapiens

----MYRVCLTHTQGCISKISKLILFWRPQPNVQCS---

>Felis_catus

----MYRACLTHTQGCISKISKLILL---

>Oryctolagus_cuniculus

----MCTDCLTHTQGCTSKISK---

>Ceratotherium_simum_simum

----MYRVCLTHTQGCISKISKLMLLWRA--

>Manis_javanica

----MCRVCLTDAGLHKQKISKCILPWRPQPSV-QRSWLEKKNCIILFFFFSYLYHYLPLYLRLLFLQETLFPTSAHQVSTSRSVWFSY

>Marmota_marmota_marmota

MSEQMYRDSLTHNQGCTSKISKSILLWRPQPSSVQRSYLEKKNCIFLMLFF--------------------------------------

HG0854 ENSG00000127249 ATP13A4

>Homo_sapiens

MGAASFETAALRECNKQGAASNSS---------------------------

>Canis_lupus_familiaris

MGAACFGTAALRQCSKQG--RNSSEQRVTRGRS-----------SVC----

>Pteropus_alecto

MGAASFGTAALREVGKQGASRKSSEQRVTRGRS-----------SIC----

>Ceratotherium_simum_simum

MGAASFGTAALRECSKQGAGRNSSEQRVTRGRS-----------SVC----

>Manis_javanica

MGAASFGTAALGEHSRQGAGRHSSKQRVTCGRS-----------SVC----

>Mus_musculus

MGAASSETAALRECRSKEQPEIPPGTKCPGGGVHFAKYCHLLKEGVCSRSF

HG0855 ENSG00000127804 METTL16

>Homo_sapiens

MAARWERIICVSRSFAMRLL

>Acinonyx_jubatus

MAAHLERNICVSRRFALRVV

>Pteropus_vampyrus

MAARWERNICVSRSFAVRLA

>Equus_caballus

MAARWERNICVSRSFALRVA

>Manis_javanica

MAARWERIICVSRSFALRVA

>Cavia_porcellus

MAARGDRSICVSRSFALRVT

HG0856 ENSG00000130055 GDPD2

>Homo_sapiens
MGKPAQRNSSPQAP
>Orcinus_orca
MGKAAQRNSSPQAP
>Dasypus_novemcinctus
MGKAAQRNSSPQAP
>Equus_asinus
MGKAAQRNSSPRAP
>Ictidomys_tridecemlineatus
MGKAAQRNSSPQAP
>Trichechus_manatus_latirostr
MGKAAQRNSSPQAR

HG0857 ENSG00000130347 RTN4IP1

>Homo_sapiens

MDRILTGTLIRAFHMRRAE

>Leptonychotes_weddellii

MDRILTGTLIGAFQMGRAE

>Balaenoptera_acutorostrata_s

MDRILTGTLIGAFQM----

>Myotis_lucifugus

MDRILTGTLIGAFPKRRAI

>Galeopterus_variegatus

MDRILTGTLTGAF------

>Equus_asinus

MDKILTGKLIGALQMRIAE

HG0858 ENSG00000132702 HAPLN2

>Homo_sapiens

MRTQAPEGRGAQVPGF---------------------------------------

>Lipotes_vexillifer

MRTQAPEGRGAQVLGLLDAWTSQGPRQLLHLEPLPPAGQKAASHSFLHLCEEAHF

>Galeopterus_variegatus

MRTQALEGRGAQVLGL---------------------------------------

>Oryctolagus_cuniculus

MPTQAPGGGGAQVPGL---------------------------------------

>Loxodonta_africana

MCTQAPEGRGLQVLGL---------------------------------------

>Heterocephalus_glaber

MRTQGPGSRGAQVPGL---------------------------------------

HG0859 ENSG00000132842 AP3B1

>Homo_sapiens
MRRGWTARSAQGAVTSPEAPARAPPYEN
>Odobenus_rosmarus_divergens
MRRGWAARSA------------------
>Orcinus_orca
MRRGWIARSALGALTSPEAPPGAPPNET
>Rousettus_aegyptiacus
MRRGWTARSA------------------
>Heterocephalus_glaber
MRRTR-APGRRVAVMSPEAAARAPPREN
>Orycteropus_afer_afer
MRRGWTARSA------------------

HG0860 ENSG00000133056 PIK3C2B

>Homo_sapiens
MASPLSPAAAAIVSPGVA
>Oryctolagus_cuniculus
MASPRSPAARAIVSPGVA
>Ceratotherium_simum_simum
MASPRSPAAPAIVSPGVA
>Manis_javanica
MASRLSPAAPAIVSPGVA
>Mesocricetus_auratus
MASPRSPAARAIVSPGAA
>Tupaia_chinensis
MASPLSPAARAIVSPGVA

HG0861 ENSG00000134001 EIF2S1

```
>Homo_sapiens
MVNDEEGPLFVQRLR------MRGGSACAVE----------------------
>Leptonychotes_weddellii
MVSEEEGPLFSWRLRDARWFRMRGGVEPSARRGGSAAGAGGLSGLCD------
>Myotis_lucifugus
MVSEEEGPRFCWRLRAARWCRMRGGTERGAR----------------------
>Galeopterus_variegatus
MISGEEAPLLSAL--------ARGGSACAVGLSEAHAEEDRWLVRLDLV----
>Condylura_cristata
---------------------MRGGSACAVGSNEAHAAEARRLLRVDHI----
>Mus_musculus
MVSGNEGSFSV------GACAVRGGSACALGWAKRTLRSFGVRGWCSESPRCH
```

HG0862 ENSG00000134245 WNT2B

>Homo_sapiens

--MAPQGGEVGAA

>Leptonychotes_weddellii

--MAPQGGEVGAA

>Orcinus_orca

--MAPQGGEVGAA

>Oryctolagus_cuniculus

---MTLGSQPPATSMAPQGGEVGAA

>Ceratotherium_simum_simum

MRARGQRGCPHTLPVAPLRAANPLTPHPAPGHRAPDPGLGAPSSASGHRSLRPSPGPCAPVPGLQVPLGPQPPASPMAPQGGEVGAA

>Manis_javanica

--MAPQGGEVGAA

HG0863 ENSG00000134313 KIDINS220

>Homo_sapiens

MAAGCGEGDALAVAVSCFPVL

>Ailuropoda_melanoleuca

MAAGFGEDEALEAAVSPFPVF

>Physeter_catodon

MAAGFGEGDSLKAAVSPFLVF

>Ceratotherium_simum_simum

MAAGCGEGDALKAGVSPLPVL

>Marmota_monax

MAAGCGEGDASKAAVFFSFVL

>Tupaia_chinensis

MAAGCGEGSPLKAAVSRFRVL

HG0864 ENSG00000134533 RERG

>Homo_sapiens

MVNPKTGTKKIYSVL-----

>Ailuropoda_melanoleuca

MVNPKAGTKKIFSALQCARQ

>Myotis_davidii

MVNPKAGTKTIFSAL-----

>Dasypus_novemcinctus

MVNPKAGTRKICSALWF---

>Oryctolagus_cuniculus

MVNPKTGTQKICSAL-----

>Ceratotherium_simum_simum

MVNPKTGTRKIFSAL-----

HG0865 ENSG00000135540 NHSL1

>Homo_sapiens

MPRRGVGLQFGMLGTRQRVGVPWLCVLQSPDKYS

>Mustela_putorius_furo

MLGTGAGSQEGMLGTHQRVGVPWFCSSPSPDKRS

>Miniopterus_natalensis

MPGRGVGLQVGMLGAHQRVGVPWFCSLQSPDKCS

>Oryctolagus_cuniculus

MPGRGAGLQFGVLGTPWSIGVPWFCSLQSTDKCA

>Manis_javanica

MPGRGVRSQCGVPGAPHRVRVPWFCSLQSPGKCS

>Cavia_porcellus

-----------MAGMHQRTPVPCFCSSQSPDKRA

HG0866 ENSG00000136011 STAB2

>Homo_sapiens

MRKNSLGVYQTKLK

>Balaenoptera_acutorostrata_s

MRKHSLGVHQTKLM

>Miniopterus_natalensis

MRENSLGVHQTKLK

>Oryctolagus_cuniculus

MRRNSLGVQQTMIK

>Equus_asinus

MRKNSLGVHQTKLK

>Peromyscus_maniculatus_baird

MRKNSPGTHQSKLK

HG0867 ENSG00000137203 TFAP2A

>Homo_sapiens
MDPSRAPALRGASAP
>Pteropus_vampyrus
MDQSRAPALGGASAR
>Oryctolagus_cuniculus
MDPSEAPALRGASAP
>Elephantulus_edwardii
MEPSGAPALGGASAP
>Tupaia_chinensis
MDPSEVPALQGASAW
>Trichechus_manatus_latirostr
MEPSRAPALGRASAP

HG0868 ENSG00000137843 PAK6

>Homo_sapiens

MPEDLMEKSQSEPGRAVEEGRAAAGA-AGP------------------------

>Ursus_maritimus

-----MEKSQSEPGCVVEEGRAAAGK-EAGARAPCSLPGSPGPAGAPRPRPRAL

>Rousettus_aegyptiacus

-----MEKSQSEPGCGVHCGGGARRDQEGG------------------------

>Equus_caballus

-----MEKSQSEPRGAVEEGRAAAE----PVARRCRLPGSPGPAGALWPRCL--

>Manis_javanica

-----MEKSLSEPGCAVEEEPSAASTGKAARARQCRLPGSPGPAGAPGPLYL--

>Microtus_ochrogaster

-----MEKSQSELGCTVEEGRAAAGA-TGP------------------------

HG0869 ENSG00000139910 NOVA1

>Homo_sapiens
MSFNGTDIASSFALESS
>Ailuropoda_melanoleuca
MSYNGTGIASSFALESS
>Physeter_catodon
MSFNGTGIASSFALESS
>Rousettus_aegyptiacus
MSYNGTGIASSFALESS
>Manis_javanica
MSYNGTGIASSFALESS
>Cavia_porcellus
MSYNGTGIASSFALESS

HG0870 ENSG00000140993 TIGD7

>Homo_sapiens

MALGSTRRTRKEYLRA-------------------

>Balaenoptera_acutorostrata_s

MALGGTRRT--------------------------

>Pteropus_vampyrus

MELGSTRRT--------------------------

>Galeopterus_variegatus

MALGSTRKTWKKYMRAQNYRRVFQQKKFMRNN---

>Ceratotherium_simum_simum

MALGSTRRTCKEYLRAQSYKGCQQKNLWETIDQKH

>Marmota_marmota_marmota

MALGSTRSS--------------------------

HG0871 ENSG00000141127 PRPSAP2

>Homo_sapiens

MPAQKGREPSRGTAEEESILEEFCCQRIKGGCP

>Ursus_maritimus

MSL-----LMALPFFCVLIQEEFCCQRIKGGCP

>Rousettus_aegyptiacus

MSL-----LMALPFFCVLIQEEFCCQRLKGGCP

>Oryctolagus_cuniculus

---------MALPFFCVLIQEEFCCQRLKGGCP

>Equus_caballus

MSL-----LMALPFFCVSIQEEFCCQSIKGVCP

>Mesocricetus_auratus

MFVMLKTPTARSPGRARVRLEEFCCQRITGGCP

HG0872 ENSG00000142583 SLC2A5

>Homo_sapiens

MLLAVIWHGQSAPRMSSLSIQCTRYFG

>Ursus_maritimus

--------------MSSLSIQRVCYFG

>Physeter_catodon

MLLAVTWLGQSTARMSSLAIQCICYFG

>Cynopterus_sphinx

MRLVVTWHGQSAVGMSSLSIQCMCYFG

>Dasypus_novemcinctus

--------------MSSLSI-------

>Ceratotherium_simum_simum

--------------MSSLSIQCICYFG

HG0873 ENSG00000142875 PRKACB

>Homo_sapiens

MLGSYALLLGASLFKTKESSFRFLCAACSSVCVYTIGSSPSRD

>Felis_catus

MLGSYALLLGAFLF-----------------------------

>Rousettus_aegyptiacus

MLGSYALLLGDFL------------------------------

>Galeopterus_variegatus

MLGSYALLLGAFLFKTKESSFHFLCAACSSVRVYTIGSSTSRD

>Manis_javanica

MLGSYALLLGAFLFKTKETAFHFLCAACSSVCVYTISSTSRD-

>Ictidomys_tridecemlineatus

MLGSYALLLGVFLFKTKESSSHFLCAACSSVCVYTIGSSTSRD

HG0874 ENSG00000143515 ATP8B2

>Homo_sapiens

MGSTGPPGRAPSAER

>Lipotes_vexillifer

MGRTWPPGRAPSAER

>Condylura_cristata

MGWTGPPGRAPGAER

>Oryctolagus_cuniculus

MGRTGPPGRAPSAER

>Ceratotherium_simum_simum

MGRTGPPGRAPSAER

>Mus_musculus

MGRTGPPGRAPRAER

HG0875 ENSG00000145284 SCD5

>Homo_sapiens

MGDTPATDAGRQDLHP

>Rhinolophus_ferrumequinum

MGNTPATDAGLPDLHP

>Condylura_cristata

MGGTPATDAGRQDLHP

>Ceratotherium_simum_simum

MGDTPATDAGRQDLHP

>Loxodonta_africana

MGGTPATDAGRLDLHL

>Orycteropus_afer_afer

MSDTPATDAGRPDLHP

HG0876 ENSG00000145908 ZNF300

>Homo_sapiens

-MAAPCGNSSREQPCCFLNKASEDAERAKPPSTERRS-------------LGSPVWCSRTGSRRVCQ-

>Ursus_maritimus

MLGSPVLCS----------PRQEAGE----------------FFNPR---------------------

>Dasypus_novemcinctus

-MAAPIRNSR--

>Equus_caballus

--------------MLLLNKAAEDAERAKPPGAEPRRLGAPRFAHPGRKRESPPVWMRRPSQVKP---

>Manis_javanica

-MAAPRRTLRPGAPSCLLDKAAEDAEWAKPPGGTSAGSRASWFAHPDRKRERWPVWM-----------

>Chinchilla_lanigera

-MAAPLRIVAWEQPYCLLNKAPLTEEYRLQPSAALSHRK----------RERAHRY----G-CDSCHL

HG0877 ENSG00000146426 TIAM2

>Homo_sapiens
-------------MKKRLTSWTHFACLLNVLCCSQDHVKPTDH
>Felis_catus
-----------------------------MLPCSQDPVKPSDY
>Physeter_catodon
-----------------------------MLPCSQDPVKSTDH
>Pteropus_vampyrus
-----------------------------MLPCSQDHVKPAEH
>Ceratotherium_simum_simum
------------MSWQSIISRTHFLHVLTMLPCSQDHVKPTEH
>Cavia_porcellus
MITLSVLYSHFPDWTASRNTWIHFPCFLNMLHCSQDHVRSTDI

HG0878 ENSG00000146802 TMEM168

>Homo_sapiens

MAVAAGPVTEKVYADTGLY--------------------------

>Felis_catus

MAAAARPVTEKVSIETGLY--------------------------

>Lipotes_vexillifer

MAAVAGPVVEKVSDETGLC--------------------------

>Dasypus_novemcinctus

MAAVAGPVTEKVSAETGLWLAPRQTPRVGKGLVAAAKIIINKNFT

>Equus_asinus

MAAAAGLVTEKVSNDTGLC--------------------------

>Fukomys_darlingi

MAAAAGPVTEKVSTETGLY--------------------------

HG0879 ENSG00000147145 LPAR4

>Homo_sapiens

MDKNMHFQRASCPFTCLLANYLQASAHSLI

>Galeopterus_variegatus

----MHFQSASCPFTCLLANCLQA------

>Ochotona_princeps

MDKNMHFQSASCPFTCLLANCLQA------

>Loxodonta_africana

MDKNMHFQSASCPFTCLLANCLQA------

>Cricetulus_griseus

MDKNMRFQSASCPFTCLLANYLQA------

>Trichechus_manatus_latirostr

MDKNMHFQSANCPFTCLLANSLQA------

HG0880 ENSG00000151789 ZNF385D

>Homo_sapiens

MGSAAGSPSALSSRRGS

>Canis_lupus_familiaris

MGSAAGSPSARSSRGS-

>Myotis_lucifugus

MGSAAGSPGARSSRRGS

>Equus_asinus

MGSAAGSPSARSCRRGS

>Ictidomys_tridecemlineatus

MGSAAGSPSARSSRRGP

>Orycteropus_afer_afer

MGSAAGSPSAHSSRRGL

HG0881 ENSG00000153822 KCNJ16

>Homo_sapiens
---MAVGADSFRRVLTGPREKGPEMLSYGALTR------
>Balaenoptera_acutorostrata_s
---MAVGADSLRESLEEQRERSPGRLVAVLLYDAKDTGV
>Rhinolophus_ferrumequinum
---MAVGADSLRESTEEQREKNSEVSSSELKRT------
>Oryctolagus_cuniculus
---MVVGADSRRQSRKRAKRKSPEMPECGAPTR------
>Equus_caballus
MGREEGDFVFILVFCKPPLARLPTCRHGNQLKKVLIHWSEAENSVAFVCGECVRKSKEEQDSPVEFLISMAVGADSFRESTKEQKEKNPEMSSYSALRR------
>Manis_javanica
---MGFLISMAVGADSLRENMKGQRGKSPRCLAAVLL--------

HG0882.1 ENSG00000154832 CXXC1

>Homo_sapiens
--MFPANIWIRPPYGYDKQDGGA-----

>Odobenus_rosmarus_divergens
MAKMPAPSWKPRLGRCHVLPVNVRTRPSYGISFQDDGICVEVAHRRGHISCNVESVQAVTVRTQPTRWRCPRESSHIGAAMFPANLWTRPPYGLSSQDGGA-----
----

>Oryctolagus_cuniculus
--MGAAMFPTGEWTLAPYVLDSQDGGT-----

>Manis_javanica
----------------------------------MTASAYKSRGRRSHVSCNVEAVQVVTVRTQPTKWRRPQDSSETGAAMFPGTVCIRPPYGLGGQDGGTWESRS
SGIYVVAVKPTHWACAVGVSGKRD

>Loxodonta_africana
--MFHGNLRTRSPYGLDSQDGGS-----

>Fukomys_damarensis
--MFPANI--------------------

HG0882.2 ENSG00000154832 CXXC1

>Homo_sapiens

MWKGKETEWRNEYNLIQVVLRKRSLYL--

>Mustela_putorius_furo

MWKGKVTEWKSECNLIQVVFREEPLFL--

>Manis_javanica

-----MILWKKERNLIQAVLRKRPIFIPTSLKVPAYGISPQDGGTYAEAGSRTLPCVACERTDSAILRNHIPR

HG0883 ENSG00000156011 PSD3

>Homo_sapiens

MLGEAAWCFLS

>Rousettus_aegyptiacus

MLGEAAWCSLS

>Oryctolagus_cuniculus

MLGEAAWCFLS

>Equus_asinus

MLGEAAWCFLS

>Microtus_ochrogaster

MLGEAAWCFLS

>Tupaia_chinensis

MLGEAAWCFLS

HG0884 ENSG00000156463 SH3RF2

>Homo_sapiens

MWTLLLQVGTGVLK

>Pteropus_alecto

MWTLPLQVRTGVLK

>Oryctolagus_cuniculus

MWTLLLHVGTGVWK

>Ceratotherium_simum_simum

MWTLPLQVRTGVLK

>Manis_javanica

MWTLPLQVRTGVLK

>Mus_musculus

MWILLLQVGTGVLK

HG0885.1 ENSG00000157502 MUM1L1

>Homo_sapiens

MPTKPWKDSAT-TEVPSWVKALLDKQLE

>Mustela_putorius_furo

MPIEPQKGSVT-SEVLSREKALLDKQLE

>Balaenoptera_acutorostrata_s

MPVEPQKVSAT-TKVPSREKALLDKQLE

>Ceratotherium_simum_simum

MPVEPQKGSAT-TEALSREKALLDKQLE

>Nannospalax_galili

-----------------MKAPLGKLLE

>Tupaia_chinensis

MPVKPQKGSSTPTAVPSRVKAVLDKQS-

HG0885.2 ENSG00000157502 MUM1L1

>Homo_sapiens
MAVVNASYFQRVLEDLE
>Canis_lupus_familiaris
MAVVKASYFRRFLETFE
>Equus_asinus
MAVVKASYFRRFLETLK
>Chinchilla_lanigera
-----MSYFQRLLEDLV

HG0886 ENSG00000158850 B4GALT3

>Homo_sapiens
MAAAMPARLLGWR
>Leptonychotes_weddellii
MAVTMPARLSGWR
>Pteropus_alecto
MAAAVPARLSGWR
>Galeopterus_variegatus
MAATMPARLSGWR
>Ochotona_princeps
MAAAKPARLLGWR
>Mus_musculus
MAAAIPARLSGWR

HG0887 ENSG00000160959

>Homo_sapiens

--MIVEICPPAELGRGLVSGTFQEPVP

>Leptonychotes_weddellii

MNRPREICLQAEQGRGLAFGASQEPVP

>Balaenoptera_acutorostrata_s

MNRPREICPQAERGRGLTSRASQEPVP

>Pteropus_alecto

MNRPREICPQAERGRGLASGASQEPVP

>Ceratotherium_simum_simum

MNRPREICPQAERGRGLASGVSQEPVP

>Heterocephalus_glaber

---------MAEPGRGWVSGVSQEPVP

HG0888 ENSG00000164023 SGMS2

>Homo_sapiens

------MEETVIGWLPSTFCIRKQSPL

>Canis_lupus_familiaris

------MEGTVIRWLPSTFCARKQNQL

>Orcinus_orca

----------------MFCTRKQNQL

>Oryctolagus_cuniculus

MYNRELNKNNGIRWLPSMFCVKKQNQV

>Equus_caballus

------MAGTIIG--------------

>Marmota_marmota_marmota

------MAGTIIRWLPSAFCIRKQNQL

HG0889 ENSG00000164091 WDR82

>Homo_sapiens
MADTMSRRRAGCP
>Canis_lupus_familiaris
MADTMSRRRAGWP
>Rousettus_aegyptiacus
MADTMSRRRAGCP
>Galeopterus_variegatus
MADTMSRRLAGWP
>Oryctolagus_cuniculus
MADTMSRCRAGCP
>Ictidomys_tridecemlineatus
MADTMSRRRAGWP

HG0890 ENSG00000165813

>Homo_sapiens
MEKLAPEPELQQPC
>Mustela_putorius_furo
MEKLGPEPELQRPR
>Equus_caballus
MEKLSPEPELQRPR
>Callospermophilus_lateralis
MERLAPEPELQRPR
>Trichechus_manatus_latirostr
MEKLAPEPELQRPR
>Orycteropus_afer_afer
MKKLVPEPELQ---

HG0891 ENSG00000166211 SPIC

>Homo_sapiens

---------MKHLYKGLKCLPGLSTYFILFSSSNNC

>Ailuropoda_melanoleuca

----MILVLMKHFYKGLKCLPELSVY----------

>Lipotes_vexillifer

---------MKQLGKGLKCLPELSIY----------

>Oryctolagus_cuniculus

MQLCQCLMLMKHLCTGLKCVPGLPVY----------

>Equus_przewalskii

---------MKHLCKGLKCLPELSIY----------

>Tupaia_chinensis

---------MKQLCKGLKCLPESSIY----------

HG0892 ENSG00000168418 KCNG4

>Homo_sapiens

MGSPCLAEKQLETGSLRQEDW----------------------------

>Balaenoptera_acutorostrata_s

MGSPCPAEKQLERGSLRQEGCSSFSGRTL--------------------

>Rousettus_aegyptiacus

MGSPCLAKKQLETGSLRQEGCSSSSGHTL--------------------

>Equus_przewalskii

MGSPCPAEKQLETGSLRQEGCSSSSGRAL--------------------

>Loxodonta_africana

MGSPCLAKEQLESGCLRQEEG----------------------------

>Mesocricetus_auratus

MGSPCLAENWLGTRFLRQSAALLPLAVCCEPSNACEIRVHCSASVKLGP

HG0893 ENSG00000168769 TET2

>Homo_sapiens

MQVTSAPSARPPRDAGPAE-----------

>Odobenus_rosmarus_divergens

MRVTSAPSARPPRDAGPAE-----------

>Orcinus_orca

MRVTSAPSPRPPRDAGPAE-----------

>Pteropus_vampyrus

MRVTSAPSARPPREAGPAE-----------

>Oryctolagus_cuniculus

MRVTSAPSARPPRDASPAE-----------

>Ceratotherium_simum_simum

MRVTSAPSARPPWDAGPPAMRTDVKVPYEY

HG0894.1 ENSG00000169282 KCNAB1

>Homo_sapiens

MLRAPVEHPACGPGDAGGASCGKPALI

>Acinonyx_jubatus

MLRVPVEHPA-----------------

>Physeter_catodon

MLRAPVEHPACGPEGAGSASCGMPAWI

>Rousettus_aegyptiacus

MLRVPVEHPACGPEGAGGASCGMPAWI

>Equus_asinus

MLHAPVEHPACGPEGAGGASCGMPAWI

>Heterocephalus_glaber

MLRAPAAS-CLRSKDAGGASCGMPELI

HG0894.2 ENSG00000069424 KCNAB2

>Homo_sapiens

MALSPVVPVQEQNFPTVKNRASLDK

>Ursus_maritimus

-----MLLSQEQNLPSVKKRAGQDQ

>Rousettus_aegyptiacus

MVLSLAVLTQEQNLPSVKNRAGGGT

>Equus_caballus

MLSSLAVLIQEQNLPSVKKRAGRDQ

>Cricetulus_griseus

-MVSSTGPVPQQDFPSVKRRAGLDK

HG0895 ENSG00000170035 UBE2E3

>Homo_sapiens

MNLRCWPWKQLEQLFSKR

>Odobenus_rosmarus_divergens

MNLRCWPWKRLEQLFSKK

>Lipotes_vexillifer

MNLLCWP-----------

>Miniopterus_natalensis

MNLRCWPWKRLEQLFSKS

>Manis_javanica

MNLRCWPCKRLEQLVS--

>Trichechus_manatus_latirostr

MNLRCWPWKRLEQLFSKK

HG0896 ENSG00000170365 SMAD1

>Homo_sapiens
----------MCFRRGGGTNACQISQLQ-------------
>Panthera_tigris
----------MYFRKGGGTNACRILQLQ-------------
>Tursiops_truncatus
MKASRESVFLMYFGKGGGTNACRILQPQ-------------
>Myotis_davidii
----------MSLGREGGTDACRISQLQ-------------
>Fukomys_damarensis
----------MIFGKGGGTNPCQISHLQ-------------
>Trichechus_manatus_latirostr
----------MCFRKGGGTNACRILQLSSCGSCLVGGTWNF

HG0897 ENSG00000171435 KSR2

>Homo_sapiens

MPCPCNWEGGKPDFF------------

>Mustela_putorius_furo

MPCHCNWEGGKADFF------------

>Myotis_lucifugus

MPCHCNWEGGKPDFFFFLHKSQPAKNT

>Equus_caballus

MPCHCNWEGGKPDFF------------

>Manis_javanica

MPCHCHREGGKPDFF------------

>Tupaia_chinensis

MPRRCSGEGGKPDFF------------

HG0898 ENSG00000171729 TMEM51

>Homo_sapiens

MKSCLLACPESPQITTGPSTAAIRTGGLAIARNHPAGVQLIFSSVGREILWSAFKGFLL

>Ailuropoda_melanoleuca

MESCFQDCPEHPHVTTDPSATAVHTGCLESA----------------------------

>Balaenoptera_acutorostrata_s

MKSCCQACLEAP---

>Manis_javanica

MKSCFQACPVPLQVSTDLS--AVCMGRLTLARSRPWRGQLLFCECGTRGFVQRV-----

>Loxodonta_africana

MKSCFQACPESPQVTTDFSAAAVHTGCLTYTWNHPSRVWLIFF----------------

>Cavia_porcellus

MKSCVQVCWEPPPVTTELSAAAVCTRHPKSVRSHPLRVQRMTF----------------

HG0899 ENSG00000173320 STOX2

>Homo_sapiens

MCRGHMRVGAPTQPSTRAHAQRPEPRQG------ED--

>Orcinus_orca

-----MRVGARAHTRTRTHARTPP--

>Pteropus_vampyrus

MCRGHMRVGAHTHARAHTHTRAGTHTTLSLGRSESDETLQPRGLLPLFLDSAEPARGSCCL---

>Elephantulus_edwardii

MCGGHMRVGTHTPTPSRASAEKEAGLTRRCRG-ERDR---GASTLPGLG--GTDCR-SRCLQSRPR-SRCTRDP-HSSLCDEDCLPRTAGEAEAACAGDCGPRD

>Equus_asinus

-----MRVGAHTRAHTRA------RTTLSLGRRKIDEALQSRGRLGLFLDSAGAGLPQ-LLSAEPARIPVHTRPRFEPL-------------------------

>Trichechus_manatus_latirostr

MCGGHMRVGTHTRTPPRASARKKEN---

HG0900 ENSG00000173517 PEAK1

>Homo_sapiens

MVVAAAACAPLRYLPKLELML--------

>Mustela_putorius_furo

MVVTTAACAPFRYLPELELML--------

>Pteropus_vampyrus

MVVAAAACSPLRYLPKLELML--------

>Dasypus_novemcinctus

MVVPAAACAPHRCLSKLGLMLLTYLGWFI

>Loxodonta_africana

MVVPAAACAPFRYLPQLDLMLKTFLCGFV

>Ictidomys_tridecemlineatus

MVVTAAACATLRYLPKLELML--------

HG0901 ENSG00000175697 GPR156

>Homo_sapiens

MRNQSEGTRTVTAIWWLTRIITLCSLMVTKMSTL----DSHGMHSQQGTS----------

>Canis_lupus_familiaris

--------------------------MVTKMPTL----DSHDMHCQQGTRGAWRVSDEGD

>Myotis_davidii

------------------------------MPTP----DSHGMHWQQGTSRAWWVTEEGA

>Oryctolagus_cuniculus

--------------------------MVAKWSSL----DSPAAGDPLDNV----------

>Equus_przewalskii

--------------------------MVTKRPTL----DSHGMH----------------

>Manis_javanica

--------------------------MIAKMPPRLTWCDSVAEHK-----ESQRVSDEEA

HG0902 ENSG00000177182 CLVS1

>Homo_sapiens

MASVLLLFWMNTTT

>Rousettus_aegyptiacus

MASVLLLLWMSTAT

>Dasypus_novemcinctus

MASILLPFWMATTT

>Condylura_cristata

MASVLLLFWMATTK

>Equus_caballus

MALTLLLFWMATTT

>Jaculus_jaculus

MASVLLLFWMATTT

HG0903 ENSG00000180616 SSTR2

>Homo_sapiens

MSHWILGLQGPLR

>Miniopterus_natalensis

MSQWILGLQDPLR

>Oryctolagus_cuniculus

MSQWILGLQGPLR

>Ceratotherium_simum_simum

MSQWILCLQDPLR

>Nannospalax_galili

MSQWILGLQGPLR

>Tupaia_chinensis

MSQWILGLQGPLR

HG0904 ENSG00000182329

>Homo_sapiens

MYLIKGPVFVVFLGLL---------------------

>Canis_lupus_familiaris

MYLIKGPVFVVFLGSLLSSGNLEV-------------

>Rousettus_aegyptiacus

MYLIKGPVFVVSLGSLLSSGNLEV-------------

>Ceratotherium_simum_simum

MYLIKGPVFVVFLGSLLSSGNLEV-------------

>Manis_javanica

MYLIKGPVFVAFLGSLLSSGNLEV-------------

>Chinchilla_lanigera

MYLIKGPVFVVFLGFCGASEILRCNQLLAFKTKMNCP

HG0905 ENSG00000183337 BCOR

>Homo_sapiens
MLESVEDGAGRPED
>Canis_lupus_familiaris
MPESVEAGAGRPED
>Balaenoptera_acutorostrata_s
MPESVEAGAGRPED
>Rousettus_aegyptiacus
MPESVEAGAGRPED
>Manis_javanica
MPESVEAGAGRPED
>Fukomys_darlingi
MPESVEAGAGRPED

HG0906 ENSG00000183773 AIFM3

>Homo_sapiens

MAAPASLRPAGGPAPWGAP

>Eptesicus_fuscus

MAASAFLRAVGGPAPRGAL

>Galeopterus_variegatus

MAAPAFLRAAGCPAPWGAP

>Equus_przewalskii

MVAPASLRAVGGPAPLGAL

>Marmota_marmota_marmota

MATPASLRAAGDPAPWGAP

>Tupaia_chinensis

MADPASPRAAGRPALWGAP

HG0907 ENSG00000186862 PDZD7

>Homo_sapiens
MELEARRAGLSRASRFLLDL
>Felis_catus
MEPGARGALCSRASRFFLDL
>Orcinus_orca
MEPGARGALLSRASRFPLDL
>Myotis_lucifugus
MEPGARRALLPRASRFLLDL
>Ceratotherium_simum_simum
MELGARRAFLLRASRFLLDL
>Ictidomys_tridecemlineatus
MELRALGAGLWRVGRFLLDL

HG0908.1 ENSG00000188554 NBR1

>Homo_sapiens

MHCGTKGLGHTVPWKL

>Felis_catus

MHCGSKGLRHSVPWKL

>Pteropus_alecto

MHCGSKGLRHCVPWKL

>Oryctolagus_cuniculus

MHCVTKGLRHSVPWKL

>Ceratotherium_simum_simum

MHCGSKGLRHSVPWKL

>Microtus_ochrogaster

MHCGTKGLHHSIPWKL

HG0908.2 ENSG00000188554 NBR1

>Homo_sapiens

MERNIQYQSGHNSHGNPVDRLETSAGLYMSTVMDWSVVMFSYLESRD

>Felis_catus

--------------------------------MDWSV-RFSHLESGE

>Rousettus_aegyptiacus

--------------------------------MDWSL-MFSYLESGE

>Oryctolagus_cuniculus

MEKSSRLWKRARSSRHVVERLEPSARLCTSTAMDWSAVRCLYLKSGR

>Ceratotherium_simum_simum

--------------------------------MDWSV-MFSHLESGE

HG0909 ENSG00000189079 ARID2

>Homo_sapiens

MTEPRRRRPRNGLRALVGSAGSGGRF-------

>Rousettus_aegyptiacus

MTEPRRRRPRNGLRALVGSAGSGGRF-------

>Erinaceus_europaeus

MTEPRRRRPRNGLRALVGSAGSGGRL-------

>Ceratotherium_simum_simum

MTEPRRRRPRNGLRALVGSAGSGGRF-------

>Manis_javanica

MTEPRRRRPRNGLRALVGGAGSGGRFWTAIGAF

>Heterocephalus_glaber

MTEPRRRRPRNGLRALVGSAGRGGRF-------

HG0910 ENSG00000197935 ZNF311

>Homo_sapiens

---MNPKSRAVFDTLSQTVAALWVKKGPLAGTTKTCVLQRRLASTGATSQLV

>Ailuropoda_melanoleuca

MLLMNPKSRALCDT--------------------------------------

>Physeter_catodon

---MNPKSRAVFDTSSQKAAALWVRRGPLAGTTKA-----------------

>Oryctolagus_cuniculus

MLLIVPKSRAVFDTPKHRAAVFWVGKGPLTGLSETCVPQRRLLSTGTT----

>Equus_asinus

MLLMNPKSRAVFDTSRQVAAAL------------------------------

>Marmota_marmota_marmota

--MMNLKSRAMFKTSSQIAAAL------------------------------

HG0911 ENSG00000198673 FAM19A2

>Homo_sapiens

MPRRAAGGLRGALPAALRGGIEGLKLARSCC

>Felis_catus

MPRGAAAGLRGALPAALRGGIEGLQLARSCC

>Rousettus_aegyptiacus

MPRGSAVGPRGALPAALLGGIEGLQLARSCC

>Oryctolagus_cuniculus

MPRGVTGELRGALPAALGGSSDWLRLAGSCC

>Equus_przewalskii

MPRGAAVGLRGAAPAALGGGISGLQLDRSCC

>Rattus_norvegicus

MPRVAAGGLREAGPAALEGEIKGLQLARSCC

HG0912 ENSG00000198822 GRM3

>Homo_sapiens

-----------------------------MPGGPCFCQESQLDATASAWSR

>Mustela_putorius_furo

-------------MREG--GKEKENEKERMPEGPRLCRESQLDATV-----

>Tursiops_truncatus

-----------------------------MPEGPCLCRESQLDAKNSARPR

>Rousettus_aegyptiacus

MKGGRKEEGEREKENEGGRKEEKENEKERMPEGPCLC--------------

>Manis_javanica

-----------------------------MPEGPCLC--------------

>Nannospalax_galili

-----------------------------MPEGPCLCWESQLDATVSA---

HG0913.1 ENSG00000204599 TRIM39

>Homo_sapiens

MRLSLKFALQLRHKE

>Acinonyx_jubatus

MRTSLKFALLLRHKE

>Physeter_catodon

MRTALKFALLLRHEE

>Equus_przewalskii

MRTSLKFALLLRHKE

>Loxodonta_africana

MRTSLEFALPLKHEE

>Ictidomys_tridecemlineatus

MRTSLKFVLLLRHEE

HG0913.2 ENSG00000204599 TRIM39

>Homo_sapiens

MPQPQIWRKKMENGGGTELWTTSQERVARWDPEQW------------------------

>Oryctolagus_cuniculus

MPLPEIWREKMETRGGRSFGRRSGE-GRQVGPGTAMDAKSGRP----------------

>Equus_caballus

----------METFGGTELWTTLRR-GSQSGT-----PDSGRNKMRTSLKFALLLRLKE

>Manis_javanica

----------METGVGLSCGARSGE-GRRWDPGQW------------------------

>Loxodonta_africana

----------METGVGQSCGQRSGE-GCKMGPWTVVETQ--------------------

HG0914 ENSG00000227877 MRLN

>Homo_sapiens

MVFFIKERDHSLDFGLRLLNPWTESRLLRDHLC

>Panthera_tigris

---MIHSRDTI----------------------

>Orcinus_orca

MVFVTKERDHLLDFGLRLSNP------------

>Myotis_davidii

MVFFTKESDHLLNFGLRFLTPEQIQGC------

>Ceratotherium_simum_simum

MVFFTEERDHLLDFGLRFLNA------------

>Nannospalax_galili

----MGKRDHSLDFGLGLLNP------------

HG0915 ENSG00000254469 AP002495.2

```
>Homo_sapiens
MYFPGCSTCHLASVGLIFKNSSFLKPQNAFLCYGRRKSSSRGQKAKVRRPNWRENRFGGPRSEA-TVAPRGPRSRE----------
>Felis_catus
MHFTGYITCLLASVGIIFQKQRLLEAPGQIPGIPEAESSATNQKSEVRGPNWREKGFCGPRSEA-AMALPGVA-------------
>Balaenoptera_acutorostrata_s
MYFPGNSTYLLTSVGVIFRKQRLLEAPKRIHGLREAEEWRFGSEGG--GVT-----------------------------------
>Equus_caballus
-----------------------MRLQNEFPGFTRRKSGAPGQKAEVRGLNWRENGFVVRDLRQRWLFKVHCPG------------
>Manis_javanica
-----MPSCL---GGGHFQKHRFLRPRNEFPGFGRRKSDAPGQKAEVRRLNWRENGLLVRELRQPAVALGGPVSWGYHKDSFSSGS
>Peromyscus_maniculatus_baird
-----MSNCCLASVGAIFQKSGFFKPQNASLSFCRRGGARARRR-------RSPDRFGGRIRLE----PRS---------------
```

HG0916 ENSG00000020633 RUNX3

>Homo_sapiens
MIHSAYASVENLWLDIPVGPGIQILGS--
>Odobenus_rosmarus_divergens
MIHSAYASVENLWLDIPVGLGIQILGS--
>Loxodonta_africana
MIHSAYASVENLWVDIPVGPGIQILGS--
>Octodon_degus
MIHSAYAAVENLWLDIPVGPGIRILGYKQKLTFLGDFFYSLTHTLSRSFLFFFFVAVGGERTKQKTNKNQHPHPFTSNF
>Orycteropus_afer_afer
MIHSVYASVENLWVDIPVGPGI---

HG0917 ENSG00000042980 ADAM28

>Homo_sapiens

MNQGVSFCLTGEEAGTDPAAPT

>Ailuropoda_melanoleuca

MKPGVSFCLT------------

>Orcinus_orca

MNPEVSFCLT------------

>Equus_asinus

MNLGVPFCLT------------

>Mus_musculus

MKQGVSFCLP------------

HG0918 ENSG00000047648 ARHGAP6

>Homo_sapiens

MDLLLLPPPGHLERTGGA

>Pteropus_vampyrus

MDLLLLSSPGHLECAGGA

>Ceratotherium_simum_simum

MDLLLLPPRGYLERAGGA

>Manis_javanica

MGLLPLSPLGHLERAGGA

>Cavia_porcellus

MDLLLLPPQGHLERVGGA

HG0919 ENSG00000051009 FAM160A2

>Homo_sapiens

MGSYWLLQTG

>Ursus_maritimus

MASYWLLQTG

>Sorex_araneus

MASYWLLQTG

>Microtus_ochrogaster

MASYWLLQTG

>Orycteropus_afer_afer

MASYWLLQTG

HG0920 ENSG00000051382 PIK3CB

>Homo_sapiens

---MGATILRAPWL

>Canis_lupus_familiaris

---MGAVILRAPWL

>Pteropus_alecto

MTEMGATILRAPWL

>Condylura_cristata

---MGAIILRAPWL

>Marmota_marmota_marmota

---MGTIILRAPWL

HG0921 ENSG00000058799 YIPF1

>Homo_sapiens
MRKLRPRGAR-IQQRSSRIGNQTQLCLTPEPVPLTTG--
>Leptonychotes_weddellii
MRKLRPREAHSPTARRSRIGNQTQVCWTPEPVPLTTG--
>Lipotes_vexillifer
MRKLRPPGAHSLTARSSRIGNQTQVCSTPEPVPLTTG--
>Loxodonta_africana
MRKLRPRGAHSHAARSSRIGSQTQFLQSFFLIIGRRPTG
>Microtus_ochrogaster
MRKLRPRGAHT-QQRSNRIGTQTQTPENSLPHPWERASG

HG0922 ENSG00000079841 RIMS1

>Homo_sapiens

MQTTMKDWVLALPGSAAAAAAAAAAAAPPPA--AAARAPL

>Odobenus_rosmarus_divergens

MQTTMKDWVLSLPGSAAAAA---AAPPPPA--AARRAPL

>Rousettus_aegyptiacus

----MKDWVRSLPGSAAAAA---AAPPPAA--AARRAPL

>Condylura_cristata

MQTTMKDWVLCLPGSAAAA----ASAPPPA--AAGRAPL

>Nannospalax_galili

----MRDWVLALPGSAAAAATA---PPPAAAAATGRAPL

HG0923 ENSG00000081138 CDH7

>Homo_sapiens

---------------------------------MTLPSCLRSSTD----HGFHDRLRYLLHHSLAKAIQQW

>Odobenus_rosmarus_divergens

---------------------------------MTLPSCLRSSID----HGFYDRLGYLLHHSLAKVIQQW

>Myotis_brandtii

MDGLRPLLLSPSSSGFSCEGLHLSGGRWRRGARGCARVCARLAAEQLPFRQPSLGLRNLLHHSLAKAIQRW

>Dasypus_novemcinctus

--MSEFRCLLQHSLAKAIQQW

>Peromyscus_maniculatus_baird

---------------------------------ML--AQLRQPV-PPASAEVLAGRRYLLHHSLAKGTQHW

HG0924 ENSG00000081307 UBA5

>Homo_sapiens

MSATHRKRLRGRPVGVSETCLSVRRWVHVPRALG------------------

>Odobenus_rosmarus_divergens

MTATYRKRYPGRPAGAAATCCLCGAGGTSPGFWARKAAATCLLTYPWRGLQA

>Galeopterus_variegatus

MTAAHRKRCREMRVGAAETCL-------------------------------

>Ceratotherium_simum_simum

MTATYRKRYRVRPAGVAETWLAVRG---------------------------

>Orycteropus_afer_afer

MTAAHRKQHCGRSAGAEETCLSVRCWRYPGLS--------------------

HG0925 ENSG00000088766 CRLS1

>Homo_sapiens

---MEAAVAQCLSGCRVSMEKRLASVPGC

>Pteropus_vampyrus

MNYKRLPLPPGAQGSPAATVSQKRREDVRPPLAAPDHLYKRTCWGLKTSMEVGEVLGGFGYSREKRLFSAPGC

>Erinaceus_europaeus

---MEEAELPPDCRNSMEQRVACVPDC

>Manis_javanica

------------------MVSQKRRADVRPWR-LQATCISGVCRGLKTGMEAAEVLRACGKSMERRSAGVPGC

>Marmota_marmota_marmota

---MEASAAVLPRHFGVSMEKRLAGDPSC

HG0926 ENSG00000095397 WHRN

>Homo_sapiens

MPRTPCRTPAPLHRLPLPGVLWD

>Balaenoptera_acutorostrata_s

MPRTPCRTPVRLHQLRFGG-LWD

>Pteropus_vampyrus

MPRTPCRTAARLHRLQFGG-VWD

>Condylura_cristata

MPRTPWRTPARLHRLRFGG-L--

>Equus_asinus

MPRTPCRTPARLHQLRFGG-LWD

HG0927 ENSG00000095951 HIVEP1

>Homo_sapiens

MAACGRPLALLGALAARE--------PSSTWIN-

>Mustela_putorius_furo

MAACGRHLPLLGALAARERPELRSCEASST----

>Rousettus_aegyptiacus

MAACGRHLPLLGALAARERPELRSCELSSKWIN-

>Marmota_monax

MAACGSHLPLLGALTAREQPELRSCDPSSTWIN-

>Tupaia_chinensis

MAACGRHLPLLGALAARERPELRSCEQSSTWSSN

HG0928 ENSG00000102057 KCND1

>Homo_sapiens

MPQSPLHHCKGIPGSSAFPETLESAENSPRVSWPL

>Mustela_putorius_furo

--------MRKLQFPNLFPRSLEGTDDPPQLSWPL

>Oryctolagus_cuniculus

MPQSPRQPCQGVPGPFSIPKNSGECDSRSRVCWPL

>Manis_javanica

MSQFPLQPC--------------------------

>Heterocephalus_glaber

MPQSPLQPY--------------------------

HG0929 ENSG00000102239 BRS3

>Homo_sapiens
MGKHLAKHAGRHTELKQRSIWMSWIFFPFCSVLFS--
>Galeopterus_variegatus
---------------------MSWIFLPFCCILFS--
>Condylura_cristata
---------------------MSWICLPFCSVLPSRY
>Oryctolagus_cuniculus
-------MLGDTEPLKERSIRMSWIFFPFSSVLFS--
>Ictidomys_tridecemlineatus
---------------------MSWIFFPFCSVLFS--

HG0930 ENSG00000102524 TNFSF13B

>Homo_sapiens

MQKGRKEKIQDNSPEG--------------------

>Balaenoptera_acutorostrata_s

MQPGREKKIQENSPEGERRAGPCSQCRTSTNTHNRK

>Pteropus_vampyrus

MQQGREKKIQENSPEGKSRAGRWSQCRTSTNR----

>Oryctolagus_cuniculus

MQRGREKRIEENSPEGQSRVLRCSQCRAHTDNRK--

>Equus_asinus

MQRGREKKIQENSPEGDSQAGRCSQCRTSTNTDNRK

HG0931 ENSG00000104951 IL4I1

>Homo_sapiens

MCFLPSPDKWPFLKFLINSYEDNTFGD

>Canis_lupus_familiaris

MRFFASPDKRPLLKFLFIK--------

>Pteropus_alecto

MCFLASPDKWPPLTFLSVN--------

>Equus_przewalskii

MRLPARPDTWPLLNFLLIN--------

>Tupaia_chinensis

MHFLASPDTWPLLKFLFIN--------

HG0932 ENSG00000105997 HOXA3

>Homo_sapiens

MLRVRAHPAPRGPGRAAKPGRGPEPVSEPEPEPALVRS-----

>Felis_catus

MLRVGAHPAPRGPGRAAKPGRGPEPEPEPKPEPEPELAALIRS

>Pteropus_vampyrus

MLRVGAHPALRGPGRAAEPGRGPEPEP--------ALAALIRS

>Ceratotherium_simum_simum

MPRVGAHPVPRGPGRAAKPGRGLEPEPEPA------LAALFRS

>Cavia_porcellus

MLRVHPHPAPRGPGRAAQPGRGPEPEPEPA------LAAIVRS

HG0933 ENSG00000106330 MOSPD3

>Homo_sapiens

MTEAVRRKYRKGLSGTWQH---------------

>Odobenus_rosmarus_divergens

MTEAVRRKCRNGLSGRWQPRSGQKLMGRCSDQKL

>Rousettus_aegyptiacus

MTAVIRRKCRNGLSGRWQPRSGHKLIGRCCDQKL

>Galeopterus_variegatus

MTDVVRRKCRKGPSMRWHPWPGHRLIGRCFDHKP

>Fukomys_damarensis

MGEAVRRKYRKGLSGRWRH---------------

HG0934 ENSG00000107443 CCNJ

>Homo_sapiens
MSGAGVRRTTAGLGL
>Tursiops_truncatus
MSGAGVPRTLAGLGL
>Rousettus_aegyptiacus
MSGAGVPRTLAGLGL
>Ceratotherium_simum_simum
MSGAGVPRTTAGLGL
>Ictidomys_tridecemlineatus
MSGAGVPRTPAGLGL

HG0935 ENSG00000108518 PFN1

>Homo_sapiens

--------------------------MGSRPRPYRPLQIKI-----------------

>Eptesicus_fuscus

--------------------------MGSRPRPYRPLQIKI-----------------

>Condylura_cristata

MGGGIFKKLHLQSSVSGQAGDGGREGMGNKPRPYRPLQIKI-----------------

>Oryctolagus_cuniculus

--------------------------MGSRPRPYRPLQIKI-----------------

>Ictidomys_tridecemlineatus

--------------------------MGSRPRPYRPLQIKIQRGGEEQEVAVRGLLHS

HG0936 ENSG00000108799 EZH1

>Homo_sapiens

MEAGHLFCCCVLPFS

>Canis_lupus_familiaris

MEAEHLFCLCVLPFL

>Pteropus_vampyrus

MEAEHLFCLYVFPFL

>Galeopterus_variegatus

MEAEHFFCLSVLPFL

>Ceratotherium_simum_simum

MEAEHLFCLCVLPFL

HG0937 ENSG00000109846 CRYAB

>Homo_sapiens

MTSHWPAQTCLCLSFLSSVSTGYVSHCQIPDHKSP

>Odobenus_rosmarus_divergens

-------------MGSSSVSTGYVSHCQIPDHKSP

>Ochotona_princeps

MTSHWLAQTCLCFSFLSSVSTGYVSRCQIPDHKSP

>Equus_asinus

--------MCLCFSFLSSVSPGYVSHCQIPNHKSP

>Marmota_monax

MTSHWPAQTCLCFSFLSSVSTGYVSHCQIPDHKSP

HG0938 ENSG00000111665 CDCA3

>Homo_sapiens

--------------MGTTSAQPIGSGQGAWREV

>Canis_lupus_familiaris

----------------------MESRQGAWREV

>Myotis_lucifugus

MASLPVGGSPIADLSEPVSAQPIGSRQGAWREV

>Galeopterus_variegatus

MASCAGGGSHTAGLWESTSAQPIGSGQRAWREV

>Mesocricetus_auratus

----------------------MGSGQGAWREV

HG0939 ENSG00000111674 ENO2

>Homo_sapiens

--------------------------MG----A---LEERPRQPILFLAPRRNASLAAIPL

>Lipotes_vexillifer

--MLFWAPRRNANLAAGPP

>Miniopterus_natalensis

--------------------------MR----AVDEGLQLCRLPTLFLAPRRRASLAAIPL

>Condylura_cristata

--------------------------MG----LLEERPHLPRQPI-FLAPT----------

>Trichechus_manatus_latirostr

MHVWERDWDLGEWVESGQAGVLSGGRMGGSHPRSRREAGQARQPLLFLGPRRNKSLAAILL

HG0940 ENSG00000115556 PLCD4

>Homo_sapiens

--------MGWTGESSHLSPSYCFPPAITCQSQQPAAVEEGRKQASARGAKEKRSQAGLPDPTASQSSGGTAHRHRKHRTAILLSCPR

>Balaenoptera_acutorostrata_s

--------MGWTEERAHTSPHPTLSIWL--

>Rousettus_aegyptiacus

--------MGWTEERAHTSPHPLLPSGCNCQSQQQTAVEEGRKPEGAKGEK---ELAGFPHPTEESV--------TEHTDTGLLSCPR

>Equus_asinus

MSWAFKNWRGWTEESSHLSPPYSFPLAVTASHSSKLR---

>Chinchilla_lanigera

--------MGWTKERAYTSPHPAPSLCL--

HG0941 ENSG00000116285 ERRFI1

>Homo_sapiens

MRGRAPRGSRRSSGGMKATG-----

>Mustela_putorius_furo

MRGRAPWGSRGSAGGMQATG-----

>Rhinolophus_ferrumequinum

MRGRAPWGSRGSAGGTKATSRPTNP

>Equus_asinus

MRGRAPWGSRGSAGGMKATG-----

>Ctenomys_sociabilis

MRGRAPPGSRRFPGGMKATG-----

HG0942 ENSG00000117477

>Homo_sapiens

MEDAEARCCHGSVVLAAAEAGAGVSFASM----------------

>Felis_catus

MEDAETRRCYGTWVLAAVGAGAGVSLASLSVRA------------

>Tursiops_truncatus

MEDAETRRCHGSRVLAAAA-RAGVSLALLSELRKRRKGELPWPTL

>Myotis_brandtii

MEDAETRRCHGSRVPAA----------------------------

>Oryctolagus_cuniculus

MEDSAPRRCHGSPVLAAAGVAAGVFHASLSELRKR-RGTLPRPSL

HG0943 ENSG00000120690 ELF1

>Homo_sapiens

--MLVRPRSLREEKFQKNCLNLTRISMNQEN-----

>Balaenoptera_acutorostrata_s

--MPLESRSSREEKFQKNCLNLSKIPVNQENLKHLP

>Rousettus_aegyptiacus

---------------MTWSSAVSFLLQPGLEIRIHFSTLKQKPLKTGSTWPRRSREEKFQKNCLRLNRISVNQEHSKFLP

>Oryctolagus_cuniculus

---MCYPRPRRLREQNFQQHCLSLSRISTNQENLKHLS

>Heterocephalus_glaber

MNEGEAEEEAAAAAGAAWSSV------------------AEVAAAFMLVRSKSLREEKV---------------------

HG0944 ENSG00000120833 SOCS2

>Homo_sapiens

MVQERR-GGGVPGIFHVYFPTPTPAAGVQFGLTQPPAFSL

>Ursus_maritimus

MVQEHR-RGGVPGIFHVSVSPPTPQPQGSSLD--------

>Miniopterus_natalensis

MVQEHR-RAGVPGIFHVAVSPPTPRPGV------------

>Equus_przewalskii

MVREHR-CGGVPGIFHVCVFPPTPQP--------------

>Chinchilla_lanigera

MVQEHRLDGGVPGIFHFSVSPARPQGSSWD----------

HG0945 ENSG00000124782 RREB1

>Homo_sapiens

MIATAGESFIVYQWVRKWR----------------

>Odobenus_rosmarus_divergens

MIAIAGESLIVYQLGQKMEVIGSRGWVLLSTFWVL

>Myotis_lucifugus

MIAIARENLIVYQLGQKWSFMAEAS----------

>Equus_przewalskii

MIAIAGENLIVYQLGQKMEFYGRGFLEA-------

>Manis_javanica

MIAIAGESLVVYQLGQKMEFHGRGFLEA-------

HG0946.1 ENSG00000127324 TSPAN8

>Homo_sapiens

MTSKGTYLPGDSLCDI

>Mustela_putorius_furo

MTSKRTYSPSDSPCDI

>Pteropus_alecto

MTSKRTYLPKDSLCDI

>Dasypus_novemcinctus

MTSKRTYPPRDSPCDI

>Heterocephalus_glaber

MTSKRTYMPGDSLCDI

HG0946.2 ENSG00000127324 TSPAN8

>Homo_sapiens

MCGLIFLSSLITFYSSLYFFAFSFCSEAVDTEISAGKLLQSILQDKPVTNS

>Rousettus_aegyptiacus

MCGLIFYPL--

>Galeopterus_variegatus

MCGLIFYPL--

>Ceratotherium_simum_simum

MRGLIFYPL--

>Manis_javanica

MCWLIFYLL--

HG0947 ENSG00000129226 CD68

>Homo_sapiens

-MTKREVARAEAPESGCGWDHLQYRK-------------------

>Physeter_catodon

-MTKTLVAGAEVPESGWGLGCLSYRKSKIYFLFFQMNEGRGYRVD

>Equus_asinus

-MTKRLVAGGEVRESGWGWDY---LKSKTFISSFP-KGGE-----

>Loxodonta_africana

-MTKRLVAGAEVSESGLGVGIFSVTGSKTFISSFP-KEGRGNRVD

>Nannospalax_galili

MTKEGPVAGAEVSESGWGWDYLSYGK-------------------

HG0948 ENSG00000129595 EPB41L4A

>Homo_sapiens

MRGDAPPPSAPSSAGASPAAPGR

>Odobenus_rosmarus_divergens

MRGDAPPPSALSSAGASPAAPGR

>Miniopterus_natalensis

MRGDAPPPSAPSSAGASPAAPGR

>Equus_asinus

MRGDAPPPSAPSSAGASPAAPGR

>Marmota_marmota_marmota

MHGDA-PPSAPSSAGASPAAPGR

HG0949 ENSG00000131381 RBSN

>Homo_sapiens

------------------------MKPLPHHLCAA---

>Canis_lupus_familiaris

------------------------MIPLPHHLCAAWLS

>Physeter_catodon

MLWTWGLAWWFVDSYSREDTSSRAVMPLPHHLCAAWLS

>Myotis_lucifugus

------------------------MMPLPHHLCAAWLS

>Equus_asinus

------------------------MMPLPHHLCAAWLS

HG0950 ENSG00000132964 CDK8

>Homo_sapiens

MSLALSRGLLLLLPHQSGWCCGRRA

>Felis_catus

MSPALLRGLLLLLPHQSGWCCGRRA

>Oryctolagus_cuniculus

MSPALSRGLLLLLPHPSRWCCGRRA

>Ceratotherium_simum_simum

MSPALSRGLLLRLPHQSGWCCGRRV

>Mus_musculus

MSPAPARGLL-PSPREAGWCCGRRV

HG0951 ENSG00000134061 CD180

>Homo_sapiens

MLSSQQHFLFQDHPSEYLSGCQIARAFTV

>Balaenoptera_acutorostrata_s

MLSSQQHFLFSDYPSEHSLAARLSRPS--

>Galeopterus_variegatus

MLSRQQYFLFQDHPLSTSLAAKLQGPL--

>Ceratotherium_simum_simum

MLSHQQHFLF-------------------

>Trichechus_manatus_latirostr

MRNSQQHFLF-------------------

HG0952 ENSG00000134453 RBM17

>Homo_sapiens

MGRVGRAGRH

>Canis_lupus_familiaris

MGRVGRAGRH

>Dasypus_novemcinctus

MGKVGRAGRH

>Condylura_cristata

MGRVGRAGRH

>Equus_caballus

MGRVGRAGRH

HG0953 ENSG00000134815 DHX34

>Homo_sapiens

MNLCPSQRSLQMS

>Orcinus_orca

MNLCPSQRSPQES

>Cynopterus_sphinx

MNLCPSQRSLQDS

>Ceratotherium_simum_simum

MNLCPSQRSLQES

>Heterocephalus_glaber

MNLCPSLRSLQES

HG0954 ENSG00000134874 DZIP1

>Homo_sapiens

MSGPSRNSPVDWGMSGFSSAY

>Odobenus_rosmarus_divergens

MVVPSRDSPVD----------

>Physeter_catodon

MVGPSRDSPVD----------

>Myotis_brandtii

MVGPSQGSLVD----------

>Ceratotherium_simum_simum

MVGPSRDSPVD----------

HG0955 ENSG00000134982 APC

>Homo_sapiens

MLFPGTVVGCW

>Acinonyx_jubatus

MLFPGSVVGYW

>Ceratotherium_simum_simum

MLFPGSLVGYW

>Ictidomys_tridecemlineatus

MLFPGSAVGCR

>Orycteropus_afer_afer

MLFPGNAVGCW

HG0956 ENSG00000136235 GPNMB

>Homo_sapiens

MILPEA-LYLIKIEREAPQMPEEHCCSWWTGPEEFRVKP

>Canis_lupus_familiaris

MICPKAPLYLIKIRSGAQQRPEEHCCS------------

>Dasypus_novemcinctus

MTCPEA---------------------------------

>Equus_asinus

MICPEA-QSFIKIRSGAQQRPEEHG--------------

>Manis_javanica

MICPKALN-------------------------------

HG0957 ENSG00000136874 STX17

>Homo_sapiens
MTRRSMVVRLGGLGARGRKPLLAKPRGGWQKVPHLGSLLLANRGSERRLYFDFSLQIRFFYMSGEDSCYQGGHTTFF
>Myotis_lucifugus
---MSREDSCCK-------
>Equus_asinus
---MSGEDSCYK-------
>Manis_javanica
---MSGEDSCFKER-----
>Ictidomys_tridecemlineatus
---MSGEDRCYK-------

HG0958 ENSG00000137497 NUMA1

>Homo_sapiens

MNNHSCPFAPSLGGN-------------

>Felis_catus

MNKHSCPFAPSLGRG-------------

>Equus_asinus

MNKHSCPFAPSLGRG-------------

>Cavia_porcellus

MNKHSCPFVPSSLGWKLIRIQPIRLSVS

>Tupaia_chinensis

MNKHSCPFASSLLKA-HSKARPLRIALP

HG0959 ENSG00000137558 PI15

>Homo_sapiens
-------------------------MLPNDVFVNSYPLALFFF----IDPNSTFLL
>Leptonychotes_weddellii
-------------------------MLPNDDFVNSYPLALFLFFSFLIDPNSTFLL
>Lipotes_vexillifer
MYRYHYRLSSGLEF--SICLDFLKKVKSSNDFVNSYPLALFFFSFHRP--------
>Ochotona_princeps
MGRFHYNCLLGLSSIFWVWRKSDQAMLPNVDFVNSSPLALFPPYILTLPF------
>Trichechus_manatus_latirostr
-------------------------MLPKDNFINSHLLSLSPFFFSFIDNNSTFLL

HG0960 ENSG00000140297 GCNT3

>Homo_sapiens

MISLSLLSYLRAGSQKESDGATCCPLLCRY--

>Myotis_lucifugus

MTYLSLLSYLRAGN--

>Equus_przewalskii

MLYLSLLFYLRTGS--

>Manis_javanica

MVGLSLLSYVRAGNQAG-DRATCCSLFSEIKEEPEA---VSWTSCEYQKIEFGAVKRRPGDMVLTGASLELNQPSPGNKYVMASGPPQ

>Mus_musculus

MVCLSSLPPLRTGSQKVSDRATCCSLLCIGLTGAWVSSLDGW--

HG0961 ENSG00000140853 NLRC5

>Homo_sapiens
MARLEIAEGRREGEKWIPVLRSGLEGEVCKESALWKQPVNPAQGT-HSPDTHETLSVGTLEHLS
>Canis_lupus_familiaris
--------------------MASVSLYPHEEPVLWKQPANPAQSAGHGSGTHETISADTLEYL-
>Myotis_brandtii
----------------------MLSEADHWRPVLWKQPANPAQGTGLGPGTHETVSTDTLEHL-
>Oryctolagus_cuniculus
--MGTLEHLP
>Nannospalax_galili
--MLKHLS

HG0962.1 ENSG00000143319 ISG20L2

>Homo_sapiens
--------------------MVSRSLRGRRTWVRCMRRLPPIPAWSQGKGMPGFVSLLVVHAADAWVAQR---LSTPYFSLFLSIPRCSFPRRSIDRTCSSLLDSE
GSSSISPSTPFILQMRLKPRAR----------
>Physeter_catodon
------------------------------------MHVEISPTWRRGKWVPEFVSVLGTRARHAWVIQR---STMPNL---------------------------
----
>Myotis_brandtii
-------------------------------------MWRWPPPGNGGKGYLELHLFVGPALGMPGKPR-------------------------------------
----
>Ceratotherium_simum_simum
-------------------------------------MWRFSPPGGEGKGCLDCVSVCGTHAGAAWGI--------------------------------------
----
>Ictidomys_tridecemlineatus
MCLLRWATSIVNPLPPGTAVVWCQRSPPGRTWVRCA--WRLLPPSRSERGVPGFVSLFVGSRLRCHLCRTPVSHSSPEH-----LRHC-LSGRITALACSGFP---
-------------DLRLRTRRAHWVLLFLWCW

HG0962.2 ENSG00000143319 ISG20L2

>Homo_sapiens
----------------MLDSRIQQRMSPTSSFHP
>Dasypus_novemcinctus
----------------MLVLGIQQRMIPTSSFHP
>Manis_javanica
----------------MLVRGIQQRMSPLSSFHR
>Dipodomys_ordii
MQVPESLWTPRFSPDHMLDNGIQQRMSSSSFFHT
>Tupaia_chinensis
----------------MLDAEIQQRMSPPASIHP

HG0963 ENSG00000143867 OSR1

>Homo_sapiens

MSESELGLSPARSQLLLVQTQRGSRERGSPSPACRIRG---C--

--

>Myotis_lucifugus

MSESELGLSPARSQPLPVQTQPGSREPRAASGTHHPRR-------PELRGR---

--

>Manis_javanica

MSESELRLYPTRSQLLRVQTQRGSRERASPSRGRGTPG---QQSAPACRAPSVP---------AAGEGPGL-----------------------------------

--

>Marmota_marmota_marmota

MSESELGLSPARSQQLRFRTSEGTRSEAHDPSAGRTPRLL---SAPACGALGVP---------AAGEGSGLQNIRRVCGLQLLWLPGSRAASPRDALSSRTTAERD

RD

>Tupaia_chinensis

MSESKLGLYPARSQAFPVQTQRGSREQGSPTPGGLDSRAVGCSRVSDSRNPRNWREIWTLALQGYPRTPALLVSPVSC-----------SESRGDALNPGTAE---

--

HG0964 ENSG00000145246 ATP10D

>Homo_sapiens

MAGVEAQPAFTLRICA--------------

>Mustela_putorius_furo

MAGVEA------------------------

>Balaenoptera_acutorostrata_s

MAGIEA------------------------

>Myotis_brandtii

MAGVEAPPVSALRICAWLLSCFLGLGIVVL

>Ceratotherium_simum_simum

MAGVEAQAASTRRICAWFFSCTLGLAIVML

HG0965 ENSG00000146006 LRRTM2

>Homo_sapiens

MKPMLSRGIS

>Leptonychotes_weddellii

MKPMLSRGIS

>Balaenoptera_acutorostrata_s

MKPMLSRGIS

>Erinaceus_europaeus

MKPMLSRGIS

>Orycteropus_afer_afer

MKPMLSRGIS

HG0966 ENSG00000148356 LRSAM1

>Homo_sapiens

MFVVGRRLRLTAGKQGTAVR-------------------------

>Canis_lupus_familiaris

MLLVGRRLMLTAGEPCSAVLRFSVSDFVILV--CMTF--------

>Rhinolophus_ferrumequinum

MLLVGRRQRLTGGEPGTALLPLEETSLWIQGSSCQVVCDWRP---

>Oryctolagus_cuniculus

MFLVGRLLRLTAGEPGASR--------------------------

>Fukomys_damarensis

MFLMGRCLKLAAGKSGTARVTPE----------CQAPKPRAPPIL

HG0967 ENSG00000149925 ALDOA

>Homo_sapiens

MSGASGFPKYRSLPEDPWREKGRGH----------------

>Lipotes_vexillifer

MSGASGFPKYRSPARGSVARKGQGSPEKVGDIGWG------

>Ceratotherium_simum_simum

MSGASGFPKYRSLPGDLWRGKGRGHWRLLGTLAGGRAGAAL

>Manis_javanica

MSGASSFPKYRSLPGDLP-----------------------

>Heterocephalus_glaber

MSGASGFPKYRSLLGDPRREKGRG-----------------

HG0968 ENSG00000154319 FAM167A

>Homo_sapiens

MRFSSLPQGLQPPALPEKRELWAFFLPVWSSSAFRTDRPWHRLPRDSCDAVCSCLLWSMAPTGFQDGS

>Rousettus_aegyptiacus

--MVPTGLGEES

>Equus_asinus

--MAPTGLWEES

>Fukomys_damarensis

--MAPTGFQD--

>Tupaia_chinensis

--MAPTGFQDES

HG0969 ENSG00000154645 CHODL

>Homo_sapiens

MRAGGRAAGLGRRE

>Physeter_catodon

MRSGGRAAGFGRPE

>Myotis_lucifugus

MRAGDRAAGLGQRE

>Dasypus_novemcinctus

MRAGGRAAGLARQE

>Chinchilla_lanigera

MRAGARATGLGRRE

HG0970 ENSG00000154727 GABPA

>Homo_sapiens

--MGRRFSRSTLTGQEASRRQSATGRV

>Miniopterus_natalensis

--MGRRFSRSALTGQEASRRQSATGRL

>Oryctolagus_cuniculus

--MGRRFSRSALTGQEASRRQSATGRL

>Manis_javanica

--MGHRFNRSTFTEQEVSRRRSATRRL

>Trichechus_manatus_latirostr

MHYGPRCTRSSLTGQEASRRRSATGRL

HG0971 ENSG00000155592 ZKSCAN2

>Homo_sapiens

MHSGKPHCAVTSCLEKRPGFCILE--YTFTFSRAWHRFD-----------------------

>Oryctolagus_cuniculus

MHPGKPSGRDTSCLGKRLGFFLDLSVPGHLFRGLG---------------------------

>Ceratotherium_simum_simum

MHPGKPHRGDTSCLEKRPSLGTLV--HRHLFKGLG---------------------------

>Loxodonta_africana

MHPGKPHCGDTSCLEKRSGFWTLG--YTVLFSRA----------------------------

>Cavia_porcellus

MHSGKPHCQVTSCLEKRPGFWIWG--THSPFQGPTIDLINQPEVLFLLKLLNCGFGVRLDSF

HG0972 ENSG00000155926 SLA

>Homo_sapiens

MKNFNRKLLLQKIRSFAANGEHLRQP---

>Canis_lupus_familiaris

-MNFNRKLLLQKIRSFSESGGFPGISGSRDAPRREACSEWLLKTGTYPSASPAHLPHF-----------

>Balaenoptera_acutorostrata_s

MKNFNRKLLLQKIRLFSANRAFPGISGSADVPRREVCSVPLLKTGTCLLQAF-LVSSRGSCNWFSNYAS

>Oryctolagus_cuniculus

MKDFNSERLLQELRSVSANAAFPRRAGCPAAPAFGRCHLRSRGS-------------------------

>Heterocephalus_glaber

MKNFNRKLLLQKIRSFPKNGALLGISSSPDALWREVCSEWLVETGTRPLPAK-RLPRF-----------

HG0973 ENSG00000156535 CD109

>Homo_sapiens

---------------------MFATAARSAIVMGMGGVEPPSPVSI

>Mustela_putorius_furo

---------------------MPAGAARSAIVMPVGGVEPPSPVSI

>Rousettus_aegyptiacus

---------------------MSARATRSAIVMRVGGVEPPSPVSI

>Marmota_marmota_marmota

--------------------------------MRVGRVEPPSPVSI

>Tupaia_chinensis

MLEPGWADRVERPLSNPGGFAIFAGAARSAIVIRVGGVEPPSPVSI

HG0974 ENSG00000157578 LCA5L

>Homo_sapiens

MQTNDHPYADHFEYLQPCFCHCLCRKATCFKKKSSWSAY--ERVGCFLQ-----------LIFPKQTIPPAISGFLKYILSLKRDQNLSKKYYRGKLINTTV

>Mustela_putorius_furo

---MMVFLKYILSLKRDQNMSKNTALETS-----

>Miniopterus_natalensis

---MSKNTTLGTS-----

>Equus_przewalskii

----------------------------------MWEFRLFVPQSFFFGFLERHQKRQVEMETDGHPCADHFASFLKYILSVKRDQNMSKNTTLETS-----

>Dipodomys_ordii

-------------------------------------MVVQFPFLCYF---------------------GALRCFLKYIF----------------------

HG0975.1 ENSG00000182158 CREB3L2

>Homo_sapiens
MRAGLLPSFWMHIPL
>Mustela_putorius_furo
MRAALLPSFWMRALL
>Equus_caballus
MRAYLLPSFWIHTLL
>Dipodomys_ordii
MRADLLPSLWMHIPR
>Tupaia_chinensis
MRADLLPLFWMLIPL

HG0975.2 ENSG00000157613 CREB3L1

>Homo_sapiens
MEASCAPGGAGGGGVG
>Odobenus_rosmarus_divergens
MEASCAPGGAGGGGVG
>Lipotes_vexillifer
MEASCAPGGAGGGGVG
>Ceratotherium_simum_simum
MEASCAPGGAGGGGVG
>Loxodonta_africana
MEASCAPGGAGGGGVG

HG0976 ENSG00000158008 EXTL1

>Homo_sapiens
---MGLSLPICTMTAQD
>Canis_lupus_familiaris
---MGLSLPICTMKALN
>Oryctolagus_cuniculus
MWPGGQRDTALWSGQGLHQPEQCPVAGALLLGLGCPEAEEAESGEQKGKSGKLGASPCLAVTCCSTLHRQPVSAGLSLPICTMRALD
>Loxodonta_africana
--MPGLSLPICTMRALD
>Chinchilla_lanigera
--MASDFLYGPRLVLMGLSLPICTMTVLD

HG0977 ENSG00000158321 AUTS2

>Homo_sapiens

MLPS-LGGLAPLGSLRSPGLPWLHFLKIWEPGSEFSPRRV

>Odobenus_rosmarus_divergens

MLPS-LGGLVPLRSLGSLGLPWMHFLKILEPGSEFSLRRV

>Rousettus_aegyptiacus

MLPS-LGGLVPLRSLGSPGLPWMHFLKIWEPGSEFSLRCV

>Galeopterus_variegatus

MLPS-LGGLAPRRSLWSPGLPWVHFLKILEPGSEFSPRRV

>Chinchilla_lanigera

MLPSQGGGLAPLRSLPSPGLPWMQFLKNFAAWE-------

HG0978 ENSG00000159023 EPB41

>Homo_sapiens

MCHCGDGSMASPGLAA

>Balaenoptera_acutorostrata_s

MCHCGDDSTASPLTAA

>Rousettus_aegyptiacus

MCHRGDGFMAPPGLAV

>Equus_przewalskii

MCHCGDGSMAFLLLAA

>Peromyscus_maniculatus_baird

MCHSDDGSTASP----

HG0979 ENSG00000159202 UBE2Z

>Homo_sapiens

MWEEVRVSGKWLFQADI

>Acinonyx_jubatus

---------MWLFQGDI

>Oryctolagus_cuniculus

MWEEVRVNGMWLFQGDI

>Equus_caballus

MWEEVRVNGMWLFRGDI

>Manis_javanica

MWEEVRVNGMWLFHGAI

HG0980 ENSG00000162670 BRINP3

>Homo_sapiens

MIQILGT-F-FCVLHRFVCHHAVKQVLRES

>Odobenus_rosmarus_divergens

MIQN-FE-I-FCMLFRFVCHHAVKQVLRES

>Equus_asinus

MIQIWGLFL-LRAI----------------

>Chinchilla_lanigera

MIRFWGLFFSFCLLCRFVCHHAVSRC----

>Tupaia_chinensis

MIQMLGTFF-FCVLFRFVCHPCS-------

HG0981 ENSG00000163629 PTPN13

>Homo_sapiens

MLTAPGDRRAGEPC-SVAPRSAS---AAVRVAYRS

>Pteropus_vampyrus

MITGPRDRRAGKQH-TLAPRSASATSAALEARSRS

>Oryctolagus_cuniculus

MITAPRDRRAGALH-SQAPGSAS---AALGVGCRS

>Ceratotherium_simum_simum

MVTVSGDRRVGEQH-SLAPRPASASRAALGAGCRS

>Chinchilla_lanigera

MVSGPGDRRAGEQQRPRAPRSASDAPAALGAGCRR

HG0982 ENSG00000164116 GUCY1A3

>Homo_sapiens

MCGFARRALELLEIRKHSPEVCEATKHPSYQCP

>Mustela_putorius_furo

MWGFAGRARELPEIRKRSRRVWEANEHPSHQYP

>Dasypus_novemcinctus

MCGFARRALELLEIGKRSREVWEAIEHSSHQCP

>Oryctolagus_cuniculus

MCGFARRAPQLLRIPQRSPEVWAVHRAPQL---

>Ictidomys_tridecemlineatus

MCGFARRAPELQQIWKHGPQVWETAKHTGQQCP

HG0983 ENSG00000164128 NPY1R

>Homo_sapiens

MDSNMGNKNKLNS

>Galeopterus_variegatus

MDSNMGNKNKLNS

>Erinaceus_europaeus

MDSNMGNKNKLNI

>Ochotona_princeps

MDSNMGYKNKLNS

>Ceratotherium_simum_simum

MDSNMGNKNKLNI

HG0984 ENSG00000164185 ZNF474

>Homo_sapiens

----------------------MLKGKIKRSWRHLSFNEDFPTFQTAVLQ--

>Ailuropoda_melanoleuca

-----------------------MTTNITRSWRHLRFNEDFPAFQTAALQ--

>Orcinus_orca

----------------------MIKAKIER----------------------

>Mus_musculus

---------------------------MSRSWRHLRFNGGFPTFRTAALQ--

>Tupaia_chinensis

MALCPHTVDSPRTAGTTRPGPTQRERGEAAGWRLLRLDGDFPTFQTCPVMRL

HG0985 ENSG00000164604 GPR85

>Homo_sapiens

MKACLLVL----GRRTGKLLLKILYHLHKQNSSEMVK

>Pteropus_alecto

MKARLLIF----WR-----------------------

>Elephantulus_edwardii

MSARIWGGFPWQGGGGGELSFKILNHPHKQNSSEMVK

>Loxodonta_africana

MSAWIFFLARWEE----EGEFKILYHPHKQNSSEMVK

>Ictidomys_tridecemlineatus

MSACF--W----GVGSGGWLLKILYHPHKKNSSEIVK

HG0986 ENSG00000164663 USP49

>Homo_sapiens

MVRGAGRDGPQNLKDEMGSKGREGAKRSWGSPDGCRTGVDCRGVGIECACYGELLQKVCKGQGRNAVVKDQV

>Acinonyx_jubatus

--------------MRWGVREENRVKGSWRSPAGGRTGLACTGVGVRCACCGEMLRKVCMGQGTGAVVKDQV

>Dasypus_novemcinctus

---MKCACCGEILQKVWKGQGTKP------

>Oryctolagus_cuniculus

----------------------------------MSNSSRARRAGIERACGRERLQKACEGQGRKAGVRDPV

>Cricetulus_griseus

----------------MGSKRREAGRALMG------------------------VEPWWAAQGWGLHMYLP-

HG0987 ENSG00000164932 CTHRC1

>Homo_sapiens

MQPAAASERGGARR

>Physeter_catodon

MQPAVASERGGAGL

>Oryctolagus_cuniculus

MQPAAVSQRGGARS

>Equus_asinus

MQPAAASERGGARL

>Dipodomys_ordii

MQPAAASERGAARH

HG0988 ENSG00000166130 IKBIP

>Homo_sapiens

--------MELAAERLSEATRMPGRGKGLGSGRCQVCEGARSPPRVPTNAKAATSCSPW

>Physeter_catodon

---------------------MAGRGRGLVSGRCQV-----------------------

>Rousettus_aegyptiacus

MGSHVSQLEEFGEGGCCEAAGMVGRKRGLVYGRR-------------------------

>Equus_asinus

--------------MRCEAARMAGRGRGLVSGRRQIREGARSPPRLPAELRRPRPDPPG

>Ctenomys_sociabilis

---------------------MAGRGKGLVSGRGRVCEEARPPRDAPVATSRESLK---

HG0989 ENSG00000166603 MC4R

>Homo_sapiens

----------------------MAASRKIILNRLECIRLKLKQK

>Balaenoptera_acutorostrata_s

------------------------------------MRLKLKWK

>Myotis_lucifugus

MLVGASERQLMWACAHTLSPNGTAAPRRIILKRPECLRL-----

>Equus_caballus

------------------------------------MRPKLK--

>Trichechus_manatus_latirostr

------------------------------------MRLKLKQK

HG0990 ENSG00000166866 MYO1A

>Homo_sapiens

MKLGHLYTCEGAWAGRGSKRENQKSGHWQEEGSLFPSFLAPWTAPIKPPLHP-GARTLMPHSCQIEISSTILQGQ-----

>Canis_lupus_familiaris

--MPPSCQIGISFTILQGTP----

>Dasypus_novemcinctus

--MPPSCQIAISSTILQGGTEVTP

>Manis_javanica

--------------------------------------MTPWTAPINQGTSQPWGLGFLCPFVTLEFYPPFSKGHSEGTP

>Chinchilla_lanigera

--MPPSCQTGISSTLLQGTLK---

HG0991 ENSG00000167654 ATCAY

>Homo_sapiens

MPSCTRAGLAFVCQ

>Odobenus_rosmarus_divergens

MSGCIRAGLAFVCA

>Myotis_brandtii

MPGCSRAGLAFVCA

>Equus_asinus

MLGCIRAGLAFVCA

>Manis_javanica

MPGCNRAGPAFVCA

HG0992 ENSG00000168000 BSCL2

>Homo_sapiens

MRHRYQRLDRHFTF

>Rousettus_aegyptiacus

MSYRYQLLDQHFVF

>Galeopterus_variegatus

MRYRYQLLDHHFTF

>Equus_asinus

MRHRYQLLDHHFMF

>Manis_javanica

MSYRYQLLDHHFMF

HG0993 ENSG00000169306 IL1RAPL1

>Homo_sapiens

------------MYSEDLQATLQFLS

>Ochotona_princeps

MLLGEQEDLKTYMYPEDLQARLQFLS

>Loxodonta_africana

------------MYSEDLQARLRFLS

>Mesocricetus_auratus

------------MYSEDLQARLRFLS

>Orycteropus_afer_afer

------------MYSEDLQARLRFLS

HG0994 ENSG00000170852 KBTBD2

>Homo_sapiens

MLQITASRFS--FISEVFT

>Acinonyx_jubatus

MLQITASRVSPFFISEAFT

>Tursiops_truncatus

MLQITASRVSPFFISEAFT

>Rousettus_aegyptiacus

MLQITASRVSPFFISEAFT

>Ceratotherium_simum_simum

MLQITASRVSPFFITEAFT

HG0995 ENSG00000171307 ZDHHC16

>Homo_sapiens
MITADTYLVLPHWQECPCWYL--
>Galeopterus_variegatus
MIAADTYLVLSNWQKCPC-----
>Oryctolagus_cuniculus
MVAADTYLVLLHWQECLCRCL--
>Equus_asinus
MIAADTYLVLSHWQECPCYLWDR
>Peromyscus_maniculatus_baird
-MRAANTLVLSHWQECPCWLL--

HG0996 ENSG00000173890 GPR160

>Homo_sapiens

----------------MKCRCIELTHIAYWYIKMKCKEPKIT

>Dasypus_novemcinctus

---------------------------------MKCKETKVR

>Galeopterus_variegatus

--------------------------MAFWYSKMKCKNQK--

>Ochotona_princeps

---------------------------------MKGKEPKIT

>Fukomys_damarensis

MQVDAGSAFSTQRALSIHQAHRYRTYIAFWHSKMKCKEPTVT

HG0997 ENSG00000177728 TMEM94

>Homo_sapiens
MAAAPGGGDVK
>Felis_catus
MAAAPGGGDVK
>Oryctolagus_cuniculus
MAAAPGGGDVK
>Nannospalax_galili
MAAAPGGGDVK
>Trichechus_manatus_latirostr
MAAAPGGGDVK

HG0998 ENSG00000178172 SPINK6

>Homo_sapiens
MYWLLGKCRLPVENDILY------------------------
>Eptesicus_fuscus
MYWLLGKFSLLVENDILY------------------------
>Galeopterus_variegatus
MYWLLGKCRLLVENNILLIGSKCFLGSVLTENLSWAKHPYSE
>Condylura_cristata
MYWLLGKCRLLVGNDRSTNKI---------------------
>Loxodonta_africana
MYWLLGKCRLPVENNILY------------------------

HG0999 ENSG00000178401 DNAJC22

>Homo_sapiens

MEVGAGRDGPFSLCGQRK

>Canis_lupus_familiaris

MEVGAE------------

>Pteropus_alecto

MEVEAE------------

>Equus_asinus

MAVGAE------------

>Marmota_monax

MEVGAE------------

HG1000 ENSG00000179083 FAM133A

>Homo_sapiens

---------------MLPIFFSATEQLPWYNKKDEYLLFIERMELA--------------------

>Myotis_davidii

MSGKPGLYPSARFANPCCRFFSATEQLPWYNPKDQCLLFIERT-----------------------

>Oryctolagus_cuniculus

---------------MLPIL-FCYRTTPLVNPKDQCLLFTERKIPRSGMAGSHEDPEKRTGKQLER

>Manis_javanica

---------MKILQMASLGFFSATEHLPWYNQKDQCLLFMKRRT----------------------

>Chinchilla_lanigera

MFGKLGLYPSTRFANPCCRFFSATELFSWYNTKDLCLLFIERIEPAWLTSR---------------

HG1001 ENSG00000179761 PIPOX

>Homo_sapiens
MTIIEPRIESHRDARVRLAAELG
>Ailuropoda_melanoleuca
MTIIEPRIES-------------
>Rousettus_aegyptiacus
MTIIEPRIES-------------
>Oryctolagus_cuniculus
MTIIEPRIES-------------
>Chinchilla_lanigera
MTIIEPRIES-------------

HG1002 ENSG00000181027 FKRP

>Homo_sapiens

--MAAAAAAGAQLSWAGTALLELPQPTT------

-

>Canis_lupus_familiaris

--MAAAAAAAMQLSWAGTTHPELPQQPQRMPWKP

S

>Pteropus_alecto

-------------------------------MPARPLSPLAGPKSPR-------------RPLSHQEPIGLHLQPAPGRDGAVQLSWAGTTPLELPQPGM------

-

>Condylura_cristata

--MTLCSIMQNWLIWDFLVFLPAALPLSWAGTAPPELPQPAT------

-

>Heterocephalus_glaber

MPQVVPKFIGLQEKGFPEGGDLPHWFGCLLRKRKDPIRSLVEKKKEKGFPDLVGVTFLGTMKLGSIVPNQLTCGFLVFLPSVEQLSWTGTALLKLPQPTA------

-

HG1003 ENSG00000184867 ARMCX2

>Homo_sapiens
---MLIHPPGFHCLTSTVYISALQSVLRMKL-
>Miniopterus_natalensis
--MSAVCISALQSVLKMKL-
>Oryctolagus_cuniculus
--MLAIYISALQSVLRMKLN
>Ceratotherium_simum_simum
MRSSRDQELTWAKVLKALTAVLRPPLGTNSNALRTAVRLTQARRRRRKFPQIRAGSQPTSAVYISALQSVLRMKP-
>Peromyscus_maniculatus_baird
---MMICPPGS---------------------

HG1004 ENSG00000185875 THNSL1

>Homo_sapiens

MFTSQWKLSTTHIN----

>Canis_lupus_familiaris

MFTSQWELSASHTC----

>Pteropus_vampyrus

MFTSQWELSTAHTS----

>Galeopterus_variegatus

MFTCQWELSTAHTS----

>Loxodonta_africana

MFTSQWELSTAHTSRKAK

HG1005 ENSG00000186472 PCLO

>Homo_sapiens
MEEAPAAGVTCERD
>Mustela_putorius_furo
MEEAPAAGVTCERD
>Myotis_lucifugus
MEEAPAAGVPCERD
>Galeopterus_variegatus
MEEAPAAGVTCERD
>Equus_asinus
MEEAPAAGVTCELD

HG1006.1 ENSG00000188227 ZNF793

>Homo_sapiens

---------------------------------------MDVTLPVVRKQIY-

>Mustela_putorius_furo

-----------------------------------MAARMDVTLPVVRKQRIY

>Balaenoptera_acutorostrata_s

MRGKWERFFLENVMTCPSVTASQESAWFLEIYSDSTAARMDVTLPVVRKQRVY

>Loxodonta_africana

-----------------------------------MAVGMDVTLPVVRKQRVY

>Cavia_porcellus

-----------------------------------MATKMDVTLPMVRKQKVY

HG1006.2 ENSG00000188227 ZNF793

>Homo_sapiens

---------------------------MGLFLAKVMTYASVPASQE

>Felis_catus

-----------------------------------MMYPSVTASQE

>Equus_asinus

MGRACPLARRRGTAIDLGLWARIQEKWERFFLAKEMTCPSVTASQE

>Cavia_porcellus

----------------------------------MMTYLSVTSFQE

HG1007 ENSG00000188910 GJB3

>Homo_sapiens

----------MATDAPISRLRKRRLRRATSPSGGWPGS--AWQRRV

>Mustela_putorius_furo

----------MAADAPISHMKKPRLRAATGPSEGGPGSGCPWH---

>Balaenoptera_acutorostrata_s

------------------MKKKPRLRRASSPGEGCRGNE-------

>Equus_asinus

MRFICSLALPMATDALISHLKTRRLRRATSPSTSGPGN--PWLRGA

>Chinchilla_lanigera

----------MAASAPTPCRSKPRLGRARIPSGAGPGA--TWLRRD

HG1008 ENSG00000198252 STYX

>Homo_sapiens

MAAGCKTPDPPLPCLRRR--RCWSHWDPVVCVC--

>Felis_catus

MAAGYEPPNLPLPRCRRR--RRRSRWDPLVCVC--

>Physeter_catodon

MAAGHEPPSPPLPGCRRRHRRRRSRWDPLVCVC--

>Eptesicus_fuscus

MAAGYEPPNPPLPLCRRR--RRRSRRDPLVCVCQL

>Sorex_araneus

MAAGHEPPNPPLPHCRRR--RRRSRWDPSVCEC--

HG1009 ENSG00000198909 MAP3K3

>Homo_sapiens

MPQRPAAGGAGAGAWGGGGGPERSPRPPRGARPAAVPAARAPGMQPRLRR

>Odobenus_rosmarus_divergens

MPQRPAAGGAGAGAWGGGGGPERSPRPPRGARPAAVPAARAPGMQLRLRR

>Dasypus_novemcinctus

MPQRPAAGGAGAGAWGGGGGPERSPRPPRGARPATVPAARAPGMQLRLRR

>Oryctolagus_cuniculus

MPQSPVAAGAGAGAWAGGGGPERSPRPPRGARPAAVPAARAPGMQLRLRR

>Rattus_norvegicus

MPQRPAR--AGAGAWGGGGGPERSPRPPRGARPAAVPAACSPGMQPRLRR

HG1010 ENSG00000203740 METTL11B

>Homo_sapiens

-------MTVSSSFRKRRLIQRNSKASFLSL--

>Galeopterus_variegatus

-------MTVSSSFKKRSLIQRKSQANFLSL--

>Ochotona_princeps

-------MTVSSSFRKRRLIHRSSQASFLSQ--

>Equus_asinus

-------MTVSSSCRKRRLIQRNSQASFLSL--

>Chinchilla_lanigera

MGQDWFKMTVSSSFRKRRLIQRNSHTSFFSLQE

HG1011 ENSG00000204439 C6orf47

>Homo_sapiens

----------------------MTIQRREEPLLNSE----------

>Lipotes_vexillifer

----------------------MTIQGSGEPLLNSE----------

>Rousettus_aegyptiacus

MCLFSLPQKLKRKQAACPTLGCMTIQGRGEPLQNSE----------

>Manis_javanica

----------------------MTIQGNGEPLLNSEQDSPSKYLFF

>Ictidomys_tridecemlineatus

----------------------MTIQGSGEPLLNSE----------

HG1012 ENSG00000213694 S1PR3

>Homo_sapiens
MNVPGAPSRGFWS
>Balaenoptera_acutorostrata_s
MDVPRAPSGGFWS
>Condylura_cristata
MDVPGAPSAGIWN
>Equus_przewalskii
MGVPRAPSAGFWS
>Microtus_ochrogaster
MNVPGAPSRGLLR

HG1013 ENSG00000214022 REPIN1

>Homo_sapiens

MDGRRVPAQPAAEVRLASLQSEV

>Felis_catus

MDARRASAHPAGEVRLASLRSEV

>Rousettus_aegyptiacus

MDARRASAHPAGEVRLASLQSGA

>Dasypus_novemcinctus

MDGRRASAHPAGEVRLASQQSEV

>Heterocephalus_glaber

MDGRRAPARPAGEVRLASLRSEG

HG1014.1 ENSG00000214595 EML6

>Homo_sapiens

MQIPDVKGGVAPPRASPGGRSPQVQ

>Mustela_putorius_furo

MQMPDLKGGVTLPRTSPGGRSPQVQ

>Pteropus_vampyrus

MQISDLKGGATPARTSPRGRSPQVQ

>Manis_javanica

MQIPDLKGGVTPPRTSPGGRSPQVQ

>Mesocricetus_auratus

MQISDFKGGVTPGRTSPAGRSPQVQ

HG1014.2 ENSG00000214595 EML6

>Homo_sapiens
--MAELTRVACQIKAIAE
>Mustela_putorius_furo
MVMAKLTRVLCQIKAVTE
>Myotis_lucifugus
MVMAKLSRVPCQIKAVTE
>Manis_javanica
--MAKLTPASCQIKAVTE
>Chinchilla_lanigera
--MAKLTRAACQIETVTE

HG1015 ENSG00000240720 LRRD1

>Homo_sapiens

MKIKIKLQFCIGIKHISK

>Ursus_maritimus

MKIKIELQSCTDVKHSSK

>Pteropus_alecto

MKIKIELQSCIGIKHISK

>Galeopterus_variegatus

MKMKIKLQSCIDRKRISK

>Tupaia_chinensis

MKIKIKLPSCIGIKYNI-

HG1016 ENSG00000254004 ZNF260

>Homo_sapiens

---MLHFIPFCNHLISG

>Balaenoptera_acutorostrata_s

-MRMLYFIPICNQLITG

>Pteropus_alecto

-MRMLYFIMFCNHLISG

>Galeopterus_variegatus

MKRMLYFIPFCNHLI--

>Equus_asinus

-MRMLYFTPFCNHLISG

HG1017 ENSG00000255154 HTD2

>Homo_sapiens
MLHSSNSCLFRL
>Felis_catus
MLHSSNSCLFQP
>Balaenoptera_acutorostrata_s
MLHSSNSCLFQP
>Pteropus_vampyrus
MLHSSNSCLFQP
>Loxodonta_africana
MLHSSNSCLFWL

HG1018 ENSG00000259120 SMIM6

```
>Homo_sapiens
----------------------------------------------MPFPLNSCFFESGCSSGHLAASSAQPSTKCEPEGPP--------------
>Ursus_maritimus
-------------------------------M--AMRSGQGYLTMPIPFSLNSCFFESGCGSGPQSVGPAGLP-----------------------
>Myotis_brandtii
MI---------A-KRDLLLPHLLAPAAHPNHL--ASSTKQG-----ADAPRVTCFSESEHRSGHLLASPARPPKCGPLRVTPSALSGLGSVNSRVR
>Manis_javanica
-------------------------------M--TMRTGQGHLAMQTRFPPNSCLFESGCSPGHLVASAARVPKAWGLKNYPDQPL----------
>Rattus_norvegicus
MPSQKGRTLMEEMREGVALPGN---QSSPPLFALVAAAKVWSENLATPWAANSNFFELWHSCGHLAVSSLWPEGQS--------------------
```

HG1019 ENSG00000019995 ZRANB1

>Homo_sapiens

MASLKLNISISGFPLISSFLDQTSL-YLLPISFAFQSSVLLNPRV---

>Odobenus_rosmarus_divergens

----------------------MSL-YLLLISFAF-------------

>Lipotes_vexillifer

MTWLRLNISVRGFLI---------------------------------

>Orycteropus_afer_afer

MSWLKLNISIGGLPILTNPFPLKTFSTDLPLSLAYYLPCTAKYNFITS

HG1020 ENSG00000023902 PLEKHO1

>Homo_sapiens

MEGAKGRGAGVRVCASANAREAGLE

>Orcinus_orca

MEGAEGRGAGVRVCASAREAG--L-

>Rhinolophus_ferrumequinum

MEGAEGRGASVRVCASAGEAG--P-

>Ochotona_princeps

MEGAEGRGAGVRVCASAREAG--L-

HG1021 ENSG00000064692 SNCAIP

>Homo_sapiens

MPTFRSPPSRVSSPSLCWNPGMVWGPMRWQRLTLGSRNL---

>Panthera_tigris

--------------------------MRWQRLTRGSRAFIKI

>Manis_javanica

---------------------MVWGPVSWQRSTRGSRAS---

>Mus_musculus

---------------------MVWGPMRWQRLIHGSRKL---

HG1022 ENSG00000065357 DGKA

>Homo_sapiens
---MPGSPVPASESLGLHLSPLLYHLHH
HPCDPKSLNDSRRDSRQGKLKG-PFTPYFWPGPSVPPAKTSRYN-----------FPKSHSGIRAKNGTQI
>Pteropus_alecto
---MPGSPVPTSRSLGLCLSPLLYQLRH
PQ----S-LDSRRDSRQKKLKGSPYTPTFGQGLLYHPPRPADLPLEAEVQAPKVLLPSCLCGLP-------
>Equus_asinus
---MKGLCLSPLLYQPHH
HPRCPKSLNNSRTDSKQEKLKGFPFTSTFDQGLLRHLPRPADLTLEVEVQAPEVLLPSCLCGHP-------
>Chinchilla_lanigera
MCISCVCVCVCVCVCVCVDRDIQTHPTYTCTGFWSEIPPRPHTHPSGVKRAIPGTQGTGTLPYFHEGQEGAHRGWGGARGQDAWVSVPTSKSVGLCLSLLLYQPQH
HPCRPRALKLLNRLEAEAE--RSSFVPLLCARAFCT---------TCKVSGPY---P--------------

HG1023 ENSG00000068001 HYAL2

>Homo_sapiens
MGGHVPLQRTWGSSSPKRGTGTEKVPELVPGR--

>Felis_catus
MDGHVPRGRIWGPWSLKRGTGCAKVGNTFPPRPQVLPQAHLRAGFLKGPRAGVGRGR---GGAAEARSLGCAWASGRSGPGWAGDGSGHGVGPGSAPREFLSWCQA
GDTVFL
>Pteropus_alecto
MDGHVPWRRIWGPWSPKRGIGG--

>Equus_caballus
MGGHVPRRRTWGPWSPKRGTGGAKVGETFPPLPQVLSRAHLRAGFLRGPRAGGRSQQRRRAGAVPGRKAG----ACRAGPGMAVAVAQVPAA----PRASS-----
------

HG1024 ENSG00000073464 CLCN4

>Homo_sapiens

MSLSVSASIRPSTQRSRRFR

>Equus_asinus

MSLSASVSIRPSTR-SRGFR

>Loxodonta_africana

MSLSGSVCISPSTQRRRGFP

>Heterocephalus_glaber

MNLSVSACIRPSTQRSGGLR

HG1025 ENSG00000074621 SLC24A1

>Homo_sapiens

MSRSEGLWIPPGWASWRNLL

>Orcinus_orca

MSRFEGLWLPPGWASWRNLL

>Myotis_davidii

MSRFEGLWISLSWASWRNLP

>Rattus_norvegicus

MSRSEGLWIPPGWASWRNLS

HG1026.1 ENSG00000075035 WSCD2

>Homo_sapiens

MCVLSLKTPLLAQRSSPSVWEGW

>Orcinus_orca

----------MAQRSSPWLREGW

>Pteropus_alecto

----------MAQRSSL------

>Manis_javanica

----------MAQRSSP------

HG1026.2 ENSG00000179314 WSCD1

>Homo_sapiens

MTPPEALASLPPGR

>Eptesicus_fuscus

MMPPEAPASLPPGR

>Galeopterus_variegatus

MTSPEALASLPPGC

>Chinchilla_lanigera

MTPPEALASLLPGC

HG1026.3 ENSG00000179314 WSCD1

>Homo_sapiens
MVPFEHLLETGQEQ------------------------
>Rousettus_aegyptiacus
MVPFKHLLETGQEEQ-----------------------
>Ceratotherium_simum_simum
MVPSEHLLETGQEEQ-----------------------
>Loxodonta_africana
MVPFKHLLKTGKRAHAGARSQDAGMTPPGVPACQPPGR

HG1027 ENSG00000075223 SEMA3C

>Homo_sapiens

MRACRAARRGSRSRAPRRCNRTPRALAPGLRPLSIHSEVLD--

>Balaenoptera_acutorostrata_s

----------------------------MRRPLSIHSVVLD--

>Ceratotherium_simum_simum

-----------MRRAPRRCNRTPRARPAPATFHSLGGAVLSAAETGSGAADSFTKKLRGPGRRNLTAGPLQKLRSSGEGTGDSLGAQPAAN

>Ictidomys_tridecemlineatus

MRAGWAARRGSPSRARRRCNRTPSARPLRLATFPFTPRCLIERR-GEGLRCHGTTGLRIPVQRNSE--------DLGEKKEYHIGTL----

HG1028 ENSG00000076356 PLXNA2

>Homo_sapiens
MNLDSMWIWSSP
>Felis_catus
MNLDSMWIWSSP
>Tursiops_truncatus
MNLDSMWIWSFP
>Ceratotherium_simum_simum
MNLDSMWLWSSP

HG1029 ENSG00000079482 OPHN1

>Homo_sapiens

MERGYVSLPPWLNPSGA--------------------

>Pteropus_alecto

MEEGDVPLPPRLNPSGA--------------------

>Equus_asinus

MEEGYVSLLPLLNSSGAYLDSKPVPLDQEELLGAGGL

>Chinchilla_lanigera

MEEGYVSLPPGLNPSGT--------------------

HG1030 ENSG00000082175 PGR

>Homo_sapiens

-----------------------------MRTSFTCHLSEIYNPRRLVLPHYWDLRSSEMTVARSTEPAEVRPFLGMGCTERSD--------------

>Mustela_putorius_furo

-----------------------------MRTSFTRHLSEICKYSFLLS--YRET---

>Oryctolagus_cuniculus

MGPVHAVTTRVKLCSTCVCEDSGVWGAFSMRTSFTCHLRLGTRGGS----------SSRNAGNRESSGDQCGPL------------------------

>Ceratotherium_simum_simum

-----------------------------MRTSFTWHLSGICNHRSFALPDSEKCESCR---------GDCRQLLGAGRNTTVLEKGLYRKVKVAPGF

HG1031 ENSG00000082269 FAM135A

>Homo_sapiens

MLAGRAGPVAEPR-----------------

>Canis_lupus_familiaris

MLAGRFGPVAEPR-----------------

>Pteropus_alecto

MLAGRAGPVAERR-----------------

>Dasypus_novemcinctus

MLGGRAGPVAEPRRRTESSAAVVAVAAMAM

HG1032 ENSG00000084453 SLCO1A2

>Homo_sapiens

MQPSEPKKLNQRFKTYINKDSFIYNSASLMNKKLQLTRLRCLIHTGYSICNFLKNLLVLQYGSKNEW-

>Ceratotherium_simum_simum

--MILQYRSSGEWS

>Manis_javanica

--MILQYGSSSD--

>Cavia_porcellus

MQPSEPKKLYQRIKSYVNKDPFIYISTSLKNKTLQFTCSQCFTCNS--TCNLF----FKKFDVLAD--

HG1033 ENSG00000089225 TBX5

>Homo_sapiens

MPAPPPHRRLPC

>Felis_catus

MPAPPPRRQLPC

>Miniopterus_natalensis

MPAPPPSRRLPC

>Heterocephalus_glaber

MPAPPPHRRLPC

HG1034 ENSG00000090487 SPG21

>Homo_sapiens

-----MQHLVSILPFMYCQTSTSVKITCLYKNLVTAEWNLSIL--

>Acinonyx_jubatus

MSVGQMQHLVSILPFMYCQTNTSVKITCLYKNLVTVGWNLSIQ--

>Equus_asinus

-----MQHLVSVLPFMYCQTSTSVKLTCLYKNLVTVGWNLSIQQD

>Manis_javanica

-----MQHLVSISPFMYC---------------------------

HG1035 ENSG00000093000 NUP50

>Homo_sapiens

MAARSVFGAVPPAVSG

>Leptonychotes_weddellii

MAAWSAFGAVPPAVSG

>Dasypus_novemcinctus

MAAQSVFAAVSPAISG

>Equus_asinus

MAAWSALGAVPPAVSG

HG1036 ENSG00000100813 ACIN1

>Homo_sapiens

-------------------MVIPGSRVSEYIRFKASARRRKPSLKAGREKGRCRADGKN

>Myotis_lucifugus

MFPENPKQGQPGSPRTSGPGPAPNTQAPQRLSFRSEESRMTPIMVMPKSRASE------

>Equus_asinus

-------------------MVMPESRASEQIRFKASVRRR-------------------

>Mesocricetus_auratus

MFPENPKQSQPSNPRTSGLKPAPNTQVPRRLSPRSEDARISPIMVMRDSRSSE------

HG1037 ENSG00000102710 SUPT20H

>Homo_sapiens
MVFTPLHQRT
>Canis_lupus_familiaris
MVFTPLHQRT
>Galeopterus_variegatus
MVFTPLHQRT
>Ceratotherium_simum_simum
MVFIPLHQRT

HG1038 ENSG00000104331 IMPAD1

>Homo_sapiens

MGRTGAGERAPV

>Orcinus_orca

MGRAGAGERAPV

>Eptesicus_fuscus

MGRAGAGERAPV

>Ictidomys_tridecemlineatus

MGRAGAGERAPV

HG1039 ENSG00000104804 TULP2

>Homo_sapiens
MFRDWPERQGTEASM
>Panthera_tigris
MFRNWPERQGTEA--
>Orcinus_orca
MFRNWPERQGTEV--
>Equus_przewalskii
MFRNWPGRQGIEA--

HG1040 ENSG00000105223 PLD3

>Homo_sapiens

MRKAHAWPPPACSLAVVRLRAARRGRQAG-------IQP--GR---------

>Odobenus_rosmarus_divergens

MRKAHA--

>Heterocephalus_glaber

MRKAQAYPPPSRSLAGVRLRAGVAGRRGQ----SLGVALRAGSEGGDAGVQF

>Tupaia_chinensis

MREAHVWHPLACRLAGVRLRKRGGGQAGVQPRQCVGLGPRVGAVECR-----

HG1041 ENSG00000106536 POU6F2

>Homo_sapiens

--MNSLGLCYPVCCLLSRC

>Acinonyx_jubatus

MGMNSLGLCYPVCCLLSRC

>Pteropus_alecto

--MNSLGLCDPVSCLLPRC

>Loxodonta_africana

--MNSLELCYPVCCLLSKC

HG1042 ENSG00000107249 GLIS3

>Homo_sapiens

MNRTGEAQSRN---

>Odobenus_rosmarus_divergens

MSRTGEAQSRNSTF

>Pteropus_vampyrus

MSRTGEAQSRNSTF

>Equus_asinus

MNRTGEAQSRNSTF

HG1043 ENSG00000107736 CDH23

>Homo_sapiens

MERGVAEPLDVWGAPSPSPRRA

>Manis_javanica

MESGGAESRDVWEALRPSPRRA

>Octodon_degus

MERGVAQRLHVWGVPRPSPRRT

>Trichechus_manatus_latirostr

MEPWGAEPRDVWGALRPSP---

HG1044 ENSG00000109381 ELF2

>Homo_sapiens

-------MIMQTYNTGNHQ

>Ailuropoda_melanoleuca

MSIDFENMIMQTCNTGNHP

>Miniopterus_natalensis

-------MIMQTYNTGNHP

>Marmota_marmota_marmota

-------MIMQTYNIGNHL

HG1045 ENSG00000110925 CSRNP2

>Homo_sapiens

---MGGLEACRIADED

>Ailuropoda_melanoleuca

MREARTGGGEPGRRWEGKESGEAWELGARRTRARTAGAARGRPMGGLEAGRIADEG

>Ceratotherium_simum_simum

---MGGLEAGRIADEG

>Heterocephalus_glaber

---MGGLEAGRIVDED

HG1046 ENSG00000112701 SENP6

>Homo_sapiens
MHSRLGGVGGRP
>Rousettus_aegyptiacus
MHSRLGGVGGRP
>Condylura_cristata
MHSRLGGVGGRP
>Marmota_monax
MHSRLGGVGGRP

HG1047 ENSG00000112769 LAMA4

>Homo_sapiens

MNVRRKPGQRSPQSRTARERRWPSAGGAHPNPSAF---------------------------

>Odobenus_rosmarus_divergens

MDVRRKARQRSPQSPPSPGQE--VAGSARPNPVCLLMLSLKLHPSIRPSARASGERESGREK

>Balaenoptera_acutorostrata_s

MGVGRKPGQRSPQSRPPGTGGGRRSRGSPQTHPPLDA---EL--------------------

>Equus_przewalskii

MGVGRKPGQRSPQSRRARDRRWPVLAGLAPTQSVF---------------------------

HG1048 ENSG00000115295 CLIP4

>Homo_sapiens
MKTPISMHSQPTR
>Lipotes_vexillifer
MNTPISIHSQPTL
>Loxodonta_africana
MKTPISIHSEPTL
>Fukomys_damarensis
MKTPISIHSQPTL

HG1049 ENSG00000116212

>Homo_sapiens
MPRKELELWS-NAALRLNSGSATY
>Physeter_catodon
MQWQELELWS-DTALRLNSGSATY
>Pteropus_alecto
MQWKELELWS-NTALRLNSGSATY
>Manis_javanica
MQWKELELWRSNTALRLNSGSATS

HG1050 ENSG00000116489 CAPZA1

>Homo_sapiens

-------------------MGNECNSLGKILDFKSPKANPTSWGAGAGAGVRRRHSRA---

>Ursus_maritimus

-----------------MNGGNECNSVRKVLDLKSFKEKSYGRPRD--VQERRLSQPPPHP

>Galeopterus_variegatus

MEPRRQEQSKHAGKRATDERGNECNSERKTLDFKSLKAKPTSWCAGCGRRHPRA-------

>Marmota_marmota_marmota

-------------------MGNDYN------------------------------------

HG1051 ENSG00000116852 KIF21B

>Homo_sapiens
MARGGARAQPRGAGRG--
>Odobenus_rosmarus_divergens
MARGGARAQPRGASPGRG
>Rousettus_aegyptiacus
MARGGARDQPRGAAPGRG
>Heterocephalus_glaber
MARGGARDQPRGAGRD--

HG1052 ENSG00000118640 VAMP8

>Homo_sapiens

--MRGGRGQAESWSKQEVN----

>Balaenoptera_acutorostrata_s

--MRGGRGRAESWTSRK------

>Rhinolophus_ferrumequinum

MGCEVSKGRAESWSKQEVN----

>Microtus_ochrogaster

----MGRRRAESWSKQEVKLWLR

HG1053 ENSG00000119522 DENND1A

>Homo_sapiens

MAFAGLERVH

>Mustela_putorius_furo

MAFAGLERVH

>Rousettus_aegyptiacus

MAFAGLERVH

>Erinaceus_europaeus

MAFAGLERVH

HG1054 ENSG00000120832 MTERF2

>Homo_sapiens

MGEGTDGLVGAAENCRELLETEDRNPGSRPRGLRTVGRLAGERGKDGAWPPGLPVPCLELTPTVYCRELTRSHFEKKLERIPSG

>Panthera_tigris

--MTRSHFENLER-SSSD

>Dasypus_novemcinctus

-----------------ML-------ERRPSGLTQA---------------------------LDEDQISRERLQGSLSTF---

>Oryctolagus_cuniculus

--MTRSHF----------

HG1055 ENSG00000122299 ZC3H7A

>Homo_sapiens

---MSLIAHLESGCVG

>Miniopterus_natalensis

---MCVLSLQIAHLESGCVG

>Galeopterus_variegatus

---MLMIAHLESGCAG

>Chinchilla_lanigera

MGSGCALPLGSREAEPLARGAPALASANTDTRSSGQGQPAQLDAGCLWTLRPSFPSIAHLESGCAG

HG1056 ENSG00000128271 ADORA2A

>Homo_sapiens

MMLLPEPLQRAWFQETQSPL

>Acinonyx_jubatus

--MLPEPLKRVWFLETQSWL

>Galeopterus_variegatus

MTPCPEPLVRVWFLETLSRL

>Equus_caballus

--MPREPLERAWFLETRSWL

HG1057 ENSG00000131097 HIGD1B

>Homo_sapiens

-----------MAQPPEMGYLGGTSFQVEAVLGHGERGS

>Erinaceus_europaeus

--------MELVVQPSETGYPGR-FFQVEAVLGID----

>Ictidomys_tridecemlineatus

-----MEEVALVAQTPETGYPGGTSFQEETVLSMDWAEV

>Tupaia_chinensis

MLGRRDGEMVLAAQPPEAGYLGRTSLQVQVMLGIARCS-

HG1058 ENSG00000131386 GALNT15

>Homo_sapiens

---------------MESLFFSQGENRGKGREAIQFEVPVNGLSEGN

>Myotis_davidii

---------MRHLSRMESLLFPKEKRGQTV-----------------

>Galeopterus_variegatus

MRRGKETHGTKHLSRMESLFFSQGENQGKGGGGKQFNLKSL------

>Marmota_marmota_marmota

MQRGKETHGTKHLSRMDSFFFPKEKNRVKGEQAIQFKVLVNGHSEGN

HG1059.1 ENSG00000132970 WASF3

>Homo_sapiens

MLLEGNFCNP

>Mustela_putorius_furo

MLPEGNFCNP

>Myotis_davidii

MLPEGNFCNP

>Equus_caballus

MLPEGNFCNP

HG1059.2 ENSG00000112290 WASF1

>Homo_sapiens

---------------MMNWNNDERKHIRSQHSRPAL----

>Galeopterus_variegatus

MPPSFDTLTPSLALAMMDWNNDERKAYPI-----------

>Equus_caballus

----------------MNWNNDEKKARLNIHNLSYSRLID

HG1060 ENSG00000134242 PTPN22

>Homo_sapiens

MLCSGGSSGFLEVSRP

>Acinonyx_jubatus

MLCSGGSSGFLVVSRP

>Orcinus_orca

MLCSGGSSGFSVLSRP

>Tupaia_chinensis

MLCSGSSSGFSAVSQP

HG1061 ENSG00000134802 SLC43A3

>Homo_sapiens

MCCLCVLWVLGVTFSLGDGIQNSSAGAPLLHLLPGSYWDPENHPLMVFAQA---------

>Balaenoptera_acutorostrata_s

---MVFTQA---------

>Marmota_marmota_marmota

---MGFAQAPKSWNYRAP

>Tupaia_chinensis

---MFFAQAENTQK----

HG1062 ENSG00000137337 MDC1

>Homo_sapiens

MSQRTPCLGGPRKGALALPD-TSDVYDTAPLSVAGSWRQTLWSRFGTARE---VGGGRASGSGL------------------SLGAAQSTCIILLAH----GGLQL

PSH---

>Mustela_putorius_furo

MSQRTPCLGGPRKVLPP---

>Loxodonta_africana

MSQQTPCLGGRRNGALAATDSSGDVYGTAPLSAARRWCRTLWSRFGNLRGKWEVGGETVVGCPFELPNRGALFCGRTEASHYSLNRSQPPCSLFELRGWGGGGLSL

LAETLPISVQQSCKGQQQEPTSGPVLFLHTHSRLPGRGRYWWRARGLKVEAVYFSLCHQRYLKNASGG

>Chinchilla_lanigera

MSQRTPCLGGPKKDALATSDAVHDIYGTALLAAASLGRQTLW--

HG1063 ENSG00000138162 TACC2

>Homo_sapiens
MELCEDAGVGAARR
>Odobenus_rosmarus_divergens
MELCEDAGVGAAWR
>Ceratotherium_simum_simum
MELCEDKGFGAAR-
>Manis_javanica
MELCEDAGVGAAR-

HG1064 ENSG00000139734 DIAPH3

>Homo_sapiens

MCWLRPRLQRLG-----

>Rousettus_aegyptiacus

MCWRRPRLQRLG-----

>Ceratotherium_simum_simum

MCWPRPRLQRLG-----

>Chinchilla_lanigera

MCWPRPRLQRLGWAGLG

HG1065 ENSG00000140030 GPR65

>Homo_sapiens

MQAQIYQAPQSTNTSPEEIWKERNFKRKYQSLCKQSLVYSCLHQSTVRFMKKNKLRTTLYVHLQMPQLMLVGCLSAFCDNEHMDFCLLNSVDPFSQLPGAWIF-TS

NC--------

>Mustela_putorius_furo

--MELLKISQRLNARKCPAVWVSFSS

RCKQIYPAPR

>Galeopterus_variegatus

---MEAKELSQLILEGSLSVFCDNEHMDLCSLNLADHFS-----------

>Oryctolagus_cuniculus

---MNTKGISQLIIQSSSSVFCDHDHMDVCSLNSTDHFS-----------

HG1066 ENSG00000140553 UNC45A

>Homo_sapiens
MGIQRR-SGPLLTGLSVGLLLLFSWVTLVQPTSLSALVHHPHLCQTTRDVHGRHYPGFFCPRLSDSPEEAYCCHLQAAGGSCCTRAEFEALYQVNLSALPPPPILR
GPGPLLVLGLYNLLVVTLMTVDLVHFCCGRGRSLGW--SHRRPPSGSSAASSLQVSAGTA
>Mustela_putorius_furo
MGFQRR-LGLLLTGLTVGLLLLFSWATLAQPTSLSALAHHPHLCQTTWDADGRRYPGFFCPRLSDTPEEAFCCHLQAAGGSCCTQAEFEALYQVNLSSLPPPPILR
GRGPLLALGLYTLLLVALMTADLVHFCRGRGGGRRR--RPSRLSSRSSAARPLQVPVGTD
>Pteropus_alecto
MGFPRR--------LPLRLLLLFGWMTLAQPASLSALAQYPHLCQTTRDADGRHYPGFFCPRLTDAPEEAYCCHLQAAGGSCCTRDEFEGLHQVNLSALPPPPILR
GPGPLLALSLYGLLLAVLVTADLVHFCRSRRRGGGW--GRRGPSFRSSAARPLQVSVGID
>Mesocricetus_auratus
MGLQRRSPGPLLTGLSVGLLLLFSQTNLAHPTDLSALAHHPNLCQTTRDAEGRHYPGFFCPRLSDTPEEAYCCHLQAAGRSCCTRDEFEALYQVNLSSLQPPPILR
GPGPLLALGLYSLLLMALMTADFVHFCRGRGRRESLNSRSRGLRFRGSAARPLRVPVGTD

HG1067 ENSG00000143340 FAM163A

>Homo_sapiens

MRLHARSRAVLGAPGRPRSLTCAAPAAGARKDALRPRGSRTRESLPERSCSAAGLMPLARRTPNCCLLRCSPRD

>Mustela_putorius_furo

---MPMAMTHPQPLSARVQPA-

>Balaenoptera_acutorostrata_s

---MTCPQLLSAQAQPA-

>Myotis_lucifugus

---MPLAMTQPQPLSAQAQPV-

HG1068 ENSG00000144791 LIMD1

>Homo_sapiens

MPFTLGRWKSLLVLGRT

>Myotis_davidii

MTLTLGRWRVLLVLGWT

>Manis_javanica

MPLTPGRWKALPVLGWT

>Microtus_ochrogaster

MTFTLGRWESLSALGWT

HG1069 ENSG00000146414 SHPRH

>Homo_sapiens

MFGGWRMRARDLLGL

>Mustela_putorius_furo

------MRARDLRGL

>Balaenoptera_acutorostrata_s

MFAGWRMRARDLRGH

>Manis_javanica

MLDGWRRRSRDLLGL

HG1070 ENSG00000147459 DOCK5

>Homo_sapiens
MAEASGARRSAGRRRPEPEEL
>Manis_javanica
MAGAPGARGSEARRRPAPQEL
>Loxodonta_africana
MAEEPGARSSAGRRRPAPEEL
>Heterocephalus_glaber
MAEEPGARKSA-RRRPQSQEL

HG1071 ENSG00000147573 TRIM55

>Homo_sapiens

-------------------MDTSAHRHVTAGC

>Oryctolagus_cuniculus

-------------------MDKSAHGHVTLGC

>Dipodomys_ordii

-------------------MDKSAHGHVTLGC

>Tupaia_chinensis

MLTPALCHLSGSILLSPAYMDKSAHRHVIPGC

HG1072 ENSG00000147874 HAUS6

>Homo_sapiens
MEGPPRPQADRREGS
>Felis_catus
MEGPPLPQAARREGA
>Myotis_lucifugus
MRGPPRPQAACREGA
>Galeopterus_variegatus
MDGPPRPQAARRGGS

HG1073 ENSG00000149187 CELF1

>Homo_sapiens

---MRRGK-DVIKCWRNTLIEAASSVAVWYGCRVLCDYTLMYSGSESL

RDLFCTLCIVGFGCLFV---

>Rousettus_aegyptiacus

---MRREKVFKCWRNTFTEAASS----CGCVVWLLS-----SVGPF

ASVF----------------

>Galeopterus_variegatus

MNSSLLINLSLNSSLFLCFTVSFTPLPHTPMSHNHACRVGNRTCQLSEDETVLCTYLSCNCGRSGEERSLNARETPPLRHLLPVAVWCGCTVLCDCSLMYSGSESL

RNLFHSVSIVGFGCVCLFRA

>Trichechus_manatus_latirostr

--MDSGSESL

RSLFCGLSIVGFGCMCLFRA

HG1074 ENSG00000150275 PCDH15

>Homo_sapiens

MRVDINASQKQEVEHVNESSWN

>Myotis_lucifugus

MRLDINASQNQEV---------

>Equus_przewalskii

----MNASQKREE---------

>Orycteropus_afer_afer

MSLDISASEKQEE---------

HG1075 ENSG00000150347 ARID5B

>Homo_sapiens
---MSNGAAGGGEL
>Mustela_putorius_furo
---MSNGAAGGGEL
>Rhinolophus_ferrumequinum
---MSNGAAGGGEL
>Chinchilla_lanigera
MKSPAPRGEVERQIPPCQSGSSSKALRAHPTHSVLVTACVYMSNGAAGGGEL

HG1076 ENSG00000150967 ABCB9

>Homo_sapiens

MSSAETA-PQTRACCAPPPRAHLVPGNRPGLALWMMMALPP

>Manis_javanica

MSCAEPAP---------------------------------

>Ictidomys_tridecemlineatus

MSCVEPAPWTT------------------------------

>Tupaia_chinensis

MSGAEPAPPTTRALCSPLPTAHLVPGKRSSPAPRTMTALPP

HG1077 ENSG00000151474 FRMD4A

>Homo_sapiens

MFLLSMLALPGAELASLLGEEK

>Myotis_brandtii

MFLGSILAPPGTKLASPLGEEK

>Oryctolagus_cuniculus

MFLVSMLALRGPEPASLLGEK-

>Fukomys_damarensis

MFPLSMLALWGAELASPPGEEE

HG1078 ENSG00000162607 USP1

>Homo_sapiens

MRAEGGAPRLRNTPYNW

>Tursiops_truncatus

MRAERGETRLRNTLYDW

>Oryctolagus_cuniculus

MRARGGEPRLRNSVYNW

>Orycteropus_afer_afer

MQAEGGERLLRNTPYNW

HG1079 ENSG00000162975 KCNF1

>Homo_sapiens
MPA--PSPAAGCMPPPPRRAPAGCCPL
>Odobenus_rosmarus_divergens
MPG--RSPAAGCMPPPPRRAPAACRPL
>Oryctolagus_cuniculus
------------MPPPPRRAPAGGRPL
>Peromyscus_maniculatus_baird
MPAGSPAAATGCMAPLRRAPACCRPL

HG1080 ENSG00000164168 TMEM184C

>Homo_sapiens

MVQAELRAIPR

>Balaenoptera_acutorostrata_s

MLQAELRAIPR

>Miniopterus_natalensis

MVQAELRAIPR

>Ochotona_princeps

MVQAELRGIPR

HG1081 ENSG00000165392 WRN

>Homo_sapiens

MPGLQALLVVALHSHPAE-EDLLDWIFSGFLSDIVLYLPMKTLFFGLCK

>Pteropus_alecto

MPGFQALLSWLSTRHPAE-EDPFNCLFSGFYN-----------------

>Oryctolagus_cuniculus

MPGLQALVLWRPTSPPAEGEVPLTWLFSGAF------------------

>Equus_asinus

MPGFRVLLPWGSTSHPAE-EDP---------------------------

HG1082 ENSG00000165434 PGM2L1

>Homo_sapiens
MPFSEVGTPGPW
>Pteropus_alecto
MPFSEKGASGPW
>Dipodomys_ordii
MPFSEEGAPGPW
>Tupaia_chinensis
MPFSEKGTSGPW

HG1083 ENSG00000165804 ZNF219

>Homo_sapiens

MSVRRTAIPGTM

>Leptonychotes_weddellii

MSVRRTPIPGTM

>Myotis_davidii

MSVRRTPIPGTM

>Mus_musculus

MNVRRTPIPGTM

HG1084 ENSG00000165923 AGBL2

>Homo_sapiens
MG----PAPWEEEAGRYAALPVSDAPLASA
>Balaenoptera_acutorostrata_s
MGPAGVLAPPEEEAGRQAALPNSSASPV--
>Rousettus_aegyptiacus
MGPAMVLAPREEKAGRHAALPNSSASPM--
>Dasypus_novemcinctus
MGPARVLAPREEEAGRHAALPSSSASPV--

HG1085 ENSG00000166532 RIMKLB

>Homo_sapiens

--MNTSRGWGKGTEQSQLFHTLGSRSSFYHLPLGSRHRDIN

>Pteropus_alecto

--MNPGRGWGKGTEQNQLSHTLGS--SLYHLPLGSRHRDLN

>Loxodonta_africana

--MNTGRGWGKRTEQIKLFHTVGSRSRFYHLPLGSRHRDLN

>Tupaia_chinensis

MDHEYCWGWGTGTERASCP-TLWETSS-FCHLLGSRHRDIH

HG1086 ENSG00000167377 ZNF23

>Homo_sapiens

MLRSGRNINLCASRVWTVCSQAPDIGS

>Felis_catus

MLKSYRDSYLYAFQVWTVCLLTPNIGS

>Galeopterus_variegatus

MLRSCRDFYLY--EVWTVCSLPPDMGS

>Equus_asinus

MLRSCRGFYLYAFHVWTVYSLALNRGS

HG1087 ENSG00000168675 LDLRAD4

>Homo_sapiens

MLPEAGPAERWTWTG

>Oryctolagus_cuniculus

MLPRAGLAERWTCTG

>Rattus_norvegicus

MLPKAGPAERWTWSG

>Tupaia_chinensis

MLLRAGPAE------

HG1088 ENSG00000169740 ZNF32

>Homo_sapiens

-----------MSRPGRPGP--RG-RPAASGEVGGPQS---RVPWTGERDAEVLRNSPWKNHMKRW

>Canis_lupus_familiaris

-------------------------------------M---LNSFQGLKDKRVFKNSPWKNHMKRW

>Ceratotherium_simum_simum

MLHQAIQISSIYLYPKLQLLLTLSFSLINSSALSCLFLLFCFVFVCLFFPPQVLKHSPWKKHMKRW

>Ictidomys_tridecemlineatus

---MQQVLRNSPWKNYMKRW

HG1089 ENSG00000169813 HNRNPF

>Homo_sapiens
MHLEFPRSPLRG
>Pteropus_alecto
MHLDFPRSPLRF
>Equus_asinus
MHLDFPRSPLRV
>Rattus_norvegicus
MHLDFPRSPLRF

HG1090.1 ENSG00000170458 CD14

>Homo_sapiens

MPCRILPVTVPLPETSFIAIFPGKEGGWLGGREVGR------------------------

>Orcinus_orca

--------MAPLLAKSFTAKFPRTADGVVGRKKCGEWQGSKRRERSDIPGLHKLSEAARG

>Rattus_norvegicus

MPLRILPVSVPPCKTFHCNIY---------------------------------------

>Tupaia_chinensis

MPCGILPVVAPLPETFFIAIFPRREVKGAAGRKRGVCVCV--------------------

HG1090.2 ENSG00000170458 CD14

>Homo_sapiens

MRFTISSTKHAVKYLRIFRDLDLVAGDQHKPRQ-GRSQRNESSRFSGI---------

>Felis_catus

MKFAIPSTKYPIIYLRIFR--------------------------------------

>Orcinus_orca

MKFAISSTKYPVKYLRIFRELDLGDRRLTVITKTREN--------------------

>Manis_javanica

MKFAISSTKYPVKYLRIFGRLDLGEGRLIVTTKTRESQGNEVGRVSGMQAGKVRVLA

HG1091 ENSG00000172183 ISG20

>Homo_sapiens

----------------------MRHLRRGRAGAKEGHPDMEPASSVSPDSARS---

>Orcinus_orca

----------------------------------MGHTDKEPASLVNSDSGH--

>Myotis_lucifugus

-----------------------------MGGAEAGQRDMEPVRSASPDSGAC-------------RPSCISRQEMATCSLENEPETEAAPGQRGQDSPLEVRGDS

PLTATDLGS

>Ceratotherium_simum_simum

MFILQAGRRVRKERKMLQSTPSTASSEEGRAGAKAGHPDMEPASFVSPDSGHRALHLPAAPGDGAAQPTCLAFRP-------------------------------

----

HG1092 ENSG00000173327 MAP3K11

>Homo_sapiens

--------------------------------------MVRRRRPRGPTPPA
>Ailuropoda_melanoleuca

--------------------------------------MVRRRRPWGPTPRA
>Myotis_lucifugus

--------------------------------------MVRRQRPRGPTPRA
>Cavia_porcellus
MGVLGILVLLSLLARLPGNSPAQPLEPPPGGSVASLPRHLMLFPAGGRRAEKSETPPSTSASRPRGQSTKGGGRRERGRSCRAKAPGPGRDSFSLTGDTLHTLDKD
PQESRHYPWQPHLTLDWCHGQPPRTYFGTQGRLGPSNAAMVRRRRPQGPTPPA

HG1093 ENSG00000174371 EXO1

>Homo_sapiens

MYTVILTIALSHSYYAKEEIIIRGYLPGPEETYVNFMNYYIRFPRSERKLFLDII--------------

>Panthera_tigris

---------------------MRAYLPSLEETSASQNVCYVSFPQSGRKLFLEALSERWEADCFSQTAA

>Dasypus_novemcinctus

----MFHMALSRSYCAEEKVFMGGYLPSRKETSEFHEMYILSVVLRVK--------GSW----------

>Fukomys_damarensis

----MCRMAHSHSCWANEEMLIRGHLPGLQETYQNFESVFLTVMVDG---FLPSVA-------------

HG1094 ENSG00000177917 ARL6IP6

>Homo_sapiens

MGEPGEECERSGEEGRRQIGSGMCLRGRERALGPGVEKMRLWGCRGARSRW---------------------------

>Panthera_tigris

--------------------------------------MQLWGLPGRAVA----------------------------

>Myotis_davidii

----------------------MLIEVHERALGPGIGKMQLGGPTVPSLAWR--------------------------

>Marmota_marmota_marmota

--------------------------------------MRLWGCRGSRSPGGNAAVPEVFGRDETLQGRVTCLQPASA

HG1095 ENSG00000179921 GPBAR1

>Homo_sapiens

MHLVHLCSSARTPDMVQTAAGLAAATP

>Ailuropoda_melanoleuca

-----MPSPARTPDSARSPAGLPAAVP

>Ceratotherium_simum_simum

-----MLSPARTPDVFRSPAGLAAATP

>Manis_javanica

-----MPSPARTPDIARSPAGLAAATP

HG1096 ENSG00000181544 FANCB

>Homo_sapiens

--MDTVSTW-GLRYVAAALRFRNINWNQFAFEN---------

>Odobenus_rosmarus_divergens

--MIL---IVPDLCIYCLTTNQFKSISWNQFAFENWTNTKT---

>Rousettus_aegyptiacus

MEKKRNMFLKNFLDAILIAFCMGIPTFIVLCFIVLHRCYFILFFYKVKARPITSKKITAGFVVILTLLWRSGTEPALSLRFKIIEWNQFAFENWTNTKT---

>Cavia_porcellus

---MLEERIKSISWNQFAFENWMNTKDLIL

HG1097 ENSG00000182481 KPNA2

>Homo_sapiens

MPSSFFFFLSPLPLAPKPYDSLPSQRVFQIHQWAHSLGSNQPIGMRSHREI

>Ursus_maritimus

--MRGHREI

>Equus_asinus

--MRSHREI

>Fukomys_damarensis

--MRSHREI

HG1098 ENSG00000183775 KCTD16

>Homo_sapiens

MKSCARKLWFEHGQFSPTVSYIHKHHMKWRSGSSVYFSQVPQ

>Odobenus_rosmarus_divergens

MENFARKFWLEHG-----------------------------

>Pteropus_alecto

MGSCARKLWFEHG-----------------------------

>Ochotona_princeps

MGNCARKLWFKRGQFSPTFSHIHKHQMKWRFGSSVYIYHIPQ

HG1099 ENSG00000186334 SLC36A3

>Homo_sapiens

MVSSSSQSHCSLCPTAARAKLGEAKSPGKSWLGKQLRVKLRTILTEMGLS---------

>Mustela_putorius_furo

MVTSSSQSHSSLCPIALRAVLGGTGSPGESWRVANQMVRGPEGHNEMRLSKVPQKLSEA

>Galeopterus_variegatus

MVPSSAQSHCSLCPRAVRAESAEAESPGKSWQVAEQL----------------------

>Oryctolagus_cuniculus

MVPSSSQSHCSLGPTKARAALGEAKRPGESWQVAAGPRVSRGSPSPGPLEPGEAP----

HG1100 ENSG00000187815 ZFP69

>Homo_sapiens

----------------------MNSNLTNWLPPKESA------------------

>Felis_catus

MCGQCFWIPPLLSFAPLEDCRGMHSRLLNWLPPKEALLNLREKSVFQSC------

>Miniopterus_natalensis

----------------------MHSNLCNRLPPKEAVLNLRQESVFQTHPSHISL

>Galeopterus_variegatus

MTG---ASGSLLSFVPLEENIDMYSNLPNWLPPKETVLNLRGESVFQSC------

HG1101 ENSG00000189334 S100A14

>Homo_sapiens
MSHQLLWGQL
>Manis_javanica
MSHQLLWGRL
>Nannospalax_galili
MSHQLLWGRL
>Tupaia_chinensis
MSHQLLWGRL

HG1102 ENSG00000196704 AMZ2

>Homo_sapiens
-----MKSHSTTFFQPGPDMSVLVS
>Galeopterus_variegatus
-------------------MPVLVR
>Ceratotherium_simum_simum
-------------------MPVLVR
>Tupaia_chinensis
MFCVRVKTPSTTRCQPDPNMPVLVR

HG1103 ENSG00000197620 CXorf40A

>Homo_sapiens

MLGCKNAAQHGEMIFCPAGWCTLWSLTQTGAEGPTAVISRTKL--------------------

>Panthera_tigris

------------MVFSAAGWGLMWLAEPTRE--------------------------------

>Rousettus_aegyptiacus

------------MIFCTARWAIMWSLTQPGKKRASAMISRTKL--------------------

>Equus_caballus

------------MCCAAGCWGVMWSHSPPGPERPTAVMSRAKACVRGFPPVGPSRVLLASSPC

HG1104 ENSG00000205155 PSENEN

>Homo_sapiens

--MR-AVLPRPKKTTISRETWGVSRKRP------------

>Leptonychotes_weddellii

----------------------MSRKRPELQVAKASKRRK

>Oryctolagus_cuniculus

--MRTALPPRPEETTNSRATCGLSRRRP------------

>Ictidomys_tridecemlineatus

MRMRTAFPLQKERTTNSSETCGTSRKRP------------

HG1105 ENSG00000213654 GPSM3

>Homo_sapiens

MRPPGVLPHHEPPPLLQLEEFFPVSVLPWGERD----------------

>Rousettus_aegyptiacus

MRRPRVLPHLETPPLLLE-EILPV-FTPLGERQRSNKQFGWEKRTGG-S

>Equus_caballus

-----------MN--PLHDCSFSQSVLPWGERN----------------

>Tupaia_chinensis

-------------------------MLPWGERGQPS----WVGKENGGS

HG1106 ENSG00000215021 PHB2

>Homo_sapiens

MEMTCCLALPS--LGGQPSRKGRDFRMRDSCARSSGP-------

>Odobenus_rosmarus_divergens

--------------------------MRNSCARSSGPLWAPGRV

>Equus_asinus

--MTCCLALPKSLWEGRVPKKGGNFRMRNSCARSSGPQWPEGRV

>Marmota_monax

--------------------------MRDSCARSSGPL------

HG1107 ENSG00000217930 PAM16

>Homo_sapiens

--MRMPSAVSRTRSLRLSLTSDPPTTLDPRPRGQEVGV-----

>Felis_catus

----MLCAASRTRSLP---------------------------

>Myotis_brandtii

----MPSVASRTRSP----------------------------

>Cavia_porcellus

MTMRMPSTPWRTRSLLSFLTSDLPAALERGPRVQEAQARGPDA

HG1108 ENSG00000251247 ZNF345

>Homo_sapiens

MAVEHPQGTCKTSNFTLELSRNMACLFPQISLGLQTKGWNFEPISRLLVTVPEERQSYGYYESKLRPRNYF

>Felis_catus

MAVENPQGSCKTVNQG---

>Pteropus_alecto

MAVEHPQGTCKNVNQG---

>Equus_asinus

MAVEHPQGTCKTVTQG---

HG1109 ENSG00000278311 GGNBP2

>Homo_sapiens

MGNAARLSRTLPLRS--LPPVANVLRSGGCLRSRGLAVAGRSWEEAAAAAAET---AAAAAAAAGRRW

>Rousettus_aegyptiacus

MGNAARLFRPLLLLS--LPPVANVLRGGGCLSSRRLAVAGRSWEEAAAAAAAAAGAAAAAAAAAGRRW

>Equus_asinus

MGNAARLSRPLLLLS--LPPVANVLRGGGCLRSRGLAVAGRSWEEAAAAAAGAAE-AAAAAAAAGRRW

>Microtus_ochrogaster

MGNAPRLSRPLLLRAPPFSPVANVLRGGGCLRSRGLAVAGRSWEEAAAAG-----AAAAAAAAAGKRW

HG1110 ENSG00000034152 MAP2K3

>Homo_sapiens
MSVLGPGGARSPLD
>Tursiops_truncatus
MSVLRPGRARRPLD
>Ctenomys_sociabilis
MSTLGPGGARRPLD

HG1111 ENSG00000040531 CTNS

>Homo_sapiens

MEGGLSFAHERSREALAAPRVSVSLAADLIFPHAGAPISAPRPDPAALCP---------SKTRGNYNSQSSSRRDPAPRVRWRRSGDSGPASPKV-----------
----

>Pteropus_vampyrus

-------------------------MACLILPHTRASIPA--KVPAPLRSS-STLHNCPAKPRENYSSHSASLRDSKS-------GDSGQMASGLRLSLVKPAGEN
GYSLKRLLHLVWSVSRASEVLSCRLNLSRGVCRLLWRGETVKAPFQELPLAC

>Ctenomys_sociabilis

---------------------------------------MSDKPPGPLCPPCTTIRLSLVKTRKNYNCQGPSRRDLASTSQVTRVR--------------------
----

HG1112 ENSG00000068885 IFT80

>Homo_sapiens

MVTG-RY-------QVFESNRHRFPGT

>Miniopterus_natalensis

MVTGGREASW-----------------

>Microtus_ochrogaster

MVTG-RSGGFLVSGQSSESNRRRFPGT

HG1113 ENSG00000072201 LNX1

>Homo_sapiens
MPSSFLQAPVHRS
>Peromyscus_maniculatus_baird
MPSFFLQPPVHRS
>Tupaia_chinensis
MPSSFLQAPVHRS

HG1114 ENSG00000073737 DHRS9

>Homo_sapiens
MGVTHRARREPEDSSHM
>Dasypus_novemcinctus
MGVTHSARREREDSSHM
>Jaculus_jaculus
MGVTHGVKRELQESSHV

HG1115 ENSG00000074219 TEAD2

>Homo_sapiens

MAGRPCPPACGSVGLSWSRPQLLP

>Odobenus_rosmarus_divergens

MAGRPCPPVCGSVGFSWSGLEFLP

>Physeter_catodon

MAGRPCPPVCGSVGLSWSWLEFVP

HG1116 ENSG00000084090 STARD7

>Homo_sapiens

MSGGSWPRDRETGSWPAI

>Oryctolagus_cuniculus

MRGGSWPRDRETLSGPAS

>Mesocricetus_auratus

MRGGSWPREREAGAGSAS

HG1117 ENSG00000089902 RCOR1

>Homo_sapiens
MRAKVALGSSLGA
>Oryctolagus_cuniculus
MRAKVALGSAPGA
>Manis_javanica
MRAKVALGTALGA

HG1118 ENSG00000090975 PITPNM2

>Homo_sapiens

MESMVPTPQLVSMLFPESPTIHLLSF

>Canis_lupus_familiaris

MEFTEPTPQLVPVLFPESPTTCPLSF

>Mus_musculus

MESKEPTPPLLSVWFPESPTTYPLSF

HG1119 ENSG00000092020 PPP2R3C

>Homo_sapiens

MKRNCERRR----FPQAGT-NELLGHVVRTRQRHTIKENAGLSWPLKSGLPRLPPSIKMVVGRPS

>Odobenus_rosmarus_divergens

MKRNCEAKKKKKSLPRAGNAIGLLNHVVHTRQSPKLKK---------------------------

>Ceratotherium_simum_simum

MKRNCEGRK---FFPRAGKA---

HG1120 ENSG00000095585 BLNK

>Homo_sapiens
MLLPRRARNC
>Dasypus_novemcinctus
MLLPRRARNC
>Marmota_monax
MLLPRRARNC

HG1121 ENSG00000100350 FOXRED2

>Homo_sapiens

MEGLGIPG-SPKSQLETVESGQMSSE-DGFWAWP-----

>Balaenoptera_acutorostrata_s

-----------------MESGQMSSEVLGFWSWLRPKSQ

>Equus_asinus

MEGFRIPGGSPRSELETMESGPMSREGFGSWSWLCPK--

HG1122 ENSG00000100410 PHF5A

>Homo_sapiens

---------------MGVKGRTEVPEASGGRLS

>Acinonyx_jubatus

---------------MEVKGRTEVP--------

>Ctenomys_sociabilis

MEASRTNHRDLEAGAIGVKGRTEVTQASGSPLG

HG1123 ENSG00000100884 CPNE6

>Homo_sapiens

MCEPVSRGCMCRHHFSV----------------------

>Manis_javanica

MRKPVSHGYMCLHHFLV----------------------

>Ctenomys_sociabilis

MCEPVSCGCMCRHHFCVDVCVHEPLQCVYLSVMPGVGAV

HG1124 ENSG00000100934 SEC23A

>Homo_sapiens

MLAGRRSSLRPGAGWRHP

>Oryctolagus_cuniculus

MLAGPRSSRRPGAGRCRP

>Tupaia_chinensis

MVAGLRSSQRPGAGRRGP

HG1125 ENSG00000101574 METTL4

>Homo_sapiens

MNDKFSSMCMSGFCTCISNLSVCMKCMFIEIQDI

>Felis_catus

MNVKFSFV--------------------------

>Dasypus_novemcinctus

MNFKFSSGYI------------------------

HG1126 ENSG00000101890 GUCY2F

>Homo_sapiens
MEFVNRLESLTGL
>Equus_przewalskii
MEFINRLQSLAGL
>Octodon_degus
MEFLNRLESLTGL

HG1127 ENSG00000103064 SLC7A6

>Homo_sapiens

MGGRAGSGAGAGPRSVGAEPCGSSLPPSGAGCGPETARGWPIGAAREALRSLCGRGRQVELGLAAGDFISFSVLDPFMRRWLMKV

>Galeopterus_variegatus

---MMRWLMKV

>Loxodonta_africana

---MNGIRSCCGDFIFFSVPDPFMMRRLMKV

HG1128 ENSG00000103852 TTC23

>Homo_sapiens

MVINLYKESPN------------

>Rousettus_aegyptiacus

MLINLFKESPNRYSGNQSVKSGC

>Fukomys_damarensis

MVINLYKESPN------------

HG1129 ENSG00000104432 IL7

>Homo_sapiens
MLMIPKIEPADQAQSRN
>Oryctolagus_cuniculus
--MNPKIEPAYQAQTRN
>Manis_javanica
--MNPKIEPADQAQSRN

HG1130 ENSG00000105559 PLEKHA4

>Homo_sapiens

MSERETGRDRDQRETQKVRVGREVV

>Equus_asinus

MSDRETGRDRD--------------

>Manis_javanica

MSDRETGRDRD--------------

HG1131 ENSG00000106780 MEGF9

>Homo_sapiens
MVRPVAVAAAVAEASLVGLQRGEDD
>Manis_javanica
MVRPVAVAA--ATASLVRVQRGEDD
>Microtus_ochrogaster
MVRPVAAAAAAATASLVGRQGGEDD

HG1132 ENSG00000109771 LRP2BP

>Homo_sapiens

MKTLIYHSTVIHLITWQSNTDRKIKLN---NS-----

>Rousettus_aegyptiacus

MKALIFHSPVIHLITWQSNTDRKIKPN---KIKCIPR

>Cricetulus_griseus

MKALTYHTVVIHLVTWQSNTDWKRKLSTTAKIQCIPR

HG1133 ENSG00000111328 CDK2AP1

>Homo_sapiens

-----MSSIGYSNPGAWPDLGVWNDLRA

>Manis_javanica

-----MSRTGCSYSGK------------

>Tupaia_chinensis

MDLKSMSGIGCSRSGAWPDPSIWNDLRA

HG1134 ENSG00000112984 KIF20A

>Homo_sapiens

----MSFFPLRQSKHPKPVTLCTPVKIVAKEGQELAAEASR-FPLGS----

>Ceratotherium_simum_simum

MAAEASGFPLGFLEK-----KYLGFK--KKC--------------------

>Chinchilla_lanigera

MAARASVFPLGFLVN-----KCLGFK--EKRWEESRAKQSKLATLGIADTR

HG1135 ENSG00000113597 TRAPPC13

>Homo_sapiens

MENKVFYAYVSKIEEMDLLKRSQFVKDTVGEFKVPPPERG

>Oryctolagus_cuniculus

---------------MGLVKRSRWVRKGRSERRI------

>Ceratotherium_simum_simum

---------------MALVKRSCWMRR-------------

HG1136 ENSG00000113749 HRH2

>Homo_sapiens
MGAGTSYGEGYSCVST
>Myotis_lucifugus
MGAAVSYGEGCSHVST
>Manis_javanica
MGAAASYGEGHRHVHT

HG1137 ENSG00000115307 AUP1

>Homo_sapiens
MPGIVVSAGREL
>Pteropus_vampyrus
MPGIVVSAGREL
>Galeopterus_variegatus
MPGIVVPAGREF

HG1138 ENSG00000116871 MAP7D1

>Homo_sapiens
MRRGTPVLATGRRRRRR
>Elephantulus_edwardii
MRRGTPVLAAGRRRRCR
>Cavia_porcellus
MRRGTPVLASGRRRRRR

HG1139.1 ENSG00000118308 LRMP

>Homo_sapiens
MQTQNCRSSGEAYHFPEKKNTFSVLPAS
>Canis_lupus_familiaris
MQTQNCRSSVEAYHFPEKKSTFSVSYA-
>Pteropus_vampyrus
MQTQNCRSSVEAYHFPEKKSTFSVSCA-
>Ceratotherium_simum_simum
MQTQNCRSAVEAYHFPEKKSTFSVSCA-
>Manis_javanica
MQTRNCRSAVEAYHFPEKKSTFSVSSA-
>Loxodonta_africana
MQTQNCRSSVEAYHFPEKKSTFSVSCA-

HG1139.2 ENSG00000118308 LRMP

>Homo_sapiens
MKEATLQKEAT
>Equus_asinus
MKEATLQKEAT
>Loxodonta_africana
MKEATLQKEAT

HG1140 ENSG00000118495 PLAGL1

>Homo_sapiens

MLPLPWSGVQLQGLC

>Tupaia_chinensis

MLPLPWSGAQLQ---

>Trichechus_manatus_latirostr

MLPLPWSGVELQWLG

HG1141 ENSG00000121964 GTDC1

>Homo_sapiens
MHREPKSVTEIVWVSLH--------
>Equus_przewalskii
MHREPKSVTEIVWVRGVCMYSRI--
>Chinchilla_lanigera
MHREPKSVTEIVCV--VCLLSPVMH

HG1142 ENSG00000124613 ZNF391

>Homo_sapiens
-----------MLDLQEAEEGSAQKYLS-
>Odobenus_rosmarus_divergens
MVIQSEGGQLWMLDLQGTEEGSEQKYLSG
>Galeopterus_variegatus
-----------MLDLQGAEEGIAPKHLSR

HG1143 ENSG00000125245 GPR18

>Homo_sapiens
MPSFQGELMLSVTS
>Rousettus_aegyptiacus
MPSFQVELMLSMTS
>Galeopterus_variegatus
MPSFQGELLLSMIS

HG1144 ENSG00000128203 ASPHD2

>Homo_sapiens

MPPPAPAAPSPPR

>Ailuropoda_melanoleuca

MPPPAPAAPSPPR

>Rhinolophus_ferrumequinum

MPPPAPAAPSPPR

HG1145 ENSG00000128585 MKLN1

>Homo_sapiens

--------MLENMGIGIRFWRRNVS---------

>Rousettus_aegyptiacus

MIGFITHSILENMGIGIRYVQRAEGAEVNQRMHR

>Ceratotherium_simum_simum

------------MGIGIRYVQRAEGAEVNHRMHC

HG1146 ENSG00000130413 STK33

>Homo_sapiens

MVISFLSAALTCEYVSSASLT

>Dasypus_novemcinctus

----------MCEYESSASLT

>Oryctolagus_cuniculus

----------MCEYKSSASLT

HG1147 ENSG00000131781 FMO5

>Homo_sapiens

---------------------------MPRPRSSLKNVNRCVLFVFCHPFFRTGDTNR

>Rhinolophus_ferrumequinum

MRSNSLLLRRQQNLASPKAPAVAGFIRMLWPRSSLKDVN-------------------

>Ceratotherium_simum_simum

---------------------------MLWPRSSLKSVN-------------------

HG1148 ENSG00000132938 MTUS2

>Homo_sapiens
MAVKLIFWGIGSHGLIGSHSAGT
>Mustela_putorius_furo
MAVKLLFWGSRSYGLIGSHSAGT
>Myotis_davidii
MSVKLVFWGSRSHGLIGSCSASP

HG1149 ENSG00000132975 GPR12

>Homo_sapiens

----------------MILDCHPAVFKH

>Canis_lupus_familiaris

MVVSEWGLASVCVSFCMILDCHPAVSKH

>Galeopterus_variegatus

----------------MILDCHPAVCKH

HG1150 ENSG00000135299 ANKRD6

>Homo_sapiens

MQTSFLCHLSVIQKCSSDL

>Leptonychotes_weddellii

MQTSSLCHLSIIQKHSAHL

>Tupaia_chinensis

MQTSLLCHPSVIQKHSSGL

HG1151 ENSG00000137168 PPIL1

>Homo_sapiens

MKFAFSSVLEPSFFSSPTPASRHWKKGPSSSLSWPPKSWYRKG

>Mustela_putorius_furo

MELALFSVLAISFFSSRIPASRPWKGDPSSSLSQSPKSWHRKG

>Chinchilla_lanigera

MNLAFSSLLALSFF-----------------------------

HG1152 ENSG00000138658 ZGRF1

>Homo_sapiens
MTSRCFAGEIRKWILCNLTGGINS
>Ursus_maritimus
MTSRSFAGEIRKWILWT--GHINN
>Chinchilla_lanigera
MTSRSFAGEIRKWMLCS--GGINT

HG1153 ENSG00000138738 PRDM5

>Homo_sapiens

--MCRLPGAKRRC

>Canis_lupus_familiaris

MSMCRLPGAERWC

>Galeopterus_variegatus

--MCRLPGAERRC

HG1154 ENSG00000139725 RHOF

>Homo_sapiens

MAKPKDSQLEDWRRLFKKIWIFPYDLESIFVLYN

>Balaenoptera_acutorostrata_s

MAKLKDSQLEE-----------------------

>Nannospalax_galili

MAKLKDSQLEE-----------------------

HG1155 ENSG00000141668 CBLN2

>Homo_sapiens

MKVVTPFNIKTWRELGKEKKESKRREATGAFKGV-----

>Erinaceus_europaeus

---MKVLKKESWREFGKEKEGSPSRGASGASRGA-----

>Cavia_porcellus

MKVVTPFKIKTWREFGKEKKESKRRDAAGAFEGLLAPSS

HG1156 ENSG00000144741 SLC25A26

>Homo_sapiens

MAAPSARGRDPLLLRLGL--

>Myotis_brandtii

MAAPSARGRDVLRLDSGL--

>Equus_asinus

MAAPSAWGRDPALLSLELYP

HG1157 ENSG00000148123 PLPPR1

>Homo_sapiens
MYLAPGSRTAQ
>Canis_lupus_familiaris
MHLAPGSRTAQ
>Pteropus_alecto
MHLAPGSRTAQ

HG1158 ENSG00000149571 KIRREL3

>Homo_sapiens

MVGANPRGPA-EAEHREPPRKRN

>Cavia_porcellus

MVGANQRGPAAEAEHREPPRKRN

>Trichechus_manatus_latirostr

MVGASQRGPAAALEESEPGRQRN

HG1159 ENSG00000152822 GRM1

>Homo_sapiens

----------MSSKQTQHLAHRCQHDFHCTLDQFTLMHYR

>Myotis_lucifugus

---MCTLSPRASTTDSFQLTAGCHHDAHCPLDPFTLVHR-

>Heterocephalus_glaber

MPGSSDLGTTALSTPTQRPAHRCQHDVQCTLDQFTLIHL-

HG1160 ENSG00000154162 CDH12

>Homo_sapiens

------------MMCQRHAVFCF--EGGEMTGGNPGPPLARRSPLQLSSSRSESIIDLKGKCGGSHREGSRLF

>Rousettus_aegyptiacus

-------------MCQRHAVFCF--EGGEMTGRESWAPIGWPLSAAVALQ------PL---------------

>Mus_musculus

MHFKYRIQLLSTTQRRVKGTLCFVLRAGR--

HG1161 ENSG00000154227 CERS3

>Homo_sapiens

MHRCGDPPPGLADPPRPRTEEEVVKQEATSHQEEPEGAIQ

>Equus_caballus

-------------MEGGRAAAEVV-KQETSHQEEPEGAIQ

>Fukomys_damarensis

------MPRGLKEESVWTQEDKVVKQEATSHQEEPESAIQ

HG1162 ENSG00000155380 SLC16A1

>Homo_sapiens

MQVFCSKYLDLTCILEFIYT

>Odobenus_rosmarus_divergens

MHSFCSKYLDLNCILEIIYT

>Ceratotherium_simum_simum

MHAVCSKYLDLNCILEFIYT

HG1163 ENSG00000157637 SLC38A10

>Homo_sapiens
MALRRPQGLAGSGG
>Odobenus_rosmarus_divergens
--MRQPQGLAGSGG
>Ctenomys_sociabilis
MAMRRPHGLAGSGG

HG1164 ENSG00000162639 HENMT1

>Homo_sapiens

MASGHAPQRPLRDAKPTPWN--------------------

>Dasypus_novemcinctus

MASGHAPPRPLGDAKPTARAGAQD----------------

>Peromyscus_maniculatus_baird

MSSGHAPQRPLRDPEPTPWSRQPGGMGGPEHGRAPHAQRGP

HG1165 ENSG00000162694 EXTL2

>Homo_sapiens

----MESPLTPTRPPTTSVPGCMGSSACPFAISSQLALNPGVVDISD

>Acinonyx_jubatus

MAGWLRACSRPPRPPTTDVPGCAGVQRLPFHHCPLLTKSRCFR----

>Pteropus_alecto

-----------------MFPDTLASSACPFAISP-ITINPGASGSKA

HG1166 ENSG00000165071 TMEM71

>Homo_sapiens

------------------------------MCVGI--CEKKEASYFSNNFVLCQLEEEAGNSLWKEHRICEV-----------------

>Panthera_tigris

MPFYSRKQPKQQEAAEDKTSLHTPAHADSLLNPGFLHRQPQEASYFSN-SPLHQWEEETGNSLWKEHKICEV-----------------

>Heterocephalus_glaber

-------------------------MPFQQLSPQFHHCQKQEALYSSKCFAIYQLEEETGNSLWKDYTYGQIIFHNTCFALMEQKICEV

HG1167 ENSG00000165934 CPSF2

>Homo_sapiens

--MGF----KLPAVLRQPINKELFRIPGVSSYIRLRYQLF

>Odobenus_rosmarus_divergens

MRLGVGKGSGRRWGRTDEIYKELFP---------------

>Pteropus_alecto

-----------MRGNCSQIHKELFL---------------

HG1168 ENSG00000166669 ATF7IP2

>Homo_sapiens

MTCDITICKTNGEDQDKDKLYHRKAIENAKMKHLNEGLITLNSAFVNLICRYVQTTS--

>Oryctolagus_cuniculus

------------------------------MKHLNEGLITLNSAFVKLSLHFAVLTLLV

>Equus_caballus

---------MGKNIQDKDNLYHRKAIENAKVKHLYEGLITLNSAFVKI-----------

HG1169 ENSG00000167637 ZNF283

>Homo_sapiens

MTARKKSMLSPIMNVSSKKDENFRSGSVRGTGSCQDLMGKF

>Physeter_catodon

------------MYVSSKKVQI-------------------

>Rousettus_aegyptiacus

------------MNVLRKKAQV-------------------

HG1170 ENSG00000167858 TEKT1

>Homo_sapiens

METALFTSLFCKSWKLQLG---------

>Myotis_lucifugus

METSLFTFLFCESRELQLGLGGSYSELV

>Equus_asinus

METALFTSQFCKSSGLPWEG-GSPPELV

HG1171 ENSG00000167968 DNASE1L2

>Homo_sapiens

MLGRECGWSYLRCLAFPEGILLREAESRRPNF

>Myotis_davidii

------MWSRNSGRCGAKEGLS----------

>Cavia_porcellus

MLGQEWGWSFLTA-CLPKRIWLKEADSR----

HG1172 ENSG00000170381 SEMA3E

>Homo_sapiens

MNSFAQVTEKYSFFLNLPA

>Rousettus_aegyptiacus

MNRLQK-------------

>Loxodonta_africana

MNRLPK-------------

HG1173 ENSG00000170464 DNAJC18

>Homo_sapiens

MGQHLNHIGHRTDLSITSKNPLQFNM--

>Odobenus_rosmarus_divergens

MGQYLNHIGHKANLSITSKNSLQFNKYF

>Balaenoptera_acutorostrata_s

MGQHLNHIGHMANLSITSKNSLQFNKFF

HG1174 ENSG00000173918 C1QTNF1

>Homo_sapiens

MGGHSRQGKKPRFRFWSQRPRRPRRGDGGAVGGGDLWLLVPPPTLLFCTAVLRKTFSPALFPSPSLCIAPDL--AGRRLGRREML---------------------

----

>Mustela_putorius_furo

------------MCAGSVGGSAPCSH------------L---------AGVCSLRVCPAFLPLPLLSRNRASASGQRDLGPEEVTVPGRQGPAAACTRPLCTFFPE

DFFCPPGSLL

>Ceratotherium_simum_simum

-------------MRWERSRLRGRSH------------L---------AGVCSLRVCPAFFVSSFTFEKP-----------------------------RFRFWPK

RL--------

HG1175 ENSG00000174473 GALNTL6

>Homo_sapiens
MVLWVRSVARKEW
>Odobenus_rosmarus_divergens
MFLWVRSIAGKEW
>Marmota_marmota_marmota
MFLWVRSVAGKEW

HG1176 ENSG00000176472 ZNF575

>Homo_sapiens

MKFNGKKGAVDAASMN

>Ailuropoda_melanoleuca

MKFSNKKEAVHAASMD

>Heterocephalus_glaber

MKFNDRKDAVDAAVID

HG1177 ENSG00000178295 GEN1

>Homo_sapiens

MTKGLLPEPRRKGCPVCFT

>Acinonyx_jubatus

MTEGRLPKPRRKGCPVF--

>Equus_asinus

MTEGRLPKPRGEGCPVCFK

HG1178 ENSG00000182670 TTC3

>Homo_sapiens
----------------MCMRNITCTSNL
>Canis_lupus_familiaris
MRYLKWHVDFLIVKNLMCMKNVSCTSNL
>Pteropus_alecto
----------------MCMKNINCTSNL

HG1179 ENSG00000185933 CALHM1

>Homo_sapiens

MRWAPSGQGGQQGPSWRQQ

>Ceratotherium_simum_simum

MRWAPSVRSGPQGPSLRQW

>Tupaia_chinensis

MGWAPSGQSGPQGPWWRRQ

HG1180 ENSG00000188674

>Homo_sapiens

MLQHASRSRERTKALEPVSGCL--------------

>Acinonyx_jubatus

MLQHASRSRERTKAPESVSGCL--------------

>Rousettus_aegyptiacus

MLKHASRSRERTKALESGSAYRLREAGCSHRISGAV

HG1181 ENSG00000189058 APOD

>Homo_sapiens

MDTTSHLCVSIQTKLLGQPS

>Ursus_maritimus

MDKTSHLCVWYRAKLLGQPS

>Pteropus_vampyrus

MDKTFHLCVLYRAKLLGQPS

HG1182 ENSG00000196155 PLEKHG4

>Homo_sapiens
MEQDNSKAPLWQPDLLMKKAPR---
>Panthera_tigris
----------------MKKAPRNLV
>Loxodonta_africana
----------------MKKAPR---

HG1183 ENSG00000198133 TMEM229B

>Homo_sapiens

-------------------MKVACRDGKCCLRRDTWKLPAPQPRIS

>Mustela_putorius_furo

------------------------------MDRDTWKPPADQPRIS

>Cricetulus_griseus

MSGIELFRPHAMALLMVADMEHASLGFFPQGDRDTWKLPANQPRIS

HG1184 ENSG00000198894 CIPC

>Homo_sapiens

MGRAVQMKRVPMNLPPAE

>Physeter_catodon

------MKRVLMNALPAQ

>Tupaia_chinensis

MGRAIQMKRVPMSALAAW

HG1185 ENSG00000198920 KIAA0753

>Homo_sapiens

--------------------------MCL--LPS---AFFMCATALEIRTLRQLSLCSQEVSVFKQI-----------------

>Balaenoptera_acutorostrata_s

MPRPQFCRLLHSAALAPGLWRCQASWDCLTRVPPPTFAFDHVSTALEIRNL----YTSTDVSVFKQIQSTILEFIQKLKLGIS-

>Manis_javanica

MEN----KIIRPLASKGKCWPHE-SCRCPGPISAGSFI--L-----QLQP-----LNSSDVSPVGI--ASLMCFLPSSFLIMSL

HG1186 ENSG00000204923

>Homo_sapiens
MGATVVLASPKT--
>Ursus_maritimus
MGATVALASPET--
>Ceratotherium_simum_simum
MGATVVLASPETYR

HG1187 ENSG00000213762 ZNF134

>Homo_sapiens
MAAAAVMGPAQILCGC
>Ailuropoda_melanoleuca
MAAAAVMDPA------
>Manis_javanica
MAAAAVTDPASILCGC

HG1188 ENSG00000214415 GNAT3

>Homo_sapiens

MSKPQLCSTFEKSEHIN

>Rousettus_aegyptiacus

MLKPELCSTFEKV----

>Condylura_cristata

MSKPELCSTFEDV----

HG1189 ENSG00000279909

>Homo_sapiens

-----MELTSTTELFTLHCKYTYVC----PPSQLSCQCLE------------------------

>Ailuropoda_melanoleuca

MTPEKHRPISNRELFTLHYKYTYDTHRPPPISQTGCECLL------------------------

>Marmota_marmota_marmota

----------------MNCKHTYNTC---LPSHTSCECFERTGCGSFNFISRMTLIRHSMFFFA

HG1190 ENSG00000123684 LPGAT1

>Homo_sapiens

MHRCAGIVVVRRLRSSVSGKSY---------------------

>Galeopterus_variegatus

MNQCAGIAVG-RPRCSVSGKSDREVPVLSFHS-GRHFGDTEGF

>Ictidomys_tridecemlineatus

MKQCAGMVVERTPSFSLSGKSDREVPASVLSLVYPRLGDTEGV

HG1191 ENSG00000164331 ANKRA2

>Homo_sapiens

MSESFFLGLFGHSWPVATERTFFPARVSPPAGDSGL-LSRDAGTPVA--YF----------

>Oryctolagus_cuniculus

MSERFPPGLSEHCWPRRRSGRPLPRVCPAASSWPGTPAAYFLKIQISRIHWISLIMKKCFN

>Tupaia_chinensis

-MSESCLGFWGRSWPLASERTSAPYKSLQPAPVLGH-LSPIFNIQIPRKHWVPLL------

HG1192 ENSG00000168758 SEMA4C

>Homo_sapiens

---MWNGVWGSAGLLSALGKGTGRDRDL-RSQPAAPAGQLA-------------------

>Oryctolagus_cuniculus

---MWNGVLGSAGLLSALGEGTGRAGTSARRQPAAPSARGRLCPLPAPHMPPAPLRGWPL

>Chinchilla_lanigera

MRPWSFPRPSHPLPASLLGNKGASGNWAPQAPGPAPAPALLMWNGVWGSAGPLSALGGGDQQ-----GPGPAAPRPRGGLCQLPPHLTPPHP------

HG1193 ENSG00000177706 FAM20C

>Homo_sapiens

MDLDPRGGAALVPSCS

>Dipodomys_ordii

MDLDPRGGAAFVPSSS

>Tupaia_chinensis

MDLDPRGGEAFVSNQG

HG1194 ENSG00000214694 ARHGEF33

>Homo_sapiens
MGTSRENKKKTSKNML
>Oryctolagus_cuniculus
MGTSREIMKKTSKNMM
>Fukomys_darlingi
MGTSREIRRKTSKNMV

Supplementary Figure S5. FASTA-formatted CPuORF peptide sequences with gaps used in Supplementary Figure S4.

Supplementary Figure S6

(a) *PTP4A1*

1 GCTAGCCTGTGTCGCCGCCGCCTCGGGACCGGCTGT**ATGATTAGGCCACAATCTTCTGTG**

M I R P Q S S V

M I R P Q S S V

61 **TCGCCGCCGCCTCGGGACCGGCTGTCAATGAGTAAACATATTCCTCAATTCTGTGGTGTT**

S P P P R D R L S M S K H I P Q F C G V

S P P P R D R L S M S K H I P Q F C G V

C

121 **CTTGGTCACACATTTATGGAGTTTCTGAAGGGCAGTGGAGATTACTGCCAGGCACAGCAC**

L G H T F M E F L K G S G D Y C Q A Q H

L G H T F M E F S E G Q W R L L P G T A R

C

181 **GACCTCTATGCAGACAAGTGA**ACTGTAGAAACTGATTACTGCTCCACCAAGAAGCCCCCA

D L Y A D K *

P L R R Q *

241 TAAGAGTGGTTATCCTGGACACAGAAGTGTTGAATTGAAATCCACAGAGCATTTTACAAG

301 AGTTCTGACCTGGATGGGGTAAACCTCAGTGCACTTCTTTTCTGTTGGCCTCAGTATTAC

361 TGGATTGAAGAATTGCTGCTTCTTGTTAGGAGGTTCATTTCACTTATCATTACTTACAAC

421 TTCATACTCAAAGCACTGAGAATTTCAAGTGGAGTATATTGAAGTAGACTTCAGTTTCTT

481 TGCATCATTTCTGTATTCAATTTTTTTAATTATTTCATAACCCTATTGAGTGTTTTTTAA

541 CTAAATTAACATGGCTCAAGCTT

(b) ***MKKS***

A

1 GCTAGCTTGAATCTGAGCTTCATATCGAAAGAAGAG**ATGAAAAATACCAGTTGGATTAGA**

M K N T S W I R

M K N T S W I R K

61 **AAGAACTGGCTTCTTGTAGCTGGGATATCTTTCATAGGTGTCCATCTTGGAACATACTTT**

K N W L L V A G I S F I G V H L G T Y F

E L A S C S W D I F H R C P S W N I L F

121 **TTGCAGAGGTCTGCAAAGCAGTCTGTAAAATTTCAGTCTCAAAGCAAACAAAAGAGTATT**

L Q R S A K Q S V K F Q S Q S K Q K S I

A E V C K A V C K I S V S K Q T K E Y

181 **GAAGAGTGA**AGTAAAATAAATATTTGGAATTACTAATTTGTCATTAAATCATTCTATGCT

E E *

E E *

241 GATTAGCTTCATAAACATTGAACTTTTTGATTTTATAGCCACAATGCTGCATATTCATAC

301 TTTAATTCCTAAAGAATAATTTTTAATGTTAAAACGTGATAATGCAATAAATAGAAAAAT

361 GTGGTTTACAAAATAAAAACGGTCTTCACTAGTTACCACCTGAAGTAAGATGTCTCGTTA

421 AGCTT

(c) ***SLC6A8***

A

1 GCTAGCCGCCCGCGCGCCCCCGGGCCCCCGACACAC**ATGAGATTCTTCAGGCTCACTTTC**

M R F F R L T F

M R I L Q A H F Q

61 **AAGTGCTTCGTGGACTGCTTCTGA**CTGCGCCGCCCGCGCCCCGCACCCCGCCGCCCGCCC

K C F V D C F *

V L R G L L *

121 GCCGCCCCGTCCCCCGGCCCGGCCGCCCCCCGGCCCCCGGCCGGCCCGCGCCCTCGGGGC

181 CCTCCCCGGTGCCGCCGGTGCCCCCCGCCTGACCGCCGCCCCCCGTGAGGCGCCGCGACC

241 CCGGCCCGGCCGTGCGGCCCGCCGAGGCCATGGAAGCTT

(d) ***FAM13B***

```
                                                             A
                                                             ⬇
  1 GCTAGCGAAGTACTGATCGAGTTCTGCATTTCTTCAATGAGGAACTACAGGCTGATCTTC
                                        M  R  N  Y  R  L  I  F
                                        M  R K  L  Q  A  D  L  L

         A
         ⬆
 61 TGCCATAATCTCAAACAGCCATAAATGACAAAAGAATGCTTGCTCAGTGAGGGTAGCTGG
    C  H  N  L  K  Q  P  *
      P   K S  Q  T  A   *
121 TGCAGAAGCCATTTTTTAAACTTAGGTATTTAAGTACTGAAAGAAAAGACAGCTTTGATT
181 TCTGGCTGCAAAAAAGATATGAAAGCTT
```

(e) ***MIEF1***

```
  1 GCTAGCGGTGTGACACCCAGCCCCTGCCAGTCCCCCATGGCCCCGTGGAGCCGAGAGGCG
                                        M  A  P  W  S  R  E  A
                                        M  A  P  W  S  R  E  A
 61 GTGCTGAGTCTCTATCGGGCTCTGTTGCGCCAGGGCCGACAGCTTCGCTACACTGATCGA
    V  L  S  L  Y  R  A  L  L  R  Q  G  R  Q  L  R  Y  T  D  R
    V  L  S  L  Y  R  A  L  L  R  Q  G  R  Q  L  R  Y  T  D  R
121 GACTTCTACTTTGCCTCCATCCGCCGTGAATTCCGAAAAAATCAGAAGCTAGAGGACGCT
    D  F  Y  F  A  S  I  R  R  E  F  R  K  N  Q  K  L  E  D  A
    D  F  Y  F  A  S  I  R  R  E  F  R  K  N  Q  K  L  E  D  A
          T
          ⬇
181 GAGGCCCGGGAGAGGCAGCTGGAGAAGGGCCTGGTCTTTCTCAACGGCAAATTGGGGAGG
    E  A  R  E  R  Q  L  E  K  G  L  V  F  L  N  G  K  L  G  R
    E  AS  G  E  A  A  G  E  G  P  G  L  S  Q  R  Q  I  G  E  N

241 ATCATTTAGGATCCTCCAAGGGAAAGAGGACAAAGGTGCCTTCTGTAGACACTCCTGCTC
    I  I  *
       I  *
301 TCTTCCATCCCCATCTTACAGATGTATTAAGAAGCCTCAGATGAGCAATGGAAGCTT
```

(f) ***EIF5***

```
                                                          C                                                       A
1   GCTAGCCAGCCATTGGTACCTGTATTGGGGAAACATAGCATACAAGCAAGAAGCTTACAG

61  CCTCAGTGGCGAAAATTTTTTCATGTCAGAGACCGAGAACTCTTGCAGTCGTTTATGTCA
                                                          M  S
                                                          M  S I
121 TCCCTTCTTCTCCAGACAGAAGATACCAAAAAGTTGCAATCAAAGATCTCTTCATCTTAT
    S  L  L  L  Q  T  E  D  T  K  K  L  Q  S  K  I  S  S  S  Y
      P  S  S  P  D  R  R  Y  Q  K  V  A  I  K  D  L  F  I  L
181 TGATAAAGCCACTAATAAGCCAAAATGTCCTCGAG
    *
    *
```

(g) ***MAPK6***

```
  1 GCTAGCGTCGGAGAAGTCCCGTTGTATCAGAGTAAGATGGACGGTAGCTTTGATTGTGAT
                                        M  D  G  S  F  D  C  D
                                        M  D   V  A  L  I  V  I
                                            A
 61 TGTGGTGAGCTGGAGCCACCTGATCACTAACAAAAGACATCTTCTGTTAACCAACAGCCG
    C  G  E  L  E  P  P  D  H  *
     V  V  S  W  S  H  L  I  T *
121 CCAGGGCTTCCTGTTGAAATAAATATATAGCAACAAAGGAAAAAAAGAAGCAAAACGGAA
181 ATAGTGCTTACCAGCACCTTAGAATGATGCTGCTCAGGACCAGTCCAACACTGAATGTAT
241 CTGCACTGTGAGGAGAATGTTCATAGAAGCCTGTTGTGTGCATATTTATTCACATTTTTG
301 TTAAATGTTAAATCGTTTAGCACGGTAATCTGAGTGCACAGTATGTCATTTCATTCCGTT
361 TGAGTTTCTTGTTTTCGTTAAATGTCTGCAGAGTTGCTGCCCCTTTCTTGAACTATGAGT
421 ACTGCAATCTTTTTAATTCTCAATATGAATAGAGCTTTTTGAGCTTTAAATCTAAGGGGA
481 ACTCGACAGGCCTGTTTGGCATATGCAATGAACATCAAGAAACCATCTTGCTGTGGAAGC
541 ATAATTATTTTTCTTCTCCCTTTTTGAAAGATCTTTCCTTTTGATGCCAGTTTTCTTCCT
601 TGTTTACACAAGTTCAATTTGAAAGGAAAAGGCAATAGTAAGGGTTTCAAAATGGAAGCT
661 T
```

(h) *MEIS2*

A C

1 GCTAGCTTTGATTGACAGCTGGAGTGGCAAAAAGCC**ATGAAACACGACAGTTCGGTTACA**

M K H D S S V T

M K T R Q F G Y L

61 **TGTGGGCTGCTGACGGGCCGCTCGTAA**CCTTCAGTTCGGGGGCTTGACAATTTTTTTCTT

C G L L T G R S *

W A A D G P L *

121 CTTTTTCTTCTTTTTCTTTTCTTTCTTTTTTTTTCCAACTGAGGGGAAGAGAAGAGAAAG

181 AGGGGGAAAGGAGGACCGAAGAGGAGGAGGAGGGGAGGGGGGAGGAGGAGGAGGTGGAGG

241 AGGAGGAGGAAGATCAGGAGGAGGAGGAAGAAGAGGAAAAAAGAGAAAAAGAAGAAATAT

301 CACAGAAAAAAAAATTCTTCGTTGTCTAGACTGGGCTTTTTTTCCCCCCTAAAAAATAGC

361 ATATTGGAGAATTGGGAGAAGTCTCTTTGGTTTGGAAAAAAAAAAAAGGAATCTTCAGCC

421 TAGATCACTTTCTTATCCGGACTGGGATATTAAATATACGACACATCCAGGAGTTTATTG

481 GAGCGCAGACTGATGGCTCGAG

(i) *KAT6A*

1 GCTAGCCAACAGGTTGTTTTGGTTTCTATAGTACAATTGGGGTGGCATTCTGTTTTGTGA

A

61 AAGGAGGAAGGACTTAGGCCAGAAAACTCATATGCT**ATGGTTAACTGGTTCCCAGCCTCC**

M V N W F P A S

M V K L V P S L R

C

121 **GAGAATCTTGTTTTCCATGGTGTAAAACTTACTCAGCATCAGGATAAGGGATAA**CGACTC

E N L V F H G V K L T Q H Q D K G *

E S C F P R C K T Y S A S G K G *

181 TATGGATATACAGAATCCTTCACCATGGTAAAACCTCGAG

(j) ***SLC35A4***

C ↑ (position 46)

1 GAGCTCTGCGCCTGCGCAACAAGTTCGGCGGGGAAG**ATGGCGGATGACAAGGATTCTCTG**

M A D D K D S L

M A T T R I L C

A ↓ (position 120)

61 **CCTAAGCTTAAGGACCTGGCATTTCTCAAGAACCAGCTGGAAAGCCTGCAGCGGCGTGTA**

P K L K D L A F L K N Q L E S L Q R R V

L S L R T W H F S R T S W K A C S G V I R

A (position 121), C ↑ (position 154)

121 **GAAGACGAAGTCAACAGTGGAGTGGGCCAGGATGGCTCGCTGTTGTCCTCCCCGTTCCTC**

E D E V N S G V G Q D G S L L S S P F L

R R S H S G V G Q D G S L L S S P F L

C ↑ (position 194)

181 **AAGGGATTCCTGGCTGGCTATGTGGTGGCCAAACTGAGGGCATCAGCAGTATTGGGCTTT**

K G F L A G Y V V A K L R A S A V L G F

K G F L A G Y V V A K L R A S A V L G F

C ↑ (position 250), C ↑ (position 260)

241 **GCTGTGGGCACCTGCACTGGCATCTATGCGGCTCAGGCATATGCTGTGCCCAACGTGGAG**

A V G T C T G I Y A A Q A Y A V P N V E

A V G T C T G I Y A A Q A Y A V P N V E

301 **AAGACATTAAGGGACTATTTGCAGTTGCTACGCAAGGGGCCCGACTAG**CTCTAGGTGCCA

K T L R D Y L Q L L R K G P D *

K T L R D Y L Q L L R K G P D *

361 TGGAAGAGGCAGGATGAGCAGCTCAGCCTTCAGGTGGAGACACTTTATCTGGATTCCCCA

421 GCTGTCATCCATTTGCTATCTCCAACTTTCCTGCCACCTTCATCCTTGCCTCCCTTCCTG

481 CAGATTGTGGACAGTAGTTCCTCAGCCTGCACCCTGGATTCCTTCTTCCCCTTCCTAGCT

541 CCATGGGACTCGCCCCAAGACTGTGGCTTCAAGGACCACCAGCCCCTTACTCTTCAAGCC

601 CTGACTGTGGAGTTGGTAGATGCCTCTGATCCTCAGTATTCTCTCTGGCAATGTTCCACG

661 GCTTCTCCTTCCTGGGAGCTGGCTCCATAACTTGATTTTCCCCAAACGTGTTGCAATCCC

721 TGCTGCCCCTTAGCCACCCAGGGTCTTGTGTGGGTATGAGCTCGAG

(k) ***LRRC8B***

```
  1 GCTAGCTCAGAAGGTGATCTCTTTAATGCTTTCTTTTTAAGAATTTTTCAAATTGAGACT
 61 AATTGCAGAGGTTCCAGTTGACCAGCATTCATAGGAATGAAGACAAACACAGAGATGGTG
                                         M  K  T  N  T  E  M  V
                                         M  K  T  N  T  E  M  V
                                        A
                                        ⬇
121 TGTCTAAGAAACTTCAAAAGGTGTAGACCTCCTGACTGAAGCATATTGGATTTATTTAAT
    C  L  R  N  F  K  R  C  R  P  P  D  *
    C  L   K  T  S  K  G  V  D  L  L  N *
181 TTTTTTCACTGTATTTCTGTCCTCCTACAAGGGAAAGTCATGATTACACAAGCTT
```

(I) ***CDH11***

A

1 GCTAGCCGTGTTGTCATTTGTTGAGTGACCAATCAG**ATGGGTGGAGTGTGTTACAGAAAT**

M G G V C Y R N

M G R S V L Q K L

C

61 **TGGCAGCAAGTATCCAATGGGTGA**AGAAGAAGCTAACTGGGGACGTGGGCAGCCCTGACG

W Q Q V S N G *

A A S I Q R *

121 TGATGAGCTCAACCAGCAGAGACATTCCATCCCAAGAGAGGTCTGCGTGACGCGTCCGGG

181 AGGCCACCCTCAGCAAGACCACCGTACAGTTGGTGGAAGGGGTGACAGCTGCATTCTCCT

241 GTGCCTACCACGTAACCAAAAATGAAAGCTT

(m) ***PNRC2***

```
  1 GCTAGCCTCTCAAACTTGTGTGCTGAGGAGACTCAGATGTTGGCCTCAGCTCCTAGGCTG
                                        M  L  A  S  A  P  R  L
                                        M  L  A  S  A  P  R  L

                                  A
                                  ⬇
 61 AACTCAGCAGATCGGCCCATGAAAACTTCTGTATTGAGACAAAGGAAGGGATCTGTCAGA
    N  S  A  D  R  P  M  K  T  S  V  L  R  Q  R  K  G  S  V  R
    N  S  A  D  R  P  M  K N  F  C  I  E  T  K  E  G  I  C  Q  K
121 AAGCAACACTTGTTATCTTGGGCTTGGCAGCAAGGAAGAGGACAGGTAGTGGAGATCCTG
    K  Q  H  L  L  S  W  A  W  Q  Q  G  R  G  Q  V  V  E  I  L
      A  T  L  V  I  L  G  L  A  A  R  K  R  T  G  S  G  D  P  A

          T         T
          ⬆         ⬆
181 CAATCTGAAAAGCAGACTGAAAGGTGACAAAGAAGCTGAAGATGGAAGCTT
    Q  S  E  K  Q  T  E  R  *
      I  L  K  A  D  L  K   *
```

(n) *BACH2*

```
                                          C                    T
1   GCTAGCGCCCCACAAAACTTTGGGGTCCATGTCTGAATGGATTGCCAGAGCCTTCTCATC
                                                M   D   C   Q   S   L   L   I
                                                M   D   C P   E   P   S   H   L
61  TCTCCCTTCGCCCAGTTCCCTGCATCCTAAGACTCGAAGGCAGCACAGGACCTGGAAAAA
    S   P   F   A   Q   F   P   A   S   *
      S   L   R   P   V   P   C   I   *
121 TTACATGGTGTGAACGGCATGTAAGCTT
```

(o) *FGF9*

```
  1 GCTAGCCTATAATAACGCCTAGGCATTTAAGTTGCTATGGTCATTCTGATCTCAAACCAA
                                        M  V  I  L  I  S  N  Q
                                        M  V  I  L  I  S  N  Q
          A                                          T
          ↓                                          ↑
 61 ATGGAGAAACTACGGATTTTTTTTCCTTATTACGGTCGGATGGGATGAAGACCTTCCTGC
    M  E  K  L  R  I  F  F  P  Y  Y  G  R  M  G  *
    M  E  KT T  D  F  F  S  L  L  R  S  M  G  *
121 CTGCTAAGAGCTGGGGATCTATCTATAGAGATACATAGATATGTTTATCAATATGTCAGT
181 GTGTGAGTATAAAGTGGTGGTTTCTTAGACTATCAGTGGTTTGACCTTGAACCTGTGCCA
241 GTGAAACAGCAGATTACTTTTATTTATGCATTTAATGGATTGAAGAAAAGAACCTTTTTT
301 TTCTCTCTCTCTCTGCAACTGCAGTAAGGGAGGGGAGTTGGATATACCTCGCCTAATATC
361 TCCTGGGTTGACACCATCATTATTGTTTATTCTTGTGCTCCAAAAGCCGAGTCCTCTGAT
421 GGAAGCTT
```

(p) *PNISR*

```
                                                               
  1 GCTAGCCTAGAGTGGGGCATACATAATCTTGCTGCTATGCTTCGAAGCTGTAGTCTGAAT
                                        M  L  R  S  C  S  L  N
                                        M  L  R  S  C  S  L  I

                A
                ⬇
 61 CAACCTAAGTTTTTAAACAGAAGGTGAACCTCTGAGAAAATCAAGTATATTTTAAAAGAAG
    Q  P  K  F  *
     N  L  S  L *

121 GGATGTGGGAAGCTT
```

(q) ***TMEM184C***

```
                                                    A
                                                    ⬇
  1 GCTAGCTTCCTGGTCTGTGCTGCTCTCCTGGAAGCCATGGTACAGGCAGAGCTCAGGGCG
                                         M  V  Q  A  E  L  R  A
                                         M  V  Q   Q  S  S  G  R

               A
               ⬇
 61 ATCCCCAGGTGAGGGCAGCGGCTCTGCCTGGGATTCCACCGCAGTACAACCGGGTAGATG
    I  P  R  *
     S  P  G *
121 CGGGGTGGAGAAGAAAGGATGTTGCCTGCACTGCTCGCCAATAGCACCCTGAGAGGCTAC
181 ATTTGCAGAAGCAGCAGCAGCAGAAGACACAGCGCCGGTCCAGGAGGCGGCTCGAGCTGT
241 TCGTAAAGTCGCCCGACAGCTTTTTCTCCGTAGTATGCGAGTTGACAAAACAGCCAGAGA
301 ACAGGGCTCCCCATTACAATCTTTTCGAGATCTTTTCCCTTGCTAACCGGATCTGATTTG
361 TGCGAAAACATGCCTTGCAAAGCTT
```

Supplementary Figure S6. Nucleotide sequences of the 5′-UTRs and amino acid sequences of the CPuORFs analyzed in this study. **(a-q)** The 5′-UTRs of *PTP4A1* **(a)**, *MKKS* **(b)**, *SLC6A8* **(c)**, *FAM13B* **(d)**, *MIEF1* **(e)**, *EIF5* **(f)**, *MAPK6* **(g)**, *MEIS2* **(h)**, *KAT6A* **(i)**, *SLC35A4* **(j)**, *LRRC8B* **(k)**, *CDH11* **(l)**, *PNRC2* **(m)**, *BACH2* **(n)**, *FGF9* **(o)**, *PNISR* **(p)**, and *TMEM184C* **(q)**. The nucleotide sequences of the CPuORFs are shown in bold. The deduced amino acid sequences of the wild-type (WT-aa) and frameshift (fs) CPuORFs are indicated. The nucleotide sequences of other uORFs are underlined with a bold line. A dotted underline indicates the nucleotide sequences of other uORFs overlapping the CPuORFs. The initiation codons of these overlapping uORFs were changed to other codons, as indicated, while keeping the CPuORF amino acid sequence unaltered. The replaced nucleotides are shown as white letters in a black background. The nucleotides that were deleted and inserted in the frameshift mutants are shaded. The main coding sequences that were contained in the reporter constructs are boxed. The shaded boxes indicate the nucleotides changed to avoid the appearance of in-frame termination codons. Cloning sites were added at either end of the nucleotide sequences in controls to be subcloned into plasmid pGL4.10 with an SV40 promoter (pSV40:5'UTR::luc2) are underlined (Fig.2b).

Supplementary Table S1. CPuORFs extracted from *D. melanogaster* , *D. rerio* , *G. gallus* , and *H. sapiens* .

HG number	Species	Gene ID	Gene symbol	Gene description[†]	uORF-mORF fusion ratio	K_a/K_s analysis before manual validation			K_a/K_s analysis after manual validation		Previous report[*]	CPuORF amino acid sequence
						Median pairwise K_a/K_s ratio	*U*-test *p* value	*q* value	Median pairwise K_a/K_s ratio	*U*-test *p* value		
HG0001	*D. melanogaster*	FBgn0024734	PRL-1	PRL-1	0.00	0.06	0.0E+00	0.0E+00	0.06	0.0E+00	Crowe	MCMAIVLVRPVPTSMEFHLDSAEYCQAQHK*
	D. rerio	ENSDARG00000006242	ptp4a1	protein tyrosine phosphatase type IVA, member 1	0.04	0.10	0.0E+00	0.0E+00	0.10	0.0E+00	Crowe	MIKLQSSMSKHIPFCGVLGHTFMEFLKGSGDYCQAQHGVYAEK*
		ENSDARG00000035676	ptp4a2b	protein tyrosine phosphatase type IVA, member 2b	0.00	0.22	0.0E+00	0.0E+00	0.22	0.0E+00	Crowe	MVGPFFCFRVDSDHTYMAFVTVCREFCQAQHLTFADK*
		ENSDARG00000039997	ptp4a3	protein tyrosine phosphatase type IVA, member 3	0.00	0.07	0.0E+00	0.0E+00	0.07	0.0E+00	Crowe	MAFLQRTGDYCHAQHLHI*
		ENSDARG00000054814	PTP4A3	protein tyrosine phosphatase 4A3b	0.00	0.08	0.0E+00	0.0E+00	0.08	0.0E+00	Crowe	MEFWQGSGDYCHAQHHTA*
		ENSDARG00000087443	ptp4a2a	protein tyrosine phosphatase type IVA, member 2a	0.00	0.14	0.0E+00	0.0E+00	0.14	0.0E+00	Crowe	MQSSQLVFCSLIEGCTFMAFVSVSGDFCQAQHLVIVDK*
	G. gallus	ENSGALG00000003265	PTP4A2	protein tyrosine phosphatase type IVA, member 2	0.00	0.24	0.0E+00	0.0E+00	0.24	0.0E+00	Crowe	MPGVFCWNLRRTFMAILSVRAEFCQAQHSALADK*
		ENSGALG00000016271	PTP4A1	protein tyrosine phosphatase type IVA, member 1	0.00	0.08	0.0E+00	0.0E+00	0.08	0.0E+00	Crowe	MIRLQSSMSNHIPQFCGVLGHTFMEFLKGSGDYCQAQHDLYADK*
	H. sapiens	ENSG00000112245	PTP4A1	protein tyrosine phosphatase type IVA, member 1	0.00	0.09	0.0E+00	0.0E+00	0.09	0.0E+00	Crowe	MIRPQSSMSKHIPQFCGVLGHTFMEFLKGSGDYCQAQHDLYADK*
		ENSG00000184007	PTP4A2	protein tyrosine phosphatase type IVA, member 2	0.00	0.22	0.0E+00	0.0E+00	0.22	0.0E+00	Crowe	MAILSVRADFCQAQHSIFADK*
HG0002	*D. rerio*	ENSDARG00000003077	gpx9	glutathione peroxidase 9	0.04	0.07	0.0E+00	0.0E+00	0.07	0.0E+00		MAANSVYDFTVETLEEKSVSLDIFRGKVLLIMNVATF*
HG0003	*D. melanogaster*	FBgn0039280	Mocs2	Molybdenum cofactor synthesis 2	0.14	0.07	0.0E+00	0.0E+00	0.07	0.0E+00	Hayden	MNADGPVVNVHVLFFAKSRELANTPRSTVEVPTEITATELLDHLVSKFGLTSIRDNLILAHNESYIDNLSDRILFKEGDELAIIPPLSGG*
	G. gallus	ENSGALG00000014906	MOCS2	molybdenum cofactor synthesis 2	0.02	0.07	0.0E+00	0.0E+00	0.07	0.0E+00	Hayden	MSCQVTVLYFARSAELVGLRSESVSVPQRITSLQLWEEIVKIHPRLAVIRDQVVFAVRQEYVLLGDQLLVLQTGDEVAIIPPISGG*
HG0004.1	*D. melanogaster*	FBgn0028494			0.00	0.01	9.6E-86	1.2E-85	0.01	9.6E-86	Mackowiak	MLRYRLVSTHFLKQP*
	D. rerio	ENSDARG00000076779	fam13b	family with sequence similarity 13, member B	0.00	0.00	0.0E+00	0.0E+00	0.00	0.0E+00	Mackowiak	MRNYRLIFCHNLKQP*
	G. gallus	ENSGALG00000041706			0.00	0.00	0.0E+00	0.0E+00	0.00	0.0E+00	Mackowiak	MRNYRLIFCHNLKQP*
	H. sapiens	ENSG00000031003	FAM13B	family with sequence similarity 13 member B	0.00	0.00	0.0E+00	0.0E+00	0.00	0.0E+00	Mackowiak	MRNYRLIFCHNLKQP*
HG0004.2	*H. sapiens*	ENSG00000138640	FAM13A	family with sequence similarity 13 member A	0.15	0.34	2.4E-05	1.3E-04	0.34	2.4E-05		MLEAALRLPWGLCIGSCFSHQRICAEWNFSILLSF*
HG0005	*D. rerio*	ENSDARG00000011055	fbxo9	F-box protein 9	0.16	0.04	0.0E+00	0.0E+00	0.04	0.0E+00	Crowe, Mackowiak	MAAVWRARKFARCVVRFSDRDL*
	G. gallus	ENSGALG00000016320			0.22	0.04	0.0E+00	0.0E+00	0.04	0.0E+00	Crowe, Mackowiak	MAAVRRARSYSRCVVRFSDRELC*
HG0006	*D. melanogaster*	FBgn0029971	CG18624	GEO13364p1	0.09	0.08	0.0E+00	0.0E+00	0.08	0.0E+00	Hayden, Mackowiak	MGSGIVVQLARKYGAVIFFPTVAVGSIYADWSHTRQWKRQQLQLAHRAQLKDQR*
HG0007	*D. melanogaster*	FBgn0034372	Gint3	GDI interacting protein 3	0.25	0.06	0.0E+00	0.0E+00	0.06	0.0E+00	Hayden	MSFNFYVESFNSTLQSIARQVEQFAGVALTAAVDLRGNLESLTGDFRWENGPIAFTDLQATLLKVLIVLLLGTCVLAGYSWSIYGKVITEKFVRPSTLKEIEELKLSVAKLKLPKEHSPRI*
HG0008	*H. sapiens*	ENSG00000175567	UCP2	uncoupling protein 2	0.00	0.30	1.3E-225	2.6E-223	0.30	1.3E-225	Crowe, Samandi	MTIRCFVSHPFSMENQGDRAMIATGSFEERDTFREA*
HG0009	*D. melanogaster*	FBgn0050100	CG30100	AT22563p2	0.07	0.05	0.0E+00	0.0E+00	0.05	0.0E+00	Hayden	MKMVQPSRQQILRLYKHLIRYGNHLKLTDKNYFLGRVRHEFRDNRSLTNPVEVEFSFKRGETLLKKGRIL*
HG0010.1	*G. gallus*	ENSGALG00000009013	MKKS	McKusick-Kaufman syndrome	0.00	0.11	1.1E-270	7.2E-269	0.11	5.4E-265	Akimoto	MMSLKTLWKDYKVLIVMGISLGLVHWSWFHIKSSPLFQVKTEEIVPEPGIVTYVMQSDHKNKEK*
	H. sapiens	ENSG00000125863	MKKS	McKusick-Kaufman syndrome	0.00	0.10	0.0E+00	0.0E+00	0.10	0.0E+00	Akimoto	MSLRNLWRDYKVLVVMVPLVGLIHLGWYRIKSSPVFQIPKNDDIPEQDSLGLSNLQKSQIQGK*
HG0010.2	*G. gallus*	ENSGALG00000009013	MKKS	McKusick-Kaufman syndrome	0.00	0.27	5.0E-187	2.3E-185	0.27	5.0E-187	Crowe, Akimoto, Mackowiak	MRKASWSKKNFLLVAGLSLIGVHFGTMLVNFVAKKSARSHSETKRDNHRE*
	H. sapiens	ENSG00000125863	MKKS	McKusick-Kaufman syndrome	0.00	0.23	3.0E-245	7.0E-243	0.23	3.0E-245	Crowe, Akimoto, Mackowiak	MKNTSWIRKNWLLVAGISFIGVHLGTYFLQRSAKQSVKFQSQSKQKSIEE*
HG0010.3	*H. sapiens*	ENSG00000125863	MKKS	McKusick-Kaufman syndrome	0.00	0.44	7.0E-15	1.3E-13	0.44	7.0E-15		MLISFINIELFDFIATMLHIHTLIPKE*
HG0011	*D. melanogaster*	FBgn0027360	Tim10	Translocase of inner membrane 10	0.03	0.05	3.5E-290	9.6E-290	0.05	3.5E-290	Hayden	MRVRERMRKANQGDYDSSAASDRRFDY*
HG0012	*D. melanogaster*	FBgn0064116	CG33713	GM05135p	0.07	0.05	5.0E-301	1.4E-300	0.05	5.0E-301	Hayden	MATAAAVAKVGKSVHRIFVGNLPWTVGHQELRGYFREFGRVVSANVIFDKRTGCSKGYGFVSFNSLTALEKIENEQKHILEGNYLNIQKS*
HG0013	*D. melanogaster*	FBgn0261381	mtTFB1	Mitochondrial Transcription Factor B1	0.21	0.05	4.3E-307	1.4E-306	0.05	4.3E-307		MSASEQGPKIKYGESAPKLDKAQLQFMKLIEEQNLDRVQKLKRIRRNNLLTAGALGVSVLAIYGYSIFSVQQEKFLDDFEEPKKVSS*
HG0014	*D. melanogaster*	FBgn0260392	CG42518	Uncharacterized protein, isoform A	0.27	0.04	0.0E+00	0.0E+00	0.04	0.0E+00		MMDLSPNNQIEDRKPILTADGLVQTSNSPFEPTISQETQTSNGIGGQCHLTVDQLDIEILPIIYDIVRCVEKDPLENAVKLRESQDCNHKIFELQKRFESAREQIRQLPGIDFNKEEQQQRLELLRNQLKLKQQLIRKYKDTEF*
HG0015	*D. rerio*	ENSDARG00000007377	odc1	ornithine decarboxylase 1	0.00	0.24	2.5E-211	4.1E-210	0.24	2.5E-211		MRFKNSCLREVHTGPGLEAYLKGFNFTGEPPWLSDIC*
HG0016	*G. gallus*	ENSGALG00000010399	SELENOT	selenoprotein T	0.00	0.14	4.1E-254	2.4E-252	0.14	4.1E-254		MAYATGPLLKFQICVS*
HG0017	*D. rerio*	ENSDARG00000043154	ucp2	uncoupling protein 2	0.04	0.30	2.3E-104	3.0E-103	0.30	2.3E-104	Crowe	MFFICTSSQRFSMEERGNKIQVESHFNQRDAFRES*
HG0018	*D. rerio*	ENSDARG00000053291	pnrc2	proline-rich nuclear receptor coactivator 2	0.00	0.47	9.8E-87	1.1E-85	0.47	9.8E-87	Crowe, Samandi	MFNSVPRQNNAHWHMKTSDLAGHTCAARSRSTTSRTWSPIGEEKLKSEKNHALNFFTTER*
	G. gallus	ENSGALG00000004122	PNRC2	proline rich nuclear receptor coactivator 2	0.00	0.34	0.0E+00	0.0E+00	0.34	0.0E+00	Crowe, Samandi	MLGSAAWLNTADRPMKTSVPSQRKESSERQHLLFQAWKQERGQELESVESEKTTER*
	H. sapiens	ENSG00000189266	PNRC2	proline rich nuclear receptor coactivator 2	0.00	0.32	0.0E+00	0.0E+00	0.34	0.0E+00	Crowe, Samandi	MLASAPRLNSADRPMKTSVLRQRKGSVRKQHLLSWAWQQGRGQVVEILQSEKQTER*
HG0019	*H. sapiens*	ENSG00000178397	FAM220A	family with sequence similarity 220 member A	0.06	0.07	8.8E-203	1.5E-200	0.07	8.8E-203		MAAALSGLAVRLSRSAAARSYGVFCKGLTRTLLIFFDLAWRLRINFPYLYIVASMMLNVRLQVHIEIH*
HG0020	*D. melanogaster*	FBgn0035436	CG12016	SD05789p2	0.07	0.07	1.1E-163	1.7E-163	0.07	1.1E-163	Hayden	MASNAQLGKIILISAIAVFFYYFFWVAVLPFMLIDEGNPIRLFFPPLKYAFIVPTVFGVIFLGGIAAFSFYHIWSLRVKRD*
HG0021.1	*D. rerio*	ENSDARG00000068708	ifrd1	interferon-related developmental regulator 1	0.05	0.49	3.6E-60	3.5E-59	0.49	3.6E-60	Zhao, Samandi	MYRSRSKSNTGIAFNTFLFTVPLEYTQTKRHYSKNWSCIG*
	G. gallus	ENSGALG00000009448	IFRD1	interferon related developmental regulator 1	0.02	0.45	2.8E-99	8.7E-98	0.45	2.8E-99	Zhao, Samandi	MHRLRSRAFTGISAIAASSSFACLRQSPPLPRPEKSASQRRWNKKRSCSSIG*
	H. sapiens	ENSG00000006652	IFRD1	interferon related developmental regulator 1	0.00	0.38	3.8E-79	2.9E-77	0.38	3.8E-79	Zhao, Samandi	MYRFRSQLFTGISAAATAHSYPRRFSTLLLAEDSPLSRPPHRRTSKKCSSIG*
HG0021.2	*D. rerio*	ENSDARG00000036811	ifrd2	interferon-related developmental regulator 2	0.00	0.27	8.4E-109	1.1E-107	0.27	8.4E-109		MVESGAATCTILRPKHFQKHLPSQPGIRHRLPGDNHR*
HG0022	*D. melanogaster*	FBgn0061359	CG33671	Mevalonate kinase	0.00	0.03	2.7E-164	4.2E-164	0.03	2.7E-164	Hayden	MSKYDSKYLEEKLKRELQTEYVSVTDESDGCGGKFSAVIVSPAFSGKTLLQKHRLVNSTLAEELKEIHAFSQKSYTPEEWEKVKAQ*
HG0023.1	*H. sapiens*	ENSG00000175197	DDIT3	DNA damage inducible transcript 3	0.22	0.27	1.3E-37	5.8E-36	0.27	1.3E-37	Crowe, Jousse, Mackowiak, Samandi	MLKMSGWQRQSQNQSWNLRRECSRRKCIFIHHHT*
HG0023.2	*D. rerio*	ENSDARG00000059836	ddit3	DNA-damage-inducible transcript 3	0.06	0.34	8.9E-49	7.8E-48	0.34	8.9E-49	Crowe, Jousse	MVNMSDQPSLQKHTQTLNQKQPRKRNNKKKRSYWDKISPTTHIQT*
HG0024.1	*D. rerio*	ENSDARG00000093406	C19H6orf62		0.00	0.48	2.9E-39	2.4E-38	0.48	2.9E-39		MGPPRCRSRRLLPERTEDFGRFVSLS*
HG0024.2	*G. gallus*	ENSGALG00000013628	C6orf62	chromosome 6 open reading frame 62	0.00	0.00	3.7E-13	2.2E-12	0.00	3.7E-13		MDWACASCFLLYT*
	H. sapiens	ENSG00000112308	C6orf62	chromosome 6 open reading frame 62	0.00	0.39	7.5E-12	1.0E-10	0.39	7.5E-12		MDFRDWSGVSCVLLHT*
HG0024.3	*G. gallus*	ENSGALG00000013628	C6orf62	chromosome 6 open reading frame 62	0.00	0.48	5.2E-47	7.4E-46	0.48	5.2E-47		MAGQLLILFLDPSNTTFP*
HG0025	*D. rerio*	ENSDARG00000060054	epc1b	enhancer of polycomb homolog 1 (Drosophila) b	0.10	0.41	8.5E-29	5.5E-28	0.41	8.5E-29		MRTLIGPAGNIRGSDRFMLHLEL*
HG0026	*D. rerio*	ENSDARG00000087059	fam213ab	family with sequence similarity 213, member Ab	0.22	0.08	8.8E-53	7.9E-52	0.07	1.6E-51		MELVTRAVGSVGLTVIEALRSFTELFLTQPVCATLTQLADTDLRTLDGDDRVFKARELWESSGAVIMAVRRPG*
HG0027	*H. sapiens*	ENSG00000077254	USP33	ubiquitin specific peptidase 33	0.00	0.47	1.0E-02	2.6E-02	0.47	1.0E-02		MERNNRYQPLQKHAKL*
HG0028.1	*D. rerio*	ENSDARG00000032103	mapk6	mitogen-activated protein kinase 6	0.00	0.07	0.0E+00	0.0E+00	0.07	0.0E+00		MEDSFDCDCGELEPPDH*
	G. gallus	ENSGALG00000031448	MAPK6	Mitogen-activated protein kinase 6	0.00	0.07	0.0E+00	0.0E+00	0.07	0.0E+00		MDGSFDCDCGELEPPDH*
	H. sapiens	ENSG00000069956	MAPK6	mitogen-activated protein kinase 6	0.00	0.07	0.0E+00	0.0E+00	0.07	0.0E+00		MDGSFDCDCGELEPPDH*
HG0028.2	*H. sapiens*	ENSG00000069956	MAPK6	mitogen-activated protein kinase 6	0.00	0.35	9.1E-15	1.6E-13	0.35	9.1E-15		MFIEACCVHIYSHFC*
HG0029	*D. rerio*	ENSDARG00000007523	kmt2e	lysine (K)-specific methyltransferase 2E	0.00	0.37	4.7E-64	4.7E-63	0.37	4.7E-64		MHLDVCNECCG*
	G. gallus	ENSGALG00000008167			0.00	0.37	1.5E-134	5.6E-133	0.37	1.5E-134		MHLDVCNEHCGWHAPVFWIPMHRTL*
	H. sapiens	ENSG00000005483	KMT2E	lysine methyltransferase 2E	0.00	0.36	7.2E-199	1.2E-196	0.36	7.2E-199		MGLWMHLDVCNEHCGWHAPVFWIPMHRTP*
		ENSG00000168137			0.00	0.30	3.0E-274	7.7E-272	0.30	3.0E-274		MSITCWMRLCSSPPLSEWSVSINWTP*
HG0030.1	*G. gallus*	ENSGALG00000011464	EIF5	eukaryotic translation initiation factor 5	0.00	0.36	4.2E-136	1.6E-134	0.36	4.2E-136		MSSRLLQTEDTKKLQSKISSSY*
	H. sapiens	ENSG00000100664	EIF5	eukaryotic translation initiation factor 5	0.00	0.42	1.0E-91	9.7E-90	0.42	1.0E-91		MSSLLLQTEDTKKLQSKISSSY*
HG0030.2	*D. melanogaster*	FBgn0030719	eIF5	eIF5	0.00	0.10	3.0E-116	4.0E-116	0.10	3.0E-116	Mackowiak	MNSKGRTKNHRTSSNRHRKLQPFRYT*
HG0031	*D. rerio*	ENSDARG00000000540	asnsd1	asparagine synthetase domain containing 1	0.00	0.09	0.0E+00	0.0E+00	0.09	0.0E+00	Mackowiak	MSSKNIQRTEPGASEERAQKEELNQKIKEQKIVVDELSNLKKNRRVYTQQPNSNIFFLADKGETLSSCKQDLDNMRKEYQDI*
	G. gallus	ENSGALG00000041666			0.00	0.04	0.0E+00	0.0E+00	0.04	0.0E+00	Mackowiak	MSRREEEETAELGYKGELSRRIKEQKVVVDELSNLKKNRKVYRQQPNSNIFFLVDRTEALSQCKNTLDQLKKAHQELENSEKTKVKK*
	H. sapiens	ENSG00000138381	ASNSD1	asparagine synthetase domain containing 1	0.00	0.07	0.0E+00	0.0E+00	0.07	0.0E+00	Mackowiak	MPSRGTRPEDSSVLIPTDNSTPHKEDLSSKIKEQKIVVDELSNLKKNRTEQKCCLRARIYWMN*
HG0032	*D. rerio*	ENSDARG00000104981	SLC45A4		0.00	0.36	0.0E+00	0.0E+00	0.36	0.0E+00		MGILRCFPSGFSRPSPLGLPSTLYSTASIQIMKLVLTEWQLIWIFTVSSRQ*
HG0033	*G. gallus*	ENSGALG00000016321	ICK	intestinal cell kinase	0.00	0.47	3.1E-52	4.9E-51	0.47	3.1E-52	Crowe	MIPATVDTVLSALCLCCHRKNWLQSEKKH*
	H. sapiens	ENSG00000112144	ICK	intestinal cell kinase	0.01	0.33	2.8E-117	3.2E-115	0.33	2.8E-117	Crowe	MYLGDSHVLLNTLCWCCHRKIWLHSGEDCYHCRTEPLRP*
HG0034	*D. rerio*	ENSDARG00000060991	mief1	mitochondrial elongation factor 1	0.00	0.12	0.0E+00	0.0E+00	0.12	0.0E+00	Andreev, Mackowiak, Samandi	MGGWSRSAVLQLYRALLRAGQHLQYTDRDYYRRVVTREFRRCQTLSAPAEREDALKRGQFFLNSGLGDLV*
	H. sapiens	ENSG00000100335	MIEF1	mitochondrial elongation factor 1	0.00	0.14	0.0E+00	0.0E+00	0.14	0.0E+00	Andreev, Mackowiak, Samandi	MAPWSREAVLSLYRALLRQGRQLRYTDRDFYFASIRREFRKNQKLEDAEARERQLEKGLVFLNGKLGRII*
HG0035	*G. gallus*	ENSGALG00000006167	LRRC8B	leucine rich repeat containing 8 family member B	0.00	0.21	5.7E-305	4.7E-303	0.21	5.7E-305	Mackowiak	MKTNVKMACPRNFKRCRPPD*
	H. sapiens	ENSG00000197147	LRRC8B	leucine rich repeat containing 8 family member B	0.00	0.20	0.0E+00	0.0E+00	0.20	0.0E+00	Mackowiak	MKTNTEMVCLRNFKRCRPPD*
HG0036	*G. gallus*	ENSGALG00000029295			0.00	0.34	2.2E-39	2.7E-38	0.34	2.2E-39		MIVGFHLLTCPFCCII*
	H. sapiens	ENSG00000155096	AZIN1	antizyme inhibitor 1	0.00	0.34	1.6E-44	7.9E-43	0.34	1.6E-44		MIGGFHLPTCPFCCIF*
HG0037	*G. gallus*	ENSGALG00000010747	FBLN5	fibulin 5	0.00	0.06	2.4E-06	9.5E-06	0.06	2.4E-06		MKCSFLTFLLQLV*
	H. sapiens	ENSG00000140092	FBLN5	fibulin 5	0.00	0.06	1.9E-04	8.1E-04	0.06	1.9E-04		MKCSFLTFLLQLV*
HG0038	*G. gallus*	ENSGALG00000006571			0.04	0.26	1.9E-221	9.8E-220	0.26	1.9E-221		MSVTCWPRLCGSSPPSERPVAALWTP*
HG0039.1	*H. sapiens*	ENSG00000077092	RARB	retinoic acid receptor beta	0.02	0.39	1.2E-126	1.5E-124	0.39	1.2E-126	Reynolds	MIIWINYRLLAGLSVIIHDSGLGKRPTAYVPKKGQSLMELGGLFYAICLHT*
HG0039.2	*H. sapiens*	ENSG00000077092	RARB	retinoic acid receptor beta	0.04	0.29	2.2E-22	5.9E-21	0.29	2.2E-22	Reynolds	MPRTRAIRAGFVWAPSG*
HG0040.1	*H. sapiens*	ENSG00000164190	NIPBL	NIPBL, cohesin loading factor	0.00	0.00	1.8E-20	4.5E-19	0.00	1.8E-20		MGSNDSWHLN*
HG0040.2	*H. sapiens*	ENSG00000164190	NIPBL	NIPBL, cohesin loading factor	0.00	0.40	3.3E-36	1.4E-34	0.40	3.3E-36	Crowe	MFPALRRRKRSRSHPPSRPGL*
HG0041	*D. rerio*	ENSDARG00000061352	bcl11aa	B-cell CLL/lymphoma 11Aa	0.00	0.14	2.1E-59	2.0E-58	0.14	2.1E-59		MTAIPQSIFPAPSLYYF*
	G. gallus	ENSGALG00000034048			0.00	0.14	4.0E-46	5.5E-45	0.14	4.0E-46		MTAIPQFIFPAPSLYYF*
	H. sapiens	ENSG00000119866	BCL11A	B-cell CLL/lymphoma 11A	0.00	0.14	3.0E-90	2.7E-88	0.14	3.0E-90		MTALPQFIFPAPSLYYF*
HG0042.1	*H. sapiens*	ENSG00000140937	CDH11	cadherin 11	0.02	0.20	2.7E-227	5.4E-225	0.20	2.7E-227		MGGVCYRNWQQVSNG*
HG0042.2	*G. gallus*	ENSGALG00000039985			0.02	0.37	1.4E-11	7.9E-11	0.37	1.4E-11		MHSRCGGSVCAEHFI*
HG0042.3	*H. sapiens*	ENSG00000140937	CDH11	cadherin 11	0.00	0.00	1.3E-02	3.2E-02	0.00	1.3E-02		MPRGPLAAAADL*
HG0042.4	*H. sapiens*	ENSG00000140937	CDH11	cadherin 11	0.00	0.29	2.1E-04	8.9E-04	0.29	2.1E-04		MSSTSRDIPSQERSA*
HG0042.5	*H. sapiens*	ENSG00000140937	CDH11	cadherin 11	0.00	0.47	1.4E-03	4.9E-03	0.47	1.4E-03		MQKSLCFQLSGPVTFLRVVIC*
HG0043	*H. sapiens*	ENSG00000172995	ARPP21	cAMP regulated phosphoprotein 21	0.00	0.17	3.1E-276	8.4E-274	0.17	2.4E-265		MLFNEGASASLSNKRSMREDRAVHGYGYWTRIFGKVDDVSHCVRESHGDGHSELLMGTWDSSCL*
HG0044	*D. rerio*	ENSDARG00000061687	atxn1a	ataxin 1a	0.00	0.33	9.6E-100	1.2E-98	0.33	9.6E-100	Crowe	MIPHELHGPLSRSRCDFNCLRLKSVSIRP*
	H. sapiens	ENSG00000124788	ATXN1	ataxin 1	0.00	0.36	2.1E-157	3.2E-155	0.36	2.1E-157	Crowe	MIPHEEPGSPTEIKCDFPFIRLKSEPSRQ*
HG0045.1	*H. sapiens*	ENSG00000083168	KAT6A	lysine acetyltransferase 6A	0.00	0.45	2.7E-102	2.8E-100	0.45	2.7E-102		MVNWFPASENLVFHGVKLTQHQDKG*

HG0045.2	*H. sapiens*	ENSG00000083168	KAT6A	lysine acetyltransferase 6A	0.04	0.37	7.0E-26	2.1E-24	0.37	7.0E-26		MAAVSVWGISAVRPGAPGSRLSFLPA*
HG0046	*D. rerio*	ENSDARG00000018817	bdnf	brain-derived neurotrophic factor	0.01	0.18	4.1E-213	7.1E-212	0.18	4.1E-213		MMTSNKTHYLPASVGVCGY*
	H. sapiens	ENSG00000176697	BDNF	brain derived neurotrophic factor	0.00	0.18	2.0E-109	2.2E-107	0.18	2.0E-109		MMTSNKTHYLPASVGETRF*
HG0047	*G. gallus*	ENSGALG00000015422	NRG1	neuregulin 1	0.00	0.34	1.3E-173	5.5E-172	0.34	1.3E-173		MLLLLSLLPLPLLLQILLLLLLPHDNCFPRLSRYQPQMLKVRVLPFALGYWFT*
HG0048	*G. gallus*	ENSGALG00000039182			0.00	0.30	2.2E-39	2.7E-38	0.30	2.2E-39		MGCVLCCDLMV*
	H. sapiens	ENSG00000177565	TBL1XR1	transducin beta like 1 X-linked receptor 1	0.00	0.30	8.0E-29	2.6E-27	0.30	8.0E-29		MGYILCCDLMV*
HG0049	*D. rerio*	ENSDARG00000044485	sall4	spalt-like transcription factor 4	0.05	0.31	2.6E-32	1.8E-31	0.31	2.6E-32	Mackowiak	MINRNALLIIIITLTCAFLLEGLISPNLENF*
	G. gallus	ENSGALG00000039238			0.00	0.46	1.7E-100	5.3E-99	0.46	1.7E-100	Mackowiak	MINRNALLIIIIIAGHMRGARRTGEAGGGRLDFRNFEKIFF*
HG0050.1	*H. sapiens*	ENSG00000074054	CLASP1	cytoplasmic linker associated protein 1	0.00	0.45	2.7E-143	3.7E-141	0.45	2.7E-143		MVHCCHSPDCIFETQALSNLQRTKRQPPRYVCWEGVIVTAL*
HG0050.2	*H. sapiens*	ENSG00000074054	CLASP1	cytoplasmic linker associated protein 1	0.00	0.45	1.9E-06	1.2E-05	0.45	1.9E-06		MKYFRPSSRTSLCHSLLYLLLVI*
HG0051	*G. gallus*	ENSGALG00000037162			0.01	0.38	4.3E-59	7.8E-58	0.38	4.3E-59	Crowe	MSCIIRAERQKIMMHDYHRRNSCL*
	H. sapiens	ENSG00000116679	IVNS1ABP	influenza virus NS1A binding protein	0.00	0.37	4.9E-63	3.3E-61	0.37	4.9E-63	Crowe	MNVSISSEMNQIMMHHYHRRNSCL*
HG0052	*H. sapiens*	ENSG00000178235	SLITRK1	SLIT and NTRK like family member 1	0.17	0.35	1.6E-124	2.0E-122	0.35	1.6E-124		MNLNCVCGEQDGRCYFVMRSGMNCSL*
HG0053.1	*H. sapiens*	ENSG00000184564	SLITRK6	SLIT and NTRK like family member 6	0.00	0.41	3.9E-06	2.3E-05	0.41	3.9E-06		MVNYMQKSQHSCEVCREF*
HG0053.2	*H. sapiens*	ENSG00000184564	SLITRK6	SLIT and NTRK like family member 6	0.00	0.31	8.6E-19	2.0E-17	0.31	8.6E-19		MLSILSLTRGFNFMKLWDLTKSDI*
HG0054	*G. gallus*	ENSGALG00000016222	NDP	NDP, norrin cystine knot growth factor	0.19	0.23	1.2E-198	5.8E-197	0.23	1.2E-198		MTAKRLKPDFSLSWRGTGSQYLEFF*
	H. sapiens	ENSG00000124479	NDP	NDP, norrin cystine knot growth factor	0.00	0.29	2.2E-38	9.9E-37	0.29	2.2E-38		MTTRKLQPDPALSLNGTGS*
HG0055.1	*G. gallus*	ENSGALG00000010533	ELAVL4	ELAV like RNA binding protein 4	0.00	0.06	3.0E-138	1.2E-136	0.06	3.0E-138		MLHIVFQRVSCHDLIKRGGGCVCCSA*
HG0055.2	*G. gallus*	ENSGALG00000010533	ELAVL4	ELAV like RNA binding protein 4	0.00	0.00	1.3E-33	1.4E-32	0.00	1.3E-33		MSILLHRWAAAL*
HG0056	*G. gallus*	ENSGALG00000011271	LUM	lumican	0.01	0.32	1.6E-51	2.5E-50	0.32	1.6E-51		MGCPCTECGIHLHFIKRAAFSYMPHHKITMMT*
	H. sapiens	ENSG00000139329	LUM	lumican	0.01	0.33	2.2E-15	4.0E-14	0.36	2.5E-09		MSWAVLSPHVHLHFVREQCSHATPQDPHNDITPFRDWRDWAGSPHPPSALVSLRIWQPVPS*
HG0057	*H. sapiens*	ENSG00000280987	MATR3	matrin 3	0.00	0.42	5.6E-41	2.7E-39	0.42	1.1E-33		MLGAQWRRNQPSRAAESCLLVLSLKKILQSLLILFLGVTIFEAKLT*
HG0058	*H. sapiens*	ENSG00000180332	KCTD4	potassium channel tetramerization domain containing 4	0.01	0.21	1.3E-146	1.8E-144	0.21	1.3E-146		MLPAFPFHHQAGAE*
HG0059.1	*D. rerio*	ENSDARG00000018060	pik3r2	phosphoinositide-3-kinase, regulatory subunit 2 (beta)	0.01	0.17	1.1E-275	2.3E-274	0.17	3.9E-270		MLHSLCSIIWLNRGFKLRKVACTANTGQV*
	G. gallus	ENSGALG00000003428			0.01	0.17	1.4E-168	5.6E-167	0.17	1.4E-168		MPTHECWIMFVSFSLCSIIWLNRGFKLRKRGCT*
HG0059.2	*H. sapiens*	ENSG00000145675	PIK3R1	phosphoinositide-3-kinase regulatory subunit 1	0.00	0.48	1.3E-11	1.7E-10	0.48	1.3E-11		MDSVTKVSERIGPRCESQPGFKVLTSC*
HG0059.3	*H. sapiens*	ENSG00000105647	PIK3R2	phosphoinositide-3-kinase regulatory subunit 2	0.13	0.50	2.5E-07	1.9E-06	0.50	2.5E-07		MVAACTLGSLVWCSQQSRQPWAPMALRVRA*
HG0059.4	*H. sapiens*	ENSG00000145675	PIK3R1	phosphoinositide-3-kinase regulatory subunit 1	0.00	0.00	2.0E-04	8.6E-04	0.00	2.0E-04		MDSRMASHLSS*
HG0059.5	*D. rerio*	ENSDARG00000038524	pik3r1	phosphoinositide-3-kinase, regulatory subunit 1 (alpha)	0.18	0.26	6.7E-06	2.3E-05	0.26	6.7E-06		MGRVCMKHLEGAPNHWTISFT*
HG0060.1	*G. gallus*	ENSGALG00000014751			0.00	0.48	1.1E-19	8.7E-19	0.48	1.1E-19		MTADIEESCWKRVPVKKWNIQ*
	H. sapiens	ENSG00000049192	ADAMTS6	ADAM metallopeptidase with thrombospondin type 1 motif	0.00	0.47	9.0E-55	5.3E-53	0.47	9.0E-55		MNPDIEESAGCWIKVALKQWNIQ*
HG0060.2	*G. gallus*	ENSGALG00000014751			0.00	0.35	1.6E-16	1.1E-15	0.35	1.6E-16		MVQFWSGVNPIAHR*
	H. sapiens	ENSG00000049192	ADAMTS6	ADAM metallopeptidase with thrombospondin type 1 motif	0.00	0.36	4.0E-29	1.3E-27	0.36	4.0E-29		MLRFWYGIDSLAHR*
HG0061.1	*G. gallus*	ENSGALG00000004949			0.00	0.27	4.3E-12	2.5E-11	0.27	4.3E-12		MPFVKPMYTVLHCYCT*
	H. sapiens	ENSG00000166398			0.02	0.27	2.2E-51	1.3E-49	0.27	2.2E-51		MVTCLMEMPFLKLRFAELHYFYT*
HG0061.2	*G. gallus*	ENSGALG00000004949			0.00	0.34	3.2E-06	1.2E-05	0.34	3.2E-06		MLQRKQLPQQTRKGEEGLQ*
HG0061.3	*H. sapiens*	ENSG00000166398			0.03	0.30	9.8E-10	1.0E-08	0.30	9.8E-10		MLQGNRYLSKQKEWRRRLTTNAI*
HG0062.1	*D. rerio*	ENSDARG00000075569	gpbp1l1	GC-rich promoter binding protein 1-like 1	0.00	0.13	9.7E-04	2.6E-03	0.13	9.7E-04		MPPSTDGRHWT*
	G. gallus	ENSGALG00000010276	GPBP1L1	GC-rich promoter binding protein 1 like 1	0.00	0.12	1.8E-02	3.7E-02	0.12	1.8E-02		MPHFIEGRHWT*
HG0062.2	*G. gallus*	ENSGALG00000010276	GPBP1L1	GC-rich promoter binding protein 1 like 1	0.00	0.38	5.3E-08	2.3E-07	0.38	5.3E-08		MSCPADLGVSVQY*
HG0062.3	*H. sapiens*	ENSG00000159592	GPBP1L1	GC-rich promoter binding protein 1 like 1	0.00	0.29	3.0E-14	5.1E-13	0.29	3.0E-14		MSYLIKQHHSAITQGS*
HG0063.1	*G. gallus*	ENSGALG00000002477			0.00	0.12	5.1E-09	2.4E-08	0.12	5.1E-09		MCACSIASFLLK*
	H. sapiens	ENSG00000162630	B3GALT2	beta-1,3-galactosyltransferase 2	0.00	0.12	5.9E-09	5.5E-08	0.12	5.9E-09		MCACSIASFLLK*
HG0063.2	*H. sapiens*	ENSG00000162630	B3GALT2	beta-1,3-galactosyltransferase 2	0.00	0.41	1.9E-11	2.4E-10	0.41	1.9E-11		MDTETRTQHCIVQPGK*
HG0064.1	*H. sapiens*	ENSG00000102678	FGF9	fibroblast growth factor 9	0.00	0.33	2.1E-133	2.7E-131	0.33	2.1E-133		MVILISNQMEKLRIFFPYYGRMG*
HG0064.2	*H. sapiens*	ENSG00000113578	FGF1	fibroblast growth factor 1	0.01	0.38	1.2E-14	2.1E-13	0.38	1.2E-14		MVCGFTQFFSWGFSELHSYQKGSRRERTSGSNSLRSRGIRASPPFLGGHSLLMVNGNSLPPAAACLQLSW*
HG0065	*G. gallus*	ENSGALG00000029927	STRBP	Spermatid perinuclear RNA-binding protein	0.00	0.36	1.6E-85	4.3E-84	0.36	1.6E-85		MKRFCQAKQLLSKTPRRREKEKERES*
HG0066	*H. sapiens*	ENSG00000112182	BACH2	BTB domain and CNC homolog 2	0.22	0.18	5.0E-206	8.9E-204	0.18	5.0E-206		MDCQSLLISPFAQFPAS*
HG0067	*H. sapiens*	ENSG00000166862	CACNG2	calcium voltage-gated channel auxiliary subunit gamma 2	0.00	0.01	2.6E-80	2.1E-78	0.01	2.6E-80		METGDQNFRKRD*
HG0068	*D. rerio*	ENSDARG00000071235			0.02	0.00	3.2E-256	6.1E-255	0.00	3.2E-256	Mackowiak	MRFFRLTFKCFVDCF*
	G. gallus	ENSGALG00000041419			0.03	0.00	1.7E-293	1.3E-291	0.00	1.7E-293	Mackowiak	MRFFRLTFKCFVDCF*
	H. sapiens	ENSG00000130821	SLC6A8	solute carrier family 6 member 8	0.02	0.00	2.0E-232	4.3E-230	0.00	2.0E-232	Mackowiak	MRFFRLTFKCFVDCF*
HG0069	*G. gallus*	ENSGALG00000010999	DICER1	dicer 1, ribonuclease III	0.00	0.42	2.9E-40	3.7E-39	0.42	2.9E-40		MMSKCSLESISTEAAEIKT*
	H. sapiens	ENSG00000100697	DICER1	dicer 1, ribonuclease III	0.03	0.27	1.1E-78	8.0E-77	0.27	1.1E-78		MSKCSSDKSNTEISNIKT*
HG0070	*D. rerio*	ENSDARG00000040926	nr2f2	nuclear receptor subfamily 2, group F, member 2	0.00	0.36	4.4E-41	3.7E-40	0.36	4.4E-41		MDDCKCPWTWPLRARLLFGLLQF*
		ENSDARG00000052695	nr2f1a	nuclear receptor subfamily 2, group F, member 1a	0.03	0.13	1.7E-126	2.3E-125	0.13	1.7E-126		MADCNLPWNWPPDNCIS*
	H. sapiens	ENSG00000175745	NR2F1	nuclear receptor subfamily 2 group F member 1	0.03	0.12	6.3E-162	9.8E-160	0.12	6.3E-162		MAGCVRPWTWPPDTRAP*
		ENSG00000185551	NR2F2	nuclear receptor subfamily 2 group F member 2	0.20	0.07	1.5E-119	1.8E-117	0.07	1.5E-119		MADCGLPWTWPPDSRLSSSSTSSFTTTSSSSSSSSSSANSSAAHQL*
HG0071.1	*D. rerio*	ENSDARG00000098240	meis2a	Meis homeobox 2a	0.00	0.39	2.0E-42	1.7E-41	0.39	2.0E-42		MRHDSLVTCRLLMGRL*
	H. sapiens	ENSG00000134138	MEIS2	Meis homeobox 2	0.00	0.39	1.1E-68	8.0E-67	0.39	1.1E-68		MKHDSSVTCGLLTGRS*
HG0071.2	*H. sapiens*	ENSG00000143995	MEIS1	Meis homeobox 1	0.00	0.00	1.2E-08	1.1E-07	0.00	1.2E-08		MLSDSGALLQVP*
HG0072	*G. gallus*	ENSGALG00000039690	STMN2	Stathmin-2	0.11	0.34	2.7E-35	3.0E-34	0.34	2.7E-35		MPGDSGSAGVMSGDL*
	H. sapiens	ENSG00000104435	STMN2	stathmin 2	0.12	0.40	1.7E-101	1.7E-99	0.40	1.7E-101		MGGADREGLISISNCILVLCTPAPLADQYLMLGDSDSAGVMSGDLGSQSA*
HG0073	*D. rerio*	ENSDARG00000041708	csrp2bp	csrp2 binding protein	0.02	0.13	2.3E-203	3.7E-202	0.13	2.3E-203		MSTASKVVLGLSIVLTISTVAGVHIKQNWDRQRLREGVVRDLERLERKRENLRALEEQIQLTRELVTERNRRQAEASETHDS*
HG0074	*D. rerio*	ENSDARG00000078624	arhgef9b	Cdc42 guanine nucleotide exchange factor (GEF) 9b	0.06	0.06	1.9E-10	8.0E-10	0.06	1.9E-10	Mackowiak	MTLDVQTVAVFTVIAILLLVNVILIFFMGSS*
	H. sapiens	ENSG00000131089	ARHGEF9	Cdc42 guanine nucleotide exchange factor 9	0.01	0.04	8.1E-218	1.5E-215	0.04	8.1E-218	Mackowiak	MDSLTEQRLTSPNLPAPHLEHYSVLHCTMTLDVQTVVVFAVIVVLLLVNVILMFFLGTR*
HG0075	*H. sapiens*	ENSG00000165699	TSC1	tuberous sclerosis 1	0.00	0.45	1.6E-49	8.8E-48	0.45	1.6E-49	Crowe	MKDTRLTALETEVPVVARTVW*
HG0076	*D. rerio*	ENSDARG00000040008	neurod6a	neuronal differentiation 6a	0.01	0.30	5.9E-120	8.1E-119	0.50	7.1E-15		MKTTWTHCLLTGFDGRTTVKNTKANSTPGAVMMDAPLWTGHDGQQAALNRHAPGRVVRSSRHMPDDQGRNTGPLRASHVTSPCHVTSAADGMGTSSGSWHQCHFS*
	H. sapiens	ENSG00000164600	NEUROD6	neuronal differentiation 6	0.00	0.47	5.9E-31	2.1E-29	0.47	5.7E-09		MGTSSGSWHQCHFLEINEIVQIYTDLETPGDDATLSLKRFGRPKMKTDY*
HG0077.1	*G. gallus*	ENSGALG00000005074			0.00	0.48	7.0E-33	7.1E-32	0.48	7.0E-33		MIPQPDPTTKEKKIVHLRLGRHCN*
HG0077.2	*G. gallus*	ENSGALG00000005074			0.00	0.08	1.3E-10	6.8E-10	0.08	1.3E-10		MPSGGVLLKLGHCNL*
HG0078.1	*G. gallus*	ENSGALG00000016633	HMBOX1	homeobox containing 1	0.00	0.01	6.7E-05	2.2E-04	0.01	6.7E-05		MVNNTEDPQEAL*
HG0078.2	*H. sapiens*	ENSG00000147421	HMBOX1	homeobox containing 1	0.00	0.37	1.4E-02	3.4E-02	0.37	1.4E-02		MVDNADHLWKGY*
HG0079	*H. sapiens*	ENSG00000204406	MBD5	methyl-CpG binding domain protein 5	0.00	0.30	4.9E-94	4.8E-92	0.31	2.0E-89		MSTIFWREKRYSLIGTRKDIEHLEINCLHYTAESHTTLVMFSSLLSRRLHYWNFEVMKTSDEPTCNTLDSDWKIKRKFKECGL*
HG0080.1	*G. gallus*	ENSGALG00000039403			0.01	0.29	4.2E-68	9.5E-67	0.29	4.2E-68		MVLYHLLGTDGSLQKVQFQMVNLNL*
	H. sapiens	ENSG00000179603	GRM8	glutamate metabotropic receptor 8	0.02	0.29	1.0E-86	8.8E-85	0.29	1.0E-86		MGPDGSLQKVQPQVVPFLLWQE*
HG0080.2	*H. sapiens*	ENSG00000179603	GRM8	glutamate metabotropic receptor 8	0.00	0.09	8.7E-03	2.3E-02	0.09	8.7E-03		MTRNNSATRLISRT*
HG0080.3	*H. sapiens*	ENSG00000179603	GRM8	glutamate metabotropic receptor 8	0.09	0.34	1.0E-06	6.9E-06	0.34	1.0E-06		MEETWRLLGQLSPLSPEISHKCGRCARLTRRRRRRRRR*
HG0081.1	*D. rerio*	ENSDARG00000028228	zbtb18	zinc finger and BTB domain containing 18	0.06	0.41	7.1E-19	3.9E-18	0.41	7.1E-19		MMSEQLTGCHPPVFSSNAQTGS*
HG0081.2	*D. rerio*	ENSDARG00000028228	zbtb18	zinc finger and BTB domain containing 18	0.08	0.35	1.2E-02	2.2E-02	0.35	1.2E-02		MIVPIVSYFFFPPPLSVVQRVSKMNDGNGAASEP*
HG0082	*D. rerio*	ENSDARG00000062379	slc35a4	solute carrier family 35, member A4	0.01	0.07	5.5E-233	9.9E-232	0.07	5.5E-233	Andreev	MAEDKDPVTKLKDLVHLKDQLEEIQKKVENEVQAGVPQGGSLLASPFLKGFLAGYVVSRLRSSAVLGVALGTLSGIFAAQNYQVPNIEATLRDILNSLKKRPGE*
	H. sapiens	ENSG00000176087	SLC35A4	solute carrier family 35 member A4	0.01	0.07	2.7E-235	6.1E-233	0.07	2.7E-235	Andreev	MADDKDSLPKLKDLAFLKNQLESLQRRVEDEVNSGVGQDGSLLSSPFLKGFLAGYVVAKLRASAVLGFAVGTCTGIYAAQAYAVPNVEKTLRDYLQLLRKGPD*
HG0083.1	*G. gallus*	ENSGALG00000008039	MFSD13A	major facilitator superfamily domain containing 13A	0.00	0.31	1.3E-53	2.2E-52	0.31	1.3E-53		MDWPRQRCNRCCEKTSHLKWR*
HG0083.2	*H. sapiens*	ENSG00000138111	MFSD13A	major facilitator superfamily domain containing 13A	0.07	0.49	1.1E-03	3.9E-03	0.49	1.1E-03		MGRPPVAMSPPRCSWG*
HG0084.1	*G. gallus*	ENSGALG00000009207	TRIM2	tripartite motif containing 2	0.00	0.50	3.2E-35	3.6E-34	0.50	3.2E-35		MMFYSFGLLRNCSSIPARILSSALH*
HG0084.2	*H. sapiens*	ENSG00000154447	SH3RF1	SH3 domain containing ring finger 1	0.00	0.12	4.4E-03	1.3E-02	0.12	4.4E-03		MSHFKGCTQL*
HG0085	*G. gallus*	ENSGALG00000002003	BMPR1A	bone morphogenetic protein receptor type 1A	0.00	0.10	5.8E-17	4.2E-16	0.10	5.8E-17		MRFHNTTFELMKWKYR*
	H. sapiens	ENSG00000107779	BMPR1A	bone morphogenetic protein receptor type 1A	0.00	0.28	4.3E-74	3.1E-72	0.28	4.3E-74		MHQFNTVLEFMRWKHRSKLFGENQKYSFI*
HG0086	*G. gallus*	ENSGALG00000009835	XPOT	exportin for tRNA	0.00	0.41	1.7E-90	4.8E-89	0.41	1.7E-90		MLVATSELWGGSRHRRDGGVTGKMIVISTLEEVGKPVSRSRKRRV*
HG0087	*H. sapiens*	ENSG00000164548	TRA2A	transformer 2 alpha homolog	0.00	0.06	5.2E-81	4.2E-79	0.06	5.2E-81		MWKINQDKRRLMKSIKDQKIKIEWGKKLNRRLVR*
HG0088	*H. sapiens*	ENSG00000273079	GRIN2B	glutamate ionotropic receptor NMDA type subunit 2B	0.02	0.35	1.5E-30	5.2E-29	0.35	1.5E-30		MQARRRDWTFPTCSLP*
HG0089.1	*G. gallus*	ENSGALG00000002294	SEMA6A	semaphorin 6A	0.00	0.36	2.0E-69	4.8E-68	0.36	2.0E-69		MTKDCKGESGSSFPERSP*
	H. sapiens	ENSG00000092421	SEMA6A	semaphorin 6A	0.00	0.40	3.1E-68	2.1E-66	0.40	3.1E-68		MTKACEGESAARGPERSPR*
HG0089.2	*H. sapiens*	ENSG00000092421	SEMA6A	semaphorin 6A	0.00	0.00	6.4E-18	1.4E-16	0.00	6.4E-18		MNTSRLHPRK*
HG0089.3	*G. gallus*	ENSGALG00000002294	SEMA6A	semaphorin 6A	0.00	0.00	1.1E-14	7.5E-14	0.00	1.1E-14		MNKLHLQFRK*
HG0090.1	*G. gallus*	ENSGALG00000008487	TMCC1	transmembrane and coiled-coil domain family 1	0.00	0.02	1.3E-53	2.3E-52	0.02	1.3E-53		MEISMRSPWHSCMCSHS*
	H. sapiens	ENSG00000172765	TMCC1	transmembrane and coiled-coil domain family 1	0.00	0.35	1.1E-02	2.9E-02	0.35	1.1E-02		MKIGMKPSSHSCMRSHC*
HG0090.2	*D. rerio*	ENSDARG00000060954	tmcc1b	transmembrane and coiled-coil domain family 1b	0.00	0.34	1.2E-37	9.5E-37	0.34	1.2E-37		MTSMTLRQTTDACTLSEST*
HG0090.3	*G. gallus*	ENSGALG00000008487	TMCC1	transmembrane and coiled-coil domain family 1	0.09	0.33	2.0E-08	9.2E-08	0.33	2.0E-08		MLSFVQWRDCCMSAMLKGK*
HG0091.1	*H. sapiens*	ENSG00000172209	GPR22	G protein-coupled receptor 22	0.00	0.37	3.4E-42	1.7E-40	0.37	3.4E-42		MAHYRHIFSLRSFSKRCL*
HG0091.2	*H. sapiens*	ENSG00000172209	GPR22	G protein-coupled receptor 22	0.00	0.47	2.3E-12	3.3E-11	0.47	2.3E-12		MSKYILEATNVMFPFLFSHILENKEILSCTPSKGNIIPII*
HG0091.3	*H. sapiens*	ENSG00000172209	GPR22	G protein-coupled receptor 22	0.00	0.35	4.9E-11	6.0E-10	0.35	4.9E-11		MYPLLMKTNKIKQQMVFIKRTWPGFII*
HG0092.1	*G. gallus*	ENSGALG00000009966	FRS2	fibroblast growth factor receptor substrate 2	0.00	0.11	8.3E-65	1.8E-63	0.11	8.3E-65		MISSVTRQHDTLKYDRSPDAATEAGNINGQI*

HG0092.2	*H. sapiens*	ENSG00000166225	FRS2	fibroblast growth factor receptor substrate 2	0.01	0.36	6.4E-07	4.4E-06	0.36	6.4E-07		MKQSICHKVRCSCGMSTSLEQNCICFSQKRIPQG*
HG0093	*G. gallus*	ENSGALG00000040465	Sip1	zinc finger E-box binding homeobox 2	0.13	0.26	1.7E-35	2.0E-34	0.26	1.7E-35	Crowe, Samandi	MLFLLTISGQNYKQDFAESACC*
	H. sapiens	ENSG00000169554	ZEB2	zinc finger E-box binding homeobox 2	0.22	0.44	2.7E-40	1.3E-38	0.44	2.7E-40	Crowe, Samandi	MLFLLTISGQNYKQDFAAPCLN*
HG0094.1	*H. sapiens*	ENSG00000112175	BMP5	bone morphogenetic protein 5	0.00	0.31	2.7E-60	1.7E-58	0.31	2.7E-60		MQQKLLTTVDNWKSEFQLS*
HG0094.2	*H. sapiens*	ENSG00000112175	BMP5	bone morphogenetic protein 5	0.00	0.15	8.4E-26	2.6E-24	0.15	8.4E-26		MDLQEGFQVNSGKHIYLNSTT*
HG0095	*D. rerio*	ENSDARG00000013708	usp9	ubiquitin specific peptidase 9	0.00	0.46	9.7E-57	8.9E-56	0.46	9.7E-57		MEERSSLILGYAMVSATYPVPQVVLSLSEVCRFSVAPEEKLISSGVPAT*
HG0096	*G. gallus*	ENSGALG00000014186	MPPED1	metallophosphoesterase domain containing 1	0.01	0.42	6.1E-03	1.4E-02	0.42	6.1E-03		MVFSCRCRKIADMIA*
HG0097	*G. gallus*	ENSGALG00000037496			0.01	0.25	1.6E-37	1.8E-36	0.25	1.6E-37		MELQPRAGAADTPSAFLRSSHHERGRFHLVKMAVWDRCNL*
HG0098	*H. sapiens*	ENSG00000198739	LRRTM3	leucine rich repeat transmembrane neuronal 3	0.00	0.00	1.2E-06	8.1E-06	0.00	1.2E-06		MQKRNIHEDPIT*
HG0099.1	*H. sapiens*	ENSG00000168575	SLC20A2	solute carrier family 20 member 2	0.00	0.01	5.3E-105	5.6E-103	0.01	5.3E-105		MRKTNGSEKVYRIIGGDITAFPDREEKYGMCFTAD*
HG0099.2	*H. sapiens*	ENSG00000144136	SLC20A1	solute carrier family 20 member 1	0.00	0.28	3.1E-03	9.5E-03	0.28	3.1E-03		MNLRPFSSPPWNSAPCF*
HG0099.3	*H. sapiens*	ENSG00000168575	SLC20A2	solute carrier family 20 member 2	0.00	0.45	1.0E-02	2.6E-02	0.45	1.0E-02		MMLCIVHQGTPLGRISH*
HG0099.4	*D. rerio*	ENSDARG00000010641	slc20a1b	solute carrier family 20 (phosphate transporter), member 1b	0.00	0.36	5.5E-07	2.1E-06	0.36	5.5E-07		MDVRCICLIVQAKTNRNRSSLFRG*
HG0100.1	*D. rerio*	ENSDARG00000104148	crebbpb	CREB binding protein b	0.00	0.48	5.8E-40	4.7E-39	0.48	5.8E-40		MRVLGVGLSWGEWRWRSGALLSRV*
		ENSDARG00000104609	crebbpa	CREB binding protein a	0.00	0.50	2.6E-33	1.8E-32	0.50	2.6E-33		MIMWVLGVGLSWGEWWWRRGALLLLF*
HG0100.2	*D. rerio*	ENSDARG00000061108	ep300b	E1A binding protein p300 b	0.00	0.47	2.3E-13	1.0E-12	0.47	2.3E-13		MAEKTDASSEDKSRENCSVAEEAT*
HG0101	*H. sapiens*	ENSG00000099250	NRP1	neuropilin 1	0.00	0.45	2.0E-19	4.7E-18	0.45	2.0E-19		MARAVAPGRGTSVGKGRGSRS*
HG0102	*H. sapiens*	ENSG00000182263	FIGN	fidgetin, microtubule severing factor	0.00	0.45	6.5E-32	2.4E-30	0.45	6.5E-32		MISNHRHLHLTLASIHQQTHRIDATLTYRVLSPRVQVK*
HG0103	*G. gallus*	ENSGALG00000016558	VEGFD	vascular endothelial growth factor D	0.00	0.21	2.2E-93	6.6E-92	0.21	2.2E-93		MHFKRDASSCMTTTA*
	H. sapiens	ENSG00000165197	VEGFD	vascular endothelial growth factor D	0.00	0.48	1.7E-02	4.1E-02	0.48	1.7E-02		MPFETIFSSCLMSTA*
HG0104.1	*G. gallus*	ENSGALG00000038097	EI24	EI24, autophagy associated transmembrane protein	0.00	0.32	3.0E-25	2.6E-24	0.32	3.0E-25		MMREAWVRLFPSPCS*
HG0104.2	*H. sapiens*	ENSG00000149547	EI24	EI24, autophagy associated transmembrane protein	0.02	0.47	5.5E-05	2.7E-04	0.47	5.5E-05		MLVLKQKSSLQSLSKNKSRPRSFMMRDLGTLLSPVCS*
HG0105	*G. gallus*	ENSGALG00000016176			0.00	0.33	6.9E-110	2.4E-108	0.35	2.0E-107		MWLDLADPEPELLFFRGAVGVREACGGYLAAFFSLLLPFFFFFFFFLVGFEPEPGKMGNGASDRRGISPSLVSGRVPVVLEV*
	H. sapiens	ENSG00000135298	ADGRB3	adhesion G protein-coupled receptor B3	0.00	0.33	1.1E-87	9.3E-86	0.33	1.1E-87		MGNGDWDRRGVSPSLLLGRVPVLVS*
HG0106	*H. sapiens*	ENSG00000147548	NSD3	nuclear receptor binding SET domain protein 3	0.00	0.11	1.8E-29	6.2E-28	0.11	1.8E-29		MEQTEGPMRERES*
HG0107.1	*G. gallus*	ENSGALG00000003601	ATP2B4	ATPase plasma membrane Ca2+ transporting 4	0.00	0.22	4.5E-81	1.1E-79	0.22	4.5E-81		MHILFTPSSGGLGLG*
	H. sapiens	ENSG00000058668	ATP2B4	ATPase plasma membrane Ca2+ transporting 4	0.00	0.20	1.7E-80	1.4E-78	0.20	1.7E-80		MHILFTPSSGQVGLS*
HG0107.2	*G. gallus*	ENSGALG00000030550			0.00	0.30	6.0E-44	7.8E-43	0.30	6.0E-44		MGRPQRYLSKMGLLLG*
	H. sapiens	ENSG00000070961	ATP2B1	ATPase plasma membrane Ca2+ transporting 1	0.00	0.31	1.2E-12	1.7E-11	0.31	1.2E-12		MIDMEKLVMGQRSRYFSGKCCC*
HG0107.3	*D. rerio*	ENSDARG00000063433	atp2b2	ATPase, Ca++ transporting, plasma membrane 2	0.01	0.28	3.5E-23	2.1E-22	0.28	3.5E-23	Crowe	MGNHVWSDAGLQPW*
	H. sapiens	ENSG00000157087	ATP2B2	ATPase plasma membrane Ca2+ transporting 2	0.00	0.41	9.9E-07	6.6E-06	0.41	9.9E-07	Crowe	MPVPGSSMGPTEWRLLGLQP*
HG0107.4	*H. sapiens*	ENSG00000070961	ATP2B1	ATPase plasma membrane Ca2+ transporting 1	0.00	0.00	6.5E-03	1.8E-02	0.00	6.5E-03		MSVWFKKDFHT*
HG0108.1	*G. gallus*	ENSGALG00000007396	TAOK3	TAO kinase 3	0.00	0.29	5.8E-53	9.5E-52	0.29	5.8E-53		MKEETDWDLKAPTSS*
HG0108.2	*H. sapiens*	ENSG00000135090	TAOK3	TAO kinase 3	0.01	0.32	8.1E-10	8.7E-09	0.32	8.1E-10		MNLSNMEYFVPHTKRY*
HG0108.3	*D. rerio*	ENSDARG00000098304	taok1b	TAO kinase 1b	0.07	0.23	1.6E-02	2.9E-02	0.23	1.6E-02		MSNQRGLTCRSRTAR*
HG0109.1	*H. sapiens*	ENSG00000048540	LMO3	LIM domain only 3	0.00	0.34	1.3E-34	5.1E-33	0.34	1.3E-34		MNGSHRQTSDCSHLQAFLKGEGCVLFPGYSGEALQSSHCAY*
HG0109.2	*H. sapiens*	ENSG00000048540	LMO3	LIM domain only 3	0.17	0.41	1.9E-23	5.3E-22	0.41	1.9E-23		MQHIFYGDYSDCCYEIQVRFMIFFILNMICYANAACDSLIKSICYQEAKRGRWDFRITGRVREACGFVIRHHFAHCEGLRVCALSCLAVLIPSPTPCRHSDPLPKWLHNSRSSAV*
HG0109.3	*H. sapiens*	ENSG00000048540	LMO3	LIM domain only 3	0.08	0.43	1.7E-15	3.1E-14	0.43	1.7E-15		MYACLDGPEKPNLRNLPMYRTSQLRGSH*
HG0110.1	*H. sapiens*	ENSG00000091656	ZFHX4	zinc finger homeobox 4	0.00	0.34	5.2E-28	1.6E-26	0.34	5.2E-28		MPPTFCSSVFIFTQ*
HG0110.2	*D. rerio*	ENSDARG00000073944	ZFHX3 (1 of many)	si:ch73-386h18.1	0.19	0.47	8.0E-08	3.1E-07	0.47	8.0E-08		MRSVLIPAIFCTKVISRNKDRTDFFSLSPPSSNRSQGASVVCSTTKKQTLLNALRNTEIPRLTSAA*
HG0111	*G. gallus*	ENSGALG00000034944			0.04	0.25	5.3E-63	1.1E-61	0.25	5.3E-63		MEVISWGGPRALWARVLLSDHWLPPE*
HG0112	*H. sapiens*	ENSG00000105997	HOXA3	homeobox A3	0.00	0.32	2.6E-50	1.4E-48	0.32	2.6E-50		MKRSARGGHWRRSVT*
HG0113	*D. rerio*	ENSDARG00000099727	ssbp2	single-stranded DNA binding protein 2	0.00	0.40	4.7E-35	3.4E-34	0.40	4.7E-35		MGGIAPAVAMVTGLCPGRL*
	G. gallus	ENSGALG00000023089	SSBP2	single stranded DNA binding protein 2	0.00	0.38	2.5E-05	8.8E-05	0.38	2.5E-05		MGCVCGGVAMATGLFPGRL*
HG0114.1	*G. gallus*	ENSGALG00000003701	SGMS1	sphingomyelin synthase 1	0.01	0.46	1.3E-14	8.5E-14	0.46	1.3E-14		MKPRRSLLMLWSRSDCCPL*
HG0114.2	*H. sapiens*	ENSG00000198964	SGMS1	sphingomyelin synthase 1	0.00	0.36	3.6E-04	1.5E-03	0.36	3.6E-04		MTRMFLNNPRKNPAQK*
HG0115.1	*G. gallus*	ENSGALG00000010976	CCDC126	coiled-coil domain containing 126	0.00	0.41	5.0E-11	2.6E-10	0.41	5.0E-11		MESRPAKPRRCVQDDPARRLLTL*
HG0115.2	*H. sapiens*	ENSG00000169193	CCDC126	coiled-coil domain containing 126	0.02	0.01	6.5E-10	7.1E-09	0.01	6.5E-10		MSANRVNRVDTTLLKMKNIQY*
HG0116	*G. gallus*	ENSGALG00000033038			0.01	0.30	4.0E-36	4.6E-35	0.30	4.0E-36		MDQHSSDGETGKGLL*
	H. sapiens	ENSG00000010270	STARD3NL	STARD3 N-terminal like	0.00	0.35	1.5E-05	8.0E-05	0.35	1.5E-05		MCLLFRDGEVGKRLL*
HG0117.1	*H. sapiens*	ENSG00000140945	CDH13	cadherin 13	0.07	0.49	2.4E-19	5.6E-18	0.49	2.4E-19		MLSAAACMNENAAGRF*
HG0117.2	*H. sapiens*	ENSG00000140945	CDH13	cadherin 13	0.00	0.00	1.1E-03	4.0E-03	0.00	1.1E-03		MPPPRGSLLARCSPRICHAKRGSVRKESVL*
HG0118.1	*H. sapiens*	ENSG00000183662	FAM19A1	family with sequence similarity 19 member A1, C-C motif ch	0.00	0.22	2.2E-18	4.8E-17	0.22	2.2E-18		MTRFPPLKPWLLF*
HG0118.2	*H. sapiens*	ENSG00000183662	FAM19A1	family with sequence similarity 19 member A1, C-C motif ch	0.00	0.38	1.3E-03	4.4E-03	0.38	1.3E-03		MHWSGDGPSATIN*
HG0119	*G. gallus*	ENSGALG00000043570	SGK3		0.04	0.20	3.5E-51	5.4E-50	0.20	3.5E-51		MMEFTFSQEELRILDQYR*
HG0120	*H. sapiens*	ENSG00000022355	GABRA1	gamma-aminobutyric acid type A receptor alpha1 subunit	0.08	0.46	1.5E-03	5.1E-03	0.46	1.5E-03		MKSPPISHPVCPGTYYYCLGKEKSFVV*
HG0121	*H. sapiens*	ENSG00000122786	CALD1	caldesmon 1	0.00	0.50	6.8E-12	9.1E-11	0.50	6.8E-12		MCSLSMDFWEGPRKGGLEFY*
HG0122	*H. sapiens*	ENSG00000169925	BRD3	bromodomain containing 3	0.00	0.40	4.5E-75	3.3E-73	0.40	4.5E-75		MLSCDRPSSCLEEAGLQPGSLPLCPDHPHIPKAVAWLGPEMGRRAFSGSVEAARASPSRCCDRK*
HG0123	*H. sapiens*	ENSG00000173276	ZBTB21	zinc finger and BTB domain containing 21	0.03	0.41	3.1E-36	1.4E-34	0.41	3.1E-36		MTSSLVWDDVKFKMDGITAAAAAAARASRV*
HG0124.1	*D. rerio*	ENSDARG00000031763	smad6b	SMAD family member 6b	0.00	0.49	2.3E-13	1.0E-12	0.49	2.3E-13		MQYSIVPSKLMLWLCHRYTVLGCARAARHSANINVARAQK*
HG0124.2	*D. rerio*	ENSDARG00000053209	smad6a	SMAD family member 6a	0.09	0.14	3.5E-04	9.9E-04	0.14	3.5E-04		MMGINQWLHCSPQGPRVCVSEEERE*
HG0125.1	*D. rerio*	ENSDARG00000051926	plag1	pleiomorphic adenoma gene 1	0.00	0.33	2.7E-38	2.1E-37	0.33	2.7E-38		MRKKLLKIREIVESQEKYRRKDVNAPAGLMSLLC*
HG0125.2	*H. sapiens*	ENSG00000181690	PLAG1	PLAG1 zinc finger	0.00	0.48	3.3E-09	3.2E-08	0.50	3.5E-04		MLKPRDWPKWEGLDSTLFHEESMGLAKIKV*
HG0126.1	*G. gallus*	ENSGALG00000010899	G6PC2	glucose-6-phosphatase catalytic subunit 2	0.00	0.30	2.5E-10	1.2E-09	0.30	2.5E-10		MNCEKIQANMFQLFIRRIA*
HG0126.2	*G. gallus*	ENSGALG00000010899	G6PC2	glucose-6-phosphatase catalytic subunit 2	0.07	0.46	1.2E-15	8.2E-15	0.46	1.2E-15		MCFPRDSEPRVPGMAGYATLIQYKAQPSGWY*
HG0127.1	*G. gallus*	ENSGALG00000029709			0.00	0.33	3.4E-34	3.7E-33	0.33	3.4E-34		MQNQSTHEEKSPLRGNVRGIAVGDNLLHSPTP*
HG0127.2	*H. sapiens*	ENSG00000109452	INPP4B	inositol polyphosphate-4-phosphatase type II B	0.00	0.40	6.2E-09	5.8E-08	0.40	6.2E-09		MVLPGKRSSVFYEKLDKGFTGGIVLEICS*
HG0128	*D. rerio*	ENSDARG00000091029	phox2bb	paired-like homeobox 2bb	0.00	0.25	4.1E-45	3.6E-44	0.25	4.1E-45		MRTICSRDLGLGGSLGGFS*
	H. sapiens	ENSG00000109132	PHOX2B	paired like homeobox 2b	0.12	0.39	5.7E-33	2.2E-31	0.39	5.7E-33		MQESMIGRLDSSVPQCQPNRRMSYCHVKSASNKTNRFAIVQVERAKFIMRTICSRDLRQGIS*
HG0129	*D. rerio*	ENSDARG00000099437			0.00	0.14	2.5E-88	3.0E-87	0.14	2.5E-88		MPAVFLLLRSLVVRLFGSRLAASGVQLLRRILTTATGHLGTVLRNIWERISSQQSKEAILGCVLCLLNMHKKVDN*
HG0130	*G. gallus*	ENSGALG00000002993			0.00	0.31	1.7E-69	4.1E-68	0.31	1.7E-69		MSCWEIRRIFFSLVEEEPCPGGWLHGLFWRAKEAKGSCLSRTQRELGHVLSAHLGNTVCKSRRKIAGIIQTAILIFVIFFRRGMHLA*
HG0131	*G. gallus*	ENSGALG00000013135	GALNT1	polypeptide N-acetylgalactosaminyltransferase 1	0.00	0.35	1.1E-14	7.4E-14	0.35	1.1E-14		MAVNLQRRYRTWSLKLD*
HG0132	*H. sapiens*	ENSG00000151067	CACNA1C	calcium voltage-gated channel subunit alpha1 C	0.00	0.32	1.0E-41	5.2E-40	0.32	1.0E-41	Crowe	MRYSFTRGGSSGKEQFLGFDAIMGIR*
HG0133.1	*D. rerio*	ENSDARG00000051886	ankrd11	ankyrin repeat domain 11	0.08	0.00	4.1E-82	4.3E-81	0.00	4.1E-82	Mackowiak	MESTVDDDAMEA*
	H. sapiens	ENSG00000167522	ANKRD11	ankyrin repeat domain 11	0.13	0.05	2.0E-09	2.0E-08	0.05	2.0E-09	Mackowiak	MESQTVDDEAPAV*
HG0133.2	*D. rerio*	ENSDARG00000051886	ankrd11	ankyrin repeat domain 11	0.00	0.17	7.2E-03	1.5E-02	0.17	7.2E-03		MGKTSIGREYQV*
HG0133.3	*H. sapiens*	ENSG00000167522	ANKRD11	ankyrin repeat domain 11	0.00	0.36	3.2E-04	1.3E-03	0.36	3.2E-04		MGSARAPPLSRRGAAQPSEHEYIQPCSGTHLHWI*
HG0134.1	*G. gallus*	ENSGALG00000041267	BMI1	Polycomb complex protein BMI-1	0.09	0.24	1.8E-02	3.7E-02	0.24	1.8E-02		MAAWLTFIFC*
HG0134.2	*H. sapiens*	ENSG00000168283	BMI1	BMI1 proto-oncogene, polycomb ring finger	0.08	0.46	7.1E-07	4.9E-06	0.46	7.1E-07		MRGRDRGETMGMWAREPRSGLAAPPSPAE*
HG0135	*D. rerio*	ENSDARG00000094132	igf1	insulin-like growth factor 1	0.00	0.31	6.6E-08	2.6E-07	0.31	6.6E-08		MLPQLFPVENVSVM*
	H. sapiens	ENSG00000017427	IGF1	insulin like growth factor 1	0.00	0.38	1.6E-13	2.6E-12	0.38	2.0E-17		MFPQLFPVYSVCVL*
HG0136.1	*H. sapiens*	ENSG00000125845	BMP2	bone morphogenetic protein 2	0.03	0.15	2.0E-30	7.1E-29	0.15	2.0E-30	Crowe	MWTLFQWTCPRVLLRRTAVS*
HG0136.2	*H. sapiens*	ENSG00000125845	BMP2	bone morphogenetic protein 2	0.15	0.30	6.3E-04	2.4E-03	0.30	6.3E-04		MRSTYCRRSGAWDALAEV*
HG0137.1	*H. sapiens*	ENSG00000181722	ZBTB20	zinc finger and BTB domain containing 20	0.00	0.32	1.1E-04	5.1E-04	0.32	1.1E-04		MNICTRPKPWSNLPEE*
HG0137.2	*H. sapiens*	ENSG00000181722	ZBTB20	zinc finger and BTB domain containing 20	0.00	0.20	6.2E-03	1.8E-02	0.20	6.2E-03		MSWKCRNLSLPSRKNL*
HG0138.1	*H. sapiens*	ENSG00000185070	FLRT2	fibronectin leucine rich transmembrane protein 2	0.00	0.13	5.3E-92	5.1E-90	0.13	5.3E-92	Mackowiak	MPRDPRCCRASVFPFHLPDPVGFG*
HG0138.2	*H. sapiens*	ENSG00000185070	FLRT2	fibronectin leucine rich transmembrane protein 2	0.00	0.00	2.6E-06	1.6E-05	0.00	2.6E-06	Mackowiak	MGRRDLGGDP*
HG0139	*G. gallus*	ENSGALG00000006379	SHH	sonic hedgehog	0.14	0.50	1.4E-02	3.0E-02	0.50	1.4E-02		MCSVISDLKAAIPAPVLLGIGG*
HG0140	*H. sapiens*	ENSG00000009307	CSDE1	cold shock domain containing E1	0.00	0.00	1.3E-05	7.3E-05	0.00	1.3E-05		MKCNRTDFTT*
HG0141.1	*G. gallus*	ENSGALG00000010435	RASGRP3	RAS guanyl releasing protein 3	0.02	0.43	2.5E-10	1.3E-09	0.43	2.5E-10		MFVLNTSAEPSTIFLEAQLRMQPTIK*
HG0141.2	*H. sapiens*	ENSG00000152689	RASGRP3	RAS guanyl releasing protein 3	0.00	0.21	3.4E-13	5.3E-12	0.21	3.4E-13		MIMEMVYLMLKMSLVF*
HG0141.3	*H. sapiens*	ENSG00000152689	RASGRP3	RAS guanyl releasing protein 3	0.00	0.28	3.0E-05	1.5E-04	0.28	3.0E-05		MLMRWLSQLRGYEVVCE*
HG0142.1	*H. sapiens*	ENSG00000204217	BMPR2	bone morphogenetic protein receptor type 2	0.00	0.10	2.5E-68	1.8E-66	0.10	2.5E-68	Mackowiak	MFSPRPGYFFDIVKLRGK*
HG0142.2	*H. sapiens*	ENSG00000204217	BMPR2	bone morphogenetic protein receptor type 2	0.00	0.32	1.5E-13	2.5E-12	0.32	1.5E-13		MKALQLGPLISHLSFQTVL*
HG0143	*H. sapiens*	ENSG00000101958	GLRA2	glycine receptor alpha 2	0.00	0.44	1.5E-32	5.6E-31	0.44	1.5E-32		MMPRTGTFSFFSANCTKPNLFLIFKETRFLPNFESGQ*
HG0144	*H. sapiens*	ENSG00000117114	ADGRL2	adhesion G protein-coupled receptor L2	0.00	0.09	1.4E-37	6.2E-36	0.09	1.4E-37		MMQTDGFDEAGHL*
HG0145	*H. sapiens*	ENSG00000162599	NFIA	nuclear factor I A	0.00	0.39	1.2E-06	7.9E-06	0.39	1.2E-06		MIPYGMDRTTCCPTCHCKCFLN*
HG0146.1	*H. sapiens*	ENSG00000163681	SLMAP	sarcolemma associated protein	0.00	0.47	1.2E-06	8.0E-06	0.47	1.2E-06		MLQCCNVSATKGEKRPRSQTKHKNWRVTHLLGYLQSPNCVPGVFLAEAARCLSLFFPEKKMD*
HG0146.2	*H. sapiens*	ENSG00000163681	SLMAP	sarcolemma associated protein	0.00	0.32	4.8E-04	1.9E-03	0.32	4.8E-04		MLRQCAVCVNLKFWVG*
HG0146.3	*H. sapiens*	ENSG00000163681	SLMAP	sarcolemma associated protein	0.00	0.40	4.6E-05	2.3E-04	0.40	4.6E-05		MAAGAGKVGKFPASSGAA*
HG0147	*G. gallus*	ENSGALG00000004370	MAP2K6	mitogen-activated protein kinase kinase 6	0.01	0.48	6.0E-03	1.4E-02	0.48	6.0E-03		MKIERLQFASLLQN*
	H. sapiens	ENSG00000108984	MAP2K6	mitogen-activated protein kinase kinase 6	0.01	0.48	1.5E-11	1.9E-10	0.48	1.5E-11		MKIARLQLASLLQN*
HG0148.1	*G. gallus*	ENSGALG00000011855	RHOJ	ras homolog family member J	0.00	0.00	2.6E-60	5.0E-59	0.00	2.6E-60		MLEIRWKMEWI*

HG0148.2	*G. gallus*	ENSGALG00000011855	RHOJ	ras homolog family member J	0.00	0.41	2.3E-08	1.1E-07	0.41	2.3E-08		MILWTALVIVRSSCDSLETLV*
HG0149.1	*G. gallus*	ENSGALG00000014645	MEF2C	myocyte enhancer factor 2C	0.00	0.10	3.0E-10	1.5E-09	0.10	3.0E-10		MILTIKRVYVQERMQGTVN*
HG0149.2	*H. sapiens*	ENSG00000081189	MEF2C	myocyte enhancer factor 2C	0.00	0.34	9.3E-08	7.3E-07	0.34	9.3E-08		MWNILKDNIKVNECRNLGTELCKC*
HG0150.1	*G. gallus*	ENSGALG00000032289	IKZF5	Zinc finger protein Pegasus	0.25	0.00	1.5E-48	2.2E-47	0.00	1.5E-48		MAAAAEGGGRGAGRRRRHQQMEHKLT*
HG0150.2	*H. sapiens*	ENSG00000095574	IKZF5	IKAROS family zinc finger 5	0.00	0.26	2.0E-48	1.1E-46	0.26	2.0E-48		MAFWSPLLYLRSKFQLLYISSMLEWQEKGGTDLG*
HG0151	*G. gallus*	ENSGALG00000035504	ER81	ETS variant 1	0.00	0.32	1.1E-04	3.5E-04	0.32	1.1E-04		MFLLNLEGNFLQPIAARGLQADWSPDPEEISRSNAH*
	H. sapiens	ENSG00000006468	ETV1	ETS variant 1	0.00	0.21	4.0E-35	1.6E-33	0.21	4.0E-35		MFLLNLEGSFCSLSPSVAFSPVRCKASLVDRHC*
HG0152	*D. rerio*	ENSDARG00000015536	sox6	SRY (sex determining region Y)-box 6	0.00	0.26	4.8E-14	2.3E-13	0.26	4.8E-14		MLHDGKCFEYLRHGRLH*
HG0153	*G. gallus*	ENSGALG00000003136	IKZF2	IKAROS family zinc finger 2	0.00	0.18	2.4E-31	2.4E-30	0.18	2.4E-31		MEEVPAGLPLATAPLPQFP*
HG0154	*H. sapiens*	ENSG00000128573	FOXP2	forkhead box P2	0.02	0.46	1.7E-04	7.4E-04	0.46	1.7E-04		MPSVWDVIGQRCTHSSVNTAVNSCLMVALTVS*
HG0155	*H. sapiens*	ENSG00000136535	TBR1	T-box, brain 1	0.00	0.44	3.9E-09	3.8E-08	0.44	3.9E-09		MAGAVSSAAQAARSKVSLF*
HG0156	*H. sapiens*	ENSG00000156113	KCNMA1	potassium calcium-activated channel subfamily M alpha 1	0.06	0.45	1.5E-11	1.9E-10	0.45	1.5E-11		MAVDGCLGWRWRREELPRAPAPAARC*
HG0157	*H. sapiens*	ENSG00000173926	MARCH3	membrane associated ring-CH-type finger 3	0.00	0.00	1.1E-30	3.9E-29	0.00	1.1E-30		MCPGPLGREQPPTGGNLALRTAAGTSVCYRDSARTMEILYECGSH*
HG0158	*H. sapiens*	ENSG00000198939	ZFP2	ZFP2 zinc finger protein	0.26	0.32	2.6E-22	7.1E-21	0.32	2.6E-22		MTVYGEVMLENYRNPVSLGNHLSKPNGTSQLERELSLMQKEPPEGGFRDFLGYCCQINLTFSPLIILSQSRGESID*
HG0159.1	*H. sapiens*	ENSG00000182197	EXT1	exostosin glycosyltransferase 1	0.01	0.08	7.8E-35	3.1E-33	0.08	7.8E-35	Crowe	MQENYSPLLARPGCEIFSPALSRALCNPKMKDRRGES*
HG0159.2	*H. sapiens*	ENSG00000182197	EXT1	exostosin glycosyltransferase 1	0.00	0.19	7.2E-08	5.7E-07	0.19	7.2E-08		MQGFVRLRRPSCKMH*
HG0160	*D. rerio*	ENSDARG00000060089	btaf1	BTAF1 RNA polymerase II, B-TFIID transcription factor-ass	0.00	0.33	9.3E-35	6.7E-34	0.33	9.3E-35		MAPHIKPAAWLLFCDLQNYFSPFVRLKYRFRGSAEG*
HG0161	*G. gallus*	ENSGALG00000017191			0.02	0.25	2.7E-20	2.2E-19	0.25	2.7E-20		MCWLFFVPYTFPNS*
HG0162	*H. sapiens*	ENSG00000138347	MYPN	myopalladin	0.01	0.37	1.9E-13	3.0E-12	0.37	1.9E-13		MFHRHMVYTAQNNLGPVS*
HG0163	*H. sapiens*	ENSG00000146285	SCML4	sex comb on midleg like 4 (Drosophila)	0.02	0.29	1.2E-25	3.6E-24	0.29	1.2E-25		MFAAGAQGCWRLTVSY*
HG0164	*H. sapiens*	ENSG00000151292	CSNK1G3	casein kinase 1 gamma 3	0.00	0.30	4.8E-39	2.3E-37	0.30	4.8E-39		MLTLPIWYSYLVMYLFSQYPVSVCLS*
HG0165	*H. sapiens*	ENSG00000177508	IRX3	iroquois homeobox 3	0.17	0.17	1.1E-58	6.7E-57	0.17	1.1E-58		MSIRARSPGNLRQSVRT*
HG0166.1	*H. sapiens*	ENSG00000148948	LRRC4C	leucine rich repeat containing 4C	0.00	0.01	2.6E-28	8.3E-27	0.01	2.6E-28		MKLQRRWREGDRI*
HG0166.2	*H. sapiens*	ENSG00000148948	LRRC4C	leucine rich repeat containing 4C	0.00	0.35	4.8E-06	2.8E-05	0.35	4.8E-06		MQDAAFSWNRTQWIN*
HG0166.3	*H. sapiens*	ENSG00000148948	LRRC4C	leucine rich repeat containing 4C	0.00	0.50	4.7E-04	1.8E-03	0.50	4.7E-04		MIGNEEPRIHKEKSMFIFLYKGESEPRRYFWNEKFGAFLVK*
HG0166.4	*H. sapiens*	ENSG00000148948	LRRC4C	leucine rich repeat containing 4C	0.01	0.31	5.8E-10	6.3E-09	0.31	5.8E-10		MEGDWGFCRAGSREPGRWR*
HG0167	*D. rerio*	ENSDARG00000104005	slc4a3		0.04	0.31	1.8E-33	1.3E-32	0.31	1.8E-33		MPPHRLTGNPAVKEKG*
HG0168	*G. gallus*	ENSGALG00000006997	BRINP1	BMP/retinoic acid inducible neural specific 1	0.00	0.15	3.1E-10	1.5E-09	0.15	3.1E-10		MPAIWIKEGDFLSEHEKDC*
HG0169	*H. sapiens*	ENSG00000114933	INO80D	INO80 complex subunit D	0.00	0.47	2.0E-08	1.7E-07	0.47	2.0E-08		MVGKLSFRRAEEQLSGVMSSQCL*
HG0170	*H. sapiens*	ENSG00000180530	NRIP1	nuclear receptor interacting protein 1	0.00	0.07	3.1E-51	1.7E-49	0.04	2.7E-48		MKALRSAEQIDYSGVPVLGTTAVRERTLIFAFNGEQKMKKEKEYIH*
HG0171.1	*H. sapiens*	ENSG00000177311	ZBTB38	zinc finger and BTB domain containing 38	0.00	0.00	2.8E-59	1.7E-57	0.00	2.8E-59		MYSKEKRTLAMVQLNQDSEADMQCHLQH*
HG0171.2	*H. sapiens*	ENSG00000177311	ZBTB38	zinc finger and BTB domain containing 38	0.00	0.10	5.2E-30	1.8E-28	0.47	2.6E-11		MRNFFFQSCFPVDKFSSVITVHSSCSAYDFCVYVTIASKTCEQEDREISWSPSLYIKMAECLVMLALH*
HG0171.3	*H. sapiens*	ENSG00000177311	ZBTB38	zinc finger and BTB domain containing 38	0.00	0.01	4.1E-18	8.9E-17	0.01	4.1E-18		MLAVLQKAFRAADLDVEEMEDSEPRISK*
HG0171.4	*H. sapiens*	ENSG00000177311	ZBTB38	zinc finger and BTB domain containing 38	0.00	0.31	9.9E-03	2.6E-02	0.31	9.9E-03		MGDIIRKICGADHKK*
HG0172.1	*H. sapiens*	ENSG00000196782	MAML3	mastermind like transcriptional coactivator 3	0.07	0.48	2.2E-12	3.1E-11	0.48	2.2E-12		MQLSSQSTLRTRGGINTKTFLWKENAISKLAVFRCGVE*
HG0172.2	*H. sapiens*	ENSG00000196782	MAML3	mastermind like transcriptional coactivator 3	0.01	0.21	3.2E-06	1.9E-05	0.21	3.2E-06		MDYKKKASKERISP*
HG0173	*H. sapiens*	ENSG00000101746	NOL4	nucleolar protein 4	0.16	0.00	1.4E-02	3.4E-02	0.00	1.4E-02		MIELACHRHWYLS*
HG0174	*H. sapiens*	ENSG00000144355	DLX1	distal-less homeobox 1	0.01	0.39	2.1E-06	1.3E-05	0.39	2.1E-06		MRLTRGRAASPALVQRSPSASDQSPVIMQDSGPISSASS*
HG0175	*H. sapiens*	ENSG00000151320	AKAP6	A-kinase anchoring protein 6	0.00	0.39	3.9E-12	5.4E-11	0.39	3.9E-12		MCCKHQKKTVGSGDAVLERSEV*
HG0176	*H. sapiens*	ENSG00000170962	PDGFD	platelet derived growth factor D	0.20	0.01	1.3E-23	3.6E-22	0.01	1.3E-23		MQEVWGELGD*
HG0177	*H. sapiens*	ENSG00000171843	MLLT3	MLLT3, super elongation complex subunit	0.09	0.46	5.3E-08	4.3E-07	0.46	5.3E-08		MLRNHLLYPGAAAAAAAFAFGAEFNKRASEQASAGGKRQRMSAIYPPLLGALSFIAASS*
HG0178.1	*G. gallus*	ENSGALG00000029047	TMEM204	transmembrane protein 204	0.00	0.15	4.4E-45	5.8E-44	0.15	4.4E-45	Crowe, Mackowiak	MDLHVFQFITGLALLIKVQEGG*
	H. sapiens	ENSG00000131634	TMEM204	transmembrane protein 204	0.05	0.24	6.4E-27	2.0E-25	0.24	6.4E-27	Crowe, Mackowiak	MGPEASSFSTGLALLQVQEGG*
HG0178.2	*G. gallus*	ENSGALG00000029047	TMEM204	transmembrane protein 204	0.02	0.49	7.3E-09	3.4E-08	0.49	7.3E-09		MFDCRGSGSVCCSRLRPEAHQNSHKNSHFL*
HG0179	*D. rerio*	ENSDARG00000036549	agpat3	1-acylglycerol-3-phosphate O-acyltransferase 3	0.00	0.09	8.0E-29	5.2E-28	0.09	8.0E-29	Mackowiak	MLSHIERLPCYNRLRDTAVAQYEREKLEKVAGFPPTGSVVLSEGPCKRGL*
	H. sapiens	ENSG00000160216	AGPAT3	1-acylglycerol-3-phosphate O-acyltransferase 3	0.12	0.08	8.3E-54	4.7E-52	0.08	8.3E-54	Mackowiak	MLRWVSVASPEFLLNEVCRVIWTAVLSEGPCTRS*
HG0180.1	*H. sapiens*	ENSG00000112246	SIM1	single-minded family bHLH transcription factor 1	0.01	0.33	7.1E-35	2.8E-33	0.33	7.1E-35		MSIFRGPGFAAGSRRKGWLLRGPTGI*
HG0180.2	*H. sapiens*	ENSG00000112246	SIM1	single-minded family bHLH transcription factor 1	0.03	0.34	1.2E-06	7.9E-06	0.34	1.2E-06		MRTVKRWAPPKPLPLVGTEGP*
HG0181.1	*H. sapiens*	ENSG00000143776	CDC42BPA	CDC42 binding protein kinase alpha	0.00	0.48	3.7E-15	6.6E-14	0.48	3.7E-15		MHSVFFLPFLFFFFPGSELLMQIGLYSLMMQPDSFQDYVARVEF*
HG0181.2	*H. sapiens*	ENSG00000143776	CDC42BPA	CDC42 binding protein kinase alpha	0.00	0.04	4.5E-06	2.7E-05	0.04	4.5E-06		MYMGEIEDASVK*
HG0182	*G. gallus*	ENSGALG00000031991			0.06	0.05	1.1E-56	1.9E-55	0.05	1.1E-56		MDVNQPKQEHLLALKGPVSH*
HG0183	*H. sapiens*	ENSG00000011465	DCN	decorin	0.02	0.41	1.8E-12	2.6E-11	0.41	1.8E-12		MWVSAGLCSLRETLFFNCAME*
HG0184	*H. sapiens*	ENSG00000136542	GALNT5	polypeptide N-acetylgalactosaminyltransferase 5	0.00	0.28	9.3E-07	6.2E-06	0.28	9.3E-07		MLCYEMPRRQRLTSGRN*
HG0185	*H. sapiens*	ENSG00000143033	MTF2	metal response element binding transcription factor 2	0.00	0.18	3.0E-04	1.2E-03	0.18	3.0E-04		MHRQSAGNQNGEGLY*
HG0186	*H. sapiens*	ENSG00000167552	TUBA1A	tubulin alpha 1a	0.08	0.41	3.3E-14	5.7E-13	0.41	3.3E-14		MASLGRLGVWTNRKRPRPIPMVTELYIGSRIRLCAAGSLTSTA*
HG0187.1	*G. gallus*	ENSGALG00000004058	GPR146	G protein-coupled receptor 146	0.25	0.40	5.2E-14	3.3E-13	0.40	5.2E-14		MDKMFLPASTGQQGTAYQKSW*
HG0187.2	*D. rerio*	ENSDARG00000059610	gpr146	G protein-coupled receptor 146	0.00	0.25	5.3E-04	1.5E-03	0.25	5.3E-04		MSMEIQQNPMPP*
HG0187.3	*G. gallus*	ENSGALG00000004058	GPR146	G protein-coupled receptor 146	0.08	0.36	4.5E-06	1.7E-05	0.36	4.5E-06		MCMYVQGKSHVNTDSVP*
HG0188	*G. gallus*	ENSGALG00000016284	TAB3	TGF-beta activated kinase 1 and MAP3K7 binding protein 3	0.03	0.02	2.4E-33	2.5E-32	0.02	2.4E-33		MPEASEDHNHWKVC*
	H. sapiens	ENSG00000157625	TAB3	TGF-beta activated kinase 1 and MAP3K7 binding protein 3	0.00	0.27	4.9E-28	1.5E-26	0.27	4.9E-28		MTVVLEDHYFWKAG*
HG0189	*D. rerio*	ENSDARG00000001807	tnfrsf21	tumor necrosis factor receptor superfamily, member 21	0.00	0.25	7.0E-24	4.3E-23	0.25	7.0E-24		MGIIAFVFQNQALIPYYLKPVHSGAQTIKRREGTEFVH*
HG0190	*G. gallus*	ENSGALG00000006949	CHD2	chromodomain helicase DNA binding protein 2	0.00	0.00	4.3E-08	1.9E-07	0.00	4.3E-08		MSFFPRTFLY*
HG0191	*G. gallus*	ENSGALG00000036097			0.00	0.42	8.6E-16	6.0E-15	0.42	8.6E-16		MNKRKNPFWTVFLNQASPSISRTF*
HG0192	*H. sapiens*	ENSG00000125107	CNOT1	CCR4-NOT transcription complex subunit 1	0.00	0.23	1.0E-18	2.3E-17	0.23	1.0E-18		MQKLPRSCQAISQLSIQKETYHSKDLRSCMPGNGMKTKCAMFQNRVRPLVKKN*
HG0193	*H. sapiens*	ENSG00000134853	PDGFRA	platelet derived growth factor receptor alpha	0.03	0.22	8.1E-06	4.6E-05	0.22	8.1E-06		MISFRRMDYNIESITKRGF*
HG0194	*H. sapiens*	ENSG00000143507	DUSP10	dual specificity phosphatase 10	0.01	0.37	5.3E-11	6.4E-10	0.37	5.3E-11		MCESIAEEERQMVEEYTYL*
HG0195	*H. sapiens*	ENSG00000165966	PDZRN4	PDZ domain containing ring finger 4	0.01	0.44	9.5E-05	4.3E-04	0.44	9.5E-05		MKNSSQVWRVIPFQFTCLSVLGKLEDLLSLSAS*
HG0196	*H. sapiens*	ENSG00000189403	HMGB1	high mobility group box 1	0.01	0.36	4.2E-09	4.0E-08	0.36	4.2E-09		MLQSGESEEAASGSRSHSHCSTLSSIETAPGQVRAGRALGDSVPR*
HG0197	*H. sapiens*	ENSG00000196132	MYT1	myelin transcription factor 1	0.00	0.00	3.4E-04	1.4E-03	0.00	3.4E-04		MIAFRDVILKEDRQ*
HG0198.1	*D. rerio*	ENSDARG00000018688	elk3	ELK3, ETS-domain protein	0.06	0.30	2.3E-03	5.6E-03	0.30	2.3E-03		MCVCVRIRVCARVPECRMVARGGKACLHRRAERLGNRARREEVSRLAVCLLQLPAHRERERKSE*
HG0198.2	*H. sapiens*	ENSG00000111145	ELK3	ELK3, ETS transcription factor	0.12	0.00	7.2E-05	3.4E-04	0.00	7.2E-05		MESRGLSPGSS*
HG0199	*G. gallus*	ENSGALG00000007989	ADNP	activity dependent neuroprotector homeobox	0.00	0.37	9.0E-03	2.0E-02	0.37	9.0E-03		MLRQKGNEQDMTLLLCPGFGLTQ*
HG0200	*G. gallus*	ENSGALG00000012015	NDST4	N-deacetylase and N-sulfotransferase 4	0.02	0.40	1.1E-17	8.0E-17	0.40	1.1E-17		MHCNGHMNVSGEESAPLRLIVIMAL*
HG0201	*G. gallus*	ENSGALG00000015256	PAM	peptidylglycine alpha-amidating monooxygenase	0.00	0.30	8.1E-14	5.0E-13	0.30	8.1E-14		MVCQKLEDACRAHNNVVMN*
HG0202	*H. sapiens*	ENSG00000124766	SOX4	SRY-box 4	0.09	0.46	6.4E-15	1.1E-13	0.46	6.4E-15		MRSDLELLIARGDYYCIGFQANGKPGGGVWHEEALVTAADWLQPRL*
HG0203	*H. sapiens*	ENSG00000128606	LRRC17	leucine rich repeat containing 17	0.09	0.21	3.0E-14	5.1E-13	0.21	3.0E-14		MSLWHPFPKFSLGTPRTPAGSHCSRTALVKITQTWISKEYFHCSVCNTK*
HG0204	*H. sapiens*	ENSG00000162599	NFIA	nuclear factor I A	0.00	0.00	2.7E-09	2.7E-08	0.00	2.7E-09		MPLTPAYSRHV*
HG0205	*H. sapiens*	ENSG00000184611	KCNH7	potassium voltage-gated channel subfamily H member 7	0.01	0.35	6.7E-04	2.5E-03	0.35	6.7E-04		MGREFWFQKRMLCHKERLARWPGL*
HG0206.1	*G. gallus*	ENSGALG00000002069			0.00	0.48	3.4E-17	2.5E-16	0.48	3.4E-17		MVGAIPRQPRRFWGVPGWQVSSASCANDRPLWDGEIDPEWSGILLQREPHTGGLTWTS*
HG0206.2	*H. sapiens*	ENSG00000005238	FAM214B	family with sequence similarity 214 member B	0.00	0.16	6.0E-30	2.1E-28	0.16	6.0E-30		MGLVLLSSGAARNGGSHLASAAGMDQTAS*
HG0207.1	*G. gallus*	ENSGALG00000007121	TNFSF8	TNF superfamily member 8	0.04	0.40	1.5E-13	9.0E-13	0.40	1.5E-13		MSQDDGENPARNRDGLRPEPDQGEIQTSTLTAI*
HG0207.2	*H. sapiens*	ENSG00000106952	TNFSF8	TNF superfamily member 8	0.02	0.24	1.3E-02	3.2E-02	0.24	1.3E-02		MGPELNQMKRDKV*
HG0208	*G. gallus*	ENSGALG00000013272	PRMT8	protein arginine methyltransferase 8	0.00	0.41	4.5E-03	1.1E-02	0.41	4.5E-03		MPSWSLHAQVQFSWCFSPV*
HG0209	*H. sapiens*	ENSG00000101040	ZMYND8	zinc finger MYND-type containing 8	0.22	0.31	1.9E-13	2.9E-12	0.31	1.9E-13		MHPQSPKSASLFSLPNCGKMRRLLEHLLAWLKRK*
HG0210	*H. sapiens*	ENSG00000155849	ELMO1	engulfment and cell motility 1	0.03	0.36	2.4E-03	7.6E-03	0.36	2.4E-03		MPCRCKMCLWPAGGRKRK*
HG0211	*H. sapiens*	ENSG00000176204	LRRTM4	leucine rich repeat transmembrane neuronal 4	0.00	0.00	1.1E-06	7.2E-06	0.00	1.1E-06		MDKPSLHSF*
HG0212	*D. rerio*	ENSDARG00000100244	ebf3a	early B-cell factor 3a	0.00	0.32	2.9E-07	1.1E-06	0.32	2.9E-07		MLPSILQRRAKGPSSSSPVSGNTSTVIIYAFICLLEPEVVDSAVSRSLF*
HG0213.1	*G. gallus*	ENSGALG00000001946	RSPO1	R-spondin 1	0.23	0.47	4.4E-05	1.5E-04	0.47	4.4E-05		MGLDPSSCAPYGDEGRTMLPAT*
HG0213.2	*G. gallus*	ENSGALG00000001946	RSPO1	R-spondin 1	0.07	0.29	3.4E-03	8.6E-03	0.29	3.4E-03		MGMESTALRPGFINVQIALI*
HG0214.1	*G. gallus*	ENSGALG00000012917	CDH6	cadherin 6	0.20	0.47	6.3E-08	2.7E-07	0.47	6.3E-08		METNQIESPLGSGCLHAGVKKCCRSSGSPSERRGG*
HG0214.2	*H. sapiens*	ENSG00000040731	CDH10	cadherin 10	0.01	0.38	1.0E-02	2.7E-02	0.38	1.0E-02		MNKTLMKCWKPVETQ*
HG0215	*D. rerio*	ENSDARG00000079280	dhrsx	dehydrogenase/reductase (SDR family) X-linked	0.12	0.01	1.2E-15	6.1E-15	0.01	1.2E-15		MQSLISPVTKAILVALFIFAILLILYVILWYICRDVDCDHGI*
	G. gallus	ENSGALG00000016681	DHRSX	dehydrogenase/reductase X-linked	0.03	0.01	1.7E-25	1.5E-24	0.01	1.7E-25		MQSLISPVTKAILVALFIFAILLILYVILWYICRDVDCDHGI*
HG0216.1	*G. gallus*	ENSGALG00000020922			0.00	0.18	2.6E-04	8.0E-04	0.18	2.6E-04		MICGSVQLELSE*
HG0216.2	*H. sapiens*	ENSG00000116667	C1orf21	chromosome 1 open reading frame 21	0.07	0.14	2.6E-09	2.6E-08	0.14	2.6E-09		MICSSARLKLTE*
HG0217.1	*H. sapiens*	ENSG00000150394	CDH8	cadherin 8	0.00	0.04	1.0E-02	2.7E-02	0.04	1.0E-02		MNFQVVDLPLRSAQ*
HG0217.2	*G. gallus*	ENSGALG00000005319	CDH8	cadherin 8	0.11	0.50	1.3E-09	6.5E-09	0.50	1.3E-09		MRRKSGESCLFTGTGEWLFYLTLSIRV*
HG0218	*D. rerio*	ENSDARG00000100149	axin2	axin 2 (conductin, axil)	0.02	0.45	9.2E-04	2.4E-03	0.45	9.2E-04		MDERSSPADLRERGDYS*
HG0219	*D. rerio*	ENSDARG00000104082	trps1	trichorhinophalangeal syndrome I	0.00	0.29	1.8E-04	5.3E-04	0.29	1.8E-04		MAHAKAPPSFRLQRLLQFNQQ*
HG0220	*G. gallus*	ENSGALG00000008418	LARGE2	LARGE xylosyl- and glucuronyltransferase 2	0.06	0.01	2.1E-19	1.6E-18	0.01	2.1E-19		MLGPAGLGLPT*
HG0221	*G. gallus*	ENSGALG00000010859	EPGN	epithelial mitogen	0.00	0.33	6.7E-14	4.2E-13	0.33	6.7E-14		MPTGNRKLGFQEKTRSQC*
HG0222	*G. gallus*	ENSGALG00000011564	RUFY3	RUN and FYVE domain containing 3	0.00	0.39	7.0E-04	2.0E-03	0.39	7.0E-04		MQKPGFLWLLRINSL*
HG0223	*H. sapiens*	ENSG00000091656	ZFHX4	zinc finger homeobox 4	0.00	0.50	1.9E-05	9.9E-05	0.50	1.9E-05		MDGRVRERLRLPLPFRVLLRLRLRLGCDWDATVTRASIASIESNAKTQPGSLTGWMK*

HG0224	*H. sapiens*	ENSG00000118007	STAG1	stromal antigen 1	0.00	0.09	9.2E-07	6.1E-06	0.09	9.2E-07		MPPDGGLGLQLR*
HG0225	*H. sapiens*	ENSG00000150051	MKX	mohawk homeobox	0.21	0.30	5.2E-10	5.7E-09	0.30	5.2E-10		MFAGWDRQSGNMRRPRYSCSRAARAGLGK*
HG0226.1	*H. sapiens*	ENSG00000180592			0.07	0.06	1.7E-05	9.1E-05	0.06	1.7E-05		MERFIYRLYYLLWEGRWQYKGMLISGRFWGEGVEYQFLLHHLSGVSNQRWL*
HG0226.2	*H. sapiens*	ENSG00000180592			0.09	0.44	1.9E-02	4.5E-02	0.44	1.9E-02		MAEQPIESADMVNKFIKETPWLLKNSTLYIWICTSGKAQKYPFILLITRMCLFLEEQGNT*
HG0226.3	*H. sapiens*	ENSG00000180592			0.26	0.40	8.1E-03	2.2E-02	0.40	8.1E-03		MRLGGPLLSFGNILDILENDTKICLNLTKKRLNHIDFHPK*
HG0227.1	*G. gallus*	ENSGALG00000031929			0.05	0.48	5.6E-04	1.6E-03	0.48	5.6E-04		MTALNNWWHFGGYGWSSVY*
HG0227.2	*H. sapiens*	ENSG00000275163			0.00	0.20	2.6E-04	1.1E-03	0.20	2.6E-04		MGKVSEEEPFLPFVAISTGLQGTSFLLGFYVCFTHCLSFLCLRHFHE*
HG0228	*D. rerio*	ENSDARG00000087887	phf21b	PHD finger protein 21B	0.05	0.39	2.1E-13	9.4E-13	0.39	2.1E-13		MSLTASKEARTCCAARVVLAIVIVAFQNTSLTPQITRIASEVEEKCS*
HG0229	*G. gallus*	ENSGALG00000005474			0.00	0.40	3.0E-03	7.7E-03	0.40	3.0E-03		MCIHFWWTLPFLGV*
HG0230	*G. gallus*	ENSGALG00000011007	OSBPL3	oxysterol binding protein like 3	0.00	0.48	2.0E-05	7.2E-05	0.48	2.0E-05		MQIMLLECIWVRDEFCITLRSLPIHLLDYLLMTF*
HG0231	*H. sapiens*	ENSG00000150636			0.13	0.48	1.3E-04	5.9E-04	0.48	1.3E-04		MQEILLFTHVAATETLEQPSKPRGS*
HG0232	*H. sapiens*	ENSG00000180875	GREM2	gremlin 2, DAN family BMP antagonist	0.20	0.34	9.4E-11	1.1E-09	0.34	9.4E-11		MGVCCSLAAVELKAAAGDQT*
HG0233	*H. sapiens*	ENSG00000206432	TMEM200C	transmembrane protein 200C	0.17	0.45	1.2E-08	1.1E-07	0.45	1.2E-08		MSGGEEGSEAGCGMRDAEDLTLLGNSWKWLPPQRRRGLRAWGGRSPPRGC*
HG0234.1	*G. gallus*	ENSGALG00000011250	ASIC4	acid sensing ion channel subunit family member 4	0.10	0.00	5.9E-03	1.4E-02	0.00	5.9E-03		MLTCLELLSIPWILLAHDA*
HG0234.2	*H. sapiens*	ENSG00000108684	ASIC2	acid sensing ion channel subunit 2	0.03	0.43	1.5E-04	6.5E-04	0.43	1.5E-04		MVNMGHYALVNRLRAAQRLVLSIFSLALAFNLVSSGVCRGEDFISIG*
HG0235.1	*G. gallus*	ENSGALG00000035927			0.00	0.48	5.7E-08	2.5E-07	0.48	5.7E-08		MLATSESLGNFTVGALRAVLELTAKEARRGQTMGCPVLGLLHEQTPRAPLGCPAKRA*
HG0235.2	*H. sapiens*	ENSG00000101638	ST8SIA5	ST8 alpha-N-acetyl-neuraminide alpha-2,8-sialyltransferase	0.03	0.37	9.2E-05	4.2E-04	0.37	9.2E-05		MPPAPAAPTRNFAPRSPPPA*
HG0236	*G. gallus*	ENSGALG00000004437	LHFPL2	lipoma HMGIC fusion partner-like 2	0.02	0.47	1.0E-07	4.6E-07	0.47	1.0E-07		MVAAGAGQGEPGGARAGGGGGSGAEPQRTGGAGERALLAGGIARLLKWLTKAKNRGHWCVMSSSGQSPRPSPPVRSVMCQYEQ*
HG0237	*G. gallus*	ENSGALG00000012123	PAX6	paired box 6	0.00	0.35	1.3E-04	4.1E-04	0.35	1.3E-04		MCGVISGFEKRAPARPHFPLWLRRREEVFCWKMMTEVRLC*
HG0238	*H. sapiens*	ENSG00000049618	ARID1B	AT-rich interaction domain 1B	0.16	0.43	1.9E-05	1.0E-04	0.43	1.9E-05		MLGPIGSASRVFTHIRAREEKRKVSFKPELKTFTMKAHSRSRPRA*
HG0239	*H. sapiens*	ENSG00000149256	TENM4	teneurin transmembrane protein 4	0.00	0.33	1.4E-02	3.5E-02	0.33	1.4E-02		MEPGRLKLPCVQIKIAYFKDYLKDYS*
HG0240	*H. sapiens*	ENSG00000166342	NETO1	neuropilin and tolloid like 1	0.06	0.42	1.0E-08	9.2E-08	0.42	1.0E-08		MTDPFFLPSMPQRKIPKGWSEERCRWTSSRGGCSDLLRGASETGD*
HG0241	*H. sapiens*	ENSG00000198561	CTNND1	catenin delta 1	0.06	0.49	1.4E-03	4.8E-03	0.49	1.4E-03		MEVSGWLCSLLPASSLLWWLGCFFHDFLNLDWAVLCVKPISCDLLLTV*
HG0242.1	*G. gallus*	ENSGALG00000007112	LINS1	lines homolog 1	0.00	0.21	3.5E-08	1.6E-07	0.21	3.5E-08		MSRGNLEEECLLACLFLLLDKNNFKTDLQQSEIIGEDLQRSVIY*
HG0242.2	*H. sapiens*	ENSG00000140471	LINS1	lines homolog 1	0.08	0.45	1.1E-02	2.7E-02	0.45	1.1E-02		MREEVPACTFCELRWPRAGICVPSNGGVKRVRLSQIPGFTEETS*
HG0243.1	*H. sapiens*	ENSG00000163697	APBB2	amyloid beta precursor protein binding family B member 2	0.04	0.43	3.8E-04	1.5E-03	0.43	3.8E-04		MGSLLSRQLIFQIITYFLHHC*
HG0243.2	*H. sapiens*	ENSG00000163697	APBB2	amyloid beta precursor protein binding family B member 2	0.00	0.13	2.9E-17	5.9E-16	0.13	2.9E-17	Crowe	MLWSDLPSRGTETFAGWKIHKRKLL*
HG0244	*G. gallus*	ENSGALG00000010837	ASB5	ankyrin repeat and SOCS box containing 5	0.00	0.22	9.7E-05	3.2E-04	0.22	9.7E-05		MLTGTLRSLKAVVMKQPKYQHFSRIDPSEQLMLMVVF*
HG0245	*H. sapiens*	ENSG00000091831	ESR1	estrogen receptor 1	0.24	0.37	5.6E-05	2.7E-04	0.37	5.6E-05		MQPHTPFYLPYLGYSVVLPRVILCTHYVFLANEEGESNRKRDKQRYIGVWHGAHKAAH*
HG0246	*H. sapiens*	ENSG00000162676	GFI1	growth factor independent 1 transcriptional repressor	0.12	0.30	6.3E-08	5.1E-07	0.30	6.3E-08		MSKPLGLGVKVSSGLGLSKPSE*
HG0247.1	*D. rerio*	ENSDARG00000014746	rbfox1	RNA binding protein, fox-1 homolog (C. elegans) 1	0.05	0.00	8.4E-07	3.1E-06	0.00	8.4E-07		MLALGRCCRDWD*
HG0247.2	*H. sapiens*	ENSG00000100320	RBFOX2	RNA binding protein, fox-1 homolog 2	0.05	0.00	1.2E-05	6.8E-05	0.00	1.2E-05		MGGSWRKKEGERLPERKRKERKKH*
HG0248.1	*G. gallus*	ENSGALG00000004570	NCOA3	nuclear receptor coactivator 3	0.00	0.40	2.1E-02	4.3E-02	0.40	2.1E-02		MVSDSGDPVKSILCLNAC*
HG0248.2	*H. sapiens*	ENSG00000124151	NCOA3	nuclear receptor coactivator 3	0.00	0.41	1.8E-09	1.8E-08	0.41	1.8E-09		MAAGGSKYLLDGGLRDQ*
HG0249	*D. rerio*	ENSDARG00000062550	rc3h1a	ring finger and CCCH-type domains 1a	0.00	0.08	7.9E-04	2.2E-03	0.08	7.9E-04		MLCFSHYVLNAHNARKYLRTVIFGRALSVPVEPLGLTAVSFYAKRT*
HG0250	*G. gallus*	ENSGALG00000014861			0.02	0.30	3.4E-03	8.7E-03	0.30	3.4E-03		MNWKHLDLCRALNQILV*
HG0251	*H. sapiens*	ENSG00000185053	SGCZ	sarcoglycan zeta	0.00	0.09	7.6E-05	3.6E-04	0.09	7.6E-05		MMLTREAAFFLGMQQFAGF*
HG0252.1	*G. gallus*	ENSGALG00000009645	ESRRG	estrogen related receptor gamma	0.00	0.03	7.2E-04	2.1E-03	0.03	7.2E-04		MSLHGVVDFPSKETGFGFFKIPDDSPD*
HG0252.2	*H. sapiens*	ENSG00000119715	ESRRB	estrogen related receptor beta	0.00	0.49	1.4E-03	4.9E-03	0.49	1.4E-03		MVVRLFFFFFFFCQLPVISITESGSIYRLGVLCP*
HG0252.3	*H. sapiens*	ENSG00000196482	ESRRG	estrogen related receptor gamma	0.00	0.22	1.1E-04	5.1E-04	0.22	1.1E-04		MCNVPAWGFCRLS*
HG0253.1	*G. gallus*	ENSGALG00000015184			0.02	0.28	6.1E-05	2.0E-04	0.28	6.1E-05		MYQNVNDEALLNLVKGVTSAERAVGAARSGGAGRRAGQGRVGQGGPGRAGHGAPSSAAAASRSLDRQRRARKPNTNKAARTVPSSSSLSLFLIF*
HG0253.2	*H. sapiens*	ENSG00000140332	TLE3	transducin like enhancer of split 3	0.03	0.41	4.8E-16	9.1E-15	0.41	4.8E-16		MSAQSQALTRSTQPREGGRPLPAEP*
HG0253.3	*H. sapiens*	ENSG00000140332	TLE3	transducin like enhancer of split 3	0.00	0.00	2.0E-03	6.5E-03	0.00	2.0E-03		MPGRLICTY*
HG0254.1	*G. gallus*	ENSGALG00000035919			0.05	0.46	3.4E-04	1.0E-03	0.46	3.4E-04		MLDGACGCQTQPEAAADGRRDPSCAPPVPPRS*
HG0254.2	*H. sapiens*	ENSG00000072134	EPN2	epsin 2	0.00	0.45	5.6E-03	1.6E-02	0.45	5.6E-03		MTIQGSYSSLITETEPS*
HG0255	*G. gallus*	ENSGALG00000012139	ARL14EP	ADP ribosylation factor like GTPase 14 effector protein	0.00	0.20	1.6E-05	5.7E-05	0.20	1.6E-05		MSATGSAAVGGAARKPGAGVPAQRRRKVHGKGDLHRNH*
HG0256	*G. gallus*	ENSGALG00000039901			0.23	0.35	7.7E-05	2.5E-04	0.35	7.7E-05		MAATTTISLRHSQPCNRFPRDVPELLEFKPPKGPRSTQKQCP*
HG0257	*H. sapiens*	ENSG00000198791	CNOT7	CCR4-NOT transcription complex subunit 7	0.00	0.41	6.7E-04	2.5E-03	0.41	6.7E-04		MLSPVMRRPLVSLRGFWDCSREREGFEISLPKNQQELCL*
HG0258.1	*G. gallus*	ENSGALG00000016797	FHL2	four and a half LIM domains 2	0.00	0.19	2.0E-02	4.1E-02	0.19	2.0E-02		MSNCLITKYIVWVIQ*
HG0258.2	*G. gallus*	ENSGALG00000016797	FHL2	four and a half LIM domains 2	0.18	0.00	1.7E-14	1.1E-13	0.00	1.7E-14		MDVTLCPWSRR*
HG0259	*G. gallus*	ENSGALG00000009936	AP4S1	adaptor related protein complex 4 sigma 1 subunit	0.00	0.15	1.5E-02	3.1E-02	0.15	1.5E-02		MLKSKMAAPGKGAQFRP*
HG0260.1	*G. gallus*	ENSGALG00000016890	MBNL2	muscleblind-like 2 isoform 7	0.00	0.36	3.6E-27	3.3E-26	0.36	3.6E-27		MLLSMIFFSQKSTSGT*
HG0260.2	*H. sapiens*	ENSG00000139793	MBNL2	muscleblind like splicing regulator 2	0.00	0.47	2.1E-16	4.0E-15	0.47	2.1E-16		MSDCLSIHSLIILFLGLDFTKQFITHLQTYMWEFSQQ*
HG0260.3	*H. sapiens*	ENSG00000152601	MBNL1	muscleblind like splicing regulator 1	0.00	0.35	3.3E-09	3.2E-08	0.35	3.3E-09		MTPTMPWALGRQWGRL*
HG0260.4	*H. sapiens*	ENSG00000152601	MBNL1	muscleblind like splicing regulator 1	0.00	0.30	7.3E-08	5.8E-07	0.30	7.3E-08		MSCGAHNAPMTRS*
HG0260.5	*H. sapiens*	ENSG00000152601	MBNL1	muscleblind like splicing regulator 1	0.10	0.38	7.5E-03	2.1E-02	0.38	7.5E-03		MFFPPVSPPGSKTVWCILRRQSHQIAGF*
HG0260.6	*H. sapiens*	ENSG00000152601	MBNL1	muscleblind like splicing regulator 1	0.03	0.06	4.1E-03	1.2E-02	0.06	4.1E-03		MGLRFICSGDNMAYLPWNIPVFQK*
HG0261.1	*G. gallus*	ENSGALG00000005867	MARCH8	membrane associated ring-CH-type finger 8	0.00	0.24	8.2E-03	1.9E-02	0.24	8.2E-03		MSAAFKPPSCK*
HG0261.2	*G. gallus*	ENSGALG00000009520	MARCH1	membrane associated ring-CH-type finger 1	0.00	0.26	1.3E-04	4.1E-04	0.26	1.3E-04		MEYLTQRQEMKPGSNVKGHVKLLHSRNLLKDCFT*
HG0261.3	*H. sapiens*	ENSG00000145416	MARCH1	membrane associated ring-CH-type finger 1	0.00	0.49	8.0E-05	3.7E-04	0.49	8.0E-05		MPAPRDAGRTQPGGRLSYFTLRR*
HG0261.4	*H. sapiens*	ENSG00000165406	MARCH8	membrane associated ring-CH-type finger 8	0.03	0.48	2.1E-02	4.8E-02	0.48	2.1E-02		MLEAGQGGSAASSEPT*
HG0262.1	*H. sapiens*	ENSG00000023445	BIRC3	baculoviral IAP repeat containing 3	0.00	0.37	1.3E-05	7.0E-05	0.35	3.2E-05		MFLLPPGNKALKSAVEKPNVCEIQETMLANHW*
HG0262.2	*H. sapiens*	ENSG00000023445	BIRC3	baculoviral IAP repeat containing 3	0.00	0.39	5.6E-03	1.6E-02	0.39	5.6E-03		MSLILNLICGNKGKVILAGAYC*
HG0262.3	*G. gallus*	ENSGALG00000017186			0.09	0.10	6.8E-03	1.6E-02	0.10	6.8E-03		MHSTRKAHNVTVAQEKVLFDIPVFVNGVDQLLH*
HG0262.4	*H. sapiens*	ENSG00000023445	BIRC3	baculoviral IAP repeat containing 3	0.00	0.14	3.9E-03	1.2E-02	0.14	3.9E-03		MNTKKIFITMCKIFGQGKEY*
HG0263.1	*H. sapiens*	ENSG00000169826	CSGALNACT2	chondroitin sulfate N-acetylgalactosaminyltransferase 2	0.14	0.48	7.1E-03	2.0E-02	0.48	7.1E-03		MEILILFLIFDNFLLKV*
HG0263.2	*G. gallus*	ENSGALG00000002543	CSGALNACT2	chondroitin sulfate N-acetylgalactosaminyltransferase 2	0.00	0.20	1.5E-04	4.8E-04	0.20	1.5E-04		MANVGTCDLWLCNDT*
HG0263.3	*G. gallus*	ENSGALG00000002543	CSGALNACT2	chondroitin sulfate N-acetylgalactosaminyltransferase 2	0.00	0.32	1.1E-04	3.6E-04	0.32	1.1E-04		MIPGWCLPFCSPSAVFFVWDKKS*
HG0263.4	*H. sapiens*	ENSG00000147408	CSGALNACT1	chondroitin sulfate N-acetylgalactosaminyltransferase 1	0.00	0.34	1.5E-02	3.7E-02	0.34	1.5E-02		MDASSSGGPKRMLPVV*
HG0264.1	*H. sapiens*	ENSG00000151458	ANKRD50	ankyrin repeat domain 50	0.00	0.45	2.0E-10	2.3E-09	0.45	2.0E-10		MELCDSLKFRKSVAELWNQAFLPQFNN*
HG0264.2	*H. sapiens*	ENSG00000151458	ANKRD50	ankyrin repeat domain 50	0.00	0.31	1.4E-16	2.8E-15	0.31	1.4E-16		MYCLISVLETSTIT*
HG0264.3	*G. gallus*	ENSGALG00000011831	ANKRD50	ankyrin repeat domain 50	0.00	0.01	3.4E-03	8.7E-03	0.01	3.4E-03		MDSFFWIYIRRD*
HG0264.4	*H. sapiens*	ENSG00000151458	ANKRD50	ankyrin repeat domain 50	0.18	0.15	3.6E-03	1.1E-02	0.15	3.6E-03		MERPVLMYPDSSK*
HG0265.1	*H. sapiens*	ENSG00000144285	SCN1A	sodium voltage-gated channel alpha subunit 1	0.00	0.30	2.0E-05	1.0E-04	0.30	2.0E-05		MELFLQINTSGVMEAGGS*
HG0265.2	*H. sapiens*	ENSG00000196876	SCN8A	sodium voltage-gated channel alpha subunit 8	0.16	0.21	1.2E-06	7.9E-06	0.21	1.2E-06		MAPTAVPPAASSAPLQSGRASRAPR*
HG0265.3	*D. rerio*	ENSDARG00000018032	scn8ab	sodium channel, voltage gated, type VIII, alpha subunit b	0.00	0.36	8.9E-04	2.4E-03	0.36	8.9E-04		MATSAVSPGSFKHP*
HG0266.1	*H. sapiens*	ENSG00000184185	KCNJ12	potassium voltage-gated channel subfamily J member 12	0.03	0.48	4.0E-10	4.5E-09	0.48	4.0E-10		MAATTNSALKTVPCPLSTIT*
		ENSG00000260458	KCNJ18	potassium voltage-gated channel subfamily J member 18	0.08	0.47	3.4E-09	3.4E-08	0.47	3.4E-09		MAATTNSALKTVPCLLSTIT*
HG0266.2	*G. gallus*	ENSGALG00000001181	KCNJ5	potassium voltage-gated channel subfamily J member 5	0.28	0.39	2.4E-04	7.3E-04	0.39	2.4E-04		MFCHAGQREGRRRRPRDQKTTTACW*
HG0267	*D. rerio*	ENSDARG00000028173	slc4a2a	solute carrier family 4 (anion exchanger), member 2a	0.00	0.33	7.2E-05	2.2E-04	0.33	7.2E-05	Mackowiak	MLPESSQLSLSKREKG*
		ENSDARG00000052330	slc4a2b	solute carrier family 4 (anion exchanger), member 2b	0.00	0.37	3.4E-14	1.6E-13	0.37	3.4E-14	Mackowiak	MLPVNSCLSLSEKEKG*
	H. sapiens	ENSG00000164889	SLC4A2	solute carrier family 4 member 2	0.00	0.06	3.1E-31	1.1E-29	0.06	3.1E-31	Mackowiak	MPPPRCPESRSDSEKG*
HG0268.1	*D. rerio*	ENSDARG00000011703	clocka	clock circadian regulator a	0.00	0.34	7.6E-07	2.8E-06	0.34	7.6E-07		MSTSRLKVLSLQLRP*
HG0268.2	*H. sapiens*	ENSG00000134852	CLOCK	clock circadian regulator	0.00	0.34	3.1E-04	1.3E-03	0.34	3.1E-04		MVGHIEEYLEPIVS*
HG0268.3	*H. sapiens*	ENSG00000134852	CLOCK	clock circadian regulator	0.01	0.40	1.5E-02	3.8E-02	0.40	1.5E-02		MITRRQLVLRRSTNVYYKTKT*
HG0269.1	*G. gallus*	ENSGALG00000028685	SEMA5A	semaphorin 5A	0.00	0.32	2.5E-06	9.7E-06	0.32	2.5E-06		MVTERCFSPMSARRVSS*
HG0269.2	*D. rerio*	ENSDARG00000061471	sema5ba	sema domain, seven thrombospondin repeats (type 1 and t	0.29	0.04	5.2E-03	1.1E-02	0.04	5.2E-03		MLVMPLCIPEAHLGS*
HG0269.3	*G. gallus*	ENSGALG00000011686	SEMA5B	semaphorin 5B	0.02	0.11	8.6E-03	2.0E-02	0.11	8.6E-03		MPLTQTEITKDFPWCLTSNMTLQ*
HG0270.1	*G. gallus*	ENSGALG00000015147	ALDH1A1	aldehyde dehydrogenase 1 family member A1	0.00	0.46	2.0E-15	1.4E-14	0.46	2.0E-15		MVNVTVFKMFHKCRSRITIFPSCFLFFFFSFLFFFFFISSLARMSELD*
HG0270.2	*G. gallus*	ENSGALG00000015147	ALDH1A1	aldehyde dehydrogenase 1 family member A1	0.00	0.32	2.3E-17	1.7E-16	0.32	2.3E-17		MISFLFLALPPPSLSLISLT*
HG0270.3	*H. sapiens*	ENSG00000128918	ALDH1A2	aldehyde dehydrogenase 1 family member A2	0.01	0.05	3.5E-05	1.8E-04	0.05	3.5E-05		MEAAPGVLGRMDQPPAPRPSRPIRRAPA*
HG0271.1	*G. gallus*	ENSGALG00000004064	GPER1	G protein-coupled estrogen receptor 1	0.00	0.29	7.8E-11	4.1E-10	0.29	7.8E-11		MELELCHLPTCTKENC*
HG0271.2	*H. sapiens*	ENSG00000164850	GPER1	G protein-coupled estrogen receptor 1	0.10	0.38	1.3E-05	7.3E-05	0.38	1.3E-05		MPGSLQGESRFFLKMDSPFKTELWEPFGKS*
HG0271.3	*D. rerio*	ENSDARG00000074661	gper1	G protein-coupled estrogen receptor 1	0.00	0.37	7.1E-04	1.9E-03	0.37	7.1E-04		MLMDWRKQDCAGLNTCEKQSVQKKKKKQDP*
HG0272.1	*D. melanogaster*	FBgn0034649	CG9865	CG9865, isoform A	0.09	0.06	5.2E-266	1.3E-265	0.06	5.2E-266	Hayden	MSDTRKDPCKPNACRIQACLTKNHYQEDKCLDVLEAMRQCCLKWHKESLCCSGIDLEKTYLPDASKEKNATGKSNK*
HG0272.2	*D. melanogaster*	FBgn0034649	CG9865	CG9865, isoform A	0.00	0.05	2.2E-230	4.4E-230	0.05	2.2E-230	Hayden	MESVRKANQRIRNYPVLLSKCADKATAYAVCVSRDLNVQHKICDTEFKEFLSCIRKTALEMKTKL*
HG0272.3	*H. sapiens*	ENSG00000143315	PIGM	phosphatidylinositol glycan anchor biosynthesis class M	0.07	0.26	1.6E-04	6.8E-04	0.26	1.6E-04		MPDVIVCSRFGWQPGGWEMRRPPAAKNRREG*
HG0273.1	*G. gallus*	ENSGALG00000003293	MBTPS1	membrane bound transcription factor peptidase, site 1	0.00	0.00	2.5E-25	2.2E-24	0.00	2.5E-25		MSLFIRLYLKRQ*
HG0273.2	*H. sapiens*	ENSG00000140943	MBTPS1	membrane bound transcription factor peptidase, site 1	0.00	0.49	1.3E-06	8.4E-06	0.49	1.3E-06		MECPGVFAAEAPLSRARRP*
HG0273.3	*H. sapiens*	ENSG00000140943	MBTPS1	membrane bound transcription factor peptidase, site 1	0.00	0.00	1.4E-03	4.7E-03	0.00	1.4E-03		MLQLLAETKIHF*
HG0274.1	*H. sapiens*	ENSG00000204256	BRD2	bromodomain containing 2	0.00	0.41	1.2E-28	4.0E-27	0.44	1.5E-19		MTGPSGQYRPPRSPWSSPFSGPAQSSESVHPLYSALKRISKMEAAAP*
HG0274.2	*D. rerio*	ENSDARG00000100129			0.13	0.43	1.2E-02	2.3E-02	0.43	1.2E-02		MLLRNKSYPKICKDNATGAARSESNKVQHIHLRLAHSDRSSRSV*
HG0275.1	*H. sapiens*	ENSG00000100239	PPP6R2	protein phosphatase 6 regulatory subunit 2	0.00	0.35	2.4E-04	1.0E-03	0.37	1.9E-03		MKKIFLKLHILQEKNHKCFSVLPEYMI*

HG0275.2	*D. rerio*	ENSDARG00000045540	ppp6r2a	protein phosphatase 6, regulatory subunit 2a	0.03	0.29	2.8E-03	6.7E-03	0.29	2.8E-03		MLGHSVPSAFCHSNACRPVEEV*
HG0276.1	*G. gallus*	ENSGALG00000008912			0.13	0.28	7.7E-04	2.2E-03	0.28	7.7E-04		MDSSSSITVTWNIQHHM*
HG0276.2	*H. sapiens*	ENSG00000073734	ABCB11	ATP binding cassette subfamily B member 11	0.12	0.30	6.9E-03	1.9E-02	0.30	6.9E-03		MMKTEVGKGCETF*
HG0277.1	*H. sapiens*	ENSG00000102531	FNDC3A	fibronectin type III domain containing 3A	0.10	0.02	5.4E-16	1.0E-14	0.02	5.4E-16		MAAAAFVVPRVKQKAGATRRGSEERG*
HG0277.2	*G. gallus*	ENSGALG00000004169			0.06	0.00	7.2E-03	1.7E-02	0.00	7.2E-03		MAERSVGGRQRRG*
HG0278.1	*H. sapiens*	ENSG00000119946	CNNM1	cyclin and CBS domain divalent metal cation transport med	0.20	0.22	2.0E-09	2.0E-08	0.22	2.0E-09		MPGHLQPPAGTSCPGGV*
HG0278.2	*D. rerio*	ENSDARG00000078733	cnnm2b	cyclin and CBS domain divalent metal cation transport med	0.00	0.19	2.7E-03	6.5E-03	0.19	2.7E-03		MHKSRRRAAEMKPDWL*
HG0279.1	*H. sapiens*	ENSG00000175928	LRRN1	leucine rich repeat neuronal 1	0.00	0.35	1.0E-13	1.6E-12	0.38	1.3E-10		MFTFSARCPTYHNIVFTFC*
HG0279.2	*G. gallus*	ENSGALG00000035099	Lrrn1	leucine rich repeat neuronal 1	0.28	0.47	2.3E-02	4.6E-02	0.47	2.3E-02		MSAPPLRGGEERLRGQRPPGCRNVFRHGFVEDPGTQRSS*
HG0280.1	*H. sapiens*	ENSG00000156299	TIAM1	T-cell lymphoma invasion and metastasis 1	0.00	0.26	2.2E-05	1.2E-04	0.26	2.2E-05	Crowe	MAHLEILTKPLLFPMGFLAHSHRLR*
HG0280.2	*D. melanogaster*	FBgn0085447	sif	still life	0.00	0.04	9.4E-08	9.7E-08	0.04	9.4E-08		MGAKESVEAAAKMNADGWGHVERSSRYHNHRHNQRSQSLNRGQALSQADVSGMW*
HG0281.1	*G. gallus*	ENSGALG00000006766	DEPDC5	DEP domain containing 5	0.01	0.12	4.8E-28	4.5E-27	0.12	4.8E-28		MCRMSSTVFTRNS*
HG0281.2	*H. sapiens*	ENSG00000100150	DEPDC5	DEP domain containing 5	0.04	0.11	1.0E-09	1.1E-08	0.11	1.0E-09		MTSLPQAWNS*
HG0282.1	*D. rerio*	ENSDARG00000061328	cdon	cell adhesion associated, oncogene regulated	0.07	0.29	2.2E-19	1.2E-18	0.29	2.2E-19		MEGPVRICHRENVFRC*
HG0282.2	*H. sapiens*	ENSG00000144857	BOC	BOC cell adhesion associated, oncogene regulated	0.05	0.27	1.8E-02	4.2E-02	0.27	1.8E-02		MKSCRHLYRLRVAARK*
HG0283.1	*G. gallus*	ENSGALG00000010270	AREL1	apoptosis resistant E3 ubiquitin protein ligase 1	0.10	0.29	3.9E-31	3.9E-30	0.29	3.9E-31		MQVPGVPFSVTAFSMASLC*
HG0283.2	*H. sapiens*	ENSG00000119682	AREL1	apoptosis resistant E3 ubiquitin protein ligase 1	0.00	0.42	7.2E-24	2.1E-22	0.42	7.2E-24		MDRPFLLTLWRFSFMWKLEDKVKGAKSYLLW*
HG0284.1	*H. sapiens*	ENSG00000007168	PAFAH1B1	platelet activating factor acetylhydrolase 1b regulatory subu	0.13	0.10	4.4E-05	2.2E-04	0.10	4.4E-05	Crowe	MGVKDGRGPAEAAVQRSGGMNLTC*
HG0284.2	*G. gallus*	ENSGALG00000005834	PAFAH1B1	platelet activating factor acetylhydrolase 1b regulatory subu	0.03	0.18	1.5E-02	3.3E-02	0.18	1.5E-02		MPPGFSVGFPIWRNICALSHCGKLNCGS*
HG0285.1	*G. gallus*	ENSGALG00000012156	DPP10	dipeptidyl peptidase like 10	0.20	0.13	9.2E-03	2.1E-02	0.13	9.2E-03		MLRGRPRAAGQEEHGGGADTGLGFPPAGAVAPRLPPAFLFLYFSSSSAAAPLRGEVGVMRLAPREGCPGPQRQ*
HG0285.2	*H. sapiens*	ENSG00000175497	DPP10	dipeptidyl peptidase like 10	0.00	0.00	9.9E-03	2.6E-02	0.00	9.9E-03		MNQVNALFRAK*
HG0286.1	*H. sapiens*	ENSG00000116698	SMG7	SMG7, nonsense mediated mRNA decay factor	0.00	0.00	5.8E-04	2.2E-03	0.00	5.8E-04		MSLQSAQYLRF*
HG0286.2	*D. rerio*	ENSDARG00000060767	smg7	SMG7 nonsense mediated mRNA decay factor	0.08	0.49	9.9E-03	2.0E-02	0.49	9.9E-03		MDNMAPGVDLACESVVV*
HG0287.1	*H. sapiens*	ENSG00000170325	PRDM10	PR/SET domain 10	0.00	0.33	1.7E-12	2.4E-11	0.33	1.7E-12		MLQTDLSVLHEHTCGRQMTVLYPVLL*
HG0287.2	*D. rerio*	ENSDARG00000104251	prdm10	PR domain containing 10	0.00	0.40	5.4E-07	2.0E-06	0.40	5.4E-07		MQGSLLCSYHSEGRPTSTFS*
HG0288.1	*G. gallus*	ENSGALG00000006655	MAP3K13	mitogen-activated protein kinase kinase kinase 13	0.20	0.44	4.8E-03	1.2E-02	0.44	4.8E-03		MIRYWLPASRLNCPVL*
HG0288.2	*H. sapiens*	ENSG00000073803	MAP3K13	mitogen-activated protein kinase kinase kinase 13	0.02	0.41	6.9E-04	2.6E-03	0.41	6.9E-04		MYCGIQILALWERFWSPLLSQNSVPKIF*
HG0289	*G. gallus*	ENSGALG00000009581	MPP5	membrane palmitoylated protein 5	0.00	0.45	1.1E-32	1.1E-31	0.45	1.1E-32		MDFTQQRNENWPNISISI*
	H. sapiens	ENSG00000072415	MPP5	membrane palmitoylated protein 5	0.00	0.30	1.5E-16	2.9E-15	0.30	1.5E-16		MDFILQNQENWLIGKIDL*
HG0290.1	*H. sapiens*	ENSG00000116539	ASH1L	ASH1 like histone lysine methyltransferase	0.00	0.48	1.9E-09	1.9E-08	0.48	1.9E-09		MLVSSFGDPCFLWNTVL*
HG0290.2	*D. rerio*	ENSDARG00000070981	ash1l	ash1 (absent, small, or homeotic)-like (Drosophila)	0.04	0.28	6.6E-06	2.3E-05	0.28	6.6E-06		MEARLTREARSDEPELH*
HG0291.1	*G. gallus*	ENSGALG00000009173	GFRA1	GDNF family receptor alpha 1	0.24	0.41	2.2E-03	5.9E-03	0.41	2.2E-03		MDRNLESLHLGGFGISTC*
HG0291.2	*H. sapiens*	ENSG00000151892	GFRA1	GDNF family receptor alpha 1	0.14	0.44	3.0E-06	1.8E-05	0.44	3.0E-06		MELNFGRPEQHSCPGIAAR*
HG0292.1	*G. gallus*	ENSGALG00000006502	PCGF5	polycomb group ring finger 5	0.17	0.24	1.0E-28	9.6E-28	0.24	1.0E-28		MPGKVPLRRAAPQLS*
HG0292.2	*H. sapiens*	ENSG00000180628	PCGF5	polycomb group ring finger 5	0.00	0.11	5.2E-06	3.0E-05	0.11	5.2E-06		MALSPLRRRWDRLGEDAAEETNSQDMGKRNHQKE*
HG0293	*G. gallus*	ENSGALG00000012187	MGAT5	mannosyl (alpha-1,6-)-glycoprotein beta-1,6-N-acetyl-gluco	0.00	0.33	4.8E-27	4.4E-26	0.33	4.8E-27	Mackowiak	MLRKAHWPTNRMELKYGYMLTKESE*
	H. sapiens	ENSG00000152127	MGAT5	mannosyl (alpha-1,6-)-glycoprotein beta-1,6-N-acetyl-gluco	0.00	0.28	1.0E-23	2.8E-22	0.28	1.0E-23	Mackowiak	MEVRKGNQLTQEPE*
HG0294.1	*G. gallus*	ENSGALG00000017036	LHFP	lipoma HMGIC fusion partner	0.05	0.41	7.5E-14	4.7E-13	0.41	7.5E-14		MMGRNILLAFRIRASPSVRICQG*
HG0294.2	*H. sapiens*	ENSG00000183722	LHFP	lipoma HMGIC fusion partner	0.12	0.43	3.7E-04	1.5E-03	0.43	3.7E-04		MFPPEKDSKLCVRPGRGREGGRRPPAMDHHL*
HG0295.1	*H. sapiens*	ENSG00000166888	STAT6	signal transducer and activator of transcription 6	0.15	0.06	9.5E-36	3.9E-34	0.06	9.5E-36		MYVCALSYFSFWWWWWKGGGASRASLELAGQSYRPMGPGSAR*
HG0295.2	*G. gallus*	ENSGALG00000003282	STAT5B	signal transducer and activator of transcription 5B	0.17	0.50	8.8E-05	2.9E-04	0.50	8.8E-05		MRAGVPSLCPQLRCAGGGRKQPEHSAARLRRQLTFWKEIAALSSFL*
HG0296	*G. gallus*	ENSGALG00000006912	CPEB3	cytoplasmic polyadenylation element binding protein 3	0.23	0.17	2.1E-03	5.6E-03	0.17	2.1E-03		MQIARGRRWRRRRVLGKTGTRISFSLQGESSEFRCKRKETWRNCCISLPYKHISHLD*
HG0297	*G. gallus*	ENSGALG00000031684			0.20	0.00	1.1E-05	4.0E-05	0.00	1.1E-05		MCFCFVLQRFFWRLIFTF*
HG0298	*G. gallus*	ENSGALG00000042308			0.00	0.01	2.0E-04	6.2E-04	0.01	2.0E-04		MESIVWECG*
HG0299	*G. gallus*	ENSGALG00000040465	Sip1	zinc finger E-box binding homeobox 2	0.00	0.39	1.2E-02	2.7E-02	0.39	1.2E-02		MLGKEKEKNVIRRGVTRQSVPRFVFPGVAERDQS*
HG0300	*D. melanogaster*	FBgn0031668	CG31917	RE52596p2	0.00	0.02	0.0E+00	0.0E+00	0.02	0.0E+00	Hayden	MVNVMKGVLVECDPAMKQFLLHLDEKLALGRKFIIQDLDENHLFISTDIVEVLQARVDDLMDRISFPLHDKDA*
HG0301	*D. melanogaster*	FBgn0036856	CG9666	CG9666, isoform A	0.15	0.05	0.0E+00	0.0E+00	0.05	0.0E+00	Hayden	MAFPTTSAQQAETNRKILEEIQTKKQLLAGGIINLGLSPPNQMPAPQLLGQPTTVNPDFQAGVGIATNATSTARSAFNPTSSTTLGFFIPQDSYFGNSFIPVLPRLEPLPSPATTPTTPNAPPSHSISK*
HG0302	*D. melanogaster*	FBgn0031454	CG9960	CG9960	0.20	0.04	1.2E-269	3.2E-269	0.04	1.2E-269	Hayden	MDSDSTVTSLEENTENFCTNPTRDILAEGITNLFKPTIERLDERVASTIQLQAELRGQLDALAAQLRDIEKAQSQIPEFADKVKELLNVKHKVTVISNVLVTSQERLTGLHKLIEKEQRRRQALLDSALSTNIS*
HG0303	*D. melanogaster*	FBgn0260464			0.00	0.05	1.4E-232	2.9E-232	0.05	1.4E-232	Hayden	MHTDLSSHLHTPACNKLIEELQACHENNAFAKFVGVCNSIDDKVVKCLKGERIARSAANRAKARERQAKYKEKLLQQESN*
HG0304	*D. melanogaster*	FBgn0260467	CG7071	CG7071, isoform A	0.03	0.06	0.0E+00	0.0E+00	0.06	0.0E+00	Hayden	MKIMISQVGRELYKVPLRILDHRVFVNGEIEAFLENFEVRRNDSEVEKLFQVTETVGSLKYDLSRCSATGRGSGAENLAQLDTEVSHLLDGVNALLAKAKVERTASTQLQEARLAREQRRAEFLTNLEHGYRRIENSFEEKEEEIAELYSDLQLKLNIAK*
HG0305	*D. melanogaster*	FBgn0050290	Ppcdc	Phosphopantothenoylcysteine decarboxylase	0.29	0.04	4.7E-246	1.1E-245	0.04	4.7E-246	Hayden	MTSQIYSQKKFVPTPPEKGSFPLDHEGLCKKQFLLYASCLRKNAQDTSQCRQDAQNYLACRMDNNLMEKTEWSKLGFHDQSTKTDQKEPEVQKQ*
HG0306	*D. melanogaster*	FBgn0259726			0.06	0.08	0.0E+00	0.0E+00	0.08	0.0E+00	Hayden	MSIPQYRPPKEIQDLLDQKEQQQANQRRRTAVLPKRTWIQRNPRLFQISFITGSLLIFFSKPLFDCFIADPLPPPENKVPPHKR*
HG0307	*D. melanogaster*	FBgn0037822	CG14683	Probable methyltransferase-like protein 15 homolog	0.12	0.06	1.1E-202	2.0E-202	0.06	1.1E-202		MSAQYQRFLKVLEKWPAEKSKVGRDLGEQIRKQVTKLTSLEGGATDKELDRQINSLERLSNNVYAKKYPRTFESTATGLTAAQCSQVLSSEFLQYLNEDSKKKK*
HG0308	*D. melanogaster*	FBgn0264743	CG44001	LP22840p	0.00	0.04	2.4E-197	4.3E-197	0.04	2.4E-197	Hayden	MPYIIIRGNLASYSHKYPWRVLVSGLKADDIEQLNKFSCGGYSDESTIVYLVHPCRILSALEILGFRVVASSSTAVKQDYNEYMWTMRKEFDEPEPLEAESVVRENLSNIGREAASLGNYHKVDSPE*
HG0309	*D. melanogaster*	FBgn0037689	CG8135	LMBR1 domain-containing protein 2 homolog	0.23	0.06	2.3E-243	5.2E-243	0.06	2.3E-243		MSSVEAEVLQKIDLIEPTTGKLFFEHKTELLFCRPHLMPLKTQALERLEEMHRDTARQLQKKRQQKKKSTEPTGSL*
HG0310	*D. melanogaster*	FBgn0039339	CG5116	CG5116, isoform A	0.08	0.05	6.5E-119	8.7E-119	0.05	6.5E-119	Hayden	MNDIKRLKDQQREKHPGFDGYMDCMTRSLFTGLATFCLSFSGTYFAQKIVQSKIRYPIKYNILISSLVATGVSYQITSTRTKACQAAWMAFEDKHSVLKEKTF*
HG0311	*D. melanogaster*	FBgn0263251	vnc	variable nurse cells	0.01	0.06	5.0E-128	6.8E-128	0.06	5.0E-128	Hayden	MSLQTGLAFIFTILASVSIVAAIILLGWFLIWKAFLSKFRLVRELLGQEEPEDQQPLAWDHQNQQPHHHGRARKARRD*
HG0312	*D. melanogaster*	FBgn0038641	CG7708	High-affinity choline transporter 1	0.07	0.05	8.1E-98	1.0E-97	0.05	8.1E-98	Mackowiak	MCGLHFRTRNSPSQKTSLRWGDCCLA*
HG0313	*D. melanogaster*	FBgn0259725			0.12	0.06	9.8E-78	1.2E-77	0.06	9.8E-78		MSDTRKDPCKPNACRIQACLTSKHRR*
HG0314	*D. melanogaster*	FBgn0260468	CG7950	CG7950, isoform A	0.15	0.06	0.0E+00	0.0E+00	0.06	0.0E+00	Hayden	MSGYQYLDVKIKLRDPDSVTLTPVFFCGCIHSSLASIFGEIGGQTILEIVKFSSSQKRSILRVPENVLDRVRVAIALIGYYQEVPCHFQVLSTSRKPLDFEESPEEFVAFN*
HG0315	*D. melanogaster*	FBgn0260234	CG42508	GH25417p	0.00	0.07	3.8E-68	4.5E-68	0.07	3.8E-68		MKPKKSAFANSSTGYGRNKHGKGHSGKDKVEGGGKGKSRDEKDKHKHEQKVDEKDDKDLNSGFGDYLRTPEAFEMMKLFVFANTIMLIVTMAWPHIKEQFYMVNQWLESFREEHQQ*
HG0316	*D. melanogaster*	FBgn0041164	armi	armitage	0.10	0.07	6.3E-165	1.0E-164	0.07	6.3E-165		MSSEASGSITIKEEASTSGTATNIPVESSETEAKKPEIGADKAESQSETTEGGKAAETTENHLKRLQNLRKELSYLSETDWMYESLDKKAAQ*
HG0317	*D. melanogaster*	FBgn0050011	gem	gemini	0.00	0.05	4.5E-17	5.0E-17	0.05	4.5E-17	Mackowiak	MEKSEIRLQRMSNEYQSQSSYMYLRTKMLLKIENTLLRSHRQRETTGIKKLYNSFFVLF*
HG0318	*D. melanogaster*	FBgn0035293	CG5687	CG5687, isoform A	0.00	0.04	1.7E-13	1.8E-13	0.04	1.7E-13	Mackowiak	MKYRNNRGTILQIQFRTFLRYSAANLIPFES*
HG0319	*D. melanogaster*	FBgn0026778	Rad1	Radiation insensitive 1	0.00	0.07	5.4E-12	5.7E-12	0.07	5.4E-12	Hayden	MASESENTTSYFVPHLVDQVFELYRSKAESNSLTKEERGQFFYDLSILLGQELVLRSLHLLDDHNFSFFHAKNNRSVCVVEISKGTEYFRLIPGVSFCKCEFFQCHVLQLPRGILYQDLPSGEPGILEDWSEESRVSYTCQHILALRLHQFLKHTGGKTTEKILKKEDIKELRTDVFRD*
HG0320	*D. melanogaster*	FBgn0040993			0.00	0.06	1.0E-02	1.0E-02	0.06	1.0E-02	Hayden	MKTPDTKAKLLNNISLSKHLSSKKGGSRTSNCGNCLEFSVLTRLSSVP*
HG0321	*D. rerio*	ENSDARG00000070426	chac1	ChaC, cation transport regulator homolog 1 (E. coli)	0.03	0.25	6.2E-160	9.3E-159	0.25	6.2E-160		MHQAKKSVLRDCTITTAKQLGSDTVRGIAVSQPIPLLLDRRKLTLAT*
HG0322	*D. rerio*	ENSDARG00000070917	kitlga	kit ligand a	0.00	0.34	5.5E-27	3.4E-26	0.34	5.5E-27		MMILDMYLWDEENPFPARRSKIK*
HG0323	*D. rerio*	ENSDARG00000071197	usp40	ubiquitin specific peptidase 40	0.02	0.03	7.8E-132	1.1E-130	0.03	7.8E-132	Mackowiak	MFIPVKSSYARVSPLIVNRELGKTKMR*
HG0324	*D. rerio*	ENSDARG00000094965	nfil3-5	nuclear factor, interleukin 3 regulated, member 5	0.00	0.10	6.6E-97	8.1E-96	0.10	6.6E-97		MTVVFVEFHLFLIFLTSLDT*
HG0325	*D. rerio*	ENSDARG00000079738	znf219	zinc finger protein 219	0.13	0.07	2.2E-12	9.8E-12	0.07	2.2E-12		MYLIQVKRNHDVCCH*
HG0326	*D. rerio*	ENSDARG00000012848	arih2	ariadne homolog 2 (Drosophila)	0.00	0.00	5.5E-89	6.6E-88	0.00	5.5E-89		MSDGECDFFCFFRCC*
HG0327	*D. rerio*	ENSDARG00000022203	gtf2ird1	GTF2I repeat domain containing 1	0.00	0.39	3.7E-20	2.2E-19	0.39	3.7E-20		MTFNFTGLQNKERGKGTHSSVKENREKTGQTHQINHTECFSTDYRPFEIMTFSKVAVAFL*
HG0328	*D. rerio*	ENSDARG00000099999	tcf3b	transcription factor 3b	0.00	0.08	2.0E-63	2.0E-62	0.08	2.0E-63		MWMEVVKMSTRRMNSPQL*
HG0329	*D. rerio*	ENSDARG00000036442	atp11c	ATPase, Class VI, type 11C	0.00	0.43	2.2E-20	1.3E-19	0.43	2.2E-20		MRMVREDSIQLIWKRCSFTAALHIARGNRLESR*
HG0330	*D. rerio*	ENSDARG00000039392	wnk1b	WNK lysine deficient protein kinase 1b	0.00	0.32	3.7E-37	2.8E-36	0.32	3.7E-37		MDEVDLTVFVCHHQCLPLSNCVLFFYVYKRTPPLLGSGPLHCKSNC*
HG0331	*D. rerio*	ENSDARG00000044899	tmem183a	transmembrane protein 183A	0.02	0.34	6.3E-58	5.9E-57	0.34	6.3E-58		MYATLSVSSIMAEDPGGAFFNFTLILTDFSLPLLERWQG*
HG0332	*D. rerio*	ENSDARG00000102893	atg7	ATG7 autophagy related 7 homolog (S. cerevisiae)	0.00	0.03	3.8E-84	4.2E-83	0.03	3.8E-84		MKRNSVYRFLSVFSCLLVCVFLFFWGFAFYSMVLAP*
HG0333	*D. rerio*	ENSDARG00000042518	hipk2	homeodomain interacting protein kinase 2	0.00	0.47	5.0E-15	2.5E-14	0.47	5.0E-15		MQEIAAILSAAQDGRFLLSCEQCCRFIFCHE*
HG0334	*D. rerio*	ENSDARG00000075349	si:ch73-138n13.1	si:ch73-138n13.1	0.05	0.46	3.0E-14	1.5E-13	0.46	3.0E-14		MTSLCGYHRNQGSTADG*
HG0335	*D. rerio*	ENSDARG00000034700	vegfab	vascular endothelial growth factor Ab	0.00	0.32	2.3E-31	1.5E-30	0.32	2.3E-31		MWIVIALEEKFDLLLNNVRGPASWILIFLDILKIIGNS*
HG0336	*D. rerio*	ENSDARG00000028676	nuak1b	NUAK family, SNF1-like kinase, 1b	0.03	0.34	1.1E-13	5.4E-13	0.34	1.1E-13		MRVIYVQNAGVVAAFSPEIFCGRLKQRNNGEIMTERATGKRTRHNGVGYVIME*
HG0337	*D. rerio*	ENSDARG00000054906			0.06	0.47	8.6E-13	3.8E-12	0.47	8.6E-13		MFLHRADLSLRPRTKYRDSD*
HG0338	*D. rerio*	ENSDARG00000000151	thraa	thyroid hormone receptor alpha a	0.04	0.00	1.3E-19	7.3E-19	0.00	1.3E-19		MLTCRGSSPPQSAVIGCWMESR*
HG0339	*D. rerio*	ENSDARG00000063144	shisa7b	shisa family member 7	0.11	0.21	2.3E-35	1.7E-34	0.21	2.3E-35	Mackowiak	MNPDLFMPVQWAQPCCPVRTGPKPPFVLFCWVELNPEQRHSRKRVQHT*
HG0340	*D. rerio*	ENSDARG00000017591	fat1a	FAT atypical cadherin 1a	0.06	0.15	2.6E-15	1.3E-14	0.15	2.6E-15		MQRLGTYKLHEKPPNKGR*
HG0341	*D. rerio*	ENSDARG00000039430	msl2b	male-specific lethal 2 homolog b (Drosophila)	0.00	0.08	5.0E-20	2.9E-19	0.08	5.0E-20		MMTRIWSYFVPWKRGKR*
HG0342	*D. rerio*	ENSDARG00000045801	fbxl14b	F-box and leucine-rich repeat protein 14b	0.08	0.30	2.2E-16	1.2E-15	0.30	2.2E-16		MYDRLDPRVERRLLSKRQTGSSAA*
HG0343	*D. rerio*	ENSDARG00000057062	si:dkey-157g16.6	si:dkey-157g16.6	0.05	0.30	1.3E-09	5.1E-09	0.30	1.3E-09		MDYSRTDANLGFPTELLP*
HG0344	*D. rerio*	ENSDARG00000032072	cdk16	cyclin-dependent kinase 16	0.19	0.16	1.8E-11	7.6E-11	0.16	1.8E-11		MAEDYWERAAALGYYYCC*
HG0345	*D. rerio*	ENSDARG00000035910	znf281b	zinc finger protein 281b	0.05	0.39	4.4E-05	1.4E-04	0.39	4.4E-05		MEEANSCKGTLFWL*
HG0346	*D. rerio*	ENSDARG00000041982	adamts6	ADAM metallopeptidase with thrombospondin type 1 motif,	0.22	0.25	1.9E-13	8.9E-13	0.25	1.9E-13		MFVCTVAASASAGKQMDSSAQVPNIDLLLWNNMKGVVMDSCPAHG*
HG0347	*D. rerio*	ENSDARG00000063194			0.00	0.00	2.2E-02	3.9E-02	0.00	2.2E-02		MADLSKSKPGLK*
HG0348	*D. rerio*	ENSDARG00000036139	lctla	lactase-like a	0.08	0.11	7.7E-15	3.9E-14	0.11	7.7E-15		MSFLLAALDKLAYLLSLVAFCVSLSYSWLK*
HG0349	*D. rerio*	ENSDARG00000028699	crkl	v-crk avian sarcoma virus CT10 oncogene homolog-like	0.00	0.36	1.4E-05	4.8E-05	0.36	1.4E-05		MHQSWLTLVAACLVKRQVAVQ*
HG0350	*D. rerio*	ENSDARG00000036194	pitx2	paired-like homeodomain 2	0.12	0.46	1.5E-03	3.9E-03	0.46	1.5E-03		MQVDIISHTCPCHSGSPCSRLKPRYLLPSRNGKKIFP*
HG0351	*D. rerio*	ENSDARG00000068096	atf5a	activating transcription factor 5a	0.00	0.42	5.5E-04	1.5E-03	0.42	5.5E-04		MRTKPCFLWLNSQTSLSCTLSSGI*
HG0352	*D. rerio*	ENSDARG00000070214	slc41a1	solute carrier family 41 (magnesium transporter), member 1	0.05	0.33	3.5E-05	1.1E-04	0.33	3.5E-05		MGNIIQVLPKFRQDLTQAWRRGTHLPLFLDL*
HG0353	*D. rerio*	ENSDARG00000078864	lats2	large tumor suppressor kinase 2	0.23	0.40	2.9E-06	1.0E-05	0.40	2.9E-06		MANMHTRLGWKKHADVNPHMSLEEGECEP*
HG0354	*D. rerio*	ENSDARG00000019897	gpat4	glycerol-3-phosphate acyltransferase 4	0.05	0.18	1.6E-06	5.6E-06	0.18	1.6E-06		MIIFPYMPMKSMFQRRLQKLRHSRGVQQSVFR*
HG0355	*D. rerio*	ENSDARG00000019930	tal1	T-cell acute lymphocytic leukemia 1	0.27	0.50	9.6E-04	2.5E-03	0.50	9.6E-04		MDDPPQNGSMHTLTSTARRR*
HG0356	*D. rerio*	ENSDARG00000020475	znrf1	zinc and ring finger 1	0.08	0.31	1.2E-02	2.3E-02	0.31	1.2E-02		MIRPGKWAVASARHSCFSQT*
HG0357	*D. rerio*	ENSDARG00000037285	mipa	major intrinsic protein of lens fiber a	0.05	0.40	2.3E-03	5.7E-03	0.40	2.3E-03		MSAGHAHHLSIRVPLLNLS*

HG0358	*D. rerio*	ENSDARG00000062506	zc3h18	zinc finger CCCH-type containing 18	0.15	0.28	4.7E-06	1.6E-05	0.28	4.7E-06		MAALTVSCRVYAFFR*
HG0359	*D. rerio*	ENSDARG00000070348	hoxc10a	homeobox C10a	0.07	0.30	3.9E-07	1.5E-06	0.30	3.9E-07		MSASYCPYPLRHKGDCFESKEKQDK*
HG0360	*D. rerio*	ENSDARG00000070801	opa1	optic atrophy 1 (autosomal dominant)	0.04	0.10	7.6E-06	2.6E-05	0.10	7.6E-06		MCPIWKDELNFILQRDPKLIYICH*
HG0361	*D. rerio*	ENSDARG00000077581	zzz3	zinc finger, ZZ-type containing 3	0.24	0.37	1.5E-03	3.9E-03	0.37	1.5E-03		MCSKRFAEVRCPHTAAEERAPR*
HG0362	*D. rerio*	ENSDARG00000078355	zc3h4	zinc finger CCCH-type containing 4	0.01	0.42	7.7E-04	2.1E-03	0.42	7.7E-04		MSFISHRNCRTENLRNVFNLFLF*
HG0363	*D. rerio*	ENSDARG00000095896	pou3f3b	POU class 3 homeobox 3b	0.22	0.31	2.3E-04	6.6E-04	0.31	2.3E-04		MRYNLPALKKFLQKGGGDQQTYS*
HG0364	*D. rerio*	ENSDARG00000004988	ppp3ca	protein phosphatase 3, catalytic subunit, alpha isozyme	0.00	0.42	9.3E-03	1.9E-02	0.42	9.3E-03		MPLHQPHLVTANTRRE*
HG0365	*D. rerio*	ENSDARG00000016132	keap1a	kelch-like ECH-associated protein 1a	0.00	0.17	3.3E-05	1.1E-04	0.17	3.3E-05		MPTWLETESCYSGLLTRQKKHGSLSLNDNQLCLYFFMARGWV*
HG0366	*D. rerio*	ENSDARG00000017803	gsk3b	glycogen synthase kinase 3 beta	0.00	0.20	8.8E-05	2.7E-04	0.20	8.8E-05		MELSGSERPRISVLKLGNWKILIRIAG*
HG0367	*D. rerio*	ENSDARG00000034056	csnk1g2b	casein kinase 1, gamma 2b	0.03	0.22	8.1E-04	2.2E-03	0.22	8.1E-04		MFTVLPRTDFKETMRSTRGRSLVGFH*
HG0368	*D. rerio*	ENSDARG00000058606	sik1	salt-inducible kinase 1	0.23	0.28	2.1E-03	5.2E-03	0.28	2.1E-03		MTCKCKCKNHLCCGRRWKLFAS*
HG0369	*D. rerio*	ENSDARG00000059276	hoxd4a	homeobox D4a	0.04	0.48	2.1E-02	3.8E-02	0.48	2.1E-02		MKSLHLDWPSWSHGRLTLFS*
HG0370	*D. rerio*	ENSDARG00000062693	nrxn3b	neurexin 3b	0.02	0.32	2.2E-02	3.8E-02	0.32	2.2E-02		MWQNNRIRSNMENLCFNPERRLKGTSSVKHF*
HG0371	*D. rerio*	ENSDARG00000062765	whsc1l1	Wolf-Hirschhorn syndrome candidate 1-like 1	0.00	0.24	9.7E-03	1.9E-02	0.24	9.7E-03		MEHAEAGPVRKRET*
HG0372	*D. rerio*	ENSDARG00000069467	igsf9bb	immunoglobulin superfamily, member 9Bb	0.26	0.50	3.0E-02	5.0E-02	0.50	3.0E-02		MQGMSKRVSAADSASNDQGLG*
HG0373	*D. rerio*	ENSDARG00000074611	wdr37	WD repeat domain 37	0.06	0.46	4.2E-03	9.4E-03	0.46	4.2E-03		MTVALRSAEAVINALGFLFEVFLFFITLSSRLRMCLLCLSFTALHGCFR*
HG0374	*D. rerio*	ENSDARG00000075147	lrrc38a	leucine rich repeat containing 38a	0.18	0.17	2.7E-03	6.5E-03	0.17	2.7E-03		MISWKIELRVFVAAFRCTSLCRLQSRRQGKMPSRQT*
HG0375	*D. rerio*	ENSDARG00000076171	znf827	zinc finger protein 827	0.00	0.18	1.6E-02	3.0E-02	0.18	1.6E-02		MGKDLFSSYP*
HG0376	*D. rerio*	ENSDARG00000077229	ano8b	anoctamin 8b	0.07	0.01	9.4E-05	2.9E-04	0.01	9.4E-05		MTHNNLHFTLTFI*
HG0377	*D. rerio*	ENSDARG00000079549	cdc42ep1a	CDC42 effector protein (Rho GTPase binding) 1a	0.00	0.28	2.5E-02	4.2E-02	0.28	2.5E-02		MQPRFSGIGVMHIFSSHLSSPNLSGYEQRPMAKTGHS*
HG0378	*D. rerio*	ENSDARG00000080009	bahcc1b	BAH domain and coiled-coil containing 1b	0.21	0.16	6.4E-05	2.0E-04	0.16	6.4E-05		MIGTYFFGRQSQNCAECKT*
HG0379	*D. rerio*	ENSDARG00000104372	gnb1b	guanine nucleotide binding protein (G protein), beta polype	0.00	0.47	1.7E-02	3.0E-02	0.47	1.7E-02		MRGFDSHTIVFFGQCHINKGHLRLRTDQNVSRQ*
HG0380	*D. rerio*	ENSDARG00000005350	dip2ba	disco-interacting protein 2 homolog Ba	0.00	0.41	9.4E-03	1.9E-02	0.41	9.4E-03		MAEGVVLRMDCYSPFNSRFMP*
HG0381	*D. rerio*	ENSDARG00000006124	kdm5c	lysine (K)-specific demethylase 5C	0.00	0.04	3.0E-03	7.1E-03	0.04	3.0E-03		MACSSDRMNGG*
HG0382	*D. rerio*	ENSDARG00000017242			0.00	0.29	4.4E-03	1.0E-02	0.29	4.4E-03		MTEPFFTADACGGGLTCRC*
HG0383	*D. rerio*	ENSDARG00000036501	rab39bb	RAB39B, member RAS oncogene family b	0.00	0.27	1.7E-02	3.1E-02	0.27	1.7E-02		MMLLLSLPFHLHKTSKHFSIFQTWIKT*
HG0384	*D. rerio*	ENSDARG00000054748	cuedc1b	CUE domain containing 1b	0.06	0.04	1.2E-02	2.2E-02	0.04	1.2E-02		MQLNKQKKRRKGFLSLQWTRFFIV*
HG0385	*D. rerio*	ENSDARG00000059549	akap10	A kinase (PRKA) anchor protein 10	0.00	0.15	3.2E-03	7.6E-03	0.15	3.2E-03		MNEGNRESSGCCL*
HG0386	*D. rerio*	ENSDARG00000062084	clcn1a	chloride channel, voltage-sensitive 1a	0.00	0.25	5.9E-03	1.3E-02	0.25	5.9E-03		MCEEETERLSVCVCMCPCHPRVGMLRQTGSLFLGQ*
HG0387	*D. rerio*	ENSDARG00000071150	cica	capicua transcriptional repressor a	0.16	0.28	1.2E-02	2.2E-02	0.28	1.2E-02		MQERNRVEDIDRLGLTGNQED*
HG0388	*D. rerio*	ENSDARG00000077228	ntrk3a	neurotrophic tyrosine kinase, receptor, type 3a	0.00	0.35	1.4E-02	2.6E-02	0.35	1.4E-02		MQPVEEDSLGALIFEPFLH*
HG0389	*D. rerio*	ENSDARG00000077361	bptf	bromodomain PHD finger transcription factor	0.08	0.36	1.5E-02	2.7E-02	0.36	1.5E-02		MAAASDLKRGAGRSF*
HG0390	*D. rerio*	ENSDARG00000090035	rtn4r	reticulon 4 receptor	0.26	0.36	6.3E-03	1.3E-02	0.36	6.3E-03		MTERGTTGRRCSPSRARTATVG*
HG0391	*D. rerio*	ENSDARG00000099634	elna	elastin a	0.00	0.15	2.5E-02	4.4E-02	0.15	2.5E-02		MIHRTKGFALS*
HG0392	*D. rerio*	ENSDARG00000100003	glulb	glutamate-ammonia ligase (glutamine synthase) b	0.00	0.04	4.1E-03	9.4E-03	0.04	4.1E-03		MRMRDKQPGLPYKTLWKYSHSYSDYLYFLIGLI*
HG0393	*G. gallus*	ENSGALG00000002090	GPSM2	G protein signaling modulator 2	0.00	0.43	5.0E-36	5.8E-35	0.43	5.0E-36		MELKGPQMKELERVSDSLRCV*
HG0394	*G. gallus*	ENSGALG00000002479	MAT1A	methionine adenosyltransferase 1A	0.00	0.39	1.6E-46	2.2E-45	0.39	1.6E-46		MRSPCMHTQIQAHADFTD*
HG0395	*G. gallus*	ENSGALG00000014936	ESCO1	establishment of sister chromatid cohesion N-acetyltransfer	0.00	0.40	5.4E-03	1.3E-02	0.40	5.4E-03		MLLAHRDKLLWPKHRLPLVKT*
HG0396.1	*G. gallus*	ENSGALG00000034253			0.00	0.43	2.9E-22	2.4E-21	0.43	2.9E-22		MKDHCKEPWTFVVSYNTVIQRV*
HG0396.2	*G. gallus*	ENSGALG00000034253			0.00	0.37	1.0E-04	3.3E-04	0.37	1.0E-04		MQKKTNKGRKDVKHDLP*
HG0397	*G. gallus*	ENSGALG00000009438	SLC39A9	solute carrier family 39 member 9	0.00	0.17	3.1E-46	4.3E-45	0.17	3.1E-46		MAESPSWDLGVCTG*
HG0398	*G. gallus*	ENSGALG00000005369	BTBD10	BTB domain containing 10	0.00	0.13	3.0E-52	4.9E-51	0.13	3.0E-52		MMEEKVRLARAALNY*
HG0399	*G. gallus*	ENSGALG00000032659			0.00	0.43	4.8E-07	2.0E-06	0.43	4.8E-07		MTNHFCVCTTRIQNS*
HG0400	*G. gallus*	ENSGALG00000004261	AQP9	aquaporin 9	0.00	0.19	1.3E-13	7.7E-13	0.19	1.3E-13		MISLAVQHEISYFSV*
HG0401	*G. gallus*	ENSGALG00000009057	TASP1	taspase 1	0.10	0.38	1.3E-16	9.5E-16	0.38	1.3E-16		MILLQNLFLNGSMLFASRRC*
HG0402	*G. gallus*	ENSGALG00000035584			0.24	0.34	3.8E-03	9.5E-03	0.34	3.8E-03		MARCWKGREQPMLLI*
HG0403.1	*G. gallus*	ENSGALG00000007717	GCNT7	glucosaminyl (N-acetyl) transferase family member 7	0.00	0.12	3.9E-50	5.9E-49	0.33	5.1E-19		MNPPPNTNPFSRMLSGKEAFIQHEIQSIPITCMPN*
HG0403.2	*G. gallus*	ENSGALG00000007717	GCNT7	glucosaminyl (N-acetyl) transferase family member 7	0.00	0.33	1.1E-02	2.4E-02	0.33	1.1E-02		MLSIAGNQVMLMFNPFQLYEISTQATE*
HG0404	*G. gallus*	ENSGALG00000008700	PDCD4	programmed cell death 4	0.00	0.32	1.4E-17	1.0E-16	0.32	1.4E-17		MEHSTRKDKTVLTR*
HG0405.1	*G. gallus*	ENSGALG00000009185			0.00	0.49	5.3E-08	2.4E-07	0.49	5.3E-08		MLLSSLWAFLDLRFFS*
HG0405.2	*G. gallus*	ENSGALG00000009185			0.00	0.40	2.9E-10	1.4E-09	0.40	2.9E-10		MPHPDIFSPELSKLPVL*
HG0406	*G. gallus*	ENSGALG00000004935	LSM14A	LSM14A, mRNA processing body assembly factor	0.23	0.34	1.4E-04	4.3E-04	0.34	1.4E-04		MKAAAMLGASGRFWCGVC*
HG0407	*G. gallus*	ENSGALG00000015027	JAK2	Janus kinase 2	0.05	0.28	2.5E-25	2.1E-24	0.28	2.5E-25		MERARVLRGSRQLALLRRAPAGYIMFYHCLCACFI*
HG0408	*G. gallus*	ENSGALG00000036255	WNT7B	Protein Wnt-7b	0.06	0.21	1.5E-10	7.7E-10	0.21	1.5E-10		MTGRSPEAAAAARTAAAAHTLPREQH*
HG0409	*G. gallus*	ENSGALG00000002315			0.00	0.32	1.1E-07	4.6E-07	0.32	1.1E-07		MVSTPCEPPLQK*
HG0410	*G. gallus*	ENSGALG00000008148	MYBPC3	myosin-binding protein C, cardiac-type	0.20	0.46	4.4E-11	2.4E-10	0.46	4.4E-11		MGASTEWFFWGSQKPCSLICV*
HG0411	*G. gallus*	ENSGALG00000012166	SLC35F5	solute carrier family 35 member F5	0.00	0.27	7.8E-27	7.1E-26	0.27	7.8E-27		MAIPTLTLLKWRLSSL*
HG0412	*G. gallus*	ENSGALG00000003446	PRLR	prolactin receptor	0.02	0.43	1.5E-05	5.4E-05	0.43	1.5E-05		MNERNKIFLCTHESFREPLM*
HG0413	*G. gallus*	ENSGALG00000005475			0.19	0.36	5.6E-13	3.2E-12	0.36	5.6E-13		MRSCCWLLLDFVSG*
HG0414	*G. gallus*	ENSGALG00000032599			0.18	0.17	2.7E-03	7.1E-03	0.17	2.7E-03		MMKALQWERAVVQG*
HG0415	*G. gallus*	ENSGALG00000034970			0.04	0.46	4.8E-07	2.0E-06	0.46	4.8E-07		MLSRTVMTREASLAKD*
HG0416	*G. gallus*	ENSGALG00000015874	TTK	TTK protein kinase	0.13	0.31	7.0E-06	2.6E-05	0.31	7.0E-06		MKDYISCRCWILGII*
HG0417	*G. gallus*	ENSGALG00000001522	PHF20	PHD finger protein 20	0.00	0.48	8.0E-06	2.9E-05	0.48	8.0E-06		MDFSALTIPDGGKHVVGGELGE*
HG0418	*G. gallus*	ENSGALG00000010337	GPR149	G protein-coupled receptor 149	0.14	0.36	1.3E-06	5.1E-06	0.36	1.3E-06		MKNCIFSSTFCTSLLPDFSRA*
HG0419	*G. gallus*	ENSGALG00000012542	RASD2	RASD family member 2	0.03	0.48	1.7E-03	4.6E-03	0.48	1.7E-03		MGGRPLEPCSPSQSC*
HG0420	*G. gallus*	ENSGALG00000000336	PPP1R12B		0.00	0.38	4.4E-04	1.3E-03	0.38	4.4E-04		MPKIIHLNRQGLITRAANSLISLF*
HG0421	*G. gallus*	ENSGALG00000003492			0.21	0.05	2.8E-20	2.2E-19	0.05	2.8E-20		MEANLKRAKKGDLKVSVHRMEIERIRYVLSSYLRCRLAKIEKFFPHVLEKEKSRAKGEPSILSPEEFAFAKEYMANTETYLKNVALKHMPPNLQKVALLKSVPKPNLDSFVFLRVLKRQENILVEPETDEQREYTIDLEEGSQHLIRYKTIAPLVASGAVQLI*
HG0422	*G. gallus*	ENSGALG00000005772	BOK	BOK, BCL2 family apoptosis regulator	0.00	0.44	7.2E-03	1.7E-02	0.44	7.2E-03		MKGKMKNRLWESSSWEL*
HG0423	*G. gallus*	ENSGALG00000006329			0.00	0.28	2.6E-03	6.9E-03	0.28	2.6E-03		MLSKLVGKKGGHLVSVL*
HG0424	*G. gallus*	ENSGALG00000039553			0.00	0.27	2.8E-04	8.4E-04	0.27	2.8E-04		MTIYLSCIRQRCCLQ*
HG0425	*G. gallus*	ENSGALG00000043044			0.00	0.26	6.2E-07	2.5E-06	0.26	6.2E-07		MIQKTVRSCDCKMSSFYLMLKGGKLKLQEL*
HG0426	*G. gallus*	ENSGALG00000002192	PTPRC	protein tyrosine phosphatase, receptor type C	0.00	0.17	1.6E-03	4.4E-03	0.17	1.6E-03		MLLRKDTPDFQK*
HG0427	*G. gallus*	ENSGALG00000005550	ST6GAL1	ST6 beta-galactoside alpha-2,6-sialyltransferase 1	0.01	0.45	1.3E-03	3.6E-03	0.45	1.3E-03		MKVFLELFSASRGCCVWGYPALNPGQKSANSQK*
HG0428	*G. gallus*	ENSGALG00000005976	TCEA2	transcription elongation factor A2	0.00	0.04	1.8E-09	8.8E-09	0.04	1.8E-09		MSHPVAGRFAAPLLK*
HG0429	*G. gallus*	ENSGALG00000006626	PDE6C	phosphodiesterase 6C	0.00	0.00	3.1E-12	1.8E-11	0.00	3.1E-12		MPLYSMYKVPFSY*
HG0430	*G. gallus*	ENSGALG00000015490			0.00	0.39	2.1E-07	9.0E-07	0.39	2.1E-07		MLSMGTSASDALTLLKTYFVAWLKRR*
HG0431	*G. gallus*	ENSGALG00000026542	FAM217B	family with sequence similarity 217 member B	0.00	0.33	6.8E-09	3.2E-08	0.33	6.8E-09		MPAEAESKDVVMFAFGTLCCVDRKEWSRWKIM*
HG0432	*G. gallus*	ENSGALG00000040546			0.05	0.44	6.9E-03	1.6E-02	0.44	6.9E-03		MLENFTSVLIVISEHPPKN*
HG0433	*G. gallus*	ENSGALG00000002952	NRG4	neuregulin 4	0.00	0.43	9.6E-05	3.1E-04	0.43	9.6E-05		MQISGGVDSADCPVRV*
HG0434	*G. gallus*	ENSGALG00000008357	TPCN1	two pore segment channel 1	0.24	0.34	5.9E-05	2.0E-04	0.34	5.9E-05		MGARTFAFWWLEDGHDGRFPGKRF*
HG0435	*G. gallus*	ENSGALG00000010778	CHRM3	cholinergic receptor muscarinic 3	0.00	0.14	2.6E-07	1.1E-06	0.14	2.6E-07		MAKSFSSVWYIQLQAG*
HG0436	*G. gallus*	ENSGALG00000026736	OGN	osteoglycin	0.10	0.16	8.1E-07	3.3E-06	0.26	2.5E-03		MLKLSTAPCSRILPLTLGTECWQSIFYPMESTFKTVNLGKLSHCSNLDVIHCWIVHNFTLLSVCMGLTYA*
HG0437	*G. gallus*	ENSGALG00000001728	NACC2	NACC family member 2	0.28	0.40	7.4E-04	2.1E-03	0.40	7.4E-04		MRGAAPARRPGPPFSPGPRRPASAAAARRKTMGRTMCPTLDTPALNA*
HG0438	*G. gallus*	ENSGALG00000008545	SENP5	SUMO1/sentrin specific peptidase 5	0.12	0.16	3.0E-05	1.0E-04	0.16	3.0E-05		MWYTARCCVTCKERQFCT*
HG0439	*G. gallus*	ENSGALG00000009172	OSBPL6	oxysterol binding protein like 6	0.14	0.04	1.6E-02	3.4E-02	0.04	1.6E-02		MQKSIPCIFVHE*
HG0440	*G. gallus*	ENSGALG00000010090			0.00	0.28	5.3E-05	1.8E-04	0.28	5.3E-05		MMLGHLLLTTGFSKEIQHGLMC*
HG0441	*G. gallus*	ENSGALG00000015069	B4GALT4	beta-1,4-galactosyltransferase 4	0.00	0.40	2.4E-04	7.5E-04	0.40	2.4E-04		MSHRTTRSAAALPTCSFPSLL*
HG0442	*G. gallus*	ENSGALG00000030250			0.00	0.40	7.3E-03	1.7E-02	0.40	7.3E-03		MTFTSMMNQEKPVKSS*
HG0443	*G. gallus*	ENSGALG00000039454			0.00	0.00	7.9E-04	2.3E-03	0.00	7.9E-04		MGWVSNHVSQDSVLL*
HG0444	*G. gallus*	ENSGALG00000000558	SLC1A6	solute carrier family 1 member 6	0.13	0.39	3.0E-03	7.7E-03	0.39	3.0E-03		MLYLPKEKILHPAFELRESRNKTTQNNPPRRKRPTVLLPDH*
HG0445	*G. gallus*	ENSGALG00000004017			0.03	0.41	1.3E-02	2.8E-02	0.41	1.3E-02		MVALPGVGLGQPPQQGGDESLYKQGRK*
HG0446	*G. gallus*	ENSGALG00000006807	PLSCR5	phospholipid scramblase family member 5	0.00	0.32	5.4E-03	1.3E-02	0.32	5.4E-03		MLPNTSLCSPARSAWALPFCSHASYLSAAHMCVISMATWALEVEI*
HG0447	*G. gallus*	ENSGALG00000008784			0.00	0.16	8.3E-03	1.9E-02	0.16	8.3E-03		MKLNFKECFNQKEN*
HG0448	*G. gallus*	ENSGALG00000011762	UBA6	ubiquitin like modifier activating enzyme 6	0.00	0.09	5.9E-05	2.0E-04	0.09	5.9E-05		MRPGLPAVPAC*
HG0449	*G. gallus*	ENSGALG00000014106	PPFIBP1	PPFIA binding protein 1	0.00	0.30	5.0E-03	1.2E-02	0.30	5.0E-03		MDQWNWLKRNGGDQH*
HG0450	*G. gallus*	ENSGALG00000015528	JAKMIP1	janus kinase and microtubule interacting protein 1	0.00	0.09	8.9E-04	2.5E-03	0.09	8.9E-04		MRDKTHMADGRDFCERFG*
HG0451	*G. gallus*	ENSGALG00000019489	CHST9	carbohydrate sulfotransferase 9	0.00	0.35	1.2E-03	3.3E-03	0.35	1.2E-03		MGLCTDFRLYRSESGQVTEEDAGTVRTSEK*
HG0452	*G. gallus*	ENSGALG00000004227	FAM20B	FAM20B, glycosaminoglycan xylosylkinase	0.00	0.14	6.2E-03	1.5E-02	0.14	6.2E-03		MLPWQSVFYSASYE*
HG0453	*G. gallus*	ENSGALG00000005995	SLC13A5	solute carrier family 13 member 5	0.07	0.08	1.4E-03	3.8E-03	0.08	1.4E-03		MQVLADSPLSARLCAGVKFPLC*
HG0454	*G. gallus*	ENSGALG00000007186	TMEM268	transmembrane protein 268	0.08	0.36	1.8E-02	3.7E-02	0.36	1.8E-02		MLPCQWRAESQGSRQWESE*
HG0455	*G. gallus*	ENSGALG00000010718	THSD7A	thrombospondin type 1 domain containing 7A	0.01	0.17	2.2E-02	4.5E-02	0.17	2.2E-02		MTAAASQRKITCSVSWSPLRTAVSLLADGKLLLTAEWKSVEQQPY*
HG0456	*G. gallus*	ENSGALG00000033256			0.00	0.20	3.1E-03	8.0E-03	0.20	3.1E-03		MHCSKLFLVFSSG*
HG0457	*G. gallus*	ENSGALG00000039538	CLDND1	claudin domain containing 1	0.00	0.28	2.3E-02	4.6E-02	0.28	2.3E-02		MEQLGPAGSWRCVLLAYCRTPVARCL*

HG0458	*H. sapiens*	ENSG00000060339	CCAR1	cell division cycle and apoptosis regulator 1	0.05	0.35	6.3E-39	2.9E-37	0.35	6.3E-39		MWRRGAAWRKRGKLAHAPKADGFEMASMLAGTRLRSML*
HG0459.1	*H. sapiens*	ENSG00000163848	ZNF148	zinc finger protein 148	0.00	0.30	5.3E-10	5.8E-09	0.30	5.3E-10		MKGRNSTMISFSTLQHSFP*
HG0459.2	*H. sapiens*	ENSG00000163848	ZNF148	zinc finger protein 148	0.03	0.47	1.3E-11	1.7E-10	0.47	1.3E-11		MGVCLTYLMEESDGISD*
HG0459.3	*H. sapiens*	ENSG00000163848	ZNF148	zinc finger protein 148	0.00	0.21	1.3E-05	7.3E-05	0.21	1.3E-05		MSASLLLWEEEEKEEEEEEEEGEEEEKGAGVGAVAEAATAKVLPPPPPFPSSQGP*
HG0460	*H. sapiens*	ENSG00000180008	SOCS4	suppressor of cytokine signaling 4	0.01	0.34	7.7E-46	4.0E-44	0.40	1.5E-38	Crowe, Samandi	MAMVMAAVTRTTPVKLRSGRYIQKVPRRNFLLEKMKKQYL*
HG0461	*H. sapiens*	ENSG00000064419	TNPO3	transportin 3	0.02	0.35	8.0E-03	2.2E-02	0.35	8.0E-03		MAPVRERPWPRQ*
HG0462	*H. sapiens*	ENSG00000125686	MED1	mediator complex subunit 1	0.08	0.32	6.4E-18	1.4E-16	0.32	6.4E-18		MAAASSTLLFLPPGTSD*
HG0463	*H. sapiens*	ENSG00000129473	BCL2L2	BCL2 like 2	0.01	0.18	2.8E-30	9.8E-29	0.18	2.8E-30		MKGPSWGLLATSAVS*
HG0464	*H. sapiens*	ENSG00000188215	DCUN1D3	defective in cullin neddylation 1 domain containing 3	0.01	0.48	1.3E-10	1.5E-09	0.48	1.3E-10		MLRYMGQRQRGHPIQRGPL*
HG0465	*H. sapiens*	ENSG00000132153	DHX30	DExH-box helicase 30	0.00	0.35	2.8E-23	7.9E-22	0.35	2.8E-23	Crowe	MRNSKERIQLQFKSLQNLRLSLLL*
HG0466	*H. sapiens*	ENSG00000139679	LPAR6	lysophosphatidic acid receptor 6	0.04	0.49	1.5E-12	2.2E-11	0.49	1.5E-12		MFLLGISEKKEIFIKPSKDPDNLQINWRL*
HG0467.1	*H. sapiens*	ENSG00000163320	CGGBP1	CGG triplet repeat binding protein 1	0.00	0.35	7.1E-15	1.3E-13	0.35	7.1E-15		MTSWHHRENRVQAQKLFPHHHLLKN*
HG0467.2	*H. sapiens*	ENSG00000163320	CGGBP1	CGG triplet repeat binding protein 1	0.01	0.32	3.0E-07	2.2E-06	0.32	3.0E-07		MRLWFRSGDHSLLSIKTKKTGN*
HG0468	*H. sapiens*	ENSG00000187778	MCRS1	microspherule protein 1	0.00	0.09	7.2E-27	2.2E-25	0.09	7.2E-27	Samandi	MLLPRVLETDLDRSPTLGVHSWENEGRRVRISSQPLSSPGQNPWMSLWDRSS*
HG0469	*H. sapiens*	ENSG00000198369	SPRED2	sprouty related EVH1 domain containing 2	0.02	0.01	2.1E-19	5.1E-18	0.01	2.1E-19		MKGAKLPAAGQASR*
HG0470	*H. sapiens*	ENSG00000224470	ATXN1L	ataxin 1 like	0.00	0.39	1.4E-20	3.4E-19	0.39	1.4E-20		MWAPQPEAERGTGKLQRSPF*
HG0471	*H. sapiens*	ENSG00000023041	ZDHHC6	zinc finger DHHC-type containing 6	0.01	0.43	2.4E-19	5.6E-18	0.43	2.4E-19		MTIKEHTWTCGTWKCAHRKRKSIILLK*
HG0472	*H. sapiens*	ENSG00000105991	HOXA1	homeobox A1	0.06	0.23	1.2E-25	3.5E-24	0.23	1.2E-25		MEEVRKLAQSRRASQDQVTQ*
HG0473.1	*H. sapiens*	ENSG00000138650	PCDH10	protocadherin 10	0.00	0.42	1.3E-02	3.2E-02	0.42	1.3E-02		MRKGDACHPSCAKI*
HG0473.2	*H. sapiens*	ENSG00000240184	PCDHGC3	protocadherin gamma subfamily C, 3	0.02	0.18	6.6E-10	7.1E-09	0.18	6.6E-10		MSDSAPSAQALTR*
HG0474	*H. sapiens*	ENSG00000139083	ETV6	ETS variant 6	0.00	0.39	1.3E-08	1.1E-07	0.39	1.3E-08		MTASGWPWSLSGLGRGKESGKNLRTS*
HG0475	*H. sapiens*	ENSG00000109118	PHF12	PHD finger protein 12	0.02	0.42	6.9E-09	6.3E-08	0.42	6.9E-09		MAAPASLPGRTGCRQCRL*
HG0476	*H. sapiens*	ENSG00000130939	UBE4B	ubiquitination factor E4B	0.00	0.15	1.8E-05	9.6E-05	0.15	1.8E-05		MNNTWWGEGERVGVEG*
HG0477	*H. sapiens*	ENSG00000166925	TSC22D4	TSC22 domain family member 4	0.01	0.17	8.4E-52	4.8E-50	0.17	8.4E-52	Mackowiak	MSDPDVPRGSDVPAMWPPCSRGA*
HG0478	*H. sapiens*	ENSG00000168214	RBPJ	recombination signal binding protein for immunoglobulin ka	0.00	0.10	1.3E-05	6.9E-05	0.10	1.3E-05		MISKRKENDTDTLAEVF*
HG0479.1	*H. sapiens*	ENSG00000168453	HR	HR, lysine demethylase and nuclear receptor corepressor	0.00	0.07	1.1E-28	3.6E-27	0.07	1.1E-28	Crowe, Mackowiak	MAQPTASAQKLVRPIRAVCRILQIPESDPSNLRP*
HG0479.2	*H. sapiens*	ENSG00000168453	HR	HR, lysine demethylase and nuclear receptor corepressor	0.00	0.38	5.9E-05	2.9E-04	0.38	5.9E-05		MAIRGPAALSAALYLH*
HG0480	*H. sapiens*	ENSG00000198018	ENTPD7	ectonucleoside triphosphate diphosphohydrolase 7	0.00	0.20	1.2E-18	2.8E-17	0.20	1.2E-18		MPGTRRPPSQGKRKRR*
HG0481	*H. sapiens*	ENSG00000198963	RORB	RAR related orphan receptor B	0.01	0.38	1.0E-06	6.7E-06	0.38	1.0E-06		MVFSAEQLFADHLLHSC*
HG0482	*H. sapiens*	ENSG00000067167	TRAM1	translocation associated membrane protein 1	0.11	0.50	1.4E-17	2.9E-16	0.50	1.4E-17	Crowe	MGSEAPASGCSGAVRSSQREAAASR*
HG0483	*H. sapiens*	ENSG00000164463	CREBRF	CREB3 regulatory factor	0.07	0.22	3.8E-17	7.7E-16	0.22	3.8E-17	Mackowiak	MERGRGPCSGTGPSP*
HG0484	*H. sapiens*	ENSG00000166444	ST5	suppression of tumorigenicity 5	0.00	0.41	9.3E-17	1.9E-15	0.44	2.7E-11		MNQLASISLTNHRKMRDCLGATTSTMSPCTG*
HG0485.1	*H. sapiens*	ENSG00000182667	NTM	neurotrimin	0.00	0.00	4.5E-16	8.6E-15	0.00	4.5E-16		MSGDNYGEVILSHTLGFLVVSFSKTVDLNLLAQA*
HG0485.2	*H. sapiens*	ENSG00000183715	OPCML	opioid binding protein/cell adhesion molecule like	0.03	0.00	2.4E-03	7.6E-03	0.00	2.4E-03		MQEGASGSARLES*
HG0486	*H. sapiens*	ENSG00000067900	ROCK1	Rho associated coiled-coil containing protein kinase 1	0.00	0.16	2.0E-25	5.9E-24	0.16	2.0E-25		MRLWASERLWVAVAPGPGSGERRSPQ*
HG0487	*H. sapiens*	ENSG00000091831	ESR1	estrogen receptor 1	0.07	0.41	1.3E-11	1.6E-10	0.41	1.3E-11		MEHFWKDVLDPAGWPAGF*
HG0488.1	*H. sapiens*	ENSG00000116194	ANGPTL1	angiopoietin like 1	0.00	0.48	1.5E-09	1.5E-08	0.48	1.5E-09		MWQTEQSHNAFSAGLKTAHRPELPLYYLKLHRWLVKFN*
HG0488.2	*H. sapiens*	ENSG00000116194	ANGPTL1	angiopoietin like 1	0.03	0.24	1.1E-04	4.9E-04	0.24	1.1E-04		MQLPLKSQMNKLLH*
HG0489	*H. sapiens*	ENSG00000143847	PPFIA4	PTPRF interacting protein alpha 4	0.00	0.48	6.5E-06	3.7E-05	0.48	6.5E-06		MLWEKPLPTCPLAWHCPL*
HG0490.1	*H. sapiens*	ENSG00000152413	HOMER1	homer scaffolding protein 1	0.00	0.48	6.0E-07	4.2E-06	0.48	6.0E-07		MAPFILFGRMNRSCLAFSSSSPPLLCSES*
HG0490.2	*H. sapiens*	ENSG00000152413	HOMER1	homer scaffolding protein 1	0.00	0.45	6.3E-04	2.4E-03	0.45	6.3E-04		MPPLFERFGIYKREIILPKRTN*
HG0491	*H. sapiens*	ENSG00000154114	TBCEL	tubulin folding cofactor E like	0.03	0.26	3.4E-12	4.8E-11	0.26	3.4E-12	Mackowiak	MQDTGLRRPEWGTR*
HG0492	*H. sapiens*	ENSG00000162670	BRINP3	BMP/retinoic acid inducible neural specific 3	0.00	0.40	1.2E-06	7.6E-06	0.40	1.2E-06		MEYLFESWMYFSHSLLAS*
HG0493	*H. sapiens*	ENSG00000164707	SLC13A4	solute carrier family 13 member 4	0.12	0.34	3.1E-17	6.3E-16	0.34	3.1E-17		MPPFIILSQFLFAGHKTMLINPT*
HG0494	*H. sapiens*	ENSG00000184226	PCDH9	protocadherin 9	0.00	0.44	1.7E-14	2.9E-13	0.44	1.7E-14		MHAAVENIPELKKCNYVLCVLNAQKNPDPVGKRDEMWRE*
HG0495.1	*H. sapiens*	ENSG00000196730	DAPK1	death associated protein kinase 1	0.00	0.13	2.1E-08	1.8E-07	0.13	2.1E-08		MHEGATEAQERW*
HG0495.2	*H. sapiens*	ENSG00000196730	DAPK1	death associated protein kinase 1	0.00	0.26	1.1E-05	6.2E-05	0.26	1.1E-05		MVWEAELKCPGLW*
HG0496	*H. sapiens*	ENSG00000135913	USP37	ubiquitin specific peptidase 37	0.01	0.42	1.1E-02	2.8E-02	0.42	1.1E-02		MSSVPRRVLCLWHKDQNKGELLLHRVFR*
HG0497	*H. sapiens*	ENSG00000147571	CRH	corticotropin releasing hormone	0.09	0.00	3.9E-04	1.5E-03	0.00	3.9E-04		MIYAGAEAARNRAVKRASAY*
HG0498	*H. sapiens*	ENSG00000005379	TSPOAP1	TSPO associated protein 1	0.00	0.00	4.8E-16	9.1E-15	0.00	4.8E-16		MRQAAHRPRPP*
HG0499	*H. sapiens*	ENSG00000011258	MBTD1	mbt domain containing 1	0.02	0.13	2.0E-03	6.6E-03	0.13	2.0E-03		MLMLLISWGHPS*
HG0500	*H. sapiens*	ENSG00000070778	PTPN21	protein tyrosine phosphatase, non-receptor type 21	0.00	0.24	6.4E-22	1.7E-20	0.24	6.4E-22		MLPSSGHWRLRCFLMAAAAAG*
HG0501	*H. sapiens*	ENSG00000110400	NECTIN1	nectin cell adhesion molecule 1	0.11	0.19	5.4E-07	3.8E-06	0.19	5.4E-07		MTRATCGGHLPSL*
HG0502	*H. sapiens*	ENSG00000126882			0.00	0.00	1.7E-22	4.6E-21	0.00	1.7E-22		MSEPHNYSLNYKLFLTQRISVIFVHCEEKKREPRAHEMQQNPYL*
HG0503	*H. sapiens*	ENSG00000141564	RPTOR	regulatory associated protein of MTOR complex 1	0.00	0.19	3.7E-18	8.2E-17	0.19	3.7E-18		MGRSWLWLQQLRRVRDPQG*
HG0504	*H. sapiens*	ENSG00000144460	NYAP2	neuronal tyrosine-phosphorylated phosphoinositide-3-kinas	0.00	0.28	2.6E-14	4.5E-13	0.36	3.7E-09		MQTDCRNREERLLLISEEYNQMRYISQLGDCFWSCNHLCSKVGKIIQFLPCWTWKERGCVFIPTH*
HG0505	*H. sapiens*	ENSG00000157470			0.05	0.31	6.7E-07	4.6E-06	0.31	6.7E-07		MGSRGAPTPRADVNY*
HG0506.1	*H. sapiens*	ENSG00000177853	ZNF518A	zinc finger protein 518A	0.00	0.18	4.0E-08	3.3E-07	0.33	1.1E-04		MAAIRDSQAMGTTTDIISSFDHCS*
HG0506.2	*H. sapiens*	ENSG00000177853	ZNF518A	zinc finger protein 518A	0.00	0.49	2.0E-02	4.7E-02	0.49	2.0E-02		MSLHSIVSCLNYR*
HG0507	*H. sapiens*	ENSG00000060237	WNK1	WNK lysine deficient protein kinase 1	0.01	0.44	1.9E-08	1.7E-07	0.44	1.9E-08		MTAAPLLPPPARPPLAAGWMRTVRR*
HG0508	*H. sapiens*	ENSG00000061936	SFSWAP	splicing factor SWAP homolog	0.16	0.38	5.4E-10	5.9E-09	0.38	5.4E-10		MAAVLRLGTGCGVFD*
HG0509	*H. sapiens*	ENSG00000068024	HDAC4	histone deacetylase 4	0.10	0.30	2.0E-08	1.8E-07	0.30	2.0E-08		MCWWEFGARWSYRFRGNFEPFRIT*
HG0510	*H. sapiens*	ENSG00000134769	DTNA	dystrobrevin alpha	0.04	0.19	7.2E-05	3.4E-04	0.19	7.2E-05		MVLKSSCCPFIEF*
HG0511	*H. sapiens*	ENSG00000143337	TOR1AIP1	torsin 1A interacting protein 1	0.25	0.45	8.1E-15	1.4E-13	0.45	8.1E-15		MAETSSSPFSSSLVPPARRRERSTAADPAQALPLSQPSRHTMGPEAGLLHSSDDAGGGPSDSQLPP*
HG0512.1	*H. sapiens*	ENSG00000112319	EYA4	EYA transcriptional coactivator and phosphatase 4	0.00	0.34	6.1E-09	5.7E-08	0.34	6.1E-09		MSCFHQRHSAKGKLRHWKERE*
HG0512.2	*H. sapiens*	ENSG00000104313	EYA1	EYA transcriptional coactivator and phosphatase 1	0.02	0.43	3.6E-13	5.6E-12	0.43	3.6E-13		MLSAAAVWWGRSAGRKAVTKQNGGS*
HG0512.3	*H. sapiens*	ENSG00000104313	EYA1	EYA transcriptional coactivator and phosphatase 1	0.00	0.00	3.4E-03	1.0E-02	0.00	3.4E-03		MPFTAAVSSFFSFG*
HG0513	*H. sapiens*	ENSG00000126860	EVI2A	ecotropic viral integration site 2A	0.02	0.20	1.5E-11	2.0E-10	0.23	1.6E-06		MCFSCSGKEMCHLWFGFKSGKLAAHILFYCRFTLRLIFSKSILL*
HG0514	*H. sapiens*	ENSG00000147570	DNAJC5B	DnaJ heat shock protein family (Hsp40) member C5 beta	0.00	0.39	6.0E-07	4.2E-06	0.39	6.0E-07		MKKRRDLKRDNQLAGKVTNEK*
HG0515	*H. sapiens*	ENSG00000170471	RALGAPB	Ral GTPase activating protein non-catalytic beta subunit	0.06	0.00	4.1E-03	1.2E-02	0.00	4.1E-03		MRAPPVASGAIWIVL*
HG0516	*H. sapiens*	ENSG00000041515	MYO16	myosin XVI	0.16	0.20	4.1E-09	3.9E-08	0.20	4.1E-09		MRMSLKLFAGSWNRASMQ*
HG0517	*H. sapiens*	ENSG00000086717	PPEF1	protein phosphatase with EF-hand domain 1	0.00	0.42	1.5E-04	6.4E-04	0.42	1.5E-04		MIDCVLSPQVRLKESTTSKVKI*
HG0518	*H. sapiens*	ENSG00000111254	AKAP3	A-kinase anchoring protein 3	0.06	0.44	2.0E-04	8.6E-04	0.44	2.0E-04		MEGHRKKQDLELSKNIPASSLTLKVYSGVFRGSLFQYYRDSLYYMAGGG*
HG0519	*H. sapiens*	ENSG00000127074	RGS13	regulator of G protein signaling 13	0.01	0.23	2.9E-08	2.4E-07	0.23	2.9E-08		MVDCSTGYICQWNVSTYYTTNMRKK*
HG0520	*H. sapiens*	ENSG00000197415	VEPH1	ventricular zone expressed PH domain containing 1	0.00	0.49	1.3E-04	5.9E-04	0.49	1.3E-04		MNNFRFTELSGFHSLCPRSLPESPAQSQTMQLSNCKGAFEE*
HG0521	*H. sapiens*	ENSG00000038274	MAT2B	methionine adenosyltransferase 2B	0.08	0.21	2.7E-04	1.1E-03	0.21	2.7E-04		MWRTARDSGIGIKMLI*
HG0522	*H. sapiens*	ENSG00000140396	NCOA2	nuclear receptor coactivator 2	0.00	0.25	1.4E-05	7.8E-05	0.25	1.4E-05		MAAAASATASAAKVSADGSRHLTA*
HG0523	*H. sapiens*	ENSG00000075213	SEMA3A	semaphorin 3A	0.10	0.04	1.1E-03	3.9E-03	0.04	1.1E-03		MGCSWYSYKVNMLLAAHIYP*
HG0524	*H. sapiens*	ENSG00000107929	LARP4B	La ribonucleoprotein domain family member 4B	0.13	0.13	1.1E-03	4.1E-03	0.13	1.1E-03		MRTGRSVGDLESRG*
HG0525	*H. sapiens*	ENSG00000136167	LCP1	lymphocyte cytosolic protein 1	0.07	0.35	1.7E-02	4.0E-02	0.35	1.7E-02		MTSPEIAVVQRKQSRGIELLQNARVSNIVSAAASYLTKVEEILYFQLRKLKIPPASDDQAPLDTEHCAFLLQVTSCLVTTHPGLTKLFCRSERRGSWSYTSTTKDSFFPARSVT*
HG0526	*H. sapiens*	ENSG00000143569	UBAP2L	ubiquitin associated protein 2 like	0.06	0.40	8.3E-04	3.0E-03	0.40	8.3E-04		MRLEPERGERERARASEAWALPEYSTL*
HG0527	*H. sapiens*	ENSG00000198382	UVRAG	UV radiation resistance associated	0.08	0.08	1.5E-04	6.6E-04	0.08	1.5E-04		MSAPGLSGVEGLHCGNMALP*
HG0528	*H. sapiens*	ENSG00000204767			0.00	0.32	7.8E-03	2.1E-02	0.32	7.8E-03		MGMFPWEHHVSSNKQTSRSFISPPVYLLWKEDAFKFPAVSPWKLMLWETKSCMF*
HG0529	*H. sapiens*	ENSG00000112851	ERBIN	erbb2 interacting protein	0.10	0.50	6.4E-03	1.8E-02	0.50	6.4E-03		MLAQLRNIREINSTQCV*
HG0530	*H. sapiens*	ENSG00000128594	LRRC4	leucine rich repeat containing 4	0.00	0.00	3.0E-11	3.7E-10	0.00	3.0E-11		MREQSAWLPPATPRARREEETKSEWERGAQLPWRKLKDTTPVLSCLPSEMERGAPSSLKPG*
HG0531	*H. sapiens*	ENSG00000146834	MEPCE	methylphosphate capping enzyme	0.09	0.47	4.0E-13	6.2E-12	0.47	4.0E-13		MRAQPGWWRSAHAWFPGEGNARSPCALAAGW*
HG0532	*H. sapiens*	ENSG00000105221	AKT2	AKT serine/threonine kinase 2	0.00	0.32	4.0E-19	9.2E-18	0.32	4.0E-19		MVPGTVLCPVHGASCMSCCPELSRAR*
HG0533	*H. sapiens*	ENSG00000114209	PDCD10	programmed cell death 10	0.00	0.28	1.1E-05	6.0E-05	0.28	1.1E-05		MKIPLHFSTAFSTVVGF*
HG0534	*H. sapiens*	ENSG00000117408	IPO13	importin 13	0.02	0.29	2.5E-36	1.1E-34	0.29	2.5E-36	Mackowiak	MPALGPPSPHHSLGTQAGV*
HG0535	*H. sapiens*	ENSG00000118407	FILIP1	filamin A interacting protein 1	0.00	0.21	1.6E-11	2.0E-10	0.21	1.6E-11		MTSAIGWSWERKWEKLFPLYSFGLDSF*
HG0536	*H. sapiens*	ENSG00000181751	C5orf30	chromosome 5 open reading frame 30	0.04	0.20	2.7E-05	1.4E-04	0.20	2.7E-05		MSVFPWDSLEIVQ*
HG0537	*H. sapiens*	ENSG00000184481	FOXO4	forkhead box O4	0.00	0.40	2.5E-16	4.8E-15	0.40	2.5E-16		MCLLALDASPLRGGIVRDCGRLH*
HG0538	*H. sapiens*	ENSG00000006116	CACNG3	calcium voltage-gated channel auxiliary subunit gamma 3	0.04	0.46	5.6E-03	1.6E-02	0.46	5.6E-03		MRTNPPEPAPFRGP*
HG0539	*H. sapiens*	ENSG00000115966	ATF2	activating transcription factor 2	0.00	0.21	1.3E-20	3.4E-19	0.21	1.3E-20		MAHQLESILAMRKRM*
HG0540	*H. sapiens*	ENSG00000120519	SLC10A7	solute carrier family 10 member 7	0.04	0.32	2.8E-37	1.2E-35	0.32	2.8E-37	Samandi	MSGGTKESVETLQGLGDRSLKRKKAKQINKTHPP*
HG0541.1	*H. sapiens*	ENSG00000122584	NXPH1	neurexophilin 1	0.00	0.28	7.3E-24	2.1E-22	0.28	7.3E-24		METERLGGHVRGAGDARPE*
HG0541.2	*H. sapiens*	ENSG00000122584	NXPH1	neurexophilin 1	0.00	0.41	7.8E-13	1.2E-11	0.41	7.8E-13		MEPAQSGPFSQDSPQVLCGC*
HG0542	*H. sapiens*	ENSG00000126581	BECN1	beclin 1	0.03	0.34	1.1E-29	3.7E-28	0.34	1.1E-29		MVVLEASLRGRPEATVPPR*
HG0543	*H. sapiens*	ENSG00000131931	THAP1	THAP domain containing 1	0.01	0.17	1.1E-04	4.8E-04	0.17	1.1E-04		MGDGRWLWSFC*
HG0544	*H. sapiens*	ENSG00000136802	LRRC8A	leucine rich repeat containing 8 family member A	0.01	0.45	8.6E-17	1.7E-15	0.45	8.6E-17		MEQKECQDPAQADAGQPQHRQPTRR*
HG0545	*H. sapiens*	ENSG00000142784	WDTC1	WD and tetratricopeptide repeats 1	0.01	0.43	3.7E-09	3.6E-08	0.43	3.7E-09	Crowe	MCILWTWAWLECSGVLKILL*
HG0546	*H. sapiens*	ENSG00000148143	ZNF462	zinc finger protein 462	0.00	0.42	6.5E-07	4.5E-06	0.42	6.5E-07		MRLPNNFHNNNPSRKRRKRKRIRRRWGWRTRSTSRRRDASSVPNVRG*
HG0547	*H. sapiens*	ENSG00000149596	JPH2	junctophilin 2	0.05	0.48	6.4E-25	1.9E-23	0.48	6.4E-25	Crowe	MALTSRPGADLAPGLFQPLLASCFPPPGVQW*

HG0548	H. sapiens	ENSG00000155744	FAM126B	family with sequence similarity 126 member B	0.00	0.30	1.5E-12	2.1E-11	0.30	1.5E-12		MNSPLELCRIPVPPPPLEN*
HG0549	H. sapiens	ENSG00000156256	USP16	ubiquitin specific peptidase 16	0.16	0.20	2.0E-04	8.5E-04	0.20	2.0E-04		MQLWALSPWIVILCQ*
HG0550.1	H. sapiens	ENSG00000156650	KAT6B	lysine acetyltransferase 6B	0.00	0.14	8.7E-24	2.5E-22	0.14	8.7E-24		MTFKCASSVKYKENLYG*
HG0550.2	H. sapiens	ENSG00000156650	KAT6B	lysine acetyltransferase 6B	0.00	0.15	1.1E-06	7.2E-06	0.15	1.1E-06		MFFTRMQSFLLVK*
HG0551	H. sapiens	ENSG00000162951	LRRTM1	leucine rich repeat transmembrane neuronal 1	0.03	0.48	1.9E-15	3.4E-14	0.48	1.9E-15		MSEPALCSCPGREWQAVSAE*
HG0552	H. sapiens	ENSG00000169641	LUZP1	leucine zipper protein 1	0.00	0.25	6.3E-16	1.2E-14	0.25	6.3E-16		MMVSSCQRRLWLPRER*
HG0553	H. sapiens	ENSG00000183475	ASB7	ankyrin repeat and SOCS box containing 7	0.00	0.49	6.0E-16	1.1E-14	0.49	6.0E-16		MVTRQSVHQEKGHSSIRLIPVT*
HG0554	H. sapiens	ENSG00000196914	ARHGEF12	Rho guanine nucleotide exchange factor 12	0.00	0.47	2.2E-09	2.2E-08	0.47	2.2E-09	Samandi	MTEWSFELDFCVPDGVGPDPRALGVGRRCYCKMQVG*
HG0555	H. sapiens	ENSG00000204310	AGPAT1	1-acylglycerol-3-phosphate O-acyltransferase 1	0.15	0.18	1.8E-16	3.5E-15	0.18	1.8E-16		MGPTSHSHLPSIGRG*
HG0556	H. sapiens	ENSG00000273841	TAF9	TATA-box binding protein associated factor 9	0.00	0.12	1.5E-59	9.5E-58	0.11	2.2E-51		MLLPNILLTGTPGVGKTTLGKELASKSGLKYINVGDLAREV*
HG0557	H. sapiens	ENSG00000012232	EXTL3	exostosin like glycosyltransferase 3	0.00	0.30	1.6E-03	5.5E-03	0.30	1.6E-03		MHICWSAKPRNKSYGI*
HG0558	H. sapiens	ENSG00000029363	BCLAF1	BCL2 associated transcription factor 1	0.01	0.35	2.9E-12	4.1E-11	0.35	2.9E-12		MVFLAFLSREMAAVWLQRR*
HG0559	H. sapiens	ENSG00000078747	ITCH	itchy E3 ubiquitin protein ligase	0.00	0.13	3.2E-20	7.8E-19	0.13	3.2E-20		MHFTVALWRQRLNPRK*
HG0560.1	H. sapiens	ENSG00000092051	JPH4	junctophilin 4	0.11	0.42	3.1E-04	1.2E-03	0.42	3.1E-04		MDAVPGRQPEPQPTCHSG*
HG0560.2	H. sapiens	ENSG00000092051	JPH4	junctophilin 4	0.00	0.00	5.0E-03	1.4E-02	0.00	5.0E-03		MEQTLICAGMESEF*
HG0561	H. sapiens	ENSG00000100393	EP300	E1A binding protein p300	0.04	0.34	1.9E-04	8.1E-04	0.34	1.9E-04		MRRRRTAPRRKRLMAAAELRETSAGQGPAVAGRGLRL*
HG0562	H. sapiens	ENSG00000105821	DNAJC2	DnaJ heat shock protein family (Hsp40) member C2	0.07	0.45	8.4E-12	1.1E-10	0.45	8.4E-12		MRRFLGVLEPRREARSER*
HG0563	H. sapiens	ENSG00000107249	GLIS3	GLIS family zinc finger 3	0.00	0.17	2.1E-08	1.8E-07	0.17	2.1E-08		MDDSIRLITHTMDNLLDFA*
HG0564	H. sapiens	ENSG00000109171	SLAIN2	SLAIN motif family member 2	0.05	0.37	4.6E-05	2.3E-04	0.37	4.6E-05		MAAGAGYWRRLPQSRSRRR*
HG0565	H. sapiens	ENSG00000120533	ENY2	ENY2, transcription and export complex 2 subunit	0.00	0.00	1.8E-05	9.5E-05	0.00	1.8E-05		MRVLAFCVLRCPSY*
HG0566	H. sapiens	ENSG00000124496	TRERF1	transcriptional regulating factor 1	0.00	0.31	4.9E-17	9.9E-16	0.31	4.9E-17		MHLLQKQPRPWLWRMLKEEDAEAGRP*
HG0567	H. sapiens	ENSG00000132155	RAF1	Raf-1 proto-oncogene, serine/threonine kinase	0.01	0.08	1.1E-35	4.6E-34	0.08	1.1E-35		MRGLLGAPSPGLGTRRM*
HG0568.1	H. sapiens	ENSG00000138271	GPR87	G protein-coupled receptor 87	0.00	0.32	1.7E-22	4.7E-21	0.32	1.7E-22		MKEIKPGITYAEPTPQSSPSVS*
HG0568.2	H. sapiens	ENSG00000138271	GPR87	G protein-coupled receptor 87	0.00	0.38	2.0E-04	8.4E-04	0.38	2.0E-04		MFRFHALPENPLPCRP*
HG0569	H. sapiens	ENSG00000139651	ZNF740	zinc finger protein 740	0.00	0.30	1.8E-18	4.0E-17	0.30	1.8E-18		MATGLMGHEGVATVIW*
HG0570	H. sapiens	ENSG00000152214	RIT2	Ras like without CAAX 2	0.02	0.38	7.9E-05	3.7E-04	0.38	7.9E-05		MRPARAEDQQPARR*
HG0571	H. sapiens	ENSG00000153266	FEZF2	FEZ family zinc finger 2	0.13	0.00	3.0E-05	1.5E-04	0.00	3.0E-05		MARNASPWWWGKET*
HG0572	H. sapiens	ENSG00000158161	EYA3	EYA transcriptional coactivator and phosphatase 3	0.00	0.45	1.1E-22	3.1E-21	0.45	1.1E-22		MRIRVTLTPRFPPLDWFYCGSGLISMSCCGAFTAFGL*
HG0573.1	H. sapiens	ENSG00000162526	TSSK3	testis specific serine kinase 3	0.01	0.39	2.8E-11	3.4E-10	0.39	2.8E-11		MKSLGRKGAGQGRWWSRAASQRQHELRG*
HG0573.2	H. sapiens	ENSG00000206203	TSSK2	testis specific serine kinase 2	0.00	0.27	1.1E-10	1.3E-09	0.27	1.1E-10		MRTMPAGPHDGGM*
HG0574	H. sapiens	ENSG00000164663	USP49	ubiquitin specific peptidase 49	0.00	0.28	1.7E-17	3.5E-16	0.28	1.7E-17		MEEDGKNLFNGLRKWRLL*
HG0575	H. sapiens	ENSG00000164663	USP49	ubiquitin specific peptidase 49	0.00	0.45	1.7E-02	4.0E-02	0.45	1.7E-02		MMAILWMGRDLERTSSLSCNSSQYPT*
HG0576	H. sapiens	ENSG00000175029	CTBP2	C-terminal binding protein 2	0.00	0.37	3.8E-09	3.7E-08	0.37	3.8E-09		MKVYSLKKTDSPSCEL*
HG0577	H. sapiens	ENSG00000250510	GPR162	G protein-coupled receptor 162	0.00	0.49	1.9E-13	3.0E-12	0.49	1.9E-13	Crowe	MLSTGVSLGAACLLTGRGLWGSWECL*
HG0578	H. sapiens	ENSG00000004897	CDC27	cell division cycle 27	0.05	0.22	6.1E-11	7.3E-10	0.22	6.1E-11		MSRGWPGRSRYRGGLRHCRKWA*
HG0579	H. sapiens	ENSG00000010818	HIVEP2	human immunodeficiency virus type I enhancer binding pro	0.08	0.25	1.5E-17	3.2E-16	0.28	9.6E-14		MPIDCGVRVEGSKEKEQSASLQTPWNMEATKL*
HG0580	H. sapiens	ENSG00000073711	PPP2R3A	protein phosphatase 2 regulatory subunit B"alpha	0.00	0.40	1.8E-04	7.9E-04	0.40	1.8E-04		MCSSYCILIVTKPQI*
HG0581	H. sapiens	ENSG00000087074	PPP1R15A	protein phosphatase 1 regulatory subunit 15A	0.14	0.39	3.3E-20	8.2E-19	0.39	3.3E-20	Crowe, Mackowiak	MNALASLTVRTCDRFWQTEPALLPPG*
HG0582	H. sapiens	ENSG00000087338	GMCL1	germ cell-less, spermatogenesis associated 1	0.01	0.39	1.2E-17	2.5E-16	0.39	1.2E-17		METVASAATVGAAREKVAV*
HG0583	H. sapiens	ENSG00000087502	ERGIC2	ERGIC and golgi 2	0.04	0.18	4.1E-12	5.6E-11	0.18	4.1E-12		MAVGWDHNTSMTI*
HG0584	H. sapiens	ENSG00000092051	JPH4	junctophilin 4	0.07	0.25	2.6E-05	1.4E-04	0.25	2.6E-05		MDAVPGRQPEPQPT*
HG0585	H. sapiens	ENSG00000106038	EVX1	even-skipped homeobox 1	0.05	0.16	8.2E-03	2.2E-02	0.16	8.2E-03		MGLPPRGGAAQRSARFTR*
HG0586.1	H. sapiens	ENSG00000108375	RNF43	ring finger protein 43	0.00	0.08	7.3E-05	3.4E-04	0.08	7.3E-05		MGENDIPAADVG*
HG0586.2	H. sapiens	ENSG00000108375	RNF43	ring finger protein 43	0.00	0.00	1.4E-09	1.4E-08	0.00	1.4E-09		MLFGEKKNFC*
HG0587	H. sapiens	ENSG00000119138	KLF9	Kruppel like factor 9	0.22	0.42	4.0E-19	9.2E-18	0.42	4.0E-19		MPERCGPAGAGVQKPSVDSGKSVS*
HG0588	H. sapiens	ENSG00000120162	MOB3B	MOB kinase activator 3B	0.00	0.07	6.6E-29	2.2E-27	0.07	6.6E-29		MRFPWKSFKRKMEGAV*
HG0589.1	H. sapiens	ENSG00000121871	SLITRK3	SLIT and NTRK like family member 3	0.01	0.45	6.5E-10	7.1E-09	0.45	6.5E-10		MMDTIQLRREFAPPAG*
HG0589.2	H. sapiens	ENSG00000121871	SLITRK3	SLIT and NTRK like family member 3	0.04	0.44	2.8E-06	1.7E-05	0.44	2.8E-06		MQRPFPCKSQRWKHLNGLQA*
HG0589.3	H. sapiens	ENSG00000121871	SLITRK3	SLIT and NTRK like family member 3	0.20	0.44	2.3E-12	3.3E-11	0.44	2.3E-12		MDLHSGSSPAFPILTPYTRPGAPV*
HG0590	H. sapiens	ENSG00000122557	HERPUD2	HERPUD family member 2	0.00	0.25	6.0E-23	1.7E-21	0.25	6.0E-23		MGFVSSSALVTDLDTEY*
HG0591.1	H. sapiens	ENSG00000127124	HIVEP3	human immunodeficiency virus type I enhancer binding pro	0.00	0.12	1.8E-37	7.9E-36	0.12	1.8E-37	Mackowiak	MNAGFQREQRFSFGHRKWCLQHRRRA*
HG0591.2	H. sapiens	ENSG00000127124	HIVEP3	human immunodeficiency virus type I enhancer binding pro	0.01	0.35	3.3E-10	3.7E-09	0.35	3.3E-10		MWPKLDTSLGHWDYS*
HG0591.3	H. sapiens	ENSG00000127124	HIVEP3	human immunodeficiency virus type I enhancer binding pro	0.00	0.49	1.4E-05	7.4E-05	0.49	1.4E-05		MEEPRGVLAKQARPSGGKSGA*
HG0591.4	H. sapiens	ENSG00000127124	HIVEP3	human immunodeficiency virus type I enhancer binding pro	0.06	0.19	1.3E-03	4.5E-03	0.19	1.3E-03		MDTDVSNLQPFA*
HG0592	H. sapiens	ENSG00000133401	PDZD2	PDZ domain containing 2	0.00	0.46	1.7E-18	3.7E-17	0.46	1.7E-18		MNTGKADDGQGPQGTWGPVGSGPQEQDL*
HG0593	H. sapiens	ENSG00000135048	TMEM2	transmembrane protein 2	0.01	0.44	1.4E-12	2.0E-11	0.44	1.4E-12		MERRSGPEDARDSSPSPWTAV*
HG0594	H. sapiens	ENSG00000136169	SETDB2	SET domain bifurcated 2	0.00	0.26	6.0E-11	7.3E-10	0.26	6.0E-11		MFHPFVDQKMELVFIFKKIMLMI*
HG0595	H. sapiens	ENSG00000141098	GFOD2	glucose-fructose oxidoreductase domain containing 2	0.00	0.40	6.8E-13	1.0E-11	0.40	6.8E-13		MPACQEPCLTRTFGRSYPCQRLL*
HG0596	H. sapiens	ENSG00000157483	MYO1E	myosin IE	0.11	0.24	1.9E-04	8.3E-04	0.24	1.9E-04		MESPLGSHWLL*
HG0597	H. sapiens	ENSG00000163032	VSNL1	visinin like 1	0.07	0.26	5.9E-24	1.7E-22	0.26	5.9E-24		MGFPICSRDLPERVAAAAGLARRDPRGI*
HG0598	H. sapiens	ENSG00000163788	SNRK	SNF related kinase	0.03	0.35	7.8E-10	8.4E-09	0.35	7.8E-10		MTTLKMNFLYSPDILI*
HG0599	H. sapiens	ENSG00000164930	FZD6	frizzled class receptor 6	0.00	0.36	4.8E-18	1.0E-16	0.36	2.2E-17		MRNLNILSLDGDLLRMQRVIHPSHVVKSGI*
HG0600	H. sapiens	ENSG00000173065	FAM222B	family with sequence similarity 222 member B	0.06	0.29	5.9E-05	2.8E-04	0.29	5.9E-05		MRAGPSHRPVSHCPMLC*
HG0601	H. sapiens	ENSG00000175893	ZDHHC21	zinc finger DHHC-type containing 21	0.00	0.42	6.9E-05	3.3E-04	0.42	6.9E-05		MNQKKISKCRFQKNYSTSQ*
HG0602	H. sapiens	ENSG00000179195	ZNF664	zinc finger protein 664	0.00	0.32	1.7E-20	4.3E-19	0.32	1.7E-20		MQEETFRRKTGREKLRLTLNLPNRASLR*
HG0603	H. sapiens	ENSG00000197329	PELI1	pellino E3 ubiquitin protein ligase 1	0.05	0.02	5.1E-21	1.3E-19	0.02	5.1E-21		MSVTQRRCEAEGPSGGAAAASTSSSGSSPSPAVRSNWSPALRERTTKQPQRLSLRPRLLSARLRGQLPLAGLGWRARRAAAERRAVCSRSPRTTLHPLASGLGGRSGIPATSNCPEIMQKVSQGS*
HG0604.1	H. sapiens	ENSG00000003147	ICA1	islet cell autoantigen 1	0.03	0.33	5.9E-04	2.2E-03	0.33	5.9E-04		MLFSCPFSPNHQQ*
HG0604.2	H. sapiens	ENSG00000003147	ICA1	islet cell autoantigen 1	0.13	0.48	1.8E-03	5.9E-03	0.42	7.4E-03		MSDPEGRSDRGREAPGGRGSEVII*
HG0604.3	H. sapiens	ENSG00000003147	ICA1	islet cell autoantigen 1	0.22	0.35	6.2E-05	3.0E-04	0.35	6.2E-05		MATWNTCRRAGAGSLWAADPSGGYPRRWGSRRDPGE*
HG0605	H. sapiens	ENSG00000053254	FOXN3	forkhead box N3	0.00	0.09	1.4E-20	3.4E-19	0.09	1.4E-20		MKKNQPLEDQHSRDYGGPSVFSVTGAMLTSVSWTHSDSGAFT*
HG0606	H. sapiens	ENSG00000055130	CUL1	cullin 1	0.00	0.48	4.4E-05	2.2E-04	0.48	4.4E-05		MRFHSLHLKDILSELL*
HG0607	H. sapiens	ENSG00000074657	ZNF532	zinc finger protein 532	0.00	0.44	4.5E-11	5.5E-10	0.49	8.0E-09		MRFQRPPCPLSHWPCLRPPPPPQTAPVFFPRGEGIWTSPPRCLKEEKGMFCLVLFLLEATKGCWGCF*
HG0608	H. sapiens	ENSG00000084731	KIF3C	kinesin family member 3C	0.01	0.30	8.5E-04	3.1E-03	0.30	8.5E-04		MNDPPAWGEDSR*
HG0609	H. sapiens	ENSG00000095539	SEMA4G	semaphorin 4G	0.00	0.16	7.5E-31	2.7E-29	0.16	7.5E-31	Crowe	MATQPCDSMSPRFQDPPWPHDSLTPMTL*
HG0610	H. sapiens	ENSG00000106069	CHN2	chimerin 2	0.22	0.38	1.2E-12	1.8E-11	0.38	1.2E-12		MGWGPRRLRAAGRGQRRRRPHRG*
HG0611	H. sapiens	ENSG00000108309	RUNDC3A	RUN domain containing 3A	0.01	0.29	2.7E-18	6.0E-17	0.29	2.7E-18		MAMEGLRGPVGQRAQP*
HG0612.1	H. sapiens	ENSG00000110422	HIPK3	homeodomain interacting protein kinase 3	0.00	0.50	5.0E-14	8.4E-13	0.50	5.0E-14		MAASRALWPRPQRRGAAGLQGVNSGDVRACV*
HG0612.2	H. sapiens	ENSG00000110422	HIPK3	homeodomain interacting protein kinase 3	0.00	0.14	6.8E-07	4.6E-06	0.14	6.8E-07		MLRAAAARRGESAGL*
HG0612.3	H. sapiens	ENSG00000110422	HIPK3	homeodomain interacting protein kinase 3	0.06	0.20	9.2E-03	2.4E-02	0.20	9.2E-03		MKMSVSLSGGRRLL*
HG0613	H. sapiens	ENSG00000114861	FOXP1	forkhead box P1	0.00	0.17	2.7E-08	2.3E-07	0.17	2.7E-08		MVRASIPSLICCQP*
HG0614	H. sapiens	ENSG00000122203	KIAA1191	KIAA1191	0.03	0.30	7.8E-29	2.6E-27	0.36	2.0E-21	Crowe	MGRGRVSRSWTTTPSMPCAPLGQVSREIPRSPEALSAVLPARWVVLLPSGSWGPSKSPPLSQWLGSRLRHSLSGDVGPRNLFSAGLRAAGSPAAAGSQNLWMDSSWEALLLCSSWTMFLINICLSA*
HG0615	H. sapiens	ENSG00000128191	DGCR8	DGCR8, microprocessor complex subunit	0.00	0.07	9.9E-11	1.2E-09	0.07	9.9E-11		MKTDSLSRQSLKLSAL*
HG0616	H. sapiens	ENSG00000128739	SNRPN	small nuclear ribonucleoprotein polypeptide N	0.00	0.06	4.5E-36	1.9E-34	0.06	4.5E-36		MERARDRLHLRRTTEQHVPEVEVQVKRRRTASLSNQECQLYPRRSQQQQVPVVDFQAELRQAFLAETPRGG*
HG0617	H. sapiens	ENSG00000135164	DMTF1	cyclin D binding myb like transcription factor 1	0.00	0.01	3.5E-34	1.4E-32	0.01	3.5E-34		MRATWVKKLPVNSHHFCPSLGNTFRECCGDRNMGEKQSG*
HG0618	H. sapiens	ENSG00000137817	PARP6	poly(ADP-ribose) polymerase family member 6	0.01	0.26	4.5E-12	6.2E-11	0.26	4.5E-12		MPISLALVRLVVWRGAA*
HG0619	H. sapiens	ENSG00000138593	SECISBP2L	SECIS binding protein 2 like	0.04	0.38	9.7E-12	1.3E-10	0.38	9.7E-12		MEKAARGAVYTD*
HG0620	H. sapiens	ENSG00000145555	MYO10	myosin X	0.05	0.47	8.6E-04	3.1E-03	0.47	8.6E-04		MCHQLFSACERCAQ*
HG0621.1	H. sapiens	ENSG00000146352	CLVS2	clavesin 2	0.03	0.40	1.8E-03	6.0E-03	0.40	1.8E-03		MPLSPPPPLLKPRLMDARECRIA*
HG0621.2	H. sapiens	ENSG00000146352	CLVS2	clavesin 2	0.02	0.37	4.3E-03	1.3E-02	0.37	4.3E-03		MAGPGENLGQRWP*
HG0621.3	H. sapiens	ENSG00000146352	CLVS2	clavesin 2	0.03	0.09	4.6E-06	2.7E-05	0.09	4.6E-06		MPPKLPSYPNEAGKGAERARELSECLRRSEICAGIIREELG*
HG0622	H. sapiens	ENSG00000148677	ANKRD1	ankyrin repeat domain 1	0.13	0.43	1.4E-13	2.3E-12	0.43	1.4E-13		MKIHLPLSWLLMGVGVLLRFPGWKIISPGPSYIS*
HG0623	H. sapiens	ENSG00000151422	FER	FER tyrosine kinase	0.00	0.45	5.5E-17	1.1E-15	0.45	5.5E-17		MTIFARLHCTFSQVSSLSLMCDTSSWTRYFS*
HG0624	H. sapiens	ENSG00000156853	ZNF689	zinc finger protein 689	0.07	0.39	9.0E-10	9.6E-09	0.39	9.0E-10	Crowe, Samandi	MALRSIKSIAGSCLCSRQRRCGSSAAIFPEGIFRCLSPKFGQEFPE*
HG0625	H. sapiens	ENSG00000164050	PLXNB1	plexin B1	0.01	0.19	1.7E-04	7.2E-04	0.19	1.7E-04		MLLSPDPAGPAR*
HG0626	H. sapiens	ENSG00000168939	SPRY3	sprouty RTK signaling antagonist 3	0.01	0.36	1.5E-05	7.9E-05	0.36	1.5E-05		MDKPFPQGFSHVP*
HG0627	H. sapiens	ENSG00000169760	NLGN1	neuroligin 1	0.00	0.39	4.2E-08	3.4E-07	0.39	4.2E-08		MRGLPLWIYLAEGYRPAANWI*
HG0628	H. sapiens	ENSG00000170677	SOCS6	suppressor of cytokine signaling 6	0.00	0.34	1.5E-14	2.6E-13	0.34	1.5E-14		MKQEKYIDAALQPLQMFGDNIPDRNIDPLD*
HG0629	H. sapiens	ENSG00000170961	HAS2	hyaluronan synthase 2	0.00	0.00	2.7E-06	1.7E-05	0.00	2.7E-06		MLFLNFLFFLAGRLKFI*
HG0630	H. sapiens	ENSG00000171552	BCL2L1	BCL2 like 1	0.00	0.25	3.2E-13	5.0E-12	0.25	3.2E-13		MIPPAGAGFFASHREAG*
HG0631	H. sapiens	ENSG00000173548	SNX33	sorting nexin 33	0.00	0.49	7.7E-13	1.2E-11	0.49	7.7E-13		MSPPCSQAYARRPDRAVSGPWTLL*
HG0632.1	H. sapiens	ENSG00000181315	ZNF322	zinc finger protein 322	0.01	0.27	1.1E-09	1.1E-08	0.27	1.1E-09		MTVRATMGQEDQRKTP*
HG0632.2	H. sapiens	ENSG00000181315	ZNF322	zinc finger protein 322	0.00	0.40	9.9E-03	2.6E-02	0.40	9.9E-03		MYVFFHLDSRVGNTK*
HG0633	H. sapiens	ENSG00000186174	BCL9L	B-cell CLL/lymphoma 9 like	0.02	0.49	2.0E-06	1.2E-05	0.49	2.0E-06		MLRGWEAGTQAPSDPRIASPGPGWY*

HG0634	*H. sapiens*	ENSG00000198026	ZNF335	zinc finger protein 335	0.04	0.23	1.0E-20	2.6E-19	0.23	1.0E-20		MPESERNVATKASE*
HG0635	*H. sapiens*	ENSG00000259332	ST20-MTHFS	ST20-MTHFS readthrough	0.05	0.48	4.1E-10	4.6E-09	0.32	1.9E-18		MAAETGYWRCLPESRTCAEEAVHLWPAVLRDPRWGNPGDGAWPCYMRGPRLE*
HG0636	*H. sapiens*	ENSG00000002587	HS3ST1	heparan sulfate-glucosamine 3-sulfotransferase 1	0.08	0.32	1.8E-19	4.4E-18	0.32	1.8E-19		MAQPRSLFRAPDGLDRGAAQ*
HG0637	*H. sapiens*	ENSG00000005102	MEOX1	mesenchyme homeobox 1	0.03	0.42	1.7E-02	4.1E-02	0.42	1.7E-02		MFSVIGDKYTYRGSQ*
HG0638	*H. sapiens*	ENSG00000013561	RNF14	ring finger protein 14	0.00	0.45	3.3E-12	4.7E-11	0.45	3.3E-12		MSSTSWPLTQLQQSQLHQLENKWDLHELHSELKYRLRC*
HG0639	*H. sapiens*	ENSG00000018236	CNTN1	contactin 1	0.00	0.00	5.2E-11	6.3E-10	0.00	5.2E-11		MGAAVPACEVTA*
HG0640	*H. sapiens*	ENSG00000062194	GPBP1	GC-rich promoter binding protein 1	0.00	0.00	5.5E-06	3.2E-05	0.00	5.5E-06		MLFRCEIFFLFLLFGS*
HG0641	*H. sapiens*	ENSG00000100359	SGSM3	small G protein signaling modulator 3	0.00	0.41	1.9E-09	1.9E-08	0.40	2.2E-07		MILDQMKPEEPSSSKIAG*
HG0642	*H. sapiens*	ENSG00000100815	TRIP11	thyroid hormone receptor interactor 11	0.00	0.44	1.1E-09	1.2E-08	0.44	1.1E-09		MAAGVELAGVTHGTEESH*
HG0643	*H. sapiens*	ENSG00000103653	CSK	CSK, non-receptor tyrosine kinase	0.00	0.48	5.0E-08	4.1E-07	0.48	5.0E-08		MLFPTALMVPSDRLALL*
HG0644	*H. sapiens*	ENSG00000104299	INTS9	integrator complex subunit 9	0.00	0.48	3.3E-10	3.7E-09	0.48	3.3E-10		MLSAGRWRRLHRKTLPGFEEFSDCY*
HG0645	*H. sapiens*	ENSG00000107282	APBA1	amyloid beta precursor protein binding family A member 1	0.03	0.42	4.7E-09	4.4E-08	0.42	4.7E-09		MVALEAAAAGAELYSQAWHQRCH*
HG0646	*H. sapiens*	ENSG00000108312	UBTF	upstream binding transcription factor, RNA polymerase I	0.28	0.00	2.4E-07	1.8E-06	0.00	2.4E-07		MEQWEREAGRFPCKSPSGLTPSRRPRQGSEDPGEQMEEWSSL*
HG0647	*H. sapiens*	ENSG00000108395	TRIM37	tripartite motif containing 37	0.00	0.34	1.6E-08	1.4E-07	0.34	1.6E-08		MGVGGAGPEEMGRAGGAVVRAP*
HG0648	*H. sapiens*	ENSG00000114331	ACAP2	ArfGAP with coiled-coil, ankyrin repeat and PH domains 2	0.02	0.35	2.9E-11	3.6E-10	0.35	2.9E-11	Mackowiak	MTSGRGALRSSAQR*
HG0649.1	*H. sapiens*	ENSG00000114439	BBX	BBX, HMG-box containing	0.00	0.16	5.9E-03	1.7E-02	0.16	5.9E-03		MWRIHWDIEKYQRRIVPL*
HG0649.2	*H. sapiens*	ENSG00000114439	BBX	BBX, HMG-box containing	0.00	0.12	4.1E-04	1.6E-03	0.12	4.1E-04		MESLDLTGTLRKTPSL*
HG0650	*H. sapiens*	ENSG00000115226	FNDC4	fibronectin type III domain containing 4	0.29	0.00	1.2E-10	1.4E-09	0.00	1.2E-10	Mackowiak	MGRPLGTEQPPEA*
HG0651	*H. sapiens*	ENSG00000115935	WIPF1	WAS/WASL interacting protein family member 1	0.01	0.45	2.0E-16	3.9E-15	0.45	2.0E-16		MIPFSGTVFSLGSCPAGPLSACVPDHGSLQYPLTIYQQDC*
HG0652	*H. sapiens*	ENSG00000118515	SGK1	serum/glucocorticoid regulated kinase 1	0.18	0.37	9.6E-14	1.6E-12	0.37	9.6E-14		MMEGEKPLSVATESGERLLQ*
HG0653	*H. sapiens*	ENSG00000120334	CENPL	centromere protein L	0.01	0.32	2.2E-04	9.4E-04	0.32	2.2E-04		MIRESGMGDSVITAFY*
HG0654	*H. sapiens*	ENSG00000133794	ARNTL	aryl hydrocarbon receptor nuclear translocator like	0.12	0.41	3.0E-03	9.5E-03	0.41	3.0E-03		MWNPGPSLVFGRS*
HG0655	*H. sapiens*	ENSG00000143061	IGSF3	immunoglobulin superfamily member 3	0.00	0.22	8.6E-23	2.4E-21	0.22	8.6E-23		MWQEPGATSELLWTLPVA*
HG0656	*H. sapiens*	ENSG00000150776	C11orf57	chromosome 11 open reading frame 57	0.00	0.36	2.8E-13	4.3E-12	0.36	2.8E-13		MGIFPLSSTWKPQPPLQAP*
HG0657	*H. sapiens*	ENSG00000151276	MAGI1	membrane associated guanylate kinase, WW and PDZ don	0.00	0.28	1.7E-04	7.3E-04	0.28	1.7E-04	Crowe	MQTKLAGACAGLAGKPRKFVL*
HG0658	*H. sapiens*	ENSG00000153786	ZDHHC7	zinc finger DHHC-type containing 7	0.00	0.39	1.3E-02	3.3E-02	0.39	1.3E-02		MHLKILKKRHKMFK*
HG0659.1	*H. sapiens*	ENSG00000158856	DMTN	dematin actin binding protein	0.00	0.24	1.2E-19	2.9E-18	0.24	1.2E-19		MEVYVTEEAAALLPHALGP*
HG0659.2	*H. sapiens*	ENSG00000158856	DMTN	dematin actin binding protein	0.01	0.46	5.3E-09	5.0E-08	0.46	5.3E-09		MRTRQPGGTRQLLSRPQARSAQQPRRA*
HG0660	*H. sapiens*	ENSG00000165861	ZFYVE1	zinc finger FYVE-type containing 1	0.00	0.30	1.4E-03	4.8E-03	0.30	1.4E-03		MEDETIKPNQRCC*
HG0661	*H. sapiens*	ENSG00000166845	C18orf54	chromosome 18 open reading frame 54	0.00	0.33	1.6E-17	3.4E-16	0.29	8.9E-16		MDFLLEIRTEACTEEMLFLREMNRNNPL*
HG0662	*H. sapiens*	ENSG00000166897	ELFN2	extracellular leucine rich repeat and fibronectin type III dom	0.00	0.40	5.3E-08	4.3E-07	0.40	5.3E-08		MVAAPGRTVLPAPASGR*
HG0663	*H. sapiens*	ENSG00000168615	ADAM9	ADAM metallopeptidase domain 9	0.09	0.42	8.9E-11	1.1E-09	0.42	8.9E-11		MSGASSDRGLSCVSAARVLVGRACSSGARASRPDLGPRQGWKMMEEAEVEATEC*
HG0664	*H. sapiens*	ENSG00000169085			0.01	0.25	1.9E-07	1.4E-06	0.25	1.9E-07		MIPEPDWIRMPRD*
HG0665	*H. sapiens*	ENSG00000169515	CCDC8	coiled-coil domain containing 8	0.07	0.19	1.1E-05	6.0E-05	0.19	1.1E-05		MSAKMWIWTSSWKP*
HG0666	*H. sapiens*	ENSG00000171943	SRGAP2C	SLIT-ROBO Rho GTPase activating protein 2C	0.00	0.00	1.8E-05	9.7E-05	0.00	1.8E-05		MSDPRPASGRCSSPG*
HG0667.1	*H. sapiens*	ENSG00000182700	IGIP	IgA inducing protein	0.00	0.00	1.3E-11	1.7E-10	0.00	1.3E-11		MCQYTVRISLICTVWGPHLRMLNFAN*
HG0667.2	*H. sapiens*	ENSG00000182700	IGIP	IgA inducing protein	0.17	0.45	5.1E-04	2.0E-03	0.45	5.1E-04		MLRKSVGFSAVREGRSSALIWVTEANMSKTLVN*
HG0668	*H. sapiens*	ENSG00000182944	EWSR1	EWS RNA binding protein 1	0.03	0.21	3.1E-20	7.7E-19	0.21	3.1E-20		MVAPGVARFAPAQCGA*
HG0669	*H. sapiens*	ENSG00000183876	ARSI	arylsulfatase family member I	0.16	0.41	8.2E-06	4.6E-05	0.41	8.2E-06		MVESTFWKLPRRVSGWPG*
HG0670	*H. sapiens*	ENSG00000187164	SHTN1	shootin 1	0.04	0.27	9.0E-07	6.1E-06	0.27	9.0E-07		MISLARSAPGGGGAGADPTSG*
HG0671	*H. sapiens*	ENSG00000189184	PCDH18	protocadherin 18	0.01	0.36	2.5E-10	2.8E-09	0.36	2.5E-10		MPKVSGSRDTVCIKQKFASC*
HG0672	*H. sapiens*	ENSG00000196369	SRGAP2B	SLIT-ROBO Rho GTPase activating protein 2B	0.00	0.00	1.8E-05	9.7E-05	0.00	1.8E-05		MSDPRPASGRCSSPG*
HG0673	*H. sapiens*	ENSG00000266028	SRGAP2	SLIT-ROBO Rho GTPase activating protein 2	0.00	0.00	1.8E-05	9.7E-05	0.00	1.8E-05		MSDPRPASGRCSSPG*
HG0674.1	*H. sapiens*	ENSG00000006576	PHTF2	putative homeodomain transcription factor 2	0.01	0.19	3.3E-04	1.3E-03	0.19	3.3E-04		MFFSLAQPKMTNV*
HG0674.2	*H. sapiens*	ENSG00000116793	PHTF1	putative homeodomain transcription factor 1	0.00	0.43	1.5E-03	5.2E-03	0.43	1.5E-03		MRPAWPAASPPASRWDPGH*
HG0675	*H. sapiens*	ENSG00000048991	R3HDM1	R3H domain containing 1	0.01	0.46	6.2E-03	1.8E-02	0.46	6.2E-03		MQSISLQVLWKEHLLNSV*
HG0676	*H. sapiens*	ENSG00000058673	ZC3H11A	zinc finger CCCH-type containing 11A	0.00	0.23	2.0E-16	3.9E-15	0.23	2.0E-16	Mackowiak	MAIMLLCLLQLAAPLCSYSITIRFYLFWLNTP*
HG0677	*H. sapiens*	ENSG00000064989	CALCRL	calcitonin receptor like receptor	0.06	0.34	3.3E-09	3.2E-08	0.34	3.3E-09		MIHQPLVLPSVKTLDGPTFRS*
HG0678.1	*H. sapiens*	ENSG00000090776	EFNB1	ephrin B1	0.07	0.40	7.1E-14	1.2E-12	0.40	7.1E-14		MGLARSAASPRSSKYTEQGPR*
HG0678.2	*H. sapiens*	ENSG00000108947	EFNB3	ephrin B3	0.08	0.06	1.2E-06	7.7E-06	0.06	1.2E-06		MPPPPGPRLPQSPT*
HG0679	*H. sapiens*	ENSG00000100281	HMGXB4	HMG-box containing 4	0.20	0.38	6.5E-06	3.7E-05	0.38	6.5E-06		MAAIGGVEAGSGRAE*
HG0680	*H. sapiens*	ENSG00000100678	SLC8A3	solute carrier family 8 member A3	0.01	0.00	1.2E-02	3.1E-02	0.00	1.2E-02		MPRTRLRPAGSPRGR*
HG0681	*H. sapiens*	ENSG00000101349	PAK5	p21 (RAC1) activated kinase 5	0.00	0.21	7.2E-03	2.0E-02	0.21	7.2E-03		MLPPLAKMNTEEK*
HG0682	*H. sapiens*	ENSG00000105829	BET1	Bet1 golgi vesicular membrane trafficking protein	0.06	0.40	9.6E-04	3.5E-03	0.40	9.6E-04		MSWRFRGRSWCFAGPWY*
HG0683	*H. sapiens*	ENSG00000111837	MAK	male germ cell associated kinase	0.02	0.39	1.2E-11	1.5E-10	0.38	2.5E-10		MKHTRRRSFTRERTRVFIRMEVLFPCPIPSCYIS*
HG0684	*H. sapiens*	ENSG00000113916	BCL6	B-cell CLL/lymphoma 6	0.00	0.21	2.8E-10	3.2E-09	0.21	2.8E-10		MRGLCHSPHCLLH*
HG0685	*H. sapiens*	ENSG00000117262	GPR89A	G protein-coupled receptor 89A	0.08	0.38	1.1E-11	1.4E-10	0.38	1.1E-11		MASGWRAAVPAAAPGRRQTV*
HG0686	*H. sapiens*	ENSG00000118985	ELL2	elongation factor for RNA polymerase II 2	0.08	0.22	1.8E-07	1.4E-06	0.22	1.8E-07		MTVRQQRDGPSSGGGCRSPSSRGRSGG*
HG0687.1	*H. sapiens*	ENSG00000120549	KIAA1217	KIAA1217	0.05	0.45	4.1E-05	2.1E-04	0.45	4.1E-05		MQFSRARETLHRSGK*
HG0687.2	*H. sapiens*	ENSG00000120549	KIAA1217	KIAA1217	0.00	0.05	5.2E-03	1.5E-02	0.05	5.2E-03		MVFIGCEHSS*
HG0688	*H. sapiens*	ENSG00000122420	PTGFR	prostaglandin F receptor	0.00	0.33	5.8E-09	5.5E-08	0.33	5.8E-09		MSGLQSCTVLRGR*
HG0689	*H. sapiens*	ENSG00000123352	SPATS2	spermatogenesis associated serine rich 2	0.00	0.25	2.3E-08	2.0E-07	0.25	2.3E-08		MNVTTLVTSYNLPGKGDMNVCNDTS*
HG0690	*H. sapiens*	ENSG00000125931	CITED1	Cbp/p300 interacting transactivator with Glu/Asp rich carbo	0.25	0.18	6.0E-08	4.8E-07	0.18	6.0E-08		MCPAAKLQKGHR*
HG0691	*H. sapiens*	ENSG00000129535	NRL	neural retina leucine zipper	0.22	0.48	5.6E-07	3.9E-06	0.48	5.6E-07		MPFGLCAHSPLELSRGTRPCSMEPSVSWEAVPVWLWH*
HG0692.1	*H. sapiens*	ENSG00000130338	TULP4	tubby like protein 4	0.00	0.41	1.5E-14	2.7E-13	0.41	1.5E-14		MALRGAPAGGGAVRWSPAFPGDTGPSFEVESFWF*
HG0692.2	*H. sapiens*	ENSG00000130338	TULP4	tubby like protein 4	0.00	0.37	6.5E-03	1.8E-02	0.37	6.5E-03		MDLILRLAFFIKMEKDL*
HG0693	*H. sapiens*	ENSG00000131653	TRAF7	TNF receptor associated factor 7	0.05	0.37	4.1E-16	7.8E-15	0.37	4.1E-16		MKRAGGAAAPGRPRAGGASQGP*
HG0694	*H. sapiens*	ENSG00000133265	HSPBP1	HSPA (Hsp70) binding protein 1	0.06	0.23	1.2E-09	1.2E-08	0.23	1.2E-09		MAAPSRVRCGRLCL*
HG0695	*H. sapiens*	ENSG00000133704	IPO8	importin 8	0.05	0.45	1.5E-07	1.1E-06	0.45	1.5E-07		MESELWGGKREDRGRGVRVAQQVAAGGDGGGGWGVR*
HG0696	*H. sapiens*	ENSG00000138380	CARF	calcium responsive transcription factor	0.11	0.32	1.6E-05	8.8E-05	0.32	1.6E-05		MLEAWNLNLSHYLKLLNNRRKWN*
HG0697	*H. sapiens*	ENSG00000143344	RGL1	ral guanine nucleotide dissociation stimulator like 1	0.00	0.32	1.4E-07	1.0E-06	0.32	1.4E-07		MDWLPDRVPCSW*
HG0698	*H. sapiens*	ENSG00000157554	ERG	ERG, ETS transcription factor	0.00	0.49	1.6E-07	1.2E-06	0.49	1.6E-07		MTHREKRWQNQGQLKPSGSEQLVDGLAY*
HG0699	*H. sapiens*	ENSG00000160785	SLC25A44	solute carrier family 25 member 44	0.09	0.43	7.7E-12	1.0E-10	0.43	7.7E-12		MEEDATGRRHSLGTTDRLGAAA*
HG0700	*H. sapiens*	ENSG00000165156	ZHX1	zinc fingers and homeoboxes 1	0.00	0.46	8.3E-06	4.7E-05	0.46	8.3E-06		MQDGGGVALRGAAAPAGSGASRLRDQRARRLWAAGGNWSAESAAGTGKEKTDCCFGPVGNDGLS*
HG0701	*H. sapiens*	ENSG00000166292	TMEM100	transmembrane protein 100	0.05	0.46	7.2E-05	3.4E-04	0.46	7.2E-05		MDCLSPSFHPGETVQI*
HG0702	*H. sapiens*	ENSG00000167377	ZNF23	zinc finger protein 23	0.02	0.00	6.8E-05	3.2E-04	0.00	6.8E-05		MELALGQQEPIVSEVGDL*
HG0703	*H. sapiens*	ENSG00000171862	PTEN	phosphatase and tensin homolog	0.19	0.42	4.1E-10	4.6E-09	0.42	4.1E-10		MRCGRIRARRWDATALSSLLSEAAAMMEV*
HG0704.1	*H. sapiens*	ENSG00000173598	NUDT4	nudix hydrolase 4	0.05	0.46	2.5E-07	1.9E-06	0.46	2.5E-07		MESGGQLGRGGAFISAPWFQ*
HG0704.2	*H. sapiens*	ENSG00000272325	NUDT3	nudix hydrolase 3	0.00	0.00	7.5E-05	3.5E-04	0.00	7.5E-05		MRRPRRASGGAV*
HG0705.1	*H. sapiens*	ENSG00000175224	ATG13	autophagy related 13	0.00	0.29	7.7E-09	7.1E-08	0.29	7.7E-09		MAAPGSDRLLGLRRE*
HG0705.2	*H. sapiens*	ENSG00000175224	ATG13	autophagy related 13	0.00	0.29	1.3E-12	1.9E-11	0.29	1.3E-12	Samandi	MRARNHSLCRSFAVERPVETCDPFSLNKSLVLLCCPGWSVVV*
HG0706	*H. sapiens*	ENSG00000178498	DTX3	deltex E3 ubiquitin ligase 3	0.05	0.00	3.8E-06	2.3E-05	0.00	3.8E-06		MGFPGHTTYK*
HG0707	*H. sapiens*	ENSG00000179796	LRRC3B	leucine rich repeat containing 3B	0.00	0.01	2.8E-12	3.9E-11	0.01	2.8E-12		MGAQARKEIMKRHMC*
HG0708	*H. sapiens*	ENSG00000184613	NELL2	neural EGFL like 2	0.15	0.41	1.1E-06	7.4E-06	0.41	1.1E-06		MMCALEGLSSSRAWGGLPFLS*
HG0709	*H. sapiens*	ENSG00000186439	TRDN	triadin	0.00	0.39	8.4E-03	2.3E-02	0.39	8.4E-03		MRLFLFEADTSS*
HG0710	*H. sapiens*	ENSG00000196505	GDAP2	ganglioside induced differentiation associated protein 2	0.00	0.36	1.2E-04	5.2E-04	0.36	1.2E-04		MGPPECSKVSSEY*
HG0711	*H. sapiens*	ENSG00000198300	PEG3	paternally expressed 3	0.01	0.35	4.2E-11	5.1E-10	0.35	4.2E-11	Mackowiak	MSTLGWWRRRAPGAMRVR*
HG0712	*H. sapiens*	ENSG00000213949	ITGA1	integrin subunit alpha 1	0.04	0.32	2.3E-12	3.2E-11	0.32	2.3E-12		MKGRCPFKVCFYSPWTLA*
HG0713	*H. sapiens*	ENSG00000008513	ST3GAL1	ST3 beta-galactoside alpha-2,3-sialyltransferase 1	0.00	0.46	2.3E-04	9.5E-04	0.46	2.3E-04		MPDSFDTLQRQRALDRREEEEEEERREKEEAM*
HG0714	*H. sapiens*	ENSG00000051825	MPHOSPH9	M-phase phosphoprotein 9	0.00	0.33	6.9E-08	5.5E-07	0.33	6.9E-08		MTRIHFSQTAQEPLFSNKRI*
HG0715	*H. sapiens*	ENSG00000071246	VASH1	vasohibin 1	0.03	0.30	6.3E-03	1.8E-02	0.30	6.3E-03		MCYLCSRCVGGLL*
HG0716	*H. sapiens*	ENSG00000075413	MARK3	microtubule affinity regulating kinase 3	0.03	0.29	1.5E-03	5.2E-03	0.29	1.5E-03		MSPGTRRGTARARPGI*
HG0717	*H. sapiens*	ENSG00000083857	FAT1	FAT atypical cadherin 1	0.00	0.50	1.4E-02	3.4E-02	0.50	1.4E-02		MLKIPGRQSWYFKPGS*
HG0718	*H. sapiens*	ENSG00000085274	MYNN	myoneurin	0.02	0.42	2.1E-07	1.5E-06	0.42	2.1E-07		MMGEDKQLQAPARSAQLASGERPAVLCCRASAQSPGRAPDADCRHQNARDEEDRRSAAS*
HG0719	*H. sapiens*	ENSG00000085998	POMGNT1	protein O-linked mannose N-acetylglucosaminyltransferase	0.03	0.29	6.6E-07	4.5E-06	0.16	1.7E-09		MTGQRPPLLQQPAHCLDALIQGHQLLQDLHIDGSQIPEVMGWPGHW*
HG0720	*H. sapiens*	ENSG00000087053	MTMR2	myotubularin related protein 2	0.19	0.18	2.9E-05	1.5E-04	0.18	2.9E-05		MAASGLPPGPALA*
HG0721	*H. sapiens*	ENSG00000095951	HIVEP1	human immunodeficiency virus type I enhancer binding pro	0.00	0.27	8.7E-05	4.0E-04	0.27	8.7E-05		MYVEFMELPFGGLLYLQFLRRKRRP*
HG0722	*H. sapiens*	ENSG00000101445	PPP1R16B	protein phosphatase 1 regulatory subunit 16B	0.16	0.42	2.1E-09	2.1E-08	0.42	2.1E-09		MRPQPHQRPRAALAPGAPC*
HG0723	*H. sapiens*	ENSG00000102974	CTCF	CCCTC-binding factor	0.00	0.32	1.5E-03	5.1E-03	0.32	1.5E-03		MITDLKPKNKMR*
HG0724	*H. sapiens*	ENSG00000104783	KCNN4	potassium calcium-activated channel subfamily N member	0.30	0.33	5.7E-12	7.7E-11	0.49	7.6E-05		MDVSFLLCPKTHPSPSLFSKGGAGEPPPTLGPPLPLPALPGRHHSPEGTGLCCASEQKSQSQQSRLTTCKPQWLPCACCEVGDPGQEAG*
HG0725	*H. sapiens*	ENSG00000112218	GPR63	G protein-coupled receptor 63	0.14	0.49	2.9E-08	2.5E-07	0.49	2.9E-08		MYFQAHQWDLILLFCVEMLEELLHLCLHLPSY*
HG0726.1	*H. sapiens*	ENSG00000115504	EHBP1	EH domain binding protein 1	0.00	0.37	4.6E-08	3.7E-07	0.37	4.6E-08		MWLRRGWRTRWRRRLLLRRRRRSGYEKGTGPDSARQRD*
HG0726.2	*H. sapiens*	ENSG00000115504	EHBP1	EH domain binding protein 1	0.00	0.13	1.5E-04	6.6E-04	0.13	1.5E-04		MQSSFALDPLHY*

HG0727	*H. sapiens*	ENSG00000115825	PRKD3	protein kinase D3	0.00	0.05	7.4E-17	1.5E-15	0.05	3.0E-11		MKYACTILKSFVFRHRKVHPPRMKMRTFEKVI*
HG0728	*H. sapiens*	ENSG00000116991	SIPA1L2	signal induced proliferation associated 1 like 2	0.00	0.28	1.2E-10	1.4E-09	0.28	1.2E-10		MLLKRTEAYNIITTTEKQVGFEETVF*
HG0729	*H. sapiens*	ENSG00000119383	PTPA	protein phosphatase 2 phosphatase activator	0.00	0.32	1.3E-08	1.1E-07	0.32	1.3E-08		MAAVFAVVTLTLGFRL*
HG0730	*H. sapiens*	ENSG00000125170	DOK4	docking protein 4	0.00	0.30	2.9E-08	2.4E-07	0.30	2.9E-08		MQARGEGAARTMLRRVR*
HG0731	*H. sapiens*	ENSG00000129255	MPDU1	mannose-P-dolichol utilization defect 1	0.06	0.47	1.0E-06	6.7E-06	0.47	1.0E-06		MAPSRRSGERQGCKSGFQTGSVNKGVGGLTAQACSFGKLYNSNSASKTWSLTETYFTPLE*
HG0732	*H. sapiens*	ENSG00000135914	HTR2B	5-hydroxytryptamine receptor 2B	0.21	0.28	4.1E-07	2.9E-06	0.28	4.1E-07		MDRCVYALILENLNFFF*
HG0733	*H. sapiens*	ENSG00000136247	ZDHHC4	zinc finger DHHC-type containing 4	0.18	0.49	1.6E-06	1.0E-05	0.49	1.6E-06		MLGRGMPELPAENYPTKRNVGWVP*
HG0734	*H. sapiens*	ENSG00000139318	DUSP6	dual specificity phosphatase 6	0.04	0.48	1.6E-04	6.8E-04	0.48	1.6E-04		MCPAPPPLPPPLLSPSSRRARCCSLFALGLIRSGNSFPFL*
HG0735	*H. sapiens*	ENSG00000139579	NABP2	nucleic acid binding protein 2	0.22	0.49	1.2E-06	7.8E-06	0.49	1.2E-06		MDRVPACRDEGSGRSGQ*
HG0736	*H. sapiens*	ENSG00000139826	ABHD13	abhydrolase domain containing 13	0.20	0.47	1.6E-02	3.9E-02	0.47	1.6E-02		MWEPAGAFAGDTRAGAGSGATARSRSSGLDRGAGSGPWAGGG*
HG0737	*H. sapiens*	ENSG00000141639	MAPK4	mitogen-activated protein kinase 4	0.00	0.40	3.2E-06	2.0E-05	0.40	3.2E-06		MLPTSKGSRGSWPGS*
HG0738	*H. sapiens*	ENSG00000143393	PI4KB	phosphatidylinositol 4-kinase beta	0.03	0.00	1.2E-08	1.1E-07	0.00	1.2E-08		MEPGSGRCC*
HG0739	*H. sapiens*	ENSG00000144331	ZNF385B	zinc finger protein 385B	0.19	0.42	1.6E-06	1.0E-05	0.42	1.6E-06		MICSEALPAGCFLAPHFTGAPG*
HG0740	*H. sapiens*	ENSG00000144331	ZNF385B	zinc finger protein 385B	0.00	0.34	1.0E-03	3.7E-03	0.34	1.0E-03		MFYISTPSNIKLMFMG*
HG0741	*H. sapiens*	ENSG00000144834	TAGLN3	transgelin 3	0.04	0.35	9.4E-06	5.2E-05	0.35	9.4E-06		MRKSNQCAILPGARQQRLQFNMKGGFLPAC*
HG0742	*H. sapiens*	ENSG00000154556	SORBS2	sorbin and SH3 domain containing 2	0.00	0.50	1.3E-04	5.8E-04	0.50	1.3E-04		MFCKFPGGHEAPDTWIGLNLKPLEFKTF*
HG0743	*H. sapiens*	ENSG00000154814	OXNAD1	oxidoreductase NAD binding domain containing 1	0.24	0.35	1.8E-07	1.4E-06	0.35	1.8E-07		MEKLSMFQLLYIQKSQCNSRTKIFCQSADHILNDS*
HG0744	*H. sapiens*	ENSG00000163053	SLC16A14	solute carrier family 16 member 14	0.04	0.35	4.7E-14	8.0E-13	0.35	4.7E-14		MIGDIATNLYTQQLRGLQAEQQVRGSGRGAE*
HG0745	*H. sapiens*	ENSG00000163145	C1QTNF7	C1q and TNF related 7	0.00	0.31	4.7E-05	2.3E-04	0.31	4.7E-05		MLRRALYFSSSYFLHCFGVASNNKHIFLL*
HG0746	*H. sapiens*	ENSG00000164741	DLC1	DLC1 Rho GTPase activating protein	0.03	0.08	5.4E-04	2.1E-03	0.08	5.4E-04		MISTTKWELRFWRN*
HG0747	*H. sapiens*	ENSG00000168036	CTNNB1	catenin beta 1	0.00	0.16	2.0E-04	8.5E-04	0.16	2.0E-04		MIFSMNLGFCRSIHLQSQNPPGI*
HG0748	*H. sapiens*	ENSG00000168830	HTR1E	5-hydroxytryptamine receptor 1E	0.03	0.46	1.7E-04	7.2E-04	0.46	1.7E-04		MLWPFPLPTENGTQETT*
HG0749	*H. sapiens*	ENSG00000169760	NLGN1	neuroligin 1	0.00	0.47	6.8E-04	2.5E-03	0.47	6.8E-04		MVGFTWRWLWVPRILDRGCIWREKRINRHVLEVSVGE*
HG0750	*H. sapiens*	ENSG00000170881	RNF139	ring finger protein 139	0.28	0.20	3.0E-06	1.8E-05	0.20	3.0E-06		MVAPRAGEAVEGGGVRP*
HG0751	*H. sapiens*	ENSG00000171791	BCL2	BCL2, apoptosis regulator	0.01	0.36	4.1E-06	2.4E-05	0.36	4.1E-06		MGSLPYAFVLVLQKGNLTEDHAVLKKYK*
HG0752	*H. sapiens*	ENSG00000172269	DPAGT1	dolichyl-phosphate N-acetylglucosaminephosphotransferas	0.05	0.44	1.6E-07	1.2E-06	0.44	1.6E-07		MKFGITLLFHFHPHPLMPILKMAAAAFRSNGCSANKIRAETVDRRVQRGRT*
HG0753	*H. sapiens*	ENSG00000173406	DAB1	DAB1, reelin adaptor protein	0.00	0.33	1.5E-04	6.7E-04	0.33	1.5E-04		MGSRFLGGCGSGGGCSGLGEHSKRVNEMCSSECHF*
HG0754	*H. sapiens*	ENSG00000175182	FAM131A	family with sequence similarity 131 member A	0.05	0.28	2.5E-05	1.3E-04	0.28	2.5E-05		MRQECSPYVTRHRFQMGRGLAQHPEPKQE*
HG0755	*H. sapiens*	ENSG00000176597	B3GNT5	UDP-GlcNAc:betaGal beta-1,3-N-acetylglucosaminyltransfe	0.00	0.49	1.2E-06	7.7E-06	0.49	1.2E-06		MPVWKNFDVPVSSFYRVFHSSAISEKWD*
HG0756	*H. sapiens*	ENSG00000177051	FBXO46	F-box protein 46	0.06	0.40	3.4E-05	1.7E-04	0.40	3.4E-05		MQRAAMVACGASHHHLGPEMR*
HG0757.1	*H. sapiens*	ENSG00000177301	KCNA2	potassium voltage-gated channel subfamily A member 2	0.00	0.11	3.3E-15	5.9E-14	0.11	3.3E-15		MLLCFLGPVAAP*
HG0757.2	*H. sapiens*	ENSG00000182255	KCNA4	potassium voltage-gated channel subfamily A member 4	0.00	0.16	5.5E-03	1.6E-02	0.16	5.5E-03		MNLKGEPGESS*
HG0758	*H. sapiens*	ENSG00000179299	NSUN7	NOP2/Sun RNA methyltransferase family member 7	0.05	0.24	2.8E-12	3.9E-11	0.24	2.8E-12		MLQMRGKPFPGTSEF*
HG0759	*H. sapiens*	ENSG00000180929	GPR62	G protein-coupled receptor 62	0.23	0.23	2.1E-10	2.3E-09	0.23	2.1E-10		MAAWASEPPPGQPMR*
HG0760	*H. sapiens*	ENSG00000182463	TSHZ2	teashirt zinc finger homeobox 2	0.22	0.36	3.9E-08	3.2E-07	0.36	3.9E-08		MFNKEIKCLNSHQKKKKPQKQNQKNSKSKNKKERGKKIQNKQTNKQGRTNLYFKAAGTSHPCLPPREGGLWPVVEELQGGSSGGQRPSDVLGATGQEAEETQRGQRDSGPQRAARGWGARSGTGAK*
HG0761	*H. sapiens*	ENSG00000185985	SLITRK2	SLIT and NTRK like family member 2	0.00	0.43	1.3E-10	1.5E-09	0.43	1.3E-10		MTWRLAATPREIRCKHVCSRGACVGGLGMGE*
HG0762	*H. sapiens*	ENSG00000187147	RNF220	ring finger protein 220	0.00	0.14	1.5E-08	1.3E-07	0.14	1.5E-08		MHHTLRLLPSVS*
HG0763	*H. sapiens*	ENSG00000188050	RNF133	ring finger protein 133	0.00	0.41	2.0E-07	1.5E-06	0.41	2.0E-07	Crowe	MLEDSGSCLYPEDFFLFVILN*
HG0764	*H. sapiens*	ENSG00000188725	SMIM15	small integral membrane protein 15	0.11	0.37	2.2E-09	2.2E-08	0.37	2.2E-09		MCTVELKKTDYQGSSEVVIGSLITPGQS*
HG0765	*H. sapiens*	ENSG00000196123			0.08	0.44	1.1E-06	7.0E-06	0.44	1.1E-06		MVKSLLQEYIAKAFLAEGPET*
HG0766	*H. sapiens*	ENSG00000196189	SEMA4A	semaphorin 4A	0.15	0.20	6.0E-13	9.1E-12	0.20	6.0E-13		MMGTWRAALPFQPG*
HG0767.1	*H. sapiens*	ENSG00000197555	SIPA1L1	signal induced proliferation associated 1 like 1	0.00	0.28	5.5E-05	2.7E-04	0.28	5.5E-05		MLDSFCNILVSRKHGIMATTESQ*
HG0767.2	*H. sapiens*	ENSG00000197555	SIPA1L1	signal induced proliferation associated 1 like 1	0.00	0.26	4.9E-03	1.4E-02	0.26	4.9E-03		MERATQSCVLFLPSS*
HG0768	*H. sapiens*	ENSG00000213023	SYT3	synaptotagmin 3	0.13	0.30	2.0E-06	1.3E-05	0.30	2.0E-06		MGCRPPPPRLPPRTRQR*
HG0769.1	*H. sapiens*	ENSG00000280165	PCDH20	protocadherin 20	0.11	0.34	3.7E-07	2.7E-06	0.34	3.7E-07		MNREGVSSAALPSRAL*
HG0769.2	*H. sapiens*	ENSG00000197991			0.00	0.46	2.3E-03	7.4E-03	0.46	2.3E-03		MNVPFCPLAFLPLFEFPSLIEEPVTIG*
HG0770	*H. sapiens*	ENSG00000010165	METTL13	methyltransferase like 13	0.00	0.45	4.3E-07	3.1E-06	0.45	4.3E-07		MWLFFRGKNSHCSVPEPACGGQAPQMVSHRE*
HG0771	*H. sapiens*	ENSG00000013306	SLC25A39	solute carrier family 25 member 39	0.00	0.37	8.4E-07	5.7E-06	0.37	8.4E-07		MGPPDPGRRAARAPA*
HG0772	*H. sapiens*	ENSG00000064309	CDON	cell adhesion associated, oncogene regulated	0.03	0.22	1.5E-11	1.9E-10	0.22	1.5E-11		MTWGWLSEPAAAQEVWRLSQPRGE*
HG0773	*H. sapiens*	ENSG00000065548	ZC3H15	zinc finger CCCH-type containing 15	0.04	0.47	8.6E-05	4.0E-04	0.47	8.6E-05		MLCDIDLSQRPRLRGQ*
HG0774	*H. sapiens*	ENSG00000066933	MYO9A	myosin IXA	0.00	0.42	1.9E-03	6.2E-03	0.42	1.9E-03		MCLLRPGGVSLTGRGMRRGRSPGP*
HG0775	*H. sapiens*	ENSG00000068001	HYAL2	hyaluronoglucosaminidase 2	0.02	0.48	1.3E-03	4.5E-03	0.48	1.3E-03		MGFIKSSASCPTGSSWRK*
HG0776	*H. sapiens*	ENSG00000082014	SMARCD3	SWI/SNF related, matrix associated, actin dependent regul	0.16	0.33	2.2E-07	1.7E-06	0.33	2.2E-07		MALGAAGAVGSGPRHRGGTG*
HG0777	*H. sapiens*	ENSG00000085644	ZNF213	zinc finger protein 213	0.20	0.25	5.9E-07	4.2E-06	0.25	5.9E-07		MPAQLPQGIPLGD*
HG0778	*H. sapiens*	ENSG00000089116	LHX5	LIM homeobox 5	0.02	0.17	1.7E-02	4.1E-02	0.17	1.7E-02		MRELRGDQTKMRIRTA*
HG0779	*H. sapiens*	ENSG00000091140	DLD	dihydrolipoamide dehydrogenase	0.09	0.45	2.8E-05	1.4E-04	0.45	2.8E-05		MRREGRPWRSGGGAQRR*
HG0780	*H. sapiens*	ENSG00000092199	HNRNPC	heterogeneous nuclear ribonucleoprotein C (C1/C2)	0.02	0.00	1.8E-04	7.8E-04	0.00	1.8E-04		MPSLEQQQPG*
HG0781	*H. sapiens*	ENSG00000100099	HPS4	HPS4, biogenesis of lysosomal organelles complex 3 subu	0.00	0.50	9.0E-05	4.2E-04	0.50	9.0E-05		MHLPLLAFLERSLKRWFCRQPQ*
HG0782	*H. sapiens*	ENSG00000100368	CSF2RB	colony stimulating factor 2 receptor beta common subunit	0.08	0.35	2.8E-06	1.7E-05	0.35	2.8E-06		MGLAWLPAGQEHRTSGH*
HG0783	*H. sapiens*	ENSG00000105053	VRK3	vaccinia related kinase 3	0.17	0.04	8.6E-07	5.8E-06	0.04	8.6E-07		MRAQRSPPSGSFRWATEKVAVKMEFPTQ*
HG0784	*H. sapiens*	ENSG00000105851	PIK3CG	phosphatidylinositol-4,5-bisphosphate 3-kinase catalytic su	0.11	0.43	7.3E-03	2.0E-02	0.43	7.3E-03		MKGAPEKRKNLDAHWVA*
HG0785	*H. sapiens*	ENSG00000107242	PIP5K1B	phosphatidylinositol-4-phosphate 5-kinase type 1 beta	0.00	0.38	1.1E-03	4.1E-03	0.38	1.1E-03		MSRPPITTNYR*
HG0786	*H. sapiens*	ENSG00000107862	GBF1	golgi brefeldin A resistant guanine nucleotide exchange fac	0.00	0.00	4.4E-05	2.2E-04	0.00	4.4E-05		MSSGCRSWD*
HG0787	*H. sapiens*	ENSG00000108592	FTSJ3	FtsJ homolog 3	0.18	0.23	9.8E-11	1.2E-09	0.23	9.8E-11		MAENLEPGTQGTQAEAEVVSCILVRFQVWRKRLWV*
HG0788	*H. sapiens*	ENSG00000113739	STC2	stanniocalcin 2	0.10	0.00	2.5E-08	2.1E-07	0.00	2.5E-08		MLSGELREGAG*
HG0789	*H. sapiens*	ENSG00000115355	CCDC88A	coiled-coil domain containing 88A	0.00	0.25	3.2E-08	2.6E-07	0.25	3.2E-08		MVSARGEGASPACWY*
HG0790.1	*H. sapiens*	ENSG00000115568	ZNF142	zinc finger protein 142	0.00	0.47	2.9E-05	1.5E-04	0.47	2.9E-05		MTMLHPKPSRLQTAFPVRWLL*
HG0790.2	*H. sapiens*	ENSG00000115568	ZNF142	zinc finger protein 142	0.00	0.20	6.9E-05	3.3E-04	0.20	6.9E-05		MWTLSSPPFYR*
HG0791	*H. sapiens*	ENSG00000131323	TRAF3	TNF receptor associated factor 3	0.00	0.32	6.1E-04	2.3E-03	0.32	6.1E-04		MRKMRPKEVMPLG*
HG0792	*H. sapiens*	ENSG00000132424	PNISR	PNN interacting serine and arginine rich protein	0.00	0.17	1.2E-05	6.7E-05	0.17	1.2E-05		MLRSCSLNQPKF*
HG0793	*H. sapiens*	ENSG00000132698	RAB25	RAB25, member RAS oncogene family	0.00	0.26	4.4E-07	3.1E-06	0.26	4.4E-07		MRGGAGPDLLRAEG*
HG0794.1	*H. sapiens*	ENSG00000135218	CD36	CD36 molecule	0.00	0.41	4.6E-04	1.8E-03	0.41	4.6E-04		MLEHLIEKSFLAILKIAFQ*
HG0794.2	*H. sapiens*	ENSG00000135218	CD36	CD36 molecule	0.00	0.17	6.0E-04	2.3E-03	0.17	6.0E-04		MSMAFRCQDNLKDR*
HG0795	*H. sapiens*	ENSG00000137601	NEK1	NIMA related kinase 1	0.00	0.35	1.9E-05	9.8E-05	0.35	1.9E-05		MSFSKCGMVPLLY*
HG0796	*H. sapiens*	ENSG00000138074	SLC5A6	solute carrier family 5 member 6	0.03	0.33	2.7E-11	3.3E-10	0.33	2.7E-11		MRPSPLSGILADPLFLFPFSEGLPRRRAASRSRLQTPSARCSPRPDAEVAASQKLGPLHPPSEKGAMLISGSTKGPS*
HG0797	*H. sapiens*	ENSG00000140526	ABHD2	abhydrolase domain containing 2	0.01	0.34	5.0E-09	4.7E-08	0.34	5.0E-09		MSAAGSRGAAGTRLGAS*
HG0798.1	*H. sapiens*	ENSG00000142949	PTPRF	protein tyrosine phosphatase, receptor type F	0.06	0.42	5.2E-05	2.5E-04	0.42	5.2E-05		MRTDGPWIGGRSGGPGARPLVLSIQQE*
HG0798.2	*H. sapiens*	ENSG00000153707	PTPRD	protein tyrosine phosphatase, receptor type D	0.00	0.47	1.6E-02	3.8E-02	0.47	1.6E-02		MLAPREAMGFGSRFLS*
HG0799	*H. sapiens*	ENSG00000143398	PIP5K1A	phosphatidylinositol-4-phosphate 5-kinase type 1 alpha	0.01	0.35	1.6E-06	1.0E-05	0.35	1.6E-06		MPGEWCGLELVQEPARRV*
HG0800	*H. sapiens*	ENSG00000143612	C1orf43	chromosome 1 open reading frame 43	0.03	0.37	1.6E-02	3.9E-02	0.37	1.6E-02		MFSGRVWLGLPP*
HG0801	*H. sapiens*	ENSG00000144278	GALNT13	polypeptide N-acetylgalactosaminyltransferase 13	0.00	0.39	6.0E-04	2.3E-03	0.39	6.0E-04		MFANHFEIAKCHIQFNWI*
HG0802	*H. sapiens*	ENSG00000144283	PKP4	plakophilin 4	0.12	0.00	7.2E-05	3.4E-04	0.00	7.2E-05		MSLIPGATTAAA*
HG0803	*H. sapiens*	ENSG00000144802	NFKBIZ	NFKB inhibitor zeta	0.05	0.40	1.8E-05	9.5E-05	0.40	1.8E-05		MLSRTWVIIADYSWKQLICSYVFV*
HG0804	*H. sapiens*	ENSG00000144815	NXPE3	neurexophilin and PC-esterase domain family member 3	0.00	0.46	2.7E-03	8.4E-03	0.46	2.7E-03		MCFLNQLQSLLCIRALKGTSRIEANETESSSAAQKSKDMEF*
HG0805.1	*H. sapiens*	ENSG00000145936	KCNMB1	potassium calcium-activated channel subfamily M regulator	0.04	0.49	8.8E-05	4.1E-04	0.49	8.8E-05		MFGHGGLPQMGTKSCFPGDGRGGQTSC*
HG0805.2	*H. sapiens*	ENSG00000145936	KCNMB1	potassium calcium-activated channel subfamily M regulator	0.06	0.49	6.4E-03	1.8E-02	0.49	6.4E-03		MEVQGLGNSCLPQGWVPALRVCVSRLCAQT*
HG0806	*H. sapiens*	ENSG00000147481	SNTG1	syntrophin gamma 1	0.00	0.42	7.5E-05	3.5E-04	0.42	7.5E-05		MGRAVGVEMLKGPHFLK*
HG0807.1	*H. sapiens*	ENSG00000147488	ST18	ST18, C2H2C-type zinc finger	0.00	0.31	6.6E-10	7.1E-09	0.31	6.6E-10	Mackowiak	MFLTECDLRGILEKIIKFLNGAAWRNPKRNSIRAFWNTSRKR*
HG0807.2	*H. sapiens*	ENSG00000147488	ST18	ST18, C2H2C-type zinc finger	0.00	0.37	3.8E-07	2.8E-06	0.37	3.8E-07		MSGQILQETQKSMIKKLSTLIYCKG*
HG0807.3	*H. sapiens*	ENSG00000186487	MYT1L	myelin transcription factor 1 like	0.00	0.00	1.5E-05	7.8E-05	0.00	1.5E-05		MEKSRHREIK*
HG0808	*H. sapiens*	ENSG00000149269	PAK1	p21 (RAC1) activated kinase 1	0.15	0.42	7.2E-03	2.0E-02	0.42	7.2E-03		MPGLAFPTSSCCWW*
HG0809	*H. sapiens*	ENSG00000151287	TEX30	testis expressed 30	0.04	0.38	5.2E-03	1.5E-02	0.38	5.2E-03		MCSFIVFVSYPCLPTHLSQQNLLFREMSFASQM*
HG0810	*H. sapiens*	ENSG00000156504	FAM122B	family with sequence similarity 122B	0.00	0.44	6.2E-03	1.8E-02	0.44	6.2E-03		MGARTRKNWPSGGSRF*
HG0811	*H. sapiens*	ENSG00000157350	ST3GAL2	ST3 beta-galactoside alpha-2,3-sialyltransferase 2	0.00	0.40	1.3E-08	1.2E-07	0.40	1.3E-08		MGSRRARPTAERISISAAEFSHTHRASCFCPLRVGVWLPSSGRGFGPGGGTTLAWNPSPLKPLCSLL*
HG0812	*H. sapiens*	ENSG00000157368	IL34	interleukin 34	0.02	0.31	1.4E-08	1.2E-07	0.31	1.4E-08		MGLAATAPRLSSTLPGR*
HG0813	*H. sapiens*	ENSG00000160199	PKNOX1	PBX/knotted 1 homeobox 1	0.00	0.32	3.4E-04	1.4E-03	0.32	3.4E-04		MKIGTQTPFAFHPR*
HG0814	*H. sapiens*	ENSG00000163071	SPATA18	spermatogenesis associated 18	0.18	0.40	8.8E-05	4.1E-04	0.40	8.8E-05		MRRGGCGPGHLPSGFPNPARSDPRGRMETPAAL*
HG0815	*H. sapiens*	ENSG00000163072	NOSTRIN	nitric oxide synthase trafficking	0.00	0.26	2.3E-03	7.5E-03	0.26	2.3E-03		MKVQGWDFLLGMFDKD*
HG0816	*H. sapiens*	ENSG00000163794	UCN	urocortin	0.20	0.15	2.3E-15	4.3E-14	0.39	1.9E-02		MFFADVSRSCPGTI*
HG0817	*H. sapiens*	ENSG00000163888	CAMK2N2	calcium/calmodulin dependent protein kinase II inhibitor 2	0.00	0.00	4.4E-04	1.7E-03	0.00	4.4E-04		MTSSFRRLPC*
HG0818	*H. sapiens*	ENSG00000164125	FAM198B	family with sequence similarity 198 member B	0.00	0.49	4.2E-03	1.3E-02	0.49	4.2E-03		MVRNKPHFSLGRLII*
HG0819	*H. sapiens*	ENSG00000164188	RANBP3L	RAN binding protein 3 like	0.00	0.36	3.5E-04	1.4E-03	0.36	3.5E-04		MKELSCVFNQMNWHLYI*
HG0820	*H. sapiens*	ENSG00000164946	FREM1	FRAS1 related extracellular matrix 1	0.00	0.37	8.9E-06	5.0E-05	0.37	8.9E-06		MLRSRGTLSGPLTLPLGL*

HG0821	H. sapiens	ENSG00000165300	SLITRK5	SLIT and NTRK like family member 5	0.04	0.07	2.5E-08	2.1E-07	0.07	2.5E-08		MMTGPWRCETGR*
HG0822	H. sapiens	ENSG00000166170	BAG5	BCL2 associated athanogene 5	0.18	0.26	7.4E-03	2.0E-02	0.26	7.4E-03		MQLGAQLRCRTP*
HG0823	H. sapiens	ENSG00000166268	MYRFL	myelin regulatory factor-like	0.00	0.27	7.7E-07	5.2E-06	0.27	7.7E-07		MAEDMLHSNKKKMKIFQEHS*
HG0824	H. sapiens	ENSG00000167100			0.04	0.26	1.7E-04	7.3E-04	0.26	1.7E-04		MERLEPARSPPRPG*
HG0825	H. sapiens	ENSG00000169180	XPO6	exportin 6	0.00	0.00	3.6E-05	1.8E-04	0.00	3.6E-05		MGGGLPESAGTMT*
HG0826	H. sapiens	ENSG00000169851	PCDH7	protocadherin 7	0.00	0.45	6.2E-04	2.3E-03	0.45	6.2E-04		MTLRRVVKKKEKPALSGVRRD*
HG0827	H. sapiens	ENSG00000170365	SMAD1	SMAD family member 1	0.00	0.35	1.0E-08	9.2E-08	0.35	1.0E-08		MCIREFAVEQLFLYSAPCL*
HG0828	H. sapiens	ENSG00000170390	DCLK2	doublecortin like kinase 2	0.00	0.17	6.8E-08	5.5E-07	0.17	6.8E-08		MTWAARRCGHF*
HG0829	H. sapiens	ENSG00000171502	COL24A1	collagen type XXIV alpha 1 chain	0.12	0.29	3.4E-04	1.4E-03	0.29	3.4E-04		MDSTCTLSSSELASVFR*
HG0830	H. sapiens	ENSG00000171631	P2RY6	pyrimidinergic receptor P2Y6	0.25	0.41	6.6E-08	5.3E-07	0.41	6.6E-08		MALEGGVQAEEMGAVLSEPLPP*
HG0831.1	H. sapiens	ENSG00000174482	LINGO2	leucine rich repeat and Ig domain containing 2	0.00	0.32	2.5E-03	8.0E-03	0.32	2.5E-03		MQLSRFVSSSWLVGL*
HG0831.2	H. sapiens	ENSG00000174482	LINGO2	leucine rich repeat and Ig domain containing 2	0.21	0.44	1.3E-02	3.3E-02	0.44	1.3E-02		MRRSSEQSTESGHYQHEEPNNSNW*
HG0832	H. sapiens	ENSG00000175048	ZDHHC14	zinc finger DHHC-type containing 14	0.13	0.37	1.6E-03	5.4E-03	0.37	1.6E-03	Crowe	MTVGLPGGLTWSSAKLSPSREPV*
HG0833	H. sapiens	ENSG00000176641	RNF152	ring finger protein 152	0.00	0.37	6.7E-06	3.8E-05	0.37	6.7E-06		MSSRRRGERDSVTDTEMNTPVSTGCL*
HG0834	H. sapiens	ENSG00000182489	XKRX	XK related, X-linked	0.00	0.33	1.7E-02	4.2E-02	0.33	1.7E-02		MLAFVLFKFHSKYVDDWNRGQSRVKFKVVLLGFPSQTPSRI*
HG0835	H. sapiens	ENSG00000182901	RGS7	regulator of G protein signaling 7	0.06	0.23	4.0E-04	1.6E-03	0.23	4.0E-04		MKLPCLAAPLAAS*
HG0836	H. sapiens	ENSG00000197461	PDGFA	platelet derived growth factor subunit A	0.27	0.12	2.3E-05	1.2E-04	0.12	2.3E-05		MSANMQNYRPGRS*
HG0837	H. sapiens	ENSG00000198690	FAN1	FANCD2 and FANCI associated nuclease 1	0.02	0.40	1.0E-06	6.8E-06	0.40	1.0E-06		MRSYRIPLLVISSQESKSKPLLSFTLNILCFIAQNIQFF*
HG0838	H. sapiens	ENSG00000198707	CEP290	centrosomal protein 290	0.01	0.41	9.8E-03	2.6E-02	0.41	9.8E-03		MVSPSSRCRFGLLGTIWLDPESAWNRDRDLSGPAAGSSLVVAVVRGLRWLPGLV*
HG0839	H. sapiens	ENSG00000198791	CNOT7	CCR4-NOT transcription complex subunit 7	0.04	0.42	5.2E-07	3.7E-06	0.42	5.2E-07		MGHPLPPPLPPPPPPPPPSAVSMARRRRRSASSATQVHK*
HG0840	H. sapiens	ENSG00000278195	SSTR3	somatostatin receptor 3	0.16	0.43	1.8E-06	1.1E-05	0.43	1.8E-06		MITSLAARREALTPWAGK*
HG0841	H. sapiens	ENSG00000278259	MYO19	myosin XIX	0.03	0.16	6.9E-09	6.3E-08	0.16	6.9E-09		MTRWIVLLVPPATQNPG*
HG0842	H. sapiens	ENSG00000002746	HECW1	HECT, C2 and WW domain containing E3 ubiquitin protein	0.03	0.37	1.1E-03	4.0E-03	0.37	1.1E-03		MKCAALPRIRRCCWSRNTVRL*
HG0843	H. sapiens	ENSG00000043093	DCUN1D1	defective in cullin neddylation 1 domain containing 1	0.12	0.44	1.0E-02	2.6E-02	0.44	1.0E-02		MRVEELRSTDPHVP*
HG0844	H. sapiens	ENSG00000091009	RBM27	RNA binding motif protein 27	0.00	0.00	1.4E-03	4.8E-03	0.00	1.4E-03		MPGGLGHREL*
HG0845	H. sapiens	ENSG00000099260	PALMD	palmdelphin	0.02	0.44	7.0E-04	2.6E-03	0.44	7.0E-04		MVALDLLPRLTVLDF*
HG0846	H. sapiens	ENSG00000100852	ARHGAP5	Rho GTPase activating protein 5	0.00	0.33	3.8E-05	1.9E-04	0.33	3.8E-05	Mackowiak	MGKREKEDDPYDLEDVSAQK*
HG0847	H. sapiens	ENSG00000106086	PLEKHA8	pleckstrin homology domain containing A8	0.28	0.13	1.8E-08	1.6E-07	0.13	1.8E-08		MALLLVLLASWGLGQ*
HG0848	H. sapiens	ENSG00000111817	DSE	dermatan sulfate epimerase	0.29	0.15	3.8E-04	1.5E-03	0.15	3.8E-04		MARRGRGGLGSEGAGSPGALE*
HG0849	H. sapiens	ENSG00000111846	GCNT2	glucosaminyl (N-acetyl) transferase 2, I-branching enzyme	0.01	0.49	3.2E-03	9.9E-03	0.49	3.2E-03		MTCRMVTSPTCNFMVGTGGPPGIRTSLRTSVFV*
HG0850	H. sapiens	ENSG00000113916	BCL6	B-cell CLL/lymphoma 6	0.00	0.41	5.7E-03	1.6E-02	0.41	5.7E-03		MCLQLSFAKEKILEVW*
HG0851	H. sapiens	ENSG00000114279	FGF12	fibroblast growth factor 12	0.00	0.19	3.9E-06	2.4E-05	0.19	3.9E-06		MHSVDLTLLGRTS*
HG0852	H. sapiens	ENSG00000114942	EEF1B2	eukaryotic translation elongation factor 1 beta 2	0.05	0.25	1.8E-07	1.4E-06	0.25	1.8E-07		MPILLIYFQTQRKVHGQFVGHLIQGPQFVRGEVGMQKYFDL*
HG0853	H. sapiens	ENSG00000121807	CCR2	C-C motif chemokine receptor 2	0.07	0.41	1.1E-04	4.9E-04	0.41	1.1E-04		MYRVCLTHTQGCISKISKLILFWRPQPNVQCS*
HG0854	H. sapiens	ENSG00000127249	ATP13A4	ATPase 13A4	0.00	0.42	5.6E-04	2.1E-03	0.42	5.6E-04		MGAASFETAALRECNKQGAASNSS*
HG0855	H. sapiens	ENSG00000127804	METTL16	methyltransferase like 16	0.10	0.49	1.6E-02	3.9E-02	0.49	1.6E-02		MAARWERIICVSRSFAMRLL*
HG0856	H. sapiens	ENSG00000130055	GDPD2	glycerophosphodiester phosphodiesterase domain containi	0.04	0.33	1.8E-02	4.3E-02	0.33	1.8E-02		MGKPAQRNSSPQAP*
HG0857	H. sapiens	ENSG00000130347	RTN4IP1	reticulon 4 interacting protein 1	0.00	0.29	3.7E-07	2.6E-06	0.29	3.7E-07		MDRILTGTLIRAFHMRRAE*
HG0858	H. sapiens	ENSG00000132702	HAPLN2	hyaluronan and proteoglycan link protein 2	0.04	0.23	1.8E-08	1.6E-07	0.23	1.8E-08		MRTQAPEGRGAQVPGF*
HG0859	H. sapiens	ENSG00000132842	AP3B1	adaptor related protein complex 3 beta 1 subunit	0.12	0.05	2.6E-05	1.4E-04	0.05	2.6E-05		MRRGWTARSAQGAVTSPEAPARAPPYEN*
HG0860	H. sapiens	ENSG00000133056	PIK3C2B	phosphatidylinositol-4-phosphate 3-kinase catalytic subunit	0.00	0.24	5.9E-07	4.1E-06	0.24	5.9E-07		MASPLSPAAAAIVSPGVA*
HG0861	H. sapiens	ENSG00000134001	EIF2S1	eukaryotic translation initiation factor 2 subunit alpha	0.00	0.13	1.2E-07	9.3E-07	0.13	1.2E-07		MVNDEEGPLFVQRLRMRGGSACAVE*
HG0862	H. sapiens	ENSG00000134245	WNT2B	Wnt family member 2B	0.02	0.00	3.0E-08	2.5E-07	0.00	3.0E-08		MAPQGGEVGAA*
HG0863	H. sapiens	ENSG00000134313	KIDINS220	kinase D interacting substrate 220	0.00	0.45	1.2E-03	4.3E-03	0.45	1.2E-03		MAAGCGEGDALAVAVSCFPVL*
HG0864	H. sapiens	ENSG00000134533	RERG	RAS like estrogen regulated growth inhibitor	0.00	0.46	1.3E-03	4.7E-03	0.46	1.3E-03		MVNPKTGTKKIYSVL*
HG0865	H. sapiens	ENSG00000135540	NHSL1	NHS like 1	0.00	0.39	1.3E-06	8.4E-06	0.39	1.3E-06		MPRRGVGLQFGMLGTRQRVGVPWLCVLQSPDKYS*
HG0866	H. sapiens	ENSG00000136011	STAB2	stabilin 2	0.09	0.18	4.8E-05	2.3E-04	0.18	4.8E-05		MRKNSLGVYQTKLK*
HG0867	H. sapiens	ENSG00000137203	TFAP2A	transcription factor AP-2 alpha	0.09	0.41	9.6E-03	2.5E-02	0.41	9.6E-03		MDPSRAPALRGASAP*
HG0868	H. sapiens	ENSG00000137843	PAK6	p21 (RAC1) activated kinase 6	0.09	0.41	3.5E-08	2.9E-07	0.41	3.5E-08		MPEDLMEKSQSEPGRAVEEGRAAAGAAGP*
HG0869	H. sapiens	ENSG00000139910	NOVA1	NOVA alternative splicing regulator 1	0.04	0.35	1.1E-03	4.1E-03	0.35	1.1E-03		MSFNGTDIASSFALESS*
HG0870	H. sapiens	ENSG00000140993	TIGD7	tigger transposable element derived 7	0.01	0.30	2.5E-05	1.3E-04	0.30	2.5E-05		MALGSTRRTRKEYLRA*
HG0871	H. sapiens	ENSG00000141127	PRPSAP2	phosphoribosyl pyrophosphate synthetase associated prote	0.04	0.37	6.8E-06	3.9E-05	0.37	6.8E-06		MPAQKGREPSRGTAEEESILEEFCCQRIKGGCP*
HG0872	H. sapiens	ENSG00000142583	SLC2A5	solute carrier family 2 member 5	0.16	0.30	1.2E-05	6.6E-05	0.30	1.2E-05		MLLAVIWHGQSAPRMSSLSIQCTRYFG*
HG0873	H. sapiens	ENSG00000142875	PRKACB	protein kinase cAMP-activated catalytic subunit beta	0.00	0.48	1.2E-04	5.5E-04	0.48	1.2E-04		MLGSYALLLGASLFKTKESSFRFLCAACSSVCVYTIGSSPSRD*
HG0874	H. sapiens	ENSG00000143515	ATP8B2	ATPase phospholipid transporting 8B2	0.22	0.24	9.2E-05	4.2E-04	0.24	9.2E-05		MGSTGPPGRAPSAER*
HG0875	H. sapiens	ENSG00000145284	SCD5	stearoyl-CoA desaturase 5	0.09	0.26	1.3E-05	7.0E-05	0.26	1.3E-05		MGDTPATDAGRQDLHP*
HG0876	H. sapiens	ENSG00000145908	ZNF300	zinc finger protein 300	0.05	0.36	1.2E-03	4.3E-03	0.36	1.2E-03		MAAPCGNSSREQPCCFLNKASEDAERAKPPSTERRSLGSPVWCSRTGSRRVCQ*
HG0877	H. sapiens	ENSG00000146426	TIAM2	T-cell lymphoma invasion and metastasis 2	0.00	0.33	3.3E-07	2.4E-06	0.33	3.3E-07		MKKRLTSWTHFACLLNVLCCSQDHVKPTDH*
HG0878	H. sapiens	ENSG00000146802	TMEM168	transmembrane protein 168	0.01	0.37	1.8E-04	7.8E-04	0.37	1.8E-04		MAVAAGPVTEKVYADTGLY*
HG0879	H. sapiens	ENSG00000147145	LPAR4	lysophosphatidic acid receptor 4	0.03	0.28	2.0E-07	1.5E-06	0.28	2.0E-07		MDKNMHFQRASCPFTCLLANYLQASAHSLI*
HG0880	H. sapiens	ENSG00000151789	ZNF385D	zinc finger protein 385D	0.01	0.33	5.7E-05	2.8E-04	0.33	5.7E-05		MGSAAGSPSALSSRRGS*
HG0881	H. sapiens	ENSG00000153822	KCNJ16	potassium voltage-gated channel subfamily J member 16	0.00	0.45	1.6E-02	3.9E-02	0.45	1.6E-02		MAVGADSFRRVLTGPREKGPEMLSYGALTR*
HG0882.1	H. sapiens	ENSG00000154832	CXXC1	CXXC finger protein 1	0.01	0.30	4.5E-06	2.6E-05	0.30	4.5E-06		MFPANIWIRPPYGYDKQDGGA*
HG0882.2	H. sapiens	ENSG00000154832	CXXC1	CXXC finger protein 1	0.01	0.26	7.0E-03	1.9E-02	0.26	7.0E-03		MWKGKETEWRNEYNLIQVVLRKRSLYL*
HG0883	H. sapiens	ENSG00000156011	PSD3	pleckstrin and Sec7 domain containing 3	0.00	0.00	1.5E-05	7.9E-05	0.00	1.5E-05		MLGEAAWCFLS*
HG0884	H. sapiens	ENSG00000156463	SH3RF2	SH3 domain containing ring finger 2	0.00	0.16	2.9E-08	2.4E-07	0.16	2.9E-08		MWTLLLQVGTGVLK*
HG0885.1	H. sapiens	ENSG00000157502	MUM1L1	MUM1 like 1	0.00	0.37	2.7E-07	2.0E-06	0.37	2.7E-07		MPTKPWKDSATTEVPSWVKALLDKQLE*
HG0885.2	H. sapiens	ENSG00000157502	MUM1L1	MUM1 like 1	0.00	0.43	3.6E-03	1.1E-02	0.43	3.6E-03		MAVVNASYFQRVLEDLE*
HG0886	H. sapiens	ENSG00000158850	B4GALT3	beta-1,4-galactosyltransferase 3	0.05	0.33	4.4E-05	2.2E-04	0.33	4.4E-05		MAAAMPARLLGWR*
HG0887	H. sapiens	ENSG00000160959			0.00	0.43	1.5E-06	9.7E-06	0.43	1.5E-06		MIVEICPPAELGRGLVSGTFQEPVP*
HG0888	H. sapiens	ENSG00000164023	SGMS2	sphingomyelin synthase 2	0.00	0.27	1.5E-05	8.2E-05	0.27	1.5E-05		MEETVIGWLPSTFCIRKQSPL*
HG0889	H. sapiens	ENSG00000164091	WDR82	WD repeat domain 82	0.08	0.24	1.4E-04	6.2E-04	0.24	1.4E-04		MADTMSRRRAGCP*
HG0890	H. sapiens	ENSG00000165813			0.11	0.18	2.5E-07	1.8E-06	0.18	2.5E-07		MEKLAPEPELQQPC*
HG0891	H. sapiens	ENSG00000166211	SPIC	Spi-C transcription factor	0.00	0.40	2.2E-05	1.1E-04	0.40	2.2E-05		MKHLYKGLKCLPGLSTYFILFSSSNNC*
HG0892	H. sapiens	ENSG00000168418	KCNG4	potassium voltage-gated channel modifier subfamily G mer	0.28	0.43	1.3E-06	8.4E-06	0.43	1.3E-06		MGSPCLAEKQLETGSLRQEDW*
HG0893	H. sapiens	ENSG00000168769	TET2	tet methylcytosine dioxygenase 2	0.26	0.38	1.0E-02	2.7E-02	0.38	1.0E-02		MQVTSAPSARPPRDAGPAE*
HG0894.1	H. sapiens	ENSG00000169282	KCNAB1	potassium voltage-gated channel subfamily A member regu	0.00	0.50	4.8E-04	1.9E-03	0.50	4.8E-04		MLRAPVEHPACGPGDAGGASCGKPALI*
HG0894.2	H. sapiens	ENSG00000069424	KCNAB2	potassium voltage-gated channel subfamily A regulatory be	0.00	0.29	2.2E-04	9.2E-04	0.29	2.2E-04		MALSPVVPVQEQNFPTVKNRASLDK*
HG0895	H. sapiens	ENSG00000170035	UBE2E3	ubiquitin conjugating enzyme E2 E3	0.00	0.45	1.2E-02	3.0E-02	0.45	1.2E-02		MNLRCWPWKQLEQLFSKR*
HG0896	H. sapiens	ENSG00000170365	SMAD1	SMAD family member 1	0.00	0.40	2.1E-03	6.9E-03	0.40	2.1E-03		MCFRRGGGTNACQISQLQ*
HG0897	H. sapiens	ENSG00000171435	KSR2	kinase suppressor of ras 2	0.10	0.20	2.9E-05	1.5E-04	0.20	2.9E-05		MPCPCNWEGGKPDFF*
HG0898	H. sapiens	ENSG00000171729	TMEM51	transmembrane protein 51	0.02	0.34	1.2E-07	9.4E-07	0.34	1.2E-07		MKSCLLACPESPQITTGPSTAAIRTGGLAIARNHPAGVQLIFSSVGREILWSAFKGFLL*
HG0899	H. sapiens	ENSG00000173320	STOX2	storkhead box 2	0.03	0.36	7.9E-05	3.7E-04	0.36	7.9E-05		MCRGHMRVGAPTQPSTRAHAQRPEPRQGED*
HG0900	H. sapiens	ENSG00000173517	PEAK1	pseudopodium enriched atypical kinase 1	0.00	0.40	7.4E-05	3.5E-04	0.40	7.4E-05		MVVAAAACAPLRYLPKLELML*
HG0901	H. sapiens	ENSG00000175697	GPR156	G protein-coupled receptor 156	0.00	0.49	5.2E-03	1.5E-02	0.49	5.2E-03		MRNQSEGTRTVTAIWWLTRIITLCSLMVTKMSTLDSHGMHSQQGTS*
HG0902	H. sapiens	ENSG00000177182	CLVS1	clavesin 1	0.00	0.20	4.2E-08	3.5E-07	0.20	4.2E-08		MASVLLLFWMNTTT*
HG0903	H. sapiens	ENSG00000180616	SSTR2	somatostatin receptor 2	0.05	0.09	7.0E-08	5.6E-07	0.09	7.0E-08		MSHWILGLQGPLR*
HG0904	H. sapiens	ENSG00000182329			0.00	0.29	3.7E-04	1.5E-03	0.29	3.7E-04		MYLIKGPVFVVFLGLL*
HG0905	H. sapiens	ENSG00000183337	BCOR	BCL6 corepressor	0.00	0.00	1.2E-05	6.4E-05	0.00	1.2E-05		MLESVEDGAGRPED*
HG0906	H. sapiens	ENSG00000183773	AIFM3	apoptosis inducing factor, mitochondria associated 3	0.11	0.19	4.4E-09	4.2E-08	0.19	4.4E-09		MAAPASLRPAGGPAPWGAP*
HG0907	H. sapiens	ENSG00000186862	PDZD7	PDZ domain containing 7	0.05	0.45	3.5E-05	1.8E-04	0.45	3.5E-05		MELEARRAGLSRASRFLLDL*
HG0908.1	H. sapiens	ENSG00000188554	NBR1	NBR1, autophagy cargo receptor	0.00	0.39	7.4E-04	2.7E-03	0.39	7.4E-04		MHCGTKGLGHTVPWKL*
HG0908.2	H. sapiens	ENSG00000188554	NBR1	NBR1, autophagy cargo receptor	0.00	0.25	4.4E-06	2.6E-05	0.25	4.4E-06		MERNIQYQSGHNSHGNPVDRLETSAGLYMSTVMDWSVVMFSYLESRD*
HG0909	H. sapiens	ENSG00000189079	ARID2	AT-rich interaction domain 2	0.27	0.18	8.0E-04	2.9E-03	0.18	8.0E-04		MTEPRRRRPRNGLRALVGSAGSGGRF*
HG0910	H. sapiens	ENSG00000197935	ZNF311	zinc finger protein 311	0.00	0.41	5.5E-06	3.2E-05	0.41	5.5E-06		MNPKSRAVFDTLSQTVAALWVKKGPLAGTTKTCVLQRRLASTGATSQLV*
HG0911	H. sapiens	ENSG00000198673	FAM19A2	family with sequence similarity 19 member A2, C-C motif cl	0.00	0.41	8.9E-04	3.2E-03	0.41	8.9E-04		MPRRAAGGLRGALPAALRGGIEGLKLARSCC*
HG0912	H. sapiens	ENSG00000198822	GRM3	glutamate metabotropic receptor 3	0.11	0.35	2.4E-04	1.0E-03	0.35	2.4E-04		MPGGPCFCQESQLDATASAWSR*
HG0913.1	H. sapiens	ENSG00000204599	TRIM39	tripartite motif containing 39	0.00	0.45	4.7E-04	1.8E-03	0.45	4.7E-04		MRLSLKFALQLRHKE*
HG0913.2	H. sapiens	ENSG00000204599	TRIM39	tripartite motif containing 39	0.01	0.10	2.7E-04	1.1E-03	0.10	2.7E-04		MPQPQIWRKKMENGGGTELWTTSQERVARWDPEQW*
HG0914	H. sapiens	ENSG00000227877	MRLN	myoregulin	0.01	0.33	8.5E-04	3.1E-03	0.33	8.5E-04		MVFFIKERDHSLDFGLRLLNPWTESRLLRDHLC*
HG0915	H. sapiens	ENSG00000254469	AP002495.2	Putative short transient receptor potential channel 2-like pro	0.10	0.47	9.2E-04	3.3E-03	0.47	9.2E-04		MYFPGCSTCHLASVGLIFKNSSFLKPQNAFLCYGRRKSSSRGQKAKVRRPNWRENRFGGPRSEATVAPRGPRSRE*
HG0916	H. sapiens	ENSG00000020633	RUNX3	runt related transcription factor 3	0.17	0.26	6.3E-04	2.4E-03	0.26	6.3E-04		MIHSAYASVENLWLDIPVGPGIQILGS*
HG0917	H. sapiens	ENSG00000042980	ADAM28	ADAM metallopeptidase domain 28	0.01	0.41	8.1E-03	2.2E-02	0.41	8.1E-03		MNQGVSFCLTGEEAGTDPAAPT*

HG0918	*H. sapiens*	ENSG00000047648	ARHGAP6	Rho GTPase activating protein 6	0.11	0.43	2.6E-03	8.3E-03	0.43	2.6E-03		MDLLLLPPPGHLERTGGA*
HG0919	*H. sapiens*	ENSG00000051009	FAM160A2	family with sequence similarity 160 member A2	0.00	0.00	4.6E-05	2.3E-04	0.00	4.6E-05		MGSYWLLQTG*
HG0920	*H. sapiens*	ENSG00000051382	PIK3CB	phosphatidylinositol-4,5-bisphosphate 3-kinase catalytic su	0.00	0.12	9.4E-06	5.2E-05	0.12	9.4E-06		MGATILRAPWL*
HG0921	*H. sapiens*	ENSG00000058799	YIPF1	Yip1 domain family member 1	0.03	0.46	1.1E-04	4.9E-04	0.46	1.1E-04		MRKLRPRGARIQQRSSRIGNQTQLCLTPEPVPLTTG*
HG0922	*H. sapiens*	ENSG00000079841	RIMS1	regulating synaptic membrane exocytosis 1	0.03	0.34	2.2E-07	1.6E-06	0.34	2.2E-07		MQTTMKDWVLALPGSAAAAAAAAAAAPPPAAAARAPL*
HG0923	*H. sapiens*	ENSG00000081138	CDH7	cadherin 7	0.14	0.30	1.7E-04	7.5E-04	0.30	1.7E-04		MTLPSCLRSSTDHGFHDRLRYLLHHSLAKAIQQW*
HG0924	*H. sapiens*	ENSG00000081307	UBA5	ubiquitin like modifier activating enzyme 5	0.21	0.50	2.8E-04	1.1E-03	0.50	2.8E-04		MSATHRKRLRGRPVGVSETCLSVRRWVHVPRALG*
HG0925	*H. sapiens*	ENSG00000088766	CRLS1	cardiolipin synthase 1	0.30	0.49	3.3E-03	1.0E-02	0.49	3.3E-03		MEAAVAQCLSGCRVSMEKRLASVPGC*
HG0926	*H. sapiens*	ENSG00000095397	WHRN	whirlin	0.00	0.34	5.8E-07	4.1E-06	0.34	5.8E-07		MPRTPCRTPAPLHRLPLPGVLWD*
HG0927	*H. sapiens*	ENSG00000095951	HIVEP1	human immunodeficiency virus type I enhancer binding pro	0.00	0.30	1.1E-05	6.1E-05	0.30	1.1E-05		MAACGRPLALLGALAAREPSSTWIN*
HG0928	*H. sapiens*	ENSG00000102057	KCND1	potassium voltage-gated channel subfamily D member 1	0.05	0.29	1.7E-03	5.7E-03	0.29	1.7E-03		MPQSPLHHCKGIPGSSAFPETLESAENSPRVSWPL*
HG0929	*H. sapiens*	ENSG00000102239	BRS3	bombesin receptor subtype 3	0.25	0.20	1.5E-03	5.1E-03	0.20	1.5E-03		MGKHLAKHAGRHTELKQRSIWMSWIFFPFCSVLFS*
HG0930	*H. sapiens*	ENSG00000102524	TNFSF13B	TNF superfamily member 13b	0.12	0.31	2.9E-04	1.2E-03	0.31	2.9E-04		MQKGRKEKIQDNSPEG*
HG0931	*H. sapiens*	ENSG00000104951	IL4I1	interleukin 4 induced 1	0.12	0.45	4.2E-03	1.3E-02	0.45	4.2E-03		MCFLPSPDKWPFLKFLINSYEDNTFGD*
HG0932	*H. sapiens*	ENSG00000105997	HOXA3	homeobox A3	0.11	0.48	9.3E-05	4.3E-04	0.48	9.3E-05		MLRVRAHPAPRGPGRAAKPGRGPEPVSEPEPEPALVRS*
HG0933	*H. sapiens*	ENSG00000106330	MOSPD3	motile sperm domain containing 3	0.05	0.34	7.1E-04	2.7E-03	0.34	7.1E-04		MTEAVRRKYRKGLSGTWQH*
HG0934	*H. sapiens*	ENSG00000107443	CCNJ	cyclin J	0.00	0.19	4.4E-05	2.2E-04	0.19	4.4E-05		MSGAGVRRTTAGLGL*
HG0935	*H. sapiens*	ENSG00000108518	PFN1	profilin 1	0.06	0.14	1.6E-03	5.5E-03	0.14	1.6E-03		MGSRPRPYRPLQIKI*
HG0936	*H. sapiens*	ENSG00000108799	EZH1	enhancer of zeste 1 polycomb repressive complex 2 subun	0.00	0.46	6.0E-03	1.7E-02	0.46	6.0E-03		MEAGHLFCCCVLPFS*
HG0937	*H. sapiens*	ENSG00000109846	CRYAB	crystallin alpha B	0.03	0.44	1.1E-04	5.1E-04	0.44	1.1E-04		MTSHWPAQTCLCLSFLSSVSTGYVSHCQIPDHKSP*
HG0938	*H. sapiens*	ENSG00000111665	CDCA3	cell division cycle associated 3	0.13	0.33	9.1E-04	3.3E-03	0.33	9.1E-04		MGTTSAQPIGSGQGAWREV*
HG0939	*H. sapiens*	ENSG00000111674	ENO2	enolase 2	0.22	0.49	4.9E-03	1.4E-02	0.49	4.9E-03		MGALEERPRQPILFLAPRRNASLAAIPL*
HG0940	*H. sapiens*	ENSG00000115556	PLCD4	phospholipase C delta 4	0.01	0.20	9.7E-03	2.5E-02	0.20	9.7E-03		MGWTGESSHLSPSYCFPPAITCQSQQPAAVEEGRKQASARGAKEKRSQAGLPDPTASQSSGGTAHRHRKHRTAILLSCPR*
HG0941	*H. sapiens*	ENSG00000116285	ERRFI1	ERBB receptor feedback inhibitor 1	0.11	0.42	8.6E-04	3.1E-03	0.42	8.6E-04		MRGRAPRGSRRSSGGMKATG*
HG0942	*H. sapiens*	ENSG00000117477			0.04	0.47	6.5E-05	3.1E-04	0.47	6.5E-05		MEDAEARCCHGSVVLAAAEAGAGVSFASM*
HG0943	*H. sapiens*	ENSG00000120690	ELF1	E74 like ETS transcription factor 1	0.00	0.38	9.3E-04	3.4E-03	0.38	9.3E-04		MLVRPRSLREEKFQKNCLNLTRISMNQEN*
HG0944	*H. sapiens*	ENSG00000120833	SOCS2	suppressor of cytokine signaling 2	0.00	0.50	4.6E-04	1.8E-03	0.50	4.6E-04		MVQERRGGGVPGIFHVYFPTPTPAAGVQFGLTQPPAFSL*
HG0945	*H. sapiens*	ENSG00000124782	RREB1	ras responsive element binding protein 1	0.00	0.43	1.4E-03	4.9E-03	0.43	1.4E-03		MIATAGESFIVYQWVRKWR*
HG0946.1	*H. sapiens*	ENSG00000127324	TSPAN8	tetraspanin 8	0.00	0.43	5.8E-03	1.7E-02	0.43	5.8E-03		MTSKGTYLPGDSLCDI*
HG0946.2	*H. sapiens*	ENSG00000127324	TSPAN8	tetraspanin 8	0.00	0.08	3.4E-06	2.0E-05	0.27	7.4E-04		MCGLIFLSSLITFYSSLYFFAFSFCSEAVDTEISAGKLLQSILQDKPVTNS*
HG0947	*H. sapiens*	ENSG00000129226	CD68	CD68 molecule	0.14	0.49	1.3E-02	3.3E-02	0.49	1.3E-02		MTKREVARAEAPESGCGWDHLQYRK*
HG0948	*H. sapiens*	ENSG00000129595	EPB41L4A	erythrocyte membrane protein band 4.1 like 4A	0.01	0.12	1.8E-02	4.3E-02	0.12	1.8E-02		MRGDAPPPSAPSSAGASPAAPGR*
HG0949	*H. sapiens*	ENSG00000131381	RBSN	rabenosyn, RAB effector	0.00	0.16	5.9E-05	2.9E-04	0.16	5.9E-05		MKPLPHHLCAA*
HG0950	*H. sapiens*	ENSG00000132964	CDK8	cyclin dependent kinase 8	0.13	0.44	6.1E-04	2.3E-03	0.44	6.1E-04		MSLALSRGLLLLLPHQSGWCCGRRA*
HG0951	*H. sapiens*	ENSG00000134061	CD180	CD180 molecule	0.00	0.29	1.1E-02	2.8E-02	0.29	1.1E-02		MLSSQQHFLFQDHPSEYLSGCQIARAFTV*
HG0952	*H. sapiens*	ENSG00000134453	RBM17	RNA binding motif protein 17	0.05	0.00	7.1E-03	2.0E-02	0.00	7.1E-03		MGRVGRAGRH*
HG0953	*H. sapiens*	ENSG00000134815	DHX34	DExH-box helicase 34	0.00	0.10	1.0E-04	4.7E-04	0.10	1.0E-04		MNLCPSQRSLQMS*
HG0954	*H. sapiens*	ENSG00000134874	DZIP1	DAZ interacting zinc finger protein 1	0.00	0.27	9.6E-05	4.4E-04	0.27	9.6E-05		MSGPSRNSPVDWGMSGFSSAY*
HG0955	*H. sapiens*	ENSG00000134982	APC	APC, WNT signaling pathway regulator	0.00	0.17	1.2E-03	4.1E-03	0.17	1.2E-03		MLFPGTVVGCW*
HG0956	*H. sapiens*	ENSG00000136235	GPNMB	glycoprotein nmb	0.09	0.14	6.4E-05	3.1E-04	0.36	1.1E-02		MILPEALYLIKIEREAPQMPEEHCCSWWTGPEEFRVKP*
HG0957	*H. sapiens*	ENSG00000136874	STX17	syntaxin 17	0.04	0.48	8.3E-03	2.2E-02	0.48	8.3E-03		MTRRSMVVRLGGLGARGRKPLLAKPRGGWQKVPHLGSLLLANRGSERRLYFDFSLQIRFFYMSGEDSCYQGGHTTFF*
HG0958	*H. sapiens*	ENSG00000137497	NUMA1	nuclear mitotic apparatus protein 1	0.00	0.10	2.2E-05	1.2E-04	0.10	2.2E-05		MNNHSCPFAPSLGGN*
HG0959	*H. sapiens*	ENSG00000137558	PI15	peptidase inhibitor 15	0.00	0.47	1.4E-03	4.9E-03	0.47	1.4E-03		MLPNDVFVNSYPLALFFFIDPNSTFLL*
HG0960	*H. sapiens*	ENSG00000140297	GCNT3	glucosaminyl (N-acetyl) transferase 3, mucin type	0.00	0.46	6.6E-04	2.5E-03	0.46	6.6E-04		MISLSLLSYLRAGSQKESDGATCCPLLCRY*
HG0961	*H. sapiens*	ENSG00000140853	NLRC5	NLR family CARD domain containing 5	0.09	0.04	4.2E-07	3.0E-06	0.04	4.2E-07		MARLEIAEGRREGEKWIPVLRSGLEGEVCKESALWKQPVNPAQGTHSPDTHETLSVGTLEHLS*
HG0962.1	*H. sapiens*	ENSG00000143319	ISG20L2	interferon stimulated exonuclease gene 20 like 2	0.11	0.11	4.8E-04	1.9E-03	0.11	4.8E-04		MVSRSLRGRRTWVRCMRRLPPIPAWSQGKGMPGFVSLLVVHAADAWVAQRLSTPYFSLFLSIPRCSFPRRSIDRTCSSLLDSEGSSSISPSTPFILQMRLKPRAR*
HG0962.2	*H. sapiens*	ENSG00000143319	ISG20L2	interferon stimulated exonuclease gene 20 like 2	0.00	0.36	5.6E-04	2.2E-03	0.36	5.6E-04		MLDSRIQQRMSPTSSFHP*
HG0963	*H. sapiens*	ENSG00000143867	OSR1	odd-skipped related transciption factor 1	0.13	0.49	6.2E-05	3.0E-04	0.49	6.2E-05		MSESELGLSPARSQLLLVQTQRGSRERGSPSPACRIRGC*
HG0964	*H. sapiens*	ENSG00000145246	ATP10D	ATPase phospholipid transporting 10D (putative)	0.04	0.23	2.5E-04	1.0E-03	0.23	2.5E-04		MAGVEAQPAFTLRICA*
HG0965	*H. sapiens*	ENSG00000146006	LRRTM2	leucine rich repeat transmembrane neuronal 2	0.00	0.00	6.2E-07	4.3E-06	0.00	6.2E-07		MKPMLSRGIS*
HG0966	*H. sapiens*	ENSG00000148356	LRSAM1	leucine rich repeat and sterile alpha motif containing 1	0.05	0.43	5.4E-05	2.6E-04	0.43	5.4E-05		MFVVGRRLRLTAGKQGTAVR*
HG0967	*H. sapiens*	ENSG00000149925	ALDOA	aldolase, fructose-bisphosphate A	0.11	0.49	8.3E-04	3.1E-03	0.49	8.3E-04		MSGASGFPKYRSLPEDPWREKGRGH*
HG0968	*H. sapiens*	ENSG00000154319	FAM167A	family with sequence similarity 167 member A	0.02	0.27	3.6E-06	2.2E-05	0.32	4.8E-04		MRFSSLPQGLQPPALPEKRELWAFFLPVWSSSAFRTDRPWHRLPRDSCDAVCSCLLWSMAPTGFQDGS*
HG0969	*H. sapiens*	ENSG00000154645	CHODL	chondrolectin	0.15	0.30	4.2E-05	2.1E-04	0.30	4.2E-05		MRAGGRAAGLGRRE*
HG0970	*H. sapiens*	ENSG00000154727	GABPA	GA binding protein transcription factor alpha subunit	0.06	0.37	2.6E-03	8.2E-03	0.37	2.6E-03		MGRRFSRSTLTGQEASRRQSATGRV*
HG0971	*H. sapiens*	ENSG00000155592	ZKSCAN2	zinc finger with KRAB and SCAN domains 2	0.15	0.47	1.9E-03	6.2E-03	0.47	1.9E-03		MHSGKPHCAVTSCLEKRPGFCILEYTFTFSRAWHRFD*
HG0972	*H. sapiens*	ENSG00000155926	SLA	Src like adaptor	0.27	0.42	9.4E-03	2.5E-02	0.42	9.4E-03		MKNFNRKLLLQKIRSFAANGEHLRQP*
HG0973	*H. sapiens*	ENSG00000156535	CD109	CD109 molecule	0.16	0.24	6.9E-06	4.0E-05	0.24	6.9E-06		MFATAARSAIVMGMGGVEPPSPVSI*
HG0974	*H. sapiens*	ENSG00000157578	LCA5L	LCA5L, lebercilin like	0.03	0.23	1.3E-02	3.3E-02	0.23	1.3E-02		MQTNDHPYADHFEYLQPCFCHCLCRKATCFKKKSSWSAYERVGCFLQLIFPKQTIPPAISGFLKYILSLKRDQNLSKKYYRGKLINTTV*
HG0975.1	*H. sapiens*	ENSG00000182158	CREB3L2	cAMP responsive element binding protein 3 like 2	0.02	0.38	1.4E-04	6.2E-04	0.38	1.4E-04		MRAGLLPSFWMHIPL*
HG0975.2	*H. sapiens*	ENSG00000157613	CREB3L1	cAMP responsive element binding protein 3 like 1	0.19	0.00	7.0E-08	5.6E-07	0.00	7.0E-08		MEASCAPGGAGGGGVG*
HG0976	*H. sapiens*	ENSG00000158008	EXTL1	exostosin like glycosyltransferase 1	0.03	0.41	7.0E-03	1.9E-02	0.41	7.0E-03		MGLSLPICTMTAQD*
HG0977	*H. sapiens*	ENSG00000158321	AUTS2	AUTS2, activator of transcription and developmental regula	0.17	0.47	3.0E-04	1.2E-03	0.47	3.0E-04		MLPSLGGLAPLGSLRSPGLPWLHFLKIWEPGSEFSPRRV*
HG0978	*H. sapiens*	ENSG00000159023	EPB41	erythrocyte membrane protein band 4.1	0.01	0.40	2.7E-03	8.6E-03	0.40	2.7E-03		MCHCGDGSMASPGLAA*
HG0979	*H. sapiens*	ENSG00000159202	UBE2Z	ubiquitin conjugating enzyme E2 Z	0.00	0.37	1.2E-02	3.0E-02	0.37	1.2E-02		MWEEVRVSGKWLFQADI*
HG0980	*H. sapiens*	ENSG00000162670	BRINP3	BMP/retinoic acid inducible neural specific 3	0.00	0.16	3.8E-06	2.3E-05	0.16	3.8E-06		MIQILGTFFCVLHRFVCHHAVKQVLRES*
HG0981	*H. sapiens*	ENSG00000163629	PTPN13	protein tyrosine phosphatase, non-receptor type 13	0.00	0.48	5.5E-03	1.6E-02	0.48	5.5E-03		MLTAPGDRRAGEPCSVAPRSASAAVRVAYRS*
HG0982	*H. sapiens*	ENSG00000164116	GUCY1A3	guanylate cyclase 1 soluble subunit alpha	0.00	0.47	3.0E-04	1.2E-03	0.47	3.0E-04		MCGFARRALELLEIRKHSPEVCEATKHPSYQCP*
[truncated: 228,968 more chars]
